# Proteomic Analysis of Endothelial Activation Induced by Adult *Angiostrongylus vasorum* Homogenate: Insights into Vascular Remodeling and Hemostatic Imbalance

**DOI:** 10.3390/ani16060926

**Published:** 2026-03-15

**Authors:** Manuel Collado-Cuadrado, Iván Rodríguez-Escolar, Alfonso Balmori-de la Puente, Ana Montero-Calle, Sara Vázquez-Ávila, Fabio Macchioni, Rodrigo Barderas, Javier Sotillo, Miguel Pericacho, Rodrigo Morchón

**Affiliations:** 1 Zoonotic Diseases and One Health Group, Faculty of Pharmacy, Centre for Environmental Studies and Rural Dynamization (CEADIR), University of Salamanca, 37007 Salamanca, Spain; ivanrodriguez@usal.es (I.R.-E.); a.balmori@usal.es (A.B.-d.l.P.); 2 Chronic Disease Programme, UFIEC, Proteomics Core UCCTs, Instituto de Salud Carlos III, 28220 Madrid, Spain; ana.monteroc@isciii.es (A.M.-C.); r.barderasm@isciii.es (R.B.); 3 Proteomics Core UCCTs, Instituto de Salud Carlos III, 28220 Madrid, Spain; 4 Reference and Research Laboratory in Parasitology, Centro Nacional de Microbiología, Instituto de Salud Carlos III, 28220 Madrid, Spain; s.vazquez@isciii.es (S.V.-Á.); javier.sotillo@isciii.es (J.S.); 5 Dipartimento di Scienze Veterinarie, Università di Pisa, 56124 Pisa, Italy; fabio.macchioni@unipi.it; 6 CIBER of Frailty and Healthy Aging (CIBERFES), 28029 Madrid, Spain; 7 Department of Physiology and Pharmacology, University of Salamanca, 37007 Salamanca, Spain; pericacho@usal.es; 8 Biomedical Research Institute of Salamanca (IBSAL), University of Salamanca, 37007 Salamanca, Spain

**Keywords:** *Angiostrongylus vasorum*, vascular endothelium, in vitro model, proteomics, endothelial activation

## Abstract

The interaction between *Angiostrongylus vasorum* and the vascular endothelium of the host plays a key role in the pathogenesis of canine angiostrongylosis. The adult stage of *A. vasorum* resides in right ventricles and pulmonary arteries of dogs and foxes and maintains close contact with the endothelium, whose activation may contribute to the hemostatic and hemorrhagic disorders observed in infected animals. However, the molecular mechanisms underlying this endothelial dysfunction remain poorly understood. To investigate this interaction, an in vitro model of vascular endothelial cells was stimulated with the adult *A. vasorum* homogenate. Quantitative proteomic analysis, combined with bioinformatic tools, identified 691 and 6011 protein groups in the cell supernatants and the cell lysates, respectively. Of these, 213 proteins in the cell supernatants (193 up-regulated and 20 down-regulated) and 564 in the cell lysates (358 up-regulated and 206 down-regulated) showed differential expression compared to control cells. Up-regulated proteins included TFPI, CD59, VWF, ANGPT2, MMRN1, and FLT1, which are involved in endothelial activation, angio-genesis, and coagulation regulation. Conversely, C3, SERPINE1, SERPINB2, PLAU, PLAUR, and ICAM1 were down-regulated, suggesting modulation of fibrinolysis, inflammation, and cell adhesion pathways. These findings indicate that adult *A. vasorum* homogenate induces a multifactorial endothelial activation characterized by dysregulation of coagulation, complement, and vascular remodelling pathways. Future studies focusing on the temporal and molecular characterization of endothelial responses to excretory/secretory antigens in both definitive and accidental hosts will further clarify the mechanisms of vascular pathology and parasite tolerance.

## Simple Summary

The parasite *Angiostrongylus vasorum* damages the vascular endothelium in dogs, leading to severe coagulation and hemorrhagic disorders. Proteomic analysis shows that its antigens trigger endothelial activation, dysregulating proteins involved in inflammation and vascular remodeling. These findings clarify the molecular mechanisms behind the vascular pathology and provide a clearer understanding of how the parasite interacts with and compromises the host’s system.

## 1. Introduction

*Angiostrongylus vasorum* (Baillet, 1866) is a nematode parasite belonging to the family *Metastrongylidae*, which mainly affects canids, both domestic and wild [1]. The parasite has an indirect life cycle in which terrestrial and aquatic gastropods act as intermediate hosts, and canids, mainly the dog (*Canis familiaris*) and the red fox (*Vulpes vulpes*), serve as definitive hosts. In these, the worms reach the adult stage and reside in the right side of the heart and in the pulmonary arteries, being in direct contact with the endothelium [2].

This parasitosis produces a broad spectrum of clinical manifestations in domestic dogs, which are commonly grouped into four categories. Respiratory and cardiorespiratory signs are the most frequently reported and include coughing, dyspnea and tachypnea, reflecting pulmonary arterial damage, inflammatory infiltration and impaired gas exchange [3]. Haemorrhagic disorders are also serious and potentially fatal manifestations of the disease, ranging from epistaxis and prolonged bleeding to intracranial and pulmonary haematomas [4]. Neurological signs are mainly attributed to the entrapment of migrating *A. vasorum* larvae in the cerebral circulation, which can induce hemorrhagic or embolic events resulting in seizures, ataxia, or acute paresis [5]. Finally, nonspecific clinical signs, such as abdominal or lumbar pain, lethargy, anorexia and weight loss, may occur and often contribute to the heterogeneous and sometimes misleading clinical picture of the disease [6,7]. Recently, this parasite has gained attention within the veterinary community due to its spread beyond traditionally endemic areas, with an increased prevalence reported mainly in the Americas and Europe [8,9,10,11]. Climate change and the increasing amount of international movement of animals to endemic regions are considered key factors contributing to the rise in case numbers [10,12,13,14].

Understanding host–parasite interactions is essential for addressing parasitic infections, but this relationship remains poorly understood in the case of *A. vasorum* and its hosts [15,16,17]. In foxes, it has been shown that *A. vasorum* antigen induces endothelial activation by modifying the expression levels of vascular adhesion molecules and stimulating a more controlled immune and inflammatory response than in dogs, suggesting that foxes are better adapted to the infection [15,18]. In contrast, several studies have associated *A. vasorum* infection in domestic dogs with hemorrhagic disorders such as disseminated intravascular coagulation (DIC), hypocoagulation, and low fibrinogen levels, which currently represent a more complex clinical picture [15,19,20,21,22]. However, other authors have reported no differences in D-dimer concentrations, a biomarker of thrombus formation, between hemorrhagic and non-hemorrhagic dogs infected with *A. vasorum*, suggesting that these alterations may result from hyperfibrinolysis and unstable clot formation [22,23,24].

Subsequent studies have shown that *A. vasorum* excretory/secretory antigens do not directly increase fibrinolysis, suggesting that the hyperfibrinolysis observed in infected dogs appears to be a multifactorial host response rather than a direct effect of the parasite on vascular endothelium [21]. Moreover, a decrease or even inhibition of the complement and coagulation cascades has been reported [25,26].

Considering these findings, our goal was to investigate the interaction between *A. vasorum* and the vascular endothelium by analyzing the differential protein expression induced by an adult *A. vasorum* homogenate in an in vitro model of vascular endothelial cells, with the aim of elucidating the molecular mechanisms underlying endothelial activation.

## 2. Materials and Methods

### 2.1. Antigen Preparation

Adult *A. vasorum* homogenate (AAvH) was obtained following the methodology previously described by Morchón et al. [27]. Thirty-six *A. vasorum* adult worms obtained from naturally infected dogs were washed in phosphate-buffered saline (PBS) pH 7.2, cut into pieces, and homogenized in PBS. The homogenate was sonicated (three cycles of 70 KHz, 30 s) and centrifuged at 10,000 rpm for 30 min at 4 °C. Protein concentration was measured in the supernatant using a detergent-compatible (DC) protein assay commercial kit (Bio-Rad Laboratories, Hercules, CA, USA). All samples were stored at −80 °C until analysis.

### 2.2. Cell Culture, Maintenance and Treatments and Samples

An in vitro model of human umbilical vein endothelial cells (HUVEC) was used under the same conditions reported by Machado et al. [28] and Collado-Cuadrado et al. [29]. Cells were maintained in a CO_2_ incubator (Thermo Fisher Scientific, Barcelona, Spain) at 37 °C, 95% humidity, and 5% CO_2_, in plates previously coated with a matrix solution containing 0.1% porcine gelatin (Sigma Chemical Co., St. Louis, MO, USA), 0.01% fibronectin (Sigma-Aldrich, St. Louis, MO, USA), and 0.01% collagen (Corning^®^, New York, NY, USA). Cells were cultured in Endothelial Basal Medium-2 (Lonza, Walkersville, MD, USA) supplemented with SingleQuots^®^ (Lonza, Walkersville, MD, USA) and with gentamicin 30 mg/mL and amphotericin B 15 μg/mL. The culture medium was changed every 3 days.

Cell expansion was performed by trypsinization (Trypsin/EDTA, Lonza, Walkersville, MD, USA) when endothelial cell monolayers reached nearly 100% confluence. Treatments were carried out in 60 mm plates containing confluent endothelial cells exposed to 1 μg/μL of AAvH for 24 h following the methodology described by Machado et al. [28] and Collado-Cuadrado et al. [29]. Non-stimulated cells were used as controls under the same conditions. All experimental conditions were performed in triplicate. The culture medium and cell lysates from treated and control cultures were collected at 4 °C.

Specifically, cell lysates were mechanically collected after two washes with sterile PBS 1X by cell scraping (Corning^®^ Falcon^®^ Cell Scraper, Corning, New York, NY, USA) in RIPA buffer (Sigma-Aldrich, St. Louis, MO, USA) supplemented with a cocktail of protease inhibitors (1 mM EDTA, 1 mM N-ethylmaleimide, 0.1 M pepstatin A, 1 mM PMSF, and 0.1 mM N-tosylamide-L-phenylalanine chloromethyl ketone: PanReac AppliChem, Barcelona, Spain) to prevent protein degradation. Samples were then centrifuged at 14,000 rpm for 10 min at 4 °C, and the supernatant was collected. All samples were stored at −80 °C until processing.

### 2.3. Cellular Viability and Cytotoxicity Assays

Subsequently, cell counts were performed using the equipment Countess^®^ Automated Cell Counter (Invitrogen, Barcelona, Spain) following the manufacturer’s instructions to analyze the cellular viability (living and dead cells after treatment). Cytotoxicity was assessed in the supernatant of the stimulated and control cell cultures by a Toxilight BioAssay Kit (Cambrex, Verviers, Belgium) following commercial instructions. This commercial kit quantitatively measures the release of adenylate kinase from damaged cells.

### 2.4. Protein Sample Processing

Sample processing was assessed as previously described by Montero-Calle et al. [30] with some modifications. In brief, a total of 20 μg of proteins of each sample in 100 μL of RIPA buffer was reduced with 11 μL of 100 mM tris(2-carboxyethyl)phosphine (TCEP) (PanReac AppliChem, Barcelona, Spain) at 600 rpm for 45 min at 37 °C in a thermomixer and alkylated with 12 μL of 400 mM chloroacetamide (PanReac AppliChem, Barcelona, Spain) for 30 min in agitation in the dark to denature proteins and improve subsequent enzymatic digestion. Next, the samples were incubated with 100 μL of a stock of Sera-Mag magnetic beads (50% hydrophilic, 50% hydrophobic) and 200 μL of 100% acetonitrile (PanReac AppliChem, Barcelona, Spain) at 600 rpm for 35 min in a thermomixer at room temperature to promote protein binding to the beads.

Then, the supernatant was discarded, and the sediment was washed twice for 30 s with 200 μL of 70% ethanol (PanReac AppliChem, Barcelona, Spain) and once for 30 s with 200 μL of 100% acetonitrile (PanReac AppliChem, Barcelona, Spain). Subsequently the supernatant was discarded and the magnetic beads were resuspended in 100 μL of a digestion solution containing 20 μg of trypsin in 2 mL of 200 mM ammonium bicarbonate, pH 8.0 (PanReac AppliChem, Barcelona, Spain), and incubated at 600 rpm for 14 h at 37 °C in a thermomixer. Next day, samples were sonicated for two minutes and the supernatants were collected, dried under vacuum and stored at −80 °C until analysis.

### 2.5. Data-Independent Acquistion Label-Free Quantification Liquid Chromatography Tandem Mass Spectometry (DIA-LFQ LC-MS/MS)

For LC-MS/MS, peptides were analyzed in an Orbitrap Astral mass spectrometer coupled to a Vanquish Neo UHPLC System (Thermo Fisher Scientific, Madrid, Spain). Peptide samples were loaded into a precolumn PepMap Trap Catridge 5 µm, 300 µm × 5 mm (Thermo Fisher Scientific, Madrid, Spain) and eluted in an Easy-Spray PepMap RSLC C18 2 µm, 50 µm × 15 cm (Thermo Fisher Scientific, Madrid, Spain) heated at 50 °C. The mobile phase flow rate was 300 nL/min, and 0.1% formic acid (FA) in H_2_O_mq_ and 0.1% FA in 80% acetonitrile (ACN) were used as elution buffers A and B, respectively. The 15 min elution gradient was: 4–10% buffer B for 2 min, 10–40% buffer B for 11 min, 40–99% buffer B for 0.5 min, and 99% buffer B for 1.5 min. Prior to injection, samples were re-suspended in 10 µL of buffer A, and 1 µL of each sample was injected, and analyzed in data independent acquisition (DIA) mode. For ionization, 1900 V of liquid junction voltage and 280 °C capillary temperature were used. The full scan method employed a *m*/*z* 380–980 mass selection, an Orbitrap resolution of 240,000 (at *m*/*z* 200), an automatic gain control (AGC) value of 500%, and a maximum injection time (IT) of 5 ms. The MS/MS was performed with the Astral mass analyzer, using an AGC of 500%, an IT of 3 ms, and a normalized collision energy (NCE) of 25 for fragmentation of precursors. The scan range was set from 380 to 980 *m*/*z*, with an isolation window of 2 *m*/*z*, and window placement optimization was enabled. Thus, a total of 299 windows were analyzed in each cycle. Prior to mass spectrometry, peptide samples were resuspended in 20 µL of 0.1% formic acid (FA) and 1 µL was injected per run.

Raw data were analyzed using Spectronaut (v.19.1.) using standardized workflows. DIA raw data and the Uniprot UP000005640_9606.fasta (March 2024) database (20,418 protein entries), the *A. vasorum* database retrieved from WormBase Parasite (release WBPS19, BioProject PRJNA663250; 13,703 entries) and a dataset of common contaminants (246 entries) were used for the construction of the spectral library by directDIA. Trypsin/P and Lys/P were selected as the digestion enzymes and a maximum of 2 missed cleavages was allowed. Carbamidomethylation of cysteines was set as a fixed modification, and methionine oxidation and N-terminal acetylation were set as variable modifications. For DIA analysis, the standard Spectronaut workflow was used. The maximum FDR for peptide spectral match (PSM), peptide, and protein identifications was set at 0.01 (0.1%). Protein interference was performed using the IDPicker algorithm, and all identified proteins were used for protein identification. Automatic cross-run normalization was enabled. Regarding missing values, a Global Imputing strategy was applied during quantification to infer signal intensities based on the global data structure. Differential abundance was performed using unpaired Student’s *t*-test. Significantly changed candidates were selected based on a Q-value ≤ 0.05 and an absolute Log2 ratio ≥ 0.58.

### 2.6. Bioinformatic Analysis

Proteomic differential analysis was performed using Spectronaut (v.19.1.) [31] based on pairwise comparisons between AAvH-stimulated and control samples. Protein abundances were log_2_-transformed, and statistical significance was assessed using Spectronaut’s built-in statistical model. *p*-values were adjusted for multiple testing using the Benjamini–Hochberg false discovery rate correction. Proteins with an absolute average Log_2_ ratio ≥ 0.58, which corresponds with a Fold Change ≥ 1.5 and a Q-value ≤ 0.05, were considered dysregulated. Principal component analysis (PCA) of dysregulated proteins and the volcano plot displaying their distribution were generated using the ggplot package [32] in R v4.4.1 [33]. The use of a 1.5-fold change threshold was chosen as a biologically meaningful cutoff commonly applied in endothelial and proteomic studies, where moderate but coordinated changes in protein abundance can reflect relevant functional activation rather than stochastic variation. Therefore, this threshold allows the identification of biologically relevant protein modulation while maintaining statistical stringency.

Gene Ontology (GO) enrichment analysis of the differentially abundant proteins was carried out using the g:Profiler web tool [34]. The resulting list of enriched GO terms was semantically reduced using the rrvgo package in R [35]. Visualization of the GO results was also performed with ggplot. Differentially abundant proteins were represented in protein–protein interaction (PPI) networks constructed through STRING-DB v.12 [36]. STRING DB allows clustering, which enables us to visualize which nodes are most interconnected with others, reflecting the functional modularity of our protein list. Markov Cluster Algorithm (MCL) clustering was applied with an inflation parameter of 1.8 to identify dysregulated interaction modules, and disconnected proteins were excluded from the final network visualization.

## 3. Results

### 3.1. Effect of AAvH on Cell Viability and Cytotoxity

We verified that the viability of cells exposed to AAvH for 24 h and cytotoxicity remained equivalent to that of untreated cultures. The number of living cells exceeded 85% in all situations, with no differences observed between stimulated cultures with AgAv compared to non-stimulated cells. At the same time, no cytotoxic effect was observed from the antigen used, with no significant differences observed between them.

### 3.2. Principal Component Analysis and Volcano Plot

In the analysis of cell supernatants, 7959 peptides corresponding to 795 protein groups were identified, while 79,698 peptides corresponding to 6100 protein groups were detected in cell lysates using a Q-value ≤ 0.01. After removal of the *A. vasorum*-related proteins and common contaminants, 691 and 6011 protein groups were retained for subsequent analyses (Supplementary Tables S1–S7).

**Figure 1:**
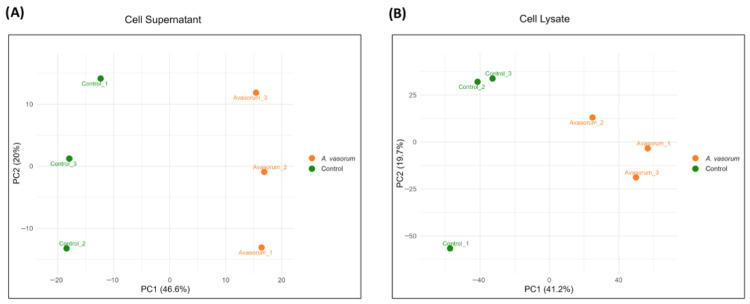
Principal Component Analysis of proteins identified in the cell supernatants (**A**) and cell lysates (**B**). Green dots correspond to control replicates and orange dots to endothelial cells treated with AAvH. The percentage of variability explained by PC1 and PC2 is indicated on the x- and y-axes, respectively.

PCA was performed using the proteins identified in both cell supernatants and cell lysates from control and AAvH-treated endothelial cells. In both datasets, PC1 accounted for the largest proportion of variance (46.6% for supernatants and 41.2% for cell lysates), while PC2 explained 20% and 19.7% of the variability, respectively. Two well-defined clusters were observed, corresponding to control and treated samples, indicating clear discrimination between experimental conditions. Variability was greater in the supernatants than in the cell lysates, where one control replicate showed a higher degree of divergence along PC2 compared to the other control replicates (Figure 1).

**Figure 2:**
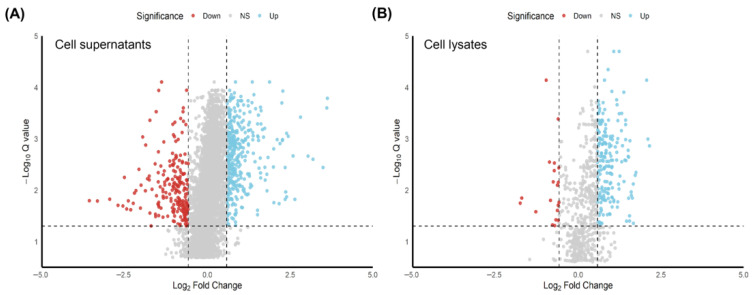
Volcano plot showing differentially abundant proteins in the cell lysates (**A**) and cell supernatants (**B**). Proteins located in the left side (red dots) and right side (blue dots) indicate down-regulated and up-regulated proteins respectively following the stimulation with AAvH in comparison with control group.

To identify differentially regulated proteins following stimulation with AAvH, an absolute AVG Log2 Ratio ≥ 0.58 (≥0.58 for up-regulated and ≤−0.58 for down-regulated proteins) and a Q-value ≤ 0.05 were applied as selection criteria (Figure 2). A total of 213 deregulated proteins were identified in the supernatants, including 193 up-regulated and 20 down-regulated proteins, whereas 564 deregulated proteins were detected in the cell lysates, comprising 358 up-regulated and 206 down-regulated proteins.

**Figure 3:**
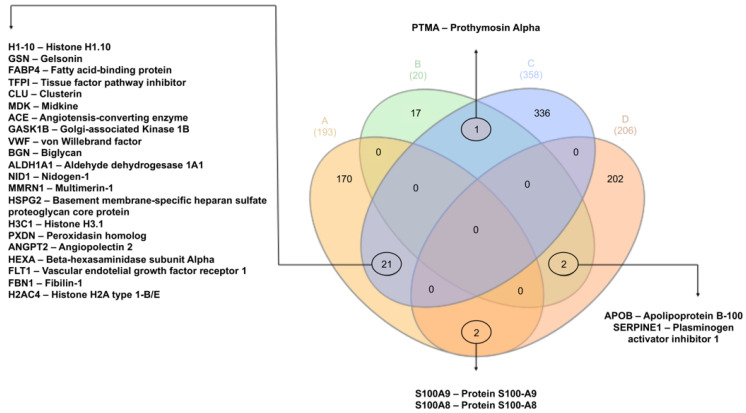
Venn diagram showing up-regulated and down-regulated proteins identified in vascular endothelial cells stimulated with AAvH compared to control cells, in four groups: up-regulated (**A**) and down-regulated (**B**) proteins in the cell supernatants, and up-regulated (**C**) and down-regulated (**D**) proteins in the cell lysates.

A Venn diagram was generated to identify common proteins between the supernatants and cell lysates (Figure 3). Twenty-one shared proteins were identified among the up-regulated groups, several of which were associated with endothelial remodeling, angiogenesis, and vascular protection: histones H1.10, H3C1, and H2AC4; Gelsolin (GSN); Angiotensin-Converting Enzyme (ACE); Midkine (MDK); Biglycan (BGN); Perlecan (Heparan Sulfate Proteoglycan 2, HSPG2); Peroxidasin (PXDN); Angiopoietin-2 (ANGPT2); VEGF receptor 1 (FLT1); Tissue Factor Pathway Inhibitor (TFPI); Clusterin (CLU); von Willebrand Factor (VWF); Multimerin-1 (MMRN1); Fatty Acid Binding Protein 4 (FABP4); Golgi-associated kinase 1B (GASK1B); Aldehyde Dehydrogenase 1A1 (ALDH1A1), Nidogen-1 (NID1); β-Hexosaminidasa subunidad alfa (HEXA); and Fibrillin-1 (FBN1). In the supernatants, additional up-regulated proteins included CD59 (Protectin), Tyrosine Kinase with Immunoglobulin-like and EGF-like Domains 1 (TIE1), and Tissue Inhibitors of Metalloproteinases 1 and 2 (TIMP1 and TIMP2), which are associated with endothelial proliferation and migration. In contrast, in the cell lysate, Interleukin 33 (IL-33) and Thrombospondin-1 (THBS1) were identified as up-regulated.

Among the down-regulated proteins, two were shared between the supernatants and cell lysates: Serpin family E member 1 (Plasminogen Activator Inhibitor-1, PAI-1; SERPINE1) and Apolipoprotein B-100 (APOB), involved in lipid metabolism. Additional down-regulated proteins detected in the cell lysates included Serpin family B member 2 (Plasminogen Activator Inhibitor type 2, PAI-2; SERPINB2), Plasminogen Activator, Urokinase (uPA; PLAU), and its receptor Urokinase Plasminogen Activator Surface Receptor (uPAR; PLAUR), all of which are related to fibrinolysis and inflammation. Furthermore, Intercellular Adhesion Molecule 1 (ICAM-1), associated with cell adhesion, and Tissue Factor Pathway Inhibitor 2 (TFPI2), linked to extracellular matrix maintenance, were down-regulated exclusively in the cell lysates.

In the supernatants, Coagulation Factor XIII A Chain (F13A1), related to clot stabilization, and Complement Component 3 (C3), a precursor of the complement cascade, were identified as up-regulated. The inflammatory proteins S100A8 (Calgranulin A) and S100A9 (Calgranulin B) were down-regulated in cell lysates but up-regulated in supernatants, whereas Prothymosin Alpha (PTMA), involved in cell proliferation, immune response, and apoptosis inhibition, showed the opposite trend—down-regulated in the supernatants and up-regulated in cell lysates.

### 3.3. Functional Annotation

**Figure 4:**
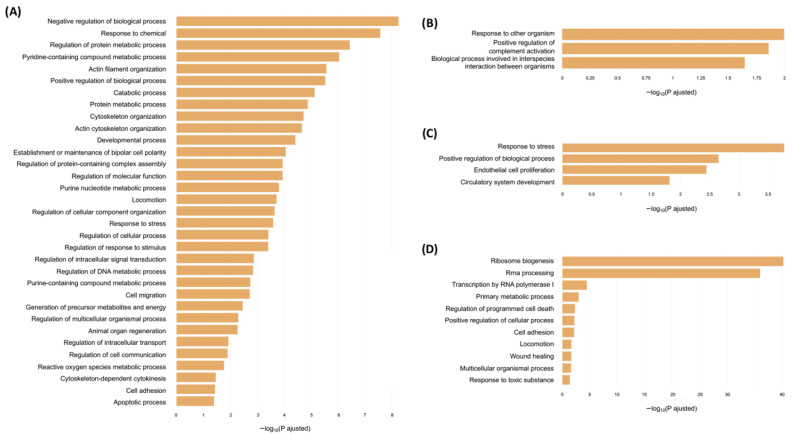
Gene Ontology enrichment analysis in the categories of Biological Process for (**A**) up-regulated proteins in the cell supernatants, (**B**) down-regulated proteins in the supernatant, (**C**) up-regulated proteins in the lysate, and (**D**) down-regulated proteins in the cell lysates identified of endothelial cells treated with AAvH. GO analyses were performed with g:Profiler and semantically reduced using rrvgo.

GO analyses were performed on the set of differentially abundant proteins using the g:Profiler tool, considering the three GO categories: Biological Process (BP), Molecular Function (MF), and Cellular Component (CC). Up-regulated and down-regulated proteins were analyzed separately to identify enriched biological terms associated with increased or decreased expression following stimulation with AAvH. To enhance data visualization, the R package rrvgo was used to semantically reduce GO terms, grouping related categories and highlighting the most representative terms within each cluster (Figure 4 and Supplementary Figures S1 and S2).

In the supernatants, enriched GO terms in the Biological Process category for up-regulated proteins were mainly associated with cellular organization and structural processes, particularly cytoskeletal organization and dynamics, including terms such as “actin cytoskeleton organization”, “actin filament organization”, and “establishment or maintenance of bipolar cell polarity”. Developmental processes were also represented by the broad term “developmental process”, which encompassed secondary terms related to endothelial activation such as “angiogenesis”, “tube development”, and “blood vessel development”. Metabolic processes were also enriched, primarily involving nitrogen-containing compound metabolism through terms such as “pyridine-containing compound metabolic process”, “purine nucleotide metabolic process”, and “purine-containing compound metabolic process”, as well as protein metabolism through “regulation of protein metabolic process” and “protein metabolic process”. Additional enriched biological processes included cell adhesion, migration, response to stimuli, and cell–cell communication. In the Molecular Function category, most enriched terms were related to binding activity, along with catalytic, structural, and antioxidant functions.

For down-regulated proteins in the supernatants, three enriched terms were identified in the Biological Process category: “response to other organism” and “biological regulation involved in interspecies interaction between organisms”, both related to responses to external stimuli or other organisms, and “positive regulation of complement activation”, linked to innate immunity. Within the Molecular Function category, only the term “platelet-derived growth factor binding” was enriched. Regarding the Cellular Component category, most up-regulated proteins were localized to extracellular exosomes, whereas down-regulated proteins were primarily associated with the extracellular space. In both analyses, the term “vesicle” ranked as the second most significant.

In the cell lysates, four GO terms within the Biological Process category were enriched among up-regulated proteins. These were mainly related to circulatory system development, cell proliferation, regulation of biological processes, and response to stress. Most enriched Molecular Function terms were associated with binding activities, while in the Cellular Component category, most proteins were localized to the cytoplasm.

Down-regulated proteins in the cell lysates were primarily related to “rRNA processing” and “ribosome biogenesis”, as well as to cell adhesion and migration within the Biological Process category. In the Molecular Function category, only two binding-related terms were enriched. The Cellular Component analysis revealed that most down-regulated proteins were components of the preribosome.

### 3.4. Protein–Protein Interaction Network Analysis

Protein–protein interaction networks were constructed using STRING-DB based on the sets of deregulated proteins identified in the supernatants and cell lysates, respectively, to identify dysregulated functional interaction modules. The analysis was performed with a high-confidence score (0.7), excluding unconnected proteins from the network.

**Figure 5:**
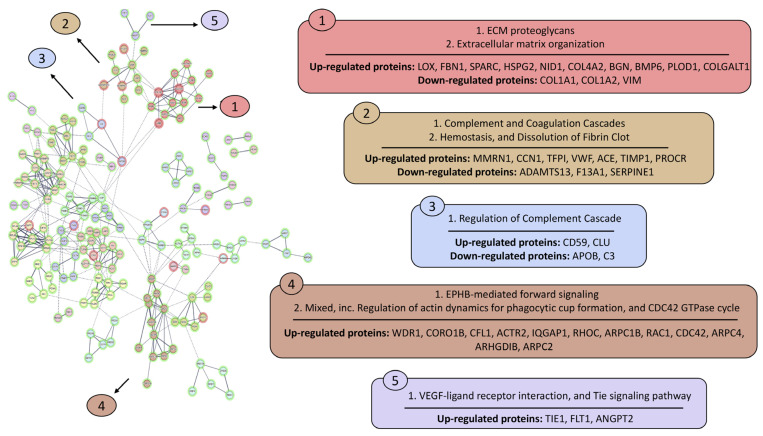
Protein–protein interaction network analysis of deregulated proteins identified in the cell supernatants using STRING-DB. The analysis was performed with high confidence (0.7), excluding unconnected nodes. Up-regulated proteins after stimulation with AAvH compared to the control group are represented by a green halo, and down-regulated proteins after stimulation are represented by red halo.

In the supernatants (Figure 5), 35 clusters containing two or more interacting proteins were obtained, each assigned to a functional term derived from STRING enrichment analysis, except for three clusters without an associated term. Cluster 1, related to extracellular matrix remodelling, contained 13 proteins (three down-regulated and nine up-regulated) with “COL1A1” as the central node, followed by “COL1A2”. Cluster 2, associated with the complement and coagulation cascade, comprised 10 proteins (three down-regulated and seven up-regulated), with VWF as the central node. Cluster 3 was linked to regulation of the complement cascade and included four proteins (two down-regulated and two up-regulated). Cluster 4, associated with actin cytoskeleton modulation, contained 12 up-regulated proteins, with “ACTR2” and “CDC42” as central nodes. Cluster 5, related to endothelial activation and vascular remodeling, contained three up-regulated proteins.

Additional clusters identified in the supernatants network were associated with metabolic pathways, including the tricarboxylic acid cycle, glycolytic processes, nucleotide di- and triphosphate interconversion, and nicotinate and nicotinamide metabolism, as well as with biological processes such as spliceosome function, reactive oxygen species detoxification, and C1q complex binding in the complement system.

**Figure 6:**
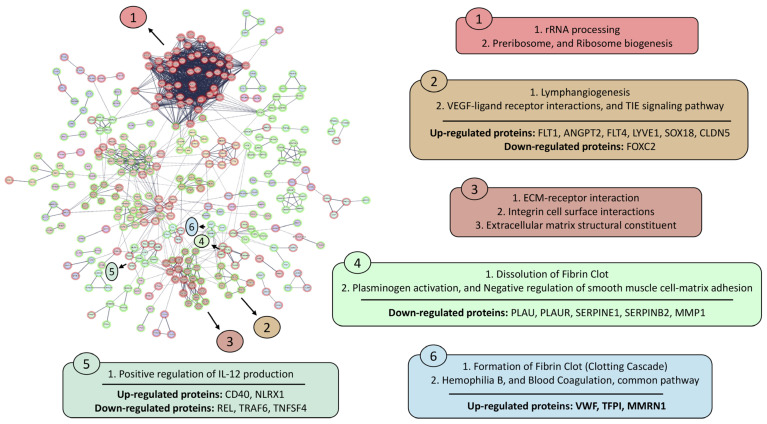
Protein–protein interaction network analysis of deregulated proteins identified in vascular endothelial cell lysates using STRING-DB. The analysis was performed with high confidence (0.7), and unconnected nodes were excluded from the network visualization. Up-regulated proteins after stimulation with AAvH compared to the control group are represented by a green halo, and down-regulated proteins after stimulation are represented by red halo.

In the cell lysate, 63 clusters containing two or more proteins were identified (Figure 6) under the established parameters. STRING-DB assigned representative biological terms to each cluster, except for 13 clusters for which no specific term was assigned. Cluster 1, related to “rRNA processing” and “ribosome biogenesis”, contained 52 proteins, all down-regulated except for two (RPL37 and TLE5), which were up-regulated. Cluster 2, associated with “endothelial activation” and “vascular remodeling”, comprised seven proteins, six of which were up-regulated and one down-regulated. Cluster 3, related to “extracellular matrix (ECM) remodeling”, was composed of 21 proteins (12 up-regulated and nine down-regulated) and had CD44 as its central node. Cluster 4, associated with “fibrin clot dissolution” and “plasminogen activation”, included five down-regulated proteins. Cluster 5, linked to “regulation of interleukin-12 production”, contained five proteins, two up-regulated and three down-regulated. Finally, cluster 6, associated with the “coagulation cascade”, comprised three up-regulated proteins. Other clusters identified in the cell lysates network were related to “galactose metabolism”, “cholesterol metabolism”, “pyrimidine metabolism”, “inositol phosphate and triphosphate metabolism”, and “glutathione metabolism and detoxification of reactive oxygen species”.

## 4. Discussion

The parasite–host interaction is a key element in understanding parasitic diseases, as it largely determines pathogenesis, clinical evolution, and immune response to infection [37]. *Angiostrongylus vasorum* is a cardiopulmonary parasite that interacts with the vascular endothelium in all its stages, making vascular endothelial cells an essential component in the definitive host’s response, capable of detecting and responding to parasitic stimuli by secreting proinflammatory mediators, adhesion molecules, and coagulation factors [15]. However, this interaction between the vascular system and *A. vasorum* is still poorly understood. Studies have observed endothelial activation in response to the parasite antigen in both dogs and foxes. They have also observed activation of the immune response and found that it is more controlled in foxes, suggesting that they have a greater tolerance to infection [15,18]. In dogs, the infection is sometimes associated with coagulopathies traditionally considered to be a phenomenon of DIC [19]. However, recent research associates bleeding with a multifactorial response, which could depend on alterations in the fibrinolytic system and in the complement and coagulation cascade [21,26].

In the present study, HUVEC was used as an in vitro model to evaluate differential protein expression and the biological processes derived from endothelial dysfunction induced by AAvH. HUVECs represent one of the most robust, accessible, and thoroughly characterized in vitro models for studying vascular biology which, at a basic cellular level, does not differ from that of other organisms and is highly conserved among mammals [38]. This model, as well as similar approaches using human endothelial cells, has been validated and successfully employed previously to investigate interactions between the endothelium and antigens from other cardiovascular parasites [27,28,29,39,40], and provides a valuable and standardized tool for dissecting specific and conserved cellular mechanisms that initiate vascular pathology.

Regarding our in vitro model, AAvH did not exert any cytotoxic effect on endothelial cells, and their viability remained unaltered; therefore, the effect of AAvH did not modify the normal behavior of vascular cells. As initial results of our study, stimulation of endothelial cells with AAvH produced a generalized increase in protein expression, with 193 proteins up-regulated in the supernatants and 358 in the cell lysate, compared to 20 and 206 down-regulated, respectively. This trend toward overexpression reflects a state of endothelial activation induced by the parasite. Functional analysis using GO and protein–protein interaction networks confirmed the involvement of pathways related to cytoskeletal remodeling, angiogenesis, cell adhesion, and metabolic processes, suggesting a general restructuring of the architecture and functionality of the endothelium.

One of the most relevant aspects of angiostrongylosis is the onset of coagulopathies and hemorrhages. Hemorrhagic symptoms in infected dogs have been described associated with hyperfibrinolysis [22,23,24]. Fibrinolysis is an essential process in vascular homeostasis that regulates the dissolution of fibrin clots and can be modulated by parasites to promote their survival [41]. In vascular helminths such as *Dirofilaria immitis*, it has been observed that excretory/secretory antigens can alter the expression of plasminogen activators and inhibitors [42,43,44]. However, in the case of *A. vasorum*, Gillis-Germitsch et al. [21] demonstrated that its excretory/secretory antigens do not induce significant changes in fibrinolytic factors and, in fact, promote an increase in SERPINE1 (PAI-1), suggesting an inhibition of fibrinolysis. In our model, on the contrary, SERPINE1 and SERPINB2 were found to be down-regulated, which could imply a potentiation of the fibrinolytic process due to a decrease in plasminogen activator inhibitors [45,46]. However, the simultaneous decrease in u-PA (PLAU) and its receptor u-PAR (PLAUR) suggests that this potentiation does not necessarily translate into active hyperfibrinolysis, supporting the hypothesis that hemorrhagic coagulopathies in dogs infected with *A. vasorum* are not the direct result of the parasite’s action on the endothelium but rather of a complex systemic response by the host.

Our results also show modulation of the complement and coagulation cascades. In the protein network analysis, modules related to coagulation and complement regulation were identified in both supernatants and cell lysates. Among the most prominent proteins are VWF, TFPI, and MMRN1, all of which are up-regulated. TFPI acts as an inhibitor of the tissue factor pathway, limiting thrombin formation and preventing excessive clot generation [47]. It has been proposed that some parasites, such as *Schistosoma* spp., can induce the release of TFPI from the endothelium through heparin-like molecules [48], suggesting a possible parallel with the modulation exerted by *A. vasorum*. In contrast, the proteins MMRN1 and VWF, both procoagulants, promote factor V stabilization and platelet adhesion, respectively. The overexpression of VWF in our model could reflect a state of endothelial activation, similar to that described in patients with schistosomiasis, where elevated VWF levels are associated with endothelial damage and vascular parasitism [49]. Furthermore, other authors identified orthologs of the VWF D domain in the excretory/secretory antigens of *A. vasorum*, reinforcing the hypothesis of a direct interaction between the parasite and the host’s hemostatic system [21].

Another noteworthy aspect is the overexpression of CD59 in the cell supernatants. This protein, which inhibits the formation of the membrane attack complex (MAC), suggests a possible negative modulation of the terminal complement pathway, reducing the damage mediated by this system. In parallel, the underexpression of factor XIIIa (F13A1), involved in the stabilization of fibrin clots, is consistent with the findings of Tritten et al. [25], who detected a decrease in the FXIIIB subunit in the serum of infected dogs. Both subunits cooperate in clot consolidation, so their reduction could contribute to the hemorrhagic state observed clinically. Likewise, the decrease in protein C3, a central component of the complement system, suggests an alteration in this pathway, possibly mediated by proteases of the excretory/secretory antigen of *A. vasorum* capable of degrading C3 [26]. Taken together, these results reinforce the hypothesis of a consumption or imbalance of coagulation and complement factors as the origin of hemostatic alterations.

In addition to hemostatic pathways, functional analysis revealed pathways associated with endothelial and extracellular matrix remodeling. Clusters related to endothelial and matrix remodeling were identified in the supernatants (cluster 5) and the cell lysates (cluster 2), with ANGPT2 protein among the most representative. ANGPT2 is an angiogenic mediator whose overexpression in pathological conditions promotes vascular destabilization and angiogenesis [50]. In patients with schistosomiasis, elevated levels of ANGPT2 and the ANGPT2/ANGPT1 ratio correlate with higher parasite load and endothelial damage [51]. In our model, ANGPT2 overexpression, together with the differential regulation of extracellular matrix and cytoskeletal proteins (COL1A1, COL1A2, CD44), supports the existence of a state of endothelial activation and remodeling induced by AAvH.

From an immunological point of view, an increase in IL-33 was detected in the cell lysate, which could reflect its release as an alarmin following endothelial damage. IL-33 acts as a key mediator in the induction of Th_2_-type immune responses, typical of helminth infections [52]. This finding is reinforced by the underexpression of proteins such as REL and TRAF6, involved in the activation of IL-12, and the overexpression of NLRX1, which acts as an inhibitor of this same cytokine [53,54,55]. Taken together, the results point to a polarization of the endothelial immune response toward a Th_2_ profile, consistent with the immunomodulation characteristic of helminths.

Among the limitations of our study, it is worth noting the use of HUVECs. These cells are a widely validated primary vascular model. HUVECs offer a solid, stable, and reproducible platform for evaluating general endothelial responses to inflammatory or parasitic stimuli. Their use is widely accepted in vascular biology and comparative parasitology studies, allowing the interpretation of results within a coherent pathophysiological context. Secondly, exposure to AAvH represents an acute and controlled approach, without considering the continuous release of excretory/secretory antigens by live parasites. However, this experimental strategy allows the specific effects of the structural components of the parasite on the endothelium to be isolated and constitutes an essential first step in unraveling the initial mechanisms of endothelial activation before moving on to more complex models. This approach provides a solid and reproducible basis on which to build future in vivo research aimed at validating the pathways and processes identified in this work. It should also be noted that AAvH contains molecules from both the soma and/or excretory/secretory products of the adult parasite, making it impossible to isolate the effect of each one individually. Further research is therefore needed in this area, with an emphasis on the excretory/secretory products that would simulate the effect of the live parasite in the circulatory system of the animal host.

## 5. Conclusions

The results of this study show that exposure of the vascular endothelium to AAvH induces a multifactorial response characterized by endothelial activation, extracellular matrix remodeling, and imbalance of hemostatic pathways. The differential regulation of various proteins related to vascular homeostasis and immune and endothelial activation suggests indirect modulation of the host’s coagulation and complement systems, contributing to the development of coagulopathies and vascular damage. These findings reinforce the idea that the hemorrhagic alterations observed in infected dogs derive from a complex endothelial response rather than direct action by the parasite, and open new perspectives for the use of endothelial biomarkers in the diagnosis and monitoring of angiostrongylosis. Future studies focusing on the temporal and molecular characterization of the endothelial response to excretory/secretory, as well as on the comparison between natural and accidental hosts, would help to understand the mechanisms of tolerance and parasitic persistence and advance toward a more complete understanding of the vascular pathogenesis induced by *A. vasorum*.

## Data Availability

Data is contained within the article or Supplementary Materials.

## Supplementary Materials

The following supporting information can be downloaded at: https://www.mdpi.com/article/10.3390/ani16060926/s1, Figure S1: Gene Ontology enrichment analysis in the categories of Molecular Function for (A) up-regulated proteins in the cell supernatants, (B) down-regulated proteins in the cell supernatants, (C) up-regulated proteins in the cell lysates, and (D) down-regulated proteins in the cell lysates identified of endothelial cells treated with AAvH. GO analyses were performed with g:Profiler and semantically reduced using rrvgo; Figure S2: Gene Ontology enrichment analysis in the categories of Cellular Component (A) up-regulated proteins in the cell supernatants, (B) down-regulated proteins in the cell supernatants, (C) up-regulated proteins in the cell lysates, and (D) down-regulated proteins in the cell lysates identified of endothelial cells treated with AAvH. GO analyses were performed with g:Profiler and semantically reduced using rrvgo; Table S1: Proteins identified in cell supernatants of cells treated with AAvH and control group. Proteins with an Absolute AVG Log2 Ratio ≥ 0.58 and Q-value ≤ 0.05 were dysregulated after stimulation over the control group; Table S2: Proteins identified in cell lysates of cells treated with AAvH and control group. Proteins with an Absolute AVG Log2 Ratio ≥ 0.58 and Q-value ≤ 0.05 were dysregulated after stimulation over the control group; Table S3: Dysregulated proteins in cell supernatants and cell lysates; Table S4: Proteins identified in the cell supernatants of the three replicates of cells treated with AAvH; Table S5: Proteins identified in the cell supernatants of the three replicates of cells used as control group; Table S6: Proteins identified in the cell lysates of the three replicates of cells treated with AAvH; Table S7: Proteins identified in the cell supernatants of the three replicates of cells treated used as a control group.

## References

[1] Blanch-Lázaro B., Mitton Z., Tudor C., Hindle J., Martineau H., Fox M., Blake D.P. (2018). Genetic diversity and population structure of *Angiostrongylus vasorum* parasites within and between local urban foxes (*Vulpes vulpes*). Vet. Parasitol..

[2] Ferdushy T., Hasan M.T. (2010). *Angiostrongylus vasorum*: The ‘French Heartworm’. Parasitol. Res..

[3] Elsheikha H.M., Holmes S.A., Wright I., Morgan E.R., Lacher D.W. (2014). Recent advances in the epidemiology, clinical and diagnostic features, and control of canine cardio-pulmonary angiostrongylosis. Vet. Res..

[4] Santifort K.M., den Toom M., Garosi L., Carrera I. (2023). Case report: Intracranial and spinal subarachnoid hemorrhage in a dog with Angiostrongylosis. Front. Vet. Sci..

[5] Gredal H., Willesen J.L., Jensen H.E., Nielsen O.L., Kristensen A.T., Koch J., Kirk R.K., E Pors S., Skerritt G.C., Berendt M. (2011). Acute neurological signs as the predominant clinical manifestation in four dogs with *Angiostrongylus vasorum* infections in Denmark. Acta Vet. Scand..

[6] Colombo M., Traversa D., Grillotti E., Pezzuto C., De Tommaso C., Pampurini F., Schaper R., Drake J., Crisi P.E., Russi I. (2021). Highly Variable Clinical Pictures in Dogs Naturally Infected with *Angiostrongylus vasorum*. Pathogens.

[7] Thomsen A.S., Petersen M.P., Willesen J.L., Bach M.B.T., Kieler I.N., Kristensen A.T., Koch J., Nielsen L.N. (2024). Clinical bleeding diathesis, laboratory haemostatic aberrations and survival in dogs infected with *Angiostrongylus vasorum*: 180 cases (2005–2019). J. Small Anim. Pract..

[8] Fuehrer H.P., Morelli S., Unterköfler M.S., Bajer A., Bakran-Lebl K., Dwużnik-Szarek D., Farkas R., Grandi G., Heddergott M., Jokelainen P. (2021). Dirofilaria spp. and *Angiostrongylus vasorum*: Current risk of spreading in Central and Northern Europe. Pathogens.

[9] Morelli S., Gori F., Colombo M., Traversa D., Sarrocco G., Simonato G., Nespeca C., Di Cesare A., di Regalbono A.F., Veronesi F. (2021). Simultaneous exposure to *Angiostrongylus vasorum* and vector-borne pathogens in dogs from Italy. Pathogens.

[10] Robleto-Quesada J., Umaña-Blanco F., Solano-Barquero A., Allen J., Levi T., Gori F., Schnyder M., Rojas A. (2024). Seek, and you will find: Cryptic diversity of the cardiopulmonary nematode *Angiostrongylus vasorum* in the Americas. Acta Trop..

[11] Quesada J., Alfaro-Segura P., Mata-Somarribas C., Alger J., Toledo M., Ramos de Souza J., Mora J., Graeff-Teixeira C., Solano-Barquero A., Rojas A. (2024). Real-time qPCR coupled with high-resolution melting curve analysis for the detection of the internal transcribed spacer 1 of Angiostrongylus costaricensis. Parasitol. Res..

[12] Cowie R.H., Malik R., Morgan E.R. (2023). Comparative biology of parasitic nematodes in the genus Angiostrongylus and related genera. Adv. Parasitol..

[13] Mechouck N., Deak G., Ionică A.M., Toma C., Negoescu A.G., Taulescu M., Bouslama Z., Mihalca A.D. (2024). First report of *Angiostrongylus vasorum* in an African golden wolf (*Canis lupaster*) in Algeria. Parasit. Vectors.

[14] Mitrea I.B., Iani A.D., Gherman C.M., Cazan C.D., Ionică A.M., Deak G., Negoescu A., Rabei Ș.O., Cernea M.S., Alexe V. (2025). Golden jackals (*Canis aureus*) as novel hosts for *Angiostrongylus vasorum* in Romania. Vet. Parasitol. Reg. Stud. Rep..

[15] Eisenhut B., Wittwer A., Schnyder M., Oehm A.W. (2025). Host-specific vascular endothelial cell responses to *Angiostrongylus vasorum*: A comparative in vitro study in red foxes (Vulpes vulpes) and domestic dogs. Front. Cell. Infect. Microbiol..

[16] Hertaeg J., Salazar U., Vom Berg J., LeibundGut-Landmann S., Oehm A.W., Schnyder M. (2025). In vitro cytokine response of circulating mononuclear cells from healthy dogs to stage-specific antigens of *Angiostrongylus vasorum*. BMC Vet. Res..

[17] Oehm A.W., Esteves B.I.O., Hetzel U., Alves M.P., Schnyder M. (2025). Establishment and validation of red fox (*Vulpes vulpes*) airway epithelial cell cultures at the air-liquid-interface. Sci. Rep..

[18] Grob D., Conejeros I., López-Osorio S., Velásquez Z.D., Segeritz L., Gärtner U., Schaper R., Hermosilla C., Taubert A. (2021). Canine *Angiostrongylus vasorum*-induced early innate immune reactions based on NETs formation and canine vascular endothelial cell activation in vitro. Biology.

[19] Ramsey I.K., Littlewood J.D., Dunn J.K., Herrtage M.E. (1996). Role of chronic disseminated intravascular coagulation in a case of canine angiostrongylosis. Vet. Rec..

[20] Adamantos S., Waters S., Boag A. (2015). Coagulation status in dogs with naturally occurring *Angiostrongylus vasorum* infection. J. Small Anim. Pract..

[21] Gillis-Germitsch N., Kockmann T., Asmis L.M., Tritten L., Schnyder M. (2021). The *Angiostrongylus vasorum* excretory/secretory and surface proteome contains putative modulators of the host coagulation. Front. Cell. Infect. Microbiol..

[22] Sigrist N.E., Tritten L., Kümmerle-Fraune C., Hofer-Inteeworn N., Jud Schefer R., Schnyder M., Kutter A.P.N. (2021). Coagulation status in dogs naturally infected with *Angiostrongylus vasorum*. Pathogens.

[23] Sigrist N.E., Hofer-Inteeworn N., Jud Schefer R., Kuemmerle-Fraune C., Schnyder M., Kutter A.P. (2017). Hyperfibrinolysis and hypofibrinogenemia diagnosed with rotational thromboelastometry in dogs naturally infected with *Angiostrongylus vasorum*. J. Vet. Intern. Med..

[24] Zoia A., Caldin M. (2015). Coagulation status in dogs with naturally occurring *Angiostrongylus vasorum* infection and primary hyperfibrinolysis. J. Small Anim. Pract..

[25] Tritten L., Gillis-Germitsch N., Kockmann T., Schnyder M. (2021). Quantitative proteomics analysis of *Angiostrongylus vasorum*-induced alterations in dog serum sheds light on the pathogenesis of canine angiostrongylosis. Sci. Rep..

[26] Germitsch N., Kockmann T., Schnyder M., Tritten L. (2025). N-terminomics profiling of host proteins targeted by excretory-secretory proteases of the nematode *Angiostrongylus vasorum* identifies points of interaction with canine coagulation and complement cascade. PLoS ONE.

[27] Morchón R., Rodríguez-Barbero A., Velasco S., López-Belmonte J., Simón F. (2008). Vascular endothelial cell activation by adult *Dirofilaria immitis* antigens. Parasitol. Int..

[28] Machado C.D.C., Alarcón-Torrecillas C., Pericacho M., Rodríguez-Escolar I., Carretón E., Montoya-Alonso J.A., Morchón R. (2023). Involvement of the excretory/secretory and surface-associated antigens of Dirofilaria immitis adult worms in the angiogenic response in an in-vitro endothelial cell model. Vet. Parasitol..

[29] Collado-Cuadrado M., Alarcón-Torrecillas C., Balmori-de la Puente A., Rodríguez-Escolar I., Infante González-Mohino E., Pericacho M., Morchón R. (2024). Angiogenesis as a Survival Mechanism in Heartworm Disease: The Role of Fructose-Bisphosphate Aldolase and Actin from Dirofilaria immitis in an In Vitro Endothelial Model. Animals.

[30] Montero-Calle A., Coronel R., Garranzo-Asensio M., Solís-Fernández G., Rábano A., de Los Ríos V., Fernández-Aceñero M.J., Mendes M.L., Martínez-Useros J., Megías D. (2023). Proteomics analysis of prefrontal cortex of Alzheimer’s disease patients revealed dysregulated proteins in the disease and novel proteins associated with amyloid-β pathology. Cell. Mol. Life Sci..

[31] Bruderer R., Bernhardt O.M., Gandhi T., Miladinović S.M., Cheng L.Y., Messner S., Ehrenberger T., Zanotelli V., Butscheid Y., Escher C. (2015). Extending the limits of quantitative proteome profiling with data-independent acquisition and application to acetaminophen-treated three-dimensional liver microtissues. Mol. Cell. Proteom..

[32] Wickham H. (2016). ggplot2: Elegant Graphics for Data Analysis.

[33] R Core Team (2024). R: A Language and Environment for Statistical Computing.

[34] Kolberg L., Raudvere U., Kuzmin I., Adler P., Vilo J., Peterson H. (2023). g:Profiler—Interoperable web service for functional enrichment analysis and gene identifier mapping (2023 update). Nucleic Acids Res..

[35] Sayols S. (2023). rrvgo: A Bioconductor package for interpreting lists of Gene Ontology terms. MicroPubl. Biol..

[36] Szklarczyk D., Nastou K., Koutrouli M., Kirsch R., Mehryary F., Hachilif R., Hu D., E Peluso M., Huang Q., Fang T. (2025). The STRING database in 2025: Protein networks with directionality of regulation. Nucleic Acids Res..

[37] Rodríguez-Pastor R., Garrido M., Knossow N., Shahar N., Flatau R., Hawlena H. (2025). Variability in infection dynamics emerges from the interplay between unique host and pathogen characteristics. Sci. Rep..

[38] Pober J.S., Sessa W.C. (2007). Evolving functions of endothelial cells in inflammation. Nat. Rev. Immunol..

[39] Morchón R., González-Miguel J., Mellado I., Velasco S., Rodríguez-Barbero A., Simón F. (2010). Adult Dirofilaria immitis excretory/secretory antigens upregulate the production of prostaglandin E2 and downregulate monocyte transmigration in an in vitro model of vascular endothelial cell cultures. Vet. Parasitol..

[40] Ponomarev D.V., Lishai E.A., Kovner A.V., Kharkova M.V., Zaparina O., Kapuschak Y.K., Mordvinov V.A., Pakharukova M.Y. (2023). Extracellular vesicles of the liver fluke Opisthorchis felineus stimulate the angiogenesis of human umbilical vein endothelial cells. Curr. Res. Parasitol. Vector Borne Dis..

[41] Figuera L., Gómez-Arreaza A., Avilán L. (2013). Parasitism in optima forma: Exploiting the host fibrinolytic system for invasion. Acta Trop..

[42] Diosdado A., Simón F., Morchón R., González-Miguel J. (2020). Pro-fibrinolytic potential of the third larval stage of Ascaris suum as a possible mechanism facilitating its migration through the host tissues. Parasit. Vectors.

[43] González-Miguel J., Morchón R., Siles-Lucas M., Simón F. (2015). Fibrinolysis and proliferative endarteritis: Two related processes in chronic infections? The model of the blood-borne pathogen *Dirofilaria immitis*. PLoS ONE.

[44] González-Miguel J., Morchón R., Siles-Lucas M., Oleaga A., Simón F. (2015). Surface-displayed glyceraldehyde 3-phosphate dehydrogenase and galectin from Dirofilaria immitis enhance the activation of the fibrinolytic system of the host. Acta Trop..

[45] Binder B.R., Christ G., Gruber F., Grubic N., Hufnagl P., Krebs M., Mihaly J., Prager G.W. (2002). Plasminogen activator inhibitor 1: Physiological and pathophysiological roles. News Physiol. Sci..

[46] Boncela J., Przygodzka P., Papiewska-Pajak I., Wyroba E., Cierniewski C.S. (2011). Plasminogen activator inhibitor type 1 interacts with α3β1 integrin at the cell surface of endothelial cells for the regulation of the uPA/uPAR system. J. Cell Sci..

[47] Mast A.E., Ruf W. (2022). Regulation of coagulation by tissue factor pathway inhibitor: Implications for hemophilia therapy. J. Thromb. Haemost..

[48] Mebius M.M., van Genderen P.J., Urbanus R.T., Tielens A.G., de Groot P.G., van Hellemond J.J. (2013). Interference with the host haemostatic system by *Schistosoma* spp.. PLoS Pathog..

[49] Mebius M.M., Adegnika A.A., Zinsou J.F., Agobe J.C.D., Honkpehedji Y.J., Yazdanbakhsh M., van Dam G., Corstjens P., Tielens A., de Groot P. (2019). Haemostatic changes in urogenital Schistosoma haematobium: A case-control study in Gabonese schoolchildren. J. Helminthol..

[50] Akwii R.G., Sajib M.S., Zahra F.T., Mikelis C.M. (2019). Role of Angiopoietin-2 in Vascular Physiology and Pathophysiology. Cells.

[51] Coimbra R.L.S., Meneses G.C., Galvão R.L.F., Pinheiro M.C.C., de Oliveira L.M., Lope N.C., Martins B.V.B., de Araújo L.M., Vieira R.Y.R., Oriá R.B. (2025). Angiopoietins as biomarkers of schistosomiasis severity: A cross-sectional study. Parasitology.

[52] McSorley H.J., Smyth D.J. (2021). IL-33: A central cytokine in helminth infections. Semin. Immunol..

[53] Sanjabi S., Hoffmann A., Liou H.C., Baltimore D., Smale S.T. (2000). Essential role of STAT6 in the development of antigen-induced airway hyperreactivity. Proc. Natl. Acad. Sci. USA.

[54] Mason N.J., Fiore J., Kobayashi T., Masek K.S., Choi Y., Hunter C.A. (2004). TRAF6-dependent mitogen-activated protein kinase activation differentially regulates the production of interleukin-12 by macrophages in response to Toxoplasma gondii. Infect. Immun..

[55] Allen I.C., Moore C.B., Schneider M., Lei Y., Davis B.K., Scull M.A., Gris D., Roney K.E., Zimmermann A.G., Bowzard J.B. (2011). NLRX1 protein attenuates inflammatory responses to infection by interfering with the RIG-I-MAVS and TRAF6-NF-κB signaling pathways. Immunity.

