# Supplementary material for: Proteomic Analysis of Endothelial Activation Induced by Adult Angiostrongylus vasorum Homogenate: Insights into Vascular Remodeling and Hemostatic Imbalance
Source: Animals (Basel). 2026 Mar 15;16(6):926. doi: 10.3390/ani16060926 (PMC13023303; doi:10.3390/ani16060926)
Supplement: Supplementary file 1 [file animals-16-00926-s001.zip › Supplmentary Figure S1.pdf]

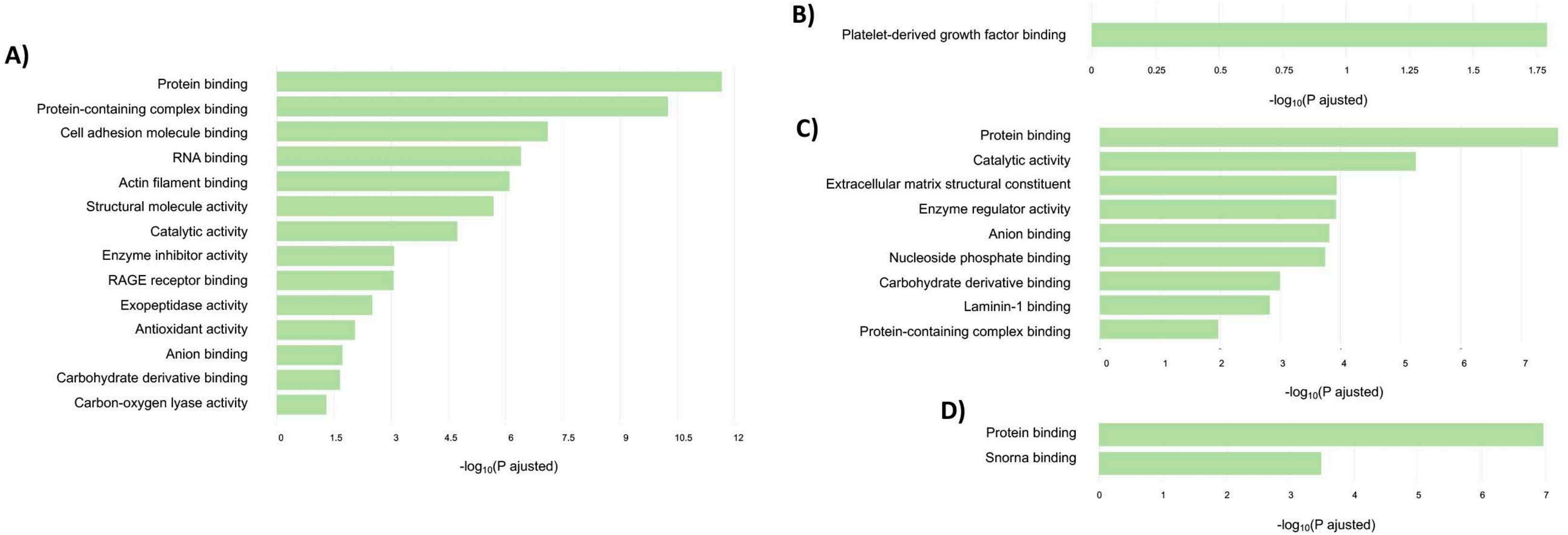

A)
Protein binding
Protein-containing complex binding
Cell adhesion molecule binding
RNA binding
Actin filament binding
Structural molecule activity
Catalytic activity
Enzyme inhibitor activity
RAGE receptor binding
Exopeptidase activity
Antioxidant activity
Anion binding
Carbohydrate derivative binding
Carbon-oxygen lyase activity
0 1.5 3 4.5 6 7.5 9 10.5 12
-log10(P ajusted)
B)
Platelet-derived growth factor binding
0 0.25 0.5 0.75 1 1.25 1.5 1.75
-log10(P ajusted)
C)
Protein binding
Catalytic activity
Extracellular matrix structural constituent
Enzyme regulator activity
Anion binding
Nucleoside phosphate binding
Carbohydrate derivative binding
Laminin-1 binding
Protein-containing complex binding
0 1 2 3 4 5 6 7
-log10(P ajusted)
D)
Protein binding
Snorna binding
0 1 2 3 4 5 6 7
-log10(P ajusted)
