# Supplementary material for: Proteomic Analysis of Endothelial Activation Induced by Adult Angiostrongylus vasorum Homogenate: Insights into Vascular Remodeling and Hemostatic Imbalance
Source: Animals (Basel). 2026 Mar 15;16(6):926. doi: 10.3390/ani16060926 (PMC13023303; doi:10.3390/ani16060926)
Supplement: Supplementary file 1 [file animals-16-00926-s001.zip › Supplmentary Table S1.pdf]

**Supplementary Table S1: Proteins identified in cell supernatants of cells treated with *A.vasorum* and control group. Proteins with an Absolute**

| Comparison (group1/group2) | ProteinGroups | AVG Log2 Ratio | Absolute AVG Log2 Ratio | Pvalue | Qvalue | # of Ratios | Genes |
|---|---|---|---|---|---|---|---|
| *A.vasorum* /Control | P00352 | 1.076345724 | 1.076345724 | 2.43E-07 | 2.01E-05 | 12 | ALDH1A1 |
| *A.vasorum* /Control | P04275 | 1.23621967 | 1.23621967 | 1.29E-07 | 2.01E-05 | 12 | VWF |
| *A.vasorum* /Control | P43121 | 0.28685962 | 0.28685962 | 3.05E-07 | 2.01E-05 | 12 | MCAM |
| *A.vasorum* /Control | P98160 | 0.903048915 | 0.903048915 | 9.13E-07 | 4.52E-05 | 12 | HSPG2 |
| *A.vasorum* /Control | P04114 | -0.96827868 | 0.96827868 | 2.21E-06 | 7.26E-05 | 12 | APOB |
| *A.vasorum* /Control | P06396 | 2.067271535 | 2.067271535 | 2.57E-06 | 7.26E-05 | 12 | GSN |
| *A.vasorum* /Control | P46940 | 0.792181744 | 0.792181744 | 2.44E-06 | 7.26E-05 | 12 | IQGAP1 |
| *A.vasorum* /Control | P04406 | 0.495404212 | 0.495404212 | 4.04E-06 | 9.84E-05 | 12 | GAPDH |
| *A.vasorum* /Control | P17096 | 0.49495264 | 0.49495264 | 4.47E-06 | 9.84E-05 | 12 | HMGA1 |
| *A.vasorum* /Control | Q13201 | 1.014412209 | 1.014412209 | 6.07E-06 | 0.00012 | 12 | MMRN1 |
| *A.vasorum* /Control | P23381 | 1.378699382 | 1.378699382 | 6.91E-06 | 0.000124 | 12 | WARS1 |
| *A.vasorum* /Control | P16402 | 1.162246852 | 1.162246852 | 1.06E-05 | 0.000176 | 12 | H1-3 |
| *A.vasorum* /Control | P31946 | 0.392700965 | 0.392700965 | 1.32E-05 | 0.000186 | 12 | YWHAB |
| *A.vasorum* /Control | P61981 | 0.61226291 | 0.61226291 | 1.29E-05 | 0.000186 | 12 | YWHAG |
| *A.vasorum* /Control | P12814 | 0.545455723 | 0.545455723 | 1.61E-05 | 0.000199 | 12 | ACTN1 |
| *A.vasorum* /Control | P68431 | 0.751556652 | 0.751556652 | 1.58E-05 | 0.000199 | 12 | H3C1 |
| *A.vasorum* /Control | P09486 | 1.17434566 | 1.17434566 | 1.94E-05 | 0.000218 | 12 | SPARC |
| *A.vasorum* /Control | P13489 | 0.621719855 | 0.621719855 | 1.98E-05 | 0.000218 | 12 | RNH1 |
| *A.vasorum* /Control | P35579 | 0.399154826 | 0.399154826 | 2.11E-05 | 0.000219 | 12 | MYH9 |
| *A.vasorum* /Control | P07355 | 0.133765059 | 0.133765059 | 2.74E-05 | 0.000259 | 12 | ANXA2 |
| *A.vasorum* /Control | Q01082 | 0.647807829 | 0.647807829 | 2.68E-05 | 0.000259 | 12 | SPTBN1 |
| *A.vasorum* /Control | P16401 | 0.804258532 | 0.804258532 | 3.23E-05 | 0.000272 | 12 | H1-5 |
| *A.vasorum* /Control | Q15149 | 0.439721581 | 0.439721581 | 3.15E-05 | 0.000272 | 12 | PLEC |
| *A.vasorum* /Control | P00491 | 0.645903185 | 0.645903185 | 3.80E-05 | 0.000289 | 12 | PNP |
| *A.vasorum* /Control | O43242 | 0.151705612 | 0.151705612 | 3.95E-05 | 0.00029 | 12 | PSMD3 |
| *A.vasorum* /Control | P15311 | 1.313578133 | 1.313578133 | 5.38E-05 | 0.000323 | 12 | EZR |
| *A.vasorum* /Control | P27348 | 0.593269685 | 0.593269685 | 5.31E-05 | 0.000323 | 12 | YWHAQ |
| *A.vasorum* /Control | P42785 | 0.614577445 | 0.614577445 | 4.93E-05 | 0.000323 | 12 | PRCP |
| *A.vasorum* /Control | P60709 | 0.062717007 | 0.062717007 | 5.00E-05 | 0.000323 | 12 | ACTB |
| *A.vasorum* /Control | Q92626 | 0.749250148 | 0.749250148 | 5.15E-05 | 0.000323 | 12 | PXDN |
| *A.vasorum* /Control | P48735 | 1.236250195 | 1.236250195 | 5.61E-05 | 0.000327 | 12 | IDH2 |
| *A.vasorum* /Control | P62081 | 0.40726163 | 0.40726163 | 6.46E-05 | 0.000366 | 12 | RPS7 |
| *A.vasorum* /Control | P01024 | -0.605521952 | 0.605521952 | 7.75E-05 | 0.000415 | 12 | C3 |

| | | | | | | | |
|---|---|---|---|---|---|---|---|
| *A.vasorum* /Control | P01033 | 0.727307259 | 0.727307259 | 9.24E-05 | 0.000435 | 12 | TIMP1 |
| *A.vasorum* /Control | P22392 | 0.580609273 | 0.580609273 | 9.04E-05 | 0.000435 | 12 | NME2 |
| *A.vasorum* /Control | P50395 | 0.431542938 | 0.431542938 | 8.85E-05 | 0.000435 | 12 | GDI2 |
| *A.vasorum* /Control | Q16666 | 0.914520905 | 0.914520905 | 9.08E-05 | 0.000435 | 12 | IFI16 |
| *A.vasorum* /Control | Q96FW1 | 1.432473086 | 1.432473086 | 8.42E-05 | 0.000435 | 12 | OTUB1 |
| *A.vasorum* /Control | P52209 | 0.530653368 | 0.530653368 | 9.79E-05 | 0.000441 | 12 | PGD |
| *A.vasorum* /Control | P60174 | 0.635132291 | 0.635132291 | 9.80E-05 | 0.000441 | 12 | TPI1 |
| *A.vasorum* /Control | P22314 | 0.693024512 | 0.693024512 | 0.000103 | 0.000445 | 12 | UBA1 |
| *A.vasorum* /Control | P26640 | 1.197485464 | 1.197485464 | 0.000102 | 0.000445 | 12 | VARS1 |
| *A.vasorum* /Control | O15123 | 0.734029513 | 0.734029513 | 0.00011 | 0.000462 | 12 | ANGPT2 |
| *A.vasorum* /Control | Q16270 | 0.50855366 | 0.50855366 | 0.000116 | 0.000476 | 12 | IGFBP7 |
| *A.vasorum* /Control | P37837 | 0.686819008 | 0.686819008 | 0.000119 | 0.000479 | 12 | TALDO1 |
| *A.vasorum* /Control | P35241 | 0.59173796 | 0.59173796 | 0.000125 | 0.000485 | 12 | RDX |
| *A.vasorum* /Control | Q16555 | 0.559038014 | 0.559038014 | 0.000123 | 0.000485 | 12 | DPYSL2 |
| *A.vasorum* /Control | P18669 | 0.654330288 | 0.654330288 | 0.000148 | 0.00052 | 12 | PGAM1 |
| *A.vasorum* /Control | P37802 | 0.166004655 | 0.166004655 | 0.00014 | 0.00052 | 12 | TAGLN2 |
| *A.vasorum* /Control | P78417 | 0.68437883 | 0.68437883 | 0.000145 | 0.00052 | 12 | GSTO1 |
| *A.vasorum* /Control | P04083 | 0.317126587 | 0.317126587 | 0.000184 | 0.00053 | 12 | ANXA1 |
| *A.vasorum* /Control | P18206 | 0.036721025 | 0.036721025 | 0.000173 | 0.00053 | 12 | VCL |
| *A.vasorum* /Control | P21741 | 1.431041639 | 1.431041639 | 0.000184 | 0.00053 | 12 | MDK |
| *A.vasorum* /Control | P62318 | 0.549772327 | 0.549772327 | 0.000165 | 0.00053 | 12 | SNRPD3 |
| *A.vasorum* /Control | P63104 | 0.406468176 | 0.406468176 | 0.000176 | 0.00053 | 12 | YWHAZ |
| *A.vasorum* /Control | Q00610 | 0.513883523 | 0.513883523 | 0.000181 | 0.00053 | 12 | CLTC |
| *A.vasorum* /Control | Q14974 | 0.824447404 | 0.824447404 | 0.000168 | 0.00053 | 12 | KPNB1 |
| *A.vasorum* /Control | Q9HB71 | 0.231973527 | 0.231973527 | 0.000171 | 0.00053 | 12 | CACYBP |
| *A.vasorum* /Control | Q9Y490 | 0.447325735 | 0.447325735 | 0.000185 | 0.00053 | 12 | TLN1 |
| *A.vasorum* /Control | Q01813 | 0.501824194 | 0.501824194 | 0.000209 | 0.000584 | 12 | PFKP |
| *A.vasorum* /Control | P08865 | 0.008081957 | 0.008081957 | 0.000224 | 0.000616 | 12 | RPSA |
| *A.vasorum* /Control | P23528 | 0.828871649 | 0.828871649 | 0.000236 | 0.00064 | 12 | CFL1 |
| *A.vasorum* /Control | O43707 | 0.587825789 | 0.587825789 | 0.000256 | 0.000675 | 12 | ACTN4 |
| *A.vasorum* /Control | Q9BZZ5 | 1.30374958 | 1.30374958 | 0.000269 | 0.0007 | 12 | API5 |
| *A.vasorum* /Control | P00568 | 0.798931792 | 0.798931792 | 0.000288 | 0.000724 | 12 | AK1 |
| *A.vasorum* /Control | P26038 | 0.599215502 | 0.599215502 | 0.000285 | 0.000724 | 12 | MSN |
| *A.vasorum* /Control | P46783 | 0.613681284 | 0.613681284 | 0.000289 | 0.000724 | 12 | RPS10 |
| *A.vasorum* /Control | O00622 | 0.635331528 | 0.635331528 | 0.000302 | 0.000747 | 12 | CCN1 |

| | | | | | | | |
|---|---|---|---|---|---|---|---|
| *A.vasorum* /Control | Q6NZI2 | 0.817761304 | 0.817761304 | 0.00031 | 0.000758 | 12 | CAVIN1 |
| *A.vasorum* /Control | P08253 | 0.545173499 | 0.545173499 | 0.000323 | 0.000771 | 12 | MMP2 |
| *A.vasorum* /Control | P55072 | 0.610627384 | 0.610627384 | 0.00032 | 0.000771 | 12 | VCP |
| *A.vasorum* /Control | P55786 | 0.758379979 | 0.758379979 | 0.000379 | 0.000884 | 12 | NPEPPS |
| *A.vasorum* /Control | P06899 | 1.038418009 | 1.038418009 | 0.000405 | 0.00089 | 12 | H2BC11 |
| *A.vasorum* /Control | P08572 | 0.867304385 | 0.867304385 | 0.0004 | 0.00089 | 12 | COL4A2 |
| *A.vasorum* /Control | P09211 | 0.81666119 | 0.81666119 | 0.0004 | 0.00089 | 12 | GSTP1 |
| *A.vasorum* /Control | Q92743 | 0.973988243 | 0.973988243 | 0.000417 | 0.000907 | 12 | HTRA1 |
| *A.vasorum* /Control | O75369 | 0.500797732 | 0.500797732 | 0.000424 | 0.00091 | 12 | FLNB |
| *A.vasorum* /Control | P07996 | 0.230805221 | 0.230805221 | 0.000427 | 0.00091 | 12 | THBS1 |
| *A.vasorum* /Control | P60866 | 0.623912561 | 0.623912561 | 0.000462 | 0.000974 | 12 | RPS20 |
| *A.vasorum* /Control | P78527 | 0.661645166 | 0.661645166 | 0.00047 | 0.000979 | 12 | PRKDC |
| *A.vasorum* /Control | P62277 | 0.446485903 | 0.446485903 | 0.000482 | 0.000995 | 12 | RPS13 |
| *A.vasorum* /Control | P21810 | 1.196051412 | 1.196051412 | 0.000493 | 0.000998 | 12 | BGN |
| *A.vasorum* /Control | Q9Y696 | 0.976175081 | 0.976175081 | 0.000498 | 0.000998 | 12 | CLIC4 |
| *A.vasorum* /Control | O00391 | 0.483083807 | 0.483083807 | 0.000517 | 0.00102 | 12 | QSOX1 |
| *A.vasorum* /Control | P49458 | 2.109689449 | 2.109689449 | 0.00052 | 0.00102 | 12 | SRP9 |
| *A.vasorum* /Control | O14980 | 0.670220685 | 0.670220685 | 0.000538 | 0.00104 | 12 | XPO1 |
| *A.vasorum* /Control | O60814 | 0.53926191 | 0.53926191 | 0.000553 | 0.00104 | 12 | H2BC12 |
| *A.vasorum* /Control | P04075 | 0.247691995 | 0.247691995 | 0.000547 | 0.00104 | 12 | ALDOA |
| *A.vasorum* /Control | P09429 | 1.032704236 | 1.032704236 | 0.000557 | 0.00104 | 12 | HMGB1 |
| *A.vasorum* /Control | P50452 | 1.430435649 | 1.430435649 | 0.000555 | 0.00104 | 12 | SERPINB8 |
| *A.vasorum* /Control | Q02809 | 0.736517269 | 0.736517269 | 0.000562 | 0.00104 | 12 | PLOD1 |
| *A.vasorum* /Control | Q99536 | 0.495720251 | 0.495720251 | 0.000578 | 0.00105 | 12 | VAT1 |
| *A.vasorum* /Control | P02545 | 0.391076743 | 0.391076743 | 0.00059 | 0.001062 | 12 | LMNA |
| *A.vasorum* /Control | P10909 | 1.562732272 | 1.562732272 | 0.000615 | 0.001097 | 12 | CLU |
| *A.vasorum* /Control | Q07954 | -0.50266977 | 0.50266977 | 0.00065 | 0.001138 | 12 | LRP1 |
| *A.vasorum* /Control | O95445 | -0.55428017 | 0.55428017 | 0.000661 | 0.001148 | 12 | APOM |
| *A.vasorum* /Control | P30153 | 1.175446661 | 1.175446661 | 0.000699 | 0.001191 | 12 | PPP2R1A |
| *A.vasorum* /Control | Q96CX2 | 0.777975957 | 0.777975957 | 0.000725 | 0.001196 | 12 | KCTD12 |
| *A.vasorum* /Control | Q96QK1 | 0.813531421 | 0.813531421 | 0.000723 | 0.001196 | 12 | VPS35 |
| *A.vasorum* /Control | P50991 | 0.646754997 | 0.646754997 | 0.00076 | 0.001214 | 12 | CCT4 |
| *A.vasorum* /Control | Q16658 | 0.821547212 | 0.821547212 | 0.000747 | 0.001214 | 12 | FSCN1 |
| *A.vasorum* /Control | Q16881 | 0.24559468 | 0.24559468 | 0.000771 | 0.001222 | 12 | TXNRD1 |
| *A.vasorum* /Control | Q08211 | 0.627575 | 0.627575 | 0.000798 | 0.001254 | 12 | DHX9 |

| | | | | | | | |
|---|---|---|---|---|---|---|---|
| *A.vasorum* /Control | P27695 | 0.944237178 | 0.944237178 | 0.000818 | 0.001258 | 12 | APEX1 |
| *A.vasorum* /Control | P35590 | 0.838187492 | 0.838187492 | 0.00082 | 0.001258 | 12 | TIE1 |
| *A.vasorum* /Control | Q12906 | 0.747148003 | 0.747148003 | 0.000843 | 0.001264 | 12 | ILF3 |
| *A.vasorum* /Control | Q13740 | 0.742283644 | 0.742283644 | 0.000837 | 0.001264 | 12 | ALCAM |
| *A.vasorum* /Control | P35237 | 0.731666176 | 0.731666176 | 0.00087 | 0.001295 | 12 | SERPINB6 |
| *A.vasorum* /Control | Q92896 | 0.499163222 | 0.499163222 | 0.000892 | 0.001319 | 12 | GLG1 |
| *A.vasorum* /Control | Q01469 | 0.994640441 | 0.994640441 | 0.000939 | 0.001368 | 12 | FABP5 |
| *A.vasorum* /Control | Q6UWH4 | 1.335242504 | 1.335242504 | 0.000948 | 0.00137 | 12 | GASK1B |
| *A.vasorum* /Control | Q92522 | 2.154171607 | 2.154171607 | 0.00096 | 0.001377 | 12 | H1-10 |
| *A.vasorum* /Control | P40925 | 0.612995108 | 0.612995108 | 0.000981 | 0.001381 | 12 | MDH1 |
| *A.vasorum* /Control | P68371 | 0.074010786 | 0.074010786 | 0.000982 | 0.001381 | 12 | TUBB4B |
| *A.vasorum* /Control | P02751 | 0.170675698 | 0.170675698 | 0.001071 | 0.001443 | 12 | FN1 |
| *A.vasorum* /Control | P15121 | 0.561795753 | 0.561795753 | 0.001066 | 0.001443 | 12 | AKR1B1 |
| *A.vasorum* /Control | P26583 | 0.941031416 | 0.941031416 | 0.001054 | 0.001443 | 12 | HMGB2 |
| *A.vasorum* /Control | Q96AG4 | 0.492791491 | 0.492791491 | 0.00107 | 0.001443 | 12 | LRRC59 |
| *A.vasorum* /Control | P63000 | 1.119057669 | 1.119057669 | 0.001079 | 0.001443 | 12 | RAC1 |
| *A.vasorum* /Control | P62805 | 0.477908744 | 0.477908744 | 0.001086 | 0.001444 | 12 | H4C1 |
| *A.vasorum* /Control | P13010 | 0.556139248 | 0.556139248 | 0.001197 | 0.00157 | 12 | XRCC5 |
| *A.vasorum* /Control | P63010 | 1.508727852 | 1.508727852 | 0.001294 | 0.001686 | 12 | AP2B1 |
| *A.vasorum* /Control | P00558 | 0.843255427 | 0.843255427 | 0.001315 | 0.001702 | 12 | PGK1 |
| *A.vasorum* /Control | P04908 | 0.591489252 | 0.591489252 | 0.001383 | 0.001779 | 12 | H2AC4 |
| *A.vasorum* /Control | Q14204 | 0.353178552 | 0.353178552 | 0.001392 | 0.001779 | 12 | DYNC1H1 |
| *A.vasorum* /Control | Q9BR76 | 1.163238897 | 1.163238897 | 0.001446 | 0.001836 | 12 | CORO1B |
| *A.vasorum* /Control | P21399 | 0.838522787 | 0.838522787 | 0.001511 | 0.001893 | 12 | ACO1 |
| *A.vasorum* /Control | P29401 | 0.395396146 | 0.395396146 | 0.001509 | 0.001893 | 12 | TKT |
| *A.vasorum* /Control | P12956 | 0.697581897 | 0.697581897 | 0.00154 | 0.001907 | 12 | XRCC6 |
| *A.vasorum* /Control | Q86VP6 | 0.831009458 | 0.831009458 | 0.001543 | 0.001907 | 12 | CAND1 |
| *A.vasorum* /Control | Q06830 | 0.70963925 | 0.70963925 | 0.001561 | 0.001908 | 12 | PRDX1 |
| *A.vasorum* /Control | P54687 | 0.956242009 | 0.956242009 | 0.001588 | 0.001929 | 12 | BCAT1 |
| *A.vasorum* /Control | Q9UBR2 | 0.697686894 | 0.697686894 | 0.001642 | 0.001946 | 12 | CTSZ |
| *A.vasorum* /Control | P26641 | 0.554685129 | 0.554685129 | 0.001658 | 0.001954 | 12 | EEF1G |
| *A.vasorum* /Control | O75083 | 0.586856188 | 0.586856188 | 0.001786 | 0.002093 | 12 | WDR1 |
| *A.vasorum* /Control | Q16531 | 0.989574944 | 0.989574944 | 0.001876 | 0.002185 | 12 | DDB1 |
| *A.vasorum* /Control | P00505 | 0.787566302 | 0.787566302 | 0.001908 | 0.002209 | 12 | GOT2 |
| *A.vasorum* /Control | P40926 | 0.723801647 | 0.723801647 | 0.001936 | 0.002216 | 12 | MDH2 |

| | | | | | | | |
|---|---|---|---|---|---|---|---|
| *A.vasorum* /Control | P49327 | 0.675704077 | 0.675704077 | 0.001933 | 0.002216 | 12 | FASN |
| *A.vasorum* /Control | Q9H7Y0 | 1.509493535 | 1.509493535 | 0.001948 | 0.002217 | 12 | DIPK2B |
| *A.vasorum* /Control | P00742 | -0.535667817 | 0.535667817 | 0.002024 | 0.002277 | 12 | F10 |
| *A.vasorum* /Control | P20062 | 1.524263529 | 1.524263529 | 0.00204 | 0.002282 | 12 | TCN2 |
| *A.vasorum* /Control | P49207 | 0.562699425 | 0.562699425 | 0.002052 | 0.002282 | 12 | RPL34 |
| *A.vasorum* /Control | P25786 | 0.390714144 | 0.390714144 | 0.002088 | 0.002309 | 12 | PSMA1 |
| *A.vasorum* /Control | P60228 | 0.807421529 | 0.807421529 | 0.002104 | 0.002315 | 12 | EIF3E |
| *A.vasorum* /Control | P52565 | 0.344546204 | 0.344546204 | 0.00213 | 0.00233 | 12 | ARHGDIA |
| *A.vasorum* /Control | Q9NPY3 | 1.411041458 | 1.411041458 | 0.002408 | 0.002605 | 12 | CD93 |
| *A.vasorum* /Control | P27635 | 0.740305205 | 0.740305205 | 0.002449 | 0.002607 | 12 | RPL10 |
| *A.vasorum* /Control | P29966 | 0.568106438 | 0.568106438 | 0.002432 | 0.002607 | 12 | MARCKS |
| *A.vasorum* /Control | P99999 | 1.159229648 | 1.159229648 | 0.002467 | 0.002612 | 12 | CYCS |
| *A.vasorum* /Control | P09960 | 0.997081203 | 0.997081203 | 0.002503 | 0.002622 | 12 | LTA4H |
| *A.vasorum* /Control | Q86T13 | -0.13468782 | 0.13468782 | 0.002517 | 0.002623 | 12 | CLEC14A |
| *A.vasorum* /Control | Q07021 | 1.036796145 | 1.036796145 | 0.002532 | 0.002624 | 12 | C1QBP |
| *A.vasorum* /Control | P30520 | 0.384499644 | 0.384499644 | 0.002583 | 0.00265 | 12 | ADSS2 |
| *A.vasorum* /Control | P61204 | 0.43433578 | 0.43433578 | 0.00257 | 0.00265 | 12 | ARF3 |
| *A.vasorum* /Control | P62826 | 0.449720242 | 0.449720242 | 0.00261 | 0.002651 | 12 | RAN |
| *A.vasorum* /Control | Q15404 | 0.536683274 | 0.536683274 | 0.002608 | 0.002651 | 12 | RSU1 |
| *A.vasorum* /Control | P28066 | 1.443566781 | 1.443566781 | 0.002639 | 0.002665 | 12 | PSMA5 |
| *A.vasorum* /Control | Q9UL46 | 0.668911388 | 0.668911388 | 0.002677 | 0.002691 | 12 | PSME2 |
| *A.vasorum* /Control | P17980 | 0.339343422 | 0.339343422 | 0.002696 | 0.002696 | 12 | PSMC3 |
| *A.vasorum* /Control | P28074 | 0.559396966 | 0.559396966 | 0.002735 | 0.002709 | 12 | PSMB5 |
| *A.vasorum* /Control | Q01105 | 0.259000256 | 0.259000256 | 0.002736 | 0.002709 | 12 | SET |
| *A.vasorum* /Control | P43490 | 0.751612264 | 0.751612264 | 0.002757 | 0.002716 | 12 | NAMPT |
| *A.vasorum* /Control | Q14103 | 0.522359476 | 0.522359476 | 0.002782 | 0.002727 | 12 | HNRNPD |
| *A.vasorum* /Control | P61158 | 0.471620721 | 0.471620721 | 0.002814 | 0.002745 | 12 | ACTR3 |
| *A.vasorum* /Control | P32119 | 0.86179387 | 0.86179387 | 0.002845 | 0.002756 | 12 | PRDX2 |
| *A.vasorum* /Control | P36871 | 0.918621779 | 0.918621779 | 0.002888 | 0.002776 | 12 | PGM1 |
| *A.vasorum* /Control | P28482 | 1.314913555 | 1.314913555 | 0.002935 | 0.002807 | 12 | MAPK1 |
| *A.vasorum* /Control | P54289 | -0.869999796 | 0.869999796 | 0.002997 | 0.00284 | 12 | CACNA2D1 |
| *A.vasorum* /Control | P17844 | 0.582243541 | 0.582243541 | 0.003071 | 0.002895 | 12 | DDX5 |
| *A.vasorum* /Control | P07195 | 0.256289347 | 0.256289347 | 0.003088 | 0.002898 | 12 | LDHB |
| *A.vasorum* /Control | P05121 | -0.717738962 | 0.717738962 | 0.003191 | 0.002981 | 12 | SERPINE1 |
| *A.vasorum* /Control | Q12805 | 0.278563173 | 0.278563173 | 0.003367 | 0.00313 | 12 | EFEMP1 |

| | | | | | | | |
|---|---|---|---|---|---|---|---|
| *A.vasorum* /Control | P00338 | 0.167466989 | 0.167466989 | 0.003584 | 0.003301 | 12 | LDHA |
| *A.vasorum* /Control | P25789 | 0.430936661 | 0.430936661 | 0.003645 | 0.003341 | 12 | PSMA4 |
| *A.vasorum* /Control | O60568 | 0.524582634 | 0.524582634 | 0.003674 | 0.003352 | 12 | PLOD3 |
| *A.vasorum* /Control | P29279 | 0.280324982 | 0.280324982 | 0.003729 | 0.003387 | 12 | CCN2 |
| *A.vasorum* /Control | P12429 | 0.676604167 | 0.676604167 | 0.003891 | 0.003439 | 12 | ANXA3 |
| *A.vasorum* /Control | P20700 | 0.846100451 | 0.846100451 | 0.003892 | 0.003439 | 12 | LMNB1 |
| *A.vasorum* /Control | P26927 | -0.535781151 | 0.535781151 | 0.003841 | 0.003439 | 12 | MST1 |
| *A.vasorum* /Control | P28300 | 0.910788613 | 0.910788613 | 0.003917 | 0.003439 | 12 | LOX |
| *A.vasorum* /Control | P49407 | 0.651630739 | 0.651630739 | 0.003818 | 0.003439 | 12 | ARRB1 |
| *A.vasorum* /Control | Q01518 | 0.433735743 | 0.433735743 | 0.00394 | 0.003439 | 12 | CAP1 |
| *A.vasorum* /Control | Q9NZN4 | 0.936228795 | 0.936228795 | 0.003943 | 0.003439 | 12 | EHD2 |
| *A.vasorum* /Control | P53999 | 1.025048407 | 1.025048407 | 0.004022 | 0.003492 | 12 | SUB1 |
| *A.vasorum* /Control | P07686 | 0.365570088 | 0.365570088 | 0.004127 | 0.003568 | 12 | HEXB |
| *A.vasorum* /Control | P07737 | 0.071058775 | 0.071058775 | 0.004189 | 0.003591 | 12 | PFN1 |
| *A.vasorum* /Control | P07951 | 0.176439615 | 0.176439615 | 0.004187 | 0.003591 | 12 | TPM2 |
| *A.vasorum* /Control | P11717 | -0.385653766 | 0.385653766 | 0.004257 | 0.00362 | 12 | IGF2R |
| *A.vasorum* /Control | P78539 | 0.339064729 | 0.339064729 | 0.00426 | 0.00362 | 12 | SRPX |
| *A.vasorum* /Control | Q8NBJ5 | 0.798589781 | 0.798589781 | 0.004302 | 0.003641 | 12 | COLGALT1 |
| *A.vasorum* /Control | P15531 | 1.011264928 | 1.011264928 | 0.004321 | 0.003641 | 12 | NME1 |
| *A.vasorum* /Control | Q76LX8 | -0.586622921 | 0.586622921 | 0.004398 | 0.00369 | 12 | ADAMTS13 |
| *A.vasorum* /Control | P67936 | 0.255950564 | 0.255950564 | 0.004418 | 0.003691 | 12 | TPM4 |
| *A.vasorum* /Control | P62263 | 0.273339455 | 0.273339455 | 0.0045 | 0.003728 | 12 | RPS14 |
| *A.vasorum* /Control | O14773 | 0.784691435 | 0.784691435 | 0.004541 | 0.003731 | 12 | TPP1 |
| *A.vasorum* /Control | P62140 | 0.161100303 | 0.161100303 | 0.00453 | 0.003731 | 12 | PPP1CB |
| *A.vasorum* /Control | P62937 | 0.334353774 | 0.334353774 | 0.004743 | 0.003849 | 12 | PPIA |
| *A.vasorum* /Control | Q13813 | 0.781833744 | 0.781833744 | 0.004786 | 0.003868 | 12 | SPTAN1 |
| *A.vasorum* /Control | P07954 | 0.693341479 | 0.693341479 | 0.005037 | 0.004022 | 12 | FH |
| *A.vasorum* /Control | Q99715 | -0.380622431 | 0.380622431 | 0.005091 | 0.004041 | 12 | COL12A1 |
| *A.vasorum* /Control | O43396 | 0.632421545 | 0.632421545 | 0.005272 | 0.004144 | 12 | TXNL1 |
| *A.vasorum* /Control | P33151 | 0.494319071 | 0.494319071 | 0.005275 | 0.004144 | 12 | CDH5 |
| *A.vasorum* /Control | P02452 | -0.726010763 | 0.726010763 | 0.00536 | 0.004195 | 12 | COL1A1 |
| *A.vasorum* /Control | P21333 | 0.177385275 | 0.177385275 | 0.005436 | 0.004238 | 12 | FLNA |
| *A.vasorum* /Control | P08134 | 0.609865504 | 0.609865504 | 0.005673 | 0.004405 | 12 | RHOC |
| *A.vasorum* /Control | P30050 | 0.380450437 | 0.380450437 | 0.00587 | 0.004522 | 12 | RPL12 |
| *A.vasorum* /Control | P30084 | 1.74561758 | 1.74561758 | 0.0059 | 0.004528 | 12 | ECHS1 |

| | | | | | | | |
|---|---|---|---|---|---|---|---|
| *A.vasorum* /Control | Q9H4M9 | 0.869159513 | 0.869159513 | 0.006094 | 0.004641 | 12 | EHD1 |
| *A.vasorum* /Control | Q12841 | 0.554539313 | 0.554539313 | 0.006146 | 0.004662 | 12 | FSTL1 |
| *A.vasorum* /Control | P49411 | 0.956716209 | 0.956716209 | 0.006217 | 0.004698 | 12 | TUFM |
| *A.vasorum* /Control | Q8IUE6 | 0.981885762 | 0.981885762 | 0.006285 | 0.004714 | 12 | H2AC21 |
| *A.vasorum* /Control | P35268 | 0.253628577 | 0.253628577 | 0.006398 | 0.004762 | 12 | RPL22 |
| *A.vasorum* /Control | P05067 | 0.46137589 | 0.46137589 | 0.006529 | 0.004841 | 12 | APP |
| *A.vasorum* /Control | P31948 | 0.663078863 | 0.663078863 | 0.006661 | 0.004903 | 12 | STIP1 |
| *A.vasorum* /Control | O15143 | 1.013785393 | 1.013785393 | 0.006691 | 0.004907 | 12 | ARPC1B |
| *A.vasorum* /Control | Q71UM5 | -0.441961872 | 0.441961872 | 0.00698 | 0.0051 | 12 | RPS27L |
| *A.vasorum* /Control | P49368 | 0.537927205 | 0.537927205 | 0.007049 | 0.005131 | 12 | CCT3 |
| *A.vasorum* /Control | P55209 | 0.40641568 | 0.40641568 | 0.007099 | 0.005149 | 12 | NAP1L1 |
| *A.vasorum* /Control | P13987 | 1.721643666 | 1.721643666 | 0.007126 | 0.005149 | 12 | CD59 |
| *A.vasorum* /Control | P25788 | 0.449333988 | 0.449333988 | 0.007173 | 0.00516 | 12 | PSMA3 |
| *A.vasorum* /Control | Q13418 | 0.529686453 | 0.529686453 | 0.007193 | 0.00516 | 12 | ILK |
| *A.vasorum* /Control | P0DPH7 | -0.345742694 | 0.345742694 | 0.007542 | 0.005354 | 12 | TUBA3C |
| *A.vasorum* /Control | Q06323 | 1.259519805 | 1.259519805 | 0.007544 | 0.005354 | 12 | PSME1 |
| *A.vasorum* /Control | P62258 | 0.362847898 | 0.362847898 | 0.007642 | 0.005404 | 12 | YWHAE |
| *A.vasorum* /Control | P60981 | 0.487695718 | 0.487695718 | 0.007746 | 0.005439 | 12 | DSTN |
| *A.vasorum* /Control | P02753 | -0.522174199 | 0.522174199 | 0.007844 | 0.005469 | 12 | RBP4 |
| *A.vasorum* /Control | P14324 | 0.585811648 | 0.585811648 | 0.007818 | 0.005469 | 12 | FDPS |
| *A.vasorum* /Control | P68363 | 0.136854205 | 0.136854205 | 0.007903 | 0.005491 | 12 | TUBA1B |
| *A.vasorum* /Control | P50502 | 0.253557348 | 0.253557348 | 0.007947 | 0.005502 | 12 | ST13 |
| *A.vasorum* /Control | P12111 | -0.36971175 | 0.36971175 | 0.008882 | 0.006085 | 12 | COL6A3 |
| *A.vasorum* /Control | O00299 | 0.626329801 | 0.626329801 | 0.00931 | 0.00635 | 12 | CLIC1 |
| *A.vasorum* /Control | P39023 | 0.144899857 | 0.144899857 | 0.009568 | 0.006488 | 12 | RPL3 |
| *A.vasorum* /Control | P12821 | 1.380616686 | 1.380616686 | 0.010225 | 0.006842 | 12 | ACE |
| *A.vasorum* /Control | P35221 | 0.568802761 | 0.568802761 | 0.010228 | 0.006842 | 12 | CTNNA1 |
| *A.vasorum* /Control | Q9H1E3 | 0.805261249 | 0.805261249 | 0.010174 | 0.006842 | 12 | NUCKS1 |
| *A.vasorum* /Control | Q02543 | 0.869182322 | 0.869182322 | 0.010277 | 0.006851 | 12 | RPL18A |
| *A.vasorum* /Control | P27816 | -0.755672614 | 0.755672614 | 0.010396 | 0.006907 | 12 | MAP4 |
| *A.vasorum* /Control | P06576 | 0.208666965 | 0.208666965 | 0.010433 | 0.006909 | 12 | ATP5F1B |
| *A.vasorum* /Control | P00488 | -0.601880557 | 0.601880557 | 0.010802 | 0.007087 | 12 | F13A1 |
| *A.vasorum* /Control | P11940 | -0.361401522 | 0.361401522 | 0.01081 | 0.007087 | 12 | PABPC1 |
| *A.vasorum* /Control | P67809 | 0.574736955 | 0.574736955 | 0.010889 | 0.007095 | 12 | YBX1 |
| *A.vasorum* /Control | P07900 | 0.172283615 | 0.172283615 | 0.011161 | 0.007198 | 12 | HSP90AA1 |

| | | | | | | | |
|---|---|---|---|---|---|---|---|
| *A.vasorum* /Control | P61604 | 0.982460433 | 0.982460433 | 0.01114 | 0.007198 | 12 | HSPE1 |
| *A.vasorum* /Control | Q07955 | 0.589799176 | 0.589799176 | 0.011144 | 0.007198 | 12 | SRSF1 |
| *A.vasorum* /Control | P16930 | -0.244089251 | 0.244089251 | 0.011457 | 0.007348 | 12 | FAH |
| *A.vasorum* /Control | Q14764 | -0.104939186 | 0.104939186 | 0.011468 | 0.007348 | 12 | MVP |
| *A.vasorum* /Control | P62249 | 0.125937278 | 0.125937278 | 0.011522 | 0.007359 | 12 | RPS16 |
| *A.vasorum* /Control | Q13308 | 0.06171584 | 0.06171584 | 0.011697 | 0.007447 | 12 | PTK7 |
| *A.vasorum* /Control | Q9UJ70 | 0.964425352 | 0.964425352 | 0.011806 | 0.007492 | 12 | NAGK |
| *A.vasorum* /Control | P19623 | -0.267532397 | 0.267532397 | 0.011942 | 0.007507 | 12 | SRM |
| *A.vasorum* /Control | Q6YHK3 | 0.455617322 | 0.455617322 | 0.011906 | 0.007507 | 12 | CD109 |
| *A.vasorum* /Control | P15090 | 1.664502538 | 1.664502538 | 0.0122 | 0.007644 | 12 | FABP4 |
| *A.vasorum* /Control | P43243 | 1.220696175 | 1.220696175 | 0.01226 | 0.007658 | 12 | MATR3 |
| *A.vasorum* /Control | O00159 | -0.626787957 | 0.626787957 | 0.012863 | 0.008009 | 12 | MYO1C |
| *A.vasorum* /Control | P62244 | 0.124494821 | 0.124494821 | 0.012913 | 0.008015 | 12 | RPS15A |
| *A.vasorum* /Control | Q96QV1 | 0.403606622 | 0.403606622 | 0.013015 | 0.008053 | 12 | HHIP |
| *A.vasorum* /Control | Q9NPH3 | -0.406076538 | 0.406076538 | 0.013368 | 0.00822 | 12 | IL1RAP |
| *A.vasorum* /Control | P01034 | 0.329333408 | 0.329333408 | 0.013527 | 0.008274 | 12 | CST3 |
| *A.vasorum* /Control | P47756 | 0.44825986 | 0.44825986 | 0.013539 | 0.008274 | 12 | CAPZB |
| *A.vasorum* /Control | O95810 | 0.544500296 | 0.544500296 | 0.014152 | 0.008595 | 12 | CAVIN2 |
| *A.vasorum* /Control | Q12905 | 0.528179706 | 0.528179706 | 0.014251 | 0.008629 | 12 | ILF2 |
| *A.vasorum* /Control | P16035 | 0.717014843 | 0.717014843 | 0.014524 | 0.008767 | 12 | TIMP2 |
| *A.vasorum* /Control | P22352 | -0.322800154 | 0.322800154 | 0.015 | 0.009027 | 12 | GPX3 |
| *A.vasorum* /Control | P14543 | 1.032119964 | 1.032119964 | 0.015126 | 0.009048 | 12 | NID1 |
| *A.vasorum* /Control | Q16363 | 0.246388002 | 0.246388002 | 0.015126 | 0.009048 | 12 | LAMA4 |
| *A.vasorum* /Control | P62736 | 0.070855236 | 0.070855236 | 0.015203 | 0.009067 | 12 | ACTA2 |
| *A.vasorum* /Control | P06744 | 0.327290423 | 0.327290423 | 0.01525 | 0.009068 | 12 | GPI |
| *A.vasorum* /Control | P62491 | 0.814402513 | 0.814402513 | 0.015828 | 0.009369 | 12 | RAB11A |
| *A.vasorum* /Control | Q14019 | 0.60411723 | 0.60411723 | 0.015851 | 0.009369 | 12 | COTL1 |
| *A.vasorum* /Control | P52566 | 0.599199856 | 0.599199856 | 0.016087 | 0.009424 | 12 | ARHGDIB |
| *A.vasorum* /Control | Q9NQ88 | 0.923517254 | 0.923517254 | 0.016051 | 0.009424 | 12 | TIGAR |
| *A.vasorum* /Control | Q9UNN8 | 0.892113016 | 0.892113016 | 0.016078 | 0.009424 | 12 | PROCR |
| *A.vasorum* /Control | P40227 | 0.269074331 | 0.269074331 | 0.016199 | 0.009442 | 12 | CCT6A |
| *A.vasorum* /Control | Q86UX7 | 0.344695188 | 0.344695188 | 0.016213 | 0.009442 | 12 | FERMT3 |
| *A.vasorum* /Control | P14618 | 0.215984321 | 0.215984321 | 0.01658 | 0.009488 | 12 | PKM |
| *A.vasorum* /Control | P22626 | -0.393180414 | 0.393180414 | 0.016445 | 0.009488 | 12 | HNRNPA2 |
| *A.vasorum* /Control | P61353 | 0.563681165 | 0.563681165 | 0.01657 | 0.009488 | 12 | RPL27 |

| | | | | | | | |
|---|---|---|---|---|---|---|---|
| *A.vasorum* /Control | Q99873 | 1.233032735 | 1.233032735 | 0.016553 | 0.009488 | 12 | PRMT1 |
| *A.vasorum* /Control | Q13765 | -0.132288369 | 0.132288369 | 0.016764 | 0.009532 | 12 | NACA |
| *A.vasorum* /Control | P13797 | 0.5231238 | 0.5231238 | 0.016801 | 0.009532 | 12 | PLS3 |
| *A.vasorum* /Control | P30041 | 0.709391716 | 0.709391716 | 0.01689 | 0.009555 | 12 | PRDX6 |
| *A.vasorum* /Control | P55084 | 0.124473785 | 0.124473785 | 0.017187 | 0.009668 | 12 | HADHB |
| *A.vasorum* /Control | P63241 | 0.537145029 | 0.537145029 | 0.017144 | 0.009668 | 12 | EIF5A |
| *A.vasorum* /Control | Q07020 | -0.2374324 | 0.2374324 | 0.017581 | 0.009861 | 12 | RPL18 |
| *A.vasorum* /Control | P00750 | 0.296598969 | 0.296598969 | 0.017742 | 0.009923 | 12 | PLAT |
| *A.vasorum* /Control | P21980 | 0.29122374 | 0.29122374 | 0.01785 | 0.009928 | 12 | TGM2 |
| *A.vasorum* /Control | Q9NY33 | 0.116540062 | 0.116540062 | 0.017848 | 0.009928 | 12 | DPP3 |
| *A.vasorum* /Control | P62269 | -0.057093167 | 0.057093167 | 0.018158 | 0.010071 | 12 | RPS18 |
| *A.vasorum* /Control | P62854 | -0.052037734 | 0.052037734 | 0.0187 | 0.010313 | 12 | RPS26 |
| *A.vasorum* /Control | P26368 | 0.75086775 | 0.75086775 | 0.019067 | 0.010487 | 12 | U2AF2 |
| *A.vasorum* /Control | P62888 | 0.345020257 | 0.345020257 | 0.019214 | 0.010491 | 12 | RPL30 |
| *A.vasorum* /Control | Q9Y4K0 | 0.398759268 | 0.398759268 | 0.019234 | 0.010491 | 12 | LOXL2 |
| *A.vasorum* /Control | Q13200 | 0.226845608 | 0.226845608 | 0.019401 | 0.010526 | 12 | PSMD2 |
| *A.vasorum* /Control | P52907 | 0.30659172 | 0.30659172 | 0.019934 | 0.010755 | 12 | CAPZA1 |
| *A.vasorum* /Control | Q9P2J5 | 0.814042716 | 0.814042716 | 0.019922 | 0.010755 | 12 | LARS1 |
| *A.vasorum* /Control | P10646 | 1.599559198 | 1.599559198 | 0.020159 | 0.010846 | 12 | TFPI |
| *A.vasorum* /Control | P00387 | 0.53426394 | 0.53426394 | 0.021025 | 0.011251 | 12 | CYB5R3 |
| *A.vasorum* /Control | Q13263 | -0.06737181 | 0.06737181 | 0.020978 | 0.011251 | 12 | TRIM28 |
| *A.vasorum* /Control | P17948 | 0.660856623 | 0.660856623 | 0.021191 | 0.01131 | 12 | FLT1 |
| *A.vasorum* /Control | P40261 | 0.884337732 | 0.884337732 | 0.021382 | 0.01138 | 12 | NNMT |
| *A.vasorum* /Control | P23284 | 0.093607577 | 0.093607577 | 0.021471 | 0.011397 | 12 | PPIB |
| *A.vasorum* /Control | O00567 | 0.435093732 | 0.435093732 | 0.021998 | 0.011646 | 12 | NOP56 |
| *A.vasorum* /Control | P28838 | 0.90582933 | 0.90582933 | 0.022137 | 0.011688 | 12 | LAP3 |
| *A.vasorum* /Control | P05305 | 0.347336607 | 0.347336607 | 0.022508 | 0.011852 | 12 | EDN1 |
| *A.vasorum* /Control | P50990 | 0.205309403 | 0.205309403 | 0.023249 | 0.01221 | 12 | CCT8 |
| *A.vasorum* /Control | P13473 | 0.10546561 | 0.10546561 | 0.0242 | 0.012643 | 12 | LAMP2 |
| *A.vasorum* /Control | O94985 | 0.657729812 | 0.657729812 | 0.024626 | 0.012681 | 12 | CLSTN1 |
| *A.vasorum* /Control | P22004 | 0.615181777 | 0.615181777 | 0.024657 | 0.012681 | 12 | BMP6 |
| *A.vasorum* /Control | P38646 | 0.54685525 | 0.54685525 | 0.024411 | 0.012681 | 12 | HSPA9 |
| *A.vasorum* /Control | P41250 | 0.346941836 | 0.346941836 | 0.024451 | 0.012681 | 12 | GARS1 |
| *A.vasorum* /Control | P61326 | 1.606210847 | 1.606210847 | 0.024578 | 0.012681 | 12 | MAGOH |
| *A.vasorum* /Control | P63173 | 0.76593119 | 0.76593119 | 0.024654 | 0.012681 | 12 | RPL38 |

| | | | | | | | |
|---|---|---|---|---|---|---|---|
| *A.vasorum* /Control | Q15233 | 0.043165508 | 0.043165508 | 0.025391 | 0.013024 | 12 | NONO |
| *A.vasorum* /Control | P05455 | 0.527009604 | 0.527009604 | 0.025544 | 0.013069 | 12 | SSB |
| *A.vasorum* /Control | O00625 | 0.181830806 | 0.181830806 | 0.025754 | 0.013109 | 12 | PIR |
| *A.vasorum* /Control | Q14767 | 0.459690522 | 0.459690522 | 0.025754 | 0.013109 | 12 | LTBP2 |
| *A.vasorum* /Control | P55060 | 0.405561864 | 0.405561864 | 0.025945 | 0.013172 | 12 | CSE1L |
| *A.vasorum* /Control | P38606 | 0.543928865 | 0.543928865 | 0.026244 | 0.01329 | 12 | ATP6V1A |
| *A.vasorum* /Control | Q8NBS9 | 0.489010109 | 0.489010109 | 0.026557 | 0.013414 | 12 | TXNDC5 |
| *A.vasorum* /Control | P23396 | 0.231637329 | 0.231637329 | 0.027037 | 0.013587 | 12 | RPS3 |
| *A.vasorum* /Control | O43684 | 1.014758824 | 1.014758824 | 0.027399 | 0.013734 | 12 | BUB3 |
| *A.vasorum* /Control | O75489 | 0.712848922 | 0.712848922 | 0.02768 | 0.01384 | 12 | NDUFS3 |
| *A.vasorum* /Control | P07998 | 1.030173108 | 1.030173108 | 0.027991 | 0.01396 | 12 | RNASE1 |
| *A.vasorum* /Control | Q92973 | 0.777599934 | 0.777599934 | 0.028156 | 0.013972 | 12 | TNPO1 |
| *A.vasorum* /Control | P81605 | -1.709103878 | 1.709103878 | 0.02876 | 0.014236 | 12 | DCD |
| *A.vasorum* /Control | P28072 | 1.635514115 | 1.635514115 | 0.029027 | 0.014297 | 12 | PSMB6 |
| *A.vasorum* /Control | Q9BWD1 | 0.701060461 | 0.701060461 | 0.028961 | 0.014297 | 12 | ACAT2 |
| *A.vasorum* /Control | P18085 | 1.521235109 | 1.521235109 | 0.029296 | 0.014394 | 12 | ARF4 |
| *A.vasorum* /Control | Q9BUF5 | 0.376526693 | 0.376526693 | 0.029421 | 0.014419 | 12 | TUBB6 |
| *A.vasorum* /Control | P48723 | 0.286405605 | 0.286405605 | 0.029496 | 0.01442 | 12 | HSPA13 |
| *A.vasorum* /Control | P08621 | 0.912902115 | 0.912902115 | 0.029885 | 0.014539 | 12 | SNRNP70 |
| *A.vasorum* /Control | P62280 | -0.085066969 | 0.085066969 | 0.029817 | 0.014539 | 12 | RPS11 |
| *A.vasorum* /Control | P55290 | -0.433791495 | 0.433791495 | 0.030623 | 0.014843 | 12 | CDH13 |
| *A.vasorum* /Control | P61160 | 0.611161848 | 0.611161848 | 0.030661 | 0.014843 | 12 | ACTR2 |
| *A.vasorum* /Control | P18124 | -0.138073887 | 0.138073887 | 0.030922 | 0.014897 | 12 | RPL7 |
| *A.vasorum* /Control | P04792 | 0.514268508 | 0.514268508 | 0.03116 | 0.014972 | 12 | HSPB1 |
| *A.vasorum* /Control | P62753 | -0.026469409 | 0.026469409 | 0.031229 | 0.014972 | 12 | RPS6 |
| *A.vasorum* /Control | Q92841 | 0.461102051 | 0.461102051 | 0.032049 | 0.015291 | 12 | DDX17 |
| *A.vasorum* /Control | Q9NVD7 | 0.739036248 | 0.739036248 | 0.032005 | 0.015291 | 12 | PARVA |
| *A.vasorum* /Control | P12109 | -0.296958365 | 0.296958365 | 0.032256 | 0.015352 | 12 | COL6A1 |
| *A.vasorum* /Control | P06733 | 0.131677346 | 0.131677346 | 0.033149 | 0.015692 | 12 | ENO1 |
| *A.vasorum* /Control | P11142 | 0.131175714 | 0.131175714 | 0.033286 | 0.015692 | 12 | HSPA8 |
| *A.vasorum* /Control | P16949 | 0.789225787 | 0.789225787 | 0.033092 | 0.015692 | 12 | STMN1 |
| *A.vasorum* /Control | P22692 | 1.079932591 | 1.079932591 | 0.033271 | 0.015692 | 12 | IGFBP4 |
| *A.vasorum* /Control | O60506 | 0.387282172 | 0.387282172 | 0.033784 | 0.015889 | 12 | SYNCRIP |
| *A.vasorum* /Control | P34096 | -0.838055919 | 0.838055919 | 0.033961 | 0.015897 | 12 | RNASE4 |
| *A.vasorum* /Control | Q13596 | 0.828510252 | 0.828510252 | 0.033928 | 0.015897 | 12 | SNX1 |

| | | | | | | | |
|---|---|---|---|---|---|---|---|
| *A.vasorum* /Control | O95084 | 0.056198497 | 0.056198497 | 0.034621 | 0.016167 | 12 | PRSS23 |
| *A.vasorum* /Control | Q96AE4 | 0.48474428 | 0.48474428 | 0.035736 | 0.01661 | 12 | FUBP1 |
| *A.vasorum* /Control | Q93088 | -0.570141544 | 0.570141544 | 0.035964 | 0.016676 | 12 | BHMT |
| *A.vasorum* /Control | P09012 | 1.075609271 | 1.075609271 | 0.036257 | 0.016773 | 12 | SNRPA |
| *A.vasorum* /Control | O14818 | 0.464236328 | 0.464236328 | 0.036732 | 0.016918 | 12 | PSMA7 |
| *A.vasorum* /Control | O15144 | 0.355230866 | 0.355230866 | 0.036826 | 0.016918 | 12 | ARPC2 |
| *A.vasorum* /Control | P12004 | 1.197091626 | 1.197091626 | 0.036766 | 0.016918 | 12 | PCNA |
| *A.vasorum* /Control | P13639 | 0.23483983 | 0.23483983 | 0.037317 | 0.017101 | 12 | EEF2 |
| *A.vasorum* /Control | P54136 | 0.966524444 | 0.966524444 | 0.037398 | 0.017101 | 12 | RARS1 |
| *A.vasorum* /Control | O00410 | 0.580278994 | 0.580278994 | 0.037943 | 0.01731 | 12 | IPO5 |
| *A.vasorum* /Control | P08123 | -0.584400777 | 0.584400777 | 0.03845 | 0.017461 | 12 | COL1A2 |
| *A.vasorum* /Control | P60953 | 0.685025468 | 0.685025468 | 0.038585 | 0.017482 | 12 | CDC42 |
| *A.vasorum* /Control | P51858 | -0.139182746 | 0.139182746 | 0.039281 | 0.017757 | 12 | HDGF |
| *A.vasorum* /Control | P48739 | -0.449380689 | 0.449380689 | 0.039798 | 0.017909 | 12 | PITPNB |
| *A.vasorum* /Control | Q09666 | -1.752467203 | 1.752467203 | 0.039781 | 0.017909 | 12 | AHNAK |
| *A.vasorum* /Control | P62899 | 0.09966138 | 0.09966138 | 0.040327 | 0.018106 | 12 | RPL31 |
| *A.vasorum* /Control | P11413 | 0.391657549 | 0.391657549 | 0.040865 | 0.018306 | 12 | G6PD |
| *A.vasorum* /Control | Q32P28 | -0.032567905 | 0.032567905 | 0.041364 | 0.018446 | 12 | P3H1 |
| *A.vasorum* /Control | P07602 | 0.105293035 | 0.105293035 | 0.042168 | 0.018763 | 12 | PSAP |
| *A.vasorum* /Control | P17301 | 0.542665635 | 0.542665635 | 0.04237 | 0.01881 | 12 | ITGA2 |
| *A.vasorum* /Control | Q15493 | -0.284168384 | 0.284168384 | 0.042907 | 0.019006 | 12 | RGN |
| *A.vasorum* /Control | P35232 | -0.604582009 | 0.604582009 | 0.044791 | 0.019796 | 12 | PHB1 |
| *A.vasorum* /Control | Q9Y3I0 | -0.205998546 | 0.205998546 | 0.045054 | 0.019868 | 12 | RTCB |
| *A.vasorum* /Control | P17655 | 0.474115319 | 0.474115319 | 0.045795 | 0.02015 | 12 | CAPN2 |
| *A.vasorum* /Control | P09651 | -0.04511532 | 0.04511532 | 0.047004 | 0.02059 | 12 | HNRNPA1 |
| *A.vasorum* /Control | Q9UQ80 | 0.169643286 | 0.169643286 | 0.04833 | 0.021124 | 12 | PA2G4 |
| *A.vasorum* /Control | Q14515 | -0.579143512 | 0.579143512 | 0.048701 | 0.02124 | 12 | SPARCL1 |
| *A.vasorum* /Control | P12268 | 1.026279586 | 1.026279586 | 0.048876 | 0.021269 | 12 | IMPDH2 |
| *A.vasorum* /Control | Q99729 | 1.183816698 | 1.183816698 | 0.049471 | 0.021481 | 12 | HNRNPAB |
| *A.vasorum* /Control | P07203 | 0.455766761 | 0.455766761 | 0.050201 | 0.02175 | 12 | GPX1 |
| *A.vasorum* /Control | Q9BXJ4 | -0.020386127 | 0.020386127 | 0.050683 | 0.021863 | 12 | C1QTNF3 |
| *A.vasorum* /Control | P08648 | -0.359205156 | 0.359205156 | 0.051451 | 0.022146 | 12 | ITGA5 |
| *A.vasorum* /Control | O00232 | 0.683732709 | 0.683732709 | 0.053093 | 0.022719 | 12 | PSMD12 |
| *A.vasorum* /Control | P55263 | 0.30894605 | 0.30894605 | 0.053569 | 0.022859 | 12 | ADK |
| *A.vasorum* /Control | O95782 | 0.302139305 | 0.302139305 | 0.053747 | 0.022886 | 12 | AP2A1 |

| | | | | | | | |
|---|---|---|---|---|---|---|---|
| *A.vasorum* /Control | P84103 | 0.553607389 | 0.553607389 | 0.05585 | 0.02373 | 12 | SRSF3 |
| *A.vasorum* /Control | O43143 | 0.519794367 | 0.519794367 | 0.056085 | 0.023779 | 12 | DHX15 |
| *A.vasorum* /Control | Q16543 | 0.515049003 | 0.515049003 | 0.057468 | 0.024314 | 12 | CDC37 |
| *A.vasorum* /Control | Q14766 | 0.562880757 | 0.562880757 | 0.058181 | 0.02451 | 12 | LTBP1 |
| *A.vasorum* /Control | P31930 | 0.702062085 | 0.702062085 | 0.058859 | 0.024743 | 12 | UQCRC1 |
| *A.vasorum* /Control | P27708 | -0.626806778 | 0.626806778 | 0.059488 | 0.024955 | 12 | CAD |
| *A.vasorum* /Control | P56537 | 0.417409054 | 0.417409054 | 0.05979 | 0.025029 | 12 | EIF6 |
| *A.vasorum* /Control | P61619 | -0.473830111 | 0.473830111 | 0.06051 | 0.025223 | 12 | SEC61A1 |
| *A.vasorum* /Control | P53621 | 0.401937746 | 0.401937746 | 0.061052 | 0.025396 | 12 | COPA |
| *A.vasorum* /Control | Q99988 | 0.423754168 | 0.423754168 | 0.062805 | 0.02607 | 12 | GDF15 |
| *A.vasorum* /Control | A6NIZ1 | -0.309518515 | 0.309518515 | 0.063508 | 0.026307 | 12 | RAP1BL |
| *A.vasorum* /Control | P15170 | -1.280032174 | 1.280032174 | 0.063853 | 0.026394 | 12 | GSPT1 |
| *A.vasorum* /Control | P49747 | -0.518076389 | 0.518076389 | 0.064255 | 0.026505 | 12 | COMP |
| *A.vasorum* /Control | P25787 | 0.5427011 | 0.5427011 | 0.065819 | 0.027038 | 12 | PSMA2 |
| *A.vasorum* /Control | Q9Y3U8 | 0.671546207 | 0.671546207 | 0.066563 | 0.027287 | 12 | RPL36 |
| *A.vasorum* /Control | P59998 | 0.949395952 | 0.949395952 | 0.071262 | 0.028973 | 12 | ARPC4 |
| *A.vasorum* /Control | P68036 | 0.565084062 | 0.565084062 | 0.071246 | 0.028973 | 12 | UBE2L3 |
| *A.vasorum* /Control | P23246 | 0.356981579 | 0.356981579 | 0.072193 | 0.029232 | 12 | SFPQ |
| *A.vasorum* /Control | Q06828 | -0.080874308 | 0.080874308 | 0.072522 | 0.029283 | 12 | FMOD |
| *A.vasorum* /Control | Q12931 | 0.594667757 | 0.594667757 | 0.072615 | 0.029283 | 12 | TRAP1 |
| *A.vasorum* /Control | O15145 | 0.960266475 | 0.960266475 | 0.073385 | 0.029533 | 12 | ARPC3 |
| *A.vasorum* /Control | Q9BXJ0 | 0.49522851 | 0.49522851 | 0.07509 | 0.030158 | 12 | C1QTNF5 |
| *A.vasorum* /Control | P07384 | 0.880741408 | 0.880741408 | 0.075262 | 0.030166 | 12 | CAPN1 |
| *A.vasorum* /Control | Q03135 | 0.566415566 | 0.566415566 | 0.075634 | 0.030254 | 12 | CAV1 |
| *A.vasorum* /Control | O75923 | 0.75836028 | 0.75836028 | 0.0759 | 0.030299 | 12 | DYSF |
| *A.vasorum* /Control | P14866 | 0.409025431 | 0.409025431 | 0.076334 | 0.030411 | 12 | HNRNPL |
| *A.vasorum* /Control | Q9NR45 | 0.539222347 | 0.539222347 | 0.076695 | 0.030493 | 12 | NANS |
| *A.vasorum* /Control | P07339 | 0.09150036 | 0.09150036 | 0.077504 | 0.030753 | 12 | CTSD |
| *A.vasorum* /Control | P35555 | 0.63698327 | 0.63698327 | 0.077939 | 0.030864 | 12 | FBN1 |
| *A.vasorum* /Control | P31943 | -0.57095157 | 0.57095157 | 0.078127 | 0.030876 | 12 | HNRNPH1 |
| *A.vasorum* /Control | P63244 | 0.065377685 | 0.065377685 | 0.079299 | 0.031277 | 12 | RACK1 |
| *A.vasorum* /Control | P48059 | 1.548359684 | 1.548359684 | 0.08073 | 0.031778 | 12 | LIMS1 |
| *A.vasorum* /Control | Q08629 | 0.208419433 | 0.208419433 | 0.084 | 0.033 | 12 | SPOCK1 |
| *A.vasorum* /Control | P15880 | -0.163543541 | 0.163543541 | 0.085031 | 0.033339 | 12 | RPS2 |
| *A.vasorum* /Control | P62917 | 0.262572585 | 0.262572585 | 0.085806 | 0.033576 | 12 | RPL8 |

| | | | | | | | | |
|---|---|---|---|---|---|---|---|---|
| *A.vasorum* /Control | P49720 | 0.352171909 | 0.352171909 | 0.086921 | 0.033946 | 12 | PSMB3 |
| *A.vasorum* /Control | B5ME19 | 0.430863456 | 0.430863456 | 0.090245 | 0.035174 | 12 | EIF3CL |
| *A.vasorum* /Control | P22234 | 0.099977863 | 0.099977863 | 0.090799 | 0.035321 | 12 | PAICS |
| *A.vasorum* /Control | Q9Y230 | -0.011674868 | 0.011674868 | 0.091768 | 0.035627 | 12 | RUVBL2 |
| *A.vasorum* /Control | O15232 | 0.47954281 | 0.47954281 | 0.094896 | 0.036626 | 12 | MATN3 |
| *A.vasorum* /Control | P55145 | 0.169354831 | 0.169354831 | 0.096296 | 0.037095 | 12 | MANF |
| *A.vasorum* /Control | P10124 | -0.676060827 | 0.676060827 | 0.099238 | 0.038011 | 12 | SRGN |
| *A.vasorum* /Control | P51148 | 0.195550496 | 0.195550496 | 0.099107 | 0.038011 | 12 | RAB5C |
| *A.vasorum* /Control | Q9BTV4 | -0.173397831 | 0.173397831 | 0.099251 | 0.038011 | 12 | TMEM43 |
| *A.vasorum* /Control | Q15257 | -0.602859015 | 0.602859015 | 0.101594 | 0.038758 | 12 | PTPA |
| *A.vasorum* /Control | Q7Z7G0 | 0.374882442 | 0.374882442 | 0.101566 | 0.038758 | 12 | ABI3BP |
| *A.vasorum* /Control | Q8IUX7 | 1.551070392 | 1.551070392 | 0.104739 | 0.039881 | 12 | AEBP1 |
| *A.vasorum* /Control | P05109 | 1.460111971 | 1.460111971 | 0.105628 | 0.040143 | 12 | S100A8 |
| *A.vasorum* /Control | Q06481 | 0.004640282 | 0.004640282 | 0.106269 | 0.040309 | 12 | APLP2 |
| *A.vasorum* /Control | Q9NTK5 | 0.176092267 | 0.176092267 | 0.111115 | 0.042066 | 12 | OLA1 |
| *A.vasorum* /Control | P15153 | 0.002604313 | 0.002604313 | 0.111479 | 0.042124 | 12 | RAC2 |
| *A.vasorum* /Control | P17858 | 0.599027978 | 0.599027978 | 0.111743 | 0.042143 | 12 | PFKL |
| *A.vasorum* /Control | Q02818 | 0.643284143 | 0.643284143 | 0.11199 | 0.042156 | 12 | NUCB1 |
| *A.vasorum* /Control | P18621 | 0.076553688 | 0.076553688 | 0.112316 | 0.042198 | 12 | RPL17 |
| *A.vasorum* /Control | Q8WUM4 | 0.68548422 | 0.68548422 | 0.117643 | 0.044116 | 12 | PDCD6IP |
| *A.vasorum* /Control | P06702 | 1.668837184 | 1.668837184 | 0.118765 | 0.044369 | 12 | S100A9 |
| *A.vasorum* /Control | P06865 | 0.714476157 | 0.714476157 | 0.118676 | 0.044369 | 12 | HEXA |
| *A.vasorum* /Control | P08238 | 0.114821174 | 0.114821174 | 0.120207 | 0.044734 | 12 | HSP90AB1 |
| *A.vasorum* /Control | P61916 | 0.140345105 | 0.140345105 | 0.120351 | 0.044734 | 12 | NPC2 |
| *A.vasorum* /Control | Q9BSJ8 | 0.482140599 | 0.482140599 | 0.120421 | 0.044734 | 12 | ESYT1 |
| *A.vasorum* /Control | P02768 | -0.576414178 | 0.576414178 | 0.120695 | 0.044752 | 12 | ALB |
| *A.vasorum* /Control | O75436 | 0.232054703 | 0.232054703 | 0.121393 | 0.044843 | 12 | VPS26A |
| *A.vasorum* /Control | P02458 | -0.376787147 | 0.376787147 | 0.12117 | 0.044843 | 12 | COL2A1 |
| *A.vasorum* /Control | P42166 | 0.523214985 | 0.523214985 | 0.121722 | 0.044881 | 12 | TMPO |
| *A.vasorum* /Control | Q04446 | 0.371857833 | 0.371857833 | 0.124589 | 0.045852 | 12 | GBE1 |
| *A.vasorum* /Control | P62847 | 0.121988776 | 0.121988776 | 0.12614 | 0.046337 | 12 | RPS24 |
| *A.vasorum* /Control | P38159 | 0.647742693 | 0.647742693 | 0.129145 | 0.047305 | 12 | RBMX |
| *A.vasorum* /Control | Q13885 | -0.310363752 | 0.310363752 | 0.129731 | 0.047305 | 12 | TUBB2A |
| *A.vasorum* /Control | Q16394 | 0.089635161 | 0.089635161 | 0.129665 | 0.047305 | 12 | EXT1 |
| *A.vasorum* /Control | Q9BT78 | -0.309840087 | 0.309840087 | 0.130132 | 0.047364 | 12 | COPS4 |

| | | | | | | | | |
|---|---|---|---|---|---|---|---|---|
| *A.vasorum* /Control | Q9Y5X9 | 0.417570628 | 0.417570628 | 0.130737 | 0.047497 | 12 | LIPG |
| *A.vasorum* /Control | Q15029 | -0.40493974 | 0.40493974 | 0.130977 | 0.047497 | 12 | EFTUD2 |
| *A.vasorum* /Control | P06454 | -0.780143314 | 0.780143314 | 0.131487 | 0.047595 | 12 | PTMA |
| *A.vasorum* /Control | O75874 | 0.570129466 | 0.570129466 | 0.132121 | 0.047678 | 12 | IDH1 |
| *A.vasorum* /Control | P19338 | -0.445557228 | 0.445557228 | 0.132198 | 0.047678 | 12 | NCL |
| *A.vasorum* /Control | P40121 | 0.409298978 | 0.409298978 | 0.133571 | 0.048086 | 12 | CAPG |
| *A.vasorum* /Control | P08670 | -0.708725878 | 0.708725878 | 0.136895 | 0.049193 | 12 | VIM |
| *A.vasorum* /Control | P46777 | 0.180548025 | 0.180548025 | 0.145008 | 0.05192 | 12 | RPL5 |
| *A.vasorum* /Control | P53618 | 0.354948114 | 0.354948114 | 0.144746 | 0.05192 | 12 | COPB1 |
| *A.vasorum* /Control | P0CG47 | -0.414099979 | 0.414099979 | 0.146049 | 0.052104 | 12 | UBB |
| *A.vasorum* /Control | Q14203 | 0.22023101 | 0.22023101 | 0.145977 | 0.052104 | 12 | DCTN1 |
| *A.vasorum* /Control | Q15393 | 0.709583058 | 0.709583058 | 0.147717 | 0.052604 | 12 | SF3B3 |
| *A.vasorum* /Control | O43390 | 0.506515866 | 0.506515866 | 0.148386 | 0.052653 | 12 | HNRNPR |
| *A.vasorum* /Control | P14868 | 0.957139385 | 0.957139385 | 0.148164 | 0.052653 | 12 | DARS1 |
| *A.vasorum* /Control | Q15365 | -0.044269157 | 0.044269157 | 0.148906 | 0.052743 | 12 | PCBP1 |
| *A.vasorum* /Control | Q9NY15 | 0.402838188 | 0.402838188 | 0.149555 | 0.052878 | 12 | STAB1 |
| *A.vasorum* /Control | O75390 | 0.762410551 | 0.762410551 | 0.15073 | 0.053199 | 12 | CS |
| *A.vasorum* /Control | P15586 | 0.423056053 | 0.423056053 | 0.153463 | 0.053875 | 12 | GNS |
| *A.vasorum* /Control | Q04917 | 0.677190454 | 0.677190454 | 0.153429 | 0.053875 | 12 | YWHAH |
| *A.vasorum* /Control | Q15582 | -0.192423547 | 0.192423547 | 0.154735 | 0.054226 | 12 | TGFBI |
| *A.vasorum* /Control | Q9NVA2 | 0.113185286 | 0.113185286 | 0.155401 | 0.054363 | 12 | SEPTIN11 |
| *A.vasorum* /Control | Q12860 | -0.505540376 | 0.505540376 | 0.155858 | 0.054426 | 12 | CNTN1 |
| *A.vasorum* /Control | P50454 | 0.164922021 | 0.164922021 | 0.158482 | 0.055246 | 12 | SERPINH1 |
| *A.vasorum* /Control | P20774 | 0.146628407 | 0.146628407 | 0.158904 | 0.055285 | 12 | OGN |
| *A.vasorum* /Control | Q9UBP4 | 0.611126789 | 0.611126789 | 0.159153 | 0.055285 | 12 | DKK3 |
| *A.vasorum* /Control | P46778 | 0.502120357 | 0.502120357 | 0.159875 | 0.055379 | 12 | RPL21 |
| *A.vasorum* /Control | P54578 | 0.352518003 | 0.352518003 | 0.159985 | 0.055379 | 12 | USP14 |
| *A.vasorum* /Control | P13073 | -0.204744919 | 0.204744919 | 0.161355 | 0.055756 | 12 | COX4I1 |
| *A.vasorum* /Control | Q7L576 | -0.244243603 | 0.244243603 | 0.161776 | 0.055804 | 12 | CYFIP1 |
| *A.vasorum* /Control | P20042 | 0.168517876 | 0.168517876 | 0.164414 | 0.056616 | 12 | EIF2S2 |
| *A.vasorum* /Control | P62906 | -0.088264492 | 0.088264492 | 0.172391 | 0.059259 | 12 | RPL10A |
| *A.vasorum* /Control | P34932 | 0.322774648 | 0.322774648 | 0.174703 | 0.059865 | 12 | HSPA4 |
| *A.vasorum* /Control | P60842 | 0.011138048 | 0.011138048 | 0.174757 | 0.059865 | 12 | EIF4A1 |
| *A.vasorum* /Control | P55285 | 0.078134068 | 0.078134068 | 0.17619 | 0.060171 | 12 | CDH6 |
| *A.vasorum* /Control | P32969 | 0.153979102 | 0.153979102 | 0.178775 | 0.06082 | 12 | RPL9 |

| | | | | | | | |
|---|---|---|---|---|---|---|---|
| *A.vasorum* /Control | P05198 | 0.019964327 | 0.019964327 | 0.183084 | 0.062179 | 12 | EIF2S1 |
| *A.vasorum* /Control | P46926 | 0.255486962 | 0.255486962 | 0.192494 | 0.065263 | 12 | GNPDA1 |
| *A.vasorum* /Control | Q7KZF4 | 0.12252764 | 0.12252764 | 0.194835 | 0.065944 | 12 | SND1 |
| *A.vasorum* /Control | P07910 | -0.044376123 | 0.044376123 | 0.196785 | 0.066377 | 12 | HNRNPC |
| *A.vasorum* /Control | Q14195 | 0.520981378 | 0.520981378 | 0.196589 | 0.066377 | 12 | DPYSL3 |
| *A.vasorum* /Control | P39656 | -0.65234343 | 0.65234343 | 0.197781 | 0.066534 | 12 | DDOST |
| *A.vasorum* /Control | P46782 | -0.132131092 | 0.132131092 | 0.197921 | 0.066534 | 12 | RPS5 |
| *A.vasorum* /Control | Q13838 | 0.1526378 | 0.1526378 | 0.20134 | 0.067568 | 12 | DDX39B |
| *A.vasorum* /Control | P61019 | 0.778467284 | 0.778467284 | 0.201959 | 0.067661 | 12 | RAB2A |
| *A.vasorum* /Control | P06732 | 0.755860036 | 0.755860036 | 0.203057 | 0.067914 | 12 | CKM |
| *A.vasorum* /Control | O14950 | 0.144473813 | 0.144473813 | 0.204509 | 0.06821 | 12 | MYL12B |
| *A.vasorum* /Control | P49721 | 1.074729429 | 1.074729429 | 0.20463 | 0.06821 | 12 | PSMB2 |
| *A.vasorum* /Control | O75131 | 0.057509449 | 0.057509449 | 0.206307 | 0.068292 | 12 | CPNE3 |
| *A.vasorum* /Control | P00403 | 0.552783164 | 0.552783164 | 0.206448 | 0.068292 | 12 | MT-CO2 |
| *A.vasorum* /Control | P11279 | 0.077457891 | 0.077457891 | 0.205298 | 0.068292 | 12 | LAMP1 |
| *A.vasorum* /Control | P62191 | 0.886492187 | 0.886492187 | 0.2066 | 0.068292 | 12 | PSMC1 |
| *A.vasorum* /Control | P62913 | -0.310882694 | 0.310882694 | 0.205673 | 0.068292 | 12 | RPL11 |
| *A.vasorum* /Control | P06703 | 0.657228768 | 0.657228768 | 0.207111 | 0.068347 | 12 | S100A6 |
| *A.vasorum* /Control | P62851 | 0.460394234 | 0.460394234 | 0.209155 | 0.068906 | 12 | RPS25 |
| *A.vasorum* /Control | P08708 | 0.029680743 | 0.029680743 | 0.212836 | 0.069886 | 12 | RPS17 |
| *A.vasorum* /Control | Q00839 | 0.124304729 | 0.124304729 | 0.21271 | 0.069886 | 12 | HNRNPU |
| *A.vasorum* /Control | P10586 | -0.124965483 | 0.124965483 | 0.215912 | 0.070779 | 12 | PTPRF |
| *A.vasorum* /Control | O00154 | 0.32678604 | 0.32678604 | 0.223344 | 0.073094 | 12 | ACOT7 |
| *A.vasorum* /Control | Q9NZV1 | 0.406204279 | 0.406204279 | 0.225199 | 0.07358 | 12 | CRIM1 |
| *A.vasorum* /Control | P27797 | -0.217488975 | 0.217488975 | 0.230357 | 0.075018 | 12 | CALR |
| *A.vasorum* /Control | P30101 | -0.082669204 | 0.082669204 | 0.230352 | 0.075018 | 12 | PDIA3 |
| *A.vasorum* /Control | P11021 | -0.3246254 | 0.3246254 | 0.233013 | 0.075758 | 12 | HSPA5 |
| *A.vasorum* /Control | Q92820 | 0.650996779 | 0.650996779 | 0.23416 | 0.076006 | 12 | GGH |
| *A.vasorum* /Control | Q07065 | -0.273457136 | 0.273457136 | 0.237251 | 0.076883 | 12 | CKAP4 |
| *A.vasorum* /Control | P10619 | 0.277170757 | 0.277170757 | 0.243738 | 0.078857 | 12 | CTSA |
| *A.vasorum* /Control | P39019 | 0.187387251 | 0.187387251 | 0.248416 | 0.080237 | 12 | RPS19 |
| *A.vasorum* /Control | P78371 | 0.126221062 | 0.126221062 | 0.248817 | 0.080237 | 12 | CCT2 |
| *A.vasorum* /Control | O00469 | -0.125894984 | 0.125894984 | 0.249659 | 0.080292 | 12 | PLOD2 |
| *A.vasorum* /Control | P35443 | -0.092013032 | 0.092013032 | 0.249796 | 0.080292 | 12 | THBS4 |
| *A.vasorum* /Control | P08758 | -0.023486699 | 0.023486699 | 0.255977 | 0.082145 | 12 | ANXA5 |

| | | | | | | | |
|---|---|---|---|---|---|---|---|
| *A.vasorum* /Control | Q15366 | 0.034543128 | 0.034543128 | 0.261057 | 0.083505 | 12 | PCBP2 |
| *A.vasorum* /Control | P11047 | 0.022256807 | 0.022256807 | 0.263257 | 0.084072 | 12 | LAMC1 |
| *A.vasorum* /Control | Q9Y266 | 0.78522027 | 0.78522027 | 0.272691 | 0.086945 | 12 | NUDC |
| *A.vasorum* /Control | P48643 | 0.199926826 | 0.199926826 | 0.274665 | 0.087433 | 12 | CCT5 |
| *A.vasorum* /Control | P07814 | 0.600391048 | 0.600391048 | 0.27913 | 0.088712 | 12 | EPRS1 |
| *A.vasorum* /Control | Q99623 | 0.011509104 | 0.011509104 | 0.280389 | 0.088969 | 12 | PHB2 |
| *A.vasorum* /Control | O60462 | -0.011088806 | 0.011088806 | 0.282633 | 0.089395 | 12 | NRP2 |
| *A.vasorum* /Control | Q9Y678 | 0.417370743 | 0.417370743 | 0.284462 | 0.08983 | 12 | COPG1 |
| *A.vasorum* /Control | Q9H0U4 | -1.048604167 | 1.048604167 | 0.287424 | 0.090621 | 12 | RAB1B |
| *A.vasorum* /Control | P07942 | -0.061743555 | 0.061743555 | 0.288728 | 0.090887 | 12 | LAMB1 |
| *A.vasorum* /Control | P20618 | 0.462203351 | 0.462203351 | 0.290115 | 0.091179 | 12 | PSMB1 |
| *A.vasorum* /Control | P05091 | -0.519580409 | 0.519580409 | 0.292317 | 0.091725 | 12 | ALDH2 |
| *A.vasorum* /Control | P16403 | -0.104307116 | 0.104307116 | 0.294862 | 0.092378 | 12 | H1-2 |
| *A.vasorum* /Control | Q53GQ0 | 0.332094722 | 0.332094722 | 0.295468 | 0.092421 | 12 | HSD17B12 |
| *A.vasorum* /Control | P62266 | 0.145322192 | 0.145322192 | 0.296465 | 0.092587 | 12 | RPS23 |
| *A.vasorum* /Control | P20908 | -0.15051118 | 0.15051118 | 0.297108 | 0.092641 | 12 | COL5A1 |
| *A.vasorum* /Control | P16284 | -0.124279359 | 0.124279359 | 0.300679 | 0.093608 | 12 | PECAM1 |
| *A.vasorum* /Control | P51149 | 0.623853116 | 0.623853116 | 0.302291 | 0.093962 | 12 | RAB7A |
| *A.vasorum* /Control | Q99714 | 0.482773404 | 0.482773404 | 0.307369 | 0.09539 | 12 | HSD17B10 |
| *A.vasorum* /Control | Q9Y5B9 | 0.366340626 | 0.366340626 | 0.308247 | 0.095513 | 12 | SUPT16H |
| *A.vasorum* /Control | P05023 | -0.0981316 | 0.0981316 | 0.310384 | 0.096025 | 12 | ATP1A1 |
| *A.vasorum* /Control | P54886 | -0.023325725 | 0.023325725 | 0.317167 | 0.097971 | 12 | ALDH18A1 |
| *A.vasorum* /Control | P04843 | -0.251012055 | 0.251012055 | 0.321246 | 0.098922 | 12 | RPN1 |
| *A.vasorum* /Control | P08133 | 0.290787273 | 0.290787273 | 0.321111 | 0.098922 | 12 | ANXA6 |
| *A.vasorum* /Control | O75915 | -0.029313287 | 0.029313287 | 0.32303 | 0.099317 | 12 | ARL6IP5 |
| *A.vasorum* /Control | P35998 | -0.184965112 | 0.184965112 | 0.3237 | 0.099368 | 12 | PSMC2 |
| *A.vasorum* /Control | Q9Y240 | -0.272311708 | 0.272311708 | 0.324201 | 0.099368 | 12 | CLEC11A |
| *A.vasorum* /Control | P62829 | 0.039767232 | 0.039767232 | 0.335334 | 0.102463 | 12 | RPL23 |
| *A.vasorum* /Control | Q9ULV4 | 1.096374628 | 1.096374628 | 0.339009 | 0.103426 | 12 | CORO1C |
| *A.vasorum* /Control | P61247 | 0.27709371 | 0.27709371 | 0.346718 | 0.105616 | 12 | RPS3A |
| *A.vasorum* /Control | P27824 | 0.210817799 | 0.210817799 | 0.350588 | 0.106631 | 12 | CANX |
| *A.vasorum* /Control | P09525 | -0.340881556 | 0.340881556 | 0.354809 | 0.107419 | 12 | ANXA4 |
| *A.vasorum* /Control | P30086 | -0.183467901 | 0.183467901 | 0.353881 | 0.107419 | 12 | PEBP1 |
| *A.vasorum* /Control | P46779 | -0.150841836 | 0.150841836 | 0.354399 | 0.107419 | 12 | RPL28 |
| *A.vasorum* /Control | P48047 | 0.139434614 | 0.139434614 | 0.356322 | 0.107712 | 12 | ATP5PO |

| | | | | | | | |
|---|---|---|---|---|---|---|---|
| *A.vasorum* /Control | P45880 | 0.086014429 | 0.086014429 | 0.362742 | 0.109486 | 12 | VDAC2 |
| *A.vasorum* /Control | Q13162 | -0.738232981 | 0.738232981 | 0.365758 | 0.110228 | 12 | PRDX4 |
| *A.vasorum* /Control | P15144 | -0.110651324 | 0.110651324 | 0.366475 | 0.110277 | 12 | ANPEP |
| *A.vasorum* /Control | Q13185 | -0.009208441 | 0.009208441 | 0.372015 | 0.111774 | 12 | CBX3 |
| *A.vasorum* /Control | P05783 | 0.06174798 | 0.06174798 | 0.372631 | 0.111789 | 12 | KRT18 |
| *A.vasorum* /Control | P25705 | -0.016691962 | 0.016691962 | 0.377086 | 0.112955 | 12 | ATP5F1A |
| *A.vasorum* /Control | O00571 | -0.076025694 | 0.076025694 | 0.37931 | 0.113449 | 12 | DDX3X |
| *A.vasorum* /Control | P47897 | 0.441670147 | 0.441670147 | 0.381361 | 0.113891 | 12 | QARS1 |
| *A.vasorum* /Control | P62750 | 0.999331364 | 0.999331364 | 0.387094 | 0.115429 | 12 | RPL23A |
| *A.vasorum* /Control | P40939 | 0.088164209 | 0.088164209 | 0.388157 | 0.115571 | 12 | HADHA |
| *A.vasorum* /Control | Q8TCT9 | -0.112005411 | 0.112005411 | 0.390494 | 0.116093 | 12 | HM13 |
| *A.vasorum* /Control | P67812 | 0.137307774 | 0.137307774 | 0.399279 | 0.118527 | 12 | SEC11A |
| *A.vasorum* /Control | Q9BRK5 | 0.304068858 | 0.304068858 | 0.40215 | 0.1192 | 12 | SDF4 |
| *A.vasorum* /Control | Q9Y4L1 | 0.043103395 | 0.043103395 | 0.403626 | 0.119459 | 12 | HYOU1 |
| *A.vasorum* /Control | P56545 | 0.021336863 | 0.021336863 | 0.406981 | 0.120272 | 12 | CTBP2 |
| *A.vasorum* /Control | Q9Y265 | 0.08269074 | 0.08269074 | 0.407887 | 0.12036 | 12 | RUVBL1 |
| *A.vasorum* /Control | P46776 | 0.49407198 | 0.49407198 | 0.418264 | 0.123159 | 12 | RPL27A |
| *A.vasorum* /Control | P48740 | -0.626680646 | 0.626680646 | 0.418617 | 0.123159 | 12 | MASP1 |
| *A.vasorum* /Control | Q00325 | 0.108279268 | 0.108279268 | 0.420237 | 0.123452 | 12 | SLC25A3 |
| *A.vasorum* /Control | P07237 | 0.104799827 | 0.104799827 | 0.421018 | 0.123499 | 12 | P4HB |
| *A.vasorum* /Control | P26599 | 0.204266046 | 0.204266046 | 0.422234 | 0.123672 | 12 | PTBP1 |
| *A.vasorum* /Control | P06748 | -0.193944649 | 0.193944649 | 0.428348 | 0.125277 | 12 | NPM1 |
| *A.vasorum* /Control | P84098 | -0.441767744 | 0.441767744 | 0.431924 | 0.126137 | 12 | RPL19 |
| *A.vasorum* /Control | O95497 | -0.194792455 | 0.194792455 | 0.438557 | 0.127886 | 12 | VNN1 |
| *A.vasorum* /Control | Q15293 | 0.071993156 | 0.071993156 | 0.442781 | 0.128927 | 12 | RCN1 |
| *A.vasorum* /Control | P51991 | -0.241285088 | 0.241285088 | 0.444292 | 0.128988 | 12 | HNRNPA3 |
| *A.vasorum* /Control | Q04637 | -0.041504499 | 0.041504499 | 0.444093 | 0.128988 | 12 | EIF4G1 |
| *A.vasorum* /Control | P22695 | 0.735428297 | 0.735428297 | 0.448215 | 0.129936 | 12 | UQCRC2 |
| *A.vasorum* /Control | Q9NQ30 | -0.111906328 | 0.111906328 | 0.456692 | 0.132007 | 12 | ESM1 |
| *A.vasorum* /Control | Q15019 | 0.391210901 | 0.391210901 | 0.461728 | 0.133269 | 12 | SEPTIN2 |
| *A.vasorum* /Control | P11233 | 1.114900689 | 1.114900689 | 0.466026 | 0.134313 | 12 | RALA |
| *A.vasorum* /Control | P07858 | -0.096174772 | 0.096174772 | 0.47424 | 0.136284 | 12 | CTSB |
| *A.vasorum* /Control | P41091 | 0.291247385 | 0.291247385 | 0.473589 | 0.136284 | 12 | EIF2S3 |
| *A.vasorum* /Control | Q12907 | 0.741709411 | 0.741709411 | 0.478739 | 0.137377 | 12 | LMAN2 |
| *A.vasorum* /Control | Q99538 | 0.025894124 | 0.025894124 | 0.483063 | 0.138417 | 12 | LGMN |

| | | | | | | | |
|---|---|---|---|---|---|---|---|
| *A.vasorum* /Control | Q02878 | 0.118561754 | 0.118561754 | 0.48479 | 0.138712 | 12 | RPL6 |
| *A.vasorum* /Control | O00231 | 0.427692247 | 0.427692247 | 0.485911 | 0.138832 | 12 | PSMD11 |
| *A.vasorum* /Control | P05141 | -0.076503785 | 0.076503785 | 0.493549 | 0.140811 | 12 | SLC25A5 |
| *A.vasorum* /Control | P14625 | -0.115230867 | 0.115230867 | 0.498235 | 0.141943 | 12 | HSP90B1 |
| *A.vasorum* /Control | P47755 | 0.414204035 | 0.414204035 | 0.499036 | 0.141967 | 12 | CAPZA2 |
| *A.vasorum* /Control | P53396 | 0.222389246 | 0.222389246 | 0.513818 | 0.145753 | 12 | ACLY |
| *A.vasorum* /Control | P35222 | 0.504280275 | 0.504280275 | 0.522585 | 0.148028 | 12 | CTNNB1 |
| *A.vasorum* /Control | Q01130 | 0.313094693 | 0.313094693 | 0.526846 | 0.149022 | 12 | SRSF2 |
| *A.vasorum* /Control | Q9BRX8 | 0.020127565 | 0.020127565 | 0.531299 | 0.149854 | 12 | PRXL2A |
| *A.vasorum* /Control | P45974 | 0.117481521 | 0.117481521 | 0.542118 | 0.152688 | 12 | USP5 |
| *A.vasorum* /Control | P03956 | -0.247794012 | 0.247794012 | 0.543623 | 0.152894 | 12 | MMP1 |
| *A.vasorum* /Control | Q99832 | 0.111712292 | 0.111712292 | 0.551113 | 0.154781 | 12 | CCT7 |
| *A.vasorum* /Control | P07437 | -0.323563105 | 0.323563105 | 0.552484 | 0.154946 | 12 | TUBB |
| *A.vasorum* /Control | P53004 | 0.91555305 | 0.91555305 | 0.56757 | 0.158952 | 12 | BLVRA |
| *A.vasorum* /Control | P27105 | 0.05126352 | 0.05126352 | 0.569978 | 0.159401 | 12 | STOM |
| *A.vasorum* /Control | P52272 | -0.134748995 | 0.134748995 | 0.576343 | 0.160953 | 12 | HNRNPM |
| *A.vasorum* /Control | P62873 | 0.300078851 | 0.300078851 | 0.579894 | 0.161717 | 12 | GNB1 |
| *A.vasorum* /Control | P61313 | -0.101173312 | 0.101173312 | 0.580927 | 0.161777 | 12 | RPL15 |
| *A.vasorum* /Control | Q07666 | 0.084293111 | 0.084293111 | 0.581905 | 0.161822 | 12 | KHDRBS1 |
| *A.vasorum* /Control | P09669 | 0.683964039 | 0.683964039 | 0.583504 | 0.162039 | 12 | COX6C |
| *A.vasorum* /Control | P17987 | 0.30937411 | 0.30937411 | 0.591669 | 0.164076 | 12 | TCP1 |
| *A.vasorum* /Control | P48444 | 0.100732998 | 0.100732998 | 0.596536 | 0.165195 | 12 | ARCN1 |
| *A.vasorum* /Control | Q14315 | 0.170538512 | 0.170538512 | 0.598535 | 0.165517 | 12 | FLNC |
| *A.vasorum* /Control | Q9Y5S9 | 0.961977204 | 0.961977204 | 0.60568 | 0.167259 | 12 | RBM8A |
| *A.vasorum* /Control | P12107 | -0.336085901 | 0.336085901 | 0.615923 | 0.169851 | 12 | COL11A1 |
| *A.vasorum* /Control | P04844 | 0.022481867 | 0.022481867 | 0.6196 | 0.17039 | 12 | RPN2 |
| *A.vasorum* /Control | Q9HDC9 | 0.055938054 | 0.055938054 | 0.619259 | 0.17039 | 12 | APMAP |
| *A.vasorum* /Control | P19367 | 0.033898987 | 0.033898987 | 0.628017 | 0.172465 | 12 | HK1 |
| *A.vasorum* /Control | P62424 | -0.049923545 | 0.049923545 | 0.630057 | 0.172786 | 12 | RPL7A |
| *A.vasorum* /Control | P55884 | 0.397450184 | 0.397450184 | 0.640559 | 0.175423 | 12 | EIF3B |
| *A.vasorum* /Control | P83881 | -0.125602583 | 0.125602583 | 0.641632 | 0.175474 | 12 | RPL36A |
| *A.vasorum* /Control | P26373 | 0.164848441 | 0.164848441 | 0.648637 | 0.177145 | 12 | RPL13 |
| *A.vasorum* /Control | P18754 | 0.611768003 | 0.611768003 | 0.652493 | 0.177953 | 12 | RCC1 |
| *A.vasorum* /Control | P56134 | 0.224920447 | 0.224920447 | 0.666168 | 0.18139 | 12 | ATP5MF |
| *A.vasorum* /Control | P68104 | -0.176699153 | 0.176699153 | 0.666929 | 0.18139 | 12 | EEF1A1 |

| | | | | | | | |
|---|---|---|---|---|---|---|---|
| *A.vasorum* /Control | P29692 | -0.095700163 | 0.095700163 | 0.670566 | 0.181919 | 12 | EEF1D |
| *A.vasorum* /Control | P53634 | 0.365511309 | 0.365511309 | 0.670711 | 0.181919 | 12 | CTSC |
| *A.vasorum* /Control | Q16853 | -0.335913881 | 0.335913881 | 0.67521 | 0.182889 | 12 | AOC3 |
| *A.vasorum* /Control | P00492 | 0.009043101 | 0.009043101 | 0.677753 | 0.183327 | 12 | HPRT1 |
| *A.vasorum* /Control | P31153 | -0.101365683 | 0.101365683 | 0.682968 | 0.184485 | 12 | MAT2A |
| *A.vasorum* /Control | P06753 | -0.170607833 | 0.170607833 | 0.687362 | 0.185167 | 12 | TPM3 |
| *A.vasorum* /Control | Q14240 | 0.40380557 | 0.40380557 | 0.686615 | 0.185167 | 12 | EIF4A2 |
| *A.vasorum* /Control | O94979 | -0.010778721 | 0.010778721 | 0.692226 | 0.186224 | 12 | SEC31A |
| *A.vasorum* /Control | P11766 | 0.062324738 | 0.062324738 | 0.696149 | 0.187025 | 12 | ADH5 |
| *A.vasorum* /Control | P61978 | -0.020622538 | 0.020622538 | 0.699535 | 0.187426 | 12 | HNRNPK |
| *A.vasorum* /Control | Q9NZM1 | 0.221906777 | 0.221906777 | 0.698688 | 0.187426 | 12 | MYOF |
| *A.vasorum* /Control | Q01638 | -0.204598899 | 0.204598899 | 0.70265 | 0.188006 | 12 | IL1RL1 |
| *A.vasorum* /Control | P13667 | -0.082021697 | 0.082021697 | 0.717874 | 0.191821 | 12 | PDIA4 |
| *A.vasorum* /Control | P62910 | 0.962125192 | 0.962125192 | 0.728532 | 0.194406 | 12 | RPL32 |
| *A.vasorum* /Control | Q16610 | -0.010038078 | 0.010038078 | 0.737814 | 0.196354 | 12 | ECM1 |
| *A.vasorum* /Control | P0DMV8 | 0.085872281 | 0.085872281 | 0.747986 | 0.198794 | 12 | HSPA1A |
| *A.vasorum* /Control | P26022 | -0.144157507 | 0.144157507 | 0.755576 | 0.200325 | 12 | PTX3 |
| *A.vasorum* /Control | Q9UJZ1 | -0.024174501 | 0.024174501 | 0.755773 | 0.200325 | 12 | STOML2 |
| *A.vasorum* /Control | P14314 | -0.031113835 | 0.031113835 | 0.767614 | 0.203192 | 12 | PRKCSH |
| *A.vasorum* /Control | O14786 | 0.108826384 | 0.108826384 | 0.770457 | 0.203401 | 12 | NRP1 |
| *A.vasorum* /Control | P18077 | 0.419569652 | 0.419569652 | 0.770109 | 0.203401 | 12 | RPL35A |
| *A.vasorum* /Control | P60900 | 0.195255053 | 0.195255053 | 0.788283 | 0.20783 | 12 | PSMA6 |
| *A.vasorum* /Control | Q99519 | 0.730136666 | 0.730136666 | 0.791317 | 0.208352 | 12 | NEU1 |
| *A.vasorum* /Control | P31949 | 0.223418364 | 0.223418364 | 0.80325 | 0.211213 | 12 | S100A11 |
| *A.vasorum* /Control | P62701 | 0.078898153 | 0.078898153 | 0.811154 | 0.213009 | 12 | RPS4X |
| *A.vasorum* /Control | P83731 | -0.016282644 | 0.016282644 | 0.819487 | 0.214912 | 12 | RPL24 |
| *A.vasorum* /Control | P46977 | 0.498179884 | 0.498179884 | 0.82961 | 0.217279 | 12 | STT3A |
| *A.vasorum* /Control | P55058 | 0.419779559 | 0.419779559 | 0.835527 | 0.218539 | 12 | PLTP |
| *A.vasorum* /Control | P05114 | -0.752976017 | 0.752976017 | 0.839657 | 0.219041 | 12 | HMGN1 |
| *A.vasorum* /Control | P40429 | -0.543170091 | 0.543170091 | 0.838775 | 0.219041 | 12 | RPL13A |
| *A.vasorum* /Control | Q00688 | 0.534141305 | 0.534141305 | 0.841345 | 0.219192 | 12 | FKBP3 |
| *A.vasorum* /Control | P46781 | -0.064508609 | 0.064508609 | 0.842818 | 0.219234 | 12 | RPS9 |
| *A.vasorum* /Control | Q15084 | -0.133020229 | 0.133020229 | 0.843719 | 0.219234 | 12 | PDIA6 |
| *A.vasorum* /Control | P62241 | -0.023327905 | 0.023327905 | 0.856495 | 0.222262 | 12 | RPS8 |
| *A.vasorum* /Control | P11586 | -1.46734488 | 1.46734488 | 0.862829 | 0.223613 | 12 | MTHFD1 |

| | | | | | | | |
|---|---|---|---|---|---|---|---|
| *A.vasorum* /Control | P39687 | 0.435671698 | 0.435671698 | 0.866864 | 0.224365 | 12 | ANP32A |
| *A.vasorum* /Control | Q14152 | -0.01948356 | 0.01948356 | 0.880326 | 0.227039 | 12 | EIF3A |
| *A.vasorum* /Control | Q16181 | 0.191667254 | 0.191667254 | 0.880637 | 0.227039 | 12 | SEPTIN7 |
| *A.vasorum* /Control | Q96KP4 | 0.873596093 | 0.873596093 | 0.883861 | 0.227278 | 12 | CNDP2 |
| *A.vasorum* /Control | Q8IV08 | 0.197121251 | 0.197121251 | 0.891833 | 0.229031 | 12 | PLD3 |
| *A.vasorum* /Control | P05556 | -0.716966729 | 0.716966729 | 0.910808 | 0.232295 | 12 | ITGB1 |
| *A.vasorum* /Control | P10809 | -0.018877686 | 0.018877686 | 0.913448 | 0.232295 | 12 | HSPD1 |
| *A.vasorum* /Control | P19021 | 0.252477891 | 0.252477891 | 0.910006 | 0.232295 | 12 | PAM |
| *A.vasorum* /Control | P28799 | 0.592596723 | 0.592596723 | 0.908758 | 0.232295 | 12 | GRN |
| *A.vasorum* /Control | P48681 | 0.381273494 | 0.381273494 | 0.915103 | 0.232295 | 12 | NES |
| *A.vasorum* /Control | Q14697 | 0.253037753 | 0.253037753 | 0.91263 | 0.232295 | 12 | GANAB |
| *A.vasorum* /Control | Q7LGC8 | -0.68763855 | 0.68763855 | 0.90983 | 0.232295 | 12 | CHST3 |
| *A.vasorum* /Control | Q9GZM7 | 0.683991058 | 0.683991058 | 0.909383 | 0.232295 | 12 | TINAGL1 |
| *A.vasorum* /Control | Q9Y277 | 0.119519842 | 0.119519842 | 0.913998 | 0.232295 | 12 | VDAC3 |
| *A.vasorum* /Control | P36578 | -0.393597568 | 0.393597568 | 0.918686 | 0.232906 | 12 | RPL4 |
| *A.vasorum* /Control | O14979 | 0.041949922 | 0.041949922 | 0.920213 | 0.232995 | 12 | HNRNPDL |
| *A.vasorum* /Control | P04439 | 0.345206086 | 0.345206086 | 0.922229 | 0.233207 | 12 | HLA-A |
| *A.vasorum* /Control | P46060 | -0.337242383 | 0.337242383 | 0.931091 | 0.235148 | 12 | RANGAP1 |
| *A.vasorum* /Control | P04899 | -0.190377312 | 0.190377312 | 0.939185 | 0.23689 | 12 | GNAI2 |
| *A.vasorum* /Control | P12236 | -0.267498213 | 0.267498213 | 0.941537 | 0.23717 | 12 | SLC25A6 |
| *A.vasorum* /Control | P12955 | 0.804588572 | 0.804588572 | 0.94858 | 0.238349 | 12 | PEPD |
| *A.vasorum* /Control | P21796 | -0.151532295 | 0.151532295 | 0.952861 | 0.239121 | 12 | VDAC1 |
| *A.vasorum* /Control | P23919 | -0.179949857 | 0.179949857 | 0.958161 | 0.240147 | 12 | DTYMK |
| *A.vasorum* /Control | P05388 | -0.072260386 | 0.072260386 | 0.959857 | 0.240162 | 12 | RPLP0 |
| *A.vasorum* /Control | P23526 | -0.203798902 | 0.203798902 | 0.960647 | 0.240162 | 12 | AHCY |
| *A.vasorum* /Control | Q15436 | -0.111151678 | 0.111151678 | 0.975859 | 0.243657 | 12 | SEC23A |
| *A.vasorum* /Control | P50914 | -0.106502539 | 0.106502539 | 0.985693 | 0.245803 | 12 | RPL14 |
| *A.vasorum* /Control | Q05682 | 0.434866492 | 0.434866492 | 0.997049 | 0.248322 | 12 | CALD1 |

**: AVG Log2 Ratio ≥ 0.58 and Qvalue ≤ 0.05 were dysregulated after stimulation over the control group.**

| ProteinDescriptions | ProteinNames | # Unique Total Peptides | % Change |
|---|---|---|---|
| Aldehyde dehydrogenase 1A1 | AL1A1_HUMAN | 5 | -52.5771 |
| von Willebrand factor | VWF_HUMAN | 98 | -57.5517 |
| Cell surface glycoprotein MUC18 | MUC18_HUMAN | 13 | -18.0316 |
| Basement membrane-specific heparan sulfate proteoglycan core protein | PGBM_HUMAN | 153 | -46.5245 |
| Apolipoprotein B-100 | APOB_HUMAN | 7 | 95.65048 |
| Gelsolin | GELS_HUMAN | 10 | -76.139 |
| Ras GTPase-activating-like protein IQGAP1 | IQGA1_HUMAN | 41 | -42.253 |
| Glyceraldehyde-3-phosphate dehydrogenase | G3P_HUMAN | 18 | -29.0637 |
| High mobility group protein HMG-I/HMG-Y | HMGA1_HUMAN | 4 | -29.0415 |
| Multimerin-1 | MMRN1_HUMAN | 42 | -50.497 |
| Tryptophan--tRNA ligase, cytoplasmic | SYWC_HUMAN | 10 | -61.5435 |
| Histone H1.3 | H13_HUMAN | 6 | -55.3184 |
| 14-3-3 protein beta/alpha | 1433B_HUMAN | 11 | -23.8298 |
| 14-3-3 protein gamma | 1433G_HUMAN | 5 | -34.583 |
| Alpha-actinin-1 | ACTN1_HUMAN | 40 | -31.4825 |
| Histone H3.1 | H31_HUMAN | 5 | -40.6038 |
| SPARC | SPRC_HUMAN | 11 | -55.6915 |
| Ribonuclease inhibitor | RINI_HUMAN | 8 | -35.0104 |
| Myosin-9 | MYH9_HUMAN | 89 | -24.1698 |
| Annexin A2 | ANXA2_HUMAN | 25 | -8.85503 |
| Spectrin beta chain, non-erythrocytic 1 | SPTB2_HUMAN | 20 | -36.1751 |
| Histone H1.5 | H15_HUMAN | 5 | -42.7344 |
| Plectin | PLEC_HUMAN | 58 | -26.2723 |
| Purine nucleoside phosphorylase | PNPH_HUMAN | 10 | -36.0907 |
| 26S proteasome non-ATPase regulatory subunit 3 | PSMD3_HUMAN | 3 | -9.98144 |
| Ezrin | EZRI_HUMAN | 4 | -59.7678 |
| 14-3-3 protein theta | 1433T_HUMAN | 8 | -33.7161 |
| Lysosomal Pro-X carboxypeptidase | PCP_HUMAN | 3 | -34.6879 |
| Actin, cytoplasmic 1 | ACTB_HUMAN | 19 | -4.25407 |
| Peroxidasin homolog | PXDN_HUMAN | 33 | -40.5087 |
| Isocitrate dehydrogenase [NADP], mitochondrial | IDHP_HUMAN | 6 | -57.5525 |
| Small ribosomal subunit protein eS7 | RS7_HUMAN | 4 | -24.5947 |
| Complement C3 | CO3_HUMAN | 13 | 52.15291 |

| | | | |
|---|---|---|---|
| Metalloproteinase inhibitor 1 | TIMP1_HUMAN | 4 | -39.597 |
| Nucleoside diphosphate kinase B | NDKB_HUMAN | 8 | -33.1319 |
| Rab GDP dissociation inhibitor beta | GDIB_HUMAN | 21 | -25.8532 |
| Gamma-interferon-inducible protein 16 | IF16_HUMAN | 6 | -46.948 |
| Ubiquitin thioesterase OTUB1 | OTUB1_HUMAN | 4 | -62.9505 |
| 6-phosphogluconate dehydrogenase, decarboxylating | 6PGD_HUMAN | 15 | -30.7759 |
| Triosephosphate isomerase | TPIS_HUMAN | 12 | -35.6118 |
| Ubiquitin-like modifier-activating enzyme 1 | UBA1_HUMAN | 20 | -38.1444 |
| Valine--tRNA ligase | SYVC_HUMAN | 2 | -56.3965 |
| Angiopoietin-2 | ANGP2_HUMAN | 12 | -39.8778 |
| Insulin-like growth factor-binding protein 7 | IBP7_HUMAN | 13 | -29.7073 |
| Transaldolase | TALDO_HUMAN | 13 | -37.8778 |
| Radixin | RADI_HUMAN | 15 | -33.6457 |
| Dihydropyrimidinase-related protein 2 | DPYL2_HUMAN | 15 | -32.1245 |
| Phosphoglycerate mutase 1 | PGAM1_HUMAN | 7 | -36.463 |
| Transgelin-2 | TAGL2_HUMAN | 3 | -10.8692 |
| Glutathione S-transferase omega-1 | GSTO1_HUMAN | 9 | -37.7726 |
| Annexin A1 | ANXA1_HUMAN | 20 | -19.7333 |
| Vinculin | VINC_HUMAN | 35 | -2.51319 |
| Midkine | MK_HUMAN | 5 | -62.9137 |
| Small nuclear ribonucleoprotein Sm D3 | SMD3_HUMAN | 2 | -31.6872 |
| 14-3-3 protein zeta/delta | 1433Z_HUMAN | 9 | -24.5532 |
| Clathrin heavy chain 1 | CLH1_HUMAN | 38 | -29.9665 |
| Importin subunit beta-1 | IMB1_HUMAN | 13 | -43.5302 |
| Calcyclin-binding protein | CYBP_HUMAN | 3 | -14.8531 |
| Talin-1 | TLN1_HUMAN | 58 | -26.6599 |
| ATP-dependent 6-phosphofructokinase, platelet type | PFKAP_HUMAN | 12 | -29.3787 |
| Small ribosomal subunit protein uS2 | RSSA_HUMAN | 2 | -0.55863 |
| Cofilin-1 | COF1_HUMAN | 15 | -43.7031 |
| Alpha-actinin-4 | ACTN4_HUMAN | 26 | -33.4655 |
| Apoptosis inhibitor 5 | API5_HUMAN | 3 | -59.4928 |
| Adenylate kinase isoenzyme 1 | KAD1_HUMAN | 4 | -42.5225 |
| Moesin | MOES_HUMAN | 24 | -33.9887 |
| Small ribosomal subunit protein eS10 | RS10_HUMAN | 2 | -34.6473 |
| CCN family member 1 | CCN1_HUMAN | 16 | -35.6207 |

| | | | |
|---|---|---|---|
| Caveolae-associated protein 1 | CAVN1_HUMAN | 9 | -43.2678 |
| 72 kDa type IV collagenase | MMP2_HUMAN | 24 | -31.4691 |
| Transitional endoplasmic reticulum ATPase | TERA_HUMAN | 29 | -34.5088 |
| Puromycin-sensitive aminopeptidase | PSA_HUMAN | 8 | -40.884 |
| Histone H2B type 1-J | H2B1J_HUMAN | 2 | -51.3139 |
| Collagen alpha-2(IV) chain | CO4A2_HUMAN | 6 | -45.183 |
| Glutathione S-transferase P | GSTP1_HUMAN | 8 | -43.2246 |
| Serine protease HTRA1 | HTRA1_HUMAN | 7 | -49.0903 |
| Filamin-B | FLNB_HUMAN | 88 | -29.3284 |
| Thrombospondin-1 | TSP1_HUMAN | 66 | -14.7841 |
| Small ribosomal subunit protein uS10 | RS20_HUMAN | 3 | -35.1091 |
| DNA-dependent protein kinase catalytic subunit | PRKDC_HUMAN | 20 | -36.7843 |
| Small ribosomal subunit protein uS15 | RS13_HUMAN | 2 | -26.6172 |
| Biglycan | PGS1_HUMAN | 13 | -56.3532 |
| Chloride intracellular channel protein 4 | CLIC4_HUMAN | 12 | -49.1674 |
| Sulfhydryl oxidase 1 | QSOX1_HUMAN | 23 | -28.4553 |
| Signal recognition particle 9 kDa protein | SRP09_HUMAN | 2 | -76.8303 |
| Exportin-1 | XPO1_HUMAN | 5 | -37.1589 |
| Histone H2B type 1-K | H2B1K_HUMAN | 6 | -31.1877 |
| Fructose-bisphosphate aldolase A | ALDOA_HUMAN | 14 | -15.7757 |
| High mobility group protein B1 | HMGB1_HUMAN | 8 | -51.1207 |
| Serpin B8 | SPB8_HUMAN | 2 | -62.8981 |
| Procollagen-lysine,2-oxoglutarate 5-dioxygenase 1 | PLOD1_HUMAN | 16 | -39.9814 |
| Synaptic vesicle membrane protein VAT-1 homolog | VAT1_HUMAN | 10 | -29.0792 |
| Prelamin-A/C | LMNA_HUMAN | 31 | -23.744 |
| Clusterin | CLUS_HUMAN | 7 | -66.1491 |
| Prolow-density lipoprotein receptor-related protein 1 | LRP1_HUMAN | 10 | 41.6833 |
| Apolipoprotein M | APOM_HUMAN | 3 | 46.84358 |
| Serine/threonine-protein phosphatase 2A 65 kDa regulatory subunit A alpha isoform | 2AAA_HUMAN | 8 | -55.7253 |
| BTB/POZ domain-containing protein KCTD12 | KCD12_HUMAN | 9 | -41.6816 |
| Vacuolar protein sorting-associated protein 35 | VPS35_HUMAN | 7 | -43.1013 |
| T-complex protein 1 subunit delta | TCPD_HUMAN | 10 | -36.1285 |
| Fascin | FSCN1_HUMAN | 16 | -43.4165 |
| Thioredoxin reductase 1, cytoplasmic | TRXR1_HUMAN | 17 | -15.6532 |
| ATP-dependent RNA helicase A | DHX9_HUMAN | 12 | -35.2737 |

| | | | |
|---|---|---|---|
| DNA-(apurinic or apyrimidinic site) endonuclease | APEX1_HUMAN | 8 | -48.0296 |
| Tyrosine-protein kinase receptor Tie-1 | TIE1_HUMAN | 11 | -44.0654 |
| Interleukin enhancer-binding factor 3 | ILF3_HUMAN | 13 | -40.422 |
| CD166 antigen | CD166_HUMAN | 4 | -40.2208 |
| Serpin B6 | SPB6_HUMAN | 2 | -39.7792 |
| Golgi apparatus protein 1 | GSLG1_HUMAN | 5 | -29.2483 |
| Fatty acid-binding protein 5 | FABP5_HUMAN | 6 | -49.8139 |
| Golgi-associated kinase 1B | GAK1B_HUMAN | 4 | -60.3675 |
| Histone H1.10 | H1X_HUMAN | 2 | -77.5338 |
| Malate dehydrogenase, cytoplasmic | MDHC_HUMAN | 9 | -34.6162 |
| Tubulin beta-4B chain | TBB4B_HUMAN | 19 | -5.00067 |
| Fibronectin | FINC_HUMAN | 78 | -11.1574 |
| Aldo-keto reductase family 1 member B1 | ALDR_HUMAN | 7 | -32.2542 |
| High mobility group protein B2 | HMGB2_HUMAN | 3 | -47.914 |
| Leucine-rich repeat-containing protein 59 | LRC59_HUMAN | 7 | -28.9351 |
| Ras-related C3 botulinum toxin substrate 1 | RAC1_HUMAN | 4 | -53.9606 |
| Histone H4 | H4_HUMAN | 8 | -28.1982 |
| X-ray repair cross-complementing protein 5 | XRCC5_HUMAN | 14 | -31.988 |
| AP-2 complex subunit beta | AP2B1_HUMAN | 6 | -64.8579 |
| Phosphoglycerate kinase 1 | PGK1_HUMAN | 22 | -44.2616 |
| Histone H2A type 1-B/E | H2A1B_HUMAN | 4 | -33.6343 |
| Cytoplasmic dynein 1 heavy chain 1 | DYHC1_HUMAN | 43 | -21.7143 |
| Coronin-1B | COR1B_HUMAN | 2 | -55.3491 |
| Cytoplasmic aconitate hydratase | ACOHC_HUMAN | 6 | -44.0784 |
| Transketolase | TKT_HUMAN | 21 | -23.9719 |
| X-ray repair cross-complementing protein 6 | XRCC6_HUMAN | 21 | -38.3395 |
| Cullin-associated NEDD8-dissociated protein 1 | CAND1_HUMAN | 14 | -43.7864 |
| Peroxiredoxin-1 | PRDX1_HUMAN | 9 | -38.8527 |
| Branched-chain-amino-acid aminotransferase, cytosolic | BCAT1_HUMAN | 4 | -48.4602 |
| Cathepsin Z | CATZ_HUMAN | 6 | -38.344 |
| Elongation factor 1-gamma | EF1G_HUMAN | 16 | -31.9194 |
| WD repeat-containing protein 1 | WDR1_HUMAN | 13 | -33.4208 |
| DNA damage-binding protein 1 | DDB1_HUMAN | 8 | -49.6374 |
| Aspartate aminotransferase, mitochondrial | AATM_HUMAN | 10 | -42.0679 |
| Malate dehydrogenase, mitochondrial | MDHM_HUMAN | 14 | -39.45 |

| | | | |
|---|---|---|---|
| Fatty acid synthase | FAS_HUMAN | 46 | -37.3973 |
| Divergent protein kinase domain 2B | DIK2B_HUMAN | 2 | -64.8766 |
| Coagulation factor X | FA10_HUMAN | 3 | 44.9613 |
| Transcobalamin-2 | TCO2_HUMAN | 3 | -65.2343 |
| Large ribosomal subunit protein eL34 | RL34_HUMAN | 3 | -32.2966 |
| Proteasome subunit alpha type-1 | PSA1_HUMAN | 8 | -23.7248 |
| Eukaryotic translation initiation factor 3 subunit E | EIF3E_HUMAN | 2 | -42.8598 |
| Rho GDP-dissociation inhibitor 1 | GDIR1_HUMAN | 4 | -21.2444 |
| Complement component C1q receptor | C1QR1_HUMAN | 4 | -62.396 |
| Large ribosomal subunit protein uL16 | RL10_HUMAN | 3 | -40.1387 |
| Myristoylated alanine-rich C-kinase substrate | MARCS_HUMAN | 5 | -32.5498 |
| Cytochrome c | CYC_HUMAN | 4 | -55.2248 |
| Leukotriene A-4 hydrolase | LKHA4_HUMAN | 8 | -49.8987 |
| C-type lectin domain family 14 member A | CLC14_HUMAN | 2 | 9.785523 |
| Complement component 1 Q subcomponent-binding protein, mitochondrial | C1QBP_HUMAN | 2 | -51.2591 |
| Adenylosuccinate synthetase isozyme 2 | PURA2_HUMAN | 3 | -23.3955 |
| ADP-ribosylation factor 3 | ARF3_HUMAN | 6 | -25.9966 |
| GTP-binding nuclear protein Ran | RAN_HUMAN | 7 | -26.7815 |
| Ras suppressor protein 1 | RSU1_HUMAN | 7 | -31.0646 |
| Proteasome subunit alpha type-5 | PSA5_HUMAN | 2 | -63.2343 |
| Proteasome activator complex subunit 2 | PSME2_HUMAN | 3 | -37.1019 |
| 26S proteasome regulatory subunit 6A | PRS6A_HUMAN | 2 | -20.9599 |
| Proteasome subunit beta type-5 | PSB5_HUMAN | 6 | -32.1414 |
| Protein SET | SET_HUMAN | 6 | -16.4333 |
| Nicotinamide phosphoribosyltransferase | NAMPT_HUMAN | 7 | -40.6061 |
| Heterogeneous nuclear ribonucleoprotein D0 | HNRPD_HUMAN | 7 | -30.3768 |
| Actin-related protein 3 | ARP3_HUMAN | 14 | -27.8846 |
| Peroxiredoxin-2 | PRDX2_HUMAN | 6 | -44.9732 |
| Phosphoglucomutase-1 | PGM1_HUMAN | 3 | -47.0986 |
| Mitogen-activated protein kinase 1 | MK01_HUMAN | 6 | -59.805 |
| Voltage-dependent calcium channel subunit alpha-2/delta-1 | CA2D1_HUMAN | 5 | 82.76626 |
| Probable ATP-dependent RNA helicase DDX5 | DDX5_HUMAN | 9 | -33.2076 |
| L-lactate dehydrogenase B chain | LDHB_HUMAN | 14 | -16.2761 |
| Plasminogen activator inhibitor 1 | PAI1_HUMAN | 21 | 64.46025 |
| EGF-containing fibulin-like extracellular matrix protein 1 | FBLN3_HUMAN | 21 | -17.5588 |

| | | | |
|---|---|---|---|
| L-lactate dehydrogenase A chain | LDHA_HUMAN | 13 | -10.9595 |
| Proteasome subunit alpha type-4 | PSA4_HUMAN | 4 | -25.822 |
| Multifunctional procollagen lysine hydroxylase and glycosyltransferase LH3 | PLOD3_HUMAN | 4 | -30.484 |
| CCN family member 2 | CCN2_HUMAN | 22 | -17.6594 |
| Annexin A3 | ANXA3_HUMAN | 7 | -37.4364 |
| Lamin-B1 | LMNB1_HUMAN | 7 | -44.3714 |
| Hepatocyte growth factor-like protein | HGFL_HUMAN | 5 | 44.97269 |
| Protein-lysine 6-oxidase | LYOX_HUMAN | 5 | -46.8106 |
| Beta-arrestin-1 | ARRB1_HUMAN | 2 | -36.344 |
| Adenylyl cyclase-associated protein 1 | CAP1_HUMAN | 13 | -25.9658 |
| EH domain-containing protein 2 | EHD2_HUMAN | 11 | -47.7403 |
| Activated RNA polymerase II transcriptional coactivator p15 | TCP4_HUMAN | 4 | -50.8606 |
| Beta-hexosaminidase subunit beta | HEXB_HUMAN | 7 | -22.3838 |
| Profilin-1 | PROF1_HUMAN | 6 | -4.80609 |
| Tropomyosin beta chain | TPM2_HUMAN | 2 | -11.5116 |
| Cation-independent mannose-6-phosphate receptor | MPRI_HUMAN | 6 | 30.64517 |
| Sushi repeat-containing protein SRPX | SRPX_HUMAN | 14 | -20.9446 |
| Procollagen galactosyltransferase 1 | GT251_HUMAN | 4 | -42.5089 |
| Nucleoside diphosphate kinase A | NDKA_HUMAN | 2 | -50.3889 |
| A disintegrin and metalloproteinase with thrombospondin motifs 13 | ATS13_HUMAN | 5 | 50.17274 |
| Tropomyosin alpha-4 chain | TPM4_HUMAN | 13 | -16.2565 |
| Small ribosomal subunit protein uS11 | RS14_HUMAN | 3 | -17.2598 |
| Tripeptidyl-peptidase 1 | TPP1_HUMAN | 8 | -41.9524 |
| Serine/threonine-protein phosphatase PP1-beta catalytic subunit | PP1B_HUMAN | 3 | -10.5657 |
| Peptidyl-prolyl cis-trans isomerase A | PPIA_HUMAN | 5 | -20.6861 |
| Spectrin alpha chain, non-erythrocytic 1 | SPTN1_HUMAN | 28 | -41.8373 |
| Fumarate hydratase, mitochondrial | FUMH_HUMAN | 3 | -38.158 |
| Collagen alpha-1(XII) chain | COCA1_HUMAN | 4 | 30.19034 |
| Thioredoxin-like protein 1 | TXNL1_HUMAN | 4 | -35.4907 |
| Cadherin-5 | CADH5_HUMAN | 12 | -29.0103 |
| Collagen alpha-1(I) chain | CO1A1_HUMAN | 10 | 65.40591 |
| Filamin-A | FLNA_HUMAN | 59 | -11.5696 |
| Rho-related GTP-binding protein RhoC | RHOC_HUMAN | 2 | -34.4742 |
| Large ribosomal subunit protein uL11 | RL12_HUMAN | 5 | -23.1802 |
| Enoyl-CoA hydratase, mitochondrial | ECHM_HUMAN | 3 | -70.1794 |

| | | | |
|---|---|---|---|
| EH domain-containing protein 1 | EHD1_HUMAN | 3 | -45.2534 |
| Follistatin-related protein 1 | FSTL1_HUMAN | 10 | -31.9126 |
| Elongation factor Tu, mitochondrial | EFTU_HUMAN | 8 | -48.4772 |
| Histone H2A type 2-B | H2A2B_HUMAN | 2 | -49.3683 |
| Large ribosomal subunit protein eL22 | RL22_HUMAN | 2 | -16.1216 |
| Amyloid-beta precursor protein | A4_HUMAN | 15 | -27.3707 |
| Stress-induced-phosphoprotein 1 | STIP1_HUMAN | 15 | -36.8471 |
| Actin-related protein 2/3 complex subunit 1B | ARC1B_HUMAN | 7 | -50.4755 |
| Ribosomal protein eS27-like | RS27L_HUMAN | 2 | 35.84504 |
| T-complex protein 1 subunit gamma | TCPG_HUMAN | 17 | -31.124 |
| Nucleosome assembly protein 1-like 1 | NP1L1_HUMAN | 5 | -24.5504 |
| CD59 glycoprotein | CD59_HUMAN | 2 | -69.6797 |
| Proteasome subunit alpha type-3 | PSA3_HUMAN | 4 | -26.7619 |
| Integrin-linked protein kinase | ILK_HUMAN | 3 | -30.7295 |
| Tubulin alpha-3C chain | TBA3C_HUMAN | 2 | 27.0805 |
| Proteasome activator complex subunit 1 | PSME1_HUMAN | 2 | -58.2317 |
| 14-3-3 protein epsilon | 1433E_HUMAN | 11 | -22.2372 |
| Destrin | DEST_HUMAN | 5 | -28.6837 |
| Retinol-binding protein 4 | RET4_HUMAN | 5 | 43.61179 |
| Farnesyl pyrophosphate synthase | FPPS_HUMAN | 5 | -33.3726 |
| Tubulin alpha-1B chain | TBA1B_HUMAN | 19 | -9.04998 |
| Hsc70-interacting protein | F10A1_HUMAN | 6 | -16.1174 |
| Collagen alpha-3(VI) chain | CO6A3_HUMAN | 2 | 29.20946 |
| Chloride intracellular channel protein 1 | CLIC1_HUMAN | 6 | -35.2178 |
| Large ribosomal subunit protein uL3 | RL3_HUMAN | 8 | -9.55578 |
| Angiotensin-converting enzyme | ACE_HUMAN | 6 | -61.5945 |
| Catenin alpha-1 | CTNA1_HUMAN | 15 | -32.5824 |
| Nuclear ubiquitous casein and cyclin-dependent kinase substrate 1 | NUCKS_HUMAN | 2 | -42.7742 |
| Large ribosomal subunit protein eL20 | RL18A_HUMAN | 2 | -45.2543 |
| Microtubule-associated protein 4 | MAP4_HUMAN | 2 | 68.84186 |
| ATP synthase subunit beta, mitochondrial | ATPB_HUMAN | 18 | -13.4664 |
| Coagulation factor XIII A chain | F13A_HUMAN | 2 | 51.76936 |
| Polyadenylate-binding protein 1 | PABP1_HUMAN | 8 | 28.46733 |
| Y-box-binding protein 1 | YBOX1_HUMAN | 2 | -32.8591 |
| Heat shock protein HSP 90-alpha | HS90A_HUMAN | 18 | -11.2563 |

| | | | | |
|---|---|---|---|---|
| 10 kDa heat shock protein, mitochondrial | CH10_HUMAN | | 5 | -49.3884 |
| Serine/arginine-rich splicing factor 1 | SRSF1_HUMAN | | 8 | -33.5565 |
| Fumarylacetoacetase | FAAA_HUMAN | | 3 | 18.43449 |
| Major vault protein | MVP_HUMAN | | 13 | 7.544906 |
| Small ribosomal subunit protein uS9 | RS16_HUMAN | | 8 | -8.35915 |
| Inactive tyrosine-protein kinase 7 | PTK7_HUMAN | | 2 | -4.18761 |
| N-acetyl-D-glucosamine kinase | NAGK_HUMAN | | 4 | -48.7517 |
| Spermidine synthase | SPEE_HUMAN | | 2 | 20.37472 |
| CD109 antigen | CD109_HUMAN | | 16 | -27.0802 |
| Fatty acid-binding protein, adipocyte | FABP4_HUMAN | | 3 | -68.4547 |
| Matrin-3 | MATR3_HUMAN | | 2 | -57.0924 |
| Unconventional myosin-Ic | MYO1C_HUMAN | | 7 | 54.41233 |
| Small ribosomal subunit protein uS8 | RS15A_HUMAN | | 4 | -8.26748 |
| Hedgehog-interacting protein | HHIP_HUMAN | | 18 | -24.4034 |
| Interleukin-1 receptor accessory protein | IL1AP_HUMAN | | 3 | 32.50773 |
| Cystatin-C | CYTC_HUMAN | | 3 | -20.4096 |
| F-actin-capping protein subunit beta | CAPZB_HUMAN | | 8 | -26.7074 |
| Caveolae-associated protein 2 | CAVN2_HUMAN | | 5 | -31.4371 |
| Interleukin enhancer-binding factor 2 | ILF2_HUMAN | | 10 | -30.6571 |
| Metalloproteinase inhibitor 2 | TIMP2_HUMAN | | 4 | -39.1645 |
| Glutathione peroxidase 3 | GPX3_HUMAN | | 2 | 25.07558 |
| Nidogen-1 | NID1_HUMAN | | 11 | -51.1009 |
| Laminin subunit alpha-4 | LAMA4_HUMAN | | 29 | -15.6996 |
| Actin, aortic smooth muscle | ACTA_HUMAN | | 2 | -4.79266 |
| Glucose-6-phosphate isomerase | G6PI_HUMAN | | 11 | -20.2968 |
| Ras-related protein Rab-11A | RB11A_HUMAN | | 5 | -43.1356 |
| Coactosin-like protein | COTL1_HUMAN | | 5 | -34.2126 |
| Rho GDP-dissociation inhibitor 2 | GDIR2_HUMAN | | 5 | -33.988 |
| Fructose-2,6-bisphosphatase TIGAR | TIGAR_HUMAN | | 3 | -47.2778 |
| Endothelial protein C receptor | EPCR_HUMAN | | 4 | -46.1176 |
| T-complex protein 1 subunit zeta | TCPZ_HUMAN | | 9 | -17.0148 |
| Fermitin family homolog 3 | URP2_HUMAN | | 9 | -21.2526 |
| Pyruvate kinase PKM | KPYM_HUMAN | | 30 | -13.9041 |
| Heterogeneous nuclear ribonucleoproteins A2/B1 | ROA2_HUMAN | | 12 | 31.32853 |
| Large ribosomal subunit protein eL27 | RL27_HUMAN | | 4 | -32.3426 |

| | | | |
|---|---|---|---|
| Protein arginine N-methyltransferase 1 | ANM1_HUMAN | 5 | -57.4578 |
| Nascent polypeptide-associated complex subunit alpha | NACA_HUMAN | 2 | 9.603082 |
| Plastin-3 | PLST_HUMAN | 25 | -30.4137 |
| Peroxiredoxin-6 | PRDX6_HUMAN | 12 | -38.8422 |
| Trifunctional enzyme subunit beta, mitochondrial | ECHB_HUMAN | 3 | -8.26614 |
| Eukaryotic translation initiation factor 5A-1 | IF5A1_HUMAN | 7 | -31.0867 |
| Large ribosomal subunit protein eL18 | RL18_HUMAN | 6 | 17.88927 |
| Tissue-type plasminogen activator | TPA_HUMAN | 10 | -18.5831 |
| Protein-glutamine gamma-glutamyltransferase 2 | TGM2_HUMAN | 23 | -18.2791 |
| Dipeptidyl peptidase 3 | DPP3_HUMAN | 3 | -7.76029 |
| Small ribosomal subunit protein uS13 | RS18_HUMAN | 6 | 4.036745 |
| Small ribosomal subunit protein eS26 | RS26_HUMAN | 2 | 3.672822 |
| Splicing factor U2AF 65 kDa subunit | U2AF2_HUMAN | 3 | -40.5754 |
| Large ribosomal subunit protein eL30 | RL30_HUMAN | 4 | -21.2703 |
| Lysyl oxidase homolog 2 | LOXL2_HUMAN | 20 | -24.149 |
| 26S proteasome non-ATPase regulatory subunit 2 | PSMD2_HUMAN | 5 | -14.5499 |
| F-actin-capping protein subunit alpha-1 | CAZA1_HUMAN | 7 | -19.145 |
| Leucine--tRNA ligase, cytoplasmic | SYLC_HUMAN | 8 | -43.1214 |
| Tissue factor pathway inhibitor | TFPI1_HUMAN | 6 | -67.0022 |
| NADH-cytochrome b5 reductase 3 | NB5R3_HUMAN | 5 | -30.9489 |
| Transcription intermediary factor 1-beta | TIF1B_HUMAN | 6 | 4.780613 |
| Vascular endothelial growth factor receptor 1 | VGFR1_HUMAN | 4 | -36.7497 |
| Nicotinamide N-methyltransferase | NNMT_HUMAN | 3 | -45.8264 |
| Peptidyl-prolyl cis-trans isomerase B | PPIB_HUMAN | 11 | -6.28237 |
| Nucleolar protein 56 | NOP56_HUMAN | 2 | -26.0354 |
| Cytosol aminopeptidase | AMPL_HUMAN | 5 | -46.6274 |
| Endothelin-1 | EDN1_HUMAN | 2 | -21.3966 |
| T-complex protein 1 subunit theta | TCPQ_HUMAN | 21 | -13.2647 |
| Lysosome-associated membrane glycoprotein 2 | LAMP2_HUMAN | 2 | -7.04951 |
| Calsyntenin-1 | CSTN1_HUMAN | 13 | -36.6125 |
| Bone morphogenetic protein 6 | BMP6_HUMAN | 2 | -34.7152 |
| Stress-70 protein, mitochondrial | GRP75_HUMAN | 13 | -31.5489 |
| Glycine--tRNA ligase | GARS_HUMAN | 5 | -21.3751 |
| Protein mago nashi homolog | MGN_HUMAN | 2 | -67.154 |
| Large ribosomal subunit protein eL38 | RL38_HUMAN | 3 | -41.1926 |

| | | | |
|---|---|---|---|
| Non-POU domain-containing octamer-binding protein | NONO_HUMAN | 5 | -2.94769 |
| Lupus La protein | LA_HUMAN | 5 | -30.6008 |
| Pirin | PIR_HUMAN | 3 | -11.8416 |
| Latent-transforming growth factor beta-binding protein 2 | LTBP2_HUMAN | 20 | -27.2858 |
| Exportin-2 | XPO2_HUMAN | 10 | -24.5058 |
| V-type proton ATPase catalytic subunit A | VATA_HUMAN | 6 | -31.41 |
| Thioredoxin domain-containing protein 5 | TXND5_HUMAN | 14 | -28.7486 |
| Small ribosomal subunit protein uS3 | RS3_HUMAN | 17 | -14.8332 |
| Mitotic checkpoint protein BUB3 | BUB3_HUMAN | 3 | -50.5089 |
| NADH dehydrogenase [ubiquinone] iron-sulfur protein 3, mitochondrial | NDUS3_HUMAN | 2 | -38.9886 |
| Ribonuclease pancreatic | RNAS1_HUMAN | 3 | -51.0349 |
| Transportin-1 | TNPO1_HUMAN | 3 | -41.6664 |
| Dermcidin | DCD_HUMAN | 3 | 226.9577 |
| Proteasome subunit beta type-6 | PSB6_HUMAN | 2 | -67.8144 |
| Acetyl-CoA acetyltransferase, cytosolic | THIC_HUMAN | 7 | -38.488 |
| ADP-ribosylation factor 4 | ARF4_HUMAN | 4 | -65.1612 |
| Tubulin beta-6 chain | TBB6_HUMAN | 9 | -22.971 |
| Heat shock 70 kDa protein 13 | HSP13_HUMAN | 7 | -18.0058 |
| U1 small nuclear ribonucleoprotein 70 kDa | RU17_HUMAN | 2 | -46.8884 |
| Small ribosomal subunit protein uS17 | RS11_HUMAN | 7 | 6.073698 |
| Cadherin-13 | CAD13_HUMAN | 3 | 35.07788 |
| Actin-related protein 2 | ARP2_HUMAN | 8 | -34.5331 |
| Large ribosomal subunit protein uL30 | RL7_HUMAN | 8 | 10.0435 |
| Heat shock protein beta-1 | HSPB1_HUMAN | 8 | -29.9852 |
| Small ribosomal subunit protein eS6 | RS6_HUMAN | 5 | 1.851654 |
| Probable ATP-dependent RNA helicase DDX17 | DDX17_HUMAN | 7 | -27.3569 |
| Alpha-parvin | PARVA_HUMAN | 4 | -40.0861 |
| Collagen alpha-1(VI) chain | CO6A1_HUMAN | 8 | 22.85515 |
| Alpha-enolase | ENOA_HUMAN | 22 | -8.72304 |
| Heat shock cognate 71 kDa protein | HSP7C_HUMAN | 32 | -8.6913 |
| Stathmin | STMN1_HUMAN | 3 | -42.1345 |
| Insulin-like growth factor-binding protein 4 | IBP4_HUMAN | 7 | -52.6949 |
| Heterogeneous nuclear ribonucleoprotein Q | HNRPQ_HUMAN | 5 | -23.5431 |
| Ribonuclease 4 | RNAS4_HUMAN | 2 | 78.76396 |
| Sorting nexin-1 | SNX1_HUMAN | 2 | -43.689 |

| | | | |
|---|---|---|---|
| Serine protease 23 | PRS23_HUMAN | 4 | -3.82049 |
| Far upstream element-binding protein 1 | FUBP1_HUMAN | 2 | -28.5376 |
| Betaine--homocysteine S-methyltransferase 1 | BHMT1_HUMAN | 3 | 48.46692 |
| U1 small nuclear ribonucleoprotein A | SNRPA_HUMAN | 2 | -52.5529 |
| Proteasome subunit alpha type-7 | PSA7_HUMAN | 9 | -27.5145 |
| Actin-related protein 2/3 complex subunit 2 | ARPC2_HUMAN | 7 | -21.8255 |
| Proliferating cell nuclear antigen | PCNA_HUMAN | 2 | -56.3846 |
| Elongation factor 2 | EF2_HUMAN | 37 | -15.0221 |
| Arginine--tRNA ligase, cytoplasmic | SYRC_HUMAN | 5 | -48.8263 |
| Importin-5 | IPO5_HUMAN | 7 | -33.1166 |
| Collagen alpha-2(I) chain | CO1A2_HUMAN | 7 | 49.94161 |
| Cell division control protein 42 homolog | CDC42_HUMAN | 3 | -37.8005 |
| Hepatoma-derived growth factor | HDGF_HUMAN | 2 | 10.12811 |
| Phosphatidylinositol transfer protein beta isoform | PIPNB_HUMAN | 3 | 36.5454 |
| Neuroblast differentiation-associated protein AHNAK | AHNK_HUMAN | 10 | 236.9343 |
| Large ribosomal subunit protein eL31 | RL31_HUMAN | 3 | -6.6748 |
| Glucose-6-phosphate 1-dehydrogenase | G6PD_HUMAN | 10 | -23.7747 |
| Prolyl 3-hydroxylase 1 | P3H1_HUMAN | 4 | 2.283108 |
| Prosaposin | SAP_HUMAN | 12 | -7.03839 |
| Integrin alpha-2 | ITA2_HUMAN | 3 | -31.3499 |
| Regucalcin | RGN_HUMAN | 2 | 21.77081 |
| Prohibitin 1 | PHB1_HUMAN | 2 | 52.05381 |
| RNA-splicing ligase RtcB homolog | RTCB_HUMAN | 5 | 15.34844 |
| Calpain-2 catalytic subunit | CAN2_HUMAN | 10 | -28.0092 |
| Heterogeneous nuclear ribonucleoprotein A1 | ROA1_HUMAN | 10 | 3.176565 |
| Proliferation-associated protein 2G4 | PA2G4_HUMAN | 12 | -11.0938 |
| SPARC-like protein 1 | SPRL1_HUMAN | 2 | 49.39621 |
| Inosine-5'-monophosphate dehydrogenase 2 | IMDH2_HUMAN | 2 | -50.9025 |
| Heterogeneous nuclear ribonucleoprotein A/B | ROAA_HUMAN | 2 | -55.9815 |
| Glutathione peroxidase 1 | GPX1_HUMAN | 2 | -27.0877 |
| Complement C1q tumor necrosis factor-related protein 3 | C1QT3_HUMAN | 2 | 1.423089 |
| Integrin alpha-5 | ITA5_HUMAN | 5 | 28.2719 |
| 26S proteasome non-ATPase regulatory subunit 12 | PSD12_HUMAN | 3 | -37.7448 |
| Adenosine kinase | ADK_HUMAN | 2 | -19.2769 |
| AP-2 complex subunit alpha-1 | AP2A1_HUMAN | 2 | -18.8951 |

| | | | |
|---|---|---|---|
| Serine/arginine-rich splicing factor 3 | SRSF3_HUMAN | 3 | -31.8686 |
| ATP-dependent RNA helicase DHX15 | DHX15_HUMAN | 7 | -30.2529 |
| Hsp90 co-chaperone Cdc37 | CDC37_HUMAN | 5 | -30.0231 |
| Latent-transforming growth factor beta-binding protein 1 | LTBP1_HUMAN | 8 | -32.3051 |
| Cytochrome b-c1 complex subunit 1, mitochondrial | QCR1_HUMAN | 3 | -38.5307 |
| Multifunctional protein CAD | PYR1_HUMAN | 3 | 54.41434 |
| Eukaryotic translation initiation factor 6 | IF6_HUMAN | 4 | -25.1232 |
| Protein transport protein Sec61 subunit alpha isoform 1 | S61A1_HUMAN | 3 | 38.87916 |
| Coatomer subunit alpha | COPA_HUMAN | 11 | -24.3159 |
| Growth/differentiation factor 15 | GDF15_HUMAN | 7 | -25.4518 |
| Ras-related protein Rap-1b-like protein | RP1BL_HUMAN | 3 | 23.9294 |
| Eukaryotic peptide chain release factor GTP-binding subunit ERF3A | ERF3A_HUMAN | 2 | 142.8444 |
| Cartilage oligomeric matrix protein | COMP_HUMAN | 4 | 43.20446 |
| Proteasome subunit alpha type-2 | PSA2_HUMAN | 6 | -31.3516 |
| Large ribosomal subunit protein eL36 | RL36_HUMAN | 2 | -37.2167 |
| Actin-related protein 2/3 complex subunit 4 | ARPC4_HUMAN | 3 | -48.2151 |
| Ubiquitin-conjugating enzyme E2 L3 | UB2L3_HUMAN | 4 | -32.4084 |
| Splicing factor, proline- and glutamine-rich | SFPQ_HUMAN | 12 | -21.9204 |
| Fibromodulin | FMOD_HUMAN | 4 | 5.765881 |
| Heat shock protein 75 kDa, mitochondrial | TRAP1_HUMAN | 2 | -33.7803 |
| Actin-related protein 2/3 complex subunit 3 | ARPC3_HUMAN | 4 | -48.6038 |
| Complement C1q tumor necrosis factor-related protein 5 | C1QT5_HUMAN | 3 | -29.0551 |
| Calpain-1 catalytic subunit | CAN1_HUMAN | 7 | -45.6912 |
| Caveolin-1 | CAV1_HUMAN | 6 | -32.4708 |
| Dysferlin | DYSF_HUMAN | 6 | -40.8832 |
| Heterogeneous nuclear ribonucleoprotein L | HNRPL_HUMAN | 5 | -24.6868 |
| Sialic acid synthase | SIAS_HUMAN | 2 | -31.1858 |
| Cathepsin D | CATD_HUMAN | 12 | -6.14538 |
| Fibrillin-1 | FBN1_HUMAN | 8 | -35.6944 |
| Heterogeneous nuclear ribonucleoprotein H | HNRH1_HUMAN | 5 | 48.55031 |
| Small ribosomal subunit protein RACK1 | RACK1_HUMAN | 2 | -4.43049 |
| LIM and senescent cell antigen-like-containing domain protein 1 | LIMS1_HUMAN | 2 | -65.8101 |
| Testican-1 | TICN1_HUMAN | 8 | -13.4515 |
| Small ribosomal subunit protein uS5 | RS2_HUMAN | 9 | 12.00348 |
| Large ribosomal subunit protein uL2 | RL8_HUMAN | 4 | -16.64 |

| | | | |
|---|---|---|---|
| Proteasome subunit beta type-3 | PSB3_HUMAN | 6 | -21.6596 |
| Eukaryotic translation initiation factor 3 subunit C-like protein;Eukaryotic translation | EIFCL_HUMAN | 2 | -25.8182 |
| Bifunctional phosphoribosylaminoimidazole carboxylase/phosphoribosylaminoimida | PUR6_HUMAN | 5 | -6.69527 |
| RuvB-like 2 | RUVB2_HUMAN | 4 | 0.812523 |
| Matrilin-3 | MATN3_HUMAN | 2 | -28.2795 |
| Mesencephalic astrocyte-derived neurotrophic factor | MANF_HUMAN | 3 | -11.076 |
| Serglycin | SRGN_HUMAN | 2 | 59.77712 |
| Ras-related protein Rab-5C | RAB5C_HUMAN | 4 | -12.676 |
| Transmembrane protein 43 | TMM43_HUMAN | 4 | 12.77113 |
| Serine/threonine-protein phosphatase 2A activator | PTPA_HUMAN | 2 | 51.87233 |
| Target of Nesh-SH3 | TARSH_HUMAN | 8 | -22.8832 |
| Adipocyte enhancer-binding protein 1 | AEBP1_HUMAN | 6 | -65.8743 |
| Protein S100-A8 | S10A8_HUMAN | 2 | -63.6535 |
| Amyloid beta precursor like protein 2 | APLP2_HUMAN | 3 | -0.32112 |
| Obg-like ATPase 1 | OLA1_HUMAN | 6 | -11.4903 |
| Ras-related C3 botulinum toxin substrate 2 | RAC2_HUMAN | 2 | -0.18035 |
| ATP-dependent 6-phosphofructokinase, liver type | PFKAL_HUMAN | 2 | -33.9801 |
| Nucleobindin-1 | NUCB1_HUMAN | 11 | -35.9746 |
| Large ribosomal subunit protein uL22 | RL17_HUMAN | 5 | -5.16797 |
| Programmed cell death 6-interacting protein | PDC6I_HUMAN | 9 | -37.8203 |
| Protein S100-A9 | S10A9_HUMAN | 2 | -68.5493 |
| Beta-hexosaminidase subunit alpha | HEXA_HUMAN | 4 | -39.0574 |
| Heat shock protein HSP 90-beta | HS90B_HUMAN | 31 | -7.65032 |
| NPC intracellular cholesterol transporter 2 | NPC2_HUMAN | 2 | -9.26979 |
| Extended synaptotagmin-1 | ESYT1_HUMAN | 7 | -28.4085 |
| Albumin | ALBU_HUMAN | 5 | 49.11384 |
| Vacuolar protein sorting-associated protein 26A | VP26A_HUMAN | 3 | -14.8579 |
| Collagen alpha-1(II) chain | CO2A1_HUMAN | 4 | 29.8447 |
| Lamina-associated polypeptide 2, isoform alpha | LAP2A_HUMAN | 2 | -30.4181 |
| 1,4-alpha-glucan-branching enzyme | GLGB_HUMAN | 3 | -22.7213 |
| Small ribosomal subunit protein eS24 | RS24_HUMAN | 3 | -8.108 |
| RNA-binding motif protein, X chromosome | RBMX_HUMAN | 6 | -36.1722 |
| Tubulin beta-2A chain | TBB2A_HUMAN | 2 | 24.00203 |
| Exostosin-1 | EXT1_HUMAN | 2 | -6.02396 |
| COP9 signalosome complex subunit 4 | CSN4_HUMAN | 2 | 23.95703 |

| | | | |
|---|---|---|---|
| Endothelial lipase | LIPG_HUMAN | 4 | -25.1316 |
| 116 kDa U5 small nuclear ribonucleoprotein component | U5S1_HUMAN | 2 | 32.40336 |
| Prothymosin alpha | PTMA_HUMAN | 4 | 71.73015 |
| Isocitrate dehydrogenase [NADP] cytoplasmic | IDHC_HUMAN | 13 | -32.6444 |
| Nucleolin | NUCL_HUMAN | 16 | 36.184 |
| Macrophage-capping protein | CAPG_HUMAN | 2 | -24.7011 |
| Vimentin | VIME_HUMAN | 32 | 63.43601 |
| Large ribosomal subunit protein uL18 | RL5_HUMAN | 6 | -11.7632 |
| Coatomer subunit beta | COPB_HUMAN | 10 | -21.8102 |
| Polyubiquitin-B | UBB_HUMAN | 4 | 33.24672 |
| Dynactin subunit 1 | DCTN1_HUMAN | 3 | -14.1572 |
| Splicing factor 3B subunit 3 | SF3B3_HUMAN | 7 | -38.8503 |
| Heterogeneous nuclear ribonucleoprotein R | HNRPR_HUMAN | 7 | -29.608 |
| Aspartate--tRNA ligase, cytoplasmic | SYDC_HUMAN | 3 | -48.4923 |
| Poly(rC)-binding protein 1 | PCBP1_HUMAN | 7 | 3.116068 |
| Stabilin-1 | STAB1_HUMAN | 4 | -24.3631 |
| Citrate synthase, mitochondrial | CISY_HUMAN | 6 | -41.0489 |
| N-acetylglucosamine-6-sulfatase | GNS_HUMAN | 5 | -25.4157 |
| 14-3-3 protein eta | 1433F_HUMAN | 4 | -37.4618 |
| Transforming growth factor-beta-induced protein ig-h3 | BGH3_HUMAN | 6 | 14.26817 |
| Septin-11 | SEP11_HUMAN | 5 | -7.54555 |
| Contactin-1 | CNTN1_HUMAN | 15 | 41.9655 |
| Serpin H1 | SERPH_HUMAN | 13 | -10.8023 |
| Mimecan | MIME_HUMAN | 3 | -9.66408 |
| Dickkopf-related protein 3 | DKK3_HUMAN | 5 | -34.5315 |
| Large ribosomal subunit protein eL21 | RL21_HUMAN | 2 | -29.3932 |
| Ubiquitin carboxyl-terminal hydrolase 14 | UBP14_HUMAN | 2 | -21.6784 |
| Cytochrome c oxidase subunit 4 isoform 1, mitochondrial | COX41_HUMAN | 2 | 15.24826 |
| Cytoplasmic FMR1-interacting protein 1 | CYFP1_HUMAN | 2 | 18.44716 |
| Eukaryotic translation initiation factor 2 subunit 2 | IF2B_HUMAN | 2 | -11.0244 |
| Large ribosomal subunit protein uL1 | RL10A_HUMAN | 5 | 6.309055 |
| Heat shock 70 kDa protein 4 | HSP74_HUMAN | 6 | -20.0469 |
| Eukaryotic initiation factor 4A-I | IF4A1_HUMAN | 16 | -0.76906 |
| Cadherin-6 | CADH6_HUMAN | 3 | -5.2718 |
| Large ribosomal subunit protein uL6 | RL9_HUMAN | 5 | -10.1232 |

| | | | |
|---|---|---|---|
| Eukaryotic translation initiation factor 2 subunit 1 | IF2A_HUMAN | 3 | -1.37429 |
| Glucosamine-6-phosphate isomerase 1 | GNPI1_HUMAN | 3 | -16.2296 |
| Staphylococcal nuclease domain-containing protein 1 | SND1_HUMAN | 19 | -8.14231 |
| Heterogeneous nuclear ribonucleoproteins C1/C2 | HNRPC_HUMAN | 13 | 3.123714 |
| Dihydropyrimidinase-related protein 3 | DPYL3_HUMAN | 6 | -30.3102 |
| Dolichyl-diphosphooligosaccharide--protein glycosyltransferase 48 kDa subunit | OST48_HUMAN | 5 | 57.17191 |
| Small ribosomal subunit protein uS7 | RS5_HUMAN | 5 | 9.591134 |
| Spliceosome RNA helicase DDX39B | DX39B_HUMAN | 8 | -10.0396 |
| Ras-related protein Rab-2A | RAB2A_HUMAN | 2 | -41.7014 |
| Creatine kinase M-type | KCRM_HUMAN | 4 | -40.7807 |
| Myosin regulatory light chain 12B | ML12B_HUMAN | 7 | -9.52907 |
| Proteasome subunit beta type-2 | PSB2_HUMAN | 3 | -52.524 |
| Copine-3 | CPNE3_HUMAN | 2 | -3.90785 |
| Cytochrome c oxidase subunit 2 | COX2_HUMAN | 2 | -31.8296 |
| Lysosome-associated membrane glycoprotein 1 | LAMP1_HUMAN | 4 | -5.22739 |
| 26S proteasome regulatory subunit 4 | PRS4_HUMAN | 3 | -45.9072 |
| Large ribosomal subunit protein uL5 | RL11_HUMAN | 4 | 24.04664 |
| Protein S100-A6 | S10A6_HUMAN | 2 | -36.5905 |
| Small ribosomal subunit protein eS25 | RS25_HUMAN | 4 | -27.3212 |
| Small ribosomal subunit protein eS17 | RS17_HUMAN | 2 | -2.03629 |
| Heterogeneous nuclear ribonucleoprotein U | HNRPU_HUMAN | 13 | -8.25539 |
| Receptor-type tyrosine-protein phosphatase F | PTPRF_HUMAN | 8 | 9.048164 |
| Cytosolic acyl coenzyme A thioester hydrolase | BACH_HUMAN | 4 | -20.2689 |
| Cysteine-rich motor neuron 1 protein | CRIM1_HUMAN | 5 | -24.5394 |
| Calreticulin | CALR_HUMAN | 12 | 16.27081 |
| Protein disulfide-isomerase A3 | PDIA3_HUMAN | 22 | 5.897549 |
| Endoplasmic reticulum chaperone BiP | BIP_HUMAN | 23 | 25.23392 |
| Gamma-glutamyl hydrolase | GGH_HUMAN | 7 | -36.316 |
| Cytoskeleton-associated protein 4 | CKAP4_HUMAN | 14 | 20.87008 |
| Lysosomal protective protein | PPGB_HUMAN | 2 | -17.4792 |
| Small ribosomal subunit protein eS19 | RS19_HUMAN | 4 | -12.1805 |
| T-complex protein 1 subunit beta | TCPB_HUMAN | 19 | -8.37718 |
| Procollagen-lysine,2-oxoglutarate 5-dioxygenase 2 | PLOD2_HUMAN | 7 | 9.118445 |
| Thrombospondin-4 | TSP4_HUMAN | 5 | 6.585636 |
| Annexin A5 | ANXA5_HUMAN | 16 | 1.641298 |

| | | | |
|---|---|---|---|
| Poly(rC)-binding protein 2 | PCBP2_HUMAN | 9 | -2.36591 |
| Laminin subunit gamma-1 | LAMC1_HUMAN | 21 | -1.53089 |
| Nuclear migration protein nudC | NUDC_HUMAN | 3 | -41.9737 |
| T-complex protein 1 subunit epsilon | TCPE_HUMAN | 12 | -12.9405 |
| Bifunctional glutamate/proline--tRNA ligase | SYEP_HUMAN | 3 | -34.0425 |
| Prohibitin-2 | PHB2_HUMAN | 5 | -0.79458 |
| Neuropilin-2 | NRP2_HUMAN | 8 | 0.771579 |
| Coatomer subunit gamma-1 | COPG1_HUMAN | 5 | -25.1212 |
| Ras-related protein Rab-1B | RAB1B_HUMAN | 4 | 106.8528 |
| Laminin subunit beta-1 | LAMB1_HUMAN | 21 | 4.372638 |
| Proteasome subunit beta type-1 | PSB1_HUMAN | 3 | -27.4123 |
| Aldehyde dehydrogenase, mitochondrial | ALDH2_HUMAN | 2 | 43.35383 |
| Histone H1.2 | H12_HUMAN | 2 | 7.497799 |
| Very-long-chain 3-oxoacyl-CoA reductase | DHB12_HUMAN | 2 | -20.5618 |
| Small ribosomal subunit protein uS12 | RS23_HUMAN | 2 | -9.58226 |
| Collagen alpha-1(V) chain | CO5A1_HUMAN | 11 | 10.99627 |
| Platelet endothelial cell adhesion molecule | PECA1_HUMAN | 8 | 8.996315 |
| Ras-related protein Rab-7a | RAB7A_HUMAN | 3 | -35.1065 |
| 3-hydroxyacyl-CoA dehydrogenase type-2 | HCD2_HUMAN | 2 | -28.4399 |
| FACT complex subunit SPT16 | SP16H_HUMAN | 2 | -22.4252 |
| Sodium/potassium-transporting ATPase subunit alpha-1 | AT1A1_HUMAN | 6 | 7.038633 |
| Delta-1-pyrroline-5-carboxylate synthase | P5CS_HUMAN | 2 | 1.629957 |
| Dolichyl-diphosphooligosaccharide--protein glycosyltransferase subunit 1 | RPN1_HUMAN | 10 | 19.00416 |
| Annexin A6 | ANXA6_HUMAN | 20 | -18.2544 |
| PRA1 family protein 3 | PRAF3_HUMAN | 3 | 2.052625 |
| 26S proteasome regulatory subunit 7 | PRS7_HUMAN | 6 | 13.67895 |
| C-type lectin domain family 11 member A | CLC11_HUMAN | 2 | 20.77415 |
| Large ribosomal subunit protein uL14 | RL23_HUMAN | 2 | -2.71881 |
| Coronin-1C | COR1C_HUMAN | 4 | -53.231 |
| Small ribosomal subunit protein eS1 | RS3A_HUMAN | 9 | -17.4748 |
| Calnexin | CALX_HUMAN | 15 | -13.5953 |
| Annexin A4 | ANXA4_HUMAN | 2 | 26.65303 |
| Phosphatidylethanolamine-binding protein 1 | PEBP1_HUMAN | 2 | 13.56103 |
| Large ribosomal subunit protein eL28 | RL28_HUMAN | 2 | 11.02171 |
| ATP synthase subunit O, mitochondrial | ATPO_HUMAN | 3 | -9.21251 |

| | | | |
|---|---|---|---|
| Voltage-dependent anion-selective channel protein 2 | VDAC2_HUMAN | 7 | -5.78781 |
| Peroxiredoxin-4 | PRDX4_HUMAN | 2 | 66.81315 |
| Aminopeptidase N | AMPN_HUMAN | 23 | 7.971558 |
| Chromobox protein homolog 3 | CBX3_HUMAN | 3 | 0.640322 |
| Keratin, type I cytoskeletal 18 | K1C18_HUMAN | 6 | -4.18974 |
| ATP synthase subunit alpha, mitochondrial | ATPA_HUMAN | 17 | 1.163718 |
| ATP-dependent RNA helicase DDX3X | DDX3X_HUMAN | 7 | 5.41102 |
| Glutamine--tRNA ligase | SYQ_HUMAN | 6 | -26.3718 |
| Large ribosomal subunit protein uL23 | RL23A_HUMAN | 3 | -49.9768 |
| Trifunctional enzyme subunit alpha, mitochondrial | ECHA_HUMAN | 13 | -5.9281 |
| Minor histocompatibility antigen H13 | HM13_HUMAN | 3 | 8.072946 |
| Signal peptidase complex catalytic subunit SEC11A | SC11A_HUMAN | 2 | -9.07857 |
| 45 kDa calcium-binding protein | CAB45_HUMAN | 2 | -19.0035 |
| Hypoxia up-regulated protein 1 | HYOU1_HUMAN | 17 | -2.94351 |
| C-terminal-binding protein 2;C-terminal-binding protein 1 | CTBP2_HUMAN | 2 | -1.46808 |
| RuvB-like 1 | RUVB1_HUMAN | 5 | -5.57052 |
| Large ribosomal subunit protein uL15 | RL27A_HUMAN | 2 | -28.9982 |
| Mannan-binding lectin serine protease 1 | MASP1_HUMAN | 2 | 54.40084 |
| Solute carrier family 25 member 3 | S25A3_HUMAN | 3 | -7.23061 |
| Protein disulfide-isomerase | PDIA1_HUMAN | 20 | -7.0066 |
| Polypyrimidine tract-binding protein 1 | PTBP1_HUMAN | 4 | -13.202 |
| Nucleophosmin | NPM_HUMAN | 7 | 14.38871 |
| Large ribosomal subunit protein eL19 | RL19_HUMAN | 2 | 35.82676 |
| Pantetheinase | VNN1_HUMAN | 3 | 14.45595 |
| Reticulocalbin-1 | RCN1_HUMAN | 3 | -4.86772 |
| Heterogeneous nuclear ribonucleoprotein A3 | ROA3_HUMAN | 7 | 18.20451 |
| Eukaryotic translation initiation factor 4 gamma 1 | IF4G1_HUMAN | 4 | 2.918654 |
| Cytochrome b-c1 complex subunit 2, mitochondrial | QCR2_HUMAN | 4 | -39.936 |
| Endothelial cell-specific molecule 1 | ESM1_HUMAN | 5 | 8.065523 |
| Septin-2 | SEPT2_HUMAN | 6 | -23.7511 |
| Ras-related protein Ral-A | RALA_HUMAN | 4 | -53.8277 |
| Cathepsin B | CATB_HUMAN | 8 | 6.893548 |
| Eukaryotic translation initiation factor 2 subunit 3 | IF2G_HUMAN | 5 | -18.2805 |
| Vesicular integral-membrane protein VIP36 | LMAN2_HUMAN | 2 | -40.197 |
| Legumain | LGMN_HUMAN | 2 | -1.77883 |

| | | | |
|---|---|---|---|
| Large ribosomal subunit protein eL6 | RL6_HUMAN | 9 | -7.88945 |
| 26S proteasome non-ATPase regulatory subunit 11 | PSD11_HUMAN | 4 | -25.655 |
| ADP/ATP translocase 2 | ADT2_HUMAN | 3 | 5.445957 |
| Endoplasmin | ENPL_HUMAN | 32 | 8.314836 |
| F-actin-capping protein subunit alpha-2 | CAZA2_HUMAN | 3 | -24.9567 |
| ATP-citrate synthase | ACLY_HUMAN | 20 | -14.2855 |
| Catenin beta-1 | CTNB1_HUMAN | 4 | -29.4988 |
| Serine/arginine-rich splicing factor 2 | SRSF2_HUMAN | 3 | -19.5087 |
| Peroxiredoxin-like 2A | PXL2A_HUMAN | 6 | -1.38545 |
| Ubiquitin carboxyl-terminal hydrolase 5 | UBP5_HUMAN | 3 | -7.82046 |
| Interstitial collagenase | MMP1_HUMAN | 22 | 18.73901 |
| T-complex protein 1 subunit eta | TCPH_HUMAN | 13 | -7.4511 |
| Tubulin beta chain | TBB5_HUMAN | 6 | 25.14174 |
| Biliverdin reductase A | BIEA_HUMAN | 2 | -46.9859 |
| Stomatin | STOM_HUMAN | 5 | -3.49093 |
| Heterogeneous nuclear ribonucleoprotein M | HNRPM_HUMAN | 22 | 9.790178 |
| Guanine nucleotide-binding protein G(I)/G(S)/G(T) subunit beta-1 | GBB1_HUMAN | 5 | -18.7792 |
| Large ribosomal subunit protein eL15 | RL15_HUMAN | 4 | 7.264547 |
| KH domain-containing, RNA-binding, signal transduction-associated protein 1 | KHDR1_HUMAN | 3 | -5.67534 |
| Cytochrome c oxidase subunit 6C | COX6C_HUMAN | 2 | -37.7547 |
| T-complex protein 1 subunit alpha | TCPA_HUMAN | 13 | -19.3008 |
| Coatomer subunit delta | COPD_HUMAN | 2 | -6.74409 |
| Filamin-C | FLNC_HUMAN | 10 | -11.1489 |
| RNA-binding protein 8A | RBM8A_HUMAN | 2 | -48.6647 |
| Collagen alpha-1(XI) chain | COBA1_HUMAN | 6 | 26.23272 |
| Dolichyl-diphosphooligosaccharide--protein glycosyltransferase subunit 2 | RPN2_HUMAN | 8 | -1.54625 |
| Adipocyte plasma membrane-associated protein | APMAP_HUMAN | 5 | -3.80312 |
| Hexokinase-1 | HXK1_HUMAN | 2 | -2.32231 |
| Large ribosomal subunit protein eL8 | RL7A_HUMAN | 7 | 3.521006 |
| Eukaryotic translation initiation factor 3 subunit B | EIF3B_HUMAN | 5 | -24.0801 |
| Large ribosomal subunit protein eL42 | RL36A_HUMAN | 2 | 9.096331 |
| Large ribosomal subunit protein eL13 | RL13_HUMAN | 4 | -10.7978 |
| Regulator of chromosome condensation | RCC1_HUMAN | 3 | -34.5606 |
| ATP synthase subunit f, mitochondrial | ATPK_HUMAN | 2 | -14.4358 |
| Elongation factor 1-alpha 1 | EF1A1_HUMAN | 18 | 13.02948 |

| | | | |
|---|---|---|---|
| Elongation factor 1-delta | EF1D_HUMAN | 6 | 6.858388 |
| Dipeptidyl peptidase 1 | CATC_HUMAN | 4 | -22.3806 |
| Membrane primary amine oxidase | AOC3_HUMAN | 2 | 26.21767 |
| Hypoxanthine-guanine phosphoribosyltransferase | HPRT_HUMAN | 4 | -0.62486 |
| S-adenosylmethionine synthase isoform type-2 | METK2_HUMAN | 4 | 7.27885 |
| Tropomyosin alpha-3 chain | TPM3_HUMAN | 3 | 12.55326 |
| Eukaryotic initiation factor 4A-II | IF4A2_HUMAN | 3 | -24.4138 |
| Protein transport protein Sec31A | SC31A_HUMAN | 5 | 0.749922 |
| Alcohol dehydrogenase class-3 | ADHX_HUMAN | 2 | -4.22804 |
| Heterogeneous nuclear ribonucleoprotein K | HNRPK_HUMAN | 13 | 1.439711 |
| Myoferlin | MYOF_HUMAN | 9 | -14.2569 |
| Interleukin-1 receptor-like 1 | ILRL1_HUMAN | 2 | 15.23659 |
| Protein disulfide-isomerase A4 | PDIA4_HUMAN | 19 | 5.850031 |
| Large ribosomal subunit protein eL32 | RL32_HUMAN | 2 | -48.67 |
| Extracellular matrix protein 1 | ECM1_HUMAN | 2 | 0.698213 |
| Heat shock 70 kDa protein 1A | HS71A_HUMAN | 11 | -5.77853 |
| Pentraxin-related protein PTX3 | PTX3_HUMAN | 12 | 10.50851 |
| Stomatin-like protein 2, mitochondrial | STML2_HUMAN | 2 | 1.689766 |
| Glucosidase 2 subunit beta | GLU2B_HUMAN | 7 | 2.18007 |
| Neuropilin-1 | NRP1_HUMAN | 7 | -7.26579 |
| Large ribosomal subunit protein eL33 | RL35A_HUMAN | 2 | -25.2352 |
| Proteasome subunit alpha type-6 | PSA6_HUMAN | 6 | -12.6582 |
| Sialidase-1 | NEUR1_HUMAN | 2 | -39.7153 |
| Protein S100-A11 | S10AB_HUMAN | 4 | -14.3466 |
| Small ribosomal subunit protein eS4, X isoform | RS4X_HUMAN | 12 | -5.32195 |
| Large ribosomal subunit protein eL24 | RL24_HUMAN | 3 | 1.13502 |
| Dolichyl-diphosphooligosaccharide--protein glycosyltransferase subunit STT3A | STT3A_HUMAN | 2 | -29.2001 |
| Phospholipid transfer protein | PLTP_HUMAN | 7 | -25.2461 |
| Non-histone chromosomal protein HMG-14 | HMGN1_HUMAN | 2 | 68.52656 |
| Large ribosomal subunit protein uL13 | RL13A_HUMAN | 3 | 45.71709 |
| Peptidyl-prolyl cis-trans isomerase FKBP3 | FKBP3_HUMAN | 2 | -30.943 |
| Small ribosomal subunit protein uS4 | RS9_HUMAN | 9 | 4.57287 |
| Protein disulfide-isomerase A6 | PDIA6_HUMAN | 9 | 9.658696 |
| Small ribosomal subunit protein eS8 | RS8_HUMAN | 6 | 1.630111 |
| C-1-tetrahydrofolate synthase, cytoplasmic | C1TC_HUMAN | 2 | 176.5125 |

| | | | |
|---|---|---|---|
| Acidic leucine-rich nuclear phosphoprotein 32 family member A | AN32A_HUMAN | 3 | -26.0651 |
| Eukaryotic translation initiation factor 3 subunit A | EIF3A_HUMAN | 9 | 1.359658 |
| Septin-7 | SEPT7_HUMAN | 5 | -12.4407 |
| Cytosolic non-specific dipeptidase | CNDP2_HUMAN | 3 | -45.4215 |
| 5'-3' exonuclease PLD3 | PLD3_HUMAN | 3 | -12.7711 |
| Integrin beta-1 | ITB1_HUMAN | 5 | 64.37225 |
| 60 kDa heat shock protein, mitochondrial | CH60_HUMAN | 20 | 1.3171 |
| Peptidyl-glycine alpha-amidating monooxygenase | AMD_HUMAN | 2 | -16.0547 |
| Progranulin | GRN_HUMAN | 2 | -33.6852 |
| Nestin | NEST_HUMAN | 5 | -23.224 |
| Neutral alpha-glucosidase AB | GANAB_HUMAN | 23 | -16.0872 |
| Carbohydrate sulfotransferase 3 | CHST3_HUMAN | 2 | 61.0645 |
| Tubulointerstitial nephritis antigen-like | TINAL_HUMAN | 6 | -37.7559 |
| Voltage-dependent anion-selective channel protein 3 | VDAC3_HUMAN | 3 | -7.9506 |
| Large ribosomal subunit protein uL4 | RL4_HUMAN | 9 | 31.36651 |
| Heterogeneous nuclear ribonucleoprotein D-like | HNRDL_HUMAN | 2 | -2.86588 |
| HLA class I histocompatibility antigen, A alpha chain | HLAA_HUMAN | 7 | -21.2804 |
| Ran GTPase-activating protein 1 | RAGP1_HUMAN | 2 | 26.33395 |
| Guanine nucleotide-binding protein G(i) subunit alpha-2 | GNAI2_HUMAN | 5 | 14.10621 |
| ADP/ATP translocase 3 | ADT3_HUMAN | 12 | 20.37186 |
| Xaa-Pro dipeptidase | PEPD_HUMAN | 2 | -42.7475 |
| Voltage-dependent anion-selective channel protein 1 | VDAC1_HUMAN | 7 | 11.07486 |
| Thymidylate kinase | KTHY_HUMAN | 2 | 13.28445 |
| Large ribosomal subunit protein uL10 | RLA0_HUMAN | 10 | 5.136265 |
| Adenosylhomocysteinase | SAHH_HUMAN | 13 | 15.17271 |
| Protein transport protein Sec23A | SC23A_HUMAN | 2 | 8.009011 |
| Large ribosomal subunit protein eL14 | RL14_HUMAN | 4 | 7.661508 |
| Caldesmon | CALD1_HUMAN | 3 | -26.0238 |

| Ratio |
|---|
| 0.474229 |
| 0.424483 |
| 0.819684 |
| 0.534755 |
| 1.956505 |
| 0.23861 |
| 0.57747 |
| 0.709363 |
| 0.709585 |
| 0.49503 |
| 0.384565 |
| 0.446816 |
| 0.761702 |
| 0.65417 |
| 0.685175 |
| 0.593962 |
| 0.443085 |
| 0.649896 |
| 0.758302 |
| 0.91145 |
| 0.638249 |
| 0.572656 |
| 0.737277 |
| 0.639093 |
| 0.900186 |
| 0.402322 |
| 0.662839 |
| 0.653121 |
| 0.957459 |
| 0.594913 |
| 0.424475 |
| 0.754053 |
| 1.521529 |

| |
|---|
| 0.60403 |
| 0.668681 |
| 0.741468 |
| 0.53052 |
| 0.370495 |
| 0.692241 |
| 0.643882 |
| 0.618556 |
| 0.436035 |
| 0.601222 |
| 0.702927 |
| 0.621222 |
| 0.663543 |
| 0.678755 |
| 0.63537 |
| 0.891308 |
| 0.622274 |
| 0.802667 |
| 0.974868 |
| 0.370863 |
| 0.683128 |
| 0.754468 |
| 0.700335 |
| 0.564698 |
| 0.851469 |
| 0.733401 |
| 0.706213 |
| 0.994414 |
| 0.562969 |
| 0.665345 |
| 0.405072 |
| 0.574775 |
| 0.660113 |
| 0.653527 |
| 0.643793 |

| |
|---|
| 0.567322 |
| 0.685309 |
| 0.654912 |
| 0.59116 |
| 0.486861 |
| 0.54817 |
| 0.567754 |
| 0.509097 |
| 0.706716 |
| 0.852159 |
| 0.648909 |
| 0.632157 |
| 0.733828 |
| 0.436468 |
| 0.508326 |
| 0.715447 |
| 0.231697 |
| 0.628411 |
| 0.688123 |
| 0.842243 |
| 0.488793 |
| 0.371019 |
| 0.600186 |
| 0.709208 |
| 0.76256 |
| 0.338509 |
| 1.416833 |
| 1.468436 |
| 0.442747 |
| 0.583184 |
| 0.568987 |
| 0.638715 |
| 0.565835 |
| 0.843468 |
| 0.647263 |

| |
|---|
| 0.519704 |
| 0.559346 |
| 0.59578 |
| 0.597792 |
| 0.602208 |
| 0.707517 |
| 0.501861 |
| 0.396325 |
| 0.224662 |
| 0.653838 |
| 0.949993 |
| 0.888426 |
| 0.677458 |
| 0.52086 |
| 0.710649 |
| 0.460394 |
| 0.718018 |
| 0.68012 |
| 0.351421 |
| 0.557384 |
| 0.663657 |
| 0.782857 |
| 0.446509 |
| 0.559216 |
| 0.760281 |
| 0.616605 |
| 0.562136 |
| 0.611473 |
| 0.515398 |
| 0.61656 |
| 0.680806 |
| 0.665792 |
| 0.503626 |
| 0.579321 |
| 0.6055 |

| |
|---|
| 0.626027 |
| 0.351234 |
| 1.449613 |
| 0.347657 |
| 0.677034 |
| 0.762752 |
| 0.571402 |
| 0.787556 |
| 0.37604 |
| 0.598613 |
| 0.674502 |
| 0.447752 |
| 0.501013 |
| 1.097855 |
| 0.487409 |
| 0.766045 |
| 0.740034 |
| 0.732185 |
| 0.689354 |
| 0.367657 |
| 0.628981 |
| 0.790401 |
| 0.678586 |
| 0.835667 |
| 0.593939 |
| 0.696232 |
| 0.721154 |
| 0.550268 |
| 0.529014 |
| 0.40195 |
| 1.827663 |
| 0.667924 |
| 0.837239 |
| 1.644603 |
| 0.824412 |

| |
|---|
| 0.890405 |
| 0.74178 |
| 0.69516 |
| 0.823406 |
| 0.625636 |
| 0.556286 |
| 1.449727 |
| 0.531894 |
| 0.63656 |
| 0.740342 |
| 0.522597 |
| 0.491394 |
| 0.776162 |
| 0.951939 |
| 0.884884 |
| 1.306452 |
| 0.790554 |
| 0.574911 |
| 0.496111 |
| 1.501727 |
| 0.837435 |
| 0.827402 |
| 0.580476 |
| 0.894343 |
| 0.793139 |
| 0.581627 |
| 0.61842 |
| 1.301903 |
| 0.645093 |
| 0.709897 |
| 1.654059 |
| 0.884304 |
| 0.655258 |
| 0.768198 |
| 0.298206 |

| |
|---|
| 0.547466 |
| 0.680874 |
| 0.515228 |
| 0.506317 |
| 0.838784 |
| 0.726293 |
| 0.631529 |
| 0.495245 |
| 1.35845 |
| 0.68876 |
| 0.754496 |
| 0.303203 |
| 0.732381 |
| 0.692705 |
| 1.270805 |
| 0.417683 |
| 0.777628 |
| 0.713163 |
| 1.436118 |
| 0.666274 |
| 0.9095 |
| 0.838826 |
| 1.292095 |
| 0.647822 |
| 0.904442 |
| 0.384055 |
| 0.674176 |
| 0.572258 |
| 0.547457 |
| 1.688419 |
| 0.865336 |
| 1.517694 |
| 1.284673 |
| 0.671409 |
| 0.887437 |

| |
|---|
| 0.506116 |
| 0.664435 |
| 1.184345 |
| 1.075449 |
| 0.916408 |
| 0.958124 |
| 0.512483 |
| 1.203747 |
| 0.729198 |
| 0.315453 |
| 0.429076 |
| 1.544123 |
| 0.917325 |
| 0.755966 |
| 1.325077 |
| 0.795904 |
| 0.732926 |
| 0.685629 |
| 0.693429 |
| 0.608355 |
| 1.250756 |
| 0.488991 |
| 0.843004 |
| 0.952073 |
| 0.797032 |
| 0.568644 |
| 0.657874 |
| 0.66012 |
| 0.527222 |
| 0.538824 |
| 0.829852 |
| 0.787474 |
| 0.860959 |
| 1.313285 |
| 0.676574 |

| |
|---|
| 0.425422 |
| 1.096031 |
| 0.695863 |
| 0.611578 |
| 0.917339 |
| 0.689133 |
| 1.178893 |
| 0.814169 |
| 0.817209 |
| 0.922397 |
| 1.040367 |
| 1.036728 |
| 0.594246 |
| 0.787297 |
| 0.75851 |
| 0.854501 |
| 0.80855 |
| 0.568786 |
| 0.329978 |
| 0.690511 |
| 1.047806 |
| 0.632503 |
| 0.541736 |
| 0.937176 |
| 0.739646 |
| 0.533726 |
| 0.786034 |
| 0.867353 |
| 0.929505 |
| 0.633875 |
| 0.652848 |
| 0.684511 |
| 0.786249 |
| 0.32846 |
| 0.588074 |

| |
|---|
| 0.970523 |
| 0.693992 |
| 0.881584 |
| 0.727142 |
| 0.754942 |
| 0.6859 |
| 0.712514 |
| 0.851668 |
| 0.494911 |
| 0.610114 |
| 0.489651 |
| 0.583336 |
| 3.269577 |
| 0.321856 |
| 0.61512 |
| 0.348388 |
| 0.77029 |
| 0.819942 |
| 0.531116 |
| 1.060737 |
| 1.350779 |
| 0.654669 |
| 1.100435 |
| 0.700148 |
| 1.018517 |
| 0.726431 |
| 0.599139 |
| 1.228552 |
| 0.91277 |
| 0.913087 |
| 0.578655 |
| 0.473051 |
| 0.764569 |
| 1.78764 |
| 0.56311 |

| |
|---|
| 0.961795 |
| 0.714624 |
| 1.484669 |
| 0.474471 |
| 0.724855 |
| 0.781745 |
| 0.436154 |
| 0.849779 |
| 0.511737 |
| 0.668834 |
| 1.499416 |
| 0.621995 |
| 1.101281 |
| 1.365454 |
| 3.369343 |
| 0.933252 |
| 0.762253 |
| 1.022831 |
| 0.929616 |
| 0.686501 |
| 1.217708 |
| 1.520538 |
| 1.153484 |
| 0.719908 |
| 1.031766 |
| 0.889062 |
| 1.493962 |
| 0.490975 |
| 0.440185 |
| 0.729123 |
| 1.014231 |
| 1.282719 |
| 0.622552 |
| 0.807231 |
| 0.811049 |

| |
|---|
| 0.681314 |
| 0.697471 |
| 0.699769 |
| 0.676949 |
| 0.614693 |
| 1.544143 |
| 0.748768 |
| 1.388792 |
| 0.756841 |
| 0.745482 |
| 1.239294 |
| 2.428444 |
| 1.432045 |
| 0.686484 |
| 0.627833 |
| 0.517849 |
| 0.675916 |
| 0.780796 |
| 1.057659 |
| 0.662197 |
| 0.513962 |
| 0.709449 |
| 0.543088 |
| 0.675292 |
| 0.591168 |
| 0.753132 |
| 0.688142 |
| 0.938546 |
| 0.643056 |
| 1.485503 |
| 0.955695 |
| 0.341899 |
| 0.865485 |
| 1.120035 |
| 0.8336 |

| |
|---|
| 0.783404 |
| 0.741818 |
| 0.933047 |
| 1.008125 |
| 0.717205 |
| 0.88924 |
| 1.597771 |
| 0.87324 |
| 1.127711 |
| 1.518723 |
| 0.771168 |
| 0.341257 |
| 0.363465 |
| 0.996789 |
| 0.885097 |
| 0.998196 |
| 0.660199 |
| 0.640254 |
| 0.94832 |
| 0.621797 |
| 0.314507 |
| 0.609426 |
| 0.923497 |
| 0.907302 |
| 0.715915 |
| 1.491138 |
| 0.851421 |
| 1.298447 |
| 0.695819 |
| 0.772787 |
| 0.91892 |
| 0.638278 |
| 1.24002 |
| 0.93976 |
| 1.23957 |

| |
|---|
| 0.748684 |
| 1.324034 |
| 1.717301 |
| 0.673556 |
| 1.36184 |
| 0.752989 |
| 1.63436 |
| 0.882368 |
| 0.781898 |
| 1.332467 |
| 0.858428 |
| 0.611497 |
| 0.70392 |
| 0.515077 |
| 1.031161 |
| 0.756369 |
| 0.589511 |
| 0.745843 |
| 0.625382 |
| 1.142682 |
| 0.924545 |
| 1.419655 |
| 0.891977 |
| 0.903359 |
| 0.654685 |
| 0.706068 |
| 0.783216 |
| 1.152483 |
| 1.184472 |
| 0.889756 |
| 1.063091 |
| 0.799531 |
| 0.992309 |
| 0.947282 |
| 0.898768 |

| |
|---|
| 0.986257 |
| 0.837704 |
| 0.918577 |
| 1.031237 |
| 0.696898 |
| 1.571719 |
| 1.095911 |
| 0.899604 |
| 0.582986 |
| 0.592193 |
| 0.904709 |
| 0.47476 |
| 0.960922 |
| 0.681704 |
| 0.947726 |
| 0.540928 |
| 1.240466 |
| 0.634095 |
| 0.726788 |
| 0.979637 |
| 0.917446 |
| 1.090482 |
| 0.797311 |
| 0.754606 |
| 1.162708 |
| 1.058975 |
| 1.252339 |
| 0.63684 |
| 1.208701 |
| 0.825208 |
| 0.878195 |
| 0.916228 |
| 1.091184 |
| 1.065856 |
| 1.016413 |

| |
|---|
| 0.976341 |
| 0.984691 |
| 0.580263 |
| 0.870595 |
| 0.659575 |
| 0.992054 |
| 1.007716 |
| 0.748788 |
| 2.068528 |
| 1.043726 |
| 0.725877 |
| 1.433538 |
| 1.074978 |
| 0.794382 |
| 0.904177 |
| 1.109963 |
| 1.089963 |
| 0.648935 |
| 0.715601 |
| 0.775748 |
| 1.070386 |
| 1.0163 |
| 1.190042 |
| 0.817456 |
| 1.020526 |
| 1.136789 |
| 1.207742 |
| 0.972812 |
| 0.46769 |
| 0.825252 |
| 0.864047 |
| 1.26653 |
| 1.13561 |
| 1.110217 |
| 0.907875 |

| |
|---|
| 0.942122 |
| 1.668131 |
| 1.079716 |
| 1.006403 |
| 0.958103 |
| 1.011637 |
| 1.05411 |
| 0.736282 |
| 0.500232 |
| 0.940719 |
| 1.080729 |
| 0.909214 |
| 0.809965 |
| 0.970565 |
| 0.985319 |
| 0.944295 |
| 0.710018 |
| 1.544008 |
| 0.927694 |
| 0.929934 |
| 0.86798 |
| 1.143887 |
| 1.358268 |
| 1.144559 |
| 0.951323 |
| 1.182045 |
| 1.029187 |
| 0.60064 |
| 1.080655 |
| 0.762489 |
| 0.461723 |
| 1.068935 |
| 0.817195 |
| 0.59803 |
| 0.982212 |

| |
|---|
| 0.921105 |
| 0.74345 |
| 1.05446 |
| 1.083148 |
| 0.750433 |
| 0.857145 |
| 0.705012 |
| 0.804913 |
| 0.986146 |
| 0.921795 |
| 1.18739 |
| 0.925489 |
| 1.251417 |
| 0.530141 |
| 0.965091 |
| 1.097902 |
| 0.812208 |
| 1.072645 |
| 0.943247 |
| 0.622453 |
| 0.806992 |
| 0.932559 |
| 0.888511 |
| 0.513353 |
| 1.262327 |
| 0.984538 |
| 0.961969 |
| 0.976777 |
| 1.03521 |
| 0.759199 |
| 1.090963 |
| 0.892022 |
| 0.654394 |
| 0.855642 |
| 1.130295 |

| |
|---|
| 1.068584 |
| 0.776194 |
| 1.262177 |
| 0.993751 |
| 1.072789 |
| 1.125533 |
| 0.755862 |
| 1.007499 |
| 0.95772 |
| 1.014397 |
| 0.857431 |
| 1.152366 |
| 1.0585 |
| 0.5133 |
| 1.006982 |
| 0.942215 |
| 1.105085 |
| 1.016898 |
| 1.021801 |
| 0.927342 |
| 0.747648 |
| 0.873418 |
| 0.602847 |
| 0.856534 |
| 0.94678 |
| 1.01135 |
| 0.707999 |
| 0.747539 |
| 1.685266 |
| 1.457171 |
| 0.69057 |
| 1.045729 |
| 1.096587 |
| 1.016301 |
| 2.765125 |

| |
|---|
| 0.739349 |
| 1.013597 |
| 0.875593 |
| 0.545785 |
| 0.872289 |
| 1.643722 |
| 1.013171 |
| 0.839453 |
| 0.663148 |
| 0.76776 |
| 0.839128 |
| 1.610645 |
| 0.622441 |
| 0.920494 |
| 1.313665 |
| 0.971341 |
| 0.787196 |
| 1.263339 |
| 1.141062 |
| 1.203719 |
| 0.572525 |
| 1.110749 |
| 1.132845 |
| 1.051363 |
| 1.151727 |
| 1.08009 |
| 1.076615 |
| 0.739762 |
