# Supplementary material for: Proteomic Analysis of Endothelial Activation Induced by Adult Angiostrongylus vasorum Homogenate: Insights into Vascular Remodeling and Hemostatic Imbalance
Source: Animals (Basel). 2026 Mar 15;16(6):926. doi: 10.3390/ani16060926 (PMC13023303; doi:10.3390/ani16060926)
Supplement: Supplementary file 1 [file animals-16-00926-s001.zip › Supplmentary Table S2.pdf]

Supplementary Table S2: Proteins identified in cell lysates of cells treated with *A.vasorum* and control

Comparison (group1/group2)	ProteinGroups	AVG Log2 Ratio	Absolute AVG Log2 Ratio	Pvalue
A.vasorum /Control	P02786	-1.384331821	1.384331821	2.86E-07
A.vasorum /Control	P21741	1.877822352	1.877822352	3.15E-07
A.vasorum /Control	Q14392	1.355083197	1.355083197	2.55E-07
A.vasorum /Control	Q9C0B1	0.206524551	0.206524551	1.46E-07
A.vasorum /Control	Q9UKU9	0.845993128	0.845993128	1.65E-07
A.vasorum /Control	Q9NP74	0.324614876	0.324614876	5.43E-07
A.vasorum /Control	P28062	0.441409026	0.441409026	6.34E-07
A.vasorum /Control	P05120	-0.630624657	0.630624657	9.00E-07
A.vasorum /Control	P17980	0.145146954	0.145146954	9.13E-07
A.vasorum /Control	P36871	0.244091975	0.244091975	8.60E-07
A.vasorum /Control	P04040	0.313212954	0.313212954	1.28E-06
A.vasorum /Control	Q6P2H3	-1.473527543	1.473527543	1.29E-06
A.vasorum /Control	Q7Z3D6	0.671764966	0.671764966	1.26E-06
A.vasorum /Control	Q9H993	0.133815272	0.133815272	1.25E-06
A.vasorum /Control	P31930	0.232282593	0.232282593	1.48E-06
A.vasorum /Control	P40121	0.233843164	0.233843164	1.41E-06
A.vasorum /Control	A6NDU8	2.281120444	2.281120444	1.72E-06
A.vasorum /Control	Q9UL46	0.498145348	0.498145348	1.80E-06
A.vasorum /Control	Q9Y3Z3	0.470982441	0.470982441	1.70E-06
A.vasorum /Control	Q9H9T3	0.24942311	0.24942311	1.93E-06
A.vasorum /Control	Q12996	0.046728255	0.046728255	2.14E-06
A.vasorum /Control	Q9ULS5	0.814879424	0.814879424	2.25E-06
A.vasorum /Control	O00418	0.305198074	0.305198074	2.54E-06
A.vasorum /Control	P21980	0.227985333	0.227985333	2.91E-06
A.vasorum /Control	P42566	0.074754942	0.074754942	2.69E-06
A.vasorum /Control	P61769	1.404742575	1.404742575	2.81E-06
A.vasorum /Control	P99999	0.399110159	0.399110159	3.12E-06
A.vasorum /Control	Q96G01	0.722398936	0.722398936	3.05E-06
A.vasorum /Control	P56377	0.169509365	0.169509365	3.36E-06
A.vasorum /Control	Q14344	0.106040884	0.106040884	3.26E-06
A.vasorum /Control	P52209	-0.135845099	0.135845099	3.97E-06
A.vasorum /Control	Q11201	1.009079992	1.009079992	3.89E-06
A.vasorum /Control	Q9NUJ1	0.128563616	0.128563616	4.04E-06
A.vasorum /Control	Q9NV96	0.500300293	0.500300293	4.10E-06
A.vasorum /Control	P63104	0.131536644	0.131536644	4.39E-06
A.vasorum /Control	Q15048	0.460508386	0.460508386	4.45E-06
A.vasorum /Control	P51003	0.111054176	0.111054176	4.98E-06
A.vasorum /Control	P56537	-0.197860991	0.197860991	5.11E-06
A.vasorum /Control	Q16363	0.793842277	0.793842277	5.24E-06
A.vasorum /Control	Q9BWT7	0.695375851	0.695375851	5.13E-06
A.vasorum /Control	Q9UBU9	0.080182327	0.080182327	5.22E-06
A.vasorum /Control	Q9UIJ7	0.322703253	0.322703253	4.83E-06
A.vasorum /Control	P05556	-0.163664634	0.163664634	5.48E-06
A.vasorum /Control	P21926	-0.300789531	0.300789531	5.75E-06
A.vasorum /Control	Q96HW7	0.263115272	0.263115272	5.73E-06
A.vasorum /Control	O60242	3.625277604	3.625277604	6.03E-06
A.vasorum /Control	Q3LXA3	0.756711489	0.756711489	6.21E-06
A.vasorum /Control	Q9H9Q2	0.198750219	0.198750219	6.43E-06

A.vasorum /Control	O95810	0.711180344	0.711180344	7.23E-06
A.vasorum /Control	P11310	0.467969674	0.467969674	6.68E-06
A.vasorum /Control	P31323	0.331572511	0.331572511	6.93E-06
A.vasorum /Control	Q9H444	0.147625344	0.147625344	6.92E-06
A.vasorum /Control	Q9H8L6	0.259676965	0.259676965	7.17E-06
A.vasorum /Control	Q9Y2Z4	0.154086125	0.154086125	7.03E-06
A.vasorum /Control	Q6ZNJ1	0.303659759	0.303659759	7.48E-06
A.vasorum /Control	Q9NZN4	0.500355195	0.500355195	7.66E-06
A.vasorum /Control	O15123	1.003139264	1.003139264	9.36E-06
A.vasorum /Control	O75146	0.311195662	0.311195662	1.17E-05
A.vasorum /Control	O75874	0.1353314	0.1353314	1.12E-05
A.vasorum /Control	P01889	0.925856848	0.925856848	9.71E-06
A.vasorum /Control	P06396	0.605403601	0.605403601	9.32E-06
A.vasorum /Control	P11047	0.682794779	0.682794779	1.18E-05
A.vasorum /Control	P17813	-0.499953154	0.499953154	8.66E-06
A.vasorum /Control	P19525	0.171404626	0.171404626	1.12E-05
A.vasorum /Control	P21291	-0.38845359	0.38845359	9.93E-06
A.vasorum /Control	P28340	0.156100512	0.156100512	8.77E-06
A.vasorum /Control	P36269	1.229373507	1.229373507	9.10E-06
A.vasorum /Control	P47755	0.254883569	0.254883569	1.05E-05
A.vasorum /Control	P52272	0.155883264	0.155883264	9.60E-06
A.vasorum /Control	Q13201	1.038060593	1.038060593	1.20E-05
A.vasorum /Control	Q14697	0.10530861	0.10530861	1.20E-05
A.vasorum /Control	Q6NZI2	0.349494506	0.349494506	1.19E-05
A.vasorum /Control	Q6UW68	0.270730492	0.270730492	1.19E-05
A.vasorum /Control	Q712K3	0.099098333	0.099098333	8.99E-06
A.vasorum /Control	Q8N1G4	0.175739135	0.175739135	9.50E-06
A.vasorum /Control	Q96CX2	0.571411444	0.571411444	1.10E-05
A.vasorum /Control	Q9H270	0.166066357	0.166066357	1.02E-05
A.vasorum /Control	Q9HCM4	0.071617702	0.071617702	1.12E-05
A.vasorum /Control	Q9NQ50	0.363392171	0.363392171	1.11E-05
A.vasorum /Control	Q9UDR5	0.292088815	0.292088815	1.18E-05
A.vasorum /Control	Q9UEY8	0.3263272	0.3263272	1.20E-05
A.vasorum /Control	Q9UNN5	0.03114767	0.03114767	1.12E-05
A.vasorum /Control	Q13423	0.231764776	0.231764776	1.24E-05
A.vasorum /Control	Q93100	0.251388062	0.251388062	1.25E-05
A.vasorum /Control	P30740	0.551636502	0.551636502	1.28E-05
A.vasorum /Control	P09110	0.099751573	0.099751573	1.32E-05
A.vasorum /Control	Q06203	0.292385711	0.292385711	1.35E-05
A.vasorum /Control	Q15126	0.212608563	0.212608563	1.34E-05
A.vasorum /Control	Q9UBR2	0.362351659	0.362351659	1.34E-05
A.vasorum /Control	P23381	0.426411033	0.426411033	1.39E-05
A.vasorum /Control	P18074	0.170970064	0.170970064	1.41E-05
A.vasorum /Control	Q9ULE6	1.346439309	1.346439309	1.46E-05
A.vasorum /Control	Q12882	2.246644584	2.246644584	1.49E-05
A.vasorum /Control	Q8ND24	0.124190835	0.124190835	1.53E-05
A.vasorum /Control	O60942	0.60018189	0.60018189	1.56E-05
A.vasorum /Control	Q14689	0.027508153	0.027508153	1.62E-05
A.vasorum /Control	O60462	0.069605875	0.069605875	1.68E-05
A.vasorum /Control	Q12981	0.135974435	0.135974435	1.65E-05

A.vasorum /Control	Q9NR31	0.141976381	0.141976381	1.67E-05
A.vasorum /Control	O00712	0.912029677	0.912029677	1.70E-05
A.vasorum /Control	Q9Y5Y7	1.091807434	1.091807434	1.77E-05
A.vasorum /Control	O75947	0.428388386	0.428388386	1.91E-05
A.vasorum /Control	P10412	0.297998339	0.297998339	1.87E-05
A.vasorum /Control	Q4G0J3	0.03057812	0.03057812	1.91E-05
A.vasorum /Control	Q8NB37	0.420002183	0.420002183	1.91E-05
A.vasorum /Control	Q9BPW8	0.235684881	0.235684881	1.85E-05
A.vasorum /Control	O60610	0.167164487	0.167164487	2.02E-05
A.vasorum /Control	P15090	0.923839842	0.923839842	2.00E-05
A.vasorum /Control	Q6P179	0.253264709	0.253264709	1.96E-05
A.vasorum /Control	Q8NHV1	1.233449269	1.233449269	2.00E-05
A.vasorum /Control	Q9NZ01	0.00362003	0.00362003	2.02E-05
A.vasorum /Control	Q9UJ83	0.264860809	0.264860809	2.05E-05
A.vasorum /Control	P22695	0.286303198	0.286303198	2.11E-05
A.vasorum /Control	P33151	0.226730328	0.226730328	2.11E-05
A.vasorum /Control	Q16774	0.31820649	0.31820649	2.27E-05
A.vasorum /Control	Q8TF65	1.605481559	1.605481559	2.25E-05
A.vasorum /Control	O14657	0.163779886	0.163779886	2.38E-05
A.vasorum /Control	O60487	0.372782661	0.372782661	2.30E-05
A.vasorum /Control	P47985	0.389083732	0.389083732	2.33E-05
A.vasorum /Control	Q86W42	0.240555483	0.240555483	2.37E-05
A.vasorum /Control	Q96BZ8	0.315195533	0.315195533	2.37E-05
A.vasorum /Control	Q63HR2	0.51833991	0.51833991	2.40E-05
A.vasorum /Control	Q8NEB9	0.245931831	0.245931831	2.45E-05
A.vasorum /Control	Q9H0U3	-0.134304284	0.134304284	2.45E-05
A.vasorum /Control	P07339	0.25252681	0.25252681	2.48E-05
A.vasorum /Control	O60830	0.36815193	0.36815193	2.98E-05
A.vasorum /Control	O75794	-0.147911914	0.147911914	2.65E-05
A.vasorum /Control	P00374	0.215064318	0.215064318	2.83E-05
A.vasorum /Control	P05362	-0.730059722	0.730059722	2.98E-05
A.vasorum /Control	P07942	0.467082185	0.467082185	2.97E-05
A.vasorum /Control	P12429	0.275418306	0.275418306	2.98E-05
A.vasorum /Control	P12694	0.490101458	0.490101458	2.64E-05
A.vasorum /Control	P25205	0.081353303	0.081353303	3.01E-05
A.vasorum /Control	P40261	0.125622707	0.125622707	2.93E-05
A.vasorum /Control	P41567	0.071990778	0.071990778	3.00E-05
A.vasorum /Control	P46063	0.239995078	0.239995078	2.70E-05
A.vasorum /Control	P47974	0.325021286	0.325021286	2.88E-05
A.vasorum /Control	P51553	0.329436467	0.329436467	3.03E-05
A.vasorum /Control	P51813	0.547675658	0.547675658	2.91E-05
A.vasorum /Control	Q2M389	0.069375633	0.069375633	3.02E-05
A.vasorum /Control	Q8TBQ9	3.60903519	3.60903519	2.81E-05
A.vasorum /Control	Q8WXH0	0.434335678	0.434335678	2.88E-05
A.vasorum /Control	Q9BUJ2	0.068131557	0.068131557	2.80E-05
A.vasorum /Control	Q9BUK6	0.265434579	0.265434579	2.69E-05
A.vasorum /Control	Q9GZM7	0.446734716	0.446734716	2.96E-05
A.vasorum /Control	Q9NWX6	0.811631551	0.811631551	2.71E-05
A.vasorum /Control	Q9NZJ7	0.264636144	0.264636144	2.89E-05
A.vasorum /Control	Q9P253	0.226272413	0.226272413	2.64E-05

A.vasorum /Control	Q9P2K8	0.170640951	0.170640951	2.64E-05
A.vasorum /Control	Q9Y2B0	0.64488084	0.64488084	2.84E-05
A.vasorum /Control	Q9Y3D5	0.558416497	0.558416497	2.99E-05
A.vasorum /Control	Q53EL6	0.508196868	0.508196868	3.05E-05
A.vasorum /Control	Q9Y6E2	0.075998195	0.075998195	3.08E-05
A.vasorum /Control	P09429	0.289408086	0.289408086	3.16E-05
A.vasorum /Control	Q9Y4K0	-0.002257811	0.002257811	3.18E-05
A.vasorum /Control	Q8IUD2	0.275533172	0.275533172	3.23E-05
A.vasorum /Control	Q9Y4K1	1.457312182	1.457312182	3.26E-05
A.vasorum /Control	P51659	0.135169819	0.135169819	3.28E-05
A.vasorum /Control	P22033	0.262550417	0.262550417	3.39E-05
A.vasorum /Control	P26583	0.44868544	0.44868544	3.39E-05
A.vasorum /Control	P09874	0.084322917	0.084322917	3.51E-05
A.vasorum /Control	Q9UP83	0.054924093	0.054924093	3.55E-05
A.vasorum /Control	P04792	0.383103253	0.383103253	3.63E-05
A.vasorum /Control	Q14146	-0.073668224	0.073668224	3.59E-05
A.vasorum /Control	Q9Y5Z0	0.659987855	0.659987855	3.63E-05
A.vasorum /Control	O95858	0.242480992	0.242480992	3.72E-05
A.vasorum /Control	P29992	0.066890605	0.066890605	3.71E-05
A.vasorum /Control	P55899	0.711169809	0.711169809	3.72E-05
A.vasorum /Control	O60784	0.080601423	0.080601423	3.79E-05
A.vasorum /Control	O43684	0.070801592	0.070801592	3.84E-05
A.vasorum /Control	Q15404	0.188967883	0.188967883	3.89E-05
A.vasorum /Control	Q8WW01	0.238885378	0.238885378	3.82E-05
A.vasorum /Control	Q9H7C9	0.572958075	0.572958075	3.90E-05
A.vasorum /Control	Q9HCE1	0.349262444	0.349262444	3.89E-05
A.vasorum /Control	P27348	0.143373855	0.143373855	3.94E-05
A.vasorum /Control	O00501	0.983127849	0.983127849	4.10E-05
A.vasorum /Control	P02792	1.623385761	1.623385761	4.07E-05
A.vasorum /Control	P07602	0.157375655	0.157375655	4.11E-05
A.vasorum /Control	Q13546	0.274864936	0.274864936	4.14E-05
A.vasorum /Control	Q6IQ22	-0.079084725	0.079084725	4.17E-05
A.vasorum /Control	Q7L3T8	0.122590304	0.122590304	4.09E-05
A.vasorum /Control	Q96HY7	0.16777322	0.16777322	4.16E-05
A.vasorum /Control	Q9NXD2	0.442278173	0.442278173	4.14E-05
A.vasorum /Control	Q9BYB0	0.363112733	0.363112733	4.29E-05
A.vasorum /Control	Q8N5C1	0.416152103	0.416152103	4.31E-05
A.vasorum /Control	Q9Y291	0.073927304	0.073927304	4.37E-05
A.vasorum /Control	O95236	0.840000631	0.840000631	4.42E-05
A.vasorum /Control	O95563	0.069257751	0.069257751	4.42E-05
A.vasorum /Control	Q92542	0.325679915	0.325679915	4.47E-05
A.vasorum /Control	P29317	-0.736842042	0.736842042	4.56E-05
A.vasorum /Control	Q9UBI1	0.648018234	0.648018234	4.54E-05
A.vasorum /Control	Q9H4F1	-1.547144244	1.547144244	4.59E-05
A.vasorum /Control	P14543	1.283600866	1.283600866	4.62E-05
A.vasorum /Control	P35241	0.184137129	0.184137129	4.69E-05
A.vasorum /Control	P61619	0.077694893	0.077694893	4.68E-05
A.vasorum /Control	Q13057	0.472223511	0.472223511	4.69E-05
A.vasorum /Control	O43395	0.097302237	0.097302237	4.83E-05
A.vasorum /Control	O75410	0.121769913	0.121769913	4.75E-05

A.vasorum /Control	P28838	0.486935528	0.486935528	4.76E-05
A.vasorum /Control	Q07812	0.303985635	0.303985635	4.80E-05
A.vasorum /Control	Q6IAN0	0.086912791	0.086912791	4.83E-05
A.vasorum /Control	Q8IYS2	0.114194286	0.114194286	4.87E-05
A.vasorum /Control	Q92900	0.026307998	0.026307998	4.90E-05
A.vasorum /Control	Q9NXH8	0.206269056	0.206269056	4.90E-05
A.vasorum /Control	Q9BPY3	0.397259652	0.397259652	4.95E-05
A.vasorum /Control	Q9H9A6	0.141247933	0.141247933	5.00E-05
A.vasorum /Control	O75964	0.117561618	0.117561618	5.03E-05
A.vasorum /Control	O75173	-0.060411412	0.060411412	5.06E-05
A.vasorum /Control	Q9C0D3	0.265211801	0.265211801	5.11E-05
A.vasorum /Control	Q9UBQ0	0.305649481	0.305649481	5.15E-05
A.vasorum /Control	P11117	0.416975731	0.416975731	5.21E-05
A.vasorum /Control	Q12802	0.122048959	0.122048959	5.21E-05
A.vasorum /Control	Q92600	-0.04262141	0.04262141	5.24E-05
A.vasorum /Control	Q9BXI6	0.447917273	0.447917273	5.27E-05
A.vasorum /Control	P16284	0.394622631	0.394622631	5.34E-05
A.vasorum /Control	O14735	0.262062133	0.262062133	5.40E-05
A.vasorum /Control	O43175	0.483641912	0.483641912	5.45E-05
A.vasorum /Control	O95361	0.357509563	0.357509563	5.45E-05
A.vasorum /Control	Q15417	0.30377015	0.30377015	5.50E-05
A.vasorum /Control	Q9HDC9	0.010919173	0.010919173	5.58E-05
A.vasorum /Control	Q9H2D6	0.174309524	0.174309524	5.62E-05
A.vasorum /Control	P17050	0.450346227	0.450346227	5.67E-05
A.vasorum /Control	P22314	0.072257977	0.072257977	5.84E-05
A.vasorum /Control	P27694	0.257492563	0.257492563	5.81E-05
A.vasorum /Control	P49593	0.180427626	0.180427626	5.78E-05
A.vasorum /Control	P51687	0.265658186	0.265658186	5.83E-05
A.vasorum /Control	P61018	0.422742103	0.422742103	5.82E-05
A.vasorum /Control	Q5JTZ9	0.109079544	0.109079544	5.82E-05
A.vasorum /Control	Q96CV9	0.015253219	0.015253219	5.81E-05
A.vasorum /Control	P15121	0.075200367	0.075200367	6.01E-05
A.vasorum /Control	P42226	0.364836172	0.364836172	5.95E-05
A.vasorum /Control	Q03135	0.227443785	0.227443785	6.01E-05
A.vasorum /Control	Q96HR3	1.106625303	1.106625303	5.96E-05
A.vasorum /Control	P56937	0.472722896	0.472722896	6.12E-05
A.vasorum /Control	P80217	0.685330605	0.685330605	6.18E-05
A.vasorum /Control	Q8NCG7	0.643412715	0.643412715	6.15E-05
A.vasorum /Control	Q8NHP8	0.306387604	0.306387604	6.17E-05
A.vasorum /Control	Q96C86	0.249366621	0.249366621	6.20E-05
A.vasorum /Control	Q9BXB5	0.309792303	0.309792303	6.08E-05
A.vasorum /Control	Q8N8R3	0.372417675	0.372417675	6.31E-05
A.vasorum /Control	Q13547	0.144734831	0.144734831	6.35E-05
A.vasorum /Control	P62491	0.109931976	0.109931976	6.44E-05
A.vasorum /Control	Q14108	0.24461843	0.24461843	6.47E-05
A.vasorum /Control	Q9BZI7	0.156706619	0.156706619	6.47E-05
A.vasorum /Control	P41214	0.043499617	0.043499617	6.56E-05
A.vasorum /Control	Q96JJ3	0.248507822	0.248507822	6.59E-05
A.vasorum /Control	O00291	0.366602294	0.366602294	6.64E-05
A.vasorum /Control	P35968	0.192710582	0.192710582	6.95E-05

A.vasorum /Control	P62714	0.06064066	0.06064066	7.07E-05
A.vasorum /Control	Q7Z7H8	1.542565399	1.542565399	7.05E-05
A.vasorum /Control	Q9H0C8	0.009525405	0.009525405	7.06E-05
A.vasorum /Control	Q9NRQ2	0.678164706	0.678164706	7.05E-05
A.vasorum /Control	Q9NUQ2	0.006268099	0.006268099	7.07E-05
A.vasorum /Control	Q9Y6D9	0.152240995	0.152240995	7.05E-05
A.vasorum /Control	Q9HAS0	0.322134909	0.322134909	7.24E-05
A.vasorum /Control	Q9BZZ5	0.056993071	0.056993071	7.33E-05
A.vasorum /Control	Q9NVE5	0.887802965	0.887802965	7.38E-05
A.vasorum /Control	Q6V0I7	0.530000043	0.530000043	7.42E-05
A.vasorum /Control	P09622	0.380415065	0.380415065	7.57E-05
A.vasorum /Control	P13804	0.24905969	0.24905969	7.59E-05
A.vasorum /Control	Q9Y2H0	-0.254222955	0.254222955	7.58E-05
A.vasorum /Control	Q9UBP0	0.333522715	0.333522715	7.63E-05
A.vasorum /Control	O00625	0.372185807	0.372185807	7.67E-05
A.vasorum /Control	Q9UKZ1	0.662950224	0.662950224	7.91E-05
A.vasorum /Control	P35222	0.009618233	0.009618233	8.00E-05
A.vasorum /Control	O00115	0.401410033	0.401410033	8.19E-05
A.vasorum /Control	O75251	0.255087447	0.255087447	8.34E-05
A.vasorum /Control	P04275	0.970225428	0.970225428	8.39E-05
A.vasorum /Control	P46939	0.1659087	0.1659087	8.41E-05
A.vasorum /Control	P55072	0.109975791	0.109975791	8.31E-05
A.vasorum /Control	P98160	0.803987897	0.803987897	8.39E-05
A.vasorum /Control	Q14999	0.489439984	0.489439984	8.36E-05
A.vasorum /Control	Q9NTX5	0.066848792	0.066848792	8.24E-05
A.vasorum /Control	Q9P0V9	0.050989529	0.050989529	8.20E-05
A.vasorum /Control	Q9Y3D6	2.816345436	2.816345436	8.25E-05
A.vasorum /Control	P51688	0.232816845	0.232816845	8.47E-05
A.vasorum /Control	Q8NBM8	0.674898244	0.674898244	8.58E-05
A.vasorum /Control	Q92896	0.055918782	0.055918782	8.57E-05
A.vasorum /Control	Q96AP7	0.308843803	0.308843803	8.57E-05
A.vasorum /Control	P61916	0.271473916	0.271473916	8.68E-05
A.vasorum /Control	Q86T13	0.256311552	0.256311552	8.66E-05
A.vasorum /Control	P11308	0.129987861	0.129987861	8.87E-05
A.vasorum /Control	Q92834	0.167951863	0.167951863	8.95E-05
A.vasorum /Control	P51572	-0.074437875	0.074437875	9.04E-05
A.vasorum /Control	P55212	0.252070831	0.252070831	9.09E-05
A.vasorum /Control	Q9BZZ2	0.817938841	0.817938841	9.12E-05
A.vasorum /Control	Q9NV88	0.148475252	0.148475252	9.10E-05
A.vasorum /Control	Q9Y606	0.071411564	0.071411564	9.03E-05
A.vasorum /Control	Q9GZQ8	-0.347772813	0.347772813	9.22E-05
A.vasorum /Control	O15111	0.185961791	0.185961791	9.24E-05
A.vasorum /Control	P14625	0.170905857	0.170905857	9.43E-05
A.vasorum /Control	Q9NXV2	-0.807998925	0.807998925	9.43E-05
A.vasorum /Control	O43447	0.197911207	0.197911207	9.55E-05
A.vasorum /Control	P35611	0.256696156	0.256696156	9.51E-05
A.vasorum /Control	Q2TAA5	0.13973464	0.13973464	9.56E-05
A.vasorum /Control	P50579	0.41332431	0.41332431	9.72E-05
A.vasorum /Control	P36551	-0.046276537	0.046276537	9.92E-05
A.vasorum /Control	O95478	-1.739896551	1.739896551	0.000105

A.vasorum /Control	P14923	0.470388065	0.470388065	0.000106
A.vasorum /Control	P39687	0.247535662	0.247535662	0.000107
A.vasorum /Control	P50454	0.129859236	0.129859236	0.000108
A.vasorum /Control	P61158	0.088708496	0.088708496	0.000107
A.vasorum /Control	Q13277	0.268734012	0.268734012	0.000108
A.vasorum /Control	Q15165	0.290044444	0.290044444	0.000106
A.vasorum /Control	Q5SW96	0.731074945	0.731074945	0.000106
A.vasorum /Control	Q9Y5Z4	1.082331203	1.082331203	0.000108
A.vasorum /Control	Q9NVC3	0.272297663	0.272297663	0.00011
A.vasorum /Control	O95302	0.186544213	0.186544213	0.000112
A.vasorum /Control	O43719	0.224749975	0.224749975	0.000112
A.vasorum /Control	Q6KC79	0.237782506	0.237782506	0.000113
A.vasorum /Control	Q92626	0.713476492	0.713476492	0.000113
A.vasorum /Control	P05121	-0.721995544	0.721995544	0.000114
A.vasorum /Control	P30419	0.188876692	0.188876692	0.000115
A.vasorum /Control	P61764	0.063435622	0.063435622	0.000114
A.vasorum /Control	Q92541	0.122760417	0.122760417	0.000115
A.vasorum /Control	Q9NVR0	0.804988483	0.804988483	0.000115
A.vasorum /Control	P51610	0.043581138	0.043581138	0.000117
A.vasorum /Control	P35555	0.689923031	0.689923031	0.000121
A.vasorum /Control	P35658	0.075571738	0.075571738	0.000121
A.vasorum /Control	P55039	0.013682435	0.013682435	0.000121
A.vasorum /Control	Q8IYI6	0.217050616	0.217050616	0.000121
A.vasorum /Control	Q96IU4	0.532335974	0.532335974	0.000121
A.vasorum /Control	P07992	1.035589546	1.035589546	0.000125
A.vasorum /Control	P13010	0.10983426	0.10983426	0.000126
A.vasorum /Control	Q8N2K0	0.39978326	0.39978326	0.000126
A.vasorum /Control	Q96G03	-0.01578576	0.01578576	0.000125
A.vasorum /Control	Q9NSK0	0.252251619	0.252251619	0.000126
A.vasorum /Control	Q9P2T1	0.142768944	0.142768944	0.000126
A.vasorum /Control	Q9UHN6	0.302311037	0.302311037	0.000125
A.vasorum /Control	P05091	0.125862818	0.125862818	0.000127
A.vasorum /Control	P07954	0.000890037	0.000890037	0.000131
A.vasorum /Control	P09601	-0.981706645	0.981706645	0.000132
A.vasorum /Control	P23284	0.148052475	0.148052475	0.000129
A.vasorum /Control	P62195	-0.019617485	0.019617485	0.000128
A.vasorum /Control	Q10589	1.268879657	1.268879657	0.00013
A.vasorum /Control	Q12768	0.094156277	0.094156277	0.000129
A.vasorum /Control	Q16698	0.109651465	0.109651465	0.00013
A.vasorum /Control	Q16762	0.400894099	0.400894099	0.00013
A.vasorum /Control	Q8IY17	0.151833771	0.151833771	0.000128
A.vasorum /Control	Q92930	0.121685288	0.121685288	0.00013
A.vasorum /Control	Q9BYK8	0.391538275	0.391538275	0.000129
A.vasorum /Control	Q9Y2H2	0.155282946	0.155282946	0.000131
A.vasorum /Control	Q9Y570	0.126598983	0.126598983	0.000132
A.vasorum /Control	Q9NT62	0.338928308	0.338928308	0.000134
A.vasorum /Control	Q13315	0.577737815	0.577737815	0.000135
A.vasorum /Control	Q8WVX9	0.122955482	0.122955482	0.000136
A.vasorum /Control	Q8WWN8	0.226534783	0.226534783	0.000136
A.vasorum /Control	Q63ZY3	0.224753574	0.224753574	0.000138

A.vasorum /Control	O15270	0.206554457	0.206554457	0.000139
A.vasorum /Control	P35590	0.504556878	0.504556878	0.00014
A.vasorum /Control	Q86UU0	0.512382788	0.512382788	0.00014
A.vasorum /Control	Q8WYA6	0.192208902	0.192208902	0.00014
A.vasorum /Control	Q8WWP7	0.919566484	0.919566484	0.000141
A.vasorum /Control	Q8TDB6	0.507670632	0.507670632	0.000141
A.vasorum /Control	Q9P2I0	0.048696777	0.048696777	0.000144
A.vasorum /Control	Q08174	0.254092109	0.254092109	0.000145
A.vasorum /Control	O94804	0.30839881	0.30839881	0.000146
A.vasorum /Control	Q14554	0.017269101	0.017269101	0.000147
A.vasorum /Control	Q96RR4	0.607605478	0.607605478	0.000148
A.vasorum /Control	O43731	-0.138106011	0.138106011	0.000149
A.vasorum /Control	P06737	-0.0606739	0.0606739	0.000149
A.vasorum /Control	P12821	1.516175714	1.516175714	0.00015
A.vasorum /Control	P27658	1.618069853	1.618069853	0.00015
A.vasorum /Control	Q12824	-0.045978826	0.045978826	0.000149
A.vasorum /Control	P61163	0.109662425	0.109662425	0.000151
A.vasorum /Control	Q13616	0.116764896	0.116764896	0.000152
A.vasorum /Control	P02795	-1.043760956	1.043760956	0.000157
A.vasorum /Control	P09960	0.368392302	0.368392302	0.000154
A.vasorum /Control	P17948	0.619277597	0.619277597	0.000155
A.vasorum /Control	P32119	0.221274065	0.221274065	0.000155
A.vasorum /Control	P50851	0.314988761	0.314988761	0.000157
A.vasorum /Control	Q709C8	0.195500753	0.195500753	0.000156
A.vasorum /Control	Q70E73	0.185135111	0.185135111	0.000157
A.vasorum /Control	Q86XA9	0.2208844	0.2208844	0.000157
A.vasorum /Control	Q8IZA0	0.467424646	0.467424646	0.000154
A.vasorum /Control	Q8NBX0	0.215108477	0.215108477	0.000155
A.vasorum /Control	O00468	0.521572492	0.521572492	0.000162
A.vasorum /Control	O75843	0.940008861	0.940008861	0.00016
A.vasorum /Control	P49821	0.441196651	0.441196651	0.000161
A.vasorum /Control	P53634	0.592910042	0.592910042	0.00016
A.vasorum /Control	Q14165	0.309000757	0.309000757	0.000162
A.vasorum /Control	Q5K651	0.456964069	0.456964069	0.000162
A.vasorum /Control	Q86VX9	0.712776406	0.712776406	0.000162
A.vasorum /Control	Q9Y4W6	0.106511119	0.106511119	0.000162
A.vasorum /Control	P20020	0.369057435	0.369057435	0.000164
A.vasorum /Control	Q04864	-0.650215441	0.650215441	0.000164
A.vasorum /Control	Q9UBG0	0.380326468	0.380326468	0.000164
A.vasorum /Control	Q9H201	1.242063974	1.242063974	0.000166
A.vasorum /Control	P15153	-0.247703689	0.247703689	0.000167
A.vasorum /Control	Q8N3P4	0.273446248	0.273446248	0.000168
A.vasorum /Control	O43242	0.077014494	0.077014494	0.000168
A.vasorum /Control	Q14249	0.031440549	0.031440549	0.000171
A.vasorum /Control	Q6P1N0	0.107968826	0.107968826	0.000172
A.vasorum /Control	Q8WUY1	0.248404798	0.248404798	0.000174
A.vasorum /Control	O43813	0.288793239	0.288793239	0.000176
A.vasorum /Control	P54577	-0.023120283	0.023120283	0.000183
A.vasorum /Control	O43148	0.113827647	0.113827647	0.000185
A.vasorum /Control	Q63HN8	0.440724317	0.440724317	0.000184

A.vasorum /Control	P10646	0.733044056	0.733044056	0.000187
A.vasorum /Control	Q6DKJ4	0.985197669	0.985197669	0.000186
A.vasorum /Control	Q9BYB4	-0.024573585	0.024573585	0.000187
A.vasorum /Control	Q9H3K2	-0.26933058	0.26933058	0.000188
A.vasorum /Control	O15372	0.057717102	0.057717102	0.000192
A.vasorum /Control	O60502	0.240257275	0.240257275	0.000192
A.vasorum /Control	P00338	-0.089235376	0.089235376	0.000192
A.vasorum /Control	Q13085	0.043178388	0.043178388	0.000191
A.vasorum /Control	Q13393	0.572935197	0.572935197	0.000191
A.vasorum /Control	Q96DX4	0.290348168	0.290348168	0.000193
A.vasorum /Control	Q96KP1	0.097830148	0.097830148	0.000192
A.vasorum /Control	Q96RU3	0.317504538	0.317504538	0.000193
A.vasorum /Control	P60983	0.441721912	0.441721912	0.000197
A.vasorum /Control	P35606	-0.00027041	0.00027041	0.000199
A.vasorum /Control	P06865	0.672019362	0.672019362	0.0002
A.vasorum /Control	Q9UJV9	0.013821795	0.013821795	0.000201
A.vasorum /Control	P05165	0.177434371	0.177434371	0.000203
A.vasorum /Control	P50895	0.659373369	0.659373369	0.000205
A.vasorum /Control	P52294	0.123532708	0.123532708	0.000206
A.vasorum /Control	Q13617	0.032161187	0.032161187	0.000205
A.vasorum /Control	Q14376	0.138852905	0.138852905	0.000204
A.vasorum /Control	Q15629	0.200779884	0.200779884	0.000204
A.vasorum /Control	Q9H330	0.085604601	0.085604601	0.000205
A.vasorum /Control	P56962	1.100100113	1.100100113	0.000206
A.vasorum /Control	Q9NRF8	0.16096865	0.16096865	0.000207
A.vasorum /Control	Q9P035	0.124178279	0.124178279	0.000208
A.vasorum /Control	Q2M1Z3	0.200475935	0.200475935	0.000209
A.vasorum /Control	Q8N668	0.415547614	0.415547614	0.000209
A.vasorum /Control	Q06323	0.223938584	0.223938584	0.000211
A.vasorum /Control	Q53EZ4	0.068783149	0.068783149	0.000213
A.vasorum /Control	Q9GZZ1	-0.113114736	0.113114736	0.000212
A.vasorum /Control	Q9NSC5	0.327934309	0.327934309	0.000213
A.vasorum /Control	Q96QC0	0.231580515	0.231580515	0.000214
A.vasorum /Control	Q8TDW0	0.169626328	0.169626328	0.000217
A.vasorum /Control	Q96ME1	0.196580011	0.196580011	0.000218
A.vasorum /Control	Q9NQR4	0.045540625	0.045540625	0.000219
A.vasorum /Control	Q9UK41	0.106953699	0.106953699	0.000218
A.vasorum /Control	Q9HAT2	1.733278394	1.733278394	0.00022
A.vasorum /Control	Q14814	0.285300665	0.285300665	0.000222
A.vasorum /Control	Q99598	0.15572322	0.15572322	0.000221
A.vasorum /Control	P30101	0.163534764	0.163534764	0.000224
A.vasorum /Control	O75832	0.077217674	0.077217674	0.000225
A.vasorum /Control	Q13427	-0.09360575	0.09360575	0.000225
A.vasorum /Control	Q8IXH7	0.237827009	0.237827009	0.000231
A.vasorum /Control	O60749	0.11087456	0.11087456	0.000233
A.vasorum /Control	O75175	0.10940303	0.10940303	0.000234
A.vasorum /Control	P06454	0.750199362	0.750199362	0.000236
A.vasorum /Control	P33176	0.023185914	0.023185914	0.000234
A.vasorum /Control	P40692	0.378819181	0.378819181	0.000234
A.vasorum /Control	P48163	-0.048861181	0.048861181	0.000236

A.vasorum /Control	Q09472	0.390784438	0.390784438	0.000235
A.vasorum /Control	Q5HYK7	0.088471567	0.088471567	0.000237
A.vasorum /Control	Q9HCS7	0.156869667	0.156869667	0.000236
A.vasorum /Control	O94973	0.097037095	0.097037095	0.000237
A.vasorum /Control	O60762	0.135037314	0.135037314	0.000239
A.vasorum /Control	P13667	0.094719247	0.094719247	0.00024
A.vasorum /Control	Q9Y490	0.011735891	0.011735891	0.00024
A.vasorum /Control	P07900	0.034777817	0.034777817	0.000247
A.vasorum /Control	P21333	-0.155041876	0.155041876	0.000247
A.vasorum /Control	P25098	0.342515133	0.342515133	0.000246
A.vasorum /Control	P28906	1.663409949	1.663409949	0.000243
A.vasorum /Control	P35232	0.182070817	0.182070817	0.000246
A.vasorum /Control	P42229	0.336038913	0.336038913	0.000244
A.vasorum /Control	Q14254	0.28219648	0.28219648	0.000246
A.vasorum /Control	Q7L5N7	0.311160481	0.311160481	0.000243
A.vasorum /Control	Q96C24	0.389015853	0.389015853	0.000245
A.vasorum /Control	Q9Y265	-0.011500806	0.011500806	0.000247
A.vasorum /Control	Q9Y3R5	1.039912598	1.039912598	0.000247
A.vasorum /Control	P01893	0.499561054	0.499561054	0.00025
A.vasorum /Control	P35251	0.081145971	0.081145971	0.00025
A.vasorum /Control	Q4G176	0.251748904	0.251748904	0.000249
A.vasorum /Control	Q9UM54	0.320037642	0.320037642	0.000249
A.vasorum /Control	O95081	0.359927781	0.359927781	0.000252
A.vasorum /Control	P51532	-0.047991635	0.047991635	0.000251
A.vasorum /Control	Q9HCC0	0.455856926	0.455856926	0.000251
A.vasorum /Control	P09758	0.240362396	0.240362396	0.000254
A.vasorum /Control	P13473	0.038395104	0.038395104	0.000253
A.vasorum /Control	Q13042	0.079154104	0.079154104	0.000253
A.vasorum /Control	Q9BWM7	0.086459756	0.086459756	0.000254
A.vasorum /Control	Q8NFI3	0.240403466	0.240403466	0.000257
A.vasorum /Control	Q5VW36	0.056771148	0.056771148	0.000259
A.vasorum /Control	O94788	0.348493204	0.348493204	0.000261
A.vasorum /Control	Q9NVJ2	1.304216962	1.304216962	0.00026
A.vasorum /Control	Q07960	0.022434019	0.022434019	0.000262
A.vasorum /Control	Q9Y6M1	0.041155427	0.041155427	0.000262
A.vasorum /Control	Q96GA7	0.315286633	0.315286633	0.000267
A.vasorum /Control	Q07157	0.296193377	0.296193377	0.000268
A.vasorum /Control	Q9P2R7	0.068213639	0.068213639	0.00027
A.vasorum /Control	A6NJ78	0.272507092	0.272507092	0.000271
A.vasorum /Control	O75578	0.760379914	0.760379914	0.000273
A.vasorum /Control	P07996	1.236996093	1.236996093	0.000273
A.vasorum /Control	Q16822	0.572684534	0.572684534	0.000272
A.vasorum /Control	Q8NCH0	0.151585306	0.151585306	0.000272
A.vasorum /Control	Q8ND71	1.244824905	1.244824905	0.000272
A.vasorum /Control	Q9UJX5	0.23193808	0.23193808	0.000274
A.vasorum /Control	Q9H2M9	-0.093783031	0.093783031	0.000275
A.vasorum /Control	O75340	0.228304682	0.228304682	0.000276
A.vasorum /Control	A1L0T0	0.098696063	0.098696063	0.00028
A.vasorum /Control	O14980	0.14617277	0.14617277	0.000282
A.vasorum /Control	O43491	-0.032692312	0.032692312	0.000278

A.vasorum /Control	O75312	0.315738915	0.315738915	0.000281
A.vasorum /Control	Q8N4P3	0.359943123	0.359943123	0.000279
A.vasorum /Control	Q96PQ7	-0.001838831	0.001838831	0.000281
A.vasorum /Control	Q96T51	0.171238249	0.171238249	0.000281
A.vasorum /Control	Q9NYB9	0.309695677	0.309695677	0.000279
A.vasorum /Control	Q9NYF8	0.064380305	0.064380305	0.000279
A.vasorum /Control	Q9Y3C6	0.150942264	0.150942264	0.000277
A.vasorum /Control	P84085	0.338642244	0.338642244	0.000282
A.vasorum /Control	Q16666	0.199158134	0.199158134	0.000283
A.vasorum /Control	Q9BQE3	-0.568266942	0.568266942	0.000284
A.vasorum /Control	P48426	0.054224141	0.054224141	0.000286
A.vasorum /Control	Q92604	0.89375844	0.89375844	0.000286
A.vasorum /Control	P39060	0.530277634	0.530277634	0.000288
A.vasorum /Control	Q8N335	0.393889278	0.393889278	0.000292
A.vasorum /Control	O94915	0.248210752	0.248210752	0.000294
A.vasorum /Control	P00813	0.362370797	0.362370797	0.000294
A.vasorum /Control	P17568	0.290971495	0.290971495	0.000294
A.vasorum /Control	P78527	0.135330225	0.135330225	0.000295
A.vasorum /Control	Q14566	0.033425209	0.033425209	0.000297
A.vasorum /Control	P49419	0.314304583	0.314304583	0.0003
A.vasorum /Control	Q00341	0.047132576	0.047132576	0.0003
A.vasorum /Control	Q15084	0.262053026	0.262053026	0.0003
A.vasorum /Control	Q6P1J9	0.048843811	0.048843811	0.000299
A.vasorum /Control	Q6P6C2	0.760247761	0.760247761	0.000299
A.vasorum /Control	O94888	0.423667905	0.423667905	0.000307
A.vasorum /Control	P06576	0.0804771	0.0804771	0.000308
A.vasorum /Control	P24723	0.351954088	0.351954088	0.000306
A.vasorum /Control	P32455	0.329318257	0.329318257	0.000305
A.vasorum /Control	P48735	0.204301648	0.204301648	0.000304
A.vasorum /Control	P98179	-0.475908011	0.475908011	0.000302
A.vasorum /Control	Q5TA45	0.25114856	0.25114856	0.000306
A.vasorum /Control	Q6P1Q9	0.523128548	0.523128548	0.000304
A.vasorum /Control	Q6P1R3	0.422345577	0.422345577	0.000303
A.vasorum /Control	Q86UP2	0.114046091	0.114046091	0.000309
A.vasorum /Control	Q8IYB5	0.455875791	0.455875791	0.000308
A.vasorum /Control	Q8TE77	0.249298511	0.249298511	0.000308
A.vasorum /Control	Q969X5	0.12230209	0.12230209	0.000309
A.vasorum /Control	Q9UBP6	0.227749474	0.227749474	0.000305
A.vasorum /Control	Q9UKS6	-0.102296375	0.102296375	0.00031
A.vasorum /Control	Q6XQN6	0.471273375	0.471273375	0.000311
A.vasorum /Control	Q00325	0.16539075	0.16539075	0.000312
A.vasorum /Control	P37059	0.849823388	0.849823388	0.000314
A.vasorum /Control	Q8NC56	0.116729844	0.116729844	0.000315
A.vasorum /Control	Q9H5Z6	0.606347348	0.606347348	0.000316
A.vasorum /Control	O43286	0.600104813	0.600104813	0.000318
A.vasorum /Control	Q96PE2	0.762205196	0.762205196	0.000321
A.vasorum /Control	Q08722	-0.072553808	0.072553808	0.000322
A.vasorum /Control	P28702	0.31873319	0.31873319	0.000323
A.vasorum /Control	Q9H1Y0	0.116864961	0.116864961	0.000324
A.vasorum /Control	Q92609	0.153955825	0.153955825	0.000329

A.vasorum /Control	P51970	0.177675086	0.177675086	0.000331
A.vasorum /Control	O43488	0.231983734	0.231983734	0.000333
A.vasorum /Control	P12956	0.157925728	0.157925728	0.000333
A.vasorum /Control	Q13451	0.21155899	0.21155899	0.000334
A.vasorum /Control	P41252	0.042389247	0.042389247	0.000335
A.vasorum /Control	O75381	0.347999611	0.347999611	0.000337
A.vasorum /Control	Q8N1I0	0.106403917	0.106403917	0.000337
A.vasorum /Control	Q9H3T3	0.393809609	0.393809609	0.000336
A.vasorum /Control	P20933	0.655371488	0.655371488	0.000338
A.vasorum /Control	Q92625	0.898552745	0.898552745	0.000338
A.vasorum /Control	P15311	0.068530297	0.068530297	0.000349
A.vasorum /Control	P15559	0.589009758	0.589009758	0.000347
A.vasorum /Control	P40939	0.085486846	0.085486846	0.00035
A.vasorum /Control	Q02952	-0.762803834	0.762803834	0.000342
A.vasorum /Control	Q0VF96	0.353436777	0.353436777	0.000349
A.vasorum /Control	Q13011	0.412324334	0.412324334	0.00034
A.vasorum /Control	Q13228	1.144263546	1.144263546	0.000344
A.vasorum /Control	Q14839	0.091334871	0.091334871	0.000346
A.vasorum /Control	Q16795	0.416447901	0.416447901	0.000348
A.vasorum /Control	Q7L311	0.040526742	0.040526742	0.000349
A.vasorum /Control	Q92562	0.852051925	0.852051925	0.00034
A.vasorum /Control	Q93063	0.388176282	0.388176282	0.00035
A.vasorum /Control	Q96N66	0.103751508	0.103751508	0.000347
A.vasorum /Control	Q9NTJ5	0.164737129	0.164737129	0.000346
A.vasorum /Control	Q9NZN3	0.158290481	0.158290481	0.000343
A.vasorum /Control	Q9P227	0.359473984	0.359473984	0.000349
A.vasorum /Control	Q9P2B2	0.342860024	0.342860024	0.000348
A.vasorum /Control	Q9UHP3	0.048960504	0.048960504	0.000346
A.vasorum /Control	Q9Y305	0.05567278	0.05567278	0.00035
A.vasorum /Control	P35916	1.371037994	1.371037994	0.000351
A.vasorum /Control	Q9NQ66	0.689308475	0.689308475	0.000354
A.vasorum /Control	Q6P9B9	0.363522713	0.363522713	0.000355
A.vasorum /Control	Q9H0Q0	0.314919873	0.314919873	0.000357
A.vasorum /Control	P38117	0.167443325	0.167443325	0.000362
A.vasorum /Control	O75923	0.401766635	0.401766635	0.000367
A.vasorum /Control	P17152	0.135858649	0.135858649	0.000365
A.vasorum /Control	Q15020	0.160315982	0.160315982	0.000364
A.vasorum /Control	Q4ZIN3	0.182647194	0.182647194	0.000364
A.vasorum /Control	Q7Z460	0.211675163	0.211675163	0.000367
A.vasorum /Control	Q9NXE4	0.184075011	0.184075011	0.000367
A.vasorum /Control	Q9NZD2	0.314354224	0.314354224	0.000366
A.vasorum /Control	Q9Y5V0	0.116449741	0.116449741	0.000368
A.vasorum /Control	O15228	0.324510434	0.324510434	0.00037
A.vasorum /Control	O75844	0.090638559	0.090638559	0.000371
A.vasorum /Control	Q15418	0.60822995	0.60822995	0.000372
A.vasorum /Control	Q8TAF3	0.583653772	0.583653772	0.00037
A.vasorum /Control	Q9BXS5	0.061936265	0.061936265	0.00037
A.vasorum /Control	Q14CX7	0.064468627	0.064468627	0.000373
A.vasorum /Control	Q9Y3A6	0.010671328	0.010671328	0.000375
A.vasorum /Control	Q9NUL5	2.392961995	2.392961995	0.000377

A.vasorum /Control	Q9P015	0.14846105	0.14846105	0.000377
A.vasorum /Control	Q9BRF8	0.261706595	0.261706595	0.00038
A.vasorum /Control	P52948	0.099209874	0.099209874	0.000382
A.vasorum /Control	Q9NPF4	0.114983746	0.114983746	0.000385
A.vasorum /Control	Q12907	0.179143454	0.179143454	0.000388
A.vasorum /Control	P49321	0.41943667	0.41943667	0.000389
A.vasorum /Control	Q9NUQ9	0.175921807	0.175921807	0.000389
A.vasorum /Control	O14618	0.47134958	0.47134958	0.000391
A.vasorum /Control	Q9H479	0.685015756	0.685015756	0.000391
A.vasorum /Control	P04181	-0.338272606	0.338272606	0.000394
A.vasorum /Control	Q5VT52	0.020986818	0.020986818	0.000396
A.vasorum /Control	Q6NY19	0.668940128	0.668940128	0.000397
A.vasorum /Control	Q9Y2V2	0.596358334	0.596358334	0.000398
A.vasorum /Control	P29590	0.249731915	0.249731915	0.000399
A.vasorum /Control	P50416	0.355742594	0.355742594	0.000405
A.vasorum /Control	P36507	0.185614479	0.185614479	0.000406
A.vasorum /Control	Q8TB22	0.445745746	0.445745746	0.000407
A.vasorum /Control	Q14738	0.017658569	0.017658569	0.000408
A.vasorum /Control	Q8WX92	0.555600195	0.555600195	0.000409
A.vasorum /Control	Q9BZL6	0.288391803	0.288391803	0.00041
A.vasorum /Control	P30520	0.042181286	0.042181286	0.000411
A.vasorum /Control	P04439	0.191792466	0.191792466	0.000419
A.vasorum /Control	O95707	0.549068025	0.549068025	0.00042
A.vasorum /Control	Q99958	-1.029195764	1.029195764	0.00042
A.vasorum /Control	P11498	0.394659421	0.394659421	0.000422
A.vasorum /Control	P62140	0.147415971	0.147415971	0.000422
A.vasorum /Control	O43520	0.200364458	0.200364458	0.000426
A.vasorum /Control	P11171	0.224241751	0.224241751	0.000425
A.vasorum /Control	Q53FP2	0.116854321	0.116854321	0.000426
A.vasorum /Control	Q6ZRP7	0.33683791	0.33683791	0.00043
A.vasorum /Control	Q96C36	0.217577433	0.217577433	0.00043
A.vasorum /Control	Q9UNH7	0.124803255	0.124803255	0.00043
A.vasorum /Control	O00442	0.33444561	0.33444561	0.000435
A.vasorum /Control	Q9Y2S2	0.170236627	0.170236627	0.000438
A.vasorum /Control	O43815	0.021046369	0.021046369	0.000439
A.vasorum /Control	P56381	0.057609538	0.057609538	0.000442
A.vasorum /Control	Q6NUM9	0.184596274	0.184596274	0.000444
A.vasorum /Control	Q9NXS2	0.010316798	0.010316798	0.000444
A.vasorum /Control	Q6AI12	0.79815095	0.79815095	0.000445
A.vasorum /Control	Q96RL7	0.426482521	0.426482521	0.000447
A.vasorum /Control	P57088	-0.185277453	0.185277453	0.000448
A.vasorum /Control	Q9NX46	0.252344133	0.252344133	0.000448
A.vasorum /Control	P48047	0.00916787	0.00916787	0.000452
A.vasorum /Control	Q3KQZ1	0.097900819	0.097900819	0.000452
A.vasorum /Control	P32456	0.757125045	0.757125045	0.000455
A.vasorum /Control	Q99418	1.382488458	1.382488458	0.000454
A.vasorum /Control	Q8TB72	0.494818793	0.494818793	0.000457
A.vasorum /Control	O60664	0.191518273	0.191518273	0.00046
A.vasorum /Control	O75592	0.268787975	0.268787975	0.000459
A.vasorum /Control	P25942	1.518870491	1.518870491	0.000461

A.vasorum /Control	P30048	0.206908445	0.206908445	0.000462
A.vasorum /Control	Q9Y5S2	0.110317573	0.110317573	0.000462
A.vasorum /Control	O60716	0.171775102	0.171775102	0.000464
A.vasorum /Control	P25686	0.309343211	0.309343211	0.000463
A.vasorum /Control	P36405	0.109164967	0.109164967	0.000465
A.vasorum /Control	Q7Z4V5	0.154985674	0.154985674	0.000466
A.vasorum /Control	P30085	0.094064375	0.094064375	0.000468
A.vasorum /Control	Q13363	0.061051832	0.061051832	0.000469
A.vasorum /Control	Q13535	0.171941923	0.171941923	0.000467
A.vasorum /Control	Q9Y2A7	0.146286014	0.146286014	0.000467
A.vasorum /Control	O14936	0.026530065	0.026530065	0.00047
A.vasorum /Control	Q96AY4	0.342086337	0.342086337	0.000471
A.vasorum /Control	Q12797	0.103801189	0.103801189	0.000474
A.vasorum /Control	O94874	0.081840105	0.081840105	0.000476
A.vasorum /Control	P11137	0.344484874	0.344484874	0.000475
A.vasorum /Control	O00203	0.046365665	0.046365665	0.00048
A.vasorum /Control	O60739	2.443920776	2.443920776	0.000483
A.vasorum /Control	O75955	-0.027102571	0.027102571	0.000481
A.vasorum /Control	P20340	0.02314374	0.02314374	0.000482
A.vasorum /Control	Q8NFQ8	0.176277025	0.176277025	0.000478
A.vasorum /Control	Q92882	0.538054731	0.538054731	0.00048
A.vasorum /Control	Q96EP0	0.090191568	0.090191568	0.000483
A.vasorum /Control	Q96IJ6	0.109863332	0.109863332	0.000481
A.vasorum /Control	Q96MW5	0.263352855	0.263352855	0.000483
A.vasorum /Control	Q9HB40	0.325534847	0.325534847	0.000479
A.vasorum /Control	P11717	0.108123771	0.108123771	0.000484
A.vasorum /Control	Q8NBL1	0.180977521	0.180977521	0.000485
A.vasorum /Control	Q96BM9	0.196439892	0.196439892	0.000486
A.vasorum /Control	O00192	0.59754083	0.59754083	0.000487
A.vasorum /Control	P36915	0.161536096	0.161536096	0.000489
A.vasorum /Control	A6NDG6	0.133953551	0.133953551	0.000491
A.vasorum /Control	P28288	0.268423758	0.268423758	0.000491
A.vasorum /Control	P14927	0.318273799	0.318273799	0.000493
A.vasorum /Control	O95807	0.166864343	0.166864343	0.000497
A.vasorum /Control	Q8WXG6	0.308922434	0.308922434	0.000497
A.vasorum /Control	Q92974	0.323819297	0.323819297	0.000497
A.vasorum /Control	O60934	0.233538642	0.233538642	0.000499
A.vasorum /Control	O95347	-0.075536544	0.075536544	0.000499
A.vasorum /Control	Q5HYI8	0.071735601	0.071735601	0.0005
A.vasorum /Control	P03956	-0.995684699	0.995684699	0.000502
A.vasorum /Control	Q15386	0.175470419	0.175470419	0.000504
A.vasorum /Control	Q15643	0.151064639	0.151064639	0.000504
A.vasorum /Control	Q96CW5	0.089038008	0.089038008	0.000503
A.vasorum /Control	Q9P0I2	0.026443474	0.026443474	0.000504
A.vasorum /Control	P21579	0.142621856	0.142621856	0.000506
A.vasorum /Control	P29084	0.222887043	0.222887043	0.000507
A.vasorum /Control	Q9ULK4	0.21952999	0.21952999	0.000508
A.vasorum /Control	O14639	0.488582013	0.488582013	0.000509
A.vasorum /Control	Q9UHB9	0.017602308	0.017602308	0.000513
A.vasorum /Control	Q9H223	0.01046628	0.01046628	0.000517

A.vasorum /Control	O43823	-0.052380441	0.052380441	0.000518
A.vasorum /Control	Q9P016	0.324702092	0.324702092	0.000519
A.vasorum /Control	P49458	0.242229196	0.242229196	0.000521
A.vasorum /Control	O43704	0.11963348	0.11963348	0.000525
A.vasorum /Control	P10909	1.533468514	1.533468514	0.000523
A.vasorum /Control	P11279	0.175029889	0.175029889	0.000524
A.vasorum /Control	P15289	1.225579149	1.225579149	0.000524
A.vasorum /Control	P48200	-1.955124953	1.955124953	0.000525
A.vasorum /Control	Q8NBS9	0.205109788	0.205109788	0.000522
A.vasorum /Control	P29466	0.614188835	0.614188835	0.000527
A.vasorum /Control	P49006	0.456778383	0.456778383	0.000531
A.vasorum /Control	P55290	0.814905781	0.814905781	0.000528
A.vasorum /Control	P61026	0.072801749	0.072801749	0.000531
A.vasorum /Control	Q567U6	0.065109114	0.065109114	0.00053
A.vasorum /Control	Q8IV36	1.695072735	1.695072735	0.00053
A.vasorum /Control	O75608	0.416044594	0.416044594	0.000532
A.vasorum /Control	P18077	0.099337238	0.099337238	0.000534
A.vasorum /Control	O95831	0.182157178	0.182157178	0.000537
A.vasorum /Control	P26572	0.126191906	0.126191906	0.000538
A.vasorum /Control	O60493	0.156721634	0.156721634	0.000539
A.vasorum /Control	Q7Z6Z7	0.107027386	0.107027386	0.00054
A.vasorum /Control	O75376	0.22272808	0.22272808	0.000541
A.vasorum /Control	Q92466	0.25863277	0.25863277	0.000542
A.vasorum /Control	O14773	0.313247639	0.313247639	0.000546
A.vasorum /Control	O15061	0.210938447	0.210938447	0.000546
A.vasorum /Control	Q32P41	0.228461451	0.228461451	0.000546
A.vasorum /Control	Q96DE0	0.677880286	0.677880286	0.000545
A.vasorum /Control	Q9NRZ9	-0.081943718	0.081943718	0.000544
A.vasorum /Control	P08581	0.268706458	0.268706458	0.000549
A.vasorum /Control	O43752	0.642854401	0.642854401	0.00055
A.vasorum /Control	Q8IVL6	0.123509624	0.123509624	0.000552
A.vasorum /Control	Q96CP2	0.139204497	0.139204497	0.000552
A.vasorum /Control	Q9NR19	0.178742126	0.178742126	0.000552
A.vasorum /Control	Q01469	-0.375813662	0.375813662	0.000554
A.vasorum /Control	Q96GD0	0.355396183	0.355396183	0.000555
A.vasorum /Control	A1A4S6	-0.163200262	0.163200262	0.00056
A.vasorum /Control	Q9H9S3	-0.024117433	0.024117433	0.000562
A.vasorum /Control	Q9NQW7	0.107611493	0.107611493	0.000564
A.vasorum /Control	Q9UDY4	0.355605274	0.355605274	0.000563
A.vasorum /Control	Q9BTE7	0.165880782	0.165880782	0.000567
A.vasorum /Control	P84090	0.516841668	0.516841668	0.000568
A.vasorum /Control	Q99798	0.562030147	0.562030147	0.000573
A.vasorum /Control	Q9HCN8	0.113489141	0.113489141	0.000574
A.vasorum /Control	Q9ULD2	0.754706475	0.754706475	0.000575
A.vasorum /Control	Q15751	0.101619508	0.101619508	0.000577
A.vasorum /Control	Q5T0D9	-0.102110999	0.102110999	0.000578
A.vasorum /Control	P40763	0.111390742	0.111390742	0.000587
A.vasorum /Control	Q00765	0.082971562	0.082971562	0.000581
A.vasorum /Control	Q15046	0.072250401	0.072250401	0.000588
A.vasorum /Control	Q15554	0.485571638	0.485571638	0.000582

A.vasorum /Control	Q75N03	-0.096116554	0.096116554	0.000588
A.vasorum /Control	Q8TB40	0.538728244	0.538728244	0.000585
A.vasorum /Control	Q96ST2	0.170138357	0.170138357	0.000586
A.vasorum /Control	Q9H5K3	0.413138836	0.413138836	0.000585
A.vasorum /Control	Q9NSI2	-0.528001918	0.528001918	0.000586
A.vasorum /Control	Q9Y2L5	0.188555181	0.188555181	0.000583
A.vasorum /Control	Q9Y315	0.285462034	0.285462034	0.000584
A.vasorum /Control	Q9BYT8	0.122028069	0.122028069	0.000592
A.vasorum /Control	P05067	0.267500783	0.267500783	0.000595
A.vasorum /Control	P49750	0.106441148	0.106441148	0.000598
A.vasorum /Control	Q14204	0.054924377	0.054924377	0.000598
A.vasorum /Control	Q96AT1	0.441323241	0.441323241	0.000598
A.vasorum /Control	Q96AC1	0.078738409	0.078738409	0.000601
A.vasorum /Control	P17542	0.732626433	0.732626433	0.000604
A.vasorum /Control	O94826	0.135853718	0.135853718	0.000607
A.vasorum /Control	Q14103	-0.160395266	0.160395266	0.000606
A.vasorum /Control	Q15291	0.063859633	0.063859633	0.000613
A.vasorum /Control	Q9NUU7	0.137780253	0.137780253	0.000615
A.vasorum /Control	P15735	0.411753762	0.411753762	0.000617
A.vasorum /Control	Q53T59	0.238393691	0.238393691	0.00062
A.vasorum /Control	P21912	0.530971558	0.530971558	0.000622
A.vasorum /Control	Q12851	0.924948423	0.924948423	0.000624
A.vasorum /Control	Q9HBH5	0.095716147	0.095716147	0.000626
A.vasorum /Control	Q9NXX6	1.147764845	1.147764845	0.000628
A.vasorum /Control	O76024	0.378435528	0.378435528	0.00063
A.vasorum /Control	P30566	0.069857815	0.069857815	0.000629
A.vasorum /Control	Q9NRF2	0.249072857	0.249072857	0.000633
A.vasorum /Control	Q9P2E9	0.117270331	0.117270331	0.000632
A.vasorum /Control	Q9UJ70	0.215560754	0.215560754	0.000631
A.vasorum /Control	Q9UQQ2	0.314896477	0.314896477	0.000633
A.vasorum /Control	P17252	-0.16741174	0.16741174	0.000639
A.vasorum /Control	P49903	0.11046027	0.11046027	0.000638
A.vasorum /Control	P54760	0.15480966	0.15480966	0.000636
A.vasorum /Control	Q9UPT5	0.167484802	0.167484802	0.000636
A.vasorum /Control	Q9Y259	0.323746354	0.323746354	0.000636
A.vasorum /Control	Q9Y450	-0.050124615	0.050124615	0.000638
A.vasorum /Control	Q16891	0.022434313	0.022434313	0.000642
A.vasorum /Control	Q4G148	0.07732746	0.07732746	0.000643
A.vasorum /Control	Q9UJX2	0.130057716	0.130057716	0.000644
A.vasorum /Control	Q92692	0.249703353	0.249703353	0.000646
A.vasorum /Control	Q16881	-0.312642565	0.312642565	0.000648
A.vasorum /Control	O00159	0.098437532	0.098437532	0.000655
A.vasorum /Control	Q5U651	0.069648475	0.069648475	0.000657
A.vasorum /Control	Q99460	-0.028700549	0.028700549	0.000657
A.vasorum /Control	Q9Y224	0.071309593	0.071309593	0.000655
A.vasorum /Control	O95671	0.066644196	0.066644196	0.000658
A.vasorum /Control	O00170	0.091351539	0.091351539	0.000661
A.vasorum /Control	P67775	-0.883643985	0.883643985	0.000662
A.vasorum /Control	P10809	0.016714879	0.016714879	0.000667
A.vasorum /Control	P43155	0.292390674	0.292390674	0.000665

A.vasorum /Control	Q6UWH4	0.779533303	0.779533303	0.000666
A.vasorum /Control	Q9HAU5	0.241220559	0.241220559	0.000665
A.vasorum /Control	P04062	0.315280244	0.315280244	0.000668
A.vasorum /Control	Q9Y6Q2	2.108726869	2.108726869	0.000669
A.vasorum /Control	Q7Z4H8	0.912184375	0.912184375	0.000671
A.vasorum /Control	P24844	2.272222609	2.272222609	0.000674
A.vasorum /Control	P35237	0.357277812	0.357277812	0.000674
A.vasorum /Control	P61160	0.103018902	0.103018902	0.000674
A.vasorum /Control	P82912	0.14436375	0.14436375	0.000673
A.vasorum /Control	P04049	0.245441507	0.245441507	0.000675
A.vasorum /Control	Q96SQ9	0.594192581	0.594192581	0.000676
A.vasorum /Control	Q13724	0.043264264	0.043264264	0.00068
A.vasorum /Control	Q99704	1.368250988	1.368250988	0.000681
A.vasorum /Control	P16435	0.135445534	0.135445534	0.000682
A.vasorum /Control	Q92620	0.26913035	0.26913035	0.000684
A.vasorum /Control	Q9Y6I3	0.233477303	0.233477303	0.000685
A.vasorum /Control	Q8IYB7	0.175187136	0.175187136	0.000688
A.vasorum /Control	Q7Z6E9	-0.040617018	0.040617018	0.000694
A.vasorum /Control	Q969S3	-0.01733503	0.01733503	0.000694
A.vasorum /Control	Q92791	0.023908066	0.023908066	0.000697
A.vasorum /Control	Q99715	1.152795888	1.152795888	0.000698
A.vasorum /Control	Q9BPX7	0.036057602	0.036057602	0.000699
A.vasorum /Control	O00767	-0.181562981	0.181562981	0.0007
A.vasorum /Control	Q9HCN4	0.125307632	0.125307632	0.000701
A.vasorum /Control	Q01130	0.013257355	0.013257355	0.000703
A.vasorum /Control	Q9NUV9	0.832442335	0.832442335	0.000703
A.vasorum /Control	Q10472	-0.029745153	0.029745153	0.000708
A.vasorum /Control	Q9H5X1	0.150897606	0.150897606	0.000708
A.vasorum /Control	Q9Y2I1	0.21321884	0.21321884	0.000709
A.vasorum /Control	Q9BY43	0.128547526	0.128547526	0.000714
A.vasorum /Control	P47756	0.081508103	0.081508103	0.000718
A.vasorum /Control	O60341	0.094026277	0.094026277	0.000727
A.vasorum /Control	P21810	1.544528622	1.544528622	0.000721
A.vasorum /Control	P30038	0.431246321	0.431246321	0.000727
A.vasorum /Control	P31948	0.025445605	0.025445605	0.000724
A.vasorum /Control	Q13308	0.487578922	0.487578922	0.00072
A.vasorum /Control	Q14232	0.034670953	0.034670953	0.000722
A.vasorum /Control	Q5JU69	2.267662756	2.267662756	0.00072
A.vasorum /Control	Q8IY67	0.123912329	0.123912329	0.000727
A.vasorum /Control	Q9H1Z4	-0.103577319	0.103577319	0.000723
A.vasorum /Control	Q9UGV2	0.132171266	0.132171266	0.000726
A.vasorum /Control	O60234	0.704559158	0.704559158	0.000733
A.vasorum /Control	Q15599	0.080898464	0.080898464	0.000734
A.vasorum /Control	Q5TEJ8	1.038675636	1.038675636	0.000734
A.vasorum /Control	Q6P1X5	0.324106475	0.324106475	0.000731
A.vasorum /Control	Q8IZ69	0.293744241	0.293744241	0.00073
A.vasorum /Control	Q9NPL8	0.212623873	0.212623873	0.000733
A.vasorum /Control	P11142	-0.12103296	0.12103296	0.000741
A.vasorum /Control	P18615	0.124490125	0.124490125	0.000747
A.vasorum /Control	P31483	0.054735081	0.054735081	0.000747

A.vasorum /Control	Q66K14	0.129387981	0.129387981	0.000746
A.vasorum /Control	Q6PCE3	0.173409295	0.173409295	0.000745
A.vasorum /Control	Q8NC44	0.462060931	0.462060931	0.000746
A.vasorum /Control	Q9BS40	0.679094009	0.679094009	0.000743
A.vasorum /Control	Q9P258	0.128936923	0.128936923	0.000744
A.vasorum /Control	Q9UID3	-0.121366413	0.121366413	0.000747
A.vasorum /Control	Q14571	0.246420253	0.246420253	0.000752
A.vasorum /Control	Q9HD20	-0.032939763	0.032939763	0.000752
A.vasorum /Control	Q9P215	0.271355743	0.271355743	0.000753
A.vasorum /Control	Q13033	0.144522114	0.144522114	0.000754
A.vasorum /Control	Q86YR5	-0.023121197	0.023121197	0.000757
A.vasorum /Control	Q92820	0.782761634	0.782761634	0.000757
A.vasorum /Control	Q08209	0.364583213	0.364583213	0.000758
A.vasorum /Control	Q9H446	0.342527733	0.342527733	0.000762
A.vasorum /Control	Q7L576	0.10460852	0.10460852	0.000766
A.vasorum /Control	P14735	0.049433283	0.049433283	0.000771
A.vasorum /Control	Q15075	-0.000507583	0.000507583	0.00077
A.vasorum /Control	Q9Y6Y8	0.019017175	0.019017175	0.000773
A.vasorum /Control	Q96ND0	1.081279151	1.081279151	0.000775
A.vasorum /Control	P37108	0.132178293	0.132178293	0.000778
A.vasorum /Control	O43795	0.080646271	0.080646271	0.000784
A.vasorum /Control	P05455	0.073376569	0.073376569	0.000784
A.vasorum /Control	P08133	0.104819033	0.104819033	0.000784
A.vasorum /Control	P11586	0.03127847	0.03127847	0.00078
A.vasorum /Control	P37235	0.161669851	0.161669851	0.000785
A.vasorum /Control	Q15797	0.081602212	0.081602212	0.000781
A.vasorum /Control	Q9P291	0.27722761	0.27722761	0.000786
A.vasorum /Control	Q9UHV9	0.318931216	0.318931216	0.000785
A.vasorum /Control	Q9BYV8	0.382428222	0.382428222	0.000789
A.vasorum /Control	P13073	0.090664904	0.090664904	0.000791
A.vasorum /Control	Q9NY15	0.226985056	0.226985056	0.000791
A.vasorum /Control	Q9BZE4	-0.953277967	0.953277967	0.000793
A.vasorum /Control	Q9NYU2	0.218775769	0.218775769	0.000793
A.vasorum /Control	Q9Y2G5	0.068257025	0.068257025	0.000794
A.vasorum /Control	Q9NU19	0.552918855	0.552918855	0.000806
A.vasorum /Control	Q14161	0.201278787	0.201278787	0.00081
A.vasorum /Control	Q96C23	0.838774733	0.838774733	0.00081
A.vasorum /Control	Q9BRX2	-0.042480755	0.042480755	0.000812
A.vasorum /Control	P57764	0.2576763	0.2576763	0.000814
A.vasorum /Control	Q5H9R7	0.006141804	0.006141804	0.000816
A.vasorum /Control	Q9Y624	0.180700494	0.180700494	0.000821
A.vasorum /Control	A2RUS2	0.004207193	0.004207193	0.000826
A.vasorum /Control	P08238	0.013265047	0.013265047	0.000829
A.vasorum /Control	Q99988	-1.316039998	1.316039998	0.000829
A.vasorum /Control	P52943	0.115406662	0.115406662	0.000832
A.vasorum /Control	Q8IWA5	0.797866263	0.797866263	0.000833
A.vasorum /Control	Q96GX2	1.083180332	1.083180332	0.000832
A.vasorum /Control	Q8N1W1	0.10589364	0.10589364	0.000834
A.vasorum /Control	P80303	0.754462143	0.754462143	0.000841
A.vasorum /Control	Q9BYN8	0.086958374	0.086958374	0.000842

A.vasorum /Control	Q9NQT5	-0.068697061	0.068697061	0.000841
A.vasorum /Control	P08962	0.632344558	0.632344558	0.000849
A.vasorum /Control	Q7Z4H7	-0.039029215	0.039029215	0.000849
A.vasorum /Control	Q9NVD7	0.029258892	0.029258892	0.000848
A.vasorum /Control	Q9H4B0	0.379398648	0.379398648	0.000853
A.vasorum /Control	P62258	0.197241306	0.197241306	0.000854
A.vasorum /Control	Q02539	0.441505915	0.441505915	0.000856
A.vasorum /Control	Q14185	0.366635992	0.366635992	0.000856
A.vasorum /Control	Q9UIQ6	0.074418274	0.074418274	0.000858
A.vasorum /Control	P28482	0.122923209	0.122923209	0.000863
A.vasorum /Control	Q14696	0.098366319	0.098366319	0.000865
A.vasorum /Control	Q92552	0.073812639	0.073812639	0.000867
A.vasorum /Control	Q9UJW0	0.014003237	0.014003237	0.000869
A.vasorum /Control	P50336	0.71884363	0.71884363	0.000872
A.vasorum /Control	Q92621	0.05995374	0.05995374	0.000872
A.vasorum /Control	Q9Y5L0	0.074428641	0.074428641	0.000873
A.vasorum /Control	Q5VTL8	0.18715541	0.18715541	0.000876
A.vasorum /Control	Q01995	0.127734134	0.127734134	0.000878
A.vasorum /Control	P42785	0.559880882	0.559880882	0.000881
A.vasorum /Control	O43679	0.705028939	0.705028939	0.000883
A.vasorum /Control	Q15648	0.116749175	0.116749175	0.000884
A.vasorum /Control	Q9NQX3	0.112505834	0.112505834	0.000885
A.vasorum /Control	O00423	0.013588373	0.013588373	0.000891
A.vasorum /Control	Q13330	-0.14001353	0.14001353	0.000892
A.vasorum /Control	Q5T4S7	-0.013728835	0.013728835	0.00089
A.vasorum /Control	Q6UWP7	0.157005853	0.157005853	0.000891
A.vasorum /Control	Q96AY3	-0.005658881	0.005658881	0.000892
A.vasorum /Control	Q9H4A4	0.172753598	0.172753598	0.000892
A.vasorum /Control	Q16851	-0.009133801	0.009133801	0.000896
A.vasorum /Control	Q9HC38	0.109794007	0.109794007	0.000897
A.vasorum /Control	Q9BZQ6	-0.012361507	0.012361507	0.000901
A.vasorum /Control	Q9UHX1	-0.006212851	0.006212851	0.000903
A.vasorum /Control	P43246	0.166560693	0.166560693	0.000909
A.vasorum /Control	Q7Z2K6	0.571356504	0.571356504	0.000911
A.vasorum /Control	Q99758	0.38464412	0.38464412	0.000911
A.vasorum /Control	Q9BTE1	0.353967642	0.353967642	0.000915
A.vasorum /Control	P46782	-0.024615125	0.024615125	0.000918
A.vasorum /Control	Q5T6V5	0.864784674	0.864784674	0.000918
A.vasorum /Control	Q8NEC7	0.464713746	0.464713746	0.000919
A.vasorum /Control	Q9Y680	0.391557923	0.391557923	0.000918
A.vasorum /Control	Q9Y285	0.105599861	0.105599861	0.000922
A.vasorum /Control	Q9UDW1	0.482071651	0.482071651	0.000923
A.vasorum /Control	Q8WW59	0.08433713	0.08433713	0.000925
A.vasorum /Control	Q9HCG8	0.390933565	0.390933565	0.000925
A.vasorum /Control	Q9Y617	-0.006022051	0.006022051	0.000927
A.vasorum /Control	Q9H3S7	0.196453758	0.196453758	0.000933
A.vasorum /Control	Q8IUF8	-0.104554474	0.104554474	0.000936
A.vasorum /Control	Q6ZNB6	0.223236304	0.223236304	0.00094
A.vasorum /Control	Q9GZP4	0.295224651	0.295224651	0.00094
A.vasorum /Control	Q9HBL8	0.160191798	0.160191798	0.000939

A.vasorum /Control	P09497	0.744365399	0.744365399	0.000945
A.vasorum /Control	P38571	2.19482142	2.19482142	0.000947
A.vasorum /Control	O60763	0.083271598	0.083271598	0.00095
A.vasorum /Control	Q92613	0.173244357	0.173244357	0.00095
A.vasorum /Control	Q9C0H6	0.034438048	0.034438048	0.00095
A.vasorum /Control	O94875	0.150000716	0.150000716	0.000955
A.vasorum /Control	P30626	0.501622594	0.501622594	0.000957
A.vasorum /Control	P45954	0.20463849	0.20463849	0.000962
A.vasorum /Control	P13747	0.94947904	0.94947904	0.000965
A.vasorum /Control	P42892	0.256019393	0.256019393	0.000971
A.vasorum /Control	P34949	0.828574372	0.828574372	0.000975
A.vasorum /Control	Q96AB3	1.238133856	1.238133856	0.000975
A.vasorum /Control	O43149	0.043164265	0.043164265	0.000978
A.vasorum /Control	Q8NBF6	0.003957357	0.003957357	0.000979
A.vasorum /Control	Q9Y2S7	0.10807307	0.10807307	0.000977
A.vasorum /Control	P78347	0.175896561	0.175896561	0.000981
A.vasorum /Control	Q8IUI8	0.040653052	0.040653052	0.000986
A.vasorum /Control	Q96SL4	0.985688757	0.985688757	0.000986
A.vasorum /Control	Q9NWZ3	0.470141124	0.470141124	0.000986
A.vasorum /Control	P41227	-0.026350186	0.026350186	0.000987
A.vasorum /Control	O95251	0.574578614	0.574578614	0.000989
A.vasorum /Control	P36969	0.124217129	0.124217129	0.000993
A.vasorum /Control	P42356	0.230581889	0.230581889	0.000993
A.vasorum /Control	Q96HP0	0.232140198	0.232140198	0.000998
A.vasorum /Control	Q9H9E3	-0.036830269	0.036830269	0.000998
A.vasorum /Control	Q9UJZ1	-0.000321875	0.000321875	0.000998
A.vasorum /Control	Q9ULZ3	0.246896084	0.246896084	0.001002
A.vasorum /Control	P49755	0.080572363	0.080572363	0.001006
A.vasorum /Control	Q13371	0.072324408	0.072324408	0.001005
A.vasorum /Control	Q9BTZ2	0.083288579	0.083288579	0.001007
A.vasorum /Control	Q9H061	0.07937411	0.07937411	0.001006
A.vasorum /Control	O95747	-0.033148552	0.033148552	0.001011
A.vasorum /Control	P13598	0.01110374	0.01110374	0.00101
A.vasorum /Control	Q03519	0.188120636	0.188120636	0.001011
A.vasorum /Control	O94851	0.086358736	0.086358736	0.001014
A.vasorum /Control	Q6STE5	0.767161274	0.767161274	0.001016
A.vasorum /Control	Q9BUH6	0.18339548	0.18339548	0.001017
A.vasorum /Control	P28065	0.59402348	0.59402348	0.001018
A.vasorum /Control	Q9Y3E0	0.525574967	0.525574967	0.00102
A.vasorum /Control	Q12769	0.193265334	0.193265334	0.001024
A.vasorum /Control	Q9NVR2	0.514687122	0.514687122	0.001023
A.vasorum /Control	Q8TB96	0.780566729	0.780566729	0.00103
A.vasorum /Control	Q6P1A2	0.356721096	0.356721096	0.001033
A.vasorum /Control	P35244	0.695942431	0.695942431	0.001041
A.vasorum /Control	P40938	0.072228327	0.072228327	0.001041
A.vasorum /Control	Q96F63	0.813884977	0.813884977	0.001047
A.vasorum /Control	Q16836	0.249116898	0.249116898	0.001049
A.vasorum /Control	O43809	0.147930141	0.147930141	0.001051
A.vasorum /Control	P14868	0.113673126	0.113673126	0.001055
A.vasorum /Control	Q15035	0.180551592	0.180551592	0.001055

A.vasorum /Control	Q9H1A4	0.113678854	0.113678854	0.001053
A.vasorum /Control	Q9UBF2	0.278557239	0.278557239	0.001057
A.vasorum /Control	P11182	0.272283684	0.272283684	0.001063
A.vasorum /Control	Q16204	0.002117129	0.002117129	0.001063
A.vasorum /Control	Q8WWH5	-0.10602144	0.10602144	0.001061
A.vasorum /Control	Q9Y6G5	0.465375198	0.465375198	0.001063
A.vasorum /Control	P41212	0.401488238	0.401488238	0.001066
A.vasorum /Control	Q9HCG7	0.146098039	0.146098039	0.001067
A.vasorum /Control	Q96PE3	0.367011291	0.367011291	0.00107
A.vasorum /Control	Q9NX00	0.149538884	0.149538884	0.001071
A.vasorum /Control	P52788	0.02681536	0.02681536	0.001073
A.vasorum /Control	O60504	0.192837987	0.192837987	0.001076
A.vasorum /Control	P04920	-1.864279413	1.864279413	0.001081
A.vasorum /Control	P35914	0.340986237	0.340986237	0.001081
A.vasorum /Control	Q9Y296	0.119346732	0.119346732	0.00108
A.vasorum /Control	Q15154	-0.009856084	0.009856084	0.001086
A.vasorum /Control	Q6PEY1	1.243720057	1.243720057	0.001084
A.vasorum /Control	Q9Y478	0.464797975	0.464797975	0.001086
A.vasorum /Control	Q02318	0.79281479	0.79281479	0.001089
A.vasorum /Control	Q9Y5Q8	0.102572001	0.102572001	0.001097
A.vasorum /Control	P61803	0.329292876	0.329292876	0.001098
A.vasorum /Control	P82663	0.011803315	0.011803315	0.001101
A.vasorum /Control	O75489	0.363172057	0.363172057	0.001107
A.vasorum /Control	P86790	0.095978941	0.095978941	0.001107
A.vasorum /Control	Q96I59	0.056247378	0.056247378	0.001113
A.vasorum /Control	Q9NVG8	0.02043322	0.02043322	0.001114
A.vasorum /Control	Q6Y1H2	0.172503707	0.172503707	0.001121
A.vasorum /Control	P63151	0.136359747	0.136359747	0.001123
A.vasorum /Control	Q9HAB8	0.314303133	0.314303133	0.001124
A.vasorum /Control	O14874	0.095597584	0.095597584	0.001129
A.vasorum /Control	P19838	0.051396498	0.051396498	0.00113
A.vasorum /Control	Q01813	0.02159669	0.02159669	0.001131
A.vasorum /Control	Q8N983	-0.103191406	0.103191406	0.001133
A.vasorum /Control	Q96QZ7	0.477697023	0.477697023	0.00114
A.vasorum /Control	P53396	0.004192271	0.004192271	0.001147
A.vasorum /Control	P53618	0.040458715	0.040458715	0.001149
A.vasorum /Control	Q14739	0.254695267	0.254695267	0.00115
A.vasorum /Control	Q9GZM5	-0.003621614	0.003621614	0.00115
A.vasorum /Control	Q9NVP2	0.049237438	0.049237438	0.001149
A.vasorum /Control	Q9Y6W5	-0.003209054	0.003209054	0.001148
A.vasorum /Control	O00401	0.325920131	0.325920131	0.001153
A.vasorum /Control	P32321	0.193179017	0.193179017	0.001157
A.vasorum /Control	P54252	0.305390363	0.305390363	0.001155
A.vasorum /Control	Q8IZ83	0.244198988	0.244198988	0.001158
A.vasorum /Control	Q9UNI6	0.05733717	0.05733717	0.001157
A.vasorum /Control	Q8NEZ3	0.732375984	0.732375984	0.001169
A.vasorum /Control	P33316	0.029658212	0.029658212	0.001176
A.vasorum /Control	Q8NFH4	0.117422704	0.117422704	0.001179
A.vasorum /Control	P61077	0.207971889	0.207971889	0.001189
A.vasorum /Control	E9PRG8	0.395326488	0.395326488	0.00119

A.vasorum /Control	Q8WZA9	0.558638422	0.558638422	0.001196
A.vasorum /Control	P55809	0.138478426	0.138478426	0.001202
A.vasorum /Control	P78536	0.092830849	0.092830849	0.001212
A.vasorum /Control	Q5VZ89	0.235061114	0.235061114	0.001205
A.vasorum /Control	Q6ICL3	0.282728229	0.282728229	0.001213
A.vasorum /Control	Q6PI48	0.306575964	0.306575964	0.001208
A.vasorum /Control	Q8TBF2	0.845859363	0.845859363	0.001214
A.vasorum /Control	Q96KA5	0.387699929	0.387699929	0.001209
A.vasorum /Control	Q9H4Z3	0.535741091	0.535741091	0.001208
A.vasorum /Control	Q9H6S0	0.122603371	0.122603371	0.001207
A.vasorum /Control	Q9NRD5	0.694028637	0.694028637	0.001209
A.vasorum /Control	Q9UNW9	0.26396793	0.26396793	0.001212
A.vasorum /Control	O95071	0.013941853	0.013941853	0.001217
A.vasorum /Control	Q13464	0.14882141	0.14882141	0.001216
A.vasorum /Control	Q9BQE5	0.295233767	0.295233767	0.001217
A.vasorum /Control	P49792	0.027104201	0.027104201	0.001229
A.vasorum /Control	Q86X76	0.00570874	0.00570874	0.001229
A.vasorum /Control	Q9NS87	0.260617187	0.260617187	0.001229
A.vasorum /Control	P24941	0.066774551	0.066774551	0.001231
A.vasorum /Control	Q9NTJ4	0.240885619	0.240885619	0.001235
A.vasorum /Control	O94989	0.103269004	0.103269004	0.001242
A.vasorum /Control	O95260	0.10618091	0.10618091	0.001237
A.vasorum /Control	P50749	0.347282034	0.347282034	0.001243
A.vasorum /Control	P57678	-0.083298759	0.083298759	0.001242
A.vasorum /Control	P57740	0.026909285	0.026909285	0.001241
A.vasorum /Control	Q14974	0.051594349	0.051594349	0.001237
A.vasorum /Control	Q9NRF9	0.154456589	0.154456589	0.001242
A.vasorum /Control	Q8NCE2	0.826083401	0.826083401	0.001249
A.vasorum /Control	Q7Z3E2	0.310823768	0.310823768	0.001253
A.vasorum /Control	Q9BRG1	0.254640191	0.254640191	0.001255
A.vasorum /Control	O15381	0.05686387	0.05686387	0.001266
A.vasorum /Control	P10586	0.102646129	0.102646129	0.001272
A.vasorum /Control	P42167	0.151612068	0.151612068	0.001268
A.vasorum /Control	Q0VDF9	-0.045468573	0.045468573	0.001271
A.vasorum /Control	Q13619	0.179610614	0.179610614	0.001269
A.vasorum /Control	Q7RTS9	0.214420766	0.214420766	0.001265
A.vasorum /Control	Q86TM6	0.548493485	0.548493485	0.001265
A.vasorum /Control	Q86X83	0.202295147	0.202295147	0.001263
A.vasorum /Control	Q96D46	-0.018596846	0.018596846	0.001271
A.vasorum /Control	Q9H857	0.20617915	0.20617915	0.001273
A.vasorum /Control	Q9NRA2	0.26492928	0.26492928	0.001271
A.vasorum /Control	Q9UKG1	0.273232764	0.273232764	0.001269
A.vasorum /Control	P19404	0.811549504	0.811549504	0.001275
A.vasorum /Control	P00352	0.988693811	0.988693811	0.001277
A.vasorum /Control	Q8WXA9	0.073859362	0.073859362	0.001277
A.vasorum /Control	Q9NUW8	0.22947281	0.22947281	0.001278
A.vasorum /Control	Q9NR28	0.581212716	0.581212716	0.001282
A.vasorum /Control	Q9P2P5	0.132210901	0.132210901	0.001282
A.vasorum /Control	O15127	0.08434771	0.08434771	0.001283
A.vasorum /Control	P56545	0.098058571	0.098058571	0.001284

A.vasorum /Control	Q86Y56	0.221342749	0.221342749	0.001288
A.vasorum /Control	Q8NF37	0.169410378	0.169410378	0.001291
A.vasorum /Control	Q5VV42	0.314211733	0.314211733	0.001293
A.vasorum /Control	Q13444	0.223350744	0.223350744	0.001294
A.vasorum /Control	Q9NZJ9	0.250321647	0.250321647	0.001297
A.vasorum /Control	O75569	0.005109664	0.005109664	0.0013
A.vasorum /Control	Q08426	1.025514516	1.025514516	0.0013
A.vasorum /Control	Q5JTD0	-0.090999196	0.090999196	0.001302
A.vasorum /Control	O15235	-0.297462982	0.297462982	0.001305
A.vasorum /Control	P42858	0.388245912	0.388245912	0.001309
A.vasorum /Control	Q15398	-0.222392734	0.222392734	0.001317
A.vasorum /Control	Q68EM7	0.09860509	0.09860509	0.001317
A.vasorum /Control	Q9Y2X9	0.647759504	0.647759504	0.001316
A.vasorum /Control	P08236	0.86639946	0.86639946	0.001321
A.vasorum /Control	Q8TAG9	0.127143156	0.127143156	0.00132
A.vasorum /Control	Q92598	-0.298148769	0.298148769	0.00132
A.vasorum /Control	Q9H089	-0.100953246	0.100953246	0.001322
A.vasorum /Control	P52566	0.116651134	0.116651134	0.00133
A.vasorum /Control	Q09161	0.040543099	0.040543099	0.001328
A.vasorum /Control	Q32MZ4	0.03579945	0.03579945	0.00133
A.vasorum /Control	Q92545	0.252533918	0.252533918	0.001329
A.vasorum /Control	Q9Y2I8	0.343806838	0.343806838	0.001329
A.vasorum /Control	Q9BS26	0.008363192	0.008363192	0.001335
A.vasorum /Control	O15382	-0.090511369	0.090511369	0.001338
A.vasorum /Control	P55036	0.038625425	0.038625425	0.001338
A.vasorum /Control	Q08257	0.169969207	0.169969207	0.001346
A.vasorum /Control	Q8NBJ7	0.681349768	0.681349768	0.001344
A.vasorum /Control	Q8TAA9	0.250550392	0.250550392	0.001345
A.vasorum /Control	Q8WVV9	0.091549322	0.091549322	0.001347
A.vasorum /Control	Q96JB2	0.000296341	0.000296341	0.001346
A.vasorum /Control	Q9HD67	0.205460309	0.205460309	0.001345
A.vasorum /Control	O00139	0.114095335	0.114095335	0.001353
A.vasorum /Control	Q9BVC5	0.311981059	0.311981059	0.001356
A.vasorum /Control	Q9BTC8	0.04824566	0.04824566	0.00136
A.vasorum /Control	Q9NXF1	0.153419471	0.153419471	0.001362
A.vasorum /Control	P53621	-0.02669687	0.02669687	0.001365
A.vasorum /Control	Q96KP4	0.086904734	0.086904734	0.001368
A.vasorum /Control	P24539	0.08945544	0.08945544	0.001371
A.vasorum /Control	P49441	0.163386477	0.163386477	0.001374
A.vasorum /Control	O14617	0.039481227	0.039481227	0.001383
A.vasorum /Control	P11172	0.038070559	0.038070559	0.001384
A.vasorum /Control	P27824	0.062796121	0.062796121	0.001388
A.vasorum /Control	P49354	0.029272086	0.029272086	0.001389
A.vasorum /Control	P53384	0.35134667	0.35134667	0.00139
A.vasorum /Control	O14678	0.264296766	0.264296766	0.001404
A.vasorum /Control	O60443	0.121458218	0.121458218	0.001405
A.vasorum /Control	Q15369	0.377082048	0.377082048	0.001397
A.vasorum /Control	Q16513	0.099054679	0.099054679	0.001402
A.vasorum /Control	Q53GS7	0.133111443	0.133111443	0.001406
A.vasorum /Control	Q7Z7E8	0.35370609	0.35370609	0.001408

A.vasorum /Control	Q86TI2	0.07123339	0.07123339	0.001397
A.vasorum /Control	Q8IXK0	0.514626494	0.514626494	0.001399
A.vasorum /Control	Q96RQ1	1.087165017	1.087165017	0.001405
A.vasorum /Control	Q9BZ29	0.04839245	0.04839245	0.001407
A.vasorum /Control	Q9H269	0.211737766	0.211737766	0.001399
A.vasorum /Control	P29279	0.263977255	0.263977255	0.001412
A.vasorum /Control	P50750	0.095117757	0.095117757	0.001413
A.vasorum /Control	Q9H267	0.264237832	0.264237832	0.001411
A.vasorum /Control	Q9Y6N5	0.370966341	0.370966341	0.001418
A.vasorum /Control	Q8IY47	0.252600322	0.252600322	0.001427
A.vasorum /Control	Q6YP21	0.094679054	0.094679054	0.001429
A.vasorum /Control	Q96PZ2	0.37780401	0.37780401	0.001445
A.vasorum /Control	Q9Y375	0.400185915	0.400185915	0.001458
A.vasorum /Control	Q86U86	-0.034189803	0.034189803	0.001461
A.vasorum /Control	Q9BXB4	0.071753965	0.071753965	0.001467
A.vasorum /Control	P63000	-0.099527947	0.099527947	0.001468
A.vasorum /Control	Q9BTC0	0.129479684	0.129479684	0.001474
A.vasorum /Control	Q0VDG4	0.676537792	0.676537792	0.001476
A.vasorum /Control	P49961	2.004912717	2.004912717	0.001478
A.vasorum /Control	Q93008	0.162245472	0.162245472	0.001477
A.vasorum /Control	O75190	0.1979042	0.1979042	0.001481
A.vasorum /Control	Q8IV63	0.402731804	0.402731804	0.001486
A.vasorum /Control	Q96L91	0.080090153	0.080090153	0.001491
A.vasorum /Control	Q9UK59	0.164353318	0.164353318	0.001493
A.vasorum /Control	P34931	0.058997294	0.058997294	0.001498
A.vasorum /Control	O00186	0.109397951	0.109397951	0.001501
A.vasorum /Control	O95716	0.388564389	0.388564389	0.001502
A.vasorum /Control	Q9Y672	0.939704475	0.939704475	0.001502
A.vasorum /Control	P52292	-0.153169234	0.153169234	0.001505
A.vasorum /Control	Q13905	0.234541876	0.234541876	0.001506
A.vasorum /Control	Q96G23	-0.062137422	0.062137422	0.001508
A.vasorum /Control	A0AVT1	-0.066057421	0.066057421	0.001509
A.vasorum /Control	P61758	0.293098509	0.293098509	0.001511
A.vasorum /Control	Q96A65	0.124845134	0.124845134	0.001514
A.vasorum /Control	O75676	0.112480695	0.112480695	0.001524
A.vasorum /Control	P46977	0.046407539	0.046407539	0.001525
A.vasorum /Control	Q16576	0.1320844	0.1320844	0.001524
A.vasorum /Control	Q96CW1	0.061926629	0.061926629	0.001537
A.vasorum /Control	Q9P2N5	0.086171718	0.086171718	0.001536
A.vasorum /Control	P08069	0.13858734	0.13858734	0.001544
A.vasorum /Control	P13693	0.230161061	0.230161061	0.001544
A.vasorum /Control	Q2VPK5	0.148070548	0.148070548	0.001543
A.vasorum /Control	Q6P2E9	0.059375802	0.059375802	0.00154
A.vasorum /Control	Q9C0E2	0.130122146	0.130122146	0.001542
A.vasorum /Control	Q6UXH1	0.370819212	0.370819212	0.001551
A.vasorum /Control	Q8IYM9	0.223889623	0.223889623	0.001553
A.vasorum /Control	P04632	0.450945144	0.450945144	0.001565
A.vasorum /Control	Q5VUA4	0.303766477	0.303766477	0.001567
A.vasorum /Control	Q969G3	-0.152485329	0.152485329	0.001577
A.vasorum /Control	Q9UN37	0.038627992	0.038627992	0.00158

A.vasorum /Control	Q8IZ81	0.179402507	0.179402507	0.001583
A.vasorum /Control	P11177	0.052003161	0.052003161	0.001585
A.vasorum /Control	Q9H8Y8	0.205253497	0.205253497	0.001586
A.vasorum /Control	Q9NPH2	0.258253294	0.258253294	0.001592
A.vasorum /Control	Q12979	0.090068016	0.090068016	0.001599
A.vasorum /Control	Q13596	0.106215043	0.106215043	0.0016
A.vasorum /Control	O75368	0.275304261	0.275304261	0.001606
A.vasorum /Control	P67812	0.063120173	0.063120173	0.001607
A.vasorum /Control	Q13164	0.753475944	0.753475944	0.001604
A.vasorum /Control	Q9P0J0	0.233789798	0.233789798	0.001603
A.vasorum /Control	Q15293	0.207245821	0.207245821	0.001613
A.vasorum /Control	Q12872	0.205608984	0.205608984	0.001618
A.vasorum /Control	P78406	-0.024835799	0.024835799	0.00162
A.vasorum /Control	Q6NZY4	0.045531557	0.045531557	0.001624
A.vasorum /Control	Q8WUA2	0.011080527	0.011080527	0.001623
A.vasorum /Control	Q96CM8	0.820285816	0.820285816	0.001624
A.vasorum /Control	Q8N1B4	0.055230167	0.055230167	0.00163
A.vasorum /Control	Q8N4T8	0.788680989	0.788680989	0.00163
A.vasorum /Control	Q96BH1	0.279497259	0.279497259	0.001628
A.vasorum /Control	Q96EP5	-0.299203455	0.299203455	0.001626
A.vasorum /Control	Q9ULA0	0.226077591	0.226077591	0.001635
A.vasorum /Control	Q99653	0.350421785	0.350421785	0.001638
A.vasorum /Control	Q9BQ95	0.173098377	0.173098377	0.001642
A.vasorum /Control	Q9HD33	0.076696764	0.076696764	0.001642
A.vasorum /Control	O75477	0.16440144	0.16440144	0.001656
A.vasorum /Control	P15374	0.300287599	0.300287599	0.001653
A.vasorum /Control	P28331	0.313962507	0.313962507	0.001657
A.vasorum /Control	P46109	-0.118013679	0.118013679	0.001656
A.vasorum /Control	Q92530	0.067109136	0.067109136	0.001663
A.vasorum /Control	P61020	0.09831949	0.09831949	0.001665
A.vasorum /Control	P08865	0.111410321	0.111410321	0.001671
A.vasorum /Control	P36404	0.342897368	0.342897368	0.001682
A.vasorum /Control	P46060	-0.261431903	0.261431903	0.001684
A.vasorum /Control	Q32P44	0.259063539	0.259063539	0.001686
A.vasorum /Control	P08237	0.102619959	0.102619959	0.001693
A.vasorum /Control	P51784	0.549736747	0.549736747	0.001691
A.vasorum /Control	P61006	0.00332932	0.00332932	0.001692
A.vasorum /Control	Q969M3	-0.151302596	0.151302596	0.001701
A.vasorum /Control	Q96L58	0.310857192	0.310857192	0.001707
A.vasorum /Control	Q2TAL8	0.150329939	0.150329939	0.001723
A.vasorum /Control	Q69YN4	0.016021592	0.016021592	0.001723
A.vasorum /Control	Q92508	-0.025200122	0.025200122	0.001723
A.vasorum /Control	O75695	0.002054474	0.002054474	0.001727
A.vasorum /Control	P49590	-0.004329113	0.004329113	0.00174
A.vasorum /Control	P49588	0.07375656	0.07375656	0.001743
A.vasorum /Control	O00499	0.028332981	0.028332981	0.00175
A.vasorum /Control	Q96GG9	0.060570694	0.060570694	0.00175
A.vasorum /Control	O14818	-0.027371085	0.027371085	0.001753
A.vasorum /Control	Q6VN20	0.209134483	0.209134483	0.001766
A.vasorum /Control	P26440	0.444485217	0.444485217	0.001772

A.vasorum /Control	P62328	0.536060116	0.536060116	0.001774
A.vasorum /Control	P42898	0.614220075	0.614220075	0.001783
A.vasorum /Control	P02545	-0.301774583	0.301774583	0.001787
A.vasorum /Control	Q02978	0.258399357	0.258399357	0.001792
A.vasorum /Control	Q9NV70	0.100798423	0.100798423	0.0018
A.vasorum /Control	Q8TCU6	0.497508559	0.497508559	0.001806
A.vasorum /Control	Q9Y3A2	-1.478827128	1.478827128	0.001807
A.vasorum /Control	Q92688	0.160252799	0.160252799	0.001813
A.vasorum /Control	Q10567	-0.040862438	0.040862438	0.001815
A.vasorum /Control	Q13825	0.174364347	0.174364347	0.001817
A.vasorum /Control	O14974	0.083503992	0.083503992	0.001826
A.vasorum /Control	P28799	0.134387549	0.134387549	0.001825
A.vasorum /Control	Q9H4I3	-0.058843831	0.058843831	0.001827
A.vasorum /Control	Q96JQ0	1.067299584	1.067299584	0.00183
A.vasorum /Control	Q6YHK3	0.418583743	0.418583743	0.001832
A.vasorum /Control	Q9GZQ3	0.19378391	0.19378391	0.001839
A.vasorum /Control	Q99805	0.056249554	0.056249554	0.001842
A.vasorum /Control	P25705	0.102068061	0.102068061	0.001845
A.vasorum /Control	Q8IUR7	0.130996384	0.130996384	0.001852
A.vasorum /Control	O14929	0.279983934	0.279983934	0.001857
A.vasorum /Control	P48651	0.182594207	0.182594207	0.001857
A.vasorum /Control	Q562E7	0.479160139	0.479160139	0.001855
A.vasorum /Control	Q9H1H9	0.40061717	0.40061717	0.001858
A.vasorum /Control	P28370	0.182041329	0.182041329	0.001864
A.vasorum /Control	P43304	0.220656374	0.220656374	0.001863
A.vasorum /Control	O15264	1.213388608	1.213388608	0.001868
A.vasorum /Control	Q8NBJ5	0.05169215	0.05169215	0.001868
A.vasorum /Control	P36959	0.184960619	0.184960619	0.00187
A.vasorum /Control	Q6ZMP0	0.255066262	0.255066262	0.001878
A.vasorum /Control	Q9Y4G8	0.333894709	0.333894709	0.001878
A.vasorum /Control	P27361	0.187844276	0.187844276	0.001885
A.vasorum /Control	P30519	0.080989321	0.080989321	0.001887
A.vasorum /Control	P62241	-0.130229343	0.130229343	0.001884
A.vasorum /Control	Q7Z401	0.470116471	0.470116471	0.00189
A.vasorum /Control	Q96RT1	0.272896773	0.272896773	0.00189
A.vasorum /Control	Q9UL26	0.083002768	0.083002768	0.00189
A.vasorum /Control	O76031	-0.157622503	0.157622503	0.001897
A.vasorum /Control	P61224	0.136751947	0.136751947	0.001896
A.vasorum /Control	P78330	-0.020474839	0.020474839	0.001897
A.vasorum /Control	Q9NVN3	0.763481674	0.763481674	0.001895
A.vasorum /Control	Q9Y263	0.024319356	0.024319356	0.0019
A.vasorum /Control	P22307	0.108602153	0.108602153	0.001905
A.vasorum /Control	P53004	0.078262084	0.078262084	0.001908
A.vasorum /Control	P49916	0.179154767	0.179154767	0.001913
A.vasorum /Control	O00471	0.183146067	0.183146067	0.001926
A.vasorum /Control	O00483	0.372514641	0.372514641	0.001925
A.vasorum /Control	P84098	0.379456331	0.379456331	0.001927
A.vasorum /Control	Q9NYP7	-0.136021843	0.136021843	0.001936
A.vasorum /Control	Q8N8S7	-0.059632536	0.059632536	0.001944
A.vasorum /Control	Q00403	0.163722699	0.163722699	0.001946

A.vasorum /Control	P19387	0.288472259	0.288472259	0.00195
A.vasorum /Control	O00743	-0.030326753	0.030326753	0.001956
A.vasorum /Control	A2RRP1	0.11977819	0.11977819	0.001961
A.vasorum /Control	Q14980	0.096341415	0.096341415	0.001972
A.vasorum /Control	O75937	0.147553975	0.147553975	0.001974
A.vasorum /Control	Q66LE6	0.065547032	0.065547032	0.001979
A.vasorum /Control	Q99615	-0.07266325	0.07266325	0.001979
A.vasorum /Control	P49642	-0.086649442	0.086649442	0.001982
A.vasorum /Control	Q8TD19	0.167231164	0.167231164	0.001983
A.vasorum /Control	Q8N7H5	0.094509386	0.094509386	0.00199
A.vasorum /Control	P29144	0.000456905	0.000456905	0.001994
A.vasorum /Control	P31939	0.086190882	0.086190882	0.001996
A.vasorum /Control	Q6ZSR9	0.040677922	0.040677922	0.001996
A.vasorum /Control	Q9Y3X0	0.336863484	0.336863484	0.001993
A.vasorum /Control	P15151	-0.371561429	0.371561429	0.002004
A.vasorum /Control	Q96DM3	0.190077182	0.190077182	0.002002
A.vasorum /Control	Q9UI26	-0.153624901	0.153624901	0.002004
A.vasorum /Control	P53041	0.019996772	0.019996772	0.002015
A.vasorum /Control	Q9UNM6	-0.010219546	0.010219546	0.002018
A.vasorum /Control	O43592	0.080535706	0.080535706	0.002022
A.vasorum /Control	Q05519	0.152489113	0.152489113	0.002025
A.vasorum /Control	Q96CG8	0.578297206	0.578297206	0.002034
A.vasorum /Control	Q9NPJ3	0.023920621	0.023920621	0.002036
A.vasorum /Control	Q8NBQ5	0.349525846	0.349525846	0.002038
A.vasorum /Control	O76094	-0.023483076	0.023483076	0.002046
A.vasorum /Control	P23467	0.02834701	0.02834701	0.002045
A.vasorum /Control	P48454	0.540618765	0.540618765	0.002045
A.vasorum /Control	P55196	0.32587541	0.32587541	0.002044
A.vasorum /Control	P78549	1.38751999	1.38751999	0.002041
A.vasorum /Control	P46459	0.049410813	0.049410813	0.002053
A.vasorum /Control	Q15061	-1.053393923	1.053393923	0.002053
A.vasorum /Control	Q4KMP7	0.307989825	0.307989825	0.002052
A.vasorum /Control	Q8WUZ0	0.71789476	0.71789476	0.002049
A.vasorum /Control	O43172	-0.1459861	0.1459861	0.002056
A.vasorum /Control	Q14258	0.233256065	0.233256065	0.002062
A.vasorum /Control	Q9P0J1	0.205906891	0.205906891	0.002064
A.vasorum /Control	P40222	-0.060432495	0.060432495	0.002067
A.vasorum /Control	P04179	0.813102087	0.813102087	0.002078
A.vasorum /Control	P17174	0.238198453	0.238198453	0.002076
A.vasorum /Control	Q9BWE0	-0.118965218	0.118965218	0.002077
A.vasorum /Control	Q8WXA3	0.313341309	0.313341309	0.002086
A.vasorum /Control	O75935	0.009238638	0.009238638	0.002095
A.vasorum /Control	P30084	0.105787877	0.105787877	0.002097
A.vasorum /Control	Q9H4L4	0.122482368	0.122482368	0.002097
A.vasorum /Control	Q8N3U4	-0.034149153	0.034149153	0.002102
A.vasorum /Control	Q14C86	-0.06580464	0.06580464	0.002109
A.vasorum /Control	Q9HB63	1.005067109	1.005067109	0.002116
A.vasorum /Control	P50583	-0.066827732	0.066827732	0.002138
A.vasorum /Control	Q7L014	0.071952802	0.071952802	0.002141
A.vasorum /Control	Q96P16	0.254688759	0.254688759	0.002148

A.vasorum /Control	O60313	0.073619986	0.073619986	0.002154
A.vasorum /Control	P61604	0.189706581	0.189706581	0.002155
A.vasorum /Control	Q13356	0.003591346	0.003591346	0.002155
A.vasorum /Control	Q9UHD2	-0.016417875	0.016417875	0.002156
A.vasorum /Control	Q9UGJ0	0.336761735	0.336761735	0.002158
A.vasorum /Control	P60033	-0.678550453	0.678550453	0.002165
A.vasorum /Control	P53680	0.11701013	0.11701013	0.002168
A.vasorum /Control	Q9BQE4	-0.335957688	0.335957688	0.002173
A.vasorum /Control	Q96Q42	0.335135034	0.335135034	0.00218
A.vasorum /Control	Q8N9M1	-0.110036468	0.110036468	0.002188
A.vasorum /Control	Q04917	0.089988947	0.089988947	0.002194
A.vasorum /Control	Q8WVM8	0.073822274	0.073822274	0.002195
A.vasorum /Control	Q9H1E5	0.657457123	0.657457123	0.002193
A.vasorum /Control	Q8NCW5	0.527454476	0.527454476	0.002203
A.vasorum /Control	Q86VP6	0.138655969	0.138655969	0.002208
A.vasorum /Control	Q8NI08	0.366236328	0.366236328	0.002209
A.vasorum /Control	Q969T4	0.177511426	0.177511426	0.002207
A.vasorum /Control	Q13496	0.169102819	0.169102819	0.002224
A.vasorum /Control	P78362	0.272661769	0.272661769	0.002237
A.vasorum /Control	P82673	0.177070204	0.177070204	0.002234
A.vasorum /Control	Q15003	-0.058450732	0.058450732	0.002235
A.vasorum /Control	O15234	0.156197485	0.156197485	0.00225
A.vasorum /Control	O43396	0.00914548	0.00914548	0.002246
A.vasorum /Control	Q14966	0.035761499	0.035761499	0.002248
A.vasorum /Control	Q9NRW7	0.174501342	0.174501342	0.00225
A.vasorum /Control	Q6PD74	0.233954965	0.233954965	0.002253
A.vasorum /Control	Q8WTW3	0.04717034	0.04717034	0.002255
A.vasorum /Control	Q9UEU0	0.248132432	0.248132432	0.002264
A.vasorum /Control	P05423	0.284709408	0.284709408	0.002269
A.vasorum /Control	O94760	0.091406305	0.091406305	0.002272
A.vasorum /Control	P29372	0.090019832	0.090019832	0.002275
A.vasorum /Control	O75449	0.068186641	0.068186641	0.002281
A.vasorum /Control	P29218	0.106400855	0.106400855	0.002286
A.vasorum /Control	Q03468	0.220798197	0.220798197	0.002283
A.vasorum /Control	Q8TC07	0.182090588	0.182090588	0.002286
A.vasorum /Control	Q9Y4D7	0.229803431	0.229803431	0.002282
A.vasorum /Control	P49815	-0.023036422	0.023036422	0.002288
A.vasorum /Control	Q00059	0.086975114	0.086975114	0.00229
A.vasorum /Control	Q92905	0.104293185	0.104293185	0.002292
A.vasorum /Control	O43847	-0.023439454	0.023439454	0.002297
A.vasorum /Control	Q32P28	0.105224296	0.105224296	0.002294
A.vasorum /Control	Q92665	-0.054862439	0.054862439	0.002296
A.vasorum /Control	O96000	0.076966796	0.076966796	0.002311
A.vasorum /Control	Q9NPY3	0.163122679	0.163122679	0.002312
A.vasorum /Control	O95571	0.152535417	0.152535417	0.002321
A.vasorum /Control	O60568	0.12094432	0.12094432	0.002335
A.vasorum /Control	Q8IYQ7	0.030332556	0.030332556	0.002335
A.vasorum /Control	O15126	-0.047912782	0.047912782	0.002349
A.vasorum /Control	P42765	0.186114793	0.186114793	0.002345
A.vasorum /Control	Q9H173	0.048888169	0.048888169	0.002347

A.vasorum /Control	Q9UMS4	0.031497445	0.031497445	0.002347
A.vasorum /Control	Q53FA7	0.054376918	0.054376918	0.002355
A.vasorum /Control	Q9H7Z7	-0.093446174	0.093446174	0.002357
A.vasorum /Control	Q9NTK5	-0.030904515	0.030904515	0.002358
A.vasorum /Control	P20337	-0.556968191	0.556968191	0.002367
A.vasorum /Control	P35249	0.118062323	0.118062323	0.002366
A.vasorum /Control	Q14691	0.083929155	0.083929155	0.002366
A.vasorum /Control	Q6NXT6	-0.816321512	0.816321512	0.002361
A.vasorum /Control	Q9UHL4	0.63989633	0.63989633	0.002364
A.vasorum /Control	O14798	0.937480023	0.937480023	0.002372
A.vasorum /Control	P18433	0.480844277	0.480844277	0.002385
A.vasorum /Control	P18858	0.063554633	0.063554633	0.002389
A.vasorum /Control	Q93034	0.100277056	0.100277056	0.00239
A.vasorum /Control	Q96AG4	-0.187932959	0.187932959	0.002387
A.vasorum /Control	Q9NUI1	0.032791651	0.032791651	0.00239
A.vasorum /Control	P42224	0.34014949	0.34014949	0.002395
A.vasorum /Control	P31641	-0.951825148	0.951825148	0.002411
A.vasorum /Control	Q9BRP4	-0.032203575	0.032203575	0.002414
A.vasorum /Control	Q9Y6M7	0.357515189	0.357515189	0.002418
A.vasorum /Control	Q2PPJ7	0.321676181	0.321676181	0.002426
A.vasorum /Control	P09972	0.306424052	0.306424052	0.002429
A.vasorum /Control	Q16512	0.0488982	0.0488982	0.002432
A.vasorum /Control	P10253	0.754105463	0.754105463	0.002441
A.vasorum /Control	Q12873	0.223277454	0.223277454	0.002441
A.vasorum /Control	Q8N8J7	0.170946954	0.170946954	0.002439
A.vasorum /Control	O43324	0.168775979	0.168775979	0.002456
A.vasorum /Control	Q96SB4	0.087827732	0.087827732	0.00246
A.vasorum /Control	Q96DB5	0.066369759	0.066369759	0.002462
A.vasorum /Control	Q8ND76	0.161826524	0.161826524	0.00247
A.vasorum /Control	Q9BT40	0.467911924	0.467911924	0.002469
A.vasorum /Control	Q9BZQ8	0.751854738	0.751854738	0.002468
A.vasorum /Control	P46531	0.229699771	0.229699771	0.002474
A.vasorum /Control	Q7Z589	0.2739543	0.2739543	0.002477
A.vasorum /Control	Q02218	0.209268132	0.209268132	0.002479
A.vasorum /Control	Q6AZY7	2.589952992	2.589952992	0.002484
A.vasorum /Control	O43837	0.263509326	0.263509326	0.002486
A.vasorum /Control	Q6ZNL6	0.383384566	0.383384566	0.002489
A.vasorum /Control	Q8NB16	0.024475574	0.024475574	0.00249
A.vasorum /Control	Q15750	-0.03356798	0.03356798	0.002496
A.vasorum /Control	Q9H792	-0.007852199	0.007852199	0.002498
A.vasorum /Control	Q9Y5K5	-0.02452455	0.02452455	0.002508
A.vasorum /Control	O15040	0.650316926	0.650316926	0.00251
A.vasorum /Control	P23368	0.076737203	0.076737203	0.002516
A.vasorum /Control	Q9Y237	0.229295864	0.229295864	0.002515
A.vasorum /Control	Q8WUJ0	3.040244068	3.040244068	0.002528
A.vasorum /Control	O94832	0.266555391	0.266555391	0.002534
A.vasorum /Control	P62191	0.001994959	0.001994959	0.002533
A.vasorum /Control	O14786	0.146693906	0.146693906	0.002543
A.vasorum /Control	P18887	0.327203797	0.327203797	0.002545
A.vasorum /Control	Q8IZ21	0.36842712	0.36842712	0.002542

A.vasorum /Control	Q9Y4A5	0.057460607	0.057460607	0.002544
A.vasorum /Control	Q9Y6K5	0.706643179	0.706643179	0.002548
A.vasorum /Control	Q9Y5B0	0.123031953	0.123031953	0.002551
A.vasorum /Control	Q969N2	0.099284776	0.099284776	0.002558
A.vasorum /Control	Q8IXQ6	1.148299494	1.148299494	0.002561
A.vasorum /Control	Q8WUY8	0.141534414	0.141534414	0.002561
A.vasorum /Control	P78539	0.744800815	0.744800815	0.002565
A.vasorum /Control	Q9UBT2	-0.038005775	0.038005775	0.002572
A.vasorum /Control	Q9NXR7	0.593444047	0.593444047	0.002576
A.vasorum /Control	Q14139	0.028881325	0.028881325	0.002583
A.vasorum /Control	Q16222	-0.3036289	0.3036289	0.002582
A.vasorum /Control	Q49A26	0.131978089	0.131978089	0.002584
A.vasorum /Control	Q9UKK9	0.299821677	0.299821677	0.002586
A.vasorum /Control	O75116	0.121434229	0.121434229	0.00259
A.vasorum /Control	Q99685	0.100405086	0.100405086	0.002591
A.vasorum /Control	Q9BX67	0.027055562	0.027055562	0.002592
A.vasorum /Control	P50502	0.104667373	0.104667373	0.002598
A.vasorum /Control	Q9H4A5	0.114753881	0.114753881	0.002596
A.vasorum /Control	Q9UBB9	-0.039902026	0.039902026	0.002598
A.vasorum /Control	O95248	-0.044362683	0.044362683	0.002607
A.vasorum /Control	P61513	-0.526039075	0.526039075	0.002605
A.vasorum /Control	Q15276	0.1879979	0.1879979	0.002605
A.vasorum /Control	P27986	0.091468382	0.091468382	0.002611
A.vasorum /Control	Q8IWA4	0.116863865	0.116863865	0.002616
A.vasorum /Control	P33992	0.050152066	0.050152066	0.002623
A.vasorum /Control	Q8IWJ2	0.043861626	0.043861626	0.002631
A.vasorum /Control	Q9H6Q4	0.666595936	0.666595936	0.00263
A.vasorum /Control	Q9NPQ8	-0.006679047	0.006679047	0.002641
A.vasorum /Control	Q14160	0.006204136	0.006204136	0.00265
A.vasorum /Control	Q16658	0.119563135	0.119563135	0.00265
A.vasorum /Control	Q9BW27	-0.020680657	0.020680657	0.002651
A.vasorum /Control	Q9Y3C0	0.374341323	0.374341323	0.002652
A.vasorum /Control	O95163	0.151032666	0.151032666	0.002661
A.vasorum /Control	Q96ME7	0.145232715	0.145232715	0.002661
A.vasorum /Control	Q9NUL3	0.112732428	0.112732428	0.00266
A.vasorum /Control	Q9BR76	0.070226503	0.070226503	0.002663
A.vasorum /Control	P07437	1.051267768	1.051267768	0.002676
A.vasorum /Control	Q14247	0.105936659	0.105936659	0.002675
A.vasorum /Control	P24390	0.287468735	0.287468735	0.00268
A.vasorum /Control	P08493	0.93377374	0.93377374	0.002688
A.vasorum /Control	Q13217	0.06133214	0.06133214	0.002693
A.vasorum /Control	Q8WXX5	-0.024713548	0.024713548	0.002692
A.vasorum /Control	Q15008	0.030754784	0.030754784	0.002721
A.vasorum /Control	Q8N5I4	-0.062927632	0.062927632	0.002726
A.vasorum /Control	Q96RK0	-0.937527552	0.937527552	0.002731
A.vasorum /Control	Q9H3K6	0.202421187	0.202421187	0.002732
A.vasorum /Control	O75976	0.264810545	0.264810545	0.002739
A.vasorum /Control	Q8IZQ5	0.262654243	0.262654243	0.002737
A.vasorum /Control	Q9H2U2	0.116717761	0.116717761	0.002739
A.vasorum /Control	O14920	0.120391421	0.120391421	0.002762

A.vasorum /Control	Q13445	0.317661408	0.317661408	0.002762
A.vasorum /Control	P02794	1.832206577	1.832206577	0.002776
A.vasorum /Control	Q9UNK0	0.893891386	0.893891386	0.002792
A.vasorum /Control	P06703	0.163562011	0.163562011	0.002797
A.vasorum /Control	P31946	0.076490073	0.076490073	0.002797
A.vasorum /Control	P49748	0.452650344	0.452650344	0.002819
A.vasorum /Control	Q8IXM2	0.276217993	0.276217993	0.002827
A.vasorum /Control	Q96F44	0.313537668	0.313537668	0.00284
A.vasorum /Control	Q96QK1	0.031369313	0.031369313	0.00284
A.vasorum /Control	Q9H9B1	-0.034415285	0.034415285	0.002837
A.vasorum /Control	Q6VY07	0.176454286	0.176454286	0.002843
A.vasorum /Control	Q8WUA7	0.114747036	0.114747036	0.002856
A.vasorum /Control	Q8N138	0.285363891	0.285363891	0.002861
A.vasorum /Control	O14907	0.213563637	0.213563637	0.002867
A.vasorum /Control	Q5VZE5	0.099573115	0.099573115	0.002871
A.vasorum /Control	O14908	-0.046030796	0.046030796	0.002884
A.vasorum /Control	Q96MM6	0.439124438	0.439124438	0.002885
A.vasorum /Control	Q9UFC0	0.041818085	0.041818085	0.002881
A.vasorum /Control	Q6ZT07	1.597494877	1.597494877	0.002896
A.vasorum /Control	P16615	-0.028709405	0.028709405	0.002906
A.vasorum /Control	Q14166	0.116017445	0.116017445	0.002905
A.vasorum /Control	Q96T88	-0.522491092	0.522491092	0.002901
A.vasorum /Control	Q9Y4P8	0.173207257	0.173207257	0.002903
A.vasorum /Control	O60256	0.109730075	0.109730075	0.002916
A.vasorum /Control	Q86UV5	0.250308891	0.250308891	0.002914
A.vasorum /Control	Q02224	-0.962122054	0.962122054	0.002933
A.vasorum /Control	O43516	-0.192470018	0.192470018	0.002943
A.vasorum /Control	P08243	-0.227956134	0.227956134	0.002946
A.vasorum /Control	Q14558	0.16537429	0.16537429	0.002949
A.vasorum /Control	Q14669	0.047433938	0.047433938	0.002949
A.vasorum /Control	Q5T1M5	-0.131360234	0.131360234	0.002948
A.vasorum /Control	Q5T5U3	0.18604267	0.18604267	0.00295
A.vasorum /Control	P10619	0.32116986	0.32116986	0.002955
A.vasorum /Control	Q99720	0.092758981	0.092758981	0.002959
A.vasorum /Control	Q9NZ43	0.160019652	0.160019652	0.00296
A.vasorum /Control	Q92696	-0.029484085	0.029484085	0.002978
A.vasorum /Control	P17706	0.019958712	0.019958712	0.00298
A.vasorum /Control	Q9BXF6	0.264395994	0.264395994	0.002984
A.vasorum /Control	O75940	-0.00580414	0.00580414	0.002988
A.vasorum /Control	Q14562	-0.114623026	0.114623026	0.002991
A.vasorum /Control	Q9H944	0.210478332	0.210478332	0.002989
A.vasorum /Control	Q9NRY6	0.380435192	0.380435192	0.002994
A.vasorum /Control	P42025	0.122896815	0.122896815	0.003012
A.vasorum /Control	Q92835	0.043222321	0.043222321	0.003021
A.vasorum /Control	Q6ZMI0	0.347016935	0.347016935	0.003029
A.vasorum /Control	Q96S44	0.076685941	0.076685941	0.003029
A.vasorum /Control	P25325	0.269063489	0.269063489	0.003035
A.vasorum /Control	Q8NCN5	0.340845229	0.340845229	0.003046
A.vasorum /Control	Q9UBS4	0.059509618	0.059509618	0.003045
A.vasorum /Control	P24666	-0.130732597	0.130732597	0.00305

A.vasorum /Control	Q9Y4L1	0.089455525	0.089455525	0.00305
A.vasorum /Control	Q6N069	0.006031351	0.006031351	0.003063
A.vasorum /Control	Q9UL03	0.24441143	0.24441143	0.003065
A.vasorum /Control	O96011	0.132303808	0.132303808	0.003072
A.vasorum /Control	Q96T60	0.130655698	0.130655698	0.003075
A.vasorum /Control	Q96PK6	0.055865883	0.055865883	0.003077
A.vasorum /Control	O14656	0.089595674	0.089595674	0.003085
A.vasorum /Control	Q9UI09	0.731921163	0.731921163	0.00309
A.vasorum /Control	Q7LBR1	0.316657966	0.316657966	0.003098
A.vasorum /Control	O94855	0.055033137	0.055033137	0.003105
A.vasorum /Control	Q7Z569	0.168577931	0.168577931	0.003104
A.vasorum /Control	O15014	0.063636387	0.063636387	0.003109
A.vasorum /Control	Q9ULT8	-0.050978177	0.050978177	0.003108
A.vasorum /Control	Q9UBM7	-0.046297402	0.046297402	0.003116
A.vasorum /Control	P18440	0.282934807	0.282934807	0.003123
A.vasorum /Control	O14972	0.443338455	0.443338455	0.003127
A.vasorum /Control	Q93045	0.385757808	0.385757808	0.003134
A.vasorum /Control	Q6IA86	0.092006058	0.092006058	0.003147
A.vasorum /Control	O75607	3.188403305	3.188403305	0.003152
A.vasorum /Control	Q96I99	0.074833067	0.074833067	0.003151
A.vasorum /Control	Q02818	0.428249626	0.428249626	0.003155
A.vasorum /Control	Q8TCS8	-0.068859589	0.068859589	0.003175
A.vasorum /Control	P53677	0.894380821	0.894380821	0.003187
A.vasorum /Control	Q9H0U4	0.037268016	0.037268016	0.003189
A.vasorum /Control	P62873	-0.535411607	0.535411607	0.003193
A.vasorum /Control	Q9BU23	0.0747968	0.0747968	0.003226
A.vasorum /Control	Q96RQ3	0.328726786	0.328726786	0.003231
A.vasorum /Control	Q9UBS8	-0.159326885	0.159326885	0.003233
A.vasorum /Control	Q07065	0.044142543	0.044142543	0.003241
A.vasorum /Control	Q86TU7	5.25066E-05	5.25E-05	0.003238
A.vasorum /Control	Q9UDX5	0.070827877	0.070827877	0.003239
A.vasorum /Control	Q9BU76	0.624089681	0.624089681	0.003249
A.vasorum /Control	Q9UBE0	-0.052201723	0.052201723	0.00325
A.vasorum /Control	P19338	0.0835681	0.0835681	0.003261
A.vasorum /Control	P62081	0.436792514	0.436792514	0.00326
A.vasorum /Control	Q15746	0.436617744	0.436617744	0.003259
A.vasorum /Control	Q6YHU6	0.08854844	0.08854844	0.003254
A.vasorum /Control	Q9UNN8	-0.025337583	0.025337583	0.003261
A.vasorum /Control	Q9HB90	0.217341001	0.217341001	0.003268
A.vasorum /Control	O75822	-0.092264719	0.092264719	0.003283
A.vasorum /Control	Q14203	0.025968534	0.025968534	0.003285
A.vasorum /Control	P01116	-0.03699904	0.03699904	0.00329
A.vasorum /Control	Q96LW7	0.306726379	0.306726379	0.003289
A.vasorum /Control	P04843	-0.002975055	0.002975055	0.003299
A.vasorum /Control	P49721	0.121031231	0.121031231	0.003304
A.vasorum /Control	Q9BV44	0.157742939	0.157742939	0.003307
A.vasorum /Control	Q9BUT1	0.216054334	0.216054334	0.003315
A.vasorum /Control	P11234	0.380951975	0.380951975	0.003318
A.vasorum /Control	Q92990	0.059851757	0.059851757	0.003327
A.vasorum /Control	Q92878	0.228871667	0.228871667	0.003334

A.vasorum /Control	P31040	0.340865347	0.340865347	0.003338
A.vasorum /Control	P46940	-0.013523973	0.013523973	0.00334
A.vasorum /Control	Q99973	0.155190672	0.155190672	0.003341
A.vasorum /Control	P51648	0.177014211	0.177014211	0.003349
A.vasorum /Control	Q8WUI4	0.218101547	0.218101547	0.003348
A.vasorum /Control	Q7Z7F7	0.019754142	0.019754142	0.003357
A.vasorum /Control	Q99541	0.384845261	0.384845261	0.003356
A.vasorum /Control	Q01543	0.826482791	0.826482791	0.003361
A.vasorum /Control	P43250	0.617697213	0.617697213	0.003366
A.vasorum /Control	Q13232	0.157466621	0.157466621	0.003365
A.vasorum /Control	Q15257	0.166451234	0.166451234	0.003379
A.vasorum /Control	Q96EY4	0.128928864	0.128928864	0.00338
A.vasorum /Control	Q9H6T3	0.090692385	0.090692385	0.003395
A.vasorum /Control	O75582	0.6699792	0.6699792	0.003401
A.vasorum /Control	Q9BUL9	0.300353515	0.300353515	0.003401
A.vasorum /Control	O00443	0.219466011	0.219466011	0.003408
A.vasorum /Control	Q10713	-0.126987145	0.126987145	0.003411
A.vasorum /Control	O75150	0.066580368	0.066580368	0.003423
A.vasorum /Control	P30044	0.273741629	0.273741629	0.003416
A.vasorum /Control	P50548	0.178143607	0.178143607	0.003423
A.vasorum /Control	Q9NP81	0.098781367	0.098781367	0.003423
A.vasorum /Control	Q9Y2W1	0.083435591	0.083435591	0.003421
A.vasorum /Control	Q15814	-0.032591796	0.032591796	0.003436
A.vasorum /Control	Q9BYG3	-1.029759	1.029759	0.003435
A.vasorum /Control	Q5VW32	0.231566675	0.231566675	0.003439
A.vasorum /Control	P54802	0.645786298	0.645786298	0.003442
A.vasorum /Control	P33981	-0.849689488	0.849689488	0.003454
A.vasorum /Control	Q9HD42	0.114896903	0.114896903	0.003452
A.vasorum /Control	Q9UNF1	0.110635377	0.110635377	0.003462
A.vasorum /Control	P52564	0.353891913	0.353891913	0.003465
A.vasorum /Control	P19022	-0.160297619	0.160297619	0.003476
A.vasorum /Control	Q13043	0.412212088	0.412212088	0.003475
A.vasorum /Control	Q86V21	0.027630178	0.027630178	0.003475
A.vasorum /Control	Q9HA64	0.25210665	0.25210665	0.003473
A.vasorum /Control	O43896	0.09772096	0.09772096	0.00348
A.vasorum /Control	Q5NDL2	0.041746474	0.041746474	0.003494
A.vasorum /Control	P57772	0.294290713	0.294290713	0.003502
A.vasorum /Control	Q9BRT9	1.137572016	1.137572016	0.003502
A.vasorum /Control	Q9BW71	0.919074018	0.919074018	0.00351
A.vasorum /Control	Q5JPH6	-0.395294589	0.395294589	0.003513
A.vasorum /Control	Q5VTR2	0.03471685	0.03471685	0.003516
A.vasorum /Control	Q7Z2W9	0.001824235	0.001824235	0.003535
A.vasorum /Control	P50225	0.628808349	0.628808349	0.003549
A.vasorum /Control	P61970	0.357630162	0.357630162	0.00355
A.vasorum /Control	P31150	0.092119247	0.092119247	0.003554
A.vasorum /Control	Q5TAQ9	0.00949481	0.00949481	0.003562
A.vasorum /Control	Q9NPD3	0.047698685	0.047698685	0.003565
A.vasorum /Control	P54578	-0.021648505	0.021648505	0.003571
A.vasorum /Control	Q6P9H5	0.08080236	0.08080236	0.003572
A.vasorum /Control	Q15024	-0.06814905	0.06814905	0.003583

A.vasorum /Control	Q9H2P0	0.075978011	0.075978011	0.003586
A.vasorum /Control	P68036	0.267323641	0.267323641	0.003589
A.vasorum /Control	Q5SRE5	-0.008897678	0.008897678	0.003602
A.vasorum /Control	Q9C000	0.259046781	0.259046781	0.003603
A.vasorum /Control	Q9NV06	-1.143190545	1.143190545	0.003602
A.vasorum /Control	O43310	-0.103483143	0.103483143	0.003617
A.vasorum /Control	O95182	0.377962588	0.377962588	0.003616
A.vasorum /Control	Q14331	0.075775395	0.075775395	0.003616
A.vasorum /Control	Q7Z3B4	0.067403818	0.067403818	0.003613
A.vasorum /Control	Q15631	0.103805853	0.103805853	0.003624
A.vasorum /Control	Q8NHP6	0.138190227	0.138190227	0.003624
A.vasorum /Control	P15848	0.541999375	0.541999375	0.003633
A.vasorum /Control	P50402	0.237923634	0.237923634	0.003631
A.vasorum /Control	P22059	0.062192029	0.062192029	0.003643
A.vasorum /Control	P48449	0.001070835	0.001070835	0.003643
A.vasorum /Control	Q13951	-0.207776388	0.207776388	0.003649
A.vasorum /Control	Q96CU9	0.190144478	0.190144478	0.003669
A.vasorum /Control	Q86UA1	0.220936987	0.220936987	0.003679
A.vasorum /Control	P17858	0.086277332	0.086277332	0.003703
A.vasorum /Control	Q9Y2I7	0.088700424	0.088700424	0.003706
A.vasorum /Control	P04424	0.101677318	0.101677318	0.003709
A.vasorum /Control	Q969R2	0.320749806	0.320749806	0.003718
A.vasorum /Control	Q13362	0.548942243	0.548942243	0.003726
A.vasorum /Control	O15446	-0.754570849	0.754570849	0.003729
A.vasorum /Control	Q14155	0.058105978	0.058105978	0.003732
A.vasorum /Control	P13987	-0.469256504	0.469256504	0.003755
A.vasorum /Control	Q01081	-0.120077634	0.120077634	0.003757
A.vasorum /Control	Q8IWZ8	0.151248847	0.151248847	0.00376
A.vasorum /Control	P49257	0.143267322	0.143267322	0.003771
A.vasorum /Control	Q00577	-0.194246437	0.194246437	0.003773
A.vasorum /Control	P0CAP1	1.437910308	1.437910308	0.003781
A.vasorum /Control	Q9NZI7	0.261596141	0.261596141	0.003781
A.vasorum /Control	Q7L592	0.156070935	0.156070935	0.003819
A.vasorum /Control	Q6PJG6	0.092016358	0.092016358	0.003824
A.vasorum /Control	P17480	0.195478911	0.195478911	0.003827
A.vasorum /Control	Q14534	0.090730039	0.090730039	0.003837
A.vasorum /Control	P36578	-0.040320551	0.040320551	0.003841
A.vasorum /Control	P83111	-0.066650361	0.066650361	0.003846
A.vasorum /Control	Q9NWT6	0.385378575	0.385378575	0.003852
A.vasorum /Control	Q9UFW8	0.035085634	0.035085634	0.00385
A.vasorum /Control	P47813	0.391495431	0.391495431	0.003867
A.vasorum /Control	Q13557	0.118113314	0.118113314	0.003869
A.vasorum /Control	P49756	0.054421487	0.054421487	0.003874
A.vasorum /Control	Q9NP79	-0.077359468	0.077359468	0.003879
A.vasorum /Control	P49591	-0.050894793	0.050894793	0.003883
A.vasorum /Control	Q15654	0.010440649	0.010440649	0.003895
A.vasorum /Control	Q8WTS6	-0.124298973	0.124298973	0.003899
A.vasorum /Control	Q9UJX3	0.08592464	0.08592464	0.003904
A.vasorum /Control	P28161	0.152571723	0.152571723	0.003916
A.vasorum /Control	P61964	0.022369621	0.022369621	0.003912

A.vasorum /Control	Q16584	0.508996529	0.508996529	0.003914
A.vasorum /Control	Q9NVZ3	0.195610231	0.195610231	0.003917
A.vasorum /Control	Q9H8T0	0.91720418	0.91720418	0.003924
A.vasorum /Control	Q9BUL5	0.003283999	0.003283999	0.003928
A.vasorum /Control	P23919	-0.076708831	0.076708831	0.003937
A.vasorum /Control	Q9GZT8	0.949876797	0.949876797	0.003937
A.vasorum /Control	Q9BYM8	0.349438744	0.349438744	0.003939
A.vasorum /Control	Q9BTV5	0.035880122	0.035880122	0.003942
A.vasorum /Control	Q9H3F6	0.169969794	0.169969794	0.003952
A.vasorum /Control	Q9NVC6	0.429051754	0.429051754	0.003951
A.vasorum /Control	Q92575	0.635271778	0.635271778	0.003966
A.vasorum /Control	Q9UQE7	-0.050469857	0.050469857	0.003967
A.vasorum /Control	Q9Y678	-0.051068644	0.051068644	0.003968
A.vasorum /Control	O75886	0.023183103	0.023183103	0.003988
A.vasorum /Control	P45880	-0.172037598	0.172037598	0.003994
A.vasorum /Control	Q9NP97	0.50486953	0.50486953	0.003993
A.vasorum /Control	Q16832	0.336684813	0.336684813	0.004006
A.vasorum /Control	Q8IXI2	0.289448859	0.289448859	0.004008
A.vasorum /Control	Q86V87	0.098679125	0.098679125	0.004014
A.vasorum /Control	P30050	-0.176494505	0.176494505	0.004025
A.vasorum /Control	Q9H7N4	-0.091753443	0.091753443	0.004023
A.vasorum /Control	A6ZKI3	0.24213524	0.24213524	0.004041
A.vasorum /Control	O60711	-0.75903601	0.75903601	0.004051
A.vasorum /Control	Q3YEC7	-0.06541404	0.06541404	0.00405
A.vasorum /Control	Q7Z3C6	0.369655607	0.369655607	0.004047
A.vasorum /Control	Q8TF05	0.023173187	0.023173187	0.00406
A.vasorum /Control	Q96KC2	0.754481519	0.754481519	0.004061
A.vasorum /Control	P26006	0.368728243	0.368728243	0.004064
A.vasorum /Control	Q13287	0.503266024	0.503266024	0.004071
A.vasorum /Control	Q9P260	0.226029362	0.226029362	0.004073
A.vasorum /Control	P05166	0.346389585	0.346389585	0.004078
A.vasorum /Control	Q8N5K1	0.408953542	0.408953542	0.004076
A.vasorum /Control	Q9BRX8	0.60928337	0.60928337	0.004088
A.vasorum /Control	Q9NZQ3	0.33064955	0.33064955	0.0041
A.vasorum /Control	O00299	-0.151517897	0.151517897	0.004107
A.vasorum /Control	Q9C0D5	0.09779397	0.09779397	0.004107
A.vasorum /Control	Q9P265	-0.040849659	0.040849659	0.004111
A.vasorum /Control	Q92797	0.151708475	0.151708475	0.004117
A.vasorum /Control	P50479	-0.38861518	0.38861518	0.004123
A.vasorum /Control	Q6Y7W6	-0.013652133	0.013652133	0.004128
A.vasorum /Control	P48643	-0.01443314	0.01443314	0.004135
A.vasorum /Control	P61587	-0.645713489	0.645713489	0.004147
A.vasorum /Control	Q5J8M3	0.138044191	0.138044191	0.00415
A.vasorum /Control	Q96JM3	0.064865093	0.064865093	0.004154
A.vasorum /Control	Q13469	0.520782403	0.520782403	0.004188
A.vasorum /Control	Q9H4A3	0.163542351	0.163542351	0.004198
A.vasorum /Control	O95785	0.028491199	0.028491199	0.004205
A.vasorum /Control	Q68D10	-1.010827894	1.010827894	0.004222
A.vasorum /Control	Q9NZN5	-0.001881237	0.001881237	0.004235
A.vasorum /Control	P16070	-0.58118684	0.58118684	0.004262

A.vasorum /Control	Q9Y312	0.202139609	0.202139609	0.004263
A.vasorum /Control	O14976	-0.054380994	0.054380994	0.004269
A.vasorum /Control	P53778	0.663873286	0.663873286	0.004268
A.vasorum /Control	A5YKK6	0.100808176	0.100808176	0.004275
A.vasorum /Control	Q9NVI7	-0.025961463	0.025961463	0.004276
A.vasorum /Control	Q9NVV4	0.083052941	0.083052941	0.004271
A.vasorum /Control	O94913	0.030897842	0.030897842	0.004281
A.vasorum /Control	Q5SSJ5	-0.00093457	0.00093457	0.004286
A.vasorum /Control	O95870	0.11422679	0.11422679	0.004296
A.vasorum /Control	Q99816	0.034159436	0.034159436	0.004296
A.vasorum /Control	O15230	0.37307651	0.37307651	0.004302
A.vasorum /Control	P07384	0.033059686	0.033059686	0.004306
A.vasorum /Control	P22102	-0.069185382	0.069185382	0.00431
A.vasorum /Control	P42696	-0.013159212	0.013159212	0.004326
A.vasorum /Control	P08758	0.068937404	0.068937404	0.00433
A.vasorum /Control	Q68CP4	0.301284143	0.301284143	0.004345
A.vasorum /Control	Q7Z6K5	0.209788146	0.209788146	0.00435
A.vasorum /Control	Q9H3H3	0.131386173	0.131386173	0.004354
A.vasorum /Control	Q99996	0.063181616	0.063181616	0.004357
A.vasorum /Control	Q9BTX1	0.056196899	0.056196899	0.00436
A.vasorum /Control	O15427	-0.174725481	0.174725481	0.004365
A.vasorum /Control	Q9UIC8	0.107320418	0.107320418	0.004367
A.vasorum /Control	A0MZ66	0.227623661	0.227623661	0.004373
A.vasorum /Control	O15042	-0.027974845	0.027974845	0.004371
A.vasorum /Control	P16885	0.612867141	0.612867141	0.004374
A.vasorum /Control	O95479	0.141965602	0.141965602	0.004399
A.vasorum /Control	P15586	0.273752676	0.273752676	0.004399
A.vasorum /Control	Q53H12	0.166805007	0.166805007	0.004395
A.vasorum /Control	Q9NRY5	0.072531492	0.072531492	0.004395
A.vasorum /Control	P10321	0.534443056	0.534443056	0.004403
A.vasorum /Control	Q96RS6	0.008424029	0.008424029	0.004405
A.vasorum /Control	Q4V339	0.560211477	0.560211477	0.004415
A.vasorum /Control	Q9H9Y2	-1.58079648	1.58079648	0.004418
A.vasorum /Control	P49796	0.422952484	0.422952484	0.004424
A.vasorum /Control	Q8TE82	0.368761629	0.368761629	0.004427
A.vasorum /Control	Q9H3P7	-0.085140912	0.085140912	0.004427
A.vasorum /Control	Q7Z3J2	-0.027159654	0.027159654	0.004431
A.vasorum /Control	Q9BYD2	-0.006697226	0.006697226	0.004436
A.vasorum /Control	Q6NUQ4	-0.015567677	0.015567677	0.004444
A.vasorum /Control	Q96NE9	0.001507779	0.001507779	0.004444
A.vasorum /Control	Q04323	0.141258338	0.141258338	0.004456
A.vasorum /Control	Q9UBP9	0.305144863	0.305144863	0.004457
A.vasorum /Control	Q9Y5R8	0.172607111	0.172607111	0.004453
A.vasorum /Control	O95864	-0.290939182	0.290939182	0.004499
A.vasorum /Control	P40925	0.077684314	0.077684314	0.004499
A.vasorum /Control	O75400	0.041998718	0.041998718	0.00453
A.vasorum /Control	O75044	0.027561473	0.027561473	0.004535
A.vasorum /Control	O75348	-0.285168427	0.285168427	0.004535
A.vasorum /Control	Q92599	0.085968738	0.085968738	0.004552
A.vasorum /Control	Q9Y6B6	0.151326539	0.151326539	0.004552

A.vasorum /Control	P28070	0.162189395	0.162189395	0.004557
A.vasorum /Control	Q6UW02	0.236033121	0.236033121	0.004561
A.vasorum /Control	Q9UKY7	-0.339082568	0.339082568	0.004562
A.vasorum /Control	O43264	0.051903493	0.051903493	0.004568
A.vasorum /Control	Q02252	0.790674075	0.790674075	0.004566
A.vasorum /Control	P49841	-0.203785649	0.203785649	0.004577
A.vasorum /Control	Q8N392	0.198234301	0.198234301	0.004577
A.vasorum /Control	Q9Y2X0	0.633982335	0.633982335	0.004574
A.vasorum /Control	P09669	0.136475924	0.136475924	0.004591
A.vasorum /Control	P49959	0.112164534	0.112164534	0.004591
A.vasorum /Control	O94885	-0.057213769	0.057213769	0.004598
A.vasorum /Control	Q6L8Q7	-0.027868767	0.027868767	0.004611
A.vasorum /Control	P24928	0.229954169	0.229954169	0.004619
A.vasorum /Control	P61923	0.028696469	0.028696469	0.004628
A.vasorum /Control	Q9NQY0	0.120553349	0.120553349	0.004625
A.vasorum /Control	Q15021	-0.044662243	0.044662243	0.004646
A.vasorum /Control	Q15645	0.039175878	0.039175878	0.004645
A.vasorum /Control	Q9BRZ2	-0.000939814	0.000939814	0.00465
A.vasorum /Control	Q8NBI5	0.311817566	0.311817566	0.004653
A.vasorum /Control	Q5PRF9	-0.011706164	0.011706164	0.004669
A.vasorum /Control	Q9UHI6	-0.118247987	0.118247987	0.004674
A.vasorum /Control	O95573	0.018149516	0.018149516	0.004692
A.vasorum /Control	Q99735	0.74332114	0.74332114	0.004698
A.vasorum /Control	Q9ULP0	-1.056088985	1.056088985	0.004697
A.vasorum /Control	O43678	0.643636991	0.643636991	0.004703
A.vasorum /Control	Q96P48	0.146830213	0.146830213	0.004721
A.vasorum /Control	O00273	0.526250446	0.526250446	0.004732
A.vasorum /Control	O95070	0.043684133	0.043684133	0.004734
A.vasorum /Control	Q6P1M0	0.148343865	0.148343865	0.004729
A.vasorum /Control	Q9Y4W2	-0.055336971	0.055336971	0.004728
A.vasorum /Control	Q7Z422	0.27318612	0.27318612	0.004739
A.vasorum /Control	Q9P2R3	0.107328445	0.107328445	0.004741
A.vasorum /Control	Q9NYL9	0.280589198	0.280589198	0.004746
A.vasorum /Control	O96006	0.990598874	0.990598874	0.004753
A.vasorum /Control	Q9Y295	0.14091538	0.14091538	0.004755
A.vasorum /Control	O94964	0.016350476	0.016350476	0.004771
A.vasorum /Control	P62330	0.126872684	0.126872684	0.004769
A.vasorum /Control	P11274	0.158932316	0.158932316	0.004775
A.vasorum /Control	Q6R327	0.278665205	0.278665205	0.00478
A.vasorum /Control	Q7LBC6	0.070978602	0.070978602	0.004785
A.vasorum /Control	Q92890	-0.086026671	0.086026671	0.004783
A.vasorum /Control	Q9UBC5	0.338315855	0.338315855	0.004792
A.vasorum /Control	O60271	0.013824528	0.013824528	0.004805
A.vasorum /Control	P62854	0.146823086	0.146823086	0.004809
A.vasorum /Control	Q5T0N5	0.161534664	0.161534664	0.004805
A.vasorum /Control	Q9Y4X4	0.865109752	0.865109752	0.004802
A.vasorum /Control	Q9Y6N7	0.392082743	0.392082743	0.004808
A.vasorum /Control	Q9Y673	-0.014008715	0.014008715	0.004826
A.vasorum /Control	Q9H0D6	0.136050706	0.136050706	0.004839
A.vasorum /Control	Q53H47	0.55433265	0.55433265	0.004847

A.vasorum /Control	Q15004	-0.017782014	0.017782014	0.004851
A.vasorum /Control	Q7L0Y3	-0.094986504	0.094986504	0.004854
A.vasorum /Control	O75792	0.294043907	0.294043907	0.004878
A.vasorum /Control	P46199	0.193236907	0.193236907	0.004871
A.vasorum /Control	Q13614	0.140303505	0.140303505	0.004875
A.vasorum /Control	Q15382	0.049080825	0.049080825	0.004875
A.vasorum /Control	Q86YV9	0.113358331	0.113358331	0.00488
A.vasorum /Control	Q9BQS8	0.205875657	0.205875657	0.004873
A.vasorum /Control	Q9UBW8	0.085046836	0.085046836	0.004865
A.vasorum /Control	Q2KHT3	0.487862169	0.487862169	0.004909
A.vasorum /Control	O95470	0.180528744	0.180528744	0.00492
A.vasorum /Control	Q96MG8	-1.035789422	1.035789422	0.004923
A.vasorum /Control	P67870	0.074943226	0.074943226	0.004935
A.vasorum /Control	P37173	0.084600556	0.084600556	0.004946
A.vasorum /Control	P30040	0.128615831	0.128615831	0.004956
A.vasorum /Control	Q13136	-0.086092083	0.086092083	0.004952
A.vasorum /Control	Q7Z4W1	0.104523946	0.104523946	0.004956
A.vasorum /Control	P23743	0.198061858	0.198061858	0.004964
A.vasorum /Control	Q03518	0.371585849	0.371585849	0.004973
A.vasorum /Control	Q9NR09	0.135675389	0.135675389	0.004986
A.vasorum /Control	Q9Y485	0.708577181	0.708577181	0.00501
A.vasorum /Control	Q9H082	-0.143731324	0.143731324	0.005016
A.vasorum /Control	O95749	0.018629553	0.018629553	0.005021
A.vasorum /Control	P13861	0.023292155	0.023292155	0.005053
A.vasorum /Control	P16949	0.495102586	0.495102586	0.005051
A.vasorum /Control	P61353	-0.009441784	0.009441784	0.005046
A.vasorum /Control	Q93009	0.124514218	0.124514218	0.005052
A.vasorum /Control	Q9ULX3	-0.024505497	0.024505497	0.005048
A.vasorum /Control	Q96I24	-0.048380184	0.048380184	0.005061
A.vasorum /Control	Q96H20	0.248031414	0.248031414	0.005065
A.vasorum /Control	P01137	0.150694138	0.150694138	0.005071
A.vasorum /Control	O95628	0.298030865	0.298030865	0.005088
A.vasorum /Control	P20839	-0.021032748	0.021032748	0.005082
A.vasorum /Control	Q86W92	0.041576896	0.041576896	0.005094
A.vasorum /Control	Q96AQ6	0.02302619	0.02302619	0.005088
A.vasorum /Control	Q9NX47	0.167611396	0.167611396	0.005092
A.vasorum /Control	Q9ULH7	0.619238798	0.619238798	0.005092
A.vasorum /Control	Q9Y580	0.129548359	0.129548359	0.005089
A.vasorum /Control	Q9BYD6	0.047933202	0.047933202	0.005102
A.vasorum /Control	P06730	-0.020068653	0.020068653	0.005122
A.vasorum /Control	Q14766	0.346888387	0.346888387	0.005121
A.vasorum /Control	Q13796	0.201487658	0.201487658	0.005131
A.vasorum /Control	Q9BRP1	0.255292432	0.255292432	0.00513
A.vasorum /Control	P12270	0.090968514	0.090968514	0.005136
A.vasorum /Control	Q8NE01	0.259390695	0.259390695	0.005142
A.vasorum /Control	O43852	0.29078179	0.29078179	0.005172
A.vasorum /Control	P0CG39	0.412957435	0.412957435	0.005167
A.vasorum /Control	P23497	-1.132228358	1.132228358	0.005162
A.vasorum /Control	P55060	0.100060121	0.100060121	0.005157
A.vasorum /Control	Q13177	0.186947998	0.186947998	0.00517

A.vasorum /Control	Q14573	0.208467255	0.208467255	0.005162
A.vasorum /Control	Q643R3	0.538667499	0.538667499	0.005157
A.vasorum /Control	Q969U7	-0.067158247	0.067158247	0.005169
A.vasorum /Control	Q9BWH6	0.212416554	0.212416554	0.005172
A.vasorum /Control	Q8NE86	0.280370195	0.280370195	0.0052
A.vasorum /Control	Q969G5	-0.24668143	0.24668143	0.005203
A.vasorum /Control	Q96I15	0.438559536	0.438559536	0.005202
A.vasorum /Control	Q5F1R6	0.106073875	0.106073875	0.005216
A.vasorum /Control	Q96FN4	0.570503259	0.570503259	0.005211
A.vasorum /Control	Q9H0U6	-0.278135167	0.278135167	0.005221
A.vasorum /Control	Q9H2H9	-1.410468435	1.410468435	0.005221
A.vasorum /Control	Q9UNS2	-0.062843546	0.062843546	0.005217
A.vasorum /Control	Q15907	0.01414926	0.01414926	0.005228
A.vasorum /Control	Q8IZ07	0.417652294	0.417652294	0.005238
A.vasorum /Control	P25490	0.105799877	0.105799877	0.005269
A.vasorum /Control	Q01970	0.076041646	0.076041646	0.005274
A.vasorum /Control	Q6NUK4	0.00969992	0.00969992	0.005273
A.vasorum /Control	Q6ZXV5	0.237480909	0.237480909	0.005272
A.vasorum /Control	Q13188	0.268492139	0.268492139	0.005277
A.vasorum /Control	O95376	0.030256256	0.030256256	0.005293
A.vasorum /Control	Q6DD88	0.015418786	0.015418786	0.005302
A.vasorum /Control	Q7Z4L5	0.682443606	0.682443606	0.005305
A.vasorum /Control	P09211	0.079270741	0.079270741	0.00532
A.vasorum /Control	Q96FS4	0.045301904	0.045301904	0.00533
A.vasorum /Control	Q9Y371	0.193432635	0.193432635	0.005337
A.vasorum /Control	Q8TCG2	0.650305345	0.650305345	0.005343
A.vasorum /Control	Q96T76	0.082142825	0.082142825	0.005344
A.vasorum /Control	P40937	0.076883749	0.076883749	0.005367
A.vasorum /Control	Q6ZRS2	0.566565698	0.566565698	0.00537
A.vasorum /Control	Q8N766	-0.089393764	0.089393764	0.005372
A.vasorum /Control	Q8N806	0.140808184	0.140808184	0.005412
A.vasorum /Control	P61088	0.124009655	0.124009655	0.00542
A.vasorum /Control	Q9HD45	-0.125695855	0.125695855	0.005418
A.vasorum /Control	Q9NS93	0.383153951	0.383153951	0.005432
A.vasorum /Control	P40306	0.500636983	0.500636983	0.005438
A.vasorum /Control	Q96P70	0.14169594	0.14169594	0.005445
A.vasorum /Control	P10398	-0.111774638	0.111774638	0.005462
A.vasorum /Control	P60468	-0.231195438	0.231195438	0.005464
A.vasorum /Control	P41226	0.947691491	0.947691491	0.005473
A.vasorum /Control	Q5TEU4	-0.054169086	0.054169086	0.005479
A.vasorum /Control	Q8TAQ2	-0.076450827	0.076450827	0.005495
A.vasorum /Control	O00178	0.130511762	0.130511762	0.005533
A.vasorum /Control	P51858	0.161666477	0.161666477	0.005539
A.vasorum /Control	Q7Z2T5	0.20178202	0.20178202	0.005534
A.vasorum /Control	Q8N6T7	-0.902597916	0.902597916	0.005539
A.vasorum /Control	Q9P266	0.209560951	0.209560951	0.005538
A.vasorum /Control	Q9Y6K8	0.409479866	0.409479866	0.005544
A.vasorum /Control	Q9P2Y5	0.112213239	0.112213239	0.005549
A.vasorum /Control	Q9BRP8	0.081808242	0.081808242	0.005553
A.vasorum /Control	Q8N9N2	-0.023543807	0.023543807	0.005565

A.vasorum /Control	Q9Y3B4	-0.232075197	0.232075197	0.005563
A.vasorum /Control	Q01105	0.147623403	0.147623403	0.00557
A.vasorum /Control	P06746	0.068418964	0.068418964	0.005575
A.vasorum /Control	Q96K76	0.084710645	0.084710645	0.005574
A.vasorum /Control	P20226	1.992625303	1.992625303	0.005585
A.vasorum /Control	Q6NXR4	0.154127818	0.154127818	0.00559
A.vasorum /Control	P27987	0.313843428	0.313843428	0.005603
A.vasorum /Control	O43615	0.068339435	0.068339435	0.005638
A.vasorum /Control	P10589	0.00467736	0.00467736	0.005631
A.vasorum /Control	P22570	0.085985751	0.085985751	0.005635
A.vasorum /Control	P39880	0.484006406	0.484006406	0.005634
A.vasorum /Control	Q8WTV0	0.01116284	0.01116284	0.005639
A.vasorum /Control	Q9BTT0	0.1182202	0.1182202	0.005625
A.vasorum /Control	Q9H1C7	0.448869947	0.448869947	0.005641
A.vasorum /Control	Q460N5	0.476856369	0.476856369	0.00566
A.vasorum /Control	Q8NBN7	0.251522847	0.251522847	0.005663
A.vasorum /Control	P98194	-0.000563756	0.000563756	0.005672
A.vasorum /Control	Q9NW08	0.157771582	0.157771582	0.005674
A.vasorum /Control	Q6IN85	0.013648134	0.013648134	0.005686
A.vasorum /Control	Q9HCJ3	0.521015604	0.521015604	0.005684
A.vasorum /Control	Q9NV56	0.335604865	0.335604865	0.005687
A.vasorum /Control	Q99543	-0.041841387	0.041841387	0.005714
A.vasorum /Control	Q9UGI8	0.060759309	0.060759309	0.005714
A.vasorum /Control	Q9Y5A7	0.261070412	0.261070412	0.005712
A.vasorum /Control	Q96F15	1.73796097	1.73796097	0.005729
A.vasorum /Control	Q9NWY4	0.4222437	0.4222437	0.005731
A.vasorum /Control	Q70CQ2	0.190717413	0.190717413	0.00574
A.vasorum /Control	P0DMV8	0.022909694	0.022909694	0.005757
A.vasorum /Control	P12814	-0.221263991	0.221263991	0.005757
A.vasorum /Control	O75694	0.042306217	0.042306217	0.005777
A.vasorum /Control	Q14152	-0.05855561	0.05855561	0.005783
A.vasorum /Control	P30153	0.004208799	0.004208799	0.005793
A.vasorum /Control	P49327	-0.014551508	0.014551508	0.005793
A.vasorum /Control	O60343	0.102290761	0.102290761	0.005801
A.vasorum /Control	P56192	-0.005781724	0.005781724	0.005819
A.vasorum /Control	Q7L1V2	0.657865439	0.657865439	0.005813
A.vasorum /Control	Q8IW45	0.32481418	0.32481418	0.005816
A.vasorum /Control	Q96HY6	0.477727338	0.477727338	0.005823
A.vasorum /Control	Q9H2G2	-0.031689023	0.031689023	0.005821
A.vasorum /Control	Q9H3M7	2.348297341	2.348297341	0.005821
A.vasorum /Control	Q9NTJ3	-0.017237803	0.017237803	0.005818
A.vasorum /Control	Q9NVM6	0.273414311	0.273414311	0.005818
A.vasorum /Control	Q9Y6G9	0.019916017	0.019916017	0.005821
A.vasorum /Control	P84077	0.072464807	0.072464807	0.005834
A.vasorum /Control	Q9UK45	3.495017972	3.495017972	0.005836
A.vasorum /Control	Q8TF74	-0.109889478	0.109889478	0.00584
A.vasorum /Control	O14730	0.257119144	0.257119144	0.005845
A.vasorum /Control	O43633	0.115682852	0.115682852	0.005865
A.vasorum /Control	Q02127	0.614322444	0.614322444	0.005866
A.vasorum /Control	P06733	0.17582665	0.17582665	0.005879

A.vasorum /Control	P07099	0.063904876	0.063904876	0.005882
A.vasorum /Control	Q6NVY1	0.467948158	0.467948158	0.005896
A.vasorum /Control	Q9NY33	0.011751272	0.011751272	0.005909
A.vasorum /Control	P49754	0.163412826	0.163412826	0.005919
A.vasorum /Control	Q9NUQ8	0.025935535	0.025935535	0.005918
A.vasorum /Control	Q9Y3C8	0.309728095	0.309728095	0.005922
A.vasorum /Control	P68402	0.776083749	0.776083749	0.005943
A.vasorum /Control	P09525	0.090685453	0.090685453	0.005951
A.vasorum /Control	Q8WWY3	0.130697613	0.130697613	0.005968
A.vasorum /Control	Q9H832	0.103737589	0.103737589	0.005974
A.vasorum /Control	Q9H8W4	0.679103838	0.679103838	0.005976
A.vasorum /Control	Q9NZ32	0.138785195	0.138785195	0.005973
A.vasorum /Control	P49902	-0.000642914	0.000642914	0.005984
A.vasorum /Control	Q16527	0.367488995	0.367488995	0.005991
A.vasorum /Control	O75970	0.134433442	0.134433442	0.006
A.vasorum /Control	P07948	0.13403338	0.13403338	0.006009
A.vasorum /Control	O00429	-0.064520048	0.064520048	0.006012
A.vasorum /Control	P08397	0.348666735	0.348666735	0.006023
A.vasorum /Control	P29597	0.125718078	0.125718078	0.006023
A.vasorum /Control	Q05048	0.006721589	0.006721589	0.006033
A.vasorum /Control	Q6P158	0.243449519	0.243449519	0.006035
A.vasorum /Control	Q02040	0.223894236	0.223894236	0.006045
A.vasorum /Control	Q96RP9	-0.089375871	0.089375871	0.006053
A.vasorum /Control	O60315	1.558978962	1.558978962	0.006059
A.vasorum /Control	O14776	0.016764257	0.016764257	0.006099
A.vasorum /Control	O95425	0.02841378	0.02841378	0.006096
A.vasorum /Control	P00519	0.457560201	0.457560201	0.006111
A.vasorum /Control	P42766	0.271181067	0.271181067	0.006115
A.vasorum /Control	Q99501	0.479976804	0.479976804	0.006118
A.vasorum /Control	Q9BUQ8	0.052164154	0.052164154	0.006122
A.vasorum /Control	Q9UNF0	0.052239099	0.052239099	0.006122
A.vasorum /Control	O14880	-0.3477138	0.3477138	0.006137
A.vasorum /Control	Q00653	-0.081898645	0.081898645	0.00614
A.vasorum /Control	Q13243	0.199944379	0.199944379	0.006142
A.vasorum /Control	Q14156	0.275283519	0.275283519	0.006129
A.vasorum /Control	Q96PU5	0.033950914	0.033950914	0.006138
A.vasorum /Control	Q9H5Z1	0.625255975	0.625255975	0.006132
A.vasorum /Control	Q99829	0.190635066	0.190635066	0.006149
A.vasorum /Control	Q9NXN4	0.103874574	0.103874574	0.006151
A.vasorum /Control	P00403	0.235900216	0.235900216	0.006166
A.vasorum /Control	Q8WYP3	0.218308272	0.218308272	0.00618
A.vasorum /Control	P50542	0.125484489	0.125484489	0.006195
A.vasorum /Control	Q8N3C0	-0.060132569	0.060132569	0.006205
A.vasorum /Control	P28072	0.210002232	0.210002232	0.00621
A.vasorum /Control	Q9NXC5	0.281047367	0.281047367	0.006223
A.vasorum /Control	P40123	0.161027421	0.161027421	0.006227
A.vasorum /Control	Q13618	0.23514012	0.23514012	0.006235
A.vasorum /Control	Q8IV48	0.377262269	0.377262269	0.006237
A.vasorum /Control	O75027	0.165049333	0.165049333	0.00627
A.vasorum /Control	P23396	-0.010042025	0.010042025	0.006287

A.vasorum /Control	O95644	0.211266321	0.211266321	0.006307
A.vasorum /Control	Q7Z4S6	-0.097314461	0.097314461	0.006315
A.vasorum /Control	Q86TX2	0.448417035	0.448417035	0.006312
A.vasorum /Control	Q96K12	-0.063555102	0.063555102	0.006303
A.vasorum /Control	Q99941	0.803481778	0.803481778	0.006309
A.vasorum /Control	Q8WU76	0.093243761	0.093243761	0.006333
A.vasorum /Control	Q96GM5	-0.007904831	0.007904831	0.006338
A.vasorum /Control	Q9NRK6	0.19878394	0.19878394	0.006361
A.vasorum /Control	Q86US8	0.121144811	0.121144811	0.00637
A.vasorum /Control	P10155	0.068215278	0.068215278	0.006391
A.vasorum /Control	Q9NX74	0.156440187	0.156440187	0.006388
A.vasorum /Control	P56134	0.184441193	0.184441193	0.006396
A.vasorum /Control	Q99933	0.848307087	0.848307087	0.006404
A.vasorum /Control	Q9Y3A5	-0.147654337	0.147654337	0.006404
A.vasorum /Control	Q9Y6K9	0.04057307	0.04057307	0.006401
A.vasorum /Control	P48637	0.110905203	0.110905203	0.006414
A.vasorum /Control	Q9BUB7	0.11991322	0.11991322	0.006417
A.vasorum /Control	P14406	0.311110475	0.311110475	0.00643
A.vasorum /Control	Q5EBL4	0.372387466	0.372387466	0.006433
A.vasorum /Control	Q9UL18	0.136904322	0.136904322	0.006427
A.vasorum /Control	P14324	0.087834824	0.087834824	0.006446
A.vasorum /Control	Q86VX2	0.051433468	0.051433468	0.006471
A.vasorum /Control	Q8N5C6	0.244579508	0.244579508	0.006472
A.vasorum /Control	Q70Z35	0.758434831	0.758434831	0.006479
A.vasorum /Control	Q9NPJ6	0.197017838	0.197017838	0.006485
A.vasorum /Control	Q9P2K5	0.294188866	0.294188866	0.006489
A.vasorum /Control	O60645	0.096734826	0.096734826	0.006505
A.vasorum /Control	P48556	0.107483908	0.107483908	0.006512
A.vasorum /Control	Q9NZV5	0.090484368	0.090484368	0.006512
A.vasorum /Control	Q9GZT4	0.391457967	0.391457967	0.006519
A.vasorum /Control	P35269	-0.11677593	0.11677593	0.006541
A.vasorum /Control	P40818	-0.0127463	0.0127463	0.006543
A.vasorum /Control	Q8TEP8	0.402038493	0.402038493	0.006558
A.vasorum /Control	P0CG29	0.172905231	0.172905231	0.006568
A.vasorum /Control	Q15435	0.003034173	0.003034173	0.00659
A.vasorum /Control	Q00013	0.159988959	0.159988959	0.006595
A.vasorum /Control	Q02809	-0.009921676	0.009921676	0.006599
A.vasorum /Control	O15460	-0.238915385	0.238915385	0.006609
A.vasorum /Control	O95365	-2.071845419	2.071845419	0.006608
A.vasorum /Control	O15063	0.152479816	0.152479816	0.006623
A.vasorum /Control	P78356	0.299039499	0.299039499	0.006629
A.vasorum /Control	P26885	0.297527732	0.297527732	0.006661
A.vasorum /Control	P39656	-0.035744821	0.035744821	0.00666
A.vasorum /Control	O94903	0.049664236	0.049664236	0.006674
A.vasorum /Control	O00255	0.466394462	0.466394462	0.006679
A.vasorum /Control	Q6UWE0	0.108433542	0.108433542	0.006686
A.vasorum /Control	Q8N1G0	0.138829151	0.138829151	0.00669
A.vasorum /Control	P16219	0.7410079	0.7410079	0.006693
A.vasorum /Control	Q10469	-0.164321041	0.164321041	0.006702
A.vasorum /Control	Q13588	0.162775066	0.162775066	0.006716

A.vasorum /Control	P41970	0.640073844	0.640073844	0.006721
A.vasorum /Control	Q9BZ67	0.073317359	0.073317359	0.006723
A.vasorum /Control	Q14118	0.269996013	0.269996013	0.00673
A.vasorum /Control	Q9Y608	0.030429288	0.030429288	0.006728
A.vasorum /Control	O75396	-0.081143929	0.081143929	0.006737
A.vasorum /Control	Q96RS0	0.40683435	0.40683435	0.006737
A.vasorum /Control	Q96S55	0.562415564	0.562415564	0.006745
A.vasorum /Control	O60701	-0.163194131	0.163194131	0.006757
A.vasorum /Control	P06241	0.78196358	0.78196358	0.006775
A.vasorum /Control	Q9NRR4	0.467794537	0.467794537	0.006774
A.vasorum /Control	Q7L5N1	-0.041768301	0.041768301	0.006787
A.vasorum /Control	Q8TCA0	0.769924603	0.769924603	0.006787
A.vasorum /Control	Q9BQA1	-0.086272622	0.086272622	0.006789
A.vasorum /Control	Q29RF7	0.014724128	0.014724128	0.006814
A.vasorum /Control	Q92783	0.224270297	0.224270297	0.006829
A.vasorum /Control	Q9Y487	0.025129314	0.025129314	0.006835
A.vasorum /Control	P55884	-0.04683423	0.04683423	0.006844
A.vasorum /Control	O75127	-0.824004349	0.824004349	0.006866
A.vasorum /Control	O15143	0.059335115	0.059335115	0.006871
A.vasorum /Control	Q9HC35	0.122771402	0.122771402	0.006894
A.vasorum /Control	Q14318	0.060651372	0.060651372	0.006902
A.vasorum /Control	P28066	0.121344089	0.121344089	0.006924
A.vasorum /Control	P50148	0.112845761	0.112845761	0.006922
A.vasorum /Control	P21964	0.1608536	0.1608536	0.006929
A.vasorum /Control	P26599	0.159793449	0.159793449	0.006932
A.vasorum /Control	Q9GZP9	0.194499048	0.194499048	0.006946
A.vasorum /Control	Q8N684	0.091524797	0.091524797	0.00695
A.vasorum /Control	P45974	0.060450636	0.060450636	0.006959
A.vasorum /Control	Q9Y613	-0.096209833	0.096209833	0.006958
A.vasorum /Control	Q9Y619	0.136215147	0.136215147	0.006967
A.vasorum /Control	Q96K83	0.568127714	0.568127714	0.006983
A.vasorum /Control	Q99614	0.533092723	0.533092723	0.007008
A.vasorum /Control	P61962	0.147158304	0.147158304	0.007017
A.vasorum /Control	O00541	-0.547503817	0.547503817	0.007037
A.vasorum /Control	P43378	0.236250661	0.236250661	0.007034
A.vasorum /Control	Q5VYK3	0.061767443	0.061767443	0.007062
A.vasorum /Control	P52701	-0.013646532	0.013646532	0.007075
A.vasorum /Control	O96019	0.065703625	0.065703625	0.007081
A.vasorum /Control	P04844	0.155114336	0.155114336	0.0071
A.vasorum /Control	Q92793	-0.421406308	0.421406308	0.0071
A.vasorum /Control	Q8TED9	0.457747564	0.457747564	0.007104
A.vasorum /Control	Q9UM00	0.300675971	0.300675971	0.007113
A.vasorum /Control	O00267	0.099085487	0.099085487	0.007123
A.vasorum /Control	O00400	0.014626058	0.014626058	0.00712
A.vasorum /Control	P60520	-0.475622917	0.475622917	0.007131
A.vasorum /Control	Q9NU22	-0.118117142	0.118117142	0.007153
A.vasorum /Control	P62891	0.164789519	0.164789519	0.007168
A.vasorum /Control	Q969H8	-0.024679333	0.024679333	0.007173
A.vasorum /Control	Q9Y5J1	-1.024810727	1.024810727	0.007203
A.vasorum /Control	Q8WY22	0.099091441	0.099091441	0.007225

A.vasorum /Control	P52815	0.766942647	0.766942647	0.007232
A.vasorum /Control	Q13490	0.13433849	0.13433849	0.007237
A.vasorum /Control	P50570	0.157568807	0.157568807	0.007259
A.vasorum /Control	O94906	-0.003220822	0.003220822	0.007263
A.vasorum /Control	P49915	-0.024927138	0.024927138	0.00727
A.vasorum /Control	P15927	0.384780134	0.384780134	0.007273
A.vasorum /Control	Q92805	0.318045503	0.318045503	0.007281
A.vasorum /Control	Q96ST3	0.279088648	0.279088648	0.007292
A.vasorum /Control	P28715	0.156966036	0.156966036	0.007305
A.vasorum /Control	P19021	0.10105744	0.10105744	0.007317
A.vasorum /Control	P49356	0.198203128	0.198203128	0.007323
A.vasorum /Control	Q9BSF4	0.146642691	0.146642691	0.007332
A.vasorum /Control	O14787	0.044526899	0.044526899	0.007362
A.vasorum /Control	Q15833	0.315845811	0.315845811	0.007363
A.vasorum /Control	Q9Y3I0	0.006298891	0.006298891	0.007366
A.vasorum /Control	P47897	0.079158434	0.079158434	0.007378
A.vasorum /Control	P49720	0.029528339	0.029528339	0.007392
A.vasorum /Control	Q7RTT2	-1.302261557	1.302261557	0.007401
A.vasorum /Control	Q8WVJ2	0.126853487	0.126853487	0.007405
A.vasorum /Control	Q92888	0.025677248	0.025677248	0.007421
A.vasorum /Control	Q8N183	0.012856122	0.012856122	0.007425
A.vasorum /Control	Q15678	-0.025245633	0.025245633	0.007431
A.vasorum /Control	Q9UKF6	0.001438673	0.001438673	0.007442
A.vasorum /Control	Q6ZUT6	0.233802252	0.233802252	0.007454
A.vasorum /Control	O15211	0.673305935	0.673305935	0.007458
A.vasorum /Control	Q99570	0.34237905	0.34237905	0.007476
A.vasorum /Control	Q9UII2	0.840324545	0.840324545	0.007473
A.vasorum /Control	Q9Y5B8	-0.114607552	0.114607552	0.007478
A.vasorum /Control	P07686	0.265460487	0.265460487	0.007495
A.vasorum /Control	Q9NZ52	0.795907642	0.795907642	0.007495
A.vasorum /Control	O43676	0.089200855	0.089200855	0.007519
A.vasorum /Control	Q9NXR1	-0.052684871	0.052684871	0.007517
A.vasorum /Control	Q9BT22	0.101223073	0.101223073	0.007536
A.vasorum /Control	Q9NVH2	0.185279862	0.185279862	0.007562
A.vasorum /Control	P00568	-0.019568072	0.019568072	0.007576
A.vasorum /Control	Q52LW3	-0.10248354	0.10248354	0.00757
A.vasorum /Control	Q53GT1	-0.024128858	0.024128858	0.007569
A.vasorum /Control	Q5JSZ5	-0.00778891	0.00778891	0.007573
A.vasorum /Control	Q16850	0.075727278	0.075727278	0.007599
A.vasorum /Control	Q8WVP5	0.055598145	0.055598145	0.007599
A.vasorum /Control	Q9NR45	0.040131924	0.040131924	0.00761
A.vasorum /Control	Q16401	0.188411785	0.188411785	0.007619
A.vasorum /Control	Q96ER3	-0.026073726	0.026073726	0.007636
A.vasorum /Control	P61313	0.102700011	0.102700011	0.007651
A.vasorum /Control	Q14011	0.31939835	0.31939835	0.007659
A.vasorum /Control	Q9H9H4	0.416087717	0.416087717	0.00767
A.vasorum /Control	Q9H814	0.07805223	0.07805223	0.007688
A.vasorum /Control	P53992	0.116005035	0.116005035	0.007692
A.vasorum /Control	Q12906	0.077125056	0.077125056	0.007714
A.vasorum /Control	P62424	-0.194212283	0.194212283	0.007719

A.vasorum /Control	Q9H8H0	-0.968661771	0.968661771	0.007733
A.vasorum /Control	P23469	0.330223386	0.330223386	0.00775
A.vasorum /Control	Q9Y2G2	0.245366875	0.245366875	0.007747
A.vasorum /Control	O60879	0.018981786	0.018981786	0.007771
A.vasorum /Control	P23526	-0.066625374	0.066625374	0.007792
A.vasorum /Control	O75323	0.246316982	0.246316982	0.007797
A.vasorum /Control	P55084	0.104404363	0.104404363	0.007814
A.vasorum /Control	Q5W0Z9	-0.035931638	0.035931638	0.007816
A.vasorum /Control	Q96P47	0.18265149	0.18265149	0.007814
A.vasorum /Control	Q7L5Y1	0.171843056	0.171843056	0.007827
A.vasorum /Control	Q92556	0.562106755	0.562106755	0.007865
A.vasorum /Control	O75155	0.98782643	0.98782643	0.007894
A.vasorum /Control	Q27J81	0.045430796	0.045430796	0.007898
A.vasorum /Control	O15498	-0.036110995	0.036110995	0.007919
A.vasorum /Control	Q5QJ74	0.422365793	0.422365793	0.007916
A.vasorum /Control	Q9H845	0.104624453	0.104624453	0.00791
A.vasorum /Control	Q9H9J2	0.042907384	0.042907384	0.007914
A.vasorum /Control	O95352	0.190887017	0.190887017	0.00793
A.vasorum /Control	Q14457	0.436564273	0.436564273	0.007935
A.vasorum /Control	Q86VS8	0.190336404	0.190336404	0.007935
A.vasorum /Control	Q9NRG9	-0.033394208	0.033394208	0.007932
A.vasorum /Control	O43617	0.206253382	0.206253382	0.007962
A.vasorum /Control	O95299	0.201852939	0.201852939	0.007959
A.vasorum /Control	P33993	0.060614687	0.060614687	0.007957
A.vasorum /Control	Q5VWZ2	0.24042903	0.24042903	0.007958
A.vasorum /Control	Q7Z4F1	0.109006152	0.109006152	0.007964
A.vasorum /Control	Q01433	0.055790327	0.055790327	0.007979
A.vasorum /Control	Q16134	0.291681804	0.291681804	0.007976
A.vasorum /Control	Q9NUJ3	0.514338461	0.514338461	0.007972
A.vasorum /Control	Q05086	-0.016436021	0.016436021	0.007994
A.vasorum /Control	Q8IVT5	0.213566994	0.213566994	0.007995
A.vasorum /Control	P00387	0.205514603	0.205514603	0.00801
A.vasorum /Control	Q8WVB6	0.111823997	0.111823997	0.008014
A.vasorum /Control	Q6UXN9	0.300640483	0.300640483	0.008029
A.vasorum /Control	Q9BVI4	-0.799189156	0.799189156	0.008061
A.vasorum /Control	P42345	0.127006101	0.127006101	0.008071
A.vasorum /Control	O14949	0.333898986	0.333898986	0.008078
A.vasorum /Control	Q8NCC3	0.045891894	0.045891894	0.008097
A.vasorum /Control	P30041	0.048666647	0.048666647	0.008134
A.vasorum /Control	Q6VMQ6	0.213706879	0.213706879	0.008137
A.vasorum /Control	P07858	-0.135925281	0.135925281	0.008143
A.vasorum /Control	P61011	-0.049189512	0.049189512	0.008184
A.vasorum /Control	Q12931	-0.036741778	0.036741778	0.00822
A.vasorum /Control	Q9NVM9	0.101466506	0.101466506	0.008218
A.vasorum /Control	C4AMC7	0.220218614	0.220218614	0.008233
A.vasorum /Control	Q9NZC3	-1.48431898	1.48431898	0.008236
A.vasorum /Control	Q9BV20	0.022735533	0.022735533	0.008251
A.vasorum /Control	Q05209	0.274993556	0.274993556	0.00826
A.vasorum /Control	Q8IVB5	0.233302826	0.233302826	0.008258
A.vasorum /Control	Q9Y6D6	0.109236866	0.109236866	0.008265

A.vasorum /Control	P27695	0.163472078	0.163472078	0.008274
A.vasorum /Control	Q9UHJ6	0.185874768	0.185874768	0.008273
A.vasorum /Control	O43164	0.443766924	0.443766924	0.008288
A.vasorum /Control	Q9Y653	0.478473218	0.478473218	0.008283
A.vasorum /Control	Q9Y6E0	0.078677867	0.078677867	0.008285
A.vasorum /Control	P00367	0.010312769	0.010312769	0.00832
A.vasorum /Control	O94925	-0.125997691	0.125997691	0.008329
A.vasorum /Control	Q969F9	0.131206061	0.131206061	0.008326
A.vasorum /Control	Q9UBQ5	0.020266787	0.020266787	0.00834
A.vasorum /Control	Q9H1D9	0.196386794	0.196386794	0.008343
A.vasorum /Control	Q1KMD3	-0.0817411	0.0817411	0.008361
A.vasorum /Control	Q15773	0.238759918	0.238759918	0.008391
A.vasorum /Control	Q2M1P5	0.030975704	0.030975704	0.008395
A.vasorum /Control	Q96II8	0.071771962	0.071771962	0.008392
A.vasorum /Control	O75746	0.108203512	0.108203512	0.008414
A.vasorum /Control	Q9BRK5	0.184834056	0.184834056	0.008415
A.vasorum /Control	Q7L523	-0.009135858	0.009135858	0.00843
A.vasorum /Control	Q16181	0.004184703	0.004184703	0.008436
A.vasorum /Control	Q5TZA2	-0.075629106	0.075629106	0.008442
A.vasorum /Control	P62899	-0.013508458	0.013508458	0.00846
A.vasorum /Control	Q8N488	-0.073743124	0.073743124	0.008463
A.vasorum /Control	Q9UL33	0.098405497	0.098405497	0.008471
A.vasorum /Control	P82921	0.238686658	0.238686658	0.008488
A.vasorum /Control	Q07889	0.170244052	0.170244052	0.00849
A.vasorum /Control	Q5H9U9	1.604615435	1.604615435	0.008486
A.vasorum /Control	Q9UBQ7	0.181002591	0.181002591	0.008488
A.vasorum /Control	P78332	0.226442995	0.226442995	0.008498
A.vasorum /Control	Q92522	0.615081128	0.615081128	0.008496
A.vasorum /Control	Q8WUB8	-0.039853888	0.039853888	0.008514
A.vasorum /Control	Q9H6U6	0.355394726	0.355394726	0.008518
A.vasorum /Control	O95721	0.194631946	0.194631946	0.008528
A.vasorum /Control	P82914	-0.112880346	0.112880346	0.00853
A.vasorum /Control	Q9NUD5	0.776934767	0.776934767	0.008546
A.vasorum /Control	O95059	1.024432078	1.024432078	0.008564
A.vasorum /Control	Q96EB6	0.136261304	0.136261304	0.00856
A.vasorum /Control	Q96PZ0	0.199334238	0.199334238	0.008566
A.vasorum /Control	Q9Y314	-0.46333303	0.46333303	0.008566
A.vasorum /Control	Q92614	0.101945071	0.101945071	0.008603
A.vasorum /Control	Q96SI1	0.847781607	0.847781607	0.008605
A.vasorum /Control	Q96SI9	0.300637209	0.300637209	0.008623
A.vasorum /Control	Q99459	0.019328169	0.019328169	0.008671
A.vasorum /Control	P56211	-0.055021347	0.055021347	0.008677
A.vasorum /Control	Q13561	0.207931924	0.207931924	0.008692
A.vasorum /Control	Q86SF2	-0.089658716	0.089658716	0.008719
A.vasorum /Control	Q9NUY8	0.02148483	0.02148483	0.00872
A.vasorum /Control	O95490	0.361484977	0.361484977	0.008739
A.vasorum /Control	Q9H8H3	0.45872135	0.45872135	0.008738
A.vasorum /Control	Q9ULH0	0.221512771	0.221512771	0.008745
A.vasorum /Control	O15347	0.167236869	0.167236869	0.008767
A.vasorum /Control	Q6PML9	0.441639018	0.441639018	0.008764

A.vasorum /Control	O95989	0.399647615	0.399647615	0.008779
A.vasorum /Control	P23510	-0.698459203	0.698459203	0.00882
A.vasorum /Control	Q9NQ89	0.705054876	0.705054876	0.008836
A.vasorum /Control	Q6PCB7	0.719615692	0.719615692	0.008877
A.vasorum /Control	Q9Y2H1	0.471644147	0.471644147	0.008892
A.vasorum /Control	P63313	0.321335584	0.321335584	0.008909
A.vasorum /Control	Q8NDV1	0.435086072	0.435086072	0.008906
A.vasorum /Control	Q9UJS0	0.18092522	0.18092522	0.008922
A.vasorum /Control	Q15642	0.003754383	0.003754383	0.00895
A.vasorum /Control	Q9P0U4	0.428212214	0.428212214	0.008956
A.vasorum /Control	P62736	-0.080534772	0.080534772	0.008969
A.vasorum /Control	Q5T5C0	-0.046236682	0.046236682	0.008967
A.vasorum /Control	Q96K21	1.260133026	1.260133026	0.008967
A.vasorum /Control	Q13200	-0.154616779	0.154616779	0.00899
A.vasorum /Control	O95363	0.188388347	0.188388347	0.009003
A.vasorum /Control	Q6PGN9	1.214884552	1.214884552	0.009012
A.vasorum /Control	Q9ULG6	-0.728120891	0.728120891	0.009021
A.vasorum /Control	Q99717	0.225524167	0.225524167	0.009035
A.vasorum /Control	Q9NZT2	0.091584007	0.091584007	0.009045
A.vasorum /Control	Q13630	0.225979836	0.225979836	0.009055
A.vasorum /Control	Q5JVS0	0.343802787	0.343802787	0.009065
A.vasorum /Control	P54886	-0.031708726	0.031708726	0.009085
A.vasorum /Control	P55210	0.123680842	0.123680842	0.00909
A.vasorum /Control	Q9NPF5	0.111158758	0.111158758	0.009096
A.vasorum /Control	Q8TCT9	0.101474032	0.101474032	0.009108
A.vasorum /Control	Q9BY89	0.029435599	0.029435599	0.009113
A.vasorum /Control	Q9BVJ7	0.229401294	0.229401294	0.009119
A.vasorum /Control	Q6P2P2	0.784127918	0.784127918	0.009132
A.vasorum /Control	Q6PL24	0.020127185	0.020127185	0.009145
A.vasorum /Control	Q5VZL5	-0.016512182	0.016512182	0.009158
A.vasorum /Control	Q9HAV4	0.135574575	0.135574575	0.009155
A.vasorum /Control	P0DP23	0.527038151	0.527038151	0.009173
A.vasorum /Control	Q9BVK6	0.011162789	0.011162789	0.009173
A.vasorum /Control	Q96SY0	0.041103477	0.041103477	0.009185
A.vasorum /Control	P37837	0.007516937	0.007516937	0.009215
A.vasorum /Control	Q9HAH7	1.796773473	1.796773473	0.009213
A.vasorum /Control	Q9UHQ4	0.042021358	0.042021358	0.009221
A.vasorum /Control	O14939	0.237315381	0.237315381	0.009234
A.vasorum /Control	Q15906	-0.737037383	0.737037383	0.00926
A.vasorum /Control	Q7L9L4	0.2087001	0.2087001	0.00927
A.vasorum /Control	Q9Y6X3	0.375575918	0.375575918	0.00928
A.vasorum /Control	Q96KG9	0.150115606	0.150115606	0.009318
A.vasorum /Control	O60306	0.061613356	0.061613356	0.009345
A.vasorum /Control	P50395	0.016158588	0.016158588	0.009344
A.vasorum /Control	Q13131	0.111482377	0.111482377	0.009345
A.vasorum /Control	Q96EY8	0.391299974	0.391299974	0.009343
A.vasorum /Control	Q08AD1	0.0588378	0.0588378	0.009355
A.vasorum /Control	Q8NFA0	0.044716662	0.044716662	0.009363
A.vasorum /Control	Q9UET6	-0.15622284	0.15622284	0.009389
A.vasorum /Control	Q9H000	0.132328634	0.132328634	0.009403

A.vasorum /Control	P61201	-0.003319317	0.003319317	0.009421
A.vasorum /Control	Q8NEY8	-0.577940147	0.577940147	0.009418
A.vasorum /Control	Q9BVG9	0.2563957	0.2563957	0.009429
A.vasorum /Control	O15144	0.102910117	0.102910117	0.009433
A.vasorum /Control	P54652	0.641921879	0.641921879	0.009438
A.vasorum /Control	Q02878	-0.028655328	0.028655328	0.009459
A.vasorum /Control	Q9BSV6	-0.218510513	0.218510513	0.009459
A.vasorum /Control	O14734	0.024329997	0.024329997	0.009464
A.vasorum /Control	P21453	0.15167364	0.15167364	0.009475
A.vasorum /Control	O60244	0.105109657	0.105109657	0.009528
A.vasorum /Control	Q15054	-0.223684513	0.223684513	0.009524
A.vasorum /Control	Q9BZ23	-0.042592326	0.042592326	0.00952
A.vasorum /Control	Q9Y6A5	-1.332281814	1.332281814	0.009518
A.vasorum /Control	P10114	0.04558518	0.04558518	0.009545
A.vasorum /Control	Q99436	0.067368113	0.067368113	0.009582
A.vasorum /Control	Q15139	0.829360225	0.829360225	0.009601
A.vasorum /Control	P24752	0.065127312	0.065127312	0.009607
A.vasorum /Control	Q6NSW5	-0.034937044	0.034937044	0.009635
A.vasorum /Control	O00587	0.991155645	0.991155645	0.009662
A.vasorum /Control	Q12800	0.11290423	0.11290423	0.00968
A.vasorum /Control	Q92841	0.001929693	0.001929693	0.009691
A.vasorum /Control	Q9H6D7	0.226276293	0.226276293	0.009702
A.vasorum /Control	P28827	0.573928437	0.573928437	0.009711
A.vasorum /Control	Q14008	-0.057091353	0.057091353	0.009717
A.vasorum /Control	Q86X10	0.209189562	0.209189562	0.009713
A.vasorum /Control	Q15019	0.066682692	0.066682692	0.009751
A.vasorum /Control	Q9P0K7	0.035648107	0.035648107	0.009777
A.vasorum /Control	O60925	0.444026835	0.444026835	0.009789
A.vasorum /Control	O95218	0.010110367	0.010110367	0.009822
A.vasorum /Control	Q14789	0.066098424	0.066098424	0.009826
A.vasorum /Control	Q8IZL8	0.021413247	0.021413247	0.009843
A.vasorum /Control	Q9UH99	0.016611395	0.016611395	0.009857
A.vasorum /Control	Q9P0M9	-0.079130315	0.079130315	0.009863
A.vasorum /Control	P21589	0.132596994	0.132596994	0.009882
A.vasorum /Control	Q9UGP8	0.000348881	0.000348881	0.009895
A.vasorum /Control	O15321	-0.017490728	0.017490728	0.009901
A.vasorum /Control	Q16798	0.310603011	0.310603011	0.009905
A.vasorum /Control	Q8NHU6	0.438823558	0.438823558	0.009931
A.vasorum /Control	P49427	-0.205942355	0.205942355	0.009951
A.vasorum /Control	P61247	-0.10054518	0.10054518	0.009949
A.vasorum /Control	Q6PGP7	0.014565387	0.014565387	0.009954
A.vasorum /Control	P55201	0.579655803	0.579655803	0.009968
A.vasorum /Control	Q8WXF1	-0.030763491	0.030763491	0.009966
A.vasorum /Control	A4D1P6	0.534784002	0.534784002	0.009988
A.vasorum /Control	Q14241	0.123239708	0.123239708	0.009987
A.vasorum /Control	Q14257	0.320683697	0.320683697	0.009987
A.vasorum /Control	Q7L266	0.282562061	0.282562061	0.010045
A.vasorum /Control	P49589	0.016886557	0.016886557	0.010076
A.vasorum /Control	O14777	-0.044149383	0.044149383	0.010088
A.vasorum /Control	Q13572	0.273668384	0.273668384	0.010095

A.vasorum /Control	Q96M96	-0.048516588	0.048516588	0.010105
A.vasorum /Control	Q8IVH4	0.371207429	0.371207429	0.010143
A.vasorum /Control	Q9UDT6	-0.160097982	0.160097982	0.010144
A.vasorum /Control	Q5K4L6	0.901296619	0.901296619	0.01016
A.vasorum /Control	O75431	0.001852285	0.001852285	0.010199
A.vasorum /Control	Q8NBT2	0.213953218	0.213953218	0.010194
A.vasorum /Control	Q8TEU7	0.292737348	0.292737348	0.010199
A.vasorum /Control	P23921	-0.041087843	0.041087843	0.010225
A.vasorum /Control	Q04206	-0.022170597	0.022170597	0.010228
A.vasorum /Control	Q96M27	0.051878101	0.051878101	0.010218
A.vasorum /Control	Q9HBL7	0.415619448	0.415619448	0.010228
A.vasorum /Control	Q9Y2V7	0.189501682	0.189501682	0.010241
A.vasorum /Control	Q92947	0.317882401	0.317882401	0.010261
A.vasorum /Control	Q12904	0.018098472	0.018098472	0.010289
A.vasorum /Control	Q7Z6J2	0.484138462	0.484138462	0.010323
A.vasorum /Control	Q9GZU8	-0.106876471	0.106876471	0.010324
A.vasorum /Control	Q00535	0.486690822	0.486690822	0.010335
A.vasorum /Control	O00750	1.267246698	1.267246698	0.01034
A.vasorum /Control	A6NED2	-1.756621098	1.756621098	0.010352
A.vasorum /Control	Q9H0P0	0.089916094	0.089916094	0.010349
A.vasorum /Control	A9UHW6	0.164567287	0.164567287	0.010385
A.vasorum /Control	P78312	0.362354397	0.362354397	0.010385
A.vasorum /Control	Q92615	-0.102042145	0.102042145	0.010372
A.vasorum /Control	Q96PU4	0.176430715	0.176430715	0.010389
A.vasorum /Control	Q99961	-0.04278122	0.04278122	0.010387
A.vasorum /Control	Q9Y316	-0.05600058	0.05600058	0.010372
A.vasorum /Control	Q6V1X1	0.441670952	0.441670952	0.010456
A.vasorum /Control	Q96JK2	0.139532349	0.139532349	0.010459
A.vasorum /Control	Q9BW91	0.203290002	0.203290002	0.010455
A.vasorum /Control	O94808	0.087883048	0.087883048	0.010481
A.vasorum /Control	P49643	0.26105057	0.26105057	0.010497
A.vasorum /Control	O95155	0.11740038	0.11740038	0.010521
A.vasorum /Control	Q9HCP0	0.0415798	0.0415798	0.01053
A.vasorum /Control	P12235	-0.009508072	0.009508072	0.010536
A.vasorum /Control	P62136	0.112268545	0.112268545	0.01055
A.vasorum /Control	P04818	-0.466336853	0.466336853	0.010563
A.vasorum /Control	Q8NI36	-0.979387412	0.979387412	0.010568
A.vasorum /Control	Q13045	0.117594189	0.117594189	0.010581
A.vasorum /Control	O94829	0.149220255	0.149220255	0.010594
A.vasorum /Control	P18206	-0.041850031	0.041850031	0.010599
A.vasorum /Control	Q9Y2H6	0.017806584	0.017806584	0.010598
A.vasorum /Control	Q8NDH3	0.265886539	0.265886539	0.010624
A.vasorum /Control	O15260	0.083767761	0.083767761	0.010641
A.vasorum /Control	Q86VR2	0.117380073	0.117380073	0.010638
A.vasorum /Control	Q86YT6	-0.047282829	0.047282829	0.010654
A.vasorum /Control	Q9Y217	0.023748924	0.023748924	0.010664
A.vasorum /Control	Q9GZL7	-0.821823415	0.821823415	0.010718
A.vasorum /Control	Q8IWY9	0.332733106	0.332733106	0.010724
A.vasorum /Control	Q9UL15	-0.046175902	0.046175902	0.010727
A.vasorum /Control	Q9NWU1	0.252292445	0.252292445	0.010734

A.vasorum /Control	Q13813	0.092219246	0.092219246	0.010739
A.vasorum /Control	P45985	0.346336773	0.346336773	0.010749
A.vasorum /Control	Q96A35	0.140073637	0.140073637	0.010748
A.vasorum /Control	P82675	-0.193923984	0.193923984	0.010757
A.vasorum /Control	Q99538	0.748773495	0.748773495	0.01077
A.vasorum /Control	Q9NWV8	0.148113236	0.148113236	0.010769
A.vasorum /Control	O94822	-0.129287288	0.129287288	0.010775
A.vasorum /Control	Q9BXP5	0.055319196	0.055319196	0.010798
A.vasorum /Control	O15013	-0.167420092	0.167420092	0.010822
A.vasorum /Control	P18084	0.07942162	0.07942162	0.010823
A.vasorum /Control	P37275	0.152071386	0.152071386	0.010815
A.vasorum /Control	Q8WTS1	0.487778634	0.487778634	0.010851
A.vasorum /Control	Q8IWX8	-0.037300337	0.037300337	0.010857
A.vasorum /Control	Q06787	0.216949147	0.216949147	0.010868
A.vasorum /Control	O43312	0.503298395	0.503298395	0.010879
A.vasorum /Control	Q9H3N1	-0.19259587	0.19259587	0.010902
A.vasorum /Control	O75182	-0.003807284	0.003807284	0.010926
A.vasorum /Control	Q16611	0.326178097	0.326178097	0.010945
A.vasorum /Control	P30876	0.013832979	0.013832979	0.010962
A.vasorum /Control	Q03426	0.067399552	0.067399552	0.010962
A.vasorum /Control	O60231	-0.017894592	0.017894592	0.011009
A.vasorum /Control	Q9Y3A3	0.036429596	0.036429596	0.011008
A.vasorum /Control	P05997	0.383350974	0.383350974	0.011031
A.vasorum /Control	A5PLN9	0.064354886	0.064354886	0.011042
A.vasorum /Control	P46778	-0.126861444	0.126861444	0.01105
A.vasorum /Control	P78417	-0.024797202	0.024797202	0.011048
A.vasorum /Control	Q8TEL6	-0.571547223	0.571547223	0.01109
A.vasorum /Control	P06400	0.043513539	0.043513539	0.011108
A.vasorum /Control	Q6PD62	-0.063479236	0.063479236	0.011116
A.vasorum /Control	Q9C0J8	-0.035289611	0.035289611	0.011137
A.vasorum /Control	Q9NSE4	0.131408968	0.131408968	0.011151
A.vasorum /Control	Q6P1L8	0.169375366	0.169375366	0.011158
A.vasorum /Control	P24385	-0.486242619	0.486242619	0.011223
A.vasorum /Control	Q8NFD5	0.285303227	0.285303227	0.011235
A.vasorum /Control	Q9NTK1	0.091605666	0.091605666	0.011259
A.vasorum /Control	P55786	-0.008110102	0.008110102	0.01128
A.vasorum /Control	Q15811	-0.032078848	0.032078848	0.011278
A.vasorum /Control	Q9UKL0	0.179683433	0.179683433	0.011274
A.vasorum /Control	Q9ULE0	0.285861922	0.285861922	0.011287
A.vasorum /Control	Q9Y4I1	0.146641862	0.146641862	0.011292
A.vasorum /Control	Q9H0S4	-0.364997484	0.364997484	0.011298
A.vasorum /Control	P47914	-0.180906597	0.180906597	0.011331
A.vasorum /Control	Q8NBJ9	0.393425036	0.393425036	0.011326
A.vasorum /Control	Q8N573	0.492875189	0.492875189	0.01134
A.vasorum /Control	Q9BQ24	-2.509889859	2.509889859	0.011352
A.vasorum /Control	O15258	0.119840973	0.119840973	0.011365
A.vasorum /Control	P49005	0.215351144	0.215351144	0.011365
A.vasorum /Control	Q8IWE2	0.047895579	0.047895579	0.011366
A.vasorum /Control	Q13416	0.269775252	0.269775252	0.011393
A.vasorum /Control	P51452	0.186536889	0.186536889	0.011415

A.vasorum /Control	P62316	-0.390626891	0.390626891	0.011415
A.vasorum /Control	Q8IVF7	0.225410114	0.225410114	0.011426
A.vasorum /Control	Q658Y4	-0.033588477	0.033588477	0.011436
A.vasorum /Control	Q96GQ5	0.279731463	0.279731463	0.011455
A.vasorum /Control	P41250	-0.071083783	0.071083783	0.011469
A.vasorum /Control	Q9BTU6	0.067498913	0.067498913	0.011468
A.vasorum /Control	O95985	-0.049058867	0.049058867	0.011482
A.vasorum /Control	Q9GZT9	0.278829973	0.278829973	0.011497
A.vasorum /Control	P07814	-0.018429378	0.018429378	0.011502
A.vasorum /Control	Q9UHD8	0.035190855	0.035190855	0.011514
A.vasorum /Control	Q15120	0.048156573	0.048156573	0.011519
A.vasorum /Control	P07332	0.638571184	0.638571184	0.011581
A.vasorum /Control	Q9HB07	0.166102479	0.166102479	0.011617
A.vasorum /Control	P10599	0.18470041	0.18470041	0.011638
A.vasorum /Control	P04083	0.005287174	0.005287174	0.011649
A.vasorum /Control	Q495W5	0.530827052	0.530827052	0.011668
A.vasorum /Control	P07902	-0.904565491	0.904565491	0.01169
A.vasorum /Control	Q99519	0.253626334	0.253626334	0.011688
A.vasorum /Control	Q9H0J9	0.306469555	0.306469555	0.011689
A.vasorum /Control	Q9UJ41	-0.055315051	0.055315051	0.011685
A.vasorum /Control	Q6RW13	0.594085022	0.594085022	0.011703
A.vasorum /Control	A1X283	-0.004825675	0.004825675	0.011721
A.vasorum /Control	P49914	1.610156887	1.610156887	0.011721
A.vasorum /Control	Q9NRX4	1.014708191	1.014708191	0.011714
A.vasorum /Control	O43933	0.418383806	0.418383806	0.011736
A.vasorum /Control	Q6Q0C0	0.273875115	0.273875115	0.011738
A.vasorum /Control	O15397	0.037447226	0.037447226	0.011752
A.vasorum /Control	Q8NFF5	0.331516542	0.331516542	0.011756
A.vasorum /Control	O14545	0.01674147	0.01674147	0.011784
A.vasorum /Control	Q96RN5	-0.158764049	0.158764049	0.011786
A.vasorum /Control	Q9UBB6	0.161510988	0.161510988	0.011785
A.vasorum /Control	P06280	0.681472569	0.681472569	0.011798
A.vasorum /Control	Q13123	-0.041110885	0.041110885	0.011806
A.vasorum /Control	Q8IZQ1	-0.16961173	0.16961173	0.011814
A.vasorum /Control	Q9NPI6	-0.238034362	0.238034362	0.011836
A.vasorum /Control	Q9Y6I9	-0.051547299	0.051547299	0.011898
A.vasorum /Control	Q8IWZ3	0.132319498	0.132319498	0.011911
A.vasorum /Control	O75170	-0.074583332	0.074583332	0.011937
A.vasorum /Control	O95782	-0.016990665	0.016990665	0.011938
A.vasorum /Control	P39748	0.03404867	0.03404867	0.011928
A.vasorum /Control	Q9HC07	-0.143980248	0.143980248	0.011943
A.vasorum /Control	O94806	0.460397521	0.460397521	0.011978
A.vasorum /Control	Q15005	-0.004824358	0.004824358	0.011981
A.vasorum /Control	Q9BXP2	0.151137001	0.151137001	0.011973
A.vasorum /Control	Q9Y383	0.123049836	0.123049836	0.011981
A.vasorum /Control	O15439	-0.119731117	0.119731117	0.011987
A.vasorum /Control	O15162	-0.057056434	0.057056434	0.012001
A.vasorum /Control	Q96J84	0.159460426	0.159460426	0.01204
A.vasorum /Control	Q9P2X3	0.029609954	0.029609954	0.012062
A.vasorum /Control	Q14997	-0.186974739	0.186974739	0.01211

A.vasorum /Control	Q9NQG5	0.007518088	0.007518088	0.012103
A.vasorum /Control	Q9NXU5	0.543561676	0.543561676	0.01211
A.vasorum /Control	O95819	0.057285509	0.057285509	0.012166
A.vasorum /Control	P08473	0.496412562	0.496412562	0.012162
A.vasorum /Control	Q96S59	0.211807077	0.211807077	0.012158
A.vasorum /Control	P98170	0.152585348	0.152585348	0.012194
A.vasorum /Control	Q7Z5K2	0.32525095	0.32525095	0.012196
A.vasorum /Control	Q9NR12	-0.498435531	0.498435531	0.012195
A.vasorum /Control	Q14694	-0.034838738	0.034838738	0.012222
A.vasorum /Control	Q96L93	0.492206227	0.492206227	0.012227
A.vasorum /Control	P32322	0.061328495	0.061328495	0.012278
A.vasorum /Control	Q8NEU8	0.102983397	0.102983397	0.012289
A.vasorum /Control	P20290	-0.180442346	0.180442346	0.012309
A.vasorum /Control	P28074	-0.043960914	0.043960914	0.012303
A.vasorum /Control	Q96PC5	-0.015167887	0.015167887	0.012307
A.vasorum /Control	Q9NQ55	-0.897515099	0.897515099	0.012335
A.vasorum /Control	Q86X27	0.284793279	0.284793279	0.012343
A.vasorum /Control	Q9NZ08	0.202721071	0.202721071	0.012348
A.vasorum /Control	Q9BZF9	0.258053814	0.258053814	0.01239
A.vasorum /Control	Q9Y5N6	-1.811033581	1.811033581	0.012389
A.vasorum /Control	Q01082	0.03635244	0.03635244	0.012443
A.vasorum /Control	Q52LJ0	0.178939365	0.178939365	0.012437
A.vasorum /Control	Q9Y3B9	-1.19693675	1.19693675	0.012445
A.vasorum /Control	Q05397	0.050741515	0.050741515	0.012488
A.vasorum /Control	Q9NYA1	-0.831724416	0.831724416	0.012489
A.vasorum /Control	P54709	0.021765765	0.021765765	0.012526
A.vasorum /Control	Q8IWA0	-1.19568245	1.19568245	0.012524
A.vasorum /Control	Q9H2V7	0.02455189	0.02455189	0.012537
A.vasorum /Control	Q53HC9	0.016506654	0.016506654	0.012565
A.vasorum /Control	Q5UIP0	-0.324333848	0.324333848	0.012564
A.vasorum /Control	O95793	0.034305481	0.034305481	0.012582
A.vasorum /Control	Q9H8K7	0.069262508	0.069262508	0.01258
A.vasorum /Control	Q8TAT6	0.075695165	0.075695165	0.012602
A.vasorum /Control	Q96SZ5	0.099387279	0.099387279	0.012613
A.vasorum /Control	Q9NZL4	-0.016721248	0.016721248	0.012619
A.vasorum /Control	O43432	-0.008752038	0.008752038	0.012632
A.vasorum /Control	Q9UHG3	0.436812258	0.436812258	0.01265
A.vasorum /Control	A7E2V4	-0.076216643	0.076216643	0.012691
A.vasorum /Control	Q08117	1.115439687	1.115439687	0.012709
A.vasorum /Control	P20908	0.317853924	0.317853924	0.012722
A.vasorum /Control	Q9H0G5	-0.184835413	0.184835413	0.012726
A.vasorum /Control	O75600	-0.098684135	0.098684135	0.012766
A.vasorum /Control	P51114	-0.067638994	0.067638994	0.012762
A.vasorum /Control	Q7Z2W4	0.050596325	0.050596325	0.012766
A.vasorum /Control	Q9BVG4	0.140172926	0.140172926	0.012804
A.vasorum /Control	P35813	0.02520555	0.02520555	0.012872
A.vasorum /Control	Q9UHA3	-1.0529284	1.0529284	0.01293
A.vasorum /Control	P43490	0.057458716	0.057458716	0.012965
A.vasorum /Control	Q9NQ29	0.056407383	0.056407383	0.01297
A.vasorum /Control	O14782	-0.079807583	0.079807583	0.013023

A.vasorum /Control	O75352	0.932538795	0.932538795	0.013027
A.vasorum /Control	Q08379	0.029172906	0.029172906	0.013044
A.vasorum /Control	O60524	-0.001763913	0.001763913	0.013058
A.vasorum /Control	Q3V6T2	0.068153571	0.068153571	0.013059
A.vasorum /Control	Q9Y3P9	-0.000262397	0.000262397	0.013074
A.vasorum /Control	Q9Y4B6	-0.093594893	0.093594893	0.013081
A.vasorum /Control	Q8NCF5	0.251355316	0.251355316	0.013091
A.vasorum /Control	P61599	-0.107842784	0.107842784	0.01311
A.vasorum /Control	Q13084	0.284107143	0.284107143	0.013105
A.vasorum /Control	Q5TA50	0.143690562	0.143690562	0.01311
A.vasorum /Control	O75828	0.156170483	0.156170483	0.013128
A.vasorum /Control	Q9UMY4	0.073614833	0.073614833	0.013135
A.vasorum /Control	P30405	-0.054122858	0.054122858	0.013154
A.vasorum /Control	P63272	0.069920878	0.069920878	0.013166
A.vasorum /Control	Q86SQ0	0.258728361	0.258728361	0.01316
A.vasorum /Control	Q96GC5	-0.038426418	0.038426418	0.013163
A.vasorum /Control	Q96QR8	0.032467905	0.032467905	0.013167
A.vasorum /Control	Q16706	0.023698061	0.023698061	0.013195
A.vasorum /Control	Q86SZ2	0.300777213	0.300777213	0.013191
A.vasorum /Control	Q9BVL2	0.329851118	0.329851118	0.013241
A.vasorum /Control	Q8IXM6	-0.039536275	0.039536275	0.013248
A.vasorum /Control	Q9NVI1	0.136199205	0.136199205	0.013258
A.vasorum /Control	Q96IV0	-0.629016303	0.629016303	0.01328
A.vasorum /Control	Q4V328	0.208988337	0.208988337	0.013303
A.vasorum /Control	Q53SF7	0.164312772	0.164312772	0.013307
A.vasorum /Control	Q6DKI1	-1.2287448	1.2287448	0.013307
A.vasorum /Control	Q8IVD9	0.092814902	0.092814902	0.013291
A.vasorum /Control	Q96GM8	0.067319228	0.067319228	0.013294
A.vasorum /Control	Q8WXW3	-1.756226509	1.756226509	0.013313
A.vasorum /Control	Q9C0C9	-0.03798166	0.03798166	0.013358
A.vasorum /Control	O75122	-0.014019184	0.014019184	0.013384
A.vasorum /Control	Q13426	-0.060651108	0.060651108	0.013409
A.vasorum /Control	Q9UK61	0.099499577	0.099499577	0.013418
A.vasorum /Control	O14562	0.097761396	0.097761396	0.013435
A.vasorum /Control	O60547	0.320860071	0.320860071	0.013445
A.vasorum /Control	Q7Z7L1	0.160329224	0.160329224	0.013453
A.vasorum /Control	Q9BYD3	0.017467248	0.017467248	0.013502
A.vasorum /Control	O14497	-0.070783077	0.070783077	0.01352
A.vasorum /Control	Q15436	0.034509583	0.034509583	0.013524
A.vasorum /Control	Q9UBV8	0.715809653	0.715809653	0.013524
A.vasorum /Control	O75167	0.173498374	0.173498374	0.013547
A.vasorum /Control	O94905	0.111136478	0.111136478	0.013547
A.vasorum /Control	O95373	-0.025578622	0.025578622	0.013537
A.vasorum /Control	P49137	0.007510725	0.007510725	0.013543
A.vasorum /Control	Q6ZS17	-0.10739897	0.10739897	0.01356
A.vasorum /Control	Q9BRJ2	0.010752506	0.010752506	0.013589
A.vasorum /Control	Q9UI36	1.745434551	1.745434551	0.013588
A.vasorum /Control	P56589	0.630713038	0.630713038	0.013601
A.vasorum /Control	Q9NSD9	-0.092058686	0.092058686	0.013606
A.vasorum /Control	O95149	0.121065801	0.121065801	0.013653

A.vasorum /Control	Q9UBC2	-0.091115932	0.091115932	0.013654
A.vasorum /Control	P84095	0.018891302	0.018891302	0.013662
A.vasorum /Control	Q9Y6Q9	0.216933744	0.216933744	0.013693
A.vasorum /Control	Q96F85	0.27709855	0.27709855	0.013731
A.vasorum /Control	Q96DG6	0.349342128	0.349342128	0.013738
A.vasorum /Control	Q9UGL1	-0.355364403	0.355364403	0.013742
A.vasorum /Control	Q96Q05	0.090593661	0.090593661	0.013788
A.vasorum /Control	Q5TBB1	0.30223972	0.30223972	0.013812
A.vasorum /Control	P17301	-0.433660649	0.433660649	0.013818
A.vasorum /Control	P49585	0.013082602	0.013082602	0.01383
A.vasorum /Control	Q9P2C4	0.110442506	0.110442506	0.013853
A.vasorum /Control	Q9NYU1	-0.48341614	0.48341614	0.013887
A.vasorum /Control	P26022	-0.620681616	0.620681616	0.013933
A.vasorum /Control	Q2NL82	-0.036411351	0.036411351	0.01393
A.vasorum /Control	Q9Y6M9	0.038703874	0.038703874	0.013938
A.vasorum /Control	P07737	0.080481939	0.080481939	0.013974
A.vasorum /Control	Q5H8A4	0.304848403	0.304848403	0.013981
A.vasorum /Control	Q96FX7	0.126008199	0.126008199	0.013998
A.vasorum /Control	Q9P2J5	0.072498831	0.072498831	0.013997
A.vasorum /Control	Q9P2Q2	-0.028239975	0.028239975	0.014006
A.vasorum /Control	Q8N6M0	-0.214297365	0.214297365	0.014026
A.vasorum /Control	O43681	0.059170105	0.059170105	0.014038
A.vasorum /Control	P04035	0.291440282	0.291440282	0.014055
A.vasorum /Control	Q5T8D3	0.165060253	0.165060253	0.01407
A.vasorum /Control	Q15025	-0.148624767	0.148624767	0.014079
A.vasorum /Control	P46108	0.195378537	0.195378537	0.014084
A.vasorum /Control	Q13523	-0.367031348	0.367031348	0.014102
A.vasorum /Control	Q9Y5X3	0.089640305	0.089640305	0.014107
A.vasorum /Control	Q96HS1	-0.070036043	0.070036043	0.014125
A.vasorum /Control	Q9H9B4	-0.03020082	0.03020082	0.014138
A.vasorum /Control	Q9Y3L5	0.095657205	0.095657205	0.014136
A.vasorum /Control	Q9BX69	0.007971454	0.007971454	0.014167
A.vasorum /Control	O00592	-0.015649723	0.015649723	0.014223
A.vasorum /Control	Q86WR0	0.234582443	0.234582443	0.014236
A.vasorum /Control	Q9HBL0	0.154235	0.154235	0.014259
A.vasorum /Control	Q9BVV7	-0.019420529	0.019420529	0.014288
A.vasorum /Control	Q15125	0.154716427	0.154716427	0.01432
A.vasorum /Control	Q9NWZ5	0.295324885	0.295324885	0.014348
A.vasorum /Control	Q15796	-0.128796948	0.128796948	0.014367
A.vasorum /Control	Q86TV6	0.172762705	0.172762705	0.01437
A.vasorum /Control	Q8IWF6	0.372701013	0.372701013	0.014387
A.vasorum /Control	P14550	0.08320786	0.08320786	0.014406
A.vasorum /Control	O15504	0.691571739	0.691571739	0.014437
A.vasorum /Control	P23786	0.108870607	0.108870607	0.014452
A.vasorum /Control	Q16531	0.034149878	0.034149878	0.014454
A.vasorum /Control	Q15477	0.08150708	0.08150708	0.014472
A.vasorum /Control	Q13443	-0.221567929	0.221567929	0.014478
A.vasorum /Control	Q14699	0.256765887	0.256765887	0.014485
A.vasorum /Control	Q01167	0.014472745	0.014472745	0.014501
A.vasorum /Control	O43181	0.42999163	0.42999163	0.014526

A.vasorum /Control	O95816	-0.017439093	0.017439093	0.014551
A.vasorum /Control	P23246	0.053939549	0.053939549	0.014552
A.vasorum /Control	Q9H1I8	-0.111432134	0.111432134	0.014547
A.vasorum /Control	O75351	0.130031792	0.130031792	0.01458
A.vasorum /Control	Q9C0B7	-0.070750329	0.070750329	0.014579
A.vasorum /Control	O43143	0.042531282	0.042531282	0.014597
A.vasorum /Control	Q75QN2	0.136215135	0.136215135	0.014597
A.vasorum /Control	P00492	-0.00228296	0.00228296	0.014636
A.vasorum /Control	Q9UQN3	0.030209816	0.030209816	0.014662
A.vasorum /Control	Q9NYY8	0.277831439	0.277831439	0.014669
A.vasorum /Control	Q9H6R7	0.302558853	0.302558853	0.014701
A.vasorum /Control	Q96RF0	-0.099960049	0.099960049	0.014715
A.vasorum /Control	Q9NRZ7	-0.050512937	0.050512937	0.014755
A.vasorum /Control	P08648	-0.284439127	0.284439127	0.014763
A.vasorum /Control	Q08AM6	0.164314556	0.164314556	0.014766
A.vasorum /Control	P18850	0.018156903	0.018156903	0.014813
A.vasorum /Control	Q9UI12	-0.044253441	0.044253441	0.014836
A.vasorum /Control	Q15388	-0.161684479	0.161684479	0.014863
A.vasorum /Control	Q96SK2	0.216859483	0.216859483	0.014867
A.vasorum /Control	P13639	-0.108068899	0.108068899	0.014881
A.vasorum /Control	Q14690	-0.851895932	0.851895932	0.014898
A.vasorum /Control	Q9NP92	-0.050704508	0.050704508	0.014904
A.vasorum /Control	Q9HBM1	-0.067946884	0.067946884	0.014914
A.vasorum /Control	P53597	-0.192680573	0.192680573	0.014934
A.vasorum /Control	Q6I9Y2	-0.025355027	0.025355027	0.01493
A.vasorum /Control	Q7Z6M4	-0.034726533	0.034726533	0.014924
A.vasorum /Control	P30536	0.283328942	0.283328942	0.014949
A.vasorum /Control	P67809	0.039054682	0.039054682	0.014956
A.vasorum /Control	Q7L775	0.196240275	0.196240275	0.014965
A.vasorum /Control	Q9H9P8	0.869556435	0.869556435	0.014982
A.vasorum /Control	Q13111	-0.213658982	0.213658982	0.015012
A.vasorum /Control	P53355	0.26093089	0.26093089	0.01503
A.vasorum /Control	Q9Y2S6	0.115059683	0.115059683	0.015034
A.vasorum /Control	Q92544	0.054439175	0.054439175	0.01507
A.vasorum /Control	Q15058	-0.063537069	0.063537069	0.015105
A.vasorum /Control	O15027	-0.026348105	0.026348105	0.015124
A.vasorum /Control	Q96TA1	-0.096847691	0.096847691	0.015146
A.vasorum /Control	Q6PI98	0.113187764	0.113187764	0.015167
A.vasorum /Control	Q15633	0.186347405	0.186347405	0.015179
A.vasorum /Control	Q8TDQ7	-0.149367092	0.149367092	0.01518
A.vasorum /Control	P30281	-1.483259292	1.483259292	0.015206
A.vasorum /Control	P18124	0.031344581	0.031344581	0.015249
A.vasorum /Control	P43897	-0.159705438	0.159705438	0.015231
A.vasorum /Control	P53609	-0.030009021	0.030009021	0.015229
A.vasorum /Control	P62312	0.139539689	0.139539689	0.015262
A.vasorum /Control	P82933	-0.104533173	0.104533173	0.015238
A.vasorum /Control	Q8IX01	0.148737892	0.148737892	0.015226
A.vasorum /Control	Q8N4C8	0.350388985	0.350388985	0.015229
A.vasorum /Control	Q8N5A5	0.0912545	0.0912545	0.015243
A.vasorum /Control	Q969M1	0.454623851	0.454623851	0.015258

A.vasorum /Control	Q9Y4P1	-0.312861676	0.312861676	0.015262
A.vasorum /Control	Q9BQ67	-0.309317206	0.309317206	0.015299
A.vasorum /Control	Q8NEJ9	-0.41556395	0.41556395	0.015309
A.vasorum /Control	P50995	0.143997409	0.143997409	0.01532
A.vasorum /Control	P07237	0.061660924	0.061660924	0.015331
A.vasorum /Control	P49454	-0.828576234	0.828576234	0.015381
A.vasorum /Control	P98082	-0.028815491	0.028815491	0.015401
A.vasorum /Control	Q8N201	0.176556366	0.176556366	0.015419
A.vasorum /Control	Q12899	0.149038281	0.149038281	0.015441
A.vasorum /Control	Q6ICG6	0.78570341	0.78570341	0.015438
A.vasorum /Control	P50453	0.017174263	0.017174263	0.015471
A.vasorum /Control	O95219	0.122630925	0.122630925	0.01548
A.vasorum /Control	Q99500	0.745500914	0.745500914	0.015493
A.vasorum /Control	Q9NRP0	0.031878267	0.031878267	0.015501
A.vasorum /Control	O43747	-0.004991116	0.004991116	0.015542
A.vasorum /Control	Q9Y4X5	-0.010866688	0.010866688	0.015537
A.vasorum /Control	P53007	0.08508715	0.08508715	0.015595
A.vasorum /Control	Q15172	0.776263404	0.776263404	0.015612
A.vasorum /Control	Q9H490	0.179226655	0.179226655	0.015606
A.vasorum /Control	Q9HCU5	0.411043937	0.411043937	0.015607
A.vasorum /Control	O15213	-1.2972028	1.2972028	0.015629
A.vasorum /Control	Q9UKA4	0.107285462	0.107285462	0.015626
A.vasorum /Control	Q9UL25	0.007672787	0.007672787	0.01566
A.vasorum /Control	A0FGR8	0.040779975	0.040779975	0.015775
A.vasorum /Control	A8MWY0	0.804320859	0.804320859	0.015778
A.vasorum /Control	P10644	-0.081058335	0.081058335	0.015791
A.vasorum /Control	Q13439	0.044073217	0.044073217	0.015788
A.vasorum /Control	Q15181	-0.022561248	0.022561248	0.015793
A.vasorum /Control	O75306	0.097465049	0.097465049	0.015831
A.vasorum /Control	O76054	0.0358492	0.0358492	0.015815
A.vasorum /Control	P51665	0.025781721	0.025781721	0.015824
A.vasorum /Control	Q13459	-0.025739235	0.025739235	0.015821
A.vasorum /Control	Q8N3X1	0.145795859	0.145795859	0.015833
A.vasorum /Control	Q93050	-0.160319819	0.160319819	0.015936
A.vasorum /Control	Q7KZI7	0.107397	0.107397	0.015948
A.vasorum /Control	Q6UW63	0.081641209	0.081641209	0.015954
A.vasorum /Control	Q96T58	-0.770161292	0.770161292	0.015985
A.vasorum /Control	P16298	0.003501572	0.003501572	0.016012
A.vasorum /Control	P51946	0.138055511	0.138055511	0.016008
A.vasorum /Control	Q9BZ76	-0.090769661	0.090769661	0.016018
A.vasorum /Control	Q68E01	0.263697737	0.263697737	0.016052
A.vasorum /Control	Q15366	-0.019015535	0.019015535	0.016074
A.vasorum /Control	Q92643	0.73264815	0.73264815	0.016071
A.vasorum /Control	P22830	0.369329659	0.369329659	0.016095
A.vasorum /Control	Q5QJE6	-0.997285108	0.997285108	0.016116
A.vasorum /Control	Q96EU7	0.49129004	0.49129004	0.016111
A.vasorum /Control	O15091	0.027404846	0.027404846	0.016138
A.vasorum /Control	Q13492	-0.01316959	0.01316959	0.016132
A.vasorum /Control	Q9HBR0	0.574884509	0.574884509	0.016146
A.vasorum /Control	Q12933	-0.05918371	0.05918371	0.016155

A.vasorum /Control	P26640	-0.052613581	0.052613581	0.016164
A.vasorum /Control	Q8IWR0	-0.118864775	0.118864775	0.016182
A.vasorum /Control	Q9NXF7	0.122374315	0.122374315	0.016186
A.vasorum /Control	Q9UNX4	-0.809740103	0.809740103	0.01619
A.vasorum /Control	P01034	-0.327146458	0.327146458	0.016211
A.vasorum /Control	Q9Y276	0.091450815	0.091450815	0.016261
A.vasorum /Control	Q9NRA8	0.060709797	0.060709797	0.0163
A.vasorum /Control	Q99447	0.297559678	0.297559678	0.016334
A.vasorum /Control	Q9UPU5	0.2514635	0.2514635	0.016336
A.vasorum /Control	P48723	0.167408254	0.167408254	0.016349
A.vasorum /Control	Q9NTZ6	-0.032656706	0.032656706	0.016406
A.vasorum /Control	Q15363	0.322958676	0.322958676	0.016416
A.vasorum /Control	Q7Z7H5	0.053520246	0.053520246	0.016418
A.vasorum /Control	Q9NZM1	0.043769017	0.043769017	0.016436
A.vasorum /Control	Q8IXB1	0.086414685	0.086414685	0.016447
A.vasorum /Control	O95453	-0.124727835	0.124727835	0.016472
A.vasorum /Control	Q99471	0.369402101	0.369402101	0.016499
A.vasorum /Control	Q5GLZ8	0.006719789	0.006719789	0.016516
A.vasorum /Control	P27707	-0.120785808	0.120785808	0.016584
A.vasorum /Control	Q00610	-0.002870532	0.002870532	0.016583
A.vasorum /Control	Q9BT78	0.051787062	0.051787062	0.016605
A.vasorum /Control	O95487	0.10792021	0.10792021	0.016626
A.vasorum /Control	P08579	-0.104414927	0.104414927	0.0167
A.vasorum /Control	Q9HBM0	0.304779303	0.304779303	0.016728
A.vasorum /Control	Q9UPW5	0.327421811	0.327421811	0.016725
A.vasorum /Control	Q6UWZ7	0.570613235	0.570613235	0.016754
A.vasorum /Control	Q68D91	-0.036552202	0.036552202	0.016764
A.vasorum /Control	Q9UEW8	-0.091789198	0.091789198	0.016768
A.vasorum /Control	P35221	0.065189293	0.065189293	0.016796
A.vasorum /Control	Q96FV2	0.65285736	0.65285736	0.016792
A.vasorum /Control	O60318	0.087893006	0.087893006	0.016809
A.vasorum /Control	O15305	-0.076714215	0.076714215	0.016881
A.vasorum /Control	Q8TBC4	0.065459467	0.065459467	0.016943
A.vasorum /Control	P08240	-0.060673731	0.060673731	0.017024
A.vasorum /Control	P49247	0.265224977	0.265224977	0.017025
A.vasorum /Control	Q13107	-0.060638152	0.060638152	0.017111
A.vasorum /Control	Q96QD8	-1.01692634	1.01692634	0.017122
A.vasorum /Control	Q9UFN0	0.053996007	0.053996007	0.017153
A.vasorum /Control	Q9BZL4	0.110588959	0.110588959	0.0172
A.vasorum /Control	Q15437	-0.097671511	0.097671511	0.017275
A.vasorum /Control	Q12965	0.132928896	0.132928896	0.017283
A.vasorum /Control	Q99759	0.498965746	0.498965746	0.017341
A.vasorum /Control	Q86Y39	0.148935922	0.148935922	0.01735
A.vasorum /Control	Q8TDN6	-0.716098769	0.716098769	0.017353
A.vasorum /Control	Q9GZZ9	-0.066515604	0.066515604	0.017369
A.vasorum /Control	O43924	0.256459355	0.256459355	0.017388
A.vasorum /Control	Q9NRY4	0.236524216	0.236524216	0.017385
A.vasorum /Control	Q9H0E2	0.132767455	0.132767455	0.017396
A.vasorum /Control	P49790	-0.065650939	0.065650939	0.017409
A.vasorum /Control	P55268	0.281583883	0.281583883	0.017466

A.vasorum /Control	Q5T447	0.207973398	0.207973398	0.017473
A.vasorum /Control	P46013	-0.827581005	0.827581005	0.017527
A.vasorum /Control	Q96JB5	0.191498472	0.191498472	0.017523
A.vasorum /Control	Q16555	0.102119511	0.102119511	0.01755
A.vasorum /Control	Q8NFH3	0.0773276	0.0773276	0.017591
A.vasorum /Control	Q14004	0.180620053	0.180620053	0.017622
A.vasorum /Control	Q16537	-0.020385579	0.020385579	0.017613
A.vasorum /Control	Q7Z3U7	-0.113960641	0.113960641	0.01763
A.vasorum /Control	Q96SB8	-0.606423815	0.606423815	0.017625
A.vasorum /Control	Q9BU61	0.633361654	0.633361654	0.017622
A.vasorum /Control	P58335	-0.197919497	0.197919497	0.017699
A.vasorum /Control	Q15006	0.007957592	0.007957592	0.017706
A.vasorum /Control	Q9UIV1	0.138166694	0.138166694	0.017697
A.vasorum /Control	Q9ULW3	-0.080655222	0.080655222	0.0177
A.vasorum /Control	P62906	-0.13897639	0.13897639	0.017746
A.vasorum /Control	P62256	0.02087942	0.02087942	0.01779
A.vasorum /Control	P35573	0.176888959	0.176888959	0.017808
A.vasorum /Control	Q9HAC8	0.079656465	0.079656465	0.017803
A.vasorum /Control	Q6RFH5	-1.146567858	1.146567858	0.01782
A.vasorum /Control	Q6NTF9	1.305604227	1.305604227	0.017851
A.vasorum /Control	P31749	0.035188299	0.035188299	0.01787
A.vasorum /Control	Q00796	-0.279615161	0.279615161	0.017879
A.vasorum /Control	O94813	0.775294194	0.775294194	0.017922
A.vasorum /Control	Q9NNW7	0.079771577	0.079771577	0.017998
A.vasorum /Control	O43808	-0.950292007	0.950292007	0.018029
A.vasorum /Control	P09001	0.007876649	0.007876649	0.018036
A.vasorum /Control	P30154	-0.034618445	0.034618445	0.018063
A.vasorum /Control	O43292	0.176916811	0.176916811	0.018116
A.vasorum /Control	P19447	0.289022418	0.289022418	0.018109
A.vasorum /Control	Q93052	0.084293318	0.084293318	0.018114
A.vasorum /Control	Q03001	-0.058048434	0.058048434	0.018162
A.vasorum /Control	Q5VUB5	-0.18636498	0.18636498	0.018163
A.vasorum /Control	P06744	-0.01287047	0.01287047	0.01818
A.vasorum /Control	Q16718	0.475454389	0.475454389	0.018193
A.vasorum /Control	Q5VSL9	0.124861446	0.124861446	0.018223
A.vasorum /Control	Q9NZZ3	0.313542781	0.313542781	0.018225
A.vasorum /Control	P62917	0.041449826	0.041449826	0.018248
A.vasorum /Control	Q7L5Y9	-0.066233128	0.066233128	0.018243
A.vasorum /Control	O43290	-0.022086855	0.022086855	0.01835
A.vasorum /Control	P53999	0.105776261	0.105776261	0.018361
A.vasorum /Control	Q9H0L4	0.15381504	0.15381504	0.018355
A.vasorum /Control	Q9P2D3	-0.099427576	0.099427576	0.01838
A.vasorum /Control	O14744	-0.00433502	0.00433502	0.018399
A.vasorum /Control	Q9H8G2	-0.126086589	0.126086589	0.018407
A.vasorum /Control	P42575	-1.292165144	1.292165144	0.018429
A.vasorum /Control	O75165	0.159618093	0.159618093	0.018478
A.vasorum /Control	Q9NPI1	-0.915675891	0.915675891	0.018502
A.vasorum /Control	Q8IY21	0.978087518	0.978087518	0.018514
A.vasorum /Control	O00213	0.140936926	0.140936926	0.018526
A.vasorum /Control	Q14651	0.601358121	0.601358121	0.018542

A.vasorum /Control	Q8IX12	0.089309423	0.089309423	0.018556
A.vasorum /Control	P16278	0.289165046	0.289165046	0.018572
A.vasorum /Control	Q13563	0.273110803	0.273110803	0.018617
A.vasorum /Control	Q92995	0.179223907	0.179223907	0.018703
A.vasorum /Control	P46100	0.000222036	0.000222036	0.018719
A.vasorum /Control	Q9UIF8	-2.004368426	2.004368426	0.018724
A.vasorum /Control	Q16630	0.039598962	0.039598962	0.018733
A.vasorum /Control	Q7LGA3	-0.249587346	0.249587346	0.018785
A.vasorum /Control	O14763	0.123324757	0.123324757	0.018799
A.vasorum /Control	Q9BWU0	0.050976845	0.050976845	0.018815
A.vasorum /Control	Q8N9Z2	-0.036242717	0.036242717	0.018879
A.vasorum /Control	Q9Y6D0	0.814848228	0.814848228	0.018886
A.vasorum /Control	O14737	0.079847136	0.079847136	0.018946
A.vasorum /Control	P07203	0.151362593	0.151362593	0.01897
A.vasorum /Control	Q15650	-0.126581642	0.126581642	0.018974
A.vasorum /Control	Q6IQ26	0.928390517	0.928390517	0.018963
A.vasorum /Control	Q9BRA2	0.195497337	0.195497337	0.018966
A.vasorum /Control	Q9Y2C4	0.077481707	0.077481707	0.01895
A.vasorum /Control	Q96S97	-0.230256072	0.230256072	0.018981
A.vasorum /Control	O95168	0.08504218	0.08504218	0.018995
A.vasorum /Control	O76003	-0.086719966	0.086719966	0.01902
A.vasorum /Control	Q9BZW5	0.131933167	0.131933167	0.019052
A.vasorum /Control	Q8IUX1	0.337923475	0.337923475	0.019078
A.vasorum /Control	P08651	0.348396336	0.348396336	0.019114
A.vasorum /Control	Q14353	-0.534039589	0.534039589	0.019111
A.vasorum /Control	P38935	0.859812176	0.859812176	0.019132
A.vasorum /Control	P54727	0.298385234	0.298385234	0.019152
A.vasorum /Control	Q562R1	-0.072886993	0.072886993	0.019155
A.vasorum /Control	Q8IXJ6	0.212956674	0.212956674	0.019149
A.vasorum /Control	P13984	-0.009865711	0.009865711	0.019209
A.vasorum /Control	Q9H3Q1	-0.264059408	0.264059408	0.01921
A.vasorum /Control	Q99873	0.081086316	0.081086316	0.019217
A.vasorum /Control	O14578	-0.000111715	0.000111715	0.019287
A.vasorum /Control	P35250	0.036140234	0.036140234	0.019332
A.vasorum /Control	Q96JH7	0.103193338	0.103193338	0.019334
A.vasorum /Control	O75879	0.119272803	0.119272803	0.019341
A.vasorum /Control	P53671	0.168496536	0.168496536	0.019352
A.vasorum /Control	Q9NVA2	0.035877791	0.035877791	0.019418
A.vasorum /Control	Q10570	0.034776922	0.034776922	0.01945
A.vasorum /Control	Q9H900	0.388779521	0.388779521	0.019444
A.vasorum /Control	O43707	-0.220208794	0.220208794	0.019461
A.vasorum /Control	P20674	1.336566101	1.336566101	0.019654
A.vasorum /Control	Q9UHQ9	0.120587251	0.120587251	0.019715
A.vasorum /Control	Q96BK5	-0.331320627	0.331320627	0.019728
A.vasorum /Control	Q9BVT8	0.049863659	0.049863659	0.01975
A.vasorum /Control	Q96EE3	0.012213643	0.012213643	0.019774
A.vasorum /Control	P09543	0.071567214	0.071567214	0.019794
A.vasorum /Control	Q12846	0.18499621	0.18499621	0.019811
A.vasorum /Control	P36542	-0.053783498	0.053783498	0.01982
A.vasorum /Control	Q9NQT8	0.06422478	0.06422478	0.019847

A.vasorum /Control	Q96EV8	0.49398667	0.49398667	0.019861
A.vasorum /Control	P43034	0.064367232	0.064367232	0.019878
A.vasorum /Control	Q96QU8	-0.037650667	0.037650667	0.0199
A.vasorum /Control	O95336	0.464614608	0.464614608	0.019921
A.vasorum /Control	Q02750	0.036145816	0.036145816	0.019928
A.vasorum /Control	A0PJW6	0.976622035	0.976622035	0.019951
A.vasorum /Control	P11766	0.083433956	0.083433956	0.020018
A.vasorum /Control	P53365	0.222250959	0.222250959	0.020023
A.vasorum /Control	Q9NX20	0.083504757	0.083504757	0.020025
A.vasorum /Control	Q15041	-0.900994641	0.900994641	0.020072
A.vasorum /Control	Q6P4A7	0.096370429	0.096370429	0.020074
A.vasorum /Control	O43913	-0.017003181	0.017003181	0.020083
A.vasorum /Control	Q8TEA7	0.552230246	0.552230246	0.020098
A.vasorum /Control	Q9BZJ0	0.000185796	0.000185796	0.020119
A.vasorum /Control	P34896	0.201409256	0.201409256	0.020128
A.vasorum /Control	Q9BSJ2	0.01081213	0.01081213	0.020165
A.vasorum /Control	Q9NR46	0.097507465	0.097507465	0.020166
A.vasorum /Control	Q8TED0	-1.039181087	1.039181087	0.0202
A.vasorum /Control	P35270	0.218831511	0.218831511	0.02021
A.vasorum /Control	P20073	0.054468098	0.054468098	0.020227
A.vasorum /Control	P78324	0.109661171	0.109661171	0.020275
A.vasorum /Control	Q5TC12	0.145372946	0.145372946	0.020288
A.vasorum /Control	Q6FIF0	-0.129218662	0.129218662	0.020284
A.vasorum /Control	Q96DA6	0.30380086	0.30380086	0.020293
A.vasorum /Control	Q9NWU5	-0.055277839	0.055277839	0.020266
A.vasorum /Control	P23634	0.359301086	0.359301086	0.020396
A.vasorum /Control	P46736	0.073289391	0.073289391	0.020449
A.vasorum /Control	P54098	-0.187766731	0.187766731	0.020468
A.vasorum /Control	Q9NZ63	-0.003768213	0.003768213	0.020467
A.vasorum /Control	P0DP91	0.053789612	0.053789612	0.020477
A.vasorum /Control	P46926	0.068677704	0.068677704	0.0205
A.vasorum /Control	Q9BSD7	0.279569264	0.279569264	0.020494
A.vasorum /Control	Q8N2U0	1.053411557	1.053411557	0.020516
A.vasorum /Control	O14745	0.009999809	0.009999809	0.020538
A.vasorum /Control	O76021	-1.031236064	1.031236064	0.02053
A.vasorum /Control	Q9HAV7	-0.163268859	0.163268859	0.020533
A.vasorum /Control	Q96GQ7	-0.873721367	0.873721367	0.020573
A.vasorum /Control	P27708	-0.008281099	0.008281099	0.020628
A.vasorum /Control	Q03936	-0.560138087	0.560138087	0.020647
A.vasorum /Control	O95625	-1.425245394	1.425245394	0.020665
A.vasorum /Control	Q8IYU8	0.456615223	0.456615223	0.020695
A.vasorum /Control	Q8NHQ9	-0.093245889	0.093245889	0.020735
A.vasorum /Control	Q6P4R8	0.032349609	0.032349609	0.020768
A.vasorum /Control	Q9BTD8	-0.060591181	0.060591181	0.020771
A.vasorum /Control	Q3ZCQ8	0.134572049	0.134572049	0.020789
A.vasorum /Control	P19388	-0.046111984	0.046111984	0.020808
A.vasorum /Control	Q13907	-0.044595912	0.044595912	0.020811
A.vasorum /Control	P53367	0.062647968	0.062647968	0.020852
A.vasorum /Control	Q53GS9	0.088282726	0.088282726	0.020851
A.vasorum /Control	Q8NFC6	-0.143346353	0.143346353	0.020834

A.vasorum /Control	Q8WUM0	0.070621781	0.070621781	0.020851
A.vasorum /Control	Q9UPY8	0.277898167	0.277898167	0.020851
A.vasorum /Control	P30043	-0.083060555	0.083060555	0.020878
A.vasorum /Control	Q6NYC1	-0.055866016	0.055866016	0.020904
A.vasorum /Control	P62380	0.258647545	0.258647545	0.020918
A.vasorum /Control	Q8NB90	-0.13017189	0.13017189	0.020924
A.vasorum /Control	Q9Y5B6	-0.031269359	0.031269359	0.020947
A.vasorum /Control	P29474	0.349926799	0.349926799	0.020972
A.vasorum /Control	P48739	-0.128015514	0.128015514	0.020986
A.vasorum /Control	O15020	0.042631101	0.042631101	0.021057
A.vasorum /Control	O43909	-0.10956933	0.10956933	0.021047
A.vasorum /Control	P27797	0.078554781	0.078554781	0.021057
A.vasorum /Control	P36776	-0.056952377	0.056952377	0.021081
A.vasorum /Control	Q9Y3Q3	0.097260017	0.097260017	0.021099
A.vasorum /Control	P54136	-0.013087833	0.013087833	0.021126
A.vasorum /Control	O75143	0.319708214	0.319708214	0.021136
A.vasorum /Control	O75110	0.233146516	0.233146516	0.021174
A.vasorum /Control	Q6P1N9	0.284642897	0.284642897	0.021209
A.vasorum /Control	Q92572	-0.028683813	0.028683813	0.021285
A.vasorum /Control	Q9BTW9	0.215118058	0.215118058	0.021357
A.vasorum /Control	O60331	0.037139381	0.037139381	0.021406
A.vasorum /Control	Q9NZB2	-0.047909016	0.047909016	0.021442
A.vasorum /Control	Q8N2R8	0.102345122	0.102345122	0.021466
A.vasorum /Control	P35713	1.291505455	1.291505455	0.021549
A.vasorum /Control	O14662	0.030424715	0.030424715	0.021576
A.vasorum /Control	Q9UH62	-0.086874498	0.086874498	0.021711
A.vasorum /Control	P15529	-0.284589319	0.284589319	0.02176
A.vasorum /Control	P22626	-0.463026959	0.463026959	0.021803
A.vasorum /Control	Q14728	0.260555535	0.260555535	0.021803
A.vasorum /Control	Q15836	-0.317598972	0.317598972	0.0218
A.vasorum /Control	Q96HE7	0.04368857	0.04368857	0.021848
A.vasorum /Control	Q96DZ1	0.100398703	0.100398703	0.021857
A.vasorum /Control	P48444	-0.050627161	0.050627161	0.021867
A.vasorum /Control	Q13190	-0.006852553	0.006852553	0.021912
A.vasorum /Control	Q9Y333	0.197038372	0.197038372	0.021911
A.vasorum /Control	Q9BUP3	0.237408984	0.237408984	0.021922
A.vasorum /Control	Q14202	-0.080242715	0.080242715	0.021939
A.vasorum /Control	Q9Y496	0.113880878	0.113880878	0.021936
A.vasorum /Control	P78345	0.001185197	0.001185197	0.02197
A.vasorum /Control	O00217	0.538006665	0.538006665	0.021987
A.vasorum /Control	Q13895	-0.410861	0.410861	0.022059
A.vasorum /Control	O94763	-0.065574804	0.065574804	0.022124
A.vasorum /Control	P49773	0.220082999	0.220082999	0.022143
A.vasorum /Control	P55265	0.060627514	0.060627514	0.022223
A.vasorum /Control	Q9Y3D8	-0.398414865	0.398414865	0.022223
A.vasorum /Control	P51812	0.105102747	0.105102747	0.022262
A.vasorum /Control	Q15651	0.85564426	0.85564426	0.022298
A.vasorum /Control	Q9Y6A4	0.120900264	0.120900264	0.022351
A.vasorum /Control	P40425	0.63326464	0.63326464	0.022385
A.vasorum /Control	O15231	-0.686677241	0.686677241	0.022424

A.vasorum /Control	O95292	-0.148645944	0.148645944	0.022471
A.vasorum /Control	P13716	0.064456939	0.064456939	0.022499
A.vasorum /Control	P33991	-0.01177003	0.01177003	0.0225
A.vasorum /Control	Q69YQ0	-0.01126525	0.01126525	0.0225
A.vasorum /Control	P51149	-0.159483372	0.159483372	0.022515
A.vasorum /Control	Q9NW15	-0.114176088	0.114176088	0.022517
A.vasorum /Control	Q9NTI5	0.113568405	0.113568405	0.022595
A.vasorum /Control	P06132	0.213361611	0.213361611	0.022616
A.vasorum /Control	Q9H4L7	0.245093141	0.245093141	0.022619
A.vasorum /Control	P15407	-0.997084957	0.997084957	0.022628
A.vasorum /Control	Q9HBF4	0.303387046	0.303387046	0.022661
A.vasorum /Control	Q9NX61	-0.017408659	0.017408659	0.022655
A.vasorum /Control	Q86WA8	0.448776689	0.448776689	0.022676
A.vasorum /Control	Q13885	0.101644065	0.101644065	0.022699
A.vasorum /Control	Q13405	0.751554579	0.751554579	0.022763
A.vasorum /Control	Q9H8H2	-0.678532494	0.678532494	0.022771
A.vasorum /Control	P49736	0.024793015	0.024793015	0.022793
A.vasorum /Control	P49757	-0.120939141	0.120939141	0.022844
A.vasorum /Control	Q9UKK3	-0.163103643	0.163103643	0.022846
A.vasorum /Control	Q8IW35	0.303260126	0.303260126	0.022913
A.vasorum /Control	Q9UEE9	0.182695315	0.182695315	0.022916
A.vasorum /Control	O15067	0.036320497	0.036320497	0.022937
A.vasorum /Control	Q9BXJ9	-0.135753428	0.135753428	0.022962
A.vasorum /Control	O00410	-0.024348044	0.024348044	0.023
A.vasorum /Control	Q16540	0.605633232	0.605633232	0.02301
A.vasorum /Control	O43150	0.245939655	0.245939655	0.023042
A.vasorum /Control	Q5VUJ6	0.138909627	0.138909627	0.023044
A.vasorum /Control	P55795	-0.208129403	0.208129403	0.023085
A.vasorum /Control	Q13263	-0.062928583	0.062928583	0.023079
A.vasorum /Control	Q8N4Q0	0.070706137	0.070706137	0.023128
A.vasorum /Control	Q8WVQ1	0.088940115	0.088940115	0.023129
A.vasorum /Control	Q8N8A6	-0.427714384	0.427714384	0.023165
A.vasorum /Control	P08123	-0.31423645	0.31423645	0.023184
A.vasorum /Control	Q96F07	-0.135458196	0.135458196	0.023188
A.vasorum /Control	P38646	-0.133199373	0.133199373	0.023213
A.vasorum /Control	Q9UQR1	-0.520795507	0.520795507	0.023271
A.vasorum /Control	Q9Y394	0.003277786	0.003277786	0.023297
A.vasorum /Control	Q9H910	-0.327185373	0.327185373	0.023321
A.vasorum /Control	Q86VI3	-0.02965475	0.02965475	0.023349
A.vasorum /Control	Q9H0V9	0.136478252	0.136478252	0.023413
A.vasorum /Control	Q7L2E3	-0.11672844	0.11672844	0.023438
A.vasorum /Control	P50552	-0.249009887	0.249009887	0.023481
A.vasorum /Control	Q12789	-0.011379604	0.011379604	0.023505
A.vasorum /Control	Q13867	0.131258109	0.131258109	0.023495
A.vasorum /Control	Q6P2C8	-0.183213057	0.183213057	0.023506
A.vasorum /Control	O95757	-0.031745804	0.031745804	0.023534
A.vasorum /Control	O15550	1.107311559	1.107311559	0.023551
A.vasorum /Control	Q8TA86	-0.020990165	0.020990165	0.023595
A.vasorum /Control	Q969Z3	0.237341946	0.237341946	0.023595
A.vasorum /Control	Q96CT7	-0.118520923	0.118520923	0.023586

A.vasorum /Control	Q96TA2	-0.0065685	0.0065685	0.02359
A.vasorum /Control	Q8N3F8	0.567469462	0.567469462	0.023623
A.vasorum /Control	O94927	0.281873529	0.281873529	0.02367
A.vasorum /Control	Q9NRS6	-0.170235289	0.170235289	0.023692
A.vasorum /Control	Q8WXI9	-0.020391527	0.020391527	0.023724
A.vasorum /Control	Q9H6U8	0.083711056	0.083711056	0.02376
A.vasorum /Control	P61221	-0.014455799	0.014455799	0.023776
A.vasorum /Control	P35520	-0.282315194	0.282315194	0.023851
A.vasorum /Control	Q7Z3K3	0.172743863	0.172743863	0.023843
A.vasorum /Control	Q9BVP2	-0.46454879	0.46454879	0.023897
A.vasorum /Control	O00330	0.00957107	0.00957107	0.02392
A.vasorum /Control	O75909	-0.001082395	0.001082395	0.023939
A.vasorum /Control	P62487	0.141096638	0.141096638	0.023944
A.vasorum /Control	Q99567	0.052631174	0.052631174	0.023934
A.vasorum /Control	O75884	0.19495292	0.19495292	0.023964
A.vasorum /Control	Q13409	0.084573102	0.084573102	0.024
A.vasorum /Control	Q96EY7	0.033200082	0.033200082	0.024008
A.vasorum /Control	Q9H6V9	0.267087238	0.267087238	0.02403
A.vasorum /Control	Q6ZU35	0.781793332	0.781793332	0.024043
A.vasorum /Control	Q96CN7	-0.108061135	0.108061135	0.024081
A.vasorum /Control	Q99426	0.101685025	0.101685025	0.024117
A.vasorum /Control	O75915	0.320822295	0.320822295	0.024165
A.vasorum /Control	P62266	0.037591758	0.037591758	0.024162
A.vasorum /Control	P62306	0.048436817	0.048436817	0.024158
A.vasorum /Control	Q9UGM6	0.16200365	0.16200365	0.02415
A.vasorum /Control	Q93096	-1.675185749	1.675185749	0.024199
A.vasorum /Control	O94952	0.339326581	0.339326581	0.024224
A.vasorum /Control	Q86T03	-0.614504706	0.614504706	0.024283
A.vasorum /Control	Q8WWC4	0.244763737	0.244763737	0.02427
A.vasorum /Control	Q96MW1	-1.347279867	1.347279867	0.024282
A.vasorum /Control	Q9Y639	0.059987416	0.059987416	0.024261
A.vasorum /Control	Q00534	0.201375919	0.201375919	0.024297
A.vasorum /Control	Q12999	-0.062545572	0.062545572	0.024303
A.vasorum /Control	P19174	0.098236465	0.098236465	0.024369
A.vasorum /Control	O15049	0.675101471	0.675101471	0.024426
A.vasorum /Control	O43293	-0.157130725	0.157130725	0.024426
A.vasorum /Control	Q9NYM9	0.076003578	0.076003578	0.024443
A.vasorum /Control	Q96SU4	0.087368828	0.087368828	0.024493
A.vasorum /Control	A4D1U4	0.902362542	0.902362542	0.024517
A.vasorum /Control	Q9GZS3	0.006292779	0.006292779	0.024567
A.vasorum /Control	O60884	-0.017078664	0.017078664	0.024622
A.vasorum /Control	Q13685	-0.264161101	0.264161101	0.024616
A.vasorum /Control	O60518	-0.177349113	0.177349113	0.024643
A.vasorum /Control	Q9BQ04	-0.194725094	0.194725094	0.024641
A.vasorum /Control	O94766	0.356717169	0.356717169	0.0247
A.vasorum /Control	Q5U5X0	-0.046852046	0.046852046	0.024696
A.vasorum /Control	Q8TEQ6	-0.106837375	0.106837375	0.024693
A.vasorum /Control	Q9P0J7	-0.283553494	0.283553494	0.024681
A.vasorum /Control	Q5R3I4	0.1191046	0.1191046	0.024712
A.vasorum /Control	P62341	-0.028120779	0.028120779	0.024723

A.vasorum /Control	Q13155	0.123336333	0.123336333	0.024741
A.vasorum /Control	Q8WUH1	0.165721595	0.165721595	0.024764
A.vasorum /Control	Q8TBA6	0.047380928	0.047380928	0.024825
A.vasorum /Control	P42126	0.15654026	0.15654026	0.024836
A.vasorum /Control	O60551	0.025073912	0.025073912	0.024846
A.vasorum /Control	Q9Y2X7	0.199443911	0.199443911	0.024863
A.vasorum /Control	Q8IX04	-0.058539769	0.058539769	0.024902
A.vasorum /Control	Q92538	-0.000912508	0.000912508	0.024931
A.vasorum /Control	O95139	0.148215971	0.148215971	0.024946
A.vasorum /Control	P55145	0.042399429	0.042399429	0.024962
A.vasorum /Control	P68366	0.336418635	0.336418635	0.024968
A.vasorum /Control	Q9NZJ4	-0.013833586	0.013833586	0.024954
A.vasorum /Control	Q9H0A8	0.193778199	0.193778199	0.024992
A.vasorum /Control	Q07617	0.010307803	0.010307803	0.025015
A.vasorum /Control	P13798	-0.019995465	0.019995465	0.025065
A.vasorum /Control	O75427	-0.216837066	0.216837066	0.025085
A.vasorum /Control	Q8NEZ5	-0.112143103	0.112143103	0.02508
A.vasorum /Control	O75533	-0.119501657	0.119501657	0.025114
A.vasorum /Control	Q96RE7	-0.137051479	0.137051479	0.025169
A.vasorum /Control	Q96EL3	0.192736117	0.192736117	0.025186
A.vasorum /Control	O75391	-0.075304771	0.075304771	0.025213
A.vasorum /Control	Q01581	-0.240405628	0.240405628	0.025206
A.vasorum /Control	Q9H7D7	0.099578841	0.099578841	0.025293
A.vasorum /Control	O00264	0.307782272	0.307782272	0.025369
A.vasorum /Control	O14683	0.452068401	0.452068401	0.025375
A.vasorum /Control	P63173	-0.095401172	0.095401172	0.025383
A.vasorum /Control	P98175	0.06574863	0.06574863	0.025442
A.vasorum /Control	Q9P273	-0.94173258	0.94173258	0.025437
A.vasorum /Control	Q9Y2W2	-0.039923251	0.039923251	0.025439
A.vasorum /Control	Q8N999	-0.270087405	0.270087405	0.02559
A.vasorum /Control	Q5JSH3	-0.032689418	0.032689418	0.025617
A.vasorum /Control	O15533	0.282462479	0.282462479	0.025636
A.vasorum /Control	Q9Y4E6	-1.063617575	1.063617575	0.02563
A.vasorum /Control	P49189	0.075574432	0.075574432	0.025644
A.vasorum /Control	O00487	0.000854028	0.000854028	0.025694
A.vasorum /Control	P12109	-0.710081021	0.710081021	0.025725
A.vasorum /Control	Q4KMQ2	0.015722113	0.015722113	0.025723
A.vasorum /Control	P38435	0.170143653	0.170143653	0.02574
A.vasorum /Control	Q9NX40	-0.055378207	0.055378207	0.025835
A.vasorum /Control	Q9Y3B2	-0.265560131	0.265560131	0.025828
A.vasorum /Control	Q00688	0.063124225	0.063124225	0.025849
A.vasorum /Control	Q9BYJ9	1.080312735	1.080312735	0.025878
A.vasorum /Control	Q9BVL4	0.006010928	0.006010928	0.025948
A.vasorum /Control	Q9BYC9	-0.028463166	0.028463166	0.025945
A.vasorum /Control	Q9BWD1	0.078629602	0.078629602	0.025983
A.vasorum /Control	Q9NRG0	0.197019417	0.197019417	0.026014
A.vasorum /Control	Q96EC8	-0.21829028	0.21829028	0.026052
A.vasorum /Control	P17535	-0.33371735	0.33371735	0.026069
A.vasorum /Control	Q15286	0.037097397	0.037097397	0.026071
A.vasorum /Control	P55011	-0.390620846	0.390620846	0.02608

A.vasorum /Control	P62857	-0.315135081	0.315135081	0.026142
A.vasorum /Control	Q9NY65	-0.056716213	0.056716213	0.026153
A.vasorum /Control	Q96A26	-0.050493039	0.050493039	0.026173
A.vasorum /Control	Q14137	-0.934212813	0.934212813	0.026294
A.vasorum /Control	Q9UMZ2	0.311528072	0.311528072	0.026295
A.vasorum /Control	Q9NV79	0.440848944	0.440848944	0.026316
A.vasorum /Control	Q8TCY9	0.308075843	0.308075843	0.026336
A.vasorum /Control	P04080	0.190056468	0.190056468	0.026358
A.vasorum /Control	P61626	-2.159405765	2.159405765	0.026364
A.vasorum /Control	Q9BSJ8	0.022820762	0.022820762	0.026369
A.vasorum /Control	Q9UKV8	0.018247154	0.018247154	0.026427
A.vasorum /Control	O95760	1.801284323	1.801284323	0.026522
A.vasorum /Control	Q15517	-1.875504275	1.875504275	0.026546
A.vasorum /Control	Q9Y223	-0.151961531	0.151961531	0.026543
A.vasorum /Control	Q16602	0.50175577	0.50175577	0.026669
A.vasorum /Control	Q8N0X7	-0.10093457	0.10093457	0.02666
A.vasorum /Control	Q8N1F7	-0.001336352	0.001336352	0.026665
A.vasorum /Control	Q9H7B4	-0.066269728	0.066269728	0.02665
A.vasorum /Control	Q9NZM3	0.08442306	0.08442306	0.02669
A.vasorum /Control	O00232	0.020762963	0.020762963	0.026745
A.vasorum /Control	O43819	0.066318253	0.066318253	0.026774
A.vasorum /Control	P21281	-0.158225825	0.158225825	0.026772
A.vasorum /Control	Q13017	-0.262284597	0.262284597	0.026758
A.vasorum /Control	Q6IQ49	0.186432421	0.186432421	0.026752
A.vasorum /Control	Q969E2	0.238772729	0.238772729	0.026744
A.vasorum /Control	Q96SB3	0.073699997	0.073699997	0.026856
A.vasorum /Control	Q9P2W9	0.199227462	0.199227462	0.026863
A.vasorum /Control	Q8NF91	0.003519942	0.003519942	0.026905
A.vasorum /Control	Q9UPN6	-0.032063008	0.032063008	0.026989
A.vasorum /Control	Q8TDY2	0.415007796	0.415007796	0.027034
A.vasorum /Control	Q9Y230	0.068529818	0.068529818	0.027067
A.vasorum /Control	P54687	0.030424546	0.030424546	0.027128
A.vasorum /Control	O96018	0.920827784	0.920827784	0.027137
A.vasorum /Control	P35580	0.57405887	0.57405887	0.027221
A.vasorum /Control	P63279	-0.061230489	0.061230489	0.027228
A.vasorum /Control	P80723	-0.404469339	0.404469339	0.027218
A.vasorum /Control	Q96EK7	0.139572951	0.139572951	0.027231
A.vasorum /Control	Q9BX70	-0.191829331	0.191829331	0.027201
A.vasorum /Control	Q9NP58	-0.885301362	0.885301362	0.027272
A.vasorum /Control	Q6ZWJ1	0.479984655	0.479984655	0.027299
A.vasorum /Control	Q13505	0.171459006	0.171459006	0.027336
A.vasorum /Control	Q9NVH0	0.17527288	0.17527288	0.027334
A.vasorum /Control	P52306	-0.042203697	0.042203697	0.027399
A.vasorum /Control	Q8IXU6	-0.177663251	0.177663251	0.027429
A.vasorum /Control	Q96GS4	-0.003206254	0.003206254	0.027474
A.vasorum /Control	Q9BZH6	-0.014118847	0.014118847	0.02754
A.vasorum /Control	Q9Y3M8	-0.285063759	0.285063759	0.027557
A.vasorum /Control	Q9H307	-0.022846074	0.022846074	0.027566
A.vasorum /Control	O15511	0.078934724	0.078934724	0.027607
A.vasorum /Control	P31943	-0.255640699	0.255640699	0.02762

A.vasorum /Control	Q01831	0.220086044	0.220086044	0.027667
A.vasorum /Control	Q00613	0.181920211	0.181920211	0.027676
A.vasorum /Control	P48506	0.106477593	0.106477593	0.027738
A.vasorum /Control	Q5SNT2	0.02360124	0.02360124	0.027744
A.vasorum /Control	Q9UH65	0.019487483	0.019487483	0.027858
A.vasorum /Control	Q96FW1	-0.007938482	0.007938482	0.027878
A.vasorum /Control	P68104	-0.195444437	0.195444437	0.027917
A.vasorum /Control	Q9UNY4	1.109408135	1.109408135	0.027946
A.vasorum /Control	Q10471	-0.155981806	0.155981806	0.027998
A.vasorum /Control	Q96EI5	0.31101143	0.31101143	0.028019
A.vasorum /Control	Q9Y221	-1.345826381	1.345826381	0.028113
A.vasorum /Control	Q96EB1	0.196520879	0.196520879	0.028134
A.vasorum /Control	Q9Y2K2	0.256299815	0.256299815	0.028161
A.vasorum /Control	Q8WUH2	0.686979574	0.686979574	0.028183
A.vasorum /Control	Q9UKJ3	0.259622326	0.259622326	0.028216
A.vasorum /Control	P26038	-0.007293214	0.007293214	0.02823
A.vasorum /Control	O95400	0.323149234	0.323149234	0.028288
A.vasorum /Control	Q92845	-0.331846168	0.331846168	0.028407
A.vasorum /Control	O00754	0.383263634	0.383263634	0.028445
A.vasorum /Control	P54278	-0.287145139	0.287145139	0.028533
A.vasorum /Control	P68431	2.36422109	2.36422109	0.028523
A.vasorum /Control	Q96FQ6	0.010613345	0.010613345	0.028538
A.vasorum /Control	Q9HB21	0.092444373	0.092444373	0.028534
A.vasorum /Control	Q96CC6	0.619567443	0.619567443	0.028559
A.vasorum /Control	P52594	0.020501235	0.020501235	0.028592
A.vasorum /Control	Q5BJD5	0.275844267	0.275844267	0.028595
A.vasorum /Control	Q969V3	-0.105904741	0.105904741	0.028579
A.vasorum /Control	Q14677	-0.031081422	0.031081422	0.028666
A.vasorum /Control	Q5R372	0.042269062	0.042269062	0.028681
A.vasorum /Control	Q6P996	0.080912953	0.080912953	0.028728
A.vasorum /Control	Q9BV38	0.110292274	0.110292274	0.028754
A.vasorum /Control	Q15942	-0.554742123	0.554742123	0.028905
A.vasorum /Control	Q02763	0.215796822	0.215796822	0.028931
A.vasorum /Control	P31153	-0.16262556	0.16262556	0.029086
A.vasorum /Control	P54819	-0.014655461	0.014655461	0.02918
A.vasorum /Control	Q9P2A4	-0.046156963	0.046156963	0.029212
A.vasorum /Control	Q9UI10	-0.10789265	0.10789265	0.029229
A.vasorum /Control	P49406	-0.037843137	0.037843137	0.029267
A.vasorum /Control	O43598	0.177092355	0.177092355	0.029299
A.vasorum /Control	O95834	0.145125934	0.145125934	0.029361
A.vasorum /Control	Q96E11	0.196258184	0.196258184	0.029399
A.vasorum /Control	Q8IWS0	-0.045158494	0.045158494	0.029414
A.vasorum /Control	Q5T4B2	0.46718616	0.46718616	0.029431
A.vasorum /Control	Q12834	-0.179573042	0.179573042	0.029458
A.vasorum /Control	Q9Y2P8	-0.840765972	0.840765972	0.029473
A.vasorum /Control	Q8NC96	-0.06361451	0.06361451	0.029518
A.vasorum /Control	Q86WX3	-1.252768907	1.252768907	0.029535
A.vasorum /Control	Q9BUR5	0.052493503	0.052493503	0.029564
A.vasorum /Control	Q5T160	0.14918425	0.14918425	0.029593
A.vasorum /Control	Q5VYS8	-0.006757151	0.006757151	0.029587

A.vasorum /Control	Q9UL63	-0.16716063	0.16716063	0.029582
A.vasorum /Control	Q96HC4	-0.349718026	0.349718026	0.02972
A.vasorum /Control	Q7Z2Z2	-0.15529373	0.15529373	0.029761
A.vasorum /Control	P11413	-0.081425545	0.081425545	0.029822
A.vasorum /Control	P19474	-0.086402246	0.086402246	0.029824
A.vasorum /Control	Q06546	0.160567748	0.160567748	0.029814
A.vasorum /Control	Q15032	0.11954925	0.11954925	0.029814
A.vasorum /Control	Q8IV38	0.372159121	0.372159121	0.029808
A.vasorum /Control	Q9H6Y2	0.11057621	0.11057621	0.029872
A.vasorum /Control	P30837	-0.202488851	0.202488851	0.029931
A.vasorum /Control	Q99797	-0.001941894	0.001941894	0.030056
A.vasorum /Control	Q9Y289	-0.964274504	0.964274504	0.030066
A.vasorum /Control	Q8NHV4	0.153204036	0.153204036	0.030124
A.vasorum /Control	Q6PKG0	-0.091356834	0.091356834	0.030142
A.vasorum /Control	P07741	0.105957254	0.105957254	0.030161
A.vasorum /Control	O00425	0.015397413	0.015397413	0.030171
A.vasorum /Control	Q13601	-0.420234009	0.420234009	0.030207
A.vasorum /Control	Q14498	0.029739775	0.029739775	0.030208
A.vasorum /Control	P51159	0.298149957	0.298149957	0.030227
A.vasorum /Control	Q8N9T8	0.033576069	0.033576069	0.030231
A.vasorum /Control	Q9Y3B7	0.061190656	0.061190656	0.030238
A.vasorum /Control	P12236	-0.127434451	0.127434451	0.030317
A.vasorum /Control	Q03701	-0.175800417	0.175800417	0.030409
A.vasorum /Control	P0DTL6	0.208300352	0.208300352	0.030425
A.vasorum /Control	O60285	0.317285325	0.317285325	0.030436
A.vasorum /Control	Q9GZT3	-0.124424549	0.124424549	0.030477
A.vasorum /Control	P12111	-2.203858437	2.203858437	0.030542
A.vasorum /Control	Q03154	0.485044182	0.485044182	0.0306
A.vasorum /Control	Q7RTP6	0.018135857	0.018135857	0.030647
A.vasorum /Control	O60704	0.319636797	0.319636797	0.030672
A.vasorum /Control	P51692	0.104812528	0.104812528	0.030847
A.vasorum /Control	Q15185	0.00822024	0.00822024	0.030872
A.vasorum /Control	Q96KN1	0.132299958	0.132299958	0.030893
A.vasorum /Control	Q99613	-0.022763774	0.022763774	0.030885
A.vasorum /Control	O95639	-0.230510693	0.230510693	0.03095
A.vasorum /Control	P55957	-0.145145393	0.145145393	0.030941
A.vasorum /Control	Q86XP3	-0.037791886	0.037791886	0.030954
A.vasorum /Control	O75414	0.033575363	0.033575363	0.031031
A.vasorum /Control	Q03112	0.400476332	0.400476332	0.031035
A.vasorum /Control	Q9BZF1	0.077726274	0.077726274	0.031026
A.vasorum /Control	O95983	0.714044682	0.714044682	0.031108
A.vasorum /Control	Q14653	0.027745116	0.027745116	0.03115
A.vasorum /Control	Q9UKV5	0.10480299	0.10480299	0.031218
A.vasorum /Control	Q8WVT3	0.078149122	0.078149122	0.031268
A.vasorum /Control	O60333	-0.015100089	0.015100089	0.031312
A.vasorum /Control	Q969Z0	-0.090390759	0.090390759	0.031382
A.vasorum /Control	Q9H9Q4	0.016231453	0.016231453	0.031415
A.vasorum /Control	Q01968	0.079948498	0.079948498	0.031533
A.vasorum /Control	Q3KQV9	0.114654269	0.114654269	0.031542
A.vasorum /Control	Q8TB03	0.010716135	0.010716135	0.031547

A.vasorum /Control	Q9BVK8	0.266129108	0.266129108	0.031521
A.vasorum /Control	Q13242	-0.609307381	0.609307381	0.031566
A.vasorum /Control	Q86V48	-0.110635246	0.110635246	0.031659
A.vasorum /Control	P42704	-0.159237171	0.159237171	0.031685
A.vasorum /Control	Q13148	0.039613559	0.039613559	0.031693
A.vasorum /Control	Q9NYV4	-0.275705674	0.275705674	0.031701
A.vasorum /Control	Q07021	-0.143977751	0.143977751	0.031764
A.vasorum /Control	P42336	-0.055960436	0.055960436	0.031927
A.vasorum /Control	O15254	0.235500995	0.235500995	0.032007
A.vasorum /Control	P26358	-0.141273014	0.141273014	0.031992
A.vasorum /Control	P41229	-0.016685867	0.016685867	0.032009
A.vasorum /Control	Q06481	-0.119350079	0.119350079	0.031973
A.vasorum /Control	Q5VUD6	0.291850576	0.291850576	0.031999
A.vasorum /Control	Q9NPA8	0.113018312	0.113018312	0.031971
A.vasorum /Control	P43307	0.021429154	0.021429154	0.032088
A.vasorum /Control	Q13162	-0.216178112	0.216178112	0.03209
A.vasorum /Control	P48730	0.006694106	0.006694106	0.032126
A.vasorum /Control	Q96HN2	0.067616046	0.067616046	0.032157
A.vasorum /Control	Q8WUX9	0.25888522	0.25888522	0.032171
A.vasorum /Control	Q14527	0.273807279	0.273807279	0.032199
A.vasorum /Control	Q7L273	0.606626471	0.606626471	0.032219
A.vasorum /Control	O75880	0.038776264	0.038776264	0.032239
A.vasorum /Control	Q8WVC0	0.092425943	0.092425943	0.032324
A.vasorum /Control	Q9HD34	0.236852957	0.236852957	0.032381
A.vasorum /Control	Q15067	-0.063031306	0.063031306	0.032394
A.vasorum /Control	O00506	-0.136949433	0.136949433	0.032477
A.vasorum /Control	Q69YN2	0.202285234	0.202285234	0.032477
A.vasorum /Control	Q8NFJ5	-1.138732561	1.138732561	0.032468
A.vasorum /Control	Q92616	-0.127420159	0.127420159	0.032478
A.vasorum /Control	Q53F19	-0.303422921	0.303422921	0.032504
A.vasorum /Control	Q13541	1.161391628	1.161391628	0.032546
A.vasorum /Control	Q9NRN7	-0.056187776	0.056187776	0.032561
A.vasorum /Control	Q9UI30	1.234686309	1.234686309	0.032575
A.vasorum /Control	Q9H078	-0.11899273	0.11899273	0.032607
A.vasorum /Control	Q9ULU4	-0.100383404	0.100383404	0.032605
A.vasorum /Control	P19784	-0.050525517	0.050525517	0.032632
A.vasorum /Control	Q9UJY5	-0.027312995	0.027312995	0.032711
A.vasorum /Control	Q9H583	-0.659528228	0.659528228	0.032753
A.vasorum /Control	Q96PU8	0.126380494	0.126380494	0.032781
A.vasorum /Control	Q9NPE3	-0.190855965	0.190855965	0.032846
A.vasorum /Control	Q6NXE6	-0.202437558	0.202437558	0.032989
A.vasorum /Control	P12268	-0.08530033	0.08530033	0.033032
A.vasorum /Control	O14925	0.228349784	0.228349784	0.033093
A.vasorum /Control	Q96T49	0.639895791	0.639895791	0.033099
A.vasorum /Control	Q9Y5Y5	0.19656871	0.19656871	0.033106
A.vasorum /Control	Q14145	-0.172412444	0.172412444	0.033198
A.vasorum /Control	P57737	0.042934837	0.042934837	0.03323
A.vasorum /Control	Q04724	0.271944111	0.271944111	0.033329
A.vasorum /Control	Q7Z6M1	-0.175554363	0.175554363	0.033337
A.vasorum /Control	Q9NVH1	0.003116602	0.003116602	0.033322

A.vasorum /Control	P33897	0.144632431	0.144632431	0.033385
A.vasorum /Control	Q9H7H0	0.517328047	0.517328047	0.033399
A.vasorum /Control	Q8TEX9	-0.058893655	0.058893655	0.033468
A.vasorum /Control	O95232	0.08267707	0.08267707	0.033693
A.vasorum /Control	P78346	-0.075564077	0.075564077	0.03368
A.vasorum /Control	Q8WYQ5	1.20315519	1.20315519	0.033687
A.vasorum /Control	Q9H074	-0.061964565	0.061964565	0.033681
A.vasorum /Control	Q8WYP5	-0.399175223	0.399175223	0.033743
A.vasorum /Control	Q9P219	0.382612482	0.382612482	0.033737
A.vasorum /Control	O43464	0.020028012	0.020028012	0.033775
A.vasorum /Control	O75663	-0.039386144	0.039386144	0.033822
A.vasorum /Control	Q8IVM0	0.090551145	0.090551145	0.033857
A.vasorum /Control	Q9H300	0.383338479	0.383338479	0.033939
A.vasorum /Control	O43920	-0.061461996	0.061461996	0.034029
A.vasorum /Control	O00116	0.215560436	0.215560436	0.034047
A.vasorum /Control	Q8N6R0	-0.074276854	0.074276854	0.034081
A.vasorum /Control	Q8N5W9	0.511495521	0.511495521	0.034105
A.vasorum /Control	Q8WW12	-0.060770117	0.060770117	0.034098
A.vasorum /Control	Q8NDA8	0.311941517	0.311941517	0.034119
A.vasorum /Control	Q9BRR6	0.005896805	0.005896805	0.034134
A.vasorum /Control	O00258	0.241139123	0.241139123	0.034154
A.vasorum /Control	P51991	-0.409407588	0.409407588	0.034185
A.vasorum /Control	Q96Q11	0.244329929	0.244329929	0.03425
A.vasorum /Control	Q9Y303	0.278448739	0.278448739	0.034259
A.vasorum /Control	P05783	-0.213432045	0.213432045	0.034351
A.vasorum /Control	O43399	0.058126395	0.058126395	0.034404
A.vasorum /Control	Q96EV2	0.249685204	0.249685204	0.034438
A.vasorum /Control	Q7L7V1	0.133932686	0.133932686	0.034465
A.vasorum /Control	Q92478	0.094476641	0.094476641	0.034471
A.vasorum /Control	Q9Y6X9	0.066037978	0.066037978	0.034481
A.vasorum /Control	Q8ND04	-0.007258642	0.007258642	0.03451
A.vasorum /Control	Q15370	0.317386942	0.317386942	0.034538
A.vasorum /Control	P63244	-0.100461156	0.100461156	0.034619
A.vasorum /Control	Q13425	-0.113326153	0.113326153	0.034621
A.vasorum /Control	Q8NI27	-0.043716366	0.043716366	0.034699
A.vasorum /Control	Q9Y2G3	0.106671532	0.106671532	0.034787
A.vasorum /Control	P56524	-0.143590359	0.143590359	0.034809
A.vasorum /Control	Q07864	0.44628917	0.44628917	0.034999
A.vasorum /Control	Q8IWI9	0.200371301	0.200371301	0.035
A.vasorum /Control	Q9ULF5	-0.081233355	0.081233355	0.035005
A.vasorum /Control	Q86U38	-0.163722159	0.163722159	0.035019
A.vasorum /Control	Q07020	0.024306718	0.024306718	0.035149
A.vasorum /Control	O95104	0.026167403	0.026167403	0.035173
A.vasorum /Control	P21127	0.028682894	0.028682894	0.035196
A.vasorum /Control	P42330	-0.212622093	0.212622093	0.035291
A.vasorum /Control	Q13586	0.044218589	0.044218589	0.035292
A.vasorum /Control	O14531	-0.512322671	0.512322671	0.035305
A.vasorum /Control	P85037	-0.060513145	0.060513145	0.035361
A.vasorum /Control	Q9NX63	0.095964771	0.095964771	0.035352
A.vasorum /Control	Q9UKM7	0.178933593	0.178933593	0.035367

A.vasorum /Control	Q9P289	0.066827181	0.066827181	0.035391
A.vasorum /Control	Q9C0D9	-0.299189661	0.299189661	0.035449
A.vasorum /Control	O95391	-0.376448296	0.376448296	0.035519
A.vasorum /Control	Q8NHS3	0.201130546	0.201130546	0.035625
A.vasorum /Control	Q9UGR2	-0.156990013	0.156990013	0.035621
A.vasorum /Control	Q03169	0.305378347	0.305378347	0.03565
A.vasorum /Control	P62841	-0.049854453	0.049854453	0.035709
A.vasorum /Control	Q92759	0.373909445	0.373909445	0.035729
A.vasorum /Control	Q92945	-0.010793398	0.010793398	0.035771
A.vasorum /Control	Q9GZZ8	-1.540891359	1.540891359	0.035807
A.vasorum /Control	Q15155	-0.075942635	0.075942635	0.035849
A.vasorum /Control	Q9UNE7	-0.226934964	0.226934964	0.035839
A.vasorum /Control	P35637	-0.089138745	0.089138745	0.035867
A.vasorum /Control	Q9UBD5	0.156731312	0.156731312	0.035983
A.vasorum /Control	Q9UIA9	0.133300694	0.133300694	0.036073
A.vasorum /Control	O94776	-0.044482179	0.044482179	0.03614
A.vasorum /Control	Q96N67	-0.238522082	0.238522082	0.036225
A.vasorum /Control	P52735	-0.562884218	0.562884218	0.036246
A.vasorum /Control	P16930	0.317022101	0.317022101	0.036267
A.vasorum /Control	Q14746	0.169003566	0.169003566	0.036283
A.vasorum /Control	P05114	0.17961033	0.17961033	0.036363
A.vasorum /Control	P60953	0.17117941	0.17117941	0.036357
A.vasorum /Control	A3KN83	-0.038659667	0.038659667	0.036401
A.vasorum /Control	P62888	-0.293947289	0.293947289	0.036416
A.vasorum /Control	Q9BYC5	-0.576822679	0.576822679	0.036536
A.vasorum /Control	Q9BQP7	0.209346139	0.209346139	0.03661
A.vasorum /Control	Q9BYD1	-0.060385565	0.060385565	0.036605
A.vasorum /Control	P20338	0.053513824	0.053513824	0.036685
A.vasorum /Control	Q92636	0.046557583	0.046557583	0.036684
A.vasorum /Control	Q9NQS7	-1.342303068	1.342303068	0.036873
A.vasorum /Control	Q9Y2Q3	0.308914001	0.308914001	0.036874
A.vasorum /Control	Q96GX9	0.187185221	0.187185221	0.036905
A.vasorum /Control	P03886	0.44283672	0.44283672	0.036968
A.vasorum /Control	Q7Z392	-0.056856871	0.056856871	0.036986
A.vasorum /Control	Q9HD40	0.626151622	0.626151622	0.036989
A.vasorum /Control	Q14D04	-0.065498545	0.065498545	0.037024
A.vasorum /Control	O94762	0.53240593	0.53240593	0.037043
A.vasorum /Control	Q5BJF2	0.023038523	0.023038523	0.037053
A.vasorum /Control	O15121	-0.025031818	0.025031818	0.037089
A.vasorum /Control	O00233	-0.092918907	0.092918907	0.037113
A.vasorum /Control	P11021	0.053674334	0.053674334	0.037155
A.vasorum /Control	Q15813	-0.036889049	0.036889049	0.037299
A.vasorum /Control	Q5SQI0	0.173309736	0.173309736	0.037362
A.vasorum /Control	Q9BVQ7	-0.240325615	0.240325615	0.037396
A.vasorum /Control	Q86WV6	0.210558162	0.210558162	0.037511
A.vasorum /Control	Q9Y697	0.117718602	0.117718602	0.037597
A.vasorum /Control	O60476	0.316795556	0.316795556	0.03763
A.vasorum /Control	Q96CB8	-0.289696312	0.289696312	0.037639
A.vasorum /Control	Q6WCQ1	0.038855112	0.038855112	0.037699
A.vasorum /Control	Q9P287	-0.021587419	0.021587419	0.037827

A.vasorum /Control	Q02241	-0.506981542	0.506981542	0.037858
A.vasorum /Control	P63092	0.053038545	0.053038545	0.037898
A.vasorum /Control	Q9UJX6	-0.074308239	0.074308239	0.037895
A.vasorum /Control	Q9H2D1	-0.089440668	0.089440668	0.037964
A.vasorum /Control	Q9UMR5	0.344803962	0.344803962	0.037973
A.vasorum /Control	Q16740	0.07183688	0.07183688	0.038016
A.vasorum /Control	O60488	-0.207065257	0.207065257	0.038048
A.vasorum /Control	O75367	2.394165987	2.394165987	0.038101
A.vasorum /Control	Q6DN90	0.862486968	0.862486968	0.038106
A.vasorum /Control	Q9NVE7	-0.031728303	0.031728303	0.03827
A.vasorum /Control	Q07866	-0.007331773	0.007331773	0.038389
A.vasorum /Control	Q8N1Q1	-0.276847045	0.276847045	0.038416
A.vasorum /Control	P04637	-0.669000863	0.669000863	0.038443
A.vasorum /Control	Q96RU2	0.175329816	0.175329816	0.038485
A.vasorum /Control	Q9H0H5	-0.211278628	0.211278628	0.038613
A.vasorum /Control	O15379	0.428048802	0.428048802	0.038669
A.vasorum /Control	O15145	0.011169125	0.011169125	0.038687
A.vasorum /Control	Q15057	-0.056042616	0.056042616	0.038733
A.vasorum /Control	Q96TC7	0.083414648	0.083414648	0.038988
A.vasorum /Control	Q9NSV4	-0.252916095	0.252916095	0.038979
A.vasorum /Control	Q9BPX5	0.191740878	0.191740878	0.03901
A.vasorum /Control	Q92576	0.247901152	0.247901152	0.03918
A.vasorum /Control	Q15819	0.290235011	0.290235011	0.039218
A.vasorum /Control	Q9H9C1	0.160119315	0.160119315	0.039224
A.vasorum /Control	Q9Y5Z9	-1.238294455	1.238294455	0.039265
A.vasorum /Control	O60927	-0.023192287	0.023192287	0.039286
A.vasorum /Control	P82664	-0.232945974	0.232945974	0.039311
A.vasorum /Control	P40227	-0.023645756	0.023645756	0.039366
A.vasorum /Control	P62745	-0.087149437	0.087149437	0.039352
A.vasorum /Control	Q15628	-0.015724153	0.015724153	0.039338
A.vasorum /Control	Q86U42	-0.053767697	0.053767697	0.039356
A.vasorum /Control	Q9UPU7	0.300099353	0.300099353	0.039384
A.vasorum /Control	O75390	-0.079135684	0.079135684	0.039417
A.vasorum /Control	P23025	1.037758487	1.037758487	0.0395
A.vasorum /Control	P60903	0.41139687	0.41139687	0.039525
A.vasorum /Control	Q96AX1	0.034036641	0.034036641	0.039571
A.vasorum /Control	Q9Y3I1	0.169707064	0.169707064	0.03965
A.vasorum /Control	Q04637	-0.103945029	0.103945029	0.039667
A.vasorum /Control	Q96Q15	0.100234555	0.100234555	0.039698
A.vasorum /Control	Q9NWB6	0.264777196	0.264777196	0.039707
A.vasorum /Control	Q8N9N7	0.214200837	0.214200837	0.039781
A.vasorum /Control	Q9NR30	-0.888183352	0.888183352	0.039771
A.vasorum /Control	Q9NZL9	-0.07722591	0.07722591	0.039767
A.vasorum /Control	Q9UN86	-0.08851238	0.08851238	0.039773
A.vasorum /Control	Q9BVA0	0.180863008	0.180863008	0.039885
A.vasorum /Control	Q99442	-0.438812087	0.438812087	0.040035
A.vasorum /Control	O15066	0.157796088	0.157796088	0.040081
A.vasorum /Control	Q9BQC3	0.302376535	0.302376535	0.040072
A.vasorum /Control	Q8IZF2	0.106824606	0.106824606	0.040108
A.vasorum /Control	P52298	-0.099476816	0.099476816	0.040201

A.vasorum /Control	Q86XZ4	-0.211184986	0.211184986	0.040397
A.vasorum /Control	P82930	0.029753122	0.029753122	0.04041
A.vasorum /Control	Q9HCE5	0.20912466	0.20912466	0.040453
A.vasorum /Control	Q6NUK1	-0.026132868	0.026132868	0.040499
A.vasorum /Control	P51798	-0.158098764	0.158098764	0.040524
A.vasorum /Control	P82650	0.030872548	0.030872548	0.040591
A.vasorum /Control	P13674	-0.141730509	0.141730509	0.040645
A.vasorum /Control	Q9P2E5	-0.025447857	0.025447857	0.040652
A.vasorum /Control	O00422	0.069761495	0.069761495	0.040666
A.vasorum /Control	Q9Y2L1	-0.093085478	0.093085478	0.040699
A.vasorum /Control	Q15070	-0.134472336	0.134472336	0.040731
A.vasorum /Control	P48509	-0.520811374	0.520811374	0.040778
A.vasorum /Control	Q96S66	-0.220716225	0.220716225	0.040787
A.vasorum /Control	P21359	0.299548877	0.299548877	0.040893
A.vasorum /Control	Q96A73	0.300117731	0.300117731	0.040891
A.vasorum /Control	Q96T37	0.131316971	0.131316971	0.040914
A.vasorum /Control	O75616	-0.005581853	0.005581853	0.040938
A.vasorum /Control	P04114	-1.330185818	1.330185818	0.040964
A.vasorum /Control	P31949	0.317679253	0.317679253	0.041013
A.vasorum /Control	Q07352	0.075332638	0.075332638	0.041107
A.vasorum /Control	Q9H553	0.099867995	0.099867995	0.0411
A.vasorum /Control	P49407	0.046570635	0.046570635	0.041333
A.vasorum /Control	Q8N7R7	0.110571739	0.110571739	0.041357
A.vasorum /Control	Q8NBP0	0.15897087	0.15897087	0.04139
A.vasorum /Control	Q8TEA8	0.010166043	0.010166043	0.041385
A.vasorum /Control	P51570	0.132315044	0.132315044	0.041447
A.vasorum /Control	P50897	0.316219194	0.316219194	0.041473
A.vasorum /Control	Q4VC31	0.089328153	0.089328153	0.04151
A.vasorum /Control	Q12874	-0.009474575	0.009474575	0.041532
A.vasorum /Control	P03915	0.311550225	0.311550225	0.041618
A.vasorum /Control	O00399	0.184900876	0.184900876	0.041654
A.vasorum /Control	P32780	0.16086833	0.16086833	0.041655
A.vasorum /Control	Q9BUF5	-0.019266191	0.019266191	0.041707
A.vasorum /Control	P20585	0.241422165	0.241422165	0.04186
A.vasorum /Control	Q9Y2R0	0.026812002	0.026812002	0.041851
A.vasorum /Control	Q16763	-1.098796138	1.098796138	0.041927
A.vasorum /Control	Q9UGQ3	0.218629414	0.218629414	0.041947
A.vasorum /Control	O75063	0.527892784	0.527892784	0.04196
A.vasorum /Control	P68371	-0.007366668	0.007366668	0.041978
A.vasorum /Control	Q9Y2Y0	0.144529032	0.144529032	0.042027
A.vasorum /Control	Q12986	-0.361335096	0.361335096	0.042058
A.vasorum /Control	P61289	0.073154161	0.073154161	0.042117
A.vasorum /Control	Q9HB71	-0.05840859	0.05840859	0.042189
A.vasorum /Control	P16152	-0.085733251	0.085733251	0.042249
A.vasorum /Control	Q9Y2Z0	-0.084231485	0.084231485	0.042261
A.vasorum /Control	Q01085	-0.074509535	0.074509535	0.042413
A.vasorum /Control	Q99081	-0.308300436	0.308300436	0.042447
A.vasorum /Control	O43169	0.727467056	0.727467056	0.042478
A.vasorum /Control	Q5T9L3	-0.347305139	0.347305139	0.042475
A.vasorum /Control	Q9BSC4	-0.85772797	0.85772797	0.042521

A.vasorum /Control	Q9Y5S1	0.706961034	0.706961034	0.042557
A.vasorum /Control	P14866	-0.375477523	0.375477523	0.042574
A.vasorum /Control	Q9Y5X1	0.120624451	0.120624451	0.042581
A.vasorum /Control	O15173	-0.00331752	0.00331752	0.042628
A.vasorum /Control	Q8WXE0	0.53421352	0.53421352	0.04262
A.vasorum /Control	Q96QV1	-0.061018226	0.061018226	0.042605
A.vasorum /Control	O95340	-0.09211931	0.09211931	0.042645
A.vasorum /Control	O43760	0.195771489	0.195771489	0.042805
A.vasorum /Control	Q09666	-0.007380801	0.007380801	0.042828
A.vasorum /Control	Q9NYK5	-0.04387375	0.04387375	0.042953
A.vasorum /Control	Q86XI2	-0.080369809	0.080369809	0.042986
A.vasorum /Control	P20645	-0.193449076	0.193449076	0.043056
A.vasorum /Control	Q9ULM3	-0.137273788	0.137273788	0.043101
A.vasorum /Control	O94830	0.372474647	0.372474647	0.043213
A.vasorum /Control	Q9NP61	-0.007670991	0.007670991	0.043221
A.vasorum /Control	Q8WVY7	-0.028951354	0.028951354	0.043243
A.vasorum /Control	P23229	-0.127069547	0.127069547	0.043265
A.vasorum /Control	O60447	0.263777816	0.263777816	0.043298
A.vasorum /Control	Q8NHQ8	-0.055225143	0.055225143	0.043496
A.vasorum /Control	Q9BTE3	-0.077455721	0.077455721	0.043556
A.vasorum /Control	P31151	-2.952203731	2.952203731	0.043686
A.vasorum /Control	O95777	0.897783255	0.897783255	0.043712
A.vasorum /Control	Q92610	0.205274603	0.205274603	0.043731
A.vasorum /Control	Q16539	0.102294123	0.102294123	0.043767
A.vasorum /Control	Q86WB0	-0.038787171	0.038787171	0.043883
A.vasorum /Control	O43772	-0.08216716	0.08216716	0.043976
A.vasorum /Control	P0C0S8	2.640201399	2.640201399	0.043967
A.vasorum /Control	Q8WV92	0.032806522	0.032806522	0.043972
A.vasorum /Control	Q9UBB4	0.005505651	0.005505651	0.043969
A.vasorum /Control	O94929	0.553814539	0.553814539	0.044036
A.vasorum /Control	Q8N543	-0.175910931	0.175910931	0.044098
A.vasorum /Control	P14209	-0.243381323	0.243381323	0.044147
A.vasorum /Control	P62805	2.348914212	2.348914212	0.044219
A.vasorum /Control	Q14767	-0.290362889	0.290362889	0.044208
A.vasorum /Control	Q13347	0.064266065	0.064266065	0.044237
A.vasorum /Control	P03905	0.233462687	0.233462687	0.044376
A.vasorum /Control	Q96PV6	0.590248195	0.590248195	0.044491
A.vasorum /Control	Q8IVF2	-0.411411686	0.411411686	0.044595
A.vasorum /Control	Q99836	0.016414093	0.016414093	0.044612
A.vasorum /Control	Q16254	-0.466697391	0.466697391	0.044642
A.vasorum /Control	Q9NRC1	0.29687103	0.29687103	0.044686
A.vasorum /Control	Q9Y3E7	0.15562052	0.15562052	0.044785
A.vasorum /Control	Q8IYH5	0.661814062	0.661814062	0.044831
A.vasorum /Control	O94979	-0.098572124	0.098572124	0.044854
A.vasorum /Control	O75448	0.231607153	0.231607153	0.044874
A.vasorum /Control	Q9NX76	0.437266076	0.437266076	0.044927
A.vasorum /Control	Q32NB8	0.750489448	0.750489448	0.045102
A.vasorum /Control	Q9C0C2	0.211190426	0.211190426	0.04511
A.vasorum /Control	P23588	-0.292511323	0.292511323	0.045213
A.vasorum /Control	Q8N1G2	0.061907859	0.061907859	0.045413

A.vasorum /Control	Q5VIR6	-0.199740999	0.199740999	0.045429
A.vasorum /Control	P41240	-0.07364197	0.07364197	0.045515
A.vasorum /Control	O00148	-0.233066981	0.233066981	0.04567
A.vasorum /Control	Q9H7E2	0.294152443	0.294152443	0.045715
A.vasorum /Control	Q9HAB3	0.315266315	0.315266315	0.045738
A.vasorum /Control	Q9NPD8	-0.359978956	0.359978956	0.045756
A.vasorum /Control	Q9NVU7	-0.11912723	0.11912723	0.045754
A.vasorum /Control	Q08AE8	-0.025097136	0.025097136	0.045839
A.vasorum /Control	Q6P2Q9	-0.042201683	0.042201683	0.04588
A.vasorum /Control	Q08378	-0.05616885	0.05616885	0.045989
A.vasorum /Control	Q96KM6	0.35758561	0.35758561	0.04599
A.vasorum /Control	Q6ZT12	-0.34655081	0.34655081	0.046061
A.vasorum /Control	Q5HYK3	0.221682852	0.221682852	0.04612
A.vasorum /Control	Q9Y2X3	-0.835105828	0.835105828	0.046157
A.vasorum /Control	Q86WR7	0.197267552	0.197267552	0.046248
A.vasorum /Control	P07311	-0.115271003	0.115271003	0.046285
A.vasorum /Control	P62753	-0.063877546	0.063877546	0.04632
A.vasorum /Control	P26368	-0.238306744	0.238306744	0.046371
A.vasorum /Control	Q86XN8	0.047485625	0.047485625	0.046374
A.vasorum /Control	Q96Q83	-0.201365376	0.201365376	0.046372
A.vasorum /Control	P12004	-0.120554987	0.120554987	0.046421
A.vasorum /Control	Q53EU6	-0.958239306	0.958239306	0.046504
A.vasorum /Control	O60869	0.081249828	0.081249828	0.046552
A.vasorum /Control	Q9H9F9	0.164568857	0.164568857	0.046586
A.vasorum /Control	O75051	-0.556589872	0.556589872	0.046614
A.vasorum /Control	Q9NW64	-0.138018069	0.138018069	0.046603
A.vasorum /Control	Q8IY37	-0.125761981	0.125761981	0.046648
A.vasorum /Control	Q08623	0.104823277	0.104823277	0.046787
A.vasorum /Control	Q96AA3	-0.067925732	0.067925732	0.046814
A.vasorum /Control	Q92734	0.07784402	0.07784402	0.046976
A.vasorum /Control	P15291	0.069785065	0.069785065	0.047215
A.vasorum /Control	Q96GY0	0.27977059	0.27977059	0.047204
A.vasorum /Control	Q9C0E8	0.062124914	0.062124914	0.047226
A.vasorum /Control	P08754	-0.645494764	0.645494764	0.047275
A.vasorum /Control	P05109	-3.567973783	3.567973783	0.047439
A.vasorum /Control	O14757	-0.371710322	0.371710322	0.047562
A.vasorum /Control	P49368	-0.067017153	0.067017153	0.047587
A.vasorum /Control	P53814	-0.548603198	0.548603198	0.047656
A.vasorum /Control	Q9H0R6	-0.085334303	0.085334303	0.047649
A.vasorum /Control	Q9Y6W3	-0.017990136	0.017990136	0.047647
A.vasorum /Control	P52630	0.072338999	0.072338999	0.04773
A.vasorum /Control	Q8TDZ2	-0.063375849	0.063375849	0.04775
A.vasorum /Control	Q99623	0.058048947	0.058048947	0.047742
A.vasorum /Control	Q9NVP1	-0.741230019	0.741230019	0.047819
A.vasorum /Control	P31944	-2.390908512	2.390908512	0.048172
A.vasorum /Control	P17302	-0.468668359	0.468668359	0.048224
A.vasorum /Control	Q0JRZ9	-0.075928662	0.075928662	0.048221
A.vasorum /Control	Q8IYD1	-0.040344466	0.040344466	0.048282
A.vasorum /Control	Q9Y4D8	-0.846312817	0.846312817	0.0483
A.vasorum /Control	Q9H2U1	-0.17762649	0.17762649	0.048329

A.vasorum /Control	P62310	0.58060856	0.58060856	0.048354
A.vasorum /Control	P07947	-0.272877402	0.272877402	0.048405
A.vasorum /Control	Q99747	-0.223485135	0.223485135	0.048396
A.vasorum /Control	Q9Y657	0.020028761	0.020028761	0.048455
A.vasorum /Control	Q01518	-0.130815928	0.130815928	0.048504
A.vasorum /Control	P20810	-0.074073071	0.074073071	0.048536
A.vasorum /Control	Q92769	-0.019844145	0.019844145	0.048525
A.vasorum /Control	Q9BUN8	0.031834873	0.031834873	0.048572
A.vasorum /Control	Q9NZT1	-3.324274279	3.324274279	0.048701
A.vasorum /Control	Q8TCC3	-0.427946055	0.427946055	0.048716
A.vasorum /Control	Q12765	0.17946208	0.17946208	0.048952
A.vasorum /Control	P35052	-1.101388555	1.101388555	0.048992
A.vasorum /Control	Q6PIJ6	-0.17325725	0.17325725	0.049024
A.vasorum /Control	O14646	0.04139643	0.04139643	0.049094
A.vasorum /Control	P62877	0.148216517	0.148216517	0.049112
A.vasorum /Control	Q12893	-0.260162069	0.260162069	0.049173
A.vasorum /Control	P50991	-0.083569084	0.083569084	0.049306
A.vasorum /Control	Q13613	-0.036661953	0.036661953	0.049313
A.vasorum /Control	P53990	0.01557204	0.01557204	0.049394
A.vasorum /Control	P63096	0.079681093	0.079681093	0.049398
A.vasorum /Control	O94967	-0.546104946	0.546104946	0.049476
A.vasorum /Control	Q9UNZ2	0.090803289	0.090803289	0.049499
A.vasorum /Control	P21283	0.036076152	0.036076152	0.049533
A.vasorum /Control	Q8WUD1	-0.074374595	0.074374595	0.049604
A.vasorum /Control	Q9NZC9	0.104390744	0.104390744	0.049722
A.vasorum /Control	P62861	0.062318589	0.062318589	0.049801
A.vasorum /Control	Q8IY22	0.521294404	0.521294404	0.04999
A.vasorum /Control	O43765	0.03520212	0.03520212	0.050032
A.vasorum /Control	Q8IWV8	-0.130112388	0.130112388	0.050023
A.vasorum /Control	Q9H9S5	-0.242100656	0.242100656	0.050126
A.vasorum /Control	Q9H7Z3	0.086934166	0.086934166	0.050184
A.vasorum /Control	Q15334	-0.241104416	0.241104416	0.050213
A.vasorum /Control	O43294	-0.416039478	0.416039478	0.050285
A.vasorum /Control	Q8IXT5	-0.09642671	0.09642671	0.050308
A.vasorum /Control	P17028	-0.867947798	0.867947798	0.050336
A.vasorum /Control	P56378	-0.097445368	0.097445368	0.050341
A.vasorum /Control	Q13888	-0.37302931	0.37302931	0.050477
A.vasorum /Control	Q147X3	0.024321536	0.024321536	0.05053
A.vasorum /Control	Q9UPQ9	0.116307931	0.116307931	0.050849
A.vasorum /Control	Q96B97	-0.540206068	0.540206068	0.050919
A.vasorum /Control	P51153	0.212352206	0.212352206	0.050969
A.vasorum /Control	Q09028	-0.030856171	0.030856171	0.051067
A.vasorum /Control	Q99633	-0.772902758	0.772902758	0.051139
A.vasorum /Control	Q99707	0.03673345	0.03673345	0.051205
A.vasorum /Control	Q01804	-0.190740561	0.190740561	0.051415
A.vasorum /Control	Q96MX6	0.031400248	0.031400248	0.051447
A.vasorum /Control	O95861	-0.134180267	0.134180267	0.051462
A.vasorum /Control	Q9Y324	-0.905793945	0.905793945	0.051532
A.vasorum /Control	O00182	0.430067782	0.430067782	0.051598
A.vasorum /Control	Q658P3	-0.372690531	0.372690531	0.05188

A.vasorum /Control	Q03393	-1.473422535	1.473422535	0.051916
A.vasorum /Control	Q86YP4	-0.23668764	0.23668764	0.05197
A.vasorum /Control	Q9HD26	0.220711383	0.220711383	0.05258
A.vasorum /Control	Q9ULH1	0.026404215	0.026404215	0.052684
A.vasorum /Control	P83731	0.050860229	0.050860229	0.052803
A.vasorum /Control	Q96JG6	0.088760018	0.088760018	0.052791
A.vasorum /Control	O96008	-0.003480605	0.003480605	0.052863
A.vasorum /Control	P04908	2.008371188	2.008371188	0.052852
A.vasorum /Control	P19634	-0.041413935	0.041413935	0.052856
A.vasorum /Control	Q9P2B4	0.070867924	0.070867924	0.052837
A.vasorum /Control	P24468	-0.233881802	0.233881802	0.052971
A.vasorum /Control	Q8TBB5	0.29668274	0.29668274	0.05299
A.vasorum /Control	P18621	-0.039519322	0.039519322	0.053084
A.vasorum /Control	O14817	0.592049501	0.592049501	0.053149
A.vasorum /Control	Q96JC1	0.050622108	0.050622108	0.053122
A.vasorum /Control	Q9NYL2	0.043415569	0.043415569	0.053148
A.vasorum /Control	P25116	0.231437217	0.231437217	0.053331
A.vasorum /Control	Q13322	0.017211848	0.017211848	0.053361
A.vasorum /Control	P63218	0.427592127	0.427592127	0.053442
A.vasorum /Control	Q5VT25	-0.112615507	0.112615507	0.053516
A.vasorum /Control	P46087	-0.704255724	0.704255724	0.053538
A.vasorum /Control	P54277	0.30701171	0.30701171	0.053743
A.vasorum /Control	Q9NUX5	0.529073422	0.529073422	0.053786
A.vasorum /Control	Q9Y4K3	-0.626210486	0.626210486	0.053865
A.vasorum /Control	Q9NZN8	0.07826226	0.07826226	0.05394
A.vasorum /Control	Q9UPN3	-0.123313197	0.123313197	0.053938
A.vasorum /Control	A6NHX0	0.594218956	0.594218956	0.054167
A.vasorum /Control	P50748	0.005630115	0.005630115	0.054159
A.vasorum /Control	Q99986	-0.080962445	0.080962445	0.054227
A.vasorum /Control	P55209	-0.149677815	0.149677815	0.05429
A.vasorum /Control	Q8TDX7	-0.31160019	0.31160019	0.054305
A.vasorum /Control	Q0VGL1	0.202955198	0.202955198	0.054321
A.vasorum /Control	Q8WUA4	-0.214747098	0.214747098	0.054391
A.vasorum /Control	P36639	0.252703722	0.252703722	0.054471
A.vasorum /Control	P09936	0.053980048	0.053980048	0.054622
A.vasorum /Control	P62304	-0.212231551	0.212231551	0.054693
A.vasorum /Control	Q14112	0.094154901	0.094154901	0.054689
A.vasorum /Control	Q9Y3D7	-0.537723846	0.537723846	0.05485
A.vasorum /Control	Q96Q06	-2.242712909	2.242712909	0.055054
A.vasorum /Control	Q9UKD2	-0.519360948	0.519360948	0.055058
A.vasorum /Control	Q9Y4C2	-0.117008838	0.117008838	0.055102
A.vasorum /Control	Q9UHY1	-0.012026406	0.012026406	0.055157
A.vasorum /Control	Q6P587	0.004847559	0.004847559	0.055193
A.vasorum /Control	O75643	-0.118713222	0.118713222	0.055212
A.vasorum /Control	Q8NE71	-0.104714266	0.104714266	0.055423
A.vasorum /Control	Q96BP3	-0.069812982	0.069812982	0.055415
A.vasorum /Control	Q96PD2	-0.61805871	0.61805871	0.05541
A.vasorum /Control	Q53H96	0.036154673	0.036154673	0.055529
A.vasorum /Control	Q969X1	-0.431826912	0.431826912	0.055691
A.vasorum /Control	Q5TBA9	0.782354774	0.782354774	0.055749

A.vasorum /Control	Q08J23	-0.11463558	0.11463558	0.055813
A.vasorum /Control	Q92520	-0.050490576	0.050490576	0.05581
A.vasorum /Control	P07199	0.125663707	0.125663707	0.056095
A.vasorum /Control	P83881	0.0102732	0.0102732	0.056083
A.vasorum /Control	Q9BT17	0.042512778	0.042512778	0.056065
A.vasorum /Control	O60814	2.164687361	2.164687361	0.056115
A.vasorum /Control	Q5JVF3	-0.107101405	0.107101405	0.056141
A.vasorum /Control	Q96JY6	-0.840519349	0.840519349	0.056187
A.vasorum /Control	Q7Z2K8	-0.132202509	0.132202509	0.056394
A.vasorum /Control	Q96BZ9	0.124807583	0.124807583	0.056482
A.vasorum /Control	Q8N122	-0.159502205	0.159502205	0.056569
A.vasorum /Control	Q99611	0.266606727	0.266606727	0.056568
A.vasorum /Control	Q15102	0.437290747	0.437290747	0.056628
A.vasorum /Control	Q8WUF8	0.145918399	0.145918399	0.056687
A.vasorum /Control	P26641	-0.136252126	0.136252126	0.05674
A.vasorum /Control	Q9NPA0	-0.041962817	0.041962817	0.056994
A.vasorum /Control	Q13835	-1.494129054	1.494129054	0.057054
A.vasorum /Control	Q13144	-0.039684377	0.039684377	0.057272
A.vasorum /Control	Q13564	0.078872929	0.078872929	0.057261
A.vasorum /Control	Q9NP72	-0.194980671	0.194980671	0.057291
A.vasorum /Control	Q6P5R6	-0.013697589	0.013697589	0.05739
A.vasorum /Control	P52758	-0.904405482	0.904405482	0.057433
A.vasorum /Control	P83436	-0.009474294	0.009474294	0.05748
A.vasorum /Control	Q8NC60	-0.074166721	0.074166721	0.057475
A.vasorum /Control	Q9UQB8	-0.033366066	0.033366066	0.057548
A.vasorum /Control	P68400	0.009380813	0.009380813	0.057649
A.vasorum /Control	Q12974	-0.050043214	0.050043214	0.057621
A.vasorum /Control	Q96JM7	0.269276428	0.269276428	0.057646
A.vasorum /Control	P38606	-0.153703292	0.153703292	0.057674
A.vasorum /Control	Q07817	-0.17114726	0.17114726	0.057719
A.vasorum /Control	Q9NZQ7	-0.01381638	0.01381638	0.05798
A.vasorum /Control	P05026	-0.561409056	0.561409056	0.058052
A.vasorum /Control	P08559	-0.060717329	0.060717329	0.058243
A.vasorum /Control	Q13509	0.297078825	0.297078825	0.058247
A.vasorum /Control	Q5JRA6	0.0425012	0.0425012	0.058657
A.vasorum /Control	Q8N0U8	0.243172217	0.243172217	0.058734
A.vasorum /Control	Q14978	-0.75182972	0.75182972	0.058823
A.vasorum /Control	Q15637	0.029479604	0.029479604	0.058825
A.vasorum /Control	Q9Y2L9	0.188399649	0.188399649	0.058821
A.vasorum /Control	P35613	-0.028476824	0.028476824	0.058844
A.vasorum /Control	P22692	0.122814714	0.122814714	0.05888
A.vasorum /Control	Q9NZV1	0.052904707	0.052904707	0.05893
A.vasorum /Control	Q12959	-0.006517023	0.006517023	0.059042
A.vasorum /Control	Q7Z333	0.164219365	0.164219365	0.05904
A.vasorum /Control	Q9H5V9	-1.018524231	1.018524231	0.059058
A.vasorum /Control	Q14149	-0.225125259	0.225125259	0.059109
A.vasorum /Control	Q2M296	0.449559872	0.449559872	0.059103
A.vasorum /Control	P43686	-0.016513651	0.016513651	0.059281
A.vasorum /Control	Q92747	-0.087187693	0.087187693	0.059289
A.vasorum /Control	P29966	0.039797516	0.039797516	0.05932

A.vasorum /Control	Q01628	0.065828675	0.065828675	0.059541
A.vasorum /Control	Q9Y6R4	0.343978205	0.343978205	0.059646
A.vasorum /Control	O95297	-0.277193112	0.277193112	0.059847
A.vasorum /Control	Q9NR48	-0.445480706	0.445480706	0.059907
A.vasorum /Control	Q8NG68	0.84327456	0.84327456	0.060001
A.vasorum /Control	Q8TD16	-0.186543547	0.186543547	0.059986
A.vasorum /Control	P49411	-0.009666653	0.009666653	0.060161
A.vasorum /Control	Q8IUR0	-0.12322385	0.12322385	0.060155
A.vasorum /Control	O75151	0.627064919	0.627064919	0.060262
A.vasorum /Control	Q4L180	-0.236884428	0.236884428	0.060297
A.vasorum /Control	Q86VN1	-0.105621238	0.105621238	0.060381
A.vasorum /Control	Q96HD1	0.748926014	0.748926014	0.060373
A.vasorum /Control	P12081	-0.090581742	0.090581742	0.060463
A.vasorum /Control	Q9NVA1	0.167194329	0.167194329	0.06052
A.vasorum /Control	Q99523	0.217077888	0.217077888	0.060579
A.vasorum /Control	Q9ULC4	-0.090970093	0.090970093	0.060587
A.vasorum /Control	P40429	-0.054837028	0.054837028	0.060717
A.vasorum /Control	Q13098	-0.068502481	0.068502481	0.060733
A.vasorum /Control	Q9Y4R8	-0.026563382	0.026563382	0.060807
A.vasorum /Control	Q8TCJ2	-0.019515442	0.019515442	0.060837
A.vasorum /Control	O15294	-0.230160838	0.230160838	0.060886
A.vasorum /Control	Q9Y5X9	-0.027192673	0.027192673	0.061
A.vasorum /Control	Q9BRQ6	-0.004369012	0.004369012	0.061068
A.vasorum /Control	O60637	-0.001588114	0.001588114	0.061123
A.vasorum /Control	Q96CW6	-0.597627802	0.597627802	0.061188
A.vasorum /Control	P20248	-0.857818676	0.857818676	0.061208
A.vasorum /Control	Q96BX8	0.27775128	0.27775128	0.061291
A.vasorum /Control	Q96GA3	-0.079751161	0.079751161	0.061486
A.vasorum /Control	P29353	-0.07331932	0.07331932	0.061629
A.vasorum /Control	Q70Z53	-1.477370663	1.477370663	0.061625
A.vasorum /Control	Q8IVS2	-0.202294124	0.202294124	0.061741
A.vasorum /Control	P06702	-2.702174405	2.702174405	0.061799
A.vasorum /Control	O00534	0.473750119	0.473750119	0.061834
A.vasorum /Control	P31937	0.313245151	0.313245151	0.061876
A.vasorum /Control	Q6NXT4	0.170614062	0.170614062	0.061868
A.vasorum /Control	O95696	-0.596786602	0.596786602	0.061894
A.vasorum /Control	Q9Y4J8	-0.799360094	0.799360094	0.061923
A.vasorum /Control	Q9ULR0	0.41826558	0.41826558	0.061955
A.vasorum /Control	Q5HYW2	0.164363104	0.164363104	0.062071
A.vasorum /Control	P51151	0.169347734	0.169347734	0.062125
A.vasorum /Control	Q15459	-0.136209825	0.136209825	0.062214
A.vasorum /Control	Q9H7F0	-0.727396896	0.727396896	0.062214
A.vasorum /Control	Q9UBU8	-0.031759078	0.031759078	0.062253
A.vasorum /Control	O96005	-0.083110978	0.083110978	0.062316
A.vasorum /Control	P23458	-0.270151305	0.270151305	0.062333
A.vasorum /Control	Q9NXA8	0.42937481	0.42937481	0.062384
A.vasorum /Control	P09417	0.076153143	0.076153143	0.062523
A.vasorum /Control	Q9Y376	0.064380289	0.064380289	0.062511
A.vasorum /Control	Q9BSH4	-0.034991698	0.034991698	0.062564
A.vasorum /Control	P62701	0.034082136	0.034082136	0.062626

A.vasorum /Control	Q9BUE0	0.434904361	0.434904361	0.062646
A.vasorum /Control	P29401	-0.034266817	0.034266817	0.062942
A.vasorum /Control	Q70UQ0	-0.032925383	0.032925383	0.063011
A.vasorum /Control	Q9BWU1	0.341444156	0.341444156	0.063128
A.vasorum /Control	Q9Y6C9	-0.006743649	0.006743649	0.063257
A.vasorum /Control	P49761	0.081250381	0.081250381	0.063385
A.vasorum /Control	Q9UHY8	-0.164896468	0.164896468	0.063405
A.vasorum /Control	Q5HYJ3	-0.367006462	0.367006462	0.063472
A.vasorum /Control	Q9Y6M5	-0.149344568	0.149344568	0.063457
A.vasorum /Control	Q5T653	-0.124050402	0.124050402	0.063592
A.vasorum /Control	P55081	-0.065710974	0.065710974	0.063756
A.vasorum /Control	Q6PJF5	-0.191597942	0.191597942	0.063741
A.vasorum /Control	Q9H334	0.293927634	0.293927634	0.063751
A.vasorum /Control	O43426	0.22639565	0.22639565	0.06393
A.vasorum /Control	O95183	0.245584305	0.245584305	0.063914
A.vasorum /Control	O60566	0.000161538	0.000161538	0.064099
A.vasorum /Control	P69905	-2.554088592	2.554088592	0.064094
A.vasorum /Control	O43865	-0.394598497	0.394598497	0.06414
A.vasorum /Control	O75818	-0.1166467	0.1166467	0.06414
A.vasorum /Control	P11233	0.060391499	0.060391499	0.064238
A.vasorum /Control	Q96JJ7	-0.129903775	0.129903775	0.064452
A.vasorum /Control	Q9H7D0	0.175483555	0.175483555	0.06446
A.vasorum /Control	Q9UBL3	0.155691004	0.155691004	0.064502
A.vasorum /Control	Q6PJ69	-0.063277802	0.063277802	0.064694
A.vasorum /Control	Q8NB46	-0.675485573	0.675485573	0.06468
A.vasorum /Control	P51531	-0.337058012	0.337058012	0.06476
A.vasorum /Control	O60293	0.260283847	0.260283847	0.064823
A.vasorum /Control	P29083	0.202281839	0.202281839	0.064895
A.vasorum /Control	Q8WUX2	-0.043191698	0.043191698	0.064938
A.vasorum /Control	P63165	-0.248473867	0.248473867	0.065047
A.vasorum /Control	Q86YS7	0.165388306	0.165388306	0.065193
A.vasorum /Control	Q14320	0.032327173	0.032327173	0.065399
A.vasorum /Control	P07738	-0.255274098	0.255274098	0.065417
A.vasorum /Control	Q15555	0.071761411	0.071761411	0.065493
A.vasorum /Control	O94923	-0.253151158	0.253151158	0.065596
A.vasorum /Control	Q9NUT2	-0.590824349	0.590824349	0.065633
A.vasorum /Control	Q9H0X4	0.020668843	0.020668843	0.065701
A.vasorum /Control	Q8IU81	0.097321916	0.097321916	0.065923
A.vasorum /Control	P35998	-0.047350005	0.047350005	0.065943
A.vasorum /Control	Q8WX93	-0.133978141	0.133978141	0.065999
A.vasorum /Control	P52434	-0.185955233	0.185955233	0.066039
A.vasorum /Control	Q9NS69	0.290708718	0.290708718	0.066145
A.vasorum /Control	P62847	0.033065161	0.033065161	0.06619
A.vasorum /Control	Q86Y07	0.016600802	0.016600802	0.066476
A.vasorum /Control	Q96J42	0.350424403	0.350424403	0.066509
A.vasorum /Control	O95801	-0.165704066	0.165704066	0.066528
A.vasorum /Control	Q08211	-0.186626605	0.186626605	0.066775
A.vasorum /Control	Q8IZH2	-0.130262997	0.130262997	0.067086
A.vasorum /Control	P23443	0.078685035	0.078685035	0.067254
A.vasorum /Control	Q86TN4	0.616609891	0.616609891	0.067242

A.vasorum /Control	P62913	-0.014253691	0.014253691	0.067345
A.vasorum /Control	Q8NAV1	-0.363330472	0.363330472	0.067353
A.vasorum /Control	Q9BWS9	0.095355958	0.095355958	0.067403
A.vasorum /Control	P35367	-1.466021881	1.466021881	0.067424
A.vasorum /Control	P62834	0.09735724	0.09735724	0.067509
A.vasorum /Control	P30260	-0.010017784	0.010017784	0.067644
A.vasorum /Control	Q9BRU9	0.113635655	0.113635655	0.067663
A.vasorum /Control	O60826	-0.000423082	0.000423082	0.067699
A.vasorum /Control	Q8NB49	-0.133222303	0.133222303	0.067703
A.vasorum /Control	Q9P0V3	-0.16380422	0.16380422	0.067752
A.vasorum /Control	Q8NCL4	-0.371185891	0.371185891	0.067852
A.vasorum /Control	P37840	0.034735498	0.034735498	0.067916
A.vasorum /Control	Q7Z417	-0.065342621	0.065342621	0.067933
A.vasorum /Control	Q5JTJ3	-0.31067492	0.31067492	0.06809
A.vasorum /Control	Q9GZY8	0.468497447	0.468497447	0.068315
A.vasorum /Control	Q9NY61	-0.741515333	0.741515333	0.068312
A.vasorum /Control	Q16775	-0.037894974	0.037894974	0.068471
A.vasorum /Control	Q9H6K4	-0.013822454	0.013822454	0.068728
A.vasorum /Control	Q9Y5B9	-0.043250574	0.043250574	0.068787
A.vasorum /Control	Q96EQ0	1.030379753	1.030379753	0.069047
A.vasorum /Control	Q9NZI8	-0.002749824	0.002749824	0.069114
A.vasorum /Control	P49840	-0.168342967	0.168342967	0.069186
A.vasorum /Control	P60510	0.062670057	0.062670057	0.069212
A.vasorum /Control	P63220	0.236269455	0.236269455	0.069314
A.vasorum /Control	Q9NRL2	-0.212573774	0.212573774	0.069332
A.vasorum /Control	Q9Y2U8	0.061645148	0.061645148	0.069337
A.vasorum /Control	Q8NEW0	-0.047138361	0.047138361	0.069358
A.vasorum /Control	Q7Z2E3	0.006908832	0.006908832	0.06941
A.vasorum /Control	Q92685	0.083647411	0.083647411	0.069545
A.vasorum /Control	Q9HAN9	0.112308923	0.112308923	0.069691
A.vasorum /Control	Q15043	-0.250535172	0.250535172	0.06992
A.vasorum /Control	P00390	-0.152664789	0.152664789	0.069975
A.vasorum /Control	Q8N442	-0.531484108	0.531484108	0.070299
A.vasorum /Control	O14964	-0.142187088	0.142187088	0.070362
A.vasorum /Control	Q8IVH8	1.057248741	1.057248741	0.070408
A.vasorum /Control	Q9NRX5	-0.201126561	0.201126561	0.070692
A.vasorum /Control	Q03113	-1.05857186	1.05857186	0.070854
A.vasorum /Control	Q9NP64	0.103294179	0.103294179	0.071123
A.vasorum /Control	Q96DI7	-0.151816196	0.151816196	0.071167
A.vasorum /Control	Q9NX14	0.515255714	0.515255714	0.071294
A.vasorum /Control	P25789	0.018925852	0.018925852	0.071476
A.vasorum /Control	Q92922	-0.469744659	0.469744659	0.071469
A.vasorum /Control	Q96CS2	0.096229456	0.096229456	0.071461
A.vasorum /Control	Q9BUE6	0.050921285	0.050921285	0.071549
A.vasorum /Control	Q15269	-0.68891269	0.68891269	0.07161
A.vasorum /Control	Q86SR1	-0.391402618	0.391402618	0.071716
A.vasorum /Control	P47929	-2.023144978	2.023144978	0.071816
A.vasorum /Control	P49917	-0.18585591	0.18585591	0.071804
A.vasorum /Control	P63241	-0.040051728	0.040051728	0.071915
A.vasorum /Control	Q08945	-0.097158452	0.097158452	0.071945

A.vasorum /Control	Q96L92	0.059460882	0.059460882	0.071943
A.vasorum /Control	Q96KC8	-0.142240441	0.142240441	0.072001
A.vasorum /Control	Q14181	-0.08670197	0.08670197	0.072044
A.vasorum /Control	Q96MX0	0.345311255	0.345311255	0.072125
A.vasorum /Control	Q9HBI1	-0.177581443	0.177581443	0.072126
A.vasorum /Control	Q8NC42	-0.377455975	0.377455975	0.072215
A.vasorum /Control	Q96BR5	-1.405174589	1.405174589	0.072243
A.vasorum /Control	Q96K37	0.112934818	0.112934818	0.072326
A.vasorum /Control	Q00587	-0.337377607	0.337377607	0.072414
A.vasorum /Control	Q9Y5Q9	0.000327768	0.000327768	0.07245
A.vasorum /Control	Q8IXQ4	-0.089381983	0.089381983	0.072474
A.vasorum /Control	Q53GQ0	-0.002776474	0.002776474	0.072529
A.vasorum /Control	P43007	-0.232852517	0.232852517	0.072664
A.vasorum /Control	Q8IYB8	0.046384467	0.046384467	0.072769
A.vasorum /Control	O43505	-0.214764728	0.214764728	0.073013
A.vasorum /Control	Q03111	-0.488192958	0.488192958	0.073014
A.vasorum /Control	Q5VWQ0	0.042321335	0.042321335	0.073026
A.vasorum /Control	Q6XZF7	-0.150078694	0.150078694	0.07292
A.vasorum /Control	Q86UE4	-0.049232646	0.049232646	0.073036
A.vasorum /Control	Q8IWT6	0.009455157	0.009455157	0.073041
A.vasorum /Control	Q8TCG1	-0.336870737	0.336870737	0.072994
A.vasorum /Control	Q92870	0.129148696	0.129148696	0.072968
A.vasorum /Control	Q9H497	0.408912541	0.408912541	0.072978
A.vasorum /Control	Q9NYR9	0.111184701	0.111184701	0.07293
A.vasorum /Control	Q9Y3D3	-0.148842834	0.148842834	0.073111
A.vasorum /Control	Q9Y294	0.184975637	0.184975637	0.073164
A.vasorum /Control	Q04726	0.058491831	0.058491831	0.073248
A.vasorum /Control	Q96KQ7	-0.56236089	0.56236089	0.073233
A.vasorum /Control	Q9Y5T5	-0.176716567	0.176716567	0.073345
A.vasorum /Control	Q9NRX2	-0.209440851	0.209440851	0.073369
A.vasorum /Control	Q8WW22	0.578627435	0.578627435	0.073522
A.vasorum /Control	Q9NX58	-0.054356024	0.054356024	0.073527
A.vasorum /Control	Q08752	-0.07301991	0.07301991	0.073632
A.vasorum /Control	P43243	-0.212775037	0.212775037	0.073806
A.vasorum /Control	Q86VW0	0.317552654	0.317552654	0.073797
A.vasorum /Control	Q99848	-0.870408871	0.870408871	0.073927
A.vasorum /Control	Q5VZ18	0.413436476	0.413436476	0.074171
A.vasorum /Control	Q92686	-0.180060145	0.180060145	0.074222
A.vasorum /Control	Q8N4V1	0.256063792	0.256063792	0.074328
A.vasorum /Control	Q9H0R4	0.270431271	0.270431271	0.074407
A.vasorum /Control	Q68DQ2	0.291270403	0.291270403	0.074629
A.vasorum /Control	Q9BWF3	-0.211658728	0.211658728	0.074646
A.vasorum /Control	O43572	0.063123441	0.063123441	0.074955
A.vasorum /Control	Q9Y5P6	0.055257802	0.055257802	0.07501
A.vasorum /Control	Q6PIU2	-0.285056066	0.285056066	0.075121
A.vasorum /Control	Q15208	0.047169831	0.047169831	0.075163
A.vasorum /Control	P09486	0.045687999	0.045687999	0.07529
A.vasorum /Control	Q68CR1	0.028358309	0.028358309	0.075293
A.vasorum /Control	Q58WW2	-0.701160727	0.701160727	0.075366
A.vasorum /Control	Q16543	-0.005729144	0.005729144	0.075418

A.vasorum /Control	Q8IY81	-0.533719127	0.533719127	0.075538
A.vasorum /Control	P20618	-0.091702784	0.091702784	0.075574
A.vasorum /Control	Q9UDY2	-0.035257142	0.035257142	0.07559
A.vasorum /Control	Q9Y5Q0	-0.17494967	0.17494967	0.075689
A.vasorum /Control	O75718	-0.009392875	0.009392875	0.075786
A.vasorum /Control	P68871	-2.43999643	2.43999643	0.075826
A.vasorum /Control	Q9H0H0	0.174261681	0.174261681	0.076206
A.vasorum /Control	P30533	-0.121961585	0.121961585	0.076257
A.vasorum /Control	P63027	0.000928008	0.000928008	0.076329
A.vasorum /Control	P56556	0.233109659	0.233109659	0.076375
A.vasorum /Control	O95848	0.36042119	0.36042119	0.076722
A.vasorum /Control	Q03405	-1.002826572	1.002826572	0.076902
A.vasorum /Control	P12931	-0.32569471	0.32569471	0.076932
A.vasorum /Control	Q96F86	-0.050255112	0.050255112	0.076988
A.vasorum /Control	Q9H0E9	-0.64081472	0.64081472	0.077094
A.vasorum /Control	P01111	-0.084448164	0.084448164	0.077119
A.vasorum /Control	O95197	-0.106577248	0.106577248	0.077207
A.vasorum /Control	O15269	-0.109213456	0.109213456	0.077343
A.vasorum /Control	O75179	-0.216342186	0.216342186	0.077443
A.vasorum /Control	P78318	0.081509228	0.081509228	0.077427
A.vasorum /Control	Q8TDD1	-0.342859578	0.342859578	0.077414
A.vasorum /Control	P38936	-0.734139125	0.734139125	0.07759
A.vasorum /Control	Q8TAD8	-0.054263779	0.054263779	0.077621
A.vasorum /Control	Q9Y3B3	0.041973267	0.041973267	0.077646
A.vasorum /Control	Q9UPM8	-0.204816561	0.204816561	0.077804
A.vasorum /Control	Q9H3U5	0.17309077	0.17309077	0.077843
A.vasorum /Control	Q8WUH6	0.404198626	0.404198626	0.077866
A.vasorum /Control	P45973	-0.586506075	0.586506075	0.078145
A.vasorum /Control	Q8TBM8	-0.314001499	0.314001499	0.078184
A.vasorum /Control	Q9Y3T9	-0.240613229	0.240613229	0.078211
A.vasorum /Control	Q14444	-0.02894367	0.02894367	0.078379
A.vasorum /Control	O75943	0.448068758	0.448068758	0.078563
A.vasorum /Control	P61927	0.588908785	0.588908785	0.078637
A.vasorum /Control	Q13636	0.141316462	0.141316462	0.078752
A.vasorum /Control	P34947	-0.290818113	0.290818113	0.078901
A.vasorum /Control	O95630	-0.070862585	0.070862585	0.079168
A.vasorum /Control	Q9BXW9	-0.080865302	0.080865302	0.079293
A.vasorum /Control	P46934	-0.014214838	0.014214838	0.07943
A.vasorum /Control	Q96E29	-2.317269263	2.317269263	0.079411
A.vasorum /Control	Q99575	-0.252064002	0.252064002	0.079415
A.vasorum /Control	Q9BRJ6	-0.05061282	0.05061282	0.079395
A.vasorum /Control	Q08499	-0.060376832	0.060376832	0.079495
A.vasorum /Control	Q15393	-0.04046595	0.04046595	0.079613
A.vasorum /Control	Q5TDH0	-0.078540501	0.078540501	0.079756
A.vasorum /Control	O94992	-0.308140795	0.308140795	0.079852
A.vasorum /Control	P52799	0.510824384	0.510824384	0.079927
A.vasorum /Control	P14678	-0.079892671	0.079892671	0.080023
A.vasorum /Control	P16333	0.161527646	0.161527646	0.080275
A.vasorum /Control	Q56VL3	-0.294259236	0.294259236	0.080462
A.vasorum /Control	Q9GZU1	0.468415423	0.468415423	0.080605

A.vasorum /Control	A4D1E9	0.053860905	0.053860905	0.080636
A.vasorum /Control	A8MXV4	0.154035698	0.154035698	0.080762
A.vasorum /Control	Q9P2D0	0.859307395	0.859307395	0.08093
A.vasorum /Control	Q05682	-0.134198153	0.134198153	0.081466
A.vasorum /Control	P0C0S5	1.515789817	1.515789817	0.081514
A.vasorum /Control	Q9Y484	-0.790596006	0.790596006	0.081628
A.vasorum /Control	Q8TED1	0.028551352	0.028551352	0.081822
A.vasorum /Control	Q9NZW5	-0.14541182	0.14541182	0.081957
A.vasorum /Control	P15056	0.109750174	0.109750174	0.082013
A.vasorum /Control	P60059	0.537914682	0.537914682	0.082034
A.vasorum /Control	P82979	-0.160400996	0.160400996	0.082177
A.vasorum /Control	Q96P11	0.19014374	0.19014374	0.082212
A.vasorum /Control	O43639	0.119078969	0.119078969	0.082355
A.vasorum /Control	O14686	-0.195652552	0.195652552	0.082465
A.vasorum /Control	Q96K19	0.059946564	0.059946564	0.082549
A.vasorum /Control	P12955	-0.164148671	0.164148671	0.08286
A.vasorum /Control	P02751	0.044411673	0.044411673	0.083005
A.vasorum /Control	P02765	-0.613554672	0.613554672	0.082972
A.vasorum /Control	Q9NWV4	0.077471541	0.077471541	0.083008
A.vasorum /Control	P00505	-0.153110582	0.153110582	0.083161
A.vasorum /Control	O60783	0.189137321	0.189137321	0.083196
A.vasorum /Control	Q7L1W4	0.198802831	0.198802831	0.083272
A.vasorum /Control	Q9UPN9	-0.141123533	0.141123533	0.083656
A.vasorum /Control	O43776	-0.065530926	0.065530926	0.083708
A.vasorum /Control	Q9NQZ5	-0.195035893	0.195035893	0.083709
A.vasorum /Control	P10768	0.032696686	0.032696686	0.083883
A.vasorum /Control	Q9BQ90	-0.365901673	0.365901673	0.083943
A.vasorum /Control	Q5W0V3	0.113976612	0.113976612	0.084011
A.vasorum /Control	P01009	0.30449508	0.30449508	0.084207
A.vasorum /Control	Q12905	0.023367088	0.023367088	0.084248
A.vasorum /Control	Q9H098	-0.227829481	0.227829481	0.084324
A.vasorum /Control	O75554	0.124029618	0.124029618	0.084403
A.vasorum /Control	Q12980	0.236814927	0.236814927	0.084505
A.vasorum /Control	Q9NQC3	-0.163975408	0.163975408	0.084516
A.vasorum /Control	P33121	-0.218052777	0.218052777	0.084557
A.vasorum /Control	Q04941	-1.019078207	1.019078207	0.084615
A.vasorum /Control	Q6IBS0	-0.074749676	0.074749676	0.084613
A.vasorum /Control	O43665	-0.180149533	0.180149533	0.084715
A.vasorum /Control	B7ZAQ6	0.143786202	0.143786202	0.084912
A.vasorum /Control	P25788	-0.011827302	0.011827302	0.085297
A.vasorum /Control	Q86SQ9	-0.010338938	0.010338938	0.085304
A.vasorum /Control	P52789	-0.357214049	0.357214049	0.085338
A.vasorum /Control	Q15029	-0.058362662	0.058362662	0.085382
A.vasorum /Control	P05141	-0.153027395	0.153027395	0.085501
A.vasorum /Control	O43402	-0.037538504	0.037538504	0.085542
A.vasorum /Control	P27144	-0.175233855	0.175233855	0.085533
A.vasorum /Control	Q13555	-0.353307991	0.353307991	0.085623
A.vasorum /Control	O00629	-0.04681183	0.04681183	0.085856
A.vasorum /Control	Q9BR61	0.020153005	0.020153005	0.08603
A.vasorum /Control	Q86YV5	-0.057619665	0.057619665	0.086116

A.vasorum /Control	Q8N5G2	0.090795063	0.090795063	0.086186
A.vasorum /Control	Q13488	-0.230155266	0.230155266	0.086306
A.vasorum /Control	Q71SY5	0.229008563	0.229008563	0.086336
A.vasorum /Control	O94827	-0.115893811	0.115893811	0.086633
A.vasorum /Control	Q9H4A6	0.123670821	0.123670821	0.086638
A.vasorum /Control	Q9Y5X2	0.214013425	0.214013425	0.086629
A.vasorum /Control	Q16739	-0.663272068	0.663272068	0.086696
A.vasorum /Control	Q8IZ73	-0.207456866	0.207456866	0.086679
A.vasorum /Control	P48307	-1.045896924	1.045896924	0.087061
A.vasorum /Control	A6NHR9	-0.188379813	0.188379813	0.087254
A.vasorum /Control	Q9UIF9	-0.742792498	0.742792498	0.087347
A.vasorum /Control	O43299	0.107365875	0.107365875	0.087536
A.vasorum /Control	Q8IYB3	-0.030480187	0.030480187	0.087559
A.vasorum /Control	Q9H0V1	0.527776248	0.527776248	0.087561
A.vasorum /Control	P51398	-0.154715092	0.154715092	0.087696
A.vasorum /Control	Q15397	-0.907857941	0.907857941	0.087741
A.vasorum /Control	Q96SZ6	0.001846176	0.001846176	0.087844
A.vasorum /Control	P61106	-0.059736111	0.059736111	0.08818
A.vasorum /Control	Q9NPG4	0.075069792	0.075069792	0.088214
A.vasorum /Control	Q13418	0.015727953	0.015727953	0.088346
A.vasorum /Control	Q7Z5G4	-0.053786184	0.053786184	0.088448
A.vasorum /Control	Q9UKR5	0.510447268	0.510447268	0.088511
A.vasorum /Control	Q5SYE7	-0.610027518	0.610027518	0.088639
A.vasorum /Control	O60921	0.206033733	0.206033733	0.088902
A.vasorum /Control	Q96J02	0.017891402	0.017891402	0.088956
A.vasorum /Control	Q01415	0.09095537	0.09095537	0.089036
A.vasorum /Control	Q13112	0.401842565	0.401842565	0.089184
A.vasorum /Control	Q5T1J5	-0.314212372	0.314212372	0.089176
A.vasorum /Control	P55010	-0.05002708	0.05002708	0.089421
A.vasorum /Control	Q9BX95	0.117939953	0.117939953	0.089519
A.vasorum /Control	P63010	-0.038286697	0.038286697	0.089737
A.vasorum /Control	Q8IWC1	-0.198092095	0.198092095	0.089789
A.vasorum /Control	P14314	0.015638801	0.015638801	0.089861
A.vasorum /Control	Q5BJH7	-0.154345171	0.154345171	0.089884
A.vasorum /Control	P22681	0.235724223	0.235724223	0.089969
A.vasorum /Control	Q8NFW8	-0.027907997	0.027907997	0.090172
A.vasorum /Control	P45983	0.155943132	0.155943132	0.090212
A.vasorum /Control	Q5JRX3	-0.130706011	0.130706011	0.090783
A.vasorum /Control	P16403	0.257337462	0.257337462	0.090847
A.vasorum /Control	P49023	-0.261253861	0.261253861	0.090886
A.vasorum /Control	Q15042	-0.202696003	0.202696003	0.090879
A.vasorum /Control	P48147	-0.036394271	0.036394271	0.091163
A.vasorum /Control	Q13247	-0.232426293	0.232426293	0.091279
A.vasorum /Control	Q86Y79	-0.128160648	0.128160648	0.091281
A.vasorum /Control	O75208	0.218442435	0.218442435	0.091332
A.vasorum /Control	A5PLL7	-0.659989651	0.659989651	0.091514
A.vasorum /Control	Q5T8P6	-0.100488854	0.100488854	0.091632
A.vasorum /Control	Q8NEY1	-0.155592526	0.155592526	0.0917
A.vasorum /Control	Q9H4K7	-0.719238948	0.719238948	0.091734
A.vasorum /Control	Q9UPY3	0.08481489	0.08481489	0.092091

A.vasorum /Control	Q8TDM6	-1.025098187	1.025098187	0.092135
A.vasorum /Control	Q92574	0.156866022	0.156866022	0.09221
A.vasorum /Control	Q6ZSZ5	0.057891716	0.057891716	0.09237
A.vasorum /Control	O76070	0.374438742	0.374438742	0.092943
A.vasorum /Control	P50443	-0.145543591	0.145543591	0.092966
A.vasorum /Control	Q06124	0.053193633	0.053193633	0.092947
A.vasorum /Control	Q9P2R6	0.870546329	0.870546329	0.093037
A.vasorum /Control	Q9H6E4	0.467622339	0.467622339	0.093142
A.vasorum /Control	Q8NBN3	0.138737254	0.138737254	0.093179
A.vasorum /Control	P16402	0.317933392	0.317933392	0.09327
A.vasorum /Control	Q9BWH2	0.001521881	0.001521881	0.093288
A.vasorum /Control	Q6UB35	-0.139305548	0.139305548	0.093393
A.vasorum /Control	O95298	0.044471845	0.044471845	0.093574
A.vasorum /Control	P60174	-0.010783708	0.010783708	0.093568
A.vasorum /Control	Q8IY95	0.153992667	0.153992667	0.093532
A.vasorum /Control	Q969Q0	0.250770296	0.250770296	0.093719
A.vasorum /Control	Q96BY6	-0.648487234	0.648487234	0.093769
A.vasorum /Control	Q9UJF2	0.11538982	0.11538982	0.094114
A.vasorum /Control	Q86UY6	0.53946143	0.53946143	0.094267
A.vasorum /Control	Q9BQ61	-0.391298381	0.391298381	0.094359
A.vasorum /Control	P46976	-0.035628245	0.035628245	0.094489
A.vasorum /Control	O60841	-0.158408405	0.158408405	0.094732
A.vasorum /Control	Q8N357	-0.012635352	0.012635352	0.09473
A.vasorum /Control	Q99700	0.011673417	0.011673417	0.094902
A.vasorum /Control	Q9UBK8	0.407221291	0.407221291	0.094894
A.vasorum /Control	Q07820	-1.736911867	1.736911867	0.095153
A.vasorum /Control	Q13823	-0.299781461	0.299781461	0.095272
A.vasorum /Control	P28300	0.115289101	0.115289101	0.095325
A.vasorum /Control	Q8WUM4	-0.066813047	0.066813047	0.09536
A.vasorum /Control	P20936	0.165868014	0.165868014	0.09617
A.vasorum /Control	Q3SY69	0.304635409	0.304635409	0.096261
A.vasorum /Control	Q96FZ7	-0.012243038	0.012243038	0.096338
A.vasorum /Control	Q15011	0.087884558	0.087884558	0.096394
A.vasorum /Control	P60981	-0.230471784	0.230471784	0.096471
A.vasorum /Control	O60885	-0.279627926	0.279627926	0.096505
A.vasorum /Control	O60573	0.101368319	0.101368319	0.096558
A.vasorum /Control	P62851	-0.039323968	0.039323968	0.096608
A.vasorum /Control	Q15059	-0.428532119	0.428532119	0.096594
A.vasorum /Control	Q9P275	0.161886059	0.161886059	0.096579
A.vasorum /Control	Q06330	-0.089041854	0.089041854	0.096743
A.vasorum /Control	P59998	0.007420383	0.007420383	0.096897
A.vasorum /Control	Q99571	-0.121135249	0.121135249	0.09692
A.vasorum /Control	Q9UBN6	-0.166794943	0.166794943	0.096981
A.vasorum /Control	Q8N2W9	0.479854784	0.479854784	0.097023
A.vasorum /Control	Q9BQ52	-0.08754521	0.08754521	0.097036
A.vasorum /Control	P34932	-0.059813016	0.059813016	0.097137
A.vasorum /Control	O95295	0.001630079	0.001630079	0.097228
A.vasorum /Control	Q5T6F2	-0.10438638	0.10438638	0.097225
A.vasorum /Control	Q9NX62	-0.147319672	0.147319672	0.097264
A.vasorum /Control	Q6NXT1	0.670645256	0.670645256	0.097538

A.vasorum /Control	Q96IY1	0.203599341	0.203599341	0.097601
A.vasorum /Control	P40616	-0.033523074	0.033523074	0.097775
A.vasorum /Control	Q86XL3	-0.054508649	0.054508649	0.09776
A.vasorum /Control	P21397	0.490429375	0.490429375	0.098261
A.vasorum /Control	P04350	0.627224787	0.627224787	0.098429
A.vasorum /Control	Q2NKX8	-0.015442421	0.015442421	0.098428
A.vasorum /Control	P62249	0.01032507	0.01032507	0.098748
A.vasorum /Control	O15530	-0.094494026	0.094494026	0.098822
A.vasorum /Control	O43314	0.203099288	0.203099288	0.098875
A.vasorum /Control	P62068	-0.49547121	0.49547121	0.09891
A.vasorum /Control	Q15691	-0.155728093	0.155728093	0.098959
A.vasorum /Control	Q9UBV7	0.195377124	0.195377124	0.099264
A.vasorum /Control	P62253	0.509165647	0.509165647	0.099316
A.vasorum /Control	P26196	-0.057819056	0.057819056	0.099446
A.vasorum /Control	P08047	-0.111083233	0.111083233	0.099568
A.vasorum /Control	P08174	-0.460950768	0.460950768	0.099563
A.vasorum /Control	P23508	-0.538237309	0.538237309	0.099585
A.vasorum /Control	Q8N5I2	-0.039417102	0.039417102	0.0996
A.vasorum /Control	Q9Y5T4	-0.49086166	0.49086166	0.099672
A.vasorum /Control	Q92804	0.424688931	0.424688931	0.099866
A.vasorum /Control	Q19T08	0.340736345	0.340736345	0.099892
A.vasorum /Control	Q8N8Z6	-0.616393194	0.616393194	0.099965
A.vasorum /Control	P23610	0.158885343	0.158885343	0.100217
A.vasorum /Control	Q08188	-1.570540112	1.570540112	0.100995
A.vasorum /Control	P61086	-0.075419508	0.075419508	0.101093
A.vasorum /Control	Q96S52	0.065468146	0.065468146	0.101158
A.vasorum /Control	Q8N6M3	0.834956042	0.834956042	0.101229
A.vasorum /Control	Q9Y3E1	-0.058447589	0.058447589	0.101215
A.vasorum /Control	Q969G6	0.261147703	0.261147703	0.101265
A.vasorum /Control	Q8TEB1	0.177017615	0.177017615	0.101413
A.vasorum /Control	O00231	-0.082498656	0.082498656	0.101613
A.vasorum /Control	P23258	0.039886232	0.039886232	0.101602
A.vasorum /Control	Q9NTW7	0.343929414	0.343929414	0.101792
A.vasorum /Control	O60336	0.085129054	0.085129054	0.102195
A.vasorum /Control	Q96LL9	-0.663726308	0.663726308	0.102261
A.vasorum /Control	Q9BZG8	0.174331576	0.174331576	0.102367
A.vasorum /Control	P26951	-0.181929985	0.181929985	0.10249
A.vasorum /Control	Q08AF3	-0.083228019	0.083228019	0.102503
A.vasorum /Control	O94919	-0.071751982	0.071751982	0.102633
A.vasorum /Control	Q9Y512	-0.101083704	0.101083704	0.102614
A.vasorum /Control	Q96LB3	-0.238182466	0.238182466	0.102754
A.vasorum /Control	O96007	0.113322377	0.113322377	0.103225
A.vasorum /Control	Q8NBI6	0.051671172	0.051671172	0.103276
A.vasorum /Control	Q8N257	1.512430792	1.512430792	0.103687
A.vasorum /Control	P49662	-0.239766922	0.239766922	0.10384
A.vasorum /Control	Q9H0B6	-0.054252408	0.054252408	0.103855
A.vasorum /Control	Q92973	-0.073177064	0.073177064	0.103933
A.vasorum /Control	Q5JTV8	-0.066181944	0.066181944	0.10398
A.vasorum /Control	Q96SW2	0.245665138	0.245665138	0.104232
A.vasorum /Control	Q99661	-0.341998677	0.341998677	0.104611

A.vasorum /Control	Q5TAT6	-0.70753122	0.70753122	0.104648
A.vasorum /Control	Q8NHH9	-0.020762119	0.020762119	0.104891
A.vasorum /Control	Q9NWB7	0.843472318	0.843472318	0.104967
A.vasorum /Control	P37023	-0.007682045	0.007682045	0.105016
A.vasorum /Control	Q99714	0.023183726	0.023183726	0.105183
A.vasorum /Control	Q7Z6J9	0.171429002	0.171429002	0.105222
A.vasorum /Control	P21796	-0.101692371	0.101692371	0.105429
A.vasorum /Control	Q8WTT2	-0.128476031	0.128476031	0.10546
A.vasorum /Control	P62750	0.084317481	0.084317481	0.105541
A.vasorum /Control	Q9UL40	0.005182489	0.005182489	0.105557
A.vasorum /Control	P14618	-0.080683174	0.080683174	0.105597
A.vasorum /Control	Q92743	-0.098614051	0.098614051	0.106267
A.vasorum /Control	O95602	-0.205033306	0.205033306	0.106371
A.vasorum /Control	O95456	-0.02599893	0.02599893	0.106732
A.vasorum /Control	Q15018	-0.078814662	0.078814662	0.106909
A.vasorum /Control	O95243	0.469437496	0.469437496	0.106979
A.vasorum /Control	P51178	0.42678814	0.42678814	0.107373
A.vasorum /Control	Q9UKU7	0.048619452	0.048619452	0.107509
A.vasorum /Control	Q9NW81	0.33630432	0.33630432	0.10768
A.vasorum /Control	Q53H82	0.030810655	0.030810655	0.108213
A.vasorum /Control	Q5T749	-1.472398513	1.472398513	0.108457
A.vasorum /Control	Q9H981	-0.074137677	0.074137677	0.108756
A.vasorum /Control	Q9P013	-0.000717585	0.000717585	0.10876
A.vasorum /Control	P62633	-0.227826415	0.227826415	0.108873
A.vasorum /Control	Q8WUP2	-0.339609689	0.339609689	0.109376
A.vasorum /Control	Q7RTV0	-0.288031723	0.288031723	0.109551
A.vasorum /Control	Q92889	0.077960471	0.077960471	0.109571
A.vasorum /Control	Q13868	-0.168739347	0.168739347	0.109806
A.vasorum /Control	Q9UG63	-0.105030837	0.105030837	0.109783
A.vasorum /Control	Q96G21	-0.504473079	0.504473079	0.109952
A.vasorum /Control	Q01844	-0.003568568	0.003568568	0.110004
A.vasorum /Control	Q9H5U6	0.135570428	0.135570428	0.110177
A.vasorum /Control	Q68CQ7	0.267910959	0.267910959	0.11022
A.vasorum /Control	P11169	0.158149355	0.158149355	0.110317
A.vasorum /Control	Q24JP5	0.529355449	0.529355449	0.110382
A.vasorum /Control	O14979	-0.43824331	0.43824331	0.110446
A.vasorum /Control	Q7Z3T8	-0.052673488	0.052673488	0.110555
A.vasorum /Control	P55327	0.102362982	0.102362982	0.110729
A.vasorum /Control	Q9NXV6	0.059996712	0.059996712	0.11092
A.vasorum /Control	Q9UHR6	0.031645123	0.031645123	0.111141
A.vasorum /Control	Q86V85	0.612701149	0.612701149	0.111171
A.vasorum /Control	Q08629	-0.447451996	0.447451996	0.111468
A.vasorum /Control	Q9H0W8	-0.201166022	0.201166022	0.11159
A.vasorum /Control	Q14667	0.258540128	0.258540128	0.111642
A.vasorum /Control	Q86U44	-0.073644688	0.073644688	0.11167
A.vasorum /Control	P39023	-0.098344324	0.098344324	0.111986
A.vasorum /Control	P48060	-0.992315757	0.992315757	0.112107
A.vasorum /Control	Q3B726	-0.299710226	0.299710226	0.112528
A.vasorum /Control	Q86WJ1	0.02133758	0.02133758	0.112545
A.vasorum /Control	P23219	-0.681624666	0.681624666	0.112614

A.vasorum /Control	P62263	-0.063441381	0.063441381	0.112853
A.vasorum /Control	P23193	-0.00705952	0.00705952	0.112916
A.vasorum /Control	Q9BPZ7	-1.200847399	1.200847399	0.113158
A.vasorum /Control	Q9H1K1	-0.089248819	0.089248819	0.113983
A.vasorum /Control	Q9NX24	-0.254800543	0.254800543	0.114028
A.vasorum /Control	Q15121	0.027193144	0.027193144	0.114054
A.vasorum /Control	Q14240	-0.142363897	0.142363897	0.114505
A.vasorum /Control	Q15233	0.071963516	0.071963516	0.114559
A.vasorum /Control	P26639	-0.124461281	0.124461281	0.114765
A.vasorum /Control	Q02413	-1.569027659	1.569027659	0.114942
A.vasorum /Control	P35268	-0.054181467	0.054181467	0.115064
A.vasorum /Control	Q16875	-0.147736316	0.147736316	0.115107
A.vasorum /Control	Q9Y388	0.419872369	0.419872369	0.115193
A.vasorum /Control	Q08170	-0.238580869	0.238580869	0.115597
A.vasorum /Control	Q15428	-0.378368935	0.378368935	0.115662
A.vasorum /Control	Q13889	-0.065136122	0.065136122	0.115754
A.vasorum /Control	O00522	-0.009380565	0.009380565	0.116289
A.vasorum /Control	P60602	-0.062848208	0.062848208	0.116308
A.vasorum /Control	Q5T3I0	0.137992965	0.137992965	0.116264
A.vasorum /Control	P00749	-0.808778574	0.808778574	0.116488
A.vasorum /Control	Q6IA69	0.092972072	0.092972072	0.116509
A.vasorum /Control	Q8N6H7	-0.097469076	0.097469076	0.116469
A.vasorum /Control	O15541	-0.320015369	0.320015369	0.116908
A.vasorum /Control	O00505	0.062137683	0.062137683	0.117402
A.vasorum /Control	P36543	-0.140825167	0.140825167	0.117419
A.vasorum /Control	P84101	-0.053969512	0.053969512	0.11738
A.vasorum /Control	Q99504	0.464585593	0.464585593	0.117595
A.vasorum /Control	P13196	-0.149080102	0.149080102	0.118118
A.vasorum /Control	Q8NBK3	0.100996529	0.100996529	0.118153
A.vasorum /Control	P07305	0.070958782	0.070958782	0.118222
A.vasorum /Control	Q96D15	0.548995918	0.548995918	0.118253
A.vasorum /Control	Q9UPY5	-1.346353915	1.346353915	0.119135
A.vasorum /Control	O95394	-0.06535641	0.06535641	0.119204
A.vasorum /Control	Q9H6Z4	-0.106660722	0.106660722	0.119283
A.vasorum /Control	Q93074	-0.001024196	0.001024196	0.119401
A.vasorum /Control	Q9Y2Q9	0.060382002	0.060382002	0.119595
A.vasorum /Control	Q9Y2Q5	0.107607315	0.107607315	0.119741
A.vasorum /Control	Q9BW60	-0.236960362	0.236960362	0.119785
A.vasorum /Control	Q8WWI5	-0.071852587	0.071852587	0.119877
A.vasorum /Control	Q9Y4P3	-0.190361043	0.190361043	0.119931
A.vasorum /Control	Q14141	0.00953243	0.00953243	0.120006
A.vasorum /Control	Q5T0F9	-0.055963945	0.055963945	0.120001
A.vasorum /Control	P61009	-0.007819213	0.007819213	0.120088
A.vasorum /Control	Q96QB1	-0.681925707	0.681925707	0.12008
A.vasorum /Control	P18754	-0.137792975	0.137792975	0.12019
A.vasorum /Control	Q9Y2Y6	0.122902205	0.122902205	0.120254
A.vasorum /Control	Q9BT25	-0.230154537	0.230154537	0.120344
A.vasorum /Control	Q9Y4F5	-0.014862341	0.014862341	0.120817
A.vasorum /Control	Q9H2J4	-0.170876878	0.170876878	0.120866
A.vasorum /Control	Q9ULG1	-0.116243234	0.116243234	0.121043

A.vasorum /Control	Q8TD55	-0.071780055	0.071780055	0.121126
A.vasorum /Control	Q9H3U1	-0.147830672	0.147830672	0.121162
A.vasorum /Control	Q8NBU5	-0.110734703	0.110734703	0.121505
A.vasorum /Control	Q9GZS1	-0.402447357	0.402447357	0.121604
A.vasorum /Control	P53582	-0.476209396	0.476209396	0.121904
A.vasorum /Control	Q86SK9	0.070320043	0.070320043	0.121949
A.vasorum /Control	P78371	-0.067832398	0.067832398	0.122362
A.vasorum /Control	Q86W34	0.361515794	0.361515794	0.122655
A.vasorum /Control	P13995	-0.577488956	0.577488956	0.122869
A.vasorum /Control	P50281	-0.292424487	0.292424487	0.122849
A.vasorum /Control	P53801	-0.499328342	0.499328342	0.122823
A.vasorum /Control	O95202	-0.064776761	0.064776761	0.123047
A.vasorum /Control	Q6PIW4	0.103948391	0.103948391	0.123247
A.vasorum /Control	Q9NUQ6	-0.275156713	0.275156713	0.123225
A.vasorum /Control	Q9BTA9	-0.210747906	0.210747906	0.12334
A.vasorum /Control	Q9NXE8	-0.103058574	0.103058574	0.123456
A.vasorum /Control	O94842	0.105997963	0.105997963	0.123581
A.vasorum /Control	Q96BW1	0.739669263	0.739669263	0.123795
A.vasorum /Control	P17612	0.033528724	0.033528724	0.124739
A.vasorum /Control	Q9ULV3	-0.367066194	0.367066194	0.124718
A.vasorum /Control	O00214	0.372122648	0.372122648	0.124827
A.vasorum /Control	Q96LJ7	-0.157743206	0.157743206	0.124923
A.vasorum /Control	P00750	-0.402880659	0.402880659	0.125063
A.vasorum /Control	Q14119	-0.690645409	0.690645409	0.125048
A.vasorum /Control	Q99569	0.002636604	0.002636604	0.125254
A.vasorum /Control	P47712	-0.184729112	0.184729112	0.125295
A.vasorum /Control	P02647	-0.34955783	0.34955783	0.125604
A.vasorum /Control	P25685	-0.231138943	0.231138943	0.12561
A.vasorum /Control	Q14186	-0.43003755	0.43003755	0.125638
A.vasorum /Control	O95169	0.189710063	0.189710063	0.125717
A.vasorum /Control	Q5BKZ1	-0.273784384	0.273784384	0.125917
A.vasorum /Control	P29692	-0.350223119	0.350223119	0.12629
A.vasorum /Control	Q92917	0.095326185	0.095326185	0.126514
A.vasorum /Control	Q16342	-0.446278521	0.446278521	0.126749
A.vasorum /Control	Q9Y2W6	0.361963437	0.361963437	0.127407
A.vasorum /Control	Q5JTH9	-0.516478406	0.516478406	0.127582
A.vasorum /Control	Q14683	-0.16428181	0.16428181	0.127666
A.vasorum /Control	Q96HA7	0.042409947	0.042409947	0.127677
A.vasorum /Control	P78316	-0.669837617	0.669837617	0.12772
A.vasorum /Control	Q14919	-0.109775559	0.109775559	0.127973
A.vasorum /Control	Q9BZG1	0.187561352	0.187561352	0.128427
A.vasorum /Control	O00268	-0.163530373	0.163530373	0.12859
A.vasorum /Control	Q9UL54	0.031275488	0.031275488	0.128645
A.vasorum /Control	P42166	-0.214133681	0.214133681	0.129071
A.vasorum /Control	Q92785	-0.280250891	0.280250891	0.129415
A.vasorum /Control	Q9UBP4	0.07316162	0.07316162	0.129386
A.vasorum /Control	Q15652	0.088761278	0.088761278	0.129498
A.vasorum /Control	O95486	-0.150930447	0.150930447	0.129718
A.vasorum /Control	P21399	-0.270227014	0.270227014	0.12969
A.vasorum /Control	O43657	0.452639005	0.452639005	0.130013

A.vasorum /Control	O95544	-0.270834571	0.270834571	0.130055
A.vasorum /Control	Q86UT6	0.784586264	0.784586264	0.130056
A.vasorum /Control	Q9HB20	-0.158213989	0.158213989	0.129971
A.vasorum /Control	Q9H1E3	0.073025456	0.073025456	0.130379
A.vasorum /Control	O43482	-0.306292283	0.306292283	0.130442
A.vasorum /Control	Q9BZE1	-0.062999415	0.062999415	0.131037
A.vasorum /Control	Q9HA77	0.01473135	0.01473135	0.131483
A.vasorum /Control	Q9BT73	0.182961529	0.182961529	0.131592
A.vasorum /Control	P42694	0.127212687	0.127212687	0.132041
A.vasorum /Control	Q9C0B5	-0.140562015	0.140562015	0.132382
A.vasorum /Control	Q9BTE6	0.01638605	0.01638605	0.132688
A.vasorum /Control	P22234	-0.153996677	0.153996677	0.132734
A.vasorum /Control	Q96DH6	0.171086156	0.171086156	0.133204
A.vasorum /Control	Q9Y5Y2	0.033769759	0.033769759	0.133333
A.vasorum /Control	Q8NBF2	0.036231485	0.036231485	0.133512
A.vasorum /Control	P62308	0.162820303	0.162820303	0.13361
A.vasorum /Control	Q49AR2	0.303989669	0.303989669	0.133553
A.vasorum /Control	Q96EM0	-0.201252479	0.201252479	0.133589
A.vasorum /Control	P25786	-0.041471164	0.041471164	0.133963
A.vasorum /Control	Q70IA6	0.216368363	0.216368363	0.134223
A.vasorum /Control	Q96HA1	-0.227217768	0.227217768	0.134437
A.vasorum /Control	Q7Z6L1	-0.379176777	0.379176777	0.134789
A.vasorum /Control	Q8NFZ0	0.608715739	0.608715739	0.13539
A.vasorum /Control	Q9H6W3	0.058506636	0.058506636	0.135461
A.vasorum /Control	Q5JPI3	-0.116441922	0.116441922	0.136041
A.vasorum /Control	P02452	0.177211428	0.177211428	0.136449
A.vasorum /Control	Q9BXL7	-0.263498455	0.263498455	0.136467
A.vasorum /Control	O14530	-0.288287964	0.288287964	0.136526
A.vasorum /Control	Q15542	-0.254772104	0.254772104	0.136781
A.vasorum /Control	Q66PJ3	-0.019710583	0.019710583	0.136788
A.vasorum /Control	Q9Y4F3	0.000610563	0.000610563	0.136737
A.vasorum /Control	Q9UPU9	-0.014312626	0.014312626	0.137028
A.vasorum /Control	L0R819	-0.220847301	0.220847301	0.13725
A.vasorum /Control	P32856	0.050624858	0.050624858	0.137484
A.vasorum /Control	P52565	0.000375358	0.000375358	0.13747
A.vasorum /Control	Q86SQ4	0.228366104	0.228366104	0.137749
A.vasorum /Control	Q12894	0.222924586	0.222924586	0.137893
A.vasorum /Control	P0CAP2	-0.479129486	0.479129486	0.137966
A.vasorum /Control	Q68CZ6	-0.057263756	0.057263756	0.138055
A.vasorum /Control	P61225	-0.292131085	0.292131085	0.138352
A.vasorum /Control	Q9H6S1	0.507415138	0.507415138	0.138384
A.vasorum /Control	P24386	-0.61328002	0.61328002	0.138563
A.vasorum /Control	Q96C19	-0.22692492	0.22692492	0.138751
A.vasorum /Control	Q9UBK9	0.176363992	0.176363992	0.138855
A.vasorum /Control	Q8TAE8	-0.067680327	0.067680327	0.138921
A.vasorum /Control	O00165	0.002480597	0.002480597	0.139143
A.vasorum /Control	Q5MNZ9	0.08987941	0.08987941	0.13913
A.vasorum /Control	Q9BW83	0.268170244	0.268170244	0.139089
A.vasorum /Control	Q92979	-0.719265787	0.719265787	0.139248
A.vasorum /Control	Q12805	-0.011720824	0.011720824	0.139426

A.vasorum /Control	Q53TN4	0.399968576	0.399968576	0.139386
A.vasorum /Control	Q6P3X3	-0.001177666	0.001177666	0.139382
A.vasorum /Control	Q8IWU5	-0.256130091	0.256130091	0.139423
A.vasorum /Control	Q9H3P2	0.037056976	0.037056976	0.139548
A.vasorum /Control	Q9NSI8	-0.76343603	0.76343603	0.139566
A.vasorum /Control	O95140	-0.008011838	0.008011838	0.139739
A.vasorum /Control	Q4G0F5	-0.153988932	0.153988932	0.139753
A.vasorum /Control	P56945	0.097319053	0.097319053	0.140214
A.vasorum /Control	Q8NHG7	0.183824036	0.183824036	0.140199
A.vasorum /Control	Q9BTY7	0.450461263	0.450461263	0.140399
A.vasorum /Control	Q9UBK7	-0.008252401	0.008252401	0.140394
A.vasorum /Control	Q9UKB1	-0.065099439	0.065099439	0.140588
A.vasorum /Control	O14828	-0.067029783	0.067029783	0.140672
A.vasorum /Control	O75503	-0.050955892	0.050955892	0.140718
A.vasorum /Control	P30530	-0.373309849	0.373309849	0.140763
A.vasorum /Control	Q9BXW7	-0.005600907	0.005600907	0.140873
A.vasorum /Control	O00411	-0.670473552	0.670473552	0.141218
A.vasorum /Control	Q9NUB1	0.215642073	0.215642073	0.141287
A.vasorum /Control	Q9Y277	0.006293441	0.006293441	0.14151
A.vasorum /Control	P30825	-0.367886457	0.367886457	0.141594
A.vasorum /Control	Q15785	0.048443018	0.048443018	0.142045
A.vasorum /Control	Q5T7W0	-0.262637017	0.262637017	0.142283
A.vasorum /Control	P09382	0.077503784	0.077503784	0.142836
A.vasorum /Control	P31942	-0.274242287	0.274242287	0.142786
A.vasorum /Control	Q96I25	-0.004102587	0.004102587	0.142834
A.vasorum /Control	Q86UY8	-0.454484962	0.454484962	0.142928
A.vasorum /Control	P16401	-0.198630566	0.198630566	0.143345
A.vasorum /Control	O00193	0.13542834	0.13542834	0.143461
A.vasorum /Control	Q7Z7M0	-0.041981383	0.041981383	0.144005
A.vasorum /Control	Q9BUI4	0.07894606	0.07894606	0.144236
A.vasorum /Control	Q86UK7	-0.212190004	0.212190004	0.144441
A.vasorum /Control	Q96EK6	-0.225401798	0.225401798	0.144421
A.vasorum /Control	P46020	-0.015955021	0.015955021	0.144489
A.vasorum /Control	Q9BX59	0.596794124	0.596794124	0.144557
A.vasorum /Control	Q99766	0.344586893	0.344586893	0.14464
A.vasorum /Control	Q92851	0.519884731	0.519884731	0.144722
A.vasorum /Control	Q8N3R9	-0.1433006	0.1433006	0.14483
A.vasorum /Control	P09104	-0.026622365	0.026622365	0.14508
A.vasorum /Control	O14640	-1.032132172	1.032132172	0.145384
A.vasorum /Control	Q9H2C0	-0.300520884	0.300520884	0.145408
A.vasorum /Control	O75534	-0.126740473	0.126740473	0.145521
A.vasorum /Control	Q9UBQ6	-0.643951076	0.643951076	0.145658
A.vasorum /Control	Q8WWK9	-0.308897931	0.308897931	0.146463
A.vasorum /Control	O75683	-0.506243771	0.506243771	0.146603
A.vasorum /Control	Q9H8M7	0.270403152	0.270403152	0.146641
A.vasorum /Control	P98172	0.070622428	0.070622428	0.146779
A.vasorum /Control	P55735	-0.325779791	0.325779791	0.146863
A.vasorum /Control	Q9UM11	0.262470547	0.262470547	0.146971
A.vasorum /Control	P29558	-0.044425711	0.044425711	0.147157
A.vasorum /Control	P48059	-0.007207727	0.007207727	0.147651

A.vasorum /Control	P67936	-0.045874496	0.045874496	0.147733
A.vasorum /Control	Q13395	0.340734441	0.340734441	0.147743
A.vasorum /Control	Q9Y281	-0.136223232	0.136223232	0.147694
A.vasorum /Control	Q9NUL7	-0.179467989	0.179467989	0.1478
A.vasorum /Control	O43854	-0.494715076	0.494715076	0.147916
A.vasorum /Control	Q9Y243	-0.218037386	0.218037386	0.148088
A.vasorum /Control	Q86TB9	-0.079756657	0.079756657	0.14833
A.vasorum /Control	P22528	-0.3093875	0.3093875	0.148787
A.vasorum /Control	P38919	-0.089625253	0.089625253	0.148856
A.vasorum /Control	Q16514	0.22997521	0.22997521	0.14899
A.vasorum /Control	A3KMH1	-0.070301793	0.070301793	0.149413
A.vasorum /Control	P15924	-1.000615284	1.000615284	0.149908
A.vasorum /Control	O43414	0.110941036	0.110941036	0.150023
A.vasorum /Control	Q8IXM3	0.042221541	0.042221541	0.149998
A.vasorum /Control	O15160	-0.140403512	0.140403512	0.150151
A.vasorum /Control	Q9UNS1	0.504826684	0.504826684	0.150425
A.vasorum /Control	O75691	-0.290617176	0.290617176	0.150503
A.vasorum /Control	P49116	-0.088633954	0.088633954	0.150626
A.vasorum /Control	P07711	-0.312067877	0.312067877	0.150724
A.vasorum /Control	Q9ULV4	-0.277911734	0.277911734	0.150793
A.vasorum /Control	Q9NQH7	0.142113093	0.142113093	0.150891
A.vasorum /Control	Q8TCT7	-0.016595254	0.016595254	0.15167
A.vasorum /Control	Q9H6H4	-0.128424321	0.128424321	0.15187
A.vasorum /Control	Q96HJ9	-0.121840404	0.121840404	0.152153
A.vasorum /Control	P54259	-0.468367186	0.468367186	0.152536
A.vasorum /Control	P14921	0.116042761	0.116042761	0.152746
A.vasorum /Control	Q5MNZ6	-0.009519073	0.009519073	0.152894
A.vasorum /Control	Q8WXD5	0.847078356	0.847078356	0.153349
A.vasorum /Control	Q9BV57	0.204153573	0.204153573	0.153398
A.vasorum /Control	Q7Z494	0.548531461	0.548531461	0.153856
A.vasorum /Control	Q6KB66	-0.274222464	0.274222464	0.154033
A.vasorum /Control	Q8NDZ4	-0.120745258	0.120745258	0.154311
A.vasorum /Control	Q7KZ85	0.11420853	0.11420853	0.155122
A.vasorum /Control	O00221	0.398768907	0.398768907	0.155308
A.vasorum /Control	Q8IWD4	-0.030006921	0.030006921	0.155297
A.vasorum /Control	P51148	0.012609989	0.012609989	0.155567
A.vasorum /Control	Q96GY3	0.27075797	0.27075797	0.15579
A.vasorum /Control	Q9H4L5	-0.093426854	0.093426854	0.155918
A.vasorum /Control	Q15800	-0.141235088	0.141235088	0.1561
A.vasorum /Control	Q13620	-0.15997064	0.15997064	0.156708
A.vasorum /Control	Q9Y4F1	0.106490809	0.106490809	0.156698
A.vasorum /Control	P38432	0.090263615	0.090263615	0.156802
A.vasorum /Control	O95084	-0.303341955	0.303341955	0.157071
A.vasorum /Control	P78537	-0.372370306	0.372370306	0.158016
A.vasorum /Control	Q12972	-0.2047889	0.2047889	0.158307
A.vasorum /Control	Q6UX04	0.082912575	0.082912575	0.158436
A.vasorum /Control	O14672	0.022104899	0.022104899	0.159464
A.vasorum /Control	Q13257	-0.28881262	0.28881262	0.160305
A.vasorum /Control	O75907	0.392291954	0.392291954	0.160368
A.vasorum /Control	Q96J01	-0.15656238	0.15656238	0.160464

A.vasorum /Control	Q9Y5K6	0.006263575	0.006263575	0.160525
A.vasorum /Control	Q9UPW0	-0.088043348	0.088043348	0.160567
A.vasorum /Control	Q9H1A3	-0.552506411	0.552506411	0.160835
A.vasorum /Control	Q5T2E6	0.281004527	0.281004527	0.161709
A.vasorum /Control	P54198	-0.199007407	0.199007407	0.161811
A.vasorum /Control	O96013	-0.052402194	0.052402194	0.162478
A.vasorum /Control	Q9Y2R9	-0.206587682	0.206587682	0.162479
A.vasorum /Control	P11216	-0.120422297	0.120422297	0.162534
A.vasorum /Control	Q6ZMZ3	-0.387947214	0.387947214	0.162774
A.vasorum /Control	Q00537	-0.141769981	0.141769981	0.162844
A.vasorum /Control	O60828	-0.547402398	0.547402398	0.163028
A.vasorum /Control	O75131	-0.145135527	0.145135527	0.163113
A.vasorum /Control	P04406	-0.055752793	0.055752793	0.163366
A.vasorum /Control	O75436	0.030084116	0.030084116	0.163417
A.vasorum /Control	Q9NXG2	-0.169111315	0.169111315	0.1635
A.vasorum /Control	Q96GK7	0.169839717	0.169839717	0.16354
A.vasorum /Control	Q12982	-0.450487681	0.450487681	0.163587
A.vasorum /Control	Q5TC82	-0.134471583	0.134471583	0.163613
A.vasorum /Control	Q6P4E1	-0.129616228	0.129616228	0.163904
A.vasorum /Control	L0R8F8	-0.482370503	0.482370503	0.164352
A.vasorum /Control	P46777	0.034044425	0.034044425	0.164405
A.vasorum /Control	Q96DV4	-0.138446222	0.138446222	0.164496
A.vasorum /Control	Q96EK9	-0.378389186	0.378389186	0.164499
A.vasorum /Control	Q9Y6D5	0.012030625	0.012030625	0.164884
A.vasorum /Control	Q96IZ6	-0.300230576	0.300230576	0.16503
A.vasorum /Control	Q9BRJ7	-0.075793407	0.075793407	0.165533
A.vasorum /Control	P32519	-0.045499777	0.045499777	0.166031
A.vasorum /Control	Q9Y6B7	-0.100093998	0.100093998	0.166139
A.vasorum /Control	P82909	0.206895446	0.206895446	0.16638
A.vasorum /Control	Q9UMX1	0.529017005	0.529017005	0.166852
A.vasorum /Control	Q9UBX3	-0.131966927	0.131966927	0.167233
A.vasorum /Control	O95825	0.023357056	0.023357056	0.167273
A.vasorum /Control	Q92997	0.433440449	0.433440449	0.16741
A.vasorum /Control	O00622	-0.037745588	0.037745588	0.167912
A.vasorum /Control	O14681	-0.305178061	0.305178061	0.168314
A.vasorum /Control	Q14790	0.466092206	0.466092206	0.168373
A.vasorum /Control	Q8N5M9	-0.237548391	0.237548391	0.168407
A.vasorum /Control	Q9UQC2	0.320123409	0.320123409	0.168536
A.vasorum /Control	P60660	0.115629977	0.115629977	0.16867
A.vasorum /Control	P15104	-0.484171974	0.484171974	0.16919
A.vasorum /Control	Q9GZR2	-0.276476267	0.276476267	0.169261
A.vasorum /Control	O75821	0.024520358	0.024520358	0.169423
A.vasorum /Control	P46019	0.519014129	0.519014129	0.169819
A.vasorum /Control	Q8N4A0	-0.521257226	0.521257226	0.169923
A.vasorum /Control	O00567	-0.666852031	0.666852031	0.170003
A.vasorum /Control	O43318	-0.065106474	0.065106474	0.169995
A.vasorum /Control	Q9GZR1	0.400855042	0.400855042	0.170352
A.vasorum /Control	Q9GZR7	-0.339558114	0.339558114	0.170576
A.vasorum /Control	Q08431	-0.286867685	0.286867685	0.170838
A.vasorum /Control	P48634	-0.244597763	0.244597763	0.170888

A.vasorum /Control	Q9NWW5	-0.889625674	0.889625674	0.170937
A.vasorum /Control	P61956	-0.606214781	0.606214781	0.170995
A.vasorum /Control	Q8NFG4	0.086179718	0.086179718	0.171054
A.vasorum /Control	Q8WU90	-0.024379302	0.024379302	0.171324
A.vasorum /Control	Q86UW7	-0.055410128	0.055410128	0.171615
A.vasorum /Control	P39019	-0.135286236	0.135286236	0.171704
A.vasorum /Control	Q9H773	-0.329017334	0.329017334	0.171982
A.vasorum /Control	P37268	-0.19106873	0.19106873	0.172333
A.vasorum /Control	Q6P9B6	-0.065349859	0.065349859	0.17233
A.vasorum /Control	Q86XK2	-0.123680751	0.123680751	0.17242
A.vasorum /Control	Q9BXS6	-0.095943712	0.095943712	0.172527
A.vasorum /Control	Q04771	0.637456807	0.637456807	0.172565
A.vasorum /Control	Q96AQ8	-0.260737809	0.260737809	0.172791
A.vasorum /Control	Q02880	0.589977865	0.589977865	0.17339
A.vasorum /Control	Q14511	-0.822888915	0.822888915	0.173908
A.vasorum /Control	O96028	-0.059885384	0.059885384	0.174067
A.vasorum /Control	P20700	-0.20664475	0.20664475	0.174392
A.vasorum /Control	O15519	0.421095303	0.421095303	0.175005
A.vasorum /Control	Q6PHR2	0.184182077	0.184182077	0.175158
A.vasorum /Control	P15880	-0.087953818	0.087953818	0.175627
A.vasorum /Control	Q9HAD4	-0.316618282	0.316618282	0.176038
A.vasorum /Control	P28290	-0.162400543	0.162400543	0.176513
A.vasorum /Control	Q9H0E3	-0.007982842	0.007982842	0.176525
A.vasorum /Control	P60900	-0.06325634	0.06325634	0.176676
A.vasorum /Control	P04183	-0.423471774	0.423471774	0.178009
A.vasorum /Control	Q13428	-0.53865332	0.53865332	0.178226
A.vasorum /Control	Q9HAU4	-0.513130229	0.513130229	0.17833
A.vasorum /Control	Q99584	-0.017067561	0.017067561	0.178567
A.vasorum /Control	O15056	-0.281277035	0.281277035	0.178647
A.vasorum /Control	Q9BTV4	-0.010214142	0.010214142	0.178666
A.vasorum /Control	Q9NRB3	-0.33720295	0.33720295	0.179274
A.vasorum /Control	Q8IUW5	0.255309232	0.255309232	0.179439
A.vasorum /Control	O43660	-0.192667808	0.192667808	0.180203
A.vasorum /Control	P54105	0.221843487	0.221843487	0.180291
A.vasorum /Control	P50213	-0.083033092	0.083033092	0.180412
A.vasorum /Control	Q9Y547	0.297067858	0.297067858	0.180725
A.vasorum /Control	Q3SXM5	-0.274605195	0.274605195	0.181166
A.vasorum /Control	P53611	-0.077579097	0.077579097	0.181635
A.vasorum /Control	P41091	-0.056598053	0.056598053	0.181936
A.vasorum /Control	Q12792	-0.043309226	0.043309226	0.181975
A.vasorum /Control	Q9Y6V7	-0.636309225	0.636309225	0.181943
A.vasorum /Control	P08708	-0.040682354	0.040682354	0.18202
A.vasorum /Control	P05198	-0.106702183	0.106702183	0.182075
A.vasorum /Control	Q9Y676	-0.072364868	0.072364868	0.182283
A.vasorum /Control	P25787	-0.070623473	0.070623473	0.183034
A.vasorum /Control	Q9C0I1	0.179961598	0.179961598	0.183027
A.vasorum /Control	Q9BXK1	0.426421138	0.426421138	0.183598
A.vasorum /Control	B2RTY4	0.853826912	0.853826912	0.183657
A.vasorum /Control	Q9UMY1	-0.8373344	0.8373344	0.184151
A.vasorum /Control	Q9UPP1	0.364793922	0.364793922	0.184219

A.vasorum /Control	O43252	-0.048431822	0.048431822	0.184363
A.vasorum /Control	Q7L8L6	-0.473371885	0.473371885	0.184412
A.vasorum /Control	Q8NDX5	0.336971032	0.336971032	0.184809
A.vasorum /Control	P10301	-0.143325683	0.143325683	0.18502
A.vasorum /Control	Q8IY18	-0.102423848	0.102423848	0.185039
A.vasorum /Control	Q92499	-0.037590314	0.037590314	0.185388
A.vasorum /Control	Q9UKN8	-0.111546579	0.111546579	0.185814
A.vasorum /Control	Q9UHA4	0.433757854	0.433757854	0.185981
A.vasorum /Control	Q86X55	-0.024230263	0.024230263	0.186329
A.vasorum /Control	P08670	-0.102793794	0.102793794	0.186394
A.vasorum /Control	Q8IVL0	-0.644768393	0.644768393	0.187228
A.vasorum /Control	Q8N5M1	-0.234336944	0.234336944	0.187263
A.vasorum /Control	Q9P032	0.045063631	0.045063631	0.187251
A.vasorum /Control	Q9HBM6	-0.051337607	0.051337607	0.187412
A.vasorum /Control	P78344	-0.138222528	0.138222528	0.187865
A.vasorum /Control	P22061	0.089478754	0.089478754	0.188286
A.vasorum /Control	P26373	-0.001706254	0.001706254	0.18832
A.vasorum /Control	Q9UJ68	0.111969507	0.111969507	0.188305
A.vasorum /Control	Q9Y4E8	-0.17817119	0.17817119	0.188373
A.vasorum /Control	Q9H469	0.059451319	0.059451319	0.188636
A.vasorum /Control	P61081	-0.111145153	0.111145153	0.188725
A.vasorum /Control	Q9NRP2	-1.704842053	1.704842053	0.189545
A.vasorum /Control	Q9NQ30	-0.312918204	0.312918204	0.189768
A.vasorum /Control	P10606	0.282472972	0.282472972	0.19018
A.vasorum /Control	Q9Y320	-0.026318513	0.026318513	0.190389
A.vasorum /Control	Q8N5D0	-0.138784668	0.138784668	0.190455
A.vasorum /Control	Q68CP9	-0.315133358	0.315133358	0.190527
A.vasorum /Control	Q9UGP4	-0.386188351	0.386188351	0.190624
A.vasorum /Control	Q93062	-0.151627984	0.151627984	0.191058
A.vasorum /Control	Q14847	-0.351084938	0.351084938	0.191447
A.vasorum /Control	O95551	0.038961396	0.038961396	0.19171
A.vasorum /Control	Q9Y2R4	-0.098639553	0.098639553	0.192574
A.vasorum /Control	O43427	0.387038047	0.387038047	0.192743
A.vasorum /Control	Q96AJ9	0.279843179	0.279843179	0.19304
A.vasorum /Control	Q96JQ2	-0.918716746	0.918716746	0.193239
A.vasorum /Control	O00154	-0.122608076	0.122608076	0.193479
A.vasorum /Control	Q8WWQ0	0.593307384	0.593307384	0.193495
A.vasorum /Control	P58546	-0.033493256	0.033493256	0.193602
A.vasorum /Control	Q9HAV0	-0.281904193	0.281904193	0.194121
A.vasorum /Control	Q9BUR4	-0.608910758	0.608910758	0.194325
A.vasorum /Control	Q04837	-0.08747755	0.08747755	0.194823
A.vasorum /Control	O00571	-0.099161116	0.099161116	0.195177
A.vasorum /Control	P46821	-0.304235546	0.304235546	0.195177
A.vasorum /Control	Q9UNH6	0.048747965	0.048747965	0.195268
A.vasorum /Control	Q12866	-0.019190417	0.019190417	0.195584
A.vasorum /Control	P41743	-0.318800917	0.318800917	0.195742
A.vasorum /Control	Q16643	-0.329034048	0.329034048	0.195736
A.vasorum /Control	O94953	-0.467730615	0.467730615	0.1958
A.vasorum /Control	Q15311	0.027377903	0.027377903	0.195887
A.vasorum /Control	Q9ULL8	0.044975462	0.044975462	0.195887

A.vasorum /Control	O14579	-0.063666586	0.063666586	0.196359
A.vasorum /Control	Q9NRL3	-0.127158919	0.127158919	0.196463
A.vasorum /Control	Q14162	0.070990126	0.070990126	0.196631
A.vasorum /Control	O43709	0.097010625	0.097010625	0.196799
A.vasorum /Control	O95772	-0.192614114	0.192614114	0.196887
A.vasorum /Control	Q9P270	-0.23147699	0.23147699	0.196868
A.vasorum /Control	Q9UJA5	-0.137550605	0.137550605	0.197232
A.vasorum /Control	O43674	-0.026986379	0.026986379	0.197438
A.vasorum /Control	Q13325	-0.232845642	0.232845642	0.197675
A.vasorum /Control	Q9NRV9	0.244009247	0.244009247	0.198669
A.vasorum /Control	Q8NDF8	-0.068063174	0.068063174	0.199849
A.vasorum /Control	Q9UIS9	-0.420466987	0.420466987	0.199857
A.vasorum /Control	Q9NRH2	0.132459536	0.132459536	0.200338
A.vasorum /Control	O95319	-0.154469793	0.154469793	0.200423
A.vasorum /Control	Q96GZ6	0.38379371	0.38379371	0.200605
A.vasorum /Control	Q5RI15	-0.177552648	0.177552648	0.200772
A.vasorum /Control	Q9UKE5	-0.124296184	0.124296184	0.201133
A.vasorum /Control	Q9H410	1.220218915	1.220218915	0.201359
A.vasorum /Control	Q12888	-0.157257023	0.157257023	0.202111
A.vasorum /Control	Q13501	-0.195109345	0.195109345	0.202579
A.vasorum /Control	O15118	-0.04845201	0.04845201	0.20324
A.vasorum /Control	P06748	0.078111306	0.078111306	0.203476
A.vasorum /Control	Q9P2J3	-0.071234092	0.071234092	0.203576
A.vasorum /Control	O60831	0.135362706	0.135362706	0.203981
A.vasorum /Control	O00481	0.28734225	0.28734225	0.204331
A.vasorum /Control	P49207	-0.024187333	0.024187333	0.204378
A.vasorum /Control	P35579	0.071082827	0.071082827	0.204832
A.vasorum /Control	Q9BY44	-0.121188231	0.121188231	0.205409
A.vasorum /Control	P50990	-0.073038226	0.073038226	0.205545
A.vasorum /Control	O75347	0.072131981	0.072131981	0.205836
A.vasorum /Control	P62979	-0.233638819	0.233638819	0.206029
A.vasorum /Control	P14649	-0.110273414	0.110273414	0.206554
A.vasorum /Control	Q09019	0.1442649	0.1442649	0.206566
A.vasorum /Control	Q7L2J0	-0.172324229	0.172324229	0.206997
A.vasorum /Control	P09496	-0.361741456	0.361741456	0.208041
A.vasorum /Control	P23528	-0.119928289	0.119928289	0.210105
A.vasorum /Control	Q9H2H8	-0.959346178	0.959346178	0.210263
A.vasorum /Control	Q7Z7K6	-0.477049787	0.477049787	0.210918
A.vasorum /Control	O00461	-0.233055887	0.233055887	0.211062
A.vasorum /Control	Q9UKI2	0.697629242	0.697629242	0.211044
A.vasorum /Control	P11217	-0.147425986	0.147425986	0.211191
A.vasorum /Control	Q8N699	0.041407989	0.041407989	0.211647
A.vasorum /Control	Q96BD8	-0.382008332	0.382008332	0.211957
A.vasorum /Control	Q14019	-0.039989644	0.039989644	0.212894
A.vasorum /Control	P09493	-0.125404565	0.125404565	0.213152
A.vasorum /Control	Q9ULP9	-0.234044765	0.234044765	0.213208
A.vasorum /Control	Q7KYR7	-0.808596573	0.808596573	0.213816
A.vasorum /Control	O43663	-0.315463318	0.315463318	0.214186
A.vasorum /Control	Q8IYJ2	0.140820378	0.140820378	0.214511
A.vasorum /Control	Q9UBW7	0.095261233	0.095261233	0.214683

A.vasorum /Control	Q96C57	-0.260388455	0.260388455	0.214774
A.vasorum /Control	Q96EY1	-0.126251386	0.126251386	0.214749
A.vasorum /Control	Q9HCD5	-0.234697005	0.234697005	0.214912
A.vasorum /Control	P14317	-0.202081731	0.202081731	0.215042
A.vasorum /Control	O00635	0.103880193	0.103880193	0.215156
A.vasorum /Control	Q13310	-0.079601969	0.079601969	0.215113
A.vasorum /Control	Q01658	0.492492869	0.492492869	0.215535
A.vasorum /Control	Q8IWT0	-0.165172109	0.165172109	0.215507
A.vasorum /Control	Q9H5N1	-0.199302884	0.199302884	0.21603
A.vasorum /Control	O75330	-0.46745075	0.46745075	0.216157
A.vasorum /Control	P08572	-0.318564705	0.318564705	0.216596
A.vasorum /Control	O95786	-0.16429289	0.16429289	0.217013
A.vasorum /Control	Q9NRN9	-0.110185263	0.110185263	0.217174
A.vasorum /Control	Q8N556	-0.402154176	0.402154176	0.217594
A.vasorum /Control	Q9Y3T6	0.114111001	0.114111001	0.217597
A.vasorum /Control	O15355	-0.119799244	0.119799244	0.21795
A.vasorum /Control	Q96QG7	-0.095045314	0.095045314	0.217964
A.vasorum /Control	P10124	-0.622473467	0.622473467	0.219058
A.vasorum /Control	P04732	-0.785343548	0.785343548	0.219352
A.vasorum /Control	Q53EP0	-0.305778682	0.305778682	0.219519
A.vasorum /Control	Q96LD4	0.161057203	0.161057203	0.21961
A.vasorum /Control	Q8NDT2	0.029891229	0.029891229	0.219662
A.vasorum /Control	Q92766	0.161319363	0.161319363	0.220329
A.vasorum /Control	P02768	-0.829484047	0.829484047	0.220697
A.vasorum /Control	Q13137	0.194108451	0.194108451	0.220656
A.vasorum /Control	Q9NQZ2	-0.62729786	0.62729786	0.221251
A.vasorum /Control	Q14671	-0.079836751	0.079836751	0.221529
A.vasorum /Control	Q8N3V7	-0.142290185	0.142290185	0.221581
A.vasorum /Control	Q8N5L8	-0.085079713	0.085079713	0.221691
A.vasorum /Control	Q8NFV4	-0.181935553	0.181935553	0.22166
A.vasorum /Control	Q9NXH9	-0.167791427	0.167791427	0.221709
A.vasorum /Control	P51571	-0.038201799	0.038201799	0.221776
A.vasorum /Control	Q8TCB0	-0.056938453	0.056938453	0.221897
A.vasorum /Control	Q96RT7	0.194395315	0.194395315	0.221864
A.vasorum /Control	Q9Y3B8	-0.041793729	0.041793729	0.22201
A.vasorum /Control	P52907	-0.06113865	0.06113865	0.223574
A.vasorum /Control	P61019	-0.067116376	0.067116376	0.224226
A.vasorum /Control	Q96FV9	-0.038634492	0.038634492	0.224278
A.vasorum /Control	Q9H4G4	-0.301630587	0.301630587	0.224338
A.vasorum /Control	Q96JP5	0.398641271	0.398641271	0.224397
A.vasorum /Control	Q09328	-0.317185096	0.317185096	0.224534
A.vasorum /Control	O75962	-0.174789984	0.174789984	0.224813
A.vasorum /Control	Q12788	-0.670160052	0.670160052	0.225027
A.vasorum /Control	O14524	-0.221339264	0.221339264	0.225262
A.vasorum /Control	O75419	-0.058534444	0.058534444	0.2254
A.vasorum /Control	Q6Y288	-0.195841177	0.195841177	0.225729
A.vasorum /Control	Q5T1C6	0.231050386	0.231050386	0.225814
A.vasorum /Control	Q6PI78	-0.302076592	0.302076592	0.225955
A.vasorum /Control	Q9Y385	0.046162504	0.046162504	0.226419
A.vasorum /Control	Q9BZD4	-0.151438043	0.151438043	0.226611

A.vasorum /Control	Q15013	-0.629052613	0.629052613	0.226658
A.vasorum /Control	Q9H3H1	-0.130188629	0.130188629	0.226761
A.vasorum /Control	O75319	-0.286946377	0.286946377	0.227564
A.vasorum /Control	P13807	-0.256986446	0.256986446	0.227647
A.vasorum /Control	P49674	-0.912351586	0.912351586	0.227794
A.vasorum /Control	P35080	-0.063130468	0.063130468	0.228443
A.vasorum /Control	Q7RTN6	0.028844709	0.028844709	0.228687
A.vasorum /Control	Q6NW34	0.129064585	0.129064585	0.228984
A.vasorum /Control	Q9NQW6	-0.501358558	0.501358558	0.229465
A.vasorum /Control	Q13642	-0.204995596	0.204995596	0.229884
A.vasorum /Control	Q13873	-0.294868737	0.294868737	0.23013
A.vasorum /Control	Q06136	0.408951828	0.408951828	0.230232
A.vasorum /Control	Q14157	-0.072765986	0.072765986	0.23039
A.vasorum /Control	O15371	-0.089953634	0.089953634	0.230601
A.vasorum /Control	P62995	-0.373246624	0.373246624	0.232214
A.vasorum /Control	P48681	-0.473467858	0.473467858	0.232424
A.vasorum /Control	Q9H974	-0.191258745	0.191258745	0.232492
A.vasorum /Control	Q9UQ80	-0.044684524	0.044684524	0.232494
A.vasorum /Control	Q8TEQ8	-0.476323186	0.476323186	0.232695
A.vasorum /Control	P40189	-0.053933678	0.053933678	0.232762
A.vasorum /Control	P51965	-0.221094468	0.221094468	0.232838
A.vasorum /Control	Q13740	-0.136331403	0.136331403	0.23298
A.vasorum /Control	Q6AWC2	-0.053470484	0.053470484	0.233893
A.vasorum /Control	Q9ULC3	-0.422110113	0.422110113	0.234006
A.vasorum /Control	Q86VM9	-0.118109194	0.118109194	0.234215
A.vasorum /Control	Q14114	-0.729219034	0.729219034	0.23434
A.vasorum /Control	P15144	-0.343985619	0.343985619	0.234467
A.vasorum /Control	P02462	-0.478850592	0.478850592	0.234581
A.vasorum /Control	Q9BQ48	-0.308450943	0.308450943	0.234919
A.vasorum /Control	Q12778	0.46540899	0.46540899	0.235012
A.vasorum /Control	Q96RG2	-0.009551871	0.009551871	0.235535
A.vasorum /Control	Q15526	-0.238118168	0.238118168	0.235715
A.vasorum /Control	Q9P2E7	-0.246329655	0.246329655	0.235768
A.vasorum /Control	Q9NXW2	-0.200575979	0.200575979	0.23597
A.vasorum /Control	Q13574	0.209205852	0.209205852	0.236104
A.vasorum /Control	P05387	-0.008744387	0.008744387	0.236244
A.vasorum /Control	Q9H7B2	-0.646698966	0.646698966	0.236209
A.vasorum /Control	Q16773	0.022288901	0.022288901	0.237405
A.vasorum /Control	O14802	-0.004513758	0.004513758	0.23753
A.vasorum /Control	P78381	0.516451736	0.516451736	0.238213
A.vasorum /Control	Q969S9	-0.049412766	0.049412766	0.2387
A.vasorum /Control	Q9H3L0	-1.196209457	1.196209457	0.239061
A.vasorum /Control	P54289	0.003171974	0.003171974	0.239253
A.vasorum /Control	Q15583	1.001020356	1.001020356	0.23928
A.vasorum /Control	Q9BYI3	-0.092676261	0.092676261	0.239214
A.vasorum /Control	Q9Y3Y2	0.124769179	0.124769179	0.239755
A.vasorum /Control	Q7Z5H3	-0.521181928	0.521181928	0.240045
A.vasorum /Control	Q9UKM9	-0.006805924	0.006805924	0.240003
A.vasorum /Control	Q01201	-0.458462693	0.458462693	0.240445
A.vasorum /Control	O43583	-0.23484029	0.23484029	0.240539

A.vasorum /Control	Q14657	-0.264187731	0.264187731	0.240983
A.vasorum /Control	Q8NCN4	0.219454909	0.219454909	0.241247
A.vasorum /Control	O15085	-0.131755047	0.131755047	0.241503
A.vasorum /Control	Q6P1X6	0.1265086	0.1265086	0.241598
A.vasorum /Control	Q8N108	-0.11003928	0.11003928	0.241549
A.vasorum /Control	O00562	0.273248025	0.273248025	0.242245
A.vasorum /Control	Q99439	-0.138218989	0.138218989	0.242261
A.vasorum /Control	Q9UNP9	0.16120377	0.16120377	0.243486
A.vasorum /Control	Q2M2I8	-0.047352912	0.047352912	0.243627
A.vasorum /Control	P06493	-0.08736091	0.08736091	0.244309
A.vasorum /Control	Q9NXW9	-0.814079788	0.814079788	0.244769
A.vasorum /Control	P50613	-0.237114965	0.237114965	0.245121
A.vasorum /Control	P51608	0.399711042	0.399711042	0.245976
A.vasorum /Control	Q04721	-0.375878427	0.375878427	0.246136
A.vasorum /Control	Q969T7	0.106166038	0.106166038	0.246125
A.vasorum /Control	Q15007	0.031726116	0.031726116	0.246217
A.vasorum /Control	O75152	-0.15920734	0.15920734	0.246537
A.vasorum /Control	Q9UHY7	-0.000925229	0.000925229	0.246969
A.vasorum /Control	P62993	-0.127316199	0.127316199	0.24712
A.vasorum /Control	Q9H8M9	-0.835559122	0.835559122	0.247281
A.vasorum /Control	Q15047	0.146592742	0.146592742	0.247352
A.vasorum /Control	Q969V6	-0.107035134	0.107035134	0.247467
A.vasorum /Control	Q13151	-0.203412279	0.203412279	0.247779
A.vasorum /Control	Q13643	-0.384401558	0.384401558	0.247791
A.vasorum /Control	Q96EY5	-0.019901327	0.019901327	0.247911
A.vasorum /Control	P42285	-0.166619778	0.166619778	0.248794
A.vasorum /Control	Q07666	-0.057030746	0.057030746	0.248857
A.vasorum /Control	P35659	-0.171271914	0.171271914	0.248956
A.vasorum /Control	Q16629	-0.089580702	0.089580702	0.249028
A.vasorum /Control	P49770	-0.179285932	0.179285932	0.249191
A.vasorum /Control	Q969X6	-1.170063736	1.170063736	0.249483
A.vasorum /Control	Q6P3W7	-0.028299008	0.028299008	0.249836
A.vasorum /Control	Q96A33	-0.120119518	0.120119518	0.24993
A.vasorum /Control	P01130	-0.484629458	0.484629458	0.250098
A.vasorum /Control	O75954	-0.008770154	0.008770154	0.250339
A.vasorum /Control	P11940	-0.037835885	0.037835885	0.250988
A.vasorum /Control	O60563	-0.183718711	0.183718711	0.251214
A.vasorum /Control	O95372	0.064213618	0.064213618	0.251788
A.vasorum /Control	Q6ZN55	0.085535725	0.085535725	0.252112
A.vasorum /Control	Q9HBU6	-0.429421076	0.429421076	0.253272
A.vasorum /Control	Q53GA4	-0.5473479	0.5473479	0.253536
A.vasorum /Control	Q8WWM7	-0.121867147	0.121867147	0.254525
A.vasorum /Control	Q14012	0.055241691	0.055241691	0.254704
A.vasorum /Control	Q9UKV3	-0.405367123	0.405367123	0.2558
A.vasorum /Control	P08253	-0.363327955	0.363327955	0.255865
A.vasorum /Control	O14548	0.321595812	0.321595812	0.256333
A.vasorum /Control	O95905	-0.20759093	0.20759093	0.256782
A.vasorum /Control	Q9UQ13	0.075042125	0.075042125	0.256863
A.vasorum /Control	P41162	0.180694077	0.180694077	0.256941
A.vasorum /Control	Q969Q5	-0.084654956	0.084654956	0.257327

A.vasorum /Control	P21266	-0.258346654	0.258346654	0.258231
A.vasorum /Control	Q15545	-0.258830775	0.258830775	0.258625
A.vasorum /Control	Q9HAU0	-0.195491131	0.195491131	0.258583
A.vasorum /Control	P27701	-0.144158788	0.144158788	0.258721
A.vasorum /Control	P48553	-0.036961859	0.036961859	0.259268
A.vasorum /Control	Q9Y6I4	-0.123396088	0.123396088	0.259257
A.vasorum /Control	P42338	-0.238205588	0.238205588	0.259634
A.vasorum /Control	P54619	0.118660593	0.118660593	0.259643
A.vasorum /Control	O14641	0.093629389	0.093629389	0.260425
A.vasorum /Control	Q96ER9	-0.194959631	0.194959631	0.260619
A.vasorum /Control	Q9NV31	-0.464647014	0.464647014	0.260685
A.vasorum /Control	P52888	-0.061637768	0.061637768	0.260988
A.vasorum /Control	Q7L2H7	-0.205485549	0.205485549	0.261085
A.vasorum /Control	Q9UHW5	-0.623737571	0.623737571	0.263272
A.vasorum /Control	Q9BQG0	-0.2708045	0.2708045	0.263349
A.vasorum /Control	Q9BQ70	0.06193857	0.06193857	0.264483
A.vasorum /Control	Q8NC51	-0.227247752	0.227247752	0.265259
A.vasorum /Control	Q8WV22	-0.462706948	0.462706948	0.265528
A.vasorum /Control	Q9H2X9	0.375502687	0.375502687	0.265827
A.vasorum /Control	Q9Y5Z7	-0.129917096	0.129917096	0.265902
A.vasorum /Control	Q9UEE5	-0.381923284	0.381923284	0.266536
A.vasorum /Control	Q8N5M4	-0.187579806	0.187579806	0.266699
A.vasorum /Control	Q14684	-0.166288514	0.166288514	0.266891
A.vasorum /Control	Q7KZN9	-0.006183327	0.006183327	0.267196
A.vasorum /Control	O43251	-0.218028353	0.218028353	0.267534
A.vasorum /Control	Q9NTG7	0.34711288	0.34711288	0.267584
A.vasorum /Control	Q2TAY7	-0.128937554	0.128937554	0.267745
A.vasorum /Control	Q9NUQ7	-0.029135779	0.029135779	0.267921
A.vasorum /Control	O15400	-0.241658497	0.241658497	0.268117
A.vasorum /Control	Q16637	-0.133559797	0.133559797	0.268572
A.vasorum /Control	Q9UKI8	-0.177620537	0.177620537	0.268635
A.vasorum /Control	P62910	-0.018789781	0.018789781	0.269435
A.vasorum /Control	Q9NSY1	-0.166893111	0.166893111	0.269685
A.vasorum /Control	P18031	-0.23417611	0.23417611	0.270484
A.vasorum /Control	Q9BZK7	0.08600995	0.08600995	0.271495
A.vasorum /Control	Q8TB37	-0.611466166	0.611466166	0.271876
A.vasorum /Control	Q9HCE0	-0.18126318	0.18126318	0.272739
A.vasorum /Control	Q9Y2D5	-0.13321959	0.13321959	0.273184
A.vasorum /Control	O15525	-0.857929559	0.857929559	0.273658
A.vasorum /Control	Q13485	-0.097693991	0.097693991	0.27383
A.vasorum /Control	Q9H4M9	-0.014025183	0.014025183	0.273786
A.vasorum /Control	P61981	-0.021599696	0.021599696	0.274073
A.vasorum /Control	Q9Y530	-0.111464912	0.111464912	0.274324
A.vasorum /Control	Q9BX40	0.11131741	0.11131741	0.27462
A.vasorum /Control	Q5XUX1	-0.320924285	0.320924285	0.274848
A.vasorum /Control	Q9BW62	-0.246289738	0.246289738	0.274911
A.vasorum /Control	Q8IWB7	-0.160768363	0.160768363	0.275597
A.vasorum /Control	O15047	-0.009959796	0.009959796	0.275682
A.vasorum /Control	P34897	-0.127186311	0.127186311	0.276357
A.vasorum /Control	A8CG34	-0.339228122	0.339228122	0.276563

A.vasorum /Control	Q9BVC6	-0.271140724	0.271140724	0.27694
A.vasorum /Control	P46779	-0.046706808	0.046706808	0.277163
A.vasorum /Control	O15037	-0.374747815	0.374747815	0.277221
A.vasorum /Control	Q9UBI6	-0.166562197	0.166562197	0.277597
A.vasorum /Control	Q9UKD1	-0.205760554	0.205760554	0.277823
A.vasorum /Control	P21675	0.2695514	0.2695514	0.278065
A.vasorum /Control	O14795	0.006968626	0.006968626	0.27879
A.vasorum /Control	P0DPH7	-0.031131031	0.031131031	0.279174
A.vasorum /Control	Q8WZ42	-0.731547504	0.731547504	0.279279
A.vasorum /Control	Q9Y5S9	-0.040732238	0.040732238	0.279366
A.vasorum /Control	Q6PK04	-0.390461688	0.390461688	0.279931
A.vasorum /Control	Q5SW79	-0.386739946	0.386739946	0.280197
A.vasorum /Control	P48507	-0.350758142	0.350758142	0.280343
A.vasorum /Control	Q9NY93	-0.635024524	0.635024524	0.280295
A.vasorum /Control	Q13610	-0.184884155	0.184884155	0.280547
A.vasorum /Control	P17026	0.347249362	0.347249362	0.281267
A.vasorum /Control	Q6YN16	0.147891443	0.147891443	0.281338
A.vasorum /Control	Q9Y520	-0.133331154	0.133331154	0.281815
A.vasorum /Control	Q6PL18	-0.042835679	0.042835679	0.282481
A.vasorum /Control	Q71UM5	0.036859661	0.036859661	0.282823
A.vasorum /Control	Q9H6R4	-0.415366865	0.415366865	0.283968
A.vasorum /Control	P08134	-0.080299298	0.080299298	0.284779
A.vasorum /Control	Q05932	-0.675790787	0.675790787	0.286369
A.vasorum /Control	P48736	0.032338933	0.032338933	0.28652
A.vasorum /Control	Q9HC21	-0.006963503	0.006963503	0.287759
A.vasorum /Control	Q96BW9	0.115833303	0.115833303	0.288027
A.vasorum /Control	Q9UKX7	-0.027746194	0.027746194	0.288127
A.vasorum /Control	Q9UHB7	-0.036005235	0.036005235	0.28842
A.vasorum /Control	Q13573	-0.149922033	0.149922033	0.289002
A.vasorum /Control	Q9Y5A9	-0.191983796	0.191983796	0.290073
A.vasorum /Control	P25440	-0.261993887	0.261993887	0.291078
A.vasorum /Control	Q96AE4	-0.04814511	0.04814511	0.291684
A.vasorum /Control	Q96Q45	-0.417905371	0.417905371	0.291745
A.vasorum /Control	Q5VY43	-0.17312507	0.17312507	0.292409
A.vasorum /Control	Q6PK18	0.449443872	0.449443872	0.292636
A.vasorum /Control	P42677	-0.025681599	0.025681599	0.292976
A.vasorum /Control	P51116	-0.041766814	0.041766814	0.29308
A.vasorum /Control	Q13158	-0.252820396	0.252820396	0.293443
A.vasorum /Control	P62244	-0.02864212	0.02864212	0.294173
A.vasorum /Control	P49810	0.154248668	0.154248668	0.294836
A.vasorum /Control	Q9Y256	0.188768603	0.188768603	0.294904
A.vasorum /Control	Q9H147	0.098574856	0.098574856	0.29521
A.vasorum /Control	P46783	-0.049426084	0.049426084	0.295287
A.vasorum /Control	P35240	-0.194917206	0.194917206	0.296954
A.vasorum /Control	Q9Y3E5	-0.17772063	0.17772063	0.297046
A.vasorum /Control	Q13625	-0.056113863	0.056113863	0.298007
A.vasorum /Control	P61966	-0.084849919	0.084849919	0.298137
A.vasorum /Control	Q6AI08	-0.200338841	0.200338841	0.299225
A.vasorum /Control	Q9Y5V3	-0.098063403	0.098063403	0.299384
A.vasorum /Control	Q13637	-0.122730687	0.122730687	0.300013

A.vasorum /Control	Q9BT09	-0.161611944	0.161611944	0.3016
A.vasorum /Control	P48382	0.05483956	0.05483956	0.301821
A.vasorum /Control	Q12770	-0.022208832	0.022208832	0.301706
A.vasorum /Control	Q8N9N8	-0.081126271	0.081126271	0.30182
A.vasorum /Control	O75688	0.030246697	0.030246697	0.301979
A.vasorum /Control	O75787	-0.347150734	0.347150734	0.302108
A.vasorum /Control	P42574	-0.183876285	0.183876285	0.30312
A.vasorum /Control	Q92503	-0.348039411	0.348039411	0.303566
A.vasorum /Control	Q9Y3Q8	0.004054172	0.004054172	0.303894
A.vasorum /Control	Q9BW92	0.040426375	0.040426375	0.305226
A.vasorum /Control	Q6UXV4	-0.118023452	0.118023452	0.306826
A.vasorum /Control	Q9UKL6	-0.105049622	0.105049622	0.307278
A.vasorum /Control	Q6KCM7	-0.075689728	0.075689728	0.307668
A.vasorum /Control	Q14192	-0.169489304	0.169489304	0.307986
A.vasorum /Control	Q8NDV7	-0.108127048	0.108127048	0.30799
A.vasorum /Control	Q01780	-0.079178946	0.079178946	0.308654
A.vasorum /Control	P15531	0.024321164	0.024321164	0.3089
A.vasorum /Control	Q99733	-0.152512308	0.152512308	0.309424
A.vasorum /Control	Q9UJX4	-0.171561835	0.171561835	0.309761
A.vasorum /Control	Q8IXW5	-0.052606602	0.052606602	0.310308
A.vasorum /Control	Q9Y2J2	-0.270855582	0.270855582	0.310291
A.vasorum /Control	Q9HC36	-0.046558968	0.046558968	0.310938
A.vasorum /Control	Q14678	-0.25921885	0.25921885	0.314251
A.vasorum /Control	Q96JA1	0.063962283	0.063962283	0.314441
A.vasorum /Control	P57081	-0.186141585	0.186141585	0.315033
A.vasorum /Control	Q9Y5M8	-0.159321401	0.159321401	0.315356
A.vasorum /Control	Q9H1P3	-0.087759737	0.087759737	0.316936
A.vasorum /Control	Q96CN9	0.102030393	0.102030393	0.317947
A.vasorum /Control	Q92925	-0.048896074	0.048896074	0.318916
A.vasorum /Control	P23470	0.014425244	0.014425244	0.321202
A.vasorum /Control	P46734	-0.024542856	0.024542856	0.321222
A.vasorum /Control	Q15438	0.234419553	0.234419553	0.321337
A.vasorum /Control	Q99666	-0.218654928	0.218654928	0.321469
A.vasorum /Control	Q9NVS2	0.007724105	0.007724105	0.321649
A.vasorum /Control	P49281	-0.435401885	0.435401885	0.322337
A.vasorum /Control	Q9NYH9	-0.746904116	0.746904116	0.322662
A.vasorum /Control	Q12830	0.018346524	0.018346524	0.32282
A.vasorum /Control	Q6NYC8	-0.041345509	0.041345509	0.323192
A.vasorum /Control	Q9BZL1	-0.261658548	0.261658548	0.323532
A.vasorum /Control	Q16594	-0.202645302	0.202645302	0.324658
A.vasorum /Control	P04075	-0.066902806	0.066902806	0.324722
A.vasorum /Control	Q15758	-0.265373913	0.265373913	0.325237
A.vasorum /Control	P61254	-0.058824992	0.058824992	0.325534
A.vasorum /Control	Q7Z6K3	9.87283E-05	9.87E-05	0.326253
A.vasorum /Control	P00395	0.403924102	0.403924102	0.326734
A.vasorum /Control	Q6ULP2	-0.201700617	0.201700617	0.327301
A.vasorum /Control	Q9H3Z4	-0.269808065	0.269808065	0.328045
A.vasorum /Control	P62879	-0.060400524	0.060400524	0.328255
A.vasorum /Control	Q9BPX3	-0.087857632	0.087857632	0.328489
A.vasorum /Control	O43822	0.532876889	0.532876889	0.329115

A.vasorum /Control	O95822	0.518442729	0.518442729	0.329427
A.vasorum /Control	Q9BWJ5	-0.066394962	0.066394962	0.330304
A.vasorum /Control	Q7Z7N9	0.156305648	0.156305648	0.330526
A.vasorum /Control	Q8WU79	-0.230856037	0.230856037	0.330796
A.vasorum /Control	P0DPI2	0.21533997	0.21533997	0.331067
A.vasorum /Control	O95758	-0.103205629	0.103205629	0.331849
A.vasorum /Control	Q96PY5	0.001632449	0.001632449	0.332876
A.vasorum /Control	P20594	-0.076030056	0.076030056	0.333635
A.vasorum /Control	Q71RC2	-0.201312126	0.201312126	0.33404
A.vasorum /Control	Q96CB9	-0.400941444	0.400941444	0.334118
A.vasorum /Control	Q96HE9	-0.346550873	0.346550873	0.334328
A.vasorum /Control	O60725	0.071378457	0.071378457	0.334653
A.vasorum /Control	Q14195	-0.06664509	0.06664509	0.334799
A.vasorum /Control	Q9H2P9	-0.111873691	0.111873691	0.334779
A.vasorum /Control	O00560	-0.101956188	0.101956188	0.335231
A.vasorum /Control	Q9UKK6	-0.078581726	0.078581726	0.335485
A.vasorum /Control	Q9Y3S2	-0.307340555	0.307340555	0.335458
A.vasorum /Control	P10515	-0.043901485	0.043901485	0.337522
A.vasorum /Control	P60484	0.268688599	0.268688599	0.338973
A.vasorum /Control	P61421	-0.369598795	0.369598795	0.339842
A.vasorum /Control	Q9BQA9	-0.155609181	0.155609181	0.340407
A.vasorum /Control	P10620	-0.232310784	0.232310784	0.341181
A.vasorum /Control	Q9NR50	-0.083034737	0.083034737	0.341988
A.vasorum /Control	P53350	-0.457465159	0.457465159	0.342255
A.vasorum /Control	Q15904	-0.315438229	0.315438229	0.34248
A.vasorum /Control	Q9NS15	0.451281061	0.451281061	0.342441
A.vasorum /Control	Q9Y6X2	-0.48961656	0.48961656	0.342975
A.vasorum /Control	O60870	0.086645122	0.086645122	0.34474
A.vasorum /Control	Q86W50	-0.01528645	0.01528645	0.344883
A.vasorum /Control	Q9H9Y6	-0.391496854	0.391496854	0.34487
A.vasorum /Control	Q8WUD4	-0.340407879	0.340407879	0.346152
A.vasorum /Control	O43670	-0.086880925	0.086880925	0.347092
A.vasorum /Control	Q96D71	-0.16132207	0.16132207	0.34838
A.vasorum /Control	Q8N5U6	-0.062075315	0.062075315	0.350652
A.vasorum /Control	Q4J6C6	-0.273605911	0.273605911	0.351079
A.vasorum /Control	O75521	-0.190120429	0.190120429	0.351153
A.vasorum /Control	P35556	-0.25538299	0.25538299	0.351942
A.vasorum /Control	Q15434	-0.198691261	0.198691261	0.352168
A.vasorum /Control	Q9Y666	-0.189747102	0.189747102	0.352189
A.vasorum /Control	O43861	-0.05949541	0.05949541	0.352646
A.vasorum /Control	Q99832	-0.096698206	0.096698206	0.353302
A.vasorum /Control	Q06830	-0.031071128	0.031071128	0.353496
A.vasorum /Control	Q9BXY0	-0.442779917	0.442779917	0.353744
A.vasorum /Control	Q9BWN1	-0.184168744	0.184168744	0.353865
A.vasorum /Control	O75223	0.009843276	0.009843276	0.353967
A.vasorum /Control	Q9BYW2	-0.236165722	0.236165722	0.354368
A.vasorum /Control	Q9Y5P4	0.23842034	0.23842034	0.354309
A.vasorum /Control	Q6UUV7	-0.010154684	0.010154684	0.355729
A.vasorum /Control	Q8TEW0	-0.092085287	0.092085287	0.355955
A.vasorum /Control	O43824	-0.123422715	0.123422715	0.356553

A.vasorum /Control	P32121	-0.122882416	0.122882416	0.35767
A.vasorum /Control	Q9Y3A4	-0.614543638	0.614543638	0.357745
A.vasorum /Control	Q9C035	-0.037469909	0.037469909	0.357915
A.vasorum /Control	Q9NZU5	0.915027817	0.915027817	0.360123
A.vasorum /Control	P00491	-0.051962833	0.051962833	0.361765
A.vasorum /Control	Q8WUK0	0.083877075	0.083877075	0.361947
A.vasorum /Control	Q96MY1	-0.25028885	0.25028885	0.362671
A.vasorum /Control	Q13595	-0.610775282	0.610775282	0.362893
A.vasorum /Control	O14727	-0.229416816	0.229416816	0.364377
A.vasorum /Control	Q9Y262	-0.029924048	0.029924048	0.364478
A.vasorum /Control	Q14692	-0.711757907	0.711757907	0.364682
A.vasorum /Control	Q66K74	-0.1062768	0.1062768	0.36483
A.vasorum /Control	Q9UK76	-0.323874544	0.323874544	0.364861
A.vasorum /Control	Q9UPR3	0.002140352	0.002140352	0.364805
A.vasorum /Control	Q9H2W6	0.310619446	0.310619446	0.364932
A.vasorum /Control	Q86UU1	-0.293781071	0.293781071	0.365352
A.vasorum /Control	Q5SQN1	-0.10460564	0.10460564	0.366419
A.vasorum /Control	Q96MU7	-0.114668807	0.114668807	0.366416
A.vasorum /Control	P05412	-0.610694682	0.610694682	0.367122
A.vasorum /Control	C9JLW8	0.198736467	0.198736467	0.367414
A.vasorum /Control	O75648	-0.056599786	0.056599786	0.367876
A.vasorum /Control	P68363	-0.039282973	0.039282973	0.370307
A.vasorum /Control	Q9Y679	-0.177929082	0.177929082	0.370575
A.vasorum /Control	Q9UBS0	0.458223319	0.458223319	0.37124
A.vasorum /Control	Q8TB52	-0.458132847	0.458132847	0.37156
A.vasorum /Control	P41223	0.1201577	0.1201577	0.372489
A.vasorum /Control	O75153	-0.223035573	0.223035573	0.373906
A.vasorum /Control	Q9NY12	-0.222454972	0.222454972	0.373853
A.vasorum /Control	Q96MG7	0.281484226	0.281484226	0.374979
A.vasorum /Control	Q9NX02	-0.103743072	0.103743072	0.375093
A.vasorum /Control	Q9BZ95	-0.068448109	0.068448109	0.376218
A.vasorum /Control	Q9BQQ3	0.000487591	0.000487591	0.376383
A.vasorum /Control	Q7Z6J0	0.035196671	0.035196671	0.376893
A.vasorum /Control	Q96H79	-0.122960356	0.122960356	0.376832
A.vasorum /Control	P55263	-0.181818132	0.181818132	0.377739
A.vasorum /Control	O60684	-0.164225999	0.164225999	0.378636
A.vasorum /Control	O75494	-0.451843097	0.451843097	0.379762
A.vasorum /Control	Q01650	-0.414508759	0.414508759	0.379722
A.vasorum /Control	Q14315	-0.175765019	0.175765019	0.379703
A.vasorum /Control	Q9P2N6	-0.15465732	0.15465732	0.380041
A.vasorum /Control	Q9HBW9	-0.174746923	0.174746923	0.380445
A.vasorum /Control	P25774	-0.24476613	0.24476613	0.380683
A.vasorum /Control	Q5ZPR3	0.025733964	0.025733964	0.381106
A.vasorum /Control	Q9Y4C8	-0.675655241	0.675655241	0.381165
A.vasorum /Control	O95619	0.014374394	0.014374394	0.381359
A.vasorum /Control	Q96SN8	-0.87443753	0.87443753	0.382186
A.vasorum /Control	P35749	0.048632532	0.048632532	0.383793
A.vasorum /Control	Q9UHD1	-0.197039904	0.197039904	0.383782
A.vasorum /Control	P49184	-0.568044591	0.568044591	0.384744
A.vasorum /Control	Q15843	-0.145944376	0.145944376	0.384701

A.vasorum /Control	Q641Q2	-0.251646471	0.251646471	0.384869
A.vasorum /Control	Q8N1F8	0.136200771	0.136200771	0.385482
A.vasorum /Control	Q9H1K0	-0.218428337	0.218428337	0.385912
A.vasorum /Control	P52732	-0.326472181	0.326472181	0.387097
A.vasorum /Control	O75531	0.374570335	0.374570335	0.387379
A.vasorum /Control	P60891	-0.041144611	0.041144611	0.387711
A.vasorum /Control	Q7KZF4	-0.051124959	0.051124959	0.387914
A.vasorum /Control	Q86WN1	-0.39244547	0.39244547	0.389211
A.vasorum /Control	Q96NB2	-1.01594669	1.01594669	0.38948
A.vasorum /Control	P11802	-0.49128966	0.49128966	0.389647
A.vasorum /Control	P09661	-0.113749582	0.113749582	0.390465
A.vasorum /Control	P48729	-0.094336183	0.094336183	0.390531
A.vasorum /Control	P51580	-0.120044264	0.120044264	0.390421
A.vasorum /Control	Q7Z3B3	-0.236420631	0.236420631	0.391119
A.vasorum /Control	Q96EL2	0.240986956	0.240986956	0.391175
A.vasorum /Control	Q8IWB1	-0.15147727	0.15147727	0.391507
A.vasorum /Control	Q86Y82	-0.128061113	0.128061113	0.392365
A.vasorum /Control	P15170	-0.251253836	0.251253836	0.392493
A.vasorum /Control	O43390	-0.219738786	0.219738786	0.393214
A.vasorum /Control	O14613	-0.070316809	0.070316809	0.393362
A.vasorum /Control	P62273	-0.121842181	0.121842181	0.394454
A.vasorum /Control	Q6NUQ1	-0.117956857	0.117956857	0.394424
A.vasorum /Control	Q9BTT6	0.098993168	0.098993168	0.395057
A.vasorum /Control	Q8N653	-0.237089639	0.237089639	0.395479
A.vasorum /Control	O95249	-0.593004672	0.593004672	0.396279
A.vasorum /Control	O95865	0.530246748	0.530246748	0.39789
A.vasorum /Control	Q96BY7	-0.299855938	0.299855938	0.398243
A.vasorum /Control	Q8IX18	0.251983388	0.251983388	0.398875
A.vasorum /Control	Q92794	-0.348133214	0.348133214	0.399071
A.vasorum /Control	Q6PJI9	0.159617869	0.159617869	0.399397
A.vasorum /Control	O00161	0.004325512	0.004325512	0.4002
A.vasorum /Control	Q8TF01	-0.13315765	0.13315765	0.400506
A.vasorum /Control	Q9H8Y5	0.076637286	0.076637286	0.40082
A.vasorum /Control	O75934	-0.279120967	0.279120967	0.401171
A.vasorum /Control	Q5T440	-0.30177952	0.30177952	0.402297
A.vasorum /Control	O75382	-0.084343362	0.084343362	0.403376
A.vasorum /Control	Q9UK23	-0.163363979	0.163363979	0.403745
A.vasorum /Control	O15164	-0.030720705	0.030720705	0.404837
A.vasorum /Control	O94916	0.408454032	0.408454032	0.405124
A.vasorum /Control	P78540	-0.423580052	0.423580052	0.405146
A.vasorum /Control	Q13472	-0.395273527	0.395273527	0.405393
A.vasorum /Control	Q4AC94	0.097894578	0.097894578	0.4059
A.vasorum /Control	Q9H9A5	-0.124746457	0.124746457	0.406209
A.vasorum /Control	P49848	-0.074040887	0.074040887	0.406678
A.vasorum /Control	Q9Y2K7	-0.418978486	0.418978486	0.406695
A.vasorum /Control	Q9Y6K1	-0.320467828	0.320467828	0.407315
A.vasorum /Control	O95433	-0.126766197	0.126766197	0.408356
A.vasorum /Control	Q9P2E3	-0.265806657	0.265806657	0.40931
A.vasorum /Control	Q5W111	-0.009799433	0.009799433	0.409389
A.vasorum /Control	P63172	0.236707129	0.236707129	0.409993

A.vasorum /Control	Q14807	-0.572530307	0.572530307	0.41013
A.vasorum /Control	Q8N163	5.90083E-05	5.90E-05	0.410415
A.vasorum /Control	Q04760	-0.086797819	0.086797819	0.410588
A.vasorum /Control	Q8NF64	-0.412349685	0.412349685	0.41125
A.vasorum /Control	Q9P0T7	-0.094946659	0.094946659	0.414058
A.vasorum /Control	Q5VTB9	0.017771887	0.017771887	0.414263
A.vasorum /Control	P26374	-0.336174221	0.336174221	0.414802
A.vasorum /Control	O95208	0.005854394	0.005854394	0.415141
A.vasorum /Control	Q9Y219	-0.422143719	0.422143719	0.415775
A.vasorum /Control	P07195	-0.030462098	0.030462098	0.416385
A.vasorum /Control	Q02297	-0.293427536	0.293427536	0.417032
A.vasorum /Control	Q9NYB0	0.056578721	0.056578721	0.417373
A.vasorum /Control	Q8N697	-0.186406819	0.186406819	0.417971
A.vasorum /Control	Q9Y618	-0.289156355	0.289156355	0.418604
A.vasorum /Control	P49768	-0.086095208	0.086095208	0.420445
A.vasorum /Control	O75113	0.338515423	0.338515423	0.420811
A.vasorum /Control	Q9NVF7	-0.179325138	0.179325138	0.421162
A.vasorum /Control	P08574	0.231029531	0.231029531	0.421581
A.vasorum /Control	Q9UPN4	-0.228944427	0.228944427	0.421631
A.vasorum /Control	P08621	-0.096008438	0.096008438	0.42208
A.vasorum /Control	Q8TEJ3	-0.194226262	0.194226262	0.423524
A.vasorum /Control	O95396	-0.135933914	0.135933914	0.425951
A.vasorum /Control	P07355	-0.002611948	0.002611948	0.425921
A.vasorum /Control	P27105	-0.030983937	0.030983937	0.426146
A.vasorum /Control	O60330	-0.015169388	0.015169388	0.426997
A.vasorum /Control	P01127	-0.464359442	0.464359442	0.427209
A.vasorum /Control	O00469	-0.127100871	0.127100871	0.428488
A.vasorum /Control	Q92989	-0.029728837	0.029728837	0.428804
A.vasorum /Control	Q13049	-0.39030508	0.39030508	0.43059
A.vasorum /Control	Q9HA65	0.256743884	0.256743884	0.430777
A.vasorum /Control	A2RUC4	-0.038690266	0.038690266	0.432317
A.vasorum /Control	O75439	-0.072396737	0.072396737	0.43258
A.vasorum /Control	Q05D32	-0.151817591	0.151817591	0.432791
A.vasorum /Control	Q9BWT3	0.111462689	0.111462689	0.432714
A.vasorum /Control	P16220	0.156338798	0.156338798	0.432991
A.vasorum /Control	P14635	-0.427691251	0.427691251	0.433331
A.vasorum /Control	Q9Y232	-0.238394122	0.238394122	0.434631
A.vasorum /Control	Q6UN15	-0.141150344	0.141150344	0.436434
A.vasorum /Control	P16383	0.091916433	0.091916433	0.43661
A.vasorum /Control	Q3KQU3	-0.16324456	0.16324456	0.437207
A.vasorum /Control	O94966	0.056946082	0.056946082	0.437342
A.vasorum /Control	P60866	0.01493139	0.01493139	0.437834
A.vasorum /Control	Q9NY27	-0.102223301	0.102223301	0.43786
A.vasorum /Control	Q5ST30	0.3273888	0.3273888	0.437991
A.vasorum /Control	Q99627	0.114742619	0.114742619	0.438262
A.vasorum /Control	Q9H0U9	-0.468006334	0.468006334	0.439024
A.vasorum /Control	Q06210	-0.121340362	0.121340362	0.440109
A.vasorum /Control	P30414	-0.205379882	0.205379882	0.442904
A.vasorum /Control	O15084	0.088388069	0.088388069	0.443421
A.vasorum /Control	Q5SY16	-0.178008649	0.178008649	0.443586

A.vasorum /Control	Q96A72	-0.02833793	0.02833793	0.444034
A.vasorum /Control	O95817	-0.607988432	0.607988432	0.444499
A.vasorum /Control	Q8N5F7	0.153406653	0.153406653	0.44482
A.vasorum /Control	P63167	0.011249979	0.011249979	0.446106
A.vasorum /Control	Q96C90	-0.182052432	0.182052432	0.446301
A.vasorum /Control	Q9BRR8	0.584877951	0.584877951	0.44789
A.vasorum /Control	O95835	-0.153779006	0.153779006	0.448313
A.vasorum /Control	P09884	-0.015279337	0.015279337	0.448408
A.vasorum /Control	Q14676	-0.790972582	0.790972582	0.449231
A.vasorum /Control	Q53HL2	-0.650661934	0.650661934	0.449235
A.vasorum /Control	Q9BPX6	0.116647426	0.116647426	0.449991
A.vasorum /Control	Q9H5Q4	-0.258795386	0.258795386	0.450304
A.vasorum /Control	Q9Y266	-0.075707657	0.075707657	0.450515
A.vasorum /Control	Q7LG56	0.004198852	0.004198852	0.450599
A.vasorum /Control	Q13163	0.897672563	0.897672563	0.451144
A.vasorum /Control	Q96CS3	-0.137744811	0.137744811	0.45111
A.vasorum /Control	Q9HA47	0.040819063	0.040819063	0.451157
A.vasorum /Control	Q15022	-0.190774783	0.190774783	0.451839
A.vasorum /Control	O43493	-0.202885596	0.202885596	0.453027
A.vasorum /Control	Q13510	0.31967311	0.31967311	0.453025
A.vasorum /Control	O60506	-0.083503033	0.083503033	0.454116
A.vasorum /Control	O00303	-0.028302595	0.028302595	0.454681
A.vasorum /Control	Q15345	-0.16693675	0.16693675	0.456165
A.vasorum /Control	Q8NI37	0.201785032	0.201785032	0.456123
A.vasorum /Control	P62820	0.006187846	0.006187846	0.457572
A.vasorum /Control	Q14764	-0.182649315	0.182649315	0.458562
A.vasorum /Control	Q01850	-0.661376987	0.661376987	0.45963
A.vasorum /Control	O00764	0.177889669	0.177889669	0.459896
A.vasorum /Control	P61586	-0.295845406	0.295845406	0.460144
A.vasorum /Control	P83876	-0.122658091	0.122658091	0.460189
A.vasorum /Control	O75665	-0.163896102	0.163896102	0.461266
A.vasorum /Control	O14950	-0.14802207	0.14802207	0.461706
A.vasorum /Control	P78310	0.046420996	0.046420996	0.461783
A.vasorum /Control	Q9H6R0	-0.456394188	0.456394188	0.462207
A.vasorum /Control	Q8IWE4	-0.089258134	0.089258134	0.462628
A.vasorum /Control	P31689	-0.229490694	0.229490694	0.462978
A.vasorum /Control	P25398	-0.332162773	0.332162773	0.46317
A.vasorum /Control	P20042	-0.141606128	0.141606128	0.463407
A.vasorum /Control	Q9H875	-0.359555443	0.359555443	0.464317
A.vasorum /Control	Q6UUV9	-0.167335344	0.167335344	0.466438
A.vasorum /Control	Q9BSU1	0.940439242	0.940439242	0.466956
A.vasorum /Control	P82932	-0.308710943	0.308710943	0.467343
A.vasorum /Control	P25445	-0.14239814	0.14239814	0.467501
A.vasorum /Control	O95067	-0.651994367	0.651994367	0.468792
A.vasorum /Control	Q96IX5	0.010410227	0.010410227	0.469135
A.vasorum /Control	Q8IZP0	-0.0096306	0.0096306	0.469499
A.vasorum /Control	Q8N531	-0.222988868	0.222988868	0.470213
A.vasorum /Control	Q86T24	-0.976511236	0.976511236	0.470519
A.vasorum /Control	O15357	-0.090989096	0.090989096	0.471928
A.vasorum /Control	P40926	-0.047589149	0.047589149	0.471845

A.vasorum /Control	Q8N2F6	-0.064502687	0.064502687	0.472167
A.vasorum /Control	P49366	-0.334924092	0.334924092	0.47289
A.vasorum /Control	Q86XI6	0.116532544	0.116532544	0.472986
A.vasorum /Control	P55789	-0.525635717	0.525635717	0.47313
A.vasorum /Control	O75475	-0.052005634	0.052005634	0.474014
A.vasorum /Control	Q04656	0.863701809	0.863701809	0.475667
A.vasorum /Control	Q06587	-0.29043157	0.29043157	0.476105
A.vasorum /Control	Q8IUH5	-0.875908699	0.875908699	0.476273
A.vasorum /Control	O14924	-0.158439044	0.158439044	0.477094
A.vasorum /Control	Q9NW97	0.309166462	0.309166462	0.477207
A.vasorum /Control	P17405	-0.512548411	0.512548411	0.47779
A.vasorum /Control	Q0PNE2	-0.117609539	0.117609539	0.47774
A.vasorum /Control	Q9NQ48	-0.062760044	0.062760044	0.478489
A.vasorum /Control	P78357	-0.432959054	0.432959054	0.478704
A.vasorum /Control	Q16563	0.074963802	0.074963802	0.478931
A.vasorum /Control	Q8IWV7	-0.194395767	0.194395767	0.479157
A.vasorum /Control	Q8N2M8	-0.501603253	0.501603253	0.479236
A.vasorum /Control	Q5TAX3	-0.308853858	0.308853858	0.479579
A.vasorum /Control	Q4G0N4	-0.070985709	0.070985709	0.481956
A.vasorum /Control	P16989	-0.177188627	0.177188627	0.483062
A.vasorum /Control	O95235	-0.444082925	0.444082925	0.483285
A.vasorum /Control	Q9P2L0	-0.277710016	0.277710016	0.483549
A.vasorum /Control	P06756	-0.238455191	0.238455191	0.483725
A.vasorum /Control	Q13275	-0.019977658	0.019977658	0.484219
A.vasorum /Control	Q16831	-0.062360473	0.062360473	0.484166
A.vasorum /Control	Q8N9F7	-0.116810793	0.116810793	0.484207
A.vasorum /Control	P49795	-0.083115568	0.083115568	0.48469
A.vasorum /Control	Q9H488	-0.174035012	0.174035012	0.484837
A.vasorum /Control	Q9BQ39	-0.454286509	0.454286509	0.485074
A.vasorum /Control	O76071	-0.158312015	0.158312015	0.485223
A.vasorum /Control	P18669	-0.066487032	0.066487032	0.486206
A.vasorum /Control	Q9C0F1	-0.517536305	0.517536305	0.487475
A.vasorum /Control	Q9BQB6	-0.295115802	0.295115802	0.488153
A.vasorum /Control	O00189	-0.08604575	0.08604575	0.489569
A.vasorum /Control	P60228	-0.05508375	0.05508375	0.489691
A.vasorum /Control	Q9H1B7	-0.719130085	0.719130085	0.490234
A.vasorum /Control	Q8TBC3	-0.230054807	0.230054807	0.490417
A.vasorum /Control	Q96PM5	0.479126898	0.479126898	0.490818
A.vasorum /Control	P42695	-0.223843096	0.223843096	0.491002
A.vasorum /Control	P62333	-0.043994922	0.043994922	0.49092
A.vasorum /Control	P32969	-0.226250716	0.226250716	0.4911
A.vasorum /Control	Q7Z434	-0.331813365	0.331813365	0.49145
A.vasorum /Control	Q9UIG0	-0.65860283	0.65860283	0.491642
A.vasorum /Control	P14859	-0.039544701	0.039544701	0.492299
A.vasorum /Control	Q13838	-0.215655441	0.215655441	0.492832
A.vasorum /Control	Q96EA4	-0.712399739	0.712399739	0.494608
A.vasorum /Control	O75369	-0.052771891	0.052771891	0.494709
A.vasorum /Control	Q96GD4	-0.473786616	0.473786616	0.49491
A.vasorum /Control	P32189	-0.160759269	0.160759269	0.495132
A.vasorum /Control	O00746	-0.117042728	0.117042728	0.495802

A.vasorum /Control	P62829	-0.048653253	0.048653253	0.497653
A.vasorum /Control	Q7L5D6	0.001536517	0.001536517	0.498402
A.vasorum /Control	P53701	-0.386040249	0.386040249	0.499738
A.vasorum /Control	Q9Y4E1	-0.011074261	0.011074261	0.500199
A.vasorum /Control	P09651	-0.195306143	0.195306143	0.502069
A.vasorum /Control	Q9BV86	0.026376785	0.026376785	0.503276
A.vasorum /Control	P51636	0.021868562	0.021868562	0.503801
A.vasorum /Control	Q9UMR2	-0.338307469	0.338307469	0.50445
A.vasorum /Control	Q92619	-0.014391122	0.014391122	0.504649
A.vasorum /Control	Q8N755	0.116252887	0.116252887	0.505069
A.vasorum /Control	Q9NRX1	-0.442759786	0.442759786	0.505428
A.vasorum /Control	Q9NW13	-0.494643159	0.494643159	0.505948
A.vasorum /Control	Q15262	-0.237300111	0.237300111	0.506853
A.vasorum /Control	Q86Y37	0.844993204	0.844993204	0.506822
A.vasorum /Control	Q8WVC6	-0.033766362	0.033766362	0.506735
A.vasorum /Control	Q96B54	-0.392471919	0.392471919	0.507044
A.vasorum /Control	P17987	-0.065490283	0.065490283	0.50731
A.vasorum /Control	Q15738	-0.011984655	0.011984655	0.50746
A.vasorum /Control	O75815	-0.051203183	0.051203183	0.507724
A.vasorum /Control	Q9Y4B5	-0.286917982	0.286917982	0.508871
A.vasorum /Control	P20929	0.004652686	0.004652686	0.509598
A.vasorum /Control	Q9UQ35	-0.139251198	0.139251198	0.510788
A.vasorum /Control	Q96RT8	-0.461747367	0.461747367	0.511011
A.vasorum /Control	Q14197	-0.330056288	0.330056288	0.511339
A.vasorum /Control	Q9NW82	-0.158628359	0.158628359	0.511924
A.vasorum /Control	Q8NBM4	0.065351781	0.065351781	0.512454
A.vasorum /Control	Q9Y508	-0.090449107	0.090449107	0.512627
A.vasorum /Control	Q6PJT7	-0.322911679	0.322911679	0.512885
A.vasorum /Control	Q8N6S5	-0.9620908	0.9620908	0.513006
A.vasorum /Control	P37802	-0.105403456	0.105403456	0.514257
A.vasorum /Control	Q13442	-0.029999965	0.029999965	0.515406
A.vasorum /Control	Q8NDI1	-0.161048235	0.161048235	0.516496
A.vasorum /Control	Q99536	-0.040608296	0.040608296	0.516937
A.vasorum /Control	P15884	-0.06132764	0.06132764	0.518377
A.vasorum /Control	Q9H2K8	-0.145702125	0.145702125	0.518347
A.vasorum /Control	Q2PZI1	-0.075700498	0.075700498	0.519568
A.vasorum /Control	O14981	-0.157725153	0.157725153	0.520452
A.vasorum /Control	Q8NAF0	-0.13723303	0.13723303	0.520721
A.vasorum /Control	Q96AB6	0.186234916	0.186234916	0.522455
A.vasorum /Control	O14653	-0.138268992	0.138268992	0.522764
A.vasorum /Control	Q15365	0.028714039	0.028714039	0.522946
A.vasorum /Control	Q69YL0	-0.870850091	0.870850091	0.524287
A.vasorum /Control	Q9BRT6	-0.232480274	0.232480274	0.52567
A.vasorum /Control	Q3L8U1	-0.023620256	0.023620256	0.525912
A.vasorum /Control	P18846	0.117611589	0.117611589	0.526427
A.vasorum /Control	Q86YQ8	0.089371236	0.089371236	0.527237
A.vasorum /Control	P05204	0.215981019	0.215981019	0.528418
A.vasorum /Control	Q9Y448	-0.720664698	0.720664698	0.528347
A.vasorum /Control	Q9Y5W7	0.20173068	0.20173068	0.529067
A.vasorum /Control	P62937	0.054585318	0.054585318	0.529273

A.vasorum /Control	Q8ND56	-0.08821568	0.08821568	0.531916
A.vasorum /Control	Q9Y2D4	-0.232631752	0.232631752	0.531911
A.vasorum /Control	P13489	-0.140472418	0.140472418	0.534025
A.vasorum /Control	P82094	-0.199464276	0.199464276	0.534789
A.vasorum /Control	Q12841	-0.204525501	0.204525501	0.534791
A.vasorum /Control	Q1ED39	0.076092803	0.076092803	0.534998
A.vasorum /Control	P17812	-0.126958451	0.126958451	0.53543
A.vasorum /Control	Q9Y399	-0.052424315	0.052424315	0.535531
A.vasorum /Control	P55199	-0.25040273	0.25040273	0.536959
A.vasorum /Control	Q86X02	-0.038377376	0.038377376	0.54132
A.vasorum /Control	Q6FI81	-0.191309545	0.191309545	0.541684
A.vasorum /Control	Q6ZVK8	0.210770242	0.210770242	0.544743
A.vasorum /Control	P11441	-0.290210447	0.290210447	0.545454
A.vasorum /Control	P53675	0.115129	0.115129	0.545835
A.vasorum /Control	P52848	-0.303077242	0.303077242	0.546442
A.vasorum /Control	O00391	-0.249649199	0.249649199	0.547312
A.vasorum /Control	Q6IBW4	0.019965826	0.019965826	0.547533
A.vasorum /Control	P50452	-0.194345814	0.194345814	0.547819
A.vasorum /Control	Q5VWQ8	0.098415236	0.098415236	0.547756
A.vasorum /Control	P61978	-0.025706421	0.025706421	0.548176
A.vasorum /Control	O43155	-0.453269768	0.453269768	0.548441
A.vasorum /Control	Q9P107	-0.191612745	0.191612745	0.548499
A.vasorum /Control	Q8WWI1	-0.176252299	0.176252299	0.549059
A.vasorum /Control	Q14643	0.171623414	0.171623414	0.549344
A.vasorum /Control	Q9NRW3	-0.610840846	0.610840846	0.549747
A.vasorum /Control	Q7L4I2	-0.053585089	0.053585089	0.550087
A.vasorum /Control	Q99497	-0.052605906	0.052605906	0.550442
A.vasorum /Control	P27816	-0.384747644	0.384747644	0.551676
A.vasorum /Control	Q5T3F8	-0.49921876	0.49921876	0.552741
A.vasorum /Control	Q96AE7	-0.150611336	0.150611336	0.554213
A.vasorum /Control	Q9NX07	0.093738345	0.093738345	0.554387
A.vasorum /Control	O75508	-0.135927355	0.135927355	0.556634
A.vasorum /Control	Q7Z6J6	-0.190353124	0.190353124	0.55781
A.vasorum /Control	Q9Y584	-0.117931096	0.117931096	0.557986
A.vasorum /Control	O43929	-0.156717475	0.156717475	0.558185
A.vasorum /Control	Q96KR1	-0.318398055	0.318398055	0.558912
A.vasorum /Control	P15408	-0.160405699	0.160405699	0.559024
A.vasorum /Control	Q8WZ75	-0.406544173	0.406544173	0.559755
A.vasorum /Control	P53985	-0.064432094	0.064432094	0.560766
A.vasorum /Control	O60287	-0.413403484	0.413403484	0.562333
A.vasorum /Control	P57076	-0.130291395	0.130291395	0.562427
A.vasorum /Control	Q9NUQ3	-0.268275202	0.268275202	0.563542
A.vasorum /Control	O75925	-0.03370338	0.03370338	0.563655
A.vasorum /Control	Q02447	-0.434569009	0.434569009	0.567324
A.vasorum /Control	Q8TC12	-0.069712657	0.069712657	0.567477
A.vasorum /Control	Q16270	-0.036067593	0.036067593	0.567588
A.vasorum /Control	O75083	-0.043554494	0.043554494	0.567983
A.vasorum /Control	P29323	-0.195067507	0.195067507	0.568838
A.vasorum /Control	Q8N6G6	0.087820327	0.087820327	0.56899
A.vasorum /Control	Q9ULJ7	-0.269403647	0.269403647	0.56932

A.vasorum /Control	Q9Y6J0	0.106239473	0.106239473	0.56926
A.vasorum /Control	P11388	-0.189215778	0.189215778	0.569431
A.vasorum /Control	P52756	-0.047088767	0.047088767	0.569554
A.vasorum /Control	Q4G0X4	0.207846122	0.207846122	0.56988
A.vasorum /Control	Q13432	-0.04220103	0.04220103	0.571648
A.vasorum /Control	Q96GN5	-0.34481687	0.34481687	0.571812
A.vasorum /Control	P0CW19	-0.187784335	0.187784335	0.572712
A.vasorum /Control	Q13952	0.061735676	0.061735676	0.573024
A.vasorum /Control	P60709	-0.013548044	0.013548044	0.573398
A.vasorum /Control	Q86YS6	-0.191342525	0.191342525	0.574331
A.vasorum /Control	Q8NBZ7	-0.55684442	0.55684442	0.574435
A.vasorum /Control	O15226	-0.386966266	0.386966266	0.574694
A.vasorum /Control	Q68CQ4	-0.490115937	0.490115937	0.574595
A.vasorum /Control	O60264	-0.321454026	0.321454026	0.575225
A.vasorum /Control	P13051	-0.369894582	0.369894582	0.575508
A.vasorum /Control	Q14934	0.334450911	0.334450911	0.575753
A.vasorum /Control	Q9H0R3	-0.509331249	0.509331249	0.576328
A.vasorum /Control	Q8WUQ7	-0.164573321	0.164573321	0.577094
A.vasorum /Control	Q8N6N3	-0.349860135	0.349860135	0.577663
A.vasorum /Control	Q96RL1	-0.279811759	0.279811759	0.578265
A.vasorum /Control	Q86V81	-0.03171116	0.03171116	0.580466
A.vasorum /Control	Q53ET0	0.231463363	0.231463363	0.581704
A.vasorum /Control	Q13641	-0.562998773	0.562998773	0.585445
A.vasorum /Control	Q9BRD0	0.053226283	0.053226283	0.58584
A.vasorum /Control	Q96LI5	-0.054604387	0.054604387	0.587119
A.vasorum /Control	Q7L1Q6	-0.10638185	0.10638185	0.587481
A.vasorum /Control	Q96KB5	-0.441716202	0.441716202	0.587701
A.vasorum /Control	Q9UG56	0.02614485	0.02614485	0.587959
A.vasorum /Control	Q96B26	-0.149177825	0.149177825	0.588338
A.vasorum /Control	O95980	-0.214925642	0.214925642	0.589353
A.vasorum /Control	Q9P2X0	0.025273436	0.025273436	0.589541
A.vasorum /Control	P04899	-0.202537585	0.202537585	0.591188
A.vasorum /Control	P19367	-0.15386094	0.15386094	0.591734
A.vasorum /Control	Q8NCA5	-0.221587143	0.221587143	0.593166
A.vasorum /Control	O60292	-0.043273198	0.043273198	0.593343
A.vasorum /Control	Q13206	-0.4209255	0.4209255	0.593702
A.vasorum /Control	P05023	-0.177162053	0.177162053	0.594139
A.vasorum /Control	Q96BN8	-0.028316999	0.028316999	0.594196
A.vasorum /Control	Q6NW29	-0.40039109	0.40039109	0.594715
A.vasorum /Control	O15344	-0.015125942	0.015125942	0.59855
A.vasorum /Control	Q2TAA2	0.131180424	0.131180424	0.5998
A.vasorum /Control	P47895	-0.391382715	0.391382715	0.601187
A.vasorum /Control	Q14693	-0.052615182	0.052615182	0.603834
A.vasorum /Control	Q9BY41	0.257765271	0.257765271	0.604982
A.vasorum /Control	Q96ES7	-0.184595209	0.184595209	0.605166
A.vasorum /Control	P08195	-0.420549918	0.420549918	0.605821
A.vasorum /Control	Q9UER7	-0.398721111	0.398721111	0.606943
A.vasorum /Control	O95714	-0.204221777	0.204221777	0.607073
A.vasorum /Control	P51809	-0.0282316	0.0282316	0.609639
A.vasorum /Control	Q96G46	0.263838939	0.263838939	0.609669

A.vasorum /Control	Q8WVM7	-0.438083812	0.438083812	0.609953
A.vasorum /Control	Q9BY42	-0.17349581	0.17349581	0.611077
A.vasorum /Control	O60678	-0.361137631	0.361137631	0.611516
A.vasorum /Control	Q969V5	0.01614842	0.01614842	0.611414
A.vasorum /Control	Q9NXG6	-0.031698586	0.031698586	0.61381
A.vasorum /Control	Q8TF42	-0.358592706	0.358592706	0.614238
A.vasorum /Control	P58004	-0.534006477	0.534006477	0.615065
A.vasorum /Control	Q9UJK0	-0.036207613	0.036207613	0.615556
A.vasorum /Control	O75717	-0.038989437	0.038989437	0.617452
A.vasorum /Control	Q86U90	-0.501065507	0.501065507	0.617353
A.vasorum /Control	O43237	-0.180011356	0.180011356	0.617743
A.vasorum /Control	Q9H936	-0.091596574	0.091596574	0.621611
A.vasorum /Control	Q9BXK5	0.157244585	0.157244585	0.621766
A.vasorum /Control	Q9H2F5	0.318473255	0.318473255	0.621999
A.vasorum /Control	Q13671	-0.315408732	0.315408732	0.622654
A.vasorum /Control	Q15390	0.017366235	0.017366235	0.622686
A.vasorum /Control	P34059	0.032695743	0.032695743	0.623436
A.vasorum /Control	Q5JTW2	0.147376026	0.147376026	0.624093
A.vasorum /Control	Q9NWT1	-0.730953928	0.730953928	0.624896
A.vasorum /Control	Q9UNQ2	-0.221425533	0.221425533	0.625911
A.vasorum /Control	Q8TCF1	-0.525932392	0.525932392	0.62616
A.vasorum /Control	Q9UPR0	0.208719745	0.208719745	0.626212
A.vasorum /Control	Q9NQT4	-0.265121293	0.265121293	0.627024
A.vasorum /Control	Q8WXE1	-0.181957401	0.181957401	0.627388
A.vasorum /Control	P19623	-0.059024382	0.059024382	0.627735
A.vasorum /Control	Q969T9	-0.142575586	0.142575586	0.627722
A.vasorum /Control	Q9UP95	-0.18863744	0.18863744	0.629151
A.vasorum /Control	Q9BZV1	-0.095940371	0.095940371	0.629311
A.vasorum /Control	Q6ZT21	-1.205436311	1.205436311	0.631099
A.vasorum /Control	P84103	-0.133906962	0.133906962	0.631689
A.vasorum /Control	P62942	-0.175599832	0.175599832	0.632336
A.vasorum /Control	Q9P1Y6	-0.217785062	0.217785062	0.63366
A.vasorum /Control	Q9BW19	-0.352718399	0.352718399	0.635498
A.vasorum /Control	Q9P2K6	-0.050899019	0.050899019	0.63612
A.vasorum /Control	O00566	-0.467740978	0.467740978	0.637229
A.vasorum /Control	O43818	-0.685856029	0.685856029	0.63732
A.vasorum /Control	P22392	-0.016530229	0.016530229	0.638244
A.vasorum /Control	Q58FG1	0.000643576	0.000643576	0.638381
A.vasorum /Control	Q7Z406	-0.662163709	0.662163709	0.639025
A.vasorum /Control	Q8N0Z6	-0.182135367	0.182135367	0.638886
A.vasorum /Control	Q9UNW1	0.026286187	0.026286187	0.638921
A.vasorum /Control	P27635	-0.056696949	0.056696949	0.639218
A.vasorum /Control	Q9NR56	-0.070348312	0.070348312	0.639452
A.vasorum /Control	Q13627	-0.450897909	0.450897909	0.640613
A.vasorum /Control	Q9H0X9	-0.385752622	0.385752622	0.641146
A.vasorum /Control	Q9Y6A9	-0.067838519	0.067838519	0.642187
A.vasorum /Control	P11908	-0.090235963	0.090235963	0.644937
A.vasorum /Control	Q05655	-0.266560188	0.266560188	0.646089
A.vasorum /Control	Q92667	-0.504221561	0.504221561	0.647315
A.vasorum /Control	Q676U5	-0.251074738	0.251074738	0.649757

A.vasorum /Control	Q9H467	0.174061673	0.174061673	0.651146
A.vasorum /Control	Q96NB3	-0.039978055	0.039978055	0.652273
A.vasorum /Control	Q08554	-0.35305235	0.35305235	0.652704
A.vasorum /Control	Q9H501	-0.707720527	0.707720527	0.653754
A.vasorum /Control	Q15717	-0.184997751	0.184997751	0.655115
A.vasorum /Control	Q9BYC8	0.043933275	0.043933275	0.656154
A.vasorum /Control	Q6P5Z2	0.27152573	0.27152573	0.656368
A.vasorum /Control	Q14147	-0.267952132	0.267952132	0.656559
A.vasorum /Control	Q9Y5K8	-0.245371038	0.245371038	0.660199
A.vasorum /Control	P22087	-0.528431952	0.528431952	0.663042
A.vasorum /Control	Q8TBZ3	-0.201209862	0.201209862	0.664353
A.vasorum /Control	Q15287	-0.129877722	0.129877722	0.66669
A.vasorum /Control	Q9HBG6	-0.098087939	0.098087939	0.666619
A.vasorum /Control	Q9Y282	-0.45281803	0.45281803	0.667102
A.vasorum /Control	Q7Z739	-0.15717115	0.15717115	0.668595
A.vasorum /Control	Q6XE24	-0.167511332	0.167511332	0.668817
A.vasorum /Control	Q71F56	-0.551731931	0.551731931	0.670485
A.vasorum /Control	Q9BV79	0.302250686	0.302250686	0.670596
A.vasorum /Control	Q9Y4K4	-0.152943465	0.152943465	0.671427
A.vasorum /Control	Q9NYJ8	-0.245906445	0.245906445	0.671835
A.vasorum /Control	P56182	-0.588457685	0.588457685	0.672046
A.vasorum /Control	Q99496	-0.2411649	0.2411649	0.673616
A.vasorum /Control	Q00169	0.003366252	0.003366252	0.675145
A.vasorum /Control	Q8NEN9	-0.451367073	0.451367073	0.676393
A.vasorum /Control	Q16656	-0.155757444	0.155757444	0.676519
A.vasorum /Control	Q15050	-0.705289551	0.705289551	0.677405
A.vasorum /Control	Q9NVN8	-0.121779953	0.121779953	0.679216
A.vasorum /Control	Q53GG5	0.074601341	0.074601341	0.679919
A.vasorum /Control	Q9BUL8	-0.002103476	0.002103476	0.679836
A.vasorum /Control	Q96GX5	-0.340385293	0.340385293	0.680925
A.vasorum /Control	Q9UGJ1	-0.050954376	0.050954376	0.681311
A.vasorum /Control	P49069	0.165167437	0.165167437	0.681691
A.vasorum /Control	Q15424	-0.308485077	0.308485077	0.681771
A.vasorum /Control	Q96JI7	-0.024178941	0.024178941	0.681641
A.vasorum /Control	Q92540	0.497728816	0.497728816	0.682492
A.vasorum /Control	Q8N954	-0.214614739	0.214614739	0.683047
A.vasorum /Control	Q7Z7K0	0.039392615	0.039392615	0.68416
A.vasorum /Control	Q9BQ75	-0.14232321	0.14232321	0.688496
A.vasorum /Control	Q06413	0.325085433	0.325085433	0.690504
A.vasorum /Control	Q9C0H2	-0.029029629	0.029029629	0.690639
A.vasorum /Control	Q15036	-0.205651177	0.205651177	0.69119
A.vasorum /Control	Q9BXJ0	0.095243438	0.095243438	0.692401
A.vasorum /Control	Q9Y2R5	-0.047078711	0.047078711	0.693442
A.vasorum /Control	Q9Y4D1	0.052943305	0.052943305	0.694704
A.vasorum /Control	O43683	-0.360055802	0.360055802	0.695882
A.vasorum /Control	Q3T906	-0.807477898	0.807477898	0.696165
A.vasorum /Control	O95881	-0.174588363	0.174588363	0.696446
A.vasorum /Control	Q4KMQ1	-0.238801054	0.238801054	0.696998
A.vasorum /Control	Q5JPI9	0.141371618	0.141371618	0.698415
A.vasorum /Control	O15484	0.280194605	0.280194605	0.699297

A.vasorum /Control	P06753	-0.037954789	0.037954789	0.699919
A.vasorum /Control	Q96KR6	-0.180866135	0.180866135	0.699939
A.vasorum /Control	Q9H6F5	-0.510526444	0.510526444	0.701308
A.vasorum /Control	Q5T013	0.391884948	0.391884948	0.701492
A.vasorum /Control	Q9NWM8	-0.662909373	0.662909373	0.701616
A.vasorum /Control	Q92551	0.231272882	0.231272882	0.701751
A.vasorum /Control	P36873	-0.108521278	0.108521278	0.703776
A.vasorum /Control	Q8TE02	0.138316154	0.138316154	0.70452
A.vasorum /Control	Q8NFH5	-0.034920839	0.034920839	0.705134
A.vasorum /Control	Q9H999	-0.541025999	0.541025999	0.706717
A.vasorum /Control	Q9NV66	0.341841106	0.341841106	0.706809
A.vasorum /Control	O43768	-0.073063943	0.073063943	0.706945
A.vasorum /Control	Q9UBV2	-0.092906994	0.092906994	0.707691
A.vasorum /Control	Q9Y3C1	-0.333642073	0.333642073	0.711233
A.vasorum /Control	P04156	-0.282598871	0.282598871	0.711742
A.vasorum /Control	P52824	0.058137159	0.058137159	0.71252
A.vasorum /Control	Q8TB61	-0.228497996	0.228497996	0.712442
A.vasorum /Control	Q05193	-0.101938728	0.101938728	0.71284
A.vasorum /Control	Q5THJ4	0.07545124	0.07545124	0.713565
A.vasorum /Control	Q8TEV9	-0.298722608	0.298722608	0.713419
A.vasorum /Control	Q9HC98	-0.233432724	0.233432724	0.713657
A.vasorum /Control	Q9H7E9	-0.196145882	0.196145882	0.714217
A.vasorum /Control	Q9NWH9	-0.32236844	0.32236844	0.714593
A.vasorum /Control	P01023	0.096701908	0.096701908	0.715295
A.vasorum /Control	Q6ZW31	0.369658696	0.369658696	0.715756
A.vasorum /Control	Q7L7X3	-0.060523451	0.060523451	0.715635
A.vasorum /Control	Q0ZGT2	-0.350079906	0.350079906	0.717787
A.vasorum /Control	A0JNW5	0.347897952	0.347897952	0.719419
A.vasorum /Control	P07910	-0.065760157	0.065760157	0.719752
A.vasorum /Control	Q9BQL6	-0.180021151	0.180021151	0.721624
A.vasorum /Control	P46776	-0.063754203	0.063754203	0.722938
A.vasorum /Control	O60427	-0.381802036	0.381802036	0.723435
A.vasorum /Control	Q86UX7	-0.315144003	0.315144003	0.724399
A.vasorum /Control	Q9GZN8	-0.074126103	0.074126103	0.724633
A.vasorum /Control	Q13526	-0.163246751	0.163246751	0.725715
A.vasorum /Control	Q9BQ69	0.20187272	0.20187272	0.725764
A.vasorum /Control	P62280	-0.093354717	0.093354717	0.725974
A.vasorum /Control	Q15653	0.084946781	0.084946781	0.726498
A.vasorum /Control	Q8IY33	-0.02863332	0.02863332	0.726902
A.vasorum /Control	P56385	-0.02643539	0.02643539	0.728099
A.vasorum /Control	Q96IZ0	0.01497001	0.01497001	0.729073
A.vasorum /Control	P16591	0.39259046	0.39259046	0.729728
A.vasorum /Control	Q14644	-0.340414899	0.340414899	0.730764
A.vasorum /Control	O60216	-0.27116103	0.27116103	0.731721
A.vasorum /Control	Q5GJ75	-0.650111101	0.650111101	0.732382
A.vasorum /Control	Q15361	-0.21369289	0.21369289	0.734553
A.vasorum /Control	Q15831	-0.107756753	0.107756753	0.735011
A.vasorum /Control	P18085	-0.088417623	0.088417623	0.736032
A.vasorum /Control	Q8IWW6	-0.290048161	0.290048161	0.735985
A.vasorum /Control	Q92543	-0.093318458	0.093318458	0.736054

A.vasorum /Control	Q5RKV6	-0.135029325	0.135029325	0.736315
A.vasorum /Control	Q5TFE4	-0.054024347	0.054024347	0.737371
A.vasorum /Control	P17544	-0.618395113	0.618395113	0.738125
A.vasorum /Control	Q9BT23	0.181155316	0.181155316	0.73969
A.vasorum /Control	P38159	-0.166616879	0.166616879	0.740246
A.vasorum /Control	Q68CZ2	-0.549667442	0.549667442	0.7412
A.vasorum /Control	Q9ULX6	-0.092347062	0.092347062	0.741468
A.vasorum /Control	O00308	-0.016878238	0.016878238	0.742579
A.vasorum /Control	Q8NG11	-0.212050306	0.212050306	0.743315
A.vasorum /Control	Q96FJ2	-0.119590455	0.119590455	0.74365
A.vasorum /Control	Q92879	-0.332856765	0.332856765	0.744037
A.vasorum /Control	Q9Y6K0	0.195728579	0.195728579	0.744398
A.vasorum /Control	A6NFQ2	-0.424040489	0.424040489	0.74473
A.vasorum /Control	Q9HA38	-0.297366844	0.297366844	0.745529
A.vasorum /Control	Q6DKK2	-0.051955891	0.051955891	0.746503
A.vasorum /Control	Q9ULX9	-0.812568447	0.812568447	0.749222
A.vasorum /Control	Q6ZW49	-0.41391004	0.41391004	0.749685
A.vasorum /Control	Q06265	-0.21769567	0.21769567	0.754558
A.vasorum /Control	O43156	0.016680152	0.016680152	0.754764
A.vasorum /Control	Q16186	-0.195866242	0.195866242	0.754887
A.vasorum /Control	Q53GL7	-0.054195653	0.054195653	0.755609
A.vasorum /Control	Q15742	-0.120609315	0.120609315	0.757313
A.vasorum /Control	Q86XP1	-0.043687852	0.043687852	0.757183
A.vasorum /Control	Q96IW7	-0.117483335	0.117483335	0.757398
A.vasorum /Control	Q14781	0.401412037	0.401412037	0.75869
A.vasorum /Control	Q5T280	-0.271720833	0.271720833	0.759316
A.vasorum /Control	Q9NQ88	-0.06876144	0.06876144	0.75911
A.vasorum /Control	Q9BZE9	-0.230292116	0.230292116	0.76149
A.vasorum /Control	Q9Y696	-0.094145382	0.094145382	0.761787
A.vasorum /Control	P41440	-0.454492324	0.454492324	0.762289
A.vasorum /Control	O14647	-0.322238958	0.322238958	0.762623
A.vasorum /Control	O15031	-0.212705644	0.212705644	0.763489
A.vasorum /Control	P62495	-0.150341546	0.150341546	0.763627
A.vasorum /Control	P53667	-0.092121243	0.092121243	0.765566
A.vasorum /Control	Q9UMX3	-0.181108889	0.181108889	0.765874
A.vasorum /Control	Q9HCK8	-0.179443543	0.179443543	0.766499
A.vasorum /Control	Q96BF6	-1.093258162	1.093258162	0.767707
A.vasorum /Control	P17096	-0.094278512	0.094278512	0.768292
A.vasorum /Control	Q5T200	-0.262396752	0.262396752	0.769277
A.vasorum /Control	Q96K17	-0.218081627	0.218081627	0.769201
A.vasorum /Control	Q9HD15	-0.24140898	0.24140898	0.769052
A.vasorum /Control	P09132	-0.227323413	0.227323413	0.770256
A.vasorum /Control	P62826	-0.029890865	0.029890865	0.770456
A.vasorum /Control	P31350	-0.211838378	0.211838378	0.771352
A.vasorum /Control	P53602	-0.193577641	0.193577641	0.772464
A.vasorum /Control	O94889	-0.940957402	0.940957402	0.773238
A.vasorum /Control	P31146	-0.458072295	0.458072295	0.774487
A.vasorum /Control	P49641	0.020071134	0.020071134	0.775059
A.vasorum /Control	Q02543	-0.119821255	0.119821255	0.777201
A.vasorum /Control	Q9UQ90	-0.012007418	0.012007418	0.777153

A.vasorum /Control	Q8WYN0	-0.209163522	0.209163522	0.777817
A.vasorum /Control	P51617	-0.123511842	0.123511842	0.7789
A.vasorum /Control	Q13404	-0.16907143	0.16907143	0.77941
A.vasorum /Control	Q9H967	-0.597609354	0.597609354	0.779238
A.vasorum /Control	Q9Y5W9	-0.104869016	0.104869016	0.779482
A.vasorum /Control	P11387	-0.169087595	0.169087595	0.779919
A.vasorum /Control	Q99755	-0.589303642	0.589303642	0.779847
A.vasorum /Control	Q8NEM2	-0.312179225	0.312179225	0.781926
A.vasorum /Control	Q9BV40	0.65325979	0.65325979	0.782004
A.vasorum /Control	P51825	-0.115592971	0.115592971	0.783845
A.vasorum /Control	Q9NWX5	-0.465205046	0.465205046	0.783948
A.vasorum /Control	Q07955	-0.124984619	0.124984619	0.784346
A.vasorum /Control	Q6PJG2	-0.340523435	0.340523435	0.786812
A.vasorum /Control	P81605	0.264226554	0.264226554	0.786991
A.vasorum /Control	Q9Y4Z0	-0.103508359	0.103508359	0.788387
A.vasorum /Control	Q9BSR8	-0.484679215	0.484679215	0.789106
A.vasorum /Control	Q9BXR0	-0.175377117	0.175377117	0.790842
A.vasorum /Control	Q7Z4Q2	-0.232202345	0.232202345	0.791196
A.vasorum /Control	P52747	0.111519809	0.111519809	0.791553
A.vasorum /Control	Q8IYA6	-0.702715041	0.702715041	0.791845
A.vasorum /Control	Q99549	-0.379051438	0.379051438	0.79209
A.vasorum /Control	P20962	0.393411152	0.393411152	0.792247
A.vasorum /Control	Q7Z3E5	-0.102631285	0.102631285	0.793221
A.vasorum /Control	O15021	-0.12161621	0.12161621	0.793589
A.vasorum /Control	Q15031	-0.224602977	0.224602977	0.794051
A.vasorum /Control	P09234	-0.521940381	0.521940381	0.799272
A.vasorum /Control	Q96EK5	-0.21393643	0.21393643	0.799348
A.vasorum /Control	Q9BV94	0.526555584	0.526555584	0.800008
A.vasorum /Control	Q96FZ2	-0.104512201	0.104512201	0.800832
A.vasorum /Control	Q9H0A0	-0.410866854	0.410866854	0.802397
A.vasorum /Control	O60832	-0.148635664	0.148635664	0.802954
A.vasorum /Control	P24534	-0.033571034	0.033571034	0.804186
A.vasorum /Control	Q9UBF8	-0.151826513	0.151826513	0.804335
A.vasorum /Control	P61244	-0.085109561	0.085109561	0.805379
A.vasorum /Control	O75530	-0.185105917	0.185105917	0.806677
A.vasorum /Control	Q8N511	-0.178041903	0.178041903	0.806837
A.vasorum /Control	Q92968	-0.364794573	0.364794573	0.808371
A.vasorum /Control	Q7Z478	-0.268223266	0.268223266	0.809616
A.vasorum /Control	Q9BVM2	0.148163241	0.148163241	0.80949
A.vasorum /Control	Q9NS00	-0.146789582	0.146789582	0.809447
A.vasorum /Control	Q96CP6	0.099765133	0.099765133	0.810848
A.vasorum /Control	Q9BZX2	-0.24402981	0.24402981	0.810921
A.vasorum /Control	P18583	-0.232462553	0.232462553	0.812716
A.vasorum /Control	Q96QD9	-0.316669626	0.316669626	0.814784
A.vasorum /Control	Q86UL3	-0.09797098	0.09797098	0.815282
A.vasorum /Control	O14733	-0.108276918	0.108276918	0.817021
A.vasorum /Control	Q8NEF9	-0.037373094	0.037373094	0.818637
A.vasorum /Control	Q14151	-0.340977775	0.340977775	0.819128
A.vasorum /Control	Q92934	-0.237982637	0.237982637	0.819339
A.vasorum /Control	Q96GW9	-0.511093803	0.511093803	0.821553

A.vasorum /Control	Q5T5Y3	-0.120389941	0.120389941	0.822225
A.vasorum /Control	Q9NYL4	-0.142570592	0.142570592	0.822559
A.vasorum /Control	Q7L099	-0.004293139	0.004293139	0.823912
A.vasorum /Control	Q8N264	-0.357646735	0.357646735	0.824643
A.vasorum /Control	Q92823	-0.577999129	0.577999129	0.825368
A.vasorum /Control	O75157	0.008182614	0.008182614	0.826251
A.vasorum /Control	P52597	-0.087907376	0.087907376	0.826118
A.vasorum /Control	Q9P0L0	-0.205424011	0.205424011	0.826988
A.vasorum /Control	P22670	-0.052164464	0.052164464	0.827875
A.vasorum /Control	Q9NVX2	-0.442923869	0.442923869	0.828216
A.vasorum /Control	Q8WUM9	-0.483699546	0.483699546	0.828861
A.vasorum /Control	Q9C037	-0.042259101	0.042259101	0.828895
A.vasorum /Control	Q12849	-0.382766444	0.382766444	0.829064
A.vasorum /Control	Q9UPN7	-0.263204063	0.263204063	0.829809
A.vasorum /Control	O95159	0.096928822	0.096928822	0.831742
A.vasorum /Control	Q9UQ53	-1.260473357	1.260473357	0.831717
A.vasorum /Control	O95685	0.046377147	0.046377147	0.832149
A.vasorum /Control	Q92567	-0.226215294	0.226215294	0.832838
A.vasorum /Control	Q9NUP9	-0.550547223	0.550547223	0.834598
A.vasorum /Control	Q9NVU0	0.058564916	0.058564916	0.835244
A.vasorum /Control	P50914	-0.05458952	0.05458952	0.837949
A.vasorum /Control	Q9H6I2	-0.462750641	0.462750641	0.838661
A.vasorum /Control	P52926	-0.130980966	0.130980966	0.83987
A.vasorum /Control	Q02790	-0.151537186	0.151537186	0.840051
A.vasorum /Control	Q9UPT8	-0.265370123	0.265370123	0.841333
A.vasorum /Control	O75586	-0.208133914	0.208133914	0.842186
A.vasorum /Control	O60245	0.333265678	0.333265678	0.843618
A.vasorum /Control	P11166	-0.382695867	0.382695867	0.843513
A.vasorum /Control	Q9Y3D9	-0.120825725	0.120825725	0.843546
A.vasorum /Control	Q8IYB1	-0.13293941	0.13293941	0.845944
A.vasorum /Control	Q15014	-0.502734481	0.502734481	0.846134
A.vasorum /Control	P51948	-0.148651711	0.148651711	0.846428
A.vasorum /Control	Q8TBX8	0.286683228	0.286683228	0.846369
A.vasorum /Control	P14174	-0.61243098	0.61243098	0.846903
A.vasorum /Control	Q8IUH4	-0.229844433	0.229844433	0.847177
A.vasorum /Control	Q96IR7	-0.027341896	0.027341896	0.847485
A.vasorum /Control	Q13126	-0.225848267	0.225848267	0.84883
A.vasorum /Control	Q9NUM4	-0.337692935	0.337692935	0.848787
A.vasorum /Control	Q92963	-0.346177055	0.346177055	0.851928
A.vasorum /Control	Q8IXI1	0.192527881	0.192527881	0.852284
A.vasorum /Control	Q92597	0.043161474	0.043161474	0.853668
A.vasorum /Control	Q13435	-0.144243403	0.144243403	0.854187
A.vasorum /Control	Q13283	-0.163817807	0.163817807	0.856032
A.vasorum /Control	Q99729	-0.018733666	0.018733666	0.856282
A.vasorum /Control	Q9BRS2	-0.171086658	0.171086658	0.857145
A.vasorum /Control	Q9BW85	-0.156500006	0.156500006	0.857599
A.vasorum /Control	O95759	-0.111895078	0.111895078	0.859713
A.vasorum /Control	O95476	-0.221287466	0.221287466	0.860318
A.vasorum /Control	Q14BN4	0.035405747	0.035405747	0.861125
A.vasorum /Control	Q96LR5	-0.580689108	0.580689108	0.862199

A.vasorum /Control	O43353	-0.346783261	0.346783261	0.864581
A.vasorum /Control	Q8WUF5	-0.453214106	0.453214106	0.864634
A.vasorum /Control	Q9Y2J4	-0.749191173	0.749191173	0.864883
A.vasorum /Control	P46781	-0.035701178	0.035701178	0.865781
A.vasorum /Control	Q9NNW5	-0.199943014	0.199943014	0.866926
A.vasorum /Control	P62070	-0.198554628	0.198554628	0.867716
A.vasorum /Control	P62314	-0.222902128	0.222902128	0.868417
A.vasorum /Control	A6NIH7	-0.779001428	0.779001428	0.869446
A.vasorum /Control	Q5T9A4	-0.386757485	0.386757485	0.869952
A.vasorum /Control	Q9NVT9	0.050515854	0.050515854	0.870996
A.vasorum /Control	P45877	-0.159422635	0.159422635	0.872928
A.vasorum /Control	P17655	-0.101854617	0.101854617	0.873821
A.vasorum /Control	Q969P0	0.278651013	0.278651013	0.874059
A.vasorum /Control	Q96MF7	-0.26608578	0.26608578	0.874937
A.vasorum /Control	Q32M88	-0.209189603	0.209189603	0.876194
A.vasorum /Control	Q32NC0	-0.049502546	0.049502546	0.876483
A.vasorum /Control	P01112	-0.048588602	0.048588602	0.876644
A.vasorum /Control	Q96BJ3	-0.053113593	0.053113593	0.877394
A.vasorum /Control	Q8NBJ4	0.161216872	0.161216872	0.878613
A.vasorum /Control	Q9Y3D0	-0.168293148	0.168293148	0.879227
A.vasorum /Control	Q9Y2G8	0.09996814	0.09996814	0.882737
A.vasorum /Control	P30086	-0.007918043	0.007918043	0.883431
A.vasorum /Control	Q9P031	-0.500536097	0.500536097	0.884931
A.vasorum /Control	P54920	-0.164026552	0.164026552	0.885539
A.vasorum /Control	Q14914	-0.222435465	0.222435465	0.886673
A.vasorum /Control	P98196	-0.288257187	0.288257187	0.88833
A.vasorum /Control	Q9BVC4	-0.354755402	0.354755402	0.888474
A.vasorum /Control	O95999	-0.110698767	0.110698767	0.888802
A.vasorum /Control	Q9UJY4	-0.135846312	0.135846312	0.888834
A.vasorum /Control	A8MW92	-0.372479105	0.372479105	0.88955
A.vasorum /Control	O75882	-0.264672741	0.264672741	0.889655
A.vasorum /Control	Q9UBB5	-0.278359628	0.278359628	0.890124
A.vasorum /Control	A0AVF1	-0.388791743	0.388791743	0.891211
A.vasorum /Control	A6NKD9	-0.118443023	0.118443023	0.891569
A.vasorum /Control	O14966	0.138688151	0.138688151	0.891464
A.vasorum /Control	P27448	-0.075433048	0.075433048	0.891142
A.vasorum /Control	P84022	-0.196485604	0.196485604	0.891165
A.vasorum /Control	Q96BP2	-0.514227169	0.514227169	0.891607
A.vasorum /Control	Q9NZD8	-0.143902139	0.143902139	0.892682
A.vasorum /Control	P55769	-0.365304697	0.365304697	0.893245
A.vasorum /Control	O00151	-0.126244646	0.126244646	0.893815
A.vasorum /Control	Q9UNL2	-0.074220769	0.074220769	0.894171
A.vasorum /Control	Q9BVS4	-0.105657321	0.105657321	0.894555
A.vasorum /Control	Q9NZE8	0.348732877	0.348732877	0.894784
A.vasorum /Control	Q9BSL1	-0.026303331	0.026303331	0.895231
A.vasorum /Control	O95239	-0.164366278	0.164366278	0.896496
A.vasorum /Control	Q9NZM5	-0.460073089	0.460073089	0.897446
A.vasorum /Control	Q99470	-0.403711188	0.403711188	0.897797
A.vasorum /Control	P20339	-0.097581336	0.097581336	0.899196
A.vasorum /Control	Q9NRZ5	0.462904047	0.462904047	0.899169

A.vasorum /Control	Q9BVS5	-0.035808529	0.035808529	0.899631
A.vasorum /Control	Q9NS86	-0.363580876	0.363580876	0.90018
A.vasorum /Control	Q9UJU6	-0.104040291	0.104040291	0.900452
A.vasorum /Control	Q5VU43	0.114425176	0.114425176	0.90217
A.vasorum /Control	Q9UM13	-0.221232559	0.221232559	0.90399
A.vasorum /Control	Q9UBN7	0.345153487	0.345153487	0.90434
A.vasorum /Control	Q6IAA8	-0.373424812	0.373424812	0.904844
A.vasorum /Control	Q96DF8	0.036181448	0.036181448	0.906445
A.vasorum /Control	Q9H299	0.070897502	0.070897502	0.907289
A.vasorum /Control	P13797	-0.029475075	0.029475075	0.90851
A.vasorum /Control	Q8WVK2	-0.11420845	0.11420845	0.910722
A.vasorum /Control	Q9BVJ6	-0.698606134	0.698606134	0.911731
A.vasorum /Control	Q96EU6	-0.802327863	0.802327863	0.913902
A.vasorum /Control	P60842	-0.0721614	0.0721614	0.914777
A.vasorum /Control	P00558	-0.049785461	0.049785461	0.914944
A.vasorum /Control	Q9UHR5	-0.156418152	0.156418152	0.915465
A.vasorum /Control	P31431	-0.767040788	0.767040788	0.916608
A.vasorum /Control	O94868	-0.23205845	0.23205845	0.916837
A.vasorum /Control	Q9UJC5	-0.457894314	0.457894314	0.917171
A.vasorum /Control	Q9Y3C4	0.131197065	0.131197065	0.917265
A.vasorum /Control	P09012	-0.164400994	0.164400994	0.91906
A.vasorum /Control	Q86W56	-0.083316809	0.083316809	0.919905
A.vasorum /Control	Q3MHD2	-0.132614399	0.132614399	0.920924
A.vasorum /Control	Q13769	-0.077448804	0.077448804	0.921398
A.vasorum /Control	Q9NZ45	0.23418884	0.23418884	0.922159
A.vasorum /Control	Q9Y6Y0	-0.170469308	0.170469308	0.923062
A.vasorum /Control	P46379	-0.164788118	0.164788118	0.923776
A.vasorum /Control	P33527	-0.209771406	0.209771406	0.924276
A.vasorum /Control	Q9UHB6	-0.292239744	0.292239744	0.924432
A.vasorum /Control	Q13185	-0.083277266	0.083277266	0.924915
A.vasorum /Control	Q15392	-0.393860573	0.393860573	0.925698
A.vasorum /Control	Q96R06	-0.266912597	0.266912597	0.926676
A.vasorum /Control	O43159	-0.429322365	0.429322365	0.927099
A.vasorum /Control	Q5C9Z4	-0.199410545	0.199410545	0.927319
A.vasorum /Control	Q7RTV5	-0.135440962	0.135440962	0.92704
A.vasorum /Control	Q9Y3F4	-0.100438568	0.100438568	0.927551
A.vasorum /Control	Q9Y5U2	-0.125964917	0.125964917	0.927512
A.vasorum /Control	P62318	-0.111504307	0.111504307	0.927965
A.vasorum /Control	Q8IV08	-0.188596989	0.188596989	0.932121
A.vasorum /Control	Q15056	-0.05308606	0.05308606	0.93261
A.vasorum /Control	Q99808	-0.157210866	0.157210866	0.933492
A.vasorum /Control	Q99590	-0.123082845	0.123082845	0.933817
A.vasorum /Control	P43487	-0.021026707	0.021026707	0.934209
A.vasorum /Control	Q9P0U3	-0.196617536	0.196617536	0.935019
A.vasorum /Control	Q6GMV2	-0.0469615	0.0469615	0.93524
A.vasorum /Control	Q9Y2T2	-0.077688512	0.077688512	0.935351
A.vasorum /Control	P83916	-0.63319237	0.63319237	0.93584
A.vasorum /Control	Q8N567	-0.120704831	0.120704831	0.936391
A.vasorum /Control	Q13438	0.104107561	0.104107561	0.937111
A.vasorum /Control	P22694	0.027615472	0.027615472	0.938303

A.vasorum /Control	P36957	-0.126865967	0.126865967	0.937758
A.vasorum /Control	P62277	-0.047621281	0.047621281	0.938133
A.vasorum /Control	P63208	0.087129002	0.087129002	0.938017
A.vasorum /Control	Q96T23	-0.156177427	0.156177427	0.938529
A.vasorum /Control	Q9BY77	-0.152498031	0.152498031	0.937931
A.vasorum /Control	Q9NP77	0.031753738	0.031753738	0.938551
A.vasorum /Control	P16035	-0.276111275	0.276111275	0.938718
A.vasorum /Control	P62269	-0.060508343	0.060508343	0.940718
A.vasorum /Control	Q9UI14	0.047498806	0.047498806	0.941585
A.vasorum /Control	Q9BT88	-0.247645268	0.247645268	0.942414
A.vasorum /Control	Q15149	-0.120319086	0.120319086	0.944818
A.vasorum /Control	P41208	0.20870568	0.20870568	0.946199
A.vasorum /Control	Q6P3S6	0.044559088	0.044559088	0.946533
A.vasorum /Control	O60508	-0.245394377	0.245394377	0.946968
A.vasorum /Control	Q8WVM0	-0.191954754	0.191954754	0.947582
A.vasorum /Control	Q6PKC3	-0.210708194	0.210708194	0.947917
A.vasorum /Control	Q8N5H7	-0.147626463	0.147626463	0.948062
A.vasorum /Control	Q9UK99	-0.273170373	0.273170373	0.948656
A.vasorum /Control	Q9NX05	-0.12696945	0.12696945	0.949957
A.vasorum /Control	O75817	-0.232313594	0.232313594	0.95013
A.vasorum /Control	Q6GQQ9	-0.276210495	0.276210495	0.952154
A.vasorum /Control	P30622	-0.220388385	0.220388385	0.952906
A.vasorum /Control	P43121	-0.096219432	0.096219432	0.953352
A.vasorum /Control	Q9NQS1	-0.478262603	0.478262603	0.954183
A.vasorum /Control	Q9NVS9	-0.51126887	0.51126887	0.954133
A.vasorum /Control	Q03252	-0.290412873	0.290412873	0.954921
A.vasorum /Control	Q7Z5L9	-0.059626874	0.059626874	0.954777
A.vasorum /Control	Q04446	-0.139283658	0.139283658	0.95534
A.vasorum /Control	Q9NTM9	-0.077679942	0.077679942	0.956477
A.vasorum /Control	Q9H939	-0.217690562	0.217690562	0.957185
A.vasorum /Control	Q5TH69	-0.027663451	0.027663451	0.958983
A.vasorum /Control	O94901	-0.673548433	0.673548433	0.962269
A.vasorum /Control	P37198	0.052052517	0.052052517	0.962183
A.vasorum /Control	Q9Y3U8	-0.111943326	0.111943326	0.9644
A.vasorum /Control	Q96BQ5	-0.569574272	0.569574272	0.966514
A.vasorum /Control	P35610	-0.299143213	0.299143213	0.968192
A.vasorum /Control	Q00839	-0.102602302	0.102602302	0.971729
A.vasorum /Control	O43826	0.085554796	0.085554796	0.972244
A.vasorum /Control	Q58EX7	-0.204063082	0.204063082	0.972332
A.vasorum /Control	O95674	0.113357724	0.113357724	0.972777
A.vasorum /Control	Q9Y6R0	-0.383554685	0.383554685	0.9728
A.vasorum /Control	P05106	-0.128302363	0.128302363	0.975981
A.vasorum /Control	P05388	-0.073904855	0.073904855	0.976934
A.vasorum /Control	Q5MIZ7	-0.210714405	0.210714405	0.977648
A.vasorum /Control	Q9NWS8	0.10682408	0.10682408	0.977692
A.vasorum /Control	Q96I51	-0.317990282	0.317990282	0.978112
A.vasorum /Control	P42684	-0.082207765	0.082207765	0.978921
A.vasorum /Control	P05161	-0.021952408	0.021952408	0.979158
A.vasorum /Control	Q9ULW0	-0.362316826	0.362316826	0.979557
A.vasorum /Control	Q8WZ82	-0.295891503	0.295891503	0.98018

A.vasorum /Control	Q8N6T3	-0.371680298	0.371680298	0.983193
A.vasorum /Control	Q7Z7A3	-0.136061045	0.136061045	0.984178
A.vasorum /Control	P51956	0.311784192	0.311784192	0.985605
A.vasorum /Control	Q7Z7C8	-1.049253307	1.049253307	0.985477
A.vasorum /Control	P62699	-0.112690682	0.112690682	0.986944
A.vasorum /Control	O75525	-0.338394126	0.338394126	0.987494
A.vasorum /Control	Q8TEY7	0.038972682	0.038972682	0.989056
A.vasorum /Control	O95359	-0.326364876	0.326364876	0.995121
A.vasorum /Control	E9PAV3	-0.164419652	0.164419652	0.998615
A.vasorum /Control	O60239	0.18733128	0.18733128	0.998931
A.vasorum /Control	P17844	-0.086677028	0.086677028	0.999166
A.vasorum /Control	Q5SWX8	-0.282771737	0.282771737	0.99919
A.vasorum /Control	Q9BV81	-0.478685154	0.478685154	0.998734

group. Proteins with an Absolute AVG Lo

Qvalue	# of Ratios	Genes
7.90E-05	12	TFRC
7.90E-05	12	MDK
7.90E-05	12	LRRC32
7.90E-05	12	FTO
7.90E-05	12	ANGPTL2
0.000113	12	PALMD
0.000114	12	PSMB8
0.000114	12	SERPINB2
0.000114	12	PSMC3
0.000114	12	PGM1
0.000115	12	CAT
0.000115	12	CEP85
0.000115	12	DGLUCY
0.000115	12	ARMT1
0.000116	12	UQCRC1
0.000116	12	CAPG
0.000119	12	RIMOC1
0.000119	12	PSME2
0.000119	12	SAMHD1
0.000121	12	ELP3
0.000128	12	CSTF3
0.000128	12	TMCC3
0.000138	12	EEF2K
0.00014	12	TGM2
0.00014	12	EPS15
0.00014	12	B2M
0.00014	12	CYCS
0.00014	12	BICD1
0.00014	12	AP1S2
0.00014	12	GNA13
0.000151	12	PGD
0.000151	12	ST3GAL1
0.000151	12	ABHD10
0.000151	12	TMEM30A
0.000155	12	YWHAZ
0.000155	12	LRRC14
0.000156	12	PAPOLA
0.000156	12	EIF6
0.000156	12	LAMA4
0.000156	12	CARD10
0.000156	12	NXF1
0.000156	12	AK3
0.00016	12	ITGB1
0.00016	12	CD9
0.00016	12	INTS4
0.000164	12	ADGRB3
0.000166	12	TKFC
0.000168	12	COPS7B

0.000168	12	CAVIN2
0.000168	12	ACADM
0.000168	12	PRKAR2B
0.000168	12	CHMP4B
0.000168	12	MMRN2
0.000168	12	YARS2
0.00017	12	NBEAL2
0.000172	12	EHD2
0.000183	12	ANGPT2
0.000183	12	HIP1R
0.000183	12	IDH1
0.000183	12	HLA-B
0.000183	12	GSN
0.000183	12	LAMC1
0.000183	12	ENG
0.000183	12	EIF2AK2
0.000183	12	CSRP1
0.000183	12	POLD1
0.000183	12	GGT5
0.000183	12	CAPZA2
0.000183	12	HNRNPM
0.000183	12	MMRN1
0.000183	12	GANAB
0.000183	12	CAVIN1
0.000183	12	TMEM205
0.000183	12	UBE2R2
0.000183	12	LRRC47
0.000183	12	KCTD12
0.000183	12	VPS11
0.000183	12	EPB41L5
0.000183	12	MRPL40
0.000183	12	AASS
0.000183	12	ADD3
0.000183	12	FAF1
0.000186	12	NNT
0.000186	12	PHKB
0.000188	12	SERPINB1
0.00019	12	ACAA1
0.00019	12	PPAT
0.00019	12	PMVK
0.00019	12	CTSZ
0.000193	12	WARS1
0.000194	12	ERCC2
0.000198	12	PALD1
0.000201	12	DPYD
0.000205	12	RNF214
0.000206	12	RNGTT
0.000211	12	DIP2A
0.000212	12	NRP2
0.000212	12	BNIP1

0.000212	12	SAR1A
0.000213	12	NFIB
0.00022	12	LYVE1
0.000226	12	ATP5PD
0.000226	12	H1-4
0.000226	12	LARP7
0.000226	12	GATD1
0.000226	12	NIPSNAP1
0.000228	12	DIAPH1
0.000228	12	FABP4
0.000228	12	ERAP2
0.000228	12	GIMAP7
0.000228	12	TECR
0.000229	12	HACL1
0.000232	12	UQCRC2
0.000232	12	CDH5
0.000245	12	GUK1
0.000245	12	GIPC2
0.000247	12	TOR1B
0.000247	12	MPZL2
0.000247	12	UQCRFS1
0.000247	12	THOC6
0.000247	12	LENG1
0.000247	12	TNS2
0.000248	12	PIK3C3
0.000248	12	MAGT1
0.000248	12	CTSD
0.000252	12	TIMM17B
0.000252	12	CDC123
0.000252	12	DHFR
0.000252	12	ICAM1
0.000252	12	LAMB1
0.000252	12	ANXA3
0.000252	12	BCKDHA
0.000252	12	MCM3
0.000252	12	NNMT
0.000252	12	EIF1
0.000252	12	RECQL
0.000252	12	ZFP36L2
0.000252	12	IDH3G
0.000252	12	BMX
0.000252	12	WASHC4
0.000252	12	TMEM167A
0.000252	12	SYNE2
0.000252	12	HNRNPUL1
0.000252	12	MSTO1
0.000252	12	TINAGL1
0.000252	12	THG1L
0.000252	12	MTCH1
0.000252	12	VPS18

0.000252	12	EIF2AK4
0.000252	12	CNPY2
0.000252	12	MRPS18C
0.000252	12	PDCD4
0.000252	12	BZW2
0.000257	12	HMGB1
0.000257	12	LOXL2
0.00026	12	ERC1
0.00026	12	CRYBG1
0.000261	12	HSD17B4
0.000266	12	MMUT
0.000266	12	HMGB2
0.000274	12	PARP1
0.000275	12	COG5
0.000276	12	HSPB1
0.000276	12	URB2
0.000276	12	BACE2
0.000278	12	TSPAN15
0.000278	12	GNA11
0.000278	12	FCGRT
0.000281	12	TOM1
0.000281	12	BUB3
0.000281	12	RSU1
0.000281	12	TSEN15
0.000281	12	AAMDC
0.000281	12	MOV10
0.000282	12	YWHAQ
0.000286	12	CLDN5
0.000286	12	FTL
0.000286	12	PSAP
0.000286	12	RIPK1
0.000286	12	RAB12
0.000286	12	PARS2
0.000286	12	DHTKD1
0.000286	12	MTMR10
0.000292	12	SHANK3
0.000292	12	CALHM5
0.000294	12	MRPS33
0.000295	12	APOL3
0.000295	12	MPC2
0.000296	12	NCSTN
0.000299	12	EPHA2
0.000299	12	COMMD3
0.0003	12	ST6GALNAC4
0.0003	12	NID1
0.0003	12	RDX
0.0003	12	SEC61A1
0.0003	12	COASY
0.000301	12	PRPF3
0.000301	12	TACC1

0.000301	12	LAP3
0.000301	12	BAX
0.000301	12	DHRS7B
0.000301	12	KIAA2013
0.000301	12	UPF1
0.000301	12	TOR4A
0.000303	12	FAM118B
0.000305	12	LRRC40
0.000305	12	ATP5MG
0.000305	12	ADAMTS4
0.000307	12	ZYG11B
0.000308	12	VPS29
0.000308	12	ACP2
0.000308	12	AKAP13
0.000309	12	CNOT9
0.000309	12	TBC1D10A
0.000311	12	PECAM1
0.000314	12	CDIPT
0.000314	12	PHGDH
0.000314	12	TRIM16
0.000315	12	CNN3
0.000318	12	APMAP
0.000319	12	TRIOBP
0.00032	12	NAGA
0.00032	12	UBA1
0.00032	12	RPA1
0.00032	12	PPM1F
0.00032	12	SUOX
0.00032	12	RAB4B
0.00032	12	AARS2
0.00032	12	OPTN
0.000324	12	AKR1B1
0.000324	12	STAT6
0.000324	12	CAV1
0.000324	12	MED30
0.000325	12	HSD17B7
0.000325	12	IFI35
0.000325	12	DAGLB
0.000325	12	PLBD2
0.000325	12	DCPS
0.000325	12	OSBPL10
0.00033	12	SLC25A29
0.00033	12	HDAC1
0.000333	12	RAB11A
0.000333	12	SCARB2
0.000333	12	UPF3B
0.000336	12	EIF2D
0.000336	12	ELMO2
0.000337	12	HIP1
0.000349	12	KDR

0.000349	12	PPP2CB
0.000349	12	MRPL10
0.000349	12	ILKAP
0.000349	12	PLSCR4
0.000349	12	AGPAT5
0.000349	12	MAD1L1
0.000356	12	C17orf75
0.000359	12	API5
0.00036	12	USP40
0.000361	12	FAT4
0.000365	12	DLD
0.000365	12	ETFA
0.000365	12	DLGAP4
0.000365	12	SPAST
0.000366	12	PIR
0.000374	12	CNOT11
0.000377	12	CTNNB1
0.000382	12	DNASE2
0.000382	12	NDUFS7
0.000382	12	VWF
0.000382	12	UTRN
0.000382	12	VCP
0.000382	12	HSPG2
0.000382	12	CUL7
0.000382	12	ECHDC1
0.000382	12	SEPTIN10
0.000382	12	FIS1
0.000383	12	SGSH
0.000384	12	PCYOX1L
0.000384	12	GLG1
0.000384	12	ESAM
0.000386	12	NPC2
0.000386	12	CLEC14A
0.000393	12	ERG
0.000395	12	RPGR
0.000396	12	BCAP31
0.000396	12	CASP6
0.000396	12	SIGLEC1
0.000396	12	INTS9
0.000396	12	PUS1
0.000398	12	MAP1LC3B
0.000398	12	CHUK
0.000404	12	HSP90B1
0.000404	12	KCTD5
0.000405	12	PPIH
0.000405	12	ADD1
0.000405	12	ALG11
0.00041	12	METAP2
0.000418	12	CPOX
0.000439	12	NSA2

0.00044	12	JUP
0.00044	12	ANP32A
0.00044	12	SERPINH1
0.00044	12	ACTR3
0.00044	12	STX3
0.00044	12	PON2
0.00044	12	LDLRAP1
0.00044	12	HEBP2
0.000449	12	SLC38A7
0.000453	12	FKBP9
0.000453	12	HTATSF1
0.000454	12	NIPBL
0.000454	12	PXDN
0.000457	12	SERPINE1
0.000457	12	NMT1
0.000457	12	STXBP1
0.000457	12	RTF1
0.000457	12	KLHL11
0.000463	12	HCFC1
0.000471	12	FBN1
0.000471	12	NUP214
0.000471	12	DRG2
0.000471	12	EXOC8
0.000471	12	ABHD14B
0.000481	12	ERCC1
0.000481	12	XRCC5
0.000481	12	ABHD12
0.000481	12	PGM2
0.000481	12	KLC4
0.000481	12	GMPR2
0.000481	12	CEMIP2
0.000481	12	ALDH2
0.000481	12	FH
0.000481	12	HMOX1
0.000481	12	PPIB
0.000481	12	PSMC5
0.000481	12	BST2
0.000481	12	WASHC5
0.000481	12	DECR1
0.000481	12	TST
0.000481	12	PNPLA6
0.000481	12	RAB8B
0.000481	12	HELZ2
0.000481	12	INPP5F
0.000481	12	PPME1
0.000488	12	ATG3
0.00049	12	ATM
0.00049	12	FAR1
0.00049	12	ARAP3
0.000494	12	KANK2

0.000497	12	SPTLC2
0.000499	12	TIE1
0.000499	12	BCL9L
0.000499	12	CTNNBL1
0.000499	12	GIMAP1
0.000499	12	DTX3L
0.000507	12	CPSF2
0.00051	12	PCDH1
0.000512	12	STK10
0.000514	12	PDIA5
0.000514	12	CAMKK2
0.000516	12	KDELR3
0.000516	12	PYGL
0.000516	12	ACE
0.000516	12	COL8A1
0.000516	12	SMARCB1
0.000517	12	ACTR1A
0.000518	12	CUL1
0.000522	12	MT2A
0.000522	12	LTA4H
0.000522	12	FLT1
0.000522	12	PRDX2
0.000522	12	LRBA
0.000522	12	VPS13C
0.000522	12	RAPH1
0.000522	12	HEATR5A
0.000522	12	KIAA0319L
0.000522	12	SCCPDH
0.000528	12	AGRN
0.000528	12	AP1G2
0.000528	12	NDUFV1
0.000528	12	CTSC
0.000528	12	MLEC
0.000528	12	SAMD9
0.000528	12	MON1A
0.000528	12	AFG3L2
0.00053	12	ATP2B1
0.00053	12	REL
0.00053	12	MRC2
0.000536	12	EPN3
0.000537	12	RAC2
0.000538	12	VPS8
0.000539	12	PSMD3
0.000547	12	ENDOG
0.000548	12	CC2D1A
0.000554	12	THEM6
0.000559	12	LANCL1
0.000579	12	YARS1
0.00058	12	RNMT
0.00058	12	RNF213

0.000583	12	TFPI
0.000583	12	NXN
0.000583	12	GNB1L
0.000585	12	GHITM
0.000589	12	EIF3H
0.000589	12	OGA
0.000589	12	LDHA
0.000589	12	ACACA
0.000589	12	PLD1
0.000589	12	RSPRY1
0.000589	12	EXOC2
0.000589	12	FNBP1
0.000599	12	GMFB
0.000603	12	COPB2
0.000605	12	HEXA
0.000606	12	DDX41
0.00061	12	PCCA
0.00061	12	BCAM
0.00061	12	KPNA1
0.00061	12	CUL2
0.00061	12	GALE
0.00061	12	TRAM1
0.00061	12	TMEM245
0.00061	12	STX17
0.000612	12	CTPS2
0.000612	12	HACD3
0.000612	12	ARHGAP31
0.000612	12	COMMD1
0.000617	12	PSME1
0.000618	12	CEP55
0.000618	12	NAA50
0.000618	12	HOMER3
0.000621	12	PPP1R10
0.000626	12	LRRC8C
0.000628	12	FBXL18
0.000628	12	NIT2
0.000628	12	VPS28
0.000628	12	SIAE
0.000632	12	MEF2D
0.000632	12	TSNAX
0.000637	12	PDIA3
0.000638	12	PSMD10
0.000638	12	PPIG
0.000653	12	NELFCD
0.000655	12	SNX2
0.000655	12	CNOT3
0.000655	12	PTMA
0.000655	12	KIF5B
0.000655	12	MLH1
0.000655	12	ME1

0.000655	12	EP300
0.000655	12	SH3D19
0.000655	12	XAB2
0.000655	12	AP2A2
0.000657	12	DPM1
0.000659	12	PDIA4
0.000659	12	TLN1
0.000662	12	HSP90AA1
0.000662	12	FLNA
0.000662	12	GRK2
0.000662	12	CD34
0.000662	12	PHB1
0.000662	12	STAT5A
0.000662	12	FLOT2
0.000662	12	LPCAT2
0.000662	12	SYTL4
0.000662	12	RUVBL1
0.000662	12	DOP1B
0.000662	12	HLA-H
0.000662	12	RFC1
0.000662	12	ACSF3
0.000662	12	MYO6
0.000663	12	AGFG2
0.000663	12	SMARCA4
0.000663	12	MCCC2
0.000664	12	TACSTD2
0.000664	12	LAMP2
0.000664	12	CDC16
0.000664	12	SFXN3
0.000671	12	ENGASE
0.000673	12	FOCAD
0.000676	12	ALDH1A2
0.000676	12	ARL8B
0.000676	12	ARHGAP1
0.000676	12	IGF2BP2
0.000689	12	SDSL
0.00069	12	TJP1
0.000691	12	SUCLA2
0.000691	12	METTL15
0.000691	12	ITGA10
0.000691	12	THBS1
0.000691	12	PCK2
0.000691	12	CHST14
0.000691	12	GIMAP8
0.000693	12	ANAPC4
0.000694	12	RAB3GAP2
0.000695	12	PDCD6
0.000695	12	ILVBL
0.000695	12	XPO1
0.000695	12	EPB41L2

0.000695	12	ZPR1
0.000695	12	HDDC3
0.000695	12	KLHL5
0.000695	12	RUFY1
0.000695	12	ABI2
0.000695	12	BCLAF1
0.000695	12	PPIL1
0.000695	12	ARF5
0.000697	12	IFI16
0.000697	12	TUBA1C
0.0007	12	PIP4K2A
0.0007	12	LPGAT1
0.000702	12	COL18A1
0.00071	12	GPD1L
0.000713	12	FRYL
0.000713	12	ADA
0.000713	12	NDUFB7
0.000714	12	PRKDC
0.000716	12	MCM6
0.000717	12	ALDH7A1
0.000717	12	HDLBP
0.000717	12	PDIA6
0.000717	12	CDC73
0.000717	12	ALKBH5
0.000719	12	UBXN7
0.000719	12	ATP5F1B
0.000719	12	PRKCH
0.000719	12	GBP1
0.000719	12	IDH2
0.000719	12	RBM3
0.000719	12	INTS11
0.000719	12	METTL2B
0.000719	12	MSANTD2
0.000719	12	KTN1
0.000719	12	SMAP1
0.000719	12	SSH3
0.000719	12	ERGIC1
0.000719	12	METTL1
0.00072	12	PACSIN3
0.00072	12	NAPRT
0.000721	12	SLC25A3
0.000725	12	HSD17B2
0.000725	12	LEMD2
0.000728	12	FAM124B
0.00073	12	B4GALT5
0.000736	12	ARHGEF17
0.000737	12	CD47
0.000738	12	RXRB
0.000739	12	ATG5
0.000749	12	TBC1D5

0.000751	12	NDUFA8
0.000754	12	AKR7A2
0.000754	12	XRCC6
0.000754	12	FKBP5
0.000756	12	IARS1
0.000756	12	PEX14
0.000756	12	DOCK4
0.000756	12	SEMA6B
0.000756	12	AGA
0.000756	12	ANKS1A
0.000757	12	EZR
0.000757	12	NQO1
0.000757	12	HADHA
0.000757	12	AKAP12
0.000757	12	CGNL1
0.000757	12	ECH1
0.000757	12	SELENBP1
0.000757	12	CHD4
0.000757	12	NDUFA9
0.000757	12	ARMCX2
0.000757	12	FIG4
0.000757	12	EXT2
0.000757	12	MBOAT7
0.000757	12	SACM1L
0.000757	12	EHD3
0.000757	12	ARHGAP23
0.000757	12	PTGFRN
0.000757	12	USP25
0.000757	12	ACOT9
0.000758	12	FLT4
0.000763	12	PLCB1
0.000764	12	INTS5
0.000766	12	CYRIA
0.000777	12	ETFB
0.000778	12	DYSF
0.000778	12	TMEM11
0.000778	12	SART3
0.000778	12	TMEM259
0.000778	12	CLASP1
0.000778	12	SMPD4
0.000778	12	GLTP
0.000779	12	ZNF706
0.000779	12	GNPAT
0.000779	12	ZMPSTE24
0.000779	12	RPS6KA1
0.000779	12	WDR48
0.000779	12	AP1M1
0.000781	12	NAA25
0.000784	12	TMED5
0.000786	12	SHFL

0.000786	12	MRPL15
0.00079	12	CPPED1
0.000792	12	NUP98
0.000798	12	OSGEP
0.000802	12	LMAN2
0.000803	12	NASP
0.000803	12	CYRIB
0.000803	12	CCS
0.000803	12	FN3K
0.000808	12	OAT
0.00081	12	RPRD2
0.000811	12	KANK3
0.000813	12	CARHSP1
0.000813	12	PML
0.000824	12	CPT1A
0.000825	12	MAP2K2
0.000825	12	SPATA20
0.000827	12	PPP2R5D
0.000827	12	NELFB
0.000828	12	PRKD2
0.000828	12	ADSS2
0.000843	12	HLA-A
0.000844	12	POP4
0.000844	12	FOXC2
0.000845	12	PC
0.000845	12	PPP1CB
0.000848	12	ATP8B1
0.000848	12	EPB41
0.000848	12	TMEM35A
0.000853	12	QSOX2
0.000853	12	PYCR2
0.000853	12	SNX6
0.00086	12	RTCA
0.000865	12	CRYL1
0.000865	12	STRN
0.00087	12	ATP5F1E
0.000871	12	RETSAT
0.000871	12	QPCTL
0.000872	12	ANKRD40
0.000874	12	VPS13A
0.000874	12	TMEM33
0.000874	12	ADPRS
0.000879	12	ATP5PO
0.000879	12	SLC25A35
0.000881	12	GBP2
0.000881	12	CYTH2
0.000885	12	PUM2
0.000887	12	PLIN3
0.000887	12	MYCBP2
0.000887	12	CD40

0.000887	12	PRDX3
0.000887	12	CDC42BPB
0.000888	12	CTNND1
0.000888	12	DNAJB2
0.000889	12	ARL3
0.000889	12	HDGFL2
0.000889	12	CMPK1
0.000889	12	CTBP1
0.000889	12	ATR
0.000889	12	NCKAP1
0.00089	12	CASK
0.000891	12	TTC28
0.000895	12	ASPH
0.000896	12	UFL1
0.000896	12	MAP2
0.000897	12	AP3B1
0.000897	12	EIF1B
0.000897	12	FLOT1
0.000897	12	RAB6A
0.000897	12	TOR1AIP2
0.000897	12	OSTF1
0.000897	12	RNF31
0.000897	12	GMPPA
0.000897	12	COG8
0.000897	12	SCPEP1
0.000897	12	IGF2R
0.000898	12	POGLUT1
0.000898	12	ARL8A
0.000898	12	ARVCF
0.000901	12	GNL1
0.000902	12	PGP
0.000902	12	ABCD3
0.000904	12	UQCRB
0.000907	12	TMEM50A
0.000907	12	MADD
0.000907	12	ARHGEF2
0.000908	12	NBN
0.000908	12	SMC2
0.000908	12	RABL3
0.00091	12	MMP1
0.00091	12	UBE3C
0.00091	12	TRIP11
0.00091	12	TUBGCP3
0.00091	12	EMC3
0.000912	12	SYT1
0.000912	12	GTF2E2
0.000913	12	MED23
0.000914	12	ABLIM1
0.000919	12	SRP68
0.000925	12	EHD4

0.000926	12	AKAP8
0.000926	12	THYN1
0.000928	12	SRP9
0.000928	12	SULT1B1
0.000928	12	CLU
0.000928	12	LAMP1
0.000928	12	ARSA
0.000928	12	IREB2
0.000928	12	TXNDC5
0.000929	12	CASP1
0.00093	12	MARCKSL1
0.00093	12	CDH13
0.00093	12	RAB10
0.00093	12	CCDC93
0.00093	12	HID1
0.000931	12	LYPLA1
0.000932	12	RPL35A
0.000937	12	AIFM1
0.000937	12	MGAT1
0.000938	12	SNX3
0.000938	12	HUWE1
0.000939	12	NCOR1
0.000939	12	DDB2
0.00094	12	TPP1
0.00094	12	SYNM
0.00094	12	TRMT5
0.00094	12	NUDT16
0.00094	12	HELLS
0.000943	12	MET
0.000944	12	STX6
0.000944	12	P3H3
0.000944	12	FLYWCH2
0.000944	12	ACSS2
0.000945	12	FABP5
0.000946	12	PDXP
0.000951	12	ARHGAP10
0.000954	12	SEC61A2
0.000954	12	XPNPEP1
0.000954	12	DNAJB4
0.000958	12	DCUN1D5
0.000958	12	ERH
0.000966	12	ACO2
0.000967	12	SDF2L1
0.000967	12	MTUS1
0.000969	12	HERC1
0.000969	12	TPRG1L
0.000972	12	STAT3
0.000972	12	REEP5
0.000972	12	KARS1
0.000972	12	TERF2

0.000972	12	CBLL1
0.000972	12	ABHD4
0.000972	12	IWS1
0.000972	12	POMK
0.000972	12	SLX9
0.000972	12	TRAPPC8
0.000972	12	DERA
0.000978	12	NLN
0.00098	12	APP
0.000982	12	YLPM1
0.000982	12	DYNC1H1
0.000982	12	KIAA1143
0.000985	12	FERMT2
0.000989	12	TAL1
0.00099	12	TOMM70
0.00099	12	HNRNPD
0.001	12	RBBP5
0.001002	12	DDX19A
0.001004	12	PHKG2
0.001007	12	HS1BP3
0.001009	12	SDHB
0.00101	12	MAP4K2
0.001014	12	RDH14
0.001014	12	NSMCE4A
0.001015	12	WFS1
0.001015	12	ADSL
0.001015	12	SH2B1
0.001015	12	RRBP1
0.001015	12	NAGK
0.001015	12	SH2B3
0.001017	12	PRKCA
0.001017	12	SEPHS1
0.001017	12	EPHB4
0.001017	12	EXOC7
0.001017	12	CHKB
0.001017	12	HBS1L
0.00102	12	IMMT
0.001021	12	GXYLT1
0.001022	12	CDC23
0.001023	12	NECTIN2
0.001025	12	TXNRD1
0.001033	12	MYO1C
0.001033	12	RASIP1
0.001033	12	PSMD1
0.001033	12	RTRAF
0.001035	12	ASMTL
0.001037	12	AIP
0.001038	12	PPP2CA
0.00104	12	HSPD1
0.00104	12	CRAT

0.00104	12	GASK1B
0.00104	12	UPF2
0.00104	12	GBA1
0.001041	12	STON1
0.001042	12	POGLUT3
0.001043	12	MYL9
0.001043	12	SERPINB6
0.001043	12	ACTR2
0.001043	12	MRPS11
0.001043	12	RAF1
0.001043	12	CYP2S1
0.001048	12	MOGS
0.001048	12	DOK1
0.001049	12	POR
0.001049	12	DHX38
0.001049	12	EPN1
0.001054	12	DIS3L2
0.00106	12	RBBP6
0.00106	12	ZNF622
0.001063	12	P3H4
0.001063	12	COL12A1
0.001063	12	C7orf25
0.001064	12	SCD
0.001064	12	GPN1
0.001065	12	SRSF2
0.001065	12	GIMAP4
0.00107	12	GALNT1
0.00107	12	CIAO2A
0.00107	12	NISCH
0.001077	12	CHMP4A
0.00108	12	CAPZB
0.001081	12	KDM1A
0.001081	12	BGN
0.001081	12	ALDH4A1
0.001081	12	STIP1
0.001081	12	PTK7
0.001081	12	EIF2B1
0.001081	12	TOR2A
0.001081	12	RAVER1
0.001081	12	WDR13
0.001081	12	NDRG3
0.001082	12	GMFG
0.001082	12	NHERF2
0.001082	12	THEMIS2
0.001082	12	TAF2
0.001082	12	TRMT2A
0.001082	12	TIMMDC1
0.001091	12	HSPA8
0.001091	12	NELFE
0.001091	12	TIA1

0.001091	12	TBC1D9B
0.001091	12	PGM2L1
0.001091	12	RETREG2
0.001091	12	LXN
0.001091	12	RCC2
0.001091	12	VPS51
0.001095	12	ITPR2
0.001095	12	ATP13A1
0.001095	12	POGK
0.001095	12	STRN3
0.001098	12	GPSM1
0.001098	12	GGH
0.001098	12	PPP3CA
0.001103	12	RWDD1
0.001107	12	CYFIP1
0.001111	12	IDE
0.001111	12	EEA1
0.001113	12	SEC23IP
0.001114	12	FAM210A
0.001117	12	SRP14
0.001119	12	MYO1B
0.001119	12	SSB
0.001119	12	ANXA6
0.001119	12	MTHFD1
0.001119	12	HPCAL1
0.001119	12	SMAD1
0.001119	12	ARMCX1
0.001119	12	PFDN2
0.001121	12	CEP41
0.001122	12	COX4I1
0.001122	12	STAB1
0.001122	12	GTPBP4
0.001122	12	UGGT1
0.001122	12	POFUT2
0.001138	12	TBC1D22B
0.001142	12	GIT2
0.001142	12	GALM
0.001143	12	PELO
0.001145	12	GSDMD
0.001146	12	PPP6R3
0.001151	12	F11R
0.001158	12	DENND3
0.001159	12	HSP90AB1
0.001159	12	GDF15
0.00116	12	CRIP2
0.00116	12	SLC44A2
0.00116	12	ATXN7L3B
0.00116	12	ARHGEF28
0.001168	12	NUCB2
0.001168	12	MRPS26

0.001168	12	EXOSC3
0.001174	12	CD63
0.001174	12	HAUS6
0.001174	12	PARVA
0.001178	12	OSGEPL1
0.001178	12	YWHAE
0.001178	12	H1-1
0.001178	12	DOCK1
0.00118	12	LNPEP
0.001185	12	MAPK1
0.001186	12	MESD
0.001188	12	MRPS27
0.001189	12	DCTN4
0.001191	12	PPOX
0.001191	12	NUP205
0.001191	12	TNPO3
0.001194	12	PRPF38B
0.001195	12	TAGLN
0.001198	12	PRCP
0.001199	12	LDB2
0.0012	12	MED1
0.0012	12	GPHN
0.001202	12	EML1
0.001202	12	MTA1
0.001202	12	UBR4
0.001202	12	LCLAT1
0.001202	12	FKBP10
0.001202	12	RNPEP
0.001205	12	UGP2
0.001205	12	GLOD4
0.00121	12	EDEM3
0.001211	12	PUF60
0.001217	12	MSH2
0.001218	12	ERMP1
0.001218	12	ABCA3
0.001222	12	DCTN5
0.001222	12	RPS5
0.001222	12	QNG1
0.001222	12	GSTCD
0.001222	12	FKBP7
0.001224	12	FARSA
0.001225	12	UQCR10
0.001225	12	SPRYD4
0.001225	12	CWC22
0.001227	12	PSAT1
0.001233	12	PTPN23
0.001235	12	RIOX2
0.001237	12	NFXL1
0.001237	12	PITHD1
0.001237	12	NMRAL1

0.001243	12	CLTB
0.001243	12	LIPA
0.001243	12	USO1
0.001243	12	JADE3
0.001243	12	KLHL4
0.001248	12	SORBS2
0.00125	12	SRI
0.001256	12	ACADSB
0.001257	12	HLA-E
0.001263	12	ECE1
0.001265	12	MPI
0.001265	12	ISOC2
0.001266	12	ZZEF1
0.001266	12	AVL9
0.001266	12	POLDIP2
0.001268	12	GTF2I
0.001271	12	CRLF3
0.001271	12	GPX7
0.001271	12	IRAK4
0.001271	12	NAA10
0.001272	12	KAT7
0.001275	12	GPX4
0.001275	12	PI4KA
0.001277	12	DOCK6
0.001277	12	COG4
0.001277	12	STOML2
0.001281	12	PYCARD
0.001282	12	TMED10
0.001282	12	PDCL
0.001282	12	DHRS4
0.001282	12	TMEM126A
0.001283	12	OXSR1
0.001283	12	ICAM2
0.001283	12	TAP2
0.001286	12	MICAL2
0.001287	12	SMARCD3
0.001287	12	PAXX
0.001287	12	PSMB9
0.001288	12	GOLT1B
0.00129	12	NUP160
0.00129	12	INTS10
0.001297	12	ITFG1
0.001299	12	LPCAT3
0.001307	12	RPA3
0.001307	12	RFC3
0.001313	12	CCDC97
0.001315	12	HADH
0.001315	12	NUDT21
0.001317	12	DARS1
0.001317	12	TRAM2

0.001317	12	ANAPC1
0.001318	12	COPG2
0.00132	12	DBT
0.00132	12	CCDC6
0.00132	12	TRUB1
0.00132	12	COMMD10
0.001322	12	ETV6
0.001322	12	GBA2
0.001325	12	INPP4A
0.001325	12	TMEM160
0.001326	12	SMS
0.001328	12	SORBS3
0.00133	12	SLC4A2
0.00133	12	HMGCL
0.00133	12	TRAPPC4
0.001332	12	PCM1
0.001332	12	TMEM88
0.001332	12	PRKAB1
0.001335	12	CYP27A1
0.001343	12	GTF3C5
0.001344	12	DAD1
0.001346	12	MRPS25
0.001351	12	NDUFS3
0.001351	12	CCZ1B
0.001356	12	NARS2
0.001356	12	TBC1D13
0.001363	12	HACD2
0.001364	12	PPP2R2A
0.001364	12	PPCS
0.001369	12	BCKDK
0.001369	12	NFKB1
0.001369	12	PFKP
0.00137	12	MRPL43
0.001377	12	MAGI1
0.001382	12	ACLY
0.001382	12	COPB1
0.001382	12	LBR
0.001382	12	YIPF3
0.001382	12	ASF1B
0.001382	12	WASF2
0.001384	12	WASL
0.001384	12	DCTD
0.001384	12	ATXN3
0.001384	12	ALDH16A1
0.001384	12	DUSP12
0.001396	12	WDR19
0.001403	12	DUT
0.001405	12	NUP37
0.001416	12	UBE2D3
0.001416	12	C11orf98

0.001422	12	IRGQ
0.001428	12	OXCT1
0.001428	12	ADAM17
0.001428	12	DENND4C
0.001428	12	TANGO2
0.001428	12	DARS2
0.001428	12	PRXL2B
0.001428	12	CLPTM1L
0.001428	12	PCIF1
0.001428	12	YTHDC2
0.001428	12	PICK1
0.001428	12	NOVA2
0.001428	12	UBR5
0.001428	12	ROCK1
0.001428	12	APOL2
0.001438	12	RANBP2
0.001438	12	NIT1
0.001438	12	KIF15
0.001438	12	CDK2
0.001442	12	MAN2C1
0.001442	12	ARHGEF15
0.001442	12	ATE1
0.001442	12	RASSF2
0.001442	12	GEMIN4
0.001442	12	NUP107
0.001442	12	KPNB1
0.001442	12	POLE3
0.001447	12	MTMR14
0.001451	12	CCDC186
0.001452	12	VPS25
0.001456	12	NVL
0.001456	12	PTPRF
0.001456	12	TMPO
0.001456	12	HSPA14
0.001456	12	CUL4A
0.001456	12	DYM
0.001456	12	SYVN1
0.001456	12	COMMD2
0.001456	12	NMD3
0.001456	12	NT5DC2
0.001456	12	SLC17A5
0.001456	12	APPL1
0.001457	12	NDUFV2
0.001457	12	ALDH1A1
0.001457	12	SREK1
0.001457	12	TDP1
0.001458	12	DIABLO
0.001458	12	HECW2
0.001459	12	SCAMP2
0.001459	12	CTBP2

0.001462	12	DNAAF5
0.001464	12	LPCAT1
0.001465	12	CDKAL1
0.001465	12	ADAM15
0.001467	12	NUDT4
0.001468	12	PRKRA
0.001468	12	EHHADH
0.001468	12	TJAP1
0.00147	12	MRPS12
0.001474	12	HTT
0.001478	12	DLGAP5
0.001478	12	ARHGAP17
0.001478	12	ZNF281
0.001479	12	GUSB
0.001479	12	EXOC6
0.001479	12	HSPH1
0.001479	12	LSG1
0.001482	12	ARHGDIB
0.001482	12	NCBP1
0.001482	12	LRRFIP1
0.001482	12	TMEM131
0.001482	12	WDR37
0.001486	12	ERP44
0.001487	12	BCAT2
0.001487	12	PSMD4
0.001488	12	CRYZ
0.001488	12	SUMF2
0.001488	12	VANGL1
0.001488	12	HNRNPLL
0.001488	12	COG3
0.001488	12	MYO10
0.001494	12	KIF2A
0.001496	12	C2orf49
0.001498	12	MTA3
0.001499	12	TEX10
0.001501	12	COPA
0.001503	12	CNDP2
0.001505	12	ATP5PB
0.001508	12	INPP1
0.001516	12	AP3D1
0.001516	12	UMPS
0.001518	12	CANX
0.001518	12	FNTA
0.001518	12	NUBP1
0.001523	12	ABCD4
0.001523	12	GSDME
0.001523	12	ELOC
0.001523	12	PKN2
0.001523	12	GLE1
0.001523	12	UBE2Q1

0.001523	12	DPP9
0.001523	12	PHC2
0.001523	12	ERGIC2
0.001523	12	DOCK9
0.001523	12	VPS16
0.001524	12	CCN2
0.001524	12	CDK9
0.001524	12	VPS33B
0.001528	12	SQOR
0.001536	12	KBTBD2
0.001537	12	KYAT3
0.001553	12	FAM111A
0.001566	12	NDUFAF1
0.001568	12	PBRM1
0.001572	12	OSBPL11
0.001573	12	RAC1
0.001577	12	DIDO1
0.001577	12	SCRN3
0.001578	12	ENTPD1
0.001578	12	USP9X
0.001579	12	DNAJB6
0.001584	12	VRK3
0.001587	12	EP400
0.001588	12	DBR1
0.001592	12	HSPA1L
0.001592	12	STXBP3
0.001592	12	RAB3D
0.001592	12	ALG6
0.001594	12	KPNA2
0.001594	12	RAPGEF1
0.001594	12	CERS2
0.001594	12	UBA6
0.001595	12	VBP1
0.001597	12	EXOC4
0.001604	12	RPS6KA4
0.001604	12	STT3A
0.001604	12	RBBP7
0.001614	12	AP2M1
0.001614	12	RBM27
0.001615	12	IGF1R
0.001615	12	TPT1
0.001615	12	CTU2
0.001615	12	EDC4
0.001615	12	XPO4
0.001621	12	CRELD2
0.001622	12	TRIM22
0.001633	12	CAPNS1
0.001633	12	ZNF318
0.001643	12	SMARCE1
0.001644	12	VPS4A

0.001646	12	ELMOD2
0.001647	12	PDHB
0.001647	12	GORASP2
0.001651	12	ISYNA1
0.001657	12	ABR
0.001657	12	SNX1
0.001658	12	SH3BGRL
0.001658	12	SEC11A
0.001658	12	MAPK7
0.001658	12	NDUFA13
0.001663	12	RCN1
0.001667	12	SFSWAP
0.001668	12	RAE1
0.001668	12	ZCCHC8
0.001668	12	PPIL4
0.001668	12	ACSF2
0.001669	12	VPS52
0.001669	12	CBR4
0.001669	12	RNF25
0.001669	12	DAZAP1
0.001672	12	DNPEP
0.001674	12	CHP1
0.001676	12	ECSIT
0.001676	12	MRPL47
0.001685	12	ERLIN1
0.001685	12	UCHL3
0.001685	12	NDUFS1
0.001685	12	CRKL
0.00169	12	PSMF1
0.001691	12	RAB5B
0.001696	12	RPSA
0.001705	12	ARL2
0.001705	12	RANGAP1
0.001706	12	EML3
0.001709	12	PFKM
0.001709	12	USP11
0.001709	12	RAB8A
0.001716	12	YIPF5
0.00172	12	B3GALT6
0.001733	12	QRICH1
0.001733	12	VIRMA
0.001733	12	PIEZO1
0.001735	12	RP2
0.001747	12	HARS2
0.001748	12	AARS1
0.001753	12	BIN1
0.001753	12	DCUN1D1
0.001754	12	PSMA7
0.001766	12	RANBP10
0.001771	12	IVD

0.001771	12	TMSB4X
0.001778	12	MTHFR
0.001781	12	LMNA
0.001785	12	SLC25A11
0.001791	12	EXOC1
0.001795	12	PREX1
0.001795	12	UTP11
0.0018	12	ANP32B
0.001801	12	AP1B1
0.001802	12	AUH
0.001807	12	PPP1R12A
0.001807	12	GRN
0.001807	12	TRABD
0.001808	12	DCHS1
0.001809	12	CD109
0.001814	12	COMMD5
0.001816	12	TM9SF2
0.001817	12	ATP5F1A
0.001823	12	ARMC8
0.001823	12	HAT1
0.001823	12	PTDSS1
0.001823	12	WDR81
0.001823	12	KIF13A
0.001826	12	SMARCA1
0.001826	12	GPD2
0.001827	12	MAPK13
0.001827	12	COLGALT1
0.001828	12	GMPR
0.001833	12	THSD4
0.001833	12	RAPGEF2
0.001836	12	MAPK3
0.001836	12	HMOX2
0.001836	12	RPS8
0.001836	12	DENND4A
0.001836	12	ERBIN
0.001836	12	RAB22A
0.001837	12	CLPX
0.001837	12	RAP1B
0.001837	12	PSPH
0.001837	12	RIC8B
0.001838	12	PLAA
0.001841	12	SCP2
0.001844	12	BLVRA
0.001847	12	LIG3
0.001856	12	EXOC5
0.001856	12	NDUFA4
0.001856	12	RPL19
0.001863	12	ELOVL5
0.001869	12	ENAH
0.00187	12	GTF2B

0.001872	12	POLR2C
0.001877	12	PPP6C
0.00188	12	NBAS
0.001889	12	NUMA1
0.00189	12	DNAJC8
0.001892	12	PPP2R2D
0.001892	12	DNAJC7
0.001892	12	PRIM1
0.001892	12	NEK9
0.001898	12	PAF1
0.001898	12	TPP2
0.001898	12	ATIC
0.001898	12	NaN
0.001898	12	CCDC9
0.001901	12	PVR
0.001901	12	RMC1
0.001901	12	IPO11
0.00191	12	PPP5C
0.001911	12	PSMD13
0.001914	12	XPOT
0.001915	12	SRSF11
0.001922	12	CTHRC1
0.001922	12	ACOT13
0.001923	12	HSD17B11
0.001923	12	SRP72
0.001923	12	PTPRB
0.001923	12	PPP3CC
0.001923	12	AFDN
0.001923	12	NTHL1
0.001925	12	NSF
0.001925	12	WDR43
0.001925	12	TBC1D10B
0.001925	12	BCL7C
0.001925	12	PRPF4
0.00193	12	TRIM25
0.001931	12	PDP1
0.001931	12	TXLNA
0.001938	12	SOD2
0.001938	12	GOT1
0.001938	12	REPIN1
0.001944	12	RUFY2
0.00195	12	DCTN3
0.00195	12	ECHS1
0.00195	12	SENP3
0.001953	12	STAG2
0.001958	12	GAPVD1
0.001963	12	NTN4
0.001982	12	NUDT2
0.001983	12	DDX46
0.001988	12	RPRD1A

0.00199	12	OPA1
0.00199	12	HSPE1
0.00199	12	PPIL2
0.00199	12	TBK1
0.00199	12	PRKAG2
0.001995	12	CD81
0.001996	12	AP2S1
0.001999	12	SELENOS
0.002004	12	ALS2
0.00201	12	C19orf47
0.002012	12	YWHAH
0.002012	12	SCFD1
0.002012	12	TMX4
0.002018	12	NAXE
0.002019	12	CAND1
0.002019	12	NCOA7
0.002019	12	UBE2E3
0.002032	12	MTM1
0.002038	12	SRPK2
0.002038	12	MRPS35
0.002038	12	NCAPH
0.002045	12	CASC3
0.002045	12	TXNL1
0.002045	12	ZNF638
0.002045	12	VPS45
0.002046	12	AAGAB
0.002046	12	COG1
0.002053	12	VTI1B
0.002056	12	POLR3D
0.002057	12	DDAH1
0.002058	12	MPG
0.002061	12	KATNA1
0.002061	12	IMPA1
0.002061	12	ERCC6
0.002061	12	TBC1D15
0.002061	12	PLXND1
0.002061	12	TSC2
0.002062	12	TFAM
0.002062	12	COPS5
0.002062	12	NRDC
0.002062	12	P3H1
0.002062	12	MRPS31
0.002072	12	NDUFB10
0.002072	12	CD93
0.002079	12	ETHE1
0.002088	12	PLOD3
0.002088	12	THNSL1
0.002095	12	SCAMP1
0.002095	12	ACAA2
0.002095	12	SIL1

0.002095	12	PRPF19
0.002099	12	TP53I3
0.002099	12	PTGES2
0.002099	12	OLA1
0.002099	12	RAB3B
0.002099	12	RFC4
0.002099	12	GINS1
0.002099	12	TAPT1
0.002099	12	DPP7
0.002102	12	TNFRSF10C
0.00211	12	PTPRA
0.00211	12	LIG1
0.00211	12	CUL5
0.00211	12	LRRC59
0.00211	12	DECR2
0.002114	12	STAT1
0.002126	12	SLC6A6
0.002128	12	PAAF1
0.002129	12	SLC4A7
0.002135	12	RALGAPA2
0.002136	12	ALDOC
0.002137	12	PKN1
0.002141	12	GAA
0.002141	12	CHD3
0.002141	12	FAM241A
0.002152	12	EEF1E1
0.002154	12	SRPK1
0.002155	12	RMDN1
0.002157	12	CCNY
0.002157	12	INPP5K
0.002157	12	NIBAN1
0.002159	12	NOTCH1
0.00216	12	EMSY
0.00216	12	OGDH
0.002164	12	SCARA3
0.002164	12	IDH3B
0.002164	12	FGD5
0.002164	12	MLKL
0.002167	12	TAB1
0.002167	12	PEAK1
0.002175	12	UCHL5
0.002175	12	TECPR2
0.002177	12	ME2
0.002177	12	PIN4
0.002186	12	STYX
0.002188	12	MYO1D
0.002188	12	PSMC1
0.002192	12	NRP1
0.002192	12	XRCC1
0.002192	12	PHACTR4

0.002192	12	TRRAP
0.002193	12	OAS3
0.002194	12	CTDP1
0.002198	12	PIGT
0.002199	12	PARP9
0.002199	12	NAT14
0.0022	12	SRPX
0.002204	12	UBA2
0.002207	12	BABAM2
0.002208	12	UBE4A
0.002208	12	UAP1
0.002208	12	GLYR1
0.002209	12	NUDT5
0.00221	12	ROCK2
0.00221	12	MGLL
0.00221	12	JAM3
0.00221	12	ST13
0.00221	12	GOLPH3L
0.00221	12	TFIP11
0.002213	12	SBF1
0.002213	12	RPL37A
0.002213	12	RABEP1
0.002215	12	PIK3R1
0.002218	12	MFN1
0.002223	12	MCM5
0.002226	12	GCC2
0.002226	12	CIAO3
0.002233	12	RIC8A
0.002237	12	SCRIB
0.002237	12	FSCN1
0.002237	12	NUP85
0.002237	12	WASHC3
0.00224	12	ELP1
0.00224	12	ZNF512
0.00224	12	STAU2
0.00224	12	CORO1B
0.002247	12	TUBB
0.002247	12	CTTN
0.00225	12	KDELR1
0.002255	12	MGP
0.002256	12	DNAJC3
0.002256	12	DNAJC9
0.002277	12	PSMD6
0.002281	12	DHRSX
0.002282	12	CIC
0.002282	12	BOLA2
0.002284	12	CPD
0.002284	12	SELENOH
0.002284	12	PPA2
0.0023	12	IKBKB

0.0023	12	TMED1
0.00231	12	FTH1
0.002322	12	STX8
0.002323	12	S100A6
0.002323	12	YWHAB
0.00234	12	ACADVL
0.002344	12	BAP18
0.00235	12	TRIM11
0.00235	12	VPS35
0.00235	12	EHMT1
0.002352	12	PACS1
0.002361	12	TBC1D22A
0.002363	12	ORMDL3
0.002367	12	TAX1BP3
0.002369	12	NAA35
0.002375	12	GIPC1
0.002375	12	HSPA12B
0.002375	12	LRWD1
0.002383	12	TBC1D9
0.002385	12	ATP2A2
0.002385	12	TTLL12
0.002385	12	UHRF1
0.002385	12	WIPI2
0.00239	12	PRPSAP2
0.00239	12	USP48
0.002402	12	CENPE
0.002407	12	WIPF1
0.002407	12	ASNS
0.002407	12	PRPSAP1
0.002407	12	TRIP12
0.002407	12	FKBP15
0.002407	12	ARHGAP21
0.002409	12	CTSA
0.00241	12	SIGMAR1
0.00241	12	USE1
0.002423	12	RABGGTA
0.002424	12	PTPN2
0.002425	12	RAB11FIP5
0.002426	12	SMNDC1
0.002426	12	DHX8
0.002426	12	MED20
0.002427	12	PLSCR3
0.00244	12	ACTR1B
0.002446	12	INPP5D
0.002449	12	PPP1R21
0.002449	12	TP53RK
0.002452	12	MPST
0.002458	12	PDPR
0.002458	12	DNAJB11
0.002458	12	ACP1

0.002458	12	HYOU1
0.002467	12	NAA16
0.002467	12	INTS6
0.002471	12	PEX11B
0.002472	12	PNKP
0.002472	12	RBM14
0.002477	12	TOR1A
0.002479	12	NDUFA12
0.002484	12	CHMP1B
0.002486	12	SEC24D
0.002486	12	BRAP
0.002486	12	ZNF609
0.002486	12	HECTD1
0.00249	12	DHCR7
0.002494	12	NAT1
0.002496	12	VPS26C
0.0025	12	STMN2
0.002509	12	ELP2
0.002509	12	NPM3
0.002509	12	SUCLG2
0.002511	12	NUCB1
0.002525	12	PNPT1
0.002533	12	AP3M2
0.002533	12	RAB1B
0.002534	12	GNB1
0.002559	12	LMF2
0.002561	12	MCCC1
0.002561	12	RNF14
0.002562	12	CKAP4
0.002562	12	SETD3
0.002562	12	MTFP1
0.002566	12	MMTAG2
0.002566	12	SAE1
0.002567	12	NCL
0.002567	12	RPS7
0.002567	12	MYLK
0.002567	12	THADA
0.002567	12	PROCR
0.002571	12	RRAGC
0.002581	12	EIF3J
0.002581	12	DCTN1
0.002582	12	KRAS
0.002582	12	CARD19
0.002587	12	RPN1
0.002589	12	PSMB2
0.00259	12	THUMPD3
0.002595	12	BDH2
0.002596	12	RALB
0.002601	12	GLMN
0.002605	12	RAD50

0.002606	12	SDHA
0.002606	12	IQGAP1
0.002606	12	TEP1
0.002609	12	ALDH3A2
0.002609	12	HDAC7
0.002612	12	MRPL55
0.002612	12	PLIN2
0.002613	12	FLI1
0.002614	12	GRK6
0.002614	12	NME3
0.00262	12	PTPA
0.00262	12	TMA16
0.002629	12	RPAP3
0.002631	12	RPS6KA5
0.002631	12	RPP25
0.002634	12	PIK3C2A
0.002635	12	PMPCA
0.002637	12	RNF40
0.002637	12	PRDX5
0.002637	12	ERF
0.002637	12	SARS2
0.002637	12	THRAP3
0.002643	12	TBCC
0.002643	12	NIFK
0.002644	12	BROX
0.002645	12	NAGLU
0.002651	12	TTK
0.002651	12	CHMP1A
0.002656	12	MAGED2
0.002656	12	MAP2K6
0.002658	12	CDH2
0.002658	12	STK4
0.002658	12	AACS
0.002658	12	FN3KRP
0.002659	12	KIF1C
0.002669	12	EOGT
0.002671	12	EEFSEC
0.002671	12	GINS4
0.002675	12	HIRIP3
0.002676	12	EARS2
0.002677	12	RNF20
0.00269	12	MRPL21
0.002698	12	SULT1A1
0.002698	12	NUTF2
0.002699	12	GDI1
0.002704	12	DCAF8
0.002705	12	EXOSC4
0.002707	12	USP14
0.002707	12	GIMAP6
0.002713	12	EXOSC7

0.002714	12	ADNP
0.002714	12	UBE2L3
0.002721	12	NUP188
0.002721	12	NLRP1
0.002721	12	DCAF13
0.002724	12	CTIF
0.002724	12	NDUFA7
0.002724	12	FRG1
0.002724	12	NUP54
0.002725	12	TSN
0.002725	12	MOSPD2
0.002728	12	ARSB
0.002728	12	EMD
0.002732	12	OSBP
0.002732	12	LSS
0.002735	12	CBFB
0.002748	12	FOXRED1
0.002755	12	PRPF39
0.002769	12	PFKL
0.002769	12	PIKFYVE
0.00277	12	ASL
0.002775	12	OSBP2
0.002778	12	PPP2R5C
0.002779	12	POLR1G
0.002779	12	ARHGEF7
0.002794	12	CD59
0.002794	12	U2AF1
0.002795	12	SUGP1
0.002801	12	LMAN1
0.002801	12	PURA
0.002804	12	MYZAP
0.002804	12	UBP1
0.002831	12	NDUFAF7
0.002832	12	BRAT1
0.002833	12	UBTF
0.002838	12	SQLE
0.00284	12	RPL4
0.002842	12	LACTB
0.002843	12	HIF1AN
0.002843	12	CGGBP1
0.002852	12	EIF1AX
0.002852	12	CAMK2D
0.002854	12	RBM25
0.002856	12	VTA1
0.002858	12	SARS1
0.002865	12	TRIP6
0.002866	12	SETD7
0.002868	12	ANAPC7
0.002871	12	GSTM2
0.002871	12	WDR5

0.002871	12	MAP3K11
0.002871	12	NECAP2
0.002874	12	AKTIP
0.002875	12	PHF23
0.002879	12	DTYMK
0.002879	12	NIF3L1
0.002879	12	RBCK1
0.002879	12	FSD1
0.002883	12	KCTD10
0.002883	12	MED17
0.002889	12	UBXN4
0.002889	12	SMC3
0.002889	12	COPG1
0.002903	12	STAM2
0.002903	12	VDAC2
0.002903	12	DYNLRB1
0.00291	12	DDR2
0.00291	12	RHOT1
0.002913	12	FHIP2B
0.002917	12	RPL12
0.002917	12	SCAF1
0.002928	12	RTL8C
0.00293	12	LPXN
0.00293	12	RABL6
0.00293	12	ATG9A
0.002934	12	PPP4R1
0.002934	12	ARL5B
0.002934	12	ITGA3
0.002937	12	NMI
0.002937	12	RELCH
0.002937	12	PCCB
0.002937	12	CISD2
0.002943	12	PRXL2A
0.00295	12	NCKIPSD
0.002952	12	CLIC1
0.002952	12	TANC1
0.002952	12	DIP2B
0.002955	12	SYMPK
0.002958	12	PDLIM4
0.002959	12	GIGYF2
0.002963	12	CCT5
0.00297	12	RND3
0.002971	12	EMC4
0.002972	12	CHAMP1
0.002994	12	NFATC2
0.002999	12	WNK1
0.003003	12	WIZ
0.003013	12	SPTY2D1
0.003021	12	ARHGEF12
0.003036	12	CD44

0.003036	12	AAR2
0.003036	12	GAK
0.003036	12	MAPK12
0.003036	12	CNOT1
0.003036	12	ATAD3A
0.003036	12	MTPAP
0.003038	12	PCF11
0.00304	12	HP1BP3
0.003044	12	ABHD16A
0.003044	12	TSG101
0.003046	12	LAMA5
0.003047	12	CAPN1
0.003049	12	GART
0.003058	12	RBM34
0.003059	12	ANXA5
0.003068	12	HGSNAT
0.00307	12	ARPIN
0.003071	12	C11orf68
0.003071	12	AKAP9
0.003072	12	NDC1
0.003073	12	SLC16A3
0.003073	12	LCMT1
0.003073	12	SHTN1
0.003073	12	U2SURP
0.003073	12	PLCG2
0.003084	12	H6PD
0.003084	12	GNS
0.003084	12	AGK
0.003084	12	FAM114A2
0.003084	12	HLA-C
0.003084	12	NUDCD1
0.003089	12	ZNG1F
0.00309	12	RPF1
0.003091	12	RGS3
0.003091	12	SH3TC1
0.003091	12	ACBD3
0.003092	12	VPS35L
0.003094	12	MRPL9
0.003096	12	TMEM214
0.003096	12	FRMD6
0.0031	12	UBXN1
0.0031	12	GULP1
0.0031	12	TRAPPC1
0.003126	12	FADS2
0.003126	12	MDH1
0.003145	12	PRPF40A
0.003146	12	SRGAP2
0.003146	12	ATP6V1G1
0.003154	12	SEPTIN8
0.003154	12	SAR1B

0.003155	12	PSMB4
0.003156	12	CYP20A1
0.003156	12	CDV3
0.003156	12	ZW10
0.003156	12	ALDH6A1
0.003157	12	GSK3B
0.003157	12	ARHGAP18
0.003157	12	MED16
0.003164	12	COX6C
0.003164	12	MRE11
0.003166	12	SASH1
0.003174	12	PDE12
0.003177	12	POLR2A
0.00318	12	COPZ1
0.00318	12	BIN3
0.003189	12	NCAPD2
0.003189	12	TRIP13
0.00319	12	TRIM56
0.00319	12	SLC43A3
0.0032	12	SAMD4B
0.003201	12	DDX20
0.003212	12	ACSL3
0.003212	12	MGST2
0.003212	12	NDRG4
0.003214	12	NDUFA2
0.003224	12	ARAP1
0.003226	12	DFFA
0.003226	12	YIF1A
0.003226	12	SLC27A4
0.003226	12	LAS1L
0.003227	12	SZRD1
0.003227	12	ANKFY1
0.003229	12	TMOD3
0.003232	12	ZBED1
0.003232	12	DRG1
0.003239	12	MTCL2
0.003239	12	ARF6
0.00324	12	BCR
0.003242	12	RICTOR
0.003242	12	KDM3B
0.003242	12	UFD1
0.003245	12	MYO1A
0.003248	12	SPAG9
0.003248	12	RPS26
0.003248	12	FNBP1L
0.003248	12	KLF12
0.003248	12	ROBO1
0.003257	12	ALG5
0.003264	12	XRN2
0.003268	12	SETMAR

0.003269	12	PCLAF
0.003269	12	TRMT10C
0.003274	12	RNASEH2A
0.003274	12	MTIF2
0.003274	12	MTMR2
0.003274	12	RHEB
0.003274	12	HPS6
0.003274	12	FYCO1
0.003274	12	COPS7A
0.003292	12	CLEC16A
0.003297	12	SGPL1
0.003298	12	PCMTD1
0.003304	12	CSNK2B
0.00331	12	TGFBR2
0.003311	12	ERP29
0.003311	12	PPFIA1
0.003311	12	DCXR
0.003315	12	DGKA
0.003319	12	TAP1
0.003326	12	BIRC6
0.00334	12	DMXL1
0.003342	12	RAB33B
0.003344	12	GGPS1
0.003356	12	PRKAR2A
0.003356	12	STMN1
0.003356	12	RPL27
0.003356	12	USP7
0.003356	12	NOB1
0.00336	12	FUBP3
0.003361	12	SNF8
0.003363	12	TGFB1
0.003365	12	CNOT4
0.003365	12	IMPDH1
0.003365	12	PPFIBP1
0.003365	12	PBXIP1
0.003365	12	MARCHF5
0.003365	12	MRTFB
0.003365	12	RBM7
0.003369	12	MRPL1
0.003379	12	EIF4E
0.003379	12	LTBP1
0.003381	12	SHROOM2
0.003381	12	PDCD2L
0.003383	12	TPR
0.003385	12	CNNM3
0.003388	12	CALU
0.003388	12	POTEJ
0.003388	12	SP100
0.003388	12	CSE1L
0.003388	12	PAK2

0.003388	12	ITPR3
0.003388	12	LPCAT4
0.003388	12	PSMG2
0.003388	12	RPAP1
0.003403	12	MCU
0.003403	12	CAVIN3
0.003403	12	SCLY
0.003407	12	DNAJC21
0.003407	12	CPNE2
0.003407	12	MRPL18
0.003407	12	SLC38A1
0.003407	12	COPS3
0.003409	12	RAB11B
0.003414	12	ANKRD13A
0.003428	12	YY1
0.003428	12	PLCB3
0.003428	12	REEP3
0.003428	12	TMTC3
0.003428	12	STK3
0.003437	12	ARIH2
0.003441	12	ATL3
0.003442	12	TTC21B
0.003449	12	GSTP1
0.003454	12	SIPA1
0.003457	12	SH3GLB1
0.003458	12	PI4K2B
0.003458	12	MMS19
0.003471	12	RFC5
0.003471	12	SRCAP
0.003471	12	EMC1
0.003495	12	UBR7
0.003497	12	UBE2N
0.003497	12	TM9SF3
0.003502	12	TM7SF3
0.003504	12	PSMB10
0.003507	12	IPO9
0.003515	12	ARAF
0.003515	12	SEC61B
0.00352	12	UBA7
0.003521	12	NDUFAF5
0.00353	12	SMARCC2
0.003549	12	GTPBP1
0.003549	12	HDGF
0.003549	12	TRMT1L
0.003549	12	SIRT6
0.003549	12	JCAD
0.003551	12	AK5
0.003552	12	UVRAG
0.003553	12	PYM1
0.003557	12	ASCC1

0.003557	12	SF3B6
0.003558	12	SET
0.003558	12	POLB
0.003558	12	USP47
0.003562	12	TBP
0.003564	12	TTI2
0.00357	12	ITPKB
0.00358	12	TIMM44
0.00358	12	NR2F1
0.00358	12	FDXR
0.00358	12	CUX1
0.00358	12	SCARB1
0.00358	12	ANP32E
0.00358	12	CYSTM1
0.00359	12	PARP14
0.00359	12	RDH13
0.003593	12	ATP2C1
0.003593	12	POLR3B
0.003596	12	PPP4R3A
0.003596	12	RAVER2
0.003596	12	MRGBP
0.003608	12	DNAJC2
0.003608	12	TES
0.003608	12	NUB1
0.003615	12	GIMAP5
0.003615	12	HPF1
0.003619	12	USP34
0.003626	12	HSPA1A
0.003626	12	ACTN1
0.003637	12	NUP155
0.003639	12	EIF3A
0.003641	12	PPP2R1A
0.003641	12	FASN
0.003642	12	TBC1D4
0.003642	12	MARS1
0.003642	12	MON1B
0.003642	12	NAXD
0.003642	12	DDRGK1
0.003642	12	SLK
0.003642	12	TXNIP
0.003642	12	SMC4
0.003642	12	DNAJC17
0.003642	12	DYNC1LI1
0.003646	12	ARF1
0.003646	12	LSM7
0.003647	12	WIPF2
0.003648	12	RIOK3
0.003658	12	CHMP2A
0.003658	12	DHODH
0.003664	12	ENO1

0.003664	12	EPHX1
0.003671	12	HIBCH
0.003678	12	DPP3
0.00368	12	VPS41
0.00368	12	ABCF3
0.00368	12	UFC1
0.003691	12	PAFAH1B2
0.003694	12	ANXA4
0.003703	12	PRPF31
0.003703	12	UBE2Z
0.003703	12	PLEKHF2
0.003703	12	ACTR10
0.003706	12	NT5C2
0.003708	12	CSRP2
0.003712	12	MPDZ
0.003715	12	LYN
0.003715	12	DNM1L
0.003719	12	HMBS
0.003719	12	TYK2
0.003723	12	CSTF1
0.003723	12	DHX57
0.003727	12	AKAP17A
0.00373	12	GFM1
0.003732	12	ZEB2
0.003753	12	TCERG1
0.003753	12	SVIL
0.003758	12	ABL1
0.003758	12	RPL35
0.003758	12	GAS2L1
0.003758	12	DDX23
0.003758	12	PACSIN2
0.003759	12	MGST3
0.003759	12	NFKB2
0.003759	12	SRSF5
0.003759	12	EFR3A
0.003759	12	NEDD4L
0.003759	12	DHX35
0.003761	12	CPNE1
0.003761	12	GDAP2
0.003768	12	MT-CO2
0.003775	12	RIN2
0.003782	12	PEX5
0.003786	12	ASCC3
0.003788	12	PSMB6
0.003794	12	MIOS
0.003794	12	CAP2
0.003797	12	CUL3
0.003797	12	ERI1
0.003815	12	ABCB7
0.003824	12	RPS3

0.003831	12	NFATC1
0.003831	12	KIF21A
0.003831	12	ACOT1
0.003831	12	FAR2
0.003831	12	ATF6B
0.00384	12	SCFD2
0.003841	12	SMARCD1
0.003854	12	ABCB10
0.003857	12	SMG6
0.003866	12	RO60
0.003866	12	DUS2
0.003867	12	ATP5MF
0.003867	12	BAG1
0.003867	12	SBDS
0.003867	12	IKBKG
0.003871	12	GSS
0.003871	12	TMEM70
0.003875	12	COX7A2
0.003875	12	RILPL1
0.003875	12	8月-01
0.00388	12	FDPS
0.003892	12	COMMD7
0.003892	12	SRBD1
0.003895	12	PREX2
0.003897	12	MED4
0.003897	12	MYEF2
0.003905	12	EXOC3
0.003906	12	PSMD8
0.003906	12	SELENON
0.003908	12	SRR
0.003918	12	GTF2F1
0.003918	12	USP8
0.003925	12	CEP192
0.00393	12	GSTT2
0.003941	12	PPP1R7
0.003942	12	MPP1
0.003943	12	PLOD1
0.003945	12	P4HA2
0.003945	12	ZBTB7A
0.003951	12	GARRE1
0.003953	12	PIP4K2B
0.003968	12	FKBP2
0.003968	12	DDOST
0.003974	12	PLPBP
0.003975	12	MEN1
0.003978	12	LRSAM1
0.003978	12	ZNF687
0.003978	12	ACADS
0.003981	12	MGAT2
0.003987	12	GRAP

0.003988	12	ELK3
0.003988	12	FRMD8
0.003988	12	DAG1
0.003988	12	LRRFIP2
0.003989	12	SEC22B
0.003989	12	TGS1
0.003992	12	WRNIP1
0.003997	12	UGDH
0.004004	12	FYN
0.004004	12	DROSHA
0.004007	12	COPS6
0.004007	12	LRRC20
0.004007	12	WDR77
0.004019	12	PDS5A
0.004026	12	STAM
0.004028	12	ATP6V0A2
0.004031	12	EIF3B
0.004042	12	PTCD1
0.004043	12	ARPC1B
0.004053	12	EML4
0.004056	12	FKBP8
0.004065	12	PSMA5
0.004065	12	GNAQ
0.004066	12	COMT
0.004066	12	PTBP1
0.004072	12	DERL2
0.004073	12	CPSF7
0.004074	12	USP5
0.004074	12	FHOD1
0.004077	12	SLC25A15
0.004084	12	ZNF521
0.004097	12	TTC1
0.0041	12	DCAF7
0.004108	12	PES1
0.004108	12	PTPN9
0.004121	12	ECPAS
0.004127	12	MSH6
0.004128	12	ACTL6A
0.004135	12	RPN2
0.004135	12	CREBBP
0.004136	12	AFAP1L1
0.004139	12	TMCO1
0.004141	12	SUPT5H
0.004141	12	SLC33A1
0.004144	12	GABARAPL2
0.004155	12	MDN1
0.004161	12	RPL39
0.004162	12	MYDGF
0.004178	12	UTP18
0.004189	12	BRI3BP

0.004191	12	MRPL12
0.004192	12	BIRC2
0.004202	12	DNM2
0.004203	12	PRPF6
0.004205	12	GMPS
0.004205	12	RPA2
0.004207	12	GOLGA1
0.004212	12	SIN3A
0.004217	12	ERCC5
0.004222	12	PAM
0.004224	12	FNTB
0.004227	12	TIMM29
0.004241	12	TNPO2
0.004241	12	STXBP2
0.004241	12	RTCB
0.004246	12	QARS1
0.004252	12	PSMB3
0.004255	12	KRT78
0.004256	12	NUDCD2
0.004263	12	ARHGEF1
0.004263	12	NDUFAF2
0.004265	12	PTPN14
0.004269	12	CPSF3
0.004274	12	CCDC9B
0.004274	12	RGL2
0.00428	12	PIK3R4
0.00428	12	ATP5IF1
0.00428	12	NME7
0.004286	12	HEXB
0.004286	12	GGA3
0.004295	12	NDUFB3
0.004295	12	NDE1
0.004303	12	ALG1
0.004316	12	INTS7
0.004317	12	AK1
0.004317	12	ARHGAP29
0.004317	12	KLHL22
0.004317	12	PRRC2B
0.004326	12	CYP51A1
0.004326	12	TNFAIP8L1
0.00433	12	NANS
0.004333	12	PSMD5
0.004341	12	SAAL1
0.004347	12	RPL15
0.00435	12	CIRBP
0.004354	12	VPS37B
0.004362	12	PHAX
0.004362	12	SEC24C
0.004373	12	ILF3
0.004374	12	RPL7A

0.00438	12	NOL11
0.004385	12	PTPRE
0.004385	12	CARD8
0.004395	12	DIAPH2
0.004406	12	AHCY
0.004406	12	NIPSNAP2
0.004411	12	HADHB
0.004411	12	ZDHHC20
0.004411	12	AGAP3
0.004415	12	ENOSF1
0.004435	12	ELMO1
0.004449	12	CAND2
0.00445	12	INF2
0.004453	12	YKT6
0.004453	12	TBCEL
0.004453	12	ACAD9
0.004453	12	MRPL44
0.004454	12	ATG7
0.004454	12	BECN1
0.004454	12	HOOK3
0.004454	12	AAAS
0.004461	12	TRAPPC3
0.004461	12	NDUFA10
0.004461	12	MCM7
0.004461	12	LYPLAL1
0.004461	12	LRP10
0.004463	12	AMPD2
0.004463	12	ETFDH
0.004463	12	TCP11L1
0.004468	12	UBE3A
0.004468	12	KSR1
0.004474	12	CYB5R3
0.004474	12	CHTF18
0.004481	12	WDR82
0.004497	12	NOC4L
0.0045	12	MTOR
0.004502	12	UQCRQ
0.004511	12	PLA2G15
0.004529	12	PRDX6
0.004529	12	ATF7IP
0.00453	12	CTSB
0.004551	12	SRP54
0.004567	12	TRAP1
0.004567	12	INTS13
0.004572	12	WASH3P
0.004572	12	GDE1
0.004578	12	MRI1
0.004579	12	PTPN12
0.004579	12	LIX1L
0.00458	12	ARFGEF1

0.004581	12	APEX1
0.004581	12	SHPK
0.004582	12	PJA2
0.004582	12	ADGRG1
0.004582	12	STK24
0.004598	12	GLUD1
0.004599	12	GLS
0.004599	12	HPS3
0.004603	12	EIF3K
0.004603	12	POLR3F
0.004611	12	HNRNPUL2
0.004623	12	MLF2
0.004623	12	KIF7
0.004623	12	LRCH3
0.00463	12	SLC25A12
0.00463	12	SDF4
0.004636	12	RRAGA
0.004638	12	SEPTIN7
0.004639	12	CROCC
0.004647	12	RPL31
0.004647	12	RYBP
0.004649	12	TRAPPC2L
0.004651	12	MRPS21
0.004651	12	SOS1
0.004651	12	DDX60L
0.004651	12	GRHPR
0.004652	12	RBM6
0.004652	12	H1-10
0.004658	12	PHF10
0.004658	12	BCAS3
0.004661	12	SNAP29
0.004661	12	MRPS15
0.004668	12	ZCCHC3
0.004671	12	RPP14
0.004671	12	SIRT1
0.004671	12	PUS7
0.004671	12	NOSIP
0.004687	12	MYO18A
0.004687	12	KCTD15
0.004695	12	STRBP
0.004719	12	CDC5L
0.004721	12	ARPP19
0.004727	12	DCTN2
0.004738	12	GALNT7
0.004738	12	TBC1D23
0.004744	12	ADGRL2
0.004744	12	TMT1A
0.004745	12	KIDINS220
0.004753	12	HMGB3
0.004753	12	SLC30A9

0.004757	12	NUDT3
0.004778	12	TNFSF4
0.004784	12	C12orf4
0.004804	12	SLC27A1
0.00481	12	STK38L
0.004815	12	TMSB10
0.004815	12	ST6GALNAC3
0.004821	12	SLC25A13
0.004833	12	TRIP10
0.004834	12	CXXC1
0.004835	12	ACTA2
0.004835	12	STXBP5
0.004835	12	ZFYVE19
0.004844	12	PSMD2
0.00485	12	FARS2
0.004852	12	PSRC1
0.004855	12	CCPG1
0.00486	12	SMAD5
0.004864	12	OGFR
0.004867	12	GFUS
0.00487	12	HABP4
0.004879	12	ALDH18A1
0.00488	12	CASP7
0.004881	12	DMAP1
0.004885	12	HM13
0.004886	12	KIAA1671
0.004887	12	DUSP23
0.004892	12	PRMT9
0.004897	12	TMED8
0.0049	12	ZMYM4
0.0049	12	XPO5
0.004903	12	CALM1
0.004903	12	TMED9
0.004907	12	INTS14
0.004919	12	TALDO1
0.004919	12	FBRS
0.00492	12	BCAP29
0.004925	12	PLD2
0.004937	12	VPS72
0.00494	12	MOB1B
0.004944	12	MAU2
0.004962	12	SCYL1
0.004968	12	AQR
0.004968	12	GDI2
0.004968	12	PRKAA1
0.004968	12	MMAB
0.004971	12	CAMSAP2
0.004973	12	USP32
0.004985	12	FTSJ1
0.00499	12	MKRN2

0.004995	12	COPS2
0.004995	12	PPHLN1
0.004998	12	PTDSS2
0.004998	12	ARPC2
0.004998	12	HSPA2
0.005005	12	RPL6
0.005005	12	TSEN34
0.005005	12	ACOT8
0.005009	12	S1PR1
0.005029	12	MED14
0.005029	12	POLD3
0.005029	12	PANK2
0.005029	12	TACC3
0.005035	12	RAP2A
0.005053	12	PSMB7
0.005061	12	PRKD1
0.005062	12	ACAT1
0.005075	12	DENND10P1
0.005086	12	MFNG
0.005094	12	TFCP2
0.005098	12	DDX17
0.005101	12	HAUS4
0.005102	12	PTPRM
0.005102	12	CKAP5
0.005102	12	RALGAPB
0.005119	12	SEPTIN2
0.00513	12	RAI14
0.005134	12	PFDN1
0.005149	12	ZRANB2
0.005149	12	GOLGB1
0.005156	12	PELP1
0.005161	12	SUN2
0.005162	12	MRPL27
0.00517	12	NT5E
0.005174	12	SEC63
0.005175	12	TM9SF1
0.005175	12	ME3
0.005187	12	TDRD7
0.005192	12	CDC34
0.005192	12	RPS3A
0.005192	12	SKIC3
0.005195	12	BRPF1
0.005195	12	PSPC1
0.005199	12	WDR91
0.005199	12	ELOA
0.005199	12	RCN2
0.005227	12	ASRGL1
0.00524	12	CARS1
0.005245	12	NDC80
0.005246	12	ITPK1

0.005249	12	FGD4
0.005265	12	MMAA
0.005265	12	CLIP2
0.005271	12	SLC27A3
0.005285	12	MTX2
0.005285	12	SPC24
0.005285	12	RAPGEF6
0.005291	12	RRM1
0.005291	12	RELA
0.005291	12	PRRC1
0.005291	12	PLGRKT
0.005296	12	COG6
0.005304	12	GCDH
0.005316	12	AIMP1
0.00533	12	TAMALIN
0.00533	12	PSME3IP1
0.005334	12	CDK5
0.005334	12	PIK3C2B
0.005336	12	RCCD1
0.005336	12	NT5C3A
0.005341	12	MIF4GD
0.005341	12	FAM193A
0.005341	12	LARP4B
0.005341	12	UHRF2
0.005341	12	SH3GL1
0.005341	12	MEMO1
0.005371	12	DPP8
0.005371	12	DCAF5
0.005371	12	NUDT9
0.00538	12	GFPT2
0.005386	12	PRIM2
0.005396	12	UBE4B
0.005399	12	CSNK1G1
0.005399	12	SLC25A4
0.005405	12	PPP1CA
0.005409	12	TYMS
0.005409	12	WDR36
0.005414	12	FLII
0.005416	12	IPO13
0.005416	12	VCL
0.005416	12	FNDC3A
0.005426	12	NPEPL1
0.005431	12	SURF4
0.005431	12	RETREG3
0.005435	12	MIB1
0.005438	12	MTMR6
0.005461	12	WDR12
0.005461	12	CDAN1
0.005461	12	BAG5
0.005463	12	OXSM

0.005463	12	SPTAN1
0.005464	12	MAP2K4
0.005464	12	MRPL24
0.005466	12	MRPS5
0.005468	12	LGMN
0.005468	12	BABAM1
0.005468	12	LTN1
0.005478	12	SRRT
0.005484	12	ARHGEF10
0.005484	12	ITGB5
0.005484	12	ZEB1
0.005495	12	ABHD5
0.005497	12	CHERP
0.0055	12	FMR1
0.005503	12	MTSS1
0.005512	12	TMX1
0.005523	12	SIN3B
0.00553	12	BAK1
0.005534	12	POLR2B
0.005534	12	MVK
0.005553	12	DHX16
0.005553	12	MOB4
0.005562	12	COL5A2
0.005565	12	TRAPPC13
0.005565	12	RPL21
0.005565	12	GSTO1
0.005583	12	TRPC4AP
0.00559	12	RB1
0.005592	12	CTR9
0.0056	12	WDR33
0.005605	12	IARS2
0.005606	12	MRPL14
0.005636	12	CCND1
0.00564	12	ARID1B
0.00565	12	DEPP1
0.005653	12	NPEPPS
0.005653	12	ITSN1
0.005653	12	RCOR1
0.005655	12	WWC3
0.005655	12	MYO5A
0.005656	12	DDX47
0.005668	12	RPL29
0.005668	12	SIDT2
0.00567	12	OXR1
0.005674	12	ZFYVE21
0.005674	12	RER1
0.005674	12	POLD2
0.005674	12	FAM114A1
0.005685	12	ORC2
0.005692	12	DUSP3

0.005692	12	SNRPD2
0.005695	12	FMNL3
0.005698	12	FAM91A1
0.005705	12	RUSF1
0.005707	12	GARS1
0.005707	12	PI4K2A
0.005711	12	TOP3B
0.005716	12	EGLN1
0.005717	12	EPRS1
0.00572	12	SEPTIN9
0.005721	12	PDK3
0.005749	12	FES
0.005765	12	MYG1
0.005773	12	TXN
0.005776	12	ANXA1
0.005783	12	FUT11
0.005785	12	GALT
0.005785	12	NEU1
0.005785	12	PARP12
0.005785	12	RABGEF1
0.005789	12	AGTRAP
0.005791	12	SH3PXD2B
0.005791	12	MTHFS
0.005791	12	PHPT1
0.005795	12	PEX1
0.005795	12	TRAF7
0.0058	12	IPO8
0.0058	12	FLAD1
0.005807	12	TRAFD1
0.005807	12	MED15
0.005807	12	NCDN
0.005811	12	GLA
0.005813	12	IK
0.005814	12	WDFY3
0.005823	12	DCP1A
0.005851	12	TEX264
0.005855	12	ANKHD1
0.005861	12	PPP6R2
0.005861	12	AP2A1
0.005861	12	FEN1
0.005862	12	TMEM165
0.005871	12	PRKD3
0.005871	12	SPCS2
0.005871	12	SLC12A9
0.005871	12	LUC7L2
0.005872	12	ABCC4
0.005876	12	PLSCR1
0.005893	12	KIRREL1
0.005902	12	IMPACT
0.005918	12	PSME4

0.005918	12	RPRD1B
0.005918	12	ARL15
0.005939	12	MAP4K4
0.005939	12	MME
0.005939	12	RANBP9
0.005946	12	XIAP
0.005946	12	WAPL
0.005946	12	PDLIM7
0.005957	12	USP10
0.005957	12	KIF16B
0.005979	12	PYCR1
0.005983	12	APPL2
0.005985	12	BTF3
0.005985	12	PSMB5
0.005985	12	MIA2
0.005995	12	PPAN
0.005997	12	RALGPS2
0.005997	12	ERAP1
0.006013	12	UACA
0.006013	12	ORC6
0.006032	12	SPTBN1
0.006032	12	FAM98B
0.006032	12	RRP15
0.006049	12	PTK2
0.006049	12	SPHK1
0.006062	12	ATP1B3
0.006062	12	WDR75
0.006065	12	SPNS1
0.006074	12	EIPR1
0.006074	12	RIF1
0.006078	12	STAU1
0.006078	12	PAAT
0.006085	12	NPLOC4
0.006088	12	ADO
0.006089	12	HSPBP1
0.006091	12	EIF4G3
0.006097	12	PCYOX1
0.006114	12	ZSWIM8
0.00612	12	TLE5
0.006124	12	COL5A1
0.006124	12	NSRP1
0.006136	12	GCAT
0.006136	12	FXR1
0.006136	12	ZC3HAV1
0.006152	12	PBDC1
0.006182	12	PPM1A
0.006207	12	RSL24D1
0.006222	12	NAMPT
0.006222	12	LUC7L
0.006242	12	KIF3C

0.006242	12	MPDU1
0.006248	12	GOLGA2
0.00625	12	NEMF
0.00625	12	CCDC88A
0.006255	12	RABGAP1
0.006256	12	DCAF1
0.006259	12	NFATC2IP
0.00626	12	NAA20
0.00626	12	MRPL28
0.00626	12	CPTP
0.006267	12	CBR3
0.006267	12	SNX12
0.006271	12	PPIF
0.006271	12	SUPT4H1
0.006271	12	PHLDB2
0.006271	12	MRPL48
0.006271	12	PURB
0.006279	12	MAN2A1
0.006279	12	TRAPPC6B
0.006299	12	NUP58
0.0063	12	NRM
0.006302	12	FANCI
0.006311	12	NGLY1
0.006311	12	GRIPAP1
0.006311	12	COBLL1
0.006311	12	RPL7L1
0.006311	12	NUDCD3
0.006311	12	TOE1
0.006312	12	PIBF1
0.006331	12	UBE2O
0.006341	12	CLASP2
0.00635	12	XRCC4
0.006352	12	TASOR
0.006358	12	UBFD1
0.00636	12	GMDS
0.006361	12	SLFN11
0.006382	12	MRPL4
0.006385	12	ARID1A
0.006385	12	SEC23A
0.006385	12	PEF1
0.006387	12	PHACTR2
0.006387	12	ERLIN2
0.006387	12	IPO7
0.006387	12	MAPKAPK2
0.00639	12	RIPOR1
0.006399	12	MRPL45
0.006399	12	DACH1
0.006402	12	PEX3
0.006402	12	FARSB
0.00642	12	SNUPN

0.00642	12	EPS15L1
0.006422	12	RHOG
0.006433	12	NCOA3
0.006449	12	CNRIP1
0.006449	12	CMBL
0.006449	12	KDM5B
0.006468	12	TRAPPC9
0.006477	12	RNASEH2B
0.006478	12	ITGA2
0.006481	12	PCYT1A
0.006489	12	TMEM181
0.006503	12	UGGT2
0.006519	12	PTX3
0.006519	12	TSR1
0.006519	12	NDUFB9
0.006534	12	PFN1
0.006535	12	PIGG
0.006538	12	TRMT61A
0.006538	12	LARS1
0.006539	12	FRMD4A
0.006546	12	OTUD6B
0.006547	12	GET3
0.006552	12	HMGCR
0.006556	12	ACBD5
0.006558	12	TNIP1
0.006558	12	CRK
0.006564	12	PRPF4B
0.006564	12	SNX5
0.00657	12	PGAM5
0.006571	12	SFXN1
0.006571	12	RAP2C
0.006582	12	CARD6
0.006606	12	PODXL
0.006609	12	CCDC25
0.006618	12	TNS1
0.006629	12	TIMM21
0.006641	12	EBP
0.006652	12	UCKL1
0.006657	12	SMAD2
0.006657	12	TTC7B
0.006662	12	DENND6A
0.006669	12	AKR1A1
0.00668	12	NUP42
0.006684	12	CPT2
0.006684	12	DDB1
0.006689	12	SKIC2
0.006689	12	ADAM9
0.00669	12	RFTN1
0.006695	12	FOXK2
0.006704	12	NDUFS4

0.006709	12	BAG2
0.006709	12	SFPQ
0.006709	12	ASCC2
0.006717	12	VPS4B
0.006717	12	TANGO6
0.00672	12	DHX15
0.00672	12	INTS8
0.006735	12	HPRT1
0.006745	12	CHMP2B
0.006746	12	FASTKD2
0.006758	12	WDCP
0.006762	12	SNX18
0.006777	12	AGPAT3
0.006778	12	ITGA5
0.006778	12	VAC14
0.006797	12	ATF6
0.006805	12	ATP6V1H
0.006814	12	TOMM20
0.006814	12	TMEM209
0.006818	12	EEF2
0.006823	12	PDCD11
0.006824	12	MRPS30
0.006825	12	SPC25
0.006827	12	SUCLG1
0.006827	12	THOC7
0.006827	12	MTERF4
0.006832	12	TSPO
0.006832	12	YBX1
0.006834	12	EPM2AIP1
0.006839	12	L2HGDH
0.006851	12	CHAF1A
0.006856	12	DAPK1
0.006856	12	TMA7
0.006869	12	TM9SF4
0.006883	12	KIF14
0.006889	12	SEC16A
0.006896	12	NIBAN2
0.006903	12	INO80C
0.006905	12	TARBP2
0.006905	12	GNPDA2
0.006914	12	CCND3
0.006914	12	RPL7
0.006914	12	TSFM
0.006914	12	PGGT1B
0.006914	12	LSM6
0.006914	12	MRPS9
0.006914	12	SUGP2
0.006914	12	MINK1
0.006914	12	ZGPAT
0.006914	12	TOMM40L

0.006914	12	ATG4B
0.006928	12	GRWD1
0.006931	12	NGDN
0.006933	12	ANXA11
0.006935	12	P4HB
0.006956	12	CENPF
0.006962	12	DAB2
0.006968	12	INTS1
0.006973	12	TRIM26
0.006973	12	KIAA0930
0.006984	12	SERPINB9
0.006985	12	SNX4
0.006989	12	S1PR3
0.00699	12	OSTC
0.007003	12	AP1G1
0.007003	12	ARIH1
0.007024	12	SLC25A1
0.007024	12	PPP2R5A
0.007024	12	PIGU
0.007024	12	PREB
0.007027	12	WDR46
0.007027	12	AKAP11
0.007039	12	RAB21
0.007086	12	ESYT2
0.007086	12	ELAPOR2
0.007086	12	PRKAR1A
0.007086	12	GOLGA4
0.007086	12	PPA1
0.007091	12	NDUFS2
0.007091	12	SEC14L2
0.007091	12	PSMD7
0.007091	12	MYO9B
0.007091	12	FNBP4
0.007134	12	ATP6V0A1
0.007137	12	MARK2
0.007138	12	POGLUT2
0.007149	12	SPEN
0.007156	12	PPP3CB
0.007156	12	CCNH
0.007156	12	CNTNAP3
0.007169	12	INTS3
0.007173	12	PCBP2
0.007173	12	PIGK
0.00718	12	FECH
0.007184	12	DNTTIP2
0.007184	12	C1GALT1C1
0.007189	12	PRORP
0.007189	12	PICALM
0.00719	12	SLC38A10
0.007191	12	TRAF2

0.007193	12	VARS1
0.007197	12	ZC3H7A
0.007197	12	DCAF16
0.007197	12	WDR3
0.007204	12	CST3
0.007223	12	BCS1L
0.007238	12	EIF4ENIF1
0.007249	12	PCYT2
0.007249	12	USP24
0.007252	12	HSPA13
0.007275	12	RBM12
0.007275	12	TMED2
0.007275	12	TMED4
0.00728	12	MYOF
0.007283	12	DNAJC10
0.007291	12	PARN
0.007301	12	PFDN5
0.007306	12	HERC4
0.007331	12	DCK
0.007331	12	CLTC
0.007337	12	COPS4
0.007344	12	SEC24B
0.007374	12	SNRPB2
0.007381	12	VEZT
0.007381	12	AGTPBP1
0.00739	12	ABRAXAS1
0.007391	12	MBLAC2
0.007391	12	STK39
0.007398	12	CTNNA1
0.007398	12	SCRN2
0.007401	12	MCM3AP
0.00743	12	PMM2
0.007455	12	UBA3
0.007486	12	SRPRA
0.007486	12	RPIA
0.007521	12	USP4
0.007523	12	SLC38A2
0.007534	12	NIPSNAP3A
0.007552	12	PPP1R12C
0.007582	12	SEC23B
0.007583	12	MYO1E
0.007606	12	MAP3K3
0.007606	12	NDUFA11
0.007606	12	BRIX1
0.00761	12	UBA5
0.007613	12	PDE6D
0.007613	12	ARHGAP35
0.007614	12	TOLLIP
0.007617	12	NUP153
0.00764	12	LAMB2

0.00764	12	HECTD3
0.007658	12	MKI67
0.007658	12	CDK5RAP3
0.007665	12	DPYSL2
0.007681	12	NUP43
0.007684	12	CDK13
0.007684	12	PPP2R5E
0.007684	12	MON2
0.007684	12	SMC6
0.007684	12	NDUFAF3
0.007707	12	ANTXR2
0.007707	12	EMC2
0.007707	12	CNOT7
0.007707	12	ABT1
0.007722	12	RPL10A
0.007738	12	UBE2H
0.007741	12	AGL
0.007741	12	UBTD1
0.007743	12	WDR74
0.007754	12	RHBDD2
0.007759	12	AKT1
0.00776	12	SORD
0.007777	12	SLIT2
0.007807	12	TXNRD2
0.007818	12	SLC25A17
0.007818	12	MRPL3
0.007827	12	PPP2R1B
0.007842	12	GPAA1
0.007842	12	ERCC3
0.007842	12	LPP
0.007857	12	DST
0.007857	12	FAM171A1
0.007861	12	GPI
0.007864	12	NDUFA5
0.007872	12	STRIP1
0.007872	12	CHMP5
0.007877	12	RPL8
0.007877	12	MAEA
0.007918	12	SART1
0.007918	12	SUB1
0.007918	12	CSTF2T
0.007923	12	HEATR5B
0.007929	12	PRMT5
0.00793	12	CAAP1
0.007936	12	CASP2
0.007954	12	DNAJC13
0.007962	12	BRD7
0.007965	12	DDX60
0.007967	12	APBB1
0.007971	12	PLS1

0.007974	12	CCAR1
0.007978	12	GLB1
0.007995	12	PKD2
0.008029	12	USP13
0.008033	12	ATRX
0.008033	12	BAZ2B
0.008034	12	CPSF6
0.008053	12	HS2ST1
0.008057	12	TNFRSF10B
0.008061	12	SLC4A1AP
0.008085	12	CCDC71L
0.008086	12	SELENOK
0.008107	12	PDCD5
0.008107	12	GPX1
0.008107	12	TRIP4
0.008107	12	DENND5A
0.008107	12	TXNDC17
0.008107	12	EXOG
0.008107	12	MYADM
0.00811	12	NDUFB4
0.008118	12	GLRX3
0.008129	12	TM6SF1
0.008137	12	TMEM126B
0.008147	12	NFIC
0.008147	12	GAMT
0.008152	12	IGHMBP2
0.008153	12	RAD23B
0.008153	12	ACTBL2
0.008153	12	SIRT2
0.008172	12	GTF2F2
0.008172	12	CDC42EP4
0.008172	12	PRMT1
0.008199	12	CIT
0.008213	12	RFC2
0.008213	12	VCPIP1
0.008213	12	GATB
0.008215	12	LIMK2
0.00824	12	SEPTIN11
0.008248	12	CPSF1
0.008248	12	ZWILCH
0.00825	12	ACTN4
0.008329	12	COX5A
0.008352	12	CYB5R1
0.008355	12	PINX1
0.008362	12	TMUB1
0.008369	12	SEH1L
0.008374	12	CNP
0.008379	12	STX4
0.00838	12	ATP5F1C
0.008388	12	KIF13B

0.008391	12	DTNBP1
0.008396	12	PAFAH1B1
0.008402	12	XPO6
0.008408	12	PGLS
0.008408	12	MAP2K1
0.008415	12	TMEM223
0.008438	12	ADH5
0.008438	12	ARFIP2
0.008438	12	MRPL16
0.008453	12	ARL6IP1
0.008453	12	SFXN4
0.008454	12	ORC5
0.008457	12	TBCK
0.008464	12	CRNKL1
0.008464	12	SHMT1
0.008475	12	TUBGCP2
0.008475	12	SH3GLB2
0.008486	12	UTP15
0.008487	12	SPR
0.008492	12	ANXA7
0.008505	12	SIRPA
0.008505	12	ATPAF1
0.008505	12	ZFAND6
0.008505	12	DNAJC19
0.008505	12	MRPL22
0.008545	12	ATP2B4
0.008565	12	BRCC3
0.008567	12	POLG
0.008567	12	C9orf78
0.008568	12	ERCC6
0.008572	12	GNPDA1
0.008572	12	NTPCR
0.008576	12	TMEM256
0.008576	12	NHERF1
0.008576	12	RSL1D1
0.008576	12	GRPEL1
0.008588	12	DDX27
0.008608	12	CAD
0.008613	12	ZNF92
0.008618	12	ZBTB11
0.008627	12	MICU2
0.008641	12	DDX55
0.00865	12	NFRKB
0.00865	12	RBM42
0.008655	12	TIMM50
0.008659	12	POLR2E
0.008659	12	IDI1
0.008661	12	ARFIP1
0.008661	12	USP39
0.008661	12	BOD1L1

0.008661	12	NUP133
0.008661	12	MAPRE3
0.008669	12	BLVRB
0.008677	12	JMJD6
0.00868	12	TBPL1
0.00868	12	AFG2A
0.008686	12	PAXBP1
0.008694	12	NOS3
0.008697	12	PITPNB
0.008717	12	SPTBN2
0.008717	12	EXTL3
0.008717	12	CALR
0.008725	12	LONP1
0.008729	12	TMED3
0.008737	12	RARS1
0.008739	12	ATG13
0.008751	12	ATP9A
0.008763	12	TATDN1
0.008792	12	AP3S1
0.008818	12	TBCD
0.008836	12	PIP5K1C
0.008848	12	FAM120A
0.008855	12	FAM43A
0.008886	12	SOX18
0.008894	12	STX16
0.008947	12	ARMCX3
0.008964	12	CD46
0.008973	12	HNRNPA2B1
0.008973	12	MFSD10
0.008973	12	VAMP3
0.008989	12	ERO1A
0.00899	12	ERLEC1
0.008991	12	ARCN1
0.009003	12	STX5
0.009003	12	LSM2
0.009004	12	HTATIP2
0.009005	12	ZMYM3
0.009005	12	KIF3A
0.009015	12	RPP38
0.009019	12	NDUFS8
0.009046	12	BYSL
0.009069	12	URI1
0.009074	12	HINT1
0.009101	12	ADAR
0.009101	12	AK6
0.009114	12	RPS6KA3
0.009126	12	HMGN3
0.009145	12	CFAP20
0.009155	12	PBX2
0.009169	12	ZNF185

0.009185	12	VAPB
0.009188	12	ALAD
0.009188	12	MCM4
0.009188	12	SPECC1L
0.009189	12	RAB7A
0.009189	12	ANO10
0.009214	12	PDS5B
0.009218	12	UROD
0.009218	12	SMARCAD1
0.009219	12	FOSL1
0.009226	12	ZFYVE1
0.009226	12	TMEM161A
0.009229	12	LONP2
0.009236	12	TUBB2A
0.009259	12	MRPL49
0.009259	12	DDX31
0.009265	12	MCM2
0.009281	12	NUMB
0.009281	12	PARP4
0.009303	12	CEP97
0.009303	12	CFDP1
0.009308	12	PFAS
0.009316	12	NAA15
0.009328	12	IPO5
0.009329	12	MRPL23
0.009337	12	ASAP2
0.009337	12	LRCH2
0.009344	12	HNRNPH2
0.009344	12	TRIM28
0.009356	12	PTGR3
0.009356	12	CANT1
0.009368	12	DDX51
0.009371	12	COL1A2
0.009371	12	CYFIP2
0.009378	12	HSPA9
0.009398	12	ZNF148
0.009406	12	DHRS7
0.009412	12	JPT2
0.009421	12	IQGAP3
0.009443	12	LMAN2L
0.009451	12	DHX30
0.009465	12	VASP
0.009466	12	GTF3C1
0.009466	12	BLMH
0.009466	12	MED27
0.009474	12	HSPA4L
0.009478	12	KDM6A
0.00948	12	RP9
0.00948	12	MTARC2
0.00948	12	CCDC124

0.00948	12	YME1L1
0.009488	12	MICALL1
0.009504	12	HAUS5
0.00951	12	SNX15
0.00952	12	GATAD2B
0.009532	12	ALG9
0.009535	12	ABCE1
0.009559	12	CBS
0.009559	12	POGZ
0.009574	12	GNL3
0.00958	12	PDHX
0.009581	12	CCNK
0.009581	12	POLR2G
0.009581	12	NUP88
0.009586	12	RBBP9
0.009597	12	DYNC1I2
0.009597	12	PTCD3
0.009603	12	LDAH
0.009605	12	CRACD
0.009617	12	ISOC1
0.009628	12	TBCB
0.009635	12	ARL6IP5
0.009635	12	RPS23
0.009635	12	SNRPF
0.009635	12	WARS2
0.009646	12	PTP4A1
0.009652	12	FBXO21
0.009664	12	PIP4P1
0.009664	12	MAIP1
0.009664	12	CCDC43
0.009664	12	NPTN
0.009666	12	CDK6
0.009666	12	TSPAN31
0.009689	12	PLCG1
0.009705	12	N4BP3
0.009705	12	DAPK3
0.009709	12	BET1L
0.009726	12	OSBPL9
0.009732	12	DENND11
0.009749	12	SKIC8
0.009765	12	DNAJA2
0.009765	12	AAMP
0.009767	12	RANBP6
0.009767	12	RBM4B
0.009777	12	B3GAT3
0.009777	12	LYRM7
0.009777	12	GEMIN5
0.009777	12	KCMF1
0.009779	12	TTC38
0.00978	12	SELENOT

0.009784	12	AIMP2
0.00979	12	CHURC1
0.009811	12	GOLGA5
0.009812	12	ECI1
0.009813	12	NMT2
0.009817	12	GIT1
0.009829	12	UEVLD
0.009838	12	GBF1
0.00984	12	NDUFB6
0.00984	12	MANF
0.00984	12	TUBA4A
0.00984	12	SACS
0.009846	12	COMMD4
0.009852	12	SPAG1
0.009869	12	APEH
0.00987	12	LRCH4
0.00987	12	FBXO22
0.009879	12	SF3B1
0.009897	12	NACC1
0.009901	12	MRPL53
0.009905	12	SPAG7
0.009905	12	HMGCS1
0.009934	12	WDR26
0.009959	12	PGRMC1
0.009959	12	TP53I11
0.00996	12	RPL38
0.009973	12	RBM10
0.009973	12	TENM3
0.009973	12	WBP11
0.010028	12	RLIG1
0.010036	12	WDR44
0.010037	12	TAPBP
0.010037	12	WDR7
0.010037	12	ALDH9A1
0.010053	12	PSMD14
0.010059	12	COL6A1
0.010059	12	ANO6
0.010062	12	GGCX
0.010093	12	OCIAD1
0.010093	12	EXOSC1
0.010095	12	FKBP3
0.010103	12	YTHDF1
0.010124	12	SELENOO
0.010124	12	MRPL20
0.010135	12	ACAT2
0.010144	12	CHRAC1
0.010155	12	YIPF6
0.010156	12	JUND
0.010156	12	RAB35
0.010157	12	SLC12A2

0.010177	12	RPS28
0.010179	12	TUBA8
0.010183	12	FAM162A
0.010224	12	BOP1
0.010224	12	SYNRG
0.010229	12	PCMTD2
0.010234	12	URGCP
0.010237	12	CSTB
0.010237	12	LYZ
0.010237	12	ESYT1
0.010257	12	8月-02
0.01029	12	IL33
0.010293	12	CDSN
0.010293	12	GNE
0.010325	12	CALCRL
0.010325	12	SPART
0.010325	12	NUP93
0.010325	12	SMYD3
0.01033	12	ITSN2
0.010343	12	PSMD12
0.010343	12	SCO2
0.010343	12	ATP6V1B2
0.010343	12	ARHGAP5
0.010343	12	SDE2
0.010343	12	SCAMP4
0.010371	12	PPP1R9B
0.010371	12	STX18
0.010384	12	SYNE1
0.010414	12	SCAF8
0.010428	12	RB1CC1
0.010437	12	RUVBL2
0.010458	12	BCAT1
0.010458	12	APBA3
0.010478	12	MYH10
0.010478	12	UBE2I
0.010478	12	BASP1
0.010478	12	FAM120B
0.010478	12	BTBD2
0.01049	12	ABCB6
0.010498	12	STXBP4
0.010505	12	MTX1
0.010505	12	EXD2
0.010526	12	RAP1GDS1
0.010535	12	SLC35F2
0.010549	12	BORCS6
0.010571	12	WDR11
0.010574	12	STARD13
0.010574	12	PNN
0.010587	12	ARPC5
0.010589	12	HNRNPH1

0.010604	12	XPC
0.010604	12	HSF1
0.010623	12	GCLC
0.010623	12	TMEM201
0.010664	12	SWAP70
0.010668	12	OTUB1
0.01068	12	EEF1A1
0.010688	12	TTF2
0.010704	12	GALNT2
0.010709	12	TCEAL4
0.010741	12	NIP7
0.010746	12	ELP4
0.010753	12	SIK3
0.010759	12	TGFBRAP1
0.010768	12	GPATCH8
0.01077	12	MSN
0.010789	12	CD2BP2
0.010831	12	KIFAP3
0.010842	12	MAN2B1
0.010864	12	PMS2
0.010864	12	H3C1
0.010864	12	S100A16
0.010864	12	PLEKHA1
0.010869	12	RHBDF1
0.010873	12	AGFG1
0.010873	12	TMEM41B
0.010873	12	NCLN
0.010897	12	CLINT1
0.010899	12	RABGAP1L
0.010913	12	PDXDC1
0.01092	12	WDR18
0.010974	12	ZYX
0.01098	12	TEK
0.011036	12	MAT2A
0.011068	12	AK2
0.011077	12	ABI3
0.01108	12	EIF2B4
0.011091	12	MRPL19
0.0111	12	DNPH1
0.01112	12	EML2
0.011131	12	MRRF
0.011133	12	PHF6
0.011136	12	CERCAM
0.011143	12	CDC20
0.011146	12	RCL1
0.011159	12	NECAP1
0.011162	12	RPS19BP1
0.01117	12	APOO
0.011171	12	RARS2
0.011171	12	TUT7

0.011171	12	MKLN1
0.011215	12	PDLIM5
0.011227	12	EFL1
0.011234	12	G6PD
0.011234	12	TRIM21
0.011234	12	GABPA
0.011234	12	R3HDM1
0.011234	12	ANKMY2
0.011249	12	WDR55
0.011268	12	ALDH1B1
0.011312	12	MIPEP
0.011312	12	SLC5A6
0.011331	12	NEDD1
0.011334	12	LARP1
0.011337	12	APRT
0.011338	12	IGF2BP3
0.011345	12	KRR1
0.011345	12	RBM39
0.011346	12	RAB27A
0.011346	12	KRI1
0.011346	12	MRPL11
0.011372	12	SLC25A6
0.011403	12	CEBPZ
0.011406	12	ZFTRAF1
0.011407	12	NUAK1
0.011419	12	SLIRP
0.011439	12	COL6A3
0.011458	12	ACY1
0.011472	12	MICAL3
0.011478	12	TPST2
0.01154	12	STAT5B
0.011546	12	PTGES3
0.011547	12	LRATD2
0.011547	12	EIF3C
0.011559	12	CPSF4
0.011559	12	BID
0.011559	12	DDX42
0.011579	12	NME6
0.011579	12	MECOM
0.011579	12	OSBPL8
0.011603	12	MBD3
0.011615	12	IRF3
0.011637	12	AMFR
0.011652	12	TRAPPC12
0.011665	12	KIF1B
0.011688	12	TBRG4
0.011697	12	NHEJ1
0.011732	12	OCRL
0.011732	12	UAP1L1
0.011732	12	CXorf38

0.011732	12	TMEM147
0.011736	12	SRSF9
0.011767	12	LUZP1
0.011772	12	LRPPRC
0.011772	12	TARDBP
0.011772	12	CDK12
0.011792	12	C1QBP
0.011849	12	PIK3CA
0.011858	12	ACOX3
0.011858	12	DNMT1
0.011858	12	KDM5C
0.011858	12	APLP2
0.011858	12	DIPK1B
0.011858	12	ENY2
0.011881	12	SSR1
0.011881	12	PRDX4
0.011891	12	CSNK1D
0.011899	12	AHCYL2
0.011901	12	CHMP7
0.011907	12	HLTF
0.011911	12	KCTD9
0.011915	12	SCO1
0.011943	12	LEO1
0.01196	12	LYRM4
0.011962	12	ACOX1
0.011979	12	STK25
0.011979	12	CWF19L1
0.011979	12	GPRC5A
0.011979	12	GCN1
0.011985	12	NCBP3
0.011997	12	EIF4EBP1
0.011999	12	AASDHPPT
0.012	12	TRMT112
0.012005	12	CLPB
0.012005	12	ZMYND8
0.012011	12	CSNK2A2
0.012036	12	GGA1
0.012048	12	HEATR1
0.012055	12	QKI
0.012075	12	NOP10
0.012124	12	ARMC6
0.012137	12	IMPDH2
0.012153	12	TIMM23
0.012153	12	PPP1R16B
0.012153	12	PEX16
0.012183	12	KEAP1
0.012191	12	CORO7
0.01222	12	TLE1
0.01222	12	RABEPK
0.01222	12	DNAJC11

0.012234	12	ABCD1
0.012236	12	METTL17
0.012257	12	IPO4
0.012325	12	LUC7L3
0.012325	12	RPP30
0.012325	12	DGCR8
0.012325	12	PAIP1
0.012337	12	AHCTF1
0.012337	12	CCDC88C
0.012345	12	HTRA2
0.012358	12	TIPRL
0.012367	12	CCDC50
0.012394	12	PARL
0.012423	12	NDUFS5
0.012426	12	AGPS
0.012435	12	METTL13
0.012436	12	RFLNB
0.012436	12	PCNP
0.012438	12	MROH1
0.012439	12	ADPGK
0.012443	12	GET1
0.012451	12	HNRNPA3
0.01247	12	TRNT1
0.01247	12	AMDHD2
0.0125	12	KRT18
0.012516	12	TPD52L2
0.012525	12	RBM33
0.012529	12	DHX32
0.012529	12	CLEC2B
0.01253	12	MORC2
0.012536	12	SMG8
0.012543	12	ELOB
0.012566	12	RACK1
0.012566	12	SNTB2
0.01259	12	THOC2
0.012619	12	ATP11B
0.012623	12	HDAC4
0.012683	12	POLE
0.012683	12	MGA
0.012683	12	SLC39A10
0.012684	12	NOP9
0.012728	12	RPL18
0.012733	12	SCAF4
0.012738	12	CDK11B
0.012765	12	AKR1C3
0.012765	12	STIM1
0.012766	12	DPYSL4
0.012777	12	FOXK1
0.012777	12	CHCHD3
0.012777	12	MAN1B1

0.012782	12	STK26
0.0128	12	SELENOI
0.012821	12	SLU7
0.012852	12	MFSD8
0.012852	12	ZC3H7B
0.012857	12	TNFAIP2
0.012875	12	RPS15
0.012878	12	GTF2H4
0.01289	12	KHSRP
0.012899	12	LACRT
0.012907	12	NOMO1
0.012907	12	STUB1
0.01291	12	FUS
0.012948	12	ORC3
0.012976	12	XPO7
0.012997	12	MTA2
0.013024	12	DOCK7
0.013027	12	VAV2
0.013031	12	FAH
0.013033	12	COG2
0.013055	12	HMGN1
0.013055	12	CDC42
0.013065	12	SBNO1
0.013066	12	RPL30
0.013105	12	FUT8
0.013124	12	MGME1
0.013124	12	MRPL13
0.013144	12	RAB4A
0.013144	12	NSMAF
0.013204	12	INCENP
0.013204	12	GSTK1
0.013211	12	APIP
0.01323	12	MT-ND1
0.01323	12	TRAPPC11
0.01323	12	SEPSECS
0.013239	12	VEPH1
0.013242	12	RECQL5
0.013242	12	TMEM97
0.013251	12	DEGS1
0.013255	12	PSMD9
0.013267	12	HSPA5
0.013314	12	TBCE
0.013333	12	ATAT1
0.013341	12	AFG2B
0.013379	12	STING1
0.013405	12	NFS1
0.013413	12	MAN1A2
0.013413	12	INTS12
0.01343	12	MPRIP
0.013472	12	BCCIP

0.013479	12	KIF23
0.013486	12	GNAS
0.013486	12	ANAPC2
0.013505	12	SLC25A32
0.013505	12	PPT2
0.013516	12	CLPP
0.013524	12	ACSL4
0.013537	12	MACROH2A1
0.013537	12	IQSEC1
0.013591	12	PANK4
0.01363	12	KLC1
0.013635	12	CA13
0.013641	12	TP53
0.013652	12	USP28
0.013694	12	RACGAP1
0.01371	12	HDAC3
0.013712	12	ARPC3
0.013725	12	ACAP2
0.013807	12	RMDN3
0.013807	12	DIAPH3
0.013811	12	ARPC5L
0.013867	12	PHF3
0.013875	12	UBE2V2
0.013875	12	VIPAS39
0.013886	12	UBIAD1
0.013889	12	PPP1R11
0.013894	12	MRPS10
0.013898	12	CCT6A
0.013898	12	RHOB
0.013898	12	TRADD
0.013898	12	PABPN1
0.0139	12	TBC1D2B
0.013908	12	CS
0.013933	12	XPA
0.013938	12	S100A10
0.01395	12	VPS33A
0.013975	12	FBXO7
0.013976	12	EIF4G1
0.013983	12	SMG1
0.013983	12	ARGLU1
0.013993	12	LRRC57
0.013993	12	DDX21
0.013993	12	MAT2B
0.013993	12	G3BP2
0.014026	12	KATNB1
0.014074	12	SEC62
0.014083	12	KIF3B
0.014083	12	DPH2
0.014088	12	ADGRF5
0.014117	12	NCBP2

0.014182	12	SPATS2
0.014182	12	MRPS34
0.014194	12	METTL14
0.014206	12	SLC25A24
0.014211	12	CLCN7
0.01423	12	MRPS22
0.014244	12	P4HA1
0.014244	12	CHPF2
0.014244	12	SAP18
0.014252	12	DIS3
0.014259	12	OXA1L
0.014271	12	CD151
0.014271	12	CLCC1
0.0143	12	NF1
0.0143	12	KIAA1191
0.014303	12	RBM15
0.014308	12	ERAL1
0.014313	12	APOB
0.014326	12	S100A11
0.014351	12	ZFP36L1
0.014351	12	ALG2
0.014426	12	ARRB1
0.01443	12	CCNYL1
0.014434	12	TTC13
0.014434	12	DTD1
0.014449	12	GALK1
0.014454	12	PPT1
0.014463	12	MIX23
0.014467	12	SF3A3
0.014493	12	MT-ND5
0.014498	12	DCTN6
0.014498	12	GTF2H1
0.014512	12	TUBB6
0.014557	12	MSH3
0.014557	12	COA3
0.014576	12	UBE2S
0.014579	12	SLC2A6
0.01458	12	FAM20B
0.014582	12	TUBB4B
0.014595	12	ARL2BP
0.014602	12	NFX1
0.014618	12	PSME3
0.014639	12	CACYBP
0.014656	12	CBR1
0.014656	12	SUGT1
0.014704	12	TIAL1
0.014712	12	TCF12
0.014715	12	CYB5B
0.014715	12	WLS
0.014725	12	NOL10

0.014734	12	TRPV2
0.014734	12	HNRNPL
0.014734	12	SNX9
0.014738	12	PGRMC2
0.014738	12	CASKIN2
0.014738	12	HHIP
0.01474	12	PAPSS2
0.014791	12	SYNGR2
0.014795	12	AHNAK
0.014834	12	MRPL39
0.014842	12	NCAPG2
0.014862	12	M6PR
0.014873	12	YEATS2
0.014906	12	DDHD2
0.014906	12	ARFGAP3
0.01491	12	UBLCP1
0.014913	12	ITGA6
0.01492	12	EVI5
0.014985	12	RASSF8
0.015001	12	MCMBP
0.015042	12	S100A7
0.015047	12	LSM8
0.015049	12	ZNF592
0.015057	12	MAPK14
0.015093	12	ZC3HC1
0.015109	12	SLC25A20
0.015109	12	H2AC11
0.015109	12	MITD1
0.015109	12	ATXN10
0.015125	12	ABLIM3
0.015142	12	OGFOD1
0.015155	12	CD99
0.015171	12	H4C1
0.015171	12	LTBP2
0.015173	12	EIF3I
0.015217	12	MT-ND4
0.015252	12	LENG8
0.015283	12	AHNAK2
0.015285	12	MYD88
0.015291	12	E2F4
0.015302	12	ST7
0.015332	12	CHMP3
0.015343	12	ZZZ3
0.015347	12	SEC31A
0.01535	12	MED24
0.015364	12	CMTM6
0.015418	12	PGS1
0.015418	12	TNKS1BP1
0.015449	12	EIF4B
0.015513	12	CMTR1

0.015514	12	VPS53
0.015539	12	CSK
0.015588	12	DDX39A
0.015599	12	TDRD3
0.0156	12	SLC52A2
0.0156	12	UBE2T
0.0156	12	SDAD1
0.015624	12	SPIRE1
0.015634	12	PRPF8
0.015663	12	GOLGA3
0.015663	12	ZNF512B
0.015683	12	UBR3
0.015699	12	COQ5
0.015707	12	NOP58
0.015734	12	PROSER2
0.015742	12	ACYP1
0.01575	12	RPS6
0.015755	12	U2AF2
0.015755	12	MEX3D
0.015755	12	ALKBH3
0.015767	12	PCNA
0.015791	12	GPAT3
0.015803	12	EDF1
0.01581	12	ACTR5
0.015811	12	PLXNA2
0.015811	12	RBM22
0.015819	12	DHX37
0.015861	12	PUDP
0.015866	12	RFT1
0.015917	12	TFG
0.015989	12	B4GALT1
0.015989	12	ZC2HC1A
0.015989	12	LNPK
0.016001	12	GNAI3
0.016052	12	S100A8
0.016089	12	CHEK1
0.016093	12	CCT3
0.016104	12	SMTN
0.016104	12	QRSL1
0.016104	12	CAPN7
0.016122	12	STAT2
0.016122	12	MICAL1
0.016122	12	PHB2
0.016141	12	DDX18
0.016256	12	CASP14
0.016265	12	GJA1
0.016265	12	FCHO2
0.01628	12	GSPT2
0.016282	12	HECTD4
0.016287	12	DHX36

0.016291	12	LSM3
0.0163	12	YES1
0.0163	12	NAPG
0.016312	12	SPIN1
0.016324	12	CAP1
0.016326	12	CAST
0.016326	12	HDAC2
0.016334	12	DERL1
0.016373	12	CALML5
0.016374	12	MRPL30
0.016449	12	SCRN1
0.016458	12	GPC1
0.016464	12	FBXO38
0.016483	12	CHD1
0.016485	12	RBX1
0.016501	12	TMEM115
0.016539	12	CCT4
0.016539	12	MTMR1
0.016558	12	IST1
0.016558	12	GNAI1
0.01658	12	WDR47
0.016584	12	NSFL1C
0.01659	12	ATP6V1C1
0.01661	12	RAB2B
0.016645	12	SMARCAL1
0.016667	12	FAU
0.016726	12	CMIP
0.016731	12	SGTA
0.016731	12	UBR2
0.016758	12	FKRP
0.016772	12	NRDE2
0.016778	12	LLGL1
0.016797	12	TGFB1I1
0.016801	12	RBM12B
0.016803	12	ZNF24
0.016803	12	ATP5MJ
0.016844	12	GTF2H2
0.016857	12	NAA30
0.016959	12	TNRC6B
0.016977	12	SH3KBP1
0.01699	12	RAB13
0.017018	12	RBBP4
0.017037	12	PRPF18
0.017055	12	MTR
0.01712	12	OTUD4
0.017126	12	DNAAF10
0.017127	12	BPNT1
0.017145	12	FCF1
0.017163	12	LGALS9
0.017252	12	STEAP3

0.017259	12	PTS
0.017273	12	GATAD2A
0.017471	12	GOPC
0.017501	12	ASAP1
0.017531	12	RPL24
0.017531	12	VPS50
0.017532	12	TOMM40
0.017532	12	H2AC4
0.017532	12	SLC9A1
0.017532	12	CTTNBP2NL
0.017564	12	NR2F2
0.017565	12	KLHDC4
0.017592	12	RPL17
0.017599	12	TSPAN4
0.017599	12	VPS39
0.017599	12	MAP3K20
0.017655	12	F2R
0.01766	12	GRB10
0.017682	12	GNG5
0.017702	12	CDC42BPA
0.017705	12	NOP2
0.017768	12	PMS1
0.017777	12	POT1
0.017799	12	TRAF6
0.017814	12	CNOT2
0.017814	12	MACF1
0.01788	12	CASTOR2
0.01788	12	KNTC1
0.017895	12	VRK1
0.017911	12	NAP1L1
0.017911	12	NEK7
0.017912	12	LAMTOR4
0.01793	12	GTF3C2
0.017952	12	NUDT1
0.017992	12	UCHL1
0.018006	12	SNRPE
0.018006	12	NID2
0.018053	12	PAM16
0.018112	12	PLIN4
0.018112	12	MRTO4
0.018122	12	TCAF1
0.018135	12	NRBP1
0.018142	12	FAHD1
0.018144	12	SNRNP200
0.018199	12	ABCF1
0.018199	12	PPWD1
0.018199	12	DCBLD2
0.018229	12	PYCR3
0.018277	12	TMBIM1
0.018291	12	FRY

0.018303	12	NSUN2
0.018303	12	FAM3C
0.018381	12	CENPB
0.018381	12	RPL36A
0.018381	12	MTG1
0.018383	12	H2BC12
0.018386	12	PCID2
0.018396	12	PDLIM2
0.018459	12	GPRIN1
0.018483	12	TBC1D20
0.018502	12	RPTOR
0.018502	12	SEPHS2
0.018512	12	PAFAH1B3
0.018526	12	ARB2A
0.018539	12	EEF1G
0.018617	12	EMC7
0.018632	12	PKP1
0.018693	12	EIF2B5
0.018693	12	NAE1
0.018694	12	RAB18
0.018722	12	RPL22L1
0.018731	12	RIDA
0.018737	12	COG7
0.018737	12	NOA1
0.018754	12	BAIAP2
0.018772	12	CSNK2A1
0.018772	12	PTP4A2
0.018772	12	L3MBTL3
0.018775	12	ATP6V1A
0.018785	12	BCL2L1
0.018865	12	CD274
0.018884	12	ATP1B1
0.018937	12	PDHA1
0.018937	12	TUBB3
0.019066	12	MIA3
0.019086	12	VKORC1L1
0.019101	12	NOLC1
0.019101	12	SF1
0.019101	12	LRCH1
0.019102	12	BSG
0.019109	12	IGFBP4
0.01912	12	CRIM1
0.019146	12	DLG1
0.019146	12	SETX
0.019146	12	STEEP1
0.019153	12	MORC3
0.019153	12	MTHFSD
0.019202	12	PSMC4
0.019202	12	ARPC1A
0.019206	12	MARCKS

0.019273	12	IFITM3
0.019302	12	MAP3K4
0.019362	12	MPZL1
0.019377	12	ASH1L
0.019397	12	TTL
0.019397	12	BICD2
0.019439	12	TUFM
0.019439	12	TRAPPC5
0.019466	12	PHF2
0.019473	12	FILIP1L
0.01949	12	VPS36
0.01949	12	CRELD1
0.019511	12	HARS1
0.019525	12	UQCC1
0.019536	12	SORT1
0.019536	12	MCTS1
0.019573	12	RPL13A
0.019573	12	GPS1
0.019592	12	TELO2
0.019597	12	STT3B
0.019607	12	OGT
0.019639	12	LIPG
0.019656	12	CHCHD6
0.019669	12	TSPAN3
0.019684	12	SLC7A6OS
0.019686	12	CCNA2
0.019707	12	MOB3A
0.01976	12	LTV1
0.019796	12	SHC1
0.019796	12	FRA10AC1
0.019827	12	MCAT
0.01984	12	S100A9
0.019847	12	VWA5A
0.01985	12	HIBADH
0.01985	12	SLC30A6
0.01985	12	BRD1
0.019854	12	DTNA
0.01986	12	ISY1
0.019892	12	NHSL2
0.019904	12	RAB9A
0.019922	12	SF3A1
0.019922	12	ATP13A3
0.01993	12	MORF4L1
0.019945	12	CLPTM1
0.019945	12	JAK1
0.019956	12	SIRT5
0.019991	12	QDPR
0.019991	12	CAB39
0.019999	12	TACO1
0.020014	12	RPS4X

0.020015	12	MED18
0.020104	12	TKT
0.020121	12	IKBIP
0.020153	12	CDK19
0.020189	12	MTCH2
0.020225	12	CLK3
0.020226	12	FEZ2
0.020238	12	FAM76B
0.020238	12	SLC30A1
0.020271	12	MRPL2
0.020307	12	MFAP1
0.020307	12	RHBDF2
0.020307	12	FOXP1
0.020352	12	SYNJ1
0.020352	12	VAMP5
0.020396	12	BUB1B
0.020396	12	HBA1
0.020399	12	AHCYL1
0.020399	12	RPP40
0.020425	12	RALA
0.020485	12	TMX3
0.020485	12	DOCK5
0.020493	12	ASH2L
0.020543	12	TRIM65
0.020543	12	ANKRD52
0.020559	12	SMARCA2
0.020574	12	ZFC3H1
0.020592	12	GTF2E1
0.0206	12	CHAC2
0.020629	12	SUMO1
0.020671	12	C2CD5
0.020731	12	FAM50A
0.020731	12	BPGM
0.02075	12	MAPRE2
0.020777	12	GLCE
0.020784	12	ABCB8
0.0208	12	FAM234A
0.020865	12	IRF2BP1
0.020866	12	PSMC2
0.020879	12	PALLD
0.020886	12	POLR2H
0.020914	12	TOMM22
0.020923	12	RPS24
0.021008	12	VRK2
0.021013	12	TXNDC15
0.021014	12	TTC4
0.021087	12	DHX9
0.02118	12	XRN1
0.021222	12	RPS6KB1
0.021222	12	TRPT1

0.021243	12	RPL11
0.021243	12	PRPF38A
0.021253	12	CHID1
0.021254	12	HRH1
0.021276	12	RAP1A
0.021313	12	CDC27
0.021314	12	UTP23
0.021316	12	CCDC22
0.021316	12	ATP11C
0.021326	12	SH3BP4
0.021352	12	GALNT6
0.021366	12	SNCA
0.021367	12	NUFIP2
0.021411	12	COA6
0.02147	12	MFF
0.02147	12	AATF
0.021514	12	HAGH
0.021589	12	OPA3
0.021603	12	SUPT16H
0.021673	12	SGTB
0.021689	12	IGF2BP1
0.021706	12	GSK3A
0.021709	12	PPP4C
0.021732	12	RPS21
0.021732	12	BAZ1A
0.021732	12	LEMD3
0.021733	12	SLC30A7
0.021744	12	APTX
0.021781	12	ALG3
0.021821	12	NMNAT1
0.021887	12	SLC39A14
0.021893	12	GSR
0.021989	12	GUF1
0.022004	12	HGS
0.022012	12	MAP4K3
0.022096	12	SERINC1
0.022141	12	GNA12
0.022219	12	ZCCHC17
0.022228	12	SNRNP40
0.022262	12	NDUFB11
0.022302	12	PSMA4
0.022302	12	SMARCC1
0.022302	12	HAUS1
0.022317	12	ISCA1
0.022327	12	PWP2
0.022354	12	GALNT10
0.022375	12	LGALS7
0.022375	12	LIG4
0.022398	12	EIF5A
0.022398	12	SSRP1

0.022398	12	SNX27
0.02241	12	DNAJC1
0.022418	12	POLA2
0.022432	12	CMTM3
0.022432	12	PARVB
0.022454	12	RNF149
0.022457	12	COA7
0.022477	12	SLC35E1
0.022499	12	CDC42EP1
0.022505	12	GTF3C3
0.022507	12	GPALPP1
0.022518	12	HSD17B12
0.022555	12	SLC1A4
0.022582	12	SUPV3L1
0.02261	12	B4GAT1
0.02261	12	MLLT1
0.02261	12	RSBN1
0.02261	12	DNMBP
0.02261	12	MTDH
0.02261	12	LRRC8A
0.02261	12	CIP2A
0.02261	12	APBB2
0.02261	12	TOR3A
0.02261	12	NKIRAS2
0.022626	12	MRPS16
0.022637	12	ASF1A
0.022646	12	TLE3
0.022646	12	EHMT2
0.022671	12	USP16
0.022673	12	MRPL17
0.02271	12	DNAJA4
0.02271	12	LYAR
0.022737	12	PPID
0.022779	12	MATR3
0.022779	12	SESTD1
0.022811	12	EBNA1BP2
0.022881	12	SHE
0.022891	12	NRGN
0.022918	12	MMGT1
0.022937	12	HDHD2
0.022999	12	CRYBG3
0.022999	12	RBM4
0.023089	12	AKAP10
0.0231	12	GMPPB
0.023128	12	NCEH1
0.023136	12	STK38
0.023164	12	SPARC
0.023164	12	SEL1L3
0.023181	12	DCAF6
0.023191	12	CDC37

0.023222	12	FTSJ3
0.023227	12	PSMB1
0.023227	12	TJP2
0.023252	12	FADS3
0.023276	12	CRTAP
0.023282	12	HBB
0.023394	12	INTS2
0.023403	12	LRPAP1
0.02342	12	VAMP2
0.023428	12	NDUFA6
0.023529	12	NUDT14
0.023578	12	PLAUR
0.023582	12	SRC
0.023593	12	EDC3
0.02362	12	BRD8
0.023622	12	NRAS
0.023643	12	RTN3
0.023679	12	SPTLC1
0.023692	12	ANKRD17
0.023692	12	IGBP1
0.023692	12	DDX54
0.023731	12	CDKN1A
0.023735	12	SNIP1
0.023737	12	TMED7
0.023779	12	AP4E1
0.023785	12	MFSD1
0.023787	12	TMEM263
0.023866	12	CBX5
0.023872	12	DNAJB14
0.023874	12	NOC2L
0.02392	12	CAPRIN1
0.02397	12	RAD17
0.023987	12	RPL37
0.024016	12	RAB31
0.02405	12	GRK5
0.024125	12	STAMBP
0.024158	12	FANCD2
0.024176	12	NEDD4
0.024176	12	MTERF3
0.024176	12	POP1
0.024176	12	C7orf50
0.02419	12	PDE4D
0.02422	12	SF3B3
0.024258	12	DDI2
0.024281	12	HEXIM1
0.024298	12	EFNB2
0.024321	12	SNRPB
0.024392	12	NCK1
0.024443	12	OCIAD2
0.02448	12	MCOLN1

0.024484	12	GTPBP10
0.024516	12	NUDT19
0.024561	12	IBTK
0.024718	12	CALD1
0.024726	12	H2AZ1
0.024755	12	WDR45
0.024808	12	GPX8
0.024843	12	PALS2
0.024854	12	BRAF
0.024854	12	SEC61G
0.024891	12	SARNP
0.024896	12	NSUN5
0.024933	12	NCK2
0.02496	12	KMT2D
0.02498	12	RNF170
0.025068	12	PEPD
0.025095	12	FN1
0.025095	12	AHSG
0.025095	12	CZIB
0.025135	12	GOT2
0.025139	12	MRPS14
0.025156	12	LRRC8D
0.025266	12	TRIM33
0.02527	12	NARS1
0.02527	12	STARD7
0.025316	12	ESD
0.025328	12	KLHDC3
0.025343	12	FHIP2A
0.025396	12	SERPINA1
0.025402	12	ILF2
0.025419	12	FAM107B
0.025436	12	WBP4
0.025458	12	NPRL3
0.025458	12	RTN4
0.025465	12	ACSL1
0.02547	12	PLP2
0.02547	12	TWF2
0.025494	12	RGS10
0.025547	12	GPR89A
0.025653	12	PSMA3
0.025653	12	DHDDS
0.025657	12	HK2
0.025664	12	EFTUD2
0.025693	12	SLC25A5
0.025693	12	EMC8
0.025693	12	AK4
0.025711	12	CAMK2G
0.025775	12	KPNA4
0.025821	12	ACBD6
0.025841	12	PRAG1

0.025856	12	MACO1
0.025886	12	TCIRG1
0.025888	12	MED25
0.02596	12	PLEKHG5
0.02596	12	GOLPH3
0.02596	12	SNX8
0.025965	12	UGCG
0.025965	12	RPUSD2
0.026068	12	TFPI2
0.026114	12	SMCHD1
0.026135	12	BAZ2A
0.026181	12	AP5Z1
0.026181	12	SRRM1
0.026181	12	TMEM168
0.026215	12	DAP3
0.026222	12	PUM3
0.026247	12	CDK5RAP1
0.026334	12	RAB14
0.026338	12	PCDH12
0.026371	12	ILK
0.026396	12	GOLGA7
0.026408	12	ERG28
0.02644	12	NHSL1
0.026512	12	HUS1
0.026522	12	ITCH
0.026539	12	GALK2
0.026571	12	CHAF1B
0.026571	12	CHCHD2P9
0.026635	12	EIF5
0.026658	12	SGPP1
0.026717	12	AP2B1
0.026726	12	MAP7D3
0.026741	12	PRKCSH
0.026741	12	YIF1B
0.02676	12	CBL
0.026814	12	CMAS
0.02682	12	MAPK8
0.026983	12	PITRM1
0.026995	12	H1-2
0.026995	12	PXN
0.026995	12	RAB3GAP1
0.02707	12	PREP
0.027093	12	SRSF6
0.027093	12	PTRH1
0.027101	12	COQ9
0.027149	12	PEDS1
0.027177	12	RBM26
0.027191	12	NAV1
0.027195	12	MTG2
0.027294	12	DICER1

0.027301	12	DLG5
0.027317	12	TSC1
0.027357	12	ARHGEF18
0.027515	12	SNCG
0.027515	12	SLC26A2
0.027515	12	PTPN11
0.027529	12	RERE
0.027554	12	CCDC134
0.027558	12	TMEM87A
0.027577	12	H1-3
0.027577	12	FUNDC2
0.027602	12	MTHFD1L
0.027636	12	NDUFC2
0.027636	12	TPI1
0.027636	12	TMEM192
0.027672	12	RPL36AL
0.02768	12	DOCK10
0.027769	12	RASAL2
0.027808	12	NAA40
0.027828	12	TRIR
0.02786	12	GYG1
0.027919	12	EIF5B
0.027919	12	SLC35F6
0.027956	12	ATXN2
0.027956	12	MTRR
0.028023	12	MCL1
0.028052	12	GNL2
0.02806	12	LOX
0.028064	12	PDCD6IP
0.028296	12	RASA1
0.028316	12	ALDH1L2
0.028332	12	CHMP6
0.028342	12	HERPUD1
0.028358	12	DSTN
0.028361	12	BRD4
0.028365	12	EIF4E2
0.028365	12	RPS25
0.028365	12	BRD3
0.028365	12	USP36
0.028398	12	RBPJ
0.028436	12	ARPC4
0.028436	12	P2RX4
0.028448	12	TNFRSF10D
0.028451	12	PIAS4
0.028451	12	ELAC2
0.028473	12	HSPA4
0.028487	12	SNAPIN
0.028487	12	UBAP2
0.028491	12	BPNT2
0.028564	12	ANKRD54

0.028576	12	NSL1
0.028614	12	ARL1
0.028614	12	ANKLE2
0.028749	12	MAOA
0.028778	12	TUBB4A
0.028778	12	ERCC6L
0.028865	12	RPS16
0.02888	12	PDPK1
0.028889	12	PPIP5K2
0.028892	12	USP46
0.0289	12	MAPRE1
0.028982	12	B4GALT7
0.02899	12	UBE2G1
0.029021	12	DDX6
0.029039	12	SP1
0.029039	12	CD55
0.029039	12	MCC
0.029039	12	ARRDC1
0.029054	12	DNAJC15
0.029103	12	TAF15
0.029104	12	ECSCR
0.029119	12	DCBLD1
0.029185	12	F8A1
0.029405	12	TGM3
0.029427	12	UBE2K
0.029439	12	PIGS
0.029446	12	FITM2
0.029446	12	HDGFL3
0.029449	12	RFK
0.029486	12	DCAF11
0.02953	12	PSMD11
0.02953	12	TUBG1
0.029575	12	ZFP64
0.029686	12	MAPKBP1
0.029698	12	DNAJC30
0.029722	12	DPH1
0.029747	12	IL3RA
0.029747	12	SLFN5
0.029771	12	ENDOD1
0.029771	12	SAMM50
0.0298	12	IFT74
0.029929	12	MOCS2
0.029937	12	XXYLT1
0.030049	12	H2BC26
0.030084	12	CASP4
0.030084	12	KLC2
0.0301	12	TNPO1
0.030106	12	TOR1AIP1
0.030172	12	CRBN
0.030275	12	KIF2C

0.030279	12	COL13A1
0.030342	12	ATL2
0.030357	12	IFT57
0.030364	12	ACVRL1
0.030406	12	HSD17B10
0.03041	12	TSEN54
0.030463	12	VDAC1
0.030465	12	NOC3L
0.030479	12	RPL23A
0.030479	12	ZNF346
0.030483	12	PKM
0.030662	12	HTRA1
0.030685	12	POLR1A
0.030782	12	PSMG1
0.030826	12	ABRAXAS2
0.030839	12	MBD4
0.030946	12	PLCD1
0.030978	12	ACAD8
0.03102	12	DMAC2
0.031167	12	LACTB2
0.03123	12	KPRP
0.031302	12	ACTR8
0.031302	12	CWC15
0.031328	12	CNBP
0.031465	12	FBLIM1
0.031507	12	PHF5A
0.031507	12	ERCC4
0.03156	12	EXOSC2
0.03156	12	ABCF2
0.031595	12	IMP4
0.031603	12	EWSR1
0.031645	12	ZCCHC4
0.03165	12	GLT8D1
0.031671	12	SLC2A3
0.031682	12	TMEM132A
0.031693	12	HNRNPDL
0.031717	12	ZFYVE16
0.03176	12	TPD52
0.031807	12	CDKN2AIP
0.031864	12	ZNHIT2
0.031865	12	GPR180
0.031943	12	SPOCK1
0.03197	12	SMG9
0.031978	12	BLTP2
0.031978	12	METTL3
0.032062	12	RPL3
0.032089	12	GLIPR1
0.0322	12	POLR1F
0.0322	12	CHD1L
0.032212	12	PTGS1

0.032273	12	RPS14
0.032284	12	TCEA1
0.032346	12	MAPKAP1
0.032574	12	ISCU
0.032579	12	NHP2
0.03258	12	PEA15
0.032686	12	EIF4A2
0.032694	12	NONO
0.032745	12	TARS1
0.032788	12	DSG1
0.032816	12	RPL22
0.03282	12	PFKFB3
0.032838	12	RBMX2
0.032945	12	SRSF4
0.032956	12	SF3A2
0.032975	12	GTF2H3
0.03311	12	KRIT1
0.03311	12	ROMO1
0.03311	12	GPATCH4
0.033145	12	PLAU
0.033145	12	NADSYN1
0.033145	12	ARFGAP2
0.033251	12	RNF113A
0.033373	12	KPNA3
0.033373	12	ATP6V1E1
0.033373	12	SERF2
0.033416	12	EYA3
0.033549	12	ALAS1
0.033551	12	SUMF1
0.033563	12	H1-0
0.033565	12	RCN3
0.033808	12	SLC7A11
0.033819	12	PGM3
0.033834	12	RANBP3
0.03386	12	MED12
0.033907	12	MRPS28
0.033941	12	LAMTOR2
0.033946	12	ELOVL1
0.033964	12	SLC44A1
0.033972	12	TBL2
0.033978	12	SEPTIN6
0.033978	12	CC2D1B
0.033986	12	SPCS3
0.033986	12	DLC1
0.034007	12	RCC1
0.034017	12	TMEM98
0.034035	12	HAUS8
0.034161	12	CEP170B
0.034167	12	PDCL3
0.03421	12	INO80

0.034225	12	PLEKHO2
0.034228	12	UNC45A
0.034317	12	ATAD1
0.034337	12	POLR1E
0.034414	12	METAP1
0.034419	12	SCD5
0.034528	12	CCT2
0.034603	12	AMZ2
0.03464	12	MTHFD2
0.03464	12	MMP14
0.03464	12	PTTG1IP
0.034682	12	LETM1
0.034723	12	FIGNL1
0.034723	12	SPATS2L
0.034741	12	WAC
0.034766	12	CWC25
0.034794	12	TOX4
0.034846	12	UPRT
0.035096	12	PRKACA
0.035096	12	CIZ1
0.035113	12	LGALS8
0.035132	12	DHRS1
0.035156	12	PLAT
0.035156	12	VEZF1
0.035201	12	PKP4
0.035205	12	PLA2G4A
0.035278	12	APOA1
0.035278	12	DNAJB1
0.035278	12	TFDP1
0.035292	12	NDUFB8
0.03534	12	ZNF326
0.035437	12	EEF1D
0.035484	12	GPKOW
0.035542	12	PDCD2
0.035719	12	TDRKH
0.035759	12	RRP12
0.03577	12	SMC1A
0.03577	12	TONSL
0.035774	12	NOP14
0.035837	12	DRAP1
0.035956	12	RAB34
0.035994	12	TAF4
0.036001	12	TAOK2
0.036104	12	TMPO
0.036176	12	DPF2
0.036176	12	DKK3
0.036191	12	JMJD1C
0.036237	12	SEC24A
0.036237	12	ACO1
0.036291	12	TSPAN6

0.036291	12	NADK
0.036291	12	NLRX1
0.036291	12	PLEKHA3
0.036373	12	NUCKS1
0.036382	12	OIP5
0.03654	12	MRPL37
0.036656	12	CARS2
0.036678	12	PSMG3
0.036795	12	HELZ
0.036882	12	ZDHHC5
0.036959	12	AARSD1
0.036964	12	PAICS
0.037086	12	MSI2
0.037114	12	NUBP2
0.037156	12	NHLRC2
0.037158	12	SNRPG
0.037158	12	C5orf22
0.037158	12	L3HYPDH
0.037248	12	PSMA1
0.037312	12	MOB2
0.037363	12	POM121
0.037453	12	TECPR1
0.037612	12	FBH1
0.037623	12	RIOX1
0.037776	12	C3orf38
0.037877	12	COL1A1
0.037877	12	CARD11
0.037885	12	TXNDC9
0.037933	12	TAF5
0.037933	12	ARL6IP4
0.037933	12	MARF1
0.037991	12	SAMD4A
0.038044	12	ASDURF
0.038092	12	STX2
0.038092	12	ARHGDIA
0.038157	12	ADGRG6
0.038189	12	IFRD2
0.0382	12	POLR2M
0.038217	12	HAUS3
0.03829	12	RAP2B
0.038291	12	AZI2
0.038332	12	CHM
0.038375	12	EFHD2
0.038395	12	UXT
0.038405	12	GADD45GIP1
0.038441	12	HAX1
0.038441	12	WIPI1
0.038441	12	IFT27
0.038462	12	EMG1
0.038477	12	EFEMP1

0.038477	12	CYBRD1
0.038477	12	TTC27
0.038477	12	SULF2
0.038499	12	NELFA
0.038499	12	SAMSN1
0.038533	12	MFN2
0.038533	12	VPS26B
0.038644	12	BCAR1
0.038644	12	SVIP
0.038678	12	HGH1
0.038678	12	RABL2A
0.038721	12	FBXW11
0.038736	12	SCAMP3
0.03874	12	CLN5
0.038744	12	AXL
0.038766	12	HDHD5
0.038852	12	POLRMT
0.038862	12	ACSS1
0.038915	12	VDAC3
0.03893	12	SLC7A1
0.039045	12	TOMM34
0.039102	12	ZNF618
0.039228	12	LGALS1
0.039228	12	HNRNPH3
0.039228	12	RBM17
0.039245	12	NT5DC3
0.039342	12	H1-5
0.039366	12	SMAP
0.039506	12	MEGF8
0.039561	12	POLR3C
0.0396	12	ZNF598
0.0396	12	GNPNAT1
0.039604	12	PHKA1
0.039614	12	TAPBPL
0.039628	12	DMAC2L
0.039642	12	CASP10
0.039663	12	PALS1
0.039723	12	ENO2
0.039795	12	DVL1
0.039795	12	GAN
0.039818	12	CSDE1
0.039846	12	EXTL2
0.040058	12	CKAP2
0.040087	12	SURF6
0.040089	12	MINDY3
0.040118	12	EFNB1
0.040132	12	SEC13
0.040153	12	FZR1
0.040195	12	RBMS1
0.04032	12	LIMS1

0.04032	12	TPM4
0.04032	12	TARBP1
0.04032	12	CFL2
0.040327	12	DDX28
0.040349	12	EDIL3
0.040388	12	AKT3
0.040445	12	PATL1
0.040561	12	SPRR1B
0.040571	12	EIF4A3
0.040598	12	TAF12
0.040705	12	VWA8
0.040831	12	DSP
0.040844	12	ERI3
0.040844	12	MRPL41
0.04087	12	POLR1C
0.040936	12	TIMELESS
0.04094	12	UTP20
0.040964	12	NR2C2
0.040982	12	CTSL
0.040992	12	CORO1C
0.041009	12	XPNPEP3
0.041212	12	SPPL2B
0.041258	12	REEP4
0.041325	12	FMC1
0.041421	12	ATN1
0.041469	12	ETS1
0.0415	12	WDR45B
0.041614	12	GEMIN6
0.041619	12	ADI1
0.041734	12	NPHP3
0.041773	12	KRT80
0.041835	12	DIPK2A
0.042032	12	SUPT6H
0.042064	12	NFKBIE
0.042064	12	CCDC117
0.042125	12	RAB5C
0.042176	12	LIN37
0.042202	12	OSBPL3
0.042242	12	MSMO1
0.042388	12	CUL4B
0.042388	12	FARP1
0.042405	12	COIL
0.042468	12	PRSS23
0.042714	12	BLOC1S1
0.042784	12	PPP1R8
0.042809	12	CWC27
0.043078	12	ADAM10
0.043296	12	MAD2L1
0.043303	12	DGAT1
0.04332	12	THOC3

0.043327	12	CD2AP
0.043329	12	FOXJ3
0.043392	12	METTL9
0.043619	12	ARMH3
0.043637	12	HIRA
0.043798	12	PAK4
0.043798	12	MRPS7
0.043803	12	PYGB
0.043859	12	SYNE3
0.043868	12	CDK17
0.043908	12	PQBP1
0.043922	12	CPNE3
0.043981	12	GAPDH
0.043985	12	VPS26A
0.043998	12	THUMPD1
0.043999	12	FAHD2A
0.044	12	BNIP2
0.044	12	RC3H1
0.044069	12	GOLM2
0.044179	12	MIEF1
0.044184	12	RPL5
0.044191	12	MRPL38
0.044191	12	KTI12
0.044285	12	ARFGEF2
0.044314	12	METTL2A
0.04444	12	NUDT16L1
0.044564	12	ELF1
0.044583	12	AP4B1
0.044638	12	KGD4
0.044756	12	SUFU
0.044848	12	SLC25A10
0.044849	12	CRYZL1
0.044876	12	DVL3
0.045001	12	CCN1
0.0451	12	EI24
0.045105	12	CASP8
0.045105	12	JAGN1
0.04513	12	GAB2
0.045147	12	MYL6
0.045276	12	GLUL
0.045286	12	REXO4
0.045319	12	EIF3G
0.045416	12	PHKA2
0.045434	12	GALNT4
0.045435	12	NOP56
0.045435	12	MAP3K7
0.045519	12	SENP6
0.045569	12	DDX24
0.045629	12	MFGE8
0.045633	12	PRRC2A

0.045637	12	CLN6
0.045642	12	SUMO2
0.045648	12	FLCN
0.045711	12	ZC3H15
0.045779	12	CADPS2
0.045793	12	RPS19
0.045857	12	DCTPP1
0.045931	12	FDFT1
0.045931	12	MEAK7
0.045944	12	FBXO11
0.045963	12	NUSAP1
0.045963	12	ACVR1
0.046014	12	MCUR1
0.046164	12	TOP2B
0.046292	12	NEDD9
0.046324	12	NSD2
0.046401	12	LMNB1
0.046554	12	CFLAR
0.046585	12	ULK3
0.0467	12	RPS2
0.046799	12	WDR41
0.046909	12	ITPRID2
0.046909	12	SAP130
0.046939	12	PSMA6
0.047283	12	TK1
0.047331	12	TCOF1
0.047348	12	SMURF2
0.047401	12	S100A13
0.047407	12	SYNJ2
0.047407	12	TMEM43
0.047559	12	CHST12
0.047592	12	RELL1
0.047785	12	PLRG1
0.047798	12	CLNS1A
0.04782	12	IDH3A
0.047893	12	IFT25
0.048	12	HSDL1
0.048114	12	RABGGTB
0.048173	12	EIF2S3
0.048173	12	TWF1
0.048173	12	DDX49
0.048175	12	RPS17
0.048179	12	EIF2S1
0.048224	12	MRPS18B
0.048403	12	PSMA2
0.048403	12	MTMR12
0.048531	12	KLF16
0.048537	12	MYO9A
0.048657	12	NOL7
0.048665	12	PHF8

0.048692	12	PAPSS1
0.048695	12	FASTKD5
0.04879	12	PHC3
0.04883	12	RRAS
0.04883	12	SMC5
0.048912	12	DDX1
0.049014	12	GTF3C4
0.049047	12	LAMTOR3
0.049129	12	CARM1
0.049136	12	VIM
0.049333	12	NAV3
0.049333	12	ATPAF2
0.049333	12	NDUFAF4
0.049362	12	TAF9B
0.049471	12	EIF4G2
0.04956	12	PCMT1
0.04956	12	RPL13
0.04956	12	MSRA
0.049563	12	USP15
0.049622	12	FBXL15
0.049635	12	UBE2M
0.049841	12	CMC2
0.049889	12	ESM1
0.049987	12	COX5B
0.050031	12	TMX2
0.050038	12	WDTC1
0.050046	12	ARID2
0.050061	12	LIMD1
0.050165	12	RBPMS
0.050256	12	LASP1
0.050315	12	TDP2
0.050531	12	DDX52
0.050565	12	FIBP
0.050632	12	VTI1A
0.050674	12	CLMN
0.05072	12	ACOT7
0.05072	12	PHIP
0.050737	12	MTPN
0.050862	12	GNB4
0.050905	12	WRAP53
0.051025	12	SSBP1
0.051096	12	DDX3X
0.051096	12	MAP1B
0.051109	12	SNX7
0.051182	12	MERTK
0.051202	12	PRKCI
0.051202	12	DBN1
0.051206	12	KDM4B
0.051207	12	RALBP1
0.051207	12	SHROOM4

0.05132	12	COPE
0.051337	12	STRN4
0.05137	12	SCARF1
0.051403	12	BUD23
0.051405	12	STARD3NL
0.051405	12	SLAIN2
0.051484	12	TRMT6
0.051527	12	NDUFB5
0.051578	12	IFIT5
0.051827	12	HEBP1
0.052115	12	TENT4B
0.052115	12	MBD1
0.052229	12	SNRK
0.052241	12	CELF2
0.052277	12	SLC41A3
0.05231	12	COX20
0.052393	12	TNIK
0.052441	12	DSN1
0.052615	12	TP53BP1
0.052726	12	SQSTM1
0.052887	12	NPC1
0.052938	12	NPM1
0.052953	12	KLHL9
0.053047	12	PRAF2
0.053127	12	BTN3A1
0.053128	12	RPL34
0.053235	12	MYH9
0.053374	12	EIF2A
0.053398	12	CCT8
0.053463	12	TBCA
0.053502	12	RPS27A
0.053619	12	MYL6B
0.053619	12	DMWD
0.05372	12	MEPCE
0.05398	12	CLTA
0.054504	12	CFL1
0.054533	12	PPIL3
0.054692	12	CENPV
0.054707	12	GOLIM4
0.054707	12	CDC42EP3
0.054729	12	PYGM
0.054836	12	MYCT1
0.054905	12	SKA1
0.055136	12	COTL1
0.055192	12	TPM1
0.055195	12	TBC1D24
0.055341	12	BTN2A1
0.055425	12	PRC1
0.055498	12	C10orf67
0.055531	12	ZMYM2

0.055531	12	CUSTOS
0.055531	12	DNAJA3
0.055555	12	NCOA5
0.055578	12	HCLS1
0.055584	12	TRIM38
0.055584	12	PABPC4
0.055659	12	DR1
0.055659	12	ZBTB8OS
0.055776	12	RABEP2
0.055797	12	HMMR
0.055899	12	COL4A2
0.055995	12	RIGI
0.056025	12	METTL5
0.056111	12	AFAP1
0.056111	12	R3HCC1
0.056182	12	PPM1G
0.056182	12	MTMR9
0.056429	12	SRGN
0.056494	12	MT1E
0.056525	12	FNDC3B
0.056537	12	TRIM47
0.056539	12	RBM15B
0.056699	12	RREB1
0.05677	12	ALB
0.05677	12	CALCOCO2
0.056901	12	UTP3
0.05696	12	PUM1
0.05696	12	SYNPO
0.05696	12	RPP25L
0.05696	12	ABHD11
0.05696	12	TRMT1
0.056966	12	SSR4
0.056974	12	IFI44
0.056974	12	TUBGCP6
0.056991	12	REXO2
0.057381	12	CAPZA1
0.05753	12	RAB2A
0.05753	12	THOC1
0.05753	12	GLIPR2
0.057533	12	ZFP91
0.057556	12	MGAT5
0.057616	12	TRIO
0.057659	12	TBL3
0.057708	12	NEMP1
0.057731	12	CDC45
0.057804	12	B3GLCT
0.057814	12	THEM4
0.057838	12	TMEM65
0.057933	12	UBE2J1
0.05797	12	NUF2

0.057971	12	MAD2L1BP
0.057985	12	TRIT1
0.058178	12	DUSP11
0.058188	12	GYS1
0.058213	12	CSNK1E
0.058368	12	PFN2
0.058418	12	STRADA
0.058482	12	NEPRO
0.058593	12	ANLN
0.058688	12	FHL1
0.058739	12	BMPR2
0.058753	12	KDSR
0.058781	12	UBAP2L
0.058823	12	EIF3D
0.059222	12	TRA2B
0.059258	12	NES
0.059258	12	QTRT2
0.059258	12	PA2G4
0.059297	12	PIGO
0.059302	12	IL6ST
0.059309	12	UBE2E1
0.059333	12	ALCAM
0.059554	12	WWC2
0.05957	12	RAB23
0.059612	12	ZC3H18
0.059631	12	LRP8
0.059651	12	ANPEP
0.059668	12	COL4A1
0.059742	12	MRPL34
0.059754	12	FOXO1
0.059875	12	PASK
0.059908	12	SURF1
0.059909	12	PCDH10
0.059949	12	DNAJB12
0.05997	12	DGKZ
0.059982	12	RPLP2
0.059982	12	RPF2
0.060264	12	KYAT1
0.060284	12	POLR3A
0.060445	12	SLC35A2
0.060556	12	GFM2
0.060636	12	MMADHC
0.060654	12	CACNA2D1
0.060654	12	TGIF1
0.060654	12	HYCC1
0.060762	12	CHTOP
0.060811	12	ARHGAP22
0.060811	12	RALY
0.060901	12	RELB
0.060912	12	DENR

0.061012	12	LAGE3
0.061067	12	RNF169
0.061118	12	ARHGEF11
0.061118	12	C8orf82
0.061118	12	MIER1
0.061261	12	PITPNM1
0.061261	12	CNN2
0.061559	12	PPIE
0.061582	12	AAK1
0.061742	12	CDK1
0.061833	12	ALKBH4
0.06191	12	CDK7
0.062113	12	MECP2
0.062129	12	NOTCH2
0.062129	12	NT5C3B
0.062136	12	WTAP
0.062205	12	ZC3H11A
0.062301	12	ENOPH1
0.062327	12	GRB2
0.062355	12	EVA1A
0.06236	12	SETDB1
0.062377	12	MRTFA
0.062433	12	HNRNPA0
0.062433	12	FHL3
0.062451	12	MVB12A
0.062648	12	MTREX
0.062651	12	KHDRBS1
0.062664	12	DEK
0.062669	12	SRSF7
0.062698	12	EIF2B2
0.062758	12	UTP4
0.062835	12	SCYL2
0.062846	12	CCDC47
0.062876	12	LDLR
0.062924	12	TSPAN9
0.063074	12	PABPC1
0.063118	12	CCNT1
0.06325	12	LYPLA2
0.063318	12	ZNF574
0.063597	12	ETNK1
0.06365	12	PHLDA2
0.063886	12	ATXN2L
0.063918	12	CAMK1
0.06418	12	ACIN1
0.064184	12	MMP2
0.064288	12	COX7A2L
0.064388	12	ECD
0.064396	12	SHOC2
0.064402	12	ETV3
0.064486	12	RAB24

0.0647	12	GSTM3
0.064772	12	TAF7
0.064772	12	PLEKHA5
0.064784	12	CD82
0.064895	12	TRAPPC10
0.064895	12	USP3
0.064963	12	PIK3CB
0.064963	12	PRKAG1
0.065145	12	DVL2
0.065181	12	CCDC51
0.065184	12	IMP3
0.065247	12	THOP1
0.065258	12	EIF3M
0.065792	12	GPN3
0.065798	12	MYBBP1A
0.066068	12	TCF25
0.066249	12	SERBP1
0.066303	12	NSMCE1
0.066364	12	SLC12A5
0.06637	12	HCFC2
0.066515	12	STK17A
0.066542	12	TTC9C
0.066577	12	RRP1B
0.06664	12	COX15
0.06671	12	RBFOX2
0.06671	12	SIRT3
0.066737	12	SMU1
0.066767	12	UFSP2
0.066803	12	STX7
0.066903	12	SMN1
0.066905	12	TLK1
0.067091	12	RPL32
0.06714	12	BMP2K
0.067326	12	PTPN1
0.067564	12	TBL1XR1
0.067645	12	NUBPL
0.067847	12	EPG5
0.067944	12	PALM2AKAP2
0.068048	12	MAFG
0.068064	12	SMAD4
0.068064	12	EHD1
0.068111	12	YWHAG
0.06816	12	OARD1
0.06822	12	LSM14B
0.068263	12	FBXW9
0.068265	12	KATNAL1
0.068422	12	WDFY1
0.068429	12	SETD1A
0.068583	12	SHMT2
0.068621	12	POM121C

0.068701	12	TMEM109
0.068743	12	RPL28
0.068743	12	KHNYN
0.068823	12	GNG12
0.068866	12	GMEB2
0.068912	12	TAF1
0.069078	12	UNC13B
0.069159	12	TUBA3C
0.069172	12	TTN
0.06918	12	RBM8A
0.069306	12	CCDC137
0.069358	12	CEP170
0.069367	12	GCLM
0.069367	12	DDX56
0.069403	12	PWP1
0.069568	12	ZNF22
0.069572	12	HSDL2
0.069676	12	PRRC2C
0.069827	12	ATAD2
0.069898	12	RPS27L
0.070167	12	NOL6
0.070353	12	RHOC
0.070732	12	FPGS
0.070756	12	PIK3CG
0.071047	12	SLC25A19
0.0711	12	TAMM41
0.07111	12	NUP50
0.071169	12	AFF4
0.071298	12	SNW1
0.071548	12	YTHDF2
0.071768	12	BRD2
0.071903	12	FUBP1
0.071904	12	TMEM237
0.072054	12	PEAR1
0.072095	12	OGFOD3
0.072165	12	RPS27
0.072176	12	FXR2
0.072252	12	FADD
0.072417	12	RPS15A
0.072566	12	PSEN2
0.072569	12	RCE1
0.07263	12	DNTTIP1
0.072634	12	RPS10
0.07303	12	NF2
0.073038	12	PTRH2
0.07326	12	TP53BP2
0.073278	12	AP1S1
0.073531	12	HEATR6
0.073556	12	MAGED1
0.073696	12	RAB32

0.074071	12	CNPY3
0.074082	12	RFX5
0.074082	12	SCAP
0.074082	12	EIF1AD
0.074106	12	PPM1B
0.074123	12	ATP6AP2
0.074357	12	CASP3
0.074452	12	SEC14L1
0.074518	12	TSC22D4
0.07483	12	TARS2
0.075207	12	APOOL
0.075303	12	PCTP
0.075384	12	SLC25A25
0.075433	12	FHL2
0.075433	12	TNRC6A
0.075581	12	EXOSC10
0.075627	12	NME1
0.07574	12	NAP1L4
0.075808	12	ANAPC5
0.075912	12	RPAP2
0.075912	12	EPB41L3
0.076052	12	MRM3
0.076847	12	KANK1
0.076878	12	LRIG1
0.077008	12	WDR4
0.077072	12	SRPRB
0.077443	12	OSBPL2
0.077675	12	GCC1
0.077896	12	SMARCD2
0.078429	12	PTPRG
0.078429	12	MAP2K3
0.078442	12	CYTH1
0.078459	12	RGPD5
0.078488	12	MRPS18A
0.07864	12	SLC11A2
0.078704	12	UTP6
0.078727	12	BPTF
0.078787	12	PPP1R18
0.078855	12	UBL5
0.079099	12	TAF9
0.079099	12	ALDOA
0.079209	12	SLC1A5
0.079266	12	RPL26
0.079426	12	PTAR1
0.079527	12	MT-CO1
0.07965	12	AFTPH
0.079815	12	DNAJC5
0.079851	12	GNB2
0.079892	12	NCAPG
0.080029	12	CFAP410

0.080089	12	MLYCD
0.080272	12	SF3B5
0.08031	12	TMEM179B
0.08036	12	SMAP2
0.08041	12	GATD3
0.080585	12	PTBP3
0.080818	12	FMNL2
0.080987	12	NPR2
0.081069	12	LARP4
0.081073	12	NSUN4
0.081108	12	PRR11
0.081171	12	ICMT
0.081175	12	DPYSL3
0.081175	12	DPH5
0.081264	12	SDCBP
0.081294	12	NXT1
0.081294	12	ZNF330
0.081772	12	DLAT
0.082108	12	PTEN
0.082303	12	ATP6V0D1
0.082407	12	CYBC1
0.082579	12	MGST1
0.082758	12	EIF2B3
0.082807	12	PLK1
0.082829	12	ATP6AP1
0.082829	12	LTBP3
0.082933	12	PIAS3
0.083344	12	KIN
0.083346	12	METTL16
0.083346	12	POLR1B
0.083637	12	CCDC12
0.083848	12	ZNF207
0.084126	12	REPS1
0.084659	12	RNF10
0.084746	12	PREPL
0.084747	12	ECI2
0.084921	12	FBN2
0.084948	12	RBMS2
0.084948	12	SLC12A7
0.085042	12	ATP9B
0.085184	12	CCT7
0.085214	12	PRDX1
0.085257	12	MAK16
0.08527	12	PRR14
0.085278	12	GGCT
0.085342	12	SETD2
0.085342	12	CERT1
0.085654	12	CRTC3
0.085692	12	PARD3
0.085819	12	GTPBP6

0.086071	12	ARRB2
0.086073	12	RRP7A
0.086097	12	TRIM5
0.086595	12	LMCD1
0.086973	12	PNP
0.087001	12	PTPMT1
0.087158	12	NOL4L
0.087194	12	TRA2A
0.087534	12	APAF1
0.087542	12	EIF3L
0.087567	12	BMS1
0.087567	12	MAP1S
0.087567	12	JPT1
0.087567	12	SMG5
0.087567	12	MRPL46
0.087634	12	PHLDB1
0.08784	12	SNAP47
0.08784	12	YTHDC1
0.087991	12	JUN
0.088045	12	MCRIP1
0.088138	12	TRMU
0.088704	12	TUBA1B
0.088751	12	AUP1
0.088893	12	RPS6KB2
0.088953	12	FBXO30
0.089158	12	BUD31
0.089464	12	CLUH
0.089464	12	GAR1
0.089703	12	NSMCE3
0.089713	12	NLRP2
0.089965	12	NSD3
0.089988	12	GORASP1
0.090075	12	SH3RF1
0.090075	12	ZC3HAV1L
0.090243	12	ADK
0.09044	12	KPNA6
0.090657	12	SRSF10
0.090657	12	SLC7A5
0.090657	12	FLNC
0.090706	12	KANSL3
0.090786	12	ADGRL4
0.090825	12	CTSS
0.090905	12	CD276
0.090905	12	RBM19
0.090934	12	YEATS4
0.091114	12	CDK5RAP2
0.091463	12	MYH11
0.091463	12	CHORDC1
0.091655	12	DNASE1L1
0.091655	12	NEDD8

0.091667	12	WASHC2A
0.091795	12	STK11IP
0.09188	12	RBSN
0.092145	12	KIF11
0.092195	12	BANF1
0.092256	12	PRPS1
0.092287	12	SND1
0.092578	12	FCHSD1
0.092624	12	SFXN2
0.092646	12	CDK4
0.092804	12	SNRPA1
0.092804	12	CSNK1A1
0.092804	12	TPMT
0.092922	12	KANSL1
0.092922	12	MRPS24
0.092983	12	ITPRIP
0.093169	12	STX12
0.093182	12	GSPT1
0.093335	12	HNRNPR
0.093353	12	CDC42EP2
0.093576	12	RPS29
0.093576	12	RINT1
0.093702	12	LRRC1
0.093784	12	LZTR1
0.093956	12	GOSR1
0.09432	12	DDAH2
0.094386	12	ATG2B
0.094518	12	DHX40
0.094547	12	KAT6A
0.0946	12	WDR59
0.09476	12	SNAP23
0.094815	12	PNISR
0.094871	12	ANKZF1
0.094937	12	BCAS2
0.095185	12	IBA57
0.095422	12	TRIM3
0.095492	12	NAGPA
0.095732	12	TRIM24
0.095769	12	NFAT5
0.095769	12	ARG2
0.095809	12	TOP3A
0.095911	12	C2CD3
0.095966	12	CNOT10
0.096044	12	TAF6
0.096044	12	KDM2A
0.096173	12	DNMT3A
0.0964	12	AHSA1
0.096607	12	ZNFX1
0.096608	12	SPRYD7
0.096732	12	DYNLT1

0.096746	12	KIF22
0.096795	12	CCAR2
0.096818	12	GLO1
0.096956	12	ZMIZ2
0.097599	12	TMEM9
0.097629	12	RNF220
0.097738	12	CHML
0.097799	12	EPN2
0.097931	12	JAG2
0.098056	12	LDHB
0.09819	12	NRG1
0.098251	12	TERF2IP
0.098374	12	SLC15A4
0.098504	12	NCOR2
0.098919	12	PSEN1
0.098987	12	N4BP1
0.09905	12	FBXO28
0.099124	12	CYC1
0.099124	12	CEP131
0.099211	12	SNRNP70
0.099531	12	SH3RF3
0.100064	12	MOCS3
0.100064	12	ANXA2
0.100091	12	STOM
0.100254	12	PCDHGA12
0.100285	12	PDGFB
0.100566	12	PLOD2
0.100621	12	CLP1
0.101022	12	TRIM32
0.101046	12	TBC1D17
0.10137	12	TYW5
0.101412	12	PMPCB
0.101424	12	CTDSPL2
0.101424	12	PAPOLG
0.101452	12	CREB1
0.101513	12	CCNB1
0.101798	12	CDYL
0.102182	12	FIP1L1
0.102204	12	GCFC2
0.102325	12	MAP7D1
0.102338	12	USP19
0.102421	12	RPS20
0.102421	12	PPP4R2
0.102432	12	VARS2
0.102476	12	COPS8
0.102635	12	TSPYL1
0.10287	12	GFPT1
0.103504	12	NKTR
0.103605	12	ANKRD28
0.103625	12	NOL9

0.10371	12	MAGOHB
0.103799	12	BAG3
0.103855	12	NKAP
0.104136	12	DYNLL1
0.104162	12	PPP1R14B
0.104513	12	GPATCH1
0.104592	12	LATS1
0.104595	12	POLA1
0.104749	12	MDC1
0.104749	12	CDCA8
0.104906	12	MICU1
0.104959	12	TFB2M
0.104989	12	NUDC
0.104989	12	RRM2B
0.105061	12	MAP2K5
0.105061	12	FAF2
0.105061	12	UCK1
0.1052	12	SUZ12
0.105437	12	TGOLN2
0.105437	12	ASAH1
0.105651	12	SYNCRIP
0.105763	12	EIF3F
0.106069	12	LRRC41
0.106069	12	PPTC7
0.106377	12	RAB1A
0.106587	12	MVP
0.106815	12	CDR2
0.106857	12	PDXK
0.106886	12	RHOA
0.106886	12	TXNL4A
0.107116	12	OFD1
0.107196	12	MYL12B
0.107196	12	CXADR
0.107275	12	DHX33
0.107353	12	DCUN1D3
0.107414	12	DNAJA1
0.107439	12	RPS12
0.107474	12	EIF2S2
0.107665	12	PRKRIP1
0.108137	12	CRTC1
0.108237	12	PHAF1
0.108307	12	MRPS6
0.108323	12	FAS
0.108603	12	CCNB2
0.108662	12	ATP5MK
0.108726	12	ABI1
0.108871	12	FBXL6
0.108922	12	ZBTB33
0.109208	12	INPPL1
0.109208	12	MDH2

0.109243	12	ARMC10
0.10939	12	DHPS
0.109392	12	PPP1R3B
0.109405	12	GFER
0.109589	12	PSIP1
0.109952	12	ATP7A
0.110032	12	RING1
0.110051	12	ZDHHC17
0.11022	12	RGS12
0.110226	12	TMEM51
0.11032	12	SMPD1
0.11032	12	ELP6
0.110461	12	LZTFL1
0.110491	12	CNTNAP1
0.110522	12	SYPL1
0.110552	12	UBR1
0.110552	12	CLASRP
0.110611	12	TUT4
0.111118	12	NADK2
0.111353	12	YBX3
0.111384	12	KIF20A
0.111424	12	WDR35
0.111444	12	ITGAV
0.111497	12	SEMA3F
0.111497	12	UPP1
0.111497	12	GDPD1
0.111585	12	RGS19
0.111598	12	POFUT1
0.111632	12	DDX50
0.111646	12	CIAO1
0.111852	12	PGAM1
0.112123	12	CEP44
0.112258	12	VKORC1
0.112563	12	AP4M1
0.11257	12	EIF3E
0.112675	12	IRF2BPL
0.112696	12	SHKBP1
0.112768	12	RCHY1
0.112769	12	NCAPD3
0.112769	12	PSMC6
0.11277	12	RPL9
0.11283	12	MAVS
0.112854	12	BAZ1B
0.112984	12	POU2F1
0.113085	12	DDX39B
0.113472	12	SPDL1
0.113474	12	FLNB
0.1135	12	AURKB
0.11353	12	GK
0.113663	12	NME4

0.114066	12	RPL23
0.114217	12	GET4
0.114502	12	HCCS
0.114587	12	WASHC2C
0.114995	12	HNRNPA1
0.11525	12	NTMT1
0.115349	12	CAV2
0.115477	12	DDX19B
0.115501	12	ARHGAP45
0.115576	12	SLC66A3
0.115637	12	PNO1
0.115735	12	RBM28
0.115879	12	PTPRK
0.115879	12	CACUL1
0.115879	12	DCAKD
0.115901	12	ZNF428
0.115941	12	TCP1
0.115954	12	NSDHL
0.115993	12	BCAR3
0.116234	12	MTCL1
0.116379	12	NEB
0.116629	12	SRRM2
0.116659	12	TUBGCP5
0.116713	12	MRPL58
0.116825	12	WDR70
0.116925	12	UBAC2
0.116943	12	RNF114
0.11698	12	ZC3H14
0.116987	12	ARL6IP6
0.117251	12	TAGLN2
0.117491	12	PDAP1
0.117718	12	EHBP1
0.117797	12	VAT1
0.118083	12	TCF4
0.118083	12	TAOK3
0.118332	12	DPY19L1
0.118512	12	BTAF1
0.118552	12	ZNF579
0.118925	12	NTAN1
0.118974	12	GOSR2
0.118994	12	PCBP1
0.119277	12	NCBP2AS2
0.11957	12	LLPH
0.119603	12	CHD9
0.119699	12	ATF1
0.119861	12	CPNE8
0.120086	12	HMGN2
0.120086	12	KNSTRN
0.120212	12	SNX14
0.120237	12	PPIA

0.120794	12	LSM14A
0.120794	12	EXOC6B
0.121251	12	RNH1
0.121381	12	TMF1
0.121381	12	FSTL1
0.121406	12	KNOP1
0.121482	12	CTPS1
0.121483	12	MRPS2
0.121785	12	ELL
0.122751	12	CDR2L
0.122812	12	CIAPIN1
0.123483	12	NUDT18
0.123622	12	UBL4A
0.123686	12	CLTCL1
0.123801	12	NDST1
0.123976	12	QSOX1
0.124003	12	NCAPH2
0.124023	12	SERPINB8
0.124023	12	DAB2IP
0.124082	12	HNRNPK
0.12411	12	FLRT2
0.12411	12	GMIP
0.124214	12	LMO7
0.124256	12	ITPR1
0.124325	12	APOBEC3C
0.124379	12	RSRC2
0.124438	12	PARK7
0.124694	12	MAP4
0.124912	12	TMEM63B
0.1252	12	TTC17
0.125216	12	TRNAU1AP
0.125701	12	CLDN11
0.125944	12	FRMD5
0.125961	12	TIMM22
0.125983	12	ORC4
0.126125	12	ZFR
0.126127	12	FOSL2
0.12627	12	ROBO4
0.126475	12	SLC16A1
0.126804	12	URB1
0.126804	12	CFAP298
0.127032	12	TXLNG
0.127035	12	PIAS1
0.127839	12	SP3
0.12785	12	RDH11
0.127853	12	IGFBP7
0.127919	12	WDR1
0.128088	12	EPHB2
0.128099	12	ADAMTSL1
0.128128	12	ANKRD50

0.128128	12	CABIN1
0.12813	12	TOP2A
0.128134	12	RBM5
0.128185	12	KCTD21
0.128559	12	UNC119
0.128573	12	CDCA7L
0.128752	12	LIMS3
0.128799	12	NFYC
0.12886	12	ACTB
0.129047	12	RAB43
0.129047	12	UXS1
0.129059	12	NKRF
0.129059	12	UTP25
0.129155	12	SMARCA5
0.129196	12	UNG
0.129204	12	NFATC4
0.12931	12	TMEM222
0.129459	12	CACTIN
0.129563	12	C1orf52
0.129675	12	UIMC1
0.130146	12	ALYREF
0.1304	12	CRTC2
0.131192	12	TPBG
0.131257	12	BUD13
0.13152	12	CNOT6L
0.131577	12	BZW1
0.131603	12	PBK
0.131637	12	PISD
0.131699	12	EXOSC8
0.131902	12	RECK
0.131921	12	DPM3
0.132266	12	GNAI2
0.132364	12	HK1
0.132637	12	FAM98A
0.132653	12	SIPA1L3
0.13271	12	DDX10
0.132773	12	ATP1A1
0.132773	12	OTULIN
0.132865	12	RWDD4
0.133698	12	MID1
0.133954	12	IAH1
0.134239	12	ALDH1A3
0.134806	12	LPIN1
0.135015	12	HDAC8
0.135032	12	SGF29
0.135154	12	SLC3A2
0.13538	12	DAXX
0.135385	12	HERC2
0.135916	12	VAMP7
0.135916	12	DUS3L

0.135955	12	STAG1
0.136181	12	RTF2
0.13623	12	PRMT3
0.13623	12	MUL1
0.136717	12	P4HTM
0.136788	12	UBASH3B
0.136948	12	SESN2
0.137033	12	TSR3
0.137406	12	WDHD1
0.137406	12	YRDC
0.137447	12	DYNC1LI2
0.138283	12	SLC25A22
0.138293	12	BCL2L13
0.13832	12	EPC1
0.138424	12	RIN1
0.138424	12	MTFR1
0.138566	12	GALNS
0.138687	12	CEP78
0.138841	12	PAK1IP1
0.139042	12	DIMT1
0.13906	12	ZFAND1
0.13906	12	PLCL2
0.139215	12	EXOSC5
0.139272	12	ATRIP
0.139299	12	SRM
0.139299	12	WBP2
0.139589	12	SLC12A4
0.139599	12	UBXN6
0.139971	12	TMPPE
0.140077	12	SRSF3
0.140196	12	FKBP1A
0.140465	12	PHRF1
0.140847	12	KIFC1
0.140936	12	KLHL42
0.141151	12	MPHOSPH10
0.141151	12	RRP9
0.141331	12	NME2
0.141336	12	HSP90AA4P
0.141404	12	MYH14
0.141404	12	TTC5
0.141404	12	MINPP1
0.141422	12	RPL10
0.141449	12	MBNL1
0.141681	12	DYRK1A
0.141773	12	OSBPL5
0.141979	12	SPCS1
0.142561	12	PRPS2
0.142791	12	PRKCD
0.143037	12	AKAP1
0.143551	12	ATG16L1

0.143832	12	CUEDC2
0.144056	12	ZNF830
0.144126	12	DSC1
0.144332	12	ESF1
0.144582	12	ELAVL1
0.144786	12	MRPL32
0.144808	12	PKN3
0.144824	12	DHX34
0.145601	12	ATP6V1D
0.146203	12	FBL
0.146466	12	WDR20
0.146904	12	RNPS1
0.146904	12	IFT122
0.146969	12	ERGIC3
0.147272	12	YTHDF3
0.147295	12	RBMS3
0.147635	12	MED13L
0.147635	12	MECR
0.147792	12	MAP4K5
0.147856	12	TAB2
0.147876	12	RRP1
0.148175	12	RNF2
0.14848	12	PITPNA
0.148728	12	PDZD8
0.14873	12	NRF1
0.148898	12	RRS1
0.14927	12	GNL3L
0.149373	12	PDLIM3
0.149373	12	PDCD10
0.149567	12	MASTL
0.149626	12	TUBGCP4
0.149648	12	CAMLG
0.149648	12	SAFB
0.149648	12	SPG11
0.14978	12	SMG7
0.149876	12	GPATCH11
0.150094	12	CMC1
0.151019	12	CMSS1
0.151433	12	MEF2C
0.151436	12	TTYH3
0.15153	12	SNX17
0.151769	12	C1QTNF5
0.151971	12	MRPS17
0.152221	12	DAAM1
0.152452	12	BUB1
0.152487	12	GNPTAB
0.152522	12	TXNDC12
0.152617	12	TPRN
0.1529	12	EEF1AKMT2
0.153067	12	CAPN5

0.153154	12	TPM3
0.153154	12	FAM210B
0.153426	12	CCDC86
0.15344	12	HYI
0.15344	12	FKBP14
0.153443	12	IP6K1
0.153832	12	PPP1CC
0.153968	12	ELP5
0.154075	12	NUP35
0.154387	12	PANK3
0.154387	12	TYW1
0.15439	12	ENSA
0.154526	12	SEL1L
0.155273	12	NOP16
0.155357	12	PRNP
0.155472	12	DGKQ
0.155472	12	SLC35B2
0.155515	12	DNM1
0.155612	12	VPS13D
0.155612	12	SMCR8
0.155612	12	NEK6
0.155707	12	C8orf33
0.155762	12	SLTM
0.155888	12	A2M
0.155934	12	SYDE1
0.155934	12	TAOK1
0.15635	12	NEXN
0.156678	12	BLTP3B
0.156723	12	HNRNPC
0.157104	12	FERMT1
0.157337	12	RPL27A
0.157416	12	FADS1
0.157598	12	FERMT3
0.157622	12	ADISSP
0.157813	12	PIN1
0.157813	12	MACROD1
0.157831	12	RPS11
0.157918	12	NFKBIB
0.157978	12	MICALL2
0.158211	12	ATP5ME
0.158395	12	PAWR
0.15851	12	FER
0.158708	12	RASA3
0.158888	12	RAD21
0.159004	12	TNFAIP8L3
0.159448	12	TTF1
0.159492	12	STK11
0.159635	12	ARF4
0.159635	12	ARHGAP12
0.159635	12	SNX19

0.159664	12	EXOSC6
0.159866	12	NT5DC1
0.160001	12	ATF7
0.160313	12	LIMD2
0.160406	12	RBMX
0.160585	12	TNS3
0.160615	12	AKAP8L
0.160828	12	WWP2
0.16096	12	TSPAN14
0.161004	12	DYNLL2
0.16106	12	CELF1
0.161111	12	CEPT1
0.161155	12	TCAF2
0.1613	12	ZMAT3
0.161483	12	TTC19
0.162043	12	MAFF
0.162115	12	PAXIP1
0.163141	12	EXOSC9
0.163156	12	TTI1
0.163156	12	ADRM1
0.163283	12	PARP10
0.163585	12	NAB2
0.163585	12	DGKH
0.163585	12	SEC22A
0.163836	12	CBX2
0.163895	12	SPOUT1
0.163895	12	TIGAR
0.164328	12	ASPSCR1
0.164363	12	CLIC4
0.164444	12	SLC19A1
0.164487	12	CHD2
0.164646	12	PLXNB2
0.164647	12	ETF1
0.165037	12	LIMK1
0.165075	12	BOK
0.165181	12	CHD8
0.165413	12	NACC2
0.165511	12	HMGA1
0.165638	12	ZC3H13
0.165638	12	BTF3L4
0.165638	12	SRA1
0.16582	12	SRP19
0.165835	12	RAN
0.165999	12	RRM2
0.16621	12	MVD
0.166348	12	KLHL18
0.166588	12	CORO1A
0.166682	12	MAN2A2
0.167085	12	RPL18A
0.167085	12	SPG7

0.167189	12	ATG4A
0.167393	12	IRAK1
0.167432	12	UBE2V1
0.167432	12	WDR76
0.167432	12	SNX11
0.167469	12	TOP1
0.167469	12	PIP5K1A
0.167859	12	SHCBP1
0.167859	12	VAMP8
0.168219	12	AFF1
0.168219	12	ASB6
0.168275	12	SRSF1
0.168776	12	MIDEAS
0.168785	12	DCD
0.169056	12	LSM4
0.169181	12	YIPF4
0.169524	12	QTRT1
0.169571	12	HEATR3
0.169618	12	ZNF143
0.169652	12	CKAP2L
0.169675	12	MPHOSPH8
0.16968	12	PTMS
0.16986	12	ARMC9
0.16991	12	MAST4
0.169979	12	LARS2
0.171055	12	SNRPC
0.171055	12	KIFBP
0.171167	12	EDEM2
0.171314	12	HMCES
0.17162	12	NAT10
0.17171	12	DKC1
0.171944	12	EEF1B2
0.171946	12	PI4KB
0.17214	12	MAX
0.172388	12	EED
0.172393	12	TMEM199
0.172691	12	PEX13
0.172869	12	DHX29
0.172869	12	DPCD
0.172869	12	C1GALT1
0.173089	12	GRAMD1A
0.173089	12	UCK2
0.173442	12	SON
0.173854	12	FYTTD1
0.17393	12	GPAT4
0.174272	12	MAP2K7
0.174587	12	SRFBP1
0.174662	12	SAFB2
0.174677	12	BAD
0.175119	12	MARS2

0.175233	12	CAMSAP1
0.175274	12	FKBP11
0.175533	12	RUFY3
0.175659	12	ARHGAP24
0.175783	12	NRCAM
0.175912	12	TSC22D2
0.175912	12	HNRNPF
0.176038	12	VAPA
0.176197	12	RFX1
0.17624	12	NLE1
0.176325	12	SLC20A1
0.176325	12	TRIM4
0.176331	12	GRSF1
0.176459	12	PPP6R1
0.17681	12	ZFPL1
0.17681	12	MGAT4B
0.176867	12	PPP1R3D
0.176983	12	FAM168A
0.177327	12	LIN7C
0.177435	12	POLR3E
0.177979	12	RPL14
0.1781	12	SOX17
0.178327	12	HMGA2
0.178335	12	FKBP4
0.178577	12	ZC3H4
0.178728	12	MED6
0.178941	12	PCDH7
0.178941	12	SLC2A1
0.178941	12	MRPS23
0.179404	12	MB21D2
0.179414	12	MORF4L2
0.179415	12	MNAT1
0.179415	12	PIP4K2C
0.179486	12	MIF
0.179513	12	ZDHHC13
0.179548	12	HPDL
0.179773	12	MTAP
0.179773	12	TMEM106B
0.180398	12	RIT1
0.180443	12	RHOT2
0.180705	12	NDRG1
0.180785	12	SF3B2
0.181145	12	G3BP1
0.181167	12	HNRNPAB
0.181319	12	RIOK1
0.181385	12	YJU2
0.181801	12	TBC1D8
0.181898	12	CTDNEP1
0.182038	12	SLMAP
0.182235	12	UBE2E2

0.182688	12	RIPK2
0.182688	12	PPP1R13L
0.18271	12	AMOTL2
0.182868	12	RPS9
0.183079	12	WDR6
0.183215	12	RRAS2
0.183333	12	SNRPD1
0.183519	12	UNC119B
0.183595	12	ATAD3B
0.183784	12	ARMC1
0.184161	12	PPIC
0.184287	12	CAPN2
0.184306	12	IGSF8
0.184461	12	NSMCE2
0.184694	12	PGGHG
0.184724	12	C18orf21
0.184727	12	HRAS
0.184854	12	AIDA
0.18508	12	GOLM1
0.185178	12	CIAO2B
0.185886	12	DNAJC16
0.186001	12	PEBP1
0.186286	12	CCDC59
0.186382	12	NAPA
0.186558	12	PTGR1
0.186875	12	ATP11A
0.186875	12	MLST8
0.186888	12	BCL10
0.186888	12	GGA2
0.186998	12	PHF20L1
0.186998	12	ATRN
0.187065	12	MBD2
0.187188	12	IFT56
0.187188	12	CCDC85C
0.187188	12	RAB29
0.187188	12	MARK3
0.187188	12	SMAD3
0.187188	12	CHCHD1
0.187383	12	SPG21
0.187469	12	SNU13
0.187558	12	PDLIM1
0.187601	12	SSR3
0.18765	12	RIOK2
0.187667	12	MRPL35
0.187729	12	UBAC1
0.187963	12	KIF4A
0.188131	12	NOP53
0.188173	12	SDF2
0.188403	12	RAB5A
0.188403	12	AGPAT4

0.188463	12	TRMT61B
0.188546	12	LANCL2
0.188572	12	DBNL
0.1889	12	PDE4DIP
0.189249	12	ANAPC10
0.189291	12	HDAC6
0.189365	12	LAMTOR1
0.189668	12	ESS2
0.189813	12	SH3BGRL3
0.190037	12	PLS3
0.190468	12	SNRNP27
0.190647	12	UTP14A
0.191069	12	RRP36
0.19122	12	EIF4A1
0.191223	12	PGK1
0.1913	12	SAP30BP
0.191507	12	SDC4
0.191523	12	FCHSD2
0.191549	12	SH3BGRL2
0.191549	12	TPRKB
0.191892	12	SNRPA
0.192036	12	PARG
0.192185	12	LSM12
0.192252	12	THOC5
0.192378	12	CISD1
0.192535	12	IVNS1ABP
0.192652	12	BAG6
0.192724	12	ABCC1
0.192725	12	LIMA1
0.192793	12	CBX3
0.192924	12	DHCR24
0.193096	12	SPAG5
0.193118	12	RRP8
0.193118	12	NOM1
0.193118	12	PRXL2C
0.193118	12	STRAP
0.193118	12	TSSC4
0.193172	12	SNRPD3
0.194005	12	PLD3
0.194075	12	EIF4H
0.194226	12	SLC29A1
0.194261	12	SCAF11
0.19431	12	RANBP1
0.194447	12	SENP1
0.194451	12	SMYD5
0.194451	12	AP3M1
0.194521	12	CBX1
0.194603	12	ZCCHC9
0.19472	12	OS9
0.194794	12	PRKACB

0.194794	12	DLST
0.194794	12	RPS13
0.194794	12	SKP1
0.194794	12	RSF1
0.194794	12	POLDIP3
0.194794	12	SSU72
0.194796	12	TIMP2
0.195179	12	RPS18
0.195326	12	RABAC1
0.195466	12	SYT11
0.195932	12	PLEC
0.196186	12	CETN2
0.196223	12	FBXO42
0.196281	12	CDC40
0.196375	12	TFB1M
0.19641	12	TXNDC11
0.19641	12	SH2D3C
0.196501	12	FBXO3
0.196738	12	FAM120C
0.196741	12	POP7
0.197127	12	OTUD7B
0.197251	12	CLIP1
0.19731	12	MCAM
0.197417	12	AVEN
0.197417	12	PNPO
0.197505	12	LMNB2
0.197505	12	IRF2BP2
0.197559	12	GBE1
0.197761	12	CUTC
0.197875	12	PSTPIP2
0.198214	12	ARFGEF3
0.198828	12	SUN1
0.198828	12	NUP62
0.199235	12	RPL36
0.199639	12	CCDC127
0.199953	12	SOAT1
0.20065	12	HNRNPU
0.200709	12	SLC37A4
0.200709	12	PLEKHG4
0.200739	12	CDS2
0.200739	12	NUMBL
0.201362	12	ITGB3
0.201526	12	RPLP0
0.201616	12	PPP4R3B
0.201616	12	RMND1
0.201669	12	RCC1L
0.201803	12	ABL2
0.201819	12	ISG15
0.201868	12	TPX2
0.201963	12	OVCA2

0.20255	12	ARFGAP1
0.20272	12	CTU1
0.202914	12	NEK3
0.202914	12	TAF8
0.203156	12	YPEL5
0.203236	12	KHDRBS3
0.203524	12	USP33
0.204739	12	TACC2
0.205407	12	NACA
0.205407	12	SH3BP5
0.205407	12	DDX5
0.205407	12	ODR4
0.205407	12	EMC6

og2 Ratio ≥ 0.58 and Qvalue ≤ 0.05 were dysregulated after stimulation over the control group.

ProteinDescriptions	ProteinNames
Transferrin receptor protein 1	TFR1_HUMAN
Midkine	MK_HUMAN
Transforming growth factor beta activator LRRC32	LRC32_HUMAN
Alpha-ketoglutarate-dependent dioxygenase FTO	FTO_HUMAN
Angiopoietin-related protein 2	ANGL2_HUMAN
Palmdelphin	PALMD_HUMAN
Proteasome subunit beta type-8	PSB8_HUMAN
Plasminogen activator inhibitor 2	PAI2_HUMAN
26S proteasome regulatory subunit 6A	PRS6A_HUMAN
Phosphoglucomutase-1	PGM1_HUMAN
Catalase	CATA_HUMAN
Centrosomal protein of 85 kDa	CEP85_HUMAN
D-glutamate cyclase, mitochondrial	GLUCM_HUMAN
Damage-control phosphatase ARMT1	ARMT1_HUMAN
Cytochrome b-c1 complex subunit 1, mitochondrial	QCR1_HUMAN
Macrophage-capping protein	CAPG_HUMAN
RAB7A-interacting MON1-CCZ1 complex subunit 1	RIMC1_HUMAN
Proteasome activator complex subunit 2	PSME2_HUMAN
Deoxynucleoside triphosphate triphosphohydrolase SAMHD1	SAMH1_HUMAN
Elongator complex protein 3	ELP3_HUMAN
Cleavage stimulation factor subunit 3	CSTF3_HUMAN
Transmembrane and coiled-coil domain protein 3	TMCC3_HUMAN
Eukaryotic elongation factor 2 kinase	EF2K_HUMAN
Protein-glutamine gamma-glutamyltransferase 2	TGM2_HUMAN
Epidermal growth factor receptor substrate 15	EPS15_HUMAN
Beta-2-microglobulin	B2MG_HUMAN
Cytochrome c	CYC_HUMAN
Protein bicaudal D homolog 1	BICD1_HUMAN
AP-1 complex subunit sigma-2	AP1S2_HUMAN
Guanine nucleotide-binding protein subunit alpha-13	GNA13_HUMAN
6-phosphogluconate dehydrogenase, decarboxylating	6PGD_HUMAN
CMP-N-acetylneuraminate-beta-galactosamide-alpha-2,3-sialyltransferase 1	SIA4A_HUMAN
Palmitoyl-protein thioesterase ABHD10, mitochondrial	ABHDA_HUMAN
Cell cycle control protein 50A	CC50A_HUMAN
14-3-3 protein zeta/delta	1433Z_HUMAN
Leucine-rich repeat-containing protein 14	LRC14_HUMAN
Poly(A) polymerase alpha	PAPOA_HUMAN
Eukaryotic translation initiation factor 6	IF6_HUMAN
Laminin subunit alpha-4	LAMA4_HUMAN
Caspase recruitment domain-containing protein 10	CAR10_HUMAN
Nuclear RNA export factor 1	NXF1_HUMAN
GTP:AMP phosphotransferase AK3, mitochondrial	KAD3_HUMAN
Integrin beta-1	ITB1_HUMAN
CD9 antigen	CD9_HUMAN
Integrator complex subunit 4	INT4_HUMAN
Adhesion G protein-coupled receptor B3	AGRB3_HUMAN
Triokinase/FMN cyclase	TKFC_HUMAN
COP9 signalosome complex subunit 7b	CSN7B_HUMAN

Caveolae-associated protein 2	CAVN2_HUMAN
Medium-chain specific acyl-CoA dehydrogenase, mitochondrial	ACADM_HUMAN
cAMP-dependent protein kinase type II-beta regulatory subunit	KAP3_HUMAN
Charged multivesicular body protein 4b	CHM4B_HUMAN
Multimerin-2	MMRN2_HUMAN
Tyrosine--tRNA ligase, mitochondrial	SYYM_HUMAN
Neurobeachin-like protein 2	NBEL2_HUMAN
EH domain-containing protein 2	EHD2_HUMAN
Angiopoietin-2	ANGP2_HUMAN
Huntingtin-interacting protein 1-related protein	HIP1R_HUMAN
Isocitrate dehydrogenase [NADP] cytoplasmic	IDHC_HUMAN
HLA class I histocompatibility antigen, B alpha chain	HLAB_HUMAN
Gelsolin	GELS_HUMAN
Laminin subunit gamma-1	LAMC1_HUMAN
Endoglin	EGLN_HUMAN
Interferon-induced, double-stranded RNA-activated protein kinase	E2AK2_HUMAN
Cysteine and glycine-rich protein 1	CSRP1_HUMAN
DNA polymerase delta catalytic subunit	DPOD1_HUMAN
Glutathione hydrolase 5 proenzyme	GGT5_HUMAN
F-actin-capping protein subunit alpha-2	CAZA2_HUMAN
Heterogeneous nuclear ribonucleoprotein M	HNRPM_HUMAN
Multimerin-1	MMRN1_HUMAN
Neutral alpha-glucosidase AB	GANAB_HUMAN
Caveolae-associated protein 1	CAVN1_HUMAN
Transmembrane protein 205	TM205_HUMAN
Ubiquitin-conjugating enzyme E2 R2	UB2R2_HUMAN
Leucine-rich repeat-containing protein 47	LRC47_HUMAN
BTB/POZ domain-containing protein KCTD12	KCD12_HUMAN
Vacuolar protein sorting-associated protein 11 homolog	VPS11_HUMAN
Band 4.1-like protein 5	E41L5_HUMAN
Large ribosomal subunit protein mL40	RM40_HUMAN
Alpha-aminoadipic semialdehyde synthase, mitochondrial	AASS_HUMAN
Gamma-adducin	ADDG_HUMAN
FAS-associated factor 1	FAF1_HUMAN
NAD(P) transhydrogenase, mitochondrial	NNTM_HUMAN
Phosphorylase b kinase regulatory subunit beta	KPBB_HUMAN
Leukocyte elastase inhibitor	ILEU_HUMAN
3-ketoacyl-CoA thiolase, peroxisomal	THIK_HUMAN
Amidophosphoribosyltransferase	PUR1_HUMAN
Phosphomevalonate kinase	PMVK_HUMAN
Cathepsin Z	CATZ_HUMAN
Tryptophan--tRNA ligase, cytoplasmic	SYWC_HUMAN
General transcription and DNA repair factor IIH helicase subunit XPD	ERCC2_HUMAN
Paladin	PALD_HUMAN
Dihydropyrimidine dehydrogenase [NADP(+)]	DPYD_HUMAN
RING finger protein 214	RN214_HUMAN
mRNA-capping enzyme	MCE1_HUMAN
Disco-interacting protein 2 homolog A	DIP2A_HUMAN
Neuropilin-2	NRP2_HUMAN
Vesicle transport protein SEC20	SEC20_HUMAN

GTP-binding protein SAR1a	SAR1A_HUMAN
Nuclear factor 1 B-type	NFIB_HUMAN
Lymphatic vessel endothelial hyaluronic acid receptor 1	LYVE1_HUMAN
ATP synthase subunit d, mitochondrial	ATP5H_HUMAN
Histone H1.4	H14_HUMAN
La-related protein 7	LARP7_HUMAN
Glutamine amidotransferase-like class 1 domain-containing protein 1	GALD1_HUMAN
Protein NipSnap homolog 1	NIPS1_HUMAN
Protein diaphanous homolog 1	DIAP1_HUMAN
Fatty acid-binding protein, adipocyte	FABP4_HUMAN
Endoplasmic reticulum aminopeptidase 2	ERAP2_HUMAN
GTPase IMAP family member 7	GIMA7_HUMAN
Very-long-chain enoyl-CoA reductase	TECR_HUMAN
2-hydroxyacyl-CoA lyase 1	HACL1_HUMAN
Cytochrome b-c1 complex subunit 2, mitochondrial	QCR2_HUMAN
Cadherin-5	CADH5_HUMAN
Guanylate kinase	KGUA_HUMAN
PDZ domain-containing protein GIPC2	GIPC2_HUMAN
Torsin-1B	TOR1B_HUMAN
Myelin protein zero-like protein 2	MPZL2_HUMAN
Cytochrome b-c1 complex subunit Rieske, mitochondrial	UCRI_HUMAN
THO complex subunit 6 homolog	THOC6_HUMAN
Leukocyte receptor cluster member 1	LENG1_HUMAN
Tensin-2	TENS2_HUMAN
Phosphatidylinositol 3-kinase catalytic subunit type 3	PK3C3_HUMAN
Magnesium transporter protein 1	MAGT1_HUMAN
Cathepsin D	CATD_HUMAN
Mitochondrial import inner membrane translocase subunit Tim17-B	TI17B_HUMAN
Translation initiation factor eIF2 assembly protein	CD123_HUMAN
Dihydrofolate reductase	DYR_HUMAN
Intercellular adhesion molecule 1	ICAM1_HUMAN
Laminin subunit beta-1	LAMB1_HUMAN
Annexin A3	ANXA3_HUMAN
2-oxoisovalerate dehydrogenase subunit alpha, mitochondrial	ODBA_HUMAN
DNA replication licensing factor MCM3	MCM3_HUMAN
Nicotinamide N-methyltransferase	NNMT_HUMAN
Eukaryotic translation initiation factor 1	EIF1_HUMAN
ATP-dependent DNA helicase Q1	RECQ1_HUMAN
mRNA decay activator protein ZFP36L2	TISD_HUMAN
Isocitrate dehydrogenase [NAD] subunit gamma, mitochondrial	IDH3G_HUMAN
Cytoplasmic tyrosine-protein kinase BMX	BMX_HUMAN
WASH complex subunit 4	WASC4_HUMAN
Protein kish-A	KISHA_HUMAN
Nesprin-2	SYNE2_HUMAN
Heterogeneous nuclear ribonucleoprotein U-like protein 1	HNRL1_HUMAN
Protein misato homolog 1	MSTO1_HUMAN
Tubulointerstitial nephritis antigen-like	TINAL_HUMAN
Probable tRNA(His) guanylyltransferase	THG1_HUMAN
Mitochondrial carrier homolog 1	MTCH1_HUMAN
Vacuolar protein sorting-associated protein 18 homolog	VPS18_HUMAN

eIF-2-alpha kinase GCN2	E2AK4_HUMAN
Protein canopy homolog 2	CNPY2_HUMAN
Small ribosomal subunit protein bS18m	RT18C_HUMAN
Programmed cell death protein 4	PDCD4_HUMAN
eIF5-mimic protein 1	5MP1_HUMAN
High mobility group protein B1	HMGB1_HUMAN
Lysyl oxidase homolog 2	LOXL2_HUMAN
ELKS/Rab6-interacting/CAST family member 1	RB6I2_HUMAN
Beta/gamma crystallin domain-containing protein 1	CRBG1_HUMAN
Peroxisomal multifunctional enzyme type 2	DHB4_HUMAN
Methylmalonyl-CoA mutase, mitochondrial	MUTA_HUMAN
High mobility group protein B2	HMGB2_HUMAN
Poly [ADP-ribose] polymerase 1	PARP1_HUMAN
Conserved oligomeric Golgi complex subunit 5	COG5_HUMAN
Heat shock protein beta-1	HSPB1_HUMAN
Unhealthy ribosome biogenesis protein 2 homolog	URB2_HUMAN
Beta-secretase 2	BACE2_HUMAN
Tetraspanin-15	TSN15_HUMAN
Guanine nucleotide-binding protein subunit alpha-11	GNA11_HUMAN
IgG receptor FcRn large subunit p51	FCGRN_HUMAN
Target of Myb1 membrane trafficking protein	TOM1_HUMAN
Mitotic checkpoint protein BUB3	BUB3_HUMAN
Ras suppressor protein 1	RSU1_HUMAN
tRNA-splicing endonuclease subunit Sen15	SEN15_HUMAN
Mth938 domain-containing protein	AAMDC_HUMAN
Helicase MOV-10	MOV10_HUMAN
14-3-3 protein theta	1433T_HUMAN
Claudin-5	CLD5_HUMAN
Ferritin light chain	FRIL_HUMAN
Prosaposin	SAP_HUMAN
Receptor-interacting serine/threonine-protein kinase 1	RIPK1_HUMAN
Ras-related protein Rab-12	RAB12_HUMAN
Probable proline--tRNA ligase, mitochondrial	SYPM_HUMAN
2-oxoadipate dehydrogenase complex component E1	DHTK1_HUMAN
Myotubularin-related protein 10	MTMRA_HUMAN
SH3 and multiple ankyrin repeat domains protein 3	SHAN3_HUMAN
Calcium homeostasis modulator protein 5	CAHM5_HUMAN
Small ribosomal subunit protein mS33	RT33_HUMAN
Apolipoprotein L3	APOL3_HUMAN
Mitochondrial pyruvate carrier 2	MPC2_HUMAN
Nicastrin	NICA_HUMAN
Ephrin type-A receptor 2	EPHA2_HUMAN
COMM domain-containing protein 3	COMD3_HUMAN
Alpha-N-acetyl-neuraminyl-2,3-beta-galactosyl-1,3-N-acetyl-galactosaminide	SIA7D_HUMAN
Nidogen-1	NID1_HUMAN
Radixin	RADI_HUMAN
Protein transport protein Sec61 subunit alpha isoform 1	S61A1_HUMAN
Bifunctional coenzyme A synthase	COASY_HUMAN
U4/U6 small nuclear ribonucleoprotein Prp3	PRPF3_HUMAN
Transforming acidic coiled-coil-containing protein 1	TACC1_HUMAN

Cytosol aminopeptidase	AMPL_HUMAN
Apoptosis regulator BAX	BAX_HUMAN
Dehydrogenase/reductase SDR family member 7B	DRS7B_HUMAN
Uncharacterized protein KIAA2013	K2013_HUMAN
Regulator of nonsense transcripts 1	RENT1_HUMAN
Torsin-4A	TOR4A_HUMAN
Protein FAM118B	F118B_HUMAN
Leucine-rich repeat-containing protein 40	LRC40_HUMAN
ATP synthase subunit g, mitochondrial	ATP5L_HUMAN
A disintegrin and metalloproteinase with thrombospondin motifs 4	ATS4_HUMAN
Protein zyg-11 homolog B	ZY11B_HUMAN
Vacuolar protein sorting-associated protein 29	VPS29_HUMAN
Lysosomal acid phosphatase	PPAL_HUMAN
A-kinase anchor protein 13	AKP13_HUMAN
CCR4-NOT transcription complex subunit 9	CNOT9_HUMAN
TBC1 domain family member 10A	TB10A_HUMAN
Platelet endothelial cell adhesion molecule	PECA1_HUMAN
CDP-diacylglycerol--inositol 3-phosphatidyltransferase	CDIPT_HUMAN
D-3-phosphoglycerate dehydrogenase	SERA_HUMAN
Tripartite motif-containing protein 16	TRI16_HUMAN
Calponin-3	CNN3_HUMAN
Adipocyte plasma membrane-associated protein	APMAP_HUMAN
TRIO and F-actin-binding protein	TARA_HUMAN
Alpha-N-acetylgalactosaminidase	NAGAB_HUMAN
Ubiquitin-like modifier-activating enzyme 1	UBA1_HUMAN
Replication protein A 70 kDa DNA-binding subunit	RFA1_HUMAN
Protein phosphatase 1F	PPM1F_HUMAN
Sulfite oxidase, mitochondrial	SUOX_HUMAN
Ras-related protein Rab-4B	RAB4B_HUMAN
Alanine--tRNA ligase, mitochondrial	SYAM_HUMAN
Optineurin	OPTN_HUMAN
Aldo-keto reductase family 1 member B1	ALDR_HUMAN
Signal transducer and activator of transcription 6	STAT6_HUMAN
Caveolin-1	CAV1_HUMAN
Mediator of RNA polymerase II transcription subunit 30	MED30_HUMAN
3-keto-steroid reductase/17-beta-hydroxysteroid dehydrogenase 7	DHB7_HUMAN
Interferon-induced 35 kDa protein	IN35_HUMAN
Diacylglycerol lipase-beta	DGLB_HUMAN
Putative phospholipase B-like 2	PLBL2_HUMAN
m7GpppX diphosphatase	DCPS_HUMAN
Oxysterol-binding protein-related protein 10	OSB10_HUMAN
Mitochondrial basic amino acids transporter	S2529_HUMAN
Histone deacetylase 1	HDAC1_HUMAN
Ras-related protein Rab-11A	RB11A_HUMAN
Lysosome membrane protein 2	SCRB2_HUMAN
Regulator of nonsense transcripts 3B	REN3B_HUMAN
Eukaryotic translation initiation factor 2D	EIF2D_HUMAN
Engulfment and cell motility protein 2	ELMO2_HUMAN
Huntingtin-interacting protein 1	HIP1_HUMAN
Vascular endothelial growth factor receptor 2	VGFR2_HUMAN

Serine/threonine-protein phosphatase 2A catalytic subunit beta isoform	PP2AB_HUMAN
Large ribosomal subunit protein uL10m	RM10_HUMAN
Integrin-linked kinase-associated serine/threonine phosphatase 2C	ILKAP_HUMAN
Phospholipid scramblase 4	PLS4_HUMAN
1-acyl-sn-glycerol-3-phosphate acyltransferase epsilon	PLCE_HUMAN
Mitotic spindle assembly checkpoint protein MAD1	MD1L1_HUMAN
Protein Njmu-R1	NJMU_HUMAN
Apoptosis inhibitor 5	API5_HUMAN
Ubiquitin carboxyl-terminal hydrolase 40	UBP40_HUMAN
Protocadherin Fat 4	FAT4_HUMAN
Dihydrolipoyl dehydrogenase, mitochondrial	DLDH_HUMAN
Electron transfer flavoprotein subunit alpha, mitochondrial	ETFA_HUMAN
Disks large-associated protein 4	DLGP4_HUMAN
Spastin	SPAST_HUMAN
Pirin	PIR_HUMAN
CCR4-NOT transcription complex subunit 11	CNO11_HUMAN
Catenin beta-1	CTNB1_HUMAN
Deoxyribonuclease-2-alpha	DNS2A_HUMAN
NADH dehydrogenase [ubiquinone] iron-sulfur protein 7, mitochondrial	NDUS7_HUMAN
von Willebrand factor	VWF_HUMAN
Utrophin	UTRN_HUMAN
Transitional endoplasmic reticulum ATPase	TERA_HUMAN
Basement membrane-specific heparan sulfate proteoglycan core protein	PGBM_HUMAN
Cullin-7	CUL7_HUMAN
Ethylmalonyl-CoA decarboxylase	ECHD1_HUMAN
Septin-10	SEP10_HUMAN
Mitochondrial fission 1 protein	FIS1_HUMAN
N-sulphoglucosamine sulphohydrolase	SPHM_HUMAN
Prenylcysteine oxidase-like	PCYXL_HUMAN
Golgi apparatus protein 1	GSLG1_HUMAN
Endothelial cell-selective adhesion molecule	ESAM_HUMAN
NPC intracellular cholesterol transporter 2	NPC2_HUMAN
C-type lectin domain family 14 member A	CLC14_HUMAN
Transcriptional regulator ERG	ERG_HUMAN
X-linked retinitis pigmentosa GTPase regulator	RPGR_HUMAN
B-cell receptor-associated protein 31	BAP31_HUMAN
Caspase-6	CASP6_HUMAN
Sialoadhesin	SN_HUMAN
Integrator complex subunit 9	INT9_HUMAN
Pseudouridylate synthase 1 homolog	PUS1_HUMAN
Microtubule-associated proteins 1A/1B light chain 3B	MLP3B_HUMAN
Inhibitor of nuclear factor kappa-B kinase subunit alpha	IKKA_HUMAN
Endoplasmin	ENPL_HUMAN
BTB/POZ domain-containing protein KCTD5	KCTD5_HUMAN
Peptidyl-prolyl cis-trans isomerase H	PPIH_HUMAN
Alpha-adducin	ADDA_HUMAN
GDP-Man:Man(3)GlcNAc(2)-PP-Dol alpha-1,2-mannosyltransferase	ALG11_HUMAN
Methionine aminopeptidase 2	MAP2_HUMAN
Oxygen-dependent coproporphyrinogen-III oxidase, mitochondrial	HEM6_HUMAN
Ribosome biogenesis protein NSA2 homolog	NSA2_HUMAN

Junction plakoglobin	PLAK_HUMAN
Acidic leucine-rich nuclear phosphoprotein 32 family member A	AN32A_HUMAN
Serpin H1	SERPH_HUMAN
Actin-related protein 3	ARP3_HUMAN
Syntaxin-3	STX3_HUMAN
Serum paraoxonase/arylesterase 2	PON2_HUMAN
Low density lipoprotein receptor adapter protein 1	ARH_HUMAN
Heme-binding protein 2	HEBP2_HUMAN
Sodium-coupled neutral amino acid transporter 7	S38A7_HUMAN
Peptidyl-prolyl cis-trans isomerase FKBP9	FKBP9_HUMAN
17S U2 SnRNP complex component HTATSF1	HTSF1_HUMAN
Nipped-B-like protein	NIPBL_HUMAN
Peroxidasin homolog	PXDN_HUMAN
Plasminogen activator inhibitor 1	PAI1_HUMAN
Glycylpeptide N-tetradecanoyltransferase 1	NMT1_HUMAN
Syntaxin-binding protein 1	STXB1_HUMAN
RNA polymerase-associated protein RTF1 homolog	RTF1_HUMAN
Kelch-like protein 11	KLH11_HUMAN
Host cell factor 1	HCFC1_HUMAN
Fibrillin-1	FBN1_HUMAN
Nuclear pore complex protein Nup214	NU214_HUMAN
Developmentally-regulated GTP-binding protein 2	DRG2_HUMAN
Exocyst complex component 8	EXOC8_HUMAN
Putative protein-lysine deacylase ABHD14B	ABHEB_HUMAN
DNA excision repair protein ERCC-1	ERCC1_HUMAN
X-ray repair cross-complementing protein 5	XRCC5_HUMAN
Lysophosphatidylserine lipase ABHD12	ABD12_HUMAN
Phosphopentomutase	PGM2_HUMAN
Kinesin light chain 4	KLC4_HUMAN
GMP reductase 2	GMPR2_HUMAN
Inactive cell surface hyaluronidase CEMIP2	CEIP2_HUMAN
Aldehyde dehydrogenase, mitochondrial	ALDH2_HUMAN
Fumarate hydratase, mitochondrial	FUMH_HUMAN
Heme oxygenase 1	HMOX1_HUMAN
Peptidyl-prolyl cis-trans isomerase B	PPIB_HUMAN
26S proteasome regulatory subunit 8	PRS8_HUMAN
Bone marrow stromal antigen 2	BST2_HUMAN
WASH complex subunit 5	WASC5_HUMAN
2,4-dienoyl-CoA reductase [(3E)-enoyl-CoA-producing], mitochondrial	DECR_HUMAN
Thiosulfate sulfurtransferase	THTR_HUMAN
Patatin-like phospholipase domain-containing protein 6	PLPL6_HUMAN
Ras-related protein Rab-8B	RAB8B_HUMAN
3'-5' exoribonuclease HELZ2	HELZ2_HUMAN
Phosphatidylinositide phosphatase SAC2	SAC2_HUMAN
Protein phosphatase methylesterase 1	PPME1_HUMAN
Ubiquitin-like-conjugating enzyme ATG3	ATG3_HUMAN
Serine-protein kinase ATM	ATM_HUMAN
Fatty acyl-CoA reductase 1	FACR1_HUMAN
Arf-GAP with Rho-GAP domain, ANK repeat and PH domain-containing protei	ARAP3_HUMAN
KN motif and ankyrin repeat domain-containing protein 2	KANK2_HUMAN

Serine palmitoyltransferase 2	SPTC2_HUMAN
Tyrosine-protein kinase receptor Tie-1	TIE1_HUMAN
B-cell CLL/lymphoma 9-like protein	BCL9L_HUMAN
Beta-catenin-like protein 1	CTBL1_HUMAN
GTPase IMAP family member 1	GIMA1_HUMAN
E3 ubiquitin-protein ligase DTX3L	DTX3L_HUMAN
Cleavage and polyadenylation specificity factor subunit 2	CPSF2_HUMAN
Protocadherin-1	PCDH1_HUMAN
Serine/threonine-protein kinase 10	STK10_HUMAN
Protein disulfide-isomerase A5	PDIA5_HUMAN
Calcium/calmodulin-dependent protein kinase kinase 2	KKCC2_HUMAN
ER lumen protein-retaining receptor 3	ERD23_HUMAN
Glycogen phosphorylase, liver form	PYGL_HUMAN
Angiotensin-converting enzyme	ACE_HUMAN
Collagen alpha-1(VIII) chain	CO8A1_HUMAN
SWI/SNF-related matrix-associated actin-dependent regulator of chromatin s	SNF5_HUMAN
Alpha-centractin	ACTZ_HUMAN
Cullin-1	CUL1_HUMAN
Metallothionein-2	MT2_HUMAN
Leukotriene A-4 hydrolase	LKHA4_HUMAN
Vascular endothelial growth factor receptor 1	VGFR1_HUMAN
Peroxiredoxin-2	PRDX2_HUMAN
Lipopolysaccharide-responsive and beige-like anchor protein	LRBA_HUMAN
Intermembrane lipid transfer protein VPS13C	VP13C_HUMAN
Ras-associated and pleckstrin homology domains-containing protein 1	RAPH1_HUMAN
HEAT repeat-containing protein 5A	HTR5A_HUMAN
Dyslexia-associated protein KIAA0319-like protein	K319L_HUMAN
Saccharopine dehydrogenase-like oxidoreductase	SCPDL_HUMAN
Agrin	AGRIN_HUMAN
AP-1 complex subunit gamma-like 2	AP1G2_HUMAN
NADH dehydrogenase [ubiquinone] flavoprotein 1, mitochondrial	NDUV1_HUMAN
Dipeptidyl peptidase 1	CATC_HUMAN
Malectin	MLEC_HUMAN
Sterile alpha motif domain-containing protein 9	SAMD9_HUMAN
Vacuolar fusion protein MON1 homolog A	MON1A_HUMAN
AFG3-like protein 2	AFG32_HUMAN
Plasma membrane calcium-transporting ATPase 1	AT2B1_HUMAN
Proto-oncogene c-Rel	REL_HUMAN
C-type mannose receptor 2	MRC2_HUMAN
Epsin-3	EPN3_HUMAN
Ras-related C3 botulinum toxin substrate 2	RAC2_HUMAN
Vacuolar protein sorting-associated protein 8 homolog	VPS8_HUMAN
26S proteasome non-ATPase regulatory subunit 3	PSMD3_HUMAN
Endonuclease G, mitochondrial	NUCG_HUMAN
Coiled-coil and C2 domain-containing protein 1A	C2D1A_HUMAN
Protein THEM6	THEM6_HUMAN
Glutathione S-transferase LANCL1	LANC1_HUMAN
Tyrosine--tRNA ligase, cytoplasmic	SYYC_HUMAN
mRNA cap guanine-N7 methyltransferase	MCES_HUMAN
E3 ubiquitin-protein ligase RNF213	RN213_HUMAN

Tissue factor pathway inhibitor	TFPI1_HUMAN
Nucleoredoxin	NXN_HUMAN
Guanine nucleotide-binding protein subunit beta-like protein 1	GNB1L_HUMAN
Growth hormone-inducible transmembrane protein	GHITM_HUMAN
Eukaryotic translation initiation factor 3 subunit H	EIF3H_HUMAN
Protein O-GlcNAcase	OGA_HUMAN
L-lactate dehydrogenase A chain	LDHA_HUMAN
Acetyl-CoA carboxylase 1	ACACA_HUMAN
Phospholipase D1	PLD1_HUMAN
RING finger and SPRY domain-containing protein 1	RSPRY_HUMAN
Exocyst complex component 2	EXOC2_HUMAN
Formin-binding protein 1	FNBP1_HUMAN
Glia maturation factor beta	GMFB_HUMAN
Coatomer subunit beta'	COPB2_HUMAN
Beta-hexosaminidase subunit alpha	HEXA_HUMAN
Probable ATP-dependent RNA helicase DDX41	DDX41_HUMAN
Propionyl-CoA carboxylase alpha chain, mitochondrial	PCCA_HUMAN
Basal cell adhesion molecule	BCAM_HUMAN
Importin subunit alpha-5	IMA5_HUMAN
Cullin-2	CUL2_HUMAN
UDP-glucose 4-epimerase	GALE_HUMAN
Translocating chain-associated membrane protein 1	TRAM1_HUMAN
Transmembrane protein 245	TM245_HUMAN
Syntaxin-17	STX17_HUMAN
CTP synthase 2	PYRG2_HUMAN
Very-long-chain (3R)-3-hydroxyacyl-CoA dehydratase 3	HACD3_HUMAN
Rho GTPase-activating protein 31	RHG31_HUMAN
COMM domain-containing protein 1	COMD1_HUMAN
Proteasome activator complex subunit 1	PSME1_HUMAN
Centrosomal protein of 55 kDa	CEP55_HUMAN
N-alpha-acetyltransferase 50	NAA50_HUMAN
Homer protein homolog 3	HOME3_HUMAN
Serine/threonine-protein phosphatase 1 regulatory subunit 10	PP1RA_HUMAN
Volume-regulated anion channel subunit LRRC8C	LRC8C_HUMAN
F-box/LRR-repeat protein 18	FXL18_HUMAN
Omega-amidase NIT2	NIT2_HUMAN
Vacuolar protein sorting-associated protein 28 homolog	VPS28_HUMAN
Sialate O-acetylesterase	SIAE_HUMAN
Myocyte-specific enhancer factor 2D	MEF2D_HUMAN
Translin-associated protein X	TSNAX_HUMAN
Protein disulfide-isomerase A3	PDIA3_HUMAN
26S proteasome non-ATPase regulatory subunit 10	PSD10_HUMAN
Peptidyl-prolyl cis-trans isomerase G	PPIG_HUMAN
Negative elongation factor C/D	NELFD_HUMAN
Sorting nexin-2	SNX2_HUMAN
CCR4-NOT transcription complex subunit 3	CNOT3_HUMAN
Prothymosin alpha	PTMA_HUMAN
Kinesin-1 heavy chain	KINH_HUMAN
DNA mismatch repair protein Mlh1	MLH1_HUMAN
NADP-dependent malic enzyme	MAOX_HUMAN

Histone acetyltransferase p300	EP300_HUMAN
SH3 domain-containing protein 19	SH319_HUMAN
Pre-mRNA-splicing factor SYF1	SYF1_HUMAN
AP-2 complex subunit alpha-2	AP2A2_HUMAN
Dolichol-phosphate mannosyltransferase subunit 1	DPM1_HUMAN
Protein disulfide-isomerase A4	PDIA4_HUMAN
Talin-1	TLN1_HUMAN
Heat shock protein HSP 90-alpha	HS90A_HUMAN
Filamin-A	FLNA_HUMAN
Beta-adrenergic receptor kinase 1	ARBK1_HUMAN
Hematopoietic progenitor cell antigen CD34	CD34_HUMAN
Prohibitin 1	PHB1_HUMAN
Signal transducer and activator of transcription 5A	STA5A_HUMAN
Flotillin-2	FLOT2_HUMAN
Lysophosphatidylcholine acyltransferase 2	PCAT2_HUMAN
Synaptotagmin-like protein 4	SYTL4_HUMAN
RuvB-like 1	RUVB1_HUMAN
Protein dopey-2	DOP2_HUMAN
Putative HLA class I histocompatibility antigen, alpha chain H	HLAH_HUMAN
Replication factor C subunit 1	RFC1_HUMAN
Malonate--CoA ligase ACSF3, mitochondrial	ACSF3_HUMAN
Unconventional myosin-VI	MYO6_HUMAN
Arf-GAP domain and FG repeat-containing protein 2	AGFG2_HUMAN
Transcription activator BRG1	SMCA4_HUMAN
Methylcrotonoyl-CoA carboxylase beta chain, mitochondrial	MCCB_HUMAN
Tumor-associated calcium signal transducer 2	TACD2_HUMAN
Lysosome-associated membrane glycoprotein 2	LAMP2_HUMAN
Cell division cycle protein 16 homolog	CDC16_HUMAN
Sideroflexin-3	SFXN3_HUMAN
Cytosolic endo-beta-N-acetylglucosaminidase	ENASE_HUMAN
Focadhesin	FOCAD_HUMAN
Retinal dehydrogenase 2	AL1A2_HUMAN
ADP-ribosylation factor-like protein 8B	ARL8B_HUMAN
Rho GTPase-activating protein 1	RHG01_HUMAN
Insulin-like growth factor 2 mRNA-binding protein 2	IF2B2_HUMAN
Serine dehydratase-like	SDSL_HUMAN
Tight junction protein ZO-1	ZO1_HUMAN
Succinate--CoA ligase [ADP-forming] subunit beta, mitochondrial	SUCB1_HUMAN
12S rRNA N4-methylcytidine (m4C) methyltransferase	MET15_HUMAN
Integrin alpha-10	ITA10_HUMAN
Thrombospondin-1	TSP1_HUMAN
Phosphoenolpyruvate carboxykinase [GTP], mitochondrial	PCKGM_HUMAN
Carbohydrate sulfotransferase 14	CHSTE_HUMAN
GTPase IMAP family member 8	GIMA8_HUMAN
Anaphase-promoting complex subunit 4	APC4_HUMAN
Rab3 GTPase-activating protein non-catalytic subunit	RBGPR_HUMAN
Programmed cell death protein 6	PDCD6_HUMAN
2-hydroxyacyl-CoA lyase 2	HACL2_HUMAN
Exportin-1	XPO1_HUMAN
Band 4.1-like protein 2	E41L2_HUMAN

Zinc finger protein ZPR1	ZPR1_HUMAN
Guanosine-3',5'-bis(diphosphate) 3'-pyrophosphohydrolase MESH1	MESH1_HUMAN
Kelch-like protein 5	KLHL5_HUMAN
RUN and FYVE domain-containing protein 1	RUFY1_HUMAN
Abl interactor 2	ABI2_HUMAN
Bcl-2-associated transcription factor 1	BCLF1_HUMAN
Peptidyl-prolyl cis-trans isomerase-like 1	PPIL1_HUMAN
ADP-ribosylation factor 5	ARF5_HUMAN
Gamma-interferon-inducible protein 16	IF16_HUMAN
Tubulin alpha-1C chain	TBA1C_HUMAN
Phosphatidylinositol 5-phosphate 4-kinase type-2 alpha	PI42A_HUMAN
Acyl-CoA:lysophosphatidylglycerol acyltransferase 1	LGAT1_HUMAN
Collagen alpha-1(XVIII) chain	COIA1_HUMAN
Glycerol-3-phosphate dehydrogenase 1-like protein	GPD1L_HUMAN
Protein furry homolog-like	FRYL_HUMAN
Adenosine deaminase	ADA_HUMAN
NADH dehydrogenase [ubiquinone] 1 beta subcomplex subunit 7	NDUB7_HUMAN
DNA-dependent protein kinase catalytic subunit	PRKDC_HUMAN
DNA replication licensing factor MCM6	MCM6_HUMAN
Alpha-aminoadipic semialdehyde dehydrogenase	AL7A1_HUMAN
Vigilin	VIGLN_HUMAN
Protein disulfide-isomerase A6	PDIA6_HUMAN
Parafibromin	CDC73_HUMAN
RNA demethylase ALKBH5	ALKB5_HUMAN
UBX domain-containing protein 7	UBXN7_HUMAN
ATP synthase subunit beta, mitochondrial	ATPB_HUMAN
Protein kinase C eta type	KPCL_HUMAN
Guanylate-binding protein 1	GBP1_HUMAN
Isocitrate dehydrogenase [NADP], mitochondrial	IDHP_HUMAN
RNA-binding protein 3	RBM3_HUMAN
Integrator complex subunit 11	INT11_HUMAN
tRNA N(3)-methylcytidine methyltransferase METTL2B	MET2B_HUMAN
Myb/SANT-like DNA-binding domain-containing protein 2	MSD2_HUMAN
Kinectin	KTN1_HUMAN
Stromal membrane-associated protein 1	SMAP1_HUMAN
Protein phosphatase Slingshot homolog 3	SSH3_HUMAN
Endoplasmic reticulum-Golgi intermediate compartment protein 1	ERGI1_HUMAN
tRNA (guanine-N(7)-)-methyltransferase	TRMB_HUMAN
Protein kinase C and casein kinase substrate in neurons protein 3	PACN3_HUMAN
Nicotinate phosphoribosyltransferase	PNCB_HUMAN
Solute carrier family 25 member 3	S25A3_HUMAN
17-beta-hydroxysteroid dehydrogenase type 2	DHB2_HUMAN
LEM domain-containing protein 2	LEMD2_HUMAN
Protein FAM124B	F124B_HUMAN
Beta-1,4-galactosyltransferase 5	B4GT5_HUMAN
Rho guanine nucleotide exchange factor 17	ARHGH_HUMAN
Leukocyte surface antigen CD47	CD47_HUMAN
Retinoic acid receptor RXR-beta	RXRB_HUMAN
Autophagy protein 5	ATG5_HUMAN
TBC1 domain family member 5	TBCD5_HUMAN

NADH dehydrogenase [ubiquinone] 1 alpha subcomplex subunit 8	NDUA8_HUMAN
Aflatoxin B1 aldehyde reductase member 2	ARK72_HUMAN
X-ray repair cross-complementing protein 6	XRCC6_HUMAN
Peptidyl-prolyl cis-trans isomerase FKBP5	FKBP5_HUMAN
Isoleucine--tRNA ligase, cytoplasmic	SYIC_HUMAN
Peroxisomal membrane protein PEX14	PEX14_HUMAN
Dedicator of cytokinesis protein 4	DOCK4_HUMAN
Semaphorin-6B	SEM6B_HUMAN
N(4)-(beta-N-acetylglucosaminyl)-L-asparaginase	ASPG_HUMAN
Ankyrin repeat and SAM domain-containing protein 1A	ANS1A_HUMAN
Ezrin	EZRI_HUMAN
NAD(P)H dehydrogenase [quinone] 1	NQO1_HUMAN
Trifunctional enzyme subunit alpha, mitochondrial	ECHA_HUMAN
A-kinase anchor protein 12	AKA12_HUMAN
Cingulin-like protein 1	CGNL1_HUMAN
Delta(3,5)-Delta(2,4)-dienoyl-CoA isomerase, mitochondrial	ECH1_HUMAN
Methanethiol oxidase	SBP1_HUMAN
Chromodomain-helicase-DNA-binding protein 4	CHD4_HUMAN
NADH dehydrogenase [ubiquinone] 1 alpha subcomplex subunit 9, mitochon	NDUA9_HUMAN
Armadillo repeat-containing X-linked protein 2	ARMX2_HUMAN
Polyphosphoinositide phosphatase	FIG4_HUMAN
Exostosin-2	EXT2_HUMAN
Lysophospholipid acyltransferase 7	MBOA7_HUMAN
Phosphatidylinositol-3-phosphatase SAC1	SAC1_HUMAN
EH domain-containing protein 3	EHD3_HUMAN
Rho GTPase-activating protein 23	RHG23_HUMAN
Prostaglandin F2 receptor negative regulator	FPRP_HUMAN
Ubiquitin carboxyl-terminal hydrolase 25	UBP25_HUMAN
Acyl-coenzyme A thioesterase 9, mitochondrial	ACOT9_HUMAN
Vascular endothelial growth factor receptor 3	VGFR3_HUMAN
1-phosphatidylinositol 4,5-bisphosphate phosphodiesterase beta-1	PLCB1_HUMAN
Integrator complex subunit 5	INT5_HUMAN
CYFIP-related Rac1 interactor A	CYRIA_HUMAN
Electron transfer flavoprotein subunit beta	ETFB_HUMAN
Dysferlin	DYSF_HUMAN
Transmembrane protein 11, mitochondrial	TMM11_HUMAN
Squamous cell carcinoma antigen recognized by T-cells 3	SART3_HUMAN
Membralin	MBRL_HUMAN
CLIP-associating protein 1	CLAP1_HUMAN
Sphingomyelin phosphodiesterase 4	NSMA3_HUMAN
Glycolipid transfer protein	GLTP_HUMAN
Zinc finger protein 706	ZN706_HUMAN
Dihydroxyacetone phosphate acyltransferase	GNPAT_HUMAN
CAAX prenyl protease 1 homolog	FACE1_HUMAN
Ribosomal protein S6 kinase alpha-1	KS6A1_HUMAN
WD repeat-containing protein 48	WDR48_HUMAN
AP-1 complex subunit mu-1	AP1M1_HUMAN
N-alpha-acetyltransferase 25, NatB auxiliary subunit	NAA25_HUMAN
Transmembrane emp24 domain-containing protein 5	TMED5_HUMAN
Shiftless antiviral inhibitor of ribosomal frameshifting protein	SHFL_HUMAN

Large ribosomal subunit protein uL15m	RM15_HUMAN
Serine/threonine-protein phosphatase CPPED1	CPPED_HUMAN
Nuclear pore complex protein Nup98-Nup96	NUP98_HUMAN
tRNA N6-adenosine threonylcarbamoyltransferase	OSGEP_HUMAN
Vesicular integral-membrane protein VIP36	LMAN2_HUMAN
Nuclear autoantigenic sperm protein	NASP_HUMAN
CYFIP-related Rac1 interactor B	CYRIB_HUMAN
Copper chaperone for superoxide dismutase	CCS_HUMAN
Fructosamine-3-kinase	FN3K_HUMAN
Ornithine aminotransferase, mitochondrial	OAT_HUMAN
Regulation of nuclear pre-mRNA domain-containing protein 2	RPRD2_HUMAN
KN motif and ankyrin repeat domain-containing protein 3	KANK3_HUMAN
Calcium-regulated heat-stable protein 1	CHSP1_HUMAN
Protein PML	PML_HUMAN
Carnitine O-palmitoyltransferase 1, liver isoform	CPT1A_HUMAN
Dual specificity mitogen-activated protein kinase kinase 2	MP2K2_HUMAN
Spermatogenesis-associated protein 20	SPT20_HUMAN
Serine/threonine-protein phosphatase 2A 56 kDa regulatory subunit delta isc	2A5D_HUMAN
Negative elongation factor B	NELFB_HUMAN
Serine/threonine-protein kinase D2	KPCD2_HUMAN
Adenylosuccinate synthetase isozyme 2	PURA2_HUMAN
HLA class I histocompatibility antigen, A alpha chain	HLAA_HUMAN
Ribonuclease P protein subunit p29	RPP29_HUMAN
Forkhead box protein C2	FOXC2_HUMAN
Pyruvate carboxylase, mitochondrial	PYC_HUMAN
Serine/threonine-protein phosphatase PP1-beta catalytic subunit	PP1B_HUMAN
Phospholipid-transporting ATPase IC	AT8B1_HUMAN
Protein 4.1	EPB41_HUMAN
Novel acetylcholine receptor chaperone	NACHO_HUMAN
Sulfhydryl oxidase 2	QSOX2_HUMAN
Pyrroline-5-carboxylate reductase 2	P5CR2_HUMAN
Sorting nexin-6	SNX6_HUMAN
RNA 3'-terminal phosphate cyclase	RTCA_HUMAN
Lambda-crystallin homolog	CRYL1_HUMAN
Striatin	STRN_HUMAN
ATP synthase subunit epsilon, mitochondrial	ATP5E_HUMAN
All-trans-retinol 13,14-reductase	RETST_HUMAN
Glutaminyl-peptide cyclotransferase-like protein	QPCTL_HUMAN
Ankyrin repeat domain-containing protein 40	ANR40_HUMAN
Intermembrane lipid transfer protein VPS13A	VP13A_HUMAN
Transmembrane protein 33	TMM33_HUMAN
ADP-ribosylhydrolase ARH3	ADPRS_HUMAN
ATP synthase subunit O, mitochondrial	ATPO_HUMAN
Solute carrier family 25 member 35	S2535_HUMAN
Guanylate-binding protein 2	GBP2_HUMAN
Cytohesin-2	CYH2_HUMAN
Pumilio homolog 2	PUM2_HUMAN
Perilipin-3	PLIN3_HUMAN
E3 ubiquitin-protein ligase MYCBP2	MYCB2_HUMAN
Tumor necrosis factor receptor superfamily member 5	TNR5_HUMAN

Thioredoxin-dependent peroxide reductase, mitochondrial	PRDX3_HUMAN
Serine/threonine-protein kinase MRCK beta	MRCKB_HUMAN
Catenin delta-1	CTND1_HUMAN
DnaJ homolog subfamily B member 2	DNJB2_HUMAN
ADP-ribosylation factor-like protein 3	ARL3_HUMAN
Hepatoma-derived growth factor-related protein 2	HDGR2_HUMAN
UMP-CMP kinase	KCY_HUMAN
C-terminal-binding protein 1	CTBP1_HUMAN
Serine/threonine-protein kinase ATR	ATR_HUMAN
Nck-associated protein 1	NCKP1_HUMAN
Peripheral plasma membrane protein CASK	CSKP_HUMAN
Tetratricopeptide repeat protein 28	TTC28_HUMAN
Aspartyl/asparaginyl beta-hydroxylase	ASPH_HUMAN
E3 UFM1-protein ligase 1	UFL1_HUMAN
Microtubule-associated protein 2	MTAP2_HUMAN
AP-3 complex subunit beta-1	AP3B1_HUMAN
Eukaryotic translation initiation factor 1b	EIF1B_HUMAN
Flotillin-1	FLOT1_HUMAN
Ras-related protein Rab-6A	RAB6A_HUMAN
Torsin-1A-interacting protein 2	TOIP2_HUMAN
Osteoclast-stimulating factor 1	OSTF1_HUMAN
E3 ubiquitin-protein ligase RNF31	RNF31_HUMAN
Mannose-1-phosphate guanyltransferase alpha	GMPPA_HUMAN
Conserved oligomeric Golgi complex subunit 8	COG8_HUMAN
Retinoid-inducible serine carboxypeptidase	RISC_HUMAN
Cation-independent mannose-6-phosphate receptor	MPRI_HUMAN
Protein O-glucosyltransferase 1	PGLT1_HUMAN
ADP-ribosylation factor-like protein 8A	ARL8A_HUMAN
Splicing regulator ARVCF	ARVC_HUMAN
Guanine nucleotide-binding protein-like 1	GNL1_HUMAN
Glycerol-3-phosphate phosphatase	PGP_HUMAN
ATP-binding cassette sub-family D member 3	ABCD3_HUMAN
Cytochrome b-c1 complex subunit 7	QCR7_HUMAN
Transmembrane protein 50A	TM50A_HUMAN
MAP kinase-activating death domain protein	MADD_HUMAN
Rho guanine nucleotide exchange factor 2	ARHG2_HUMAN
Nibrin	NBN_HUMAN
Structural maintenance of chromosomes protein 2	SMC2_HUMAN
Rab-like protein 3	RABL3_HUMAN
Interstitial collagenase	MMP1_HUMAN
Ubiquitin-protein ligase E3C	UBE3C_HUMAN
Thyroid receptor-interacting protein 11	TRIPB_HUMAN
Gamma-tubulin complex component 3	GCP3_HUMAN
ER membrane protein complex subunit 3	EMC3_HUMAN
Synaptotagmin-1	SYT1_HUMAN
Transcription initiation factor IIE subunit beta	T2EB_HUMAN
Mediator of RNA polymerase II transcription subunit 23	MED23_HUMAN
Actin-binding LIM protein 1	ABLM1_HUMAN
Signal recognition particle subunit SRP68	SRP68_HUMAN
EH domain-containing protein 4	EHD4_HUMAN

A-kinase anchor protein 8	AKAP8_HUMAN
Thymocyte nuclear protein 1	THYN1_HUMAN
Signal recognition particle 9 kDa protein	SRP09_HUMAN
Sulfotransferase 1B1	ST1B1_HUMAN
Clusterin	CLUS_HUMAN
Lysosome-associated membrane glycoprotein 1	LAMP1_HUMAN
Arylsulfatase A	ARSA_HUMAN
Iron-responsive element-binding protein 2	IREB2_HUMAN
Thioredoxin domain-containing protein 5	TXND5_HUMAN
Caspase-1	CASP1_HUMAN
MARCKS-related protein	MRP_HUMAN
Cadherin-13	CAD13_HUMAN
Ras-related protein Rab-10	RAB10_HUMAN
Coiled-coil domain-containing protein 93	CCD93_HUMAN
Protein HID1	HID1_HUMAN
Acyl-protein thioesterase 1	LYPA1_HUMAN
Large ribosomal subunit protein eL33	RL35A_HUMAN
Apoptosis-inducing factor 1, mitochondrial	AIFM1_HUMAN
Alpha-1,3-mannosyl-glycoprotein 2-beta-N-acetylglucosaminyltransferase	MGAT1_HUMAN
Sorting nexin-3	SNX3_HUMAN
E3 ubiquitin-protein ligase HUWE1	HUWE1_HUMAN
Nuclear receptor corepressor 1	NCOR1_HUMAN
DNA damage-binding protein 2	DDB2_HUMAN
Tripeptidyl-peptidase 1	TPP1_HUMAN
Synemin	SYNEM_HUMAN
tRNA (guanine(37)-N1)-methyltransferase	TRM5_HUMAN
U8 snoRNA-decapping enzyme	NUD16_HUMAN
Lymphoid-specific helicase	HELLS_HUMAN
Hepatocyte growth factor receptor	MET_HUMAN
Syntaxin-6	STX6_HUMAN
Prolyl 3-hydroxylase 3	P3H3_HUMAN
FLYWCH family member 2	FWCH2_HUMAN
Acetyl-coenzyme A synthetase, cytoplasmic	ACSA_HUMAN
Fatty acid-binding protein 5	FABP5_HUMAN
Chronophin	PLPP_HUMAN
Rho GTPase-activating protein 10	RHG10_HUMAN
Protein transport protein Sec61 subunit alpha isoform 2	S61A2_HUMAN
Xaa-Pro aminopeptidase 1	XPP1_HUMAN
DnaJ homolog subfamily B member 4	DNJB4_HUMAN
DCN1-like protein 5	DCNL5_HUMAN
Enhancer of rudimentary homolog	ERH_HUMAN
Aconitate hydratase, mitochondrial	ACON_HUMAN
Stromal cell-derived factor 2-like protein 1	SDF2L_HUMAN
Microtubule-associated tumor suppressor 1	MTUS1_HUMAN
Probable E3 ubiquitin-protein ligase HERC1	HERC1_HUMAN
Tumor protein p63-regulated gene 1-like protein	TPRGL_HUMAN
Signal transducer and activator of transcription 3	STAT3_HUMAN
Receptor expression-enhancing protein 5	REEP5_HUMAN
Lysine--tRNA ligase	SYK_HUMAN
Telomeric repeat-binding factor 2	TERF2_HUMAN

E3 ubiquitin-protein ligase Hakai	HAKAI_HUMAN
(Lyso)-N-acylphosphatidylethanolamine lipase	ABHD4_HUMAN
Protein IWS1 homolog	IWS1_HUMAN
Protein O-mannose kinase	SG196_HUMAN
Ribosome biogenesis protein SLX9 homolog	SLX9_HUMAN
Trafficking protein particle complex subunit 8	TPPC8_HUMAN
Deoxyribose-phosphate aldolase	DEOC_HUMAN
Neurolysin, mitochondrial	NEUL_HUMAN
Amyloid-beta precursor protein	A4_HUMAN
YLP motif-containing protein 1	YLPM1_HUMAN
Cytoplasmic dynein 1 heavy chain 1	DYHC1_HUMAN
Uncharacterized protein KIAA1143	K1143_HUMAN
Fermitin family homolog 2	FERM2_HUMAN
T-cell acute lymphocytic leukemia protein 1	TAL1_HUMAN
Mitochondrial import receptor subunit TOM70	TOM70_HUMAN
Heterogeneous nuclear ribonucleoprotein D0	HNRPD_HUMAN
Retinoblastoma-binding protein 5	RBBP5_HUMAN
ATP-dependent RNA helicase DDX19A	DD19A_HUMAN
Phosphorylase b kinase gamma catalytic chain, liver/testis isoform	PHKG2_HUMAN
HCLS1-binding protein 3	H1BP3_HUMAN
Succinate dehydrogenase [ubiquinone] iron-sulfur subunit, mitochondrial	SDHB_HUMAN
Mitogen-activated protein kinase kinase kinase kinase 2	M4K2_HUMAN
Retinol dehydrogenase 14	RDH14_HUMAN
Non-structural maintenance of chromosomes element 4 homolog A	NSE4A_HUMAN
Wolframin	WFS1_HUMAN
Adenylosuccinate lyase	PUR8_HUMAN
SH2B adapter protein 1	SH2B1_HUMAN
Ribosome-binding protein 1	RRBP1_HUMAN
N-acetyl-D-glucosamine kinase	NAGK_HUMAN
SH2B adapter protein 3	SH2B3_HUMAN
Protein kinase C alpha type	KPCA_HUMAN
Selenide, water dikinase 1	SPS1_HUMAN
Ephrin type-B receptor 4	EPHB4_HUMAN
Exocyst complex component 7	EXOC7_HUMAN
Choline/ethanolamine kinase	CHKB_HUMAN
HBS1-like protein	HBS1L_HUMAN
MICOS complex subunit MIC60	MIC60_HUMAN
Glucoside xylosyltransferase 1	GXLT1_HUMAN
Cell division cycle protein 23 homolog	CDC23_HUMAN
Nectin-2	NECT2_HUMAN
Thioredoxin reductase 1, cytoplasmic	TRXR1_HUMAN
Unconventional myosin-Ic	MYO1C_HUMAN
Ras-interacting protein 1	RAIN_HUMAN
26S proteasome non-ATPase regulatory subunit 1	PSMD1_HUMAN
RNA transcription, translation and transport factor protein	RTRAF_HUMAN
Probable bifunctional dTTP/UTP pyrophosphatase/methyltransferase protein	ASML_HUMAN
AH receptor-interacting protein	AIP_HUMAN
Serine/threonine-protein phosphatase 2A catalytic subunit alpha isoform	PP2AA_HUMAN
60 kDa heat shock protein, mitochondrial	CH60_HUMAN
Carnitine O-acetyltransferase	CACP_HUMAN

Golgi-associated kinase 1B	GAK1B_HUMAN
Regulator of nonsense transcripts 2	RENT2_HUMAN
Lysosomal acid glucosylceramidase	GBA1_HUMAN
Stonin-1	STON1_HUMAN
Protein O-glucosyltransferase 3	PLGT3_HUMAN
Myosin regulatory light polypeptide 9	MYL9_HUMAN
Serpin B6	SPB6_HUMAN
Actin-related protein 2	ARP2_HUMAN
Small ribosomal subunit protein uS11m	RT11_HUMAN
RAF proto-oncogene serine/threonine-protein kinase	RAF1_HUMAN
Cytochrome P450 2S1	CP2S1_HUMAN
Mannosyl-oligosaccharide glucosidase	MOGS_HUMAN
Docking protein 1	DOK1_HUMAN
NADPH--cytochrome P450 reductase	NCPR_HUMAN
Pre-mRNA-splicing factor ATP-dependent RNA helicase PRP16	PRP16_HUMAN
Epsin-1	EPN1_HUMAN
DIS3-like exonuclease 2	DI3L2_HUMAN
E3 ubiquitin-protein ligase RBBP6	RBBP6_HUMAN
Cytoplasmic 60S subunit biogenesis factor ZNF622	ZN622_HUMAN
Endoplasmic reticulum protein SC65	SC65_HUMAN
Collagen alpha-1(XII) chain	COCA1_HUMAN
UPF0415 protein C7orf25	CG025_HUMAN
Stearoyl-CoA desaturase	SCD_HUMAN
GPN-loop GTPase 1	GPN1_HUMAN
Serine/arginine-rich splicing factor 2	SRSF2_HUMAN
GTPase IMAP family member 4	GIMA4_HUMAN
Polypeptide N-acetylgalactosaminyltransferase 1	GALT1_HUMAN
Cytosolic iron-sulfur assembly component 2A	CIA2A_HUMAN
Nischarin	NISCH_HUMAN
Charged multivesicular body protein 4a	CHM4A_HUMAN
F-actin-capping protein subunit beta	CAPZB_HUMAN
Lysine-specific histone demethylase 1A	KDM1A_HUMAN
Biglycan	PGS1_HUMAN
Delta-1-pyrroline-5-carboxylate dehydrogenase, mitochondrial	AL4A1_HUMAN
Stress-induced-phosphoprotein 1	STIP1_HUMAN
Inactive tyrosine-protein kinase 7	PTK7_HUMAN
Translation initiation factor eIF2B subunit alpha	EI2BA_HUMAN
Torsin-2A	TOR2A_HUMAN
Ribonucleoprotein PTB-binding 1	RAVR1_HUMAN
WD repeat-containing protein 13	WDR13_HUMAN
Protein NDRG3	NDRG3_HUMAN
Glia maturation factor gamma	GMFG_HUMAN
Na(+)/H(+) exchange regulatory cofactor NHE-RF2	NHRF2_HUMAN
Protein THEMIS2	THMS2_HUMAN
Transcription initiation factor TFIID subunit 2	TAF2_HUMAN
tRNA (uracil-5-)-methyltransferase homolog A	TRM2A_HUMAN
Complex I assembly factor TIMMDC1, mitochondrial	TIDC1_HUMAN
Heat shock cognate 71 kDa protein	HSP7C_HUMAN
Negative elongation factor E	NELFE_HUMAN
Cytotoxic granule associated RNA binding protein TIA1	TIA1_HUMAN

TBC1 domain family member 9B	TBC9B_HUMAN
Glucose 1,6-bisphosphate synthase	PGM2L_HUMAN
Reticulophagy regulator 2	RETR2_HUMAN
Latexin	LXN_HUMAN
Protein RCC2	RCC2_HUMAN
Vacuolar protein sorting-associated protein 51 homolog	VPS51_HUMAN
Inositol 1,4,5-trisphosphate receptor type 2	ITPR2_HUMAN
Endoplasmic reticulum transmembrane helix translocase	AT131_HUMAN
Pogo transposable element with KRAB domain	POGK_HUMAN
Striatin-3	STRN3_HUMAN
G-protein-signaling modulator 1	GPSM1_HUMAN
Gamma-glutamyl hydrolase	GGH_HUMAN
Protein phosphatase 3 catalytic subunit alpha	PP2BA_HUMAN
RWD domain-containing protein 1	RWDD1_HUMAN
Cytoplasmic FMR1-interacting protein 1	CYFP1_HUMAN
Insulin-degrading enzyme	IDE_HUMAN
Early endosome antigen 1	EEA1_HUMAN
SEC23-interacting protein	S23IP_HUMAN
Protein FAM210A	F210A_HUMAN
Signal recognition particle 14 kDa protein	SRP14_HUMAN
Unconventional myosin-Ib	MYO1B_HUMAN
Lupus La protein	LA_HUMAN
Annexin A6	ANXA6_HUMAN
C-1-tetrahydrofolate synthase, cytoplasmic	C1TC_HUMAN
Hippocalcin-like protein 1	HPCL1_HUMAN
Mothers against decapentaplegic homolog 1	SMAD1_HUMAN
Armadillo repeat-containing X-linked protein 1	ARMX1_HUMAN
Prefoldin subunit 2	PFD2_HUMAN
Centrosomal protein of 41 kDa	CEP41_HUMAN
Cytochrome c oxidase subunit 4 isoform 1, mitochondrial	COX41_HUMAN
Stabilin-1	STAB1_HUMAN
GTP-binding protein 4	GTPB4_HUMAN
UDP-glucose:glycoprotein glucosyltransferase 1	UGGG1_HUMAN
GDP-fucose protein O-fucosyltransferase 2	OFUT2_HUMAN
TBC1 domain family member 22B	TB22B_HUMAN
ARF GTPase-activating protein GIT2	GIT2_HUMAN
Galactose mutarotase	GALM_HUMAN
Protein pelota homolog	PELO_HUMAN
Gasdermin-D	GSDMD_HUMAN
Serine/threonine-protein phosphatase 6 regulatory subunit 3	PP6R3_HUMAN
Junctional adhesion molecule A	JAM1_HUMAN
DENN domain-containing protein 3	DEND3_HUMAN
Heat shock protein HSP 90-beta	HS90B_HUMAN
Growth/differentiation factor 15	GDF15_HUMAN
Cysteine-rich protein 2	CRIP2_HUMAN
Choline transporter-like protein 2	CTL2_HUMAN
Ataxin-7-like protein 3B	A7L3B_HUMAN
Rho guanine nucleotide exchange factor 28	ARG28_HUMAN
Nucleobindin-2	NUCB2_HUMAN
Small ribosomal subunit protein mS26	RT26_HUMAN

Exosome complex component RRP40	EXOS3_HUMAN
CD63 antigen	CD63_HUMAN
HAUS augmin-like complex subunit 6	HAUS6_HUMAN
Alpha-parvin	PARVA_HUMAN
tRNA N6-adenosine threonylcarbamoyltransferase, mitochondrial	OSGL1_HUMAN
14-3-3 protein epsilon	1433E_HUMAN
Histone H1.1	H11_HUMAN
Dedicator of cytokinesis protein 1	DOCK1_HUMAN
Leucyl-cystinyl aminopeptidase	LCAP_HUMAN
Mitogen-activated protein kinase 1	MK01_HUMAN
LRP chaperone MESD	MESD_HUMAN
Small ribosomal subunit protein mS27	RT27_HUMAN
Dynactin subunit 4	DCTN4_HUMAN
Protoporphyrinogen oxidase	PPOX_HUMAN
Nuclear pore complex protein Nup205	NU205_HUMAN
Transportin-3	TNPO3_HUMAN
Pre-mRNA-splicing factor 38B	PR38B_HUMAN
Transgelin	TAGL_HUMAN
Lysosomal Pro-X carboxypeptidase	PCP_HUMAN
LIM domain-binding protein 2	LDB2_HUMAN
Mediator of RNA polymerase II transcription subunit 1	MED1_HUMAN
Gephyrin	GEPH_HUMAN
Echinoderm microtubule-associated protein-like 1	EMAL1_HUMAN
Metastasis-associated protein MTA1	MTA1_HUMAN
E3 ubiquitin-protein ligase UBR4	UBR4_HUMAN
Lysocardiolipin acyltransferase 1	LCLT1_HUMAN
Peptidyl-prolyl cis-trans isomerase FKBP10	FKB10_HUMAN
Aminopeptidase B	AMPB_HUMAN
UTP--glucose-1-phosphate uridylyltransferase	UGPA_HUMAN
Glyoxalase domain-containing protein 4	GLOD4_HUMAN
ER degradation-enhancing alpha-mannosidase-like protein 3	EDEM3_HUMAN
Poly(U)-binding-splicing factor PUF60	PUF60_HUMAN
DNA mismatch repair protein Msh2	MSH2_HUMAN
Endoplasmic reticulum metallopeptidase 1	ERMP1_HUMAN
Phospholipid-transporting ATPase ABCA3	ABCA3_HUMAN
Dynactin subunit 5	DCTN5_HUMAN
Small ribosomal subunit protein uS7	RS5_HUMAN
Queuosine 5'-phosphate N-glycosylase/hydrolase	QNG1_HUMAN
Glutathione S-transferase C-terminal domain-containing protein	GSTCD_HUMAN
Peptidyl-prolyl cis-trans isomerase FKBP7	FKBP7_HUMAN
Phenylalanine--tRNA ligase alpha subunit	SYFA_HUMAN
Cytochrome b-c1 complex subunit 9	QCR9_HUMAN
SPRY domain-containing protein 4	SPRY4_HUMAN
Pre-mRNA-splicing factor CWC22 homolog	CWC22_HUMAN
Phosphoserine aminotransferase	SERC_HUMAN
Tyrosine-protein phosphatase non-receptor type 23	PTN23_HUMAN
Ribosomal oxygenase 2	RIOX2_HUMAN
NF-X1-type zinc finger protein NFXL1	NFXL1_HUMAN
PITH domain-containing protein 1	PITH1_HUMAN
NmrA-like family domain-containing protein 1	NMRL1_HUMAN

Clathrin light chain B	CLCB_HUMAN
Lysosomal acid lipase/cholesteryl ester hydrolase	LICH_HUMAN
General vesicular transport factor p115	USO1_HUMAN
Protein Jade-3	JADE3_HUMAN
Kelch-like protein 4	KLHL4_HUMAN
Sorbin and SH3 domain-containing protein 2	SRBS2_HUMAN
Sorcin	SORCN_HUMAN
Short/branched chain specific acyl-CoA dehydrogenase, mitochondrial	ACDSB_HUMAN
HLA class I histocompatibility antigen, alpha chain E	HLAE_HUMAN
Endothelin-converting enzyme 1	ECE1_HUMAN
Mannose-6-phosphate isomerase	MPI_HUMAN
Isochorismatase domain-containing protein 2	ISOC2_HUMAN
Zinc finger ZZ-type and EF-hand domain-containing protein 1	ZZEF1_HUMAN
Late secretory pathway protein AVL9 homolog	AVL9_HUMAN
Polymerase delta-interacting protein 2	PDIP2_HUMAN
General transcription factor II-I	GTF2I_HUMAN
Cytokine receptor-like factor 3	CRLF3_HUMAN
Glutathione peroxidase 7	GPX7_HUMAN
Interleukin-1 receptor-associated kinase 4	IRAK4_HUMAN
N-alpha-acetyltransferase 10	NAA10_HUMAN
Histone acetyltransferase KAT7	KAT7_HUMAN
Phospholipid hydroperoxide glutathione peroxidase GPX4	GPX4_HUMAN
Phosphatidylinositol 4-kinase alpha	PI4KA_HUMAN
Dedicator of cytokinesis protein 6	DOCK6_HUMAN
Conserved oligomeric Golgi complex subunit 4	COG4_HUMAN
Stomatin-like protein 2, mitochondrial	STML2_HUMAN
Apoptosis-associated speck-like protein containing a CARD	ASC_HUMAN
Transmembrane emp24 domain-containing protein 10	TMEDA_HUMAN
Phosducin-like protein	PHLP_HUMAN
Dehydrogenase/reductase SDR family member 4	DHRS4_HUMAN
Transmembrane protein 126A	T126A_HUMAN
Serine/threonine-protein kinase OSR1	OXSR1_HUMAN
Intercellular adhesion molecule 2	ICAM2_HUMAN
Antigen peptide transporter 2	TAP2_HUMAN
[F-actin]-monooxygenase MICAL2	MICA2_HUMAN
SWI/SNF-related matrix-associated actin-dependent regulator of chromatin s	SMRD3_HUMAN
Protein PAXX	PAXX_HUMAN
Proteasome subunit beta type-9	PSB9_HUMAN
Vesicle transport protein GOT1B	GOT1B_HUMAN
Nuclear pore complex protein Nup160	NU160_HUMAN
Integrator complex subunit 10	INT10_HUMAN
T-cell immunomodulatory protein	TIP_HUMAN
Lysophospholipid acyltransferase 5	MBOA5_HUMAN
Replication protein A 14 kDa subunit	RFA3_HUMAN
Replication factor C subunit 3	RFC3_HUMAN
Coiled-coil domain-containing protein 97	CCD97_HUMAN
Hydroxyacyl-coenzyme A dehydrogenase, mitochondrial	HCDH_HUMAN
Cleavage and polyadenylation specificity factor subunit 5	CPSF5_HUMAN
Aspartate--tRNA ligase, cytoplasmic	SYDC_HUMAN
Translocating chain-associated membrane protein 2	TRAM2_HUMAN

Anaphase-promoting complex subunit 1	APC1_HUMAN
Coatomer subunit gamma-2	COPG2_HUMAN
Lipoamide acyltransferase component of branched-chain alpha-keto acid deh	ODB2_HUMAN
Coiled-coil domain-containing protein 6	CCDC6_HUMAN
Pseudouridylate synthase TRUB1	TRUB1_HUMAN
COMM domain-containing protein 10	COMDA_HUMAN
Transcription factor ETV6	ETV6_HUMAN
Non-lysosomal glucosylceramidase	GBA2_HUMAN
Inositol polyphosphate-4-phosphatase type I A	INP4A_HUMAN
Transmembrane protein 160	TM160_HUMAN
Spermine synthase	SPSY_HUMAN
Vinexin	VINEX_HUMAN
Anion exchange protein 2	B3A2_HUMAN
Hydroxymethylglutaryl-CoA lyase, mitochondrial	HMGCL_HUMAN
Trafficking protein particle complex subunit 4	TPPC4_HUMAN
Pericentriolar material 1 protein	PCM1_HUMAN
Transmembrane protein 88	TMM88_HUMAN
5'-AMP-activated protein kinase subunit beta-1	AAKB1_HUMAN
Sterol 26-hydroxylase, mitochondrial	CP27A_HUMAN
General transcription factor 3C polypeptide 5	TF3C5_HUMAN
Dolichyl-diphosphooligosaccharide--protein glycosyltransferase subunit DAD	DAD1_HUMAN
Small ribosomal subunit protein mS25	RT25_HUMAN
NADH dehydrogenase [ubiquinone] iron-sulfur protein 3, mitochondrial	NDUS3_HUMAN
Vacuolar fusion protein CCZ1 homolog B	CCZ1B_HUMAN
Asparaginyl-tRNA synthetase	SYNM_HUMAN
TBC1 domain family member 13	TBC13_HUMAN
Very-long-chain (3R)-3-hydroxyacyl-CoA dehydratase 2	HACD2_HUMAN
Serine/threonine-protein phosphatase 2A 55 kDa regulatory subunit B alpha	2ABA_HUMAN
Phosphopantothenate--cysteine ligase	PPCS_HUMAN
Branched-chain alpha-ketoacid dehydrogenase kinase	BCKD_HUMAN
Nuclear factor NF-kappa-B p105 subunit	NFKB1_HUMAN
ATP-dependent 6-phosphofructokinase, platelet type	PFKAP_HUMAN
Large ribosomal subunit protein mL43	RM43_HUMAN
Membrane-associated guanylate kinase, WW and PDZ domain-containing pro	MAGI1_HUMAN
ATP-citrate synthase	ACLY_HUMAN
Coatomer subunit beta	COPB_HUMAN
Delta(14)-sterol reductase LBR	LBR_HUMAN
Protein YIPF3	YIPF3_HUMAN
Histone chaperone ASF1B	ASF1B_HUMAN
Actin-binding protein WASF2	WASF2_HUMAN
Actin nucleation-promoting factor WASL	WASL_HUMAN
Deoxycytidylate deaminase	DCTD_HUMAN
Ataxin-3	ATX3_HUMAN
Aldehyde dehydrogenase family 16 member A1	A16A1_HUMAN
Dual specificity protein phosphatase 12	DUS12_HUMAN
WD repeat-containing protein 19	WDR19_HUMAN
Deoxyuridine 5'-triphosphate nucleotidohydrolase, mitochondrial	DUT_HUMAN
Nucleoporin Nup37	NUP37_HUMAN
Ubiquitin-conjugating enzyme E2 D3	UB2D3_HUMAN
Uncharacterized protein C11orf98	CK098_HUMAN

Immunity-related GTPase family Q protein	IRGQ_HUMAN
Succinyl-CoA:3-ketoacid coenzyme A transferase 1, mitochondrial	SCOT1_HUMAN
Disintegrin and metalloproteinase domain-containing protein 17	ADA17_HUMAN
DENN domain-containing protein 4C	DEN4C_HUMAN
Transport and Golgi organization protein 2 homolog	TNG2_HUMAN
Aspartate--tRNA ligase, mitochondrial	SYDM_HUMAN
Prostamide/prostaglandin F synthase	PXL2B_HUMAN
Lipid scramblase CLPTM1L	CLP1L_HUMAN
mRNA (2'-O-methyladenosine-N(6)-)-methyltransferase	CAPAM_HUMAN
3'-5' RNA helicase YTHDC2	YTDC2_HUMAN
PRKCA-binding protein	PICK1_HUMAN
RNA-binding protein Nova-2	NOVA2_HUMAN
E3 ubiquitin-protein ligase UBR5	UBR5_HUMAN
Rho-associated protein kinase 1	ROCK1_HUMAN
Apolipoprotein L2	APOL2_HUMAN
E3 SUMO-protein ligase RanBP2	RBP2_HUMAN
Deaminated glutathione amidase	NIT1_HUMAN
Kinesin-like protein KIF15	KIF15_HUMAN
Cyclin-dependent kinase 2	CDK2_HUMAN
Alpha-mannosidase 2C1	MA2C1_HUMAN
Rho guanine nucleotide exchange factor 15	ARHGF_HUMAN
Arginyl-tRNA--protein transferase 1	ATE1_HUMAN
Ras association domain-containing protein 2	RASF2_HUMAN
Gem-associated protein 4	GEMI4_HUMAN
Nuclear pore complex protein Nup107	NU107_HUMAN
Importin subunit beta-1	IMB1_HUMAN
DNA polymerase epsilon subunit 3	DPOE3_HUMAN
Myotubularin-related protein 14	MTMRE_HUMAN
Coiled-coil domain-containing protein 186	CC186_HUMAN
Vacuolar protein-sorting-associated protein 25	VPS25_HUMAN
Nuclear valosin-containing protein-like	NVL_HUMAN
Receptor-type tyrosine-protein phosphatase F	PTPRF_HUMAN
Lamina-associated polypeptide 2, isoforms beta/gamma	LAP2B_HUMAN
Heat shock 70 kDa protein 14	HSP7E_HUMAN
Cullin-4A	CUL4A_HUMAN
Dymeclin	DYM_HUMAN
E3 ubiquitin-protein ligase synoviolin	SYVN1_HUMAN
COMM domain-containing protein 2	COMD2_HUMAN
60S ribosomal export protein NMD3	NMD3_HUMAN
5'-nucleotidase domain-containing protein 2	NT5D2_HUMAN
Sialin	S17A5_HUMAN
DCC-interacting protein 13-alpha	DP13A_HUMAN
NADH dehydrogenase [ubiquinone] flavoprotein 2, mitochondrial	NDUV2_HUMAN
Aldehyde dehydrogenase 1A1	AL1A1_HUMAN
Splicing regulatory glutamine/lysine-rich protein 1	SREK1_HUMAN
Tyrosyl-DNA phosphodiesterase 1	TYDP1_HUMAN
Diablo IAP-binding mitochondrial protein	DBLOH_HUMAN
E3 ubiquitin-protein ligase HECW2	HECW2_HUMAN
Secretory carrier-associated membrane protein 2	SCAM2_HUMAN
C-terminal-binding protein 2	CTBP2_HUMAN

Dynein axonemal assembly factor 5	DAAF5_HUMAN
Lysophosphatidylcholine acyltransferase 1	PCAT1_HUMAN
Threonylcarbamoyladenosine tRNA methylthiotransferase	CDKAL_HUMAN
Disintegrin and metalloproteinase domain-containing protein 15	ADA15_HUMAN
Diphosphoinositol polyphosphate phosphohydrolase 2	NUDT4_HUMAN
Interferon-inducible double-stranded RNA-dependent protein kinase activato	PRKRA_HUMAN
Peroxisomal bifunctional enzyme	ECHP_HUMAN
Tight junction-associated protein 1	TJAP1_HUMAN
Small ribosomal subunit protein uS12m	RT12_HUMAN
Huntingtin	HD_HUMAN
Disks large-associated protein 5	DLGP5_HUMAN
Rho GTPase-activating protein 17	RHG17_HUMAN
Zinc finger protein 281	ZN281_HUMAN
Beta-glucuronidase	BGLR_HUMAN
Exocyst complex component 6	EXOC6_HUMAN
Heat shock protein 105 kDa	HS105_HUMAN
Large subunit GTPase 1 homolog	LSG1_HUMAN
Rho GDP-dissociation inhibitor 2	GDIR2_HUMAN
Nuclear cap-binding protein subunit 1	NCBP1_HUMAN
Leucine-rich repeat flightless-interacting protein 1	LRRF1_HUMAN
Transmembrane protein 131	TM131_HUMAN
WD repeat-containing protein 37	WDR37_HUMAN
Endoplasmic reticulum resident protein 44	ERP44_HUMAN
Branched-chain-amino-acid aminotransferase, mitochondrial	BCAT2_HUMAN
26S proteasome non-ATPase regulatory subunit 4	PSMD4_HUMAN
Quinone oxidoreductase	QOR_HUMAN
Inactive C-alpha-formylglycine-generating enzyme 2	SUMF2_HUMAN
Vang-like protein 1	VANG1_HUMAN
Heterogeneous nuclear ribonucleoprotein L-like	HNRLL_HUMAN
Conserved oligomeric Golgi complex subunit 3	COG3_HUMAN
Unconventional myosin-X	MYO10_HUMAN
Kinesin-like protein KIF2A	KIF2A_HUMAN
Ashwin	ASHWN_HUMAN
Metastasis-associated protein MTA3	MTA3_HUMAN
Testis-expressed protein 10	TEX10_HUMAN
Coatomer subunit alpha	COPA_HUMAN
Cytosolic non-specific dipeptidase	CNDP2_HUMAN
ATP synthase F(0) complex subunit B1, mitochondrial	AT5F1_HUMAN
Inositol polyphosphate 1-phosphatase	INPP_HUMAN
AP-3 complex subunit delta-1	AP3D1_HUMAN
Uridine 5'-monophosphate synthase	UMPS_HUMAN
Calnexin	CALX_HUMAN
Protein farnesyltransferase/geranylgeranyltransferase type-1 subunit alpha	FNTA_HUMAN
Cytosolic Fe-S cluster assembly factor NUBP1	NUBP1_HUMAN
Lysosomal cobalamin transporter ABCD4	ABCD4_HUMAN
Gasdermin-E	GSDME_HUMAN
Elongin-C	ELOC_HUMAN
Serine/threonine-protein kinase N2	PKN2_HUMAN
mRNA export factor GLE1	GLE1_HUMAN
Ubiquitin-conjugating enzyme E2 Q1	UB2Q1_HUMAN

Dipeptidyl peptidase 9	DPP9_HUMAN
Polyhomeotic-like protein 2	PHC2_HUMAN
Endoplasmic reticulum-Golgi intermediate compartment protein 2	ERGI2_HUMAN
Dedicator of cytokinesis protein 9	DOCK9_HUMAN
Vacuolar protein sorting-associated protein 16 homolog	VPS16_HUMAN
CCN family member 2	CCN2_HUMAN
Cyclin-dependent kinase 9	CDK9_HUMAN
Vacuolar protein sorting-associated protein 33B	VP33B_HUMAN
Sulfide:quinone oxidoreductase, mitochondrial	SQOR_HUMAN
Kelch repeat and BTB domain-containing protein 2	KBTB2_HUMAN
Kynurenine--oxoglutarate transaminase 3	KAT3_HUMAN
Serine protease FAM111A	F111A_HUMAN
Complex I intermediate-associated protein 30, mitochondrial	CIA30_HUMAN
Protein polybromo-1	PB1_HUMAN
Oxysterol-binding protein-related protein 11	OSB11_HUMAN
Ras-related C3 botulinum toxin substrate 1	RAC1_HUMAN
Death-inducer obliterator 1	DIDO1_HUMAN
Secernin-3	SCRN3_HUMAN
Ectonucleoside triphosphate diphosphohydrolase 1	ENTP1_HUMAN
Probable ubiquitin carboxyl-terminal hydrolase FAF-X	USP9X_HUMAN
DnaJ homolog subfamily B member 6	DNJB6_HUMAN
Inactive serine/threonine-protein kinase VRK3	VRK3_HUMAN
E1A-binding protein p400	EP400_HUMAN
Lariat debranching enzyme	DBR1_HUMAN
Heat shock 70 kDa protein 1-like	HS71L_HUMAN
Syntaxin-binding protein 3	STXB3_HUMAN
Ras-related protein Rab-3D	RAB3D_HUMAN
Dolichyl pyrophosphate Man9GlcNAc2 alpha-1,3-glucosyltransferase	ALG6_HUMAN
Importin subunit alpha-1	IMA1_HUMAN
Rap guanine nucleotide exchange factor 1	RPGF1_HUMAN
Ceramide synthase 2	CERS2_HUMAN
Ubiquitin-like modifier-activating enzyme 6	UBA6_HUMAN
Prefoldin subunit 3	PFD3_HUMAN
Exocyst complex component 4	EXOC4_HUMAN
Ribosomal protein S6 kinase alpha-4	KS6A4_HUMAN
Dolichyl-diphosphooligosaccharide--protein glycosyltransferase subunit STT3	STT3A_HUMAN
Histone-binding protein RBBP7	RBBP7_HUMAN
AP-2 complex subunit mu	AP2M1_HUMAN
RNA-binding protein 27	RBM27_HUMAN
Insulin-like growth factor 1 receptor	IGF1R_HUMAN
Translationally-controlled tumor protein	TCTP_HUMAN
Cytoplasmic tRNA 2-thiolation protein 2	CTU2_HUMAN
Enhancer of mRNA-decapping protein 4	EDC4_HUMAN
Exportin-4	XPO4_HUMAN
Protein disulfide isomerase CRELD2	CREL2_HUMAN
E3 ubiquitin-protein ligase TRIM22	TRI22_HUMAN
Calpain small subunit 1	CPNS1_HUMAN
Zinc finger protein 318	ZN318_HUMAN
SWI/SNF-related matrix-associated actin-dependent regulator of chromatin s	SMCE1_HUMAN
Vacuolar protein sorting-associated protein 4A	VPS4A_HUMAN

ELMO domain-containing protein 2	ELMD2_HUMAN
Pyruvate dehydrogenase E1 component subunit beta, mitochondrial	ODPB_HUMAN
Golgi reassembly-stacking protein 2	GORS2_HUMAN
Inositol-3-phosphate synthase 1	INO1_HUMAN
Active breakpoint cluster region-related protein	ABR_HUMAN
Sorting nexin-1	SNX1_HUMAN
Adapter SH3BGRL	SH3L1_HUMAN
Signal peptidase complex catalytic subunit SEC11A	SC11A_HUMAN
Mitogen-activated protein kinase 7	MK07_HUMAN
NADH dehydrogenase [ubiquinone] 1 alpha subcomplex subunit 13	NDUAD_HUMAN
Reticulocalbin-1	RCN1_HUMAN
Splicing factor, suppressor of white-apricot homolog	SFSWA_HUMAN
mRNA export factor RAE1	RAE1L_HUMAN
Zinc finger CCHC domain-containing protein 8	ZCHC8_HUMAN
Peptidyl-prolyl cis-trans isomerase-like 4	PPIL4_HUMAN
Medium-chain acyl-CoA ligase ACSF2, mitochondrial	ACSF2_HUMAN
Vacuolar protein sorting-associated protein 52 homolog	VPS52_HUMAN
3-oxoacyl-[acyl-carrier-protein] reductase	CBR4_HUMAN
E3 ubiquitin-protein ligase RNF25	RNF25_HUMAN
DAZ-associated protein 1	DAZP1_HUMAN
Aspartyl aminopeptidase	DNPEP_HUMAN
Calcineurin B homologous protein 1	CHP1_HUMAN
Evolutionarily conserved signaling intermediate in Toll pathway, mitochondri	ECSIT_HUMAN
Large ribosomal subunit protein uL29m	RM47_HUMAN
Erlin-1	ERLN1_HUMAN
Ubiquitin carboxyl-terminal hydrolase isozyme L3	UCHL3_HUMAN
NADH-ubiquinone oxidoreductase 75 kDa subunit, mitochondrial	NDUS1_HUMAN
Crk-like protein	CRKL_HUMAN
Proteasome inhibitor PI31 subunit	PSMF1_HUMAN
Ras-related protein Rab-5B	RAB5B_HUMAN
Small ribosomal subunit protein uS2	RSSA_HUMAN
ADP-ribosylation factor-like protein 2	ARL2_HUMAN
Ran GTPase-activating protein 1	RAGP1_HUMAN
Echinoderm microtubule-associated protein-like 3	EMAL3_HUMAN
ATP-dependent 6-phosphofructokinase, muscle type	PFKAM_HUMAN
Ubiquitin carboxyl-terminal hydrolase 11	UBP11_HUMAN
Ras-related protein Rab-8A	RAB8A_HUMAN
Protein YIPF5	YIPF5_HUMAN
Beta-1,3-galactosyltransferase 6	B3GT6_HUMAN
Transcriptional regulator QRICH1	QRIC1_HUMAN
Protein virilizer homolog	VIR_HUMAN
Piezo-type mechanosensitive ion channel component 1	PIEZ1_HUMAN
Protein XRP2	XRP2_HUMAN
Histidine--tRNA ligase, mitochondrial	SYHM_HUMAN
Alanine--tRNA ligase, cytoplasmic	SYAC_HUMAN
Myc box-dependent-interacting protein 1	BIN1_HUMAN
DCN1-like protein 1	DCNL1_HUMAN
Proteasome subunit alpha type-7	PSA7_HUMAN
Ran-binding protein 10	RBP10_HUMAN
Isovaleryl-CoA dehydrogenase, mitochondrial	IVD_HUMAN

Thymosin beta-4	TYB4_HUMAN
Methylenetetrahydrofolate reductase (NADPH)	MTHR_HUMAN
Prelamin-A/C	LMNA_HUMAN
Mitochondrial 2-oxoglutarate/malate carrier protein	M2OM_HUMAN
Exocyst complex component 1	EXOC1_HUMAN
Phosphatidylinositol 3,4,5-trisphosphate-dependent Rac exchanger 1 protein	PREX1_HUMAN
Probable U3 small nucleolar RNA-associated protein 11	UTP11_HUMAN
Acidic leucine-rich nuclear phosphoprotein 32 family member B	AN32B_HUMAN
AP-1 complex subunit beta-1	AP1B1_HUMAN
Methylglutaconyl-CoA hydratase, mitochondrial	AUHM_HUMAN
Protein phosphatase 1 regulatory subunit 12A	MYPT1_HUMAN
Progranulin	GRN_HUMAN
TraB domain-containing protein	TRABD_HUMAN
Protocadherin-16	PCD16_HUMAN
CD109 antigen	CD109_HUMAN
COMM domain-containing protein 5	COMD5_HUMAN
Transmembrane 9 superfamily member 2	TM9S2_HUMAN
ATP synthase subunit alpha, mitochondrial	ATPA_HUMAN
Armadillo repeat-containing protein 8	ARMC8_HUMAN
Histone acetyltransferase type B catalytic subunit	HAT1_HUMAN
Phosphatidylserine synthase 1	PTSS1_HUMAN
WD repeat-containing protein 81	WDR81_HUMAN
Kinesin-like protein KIF13A	KI13A_HUMAN
Probable global transcription activator SNF2L1	SMCA1_HUMAN
Glycerol-3-phosphate dehydrogenase, mitochondrial	GPDM_HUMAN
Mitogen-activated protein kinase 13	MK13_HUMAN
Procollagen galactosyltransferase 1	GT251_HUMAN
GMP reductase 1	GMPR1_HUMAN
Thrombospondin type-1 domain-containing protein 4	THSD4_HUMAN
Rap guanine nucleotide exchange factor 2	RPGF2_HUMAN
Mitogen-activated protein kinase 3	MK03_HUMAN
Heme oxygenase 2	HMOX2_HUMAN
Small ribosomal subunit protein eS8	RS8_HUMAN
C-myc promoter-binding protein	MYCPP_HUMAN
Erbin	ERBIN_HUMAN
Ras-related protein Rab-22A	RB22A_HUMAN
ATP-dependent Clp protease ATP-binding subunit clpX-like, mitochondrial	CLPX_HUMAN
Ras-related protein Rap-1b	RAP1B_HUMAN
Phosphoserine phosphatase	SERB_HUMAN
Synembryn-B	RIC8B_HUMAN
Phospholipase A-2-activating protein	PLAP_HUMAN
Sterol carrier protein 2	SCP2_HUMAN
Biliverdin reductase A	BIEA_HUMAN
DNA ligase 3	DNLI3_HUMAN
Exocyst complex component 5	EXOC5_HUMAN
Cytochrome c oxidase subunit NDUFA4	NDUA4_HUMAN
Large ribosomal subunit protein eL19	RL19_HUMAN
Very long chain fatty acid elongase 5	ELOV5_HUMAN
Protein enabled homolog	ENAH_HUMAN
Transcription initiation factor IIB	TF2B_HUMAN

DNA-directed RNA polymerase II subunit RPB3	RPB3_HUMAN
Serine/threonine-protein phosphatase 6 catalytic subunit	PPP6_HUMAN
NBAS subunit of NRZ tethering complex	NBAS_HUMAN
Nuclear mitotic apparatus protein 1	NUMA1_HUMAN
DnaJ homolog subfamily C member 8	DNJC8_HUMAN
Serine/threonine-protein phosphatase 2A 55 kDa regulatory subunit B delta i	2ABD_HUMAN
DnaJ homolog subfamily C member 7	DNJC7_HUMAN
DNA primase small subunit	PRI1_HUMAN
Serine/threonine-protein kinase Nek9	NEK9_HUMAN
RNA polymerase II-associated factor 1 homolog	PAF1_HUMAN
Tripeptidyl-peptidase 2	TPP2_HUMAN
Bifunctional purine biosynthesis protein ATIC	PUR9_HUMAN
Uncharacterized protein FLJ45252	YJ005_HUMAN
Coiled-coil domain-containing protein 9	CCDC9_HUMAN
Poliovirus receptor	PVR_HUMAN
Regulator of MON1-CCZ1 complex	RMC1_HUMAN
Importin-11	IPO11_HUMAN
Serine/threonine-protein phosphatase 5	PPP5_HUMAN
26S proteasome non-ATPase regulatory subunit 13	PSD13_HUMAN
Exportin-T	XPOT_HUMAN
Serine/arginine-rich splicing factor 11	SRS11_HUMAN
Collagen triple helix repeat-containing protein 1	CTHR1_HUMAN
Acyl-coenzyme A thioesterase 13	ACO13_HUMAN
Estradiol 17-beta-dehydrogenase 11	DHB11_HUMAN
Signal recognition particle subunit SRP72	SRP72_HUMAN
Receptor-type tyrosine-protein phosphatase beta	PTPRB_HUMAN
Serine/threonine-protein phosphatase 2B catalytic subunit gamma isoform	PP2BC_HUMAN
Afadin	AFAD_HUMAN
Endonuclease III-like protein 1	NTH_HUMAN
Vesicle-fusing ATPase	NSF_HUMAN
WD repeat-containing protein 43	WDR43_HUMAN
TBC1 domain family member 10B	TB10B_HUMAN
B-cell CLL/lymphoma 7 protein family member C	BCL7C_HUMAN
U4/U6 small nuclear ribonucleoprotein Prp4	PRP4_HUMAN
E3 ubiquitin/ISG15 ligase TRIM25	TRI25_HUMAN
[Pyruvate dehydrogenase [acetyl-transferring]]-phosphatase 1, mitochondria	PDP1_HUMAN
Alpha-taxilin	TXLNA_HUMAN
Superoxide dismutase [Mn], mitochondrial	SODM_HUMAN
Aspartate aminotransferase, cytoplasmic	AATC_HUMAN
Replication initiator 1	REPI1_HUMAN
RUN and FYVE domain-containing protein 2	RUFY2_HUMAN
Dynactin subunit 3	DCTN3_HUMAN
Enoyl-CoA hydratase, mitochondrial	ECHM_HUMAN
Sentrin-specific protease 3	SENP3_HUMAN
Cohesin subunit SA-2	STAG2_HUMAN
GTPase-activating protein and VPS9 domain-containing protein 1	GAPD1_HUMAN
Netrin-4	NET4_HUMAN
Bis(5'-nucleosyl)-tetraphosphatase [asymmetrical]	AP4A_HUMAN
Probable ATP-dependent RNA helicase DDX46	DDX46_HUMAN
Regulation of nuclear pre-mRNA domain-containing protein 1A	RPR1A_HUMAN

Dynamin-like 120 kDa protein, mitochondrial	OPA1_HUMAN
10 kDa heat shock protein, mitochondrial	CH10_HUMAN
RING-type E3 ubiquitin-protein ligase PPIL2	PPIL2_HUMAN
Serine/threonine-protein kinase TBK1	TBK1_HUMAN
5'-AMP-activated protein kinase subunit gamma-2	AAKG2_HUMAN
CD81 antigen	CD81_HUMAN
AP-2 complex subunit sigma	AP2S1_HUMAN
Selenoprotein S	SELS_HUMAN
Alsin	ALS2_HUMAN
Uncharacterized protein C19orf47	CS047_HUMAN
14-3-3 protein eta	1433F_HUMAN
Sec1 family domain-containing protein 1	SCFD1_HUMAN
Thioredoxin-related transmembrane protein 4	TMX4_HUMAN
NAD(P)H-hydrate epimerase	NNRE_HUMAN
Cullin-associated NEDD8-dissociated protein 1	CAND1_HUMAN
Nuclear receptor coactivator 7	NCOA7_HUMAN
Ubiquitin-conjugating enzyme E2 E3	UB2E3_HUMAN
Myotubularin	MTM1_HUMAN
SRSF protein kinase 2	SRPK2_HUMAN
Small ribosomal subunit protein mS35	RT35_HUMAN
Condensin complex subunit 2	CND2_HUMAN
Protein CASC3	CASC3_HUMAN
Thioredoxin-like protein 1	TXNL1_HUMAN
Zinc finger protein 638	ZN638_HUMAN
Vacuolar protein sorting-associated protein 45	VPS45_HUMAN
Alpha- and gamma-adaptin-binding protein p34	AAGAB_HUMAN
Conserved oligomeric Golgi complex subunit 1	COG1_HUMAN
Vesicle transport through interaction with t-SNAREs homolog 1B	VTI1B_HUMAN
DNA-directed RNA polymerase III subunit RPC4	RPC4_HUMAN
N(G),N(G)-dimethylarginine dimethylaminohydrolase 1	DDAH1_HUMAN
DNA-3-methyladenine glycosylase	3MG_HUMAN
Katanin p60 ATPase-containing subunit A1	KTNA1_HUMAN
Inositol monophosphatase 1	IMPA1_HUMAN
DNA excision repair protein ERCC-6	ERCC6_HUMAN
TBC1 domain family member 15	TBC15_HUMAN
Plexin-D1	PLXD1_HUMAN
Tuberin	TSC2_HUMAN
Transcription factor A, mitochondrial	TFAM_HUMAN
COP9 signalosome complex subunit 5	CSN5_HUMAN
Nardilysin	NRDC_HUMAN
Prolyl 3-hydroxylase 1	P3H1_HUMAN
Small ribosomal subunit protein mS31	RT31_HUMAN
NADH dehydrogenase [ubiquinone] 1 beta subcomplex subunit 10	NDUBA_HUMAN
Complement component C1q receptor	C1QR1_HUMAN
Persulfide dioxygenase ETHE1, mitochondrial	ETHE1_HUMAN
Multifunctional procollagen lysine hydroxylase and glycosyltransferase LH3	PLOD3_HUMAN
Threonine synthase-like 1	THNS1_HUMAN
Secretory carrier-associated membrane protein 1	SCAM1_HUMAN
3-ketoacyl-CoA thiolase, mitochondrial	THIM_HUMAN
Nucleotide exchange factor SIL1	SIL1_HUMAN

Pre-mRNA-processing factor 19	PRP19_HUMAN
Quinone oxidoreductase PIG3	QORX_HUMAN
Prostaglandin E synthase 2	PGES2_HUMAN
Obg-like ATPase 1	OLA1_HUMAN
Ras-related protein Rab-3B	RAB3B_HUMAN
Replication factor C subunit 4	RFC4_HUMAN
DNA replication complex GINS protein PSF1	PSF1_HUMAN
Transmembrane anterior posterior transformation protein 1 homolog	TAPT1_HUMAN
Dipeptidyl peptidase 2	DPP2_HUMAN
Tumor necrosis factor receptor superfamily member 10C	TR10C_HUMAN
Receptor-type tyrosine-protein phosphatase alpha	PTPRA_HUMAN
DNA ligase 1	DNLI1_HUMAN
Cullin-5	CUL5_HUMAN
Leucine-rich repeat-containing protein 59	LRC59_HUMAN
Peroxisomal 2,4-dienoyl-CoA reductase [(3E)-enoyl-CoA-producing]	DECR2_HUMAN
Signal transducer and activator of transcription 1-alpha/beta	STAT1_HUMAN
Sodium- and chloride-dependent taurine transporter	SC6A6_HUMAN
Proteasomal ATPase-associated factor 1	PAAF1_HUMAN
Sodium bicarbonate cotransporter 3	S4A7_HUMAN
Ral GTPase-activating protein subunit alpha-2	RGPA2_HUMAN
Fructose-bisphosphate aldolase C	ALDOC_HUMAN
Serine/threonine-protein kinase N1	PKN1_HUMAN
Lysosomal alpha-glucosidase	LYAG_HUMAN
Chromodomain-helicase-DNA-binding protein 3	CHD3_HUMAN
Uncharacterized protein FAM241A	F241A_HUMAN
Eukaryotic translation elongation factor 1 epsilon-1	MCA3_HUMAN
SRSF protein kinase 1	SRPK1_HUMAN
Regulator of microtubule dynamics protein 1	RMD1_HUMAN
Cyclin-Y	CCNY_HUMAN
Inositol polyphosphate 5-phosphatase K	INP5K_HUMAN
Protein Niban 1	NIBA1_HUMAN
Neurogenic locus notch homolog protein 1	NOTC1_HUMAN
BRCA2-interacting transcriptional repressor EMSY	EMSY_HUMAN
2-oxoglutarate dehydrogenase complex component E1	ODO1_HUMAN
Scavenger receptor class A member 3	SCAR3_HUMAN
Isocitrate dehydrogenase [NAD] subunit beta, mitochondrial	IDH3B_HUMAN
FYVE, RhoGEF and PH domain-containing protein 5	FGD5_HUMAN
Mixed lineage kinase domain-like protein	MLKL_HUMAN
TGF-beta-activated kinase 1 and MAP3K7-binding protein 1	TAB1_HUMAN
Inactive tyrosine-protein kinase PEAK1	PEAK1_HUMAN
Ubiquitin carboxyl-terminal hydrolase isozyme L5	UCHL5_HUMAN
Tectonin beta-propeller repeat-containing protein 2	TCPR2_HUMAN
NAD-dependent malic enzyme, mitochondrial	MAOM_HUMAN
Peptidyl-prolyl cis-trans isomerase NIMA-interacting 4	PIN4_HUMAN
Serine/threonine/tyrosine-interacting protein	STYX_HUMAN
Unconventional myosin-Id	MYO1D_HUMAN
26S proteasome regulatory subunit 4	PRS4_HUMAN
Neuropilin-1	NRP1_HUMAN
DNA repair protein XRCC1	XRCC1_HUMAN
Phosphatase and actin regulator 4	PHAR4_HUMAN

Transformation/transcription domain-associated protein	TRRAP_HUMAN
2'-5'-oligoadenylate synthase 3	OAS3_HUMAN
RNA polymerase II subunit A C-terminal domain phosphatase	CTDP1_HUMAN
GPI transamidase component PIG-T	PIGT_HUMAN
Protein mono-ADP-ribosyltransferase PARP9	PARP9_HUMAN
Probable N-acetyltransferase 14	NAT14_HUMAN
Sushi repeat-containing protein SRPX	SRPX_HUMAN
SUMO-activating enzyme subunit 2	SAE2_HUMAN
BRISC and BRCA1-A complex member 2	BABA2_HUMAN
Ubiquitin conjugation factor E4 A	UBE4A_HUMAN
UDP-N-acetylhexosamine pyrophosphorylase	UAP1_HUMAN
Cytokine-like nuclear factor N-PAC	GLYR1_HUMAN
ADP-sugar pyrophosphatase	NUDT5_HUMAN
Rho-associated protein kinase 2	ROCK2_HUMAN
Monoglyceride lipase	MGLL_HUMAN
Junctional adhesion molecule C	JAM3_HUMAN
Hsc70-interacting protein	F10A1_HUMAN
Golgi phosphoprotein 3-like	GLP3L_HUMAN
Tuftelin-interacting protein 11	TFP11_HUMAN
Myotubularin-related protein 5	MTMR5_HUMAN
Large ribosomal subunit protein eL43	RL37A_HUMAN
Rab GTPase-binding effector protein 1	RABE1_HUMAN
Phosphatidylinositol 3-kinase regulatory subunit alpha	P85A_HUMAN
Mitofusin-1	MFN1_HUMAN
DNA replication licensing factor MCM5	MCM5_HUMAN
GRIP and coiled-coil domain-containing protein 2	GCC2_HUMAN
Cytosolic iron-sulfur assembly component 3	CIAO3_HUMAN
Synembryn-A	RIC8A_HUMAN
Protein scribble homolog	SCRIB_HUMAN
Fascin	FSCN1_HUMAN
Nuclear pore complex protein Nup85	NUP85_HUMAN
WASH complex subunit 3	WASC3_HUMAN
Elongator complex protein 1	ELP1_HUMAN
Zinc finger protein 512	ZN512_HUMAN
Double-stranded RNA-binding protein Staufen homolog 2	STAU2_HUMAN
Coronin-1B	COR1B_HUMAN
Tubulin beta chain	TBB5_HUMAN
Src substrate cortactin	SRC8_HUMAN
ER lumen protein-retaining receptor 1	ERD21_HUMAN
Matrix Gla protein	MGP_HUMAN
DnaJ homolog subfamily C member 3	DNJC3_HUMAN
DnaJ homolog subfamily C member 9	DNJC9_HUMAN
26S proteasome non-ATPase regulatory subunit 6	PSMD6_HUMAN
Dehydrogenase/reductase SDR family member on chromosome X	DHRSX_HUMAN
Protein capicua homolog	CIC_HUMAN
BolA-like protein 2	BOLA2_HUMAN
Carboxypeptidase D	CBPD_HUMAN
Selenoprotein H	SELH_HUMAN
Inorganic pyrophosphatase 2, mitochondrial	IPYR2_HUMAN
Inhibitor of nuclear factor kappa-B kinase subunit beta	IKKB_HUMAN

Transmembrane emp24 domain-containing protein 1	TMED1_HUMAN
Ferritin heavy chain	FRIH_HUMAN
Syntaxin-8	STX8_HUMAN
Protein S100-A6	S10A6_HUMAN
14-3-3 protein beta/alpha	1433B_HUMAN
Very long-chain specific acyl-CoA dehydrogenase, mitochondrial	ACADV_HUMAN
Chromatin complexes subunit BAP18	BAP18_HUMAN
E3 ubiquitin-protein ligase TRIM11	TRI11_HUMAN
Vacuolar protein sorting-associated protein 35	VPS35_HUMAN
Histone-lysine N-methyltransferase EHMT1	EHMT1_HUMAN
Phosphofurin acidic cluster sorting protein 1	PACS1_HUMAN
TBC1 domain family member 22A	TB22A_HUMAN
ORM1-like protein 3	ORML3_HUMAN
Tax1-binding protein 3	TX1B3_HUMAN
N-alpha-acetyltransferase 35, NatC auxiliary subunit	NAA35_HUMAN
PDZ domain-containing protein GIPC1	GIPC1_HUMAN
Heat shock 70 kDa protein 12B	HS12B_HUMAN
Leucine-rich repeat and WD repeat-containing protein 1	LRWD1_HUMAN
TBC1 domain family member 9	TBCD9_HUMAN
Sarcoplasmic/endoplasmic reticulum calcium ATPase 2	AT2A2_HUMAN
Tubulin--tyrosine ligase-like protein 12	TTL12_HUMAN
E3 ubiquitin-protein ligase UHRF1	UHRF1_HUMAN
WD repeat domain phosphoinositide-interacting protein 2	WIPI2_HUMAN
Phosphoribosyl pyrophosphate synthase-associated protein 2	KPRB_HUMAN
Ubiquitin carboxyl-terminal hydrolase 48	UBP48_HUMAN
Centromere-associated protein E	CENPE_HUMAN
WAS/WASL-interacting protein family member 1	WIPF1_HUMAN
Asparagine synthetase [glutamine-hydrolyzing]	ASNS_HUMAN
Phosphoribosyl pyrophosphate synthase-associated protein 1	KPRA_HUMAN
E3 ubiquitin-protein ligase TRIP12	TRIPC_HUMAN
FK506-binding protein 15	FKB15_HUMAN
Rho GTPase-activating protein 21	RHG21_HUMAN
Lysosomal protective protein	PPGB_HUMAN
Sigma non-opioid intracellular receptor 1	SGMR1_HUMAN
Vesicle transport protein USE1	USE1_HUMAN
Geranylgeranyl transferase type-2 subunit alpha	PGTA_HUMAN
Tyrosine-protein phosphatase non-receptor type 2	PTN2_HUMAN
Rab11 family-interacting protein 5	RFIP5_HUMAN
Survival of motor neuron-related-splicing factor 30	SPF30_HUMAN
ATP-dependent RNA helicase DHX8	DHX8_HUMAN
Mediator of RNA polymerase II transcription subunit 20	MED20_HUMAN
Phospholipid scramblase 3	PLS3_HUMAN
Beta-centractin	ACTY_HUMAN
Phosphatidylinositol 3,4,5-trisphosphate 5-phosphatase 1	SHIP1_HUMAN
Protein phosphatase 1 regulatory subunit 21	PPR21_HUMAN
EKC/KEOPS complex subunit TP53RK	PRPK_HUMAN
3-mercaptopyruvate sulfurtransferase	THTM_HUMAN
Pyruvate dehydrogenase phosphatase regulatory subunit, mitochondrial	PDPR_HUMAN
DnaJ homolog subfamily B member 11	DJB11_HUMAN
Low molecular weight phosphotyrosine protein phosphatase	PPAC_HUMAN

Hypoxia up-regulated protein 1	HYOU1_HUMAN
N-alpha-acetyltransferase 16, NatA auxiliary subunit	NAA16_HUMAN
Integrator complex subunit 6	INT6_HUMAN
Peroxisomal membrane protein 11B	PX11B_HUMAN
Bifunctional polynucleotide phosphatase/kinase	PNKP_HUMAN
RNA-binding protein 14	RBM14_HUMAN
Torsin-1A	TOR1A_HUMAN
NADH dehydrogenase [ubiquinone] 1 alpha subcomplex subunit 12	NDUAC_HUMAN
Charged multivesicular body protein 1b	CHM1B_HUMAN
Protein transport protein Sec24D	SC24D_HUMAN
BRCA1-associated protein	BRAP_HUMAN
Zinc finger protein 609	ZN609_HUMAN
E3 ubiquitin-protein ligase HECTD1	HECD1_HUMAN
7-dehydrocholesterol reductase	DHCR7_HUMAN
Arylamine N-acetyltransferase 1	ARY1_HUMAN
Vacuolar protein sorting-associated protein 26C	VP26C_HUMAN
Stathmin-2	STMN2_HUMAN
Elongator complex protein 2	ELP2_HUMAN
Nucleoplasmin-3	NPM3_HUMAN
Succinate--CoA ligase [GDP-forming] subunit beta, mitochondrial	SUCB2_HUMAN
Nucleobindin-1	NUCB1_HUMAN
Polyribonucleotide nucleotidyltransferase 1, mitochondrial	PNPT1_HUMAN
AP-3 complex subunit mu-2	AP3M2_HUMAN
Ras-related protein Rab-1B	RAB1B_HUMAN
Guanine nucleotide-binding protein G(I)/G(S)/G(T) subunit beta-1	GBB1_HUMAN
Lipase maturation factor 2	LMF2_HUMAN
Methylcrotonoyl-CoA carboxylase subunit alpha, mitochondrial	MCCA_HUMAN
E3 ubiquitin-protein ligase RNF14	RNF14_HUMAN
Cytoskeleton-associated protein 4	CKAP4_HUMAN
Actin-histidine N-methyltransferase	SETD3_HUMAN
Mitochondrial fission process protein 1	MTFP1_HUMAN
Multiple myeloma tumor-associated protein 2	MMTA2_HUMAN
SUMO-activating enzyme subunit 1	SAE1_HUMAN
Nucleolin	NUCL_HUMAN
Small ribosomal subunit protein eS7	RS7_HUMAN
Myosin light chain kinase, smooth muscle	MYLK_HUMAN
tRNA (32-2'-O)-methyltransferase regulator THADA	THADA_HUMAN
Endothelial protein C receptor	EPCR_HUMAN
Ras-related GTP-binding protein C	RRAGC_HUMAN
Eukaryotic translation initiation factor 3 subunit J	EIF3J_HUMAN
Dynactin subunit 1	DCTN1_HUMAN
GTPase KRas	RASK_HUMAN
Caspase recruitment domain-containing protein 19	CAR19_HUMAN
Dolichyl-diphosphooligosaccharide--protein glycosyltransferase subunit 1	RPN1_HUMAN
Proteasome subunit beta type-2	PSB2_HUMAN
tRNA (guanine(6)-N2)-methyltransferase THUMP3	THUM3_HUMAN
Dehydrogenase/reductase SDR family member 6	DHRS6_HUMAN
Ras-related protein Ral-B	RALB_HUMAN
Glomulin	GLMN_HUMAN
DNA repair protein RAD50	RAD50_HUMAN

Succinate dehydrogenase [ubiquinone] flavoprotein subunit, mitochondrial	SDHA_HUMAN
Ras GTPase-activating-like protein IQGAP1	IQGA1_HUMAN
Telomerase protein component 1	TEP1_HUMAN
Aldehyde dehydrogenase family 3 member A2	AL3A2_HUMAN
Histone deacetylase 7	HDAC7_HUMAN
Large ribosomal subunit protein mL55	RM55_HUMAN
Perilipin-2	PLIN2_HUMAN
Friend leukemia integration 1 transcription factor	FLI1_HUMAN
G protein-coupled receptor kinase 6	GRK6_HUMAN
Nucleoside diphosphate kinase 3	NDK3_HUMAN
Serine/threonine-protein phosphatase 2A activator	PTPA_HUMAN
Translation machinery-associated protein 16	TMA16_HUMAN
RNA polymerase II-associated protein 3	RPAP3_HUMAN
Ribosomal protein S6 kinase alpha-5	KS6A5_HUMAN
Ribonuclease P protein subunit p25	RPP25_HUMAN
Phosphatidylinositol 4-phosphate 3-kinase C2 domain-containing subunit alp	P3C2A_HUMAN
Mitochondrial-processing peptidase subunit alpha	MPPA_HUMAN
E3 ubiquitin-protein ligase BRE1B	BRE1B_HUMAN
Peroxiredoxin-5, mitochondrial	PRDX5_HUMAN
ETS domain-containing transcription factor ERF	ERF_HUMAN
Serine--tRNA ligase, mitochondrial	SYSM_HUMAN
Thyroid hormone receptor-associated protein 3	TR150_HUMAN
Tubulin-specific chaperone C	TBCC_HUMAN
MKI67 FHA domain-interacting nucleolar phosphoprotein	MK67I_HUMAN
BRO1 domain-containing protein BROX	BROX_HUMAN
Alpha-N-acetylglucosaminidase	ANAG_HUMAN
Dual specificity protein kinase TTK	TTK_HUMAN
Charged multivesicular body protein 1a	CHM1A_HUMAN
Melanoma-associated antigen D2	MAGD2_HUMAN
Dual specificity mitogen-activated protein kinase kinase 6	MP2K6_HUMAN
Cadherin-2	CADH2_HUMAN
Serine/threonine-protein kinase 4	STK4_HUMAN
Acetoacetyl-CoA synthetase	AACS_HUMAN
Ketosamine-3-kinase	KT3K_HUMAN
Kinesin-like protein KIF1C	KIF1C_HUMAN
EGF domain-specific O-linked N-acetylglucosamine transferase	EOGT_HUMAN
Selenocysteine-specific elongation factor	SELB_HUMAN
DNA replication complex GINS protein SLD5	SLD5_HUMAN
HIRA-interacting protein 3	HIRP3_HUMAN
Nondiscriminating glutamyl-tRNA synthetase EARS2, mitochondrial	SYEM_HUMAN
E3 ubiquitin-protein ligase BRE1A	BRE1A_HUMAN
Large ribosomal subunit protein bL21m	RM21_HUMAN
Sulfotransferase 1A1	ST1A1_HUMAN
Nuclear transport factor 2	NTF2_HUMAN
Rab GDP dissociation inhibitor alpha	GDIA_HUMAN
DDB1- and CUL4-associated factor 8	DCAF8_HUMAN
Exosome complex component RRP41	EXOS4_HUMAN
Ubiquitin carboxyl-terminal hydrolase 14	UBP14_HUMAN
GTPase IMAP family member 6	GIMA6_HUMAN
Exosome complex component RRP42	EXOS7_HUMAN

Activity-dependent neuroprotector homeobox protein	ADNP_HUMAN
Ubiquitin-conjugating enzyme E2 L3	UB2L3_HUMAN
Nucleoporin NUP188	NU188_HUMAN
NACHT, LRR and PYD domains-containing protein 1	NLRP1_HUMAN
DDB1- and CUL4-associated factor 13	DCA13_HUMAN
CBP80/20-dependent translation initiation factor	CTIF_HUMAN
NADH dehydrogenase [ubiquinone] 1 alpha subcomplex subunit 7	NDUA7_HUMAN
Protein FRG1	FRG1_HUMAN
Nucleoporin p54	NUP54_HUMAN
Translin	TSN_HUMAN
Motile sperm domain-containing protein 2	MSPD2_HUMAN
Arylsulfatase B	ARSB_HUMAN
Emerin	EMD_HUMAN
Oxysterol-binding protein 1	OSBP1_HUMAN
Lanosterol synthase	LSS_HUMAN
Core-binding factor subunit beta	PEBB_HUMAN
FAD-dependent oxidoreductase domain-containing protein 1	FXRD1_HUMAN
Pre-mRNA-processing factor 39	PRP39_HUMAN
ATP-dependent 6-phosphofructokinase, liver type	PFKAL_HUMAN
1-phosphatidylinositol 3-phosphate 5-kinase	FYV1_HUMAN
Argininosuccinate lyase	ARLY_HUMAN
Oxysterol-binding protein 2	OSBP2_HUMAN
Serine/threonine-protein phosphatase 2A 56 kDa regulatory subunit gamma	2A5G_HUMAN
DNA-directed RNA polymerase I subunit RPA34	RPA34_HUMAN
Rho guanine nucleotide exchange factor 7	ARHG7_HUMAN
CD59 glycoprotein	CD59_HUMAN
Splicing factor U2AF 35 kDa subunit	U2AF1_HUMAN
SURP and G-patch domain-containing protein 1	SUGP1_HUMAN
Protein ERGIC-53	LMAN1_HUMAN
Transcriptional activator protein Pur-alpha	PURA_HUMAN
Myocardial zonula adherens protein	MYZAP_HUMAN
Upstream-binding protein 1	UBIP1_HUMAN
Protein arginine methyltransferase NDUFAF7, mitochondrial	NDUF7_HUMAN
BRCA1-associated ATM activator 1	BRAT1_HUMAN
Nucleolar transcription factor 1	UBF1_HUMAN
Squalene monooxygenase	ERG1_HUMAN
Large ribosomal subunit protein uL4	RL4_HUMAN
Serine beta-lactamase-like protein LACTB, mitochondrial	LACTB_HUMAN
Hypoxia-inducible factor 1-alpha inhibitor	HIF1N_HUMAN
CGG triplet repeat-binding protein 1	CGBP1_HUMAN
Eukaryotic translation initiation factor 1A, X-chromosomal	IF1AX_HUMAN
Calcium/calmodulin-dependent protein kinase type II subunit delta	KCC2D_HUMAN
RNA-binding protein 25	RBM25_HUMAN
Vacuolar protein sorting-associated protein VTA1 homolog	VTA1_HUMAN
Serine--tRNA ligase, cytoplasmic	SYSC_HUMAN
Thyroid receptor-interacting protein 6	TRIP6_HUMAN
Histone-lysine N-methyltransferase SETD7	SETD7_HUMAN
Anaphase-promoting complex subunit 7	APC7_HUMAN
Glutathione S-transferase Mu 2	GSTM2_HUMAN
WD repeat-containing protein 5	WDR5_HUMAN

Mitogen-activated protein kinase kinase kinase 11	M3K11_HUMAN
Adaptin ear-binding coat-associated protein 2	NECP2_HUMAN
AKT-interacting protein	AKTIP_HUMAN
PHD finger protein 23	PHF23_HUMAN
Thymidylate kinase	KTHY_HUMAN
NIF3-like protein 1	NIF3L_HUMAN
RanBP-type and C3HC4-type zinc finger-containing protein 1	HOIL1_HUMAN
Fibronectin type III and SPRY domain-containing protein 1	FSD1_HUMAN
BTB/POZ domain-containing adapter for CUL3-mediated RhoA degradation p	BACD3_HUMAN
Mediator of RNA polymerase II transcription subunit 17	MED17_HUMAN
UBX domain-containing protein 4	UBXN4_HUMAN
Structural maintenance of chromosomes protein 3	SMC3_HUMAN
Coatomer subunit gamma-1	COPG1_HUMAN
Signal transducing adapter molecule 2	STAM2_HUMAN
Voltage-dependent anion-selective channel protein 2	VDAC2_HUMAN
Dynein light chain roadblock-type 1	DLRB1_HUMAN
Discoidin domain-containing receptor 2	DDR2_HUMAN
Mitochondrial Rho GTPase 1	MIRO1_HUMAN
FHF complex subunit HOOK-interacting protein 2B	FHI2B_HUMAN
Large ribosomal subunit protein uL11	RL12_HUMAN
Splicing factor, arginine/serine-rich 19	SFR19_HUMAN
Retrotransposon Gag-like protein 8C	RTL8C_HUMAN
Leupaxin	LPXN_HUMAN
Rab-like protein 6	RABL6_HUMAN
Autophagy-related protein 9A	ATG9A_HUMAN
Serine/threonine-protein phosphatase 4 regulatory subunit 1	PP4R1_HUMAN
ADP-ribosylation factor-like protein 5B	ARL5B_HUMAN
Integrin alpha-3	ITA3_HUMAN
N-myc-interactor	NMI_HUMAN
RAB11-binding protein RELCH	RELCH_HUMAN
Propionyl-CoA carboxylase beta chain, mitochondrial	PCCB_HUMAN
CDGSH iron-sulfur domain-containing protein 2	CISD2_HUMAN
Peroxiredoxin-like 2A	PXL2A_HUMAN
NCK-interacting protein with SH3 domain	SPN90_HUMAN
Chloride intracellular channel protein 1	CLIC1_HUMAN
Protein TANC1	TANC1_HUMAN
Disco-interacting protein 2 homolog B	DIP2B_HUMAN
Symplekin	SYMPK_HUMAN
PDZ and LIM domain protein 4	PDLI4_HUMAN
GRB10-interacting GYF protein 2	GGYF2_HUMAN
T-complex protein 1 subunit epsilon	TCPE_HUMAN
Rho-related GTP-binding protein RhoE	RND3_HUMAN
ER membrane protein complex subunit 4	EMC4_HUMAN
Chromosome alignment-maintaining phosphoprotein 1	CHAP1_HUMAN
Nuclear factor of activated T-cells, cytoplasmic 2	NFAC2_HUMAN
Serine/threonine-protein kinase WNK1	WNK1_HUMAN
Protein Wiz	WIZ_HUMAN
Protein SPT2 homolog	SPT2_HUMAN
Rho guanine nucleotide exchange factor 12	ARHGC_HUMAN
CD44 antigen	CD44_HUMAN

Protein AAR2 homolog	AAR2_HUMAN
Cyclin-G-associated kinase	GAK_HUMAN
Mitogen-activated protein kinase 12	MK12_HUMAN
CCR4-NOT transcription complex subunit 1	CNOT1_HUMAN
ATPase family AAA domain-containing protein 3A	ATD3A_HUMAN
Poly(A) RNA polymerase, mitochondrial	PAPD1_HUMAN
Pre-mRNA cleavage complex 2 protein Pcf11	PCF11_HUMAN
Heterochromatin protein 1-binding protein 3	HP1B3_HUMAN
Phosphatidylserine lipase ABHD16A	ABHGA_HUMAN
Tumor susceptibility gene 101 protein	TS101_HUMAN
Laminin subunit alpha-5	LAMA5_HUMAN
Calpain-1 catalytic subunit	CAN1_HUMAN
Trifunctional purine biosynthetic protein adenosine-3	PUR2_HUMAN
RNA-binding protein 34	RBM34_HUMAN
Annexin A5	ANXA5_HUMAN
Heparan-alpha-glucosaminide N-acetyltransferase	HGNAT_HUMAN
Arpin	ARPIN_HUMAN
UPF0696 protein C11orf68	CK068_HUMAN
A-kinase anchor protein 9	AKAP9_HUMAN
Nucleoporin NDC1	NDC1_HUMAN
Monocarboxylate transporter 4	MOT4_HUMAN
Leucine carboxyl methyltransferase 1	LCMT1_HUMAN
Shootin-1	SHOT1_HUMAN
U2 snRNP-associated SURP motif-containing protein	SR140_HUMAN
1-phosphatidylinositol 4,5-bisphosphate phosphodiesterase gamma-2	PLCG2_HUMAN
GDH/6PGL endoplasmic bifunctional protein	G6PE_HUMAN
N-acetylglucosamine-6-sulfatase	GNS_HUMAN
Acylglycerol kinase, mitochondrial	AGK_HUMAN
Protein FAM114A2	F1142_HUMAN
HLA class I histocompatibility antigen, C alpha chain	HLAC_HUMAN
NudC domain-containing protein 1	NUDC1_HUMAN
Zinc-regulated GTPase metalloprotein activator 1F	ZNG1F_HUMAN
Ribosome production factor 1	RPF1_HUMAN
Regulator of G-protein signaling 3	RGS3_HUMAN
SH3 domain and tetratricopeptide repeat-containing protein 1	S3TC1_HUMAN
Golgi resident protein GCP60	GCP60_HUMAN
VPS35 endosomal protein-sorting factor-like	VP35L_HUMAN
Large ribosomal subunit protein bL9m	RM09_HUMAN
Transmembrane protein 214	TM214_HUMAN
FERM domain-containing protein 6	FRMD6_HUMAN
UBX domain-containing protein 1	UBXN1_HUMAN
PTB domain-containing engulfment adapter protein 1	GULP1_HUMAN
Trafficking protein particle complex subunit 1	TPPC1_HUMAN
Acyl-CoA 6-desaturase	FADS2_HUMAN
Malate dehydrogenase, cytoplasmic	MDHC_HUMAN
Pre-mRNA-processing factor 40 homolog A	PR40A_HUMAN
SLIT-ROBO Rho GTPase-activating protein 2	SRGP2_HUMAN
V-type proton ATPase subunit G 1	VATG1_HUMAN
Septin-8	SEPT8_HUMAN
GTP-binding protein SAR1b	SAR1B_HUMAN

Proteasome subunit beta type-4	PSB4_HUMAN
Cytochrome P450 20A1	CP20A_HUMAN
Protein CDV3 homolog	CDV3_HUMAN
Centromere/kinetochore protein zw10 homolog	ZW10_HUMAN
Methylmalonate-semialdehyde/malonate-semialdehyde dehydrogenase [acy	MMSA_HUMAN
Glycogen synthase kinase-3 beta	GSK3B_HUMAN
Rho GTPase-activating protein 18	RHG18_HUMAN
Mediator of RNA polymerase II transcription subunit 16	MED16_HUMAN
Cytochrome c oxidase subunit 6C	COX6C_HUMAN
Double-strand break repair protein MRE11	MRE11_HUMAN
SAM and SH3 domain-containing protein 1	SASH1_HUMAN
2',5'-phosphodiesterase 12	PDE12_HUMAN
DNA-directed RNA polymerase II subunit RPB1	RPB1_HUMAN
Coatomer subunit zeta-1	COPZ1_HUMAN
Bridging integrator 3	BIN3_HUMAN
Condensin complex subunit 1	CND1_HUMAN
Pachytene checkpoint protein 2 homolog	PCH2_HUMAN
E3 ubiquitin-protein ligase TRIM56	TRI56_HUMAN
Equilibrative nucleobase transporter 1	S43A3_HUMAN
Protein Smaug homolog 2	SMAG2_HUMAN
Probable ATP-dependent RNA helicase DDX20	DDX20_HUMAN
Fatty acid CoA ligase Acsl3	ACSL3_HUMAN
Microsomal glutathione S-transferase 2	MGST2_HUMAN
Protein NDRG4	NDRG4_HUMAN
NADH dehydrogenase [ubiquinone] 1 alpha subcomplex subunit 2	NDUA2_HUMAN
Arf-GAP with Rho-GAP domain, ANK repeat and PH domain-containing protei	ARAP1_HUMAN
DNA fragmentation factor subunit alpha	DFFA_HUMAN
Protein YIF1A	YIF1A_HUMAN
Long-chain fatty acid transport protein 4	S27A4_HUMAN
Ribosomal biogenesis protein LAS1L	LAS1L_HUMAN
SUZ domain-containing protein 1	SZRD1_HUMAN
Rabankyrin-5	ANFY1_HUMAN
Tropomodulin-3	TMOD3_HUMAN
E3 SUMO-protein ligase ZBED1	ZBED1_HUMAN
Developmentally-regulated GTP-binding protein 1	DRG1_HUMAN
Microtubule cross-linking factor 2	MTCL2_HUMAN
ADP-ribosylation factor 6	ARF6_HUMAN
Breakpoint cluster region protein	BCR_HUMAN
Rapamycin-insensitive companion of mTOR	RICTR_HUMAN
Lysine-specific demethylase 3B	KDM3B_HUMAN
Ubiquitin recognition factor in ER-associated degradation protein 1	UFD1_HUMAN
Unconventional myosin-Ia	MYO1A_HUMAN
C-Jun-amino-terminal kinase-interacting protein 4	JIP4_HUMAN
Small ribosomal subunit protein eS26	RS26_HUMAN
Formin-binding protein 1-like	FBP1L_HUMAN
Krueppel-like factor 12	KLF12_HUMAN
Roundabout homolog 1	ROBO1_HUMAN
Dolichyl-phosphate beta-glucosyltransferase	ALG5_HUMAN
5'-3' exoribonuclease 2	XRN2_HUMAN
Histone-lysine N-methyltransferase SETMAR	SETMR_HUMAN

PCNA-associated factor	PAF15_HUMAN
tRNA methyltransferase 10 homolog C	TM10C_HUMAN
Ribonuclease H2 subunit A	RNH2A_HUMAN
Translation initiation factor IF-2, mitochondrial	IF2M_HUMAN
Myotubularin-related protein 2	MTMR2_HUMAN
GTP-binding protein Rheb	RHEB_HUMAN
BLOC-2 complex member HPS6	HPS6_HUMAN
FYVE and coiled-coil domain-containing protein 1	FYCO1_HUMAN
COP9 signalosome complex subunit 7a	CSN7A_HUMAN
Protein CLEC16A	CL16A_HUMAN
Sphingosine-1-phosphate lyase 1	SGPL1_HUMAN
Protein-L-isoaspartate O-methyltransferase domain-containing protein 1	PCMD1_HUMAN
Casein kinase II subunit beta	CSK2B_HUMAN
TGF-beta receptor type-2	TGFR2_HUMAN
Endoplasmic reticulum resident protein 29	ERP29_HUMAN
Liprin-alpha-1	LIPA1_HUMAN
L-xylulose reductase	DCXR_HUMAN
Diacylglycerol kinase alpha	DGKA_HUMAN
Antigen peptide transporter 1	TAP1_HUMAN
Baculoviral IAP repeat-containing protein 6	BIRC6_HUMAN
DmX-like protein 1	DMXL1_HUMAN
Ras-related protein Rab-33B	RB33B_HUMAN
Geranylgeranyl pyrophosphate synthase	GGPPS_HUMAN
cAMP-dependent protein kinase type II-alpha regulatory subunit	KAP2_HUMAN
Stathmin	STMN1_HUMAN
Large ribosomal subunit protein eL27	RL27_HUMAN
Ubiquitin carboxyl-terminal hydrolase 7	UBP7_HUMAN
RNA-binding protein NOB1	NOB1_HUMAN
Far upstream element-binding protein 3	FUBP3_HUMAN
Vacuolar-sorting protein SNF8	SNF8_HUMAN
Transforming growth factor beta-1 proprotein	TGFB1_HUMAN
CCR4-NOT transcription complex subunit 4	CNOT4_HUMAN
Inosine-5'-monophosphate dehydrogenase 1	IMDH1_HUMAN
Liprin-beta-1	LIPB1_HUMAN
Pre-B-cell leukemia transcription factor-interacting protein 1	PBIP1_HUMAN
E3 ubiquitin-protein ligase MARCHF5	MARH5_HUMAN
Myocardin-related transcription factor B	MRTFB_HUMAN
RNA-binding protein 7	RBM7_HUMAN
Large ribosomal subunit protein uL1m	RM01_HUMAN
Eukaryotic translation initiation factor 4E	IF4E_HUMAN
Latent-transforming growth factor beta-binding protein 1	LTBP1_HUMAN
Protein Shroom2	SHRM2_HUMAN
Programmed cell death protein 2-like	PDD2L_HUMAN
Nucleoprotein TPR	TPR_HUMAN
Metal transporter CNNM3	CNNM3_HUMAN
Calumenin	CALU_HUMAN
POTE ankyrin domain family member J	POTEJ_HUMAN
Nuclear autoantigen Sp-100	SP100_HUMAN
Exportin-2	XPO2_HUMAN
Serine/threonine-protein kinase PAK 2	PAK2_HUMAN

Inositol 1,4,5-trisphosphate receptor type 3	ITPR3_HUMAN
Lysophospholipid acyltransferase LPCAT4	LPCT4_HUMAN
Proteasome assembly chaperone 2	PSMG2_HUMAN
RNA polymerase II-associated protein 1	RPAP1_HUMAN
Calcium uniporter protein, mitochondrial	MCU_HUMAN
Caveolae-associated protein 3	CAVN3_HUMAN
Selenocysteine lyase	SCLY_HUMAN
DnaJ homolog subfamily C member 21	DJC21_HUMAN
Copine-2	CPNE2_HUMAN
Large ribosomal subunit protein uL18m	RM18_HUMAN
Sodium-coupled neutral amino acid symporter 1	S38A1_HUMAN
COP9 signalosome complex subunit 3	CSN3_HUMAN
Ras-related protein Rab-11B	RB11B_HUMAN
Ankyrin repeat domain-containing protein 13A	AN13A_HUMAN
Transcriptional repressor protein YY1	TYY1_HUMAN
1-phosphatidylinositol 4,5-bisphosphate phosphodiesterase beta-3	PLCB3_HUMAN
Receptor expression-enhancing protein 3	REEP3_HUMAN
Protein O-mannosyl-transferase TMTC3	TMTC3_HUMAN
Serine/threonine-protein kinase 3	STK3_HUMAN
E3 ubiquitin-protein ligase ARIH2	ARI2_HUMAN
Atlastin-3	ATLA3_HUMAN
Tetratricopeptide repeat protein 21B	TT21B_HUMAN
Glutathione S-transferase P	GSTP1_HUMAN
Signal-induced proliferation-associated protein 1	SIPA1_HUMAN
Endophilin-B1	SHLB1_HUMAN
Phosphatidylinositol 4-kinase type 2-beta	P4K2B_HUMAN
MMS19 nucleotide excision repair protein homolog	MMS19_HUMAN
Replication factor C subunit 5	RFC5_HUMAN
Helicase SRCAP	SRCAP_HUMAN
ER membrane protein complex subunit 1	EMC1_HUMAN
Putative E3 ubiquitin-protein ligase UBR7	UBR7_HUMAN
Ubiquitin-conjugating enzyme E2 N	UBE2N_HUMAN
Transmembrane 9 superfamily member 3	TM9S3_HUMAN
Transmembrane 7 superfamily member 3	TM7S3_HUMAN
Proteasome subunit beta type-10	PSB10_HUMAN
Importin-9	IPO9_HUMAN
Serine/threonine-protein kinase A-Raf	ARAF_HUMAN
Protein transport protein Sec61 subunit beta	SC61B_HUMAN
Ubiquitin-like modifier-activating enzyme 7	UBA7_HUMAN
Arginine-hydroxylase NDUFAF5, mitochondrial	NDUF5_HUMAN
SWI/SNF complex subunit SMARCC2	SMRC2_HUMAN
GTP-binding protein 1	GTPB1_HUMAN
Hepatoma-derived growth factor	HDGF_HUMAN
TRMT1-like protein	TRM1L_HUMAN
NAD-dependent protein deacylase sirtuin-6	SIR6_HUMAN
Junctional cadherin 5-associated protein	JCAD_HUMAN
Adenylate kinase isoenzyme 5	KAD5_HUMAN
UV radiation resistance-associated gene protein	UVRAG_HUMAN
Partner of Y14 and mago	PYM1_HUMAN
Activating signal cointegrator 1 complex subunit 1	ASCC1_HUMAN

Splicing factor 3B subunit 6	SF3B6_HUMAN
Protein SET	SET_HUMAN
DNA polymerase beta	DPOLB_HUMAN
Ubiquitin carboxyl-terminal hydrolase 47	UBP47_HUMAN
TATA-box-binding protein	TBP_HUMAN
TELO2-interacting protein 2	TTI2_HUMAN
Inositol-trisphosphate 3-kinase B	IP3KB_HUMAN
Mitochondrial import inner membrane translocase subunit TIM44	TIM44_HUMAN
COUP transcription factor 1	COT1_HUMAN
NADPH:adrenodoxin oxidoreductase, mitochondrial	ADRO_HUMAN
Homeobox protein cut-like 1	CUX1_HUMAN
Scavenger receptor class B member 1	SCRB1_HUMAN
Acidic leucine-rich nuclear phosphoprotein 32 family member E	AN32E_HUMAN
Cysteine-rich and transmembrane domain-containing protein 1	CYTM1_HUMAN
Protein mono-ADP-ribosyltransferase PARP14	PAR14_HUMAN
Retinol dehydrogenase 13	RDH13_HUMAN
Calcium-transporting ATPase type 2C member 1	AT2C1_HUMAN
DNA-directed RNA polymerase III subunit RPC2	RPC2_HUMAN
Serine/threonine-protein phosphatase 4 regulatory subunit 3A	P4R3A_HUMAN
Ribonucleoprotein PTB-binding 2	RAVR2_HUMAN
MRG/MORF4L-binding protein	MRGBP_HUMAN
DnaJ homolog subfamily C member 2	DNJC2_HUMAN
Testin	TES_HUMAN
NEDD8 ultimate buster 1	NUB1_HUMAN
GTPase IMAP family member 5	GIMA5_HUMAN
Histone PARylation factor 1	HPF1_HUMAN
Ubiquitin carboxyl-terminal hydrolase 34	UBP34_HUMAN
Heat shock 70 kDa protein 1A	HS71A_HUMAN
Alpha-actinin-1	ACTN1_HUMAN
Nuclear pore complex protein Nup155	NU155_HUMAN
Eukaryotic translation initiation factor 3 subunit A	EIF3A_HUMAN
Serine/threonine-protein phosphatase 2A 65 kDa regulatory subunit A alpha	2AAA_HUMAN
Fatty acid synthase	FAS_HUMAN
TBC1 domain family member 4	TBCD4_HUMAN
Methionine--tRNA ligase, cytoplasmic	SYMC_HUMAN
Vacuolar fusion protein MON1 homolog B	MON1B_HUMAN
ATP-dependent (S)-NAD(P)H-hydrate dehydratase	NNRD_HUMAN
DDRGK domain-containing protein 1	DDRGK_HUMAN
STE20-like serine/threonine-protein kinase	SLK_HUMAN
Thioredoxin-interacting protein	TXNIP_HUMAN
Structural maintenance of chromosomes protein 4	SMC4_HUMAN
DnaJ homolog subfamily C member 17	DJC17_HUMAN
Cytoplasmic dynein 1 light intermediate chain 1	DC1L1_HUMAN
ADP-ribosylation factor 1	ARF1_HUMAN
U6 snRNA-associated Sm-like protein LSm7	LSM7_HUMAN
WAS/WASL-interacting protein family member 2	WIPF2_HUMAN
Serine/threonine-protein kinase RIO3	RIOK3_HUMAN
Charged multivesicular body protein 2a	CHM2A_HUMAN
Dihydroorotate dehydrogenase (quinone), mitochondrial	PYRD_HUMAN
Alpha-enolase	ENOA_HUMAN

Epoxide hydrolase 1	HYEP_HUMAN
3-hydroxyisobutyryl-CoA hydrolase, mitochondrial	HIBCH_HUMAN
Dipeptidyl peptidase 3	DPP3_HUMAN
Vacuolar protein sorting-associated protein 41 homolog	VPS41_HUMAN
ATP-binding cassette sub-family F member 3	ABCF3_HUMAN
Ubiquitin-fold modifier-conjugating enzyme 1	UFC1_HUMAN
Platelet-activating factor acetylhydrolase IB subunit alpha2	PA1B2_HUMAN
Annexin A4	ANXA4_HUMAN
U4/U6 small nuclear ribonucleoprotein Prp31	PRP31_HUMAN
Ubiquitin-conjugating enzyme E2 Z	UBE2Z_HUMAN
Pleckstrin homology domain-containing family F member 2	PKHF2_HUMAN
Actin-related protein 10	ARP10_HUMAN
Cytosolic purine 5'-nucleotidase	5NTC_HUMAN
Cysteine and glycine-rich protein 2	CSRP2_HUMAN
Multiple PDZ domain protein	MPDZ_HUMAN
Tyrosine-protein kinase Lyn	LYN_HUMAN
Dynamin-1-like protein	DNM1L_HUMAN
Porphobilinogen deaminase	HEM3_HUMAN
Non-receptor tyrosine-protein kinase TYK2	TYK2_HUMAN
Cleavage stimulation factor subunit 1	CSTF1_HUMAN
Putative ATP-dependent RNA helicase DHX57	DHX57_HUMAN
A-kinase anchor protein 17A	AK17A_HUMAN
Elongation factor G, mitochondrial	EFGM_HUMAN
Zinc finger E-box-binding homeobox 2	ZEB2_HUMAN
Transcription elongation regulator 1	TCRG1_HUMAN
Supervillin	SVIL_HUMAN
Tyrosine-protein kinase ABL1	ABL1_HUMAN
Large ribosomal subunit protein uL29	RL35_HUMAN
GAS2-like protein 1	GA2L1_HUMAN
Probable ATP-dependent RNA helicase DDX23	DDX23_HUMAN
Protein kinase C and casein kinase substrate in neurons protein 2	PACN2_HUMAN
Glutathione S-transferase 3, mitochondrial	MGST3_HUMAN
Nuclear factor NF-kappa-B p100 subunit	NFKB2_HUMAN
Serine/arginine-rich splicing factor 5	SRSF5_HUMAN
Protein EFR3 homolog A	EFR3A_HUMAN
E3 ubiquitin-protein ligase NEDD4-like	NED4L_HUMAN
Probable ATP-dependent RNA helicase DHX35	DHX35_HUMAN
Copine-1	CPNE1_HUMAN
Ganglioside-induced differentiation-associated protein 2	GDAP2_HUMAN
Cytochrome c oxidase subunit 2	COX2_HUMAN
Ras and Rab interactor 2	RIN2_HUMAN
Peroxisomal targeting signal 1 receptor	PEX5_HUMAN
Activating signal cointegrator 1 complex subunit 3	ASCC3_HUMAN
Proteasome subunit beta type-6	PSB6_HUMAN
GATOR2 complex protein MIOS	MIOS_HUMAN
Adenylyl cyclase-associated protein 2	CAP2_HUMAN
Cullin-3	CUL3_HUMAN
3'-5' exoribonuclease 1	ERI1_HUMAN
Iron-sulfur clusters transporter ABCB7, mitochondrial	ABCB7_HUMAN
Small ribosomal subunit protein uS3	RS3_HUMAN

Nuclear factor of activated T-cells, cytoplasmic 1	NFAC1_HUMAN
Kinesin-like protein KIF21A	KI21A_HUMAN
Acyl-coenzyme A thioesterase 1	ACOT1_HUMAN
Fatty acyl-CoA reductase 2	FACR2_HUMAN
Cyclic AMP-dependent transcription factor ATF-6 beta	ATF6B_HUMAN
Sec1 family domain-containing protein 2	SCFD2_HUMAN
SWI/SNF-related matrix-associated actin-dependent regulator of chromatin s	SMRD1_HUMAN
ATP-binding cassette sub-family B member 10, mitochondrial	ABCBA_HUMAN
Telomerase-binding protein EST1A	EST1A_HUMAN
RNA-binding protein RO60	RO60_HUMAN
tRNA-dihydrouridine(20) synthase [NAD(P)+]-like	DUS2L_HUMAN
ATP synthase subunit f, mitochondrial	ATPK_HUMAN
BAG family molecular chaperone regulator 1	BAG1_HUMAN
Ribosome maturation protein SBDS	SBDS_HUMAN
NF-kappa-B essential modulator	NEMO_HUMAN
Glutathione synthetase	GSHB_HUMAN
Transmembrane protein 70, mitochondrial	TMM70_HUMAN
Cytochrome c oxidase subunit 7A2, mitochondrial	CX7A2_HUMAN
RILP-like protein 1	RIPL1_HUMAN
Protein argonaute-1	AGO1_HUMAN
Farnesyl pyrophosphate synthase	FPPS_HUMAN
COMM domain-containing protein 7	COMD7_HUMAN
S1 RNA-binding domain-containing protein 1	SRBD1_HUMAN
Phosphatidylinositol 3,4,5-trisphosphate-dependent Rac exchanger 2 protein	PREX2_HUMAN
Mediator of RNA polymerase II transcription subunit 4	MED4_HUMAN
Myelin expression factor 2	MYEF2_HUMAN
Exocyst complex component 3	EXOC3_HUMAN
26S proteasome non-ATPase regulatory subunit 8	PSMD8_HUMAN
Selenoprotein N	SELN_HUMAN
Serine racemase	SRR_HUMAN
General transcription factor IIF subunit 1	T2FA_HUMAN
Ubiquitin carboxyl-terminal hydrolase 8	UBP8_HUMAN
Centrosomal protein of 192 kDa	CE192_HUMAN
Glutathione S-transferase theta-2	GST2_HUMAN
Protein phosphatase 1 regulatory subunit 7	PP1R7_HUMAN
55 kDa erythrocyte membrane protein	EM55_HUMAN
Procollagen-lysine,2-oxoglutarate 5-dioxygenase 1	PLOD1_HUMAN
Prolyl 4-hydroxylase subunit alpha-2	P4HA2_HUMAN
Zinc finger and BTB domain-containing protein 7A	ZBT7A_HUMAN
Granule associated Rac and RHOG effector protein 1	GRRE1_HUMAN
Phosphatidylinositol 5-phosphate 4-kinase type-2 beta	PI42B_HUMAN
Peptidyl-prolyl cis-trans isomerase FKBP2	FKBP2_HUMAN
Dolichyl-diphosphooligosaccharide--protein glycosyltransferase 48 kDa subu	OST48_HUMAN
Pyridoxal phosphate homeostasis protein	PLPHP_HUMAN
Menin	MEN1_HUMAN
E3 ubiquitin-protein ligase LRSAM1	LRSM1_HUMAN
Zinc finger protein 687	ZN687_HUMAN
Short-chain specific acyl-CoA dehydrogenase, mitochondrial	ACADS_HUMAN
Alpha-1,6-mannosyl-glycoprotein 2-beta-N-acetylglucosaminyltransferase	MGAT2_HUMAN
GRB2-related adapter protein	GRAP_HUMAN

ETS domain-containing protein Elk-3	ELK3_HUMAN
FERM domain-containing protein 8	FRMD8_HUMAN
Dystroglycan 1	DAG1_HUMAN
Leucine-rich repeat flightless-interacting protein 2	LRRF2_HUMAN
Vesicle-trafficking protein SEC22b	SC22B_HUMAN
Trimethylguanosine synthase	TGS1_HUMAN
ATPase WRNIP1	WRIP1_HUMAN
UDP-glucose 6-dehydrogenase	UGDH_HUMAN
Tyrosine-protein kinase Fyn	FYN_HUMAN
Ribonuclease 3	RNC_HUMAN
COP9 signalosome complex subunit 6	CSN6_HUMAN
Leucine-rich repeat-containing protein 20	LRC20_HUMAN
Methylosome protein WDR77	MEP50_HUMAN
Sister chromatid cohesion protein PDS5 homolog A	PDS5A_HUMAN
Signal transducing adapter molecule 1	STAM1_HUMAN
V-type proton ATPase 116 kDa subunit a 2	VPP2_HUMAN
Eukaryotic translation initiation factor 3 subunit B	EIF3B_HUMAN
Pentatricopeptide repeat-containing protein 1, mitochondrial	PTCD1_HUMAN
Actin-related protein 2/3 complex subunit 1B	ARC1B_HUMAN
Echinoderm microtubule-associated protein-like 4	EMAL4_HUMAN
Peptidyl-prolyl cis-trans isomerase FKBP8	FKBP8_HUMAN
Proteasome subunit alpha type-5	PSA5_HUMAN
Guanine nucleotide-binding protein G(q) subunit alpha	GNAQ_HUMAN
Catechol O-methyltransferase	COMT_HUMAN
Polypyrimidine tract-binding protein 1	PTBP1_HUMAN
Derlin-2	DERL2_HUMAN
Cleavage and polyadenylation specificity factor subunit 7	CPSF7_HUMAN
Ubiquitin carboxyl-terminal hydrolase 5	UBP5_HUMAN
FH1/FH2 domain-containing protein 1	FHOD1_HUMAN
Mitochondrial ornithine transporter 1	ORNT1_HUMAN
Zinc finger protein 521	ZN521_HUMAN
Tetratricopeptide repeat protein 1	TTC1_HUMAN
DDB1- and CUL4-associated factor 7	DCAF7_HUMAN
Pescadillo homolog	PESC_HUMAN
Tyrosine-protein phosphatase non-receptor type 9	PTN9_HUMAN
Proteasome adapter and scaffold protein ECM29	ECM29_HUMAN
DNA mismatch repair protein Msh6	MSH6_HUMAN
Actin-like protein 6A	ACL6A_HUMAN
Dolichyl-diphosphooligosaccharide--protein glycosyltransferase subunit 2	RPN2_HUMAN
CREB-binding protein	CBP_HUMAN
Actin filament-associated protein 1-like 1	AF1L1_HUMAN
Calcium load-activated calcium channel	TMCO1_HUMAN
Transcription elongation factor SPT5	SPT5H_HUMAN
Acetyl-coenzyme A transporter 1	ACATN_HUMAN
Gamma-aminobutyric acid receptor-associated protein-like 2	GBRL2_HUMAN
Midasin	MDN1_HUMAN
Large ribosomal subunit protein eL39	RL39_HUMAN
Myeloid-derived growth factor	MYDGF_HUMAN
U3 small nucleolar RNA-associated protein 18 homolog	UTP18_HUMAN
BRI3-binding protein	BRI3B_HUMAN

Large ribosomal subunit protein bL12m	RM12_HUMAN
Baculoviral IAP repeat-containing protein 2	BIRC2_HUMAN
Dynamin-2	DYN2_HUMAN
Pre-mRNA-processing factor 6	PRP6_HUMAN
GMP synthase [glutamine-hydrolyzing]	GUAA_HUMAN
Replication protein A 32 kDa subunit	RFA2_HUMAN
Golgin subfamily A member 1	GOGA1_HUMAN
Paired amphipathic helix protein Sin3a	SIN3A_HUMAN
DNA excision repair protein ERCC-5	ERCC5_HUMAN
Peptidyl-glycine alpha-amidating monooxygenase	AMD_HUMAN
Protein farnesyltransferase subunit beta	FNTB_HUMAN
Mitochondrial import inner membrane translocase subunit Tim29	TIM29_HUMAN
Transportin-2	TNPO2_HUMAN
Syntaxin-binding protein 2	STXB2_HUMAN
RNA-splicing ligase RtcB homolog	RTCB_HUMAN
Glutamine--tRNA ligase	SYQ_HUMAN
Proteasome subunit beta type-3	PSB3_HUMAN
Keratin-78	K2C78_HUMAN
NudC domain-containing protein 2	NUDC2_HUMAN
Rho guanine nucleotide exchange factor 1	ARHG1_HUMAN
NADH dehydrogenase [ubiquinone] 1 alpha subcomplex assembly factor 2	NDUF2_HUMAN
Tyrosine-protein phosphatase non-receptor type 14	PTN14_HUMAN
Cleavage and polyadenylation specificity factor subunit 3	CPSF3_HUMAN
Coiled-coil domain-containing protein 9B	CCD9B_HUMAN
Ral guanine nucleotide dissociation stimulator-like 2	RGL2_HUMAN
Phosphoinositide 3-kinase regulatory subunit 4	PI3R4_HUMAN
ATPase inhibitor, mitochondrial	ATIF1_HUMAN
Nucleoside diphosphate kinase 7	NDK7_HUMAN
Beta-hexosaminidase subunit beta	HEXB_HUMAN
ADP-ribosylation factor-binding protein GGA3	GGA3_HUMAN
NADH dehydrogenase [ubiquinone] 1 beta subcomplex subunit 3	NDUB3_HUMAN
Nuclear distribution protein nudE homolog 1	NDE1_HUMAN
Chitobiosyldiphosphodolichol beta-mannosyltransferase	ALG1_HUMAN
Integrator complex subunit 7	INT7_HUMAN
Adenylate kinase isoenzyme 1	KAD1_HUMAN
Rho GTPase-activating protein 29	RHG29_HUMAN
Kelch-like protein 22	KLH22_HUMAN
Protein PRRC2B	PRC2B_HUMAN
Lanosterol 14-alpha demethylase	CP51A_HUMAN
Tumor necrosis factor alpha-induced protein 8-like protein 1	TP8L1_HUMAN
Sialic acid synthase	SIAS_HUMAN
26S proteasome non-ATPase regulatory subunit 5	PSMD5_HUMAN
Protein SAAL1	SAAL1_HUMAN
Large ribosomal subunit protein eL15	RL15_HUMAN
Cold-inducible RNA-binding protein	CIRBP_HUMAN
Vacuolar protein sorting-associated protein 37B	VP37B_HUMAN
Phosphorylated adapter RNA export protein	PHAX_HUMAN
Protein transport protein Sec24C	SC24C_HUMAN
Interleukin enhancer-binding factor 3	ILF3_HUMAN
Large ribosomal subunit protein eL8	RL7A_HUMAN

Nucleolar protein 11	NOL11_HUMAN
Receptor-type tyrosine-protein phosphatase epsilon	PTPRE_HUMAN
Caspase recruitment domain-containing protein 8	CARD8_HUMAN
Protein diaphanous homolog 2	DIAP2_HUMAN
Adenosylhomocysteinase	SAHH_HUMAN
Protein NipSnap homolog 2	NIPS2_HUMAN
Trifunctional enzyme subunit beta, mitochondrial	ECHB_HUMAN
Palmitoyltransferase ZDHHC20	ZDH20_HUMAN
Arf-GAP with GTPase, ANK repeat and PH domain-containing protein 3	AGAP3_HUMAN
Mitochondrial enolase superfamily member 1	ENOF1_HUMAN
Engulfment and cell motility protein 1	ELMO1_HUMAN
Cullin-associated NEDD8-dissociated protein 2	CAND2_HUMAN
Inverted formin-2	INF2_HUMAN
Synaptobrevin homolog YKT6	YKT6_HUMAN
Tubulin-specific chaperone cofactor E-like protein	TBCEL_HUMAN
Complex I assembly factor ACAD9, mitochondrial	ACAD9_HUMAN
Large ribosomal subunit protein mL44	RM44_HUMAN
Ubiquitin-like modifier-activating enzyme ATG7	ATG7_HUMAN
Beclin-1	BECN1_HUMAN
Protein Hook homolog 3	HOOK3_HUMAN
Aladin	AAAS_HUMAN
Trafficking protein particle complex subunit 3	TPPC3_HUMAN
NADH dehydrogenase [ubiquinone] 1 alpha subcomplex subunit 10, mitocho	NDUAA_HUMAN
DNA replication licensing factor MCM7	MCM7_HUMAN
Lysophospholipase-like protein 1	LYPL1_HUMAN
Low-density lipoprotein receptor-related protein 10	LRP10_HUMAN
AMP deaminase 2	AMPD2_HUMAN
Electron transfer flavoprotein-ubiquinone oxidoreductase, mitochondrial	ETFD_HUMAN
T-complex protein 11-like protein 1	T11L1_HUMAN
Ubiquitin-protein ligase E3A	UBE3A_HUMAN
Kinase suppressor of Ras 1	KSR1_HUMAN
NADH-cytochrome b5 reductase 3	NB5R3_HUMAN
Chromosome transmission fidelity protein 18 homolog	CTF18_HUMAN
WD repeat-containing protein 82	WDR82_HUMAN
Nucleolar complex protein 4 homolog	NOC4L_HUMAN
Serine/threonine-protein kinase mTOR	MTOR_HUMAN
Cytochrome b-c1 complex subunit 8	QCR8_HUMAN
Phospholipase A2 group XV	PAG15_HUMAN
Peroxiredoxin-6	PRDX6_HUMAN
Activating transcription factor 7-interacting protein 1	MCAF1_HUMAN
Cathepsin B	CATB_HUMAN
Signal recognition particle subunit SRP54	SRP54_HUMAN
Heat shock protein 75 kDa, mitochondrial	TRAP1_HUMAN
Integrator complex subunit 13	INT13_HUMAN
Putative WAS protein family homolog 3	WASH3_HUMAN
Glycerophosphodiester phosphodiesterase 1	GDE1_HUMAN
Methylthioribose-1-phosphate isomerase	MTNA_HUMAN
Tyrosine-protein phosphatase non-receptor type 12	PTN12_HUMAN
LIX1-like protein	LIX1L_HUMAN
Brefeldin A-inhibited guanine nucleotide-exchange protein 1	BIG1_HUMAN

DNA-(apurinic or apyrimidinic site) endonuclease	APEX1_HUMAN
Sedoheptulokinase	SHPK_HUMAN
E3 ubiquitin-protein ligase Praja-2	PJA2_HUMAN
Adhesion G-protein coupled receptor G1	AGRG1_HUMAN
Serine/threonine-protein kinase 24	STK24_HUMAN
Glutamate dehydrogenase 1, mitochondrial	DHE3_HUMAN
Glutaminase kidney isoform, mitochondrial	GLSK_HUMAN
BLOC-2 complex member HPS3	HPS3_HUMAN
Eukaryotic translation initiation factor 3 subunit K	EIF3K_HUMAN
DNA-directed RNA polymerase III subunit RPC6	RPC6_HUMAN
Heterogeneous nuclear ribonucleoprotein U-like protein 2	HNRL2_HUMAN
Myeloid leukemia factor 2	MLF2_HUMAN
Kinesin-like protein KIF7	KIF7_HUMAN
DISP complex protein LRCH3	LRCH3_HUMAN
Electrogenic aspartate/glutamate antiporter SLC25A12, mitochondrial	S2512_HUMAN
45 kDa calcium-binding protein	CAB45_HUMAN
Ras-related GTP-binding protein A	RRAGA_HUMAN
Septin-7	SEPT7_HUMAN
Rootletin	CROCC_HUMAN
Large ribosomal subunit protein eL31	RL31_HUMAN
RING1 and YY1-binding protein	RYBP_HUMAN
Trafficking protein particle complex subunit 2-like protein	TPC2L_HUMAN
Small ribosomal subunit protein bS21m	RT21_HUMAN
Son of sevenless homolog 1	SOS1_HUMAN
Probable ATP-dependent RNA helicase DDX60-like	DDX6L_HUMAN
Glyoxylate reductase/hydroxypyruvate reductase	GRHPR_HUMAN
RNA-binding protein 6	RBM6_HUMAN
Histone H1.10	H1X_HUMAN
PHD finger protein 10	PHF10_HUMAN
BCAS3 microtubule associated cell migration factor	BCAS3_HUMAN
Synaptosomal-associated protein 29	SNP29_HUMAN
Small ribosomal subunit protein uS15m	RT15_HUMAN
Zinc finger CCHC domain-containing protein 3	ZCHC3_HUMAN
Ribonuclease P protein subunit p14	RPP14_HUMAN
NAD-dependent protein deacetylase sirtuin-1	SIR1_HUMAN
Pseudouridylate synthase 7 homolog	PUS7_HUMAN
Nitric oxide synthase-interacting protein	NOSIP_HUMAN
Unconventional myosin-XVIIIa	MY18A_HUMAN
BTB/POZ domain-containing protein KCTD15	KCD15_HUMAN
Spermatid perinuclear RNA-binding protein	STRBP_HUMAN
Cell division cycle 5-like protein	CDC5L_HUMAN
cAMP-regulated phosphoprotein 19	ARP19_HUMAN
Dynactin subunit 2	DCTN2_HUMAN
N-acetylgalactosaminyltransferase 7	GALT7_HUMAN
TBC1 domain family member 23	TBC23_HUMAN
Adhesion G protein-coupled receptor L2	AGRL2_HUMAN
Thiol S-methyltransferase TMT1A	TMT1A_HUMAN
Kinase D-interacting substrate of 220 kDa	KDIS_HUMAN
High mobility group protein B3	HMGB3_HUMAN
Proton-coupled zinc antiporter SLC30A9, mitochondrial	ZNT9_HUMAN

Diphosphoinositol polyphosphate phosphohydrolase 1	NUDT3_HUMAN
Tumor necrosis factor ligand superfamily member 4	TNFL4_HUMAN
Protein C12orf4	CL004_HUMAN
Long-chain fatty acid transport protein 1	S27A1_HUMAN
Serine/threonine-protein kinase 38-like	ST38L_HUMAN
Thymosin beta-10	TYB10_HUMAN
Alpha-N-acetylgalactosaminide alpha-2,6-sialyltransferase 3	SIA7C_HUMAN
Electrogenic aspartate/glutamate antiporter SLC25A13, mitochondrial	S2513_HUMAN
Cdc42-interacting protein 4	CIP4_HUMAN
CXXC-type zinc finger protein 1	CXXC1_HUMAN
Actin, aortic smooth muscle	ACTA_HUMAN
Syntaxin-binding protein 5	STXB5_HUMAN
Abscission/NoCut checkpoint regulator	ANCHR_HUMAN
26S proteasome non-ATPase regulatory subunit 2	PSMD2_HUMAN
Phenylalanine--tRNA ligase, mitochondrial	SYFM_HUMAN
Proline/serine-rich coiled-coil protein 1	PSRC1_HUMAN
Cell cycle progression protein 1	CCPG1_HUMAN
Mothers against decapentaplegic homolog 5	SMAD5_HUMAN
Opioid growth factor receptor	OGFR_HUMAN
GDP-L-fucose synthase	FCL_HUMAN
Intracellular hyaluronan-binding protein 4	HABP4_HUMAN
Delta-1-pyrroline-5-carboxylate synthase	P5CS_HUMAN
Caspase-7	CASP7_HUMAN
DNA methyltransferase 1-associated protein 1	DMAP1_HUMAN
Minor histocompatibility antigen H13	HM13_HUMAN
Uncharacterized protein KIAA1671	K1671_HUMAN
Dual specificity protein phosphatase 23	DUS23_HUMAN
Protein arginine N-methyltransferase 9	ANM9_HUMAN
Protein TMED8	TMED8_HUMAN
Zinc finger MYM-type protein 4	ZMYM4_HUMAN
Exportin-5	XPO5_HUMAN
Calmodulin-1	CALM1_HUMAN
Transmembrane emp24 domain-containing protein 9	TMED9_HUMAN
Integrator complex subunit 14	INT14_HUMAN
Transaldolase	TALDO_HUMAN
Probable fibrosin-1	FBRS_HUMAN
B-cell receptor-associated protein 29	BAP29_HUMAN
Phospholipase D2	PLD2_HUMAN
Vacuolar protein sorting-associated protein 72 homolog	VPS72_HUMAN
MOB kinase activator 1B	MOB1B_HUMAN
MAU2 chromatid cohesion factor homolog	SCC4_HUMAN
N-terminal kinase-like protein	SCYL1_HUMAN
RNA helicase aquarius	AQR_HUMAN
Rab GDP dissociation inhibitor beta	GDIB_HUMAN
5'-AMP-activated protein kinase catalytic subunit alpha-1	AAPK1_HUMAN
Corrinoid adenosyltransferase MMAB	MMAB_HUMAN
Calmodulin-regulated spectrin-associated protein 2	CAMP2_HUMAN
Ubiquitin carboxyl-terminal hydrolase 32	UBP32_HUMAN
tRNA (cytidine(32)/guanosine(34)-2'-O)-methyltransferase	TRM7_HUMAN
E3 ubiquitin-protein ligase makorin-2	MKRN2_HUMAN

COP9 signalosome complex subunit 2	CSN2_HUMAN
Periphilin-1	PPHLN_HUMAN
Phosphatidylserine synthase 2	PTSS2_HUMAN
Actin-related protein 2/3 complex subunit 2	ARPC2_HUMAN
Heat shock-related 70 kDa protein 2	HSP72_HUMAN
Large ribosomal subunit protein eL6	RL6_HUMAN
tRNA-splicing endonuclease subunit Sen34	SEN34_HUMAN
Acyl-coenzyme A thioesterase 8	ACOT8_HUMAN
Sphingosine 1-phosphate receptor 1	S1PR1_HUMAN
Mediator of RNA polymerase II transcription subunit 14	MED14_HUMAN
DNA polymerase delta subunit 3	DPOD3_HUMAN
Pantothenate kinase 2, mitochondrial	PANK2_HUMAN
Transforming acidic coiled-coil-containing protein 3	TACC3_HUMAN
Ras-related protein Rap-2a	RAP2A_HUMAN
Proteasome subunit beta type-7	PSB7_HUMAN
Serine/threonine-protein kinase D1	KPCD1_HUMAN
Acetyl-CoA acetyltransferase, mitochondrial	THIL_HUMAN
Putative DENN domain-containing protein 10 B	DE10B_HUMAN
Beta-1,3-N-acetylglucosaminyltransferase manic fringe	MFNG_HUMAN
Alpha-globin transcription factor CP2	TFCP2_HUMAN
Probable ATP-dependent RNA helicase DDX17	DDX17_HUMAN
HAUS augmin-like complex subunit 4	HAUS4_HUMAN
Receptor-type tyrosine-protein phosphatase mu	PTPRM_HUMAN
Cytoskeleton-associated protein 5	CKAP5_HUMAN
Ral GTPase-activating protein subunit beta	RLGPB_HUMAN
Septin-2	SEPT2_HUMAN
Ankycorbin	RAI14_HUMAN
Prefoldin subunit 1	PFD1_HUMAN
Zinc finger Ran-binding domain-containing protein 2	ZRAB2_HUMAN
Golgin subfamily B member 1	GOGB1_HUMAN
Proline-, glutamic acid- and leucine-rich protein 1	PELP1_HUMAN
SUN domain-containing protein 2	SUN2_HUMAN
Large ribosomal subunit protein bL27m	RM27_HUMAN
5'-nucleotidase	5NTD_HUMAN
Translocation protein SEC63 homolog	SEC63_HUMAN
Transmembrane 9 superfamily member 1	TM9S1_HUMAN
NADP-dependent malic enzyme, mitochondrial	MAON_HUMAN
Tudor domain-containing protein 7	TDRD7_HUMAN
Ubiquitin-conjugating enzyme E2 R1	UB2R1_HUMAN
Small ribosomal subunit protein eS1	RS3A_HUMAN
Superkiller complex protein 3	SKI3_HUMAN
Peregrin	BRPF1_HUMAN
Paraspeckle component 1	PSPC1_HUMAN
WD repeat-containing protein 91	WDR91_HUMAN
Elongin-A	ELOA1_HUMAN
Reticulocalbin-2	RCN2_HUMAN
Isoaspartyl peptidase/L-asparaginase	ASGL1_HUMAN
Cysteine--tRNA ligase, cytoplasmic	SYCC_HUMAN
Kinetochore protein NDC80 homolog	NDC80_HUMAN
Inositol-tetrakisphosphate 1-kinase	ITPK1_HUMAN

FYVE, RhoGEF and PH domain-containing protein 4	FGD4_HUMAN
Methylmalonic aciduria type A protein, mitochondrial	MMAA_HUMAN
CAP-Gly domain-containing linker protein 2	CLIP2_HUMAN
Long-chain fatty acid transport protein 3	S27A3_HUMAN
Metaxin-2	MTX2_HUMAN
Kinetochore protein Spc24	SPC24_HUMAN
Rap guanine nucleotide exchange factor 6	RPGF6_HUMAN
Ribonucleoside-diphosphate reductase large subunit	RIR1_HUMAN
Transcription factor p65	TF65_HUMAN
Protein PRRC1	PRRC1_HUMAN
Plasminogen receptor (KT)	PLRKT_HUMAN
Conserved oligomeric Golgi complex subunit 6	COG6_HUMAN
Glutaryl-CoA dehydrogenase, mitochondrial	GCDH_HUMAN
Aminoacyl tRNA synthase complex-interacting multifunctional protein 1	AIMP1_HUMAN
Protein TAMALIN	GRASP_HUMAN
PSME3-interacting protein	PIP30_HUMAN
Cyclin-dependent kinase 5	CDK5_HUMAN
Phosphatidylinositol 4-phosphate 3-kinase C2 domain-containing subunit bet	P3C2B_HUMAN
RCC1 domain-containing protein 1	RCCD1_HUMAN
Cytosolic 5'-nucleotidase 3A	5NT3A_HUMAN
MIF4G domain-containing protein	MI4GD_HUMAN
Protein FAM193A	F193A_HUMAN
La-related protein 4B	LAR4B_HUMAN
E3 ubiquitin-protein ligase UHRF2	UHRF2_HUMAN
Endophilin-A2	SH3G1_HUMAN
Protein MEMO1	MEMO1_HUMAN
Dipeptidyl peptidase 8	DPP8_HUMAN
DDB1- and CUL4-associated factor 5	DCAF5_HUMAN
ADP-ribose pyrophosphatase, mitochondrial	NUDT9_HUMAN
Glutamine--fructose-6-phosphate aminotransferase [isomerizing] 2	GFPT2_HUMAN
DNA primase large subunit	PRI2_HUMAN
Ubiquitin conjugation factor E4 B	UBE4B_HUMAN
Casein kinase I isoform gamma-1	KC1G1_HUMAN
ADP/ATP translocase 1	ADT1_HUMAN
Serine/threonine-protein phosphatase PP1-alpha catalytic subunit	PP1A_HUMAN
Thymidylate synthase	TYSY_HUMAN
WD repeat-containing protein 36	WDR36_HUMAN
Protein flightless-1 homolog	FLII_HUMAN
Importin-13	IPO13_HUMAN
Vinculin	VINC_HUMAN
Fibronectin type-III domain-containing protein 3A	FND3A_HUMAN
Probable aminopeptidase NPEPL1	PEPL1_HUMAN
Surfeit locus protein 4	SURF4_HUMAN
Reticulophagy regulator 3	RETR3_HUMAN
E3 ubiquitin-protein ligase MIB1	MIB1_HUMAN
Myotubularin-related protein 6	MTMR6_HUMAN
Ribosome biogenesis protein WDR12	WDR12_HUMAN
Codanin-1	CDAN1_HUMAN
BAG family molecular chaperone regulator 5	BAG5_HUMAN
3-oxoacyl-[acyl-carrier-protein] synthase, mitochondrial	OXSM_HUMAN

Spectrin alpha chain, non-erythrocytic 1	SPTN1_HUMAN
Dual specificity mitogen-activated protein kinase kinase 4	MP2K4_HUMAN
Large ribosomal subunit protein uL24m	RM24_HUMAN
Small ribosomal subunit protein uS5m	RT05_HUMAN
Legumain	LGMN_HUMAN
BRISC and BRCA1-A complex member 1	BABA1_HUMAN
E3 ubiquitin-protein ligase listerin	LTN1_HUMAN
Serrate RNA effector molecule homolog	SRRT_HUMAN
Rho guanine nucleotide exchange factor 10	ARHGA_HUMAN
Integrin beta-5	ITB5_HUMAN
Zinc finger E-box-binding homeobox 1	ZEB1_HUMAN
1-acylglycerol-3-phosphate O-acyltransferase ABHD5	ABHD5_HUMAN
Calcium homeostasis endoplasmic reticulum protein	CHERP_HUMAN
Fragile X messenger ribonucleoprotein 1	FMR1_HUMAN
Protein MTSS 1	MTSS1_HUMAN
Thioredoxin-related transmembrane protein 1	TMX1_HUMAN
Paired amphipathic helix protein Sin3b	SIN3B_HUMAN
Bcl-2 homologous antagonist/killer	BAK_HUMAN
DNA-directed RNA polymerase II subunit RPB2	RPB2_HUMAN
Mevalonate kinase	KIME_HUMAN
Pre-mRNA-splicing factor ATP-dependent RNA helicase DHX16	DHX16_HUMAN
MOB-like protein phocein	PHOCN_HUMAN
Collagen alpha-2(V) chain	CO5A2_HUMAN
Trafficking protein particle complex subunit 13	TPC13_HUMAN
Large ribosomal subunit protein eL21	RL21_HUMAN
Glutathione S-transferase omega-1	GSTO1_HUMAN
Short transient receptor potential channel 4-associated protein	TP4AP_HUMAN
Retinoblastoma-associated protein	RB_HUMAN
RNA polymerase-associated protein CTR9 homolog	CTR9_HUMAN
pre-mRNA 3' end processing protein WDR33	WDR33_HUMAN
Isoleucine--tRNA ligase, mitochondrial	SYIM_HUMAN
Large ribosomal subunit protein uL14m	RM14_HUMAN
G1/S-specific cyclin-D1	CCND1_HUMAN
AT-rich interactive domain-containing protein 1B	ARI1B_HUMAN
Protein DEPP1	DEPP1_HUMAN
Puromycin-sensitive aminopeptidase	PSA_HUMAN
Intersectin-1	ITSN1_HUMAN
REST corepressor 1	RCOR1_HUMAN
Protein WWC3	WWC3_HUMAN
Unconventional myosin-Va	MYO5A_HUMAN
Probable ATP-dependent RNA helicase DDX47	DDX47_HUMAN
Large ribosomal subunit protein eL29	RL29_HUMAN
SID1 transmembrane family member 2	SIDT2_HUMAN
Oxidation resistance protein 1	OXR1_HUMAN
Zinc finger FYVE domain-containing protein 21	ZFY21_HUMAN
Protein RER1	RER1_HUMAN
DNA polymerase delta subunit 2	DPOD2_HUMAN
Protein NOXP20	NXP20_HUMAN
Origin recognition complex subunit 2	ORC2_HUMAN
Dual specificity protein phosphatase 3	DUS3_HUMAN

Small nuclear ribonucleoprotein Sm D2	SMD2_HUMAN
Formin-like protein 3	FMNL3_HUMAN
Protein FAM91A1	F91A1_HUMAN
RUS family member 1	RUSF1_HUMAN
Glycine--tRNA ligase	GARS_HUMAN
Phosphatidylinositol 4-kinase type 2-alpha	P4K2A_HUMAN
DNA topoisomerase 3-beta-1	TOP3B_HUMAN
Egl nine homolog 1	EGLN1_HUMAN
Bifunctional glutamate/proline--tRNA ligase	SYEP_HUMAN
Septin-9	SEPT9_HUMAN
[Pyruvate dehydrogenase (acetyl-transferring)] kinase isozyme 3, mitochond	PDK3_HUMAN
Tyrosine-protein kinase Fes/Fps	FES_HUMAN
MYG1 exonuclease	MYG1_HUMAN
Thioredoxin	THIO_HUMAN
Annexin A1	ANXA1_HUMAN
Alpha-(1,3)-fucosyltransferase 11	FUT11_HUMAN
Galactose-1-phosphate uridylyltransferase	GALT_HUMAN
Sialidase-1	NEUR1_HUMAN
Protein mono-ADP-ribosyltransferase PARP12	PAR12_HUMAN
Rab5 GDP/GTP exchange factor	RABX5_HUMAN
Type-1 angiotensin II receptor-associated protein	ATRAP_HUMAN
SH3 and PX domain-containing protein 2B	SPD2B_HUMAN
5-formyltetrahydrofolate cyclo-ligase	MTHFS_HUMAN
14 kDa phosphohistidine phosphatase	PHP14_HUMAN
Peroxisomal ATPase PEX1	PEX1_HUMAN
E3 ubiquitin-protein ligase TRAF7	TRAF7_HUMAN
Importin-8	IPO8_HUMAN
FAD synthase	FAD1_HUMAN
TRAF-type zinc finger domain-containing protein 1	TRAD1_HUMAN
Mediator of RNA polymerase II transcription subunit 15	MED15_HUMAN
Neurochondrin	NCDN_HUMAN
Alpha-galactosidase A	AGAL_HUMAN
Protein Red	RED_HUMAN
WD repeat and FYVE domain-containing protein 3	WDFY3_HUMAN
mRNA-decapping enzyme 1A	DCP1A_HUMAN
Testis-expressed protein 264	TX264_HUMAN
Ankyrin repeat and KH domain-containing protein 1	ANKH1_HUMAN
Serine/threonine-protein phosphatase 6 regulatory subunit 2	PP6R2_HUMAN
AP-2 complex subunit alpha-1	AP2A1_HUMAN
Flap endonuclease 1	FEN1_HUMAN
Putative divalent cation/proton antiporter TMEM165	TM165_HUMAN
Serine/threonine-protein kinase D3	KPCD3_HUMAN
Signal peptidase complex subunit 2	SPCS2_HUMAN
Solute carrier family 12 member 9	S12A9_HUMAN
Putative RNA-binding protein Luc7-like 2	LC7L2_HUMAN
ATP-binding cassette sub-family C member 4	MRP4_HUMAN
Phospholipid scramblase 1	PLS1_HUMAN
Kin of IRRE-like protein 1	KIRR1_HUMAN
Protein IMPACT	IMPCT_HUMAN
Proteasome activator complex subunit 4	PSME4_HUMAN

Regulation of nuclear pre-mRNA domain-containing protein 1B	RPR1B_HUMAN
ADP-ribosylation factor-like protein 15	ARL15_HUMAN
Mitogen-activated protein kinase kinase kinase kinase 4	M4K4_HUMAN
Neprilysin	NEP_HUMAN
Ran-binding protein 9	RANB9_HUMAN
E3 ubiquitin-protein ligase XIAP	XIAP_HUMAN
Wings apart-like protein homolog	WAPL_HUMAN
PDZ and LIM domain protein 7	PDLI7_HUMAN
Ubiquitin carboxyl-terminal hydrolase 10	UBP10_HUMAN
Kinesin-like protein KIF16B	KI16B_HUMAN
Pyrroline-5-carboxylate reductase 1, mitochondrial	P5CR1_HUMAN
DCC-interacting protein 13-beta	DP13B_HUMAN
Transcription factor BTF3	BTF3_HUMAN
Proteasome subunit beta type-5	PSB5_HUMAN
Melanoma inhibitory activity protein 2	MIA2_HUMAN
Suppressor of SWI4 1 homolog	SSF1_HUMAN
Ras-specific guanine nucleotide-releasing factor RalGPS2	RGPS2_HUMAN
Endoplasmic reticulum aminopeptidase 1	ERAP1_HUMAN
Uveal autoantigen with coiled-coil domains and ankyrin repeats	UACA_HUMAN
Origin recognition complex subunit 6	ORC6_HUMAN
Spectrin beta chain, non-erythrocytic 1	SPTB2_HUMAN
Protein FAM98B	FA98B_HUMAN
RRP15-like protein	RRP15_HUMAN
Focal adhesion kinase 1	FAK1_HUMAN
Sphingosine kinase 1	SPHK1_HUMAN
Sodium/potassium-transporting ATPase subunit beta-3	AT1B3_HUMAN
WD repeat-containing protein 75	WDR75_HUMAN
Protein spinster homolog 1	SPNS1_HUMAN
EARP and GARP complex-interacting protein 1	EIPR1_HUMAN
Telomere-associated protein RIF1	RIF1_HUMAN
Double-stranded RNA-binding protein Staufen homolog 1	STAU1_HUMAN
ATPase PAAT	PAAT_HUMAN
Nuclear protein localization protein 4 homolog	NPL4_HUMAN
2-aminoethanethiol dioxygenase	AEDO_HUMAN
Hsp70-binding protein 1	HPBP1_HUMAN
Eukaryotic translation initiation factor 4 gamma 3	IF4G3_HUMAN
Prenylcysteine oxidase 1	PCYOX_HUMAN
Zinc finger SWIM domain-containing protein 8	ZSWM8_HUMAN
TLE family member 5	TLE5_HUMAN
Collagen alpha-1(V) chain	CO5A1_HUMAN
Nuclear speckle splicing regulatory protein 1	NSRP1_HUMAN
2-amino-3-ketobutyrate coenzyme A ligase, mitochondrial	KBL_HUMAN
RNA-binding protein FXR1	FXR1_HUMAN
Zinc finger CCCH-type antiviral protein 1	ZCCHV_HUMAN
Protein PBDC1	PBDC1_HUMAN
Protein phosphatase 1A	PPM1A_HUMAN
Probable ribosome biogenesis protein RLP24	RLP24_HUMAN
Nicotinamide phosphoribosyltransferase	NAMPT_HUMAN
Putative RNA-binding protein Luc7-like 1	LUC7L_HUMAN
Kinesin-like protein KIF3C	KIF3C_HUMAN

Mannose-P-dolichol utilization defect 1 protein	MPU1_HUMAN
Golgin subfamily A member 2	GOGA2_HUMAN
Ribosome quality control complex subunit NEMF	NEMF_HUMAN
Girdin	GRDN_HUMAN
Rab GTPase-activating protein 1	RBGP1_HUMAN
DDB1- and CUL4-associated factor 1	DCAF1_HUMAN
NFATC2-interacting protein	NF2IP_HUMAN
N-alpha-acetyltransferase 20	NAA20_HUMAN
Large ribosomal subunit protein bL28m	RM28_HUMAN
Ceramide-1-phosphate transfer protein	CPTP_HUMAN
Carbonyl reductase [NADPH] 3	CBR3_HUMAN
Sorting nexin-12	SNX12_HUMAN
Peptidyl-prolyl cis-trans isomerase F, mitochondrial	PPIF_HUMAN
Transcription elongation factor SPT4	SPT4H_HUMAN
Pleckstrin homology-like domain family B member 2	PHLB2_HUMAN
Large ribosomal subunit protein mL48	RM48_HUMAN
Transcriptional activator protein Pur-beta	PURB_HUMAN
Alpha-mannosidase 2	MA2A1_HUMAN
Trafficking protein particle complex subunit 6B	TPC6B_HUMAN
Nucleoporin p58/p45	NUP58_HUMAN
Nurim	NRM_HUMAN
Fanconi anemia group I protein	FANCI_HUMAN
Peptide-N(4)-(N-acetyl-beta-glucosaminyl)asparagine amidase	NGLY1_HUMAN
GRIP1-associated protein 1	GRAP1_HUMAN
Cordon-bleu protein-like 1	COBL1_HUMAN
Ribosomal protein uL30-like	RL7L_HUMAN
NudC domain-containing protein 3	NUDC3_HUMAN
Target of EGR1 protein 1	TOE1_HUMAN
Progesterone-induced-blocking factor 1	PIBF1_HUMAN
(E3-independent) E2 ubiquitin-conjugating enzyme	UBE2O_HUMAN
CLIP-associating protein 2	CLAP2_HUMAN
DNA repair protein XRCC4	XRCC4_HUMAN
Protein TASOR	TASOR_HUMAN
Ubiquitin domain-containing protein UBFD1	UBFD1_HUMAN
GDP-mannose 4,6 dehydratase	GMDS_HUMAN
Schlafen family member 11	SLN11_HUMAN
Large ribosomal subunit protein uL4m	RM04_HUMAN
AT-rich interactive domain-containing protein 1A	ARI1A_HUMAN
Protein transport protein Sec23A	SC23A_HUMAN
Peflin	PEF1_HUMAN
Phosphatase and actin regulator 2	PHAR2_HUMAN
Erlin-2	ERLN2_HUMAN
Importin-7	IPO7_HUMAN
MAP kinase-activated protein kinase 2	MAPK2_HUMAN
Rho family-interacting cell polarization regulator 1	RIPR1_HUMAN
Large ribosomal subunit protein mL45	RM45_HUMAN
Dachshund homolog 1	DACH1_HUMAN
Peroxisomal biogenesis factor 3	PEX3_HUMAN
Phenylalanine--tRNA ligase beta subunit	SYFB_HUMAN
Snurportin-1	SPN1_HUMAN

Epidermal growth factor receptor substrate 15-like 1	EP15R_HUMAN
Rho-related GTP-binding protein RhoG	RHOG_HUMAN
Nuclear receptor coactivator 3	NCOA3_HUMAN
CB1 cannabinoid receptor-interacting protein 1	CNRP1_HUMAN
Carboxymethylenebutenolidase homolog	CMBL_HUMAN
Lysine-specific demethylase 5B	KDM5B_HUMAN
Trafficking protein particle complex subunit 9	TPPC9_HUMAN
Ribonuclease H2 subunit B	RNH2B_HUMAN
Integrin alpha-2	ITA2_HUMAN
Choline-phosphate cytidylyltransferase A	PCY1A_HUMAN
Transmembrane protein 181	TM181_HUMAN
UDP-glucose:glycoprotein glucosyltransferase 2	UGGG2_HUMAN
Pentraxin-related protein PTX3	PTX3_HUMAN
Pre-rRNA-processing protein TSR1 homolog	TSR1_HUMAN
NADH dehydrogenase [ubiquinone] 1 beta subcomplex subunit 9	NDUB9_HUMAN
Profilin-1	PROF1_HUMAN
GPI ethanolamine phosphate transferase 2	PIGG_HUMAN
tRNA (adenine(58)-N(1))-methyltransferase catalytic subunit TRMT61A	TRM61_HUMAN
Leucine--tRNA ligase, cytoplasmic	SYLC_HUMAN
FERM domain-containing protein 4A	FRM4A_HUMAN
Deubiquitinase OTUD6B	OTU6B_HUMAN
ATPase GET3	GET3_HUMAN
3-hydroxy-3-methylglutaryl-coenzyme A reductase	HMDH_HUMAN
Acyl-CoA-binding domain-containing protein 5	ACBD5_HUMAN
TNFAIP3-interacting protein 1	TNIP1_HUMAN
Adapter molecule crk	CRK_HUMAN
Serine/threonine-protein kinase PRP4 homolog	PRP4B_HUMAN
Sorting nexin-5	SNX5_HUMAN
Serine/threonine-protein phosphatase PGAM5, mitochondrial	PGAM5_HUMAN
Sideroflexin-1	SFXN1_HUMAN
Ras-related protein Rap-2c	RAP2C_HUMAN
Caspase recruitment domain-containing protein 6	CARD6_HUMAN
Podocalyxin	PODXL_HUMAN
Coiled-coil domain-containing protein 25	CCD25_HUMAN
Tensin-1	TENS1_HUMAN
Mitochondrial import inner membrane translocase subunit Tim21	TIM21_HUMAN
3-beta-hydroxysteroid-Delta(8),Delta(7)-isomerase	EBP_HUMAN
Uridine-cytidine kinase-like 1	UCKL1_HUMAN
Mothers against decapentaplegic homolog 2	SMAD2_HUMAN
Tetratricopeptide repeat protein 7B	TTC7B_HUMAN
Protein DENND6A	DEN6A_HUMAN
Aldo-keto reductase family 1 member A1	AK1A1_HUMAN
Nucleoporin NUP42	NUP42_HUMAN
Carnitine O-palmitoyltransferase 2, mitochondrial	CPT2_HUMAN
DNA damage-binding protein 1	DDB1_HUMAN
Superkiller complex protein 2	SKI2_HUMAN
Disintegrin and metalloproteinase domain-containing protein 9	ADAM9_HUMAN
Raftlin	RFTN1_HUMAN
Forkhead box protein K2	FOXK2_HUMAN
NADH dehydrogenase [ubiquinone] iron-sulfur protein 4, mitochondrial	NDUS4_HUMAN

BAG family molecular chaperone regulator 2	BAG2_HUMAN
Splicing factor, proline- and glutamine-rich	SFPQ_HUMAN
Activating signal cointegrator 1 complex subunit 2	ASCC2_HUMAN
Vacuolar protein sorting-associated protein 4B	VPS4B_HUMAN
Transport and Golgi organization protein 6 homolog	TNG6_HUMAN
ATP-dependent RNA helicase DHX15	DHX15_HUMAN
Integrator complex subunit 8	INT8_HUMAN
Hypoxanthine-guanine phosphoribosyltransferase	HPRT_HUMAN
Charged multivesicular body protein 2b	CHM2B_HUMAN
FAST kinase domain-containing protein 2, mitochondrial	FAKD2_HUMAN
WD repeat and coiled-coil-containing protein	WDCP_HUMAN
Sorting nexin-18	SNX18_HUMAN
1-acyl-sn-glycerol-3-phosphate acyltransferase gamma	PLCC_HUMAN
Integrin alpha-5	ITA5_HUMAN
Protein VAC14 homolog	VAC14_HUMAN
Cyclic AMP-dependent transcription factor ATF-6 alpha	ATF6A_HUMAN
V-type proton ATPase subunit H	VATH_HUMAN
Mitochondrial import receptor subunit TOM20 homolog	TOM20_HUMAN
Transmembrane protein 209	TM209_HUMAN
Elongation factor 2	EF2_HUMAN
Protein RRP5 homolog	RRP5_HUMAN
Large ribosomal subunit protein mL65	RT30_HUMAN
Kinetochore protein Spc25	SPC25_HUMAN
Succinate--CoA ligase [ADP/GDP-forming] subunit alpha, mitochondrial	SUCA_HUMAN
THO complex subunit 7 homolog	THOC7_HUMAN
Transcription termination factor 4, mitochondrial	MTEF4_HUMAN
Translocator protein	TSPO_HUMAN
Y-box-binding protein 1	YBOX1_HUMAN
EPM2A-interacting protein 1	EPMIP_HUMAN
L-2-hydroxyglutarate dehydrogenase, mitochondrial	L2HDH_HUMAN
Chromatin assembly factor 1 subunit A	CAF1A_HUMAN
Death-associated protein kinase 1	DAPK1_HUMAN
Translation machinery-associated protein 7	TMA7_HUMAN
Transmembrane 9 superfamily member 4	TM9S4_HUMAN
Kinesin-like protein KIF14	KIF14_HUMAN
Protein transport protein Sec16A	SC16A_HUMAN
Protein Niban 2	NIBA2_HUMAN
INO80 complex subunit C	IN80C_HUMAN
RISC-loading complex subunit TARBP2	TRBP2_HUMAN
Glucosamine-6-phosphate isomerase 2	GNPI2_HUMAN
G1/S-specific cyclin-D3	CCND3_HUMAN
Large ribosomal subunit protein uL30	RL7_HUMAN
Elongation factor Ts, mitochondrial	EFTS_HUMAN
Geranylgeranyl transferase type-1 subunit beta	PGTB1_HUMAN
U6 snRNA-associated Sm-like protein LSm6	LSM6_HUMAN
Small ribosomal subunit protein uS9m	RT09_HUMAN
SURP and G-patch domain-containing protein 2	SUGP2_HUMAN
Misshapen-like kinase 1	MINK1_HUMAN
Zinc finger CCCH-type with G patch domain-containing protein	ZGPAT_HUMAN
Mitochondrial import receptor subunit TOM40B	TM40L_HUMAN

Cysteine protease ATG4B	ATG4B_HUMAN
Glutamate-rich WD repeat-containing protein 1	GRWD1_HUMAN
Neuroguidin	NGDN_HUMAN
Annexin A11	ANX11_HUMAN
Protein disulfide-isomerase	PDIA1_HUMAN
Centromere protein F	CENPF_HUMAN
Disabled homolog 2	DAB2_HUMAN
Integrator complex subunit 1	INT1_HUMAN
Tripartite motif-containing protein 26	TRI26_HUMAN
Uncharacterized protein KIAA0930	K0930_HUMAN
Serpin B9	SPB9_HUMAN
Sorting nexin-4	SNX4_HUMAN
Sphingosine 1-phosphate receptor 3	S1PR3_HUMAN
Oligosaccharyltransferase complex subunit OSTC	OSTC_HUMAN
AP-1 complex subunit gamma-1	AP1G1_HUMAN
E3 ubiquitin-protein ligase ARIH1	ARI1_HUMAN
Tricarboxylate transport protein, mitochondrial	TXTP_HUMAN
Serine/threonine-protein phosphatase 2A 56 kDa regulatory subunit alpha is	2A5A_HUMAN
Phosphatidylinositol glycan anchor biosynthesis class U protein	PIGU_HUMAN
Prolactin regulatory element-binding protein	PREB_HUMAN
WD repeat-containing protein 46	WDR46_HUMAN
A-kinase anchor protein 11	AKA11_HUMAN
Ras-related protein Rab-21	RAB21_HUMAN
Extended synaptotagmin-2	ESYT2_HUMAN
Endosome/lysosome-associated apoptosis and autophagy regulator family m	ELAP2_HUMAN
cAMP-dependent protein kinase type I-alpha regulatory subunit	KAP0_HUMAN
Golgin subfamily A member 4	GOGA4_HUMAN
Inorganic pyrophosphatase	IPYR_HUMAN
NADH dehydrogenase [ubiquinone] iron-sulfur protein 2, mitochondrial	NDUS2_HUMAN
SEC14-like protein 2	S14L2_HUMAN
26S proteasome non-ATPase regulatory subunit 7	PSMD7_HUMAN
Unconventional myosin-IXb	MYO9B_HUMAN
Formin-binding protein 4	FNBP4_HUMAN
V-type proton ATPase 116 kDa subunit a 1	VPP1_HUMAN
Serine/threonine-protein kinase MARK2	MARK2_HUMAN
Protein O-glucosyltransferase 2	PLGT2_HUMAN
Msx2-interacting protein	MINT_HUMAN
Serine/threonine-protein phosphatase 2B catalytic subunit beta isoform	PP2BB_HUMAN
Cyclin-H	CCNH_HUMAN
Contactin-associated protein-like 3	CNTP3_HUMAN
Integrator complex subunit 3	INT3_HUMAN
Poly(rC)-binding protein 2	PCBP2_HUMAN
GPI-anchor transamidase	GPI8_HUMAN
Ferrochelatase, mitochondrial	HEMH_HUMAN
Deoxynucleotidyltransferase terminal-interacting protein 2	TDIF2_HUMAN
C1GALT1-specific chaperone 1	C1GLC_HUMAN
Mitochondrial ribonuclease P catalytic subunit	MRPP3_HUMAN
Phosphatidylinositol-binding clathrin assembly protein	PICAL_HUMAN
Solute carrier family 38 member 10	S38AA_HUMAN
TNF receptor-associated factor 2	TRAF2_HUMAN

Valine--tRNA ligase	SYVC_HUMAN
Zinc finger CCCH domain-containing protein 7A	Z3H7A_HUMAN
DDB1- and CUL4-associated factor 16	DCA16_HUMAN
WD repeat-containing protein 3	WDR3_HUMAN
Cystatin-C	CYTC_HUMAN
Mitochondrial chaperone BCS1	BCS1_HUMAN
Eukaryotic translation initiation factor 4E transporter	4ET_HUMAN
Ethanolamine-phosphate cytidylyltransferase	PCY2_HUMAN
Ubiquitin carboxyl-terminal hydrolase 24	UBP24_HUMAN
Heat shock 70 kDa protein 13	HSP13_HUMAN
RNA-binding protein 12	RBM12_HUMAN
Transmembrane emp24 domain-containing protein 2	TMED2_HUMAN
Transmembrane emp24 domain-containing protein 4	TMED4_HUMAN
Myoferlin	MYOF_HUMAN
DnaJ homolog subfamily C member 10	DJC10_HUMAN
Poly(A)-specific ribonuclease PARN	PARN_HUMAN
Prefoldin subunit 5	PFD5_HUMAN
Probable E3 ubiquitin-protein ligase HERC4	HERC4_HUMAN
Deoxycytidine kinase	DCK_HUMAN
Clathrin heavy chain 1	CLH1_HUMAN
COP9 signalosome complex subunit 4	CSN4_HUMAN
Protein transport protein Sec24B	SC24B_HUMAN
U2 small nuclear ribonucleoprotein B''	RU2B_HUMAN
Vezatin	VEZA_HUMAN
Cytosolic carboxypeptidase 1	CBPC1_HUMAN
BRCA1-A complex subunit Abraxas 1	ABRX1_HUMAN
Acyl-coenzyme A thioesterase MBLAC2	MBLC2_HUMAN
STE20/SPS1-related proline-alanine-rich protein kinase	STK39_HUMAN
Catenin alpha-1	CTNA1_HUMAN
Secernin-2	SCRN2_HUMAN
Germinal-center associated nuclear protein	GANP_HUMAN
Phosphomannomutase 2	PMM2_HUMAN
NEDD8-activating enzyme E1 catalytic subunit	UBA3_HUMAN
Signal recognition particle receptor subunit alpha	SRPRA_HUMAN
Ribose-5-phosphate isomerase	RPIA_HUMAN
Ubiquitin carboxyl-terminal hydrolase 4	UBP4_HUMAN
Sodium-coupled neutral amino acid symporter 2	S38A2_HUMAN
Protein NipSnap homolog 3A	NPS3A_HUMAN
Protein phosphatase 1 regulatory subunit 12C	PP12C_HUMAN
Protein transport protein Sec23B	SC23B_HUMAN
Unconventional myosin-Ie	MYO1E_HUMAN
Mitogen-activated protein kinase kinase kinase 3	M3K3_HUMAN
NADH dehydrogenase [ubiquinone] 1 alpha subcomplex subunit 11	NDUAB_HUMAN
Ribosome biogenesis protein BRX1 homolog	BRX1_HUMAN
Ubiquitin-like modifier-activating enzyme 5	UBA5_HUMAN
Retinal rod rhodopsin-sensitive cGMP 3',5'-cyclic phosphodiesterase subunit	PDE6D_HUMAN
Rho GTPase-activating protein 35	RHG35_HUMAN
Toll-interacting protein	TOLIP_HUMAN
Nuclear pore complex protein Nup153	NU153_HUMAN
Laminin subunit beta-2	LAMB2_HUMAN

E3 ubiquitin-protein ligase HECTD3	HECD3_HUMAN
Proliferation marker protein Ki-67	KI67_HUMAN
CDK5 regulatory subunit-associated protein 3	CK5P3_HUMAN
Dihydropyrimidinase-related protein 2	DPYL2_HUMAN
Nucleoporin Nup43	NUP43_HUMAN
Cyclin-dependent kinase 13	CDK13_HUMAN
Serine/threonine-protein phosphatase 2A 56 kDa regulatory subunit epsilon	2A5E_HUMAN
Protein MON2 homolog	MON2_HUMAN
Structural maintenance of chromosomes protein 6	SMC6_HUMAN
NADH dehydrogenase [ubiquinone] 1 alpha subcomplex assembly factor 3	NDUF3_HUMAN
Anthrax toxin receptor 2	ANTR2_HUMAN
ER membrane protein complex subunit 2	EMC2_HUMAN
CCR4-NOT transcription complex subunit 7	CNOT7_HUMAN
Activator of basal transcription 1	ABT1_HUMAN
Large ribosomal subunit protein uL1	RL10A_HUMAN
Ubiquitin-conjugating enzyme E2 H	UBE2H_HUMAN
Glycogen debranching enzyme	GDE_HUMAN
Ubiquitin domain-containing protein 1	UBTD1_HUMAN
WD repeat-containing protein 74	WDR74_HUMAN
Rhomboid domain-containing protein 2	RHBD2_HUMAN
RAC-alpha serine/threonine-protein kinase	AKT1_HUMAN
Sorbitol dehydrogenase	DHSO_HUMAN
Slit homolog 2 protein	SLIT2_HUMAN
Thioredoxin reductase 2, mitochondrial	TRXR2_HUMAN
Peroxisomal membrane protein PMP34	PM34_HUMAN
Large ribosomal subunit protein uL3m	RM03_HUMAN
Serine/threonine-protein phosphatase 2A 65 kDa regulatory subunit A beta i	2AAB_HUMAN
Glycosylphosphatidylinositol anchor attachment 1 protein	GPAA1_HUMAN
General transcription and DNA repair factor IIH helicase subunit XPB	ERCC3_HUMAN
Lipoma-preferred partner	LPP_HUMAN
Dystonin	DYST_HUMAN
Protein FAM171A1	F1711_HUMAN
Glucose-6-phosphate isomerase	G6PI_HUMAN
NADH dehydrogenase [ubiquinone] 1 alpha subcomplex subunit 5	NDUA5_HUMAN
Striatin-interacting protein 1	STRP1_HUMAN
Charged multivesicular body protein 5	CHMP5_HUMAN
Large ribosomal subunit protein uL2	RL8_HUMAN
E3 ubiquitin-protein transferase MAEA	MAEA_HUMAN
U4/U6.U5 tri-snRNP-associated protein 1	SNUT1_HUMAN
Activated RNA polymerase II transcriptional coactivator p15	TCP4_HUMAN
Cleavage stimulation factor subunit 2 tau variant	CSTFT_HUMAN
HEAT repeat-containing protein 5B	HTR5B_HUMAN
Protein arginine N-methyltransferase 5	ANM5_HUMAN
Caspase activity and apoptosis inhibitor 1	CAAP1_HUMAN
Caspase-2	CASP2_HUMAN
DnaJ homolog subfamily C member 13	DJC13_HUMAN
Bromodomain-containing protein 7	BRD7_HUMAN
Probable ATP-dependent RNA helicase DDX60	DDX60_HUMAN
Amyloid beta precursor protein binding family B member 1	APBB1_HUMAN
Plastin-1	PLSI_HUMAN

Cell division cycle and apoptosis regulator protein 1	CCAR1_HUMAN
Beta-galactosidase	BGAL_HUMAN
Polycystin-2	PKD2_HUMAN
Ubiquitin carboxyl-terminal hydrolase 13	UBP13_HUMAN
Transcriptional regulator ATRX	ATRX_HUMAN
Bromodomain adjacent to zinc finger domain protein 2B	BAZ2B_HUMAN
Cleavage and polyadenylation specificity factor subunit 6	CPSF6_HUMAN
Heparan sulfate 2-O-sulfotransferase 1	HS2ST_HUMAN
Tumor necrosis factor receptor superfamily member 10B	TR10B_HUMAN
Kanadaptin	NADAP_HUMAN
Coiled-coil domain-containing protein 71L	CC71L_HUMAN
Selenoprotein K	SELK_HUMAN
Programmed cell death protein 5	PDCD5_HUMAN
Glutathione peroxidase 1	GPX1_HUMAN
Activating signal cointegrator 1	TRIP4_HUMAN
DENN domain-containing protein 5A	DEN5A_HUMAN
Thioredoxin domain-containing protein 17	TXD17_HUMAN
Nuclease EXOG, mitochondrial	EXOG_HUMAN
Myeloid-associated differentiation marker	MYADM_HUMAN
NADH dehydrogenase [ubiquinone] 1 beta subcomplex subunit 4	NDUB4_HUMAN
Glutaredoxin-3	GLRX3_HUMAN
Transmembrane 6 superfamily member 1	TM6S1_HUMAN
Complex I assembly factor TMEM126B, mitochondrial	T126B_HUMAN
Nuclear factor 1 C-type	NFIC_HUMAN
Guanidinoacetate N-methyltransferase	GAMT_HUMAN
DNA-binding protein SMUBP-2	SMBP2_HUMAN
UV excision repair protein RAD23 homolog B	RD23B_HUMAN
Beta-actin-like protein 2	ACTBL_HUMAN
NAD-dependent protein deacetylase sirtuin-2	SIR2_HUMAN
General transcription factor IIF subunit 2	T2FB_HUMAN
Cdc42 effector protein 4	BORG4_HUMAN
Protein arginine N-methyltransferase 1	ANM1_HUMAN
Citron Rho-interacting kinase	CTRO_HUMAN
Replication factor C subunit 2	RFC2_HUMAN
Deubiquitinating protein VCPIP1	VCIP1_HUMAN
Glutamyl-tRNA(Gln) amidotransferase subunit B, mitochondrial	GATB_HUMAN
LIM domain kinase 2	LIMK2_HUMAN
Septin-11	SEP11_HUMAN
Cleavage and polyadenylation specificity factor subunit 1	CPSF1_HUMAN
Protein zwilch homolog	ZWILC_HUMAN
Alpha-actinin-4	ACTN4_HUMAN
Cytochrome c oxidase subunit 5A, mitochondrial	COX5A_HUMAN
NADH-cytochrome b5 reductase 1	NB5R1_HUMAN
PIN2/TERF1-interacting telomerase inhibitor 1	PINX1_HUMAN
Transmembrane and ubiquitin-like domain-containing protein 1	TMUB1_HUMAN
Nucleoporin SEH1	SEH1_HUMAN
2',3'-cyclic-nucleotide 3'-phosphodiesterase	CN37_HUMAN
Syntaxin-4	STX4_HUMAN
ATP synthase subunit gamma, mitochondrial	ATPG_HUMAN
Kinesin-like protein KIF13B	KI13B_HUMAN

Dysbindin	DTBP1_HUMAN
Platelet-activating factor acetylhydrolase IB subunit beta	LIS1_HUMAN
Exportin-6	XPO6_HUMAN
6-phosphogluconolactonase	6PGL_HUMAN
Dual specificity mitogen-activated protein kinase kinase 1	MP2K1_HUMAN
Transmembrane protein 223	TM223_HUMAN
Alcohol dehydrogenase class-3	ADHX_HUMAN
Arfaptin-2	ARFP2_HUMAN
Large ribosomal subunit protein uL16m	RM16_HUMAN
ADP-ribosylation factor-like protein 6-interacting protein 1	AR6P1_HUMAN
Sideroflexin-4	SFXN4_HUMAN
Origin recognition complex subunit 5	ORC5_HUMAN
TBC domain-containing protein kinase-like protein	TBCK_HUMAN
Crooked neck-like protein 1	CRNL1_HUMAN
Serine hydroxymethyltransferase, cytosolic	GLYC_HUMAN
Gamma-tubulin complex component 2	GCP2_HUMAN
Endophilin-B2	SHLB2_HUMAN
U3 small nucleolar RNA-associated protein 15 homolog	UTP15_HUMAN
Sepiapterin reductase	SPRE_HUMAN
Annexin A7	ANXA7_HUMAN
Tyrosine-protein phosphatase non-receptor type substrate 1	SHPS1_HUMAN
ATP synthase mitochondrial F1 complex assembly factor 1	ATPF1_HUMAN
AN1-type zinc finger protein 6	ZFAN6_HUMAN
Mitochondrial import inner membrane translocase subunit TIM14	TIM14_HUMAN
Large ribosomal subunit protein uL22m	RM22_HUMAN
Plasma membrane calcium-transporting ATPase 4	AT2B4_HUMAN
Lys-63-specific deubiquitinase BRCC36	BRCC3_HUMAN
DNA polymerase subunit gamma-1	DPOG1_HUMAN
Splicing factor C9orf78	TLS1_HUMAN
Chimeric ERCC6-PGBD3 protein	ERPG3_HUMAN
Glucosamine-6-phosphate isomerase 1	GNPI1_HUMAN
Cancer-related nucleoside-triphosphatase	NTPCR_HUMAN
Transmembrane protein 256	TM256_HUMAN
Na(+)/H(+) exchange regulatory cofactor NHE-RF1	NHRF1_HUMAN
Ribosomal L1 domain-containing protein 1	RL1D1_HUMAN
GrpE protein homolog 1, mitochondrial	GRPE1_HUMAN
Probable ATP-dependent RNA helicase DDX27	DDX27_HUMAN
Multifunctional protein CAD	PYR1_HUMAN
Zinc finger protein 92	ZNF92_HUMAN
Zinc finger and BTB domain-containing protein 11	ZBT11_HUMAN
Calcium uptake protein 2, mitochondrial	MICU2_HUMAN
ATP-dependent RNA helicase DDX55	DDX55_HUMAN
Nuclear factor related to kappa-B-binding protein	NFRKB_HUMAN
RNA-binding protein 42	RBM42_HUMAN
Mitochondrial import inner membrane translocase subunit TIM50	TIM50_HUMAN
DNA-directed RNA polymerases I, II, and III subunit RPABC1	RPAB1_HUMAN
Isopentenyl-diphosphate Delta-isomerase 1	IDI1_HUMAN
Arfaptin-1	ARFP1_HUMAN
Ubiquitin carboxyl-terminal hydrolase 39	UBP39_HUMAN
Biorientation of chromosomes in cell division protein 1-like 1	BD1L1_HUMAN

Nuclear pore complex protein Nup133	NU133_HUMAN
Microtubule-associated protein RP/EB family member 3	MARE3_HUMAN
Flavin reductase (NADPH)	BLVRB_HUMAN
Bifunctional arginine demethylase and lysyl-hydroxylase JMJD6	JMJD6_HUMAN
TATA box-binding protein-like 1	TBPL1_HUMAN
ATPase family gene 2 protein homolog A	AFG2A_HUMAN
PAX3- and PAX7-binding protein 1	PAXB1_HUMAN
Nitric oxide synthase 3	NOS3_HUMAN
Phosphatidylinositol transfer protein beta isoform	PIPNB_HUMAN
Spectrin beta chain, non-erythrocytic 2	SPTN2_HUMAN
Exostosin-like 3	EXTL3_HUMAN
Calreticulin	CALR_HUMAN
Lon protease homolog, mitochondrial	LONM_HUMAN
Transmembrane emp24 domain-containing protein 3	TMED3_HUMAN
Arginine--tRNA ligase, cytoplasmic	SYRC_HUMAN
Autophagy-related protein 13	ATG13_HUMAN
Probable phospholipid-transporting ATPase IIA	ATP9A_HUMAN
Deoxyribonuclease TATDN1	TATD1_HUMAN
AP-3 complex subunit sigma-1	AP3S1_HUMAN
Tubulin-specific chaperone D	TBCD_HUMAN
Phosphatidylinositol 4-phosphate 5-kinase type-1 gamma	PI51C_HUMAN
Constitutive coactivator of PPAR-gamma-like protein 1	F120A_HUMAN
Protein FAM43A	FA43A_HUMAN
Transcription factor SOX-18	SOX18_HUMAN
Syntaxin-16	STX16_HUMAN
Armadillo repeat-containing X-linked protein 3	ARMX3_HUMAN
Membrane cofactor protein	MCP_HUMAN
Heterogeneous nuclear ribonucleoproteins A2/B1	ROA2_HUMAN
Major facilitator superfamily domain-containing protein 10	MFS10_HUMAN
Vesicle-associated membrane protein 3	VAMP3_HUMAN
ERO1-like protein alpha	ERO1A_HUMAN
Endoplasmic reticulum lectin 1	ERLEC_HUMAN
Coatomer subunit delta	COPD_HUMAN
Syntaxin-5	STX5_HUMAN
U6 snRNA-associated Sm-like protein LSm2	LSM2_HUMAN
Oxidoreductase HTATIP2	HTAI2_HUMAN
Zinc finger MYM-type protein 3	ZMYM3_HUMAN
Kinesin-like protein KIF3A	KIF3A_HUMAN
Ribonuclease P protein subunit p38	RPP38_HUMAN
NADH dehydrogenase [ubiquinone] iron-sulfur protein 8, mitochondrial	NDUS8_HUMAN
Bystin	BYST_HUMAN
Unconventional prefoldin RPB5 interactor 1	RMP_HUMAN
Adenosine 5'-monophosphoramidase HINT1	HINT1_HUMAN
Double-stranded RNA-specific adenosine deaminase	DSRAD_HUMAN
Adenylate kinase isoenzyme 6	KAD6_HUMAN
Ribosomal protein S6 kinase alpha-3	KS6A3_HUMAN
High mobility group nucleosome-binding domain-containing protein 3	HMGN3_HUMAN
Cilia- and flagella-associated protein 20	CFA20_HUMAN
Pre-B-cell leukemia transcription factor 2	PBX2_HUMAN
Zinc finger protein 185	ZN185_HUMAN

Vesicle-associated membrane protein-associated protein B/C	VAPB_HUMAN
Delta-aminolevulinic acid dehydratase	HEM2_HUMAN
DNA replication licensing factor MCM4	MCM4_HUMAN
Cytospin-A	CYTSA_HUMAN
Ras-related protein Rab-7a	RAB7A_HUMAN
Anoctamin-10	ANO10_HUMAN
Sister chromatid cohesion protein PDS5 homolog B	PDS5B_HUMAN
Uroporphyrinogen decarboxylase	DCUP_HUMAN
SWI/SNF-related matrix-associated actin-dependent regulator of chromatin s	SMRCD_HUMAN
Fos-related antigen 1	FOSL1_HUMAN
Zinc finger FYVE domain-containing protein 1	ZFYV1_HUMAN
Transmembrane protein 161A	T161A_HUMAN
Lon protease homolog 2, peroxisomal	LONP2_HUMAN
Tubulin beta-2A chain	TBB2A_HUMAN
Large ribosomal subunit protein mL49	RM49_HUMAN
Probable ATP-dependent RNA helicase DDX31	DDX31_HUMAN
DNA replication licensing factor MCM2	MCM2_HUMAN
Protein numb homolog	NUMB_HUMAN
Protein mono-ADP-ribosyltransferase PARP4	PARP4_HUMAN
Centrosomal protein of 97 kDa	CEP97_HUMAN
Craniofacial development protein 1	CFDP1_HUMAN
Phosphoribosylformylglycinamidine synthase	PUR4_HUMAN
N-alpha-acetyltransferase 15, NatA auxiliary subunit	NAA15_HUMAN
Importin-5	IPO5_HUMAN
Large ribosomal subunit protein uL23m	RM23_HUMAN
Arf-GAP with SH3 domain, ANK repeat and PH domain-containing protein 2	ASAP2_HUMAN
Leucine-rich repeat and calponin homology domain-containing protein 2	LRCH2_HUMAN
Heterogeneous nuclear ribonucleoprotein H2	HNRH2_HUMAN
Transcription intermediary factor 1-beta	TIF1B_HUMAN
Prostaglandin reductase 3	PTGR3_HUMAN
Soluble calcium-activated nucleotidase 1	CANT1_HUMAN
ATP-dependent RNA helicase DDX51	DDX51_HUMAN
Collagen alpha-2(I) chain	CO1A2_HUMAN
Cytoplasmic FMR1-interacting protein 2	CYFP2_HUMAN
Stress-70 protein, mitochondrial	GRP75_HUMAN
Zinc finger protein 148	ZN148_HUMAN
Dehydrogenase/reductase SDR family member 7	DHRS7_HUMAN
Jupiter microtubule associated homolog 2	JUPI2_HUMAN
Ras GTPase-activating-like protein IQGAP3	IQGA3_HUMAN
VIP36-like protein	LMA2L_HUMAN
ATP-dependent RNA helicase DHX30	DHX30_HUMAN
Vasodilator-stimulated phosphoprotein	VASP_HUMAN
General transcription factor 3C polypeptide 1	TF3C1_HUMAN
Bleomycin hydrolase	BLMH_HUMAN
Mediator of RNA polymerase II transcription subunit 27	MED27_HUMAN
Heat shock 70 kDa protein 4L	HS74L_HUMAN
Lysine-specific demethylase 6A	KDM6A_HUMAN
Retinitis pigmentosa 9 protein	RP9_HUMAN
Mitochondrial amidoxime reducing component 2	MARC2_HUMAN
Coiled-coil domain-containing protein 124	CC124_HUMAN

ATP-dependent zinc metalloprotease YME1L1	YMEL1_HUMAN
MICAL-like protein 1	MILK1_HUMAN
HAUS augmin-like complex subunit 5	HAUS5_HUMAN
Sorting nexin-15	SNX15_HUMAN
Transcriptional repressor p66-beta	P66B_HUMAN
Alpha-1,2-mannosyltransferase ALG9	ALG9_HUMAN
ATP-binding cassette sub-family E member 1	ABCE1_HUMAN
Cystathionine beta-synthase	CBS_HUMAN
Pogo transposable element with ZNF domain	POGZ_HUMAN
Guanine nucleotide-binding protein-like 3	GNL3_HUMAN
Pyruvate dehydrogenase protein X component, mitochondrial	ODPX_HUMAN
Cyclin-K	CCNK_HUMAN
DNA-directed RNA polymerase II subunit RPB7	RPB7_HUMAN
Nuclear pore complex protein Nup88	NUP88_HUMAN
Serine hydrolase RBBP9	RBBP9_HUMAN
Cytoplasmic dynein 1 intermediate chain 2	DC1I2_HUMAN
Small ribosomal subunit protein mS39	PTCD3_HUMAN
Lipid droplet-associated hydrolase	LDAH_HUMAN
Capping protein-inhibiting regulator of actin dynamics	CRACD_HUMAN
Isochorismatase domain-containing protein 1	ISOC1_HUMAN
Tubulin-folding cofactor B	TBCB_HUMAN
PRA1 family protein 3	PRAF3_HUMAN
Small ribosomal subunit protein uS12	RS23_HUMAN
Small nuclear ribonucleoprotein F	RUXF_HUMAN
Tryptophan--tRNA ligase, mitochondrial	SYWM_HUMAN
Protein tyrosine phosphatase type IVA 1	TP4A1_HUMAN
F-box only protein 21	FBX21_HUMAN
Type 1 phosphatidylinositol 4,5-bisphosphate 4-phosphatase	PP4P1_HUMAN
m-AAA protease-interacting protein 1, mitochondrial	MAIP1_HUMAN
Coiled-coil domain-containing protein 43	CCD43_HUMAN
Neuroplastin	NPTN_HUMAN
Cyclin-dependent kinase 6	CDK6_HUMAN
Tetraspanin-31	TSN31_HUMAN
1-phosphatidylinositol 4,5-bisphosphate phosphodiesterase gamma-1	PLCG1_HUMAN
NEDD4-binding protein 3	N4BP3_HUMAN
Death-associated protein kinase 3	DAPK3_HUMAN
BET1-like protein	BET1L_HUMAN
Oxysterol-binding protein-related protein 9	OSBL9_HUMAN
DENN domain-containing protein 11	DEN11_HUMAN
Superkiller complex protein 8	SKI8_HUMAN
DnaJ homolog subfamily A member 2	DNJA2_HUMAN
Angio-associated migratory cell protein	AAMP_HUMAN
Ran-binding protein 6	RNBP6_HUMAN
RNA-binding protein 4B	RBM4B_HUMAN
Galactosylgalactosylxylosylprotein 3-beta-glucuronosyltransferase 3	B3GA3_HUMAN
Complex III assembly factor LYRM7	LYRM7_HUMAN
Gem-associated protein 5	GEMI5_HUMAN
E3 ubiquitin-protein ligase KCMF1	KCMF1_HUMAN
Tetratricopeptide repeat protein 38	TTC38_HUMAN
Thioredoxin reductase-like selenoprotein T	SELT_HUMAN

Aminoacyl tRNA synthase complex-interacting multifunctional protein 2	AIMP2_HUMAN
Protein Churchill	CHUR_HUMAN
Golgin subfamily A member 5	GOGA5_HUMAN
Enoyl-CoA delta isomerase 1, mitochondrial	ECI1_HUMAN
Glycylpeptide N-tetradecanoyltransferase 2	NMT2_HUMAN
ARF GTPase-activating protein GIT1	GIT1_HUMAN
Ubiquitin-conjugating enzyme E2 variant 3	UEVLD_HUMAN
Golgi-specific brefeldin A-resistance guanine nucleotide exchange factor 1	GBF1_HUMAN
NADH dehydrogenase [ubiquinone] 1 beta subcomplex subunit 6	NDUB6_HUMAN
Mesencephalic astrocyte-derived neurotrophic factor	MANF_HUMAN
Tubulin alpha-4A chain	TBA4A_HUMAN
Sacsin	SACS_HUMAN
COMM domain-containing protein 4	COMD4_HUMAN
Sperm-associated antigen 1	SPAG1_HUMAN
Acylamino-acid-releasing enzyme	ACPH_HUMAN
Leucine-rich repeat and calponin homology domain-containing protein 4	LRCH4_HUMAN
F-box only protein 22	FBX22_HUMAN
Splicing factor 3B subunit 1	SF3B1_HUMAN
Nucleus accumbens-associated protein 1	NACC1_HUMAN
Large ribosomal subunit protein mL53	RM53_HUMAN
Sperm-associated antigen 7	SPAG7_HUMAN
Hydroxymethylglutaryl-CoA synthase, cytoplasmic	HMCS1_HUMAN
WD repeat-containing protein 26	WDR26_HUMAN
Membrane-associated progesterone receptor component 1	PGRC1_HUMAN
Tumor protein p53-inducible protein 11	P5I11_HUMAN
Large ribosomal subunit protein eL38	RL38_HUMAN
RNA-binding protein 10	RBM10_HUMAN
Teneurin-3	TEN3_HUMAN
WW domain-binding protein 11	WBP11_HUMAN
RNA ligase 1	RLIG1_HUMAN
WD repeat-containing protein 44	WDR44_HUMAN
Tapasin	TPSN_HUMAN
WD repeat-containing protein 7	WDR7_HUMAN
4-trimethylaminobutyraldehyde dehydrogenase	AL9A1_HUMAN
26S proteasome non-ATPase regulatory subunit 14	PSDE_HUMAN
Collagen alpha-1(VI) chain	CO6A1_HUMAN
Anoctamin-6	ANO6_HUMAN
Vitamin K-dependent gamma-carboxylase	VKGC_HUMAN
OCIA domain-containing protein 1	OCAD1_HUMAN
Exosome complex component CSL4	EXOS1_HUMAN
Peptidyl-prolyl cis-trans isomerase FKBP3	FKBP3_HUMAN
YTH domain-containing family protein 1	YTHD1_HUMAN
Protein adenylyltransferase SelO, mitochondrial	SELO_HUMAN
Large ribosomal subunit protein bL20m	RM20_HUMAN
Acetyl-CoA acetyltransferase, cytosolic	THIC_HUMAN
Chromatin accessibility complex protein 1	CHRC1_HUMAN
Protein YIPF6	YIPF6_HUMAN
Transcription factor JunD	JUND_HUMAN
Ras-related protein Rab-35	RAB35_HUMAN
Solute carrier family 12 member 2	S12A2_HUMAN

Small ribosomal subunit protein eS28	RS28_HUMAN
Tubulin alpha-8 chain	TBA8_HUMAN
Protein FAM162A	F162A_HUMAN
Ribosome biogenesis protein BOP1	BOP1_HUMAN
Synergin gamma	SYNRG_HUMAN
Protein-L-isoaspartate O-methyltransferase domain-containing protein 2	PCMD2_HUMAN
Up-regulator of cell proliferation	URGCP_HUMAN
Cystatin-B	CYTB_HUMAN
Lysozyme C	LYSC_HUMAN
Extended synaptotagmin-1	ESYT1_HUMAN
Protein argonaute-2	AGO2_HUMAN
Interleukin-33	IL33_HUMAN
Corneodesmosin	CDSN_HUMAN
Bifunctional UDP-N-acetylglucosamine 2-epimerase/N-acetylmannosamine k	GLCNE_HUMAN
Calcitonin gene-related peptide type 1 receptor	CALRL_HUMAN
Spartin	SPART_HUMAN
Nuclear pore complex protein Nup93	NUP93_HUMAN
Histone-lysine N-methyltransferase SMYD3	SMYD3_HUMAN
Intersectin-2	ITSN2_HUMAN
26S proteasome non-ATPase regulatory subunit 12	PSD12_HUMAN
Protein SCO2 homolog, mitochondrial	SCO2_HUMAN
V-type proton ATPase subunit B, brain isoform	VATB2_HUMAN
Rho GTPase-activating protein 5	RHG05_HUMAN
Splicing regulator SDE2	SDE2_HUMAN
Secretory carrier-associated membrane protein 4	SCAM4_HUMAN
Neurabin-2	NEB2_HUMAN
Syntaxin-18	STX18_HUMAN
Nesprin-1	SYNE1_HUMAN
SR-related and CTD-associated factor 8	SCAF8_HUMAN
RB1-inducible coiled-coil protein 1	RBCC1_HUMAN
RuvB-like 2	RUVB2_HUMAN
Branched-chain-amino-acid aminotransferase, cytosolic	BCAT1_HUMAN
Amyloid-beta A4 precursor protein-binding family A member 3	APBA3_HUMAN
Myosin-10	MYH10_HUMAN
SUMO-conjugating enzyme UBC9	UBC9_HUMAN
Brain acid soluble protein 1	BASP1_HUMAN
Constitutive coactivator of peroxisome proliferator-activated receptor gamm	F120B_HUMAN
BTB/POZ domain-containing protein 2	BTBD2_HUMAN
ATP-binding cassette sub-family B member 6	ABCB6_HUMAN
Syntaxin-binding protein 4	STXB4_HUMAN
Metaxin-1	MTX1_HUMAN
Exonuclease 3'-5' domain-containing protein 2	EXD2_HUMAN
Rap1 GTPase-GDP dissociation stimulator 1	GDS1_HUMAN
Solute carrier family 35 member F2	S35F2_HUMAN
BLOC-1-related complex subunit 6	BORC6_HUMAN
WD repeat-containing protein 11	WDR11_HUMAN
StAR-related lipid transfer protein 13	STA13_HUMAN
Pinin	PININ_HUMAN
Actin-related protein 2/3 complex subunit 5	ARPC5_HUMAN
Heterogeneous nuclear ribonucleoprotein H	HNRH1_HUMAN

DNA repair protein complementing XP-C cells	XPC_HUMAN
Heat shock factor protein 1	HSF1_HUMAN
Glutamate--cysteine ligase catalytic subunit	GSH1_HUMAN
Transmembrane protein 201	TM201_HUMAN
Switch-associated protein 70	SWP70_HUMAN
Ubiquitin thioesterase OTUB1	OTUB1_HUMAN
Elongation factor 1-alpha 1	EF1A1_HUMAN
Transcription termination factor 2	TTF2_HUMAN
Polypeptide N-acetylgalactosaminyltransferase 2	GALT2_HUMAN
Transcription elongation factor A protein-like 4	TCAL4_HUMAN
60S ribosome subunit biogenesis protein NIP7 homolog	NIP7_HUMAN
Elongator complex protein 4	ELP4_HUMAN
Serine/threonine-protein kinase SIK3	SIK3_HUMAN
Transforming growth factor-beta receptor-associated protein 1	TGFA1_HUMAN
G patch domain-containing protein 8	GPTC8_HUMAN
Moesin	MOES_HUMAN
CD2 antigen cytoplasmic tail-binding protein 2	CD2B2_HUMAN
Kinesin-associated protein 3	KIFA3_HUMAN
Lysosomal alpha-mannosidase	MA2B1_HUMAN
Mismatch repair endonuclease PMS2	PMS2_HUMAN
Histone H3.1	H31_HUMAN
Protein S100-A16	S10AG_HUMAN
Pleckstrin homology domain-containing family A member 1	PKHA1_HUMAN
Inactive rhomboid protein 1	RHDF1_HUMAN
Arf-GAP domain and FG repeat-containing protein 1	AGFG1_HUMAN
Transmembrane protein 41B	TM41B_HUMAN
BOS complex subunit NCLN	NCLN_HUMAN
Clathrin interactor 1	EPN4_HUMAN
Rab GTPase-activating protein 1-like	RBG1L_HUMAN
Pyridoxal-dependent decarboxylase domain-containing protein 1	PDXD1_HUMAN
WD repeat-containing protein 18	WDR18_HUMAN
Zyxin	ZYX_HUMAN
Angiopoietin-1 receptor	TIE2_HUMAN
S-adenosylmethionine synthase isoform type-2	METK2_HUMAN
Adenylate kinase 2, mitochondrial	KAD2_HUMAN
ABI gene family member 3	ABI3_HUMAN
Translation initiation factor eIF2B subunit delta	EI2BD_HUMAN
Large ribosomal subunit protein bL19m	RM19_HUMAN
5-hydroxymethyl-dUMP N-hydrolase	DNPH1_HUMAN
Echinoderm microtubule-associated protein-like 2	EMAL2_HUMAN
Ribosome-recycling factor, mitochondrial	RRFM_HUMAN
PHD finger protein 6	PHF6_HUMAN
Inactive glycosyltransferase 25 family member 3	GT253_HUMAN
Cell division cycle protein 20 homolog	CDC20_HUMAN
RNA 3'-terminal phosphate cyclase-like protein	RCL1_HUMAN
Adaptin ear-binding coat-associated protein 1	NECP1_HUMAN
Active regulator of SIRT1	AROS_HUMAN
MICOS complex subunit MIC26	MIC26_HUMAN
Probable arginine--tRNA ligase, mitochondrial	SYRM_HUMAN
Terminal uridylyltransferase 7	TUT7_HUMAN

Muskelin	MKLN1_HUMAN
PDZ and LIM domain protein 5	PDLI5_HUMAN
Elongation factor-like GTPase 1	EFL1_HUMAN
Glucose-6-phosphate 1-dehydrogenase	G6PD_HUMAN
E3 ubiquitin-protein ligase TRIM21	RO52_HUMAN
GA-binding protein alpha chain	GABPA_HUMAN
R3H domain-containing protein 1	R3HD1_HUMAN
Ankyrin repeat and MYND domain-containing protein 2	ANKY2_HUMAN
WD repeat-containing protein 55	WDR55_HUMAN
Aldehyde dehydrogenase X, mitochondrial	AL1B1_HUMAN
Mitochondrial intermediate peptidase	MIPEP_HUMAN
Sodium-dependent multivitamin transporter	SC5A6_HUMAN
Protein NEDD1	NEDD1_HUMAN
La-related protein 1	LARP1_HUMAN
Adenine phosphoribosyltransferase	APT_HUMAN
Insulin-like growth factor 2 mRNA-binding protein 3	IF2B3_HUMAN
KRR1 small subunit processome component homolog	KRR1_HUMAN
RNA-binding protein 39	RBM39_HUMAN
Ras-related protein Rab-27A	RB27A_HUMAN
Protein KRI1 homolog	KRI1_HUMAN
Large ribosomal subunit protein uL11m	RM11_HUMAN
ADP/ATP translocase 3	ADT3_HUMAN
CCAAT/enhancer-binding protein zeta	CEBPZ_HUMAN
Zinc finger TRAF-type-containing protein 1	ZTRF1_HUMAN
NUAK family SNF1-like kinase 1	NUAK1_HUMAN
SRA stem-loop-interacting RNA-binding protein, mitochondrial	SLIRP_HUMAN
Collagen alpha-3(VI) chain	CO6A3_HUMAN
Aminoacylase-1	ACY1_HUMAN
[F-actin]-monooxygenase MICAL3	MICA3_HUMAN
Protein-tyrosine sulfotransferase 2	TPST2_HUMAN
Signal transducer and activator of transcription 5B	STA5B_HUMAN
Prostaglandin E synthase 3	TEBP_HUMAN
Protein LRATD2	LRAT2_HUMAN
Eukaryotic translation initiation factor 3 subunit C	EIF3C_HUMAN
Cleavage and polyadenylation specificity factor subunit 4	CPSF4_HUMAN
BH3-interacting domain death agonist	BID_HUMAN
ATP-dependent RNA helicase DDX42	DDX42_HUMAN
Nucleoside diphosphate kinase 6	NDK6_HUMAN
Histone-lysine N-methyltransferase MECOM	MECOM_HUMAN
Oxysterol-binding protein-related protein 8	OSBL8_HUMAN
Methyl-CpG-binding domain protein 3	MBD3_HUMAN
Interferon regulatory factor 3	IRF3_HUMAN
E3 ubiquitin-protein ligase AMFR	AMFR_HUMAN
Trafficking protein particle complex subunit 12	TPC12_HUMAN
Kinesin-like protein KIF1B	KIF1B_HUMAN
FAST kinase domain-containing protein 4	FAKD4_HUMAN
Non-homologous end-joining factor 1	NHEJ1_HUMAN
Inositol polyphosphate 5-phosphatase OCRL	OCRL_HUMAN
UDP-N-acetylhexosamine pyrophosphorylase-like protein 1	UAP1L_HUMAN
Uncharacterized protein CXorf38	CX038_HUMAN

BOS complex subunit TMEM147	TM147_HUMAN
Serine/arginine-rich splicing factor 9	SRSF9_HUMAN
Leucine zipper protein 1	LUZP1_HUMAN
Leucine-rich PPR motif-containing protein, mitochondrial	LPPRC_HUMAN
TAR DNA-binding protein 43	TADBP_HUMAN
Cyclin-dependent kinase 12	CDK12_HUMAN
Complement component 1 Q subcomponent-binding protein, mitochondrial	C1QBP_HUMAN
Phosphatidylinositol 4,5-bisphosphate 3-kinase catalytic subunit alpha isofor	PK3CA_HUMAN
Peroxisomal acyl-coenzyme A oxidase 3	ACOX3_HUMAN
DNA (cytosine-5)-methyltransferase 1	DNMT1_HUMAN
Lysine-specific demethylase 5C	KDM5C_HUMAN
Amyloid beta precursor like protein 2	APLP2_HUMAN
Divergent protein kinase domain 1B	DIK1B_HUMAN
Transcription and mRNA export factor ENY2	ENY2_HUMAN
Translocon-associated protein subunit alpha	SSRA_HUMAN
Peroxiredoxin-4	PRDX4_HUMAN
Casein kinase I isoform delta	KC1D_HUMAN
Adenosylhomocysteinase 3	SAHH3_HUMAN
Charged multivesicular body protein 7	CHMP7_HUMAN
Helicase-like transcription factor	HLTF_HUMAN
BTB/POZ domain-containing protein KCTD9	KCTD9_HUMAN
Protein SCO1 homolog, mitochondrial	SCO1_HUMAN
RNA polymerase-associated protein LEO1	LEO1_HUMAN
LYR motif-containing protein 4	LYRM4_HUMAN
Peroxisomal acyl-coenzyme A oxidase 1	ACOX1_HUMAN
Serine/threonine-protein kinase 25	STK25_HUMAN
CWF19-like protein 1	C19L1_HUMAN
Retinoic acid-induced protein 3	RAI3_HUMAN
Stalled ribosome sensor GCN1	GCN1_HUMAN
Nuclear cap-binding protein subunit 3	NCBP3_HUMAN
Eukaryotic translation initiation factor 4E-binding protein 1	4EBP1_HUMAN
L-aminoadipate-semialdehyde dehydrogenase-phosphopantetheinyl transfer	ADPPT_HUMAN
Multifunctional methyltransferase subunit TRM112-like protein	TR112_HUMAN
Mitochondrial disaggregase	CLPB_HUMAN
MYND-type zinc finger-containing chromatin reader ZMYND8	ZMYD8_HUMAN
Casein kinase II subunit alpha'	CSK22_HUMAN
ADP-ribosylation factor-binding protein GGA1	GGA1_HUMAN
HEAT repeat-containing protein 1	HEAT1_HUMAN
KH domain-containing RNA-binding protein QKI	QKI_HUMAN
H/ACA ribonucleoprotein complex subunit 3	NOP10_HUMAN
Armadillo repeat-containing protein 6	ARMC6_HUMAN
Inosine-5'-monophosphate dehydrogenase 2	IMDH2_HUMAN
Mitochondrial import inner membrane translocase subunit Tim23	TIM23_HUMAN
Protein phosphatase 1 regulatory inhibitor subunit 16B	PP16B_HUMAN
Peroxisomal membrane protein PEX16	PEX16_HUMAN
Kelch-like ECH-associated protein 1	KEAP1_HUMAN
Coronin-7	CORO7_HUMAN
Transducin-like enhancer protein 1	TLE1_HUMAN
Rab9 effector protein with kelch motifs	RABEK_HUMAN
DnaJ homolog subfamily C member 11	DJC11_HUMAN

ATP-binding cassette sub-family D member 1	ABCD1_HUMAN
Methyltransferase-like protein 17, mitochondrial	MET17_HUMAN
Importin-4	IPO4_HUMAN
Luc7-like protein 3	LC7L3_HUMAN
Ribonuclease P protein subunit p30	RPP30_HUMAN
Microprocessor complex subunit DGCR8	DGCR8_HUMAN
Polyadenylate-binding protein-interacting protein 1	PAIP1_HUMAN
Protein ELYS	ELYS_HUMAN
Protein Daple	DAPLE_HUMAN
Serine protease HTRA2, mitochondrial	HTRA2_HUMAN
TIP41-like protein	TIPRL_HUMAN
Coiled-coil domain-containing protein 50	CCD50_HUMAN
Presenilin-associated rhomboid-like protein, mitochondrial	PARL_HUMAN
NADH dehydrogenase [ubiquinone] iron-sulfur protein 5	NDUS5_HUMAN
Alkyldihydroxyacetonephosphate synthase, peroxisomal	ADAS_HUMAN
eEF1A lysine and N-terminal methyltransferase	EFNMT_HUMAN
Refilin-B	RFLB_HUMAN
PEST proteolytic signal-containing nuclear protein	PCNP_HUMAN
Maestro heat-like repeat-containing protein family member 1	MROH1_HUMAN
ADP-dependent glucokinase	ADPGK_HUMAN
Guided entry of tail-anchored proteins factor 1	GET1_HUMAN
Heterogeneous nuclear ribonucleoprotein A3	ROA3_HUMAN
CCA tRNA nucleotidyltransferase 1, mitochondrial	TRNT1_HUMAN
N-acetylglucosamine-6-phosphate deacetylase	NAGA_HUMAN
Keratin, type I cytoskeletal 18	K1C18_HUMAN
Tumor protein D54	TPD54_HUMAN
RNA-binding protein 33	RBM33_HUMAN
Putative pre-mRNA-splicing factor ATP-dependent RNA helicase DHX32	DHX32_HUMAN
C-type lectin domain family 2 member B	CLC2B_HUMAN
ATPase MORC2	MORC2_HUMAN
Nonsense-mediated mRNA decay factor SMG8	SMG8_HUMAN
Elongin-B	ELOB_HUMAN
Small ribosomal subunit protein RACK1	RACK1_HUMAN
Beta-2-syntrophin	SNTB2_HUMAN
THO complex subunit 2	THOC2_HUMAN
Phospholipid-transporting ATPase IF	AT11B_HUMAN
Histone deacetylase 4	HDAC4_HUMAN
DNA polymerase epsilon catalytic subunit A	DPOE1_HUMAN
MAX gene-associated protein	MGAP_HUMAN
Zinc transporter ZIP10	S39AA_HUMAN
Nucleolar protein 9	NOP9_HUMAN
Large ribosomal subunit protein eL18	RL18_HUMAN
SR-related and CTD-associated factor 4	SCAF4_HUMAN
Cyclin-dependent kinase 11B	CD11B_HUMAN
Aldo-keto reductase family 1 member C3	AK1C3_HUMAN
Stromal interaction molecule 1	STIM1_HUMAN
Dihydropyrimidinase-related protein 4	DPYL4_HUMAN
Forkhead box protein K1	FOXK1_HUMAN
MICOS complex subunit MIC19	MIC19_HUMAN
Endoplasmic reticulum mannosyl-oligosaccharide 1,2-alpha-mannosidase	MA1B1_HUMAN

Serine/threonine-protein kinase 26	STK26_HUMAN
Ethanolaminephosphotransferase 1	EPT1_HUMAN
Pre-mRNA-splicing factor SLU7	SLU7_HUMAN
Major facilitator superfamily domain-containing protein 8	MFSD8_HUMAN
Zinc finger CCCH domain-containing protein 7B	Z3H7B_HUMAN
Tumor necrosis factor alpha-induced protein 2	TNAP2_HUMAN
Small ribosomal subunit protein uS19	RS15_HUMAN
General transcription factor IIH subunit 4	TF2H4_HUMAN
Far upstream element-binding protein 2	FUBP2_HUMAN
Extracellular glycoprotein lacritin	LACRT_HUMAN
BOS complex subunit NOMO1	NOMO1_HUMAN
E3 ubiquitin-protein ligase CHIP	CHIP_HUMAN
RNA-binding protein FUS	FUS_HUMAN
Origin recognition complex subunit 3	ORC3_HUMAN
Exportin-7	XPO7_HUMAN
Metastasis-associated protein MTA2	MTA2_HUMAN
Dedicator of cytokinesis protein 7	DOCK7_HUMAN
Guanine nucleotide exchange factor VAV2	VAV2_HUMAN
Fumarylacetoacetase	FAAA_HUMAN
Conserved oligomeric Golgi complex subunit 2	COG2_HUMAN
Non-histone chromosomal protein HMG-14	HMGN1_HUMAN
Cell division control protein 42 homolog	CDC42_HUMAN
Protein strawberry notch homolog 1	SBNO1_HUMAN
Large ribosomal subunit protein eL30	RL30_HUMAN
Alpha-(1,6)-fucosyltransferase	FUT8_HUMAN
Mitochondrial genome maintenance exonuclease 1	MGME1_HUMAN
Large ribosomal subunit protein uL13m	RM13_HUMAN
Ras-related protein Rab-4A	RAB4A_HUMAN
Protein FAN	FAN_HUMAN
Inner centromere protein	INCE_HUMAN
Glutathione S-transferase kappa 1	GSTK1_HUMAN
Methylthioribulose-1-phosphate dehydratase	MTNB_HUMAN
NADH-ubiquinone oxidoreductase chain 1	NU1M_HUMAN
Trafficking protein particle complex subunit 11	TPC11_HUMAN
O-phosphoseryl-tRNA(Sec) selenium transferase	SPCS_HUMAN
Ventricular zone-expressed PH domain-containing protein homolog 1	MELT_HUMAN
ATP-dependent DNA helicase Q5	RECQ5_HUMAN
Sigma intracellular receptor 2	SGMR2_HUMAN
Sphingolipid delta(4)-desaturase DES1	DEGS1_HUMAN
26S proteasome non-ATPase regulatory subunit 9	PSMD9_HUMAN
Endoplasmic reticulum chaperone BiP	BIP_HUMAN
Tubulin-specific chaperone E	TBCE_HUMAN
Alpha-tubulin N-acetyltransferase 1	ATAT_HUMAN
ATPase family gene 2 protein homolog B	AFG2B_HUMAN
Stimulator of interferon genes protein	STING_HUMAN
Cysteine desulfurase	NFS1_HUMAN
Mannosyl-oligosaccharide 1,2-alpha-mannosidase IB	MA1A2_HUMAN
Integrator complex subunit 12	INT12_HUMAN
Myosin phosphatase Rho-interacting protein	MPRIP_HUMAN
BRCA2 and CDKN1A-interacting protein	BCCIP_HUMAN

Kinesin-like protein KIF23	KIF23_HUMAN
Guanine nucleotide-binding protein G(s) subunit alpha isoforms short	GNAS2_HUMAN
Anaphase-promoting complex subunit 2	ANC2_HUMAN
Solute carrier family 25 member 32	S2532_HUMAN
Lysosomal thioesterase PPT2	PPT2_HUMAN
ATP-dependent Clp protease proteolytic subunit, mitochondrial	CLPP_HUMAN
Long-chain-fatty-acid--CoA ligase 4	ACSL4_HUMAN
Core histone macro-H2A.1	H2AY_HUMAN
IQ motif and SEC7 domain-containing protein 1	IQEC1_HUMAN
4'-phosphopantetheine phosphatase	PANK4_HUMAN
Kinesin light chain 1	KLC1_HUMAN
Carbonic anhydrase 13	CAH13_HUMAN
Cellular tumor antigen p53	P53_HUMAN
Ubiquitin carboxyl-terminal hydrolase 28	UBP28_HUMAN
Rac GTPase-activating protein 1	RGAP1_HUMAN
Histone deacetylase 3	HDAC3_HUMAN
Actin-related protein 2/3 complex subunit 3	ARPC3_HUMAN
Arf-GAP with coiled-coil, ANK repeat and PH domain-containing protein 2	ACAP2_HUMAN
Regulator of microtubule dynamics protein 3	RMD3_HUMAN
Protein diaphanous homolog 3	DIAP3_HUMAN
Actin-related protein 2/3 complex subunit 5-like protein	ARP5L_HUMAN
PHD finger protein 3	PHF3_HUMAN
Ubiquitin-conjugating enzyme E2 variant 2	UB2V2_HUMAN
Spermatogenesis-defective protein 39 homolog	SPE39_HUMAN
UbiA prenyltransferase domain-containing protein 1	UBIA1_HUMAN
E3 ubiquitin-protein ligase PPP1R11	PP1RB_HUMAN
Small ribosomal subunit protein uS10m	RT10_HUMAN
T-complex protein 1 subunit zeta	TCPZ_HUMAN
Rho-related GTP-binding protein RhoB	RHOB_HUMAN
Tumor necrosis factor receptor type 1-associated DEATH domain protein	TRADD_HUMAN
Polyadenylate-binding protein 2	PABP2_HUMAN
TBC1 domain family member 2B	TBD2B_HUMAN
Citrate synthase, mitochondrial	CISY_HUMAN
DNA repair protein complementing XP-A cells	XPA_HUMAN
Protein S100-A10	S10AA_HUMAN
Vacuolar protein sorting-associated protein 33A	VP33A_HUMAN
F-box only protein 7	FBX7_HUMAN
Eukaryotic translation initiation factor 4 gamma 1	IF4G1_HUMAN
Serine/threonine-protein kinase SMG1	SMG1_HUMAN
Arginine and glutamate-rich protein 1	ARGL1_HUMAN
Leucine-rich repeat-containing protein 57	LRC57_HUMAN
Nucleolar RNA helicase 2	DDX21_HUMAN
Methionine adenosyltransferase 2 subunit beta	MAT2B_HUMAN
Ras GTPase-activating protein-binding protein 2	G3BP2_HUMAN
Katanin p80 WD40 repeat-containing subunit B1	KTNB1_HUMAN
Translocation protein SEC62	SEC62_HUMAN
Kinesin-like protein KIF3B	KIF3B_HUMAN
2-(3-amino-3-carboxypropyl)histidine synthase subunit 2	DPH2_HUMAN
Adhesion G protein-coupled receptor F5	AGRF5_HUMAN
Nuclear cap-binding protein subunit 2	NCBP2_HUMAN

Spermatogenesis-associated serine-rich protein 2	SPAS2_HUMAN
Small ribosomal subunit protein mS34	RT34_HUMAN
N6-adenosine-methyltransferase non-catalytic subunit	MET14_HUMAN
Mitochondrial adenyl nucleotide antiporter SLC25A24	SCMC1_HUMAN
H(+)/Cl(-) exchange transporter 7	CLCN7_HUMAN
Small ribosomal subunit protein mS22	RT22_HUMAN
Prolyl 4-hydroxylase subunit alpha-1	P4HA1_HUMAN
Chondroitin sulfate glucuronyltransferase	CHPF2_HUMAN
Histone deacetylase complex subunit SAP18	SAP18_HUMAN
Exosome complex exonuclease RRP44	RRP44_HUMAN
Mitochondrial inner membrane protein OXA1L	OXA1L_HUMAN
CD151 antigen	CD151_HUMAN
Chloride channel CLIC-like protein 1	CLCC1_HUMAN
Neurofibromin	NF1_HUMAN
Putative monooxygenase p33MONOX	P33MX_HUMAN
RNA-binding protein 15	RBM15_HUMAN
GTPase Era, mitochondrial	ERAL1_HUMAN
Apolipoprotein B-100	APOB_HUMAN
Protein S100-A11	S10AB_HUMAN
mRNA decay activator protein ZFP36L1	TISB_HUMAN
Alpha-1,3/1,6-mannosyltransferase ALG2	ALG2_HUMAN
Beta-arrestin-1	ARRB1_HUMAN
Cyclin-Y-like protein 1	CCYL1_HUMAN
Tetratricopeptide repeat protein 13	TTC13_HUMAN
D-aminoacyl-tRNA deacylase 1	DTD1_HUMAN
Galactokinase	GALK1_HUMAN
Palmitoyl-protein thioesterase 1	PPT1_HUMAN
Protein MIX23	MIX23_HUMAN
Splicing factor 3A subunit 3	SF3A3_HUMAN
NADH-ubiquinone oxidoreductase chain 5	NU5M_HUMAN
Dynactin subunit 6	DCTN6_HUMAN
General transcription factor IIH subunit 1	TF2H1_HUMAN
Tubulin beta-6 chain	TBB6_HUMAN
DNA mismatch repair protein Msh3	MSH3_HUMAN
Cytochrome c oxidase assembly factor 3 homolog, mitochondrial	COA3_HUMAN
Ubiquitin-conjugating enzyme E2 S	UBE2S_HUMAN
Solute carrier family 2, facilitated glucose transporter member 6	GTR6_HUMAN
Glycosaminoglycan xylosylkinase	XYLK_HUMAN
Tubulin beta-4B chain	TBB4B_HUMAN
ADP-ribosylation factor-like protein 2-binding protein	AR2BP_HUMAN
Transcriptional repressor NF-X1	NFX1_HUMAN
Proteasome activator complex subunit 3	PSME3_HUMAN
Calcyclin-binding protein	CYBP_HUMAN
Carbonyl reductase [NADPH] 1	CBR1_HUMAN
Protein SGT1 homolog	SGT1_HUMAN
Nucleolysin TIAR	TIAR_HUMAN
Transcription factor 12	HTF4_HUMAN
Cytochrome b5 type B	CYB5B_HUMAN
Protein wntless homolog	WLS_HUMAN
Nucleolar protein 10	NOL10_HUMAN

Transient receptor potential cation channel subfamily V member 2	TRPV2_HUMAN
Heterogeneous nuclear ribonucleoprotein L	HNRPL_HUMAN
Sorting nexin-9	SNX9_HUMAN
Membrane-associated progesterone receptor component 2	PGRC2_HUMAN
Caskin-2	CSKI2_HUMAN
Hedgehog-interacting protein	HHIP_HUMAN
Bifunctional 3'-phosphoadenosine 5'-phosphosulfate synthase 2	PAPS2_HUMAN
Synaptogyrin-2	SNG2_HUMAN
Neuroblast differentiation-associated protein AHNAK	AHNK_HUMAN
Large ribosomal subunit protein mL39	RM39_HUMAN
Condensin-2 complex subunit G2	CNDG2_HUMAN
Cation-dependent mannose-6-phosphate receptor	MPRD_HUMAN
YEATS domain-containing protein 2	YETS2_HUMAN
Phospholipase DDHD2	DDHD2_HUMAN
ADP-ribosylation factor GTPase-activating protein 3	ARFG3_HUMAN
Ubiquitin-like domain-containing CTD phosphatase 1	UBCP1_HUMAN
Integrin alpha-6	ITA6_HUMAN
Ecotropic viral integration site 5 protein homolog	EVI5_HUMAN
Ras association domain-containing protein 8	RASF8_HUMAN
Mini-chromosome maintenance complex-binding protein	MCMBP_HUMAN
Protein S100-A7	S10A7_HUMAN
U6 snRNA-associated Sm-like protein LSm8	LSM8_HUMAN
Zinc finger protein 592	ZN592_HUMAN
Mitogen-activated protein kinase 14	MK14_HUMAN
Zinc finger C3HC-type protein 1	ZC3C1_HUMAN
Mitochondrial carnitine/acylcarnitine carrier protein	MCAT_HUMAN
Histone H2A type 1	H2A1_HUMAN
MIT domain-containing protein 1	MITD1_HUMAN
Ataxin-10	ATX10_HUMAN
Actin-binding LIM protein 3	ABLM3_HUMAN
Prolyl 3-hydroxylase OGFOD1	OGFD1_HUMAN
CD99 antigen	CD99_HUMAN
Histone H4	H4_HUMAN
Latent-transforming growth factor beta-binding protein 2	LTBP2_HUMAN
Eukaryotic translation initiation factor 3 subunit I	EIF3I_HUMAN
NADH-ubiquinone oxidoreductase chain 4	NU4M_HUMAN
Leukocyte receptor cluster member 8	LENG8_HUMAN
Protein AHNAK2	AHNK2_HUMAN
Myeloid differentiation primary response protein MyD88	MYD88_HUMAN
Transcription factor E2F4	E2F4_HUMAN
Suppressor of tumorigenicity 7 protein	ST7_HUMAN
Charged multivesicular body protein 3	CHMP3_HUMAN
ZZ-type zinc finger-containing protein 3	ZZZ3_HUMAN
Protein transport protein Sec31A	SC31A_HUMAN
Mediator of RNA polymerase II transcription subunit 24	MED24_HUMAN
CKLF-like MARVEL transmembrane domain-containing protein 6	CKLF6_HUMAN
CDP-diacylglycerol--glycerol-3-phosphate 3-phosphatidyltransferase, mitocho	PGPS1_HUMAN
182 kDa tankyrase-1-binding protein	TB182_HUMAN
Eukaryotic translation initiation factor 4B	IF4B_HUMAN
Cap-specific mRNA (nucleoside-2'-O-)-methyltransferase 1	CMTR1_HUMAN

Vacuolar protein sorting-associated protein 53 homolog	VPS53_HUMAN
Tyrosine-protein kinase CSK	CSK_HUMAN
ATP-dependent RNA helicase DDX39A	DX39A_HUMAN
Tudor domain-containing protein 3	TDRD3_HUMAN
Solute carrier family 52, riboflavin transporter, member 2	S52A2_HUMAN
Ubiquitin-conjugating enzyme E2 T	UBE2T_HUMAN
Protein SDA1 homolog	SDA1_HUMAN
Protein spire homolog 1	SPIR1_HUMAN
Pre-mRNA-processing-splicing factor 8	PRP8_HUMAN
Golgin subfamily A member 3	GOGA3_HUMAN
Zinc finger protein 512B	Z512B_HUMAN
E3 ubiquitin-protein ligase UBR3	UBR3_HUMAN
2-methoxy-6-polyprenyl-1,4-benzoquinol methylase, mitochondrial	COQ5_HUMAN
Nucleolar protein 58	NOP58_HUMAN
Proline and serine-rich protein 2	PRSR2_HUMAN
Acylphosphatase-1	ACYP1_HUMAN
Small ribosomal subunit protein eS6	RS6_HUMAN
Splicing factor U2AF 65 kDa subunit	U2AF2_HUMAN
RNA-binding protein MEX3D	MEX3D_HUMAN
Alpha-ketoglutarate-dependent dioxygenase alkB homolog 3	ALKB3_HUMAN
Proliferating cell nuclear antigen	PCNA_HUMAN
Glycerol-3-phosphate acyltransferase 3	GPAT3_HUMAN
Endothelial differentiation-related factor 1	EDF1_HUMAN
Actin-related protein 5	ARP5_HUMAN
Plexin-A2	PLXA2_HUMAN
Pre-mRNA-splicing factor RBM22	RBM22_HUMAN
Probable ATP-dependent RNA helicase DHX37	DHX37_HUMAN
Pseudouridine-5'-phosphatase	HDHD1_HUMAN
Protein RFT1 homolog	RFT1_HUMAN
Protein TFG	TFG_HUMAN
Beta-1,4-galactosyltransferase 1	B4GT1_HUMAN
Zinc finger C2HC domain-containing protein 1A	ZC21A_HUMAN
Endoplasmic reticulum junction formation protein lunapark	LNP_HUMAN
Guanine nucleotide-binding protein G(i) subunit alpha-3	GNAI3_HUMAN
Protein S100-A8	S10A8_HUMAN
Serine/threonine-protein kinase Chk1	CHK1_HUMAN
T-complex protein 1 subunit gamma	TCPG_HUMAN
Smoothelin	SMTN_HUMAN
Glutamyl-tRNA(Gln) amidotransferase subunit A, mitochondrial	GATA_HUMAN
Calpain-7	CAN7_HUMAN
Signal transducer and activator of transcription 2	STAT2_HUMAN
[F-actin]-monooxygenase MICAL1	MICA1_HUMAN
Prohibitin-2	PHB2_HUMAN
ATP-dependent RNA helicase DDX18	DDX18_HUMAN
Caspase-14	CASPE_HUMAN
Gap junction alpha-1 protein	CXA1_HUMAN
F-BAR domain only protein 2	FCHO2_HUMAN
Eukaryotic peptide chain release factor GTP-binding subunit ERF3B	ERF3B_HUMAN
Probable E3 ubiquitin-protein ligase HECTD4	HECD4_HUMAN
ATP-dependent DNA/RNA helicase DHX36	DHX36_HUMAN

U6 snRNA-associated Sm-like protein LSm3	LSM3_HUMAN
Tyrosine-protein kinase Yes	YES_HUMAN
Gamma-soluble NSF attachment protein	SNAG_HUMAN
Spindlin-1	SPIN1_HUMAN
Adenylyl cyclase-associated protein 1	CAP1_HUMAN
Calpastatin	ICAL_HUMAN
Histone deacetylase 2	HDAC2_HUMAN
Derlin-1	DERL1_HUMAN
Calmodulin-like protein 5	CALL5_HUMAN
Large ribosomal subunit protein uL30m	RM30_HUMAN
Secernin-1	SCRN1_HUMAN
Glypican-1	GPC1_HUMAN
F-box only protein 38	FBX38_HUMAN
Chromodomain-helicase-DNA-binding protein 1	CHD1_HUMAN
E3 ubiquitin-protein ligase RBX1	RBX1_HUMAN
Transmembrane protein 115	TM115_HUMAN
T-complex protein 1 subunit delta	TCPD_HUMAN
Myotubularin-related protein 1	MTMR1_HUMAN
IST1 homolog	IST1_HUMAN
Guanine nucleotide-binding protein G(i) subunit alpha-1	GNAI1_HUMAN
WD repeat-containing protein 47	WDR47_HUMAN
NSFL1 cofactor p47	NSF1C_HUMAN
V-type proton ATPase subunit C 1	VATC1_HUMAN
Ras-related protein Rab-2B	RAB2B_HUMAN
SWI/SNF-related matrix-associated actin-dependent regulator of chromatin s	SMAL1_HUMAN
Ubiquitin-like FUBI-ribosomal protein eS30 fusion protein	RS30_HUMAN
C-Maf-inducing protein	CMIP_HUMAN
Small glutamine-rich tetratricopeptide repeat-containing protein alpha	SGTA_HUMAN
E3 ubiquitin-protein ligase UBR2	UBR2_HUMAN
Ribitol 5-phosphate transferase FKRP	FKRP_HUMAN
Nuclear exosome regulator NRDE2	NRDE2_HUMAN
Lethal(2) giant larvae protein homolog 1	L2GL1_HUMAN
Transforming growth factor beta-1-induced transcript 1 protein	TGFI1_HUMAN
RNA-binding protein 12B	RB12B_HUMAN
Zinc finger protein 24	ZNF24_HUMAN
ATP synthase subunit ATP5MJ, mitochondrial	ATP68_HUMAN
General transcription factor IIH subunit 2	TF2H2_HUMAN
N-alpha-acetyltransferase 30	NAA30_HUMAN
Trinucleotide repeat-containing gene 6B protein	TNR6B_HUMAN
SH3 domain-containing kinase-binding protein 1	SH3K1_HUMAN
Ras-related protein Rab-13	RAB13_HUMAN
Histone-binding protein RBBP4	RBBP4_HUMAN
Pre-mRNA-splicing factor 18	PRP18_HUMAN
Methionine synthase	METH_HUMAN
OTU domain-containing protein 4	OTUD4_HUMAN
Dynein axonemal assembly factor 10	DAA10_HUMAN
3'(2'),5'-bisphosphate nucleotidase 1	BPNT1_HUMAN
rRNA-processing protein FCF1 homolog	FCF1_HUMAN
Galectin-9	LEG9_HUMAN
Metalloreductase STEAP3	STEA3_HUMAN

6-pyruvoyl tetrahydrobiopterin synthase	PTPS_HUMAN
Transcriptional repressor p66-alpha	P66A_HUMAN
Golgi-associated PDZ and coiled-coil motif-containing protein	GOPC_HUMAN
Arf-GAP with SH3 domain, ANK repeat and PH domain-containing protein 1	ASAP1_HUMAN
Large ribosomal subunit protein eL24	RL24_HUMAN
Syndetin	VPS50_HUMAN
Mitochondrial import receptor subunit TOM40 homolog	TOM40_HUMAN
Histone H2A type 1-B/E	H2A1B_HUMAN
Sodium/hydrogen exchanger 1	SL9A1_HUMAN
CTTNBP2 N-terminal-like protein	CT2NL_HUMAN
COUP transcription factor 2	COT2_HUMAN
Kelch domain-containing protein 4	KLDC4_HUMAN
Large ribosomal subunit protein uL22	RL17_HUMAN
Tetraspanin-4	TSN4_HUMAN
Vam6/Vps39-like protein	VPS39_HUMAN
Mitogen-activated protein kinase kinase kinase 20	M3K20_HUMAN
Proteinase-activated receptor 1	PAR1_HUMAN
Growth factor receptor-bound protein 10	GRB10_HUMAN
Guanine nucleotide-binding protein G(I)/G(S)/G(O) subunit gamma-5	GBG5_HUMAN
Serine/threonine-protein kinase MRCK alpha	MRCKA_HUMAN
Probable 28S rRNA (cytosine(4447)-C(5))-methyltransferase	NOP2_HUMAN
PMS1 protein homolog 1	PMS1_HUMAN
Protection of telomeres protein 1	POTE1_HUMAN
TNF receptor-associated factor 6	TRAF6_HUMAN
CCR4-NOT transcription complex subunit 2	CNOT2_HUMAN
Microtubule-actin cross-linking factor 1, isoforms 1/2/3/4/5	MACF1_HUMAN
Cytosolic arginine sensor for mTORC1 subunit 2	CAST2_HUMAN
Kinetochore-associated protein 1	KNTC1_HUMAN
Serine/threonine-protein kinase VRK1	VRK1_HUMAN
Nucleosome assembly protein 1-like 1	NP1L1_HUMAN
Serine/threonine-protein kinase Nek7	NEK7_HUMAN
Ragulator complex protein LAMTOR4	LTOR4_HUMAN
General transcription factor 3C polypeptide 2	TF3C2_HUMAN
Oxidized purine nucleoside triphosphate hydrolase	8ODP_HUMAN
Ubiquitin carboxyl-terminal hydrolase isozyme L1	UCHL1_HUMAN
Small nuclear ribonucleoprotein E	RUXE_HUMAN
Nidogen-2	NID2_HUMAN
Mitochondrial import inner membrane translocase subunit TIM16	TIM16_HUMAN
Perilipin-4	PLIN4_HUMAN
mRNA turnover protein 4 homolog	MRT4_HUMAN
TRPM8 channel-associated factor 1	TCAF1_HUMAN
Nuclear receptor-binding protein	NRBP_HUMAN
Acylpyruvase FAHD1, mitochondrial	FAHD1_HUMAN
U5 small nuclear ribonucleoprotein 200 kDa helicase	U520_HUMAN
ATP-binding cassette sub-family F member 1	ABCF1_HUMAN
Peptidylprolyl isomerase domain and WD repeat-containing protein 1	PPWD1_HUMAN
Discoidin, CUB and LCCL domain-containing protein 2	DCBD2_HUMAN
Pyrroline-5-carboxylate reductase 3	P5CR3_HUMAN
Protein lifeguard 3	LFG3_HUMAN
Protein furry homolog	FRY_HUMAN

RNA cytosine C(5)-methyltransferase NSUN2	NSUN2_HUMAN
Protein FAM3C	FAM3C_HUMAN
Major centromere autoantigen B	CENPB_HUMAN
Large ribosomal subunit protein eL42	RL36A_HUMAN
Mitochondrial ribosome-associated GTPase 1	MTG1_HUMAN
Histone H2B type 1-K	H2B1K_HUMAN
PCI domain-containing protein 2	PCID2_HUMAN
PDZ and LIM domain protein 2	PDLI2_HUMAN
G protein-regulated inducer of neurite outgrowth 1	GRIN1_HUMAN
TBC1 domain family member 20	TBC20_HUMAN
Regulatory-associated protein of mTOR	RPTOR_HUMAN
Selenide, water dikinase 2	SPS2_HUMAN
Platelet-activating factor acetylhydrolase IB subunit alpha1	PA1B3_HUMAN
Cotranscriptional regulator ARB2A	ARB2A_HUMAN
Elongation factor 1-gamma	EF1G_HUMAN
ER membrane protein complex subunit 7	EMC7_HUMAN
Plakophilin-1	PKP1_HUMAN
Translation initiation factor eIF2B subunit epsilon	EI2BE_HUMAN
NEDD8-activating enzyme E1 regulatory subunit	ULA1_HUMAN
Ras-related protein Rab-18	RAB18_HUMAN
Ribosomal protein eL22-like	RL22L_HUMAN
2-iminobutanoate/2-iminopropanoate deaminase	RIDA_HUMAN
Conserved oligomeric Golgi complex subunit 7	COG7_HUMAN
Nitric oxide-associated protein 1	NOA1_HUMAN
Brain-specific angiogenesis inhibitor 1-associated protein 2	BAIP2_HUMAN
Casein kinase II subunit alpha	CSK21_HUMAN
Protein tyrosine phosphatase type IVA 2	TP4A2_HUMAN
Lethal(3)malignant brain tumor-like protein 3	LMBL3_HUMAN
V-type proton ATPase catalytic subunit A	VATA_HUMAN
Bcl-2-like protein 1	B2CL1_HUMAN
Programmed cell death 1 ligand 1	PD1L1_HUMAN
Sodium/potassium-transporting ATPase subunit beta-1	AT1B1_HUMAN
Pyruvate dehydrogenase E1 component subunit alpha, somatic form, mitoch	ODPA_HUMAN
Tubulin beta-3 chain	TBB3_HUMAN
Transport and Golgi organization protein 1 homolog	TGO1_HUMAN
Vitamin K epoxide reductase complex subunit 1-like protein 1	VKORL_HUMAN
Nucleolar and coiled-body phosphoprotein 1	NOLC1_HUMAN
Splicing factor 1	SF01_HUMAN
Leucine-rich repeat and calponin homology domain-containing protein 1	LRCH1_HUMAN
Basigin	BASI_HUMAN
Insulin-like growth factor-binding protein 4	IBP4_HUMAN
Cysteine-rich motor neuron 1 protein	CRIM1_HUMAN
Disks large homolog 1	DLG1_HUMAN
Probable helicase senataxin	SETX_HUMAN
STING ER exit protein	STEEP_HUMAN
MORC family CW-type zinc finger protein 3	MORC3_HUMAN
Methenyltetrahydrofolate synthase domain-containing protein	MTHSD_HUMAN
26S proteasome regulatory subunit 6B	PRS6B_HUMAN
Actin-related protein 2/3 complex subunit 1A	ARC1A_HUMAN
Myristoylated alanine-rich C-kinase substrate	MARCS_HUMAN

Interferon-induced transmembrane protein 3	IFM3_HUMAN
Mitogen-activated protein kinase kinase kinase 4	M3K4_HUMAN
Myelin protein zero-like protein 1	MPZL1_HUMAN
Histone-lysine N-methyltransferase ASH1L	ASH1L_HUMAN
Tubulin--tyrosine ligase	TTL_HUMAN
Protein bicaudal D homolog 2	BICD2_HUMAN
Elongation factor Tu, mitochondrial	EFTU_HUMAN
Trafficking protein particle complex subunit 5	TPPC5_HUMAN
Lysine-specific demethylase PHF2	PHF2_HUMAN
Filamin A-interacting protein 1-like	FIL1L_HUMAN
Vacuolar protein-sorting-associated protein 36	VPS36_HUMAN
Protein disulfide isomerase CRELD1	CREL1_HUMAN
Histidine--tRNA ligase, cytoplasmic	HARS1_HUMAN
Ubiquinol-cytochrome c reductase complex assembly factor 1	UQCC1_HUMAN
Sortilin	SORT_HUMAN
Malignant T-cell-amplified sequence 1	MCTS1_HUMAN
Large ribosomal subunit protein uL13	RL13A_HUMAN
COP9 signalosome complex subunit 1	CSN1_HUMAN
Telomere length regulation protein TEL2 homolog	TELO2_HUMAN
Dolichyl-diphosphooligosaccharide--protein glycosyltransferase subunit STT3	STT3B_HUMAN
UDP-N-acetylglucosamine--peptide N-acetylglucosaminyltransferase 110 kDa	OGT1_HUMAN
Endothelial lipase	LIPG_HUMAN
MICOS complex subunit MIC25	MIC25_HUMAN
Tetraspanin-3	TSN3_HUMAN
Probable RNA polymerase II nuclear localization protein SLC7A6OS	S7A6O_HUMAN
Cyclin-A2	CCNA2_HUMAN
MOB kinase activator 3A	MOB3A_HUMAN
Protein LTV1 homolog	LTV1_HUMAN
SHC-transforming protein 1	SHC1_HUMAN
Protein FRA10AC1	F10C1_HUMAN
Malonyl-CoA-acyl carrier protein transacylase, mitochondrial	FABD_HUMAN
Protein S100-A9	S10A9_HUMAN
von Willebrand factor A domain-containing protein 5A	VMA5A_HUMAN
3-hydroxyisobutyrate dehydrogenase, mitochondrial	3HIDH_HUMAN
Zinc transporter 6	ZNT6_HUMAN
Bromodomain-containing protein 1	BRD1_HUMAN
Dystrobrevin alpha	DTNA_HUMAN
Pre-mRNA-splicing factor ISY1 homolog	ISY1_HUMAN
NHS-like protein 2	NHSL2_HUMAN
Ras-related protein Rab-9A	RAB9A_HUMAN
Splicing factor 3A subunit 1	SF3A1_HUMAN
Polyamine-transporting ATPase 13A3	AT133_HUMAN
Mortality factor 4-like protein 1	MO4L1_HUMAN
Putative lipid scramblase CLPTM1	CLPT1_HUMAN
Tyrosine-protein kinase JAK1	JAK1_HUMAN
NAD-dependent protein deacylase sirtuin-5, mitochondrial	SIR5_HUMAN
Dihydropteridine reductase	DHPR_HUMAN
Calcium-binding protein 39	CAB39_HUMAN
Translational activator of cytochrome c oxidase 1	TACO1_HUMAN
Small ribosomal subunit protein eS4, X isoform	RS4X_HUMAN

Mediator of RNA polymerase II transcription subunit 18	MED18_HUMAN
Transketolase	TKT_HUMAN
Inhibitor of nuclear factor kappa-B kinase-interacting protein	IKIP_HUMAN
Cyclin-dependent kinase 19	CDK19_HUMAN
Mitochondrial carrier homolog 2	MTCH2_HUMAN
Dual specificity protein kinase CLK3	CLK3_HUMAN
Fasciculation and elongation protein zeta-2	FEZ2_HUMAN
Protein FAM76B	FA76B_HUMAN
Proton-coupled zinc antiporter SLC30A1	ZNT1_HUMAN
Large ribosomal subunit protein uL2m	RM02_HUMAN
Microfibrillar-associated protein 1	MFAP1_HUMAN
Inactive rhomboid protein 2	RHDF2_HUMAN
Forkhead box protein P1	FOXP1_HUMAN
Synaptojanin-1	SYNJ1_HUMAN
Vesicle-associated membrane protein 5	VAMP5_HUMAN
Mitotic checkpoint serine/threonine-protein kinase BUB1 beta	BUB1B_HUMAN
Hemoglobin subunit alpha	HBA_HUMAN
S-adenosylhomocysteine hydrolase-like protein 1	SAHH2_HUMAN
Ribonuclease P protein subunit p40	RPP40_HUMAN
Ras-related protein Ral-A	RALA_HUMAN
Protein disulfide-isomerase TMX3	TMX3_HUMAN
Dedicator of cytokinesis protein 5	DOCK5_HUMAN
Set1/Ash2 histone methyltransferase complex subunit ASH2	ASH2L_HUMAN
E3 ubiquitin-protein ligase TRIM65	TRI65_HUMAN
Serine/threonine-protein phosphatase 6 regulatory ankyrin repeat subunit C	ANR52_HUMAN
Probable global transcription activator SNF2L2	SMCA2_HUMAN
Zinc finger C3H1 domain-containing protein	ZC3H1_HUMAN
General transcription factor IIE subunit 1	T2EA_HUMAN
Glutathione-specific gamma-glutamylcyclotransferase 2	CHAC2_HUMAN
Small ubiquitin-related modifier 1	SUMO1_HUMAN
C2 domain-containing protein 5	C2CD5_HUMAN
Protein FAM50A	FA50A_HUMAN
Bisphosphoglycerate mutase	PMGE_HUMAN
Microtubule-associated protein RP/EB family member 2	MARE2_HUMAN
D-glucuronyl C5-epimerase	GLCE_HUMAN
Mitochondrial potassium channel ATP-binding subunit	MITOS_HUMAN
Protein FAM234A	F234A_HUMAN
Interferon regulatory factor 2-binding protein 1	I2BP1_HUMAN
26S proteasome regulatory subunit 7	PRS7_HUMAN
Palladin	PALLD_HUMAN
DNA-directed RNA polymerases I, II, and III subunit RPABC3	RPAB3_HUMAN
Mitochondrial import receptor subunit TOM22 homolog	TOM22_HUMAN
Small ribosomal subunit protein eS24	RS24_HUMAN
Serine/threonine-protein kinase VRK2	VRK2_HUMAN
Thioredoxin domain-containing protein 15	TXD15_HUMAN
Tetratricopeptide repeat protein 4	TTC4_HUMAN
ATP-dependent RNA helicase A	DHX9_HUMAN
5'-3' exoribonuclease 1	XRN1_HUMAN
Ribosomal protein S6 kinase beta-1	KS6B1_HUMAN
tRNA 2'-phosphotransferase 1	TRPT1_HUMAN

Large ribosomal subunit protein uL5	RL11_HUMAN
Pre-mRNA-splicing factor 38A	PR38A_HUMAN
Chitinase domain-containing protein 1	CHID1_HUMAN
Histamine H1 receptor	HRH1_HUMAN
Ras-related protein Rap-1A	RAP1A_HUMAN
Cell division cycle protein 27 homolog	CDC27_HUMAN
rRNA-processing protein UTP23 homolog	UTP23_HUMAN
Coiled-coil domain-containing protein 22	CCD22_HUMAN
Phospholipid-transporting ATPase IG	AT11C_HUMAN
SH3 domain-binding protein 4	SH3B4_HUMAN
Polypeptide N-acetylgalactosaminyltransferase 6	GALT6_HUMAN
Alpha-synuclein	SYUA_HUMAN
FMR1-interacting protein NUFIP2	NUFP2_HUMAN
Cytochrome c oxidase assembly factor 6 homolog	COA6_HUMAN
Mitochondrial fission factor	MFF_HUMAN
Protein AATF	AATF_HUMAN
Hydroxyacylglutathione hydrolase, mitochondrial	GLO2_HUMAN
Optic atrophy 3 protein	OPA3_HUMAN
FACT complex subunit SPT16	SP16H_HUMAN
Small glutamine-rich tetratricopeptide repeat-containing protein beta	SGTB_HUMAN
Insulin-like growth factor 2 mRNA-binding protein 1	IF2B1_HUMAN
Glycogen synthase kinase-3 alpha	GSK3A_HUMAN
Serine/threonine-protein phosphatase 4 catalytic subunit	PP4C_HUMAN
Small ribosomal subunit protein eS21	RS21_HUMAN
Bromodomain adjacent to zinc finger domain protein 1A	BAZ1A_HUMAN
Inner nuclear membrane protein Man1	MAN1_HUMAN
Zinc transporter 7	ZNT7_HUMAN
Aprataxin	APTX_HUMAN
Dol-P-Man:Man(5)GlcNAc(2)-PP-Dol alpha-1,3-mannosyltransferase	ALG3_HUMAN
Nicotinamide/nicotinic acid mononucleotide adenylyltransferase 1	NMNA1_HUMAN
Metal cation symporter ZIP14	S39AE_HUMAN
Glutathione reductase, mitochondrial	GSHR_HUMAN
Translation factor GUF1, mitochondrial	GUF1_HUMAN
Hepatocyte growth factor-regulated tyrosine kinase substrate	HGS_HUMAN
Mitogen-activated protein kinase kinase kinase kinase 3	M4K3_HUMAN
Serine incorporator 1	SERC1_HUMAN
Guanine nucleotide-binding protein subunit alpha-12	GNA12_HUMAN
Zinc finger CCHC domain-containing protein 17	ZCC17_HUMAN
U5 small nuclear ribonucleoprotein 40 kDa protein	SNR40_HUMAN
NADH dehydrogenase [ubiquinone] 1 beta subcomplex subunit 11, mitochon	NDUBB_HUMAN
Proteasome subunit alpha type-4	PSA4_HUMAN
SWI/SNF complex subunit SMARCC1	SMRC1_HUMAN
HAUS augmin-like complex subunit 1	HAUS1_HUMAN
Iron-sulfur cluster assembly 1 homolog, mitochondrial	ISCA1_HUMAN
Periodic tryptophan protein 2 homolog	PWP2_HUMAN
Polypeptide N-acetylgalactosaminyltransferase 10	GLT10_HUMAN
Galectin-7	LEG7_HUMAN
DNA ligase 4	DNLI4_HUMAN
Eukaryotic translation initiation factor 5A-1	IF5A1_HUMAN
FACT complex subunit SSRP1	SSRP1_HUMAN

Sorting nexin-27	SNX27_HUMAN
DnaJ homolog subfamily C member 1	DNJC1_HUMAN
DNA polymerase alpha subunit B	DPOA2_HUMAN
CKLF-like MARVEL transmembrane domain-containing protein 3	CKLF3_HUMAN
Beta-parvin	PARVB_HUMAN
E3 ubiquitin-protein ligase RNF149	RN149_HUMAN
Cytochrome c oxidase assembly factor 7	COA7_HUMAN
Solute carrier family 35 member E1	S35E1_HUMAN
Cdc42 effector protein 1	BORG5_HUMAN
General transcription factor 3C polypeptide 3	TF3C3_HUMAN
GPALPP motifs-containing protein 1	GPAM1_HUMAN
Very-long-chain 3-oxoacyl-CoA reductase	DHB12_HUMAN
Neutral amino acid transporter A	SATT_HUMAN
ATP-dependent RNA helicase SUPV3L1, mitochondrial	SUV3_HUMAN
Beta-1,4-glucuronyltransferase 1	B4GA1_HUMAN
Protein ENL	ENL_HUMAN
Lysine-specific demethylase 9	RSBN1_HUMAN
Dynamin-binding protein	DNMBP_HUMAN
Protein LYRIC	LYRIC_HUMAN
Volume-regulated anion channel subunit LRRC8A	LRC8A_HUMAN
Protein CIP2A	CIP2A_HUMAN
Amyloid beta precursor protein binding family B member 2	APBB2_HUMAN
Torsin-3A	TOR3A_HUMAN
NF-kappa-B inhibitor-interacting Ras-like protein 2	KBRS2_HUMAN
Small ribosomal subunit protein bS16m	RT16_HUMAN
Histone chaperone ASF1A	ASF1A_HUMAN
Transducin-like enhancer protein 3	TLE3_HUMAN
Histone-lysine N-methyltransferase EHMT2	EHMT2_HUMAN
Ubiquitin carboxyl-terminal hydrolase 16	UBP16_HUMAN
Large ribosomal subunit protein bL17m	RM17_HUMAN
DnaJ homolog subfamily A member 4	DNJA4_HUMAN
Cell growth-regulating nucleolar protein	LYAR_HUMAN
Peptidyl-prolyl cis-trans isomerase D	PPID_HUMAN
Matrin-3	MATR3_HUMAN
SEC14 domain and spectrin repeat-containing protein 1	SESD1_HUMAN
Probable rRNA-processing protein EBP2	EBP2_HUMAN
SH2 domain-containing adapter protein E	SHE_HUMAN
Neurogranin	NEUG_HUMAN
ER membrane protein complex subunit 5	EMC5_HUMAN
Haloacid dehalogenase-like hydrolase domain-containing protein 2	HDHD2_HUMAN
Very large A-kinase anchor protein	CRBG3_HUMAN
RNA-binding protein 4	RBM4_HUMAN
A-kinase anchor protein 10, mitochondrial	AKA10_HUMAN
Mannose-1-phosphate guanyltransferase beta	GMPPB_HUMAN
Neutral cholesterol ester hydrolase 1	NCEH1_HUMAN
Serine/threonine-protein kinase 38	STK38_HUMAN
SPARC	SPRC_HUMAN
Protein sel-1 homolog 3	SE1L3_HUMAN
DDB1- and CUL4-associated factor 6	DCAF6_HUMAN
Hsp90 co-chaperone Cdc37	CDC37_HUMAN

pre-rRNA 2'-O-ribose RNA methyltransferase FTSJ3	SPB1_HUMAN
Proteasome subunit beta type-1	PSB1_HUMAN
Tight junction protein ZO-2	ZO2_HUMAN
Fatty acid desaturase 3	FADS3_HUMAN
Cartilage-associated protein	CRTAP_HUMAN
Hemoglobin subunit beta	HBB_HUMAN
Integrator complex subunit 2	INT2_HUMAN
Alpha-2-macroglobulin receptor-associated protein	AMRP_HUMAN
Vesicle-associated membrane protein 2	VAMP2_HUMAN
NADH dehydrogenase [ubiquinone] 1 alpha subcomplex subunit 6	NDUA6_HUMAN
Uridine diphosphate glucose pyrophosphatase NUDT14	NUD14_HUMAN
Urokinase plasminogen activator surface receptor	UPAR_HUMAN
Proto-oncogene tyrosine-protein kinase Src	SRC_HUMAN
Enhancer of mRNA-decapping protein 3	EDC3_HUMAN
Bromodomain-containing protein 8	BRD8_HUMAN
GTPase NRas	RASN_HUMAN
Reticulon-3	RTN3_HUMAN
Serine palmitoyltransferase 1	SPTC1_HUMAN
Ankyrin repeat domain-containing protein 17	ANR17_HUMAN
Immunoglobulin-binding protein 1	IGBP1_HUMAN
ATP-dependent RNA helicase DDX54	DDX54_HUMAN
Cyclin-dependent kinase inhibitor 1	CDN1A_HUMAN
Smad nuclear-interacting protein 1	SNIP1_HUMAN
Transmembrane emp24 domain-containing protein 7	TMED7_HUMAN
AP-4 complex subunit epsilon-1	AP4E1_HUMAN
Major facilitator superfamily domain-containing protein 1	MFSD1_HUMAN
Transmembrane protein 263	TM263_HUMAN
Chromobox protein homolog 5	CBX5_HUMAN
DnaJ homolog subfamily B member 14	DJB14_HUMAN
Nucleolar complex protein 2 homolog	NOC2L_HUMAN
Caprin-1	CAPR1_HUMAN
Cell cycle checkpoint protein RAD17	RAD17_HUMAN
Large ribosomal subunit protein eL37	RL37_HUMAN
Ras-related protein Rab-31	RAB31_HUMAN
G protein-coupled receptor kinase 5	GRK5_HUMAN
STAM-binding protein	STABP_HUMAN
Fanconi anemia group D2 protein	FACD2_HUMAN
E3 ubiquitin-protein ligase NEDD4	NEDD4_HUMAN
Transcription termination factor 3, mitochondrial	MTEF3_HUMAN
Ribonucleases P/MRP protein subunit POP1	POP1_HUMAN
Uncharacterized protein C7orf50	CG050_HUMAN
3',5'-cyclic-AMP phosphodiesterase 4D	PDE4D_HUMAN
Splicing factor 3B subunit 3	SF3B3_HUMAN
Protein DDI1 homolog 2	DDI2_HUMAN
Protein HEXIM1	HEXI1_HUMAN
Ephrin-B2	EFNB2_HUMAN
Small nuclear ribonucleoprotein-associated proteins B and B'	RSMB_HUMAN
SH2/SH3 adapter protein NCK1	NCK1_HUMAN
OCIA domain-containing protein 2	OCAD2_HUMAN
Mucolipin-1	MCLN1_HUMAN

GTP-binding protein 10	GTPBA_HUMAN
Acyl-coenzyme A diphosphatase NUDT19	NUD19_HUMAN
Inhibitor of Bruton tyrosine kinase	IBTK_HUMAN
Caldesmon	CALD1_HUMAN
Histone H2A.Z	H2AZ_HUMAN
WD repeat domain phosphoinositide-interacting protein 4	WIPI4_HUMAN
Probable glutathione peroxidase 8	GPX8_HUMAN
Protein PALS2	PALS2_HUMAN
Serine/threonine-protein kinase B-raf	BRAF_HUMAN
Protein transport protein Sec61 subunit gamma	SC61G_HUMAN
SAP domain-containing ribonucleoprotein	SARNP_HUMAN
28S rRNA (cytosine-C(5))-methyltransferase	NSUN5_HUMAN
Cytoplasmic protein NCK2	NCK2_HUMAN
Histone-lysine N-methyltransferase 2D	KMT2D_HUMAN
E3 ubiquitin-protein ligase RNF170	RN170_HUMAN
Xaa-Pro dipeptidase	PEPD_HUMAN
Fibronectin	FINC_HUMAN
Alpha-2-HS-glycoprotein	FETUA_HUMAN
CXXC motif containing zinc binding protein	CZIB_HUMAN
Aspartate aminotransferase, mitochondrial	AATM_HUMAN
Small ribosomal subunit protein uS14m	RT14_HUMAN
Volume-regulated anion channel subunit LRRC8D	LRC8D_HUMAN
E3 ubiquitin-protein ligase TRIM33	TRI33_HUMAN
Asparagine--tRNA ligase, cytoplasmic	SYNC_HUMAN
StAR-related lipid transfer protein 7, mitochondrial	STAR7_HUMAN
S-formylglutathione hydrolase	ESTD_HUMAN
Kelch domain-containing protein 3	KLDC3_HUMAN
FHF complex subunit HOOK interacting protein 2A	FHI2A_HUMAN
Alpha-1-antitrypsin	A1AT_HUMAN
Interleukin enhancer-binding factor 2	ILF2_HUMAN
Protein FAM107B	F107B_HUMAN
WW domain-binding protein 4	WBP4_HUMAN
GATOR1 complex protein NPRL3	NPRL3_HUMAN
Reticulon-4	RTN4_HUMAN
Long-chain-fatty-acid--CoA ligase 1	ACSL1_HUMAN
Proteolipid protein 2	PLP2_HUMAN
Twinfilin-2	TWF2_HUMAN
Regulator of G-protein signaling 10	RGS10_HUMAN
Golgi pH regulator A	GPHRA_HUMAN
Proteasome subunit alpha type-3	PSA3_HUMAN
Dehydrodolichyl diphosphate synthase complex subunit DHDDS	DHDDS_HUMAN
Hexokinase-2	HXK2_HUMAN
116 kDa U5 small nuclear ribonucleoprotein component	U5S1_HUMAN
ADP/ATP translocase 2	ADT2_HUMAN
ER membrane protein complex subunit 8	EMC8_HUMAN
Adenylate kinase 4, mitochondrial	KAD4_HUMAN
Calcium/calmodulin-dependent protein kinase type II subunit gamma	KCC2G_HUMAN
Importin subunit alpha-3	IMA3_HUMAN
Acyl-CoA-binding domain-containing protein 6	ACBD6_HUMAN
Inactive tyrosine-protein kinase PRAG1	PRAG1_HUMAN

Macoilin	MACOI_HUMAN
V-type proton ATPase 116 kDa subunit a 3	VPP3_HUMAN
Mediator of RNA polymerase II transcription subunit 25	MED25_HUMAN
Pleckstrin homology domain-containing family G member 5	PKHG5_HUMAN
Golgi phosphoprotein 3	GOLP3_HUMAN
Sorting nexin-8	SNX8_HUMAN
Ceramide glucosyltransferase	CEGT_HUMAN
Pseudouridylate synthase RPUSD2	RUSD2_HUMAN
Tissue factor pathway inhibitor 2	TFPI2_HUMAN
Structural maintenance of chromosomes flexible hinge domain-containing pr	SMHD1_HUMAN
Bromodomain adjacent to zinc finger domain protein 2A	BAZ2A_HUMAN
AP-5 complex subunit zeta-1	AP5Z1_HUMAN
Serine/arginine repetitive matrix protein 1	SRRM1_HUMAN
Transmembrane protein 168	TM168_HUMAN
Small ribosomal subunit protein mS29	RT29_HUMAN
Pumilio homolog 3	PUM3_HUMAN
Mitochondrial tRNA methylthiotransferase CDK5RAP1	CK5P1_HUMAN
Ras-related protein Rab-14	RAB14_HUMAN
Protocadherin-12	PCD12_HUMAN
Integrin-linked protein kinase	ILK_HUMAN
Golgin subfamily A member 7	GOGA7_HUMAN
Ergosterol biosynthetic protein 28 homolog	ERG28_HUMAN
NHS-like protein 1	NHSL1_HUMAN
Checkpoint protein HUS1	HUS1_HUMAN
E3 ubiquitin-protein ligase Itchy homolog	ITCH_HUMAN
N-acetylgalactosamine kinase	GALK2_HUMAN
Chromatin assembly factor 1 subunit B	CAF1B_HUMAN
Putative coiled-coil-helix-coiled-coil-helix domain-containing protein CHCHD	CHCH9_HUMAN
Eukaryotic translation initiation factor 5	IF5_HUMAN
Sphingosine-1-phosphate phosphatase 1	SGPP1_HUMAN
AP-2 complex subunit beta	AP2B1_HUMAN
MAP7 domain-containing protein 3	MA7D3_HUMAN
Glucosidase 2 subunit beta	GLU2B_HUMAN
Protein YIF1B	YIF1B_HUMAN
E3 ubiquitin-protein ligase CBL	CBL_HUMAN
N-acylneuraminate cytidylyltransferase	NEUA_HUMAN
Mitogen-activated protein kinase 8	MK08_HUMAN
Presequence protease, mitochondrial	PREP_HUMAN
Histone H1.2	H12_HUMAN
Paxillin	PAXI_HUMAN
Rab3 GTPase-activating protein catalytic subunit	RB3GP_HUMAN
Prolyl endopeptidase	PPCE_HUMAN
Serine/arginine-rich splicing factor 6	SRSF6_HUMAN
Peptidyl-tRNA hydrolase	PTH_HUMAN
Ubiquinone biosynthesis protein COQ9, mitochondrial	COQ9_HUMAN
Plasmanylethanolamine desaturase 1	PDES1_HUMAN
RNA-binding protein 26	RBM26_HUMAN
Neuron navigator 1	NAV1_HUMAN
Mitochondrial ribosome-associated GTPase 2	MTG2_HUMAN
Endoribonuclease Dicer	DICER_HUMAN

Disks large homolog 5	DLG5_HUMAN
Hamartin	TSC1_HUMAN
Rho guanine nucleotide exchange factor 18	ARHGI_HUMAN
Gamma-synuclein	SYUG_HUMAN
Sulfate transporter	S26A2_HUMAN
Tyrosine-protein phosphatase non-receptor type 11	PTN11_HUMAN
Arginine-glutamic acid dipeptide repeats protein	RERE_HUMAN
Coiled-coil domain-containing protein 134	CC134_HUMAN
Transmembrane protein 87A	TM87A_HUMAN
Histone H1.3	H13_HUMAN
FUN14 domain-containing protein 2	FUND2_HUMAN
Monofunctional C1-tetrahydrofolate synthase, mitochondrial	C1TM_HUMAN
NADH dehydrogenase [ubiquinone] 1 subunit C2	NDUC2_HUMAN
Triosephosphate isomerase	TPIS_HUMAN
Transmembrane protein 192	TM192_HUMAN
Ribosomal protein eL42-like	RL36L_HUMAN
Dedicator of cytokinesis protein 10	DOC10_HUMAN
Ras GTPase-activating protein nGAP	NGAP_HUMAN
N-alpha-acetyltransferase 40	NAA40_HUMAN
Telomerase RNA component interacting RNase	TRIR_HUMAN
Glycogenin-1	GLYG_HUMAN
Eukaryotic translation initiation factor 5B	IF2P_HUMAN
Solute carrier family 35 member F6	S35F6_HUMAN
Ataxin-2	ATX2_HUMAN
Methionine synthase reductase	MTRR_HUMAN
Induced myeloid leukemia cell differentiation protein Mcl-1	MCL1_HUMAN
Nucleolar GTP-binding protein 2	NOG2_HUMAN
Protein-lysine 6-oxidase	LYOX_HUMAN
Programmed cell death 6-interacting protein	PDC6I_HUMAN
Ras GTPase-activating protein 1	RASA1_HUMAN
Mitochondrial 10-formyltetrahydrofolate dehydrogenase	AL1L2_HUMAN
Charged multivesicular body protein 6	CHMP6_HUMAN
Homocysteine-responsive endoplasmic reticulum-resident ubiquitin-like dom	HERP1_HUMAN
Destrin	DEST_HUMAN
Bromodomain-containing protein 4	BRD4_HUMAN
Eukaryotic translation initiation factor 4E type 2	IF4E2_HUMAN
Small ribosomal subunit protein eS25	RS25_HUMAN
Bromodomain-containing protein 3	BRD3_HUMAN
Ubiquitin carboxyl-terminal hydrolase 36	UBP36_HUMAN
Recombining binding protein suppressor of hairless	SUH_HUMAN
Actin-related protein 2/3 complex subunit 4	ARPC4_HUMAN
P2X purinoceptor 4	P2RX4_HUMAN
Tumor necrosis factor receptor superfamily member 10D	TR10D_HUMAN
E3 SUMO-protein ligase PIAS4	PIAS4_HUMAN
Zinc phosphodiesterase ELAC protein 2	RNZ2_HUMAN
Heat shock 70 kDa protein 4	HSP74_HUMAN
SNARE-associated protein Snapin	SNAPN_HUMAN
Ubiquitin-associated protein 2	UBAP2_HUMAN
Golgi-resident adenosine 3',5'-bisphosphate 3'-phosphatase	IMPA3_HUMAN
Ankyrin repeat domain-containing protein 54	ANR54_HUMAN

Kinetochore-associated protein NSL1 homolog	NSL1_HUMAN
ADP-ribosylation factor-like protein 1	ARL1_HUMAN
Ankyrin repeat and LEM domain-containing protein 2	ANKL2_HUMAN
Amine oxidase [flavin-containing] A	AOFA_HUMAN
Tubulin beta-4A chain	TBB4A_HUMAN
DNA excision repair protein ERCC-6-like	ERC6L_HUMAN
Small ribosomal subunit protein uS9	RS16_HUMAN
3-phosphoinositide-dependent protein kinase 1	PDPK1_HUMAN
Inositol hexakisphosphate and diphosphoinositol-pentakisphosphate kinase 2	VIP2_HUMAN
Ubiquitin carboxyl-terminal hydrolase 46	UBP46_HUMAN
Microtubule-associated protein RP/EB family member 1	MARE1_HUMAN
Beta-1,4-galactosyltransferase 7	B4GT7_HUMAN
Ubiquitin-conjugating enzyme E2 G1	UB2G1_HUMAN
Probable ATP-dependent RNA helicase DDX6	DDX6_HUMAN
Transcription factor Sp1	SP1_HUMAN
Complement decay-accelerating factor	DAF_HUMAN
Colorectal mutant cancer protein	CRCM_HUMAN
Arrestin domain-containing protein 1	ARRD1_HUMAN
DnaJ homolog subfamily C member 15	DJC15_HUMAN
TATA-binding protein-associated factor 2N	RBP56_HUMAN
Endothelial cell-specific chemotaxis regulator	ECSCR_HUMAN
Discoidin, CUB and LCCL domain-containing protein 1	DCBD1_HUMAN
40-kDa huntingtin-associated protein	HAP40_HUMAN
Protein-glutamine gamma-glutamyltransferase E	TGM3_HUMAN
Ubiquitin-conjugating enzyme E2 K	UBE2K_HUMAN
GPI transamidase component PIG-S	PIGS_HUMAN
Acyl-coenzyme A diphosphatase FITM2	FITM2_HUMAN
Hepatoma-derived growth factor-related protein 3	HDGR3_HUMAN
Riboflavin kinase	RIFK_HUMAN
DDB1- and CUL4-associated factor 11	DCA11_HUMAN
26S proteasome non-ATPase regulatory subunit 11	PSD11_HUMAN
Tubulin gamma-1 chain	TBG1_HUMAN
Zinc finger protein 64	ZF64B_HUMAN
Mitogen-activated protein kinase-binding protein 1	MABP1_HUMAN
DnaJ homolog subfamily C member 30, mitochondrial	DJC30_HUMAN
2-(3-amino-3-carboxypropyl)histidine synthase subunit 1	DPH1_HUMAN
Interleukin-3 receptor subunit alpha	IL3RA_HUMAN
Schlafen family member 5	SLFN5_HUMAN
Endonuclease domain-containing 1 protein	ENDD1_HUMAN
Sorting and assembly machinery component 50 homolog	SAM50_HUMAN
Intraflagellar transport protein 74 homolog	IFT74_HUMAN
Molybdopterin synthase catalytic subunit	MOC2B_HUMAN
Xyloside xylosyltransferase 1	XXLT1_HUMAN
Histone H2B type 3-B	H2B3B_HUMAN
Caspase-4	CASP4_HUMAN
Kinesin light chain 2	KLC2_HUMAN
Transportin-1	TNPO1_HUMAN
Torsin-1A-interacting protein 1	TOIP1_HUMAN
Protein cereblon	CRBN_HUMAN
Kinesin-like protein KIF2C	KIF2C_HUMAN

Collagen alpha-1(XIII) chain	CODA1_HUMAN
Atlastin-2	ATLA2_HUMAN
Intraflagellar transport protein 57 homolog	IFT57_HUMAN
Serine/threonine-protein kinase receptor R3	ACVL1_HUMAN
3-hydroxyacyl-CoA dehydrogenase type-2	HCD2_HUMAN
tRNA-splicing endonuclease subunit Sen54	SEN54_HUMAN
Voltage-dependent anion-selective channel protein 1	VDAC1_HUMAN
Nucleolar complex protein 3 homolog	NOC3L_HUMAN
Large ribosomal subunit protein uL23	RL23A_HUMAN
Zinc finger protein 346	ZN346_HUMAN
Pyruvate kinase PKM	KPYM_HUMAN
Serine protease HTRA1	HTRA1_HUMAN
DNA-directed RNA polymerase I subunit RPA1	RPA1_HUMAN
Proteasome assembly chaperone 1	PSMG1_HUMAN
BRISC complex subunit Abraxas 2	ABRX2_HUMAN
Methyl-CpG-binding domain protein 4	MBD4_HUMAN
1-phosphatidylinositol 4,5-bisphosphate phosphodiesterase delta-1	PLCD1_HUMAN
Isobutyryl-CoA dehydrogenase, mitochondrial	ACAD8_HUMAN
Distal membrane-arm assembly complex protein 2	DMAC2_HUMAN
Endoribonuclease LACTB2	LACB2_HUMAN
Keratinocyte proline-rich protein	KPRP_HUMAN
Actin-related protein 8	ARP8_HUMAN
Spliceosome-associated protein CWC15 homolog	CWC15_HUMAN
CCHC-type zinc finger nucleic acid binding protein	CNBP_HUMAN
Filamin-binding LIM protein 1	FBLI1_HUMAN
PHD finger-like domain-containing protein 5A	PHF5A_HUMAN
DNA repair endonuclease XPF	XPF_HUMAN
Exosome complex component RRP4	EXOS2_HUMAN
ATP-binding cassette sub-family F member 2	ABCF2_HUMAN
U3 small nucleolar ribonucleoprotein protein IMP4	IMP4_HUMAN
RNA-binding protein EWS	EWS_HUMAN
rRNA N6-adenosine-methyltransferase ZCCHC4	ZCHC4_HUMAN
Glycosyltransferase 8 domain-containing protein 1	GL8D1_HUMAN
Solute carrier family 2, facilitated glucose transporter member 3	GTR3_HUMAN
Transmembrane protein 132A	T132A_HUMAN
Heterogeneous nuclear ribonucleoprotein D-like	HNRDL_HUMAN
Zinc finger FYVE domain-containing protein 16	ZFY16_HUMAN
Tumor protein D52	TPD52_HUMAN
CDKN2A-interacting protein	CARF_HUMAN
Zinc finger HIT domain-containing protein 2	ZNHI2_HUMAN
Integral membrane protein GPR180	GP180_HUMAN
Testican-1	TICN1_HUMAN
Nonsense-mediated mRNA decay factor SMG9	SMG9_HUMAN
Bridge-like lipid transfer protein family member 2	BLTP2_HUMAN
N6-adenosine-methyltransferase catalytic subunit	MTA70_HUMAN
Large ribosomal subunit protein uL3	RL3_HUMAN
Glioma pathogenesis-related protein 1	GLIP1_HUMAN
DNA-directed RNA polymerase I subunit RPA43	RPA43_HUMAN
Chromodomain-helicase-DNA-binding protein 1-like	CHD1L_HUMAN
Prostaglandin G/H synthase 1	PGH1_HUMAN

Small ribosomal subunit protein uS11	RS14_HUMAN
Transcription elongation factor A protein 1	TCEA1_HUMAN
Target of rapamycin complex 2 subunit MAPKAP1	SIN1_HUMAN
Iron-sulfur cluster assembly enzyme ISCU	ISCU_HUMAN
H/ACA ribonucleoprotein complex subunit 2	NHP2_HUMAN
Astrocytic phosphoprotein PEA-15	PEA15_HUMAN
Eukaryotic initiation factor 4A-II	IF4A2_HUMAN
Non-POU domain-containing octamer-binding protein	NONO_HUMAN
Threonine--tRNA ligase 1, cytoplasmic	SYTC_HUMAN
Desmoglein-1	DSG1_HUMAN
Large ribosomal subunit protein eL22	RL22_HUMAN
6-phosphofructo-2-kinase/fructose-2,6-bisphosphatase 3	F263_HUMAN
RNA-binding motif protein, X-linked 2	RBMX2_HUMAN
Serine/arginine-rich splicing factor 4	SRSF4_HUMAN
Splicing factor 3A subunit 2	SF3A2_HUMAN
General transcription factor IIH subunit 3	TF2H3_HUMAN
Krev interaction trapped protein 1	KRIT1_HUMAN
Reactive oxygen species modulator 1	ROMO1_HUMAN
G patch domain-containing protein 4	GPTC4_HUMAN
Urokinase-type plasminogen activator	UROK_HUMAN
Glutamine-dependent NAD(+) synthetase	NADE_HUMAN
ADP-ribosylation factor GTPase-activating protein 2	ARFG2_HUMAN
E3 ubiquitin-protein ligase RNF113A	R113A_HUMAN
Importin subunit alpha-4	IMA4_HUMAN
V-type proton ATPase subunit E 1	VATE1_HUMAN
Small EDRK-rich factor 2	SERF2_HUMAN
Eyes absent homolog 3	EYA3_HUMAN
5-aminolevulinate synthase, non-specific, mitochondrial	HEM1_HUMAN
Formylglycine-generating enzyme	SUMF1_HUMAN
Histone H1.0	H10_HUMAN
Reticulocalbin-3	RCN3_HUMAN
Cystine/glutamate transporter	XCT_HUMAN
Phosphoacetylglucosamine mutase	AGM1_HUMAN
Ran-binding protein 3	RANB3_HUMAN
Mediator of RNA polymerase II transcription subunit 12	MED12_HUMAN
Small ribosomal subunit protein bS1m	RT28_HUMAN
Ragulator complex protein LAMTOR2	LTOR2_HUMAN
Very long chain fatty acid elongase 1	ELOV1_HUMAN
Choline transporter-like protein 1	CTL1_HUMAN
Transducin beta-like protein 2	TBL2_HUMAN
Septin-6	SEPT6_HUMAN
Coiled-coil and C2 domain-containing protein 1B	C2D1B_HUMAN
Signal peptidase complex subunit 3	SPCS3_HUMAN
Rho GTPase-activating protein 7	RHG07_HUMAN
Regulator of chromosome condensation	RCC1_HUMAN
Transmembrane protein 98	TMM98_HUMAN
HAUS augmin-like complex subunit 8	HAUS8_HUMAN
Centrosomal protein of 170 kDa protein B	C170B_HUMAN
Phosducin-like protein 3	PDCL3_HUMAN
Chromatin-remodeling ATPase INO80	INO80_HUMAN

Pleckstrin homology domain-containing family O member 2	PKHO2_HUMAN
Protein unc-45 homolog A	UN45A_HUMAN
Outer mitochondrial transmembrane helix translocase	ATAD1_HUMAN
DNA-directed RNA polymerase I subunit RPA49	RPA49_HUMAN
Methionine aminopeptidase 1	MAP11_HUMAN
Stearoyl-CoA desaturase 5	SCD5_HUMAN
T-complex protein 1 subunit beta	TCPB_HUMAN
Archaemetzincin-2	AMZ2_HUMAN
Bifunctional methylenetetrahydrofolate dehydrogenase/cyclohydrolase, mito	MTDC_HUMAN
Matrix metalloproteinase-14	MMP14_HUMAN
Pituitary tumor-transforming gene 1 protein-interacting protein	PTTG_HUMAN
Mitochondrial proton/calcium exchanger protein	LETM1_HUMAN
Fidgetin-like protein 1	FIGL1_HUMAN
SPATS2-like protein	SPS2L_HUMAN
WW domain-containing adapter protein with coiled-coil	WAC_HUMAN
Pre-mRNA-splicing factor CWC25 homolog	CWC25_HUMAN
TOX high mobility group box family member 4	TOX4_HUMAN
Uracil phosphoribosyltransferase homolog	UPP_HUMAN
cAMP-dependent protein kinase catalytic subunit alpha	KAPCA_HUMAN
Cip1-interacting zinc finger protein	CIZ1_HUMAN
Galectin-8	LEG8_HUMAN
Dehydrogenase/reductase SDR family member 1	DHRS1_HUMAN
Tissue-type plasminogen activator	TPA_HUMAN
Vascular endothelial zinc finger 1	VEZF1_HUMAN
Plakophilin-4	PKP4_HUMAN
Cytosolic phospholipase A2	PA24A_HUMAN
Apolipoprotein A-I	APOA1_HUMAN
DnaJ homolog subfamily B member 1	DNJB1_HUMAN
Transcription factor Dp-1	TFDP1_HUMAN
NADH dehydrogenase [ubiquinone] 1 beta subcomplex subunit 8, mitochond	NDUB8_HUMAN
DBIRD complex subunit ZNF326	ZN326_HUMAN
Elongation factor 1-delta	EF1D_HUMAN
G-patch domain and KOW motifs-containing protein	GPKOW_HUMAN
Programmed cell death protein 2	PDCD2_HUMAN
Tudor and KH domain-containing protein	TDRKH_HUMAN
RRP12-like protein	RRP12_HUMAN
Structural maintenance of chromosomes protein 1A	SMC1A_HUMAN
Tonsoku-like protein	TONSL_HUMAN
Nucleolar protein 14	NOP14_HUMAN
Dr1-associated corepressor	NC2A_HUMAN
Ras-related protein Rab-34	RAB34_HUMAN
Transcription initiation factor TFIID subunit 4	TAF4_HUMAN
Serine/threonine-protein kinase TAO2	TAOK2_HUMAN
Lamina-associated polypeptide 2, isoform alpha	LAP2A_HUMAN
Zinc finger protein ubi-d4	REQU_HUMAN
Dickkopf-related protein 3	DKK3_HUMAN
Probable JmjC domain-containing histone demethylation protein 2C	JHD2C_HUMAN
Protein transport protein Sec24A	SC24A_HUMAN
Cytoplasmic aconitate hydratase	ACOHC_HUMAN
Tetraspanin-6	TSN6_HUMAN

NAD kinase	NADK_HUMAN
NLR family member X1	NLRX1_HUMAN
Pleckstrin homology domain-containing family A member 3	PKHA3_HUMAN
Nuclear ubiquitous casein and cyclin-dependent kinase substrate 1	NUCKS_HUMAN
Protein Mis18-beta	MS18B_HUMAN
Large ribosomal subunit protein mL37	RM37_HUMAN
Probable cysteine--tRNA ligase, mitochondrial	SYCM_HUMAN
Proteasome assembly chaperone 3	PSMG3_HUMAN
Probable helicase with zinc finger domain	HELZ_HUMAN
Palmitoyltransferase ZDHHC5	ZDHC5_HUMAN
Alanyl-tRNA editing protein Aarsd1	AASD1_HUMAN
Bifunctional phosphoribosylaminoimidazole carboxylase/phosphoribosylami	PUR6_HUMAN
RNA-binding protein Musashi homolog 2	MSI2H_HUMAN
Cytosolic Fe-S cluster assembly factor NUBP2	NUBP2_HUMAN
NHL repeat-containing protein 2	NHLC2_HUMAN
Small nuclear ribonucleoprotein G	RUXG_HUMAN
UPF0489 protein C5orf22	CE022_HUMAN
Trans-3-hydroxy-L-proline dehydratase	T3HPD_HUMAN
Proteasome subunit alpha type-1	PSA1_HUMAN
MOB kinase activator 2	MOB2_HUMAN
Nuclear envelope pore membrane protein POM 121	P121A_HUMAN
Tectonin beta-propeller repeat-containing protein 1	TCPR1_HUMAN
F-box DNA helicase 1	FBH1_HUMAN
Ribosomal oxygenase 1	RIOX1_HUMAN
Uncharacterized protein C3orf38	CC038_HUMAN
Collagen alpha-1(I) chain	CO1A1_HUMAN
Caspase recruitment domain-containing protein 11	CAR11_HUMAN
Thioredoxin domain-containing protein 9	TXND9_HUMAN
Transcription initiation factor TFIID subunit 5	TAF5_HUMAN
ADP-ribosylation factor-like protein 6-interacting protein 4	AR6P4_HUMAN
Meiosis regulator and mRNA stability factor 1	MARF1_HUMAN
Protein Smaug homolog 1	SMAG1_HUMAN
ASNSD1 upstream open reading frame protein	ASURF_HUMAN
Syntaxin-2	STX2_HUMAN
Rho GDP-dissociation inhibitor 1	GDIR1_HUMAN
Adhesion G-protein coupled receptor G6	AGRG6_HUMAN
Interferon-related developmental regulator 2	IFRD2_HUMAN
DNA-directed RNA polymerase II subunit GRINL1A	GRL1A_HUMAN
HAUS augmin-like complex subunit 3	HAUS3_HUMAN
Ras-related protein Rap-2b	RAP2B_HUMAN
5-azacytidine-induced protein 2	AZI2_HUMAN
Rab proteins geranylgeranyltransferase component A 1	RAE1_HUMAN
EF-hand domain-containing protein D2	EFHD2_HUMAN
Protein UXT	UXT_HUMAN
Large ribosomal subunit protein mL64	G45IP_HUMAN
HCLS1-associated protein X-1	HAX1_HUMAN
WD repeat domain phosphoinositide-interacting protein 1	WIPI1_HUMAN
Intraflagellar transport protein 27 homolog	IFT27_HUMAN
Ribosomal RNA small subunit methyltransferase NEP1	NEP1_HUMAN
EGF-containing fibulin-like extracellular matrix protein 1	FBLN3_HUMAN

Plasma membrane ascorbate-dependent reductase CYBRD1	CYBR1_HUMAN
Tetratricopeptide repeat protein 27	TTC27_HUMAN
Extracellular sulfatase Sulf-2	SULF2_HUMAN
Negative elongation factor A	NELFA_HUMAN
SAM domain-containing protein SAMSN-1	SAMN1_HUMAN
Mitofusin-2	MFN2_HUMAN
Vacuolar protein sorting-associated protein 26B	VP26B_HUMAN
Breast cancer anti-estrogen resistance protein 1	BCAR1_HUMAN
Small VCP/p97-interacting protein	SVIP_HUMAN
Protein HGH1 homolog	HGH1_HUMAN
Rab-like protein 2A	RBL2A_HUMAN
F-box/WD repeat-containing protein 11	FBW1B_HUMAN
Secretory carrier-associated membrane protein 3	SCAM3_HUMAN
Ceroid-lipofuscinosis neuronal protein 5	CLN5_HUMAN
Tyrosine-protein kinase receptor UFO	UFO_HUMAN
Haloacid dehalogenase-like hydrolase domain-containing 5	HDHD5_HUMAN
DNA-directed RNA polymerase, mitochondrial	RPOM_HUMAN
Acetyl-coenzyme A synthetase 2-like, mitochondrial	ACS2L_HUMAN
Voltage-dependent anion-selective channel protein 3	VDAC3_HUMAN
High affinity cationic amino acid transporter 1	CTR1_HUMAN
Mitochondrial import receptor subunit TOM34	TOM34_HUMAN
Zinc finger protein 618	ZN618_HUMAN
Galectin-1	LEG1_HUMAN
Heterogeneous nuclear ribonucleoprotein H3	HNRH3_HUMAN
Splicing factor 45	SPF45_HUMAN
5'-nucleotidase domain-containing protein 3	NT5D3_HUMAN
Histone H1.5	H15_HUMAN
Small acidic protein	SMAP_HUMAN
Multiple epidermal growth factor-like domains protein 8	MEGF8_HUMAN
DNA-directed RNA polymerase III subunit RPC3	RPC3_HUMAN
E3 ubiquitin-protein ligase ZNF598	ZN598_HUMAN
Glucosamine 6-phosphate N-acetyltransferase	GNA1_HUMAN
Phosphorylase b kinase regulatory subunit alpha, skeletal muscle isoform	KPB1_HUMAN
Tapasin-related protein	TPSNR_HUMAN
ATP synthase subunit s, mitochondrial	ATP5S_HUMAN
Caspase-10	CASPA_HUMAN
Protein PALS1	PALS1_HUMAN
Gamma-enolase	ENOG_HUMAN
Segment polarity protein dishevelled homolog DVL-1	DVL1_HUMAN
Gigaxonin	GAN_HUMAN
Cold shock domain-containing protein E1	CSDE1_HUMAN
Exostosin-like 2	EXTL2_HUMAN
Cytoskeleton-associated protein 2	CKAP2_HUMAN
Surfeit locus protein 6	SURF6_HUMAN
Ubiquitin carboxyl-terminal hydrolase MINDY-3	MINY3_HUMAN
Ephrin-B1	EFNB1_HUMAN
Protein SEC13 homolog	SEC13_HUMAN
Fizzy-related protein homolog	FZR1_HUMAN
RNA-binding motif, single-stranded-interacting protein 1	RBMS1_HUMAN
LIM and senescent cell antigen-like-containing domain protein 1	LIMS1_HUMAN

Tropomyosin alpha-4 chain	TPM4_HUMAN
Probable methyltransferase TARBP1	TARB1_HUMAN
Cofilin-2	COF2_HUMAN
Probable ATP-dependent RNA helicase DDX28	DDX28_HUMAN
EGF-like repeat and discoidin I-like domain-containing protein 3	EDIL3_HUMAN
RAC-gamma serine/threonine-protein kinase	AKT3_HUMAN
Protein PAT1 homolog 1	PATL1_HUMAN
Cornifin-B	SPR1B_HUMAN
Eukaryotic initiation factor 4A-III	IF4A3_HUMAN
Transcription initiation factor TFIID subunit 12	TAF12_HUMAN
von Willebrand factor A domain-containing protein 8	VWA8_HUMAN
Desmoplakin	DESP_HUMAN
ERI1 exoribonuclease 3	ERI3_HUMAN
Large ribosomal subunit protein mL41	RM41_HUMAN
DNA-directed RNA polymerases I and III subunit RPAC1	RPAC1_HUMAN
Protein timeless homolog	TIM_HUMAN
Small subunit processome component 20 homolog	UTP20_HUMAN
Nuclear receptor subfamily 2 group C member 2	NR2C2_HUMAN
Procathepsin L	CATL1_HUMAN
Coronin-1C	COR1C_HUMAN
Xaa-Pro aminopeptidase 3	XPP3_HUMAN
Signal peptide peptidase-like 2B	SPP2B_HUMAN
Receptor expression-enhancing protein 4	REEP4_HUMAN
Protein FMC1 homolog	FMC1_HUMAN
Atrophin-1	ATN1_HUMAN
Protein C-ets-1	ETS1_HUMAN
WD repeat domain phosphoinositide-interacting protein 3	WIPI3_HUMAN
Gem-associated protein 6	GEMI6_HUMAN
Acireductone dioxygenase	MTND_HUMAN
Nephrocystin-3	NPHP3_HUMAN
Keratin, type II cytoskeletal 80	K2C80_HUMAN
Divergent protein kinase domain 2A	DIK2A_HUMAN
Transcription elongation factor SPT6	SPT6H_HUMAN
NF-kappa-B inhibitor epsilon	IKBE_HUMAN
Coiled-coil domain-containing protein 117	CC117_HUMAN
Ras-related protein Rab-5C	RAB5C_HUMAN
Protein lin-37 homolog	LIN37_HUMAN
Oxysterol-binding protein-related protein 3	OSBL3_HUMAN
Methylsterol monooxygenase 1	MSMO1_HUMAN
Cullin-4B	CUL4B_HUMAN
FERM, ARHGEF and pleckstrin domain-containing protein 1	FARP1_HUMAN
Coilin	COIL_HUMAN
Serine protease 23	PRS23_HUMAN
Biogenesis of lysosome-related organelles complex 1 subunit 1	BL1S1_HUMAN
Nuclear inhibitor of protein phosphatase 1	PP1R8_HUMAN
Spliceosome-associated protein CWC27 homolog	CWC27_HUMAN
Disintegrin and metalloproteinase domain-containing protein 10	ADA10_HUMAN
Mitotic spindle assembly checkpoint protein MAD2A	MD2L1_HUMAN
Diacylglycerol O-acyltransferase 1	DGAT1_HUMAN
THO complex subunit 3	THOC3_HUMAN

CD2-associated protein	CD2AP_HUMAN
Forkhead box protein J3	FOXJ3_HUMAN
Protein-L-histidine N-pros-methyltransferase	METL9_HUMAN
Armadillo-like helical domain-containing protein 3	ARMD3_HUMAN
Protein HIRA	HIRA_HUMAN
Serine/threonine-protein kinase PAK 4	PAK4_HUMAN
Small ribosomal subunit protein uS7m	RT07_HUMAN
Glycogen phosphorylase, brain form	PYGB_HUMAN
Nesprin-3	SYNE3_HUMAN
Cyclin-dependent kinase 17	CDK17_HUMAN
Polyglutamine-binding protein 1	PQBP1_HUMAN
Copine-3	CPNE3_HUMAN
Glyceraldehyde-3-phosphate dehydrogenase	G3P_HUMAN
Vacuolar protein sorting-associated protein 26A	VP26A_HUMAN
THUMP domain-containing protein 1	THUM1_HUMAN
Fumarylacetoacetate hydrolase domain-containing protein 2A	FAH2A_HUMAN
BCL2/adenovirus E1B 19 kDa protein-interacting protein 2	BNIP2_HUMAN
Roquin-1	RC3H1_HUMAN
Protein GOLM2	GOLM2_HUMAN
Mitochondrial ribosome and complex I assembly factor AltMIEF1	MIDUO_HUMAN
Large ribosomal subunit protein uL18	RL5_HUMAN
Large ribosomal subunit protein mL38	RM38_HUMAN
Protein KTI12 homolog	KTI12_HUMAN
Brefeldin A-inhibited guanine nucleotide-exchange protein 2	BIG2_HUMAN
tRNA N(3)-methylcytidine methyltransferase METTL2A	MET2A_HUMAN
Tudor-interacting repair regulator protein	TIRR_HUMAN
ETS-related transcription factor Elf-1	ELF1_HUMAN
AP-4 complex subunit beta-1	AP4B1_HUMAN
Alpha-ketoglutarate dehydrogenase component 4	KGD4_HUMAN
Suppressor of fused homolog	SUFU_HUMAN
Mitochondrial dicarboxylate carrier	DIC_HUMAN
Quinone oxidoreductase-like protein 1	QORL1_HUMAN
Segment polarity protein dishevelled homolog DVL-3	DVL3_HUMAN
CCN family member 1	CCN1_HUMAN
Etoposide-induced protein 2.4 homolog	EI24_HUMAN
Caspase-8	CASP8_HUMAN
Protein jagunal homolog 1	JAGN1_HUMAN
GRB2-associated-binding protein 2	GAB2_HUMAN
Myosin light polypeptide 6	MYL6_HUMAN
Glutamine synthetase	GLNA_HUMAN
RNA exonuclease 4	REXO4_HUMAN
Eukaryotic translation initiation factor 3 subunit G	EIF3G_HUMAN
Phosphorylase b kinase regulatory subunit alpha, liver isoform	KPB2_HUMAN
Polypeptide N-acetylgalactosaminyltransferase 4	GALT4_HUMAN
Nucleolar protein 56	NOP56_HUMAN
Mitogen-activated protein kinase kinase kinase 7	M3K7_HUMAN
Sentrin-specific protease 6	SENP6_HUMAN
ATP-dependent RNA helicase DDX24	DDX24_HUMAN
Lactadherin	MFGM_HUMAN
Protein PRRC2A	PRC2A_HUMAN

Ceroid-lipofuscinosis neuronal protein 6	CLN6_HUMAN
Small ubiquitin-related modifier 2	SUMO2_HUMAN
Folliculin	FLCN_HUMAN
Zinc finger CCCH domain-containing protein 15	ZC3HF_HUMAN
Calcium-dependent secretion activator 2	CAPS2_HUMAN
Small ribosomal subunit protein eS19	RS19_HUMAN
dCTP pyrophosphatase 1	DCTP1_HUMAN
Squalene synthase	FDFT_HUMAN
MTOR-associated protein MEAK7	MEAK7_HUMAN
F-box only protein 11	FBX11_HUMAN
Nucleolar and spindle-associated protein 1	NUSAP_HUMAN
Activin receptor type-1	ACVR1_HUMAN
Mitochondrial calcium uniporter regulator 1	MCUR1_HUMAN
DNA topoisomerase 2-beta	TOP2B_HUMAN
Enhancer of filamentation 1	CASL_HUMAN
Histone-lysine N-methyltransferase NSD2	NSD2_HUMAN
Lamin-B1	LMNB1_HUMAN
CASP8 and FADD-like apoptosis regulator	CFLAR_HUMAN
Serine/threonine-protein kinase ULK3	ULK3_HUMAN
Small ribosomal subunit protein uS5	RS2_HUMAN
WD repeat-containing protein 41	WDR41_HUMAN
Protein ITPRID2	ITPI2_HUMAN
Histone deacetylase complex subunit SAP130	SP130_HUMAN
Proteasome subunit alpha type-6	PSA6_HUMAN
Thymidine kinase, cytosolic	KITH_HUMAN
Treacle protein	TCOF_HUMAN
E3 ubiquitin-protein ligase SMURF2	SMUF2_HUMAN
Protein S100-A13	S10AD_HUMAN
Synaptojanin-2	SYNJ2_HUMAN
Transmembrane protein 43	TMM43_HUMAN
Carbohydrate sulfotransferase 12	CHSTC_HUMAN
RELT-like protein 1	RELL1_HUMAN
Pleiotropic regulator 1	PLRG1_HUMAN
Methylosome subunit pICln	ICLN_HUMAN
Isocitrate dehydrogenase [NAD] subunit alpha, mitochondrial	IDH3A_HUMAN
Intraflagellar transport protein 25 homolog	IFT25_HUMAN
Inactive hydroxysteroid dehydrogenase-like protein 1	HSDL1_HUMAN
Geranylgeranyl transferase type-2 subunit beta	PGTB2_HUMAN
Eukaryotic translation initiation factor 2 subunit 3	IF2G_HUMAN
Twinfilin-1	TWF1_HUMAN
Probable ATP-dependent RNA helicase DDX49	DDX49_HUMAN
Small ribosomal subunit protein eS17	RS17_HUMAN
Eukaryotic translation initiation factor 2 subunit 1	IF2A_HUMAN
Small ribosomal subunit protein mS40	RT18B_HUMAN
Proteasome subunit alpha type-2	PSA2_HUMAN
Myotubularin-related protein 12	MTMRC_HUMAN
Krueppel-like factor 16	KLF16_HUMAN
Unconventional myosin-IXa	MYO9A_HUMAN
Nucleolar protein 7	NOL7_HUMAN
Histone lysine demethylase PHF8	PHF8_HUMAN

Bifunctional 3'-phosphoadenosine 5'-phosphosulfate synthase 1	PAPS1_HUMAN
FAST kinase domain-containing protein 5, mitochondrial	FAKD5_HUMAN
Polyhomeotic-like protein 3	PHC3_HUMAN
Ras-related protein R-Ras	RRAS_HUMAN
Structural maintenance of chromosomes protein 5	SMC5_HUMAN
ATP-dependent RNA helicase DDX1	DDX1_HUMAN
General transcription factor 3C polypeptide 4	TF3C4_HUMAN
Ragulator complex protein LAMTOR3	LTOR3_HUMAN
Histone-arginine methyltransferase CARM1	CARM1_HUMAN
Vimentin	VIME_HUMAN
Neuron navigator 3	NAV3_HUMAN
ATP synthase mitochondrial F1 complex assembly factor 2	ATPF2_HUMAN
NADH dehydrogenase [ubiquinone] 1 alpha subcomplex assembly factor 4	NDUF4_HUMAN
Transcription initiation factor TFIID subunit 9B	TAF9B_HUMAN
Eukaryotic translation initiation factor 4 gamma 2	IF4G2_HUMAN
Protein-L-isoaspartate(D-aspartate) O-methyltransferase	PIMT_HUMAN
Large ribosomal subunit protein eL13	RL13_HUMAN
Mitochondrial peptide methionine sulfoxide reductase	MSRA_HUMAN
Ubiquitin carboxyl-terminal hydrolase 15	UBP15_HUMAN
F-box/LRR-repeat protein 15	FXL15_HUMAN
NEDD8-conjugating enzyme Ubc12	UBC12_HUMAN
COX assembly mitochondrial protein 2 homolog	COXM2_HUMAN
Endothelial cell-specific molecule 1	ESM1_HUMAN
Cytochrome c oxidase subunit 5B, mitochondrial	COX5B_HUMAN
Thioredoxin-related transmembrane protein 2	TMX2_HUMAN
WD and tetratricopeptide repeats protein 1	WDTC1_HUMAN
AT-rich interactive domain-containing protein 2	ARID2_HUMAN
LIM domain-containing protein 1	LIMD1_HUMAN
RNA-binding protein with multiple splicing	RBPMS_HUMAN
LIM and SH3 domain protein 1	LASP1_HUMAN
Tyrosyl-DNA phosphodiesterase 2	TYDP2_HUMAN
Probable ATP-dependent RNA helicase DDX52	DDX52_HUMAN
Acidic fibroblast growth factor intracellular-binding protein	FIBP_HUMAN
Vesicle transport through interaction with t-SNAREs homolog 1A	VTI1A_HUMAN
Calmin	CLMN_HUMAN
Cytosolic acyl coenzyme A thioester hydrolase	BACH_HUMAN
PH-interacting protein	PHIP_HUMAN
Myotrophin	MTPN_HUMAN
Guanine nucleotide-binding protein subunit beta-4	GBB4_HUMAN
Telomerase Cajal body protein 1	TCAB1_HUMAN
Single-stranded DNA-binding protein, mitochondrial	SSBP_HUMAN
ATP-dependent RNA helicase DDX3X	DDX3X_HUMAN
Microtubule-associated protein 1B	MAP1B_HUMAN
Sorting nexin-7	SNX7_HUMAN
Tyrosine-protein kinase Mer	MERTK_HUMAN
Protein kinase C iota type	KPCI_HUMAN
Drebrin	DREB_HUMAN
Lysine-specific demethylase 4B	KDM4B_HUMAN
RalA-binding protein 1	RBP1_HUMAN
Protein Shroom4	SHRM4_HUMAN

Coatomer subunit epsilon	COPE_HUMAN
Striatin-4	STRN4_HUMAN
Scavenger receptor class F member 1	SREC_HUMAN
Probable 18S rRNA (guanine-N(7))-methyltransferase	BUD23_HUMAN
STARD3 N-terminal-like protein	STR3N_HUMAN
SLAIN motif-containing protein 2	SLAI2_HUMAN
tRNA (adenine(58)-N(1))-methyltransferase non-catalytic subunit TRM6	TRM6_HUMAN
NADH dehydrogenase [ubiquinone] 1 beta subcomplex subunit 5, mitochond	NDUB5_HUMAN
Interferon-induced protein with tetratricopeptide repeats 5	IFIT5_HUMAN
Heme-binding protein 1	HEBP1_HUMAN
Terminal nucleotidyltransferase 4B	PAPD5_HUMAN
Methyl-CpG-binding domain protein 1	MBD1_HUMAN
SNF-related serine/threonine-protein kinase	SNRK_HUMAN
CUGBP Elav-like family member 2	CELF2_HUMAN
Solute carrier family 41 member 3	S41A3_HUMAN
Cytochrome c oxidase assembly protein COX20, mitochondrial	COX20_HUMAN
TRAF2 and NCK-interacting protein kinase	TNIK_HUMAN
Kinetochore-associated protein DSN1 homolog	DSN1_HUMAN
TP53-binding protein 1	TP53B_HUMAN
Sequestosome-1	SQSTM_HUMAN
NPC intracellular cholesterol transporter 1	NPC1_HUMAN
Nucleophosmin	NPM_HUMAN
Kelch-like protein 9	KLHL9_HUMAN
PRA1 family protein 2	PRAF2_HUMAN
Butyrophilin subfamily 3 member A1	BT3A1_HUMAN
Large ribosomal subunit protein eL34	RL34_HUMAN
Myosin-9	MYH9_HUMAN
Eukaryotic translation initiation factor 2A	EIF2A_HUMAN
T-complex protein 1 subunit theta	TCPQ_HUMAN
Tubulin-specific chaperone A	TBCA_HUMAN
Ubiquitin-ribosomal protein eS31 fusion protein	RS27A_HUMAN
Myosin light chain 6B	MYL6B_HUMAN
Dystrophia myotonica WD repeat-containing protein	DMWD_HUMAN
7SK snRNA methylphosphate capping enzyme	MEPCE_HUMAN
Clathrin light chain A	CLCA_HUMAN
Cofilin-1	COF1_HUMAN
Peptidyl-prolyl cis-trans isomerase-like 3	PPIL3_HUMAN
Centromere protein V	CENPV_HUMAN
Golgi integral membrane protein 4	GOLI4_HUMAN
Cdc42 effector protein 3	BORG2_HUMAN
Glycogen phosphorylase, muscle form	PYGM_HUMAN
Myc target protein 1	MYCT1_HUMAN
Spindle and kinetochore-associated protein 1	SKA1_HUMAN
Coactosin-like protein	COTL1_HUMAN
Tropomyosin alpha-1 chain	TPM1_HUMAN
TBC1 domain family member 24	TBC24_HUMAN
Butyrophilin subfamily 2 member A1	BT2A1_HUMAN
Protein regulator of cytokinesis 1	PRC1_HUMAN
Uncharacterized protein C10orf67, mitochondrial	CJ067_HUMAN
Zinc finger MYM-type protein 2	ZMYM2_HUMAN

Protein CUSTOS	CSTOS_HUMAN
DnaJ homolog subfamily A member 3, mitochondrial	DNJA3_HUMAN
Nuclear receptor coactivator 5	NCOA5_HUMAN
Hematopoietic lineage cell-specific protein	HCLS1_HUMAN
E3 ubiquitin-protein ligase TRIM38	TRI38_HUMAN
Polyadenylate-binding protein 4	PABP4_HUMAN
Protein Dr1	NC2B_HUMAN
Protein archease	ARCH_HUMAN
Rab GTPase-binding effector protein 2	RABE2_HUMAN
Hyaluronan mediated motility receptor	HMMR_HUMAN
Collagen alpha-2(IV) chain	CO4A2_HUMAN
Antiviral innate immune response receptor RIG-I	RIGI_HUMAN
rRNA N6-adenosine-methyltransferase METTL5	METL5_HUMAN
Actin filament-associated protein 1	AFAP1_HUMAN
R3H and coiled-coil domain-containing protein 1	R3HC1_HUMAN
Protein phosphatase 1G	PPM1G_HUMAN
Myotubularin-related protein 9	MTMR9_HUMAN
Serglycin	SRGN_HUMAN
Metallothionein-1E	MT1E_HUMAN
Fibronectin type III domain-containing protein 3B	FND3B_HUMAN
E3 ubiquitin-protein ligase TRIM47	TRI47_HUMAN
Putative RNA-binding protein 15B	RB15B_HUMAN
Ras-responsive element-binding protein 1	RREB1_HUMAN
Albumin	ALBU_HUMAN
Calcium-binding and coiled-coil domain-containing protein 2	CACO2_HUMAN
Something about silencing protein 10	SAS10_HUMAN
Pumilio homolog 1	PUM1_HUMAN
Synaptopodin	SYNPO_HUMAN
Ribonuclease P protein subunit p25-like protein	RP25L_HUMAN
sn-1-specific diacylglycerol lipase ABHD11	ABHDB_HUMAN
tRNA (guanine(26)-N(2))-dimethyltransferase	TRM1_HUMAN
Translocon-associated protein subunit delta	SSRD_HUMAN
Interferon-induced protein 44	IFI44_HUMAN
Gamma-tubulin complex component 6	GCP6_HUMAN
Oligoribonuclease, mitochondrial	ORN_HUMAN
F-actin-capping protein subunit alpha-1	CAZA1_HUMAN
Ras-related protein Rab-2A	RAB2A_HUMAN
THO complex subunit 1	THOC1_HUMAN
Golgi-associated plant pathogenesis-related protein 1	GAPR1_HUMAN
E3 ubiquitin-protein ligase ZFP91	ZFP91_HUMAN
Alpha-1,6-mannosylglycoprotein 6-beta-N-acetylglucosaminyltransferase A	MGT5A_HUMAN
Triple functional domain protein	TRIO_HUMAN
Transducin beta-like protein 3	TBL3_HUMAN
Nuclear envelope integral membrane protein 1	NEMP1_HUMAN
Cell division control protein 45 homolog	CDC45_HUMAN
Beta-1,3-glucosyltransferase	B3GLT_HUMAN
Acyl-coenzyme A thioesterase THEM4	THEM4_HUMAN
Transmembrane protein 65	TMM65_HUMAN
Ubiquitin-conjugating enzyme E2 J1	UB2J1_HUMAN
Kinetochore protein Nuf2	NUF2_HUMAN

MAD2L1-binding protein	MD2BP_HUMAN
tRNA dimethylallyltransferase	MOD5_HUMAN
RNA/RNP complex-1-interacting phosphatase	DUS11_HUMAN
Glycogen [starch] synthase, muscle	GYS1_HUMAN
Casein kinase I isoform epsilon	KC1E_HUMAN
Profilin-2	PROF2_HUMAN
STE20-related kinase adapter protein alpha	STRAA_HUMAN
Nucleolus and neural progenitor protein	NEPRO_HUMAN
Anillin	ANLN_HUMAN
Four and a half LIM domains protein 1	FHL1_HUMAN
Bone morphogenetic protein receptor type-2	BMPR2_HUMAN
3-ketodihydrosphingosine reductase	KDSR_HUMAN
Ubiquitin-associated protein 2-like	UBP2L_HUMAN
Eukaryotic translation initiation factor 3 subunit D	EIF3D_HUMAN
Transformer-2 protein homolog beta	TRA2B_HUMAN
Nestin	NEST_HUMAN
Queuine tRNA-ribosyltransferase accessory subunit 2	QTRT2_HUMAN
Proliferation-associated protein 2G4	PA2G4_HUMAN
GPI ethanolamine phosphate transferase 3	PIGO_HUMAN
Interleukin-6 receptor subunit beta	IL6RB_HUMAN
Ubiquitin-conjugating enzyme E2 E1	UB2E1_HUMAN
CD166 antigen	CD166_HUMAN
Protein WWC2	WWC2_HUMAN
Ras-related protein Rab-23	RAB23_HUMAN
Zinc finger CCCH domain-containing protein 18	ZCH18_HUMAN
Low-density lipoprotein receptor-related protein 8	LRP8_HUMAN
Aminopeptidase N	AMPN_HUMAN
Collagen alpha-1(IV) chain	CO4A1_HUMAN
Large ribosomal subunit protein bL34m	RM34_HUMAN
Forkhead box protein O1	FOXO1_HUMAN
PAS domain-containing serine/threonine-protein kinase	PASK_HUMAN
Surfeit locus protein 1	SURF1_HUMAN
Protocadherin-10	PCD10_HUMAN
DnaJ homolog subfamily B member 12	DJB12_HUMAN
Diacylglycerol kinase zeta	DGKZ_HUMAN
Large ribosomal subunit protein P2	RLA2_HUMAN
Ribosome production factor 2 homolog	RPF2_HUMAN
Kynurenine--oxoglutarate transaminase 1	KAT1_HUMAN
DNA-directed RNA polymerase III subunit RPC1	RPC1_HUMAN
UDP-galactose translocator	S35A2_HUMAN
Ribosome-releasing factor 2, mitochondrial	RRF2M_HUMAN
Cobalamin trafficking protein CblD	MMAD_HUMAN
Voltage-dependent calcium channel subunit alpha-2/delta-1	CA2D1_HUMAN
Homeobox protein TGIF1	TGIF1_HUMAN
Hyccin	HYCCI_HUMAN
Chromatin target of PRMT1 protein	CHTOP_HUMAN
Rho GTPase-activating protein 22	RHG22_HUMAN
RNA-binding protein Raly	RALY_HUMAN
Transcription factor RelB	RELB_HUMAN
Density-regulated protein	DENR_HUMAN

EKC/KEOPS complex subunit LAGE3	LAGE3_HUMAN
E3 ubiquitin-protein ligase RNF169	RN169_HUMAN
Rho guanine nucleotide exchange factor 11	ARHGB_HUMAN
UPF0598 protein C8orf82	CH082_HUMAN
Mesoderm induction early response protein 1	MIER1_HUMAN
Membrane-associated phosphatidylinositol transfer protein 1	PITM1_HUMAN
Calponin-2	CNN2_HUMAN
Peptidyl-prolyl cis-trans isomerase E	PPIE_HUMAN
AP2-associated protein kinase 1	AAK1_HUMAN
Cyclin-dependent kinase 1	CDK1_HUMAN
Alpha-ketoglutarate-dependent dioxygenase alkB homolog 4	ALKB4_HUMAN
Cyclin-dependent kinase 7	CDK7_HUMAN
Methyl-CpG-binding protein 2	MECP2_HUMAN
Neurogenic locus notch homolog protein 2	NOTC2_HUMAN
7-methylguanosine phosphate-specific 5'-nucleotidase	5NT3B_HUMAN
Pre-mRNA-splicing regulator WTAP	FL2D_HUMAN
Zinc finger CCCH domain-containing protein 11A	ZC11A_HUMAN
Enolase-phosphatase E1	ENOPH_HUMAN
Growth factor receptor-bound protein 2	GRB2_HUMAN
Protein eva-1 homolog A	EVA1A_HUMAN
Histone-lysine N-methyltransferase SETDB1	SETB1_HUMAN
Myocardin-related transcription factor A	MRTFA_HUMAN
Heterogeneous nuclear ribonucleoprotein A0	ROA0_HUMAN
Four and a half LIM domains protein 3	FHL3_HUMAN
Multivesicular body subunit 12A	MB12A_HUMAN
Exosome RNA helicase MTR4	MTREX_HUMAN
KH domain-containing, RNA-binding, signal transduction-associated protein 1	KHDR1_HUMAN
Protein DEK	DEK_HUMAN
Serine/arginine-rich splicing factor 7	SRSF7_HUMAN
Translation initiation factor eIF2B subunit beta	EI2BB_HUMAN
U3 small nucleolar RNA-associated protein 4 homolog	UTP4_HUMAN
SCY1-like protein 2	SCYL2_HUMAN
PAT complex subunit CCDC47	CCD47_HUMAN
Low-density lipoprotein receptor	LDLR_HUMAN
Tetraspanin-9	TSN9_HUMAN
Polyadenylate-binding protein 1	PABP1_HUMAN
Cyclin-T1	CCNT1_HUMAN
Acyl-protein thioesterase 2	LYPA2_HUMAN
Zinc finger protein 574	ZN574_HUMAN
Ethanolamine kinase 1	EKI1_HUMAN
Pleckstrin homology-like domain family A member 2	PHLA2_HUMAN
Ataxin-2-like protein	ATX2L_HUMAN
Calcium/calmodulin-dependent protein kinase type 1	KCC1A_HUMAN
Apoptotic chromatin condensation inducer in the nucleus	ACINU_HUMAN
72 kDa type IV collagenase	MMP2_HUMAN
Cytochrome c oxidase subunit 7A-related protein, mitochondrial	COX7R_HUMAN
Protein ecdysoneless homolog	ECD_HUMAN
Leucine-rich repeat protein SHOC-2	SHOC2_HUMAN
ETS translocation variant 3	ETV3_HUMAN
Ras-related protein Rab-24	RAB24_HUMAN

Glutathione S-transferase Mu 3	GSTM3_HUMAN
Transcription initiation factor TFIID subunit 7	TAF7_HUMAN
Pleckstrin homology domain-containing family A member 5	PKHA5_HUMAN
CD82 antigen	CD82_HUMAN
Trafficking protein particle complex subunit 10	TPC10_HUMAN
Ubiquitin carboxyl-terminal hydrolase 3	UBP3_HUMAN
Phosphatidylinositol 4,5-bisphosphate 3-kinase catalytic subunit beta isoform	PK3CB_HUMAN
5'-AMP-activated protein kinase subunit gamma-1	AAKG1_HUMAN
Segment polarity protein dishevelled homolog DVL-2	DVL2_HUMAN
Mitochondrial potassium channel	MITOK_HUMAN
U3 small nucleolar ribonucleoprotein protein IMP3	IMP3_HUMAN
Thimet oligopeptidase	THOP1_HUMAN
Eukaryotic translation initiation factor 3 subunit M	EIF3M_HUMAN
GPN-loop GTPase 3	GPN3_HUMAN
Myb-binding protein 1A	MBB1A_HUMAN
Ribosome quality control complex subunit TCF25	TCF25_HUMAN
SERPINE1 mRNA-binding protein 1	SERB1_HUMAN
Non-structural maintenance of chromosomes element 1 homolog	NSE1_HUMAN
Solute carrier family 12 member 5	S12A5_HUMAN
Host cell factor 2	HCFC2_HUMAN
Serine/threonine-protein kinase 17A	ST17A_HUMAN
Tetratricopeptide repeat protein 9C	TTC9C_HUMAN
Ribosomal RNA processing protein 1 homolog B	RRP1B_HUMAN
Cytochrome c oxidase assembly protein COX15 homolog	COX15_HUMAN
RNA binding protein fox-1 homolog 2	RFOX2_HUMAN
NAD-dependent protein deacetylase sirtuin-3, mitochondrial	SIR3_HUMAN
WD40 repeat-containing protein SMU1	SMU1_HUMAN
Ufm1-specific protease 2	UFSP2_HUMAN
Syntaxin-7	STX7_HUMAN
Survival motor neuron protein	SMN_HUMAN
Serine/threonine-protein kinase tousled-like 1	TLK1_HUMAN
Large ribosomal subunit protein eL32	RL32_HUMAN
BMP-2-inducible protein kinase	BMP2K_HUMAN
Tyrosine-protein phosphatase non-receptor type 1	PTN1_HUMAN
F-box-like/WD repeat-containing protein TBL1XR1	TBL1R_HUMAN
Iron-sulfur cluster transfer protein NUBPL	NUBPL_HUMAN
Ectopic P granules protein 5 homolog	EPG5_HUMAN
PALM2-AKAP2 fusion protein	PLAK2_HUMAN
Transcription factor MafG	MAFG_HUMAN
Mothers against decapentaplegic homolog 4	SMAD4_HUMAN
EH domain-containing protein 1	EHD1_HUMAN
14-3-3 protein gamma	1433G_HUMAN
ADP-ribose glycohydrolase OARD1	OARD1_HUMAN
Protein LSM14 homolog B	LS14B_HUMAN
F-box/WD repeat-containing protein 9	FBXW9_HUMAN
Katanin p60 ATPase-containing subunit A-like 1	KATL1_HUMAN
WD repeat and FYVE domain-containing protein 1	WDFY1_HUMAN
Histone-lysine N-methyltransferase SETD1A	SET1A_HUMAN
Serine hydroxymethyltransferase, mitochondrial	GLYM_HUMAN
Nuclear envelope pore membrane protein POM 121C	P121C_HUMAN

Voltage-gated monoatomic cation channel TMEM109	TM109_HUMAN
Large ribosomal subunit protein eL28	RL28_HUMAN
Protein KHNYN	KHNYN_HUMAN
Guanine nucleotide-binding protein G(I)/G(S)/G(O) subunit gamma-12	GBG12_HUMAN
Glucocorticoid modulatory element-binding protein 2	GMEB2_HUMAN
Transcription initiation factor TFIID subunit 1	TAF1_HUMAN
Protein unc-13 homolog B	UN13B_HUMAN
Tubulin alpha-3C chain	TBA3C_HUMAN
Titin	TITIN_HUMAN
RNA-binding protein 8A	RBM8A_HUMAN
Coiled-coil domain-containing protein 137	CC137_HUMAN
Centrosomal protein of 170 kDa	CE170_HUMAN
Glutamate--cysteine ligase regulatory subunit	GSH0_HUMAN
Probable ATP-dependent RNA helicase DDX56	DDX56_HUMAN
Periodic tryptophan protein 1 homolog	PWP1_HUMAN
Zinc finger protein 22	ZNF22_HUMAN
Hydroxysteroid dehydrogenase-like protein 2	HSDL2_HUMAN
Protein PRRC2C	PRC2C_HUMAN
ATPase family AAA domain-containing protein 2	ATAD2_HUMAN
Ribosomal protein eS27-like	RS27L_HUMAN
Nucleolar protein 6	NOL6_HUMAN
Rho-related GTP-binding protein RhoC	RHOC_HUMAN
Folylpolyglutamate synthase, mitochondrial	FOLC_HUMAN
Phosphatidylinositol 4,5-bisphosphate 3-kinase catalytic subunit gamma isof	PK3CG_HUMAN
Mitochondrial thiamine pyrophosphate carrier	TPC_HUMAN
Phosphatidate cytidylyltransferase, mitochondrial	TAM41_HUMAN
Nuclear pore complex protein Nup50	NUP50_HUMAN
AF4/FMR2 family member 4	AFF4_HUMAN
SNW domain-containing protein 1	SNW1_HUMAN
YTH domain-containing family protein 2	YTHD2_HUMAN
Bromodomain-containing protein 2	BRD2_HUMAN
Far upstream element-binding protein 1	FUBP1_HUMAN
Transmembrane protein 237	TM237_HUMAN
Platelet endothelial aggregation receptor 1	PEAR1_HUMAN
2-oxoglutarate and iron-dependent oxygenase domain-containing protein 3	OGFD3_HUMAN
Small ribosomal subunit protein eS27	RS27_HUMAN
RNA-binding protein FXR2	FXR2_HUMAN
FAS-associated death domain protein	FADD_HUMAN
Small ribosomal subunit protein uS8	RS15A_HUMAN
Presenilin-2	PSN2_HUMAN
CAAX prenyl protease 2	FACE2_HUMAN
Deoxynucleotidyltransferase terminal-interacting protein 1	TDIF1_HUMAN
Small ribosomal subunit protein eS10	RS10_HUMAN
Merlin	MERL_HUMAN
Peptidyl-tRNA hydrolase 2, mitochondrial	PTH2_HUMAN
Apoptosis-stimulating of p53 protein 2	ASPP2_HUMAN
AP-1 complex subunit sigma-1A	AP1S1_HUMAN
HEAT repeat-containing protein 6	HEAT6_HUMAN
Melanoma-associated antigen D1	MAGD1_HUMAN
Ras-related protein Rab-32	RAB32_HUMAN

Protein canopy homolog 3	CNPY3_HUMAN
DNA-binding protein RFX5	RFX5_HUMAN
Sterol regulatory element-binding protein cleavage-activating protein	SCAP_HUMAN
Probable RNA-binding protein EIF1AD	EIF1A_HUMAN
Protein phosphatase 1B	PPM1B_HUMAN
Renin receptor	RENR_HUMAN
Caspase-3	CASP3_HUMAN
SEC14-like protein 1	S14L1_HUMAN
TSC22 domain family protein 4	T22D4_HUMAN
Threonine--tRNA ligase, mitochondrial	SYTM_HUMAN
MICOS complex subunit MIC27	MIC27_HUMAN
Phosphatidylcholine transfer protein	PPCT_HUMAN
Mitochondrial adenyl nucleotide antiporter SLC25A25	SCMC2_HUMAN
Four and a half LIM domains protein 2	FHL2_HUMAN
Trinucleotide repeat-containing gene 6A protein	TNR6A_HUMAN
Exosome complex component 10	EXOSX_HUMAN
Nucleoside diphosphate kinase A	NDKA_HUMAN
Nucleosome assembly protein 1-like 4	NP1L4_HUMAN
Anaphase-promoting complex subunit 5	APC5_HUMAN
Putative RNA polymerase II subunit B1 CTD phosphatase RPAP2	RPAP2_HUMAN
Band 4.1-like protein 3	E41L3_HUMAN
rRNA methyltransferase 3, mitochondrial	MRM3_HUMAN
KN motif and ankyrin repeat domain-containing protein 1	KANK1_HUMAN
Leucine-rich repeats and immunoglobulin-like domains protein 1	LRIG1_HUMAN
tRNA (guanine-N(7)-)-methyltransferase non-catalytic subunit WDR4	WDR4_HUMAN
Signal recognition particle receptor subunit beta	SRPRB_HUMAN
Oxysterol-binding protein-related protein 2	OSBL2_HUMAN
GRIP and coiled-coil domain-containing protein 1	GCC1_HUMAN
SWI/SNF-related matrix-associated actin-dependent regulator of chromatin s	SMRD2_HUMAN
Receptor-type tyrosine-protein phosphatase gamma	PTPRG_HUMAN
Dual specificity mitogen-activated protein kinase kinase 3	MP2K3_HUMAN
Cytohesin-1	CYH1_HUMAN
RANBP2-like and GRIP domain-containing protein 5/6	RGPD5_HUMAN
Large ribosomal subunit protein mL66	RT18A_HUMAN
Natural resistance-associated macrophage protein 2	NRAM2_HUMAN
U3 small nucleolar RNA-associated protein 6 homolog	UTP6_HUMAN
Nucleosome-remodeling factor subunit BPTF	BPTF_HUMAN
Phostensin	PPR18_HUMAN
Ubiquitin-like protein 5	UBL5_HUMAN
Transcription initiation factor TFIID subunit 9	TAF9_HUMAN
Fructose-bisphosphate aldolase A	ALDOA_HUMAN
Neutral amino acid transporter B(0)	AAAT_HUMAN
Large ribosomal subunit protein uL24	RL26_HUMAN
Protein prenyltransferase alpha subunit repeat-containing protein 1	PTAR1_HUMAN
Cytochrome c oxidase subunit 1	COX1_HUMAN
Aftiphilin	AFTIN_HUMAN
DnaJ homolog subfamily C member 5	DNJC5_HUMAN
Guanine nucleotide-binding protein G(I)/G(S)/G(T) subunit beta-2	GBB2_HUMAN
Condensin complex subunit 3	CND3_HUMAN
Cilia- and flagella-associated protein 410	CF410_HUMAN

Malonyl-CoA decarboxylase, mitochondrial	DCMC_HUMAN
Splicing factor 3B subunit 5	SF3B5_HUMAN
Transmembrane protein 179B	T179B_HUMAN
Stromal membrane-associated protein 2	SMAP2_HUMAN
Glutamine amidotransferase-like class 1 domain-containing protein 3, mitoch	GAL3A_HUMAN
Polypyrimidine tract-binding protein 3	PTBP3_HUMAN
Formin-like protein 2	FMNL2_HUMAN
Atrial natriuretic peptide receptor 2	ANPRB_HUMAN
La-related protein 4	LARP4_HUMAN
5-methylcytosine rRNA methyltransferase NSUN4	NSUN4_HUMAN
Proline-rich protein 11	PRR11_HUMAN
Protein-S-isoprenylcysteine O-methyltransferase	ICMT_HUMAN
Dihydropyrimidinase-related protein 3	DPYL3_HUMAN
Diphthine methyl ester synthase	DPH5_HUMAN
Syntenin-1	SDCB1_HUMAN
NTF2-related export protein 1	NXT1_HUMAN
Zinc finger protein 330	ZN330_HUMAN
Dihydrolipoyllysine-residue acetyltransferase component of pyruvate dehydr	ODP2_HUMAN
Phosphatidylinositol 3,4,5-trisphosphate 3-phosphatase and dual-specificity	PTEN_HUMAN
V-type proton ATPase subunit d 1	VA0D1_HUMAN
Cytochrome b-245 chaperone 1	CYBC1_HUMAN
Microsomal glutathione S-transferase 1	MGST1_HUMAN
Translation initiation factor eIF2B subunit gamma	EI2BG_HUMAN
Serine/threonine-protein kinase PLK1	PLK1_HUMAN
V-type proton ATPase subunit S1	VAS1_HUMAN
Latent-transforming growth factor beta-binding protein 3	LTBP3_HUMAN
E3 SUMO-protein ligase PIAS3	PIAS3_HUMAN
DNA/RNA-binding protein KIN17	KIN17_HUMAN
RNA N6-adenosine-methyltransferase METTL16	MET16_HUMAN
DNA-directed RNA polymerase I subunit RPA2	RPA2_HUMAN
Coiled-coil domain-containing protein 12	CCD12_HUMAN
BUB3-interacting and GLEBS motif-containing protein ZNF207	ZN207_HUMAN
RalBP1-associated Eps domain-containing protein 1	REPS1_HUMAN
E3 ubiquitin-protein ligase RNF10	RNF10_HUMAN
Prolyl endopeptidase-like	PPCEL_HUMAN
Enoyl-CoA delta isomerase 2	ECI2_HUMAN
Fibrillin-2	FBN2_HUMAN
RNA-binding motif, single-stranded-interacting protein 2	RBMS2_HUMAN
Solute carrier family 12 member 7	S12A7_HUMAN
Probable phospholipid-transporting ATPase IIB	ATP9B_HUMAN
T-complex protein 1 subunit eta	TCPH_HUMAN
Peroxiredoxin-1	PRDX1_HUMAN
Protein MAK16 homolog	MAK16_HUMAN
Proline-rich protein 14	PRR14_HUMAN
Gamma-glutamylcyclotransferase	GGCT_HUMAN
Histone-lysine N-methyltransferase SETD2	SETD2_HUMAN
Ceramide transfer protein	CERT_HUMAN
CREB-regulated transcription coactivator 3	CRTC3_HUMAN
Partitioning defective 3 homolog	PARD3_HUMAN
Putative GTP-binding protein 6	GTPB6_HUMAN

Beta-arrestin-2	ARRB2_HUMAN
Ribosomal RNA-processing protein 7 homolog A	RRP7A_HUMAN
Tripartite motif-containing protein 5	TRIM5_HUMAN
LIM and cysteine-rich domains protein 1	LMCD1_HUMAN
Purine nucleoside phosphorylase	PNPH_HUMAN
Phosphatidylglycerophosphatase and protein-tyrosine phosphatase 1	PTPM1_HUMAN
Nucleolar protein 4-like	NOL4L_HUMAN
Transformer-2 protein homolog alpha	TRA2A_HUMAN
Apoptotic protease-activating factor 1	APAF_HUMAN
Eukaryotic translation initiation factor 3 subunit L	EIF3L_HUMAN
Ribosome biogenesis protein BMS1 homolog	BMS1_HUMAN
Microtubule-associated protein 1S	MAP1S_HUMAN
Jupiter microtubule associated homolog 1	JUPI1_HUMAN
Nonsense-mediated mRNA decay factor SMG5	SMG5_HUMAN
Large ribosomal subunit protein mL46	RM46_HUMAN
Pleckstrin homology-like domain family B member 1	PHLB1_HUMAN
Synaptosomal-associated protein 47	SNP47_HUMAN
YTH domain-containing protein 1	YTDC1_HUMAN
Transcription factor Jun	JUN_HUMAN
Mapk-regulated corepressor-interacting protein 1	MCRI1_HUMAN
Mitochondrial tRNA-specific 2-thiouridylase 1	MTU1_HUMAN
Tubulin alpha-1B chain	TBA1B_HUMAN
Lipid droplet-regulating VLDL assembly factor AUP1	AUP1_HUMAN
Ribosomal protein S6 kinase beta-2	KS6B2_HUMAN
F-box only protein 30	FBX30_HUMAN
Protein BUD31 homolog	BUD31_HUMAN
Clustered mitochondria protein homolog	CLU_HUMAN
H/ACA ribonucleoprotein complex subunit 1	GAR1_HUMAN
Non-structural maintenance of chromosomes element 3 homolog	NSE3_HUMAN
NACHT, LRR and PYD domains-containing protein 2	NALP2_HUMAN
Histone-lysine N-methyltransferase NSD3	NSD3_HUMAN
Golgi reassembly-stacking protein 1	GORS1_HUMAN
E3 ubiquitin-protein ligase SH3RF1	SH3R1_HUMAN
Zinc finger CCCH-type antiviral protein 1-like	ZCCHL_HUMAN
Adenosine kinase	ADK_HUMAN
Importin subunit alpha-7	IMA7_HUMAN
Serine/arginine-rich splicing factor 10	SRS10_HUMAN
Large neutral amino acids transporter small subunit 1	LAT1_HUMAN
Filamin-C	FLNC_HUMAN
KAT8 regulatory NSL complex subunit 3	KANL3_HUMAN
Adhesion G protein-coupled receptor L4	AGRL4_HUMAN
Cathepsin S	CATS_HUMAN
CD276 antigen	CD276_HUMAN
Probable RNA-binding protein 19	RBM19_HUMAN
YEATS domain-containing protein 4	YETS4_HUMAN
CDK5 regulatory subunit-associated protein 2	CK5P2_HUMAN
Myosin-11	MYH11_HUMAN
Cysteine and histidine-rich domain-containing protein 1	CHRD1_HUMAN
Deoxyribonuclease-1-like 1	DNSL1_HUMAN
NEDD8	NEDD8_HUMAN

WASH complex subunit 2A	WAC2A_HUMAN
Serine/threonine-protein kinase 11-interacting protein	S11IP_HUMAN
Rabenosyn-5	RBNS5_HUMAN
Kinesin-like protein KIF11	KIF11_HUMAN
Barrier-to-autointegration factor	BAF_HUMAN
Ribose-phosphate pyrophosphokinase 1	PRPS1_HUMAN
Staphylococcal nuclease domain-containing protein 1	SND1_HUMAN
F-BAR and double SH3 domains protein 1	FCSD1_HUMAN
Sideroflexin-2	SFXN2_HUMAN
Cyclin-dependent kinase 4	CDK4_HUMAN
U2 small nuclear ribonucleoprotein A'	RU2A_HUMAN
Casein kinase I isoform alpha	KC1A_HUMAN
Thiopurine S-methyltransferase	TPMT_HUMAN
KAT8 regulatory NSL complex subunit 1	KANL1_HUMAN
Small ribosomal subunit protein uS3m	RT24_HUMAN
Inositol 1,4,5-trisphosphate receptor-interacting protein	IPRI_HUMAN
Syntaxin-12	STX12_HUMAN
Eukaryotic peptide chain release factor GTP-binding subunit ERF3A	ERF3A_HUMAN
Heterogeneous nuclear ribonucleoprotein R	HNRPR_HUMAN
Cdc42 effector protein 2	BORG1_HUMAN
Small ribosomal subunit protein uS14	RS29_HUMAN
RAD50-interacting protein 1	RINT1_HUMAN
Leucine-rich repeat-containing protein 1	LRRC1_HUMAN
Leucine-zipper-like transcriptional regulator 1	LZTR1_HUMAN
Golgi SNAP receptor complex member 1	GOSR1_HUMAN
Putative hydrolase DDAH2	DDAH2_HUMAN
Autophagy-related protein 2 homolog B	ATG2B_HUMAN
Probable ATP-dependent RNA helicase DHX40	DHX40_HUMAN
Histone acetyltransferase KAT6A	KAT6A_HUMAN
GATOR2 complex protein WDR59	WDR59_HUMAN
Synaptosomal-associated protein 23	SNP23_HUMAN
Arginine/serine-rich protein PNISR	PNISR_HUMAN
tRNA endonuclease ANKZF1	ANKZ1_HUMAN
Pre-mRNA-splicing factor SPF27	SPF27_HUMAN
Putative transferase CAF17, mitochondrial	CAF17_HUMAN
Tripartite motif-containing protein 3	TRIM3_HUMAN
N-acetylglucosamine-1-phosphodiester alpha-N-acetylglucosaminidase	NAGPA_HUMAN
Transcription intermediary factor 1-alpha	TIF1A_HUMAN
Nuclear factor of activated T-cells 5	NFAT5_HUMAN
Arginase-2, mitochondrial	ARGI2_HUMAN
DNA topoisomerase 3-alpha	TOP3A_HUMAN
C2 domain-containing protein 3	C2CD3_HUMAN
CCR4-NOT transcription complex subunit 10	CNO10_HUMAN
Transcription initiation factor TFIID subunit 6	TAF6_HUMAN
Lysine-specific demethylase 2A	KDM2A_HUMAN
DNA (cytosine-5)-methyltransferase 3A	DNM3A_HUMAN
Activator of 90 kDa heat shock protein ATPase homolog 1	AHSA1_HUMAN
NFX1-type zinc finger-containing protein 1	ZNFX1_HUMAN
SPRY domain-containing protein 7	SPRY7_HUMAN
Dynein light chain Tctex-type 1	DYLT1_HUMAN

Kinesin-like protein KIF22	KIF22_HUMAN
Cell cycle and apoptosis regulator protein 2	CCAR2_HUMAN
Lactoylglutathione lyase	LGUL_HUMAN
Zinc finger MIZ domain-containing protein 2	ZMIZ2_HUMAN
Proton-transporting V-type ATPase complex assembly regulator TMEM9	TMEM9_HUMAN
E3 ubiquitin-protein ligase RNF220	RN220_HUMAN
Rab proteins geranylgeranyltransferase component A 2	RAE2_HUMAN
Epsin-2	EPN2_HUMAN
Protein jagged-2	JAG2_HUMAN
L-lactate dehydrogenase B chain	LDHB_HUMAN
Pro-neuregulin-1, membrane-bound isoform	NRG1_HUMAN
Telomeric repeat-binding factor 2-interacting protein 1	TE2IP_HUMAN
Solute carrier family 15 member 4	S15A4_HUMAN
Nuclear receptor corepressor 2	NCOR2_HUMAN
Presenilin-1	PSN1_HUMAN
NEDD4-binding protein 1	N4BP1_HUMAN
F-box only protein 28	FBX28_HUMAN
Cytochrome c1, heme protein, mitochondrial	CY1_HUMAN
Centrosomal protein of 131 kDa	CP131_HUMAN
U1 small nuclear ribonucleoprotein 70 kDa	RU17_HUMAN
E3 ubiquitin-protein ligase SH3RF3	SH3R3_HUMAN
Adenylyltransferase and sulfurtransferase MOCS3	MOCS3_HUMAN
Annexin A2	ANXA2_HUMAN
Stomatin	STOM_HUMAN
Protocadherin gamma-A12	PCDGC_HUMAN
Platelet-derived growth factor subunit B	PDGFB_HUMAN
Procollagen-lysine,2-oxoglutarate 5-dioxygenase 2	PLOD2_HUMAN
Polyribonucleotide 5'-hydroxyl-kinase Clp1	CLP1_HUMAN
E3 ubiquitin-protein ligase TRIM32	TRI32_HUMAN
TBC1 domain family member 17	TBC17_HUMAN
tRNA wybutosine-synthesizing protein 5	TYW5_HUMAN
Mitochondrial-processing peptidase subunit beta	MPPB_HUMAN
CTD small phosphatase-like protein 2	CTSL2_HUMAN
Poly(A) polymerase gamma	PAPOG_HUMAN
Cyclic AMP-responsive element-binding protein 1	CREB1_HUMAN
G2/mitotic-specific cyclin-B1	CCNB1_HUMAN
Chromodomain Y-like protein	CDYL_HUMAN
Pre-mRNA 3'-end-processing factor FIP1	FIP1_HUMAN
Intron Large complex component GCFC2	GCFC2_HUMAN
MAP7 domain-containing protein 1	MA7D1_HUMAN
Ubiquitin carboxyl-terminal hydrolase 19	UBP19_HUMAN
Small ribosomal subunit protein uS10	RS20_HUMAN
Serine/threonine-protein phosphatase 4 regulatory subunit 2	PP4R2_HUMAN
Valine--tRNA ligase, mitochondrial	SYVM_HUMAN
COP9 signalosome complex subunit 8	CSN8_HUMAN
Testis-specific Y-encoded-like protein 1	TSYL1_HUMAN
Glutamine--fructose-6-phosphate aminotransferase [isomerizing] 1	GFPT1_HUMAN
NK-tumor recognition protein	NKTR_HUMAN
Serine/threonine-protein phosphatase 6 regulatory ankyrin repeat subunit A	ANR28_HUMAN
Polynucleotide 5'-hydroxyl-kinase NOL9	NOL9_HUMAN

Protein mago nashi homolog 2	MGN2_HUMAN
BAG family molecular chaperone regulator 3	BAG3_HUMAN
NF-kappa-B-activating protein	NKAP_HUMAN
Dynein light chain 1, cytoplasmic	DYL1_HUMAN
Protein phosphatase 1 regulatory subunit 14B	PP14B_HUMAN
G patch domain-containing protein 1	GPTC1_HUMAN
Serine/threonine-protein kinase LATS1	LATS1_HUMAN
DNA polymerase alpha catalytic subunit	DPOLA_HUMAN
Mediator of DNA damage checkpoint protein 1	MDC1_HUMAN
Borealin	BOREA_HUMAN
Calcium uptake protein 1, mitochondrial	MICU1_HUMAN
Dimethyladenosine transferase 2, mitochondrial	TFB2M_HUMAN
Nuclear migration protein nudC	NUDC_HUMAN
Ribonucleoside-diphosphate reductase subunit M2 B	RIR2B_HUMAN
Dual specificity mitogen-activated protein kinase kinase 5	MP2K5_HUMAN
FAS-associated factor 2	FAF2_HUMAN
Uridine-cytidine kinase 1	UCK1_HUMAN
Polycomb protein SUZ12	SUZ12_HUMAN
Trans-Golgi network integral membrane protein 2	TGON2_HUMAN
Acid ceramidase	ASAH1_HUMAN
Heterogeneous nuclear ribonucleoprotein Q	HNRPQ_HUMAN
Eukaryotic translation initiation factor 3 subunit F	EIF3F_HUMAN
Leucine-rich repeat-containing protein 41	LRC41_HUMAN
Protein phosphatase PTC7 homolog	PPTC7_HUMAN
Ras-related protein Rab-1A	RAB1A_HUMAN
Major vault protein	MVP_HUMAN
Cerebellar degeneration-related protein 2	CDR2_HUMAN
Pyridoxal kinase	PDXK_HUMAN
Transforming protein RhoA	RHOA_HUMAN
Thioredoxin-like protein 4A	TXN4A_HUMAN
Centriole and centriolar satellite protein OFD1	OFD1_HUMAN
Myosin regulatory light chain 12B	ML12B_HUMAN
Coxsackievirus and adenovirus receptor	CXAR_HUMAN
ATP-dependent RNA helicase DHX33	DHX33_HUMAN
DCN1-like protein 3	DCNL3_HUMAN
DnaJ homolog subfamily A member 1	DNJA1_HUMAN
Small ribosomal subunit protein eS12	RS12_HUMAN
Eukaryotic translation initiation factor 2 subunit 2	IF2B_HUMAN
PRKR-interacting protein 1	PKRI1_HUMAN
CREB-regulated transcription coactivator 1	CRTC1_HUMAN
Phagosome assembly factor 1	PHAF1_HUMAN
Small ribosomal subunit protein bS6m	RT06_HUMAN
Tumor necrosis factor receptor superfamily member 6	TNR6_HUMAN
G2/mitotic-specific cyclin-B2	CCNB2_HUMAN
ATP synthase membrane subunit K, mitochondrial	ATPMK_HUMAN
Abl interactor 1	ABI1_HUMAN
F-box/LRR-repeat protein 6	FBXL6_HUMAN
Transcriptional regulator Kaiso	KAISO_HUMAN
Phosphatidylinositol 3,4,5-trisphosphate 5-phosphatase 2	SHIP2_HUMAN
Malate dehydrogenase, mitochondrial	MDHM_HUMAN

Armadillo repeat-containing protein 10	ARM10_HUMAN
Deoxyhypusine synthase	DHYS_HUMAN
Protein phosphatase 1 regulatory subunit 3B	PPR3B_HUMAN
FAD-linked sulfhydryl oxidase ALR	ALR_HUMAN
PC4 and SFRS1-interacting protein	PSIP1_HUMAN
Copper-transporting ATPase 1	ATP7A_HUMAN
E3 ubiquitin-protein ligase RING1	RING1_HUMAN
Palmitoyltransferase ZDHHC17	ZDH17_HUMAN
Regulator of G-protein signaling 12	RGS12_HUMAN
Transmembrane protein 51	TMM51_HUMAN
Sphingomyelin phosphodiesterase	ASM_HUMAN
Elongator complex protein 6	ELP6_HUMAN
Leucine zipper transcription factor-like protein 1	LZTL1_HUMAN
Contactin-associated protein 1	CNTP1_HUMAN
Synaptophysin-like protein 1	SYPL1_HUMAN
E3 ubiquitin-protein ligase UBR1	UBR1_HUMAN
CLK4-associating serine/arginine rich protein	CLASR_HUMAN
Terminal uridylyltransferase 4	TUT4_HUMAN
NAD kinase 2, mitochondrial	NAKD2_HUMAN
Y-box-binding protein 3	YBOX3_HUMAN
Kinesin-like protein KIF20A	KI20A_HUMAN
WD repeat-containing protein 35	WDR35_HUMAN
Integrin alpha-V	ITAV_HUMAN
Semaphorin-3F	SEM3F_HUMAN
Uridine phosphorylase 1	UPP1_HUMAN
Lysophospholipase D GDPD1	GDPD1_HUMAN
Regulator of G-protein signaling 19	RGS19_HUMAN
GDP-fucose protein O-fucosyltransferase 1	OFUT1_HUMAN
ATP-dependent RNA helicase DDX50	DDX50_HUMAN
Probable cytosolic iron-sulfur protein assembly protein CIAO1	CIAO1_HUMAN
Phosphoglycerate mutase 1	PGAM1_HUMAN
Centrosomal protein of 44 kDa	CEP44_HUMAN
Vitamin K epoxide reductase complex subunit 1	VKOR1_HUMAN
AP-4 complex subunit mu-1	AP4M1_HUMAN
Eukaryotic translation initiation factor 3 subunit E	EIF3E_HUMAN
Probable E3 ubiquitin-protein ligase IRF2BPL	I2BPL_HUMAN
SH3KBP1-binding protein 1	SHKB1_HUMAN
RING finger and CHY zinc finger domain-containing protein 1	ZN363_HUMAN
Condensin-2 complex subunit D3	CNDD3_HUMAN
26S proteasome regulatory subunit 10B	PRS10_HUMAN
Large ribosomal subunit protein uL6	RL9_HUMAN
Mitochondrial antiviral-signaling protein	MAVS_HUMAN
Tyrosine-protein kinase BAZ1B	BAZ1B_HUMAN
POU domain, class 2, transcription factor 1	PO2F1_HUMAN
Spliceosome RNA helicase DDX39B	DX39B_HUMAN
Protein Spindly	SPDLY_HUMAN
Filamin-B	FLNB_HUMAN
Aurora kinase B	AURKB_HUMAN
Glycerol kinase	GLPK_HUMAN
Nucleoside diphosphate kinase, mitochondrial	NDKM_HUMAN

Large ribosomal subunit protein uL14	RL23_HUMAN
Golgi to ER traffic protein 4 homolog	GET4_HUMAN
Holocytochrome c-type synthase	CCHL_HUMAN
WASH complex subunit 2C	WAC2C_HUMAN
Heterogeneous nuclear ribonucleoprotein A1	ROA1_HUMAN
N-terminal Xaa-Pro-Lys N-methyltransferase 1	NTM1A_HUMAN
Caveolin-2	CAV2_HUMAN
ATP-dependent RNA helicase DDX19B	DD19B_HUMAN
Rho GTPase-activating protein 45	HMHA1_HUMAN
Solute carrier family 66 member 3	S66A3_HUMAN
RNA-binding protein PNO1	PNO1_HUMAN
RNA-binding protein 28	RBM28_HUMAN
Receptor-type tyrosine-protein phosphatase kappa	PTPRK_HUMAN
CDK2-associated and cullin domain-containing protein 1	CACL1_HUMAN
Dephospho-CoA kinase domain-containing protein	DCAKD_HUMAN
Zinc finger protein 428	ZN428_HUMAN
T-complex protein 1 subunit alpha	TCPA_HUMAN
Sterol-4-alpha-carboxylate 3-dehydrogenase, decarboxylating	NSDHL_HUMAN
Breast cancer anti-estrogen resistance protein 3	BCAR3_HUMAN
Microtubule cross-linking factor 1	MTCL1_HUMAN
Nebulin	NEBU_HUMAN
Serine/arginine repetitive matrix protein 2	SRRM2_HUMAN
Gamma-tubulin complex component 5	GCP5_HUMAN
Large ribosomal subunit protein mL62	ICT1_HUMAN
WD repeat-containing protein 70	WDR70_HUMAN
Ubiquitin-associated domain-containing protein 2	UBAC2_HUMAN
E3 ubiquitin-protein ligase RNF114	RN114_HUMAN
Zinc finger CCCH domain-containing protein 14	ZC3HE_HUMAN
ADP-ribosylation factor-like protein 6-interacting protein 6	AR6P6_HUMAN
Transgelin-2	TAGL2_HUMAN
28 kDa heat- and acid-stable phosphoprotein	HAP28_HUMAN
EH domain-binding protein 1	EHBP1_HUMAN
Synaptic vesicle membrane protein VAT-1 homolog	VAT1_HUMAN
Transcription factor 4	ITF2_HUMAN
Serine/threonine-protein kinase TAO3	TAOK3_HUMAN
Protein C-mannosyl-transferase DPY19L1	D19L1_HUMAN
TATA-binding protein-associated factor 172	BTAF1_HUMAN
Zinc finger protein 579	ZN579_HUMAN
Protein N-terminal asparagine amidohydrolase	NTAN1_HUMAN
Golgi SNAP receptor complex member 2	GOSR2_HUMAN
Poly(rC)-binding protein 1	PCBP1_HUMAN
Protein NCBP2AS2	NCAS2_HUMAN
Protein LLP homolog	LLPH_HUMAN
Chromodomain-helicase-DNA-binding protein 9	CHD9_HUMAN
Cyclic AMP-dependent transcription factor ATF-1	ATF1_HUMAN
Copine-8	CPNE8_HUMAN
Non-histone chromosomal protein HMG-17	HMGN2_HUMAN
Small kinetochore-associated protein	SKAP_HUMAN
Sorting nexin-14	SNX14_HUMAN
Peptidyl-prolyl cis-trans isomerase A	PPIA_HUMAN

Protein LSM14 homolog A	LS14A_HUMAN
Exocyst complex component 6B	EXC6B_HUMAN
Ribonuclease inhibitor	RINI_HUMAN
TATA element modulatory factor	TMF1_HUMAN
Follistatin-related protein 1	FSTL1_HUMAN
Lysine-rich nucleolar protein 1	KNOP1_HUMAN
CTP synthase 1	PYRG1_HUMAN
Small ribosomal subunit protein uS2m	RT02_HUMAN
RNA polymerase II elongation factor ELL	ELL_HUMAN
Cerebellar degeneration-related protein 2-like	CDR2L_HUMAN
Anamorsin	CPIN1_HUMAN
8-oxo-dGDP phosphatase NUDT18	NUD18_HUMAN
Ubiquitin-like protein 4A	UBL4A_HUMAN
Clathrin heavy chain 2	CLH2_HUMAN
Bifunctional heparan sulfate N-deacetylase/N-sulfotransferase 1	NDST1_HUMAN
Sulfhydryl oxidase 1	QSOX1_HUMAN
Condensin-2 complex subunit H2	CNDH2_HUMAN
Serpin B8	SPB8_HUMAN
Disabled homolog 2-interacting protein	DAB2P_HUMAN
Heterogeneous nuclear ribonucleoprotein K	HNRPK_HUMAN
Leucine-rich repeat transmembrane protein FLRT2	FLRT2_HUMAN
GEM-interacting protein	GMIP_HUMAN
LIM domain only protein 7	LMO7_HUMAN
Inositol 1,4,5-trisphosphate receptor type 1	ITPR1_HUMAN
DNA dC->dU-editing enzyme APOBEC-3C	ABC3C_HUMAN
Arginine/serine-rich coiled-coil protein 2	RSRC2_HUMAN
Parkinson disease protein 7	PARK7_HUMAN
Microtubule-associated protein 4	MAP4_HUMAN
CSC1-like protein 2	CSCL2_HUMAN
Tetratricopeptide repeat protein 17	TTC17_HUMAN
tRNA selenocysteine 1-associated protein 1	TSAP1_HUMAN
Claudin-11	CLD11_HUMAN
FERM domain-containing protein 5	FRMD5_HUMAN
Mitochondrial import inner membrane translocase subunit Tim22	TIM22_HUMAN
Origin recognition complex subunit 4	ORC4_HUMAN
Zinc finger RNA-binding protein	ZFR_HUMAN
Fos-related antigen 2	FOSL2_HUMAN
Roundabout homolog 4	ROBO4_HUMAN
Monocarboxylate transporter 1	MOT1_HUMAN
Nucleolar pre-ribosomal-associated protein 1	NPA1P_HUMAN
Cilia- and flagella-associated protein 298	CF298_HUMAN
Gamma-taxilin	TXLNG_HUMAN
E3 SUMO-protein ligase PIAS1	PIAS1_HUMAN
Transcription factor Sp3	SP3_HUMAN
Retinol dehydrogenase 11	RDH11_HUMAN
Insulin-like growth factor-binding protein 7	IBP7_HUMAN
WD repeat-containing protein 1	WDR1_HUMAN
Ephrin type-B receptor 2	EPHB2_HUMAN
ADAMTS-like protein 1	ATL1_HUMAN
Ankyrin repeat domain-containing protein 50	ANR50_HUMAN

Calcineurin-binding protein cabin-1	CABIN_HUMAN
DNA topoisomerase 2-alpha	TOP2A_HUMAN
RNA-binding protein 5	RBM5_HUMAN
BTB/POZ domain-containing protein KCTD21	KCD21_HUMAN
Protein unc-119 homolog A	U119A_HUMAN
Cell division cycle-associated 7-like protein	CDA7L_HUMAN
LIM and senescent cell antigen-like-containing domain protein 3	LIMS3_HUMAN
Nuclear transcription factor Y subunit gamma	NFYC_HUMAN
Actin, cytoplasmic 1	ACTB_HUMAN
Ras-related protein Rab-43	RAB43_HUMAN
UDP-glucuronic acid decarboxylase 1	UXS1_HUMAN
NF-kappa-B-repressing factor	NKRF_HUMAN
U3 small nucleolar RNA-associated protein 25 homolog	UTP25_HUMAN
SWI/SNF-related matrix-associated actin-dependent regulator of chromatin s	SMCA5_HUMAN
Uracil-DNA glycosylase	UNG_HUMAN
Nuclear factor of activated T-cells, cytoplasmic 4	NFAC4_HUMAN
Transmembrane protein 222	TM222_HUMAN
Splicing factor Cactin	CATIN_HUMAN
UPF0690 protein C1orf52	CA052_HUMAN
BRCA1-A complex subunit RAP80	UIMC1_HUMAN
THO complex subunit 4	THOC4_HUMAN
CREB-regulated transcription coactivator 2	CRTC2_HUMAN
Trophoblast glycoprotein	TPBG_HUMAN
BUD13 homolog	BUD13_HUMAN
CCR4-NOT transcription complex subunit 6-like	CNO6L_HUMAN
eIF5-mimic protein 2	5MP2_HUMAN
Lymphokine-activated killer T-cell-originated protein kinase	TOPK_HUMAN
Phosphatidylserine decarboxylase proenzyme, mitochondrial	PISD_HUMAN
Exosome complex component RRP43	EXOS8_HUMAN
Reversion-inducing cysteine-rich protein with Kazal motifs	RECK_HUMAN
Dolichol-phosphate mannosyltransferase subunit 3	DPM3_HUMAN
Guanine nucleotide-binding protein G(i) subunit alpha-2	GNAI2_HUMAN
Hexokinase-1	HXK1_HUMAN
Protein FAM98A	FA98A_HUMAN
Signal-induced proliferation-associated 1-like protein 3	SI1L3_HUMAN
Probable ATP-dependent RNA helicase DDX10	DDX10_HUMAN
Sodium/potassium-transporting ATPase subunit alpha-1	AT1A1_HUMAN
Ubiquitin thioesterase otulin	OTUL_HUMAN
RWD domain-containing protein 4	RWDD4_HUMAN
E3 ubiquitin-protein ligase Midline-1	TRI18_HUMAN
Isoamyl acetate-hydrolyzing esterase 1 homolog	IAH1_HUMAN
Retinaldehyde dehydrogenase 3	AL1A3_HUMAN
Phosphatidate phosphatase LPIN1	LPIN1_HUMAN
Histone deacetylase 8	HDAC8_HUMAN
SAGA-associated factor 29	SGF29_HUMAN
Amino acid transporter heavy chain SLC3A2	4F2_HUMAN
Death domain-associated protein 6	DAXX_HUMAN
E3 ubiquitin-protein ligase HERC2	HERC2_HUMAN
Vesicle-associated membrane protein 7	VAMP7_HUMAN
tRNA-dihydrouridine(47) synthase [NAD(P)(+)]-like	DUS3L_HUMAN

Cohesin subunit SA-1	STAG1_HUMAN
Replication termination factor 2	RTF2_HUMAN
Protein arginine N-methyltransferase 3	ANM3_HUMAN
Mitochondrial ubiquitin ligase activator of NFKB 1	MUL1_HUMAN
Transmembrane prolyl 4-hydroxylase	P4HTM_HUMAN
Ubiquitin-associated and SH3 domain-containing protein B	UBS3B_HUMAN
Sestrin-2	SESN2_HUMAN
18S rRNA aminocarboxypropyltransferase	TSR3_HUMAN
WD repeat and HMG-box DNA-binding protein 1	WDHD1_HUMAN
Threonylcarbamoyl-AMP synthase	YRDC_HUMAN
Cytoplasmic dynein 1 light intermediate chain 2	DC1L2_HUMAN
Mitochondrial glutamate carrier 1	GHC1_HUMAN
Bcl-2-like protein 13	B2L13_HUMAN
Enhancer of polycomb homolog 1	EPC1_HUMAN
Ras and Rab interactor 1	RIN1_HUMAN
Mitochondrial fission regulator 1	MTFR1_HUMAN
N-acetylgalactosamine-6-sulfatase	GALNS_HUMAN
Centrosomal protein of 78 kDa	CEP78_HUMAN
p21-activated protein kinase-interacting protein 1	PK1IP_HUMAN
Probable dimethyladenosine transferase	DIM1_HUMAN
AN1-type zinc finger protein 1	ZFAN1_HUMAN
Inactive phospholipase C-like protein 2	PLCL2_HUMAN
Exosome complex component RRP46	EXOS5_HUMAN
ATR-interacting protein	ATRIP_HUMAN
Spermidine synthase	SPEE_HUMAN
WW domain-binding protein 2	WBP2_HUMAN
Solute carrier family 12 member 4	S12A4_HUMAN
UBX domain-containing protein 6	UBXN6_HUMAN
Transmembrane protein with metallophosphoesterase domain	TMPPE_HUMAN
Serine/arginine-rich splicing factor 3	SRSF3_HUMAN
Peptidyl-prolyl cis-trans isomerase FKBP1A	FKB1A_HUMAN
PHD and RING finger domain-containing protein 1	PHRF1_HUMAN
Kinesin-like protein KIFC1	KIFC1_HUMAN
Kelch-like protein 42	KLH42_HUMAN
U3 small nucleolar ribonucleoprotein protein MPP10	MPP10_HUMAN
U3 small nucleolar RNA-interacting protein 2	U3IP2_HUMAN
Nucleoside diphosphate kinase B	NDKB_HUMAN
Putative heat shock protein HSP 90-alpha A4	HS904_HUMAN
Myosin-14	MYH14_HUMAN
Tetratricopeptide repeat protein 5	TTC5_HUMAN
Multiple inositol polyphosphate phosphatase 1	MINP1_HUMAN
Large ribosomal subunit protein uL16	RL10_HUMAN
Muscleblind-like protein 1	MBNL1_HUMAN
Dual specificity tyrosine-phosphorylation-regulated kinase 1A	DYR1A_HUMAN
Oxysterol-binding protein-related protein 5	OSBL5_HUMAN
Signal peptidase complex subunit 1	SPCS1_HUMAN
Ribose-phosphate pyrophosphokinase 2	PRPS2_HUMAN
Protein kinase C delta type	KPCD_HUMAN
A-kinase anchor protein 1, mitochondrial	AKAP1_HUMAN
Autophagy-related protein 16-1	A16L1_HUMAN

CUE domain-containing protein 2	CUED2_HUMAN
Zinc finger protein 830	ZN830_HUMAN
Desmocollin-1	DSC1_HUMAN
ESF1 homolog	ESF1_HUMAN
ELAV-like protein 1	ELAV1_HUMAN
Large ribosomal subunit protein bL32m	RM32_HUMAN
Serine/threonine-protein kinase N3	PKN3_HUMAN
Probable ATP-dependent RNA helicase DHX34	DHX34_HUMAN
V-type proton ATPase subunit D	VATD_HUMAN
rRNA 2'-O-methyltransferase fibrillarin	FBRL_HUMAN
WD repeat-containing protein 20	WDR20_HUMAN
RNA-binding protein with serine-rich domain 1	RNPS1_HUMAN
Intraflagellar transport protein 122 homolog	IF122_HUMAN
Endoplasmic reticulum-Golgi intermediate compartment protein 3	ERGI3_HUMAN
YTH domain-containing family protein 3	YTHD3_HUMAN
RNA-binding motif, single-stranded-interacting protein 3	RBMS3_HUMAN
Mediator of RNA polymerase II transcription subunit 13-like	MD13L_HUMAN
Enoyl-[acyl-carrier-protein] reductase, mitochondrial	MECR_HUMAN
Mitogen-activated protein kinase kinase kinase kinase 5	M4K5_HUMAN
TGF-beta-activated kinase 1 and MAP3K7-binding protein 2	TAB2_HUMAN
Ribosomal RNA processing protein 1 homolog A	RRP1_HUMAN
E3 ubiquitin-protein ligase RING2	RING2_HUMAN
Phosphatidylinositol transfer protein alpha isoform	PIPNA_HUMAN
PDZ domain-containing protein 8	PDZD8_HUMAN
Nuclear respiratory factor 1	NRF1_HUMAN
Ribosome biogenesis regulatory protein homolog	RRS1_HUMAN
Guanine nucleotide-binding protein-like 3-like protein	GNL3L_HUMAN
PDZ and LIM domain protein 3	PDLI3_HUMAN
Programmed cell death protein 10	PDC10_HUMAN
Serine/threonine-protein kinase greatwall	GWL_HUMAN
Gamma-tubulin complex component 4	GCP4_HUMAN
Guided entry of tail-anchored proteins factor CAMLG	CAMLG_HUMAN
Scaffold attachment factor B1	SAFB1_HUMAN
Spatacsin	SPTCS_HUMAN
Nonsense-mediated mRNA decay factor SMG7	SMG7_HUMAN
G patch domain-containing protein 11	GPT11_HUMAN
COX assembly mitochondrial protein homolog	COXM1_HUMAN
Protein CMSS1	CMS1_HUMAN
Myocyte-specific enhancer factor 2C	MEF2C_HUMAN
Protein tweety homolog 3	TTYH3_HUMAN
Sorting nexin-17	SNX17_HUMAN
Complement C1q tumor necrosis factor-related protein 5	C1QT5_HUMAN
Small ribosomal subunit protein uS17m	RT17_HUMAN
Disheveled-associated activator of morphogenesis 1	DAAM1_HUMAN
Mitotic checkpoint serine/threonine-protein kinase BUB1	BUB1_HUMAN
N-acetylglucosamine-1-phosphotransferase subunits alpha/beta	GNPTA_HUMAN
Thioredoxin domain-containing protein 12	TXD12_HUMAN
Taperin	TPRN_HUMAN
EEF1A lysine methyltransferase 2	EFMT2_HUMAN
Calpain-5	CAN5_HUMAN

Tropomyosin alpha-3 chain	TPM3_HUMAN
Protein FAM210B, mitochondrial	F210B_HUMAN
Coiled-coil domain-containing protein 86	CCD86_HUMAN
Putative hydroxypyruvate isomerase	HYI_HUMAN
Peptidyl-prolyl cis-trans isomerase FKBP14	FKB14_HUMAN
Inositol hexakisphosphate kinase 1	IP6K1_HUMAN
Serine/threonine-protein phosphatase PP1-gamma catalytic subunit	PP1G_HUMAN
Elongator complex protein 5	ELP5_HUMAN
Nucleoporin NUP35	NUP35_HUMAN
Pantothenate kinase 3	PANK3_HUMAN
S-adenosyl-L-methionine-dependent tRNA 4-demethylwyosine synthase TYW	TYW1_HUMAN
Alpha-endosulfine	ENSA_HUMAN
Protein sel-1 homolog 1	SE1L1_HUMAN
Nucleolar protein 16	NOP16_HUMAN
Major prion protein	PRIO_HUMAN
Diacylglycerol kinase theta	DGKQ_HUMAN
Adenosine 3'-phospho 5'-phosphosulfate transporter 1	S35B2_HUMAN
Dynamin-1	DYN1_HUMAN
Intermembrane lipid transfer protein VPS13D	VP13D_HUMAN
Guanine nucleotide exchange protein SMCR8	SMCR8_HUMAN
Serine/threonine-protein kinase Nek6	NEK6_HUMAN
UPF0488 protein C8orf33	CH033_HUMAN
SAFB-like transcription modulator	SLTM_HUMAN
Alpha-2-macroglobulin	A2MG_HUMAN
Rho GTPase-activating protein SYDE1	SYDE1_HUMAN
Serine/threonine-protein kinase TAO1	TAOK1_HUMAN
Nexilin	NEXN_HUMAN
Bridge-like lipid transfer protein family member 3B	BLT3B_HUMAN
Heterogeneous nuclear ribonucleoproteins C1/C2	HNRPC_HUMAN
Fermitin family homolog 1	FERM1_HUMAN
Large ribosomal subunit protein uL15	RL27A_HUMAN
Acyl-CoA (8-3)-desaturase	FADS1_HUMAN
Fermitin family homolog 3	URP2_HUMAN
Adipose-secreted signaling protein	ADSSP_HUMAN
Peptidyl-prolyl cis-trans isomerase NIMA-interacting 1	PIN1_HUMAN
ADP-ribose glycohydrolase MACROD1	MACD1_HUMAN
Small ribosomal subunit protein uS17	RS11_HUMAN
NF-kappa-B inhibitor beta	IKBB_HUMAN
MICAL-like protein 2	MILK2_HUMAN
ATP synthase subunit e, mitochondrial	ATP5I_HUMAN
PRKC apoptosis WT1 regulator protein	PAWR_HUMAN
Tyrosine-protein kinase Fer	FER_HUMAN
Ras GTPase-activating protein 3	RASA3_HUMAN
Double-strand-break repair protein rad21 homolog	RAD21_HUMAN
Tumor necrosis factor alpha-induced protein 8-like protein 3	TP8L3_HUMAN
Transcription termination factor 1	TTF1_HUMAN
Serine/threonine-protein kinase STK11	STK11_HUMAN
ADP-ribosylation factor 4	ARF4_HUMAN
Rho GTPase-activating protein 12	RHG12_HUMAN
Sorting nexin-19	SNX19_HUMAN

Exosome complex component MTR3	EXOS6_HUMAN
5'-nucleotidase domain-containing protein 1	NT5D1_HUMAN
Cyclic AMP-dependent transcription factor ATF-7	ATF7_HUMAN
LIM domain-containing protein 2	LIMD2_HUMAN
RNA-binding motif protein, X chromosome	RBMX_HUMAN
Tensin-3	TENS3_HUMAN
A-kinase anchor protein 8-like	AKP8L_HUMAN
NEDD4-like E3 ubiquitin-protein ligase WWP2	WWP2_HUMAN
Tetraspanin-14	TSN14_HUMAN
Dynein light chain 2, cytoplasmic	DYL2_HUMAN
CUGBP Elav-like family member 1	CELF1_HUMAN
Choline/ethanolaminephosphotransferase 1	CEPT1_HUMAN
TRPM8 channel-associated factor 2	TCAF2_HUMAN
Zinc finger matrin-type protein 3	ZMAT3_HUMAN
Tetratricopeptide repeat protein 19, mitochondrial	TTC19_HUMAN
Transcription factor MafF	MAFF_HUMAN
PAX-interacting protein 1	PAXI1_HUMAN
Exosome complex component RRP45	EXOS9_HUMAN
TELO2-interacting protein 1 homolog	TTI1_HUMAN
Proteasomal ubiquitin receptor ADRM1	ADRM1_HUMAN
Protein mono-ADP-ribosyltransferase PARP10	PAR10_HUMAN
NGFI-A-binding protein 2	NAB2_HUMAN
Diacylglycerol kinase eta	DGKH_HUMAN
Vesicle-trafficking protein SEC22a	SC22A_HUMAN
Chromobox protein homolog 2	CBX2_HUMAN
Putative methyltransferase C9orf114	CI114_HUMAN
Fructose-2,6-bisphosphatase TIGAR	TIGAR_HUMAN
Tether containing UBX domain for GLUT4	ASPC1_HUMAN
Chloride intracellular channel protein 4	CLIC4_HUMAN
Reduced folate transporter	S19A1_HUMAN
Chromodomain-helicase-DNA-binding protein 2	CHD2_HUMAN
Plexin-B2	PLXB2_HUMAN
Eukaryotic peptide chain release factor subunit 1	ERF1_HUMAN
LIM domain kinase 1	LIMK1_HUMAN
Bcl-2-related ovarian killer protein	BOK_HUMAN
Chromodomain-helicase-DNA-binding protein 8	CHD8_HUMAN
Nucleus accumbens-associated protein 2	NACC2_HUMAN
High mobility group protein HMG-I/HMG-Y	HMGA1_HUMAN
Zinc finger CCCH domain-containing protein 13	ZC3HD_HUMAN
Transcription factor BTF3 homolog 4	BT3L4_HUMAN
Steroid receptor RNA activator 1	SRA1_HUMAN
Signal recognition particle 19 kDa protein	SRP19_HUMAN
GTP-binding nuclear protein Ran	RAN_HUMAN
Ribonucleoside-diphosphate reductase subunit M2	RIR2_HUMAN
Diphosphomevalonate decarboxylase	MVD1_HUMAN
Kelch-like protein 18	KLH18_HUMAN
Coronin-1A	COR1A_HUMAN
Alpha-mannosidase 2x	MA2A2_HUMAN
Large ribosomal subunit protein eL20	RL18A_HUMAN
Paraplegin	SPG7_HUMAN

Cysteine protease ATG4A	ATG4A_HUMAN
Interleukin-1 receptor-associated kinase 1	IRAK1_HUMAN
Ubiquitin-conjugating enzyme E2 variant 1	UB2V1_HUMAN
WD repeat-containing protein 76	WDR76_HUMAN
Sorting nexin-11	SNX11_HUMAN
DNA topoisomerase 1	TOP1_HUMAN
Phosphatidylinositol 4-phosphate 5-kinase type-1 alpha	PI51A_HUMAN
SHC SH2 domain-binding protein 1	SHCBP_HUMAN
Vesicle-associated membrane protein 8	VAMP8_HUMAN
AF4/FMR2 family member 1	AFF1_HUMAN
Ankyrin repeat and SOCS box protein 6	ASB6_HUMAN
Serine/arginine-rich splicing factor 1	SRSF1_HUMAN
Mitotic deacetylase-associated SANT domain protein	MDEAS_HUMAN
Dermcidin	DCD_HUMAN
U6 snRNA-associated Sm-like protein LSm4	LSM4_HUMAN
Protein YIPF4	YIPF4_HUMAN
Queuine tRNA-ribosyltransferase catalytic subunit 1	TGT_HUMAN
HEAT repeat-containing protein 3	HEAT3_HUMAN
Zinc finger protein 143	ZN143_HUMAN
Cytoskeleton-associated protein 2-like	CKP2L_HUMAN
M-phase phosphoprotein 8	MPP8_HUMAN
Parathymosin	PTMS_HUMAN
LisH domain-containing protein ARMC9	ARMC9_HUMAN
Microtubule-associated serine/threonine-protein kinase 4	MAST4_HUMAN
Leucine--tRNA ligase, mitochondrial	SYLM_HUMAN
U1 small nuclear ribonucleoprotein C	RU1C_HUMAN
KIF-binding protein	KBP_HUMAN
ER degradation-enhancing alpha-mannosidase-like protein 2	EDEM2_HUMAN
Abasic site processing protein HMCES	HMCES_HUMAN
RNA cytidine acetyltransferase	NAT10_HUMAN
H/ACA ribonucleoprotein complex subunit DKC1	DKC1_HUMAN
Elongation factor 1-beta	EF1B_HUMAN
Phosphatidylinositol 4-kinase beta	PI4KB_HUMAN
Protein max	MAX_HUMAN
Polycomb protein EED	EED_HUMAN
Transmembrane protein 199	TM199_HUMAN
Peroxisomal membrane protein PEX13	PEX13_HUMAN
ATP-dependent RNA helicase DHX29	DHX29_HUMAN
Protein DPCD	DPCD_HUMAN
Glycoprotein-N-acetylgalactosamine 3-beta-galactosyltransferase 1	C1GLT_HUMAN
Protein Aster-A	ASTRA_HUMAN
Uridine-cytidine kinase 2	UCK2_HUMAN
Protein SON	SON_HUMAN
UAP56-interacting factor	UIF_HUMAN
Glycerol-3-phosphate acyltransferase 4	GPAT4_HUMAN
Dual specificity mitogen-activated protein kinase kinase 7	MP2K7_HUMAN
Serum response factor-binding protein 1	SRFB1_HUMAN
Scaffold attachment factor B2	SAFB2_HUMAN
Bcl2-associated agonist of cell death	BAD_HUMAN
Methionine--tRNA ligase, mitochondrial	SYMM_HUMAN

Calmodulin-regulated spectrin-associated protein 1	CAMP1_HUMAN
Peptidyl-prolyl cis-trans isomerase FKBP11	FKB11_HUMAN
Protein RUFY3	RUFY3_HUMAN
Rho GTPase-activating protein 24	RHG24_HUMAN
Neuronal cell adhesion molecule	NRCAM_HUMAN
TSC22 domain family protein 2	T22D2_HUMAN
Heterogeneous nuclear ribonucleoprotein F	HNRPF_HUMAN
Vesicle-associated membrane protein-associated protein A	VAPA_HUMAN
MHC class II regulatory factor RFX1	RFX1_HUMAN
Notchless protein homolog 1	NLE1_HUMAN
Sodium-dependent phosphate transporter 1	S20A1_HUMAN
E3 ubiquitin-protein ligase TRIM4	TRIM4_HUMAN
G-rich sequence factor 1	GRSF1_HUMAN
Serine/threonine-protein phosphatase 6 regulatory subunit 1	PP6R1_HUMAN
Zinc finger protein-like 1	ZFPL1_HUMAN
Alpha-1,3-mannosyl-glycoprotein 4-beta-N-acetylglucosaminyltransferase B	MGT4B_HUMAN
Protein phosphatase 1 regulatory subunit 3D	PPR3D_HUMAN
Protein FAM168A	F168A_HUMAN
Protein lin-7 homolog C	LIN7C_HUMAN
DNA-directed RNA polymerase III subunit RPC5	RPC5_HUMAN
Large ribosomal subunit protein eL14	RL14_HUMAN
Transcription factor SOX-17	SOX17_HUMAN
High mobility group protein HMGI-C	HMGA2_HUMAN
Peptidyl-prolyl cis-trans isomerase FKBP4	FKBP4_HUMAN
Zinc finger CCCH domain-containing protein 4	ZC3H4_HUMAN
Mediator of RNA polymerase II transcription subunit 6	MED6_HUMAN
Protocadherin-7	PCDH7_HUMAN
Solute carrier family 2, facilitated glucose transporter member 1	GTR1_HUMAN
Small ribosomal subunit protein mS23	RT23_HUMAN
Nucleotidyltransferase MB21D2	M21D2_HUMAN
Mortality factor 4-like protein 2	MO4L2_HUMAN
CDK-activating kinase assembly factor MAT1	MAT1_HUMAN
Phosphatidylinositol 5-phosphate 4-kinase type-2 gamma	PI42C_HUMAN
Macrophage migration inhibitory factor	MIF_HUMAN
Palmitoyltransferase ZDHHC13	ZDH13_HUMAN
4-hydroxyphenylpyruvate dioxygenase-like protein	HPDL_HUMAN
S-methyl-5'-thioadenosine phosphorylase	MTAP_HUMAN
Transmembrane protein 106B	T106B_HUMAN
GTP-binding protein Rit1	RIT1_HUMAN
Mitochondrial Rho GTPase 2	MIRO2_HUMAN
Protein NDRG1	NDRG1_HUMAN
Splicing factor 3B subunit 2	SF3B2_HUMAN
Ras GTPase-activating protein-binding protein 1	G3BP1_HUMAN
Heterogeneous nuclear ribonucleoprotein A/B	ROAA_HUMAN
Serine/threonine-protein kinase RIO1	RIOK1_HUMAN
Splicing factor YJU2	YJU2_HUMAN
TBC1 domain family member 8	TBCD8_HUMAN
CTD nuclear envelope phosphatase 1	CNEP1_HUMAN
Sarcolemmal membrane-associated protein	SLMAP_HUMAN
Ubiquitin-conjugating enzyme E2 E2	UB2E2_HUMAN

Receptor-interacting serine/threonine-protein kinase 2	RIPK2_HUMAN
RelA-associated inhibitor	IASPP_HUMAN
Angiomotin-like protein 2	AMOL2_HUMAN
Small ribosomal subunit protein uS4	RS9_HUMAN
tRNA (34-2'-O)-methyltransferase regulator WDR6	WDR6_HUMAN
Ras-related protein R-Ras2	RRAS2_HUMAN
Small nuclear ribonucleoprotein Sm D1	SMD1_HUMAN
Protein unc-119 homolog B	U119B_HUMAN
ATPase family AAA domain-containing protein 3B	ATD3B_HUMAN
Armadillo repeat-containing protein 1	ARMC1_HUMAN
Peptidyl-prolyl cis-trans isomerase C	PPIC_HUMAN
Calpain-2 catalytic subunit	CAN2_HUMAN
Immunoglobulin superfamily member 8	IGSF8_HUMAN
E3 SUMO-protein ligase NSE2	NSE2_HUMAN
Protein-glucosylgalactosylhydroxylysine glucosidase	PGGHG_HUMAN
UPF0711 protein C18orf21	CR021_HUMAN
GTPase HRas	RASH_HUMAN
Axin interactor, dorsalization-associated protein	AIDA_HUMAN
Golgi membrane protein 1	GOLM1_HUMAN
Cytosolic iron-sulfur assembly component 2B	CIA2B_HUMAN
DnaJ homolog subfamily C member 16	DJC16_HUMAN
Phosphatidylethanolamine-binding protein 1	PEBP1_HUMAN
Thyroid transcription factor 1-associated protein 26	TAP26_HUMAN
Alpha-soluble NSF attachment protein	SNAA_HUMAN
Prostaglandin reductase 1	PTGR1_HUMAN
Phospholipid-transporting ATPase IH	AT11A_HUMAN
Target of rapamycin complex subunit LST8	LST8_HUMAN
B-cell lymphoma/leukemia 10	BCL10_HUMAN
ADP-ribosylation factor-binding protein GGA2	GGA2_HUMAN
PHD finger protein 20-like protein 1	P20L1_HUMAN
Attractin	ATRN_HUMAN
Methyl-CpG-binding domain protein 2	MBD2_HUMAN
Intraflagellar transport protein 56	IFT56_HUMAN
Coiled-coil domain-containing protein 85C	CC85C_HUMAN
Ras-related protein Rab-7L1	RAB7L_HUMAN
MAP/microtubule affinity-regulating kinase 3	MARK3_HUMAN
Mothers against decapentaplegic homolog 3	SMAD3_HUMAN
Small ribosomal subunit protein mS37	CHCH1_HUMAN
Maspardin	SPG21_HUMAN
NHP2-like protein 1	NH2L1_HUMAN
PDZ and LIM domain protein 1	PDLI1_HUMAN
Translocon-associated protein subunit gamma	SSRG_HUMAN
Serine/threonine-protein kinase RIO2	RIOK2_HUMAN
Large ribosomal subunit protein bL35m	RM35_HUMAN
Ubiquitin-associated domain-containing protein 1	UBAC1_HUMAN
Chromosome-associated kinesin KIF4A	KIF4A_HUMAN
Ribosome biogenesis protein NOP53	NOP53_HUMAN
Stromal cell-derived factor 2	SDF2_HUMAN
Ras-related protein Rab-5A	RAB5A_HUMAN
1-acyl-sn-glycerol-3-phosphate acyltransferase delta	PLCD_HUMAN

tRNA (adenine(58)-N(1))-methyltransferase, mitochondrial	TR61B_HUMAN
LanC-like protein 2	LANC2_HUMAN
Drebrin-like protein	DBNL_HUMAN
Myomegalin	MYOME_HUMAN
Anaphase-promoting complex subunit 10	APC10_HUMAN
Histone deacetylase 6	HDAC6_HUMAN
Ragulator complex protein LAMTOR1	LTOR1_HUMAN
Splicing factor ESS-2 homolog	ESS2_HUMAN
SH3 domain-binding glutamic acid-rich-like protein 3	SH3L3_HUMAN
Plastin-3	PLST_HUMAN
U4/U6.U5 small nuclear ribonucleoprotein 27 kDa protein	SNR27_HUMAN
U3 small nucleolar RNA-associated protein 14 homolog A	UT14A_HUMAN
Ribosomal RNA processing protein 36 homolog	RRP36_HUMAN
Eukaryotic initiation factor 4A-I	IF4A1_HUMAN
Phosphoglycerate kinase 1	PGK1_HUMAN
SAP30-binding protein	S30BP_HUMAN
Syndecan-4	SDC4_HUMAN
F-BAR and double SH3 domains protein 2	FCSD2_HUMAN
SH3 domain-binding glutamic acid-rich-like protein 2	SH3L2_HUMAN
EKC/KEOPS complex subunit TPRKB	TPRKB_HUMAN
U1 small nuclear ribonucleoprotein A	SNRPA_HUMAN
Poly(ADP-ribose) glycohydrolase	PARG_HUMAN
Protein LSM12	LSM12_HUMAN
THO complex subunit 5 homolog	THOC5_HUMAN
CDGSH iron-sulfur domain-containing protein 1	CISD1_HUMAN
Influenza virus NS1A-binding protein	NS1BP_HUMAN
Large proline-rich protein BAG6	BAG6_HUMAN
Multidrug resistance-associated protein 1	MRP1_HUMAN
LIM domain and actin-binding protein 1	LIMA1_HUMAN
Chromobox protein homolog 3	CBX3_HUMAN
Delta(24)-sterol reductase	DHC24_HUMAN
Sperm-associated antigen 5	SPAG5_HUMAN
Ribosomal RNA-processing protein 8	RRP8_HUMAN
Nucleolar MIF4G domain-containing protein 1	NOM1_HUMAN
Peroxiredoxin-like 2C	PXL2C_HUMAN
Serine-threonine kinase receptor-associated protein	STRAP_HUMAN
U5 small nuclear ribonucleoprotein TSSC4	TSSC4_HUMAN
Small nuclear ribonucleoprotein Sm D3	SMD3_HUMAN
5'-3' exonuclease PLD3	PLD3_HUMAN
Eukaryotic translation initiation factor 4H	IF4H_HUMAN
Equilibrative nucleoside transporter 1	S29A1_HUMAN
Protein SCAF11	SCAFB_HUMAN
Ran-specific GTPase-activating protein	RANG_HUMAN
Sentrin-specific protease 1	SENP1_HUMAN
Histone-lysine N-trimethyltransferase SMYD5	SMYD5_HUMAN
AP-3 complex subunit mu-1	AP3M1_HUMAN
Chromobox protein homolog 1	CBX1_HUMAN
Zinc finger CCHC domain-containing protein 9	ZCHC9_HUMAN
Protein OS-9	OS9_HUMAN
cAMP-dependent protein kinase catalytic subunit beta	KAPCB_HUMAN

Dihydrolipoyllysine-residue succinyltransferase component of 2-oxoglutarate	ODO2_HUMAN
Small ribosomal subunit protein uS15	RS13_HUMAN
S-phase kinase-associated protein 1	SKP1_HUMAN
Remodeling and spacing factor 1	RSF1_HUMAN
Polymerase delta-interacting protein 3	PDIP3_HUMAN
RNA polymerase II subunit A C-terminal domain phosphatase SSU72	SSU72_HUMAN
Metalloproteinase inhibitor 2	TIMP2_HUMAN
Small ribosomal subunit protein uS13	RS18_HUMAN
Prenylated Rab acceptor protein 1	PRAF1_HUMAN
Synaptotagmin-11	SYT11_HUMAN
Plectin	PLEC_HUMAN
Centrin-2	CETN2_HUMAN
F-box only protein 42	FBX42_HUMAN
Pre-mRNA-processing factor 17	PRP17_HUMAN
Dimethyladenosine transferase 1, mitochondrial	TFB1M_HUMAN
Thioredoxin domain-containing protein 11	TXD11_HUMAN
SH2 domain-containing protein 3C	SH2D3_HUMAN
F-box only protein 3	FBX3_HUMAN
Constitutive coactivator of PPAR-gamma-like protein 2	F120C_HUMAN
Ribonuclease P protein subunit p20	POP7_HUMAN
OTU domain-containing protein 7B	OTU7B_HUMAN
CAP-Gly domain-containing linker protein 1	CLIP1_HUMAN
Cell surface glycoprotein MUC18	MUC18_HUMAN
Cell death regulator Aven	AVEN_HUMAN
Pyridoxine-5'-phosphate oxidase	PNPO_HUMAN
Lamin-B2	LMNB2_HUMAN
Interferon regulatory factor 2-binding protein 2	I2BP2_HUMAN
1,4-alpha-glucan-branching enzyme	GLGB_HUMAN
Copper homeostasis protein cutC homolog	CUTC_HUMAN
Proline-serine-threonine phosphatase-interacting protein 2	PPIP2_HUMAN
Brefeldin A-inhibited guanine nucleotide-exchange protein 3	BIG3_HUMAN
SUN domain-containing protein 1	SUN1_HUMAN
Nuclear pore glycoprotein p62	NUP62_HUMAN
Large ribosomal subunit protein eL36	RL36_HUMAN
Coiled-coil domain-containing protein 127	CC127_HUMAN
Sterol O-acyltransferase 1	SOAT1_HUMAN
Heterogeneous nuclear ribonucleoprotein U	HNRPU_HUMAN
Glucose-6-phosphate exchanger SLC37A4	G6PT1_HUMAN
Puratrophin-1	PKHG4_HUMAN
Phosphatidate cytidylyltransferase 2	CDS2_HUMAN
Numb-like protein	NUMBL_HUMAN
Integrin beta-3	ITB3_HUMAN
Large ribosomal subunit protein uL10	RLA0_HUMAN
Serine/threonine-protein phosphatase 4 regulatory subunit 3B	P4R3B_HUMAN
Required for meiotic nuclear division protein 1 homolog	RMND1_HUMAN
RCC1-like G exchanging factor-like protein	RCC1L_HUMAN
Tyrosine-protein kinase ABL2	ABL2_HUMAN
Ubiquitin-like protein ISG15	ISG15_HUMAN
Targeting protein for Xklp2	TPX2_HUMAN
Esterase OVCA2	OVCA2_HUMAN

ADP-ribosylation factor GTPase-activating protein 1	ARFG1_HUMAN
Cytoplasmic tRNA 2-thiolation protein 1	CTU1_HUMAN
Serine/threonine-protein kinase Nek3	NEK3_HUMAN
Transcription initiation factor TFIID subunit 8	TAF8_HUMAN
Protein yippee-like 5	YPEL5_HUMAN
KH domain-containing, RNA-binding, signal transduction-associated protein 3	KHDR3_HUMAN
Ubiquitin carboxyl-terminal hydrolase 33	UBP33_HUMAN
Transforming acidic coiled-coil-containing protein 2	TACC2_HUMAN
Nascent polypeptide-associated complex subunit alpha	NACAM_HUMAN
SH3 domain-binding protein 5	3BP5_HUMAN
Probable ATP-dependent RNA helicase DDX5	DDX5_HUMAN
Protein odr-4 homolog	ODR4_HUMAN
ER membrane protein complex subunit 6	EMC6_HUMAN

# Unique T	% Change	Ratio
32	161.051	2.61051
6	-72.7906	0.272094
6	-60.9088	0.390912
17	-13.3378	0.866622
11	-44.3672	0.556328
17	-20.1488	0.798512
9	-26.3585	0.736415
16	54.82352	1.548235
26	-9.57127	0.904287
23	-15.5653	0.844347
22	-19.5153	0.804847
2	177.7001	2.777001
11	-37.2262	0.627738
11	-8.8582	0.911418
20	-14.8713	0.851287
12	-14.9633	0.850367
2	-79.4262	0.205738
15	-29.1984	0.708016
23	-27.8527	0.721473
14	-15.8767	0.841233
22	-3.18706	0.968129
9	-43.1544	0.568456
21	-19.0669	0.809331
34	-14.6174	0.853826
27	-5.04966	0.949503
3	-62.2314	0.377686
8	-24.1674	0.758326
3	-39.3911	0.606089
6	-11.0855	0.889145
14	-7.08657	0.929134
28	9.873624	1.098736
4	-50.3137	0.496863
11	-8.52583	0.914742
12	-29.304	0.70696
15	-8.71414	0.912859
7	-27.327	0.72673
22	-7.40887	0.925911
9	14.69965	1.146997
52	-42.3194	0.576806
5	-38.2452	0.617548
24	-5.40619	0.945938
16	-20.043	0.79957
28	12.01288	1.120129
6	23.18184	1.231818
23	-16.6713	0.833287
2	-91.8963	0.081037
12	-40.8156	0.591844
7	-12.8695	0.871305

18	-38.918	0.61082
15	-27.7019	0.722981
14	-20.533	0.79467
7	-9.72649	0.902735
24	-16.4725	0.835275
14	-10.1299	0.898701
23	-18.9805	0.810195
30	-29.3067	0.706933
15	-50.1087	0.498913
45	-19.4026	0.805974
25	-8.95393	0.910461
5	-47.3632	0.526368
13	-34.2713	0.657287
49	-37.7043	0.622957
21	41.41676	1.414168
18	-11.2022	0.887978
10	30.89896	1.30899
30	-10.2552	0.897448
3	-57.3497	0.426503
11	-16.1945	0.838055
53	-10.2417	0.897583
40	-51.3018	0.486982
46	-7.03939	0.929606
21	-21.5141	0.784859
4	-17.11	0.8289
5	-6.63837	0.933616
26	-11.4686	0.885314
18	-32.7042	0.672958
20	-10.873	0.89127
6	-4.84296	0.95157
5	-22.2665	0.777335
26	-18.3281	0.816719
15	-20.2436	0.797564
19	-2.13585	0.978641
34	-14.8407	0.851593
15	-15.9912	0.840088
14	-31.7754	0.682246
12	-6.68063	0.933194
19	-18.3449	0.816551
11	-13.7025	0.862975
9	-22.2104	0.777896
27	-25.5889	0.744111
12	-11.1755	0.888245
20	-60.6739	0.393261
6	-78.9286	0.210714
17	-8.24815	0.917519
7	-34.0329	0.659671
15	-1.88866	0.981113
21	-4.71017	0.952898
10	-8.9945	0.910055

10	-9.37232	0.906277
8	-46.8563	0.531437
4	-53.0827	0.469173
7	-25.6909	0.743091
11	-18.662	0.81338
16	-2.09721	0.979028
4	-25.2577	0.747423
5	-15.0718	0.849282
42	-10.9409	0.890591
6	-47.2896	0.527104
31	-16.1004	0.838996
7	-57.4701	0.425299
11	-0.25061	0.997494
9	-16.7721	0.832279
15	-17.9999	0.820001
19	-14.5431	0.854569
9	-19.7934	0.802066
2	-67.1374	0.328626
6	-10.7317	0.892683
3	-22.7709	0.772291
12	-23.6386	0.763614
11	-15.3581	0.846419
7	-19.6258	0.803742
20	-30.1825	0.698175
22	-15.6729	0.843271
8	9.75634	1.097563
15	-16.0575	0.839425
2	-22.5226	0.774774
9	10.79647	1.107965
11	-13.8492	0.861508
11	65.87078	1.658708
48	-27.6574	0.723426
21	-17.3789	0.826211
10	-28.8025	0.711975
39	-5.48294	0.945171
9	-8.33917	0.916608
7	-4.86756	0.951324
29	-15.3252	0.846748
2	-20.1713	0.798287
9	-20.4153	0.795847
27	-31.5879	0.684121
30	-4.69496	0.95305
2	-91.8046	0.081954
71	-25.9966	0.740034
24	-4.61274	0.953873
14	-16.8052	0.831948
9	-26.6298	0.733702
2	-43.0263	0.569737
12	-16.7591	0.832409
25	-14.5159	0.854841

27	-11.1552	0.888448
8	-36.0454	0.639546
4	-32.0953	0.679047
18	-29.6899	0.703101
22	-5.13145	0.948686
15	-18.1762	0.818238
17	0.156622	1.001566
34	-17.3855	0.826145
25	-63.5829	0.364171
31	-8.94374	0.910563
16	-16.6387	0.833613
10	-26.729	0.73271
56	-5.67729	0.943227
15	-3.73549	0.962645
18	-23.3214	0.766786
18	5.238911	1.052389
3	-36.7116	0.632884
3	-15.471	0.84529
14	-4.53066	0.954693
5	-38.9175	0.610825
10	-5.43367	0.945663
12	-4.78912	0.952109
12	-12.2767	0.877233
3	-15.26	0.8474
8	-32.7763	0.672237
32	-21.5015	0.784985
14	-9.46007	0.905399
4	-49.4118	0.505882
4	-67.5427	0.324573
11	-10.3345	0.896655
15	-17.3472	0.826528
15	5.634766	1.056348
3	-8.1463	0.918537
4	-10.9784	0.890216
14	-26.4028	0.735972
38	-22.2515	0.777485
3	-25.0579	0.749421
3	-4.99517	0.950048
4	-44.1357	0.558643
3	-4.68718	0.953128
10	-20.2078	0.797922
27	66.65239	1.666524
6	-36.1844	0.638156
2	192.2381	2.922381
34	-58.9231	0.410769
45	-11.9825	0.880175
5	-5.24296	0.94757
9	-27.9147	0.720853
22	-6.52207	0.934779
17	-8.09406	0.919059

30	-28.6461	0.713539
9	-18.9988	0.810012
6	-5.84646	0.941535
9	-7.61019	0.923898
48	-1.80701	0.98193
13	-13.3224	0.866776
6	-24.0701	0.759299
28	-9.32655	0.906734
9	-7.82558	0.921744
8	4.276308	1.042763
7	-16.7923	0.832077
9	-19.0922	0.809078
12	-25.1007	0.748993
27	-8.11183	0.918882
10	2.998363	1.029984
2	-26.69	0.7331
39	-23.9312	0.760688
4	-16.6105	0.833895
17	-28.483	0.71517
13	-21.9489	0.780511
13	-18.9867	0.810133
25	-0.754	0.99246
26	-11.3808	0.886192
6	-26.8133	0.731867
50	-4.88518	0.951148
31	-16.3459	0.836541
16	-11.7559	0.882441
9	-16.8181	0.831819
5	-25.3995	0.746005
19	-7.28206	0.927179
21	-1.0517	0.989483
18	-5.07897	0.94921
21	-22.3443	0.776557
10	-14.5853	0.854147
3	-53.5621	0.464379
6	-27.9397	0.720603
4	-37.8137	0.621863
3	-35.9803	0.640197
12	-19.1336	0.808664
15	-15.8734	0.841266
12	-19.3242	0.806758
7	-22.7513	0.772487
13	-9.54544	0.904546
13	-7.33682	0.926632
14	-15.5961	0.844039
10	-10.2929	0.897071
13	-2.97016	0.970298
26	-15.8233	0.841767
15	-22.4393	0.775607
18	-12.504	0.87496

18	-4.11618	0.958838
3	-65.6725	0.343275
16	-0.65808	0.993419
3	-37.504	0.62496
8	-0.43353	0.995665
23	-10.0148	0.899852
6	-20.0115	0.799885
21	-3.87345	0.961266
4	-45.9563	0.540437
18	-30.7445	0.692555
13	-23.1783	0.768217
13	-15.8555	0.841445
6	19.26932	1.192693
13	-20.6404	0.793596
13	-22.7389	0.772611
4	-36.8415	0.631585
38	-0.66447	0.993355
4	-24.2882	0.757118
5	-16.2064	0.837936
111	-48.9574	0.510426
107	-10.8633	0.891367
58	-7.33964	0.926604
135	-42.7236	0.572764
9	-28.7698	0.712302
10	-4.52789	0.954721
10	-3.4726	0.965274
2	-85.803	0.14197
8	-14.9028	0.850972
4	-37.3624	0.626376
43	-3.80184	0.961982
9	-19.2712	0.807288
7	-17.1527	0.828473
17	-16.2774	0.837226
16	-8.61609	0.913839
4	-10.9895	0.890105
17	5.295069	1.052951
7	-16.031	0.83969
2	-43.2748	0.567252
5	-9.77965	0.902203
15	-4.82936	0.951706
3	27.25945	1.272595
6	-12.0937	0.879063
59	-11.1715	0.888285
4	75.07814	1.750781
5	-12.8188	0.871812
21	-16.2997	0.837003
6	-9.23139	0.907686
18	-24.9109	0.750891
16	3.259645	1.032596
8	234.0112	3.340112

26	-27.823	0.72177
12	-15.7666	0.842334
23	-8.60794	0.913921
24	-5.96358	0.940364
3	-16.9952	0.830048
5	-18.2123	0.817877
4	-39.7545	0.602455
3	-52.7735	0.472265
5	-17.2	0.828
18	-12.1292	0.878708
15	-14.4257	0.855743
22	-15.1952	0.848048
46	-39.0151	0.609849
21	64.9462	1.649462
16	-12.2711	0.877289
21	-4.30175	0.956982
20	-8.15713	0.918429
4	-42.7633	0.572367
41	-2.97564	0.970244
23	-38.0113	0.619887
38	-5.1034	0.948966
14	-0.94391	0.990561
22	-13.9678	0.860322
7	-30.8566	0.691434
3	-51.2184	0.487816
35	-7.33055	0.926695
12	-24.2028	0.757972
29	1.100194	1.011002
13	-16.0415	0.839585
16	-9.4221	0.905779
25	-18.9048	0.810952
23	-8.35442	0.916456
21	-0.06167	0.999383
12	97.48001	1.9748
15	-9.75321	0.902468
27	1.369067	1.013691
2	-58.5018	0.414982
33	-6.318	0.93682
16	-7.31881	0.926812
13	-24.2611	0.757389
32	-9.98944	0.900106
8	-8.08866	0.919113
16	-23.7684	0.762316
15	-10.2044	0.897956
15	-8.40117	0.915988
8	-20.9372	0.790628
29	-32.9986	0.670014
17	-8.16955	0.918304
30	-14.5315	0.854685
8	-14.4259	0.855741

19	-13.3396	0.866604
26	-29.5123	0.704877
2	-29.8936	0.701064
17	-12.4735	0.875265
12	-47.1332	0.528668
13	-29.6643	0.703357
17	-3.31907	0.966809
16	-16.1485	0.838515
18	-19.2462	0.807538
21	-1.18987	0.988101
7	-34.3715	0.656285
3	10.04595	1.100459
28	4.295282	1.042953
31	-65.0389	0.349611
3	-67.4229	0.325771
9	3.238338	1.032383
11	-7.31951	0.926805
29	-7.77466	0.922253
4	106.1595	2.061595
34	-22.5355	0.774645
15	-34.9003	0.650997
13	-14.2192	0.857808
41	-19.6143	0.803857
76	-12.673	0.87327
19	-12.0433	0.879567
13	-14.1961	0.858039
5	-27.6745	0.723255
12	-13.8519	0.861481
27	-30.3388	0.696612
6	-47.877	0.52123
17	-26.3477	0.736523
9	-33.6996	0.663004
12	-19.2799	0.807201
12	-27.1482	0.728518
2	-38.9855	0.610145
26	-7.11685	0.928832
24	-22.5712	0.774288
3	56.94025	1.569403
16	-23.1736	0.768264
4	-57.7233	0.422767
3	18.73158	1.187316
17	-17.2659	0.827341
34	-5.19826	0.948017
5	-2.15572	0.978443
25	-7.21065	0.927894
12	-15.8173	0.841827
11	-18.1414	0.818586
40	1.615486	1.016155
9	-7.5867	0.924133
91	-26.3235	0.736765

8	-39.8367	0.601633
8	-49.4843	0.505157
9	1.7179	1.017179
6	20.52485	1.205248
17	-3.92168	0.960783
21	-15.3406	0.846594
22	6.380622	1.063806
79	-2.94855	0.970514
21	-32.7752	0.672248
5	-18.2295	0.817705
27	-6.55626	0.934437
10	-19.7543	0.802457
5	-26.3745	0.736255
45	0.018745	1.000187
12	-37.2372	0.627628
17	-0.95348	0.990465
16	-11.5726	0.884274
11	-36.6847	0.633153
8	-8.20628	0.917937
28	-2.20458	0.977954
9	-9.1759	0.908241
5	-12.992	0.87008
4	-5.76105	0.94239
4	-53.3516	0.466484
17	-10.5576	0.894424
10	-8.24735	0.917526
25	-12.9737	0.870263
3	-25.0265	0.749735
17	-14.3775	0.856225
6	-4.65582	0.953442
15	8.156078	1.081561
12	-20.3324	0.796676
17	-14.8299	0.851701
23	-11.0927	0.889073
14	-12.7383	0.872617
17	-3.10733	0.968927
12	-7.14533	0.928547
2	-69.9232	0.300768
4	-17.9429	0.820571
12	-10.2318	0.897682
42	-10.7165	0.892835
8	-5.21161	0.947884
8	6.703371	1.067034
14	-15.1978	0.848022
21	-7.39735	0.926027
16	-7.30284	0.926972
5	-40.5479	0.594521
60	-1.59428	0.984057
12	-23.0933	0.769067
15	3.444804	1.034448

15	-23.7285	0.762715
16	-5.94814	0.940519
24	-10.3031	0.896969
35	-6.50488	0.934951
9	-8.93537	0.910646
52	-6.35455	0.936454
137	-0.81017	0.991898
33	-2.38179	0.976182
131	11.34539	1.113454
14	-21.1335	0.788665
5	-68.4308	0.315692
21	-11.8563	0.881437
17	-20.7787	0.792213
18	-17.7662	0.822338
18	-19.4007	0.805993
9	-23.635	0.76365
22	0.800361	1.008004
2	-51.3643	0.486357
10	-29.2678	0.707322
27	-5.46935	0.945306
14	-16.0122	0.839878
39	-19.8951	0.801049
10	-22.0796	0.779204
31	3.382474	1.033825
15	-27.0923	0.729077
7	-15.3467	0.846533
6	-2.62624	0.973738
8	-5.33875	0.946613
15	-5.81689	0.941831
6	-15.3491	0.846509
32	-3.85866	0.961413
15	-21.4596	0.785404
3	-59.5059	0.404941
18	-1.54298	0.98457
23	-2.81237	0.971876
8	-19.6309	0.803691
48	-18.5602	0.814398
16	-4.61817	0.953818
8	-17.212	0.82788
12	-40.9659	0.590341
66	-57.5745	0.424255
20	-32.7635	0.672365
11	-9.97393	0.900261
32	-57.8041	0.421959
11	-14.851	0.85149
34	6.716483	1.067165
9	-14.6363	0.853637
17	-6.61233	0.933877
40	-9.63555	0.903645
30	2.291929	1.022919

12	-19.6561	0.803439
3	-22.0805	0.779195
6	0.127539	1.001275
23	-11.192	0.88808
6	-19.3188	0.806812
25	-4.3644	0.956356
5	-9.9338	0.900662
6	-20.9215	0.790785
37	-12.8941	0.871059
2	48.27413	1.482741
10	-3.68877	0.963112
4	-46.179	0.53821
19	-30.7579	0.692421
10	-23.8925	0.761075
40	-15.806	0.84194
9	-22.2115	0.777885
3	-18.2649	0.817351
190	-8.95386	0.910461
34	-2.29023	0.977098
22	-19.5761	0.804239
59	-3.21419	0.967858
20	-16.61	0.8339
25	-3.32893	0.966711
6	-40.9605	0.590395
8	-25.4473	0.745527
25	-5.42552	0.945745
8	-21.6478	0.783522
12	-20.4087	0.795913
26	-13.2041	0.867959
7	39.07933	1.390793
7	-15.9773	0.840227
2	-30.4139	0.695861
4	-25.379	0.74621
74	-7.60069	0.923993
3	-27.0933	0.729067
6	-15.8695	0.841305
8	-8.12795	0.91872
11	-14.6034	0.853966
8	7.348079	1.073481
13	-27.8672	0.721328
16	-10.8313	0.891687
3	-44.5147	0.554853
16	-7.77242	0.922276
9	-34.3142	0.656858
2	-34.0294	0.659706
19	-41.0406	0.589594
3	5.15765	1.051577
7	-19.8226	0.801774
11	-7.78106	0.922189
18	-10.1217	0.898783

8	-11.5873	0.884127
11	-14.8537	0.851463
44	-10.3687	0.896313
25	-13.6396	0.863604
49	-2.89545	0.971045
9	-21.4327	0.785673
49	-7.10994	0.928901
10	-23.8883	0.761117
3	-36.5088	0.634912
8	-46.3575	0.536425
27	-4.6391	0.953609
13	-33.5201	0.664799
38	-5.75336	0.942466
79	69.67851	1.696785
15	-21.7283	0.782717
10	-24.8588	0.751412
11	-54.7579	0.452421
68	-6.13462	0.938654
17	-25.0733	0.749267
7	-2.77001	0.9723
4	-44.6004	0.553996
6	-23.5905	0.764095
9	-6.93901	0.93061
25	-10.7909	0.892091
19	-10.3914	0.896086
5	-22.0551	0.779449
15	-21.1523	0.788477
24	-3.33674	0.966633
20	-3.78543	0.962146
9	-61.3387	0.386613
14	-37.9849	0.620151
11	-22.2736	0.777264
12	-19.6104	0.803896
16	-10.9581	0.890419
78	-24.3069	0.756931
5	-8.9872	0.910128
38	-10.5171	0.894829
3	-11.8915	0.881085
45	-13.6466	0.863534
22	-11.9787	0.880213
8	-19.5789	0.804211
3	-7.75451	0.922455
11	-20.1431	0.798569
12	-6.0893	0.939107
7	-34.3999	0.656001
10	-33.2728	0.667272
17	-4.20225	0.957978
23	-4.37025	0.956297
4	-0.73695	0.99263
3	-80.9609	0.190391

15	-9.77876	0.902212
7	-16.5899	0.834101
40	-6.64559	0.933544
7	-7.66073	0.923393
13	-11.6773	0.883227
23	-25.2283	0.747717
11	-11.4798	0.885202
3	-27.871	0.72129
5	-37.8001	0.621999
17	26.4242	1.264242
23	-1.44417	0.985558
15	-37.1031	0.628969
4	-33.8579	0.661421
34	-15.8947	0.841053
18	-21.8533	0.781467
18	-12.0726	0.879274
7	-26.5795	0.734205
19	-1.21654	0.987835
15	-31.9626	0.680374
17	-18.1186	0.818814
20	-2.88145	0.971185
12	-12.4483	0.875517
4	-31.6539	0.683461
3	104.0886	2.040886
26	-23.9331	0.760669
6	-9.71339	0.902866
12	-12.9669	0.870331
15	-14.3955	0.856045
2	-7.78038	0.922196
12	-20.8225	0.791775
11	-13.9992	0.860008
20	-8.28709	0.917129
10	-20.6911	0.793089
9	-11.1303	0.888697
21	-1.44823	0.985518
3	-3.91451	0.960855
11	-12.0105	0.879895
4	-0.71256	0.992874
3	-42.4914	0.575086
17	-25.5926	0.744074
9	13.70356	1.137036
12	-16.0469	0.839531
11	-0.63345	0.993665
2	-6.56084	0.934392
9	-40.8326	0.591674
4	-61.6443	0.383557
7	-29.0349	0.709651
21	-12.4316	0.875684
47	-16.9983	0.830017
4	-65.1041	0.348959

9	-13.3608	0.866392
47	-7.36159	0.926384
45	-11.225	0.88775
4	-19.2991	0.807009
11	-7.28755	0.927125
16	-10.1859	0.898141
10	-6.31204	0.93688
9	-4.1435	0.958565
15	-11.2353	0.887647
43	-9.64264	0.903574
9	-1.82212	0.981779
30	-21.11	0.7889
24	-6.94221	0.930578
27	-5.51482	0.944852
16	-21.2411	0.787589
35	-3.16273	0.968373
2	-81.6216	0.183784
18	1.896364	1.018964
12	-1.5914	0.984086
12	-11.5016	0.884984
4	-31.1301	0.688699
13	-6.0602	0.939398
12	-7.33242	0.926676
14	-16.6851	0.833149
8	-20.1998	0.798002
41	-7.22061	0.927794
13	-11.7895	0.882105
7	-12.7299	0.872701
21	-33.912	0.66088
17	-10.5927	0.894073
10	-8.86694	0.911331
16	-16.9774	0.830226
3	-19.7971	0.802029
2	-10.9223	0.890777
7	-19.2756	0.807244
31	-20.1048	0.798952
19	-14.9454	0.850546
50	5.375286	1.053753
5	-4.85074	0.951493
20	99.40267	1.994027
28	-11.4521	0.885479
40	-9.94144	0.900586
25	-5.98506	0.940149
6	-1.81623	0.981838
8	-9.41286	0.905871
8	-14.3151	0.856849
18	-14.1155	0.858845
17	-28.7275	0.712725
36	-1.21269	0.987873
23	-0.72284	0.992772

14	3.697452	1.036975
8	-20.1537	0.798463
7	-15.4562	0.845438
12	-7.95785	0.920421
6	-65.4554	0.345446
11	-11.4251	0.885749
4	-57.2374	0.427626
8	287.7495	3.877495
23	-13.2527	0.867473
6	-34.6703	0.653297
2	-27.1389	0.728611
9	-43.1554	0.568446
13	-4.92103	0.95079
24	-4.4127	0.955873
4	-69.1161	0.308839
5	-25.0523	0.749477
10	-6.65383	0.933462
20	-11.8616	0.881384
11	-8.37532	0.916247
13	-10.2939	0.897061
104	-7.15008	0.928499
19	-14.3057	0.856943
13	-16.412	0.83588
9	-19.5172	0.804828
30	-13.6025	0.863975
8	-14.6455	0.853545
8	-37.4917	0.625083
15	5.84431	1.058443
16	-16.9937	0.830063
6	-35.9555	0.640445
19	-8.20482	0.917952
6	-9.19803	0.90802
19	-11.6527	0.883473
13	29.75712	1.297571
4	-21.8345	0.781655
8	11.97683	1.119768
8	1.685744	1.016857
23	-7.18766	0.928123
17	-21.8458	0.781542
10	-10.8616	0.891384
2	-30.11	0.6989
36	-32.2652	0.677348
5	-7.56502	0.92435
7	-40.7333	0.592667
19	-6.80138	0.931986
4	7.334286	1.073343
30	-7.43047	0.925695
8	-5.5889	0.944111
31	-4.88468	0.951153
9	-28.5786	0.714214

4	6.889234	1.068892
2	-31.1623	0.688377
15	-11.1243	0.888757
4	-24.9012	0.750988
7	44.19308	1.441931
16	-12.2516	0.877484
12	-17.9521	0.820479
27	-8.1105	0.918895
22	-16.9243	0.830757
27	-7.11234	0.928877
251	-3.73551	0.962645
3	-26.3541	0.736459
28	-5.31147	0.946885
2	-39.8193	0.601807
26	-8.98689	0.910131
21	11.75933	1.117593
12	-4.32988	0.956701
20	-9.10835	0.908917
6	-24.8291	0.751709
10	-15.2311	0.847689
11	-30.7911	0.692089
6	-47.3301	0.526699
5	-6.41924	0.935808
3	-54.8676	0.451324
17	-23.0729	0.769271
16	-4.72681	0.952732
6	-15.8563	0.841437
76	-7.80696	0.92193
18	-13.8789	0.861211
6	-19.6091	0.803909
16	12.30419	1.123042
10	-7.37075	0.926292
21	-10.1749	0.898251
27	-10.9606	0.890394
5	-20.1008	0.798992
18	3.535435	1.035354
44	-1.543	0.98457
4	-5.21882	0.947812
15	-8.62051	0.913795
12	-15.8931	0.841069
27	24.19805	1.241981
44	-6.5956	0.934044
33	-4.71299	0.95287
40	2.00929	1.020093
14	-4.82264	0.951774
17	-4.51435	0.954856
15	-6.1357	0.938643
2	84.50296	1.84503
44	-1.1519	0.988481
11	-18.3452	0.816548

9	-41.7445	0.582555
17	-15.3971	0.846029
15	-19.6305	0.803695
5	-76.8148	0.231852
16	-46.862	0.53138
2	-79.2989	0.207011
16	-21.9364	0.780636
20	-6.89174	0.931083
3	-9.52217	0.904778
7	-15.6442	0.843558
4	-33.7585	0.662415
29	-2.95433	0.970457
3	-61.2639	0.387361
22	-8.96114	0.910389
27	-17.018	0.82982
10	-14.9418	0.850582
21	-11.4347	0.885653
15	2.855363	1.028554
12	1.208821	1.012088
12	-1.64353	0.983565
58	-55.0247	0.449753
5	-2.46835	0.975317
2	13.41119	1.134112
6	-8.31915	0.916809
7	-0.91472	0.990853
16	-43.8422	0.561578
16	2.083178	1.020832
3	-9.93101	0.90069
8	-13.739	0.86261
7	-8.52481	0.914752
22	-5.49308	0.945069
17	-6.30956	0.936904
9	-65.7192	0.342808
11	-25.8379	0.741621
54	-1.74829	0.982517
21	-28.6779	0.713221
16	-2.37456	0.976254
2	-79.2334	0.207666
13	-8.23044	0.917696
13	7.443434	1.074434
8	-8.75428	0.912457
5	-38.637	0.61363
16	-5.45313	0.945469
2	-51.3226	0.486774
9	-20.1207	0.798793
6	-18.4218	0.815782
2	-13.7034	0.862966
51	8.751324	1.087513
14	-8.26718	0.917328
11	-3.72288	0.962771

27	-8.57808	0.914219
22	-11.3255	0.886745
3	-27.4052	0.725948
5	-37.5443	0.624557
25	-8.54949	0.914505
18	8.776462	1.087765
43	-15.7014	0.842986
25	2.309475	1.023095
4	-17.1459	0.828541
13	-9.5321	0.904679
10	1.61555	1.016156
10	-41.8747	0.581253
5	-22.3307	0.776693
5	-21.1342	0.788658
42	-6.99427	0.930057
38	-3.36842	0.966316
75	0.035189	1.000352
23	-1.30952	0.986905
2	-52.739	0.47261
9	-8.75473	0.912453
31	-5.43661	0.945634
29	-4.9589	0.950411
52	-7.00784	0.929922
47	-2.14472	0.978553
12	-10.601	0.89399
9	-5.49924	0.945008
11	-17.4825	0.825175
8	-19.8336	0.801664
10	-23.2855	0.767145
10	-6.09102	0.93909
47	-14.5581	0.854419
27	93.62671	1.936267
51	-14.0706	0.859294
12	-4.62104	0.95379
5	-31.836	0.68164
13	-13.0221	0.869779
3	-44.0882	0.559118
12	2.988322	1.029883
8	-16.3566	0.836434
21	-0.42481	0.995752
13	-11.7725	0.882275
14	-0.2912	0.997088
56	-0.91525	0.990848
9	148.9817	2.489817
10	-7.68779	0.923122
10	-42.4801	0.575199
2	-52.8013	0.471987
31	-7.07708	0.929229
4	-40.7233	0.592767
6	-5.84944	0.941506

8	4.876908	1.048769
3	-35.4873	0.645127
12	2.742224	1.027422
12	-2.00764	0.979924
2	-23.1242	0.768758
24	-12.7783	0.872217
2	-26.3634	0.736366
25	-22.4411	0.775589
22	-5.0275	0.949725
20	-8.1675	0.918325
9	-6.59099	0.93409
15	-4.98762	0.950124
15	-0.96593	0.990341
7	-39.2416	0.607584
51	-4.07051	0.959295
19	-5.02818	0.949718
10	-12.1664	0.878336
16	-8.47322	0.915268
15	-32.1642	0.678358
5	-38.657	0.61343
20	-7.77366	0.922263
14	-7.50199	0.92498
16	-0.93745	0.990625
13	10.19155	1.101915
107	0.956153	1.009562
7	-10.3115	0.896885
22	0.393014	1.00393
25	-11.2852	0.887148
25	0.635115	1.006351
17	-7.32796	0.92672
18	0.860516	1.008605
24	0.431571	1.004316
31	-10.9036	0.890964
15	-32.7016	0.672984
13	-23.4032	0.765968
4	-21.7571	0.782429
14	1.720829	1.017208
9	-45.0872	0.549128
5	-27.5385	0.724615
7	-23.7694	0.762306
19	-7.05816	0.929418
3	-28.4051	0.715949
9	-5.67822	0.943218
12	-23.7364	0.762636
17	0.418289	1.004183
38	-12.7307	0.872693
8	7.516231	1.075162
6	-14.3358	0.856642
9	-18.5055	0.814945
4	-10.5094	0.894906

5	-40.307	0.59693
2	-78.158	0.21842
36	-5.60853	0.943915
2	-11.3154	0.886846
15	-2.3588	0.976412
18	-9.875	0.90125
6	-29.3688	0.706312
6	-13.2244	0.867756
5	-48.2181	0.517819
32	-16.2605	0.837395
9	-43.6915	0.563085
4	-57.6079	0.423921
35	-2.9476	0.970524
9	-0.27393	0.997261
12	-7.21735	0.927826
36	-11.4783	0.885217
11	-2.77852	0.972215
4	-49.5015	0.504985
11	-27.8106	0.721894
17	1.843237	1.018432
4	-32.8518	0.671482
12	-8.24982	0.917502
35	-14.7709	0.852291
58	-14.8629	0.851371
22	2.585745	1.025857
12	0.022313	1.000223
5	-15.7292	0.842708
10	-5.43176	0.945682
9	-4.88956	0.951104
6	-5.60964	0.943904
7	-5.35318	0.946468
17	2.324283	1.023243
9	-0.7667	0.992333
10	-12.2252	0.877748
19	-5.8103	0.941897
6	-41.2428	0.587572
4	-11.9372	0.880628
3	-33.7507	0.662493
5	-30.5318	0.694682
28	-12.5376	0.874624
12	-30.0055	0.699945
4	-41.7862	0.582138
5	-21.9063	0.780937
6	-38.2694	0.617306
13	-4.88323	0.951168
4	-43.1152	0.568848
10	-15.8589	0.841411
10	-9.74556	0.902544
33	-7.5768	0.924232
3	-11.7634	0.882366

31	-7.57717	0.924228
23	-17.5585	0.824415
9	-17.1992	0.828008
14	-0.14664	0.998534
8	7.625612	1.076256
5	-27.5717	0.724283
5	-24.2923	0.757077
23	-9.63087	0.903691
11	-22.4613	0.775387
3	-9.84614	0.901539
17	-1.84153	0.981585
8	-12.5117	0.874883
4	264.086	3.64086
9	-21.0499	0.789501
8	-7.93956	0.920604
35	0.685511	1.006855
2	-57.7718	0.422282
4	-27.5427	0.724573
5	-42.2783	0.577217
18	-6.86289	0.931371
4	-20.4073	0.795927
8	-0.81481	0.991852
16	-22.2547	0.777453
14	-6.43628	0.935637
5	-3.82375	0.961763
15	-1.40634	0.985937
3	-11.2699	0.887301
14	-9.01881	0.909812
12	-19.5761	0.804239
4	-6.41155	0.935885
22	-3.49982	0.965002
34	-1.48582	0.985142
7	7.414697	1.074147
9	-28.1877	0.718123
58	-0.29016	0.997098
45	-2.76543	0.972346
15	-16.1836	0.838164
8	0.251346	1.002513
4	-3.3553	0.966447
9	0.222682	1.002227
11	-20.2211	0.797789
8	-12.5324	0.874676
5	-19.0777	0.809223
15	-15.5716	0.844284
9	-3.89637	0.961036
4	-39.8088	0.601912
10	-2.03476	0.979652
8	-7.8167	0.921833
3	-13.4247	0.865753
4	-23.9683	0.760317

4	-32.1057	0.678943
16	-9.15232	0.908477
12	-6.2319	0.937681
20	-15.0351	0.849649
3	-17.7965	0.822035
13	-19.1442	0.808558
5	-44.3621	0.556379
7	-23.5653	0.764347
4	-31.0196	0.689804
34	-8.14713	0.918529
5	-38.1875	0.618125
15	-16.7206	0.832794
38	-0.96172	0.990383
41	-9.8013	0.901987
8	-18.506	0.81494
103	-1.86118	0.981388
8	-0.39492	0.996051
20	-16.5269	0.834731
11	-4.52298	0.95477
9	-15.3774	0.846226
14	-6.90788	0.930921
6	-7.09558	0.929044
10	-21.3936	0.786064
20	5.94377	1.059438
25	-1.84792	0.981521
33	-3.51306	0.964869
4	-10.1529	0.898471
8	-43.5942	0.564058
7	-19.3819	0.806181
5	-16.1804	0.838196
14	-3.86484	0.961352
43	-6.86768	0.931323
20	-9.9756	0.900244
14	3.201831	1.032018
17	-11.7059	0.882941
5	-13.8108	0.861892
3	-31.6266	0.683734
4	-13.0833	0.869167
14	1.297379	1.012974
21	-13.317	0.86683
6	-16.776	0.83224
17	-17.2537	0.827463
3	-43.023	0.56977
20	-49.6066	0.503934
4	-4.9907	0.950093
14	-14.7053	0.852947
6	-33.1598	0.668402
24	-8.75679	0.912432
3	-5.67891	0.943211
13	-6.57106	0.934289

25	-14.2233	0.857767
9	-11.0794	0.889206
10	-19.571	0.80429
15	-14.3426	0.856574
6	-15.9291	0.840709
13	-0.35355	0.996465
7	-50.8765	0.491235
9	6.510761	1.065108
3	22.89813	1.228981
35	-23.5942	0.764058
26	16.66669	1.166667
19	-6.60644	0.933936
2	-36.1729	0.638271
6	-45.1486	0.548514
20	-8.43572	0.915643
42	22.95657	1.229566
24	7.248186	1.072482
16	-7.76739	0.922326
24	-2.77111	0.972289
24	-2.45089	0.975491
9	-16.0579	0.839421
8	-21.2041	0.787959
15	-0.57802	0.99422
15	6.474752	1.064748
14	-2.64179	0.973582
15	-11.1138	0.888862
8	-37.6418	0.623582
7	-15.9424	0.840576
16	-6.14857	0.938514
12	-0.02054	0.999795
29	-13.2738	0.867262
23	-7.60385	0.923962
3	-19.4465	0.805535
15	-3.28884	0.967112
22	-10.0883	0.899117
62	1.867714	1.018677
22	-5.84594	0.941541
13	-6.01226	0.939877
9	-10.7073	0.892927
33	-2.69952	0.973005
23	-2.60434	0.973957
36	-4.25933	0.957407
11	-2.00854	0.979915
7	-21.6148	0.783852
6	-16.7396	0.832604
15	-8.0742	0.919258
6	-23.0007	0.769993
35	-6.63554	0.933645
11	-8.81373	0.911863
5	-21.7429	0.782571

20	-4.81761	0.951824
7	-30.0026	0.699974
5	-52.9315	0.470685
53	-3.29868	0.967013
18	-13.6504	0.863496
21	-16.7211	0.832789
11	-6.38042	0.936196
25	-16.7362	0.832638
11	-22.6736	0.773264
11	-16.0618	0.839382
15	-6.35194	0.936481
4	-23.0392	0.769608
3	-24.2239	0.757761
22	2.398161	1.023982
12	-4.85195	0.951481
10	7.142283	1.071423
31	-8.58389	0.914161
4	-37.4335	0.625665
2	-75.085	0.24915
63	-10.6367	0.893633
7	-12.8184	0.871816
2	-24.3575	0.756425
15	-5.40015	0.945999
7	-10.7672	0.892328
3	-4.00689	0.959931
15	-7.30252	0.926975
5	-23.6111	0.763889
2	-47.866	0.52134
16	11.20096	1.11201
14	-15.0045	0.849955
4	4.401137	1.044011
39	4.685195	1.046852
13	-18.3853	0.816147
32	-8.28975	0.917102
8	-7.50038	0.924996
19	-3.16554	0.968345
8	-8.74879	0.912512
21	-4.20161	0.957984
17	-5.79809	0.942019
13	-9.15918	0.908408
10	-14.746	0.85254
12	-9.75434	0.902457
36	-4.03208	0.959679
20	-8.62459	0.913754
7	-22.6657	0.773343
10	-14.3746	0.856254
7	-26.8437	0.731563
12	-18.9865	0.810135
15	11.14826	1.111483
13	-2.64196	0.97358

7	-11.6931	0.883069
12	-3.54039	0.964596
9	-13.2614	0.867386
14	-16.39	0.8361
11	-6.05215	0.939478
17	-7.09778	0.929022
4	-17.3724	0.826276
6	-4.28083	0.957192
4	-40.6827	0.593173
7	-14.9602	0.850398
12	-13.3811	0.866189
4	-13.2827	0.867173
11	1.736389	1.017364
16	-3.10672	0.968933
12	-0.7651	0.992349
10	-43.367	0.56633
16	-3.75591	0.962441
3	-42.1127	0.578873
9	-17.6122	0.823878
9	23.04649	1.230465
12	-14.5044	0.854956
8	-21.5645	0.784355
6	-11.3064	0.886936
12	-5.17738	0.948226
12	-10.7701	0.892299
6	-18.791	0.81209
31	-19.5571	0.804429
10	8.523966	1.08524
10	-4.54512	0.954549
6	-6.58795	0.93412
18	-7.43173	0.925683
8	-21.1544	0.788456
34	19.86678	1.198668
21	-16.437	0.83563
20	-6.86599	0.93134
7	-31.6855	0.683145
8	-0.2305	0.997695
2	11.05717	1.110572
4	-19.3837	0.806163
11	-9.89556	0.901044
16	-1.10439	0.988956
19	1.762084	1.017621
10	-0.1423	0.998577
11	0.300522	1.003005
45	-4.98393	0.950161
17	-1.94473	0.980553
7	-4.11153	0.958885
15	1.915331	1.019153
6	-13.4944	0.865056
6	-26.5154	0.734846

6	-31.0348	0.689652
5	-34.6717	0.653283
61	23.26597	1.23266
19	-16.3985	0.836015
22	-6.74832	0.932517
22	-29.1671	0.708329
5	178.722	2.78722
6	-10.5132	0.894868
42	2.872861	1.028729
3	-11.3842	0.886158
34	-5.62373	0.943763
12	-8.89435	0.911057
5	4.163067	1.041631
32	-52.2789	0.477211
32	-25.1841	0.748159
6	-12.569	0.87431
10	-3.82389	0.961761
33	-6.83035	0.931696
15	-8.67995	0.913201
8	-17.64	0.8236
8	-11.8883	0.881117
10	-28.2605	0.717395
9	-24.2466	0.757534
14	-11.8545	0.881455
31	-14.1825	0.858175
3	-56.8746	0.431254
20	-3.5196	0.964804
7	-12.0327	0.879673
12	-16.2051	0.837949
18	-20.6608	0.793392
13	-12.2083	0.877917
19	-5.45909	0.945409
13	9.446767	1.094468
5	-27.8094	0.721906
19	-17.2344	0.827656
9	-5.59094	0.944091
19	11.54474	1.115447
12	-9.04354	0.909565
8	1.429326	1.014293
3	-41.0927	0.589073
32	-1.67156	0.983284
20	-7.25137	0.927486
14	-5.2802	0.947198
27	-11.678	0.88322
21	-11.922	0.88078
4	-22.7565	0.772435
9	-23.1273	0.768727
3	9.887085	1.098871
15	4.220027	1.0422
11	-10.7281	0.892719

8	-18.1231	0.818769
11	2.12434	1.021243
39	-7.96709	0.920329
96	-6.45979	0.935402
12	-9.72202	0.90278
3	-4.44171	0.955583
29	5.165628	1.051656
7	6.190113	1.061901
18	-10.945	0.89055
16	-6.34093	0.936591
49	-0.03167	0.999683
33	-5.79934	0.942007
12	-2.7802	0.972198
5	-20.8239	0.791761
5	29.37523	1.293752
8	-12.3441	0.876559
16	11.23609	1.112361
22	-1.37651	0.986235
24	0.71088	1.007109
24	-5.42936	0.945706
7	-10.0303	0.899697
4	-33.0246	0.669754
5	-1.64438	0.983556
6	-21.5158	0.784842
30	1.641042	1.01641
64	-1.94569	0.980543
3	-31.2524	0.687476
42	-20.2186	0.797814
6	-61.7779	0.382221
37	-3.36691	0.966331
9	107.5406	2.075406
10	-19.2234	0.807766
5	-39.2016	0.607984
20	10.64867	1.106487
27	-14.9287	0.850713
14	-13.3006	0.866994
24	4.277832	1.042778
3	-43.0843	0.569157
19	-15.2197	0.847803
2	8.595567	1.085956
6	-19.5224	0.804776
8	-0.63833	0.993617
11	-7.07027	0.929297
10	-8.13943	0.918606
18	2.395276	1.023953
34	4.666854	1.046669
9	-50.1753	0.498247
7	4.741105	1.047411
41	-4.86506	0.951349
10	-16.1832	0.838168

34	-4.97493	0.950251
8	-12.3216	0.876784
10	-0.24862	0.997514
21	1.1445	1.011445
5	-20.8183	0.791817
3	60.05308	1.600531
8	-7.79034	0.922097
6	26.2215	1.262215
6	-20.729	0.79271
5	7.925552	1.079256
17	-6.04701	0.93953
24	-4.98826	0.950117
4	-36.6005	0.633995
5	-30.6222	0.693778
55	-9.1635	0.908365
15	-22.4196	0.775804
4	-11.5773	0.884227
12	-11.0604	0.889396
10	-17.2209	0.827791
9	-11.5503	0.884497
21	4.134689	1.041347
4	-10.2613	0.897387
19	-0.63191	0.993681
33	-2.44833	0.975517
17	-11.3926	0.886074
4	-14.9699	0.850301
23	-3.21673	0.967833
8	-15.8014	0.841986
6	-17.9093	0.820907
16	-6.13926	0.938607
11	-6.04902	0.93951
6	-4.61639	0.953836
9	-7.10975	0.928903
2	-14.1909	0.858091
18	-11.8575	0.881425
48	-14.7249	0.852751
26	1.609579	1.016096
13	-5.85053	0.941495
16	-6.97394	0.930261
34	1.637969	1.01638
23	-7.03396	0.92966
10	3.876005	1.03876
11	-5.19512	0.948049
13	-10.691	0.89309
8	-10.0332	0.899668
23	-8.04145	0.919586
6	-2.08054	0.979195
7	3.376824	1.033768
18	-12.103	0.87897
7	-3.3319	0.966681

17	-2.15958	0.978404
14	-3.69897	0.96301
17	6.691569	1.066916
20	2.165246	1.021652
5	47.11743	1.471174
11	-7.85756	0.921424
6	-5.65154	0.943485
3	76.09104	1.76091
7	-35.8241	0.641759
3	-47.7856	0.522144
12	-28.3442	0.716558
22	-4.30965	0.956904
24	-6.71462	0.932854
16	13.91304	1.13913
11	-2.24731	0.977527
31	-21.0041	0.789959
3	93.43182	1.934318
5	2.257281	1.022573
15	-21.9492	0.780508
15	-19.986	0.80014
10	-19.1356	0.808644
22	-3.33257	0.966674
16	-40.7086	0.592914
11	-14.3383	0.856617
5	-11.1741	0.888259
10	-11.0403	0.889597
16	-5.90615	0.940938
10	-4.49619	0.955038
8	-10.6107	0.893893
6	-27.699	0.72301
6	-40.616	0.59384
24	-14.7188	0.852812
9	-17.295	0.82705
42	-13.5024	0.864976
3	-83.3909	0.166091
14	-16.6941	0.833059
22	-23.3363	0.766637
14	-1.68221	0.983178
16	2.354035	1.02354
24	0.545757	1.005458
12	1.714443	1.017144
7	-36.286	0.63714
20	-5.18003	0.9482
6	-14.6949	0.853051
2	-87.8439	0.121561
28	-16.8698	0.831302
30	-0.13818	0.998618
30	-9.66819	0.903318
11	-20.292	0.79708
7	-22.5373	0.774627

41	-3.90459	0.960954
14	-38.7256	0.612744
15	-8.17442	0.918256
9	-6.65043	0.933496
8	-54.8843	0.451157
5	-9.34455	0.906554
8	-40.325	0.59675
28	2.669366	1.026694
10	-33.7241	0.662759
20	-1.982	0.98018
25	23.42451	1.234245
14	-8.74207	0.912579
13	-18.7647	0.812353
46	-8.07267	0.919273
12	-6.7229	0.932771
7	-1.85787	0.981421
12	-6.99807	0.930019
5	-7.64602	0.92354
26	2.804401	1.028044
25	3.122753	1.031228
5	43.99703	1.43997
16	-12.2177	0.877823
11	-6.1433	0.938567
17	-7.78099	0.92219
37	-3.41655	0.965835
24	-2.99451	0.970055
4	-37.0009	0.629991
21	0.46403	1.00464
39	-0.42911	0.995709
32	-7.95337	0.920466
15	1.443797	1.014438
5	-22.8542	0.771458
35	-9.93944	0.900606
9	-9.57665	0.904234
20	-7.51652	0.924835
17	-4.75116	0.952488
5	-51.7456	0.482544
30	-7.07985	0.929201
3	-18.0662	0.819338
2	-47.6513	0.523487
21	-4.16212	0.958379
15	1.727769	1.017278
24	-2.1092	0.978908
3	4.458336	1.044583
4	91.52431	1.915243
5	-13.0909	0.869091
33	-16.7692	0.832308
6	-16.6447	0.833553
12	-7.77165	0.922284
17	-8.0062	0.919938

6	-19.7631	0.802369
7	-71.9165	0.280835
4	-46.1839	0.538161
3	-10.7182	0.892818
17	-5.16379	0.948362
26	-26.9301	0.730699
5	-17.4247	0.825753
8	-19.5334	0.804666
31	-2.15089	0.978491
14	2.414166	1.024142
18	-11.5125	0.884875
10	-7.64558	0.923544
3	-17.9465	0.820535
4	-13.7596	0.862404
16	-6.66909	0.933309
15	3.242057	1.032421
17	-26.2418	0.737582
9	-2.857	0.97143
2	-66.955	0.33045
47	2.009917	1.020099
26	-7.72687	0.922731
19	43.64334	1.436433
7	-11.3131	0.886869
13	-7.32386	0.926761
21	-15.9284	0.840716
4	94.81733	1.948173
8	14.27185	1.142718
13	17.11746	1.171175
10	-10.8303	0.891697
46	-3.23441	0.967656
21	9.532593	1.095326
10	-12.0986	0.879014
9	-19.9579	0.800421
4	-6.22723	0.937728
7	-10.4987	0.895013
25	2.064707	1.020647
11	-1.37391	0.986261
17	-16.7453	0.832547
7	0.403123	1.004031
25	8.26921	1.082692
4	-13.5749	0.864251
3	-23.1794	0.768206
14	-8.16582	0.918342
22	-2.95151	0.970485
16	-21.3792	0.786208
7	-5.17666	0.948233
13	-17.0142	0.829858
21	-21.0421	0.789579
14	-4.04098	0.95959
5	9.484952	1.09485

53	-6.01226	0.939877
11	-0.41719	0.995828
18	-15.584	0.84416
13	-8.76267	0.912373
15	-8.65838	0.913416
21	-3.79831	0.962017
8	-6.02139	0.939786
9	-39.7898	0.602102
5	-19.7072	0.802928
28	-3.74277	0.962572
11	-11.0281	0.889719
17	-4.31507	0.956849
58	3.596709	1.035967
9	3.261138	1.032611
7	-17.8083	0.821917
9	-26.4569	0.735431
4	-23.4623	0.765377
13	-6.17827	0.938217
3	-89.0303	0.109697
20	-5.0548	0.949452
11	-25.6837	0.743163
27	4.888724	1.048887
3	-46.2022	0.537978
14	-2.55014	0.974499
8	44.93556	1.449356
13	-5.05242	0.949476
8	-20.3761	0.796239
9	11.6766	1.116766
42	-3.01339	0.969866
23	-0.00364	0.999964
6	-4.79085	0.952091
5	-35.1171	0.648829
17	3.684607	1.036846
40	-5.62793	0.943721
17	-26.1225	0.738775
8	-26.1135	0.738865
26	-5.95315	0.940469
6	1.77178	1.017718
7	-13.9851	0.860149
14	6.604233	1.066042
58	-1.7839	0.982161
2	2.597746	1.025977
5	-19.1526	0.808474
37	0.206428	1.002064
12	-8.04699	0.91953
7	-10.3574	0.896426
7	-13.9083	0.860917
13	-23.2069	0.767931
9	-4.06373	0.959363
39	-14.6698	0.853302

16	-21.0432	0.789568
93	0.941818	1.009418
21	-10.1986	0.898014
11	-11.5468	0.884532
22	-14.0304	0.859696
2	-1.35992	0.986401
5	-23.4139	0.765861
12	-43.6098	0.563902
5	-34.829	0.65171
5	-10.3402	0.896598
11	-10.8968	0.891032
7	-8.54898	0.91451
21	-6.0928	0.939072
5	-37.1484	0.628516
4	-18.7947	0.812053
47	-14.1117	0.858883
18	9.201082	1.092011
23	-4.51013	0.954899
8	-17.2829	0.827171
5	-11.616	0.88384
18	-6.61785	0.933821
26	-5.61926	0.943807
13	2.284802	1.022848
10	104.1683	2.041683
8	-14.8291	0.851709
9	-36.0856	0.639144
5	80.2113	1.802113
6	-7.65517	0.923448
24	-7.38199	0.92618
13	-21.753	0.78247
18	11.75177	1.117518
15	-24.853	0.75147
15	-1.89695	0.98103
9	-16.0331	0.839669
30	-6.54919	0.934508
16	-2.85218	0.971478
13	-18.4527	0.815473
6	-54.5476	0.454524
3	-47.1152	0.528848
9	31.52113	1.315211
25	-2.37767	0.976223
10	-0.12637	0.998736
7	-35.329	0.64671
5	-21.9554	0.780446
14	-6.18563	0.938144
11	-0.65597	0.99344
7	-3.25216	0.967478
29	1.511875	1.015119
4	-5.44684	0.945532
8	4.837078	1.048371

22	-5.13012	0.948699
11	-16.9141	0.830859
37	0.618646	1.006186
13	-16.436	0.83564
10	120.8689	2.208689
13	7.43642	1.074364
6	-23.0476	0.769524
5	-5.1168	0.948832
17	-4.56462	0.954354
14	-6.94251	0.930575
6	-9.13417	0.908658
7	-31.3182	0.686818
11	-15.2035	0.847965
22	-4.21923	0.957808
30	-0.0742	0.999258
6	15.49068	1.154907
9	-12.3482	0.876518
7	-14.1992	0.858008
25	-5.80498	0.94195
7	-5.96306	0.940369
16	-6.80511	0.931949
3	-19.9346	0.800654
7	-31.6479	0.683521
9	68.71297	1.68713
29	-3.94757	0.960524
4	38.43958	1.384396
9	8.679334	1.086793
16	-9.95294	0.900471
16	-9.45338	0.905466
6	14.41264	1.144126
2	-63.0898	0.369102
6	-16.5835	0.834165
10	-10.2534	0.897466
17	-6.17894	0.938211
25	-12.6717	0.873283
9	-6.09526	0.939047
27	2.834229	1.028342
11	4.728229	1.047282
5	-23.4422	0.765578
6	-2.40262	0.975974
9	-23.7661	0.762339
13	-7.86082	0.921392
26	-3.70195	0.962981
8	5.508517	1.055085
24	3.590722	1.035907
11	-0.72108	0.992789
6	8.997797	1.089978
19	-5.78195	0.94218
5	-10.0355	0.899645
10	-1.53858	0.984614

6	-29.7289	0.702711
9	-12.6797	0.873203
3	-47.0466	0.529534
2	-0.22737	0.997726
12	5.460945	1.054609
6	-48.2323	0.517677
12	-21.5111	0.784889
9	-2.45635	0.975437
7	-11.1139	0.888861
13	-25.725	0.74275
10	-35.618	0.64382
43	3.560214	1.035602
39	3.603206	1.036032
8	-1.59409	0.984059
17	12.66486	1.126649
4	-29.5276	0.704724
7	-20.8141	0.791859
9	-18.1785	0.818215
5	-6.61124	0.933888
10	13.01345	1.130135
14	6.56646	1.065665
4	-15.4507	0.845493
5	69.23594	1.692359
18	4.63852	1.046385
11	-22.6033	0.773967
16	-1.59341	0.984066
3	-40.7241	0.592759
17	-22.5535	0.774465
10	-29.4492	0.705508
11	-14.5015	0.854985
15	-21.345	0.78655
6	-24.6831	0.753169
15	-34.4478	0.655522
8	-20.4822	0.795178
15	11.07375	1.110737
55	-6.55392	0.934461
24	2.87195	1.02872
31	-9.98162	0.900184
12	30.91362	1.309136
28	0.950785	1.009508
36	1.00545	1.010055
4	56.45128	1.564513
5	-9.12497	0.90875
15	-4.39653	0.956035
7	-30.3006	0.696994
19	-10.717	0.89283
19	-1.95549	0.980445
4	101.5067	2.015067
27	0.130482	1.001305
9	49.60795	1.496079

8	-13.074	0.86926
28	3.841346	1.038413
4	-36.8819	0.631181
64	-6.74895	0.93251
30	1.8158	1.018158
14	-5.59422	0.944058
7	-2.1189	0.978811
25	0.0648	1.000648
13	-7.61227	0.923877
14	-2.33994	0.976601
17	-22.7866	0.772134
36	-2.26547	0.977345
39	4.912413	1.049124
12	0.9163	1.009163
29	-4.66601	0.95334
4	-18.847	0.81153
3	-13.5336	0.864664
10	-8.70462	0.912954
45	-4.2849	0.957151
10	-3.82038	0.961796
8	12.87496	1.12875
9	-7.16893	0.928311
18	-14.596	0.85404
30	1.957991	1.01958
8	-34.6104	0.653896
27	-9.37165	0.906284
13	-17.2835	0.827165
18	-10.9187	0.890813
20	-4.90321	0.950968
3	-30.9575	0.690425
20	-0.58221	0.994178
3	-32.1797	0.678203
7	199.1349	2.991349
18	-25.4103	0.745897
7	-22.5553	0.774447
15	6.079135	1.060791
24	1.900396	1.019004
11	0.465295	1.004653
26	1.084912	1.010849
8	-0.10446	0.998955
11	-9.32721	0.906728
5	-19.0639	0.809361
7	-11.2762	0.887238
12	22.34365	1.223436
17	-5.24226	0.947577
21	-2.86916	0.971308
20	-1.89228	0.981077
7	21.85525	1.218553
10	-5.78483	0.942152
3	-9.95778	0.900422

7	-10.6332	0.893668
10	-15.0923	0.849077
11	26.49519	1.264952
19	-3.53373	0.964663
13	-42.1926	0.578074
15	15.17165	1.151717
33	-12.8383	0.871617
8	-35.5605	0.644395
9	-9.02613	0.909739
18	-7.48011	0.925199
26	4.045442	1.040454
13	1.950494	1.019505
36	-14.7338	0.852662
6	-1.96944	0.980306
12	-8.01652	0.919835
41	3.144167	1.031442
17	-2.67893	0.973211
12	0.065164	1.000652
4	-19.4374	0.805626
3	0.81471	1.008147
20	8.541593	1.085416
28	-1.25015	0.987499
2	-40.2637	0.597363
2	107.9287	2.079287
3	-35.9903	0.640097
18	-9.67672	0.903233
6	-30.5643	0.694357
5	-2.98257	0.970174
22	-9.77144	0.902286
15	3.910178	1.039102
6	-17.251	0.82749
25	-7.16945	0.928305
15	-17.6745	0.823255
5	-49.6731	0.503269
17	-9.30565	0.906944
27	-1.12693	0.988731
4	-8.41855	0.915815
28	-10.4312	0.895688
18	-17.5647	0.824353
25	-4.8008	0.951992
12	6.144283	1.061443
4	-20.9036	0.790964
37	-0.95367	0.990463
3	-9.67627	0.903237
15	-10.5927	0.894073
2	-45.0995	0.549005
4	-23.7971	0.762029
8	0.97574	1.009757
30	-8.99932	0.910007
2	-31.9028	0.680972

3	1.240183	1.012402
21	6.805542	1.068055
8	-18.4387	0.815613
6	-12.5359	0.874641
15	-9.26717	0.907328
9	-3.34481	0.966552
9	-7.55664	0.924434
30	-13.2988	0.867012
10	-5.72461	0.942754
9	-28.6919	0.713081
13	-11.762	0.88238
3	105.0235	2.050235
6	-5.06205	0.949379
17	-5.69544	0.943046
10	-8.52914	0.914709
12	6.149096	1.061491
8	-6.98882	0.930112
16	-12.8279	0.871721
7	-22.7068	0.772932
49	-8.97564	0.910244
6	-38.8077	0.611923
4	10.47587	1.104759
3	-1.283	0.98717
21	-1.60153	0.983985
9	-29.0489	0.709511
8	0.656601	1.006566
46	-8.26871	0.917313
11	1.7131	1.017131
26	3.410321	1.034103
3	-15.7955	0.842045
6	-9.91831	0.900817
5	-18.6638	0.813362
18	1.468558	1.014686
36	-2.84076	0.971592
21	-1.58338	0.984166
11	-10.9684	0.890316
9	-34.8986	0.651014
7	-8.58824	0.914118
10	-3.26789	0.967321
9	1.400773	1.014008
20	-21.3722	0.786278
17	-13.0347	0.869653
5	-16.2183	0.837817
96	-6.11078	0.938892
10	-16.4559	0.835441
18	-18.2541	0.817459
5	-24.8918	0.751082
13	119.197	2.19197
42	-6.70059	0.932994
29	-12.1538	0.878462

50	-13.4544	0.865456
8	-31.1594	0.688406
7	4.765104	1.047651
15	-13.691	0.86309
8	-17.662	0.82338
9	18.64748	1.186475
6	-26.2129	0.737871
8	-7.08869	0.929113
15	-32.6618	0.673382
5	21.26264	1.212626
5	165.8235	2.658235
13	4.452248	1.044522
2	-0.97596	0.99024
6	-25.1358	0.748642
7	-7.07104	0.92929
31	-5.13431	0.948657
5	-0.67009	0.993299
14	-15.1775	0.848225
5	-16.9813	0.830187
8	-2.07537	0.979246
16	-1.06306	0.989369
5	-37.6891	0.623109
15	-5.3464	0.946536
13	-3.0913	0.969087
12	-12.5478	0.874522
4	-36.2855	0.637145
27	-5.53465	0.944654
17	-5.18966	0.948103
7	-32.4778	0.675222
28	6.392302	1.063923
9	-9.29891	0.907011
11	-8.23663	0.917634
12	9.103384	1.091034
2	-23.324	0.76676
4	-29.3205	0.706795
29	-9.3547	0.906453
7	8.05566	1.080557
3	17.38072	1.173807
10	-48.1539	0.518461
5	3.826095	1.038261
24	5.442087	1.054421
15	-8.64927	0.913507
20	-10.6008	0.893992
11	-13.0524	0.869476
3	86.94293	1.869429
23	-13.52	0.8648
12	-24.7105	0.752895
13	-7.48323	0.925168
12	-5.51274	0.944873
6	1.645321	1.016453

6	17.45232	1.174523
10	-9.72637	0.902736
6	-4.63174	0.953683
32	-5.70264	0.942974
2	-74.8719	0.251281
9	-10.1324	0.898676
9	-19.5504	0.804496
25	-4.62649	0.953735
7	-0.32368	0.996763
21	-5.78594	0.942141
11	-28.5011	0.714989
10	-0.77076	0.992292
6	-7.86764	0.921324
2	-26.7384	0.732616
30	-28.1458	0.718542
8	-15.9991	0.840009
20	0.039084	1.000391
12	-10.3591	0.896409
12	-0.94156	0.990584
4	-30.3119	0.696881
2	-20.7548	0.792452
15	2.94269	1.029427
20	-4.12406	0.958759
13	-16.5531	0.834469
5	-70.0207	0.299793
12	-25.3737	0.746263
17	-12.383	0.87617
32	-1.57544	0.984246
62	16.57545	1.165754
34	-2.88986	0.971101
82	4.142259	1.041423
30	-0.29131	0.997087
114	1.013738	1.010137
34	-6.84473	0.931553
33	0.401563	1.004016
7	-36.6185	0.633815
7	-20.1599	0.798401
8	-28.1892	0.718108
40	2.220817	1.022208
3	-80.3622	0.196378
42	1.202	1.01202
7	-17.2641	0.827359
23	-1.37099	0.98629
11	-4.89882	0.951012
2	-91.1306	0.088694
7	7.914556	1.079146
4	-16.3243	0.836757
9	-7.70546	0.922945
6	-34.6763	0.653237
33	-11.474	0.88526

17	-4.33288	0.956671
14	-27.7008	0.722992
32	-0.81123	0.991888
13	-10.709	0.89291
19	-1.78165	0.982183
4	-19.3206	0.806794
5	-41.605	0.58395
21	-6.09235	0.939076
18	-8.66103	0.91339
11	-6.93811	0.930619
4	-37.5447	0.624553
14	-9.17164	0.908284
12	0.044573	1.000446
6	-22.487	0.77513
18	-8.89725	0.911028
13	-8.87198	0.91128
39	4.573699	1.045737
5	-21.469	0.78531
9	-8.34523	0.916548
13	-0.46482	0.995352
30	-15.5277	0.844723
12	-14.3749	0.856251
25	6.390982	1.06391
2	-66.0609	0.339391
32	-1.15528	0.988447
53	-1.95023	0.980498
8	-27.1783	0.728217
4	-17.1359	0.828641
3	-28.3011	0.716989
31	-3.55116	0.964488
17	-3.55617	0.964438
4	27.25425	1.272542
17	5.841003	1.05841
10	-12.9416	0.870584
19	-17.3712	0.826288
6	-2.32582	0.976742
4	-35.1695	0.648305
17	-12.378	0.87622
2	-6.94694	0.930531
7	-15.0845	0.849155
8	-14.0427	0.859573
7	-8.33039	0.916696
58	4.256156	1.042562
8	-13.5464	0.864536
10	-17.7007	0.822993
15	-10.5612	0.894388
28	-15.0398	0.849602
6	-23.0103	0.769897
11	-10.8102	0.891898
23	0.698488	1.006985

8	-13.6221	0.863779
9	6.978024	1.06978
11	-26.7153	0.732847
11	4.503778	1.045038
5	-42.7035	0.572965
11	-6.25873	0.937413
7	0.549425	1.005494
14	-12.8715	0.871285
15	-8.05422	0.919458
20	-4.61828	0.953817
4	-10.2764	0.897236
4	-12.001	0.87999
8	-44.4564	0.555436
19	10.77669	1.107767
10	-2.77313	0.972269
24	-7.39931	0.926007
4	-7.9757	0.920243
2	-19.3979	0.806021
9	-22.7497	0.772503
13	-9.05314	0.909469
11	-5.90662	0.940934
7	-3.5023	0.964977
13	-15.5938	0.844062
10	-40.8863	0.591137
5	-12.7648	0.872352
15	-18.4469	0.815531
22	-6.48529	0.935147
15	-7.17945	0.928205
8	-6.07926	0.939207
5	-23.7641	0.762359
16	8.430899	1.084309
16	0.887421	1.008874
6	-24.3212	0.756788
3	-11.2945	0.887055
17	-0.21009	0.997899
9	-10.4968	0.895032
27	0.690088	1.006901
17	18.01051	1.180105
3	320.4241	4.204241
5	-10.0297	0.899703
9	-18.7207	0.812793
4	-18.6354	0.813646
13	2.508591	1.025086
9	-3.38388	0.966161
4	-27.6229	0.723771
19	-7.24053	0.927595
7	-9.1744	0.908256
7	-40.1679	0.598321
10	12.06386	1.120639
7	-10.6695	0.893305

5	-35.832	0.64168
9	-4.955	0.95045
12	-17.0678	0.829322
13	-2.08711	0.979129
13	5.785649	1.057856
4	-24.5723	0.754277
9	-32.2833	0.677167
28	11.97636	1.119764
5	-41.8425	0.581575
3	-27.6931	0.723069
11	2.937475	1.029375
4	-41.3552	0.586448
9	6.16238	1.061624
43	-1.01541	0.989846
10	-14.3972	0.856028
14	-1.72675	0.982733
41	3.299569	1.032996
3	77.03129	1.770313
19	-4.02937	0.959706
23	-8.15783	0.918422
12	-4.11689	0.958831
10	-8.06692	0.919331
6	-7.52379	0.924762
14	-10.5504	0.894496
15	-10.4847	0.895153
2	-12.6124	0.873876
9	-6.14697	0.93853
30	-4.10355	0.958965
39	6.896146	1.068961
6	-9.00969	0.909903
5	-32.5508	0.674492
3	-30.8928	0.691072
8	-9.69726	0.903027
18	46.15547	1.461555
11	-15.1051	0.848949
59	-4.19104	0.95809
35	0.950393	1.009504
16	-4.45208	0.955479
18	-10.1939	0.898061
5	33.92324	1.339232
9	-27.1878	0.728122
4	-18.8128	0.811872
20	-6.63754	0.933625
9	-1.00868	0.989913
5	39.05185	1.390518
64	8.531749	1.085317
2	-10.7941	0.892059
6	1.725356	1.017254
9	103.4692	2.034692
3	-6.63792	0.933621

2	-41.2338	0.587662
8	-8.89125	0.911087
37	-10.3465	0.896535
39	0.2235	1.002235
29	1.742831	1.017428
8	-23.4104	0.765896
16	-19.7844	0.802156
14	-17.5889	0.824111
11	-10.3091	0.896909
6	-6.76506	0.932349
5	-12.8364	0.871636
7	-9.66498	0.90335
8	-3.03923	0.969608
20	-19.662	0.80338
27	-0.43565	0.995643
38	-5.33903	0.94661
10	-2.02594	0.979741
8	146.6152	2.466152
6	-8.41733	0.915827
27	-1.76407	0.982359
9	-0.88716	0.991128
20	1.765294	1.017653
17	-0.09967	0.999003
8	-14.9609	0.850391
14	-37.2932	0.627068
27	-21.126	0.78874
2	-44.1482	0.558518
10	8.268049	1.08268
18	-16.8067	0.831933
6	-42.4019	0.575981
5	-5.99567	0.940043
8	3.719336	1.037193
7	-6.77577	0.932242
13	-12.0522	0.879478
14	1.365596	1.013656
39	7.362007	1.07362
10	1.686549	1.016865
26	0.541346	1.005413
17	-5.11363	0.948864
2	-3.78046	0.962195
17	-2.7434	0.972566
19	-12.2429	0.877571
6	1.823723	1.018237
10	-6.87116	0.931288
7	-19.8596	0.801404
5	-25.0546	0.749454
10	-5.26642	0.947336
23	-7.72607	0.922739
39	-5.20552	0.947945
21	14.40993	1.144099

15	95.70244	1.957024
10	-20.4587	0.795413
6	-15.6399	0.843601
21	-1.3071	0.986929
25	4.726415	1.047264
7	-15.6954	0.843046
28	-6.98111	0.930189
6	2.521866	1.025219
13	-11.8918	0.881082
8	-11.2292	0.887708
12	-32.2688	0.677312
13	-49.5763	0.504237
30	-3.09996	0.969
12	2.534612	1.025346
9	-25.38	0.7462
22	-6.9953	0.930047
13	-2.93032	0.970697
26	-12.3933	0.876067
7	-26.1108	0.738892
21	-12.3599	0.876401
15	2.341707	1.023417
9	-13.3215	0.866785
14	-13.0567	0.869433
38	-4.11445	0.958855
6	-15.3506	0.846494
2	-7.27734	0.927227
28	-3.79327	0.962067
12	-18.3051	0.816949
6	-29.9886	0.700114
23	1.145772	1.011458
8	-13.7598	0.862402
15	-13.2771	0.867229
7	-7.45827	0.925417
9	-18.8108	0.811892
13	74.01228	1.740123
44	-8.42702	0.91573
5	-20.6611	0.793389
8	-3.13092	0.968691
21	-3.31705	0.966829
11	-13.7681	0.862319
14	9.879731	1.098797
24	3.468349	1.034683
31	2.579453	1.025795
17	-6.7915	0.932085
9	-14.1565	0.858435
4	179.7851	2.797851
11	-1.56355	0.984364
23	-17.3546	0.826454
8	-14.9315	0.850685
42	-7.29217	0.927078

21	-10.7126	0.892874
11	-12.0884	0.879116
5	-26.4788	0.735212
4	-28.2263	0.717737
19	-5.3075	0.946925
26	-0.71228	0.992877
22	9.126213	1.091262
9	-8.69322	0.913068
8	-1.39497	0.98605
8	-12.7266	0.872734
33	5.829446	1.058294
4	-15.2527	0.847473
8	-2.12419	0.978758
14	-4.85313	0.951469
19	-7.22574	0.927743
10	-12.025	0.87975
5	0.635259	1.006353
20	-0.28964	0.997104
7	5.382047	1.05382
7	0.940732	1.009407
2	5.244375	1.052444
5	-6.59352	0.934065
3	-15.2484	0.847516
15	-11.1308	0.888692
2	-67.1177	0.328823
12	-11.791	0.88209
16	-14.526	0.85474
7	-34.7107	0.652893
7	2.800971	1.02801
6	-21.8344	0.781656
10	-12.6204	0.873796
9	8.138507	1.081385
9	-41.6395	0.583605
2	-50.8396	0.491604
5	-9.0126	0.909874
21	-12.9048	0.870952
13	37.87234	1.378723
11	-6.82241	0.931776
5	-44.4362	0.555638
12	-18.8106	0.811894
30	-1.33079	0.986692
9	3.887448	1.038874
15	-13.4223	0.865777
13	6.411842	1.064118
9	-1.47818	0.985218
12	-22.1637	0.778363
5	-27.2369	0.727631
11	-14.2334	0.857666
12	-10.9453	0.890547
5	-26.3702	0.736298

5	-24.1957	0.758043
6	62.27707	1.622771
3	-38.6581	0.613419
2	-39.2741	0.607259
5	-27.8858	0.721142
2	-19.9671	0.800329
5	-26.035	0.73965
27	-11.7863	0.882137
17	-0.2599	0.997401
3	-25.6818	0.743182
8	5.740992	1.05741
6	3.256792	1.032568
3	-58.2495	0.417505
47	11.31259	1.113126
5	-12.2414	0.877586
2	-56.9192	0.430808
6	65.64801	1.65648
2	-14.4716	0.855284
14	-6.15082	0.938492
10	-14.4986	0.855014
5	-21.2038	0.787962
31	2.222213	1.022222
8	-8.21571	0.917843
6	-7.41559	0.925844
11	-6.79198	0.93208
26	-2.01965	0.979804
8	-14.7011	0.852989
3	-41.9297	0.580703
5	-1.38542	0.986146
14	1.151112	1.011511
31	-8.96928	0.910307
6	-30.6022	0.693978
10	-0.77076	0.992292
7	-2.80887	0.971911
22	-0.51968	0.994803
2	-71.2182	0.287818
8	-2.87069	0.971293
8	-15.1678	0.848322
4	66.67496	1.66675
6	-13.4683	0.865317
5	-22.9202	0.770798
22	-9.88218	0.901178
30	-4.1808	0.958192
39	-1.11378	0.988862
15	-7.43635	0.925636
6	-23.7558	0.762442
20	-3.99628	0.960037
6	-3.05198	0.96948
9	11.43658	1.114366
9	-8.76424	0.912358

21	0.230342	1.002303
3	49.27165	1.492716
8	-16.2823	0.837177
19	-6.88472	0.931153
6	-35.9141	0.640859
15	2.006093	1.020061
7	16.35317	1.163532
6	-1.67229	0.983277
2	-9.97945	0.900206
16	-7.02657	0.929734
9	16.7712	1.167712
7	2.996287	1.029963
2	151.8006	2.518006
6	-3.11033	0.968897
7	-4.56225	0.954377
4	-43.7221	0.562779
19	-4.4139	0.955861
6	2.451211	1.024512
3	-49.6925	0.503075
8	-7.52753	0.924725
34	-0.13367	0.998663
8	-14.5162	0.854838
9	-32.8215	0.671785
79	4.036614	1.040366
14	-13.4977	0.865023
17	-4.5169	0.954831
39	-2.44066	0.975593
3	-26.492	0.73508
14	-0.69835	0.993017
65	-4.47822	0.955218
22	-1.47329	0.985267
18	-1.14481	0.988552
2	5.638104	1.056381
17	-8.78121	0.912188
27	-0.02418	0.999758
4	1.219744	1.012197
3	-19.3695	0.806305
12	-26.2264	0.737736
7	15.34395	1.15344
24	7.217855	1.072179
42	-1.00452	0.989955
4	-33.0877	0.669123
18	2.15526	1.021553
9	-30.9738	0.690262
10	-8.18764	0.918124
12	-19.931	0.80069
9	-17.787	0.82213
26	-1.16366	0.988363
22	3.107508	1.031075
3	-17.2787	0.827213

28	3.420099	1.034201
6	-22.6865	0.773135
16	11.7363	1.117363
4	-46.4595	0.535405
7	-0.12831	0.998717
2	-13.7828	0.862172
7	-18.3648	0.816352
33	2.888935	1.028889
13	1.548617	1.015486
8	-3.53203	0.96468
4	-25.0302	0.749698
11	-12.3091	0.876909
8	-19.7753	0.802247
20	-1.24665	0.987533
8	-28.5076	0.714924
7	7.689416	1.076894
10	-28.634	0.71366
8	-58.4548	0.415452
2	237.9058	3.379058
8	-6.04226	0.939577
6	-10.7804	0.892196
2	-22.2106	0.777894
17	7.329164	1.073292
17	-11.511	0.88489
17	3.009773	1.030098
10	3.957985	1.03958
6	-26.3719	0.736281
7	-9.21866	0.907813
11	-13.1432	0.868568
5	-5.90976	0.940902
13	-16.552	0.83448
23	-7.81528	0.921847
4	-2.84096	0.97159
19	0.661226	1.006612
7	-7.48678	0.925132
10	38.1597	1.381597
21	97.16281	1.971628
45	-7.82766	0.921723
7	-9.82623	0.901738
77	2.943307	1.029433
23	-1.22667	0.987733
7	-16.8312	0.831688
5	-5.64099	0.94359
6	-7.81398	0.92186
10	3.331694	1.033317
19	-1.63267	0.983673
11	76.76387	1.767639
8	-20.5969	0.794031
14	3.252442	1.032524
6	-16.0439	0.839561

147	-6.19214	0.938079
9	-21.3421	0.786579
11	-9.25272	0.907473
10	14.38707	1.143871
4	-40.4891	0.595109
9	-9.75701	0.90243
24	9.375324	1.093753
33	-3.76185	0.962381
24	12.30484	1.123048
19	-5.3563	0.946437
8	-10.0043	0.899957
2	-28.6878	0.713122
19	2.619175	1.026192
11	-13.9617	0.860383
5	-29.4508	0.705492
9	14.28182	1.142818
9	0.264249	1.002642
3	-20.2353	0.797647
32	-0.95425	0.990458
11	-4.56433	0.954357
30	1.248083	1.012481
6	-2.49349	0.975065
7	-23.3345	0.766655
5	-4.36271	0.956373
8	9.191567	1.091916
15	1.733668	1.017337
4	48.61165	1.486117
13	-2.9711	0.970289
13	4.498283	1.044983
17	2.476252	1.024763
32	-8.70606	0.912939
6	-11.0772	0.889228
4	40.07919	1.400792
12	-17.9431	0.820569
2	-6.15223	0.938478
44	0.563732	1.005637
22	2.248441	1.022484
9	-11.7103	0.882897
8	-17.9749	0.820251
39	-9.66493	0.903351
15	28.78794	1.287879
3	13.3596	1.133596
2	-23.868	0.76132
9	-28.9393	0.710607
3	469.5766	5.695766
7	-7.97109	0.920289
12	-13.8664	0.861336
19	-3.26537	0.967346
7	-17.0551	0.829449
7	-12.1288	0.878712

9	31.09629	1.310963
23	-14.4648	0.855352
17	2.355489	1.023555
5	-17.6256	0.823744
33	5.050555	1.050506
20	-4.57091	0.954291
5	3.45898	1.03459
10	-17.5741	0.824259
68	1.285621	1.012856
23	-2.40974	0.975903
7	-3.28286	0.967171
4	-35.7651	0.642349
11	-10.8753	0.891247
4	-12.0168	0.879832
29	-0.36581	0.996342
6	-30.7842	0.692158
4	87.19806	1.871981
7	-16.1215	0.838785
11	-19.1382	0.808618
11	3.908599	1.039086
3	-33.7536	0.662464
26	0.33505	1.003351
2	-67.2437	0.327563
4	-50.5072	0.494928
9	-25.1738	0.748262
5	-17.2905	0.827095
8	-2.56225	0.974378
10	-20.5299	0.794701
9	-1.15372	0.988463
8	11.63304	1.11633
11	-10.5912	0.894088
6	-37.6471	0.623529
18	2.890579	1.028906
50	12.47557	1.124756
13	17.93847	1.179385
7	3.637585	1.036376
21	-8.76366	0.912363
17	5.305686	1.053057
29	1.184665	1.011847
13	-2.33244	0.976676
5	10.49494	1.104949
4	-27.3214	0.726786
15	0.334959	1.00335
10	-9.94595	0.90054
11	-8.17556	0.918244
13	8.653234	1.086532
3	4.034096	1.040341
9	-10.464	0.89536
9	-2.03149	0.979685
32	13.83741	1.138374

8	-0.51976	0.994802
3	-31.3925	0.686075
32	-3.89293	0.961071
9	-29.1133	0.708867
10	-13.6545	0.863455
13	-10.0363	0.899637
14	-20.184	0.79816
17	41.26808	1.412681
17	2.444231	1.024442
5	-28.9063	0.710937
12	-4.16188	0.958381
13	-6.88945	0.931106
8	13.32313	1.133231
15	3.094039	1.03094
15	1.056904	1.010569
12	86.28546	1.862855
5	-17.9141	0.820859
37	-13.109	0.86891
31	-16.3785	0.836215
2	250.8936	3.508936
123	-2.48828	0.975117
8	-11.6648	0.883352
7	129.2524	2.292524
29	-3.456	0.96544
3	77.98115	1.779811
14	-1.49736	0.985026
11	129.0532	2.290532
4	-1.68741	0.983126
8	-1.13763	0.988624
30	25.20862	1.252086
21	-2.34983	0.976502
4	-4.68749	0.953125
24	-5.11152	0.948885
6	-6.65707	0.933429
14	1.165771	1.011658
35	0.608489	1.006085
14	-26.1235	0.738765
14	5.424972	1.05425
2	-53.845	0.46155
9	-19.7738	0.802262
3	13.66873	1.136687
5	7.079636	1.070796
23	4.80002	1.048
34	-3.44628	0.965537
9	-9.25896	0.90741
12	-1.73194	0.982681
2	107.4737	2.074737
29	-3.90447	0.960955
14	-3.83441	0.961656
2	5.687707	1.056877

3	-47.6064	0.523936
27	-2.0018	0.979982
20	0.12234	1.001223
24	-4.6142	0.953858
26	0.01819	1.000182
9	6.702568	1.067026
3	-15.9893	0.840107
5	7.761571	1.077616
7	-17.875	0.82125
3	-9.47994	0.905201
13	-10.2596	0.897404
10	-4.9746	0.950254
6	3.822768	1.038228
2	-4.73098	0.95269
21	-16.4176	0.835824
4	2.699305	1.026993
9	-2.22537	0.977746
31	-1.62921	0.983708
6	-18.8185	0.811815
7	-20.4381	0.795619
5	2.778341	1.027783
22	-9.00868	0.909913
4	54.65102	1.54651
20	-13.4856	0.865144
16	-10.7647	0.892353
8	134.363	2.34363
15	-6.23086	0.937691
12	-4.55902	0.95441
3	237.8134	3.378134
30	2.667649	1.026676
31	0.976472	1.009765
8	4.293635	1.042936
12	-6.66433	0.933357
7	-6.55181	0.934482
12	-19.9408	0.800592
21	-10.5179	0.894821
10	-1.20344	0.987966
26	5.028661	1.050287
30	-2.36364	0.976364
4	-39.1137	0.608863
9	-11.331	0.88669
14	-7.41416	0.925858
26	1.788785	1.017888
13	-0.51925	0.994807
26	7.728425	1.077284
11	-0.74254	0.992575
2	-70.1756	0.298244
3	-35.4143	0.645857
29	6.589009	1.06589
5	-8.04919	0.919508

27	6.51938	1.065194
10	-1.30091	0.986991
7	-13.9608	0.860392
6	-17.4751	0.825249
8	-21.5058	0.784942
3	27.93087	1.279309
11	-6.08638	0.939136
8	-18.9008	0.810992
32	35.06563	1.350656
14	-0.90272	0.990973
4	-7.36961	0.926304
11	39.80502	1.39805
7	53.76015	1.537601
22	2.555961	1.02556
10	-2.64708	0.973529
12	-5.42583	0.945742
6	-19.0473	0.809527
7	-8.36366	0.916363
58	-4.90106	0.950989
15	1.97673	1.019767
5	16.01388	1.160139
12	-4.01839	0.959816
4	-18.2914	0.817086
8	-10.8109	0.891891
6	10.85123	1.108512
14	-12.6656	0.873344
16	28.96963	1.289696
17	-6.0243	0.939757
13	4.974291	1.049743
12	2.115426	1.021154
3	-6.41542	0.935846
15	-0.55102	0.99449
9	1.090661	1.010907
10	-15.0069	0.849931
15	-10.1391	0.898609
5	1.35523	1.013552
3	-10.1691	0.898309
8	-18.5111	0.814889
11	9.338156	1.093382
9	-11.2858	0.887142
7	-22.7665	0.772335
17	-5.60436	0.943956
2	-38.0821	0.619179
15	-7.26863	0.927314
46	-2.33929	0.976607
35	-5.49301	0.94507
19	16.60001	1.166
14	-16.3038	0.836962
6	-0.99816	0.990018
6	-25.7734	0.742266

10	1.216121	1.012161
30	-3.66977	0.963302
15	8.03001	1.0803
18	-8.61887	0.913811
10	5.026277	1.050263
35	-2.90501	0.97095
6	-9.00969	0.909903
10	0.158368	1.001584
6	-2.07221	0.979278
10	-17.517	0.82483
4	-18.9187	0.810813
5	7.174378	1.071744
5	3.563307	1.035633
27	21.79367	1.217937
15	-10.7648	0.892352
11	-1.25065	0.987493
15	3.114945	1.031149
3	11.85924	1.118592
9	-13.9564	0.860436
56	7.778462	1.077785
48	80.48713	1.804871
5	3.57706	1.035771
7	4.822388	1.048224
7	14.28853	1.142885
8	1.773011	1.01773
5	2.436263	1.024363
3	-17.8307	0.821693
18	-2.67075	0.973292
9	-12.7178	0.872822
6	-45.2685	0.547315
6	15.96255	1.159626
12	-16.5451	0.834549
3	-7.66559	0.923344
13	-3.70313	0.962969
11	4.502472	1.045025
36	1.84309	1.018431
25	6.943418	1.069434
3	-7.54571	0.924543
6	-12.1172	0.878828
5	10.90828	1.109083
3	179.5796	2.795796
18	-2.14921	0.978508
12	11.7059	1.117059
9	2.101851	1.021019
5	-9.21912	0.907809
16	7.514644	1.075146
18	-9.79608	0.902039
8	-21.5627	0.784373
8	-6.12939	0.938706
2	-27.03	0.7297

7	24.21692	1.242169
11	23.91211	1.239121
7	33.382	1.33382
19	-9.49919	0.905008
41	-4.18396	0.95816
8	77.59319	1.775932
22	2.017418	1.020174
37	-11.5188	0.884812
6	-9.81486	0.901851
2	-41.9931	0.580069
21	-1.18337	0.988166
13	-8.14889	0.918511
2	-40.3539	0.596461
4	-2.1854	0.978146
22	0.346557	1.003466
8	0.756065	1.007561
10	-5.72724	0.942728
9	-41.6123	0.583877
3	-11.6824	0.883176
3	-24.7921	0.752079
8	145.7519	2.457519
9	-7.16669	0.928333
8	-0.53043	0.994696
21	-2.78708	0.972129
6	-42.7368	0.572632
20	5.779373	1.057794
44	-3.00873	0.969913
20	1.576118	1.015761
16	-6.53261	0.934674
9	-2.45426	0.975457
13	-1.77118	0.982288
44	1.800118	1.018001
8	-9.61194	0.903881
14	11.75348	1.117535
15	-7.17386	0.928261
13	-5.5018	0.944982
19	70.54604	1.70546
10	-0.24242	0.997576
5	-9.12569	0.908743
8	6.493816	1.064938
18	-16.705	0.83295
18	1.326781	1.013268
8	-39.8202	0.601798
9	-22.5858	0.774142
14	99.62399	1.99624
5	-28.8611	0.711389
12	-1.88163	0.981184
16	0.917026	1.00917
5	-32.866	0.67134
9	4.187609	1.041876

55	3.714211	1.037142
18	8.588007	1.08588
3	-8.13255	0.918674
15	75.28956	1.752896
2	25.45296	1.25453
14	-6.14216	0.938578
8	-4.12077	0.958792
15	-18.6373	0.813627
30	-15.9956	0.840044
10	-10.9559	0.890441
24	2.289404	1.022894
8	-20.0571	0.799429
4	-3.64177	0.963582
124	-2.98828	0.970117
16	-5.81395	0.941861
10	9.030203	1.090302
3	-22.5897	0.774103
25	-0.4647	0.995353
8	8.732695	1.087327
88	0.199168	1.001992
22	-3.52594	0.964741
22	-7.20752	0.927925
8	7.505832	1.075058
6	-19.0434	0.809566
12	-20.3041	0.796959
2	-32.6669	0.673331
8	2.565974	1.02566
7	6.569101	1.065691
51	-4.41801	0.95582
5	-36.3981	0.636019
11	-5.91041	0.940896
12	5.461339	1.054613
19	-4.43591	0.955641
25	4.29527	1.042953
8	-16.7931	0.832069
20	4.292698	1.042927
3	102.3603	2.023603
12	-3.67354	0.963265
23	-7.37901	0.92621
20	7.004503	1.070045
36	-8.80219	0.911978
6	-29.2386	0.707614
6	-9.80846	0.901915
15	64.27339	1.642734
7	4.718447	1.047184
4	-16.286	0.83714
16	-15.1212	0.848788
9	-8.79198	0.91208
44	4.655704	1.046557
22	-17.7313	0.822687

22	-13.4247	0.865753
72	77.47072	1.774707
19	-12.4304	0.875696
26	-6.83368	0.931663
6	-5.21883	0.947812
5	-11.7676	0.882324
12	1.423051	1.014231
28	8.219512	1.082195
11	52.24806	1.522481
3	-35.5328	0.644672
10	14.7043	1.147043
10	-0.55006	0.994499
6	-9.13269	0.908673
7	5.749821	1.057498
14	10.11236	1.101124
6	-1.43683	0.985632
24	-11.5391	0.884609
4	-5.37171	0.946283
8	121.3866	2.213866
2	-59.5448	0.404552
20	-2.40956	0.975904
15	21.3871	1.213871
6	-41.5731	0.584269
10	-5.37926	0.946207
2	93.22637	1.932264
7	-0.54448	0.994555
15	2.428589	1.024286
7	-11.5409	0.884591
10	-18.1544	0.818456
18	-5.67535	0.943246
129	4.105655	1.041057
10	13.78931	1.137893
23	0.896104	1.008961
4	-28.076	0.71924
15	-8.29079	0.917092
5	-19.5337	0.804663
15	-2.8322	0.971678
4	4.697945	1.046979
29	1.542723	1.015427
7	-7.06952	0.929305
10	-10.113	0.89887
22	7.134829	1.071348
23	0.300933	1.003009
2	9.132938	1.091329
4	144.8953	2.448953
65	-10.4738	0.895262
4	88.64527	1.886453
9	-49.2348	0.507652
5	-9.307	0.90693
3	-34.0867	0.659133

22	-6.00274	0.939973
14	-18.1624	0.818376
4	-17.2467	0.827533
11	-11.6822	0.883178
22	-0.01539	0.999846
3	301.213	4.01213
15	-2.70746	0.972925
7	18.8867	1.188867
7	-8.19305	0.918069
15	-3.47175	0.965283
6	2.543974	1.02544
3	-43.1532	0.568468
7	-5.38421	0.946158
11	-9.96003	0.9004
12	9.170392	1.091704
4	-47.4556	0.525444
3	-12.6728	0.873272
5	-5.22895	0.94771
3	17.30431	1.173043
6	-5.7243	0.942757
17	6.195304	1.061953
2	-8.73922	0.912608
3	-20.8821	0.791179
5	-21.4543	0.785457
2	44.79779	1.447978
4	-44.8976	0.551024
6	-18.6838	0.813162
3	5.181939	1.051819
8	-13.7233	0.862767
13	0.686182	1.006862
5	20.08529	1.200853
21	-5.46544	0.945346
30	0.007744	1.000077
16	-2.47393	0.975261
20	-6.90299	0.93097
6	-7.93484	0.920652
2	-11.0231	0.889769
20	-2.45619	0.975438
32	-2.38173	0.976183
6	-23.6225	0.763775
48	16.49022	1.164902
2	-60.4038	0.395962
8	-8.01868	0.919813
5	25.81646	1.258165
2	-3.39724	0.966028
11	-0.84301	0.99157
22	-4.83963	0.951604
7	-12.0349	0.879651
13	3.798349	1.037983
33	-4.35409	0.956459

6	-28.994	0.71006
19	-4.36353	0.956365
13	2.644097	1.026441
12	-27.5335	0.724665
10	-2.47431	0.975257
2	-49.1832	0.508168
16	-5.61915	0.943808
8	-14.2773	0.857227
9	-5.62378	0.943762
2	86.7353	1.867353
5	-6.46167	0.935383
8	1.185543	1.011855
5	-31.8035	0.681965
22	-0.01288	0.999871
14	-13.0299	0.869701
22	-0.74664	0.992534
13	-6.53536	0.934646
12	105.5061	2.055061
9	-14.0739	0.859261
25	-3.70506	0.962949
7	-7.31943	0.926806
3	-9.58544	0.904146
4	9.370121	1.093701
7	-18.9885	0.810115
8	3.905919	1.039059
16	-22.0458	0.779542
9	-4.95316	0.950468
12	13.89992	1.138999
5	0.261534	1.002615
11	-3.65976	0.963402
10	-4.64885	0.953512
9	-17.6163	0.823837
2	-51.8173	0.481827
8	-0.69074	0.993093
26	104.3775	2.043775
11	11.98216	1.119822
24	83.23834	1.832383
81	0.575653	1.005757
2	47.44103	1.47441
3	168.5602	2.685602
8	-27.1306	0.728694
8	6.676758	1.066768
9	-2.21735	0.977826
10	4.289303	1.042893
11	-8.906	0.91094
7	3.247867	1.032479
10	3.139425	1.031394
11	-4.24949	0.957505
15	-5.93582	0.940642
23	10.4464	1.104464

33	-4.77725	0.952228
4	-17.5208	0.824792
6	5.926279	1.059263
9	3.948289	1.039483
4	-16.4129	0.835871
24	9.442409	1.094424
11	2.191086	1.021911
25	-21.5376	0.784624
20	9.278949	1.092789
22	-2.91173	0.970883
4	7.890612	1.078906
28	-5.29942	0.947006
32	4.026593	1.040266
3	-6.51933	0.934807
36	0.911307	1.009113
6	-19.8768	0.801232
13	-14.9223	0.850777
7	-17.9055	0.820945
5	2.008107	1.020081
28	-13.8524	0.861476
11	-2.54145	0.974585
41	3.376554	1.033766
6	-6.84824	0.931518
3	-59.1475	0.408525
8	-2.0868	0.979132
12	6.206679	1.062067
7	21.80635	1.218063
25	37.84309	1.378431
8	-16.5234	0.834766
3	24.62547	1.246255
19	-2.98287	0.970171
9	-6.72248	0.932775
33	3.571507	1.035715
8	0.476113	1.004761
6	-12.766	0.87234
10	-15.1733	0.848267
6	5.719589	1.057196
11	-7.59011	0.924099
7	-0.08212	0.999179
4	-31.1278	0.688722
16	32.9479	1.329479
9	4.650181	1.046502
5	-14.1484	0.858516
43	-4.1153	0.958847
5	31.80589	1.318059
26	-7.02613	0.929739
2	-44.7382	0.552618
6	-8.03864	0.919614
5	-35.5284	0.644716
31	60.95721	1.609572

11	10.85286	1.108529
12	-4.36948	0.956305
38	0.819173	1.008192
32	0.783904	1.007839
16	11.68871	1.116887
10	8.235674	1.082357
34	-7.5701	0.924299
13	-13.7475	0.862525
15	-15.6239	0.843761
3	99.5963	1.995963
8	-18.9652	0.810348
4	1.213986	1.01214
7	-26.7336	0.732664
8	-6.80297	0.93197
4	-40.6037	0.593963
9	60.05109	1.600511
32	-1.70384	0.982962
14	8.744252	1.087443
45	11.96933	1.119693
6	-18.9581	0.810419
4	-11.8945	0.881055
42	-2.48612	0.975139
41	9.866643	1.098666
41	1.702	1.01702
8	-34.2817	0.657183
9	-15.6734	0.843266
6	-9.17947	0.908205
7	15.51894	1.155189
29	4.458405	1.044584
4	-4.78282	0.952172
8	-5.97868	0.940213
14	34.51009	1.345101
4	24.33534	1.243353
4	9.844162	1.098442
47	9.672314	1.096723
8	43.47462	1.434746
7	-0.22694	0.997731
8	25.45634	1.254563
31	2.076782	1.020768
8	-9.02628	0.909737
38	8.42733	1.084273
20	18.83912	1.188391
37	0.791893	1.007919
15	-8.69651	0.913035
5	13.54098	1.13541
23	2.22484	1.022248
3	-53.5842	0.464158
4	1.465563	1.014656
11	-15.1693	0.848307
14	8.562129	1.085621

17	0.456332	1.004563
6	-32.5201	0.674799
9	-17.7478	0.822522
3	12.5242	1.125242
14	1.423469	1.014235
4	-5.63728	0.943627
28	1.007036	1.01007
6	21.6145	1.216145
16	-11.2846	0.887154
23	37.98857	1.379886
9	-0.66122	0.993388
5	0.075054	1.000751
6	-9.31704	0.90683
16	-3.58237	0.964176
6	-12.6399	0.873601
21	-5.69364	0.943064
15	-2.27498	0.97725
5	-16.9004	0.830996
7	-41.8357	0.581643
7	7.777882	1.077779
9	-6.80561	0.931944
7	-19.9387	0.800613
5	-2.57201	0.97428
6	-3.30165	0.966984
6	-10.6217	0.893783
2	219.3605	3.193605
4	-20.959	0.79041
5	53.10323	1.531032
5	-15.6046	0.843954
2	154.432	2.54432
6	-4.07275	0.959272
5	-13.0279	0.869721
3	4.430677	1.044307
33	-6.58258	0.934174
6	-37.3712	0.626288
12	11.50673	1.115067
2	-5.1318	0.948682
12	-5.87622	0.941238
2	-46.499	0.53501
9	-0.43523	0.995648
17	1.190837	1.011908
9	20.09375	1.200938
23	13.08042	1.130804
18	14.45061	1.144506
5	-21.906	0.78094
4	3.300844	1.033008
35	7.686498	1.076865
2	21.71892	1.217189
9	-7.92411	0.920759
3	1.968304	1.019683

12	-8.19379	0.918062
3	-10.8517	0.891483
14	-3.23085	0.967691
7	-10.2826	0.897174
9	-1.72298	0.98277
21	-12.9114	0.870886
9	4.141116	1.041411
45	0.06327	1.000633
8	-9.76344	0.902366
13	-2.89614	0.971039
3	-20.7995	0.792005
72	0.963483	1.009635
5	-12.5687	0.874313
7	-0.71194	0.992881
19	1.395629	1.013956
12	16.21828	1.162183
9	8.083261	1.080833
57	8.635954	1.08636
9	9.965539	1.099655
4	-12.5055	0.874945
10	5.358358	1.053584
19	18.13248	1.181325
12	-6.66946	0.933305
4	-19.2117	0.807883
3	-26.9006	0.730994
7	6.836245	1.068362
19	-4.45506	0.955449
16	92.08336	1.920834
17	2.805913	1.028059
5	20.58809	1.205881
25	2.291723	1.022917
4	-17.7814	0.822186
4	109.0166	2.090166
20	-5.10358	0.948964
8	-0.05918	0.999408
7	63.5896	1.635896
19	-1.08386	0.989161
9	-11.1246	0.888754
9	3.913148	1.039131
7	20.21027	1.202103
13	-4.2811	0.957189
2	-52.7074	0.472926
13	-0.41578	0.995842
5	1.992507	1.019925
16	-5.30433	0.946957
3	-12.7649	0.872351
3	16.33541	1.163354
4	26.02565	1.260256
8	-2.53862	0.974614
21	31.09574	1.310957

3	24.41281	1.244128
4	4.009565	1.040096
4	3.561878	1.035619
15	91.08477	1.910848
12	-19.4212	0.805788
3	-26.3299	0.736701
8	-19.2282	0.807718
4	-12.3429	0.876571
2	346.7308	4.467308
40	-1.56937	0.984306
15	-1.25683	0.987432
3	-71.3081	0.286919
2	266.9299	3.669299
11	11.10791	1.111079
6	-29.3753	0.706247
21	7.246798	1.072468
44	0.092672	1.000927
8	4.700601	1.047006
19	-5.68384	0.943162
24	-1.42887	0.985711
5	-4.49278	0.955072
19	11.5914	1.115914
5	19.93765	1.199376
4	-12.1224	0.878776
3	-15.2534	0.847466
11	-4.9802	0.950198
5	-12.8983	0.871017
29	-0.24369	0.997563
10	2.247319	1.022473
11	-24.9985	0.750015
28	-4.63907	0.953609
12	-2.08679	0.979132
2	-47.1794	0.528206
106	-32.8276	0.671724
6	4.335527	1.043355
14	32.3602	1.323602
15	-9.22122	0.907788
4	14.22111	1.142211
4	84.71504	1.84715
2	-28.3015	0.716985
8	-11.2056	0.887944
13	-11.44	0.8856
20	2.968546	1.029685
3	13.10504	1.13105
8	0.222488	1.002225
24	0.983448	1.009834
15	21.84641	1.218464
20	1.596174	1.015962
10	-5.32435	0.946756
18	19.38658	1.193866

10	-14.1486	0.858514
4	-11.8471	0.881529
11	-7.11469	0.928853
5	-1.6226	0.983774
40	-1.34169	0.986583
14	0.55177	1.005518
35	14.50769	1.145077
5	-53.6516	0.463484
20	11.41796	1.11418
8	-19.3924	0.806076
5	154.1757	2.541757
5	-12.7348	0.872652
2	-16.2768	0.837232
6	-37.8847	0.621153
15	-16.4693	0.835307
47	0.506807	1.005068
5	-20.0677	0.799323
6	25.8623	1.258623
11	-23.3299	0.766701
8	22.02233	1.220223
7	-80.5778	0.194222
5	-0.73296	0.99267
8	-6.20677	0.937932
2	-34.9134	0.650866
11	-1.41099	0.98589
2	-17.4033	0.825967
17	7.616906	1.076169
15	2.177775	1.021778
8	-2.88736	0.971126
18	-5.45408	0.945459
10	-7.35996	0.9264
11	46.8906	1.468906
8	-13.893	0.86107
19	11.93223	1.119322
10	1.021016	1.01021
10	3.251086	1.032511
17	7.765295	1.077653
12	2.657792	1.026578
6	-11.5516	0.884484
5	-9.56996	0.9043
7	-12.7189	0.872811
9	3.179653	1.031797
2	-27.6626	0.723374
5	13.25487	1.132549
9	79.10008	1.791001
5	4.508082	1.045081
3	138.2983	2.382983
4	-3.57317	0.964268
10	-9.82398	0.90176
9	0.469469	1.004695

12	12.28464	1.122846
28	27.43115	1.274312
22	11.36483	1.113648
39	5.806301	1.058063
11	6.171919	1.061719
10	-10.5327	0.894673
3	-7.95248	0.920475
6	-22.7375	0.772625
9	-7.37819	0.926218
16	15.06817	1.150682
25	0.134692	1.001347
2	95.10821	1.951082
6	-10.0749	0.899251
33	6.537168	1.065372
9	-7.08118	0.929188
30	-1.06159	0.989384
7	33.81446	1.338145
19	-2.0403	0.979597
10	-18.6705	0.813295
11	-2.30044	0.976996
12	-4.15272	0.958473
2	9.234944	1.092349
15	12.95909	1.129591
5	-13.4444	0.865556
3	-19.7421	0.802579
6	9.007285	1.090073
5	360.7098	4.607098
8	-28.5525	0.714475
11	-1.24921	0.987508
4	-19.8728	0.801272
8	-7.00742	0.929926
10	-0.56816	0.994318
2	-8.76242	0.912376
34	1.590379	1.015904
8	17.32502	1.17325
3	10.58421	1.105842
26	2.654145	1.026541
6	-2.30039	0.976996
6	-24.2392	0.757608
18	-5.24502	0.94755
4	-39.0391	0.609609
8	-1.90477	0.980952
7	-7.00681	0.929932
13	-5.27278	0.947272
22	1.052155	1.010522
13	6.465851	1.064659
2	-1.11877	0.988812
17	-5.39086	0.946091
13	-7.63964	0.923604
8	-0.74003	0.9926

3	-16.8452	0.831548
14	52.55266	1.525527
51	7.970355	1.079704
73	11.66965	1.116697
12	-2.70845	0.972916
12	21.05861	1.210586
7	10.49474	1.104947
10	3.955093	1.039551
13	-15.061	0.84939
36	10.28779	1.102878
7	1.16329	1.011633
21	8.624541	1.086245
7	-18.3146	0.816854
3	-7.53485	0.924652
3	-1.47438	0.985256
12	16.16521	1.161652
10	-0.46293	0.995371
13	-4.57865	0.954213
11	-16.4267	0.835733
6	-17.2866	0.827134
4	-34.3269	0.656731
6	-2.65197	0.97348
7	-6.20558	0.937944
3	-15.1406	0.848594
19	4.465843	1.044658
4	9.957761	1.099578
15	-13.0827	0.869173
3	120.1875	2.201875
121	9.233862	1.092339
9	23.40689	1.234069
2	-55.2919	0.447081
10	3.971475	1.039715
3	-57.5065	0.424935
17	8.597638	1.085976
12	7.205833	1.072058
14	3.56421	1.035642
10	1.911227	1.019112
38	57.9566	1.579566
14	-8.3873	0.916127
4	14.14407	1.141441
9	15.06408	1.150641
24	6.090857	1.060909
4	-14.6389	0.853611
3	-35.8241	0.641759
4	-12.7376	0.872624
11	12.69414	1.126941
21	-2.93217	0.970678
7	-17.1797	0.828203
7	12.93983	1.129398
17	-0.21579	0.997842

8	-9.53902	0.90461
3	-30.1335	0.698665
21	4.166664	1.041667
14	-5.56962	0.944304
9	5.377297	1.053773
2	-56.5676	0.434324
12	4.388629	1.043886
43	31.87538	1.318754
6	-23.2953	0.767047
4	-1.37864	0.986214
12	2.767647	1.027676
14	-6.08361	0.939164
3	-23.3339	0.766661
6	4.352271	1.043523
23	-13.8788	0.861212
12	5.283317	1.052833
3	-29.8505	0.701495
8	4.302238	1.043022
18	-19.4443	0.805557
11	-0.4079	0.995921
5	-15.3923	0.846077
18	32.81403	1.32814
14	-15.5792	0.844208
7	-17.5523	0.824477
27	15.94431	1.159443
13	-3.94893	0.960511
13	-15.892	0.84108
8	-8.86562	0.911344
2	-6.3388	0.936612
7	-4.47422	0.955258
15	0.504399	1.005044
6	-19.7478	0.802522
22	7.211611	1.072116
23	8.171928	1.081719
21	3.076565	1.030766
2	-7.12717	0.928728
9	10.46508	1.104651
11	-26.6072	0.733928
8	-12.9673	0.870327
4	5.792207	1.057922
12	12.01735	1.120173
9	-1.6707	0.983293
6	-1.79744	0.982026
22	-1.96851	0.980315
5	15.87924	1.158792
10	-3.0185	0.969815
18	42.63447	1.426345
18	4.283662	1.042837
10	-6.43537	0.935646
7	-11.6644	0.883356

5	-4.52646	0.954735
2	23.04531	1.230453
6	29.81421	1.298142
2	-13.0131	0.869869
20	11.49585	1.114959
7	-19.077	0.80923
5	3.516049	1.03516
7	-22.8311	0.771689
26	0.750947	1.007509
2	190.9742	2.909742
35	5.404951	1.05405
16	17.03459	1.170346
11	6.373497	1.063735
7	-10.2945	0.897055
33	-8.82569	0.911743
20	3.131295	1.031313
35	17.97835	1.179783
9	47.72195	1.47722
10	-19.7275	0.802725
12	-11.0543	0.889457
9	-11.7059	0.882941
10	-11.1884	0.888116
18	2.71591	1.027159
9	22.59901	1.22599
11	49.15607	1.491561
6	-13.5071	0.864929
10	4.27444	1.042744
9	-3.64134	0.963587
6	-3.17561	0.968244
4	153.5558	2.535558
12	-19.2751	0.807249
8	-12.1682	0.878318
2	-26.4313	0.735687
16	4.019706	1.040197
2	-35.2098	0.647902
15	4.644649	1.046446
2	-30.8599	0.691401
2	-1.58423	0.984158
3	1.750213	1.017502
7	6.652583	1.066526
49	-3.65206	0.963479
23	2.589924	1.025899
3	-11.3194	0.886806
15	18.12592	1.181259
8	-13.5797	0.864203
12	-7.83561	0.921644
4	-19.7149	0.802851
5	22.23829	1.222383
32	-2.65729	0.973427
15	1.507577	1.015076

10	42.10739	1.421074
14	-3.60959	0.963904
17	5.285608	1.052856
3	6.395761	1.063958
3	-21.2585	0.787415
10	-4.85742	0.951426
24	15.43376	1.154338
11	-80.9768	0.190232
6	-44.9996	0.550004
22	2.2236	1.022236
30	0.509493	1.005095
5	21.15442	1.211544
3	58.99715	1.589971
8	-11.4435	0.885565
9	15.77138	1.157714
4	-25.6734	0.743266
11	-0.7712	0.992288
18	3.961014	1.03961
7	-5.61789	0.943821
14	19.16133	1.191613
3	-12.4451	0.875549
9	-15.7879	0.842121
4	-18.2231	0.817769
12	-10.5049	0.894951
3	135.9195	2.359195
4	1.620558	1.016206
4	17.52323	1.175232
23	1.652504	1.016525
5	6.226921	1.062269
11	1.095876	1.010959
9	3.797212	1.037972
13	-18.7804	0.812196
19	5.638497	1.056385
4	-51.2916	0.487084
4	-24.8105	0.751895
14	-2.33163	0.976684
8	-11.0977	0.889023
55	7.470822	1.074708
34	-6.71187	0.932881
3	-16.7673	0.832327
9	-13.7976	0.862024
41	85.08441	1.850844
18	5.49875	1.054988
16	6.327323	1.063273
11	-11.7825	0.882175
8	35.54878	1.355488
10	-10.3607	0.896393
6	-18.9085	0.810915
7	-7.13703	0.92863
4	7.138486	1.071385

18	15.76386	1.157639
9	-2.04121	0.979588
5	-13.4938	0.865062
25	1.827898	1.018279
7	11.58157	1.115816
21	-2.11719	0.978828
19	10.32276	1.103228
16	1.77956	1.017796
10	-4.72045	0.952796
25	6.664898	1.066649
9	9.769126	1.097691
5	43.47619	1.434762
7	16.5312	1.165312
9	-18.7494	0.812506
4	-18.7814	0.812186
17	-8.70024	0.912998
10	0.387654	1.003877
5	151.4351	2.514351
5	-19.764	0.80236
4	-5.08767	0.949123
9	-6.68816	0.933118
13	-3.17649	0.968235
3	-7.37791	0.926221
10	-10.4336	0.895664
5	-0.70218	0.992978
16	-8.76338	0.912366
9	-19.6828	0.803172
5	-6.00396	0.93996
18	0.658889	1.006589
4	-19.4225	0.805775
3	-12.029	0.87971
5	-10.5513	0.894487
19	1.344387	1.013444
16	-15.4089	0.845911
4	-1.8413	0.981587
5	114.1759	2.141759
3	-14.0619	0.859381
4	-30.6433	0.693567
28	0.511924	1.005119
5	-9.53253	0.904675
3	28.46142	1.284614
14	-4.94425	0.950558
20	4.131647	1.041316
16	6.122697	1.061227
21	6.012287	1.060123
7	5.300299	1.053003
9	23.82481	1.238248
5	-39.6037	0.603963
8	27.21821	1.272182
14	81.21821	1.812182

4	-38.7391	0.612609
25	29.72689	1.297269
15	-8.02106	0.919789
9	0.230218	1.002302
10	-30.9465	0.690535
30	4.320177	1.043202
23	6.593488	1.065935
5	-12.6894	0.873106
351	0.512909	1.005129
13	3.08781	1.030878
12	5.728902	1.057289
8	14.34942	1.143494
10	9.982485	1.099825
5	-22.7544	0.772456
16	0.533129	1.005331
11	2.027026	1.02027
36	9.207319	1.092073
4	-16.7096	0.832904
5	3.902124	1.039021
20	5.515557	1.055156
4	673.9303	7.739303
2	-46.3289	0.536711
2	-13.2626	0.867374
12	-6.84495	0.93155
18	2.724989	1.02725
8	5.860705	1.058607
2	-83.9594	0.160406
4	-2.24831	0.977517
19	-0.3809	0.996191
4	-31.8783	0.681217
12	12.96775	1.129677
3	18.37639	1.183764
13	-80.3706	0.196294
14	22.29479	1.222948
15	-4.35682	0.956432
5	-14.9409	0.850591
2	-33.5771	0.664229
139	32.99866	1.329987
12	-1.13129	0.988687
5	38.19423	1.381942
3	-18.5984	0.814016
7	-10.2254	0.897746
2	-36.7917	0.632083
36	7.071322	1.070713
14	-14.8314	0.851686
2	-26.1467	0.738533
3	-40.5598	0.594402
38	-13.6176	0.863824
22	22.47704	1.22477
18	-4.20036	0.957996

21	14.84922	1.148492
19	5.236996	1.05237
21	17.53309	1.175331
7	-18.4449	0.815551
2	-19.6297	0.803703
7	28.34072	1.283407
12	8.607763	1.086078
7	1.75482	1.017548
66	2.968402	1.029684
46	3.970111	1.039701
7	-21.953	0.78047
7	27.15171	1.271517
2	-14.2435	0.857565
16	78.39879	1.783988
5	-12.7799	0.872201
4	8.31785	1.083178
14	4.527138	1.045271
13	17.96074	1.179607
8	-3.23787	0.967621
2	14.9786	1.149786
15	8.7153	1.087153
4	94.29372	1.942937
6	-5.47616	0.945238
5	-10.7805	0.892195
52	47.07886	1.470789
9	10.03924	1.100392
23	9.108385	1.091084
5	-7.00812	0.929919
5	4.820851	1.048209
14	-5.25275	0.947473
6	-4.72201	0.95278
4	-17.6278	0.823722
6	-4.21477	0.957852
5	56.42757	1.564276
4	1085.952	11.85952
11	29.38858	1.293886
40	4.754858	1.047549
22	46.26689	1.462669
9	6.093355	1.060934
9	1.254788	1.012548
14	-4.89052	0.951095
18	4.490795	1.044908
18	-3.94377	0.960562
16	67.16004	1.6716
5	424.4875	5.244875
9	38.38316	1.383832
9	5.40393	1.054039
19	2.835933	1.028359
5	79.79	1.7979
27	13.10216	1.131022

2	-33.1318	0.668682
18	20.82152	1.208215
10	16.75506	1.167551
5	-1.3787	0.986213
25	9.491276	1.094913
27	5.268447	1.052684
10	1.384995	1.01385
6	-2.18246	0.978175
4	901.6276	10.01628
3	34.53169	1.345317
18	-11.6968	0.883032
3	114.5611	2.145611
7	12.76015	1.127601
14	-2.82861	0.971714
4	-9.76347	0.902365
3	19.76132	1.197613
32	5.963624	1.059636
9	2.573777	1.025738
10	-1.07357	0.989264
3	-5.37332	0.946267
8	46.01382	1.460138
18	-6.10002	0.939
19	-2.4696	0.975304
5	5.290451	1.052905
11	-6.98023	0.930198
3	-4.22763	0.957724
5	-30.3254	0.696746
5	-2.4105	0.975895
9	9.437895	1.094379
6	18.27135	1.182714
2	-5.84786	0.941521
17	18.18971	1.181897
8	33.42597	1.33426
14	6.912216	1.069122
2	82.50649	1.825065
3	6.987731	1.069877
5	29.50693	1.295069
8	-1.67171	0.983283
10	-7.74544	0.922546
12	45.41802	1.45418
6	-13.6871	0.863129
13	2.161823	1.021618
2	70.87043	1.708704
24	-2.51403	0.97486
11	14.13494	1.141349
10	-2.15298	0.97847
14	9.746906	1.097469
2	87.35753	1.873575
3	-25.7773	0.742227
6	29.47652	1.294765

2	177.6799	2.776799
18	17.82843	1.178284
9	-14.1858	0.858142
16	-1.81355	0.981864
9	-3.46395	0.965361
16	-5.96694	0.940331
9	0.241548	1.002415
7	-75.1446	0.248554
6	2.912194	1.029122
22	-4.79349	0.952065
5	17.59949	1.175995
7	-18.5878	0.814122
10	2.777134	1.027771
3	-33.66	0.6634
15	-3.44801	0.96552
12	-2.96451	0.970355
5	-14.8214	0.851786
15	-1.18595	0.988141
3	-25.6498	0.743502
43	8.118658	1.081187
19	62.93039	1.629304
11	-19.1686	0.808314
2	-30.7	0.693
4	54.35054	1.543505
10	-5.28021	0.947198
324	8.923345	1.089233
3	-33.7597	0.662403
27	-0.38949	0.996105
11	5.772343	1.057723
8	10.93217	1.109322
8	24.10835	1.241084
3	-13.1231	0.868769
10	16.05005	1.1605
4	-16.0678	0.839322
9	-3.67248	0.963275
3	15.84787	1.158479
6	-6.31791	0.936821
4	45.16804	1.45168
4	373.2862	4.732862
14	43.33202	1.43332
18	8.448405	1.084484
13	0.837091	1.008371
7	-0.33544	0.996646
80	8.576601	1.085766
32	7.52814	1.075281
17	4.958062	1.049581
6	53.48086	1.534809
7	-2.47491	0.975251
3	34.89407	1.348941
5	-41.8583	0.581417

32	8.270152	1.082702
8	3.561702	1.035617
5	-8.34177	0.916582
6	-0.70955	0.992904
7	-2.90377	0.970962
7	-77.697	0.22303
15	7.706208	1.077062
3	79.06946	1.790695
7	9.596559	1.095966
6	-8.28736	0.917126
15	11.69017	1.116902
5	-16.8728	0.831272
4	-26.148	0.73852
5	-9.61962	0.903804
26	9.904627	1.099046
7	2.951355	1.029514
6	181.694	2.81694
17	2.788893	1.027889
15	-5.3203	0.946797
10	14.47088	1.144709
3	0.953966	1.00954
3	87.1773	1.871773
17	0.658869	1.006589
12	5.275281	1.052753
8	2.339711	1.023397
15	-0.64812	0.993519
6	3.529593	1.035296
11	-17.0264	0.829736
31	11.24213	1.112421
3	12.59535	1.125954
5	0.962279	1.009623
10	47.57098	1.47571
19	4.298422	1.042984
9	-18.6101	0.813899
32	-2.90299	0.97097
5	-15.5114	0.844886
12	68.39271	1.683927
15	-2.02264	0.979774
5	-12.2421	0.877579
10	1.993473	1.019935
2	-8.16059	0.918394
19	-3.60065	0.963993
17	0.452747	1.004527
10	-10.7589	0.892411
3	102.5846	2.025846
11	16.88787	1.168879
4	-26.7734	0.732266
26	1.151215	1.011512
16	6.229738	1.062297
14	-2.72085	0.972791

2	-4.46036	0.955396
9	-21.2134	0.787866
5	21.18349	1.211835
2	36.17678	1.361768
3	-44.2623	0.557377
24	13.80339	1.138034
27	0.672291	1.006723
5	8.9166	1.089166
2	-35.2508	0.647492
17	17.8445	1.178445
12	7.595761	1.075958
6	-40.4954	0.595046
25	6.479946	1.064799
6	-10.9427	0.890573
3	-13.9694	0.860306
7	6.508612	1.065086
15	3.874176	1.038742
16	4.862764	1.048628
20	1.858289	1.018583
17	1.361898	1.013619
26	17.29657	1.172966
9	1.902728	1.019027
5	0.303296	1.003033
4	0.11014	1.001101
3	51.32263	1.513226
3	81.22961	1.812296
6	-17.5124	0.824876
9	5.683574	1.056836
14	5.213463	1.052135
4	178.4408	2.784408
8	15.05264	1.150526
3	550.782	6.50782
2	-27.991	0.72009
8	-19.5171	0.804829
2	-11.1536	0.888464
3	51.23443	1.512344
5	74.0329	1.740329
4	-25.1676	0.748324
16	-10.7678	0.892322
8	-11.0755	0.889245
33	9.901405	1.099014
4	65.56491	1.655649
6	2.225781	1.022258
14	5.929982	1.0593
33	20.59343	1.205934
5	-25.7416	0.742584
12	-5.14164	0.948584
16	-4.3644	0.956356
7	2.455093	1.024551
21	-2.33471	0.976653

2	-26.0257	0.739743
31	2.403627	1.024036
16	2.308455	1.023085
3	-21.0749	0.789251
11	0.468528	1.004685
3	-5.47619	0.945238
6	12.10856	1.121086
3	28.9674	1.289674
8	10.90655	1.109065
7	8.979018	1.08979
5	4.660059	1.046601
5	14.20279	1.142028
3	-18.4322	0.815678
10	-14.5232	0.854768
4	-15.6526	0.843474
8	-0.0112	0.999888
2	487.2963	5.872963
9	31.45769	1.314577
5	8.421186	1.084212
7	-4.09962	0.959004
18	9.422072	1.094221
6	-11.4529	0.885471
8	-10.2298	0.897702
6	4.483693	1.044837
3	59.71342	1.597134
11	26.31781	1.263178
8	-16.5076	0.834924
7	-13.0825	0.869175
4	3.039086	1.030391
4	18.79498	1.18795
17	-10.8312	0.891688
12	-2.21583	0.977842
8	19.35625	1.193562
11	-4.85244	0.951476
15	19.18074	1.191807
11	50.61071	1.506107
2	-1.42244	0.985776
3	-6.52334	0.934767
33	3.336506	1.033365
23	9.731531	1.097315
5	13.75699	1.13757
6	-18.25	0.8175
7	-2.26584	0.977342
11	-1.14408	0.988559
4	-21.5647	0.784353
10	12.17134	1.121713
52	13.80994	1.138099
27	9.44932	1.094493
5	-5.30797	0.94692
3	-34.7798	0.652202

10	0.992887	1.009929
7	28.63921	1.286392
8	-6.39587	0.936041
2	176.2591	2.762591
2	-6.52563	0.934744
18	0.696796	1.006968
2	-7.5744	0.924256
22	0.02933	1.000293
14	9.674057	1.096741
25	12.02372	1.120237
13	29.34156	1.293416
7	-2.37893	0.976211
20	4.63334	1.046333
2	24.02878	1.240288
5	-27.7283	0.722717
10	67.1931	1.671931
9	2.661481	1.026615
3	0.962704	1.009627
36	3.043291	1.030433
2	-51.0419	0.489581
20	0.190785	1.001908
8	12.3767	1.123767
9	-4.25096	0.95749
5	-15.1062	0.848938
28	15.87536	1.158754
19	-4.18291	0.958171
6	3.321347	1.033213
5	-0.47774	0.995223
4	-5.63311	0.943669
4	-7.48937	0.925106
4	18.96483	1.189648
14	11.16208	1.111621
7	44.54153	1.445415
15	10.35768	1.103577
2	-51.9452	0.480548
4	14.95957	1.149596
2	108.2869	2.082869
6	-6.9095	0.930905
13	11.09672	1.110967
3	-30.0331	0.699669
14	-1.30327	0.986967
14	38.48643	1.384864
3	-6.45253	0.935475
2	-3.46803	0.96532
15	61.20681	1.612068
7	31.1668	1.311668
2	306.4689	4.064689
11	13.74916	1.137492
14	2.815069	1.028151
21	6.966456	1.069665

14	-4.03773	0.959623
7	10.36177	1.103618
8	6.193979	1.06194
2	-21.2862	0.787138
8	13.09863	1.130986
4	29.90491	1.299049
2	164.8498	2.648498
4	-7.52949	0.924705
8	26.34579	1.263458
12	-0.02272	0.999773
6	6.391433	1.063914
14	0.192636	1.001926
3	17.51562	1.175156
18	-3.16399	0.96836
5	16.05146	1.160515
3	40.26868	1.402687
7	-2.89088	0.971091
37	10.963	1.10963
18	3.471442	1.034714
25	-0.65324	0.993468
16	26.30141	1.263014
11	-8.56292	0.914371
6	-24.6809	0.753191
3	-7.41725	0.925827
3	10.86799	1.10868
2	-12.0336	0.879664
7	-3.97326	0.960267
16	47.66838	1.476684
11	13.03085	1.130308
10	15.624	1.15624
5	-33.0399	0.669601
16	3.839549	1.038395
22	5.19163	1.051916
37	15.89152	1.158915
4	-19.757	0.80243
13	82.81809	1.828181
5	-24.9167	0.750833
2	13.29311	1.132931
3	-16.2631	0.837369
3	-17.0928	0.829072
9	-18.2818	0.817182
4	15.80188	1.158019
7	-4.28104	0.95719
10	-3.75776	0.962422
15	21.84576	1.218458
10	-3.21669	0.967833
6	-3.11723	0.968828
5	-1.94646	0.980535
6	62.58123	1.625812
21	0.397904	1.003979

19	44.76563	1.447656
12	6.562718	1.065627
54	2.473945	1.024739
5	12.8925	1.128925
17	0.653189	1.006532
7	442.6404	5.426404
11	-11.3779	0.886221
18	8.821347	1.088213
6	-0.0643	0.999357
5	-14.9201	0.850799
4	-22.1063	0.778937
4	100.3922	2.003922
10	25.32678	1.253268
12	3.544801	1.035448
8	55.92094	1.559209
7	6.02821	1.060282
4	7.667083	1.076671
16	7.864001	1.07864
32	16.17843	1.161784
11	-5.49315	0.945068
20	26.82679	1.268268
4	66.34046	1.663405
4	3.83291	1.038329
8	-2.86745	0.971325
8	15.25398	1.15254
2	-11.306	0.88694
4	-24.4344	0.755656
9	50.16057	1.501606
6	24.3151	1.243151
13	18.14948	1.181495
18	2.026482	1.020265
3	-26.6977	0.733023
3	-33.5154	0.664846
2	-9.33086	0.906691
3	22.33338	1.223334
8	5.03445	1.050344
7	5.765221	1.057652
17	0.990168	1.009902
2	398.388	4.98388
30	19.09097	1.19091
8	3.570477	1.035705
11	4.273809	1.042738
41	2.844593	1.028446
8	5.594925	1.055949
5	23.81111	1.238111
7	-29.8179	0.701821
7	5.693941	1.056939
15	-10.5922	0.894078
8	22.62552	1.226255
2	-27.7242	0.722758

7	-3.66452	0.963355
7	-10.1267	0.898733
3	-44.8783	0.551217
35	9.748267	1.097483
3	-65.0295	0.349705
2	72.97889	1.729789
11	-1.95957	0.980404
10	10.60463	1.106046
6	-7.32515	0.926749
2	-31.1234	0.688766
10	11.75977	1.117598
4	-12.3482	0.876518
5	-7.92247	0.920775
4	14.52421	1.145242
3	-4.07003	0.9593
12	12.05047	1.120505
69	-3.03148	0.969685
5	53.00244	1.530024
7	-5.22828	0.947717
23	11.19644	1.111964
2	-12.287	0.87713
2	-12.8727	0.871273
11	10.27636	1.102764
26	4.646998	1.04647
3	14.47526	1.144753
10	-2.24087	0.977591
3	28.86868	1.288687
11	-7.59624	0.924038
2	-19.0274	0.809726
17	-1.60664	0.983934
8	17.10718	1.171072
4	-8.2379	0.917621
3	-15.1383	0.848617
10	12.03701	1.12037
22	16.31626	1.163163
2	102.6624	2.026624
17	5.317828	1.053178
6	13.30013	1.133001
4	-9.48594	0.905141
12	0.823176	1.008232
2	0.719215	1.007192
31	28.09499	1.28095
40	4.128332	1.041283
9	11.19003	1.1119
6	2.636118	1.026361
2	12.91474	1.129147
7	27.74865	1.277486
9	3.297965	1.03298
5	-1.38719	0.986128
10	4.074719	1.040747

6	-6.09949	0.939005
17	17.29612	1.172961
6	-14.6779	0.853221
8	8.36462	1.083646
6	-8.21507	0.917849
8	-13.7864	0.862136
3	58.36704	1.58367
9	15.4651	1.154651
9	106.465	2.06465
44	13.94833	1.139483
16	67.34118	1.673412
5	-7.17186	0.928281
14	2.135202	1.021352
3	-30.6377	0.693623
13	11.32018	1.113202
14	87.62576	1.876258
8	-0.12789	0.998721
15	4.22751	1.042275
8	-5.07038	0.949296
23	-1.08426	0.989157
5	3.798542	1.037985
3	-29.7995	0.702005
15	52.62883	1.526288
3	-13.3083	0.866917
14	-1.23248	0.987675
7	-6.10992	0.938901
4	-24.3109	0.756891
2	24.33327	1.243333
19	3.528436	1.035284
2	-7.84975	0.921503
20	2.689359	1.026894
19	14.71803	1.14718
21	-1.07815	0.989219
5	11.29164	1.112916
21	-15.0741	0.849259
12	1.953266	1.019533
8	-10.2455	0.897545
40	9.482935	1.094829
5	-16.3369	0.836631
18	19.85199	1.19852
29	15.0847	1.150847
32	2.554747	1.025547
18	17.48091	1.174809
4	9.289943	1.092899
2	-14.0507	0.859493
4	58.00713	1.580071
24	7.213669	1.072137
26	11.3879	1.113879
2	64.63133	1.646313
16	-5.70945	0.942906

5	103.5098	2.035098
2	-10.3029	0.896971
7	-3.9333	0.960667
6	-22.8595	0.771405
3	10.61474	1.106147
25	-3.61996	0.9638
2	-45.306	0.54694
5	-27.6845	0.723155
7	-9.16862	0.908314
3	-19.7782	0.802218
3	-0.10543	0.998946
28	10.13748	1.101375
6	-3.03553	0.969645
20	0.75027	1.007503
5	-10.124	0.89876
2	-15.9552	0.840448
27	56.75237	1.567524
11	-7.68672	0.923133
4	-31.1972	0.688028
6	31.15732	1.311573
9	2.500308	1.025003
44	11.60552	1.116055
4	0.879662	1.008797
20	-0.80587	0.991941
3	-24.5926	0.754074
2	233.3209	3.333209
14	23.09579	1.230958
4	-7.68027	0.923197
46	4.740039	1.0474
24	-10.8608	0.891392
2	-19.0353	0.809647
6	0.852234	1.008522
3	-5.90986	0.940901
12	17.32185	1.173219
21	21.38818	1.213882
9	-6.78515	0.932148
7	2.763218	1.027632
5	34.58635	1.345864
4	-10.6144	0.893856
5	6.366353	1.063664
8	-0.51302	0.99487
6	8.759034	1.08759
3	12.25619	1.122562
2	-28.295	0.71705
24	6.256066	1.062561
52	4.233066	1.042331
5	-0.11292	0.998871
17	7.503705	1.075037
10	10.751	1.10751
2	-37.1774	0.628226

4	-13.1619	0.868381
8	2.350849	1.023508
25	3.850535	1.038505
4	-28.8187	0.711813
2	-35.2579	0.647421
14	1.076136	1.010761
16	-0.71312	0.992869
10	6.769089	1.067691
18	-13.1318	0.868682
2	40.97811	1.409781
14	11.39837	1.113984
6	-12.6655	0.873345
4	-29.7371	0.702629
19	4.089104	1.040891
5	8.003887	1.080039
8	37.64486	1.376449
7	45.21971	1.452197
4	2.769852	1.027699
3	40.52839	1.405284
6	-25.5001	0.744999
2	-21.0362	0.789638
6	53.30377	1.533038
2	-10.4283	0.895717
8	197.0159	2.970159
11	5.366738	1.053667
13	-4.43648	0.955635
2	-43.94	0.5606
3	4.134462	1.041345
2	-16.5576	0.834424
7	-11.547	0.88453
29	5.885031	1.05885
13	-2.72683	0.972732
2	-21.2108	0.787892
4	-5.72998	0.9427
3	58.41691	1.584169
6	-11.3822	0.886178
2	13.44004	1.1344
12	5.938576	1.059386
4	5.099221	1.050992
16	7.257885	1.072579
8	17.95058	1.179506
5	-7.55433	0.924457
11	-3.51819	0.964818
3	-64.948	0.35052
9	18.08019	1.180802
21	3.832092	1.038321
29	5.203089	1.052031
16	4.694231	1.046942
5	-15.6573	0.843427
14	26.75114	1.267511

4	63.30073	1.633007
9	1.449526	1.014495
3	-44.2699	0.557301
12	0.533899	1.005339
14	-1.59413	0.984059
3	-11.2037	0.887963
18	7.303146	1.073031
15	9.313837	1.093138
12	-5.67693	0.943231
6	-0.35858	0.996414
47	5.75187	1.057519
13	7.074434	1.070744
41	15.2713	1.152713
11	1.818444	1.018184
14	5.614994	1.05615
4	-27.7754	0.722246
6	-25.6084	0.743916
8	-3.31389	0.966861
3	-20.7932	0.792068
4	-2.11299	0.97887
5	177.4828	2.774828
7	5.273161	1.052732
3	0.049752	1.000498
12	17.10693	1.171069
23	26.54142	1.265414
6	22.09734	1.220973
8	-5.2604	0.947396
8	12.40758	1.124076
26	7.551738	1.075517
7	41.86051	1.418605
8	0.24766	1.002477
2	-8.96902	0.91031
8	-16.9479	0.830521
5	-10.3826	0.896174
5	-30.7136	0.692864
11	35.49535	1.354953
16	3.718517	1.037185
2	-6.8494	0.931506
22	-4.07337	0.959266
7	-2.16959	0.978304
5	-34.6029	0.653971
5	36.36298	1.36363
7	14.96271	1.149627
9	-16.4067	0.835933
8	5.237194	1.052372
26	7.054417	1.070544
5	98.93757	1.989376
5	23.08972	1.230897
12	-1.46812	0.985319
3	60.3945	1.603945

10	4.495541	1.044955
18	0.490528	1.004905
2	129.8747	2.298747
5	6.381613	1.063816
6	19.31708	1.193171
5	-1.86723	0.981328
9	10.37121	1.103712
28	-4.86577	0.951342
37	9.01006	1.090101
13	196.7047	2.967047
7	3.826986	1.03827
7	10.78298	1.10783
4	-25.2509	0.747491
11	17.98315	1.179832
9	29.98714	1.299871
3	4.618365	1.046184
2	0.65233	1.006523
2	4.452586	1.044526
7	-9.12175	0.908783
7	75.17278	1.751728
10	-6.24108	0.937589
13	6.98949	1.069895
4	24.83438	1.248344
12	-4.21562	0.957844
9	10.25355	1.102536
2	3.811733	1.038117
2	-27.5321	0.724679
5	10.88622	1.108862
9	-6.76113	0.932389
5	-4.79949	0.952005
5	-31.6504	0.683496
4	154.2687	2.542687
21	4.63434	1.046343
12	7.673313	1.076733
15	0.071017	1.00071
7	-4.09898	0.95901
5	-7.18739	0.928126
2	17.8507	1.178507
8	5.106551	1.051066
17	14.10492	1.141049
6	-0.65856	0.993414
8	3.955345	1.039553
4	0.543458	1.005435
12	60.42797	1.60428
15	10.02207	1.100221
2	-8.16616	0.918338
2	17.29606	1.172961
12	1.035504	1.010355
12	12.57425	1.125743
2	8.39087	1.083909

14	5.101267	1.051013
41	10.79023	1.107902
9	7.977798	1.079778
12	32.17482	1.321748
9	39.10839	1.391084
6	-4.75733	0.952427
37	4.81407	1.048141
4	-22.1654	0.778346
7	49.22497	1.49225
20	22.46967	1.224697
4	41.35553	1.413555
26	4.592308	1.045923
4	-6.95171	0.930483
16	21.01255	1.210126
8	15.7288	1.157288
2	7.404808	1.074048
8	-7.0838	0.929162
4	-40.1123	0.598877
14	-2.29724	0.977028
3	28.97274	1.289727
3	-22.7355	0.772645
5	11.55407	1.115541
7	32.21452	1.322145
4	61.40054	1.614005
4	-0.18259	0.998174
14	13.66035	1.136604
2	27.417	1.27417
13	17.37612	1.173761
2	34.72686	1.347269
2	-12.3218	0.876782
15	20.8975	1.208975
11	27.47578	1.274758
15	-6.39394	0.936061
4	36.25211	1.362521
4	-22.1895	0.778105
33	43.04593	1.430459
46	12.06081	1.120608
4	-2.89685	0.971032
16	59.08939	1.590894
4	7.906035	1.07906
10	-12.1911	0.878089
5	12.00246	1.120025
12	-2.14452	0.978555
20	16.00071	1.160007
10	21.44061	1.214406
3	-4.94474	0.950553
9	-5.96702	0.94033
20	11.02853	1.110285
34	20.59976	1.205998
6	-26.9295	0.730705

5	20.65056	1.206506
5	-41.9482	0.580518
3	11.59048	1.115905
7	-4.93577	0.950642
2	23.65257	1.236526
19	4.463534	1.044635
11	-1.0159	0.989841
4	-11.9107	0.880893
9	-8.44013	0.915599
2	10.23345	1.102334
11	-1.12937	0.988706
26	11.26476	1.112648
5	-11.1826	0.888174
7	-2.31356	0.976864
13	-2.4801	0.975199
4	-10.6723	0.893277
7	-18.9991	0.810009
10	14.9696	1.149696
20	2.916276	1.029163
3	-13.9271	0.860729
3	17.05753	1.170575
9	30.05995	1.3006
2	-34.422	0.65578
9	-3.97424	0.960258
4	8.405798	1.084058
10	-11.5589	0.884411
10	20.0386	1.200386
10	22.11902	1.22119
3	19.31473	1.193147
8	1.375609	1.013756
7	-0.04231	0.999577
10	0.997013	1.00997
5	16.54178	1.165418
3	-3.44819	0.965518
11	-0.02601	0.99974
9	-14.6399	0.853601
2	-14.3173	0.856827
3	39.39023	1.393902
5	4.049047	1.04049
6	22.44476	1.224448
3	-29.6518	0.703482
5	52.97332	1.529733
17	17.03377	1.170338
3	-11.507	0.88493
8	4.803023	1.04803
4	-0.17179	0.998282
4	-6.03987	0.939601
5	-16.9628	0.830372
11	64.6344	1.646344
17	0.815735	1.008157

2	-24.2125	0.757875
14	0.081663	1.000817
10	19.42709	1.194271
6	-2.53589	0.974641
4	69.75288	1.697529
17	0.556883	1.005569
11	11.26416	1.112642
27	-6.52316	0.934768
2	-11.9634	0.880366
4	-26.8191	0.731809
3	0.573652	1.005737
6	4.615704	1.046157
7	4.755775	1.047558
5	3.595109	1.035951
7	29.53212	1.295321
8	0.38898	1.00389
6	59.15953	1.591595
8	-13.8837	0.861163
13	-0.43528	0.995647
5	29.04609	1.290461
20	-3.30207	0.966979
20	19.96695	1.19967
8	-5.2304	0.947696
13	20.93588	1.209359
14	0.284774	1.002848
4	37.02935	1.370294
8	14.76085	1.147609
2	-8.96005	0.910399
4	2.95268	1.029527
11	-5.3251	0.946749
20	15.84454	1.158445
7	16.91028	1.169103
4	1.112056	1.011121
3	-33.8778	0.661222
4	-21.2467	0.787533
8	-30.2572	0.697428
8	10.44289	1.104429
11	1.862453	1.018625
2	104.5044	2.045044
5	23.1589	1.231589
44	9.182412	1.091824
3	56.26028	1.562603
6	23.87611	1.238761
8	42.03473	1.420347
4	-17.0912	0.829088
8	-4.77729	0.952227
12	25.33417	1.253342
4	-16.6341	0.833659
5	3.127258	1.031273
11	0.500852	1.005009

22	3.230873	1.032309
6	-21.0361	0.789639
8	9.902426	1.099024
9	13.24662	1.132466
16	40.90425	1.409042
5	16.31502	1.16315
12	5.683977	1.05684
2	23.91815	1.239181
19	6.409374	1.064094
2	-14.735	0.85265
27	4.993629	1.049936
35	100.0853	2.000853
3	-7.40161	0.925984
6	-2.88416	0.971158
12	10.22134	1.102213
2	-29.5255	0.704745
49	22.31634	1.223163
9	6.336284	1.063363
5	24.14859	1.241486
22	21.24386	1.212439
4	-9.38091	0.906191
4	1.156937	1.011569
3	9.309919	1.093099
4	8.812206	1.088122
2	38.35427	1.383543
4	-7.72849	0.922715
4	0.661993	1.00662
2	-44.4091	0.555909
8	-13.1952	0.868048
4	-31.6284	0.683716
9	20.93421	1.209342
9	8.729639	1.087296
33	-7.6111	0.923889
3	-24.1495	0.758505
2	2.101702	1.021017
8	-0.87025	0.991298
2	-17.1116	0.828884
21	6.69014	1.066901
4	10.28489	1.102849
42	11.72644	1.117264
8	-7.11554	0.928845
11	-6.06489	0.939351
7	23.39996	1.234
3	29.44779	1.294478
10	15.25177	1.152518
6	-5.58504	0.94415
18	-1.52052	0.984795
7	22.16344	1.221634
3	-23.8082	0.761918
6	11.46281	1.114628

23	-0.43322	0.995668
2	6.292761	1.062928
4	46.66315	1.466631
5	-17.6982	0.823018
11	14.79083	1.147908
10	3.699015	1.03699
12	15.39556	1.153956
36	8.705301	1.087053
23	30.85302	1.30853
16	10.32578	1.103258
9	46.1452	1.461452
19	10.58345	1.105835
24	3.940132	1.039401
17	-2.06368	0.979363
11	12.43657	1.124366
11	-11.1059	0.888941
3	36.65021	1.366502
6	9.769069	1.097691
6	9.400265	1.094003
2	39.70373	1.397037
17	-2.33215	0.976678
16	10.0719	1.100719
11	29.98897	1.29989
18	-0.83043	0.991696
3	23.13412	1.231341
8	5.394049	1.05394
8	3.204064	1.032041
7	7.18433	1.071843
2	-13.36	0.8664
3	-30.6973	0.693027
10	9.578665	1.095787
8	-1.60595	0.98394
3	-25.9506	0.740494
23	2.650851	1.026509
5	23.55711	1.235571
4	-27.6077	0.723923
2	17.89875	1.178987
4	-19.8999	0.801001
7	-7.70208	0.922979
5	39.87828	1.398783
9	21.12329	1.211233
18	-1.68526	0.983147
2	-30.2151	0.697849
9	43.52054	1.435205
24	58.7605	1.587605
10	4.616215	1.046162
8	-24.2591	0.757409
22	26.5369	1.265369
8	21.99886	1.219989
41	18.47624	1.184762

2	85.26954	1.852695
2	52.226	1.52226
7	-5.79861	0.942014
14	1.704203	1.017042
7	3.915447	1.039154
16	9.83107	1.098311
3	25.61575	1.256157
19	14.16091	1.141609
11	4.633865	1.046339
4	8.951099	1.089511
8	6.876429	1.068764
2	-35.7155	0.642845
2	19.80913	1.198091
18	-33.5647	0.664353
2	76.89447	1.768945
14	4.238294	1.042383
47	15.40012	1.154001
2	-25.3143	0.746857
6	-11.9852	0.880148
17	6.286165	1.062862
4	24.54079	1.245408
36	11.91478	1.119148
7	0.554862	1.005549
14	4.482139	1.044821
11	34.11511	1.341151
24	45.26159	1.452616
22	42.71433	1.427143
5	1.190059	1.011901
24	21.52701	1.21527
18	0.710503	1.007105
2	26.3305	1.263305
5	-16.2192	0.837808
10	14.28751	1.142875
3	-14.2531	0.857469
14	5.924263	1.059243
3	-18.6095	0.813905
5	20.9663	1.209663
5	5.524581	1.055246
24	4.001047	1.04001
13	3.04748	1.030475
7	55.43477	1.554348
8	2.860021	1.0286
26	7.676408	1.076764
7	5.143879	1.051439
13	5.017043	1.05017
8	-11.7274	0.882726
2	-25.5895	0.744105
3	-44.6685	0.553315
4	78.67458	1.786746
5	-22.342	0.77658

20	3.414023	1.03414
8	38.83505	1.388351
3	-20.8298	0.791702
8	10.44481	1.104448
11	7.357564	1.073576
36	2.639804	1.026398
22	8.03858	1.080386
3	-25.9669	0.740331
15	1.693697	1.016937
63	7.385097	1.073851
7	56.34883	1.563488
7	17.6366	1.176366
6	-3.07529	0.969247
3	3.622522	1.036225
41	10.05483	1.100548
7	-6.01377	0.939862
15	0.118338	1.001183
6	-7.4676	0.925324
32	13.14487	1.131449
4	-4.0371	0.959629
8	8.008522	1.080085
2	225.9932	3.259932
6	24.22178	1.242218
3	-17.782	0.82218
9	1.841002	1.01841
3	10.09773	1.100977
5	24.41267	1.244127
8	30.69359	1.306936
2	11.08223	1.110822
17	27.55195	1.275519
7	-2.66446	0.973355
7	7.076327	1.070763
10	-23.5302	0.764698
3	-17.6319	0.823681
3	89.0433	1.890433
16	8.870122	1.088701
3	-33.7178	0.662822
2	2.348734	1.023487
6	21.57985	1.215799
3	52.51073	1.525107
7	6.251083	1.062511
43	7.115044	1.07115
90	23.47642	1.234764
6	-3.3225	0.966775
4	1.339065	1.013391
5	24.72934	1.247293
22	25.6172	1.256172
3	38.29324	1.382932
4	-1.8798	0.981202
23	-3.06937	0.969306

12	4.511854	1.045119
9	9.214084	1.092141
9	-4.80156	0.951984
4	-6.50317	0.934968
3	14.28326	1.142833
12	17.40363	1.174036
11	10.00359	1.100036
3	1.888158	1.018882
2	17.51506	1.175151
5	-15.5605	0.844395
5	4.830838	1.048308
4	33.83607	1.338361
14	-8.77251	0.912275
3	11.30125	1.113012
2	-23.358	0.76642
5	13.09637	1.130964
15	8.997586	1.089976
3	-57.0782	0.429218
19	11.51649	1.115165
9	14.48109	1.144811
11	3.41547	1.034155
11	-5.2703	0.947297
10	5.0615	1.050615
2	-8.95591	0.910441
2	-18.059	0.81941
7	1.690671	1.016907
149	-4.80767	0.951923
24	8.763029	1.08763
43	5.192965	1.05193
11	-4.87687	0.951231
8	17.57969	1.175797
4	7.943279	1.079433
4	-9.51597	0.90484
10	12.68725	1.126872
6	28.4976	1.284976
19	8.668085	1.086681
4	94.44285	1.944428
5	39.18944	1.391894
14	17.53219	1.175322
2	-38.3415	0.616585
11	10.75916	1.107592
4	-2.82938	0.971706
2	30.31547	1.303155
11	2.810645	1.028106
8	9.081358	1.090814
9	17.61277	1.176128
3	75.15068	1.751507
4	24.44112	1.244411
2	-9.29968	0.907003
8	-6.38973	0.936103

6	19.78012	1.197801
7	9.145404	1.091454
14	17.66596	1.17666
16	15.03571	1.150357
5	-6.94731	0.930527
17	5.672646	1.056726
3	-28.9204	0.710796
2	12.12998	1.1213
15	14.81434	1.148143
9	38.26641	1.382664
14	24.70892	1.247089
31	12.06167	1.120617
4	7.936683	1.079367
12	32.14796	1.32148
3	-7.60485	0.923951
20	8.658365	1.086584
11	6.809896	1.068099
2	53.95124	1.539512
2	72.35027	1.723503
27	23.60856	1.236086
15	-10.5631	0.894369
12	-2.05059	0.979494
3	-10.5793	0.894207
17	77.70497	1.77705
2	-12.5887	0.874113
5	54.46691	1.544669
21	5.689844	1.056898
21	10.36557	1.103656
3	6.074635	1.060746
9	13.44048	1.134405
15	12.33375	1.123337
5	2.683317	1.026833
2	4.025589	1.040256
11	-12.6061	0.873939
11	2.939289	1.029393
14	4.328885	1.043289
15	4.762063	1.047621
16	2.714118	1.027141
5	23.25367	1.232537
2	-24.1428	0.758572
11	24.58973	1.245897
36	12.88001	1.1288
20	59.12495	1.591249
4	16.58153	1.165815
5	4.140731	1.041407
3	14.53918	1.145392
3	-14.7986	0.852014
6	23.29178	1.232918
7	-3.14909	0.968509
9	11.0676	1.110676

2	54.65491	1.546549
3	9.443679	1.094437
3	22.00552	1.220055
15	19.498	1.19498
3	88.21108	1.882111
5	4.473024	1.04473
4	-1.97951	0.980205
7	-8.55758	0.914424
28	41.55459	1.415546
7	15.26828	1.152683
10	22.67734	1.226773
3	-24.683	0.75317
26	5.173117	1.051731
19	6.433598	1.064336
10	29.52644	1.295264
76	38.84429	1.388443
7	14.17595	1.141759
24	3.14576	1.031458
4	39.11936	1.391194
23	3.809155	1.038092
3	16.56175	1.165618
17	9.910667	1.099107
13	3.775831	1.037758
5	33.98859	1.339886
12	8.531151	1.085312
4	65.77415	1.657741
41	26.92582	1.269258
4	39.36329	1.393633
4	23.83773	1.238377
4	-27.5734	0.724266
3	0.664282	1.006643
4	17.94532	1.179453
7	18.61855	1.186186
12	14.91571	1.149157
8	-13.4987	0.865013
5	0.607955	1.00608
7	56.55819	1.565582
4	-1.53308	0.984669
15	0.31336	1.003134
2	-30.0911	0.699089
12	3.484362	1.034844
5	129.1368	2.291368
14	-0.21962	0.997804
2	-50.0354	0.499646
7	6.634647	1.066346
6	-8.28492	0.917151
8	43.51305	1.43513
15	0.472865	1.004729
2	37.40768	1.374077
10	17.67765	1.176776

3	20.09597	1.20096
5	-14.111	0.85889
8	9.562573	1.095626
2	-8.39544	0.916046
5	7.925762	1.079258
2	-17.2545	0.827455
15	10.05457	1.100546
3	-10.5721	0.894279
17	3.336714	1.033367
15	6.242493	1.062425
4	75.81764	1.758176
8	17.86333	1.178633
10	-24.199	0.75801
7	29.76294	1.297629
8	-7.09463	0.929054
10	-2.17508	0.978249
19	11.66734	1.116673
7	0.064153	1.000642
9	9.225991	1.09226
2	78.45485	1.784549
11	-9.66185	0.903381
4	7.70126	1.077013
7	15.14185	1.151418
5	30.53182	1.305318
7	1.389013	1.01389
30	12.24256	1.122426
11	4.032244	1.040322
16	12.60508	1.126051
9	6.406088	1.064061
10	13.23233	1.132323
10	125.0216	2.250216
15	1.980902	1.019809
18	8.68249	1.086825
18	39.92264	1.399226
3	0.609752	1.006098
42	2.657276	1.026573
4	13.58078	1.135808
5	-4.35335	0.956467
3	-5.75655	0.942435
3	34.66931	1.346693
2	46.13967	1.461397
33	8.814223	1.088142
3	-3.75668	0.962433
37	32.44259	1.324426
24	28.63899	1.28639
2	-19.9816	0.800184
10	15.47583	1.154758
8	-5.06856	0.949314
2	-11.7722	0.882278
4	6.043409	1.060434

7	19.61072	1.196107
5	19.65086	1.196509
12	14.51139	1.145114
3	10.50861	1.105086
8	2.595102	1.025951
4	8.929604	1.089296
4	17.95247	1.179525
5	-7.89576	0.921042
7	-6.28378	0.937162
5	14.46921	1.144692
5	37.99797	1.37998
22	4.364985	1.04365
14	15.30744	1.153074
3	54.08619	1.540862
48	20.64804	1.20648
10	-4.2024	0.957976
24	17.05996	1.1706
2	37.81252	1.378125
2	-22.9163	0.770837
4	9.423082	1.094231
5	30.30779	1.303078
6	13.88516	1.138852
22	12.21679	1.122168
5	0.429515	1.004295
4	16.31429	1.163143
2	-21.3844	0.786156
16	9.348813	1.093488
13	2.040069	1.020401
8	18.23511	1.182351
5	9.699716	1.096997
4	13.10169	1.131017
10	1.310927	1.013109
4	12.26382	1.122638
20	17.62348	1.176235
12	-5.78752	0.942125
3	52.78111	1.527811
10	13.38762	1.133876
29	9.673851	1.096739
2	81.24354	1.812435
6	7.00617	1.070062
20	0.976892	1.009769
11	1.508441	1.015084
4	8.032464	1.080325
5	-7.42577	0.925742
3	24.91306	1.249131
6	18.61527	1.186153
14	11.78824	1.117882
9	0.692749	1.006927
25	9.216158	1.092162
11	26.50796	1.26508

5	20.67616	1.206762
10	3.290445	1.032904
8	29.66129	1.296613
4	12.23808	1.122381
4	15.32942	1.153294
4	-17.0423	0.829577
10	-0.48186	0.995181
3	2.181288	1.021813
2	66.04192	1.660419
7	2.863578	1.028636
6	31.08128	1.310813
52	30.74357	1.307436
8	27.52306	1.275231
13	55.29641	1.552964
14	13.67257	1.136726
3	-21.3919	0.786081
8	-9.74314	0.902569
57	9.682332	1.096823
2	3.013662	1.030137
6	-2.52256	0.974774
20	33.36378	1.333638
12	5.723735	1.057237
3	59.74722	1.597472
3	-2.21663	0.977834
4	0.48384	1.004838
4	-7.71509	0.922849
11	1.941833	1.019418
8	2.527096	1.025271
25	10.95095	1.10951
9	14.23334	1.142333
10	19.91348	1.199135
30	3.393473	1.033935
3	33.59864	1.335986
10	12.74982	1.127498
2	-26.7675	0.732325
3	1.796051	1.017961
19	2.937369	1.029374
2	19.15342	1.191534
9	2.005159	1.020052
2	-10.14	0.8986
2	-12.2646	0.877354
6	-6.60449	0.933955
14	3.485317	1.034853
9	14.46585	1.144658
8	13.10954	1.131095
6	3.966149	1.039661
4	6.057741	1.060577
9	14.89682	1.148968
7	7.033574	1.070336
11	8.879375	1.088794

4	11.85362	1.118536
5	-3.72985	0.962701
5	1.551309	1.015513
4	5.784355	1.057844
9	-2.07472	0.979253
6	27.20459	1.272046
13	13.59319	1.135932
28	27.28297	1.27283
6	-0.28062	0.997194
14	-2.76325	0.972368
7	8.524701	1.085247
3	7.553138	1.075531
3	5.386475	1.053865
17	12.46603	1.12466
13	7.782806	1.077828
32	5.641665	1.056417
6	-1.67168	0.983283
11	11.15034	1.111503
11	12.62771	1.126277
4	3.713709	1.037137
36	20.65231	1.206523
7	3.279861	1.032799
16	19.68305	1.196831
4	-4.33668	0.956633
6	13.77169	1.137717
16	11.67617	1.116762
2	6.271868	1.062719
8	-6.82792	0.931721
10	3.447306	1.034473
11	-0.9949	0.990051
10	1.715734	1.017157
7	-14.9973	0.850027
3	16.36482	1.163648
6	-0.53396	0.99466
2	35.22875	1.352287
15	67.81877	1.678188
18	-1.26363	0.987364
24	2.907313	1.029073
3	19.88561	1.198856
8	15.08065	1.150807
35	4.746556	1.047466
11	20.19475	1.201948
9	4.161707	1.041617
3	-0.00684	0.999932
2	-24.42	0.7558
4	15.00532	1.150053
2	20.56474	1.205647
15	4.275521	1.042755
23	6.279079	1.062791
2	-30.8825	0.691175

4	-30.1875	0.698125
2	4.70969	1.047097
3	-10.268	0.89732
2	17.35311	1.173531
8	-13.8657	0.861343
11	7.415756	1.074158
9	-0.11309	0.998869
4	5.411338	1.054113
19	14.97436	1.149744
4	32.03692	1.320369
4	27.15171	1.271517
2	-4.82718	0.951728
30	4.727846	1.047278
5	8.063079	1.080631
12	7.322769	1.073228
3	5.597943	1.055979
6	23.74245	1.237425
15	3.089792	1.030898
3	-16.9926	0.830074
9	29.19935	1.291993
3	11.38919	1.113892
5	17.4715	1.174715
18	5.924384	1.059244
13	37.31271	1.373127
11	24.43896	1.24439
3	-26.8607	0.731393
2	40.40717	1.404072
7	-5.82899	0.94171
7	1.065209	1.010652
15	31.17537	1.311754
5	26.61145	1.266114
7	6.207152	1.062072
5	11.83115	1.118311
3	4.396643	1.043966
6	20.88254	1.208825
19	14.08589	1.140859
9	19.36526	1.193653
7	14.76568	1.147657
13	14.05638	1.140564
2	4.210122	1.042101
38	6.932337	1.069323
20	2.177046	1.02177
7	35.92209	1.359221
2	13.61621	1.136162
7	-0.67996	0.9932
12	17.78581	1.177858
11	-15.2327	0.847673
5	0.706352	1.007064
9	6.590975	1.06591
7	8.931614	1.089316

2	8.890826	1.088908
8	53.10736	1.531074
6	2.631238	1.026312
5	-46.9666	0.530334
18	3.667439	1.036674
6	-5.64814	0.943519
7	18.94452	1.189445
6	52.70796	1.52708
12	17.23609	1.172361
24	2.095837	1.020958
24	63.77985	1.637799
20	7.644664	1.076447
2	25.16876	1.251688
8	-0.14825	0.998518
4	-19.3705	0.806295
35	22.58488	1.225849
2	7.520044	1.0752
5	8.272646	1.082726
5	52.69943	1.526994
2	-12.8687	0.871313
4	4.001172	1.040012
26	2.760298	1.027603
11	13.12589	1.131259
3	-27.2118	0.727882
7	37.37627	1.373763
9	-7.99129	0.920087
39	16.71869	1.167187
5	16.67173	1.166717
5	-17.7256	0.822744
11	7.455779	1.074558
5	4.858812	1.048588
3	-0.03379	0.999662
4	-2.41013	0.975899
7	8.896709	1.088967
14	13.43125	1.134312
15	12.05647	1.120565
10	36.77865	1.367787
4	33.28448	1.332845
92	12.95632	1.129563
6	11.31572	1.113157
12	12.87664	1.128766
3	18.49007	1.184901
5	-1.76793	0.982321
12	59.73221	1.597322
5	-0.99141	0.990086
5	83.32932	1.833293
3	-3.31477	0.966852
13	14.63439	1.146344
4	48.25128	1.482513
3	10.64547	1.106455

16	19.05651	1.190565
6	-9.00878	0.909912
3	16.34654	1.163465
17	25.39434	1.253943
3	-22.8665	0.771335
6	2.892984	1.02893
51	3.60725	1.036072
5	31.26165	1.312616
2	102.2229	2.022229
7	40.57009	1.405701
11	8.203681	1.082037
11	6.757408	1.067574
10	8.676821	1.086768
2	17.80662	1.178066
2	-15.3834	0.846166
10	11.07062	1.110706
10	9.282403	1.092824
11	19.02411	1.190241
26	16.45227	1.164523
3	4.994722	1.049947
5	8.81234	1.088123
10	8.519692	1.085197
5	-6.63156	0.933684
6	17.86126	1.178613
7	50.8385	1.508385
5	-30.7564	0.692436
13	23.10215	1.231021
4	-16.0259	0.839741
4	27.29125	1.272912
3	-10.4738	0.895262
13	-0.29937	0.997006
4	9.669142	1.096691
7	-5.17347	0.948265
4	21.34553	1.213455
4	23.26639	1.232664
11	6.020508	1.060205
5	11.98954	1.119895
7	2.15223	1.021522
5	-24.657	0.75343
5	34.12517	1.341252
5	31.51921	1.315192
3	-6.56044	0.934396
11	9.03161	1.090316
11	5.266099	1.052661
10	33.69806	1.336981
6	24.87354	1.248735
19	9.184359	1.091844
14	20.23081	1.202308
4	0.681557	1.006816
2	-15.132	0.84868

9	48.71295	1.48713
30	-0.00409	0.999959
9	6.201034	1.06201
5	33.08516	1.330852
2	6.802592	1.068026
2	-1.2243	0.987757
6	26.24045	1.262404
9	-0.40497	0.99595
11	33.99171	1.339917
18	2.133921	1.021339
3	22.55485	1.225548
3	-3.84583	0.961542
3	13.79261	1.137926
29	22.19255	1.221926
3	6.149326	1.061493
6	-20.9145	0.790855
3	13.23541	1.132354
10	-14.7973	0.852027
18	17.19771	1.171977
16	6.881224	1.068812
2	14.41104	1.14411
9	9.880388	1.098804
34	0.18121	1.001812
12	2.170871	1.021709
3	1.057009	1.01057
2	37.97046	1.379705
30	9.20969	1.092097
3	2.082024	1.02082
8	31.06705	1.310671
5	-16.3025	0.836975
3	2.718089	1.027181
19	5.146202	1.051462
6	11.09683	1.110968
6	-7.43509	0.925649
3	-10.2701	0.897299
6	34.50793	1.345079
7	17.96788	1.179679
13	10.27841	1.102784
8	-6.17245	0.938276
21	11.98027	1.119803
12	-3.87031	0.961297
5	-1.02963	0.989704
11	7.342642	1.073426
6	-20.3022	0.796978
3	-7.64529	0.923547
7	38.31967	1.383197
27	8.774498	1.087745
7	15.29899	1.15299
7	-5.94269	0.940573
11	13.13212	1.131321

11	1.983654	1.019837
17	52.41326	1.524133
5	-10.0875	0.899125
2	-0.77676	0.992232
2	13.44967	1.134497
2	-33.3294	0.666706
7	11.24797	1.11248
20	1.064711	1.010647
7	73.02405	1.73024
5	56.98883	1.569888
9	-7.76715	0.922328
10	19.64793	1.196479
21	5.387785	1.053878
9	-0.29062	0.997094
2	-46.3248	0.536752
14	10.0184	1.100184
3	-2.78971	0.972103
6	14.13765	1.141377
9	15.09982	1.150998
3	-19.8749	0.801251
24	5.958772	1.059588
12	1.981156	1.019812
2	12.26722	1.122672
5	-13.0526	0.869474
7	-0.42799	0.99572
51	13.49662	1.134966
2	58.15915	1.581591
5	-11.6005	0.883995
3	22.76041	1.227604
3	8.873896	1.088739
3	12.03085	1.120309
10	10.80493	1.108049
6	-3.16644	0.968336
6	37.21081	1.372108
4	6.3823	1.063823
19	17.2421	1.172421
7	25.88992	1.258899
23	10.31325	1.103133
2	28.30305	1.28303
3	12.29824	1.122982
3	-47.8926	0.521074
5	23.86005	1.238601
2	10.37383	1.103738
2	57.13389	1.571339
3	-0.71898	0.99281
10	0.669775	1.006698
2	16.71491	1.167149
3	96.77013	1.967701
20	6.510015	1.0651
19	3.353636	1.033536

6	4.572441	1.045724
7	26.1311	1.261311
2	-7.75981	0.922402
4	43.95678	1.439568
13	3.670515	1.036705
3	-45.0459	0.549541
4	22.30061	1.223006
2	83.51636	1.835164
8	11.60789	1.116079
2	-19.2892	0.807108
4	42.65679	1.426568
5	8.49357	1.084936
9	4.446203	1.044462
28	34.99997	1.35
3	-5.06341	0.949366
24	14.42448	1.144245
2	41.5786	1.415786
4	23.87232	1.238723
8	5.043414	1.050434
11	13.06784	1.130678
10	36.0449	1.360449
2	21.22691	1.212269
44	17.97288	1.179729
3	1.394378	1.013944
9	4.417279	1.044173
3	8.433519	1.084335
2	5.930319	1.059303
10	12.82095	1.12821
15	37.0105	1.370105
8	11.59807	1.115981
16	4.716373	1.047164
4	43.15086	1.431509
3	22.69835	1.226983
5	6.145687	1.061457
20	3.891941	1.038919
2	64.61891	1.646189
9	17.28795	1.17288
2	-28.2588	0.717412
12	16.78404	1.16784
22	3.096469	1.030965
9	16.97909	1.169791
7	25.85943	1.258594
33	57.85531	1.578553
4	2.778942	1.027789
9	16.12314	1.161231
11	63.85273	1.638527
161	3.725592	1.037256
7	38.87497	1.38875
3	11.78753	1.117875
4	8.450953	1.08451

9	3.429896	1.034299
8	-0.10645	0.998936
13	30.68017	1.306802
2	0.770563	1.007706
25	14.49671	1.144967
8	-1.81169	0.981883
3	-1.50438	0.984956
3	26.42725	1.264273
5	1.002508	1.010025
2	-7.74192	0.922581
10	35.92019	1.359202
17	40.89722	1.408972
26	17.87846	1.178785
3	-44.3287	0.556713
2	2.368111	1.023681
2	31.26406	1.312641
32	4.64405	1.046441
16	0.834173	1.008342
8	3.612868	1.036129
18	22.00311	1.220031
4	-0.32198	0.99678
56	10.13333	1.101333
4	37.72089	1.377209
10	25.70624	1.257062
17	11.62254	1.116225
6	-4.42877	0.955712
6	6.470157	1.064702
18	25.08525	1.250853
4	94.81311	1.948131
14	7.57952	1.075795
9	2.10121	1.021012
10	11.80992	1.118099
21	2.854741	1.028547
4	4.342553	1.043426
13	10.62689	1.106269
9	5.387262	1.053873
16	11.55268	1.115527
6	9.979378	1.099794
7	-12.1104	0.878896
5	10.05838	1.100584
12	-1.97063	0.980294
2	82.87401	1.82874
3	17.4853	1.174853
9	1.650707	1.016507
3	-7.82877	0.921712
6	-6.00677	0.939932
5	-13.9039	0.860961
3	64.79411	1.647941
3	-13.0493	0.869507
13	-3.71288	0.962871

10	6.305459	1.063055
12	17.49764	1.174976
25	10.2266	1.102266
22	14.82719	1.148272
11	15.23073	1.152307
8	-5.13767	0.948623
18	9.19891	1.091989
8	3.700605	1.037006
9	18.95391	1.189539
2	2.695814	1.026958
14	14.17997	1.1418
3	-13.5924	0.864076
6	22.28186	1.222819
2	-7.67003	0.9233
4	23.37732	1.233773
12	18.8918	1.188918
2	-1.37439	0.986256
19	14.42052	1.144205
6	-6.59415	0.934058
27	1.797803	1.017978
7	36.91398	1.36914
9	14.20397	1.14204
22	12.99448	1.129945
7	-11.2157	0.887843
2	52.7149	1.527149
6	3.784075	1.037841
10	3.713659	1.037137
55	30.56314	1.305631
4	41.3448	1.413448
5	11.00397	1.11004
3	-6.29086	0.937091
3	9.879889	1.098799
2	14.1043	1.141043
3	8.517754	1.085178
9	11.47479	1.114748
24	24.69452	1.246945
3	11.76014	1.117601
15	32.55069	1.325507
8	4.567324	1.045673
17	33.1824	1.331824
3	9.451475	1.094515
14	20.43671	1.204367
2	2.363642	1.023636
3	35.1507	1.351507
10	4.950763	1.049508
11	2.531527	1.025315
32	3.065	1.03065
23	14.47777	1.144778
7	-5.90567	0.940943
31	20.53095	1.205309

2	-7.09935	0.929006
7	14.01438	1.140144
11	3.317796	1.033178
2	-13.4171	0.865829
3	2.968355	1.029684
4	26.99898	1.26999
4	13.90131	1.139013
3	-4.18893	0.958111
32	0.943502	1.009435
5	14.18258	1.141826
3	47.10481	1.471048
16	30.76408	1.307641
15	40.45577	1.404558
47	24.95893	1.249589
3	29.22584	1.292258
5	-20.6914	0.793086
3	42.33902	1.42339
8	12.08345	1.120835
2	27.44371	1.274437
7	21.40365	1.214036
11	2.222385	1.022224
3	-14.823	0.85177
2	47.73368	1.477337
8	-3.62214	0.963779
5	3.857427	1.038574
21	7.652502	1.076525
10	35.82191	1.358219
3	-1.7959	0.982041
7	10.89373	1.108937
3	16.06441	1.160644
2	-1.73657	0.982634
17	15.07206	1.150721
43	11.25429	1.112543
9	16.60156	1.166016
2	3.044907	1.030449
14	33.87861	1.338786
47	13.06576	1.130658
9	1.982174	1.019822
5	31.98657	1.319866
2	1.053966	1.01054
4	-8.69159	0.913084
7	31.16499	1.31165
9	3.714326	1.037143
3	-16.3618	0.836382
2	13.64981	1.136498
24	33.84376	1.338438
3	31.83387	1.318339
27	15.20647	1.152065
6	1.976138	1.019761
5	-16.7131	0.832869

12	35.48037	1.354804
8	12.77879	1.127788
8	28.44383	1.284438
3	-1.11308	0.988869
2	2.221494	1.022215
7	28.21746	1.282175
3	44.79447	1.447945
2	2.541479	1.025415
24	2.739392	1.027394
6	41.52584	1.415258
14	13.28928	1.132893
9	6.554873	1.065549
6	-10.3264	0.896736
3	-19.8082	0.801918
7	24.43641	1.244364
2	-1.19652	0.988035
5	-2.24081	0.977592
6	-9.71089	0.902891
8	65.97362	1.659736
13	16.5885	1.165885
3	43.98638	1.439864
5	-13.4695	0.865305
4	20.17371	1.201737
2	13.4422	1.134422
14	4.176104	1.041761
6	10.38741	1.103874
21	13.96868	1.139687
13	6.876182	1.068762
3	130.607	2.30607
10	9.726117	1.097261
2	12.94339	1.129434
11	16.29468	1.162947
11	27.69645	1.276964
6	3.591025	1.03591
6	38.29423	1.382942
9	60.86562	1.608656
13	1.152377	1.011524
2	-0.0446	0.999554
3	58.24542	1.582454
10	13.45619	1.134562
7	-1.80552	0.981945
9	4.008177	1.040082
9	4.997015	1.04997
2	36.68907	1.366891
4	30.65412	1.306541
2	4.814515	1.048145
12	6.454428	1.064544
11	20.29362	1.202936
8	41.83578	1.418358
12	19.00933	1.190093

3	-11.3656	0.886344
2	2.809819	1.028098
8	27.72601	1.27726
8	63.32216	1.633222
15	13.68152	1.136815
2	-2.99932	0.970007
9	-17.1557	0.828443
2	20.40974	1.204097
8	18.53976	1.185398
14	44.23607	1.442361
3	14.96621	1.149662
6	9.420096	1.094201
4	7.035394	1.070354
12	36.87112	1.368711
11	11.50985	1.115098
2	12.31194	1.123119
2	46.58444	1.465844
4	-18.9014	0.810986
14	11.18356	1.111836
4	18.58376	1.185838
18	50.36384	1.503638
6	18.19466	1.181947
11	-0.23306	0.997669
8	36.73353	1.367335
4	11.40063	1.114006
13	63.04719	1.630472
12	8.807647	1.088076
4	-5.03955	0.949604
11	0.145908	1.001459
4	26.60947	1.266095
8	3.595	1.03595
2	-10.8175	0.891825
16	23.84066	1.238407
6	1.690079	1.016901
2	-29.1779	0.708221
3	16.0394	1.160394
3	-2.69355	0.973065
5	10.3681	1.103681
2	-20.1749	0.798251
8	2.032561	1.020326
13	15.32067	1.153207
4	-6.38857	0.936114
3	3.317075	1.033171
6	-3.60323	0.963968
3	28.34755	1.283476
5	75.01492	1.750149
2	12.86423	1.128642
3	18.00116	1.180012
3	-9.33432	0.906657
6	-17.652	0.82348

14	2.665737	1.026657
2	13.35642	1.133564
8	42.45699	1.42457
3	-23.7867	0.762133
4	58.32723	1.583272
2	-14.8117	0.851883
16	7.812262	1.078123
2	-9.1421	0.908579
8	2.450061	1.024501
2	45.50069	1.455007
3	-21.0966	0.789034
12	5.19484	1.051948
9	6.651702	1.066517
6	26.01907	1.260191
5	21.63841	1.216384
6	-3.94965	0.960504
9	17.16145	1.171615
2	7.321471	1.073215
3	-5.09548	0.949045
6	23.00548	1.230055
6	17.56289	1.175629
5	14.56337	1.145634
12	25.03816	1.250382
3	-6.48316	0.935168
3	-22.6034	0.773966
14	4.284407	1.042844
11	27.46312	1.274631
3	-21.4272	0.785728
20	4.663627	1.046636
3	13.29005	1.1329
8	4.518201	1.045182
7	30.29683	1.302968
31	24.41358	1.244136
3	5.272317	1.052723
9	11.98044	1.119804
2	-13.0579	0.869421
14	6.684806	1.066848
3	-5.71807	0.942819
9	2.004537	1.020045
5	1.849252	1.018493
10	-1.03228	0.989677
2	-23.8239	0.761761
21	26.61207	1.266121
16	20.67786	1.206779
5	56.9289	1.569289
7	15.96528	1.159653
4	7.755145	1.077551
6	6.32034	1.063203
5	22.26811	1.222681
2	6.682124	1.066821

10	9.811513	1.098115
7	3.815679	1.038157
2	53.51665	1.535166
3	-11.8004	0.881996
18	12.24233	1.122423
23	46.37482	1.463748
13	6.610317	1.066103
7	1.176781	1.011768
6	15.83332	1.158333
4	8.642641	1.086426
10	25.95049	1.259505
3	-12.6868	0.873132
18	34.16799	1.34168
3	22.88994	1.228899
4	3.666941	1.036669
4	75.63355	1.756335
5	33.22917	1.332292
7	16.28747	1.162875
7	-1.14952	0.988505
11	14.54117	1.145412
3	3.828007	1.03828
2	8.719394	1.087194
3	3.074528	1.030745
2	8.484079	1.084841
3	-24.2883	0.757117
5	20.7247	1.207247
8	4.881588	1.048816
13	17.30724	1.173072
17	6.74329	1.067433
7	37.03005	1.370301
13	25.02694	1.250269
21	15.88595	1.158859
20	10.98322	1.109832
2	6.593631	1.065936
2	13.3755	1.133755
13	13.2447	1.132447
2	113.3553	2.133553
8	6.75314	1.067531
10	19.94697	1.19947
6	16.31859	1.163186
6	18.21466	1.182147
9	17.0661	1.170661
12	2.093489	1.020935
18	15.81631	1.158163
14	14.35961	1.143596
2	91.98018	1.919802
8	37.37051	1.373705
11	-1.38159	0.986184
11	8.660023	1.0866
7	0.835764	1.008358

5	15.60177	1.156018
6	8.938344	1.089383
9	12.43346	1.124335
2	51.3207	1.513207
3	7.539675	1.075397
30	12.43472	1.124347
7	50.45204	1.50452
12	24.15817	1.241582
2	-36.4158	0.635842
2	8.342026	1.08342
2	38.05135	1.380514
20	9.049611	1.090496
17	26.62159	1.266216
2	-16.7355	0.832645
6	7.438298	1.074383
3	39.92747	1.399275
7	12.92596	1.12926
19	17.46267	1.174627
2	-7.43875	0.925612
3	62.75649	1.627565
5	30.04865	1.300487
2	-23.8673	0.761327
13	7.373002	1.07373
19	8.795298	1.087953
15	16.84557	1.168456
3	43.58852	1.435885
17	15.98485	1.159849
2	-30.579	0.69421
6	7.513081	1.075131
33	32.94844	1.329484
21	10.85207	1.108521
8	2.354252	1.023543
8	11.09751	1.110975
3	6.07683	1.060768
11	13.69004	1.1369
5	13.13473	1.131347
5	28.76983	1.287698
36	20.43237	1.204324
5	-9.76014	0.902399
6	10.71031	1.107103
9	-6.68151	0.933185
6	18.42961	1.184296
45	17.48386	1.174839
9	24.54522	1.245452
7	7.026717	1.070267
7	7.794003	1.07794
6	2.624351	1.026244
23	26.66147	1.266615
3	17.93424	1.179342
3	42.51303	1.42513

12	8.702863	1.087029
5	10.38702	1.10387
3	0.298021	1.00298
18	28.13341	1.281334
17	49.27775	1.492777
3	-0.56557	0.994344
15	6.282744	1.062827
14	15.30252	1.153025
11	3.681929	1.036819
10	35.93565	1.359357
3	39.83248	1.398325
6	2.9725	1.029725
10	30.38396	1.30384
12	20.01411	1.200141
5	-6.49787	0.935021
2	139.5743	2.395743
6	-3.1635	0.968365
2	16.97622	1.169762
5	46.46411	1.464641
8	-3.97812	0.960219
7	3.856357	1.038564
4	37.81669	1.378167
4	9.503802	1.095038
32	11.07523	1.110752
9	20.19444	1.201944
2	15.5193	1.155193
2	-20.6262	0.793738
5	30.37759	1.303776
8	8.735703	1.087357
9	9.652554	1.096526
4	41.68966	1.416897
5	10.8533	1.108533
5	-18.0215	0.819785
2	52.88332	1.528833
12	17.27085	1.172708
7	1.913269	1.019133
15	16.94647	1.169465
8	26.37341	1.263734
2	27.11877	1.271188
9	-12.4929	0.875071
6	-2.94742	0.970526
39	10.51509	1.105151
16	12.02477	1.120248
10	1.306986	1.01307
8	12.59062	1.125906
6	11.45799	1.11458
6	8.064681	1.080647
2	16.57735	1.165773
8	-2.42427	0.975757
3	49.55634	1.495563

10	27.17219	1.271722
21	36.9087	1.369087
4	68.08502	1.68085
20	2.50549	1.025055
16	14.8653	1.148653
12	14.75481	1.147548
4	16.70789	1.167079
6	71.59428	1.715943
9	30.74515	1.307452
9	-3.4409	0.965591
2	11.68401	1.11684
28	7.315214	1.073152
2	-17.5639	0.824361
4	20.25407	1.202541
2	15.60386	1.156039
2	3.490802	1.034908
4	3.425261	1.034253
10	3.750162	1.037502
6	-10.573	0.89427
2	12.37282	1.123728
4	-6.69464	0.933054
9	0.550346	1.005503
4	41.47392	1.414739
19	12.04098	1.12041
14	16.67015	1.166701
5	22.11642	1.221164
5	27.87688	1.278769
8	7.975109	1.079751
8	9.873716	1.098737
2	29.45755	1.294575
7	20.13635	1.201364
6	21.28151	1.212815
2	30.92964	1.309296
4	8.556268	1.085563
6	-9.16553	0.908345
17	5.367727	1.053677
7	14.59035	1.145904
3	42.82289	1.428229
7	10.48895	1.10489
7	28.81537	1.288154
12	9.144894	1.091449
2	5.279225	1.052792
9	7.598452	1.075985
2	-21.4726	0.785274
7	1.83993	1.018399
26	12.06737	1.120674
6	37.56115	1.375612
6	32.29066	1.322907
10	6.997815	1.069978
3	-27.4476	0.725524

3	2.513118	1.025131
8	28.66154	1.286615
19	7.477919	1.074779
6	-7.62497	0.92375
4	16.57291	1.165729
5	-21.2776	0.787224
7	29.54244	1.295424
8	-2.47672	0.975233
4	-4.79545	0.952046
43	2.06407	1.020641
5	8.238102	1.082381
23	62.2936	1.622936
5	74.39128	1.743913
32	5.129051	1.051291
35	3.511098	1.035111
7	11.45166	1.114517
3	70.17756	1.701776
6	17.45096	1.17451
2	37.35356	1.373536
3	-8.69265	0.913074
12	12.07007	1.120701
8	5.945096	1.059451
5	9.627854	1.096279
7	5.515051	1.055151
2	-14.9837	0.850163
8	12.54245	1.125425
16	12.10014	1.121001
33	15.65049	1.156505
29	22.45399	1.22454
9	5.942192	1.059422
12	31.39046	1.313905
10	20.32301	1.20323
12	34.66009	1.346601
12	14.82291	1.148229
4	9.84285	1.098428
20	7.209932	1.072099
2	9.123734	1.091237
5	8.035414	1.080354
12	13.96549	1.139655
16	3.748182	1.037482
9	11.51292	1.115129
17	8.905955	1.08906
7	1.468133	1.014681
5	14.60083	1.146008
5	3.308682	1.033087
14	5.532584	1.055326
3	55.09932	1.550993
2	8.726592	1.087266
5	-6.96197	0.93038
5	-1.89595	0.98104

15	9.19191	1.091919
15	3.355938	1.033559
5	-5.86057	0.941394
5	11.43307	1.114331
16	11.14924	1.111492
6	-2.17696	0.97823
7	21.09265	1.210926
16	4.283315	1.042833
4	-3.23876	0.967612
4	18.72677	1.187268
332	8.697525	1.086975
4	-13.4687	0.865313
2	-3.04139	0.969586
11	18.54168	1.185417
11	14.23104	1.14231
5	15.72561	1.157256
17	10.77455	1.107745
6	20.84605	1.208461
7	9.199742	1.091997
2	17.47173	1.174717
7	21.10098	1.21101
38	16.50472	1.165047
33	6.896857	1.068969
4	39.3065	1.393065
2	42.53032	1.425303
40	22.29902	1.22299
10	4.219618	1.042196
29	10.13581	1.101358
3	5.531957	1.05532
10	16.28706	1.162871
13	1.935986	1.01936
15	59.49912	1.594991
5	-3.54369	0.964563
6	8.068295	1.080683
2	48.40856	1.484086
8	23.04135	1.230413
40	7.370845	1.073708
2	-5.75779	0.942422
6	15.1938	1.151938
5	-7.5566	0.924434
6	30.45522	1.304552
21	9.300679	1.093007
17	5.256174	1.052562
10	15.72611	1.157261
5	-7.13699	0.92863
7	24.65928	1.246593
9	5.863684	1.058637
8	1.533261	1.015333
15	28.54886	1.285489
7	22.76434	1.227643

10	29.38589	1.293859
6	9.890071	1.098901
2	-19.4355	0.805645
2	106.9458	2.069458
4	8.124292	1.081243
2	26.43485	1.264348
2	-2.66522	0.973348
10	25.38501	1.25385
5	12.07152	1.120715
2	-12.1771	0.878229
26	6.192143	1.061921
11	21.65299	1.21653
2	39.34731	1.393473
