# Supplementary material for: Proteomic Analysis of Endothelial Activation Induced by Adult Angiostrongylus vasorum Homogenate: Insights into Vascular Remodeling and Hemostatic Imbalance
Source: Animals (Basel). 2026 Mar 15;16(6):926. doi: 10.3390/ani16060926 (PMC13023303; doi:10.3390/ani16060926)
Supplement: Supplementary file 1 [file animals-16-00926-s001.zip › Supplmentary Table S3.pdf]

Supplementary Table S3: Proteins identified in the sup

R.Condition	R.Replicat	PG.ProteinAccessions
Avasorum	1	A0A8I5KQE6
Avasorum	1	A6NIZ1
Avasorum	1	P08134
Avasorum	1	B5ME19
Avasorum	1	E9PAV3
Avasorum	1	O00154
Avasorum	1	O00159
Avasorum	1	O00231
Avasorum	1	O00232
Avasorum	1	O00299
Avasorum	1	O00391
Avasorum	1	O00410
Avasorum	1	O00469
Avasorum	1	O00567
Avasorum	1	O00571
Avasorum	1	O00622
Avasorum	1	O00625
Avasorum	1	O14773
Avasorum	1	O14786
Avasorum	1	O14818
Avasorum	1	O14950
Avasorum	1	O14979
Avasorum	1	O14980
Avasorum	1	O15123
Avasorum	1	O15143
Avasorum	1	O15144
Avasorum	1	O15145
Avasorum	1	O15232
Avasorum	1	O43143
Avasorum	1	O43242
Avasorum	1	O43390
Avasorum	1	O43396
Avasorum	1	O43684
Avasorum	1	O43707
Avasorum	1	O60462
Avasorum	1	O60506
Avasorum	1	O60568
Avasorum	1	O60814
Avasorum	1	O75083
Avasorum	1	O75131
Avasorum	1	O75369
Avasorum	1	O75390
Avasorum	1	O75436
Avasorum	1	O75489
Avasorum	1	O75874
Avasorum	1	O75915
Avasorum	1	O75923
Avasorum	1	O94979

Avasorum	1	O94985
Avasorum	1	O95084
Avasorum	1	O95445
Avasorum	1	O95497
Avasorum	1	O95782
Avasorum	1	O95810
Avasorum	1	P00338
Avasorum	1	P00352
Avasorum	1	P00387
Avasorum	1	P00403
Avasorum	1	P00488
Avasorum	1	P00491
Avasorum	1	P00492
Avasorum	1	P00505
Avasorum	1	P00558
Avasorum	1	P00568
Avasorum	1	P00742
Avasorum	1	P00750
Avasorum	1	P01024
Avasorum	1	P01033
Avasorum	1	P01034
Avasorum	1	P02452
Avasorum	1	P02458
Avasorum	1	P02545
Avasorum	1	P02751
Avasorum	1	P02753
Avasorum	1	P02768
Avasorum	1	P03956
Avasorum	1	P04075
Avasorum	1	P04083
Avasorum	1	P04114
Avasorum	1	P04275
Avasorum	1	P04406
Avasorum	1	P04439
Avasorum	1	P04792
Avasorum	1	P04843
Avasorum	1	P04844
Avasorum	1	P04899
Avasorum	1	P04908
Avasorum	1	P05023
Avasorum	1	P05067
Avasorum	1	P05091
Avasorum	1	P05109
Avasorum	1	P05114
Avasorum	1	P05121
Avasorum	1	P05141
Avasorum	1	P05198
Avasorum	1	P05305
Avasorum	1	P05388
Avasorum	1	P05455

Avasorum	1	P05556
Avasorum	1	P05783
Avasorum	1	P06396
Avasorum	1	P06454
Avasorum	1	P06576
Avasorum	1	P06702
Avasorum	1	P06703
Avasorum	1	P06732
Avasorum	1	P06733
Avasorum	1	P06744
Avasorum	1	P06748
Avasorum	1	P06753
Avasorum	1	P06865
Avasorum	1	P06899
Avasorum	1	P07195
Avasorum	1	P07203
Avasorum	1	P07237
Avasorum	1	P07339
Avasorum	1	P07355
Avasorum	1	P07384
Avasorum	1	P07437
Avasorum	1	P07602
Avasorum	1	P07686
Avasorum	1	P07737
Avasorum	1	P07814
Avasorum	1	P07858
Avasorum	1	P07900
Avasorum	1	P07910
Avasorum	1	P07942
Avasorum	1	P07951
Avasorum	1	P07954
Avasorum	1	P07996
Avasorum	1	P07998
Avasorum	1	P08123
Avasorum	1	P08133
Avasorum	1	P08238
Avasorum	1	P08253
Avasorum	1	P08572
Avasorum	1	P08621
Avasorum	1	P08648
Avasorum	1	P08670
Avasorum	1	P08708
Avasorum	1	P08758
Avasorum	1	P09012
Avasorum	1	P09211
Avasorum	1	P09429
Avasorum	1	P09486
Avasorum	1	P09525
Avasorum	1	P09651
Avasorum	1	P09669

Avasorum	1	P09960
Avasorum	1	P0CG47
Avasorum	1	P0DMV8
Avasorum	1	P0DPH7
Avasorum	1	P10124
Avasorum	1	P10586
Avasorum	1	P10619
Avasorum	1	P10646
Avasorum	1	P10809
Avasorum	1	P10909
Avasorum	1	P11021
Avasorum	1	P11047
Avasorum	1	P11142
Avasorum	1	P11233
Avasorum	1	P11279
Avasorum	1	P11413
Avasorum	1	P11586
Avasorum	1	P11717
Avasorum	1	P11766
Avasorum	1	P11940
Avasorum	1	P12004
Avasorum	1	P12107
Avasorum	1	P12109
Avasorum	1	P12111
Avasorum	1	P12236
Avasorum	1	P12268
Avasorum	1	P12429
Avasorum	1	P12814
Avasorum	1	P12821
Avasorum	1	P12955
Avasorum	1	P12956
Avasorum	1	P13010
Avasorum	1	P13073
Avasorum	1	P13473
Avasorum	1	P13489
Avasorum	1	P13639
Avasorum	1	P13667
Avasorum	1	P13797
Avasorum	1	P13987
Avasorum	1	P14314
Avasorum	1	P14324
Avasorum	1	P14543
Avasorum	1	P14618
Avasorum	1	P14625
Avasorum	1	P14866
Avasorum	1	P14868
Avasorum	1	P15090
Avasorum	1	P15121
Avasorum	1	P15144
Avasorum	1	P15153

Avasorum	1	P15170
Avasorum	1	P15311
Avasorum	1	P15531
Avasorum	1	P15586
Avasorum	1	P15880
Avasorum	1	P16035
Avasorum	1	P16284
Avasorum	1	P16401
Avasorum	1	P16402
Avasorum	1	P16403
Avasorum	1	P16930
Avasorum	1	P16949
Avasorum	1	P17096
Avasorum	1	P17301
Avasorum	1	P17655
Avasorum	1	P17844
Avasorum	1	P17858
Avasorum	1	P17948
Avasorum	1	P17980
Avasorum	1	P17987
Avasorum	1	P18077
Avasorum	1	P18085
Avasorum	1	P18124
Avasorum	1	P18206
Avasorum	1	P18621
Avasorum	1	P18669
Avasorum	1	P18754
Avasorum	1	P19021
Avasorum	1	P19338
Avasorum	1	P19367
Avasorum	1	P19623
Avasorum	1	P20042
Avasorum	1	P20062
Avasorum	1	P20618
Avasorum	1	P20700
Avasorum	1	P20774
Avasorum	1	P20908
Avasorum	1	P21333
Avasorum	1	P21399
Avasorum	1	P21741
Avasorum	1	P21796
Avasorum	1	P21810
Avasorum	1	P21980
Avasorum	1	P22004
Avasorum	1	P22234
Avasorum	1	P22314
Avasorum	1	P22352
Avasorum	1	P22392
Avasorum	1	P22626
Avasorum	1	P22692

Avasorum	1	P22695
Avasorum	1	P23246
Avasorum	1	P23284
Avasorum	1	P23381
Avasorum	1	P23396
Avasorum	1	P23526
Avasorum	1	P23528
Avasorum	1	P25705
Avasorum	1	P25786
Avasorum	1	P25787
Avasorum	1	P25788
Avasorum	1	P25789
Avasorum	1	P26022
Avasorum	1	P26038
Avasorum	1	P26368
Avasorum	1	P26373
Avasorum	1	P26583
Avasorum	1	P26599
Avasorum	1	P26640
Avasorum	1	P26641
Avasorum	1	P26927
Avasorum	1	P27105
Avasorum	1	P27348
Avasorum	1	P27635
Avasorum	1	P27695
Avasorum	1	P27708
Avasorum	1	P27797
Avasorum	1	P27816
Avasorum	1	P27824
Avasorum	1	P28066
Avasorum	1	P28072
Avasorum	1	P28074
Avasorum	1	P28300
Avasorum	1	P28482
Avasorum	1	P28799
Avasorum	1	P28838
Avasorum	1	P29279
Avasorum	1	P29401
Avasorum	1	P29692
Avasorum	1	P29966
Avasorum	1	P30041
Avasorum	1	P30050
Avasorum	1	P30084
Avasorum	1	P30086
Avasorum	1	P30101
Avasorum	1	P30153
Avasorum	1	P30520
Avasorum	1	P31153
Avasorum	1	P31930
Avasorum	1	P31943

Avasorum	1	P31946
Avasorum	1	P31948
Avasorum	1	P31949
Avasorum	1	P32119
Avasorum	1	P32969
Avasorum	1	P33151
Avasorum	1	P34096
Avasorum	1	P34932
Avasorum	1	P35221
Avasorum	1	P35222
Avasorum	1	P35232
Avasorum	1	P35237
Avasorum	1	P35241
Avasorum	1	P35268
Avasorum	1	P35443
Avasorum	1	P35555
Avasorum	1	P35579
Avasorum	1	P35590
Avasorum	1	P35998
Avasorum	1	P36578
Avasorum	1	P36871
Avasorum	1	P37802
Avasorum	1	P37837
Avasorum	1	P38159
Avasorum	1	P38606
Avasorum	1	P38646
Avasorum	1	P39019
Avasorum	1	P39023
Avasorum	1	P39656
Avasorum	1	P39687
Avasorum	1	P40121
Avasorum	1	P40227
Avasorum	1	P40261
Avasorum	1	P40429
Avasorum	1	P40925
Avasorum	1	P40926
Avasorum	1	P40939
Avasorum	1	P41091
Avasorum	1	P41250
Avasorum	1	P42166
Avasorum	1	P42785
Avasorum	1	P43121
Avasorum	1	P43243
Avasorum	1	P43490
Avasorum	1	P45880
Avasorum	1	P45974
Avasorum	1	P46060
Avasorum	1	P46776
Avasorum	1	P46777
Avasorum	1	P46778

Avasorum	1	P46779
Avasorum	1	P46781
Avasorum	1	P46782
Avasorum	1	P46783
Avasorum	1	P46926
Avasorum	1	P46940
Avasorum	1	P46977
Avasorum	1	P47755
Avasorum	1	P47756
Avasorum	1	P47897
Avasorum	1	P48047
Avasorum	1	P48059
Avasorum	1	P48444
Avasorum	1	P48643
Avasorum	1	P48681
Avasorum	1	P48723
Avasorum	1	P48735
Avasorum	1	P48739
Avasorum	1	P48740
Avasorum	1	P49207
Avasorum	1	P49327
Avasorum	1	P49368
Avasorum	1	P49407
Avasorum	1	P49411
Avasorum	1	P49458
Avasorum	1	P49720
Avasorum	1	P49721
Avasorum	1	P49747
Avasorum	1	P50395
Avasorum	1	P50452
Avasorum	1	P50454
Avasorum	1	P50502
Avasorum	1	P50914
Avasorum	1	P50990
Avasorum	1	P50991
Avasorum	1	P51148
Avasorum	1	P51149
Avasorum	1	P51858
Avasorum	1	P51991
Avasorum	1	P52209
Avasorum	1	P52272
Avasorum	1	P52565
Avasorum	1	P52566
Avasorum	1	P52907
Avasorum	1	P53004
Avasorum	1	P53396
Avasorum	1	P53618
Avasorum	1	P53621
Avasorum	1	P53634
Avasorum	1	P53999

Avasorum	1	P54136
Avasorum	1	P54289
Avasorum	1	P54578
Avasorum	1	P54687
Avasorum	1	P54886
Avasorum	1	P55058
Avasorum	1	P55060
Avasorum	1	P55072
Avasorum	1	P55084
Avasorum	1	P55145
Avasorum	1	P55209
Avasorum	1	P55263
Avasorum	1	P55285
Avasorum	1	P55290
Avasorum	1	P55786
Avasorum	1	P55884
Avasorum	1	P56134
Avasorum	1	P56537
Avasorum	1	P56545
Avasorum	1	P59998
Avasorum	1	P60174
Avasorum	1	P60228
Avasorum	1	P60709
Avasorum	1	P60842
Avasorum	1	P60866
Avasorum	1	P60900
Avasorum	1	P60953
Avasorum	1	P60981
Avasorum	1	P61019
Avasorum	1	P61158
Avasorum	1	P61160
Avasorum	1	P61204
Avasorum	1	P61247
Avasorum	1	P61313
Avasorum	1	P61326
Avasorum	1	P61353
Avasorum	1	P61604
Avasorum	1	P61619
Avasorum	1	P61916
Avasorum	1	P61978
Avasorum	1	P61981
Avasorum	1	P62081
Avasorum	1	P62140
Avasorum	1	P62191
Avasorum	1	P62241
Avasorum	1	P62244
Avasorum	1	P62249
Avasorum	1	P62258
Avasorum	1	P62263
Avasorum	1	P62266

Avasorum	1	P62269
Avasorum	1	P62277
Avasorum	1	P62280
Avasorum	1	P62318
Avasorum	1	P62424
Avasorum	1	P62491
Avasorum	1	P62701
Avasorum	1	P62736
Avasorum	1	P62750
Avasorum	1	P62753
Avasorum	1	P62805
Avasorum	1	P62826
Avasorum	1	P62829
Avasorum	1	P62847
Avasorum	1	P62851
Avasorum	1	P62854
Avasorum	1	P62873
Avasorum	1	P62888
Avasorum	1	P62899
Avasorum	1	P62906
Avasorum	1	P62910
Avasorum	1	P62913
Avasorum	1	P62917
Avasorum	1	P62937
Avasorum	1	P63000
Avasorum	1	P63010
Avasorum	1	P63104
Avasorum	1	P63173
Avasorum	1	P63241
Avasorum	1	P63244
Avasorum	1	P67809
Avasorum	1	P67812
Avasorum	1	P67936
Avasorum	1	P68036
Avasorum	1	P68104
Avasorum	1	P68363
Avasorum	1	P68371
Avasorum	1	P68431
Avasorum	1	P78371
Avasorum	1	P78417
Avasorum	1	P78527
Avasorum	1	P78539
Avasorum	1	P81605
Avasorum	1	P83731
Avasorum	1	P83881
Avasorum	1	P84098
Avasorum	1	P84103
Avasorum	1	P98160
Avasorum	1	P99999
Avasorum	1	Q00325

Avasorum	1	Q00610
Avasorum	1	Q00688
Avasorum	1	Q00839
Avasorum	1	Q01082
Avasorum	1	Q01105
Avasorum	1	Q01130
Avasorum	1	Q01469
Avasorum	1	Q01518
Avasorum	1	Q01638
Avasorum	1	Q01813
Avasorum	1	Q02543
Avasorum	1	Q02809
Avasorum	1	Q02818
Avasorum	1	Q02878
Avasorum	1	Q03135
Avasorum	1	Q04446
Avasorum	1	Q04637
Avasorum	1	Q04917
Avasorum	1	Q05682
Avasorum	1	Q06323
Avasorum	1	Q06481
Avasorum	1	Q06828
Avasorum	1	Q06830
Avasorum	1	Q07020
Avasorum	1	Q07021
Avasorum	1	Q07065
Avasorum	1	Q07666
Avasorum	1	Q07954
Avasorum	1	Q07955
Avasorum	1	Q08211
Avasorum	1	Q08629
Avasorum	1	Q09666
Avasorum	1	Q12805
Avasorum	1	Q12841
Avasorum	1	Q12860
Avasorum	1	Q12905
Avasorum	1	Q12906
Avasorum	1	Q12907
Avasorum	1	Q12931
Avasorum	1	Q13162
Avasorum	1	Q13185
Avasorum	1	Q13200
Avasorum	1	Q13201
Avasorum	1	Q13263
Avasorum	1	Q13308
Avasorum	1	Q13418
Avasorum	1	Q13596
Avasorum	1	Q13740
Avasorum	1	Q13813
Avasorum	1	Q13838

Avasorum	1	Q13885
Avasorum	1	Q14019
Avasorum	1	Q14103
Avasorum	1	Q14152
Avasorum	1	Q14195
Avasorum	1	Q14203
Avasorum	1	Q14204
Avasorum	1	Q14240
Avasorum	1	Q14315
Avasorum	1	Q14515
Avasorum	1	Q14697
Avasorum	1	Q14764
Avasorum	1	Q14766
Avasorum	1	Q14767
Avasorum	1	Q14974
Avasorum	1	Q15019
Avasorum	1	Q15029
Avasorum	1	Q15084
Avasorum	1	Q15149
Avasorum	1	Q15233
Avasorum	1	Q15257
Avasorum	1	Q15293
Avasorum	1	Q15365
Avasorum	1	Q15366
Avasorum	1	Q15393
Avasorum	1	Q15404
Avasorum	1	Q15436
Avasorum	1	Q15493
Avasorum	1	Q15582
Avasorum	1	Q16181
Avasorum	1	Q16270
Avasorum	1	Q16363
Avasorum	1	Q16394
Avasorum	1	Q16531
Avasorum	1	Q16543
Avasorum	1	Q16555
Avasorum	1	Q16610
Avasorum	1	Q16658
Avasorum	1	Q16666
Avasorum	1	Q16853
Avasorum	1	Q16881
Avasorum	1	Q32P28
Avasorum	1	Q53GQ0
Avasorum	1	Q6NZI2
Avasorum	1	Q6UWH4
Avasorum	1	Q6YHK3
Avasorum	1	Q71UM5
Avasorum	1	Q76LX8
Avasorum	1	Q7KZF4
Avasorum	1	Q7L576

Avasorum	1	Q7LGC8
Avasorum	1	Q7Z7G0
Avasorum	1	Q86T13
Avasorum	1	Q86UX7
Avasorum	1	Q86VP6
Avasorum	1	Q8IUE6
Avasorum	1	Q8IUX7
Avasorum	1	Q8IV08
Avasorum	1	Q8NBJ5
Avasorum	1	Q8NBS9
Avasorum	1	Q8TCT9
Avasorum	1	Q8WUM4
Avasorum	1	Q92522
Avasorum	1	Q92626
Avasorum	1	Q92743
Avasorum	1	Q92820
Avasorum	1	Q92841
Avasorum	1	Q92896
Avasorum	1	Q92973
Avasorum	1	Q93088
Avasorum	1	Q96AE4
Avasorum	1	Q96AG4
Avasorum	1	Q96CX2
Avasorum	1	Q96FW1
Avasorum	1	Q96KP4
Avasorum	1	Q96QK1
Avasorum	1	Q96QV1
Avasorum	1	Q99519
Avasorum	1	Q99536
Avasorum	1	Q99538
Avasorum	1	Q99623
Avasorum	1	Q99714
Avasorum	1	Q99715
Avasorum	1	Q99729
Avasorum	1	Q99832
Avasorum	1	Q99873
Avasorum	1	Q99988
Avasorum	1	Q9BR76
Avasorum	1	Q9BRK5
Avasorum	1	Q9BRX8
Avasorum	1	Q9BSJ8
Avasorum	1	Q9BT78
Avasorum	1	Q9BTV4
Avasorum	1	Q9BUF5
Avasorum	1	Q9BWD1
Avasorum	1	Q9BXJ0
Avasorum	1	Q9BXJ4
Avasorum	1	Q9BZZ5
Avasorum	1	Q9GZM7
Avasorum	1	Q9H0U4

Avasorum	1	Q9H1E3
Avasorum	1	Q9H4M9
Avasorum	1	Q9H7Y0
Avasorum	1	Q9HB71
Avasorum	1	Q9HDC9
Avasorum	1	Q9NPH3
Avasorum	1	Q9NPY3
Avasorum	1	Q9NQ30
Avasorum	1	Q9NQ88
Avasorum	1	Q9NR45
Avasorum	1	Q9NTK5
Avasorum	1	Q9NVA2
Avasorum	1	Q9NVD7
Avasorum	1	Q9NY15
Avasorum	1	Q9NY33
Avasorum	1	Q9NZM1
Avasorum	1	Q9NZN4
Avasorum	1	Q9NZV1
Avasorum	1	Q9P2J5
Avasorum	1	Q9UBP4
Avasorum	1	Q9UBR2
Avasorum	1	Q9UJ70
Avasorum	1	Q9UJZ1
Avasorum	1	Q9UL46
Avasorum	1	Q9ULV4
Avasorum	1	Q9UNN8
Avasorum	1	Q9UQ80
Avasorum	1	Q9Y230
Avasorum	1	Q9Y240
Avasorum	1	Q9Y265
Avasorum	1	Q9Y266
Avasorum	1	Q9Y277
Avasorum	1	Q9Y3I0
Avasorum	1	Q9Y3U8
Avasorum	1	Q9Y490
Avasorum	1	Q9Y4K0
Avasorum	1	Q9Y4L1
Avasorum	1	Q9Y5B9
Avasorum	1	Q9Y5S9
Avasorum	1	Q9Y5X9
Avasorum	1	Q9Y678
Avasorum	1	Q9Y696
Avasorum	2	A0A8I5KQE6
Avasorum	2	A6NIZ1
Avasorum	2	P08134
Avasorum	2	B5ME19
Avasorum	2	E9PAV3
Avasorum	2	O00154
Avasorum	2	O00159
Avasorum	2	O00231

Avasorum	2	O00232
Avasorum	2	O00299
Avasorum	2	O00391
Avasorum	2	O00410
Avasorum	2	O00469
Avasorum	2	O00567
Avasorum	2	O00571
Avasorum	2	O00622
Avasorum	2	O00625
Avasorum	2	O14773
Avasorum	2	O14786
Avasorum	2	O14818
Avasorum	2	O14950
Avasorum	2	O14979
Avasorum	2	O14980
Avasorum	2	O15123
Avasorum	2	O15143
Avasorum	2	O15144
Avasorum	2	O15145
Avasorum	2	O15232
Avasorum	2	O43143
Avasorum	2	O43242
Avasorum	2	O43390
Avasorum	2	O43396
Avasorum	2	O43684
Avasorum	2	O43707
Avasorum	2	O60462
Avasorum	2	O60506
Avasorum	2	O60568
Avasorum	2	O60814
Avasorum	2	O75083
Avasorum	2	O75131
Avasorum	2	O75369
Avasorum	2	O75390
Avasorum	2	O75436
Avasorum	2	O75489
Avasorum	2	O75874
Avasorum	2	O75915
Avasorum	2	O75923
Avasorum	2	O94979
Avasorum	2	O94985
Avasorum	2	O95084
Avasorum	2	O95445
Avasorum	2	O95497
Avasorum	2	O95782
Avasorum	2	O95810
Avasorum	2	P00338
Avasorum	2	P00352
Avasorum	2	P00387
Avasorum	2	P00403

Avasorum	2	P00488
Avasorum	2	P00491
Avasorum	2	P00492
Avasorum	2	P00505
Avasorum	2	P00558
Avasorum	2	P00568
Avasorum	2	P00742
Avasorum	2	P00750
Avasorum	2	P01024
Avasorum	2	P01033
Avasorum	2	P01034
Avasorum	2	P02452
Avasorum	2	P02458
Avasorum	2	P02545
Avasorum	2	P02751
Avasorum	2	P02753
Avasorum	2	P02768
Avasorum	2	P03956
Avasorum	2	P04075
Avasorum	2	P04083
Avasorum	2	P04114
Avasorum	2	P04275
Avasorum	2	P04406
Avasorum	2	P04439
Avasorum	2	P04792
Avasorum	2	P04843
Avasorum	2	P04844
Avasorum	2	P04899
Avasorum	2	P04908
Avasorum	2	P05023
Avasorum	2	P05067
Avasorum	2	P05091
Avasorum	2	P05114
Avasorum	2	P05121
Avasorum	2	P05141
Avasorum	2	P05198
Avasorum	2	P05305
Avasorum	2	P05388
Avasorum	2	P05455
Avasorum	2	P05556
Avasorum	2	P05783
Avasorum	2	P06396
Avasorum	2	P06454
Avasorum	2	P06576
Avasorum	2	P06703
Avasorum	2	P06732
Avasorum	2	P06733
Avasorum	2	P06744
Avasorum	2	P06748
Avasorum	2	P06753

Avasorum	2	P06865
Avasorum	2	P06899
Avasorum	2	P07195
Avasorum	2	P07203
Avasorum	2	P07237
Avasorum	2	P07339
Avasorum	2	P07355
Avasorum	2	P07384
Avasorum	2	P07437
Avasorum	2	P07602
Avasorum	2	P07686
Avasorum	2	P07737
Avasorum	2	P07814
Avasorum	2	P07858
Avasorum	2	P07900
Avasorum	2	P07910
Avasorum	2	P07942
Avasorum	2	P07951
Avasorum	2	P07954
Avasorum	2	P07996
Avasorum	2	P07998
Avasorum	2	P08123
Avasorum	2	P08133
Avasorum	2	P08238
Avasorum	2	P08253
Avasorum	2	P08572
Avasorum	2	P08621
Avasorum	2	P08648
Avasorum	2	P08670
Avasorum	2	P08708
Avasorum	2	P08758
Avasorum	2	P09012
Avasorum	2	P09211
Avasorum	2	P09429
Avasorum	2	P09486
Avasorum	2	P09525
Avasorum	2	P09651
Avasorum	2	P09669
Avasorum	2	P09960
Avasorum	2	P0CG47
Avasorum	2	P0DMV8
Avasorum	2	P0DPH7
Avasorum	2	P10124
Avasorum	2	P10586
Avasorum	2	P10619
Avasorum	2	P10646
Avasorum	2	P10809
Avasorum	2	P10909
Avasorum	2	P11021
Avasorum	2	P11047

Avasorum	2	P11142
Avasorum	2	P11233
Avasorum	2	P11279
Avasorum	2	P11413
Avasorum	2	P11586
Avasorum	2	P11717
Avasorum	2	P11766
Avasorum	2	P11940
Avasorum	2	P12004
Avasorum	2	P12107
Avasorum	2	P12109
Avasorum	2	P12111
Avasorum	2	P12236
Avasorum	2	P12268
Avasorum	2	P12429
Avasorum	2	P12814
Avasorum	2	P12821
Avasorum	2	P12955
Avasorum	2	P12956
Avasorum	2	P13010
Avasorum	2	P13073
Avasorum	2	P13473
Avasorum	2	P13489
Avasorum	2	P13639
Avasorum	2	P13667
Avasorum	2	P13797
Avasorum	2	P13987
Avasorum	2	P14314
Avasorum	2	P14324
Avasorum	2	P14543
Avasorum	2	P14618
Avasorum	2	P14625
Avasorum	2	P14866
Avasorum	2	P14868
Avasorum	2	P15090
Avasorum	2	P15121
Avasorum	2	P15144
Avasorum	2	P15153
Avasorum	2	P15170
Avasorum	2	P15311
Avasorum	2	P15531
Avasorum	2	P15586
Avasorum	2	P15880
Avasorum	2	P16035
Avasorum	2	P16284
Avasorum	2	P16401
Avasorum	2	P16402
Avasorum	2	P16403
Avasorum	2	P16930
Avasorum	2	P16949

Avasorum	2	P17096
Avasorum	2	P17301
Avasorum	2	P17655
Avasorum	2	P17844
Avasorum	2	P17858
Avasorum	2	P17948
Avasorum	2	P17980
Avasorum	2	P17987
Avasorum	2	P18077
Avasorum	2	P18085
Avasorum	2	P18124
Avasorum	2	P18206
Avasorum	2	P18621
Avasorum	2	P18669
Avasorum	2	P18754
Avasorum	2	P19021
Avasorum	2	P19338
Avasorum	2	P19367
Avasorum	2	P19623
Avasorum	2	P20042
Avasorum	2	P20062
Avasorum	2	P20618
Avasorum	2	P20700
Avasorum	2	P20774
Avasorum	2	P20908
Avasorum	2	P21333
Avasorum	2	P21399
Avasorum	2	P21741
Avasorum	2	P21796
Avasorum	2	P21810
Avasorum	2	P21980
Avasorum	2	P22004
Avasorum	2	P22234
Avasorum	2	P22314
Avasorum	2	P22352
Avasorum	2	P22392
Avasorum	2	P22626
Avasorum	2	P22692
Avasorum	2	P22695
Avasorum	2	P23246
Avasorum	2	P23284
Avasorum	2	P23381
Avasorum	2	P23396
Avasorum	2	P23526
Avasorum	2	P23528
Avasorum	2	P23919
Avasorum	2	P25705
Avasorum	2	P25786
Avasorum	2	P25787
Avasorum	2	P25788

Avasorum	2	P25789
Avasorum	2	P26022
Avasorum	2	P26038
Avasorum	2	P26368
Avasorum	2	P26373
Avasorum	2	P26583
Avasorum	2	P26599
Avasorum	2	P26640
Avasorum	2	P26641
Avasorum	2	P26927
Avasorum	2	P27105
Avasorum	2	P27348
Avasorum	2	P27635
Avasorum	2	P27695
Avasorum	2	P27708
Avasorum	2	P27797
Avasorum	2	P27816
Avasorum	2	P27824
Avasorum	2	P28066
Avasorum	2	P28072
Avasorum	2	P28074
Avasorum	2	P28300
Avasorum	2	P28482
Avasorum	2	P28799
Avasorum	2	P28838
Avasorum	2	P29279
Avasorum	2	P29401
Avasorum	2	P29692
Avasorum	2	P29966
Avasorum	2	P30041
Avasorum	2	P30050
Avasorum	2	P30084
Avasorum	2	P30086
Avasorum	2	P30101
Avasorum	2	P30153
Avasorum	2	P30520
Avasorum	2	P31153
Avasorum	2	P31930
Avasorum	2	P31943
Avasorum	2	P31946
Avasorum	2	P31948
Avasorum	2	P31949
Avasorum	2	P32119
Avasorum	2	P32969
Avasorum	2	P33151
Avasorum	2	P34096
Avasorum	2	P34932
Avasorum	2	P35221
Avasorum	2	P35222
Avasorum	2	P35232

Avasorum	2	P35237
Avasorum	2	P35241
Avasorum	2	P35268
Avasorum	2	P35443
Avasorum	2	P35555
Avasorum	2	P35579
Avasorum	2	P35590
Avasorum	2	P35998
Avasorum	2	P36578
Avasorum	2	P36871
Avasorum	2	P37802
Avasorum	2	P37837
Avasorum	2	P38159
Avasorum	2	P38606
Avasorum	2	P38646
Avasorum	2	P39019
Avasorum	2	P39023
Avasorum	2	P39656
Avasorum	2	P39687
Avasorum	2	P40121
Avasorum	2	P40227
Avasorum	2	P40261
Avasorum	2	P40429
Avasorum	2	P40925
Avasorum	2	P40926
Avasorum	2	P40939
Avasorum	2	P41091
Avasorum	2	P41250
Avasorum	2	P42166
Avasorum	2	P42785
Avasorum	2	P43121
Avasorum	2	P43243
Avasorum	2	P43490
Avasorum	2	P45880
Avasorum	2	P45974
Avasorum	2	P46060
Avasorum	2	P46776
Avasorum	2	P46777
Avasorum	2	P46778
Avasorum	2	P46779
Avasorum	2	P46781
Avasorum	2	P46782
Avasorum	2	P46783
Avasorum	2	P46926
Avasorum	2	P46940
Avasorum	2	P46977
Avasorum	2	P47755
Avasorum	2	P47756
Avasorum	2	P47897
Avasorum	2	P48047

Avasorum	2	P48059
Avasorum	2	P48444
Avasorum	2	P48643
Avasorum	2	P48681
Avasorum	2	P48723
Avasorum	2	P48735
Avasorum	2	P48739
Avasorum	2	P48740
Avasorum	2	P49207
Avasorum	2	P49327
Avasorum	2	P49368
Avasorum	2	P49407
Avasorum	2	P49411
Avasorum	2	P49458
Avasorum	2	P49720
Avasorum	2	P49721
Avasorum	2	P49747
Avasorum	2	P50395
Avasorum	2	P50452
Avasorum	2	P50454
Avasorum	2	P50502
Avasorum	2	P50914
Avasorum	2	P50990
Avasorum	2	P50991
Avasorum	2	P51148
Avasorum	2	P51149
Avasorum	2	P51858
Avasorum	2	P51991
Avasorum	2	P52209
Avasorum	2	P52272
Avasorum	2	P52565
Avasorum	2	P52566
Avasorum	2	P52907
Avasorum	2	P53004
Avasorum	2	P53396
Avasorum	2	P53618
Avasorum	2	P53621
Avasorum	2	P53634
Avasorum	2	P53999
Avasorum	2	P54136
Avasorum	2	P54289
Avasorum	2	P54578
Avasorum	2	P54687
Avasorum	2	P54886
Avasorum	2	P55058
Avasorum	2	P55060
Avasorum	2	P55072
Avasorum	2	P55084
Avasorum	2	P55145
Avasorum	2	P55209

Avasorum	2	P55263
Avasorum	2	P55285
Avasorum	2	P55290
Avasorum	2	P55786
Avasorum	2	P55884
Avasorum	2	P56134
Avasorum	2	P56537
Avasorum	2	P56545
Avasorum	2	P59998
Avasorum	2	P60174
Avasorum	2	P60228
Avasorum	2	P60709
Avasorum	2	P60842
Avasorum	2	P60866
Avasorum	2	P60900
Avasorum	2	P60953
Avasorum	2	P60981
Avasorum	2	P61019
Avasorum	2	P61158
Avasorum	2	P61160
Avasorum	2	P61204
Avasorum	2	P61247
Avasorum	2	P61313
Avasorum	2	P61326
Avasorum	2	P61353
Avasorum	2	P61604
Avasorum	2	P61619
Avasorum	2	P61916
Avasorum	2	P61978
Avasorum	2	P61981
Avasorum	2	P62081
Avasorum	2	P62140
Avasorum	2	P62191
Avasorum	2	P62241
Avasorum	2	P62244
Avasorum	2	P62249
Avasorum	2	P62258
Avasorum	2	P62263
Avasorum	2	P62266
Avasorum	2	P62269
Avasorum	2	P62277
Avasorum	2	P62280
Avasorum	2	P62318
Avasorum	2	P62424
Avasorum	2	P62491
Avasorum	2	P62701
Avasorum	2	P62736
Avasorum	2	P62750
Avasorum	2	P62753
Avasorum	2	P62805

Avasorum	2	P62826
Avasorum	2	P62829
Avasorum	2	P62847
Avasorum	2	P62851
Avasorum	2	P62854
Avasorum	2	P62873
Avasorum	2	P62888
Avasorum	2	P62899
Avasorum	2	P62906
Avasorum	2	P62910
Avasorum	2	P62913
Avasorum	2	P62917
Avasorum	2	P62937
Avasorum	2	P63000
Avasorum	2	P63010
Avasorum	2	P63104
Avasorum	2	P63173
Avasorum	2	P63241
Avasorum	2	P63244
Avasorum	2	P67809
Avasorum	2	P67812
Avasorum	2	P67936
Avasorum	2	P68036
Avasorum	2	P68104
Avasorum	2	P68363
Avasorum	2	P68371
Avasorum	2	P68431
Avasorum	2	P78371
Avasorum	2	P78417
Avasorum	2	P78527
Avasorum	2	P78539
Avasorum	2	P81605
Avasorum	2	P83731
Avasorum	2	P83881
Avasorum	2	P84098
Avasorum	2	P84103
Avasorum	2	P98160
Avasorum	2	P99999
Avasorum	2	Q00325
Avasorum	2	Q00610
Avasorum	2	Q00688
Avasorum	2	Q00839
Avasorum	2	Q01082
Avasorum	2	Q01105
Avasorum	2	Q01130
Avasorum	2	Q01469
Avasorum	2	Q01518
Avasorum	2	Q01638
Avasorum	2	Q01813
Avasorum	2	Q02543

Avasorum	2	Q02809
Avasorum	2	Q02818
Avasorum	2	Q02878
Avasorum	2	Q03135
Avasorum	2	Q04446
Avasorum	2	Q04637
Avasorum	2	Q04917
Avasorum	2	Q05682
Avasorum	2	Q06323
Avasorum	2	Q06481
Avasorum	2	Q06828
Avasorum	2	Q06830
Avasorum	2	Q07020
Avasorum	2	Q07021
Avasorum	2	Q07065
Avasorum	2	Q07666
Avasorum	2	Q07954
Avasorum	2	Q07955
Avasorum	2	Q08211
Avasorum	2	Q08629
Avasorum	2	Q09666
Avasorum	2	Q12805
Avasorum	2	Q12841
Avasorum	2	Q12860
Avasorum	2	Q12905
Avasorum	2	Q12906
Avasorum	2	Q12907
Avasorum	2	Q12931
Avasorum	2	Q13162
Avasorum	2	Q13185
Avasorum	2	Q13200
Avasorum	2	Q13201
Avasorum	2	Q13263
Avasorum	2	Q13308
Avasorum	2	Q13418
Avasorum	2	Q13596
Avasorum	2	Q13740
Avasorum	2	Q13813
Avasorum	2	Q13838
Avasorum	2	Q13885
Avasorum	2	Q14019
Avasorum	2	Q14103
Avasorum	2	Q14152
Avasorum	2	Q14195
Avasorum	2	Q14203
Avasorum	2	Q14204
Avasorum	2	Q14240
Avasorum	2	Q14315
Avasorum	2	Q14515
Avasorum	2	Q14697

Avasorum	2	Q14764
Avasorum	2	Q14766
Avasorum	2	Q14767
Avasorum	2	Q14974
Avasorum	2	Q15019
Avasorum	2	Q15029
Avasorum	2	Q15084
Avasorum	2	Q15149
Avasorum	2	Q15233
Avasorum	2	Q15257
Avasorum	2	Q15293
Avasorum	2	Q15365
Avasorum	2	Q15366
Avasorum	2	Q15393
Avasorum	2	Q15404
Avasorum	2	Q15436
Avasorum	2	Q15493
Avasorum	2	Q15582
Avasorum	2	Q16181
Avasorum	2	Q16270
Avasorum	2	Q16363
Avasorum	2	Q16394
Avasorum	2	Q16531
Avasorum	2	Q16543
Avasorum	2	Q16555
Avasorum	2	Q16610
Avasorum	2	Q16658
Avasorum	2	Q16666
Avasorum	2	Q16853
Avasorum	2	Q16881
Avasorum	2	Q32P28
Avasorum	2	Q53GQ0
Avasorum	2	Q6NZI2
Avasorum	2	Q6UWH4
Avasorum	2	Q6YHK3
Avasorum	2	Q71UM5
Avasorum	2	Q76LX8
Avasorum	2	Q7KZF4
Avasorum	2	Q7L576
Avasorum	2	Q7LGC8
Avasorum	2	Q7Z7G0
Avasorum	2	Q86T13
Avasorum	2	Q86UX7
Avasorum	2	Q86VP6
Avasorum	2	Q8IUE6
Avasorum	2	Q8IUX7
Avasorum	2	Q8IV08
Avasorum	2	Q8NBJ5
Avasorum	2	Q8NBS9
Avasorum	2	Q8TCT9

Avasorum	2	Q8WUM4
Avasorum	2	Q92522
Avasorum	2	Q92626
Avasorum	2	Q92743
Avasorum	2	Q92820
Avasorum	2	Q92841
Avasorum	2	Q92896
Avasorum	2	Q92973
Avasorum	2	Q93088
Avasorum	2	Q96AE4
Avasorum	2	Q96AG4
Avasorum	2	Q96CX2
Avasorum	2	Q96FW1
Avasorum	2	Q96KP4
Avasorum	2	Q96QK1
Avasorum	2	Q96QV1
Avasorum	2	Q99519
Avasorum	2	Q99536
Avasorum	2	Q99538
Avasorum	2	Q99623
Avasorum	2	Q99714
Avasorum	2	Q99715
Avasorum	2	Q99729
Avasorum	2	Q99832
Avasorum	2	Q99873
Avasorum	2	Q99988
Avasorum	2	Q9BR76
Avasorum	2	Q9BRK5
Avasorum	2	Q9BRX8
Avasorum	2	Q9BSJ8
Avasorum	2	Q9BT78
Avasorum	2	Q9BTV4
Avasorum	2	Q9BUF5
Avasorum	2	Q9BWD1
Avasorum	2	Q9BXJ0
Avasorum	2	Q9BXJ4
Avasorum	2	Q9BZZ5
Avasorum	2	Q9GZM7
Avasorum	2	Q9H0U4
Avasorum	2	Q9H1E3
Avasorum	2	Q9H4M9
Avasorum	2	Q9H7Y0
Avasorum	2	Q9HB71
Avasorum	2	Q9HDC9
Avasorum	2	Q9NPH3
Avasorum	2	Q9NPY3
Avasorum	2	Q9NQ30
Avasorum	2	Q9NQ88
Avasorum	2	Q9NR45
Avasorum	2	Q9NTK5

Avasorum	2	Q9NVA2
Avasorum	2	Q9NVD7
Avasorum	2	Q9NY15
Avasorum	2	Q9NY33
Avasorum	2	Q9NZM1
Avasorum	2	Q9NZN4
Avasorum	2	Q9NZV1
Avasorum	2	Q9P2J5
Avasorum	2	Q9UBP4
Avasorum	2	Q9UBR2
Avasorum	2	Q9UJ70
Avasorum	2	Q9UJZ1
Avasorum	2	Q9UL46
Avasorum	2	Q9ULV4
Avasorum	2	Q9UNN8
Avasorum	2	Q9UQ80
Avasorum	2	Q9Y230
Avasorum	2	Q9Y240
Avasorum	2	Q9Y265
Avasorum	2	Q9Y266
Avasorum	2	Q9Y277
Avasorum	2	Q9Y3I0
Avasorum	2	Q9Y3U8
Avasorum	2	Q9Y490
Avasorum	2	Q9Y4K0
Avasorum	2	Q9Y4L1
Avasorum	2	Q9Y5B9
Avasorum	2	Q9Y5S9
Avasorum	2	Q9Y5X9
Avasorum	2	Q9Y678
Avasorum	2	Q9Y696
Avasorum	3	A0A8I5KQE6
Avasorum	3	A6NIZ1
Avasorum	3	P08134
Avasorum	3	B5ME19
Avasorum	3	E9PAV3
Avasorum	3	O00154
Avasorum	3	O00159
Avasorum	3	O00231
Avasorum	3	O00232
Avasorum	3	O00299
Avasorum	3	O00391
Avasorum	3	O00410
Avasorum	3	O00469
Avasorum	3	O00567
Avasorum	3	O00571
Avasorum	3	O00622
Avasorum	3	O00625
Avasorum	3	O14773
Avasorum	3	O14786

Avasorum	3	O14818
Avasorum	3	O14950
Avasorum	3	O14979
Avasorum	3	O14980
Avasorum	3	O15123
Avasorum	3	O15143
Avasorum	3	O15144
Avasorum	3	O15145
Avasorum	3	O15232
Avasorum	3	O43143
Avasorum	3	O43242
Avasorum	3	O43390
Avasorum	3	O43396
Avasorum	3	O43684
Avasorum	3	O43707
Avasorum	3	O60462
Avasorum	3	O60506
Avasorum	3	O60568
Avasorum	3	O60814
Avasorum	3	O75083
Avasorum	3	O75369
Avasorum	3	O75390
Avasorum	3	O75436
Avasorum	3	O75489
Avasorum	3	O75874
Avasorum	3	O75915
Avasorum	3	O75923
Avasorum	3	O94979
Avasorum	3	O94985
Avasorum	3	O95084
Avasorum	3	O95445
Avasorum	3	O95497
Avasorum	3	O95782
Avasorum	3	O95810
Avasorum	3	P00338
Avasorum	3	P00352
Avasorum	3	P00387
Avasorum	3	P00403
Avasorum	3	P00488
Avasorum	3	P00491
Avasorum	3	P00492
Avasorum	3	P00505
Avasorum	3	P00558
Avasorum	3	P00568
Avasorum	3	P00742
Avasorum	3	P00750
Avasorum	3	P01024
Avasorum	3	P01033
Avasorum	3	P01034
Avasorum	3	P02452

Avasorum	3	P02458
Avasorum	3	P02545
Avasorum	3	P02751
Avasorum	3	P02753
Avasorum	3	P02768
Avasorum	3	P03956
Avasorum	3	P04075
Avasorum	3	P04083
Avasorum	3	P04114
Avasorum	3	P04275
Avasorum	3	P04406
Avasorum	3	P04439
Avasorum	3	P04792
Avasorum	3	P04843
Avasorum	3	P04844
Avasorum	3	P04899
Avasorum	3	P04908
Avasorum	3	P05023
Avasorum	3	P05067
Avasorum	3	P05091
Avasorum	3	P05109
Avasorum	3	P05114
Avasorum	3	P05121
Avasorum	3	P05141
Avasorum	3	P05198
Avasorum	3	P05305
Avasorum	3	P05388
Avasorum	3	P05455
Avasorum	3	P05556
Avasorum	3	P05783
Avasorum	3	P06396
Avasorum	3	P06454
Avasorum	3	P06576
Avasorum	3	P06703
Avasorum	3	P06732
Avasorum	3	P06733
Avasorum	3	P06744
Avasorum	3	P06748
Avasorum	3	P06753
Avasorum	3	P06865
Avasorum	3	P06899
Avasorum	3	P07195
Avasorum	3	P07203
Avasorum	3	P07237
Avasorum	3	P07339
Avasorum	3	P07355
Avasorum	3	P07384
Avasorum	3	P07437
Avasorum	3	P07602
Avasorum	3	P07686

Avasorum	3	P07737
Avasorum	3	P07814
Avasorum	3	P07858
Avasorum	3	P07900
Avasorum	3	P07910
Avasorum	3	P07942
Avasorum	3	P07951
Avasorum	3	P07954
Avasorum	3	P07996
Avasorum	3	P07998
Avasorum	3	P08123
Avasorum	3	P08133
Avasorum	3	P08238
Avasorum	3	P08253
Avasorum	3	P08572
Avasorum	3	P08621
Avasorum	3	P08648
Avasorum	3	P08670
Avasorum	3	P08708
Avasorum	3	P08758
Avasorum	3	P09012
Avasorum	3	P09211
Avasorum	3	P09429
Avasorum	3	P09486
Avasorum	3	P09525
Avasorum	3	P09651
Avasorum	3	P09669
Avasorum	3	P09960
Avasorum	3	P0CG47
Avasorum	3	P0DMV8
Avasorum	3	P0DPH7
Avasorum	3	P10124
Avasorum	3	P10586
Avasorum	3	P10619
Avasorum	3	P10646
Avasorum	3	P10809
Avasorum	3	P10909
Avasorum	3	P11021
Avasorum	3	P11047
Avasorum	3	P11142
Avasorum	3	P11233
Avasorum	3	P11279
Avasorum	3	P11413
Avasorum	3	P11586
Avasorum	3	P11717
Avasorum	3	P11766
Avasorum	3	P11940
Avasorum	3	P12004
Avasorum	3	P12107
Avasorum	3	P12109

Avasorum	3	P12111
Avasorum	3	P12236
Avasorum	3	P12268
Avasorum	3	P12429
Avasorum	3	P12814
Avasorum	3	P12821
Avasorum	3	P12956
Avasorum	3	P13010
Avasorum	3	P13073
Avasorum	3	P13473
Avasorum	3	P13489
Avasorum	3	P13639
Avasorum	3	P13667
Avasorum	3	P13797
Avasorum	3	P13987
Avasorum	3	P14314
Avasorum	3	P14324
Avasorum	3	P14543
Avasorum	3	P14618
Avasorum	3	P14625
Avasorum	3	P14866
Avasorum	3	P14868
Avasorum	3	P15090
Avasorum	3	P15121
Avasorum	3	P15144
Avasorum	3	P15153
Avasorum	3	P15170
Avasorum	3	P15311
Avasorum	3	P15531
Avasorum	3	P15586
Avasorum	3	P15880
Avasorum	3	P16035
Avasorum	3	P16284
Avasorum	3	P16401
Avasorum	3	P16402
Avasorum	3	P16403
Avasorum	3	P16930
Avasorum	3	P16949
Avasorum	3	P17096
Avasorum	3	P17301
Avasorum	3	P17655
Avasorum	3	P17844
Avasorum	3	P17858
Avasorum	3	P17948
Avasorum	3	P17980
Avasorum	3	P17987
Avasorum	3	P18077
Avasorum	3	P18085
Avasorum	3	P18124
Avasorum	3	P18206

Avasorum	3	P18621
Avasorum	3	P18669
Avasorum	3	P18754
Avasorum	3	P19021
Avasorum	3	P19338
Avasorum	3	P19367
Avasorum	3	P19623
Avasorum	3	P20042
Avasorum	3	P20062
Avasorum	3	P20618
Avasorum	3	P20700
Avasorum	3	P20774
Avasorum	3	P20908
Avasorum	3	P21333
Avasorum	3	P21399
Avasorum	3	P21741
Avasorum	3	P21796
Avasorum	3	P21810
Avasorum	3	P21980
Avasorum	3	P22004
Avasorum	3	P22234
Avasorum	3	P22314
Avasorum	3	P22352
Avasorum	3	P22392
Avasorum	3	P22626
Avasorum	3	P22692
Avasorum	3	P22695
Avasorum	3	P23246
Avasorum	3	P23284
Avasorum	3	P23381
Avasorum	3	P23396
Avasorum	3	P23526
Avasorum	3	P23528
Avasorum	3	P23919
Avasorum	3	P25705
Avasorum	3	P25786
Avasorum	3	P25787
Avasorum	3	P25788
Avasorum	3	P25789
Avasorum	3	P26022
Avasorum	3	P26038
Avasorum	3	P26368
Avasorum	3	P26373
Avasorum	3	P26583
Avasorum	3	P26599
Avasorum	3	P26640
Avasorum	3	P26641
Avasorum	3	P26927
Avasorum	3	P27105
Avasorum	3	P27348

Avasorum	3	P27635
Avasorum	3	P27695
Avasorum	3	P27708
Avasorum	3	P27797
Avasorum	3	P27816
Avasorum	3	P27824
Avasorum	3	P28066
Avasorum	3	P28072
Avasorum	3	P28074
Avasorum	3	P28300
Avasorum	3	P28482
Avasorum	3	P28799
Avasorum	3	P28838
Avasorum	3	P29279
Avasorum	3	P29401
Avasorum	3	P29692
Avasorum	3	P29966
Avasorum	3	P30041
Avasorum	3	P30050
Avasorum	3	P30084
Avasorum	3	P30086
Avasorum	3	P30101
Avasorum	3	P30153
Avasorum	3	P30520
Avasorum	3	P31153
Avasorum	3	P31930
Avasorum	3	P31943
Avasorum	3	P31946
Avasorum	3	P31948
Avasorum	3	P31949
Avasorum	3	P32119
Avasorum	3	P32969
Avasorum	3	P33151
Avasorum	3	P34096
Avasorum	3	P34932
Avasorum	3	P35221
Avasorum	3	P35222
Avasorum	3	P35232
Avasorum	3	P35237
Avasorum	3	P35241
Avasorum	3	P35268
Avasorum	3	P35443
Avasorum	3	P35555
Avasorum	3	P35579
Avasorum	3	P35590
Avasorum	3	P35998
Avasorum	3	P36578
Avasorum	3	P36871
Avasorum	3	P37802
Avasorum	3	P37837

Avasorum	3	P38159
Avasorum	3	P38606
Avasorum	3	P38646
Avasorum	3	P39019
Avasorum	3	P39023
Avasorum	3	P39656
Avasorum	3	P39687
Avasorum	3	P40121
Avasorum	3	P40227
Avasorum	3	P40261
Avasorum	3	P40429
Avasorum	3	P40925
Avasorum	3	P40926
Avasorum	3	P40939
Avasorum	3	P41091
Avasorum	3	P41250
Avasorum	3	P42166
Avasorum	3	P42785
Avasorum	3	P43121
Avasorum	3	P43243
Avasorum	3	P43490
Avasorum	3	P45880
Avasorum	3	P45974
Avasorum	3	P46060
Avasorum	3	P46776
Avasorum	3	P46777
Avasorum	3	P46778
Avasorum	3	P46779
Avasorum	3	P46781
Avasorum	3	P46782
Avasorum	3	P46783
Avasorum	3	P46926
Avasorum	3	P46940
Avasorum	3	P46977
Avasorum	3	P47755
Avasorum	3	P47756
Avasorum	3	P47897
Avasorum	3	P48047
Avasorum	3	P48059
Avasorum	3	P48444
Avasorum	3	P48643
Avasorum	3	P48681
Avasorum	3	P48723
Avasorum	3	P48735
Avasorum	3	P48739
Avasorum	3	P48740
Avasorum	3	P49207
Avasorum	3	P49327
Avasorum	3	P49368
Avasorum	3	P49407

Avasorum	3	P49411
Avasorum	3	P49458
Avasorum	3	P49720
Avasorum	3	P49721
Avasorum	3	P49747
Avasorum	3	P50395
Avasorum	3	P50452
Avasorum	3	P50454
Avasorum	3	P50502
Avasorum	3	P50914
Avasorum	3	P50990
Avasorum	3	P50991
Avasorum	3	P51148
Avasorum	3	P51149
Avasorum	3	P51858
Avasorum	3	P51991
Avasorum	3	P52209
Avasorum	3	P52272
Avasorum	3	P52565
Avasorum	3	P52566
Avasorum	3	P52907
Avasorum	3	P53004
Avasorum	3	P53396
Avasorum	3	P53618
Avasorum	3	P53621
Avasorum	3	P53634
Avasorum	3	P53999
Avasorum	3	P54136
Avasorum	3	P54289
Avasorum	3	P54578
Avasorum	3	P54687
Avasorum	3	P54886
Avasorum	3	P55058
Avasorum	3	P55060
Avasorum	3	P55072
Avasorum	3	P55084
Avasorum	3	P55145
Avasorum	3	P55209
Avasorum	3	P55263
Avasorum	3	P55285
Avasorum	3	P55290
Avasorum	3	P55786
Avasorum	3	P55884
Avasorum	3	P56134
Avasorum	3	P56537
Avasorum	3	P56545
Avasorum	3	P59998
Avasorum	3	P60174
Avasorum	3	P60228
Avasorum	3	P60709

Avasorum	3	P60842
Avasorum	3	P60866
Avasorum	3	P60900
Avasorum	3	P60953
Avasorum	3	P60981
Avasorum	3	P61019
Avasorum	3	P61158
Avasorum	3	P61160
Avasorum	3	P61204
Avasorum	3	P61247
Avasorum	3	P61313
Avasorum	3	P61326
Avasorum	3	P61353
Avasorum	3	P61604
Avasorum	3	P61619
Avasorum	3	P61916
Avasorum	3	P61978
Avasorum	3	P61981
Avasorum	3	P62081
Avasorum	3	P62140
Avasorum	3	P62191
Avasorum	3	P62241
Avasorum	3	P62244
Avasorum	3	P62249
Avasorum	3	P62258
Avasorum	3	P62263
Avasorum	3	P62266
Avasorum	3	P62269
Avasorum	3	P62277
Avasorum	3	P62280
Avasorum	3	P62318
Avasorum	3	P62424
Avasorum	3	P62491
Avasorum	3	P62701
Avasorum	3	P62736
Avasorum	3	P62750
Avasorum	3	P62753
Avasorum	3	P62805
Avasorum	3	P62826
Avasorum	3	P62829
Avasorum	3	P62847
Avasorum	3	P62851
Avasorum	3	P62854
Avasorum	3	P62873
Avasorum	3	P62888
Avasorum	3	P62899
Avasorum	3	P62906
Avasorum	3	P62910
Avasorum	3	P62913
Avasorum	3	P62917

Avasorum	3	P62937
Avasorum	3	P63000
Avasorum	3	P63010
Avasorum	3	P63104
Avasorum	3	P63173
Avasorum	3	P63241
Avasorum	3	P63244
Avasorum	3	P67809
Avasorum	3	P67812
Avasorum	3	P67936
Avasorum	3	P68036
Avasorum	3	P68104
Avasorum	3	P68363
Avasorum	3	P68371
Avasorum	3	P68431
Avasorum	3	P78371
Avasorum	3	P78417
Avasorum	3	P78527
Avasorum	3	P78539
Avasorum	3	P81605
Avasorum	3	P83731
Avasorum	3	P83881
Avasorum	3	P84098
Avasorum	3	P84103
Avasorum	3	P98160
Avasorum	3	P99999
Avasorum	3	Q00325
Avasorum	3	Q00610
Avasorum	3	Q00688
Avasorum	3	Q00839
Avasorum	3	Q01082
Avasorum	3	Q01105
Avasorum	3	Q01130
Avasorum	3	Q01469
Avasorum	3	Q01518
Avasorum	3	Q01638
Avasorum	3	Q01813
Avasorum	3	Q02543
Avasorum	3	Q02809
Avasorum	3	Q02818
Avasorum	3	Q02878
Avasorum	3	Q03135
Avasorum	3	Q04446
Avasorum	3	Q04637
Avasorum	3	Q04917
Avasorum	3	Q05682
Avasorum	3	Q06323
Avasorum	3	Q06481
Avasorum	3	Q06828
Avasorum	3	Q06830

Avasorum	3	Q07020
Avasorum	3	Q07021
Avasorum	3	Q07065
Avasorum	3	Q07666
Avasorum	3	Q07954
Avasorum	3	Q07955
Avasorum	3	Q08211
Avasorum	3	Q08629
Avasorum	3	Q09666
Avasorum	3	Q12805
Avasorum	3	Q12841
Avasorum	3	Q12860
Avasorum	3	Q12905
Avasorum	3	Q12906
Avasorum	3	Q12907
Avasorum	3	Q12931
Avasorum	3	Q13162
Avasorum	3	Q13185
Avasorum	3	Q13200
Avasorum	3	Q13201
Avasorum	3	Q13263
Avasorum	3	Q13308
Avasorum	3	Q13418
Avasorum	3	Q13596
Avasorum	3	Q13740
Avasorum	3	Q13813
Avasorum	3	Q13838
Avasorum	3	Q13885
Avasorum	3	Q14019
Avasorum	3	Q14103
Avasorum	3	Q14152
Avasorum	3	Q14195
Avasorum	3	Q14203
Avasorum	3	Q14204
Avasorum	3	Q14240
Avasorum	3	Q14315
Avasorum	3	Q14515
Avasorum	3	Q14697
Avasorum	3	Q14764
Avasorum	3	Q14766
Avasorum	3	Q14767
Avasorum	3	Q14974
Avasorum	3	Q15019
Avasorum	3	Q15029
Avasorum	3	Q15084
Avasorum	3	Q15149
Avasorum	3	Q15233
Avasorum	3	Q15257
Avasorum	3	Q15293
Avasorum	3	Q15365

Avasorum	3	Q15366
Avasorum	3	Q15393
Avasorum	3	Q15404
Avasorum	3	Q15436
Avasorum	3	Q15493
Avasorum	3	Q15582
Avasorum	3	Q16181
Avasorum	3	Q16270
Avasorum	3	Q16363
Avasorum	3	Q16394
Avasorum	3	Q16531
Avasorum	3	Q16543
Avasorum	3	Q16555
Avasorum	3	Q16610
Avasorum	3	Q16658
Avasorum	3	Q16666
Avasorum	3	Q16853
Avasorum	3	Q16881
Avasorum	3	Q32P28
Avasorum	3	Q6NZI2
Avasorum	3	Q6UWH4
Avasorum	3	Q6YHK3
Avasorum	3	Q71UM5
Avasorum	3	Q76LX8
Avasorum	3	Q7KZF4
Avasorum	3	Q7L576
Avasorum	3	Q7LGC8
Avasorum	3	Q7Z7G0
Avasorum	3	Q86T13
Avasorum	3	Q86UX7
Avasorum	3	Q86VP6
Avasorum	3	Q8IUE6
Avasorum	3	Q8IUX7
Avasorum	3	Q8IV08
Avasorum	3	Q8NBJ5
Avasorum	3	Q8NBS9
Avasorum	3	Q8TCT9
Avasorum	3	Q8WUM4
Avasorum	3	Q92522
Avasorum	3	Q92626
Avasorum	3	Q92743
Avasorum	3	Q92820
Avasorum	3	Q92841
Avasorum	3	Q92896
Avasorum	3	Q92973
Avasorum	3	Q93088
Avasorum	3	Q96AE4
Avasorum	3	Q96AG4
Avasorum	3	Q96CX2
Avasorum	3	Q96FW1

Avasorum	3	Q96KP4
Avasorum	3	Q96QK1
Avasorum	3	Q96QV1
Avasorum	3	Q99519
Avasorum	3	Q99536
Avasorum	3	Q99538
Avasorum	3	Q99623
Avasorum	3	Q99714
Avasorum	3	Q99715
Avasorum	3	Q99729
Avasorum	3	Q99832
Avasorum	3	Q99873
Avasorum	3	Q99988
Avasorum	3	Q9BR76
Avasorum	3	Q9BRK5
Avasorum	3	Q9BRX8
Avasorum	3	Q9BSJ8
Avasorum	3	Q9BT78
Avasorum	3	Q9BTV4
Avasorum	3	Q9BUF5
Avasorum	3	Q9BWD1
Avasorum	3	Q9BXJ0
Avasorum	3	Q9BXJ4
Avasorum	3	Q9BZZ5
Avasorum	3	Q9GZM7
Avasorum	3	Q9H0U4
Avasorum	3	Q9H1E3
Avasorum	3	Q9H4M9
Avasorum	3	Q9H7Y0
Avasorum	3	Q9HB71
Avasorum	3	Q9HDC9
Avasorum	3	Q9NPH3
Avasorum	3	Q9NPY3
Avasorum	3	Q9NQ30
Avasorum	3	Q9NQ88
Avasorum	3	Q9NR45
Avasorum	3	Q9NTK5
Avasorum	3	Q9NVA2
Avasorum	3	Q9NVD7
Avasorum	3	Q9NY15
Avasorum	3	Q9NY33
Avasorum	3	Q9NZM1
Avasorum	3	Q9NZN4
Avasorum	3	Q9NZV1
Avasorum	3	Q9P2J5
Avasorum	3	Q9UBP4
Avasorum	3	Q9UBR2
Avasorum	3	Q9UJ70
Avasorum	3	Q9UJZ1
Avasorum	3	Q9UL46

Avasorum	3	Q9ULV4
Avasorum	3	Q9UNN8
Avasorum	3	Q9UQ80
Avasorum	3	Q9Y230
Avasorum	3	Q9Y240
Avasorum	3	Q9Y265
Avasorum	3	Q9Y266
Avasorum	3	Q9Y277
Avasorum	3	Q9Y3I0
Avasorum	3	Q9Y3U8
Avasorum	3	Q9Y490
Avasorum	3	Q9Y4K0
Avasorum	3	Q9Y4L1
Avasorum	3	Q9Y5B9
Avasorum	3	Q9Y5S9
Avasorum	3	Q9Y5X9
Avasorum	3	Q9Y678
Avasorum	3	Q9Y696

ernatants of the three replicates of cells treated with *A.vasorum* somatic antigen.

PG.ProteinDescriptions
Small ribosomal subunit protein uS2B;Small ribosomal subunit protein uS2
Ras-related protein Rap-1b-like protein
Rho-related GTP-binding protein RhoC
Eukaryotic translation initiation factor 3 subunit C-like protein
Nascent polypeptide-associated complex subunit alpha, muscle-specific form
Cytosolic acyl coenzyme A thioester hydrolase
Unconventional myosin-Ic
26S proteasome non-ATPase regulatory subunit 11
26S proteasome non-ATPase regulatory subunit 12
Chloride intracellular channel protein 1
Sulfhydryl oxidase 1
Importin-5
Procollagen-lysine,2-oxoglutarate 5-dioxygenase 2
Nucleolar protein 56
ATP-dependent RNA helicase DDX3X
CCN family member 1
Pirin
Tripeptidyl-peptidase 1
Neuropilin-1
Proteasome subunit alpha type-7
Myosin regulatory light chain 12B
Heterogeneous nuclear ribonucleoprotein D-like
Exportin-1
Angiopoietin-2
Actin-related protein 2/3 complex subunit 1B
Actin-related protein 2/3 complex subunit 2
Actin-related protein 2/3 complex subunit 3
Matrilin-3
ATP-dependent RNA helicase DHX15
26S proteasome non-ATPase regulatory subunit 3
Heterogeneous nuclear ribonucleoprotein R
Thioredoxin-like protein 1
Mitotic checkpoint protein BUB3
Alpha-actinin-4
Neuropilin-2
Heterogeneous nuclear ribonucleoprotein Q
Multifunctional procollagen lysine hydroxylase and glycosyltransferase LH3
Histone H2B type 1-K
WD repeat-containing protein 1
Copine-3
Filamin-B
Citrate synthase, mitochondrial
Vacuolar protein sorting-associated protein 26A
NADH dehydrogenase [ubiquinone] iron-sulfur protein 3, mitochondrial
Isocitrate dehydrogenase [NADP] cytoplasmic
PRA1 family protein 3
Dysferlin
Protein transport protein Sec31A

Calsyntenin-1
Serine protease 23
Apolipoprotein M
Pantetheinase
AP-2 complex subunit alpha-1
Caveolae-associated protein 2
L-lactate dehydrogenase A chain
Aldehyde dehydrogenase 1A1
NADH-cytochrome b5 reductase 3
Cytochrome c oxidase subunit 2
Coagulation factor XIII A chain
Purine nucleoside phosphorylase
Hypoxanthine-guanine phosphoribosyltransferase
Aspartate aminotransferase, mitochondrial
Phosphoglycerate kinase 1
Adenylate kinase isoenzyme 1
Coagulation factor X
Tissue-type plasminogen activator
Complement C3
Metalloproteinase inhibitor 1
Cystatin-C
Collagen alpha-1(I) chain
Collagen alpha-1(II) chain
Prelamin-A/C
Fibronectin
Retinol-binding protein 4
Albumin
Interstitial collagenase
Fructose-bisphosphate aldolase A
Annexin A1
Apolipoprotein B-100
von Willebrand factor
Glyceraldehyde-3-phosphate dehydrogenase
HLA class I histocompatibility antigen, A alpha chain
Heat shock protein beta-1
Dolichyl-diphosphooligosaccharide--protein glycosyltransferase subunit 1
Dolichyl-diphosphooligosaccharide--protein glycosyltransferase subunit 2
Guanine nucleotide-binding protein G(i) subunit alpha-2
Histone H2A type 1-B/E
Sodium/potassium-transporting ATPase subunit alpha-1
Amyloid-beta precursor protein
Aldehyde dehydrogenase, mitochondrial
Protein S100-A8
Non-histone chromosomal protein HMG-14
Plasminogen activator inhibitor 1
ADP/ATP translocase 2
Eukaryotic translation initiation factor 2 subunit 1
Endothelin-1
Large ribosomal subunit protein uL10
Lupus La protein

Integrin beta-1
Keratin, type I cytoskeletal 18
Gelsolin
Prothymosin alpha
ATP synthase subunit beta, mitochondrial
Protein S100-A9
Protein S100-A6
Creatine kinase M-type
Alpha-enolase
Glucose-6-phosphate isomerase
Nucleophosmin
Tropomyosin alpha-3 chain
Beta-hexosaminidase subunit alpha
Histone H2B type 1-J
L-lactate dehydrogenase B chain
Glutathione peroxidase 1
Protein disulfide-isomerase
Cathepsin D
Annexin A2
Calpain-1 catalytic subunit
Tubulin beta chain
Prosaposin
Beta-hexosaminidase subunit beta
Profilin-1
Bifunctional glutamate/proline--tRNA ligase
Cathepsin B
Heat shock protein HSP 90-alpha
Heterogeneous nuclear ribonucleoproteins C1/C2
Laminin subunit beta-1
Tropomyosin beta chain
Fumarate hydratase, mitochondrial
Thrombospondin-1
Ribonuclease pancreatic
Collagen alpha-2(I) chain
Annexin A6
Heat shock protein HSP 90-beta
72 kDa type IV collagenase
Collagen alpha-2(IV) chain
U1 small nuclear ribonucleoprotein 70 kDa
Integrin alpha-5
Vimentin
Small ribosomal subunit protein eS17
Annexin A5
U1 small nuclear ribonucleoprotein A
Glutathione S-transferase P
High mobility group protein B1
SPARC
Annexin A4
Heterogeneous nuclear ribonucleoprotein A1
Cytochrome c oxidase subunit 6C

Leukotriene A-4 hydrolase
Polyubiquitin-B
Heat shock 70 kDa protein 1A
Tubulin alpha-3C chain
Serglycin
Receptor-type tyrosine-protein phosphatase F
Lysosomal protective protein
Tissue factor pathway inhibitor
60 kDa heat shock protein, mitochondrial
Clusterin
Endoplasmic reticulum chaperone BiP
Laminin subunit gamma-1
Heat shock cognate 71 kDa protein
Ras-related protein Ral-A
Lysosome-associated membrane glycoprotein 1
Glucose-6-phosphate 1-dehydrogenase
C-1-tetrahydrofolate synthase, cytoplasmic
Cation-independent mannose-6-phosphate receptor
Alcohol dehydrogenase class-3
Polyadenylate-binding protein 1
Proliferating cell nuclear antigen
Collagen alpha-1(XI) chain
Collagen alpha-1(VI) chain
Collagen alpha-3(VI) chain
ADP/ATP translocase 3
Inosine-5'-monophosphate dehydrogenase 2
Annexin A3
Alpha-actinin-1
Angiotensin-converting enzyme
Xaa-Pro dipeptidase
X-ray repair cross-complementing protein 6
X-ray repair cross-complementing protein 5
Cytochrome c oxidase subunit 4 isoform 1, mitochondrial
Lysosome-associated membrane glycoprotein 2
Ribonuclease inhibitor
Elongation factor 2
Protein disulfide-isomerase A4
Plastin-3
CD59 glycoprotein
Glucosidase 2 subunit beta
Farnesyl pyrophosphate synthase
Nidogen-1
Pyruvate kinase PKM
Endoplasmin
Heterogeneous nuclear ribonucleoprotein L
Aspartate--tRNA ligase, cytoplasmic
Fatty acid-binding protein, adipocyte
Aldo-keto reductase family 1 member B1
Aminopeptidase N
Ras-related C3 botulinum toxin substrate 2

Eukaryotic peptide chain release factor GTP-binding subunit ERF3A
Ezrin
Nucleoside diphosphate kinase A
N-acetylglucosamine-6-sulfatase
Small ribosomal subunit protein uS5
Metalloproteinase inhibitor 2
Platelet endothelial cell adhesion molecule
Histone H1.5
Histone H1.3
Histone H1.2
Fumarylacetoacetase
Stathmin
High mobility group protein HMG-I/HMG-Y
Integrin alpha-2
Calpain-2 catalytic subunit
Probable ATP-dependent RNA helicase DDX5
ATP-dependent 6-phosphofructokinase, liver type
Vascular endothelial growth factor receptor 1
26S proteasome regulatory subunit 6A
T-complex protein 1 subunit alpha
Large ribosomal subunit protein eL33
ADP-ribosylation factor 4
Large ribosomal subunit protein uL30
Vinculin
Large ribosomal subunit protein uL22
Phosphoglycerate mutase 1
Regulator of chromosome condensation
Peptidyl-glycine alpha-amidating monooxygenase
Nucleolin
Hexokinase-1
Spermidine synthase
Eukaryotic translation initiation factor 2 subunit 2
Transcobalamin-2
Proteasome subunit beta type-1
Lamin-B1
Mimecan
Collagen alpha-1(V) chain
Filamin-A
Cytoplasmic aconitate hydratase
Midkine
Voltage-dependent anion-selective channel protein 1
Biglycan
Protein-glutamine gamma-glutamyltransferase 2
Bone morphogenetic protein 6
Bifunctional phosphoribosylaminoimidazole carboxylase/phosphoribosylaminoimi[illegible]
Ubiquitin-like modifier-activating enzyme 1
Glutathione peroxidase 3
Nucleoside diphosphate kinase B
Heterogeneous nuclear ribonucleoproteins A2/B1
Insulin-like growth factor-binding protein 4

Cytochrome b-c1 complex subunit 2, mitochondrial
Splicing factor, proline- and glutamine-rich
Peptidyl-prolyl cis-trans isomerase B
Tryptophan--tRNA ligase, cytoplasmic
Small ribosomal subunit protein uS3
Adenosylhomocysteinase
Cofilin-1
ATP synthase subunit alpha, mitochondrial
Proteasome subunit alpha type-1
Proteasome subunit alpha type-2
Proteasome subunit alpha type-3
Proteasome subunit alpha type-4
Pentraxin-related protein PTX3
Moesin
Splicing factor U2AF 65 kDa subunit
Large ribosomal subunit protein eL13
High mobility group protein B2
Polypyrimidine tract-binding protein 1
Valine--tRNA ligase
Elongation factor 1-gamma
Hepatocyte growth factor-like protein
Stomatin
14-3-3 protein theta
Large ribosomal subunit protein uL16
DNA-(apurinic or apyrimidinic site) endonuclease
Multifunctional protein CAD
Calreticulin
Microtubule-associated protein 4
Calnexin
Proteasome subunit alpha type-5
Proteasome subunit beta type-6
Proteasome subunit beta type-5
Protein-lysine 6-oxidase
Mitogen-activated protein kinase 1
Progranulin
Cytosol aminopeptidase
CCN family member 2
Transketolase
Elongation factor 1-delta
Myristoylated alanine-rich C-kinase substrate
Peroxiredoxin-6
Large ribosomal subunit protein uL11
Enoyl-CoA hydratase, mitochondrial
Phosphatidylethanolamine-binding protein 1
Protein disulfide-isomerase A3
Serine/threonine-protein phosphatase 2A 65 kDa regulatory subunit A alpha isofor
Adenylosuccinate synthetase isozyme 2
S-adenosylmethionine synthase isoform type-2
Cytochrome b-c1 complex subunit 1, mitochondrial
Heterogeneous nuclear ribonucleoprotein H

14-3-3 protein beta/alpha
Stress-induced-phosphoprotein 1
Protein S100-A11
Peroxiredoxin-2
Large ribosomal subunit protein uL6
Cadherin-5
Ribonuclease 4
Heat shock 70 kDa protein 4
Catenin alpha-1
Catenin beta-1
Prohibitin 1
Serpin B6
Radixin
Large ribosomal subunit protein eL22
Thrombospondin-4
Fibrillin-1
Myosin-9
Tyrosine-protein kinase receptor Tie-1
26S proteasome regulatory subunit 7
Large ribosomal subunit protein uL4
Phosphoglucomutase-1
Transgelin-2
Transaldolase
RNA-binding motif protein, X chromosome
V-type proton ATPase catalytic subunit A
Stress-70 protein, mitochondrial
Small ribosomal subunit protein eS19
Large ribosomal subunit protein uL3
Dolichyl-diphosphooligosaccharide--protein glycosyltransferase 48 kDa subunit
Acidic leucine-rich nuclear phosphoprotein 32 family member A
Macrophage-capping protein
T-complex protein 1 subunit zeta
Nicotinamide N-methyltransferase
Large ribosomal subunit protein uL13
Malate dehydrogenase, cytoplasmic
Malate dehydrogenase, mitochondrial
Trifunctional enzyme subunit alpha, mitochondrial
Eukaryotic translation initiation factor 2 subunit 3
Glycine--tRNA ligase
Lamina-associated polypeptide 2, isoform alpha
Lysosomal Pro-X carboxypeptidase
Cell surface glycoprotein MUC18
Matrin-3
Nicotinamide phosphoribosyltransferase
Voltage-dependent anion-selective channel protein 2
Ubiquitin carboxyl-terminal hydrolase 5
Ran GTPase-activating protein 1
Large ribosomal subunit protein uL15
Large ribosomal subunit protein uL18
Large ribosomal subunit protein eL21

Large ribosomal subunit protein eL28
Small ribosomal subunit protein uS4
Small ribosomal subunit protein uS7
Small ribosomal subunit protein eS10
Glucosamine-6-phosphate isomerase 1
Ras GTPase-activating-like protein IQGAP1
Dolichyl-diphosphooligosaccharide--protein glycosyltransferase subunit STT3A
F-actin-capping protein subunit alpha-2
F-actin-capping protein subunit beta
Glutamine--tRNA ligase
ATP synthase subunit O, mitochondrial
LIM and senescent cell antigen-like-containing domain protein 1
Coatomer subunit delta
T-complex protein 1 subunit epsilon
Nestin
Heat shock 70 kDa protein 13
Isocitrate dehydrogenase [NADP], mitochondrial
Phosphatidylinositol transfer protein beta isoform
Mannan-binding lectin serine protease 1
Large ribosomal subunit protein eL34
Fatty acid synthase
T-complex protein 1 subunit gamma
Beta-arrestin-1
Elongation factor Tu, mitochondrial
Signal recognition particle 9 kDa protein
Proteasome subunit beta type-3
Proteasome subunit beta type-2
Cartilage oligomeric matrix protein
Rab GDP dissociation inhibitor beta
Serpin B8
Serpin H1
Hsc70-interacting protein
Large ribosomal subunit protein eL14
T-complex protein 1 subunit theta
T-complex protein 1 subunit delta
Ras-related protein Rab-5C
Ras-related protein Rab-7a
Hepatoma-derived growth factor
Heterogeneous nuclear ribonucleoprotein A3
6-phosphogluconate dehydrogenase, decarboxylating
Heterogeneous nuclear ribonucleoprotein M
Rho GDP-dissociation inhibitor 1
Rho GDP-dissociation inhibitor 2
F-actin-capping protein subunit alpha-1
Biliverdin reductase A
ATP-citrate synthase
Coatomer subunit beta
Coatomer subunit alpha
Dipeptidyl peptidase 1
Activated RNA polymerase II transcriptional coactivator p15

Arginine--tRNA ligase, cytoplasmic
Voltage-dependent calcium channel subunit alpha-2/delta-1
Ubiquitin carboxyl-terminal hydrolase 14
Branched-chain-amino-acid aminotransferase, cytosolic
Delta-1-pyrroline-5-carboxylate synthase
Phospholipid transfer protein
Exportin-2
Transitional endoplasmic reticulum ATPase
Trifunctional enzyme subunit beta, mitochondrial
Mesencephalic astrocyte-derived neurotrophic factor
Nucleosome assembly protein 1-like 1
Adenosine kinase
Cadherin-6
Cadherin-13
Puromycin-sensitive aminopeptidase
Eukaryotic translation initiation factor 3 subunit B
ATP synthase subunit f, mitochondrial
Eukaryotic translation initiation factor 6
C-terminal-binding protein 2
Actin-related protein 2/3 complex subunit 4
Triosephosphate isomerase
Eukaryotic translation initiation factor 3 subunit E
Actin, cytoplasmic 1
Eukaryotic initiation factor 4A-I
Small ribosomal subunit protein uS10
Proteasome subunit alpha type-6
Cell division control protein 42 homolog
Destrin
Ras-related protein Rab-2A
Actin-related protein 3
Actin-related protein 2
ADP-ribosylation factor 3
Small ribosomal subunit protein eS1
Large ribosomal subunit protein eL15
Protein mago nashi homolog
Large ribosomal subunit protein eL27
10 kDa heat shock protein, mitochondrial
Protein transport protein Sec61 subunit alpha isoform 1
NPC intracellular cholesterol transporter 2
Heterogeneous nuclear ribonucleoprotein K
14-3-3 protein gamma
Small ribosomal subunit protein eS7
Serine/threonine-protein phosphatase PP1-beta catalytic subunit
26S proteasome regulatory subunit 4
Small ribosomal subunit protein eS8
Small ribosomal subunit protein uS8
Small ribosomal subunit protein uS9
14-3-3 protein epsilon
Small ribosomal subunit protein uS11
Small ribosomal subunit protein uS12

Small ribosomal subunit protein uS13
Small ribosomal subunit protein uS15
Small ribosomal subunit protein uS17
Small nuclear ribonucleoprotein Sm D3
Large ribosomal subunit protein eL8
Ras-related protein Rab-11A
Small ribosomal subunit protein eS4, X isoform
Actin, aortic smooth muscle
Large ribosomal subunit protein uL23
Small ribosomal subunit protein eS6
Histone H4
GTP-binding nuclear protein Ran
Large ribosomal subunit protein uL14
Small ribosomal subunit protein eS24
Small ribosomal subunit protein eS25
Small ribosomal subunit protein eS26
Guanine nucleotide-binding protein G(I)/G(S)/G(T) subunit beta-1
Large ribosomal subunit protein eL30
Large ribosomal subunit protein eL31
Large ribosomal subunit protein uL1
Large ribosomal subunit protein eL32
Large ribosomal subunit protein uL5
Large ribosomal subunit protein uL2
Peptidyl-prolyl cis-trans isomerase A
Ras-related C3 botulinum toxin substrate 1
AP-2 complex subunit beta
14-3-3 protein zeta/delta
Large ribosomal subunit protein eL38
Eukaryotic translation initiation factor 5A-1
Small ribosomal subunit protein RACK1
Y-box-binding protein 1
Signal peptidase complex catalytic subunit SEC11A
Tropomyosin alpha-4 chain
Ubiquitin-conjugating enzyme E2 L3
Elongation factor 1-alpha 1
Tubulin alpha-1B chain
Tubulin beta-4B chain
Histone H3.1
T-complex protein 1 subunit beta
Glutathione S-transferase omega-1
DNA-dependent protein kinase catalytic subunit
Sushi repeat-containing protein SRPX
Dermcidin
Large ribosomal subunit protein eL24
Large ribosomal subunit protein El42
Large ribosomal subunit protein eL19
Serine/arginine-rich splicing factor 3
Basement membrane-specific heparan sulfate proteoglycan core protein
Cytochrome c
Solute carrier family 25 member 3

Clathrin heavy chain 1
Peptidyl-prolyl cis-trans isomerase FKBP3
Heterogeneous nuclear ribonucleoprotein U
Spectrin beta chain, non-erythrocytic 1
Protein SET
Serine/arginine-rich splicing factor 2
Fatty acid-binding protein 5
Adenylyl cyclase-associated protein 1
Interleukin-1 receptor-like 1
ATP-dependent 6-phosphofructokinase, platelet type
Large ribosomal subunit protein eL20
Procollagen-lysine,2-oxoglutarate 5-dioxygenase 1
Nucleobindin-1
Large ribosomal subunit protein eL6
Caveolin-1
1,4-alpha-glucan-branching enzyme
Eukaryotic translation initiation factor 4 gamma 1
14-3-3 protein eta
Caldesmon
Proteasome activator complex subunit 1
Amyloid beta precursor like protein 2
Fibromodulin
Peroxiredoxin-1
Large ribosomal subunit protein eL18
Complement component 1 Q subcomponent-binding protein, mitochondrial
Cytoskeleton-associated protein 4
KH domain-containing, RNA-binding, signal transduction-associated protein 1
Prolow-density lipoprotein receptor-related protein 1
Serine/arginine-rich splicing factor 1
ATP-dependent RNA helicase A
Testican-1
Neuroblast differentiation-associated protein AHNAK
EGF-containing fibulin-like extracellular matrix protein 1
Follistatin-related protein 1
Contactin-1
Interleukin enhancer-binding factor 2
Interleukin enhancer-binding factor 3
Vesicular integral-membrane protein VIP36
Heat shock protein 75 kDa, mitochondrial
Peroxiredoxin-4
Chromobox protein homolog 3
26S proteasome non-ATPase regulatory subunit 2
Multimerin-1
Transcription intermediary factor 1-beta
Inactive tyrosine-protein kinase 7
Integrin-linked protein kinase
Sorting nexin-1
CD166 antigen
Spectrin alpha chain, non-erythrocytic 1
Spliceosome RNA helicase DDX39B

Tubulin beta-2A chain
Coactosin-like protein
Heterogeneous nuclear ribonucleoprotein D0
Eukaryotic translation initiation factor 3 subunit A
Dihydropyrimidinase-related protein 3
Dynactin subunit 1
Cytoplasmic dynein 1 heavy chain 1
Eukaryotic initiation factor 4A-II
Filamin-C
SPARC-like protein 1
Neutral alpha-glucosidase AB
Major vault protein
Latent-transforming growth factor beta-binding protein 1
Latent-transforming growth factor beta-binding protein 2
Importin subunit beta-1
Septin-2
116 kDa U5 small nuclear ribonucleoprotein component
Protein disulfide-isomerase A6
Plectin
Non-POU domain-containing octamer-binding protein
Serine/threonine-protein phosphatase 2A activator
Reticulocalbin-1
Poly(rC)-binding protein 1
Poly(rC)-binding protein 2
Splicing factor 3B subunit 3
Ras suppressor protein 1
Protein transport protein Sec23A
Regucalcin
Transforming growth factor-beta-induced protein ig-h3
Septin-7
Insulin-like growth factor-binding protein 7
Laminin subunit alpha-4
Exostosin-1
DNA damage-binding protein 1
Hsp90 co-chaperone Cdc37
Dihydropyrimidinase-related protein 2
Extracellular matrix protein 1
Fascin
Gamma-interferon-inducible protein 16
Membrane primary amine oxidase
Thioredoxin reductase 1, cytoplasmic
Prolyl 3-hydroxylase 1
Very-long-chain 3-oxoacyl-CoA reductase
Caveolae-associated protein 1
Golgi-associated kinase 1B
CD109 antigen
Ribosomal protein eS27-like
A disintegrin and metalloproteinase with thrombospondin motifs 13
Staphylococcal nuclease domain-containing protein 1
Cytoplasmic FMR1-interacting protein 1

Carbohydrate sulfotransferase 3
Target of Nesh-SH3
C-type lectin domain family 14 member A
Fermitin family homolog 3
Cullin-associated NEDD8-dissociated protein 1
Histone H2A type 2-B
Adipocyte enhancer-binding protein 1
5'-3' exonuclease PLD3
Procollagen galactosyltransferase 1
Thioredoxin domain-containing protein 5
Minor histocompatibility antigen H13
Programmed cell death 6-interacting protein
Histone H1.10
Peroxidasin homolog
Serine protease HTRA1
Gamma-glutamyl hydrolase
Probable ATP-dependent RNA helicase DDX17
Golgi apparatus protein 1
Transportin-1
Betaine--homocysteine S-methyltransferase 1
Far upstream element-binding protein 1
Leucine-rich repeat-containing protein 59
BTB/POZ domain-containing protein KCTD12
Ubiquitin thioesterase OTUB1
Cytosolic non-specific dipeptidase
Vacuolar protein sorting-associated protein 35
Hedgehog-interacting protein
Sialidase-1
Synaptic vesicle membrane protein VAT-1 homolog
Legumain
Prohibitin-2
3-hydroxyacyl-CoA dehydrogenase type-2
Collagen alpha-1(XII) chain
Heterogeneous nuclear ribonucleoprotein A/B
T-complex protein 1 subunit eta
Protein arginine N-methyltransferase 1
Growth/differentiation factor 15
Coronin-1B
45 kDa calcium-binding protein
Peroxiredoxin-like 2A
Extended synaptotagmin-1
COP9 signalosome complex subunit 4
Transmembrane protein 43
Tubulin beta-6 chain
Acetyl-CoA acetyltransferase, cytosolic
Complement C1q tumor necrosis factor-related protein 5
Complement C1q tumor necrosis factor-related protein 3
Apoptosis inhibitor 5
Tubulointerstitial nephritis antigen-like
Ras-related protein Rab-1B

Nuclear ubiquitous casein and cyclin-dependent kinase substrate 1
EH domain-containing protein 1
Divergent protein kinase domain 2B
Calcyclin-binding protein
Adipocyte plasma membrane-associated protein
Interleukin-1 receptor accessory protein
Complement component C1q receptor
Endothelial cell-specific molecule 1
Fructose-2,6-bisphosphatase TIGAR
Sialic acid synthase
Obg-like ATPase 1
Septin-11
Alpha-parvin
Stabilin-1
Dipeptidyl peptidase 3
Myoferlin
EH domain-containing protein 2
Cysteine-rich motor neuron 1 protein
Leucine--tRNA ligase, cytoplasmic
Dickkopf-related protein 3
Cathepsin Z
N-acetyl-D-glucosamine kinase
Stomatin-like protein 2, mitochondrial
Proteasome activator complex subunit 2
Coronin-1C
Endothelial protein C receptor
Proliferation-associated protein 2G4
RuvB-like 2
C-type lectin domain family 11 member A
RuvB-like 1
Nuclear migration protein nudC
Voltage-dependent anion-selective channel protein 3
RNA-splicing ligase RtcB homolog
Large ribosomal subunit protein eL36
Talin-1
Lysyl oxidase homolog 2
Hypoxia up-regulated protein 1
FACT complex subunit SPT16
RNA-binding protein 8A
Endothelial lipase
Coatomer subunit gamma-1
Chloride intracellular channel protein 4
Small ribosomal subunit protein uS2B
Ras-related protein Rap-1b-like protein
Rho-related GTP-binding protein RhoC
Eukaryotic translation initiation factor 3 subunit C-like protein
Nascent polypeptide-associated complex subunit alpha, muscle-specific form
Cytosolic acyl coenzyme A thioester hydrolase
Unconventional myosin-Ic
26S proteasome non-ATPase regulatory subunit 11

26S proteasome non-ATPase regulatory subunit 12
Chloride intracellular channel protein 1
Sulfhydryl oxidase 1
Importin-5
Procollagen-lysine,2-oxoglutarate 5-dioxygenase 2
Nucleolar protein 56
ATP-dependent RNA helicase DDX3X
CCN family member 1
Pirin
Tripeptidyl-peptidase 1
Neuropilin-1
Proteasome subunit alpha type-7
Myosin regulatory light chain 12B
Heterogeneous nuclear ribonucleoprotein D-like
Exportin-1
Angiopoietin-2
Actin-related protein 2/3 complex subunit 1B
Actin-related protein 2/3 complex subunit 2
Actin-related protein 2/3 complex subunit 3
Matrilin-3
ATP-dependent RNA helicase DHX15
26S proteasome non-ATPase regulatory subunit 3
Heterogeneous nuclear ribonucleoprotein R
Thioredoxin-like protein 1
Mitotic checkpoint protein BUB3
Alpha-actinin-4
Neuropilin-2
Heterogeneous nuclear ribonucleoprotein Q
Multifunctional procollagen lysine hydroxylase and glycosyltransferase LH3
Histone H2B type 1-K
WD repeat-containing protein 1
Copine-3
Filamin-B
Citrate synthase, mitochondrial
Vacuolar protein sorting-associated protein 26A
NADH dehydrogenase [ubiquinone] iron-sulfur protein 3, mitochondrial
Isocitrate dehydrogenase [NADP] cytoplasmic
PRA1 family protein 3
Dysferlin
Protein transport protein Sec31A
Calsyntenin-1
Serine protease 23
Apolipoprotein M
Pantetheinase
AP-2 complex subunit alpha-1
Caveolae-associated protein 2
L-lactate dehydrogenase A chain
Aldehyde dehydrogenase 1A1
NADH-cytochrome b5 reductase 3
Cytochrome c oxidase subunit 2

Coagulation factor XIII A chain
Purine nucleoside phosphorylase
Hypoxanthine-guanine phosphoribosyltransferase
Aspartate aminotransferase, mitochondrial
Phosphoglycerate kinase 1
Adenylate kinase isoenzyme 1
Coagulation factor X
Tissue-type plasminogen activator
Complement C3
Metalloproteinase inhibitor 1
Cystatin-C
Collagen alpha-1(I) chain
Collagen alpha-1(II) chain
Prelamin-A/C
Fibronectin
Retinol-binding protein 4
Albumin
Interstitial collagenase
Fructose-bisphosphate aldolase A
Annexin A1
Apolipoprotein B-100
von Willebrand factor
Glyceraldehyde-3-phosphate dehydrogenase
HLA class I histocompatibility antigen, A alpha chain
Heat shock protein beta-1
Dolichyl-diphosphooligosaccharide--protein glycosyltransferase subunit 1
Dolichyl-diphosphooligosaccharide--protein glycosyltransferase subunit 2
Guanine nucleotide-binding protein G(i) subunit alpha-2
Histone H2A type 1-B/E
Sodium/potassium-transporting ATPase subunit alpha-1
Amyloid-beta precursor protein
Aldehyde dehydrogenase, mitochondrial
Non-histone chromosomal protein HMG-14
Plasminogen activator inhibitor 1
ADP/ATP translocase 2
Eukaryotic translation initiation factor 2 subunit 1
Endothelin-1
Large ribosomal subunit protein uL10
Lupus La protein
Integrin beta-1
Keratin, type I cytoskeletal 18
Gelsolin
Prothymosin alpha
ATP synthase subunit beta, mitochondrial
Protein S100-A6
Creatine kinase M-type
Alpha-enolase
Glucose-6-phosphate isomerase
Nucleophosmin
Tropomyosin alpha-3 chain

Beta-hexosaminidase subunit alpha
Histone H2B type 1-J
L-lactate dehydrogenase B chain
Glutathione peroxidase 1
Protein disulfide-isomerase
Cathepsin D
Annexin A2
Calpain-1 catalytic subunit
Tubulin beta chain
Prosaposin
Beta-hexosaminidase subunit beta
Profilin-1
Bifunctional glutamate/proline--tRNA ligase
Cathepsin B
Heat shock protein HSP 90-alpha
Heterogeneous nuclear ribonucleoproteins C1/C2
Laminin subunit beta-1
Tropomyosin beta chain
Fumarate hydratase, mitochondrial
Thrombospondin-1
Ribonuclease pancreatic
Collagen alpha-2(I) chain
Annexin A6
Heat shock protein HSP 90-beta
72 kDa type IV collagenase
Collagen alpha-2(IV) chain
U1 small nuclear ribonucleoprotein 70 kDa
Integrin alpha-5
Vimentin
Small ribosomal subunit protein eS17
Annexin A5
U1 small nuclear ribonucleoprotein A
Glutathione S-transferase P
High mobility group protein B1
SPARC
Annexin A4
Heterogeneous nuclear ribonucleoprotein A1
Cytochrome c oxidase subunit 6C
Leukotriene A-4 hydrolase
Polyubiquitin-B
Heat shock 70 kDa protein 1A
Tubulin alpha-3C chain
Serglycin
Receptor-type tyrosine-protein phosphatase F
Lysosomal protective protein
Tissue factor pathway inhibitor
60 kDa heat shock protein, mitochondrial
Clusterin
Endoplasmic reticulum chaperone BiP
Laminin subunit gamma-1

Heat shock cognate 71 kDa protein
Ras-related protein Ral-A
Lysosome-associated membrane glycoprotein 1
Glucose-6-phosphate 1-dehydrogenase
C-1-tetrahydrofolate synthase, cytoplasmic
Cation-independent mannose-6-phosphate receptor
Alcohol dehydrogenase class-3
Polyadenylate-binding protein 1
Proliferating cell nuclear antigen
Collagen alpha-1(XI) chain
Collagen alpha-1(VI) chain
Collagen alpha-3(VI) chain
ADP/ATP translocase 3
Inosine-5'-monophosphate dehydrogenase 2
Annexin A3
Alpha-actinin-1
Angiotensin-converting enzyme
Xaa-Pro dipeptidase
X-ray repair cross-complementing protein 6
X-ray repair cross-complementing protein 5
Cytochrome c oxidase subunit 4 isoform 1, mitochondrial
Lysosome-associated membrane glycoprotein 2
Ribonuclease inhibitor
Elongation factor 2
Protein disulfide-isomerase A4
Plastin-3
CD59 glycoprotein
Glucosidase 2 subunit beta
Farnesyl pyrophosphate synthase
Nidogen-1
Pyruvate kinase PKM
Endoplasmin
Heterogeneous nuclear ribonucleoprotein L
Aspartate--tRNA ligase, cytoplasmic
Fatty acid-binding protein, adipocyte
Aldo-keto reductase family 1 member B1
Aminopeptidase N
Ras-related C3 botulinum toxin substrate 2
Eukaryotic peptide chain release factor GTP-binding subunit ERF3A
Ezrin
Nucleoside diphosphate kinase A
N-acetylglucosamine-6-sulfatase
Small ribosomal subunit protein uS5
Metalloproteinase inhibitor 2
Platelet endothelial cell adhesion molecule
Histone H1.5
Histone H1.3
Histone H1.2
Fumarylacetoacetase
Stathmin

High mobility group protein HMG-I/HMG-Y
Integrin alpha-2
Calpain-2 catalytic subunit
Probable ATP-dependent RNA helicase DDX5
ATP-dependent 6-phosphofructokinase, liver type
Vascular endothelial growth factor receptor 1
26S proteasome regulatory subunit 6A
T-complex protein 1 subunit alpha
Large ribosomal subunit protein eL33
ADP-ribosylation factor 4
Large ribosomal subunit protein uL30
Vinculin
Large ribosomal subunit protein uL22
Phosphoglycerate mutase 1
Regulator of chromosome condensation
Peptidyl-glycine alpha-amidating monooxygenase
Nucleolin
Hexokinase-1
Spermidine synthase
Eukaryotic translation initiation factor 2 subunit 2
Transcobalamin-2
Proteasome subunit beta type-1
Lamin-B1
Mimecan
Collagen alpha-1(V) chain
Filamin-A
Cytoplasmic aconitate hydratase
Midkine
Voltage-dependent anion-selective channel protein 1
Biglycan
Protein-glutamine gamma-glutamyltransferase 2
Bone morphogenetic protein 6
Bifunctional phosphoribosylaminoimidazole carboxylase/phosphoribosylaminoimi
Ubiquitin-like modifier-activating enzyme 1
Glutathione peroxidase 3
Nucleoside diphosphate kinase B
Heterogeneous nuclear ribonucleoproteins A2/B1
Insulin-like growth factor-binding protein 4
Cytochrome b-c1 complex subunit 2, mitochondrial
Splicing factor, proline- and glutamine-rich
Peptidyl-prolyl cis-trans isomerase B
Tryptophan--tRNA ligase, cytoplasmic
Small ribosomal subunit protein uS3
Adenosylhomocysteinase
Cofilin-1
Thymidylate kinase
ATP synthase subunit alpha, mitochondrial
Proteasome subunit alpha type-1
Proteasome subunit alpha type-2
Proteasome subunit alpha type-3

Proteasome subunit alpha type-4
Pentraxin-related protein PTX3
Moesin
Splicing factor U2AF 65 kDa subunit
Large ribosomal subunit protein eL13
High mobility group protein B2
Polypyrimidine tract-binding protein 1
Valine--tRNA ligase
Elongation factor 1-gamma
Hepatocyte growth factor-like protein
Stomatin
14-3-3 protein theta
Large ribosomal subunit protein uL16
DNA-(apurinic or apyrimidinic site) endonuclease
Multifunctional protein CAD
Calreticulin
Microtubule-associated protein 4
Calnexin
Proteasome subunit alpha type-5
Proteasome subunit beta type-6
Proteasome subunit beta type-5
Protein-lysine 6-oxidase
Mitogen-activated protein kinase 1
Progranulin
Cytosol aminopeptidase
CCN family member 2
Transketolase
Elongation factor 1-delta
Myristoylated alanine-rich C-kinase substrate
Peroxiredoxin-6
Large ribosomal subunit protein uL11
Enoyl-CoA hydratase, mitochondrial
Phosphatidylethanolamine-binding protein 1
Protein disulfide-isomerase A3
Serine/threonine-protein phosphatase 2A 65 kDa regulatory subunit A alpha isofor
Adenylosuccinate synthetase isozyme 2
S-adenosylmethionine synthase isoform type-2
Cytochrome b-c1 complex subunit 1, mitochondrial
Heterogeneous nuclear ribonucleoprotein H
14-3-3 protein beta/alpha
Stress-induced-phosphoprotein 1
Protein S100-A11
Peroxiredoxin-2
Large ribosomal subunit protein uL6
Cadherin-5
Ribonuclease 4
Heat shock 70 kDa protein 4
Catenin alpha-1
Catenin beta-1
Prohibitin 1

Serpin B6
Radixin
Large ribosomal subunit protein eL22
Thrombospondin-4
Fibrillin-1
Myosin-9
Tyrosine-protein kinase receptor Tie-1
26S proteasome regulatory subunit 7
Large ribosomal subunit protein uL4
Phosphoglucomutase-1
Transgelin-2
Transaldolase
RNA-binding motif protein, X chromosome
V-type proton ATPase catalytic subunit A
Stress-70 protein, mitochondrial
Small ribosomal subunit protein eS19
Large ribosomal subunit protein uL3
Dolichyl-diphosphooligosaccharide--protein glycosyltransferase 48 kDa subunit
Acidic leucine-rich nuclear phosphoprotein 32 family member A
Macrophage-capping protein
T-complex protein 1 subunit zeta
Nicotinamide N-methyltransferase
Large ribosomal subunit protein uL13
Malate dehydrogenase, cytoplasmic
Malate dehydrogenase, mitochondrial
Trifunctional enzyme subunit alpha, mitochondrial
Eukaryotic translation initiation factor 2 subunit 3
Glycine--tRNA ligase
Lamina-associated polypeptide 2, isoform alpha
Lysosomal Pro-X carboxypeptidase
Cell surface glycoprotein MUC18
Matrin-3
Nicotinamide phosphoribosyltransferase
Voltage-dependent anion-selective channel protein 2
Ubiquitin carboxyl-terminal hydrolase 5
Ran GTPase-activating protein 1
Large ribosomal subunit protein uL15
Large ribosomal subunit protein uL18
Large ribosomal subunit protein eL21
Large ribosomal subunit protein eL28
Small ribosomal subunit protein uS4
Small ribosomal subunit protein uS7
Small ribosomal subunit protein eS10
Glucosamine-6-phosphate isomerase 1
Ras GTPase-activating-like protein IQGAP1
Dolichyl-diphosphooligosaccharide--protein glycosyltransferase subunit STT3A
F-actin-capping protein subunit alpha-2
F-actin-capping protein subunit beta
Glutamine--tRNA ligase
ATP synthase subunit O, mitochondrial

LIM and senescent cell antigen-like-containing domain protein 1
Coatomer subunit delta
T-complex protein 1 subunit epsilon
Nestin
Heat shock 70 kDa protein 13
Isocitrate dehydrogenase [NADP], mitochondrial
Phosphatidylinositol transfer protein beta isoform
Mannan-binding lectin serine protease 1
Large ribosomal subunit protein eL34
Fatty acid synthase
T-complex protein 1 subunit gamma
Beta-arrestin-1
Elongation factor Tu, mitochondrial
Signal recognition particle 9 kDa protein
Proteasome subunit beta type-3
Proteasome subunit beta type-2
Cartilage oligomeric matrix protein
Rab GDP dissociation inhibitor beta
Serpin B8
Serpin H1
Hsc70-interacting protein
Large ribosomal subunit protein eL14
T-complex protein 1 subunit theta
T-complex protein 1 subunit delta
Ras-related protein Rab-5C
Ras-related protein Rab-7a
Hepatoma-derived growth factor
Heterogeneous nuclear ribonucleoprotein A3
6-phosphogluconate dehydrogenase, decarboxylating
Heterogeneous nuclear ribonucleoprotein M
Rho GDP-dissociation inhibitor 1
Rho GDP-dissociation inhibitor 2
F-actin-capping protein subunit alpha-1
Biliverdin reductase A
ATP-citrate synthase
Coatomer subunit beta
Coatomer subunit alpha
Dipeptidyl peptidase 1
Activated RNA polymerase II transcriptional coactivator p15
Arginine--tRNA ligase, cytoplasmic
Voltage-dependent calcium channel subunit alpha-2/delta-1
Ubiquitin carboxyl-terminal hydrolase 14
Branched-chain-amino-acid aminotransferase, cytosolic
Delta-1-pyrroline-5-carboxylate synthase
Phospholipid transfer protein
Exportin-2
Transitional endoplasmic reticulum ATPase
Trifunctional enzyme subunit beta, mitochondrial
Mesencephalic astrocyte-derived neurotrophic factor
Nucleosome assembly protein 1-like 1

Adenosine kinase
Cadherin-6
Cadherin-13
Puromycin-sensitive aminopeptidase
Eukaryotic translation initiation factor 3 subunit B
ATP synthase subunit f, mitochondrial
Eukaryotic translation initiation factor 6
C-terminal-binding protein 2
Actin-related protein 2/3 complex subunit 4
Triosephosphate isomerase
Eukaryotic translation initiation factor 3 subunit E
Actin, cytoplasmic 1
Eukaryotic initiation factor 4A-I
Small ribosomal subunit protein uS10
Proteasome subunit alpha type-6
Cell division control protein 42 homolog
Destrin
Ras-related protein Rab-2A
Actin-related protein 3
Actin-related protein 2
ADP-ribosylation factor 3
Small ribosomal subunit protein eS1
Large ribosomal subunit protein eL15
Protein mago nashi homolog
Large ribosomal subunit protein eL27
10 kDa heat shock protein, mitochondrial
Protein transport protein Sec61 subunit alpha isoform 1
NPC intracellular cholesterol transporter 2
Heterogeneous nuclear ribonucleoprotein K
14-3-3 protein gamma
Small ribosomal subunit protein eS7
Serine/threonine-protein phosphatase PP1-beta catalytic subunit
26S proteasome regulatory subunit 4
Small ribosomal subunit protein eS8
Small ribosomal subunit protein uS8
Small ribosomal subunit protein uS9
14-3-3 protein epsilon
Small ribosomal subunit protein uS11
Small ribosomal subunit protein uS12
Small ribosomal subunit protein uS13
Small ribosomal subunit protein uS15
Small ribosomal subunit protein uS17
Small nuclear ribonucleoprotein Sm D3
Large ribosomal subunit protein eL8
Ras-related protein Rab-11A
Small ribosomal subunit protein eS4, X isoform
Actin, aortic smooth muscle
Large ribosomal subunit protein uL23
Small ribosomal subunit protein eS6
Histone H4

GTP-binding nuclear protein Ran
Large ribosomal subunit protein uL14
Small ribosomal subunit protein eS24
Small ribosomal subunit protein eS25
Small ribosomal subunit protein eS26
Guanine nucleotide-binding protein G(I)/G(S)/G(T) subunit beta-1
Large ribosomal subunit protein eL30
Large ribosomal subunit protein eL31
Large ribosomal subunit protein uL1
Large ribosomal subunit protein eL32
Large ribosomal subunit protein uL5
Large ribosomal subunit protein uL2
Peptidyl-prolyl cis-trans isomerase A
Ras-related C3 botulinum toxin substrate 1
AP-2 complex subunit beta
14-3-3 protein zeta/delta
Large ribosomal subunit protein eL38
Eukaryotic translation initiation factor 5A-1
Small ribosomal subunit protein RACK1
Y-box-binding protein 1
Signal peptidase complex catalytic subunit SEC11A
Tropomyosin alpha-4 chain
Ubiquitin-conjugating enzyme E2 L3
Elongation factor 1-alpha 1
Tubulin alpha-1B chain
Tubulin beta-4B chain
Histone H3.1
T-complex protein 1 subunit beta
Glutathione S-transferase omega-1
DNA-dependent protein kinase catalytic subunit
Sushi repeat-containing protein SRPX
Dermcidin
Large ribosomal subunit protein eL24
Large ribosomal subunit protein eL42
Large ribosomal subunit protein eL19
Serine/arginine-rich splicing factor 3
Basement membrane-specific heparan sulfate proteoglycan core protein
Cytochrome c
Solute carrier family 25 member 3
Clathrin heavy chain 1
Peptidyl-prolyl cis-trans isomerase FKBP3
Heterogeneous nuclear ribonucleoprotein U
Spectrin beta chain, non-erythrocytic 1
Protein SET
Serine/arginine-rich splicing factor 2
Fatty acid-binding protein 5
Adenylyl cyclase-associated protein 1
Interleukin-1 receptor-like 1
ATP-dependent 6-phosphofructokinase, platelet type
Large ribosomal subunit protein eL20

Procollagen-lysine,2-oxoglutarate 5-dioxygenase 1
Nucleobindin-1
Large ribosomal subunit protein eL6
Caveolin-1
1,4-alpha-glucan-branching enzyme
Eukaryotic translation initiation factor 4 gamma 1
14-3-3 protein eta
Caldesmon
Proteasome activator complex subunit 1
Amyloid beta precursor like protein 2
Fibromodulin
Peroxiredoxin-1
Large ribosomal subunit protein eL18
Complement component 1 Q subcomponent-binding protein, mitochondrial
Cytoskeleton-associated protein 4
KH domain-containing, RNA-binding, signal transduction-associated protein 1
Prolow-density lipoprotein receptor-related protein 1
Serine/arginine-rich splicing factor 1
ATP-dependent RNA helicase A
Testican-1
Neuroblast differentiation-associated protein AHNAK
EGF-containing fibulin-like extracellular matrix protein 1
Follistatin-related protein 1
Contactin-1
Interleukin enhancer-binding factor 2
Interleukin enhancer-binding factor 3
Vesicular integral-membrane protein VIP36
Heat shock protein 75 kDa, mitochondrial
Peroxiredoxin-4
Chromobox protein homolog 3
26S proteasome non-ATPase regulatory subunit 2
Multimerin-1
Transcription intermediary factor 1-beta
Inactive tyrosine-protein kinase 7
Integrin-linked protein kinase
Sorting nexin-1
CD166 antigen
Spectrin alpha chain, non-erythrocytic 1
Spliceosome RNA helicase DDX39B
Tubulin beta-2A chain
Coactosin-like protein
Heterogeneous nuclear ribonucleoprotein D0
Eukaryotic translation initiation factor 3 subunit A
Dihydropyrimidinase-related protein 3
Dynactin subunit 1
Cytoplasmic dynein 1 heavy chain 1
Eukaryotic initiation factor 4A-II
Filamin-C
SPARC-like protein 1
Neutral alpha-glucosidase AB

Major vault protein
Latent-transforming growth factor beta-binding protein 1
Latent-transforming growth factor beta-binding protein 2
Importin subunit beta-1
Septin-2
116 kDa U5 small nuclear ribonucleoprotein component
Protein disulfide-isomerase A6
Plectin
Non-POU domain-containing octamer-binding protein
Serine/threonine-protein phosphatase 2A activator
Reticulocalbin-1
Poly(rC)-binding protein 1
Poly(rC)-binding protein 2
Splicing factor 3B subunit 3
Ras suppressor protein 1
Protein transport protein Sec23A
Regucalcin
Transforming growth factor-beta-induced protein ig-h3
Septin-7
Insulin-like growth factor-binding protein 7
Laminin subunit alpha-4
Exostosin-1
DNA damage-binding protein 1
Hsp90 co-chaperone Cdc37
Dihydropyrimidinase-related protein 2
Extracellular matrix protein 1
Fascin
Gamma-interferon-inducible protein 16
Membrane primary amine oxidase
Thioredoxin reductase 1, cytoplasmic
Prolyl 3-hydroxylase 1
Very-long-chain 3-oxoacyl-CoA reductase
Caveolae-associated protein 1
Golgi-associated kinase 1B
CD109 antigen
Ribosomal protein eS27-like
A disintegrin and metalloproteinase with thrombospondin motifs 13
Staphylococcal nuclease domain-containing protein 1
Cytoplasmic FMR1-interacting protein 1
Carbohydrate sulfotransferase 3
Target of Nesh-SH3
C-type lectin domain family 14 member A
Fermitin family homolog 3
Cullin-associated NEDD8-dissociated protein 1
Histone H2A type 2-B
Adipocyte enhancer-binding protein 1
5'-3' exonuclease PLD3
Procollagen galactosyltransferase 1
Thioredoxin domain-containing protein 5
Minor histocompatibility antigen H13

Programmed cell death 6-interacting protein
Histone H1.10
Peroxidasin homolog
Serine protease HTRA1
Gamma-glutamyl hydrolase
Probable ATP-dependent RNA helicase DDX17
Golgi apparatus protein 1
Transportin-1
Betaine--homocysteine S-methyltransferase 1
Far upstream element-binding protein 1
Leucine-rich repeat-containing protein 59
BTB/POZ domain-containing protein KCTD12
Ubiquitin thioesterase OTUB1
Cytosolic non-specific dipeptidase
Vacuolar protein sorting-associated protein 35
Hedgehog-interacting protein
Sialidase-1
Synaptic vesicle membrane protein VAT-1 homolog
Legumain
Prohibitin-2
3-hydroxyacyl-CoA dehydrogenase type-2
Collagen alpha-1(XII) chain
Heterogeneous nuclear ribonucleoprotein A/B
T-complex protein 1 subunit eta
Protein arginine N-methyltransferase 1
Growth/differentiation factor 15
Coronin-1B
45 kDa calcium-binding protein
Peroxiredoxin-like 2A
Extended synaptotagmin-1
COP9 signalosome complex subunit 4
Transmembrane protein 43
Tubulin beta-6 chain
Acetyl-CoA acetyltransferase, cytosolic
Complement C1q tumor necrosis factor-related protein 5
Complement C1q tumor necrosis factor-related protein 3
Apoptosis inhibitor 5
Tubulointerstitial nephritis antigen-like
Ras-related protein Rab-1B
Nuclear ubiquitous casein and cyclin-dependent kinase substrate 1
EH domain-containing protein 1
Divergent protein kinase domain 2B
Calcyclin-binding protein
Adipocyte plasma membrane-associated protein
Interleukin-1 receptor accessory protein
Complement component C1q receptor
Endothelial cell-specific molecule 1
Fructose-2,6-bisphosphatase TIGAR
Sialic acid synthase
Obg-like ATPase 1

Septin-11
Alpha-parvin
Stabilin-1
Dipeptidyl peptidase 3
Myoferlin
EH domain-containing protein 2
Cysteine-rich motor neuron 1 protein
Leucine--tRNA ligase, cytoplasmic
Dickkopf-related protein 3
Cathepsin Z
N-acetyl-D-glucosamine kinase
Stomatin-like protein 2, mitochondrial
Proteasome activator complex subunit 2
Coronin-1C
Endothelial protein C receptor
Proliferation-associated protein 2G4
RuvB-like 2
C-type lectin domain family 11 member A
RuvB-like 1
Nuclear migration protein nudC
Voltage-dependent anion-selective channel protein 3
RNA-splicing ligase RtcB homolog
Large ribosomal subunit protein eL36
Talin-1
Lysyl oxidase homolog 2
Hypoxia up-regulated protein 1
FACT complex subunit SPT16
RNA-binding protein 8A
Endothelial lipase
Coatomer subunit gamma-1
Chloride intracellular channel protein 4
Small ribosomal subunit protein uS2B
Ras-related protein Rap-1b-like protein
Rho-related GTP-binding protein RhoC
Eukaryotic translation initiation factor 3 subunit C-like protein
Nascent polypeptide-associated complex subunit alpha, muscle-specific form
Cytosolic acyl coenzyme A thioester hydrolase
Unconventional myosin-Ic
26S proteasome non-ATPase regulatory subunit 11
26S proteasome non-ATPase regulatory subunit 12
Chloride intracellular channel protein 1
Sulfhydryl oxidase 1
Importin-5
Procollagen-lysine,2-oxoglutarate 5-dioxygenase 2
Nucleolar protein 56
ATP-dependent RNA helicase DDX3X
CCN family member 1
Pirin
Tripeptidyl-peptidase 1
Neuropilin-1

Proteasome subunit alpha type-7
Myosin regulatory light chain 12B
Heterogeneous nuclear ribonucleoprotein D-like
Exportin-1
Angiopoietin-2
Actin-related protein 2/3 complex subunit 1B
Actin-related protein 2/3 complex subunit 2
Actin-related protein 2/3 complex subunit 3
Matrilin-3
ATP-dependent RNA helicase DHX15
26S proteasome non-ATPase regulatory subunit 3
Heterogeneous nuclear ribonucleoprotein R
Thioredoxin-like protein 1
Mitotic checkpoint protein BUB3
Alpha-actinin-4
Neuropilin-2
Heterogeneous nuclear ribonucleoprotein Q
Multifunctional procollagen lysine hydroxylase and glycosyltransferase LH3
Histone H2B type 1-K
WD repeat-containing protein 1
Filamin-B
Citrate synthase, mitochondrial
Vacuolar protein sorting-associated protein 26A
NADH dehydrogenase [ubiquinone] iron-sulfur protein 3, mitochondrial
Isocitrate dehydrogenase [NADP] cytoplasmic
PRA1 family protein 3
Dysferlin
Protein transport protein Sec31A
Calsyntenin-1
Serine protease 23
Apolipoprotein M
Pantetheinase
AP-2 complex subunit alpha-1
Caveolae-associated protein 2
L-lactate dehydrogenase A chain
Aldehyde dehydrogenase 1A1
NADH-cytochrome b5 reductase 3
Cytochrome c oxidase subunit 2
Coagulation factor XIII A chain
Purine nucleoside phosphorylase
Hypoxanthine-guanine phosphoribosyltransferase
Aspartate aminotransferase, mitochondrial
Phosphoglycerate kinase 1
Adenylate kinase isoenzyme 1
Coagulation factor X
Tissue-type plasminogen activator
Complement C3
Metalloproteinase inhibitor 1
Cystatin-C
Collagen alpha-1(I) chain

Collagen alpha-1(II) chain
Prelamin-A/C
Fibronectin
Retinol-binding protein 4
Albumin
Interstitial collagenase
Fructose-bisphosphate aldolase A
Annexin A1
Apolipoprotein B-100
von Willebrand factor
Glyceraldehyde-3-phosphate dehydrogenase
HLA class I histocompatibility antigen, A alpha chain
Heat shock protein beta-1
Dolichyl-diphosphooligosaccharide--protein glycosyltransferase subunit 1
Dolichyl-diphosphooligosaccharide--protein glycosyltransferase subunit 2
Guanine nucleotide-binding protein G(i) subunit alpha-2
Histone H2A type 1-B/E
Sodium/potassium-transporting ATPase subunit alpha-1
Amyloid-beta precursor protein
Aldehyde dehydrogenase, mitochondrial
Protein S100-A8
Non-histone chromosomal protein HMG-14
Plasminogen activator inhibitor 1
ADP/ATP translocase 2
Eukaryotic translation initiation factor 2 subunit 1
Endothelin-1
Large ribosomal subunit protein uL10
Lupus La protein
Integrin beta-1
Keratin, type I cytoskeletal 18
Gelsolin
Prothymosin alpha
ATP synthase subunit beta, mitochondrial
Protein S100-A6
Creatine kinase M-type
Alpha-enolase
Glucose-6-phosphate isomerase
Nucleophosmin
Tropomyosin alpha-3 chain
Beta-hexosaminidase subunit alpha
Histone H2B type 1-J
L-lactate dehydrogenase B chain
Glutathione peroxidase 1
Protein disulfide-isomerase
Cathepsin D
Annexin A2
Calpain-1 catalytic subunit
Tubulin beta chain
Prosaposin
Beta-hexosaminidase subunit beta

Profilin-1
Bifunctional glutamate/proline--tRNA ligase
Cathepsin B
Heat shock protein HSP 90-alpha
Heterogeneous nuclear ribonucleoproteins C1/C2
Laminin subunit beta-1
Tropomyosin beta chain
Fumarate hydratase, mitochondrial
Thrombospondin-1
Ribonuclease pancreatic
Collagen alpha-2(I) chain
Annexin A6
Heat shock protein HSP 90-beta
72 kDa type IV collagenase
Collagen alpha-2(IV) chain
U1 small nuclear ribonucleoprotein 70 kDa
Integrin alpha-5
Vimentin
Small ribosomal subunit protein eS17
Annexin A5
U1 small nuclear ribonucleoprotein A
Glutathione S-transferase P
High mobility group protein B1
SPARC
Annexin A4
Heterogeneous nuclear ribonucleoprotein A1
Cytochrome c oxidase subunit 6C
Leukotriene A-4 hydrolase
Polyubiquitin-B
Heat shock 70 kDa protein 1A
Tubulin alpha-3C chain
Serglycin
Receptor-type tyrosine-protein phosphatase F
Lysosomal protective protein
Tissue factor pathway inhibitor
60 kDa heat shock protein, mitochondrial
Clusterin
Endoplasmic reticulum chaperone BiP
Laminin subunit gamma-1
Heat shock cognate 71 kDa protein
Ras-related protein Ral-A
Lysosome-associated membrane glycoprotein 1
Glucose-6-phosphate 1-dehydrogenase
C-1-tetrahydrofolate synthase, cytoplasmic
Cation-independent mannose-6-phosphate receptor
Alcohol dehydrogenase class-3
Polyadenylate-binding protein 1
Proliferating cell nuclear antigen
Collagen alpha-1(XI) chain
Collagen alpha-1(VI) chain

Collagen alpha-3(VI) chain
ADP/ATP translocase 3
Inosine-5'-monophosphate dehydrogenase 2
Annexin A3
Alpha-actinin-1
Angiotensin-converting enzyme
X-ray repair cross-complementing protein 6
X-ray repair cross-complementing protein 5
Cytochrome c oxidase subunit 4 isoform 1, mitochondrial
Lysosome-associated membrane glycoprotein 2
Ribonuclease inhibitor
Elongation factor 2
Protein disulfide-isomerase A4
Plastin-3
CD59 glycoprotein
Glucosidase 2 subunit beta
Farnesyl pyrophosphate synthase
Nidogen-1
Pyruvate kinase PKM
Endoplasmin
Heterogeneous nuclear ribonucleoprotein L
Aspartate--tRNA ligase, cytoplasmic
Fatty acid-binding protein, adipocyte
Aldo-keto reductase family 1 member B1
Aminopeptidase N
Ras-related C3 botulinum toxin substrate 2
Eukaryotic peptide chain release factor GTP-binding subunit ERF3A
Ezrin
Nucleoside diphosphate kinase A
N-acetylglucosamine-6-sulfatase
Small ribosomal subunit protein uS5
Metalloproteinase inhibitor 2
Platelet endothelial cell adhesion molecule
Histone H1.5
Histone H1.3
Histone H1.2
Fumarylacetoacetase
Stathmin
High mobility group protein HMG-I/HMG-Y
Integrin alpha-2
Calpain-2 catalytic subunit
Probable ATP-dependent RNA helicase DDX5
ATP-dependent 6-phosphofructokinase, liver type
Vascular endothelial growth factor receptor 1
26S proteasome regulatory subunit 6A
T-complex protein 1 subunit alpha
Large ribosomal subunit protein eL33
ADP-ribosylation factor 4
Large ribosomal subunit protein uL30
Vinculin

Large ribosomal subunit protein uL22
Phosphoglycerate mutase 1
Regulator of chromosome condensation
Peptidyl-glycine alpha-amidating monooxygenase
Nucleolin
Hexokinase-1
Spermidine synthase
Eukaryotic translation initiation factor 2 subunit 2
Transcobalamin-2
Proteasome subunit beta type-1
Lamin-B1
Mimecan
Collagen alpha-1(V) chain
Filamin-A
Cytoplasmic aconitate hydratase
Midkine
Voltage-dependent anion-selective channel protein 1
Biglycan
Protein-glutamine gamma-glutamyltransferase 2
Bone morphogenetic protein 6
Bifunctional phosphoribosylaminoimidazole carboxylase/phosphoribosylaminoimic
Ubiquitin-like modifier-activating enzyme 1
Glutathione peroxidase 3
Nucleoside diphosphate kinase B
Heterogeneous nuclear ribonucleoproteins A2/B1
Insulin-like growth factor-binding protein 4
Cytochrome b-c1 complex subunit 2, mitochondrial
Splicing factor, proline- and glutamine-rich
Peptidyl-prolyl cis-trans isomerase B
Tryptophan--tRNA ligase, cytoplasmic
Small ribosomal subunit protein uS3
Adenosylhomocysteinase
Cofilin-1
Thymidylate kinase
ATP synthase subunit alpha, mitochondrial
Proteasome subunit alpha type-1
Proteasome subunit alpha type-2
Proteasome subunit alpha type-3
Proteasome subunit alpha type-4
Pentraxin-related protein PTX3
Moesin
Splicing factor U2AF 65 kDa subunit
Large ribosomal subunit protein eL13
High mobility group protein B2
Polypyrimidine tract-binding protein 1
Valine--tRNA ligase
Elongation factor 1-gamma
Hepatocyte growth factor-like protein
Stomatin
14-3-3 protein theta

Large ribosomal subunit protein uL16
DNA-(apurinic or apyrimidinic site) endonuclease
Multifunctional protein CAD
Calreticulin
Microtubule-associated protein 4
Calnexin
Proteasome subunit alpha type-5
Proteasome subunit beta type-6
Proteasome subunit beta type-5
Protein-lysine 6-oxidase
Mitogen-activated protein kinase 1
Progranulin
Cytosol aminopeptidase
CCN family member 2
Transketolase
Elongation factor 1-delta
Myristoylated alanine-rich C-kinase substrate
Peroxiredoxin-6
Large ribosomal subunit protein uL11
Enoyl-CoA hydratase, mitochondrial
Phosphatidylethanolamine-binding protein 1
Protein disulfide-isomerase A3
Serine/threonine-protein phosphatase 2A 65 kDa regulatory subunit A alpha isofor
Adenylosuccinate synthetase isozyme 2
S-adenosylmethionine synthase isoform type-2
Cytochrome b-c1 complex subunit 1, mitochondrial
Heterogeneous nuclear ribonucleoprotein H
14-3-3 protein beta/alpha
Stress-induced-phosphoprotein 1
Protein S100-A11
Peroxiredoxin-2
Large ribosomal subunit protein uL6
Cadherin-5
Ribonuclease 4
Heat shock 70 kDa protein 4
Catenin alpha-1
Catenin beta-1
Prohibitin 1
Serpin B6
Radixin
Large ribosomal subunit protein eL22
Thrombospondin-4
Fibrillin-1
Myosin-9
Tyrosine-protein kinase receptor Tie-1
26S proteasome regulatory subunit 7
Large ribosomal subunit protein uL4
Phosphoglucomutase-1
Transgelin-2
Transaldolase

RNA-binding motif protein, X chromosome
V-type proton ATPase catalytic subunit A
Stress-70 protein, mitochondrial
Small ribosomal subunit protein eS19
Large ribosomal subunit protein uL3
Dolichyl-diphosphooligosaccharide--protein glycosyltransferase 48 kDa subunit
Acidic leucine-rich nuclear phosphoprotein 32 family member A
Macrophage-capping protein
T-complex protein 1 subunit zeta
Nicotinamide N-methyltransferase
Large ribosomal subunit protein uL13
Malate dehydrogenase, cytoplasmic
Malate dehydrogenase, mitochondrial
Trifunctional enzyme subunit alpha, mitochondrial
Eukaryotic translation initiation factor 2 subunit 3
Glycine--tRNA ligase
Lamina-associated polypeptide 2, isoform alpha
Lysosomal Pro-X carboxypeptidase
Cell surface glycoprotein MUC18
Matrin-3
Nicotinamide phosphoribosyltransferase
Voltage-dependent anion-selective channel protein 2
Ubiquitin carboxyl-terminal hydrolase 5
Ran GTPase-activating protein 1
Large ribosomal subunit protein uL15
Large ribosomal subunit protein uL18
Large ribosomal subunit protein eL21
Large ribosomal subunit protein eL28
Small ribosomal subunit protein uS4
Small ribosomal subunit protein uS7
Small ribosomal subunit protein eS10
Glucosamine-6-phosphate isomerase 1
Ras GTPase-activating-like protein IQGAP1
Dolichyl-diphosphooligosaccharide--protein glycosyltransferase subunit STT3A
F-actin-capping protein subunit alpha-2
F-actin-capping protein subunit beta
Glutamine--tRNA ligase
ATP synthase subunit O, mitochondrial
LIM and senescent cell antigen-like-containing domain protein 1
Coatomer subunit delta
T-complex protein 1 subunit epsilon
Nestin
Heat shock 70 kDa protein 13
Isocitrate dehydrogenase [NADP], mitochondrial
Phosphatidylinositol transfer protein beta isoform
Mannan-binding lectin serine protease 1
Large ribosomal subunit protein eL34
Fatty acid synthase
T-complex protein 1 subunit gamma
Beta-arrestin-1

Elongation factor Tu, mitochondrial
Signal recognition particle 9 kDa protein
Proteasome subunit beta type-3
Proteasome subunit beta type-2
Cartilage oligomeric matrix protein
Rab GDP dissociation inhibitor beta
Serpin B8
Serpin H1
Hsc70-interacting protein
Large ribosomal subunit protein eL14
T-complex protein 1 subunit theta
T-complex protein 1 subunit delta
Ras-related protein Rab-5C
Ras-related protein Rab-7a
Hepatoma-derived growth factor
Heterogeneous nuclear ribonucleoprotein A3
6-phosphogluconate dehydrogenase, decarboxylating
Heterogeneous nuclear ribonucleoprotein M
Rho GDP-dissociation inhibitor 1
Rho GDP-dissociation inhibitor 2
F-actin-capping protein subunit alpha-1
Biliverdin reductase A
ATP-citrate synthase
Coatomer subunit beta
Coatomer subunit alpha
Dipeptidyl peptidase 1
Activated RNA polymerase II transcriptional coactivator p15
Arginine--tRNA ligase, cytoplasmic
Voltage-dependent calcium channel subunit alpha-2/delta-1
Ubiquitin carboxyl-terminal hydrolase 14
Branched-chain-amino-acid aminotransferase, cytosolic
Delta-1-pyrroline-5-carboxylate synthase
Phospholipid transfer protein
Exportin-2
Transitional endoplasmic reticulum ATPase
Trifunctional enzyme subunit beta, mitochondrial
Mesencephalic astrocyte-derived neurotrophic factor
Nucleosome assembly protein 1-like 1
Adenosine kinase
Cadherin-6
Cadherin-13
Puromycin-sensitive aminopeptidase
Eukaryotic translation initiation factor 3 subunit B
ATP synthase subunit f, mitochondrial
Eukaryotic translation initiation factor 6
C-terminal-binding protein 2
Actin-related protein 2/3 complex subunit 4
Triosephosphate isomerase
Eukaryotic translation initiation factor 3 subunit E
Actin, cytoplasmic 1

Eukaryotic initiation factor 4A-I
Small ribosomal subunit protein uS10
Proteasome subunit alpha type-6
Cell division control protein 42 homolog
Destrin
Ras-related protein Rab-2A
Actin-related protein 3
Actin-related protein 2
ADP-ribosylation factor 3
Small ribosomal subunit protein eS1
Large ribosomal subunit protein eL15
Protein mago nashi homolog
Large ribosomal subunit protein eL27
10 kDa heat shock protein, mitochondrial
Protein transport protein Sec61 subunit alpha isoform 1
NPC intracellular cholesterol transporter 2
Heterogeneous nuclear ribonucleoprotein K
14-3-3 protein gamma
Small ribosomal subunit protein eS7
Serine/threonine-protein phosphatase PP1-beta catalytic subunit
26S proteasome regulatory subunit 4
Small ribosomal subunit protein eS8
Small ribosomal subunit protein uS8
Small ribosomal subunit protein uS9
14-3-3 protein epsilon
Small ribosomal subunit protein uS11
Small ribosomal subunit protein uS12
Small ribosomal subunit protein uS13
Small ribosomal subunit protein uS15
Small ribosomal subunit protein uS17
Small nuclear ribonucleoprotein Sm D3
Large ribosomal subunit protein eL8
Ras-related protein Rab-11A
Small ribosomal subunit protein eS4, X isoform
Actin, aortic smooth muscle
Large ribosomal subunit protein uL23
Small ribosomal subunit protein eS6
Histone H4
GTP-binding nuclear protein Ran
Large ribosomal subunit protein uL14
Small ribosomal subunit protein eS24
Small ribosomal subunit protein eS25
Small ribosomal subunit protein eS26
Guanine nucleotide-binding protein G(I)/G(S)/G(T) subunit beta-1
Large ribosomal subunit protein eL30
Large ribosomal subunit protein eL31
Large ribosomal subunit protein uL1
Large ribosomal subunit protein eL32
Large ribosomal subunit protein uL5
Large ribosomal subunit protein uL2

Peptidyl-prolyl cis-trans isomerase A
Ras-related C3 botulinum toxin substrate 1
AP-2 complex subunit beta
14-3-3 protein zeta/delta
Large ribosomal subunit protein eL38
Eukaryotic translation initiation factor 5A-1
Small ribosomal subunit protein RACK1
Y-box-binding protein 1
Signal peptidase complex catalytic subunit SEC11A
Tropomyosin alpha-4 chain
Ubiquitin-conjugating enzyme E2 L3
Elongation factor 1-alpha 1
Tubulin alpha-1B chain
Tubulin beta-4B chain
Histone H3.1
T-complex protein 1 subunit beta
Glutathione S-transferase omega-1
DNA-dependent protein kinase catalytic subunit
Sushi repeat-containing protein SRPX
Dermcidin
Large ribosomal subunit protein eL24
Large ribosomal subunit protein eL42
Large ribosomal subunit protein eL19
Serine/arginine-rich splicing factor 3
Basement membrane-specific heparan sulfate proteoglycan core protein
Cytochrome c
Solute carrier family 25 member 3
Clathrin heavy chain 1
Peptidyl-prolyl cis-trans isomerase FKBP3
Heterogeneous nuclear ribonucleoprotein U
Spectrin beta chain, non-erythrocytic 1
Protein SET
Serine/arginine-rich splicing factor 2
Fatty acid-binding protein 5
Adenylyl cyclase-associated protein 1
Interleukin-1 receptor-like 1
ATP-dependent 6-phosphofructokinase, platelet type
Large ribosomal subunit protein eL20
Procollagen-lysine,2-oxoglutarate 5-dioxygenase 1
Nucleobindin-1
Large ribosomal subunit protein eL6
Caveolin-1
1,4-alpha-glucan-branching enzyme
Eukaryotic translation initiation factor 4 gamma 1
14-3-3 protein eta
Caldesmon
Proteasome activator complex subunit 1
Amyloid beta precursor like protein 2
Fibromodulin
Peroxiredoxin-1

Large ribosomal subunit protein eL18
Complement component 1 Q subcomponent-binding protein, mitochondrial
Cytoskeleton-associated protein 4
KH domain-containing, RNA-binding, signal transduction-associated protein 1
Prolow-density lipoprotein receptor-related protein 1
Serine/arginine-rich splicing factor 1
ATP-dependent RNA helicase A
Testican-1
Neuroblast differentiation-associated protein AHNAK
EGF-containing fibulin-like extracellular matrix protein 1
Follistatin-related protein 1
Contactin-1
Interleukin enhancer-binding factor 2
Interleukin enhancer-binding factor 3
Vesicular integral-membrane protein VIP36
Heat shock protein 75 kDa, mitochondrial
Peroxiredoxin-4
Chromobox protein homolog 3
26S proteasome non-ATPase regulatory subunit 2
Multimerin-1
Transcription intermediary factor 1-beta
Inactive tyrosine-protein kinase 7
Integrin-linked protein kinase
Sorting nexin-1
CD166 antigen
Spectrin alpha chain, non-erythrocytic 1
Spliceosome RNA helicase DDX39B
Tubulin beta-2A chain
Coactosin-like protein
Heterogeneous nuclear ribonucleoprotein D0
Eukaryotic translation initiation factor 3 subunit A
Dihydropyrimidinase-related protein 3
Dynactin subunit 1
Cytoplasmic dynein 1 heavy chain 1
Eukaryotic initiation factor 4A-II
Filamin-C
SPARC-like protein 1
Neutral alpha-glucosidase AB
Major vault protein
Latent-transforming growth factor beta-binding protein 1
Latent-transforming growth factor beta-binding protein 2
Importin subunit beta-1
Septin-2
116 kDa U5 small nuclear ribonucleoprotein component
Protein disulfide-isomerase A6
Plectin
Non-POU domain-containing octamer-binding protein
Serine/threonine-protein phosphatase 2A activator
Reticulocalbin-1
Poly(rC)-binding protein 1

Poly(rC)-binding protein 2
Splicing factor 3B subunit 3
Ras suppressor protein 1
Protein transport protein Sec23A
Regucalcin
Transforming growth factor-beta-induced protein ig-h3
Septin-7
Insulin-like growth factor-binding protein 7
Laminin subunit alpha-4
Exostosin-1
DNA damage-binding protein 1
Hsp90 co-chaperone Cdc37
Dihydropyrimidinase-related protein 2
Extracellular matrix protein 1
Fascin
Gamma-interferon-inducible protein 16
Membrane primary amine oxidase
Thioredoxin reductase 1, cytoplasmic
Prolyl 3-hydroxylase 1
Caveolae-associated protein 1
Golgi-associated kinase 1B
CD109 antigen
Ribosomal protein eS27-like
A disintegrin and metalloproteinase with thrombospondin motifs 13
Staphylococcal nuclease domain-containing protein 1
Cytoplasmic FMR1-interacting protein 1
Carbohydrate sulfotransferase 3
Target of Nesh-SH3
C-type lectin domain family 14 member A
Fermitin family homolog 3
Cullin-associated NEDD8-dissociated protein 1
Histone H2A type 2-B
Adipocyte enhancer-binding protein 1
5'-3' exonuclease PLD3
Procollagen galactosyltransferase 1
Thioredoxin domain-containing protein 5
Minor histocompatibility antigen H13
Programmed cell death 6-interacting protein
Histone H1.10
Peroxidasin homolog
Serine protease HTRA1
Gamma-glutamyl hydrolase
Probable ATP-dependent RNA helicase DDX17
Golgi apparatus protein 1
Transportin-1
Betaine--homocysteine S-methyltransferase 1
Far upstream element-binding protein 1
Leucine-rich repeat-containing protein 59
BTB/POZ domain-containing protein KCTD12
Ubiquitin thioesterase OTUB1

Cytosolic non-specific dipeptidase
Vacuolar protein sorting-associated protein 35
Hedgehog-interacting protein
Sialidase-1
Synaptic vesicle membrane protein VAT-1 homolog
Legumain
Prohibitin-2
3-hydroxyacyl-CoA dehydrogenase type-2
Collagen alpha-1(XII) chain
Heterogeneous nuclear ribonucleoprotein A/B
T-complex protein 1 subunit eta
Protein arginine N-methyltransferase 1
Growth/differentiation factor 15
Coronin-1B
45 kDa calcium-binding protein
Peroxiredoxin-like 2A
Extended synaptotagmin-1
COP9 signalosome complex subunit 4
Transmembrane protein 43
Tubulin beta-6 chain
Acetyl-CoA acetyltransferase, cytosolic
Complement C1q tumor necrosis factor-related protein 5
Complement C1q tumor necrosis factor-related protein 3
Apoptosis inhibitor 5
Tubulointerstitial nephritis antigen-like
Ras-related protein Rab-1B
Nuclear ubiquitous casein and cyclin-dependent kinase substrate 1
EH domain-containing protein 1
Divergent protein kinase domain 2B
Calcyclin-binding protein
Adipocyte plasma membrane-associated protein
Interleukin-1 receptor accessory protein
Complement component C1q receptor
Endothelial cell-specific molecule 1
Fructose-2,6-bisphosphatase TIGAR
Sialic acid synthase
Obg-like ATPase 1
Septin-11
Alpha-parvin
Stabilin-1
Dipeptidyl peptidase 3
Myoferlin
EH domain-containing protein 2
Cysteine-rich motor neuron 1 protein
Leucine--tRNA ligase, cytoplasmic
Dickkopf-related protein 3
Cathepsin Z
N-acetyl-D-glucosamine kinase
Stomatin-like protein 2, mitochondrial
Proteasome activator complex subunit 2

Coronin-1C
Endothelial protein C receptor
Proliferation-associated protein 2G4
RuvB-like 2
C-type lectin domain family 11 member A
RuvB-like 1
Nuclear migration protein nudC
Voltage-dependent anion-selective channel protein 3
RNA-splicing ligase RtcB homolog
Large ribosomal subunit protein eL36
Talin-1
Lysyl oxidase homolog 2
Hypoxia up-regulated protein 1
FACT complex subunit SPT16
RNA-binding protein 8A
Endothelial lipase
Coatomer subunit gamma-1
Chloride intracellular channel protein 4

PG.ProteinNames	PG.Quantity
RPSA2_HUMAN	1844.098267
RP1BL_HUMAN	640.0415039
RHOC_HUMAN	1702.449341
EIFCL_HUMAN	220.9899445
NACAM_HUMAN	17094.98633
BACH_HUMAN	1250.216797
MYO1C_HUMAN	2330.265625
PSD11_HUMAN	302.4012756
PSD12_HUMAN	624.5145264
CLIC1_HUMAN	10599.36719
QSOX1_HUMAN	13564.11816
IPO5_HUMAN	622.6771851
PLOD2_HUMAN	1203.002075
NOP56_HUMAN	151.8658752
DDX3X_HUMAN	737.4168701
CCN1_HUMAN	10497.52344
PIR_HUMAN	375.7730103
TPP1_HUMAN	2401.424561
NRP1_HUMAN	1016.196289
PSA7_HUMAN	2705.369873
ML12B_HUMAN	3109.973633
HNRDL_HUMAN	1199.72229
XPO1_HUMAN	255.4656525
ANGP2_HUMAN	2955.552979
ARC1B_HUMAN	992.9974976
ARPC2_HUMAN	1564.323486
ARPC3_HUMAN	439.3560791
MATN3_HUMAN	605.0067139
DHX15_HUMAN	586.8050537
PSMD3_HUMAN	425.8274841
HNRPR_HUMAN	3443.254639
TXNL1_HUMAN	922.3607788
BUB3_HUMAN	550.0236206
ACTN4_HUMAN	10964.35449
NRP2_HUMAN	530.4696045
HNRPQ_HUMAN	1850.53064
PLOD3_HUMAN	906.7754517
H2B1K_HUMAN	38830.63672
WDR1_HUMAN	4550.316406
CPNE3_HUMAN	240.9827576
FLNB_HUMAN	5983.01123
CISY_HUMAN	1981.990356
VP26A_HUMAN	967.4119263
NDUS3_HUMAN	752.6850586
IDHC_HUMAN	2395.849609
PRAF3_HUMAN	782.8834839
DYSF_HUMAN	536.3670044
SC31A_HUMAN	417.9491577

CSTN1_HUMAN	2824.039307
PRS23_HUMAN	941.8334961
APOM_HUMAN	4748.103027
VNN1_HUMAN	1139.022461
AP2A1_HUMAN	575.8018188
CAVN2_HUMAN	180.6925659
LDHA_HUMAN	7808.717285
AL1A1_HUMAN	1023.774902
NB5R3_HUMAN	2456.063965
COX2_HUMAN	2448.601074
F13A_HUMAN	1358.052002
PNPH_HUMAN	4091.94458
HPRT_HUMAN	448.9337769
AATM_HUMAN	1457.536255
PGK1_HUMAN	8733.560547
KAD1_HUMAN	1186.613403
FA10_HUMAN	7081.913086
TPA_HUMAN	2539.03833
CO3_HUMAN	112801.7422
TIMP1_HUMAN	3868.947021
CYTC_HUMAN	2224.388428
CO1A1_HUMAN	7194.182129
CO2A1_HUMAN	1885.412842
LMNA_HUMAN	4616.80957
FINC_HUMAN	39773.41406
RET4_HUMAN	13682.20215
ALBU_HUMAN	8241.675781
MMP1_HUMAN	59423.40625
ALDOA_HUMAN	8126.851074
ANXA1_HUMAN	5212.901367
APOB_HUMAN	14251.79395
VWF_HUMAN	26085.40039
G3P_HUMAN	12220.96191
HLAA_HUMAN	1445.847778
HSPB1_HUMAN	7728.162109
RPN1_HUMAN	1944.083008
RPN2_HUMAN	1035.419189
GNAI2_HUMAN	1238.177979
H2A1B_HUMAN	65306.30859
AT1A1_HUMAN	967.7316895
A4_HUMAN	2959.074707
ALDH2_HUMAN	423.0088501
S10A8_HUMAN	773.0750732
HMGN1_HUMAN	67.17724609
PAI1_HUMAN	34510.32031
ADT2_HUMAN	1972.583008
IF2A_HUMAN	770.6980591
EDN1_HUMAN	825.7872314
RLA0_HUMAN	3517.581543
LA_HUMAN	389.6157837

ITB1_HUMAN	1754.632202
K1C18_HUMAN	847.4841919
GELS_HUMAN	3184.578369
PTMA_HUMAN	1011.998779
ATPB_HUMAN	4763.869629
S10A9_HUMAN	901.732605
S10A6_HUMAN	1230.814209
KCRM_HUMAN	96.29628754
ENOA_HUMAN	29013.38281
G6PI_HUMAN	2994.006836
NPM_HUMAN	5004.797852
TPM3_HUMAN	1868.008911
HEXA_HUMAN	991.065918
H2B1J_HUMAN	4627.339844
LDHB_HUMAN	9941.15918
GPX1_HUMAN	837.901062
PDIA1_HUMAN	5909.858887
CATD_HUMAN	4774.262695
ANXA2_HUMAN	14960.2002
CAN1_HUMAN	741.1672363
TBB5_HUMAN	8236.939453
SAP_HUMAN	4651.687988
HEXB_HUMAN	1955.310547
PROF1_HUMAN	4425.577637
SYEP_HUMAN	538.6950684
CATB_HUMAN	6782.711426
HS90A_HUMAN	15622.23926
HNRPC_HUMAN	5631.190918
LAMB1_HUMAN	3959.273193
TPM2_HUMAN	1949.431885
FUMH_HUMAN	427.2817688
TSP1_HUMAN	247086.5781
RNAS1_HUMAN	826.0817871
CO1A2_HUMAN	2718.031494
ANXA6_HUMAN	4414.411621
HS90B_HUMAN	25522.55859
MMP2_HUMAN	9583.256836
CO4A2_HUMAN	1961.730103
RU17_HUMAN	1464.538208
ITA5_HUMAN	1514.711304
VIME_HUMAN	19910.19531
RS17_HUMAN	941.6808472
ANXA5_HUMAN	11819.05762
SNRPA_HUMAN	338.5396729
GSTP1_HUMAN	2097.416016
HMGB1_HUMAN	1943.518921
SPRC_HUMAN	6581.40918
ANXA4_HUMAN	405.4483032
ROA1_HUMAN	3770.39502
COX6C_HUMAN	1063.389648

LKHA4_HUMAN	712.3540649
UBB_HUMAN	3601.247803
HS71A_HUMAN	2170.152832
TBA3C_HUMAN	2471.935059
SRGN_HUMAN	1775.933716
PTPRF_HUMAN	762.1446533
PPGB_HUMAN	957.8234863
TFPI1_HUMAN	1022.394043
CH60_HUMAN	4741.374512
CLUS_HUMAN	2658.814941
BIP_HUMAN	7622.82666
LAMC1_HUMAN	1650.291016
HSP7C_HUMAN	11043.91309
RALA_HUMAN	1883.727173
LAMP1_HUMAN	2268.743164
G6PD_HUMAN	1316.985229
C1TC_HUMAN	527.505249
MPRI_HUMAN	1604.27002
ADHX_HUMAN	2179.710449
PABP1_HUMAN	2546.90625
PCNA_HUMAN	820.6604614
COBA1_HUMAN	1512.574951
CO6A1_HUMAN	11523.375
CO6A3_HUMAN	753.4141235
ADT3_HUMAN	5217.022949
IMDH2_HUMAN	416.2038574
ANXA3_HUMAN	1208.523193
ACTN1_HUMAN	8496.427734
ACE_HUMAN	605.0228271
PEPD_HUMAN	245.7902832
XRCC6_HUMAN	2378.575439
XRCC5_HUMAN	1274.749268
COX41_HUMAN	1294.377197
LAMP2_HUMAN	2629.861572
RINI_HUMAN	1857.924316
EF2_HUMAN	7043.630859
PDIA4_HUMAN	3654.610107
PLST_HUMAN	8925.271484
CD59_HUMAN	1782.285645
GLU2B_HUMAN	880.9775391
FPPS_HUMAN	2393.386719
NID1_HUMAN	1091.568848
KPYM_HUMAN	25002.20117
ENPL_HUMAN	8403.308594
HNRPL_HUMAN	368.3974609
SYDC_HUMAN	676.0787354
FABP4_HUMAN	1126.483887
ALDR_HUMAN	1548.278687
AMPN_HUMAN	3098.080566
RAC2_HUMAN	375.6391907

ERF3A_HUMAN	237.8428192
EZRI_HUMAN	803.9251709
NDKA_HUMAN	567.2168579
GNS_HUMAN	1925.890869
RS2_HUMAN	2225.72998
TIMP2_HUMAN	1203.590942
PECA1_HUMAN	1267.148315
H15_HUMAN	11309.78711
H13_HUMAN	11040.73535
H12_HUMAN	1798.089355
FAAA_HUMAN	143.5692139
STMN1_HUMAN	1515.690063
HMGA1_HUMAN	3065.765137
ITA2_HUMAN	274.1769409
CAN2_HUMAN	1106.940796
DDX5_HUMAN	2214.743652
PFKAL_HUMAN	390.0302429
VGFR1_HUMAN	983.4257202
PRS6A_HUMAN	1105.760864
TCPA_HUMAN	1144.707642
RL35A_HUMAN	1496.260742
ARF4_HUMAN	2136.079834
RL7_HUMAN	2137.55127
VINC_HUMAN	3611.820801
RL17_HUMAN	1566.776001
PGAM1_HUMAN	3631.327393
RCC1_HUMAN	607.3346558
AMD_HUMAN	842.0941162
NUCL_HUMAN	4126.65332
HXK1_HUMAN	581.5777588
SPEE_HUMAN	548.5709839
IF2B_HUMAN	366.7622986
TCO2_HUMAN	511.880127
PSB1_HUMAN	1826.815552
LMNB1_HUMAN	577.6251831
MIME_HUMAN	1258.99939
CO5A1_HUMAN	2256.872314
FLNA_HUMAN	4901.860352
ACOHC_HUMAN	626.9365234
MK_HUMAN	2330.178711
VDAC1_HUMAN	1560.279541
PGS1_HUMAN	10357.57129
TGM2_HUMAN	8079.729004
BMP6_HUMAN	747.262207
PUR6_HUMAN	958.8358765
UBA1_HUMAN	2772.674561
GPX3_HUMAN	2179.950195
NDKB_HUMAN	7367.836426
ROA2_HUMAN	8592.023438
IBP4_HUMAN	1797.670898

QCR2_HUMAN	357.4637146
SFPQ_HUMAN	3237.74707
PPIB_HUMAN	11238.76465
SYWC_HUMAN	1285.14917
RS3_HUMAN	4872.914063
SAHH_HUMAN	3367.389893
COF1_HUMAN	23419.0293
ATPA_HUMAN	4749.984375
PSA1_HUMAN	3532.699707
PSA2_HUMAN	862.7363892
PSA3_HUMAN	1682.976563
PSA4_HUMAN	2447.847168
PTX3_HUMAN	13480.02734
MOES_HUMAN	7919.770508
U2AF2_HUMAN	692.0813599
RL13_HUMAN	1532.99707
HMGB2_HUMAN	1081.182373
PTBP1_HUMAN	766.5961304
SYVC_HUMAN	333.8906555
EF1G_HUMAN	4804.425781
HGFL_HUMAN	2091.61792
STOM_HUMAN	987.9219971
1433T_HUMAN	2303.858887
RL10_HUMAN	1057.252075
APEX1_HUMAN	1322.663086
PYR1_HUMAN	314.9003601
CALR_HUMAN	5664.394043
MAP4_HUMAN	145.24646
CALX_HUMAN	4762.522461
PSA5_HUMAN	709.5202026
PSB6_HUMAN	757.0209961
PSB5_HUMAN	694.6671143
LYOX_HUMAN	1835.159302
MK01_HUMAN	1194.400757
GRN_HUMAN	445.5316772
AMPL_HUMAN	187.5192719
CCN2_HUMAN	69083.83594
TKT_HUMAN	5238.521973
EF1D_HUMAN	1025.82666
MARCS_HUMAN	1247.305176
PRDX6_HUMAN	3343.74585
RL12_HUMAN	2372.544678
ECHM_HUMAN	407.9160767
PEBP1_HUMAN	602.6357422
PDIA3_HUMAN	8262.323242
2AAA_HUMAN	1308.575806
PURA2_HUMAN	504.5114136
METK2_HUMAN	1271.643921
QCR1_HUMAN	695.3753662
HNRH1_HUMAN	972.199585

1433B_HUMAN	16657.22266
STIP1_HUMAN	3761.374756
S10AB_HUMAN	881.8095703
PRDX2_HUMAN	1752.052002
RL9_HUMAN	1141.570435
CADH5_HUMAN	8372.353516
RNAS4_HUMAN	150.1255035
HSP74_HUMAN	877.8461304
CTNA1_HUMAN	828.6901855
CTNB1_HUMAN	342.9686584
PHB1_HUMAN	645.8283691
SPB6_HUMAN	710.6864624
RADI_HUMAN	19323.47461
RL22_HUMAN	3434.230469
TSP4_HUMAN	1224.040283
FBN1_HUMAN	1106.331543
MYH9_HUMAN	44247.14844
TIE1_HUMAN	1631.337036
PRS7_HUMAN	454.4632874
RL4_HUMAN	16728.05664
PGM1_HUMAN	5461.428711
TAGL2_HUMAN	645.6703491
TALDO_HUMAN	4012.951416
RBMX_HUMAN	678.1087646
VATA_HUMAN	892.0975952
GRP75_HUMAN	2501.669922
RS19_HUMAN	2051.870361
RL3_HUMAN	1618.454712
OST48_HUMAN	717.2646484
AN32A_HUMAN	1398.948975
CAPG_HUMAN	258.5143738
TCPZ_HUMAN	1472.724854
NNMT_HUMAN	45770.15234
RL13A_HUMAN	1621.822266
MDHC_HUMAN	2118.123779
MDHM_HUMAN	4052.199951
ECHA_HUMAN	559.7218628
IF2G_HUMAN	1419.539063
GARS_HUMAN	522.6192017
LAP2A_HUMAN	518.6633301
PCP_HUMAN	425.6217346
MUC18_HUMAN	3944.790283
MATR3_HUMAN	465.2714233
NAMPT_HUMAN	2114.456055
VDAC2_HUMAN	1936.172241
UBP5_HUMAN	334.6864929
RAGP1_HUMAN	143.3375549
RL27A_HUMAN	2283.717529
RL5_HUMAN	1291.921875
RL21_HUMAN	143.1222229

RL28_HUMAN	774.2699585
RS9_HUMAN	3566.629883
RS5_HUMAN	6285.675293
RS10_HUMAN	4429.285645
GNPI1_HUMAN	503.7704468
IQGA1_HUMAN	3398.136475
STT3A_HUMAN	201.4693298
CAZA2_HUMAN	709.9742432
CAPZB_HUMAN	3425.997559
SYQ_HUMAN	318.5953674
ATPO_HUMAN	1024.852661
LIMS1_HUMAN	651.0889282
COPD_HUMAN	146.5967255
TCPE_HUMAN	1646.811523
NEST_HUMAN	1184.985962
HSP13_HUMAN	2109.549316
IDHP_HUMAN	513.977356
PIPNB_HUMAN	231.604248
MASP1_HUMAN	684.8086548
RL34_HUMAN	202.9716187
FAS_HUMAN	2246.230713
TCPG_HUMAN	2177.582031
ARRB1_HUMAN	240.5150452
EFTU_HUMAN	1286.420776
SRP09_HUMAN	931.2319946
PSB3_HUMAN	1360.917358
PSB2_HUMAN	1310.368408
COMP_HUMAN	1142.117188
GDIB_HUMAN	4044.527832
SPB8_HUMAN	250.8446808
SERPH_HUMAN	16951.98047
F10A1_HUMAN	2948.432617
RL14_HUMAN	1952.84021
TCPQ_HUMAN	3285.5
TCPD_HUMAN	1813.149536
RAB5C_HUMAN	1724.177612
RAB7A_HUMAN	901.2059326
HDGF_HUMAN	217.2958832
ROA3_HUMAN	2196.457764
6PGD_HUMAN	3992.950439
HNRPM_HUMAN	3313.685059
GDIR1_HUMAN	3681.90332
GDIR2_HUMAN	1254.691895
CAZA1_HUMAN	3658.196533
BIEA_HUMAN	518.1607056
ACLY_HUMAN	1860.060303
COPB_HUMAN	2014.219604
COPA_HUMAN	1521.422363
CATC_HUMAN	2276.587402
TCP4_HUMAN	670.8137817

SYRC_HUMAN	573.2196045
CA2D1_HUMAN	1780.369751
UBP14_HUMAN	368.0563049
BCAT1_HUMAN	486.3043518
P5CS_HUMAN	717.34729
PLTP_HUMAN	2952.204346
XPO2_HUMAN	1246.939697
TERA_HUMAN	5014.561523
ECHB_HUMAN	625.7080078
MANF_HUMAN	252.4192657
NP1L1_HUMAN	2263.386963
ADK_HUMAN	596.7590332
CADH6_HUMAN	643.5512695
CAD13_HUMAN	682.9911499
PSA_HUMAN	781.8546753
EIF3B_HUMAN	698.3973389
ATPK_HUMAN	1491.598145
IF6_HUMAN	1048.668457
CTBP2_HUMAN	682.9295044
ARPC4_HUMAN	2441.685791
TPIS_HUMAN	3686.081787
EIF3E_HUMAN	673.2631226
ACTB_HUMAN	74910.16406
IF4A1_HUMAN	6638.656738
RS20_HUMAN	2070.122803
PSA6_HUMAN	1592.51123
CDC42_HUMAN	595.9003296
DEST_HUMAN	1607.327759
RAB2A_HUMAN	646.4853516
ARP3_HUMAN	2456.18335
ARP2_HUMAN	3114.822754
ARF3_HUMAN	4776.297852
RS3A_HUMAN	1567.231201
RL15_HUMAN	1154.749756
MGN_HUMAN	2444.064209
RL27_HUMAN	1706.076416
CH10_HUMAN	1562.153198
S61A1_HUMAN	277.1483154
NPC2_HUMAN	746.2003174
HNRPK_HUMAN	5118.892578
1433G_HUMAN	2288.812988
RS7_HUMAN	3002.341064
PP1B_HUMAN	1367.522827
PRS4_HUMAN	586.8946533
RS8_HUMAN	3220.476318
RS15A_HUMAN	1550.380981
RS16_HUMAN	3174.107666
1433E_HUMAN	4961.148438
RS14_HUMAN	2308.361328
RS23_HUMAN	1534.780884

RS18_HUMAN	2429.645996
RS13_HUMAN	1704.047241
RS11_HUMAN	1010.15332
SMD3_HUMAN	2418.601563
RL7A_HUMAN	1570.477295
RB11A_HUMAN	1002.168823
RS4X_HUMAN	2274.877686
ACTA_HUMAN	8130.39502
RL23A_HUMAN	2930.824951
RS6_HUMAN	2798.771973
H4_HUMAN	107918.4922
RAN_HUMAN	4810.964355
RL23_HUMAN	911.9970093
RS24_HUMAN	1136.307251
RS25_HUMAN	3562.525635
RS26_HUMAN	2321.709961
GBB1_HUMAN	2050.630859
RL30_HUMAN	1608.08606
RL31_HUMAN	1625.650879
RL10A_HUMAN	2322.802734
RL32_HUMAN	605.5106201
RL11_HUMAN	1287.492188
RL8_HUMAN	890.3021851
PPIA_HUMAN	3584.054688
RAC1_HUMAN	882.2272949
AP2B1_HUMAN	507.9927979
1433Z_HUMAN	6911.69043
RL38_HUMAN	1083.749878
IF5A1_HUMAN	2107.104248
RACK1_HUMAN	2123.517578
YBOX1_HUMAN	145.6549835
SC11A_HUMAN	940.2307739
TPM4_HUMAN	6636.42334
UB2L3_HUMAN	460.407074
EF1A1_HUMAN	37391.52344
TBA1B_HUMAN	28461.94727
TBB4B_HUMAN	15593.62891
H31_HUMAN	3859.687012
TCPB_HUMAN	1741.488892
GSTO1_HUMAN	4860.239746
PRKDC_HUMAN	973.2351074
SRPX_HUMAN	6940.002441
DCD_HUMAN	434.2346802
RL24_HUMAN	1300.584473
RL36A_HUMAN	87.23885345
RL19_HUMAN	1153.655884
SRSF3_HUMAN	1651.867676
PGBM_HUMAN	48887.28125
CYC_HUMAN	1585.406494
S25A3_HUMAN	2194.707764

CLH1_HUMAN	5576.322266
FKBP3_HUMAN	913.5932617
HNRPU_HUMAN	5485.507324
SPTB2_HUMAN	956.5684814
SET_HUMAN	2876.521973
SRSF2_HUMAN	1279.895874
FABP5_HUMAN	3263.029053
CAP1_HUMAN	4137.807129
ILRL1_HUMAN	787.4574585
PFKAP_HUMAN	3751.358398
RL18A_HUMAN	1259.88147
PLOD1_HUMAN	2443.576904
NUCB1_HUMAN	426.6174316
RL6_HUMAN	3846.854248
CAV1_HUMAN	4904.362305
GLGB_HUMAN	192.9176025
IF4G1_HUMAN	178.0554962
1433F_HUMAN	1314.542969
CALD1_HUMAN	326.7132874
PSME1_HUMAN	1072.170166
APLP2_HUMAN	876.6482544
FMOD_HUMAN	2911.339111
PRDX1_HUMAN	5794.03125
RL18_HUMAN	2739.336914
C1QBP_HUMAN	967.9786377
CKAP4_HUMAN	2192.125732
KHDR1_HUMAN	1557.224976
LRP1_HUMAN	997.7706909
SRSF1_HUMAN	2754.348877
DHX9_HUMAN	2110.730713
TICN1_HUMAN	4133.022949
AHNK_HUMAN	906.7470703
FBLN3_HUMAN	47802.65625
FSTL1_HUMAN	1890.388428
CNTN1_HUMAN	3146.474365
ILF2_HUMAN	3415.673828
ILF3_HUMAN	2748.255371
LMAN2_HUMAN	633.218811
TRAP1_HUMAN	20673.27148
PRDX4_HUMAN	868.7060547
CBX3_HUMAN	389.2430725
PSMD2_HUMAN	252.1143341
MMRN1_HUMAN	9858.632813
TIF1B_HUMAN	468.4484558
PTK7_HUMAN	871.8302002
ILK_HUMAN	278.5115356
SNX1_HUMAN	229.54422
CD166_HUMAN	1126.811035
SPTN1_HUMAN	1876.872192
DX39B_HUMAN	2184.862305

TBB2A_HUMAN	1751.82312
COTL1_HUMAN	1156.802124
HNRPD_HUMAN	5521.229004
EIF3A_HUMAN	663.3816528
DPYL3_HUMAN	1323.684448
DCTN1_HUMAN	269.1846313
DYHC1_HUMAN	1622.148682
IF4A2_HUMAN	328.2593994
FLNC_HUMAN	927.8311157
SPRL1_HUMAN	3083.059814
GANAB_HUMAN	2446.882813
MVP_HUMAN	1759.596313
LTBP1_HUMAN	251.1990204
LTBP2_HUMAN	3395.956299
IMB1_HUMAN	1002.419861
SEPT2_HUMAN	1104.201782
U5S1_HUMAN	318.6152649
PDIA6_HUMAN	4706.037598
PLEC_HUMAN	1759.881348
NONO_HUMAN	2145.684326
PTPA_HUMAN	188.0791168
RCN1_HUMAN	274.7307739
PCBP1_HUMAN	1704.140869
PCBP2_HUMAN	2304.192627
SF3B3_HUMAN	524.0812378
RSU1_HUMAN	954.2215576
SC23A_HUMAN	382.6600647
RGN_HUMAN	919.1051636
BGH3_HUMAN	2764.313477
SEPT7_HUMAN	497.9802246
IBP7_HUMAN	12688.47461
LAMA4_HUMAN	5201.55957
EXT1_HUMAN	329.1075134
DDB1_HUMAN	1160.596191
CDC37_HUMAN	1526.764038
DPYL2_HUMAN	2324.181152
ECM1_HUMAN	883.171814
FSCN1_HUMAN	5270.041992
IF16_HUMAN	1559.67749
AOC3_HUMAN	1589.55127
TRXR1_HUMAN	4281.796875
P3H1_HUMAN	238.3228455
DHB12_HUMAN	587.6477051
CAVN1_HUMAN	2365.832764
GAK1B_HUMAN	1028.852173
CD109_HUMAN	3540.056152
RS27L_HUMAN	652.3810425
ATS13_HUMAN	1620.047363
SND1_HUMAN	2270.1521
CYFP1_HUMAN	107.1356201

CHST3_HUMAN	559.288269
TARSH_HUMAN	1036.57666
CLC14_HUMAN	120.1778336
URP2_HUMAN	1035.525635
CAND1_HUMAN	1161.658813
H2A2B_HUMAN	1103.688843
AEBP1_HUMAN	652.722168
PLD3_HUMAN	667.2525024
GT251_HUMAN	587.8045654
TXND5_HUMAN	6450.131836
HM13_HUMAN	1111.910889
PDC6I_HUMAN	526.9851685
H1X_HUMAN	272.1463318
PXDN_HUMAN	3802.025391
HTRA1_HUMAN	3909.873779
GGH_HUMAN	3137.996094
DDX17_HUMAN	1169.358032
GSLG1_HUMAN	745.251709
TNPO1_HUMAN	210.2416077
BHMT1_HUMAN	602.5490112
FUBP1_HUMAN	406.3399658
LRC59_HUMAN	6468.17334
KCD12_HUMAN	1831.280762
OTUB1_HUMAN	592.4775391
CNDP2_HUMAN	239.4098511
VPS35_HUMAN	637.15448
HHIP_HUMAN	4042.521484
NEUR1_HUMAN	777.3425903
VAT1_HUMAN	4091.119629
LGMN_HUMAN	1086.288086
PHB2_HUMAN	1769.616699
HCD2_HUMAN	596.6565552
COCA1_HUMAN	259.5177002
ROAA_HUMAN	1008.172363
TCPH_HUMAN	1385.699951
ANM1_HUMAN	523.4519043
GDF15_HUMAN	2860.976563
COR1B_HUMAN	719.0836182
CAB45_HUMAN	309.4446411
PXL2A_HUMAN	2656.605469
ESYT1_HUMAN	744.6146851
CSN4_HUMAN	276.6853943
TMM43_HUMAN	319.7416992
TBB6_HUMAN	4203.426758
THIC_HUMAN	1096.418945
C1QT5_HUMAN	1778.86438
C1QT3_HUMAN	1060.070557
API5_HUMAN	556.8033447
TINAL_HUMAN	999.2744141
RAB1B_HUMAN	964.7872925

NUCKS_HUMAN	246.0427246
EHD1_HUMAN	811.746582
DIK2B_HUMAN	289.8409729
CYBP_HUMAN	287.5618896
APMAP_HUMAN	13307.66797
IL1AP_HUMAN	3251.501709
C1QR1_HUMAN	2017.838257
ESM1_HUMAN	1062.947754
TIGAR_HUMAN	307.9199524
SIAS_HUMAN	329.0744934
OLA1_HUMAN	784.4473877
SEP11_HUMAN	1103.5448
PARVA_HUMAN	361.8564148
STAB1_HUMAN	3486.442871
DPP3_HUMAN	289.9749756
MYOF_HUMAN	1073.747803
EHD2_HUMAN	1515.673828
CRIM1_HUMAN	334.331665
SYLC_HUMAN	652.6430054
DKK3_HUMAN	1027.076172
CATZ_HUMAN	2507.487305
NAGK_HUMAN	627.6259766
STML2_HUMAN	435.3933105
PSME2_HUMAN	388.52005
COR1C_HUMAN	1000.042969
EPCR_HUMAN	3488.496582
PA2G4_HUMAN	876.4992676
RUVB2_HUMAN	363.6003113
CLC11_HUMAN	1077.956543
RUVB1_HUMAN	775.083374
NUDC_HUMAN	594.4725952
VDAC3_HUMAN	995.7809448
RTCB_HUMAN	256.0928955
RL36_HUMAN	822.9539795
TLN1_HUMAN	3298.949219
LOXL2_HUMAN	4919.504395
HYOU1_HUMAN	2374.408203
SP16H_HUMAN	456.7840271
RBM8A_HUMAN	491.6063843
LIPG_HUMAN	1310.789917
COPG1_HUMAN	506.500946
CLIC4_HUMAN	954.2756958
RPSA2_HUMAN	1922.552002
RP1BL_HUMAN	552.7509766
RHOC_HUMAN	1251.800781
EIFCL_HUMAN	307.6864624
NACAM_HUMAN	19703.97461
BACH_HUMAN	1668.040649
MYO1C_HUMAN	1386.34436
PSD11_HUMAN	256.4771118

PSD12_HUMAN	362.6842346
CLIC1_HUMAN	11788.65723
QSOX1_HUMAN	12080.70215
IPO5_HUMAN	597.0057373
PLOD2_HUMAN	848.4904175
NOP56_HUMAN	161.5484009
DDX3X_HUMAN	570.5502319
CCN1_HUMAN	7851.317383
PIR_HUMAN	436.2197266
TPP1_HUMAN	1575.879517
NRP1_HUMAN	1170.229858
PSA7_HUMAN	2423.289307
ML12B_HUMAN	4743.791016
HNRDL_HUMAN	821.993042
XPO1_HUMAN	260.8776245
ANGP2_HUMAN	3265.289551
ARC1B_HUMAN	953.1622314
ARPC2_HUMAN	1769.696777
ARPC3_HUMAN	500.8414917
MATN3_HUMAN	740.4445801
DHX15_HUMAN	729.6083374
PSMD3_HUMAN	452.8480835
HNRPR_HUMAN	3281.720703
TXNL1_HUMAN	1088.055542
BUB3_HUMAN	681.9842529
ACTN4_HUMAN	12784.11035
NRP2_HUMAN	646.4746094
HNRPQ_HUMAN	2084.219727
PLOD3_HUMAN	675.6134033
H2B1K_HUMAN	44414.68359
WDR1_HUMAN	4906.321289
CPNE3_HUMAN	199.5181122
FLNB_HUMAN	6477.145508
CISY_HUMAN	2145.973145
VP26A_HUMAN	761.8987427
NDUS3_HUMAN	559.3408813
IDHC_HUMAN	2815.001465
PRAF3_HUMAN	515.3483276
DYSF_HUMAN	254.697998
SC31A_HUMAN	246.1457672
CSTN1_HUMAN	3182.247803
PRS23_HUMAN	630.9833374
APOM_HUMAN	3602.388672
VNN1_HUMAN	1297.414673
AP2A1_HUMAN	527.7005615
CAVN2_HUMAN	329.9605103
LDHA_HUMAN	8391.852539
AL1A1_HUMAN	1079.7677
NB5R3_HUMAN	2390.724854
COX2_HUMAN	1172.855713

F13A_HUMAN	1561.340576
PNPH_HUMAN	3796.7771
HPRT_HUMAN	412.847229
AATM_HUMAN	1525.232178
PGK1_HUMAN	9905.762695
KAD1_HUMAN	1317.163452
FA10_HUMAN	7500.963867
TPA_HUMAN	2290.664551
CO3_HUMAN	121317.3672
TIMP1_HUMAN	3359.117188
CYTC_HUMAN	2276.336182
CO1A1_HUMAN	6964.51416
CO2A1_HUMAN	1235.571045
LMNA_HUMAN	4787.712891
FINC_HUMAN	41352.58984
RET4_HUMAN	15712.14648
ALBU_HUMAN	8476.777344
MMP1_HUMAN	63468.40234
ALDOA_HUMAN	9111.066406
ANXA1_HUMAN	5659.596191
APOB_HUMAN	17964.26758
VWF_HUMAN	25778.57227
G3P_HUMAN	12490.96875
HLAA_HUMAN	1227.813599
HSPB1_HUMAN	6389.558105
RPN1_HUMAN	1184.672607
RPN2_HUMAN	544.5977173
GNAI2_HUMAN	1233.748291
H2A1B_HUMAN	82574.33594
AT1A1_HUMAN	737.5593262
A4_HUMAN	2953.990479
ALDH2_HUMAN	465.7444153
HMGN1_HUMAN	135.3628387
PAI1_HUMAN	34051.60938
ADT2_HUMAN	941.4909668
IF2A_HUMAN	749.7401733
EDN1_HUMAN	1316.650269
RLA0_HUMAN	3062.02124
LA_HUMAN	175.4611053
ITB1_HUMAN	1290.545166
K1C18_HUMAN	582.7918701
GELS_HUMAN	3762.401123
PTMA_HUMAN	1994.896118
ATPB_HUMAN	4801.287598
S10A6_HUMAN	1937.33606
KCRM_HUMAN	831.5899048
ENOA_HUMAN	27477.76758
G6PI_HUMAN	3257.968994
NPM_HUMAN	5295.5
TPM3_HUMAN	2248.122803

HEXA_HUMAN	1055.084717
H2B1J_HUMAN	5169.744141
LDHB_HUMAN	9982.571289
GPX1_HUMAN	558.0519409
PDIA1_HUMAN	6111.949707
CATD_HUMAN	3677.358887
ANXA2_HUMAN	14407.73242
CAN1_HUMAN	668.8252563
TBB5_HUMAN	8760.314453
SAP_HUMAN	4086.426025
HEXB_HUMAN	1561.754395
PROF1_HUMAN	4823.980469
SYEP_HUMAN	515.2694702
CATB_HUMAN	5764.101074
HS90A_HUMAN	10513.78613
HNRPC_HUMAN	5129.844238
LAMB1_HUMAN	4275.98291
TPM2_HUMAN	2584.906738
FUMH_HUMAN	511.1719971
TSP1_HUMAN	270520.2188
RNAS1_HUMAN	718.0691528
CO1A2_HUMAN	2735.147217
ANXA6_HUMAN	3299.453125
HS90B_HUMAN	20644.29492
MMP2_HUMAN	12201.5127
CO4A2_HUMAN	1392.918091
RU17_HUMAN	1576.176147
ITA5_HUMAN	888.3565063
VIME_HUMAN	22132.43164
RS17_HUMAN	966.2366333
ANXA5_HUMAN	9908.006836
SNRPA_HUMAN	308.1786194
GSTP1_HUMAN	2073.19873
HMGB1_HUMAN	1501.508301
SPRC_HUMAN	5874.862305
ANXA4_HUMAN	138.8062897
ROA1_HUMAN	4300.682129
COX6C_HUMAN	794.3567505
LKHA4_HUMAN	978.0717163
UBB_HUMAN	3883.087158
HS71A_HUMAN	2257.733643
TBA3C_HUMAN	2958.606201
SRGN_HUMAN	3330.513672
PTPRF_HUMAN	548.4180908
PPGB_HUMAN	609.5844116
TFPI1_HUMAN	913.3850708
CH60_HUMAN	4099.367676
CLUS_HUMAN	3358.904785
BIP_HUMAN	6592.300781
LAMC1_HUMAN	1867.068115

HSP7C_HUMAN	12150.23535
RALA_HUMAN	2081.885742
LAMP1_HUMAN	1958.637451
G6PD_HUMAN	1398.80249
C1TC_HUMAN	491.059967
MPRI_HUMAN	1400.699585
ADHX_HUMAN	2774.054199
PABP1_HUMAN	2660.456055
PCNA_HUMAN	916.7427368
COBA1_HUMAN	1614.42041
CO6A1_HUMAN	11610.55469
CO6A3_HUMAN	871.4937134
ADT3_HUMAN	3599.580811
IMDH2_HUMAN	308.16922
ANXA3_HUMAN	1021.940918
ACTN1_HUMAN	9269.896484
ACE_HUMAN	764.2771606
PEPD_HUMAN	844.8590088
XRCC6_HUMAN	2644.010742
XRCC5_HUMAN	1479.611328
COX41_HUMAN	591.5198975
LAMP2_HUMAN	3047.662109
RINI_HUMAN	2066.518311
EF2_HUMAN	7544.29248
PDIA4_HUMAN	3368.158936
PLST_HUMAN	10651.62109
CD59_HUMAN	1507.050903
GLU2B_HUMAN	806.3204956
FPPS_HUMAN	2379.815674
NID1_HUMAN	1175.616699
KPYM_HUMAN	27932.79102
ENPL_HUMAN	7762.747559
HNRPL_HUMAN	367.3139343
SYDC_HUMAN	676.765686
FABP4_HUMAN	1462.386841
ALDR_HUMAN	1803.929443
AMPN_HUMAN	2372.132568
RAC2_HUMAN	152.7691193
ERF3A_HUMAN	148.1189728
EZRI_HUMAN	749.8724976
NDKA_HUMAN	1024.896484
GNS_HUMAN	1003.645447
RS2_HUMAN	2223.559814
TIMP2_HUMAN	1281.809204
PECA1_HUMAN	1240.137939
H15_HUMAN	10944.02344
H13_HUMAN	11926.12988
H12_HUMAN	773.112793
FAAA_HUMAN	396.1845703
STMN1_HUMAN	1739.656494

HMGA1_HUMAN	2860.062988
ITA2_HUMAN	188.0005798
CAN2_HUMAN	1035.690063
DDX5_HUMAN	2257.171387
PFKAL_HUMAN	215.8598328
VGFR1_HUMAN	773.8002319
PRS6A_HUMAN	1062.396729
TCPA_HUMAN	1070.321167
RL35A_HUMAN	1560.38916
ARF4_HUMAN	1767.769287
RL7_HUMAN	1933.264038
VINC_HUMAN	3718.074463
RL17_HUMAN	1719.693481
PGAM1_HUMAN	3926.485107
RCC1_HUMAN	742.8728027
AMD_HUMAN	636.291748
NUCL_HUMAN	3989.332031
HXK1_HUMAN	338.1289673
SPEE_HUMAN	1464.307373
IF2B_HUMAN	341.4937439
TCO2_HUMAN	559.9267578
PSB1_HUMAN	1555.824341
LMNB1_HUMAN	747.8452148
MIME_HUMAN	1952.910522
CO5A1_HUMAN	2661.856201
FLNA_HUMAN	5615.087891
ACOHC_HUMAN	550.2821045
MK_HUMAN	2240.612061
VDAC1_HUMAN	965.1209717
PGS1_HUMAN	15115.17188
TGM2_HUMAN	6527.671875
BMP6_HUMAN	895.8165283
PUR6_HUMAN	988.4581909
UBA1_HUMAN	2961.11084
GPX3_HUMAN	2641.558105
NDKB_HUMAN	8014.159668
ROA2_HUMAN	7584.995605
IBP4_HUMAN	3204.335938
QCR2_HUMAN	242.0672302
SFPQ_HUMAN	2892.043701
PPIB_HUMAN	10764.55664
SYWC_HUMAN	1158.107178
RS3_HUMAN	5307.214355
SAHH_HUMAN	4180.320313
COF1_HUMAN	25750.53906
KTHY_HUMAN	98.80123138
ATPA_HUMAN	4738.879395
PSA1_HUMAN	3546.205566
PSA2_HUMAN	688.6635742
PSA3_HUMAN	1217.680664

PSA4_HUMAN	2192.897461
PTX3_HUMAN	10363.45898
MOES_HUMAN	6448.583984
U2AF2_HUMAN	778.2843018
RL13_HUMAN	1490.282837
HMGB2_HUMAN	839.958374
PTBP1_HUMAN	771.7735596
SYVC_HUMAN	377.1485291
EF1G_HUMAN	4113.415527
HGFL_HUMAN	2274.563232
STOM_HUMAN	634.5197144
1433T_HUMAN	2920.787354
RL10_HUMAN	1041.434082
APEX1_HUMAN	1612.204834
PYR1_HUMAN	397.2430115
CALR_HUMAN	5421.804199
MAP4_HUMAN	452.0488892
CALX_HUMAN	3685.586182
PSA5_HUMAN	756.1688232
PSB6_HUMAN	914.9499512
PSB5_HUMAN	530.8051758
LYOX_HUMAN	1656.356567
MK01_HUMAN	1216.682617
GRN_HUMAN	349.4300842
AMPL_HUMAN	294.0260315
CCN2_HUMAN	68956.75
TKT_HUMAN	5388.532715
EF1D_HUMAN	1192.913208
MARCS_HUMAN	1709.028442
PRDX6_HUMAN	3383.402832
RL12_HUMAN	2739.417969
ECHM_HUMAN	264.8078308
PEBP1_HUMAN	1168.793945
PDIA3_HUMAN	7071.313477
2AAA_HUMAN	1370.908936
PURA2_HUMAN	614.0140991
METK2_HUMAN	1366.821167
QCR1_HUMAN	719.1900635
HNRH1_HUMAN	1021.734131
1433B_HUMAN	18229.2832
STIP1_HUMAN	3947.336182
S10AB_HUMAN	621.7335815
PRDX2_HUMAN	1441.85022
RL9_HUMAN	1076.00415
CADH5_HUMAN	9197.5625
RNAS4_HUMAN	206.9338074
HSP74_HUMAN	811.0973511
CTNA1_HUMAN	695.5542603
CTNB1_HUMAN	263.5911865
PHB1_HUMAN	212.3898315

SPB6_HUMAN	733.1115723
RADI_HUMAN	15279.61035
RL22_HUMAN	3474.647705
TSP4_HUMAN	1208.898193
FBN1_HUMAN	1644.967529
MYH9_HUMAN	45184.16016
TIE1_HUMAN	1814.181274
PRS7_HUMAN	593.8112793
RL4_HUMAN	13701.16699
PGM1_HUMAN	7447.887207
TAGL2_HUMAN	677.0535278
TALDO_HUMAN	4543.232422
RBMX_HUMAN	834.1369629
VATA_HUMAN	992.394104
GRP75_HUMAN	2184.354736
RS19_HUMAN	2201.583252
RL3_HUMAN	1309.573975
OST48_HUMAN	478.840271
AN32A_HUMAN	2130.333984
CAPG_HUMAN	186.186142
TCPZ_HUMAN	1518.561646
NNMT_HUMAN	45490.77734
RL13A_HUMAN	1361.375
MDHC_HUMAN	2251.384521
MDHM_HUMAN	4074.289795
ECHA_HUMAN	502.7123413
IF2G_HUMAN	1330.2948
GARS_HUMAN	595.4487305
LAP2A_HUMAN	553.3963013
PCP_HUMAN	375.5615234
MUC18_HUMAN	4449.22998
MATR3_HUMAN	452.6784058
NAMPT_HUMAN	1879.171875
VDAC2_HUMAN	1408.273193
UBP5_HUMAN	286.5124512
RAGP1_HUMAN	93.00951385
RL27A_HUMAN	2417.009766
RL5_HUMAN	1678.024292
RL21_HUMAN	221.2735901
RL28_HUMAN	578.6210938
RS9_HUMAN	3566.602783
RS5_HUMAN	7267.993164
RS10_HUMAN	4727.129395
GNPI1_HUMAN	570.7753906
IQGA1_HUMAN	3466.244629
STT3A_HUMAN	180.7371063
CAZA2_HUMAN	770.381897
CAPZB_HUMAN	3115.936523
SYQ_HUMAN	320.1565552
ATPO_HUMAN	993.5374146

LIMS1_HUMAN	585.3228149
COPD_HUMAN	141.1434784
TCPE_HUMAN	1202.121094
NEST_HUMAN	901.9248047
HSP13_HUMAN	915.0271606
IDHP_HUMAN	458.3298645
PIPNB_HUMAN	368.610199
MASP1_HUMAN	626.4691772
RL34_HUMAN	105.208374
FAS_HUMAN	2325.444092
TCPG_HUMAN	1951.657715
ARRB1_HUMAN	277.351532
EFTU_HUMAN	1208.135132
SRP09_HUMAN	1194.580933
PSB3_HUMAN	1031.988647
PSB2_HUMAN	1136.168701
COMP_HUMAN	1496.648071
GDIB_HUMAN	4352.718262
SPB8_HUMAN	240.1139221
SERPH_HUMAN	14735.83789
F10A1_HUMAN	2267.108154
RL14_HUMAN	1923.706665
TCPQ_HUMAN	3521.788086
TCPD_HUMAN	1774.880737
RAB5C_HUMAN	2055.151611
RAB7A_HUMAN	725.0809937
HDGF_HUMAN	669.4780884
ROA3_HUMAN	1864.93396
6PGD_HUMAN	4280.137695
HNRPM_HUMAN	2529.685791
GDIR1_HUMAN	4626.544434
GDIR2_HUMAN	1516.360107
CAZA1_HUMAN	3626.35376
BIEA_HUMAN	428.6748047
ACLY_HUMAN	1940.776855
COPB_HUMAN	2200.998047
COPA_HUMAN	1494.477417
CATC_HUMAN	1788.911987
TCP4_HUMAN	789.8293457
SYRC_HUMAN	585.5637817
CA2D1_HUMAN	1556.364746
UBP14_HUMAN	545.6785889
BCAT1_HUMAN	638.6177368
P5CS_HUMAN	257.7705383
PLTP_HUMAN	3019.031006
XPO2_HUMAN	1109.261719
TERA_HUMAN	6057.299805
ECHB_HUMAN	535.8422852
MANF_HUMAN	224.9619598
NP1L1_HUMAN	2839.028809

ADK_HUMAN	1013.824524
CADH6_HUMAN	688.5424805
CAD13_HUMAN	1337.051392
PSA_HUMAN	672.1749268
EIF3B_HUMAN	682.5714722
ATPK_HUMAN	877.954834
IF6_HUMAN	1425.834106
CTBP2_HUMAN	407.9659729
ARPC4_HUMAN	2453.244873
TPIS_HUMAN	3611.51001
EIF3E_HUMAN	500.815155
ACTB_HUMAN	74195.96094
IF4A1_HUMAN	7162.877441
RS20_HUMAN	2535.436523
PSA6_HUMAN	1077.769287
CDC42_HUMAN	606.3672485
DEST_HUMAN	1602.795776
RAB2A_HUMAN	681.887207
ARP3_HUMAN	2854.320068
ARP2_HUMAN	3046.686768
ARF3_HUMAN	5363.136719
RS3A_HUMAN	1683.458252
RL15_HUMAN	864.0339966
MGN_HUMAN	889.6328735
RL27_HUMAN	1977.999878
CH10_HUMAN	840.1905518
S61A1_HUMAN	212.8427429
NPC2_HUMAN	460.240509
HNRPK_HUMAN	4948.480469
1433G_HUMAN	2715.807861
RS7_HUMAN	3346.042725
PP1B_HUMAN	1470.620239
PRS4_HUMAN	537.3959961
RS8_HUMAN	2595.803711
RS15A_HUMAN	1620.625
RS16_HUMAN	3647.248535
1433E_HUMAN	5835.319824
RS14_HUMAN	2515.934814
RS23_HUMAN	1756.751587
RS18_HUMAN	2097.154785
RS13_HUMAN	2534.985352
RS11_HUMAN	1135.108765
SMD3_HUMAN	2080.646484
RL7A_HUMAN	1473.174561
RB11A_HUMAN	982.4891968
RS4X_HUMAN	2765.604736
ACTA_HUMAN	8372.207031
RL23A_HUMAN	3569.197266
RS6_HUMAN	2677.70166
H4_HUMAN	136300.1094

RAN_HUMAN	4938.024414
RL23_HUMAN	571.3800049
RS24_HUMAN	1102.718628
RS25_HUMAN	4357.880859
RS26_HUMAN	2385.605713
GBB1_HUMAN	1884.889526
RL30_HUMAN	2023.273438
RL31_HUMAN	1672.901855
RL10A_HUMAN	2258.2146
RL32_HUMAN	681.0450439
RL11_HUMAN	1328.144653
RL8_HUMAN	1093.359009
PPIA_HUMAN	3896.007568
RAC1_HUMAN	618.3418579
AP2B1_HUMAN	529.2938232
1433Z_HUMAN	7007.510254
RL38_HUMAN	1073.637451
IF5A1_HUMAN	2190.089111
RACK1_HUMAN	2461.328125
YBOX1_HUMAN	173.2458191
SC11A_HUMAN	573.0126343
TPM4_HUMAN	6292.134766
UB2L3_HUMAN	821.7375488
EF1A1_HUMAN	43417.32422
TBA1B_HUMAN	29526.64453
TBB4B_HUMAN	16700.56641
H31_HUMAN	4630.393555
TCPB_HUMAN	1548.679565
GSTO1_HUMAN	5585.90918
PRKDC_HUMAN	811.8950195
SRPX_HUMAN	8056.364746
DCD_HUMAN	511.3421326
RL24_HUMAN	1339.97998
RL36A_HUMAN	59.27401352
RL19_HUMAN	859.2910767
SRSF3_HUMAN	1403.202148
PGBM_HUMAN	51665.09766
CYC_HUMAN	2056.623779
S25A3_HUMAN	1421.608398
CLH1_HUMAN	4980.327148
FKBP3_HUMAN	1003.154846
HNRPU_HUMAN	4215.189453
SPTB2_HUMAN	1059.289307
SET_HUMAN	4417.904297
SRSF2_HUMAN	889.7253418
FABP5_HUMAN	3292.590332
CAP1_HUMAN	4125.27002
ILRL1_HUMAN	635.4550171
PFKAP_HUMAN	4080.243652
RL18A_HUMAN	1017.688538

PLOD1_HUMAN	2369.762207
NUCB1_HUMAN	1091.538452
RL6_HUMAN	3057.353027
CAV1_HUMAN	4321.51709
GLGB_HUMAN	247.6581879
IF4G1_HUMAN	217.448288
1433F_HUMAN	1258.485229
CALD1_HUMAN	319.6752625
PSME1_HUMAN	777.699585
APLP2_HUMAN	726.8964844
FMOD_HUMAN	2693.206055
PRDX1_HUMAN	4414.496582
RL18_HUMAN	2292.010498
C1QBP_HUMAN	1122.166016
CKAP4_HUMAN	1664.407837
KHDR1_HUMAN	1418.077271
LRP1_HUMAN	1073.947876
SRSF1_HUMAN	1651.242676
DHX9_HUMAN	2006.460327
TICN1_HUMAN	5104.82959
AHNK_HUMAN	1054.466675
FBLN3_HUMAN	48322.74609
FSTL1_HUMAN	2485.695801
CNTN1_HUMAN	3581.474121
ILF2_HUMAN	3074.621826
ILF3_HUMAN	3058.101074
LMAN2_HUMAN	573.4150391
TRAP1_HUMAN	19838.11914
PRDX4_HUMAN	993.2422485
CBX3_HUMAN	383.3034058
PSMD2_HUMAN	270.5742798
MMRN1_HUMAN	11059.52441
TIF1B_HUMAN	512.6724243
PTK7_HUMAN	1070.177734
ILK_HUMAN	225.3691101
SNX1_HUMAN	293.4241943
CD166_HUMAN	1040.872803
SPTN1_HUMAN	2415.401611
DX39B_HUMAN	2632.05127
TBB2A_HUMAN	2479.328125
COTL1_HUMAN	1253.177856
HNRPD_HUMAN	5867.060547
EIF3A_HUMAN	653.3885498
DPYL3_HUMAN	1418.193359
DCTN1_HUMAN	222.6083832
DYHC1_HUMAN	1630.12854
IF4A2_HUMAN	262.2170715
FLNC_HUMAN	816.338562
SPRL1_HUMAN	3229.28418
GANAB_HUMAN	2021.203003

MVP_HUMAN	1562.652222
LTBP1_HUMAN	214.2750244
LTBP2_HUMAN	3482.905029
IMB1_HUMAN	967.0493164
SEPT2_HUMAN	953.3602295
U5S1_HUMAN	179.4870911
PDIA6_HUMAN	4329.152344
PLEC_HUMAN	1886.625488
NONO_HUMAN	1833.953247
PTPA_HUMAN	220.9613647
RCN1_HUMAN	463.6332703
PCBP1_HUMAN	1641.000366
PCBP2_HUMAN	1714.684204
SF3B3_HUMAN	422.741394
RSU1_HUMAN	1254.309937
SC23A_HUMAN	121.3386459
RGN_HUMAN	1091.600952
BGH3_HUMAN	2438.272705
SEPT7_HUMAN	634.5480347
IBP7_HUMAN	15272.92871
LAMA4_HUMAN	5629.945801
EXT1_HUMAN	434.2246399
DDB1_HUMAN	880.847168
CDC37_HUMAN	1148.404053
DPYL2_HUMAN	2422.387207
ECM1_HUMAN	1058.160522
FSCN1_HUMAN	5565.726563
IF16_HUMAN	1121.609375
AOC3_HUMAN	1805.012207
TRXR1_HUMAN	3884.148438
P3H1_HUMAN	208.9597168
DHB12_HUMAN	283.4673157
CAVN1_HUMAN	1996.195557
GAK1B_HUMAN	1299.057739
CD109_HUMAN	3534.380615
RS27L_HUMAN	766.0921021
ATS13_HUMAN	1769.823608
SND1_HUMAN	2484.268066
CYFP1_HUMAN	131.4838715
CHST3_HUMAN	307.0213623
TARSH_HUMAN	953.7067261
CLC14_HUMAN	115.482338
URP2_HUMAN	981.6450806
CAND1_HUMAN	1245.391357
H2A2B_HUMAN	2150.921631
AEBP1_HUMAN	362.2242432
PLD3_HUMAN	668.5517578
GT251_HUMAN	445.6714783
TXND5_HUMAN	4274.617188
HM13_HUMAN	880.5032959

PDC6I_HUMAN	576.7105103
H1X_HUMAN	352.7082214
PXDN_HUMAN	4500.405762
HTRA1_HUMAN	4292.528809
GGH_HUMAN	2046.927124
DDX17_HUMAN	1315.459717
GSLG1_HUMAN	518.2296143
TNPO1_HUMAN	162.1396637
BHMT1_HUMAN	934.9326782
FUBP1_HUMAN	305.5662537
LRC59_HUMAN	7309.14209
KCD12_HUMAN	1819.923828
OTUB1_HUMAN	603.4699097
CNDP2_HUMAN	259.2193298
VPS35_HUMAN	558.8399048
HHIP_HUMAN	4185.474609
NEUR1_HUMAN	503.4600525
VAT1_HUMAN	3910.24292
LGMN_HUMAN	335.3608093
PHB2_HUMAN	1155.866699
HCD2_HUMAN	450.5396423
COCA1_HUMAN	220.3792877
ROAA_HUMAN	1238.569092
TCPH_HUMAN	1538.155884
ANM1_HUMAN	599.2438354
GDF15_HUMAN	2273.673584
COR1B_HUMAN	839.7717896
CAB45_HUMAN	634.7739258
PXL2A_HUMAN	1737.937866
ESYT1_HUMAN	502.1027832
CSN4_HUMAN	328.1382141
TMM43_HUMAN	176.897049
TBB6_HUMAN	4812.718262
THIC_HUMAN	1114.825684
C1QT5_HUMAN	1921.283569
C1QT3_HUMAN	901.8514404
API5_HUMAN	522.1972046
TINAL_HUMAN	765.6850586
RAB1B_HUMAN	676.2796631
NUCKS_HUMAN	195.0258636
EHD1_HUMAN	695.7759399
DIK2B_HUMAN	427.8834839
CYBP_HUMAN	175.9997559
APMAP_HUMAN	5218.399902
IL1AP_HUMAN	3803.253174
C1QR1_HUMAN	2941.545898
ESM1_HUMAN	2561.963867
TIGAR_HUMAN	391.1263123
SIAS_HUMAN	357.4997253
OLA1_HUMAN	785.989563

SEP11_HUMAN	947.9644775
PARVA_HUMAN	445.193512
STAB1_HUMAN	1444.833496
DPP3_HUMAN	436.3122864
MYOF_HUMAN	678.2793579
EHD2_HUMAN	1774.867798
CRIM1_HUMAN	360.1984863
SYLC_HUMAN	679.6157227
DKK3_HUMAN	1374.709106
CATZ_HUMAN	2365.162354
NAGK_HUMAN	504.6655884
STML2_HUMAN	217.7376099
PSME2_HUMAN	585.9897461
COR1C_HUMAN	782.5702515
EPCR_HUMAN	2343.961914
PA2G4_HUMAN	1175.267822
RUVB2_HUMAN	400.14505
CLC11_HUMAN	1666.937012
RUVB1_HUMAN	605.6737061
NUDC_HUMAN	680.6525269
VDAC3_HUMAN	403.9078369
RTCB_HUMAN	272.5708618
RL36_HUMAN	1205.440063
TLN1_HUMAN	3019.156494
LOXL2_HUMAN	4299.416504
HYOU1_HUMAN	2056.465332
SP16H_HUMAN	206.7524414
RBM8A_HUMAN	637.382019
LIPG_HUMAN	1113.102173
COPG1_HUMAN	371.5137024
CLIC4_HUMAN	1307.227539
RPSA2_HUMAN	1693.745728
RP1BL_HUMAN	511.6100464
RHOC_HUMAN	1928.784546
EIFCL_HUMAN	401.2789917
NACAM_HUMAN	19012.28125
BACH_HUMAN	1614.703247
MYO1C_HUMAN	1557.118042
PSD11_HUMAN	278.9949036
PSD12_HUMAN	340.1757813
CLIC1_HUMAN	9516.397461
QSOX1_HUMAN	13901.29199
IPO5_HUMAN	641.9438477
PLOD2_HUMAN	719.6671143
NOP56_HUMAN	42.91609573
DDX3X_HUMAN	449.4296265
CCN1_HUMAN	9145.09668
PIR_HUMAN	428.0256653
TPP1_HUMAN	1576.157227
NRP1_HUMAN	1069.04834

PSA7_HUMAN	2598.384521
ML12B_HUMAN	3696.347656
HNRDL_HUMAN	1088.557617
XPO1_HUMAN	211.3224182
ANGP2_HUMAN	3376.962891
ARC1B_HUMAN	909.0942993
ARPC2_HUMAN	1820.521729
ARPC3_HUMAN	578.5040894
MATN3_HUMAN	616.4494629
DHX15_HUMAN	454.1141052
PSMD3_HUMAN	393.4101257
HNRPR_HUMAN	3119.926514
TXNL1_HUMAN	1427.557861
BUB3_HUMAN	580.9683838
ACTN4_HUMAN	12613.08203
NRP2_HUMAN	665.6464233
HNRPQ_HUMAN	1880.242432
PLOD3_HUMAN	761.592041
H2B1K_HUMAN	48830.62109
WDR1_HUMAN	4519.055176
FLNB_HUMAN	7742.322266
CISY_HUMAN	2186.772217
VP26A_HUMAN	870.9400635
NDUS3_HUMAN	225.6147156
IDHC_HUMAN	2766.864258
PRAF3_HUMAN	202.6333771
DYSF_HUMAN	214.6971588
SC31A_HUMAN	352.4536743
CSTN1_HUMAN	3665.467529
PRS23_HUMAN	931.395874
APOM_HUMAN	2594.989502
VNN1_HUMAN	1089.361084
AP2A1_HUMAN	312.3070374
CAVN2_HUMAN	262.2897949
LDHA_HUMAN	8877.400391
AL1A1_HUMAN	1020.053467
NB5R3_HUMAN	1131.704468
COX2_HUMAN	554.0586548
F13A_HUMAN	1501.969971
PNPH_HUMAN	3929.82373
HPRT_HUMAN	469.4165649
AATM_HUMAN	1564.03418
PGK1_HUMAN	11351.7207
KAD1_HUMAN	1412.563599
FA10_HUMAN	8157.938477
TPA_HUMAN	2372.815918
CO3_HUMAN	143975.3281
TIMP1_HUMAN	3280.719482
CYTC_HUMAN	1649.90332
CO1A1_HUMAN	9336.769531

CO2A1_HUMAN	1769.202637
LMNA_HUMAN	4347.17041
FINC_HUMAN	47320.44922
RET4_HUMAN	17750.88477
ALBU_HUMAN	11505.3125
MMP1_HUMAN	68536.1875
ALDOA_HUMAN	9725.091797
ANXA1_HUMAN	5347.162598
APOB_HUMAN	18001.00391
VWF_HUMAN	29636.53906
G3P_HUMAN	13987.75879
HLAA_HUMAN	1042.38501
HSPB1_HUMAN	3831.208252
RPN1_HUMAN	778.68927
RPN2_HUMAN	359.2077942
GNAI2_HUMAN	1116.97583
H2A1B_HUMAN	94757.78125
AT1A1_HUMAN	447.2116699
A4_HUMAN	2885.596191
ALDH2_HUMAN	495.6046753
S10A8_HUMAN	98.35312653
HMGN1_HUMAN	108.1361084
PAI1_HUMAN	41779.78906
ADT2_HUMAN	932.2803345
IF2A_HUMAN	689.605896
EDN1_HUMAN	1232.344604
RLA0_HUMAN	4182.736328
LA_HUMAN	274.1867065
ITB1_HUMAN	1127.096802
K1C18_HUMAN	320.9403076
GELS_HUMAN	4124.463379
PTMA_HUMAN	1417.502563
ATPB_HUMAN	3898.630615
S10A6_HUMAN	1738.505737
KCRM_HUMAN	137.5260162
ENOA_HUMAN	33376.46094
G6PI_HUMAN	3568.492432
NPM_HUMAN	5034.68457
TPM3_HUMAN	2432.756348
HEXA_HUMAN	622.6618652
H2B1J_HUMAN	3779.055664
LDHB_HUMAN	10511.79102
GPX1_HUMAN	358.4765625
PDIA1_HUMAN	2931.292236
CATD_HUMAN	3709.961426
ANXA2_HUMAN	14193.14551
CAN1_HUMAN	720.161438
TBB5_HUMAN	7530.641113
SAP_HUMAN	4827.777832
HEXB_HUMAN	1725.936035

PROF1_HUMAN	5065.355957
SYEP_HUMAN	577.8734741
CATB_HUMAN	6509.582031
HS90A_HUMAN	11775.22266
HNRPC_HUMAN	4622.687988
LAMB1_HUMAN	4053.396729
TPM2_HUMAN	1630.079468
FUMH_HUMAN	408.0806274
TSP1_HUMAN	317516.0313
RNAS1_HUMAN	576.911438
CO1A2_HUMAN	3075.531738
ANXA6_HUMAN	2232.839355
HS90B_HUMAN	23015.58398
MMP2_HUMAN	12532.05762
CO4A2_HUMAN	1978.50415
RU17_HUMAN	1143.968872
ITA5_HUMAN	834.2460938
VIME_HUMAN	13645.94824
RS17_HUMAN	1064.509033
ANXA5_HUMAN	5970.687012
SNRPA_HUMAN	339.6967163
GSTP1_HUMAN	1484.085083
HMGB1_HUMAN	1531.287476
SPRC_HUMAN	5170.64209
ANXA4_HUMAN	104.9907913
ROA1_HUMAN	3648.148193
COX6C_HUMAN	435.4682617
LKHA4_HUMAN	1130.832886
UBB_HUMAN	2900.016113
HS71A_HUMAN	2487.615723
TBA3C_HUMAN	3484.834229
SRGN_HUMAN	4511.373047
PTPRF_HUMAN	762.7776489
PPGB_HUMAN	484.8363037
TFPI1_HUMAN	990.1272583
CH60_HUMAN	3070.749268
CLUS_HUMAN	3364.718994
BIP_HUMAN	4386.425781
LAMC1_HUMAN	1857.135254
HSP7C_HUMAN	12862.22852
RALA_HUMAN	1683.246582
LAMP1_HUMAN	1743.950928
G6PD_HUMAN	1357.657715
C1TC_HUMAN	583.8678589
MPRI_HUMAN	1701.296021
ADHX_HUMAN	1798.539795
PABP1_HUMAN	2186.521484
PCNA_HUMAN	601.6026001
COBA1_HUMAN	2183.834717
CO6A1_HUMAN	12806.60449

CO6A3_HUMAN	751.9838867
ADT3_HUMAN	1824.965088
IMDH2_HUMAN	184.0119476
ANXA3_HUMAN	734.8731079
ACTN1_HUMAN	9233.276367
ACE_HUMAN	508.6287842
XRCC6_HUMAN	2764.541748
XRCC5_HUMAN	1213.912598
COX41_HUMAN	704.9297485
LAMP2_HUMAN	2823.036865
RINI_HUMAN	2097.933838
EF2_HUMAN	8534.604492
PDIA4_HUMAN	1964.775635
PLST_HUMAN	10387.29883
CD59_HUMAN	686.8573608
GLU2B_HUMAN	411.5352783
FPPS_HUMAN	1982.035522
NID1_HUMAN	1170.626709
KPYM_HUMAN	30898.75
ENPL_HUMAN	4636.650391
HNRPL_HUMAN	386.1684265
SYDC_HUMAN	417.3891907
FABP4_HUMAN	1830.699585
ALDR_HUMAN	2048.579346
AMPN_HUMAN	1773.11731
RAC2_HUMAN	168.9186707
ERF3A_HUMAN	182.7307434
EZRI_HUMAN	541.2485352
NDKA_HUMAN	965.8809814
GNS_HUMAN	845.4101563
RS2_HUMAN	2089.415527
TIMP2_HUMAN	1120.56958
PECA1_HUMAN	864.0609131
H15_HUMAN	13772.28418
H13_HUMAN	14174.92969
H12_HUMAN	2305.976318
FAAA_HUMAN	394.4452515
STMN1_HUMAN	2149.07959
HMGA1_HUMAN	3319.036377
ITA2_HUMAN	249.6974335
CAN2_HUMAN	1090.188965
DDX5_HUMAN	2316.052002
PFKAL_HUMAN	162.948349
VGFR1_HUMAN	925.2145386
PRS6A_HUMAN	950.4815063
TCPA_HUMAN	1171.395996
RL35A_HUMAN	1462.550659
ARF4_HUMAN	1158.325195
RL7_HUMAN	1854.690186
VINC_HUMAN	4093.808594

RL17_HUMAN	1749.774414
PGAM1_HUMAN	3860.130127
RCC1_HUMAN	321.671051
AMD_HUMAN	1076.913574
NUCL_HUMAN	4318.154785
HXK1_HUMAN	571.5296021
SPEE_HUMAN	2048.436768
IF2B_HUMAN	233.9321747
TCO2_HUMAN	611.5981445
PSB1_HUMAN	1931.701538
LMNB1_HUMAN	585.6716919
MIME_HUMAN	1806.49292
CO5A1_HUMAN	2858.507568
FLNA_HUMAN	5681.981934
ACOHC_HUMAN	662.2322388
MK_HUMAN	2971.597656
VDAC1_HUMAN	523.555542
PGS1_HUMAN	15044.20117
TGM2_HUMAN	5636.960938
BMP6_HUMAN	689.5809326
PUR6_HUMAN	883.8922119
UBA1_HUMAN	3264.900146
GPX3_HUMAN	2487.106689
NDKB_HUMAN	9249.026367
ROA2_HUMAN	7330.932617
IBP4_HUMAN	6137.099609
QCR2_HUMAN	129.7743073
SFPQ_HUMAN	2591.067871
PPIB_HUMAN	8393.232422
SYWC_HUMAN	1347.703979
RS3_HUMAN	5531.327148
SAHH_HUMAN	4240.98877
COF1_HUMAN	31159.05078
KTHY_HUMAN	135.0881042
ATPA_HUMAN	3649.338379
PSA1_HUMAN	2828.261719
PSA2_HUMAN	694.8858643
PSA3_HUMAN	1324.917725
PSA4_HUMAN	2087.784668
PTX3_HUMAN	12813.51367
MOES_HUMAN	7183.614258
U2AF2_HUMAN	437.6144714
RL13_HUMAN	1497.513794
HMGB2_HUMAN	693.9642334
PTBP1_HUMAN	804.4356079
SYVC_HUMAN	513.4711914
EF1G_HUMAN	4363.640625
HGFL_HUMAN	1974.596069
STOM_HUMAN	484.9358521
1433T_HUMAN	2608.231689

RL10_HUMAN	619.8775024
APEX1_HUMAN	1548.106201
PYR1_HUMAN	335.0799255
CALR_HUMAN	2763.690186
MAP4_HUMAN	400.8963928
CALX_HUMAN	1926.006226
PSA5_HUMAN	750.3946533
PSB6_HUMAN	431.9717712
PSB5_HUMAN	570.2929077
LYOX_HUMAN	1822.344971
MK01_HUMAN	1032.737549
GRN_HUMAN	367.4227295
AMPL_HUMAN	352.7225037
CCN2_HUMAN	93298.01563
TKT_HUMAN	6990.251465
EF1D_HUMAN	1136.636841
MARCS_HUMAN	1723.879639
PRDX6_HUMAN	2925.083496
RL12_HUMAN	3318.59082
ECHM_HUMAN	173.8428802
PEBP1_HUMAN	1633.58606
PDIA3_HUMAN	5151.748535
2AAA_HUMAN	1124.930786
PURA2_HUMAN	679.2930298
METK2_HUMAN	1376.685913
QCR1_HUMAN	237.022522
HNRH1_HUMAN	599.7583618
1433B_HUMAN	19086.42969
STIP1_HUMAN	3357.137695
S10AB_HUMAN	736.1329956
PRDX2_HUMAN	1206.715454
RL9_HUMAN	1199.351685
CADH5_HUMAN	9961.628906
RNAS4_HUMAN	133.9893951
HSP74_HUMAN	819.9574585
CTNA1_HUMAN	741.1565552
CTNB1_HUMAN	254.1412811
PHB1_HUMAN	313.9410095
SPB6_HUMAN	427.9250793
RADI_HUMAN	18096.0625
RL22_HUMAN	4405.510742
TSP4_HUMAN	1209.904907
FBN1_HUMAN	1167.341309
MYH9_HUMAN	40231.80469
TIE1_HUMAN	1897.354248
PRS7_HUMAN	365.7637329
RL4_HUMAN	13289.03711
PGM1_HUMAN	6717.69043
TAGL2_HUMAN	478.7745056
TALDO_HUMAN	4346.354004

RBMX_HUMAN	793.9509888
VATA_HUMAN	661.8981323
GRP75_HUMAN	2066.738037
RS19_HUMAN	2621.620361
RL3_HUMAN	1074.552368
OST48_HUMAN	341.2605591
AN32A_HUMAN	2435.405762
CAPG_HUMAN	142.0580444
TCPZ_HUMAN	1581.952637
NNMT_HUMAN	31156.54883
RL13A_HUMAN	1548.093628
MDHC_HUMAN	2483.400879
MDHM_HUMAN	5076.902832
ECHA_HUMAN	450.1549072
IF2G_HUMAN	1493.625732
GARS_HUMAN	661.1656494
LAP2A_HUMAN	760.4229126
PCP_HUMAN	395.1173401
MUC18_HUMAN	4420.805176
MATR3_HUMAN	355.7608032
NAMPT_HUMAN	2174.79834
VDAC2_HUMAN	783.0380249
UBP5_HUMAN	222.3736115
RAGP1_HUMAN	114.5125885
RL27A_HUMAN	1743.296143
RL5_HUMAN	1295.244751
RL21_HUMAN	171.6714935
RL28_HUMAN	447.9672852
RS9_HUMAN	2926.651123
RS5_HUMAN	9457.665039
RS10_HUMAN	6174.353516
GNPI1_HUMAN	423.9930115
IQGA1_HUMAN	3692.812988
STT3A_HUMAN	63.94773865
CAZA2_HUMAN	873.468689
CAPZB_HUMAN	3366.405518
SYQ_HUMAN	217.2220459
ATPO_HUMAN	437.6189575
LIMS1_HUMAN	608.4707642
COPD_HUMAN	148.1724701
TCPE_HUMAN	1472.901733
NEST_HUMAN	345.1431274
HSP13_HUMAN	1336.953857
IDHP_HUMAN	346.7144165
PIPNB_HUMAN	240.2961426
MASP1_HUMAN	705.4622192
RL34_HUMAN	91.48058319
FAS_HUMAN	2086.199463
TCPG_HUMAN	1884.067993
ARRB1_HUMAN	341.1914978

EFTU_HUMAN	964.8968506
SRP09_HUMAN	976.9663696
PSB3_HUMAN	1201.801147
PSB2_HUMAN	1149.916748
COMP_HUMAN	1550.075195
GDIB_HUMAN	5198.436523
SPB8_HUMAN	325.3709717
SERPH_HUMAN	7942.367676
F10A1_HUMAN	2924.536133
RL14_HUMAN	1682.439941
TCPQ_HUMAN	3708.264404
TCPD_HUMAN	1875.660522
RAB5C_HUMAN	1229.959839
RAB7A_HUMAN	514.4146729
HDGF_HUMAN	220.2229919
ROA3_HUMAN	1679.059326
6PGD_HUMAN	4438.503906
HNRPM_HUMAN	1607.17688
GDIR1_HUMAN	4548.513184
GDIR2_HUMAN	1781.548706
CAZA1_HUMAN	3991.929443
BIEA_HUMAN	129.5679779
ACLY_HUMAN	1911.202393
COPB_HUMAN	1672.977539
COPA_HUMAN	1425.690674
CATC_HUMAN	1170.070679
TCP4_HUMAN	591.1845093
SYRC_HUMAN	600.9719849
CA2D1_HUMAN	2008.658691
UBP14_HUMAN	1183.650635
BCAT1_HUMAN	471.1759949
P5CS_HUMAN	157.01474
PLTP_HUMAN	2872.899658
XPO2_HUMAN	1158.993042
TERA_HUMAN	6660.069336
ECHB_HUMAN	397.5306702
MANF_HUMAN	238.4068604
NP1L1_HUMAN	3101.882324
ADK_HUMAN	837.5783691
CADH6_HUMAN	421.7506104
CAD13_HUMAN	1026.747925
PSA_HUMAN	883.0192261
EIF3B_HUMAN	820.7434692
ATPK_HUMAN	466.7922363
IF6_HUMAN	1255.864502
CTBP2_HUMAN	191.5936279
ARPC4_HUMAN	2913.656006
TPIS_HUMAN	4753.880371
EIF3E_HUMAN	555.1511841
ACTB_HUMAN	70365.05469

IF4A1_HUMAN	6516.351563
RS20_HUMAN	2744.744385
PSA6_HUMAN	1100.188477
CDC42_HUMAN	422.9693604
DEST_HUMAN	2109.752197
RAB2A_HUMAN	627.352478
ARP3_HUMAN	3240.558838
ARP2_HUMAN	2970.054932
ARF3_HUMAN	5047.631348
RS3A_HUMAN	1602.098633
RL15_HUMAN	506.8128967
MGN_HUMAN	1841.693604
RL27_HUMAN	1948.335571
CH10_HUMAN	672.8605347
S61A1_HUMAN	101.6195831
NPC2_HUMAN	855.9363403
HNRPK_HUMAN	4473.815918
1433G_HUMAN	3026.237793
RS7_HUMAN	3641.537109
PP1B_HUMAN	1687.274536
PRS4_HUMAN	615.394165
RS8_HUMAN	3314.647949
RS15A_HUMAN	1825.093506
RS16_HUMAN	3327.010986
1433E_HUMAN	6486.841309
RS14_HUMAN	2823.34668
RS23_HUMAN	1281.444946
RS18_HUMAN	1447.949341
RS13_HUMAN	2340.981934
RS11_HUMAN	853.9429932
SMD3_HUMAN	2208.277344
RL7A_HUMAN	1115.450684
RB11A_HUMAN	693.2764282
RS4X_HUMAN	2601.763916
ACTA_HUMAN	8822.827148
RL23A_HUMAN	2987.629883
RS6_HUMAN	2118.641846
H4_HUMAN	147665.1719
RAN_HUMAN	4744.077148
RL23_HUMAN	353.7181091
RS24_HUMAN	1315.904419
RS25_HUMAN	3726.214355
RS26_HUMAN	2031.751343
GBB1_HUMAN	1660.005981
RL30_HUMAN	2212.008301
RL31_HUMAN	1749.023193
RL10A_HUMAN	2573.772217
RL32_HUMAN	489.6262512
RL11_HUMAN	1559.17041
RL8_HUMAN	1038.349487

PPIA_HUMAN	3271.435059
RAC1_HUMAN	741.8184814
AP2B1_HUMAN	506.9502563
1433Z_HUMAN	7822.038574
RL38_HUMAN	822.0158081
IF5A1_HUMAN	2405.428223
RACK1_HUMAN	2445.724609
YBOX1_HUMAN	152.6263123
SC11A_HUMAN	238.0301819
TPM4_HUMAN	6568.649902
UB2L3_HUMAN	977.680481
EF1A1_HUMAN	49716.39453
TBA1B_HUMAN	27993.58008
TBB4B_HUMAN	14834.50977
H31_HUMAN	4934.140625
TCPB_HUMAN	1710.073242
GSTO1_HUMAN	6254.086426
PRKDC_HUMAN	677.7877197
SRPX_HUMAN	7825.868652
DCD_HUMAN	1037.703979
RL24_HUMAN	1133.987671
RL36A_HUMAN	92.61239624
RL19_HUMAN	686.9300537
SRSF3_HUMAN	1493.122925
PGBM_HUMAN	58741.49609
CYC_HUMAN	2749.305176
S25A3_HUMAN	547.6260986
CLH1_HUMAN	4710.623535
FKBP3_HUMAN	590.8217773
HNRPU_HUMAN	3002.807129
SPTB2_HUMAN	950.0956421
SET_HUMAN	4702.792969
SRSF2_HUMAN	773.1519775
FABP5_HUMAN	4530.266602
CAP1_HUMAN	4000.218018
ILRL1_HUMAN	587.6832275
PFKAP_HUMAN	4287.577637
RL18A_HUMAN	653.1313477
PLOD1_HUMAN	2180.091309
NUCB1_HUMAN	405.7843628
RL6_HUMAN	3791.693115
CAV1_HUMAN	2317.5625
GLGB_HUMAN	307.8185425
IF4G1_HUMAN	291.9194336
1433F_HUMAN	1515.944092
CALD1_HUMAN	262.37677
PSME1_HUMAN	1173.514526
APLP2_HUMAN	345.5240784
FMOD_HUMAN	2652.697021
PRDX1_HUMAN	3811.711914

RL18_HUMAN	2272.040527
C1QBP_HUMAN	681.8895874
CKAP4_HUMAN	899.4830322
KHDR1_HUMAN	1416.190552
LRP1_HUMAN	1112.782349
SRSF1_HUMAN	1565.921997
DHX9_HUMAN	1826.800049
TICN1_HUMAN	5245.485352
AHNK_HUMAN	845.2067261
FBLN3_HUMAN	68450.6875
FSTL1_HUMAN	2287.068115
CNTN1_HUMAN	3427.460449
ILF2_HUMAN	3622.490967
ILF3_HUMAN	3391.987793
LMAN2_HUMAN	317.6166992
TRAP1_HUMAN	17901.06836
PRDX4_HUMAN	648.026123
CBX3_HUMAN	308.7340393
PSMD2_HUMAN	215.81633
MMRN1_HUMAN	11835.23145
TIF1B_HUMAN	324.3685608
PTK7_HUMAN	985.8502808
ILK_HUMAN	221.2397156
SNX1_HUMAN	198.0075073
CD166_HUMAN	1058.014038
SPTN1_HUMAN	2370.445313
DX39B_HUMAN	2211.576904
TBB2A_HUMAN	1150.372803
COTL1_HUMAN	1279.341064
HNRPD_HUMAN	5343.050781
EIF3A_HUMAN	626.8852539
DPYL3_HUMAN	1247.424927
DCTN1_HUMAN	190.1305542
DYHC1_HUMAN	1195.528687
IF4A2_HUMAN	320.3069458
FLNC_HUMAN	792.6472168
SPRL1_HUMAN	3743.700195
GANAB_HUMAN	1350.807373
MVP_HUMAN	1409.644409
LTBP1_HUMAN	317.0699158
LTBP2_HUMAN	3920.710205
IMB1_HUMAN	1106.487549
SEPT2_HUMAN	977.62323
U5S1_HUMAN	172.7133636
PDIA6_HUMAN	2929.40625
PLEC_HUMAN	1646.303345
NONO_HUMAN	1537.688599
PTPA_HUMAN	376.9091187
RCN1_HUMAN	110.7723007
PCBP1_HUMAN	1686.564819

PCBP2_HUMAN	1734.583618
SF3B3_HUMAN	498.5174561
RSU1_HUMAN	737.2111816
SC23A_HUMAN	423.1803284
RGN_HUMAN	1228.755127
BGH3_HUMAN	2617.532471
SEPT7_HUMAN	385.7767944
IBP7_HUMAN	17587.08594
LAMA4_HUMAN	5577.243652
EXT1_HUMAN	157.0110931
DDB1_HUMAN	846.0720215
CDC37_HUMAN	1230.521606
DPYL2_HUMAN	2154.186035
ECM1_HUMAN	998.8703613
FSCN1_HUMAN	7246.993652
IF16_HUMAN	1560.517822
AOC3_HUMAN	1560.441406
TRXR1_HUMAN	4568.631836
P3H1_HUMAN	151.0261536
CAVN1_HUMAN	1609.355713
GAK1B_HUMAN	1359.871338
CD109_HUMAN	3900.651855
RS27L_HUMAN	206.5479431
ATS13_HUMAN	1687.759766
SND1_HUMAN	2532.438721
CYFP1_HUMAN	225.1672668
CHST3_HUMAN	459.5351563
TARSH_HUMAN	963.1636963
CLC14_HUMAN	161.1808014
URP2_HUMAN	1192.254639
CAND1_HUMAN	1327.721436
H2A2B_HUMAN	2833.568604
AEBP1_HUMAN	470.9389038
PLD3_HUMAN	465.775177
GT251_HUMAN	279.8321228
TXND5_HUMAN	2391.860107
HM13_HUMAN	377.0223694
PDC6I_HUMAN	580.3969727
H1X_HUMAN	471.5856628
PXDN_HUMAN	4465.717773
HTRA1_HUMAN	4736.951172
GGH_HUMAN	2043.717407
DDX17_HUMAN	1223.184814
GSLG1_HUMAN	432.3897095
TNPO1_HUMAN	282.9736633
BHMT1_HUMAN	739.8029175
FUBP1_HUMAN	309.5687866
LRC59_HUMAN	9063.054688
KCD12_HUMAN	2016.481445
OTUB1_HUMAN	709.7212524

CNDP2_HUMAN	360.8469849
VPS35_HUMAN	459.551178
HHIP_HUMAN	4381.407715
NEUR1_HUMAN	763.1799316
VAT1_HUMAN	4149.863281
LGMN_HUMAN	571.75354
PHB2_HUMAN	673.6376953
HCD2_HUMAN	304.0643616
COCA1_HUMAN	366.4337463
ROAA_HUMAN	1431.698242
TCPH_HUMAN	1326.078857
ANM1_HUMAN	584.2424927
GDF15_HUMAN	2268.174316
COR1B_HUMAN	489.5762024
CAB45_HUMAN	334.471283
PXL2A_HUMAN	859.7903442
ESYT1_HUMAN	585.3363037
CSN4_HUMAN	240.1428833
TMM43_HUMAN	223.4441528
TBB6_HUMAN	3339.896484
THIC_HUMAN	1298.972656
C1QT5_HUMAN	1734.542847
C1QT3_HUMAN	887.8973999
API5_HUMAN	757.8132324
TINAL_HUMAN	727.449707
RAB1B_HUMAN	289.1043396
NUCKS_HUMAN	229.7606354
EHD1_HUMAN	744.3656006
DIK2B_HUMAN	442.5183411
CYBP_HUMAN	180.0259705
APMAP_HUMAN	5884.98877
IL1AP_HUMAN	3430.905762
C1QR1_HUMAN	1073.662476
ESM1_HUMAN	830.2174072
TIGAR_HUMAN	282.7289734
SIAS_HUMAN	178.3443909
OLA1_HUMAN	778.8261719
SEP11_HUMAN	1074.45813
PARVA_HUMAN	488.9445496
STAB1_HUMAN	2803.579346
DPP3_HUMAN	300.2736206
MYOF_HUMAN	352.1542358
EHD2_HUMAN	1470.692383
CRIM1_HUMAN	283.880188
SYLC_HUMAN	655.5585938
DKK3_HUMAN	935.6370239
CATZ_HUMAN	2862.098389
NAGK_HUMAN	477.7970276
STML2_HUMAN	99.53927612
PSME2_HUMAN	390.1514587

COR1C_HUMAN	799.4575195
EPCR_HUMAN	1995.199463
PA2G4_HUMAN	1112.202515
RUVB2_HUMAN	249.09729
CLC11_HUMAN	1197.105713
RUVB1_HUMAN	540.4093628
NUDC_HUMAN	709.9041138
VDAC3_HUMAN	410.2714233
RTCB_HUMAN	131.9824066
RL36_HUMAN	994.671936
TLN1_HUMAN	3245.521484
LOXL2_HUMAN	4606.319336
HYOU1_HUMAN	1214.915771
SP16H_HUMAN	481.586731
RBM8A_HUMAN	459.4204407
LIPG_HUMAN	1444.34375
COPG1_HUMAN	342.5568542
CLIC4_HUMAN	1074.739136
