# Supplementary material for: Proteomic Analysis of Endothelial Activation Induced by Adult Angiostrongylus vasorum Homogenate: Insights into Vascular Remodeling and Hemostatic Imbalance
Source: Animals (Basel). 2026 Mar 15;16(6):926. doi: 10.3390/ani16060926 (PMC13023303; doi:10.3390/ani16060926)
Supplement: Supplementary file 1 [file animals-16-00926-s001.zip › Supplmentary Table S4.pdf]

Supplementary Table S4: Proteins identified in the supernatants of the thre

R.Condition	R.Replicate	PG.ProteinAccessions
Control	1	A0A8I5KQE6
Control	1	A6NIZ1
Control	1	P08134
Control	1	B5ME19
Control	1	E9PAV3
Control	1	O00154
Control	1	O00159
Control	1	O00231
Control	1	O00232
Control	1	O00299
Control	1	O00391
Control	1	O00410
Control	1	O00469
Control	1	O00571
Control	1	O00622
Control	1	O00625
Control	1	O14773
Control	1	O14786
Control	1	O14818
Control	1	O14950
Control	1	O14979
Control	1	O14980
Control	1	O15123
Control	1	O15143
Control	1	O15144
Control	1	O15145
Control	1	O15232
Control	1	O43143
Control	1	O43242
Control	1	O43390
Control	1	O43396
Control	1	O43684
Control	1	O43707
Control	1	O60462
Control	1	O60506
Control	1	O60568
Control	1	O60814
Control	1	O75083
Control	1	O75131
Control	1	O75369
Control	1	O75390
Control	1	O75436
Control	1	O75489
Control	1	O75874
Control	1	O75915
Control	1	O75923
Control	1	O94979
Control	1	O94985

Control	1	O95084
Control	1	O95445
Control	1	O95497
Control	1	O95782
Control	1	O95810
Control	1	P00338
Control	1	P00352
Control	1	P00387
Control	1	P00403
Control	1	P00488
Control	1	P00491
Control	1	P00492
Control	1	P00505
Control	1	P00558
Control	1	P00568
Control	1	P00742
Control	1	P00750
Control	1	P01024
Control	1	P01033
Control	1	P01034
Control	1	P02452
Control	1	P02458
Control	1	P02545
Control	1	P02751
Control	1	P02753
Control	1	P02768
Control	1	P03956
Control	1	P04075
Control	1	P04083
Control	1	P04114
Control	1	P04275
Control	1	P04406
Control	1	P04439
Control	1	P04792
Control	1	P04843
Control	1	P04844
Control	1	P04899
Control	1	P04908
Control	1	P05023
Control	1	P05067
Control	1	P05091
Control	1	P05109
Control	1	P05114
Control	1	P05121
Control	1	P05141
Control	1	P05198
Control	1	P05305
Control	1	P05388
Control	1	P05455
Control	1	P05556

Control	1	P05783
Control	1	P06396
Control	1	P06454
Control	1	P06576
Control	1	P06703
Control	1	P06732
Control	1	P06733
Control	1	P06744
Control	1	P06748
Control	1	P06753
Control	1	P06865
Control	1	P06899
Control	1	P07195
Control	1	P07203
Control	1	P07237
Control	1	P07339
Control	1	P07355
Control	1	P07384
Control	1	P07437
Control	1	P07602
Control	1	P07686
Control	1	P07737
Control	1	P07814
Control	1	P07858
Control	1	P07900
Control	1	P07910
Control	1	P07942
Control	1	P07951
Control	1	P07954
Control	1	P07996
Control	1	P07998
Control	1	P08123
Control	1	P08133
Control	1	P08238
Control	1	P08253
Control	1	P08572
Control	1	P08621
Control	1	P08648
Control	1	P08670
Control	1	P08708
Control	1	P08758
Control	1	P09012
Control	1	P09211
Control	1	P09429
Control	1	P09486
Control	1	P09525
Control	1	P09651
Control	1	P09669
Control	1	P09960
Control	1	P0CG47

Control	1	P0DMV8
Control	1	P0DPH7
Control	1	P10124
Control	1	P10586
Control	1	P10619
Control	1	P10646
Control	1	P10809
Control	1	P10909
Control	1	P11021
Control	1	P11047
Control	1	P11142
Control	1	P11233
Control	1	P11279
Control	1	P11413
Control	1	P11586
Control	1	P11717
Control	1	P11766
Control	1	P11940
Control	1	P12004
Control	1	P12107
Control	1	P12109
Control	1	P12111
Control	1	P12236
Control	1	P12268
Control	1	P12429
Control	1	P12814
Control	1	P12821
Control	1	P12956
Control	1	P13010
Control	1	P13073
Control	1	P13473
Control	1	P13489
Control	1	P13639
Control	1	P13667
Control	1	P13797
Control	1	P13987
Control	1	P14314
Control	1	P14324
Control	1	P14543
Control	1	P14618
Control	1	P14625
Control	1	P14866
Control	1	P14868
Control	1	P15090
Control	1	P15121
Control	1	P15144
Control	1	P15153
Control	1	P15170
Control	1	P15311
Control	1	P15531

Control	1	P15586
Control	1	P15880
Control	1	P16035
Control	1	P16284
Control	1	P16401
Control	1	P16402
Control	1	P16403
Control	1	P16930
Control	1	P16949
Control	1	P17096
Control	1	P17301
Control	1	P17655
Control	1	P17844
Control	1	P17858
Control	1	P17948
Control	1	P17980
Control	1	P17987
Control	1	P18077
Control	1	P18085
Control	1	P18124
Control	1	P18206
Control	1	P18621
Control	1	P18669
Control	1	P18754
Control	1	P19021
Control	1	P19338
Control	1	P19367
Control	1	P19623
Control	1	P20042
Control	1	P20062
Control	1	P20618
Control	1	P20700
Control	1	P20774
Control	1	P20908
Control	1	P21333
Control	1	P21399
Control	1	P21741
Control	1	P21796
Control	1	P21810
Control	1	P21980
Control	1	P22004
Control	1	P22234
Control	1	P22314
Control	1	P22352
Control	1	P22392
Control	1	P22626
Control	1	P22692
Control	1	P22695
Control	1	P23246
Control	1	P23284

Control	1	P23381
Control	1	P23396
Control	1	P23526
Control	1	P23528
Control	1	P23919
Control	1	P25705
Control	1	P25786
Control	1	P25787
Control	1	P25788
Control	1	P25789
Control	1	P26022
Control	1	P26038
Control	1	P26368
Control	1	P26373
Control	1	P26583
Control	1	P26599
Control	1	P26640
Control	1	P26641
Control	1	P26927
Control	1	P27105
Control	1	P27348
Control	1	P27635
Control	1	P27695
Control	1	P27708
Control	1	P27797
Control	1	P27816
Control	1	P27824
Control	1	P28066
Control	1	P28072
Control	1	P28074
Control	1	P28300
Control	1	P28482
Control	1	P28838
Control	1	P29279
Control	1	P29401
Control	1	P29692
Control	1	P29966
Control	1	P30041
Control	1	P30050
Control	1	P30086
Control	1	P30101
Control	1	P30153
Control	1	P30520
Control	1	P31153
Control	1	P31930
Control	1	P31943
Control	1	P31946
Control	1	P31948
Control	1	P31949
Control	1	P32119

Control	1	P32969
Control	1	P33151
Control	1	P34096
Control	1	P34932
Control	1	P35221
Control	1	P35222
Control	1	P35232
Control	1	P35237
Control	1	P35241
Control	1	P35268
Control	1	P35443
Control	1	P35555
Control	1	P35579
Control	1	P35590
Control	1	P35998
Control	1	P36578
Control	1	P36871
Control	1	P37802
Control	1	P37837
Control	1	P38159
Control	1	P38606
Control	1	P38646
Control	1	P39019
Control	1	P39023
Control	1	P39656
Control	1	P39687
Control	1	P40227
Control	1	P40261
Control	1	P40429
Control	1	P40925
Control	1	P40926
Control	1	P40939
Control	1	P41091
Control	1	P41250
Control	1	P42166
Control	1	P42785
Control	1	P43121
Control	1	P43243
Control	1	P43490
Control	1	P45880
Control	1	P45974
Control	1	P46060
Control	1	P46776
Control	1	P46777
Control	1	P46778
Control	1	P46779
Control	1	P46781
Control	1	P46782
Control	1	P46783
Control	1	P46926

Control	1	P46940
Control	1	P46977
Control	1	P47755
Control	1	P47756
Control	1	P47897
Control	1	P48047
Control	1	P48059
Control	1	P48444
Control	1	P48643
Control	1	P48681
Control	1	P48723
Control	1	P48735
Control	1	P48739
Control	1	P48740
Control	1	P49207
Control	1	P49327
Control	1	P49368
Control	1	P49407
Control	1	P49411
Control	1	P49458
Control	1	P49720
Control	1	P49721
Control	1	P49747
Control	1	P50395
Control	1	P50454
Control	1	P50502
Control	1	P50914
Control	1	P50990
Control	1	P50991
Control	1	P51148
Control	1	P51149
Control	1	P51858
Control	1	P51991
Control	1	P52209
Control	1	P52272
Control	1	P52565
Control	1	P52566
Control	1	P52907
Control	1	P53004
Control	1	P53396
Control	1	P53618
Control	1	P53621
Control	1	P53634
Control	1	P53999
Control	1	P54136
Control	1	P54289
Control	1	P54578
Control	1	P54687
Control	1	P54886
Control	1	P55058

Control	1	P55060
Control	1	P55072
Control	1	P55084
Control	1	P55145
Control	1	P55209
Control	1	P55263
Control	1	P55285
Control	1	P55290
Control	1	P55786
Control	1	P55884
Control	1	P56134
Control	1	P56537
Control	1	P56545
Control	1	P59998
Control	1	P60174
Control	1	P60228
Control	1	P60709
Control	1	P60842
Control	1	P60866
Control	1	P60900
Control	1	P60953
Control	1	P60981
Control	1	P61019
Control	1	P61158
Control	1	P61160
Control	1	P61204
Control	1	P61247
Control	1	P61313
Control	1	P61326
Control	1	P61353
Control	1	P61604
Control	1	P61619
Control	1	P61916
Control	1	P61978
Control	1	P61981
Control	1	P62081
Control	1	P62140
Control	1	P62191
Control	1	P62241
Control	1	P62244
Control	1	P62249
Control	1	P62258
Control	1	P62263
Control	1	P62266
Control	1	P62269
Control	1	P62277
Control	1	P62280
Control	1	P62318
Control	1	P62424
Control	1	P62491

Control	1	P62701
Control	1	P62736
Control	1	P62750
Control	1	P62753
Control	1	P62805
Control	1	P62826
Control	1	P62829
Control	1	P62847
Control	1	P62851
Control	1	P62854
Control	1	P62873
Control	1	P62888
Control	1	P62899
Control	1	P62906
Control	1	P62910
Control	1	P62913
Control	1	P62917
Control	1	P62937
Control	1	P63000
Control	1	P63010
Control	1	P63104
Control	1	P63173
Control	1	P63241
Control	1	P63244
Control	1	P67809
Control	1	P67812
Control	1	P67936
Control	1	P68036
Control	1	P68104
Control	1	P68363
Control	1	P68371
Control	1	P68431
Control	1	P78371
Control	1	P78417
Control	1	P78527
Control	1	P78539
Control	1	P81605
Control	1	P83731
Control	1	P83881
Control	1	P84098
Control	1	P84103
Control	1	P98160
Control	1	P99999
Control	1	Q00325
Control	1	Q00610
Control	1	Q00688
Control	1	Q00839
Control	1	Q01082
Control	1	Q01105
Control	1	Q01130

Control	1	Q01469
Control	1	Q01518
Control	1	Q01638
Control	1	Q01813
Control	1	Q02543
Control	1	Q02809
Control	1	Q02818
Control	1	Q02878
Control	1	Q03135
Control	1	Q04446
Control	1	Q04637
Control	1	Q04917
Control	1	Q05682
Control	1	Q06323
Control	1	Q06481
Control	1	Q06828
Control	1	Q06830
Control	1	Q07020
Control	1	Q07021
Control	1	Q07065
Control	1	Q07666
Control	1	Q07954
Control	1	Q07955
Control	1	Q08211
Control	1	Q08629
Control	1	Q09666
Control	1	Q12805
Control	1	Q12841
Control	1	Q12860
Control	1	Q12905
Control	1	Q12906
Control	1	Q12907
Control	1	Q12931
Control	1	Q13162
Control	1	Q13185
Control	1	Q13200
Control	1	Q13201
Control	1	Q13263
Control	1	Q13308
Control	1	Q13418
Control	1	Q13740
Control	1	Q13813
Control	1	Q13838
Control	1	Q13885
Control	1	Q14019
Control	1	Q14103
Control	1	Q14152
Control	1	Q14195
Control	1	Q14203
Control	1	Q14204

Control	1	Q14240
Control	1	Q14315
Control	1	Q14515
Control	1	Q14697
Control	1	Q14764
Control	1	Q14766
Control	1	Q14767
Control	1	Q14974
Control	1	Q15019
Control	1	Q15029
Control	1	Q15084
Control	1	Q15149
Control	1	Q15233
Control	1	Q15257
Control	1	Q15293
Control	1	Q15365
Control	1	Q15366
Control	1	Q15393
Control	1	Q15404
Control	1	Q15436
Control	1	Q15493
Control	1	Q15582
Control	1	Q16181
Control	1	Q16270
Control	1	Q16363
Control	1	Q16394
Control	1	Q16531
Control	1	Q16543
Control	1	Q16555
Control	1	Q16610
Control	1	Q16658
Control	1	Q16666
Control	1	Q16853
Control	1	Q16881
Control	1	Q32P28
Control	1	Q53GQ0
Control	1	Q6NZI2
Control	1	Q6UWH4
Control	1	Q6YHK3
Control	1	Q71UM5
Control	1	Q76LX8
Control	1	Q7KZF4
Control	1	Q7L576
Control	1	Q7LGC8
Control	1	Q7Z7G0
Control	1	Q86UX7
Control	1	Q86VP6
Control	1	Q8IUE6
Control	1	Q8IUX7
Control	1	Q8IV08

Control	1	Q8NBJ5
Control	1	Q8NBS9
Control	1	Q8TCT9
Control	1	Q8WUM4
Control	1	Q92522
Control	1	Q92626
Control	1	Q92743
Control	1	Q92820
Control	1	Q92841
Control	1	Q92896
Control	1	Q92973
Control	1	Q93088
Control	1	Q96AE4
Control	1	Q96AG4
Control	1	Q96CX2
Control	1	Q96FW1
Control	1	Q96KP4
Control	1	Q96QK1
Control	1	Q96QV1
Control	1	Q99536
Control	1	Q99538
Control	1	Q99623
Control	1	Q99714
Control	1	Q99715
Control	1	Q99729
Control	1	Q99832
Control	1	Q99873
Control	1	Q99988
Control	1	Q9BR76
Control	1	Q9BRK5
Control	1	Q9BRX8
Control	1	Q9BSJ8
Control	1	Q9BT78
Control	1	Q9BTV4
Control	1	Q9BUF5
Control	1	Q9BWD1
Control	1	Q9BXJ0
Control	1	Q9BXJ4
Control	1	Q9BZZ5
Control	1	Q9GZM7
Control	1	Q9H0U4
Control	1	Q9H1E3
Control	1	Q9H4M9
Control	1	Q9H7Y0
Control	1	Q9HB71
Control	1	Q9HDC9
Control	1	Q9NPH3
Control	1	Q9NPY3
Control	1	Q9NQ30
Control	1	Q9NQ88

Control	1	Q9NR45
Control	1	Q9NTK5
Control	1	Q9NVA2
Control	1	Q9NVD7
Control	1	Q9NY15
Control	1	Q9NY33
Control	1	Q9NZM1
Control	1	Q9NZN4
Control	1	Q9NZV1
Control	1	Q9P2J5
Control	1	Q9UBR2
Control	1	Q9UJ70
Control	1	Q9UJZ1
Control	1	Q9UL46
Control	1	Q9ULV4
Control	1	Q9UNN8
Control	1	Q9UQ80
Control	1	Q9Y230
Control	1	Q9Y240
Control	1	Q9Y265
Control	1	Q9Y266
Control	1	Q9Y277
Control	1	Q9Y3I0
Control	1	Q9Y3U8
Control	1	Q9Y490
Control	1	Q9Y4K0
Control	1	Q9Y4L1
Control	1	Q9Y5B9
Control	1	Q9Y5S9
Control	1	Q9Y5X9
Control	1	Q9Y678
Control	1	Q9Y696
Control	2	A0A8I5KQE6
Control	2	A6NIZ1
Control	2	P08134
Control	2	B5ME19
Control	2	E9PAV3
Control	2	O00154
Control	2	O00159
Control	2	O00231
Control	2	O00232
Control	2	O00299
Control	2	O00391
Control	2	O00410
Control	2	O00469
Control	2	O00567
Control	2	O00571
Control	2	O00622
Control	2	O00625
Control	2	O14773

Control	2	O14786
Control	2	O14818
Control	2	O14950
Control	2	O14979
Control	2	O14980
Control	2	O15123
Control	2	O15143
Control	2	O15144
Control	2	O15145
Control	2	O15232
Control	2	O43143
Control	2	O43242
Control	2	O43390
Control	2	O43396
Control	2	O43684
Control	2	O43707
Control	2	O60462
Control	2	O60506
Control	2	O60568
Control	2	O60814
Control	2	O75083
Control	2	O75131
Control	2	O75369
Control	2	O75390
Control	2	O75436
Control	2	O75489
Control	2	O75874
Control	2	O75915
Control	2	O75923
Control	2	O94979
Control	2	O94985
Control	2	O95084
Control	2	O95445
Control	2	O95497
Control	2	O95782
Control	2	O95810
Control	2	P00338
Control	2	P00352
Control	2	P00387
Control	2	P00403
Control	2	P00488
Control	2	P00491
Control	2	P00492
Control	2	P00505
Control	2	P00558
Control	2	P00568
Control	2	P00742
Control	2	P00750
Control	2	P01024
Control	2	P01033

Control	2	P01034
Control	2	P02452
Control	2	P02458
Control	2	P02545
Control	2	P02751
Control	2	P02753
Control	2	P02768
Control	2	P03956
Control	2	P04075
Control	2	P04083
Control	2	P04114
Control	2	P04275
Control	2	P04406
Control	2	P04439
Control	2	P04792
Control	2	P04843
Control	2	P04844
Control	2	P04899
Control	2	P04908
Control	2	P05023
Control	2	P05067
Control	2	P05091
Control	2	P05109
Control	2	P05121
Control	2	P05141
Control	2	P05198
Control	2	P05305
Control	2	P05388
Control	2	P05455
Control	2	P05556
Control	2	P05783
Control	2	P06396
Control	2	P06454
Control	2	P06576
Control	2	P06702
Control	2	P06703
Control	2	P06732
Control	2	P06733
Control	2	P06744
Control	2	P06748
Control	2	P06753
Control	2	P06865
Control	2	P06899
Control	2	P07195
Control	2	P07203
Control	2	P07237
Control	2	P07339
Control	2	P07355
Control	2	P07384
Control	2	P07437

Control	2	P07602
Control	2	P07686
Control	2	P07737
Control	2	P07814
Control	2	P07858
Control	2	P07900
Control	2	P07910
Control	2	P07942
Control	2	P07951
Control	2	P07954
Control	2	P07996
Control	2	P07998
Control	2	P08123
Control	2	P08133
Control	2	P08238
Control	2	P08253
Control	2	P08572
Control	2	P08621
Control	2	P08648
Control	2	P08670
Control	2	P08708
Control	2	P08758
Control	2	P09012
Control	2	P09211
Control	2	P09429
Control	2	P09486
Control	2	P09525
Control	2	P09651
Control	2	P09669
Control	2	P09960
Control	2	P0CG47
Control	2	P0DMV8
Control	2	P0DPH7
Control	2	P10124
Control	2	P10586
Control	2	P10619
Control	2	P10646
Control	2	P10809
Control	2	P10909
Control	2	P11021
Control	2	P11047
Control	2	P11142
Control	2	P11233
Control	2	P11279
Control	2	P11413
Control	2	P11586
Control	2	P11717
Control	2	P11766
Control	2	P11940
Control	2	P12004

Control	2	P12107
Control	2	P12109
Control	2	P12111
Control	2	P12236
Control	2	P12268
Control	2	P12429
Control	2	P12814
Control	2	P12821
Control	2	P12955
Control	2	P12956
Control	2	P13010
Control	2	P13073
Control	2	P13473
Control	2	P13489
Control	2	P13639
Control	2	P13667
Control	2	P13797
Control	2	P13987
Control	2	P14314
Control	2	P14324
Control	2	P14543
Control	2	P14618
Control	2	P14625
Control	2	P14866
Control	2	P14868
Control	2	P15090
Control	2	P15121
Control	2	P15144
Control	2	P15153
Control	2	P15170
Control	2	P15311
Control	2	P15531
Control	2	P15586
Control	2	P15880
Control	2	P16035
Control	2	P16284
Control	2	P16401
Control	2	P16402
Control	2	P16403
Control	2	P16930
Control	2	P16949
Control	2	P17096
Control	2	P17301
Control	2	P17655
Control	2	P17844
Control	2	P17858
Control	2	P17948
Control	2	P17980
Control	2	P17987
Control	2	P18077

Control	2	P18085
Control	2	P18124
Control	2	P18206
Control	2	P18621
Control	2	P18669
Control	2	P18754
Control	2	P19021
Control	2	P19338
Control	2	P19367
Control	2	P19623
Control	2	P20042
Control	2	P20062
Control	2	P20618
Control	2	P20700
Control	2	P20774
Control	2	P20908
Control	2	P21333
Control	2	P21399
Control	2	P21741
Control	2	P21796
Control	2	P21810
Control	2	P21980
Control	2	P22004
Control	2	P22234
Control	2	P22314
Control	2	P22352
Control	2	P22392
Control	2	P22626
Control	2	P22692
Control	2	P22695
Control	2	P23246
Control	2	P23284
Control	2	P23381
Control	2	P23396
Control	2	P23526
Control	2	P23528
Control	2	P23919
Control	2	P25705
Control	2	P25786
Control	2	P25787
Control	2	P25788
Control	2	P25789
Control	2	P26022
Control	2	P26038
Control	2	P26368
Control	2	P26373
Control	2	P26583
Control	2	P26599
Control	2	P26640
Control	2	P26641

Control	2	P26927
Control	2	P27105
Control	2	P27348
Control	2	P27635
Control	2	P27695
Control	2	P27708
Control	2	P27797
Control	2	P27816
Control	2	P27824
Control	2	P28066
Control	2	P28072
Control	2	P28074
Control	2	P28300
Control	2	P28482
Control	2	P28799
Control	2	P28838
Control	2	P29279
Control	2	P29401
Control	2	P29692
Control	2	P29966
Control	2	P30041
Control	2	P30050
Control	2	P30084
Control	2	P30086
Control	2	P30101
Control	2	P30153
Control	2	P30520
Control	2	P31153
Control	2	P31930
Control	2	P31943
Control	2	P31946
Control	2	P31948
Control	2	P31949
Control	2	P32119
Control	2	P32969
Control	2	P33151
Control	2	P34096
Control	2	P34932
Control	2	P35221
Control	2	P35222
Control	2	P35232
Control	2	P35237
Control	2	P35241
Control	2	P35268
Control	2	P35443
Control	2	P35555
Control	2	P35579
Control	2	P35590
Control	2	P35998
Control	2	P36578

Control	2	P36871
Control	2	P37802
Control	2	P37837
Control	2	P38159
Control	2	P38606
Control	2	P38646
Control	2	P39019
Control	2	P39023
Control	2	P39656
Control	2	P39687
Control	2	P40121
Control	2	P40227
Control	2	P40261
Control	2	P40429
Control	2	P40925
Control	2	P40926
Control	2	P40939
Control	2	P41091
Control	2	P41250
Control	2	P42166
Control	2	P42785
Control	2	P43121
Control	2	P43243
Control	2	P43490
Control	2	P45880
Control	2	P45974
Control	2	P46060
Control	2	P46776
Control	2	P46777
Control	2	P46778
Control	2	P46779
Control	2	P46781
Control	2	P46782
Control	2	P46783
Control	2	P46926
Control	2	P46940
Control	2	P46977
Control	2	P47755
Control	2	P47756
Control	2	P47897
Control	2	P48047
Control	2	P48059
Control	2	P48444
Control	2	P48643
Control	2	P48681
Control	2	P48723
Control	2	P48735
Control	2	P48739
Control	2	P48740
Control	2	P49207

Control	2	P49327
Control	2	P49368
Control	2	P49407
Control	2	P49411
Control	2	P49458
Control	2	P49720
Control	2	P49721
Control	2	P49747
Control	2	P50395
Control	2	P50452
Control	2	P50454
Control	2	P50502
Control	2	P50914
Control	2	P50990
Control	2	P50991
Control	2	P51148
Control	2	P51149
Control	2	P51991
Control	2	P52209
Control	2	P52272
Control	2	P52565
Control	2	P52566
Control	2	P52907
Control	2	P53004
Control	2	P53396
Control	2	P53618
Control	2	P53621
Control	2	P53634
Control	2	P53999
Control	2	P54136
Control	2	P54289
Control	2	P54578
Control	2	P54687
Control	2	P54886
Control	2	P55058
Control	2	P55060
Control	2	P55072
Control	2	P55084
Control	2	P55145
Control	2	P55209
Control	2	P55263
Control	2	P55285
Control	2	P55290
Control	2	P55786
Control	2	P55884
Control	2	P56134
Control	2	P56537
Control	2	P56545
Control	2	P59998
Control	2	P60174

Control	2	P60228
Control	2	P60709
Control	2	P60842
Control	2	P60866
Control	2	P60900
Control	2	P60953
Control	2	P60981
Control	2	P61019
Control	2	P61158
Control	2	P61160
Control	2	P61204
Control	2	P61247
Control	2	P61313
Control	2	P61326
Control	2	P61353
Control	2	P61604
Control	2	P61619
Control	2	P61916
Control	2	P61978
Control	2	P61981
Control	2	P62081
Control	2	P62140
Control	2	P62191
Control	2	P62241
Control	2	P62244
Control	2	P62249
Control	2	P62258
Control	2	P62263
Control	2	P62266
Control	2	P62269
Control	2	P62277
Control	2	P62280
Control	2	P62318
Control	2	P62424
Control	2	P62491
Control	2	P62701
Control	2	P62736
Control	2	P62750
Control	2	P62753
Control	2	P62805
Control	2	P62826
Control	2	P62829
Control	2	P62847
Control	2	P62851
Control	2	P62854
Control	2	P62873
Control	2	P62888
Control	2	P62899
Control	2	P62906
Control	2	P62910

Control	2	P62913
Control	2	P62917
Control	2	P62937
Control	2	P63000
Control	2	P63010
Control	2	P63104
Control	2	P63173
Control	2	P63241
Control	2	P63244
Control	2	P67809
Control	2	P67812
Control	2	P67936
Control	2	P68036
Control	2	P68104
Control	2	P68363
Control	2	P68371
Control	2	P68431
Control	2	P78371
Control	2	P78417
Control	2	P78527
Control	2	P78539
Control	2	P81605
Control	2	P83731
Control	2	P83881
Control	2	P84098
Control	2	P84103
Control	2	P98160
Control	2	P99999
Control	2	Q00325
Control	2	Q00610
Control	2	Q00688
Control	2	Q00839
Control	2	Q01082
Control	2	Q01105
Control	2	Q01130
Control	2	Q01469
Control	2	Q01518
Control	2	Q01638
Control	2	Q01813
Control	2	Q02543
Control	2	Q02809
Control	2	Q02818
Control	2	Q02878
Control	2	Q03135
Control	2	Q04446
Control	2	Q04637
Control	2	Q04917
Control	2	Q05682
Control	2	Q06323
Control	2	Q06481

Control	2	Q06828
Control	2	Q06830
Control	2	Q07020
Control	2	Q07021
Control	2	Q07065
Control	2	Q07666
Control	2	Q07954
Control	2	Q07955
Control	2	Q08211
Control	2	Q08629
Control	2	Q09666
Control	2	Q12805
Control	2	Q12841
Control	2	Q12860
Control	2	Q12905
Control	2	Q12906
Control	2	Q12907
Control	2	Q12931
Control	2	Q13162
Control	2	Q13185
Control	2	Q13200
Control	2	Q13201
Control	2	Q13263
Control	2	Q13308
Control	2	Q13418
Control	2	Q13596
Control	2	Q13740
Control	2	Q13813
Control	2	Q13838
Control	2	Q13885
Control	2	Q14019
Control	2	Q14103
Control	2	Q14152
Control	2	Q14195
Control	2	Q14203
Control	2	Q14204
Control	2	Q14240
Control	2	Q14315
Control	2	Q14515
Control	2	Q14697
Control	2	Q14764
Control	2	Q14766
Control	2	Q14767
Control	2	Q14974
Control	2	Q15019
Control	2	Q15029
Control	2	Q15084
Control	2	Q15149
Control	2	Q15233
Control	2	Q15257

Control	2	Q15293
Control	2	Q15365
Control	2	Q15366
Control	2	Q15393
Control	2	Q15404
Control	2	Q15436
Control	2	Q15493
Control	2	Q15582
Control	2	Q16181
Control	2	Q16270
Control	2	Q16363
Control	2	Q16394
Control	2	Q16531
Control	2	Q16543
Control	2	Q16555
Control	2	Q16610
Control	2	Q16658
Control	2	Q16666
Control	2	Q16853
Control	2	Q16881
Control	2	Q32P28
Control	2	Q6NZI2
Control	2	Q6UWH4
Control	2	Q6YHK3
Control	2	Q71UM5
Control	2	Q76LX8
Control	2	Q7KZF4
Control	2	Q7L576
Control	2	Q7LGC8
Control	2	Q7Z7G0
Control	2	Q86UX7
Control	2	Q86VP6
Control	2	Q8IUE6
Control	2	Q8IUX7
Control	2	Q8IV08
Control	2	Q8NBJ5
Control	2	Q8NBS9
Control	2	Q8TCT9
Control	2	Q8WUM4
Control	2	Q92626
Control	2	Q92743
Control	2	Q92820
Control	2	Q92841
Control	2	Q92896
Control	2	Q93088
Control	2	Q96AE4
Control	2	Q96AG4
Control	2	Q96CX2
Control	2	Q96FW1
Control	2	Q96KP4

Control	2	Q96QK1
Control	2	Q96QV1
Control	2	Q99519
Control	2	Q99536
Control	2	Q99538
Control	2	Q99623
Control	2	Q99714
Control	2	Q99715
Control	2	Q99729
Control	2	Q99832
Control	2	Q99873
Control	2	Q99988
Control	2	Q9BR76
Control	2	Q9BRK5
Control	2	Q9BRX8
Control	2	Q9BSJ8
Control	2	Q9BT78
Control	2	Q9BTV4
Control	2	Q9BUF5
Control	2	Q9BWD1
Control	2	Q9BXJ0
Control	2	Q9BXJ4
Control	2	Q9BZZ5
Control	2	Q9GZM7
Control	2	Q9H0U4
Control	2	Q9H1E3
Control	2	Q9H4M9
Control	2	Q9HB71
Control	2	Q9HDC9
Control	2	Q9NPH3
Control	2	Q9NPY3
Control	2	Q9NQ30
Control	2	Q9NQ88
Control	2	Q9NR45
Control	2	Q9NTK5
Control	2	Q9NVA2
Control	2	Q9NVD7
Control	2	Q9NY15
Control	2	Q9NY33
Control	2	Q9NZM1
Control	2	Q9NZN4
Control	2	Q9NZV1
Control	2	Q9P2J5
Control	2	Q9UBP4
Control	2	Q9UBR2
Control	2	Q9UJ70
Control	2	Q9UJZ1
Control	2	Q9UL46
Control	2	Q9ULV4
Control	2	Q9UNN8

Control	2	Q9UQ80
Control	2	Q9Y230
Control	2	Q9Y240
Control	2	Q9Y265
Control	2	Q9Y266
Control	2	Q9Y277
Control	2	Q9Y3I0
Control	2	Q9Y3U8
Control	2	Q9Y490
Control	2	Q9Y4K0
Control	2	Q9Y4L1
Control	2	Q9Y5S9
Control	2	Q9Y5X9
Control	2	Q9Y678
Control	2	Q9Y696
Control	3	A0A8I5KQE6
Control	3	A6NIZ1
Control	3	P08134
Control	3	E9PAV3
Control	3	O00154
Control	3	O00159
Control	3	O00231
Control	3	O00232
Control	3	O00299
Control	3	O00391
Control	3	O00410
Control	3	O00469
Control	3	O00567
Control	3	O00571
Control	3	O00622
Control	3	O00625
Control	3	O14773
Control	3	O14786
Control	3	O14818
Control	3	O14950
Control	3	O14979
Control	3	O14980
Control	3	O15123
Control	3	O15143
Control	3	O15144
Control	3	O15145
Control	3	O15232
Control	3	O43143
Control	3	O43242
Control	3	O43390
Control	3	O43396
Control	3	O43684
Control	3	O43707
Control	3	O60462
Control	3	O60506

Control	3	O60568
Control	3	O60814
Control	3	O75083
Control	3	O75369
Control	3	O75390
Control	3	O75436
Control	3	O75489
Control	3	O75874
Control	3	O75915
Control	3	O75923
Control	3	O94979
Control	3	O94985
Control	3	O95084
Control	3	O95445
Control	3	O95497
Control	3	O95782
Control	3	O95810
Control	3	P00338
Control	3	P00352
Control	3	P00387
Control	3	P00403
Control	3	P00488
Control	3	P00491
Control	3	P00492
Control	3	P00505
Control	3	P00558
Control	3	P00568
Control	3	P00742
Control	3	P00750
Control	3	P01024
Control	3	P01033
Control	3	P01034
Control	3	P02452
Control	3	P02458
Control	3	P02545
Control	3	P02751
Control	3	P02753
Control	3	P02768
Control	3	P03956
Control	3	P04075
Control	3	P04083
Control	3	P04114
Control	3	P04275
Control	3	P04406
Control	3	P04439
Control	3	P04792
Control	3	P04843
Control	3	P04844
Control	3	P04899
Control	3	P04908

Control	3	P05023
Control	3	P05067
Control	3	P05091
Control	3	P05114
Control	3	P05121
Control	3	P05141
Control	3	P05198
Control	3	P05305
Control	3	P05388
Control	3	P05455
Control	3	P05556
Control	3	P05783
Control	3	P06396
Control	3	P06454
Control	3	P06576
Control	3	P06703
Control	3	P06732
Control	3	P06733
Control	3	P06744
Control	3	P06748
Control	3	P06753
Control	3	P06865
Control	3	P06899
Control	3	P07195
Control	3	P07203
Control	3	P07237
Control	3	P07339
Control	3	P07355
Control	3	P07384
Control	3	P07437
Control	3	P07602
Control	3	P07686
Control	3	P07737
Control	3	P07814
Control	3	P07858
Control	3	P07900
Control	3	P07910
Control	3	P07942
Control	3	P07951
Control	3	P07954
Control	3	P07996
Control	3	P07998
Control	3	P08123
Control	3	P08133
Control	3	P08238
Control	3	P08253
Control	3	P08572
Control	3	P08621
Control	3	P08648
Control	3	P08670

Control	3	P08708
Control	3	P08758
Control	3	P09012
Control	3	P09211
Control	3	P09429
Control	3	P09486
Control	3	P09525
Control	3	P09651
Control	3	P09669
Control	3	P09960
Control	3	P0CG47
Control	3	P0DMV8
Control	3	P0DPH7
Control	3	P10124
Control	3	P10586
Control	3	P10646
Control	3	P10809
Control	3	P10909
Control	3	P11021
Control	3	P11047
Control	3	P11142
Control	3	P11233
Control	3	P11279
Control	3	P11413
Control	3	P11586
Control	3	P11717
Control	3	P11766
Control	3	P11940
Control	3	P12004
Control	3	P12107
Control	3	P12109
Control	3	P12111
Control	3	P12236
Control	3	P12268
Control	3	P12429
Control	3	P12814
Control	3	P12821
Control	3	P12955
Control	3	P12956
Control	3	P13010
Control	3	P13073
Control	3	P13473
Control	3	P13489
Control	3	P13639
Control	3	P13667
Control	3	P13797
Control	3	P13987
Control	3	P14314
Control	3	P14324
Control	3	P14543

Control	3	P14618
Control	3	P14625
Control	3	P14866
Control	3	P14868
Control	3	P15090
Control	3	P15121
Control	3	P15144
Control	3	P15153
Control	3	P15170
Control	3	P15311
Control	3	P15531
Control	3	P15586
Control	3	P15880
Control	3	P16035
Control	3	P16284
Control	3	P16401
Control	3	P16402
Control	3	P16403
Control	3	P16930
Control	3	P16949
Control	3	P17096
Control	3	P17301
Control	3	P17655
Control	3	P17844
Control	3	P17948
Control	3	P17980
Control	3	P17987
Control	3	P18077
Control	3	P18085
Control	3	P18124
Control	3	P18206
Control	3	P18621
Control	3	P18669
Control	3	P18754
Control	3	P19021
Control	3	P19338
Control	3	P19367
Control	3	P19623
Control	3	P20042
Control	3	P20062
Control	3	P20618
Control	3	P20700
Control	3	P20774
Control	3	P20908
Control	3	P21333
Control	3	P21399
Control	3	P21741
Control	3	P21796
Control	3	P21810
Control	3	P21980

Control	3	P22004
Control	3	P22234
Control	3	P22314
Control	3	P22352
Control	3	P22392
Control	3	P22626
Control	3	P22692
Control	3	P22695
Control	3	P23246
Control	3	P23284
Control	3	P23381
Control	3	P23396
Control	3	P23526
Control	3	P23528
Control	3	P23919
Control	3	P25705
Control	3	P25786
Control	3	P25787
Control	3	P25788
Control	3	P25789
Control	3	P26022
Control	3	P26038
Control	3	P26368
Control	3	P26373
Control	3	P26583
Control	3	P26599
Control	3	P26641
Control	3	P26927
Control	3	P27105
Control	3	P27348
Control	3	P27635
Control	3	P27695
Control	3	P27708
Control	3	P27797
Control	3	P27816
Control	3	P27824
Control	3	P28066
Control	3	P28072
Control	3	P28074
Control	3	P28300
Control	3	P28482
Control	3	P28799
Control	3	P28838
Control	3	P29279
Control	3	P29401
Control	3	P29692
Control	3	P29966
Control	3	P30041
Control	3	P30050
Control	3	P30086

Control	3	P30101
Control	3	P30153
Control	3	P30520
Control	3	P31153
Control	3	P31930
Control	3	P31943
Control	3	P31946
Control	3	P31948
Control	3	P31949
Control	3	P32119
Control	3	P32969
Control	3	P33151
Control	3	P34096
Control	3	P34932
Control	3	P35221
Control	3	P35222
Control	3	P35232
Control	3	P35237
Control	3	P35241
Control	3	P35268
Control	3	P35443
Control	3	P35555
Control	3	P35579
Control	3	P35590
Control	3	P35998
Control	3	P36578
Control	3	P36871
Control	3	P37802
Control	3	P37837
Control	3	P38159
Control	3	P38606
Control	3	P38646
Control	3	P39019
Control	3	P39023
Control	3	P39656
Control	3	P39687
Control	3	P40121
Control	3	P40227
Control	3	P40261
Control	3	P40429
Control	3	P40925
Control	3	P40926
Control	3	P40939
Control	3	P41091
Control	3	P41250
Control	3	P42166
Control	3	P42785
Control	3	P43121
Control	3	P43243
Control	3	P43490

Control	3	P45880
Control	3	P45974
Control	3	P46776
Control	3	P46777
Control	3	P46778
Control	3	P46779
Control	3	P46781
Control	3	P46782
Control	3	P46783
Control	3	P46926
Control	3	P46940
Control	3	P47755
Control	3	P47756
Control	3	P48047
Control	3	P48059
Control	3	P48444
Control	3	P48643
Control	3	P48681
Control	3	P48723
Control	3	P48735
Control	3	P48739
Control	3	P48740
Control	3	P49207
Control	3	P49327
Control	3	P49368
Control	3	P49411
Control	3	P49458
Control	3	P49720
Control	3	P49721
Control	3	P49747
Control	3	P50395
Control	3	P50454
Control	3	P50502
Control	3	P50914
Control	3	P50990
Control	3	P50991
Control	3	P51148
Control	3	P51149
Control	3	P51858
Control	3	P51991
Control	3	P52209
Control	3	P52272
Control	3	P52565
Control	3	P52566
Control	3	P52907
Control	3	P53004
Control	3	P53396
Control	3	P53618
Control	3	P53621
Control	3	P53634

Control	3	P53999
Control	3	P54136
Control	3	P54289
Control	3	P54578
Control	3	P54687
Control	3	P54886
Control	3	P55058
Control	3	P55060
Control	3	P55072
Control	3	P55084
Control	3	P55145
Control	3	P55209
Control	3	P55263
Control	3	P55285
Control	3	P55290
Control	3	P55786
Control	3	P55884
Control	3	P56134
Control	3	P56537
Control	3	P56545
Control	3	P59998
Control	3	P60174
Control	3	P60228
Control	3	P60709
Control	3	P60842
Control	3	P60866
Control	3	P60900
Control	3	P60953
Control	3	P60981
Control	3	P61019
Control	3	P61158
Control	3	P61160
Control	3	P61204
Control	3	P61247
Control	3	P61313
Control	3	P61353
Control	3	P61604
Control	3	P61619
Control	3	P61916
Control	3	P61978
Control	3	P61981
Control	3	P62081
Control	3	P62140
Control	3	P62191
Control	3	P62241
Control	3	P62244
Control	3	P62249
Control	3	P62258
Control	3	P62263
Control	3	P62266

Control	3	P62269
Control	3	P62277
Control	3	P62280
Control	3	P62318
Control	3	P62424
Control	3	P62491
Control	3	P62701
Control	3	P62736
Control	3	P62750
Control	3	P62753
Control	3	P62805
Control	3	P62826
Control	3	P62829
Control	3	P62847
Control	3	P62851
Control	3	P62854
Control	3	P62873
Control	3	P62888
Control	3	P62899
Control	3	P62906
Control	3	P62910
Control	3	P62913
Control	3	P62917
Control	3	P62937
Control	3	P63000
Control	3	P63010
Control	3	P63104
Control	3	P63173
Control	3	P63241
Control	3	P63244
Control	3	P67809
Control	3	P67812
Control	3	P67936
Control	3	P68036
Control	3	P68104
Control	3	P68363
Control	3	P68371
Control	3	P68431
Control	3	P78371
Control	3	P78417
Control	3	P78527
Control	3	P78539
Control	3	P81605
Control	3	P83731
Control	3	P83881
Control	3	P84098
Control	3	P84103
Control	3	P98160
Control	3	P99999
Control	3	Q00325

Control	3	Q00610
Control	3	Q00688
Control	3	Q00839
Control	3	Q01082
Control	3	Q01105
Control	3	Q01130
Control	3	Q01469
Control	3	Q01518
Control	3	Q01638
Control	3	Q01813
Control	3	Q02543
Control	3	Q02809
Control	3	Q02818
Control	3	Q02878
Control	3	Q03135
Control	3	Q04446
Control	3	Q04637
Control	3	Q04917
Control	3	Q05682
Control	3	Q06323
Control	3	Q06481
Control	3	Q06828
Control	3	Q06830
Control	3	Q07020
Control	3	Q07021
Control	3	Q07065
Control	3	Q07666
Control	3	Q07954
Control	3	Q07955
Control	3	Q08211
Control	3	Q08629
Control	3	Q09666
Control	3	Q12805
Control	3	Q12841
Control	3	Q12860
Control	3	Q12905
Control	3	Q12906
Control	3	Q12907
Control	3	Q12931
Control	3	Q13162
Control	3	Q13185
Control	3	Q13200
Control	3	Q13201
Control	3	Q13263
Control	3	Q13308
Control	3	Q13418
Control	3	Q13740
Control	3	Q13813
Control	3	Q13838
Control	3	Q13885

Control	3	Q14019
Control	3	Q14103
Control	3	Q14152
Control	3	Q14195
Control	3	Q14203
Control	3	Q14204
Control	3	Q14240
Control	3	Q14315
Control	3	Q14515
Control	3	Q14697
Control	3	Q14764
Control	3	Q14766
Control	3	Q14767
Control	3	Q14974
Control	3	Q15019
Control	3	Q15029
Control	3	Q15084
Control	3	Q15149
Control	3	Q15233
Control	3	Q15257
Control	3	Q15293
Control	3	Q15365
Control	3	Q15366
Control	3	Q15393
Control	3	Q15404
Control	3	Q15436
Control	3	Q15493
Control	3	Q15582
Control	3	Q16181
Control	3	Q16270
Control	3	Q16363
Control	3	Q16394
Control	3	Q16531
Control	3	Q16543
Control	3	Q16555
Control	3	Q16610
Control	3	Q16658
Control	3	Q16666
Control	3	Q16853
Control	3	Q16881
Control	3	Q32P28
Control	3	Q53GQ0
Control	3	Q6NZI2
Control	3	Q6UWH4
Control	3	Q6YHK3
Control	3	Q71UM5
Control	3	Q76LX8
Control	3	Q7KZF4
Control	3	Q7L576
Control	3	Q7LGC8

Control	3	Q7Z7G0
Control	3	Q86UX7
Control	3	Q86VP6
Control	3	Q8IUE6
Control	3	Q8IUX7
Control	3	Q8IV08
Control	3	Q8NBJ5
Control	3	Q8NBS9
Control	3	Q8TCT9
Control	3	Q8WUM4
Control	3	Q92626
Control	3	Q92743
Control	3	Q92820
Control	3	Q92841
Control	3	Q92896
Control	3	Q92973
Control	3	Q93088
Control	3	Q96AE4
Control	3	Q96AG4
Control	3	Q96CX2
Control	3	Q96FW1
Control	3	Q96KP4
Control	3	Q96QK1
Control	3	Q96QV1
Control	3	Q99519
Control	3	Q99536
Control	3	Q99538
Control	3	Q99623
Control	3	Q99714
Control	3	Q99715
Control	3	Q99729
Control	3	Q99832
Control	3	Q99873
Control	3	Q99988
Control	3	Q9BR76
Control	3	Q9BRK5
Control	3	Q9BRX8
Control	3	Q9BSJ8
Control	3	Q9BT78
Control	3	Q9BTV4
Control	3	Q9BUF5
Control	3	Q9BWD1
Control	3	Q9BXJ0
Control	3	Q9BXJ4
Control	3	Q9BZZ5
Control	3	Q9GZM7
Control	3	Q9H0U4
Control	3	Q9H4M9
Control	3	Q9HB71
Control	3	Q9HDC9

Control	3	Q9NPH3
Control	3	Q9NPY3
Control	3	Q9NQ30
Control	3	Q9NQ88
Control	3	Q9NR45
Control	3	Q9NTK5
Control	3	Q9NVA2
Control	3	Q9NVD7
Control	3	Q9NY15
Control	3	Q9NY33
Control	3	Q9NZM1
Control	3	Q9NZN4
Control	3	Q9NZV1
Control	3	Q9P2J5
Control	3	Q9UBP4
Control	3	Q9UBR2
Control	3	Q9UJ70
Control	3	Q9UJZ1
Control	3	Q9UL46
Control	3	Q9UNN8
Control	3	Q9UQ80
Control	3	Q9Y230
Control	3	Q9Y240
Control	3	Q9Y265
Control	3	Q9Y266
Control	3	Q9Y277
Control	3	Q9Y3I0
Control	3	Q9Y3U8
Control	3	Q9Y490
Control	3	Q9Y4K0
Control	3	Q9Y4L1
Control	3	Q9Y5B9
Control	3	Q9Y5S9
Control	3	Q9Y5X9
Control	3	Q9Y678
Control	3	Q9Y696

e replicates of cells used as control group.

PG.ProteinDescriptions
Small ribosomal subunit protein Us2b
Ras-related protein Rap-1b-like protein
Rho-related GTP-binding protein RhoC
Eukaryotic translation initiation factor 3 subunit C-like protein
Nascent polypeptide-associated complex subunit alpha, muscle-specific form
Cytosolic acyl coenzyme A thioester hydrolase
Unconventional myosin-Ic
26S proteasome non-ATPase regulatory subunit 11
26S proteasome non-ATPase regulatory subunit 12
Chloride intracellular channel protein 1
Sulfhydryl oxidase 1
Importin-5
Procollagen-lysine,2-oxoglutarate 5-dioxygenase 2
ATP-dependent RNA helicase DDX3X
CCN family member 1
Pirin
Tripeptidyl-peptidase 1
Neuropilin-1
Proteasome subunit alpha type-7
Myosin regulatory light chain 12B
Heterogeneous nuclear ribonucleoprotein D-like
Exportin-1
Angiopoietin-2
Actin-related protein 2/3 complex subunit 1B
Actin-related protein 2/3 complex subunit 2
Actin-related protein 2/3 complex subunit 3
Matrilin-3
ATP-dependent RNA helicase DHX15
26S proteasome non-ATPase regulatory subunit 3
Heterogeneous nuclear ribonucleoprotein R
Thioredoxin-like protein 1
Mitotic checkpoint protein BUB3
Alpha-actinin-4
Neuropilin-2
Heterogeneous nuclear ribonucleoprotein Q
Multifunctional procollagen lysine hydroxylase and glycosyltransferase LH3
Histone H2B type 1-K
WD repeat-containing protein 1
Copine-3
Filamin-B
Citrate synthase, mitochondrial
Vacuolar protein sorting-associated protein 26A
NADH dehydrogenase [ubiquinone] iron-sulfur protein 3, mitochondrial
Isocitrate dehydrogenase [NADP] cytoplasmic
PRA1 family protein 3
Dysferlin
Protein transport protein Sec31A
Calsyntenin-1

Serine protease 23
Apolipoprotein M
Pantetheinase
AP-2 complex subunit alpha-1
Caveolae-associated protein 2
L-lactate dehydrogenase A chain
Aldehyde dehydrogenase 1A1
NADH-cytochrome b5 reductase 3
Cytochrome c oxidase subunit 2
Coagulation factor XIII A chain
Purine nucleoside phosphorylase
Hypoxanthine-guanine phosphoribosyltransferase
Aspartate aminotransferase, mitochondrial
Phosphoglycerate kinase 1
Adenylate kinase isoenzyme 1
Coagulation factor X
Tissue-type plasminogen activator
Complement C3
Metalloproteinase inhibitor 1
Cystatin-C
Collagen alpha-1(I) chain
Collagen alpha-1(II) chain
Prelamin-A/C
Fibronectin
Retinol-binding protein 4
Albumin
Interstitial collagenase
Fructose-bisphosphate aldolase A
Annexin A1
Apolipoprotein B-100
von Willebrand factor
Glyceraldehyde-3-phosphate dehydrogenase
HLA class I histocompatibility antigen, A alpha chain
Heat shock protein beta-1
Dolichyl-diphosphooligosaccharide--protein glycosyltransferase subunit 1
Dolichyl-diphosphooligosaccharide--protein glycosyltransferase subunit 2
Guanine nucleotide-binding protein G(i) subunit alpha-2
Histone H2A type 1-B/E
Sodium/potassium-transporting ATPase subunit alpha-1
Amyloid-beta precursor protein
Aldehyde dehydrogenase, mitochondrial
Protein S100-A8
Non-histone chromosomal protein HMG-14
Plasminogen activator inhibitor 1
ADP/ATP translocase 2
Eukaryotic translation initiation factor 2 subunit 1
Endothelin-1
Large ribosomal subunit protein uL10
Lupus La protein
Integrin beta-1

Keratin, type I cytoskeletal 18
Gelsolin
Prothymosin alpha
ATP synthase subunit beta, mitochondrial
Protein S100-A6
Creatine kinase M-type
Alpha-enolase
Glucose-6-phosphate isomerase
Nucleophosmin
Tropomyosin alpha-3 chain
Beta-hexosaminidase subunit alpha
Histone H2B type 1-J
L-lactate dehydrogenase B chain
Glutathione peroxidase 1
Protein disulfide-isomerase
Cathepsin D
Annexin A2
Calpain-1 catalytic subunit
Tubulin beta chain
Prosaposin
Beta-hexosaminidase subunit beta
Profilin-1
Bifunctional glutamate/proline--tRNA ligase
Cathepsin B
Heat shock protein HSP 90-alpha
Heterogeneous nuclear ribonucleoproteins C1/C2
Laminin subunit beta-1
Tropomyosin beta chain
Fumarate hydratase, mitochondrial
Thrombospondin-1
Ribonuclease pancreatic
Collagen alpha-2(I) chain
Annexin A6
Heat shock protein HSP 90-beta
72 kDa type IV collagenase
Collagen alpha-2(IV) chain
U1 small nuclear ribonucleoprotein 70 kDa
Integrin alpha-5
Vimentin
Small ribosomal subunit protein eS17
Annexin A5
U1 small nuclear ribonucleoprotein A
Glutathione S-transferase P
High mobility group protein B1
SPARC
Annexin A4
Heterogeneous nuclear ribonucleoprotein A1
Cytochrome c oxidase subunit 6C
Leukotriene A-4 hydrolase
Polyubiquitin-B

Heat shock 70 kDa protein 1A
Tubulin alpha-3C chain
Serglycin
Receptor-type tyrosine-protein phosphatase F
Lysosomal protective protein
Tissue factor pathway inhibitor
60 kDa heat shock protein, mitochondrial
Clusterin
Endoplasmic reticulum chaperone BiP
Laminin subunit gamma-1
Heat shock cognate 71 kDa protein
Ras-related protein Ral-A
Lysosome-associated membrane glycoprotein 1
Glucose-6-phosphate 1-dehydrogenase
C-1-tetrahydrofolate synthase, cytoplasmic
Cation-independent mannose-6-phosphate receptor
Alcohol dehydrogenase class-3
Polyadenylate-binding protein 1
Proliferating cell nuclear antigen
Collagen alpha-1(XI) chain
Collagen alpha-1(VI) chain
Collagen alpha-3(VI) chain
ADP/ATP translocase 3
Inosine-5'-monophosphate dehydrogenase 2
Annexin A3
Alpha-actinin-1
Angiotensin-converting enzyme
X-ray repair cross-complementing protein 6
X-ray repair cross-complementing protein 5
Cytochrome c oxidase subunit 4 isoform 1, mitochondrial
Lysosome-associated membrane glycoprotein 2
Ribonuclease inhibitor
Elongation factor 2
Protein disulfide-isomerase A4
Plastin-3
CD59 glycoprotein
Glucosidase 2 subunit beta
Farnesyl pyrophosphate synthase
Nidogen-1
Pyruvate kinase PKM
Endoplasmin
Heterogeneous nuclear ribonucleoprotein L
Aspartate--tRNA ligase, cytoplasmic
Fatty acid-binding protein, adipocyte
Aldo-keto reductase family 1 member B1
Aminopeptidase N
Ras-related C3 botulinum toxin substrate 2
Eukaryotic peptide chain release factor GTP-binding subunit ERF3A
Ezrin
Nucleoside diphosphate kinase A

N-acetylglucosamine-6-sulfatase
Small ribosomal subunit protein uS5
Metalloproteinase inhibitor 2
Platelet endothelial cell adhesion molecule
Histone H1.5
Histone H1.3
Histone H1.2
Fumarylacetoacetase
Stathmin
High mobility group protein HMG-I/HMG-Y
Integrin alpha-2
Calpain-2 catalytic subunit
Probable ATP-dependent RNA helicase DDX5
ATP-dependent 6-phosphofructokinase, liver type
Vascular endothelial growth factor receptor 1
26S proteasome regulatory subunit 6A
T-complex protein 1 subunit alpha
Large ribosomal subunit protein eL33
ADP-ribosylation factor 4
Large ribosomal subunit protein uL30
Vinculin
Large ribosomal subunit protein uL22
Phosphoglycerate mutase 1
Regulator of chromosome condensation
Peptidyl-glycine alpha-amidating monooxygenase
Nucleolin
Hexokinase-1
Spermidine synthase
Eukaryotic translation initiation factor 2 subunit 2
Transcobalamin-2
Proteasome subunit beta type-1
Lamin-B1
Mimecan
Collagen alpha-1(V) chain
Filamin-A
Cytoplasmic aconitate hydratase
Midkine
Voltage-dependent anion-selective channel protein 1
Biglycan
Protein-glutamine gamma-glutamyltransferase 2
Bone morphogenetic protein 6
Bifunctional phosphoribosylaminoimidazole carboxylase/phosphoribosylaminoimidazole succ
Ubiquitin-like modifier-activating enzyme 1
Glutathione peroxidase 3
Nucleoside diphosphate kinase B
Heterogeneous nuclear ribonucleoproteins A2/B1
Insulin-like growth factor-binding protein 4
Cytochrome b-c1 complex subunit 2, mitochondrial
Splicing factor, proline- and glutamine-rich
Peptidyl-prolyl cis-trans isomerase B

Tryptophan--tRNA ligase, cytoplasmic
Small ribosomal subunit protein uS3
Adenosylhomocysteinase
Cofilin-1
Thymidylate kinase
ATP synthase subunit alpha, mitochondrial
Proteasome subunit alpha type-1
Proteasome subunit alpha type-2
Proteasome subunit alpha type-3
Proteasome subunit alpha type-4
Pentraxin-related protein PTX3
Moesin
Splicing factor U2AF 65 kDa subunit
Large ribosomal subunit protein eL13
High mobility group protein B2
Polypyrimidine tract-binding protein 1
Valine--tRNA ligase
Elongation factor 1-gamma
Hepatocyte growth factor-like protein
Stomatin
14-3-3 protein theta
Large ribosomal subunit protein uL16
DNA-(apurinic or apyrimidinic site) endonuclease
Multifunctional protein CAD
Calreticulin
Microtubule-associated protein 4
Calnexin
Proteasome subunit alpha type-5
Proteasome subunit beta type-6
Proteasome subunit beta type-5
Protein-lysine 6-oxidase
Mitogen-activated protein kinase 1
Cytosol aminopeptidase
CCN family member 2
Transketolase
Elongation factor 1-delta
Myristoylated alanine-rich C-kinase substrate
Peroxiredoxin-6
Large ribosomal subunit protein uL11
Phosphatidylethanolamine-binding protein 1
Protein disulfide-isomerase A3
Serine/threonine-protein phosphatase 2A 65 kDa regulatory subunit A alpha isoform
Adenylosuccinate synthetase isozyme 2
S-adenosylmethionine synthase isoform type-2
Cytochrome b-c1 complex subunit 1, mitochondrial
Heterogeneous nuclear ribonucleoprotein H
14-3-3 protein beta/alpha
Stress-induced-phosphoprotein 1
Protein S100-A11
Peroxiredoxin-2

Large ribosomal subunit protein uL6
Cadherin-5
Ribonuclease 4
Heat shock 70 kDa protein 4
Catenin alpha-1
Catenin beta-1
Prohibitin 1
Serpin B6
Radixin
Large ribosomal subunit protein eL22
Thrombospondin-4
Fibrillin-1
Myosin-9
Tyrosine-protein kinase receptor Tie-1
26S proteasome regulatory subunit 7
Large ribosomal subunit protein uL4
Phosphoglucomutase-1
Transgelin-2
Transaldolase
RNA-binding motif protein, X chromosome
V-type proton ATPase catalytic subunit A
Stress-70 protein, mitochondrial
Small ribosomal subunit protein eS19
Large ribosomal subunit protein uL3
Dolichyl-diphosphooligosaccharide--protein glycosyltransferase 48 kDa subunit
Acidic leucine-rich nuclear phosphoprotein 32 family member A
T-complex protein 1 subunit zeta
Nicotinamide N-methyltransferase
Large ribosomal subunit protein uL13
Malate dehydrogenase, cytoplasmic
Malate dehydrogenase, mitochondrial
Trifunctional enzyme subunit alpha, mitochondrial
Eukaryotic translation initiation factor 2 subunit 3
Glycine--tRNA ligase
Lamina-associated polypeptide 2, isoform alpha
Lysosomal Pro-X carboxypeptidase
Cell surface glycoprotein MUC18
Matrin-3
Nicotinamide phosphoribosyltransferase
Voltage-dependent anion-selective channel protein 2
Ubiquitin carboxyl-terminal hydrolase 5
Ran GTPase-activating protein 1
Large ribosomal subunit protein uL15
Large ribosomal subunit protein uL18
Large ribosomal subunit protein eL21
Large ribosomal subunit protein eL28
Small ribosomal subunit protein uS4
Small ribosomal subunit protein uS7
Small ribosomal subunit protein eS10
Glucosamine-6-phosphate isomerase 1

Ras GTPase-activating-like protein IQGAP1
Dolichyl-diphosphooligosaccharide--protein glycosyltransferase subunit STT3A
F-actin-capping protein subunit alpha-2
F-actin-capping protein subunit beta
Glutamine--tRNA ligase
ATP synthase subunit O, mitochondrial
LIM and senescent cell antigen-like-containing domain protein 1
Coatomer subunit delta
T-complex protein 1 subunit epsilon
Nestin
Heat shock 70 kDa protein 13
Isocitrate dehydrogenase [NADP], mitochondrial
Phosphatidylinositol transfer protein beta isoform
Mannan-binding lectin serine protease 1
Large ribosomal subunit protein eL34
Fatty acid synthase
T-complex protein 1 subunit gamma
Beta-arrestin-1
Elongation factor Tu, mitochondrial
Signal recognition particle 9 kDa protein
Proteasome subunit beta type-3
Proteasome subunit beta type-2
Cartilage oligomeric matrix protein
Rab GDP dissociation inhibitor beta
Serpin H1
Hsc70-interacting protein
Large ribosomal subunit protein eL14
T-complex protein 1 subunit theta
T-complex protein 1 subunit delta
Ras-related protein Rab-5C
Ras-related protein Rab-7a
Hepatoma-derived growth factor
Heterogeneous nuclear ribonucleoprotein A3
6-phosphogluconate dehydrogenase, decarboxylating
Heterogeneous nuclear ribonucleoprotein M
Rho GDP-dissociation inhibitor 1
Rho GDP-dissociation inhibitor 2
F-actin-capping protein subunit alpha-1
Biliverdin reductase A
ATP-citrate synthase
Coatomer subunit beta
Coatomer subunit alpha
Dipeptidyl peptidase 1
Activated RNA polymerase II transcriptional coactivator p15
Arginine--tRNA ligase, cytoplasmic
Voltage-dependent calcium channel subunit alpha-2/delta-1
Ubiquitin carboxyl-terminal hydrolase 14
Branched-chain-amino-acid aminotransferase, cytosolic
Delta-1-pyrroline-5-carboxylate synthase
Phospholipid transfer protein

Exportin-2
Transitional endoplasmic reticulum ATPase
Trifunctional enzyme subunit beta, mitochondrial
Mesencephalic astrocyte-derived neurotrophic factor
Nucleosome assembly protein 1-like 1
Adenosine kinase
Cadherin-6
Cadherin-13
Puromycin-sensitive aminopeptidase
Eukaryotic translation initiation factor 3 subunit B
ATP synthase subunit f, mitochondrial
Eukaryotic translation initiation factor 6
C-terminal-binding protein 2
Actin-related protein 2/3 complex subunit 4
Triosephosphate isomerase
Eukaryotic translation initiation factor 3 subunit E
Actin, cytoplasmic 1
Eukaryotic initiation factor 4A-I
Small ribosomal subunit protein uS10
Proteasome subunit alpha type-6
Cell division control protein 42 homolog
Destrin
Ras-related protein Rab-2A
Actin-related protein 3
Actin-related protein 2
ADP-ribosylation factor 3
Small ribosomal subunit protein eS1
Large ribosomal subunit protein eL15
Protein mago nashi homolo
Large ribosomal subunit protein eL27
10 kDa heat shock protein, mitochondrial
Protein transport protein Sec61 subunit alpha isoform 1
NPC intracellular cholesterol transporter 2
Heterogeneous nuclear ribonucleoprotein K
14-3-3 protein gamma
Small ribosomal subunit protein eS7
Serine/threonine-protein phosphatase PP1-beta catalytic subunit
26S proteasome regulatory subunit 4
Small ribosomal subunit protein eS8
Small ribosomal subunit protein uS8
Small ribosomal subunit protein uS9
14-3-3 protein epsilon
Small ribosomal subunit protein uS11
Small ribosomal subunit protein uS12
Small ribosomal subunit protein uS13
Small ribosomal subunit protein uS15
Small ribosomal subunit protein uS17
Small nuclear ribonucleoprotein Sm D3
Large ribosomal subunit protein eL8
Ras-related protein Rab-11A

Small ribosomal subunit protein eS4, X isoform
Actin, aortic smooth muscle
Large ribosomal subunit protein uL23
Small ribosomal subunit protein eS6
Histone H4
GTP-binding nuclear protein Ran
Large ribosomal subunit protein uL14
Small ribosomal subunit protein eS24
Small ribosomal subunit protein eS25
Small ribosomal subunit protein eS26
Guanine nucleotide-binding protein G(I)/G(S)/G(T) subunit beta-1
Large ribosomal subunit protein eL30
Large ribosomal subunit protein eL31
Large ribosomal subunit protein uL1
Large ribosomal subunit protein eL32
Large ribosomal subunit protein uL5
Large ribosomal subunit protein uL2
Peptidyl-prolyl cis-trans isomerase A
Ras-related C3 botulinum toxin substrate 1
AP-2 complex subunit beta
14-3-3 protein zeta/delta
Large ribosomal subunit protein eL38
Eukaryotic translation initiation factor 5A-1
Small ribosomal subunit protein RACK1
Y-box-binding protein 1
Signal peptidase complex catalytic subunit SEC11A
Tropomyosin alpha-4 chain
Ubiquitin-conjugating enzyme E2 L3
Elongation factor 1-alpha 1
Tubulin alpha-1B chain
Tubulin beta-4B chain
Histone H3.1
T-complex protein 1 subunit beta
Glutathione S-transferase omega-1
DNA-dependent protein kinase catalytic subunit
Sushi repeat-containing protein SRPX
Dermcidin
Large ribosomal subunit protein eL24
Large ribosomal subunit protein eL42
Large ribosomal subunit protein eL19
Serine/arginine-rich splicing factor 3
Basement membrane-specific heparan sulfate proteoglycan core protein
Cytochrome c
Solute carrier family 25 member 3
Clathrin heavy chain 1
Peptidyl-prolyl cis-trans isomerase FKBP3
Heterogeneous nuclear ribonucleoprotein U
Spectrin beta chain, non-erythrocytic 1
Protein SET
Serine/arginine-rich splicing factor 2

Fatty acid-binding protein 5
Adenylyl cyclase-associated protein 1
Interleukin-1 receptor-like 1
ATP-dependent 6-phosphofructokinase, platelet type
Large ribosomal subunit protein eL20
Procollagen-lysine,2-oxoglutarate 5-dioxygenase 1
Nucleobindin-1
Large ribosomal subunit protein eL6
Caveolin-1
1,4-alpha-glucan-branching enzyme
Eukaryotic translation initiation factor 4 gamma 1
14-3-3 protein eta
Caldesmon
Proteasome activator complex subunit 1
Amyloid beta precursor like protein 2
Fibromodulin
Peroxiredoxin-1
Large ribosomal subunit protein eL18
Complement component 1 Q subcomponent-binding protein, mitochondrial
Cytoskeleton-associated protein 4
KH domain-containing, RNA-binding, signal transduction-associated protein 1
Prolow-density lipoprotein receptor-related protein 1
Serine/arginine-rich splicing factor 1
ATP-dependent RNA helicase A
Testican-1
Neuroblast differentiation-associated protein AHNAK
EGF-containing fibulin-like extracellular matrix protein 1
Follistatin-related protein 1
Contactin-1
Interleukin enhancer-binding factor 2
Interleukin enhancer-binding factor 3
Vesicular integral-membrane protein VIP36
Heat shock protein 75 kDa, mitochondrial
Peroxiredoxin-4
Chromobox protein homolog 3
26S proteasome non-ATPase regulatory subunit 2
Multimerin-1
Transcription intermediary factor 1-beta
Inactive tyrosine-protein kinase 7
Integrin-linked protein kinase
CD166 antigen
Spectrin alpha chain, non-erythrocytic 1
Spliceosome RNA helicase DDX39B
Tubulin beta-2A chain
Coactosin-like protein
Heterogeneous nuclear ribonucleoprotein D0
Eukaryotic translation initiation factor 3 subunit A
Dihydropyrimidinase-related protein 3
Dynactin subunit 1
Cytoplasmic dynein 1 heavy chain 1

Eukaryotic initiation factor 4A-II
Filamin-C
SPARC-like protein 1
Neutral alpha-glucosidase AB
Major vault protein
Latent-transforming growth factor beta-binding protein 1
Latent-transforming growth factor beta-binding protein 2
Importin subunit beta-1
Septin-2
116 kDa U5 small nuclear ribonucleoprotein component
Protein disulfide-isomerase A6
Plectin
Non-POU domain-containing octamer-binding protein
Serine/threonine-protein phosphatase 2A activator
Reticulocalbin-1
Poly(rC)-binding protein 1
Poly(rC)-binding protein 2
Splicing factor 3B subunit 3
Ras suppressor protein 1
Protein transport protein Sec23A
Regucalcin
Transforming growth factor-beta-induced protein ig-h3
Septin-7
Insulin-like growth factor-binding protein 7
Laminin subunit alpha-4
Exostosin-1
DNA damage-binding protein 1
Hsp90 co-chaperone Cdc37
Dihydropyrimidinase-related protein 2
Extracellular matrix protein 1
Fascin
Gamma-interferon-inducible protein 16
Membrane primary amine oxidase
Thioredoxin reductase 1, cytoplasmic
Prolyl 3-hydroxylase 1
Very-long-chain 3-oxoacyl-CoA reductase
Caveolae-associated protein 1
Golgi-associated kinase 1B
CD109 antigen
Ribosomal protein eS27-like
A disintegrin and metalloproteinase with thrombospondin motifs 13
Staphylococcal nuclease domain-containing protein 1
Cytoplasmic FMR1-interacting protein 1
Carbohydrate sulfotransferase 3
Target of Nesh-SH3
Fermitin family homolog 3
Cullin-associated NEDD8-dissociated protein 1
Histone H2A type 2-B
Adipocyte enhancer-binding protein 1
5'-3' exonuclease PLD3

Procollagen galactosyltransferase 1
Thioredoxin domain-containing protein 5
Minor histocompatibility antigen H13
Programmed cell death 6-interacting protein
Histone H1.10
Peroxidasin homolog
Serine protease HTRA1
Gamma-glutamyl hydrolase
Probable ATP-dependent RNA helicase DDX17
Golgi apparatus protein 1
Transportin-1
Betaine--homocysteine S-methyltransferase 1
Far upstream element-binding protein 1
Leucine-rich repeat-containing protein 59
BTB/POZ domain-containing protein KCTD12
Ubiquitin thioesterase OTUB1
Cytosolic non-specific dipeptidase
Vacuolar protein sorting-associated protein 35
Hedgehog-interacting protein
Synaptic vesicle membrane protein VAT-1 homolog
Legumain
Prohibitin-2
3-hydroxyacyl-CoA dehydrogenase type-2
Collagen alpha-1(XII) chain
Heterogeneous nuclear ribonucleoprotein A/B
T-complex protein 1 subunit eta
Protein arginine N-methyltransferase 1
Growth/differentiation factor 15
Coronin-1B
45 kDa calcium-binding protein
Peroxiredoxin-like 2A
Extended synaptotagmin-1
COP9 signalosome complex subunit 4
Transmembrane protein 43
Tubulin beta-6 chain
Acetyl-CoA acetyltransferase, cytosolic
Complement C1q tumor necrosis factor-related protein 5
Complement C1q tumor necrosis factor-related protein 3
Apoptosis inhibitor 5
Tubulointerstitial nephritis antigen-like
Ras-related protein Rab-1B
Nuclear ubiquitous casein and cyclin-dependent kinase substrate 1
EH domain-containing protein 1
Divergent protein kinase domain 2B
Calcyclin-binding protein
Adipocyte plasma membrane-associated protein
Interleukin-1 receptor accessory protein
Complement component C1q receptor
Endothelial cell-specific molecule 1
Fructose-2,6-bisphosphatase TIGAR

Sialic acid synthase
Obg-like ATPase 1
Septin-11
Alpha-parvin
Stabilin-1
Dipeptidyl peptidase 3
Myoferlin
EH domain-containing protein 2
Cysteine-rich motor neuron 1 protein
Leucine--tRNA ligase, cytoplasmic
Cathepsin Z
N-acetyl-D-glucosamine kinase
Stomatin-like protein 2, mitochondrial
Proteasome activator complex subunit 2
Coronin-1C
Endothelial protein C receptor
Proliferation-associated protein 2G4
RuvB-like 2
C-type lectin domain family 11 member A
RuvB-like 1
Nuclear migration protein nudC
Voltage-dependent anion-selective channel protein 3
RNA-splicing ligase RtcB homolog
Large ribosomal subunit protein eL36
Talin-1
Lysyl oxidase homolog 2
Hypoxia up-regulated protein 1
FACT complex subunit SPT16
RNA-binding protein 8A
Endothelial lipase
Coatomer subunit gamma-1
Chloride intracellular channel protein 4
Small ribosomal subunit protein uS2B
Ras-related protein Rap-1b-like protein
Rho-related GTP-binding protein RhoC
Eukaryotic translation initiation factor 3 subunit C-like protein
Nascent polypeptide-associated complex subunit alpha, muscle-specific form
Cytosolic acyl coenzyme A thioester hydrolase
Unconventional myosin-Ic
26S proteasome non-ATPase regulatory subunit 11
26S proteasome non-ATPase regulatory subunit 12
Chloride intracellular channel protein 1
Sulfhydryl oxidase 1
Importin-5
Procollagen-lysine,2-oxoglutarate 5-dioxygenase 2
Nucleolar protein 56
ATP-dependent RNA helicase DDX3X
CCN family member 1
Pirin
Tripeptidyl-peptidase 1

Neuropilin-1
Proteasome subunit alpha type-7
Myosin regulatory light chain 12B
Heterogeneous nuclear ribonucleoprotein D-like
Exportin-1
Angiopoietin-2
Actin-related protein 2/3 complex subunit 1B
Actin-related protein 2/3 complex subunit 2
Actin-related protein 2/3 complex subunit 3
Matrilin-3
ATP-dependent RNA helicase DHX15
26S proteasome non-ATPase regulatory subunit 3
Heterogeneous nuclear ribonucleoprotein R
Thioredoxin-like protein 1
Mitotic checkpoint protein BUB3
Alpha-actinin-4
Neuropilin-2
Heterogeneous nuclear ribonucleoprotein Q
Multifunctional procollagen lysine hydroxylase and glycosyltransferase LH3
Histone H2B type 1-K
WD repeat-containing protein 1
Copine-3
Filamin-B
Citrate synthase, mitochondrial
Vacuolar protein sorting-associated protein 26A
NADH dehydrogenase [ubiquinone] iron-sulfur protein 3, mitochondrial
Isocitrate dehydrogenase [NADP] cytoplasmic
PRA1 family protein 3
Dysferlin
Protein transport protein Sec31A
Calsyntenin-1
Serine protease 23
Apolipoprotein M
Pantetheinase
AP-2 complex subunit alpha-1
Caveolae-associated protein 2
L-lactate dehydrogenase A chain
Aldehyde dehydrogenase 1A1
NADH-cytochrome b5 reductase 3
Cytochrome c oxidase subunit 2
Coagulation factor XIII A chain
Purine nucleoside phosphorylase
Hypoxanthine-guanine phosphoribosyltransferase
Aspartate aminotransferase, mitochondrial
Phosphoglycerate kinase 1
Adenylate kinase isoenzyme 1
Coagulation factor X
Tissue-type plasminogen activator
Complement C3
Metalloproteinase inhibitor 1

Cystatin-C
Collagen alpha-1(I) chain
Collagen alpha-1(II) chain
Prelamin-A/C
Fibronectin
Retinol-binding protein 4
Albumin
Interstitial collagenase
Fructose-bisphosphate aldolase A
Annexin A1
Apolipoprotein B-100
von Willebrand factor
Glyceraldehyde-3-phosphate dehydrogenase
HLA class I histocompatibility antigen, A alpha chain
Heat shock protein beta-1
Dolichyl-diphosphooligosaccharide--protein glycosyltransferase subunit 1
Dolichyl-diphosphooligosaccharide--protein glycosyltransferase subunit 2
Guanine nucleotide-binding protein G(i) subunit alpha-2
Histone H2A type 1-B/E
Sodium/potassium-transporting ATPase subunit alpha-1
Amyloid-beta precursor protein
Aldehyde dehydrogenase, mitochondrial
Protein S100-A8
Plasminogen activator inhibitor 1
ADP/ATP translocase 2
Eukaryotic translation initiation factor 2 subunit 1
Endothelin-1
Large ribosomal subunit protein uL10
Lupus La protein
Integrin beta-1
Keratin, type I cytoskeletal 18
Gelsolin
Prothymosin alpha
ATP synthase subunit beta, mitochondrial
Protein S100-A9
Protein S100-A6
Creatine kinase M-type
Alpha-enolase
Glucose-6-phosphate isomerase
Nucleophosmin
Tropomyosin alpha-3 chain
Beta-hexosaminidase subunit alpha
Histone H2B type 1-J
L-lactate dehydrogenase B chain
Glutathione peroxidase 1
Protein disulfide-isomerase
Cathepsin D
Annexin A2
Calpain-1 catalytic subunit
Tubulin beta chain

Prosaposin
Beta-hexosaminidase subunit beta
Profilin-1
Bifunctional glutamate/proline--tRNA ligase
Cathepsin B
Heat shock protein HSP 90-alpha
Heterogeneous nuclear ribonucleoproteins C1/C2
Laminin subunit beta-1
Tropomyosin beta chain
Fumarate hydratase, mitochondrial
Thrombospondin-1
Ribonuclease pancreatic
Collagen alpha-2(I) chain
Annexin A6
Heat shock protein HSP 90-beta
72 kDa type IV collagenase
Collagen alpha-2(IV) chain
U1 small nuclear ribonucleoprotein 70 kDa
Integrin alpha-5
Vimentin
Small ribosomal subunit protein eS17
Annexin A5
U1 small nuclear ribonucleoprotein A
Glutathione S-transferase P
High mobility group protein B1
SPARC
Annexin A4
Heterogeneous nuclear ribonucleoprotein A1
Cytochrome c oxidase subunit 6C
Leukotriene A-4 hydrolase
Polyubiquitin-B
Heat shock 70 kDa protein 1A
Tubulin alpha-3C chain
Serglycin
Receptor-type tyrosine-protein phosphatase F
Lysosomal protective protein
Tissue factor pathway inhibitor
60 kDa heat shock protein, mitochondrial
Clusterin
Endoplasmic reticulum chaperone BiP
Laminin subunit gamma-1
Heat shock cognate 71 kDa protein
Ras-related protein Ral-A
Lysosome-associated membrane glycoprotein 1
Glucose-6-phosphate 1-dehydrogenase
C-1-tetrahydrofolate synthase, cytoplasmic
Cation-independent mannose-6-phosphate receptor
Alcohol dehydrogenase class-3
Polyadenylate-binding protein 1
Proliferating cell nuclear antigen

Collagen alpha-1(XI) chain
Collagen alpha-1(VI) chain
Collagen alpha-3(VI) chain
ADP/ATP translocase 3
Inosine-5'-monophosphate dehydrogenase 2
Annexin A3
Alpha-actinin-1
Angiotensin-converting enzyme
Xaa-Pro dipeptidase
X-ray repair cross-complementing protein 6
X-ray repair cross-complementing protein 5
Cytochrome c oxidase subunit 4 isoform 1, mitochondrial
Lysosome-associated membrane glycoprotein 2
Ribonuclease inhibitor
Elongation factor 2
Protein disulfide-isomerase A4
Plastin-3
CD59 glycoprotein
Glucosidase 2 subunit beta
Farnesyl pyrophosphate synthase
Nidogen-1
Pyruvate kinase PKM
Endoplasmin
Heterogeneous nuclear ribonucleoprotein L
Aspartate--tRNA ligase, cytoplasmic
Fatty acid-binding protein, adipocyte
Aldo-keto reductase family 1 member B1
Aminopeptidase N
Ras-related C3 botulinum toxin substrate 2
Eukaryotic peptide chain release factor GTP-binding subunit ERF3A
Ezrin
Nucleoside diphosphate kinase A
N-acetylglucosamine-6-sulfatase
Small ribosomal subunit protein uS5
Metalloproteinase inhibitor 2
Platelet endothelial cell adhesion molecule
Histone H1.5
Histone H1.3
Histone H1.2
Fumarylacetoacetase
Stathmin
High mobility group protein HMG-I/HMG-Y
Integrin alpha-2
Calpain-2 catalytic subunit
Probable ATP-dependent RNA helicase DDX5
ATP-dependent 6-phosphofructokinase, liver type
Vascular endothelial growth factor receptor 1
26S proteasome regulatory subunit 6A
T-complex protein 1 subunit alpha
Large ribosomal subunit protein eL33

ADP-ribosylation factor 4
Large ribosomal subunit protein uL30
Vinculin
Large ribosomal subunit protein uL22
Phosphoglycerate mutase 1
Regulator of chromosome condensation
Peptidyl-glycine alpha-amidating monooxygenase
Nucleolin
Hexokinase-1
Spermidine synthase
Eukaryotic translation initiation factor 2 subunit 2
Transcobalamin-2
Proteasome subunit beta type-1
Lamin-B1
Mimecan
Collagen alpha-1(V) chain
Filamin-A
Cytoplasmic aconitate hydratase
Midkine
Voltage-dependent anion-selective channel protein 1
Biglycan
Protein-glutamine gamma-glutamyltransferase 2
Bone morphogenetic protein 6
Bifunctional phosphoribosylaminoimidazole carboxylase/phosphoribosylaminoimidazole succ
Ubiquitin-like modifier-activating enzyme 1
Glutathione peroxidase 3
Nucleoside diphosphate kinase B
Heterogeneous nuclear ribonucleoproteins A2/B1
Insulin-like growth factor-binding protein 4
Cytochrome b-c1 complex subunit 2, mitochondrial
Splicing factor, proline- and glutamine-rich
Peptidyl-prolyl cis-trans isomerase B
Tryptophan--tRNA ligase, cytoplasmic
Small ribosomal subunit protein uS3
Adenosylhomocysteinase
Cofilin-1
Thymidylate kinase
ATP synthase subunit alpha, mitochondrial
Proteasome subunit alpha type-1
Proteasome subunit alpha type-2
Proteasome subunit alpha type-3
Proteasome subunit alpha type-4
Pentraxin-related protein PTX3
Moesin
Splicing factor U2AF 65 kDa subunit
Large ribosomal subunit protein eL13
High mobility group protein B2
Polypyrimidine tract-binding protein 1
Valine--tRNA ligase
Elongation factor 1-gamma

Hepatocyte growth factor-like protein
Stomatin
14-3-3 protein theta
Large ribosomal subunit protein uL16
DNA-(apurinic or apyrimidinic site) endonuclease
Multifunctional protein CAD
Calreticulin
Microtubule-associated protein 4
Calnexin
Proteasome subunit alpha type-5
Proteasome subunit beta type-6
Proteasome subunit beta type-5
Protein-lysine 6-oxidase
Mitogen-activated protein kinase 1
Progranulin
Cytosol aminopeptidase
CCN family member 2
Transketolase
Elongation factor 1-delta
Myristoylated alanine-rich C-kinase substrate
Peroxiredoxin-6
Large ribosomal subunit protein uL11
Enoyl-CoA hydratase, mitochondrial
Phosphatidylethanolamine-binding protein 1
Protein disulfide-isomerase A3
Serine/threonine-protein phosphatase 2A 65 kDa regulatory subunit A alpha isoform
Adenylosuccinate synthetase isozyme 2
S-adenosylmethionine synthase isoform type-2
Cytochrome b-c1 complex subunit 1, mitochondrial
Heterogeneous nuclear ribonucleoprotein H
14-3-3 protein beta/alpha
Stress-induced-phosphoprotein 1
Protein S100-A11
Peroxiredoxin-2
Large ribosomal subunit protein uL6
Cadherin-5
Ribonuclease 4
Heat shock 70 kDa protein 4
Catenin alpha-1
Catenin beta-1
Prohibitin 1
Serpin B6
Radixin
Large ribosomal subunit protein eL22
Thrombospondin-4
Fibrillin-1
Myosin-9
Tyrosine-protein kinase receptor Tie-1
26S proteasome regulatory subunit 7
Large ribosomal subunit protein uL4

Phosphoglucomutase-1
Transgelin-2
Transaldolase
RNA-binding motif protein, X chromosome
V-type proton ATPase catalytic subunit A
Stress-70 protein, mitochondrial
Small ribosomal subunit protein eS19
Large ribosomal subunit protein uL3
Dolichyl-diphosphooligosaccharide--protein glycosyltransferase 48 kDa subunit
Acidic leucine-rich nuclear phosphoprotein 32 family member A
Macrophage-capping protein
T-complex protein 1 subunit zeta
Nicotinamide N-methyltransferase
Large ribosomal subunit protein uL13
Malate dehydrogenase, cytoplasmic
Malate dehydrogenase, mitochondrial
Trifunctional enzyme subunit alpha, mitochondrial
Eukaryotic translation initiation factor 2 subunit 3
Glycine--tRNA ligase
Lamina-associated polypeptide 2, isoform alpha
Lysosomal Pro-X carboxypeptidase
Cell surface glycoprotein MUC18
Matrin-3
Nicotinamide phosphoribosyltransferase
Voltage-dependent anion-selective channel protein 2
Ubiquitin carboxyl-terminal hydrolase 5
Ran GTPase-activating protein 1
Large ribosomal subunit protein uL15
Large ribosomal subunit protein uL18
Large ribosomal subunit protein eL21
Large ribosomal subunit protein eL28
Small ribosomal subunit protein uS4
Small ribosomal subunit protein uS7
Small ribosomal subunit protein eS10
Glucosamine-6-phosphate isomerase 1
Ras GTPase-activating-like protein IQGAP1
Dolichyl-diphosphooligosaccharide--protein glycosyltransferase subunit STT3A
F-actin-capping protein subunit alpha-2
F-actin-capping protein subunit beta
Glutamine--tRNA ligase
ATP synthase subunit O, mitochondrial
LIM and senescent cell antigen-like-containing domain protein 1
Coatomer subunit delta
T-complex protein 1 subunit epsilon
Nestin
Heat shock 70 kDa protein 13
Isocitrate dehydrogenase [NADP], mitochondrial
Phosphatidylinositol transfer protein beta isoform
Mannan-binding lectin serine protease 1
Large ribosomal subunit protein eL34

Fatty acid synthase
T-complex protein 1 subunit gamma
Beta-arrestin-1
Elongation factor Tu, mitochondrial
Signal recognition particle 9 kDa protein
Proteasome subunit beta type-3
Proteasome subunit beta type-2
Cartilage oligomeric matrix protein
Rab GDP dissociation inhibitor beta
Serpin B8
Serpin H1
Hsc70-interacting protein
Large ribosomal subunit protein eL14
T-complex protein 1 subunit theta
T-complex protein 1 subunit delta
Ras-related protein Rab-5C
Ras-related protein Rab-7a
Heterogeneous nuclear ribonucleoprotein A3
6-phosphogluconate dehydrogenase, decarboxylating
Heterogeneous nuclear ribonucleoprotein M
Rho GDP-dissociation inhibitor 1
Rho GDP-dissociation inhibitor 2
F-actin-capping protein subunit alpha-1
Biliverdin reductase A
ATP-citrate synthase
Coatomer subunit beta
Coatomer subunit alpha
Dipeptidyl peptidase 1
Activated RNA polymerase II transcriptional coactivator p15
Arginine--tRNA ligase, cytoplasmic
Voltage-dependent calcium channel subunit alpha-2/delta-1
Ubiquitin carboxyl-terminal hydrolase 14
Branched-chain-amino-acid aminotransferase, cytosolic
Delta-1-pyrroline-5-carboxylate synthase
Phospholipid transfer protein
Exportin-2
Transitional endoplasmic reticulum ATPase
Trifunctional enzyme subunit beta, mitochondrial
Mesencephalic astrocyte-derived neurotrophic factor
Nucleosome assembly protein 1-like 1
Adenosine kinase
Cadherin-6
Cadherin-13
Puromycin-sensitive aminopeptidase
Eukaryotic translation initiation factor 3 subunit B
ATP synthase subunit f, mitochondrial
Eukaryotic translation initiation factor 6
C-terminal-binding protein 2
Actin-related protein 2/3 complex subunit 4
Triosephosphate isomerase

Eukaryotic translation initiation factor 3 subunit E
Actin, cytoplasmic 1
Eukaryotic initiation factor 4A-I
Small ribosomal subunit protein uS10
Proteasome subunit alpha type-6
Cell division control protein 42 homolog
Destrin
Ras-related protein Rab-2A
Actin-related protein 3
Actin-related protein 2
ADP-ribosylation factor 3
Small ribosomal subunit protein eS1
Large ribosomal subunit protein eL15
Protein mago nashi homolog
Large ribosomal subunit protein eL27
10 kDa heat shock protein, mitochondrial
Protein transport protein Sec61 subunit alpha isoform 1
NPC intracellular cholesterol transporter 2
Heterogeneous nuclear ribonucleoprotein K
14-3-3 protein gamma
Small ribosomal subunit protein eS7
Serine/threonine-protein phosphatase PP1-beta catalytic subunit
26S proteasome regulatory subunit 4
Small ribosomal subunit protein eS8
Small ribosomal subunit protein uS8
Small ribosomal subunit protein uS9
14-3-3 protein epsilon
Small ribosomal subunit protein uS11
Small ribosomal subunit protein uS12
Small ribosomal subunit protein uS13
Small ribosomal subunit protein uS15
Small ribosomal subunit protein uS17
Small nuclear ribonucleoprotein Sm D3
Large ribosomal subunit protein eL8
Ras-related protein Rab-11A
Small ribosomal subunit protein eS4, X isoform
Actin, aortic smooth muscle
Large ribosomal subunit protein uL23
Small ribosomal subunit protein eS6
Histone H4
GTP-binding nuclear protein Ran
Large ribosomal subunit protein uL14
Small ribosomal subunit protein eS24
Small ribosomal subunit protein eS25
Small ribosomal subunit protein eS26
Guanine nucleotide-binding protein G(I)/G(S)/G(T) subunit beta-1
Large ribosomal subunit protein eL30
Large ribosomal subunit protein eL31
Large ribosomal subunit protein uL1
Large ribosomal subunit protein eL32

Large ribosomal subunit protein uL5
Large ribosomal subunit protein uL2
Peptidyl-prolyl cis-trans isomerase A
Ras-related C3 botulinum toxin substrate 1
AP-2 complex subunit beta
14-3-3 protein zeta/delta
Large ribosomal subunit protein eL38
Eukaryotic translation initiation factor 5A-1
Small ribosomal subunit protein RACK1
Y-box-binding protein 1
Signal peptidase complex catalytic subunit SEC11A
Tropomyosin alpha-4 chain
Ubiquitin-conjugating enzyme E2 L3
Elongation factor 1-alpha 1
Tubulin alpha-1B chain
Tubulin beta-4B chain
Histone H3.1
T-complex protein 1 subunit beta
Glutathione S-transferase omega-1
DNA-dependent protein kinase catalytic subunit
Sushi repeat-containing protein SRPX
Dermcidin
Large ribosomal subunit protein eL24
Large ribosomal subunit protein eL42
Large ribosomal subunit protein eL19
Serine/arginine-rich splicing factor 3
Basement membrane-specific heparan sulfate proteoglycan core protein
Cytochrome c
Solute carrier family 25 member 3
Clathrin heavy chain 1
Peptidyl-prolyl cis-trans isomerase FKBP3
Heterogeneous nuclear ribonucleoprotein U
Spectrin beta chain, non-erythrocytic 1
Protein SET
Serine/arginine-rich splicing factor 2
Fatty acid-binding protein 5
Adenylyl cyclase-associated protein 1
Interleukin-1 receptor-like 1
ATP-dependent 6-phosphofructokinase, platelet type
Large ribosomal subunit protein eL20
Procollagen-lysine,2-oxoglutarate 5-dioxygenase 1
Nucleobindin-1
Large ribosomal subunit protein eL6
Caveolin-1
1,4-alpha-glucan-branching enzyme
Eukaryotic translation initiation factor 4 gamma 1
14-3-3 protein eta
Caldesmon
Proteasome activator complex subunit 1
Amyloid beta precursor like protein 2

Fibromodulin
Peroxiredoxin-1
Large ribosomal subunit protein eL18
Complement component 1 Q subcomponent-binding protein, mitochondrial
Cytoskeleton-associated protein 4
KH domain-containing, RNA-binding, signal transduction-associated protein 1
Prolow-density lipoprotein receptor-related protein 1
Serine/arginine-rich splicing factor 1
ATP-dependent RNA helicase A
Testican-1
Neuroblast differentiation-associated protein AHNAK
EGF-containing fibulin-like extracellular matrix protein 1
Follistatin-related protein 1
Contactin-1
Interleukin enhancer-binding factor 2
Interleukin enhancer-binding factor 3
Vesicular integral-membrane protein VIP36
Heat shock protein 75 kDa, mitochondrial
Peroxiredoxin-4
Chromobox protein homolog 3
26S proteasome non-ATPase regulatory subunit 2
Multimerin-1
Transcription intermediary factor 1-beta
Inactive tyrosine-protein kinase 7
Integrin-linked protein kinase
Sorting nexin-1
CD166 antigen
Spectrin alpha chain, non-erythrocytic 1
Spliceosome RNA helicase DDX39B
Tubulin beta-2A chain
Coactosin-like protein
Heterogeneous nuclear ribonucleoprotein D0
Eukaryotic translation initiation factor 3 subunit A
Dihydropyrimidinase-related protein 3
Dynactin subunit 1
Cytoplasmic dynein 1 heavy chain 1
Eukaryotic initiation factor 4A-II
Filamin-C
SPARC-like protein 1
Neutral alpha-glucosidase AB
Major vault protein
Latent-transforming growth factor beta-binding protein 1
Latent-transforming growth factor beta-binding protein 2
Importin subunit beta-1
Septin-2
116 kDa U5 small nuclear ribonucleoprotein component
Protein disulfide-isomerase A6
Plectin
Non-POU domain-containing octamer-binding protein
Serine/threonine-protein phosphatase 2A activator

Reticulocalbin-1
Poly(rC)-binding protein 1
Poly(rC)-binding protein 2
Splicing factor 3B subunit 3
Ras suppressor protein 1
Protein transport protein Sec23A
Regucalcin
Transforming growth factor-beta-induced protein ig-h3
Septin-7
Insulin-like growth factor-binding protein 7
Laminin subunit alpha-4
Exostosin-1
DNA damage-binding protein 1
Hsp90 co-chaperone Cdc37
Dihydropyrimidinase-related protein 2
Extracellular matrix protein 1
Fascin
Gamma-interferon-inducible protein 16
Membrane primary amine oxidase
Thioredoxin reductase 1, cytoplasmic
Prolyl 3-hydroxylase 1
Caveolae-associated protein 1
Golgi-associated kinase 1B
CD109 antigen
Ribosomal protein eS27-like
A disintegrin and metalloproteinase with thrombospondin motifs 13
Staphylococcal nuclease domain-containing protein 1
Cytoplasmic FMR1-interacting protein 1
Carbohydrate sulfotransferase 3
Target of Nesh-SH3
Fermitin family homolog 3
Cullin-associated NEDD8-dissociated protein 1
Histone H2A type 2-B
Adipocyte enhancer-binding protein 1
5'-3' exonuclease PLD3
Procollagen galactosyltransferase 1
Thioredoxin domain-containing protein 5
Minor histocompatibility antigen H13
Programmed cell death 6-interacting protein
Peroxidasin homolog
Serine protease HTRA1
Gamma-glutamyl hydrolase
Probable ATP-dependent RNA helicase DDX17
Golgi apparatus protein 1
Betaine--homocysteine S-methyltransferase 1
Far upstream element-binding protein 1
Leucine-rich repeat-containing protein 59
BTB/POZ domain-containing protein KCTD12
Ubiquitin thioesterase OTUB1
Cytosolic non-specific dipeptidase

Vacuolar protein sorting-associated protein 35
Hedgehog-interacting protein
Sialidase-1
Synaptic vesicle membrane protein VAT-1 homolog
Legumain
Prohibitin-2
3-hydroxyacyl-CoA dehydrogenase type-2
Collagen alpha-1(XII) chain
Heterogeneous nuclear ribonucleoprotein A/B
T-complex protein 1 subunit eta
Protein arginine N-methyltransferase 1
Growth/differentiation factor 15
Coronin-1B
45 kDa calcium-binding protein
Peroxiredoxin-like 2A
Extended synaptotagmin-1
COP9 signalosome complex subunit 4
Transmembrane protein 43
Tubulin beta-6 chain
Acetyl-CoA acetyltransferase, cytosolic
Complement C1q tumor necrosis factor-related protein 5
Complement C1q tumor necrosis factor-related protein 3
Apoptosis inhibitor 5
Tubulointerstitial nephritis antigen-like
Ras-related protein Rab-1B
Nuclear ubiquitous casein and cyclin-dependent kinase substrate 1
EH domain-containing protein 1
Calcyclin-binding protein
Adipocyte plasma membrane-associated protein
Interleukin-1 receptor accessory protein
Complement component C1q receptor
Endothelial cell-specific molecule 1
Fructose-2,6-bisphosphatase TIGAR
Sialic acid synthase
Obg-like ATPase 1
Septin-11
Alpha-parvin
Stabilin-1
Dipeptidyl peptidase 3
Myoferlin
EH domain-containing protein 2
Cysteine-rich motor neuron 1 protein
Leucine--tRNA ligase, cytoplasmic
Dickkopf-related protein 3
Cathepsin Z
N-acetyl-D-glucosamine kinase
Stomatin-like protein 2, mitochondrial
Proteasome activator complex subunit 2
Coronin-1C
Endothelial protein C receptor

Proliferation-associated protein 2G4
RuvB-like 2
C-type lectin domain family 11 member A
RuvB-like 1
Nuclear migration protein nudC
Voltage-dependent anion-selective channel protein 3
RNA-splicing ligase RtcB homolog
Large ribosomal subunit protein eL36
Talin-1
Lysyl oxidase homolog 2
Hypoxia up-regulated protein 1
RNA-binding protein 8A
Endothelial lipase
Coatomer subunit gamma-1
Chloride intracellular channel protein 4
Small ribosomal subunit protein uS2B
Ras-related protein Rap-1b-like protein
Rho-related GTP-binding protein RhoC
Nascent polypeptide-associated complex subunit alpha, muscle-specific form
Cytosolic acyl coenzyme A thioester hydrolase
Unconventional myosin-Ic
26S proteasome non-ATPase regulatory subunit 11
26S proteasome non-ATPase regulatory subunit 12
Chloride intracellular channel protein 1
Sulfhydryl oxidase 1
Importin-5
Procollagen-lysine,2-oxoglutarate 5-dioxygenase 2
Nucleolar protein 56
ATP-dependent RNA helicase DDX3X
CCN family member 1
Pirin
Tripeptidyl-peptidase 1
Neuropilin-1
Proteasome subunit alpha type-7
Myosin regulatory light chain 12B
Heterogeneous nuclear ribonucleoprotein D-like
Exportin-1
Angiopoietin-2
Actin-related protein 2/3 complex subunit 1B
Actin-related protein 2/3 complex subunit 2
Actin-related protein 2/3 complex subunit 3
Matrilin-3
ATP-dependent RNA helicase DHX15
26S proteasome non-ATPase regulatory subunit 3
Heterogeneous nuclear ribonucleoprotein R
Thioredoxin-like protein 1
Mitotic checkpoint protein BUB3
Alpha-actinin-4
Neuropilin-2
Heterogeneous nuclear ribonucleoprotein Q

Multifunctional procollagen lysine hydroxylase and glycosyltransferase LH3
Histone H2B type 1-K
WD repeat-containing protein 1
Filamin-B
Citrate synthase, mitochondrial
Vacuolar protein sorting-associated protein 26A
NADH dehydrogenase [ubiquinone] iron-sulfur protein 3, mitochondrial
Isocitrate dehydrogenase [NADP] cytoplasmic
PRA1 family protein 3
Dysferlin
Protein transport protein Sec31A
Calsyntenin-1
Serine protease 23
Apolipoprotein M
Pantetheinase
AP-2 complex subunit alpha-1
Caveolae-associated protein 2
L-lactate dehydrogenase A chain
Aldehyde dehydrogenase 1A1
NADH-cytochrome b5 reductase 3
Cytochrome c oxidase subunit 2
Coagulation factor XIII A chain
Purine nucleoside phosphorylase
Hypoxanthine-guanine phosphoribosyltransferase
Aspartate aminotransferase, mitochondrial
Phosphoglycerate kinase 1
Adenylate kinase isoenzyme 1
Coagulation factor X
Tissue-type plasminogen activator
Complement C3
Metalloproteinase inhibitor 1
Cystatin-C
Collagen alpha-1(I) chain
Collagen alpha-1(II) chain
Prelamin-A/C
Fibronectin
Retinol-binding protein 4
Albumin
Interstitial collagenase
Fructose-bisphosphate aldolase A
Annexin A1
Apolipoprotein B-100
von Willebrand factor
Glyceraldehyde-3-phosphate dehydrogenase
HLA class I histocompatibility antigen, A alpha chain
Heat shock protein beta-1
Dolichyl-diphosphooligosaccharide--protein glycosyltransferase subunit 1
Dolichyl-diphosphooligosaccharide--protein glycosyltransferase subunit 2
Guanine nucleotide-binding protein G(i) subunit alpha-2
Histone H2A type 1-B/E

Sodium/potassium-transporting ATPase subunit alpha-1
Amyloid-beta precursor protein
Aldehyde dehydrogenase, mitochondrial
Non-histone chromosomal protein HMG-14
Plasminogen activator inhibitor 1
ADP/ATP translocase 2
Eukaryotic translation initiation factor 2 subunit 1
Endothelin-1
Large ribosomal subunit protein uL10
Lupus La protein
Integrin beta-1
Keratin, type I cytoskeletal 18
Gelsolin
Prothymosin alpha
ATP synthase subunit beta, mitochondrial
Protein S100-A6
Creatine kinase M-type
Alpha-enolase
Glucose-6-phosphate isomerase
Nucleophosmin
Tropomyosin alpha-3 chain
Beta-hexosaminidase subunit alpha
Histone H2B type 1-J
L-lactate dehydrogenase B chain
Glutathione peroxidase 1
Protein disulfide-isomerase
Cathepsin D
Annexin A2
Calpain-1 catalytic subunit
Tubulin beta chain
Prosaposin
Beta-hexosaminidase subunit beta
Profilin-1
Bifunctional glutamate/proline--tRNA ligase
Cathepsin B
Heat shock protein HSP 90-alpha
Heterogeneous nuclear ribonucleoproteins C1/C2
Laminin subunit beta-1
Tropomyosin beta chain
Fumarate hydratase, mitochondrial
Thrombospondin-1
Ribonuclease pancreatic
Collagen alpha-2(I) chain
Annexin A6
Heat shock protein HSP 90-beta
72 kDa type IV collagenase
Collagen alpha-2(IV) chain
U1 small nuclear ribonucleoprotein 70 kDa
Integrin alpha-5
Vimentin

Small ribosomal subunit protein eS17
Annexin A5
U1 small nuclear ribonucleoprotein A
Glutathione S-transferase P
High mobility group protein B1
SPARC
Annexin A4
Heterogeneous nuclear ribonucleoprotein A1
Cytochrome c oxidase subunit 6C
Leukotriene A-4 hydrolase
Polyubiquitin-B
Heat shock 70 kDa protein 1A
Tubulin alpha-3C chain
Serglycin
Receptor-type tyrosine-protein phosphatase F
Tissue factor pathway inhibitor
60 kDa heat shock protein, mitochondrial
Clusterin
Endoplasmic reticulum chaperone BiP
Laminin subunit gamma-1
Heat shock cognate 71 kDa protein
Ras-related protein Ral-A
Lysosome-associated membrane glycoprotein 1
Glucose-6-phosphate 1-dehydrogenase
C-1-tetrahydrofolate synthase, cytoplasmic
Cation-independent mannose-6-phosphate receptor
Alcohol dehydrogenase class-3
Polyadenylate-binding protein 1
Proliferating cell nuclear antigen
Collagen alpha-1(XI) chain
Collagen alpha-1(VI) chain
Collagen alpha-3(VI) chain
ADP/ATP translocase 3
Inosine-5'-monophosphate dehydrogenase 2
Annexin A3
Alpha-actinin-1
Angiotensin-converting enzyme
Xaa-Pro dipeptidase
X-ray repair cross-complementing protein 6
X-ray repair cross-complementing protein 5
Cytochrome c oxidase subunit 4 isoform 1, mitochondrial
Lysosome-associated membrane glycoprotein 2
Ribonuclease inhibitor
Elongation factor 2
Protein disulfide-isomerase A4
Plastin-3
CD59 glycoprotein
Glucosidase 2 subunit beta
Farnesyl pyrophosphate synthase
Nidogen-1

Pyruvate kinase PKM
Endoplasmin
Heterogeneous nuclear ribonucleoprotein L
Aspartate--tRNA ligase, cytoplasmic
Fatty acid-binding protein, adipocyte
Aldo-keto reductase family 1 member B1
Aminopeptidase N
Ras-related C3 botulinum toxin substrate 2
Eukaryotic peptide chain release factor GTP-binding subunit ERF3A
Ezrin
Nucleoside diphosphate kinase A
N-acetylglucosamine-6-sulfatase
Small ribosomal subunit protein uS5
Metalloproteinase inhibitor 2
Platelet endothelial cell adhesion molecule
Histone H1.5
Histone H1.3
Histone H1.2
Fumarylacetoacetase
Stathmin
High mobility group protein HMG-I/HMG-Y
Integrin alpha-2
Calpain-2 catalytic subunit
Probable ATP-dependent RNA helicase DDX5
Vascular endothelial growth factor receptor 1
26S proteasome regulatory subunit 6A
T-complex protein 1 subunit alpha
Large ribosomal subunit protein eL33
ADP-ribosylation factor 4
Large ribosomal subunit protein uL30
Vinculin
Large ribosomal subunit protein uL22
Phosphoglycerate mutase 1
Regulator of chromosome condensation
Peptidyl-glycine alpha-amidating monooxygenase
Nucleolin
Hexokinase-1
Spermidine synthase
Eukaryotic translation initiation factor 2 subunit 2
Transcobalamin-2
Proteasome subunit beta type-1
Lamin-B1
Mimecan
Collagen alpha-1(V) chain
Filamin-A
Cytoplasmic aconitate hydratase
Midkine
Voltage-dependent anion-selective channel protein 1
Biglycan
Protein-glutamine gamma-glutamyltransferase 2

Bone morphogenetic protein 6
Bifunctional phosphoribosylaminoimidazole carboxylase/phosphoribosylaminoimidazole succ
Ubiquitin-like modifier-activating enzyme 1
Glutathione peroxidase 3
Nucleoside diphosphate kinase B
Heterogeneous nuclear ribonucleoproteins A2/B1
Insulin-like growth factor-binding protein 4
Cytochrome b-c1 complex subunit 2, mitochondrial
Splicing factor, proline- and glutamine-rich
Peptidyl-prolyl cis-trans isomerase B
Tryptophan--tRNA ligase, cytoplasmic
Small ribosomal subunit protein uS3
Adenosylhomocysteinase
Cofilin-1
Thymidylate kinase
ATP synthase subunit alpha, mitochondrial
Proteasome subunit alpha type-1
Proteasome subunit alpha type-2
Proteasome subunit alpha type-3
Proteasome subunit alpha type-4
Pentraxin-related protein PTX3
Moesin
Splicing factor U2AF 65 kDa subunit
Large ribosomal subunit protein eL13
High mobility group protein B2
Polypyrimidine tract-binding protein 1
Elongation factor 1-gamma
Hepatocyte growth factor-like protein
Stomatin
14-3-3 protein theta
Large ribosomal subunit protein uL16
DNA-(apurinic or apyrimidinic site) endonuclease
Multifunctional protein CAD
Calreticulin
Microtubule-associated protein 4
Calnexin
Proteasome subunit alpha type-5
Proteasome subunit beta type-6
Proteasome subunit beta type-5
Protein-lysine 6-oxidase
Mitogen-activated protein kinase 1
Progranulin
Cytosol aminopeptidase
CCN family member 2
Transketolase
Elongation factor 1-delta
Myristoylated alanine-rich C-kinase substrate
Peroxiredoxin-6
Large ribosomal subunit protein uL11
Phosphatidylethanolamine-binding protein 1

Protein disulfide-isomerase A3
Serine/threonine-protein phosphatase 2A 65 kDa regulatory subunit A alpha isoform
Adenylosuccinate synthetase isozyme 2
S-adenosylmethionine synthase isoform type-2
Cytochrome b-c1 complex subunit 1, mitochondrial
Heterogeneous nuclear ribonucleoprotein H
14-3-3 protein beta/alpha
Stress-induced-phosphoprotein 1
Protein S100-A11
Peroxiredoxin-2
Large ribosomal subunit protein uL6
Cadherin-5
Ribonuclease 4
Heat shock 70 kDa protein 4
Catenin alpha-1
Catenin beta-1
Prohibitin 1
Serpin B6
Radixin
Large ribosomal subunit protein eL22
Thrombospondin-4
Fibrillin-1
Myosin-9
Tyrosine-protein kinase receptor Tie-1
26S proteasome regulatory subunit 7
Large ribosomal subunit protein uL4
Phosphoglucomutase-1
Transgelin-2
Transaldolase
RNA-binding motif protein, X chromosome
V-type proton ATPase catalytic subunit A
Stress-70 protein, mitochondrial
Small ribosomal subunit protein eS19
Large ribosomal subunit protein uL3
Dolichyl-diphosphooligosaccharide--protein glycosyltransferase 48 kDa subunit
Acidic leucine-rich nuclear phosphoprotein 32 family member A
Macrophage-capping protein
T-complex protein 1 subunit zeta
Nicotinamide N-methyltransferase
Large ribosomal subunit protein uL13
Malate dehydrogenase, cytoplasmic
Malate dehydrogenase, mitochondrial
Trifunctional enzyme subunit alpha, mitochondrial
Eukaryotic translation initiation factor 2 subunit 3
Glycine--tRNA ligase
Lamina-associated polypeptide 2, isoform alpha
Lysosomal Pro-X carboxypeptidase
Cell surface glycoprotein MUC18
Matrin-3
Nicotinamide phosphoribosyltransferase

Voltage-dependent anion-selective channel protein 2
Ubiquitin carboxyl-terminal hydrolase 5
Large ribosomal subunit protein uL15
Large ribosomal subunit protein uL18
Large ribosomal subunit protein eL21
Large ribosomal subunit protein eL28
Small ribosomal subunit protein uS4
Small ribosomal subunit protein uS7
Small ribosomal subunit protein eS10
Glucosamine-6-phosphate isomerase 1
Ras GTPase-activating-like protein IQGAP1
F-actin-capping protein subunit alpha-2
F-actin-capping protein subunit beta
ATP synthase subunit O, mitochondrial
LIM and senescent cell antigen-like-containing domain protein 1
Coatomer subunit delta
T-complex protein 1 subunit epsilon
Nestin
Heat shock 70 kDa protein 13
Isocitrate dehydrogenase [NADP], mitochondrial
Phosphatidylinositol transfer protein beta isoform
Mannan-binding lectin serine protease 1
Large ribosomal subunit protein eL34
Fatty acid synthase
T-complex protein 1 subunit gamma
Elongation factor Tu, mitochondrial
Signal recognition particle 9 kDa protein
Proteasome subunit beta type-3
Proteasome subunit beta type-2
Cartilage oligomeric matrix protein
Rab GDP dissociation inhibitor beta
Serpin H1
Hsc70-interacting protein
Large ribosomal subunit protein eL14
T-complex protein 1 subunit theta
T-complex protein 1 subunit delta
Ras-related protein Rab-5C
Ras-related protein Rab-7a
Hepatoma-derived growth factor
Heterogeneous nuclear ribonucleoprotein A3
6-phosphogluconate dehydrogenase, decarboxylating
Heterogeneous nuclear ribonucleoprotein M
Rho GDP-dissociation inhibitor 1
Rho GDP-dissociation inhibitor 2
F-actin-capping protein subunit alpha-1
Biliverdin reductase A
ATP-citrate synthase
Coatomer subunit beta
Coatomer subunit alpha
Dipeptidyl peptidase 1

Activated RNA polymerase II transcriptional coactivator p15
Arginine--tRNA ligase, cytoplasmic
Voltage-dependent calcium channel subunit alpha-2/delta-1
Ubiquitin carboxyl-terminal hydrolase 14
Branched-chain-amino-acid aminotransferase, cytosolic
Delta-1-pyrroline-5-carboxylate synthase
Phospholipid transfer protein
Exportin-2
Transitional endoplasmic reticulum ATPase
Trifunctional enzyme subunit beta, mitochondrial
Mesencephalic astrocyte-derived neurotrophic factor
Nucleosome assembly protein 1-like 1
Adenosine kinase
Cadherin-6
Cadherin-13
Puromycin-sensitive aminopeptidase
Eukaryotic translation initiation factor 3 subunit B
ATP synthase subunit f, mitochondrial
Eukaryotic translation initiation factor 6
C-terminal-binding protein 2
Actin-related protein 2/3 complex subunit 4
Triosephosphate isomerase
Eukaryotic translation initiation factor 3 subunit E
Actin, cytoplasmic 1
Eukaryotic initiation factor 4A-I
Small ribosomal subunit protein uS10
Proteasome subunit alpha type-6
Cell division control protein 42 homolog
Destrin
Ras-related protein Rab-2A
Actin-related protein 3
Actin-related protein 2
ADP-ribosylation factor 3
Small ribosomal subunit protein eS1
Large ribosomal subunit protein eL15
Large ribosomal subunit protein eL27
10 kDa heat shock protein, mitochondrial
Protein transport protein Sec61 subunit alpha isoform 1
NPC intracellular cholesterol transporter 2
Heterogeneous nuclear ribonucleoprotein K
14-3-3 protein gamma
Small ribosomal subunit protein eS7
Serine/threonine-protein phosphatase PP1-beta catalytic subunit
26S proteasome regulatory subunit 4
Small ribosomal subunit protein eS8
Small ribosomal subunit protein uS8
Small ribosomal subunit protein uS9
14-3-3 protein epsilon
Small ribosomal subunit protein uS11
Small ribosomal subunit protein uS12

Small ribosomal subunit protein uS13
Small ribosomal subunit protein uS15
Small ribosomal subunit protein uS17
Small nuclear ribonucleoprotein Sm D3
Large ribosomal subunit protein eL8
Ras-related protein Rab-11A
Small ribosomal subunit protein eS4, X isoform
Actin, aortic smooth muscle
Large ribosomal subunit protein uL23
Small ribosomal subunit protein eS6
Histone H4
GTP-binding nuclear protein Ran
Large ribosomal subunit protein uL14
Small ribosomal subunit protein eS24
Small ribosomal subunit protein eS25
Small ribosomal subunit protein eS26
Guanine nucleotide-binding protein G(I)/G(S)/G(T) subunit beta-1
Large ribosomal subunit protein eL30
Large ribosomal subunit protein eL31
Large ribosomal subunit protein uL1
Large ribosomal subunit protein eL32
Large ribosomal subunit protein uL5
Large ribosomal subunit protein uL2
Peptidyl-prolyl cis-trans isomerase A
Ras-related C3 botulinum toxin substrate 1
AP-2 complex subunit beta
14-3-3 protein zeta/delta
Large ribosomal subunit protein eL38
Eukaryotic translation initiation factor 5A-1
Small ribosomal subunit protein RACK1
Y-box-binding protein 1
Signal peptidase complex catalytic subunit SEC11A
Tropomyosin alpha-4 chain
Ubiquitin-conjugating enzyme E2 L3
Elongation factor 1-alpha 1
Tubulin alpha-1B chain
Tubulin beta-4B chain
Histone H3.1
T-complex protein 1 subunit beta
Glutathione S-transferase omega-1
DNA-dependent protein kinase catalytic subunit
Sushi repeat-containing protein SRPX
Dermcidin
Large ribosomal subunit protein eL24
Large ribosomal subunit protein eL42
Large ribosomal subunit protein eL19
Serine/arginine-rich splicing factor 3
Basement membrane-specific heparan sulfate proteoglycan core protein
Cytochrome c
Solute carrier family 25 member 3

Clathrin heavy chain 1
Peptidyl-prolyl cis-trans isomerase FKBP3
Heterogeneous nuclear ribonucleoprotein U
Spectrin beta chain, non-erythrocytic 1
Protein SET
Serine/arginine-rich splicing factor 2
Fatty acid-binding protein 5
Adenylyl cyclase-associated protein 1
Interleukin-1 receptor-like 1
ATP-dependent 6-phosphofructokinase, platelet type
Large ribosomal subunit protein eL20
Procollagen-lysine,2-oxoglutarate 5-dioxygenase 1
Nucleobindin-1
Large ribosomal subunit protein eL6
Caveolin-1
1,4-alpha-glucan-branching enzyme
Eukaryotic translation initiation factor 4 gamma 1
14-3-3 protein eta
Caldesmon
Proteasome activator complex subunit 1
Amyloid beta precursor like protein 2
Fibromodulin
Peroxiredoxin-1
Large ribosomal subunit protein eL18
Complement component 1 Q subcomponent-binding protein, mitochondrial
Cytoskeleton-associated protein 4
KH domain-containing, RNA-binding, signal transduction-associated protein 1
Prolow-density lipoprotein receptor-related protein 1
Serine/arginine-rich splicing factor 1
ATP-dependent RNA helicase A
Testican-1
Neuroblast differentiation-associated protein AHNAK
EGF-containing fibulin-like extracellular matrix protein 1
Follistatin-related protein 1
Contactin-1
Interleukin enhancer-binding factor 2
Interleukin enhancer-binding factor 3
Vesicular integral-membrane protein VIP36
Heat shock protein 75 kDa, mitochondrial
Peroxiredoxin-4
Chromobox protein homolog 3
26S proteasome non-ATPase regulatory subunit 2
Multimerin-1
Transcription intermediary factor 1-beta
Inactive tyrosine-protein kinase 7
Integrin-linked protein kinase
CD166 antigen
Spectrin alpha chain, non-erythrocytic 1
Spliceosome RNA helicase DDX39B
Tubulin beta-2A chain

Coactosin-like protein
Heterogeneous nuclear ribonucleoprotein D0
Eukaryotic translation initiation factor 3 subunit A
Dihydropyrimidinase-related protein 3
Dynactin subunit 1
Cytoplasmic dynein 1 heavy chain 1
Eukaryotic initiation factor 4A-II
Filamin-C
SPARC-like protein 1
Neutral alpha-glucosidase AB
Major vault protein
Latent-transforming growth factor beta-binding protein 1
Latent-transforming growth factor beta-binding protein 2
Importin subunit beta-1
Septin-2
116 kDa U5 small nuclear ribonucleoprotein component
Protein disulfide-isomerase A6
Plectin
Non-POU domain-containing octamer-binding protein
Serine/threonine-protein phosphatase 2A activator
Reticulocalbin-1
Poly(rC)-binding protein 1
Poly(rC)-binding protein 2
Splicing factor 3B subunit 3
Ras suppressor protein 1
Protein transport protein Sec23A
Regucalcin
Transforming growth factor-beta-induced protein ig-h3
Septin-7
Insulin-like growth factor-binding protein 7
Laminin subunit alpha-4
Exostosin-1
DNA damage-binding protein 1
Hsp90 co-chaperone Cdc37
Dihydropyrimidinase-related protein 2
Extracellular matrix protein 1
Fascin
Gamma-interferon-inducible protein 16
Membrane primary amine oxidase
Thioredoxin reductase 1, cytoplasmic
Prolyl 3-hydroxylase 1
Very-long-chain 3-oxoacyl-CoA reductase
Caveolae-associated protein 1
Golgi-associated kinase 1B
CD109 antigen
Ribosomal protein eS27-like
A disintegrin and metalloproteinase with thrombospondin motifs 13
Staphylococcal nuclease domain-containing protein 1
Cytoplasmic FMR1-interacting protein 1
Carbohydrate sulfotransferase 3

Target of Nesh-SH3
Fermitin family homolog 3
Cullin-associated NEDD8-dissociated protein 1
Histone H2A type 2-B
Adipocyte enhancer-binding protein 1
5'-3' exonuclease PLD3
Procollagen galactosyltransferase 1
Thioredoxin domain-containing protein 5
Minor histocompatibility antigen H13
Programmed cell death 6-interacting protein
Peroxidasin homolog
Serine protease HTRA1
Gamma-glutamyl hydrolase
Probable ATP-dependent RNA helicase DDX17
Golgi apparatus protein 1
Transportin-1
Betaine--homocysteine S-methyltransferase 1
Far upstream element-binding protein 1
Leucine-rich repeat-containing protein 59
BTB/POZ domain-containing protein KCTD12
Ubiquitin thioesterase OTUB1
Cytosolic non-specific dipeptidase
Vacuolar protein sorting-associated protein 35
Hedgehog-interacting protein
Sialidase-1
Synaptic vesicle membrane protein VAT-1 homolog
Legumain
Prohibitin-2
3-hydroxyacyl-CoA dehydrogenase type-2
Collagen alpha-1(XII) chain
Heterogeneous nuclear ribonucleoprotein A/B
T-complex protein 1 subunit eta
Protein arginine N-methyltransferase 1
Growth/differentiation factor 15
Coronin-1B
45 kDa calcium-binding protein
Peroxiredoxin-like 2A
Extended synaptotagmin-1
COP9 signalosome complex subunit 4
Transmembrane protein 43
Tubulin beta-6 chain
Acetyl-CoA acetyltransferase, cytosolic
Complement C1q tumor necrosis factor-related protein 5
Complement C1q tumor necrosis factor-related protein 3
Apoptosis inhibitor 5
Tubulointerstitial nephritis antigen-like
Ras-related protein Rab-1B
EH domain-containing protein 1
Calcyclin-binding protein
Adipocyte plasma membrane-associated protein

Interleukin-1 receptor accessory protein
Complement component C1q receptor
Endothelial cell-specific molecule 1
Fructose-2,6-bisphosphatase TIGAR
Sialic acid synthase
Obg-like ATPase 1
Septin-11
Alpha-parvin
Stabilin-1
Dipeptidyl peptidase 3
Myoferlin
EH domain-containing protein 2
Cysteine-rich motor neuron 1 protein
Leucine--tRNA ligase, cytoplasmic
Dickkopf-related protein 3
Cathepsin Z
N-acetyl-D-glucosamine kinase
Stomatin-like protein 2, mitochondrial
Proteasome activator complex subunit 2
Endothelial protein C receptor
Proliferation-associated protein 2G4
RuvB-like 2
C-type lectin domain family 11 member A
RuvB-like 1
Nuclear migration protein nudC
Voltage-dependent anion-selective channel protein 3
RNA-splicing ligase RtcB homolog
Large ribosomal subunit protein eL36
Talin-1
Lysyl oxidase homolog 2
Hypoxia up-regulated protein 1
FACT complex subunit SPT16
RNA-binding protein 8A
Endothelial lipase
Coatomer subunit gamma-1
Chloride intracellular channel protein 4

PG.ProteinNames	PG.Quantity
RPSA2_HUMAN	1824.032104
RP1BL_HUMAN	612.8527832
RHOC_HUMAN	1030.880493
EIFCL_HUMAN	434.4083252
NACAM_HUMAN	20425.30664
BACH_HUMAN	1466.856201
MYO1C_HUMAN	2608.766602
PSD11_HUMAN	285.1694946
PSD12_HUMAN	297.1783447
CLIC1_HUMAN	6245.230957
QSOX1_HUMAN	8152.383789
IPO5_HUMAN	446.3264771
PLOD2_HUMAN	950.9047852
DDX3X_HUMAN	603.6932983
CCN1_HUMAN	4882.240234
PIR_HUMAN	465.7383118
TPP1_HUMAN	964.8692017
NRP1_HUMAN	885.8753052
PSA7_HUMAN	1773.725708
ML12B_HUMAN	3856.395508
HNRDL_HUMAN	1485.252441
XPO1_HUMAN	184.808075
ANGP2_HUMAN	1495.071045
ARC1B_HUMAN	519.9172363
ARPC2_HUMAN	1181.830444
ARPC3_HUMAN	320.0768433
MATN3_HUMAN	373.5247803
DHX15_HUMAN	410.7041626
PSMD3_HUMAN	462.6618042
HNRPR_HUMAN	2620.611816
TXNL1_HUMAN	901.6405029
BUB3_HUMAN	342.2250977
ACTN4_HUMAN	8458.182617
NRP2_HUMAN	451.2782593
HNRPQ_HUMAN	1702.168213
PLOD3_HUMAN	469.0793762
H2B1K_HUMAN	33271.91797
WDR1_HUMAN	3347.585693
CPNE3_HUMAN	157.5592804
FLNB_HUMAN	5185.497559
CISY_HUMAN	1134.947876
VP26A_HUMAN	903.6314697
NDUS3_HUMAN	289.4248047
IDHC_HUMAN	1861.891235
PRAF3_HUMAN	410.7059326
DYSF_HUMAN	175.2790222
SC31A_HUMAN	504.2483215
CSTN1_HUMAN	1592.909302

PRS23_HUMAN	853.7302856
APOM_HUMAN	4126.481445
VNN1_HUMAN	425.0233154
AP2A1_HUMAN	457.6203003
CAVN2_HUMAN	217.0490723
LDHA_HUMAN	8661.518555
AL1A1_HUMAN	464.4216614
NB5R3_HUMAN	1394.585449
COX2_HUMAN	549.8632813
F13A_HUMAN	2566.293701
PNPH_HUMAN	2572.568604
HPRT_HUMAN	495.9530945
AATM_HUMAN	1055.620728
PGK1_HUMAN	6090.916016
KAD1_HUMAN	784.3829346
FA10_HUMAN	11563.01855
TPA_HUMAN	1544.251831
CO3_HUMAN	183918.125
TIMP1_HUMAN	1839.03064
CYTC_HUMAN	1207.791382
CO1A1_HUMAN	12185.5332
CO2A1_HUMAN	2677.601807
LMNA_HUMAN	3729.746582
FINC_HUMAN	33138.63672
RET4_HUMAN	20297.4082
ALBU_HUMAN	15821.27441
MMP1_HUMAN	61955.06641
ALDOA_HUMAN	8827.250977
ANXA1_HUMAN	4875.69873
APOB_HUMAN	34551.30859
VWF_HUMAN	10569.79297
G3P_HUMAN	10212.17383
HLAA_HUMAN	1258.16394
HSPB1_HUMAN	4104.437012
RPN1_HUMAN	1609.341431
RPN2_HUMAN	574.991272
GNAI2_HUMAN	1452.352539
H2A1B_HUMAN	55597.05469
AT1A1_HUMAN	700.4694214
A4_HUMAN	1836.767578
ALDH2_HUMAN	1400.211548
S10A8_HUMAN	150.3973694
HMGN1_HUMAN	99.14880371
PAI1_HUMAN	52348.73047
ADT2_HUMAN	513.5028076
IF2A_HUMAN	991.6330566
EDN1_HUMAN	823.5776367
RLA0_HUMAN	5332.316406
LA_HUMAN	243.099884
ITB1_HUMAN	2026.97229

K1C18_HUMAN	586.3391113
GELS_HUMAN	1000.214478
PTMA_HUMAN	2302.835693
ATPB_HUMAN	3438.486816
S10A6_HUMAN	1112.655151
KCRM_HUMAN	192.8117218
ENOA_HUMAN	32796.62109
G6PI_HUMAN	2756.609131
NPM_HUMAN	6783.168945
TPM3_HUMAN	3167.21875
HEXA_HUMAN	244.3302917
H2B1J_HUMAN	1846.83728
LDHB_HUMAN	9914.040039
GPX1_HUMAN	275.8464966
PDIA1_HUMAN	4121.827148
CATD_HUMAN	3739.880859
ANXA2_HUMAN	14312.7998
CAN1_HUMAN	335.2857056
TBB5_HUMAN	12267.04688
SAP_HUMAN	4307.659668
HEXB_HUMAN	1165.700317
PROF1_HUMAN	4921.699707
SYEP_HUMAN	453.5361328
CATB_HUMAN	6165.101074
HS90A_HUMAN	14218.19824
HNRPC_HUMAN	6633.246582
LAMB1_HUMAN	4205.754883
TPM2_HUMAN	1993.449585
FUMH_HUMAN	339.0136719
TSP1_HUMAN	207753.3906
RNAS1_HUMAN	231.3851776
CO1A2_HUMAN	3833.571533
ANXA6_HUMAN	2742.643555
HS90B_HUMAN	28485.94922
MMP2_HUMAN	6289.118164
CO4A2_HUMAN	836.2284546
RU17_HUMAN	1325.345825
ITA5_HUMAN	1202.757202
VIME_HUMAN	47748.16797
RS17_HUMAN	919.4768066
ANXA5_HUMAN	9430.456055
SNRPA_HUMAN	143.170639
GSTP1_HUMAN	1179.601318
HMGB1_HUMAN	930.1287231
SPRC_HUMAN	1998.241455
ANXA4_HUMAN	247.092514
ROA1_HUMAN	4887.452637
COX6C_HUMAN	450.6929321
LKHA4_HUMAN	437.660675
UBB_HUMAN	5852.944824

HS71A_HUMAN	2510.940918
TBA3C_HUMAN	4470.070313
SRGN_HUMAN	5877.393066
PTPRF_HUMAN	737.4737549
PPGB_HUMAN	505.2848511
TFPI1_HUMAN	243.9641418
CH60_HUMAN	4331.891113
CLUS_HUMAN	832.1113281
BIP_HUMAN	8179.721191
LAMC1_HUMAN	1551.407593
HSP7C_HUMAN	12431.48438
RALA_HUMAN	960.6868286
LAMP1_HUMAN	1843.515625
G6PD_HUMAN	1297.057495
C1TC_HUMAN	1986.242798
MPRI_HUMAN	1908.544189
ADHX_HUMAN	2412.202148
PABP1_HUMAN	3205.727783
PCNA_HUMAN	348.6903687
COBA1_HUMAN	2103.703857
CO6A1_HUMAN	13575.16699
CO6A3_HUMAN	866.4106445
ADT3_HUMAN	4126.422363
IMDH2_HUMAN	180.5165253
ANXA3_HUMAN	625.5281982
ACTN1_HUMAN	6546.855469
ACE_HUMAN	245.5241852
XRCC6_HUMAN	1955.004883
XRCC5_HUMAN	1071.29248
COX41_HUMAN	874.427063
LAMP2_HUMAN	2994.779053
RINI_HUMAN	1339.628418
EF2_HUMAN	7980.442871
PDIA4_HUMAN	3000.256592
PLST_HUMAN	7132.861816
CD59_HUMAN	413.5379639
GLU2B_HUMAN	526.5719604
FPPS_HUMAN	1609.526001
NID1_HUMAN	614.3972168
KPYM_HUMAN	28172.00781
ENPL_HUMAN	8496.260742
HNRPL_HUMAN	259.3458862
SYDC_HUMAN	436.5351257
FABP4_HUMAN	403.3596802
ALDR_HUMAN	1006.704468
AMPN_HUMAN	2437.098877
RAC2_HUMAN	216.673172
ERF3A_HUMAN	554.7234497
EZRI_HUMAN	332.8883667
NDKA_HUMAN	481.4385376

GNS_HUMAN	975.3040771
RS2_HUMAN	3429.914063
TIMP2_HUMAN	485.5903931
PECA1_HUMAN	1229.494141
H15_HUMAN	7766.279785
H13_HUMAN	6692.366699
H12_HUMAN	1634.141113
FAAA_HUMAN	431.7940979
STMN1_HUMAN	1045.912964
HMGA1_HUMAN	2457.136475
ITA2_HUMAN	106.3009338
CAN2_HUMAN	1008.368225
DDX5_HUMAN	1489.610352
PFKAL_HUMAN	179.235321
VGFR1_HUMAN	564.2452393
PRS6A_HUMAN	1097.701538
TCPA_HUMAN	1116.734863
RL35A_HUMAN	975.1361084
ARF4_HUMAN	528.9936523
RL7_HUMAN	2663.434082
VINC_HUMAN	4015.9375
RL17_HUMAN	1797.712646
PGAM1_HUMAN	2647.750977
RCC1_HUMAN	345.9108276
AMD_HUMAN	415.5853271
NUCL_HUMAN	8323.823242
HXK1_HUMAN	623.1743164
SPEE_HUMAN	1947.635864
IF2B_HUMAN	304.7086487
TCO2_HUMAN	116.4928436
PSB1_HUMAN	1219.053467
LMNB1_HUMAN	381.113678
MIME_HUMAN	1766.546387
CO5A1_HUMAN	3148.443359
FLNA_HUMAN	5455.680664
ACOHC_HUMAN	331.3726807
MK_HUMAN	839.5957031
VDAC1_HUMAN	1115.75415
PGS1_HUMAN	4604.250488
TGM2_HUMAN	6056.066406
BMP6_HUMAN	368.2325439
PUR6_HUMAN	1061.663696
UBA1_HUMAN	2228.734131
GPX3_HUMAN	2603.398438
NDKB_HUMAN	5467.918457
ROA2_HUMAN	11979.54395
IBP4_HUMAN	1751.344727
QCR2_HUMAN	123.6163177
SFPQ_HUMAN	2492.519043
PPIB_HUMAN	8786.981445

SYWC_HUMAN	366.3547668
RS3_HUMAN	5297.850586
SAHH_HUMAN	4807.29248
COF1_HUMAN	16373.57617
KTHY_HUMAN	131.9526978
ATPA_HUMAN	4341.850098
PSA1_HUMAN	2817.202148
PSA2_HUMAN	631.7896729
PSA3_HUMAN	1145.992188
PSA4_HUMAN	1938.894897
PTX3_HUMAN	11354.23633
MOES_HUMAN	5413.625488
U2AF2_HUMAN	482.4472046
RL13_HUMAN	1702.44104
HMGB2_HUMAN	505.1741638
PTBP1_HUMAN	854.3616333
SYVC_HUMAN	170.957901
EF1G_HUMAN	3260.519531
HGFL_HUMAN	3024.859375
STOM_HUMAN	600.7783203
1433T_HUMAN	1782.880005
RL10_HUMAN	323.9995728
APEX1_HUMAN	880.6367188
PYR1_HUMAN	685.9694214
CALR_HUMAN	4978.004395
MAP4_HUMAN	901.1341553
CALX_HUMAN	2841.016113
PSA5_HUMAN	143.7033997
PSB6_HUMAN	274.489502
PSB5_HUMAN	397.6742859
LYOX_HUMAN	667.7030029
MK01_HUMAN	355.1748352
AMPL_HUMAN	223.1675415
CCN2_HUMAN	52392.16406
TKT_HUMAN	4970.875977
EF1D_HUMAN	1551.436768
MARCS_HUMAN	882.8748779
PRDX6_HUMAN	2301.901367
RL12_HUMAN	2229.418457
PEBP1_HUMAN	1525.22644
PDIA3_HUMAN	7355.059082
2AAA_HUMAN	687.4008179
PURA2_HUMAN	535.8730469
METK2_HUMAN	1960.881104
QCR1_HUMAN	194.6476135
HNRH1_HUMAN	1966.301392
1433B_HUMAN	15238.2334
STIP1_HUMAN	2197.887207
S10AB_HUMAN	527.6528931
PRDX2_HUMAN	856.9963989

RL9_HUMAN	1049.859863
CADH5_HUMAN	5654.416992
RNAS4_HUMAN	200.2744446
HSP74_HUMAN	721.2494507
CTNA1_HUMAN	587.0490112
CTNB1_HUMAN	194.6491699
PHB1_HUMAN	436.7400208
SPB6_HUMAN	391.1347656
RADI_HUMAN	10204.77148
RL22_HUMAN	4111.087891
TSP4_HUMAN	1451.127319
FBN1_HUMAN	765.3453369
MYH9_HUMAN	35209.01563
TIE1_HUMAN	964.0340576
PRS7_HUMAN	682.0594482
RL4_HUMAN	22616.76563
PGM1_HUMAN	3874.448975
TAGL2_HUMAN	448.7955933
TALDO_HUMAN	3076.787598
RBMX_HUMAN	456.6507263
VATA_HUMAN	600.1953125
GRP75_HUMAN	1505.234497
RS19_HUMAN	2409.944336
RL3_HUMAN	1505.745483
OST48_HUMAN	954.1627197
AN32A_HUMAN	2094.808105
TCPZ_HUMAN	1673.902832
NNMT_HUMAN	29463.72852
RL13A_HUMAN	2754.92041
MDHC_HUMAN	1531.372437
MDHM_HUMAN	2152.979492
ECHA_HUMAN	443.880188
IF2G_HUMAN	1431.265869
GARS_HUMAN	606.3781738
LAP2A_HUMAN	672.9431152
PCP_HUMAN	277.1314392
MUC18_HUMAN	3354.52417
MATR3_HUMAN	279.5576172
NAMPT_HUMAN	1258.127075
VDAC2_HUMAN	833.579834
UBP5_HUMAN	291.569458
RAGP1_HUMAN	216.6373596
RL27A_HUMAN	1321.8302
RL5_HUMAN	1706.536621
RL21_HUMAN	74.18722534
RL28_HUMAN	748.2856445
RS9_HUMAN	4596.914063
RS5_HUMAN	8937.120117
RS10_HUMAN	4308.367676
GNPI1_HUMAN	506.7244568

IQGA1_HUMAN	2145.07959
STT3A_HUMAN	128.7759857
CAZA2_HUMAN	704.6777954
CAPZB_HUMAN	2400.523682
SYQ_HUMAN	180.2309113
ATPO_HUMAN	649.3484497
LIMS1_HUMAN	162.2926483
COPD_HUMAN	155.2679138
TCPE_HUMAN	1292.435059
NEST_HUMAN	440.7417297
HSP13_HUMAN	1016.331665
IDHP_HUMAN	136.1196747
PIPNB_HUMAN	381.0359802
MASP1_HUMAN	848.6633911
RL34_HUMAN	79.38636017
FAS_HUMAN	1672.035889
TCPG_HUMAN	1614.13501
ARRB1_HUMAN	167.1033783
EFTU_HUMAN	684.684082
SRP09_HUMAN	184.4459991
PSB3_HUMAN	1052.682617
PSB2_HUMAN	462.5543518
COMP_HUMAN	2045.75708
GDIB_HUMAN	3694.990967
SERPH_HUMAN	11362.9834
F10A1_HUMAN	2513.252197
RL14_HUMAN	1945.088501
TCPQ_HUMAN	3891.393799
TCPD_HUMAN	1500.40918
RAB5C_HUMAN	1498.071777
RAB7A_HUMAN	90.77722931
HDGF_HUMAN	776.4538574
ROA3_HUMAN	2807.407715
6PGD_HUMAN	3064.943604
HNRPM_HUMAN	3204.86084
GDIR1_HUMAN	3824.388916
GDIR2_HUMAN	1166.727417
CAZA1_HUMAN	3246.242432
BIEA_HUMAN	244.9744568
ACLY_HUMAN	1911.954956
COPB_HUMAN	1742.524658
COPA_HUMAN	1301.541992
CATC_HUMAN	1132.628296
TCP4_HUMAN	264.3222656
SYRC_HUMAN	303.6939392
CA2D1_HUMAN	3297.982666
UBP14_HUMAN	963.2527466
BCAT1_HUMAN	288.8228149
P5CS_HUMAN	220.4190216
PLTP_HUMAN	1884.630127

XPO2_HUMAN	926.6765747
TERA_HUMAN	4196.430664
ECHB_HUMAN	470.2079468
MANF_HUMAN	228.6829376
NP1L1_HUMAN	2951.205322
ADK_HUMAN	492.1035156
CADH6_HUMAN	383.2825928
CAD13_HUMAN	1449.503662
PSA_HUMAN	467.1076965
EIF3B_HUMAN	794.1036987
ATPK_HUMAN	638.4593506
IF6_HUMAN	985.0061646
CTBP2_HUMAN	381.5473022
ARPC4_HUMAN	1162.810303
TPIS_HUMAN	2898.582031
EIF3E_HUMAN	466.9959106
ACTB_HUMAN	72843.96875
IF4A1_HUMAN	7935.583496
RS20_HUMAN	1663.119263
PSA6_HUMAN	1024.606812
CDC42_HUMAN	409.7521362
DEST_HUMAN	1416.16333
RAB2A_HUMAN	453.6515198
ARP3_HUMAN	2337.060791
ARP2_HUMAN	1922.401367
ARF3_HUMAN	4141.398926
RS3A_HUMAN	1660.320679
RL15_HUMAN	1119.692505
MGN_HUMAN	532.7443237
RL27_HUMAN	1349.069092
CH10_HUMAN	306.4736633
S61A1_HUMAN	328.4558716
NPC2_HUMAN	149.9640656
HNRPK_HUMAN	5912.518555
1433G_HUMAN	1426.655029
RS7_HUMAN	3166.031494
PP1B_HUMAN	1229.189087
PRS4_HUMAN	431.7938232
RS8_HUMAN	3733.283691
RS15A_HUMAN	1811.73877
RS16_HUMAN	3833.70459
1433E_HUMAN	5122.51123
RS14_HUMAN	2763.302734
RS23_HUMAN	1680.902466
RS18_HUMAN	2309.905273
RS13_HUMAN	2086.557617
RS11_HUMAN	1304.565186
SMD3_HUMAN	1847.982422
RL7A_HUMAN	1823.855225
RB11A_HUMAN	554.8582764

RS4X_HUMAN	2494.145996
ACTA_HUMAN	10893.85547
RL23A_HUMAN	1950.909912
RS6_HUMAN	2788.902344
H4_HUMAN	104540.8281
RAN_HUMAN	4481.255859
RL23_HUMAN	908.6317749
RS24_HUMAN	1315.7323
RS25_HUMAN	3166.501465
RS26_HUMAN	2672.871582
GBB1_HUMAN	1613.698364
RL30_HUMAN	1407.615234
RL31_HUMAN	1829.87561
RL10A_HUMAN	2968.499512
RL32_HUMAN	210.8569336
RL11_HUMAN	1976.532593
RL8_HUMAN	1096.442261
PPIA_HUMAN	2863.584717
RAC1_HUMAN	182.5877228
AP2B1_HUMAN	217.0917969
1433Z_HUMAN	5455.706055
RL38_HUMAN	664.4590454
IF5A1_HUMAN	1906.033081
RACK1_HUMAN	2155.068604
YBOX1_HUMAN	113.3104782
SC11A_HUMAN	445.1855469
TPM4_HUMAN	5757.477539
UB2L3_HUMAN	525.6746216
EF1A1_HUMAN	62730.20313
TBA1B_HUMAN	30883.14648
TBB4B_HUMAN	16072.48633
H31_HUMAN	2981.244629
TCPB_HUMAN	1979.914063
GSTO1_HUMAN	2990.508301
PRKDC_HUMAN	439.2425537
SRPX_HUMAN	4350.551758
DCD_HUMAN	3245.657227
RL24_HUMAN	1495.744385
RL36A_HUMAN	76.39167023
RL19_HUMAN	1425.895752
SRSF3_HUMAN	1074.419312
PGBM_HUMAN	24639.93164
CYC_HUMAN	993.3308716
S25A3_HUMAN	1578.289063
CLH1_HUMAN	4017.161621
FKBP3_HUMAN	613.2238159
HNRPU_HUMAN	4452.473633
SPTB2_HUMAN	658.0318604
SET_HUMAN	4206.091309
SRSF2_HUMAN	955.6976929

FABP5_HUMAN	1880.738525
CAP1_HUMAN	3606.997314
ILRL1_HUMAN	539.809082
PFKAP_HUMAN	3201.886963
RL18A_HUMAN	181.9008026
PLOD1_HUMAN	1185.240356
NUCB1_HUMAN	230.864502
RL6_HUMAN	3777.739746
CAV1_HUMAN	2764.600098
GLGB_HUMAN	226.6215668
IF4G1_HUMAN	377.1027527
1433F_HUMAN	938.1810913
CALD1_HUMAN	267.5254822
PSME1_HUMAN	613.4686279
APLP2_HUMAN	531.9423828
FMOD_HUMAN	2726.625488
PRDX1_HUMAN	2170.171387
RL18_HUMAN	3113.256592
C1QBP_HUMAN	397.351532
CKAP4_HUMAN	2062.435059
KHDR1_HUMAN	1999.909302
LRP1_HUMAN	1384.921631
SRSF1_HUMAN	1354.110474
DHX9_HUMAN	1497.580444
TICN1_HUMAN	3486.895508
AHNK_HUMAN	5091.043457
FBLN3_HUMAN	38582.87891
FSTL1_HUMAN	1304.19043
CNTN1_HUMAN	3497.166992
ILF2_HUMAN	2475.110596
ILF3_HUMAN	2024.227417
LMAN2_HUMAN	354.1747131
TRAP1_HUMAN	22792.81836
PRDX4_HUMAN	1443.734619
CBX3_HUMAN	444.8496704
PSMD2_HUMAN	229.1064453
MMRN1_HUMAN	4681.035156
TIF1B_HUMAN	458.7904358
PTK7_HUMAN	802.2664795
ILK_HUMAN	147.9631348
CD166_HUMAN	1026.939941
SPTN1_HUMAN	1316.990234
DX39B_HUMAN	2426.465088
TBB2A_HUMAN	1409.971802
COTL1_HUMAN	1080.489868
HNRPD_HUMAN	4517.024902
EIF3A_HUMAN	746.1581421
DPYL3_HUMAN	873.1096191
DCTN1_HUMAN	210.9230042
DYHC1_HUMAN	1309.483276

IF4A2_HUMAN	252.8213501
FLNC_HUMAN	1035.903809
SPRL1_HUMAN	4939.412109
GANAB_HUMAN	1480.697998
MVP_HUMAN	1941.630859
LTBP1_HUMAN	120.4704514
LTBP2_HUMAN	2203.227783
IMB1_HUMAN	468.4552002
SEPT2_HUMAN	784.9068604
U5S1_HUMAN	441.5927124
PDIA6_HUMAN	4150.958496
PLEC_HUMAN	1355.501465
NONO_HUMAN	1991.996094
PTPA_HUMAN	441.474823
RCN1_HUMAN	193.8973694
PCBP1_HUMAN	1541.116943
PCBP2_HUMAN	2290.344727
SF3B3_HUMAN	367.7225037
RSU1_HUMAN	613.0899048
SC23A_HUMAN	372.8241577
RGN_HUMAN	1213.224731
BGH3_HUMAN	2990.953857
SEPT7_HUMAN	563.6003418
IBP7_HUMAN	8474.450195
LAMA4_HUMAN	3997.783447
EXT1_HUMAN	278.2529602
DDB1_HUMAN	591.2893066
CDC37_HUMAN	1189.196655
DPYL2_HUMAN	1804.57251
ECM1_HUMAN	1226.180054
FSCN1_HUMAN	3427.72168
IF16_HUMAN	849.5611572
AOC3_HUMAN	2240.66626
TRXR1_HUMAN	3908.989014
P3H1_HUMAN	181.2855377
DHB12_HUMAN	344.5613098
CAVN1_HUMAN	1151.750488
GAK1B_HUMAN	274.1586609
CD109_HUMAN	2110.896484
RS27L_HUMAN	829.9456177
ATS13_HUMAN	2178.769775
SND1_HUMAN	2683.944336
CYFP1_HUMAN	103.050705
CHST3_HUMAN	607.7114258
TARSH_HUMAN	646.1106567
URP2_HUMAN	1142.763184
CAND1_HUMAN	798.0740356
H2A2B_HUMAN	1231.714355
AEBP1_HUMAN	92.10121918
PLD3_HUMAN	457.4320984

GT251_HUMAN	71.39881897
TXND5_HUMAN	2442.855713
HM13_HUMAN	851.2290649
PDC6I_HUMAN	451.8025818
H1X_HUMAN	86.30536652
PXDN_HUMAN	2220.27002
HTRA1_HUMAN	1574.886108
GGH_HUMAN	1342.032227
DDX17_HUMAN	983.9240723
GSLG1_HUMAN	472.9768372
TNPO1_HUMAN	133.7344818
BHMT1_HUMAN	971.2966309
FUBP1_HUMAN	283.8121033
LRC59_HUMAN	7275.714355
KCD12_HUMAN	1246.15332
OTUB1_HUMAN	236.8211975
CNDP2_HUMAN	138.0900269
VPS35_HUMAN	276.2492981
HHIP_HUMAN	2640.200928
VAT1_HUMAN	2988.787598
LGMN_HUMAN	645.394043
PHB2_HUMAN	1396.814209
HCD2_HUMAN	457.0718079
COCA1_HUMAN	395.8888855
ROAA_HUMAN	246.4756317
TCPH_HUMAN	1597.911621
ANM1_HUMAN	188.3956909
GDF15_HUMAN	1281.499268
COR1B_HUMAN	306.7653503
CAB45_HUMAN	95.77348328
PXL2A_HUMAN	1529.121826
ESYT1_HUMAN	385.102417
CSN4_HUMAN	240.6543579
TMM43_HUMAN	299.6790771
TBB6_HUMAN	3435.159668
THIC_HUMAN	770.6704712
C1QT5_HUMAN	1352.047607
C1QT3_HUMAN	1136.764038
API5_HUMAN	213.8803253
TINAL_HUMAN	283.9194031
RAB1B_HUMAN	2271.05249
NUCKS_HUMAN	197.6905975
EHD1_HUMAN	477.8460388
DIK2B_HUMAN	207.5149384
CYBP_HUMAN	235.7365723
APMAP_HUMAN	6943.727539
IL1AP_HUMAN	4579.015137
C1QR1_HUMAN	674.1138916
ESM1_HUMAN	892.8065796
TIGAR_HUMAN	168.1151276

SIAS_HUMAN	186.7291718
OLA1_HUMAN	671.855835
SEP11_HUMAN	1461.136597
PARVA_HUMAN	285.237793
STAB1_HUMAN	1369.441284
DPP3_HUMAN	392.3539124
MYOF_HUMAN	635.9956055
EHD2_HUMAN	810.8588867
CRIM1_HUMAN	230.9386597
SYLC_HUMAN	341.5745544
CATZ_HUMAN	1528.926758
NAGK_HUMAN	257.1359863
STML2_HUMAN	251.8646545
PSME2_HUMAN	262.061676
COR1C_HUMAN	582.1254883
EPCR_HUMAN	1123.005371
PA2G4_HUMAN	1291.618896
RUVB2_HUMAN	285.3389893
CLC11_HUMAN	1045.927979
RUVB1_HUMAN	607.1366577
NUDC_HUMAN	524.9992676
VDAC3_HUMAN	540.3890381
RTCB_HUMAN	308.1592407
RL36_HUMAN	733.189209
TLN1_HUMAN	2359.355957
LOXL2_HUMAN	3034.538086
HYOU1_HUMAN	1554.995361
SP16H_HUMAN	281.9714966
RBM8A_HUMAN	429.3356018
LIPG_HUMAN	879.5151367
COPG1_HUMAN	290.8643188
CLIC4_HUMAN	528.682312
RPSA2_HUMAN	2082.88208
RP1BL_HUMAN	838.7059326
RHOC_HUMAN	1211.478882
EIFCL_HUMAN	176.8553925
NACAM_HUMAN	17231.50781
BACH_HUMAN	825.4876099
MYO1C_HUMAN	2966.012207
PSD11_HUMAN	167.8001404
PSD12_HUMAN	225.2526245
CLIC1_HUMAN	7738.101074
QSOX1_HUMAN	11074.80371
IPO5_HUMAN	380.6049805
PLOD2_HUMAN	1217.193726
NOP56_HUMAN	72.38838196
DDX3X_HUMAN	510.3246155
CCN1_HUMAN	7843.81543
PIR_HUMAN	354.3358459
TPP1_HUMAN	1167.34436

NRP1_HUMAN	1064.938477
PSA7_HUMAN	1704.14856
ML12B_HUMAN	3573.129639
HNRDL_HUMAN	726.2230835
XPO1_HUMAN	121.4569626
ANGP2_HUMAN	2558.581543
ARC1B_HUMAN	421.4531555
ARPC2_HUMAN	1229.148926
ARPC3_HUMAN	210.652832
MATN3_HUMAN	143.4120178
DHX15_HUMAN	421.5735474
PSMD3_HUMAN	329.8792419
HNRPR_HUMAN	1894.234985
TXNL1_HUMAN	453.6097107
BUB3_HUMAN	370.6419678
ACTN4_HUMAN	8304.386719
NRP2_HUMAN	827.34021
HNRPQ_HUMAN	1195.158691
PLOD3_HUMAN	610.1419678
H2B1K_HUMAN	27804.375
WDR1_HUMAN	2772.358398
CPNE3_HUMAN	268.4838867
FLNB_HUMAN	4574.010742
CISY_HUMAN	1458.241211
VP26A_HUMAN	839.5801392
NDUS3_HUMAN	423.7444458
IDHC_HUMAN	1700.601563
PRAF3_HUMAN	752.3259888
DYSF_HUMAN	218.4864197
SC31A_HUMAN	288.9238586
CSTN1_HUMAN	2445.683594
PRS23_HUMAN	801.7210693
APOM_HUMAN	6179.200195
VNN1_HUMAN	1960.280396
AP2A1_HUMAN	322.8055725
CAVN2_HUMAN	107.0178909
LDHA_HUMAN	6623.87207
AL1A1_HUMAN	588.6146851
NB5R3_HUMAN	1506.356689
COX2_HUMAN	1663.528564
F13A_HUMAN	2298.113037
PNPH_HUMAN	2641.974121
HPRT_HUMAN	425.8704834
AATM_HUMAN	647.515686
PGK1_HUMAN	4997.761719
KAD1_HUMAN	806.6686401
FA10_HUMAN	10719.52051
TPA_HUMAN	2689.00415
CO3_HUMAN	188525.75
TIMP1_HUMAN	2362.622803

CYTC_HUMAN	2414.070801
CO1A1_HUMAN	14845.07227
CO2A1_HUMAN	1778.909058
LMNA_HUMAN	3430.817383
FINC_HUMAN	42854.75781
RET4_HUMAN	25728.68945
ALBU_HUMAN	15211.36328
MMP1_HUMAN	89738.95313
ALDOA_HUMAN	6432.629395
ANXA1_HUMAN	4156.027344
APOB_HUMAN	32623.2168
VWF_HUMAN	13150.15625
G3P_HUMAN	8214.408203
HLAA_HUMAN	875.5493164
HSPB1_HUMAN	4703.403809
RPN1_HUMAN	1634.946655
RPN2_HUMAN	744.9732666
GNAI2_HUMAN	1387.074829
H2A1B_HUMAN	47797.78516
AT1A1_HUMAN	942.1290283
A4_HUMAN	2674.291992
ALDH2_HUMAN	309.3006897
S10A8_HUMAN	101.8802948
PAI1_HUMAN	72654.45313
ADT2_HUMAN	1683.293701
IF2A_HUMAN	486.3010559
EDN1_HUMAN	985.9848633
RLA0_HUMAN	2601.975586
LA_HUMAN	172.3965149
ITB1_HUMAN	2718.72998
K1C18_HUMAN	519.3613281
GELS_HUMAN	907.15448
PTMA_HUMAN	3247.382568
ATPB_HUMAN	4619.571289
S10A9_HUMAN	167.2181702
S10A6_HUMAN	1173.598389
KCRM_HUMAN	288.1602478
ENOA_HUMAN	22036.28906
G6PI_HUMAN	2284.5
NPM_HUMAN	4955.373535
TPM3_HUMAN	2154.439209
HEXA_HUMAN	717.3933105
H2B1J_HUMAN	2899.581055
LDHB_HUMAN	7561.504395
GPX1_HUMAN	829.6950684
PDIA1_HUMAN	5969.362305
CATD_HUMAN	4375.102539
ANXA2_HUMAN	12397.16504
CAN1_HUMAN	426.1655273
TBB5_HUMAN	9377.098633

SAP_HUMAN	4986.274902
HEXB_HUMAN	1550.415039
PROF1_HUMAN	3986.771484
SYEP_HUMAN	302.3007507
CATB_HUMAN	7582.533691
HS90A_HUMAN	8552.348633
HNRPC_HUMAN	4055.293701
LAMB1_HUMAN	4667.415039
TPM2_HUMAN	1635.069946
FUMH_HUMAN	186.4696655
TSP1_HUMAN	278701.375
RNAS1_HUMAN	548.322937
CO1A2_HUMAN	4824.636719
ANXA6_HUMAN	2772.928223
HS90B_HUMAN	15309.83203
MMP2_HUMAN	9537.756836
CO4A2_HUMAN	1081.264526
RU17_HUMAN	498.6173096
ITA5_HUMAN	1050.542969
VIME_HUMAN	17964.22852
RS17_HUMAN	1011.037964
ANXA5_HUMAN	9518.120117
SNRPA_HUMAN	116.6339417
GSTP1_HUMAN	1329.018677
HMGB1_HUMAN	775.3338623
SPRC_HUMAN	3212.354004
ANXA4_HUMAN	312.8695068
ROA1_HUMAN	3411.925781
COX6C_HUMAN	718.2199707
LKHA4_HUMAN	523.0095215
UBB_HUMAN	4099.473145
HS71A_HUMAN	1874.008789
TBA3C_HUMAN	3307.47876
SRGN_HUMAN	5964.659668
PTPRF_HUMAN	863.963501
PPGB_HUMAN	965.526062
TFPI1_HUMAN	476.3365173
CH60_HUMAN	4100.150391
CLUS_HUMAN	1707.053955
BIP_HUMAN	7855.399414
LAMC1_HUMAN	2087.938232
HSP7C_HUMAN	9657.605469
RALA_HUMAN	630.0303955
LAMP1_HUMAN	2148.782471
G6PD_HUMAN	978.6691895
C1TC_HUMAN	2200.074951
MPRI_HUMAN	2001.731323
ADHX_HUMAN	1692.411621
PABP1_HUMAN	2786.150635
PCNA_HUMAN	321.7489929

COBA1_HUMAN	2239.570313
CO6A1_HUMAN	15296.76172
CO6A3_HUMAN	983.7169189
ADT3_HUMAN	4884.645996
IMDH2_HUMAN	124.0671005
ANXA3_HUMAN	568.4515991
ACTN1_HUMAN	6029.401367
ACE_HUMAN	247.4976349
PEPD_HUMAN	206.7453461
XRCC6_HUMAN	1478.296753
XRCC5_HUMAN	799.409668
COX41_HUMAN	1268.748047
LAMP2_HUMAN	3162.977783
RINI_HUMAN	1253.315063
EF2_HUMAN	4849.236816
PDIA4_HUMAN	3431.161621
PLST_HUMAN	6725.816406
CD59_HUMAN	642.6193848
GLU2B_HUMAN	994.1011963
FPPS_HUMAN	1423.690796
NID1_HUMAN	523.102478
KPYM_HUMAN	21144.49414
ENPL_HUMAN	7191.437988
HNRPL_HUMAN	220.0429535
SYDC_HUMAN	253.3217926
FABP4_HUMAN	581.1046753
ALDR_HUMAN	1116.009521
AMPN_HUMAN	3057.777344
RAC2_HUMAN	236.9705048
ERF3A_HUMAN	378.412384
EZRI_HUMAN	282.1612549
NDKA_HUMAN	378.7217407
GNS_HUMAN	872.401123
RS2_HUMAN	1597.813599
TIMP2_HUMAN	931.1416016
PECA1_HUMAN	1351.539185
H15_HUMAN	6403.333008
H13_HUMAN	4715.31543
H12_HUMAN	2139.063232
FAAA_HUMAN	257.6256714
STMN1_HUMAN	983.0638428
HMGA1_HUMAN	1949.590454
ITA2_HUMAN	178.4169159
CAN2_HUMAN	668.03125
DDX5_HUMAN	1749.762085
PFKAL_HUMAN	183.7722931
VGFR1_HUMAN	498.0848694
PRS6A_HUMAN	558.6077881
TCPA_HUMAN	714.6744385
RL35A_HUMAN	1039.429321

ARF4_HUMAN	900.0915527
RL7_HUMAN	1987.421143
VINC_HUMAN	3366.476074
RL17_HUMAN	1399.389771
PGAM1_HUMAN	2451.966309
RCC1_HUMAN	336.0324402
AMD_HUMAN	338.1713867
NUCL_HUMAN	3533.560059
HXK1_HUMAN	352.2580872
SPEE_HUMAN	1590.744629
IF2B_HUMAN	290.3715515
TCO2_HUMAN	150.0230865
PSB1_HUMAN	1077.508911
LMNB1_HUMAN	426.6582031
MIME_HUMAN	1283.537964
CO5A1_HUMAN	2589.599365
FLNA_HUMAN	4229.57666
ACOHC_HUMAN	264.8450012
MK_HUMAN	1133.24353
VDAC1_HUMAN	1251.995117
PGS1_HUMAN	7127.065918
TGM2_HUMAN	4874.500488
BMP6_HUMAN	877.3912964
PUR6_HUMAN	716.1629639
UBA1_HUMAN	1666.935059
GPX3_HUMAN	3560.643555
NDKB_HUMAN	5908.128906
ROA2_HUMAN	8510.791992
IBP4_HUMAN	2176.33374
QCR2_HUMAN	139.1104431
SFPQ_HUMAN	2059.642578
PPIB_HUMAN	10183.40723
SYWC_HUMAN	418.0725403
RS3_HUMAN	3658.505127
SAHH_HUMAN	3851.621826
COF1_HUMAN	14490.78906
KTHY_HUMAN	154.8204498
ATPA_HUMAN	4574.749512
PSA1_HUMAN	2125.677979
PSA2_HUMAN	425.8175964
PSA3_HUMAN	900.8310547
PSA4_HUMAN	1313.196777
PTX3_HUMAN	16265.13965
MOES_HUMAN	4092.405762
U2AF2_HUMAN	353.5735168
RL13_HUMAN	1186.791382
HMGB2_HUMAN	390.6855469
PTBP1_HUMAN	481.1873779
SYVC_HUMAN	242.4733276
EF1G_HUMAN	2826.821533

HGFL_HUMAN	3025.836914
STOM_HUMAN	858.2438965
1433T_HUMAN	1816.031006
RL10_HUMAN	744.43573
APEX1_HUMAN	586.9058228
PYR1_HUMAN	450.0375366
CALR_HUMAN	5971.809082
MAP4_HUMAN	286.1274109
CALX_HUMAN	3656.713623
PSA5_HUMAN	275.2619934
PSB6_HUMAN	168.2826691
PSB5_HUMAN	263.6199036
LYOX_HUMAN	1155.864136
MK01_HUMAN	405.6024475
GRN_HUMAN	364.0031128
AMPL_HUMAN	108.8807678
CCN2_HUMAN	77426.15625
TKT_HUMAN	4270.10791
EF1D_HUMAN	876.1338501
MARCS_HUMAN	1174.655884
PRDX6_HUMAN	2249.625977
RL12_HUMAN	1860.422363
ECHM_HUMAN	93.16148376
PEBP1_HUMAN	1581.443115
PDIA3_HUMAN	7631.039551
2AAA_HUMAN	591.635498
PURA2_HUMAN	439.025238
METK2_HUMAN	946.0617676
QCR1_HUMAN	238.2988281
HNRH1_HUMAN	718.9849243
1433B_HUMAN	12533.24805
STIP1_HUMAN	2186.010986
S10AB_HUMAN	779.9225464
PRDX2_HUMAN	929.2602539
RL9_HUMAN	860.7841797
CADH5_HUMAN	7849.276855
RNAS4_HUMAN	347.8139954
HSP74_HUMAN	568.1097412
CTNA1_HUMAN	511.0005188
CTNB1_HUMAN	216.1629181
PHB1_HUMAN	692.0738525
SPB6_HUMAN	352.5556641
RADI_HUMAN	12117.76758
RL22_HUMAN	2419.847412
TSP4_HUMAN	1288.860962
FBN1_HUMAN	944.5894165
MYH9_HUMAN	32536.79688
TIE1_HUMAN	818.3950195
PRS7_HUMAN	462.2348938
RL4_HUMAN	18224.58203

PGM1_HUMAN	4686.265625
TAGL2_HUMAN	543.1973877
TALDO_HUMAN	2718.606934
RBMX_HUMAN	477.1239014
VATA_HUMAN	544.1209717
GRP75_HUMAN	1658.091064
RS19_HUMAN	1730.840332
RL3_HUMAN	1054.87915
OST48_HUMAN	728.6159668
AN32A_HUMAN	1304.396851
CAPG_HUMAN	113.24366
TCPZ_HUMAN	1004.164978
NNMT_HUMAN	22548.00781
RL13A_HUMAN	1755.106323
MDHC_HUMAN	1388.378906
MDHM_HUMAN	3139.545898
ECHA_HUMAN	531.8242188
IF2G_HUMAN	907.4703979
GARS_HUMAN	389.9627686
LAP2A_HUMAN	296.3669128
PCP_HUMAN	206.6313171
MUC18_HUMAN	3771.96167
MATR3_HUMAN	155.267334
NAMPT_HUMAN	1226.729126
VDAC2_HUMAN	1524.534668
UBP5_HUMAN	236.855835
RAGP1_HUMAN	49.69713211
RL27A_HUMAN	554.8604126
RL5_HUMAN	939.392334
RL21_HUMAN	166.5647888
RL28_HUMAN	575.7668457
RS9_HUMAN	2599.108398
RS5_HUMAN	6217.323242
RS10_HUMAN	2645.640137
GNPI1_HUMAN	541.2971191
IQGA1_HUMAN	2172.161621
STT3A_HUMAN	62.8791008
CAZA2_HUMAN	447.4235535
CAPZB_HUMAN	2261.506836
SYQ_HUMAN	203.5935974
ATPO_HUMAN	883.4523315
LIMS1_HUMAN	273.104248
COPD_HUMAN	98.46429443
TCPE_HUMAN	1111.070435
NEST_HUMAN	796.4888306
HSP13_HUMAN	1329.732422
IDHP_HUMAN	266.8352661
PIPNB_HUMAN	291.6514282
MASP1_HUMAN	1049.133179
RL34_HUMAN	95.39186096

FAS_HUMAN	1295.338867
TCPG_HUMAN	1105.695313
ARRB1_HUMAN	269.3992004
EFTU_HUMAN	664.1226807
SRP09_HUMAN	267.5229187
PSB3_HUMAN	760.7424927
PSB2_HUMAN	823.1750488
COMP_HUMAN	2157.635986
GDIB_HUMAN	3113.178955
SPB8_HUMAN	114.6249313
SERPH_HUMAN	12370.33301
F10A1_HUMAN	2157.224854
RL14_HUMAN	2057.977539
TCPQ_HUMAN	2439.864014
TCPD_HUMAN	1196.935425
RAB5C_HUMAN	1508.036255
RAB7A_HUMAN	651.9746704
ROA3_HUMAN	2059.788574
6PGD_HUMAN	2632.801514
HNRPM_HUMAN	2455.060303
GDIR1_HUMAN	3123.418701
GDIR2_HUMAN	1039.039307
CAZA1_HUMAN	2875.323486
BIEA_HUMAN	131.9691772
ACLY_HUMAN	1275.793091
COPB_HUMAN	1496.667969
COPA_HUMAN	1060.04248
CATC_HUMAN	1174.914551
TCP4_HUMAN	427.7063599
SYRC_HUMAN	386.0126038
CA2D1_HUMAN	2976.506104
UBP14_HUMAN	479.0610046
BCAT1_HUMAN	212.8029938
P5CS_HUMAN	564.1859131
PLTP_HUMAN	2731.438721
XPO2_HUMAN	746.6610107
TERA_HUMAN	3624.89624
ECHB_HUMAN	407.62323
MANF_HUMAN	201.6230774
NP1L1_HUMAN	1570.80127
ADK_HUMAN	994.6757202
CADH6_HUMAN	614.4475098
CAD13_HUMAN	1617.978882
PSA_HUMAN	374.9846497
EIF3B_HUMAN	422.2979736
ATPK_HUMAN	1065.133301
IF6_HUMAN	908.4448242
CTBP2_HUMAN	413.2871094
ARPC4_HUMAN	1204.026978
TPIS_HUMAN	2308.165283

EIF3E_HUMAN	148.646759
ACTB_HUMAN	66547.73438
IF4A1_HUMAN	5736.86377
RS20_HUMAN	1357.532593
PSA6_HUMAN	1037.791016
CDC42_HUMAN	316.3653259
DEST_HUMAN	1230.873291
RAB2A_HUMAN	361.3817139
ARP3_HUMAN	1780.613037
ARP2_HUMAN	1685.318604
ARF3_HUMAN	3192.591309
RS3A_HUMAN	1211.612305
RL15_HUMAN	809.5307617
MGN_HUMAN	884.0217285
RL27_HUMAN	847.3405151
CH10_HUMAN	797.2948608
S61A1_HUMAN	244.8459473
NPC2_HUMAN	854.7524414
HNRPK_HUMAN	4083.272705
1433G_HUMAN	1606.325317
RS7_HUMAN	2152.051758
PP1B_HUMAN	1478.204346
PRS4_HUMAN	345.9859924
RS8_HUMAN	2319.814941
RS15A_HUMAN	1419.993164
RS16_HUMAN	2536.716064
1433E_HUMAN	3919.624023
RS14_HUMAN	1692.523804
RS23_HUMAN	1376.177002
RS18_HUMAN	1702.887207
RS13_HUMAN	1408.593994
RS11_HUMAN	887.2014771
SMD3_HUMAN	1336.835815
RL7A_HUMAN	1133.337769
RB11A_HUMAN	496.2575378
RS4X_HUMAN	2168.24292
ACTA_HUMAN	6221.199219
RL23A_HUMAN	295.0083618
RS6_HUMAN	2534.757813
H4_HUMAN	86443.67969
RAN_HUMAN	2954.550537
RL23_HUMAN	414.6465149
RS24_HUMAN	992.5005493
RS25_HUMAN	1980.128784
RS26_HUMAN	1983.386475
GBB1_HUMAN	1385.60498
RL30_HUMAN	1266.385254
RL31_HUMAN	1281.919312
RL10A_HUMAN	2207.736816
RL32_HUMAN	250.6630859

RL11_HUMAN	1514.614136
RL8_HUMAN	744.9536133
PPIA_HUMAN	2872.262939
RAC1_HUMAN	403.4717407
AP2B1_HUMAN	175.9145813
1433Z_HUMAN	5438.91748
RL38_HUMAN	457.992157
IF5A1_HUMAN	1505.410645
RACK1_HUMAN	2273.182617
YBOX1_HUMAN	139.2706757
SC11A_HUMAN	715.4727173
TPM4_HUMAN	5355.755859
UB2L3_HUMAN	405.1382751
EF1A1_HUMAN	35469.21875
TBA1B_HUMAN	22714.8457
TBB4B_HUMAN	13527.72363
H31_HUMAN	2067.758057
TCPB_HUMAN	1295.156616
GSTO1_HUMAN	3583.826904
PRKDC_HUMAN	536.7487183
SRPX_HUMAN	7223.687012
DCD_HUMAN	2220.593018
RL24_HUMAN	1025.534424
RL36A_HUMAN	95.04431915
RL19_HUMAN	578.9584961
SRSF3_HUMAN	948.3082886
PGBM_HUMAN	33872.375
CYC_HUMAN	971.5006104
S25A3_HUMAN	1359.7771
CLH1_HUMAN	3521.652344
FKBP3_HUMAN	411.195343
HNRPU_HUMAN	3680.044434
SPTB2_HUMAN	663.8532104
SET_HUMAN	2772.15625
SRSF2_HUMAN	784.7769775
FABP5_HUMAN	1983.811035
CAP1_HUMAN	2389.311035
ILRL1_HUMAN	1415.193115
PFKAP_HUMAN	2816.969971
RL18A_HUMAN	277.2419434
PLOD1_HUMAN	1670.601318
NUCB1_HUMAN	792.1300659
RL6_HUMAN	2815.609131
CAV1_HUMAN	2993.22998
GLGB_HUMAN	190.5362091
IF4G1_HUMAN	200.5141144
1433F_HUMAN	799.93396
CALD1_HUMAN	221.1822662
PSME1_HUMAN	516.2376709
APLP2_HUMAN	798.8406372

FMOD_HUMAN	3007.083496
PRDX1_HUMAN	3747.068604
RL18_HUMAN	2457.405029
C1QBP_HUMAN	760.6785278
CKAP4_HUMAN	2124.041016
KHDR1_HUMAN	1046.848145
LRP1_HUMAN	1687.839722
SRSF1_HUMAN	1407.283569
DHX9_HUMAN	1116.909546
TICN1_HUMAN	5269.454102
AHNK_HUMAN	1453.213379
FBLN3_HUMAN	53010.05859
FSTL1_HUMAN	2213.786377
CNTN1_HUMAN	5313.133301
ILF2_HUMAN	2386.021729
ILF3_HUMAN	1573.317749
LMAN2_HUMAN	342.6651917
TRAP1_HUMAN	7486.55127
PRDX4_HUMAN	1758.418457
CBX3_HUMAN	371.1550293
PSMD2_HUMAN	205.0105133
MMRN1_HUMAN	6828.888184
TIF1B_HUMAN	329.9299316
PTK7_HUMAN	734.3387451
ILK_HUMAN	200.0018616
SNX1_HUMAN	162.414917
CD166_HUMAN	424.0924072
SPTN1_HUMAN	1321.959106
DX39B_HUMAN	1857.115845
TBB2A_HUMAN	2371.650146
COTL1_HUMAN	633.461731
HNRPD_HUMAN	3388.344971
EIF3A_HUMAN	561.8356323
DPYL3_HUMAN	898.2876587
DCTN1_HUMAN	190.6422577
DYHC1_HUMAN	1049.561768
IF4A2_HUMAN	251.0744324
FLNC_HUMAN	709.0251465
SPRL1_HUMAN	6788.285156
GANAB_HUMAN	1780.359619
MVP_HUMAN	1514.23584
LTBP1_HUMAN	224.8945618
LTBP2_HUMAN	3298.265625
IMB1_HUMAN	655.8678589
SEPT2_HUMAN	840.1710815
U5S1_HUMAN	203.8511353
PDIA6_HUMAN	5099.932129
PLEC_HUMAN	1354.040161
NONO_HUMAN	1834.349731
PTPA_HUMAN	368.7888489

RCN1_HUMAN	469.32724
PCBP1_HUMAN	1814.799927
PCBP2_HUMAN	1474.785767
SF3B3_HUMAN	218.1125641
RSU1_HUMAN	791.081604
SC23A_HUMAN	203.3369751
RGN_HUMAN	1391.441406
BGH3_HUMAN	3326.601318
SEPT7_HUMAN	404.8501587
IBP7_HUMAN	13449.52637
LAMA4_HUMAN	5096.203125
EXT1_HUMAN	297.1888733
DDB1_HUMAN	503.5969238
CDC37_HUMAN	581.9771118
DPYL2_HUMAN	1527.432251
ECM1_HUMAN	615.8689575
FSCN1_HUMAN	3391.303711
IF16_HUMAN	771.7915039
AOC3_HUMAN	1939.403931
TRXR1_HUMAN	3036.476807
P3H1_HUMAN	251.3001099
CAVN1_HUMAN	1475.108887
GAK1B_HUMAN	673.2885742
CD109_HUMAN	3253.955322
RS27L_HUMAN	568.4329224
ATS13_HUMAN	2620.947266
SND1_HUMAN	1938.957275
CYFP1_HUMAN	173.9369049
CHST3_HUMAN	972.6523438
TARSH_HUMAN	781.6577759
URP2_HUMAN	788.2293701
CAND1_HUMAN	556.2902222
H2A2B_HUMAN	955.7943115
AEBP1_HUMAN	328.7350769
PLD3_HUMAN	766.3127441
GT251_HUMAN	402.7020569
TXND5_HUMAN	4306.844238
HM13_HUMAN	885.4865723
PDC6I_HUMAN	216.1703033
PXDN_HUMAN	2849.009521
HTRA1_HUMAN	2748.283936
GGH_HUMAN	1628.158081
DDX17_HUMAN	945.6288452
GSLG1_HUMAN	229.2214966
BHMT1_HUMAN	1130.453369
FUBP1_HUMAN	148.0165253
LRC59_HUMAN	4666.554199
KCD12_HUMAN	1096.523438
OTUB1_HUMAN	203.769165
CNDP2_HUMAN	152.7479858

VPS35_HUMAN	331.666626
HHIP_HUMAN	3552.195801
NEUR1_HUMAN	399.2303467
VAT1_HUMAN	2836.086914
LGMN_HUMAN	798.8808594
PHB2_HUMAN	1324.188232
HCD2_HUMAN	359.0883179
COCA1_HUMAN	314.7036438
ROAA_HUMAN	542.7914429
TCPH_HUMAN	917.5675659
ANM1_HUMAN	291.2171631
GDF15_HUMAN	2169.135498
COR1B_HUMAN	401.374115
CAB45_HUMAN	695.8734741
PXL2A_HUMAN	1927.451904
ESYT1_HUMAN	590.5767212
CSN4_HUMAN	391.3826599
TMM43_HUMAN	261.5491028
TBB6_HUMAN	2978.882568
THIC_HUMAN	478.6714783
C1QT5_HUMAN	1566.433105
C1QT3_HUMAN	726.2249756
API5_HUMAN	297.5409851
TINAL_HUMAN	771.7870483
RAB1B_HUMAN	1361.044067
NUCKS_HUMAN	98.52115631
EHD1_HUMAN	488.080719
CYBP_HUMAN	116.3508072
APMAP_HUMAN	7502.220703
IL1AP_HUMAN	4360.668457
C1QR1_HUMAN	703.3128052
ESM1_HUMAN	3200.796143
TIGAR_HUMAN	182.2737579
SIAS_HUMAN	239.5258484
OLA1_HUMAN	691.9815674
SEP11_HUMAN	453.0801086
PARVA_HUMAN	279.2585144
STAB1_HUMAN	2054.516846
DPP3_HUMAN	226.4230347
MYOF_HUMAN	637.3279419
EHD2_HUMAN	922.774292
CRIM1_HUMAN	280.1637573
SYLC_HUMAN	407.3641357
DKK3_HUMAN	1278.74353
CATZ_HUMAN	1652.490601
NAGK_HUMAN	204.4019623
STML2_HUMAN	304.3312683
PSME2_HUMAN	330.6890869
COR1C_HUMAN	447.8368225
EPCR_HUMAN	1966.119629

PA2G4_HUMAN	671.1289063
RUVB2_HUMAN	260.5184326
CLC11_HUMAN	1658.287598
RUVB1_HUMAN	582.9212036
NUDC_HUMAN	450.7521973
VDAC3_HUMAN	591.9934692
RTCB_HUMAN	201.8566437
RL36_HUMAN	751.2254028
TLN1_HUMAN	2267.324707
LOXL2_HUMAN	4430.259766
HYOU1_HUMAN	2259.597656
RBM8A_HUMAN	68.34227753
LIPG_HUMAN	1083.072266
COPG1_HUMAN	330.9985962
CLIC4_HUMAN	591.5204468
RPSA2_HUMAN	1522.978271
RP1BL_HUMAN	660.6970215
RHOC_HUMAN	957.2871094
NACAM_HUMAN	23514.0293
BACH_HUMAN	1321.834229
MYO1C_HUMAN	2568.507568
PSD11_HUMAN	169.9473572
PSD12_HUMAN	303.9293213
CLIC1_HUMAN	6685.066895
QSOX1_HUMAN	9065.948242
IPO5_HUMAN	418.1885376
PLOD2_HUMAN	855.7476807
NOP56_HUMAN	59.25125885
DDX3X_HUMAN	738.4719238
CCN1_HUMAN	4974.34375
PIR_HUMAN	273.1057129
TPP1_HUMAN	1091.438232
NRP1_HUMAN	1068.125
PSA7_HUMAN	2123.109131
ML12B_HUMAN	3019.968506
HNRDL_HUMAN	809.6609497
XPO1_HUMAN	151.0077667
ANGP2_HUMAN	1716.762329
ARC1B_HUMAN	472.6801453
ARPC2_HUMAN	1618.555786
ARPC3_HUMAN	249.8252258
MATN3_HUMAN	890.1480103
DHX15_HUMAN	402.6142883
PSMD3_HUMAN	352.5722046
HNRPR_HUMAN	2415.18042
TXNL1_HUMAN	862.5621338
BUB3_HUMAN	184.3949432
ACTN4_HUMAN	7430.398926
NRP2_HUMAN	578.1893311
HNRPQ_HUMAN	1548.634033

PLOD3_HUMAN	550.2208862
H2B1K_HUMAN	29808.17969
WDR1_HUMAN	3184.963135
FLNB_HUMAN	4517.905273
CISY_HUMAN	1129.413818
VP26A_HUMAN	470.6975708
NDUS3_HUMAN	224.967041
IDHC_HUMAN	1810.948242
PRAF3_HUMAN	368.6403503
DYSF_HUMAN	200.8088074
SC31A_HUMAN	230.9997864
CSTN1_HUMAN	2092.090088
PRS23_HUMAN	753.0881348
APOM_HUMAN	5767.054688
VNN1_HUMAN	1650.182129
AP2A1_HUMAN	367.8646851
CAVN2_HUMAN	205.884964
LDHA_HUMAN	7044.150391
AL1A1_HUMAN	428.2619629
NB5R3_HUMAN	1227.272461
COX2_HUMAN	633.0728149
F13A_HUMAN	1845.866821
PNPH_HUMAN	2338.602051
HPRT_HUMAN	401.0558777
AATM_HUMAN	930.9195557
PGK1_HUMAN	5627.862305
KAD1_HUMAN	659.9614258
FA10_HUMAN	10682.84375
TPA_HUMAN	1630.815063
CO3_HUMAN	202837.8281
TIMP1_HUMAN	2145.969727
CYTC_HUMAN	1273.447998
CO1A1_HUMAN	11832.28125
CO2A1_HUMAN	1893.136963
LMNA_HUMAN	3325.929932
FINC_HUMAN	38121.83984
RET4_HUMAN	21680.01758
ALBU_HUMAN	11052.90332
MMP1_HUMAN	75605.69531
ALDOA_HUMAN	7449.519043
ANXA1_HUMAN	3987.258789
APOB_HUMAN	31075.40234
VWF_HUMAN	10875.67285
G3P_HUMAN	9025.541016
HLAA_HUMAN	791.5418701
HSPB1_HUMAN	3759.061768
RPN1_HUMAN	1405.734375
RPN2_HUMAN	589.2749634
GNAI2_HUMAN	1255.732788
H2A1B_HUMAN	57633.97266

AT1A1_HUMAN	661.4111328
A4_HUMAN	1879.349121
ALDH2_HUMAN	275.0178833
HMGN1_HUMAN	197.5965881
PAI1_HUMAN	56465.09375
ADT2_HUMAN	1859.028809
IF2A_HUMAN	701.7377319
EDN1_HUMAN	843.1306152
RLA0_HUMAN	3380.829102
LA_HUMAN	166.9455566
ITB1_HUMAN	2112.358398
K1C18_HUMAN	572.1444092
GELS_HUMAN	734.3918457
PTMA_HUMAN	2047.806519
ATPB_HUMAN	3592.647705
S10A6_HUMAN	825.0331421
KCRM_HUMAN	149.9579315
ENOA_HUMAN	27195.51758
G6PI_HUMAN	2786.118408
NPM_HUMAN	5802.945801
TPM3_HUMAN	2049.329102
HEXA_HUMAN	664.7211914
H2B1J_HUMAN	1863.275513
LDHB_HUMAN	8006.247559
GPX1_HUMAN	173.6526337
PDIA1_HUMAN	3814.207275
CATD_HUMAN	3299.224365
ANXA2_HUMAN	12993.76758
CAN1_HUMAN	395.4103699
TBB5_HUMAN	9050.488281
SAP_HUMAN	3317.136475
HEXB_HUMAN	1353.303467
PROF1_HUMAN	4718.456543
SYEP_HUMAN	320.4829102
CATB_HUMAN	6622.421387
HS90A_HUMAN	10873.29395
HNRPC_HUMAN	5175.726563
LAMB1_HUMAN	3952.821777
TPM2_HUMAN	1826.276123
FUMH_HUMAN	307.2402649
TSP1_HUMAN	225202.7969
RNAS1_HUMAN	258.8730469
CO1A2_HUMAN	4129.876953
ANXA6_HUMAN	2615.420166
HS90B_HUMAN	20093.97852
MMP2_HUMAN	7690.754883
CO4A2_HUMAN	1005.98175
RU17_HUMAN	398.5874939
ITA5_HUMAN	1899.263916
VIME_HUMAN	25302.79492

RS17_HUMAN	981.3843994
ANXA5_HUMAN	9203.777344
SNRPA_HUMAN	208.2203827
GSTP1_HUMAN	701.8602905
HMGB1_HUMAN	726.9256592
SPRC_HUMAN	2599.619873
ANXA4_HUMAN	262.3269348
ROA1_HUMAN	3792.115479
COX6C_HUMAN	258.5046387
LKHA4_HUMAN	452.8159485
UBB_HUMAN	3884.388916
HS71A_HUMAN	2130.937988
TBA3C_HUMAN	3552.154297
SRGN_HUMAN	3525.023438
PTPRF_HUMAN	659.5023804
TFPI1_HUMAN	245.1834564
CH60_HUMAN	3636.335938
CLUS_HUMAN	636.8782349
BIP_HUMAN	7260.333496
LAMC1_HUMAN	1652.87146
HSP7C_HUMAN	10833.52051
RALA_HUMAN	1017.490723
LAMP1_HUMAN	1666.888672
G6PD_HUMAN	829.270813
C1TC_HUMAN	244.6103668
MPRI_HUMAN	2238.233398
ADHX_HUMAN	2362.200684
PABP1_HUMAN	3506.846436
PCNA_HUMAN	349.7265625
COBA1_HUMAN	2360.730957
CO6A1_HUMAN	15282.86816
CO6A3_HUMAN	1221.041504
ADT3_HUMAN	3798.386475
IMDH2_HUMAN	141.4103699
ANXA3_HUMAN	661.2424316
ACTN1_HUMAN	5923.193848
ACE_HUMAN	228.2053375
PEPD_HUMAN	345.0817261
XRCC6_HUMAN	1368.279053
XRCC5_HUMAN	828.1987915
COX41_HUMAN	842.7077026
LAMP2_HUMAN	1743.555908
RINI_HUMAN	1320.973389
EF2_HUMAN	6819.366699
PDIA4_HUMAN	3081.900635
PLST_HUMAN	6992.307617
CD59_HUMAN	149.4368896
GLU2B_HUMAN	623.9161377
FPPS_HUMAN	1467.62561
NID1_HUMAN	543.5596924

KPYM_HUMAN	22860.87109
ENPL_HUMAN	6844.71875
HNRPL_HUMAN	365.534668
SYDC_HUMAN	221.9500885
FABP4_HUMAN	409.7028198
ALDR_HUMAN	1536.094604
AMPN_HUMAN	2325.860107
RAC2_HUMAN	242.4256287
ERF3A_HUMAN	447.9020691
EZRI_HUMAN	227.8332214
NDKA_HUMAN	408.8890991
GNS_HUMAN	967.8122559
RS2_HUMAN	2295.849121
TIMP2_HUMAN	776.9772949
PECA1_HUMAN	1093.610718
H15_HUMAN	6460.958008
H13_HUMAN	5187.871094
H12_HUMAN	1469.655273
FAAA_HUMAN	416.9940796
STMN1_HUMAN	1098.318848
HMGA1_HUMAN	2153.290527
ITA2_HUMAN	203.98526
CAN2_HUMAN	650.9336548
DDX5_HUMAN	1294.47583
VGFR1_HUMAN	634.3205566
PRS6A_HUMAN	808.6660156
TCPA_HUMAN	901.4075317
RL35A_HUMAN	1364.204102
ARF4_HUMAN	334.5132141
RL7_HUMAN	1869.778442
VINC_HUMAN	3754.191162
RL17_HUMAN	1578.869629
PGAM1_HUMAN	2154.905029
RCC1_HUMAN	412.1244507
AMD_HUMAN	1391.297974
NUCL_HUMAN	5075.926758
HXK1_HUMAN	481.1727905
SPEE_HUMAN	1350.416016
IF2B_HUMAN	243.2376862
TCO2_HUMAN	318.7315674
PSB1_HUMAN	1560.994873
LMNB1_HUMAN	255.3703156
MIME_HUMAN	1483.335693
CO5A1_HUMAN	2894.399414
FLNA_HUMAN	4639.526367
ACOHC_HUMAN	432.4324341
MK_HUMAN	824.3536377
VDAC1_HUMAN	1018.874268
PGS1_HUMAN	5953.043945
TGM2_HUMAN	5613.299316

BMP6_HUMAN	277.2474976
PUR6_HUMAN	863.8041382
UBA1_HUMAN	1670.519043
GPX3_HUMAN	2977.250732
NDKB_HUMAN	5094.256836
ROA2_HUMAN	10382.31348
IBP4_HUMAN	1341.686035
QCR2_HUMAN	175.3228912
SFPQ_HUMAN	2257.054199
PPIB_HUMAN	9516.541016
SYWC_HUMAN	673.4446411
RS3_HUMAN	4424.585449
SAHH_HUMAN	4918.449707
COF1_HUMAN	14358.1875
KTHY_HUMAN	78.93184662
ATPA_HUMAN	4374.495117
PSA1_HUMAN	2613.830322
PSA2_HUMAN	484.4329224
PSA3_HUMAN	1047.907227
PSA4_HUMAN	1738.996948
PTX3_HUMAN	12889.72852
MOES_HUMAN	4720.699707
U2AF2_HUMAN	297.7889709
RL13_HUMAN	1143.41626
HMGB2_HUMAN	466.244812
PTBP1_HUMAN	697.9595337
EF1G_HUMAN	2954.766357
HGFL_HUMAN	3141.698242
STOM_HUMAN	574.7883301
1433T_HUMAN	1593.025513
RL10_HUMAN	558.9313354
APEX1_HUMAN	862.2783203
PYR1_HUMAN	481.0560303
CALR_HUMAN	5153.564453
MAP4_HUMAN	498.1039429
CALX_HUMAN	2465.996338
PSA5_HUMAN	395.7937927
PSB6_HUMAN	234.3937225
PSB5_HUMAN	557.286438
LYOX_HUMAN	1002.844971
MK01_HUMAN	623.4650879
GRN_HUMAN	304.9951782
AMPL_HUMAN	113.2219467
CCN2_HUMAN	60667.15625
TKT_HUMAN	4153.111816
EF1D_HUMAN	1157.930786
MARCS_HUMAN	1099.279907
PRDX6_HUMAN	1351.564697
RL12_HUMAN	2386.491455
PEBP1_HUMAN	760.1015015

PDIA3_HUMAN	6707.422363
2AAA_HUMAN	405.3560486
PURA2_HUMAN	402.3109436
METK2_HUMAN	1400.465088
QCR1_HUMAN	582.2730103
HNRH1_HUMAN	1167.651245
1433B_HUMAN	13339.8252
STIP1_HUMAN	2604.507568
S10AB_HUMAN	610.7822876
PRDX2_HUMAN	635.2620239
RL9_HUMAN	1160.380493
CADH5_HUMAN	6040.85791
RNAS4_HUMAN	329.7296753
HSP74_HUMAN	716.5840454
CTNA1_HUMAN	429.2294922
CTNB1_HUMAN	195.9925842
PHB1_HUMAN	653.4987793
SPB6_HUMAN	383.4762573
RADI_HUMAN	12645.61719
RL22_HUMAN	2959.393799
TSP4_HUMAN	1142.759399
FBN1_HUMAN	809.9713745
MYH9_HUMAN	30578.04297
TIE1_HUMAN	1206.084595
PRS7_HUMAN	463.1695251
RL4_HUMAN	16589.80859
PGM1_HUMAN	1822.249512
TAGL2_HUMAN	613.696228
TALDO_HUMAN	2219.946533
RBMX_HUMAN	538.220459
VATA_HUMAN	602.2537842
GRP75_HUMAN	1459.011841
RS19_HUMAN	1896.869263
RL3_HUMAN	1059.478516
OST48_HUMAN	733.5278931
AN32A_HUMAN	1010.784485
CAPG_HUMAN	212.9326782
TCPZ_HUMAN	1117.043091
NNMT_HUMAN	14306.24023
RL13A_HUMAN	2092.838867
MDHC_HUMAN	1560.940308
MDHM_HUMAN	2702.125732
ECHA_HUMAN	447.2170105
IF2G_HUMAN	1128.998535
GARS_HUMAN	402.5797424
LAP2A_HUMAN	305.7670288
PCP_HUMAN	297.5664978
MUC18_HUMAN	3377.626221
MATR3_HUMAN	111.6932602
NAMPT_HUMAN	1178.81543

VDAC2_HUMAN	1530.478149
UBP5_HUMAN	249.1760712
RL27A_HUMAN	2698.683838
RL5_HUMAN	1117.538086
RL21_HUMAN	137.7481232
RL28_HUMAN	675.2912598
RS9_HUMAN	3323.887207
RS5_HUMAN	10063.93652
RS10_HUMAN	3065.063477
GNPI1_HUMAN	207.3108521
IQGA1_HUMAN	1779.223022
CAZA2_HUMAN	614.2874146
CAPZB_HUMAN	2600.05249
ATPO_HUMAN	696.9480591
LIMS1_HUMAN	195.3657684
COPD_HUMAN	152.782135
TCPE_HUMAN	1359.060791
NEST_HUMAN	630.0021362
HSP13_HUMAN	1230.139648
IDHP_HUMAN	156.9361267
PIPNB_HUMAN	474.9911194
MASP1_HUMAN	1216.067261
RL34_HUMAN	95.80562592
FAS_HUMAN	1200.631958
TCPG_HUMAN	1421.894287
EFTU_HUMAN	433.6012878
SRP09_HUMAN	266.9353943
PSB3_HUMAN	1002.68219
PSB2_HUMAN	421.7234497
COMP_HUMAN	1795.213257
GDIB_HUMAN	3272.598145
SERPH_HUMAN	11615.88867
F10A1_HUMAN	2157.627197
RL14_HUMAN	1981.822876
TCPQ_HUMAN	2789.434326
TCPD_HUMAN	792.3983765
RAB5C_HUMAN	1368.201782
RAB7A_HUMAN	646.425293
HDGF_HUMAN	301.4282837
ROA3_HUMAN	1918.275879
6PGD_HUMAN	3101.741943
HNRPM_HUMAN	2520.048584
GDIR1_HUMAN	3177.765137
GDIR2_HUMAN	799.4959717
CAZA1_HUMAN	2996.028076
BIEA_HUMAN	193.7015381
ACLY_HUMAN	1708.296509
COPB_HUMAN	1364.773804
COPA_HUMAN	999.9935303
CATC_HUMAN	1756.273926

TCP4_HUMAN	316.2268066
SYRC_HUMAN	210.8260803
CA2D1_HUMAN	3495.086426
UBP14_HUMAN	200.3920288
BCAT1_HUMAN	320.9994812
P5CS_HUMAN	365.980957
PLTP_HUMAN	1995.265625
XPO2_HUMAN	980.4309692
TERA_HUMAN	3791.524658
ECHB_HUMAN	552.3739624
MANF_HUMAN	206.2015686
NP1L1_HUMAN	1668.099976
ADK_HUMAN	489.4536438
CADH6_HUMAN	663.6552124
CAD13_HUMAN	1048.057373
PSA_HUMAN	539.4769897
EIF3B_HUMAN	455.1360168
ATPK_HUMAN	723.3040771
IF6_HUMAN	899.7290649
CTBP2_HUMAN	468.8267517
ARPC4_HUMAN	1676.833374
TPIS_HUMAN	2552.97583
EIF3E_HUMAN	372.442749
ACTB_HUMAN	70743.02344
IF4A1_HUMAN	6489.181641
RS20_HUMAN	1749.024414
PSA6_HUMAN	1230.799316
CDC42_HUMAN	284.7715454
DEST_HUMAN	1146.903076
RAB2A_HUMAN	325.126709
ARP3_HUMAN	2048.95874
ARP2_HUMAN	2370.43457
ARF3_HUMAN	3904.961182
RS3A_HUMAN	1132.839233
RL15_HUMAN	779.8464966
RL27_HUMAN	1614.331543
CH10_HUMAN	452.6411438
S61A1_HUMAN	248.3220978
NPC2_HUMAN	866.4825439
HNRPK_HUMAN	4754.749023
1433G_HUMAN	2220.564941
RS7_HUMAN	2214.849609
PP1B_HUMAN	1339.880859
PRS4_HUMAN	163.2640533
RS8_HUMAN	3226.673584
RS15A_HUMAN	1351.315796
RS16_HUMAN	2929.629395
1433E_HUMAN	4397.850098
RS14_HUMAN	1871.849243
RS23_HUMAN	1077.703491

RS18_HUMAN	2203.143311
RS13_HUMAN	1333.447876
RS11_HUMAN	989.6011963
SMD3_HUMAN	1397.279663
RL7A_HUMAN	1348.352051
RB11A_HUMAN	471.675415
RS4X_HUMAN	2573.140625
ACTA_HUMAN	6996.613281
RL23A_HUMAN	2500.106934
RS6_HUMAN	2412.090088
H4_HUMAN	90394.96875
RAN_HUMAN	3175.796387
RL23_HUMAN	463.8696899
RS24_HUMAN	958.4639893
RS25_HUMAN	3317.990234
RS26_HUMAN	2330.322998
GBB1_HUMAN	1545.427856
RL30_HUMAN	1926.46521
RL31_HUMAN	1598.865356
RL10A_HUMAN	2429.952881
RL32_HUMAN	450.1946106
RL11_HUMAN	1687.561646
RL8_HUMAN	677.7527466
PPIA_HUMAN	2791.587891
RAC1_HUMAN	446.3233032
AP2B1_HUMAN	149.6708069
1433Z_HUMAN	5508.447754
RL38_HUMAN	629.6574097
IF5A1_HUMAN	1207.556152
RACK1_HUMAN	2290.830078
YBOX1_HUMAN	64.00623322
SC11A_HUMAN	431.6246948
TPM4_HUMAN	5214.414063
UB2L3_HUMAN	596.6390991
EF1A1_HUMAN	49332.58594
TBA1B_HUMAN	24602.80859
TBB4B_HUMAN	15171.74219
H31_HUMAN	2924.47876
TCPB_HUMAN	1306.29187
GSTO1_HUMAN	3817.781982
PRKDC_HUMAN	580.9595337
SRPX_HUMAN	6467.962891
DCD_HUMAN	1018.238525
RL24_HUMAN	1296.115356
RL36A_HUMAN	89.44089508
RL19_HUMAN	1662.30127
SRSF3_HUMAN	1076.021606
PGBM_HUMAN	26670.95117
CYC_HUMAN	896.8988037
S25A3_HUMAN	924.7973633

CLH1_HUMAN	3153.386963
FKBP3_HUMAN	707.2322998
HNRPU_HUMAN	3522.262451
SPTB2_HUMAN	571.1329346
SET_HUMAN	3047.430176
SRSF2_HUMAN	628.2025146
FABP5_HUMAN	1699.02356
CAP1_HUMAN	3082.727295
ILRL1_HUMAN	361.9397278
PFKAP_HUMAN	2539.868408
RL18A_HUMAN	1145.290283
PLOD1_HUMAN	1341.520996
NUCB1_HUMAN	208.8154907
RL6_HUMAN	3258.703125
CAV1_HUMAN	2037.369263
GLGB_HUMAN	161.1914215
IF4G1_HUMAN	129.8698425
1433F_HUMAN	819.0545044
CALD1_HUMAN	183.5625
PSME1_HUMAN	133.1097412
APLP2_HUMAN	612.0267944
FMOD_HUMAN	2999.635742
PRDX1_HUMAN	2655.758301
RL18_HUMAN	3039.249023
C1QBP_HUMAN	193.0834961
CKAP4_HUMAN	1562.125
KHDR1_HUMAN	1095.503052
LRP1_HUMAN	1439.144653
SRSF1_HUMAN	1206.291016
DHX9_HUMAN	1232.838135
TICN1_HUMAN	3778.761475
AHNK_HUMAN	2911.536133
FBLN3_HUMAN	44085.50781
FSTL1_HUMAN	1018.793396
CNTN1_HUMAN	5606.876465
ILF2_HUMAN	2151.368164
ILF3_HUMAN	1882.645874
LMAN2_HUMAN	214.7082062
TRAP1_HUMAN	8401.18457
PRDX4_HUMAN	984.8143311
CBX3_HUMAN	272.1995239
PSMD2_HUMAN	196.9364166
MMRN1_HUMAN	4703.985352
TIF1B_HUMAN	579.1795044
PTK7_HUMAN	1268.645874
ILK_HUMAN	154.3296814
CD166_HUMAN	477.2652283
SPTN1_HUMAN	1236.268188
DX39B_HUMAN	2039.277954
TBB2A_HUMAN	2891.577148

COTL1_HUMAN	713.1560059
HNRPD_HUMAN	3743.528564
EIF3A_HUMAN	662.0886841
DPYL3_HUMAN	1008.738281
DCTN1_HUMAN	183.8170166
DYHC1_HUMAN	1122.952637
IF4A2_HUMAN	184.5306396
FLNC_HUMAN	509.0606995
SPRL1_HUMAN	3295.651367
GANAB_HUMAN	1621.736938
MVP_HUMAN	1633.042969
LTBP1_HUMAN	184.3774261
LTBP2_HUMAN	2351.331299
IMB1_HUMAN	612.664856
SEPT2_HUMAN	689.2183838
U5S1_HUMAN	242.7387543
PDIA6_HUMAN	3869.329346
PLEC_HUMAN	1192.724854
NONO_HUMAN	1528.346802
PTPA_HUMAN	383.3763733
RCN1_HUMAN	144.5781403
PCBP1_HUMAN	1832.580688
PCBP2_HUMAN	1852.208252
SF3B3_HUMAN	297.9858398
RSU1_HUMAN	626.487793
SC23A_HUMAN	425.2757874
RGN_HUMAN	1340.052368
BGH3_HUMAN	2618.351318
SEPT7_HUMAN	360.9671326
IBP7_HUMAN	10093.27637
LAMA4_HUMAN	4738.660156
EXT1_HUMAN	289.4602051
DDB1_HUMAN	359.3419495
CDC37_HUMAN	961.9075928
DPYL2_HUMAN	1351.914185
ECM1_HUMAN	1118.682617
FSCN1_HUMAN	3412.830811
IF16_HUMAN	629.0097046
AOC3_HUMAN	2074.021973
TRXR1_HUMAN	3795.742432
P3H1_HUMAN	179.3830719
DHB12_HUMAN	287.1041565
CAVN1_HUMAN	760.8355103
GAK1B_HUMAN	514.1142578
CD109_HUMAN	2638.161865
RS27L_HUMAN	809.1320801
ATS13_HUMAN	2825.500244
SND1_HUMAN	2070.638428
CYFP1_HUMAN	272.3546143
CHST3_HUMAN	555.1015625

TARSH_HUMAN	849.8361206
URP2_HUMAN	596.3476563
CAND1_HUMAN	745.0843506
H2A2B_HUMAN	895.0430298
AEBP1_HUMAN	86.23213196
PLD3_HUMAN	347.7537537
GT251_HUMAN	280.9342651
TXND5_HUMAN	2596.065186
HM13_HUMAN	824.0042725
PDC6I_HUMAN	379.190979
PXDN_HUMAN	2526.654297
HTRA1_HUMAN	2264.213623
GGH_HUMAN	1633.29834
DDX17_HUMAN	764.055542
GSLG1_HUMAN	497.6593323
TNPO1_HUMAN	137.9929047
BHMT1_HUMAN	1279.264282
FUBP1_HUMAN	298.1416321
LRC59_HUMAN	4289.210449
KCD12_HUMAN	962.6291504
OTUB1_HUMAN	265.4508057
CNDP2_HUMAN	178.2509766
VPS35_HUMAN	334.068573
HHIP_HUMAN	3339.884766
NEUR1_HUMAN	745.7010498
VAT1_HUMAN	2792.866455
LGMN_HUMAN	513.6682129
PHB2_HUMAN	849.520874
HCD2_HUMAN	150.8028564
COCA1_HUMAN	391.2483215
ROAA_HUMAN	829.9284668
TCPH_HUMAN	1417.788574
ANM1_HUMAN	246.5565948
GDF15_HUMAN	2068.039063
COR1B_HUMAN	206.5036163
CAB45_HUMAN	244.0468445
PXL2A_HUMAN	1724.963867
ESYT1_HUMAN	335.9148254
CSN4_HUMAN	415.3582764
TMM43_HUMAN	250.8174591
TBB6_HUMAN	3103.691406
THIC_HUMAN	909.8624878
C1QT5_HUMAN	937.1567993
C1QT3_HUMAN	1027.385864
API5_HUMAN	232.6205597
TINAL_HUMAN	495.6711731
RAB1B_HUMAN	360.5158691
EHD1_HUMAN	266.9047852
CYBP_HUMAN	195.9077301
APMAP_HUMAN	9036.725586

IL1AP_HUMAN	4954.626953
C1QR1_HUMAN	891.2409668
ESM1_HUMAN	720.8555908
TIGAR_HUMAN	167.2247467
SIAS_HUMAN	168.9316101
OLA1_HUMAN	715.4886475
SEP11_HUMAN	975.8793335
PARVA_HUMAN	211.9851074
STAB1_HUMAN	2426.445557
DPP3_HUMAN	328.1198425
MYOF_HUMAN	530.8676758
EHD2_HUMAN	754.5741577
CRIM1_HUMAN	227.2120209
SYLC_HUMAN	381.7035217
DKK3_HUMAN	699.7437134
CATZ_HUMAN	1587.518311
NAGK_HUMAN	363.6042175
STML2_HUMAN	209.192627
PSME2_HUMAN	265.5953674
EPCR_HUMAN	1128.607666
PA2G4_HUMAN	850.218689
RUVB2_HUMAN	475.2148438
CLC11_HUMAN	2056.700439
RUVB1_HUMAN	624.0895996
NUDC_HUMAN	176.0881958
VDAC3_HUMAN	533.6748657
RTCB_HUMAN	252.0292206
RL36_HUMAN	413.5672607
TLN1_HUMAN	2387.293701
LOXL2_HUMAN	3021.790039
HYOU1_HUMAN	1665.012207
SP16H_HUMAN	423.1607971
RBM8A_HUMAN	317.7363281
LIPG_HUMAN	933.5
COPG1_HUMAN	292.0863953
CLIC4_HUMAN	575.6948242
