# Supplementary material for: Proteomic Analysis of Endothelial Activation Induced by Adult Angiostrongylus vasorum Homogenate: Insights into Vascular Remodeling and Hemostatic Imbalance
Source: Animals (Basel). 2026 Mar 15;16(6):926. doi: 10.3390/ani16060926 (PMC13023303; doi:10.3390/ani16060926)
Supplement: Supplementary file 1 [file animals-16-00926-s001.zip › Supplmentary Table S5.pdf]

Supplementary Table S5: Proteins identifie

R.Condition	R.Replicat	PG.ProteinAcces
Avasorum	1	A0A0B4J2D5
Avasorum	1	A0AVF1
Avasorum	1	A0AVT1
Avasorum	1	A0FGR8
Avasorum	1	A0JNW5
Avasorum	1	A0MZ66
Avasorum	1	A0PJW6
Avasorum	1	A1A4S6
Avasorum	1	A1L0T0
Avasorum	1	A1X283
Avasorum	1	A2RRP1
Avasorum	1	A2RUC4
Avasorum	1	A2RUS2
Avasorum	1	A3KMH1
Avasorum	1	A3KN83
Avasorum	1	A4D1E9
Avasorum	1	A4D1P6
Avasorum	1	A4D1U4
Avasorum	1	A5PLL7
Avasorum	1	A5PLN9
Avasorum	1	A5YKK6
Avasorum	1	A6NCE7
Avasorum	1	A6NDG6
Avasorum	1	A6NDU8
Avasorum	1	A6NED2
Avasorum	1	A6NFQ2
Avasorum	1	A6NHR9
Avasorum	1	A6NHX0
Avasorum	1	A6NIH7
Avasorum	1	A6NJ78
Avasorum	1	A6NKD9
Avasorum	1	A6ZKI3
Avasorum	1	A7E2V4
Avasorum	1	A8CG34
Avasorum	1	A8MW92
Avasorum	1	A8MWD9
Avasorum	1	A8MWY0
Avasorum	1	A8MXV4
Avasorum	1	A9UHW6
Avasorum	1	P0DP23
Avasorum	1	B2RTY4
Avasorum	1	B7ZAQ6
Avasorum	1	C4AMC7
Avasorum	1	C9JLW8
Avasorum	1	E9PAV3
Avasorum	1	E9PRG8
Avasorum	1	L0R819
Avasorum	1	L0R8F8

Avasorum	1	O00115
Avasorum	1	O00116
Avasorum	1	O00139
Avasorum	1	O00148
Avasorum	1	O00151
Avasorum	1	O00154
Avasorum	1	O00159
Avasorum	1	O00161
Avasorum	1	O00165
Avasorum	1	O00170
Avasorum	1	O00178
Avasorum	1	O00182
Avasorum	1	O00186
Avasorum	1	O00189
Avasorum	1	O00192
Avasorum	1	O00193
Avasorum	1	O00203
Avasorum	1	O00213
Avasorum	1	O00214
Avasorum	1	O00217
Avasorum	1	O00221
Avasorum	1	O00231
Avasorum	1	O00232
Avasorum	1	O00233
Avasorum	1	O00255
Avasorum	1	O00258
Avasorum	1	O00264
Avasorum	1	O00267
Avasorum	1	O00268
Avasorum	1	O00273
Avasorum	1	O00291
Avasorum	1	O00299
Avasorum	1	O00303
Avasorum	1	O00308
Avasorum	1	O00330
Avasorum	1	O00391
Avasorum	1	O00399
Avasorum	1	O00400
Avasorum	1	O00401
Avasorum	1	O00410
Avasorum	1	O00411
Avasorum	1	O00418
Avasorum	1	O00422
Avasorum	1	O00423
Avasorum	1	O00425
Avasorum	1	O00429
Avasorum	1	O00442
Avasorum	1	O00443
Avasorum	1	O00461
Avasorum	1	O00468

Avasorum	1	O00469
Avasorum	1	O00471
Avasorum	1	O00481
Avasorum	1	O00483
Avasorum	1	O00487
Avasorum	1	O00499
Avasorum	1	O00501
Avasorum	1	O00505
Avasorum	1	O00506
Avasorum	1	O00522
Avasorum	1	O00534
Avasorum	1	O00541
Avasorum	1	O00560
Avasorum	1	O00562
Avasorum	1	O00566
Avasorum	1	O00567
Avasorum	1	O00571
Avasorum	1	O00587
Avasorum	1	O00592
Avasorum	1	O00622
Avasorum	1	O00625
Avasorum	1	O00629
Avasorum	1	O00635
Avasorum	1	O00712
Avasorum	1	O00743
Avasorum	1	O00746
Avasorum	1	O00750
Avasorum	1	O00754
Avasorum	1	O00764
Avasorum	1	O00767
Avasorum	1	O14497
Avasorum	1	O14524
Avasorum	1	O14530
Avasorum	1	O14531
Avasorum	1	O14545
Avasorum	1	O14548
Avasorum	1	O14562
Avasorum	1	O14578
Avasorum	1	O14579
Avasorum	1	O14613
Avasorum	1	O14617
Avasorum	1	O14618
Avasorum	1	O14639
Avasorum	1	O14641
Avasorum	1	O14646
Avasorum	1	O14647
Avasorum	1	O14653
Avasorum	1	O14656
Avasorum	1	O14657
Avasorum	1	O14662

Avasorum	1	O14672
Avasorum	1	O14678
Avasorum	1	O14681
Avasorum	1	O14683
Avasorum	1	O14686
Avasorum	1	O14727
Avasorum	1	O14730
Avasorum	1	O14733
Avasorum	1	O14734
Avasorum	1	O14735
Avasorum	1	O14737
Avasorum	1	O14744
Avasorum	1	O14745
Avasorum	1	O14757
Avasorum	1	O14763
Avasorum	1	O14773
Avasorum	1	O14776
Avasorum	1	O14777
Avasorum	1	O14782
Avasorum	1	O14786
Avasorum	1	O14787
Avasorum	1	O14795
Avasorum	1	O14798
Avasorum	1	O14802
Avasorum	1	O14817
Avasorum	1	O14818
Avasorum	1	O14828
Avasorum	1	O14874
Avasorum	1	O14880
Avasorum	1	O14907
Avasorum	1	O14908
Avasorum	1	O14920
Avasorum	1	O14924
Avasorum	1	O14925
Avasorum	1	O14929
Avasorum	1	O14936
Avasorum	1	O14939
Avasorum	1	O14949
Avasorum	1	O14950
Avasorum	1	O14964
Avasorum	1	O14966
Avasorum	1	O14972
Avasorum	1	O14974
Avasorum	1	O14976
Avasorum	1	O14979
Avasorum	1	O14980
Avasorum	1	O14981
Avasorum	1	O15013
Avasorum	1	O15014
Avasorum	1	O15020

Avasorum	1	O15021
Avasorum	1	O15027
Avasorum	1	O15031
Avasorum	1	O15037
Avasorum	1	O15040
Avasorum	1	O15042
Avasorum	1	O15047
Avasorum	1	O15049
Avasorum	1	O15056
Avasorum	1	O15061
Avasorum	1	O15063
Avasorum	1	O15066
Avasorum	1	O15067
Avasorum	1	O15084
Avasorum	1	O15085
Avasorum	1	O15091
Avasorum	1	O15111
Avasorum	1	O15118
Avasorum	1	O15121
Avasorum	1	O15123
Avasorum	1	O15126
Avasorum	1	O15127
Avasorum	1	O15143
Avasorum	1	O15144
Avasorum	1	O15145
Avasorum	1	O15160
Avasorum	1	O15162
Avasorum	1	O15164
Avasorum	1	O15173
Avasorum	1	O15211
Avasorum	1	O15213
Avasorum	1	O15226
Avasorum	1	O15228
Avasorum	1	O15230
Avasorum	1	O15231
Avasorum	1	O15234
Avasorum	1	O15235
Avasorum	1	O15254
Avasorum	1	O15258
Avasorum	1	O15260
Avasorum	1	O15264
Avasorum	1	O15269
Avasorum	1	O15270
Avasorum	1	O15294
Avasorum	1	O15305
Avasorum	1	O15321
Avasorum	1	O15347
Avasorum	1	O15355
Avasorum	1	O15357
Avasorum	1	O15371

Avasorum	1	O15372
Avasorum	1	O15379
Avasorum	1	O15381
Avasorum	1	O15382
Avasorum	1	O15397
Avasorum	1	O15400
Avasorum	1	O15427
Avasorum	1	O15439
Avasorum	1	O15446
Avasorum	1	O15460
Avasorum	1	O15484
Avasorum	1	O15498
Avasorum	1	O15504
Avasorum	1	O15511
Avasorum	1	O15519
Avasorum	1	O15530
Avasorum	1	O15533
Avasorum	1	O15541
Avasorum	1	O15550
Avasorum	1	O43143
Avasorum	1	O43148
Avasorum	1	O43149
Avasorum	1	O43150
Avasorum	1	O43155
Avasorum	1	O43156
Avasorum	1	O43159
Avasorum	1	O43164
Avasorum	1	O43169
Avasorum	1	O43172
Avasorum	1	O43175
Avasorum	1	O43181
Avasorum	1	O43237
Avasorum	1	O43242
Avasorum	1	O43251
Avasorum	1	O43252
Avasorum	1	O43264
Avasorum	1	O43286
Avasorum	1	O43290
Avasorum	1	O43292
Avasorum	1	O43293
Avasorum	1	O43294
Avasorum	1	O43299
Avasorum	1	O43310
Avasorum	1	O43312
Avasorum	1	O43314
Avasorum	1	O43318
Avasorum	1	O43324
Avasorum	1	O43353
Avasorum	1	O43390
Avasorum	1	O43395

Avasorum	1	O43396
Avasorum	1	O43399
Avasorum	1	O43402
Avasorum	1	O43414
Avasorum	1	O43426
Avasorum	1	O43427
Avasorum	1	O43432
Avasorum	1	O43447
Avasorum	1	O43464
Avasorum	1	O43482
Avasorum	1	O43488
Avasorum	1	O43491
Avasorum	1	O43493
Avasorum	1	O43505
Avasorum	1	O43516
Avasorum	1	O43520
Avasorum	1	O43572
Avasorum	1	O43583
Avasorum	1	O43592
Avasorum	1	O43598
Avasorum	1	O43615
Avasorum	1	O43617
Avasorum	1	O43633
Avasorum	1	O43639
Avasorum	1	O43657
Avasorum	1	O43660
Avasorum	1	O43663
Avasorum	1	O43665
Avasorum	1	O43670
Avasorum	1	O43674
Avasorum	1	O43676
Avasorum	1	O43678
Avasorum	1	O43679
Avasorum	1	O43681
Avasorum	1	O43683
Avasorum	1	O43684
Avasorum	1	O43704
Avasorum	1	O43707
Avasorum	1	O43709
Avasorum	1	O43719
Avasorum	1	O43731
Avasorum	1	O43747
Avasorum	1	O43752
Avasorum	1	O43760
Avasorum	1	O43765
Avasorum	1	O43768
Avasorum	1	O43772
Avasorum	1	O43776
Avasorum	1	O43795
Avasorum	1	O43808

Avasorum	1	O43809
Avasorum	1	O43813
Avasorum	1	O43815
Avasorum	1	O43818
Avasorum	1	O43819
Avasorum	1	O43822
Avasorum	1	O43823
Avasorum	1	O43824
Avasorum	1	O43826
Avasorum	1	O43837
Avasorum	1	O43847
Avasorum	1	O43852
Avasorum	1	O43854
Avasorum	1	O43865
Avasorum	1	O43896
Avasorum	1	O43909
Avasorum	1	O43913
Avasorum	1	O43920
Avasorum	1	O43924
Avasorum	1	O43929
Avasorum	1	O43933
Avasorum	1	O60216
Avasorum	1	O60231
Avasorum	1	O60234
Avasorum	1	O60239
Avasorum	1	O60242
Avasorum	1	O60244
Avasorum	1	O60245
Avasorum	1	O60256
Avasorum	1	O60264
Avasorum	1	O60271
Avasorum	1	O60285
Avasorum	1	O60287
Avasorum	1	O60292
Avasorum	1	O60293
Avasorum	1	O60306
Avasorum	1	O60313
Avasorum	1	O60315
Avasorum	1	O60318
Avasorum	1	O60330
Avasorum	1	O60331
Avasorum	1	O60333
Avasorum	1	O60336
Avasorum	1	O60341
Avasorum	1	O60343
Avasorum	1	O60427
Avasorum	1	O60443
Avasorum	1	O60447
Avasorum	1	O60462
Avasorum	1	O60476

Avasorum	1	O60487
Avasorum	1	O60488
Avasorum	1	O60493
Avasorum	1	O60502
Avasorum	1	O60504
Avasorum	1	O60506
Avasorum	1	O60508
Avasorum	1	O60518
Avasorum	1	O60524
Avasorum	1	O60547
Avasorum	1	O60551
Avasorum	1	O60563
Avasorum	1	O60566
Avasorum	1	O60568
Avasorum	1	O60573
Avasorum	1	O60610
Avasorum	1	O60637
Avasorum	1	O60645
Avasorum	1	O60664
Avasorum	1	O60678
Avasorum	1	O60684
Avasorum	1	O60701
Avasorum	1	O60704
Avasorum	1	O60711
Avasorum	1	O60716
Avasorum	1	O60725
Avasorum	1	O60739
Avasorum	1	O60749
Avasorum	1	O60762
Avasorum	1	O60763
Avasorum	1	O60783
Avasorum	1	O60784
Avasorum	1	O60814
Avasorum	1	O60826
Avasorum	1	O60828
Avasorum	1	O60830
Avasorum	1	O60831
Avasorum	1	O60832
Avasorum	1	O60841
Avasorum	1	O60869
Avasorum	1	O60870
Avasorum	1	O60879
Avasorum	1	O60884
Avasorum	1	O60885
Avasorum	1	O60921
Avasorum	1	O60925
Avasorum	1	O60927
Avasorum	1	O60934
Avasorum	1	O60942
Avasorum	1	O75027

Avasorum	1	O75044
Avasorum	1	O75051
Avasorum	1	O75063
Avasorum	1	O75083
Avasorum	1	O75110
Avasorum	1	O75113
Avasorum	1	O75116
Avasorum	1	O75122
Avasorum	1	O75127
Avasorum	1	O75131
Avasorum	1	O75143
Avasorum	1	O75146
Avasorum	1	O75150
Avasorum	1	O75151
Avasorum	1	O75152
Avasorum	1	O75153
Avasorum	1	O75155
Avasorum	1	O75157
Avasorum	1	O75165
Avasorum	1	O75167
Avasorum	1	O75170
Avasorum	1	O75173
Avasorum	1	O75175
Avasorum	1	O75179
Avasorum	1	O75182
Avasorum	1	O75190
Avasorum	1	O75208
Avasorum	1	O75223
Avasorum	1	O75251
Avasorum	1	O75306
Avasorum	1	O75312
Avasorum	1	O75319
Avasorum	1	O75323
Avasorum	1	O75330
Avasorum	1	O75340
Avasorum	1	O75347
Avasorum	1	O75348
Avasorum	1	O75351
Avasorum	1	O75352
Avasorum	1	O75367
Avasorum	1	O75368
Avasorum	1	O75369
Avasorum	1	O75376
Avasorum	1	O75381
Avasorum	1	O75382
Avasorum	1	O75390
Avasorum	1	O75391
Avasorum	1	O75396
Avasorum	1	O75400
Avasorum	1	O75410

Avasorum	1	O75414
Avasorum	1	O75419
Avasorum	1	O75427
Avasorum	1	O75431
Avasorum	1	O75436
Avasorum	1	O75439
Avasorum	1	O75448
Avasorum	1	O75449
Avasorum	1	O75475
Avasorum	1	O75477
Avasorum	1	O75489
Avasorum	1	O75494
Avasorum	1	O75503
Avasorum	1	O75508
Avasorum	1	O75521
Avasorum	1	O75525
Avasorum	1	O75530
Avasorum	1	O75531
Avasorum	1	O75533
Avasorum	1	O75534
Avasorum	1	O75554
Avasorum	1	O75569
Avasorum	1	O75578
Avasorum	1	O75582
Avasorum	1	O75586
Avasorum	1	O75592
Avasorum	1	O75600
Avasorum	1	O75607
Avasorum	1	O75608
Avasorum	1	O75616
Avasorum	1	O75643
Avasorum	1	O75648
Avasorum	1	O75663
Avasorum	1	O75665
Avasorum	1	O75676
Avasorum	1	O75683
Avasorum	1	O75688
Avasorum	1	O75691
Avasorum	1	O75694
Avasorum	1	O75695
Avasorum	1	O75717
Avasorum	1	O75718
Avasorum	1	O75746
Avasorum	1	O75787
Avasorum	1	O75792
Avasorum	1	O75794
Avasorum	1	O75815
Avasorum	1	O75817
Avasorum	1	O75818
Avasorum	1	O75821

Avasorum	1	O75822
Avasorum	1	O75828
Avasorum	1	O75832
Avasorum	1	O75843
Avasorum	1	O75844
Avasorum	1	O75874
Avasorum	1	O75879
Avasorum	1	O75880
Avasorum	1	O75882
Avasorum	1	O75884
Avasorum	1	O75886
Avasorum	1	O75909
Avasorum	1	O75915
Avasorum	1	O75923
Avasorum	1	O75925
Avasorum	1	O75934
Avasorum	1	O75935
Avasorum	1	O75937
Avasorum	1	O75940
Avasorum	1	O75943
Avasorum	1	O75947
Avasorum	1	O75954
Avasorum	1	O75955
Avasorum	1	O75962
Avasorum	1	O75964
Avasorum	1	O75970
Avasorum	1	O75976
Avasorum	1	O76003
Avasorum	1	O76021
Avasorum	1	O76024
Avasorum	1	O76031
Avasorum	1	O76054
Avasorum	1	O76070
Avasorum	1	O76071
Avasorum	1	O76094
Avasorum	1	O94760
Avasorum	1	O94762
Avasorum	1	O94763
Avasorum	1	O94766
Avasorum	1	O94776
Avasorum	1	O94788
Avasorum	1	O94804
Avasorum	1	O94806
Avasorum	1	O94808
Avasorum	1	O94813
Avasorum	1	O94822
Avasorum	1	O94826
Avasorum	1	O94827
Avasorum	1	O94829
Avasorum	1	O94830

Avasorum	1	O94832
Avasorum	1	O94842
Avasorum	1	O94851
Avasorum	1	O94855
Avasorum	1	O94868
Avasorum	1	O94874
Avasorum	1	O94875
Avasorum	1	O94885
Avasorum	1	O94888
Avasorum	1	O94889
Avasorum	1	O94901
Avasorum	1	O94903
Avasorum	1	O94905
Avasorum	1	O94906
Avasorum	1	O94913
Avasorum	1	O94915
Avasorum	1	O94916
Avasorum	1	O94919
Avasorum	1	O94923
Avasorum	1	O94925
Avasorum	1	O94927
Avasorum	1	O94929
Avasorum	1	O94952
Avasorum	1	O94964
Avasorum	1	O94966
Avasorum	1	O94967
Avasorum	1	O94973
Avasorum	1	O94979
Avasorum	1	O94989
Avasorum	1	O94992
Avasorum	1	O95059
Avasorum	1	O95067
Avasorum	1	O95070
Avasorum	1	O95071
Avasorum	1	O95081
Avasorum	1	O95084
Avasorum	1	O95104
Avasorum	1	O95139
Avasorum	1	O95140
Avasorum	1	O95149
Avasorum	1	O95155
Avasorum	1	O95159
Avasorum	1	O95163
Avasorum	1	O95168
Avasorum	1	O95169
Avasorum	1	O95182
Avasorum	1	O95183
Avasorum	1	O95197
Avasorum	1	O95202
Avasorum	1	O95208

Avasorum	1	O95218
Avasorum	1	O95219
Avasorum	1	O95232
Avasorum	1	O95235
Avasorum	1	O95236
Avasorum	1	O95239
Avasorum	1	O95243
Avasorum	1	O95248
Avasorum	1	O95249
Avasorum	1	O95251
Avasorum	1	O95260
Avasorum	1	O95292
Avasorum	1	O95295
Avasorum	1	O95297
Avasorum	1	O95298
Avasorum	1	O95299
Avasorum	1	O95302
Avasorum	1	O95319
Avasorum	1	O95336
Avasorum	1	O95340
Avasorum	1	O95347
Avasorum	1	O95352
Avasorum	1	O95359
Avasorum	1	O95361
Avasorum	1	O95363
Avasorum	1	O95365
Avasorum	1	O95372
Avasorum	1	O95373
Avasorum	1	O95376
Avasorum	1	O95391
Avasorum	1	O95394
Avasorum	1	O95396
Avasorum	1	O95400
Avasorum	1	O95425
Avasorum	1	O95433
Avasorum	1	O95453
Avasorum	1	O95456
Avasorum	1	O95470
Avasorum	1	O95476
Avasorum	1	O95478
Avasorum	1	O95479
Avasorum	1	O95486
Avasorum	1	O95487
Avasorum	1	O95490
Avasorum	1	O95544
Avasorum	1	O95551
Avasorum	1	O95563
Avasorum	1	O95571
Avasorum	1	O95573
Avasorum	1	O95602

Avasorum	1	O95619
Avasorum	1	O95628
Avasorum	1	O95630
Avasorum	1	O95639
Avasorum	1	O95644
Avasorum	1	O95671
Avasorum	1	O95674
Avasorum	1	O95685
Avasorum	1	O95696
Avasorum	1	O95707
Avasorum	1	O95714
Avasorum	1	O95716
Avasorum	1	O95721
Avasorum	1	O95747
Avasorum	1	O95749
Avasorum	1	O95757
Avasorum	1	O95758
Avasorum	1	O95759
Avasorum	1	O95760
Avasorum	1	O95772
Avasorum	1	O95777
Avasorum	1	O95782
Avasorum	1	O95785
Avasorum	1	O95786
Avasorum	1	O95793
Avasorum	1	O95801
Avasorum	1	O95807
Avasorum	1	O95810
Avasorum	1	O95816
Avasorum	1	O95817
Avasorum	1	O95819
Avasorum	1	O95822
Avasorum	1	O95825
Avasorum	1	O95831
Avasorum	1	O95834
Avasorum	1	O95835
Avasorum	1	O95848
Avasorum	1	O95858
Avasorum	1	O95861
Avasorum	1	O95864
Avasorum	1	O95865
Avasorum	1	O95870
Avasorum	1	O95881
Avasorum	1	O95905
Avasorum	1	O95980
Avasorum	1	O95983
Avasorum	1	O95985
Avasorum	1	O95989
Avasorum	1	O95999
Avasorum	1	O96000

Avasorum	1	O96005
Avasorum	1	O96006
Avasorum	1	O96007
Avasorum	1	O96008
Avasorum	1	O96011
Avasorum	1	O96013
Avasorum	1	O96018
Avasorum	1	O96019
Avasorum	1	O96028
Avasorum	1	P00338
Avasorum	1	P00352
Avasorum	1	P00367
Avasorum	1	P00374
Avasorum	1	P00387
Avasorum	1	P00390
Avasorum	1	P00395
Avasorum	1	P00403
Avasorum	1	P00491
Avasorum	1	P00492
Avasorum	1	P00505
Avasorum	1	P00519
Avasorum	1	P00558
Avasorum	1	P00568
Avasorum	1	P00749
Avasorum	1	P00750
Avasorum	1	P00813
Avasorum	1	P01009
Avasorum	1	P01023
Avasorum	1	P01034
Avasorum	1	P01111
Avasorum	1	P01112
Avasorum	1	P01116
Avasorum	1	P01127
Avasorum	1	P01130
Avasorum	1	P01137
Avasorum	1	P01889
Avasorum	1	P01893
Avasorum	1	P02452
Avasorum	1	P02462
Avasorum	1	P02545
Avasorum	1	P02647
Avasorum	1	P02751
Avasorum	1	P02765
Avasorum	1	P02768
Avasorum	1	P02786
Avasorum	1	P02792
Avasorum	1	P02794
Avasorum	1	P02795
Avasorum	1	P03886
Avasorum	1	P03905

Avasorum	1	P03915
Avasorum	1	P03956
Avasorum	1	P04035
Avasorum	1	P04040
Avasorum	1	P04049
Avasorum	1	P04062
Avasorum	1	P04075
Avasorum	1	P04080
Avasorum	1	P04083
Avasorum	1	P04114
Avasorum	1	P04156
Avasorum	1	P04179
Avasorum	1	P04181
Avasorum	1	P04183
Avasorum	1	P04275
Avasorum	1	P04350
Avasorum	1	P04406
Avasorum	1	P04424
Avasorum	1	P04439
Avasorum	1	P04632
Avasorum	1	P04732
Avasorum	1	P04792
Avasorum	1	P04818
Avasorum	1	P04843
Avasorum	1	P04844
Avasorum	1	P04899
Avasorum	1	P04908
Avasorum	1	P05023
Avasorum	1	P05026
Avasorum	1	P05067
Avasorum	1	P05091
Avasorum	1	P05106
Avasorum	1	P05109
Avasorum	1	P05114
Avasorum	1	P05120
Avasorum	1	P05121
Avasorum	1	P05141
Avasorum	1	P05161
Avasorum	1	P05165
Avasorum	1	P05166
Avasorum	1	P05198
Avasorum	1	P05204
Avasorum	1	P05362
Avasorum	1	P05387
Avasorum	1	P05388
Avasorum	1	P05412
Avasorum	1	P05423
Avasorum	1	P05455
Avasorum	1	P05556
Avasorum	1	P05783

Avasorum	1	P05997
Avasorum	1	P06132
Avasorum	1	P06241
Avasorum	1	P06280
Avasorum	1	P06396
Avasorum	1	P06400
Avasorum	1	P06454
Avasorum	1	P06493
Avasorum	1	P06576
Avasorum	1	P06703
Avasorum	1	P06730
Avasorum	1	P06733
Avasorum	1	P06737
Avasorum	1	P06744
Avasorum	1	P06746
Avasorum	1	P06748
Avasorum	1	P06753
Avasorum	1	P06756
Avasorum	1	P06865
Avasorum	1	P07099
Avasorum	1	P07195
Avasorum	1	P07199
Avasorum	1	P07203
Avasorum	1	P07237
Avasorum	1	P07305
Avasorum	1	P07311
Avasorum	1	P07332
Avasorum	1	P07339
Avasorum	1	P07355
Avasorum	1	P07384
Avasorum	1	P07437
Avasorum	1	P07602
Avasorum	1	P07686
Avasorum	1	P07711
Avasorum	1	P07737
Avasorum	1	P07738
Avasorum	1	P07741
Avasorum	1	P07814
Avasorum	1	P07858
Avasorum	1	P07900
Avasorum	1	P07902
Avasorum	1	P07910
Avasorum	1	P07942
Avasorum	1	P07947
Avasorum	1	P07948
Avasorum	1	P07954
Avasorum	1	P07992
Avasorum	1	P07996
Avasorum	1	P08047
Avasorum	1	P08069

Avasorum	1	P08123
Avasorum	1	P08133
Avasorum	1	P08134
Avasorum	1	P08174
Avasorum	1	P08195
Avasorum	1	P08236
Avasorum	1	P08237
Avasorum	1	P08238
Avasorum	1	P08240
Avasorum	1	P08243
Avasorum	1	P08253
Avasorum	1	P08397
Avasorum	1	P08473
Avasorum	1	P08493
Avasorum	1	P08559
Avasorum	1	P08572
Avasorum	1	P08574
Avasorum	1	P08579
Avasorum	1	P08581
Avasorum	1	P08621
Avasorum	1	P08648
Avasorum	1	P08651
Avasorum	1	P08670
Avasorum	1	P08708
Avasorum	1	P08754
Avasorum	1	P08758
Avasorum	1	P08865
Avasorum	1	P08962
Avasorum	1	P09001
Avasorum	1	P09012
Avasorum	1	P09104
Avasorum	1	P09110
Avasorum	1	P09132
Avasorum	1	P09211
Avasorum	1	P09234
Avasorum	1	P09382
Avasorum	1	P09417
Avasorum	1	P09429
Avasorum	1	P09486
Avasorum	1	P09493
Avasorum	1	P09496
Avasorum	1	P09497
Avasorum	1	P09525
Avasorum	1	P09543
Avasorum	1	P09601
Avasorum	1	P09622
Avasorum	1	P09651
Avasorum	1	P09661
Avasorum	1	P09669
Avasorum	1	P09758

Avasorum	1	P09874
Avasorum	1	P09884
Avasorum	1	P09936
Avasorum	1	P09960
Avasorum	1	P09972
Avasorum	1	P0C0S5
Avasorum	1	P0C0S8
Avasorum	1	P0CAP1
Avasorum	1	P0CAP2
Avasorum	1	P0CG29
Avasorum	1	P0CG39
Avasorum	1	P0CW19
Avasorum	1	P0DMV8
Avasorum	1	P0DP91
Avasorum	1	P0DPH7
Avasorum	1	P0DTL6
Avasorum	1	P10114
Avasorum	1	P10124
Avasorum	1	P10155
Avasorum	1	P10253
Avasorum	1	P10301
Avasorum	1	P10321
Avasorum	1	P10398
Avasorum	1	P10412
Avasorum	1	P10515
Avasorum	1	P10586
Avasorum	1	P10589
Avasorum	1	P10599
Avasorum	1	P10606
Avasorum	1	P10619
Avasorum	1	P10620
Avasorum	1	P10644
Avasorum	1	P10646
Avasorum	1	P10768
Avasorum	1	P10809
Avasorum	1	P10909
Avasorum	1	P11021
Avasorum	1	P11047
Avasorum	1	P11117
Avasorum	1	P11137
Avasorum	1	P11142
Avasorum	1	P11166
Avasorum	1	P11169
Avasorum	1	P11171
Avasorum	1	P11172
Avasorum	1	P11177
Avasorum	1	P11182
Avasorum	1	P11216
Avasorum	1	P11217
Avasorum	1	P11233

Avasorum	1	P11234
Avasorum	1	P11274
Avasorum	1	P11279
Avasorum	1	P11308
Avasorum	1	P11310
Avasorum	1	P11387
Avasorum	1	P11388
Avasorum	1	P11413
Avasorum	1	P11441
Avasorum	1	P11498
Avasorum	1	P11586
Avasorum	1	P11717
Avasorum	1	P11766
Avasorum	1	P11802
Avasorum	1	P11908
Avasorum	1	P11940
Avasorum	1	P12004
Avasorum	1	P12081
Avasorum	1	P12109
Avasorum	1	P12235
Avasorum	1	P12236
Avasorum	1	P12268
Avasorum	1	P12270
Avasorum	1	P12429
Avasorum	1	P12694
Avasorum	1	P12814
Avasorum	1	P12821
Avasorum	1	P12931
Avasorum	1	P12955
Avasorum	1	P12956
Avasorum	1	P13010
Avasorum	1	P13051
Avasorum	1	P13073
Avasorum	1	P13196
Avasorum	1	P13473
Avasorum	1	P13489
Avasorum	1	P13598
Avasorum	1	P13639
Avasorum	1	P13667
Avasorum	1	P13674
Avasorum	1	P13693
Avasorum	1	P13716
Avasorum	1	P13747
Avasorum	1	P13797
Avasorum	1	P13798
Avasorum	1	P13804
Avasorum	1	P13807
Avasorum	1	P13861
Avasorum	1	P13984
Avasorum	1	P13987

Avasorum	1	P13995
Avasorum	1	P14174
Avasorum	1	P14209
Avasorum	1	P14314
Avasorum	1	P14317
Avasorum	1	P14324
Avasorum	1	P14406
Avasorum	1	P14543
Avasorum	1	P14550
Avasorum	1	P14618
Avasorum	1	P14625
Avasorum	1	P14635
Avasorum	1	P14649
Avasorum	1	P14678
Avasorum	1	P14735
Avasorum	1	P14859
Avasorum	1	P14866
Avasorum	1	P14868
Avasorum	1	P14921
Avasorum	1	P14923
Avasorum	1	P14927
Avasorum	1	P15056
Avasorum	1	P15090
Avasorum	1	P15104
Avasorum	1	P15121
Avasorum	1	P15144
Avasorum	1	P15151
Avasorum	1	P15153
Avasorum	1	P15170
Avasorum	1	P15289
Avasorum	1	P15291
Avasorum	1	P15311
Avasorum	1	P15374
Avasorum	1	P15407
Avasorum	1	P15408
Avasorum	1	P15529
Avasorum	1	P15531
Avasorum	1	P15559
Avasorum	1	P15586
Avasorum	1	P15735
Avasorum	1	P15848
Avasorum	1	P15880
Avasorum	1	P15884
Avasorum	1	P15924
Avasorum	1	P15927
Avasorum	1	P16035
Avasorum	1	P16070
Avasorum	1	P16152
Avasorum	1	P16219
Avasorum	1	P16220

Avasorum	1	P16278
Avasorum	1	P16284
Avasorum	1	P16298
Avasorum	1	P16333
Avasorum	1	P16383
Avasorum	1	P16401
Avasorum	1	P16402
Avasorum	1	P16403
Avasorum	1	P16435
Avasorum	1	P16591
Avasorum	1	P16615
Avasorum	1	P16885
Avasorum	1	P16930
Avasorum	1	P16949
Avasorum	1	P16989
Avasorum	1	P17026
Avasorum	1	P17050
Avasorum	1	P17096
Avasorum	1	P17152
Avasorum	1	P17174
Avasorum	1	P17252
Avasorum	1	P17301
Avasorum	1	P17302
Avasorum	1	P17405
Avasorum	1	P17480
Avasorum	1	P17535
Avasorum	1	P17542
Avasorum	1	P17544
Avasorum	1	P17568
Avasorum	1	P17612
Avasorum	1	P17655
Avasorum	1	P17706
Avasorum	1	P17812
Avasorum	1	P17813
Avasorum	1	P17844
Avasorum	1	P17858
Avasorum	1	P17948
Avasorum	1	P17980
Avasorum	1	P17987
Avasorum	1	P18031
Avasorum	1	P18074
Avasorum	1	P18077
Avasorum	1	P18084
Avasorum	1	P18085
Avasorum	1	P18124
Avasorum	1	P18206
Avasorum	1	P18433
Avasorum	1	P18440
Avasorum	1	P18583
Avasorum	1	P18615

Avasorum	1	P18621
Avasorum	1	P18669
Avasorum	1	P18754
Avasorum	1	P18846
Avasorum	1	P18850
Avasorum	1	P18858
Avasorum	1	P18887
Avasorum	1	P19021
Avasorum	1	P19022
Avasorum	1	P19174
Avasorum	1	P19338
Avasorum	1	P19367
Avasorum	1	P19387
Avasorum	1	P19388
Avasorum	1	P19404
Avasorum	1	P19447
Avasorum	1	P19474
Avasorum	1	P19525
Avasorum	1	P19623
Avasorum	1	P19634
Avasorum	1	P19784
Avasorum	1	P19838
Avasorum	1	P20020
Avasorum	1	P20042
Avasorum	1	P20073
Avasorum	1	P20226
Avasorum	1	P20248
Avasorum	1	P20290
Avasorum	1	P20337
Avasorum	1	P20338
Avasorum	1	P20339
Avasorum	1	P20340
Avasorum	1	P20585
Avasorum	1	P20594
Avasorum	1	P20618
Avasorum	1	P20645
Avasorum	1	P20674
Avasorum	1	P20700
Avasorum	1	P20810
Avasorum	1	P20839
Avasorum	1	P20908
Avasorum	1	P20929
Avasorum	1	P20933
Avasorum	1	P20936
Avasorum	1	P20962
Avasorum	1	P21127
Avasorum	1	P21266
Avasorum	1	P21281
Avasorum	1	P21283
Avasorum	1	P21291

Avasorum	1	P21333
Avasorum	1	P21359
Avasorum	1	P21397
Avasorum	1	P21399
Avasorum	1	P21453
Avasorum	1	P21579
Avasorum	1	P21589
Avasorum	1	P21675
Avasorum	1	P21741
Avasorum	1	P21796
Avasorum	1	P21810
Avasorum	1	P21912
Avasorum	1	P21926
Avasorum	1	P21964
Avasorum	1	P21980
Avasorum	1	P22033
Avasorum	1	P22059
Avasorum	1	P22061
Avasorum	1	P22087
Avasorum	1	P22102
Avasorum	1	P22234
Avasorum	1	P22307
Avasorum	1	P22314
Avasorum	1	P22392
Avasorum	1	P22570
Avasorum	1	P22626
Avasorum	1	P22670
Avasorum	1	P22681
Avasorum	1	P22692
Avasorum	1	P22694
Avasorum	1	P22695
Avasorum	1	P22830
Avasorum	1	P23025
Avasorum	1	P23193
Avasorum	1	P23219
Avasorum	1	P23229
Avasorum	1	P23246
Avasorum	1	P23258
Avasorum	1	P23284
Avasorum	1	P23368
Avasorum	1	P23381
Avasorum	1	P23396
Avasorum	1	P23443
Avasorum	1	P23458
Avasorum	1	P23467
Avasorum	1	P23469
Avasorum	1	P23470
Avasorum	1	P23497
Avasorum	1	P23508
Avasorum	1	P23510

Avasorum	1	P23526
Avasorum	1	P23528
Avasorum	1	P23588
Avasorum	1	P23610
Avasorum	1	P23634
Avasorum	1	P23743
Avasorum	1	P23786
Avasorum	1	P23919
Avasorum	1	P23921
Avasorum	1	P24385
Avasorum	1	P24386
Avasorum	1	P24390
Avasorum	1	P24468
Avasorum	1	P24534
Avasorum	1	P24539
Avasorum	1	P24666
Avasorum	1	P24723
Avasorum	1	P24752
Avasorum	1	P24844
Avasorum	1	P24928
Avasorum	1	P24941
Avasorum	1	P25098
Avasorum	1	P25116
Avasorum	1	P25205
Avasorum	1	P25325
Avasorum	1	P25398
Avasorum	1	P25440
Avasorum	1	P25445
Avasorum	1	P25490
Avasorum	1	P25685
Avasorum	1	P25686
Avasorum	1	P25705
Avasorum	1	P25774
Avasorum	1	P25786
Avasorum	1	P25787
Avasorum	1	P25788
Avasorum	1	P25789
Avasorum	1	P25942
Avasorum	1	P26006
Avasorum	1	P26022
Avasorum	1	P26038
Avasorum	1	P26196
Avasorum	1	P26358
Avasorum	1	P26368
Avasorum	1	P26373
Avasorum	1	P26374
Avasorum	1	P26440
Avasorum	1	P26572
Avasorum	1	P26583
Avasorum	1	P26599

Avasorum	1	P26639
Avasorum	1	P26640
Avasorum	1	P26641
Avasorum	1	P26885
Avasorum	1	P26951
Avasorum	1	P27105
Avasorum	1	P27144
Avasorum	1	P27348
Avasorum	1	P27361
Avasorum	1	P27448
Avasorum	1	P27635
Avasorum	1	P27658
Avasorum	1	P27694
Avasorum	1	P27695
Avasorum	1	P27701
Avasorum	1	P27707
Avasorum	1	P27708
Avasorum	1	P27797
Avasorum	1	P27816
Avasorum	1	P27824
Avasorum	1	P27986
Avasorum	1	P27987
Avasorum	1	P28062
Avasorum	1	P28065
Avasorum	1	P28066
Avasorum	1	P28070
Avasorum	1	P28072
Avasorum	1	P28074
Avasorum	1	P28161
Avasorum	1	P28288
Avasorum	1	P28290
Avasorum	1	P28300
Avasorum	1	P28331
Avasorum	1	P28340
Avasorum	1	P28370
Avasorum	1	P28482
Avasorum	1	P28702
Avasorum	1	P28715
Avasorum	1	P28799
Avasorum	1	P28827
Avasorum	1	P28838
Avasorum	1	P28906
Avasorum	1	P29083
Avasorum	1	P29084
Avasorum	1	P29144
Avasorum	1	P29218
Avasorum	1	P29279
Avasorum	1	P29317
Avasorum	1	P29323
Avasorum	1	P29353

Avasorum	1	P29372
Avasorum	1	P29401
Avasorum	1	P29466
Avasorum	1	P29474
Avasorum	1	P29558
Avasorum	1	P29590
Avasorum	1	P29597
Avasorum	1	P29692
Avasorum	1	P29966
Avasorum	1	P29992
Avasorum	1	P30038
Avasorum	1	P30040
Avasorum	1	P30041
Avasorum	1	P30043
Avasorum	1	P30044
Avasorum	1	P30048
Avasorum	1	P30050
Avasorum	1	P30084
Avasorum	1	P30085
Avasorum	1	P30086
Avasorum	1	P30101
Avasorum	1	P30153
Avasorum	1	P30154
Avasorum	1	P30260
Avasorum	1	P30405
Avasorum	1	P30414
Avasorum	1	P30419
Avasorum	1	P30519
Avasorum	1	P30520
Avasorum	1	P30530
Avasorum	1	P30533
Avasorum	1	P30536
Avasorum	1	P30566
Avasorum	1	P30622
Avasorum	1	P30626
Avasorum	1	P30740
Avasorum	1	P30825
Avasorum	1	P30837
Avasorum	1	P30876
Avasorum	1	P31040
Avasorum	1	P31146
Avasorum	1	P31150
Avasorum	1	P31153
Avasorum	1	P31323
Avasorum	1	P31350
Avasorum	1	P31431
Avasorum	1	P31483
Avasorum	1	P31641
Avasorum	1	P31689
Avasorum	1	P31749

Avasorum	1	P31930
Avasorum	1	P31937
Avasorum	1	P31939
Avasorum	1	P31942
Avasorum	1	P31943
Avasorum	1	P31946
Avasorum	1	P31948
Avasorum	1	P31949
Avasorum	1	P32119
Avasorum	1	P32121
Avasorum	1	P32189
Avasorum	1	P32321
Avasorum	1	P32322
Avasorum	1	P32455
Avasorum	1	P32456
Avasorum	1	P32519
Avasorum	1	P32780
Avasorum	1	P32856
Avasorum	1	P32969
Avasorum	1	P33121
Avasorum	1	P33151
Avasorum	1	P33176
Avasorum	1	P33316
Avasorum	1	P33527
Avasorum	1	P33897
Avasorum	1	P33981
Avasorum	1	P33991
Avasorum	1	P33992
Avasorum	1	P33993
Avasorum	1	P34896
Avasorum	1	P34897
Avasorum	1	P34931
Avasorum	1	P34932
Avasorum	1	P34947
Avasorum	1	P34949
Avasorum	1	P35052
Avasorum	1	P35080
Avasorum	1	P35221
Avasorum	1	P35222
Avasorum	1	P35232
Avasorum	1	P35237
Avasorum	1	P35240
Avasorum	1	P35241
Avasorum	1	P35244
Avasorum	1	P35249
Avasorum	1	P35250
Avasorum	1	P35251
Avasorum	1	P35268
Avasorum	1	P35269
Avasorum	1	P35270

Avasorum	1	P35367
Avasorum	1	P35520
Avasorum	1	P35555
Avasorum	1	P35556
Avasorum	1	P35573
Avasorum	1	P35579
Avasorum	1	P35580
Avasorum	1	P35590
Avasorum	1	P35606
Avasorum	1	P35610
Avasorum	1	P35611
Avasorum	1	P35613
Avasorum	1	P35637
Avasorum	1	P35658
Avasorum	1	P35659
Avasorum	1	P35713
Avasorum	1	P35749
Avasorum	1	P35813
Avasorum	1	P35914
Avasorum	1	P35916
Avasorum	1	P35968
Avasorum	1	P35998
Avasorum	1	P36269
Avasorum	1	P36404
Avasorum	1	P36405
Avasorum	1	P36507
Avasorum	1	P36542
Avasorum	1	P36543
Avasorum	1	P36551
Avasorum	1	P36578
Avasorum	1	P36639
Avasorum	1	P36776
Avasorum	1	P36871
Avasorum	1	P36873
Avasorum	1	P36915
Avasorum	1	P36957
Avasorum	1	P36959
Avasorum	1	P36969
Avasorum	1	P37023
Avasorum	1	P37059
Avasorum	1	P37108
Avasorum	1	P37173
Avasorum	1	P37198
Avasorum	1	P37235
Avasorum	1	P37268
Avasorum	1	P37275
Avasorum	1	P37802
Avasorum	1	P37837
Avasorum	1	P37840
Avasorum	1	P38117

Avasorum	1	P38159
Avasorum	1	P38432
Avasorum	1	P38435
Avasorum	1	P38571
Avasorum	1	P38606
Avasorum	1	P38646
Avasorum	1	P38919
Avasorum	1	P38935
Avasorum	1	P38936
Avasorum	1	P39019
Avasorum	1	P39023
Avasorum	1	P39060
Avasorum	1	P39656
Avasorum	1	P39687
Avasorum	1	P39748
Avasorum	1	P39880
Avasorum	1	P40121
Avasorum	1	P40123
Avasorum	1	P40189
Avasorum	1	P40222
Avasorum	1	P40227
Avasorum	1	P40261
Avasorum	1	P40306
Avasorum	1	P40425
Avasorum	1	P40429
Avasorum	1	P40616
Avasorum	1	P40692
Avasorum	1	P40763
Avasorum	1	P40818
Avasorum	1	P40925
Avasorum	1	P40926
Avasorum	1	P40937
Avasorum	1	P40938
Avasorum	1	P40939
Avasorum	1	P41091
Avasorum	1	P41208
Avasorum	1	P41212
Avasorum	1	P41214
Avasorum	1	P41223
Avasorum	1	P41226
Avasorum	1	P41227
Avasorum	1	P41229
Avasorum	1	P41240
Avasorum	1	P41250
Avasorum	1	P41252
Avasorum	1	P41440
Avasorum	1	P41567
Avasorum	1	P41743
Avasorum	1	P41970
Avasorum	1	P42025

Avasorum	1	P42126
Avasorum	1	P42166
Avasorum	1	P42167
Avasorum	1	P42224
Avasorum	1	P42226
Avasorum	1	P42229
Avasorum	1	P42285
Avasorum	1	P42330
Avasorum	1	P42336
Avasorum	1	P42338
Avasorum	1	P42345
Avasorum	1	P42356
Avasorum	1	P42566
Avasorum	1	P42574
Avasorum	1	P42575
Avasorum	1	P42677
Avasorum	1	P42684
Avasorum	1	P42694
Avasorum	1	P42695
Avasorum	1	P42696
Avasorum	1	P42704
Avasorum	1	P42765
Avasorum	1	P42766
Avasorum	1	P42785
Avasorum	1	P42858
Avasorum	1	P42892
Avasorum	1	P42898
Avasorum	1	P43007
Avasorum	1	P43034
Avasorum	1	P43121
Avasorum	1	P43155
Avasorum	1	P43243
Avasorum	1	P43246
Avasorum	1	P43250
Avasorum	1	P43304
Avasorum	1	P43307
Avasorum	1	P43378
Avasorum	1	P43487
Avasorum	1	P43490
Avasorum	1	P43686
Avasorum	1	P43897
Avasorum	1	P45877
Avasorum	1	P45880
Avasorum	1	P45954
Avasorum	1	P45973
Avasorum	1	P45974
Avasorum	1	P45983
Avasorum	1	P45985
Avasorum	1	P46013
Avasorum	1	P46019

Avasorum	1	P46020
Avasorum	1	P46060
Avasorum	1	P46063
Avasorum	1	P46087
Avasorum	1	P46100
Avasorum	1	P46108
Avasorum	1	P46109
Avasorum	1	P46199
Avasorum	1	P46379
Avasorum	1	P46459
Avasorum	1	P46531
Avasorum	1	P46734
Avasorum	1	P46736
Avasorum	1	P46776
Avasorum	1	P46777
Avasorum	1	P46778
Avasorum	1	P46779
Avasorum	1	P46781
Avasorum	1	P46782
Avasorum	1	P46783
Avasorum	1	P46821
Avasorum	1	P46926
Avasorum	1	P46934
Avasorum	1	P46939
Avasorum	1	P46940
Avasorum	1	P46976
Avasorum	1	P46977
Avasorum	1	P47712
Avasorum	1	P47755
Avasorum	1	P47756
Avasorum	1	P47813
Avasorum	1	P47895
Avasorum	1	P47897
Avasorum	1	P47914
Avasorum	1	P47974
Avasorum	1	P47985
Avasorum	1	P48047
Avasorum	1	P48059
Avasorum	1	P48060
Avasorum	1	P48147
Avasorum	1	P48163
Avasorum	1	P48200
Avasorum	1	P48307
Avasorum	1	P48382
Avasorum	1	P48426
Avasorum	1	P48444
Avasorum	1	P48449
Avasorum	1	P48454
Avasorum	1	P48506
Avasorum	1	P48507

Avasorum	1	P48509
Avasorum	1	P48553
Avasorum	1	P48556
Avasorum	1	P48634
Avasorum	1	P48637
Avasorum	1	P48643
Avasorum	1	P48651
Avasorum	1	P48681
Avasorum	1	P48723
Avasorum	1	P48729
Avasorum	1	P48730
Avasorum	1	P48735
Avasorum	1	P48736
Avasorum	1	P48739
Avasorum	1	P49005
Avasorum	1	P49006
Avasorum	1	P49023
Avasorum	1	P49069
Avasorum	1	P49116
Avasorum	1	P49137
Avasorum	1	P49184
Avasorum	1	P49189
Avasorum	1	P49207
Avasorum	1	P49247
Avasorum	1	P49257
Avasorum	1	P49281
Avasorum	1	P49321
Avasorum	1	P49327
Avasorum	1	P49354
Avasorum	1	P49356
Avasorum	1	P49366
Avasorum	1	P49368
Avasorum	1	P49406
Avasorum	1	P49407
Avasorum	1	P49411
Avasorum	1	P49419
Avasorum	1	P49427
Avasorum	1	P49441
Avasorum	1	P49458
Avasorum	1	P49585
Avasorum	1	P49588
Avasorum	1	P49589
Avasorum	1	P49590
Avasorum	1	P49591
Avasorum	1	P49593
Avasorum	1	P49641
Avasorum	1	P49642
Avasorum	1	P49643
Avasorum	1	P49662
Avasorum	1	P49674

Avasorum	1	P49720
Avasorum	1	P49721
Avasorum	1	P49736
Avasorum	1	P49748
Avasorum	1	P49750
Avasorum	1	P49754
Avasorum	1	P49755
Avasorum	1	P49756
Avasorum	1	P49757
Avasorum	1	P49761
Avasorum	1	P49768
Avasorum	1	P49770
Avasorum	1	P49773
Avasorum	1	P49790
Avasorum	1	P49792
Avasorum	1	P49795
Avasorum	1	P49796
Avasorum	1	P49810
Avasorum	1	P49815
Avasorum	1	P49821
Avasorum	1	P49840
Avasorum	1	P49841
Avasorum	1	P49848
Avasorum	1	P49902
Avasorum	1	P49903
Avasorum	1	P49914
Avasorum	1	P49915
Avasorum	1	P49916
Avasorum	1	P49917
Avasorum	1	P49959
Avasorum	1	P49961
Avasorum	1	P50148
Avasorum	1	P50213
Avasorum	1	P50225
Avasorum	1	P50281
Avasorum	1	P50336
Avasorum	1	P50395
Avasorum	1	P50402
Avasorum	1	P50416
Avasorum	1	P50443
Avasorum	1	P50452
Avasorum	1	P50453
Avasorum	1	P50454
Avasorum	1	P50479
Avasorum	1	P50502
Avasorum	1	P50542
Avasorum	1	P50548
Avasorum	1	P50552
Avasorum	1	P50570
Avasorum	1	P50579

Avasorum	1	P50583
Avasorum	1	P50613
Avasorum	1	P50748
Avasorum	1	P50749
Avasorum	1	P50750
Avasorum	1	P50851
Avasorum	1	P50895
Avasorum	1	P50897
Avasorum	1	P50914
Avasorum	1	P50990
Avasorum	1	P50991
Avasorum	1	P50995
Avasorum	1	P51003
Avasorum	1	P51114
Avasorum	1	P51116
Avasorum	1	P51148
Avasorum	1	P51149
Avasorum	1	P51151
Avasorum	1	P51153
Avasorum	1	P51159
Avasorum	1	P51178
Avasorum	1	P51398
Avasorum	1	P51452
Avasorum	1	P51532
Avasorum	1	P51553
Avasorum	1	P51570
Avasorum	1	P51571
Avasorum	1	P51572
Avasorum	1	P51580
Avasorum	1	P51608
Avasorum	1	P51610
Avasorum	1	P51617
Avasorum	1	P51636
Avasorum	1	P51648
Avasorum	1	P51659
Avasorum	1	P51665
Avasorum	1	P51687
Avasorum	1	P51688
Avasorum	1	P51692
Avasorum	1	P51784
Avasorum	1	P51798
Avasorum	1	P51809
Avasorum	1	P51812
Avasorum	1	P51813
Avasorum	1	P51825
Avasorum	1	P51858
Avasorum	1	P51946
Avasorum	1	P51948
Avasorum	1	P51956
Avasorum	1	P51965

Avasorum	1	P51970
Avasorum	1	P51991
Avasorum	1	P52209
Avasorum	1	P52272
Avasorum	1	P52292
Avasorum	1	P52294
Avasorum	1	P52298
Avasorum	1	P52306
Avasorum	1	P52434
Avasorum	1	P52564
Avasorum	1	P52565
Avasorum	1	P52566
Avasorum	1	P52594
Avasorum	1	P52597
Avasorum	1	P52630
Avasorum	1	P52701
Avasorum	1	P52732
Avasorum	1	P52735
Avasorum	1	P52747
Avasorum	1	P52756
Avasorum	1	P52758
Avasorum	1	P52788
Avasorum	1	P52789
Avasorum	1	P52799
Avasorum	1	P52815
Avasorum	1	P52824
Avasorum	1	P52848
Avasorum	1	P52888
Avasorum	1	P52907
Avasorum	1	P52926
Avasorum	1	P52943
Avasorum	1	P52948
Avasorum	1	P53004
Avasorum	1	P53007
Avasorum	1	P53041
Avasorum	1	P53350
Avasorum	1	P53355
Avasorum	1	P53365
Avasorum	1	P53367
Avasorum	1	P53384
Avasorum	1	P53396
Avasorum	1	P53582
Avasorum	1	P53597
Avasorum	1	P53602
Avasorum	1	P53609
Avasorum	1	P53611
Avasorum	1	P53618
Avasorum	1	P53621
Avasorum	1	P53634
Avasorum	1	P53667

Avasorum	1	P53671
Avasorum	1	P53675
Avasorum	1	P53677
Avasorum	1	P53680
Avasorum	1	P53701
Avasorum	1	P53778
Avasorum	1	P53801
Avasorum	1	P53814
Avasorum	1	P53985
Avasorum	1	P53990
Avasorum	1	P53992
Avasorum	1	P53999
Avasorum	1	P54098
Avasorum	1	P54105
Avasorum	1	P54136
Avasorum	1	P54198
Avasorum	1	P54252
Avasorum	1	P54259
Avasorum	1	P54277
Avasorum	1	P54278
Avasorum	1	P54289
Avasorum	1	P54577
Avasorum	1	P54578
Avasorum	1	P54619
Avasorum	1	P54652
Avasorum	1	P54687
Avasorum	1	P54709
Avasorum	1	P54727
Avasorum	1	P54760
Avasorum	1	P54802
Avasorum	1	P54819
Avasorum	1	P54886
Avasorum	1	P54920
Avasorum	1	P55010
Avasorum	1	P55011
Avasorum	1	P55036
Avasorum	1	P55039
Avasorum	1	P55060
Avasorum	1	P55072
Avasorum	1	P55081
Avasorum	1	P55084
Avasorum	1	P55145
Avasorum	1	P55196
Avasorum	1	P55199
Avasorum	1	P55201
Avasorum	1	P55209
Avasorum	1	P55210
Avasorum	1	P55212
Avasorum	1	P55263
Avasorum	1	P55265

Avasorum	1	P55268
Avasorum	1	P55290
Avasorum	1	P55327
Avasorum	1	P55735
Avasorum	1	P55769
Avasorum	1	P55786
Avasorum	1	P55789
Avasorum	1	P55795
Avasorum	1	P55809
Avasorum	1	P55884
Avasorum	1	P55899
Avasorum	1	P55957
Avasorum	1	P56134
Avasorum	1	P56182
Avasorum	1	P56192
Avasorum	1	P56211
Avasorum	1	P56377
Avasorum	1	P56378
Avasorum	1	P56381
Avasorum	1	P56385
Avasorum	1	P56524
Avasorum	1	P56537
Avasorum	1	P56545
Avasorum	1	P56556
Avasorum	1	P56589
Avasorum	1	P56937
Avasorum	1	P56945
Avasorum	1	P56962
Avasorum	1	P57076
Avasorum	1	P57081
Avasorum	1	P57088
Avasorum	1	P57678
Avasorum	1	P57737
Avasorum	1	P57740
Avasorum	1	P57764
Avasorum	1	P57772
Avasorum	1	P58004
Avasorum	1	P58335
Avasorum	1	P58546
Avasorum	1	P59998
Avasorum	1	P60033
Avasorum	1	P60059
Avasorum	1	P60174
Avasorum	1	P60228
Avasorum	1	P60468
Avasorum	1	P60484
Avasorum	1	P60510
Avasorum	1	P60520
Avasorum	1	P60602
Avasorum	1	P60660

Avasorum	1	P60709
Avasorum	1	P60842
Avasorum	1	P60866
Avasorum	1	P60891
Avasorum	1	P60900
Avasorum	1	P60903
Avasorum	1	P60953
Avasorum	1	P60981
Avasorum	1	P60983
Avasorum	1	P61006
Avasorum	1	P61009
Avasorum	1	P61011
Avasorum	1	P61018
Avasorum	1	P61019
Avasorum	1	P61020
Avasorum	1	P61026
Avasorum	1	P61077
Avasorum	1	P61081
Avasorum	1	P61086
Avasorum	1	P61088
Avasorum	1	P61106
Avasorum	1	P61158
Avasorum	1	P61160
Avasorum	1	P61163
Avasorum	1	P61201
Avasorum	1	P61221
Avasorum	1	P61224
Avasorum	1	P61225
Avasorum	1	P61244
Avasorum	1	P61247
Avasorum	1	P61254
Avasorum	1	P61289
Avasorum	1	P61313
Avasorum	1	P61353
Avasorum	1	P61421
Avasorum	1	P61513
Avasorum	1	P61586
Avasorum	1	P61587
Avasorum	1	P61599
Avasorum	1	P61604
Avasorum	1	P61619
Avasorum	1	P61758
Avasorum	1	P61764
Avasorum	1	P61769
Avasorum	1	P61803
Avasorum	1	P61916
Avasorum	1	P61923
Avasorum	1	P61927
Avasorum	1	P61956
Avasorum	1	P61962

Avasorum	1	P61964
Avasorum	1	P61966
Avasorum	1	P61970
Avasorum	1	P61978
Avasorum	1	P61981
Avasorum	1	P62068
Avasorum	1	P62070
Avasorum	1	P62081
Avasorum	1	P62136
Avasorum	1	P62140
Avasorum	1	P62191
Avasorum	1	P62195
Avasorum	1	P62241
Avasorum	1	P62244
Avasorum	1	P62249
Avasorum	1	P62253
Avasorum	1	P62256
Avasorum	1	P62258
Avasorum	1	P62263
Avasorum	1	P62266
Avasorum	1	P62269
Avasorum	1	P62273
Avasorum	1	P62277
Avasorum	1	P62280
Avasorum	1	P62304
Avasorum	1	P62306
Avasorum	1	P62310
Avasorum	1	P62312
Avasorum	1	P62314
Avasorum	1	P62316
Avasorum	1	P62318
Avasorum	1	P62328
Avasorum	1	P62330
Avasorum	1	P62333
Avasorum	1	P62341
Avasorum	1	P62380
Avasorum	1	P62424
Avasorum	1	P62487
Avasorum	1	P62491
Avasorum	1	P62495
Avasorum	1	P62633
Avasorum	1	P62699
Avasorum	1	P62701
Avasorum	1	P62714
Avasorum	1	P62736
Avasorum	1	P62745
Avasorum	1	P62750
Avasorum	1	P62753
Avasorum	1	P62805
Avasorum	1	P62820

Avasorum	1	P62826
Avasorum	1	P62829
Avasorum	1	P62834
Avasorum	1	P62841
Avasorum	1	P62847
Avasorum	1	P62851
Avasorum	1	P62854
Avasorum	1	P62857
Avasorum	1	P62861
Avasorum	1	P62873
Avasorum	1	P62877
Avasorum	1	P62879
Avasorum	1	P62888
Avasorum	1	P62891
Avasorum	1	P62899
Avasorum	1	P62906
Avasorum	1	P62910
Avasorum	1	P62913
Avasorum	1	P62917
Avasorum	1	P62937
Avasorum	1	P62942
Avasorum	1	P62979
Avasorum	1	P62993
Avasorum	1	P62995
Avasorum	1	P63000
Avasorum	1	P63010
Avasorum	1	P63027
Avasorum	1	P63092
Avasorum	1	P63096
Avasorum	1	P63104
Avasorum	1	P63151
Avasorum	1	P63165
Avasorum	1	P63167
Avasorum	1	P63172
Avasorum	1	P63173
Avasorum	1	P63208
Avasorum	1	P63218
Avasorum	1	P63220
Avasorum	1	P63241
Avasorum	1	P63244
Avasorum	1	P63272
Avasorum	1	P63279
Avasorum	1	P63313
Avasorum	1	P67775
Avasorum	1	P67809
Avasorum	1	P67812
Avasorum	1	P67870
Avasorum	1	P67936
Avasorum	1	P68036
Avasorum	1	P68104

Avasorum	1	P68363
Avasorum	1	P68366
Avasorum	1	P68371
Avasorum	1	P68400
Avasorum	1	P68402
Avasorum	1	P68431
Avasorum	1	P68871
Avasorum	1	P69905
Avasorum	1	P78310
Avasorum	1	P78312
Avasorum	1	P78316
Avasorum	1	P78318
Avasorum	1	P78324
Avasorum	1	P78330
Avasorum	1	P78332
Avasorum	1	P78344
Avasorum	1	P78345
Avasorum	1	P78346
Avasorum	1	P78347
Avasorum	1	P78356
Avasorum	1	P78357
Avasorum	1	P78362
Avasorum	1	P78371
Avasorum	1	P78381
Avasorum	1	P78406
Avasorum	1	P78417
Avasorum	1	P78527
Avasorum	1	P78536
Avasorum	1	P78537
Avasorum	1	P78539
Avasorum	1	P78540
Avasorum	1	P78549
Avasorum	1	P80217
Avasorum	1	P80303
Avasorum	1	P80723
Avasorum	1	P81605
Avasorum	1	P82094
Avasorum	1	P82650
Avasorum	1	P82663
Avasorum	1	P82664
Avasorum	1	P82673
Avasorum	1	P82675
Avasorum	1	P82909
Avasorum	1	P82912
Avasorum	1	P82914
Avasorum	1	P82921
Avasorum	1	P82930
Avasorum	1	P82932
Avasorum	1	P82933
Avasorum	1	P82979

Avasorum	1	P83111
Avasorum	1	P83436
Avasorum	1	P83731
Avasorum	1	P83876
Avasorum	1	P83881
Avasorum	1	P83916
Avasorum	1	P84022
Avasorum	1	P84077
Avasorum	1	P84085
Avasorum	1	P84090
Avasorum	1	P84095
Avasorum	1	P84098
Avasorum	1	P84101
Avasorum	1	P84103
Avasorum	1	P85037
Avasorum	1	P86790
Avasorum	1	P98082
Avasorum	1	P98160
Avasorum	1	P98170
Avasorum	1	P98172
Avasorum	1	P98175
Avasorum	1	P98179
Avasorum	1	P98194
Avasorum	1	P98196
Avasorum	1	P99999
Avasorum	1	Q00013
Avasorum	1	Q00059
Avasorum	1	Q00169
Avasorum	1	Q00325
Avasorum	1	Q00341
Avasorum	1	Q00403
Avasorum	1	Q00534
Avasorum	1	Q00535
Avasorum	1	Q00537
Avasorum	1	Q00577
Avasorum	1	Q00587
Avasorum	1	Q00610
Avasorum	1	Q00613
Avasorum	1	Q00653
Avasorum	1	Q00688
Avasorum	1	Q00765
Avasorum	1	Q00796
Avasorum	1	Q00839
Avasorum	1	Q01081
Avasorum	1	Q01082
Avasorum	1	Q01085
Avasorum	1	Q01105
Avasorum	1	Q01130
Avasorum	1	Q01167
Avasorum	1	Q01201

Avasorum	1	Q01415
Avasorum	1	Q01433
Avasorum	1	Q01469
Avasorum	1	Q01518
Avasorum	1	Q01543
Avasorum	1	Q01581
Avasorum	1	Q01628
Avasorum	1	Q01650
Avasorum	1	Q01658
Avasorum	1	Q01780
Avasorum	1	Q01804
Avasorum	1	Q01813
Avasorum	1	Q01831
Avasorum	1	Q01844
Avasorum	1	Q01850
Avasorum	1	Q01968
Avasorum	1	Q01970
Avasorum	1	Q01995
Avasorum	1	Q02040
Avasorum	1	Q02127
Avasorum	1	Q02218
Avasorum	1	Q02241
Avasorum	1	Q02252
Avasorum	1	Q02297
Avasorum	1	Q02318
Avasorum	1	Q02413
Avasorum	1	Q02447
Avasorum	1	Q02539
Avasorum	1	Q02543
Avasorum	1	Q02750
Avasorum	1	Q02763
Avasorum	1	Q02790
Avasorum	1	Q02809
Avasorum	1	Q02818
Avasorum	1	Q02878
Avasorum	1	Q02880
Avasorum	1	Q02952
Avasorum	1	Q02978
Avasorum	1	Q03001
Avasorum	1	Q03111
Avasorum	1	Q03112
Avasorum	1	Q03113
Avasorum	1	Q03135
Avasorum	1	Q03154
Avasorum	1	Q03169
Avasorum	1	Q03252
Avasorum	1	Q03393
Avasorum	1	Q03405
Avasorum	1	Q03426
Avasorum	1	Q03468

Avasorum	1	Q03518
Avasorum	1	Q03519
Avasorum	1	Q03701
Avasorum	1	Q03936
Avasorum	1	Q04206
Avasorum	1	Q04323
Avasorum	1	Q04446
Avasorum	1	Q04637
Avasorum	1	Q04721
Avasorum	1	Q04724
Avasorum	1	Q04726
Avasorum	1	Q04760
Avasorum	1	Q04771
Avasorum	1	Q04837
Avasorum	1	Q04864
Avasorum	1	Q04917
Avasorum	1	Q04941
Avasorum	1	Q05048
Avasorum	1	Q05086
Avasorum	1	Q05193
Avasorum	1	Q05209
Avasorum	1	Q05397
Avasorum	1	Q05519
Avasorum	1	Q05655
Avasorum	1	Q05682
Avasorum	1	Q05932
Avasorum	1	Q05D32
Avasorum	1	Q06124
Avasorum	1	Q06136
Avasorum	1	Q06203
Avasorum	1	Q06210
Avasorum	1	Q06265
Avasorum	1	Q06323
Avasorum	1	Q06330
Avasorum	1	Q06413
Avasorum	1	Q06481
Avasorum	1	Q06546
Avasorum	1	Q06587
Avasorum	1	Q06787
Avasorum	1	Q06830
Avasorum	1	Q07020
Avasorum	1	Q07021
Avasorum	1	Q07065
Avasorum	1	Q07157
Avasorum	1	Q07352
Avasorum	1	Q07617
Avasorum	1	Q07666
Avasorum	1	Q07812
Avasorum	1	Q07817
Avasorum	1	Q07820

Avasorum	1	Q07864
Avasorum	1	Q07866
Avasorum	1	Q07889
Avasorum	1	Q07955
Avasorum	1	Q07960
Avasorum	1	Q08117
Avasorum	1	Q08170
Avasorum	1	Q08174
Avasorum	1	Q08209
Avasorum	1	Q08211
Avasorum	1	Q08257
Avasorum	1	Q08378
Avasorum	1	Q08379
Avasorum	1	Q08426
Avasorum	1	Q08431
Avasorum	1	Q08499
Avasorum	1	Q08554
Avasorum	1	Q08623
Avasorum	1	Q08629
Avasorum	1	Q08722
Avasorum	1	Q08752
Avasorum	1	Q08945
Avasorum	1	Q08AD1
Avasorum	1	Q08AE8
Avasorum	1	Q08AF3
Avasorum	1	Q08AM6
Avasorum	1	Q08J23
Avasorum	1	Q09019
Avasorum	1	Q09028
Avasorum	1	Q09161
Avasorum	1	Q09328
Avasorum	1	Q09472
Avasorum	1	Q09666
Avasorum	1	Q0JRZ9
Avasorum	1	Q0PNE2
Avasorum	1	Q0VDF9
Avasorum	1	Q0VDG4
Avasorum	1	Q0VF96
Avasorum	1	Q0VGL1
Avasorum	1	Q0ZGT2
Avasorum	1	Q10469
Avasorum	1	Q10471
Avasorum	1	Q10472
Avasorum	1	Q10567
Avasorum	1	Q10570
Avasorum	1	Q10589
Avasorum	1	Q10713
Avasorum	1	Q11201
Avasorum	1	Q12765
Avasorum	1	Q12768

Avasorum	1	Q12769
Avasorum	1	Q12770
Avasorum	1	Q12778
Avasorum	1	Q12788
Avasorum	1	Q12789
Avasorum	1	Q12792
Avasorum	1	Q12797
Avasorum	1	Q12800
Avasorum	1	Q12802
Avasorum	1	Q12805
Avasorum	1	Q12824
Avasorum	1	Q12830
Avasorum	1	Q12834
Avasorum	1	Q12841
Avasorum	1	Q12846
Avasorum	1	Q12849
Avasorum	1	Q12851
Avasorum	1	Q12866
Avasorum	1	Q12872
Avasorum	1	Q12873
Avasorum	1	Q12874
Avasorum	1	Q12882
Avasorum	1	Q12888
Avasorum	1	Q12893
Avasorum	1	Q12894
Avasorum	1	Q12899
Avasorum	1	Q12904
Avasorum	1	Q12905
Avasorum	1	Q12906
Avasorum	1	Q12907
Avasorum	1	Q12931
Avasorum	1	Q12933
Avasorum	1	Q12959
Avasorum	1	Q12965
Avasorum	1	Q12972
Avasorum	1	Q12974
Avasorum	1	Q12979
Avasorum	1	Q12980
Avasorum	1	Q12981
Avasorum	1	Q12982
Avasorum	1	Q12986
Avasorum	1	Q12996
Avasorum	1	Q12999
Avasorum	1	Q13011
Avasorum	1	Q13017
Avasorum	1	Q13033
Avasorum	1	Q13042
Avasorum	1	Q13043
Avasorum	1	Q13045
Avasorum	1	Q13049

Avasorum	1	Q13057
Avasorum	1	Q13084
Avasorum	1	Q13085
Avasorum	1	Q13098
Avasorum	1	Q13107
Avasorum	1	Q13111
Avasorum	1	Q13112
Avasorum	1	Q13123
Avasorum	1	Q13126
Avasorum	1	Q13131
Avasorum	1	Q13136
Avasorum	1	Q13137
Avasorum	1	Q13144
Avasorum	1	Q13148
Avasorum	1	Q13151
Avasorum	1	Q13155
Avasorum	1	Q13158
Avasorum	1	Q13162
Avasorum	1	Q13163
Avasorum	1	Q13164
Avasorum	1	Q13177
Avasorum	1	Q13185
Avasorum	1	Q13188
Avasorum	1	Q13190
Avasorum	1	Q13200
Avasorum	1	Q13201
Avasorum	1	Q13206
Avasorum	1	Q13217
Avasorum	1	Q13228
Avasorum	1	Q13232
Avasorum	1	Q13242
Avasorum	1	Q13243
Avasorum	1	Q13247
Avasorum	1	Q13257
Avasorum	1	Q13263
Avasorum	1	Q13275
Avasorum	1	Q13277
Avasorum	1	Q13283
Avasorum	1	Q13287
Avasorum	1	Q13308
Avasorum	1	Q13310
Avasorum	1	Q13315
Avasorum	1	Q13322
Avasorum	1	Q13325
Avasorum	1	Q13330
Avasorum	1	Q13347
Avasorum	1	Q13356
Avasorum	1	Q13362
Avasorum	1	Q13363
Avasorum	1	Q13371

Avasorum	1	Q13393
Avasorum	1	Q13395
Avasorum	1	Q13404
Avasorum	1	Q13405
Avasorum	1	Q13409
Avasorum	1	Q13416
Avasorum	1	Q13418
Avasorum	1	Q13423
Avasorum	1	Q13425
Avasorum	1	Q13426
Avasorum	1	Q13427
Avasorum	1	Q13428
Avasorum	1	Q13432
Avasorum	1	Q13435
Avasorum	1	Q13438
Avasorum	1	Q13439
Avasorum	1	Q13442
Avasorum	1	Q13443
Avasorum	1	Q13444
Avasorum	1	Q13445
Avasorum	1	Q13451
Avasorum	1	Q13459
Avasorum	1	Q13464
Avasorum	1	Q13469
Avasorum	1	Q13472
Avasorum	1	Q13485
Avasorum	1	Q13488
Avasorum	1	Q13490
Avasorum	1	Q13492
Avasorum	1	Q13496
Avasorum	1	Q13501
Avasorum	1	Q13505
Avasorum	1	Q13509
Avasorum	1	Q13510
Avasorum	1	Q13523
Avasorum	1	Q13526
Avasorum	1	Q13535
Avasorum	1	Q13541
Avasorum	1	Q13546
Avasorum	1	Q13547
Avasorum	1	Q13555
Avasorum	1	Q13557
Avasorum	1	Q13561
Avasorum	1	Q13563
Avasorum	1	Q13564
Avasorum	1	Q13572
Avasorum	1	Q13573
Avasorum	1	Q13574
Avasorum	1	Q13586
Avasorum	1	Q13588

Avasorum	1	Q13595
Avasorum	1	Q13596
Avasorum	1	Q13601
Avasorum	1	Q13610
Avasorum	1	Q13613
Avasorum	1	Q13614
Avasorum	1	Q13616
Avasorum	1	Q13617
Avasorum	1	Q13618
Avasorum	1	Q13619
Avasorum	1	Q13620
Avasorum	1	Q13625
Avasorum	1	Q13630
Avasorum	1	Q13636
Avasorum	1	Q13637
Avasorum	1	Q13641
Avasorum	1	Q13642
Avasorum	1	Q13643
Avasorum	1	Q13671
Avasorum	1	Q13685
Avasorum	1	Q13724
Avasorum	1	Q13740
Avasorum	1	Q13769
Avasorum	1	Q13796
Avasorum	1	Q13813
Avasorum	1	Q13823
Avasorum	1	Q13825
Avasorum	1	Q13838
Avasorum	1	Q13867
Avasorum	1	Q13868
Avasorum	1	Q13873
Avasorum	1	Q13885
Avasorum	1	Q13888
Avasorum	1	Q13889
Avasorum	1	Q13895
Avasorum	1	Q13905
Avasorum	1	Q13907
Avasorum	1	Q13951
Avasorum	1	Q13952
Avasorum	1	Q14004
Avasorum	1	Q14008
Avasorum	1	Q14011
Avasorum	1	Q14012
Avasorum	1	Q14019
Avasorum	1	Q14103
Avasorum	1	Q14108
Avasorum	1	Q14112
Avasorum	1	Q14114
Avasorum	1	Q14118
Avasorum	1	Q14119

Avasorum	1	Q14137
Avasorum	1	Q14139
Avasorum	1	Q14141
Avasorum	1	Q14145
Avasorum	1	Q14146
Avasorum	1	Q14147
Avasorum	1	Q14149
Avasorum	1	Q14151
Avasorum	1	Q14152
Avasorum	1	Q14155
Avasorum	1	Q14156
Avasorum	1	Q14157
Avasorum	1	Q14160
Avasorum	1	Q14161
Avasorum	1	Q14162
Avasorum	1	Q14165
Avasorum	1	Q14166
Avasorum	1	Q14181
Avasorum	1	Q14185
Avasorum	1	Q14186
Avasorum	1	Q14192
Avasorum	1	Q14195
Avasorum	1	Q14197
Avasorum	1	Q14202
Avasorum	1	Q14203
Avasorum	1	Q14204
Avasorum	1	Q14232
Avasorum	1	Q14240
Avasorum	1	Q14241
Avasorum	1	Q14247
Avasorum	1	Q14249
Avasorum	1	Q14254
Avasorum	1	Q14257
Avasorum	1	Q14258
Avasorum	1	Q14315
Avasorum	1	Q14318
Avasorum	1	Q14320
Avasorum	1	Q14331
Avasorum	1	Q14344
Avasorum	1	Q14353
Avasorum	1	Q14376
Avasorum	1	Q14392
Avasorum	1	Q14444
Avasorum	1	Q14457
Avasorum	1	Q14498
Avasorum	1	Q14511
Avasorum	1	Q14527
Avasorum	1	Q14534
Avasorum	1	Q14554
Avasorum	1	Q14558

Avasorum	1	Q14562
Avasorum	1	Q14566
Avasorum	1	Q14571
Avasorum	1	Q14573
Avasorum	1	Q14643
Avasorum	1	Q14644
Avasorum	1	Q14651
Avasorum	1	Q14653
Avasorum	1	Q14657
Avasorum	1	Q14667
Avasorum	1	Q14669
Avasorum	1	Q14671
Avasorum	1	Q14676
Avasorum	1	Q14677
Avasorum	1	Q14678
Avasorum	1	Q14683
Avasorum	1	Q14684
Avasorum	1	Q14689
Avasorum	1	Q14690
Avasorum	1	Q14691
Avasorum	1	Q14692
Avasorum	1	Q14693
Avasorum	1	Q14694
Avasorum	1	Q14696
Avasorum	1	Q14697
Avasorum	1	Q14699
Avasorum	1	Q14728
Avasorum	1	Q14738
Avasorum	1	Q14739
Avasorum	1	Q14746
Avasorum	1	Q14764
Avasorum	1	Q14766
Avasorum	1	Q14767
Avasorum	1	Q14781
Avasorum	1	Q14789
Avasorum	1	Q14790
Avasorum	1	Q147X3
Avasorum	1	Q14807
Avasorum	1	Q14814
Avasorum	1	Q14839
Avasorum	1	Q14847
Avasorum	1	Q14914
Avasorum	1	Q14919
Avasorum	1	Q14934
Avasorum	1	Q14966
Avasorum	1	Q14974
Avasorum	1	Q14978
Avasorum	1	Q14980
Avasorum	1	Q14997
Avasorum	1	Q14999

Avasorum	1	Q14BN4
Avasorum	1	Q14C86
Avasorum	1	Q14CX7
Avasorum	1	Q14D04
Avasorum	1	Q15003
Avasorum	1	Q15004
Avasorum	1	Q15005
Avasorum	1	Q15006
Avasorum	1	Q15007
Avasorum	1	Q15008
Avasorum	1	Q15011
Avasorum	1	Q15013
Avasorum	1	Q15014
Avasorum	1	Q15018
Avasorum	1	Q15019
Avasorum	1	Q15020
Avasorum	1	Q15021
Avasorum	1	Q15022
Avasorum	1	Q15024
Avasorum	1	Q15025
Avasorum	1	Q15029
Avasorum	1	Q15031
Avasorum	1	Q15032
Avasorum	1	Q15035
Avasorum	1	Q15036
Avasorum	1	Q15041
Avasorum	1	Q15042
Avasorum	1	Q15043
Avasorum	1	Q15046
Avasorum	1	Q15047
Avasorum	1	Q15048
Avasorum	1	Q15050
Avasorum	1	Q15054
Avasorum	1	Q15056
Avasorum	1	Q15057
Avasorum	1	Q15058
Avasorum	1	Q15059
Avasorum	1	Q15061
Avasorum	1	Q15067
Avasorum	1	Q15070
Avasorum	1	Q15075
Avasorum	1	Q15084
Avasorum	1	Q15102
Avasorum	1	Q15120
Avasorum	1	Q15121
Avasorum	1	Q15125
Avasorum	1	Q15126
Avasorum	1	Q15139
Avasorum	1	Q15149
Avasorum	1	Q15154

Avasorum	1	Q15155
Avasorum	1	Q15165
Avasorum	1	Q15172
Avasorum	1	Q15181
Avasorum	1	Q15185
Avasorum	1	Q15208
Avasorum	1	Q15233
Avasorum	1	Q15257
Avasorum	1	Q15262
Avasorum	1	Q15269
Avasorum	1	Q15276
Avasorum	1	Q15286
Avasorum	1	Q15287
Avasorum	1	Q15291
Avasorum	1	Q15293
Avasorum	1	Q15311
Avasorum	1	Q15334
Avasorum	1	Q15345
Avasorum	1	Q15361
Avasorum	1	Q15363
Avasorum	1	Q15365
Avasorum	1	Q15366
Avasorum	1	Q15369
Avasorum	1	Q15370
Avasorum	1	Q15382
Avasorum	1	Q15386
Avasorum	1	Q15388
Avasorum	1	Q15390
Avasorum	1	Q15392
Avasorum	1	Q15393
Avasorum	1	Q15397
Avasorum	1	Q15398
Avasorum	1	Q15404
Avasorum	1	Q15417
Avasorum	1	Q15418
Avasorum	1	Q15424
Avasorum	1	Q15428
Avasorum	1	Q15434
Avasorum	1	Q15435
Avasorum	1	Q15436
Avasorum	1	Q15437
Avasorum	1	Q15438
Avasorum	1	Q15459
Avasorum	1	Q15477
Avasorum	1	Q15517
Avasorum	1	Q15526
Avasorum	1	Q15542
Avasorum	1	Q15545
Avasorum	1	Q15554
Avasorum	1	Q15555

Avasorum	1	Q15583
Avasorum	1	Q15599
Avasorum	1	Q15628
Avasorum	1	Q15629
Avasorum	1	Q15631
Avasorum	1	Q15633
Avasorum	1	Q15637
Avasorum	1	Q15642
Avasorum	1	Q15643
Avasorum	1	Q15645
Avasorum	1	Q15648
Avasorum	1	Q15650
Avasorum	1	Q15652
Avasorum	1	Q15653
Avasorum	1	Q15654
Avasorum	1	Q15678
Avasorum	1	Q15691
Avasorum	1	Q15717
Avasorum	1	Q15738
Avasorum	1	Q15742
Avasorum	1	Q15746
Avasorum	1	Q15750
Avasorum	1	Q15751
Avasorum	1	Q15758
Avasorum	1	Q15773
Avasorum	1	Q15785
Avasorum	1	Q15796
Avasorum	1	Q15797
Avasorum	1	Q15800
Avasorum	1	Q15811
Avasorum	1	Q15813
Avasorum	1	Q15814
Avasorum	1	Q15819
Avasorum	1	Q15831
Avasorum	1	Q15833
Avasorum	1	Q15836
Avasorum	1	Q15843
Avasorum	1	Q15904
Avasorum	1	Q15906
Avasorum	1	Q15907
Avasorum	1	Q15942
Avasorum	1	Q16134
Avasorum	1	Q16181
Avasorum	1	Q16186
Avasorum	1	Q16204
Avasorum	1	Q16222
Avasorum	1	Q16254
Avasorum	1	Q16270
Avasorum	1	Q16342
Avasorum	1	Q16363

Avasorum	1	Q16401
Avasorum	1	Q16512
Avasorum	1	Q16513
Avasorum	1	Q16514
Avasorum	1	Q16527
Avasorum	1	Q16531
Avasorum	1	Q16537
Avasorum	1	Q16539
Avasorum	1	Q16540
Avasorum	1	Q16543
Avasorum	1	Q16555
Avasorum	1	Q16563
Avasorum	1	Q16576
Avasorum	1	Q16584
Avasorum	1	Q16594
Avasorum	1	Q16602
Avasorum	1	Q16611
Avasorum	1	Q16629
Avasorum	1	Q16630
Avasorum	1	Q16637
Avasorum	1	Q16643
Avasorum	1	Q16656
Avasorum	1	Q16658
Avasorum	1	Q16666
Avasorum	1	Q16698
Avasorum	1	Q16706
Avasorum	1	Q16718
Avasorum	1	Q16739
Avasorum	1	Q16740
Avasorum	1	Q16762
Avasorum	1	Q16763
Avasorum	1	Q16773
Avasorum	1	Q16774
Avasorum	1	Q16775
Avasorum	1	Q16795
Avasorum	1	Q16798
Avasorum	1	Q16822
Avasorum	1	Q16831
Avasorum	1	Q16832
Avasorum	1	Q16836
Avasorum	1	Q16850
Avasorum	1	Q16851
Avasorum	1	Q16875
Avasorum	1	Q16881
Avasorum	1	Q16891
Avasorum	1	Q19T08
Avasorum	1	Q1ED39
Avasorum	1	Q1KMD3
Avasorum	1	Q24JP5
Avasorum	1	Q27J81

Avasorum	1	Q29RF7
Avasorum	1	Q2KHT3
Avasorum	1	Q2M1P5
Avasorum	1	Q2M1Z3
Avasorum	1	Q2M296
Avasorum	1	Q2M2I8
Avasorum	1	Q2M389
Avasorum	1	Q2NKX8
Avasorum	1	Q2NL82
Avasorum	1	Q2PPJ7
Avasorum	1	Q2PZI1
Avasorum	1	Q2TAA2
Avasorum	1	Q2TAA5
Avasorum	1	Q2TAL8
Avasorum	1	Q2TAY7
Avasorum	1	Q2VPK5
Avasorum	1	Q32M88
Avasorum	1	Q32MZ4
Avasorum	1	Q32NB8
Avasorum	1	Q32NC0
Avasorum	1	Q32P28
Avasorum	1	Q32P41
Avasorum	1	Q32P44
Avasorum	1	Q3B726
Avasorum	1	Q3KQU3
Avasorum	1	Q3KQV9
Avasorum	1	Q3KQZ1
Avasorum	1	Q3L8U1
Avasorum	1	Q3LXA3
Avasorum	1	Q3MHD2
Avasorum	1	Q3SXM5
Avasorum	1	Q3SY69
Avasorum	1	Q3T906
Avasorum	1	Q3V6T2
Avasorum	1	Q3YEC7
Avasorum	1	Q3ZCQ8
Avasorum	1	Q460N5
Avasorum	1	Q495W5
Avasorum	1	Q49A26
Avasorum	1	Q49AR2
Avasorum	1	Q4AC94
Avasorum	1	Q4G0F5
Avasorum	1	Q4G0J3
Avasorum	1	Q4G0N4
Avasorum	1	Q4G0X4
Avasorum	1	Q4G148
Avasorum	1	Q4G176
Avasorum	1	Q4J6C6
Avasorum	1	Q4KMP7
Avasorum	1	Q4KMQ1

Avasorum	1	Q4KMQ2
Avasorum	1	Q4L180
Avasorum	1	Q4V328
Avasorum	1	Q4V339
Avasorum	1	Q4VC31
Avasorum	1	Q4ZIN3
Avasorum	1	Q52LJ0
Avasorum	1	Q52LW3
Avasorum	1	Q53EL6
Avasorum	1	Q53EP0
Avasorum	1	Q53ET0
Avasorum	1	Q53EU6
Avasorum	1	Q53EZ4
Avasorum	1	Q53F19
Avasorum	1	Q53FA7
Avasorum	1	Q53FP2
Avasorum	1	Q53GA4
Avasorum	1	Q53GG5
Avasorum	1	Q53GL7
Avasorum	1	Q53GQ0
Avasorum	1	Q53GS7
Avasorum	1	Q53GS9
Avasorum	1	Q53GT1
Avasorum	1	Q53H12
Avasorum	1	Q53H47
Avasorum	1	Q53H82
Avasorum	1	Q53H96
Avasorum	1	Q53HC9
Avasorum	1	Q53HL2
Avasorum	1	Q53SF7
Avasorum	1	Q53T59
Avasorum	1	Q53TN4
Avasorum	1	Q562E7
Avasorum	1	Q562R1
Avasorum	1	Q567U6
Avasorum	1	Q56VL3
Avasorum	1	Q58EX7
Avasorum	1	Q58FG1
Avasorum	1	Q58WW2
Avasorum	1	Q5BJD5
Avasorum	1	Q5BJF2
Avasorum	1	Q5BJH7
Avasorum	1	Q5BKZ1
Avasorum	1	Q5C9Z4
Avasorum	1	Q5EBL4
Avasorum	1	Q5F1R6
Avasorum	1	Q5GJ75
Avasorum	1	Q5GLZ8
Avasorum	1	Q5H8A4
Avasorum	1	Q5H9R7

Avasorum	1	Q5H9U9
Avasorum	1	Q5HYI8
Avasorum	1	Q5HYJ3
Avasorum	1	Q5HYK3
Avasorum	1	Q5HYK7
Avasorum	1	Q5HYW2
Avasorum	1	Q5J8M3
Avasorum	1	Q5JPH6
Avasorum	1	Q5JPI3
Avasorum	1	Q5JPI9
Avasorum	1	Q5JRA6
Avasorum	1	Q5JRX3
Avasorum	1	Q5JSH3
Avasorum	1	Q5JSZ5
Avasorum	1	Q5JTD0
Avasorum	1	Q5JTH9
Avasorum	1	Q5JTJ3
Avasorum	1	Q5JTV8
Avasorum	1	Q5JTW2
Avasorum	1	Q5JTZ9
Avasorum	1	Q5JU69
Avasorum	1	Q5JVF3
Avasorum	1	Q5JVS0
Avasorum	1	Q5K4L6
Avasorum	1	Q5K651
Avasorum	1	Q5MIZ7
Avasorum	1	Q5MNZ6
Avasorum	1	Q5MNZ9
Avasorum	1	Q5NDL2
Avasorum	1	Q5PRF9
Avasorum	1	Q5QJ74
Avasorum	1	Q5QJE6
Avasorum	1	Q5R372
Avasorum	1	Q5R3I4
Avasorum	1	Q5RI15
Avasorum	1	Q5RKV6
Avasorum	1	Q5SNT2
Avasorum	1	Q5SQI0
Avasorum	1	Q5SQN1
Avasorum	1	Q5SRE5
Avasorum	1	Q5SSJ5
Avasorum	1	Q5ST30
Avasorum	1	Q5SW79
Avasorum	1	Q5SW96
Avasorum	1	Q5SWX8
Avasorum	1	Q5SY16
Avasorum	1	Q5SYE7
Avasorum	1	Q5T013
Avasorum	1	Q5T0D9
Avasorum	1	Q5T0F9

Avasorum	1	Q5T0N5
Avasorum	1	Q5T160
Avasorum	1	Q5T1C6
Avasorum	1	Q5T1J5
Avasorum	1	Q5T1M5
Avasorum	1	Q5T200
Avasorum	1	Q5T280
Avasorum	1	Q5T2E6
Avasorum	1	Q5T3F8
Avasorum	1	Q5T3I0
Avasorum	1	Q5T440
Avasorum	1	Q5T447
Avasorum	1	Q5T4B2
Avasorum	1	Q5T4S7
Avasorum	1	Q5T5C0
Avasorum	1	Q5T5U3
Avasorum	1	Q5T5Y3
Avasorum	1	Q5T653
Avasorum	1	Q5T6F2
Avasorum	1	Q5T6V5
Avasorum	1	Q5T749
Avasorum	1	Q5T7W0
Avasorum	1	Q5T8D3
Avasorum	1	Q5T8P6
Avasorum	1	Q5T9A4
Avasorum	1	Q5T9L3
Avasorum	1	Q5TA45
Avasorum	1	Q5TA50
Avasorum	1	Q5TAQ9
Avasorum	1	Q5TAT6
Avasorum	1	Q5TAX3
Avasorum	1	Q5TBA9
Avasorum	1	Q5TBB1
Avasorum	1	Q5TC12
Avasorum	1	Q5TC82
Avasorum	1	Q5TDH0
Avasorum	1	Q5TEJ8
Avasorum	1	Q5TEU4
Avasorum	1	Q5TFE4
Avasorum	1	Q5TH69
Avasorum	1	Q5THJ4
Avasorum	1	Q5TZA2
Avasorum	1	Q5U5X0
Avasorum	1	Q5U651
Avasorum	1	Q5UIP0
Avasorum	1	Q5VIR6
Avasorum	1	Q5VSL9
Avasorum	1	Q5VT25
Avasorum	1	Q5VT52
Avasorum	1	Q5VTB9

Avasorum	1	Q5VTL8
Avasorum	1	Q5VTR2
Avasorum	1	Q5VU43
Avasorum	1	Q5VUA4
Avasorum	1	Q5VUB5
Avasorum	1	Q5VUD6
Avasorum	1	Q5VUJ6
Avasorum	1	Q5VV42
Avasorum	1	Q5VW32
Avasorum	1	Q5VW36
Avasorum	1	Q5VWQ0
Avasorum	1	Q5VWQ8
Avasorum	1	Q5VWZ2
Avasorum	1	Q5VY43
Avasorum	1	Q5VYK3
Avasorum	1	Q5VYS8
Avasorum	1	Q5VZ18
Avasorum	1	Q5VZ89
Avasorum	1	Q5VZE5
Avasorum	1	Q5VZL5
Avasorum	1	Q5W0V3
Avasorum	1	Q5W0Z9
Avasorum	1	Q5W111
Avasorum	1	Q5XUX1
Avasorum	1	Q5ZPR3
Avasorum	1	Q63HN8
Avasorum	1	Q63HR2
Avasorum	1	Q63ZY3
Avasorum	1	Q641Q2
Avasorum	1	Q643R3
Avasorum	1	Q658P3
Avasorum	1	Q658Y4
Avasorum	1	Q66K14
Avasorum	1	Q66K74
Avasorum	1	Q66LE6
Avasorum	1	Q66PJ3
Avasorum	1	Q676U5
Avasorum	1	Q68CP4
Avasorum	1	Q68CP9
Avasorum	1	Q68CQ4
Avasorum	1	Q68CQ7
Avasorum	1	Q68CR1
Avasorum	1	Q68CZ2
Avasorum	1	Q68CZ6
Avasorum	1	Q68D10
Avasorum	1	Q68D91
Avasorum	1	Q68DQ2
Avasorum	1	Q68E01
Avasorum	1	Q68EM7
Avasorum	1	Q69YN2

Avasorum	1	Q69YN4
Avasorum	1	Q69YQ0
Avasorum	1	Q6AI08
Avasorum	1	Q6AI12
Avasorum	1	Q6AWC2
Avasorum	1	Q6AZY7
Avasorum	1	Q6DD88
Avasorum	1	Q6DKI1
Avasorum	1	Q6DKJ4
Avasorum	1	Q6DKK2
Avasorum	1	Q6DN90
Avasorum	1	Q6FI81
Avasorum	1	Q6FIF0
Avasorum	1	Q6GMV2
Avasorum	1	Q6GQQ9
Avasorum	1	Q6I9Y2
Avasorum	1	Q6IA69
Avasorum	1	Q6IA86
Avasorum	1	Q6IAA8
Avasorum	1	Q6IAN0
Avasorum	1	Q6IBS0
Avasorum	1	Q6IBW4
Avasorum	1	Q6ICG6
Avasorum	1	Q6ICL3
Avasorum	1	Q6IN85
Avasorum	1	Q6IQ22
Avasorum	1	Q6IQ26
Avasorum	1	Q6IQ49
Avasorum	1	Q6KB66
Avasorum	1	Q6KC79
Avasorum	1	Q6KCM7
Avasorum	1	Q6L8Q7
Avasorum	1	Q6N069
Avasorum	1	Q6NSW5
Avasorum	1	Q6NTF9
Avasorum	1	Q6NUK1
Avasorum	1	Q6NUK4
Avasorum	1	Q6NUM9
Avasorum	1	Q6NUQ1
Avasorum	1	Q6NUQ4
Avasorum	1	Q6NVY1
Avasorum	1	Q6NW29
Avasorum	1	Q6NW34
Avasorum	1	Q6NXE6
Avasorum	1	Q6NXR4
Avasorum	1	Q6NXT1
Avasorum	1	Q6NXT4
Avasorum	1	Q6NXT6
Avasorum	1	Q6NY19
Avasorum	1	Q6NYC1

Avasorum	1	Q6NYC8
Avasorum	1	Q6NZI2
Avasorum	1	Q6NZY4
Avasorum	1	Q6P158
Avasorum	1	Q6P179
Avasorum	1	Q6P1A2
Avasorum	1	Q6P1J9
Avasorum	1	Q6P1L8
Avasorum	1	Q6P1M0
Avasorum	1	Q6P1N0
Avasorum	1	Q6P1N9
Avasorum	1	Q6P1Q9
Avasorum	1	Q6P1R3
Avasorum	1	Q6P1X5
Avasorum	1	Q6P1X6
Avasorum	1	Q6P2C8
Avasorum	1	Q6P2E9
Avasorum	1	Q6P2P2
Avasorum	1	Q6P2Q9
Avasorum	1	Q6P3S6
Avasorum	1	Q6P3W7
Avasorum	1	Q6P3X3
Avasorum	1	Q6P4A7
Avasorum	1	Q6P4E1
Avasorum	1	Q6P4R8
Avasorum	1	Q6P587
Avasorum	1	Q6P5R6
Avasorum	1	Q6P5Z2
Avasorum	1	Q6P6C2
Avasorum	1	Q6P996
Avasorum	1	Q6P9B6
Avasorum	1	Q6P9B9
Avasorum	1	Q6P9H5
Avasorum	1	Q6PCB7
Avasorum	1	Q6PCE3
Avasorum	1	Q6PD62
Avasorum	1	Q6PD74
Avasorum	1	Q6PEY1
Avasorum	1	Q6PGN9
Avasorum	1	Q6PGP7
Avasorum	1	Q6PHR2
Avasorum	1	Q6PI48
Avasorum	1	Q6PI78
Avasorum	1	Q6PI98
Avasorum	1	Q6PIJ6
Avasorum	1	Q6PIU2
Avasorum	1	Q6PIW4
Avasorum	1	Q6PJ69
Avasorum	1	Q6PJF5
Avasorum	1	Q6PJG2

Avasorum	1	Q6PJG6
Avasorum	1	Q6PJI9
Avasorum	1	Q6PJT7
Avasorum	1	Q6PK04
Avasorum	1	Q6PK18
Avasorum	1	Q6PKC3
Avasorum	1	Q6PKG0
Avasorum	1	Q6PL18
Avasorum	1	Q6PL24
Avasorum	1	Q6PML9
Avasorum	1	Q6Q0C0
Avasorum	1	Q6R327
Avasorum	1	Q6RFH5
Avasorum	1	Q6RW13
Avasorum	1	Q6STE5
Avasorum	1	Q6UB35
Avasorum	1	Q6ULP2
Avasorum	1	Q6UN15
Avasorum	1	Q6UUV7
Avasorum	1	Q6UUV9
Avasorum	1	Q6UW02
Avasorum	1	Q6UW63
Avasorum	1	Q6UW68
Avasorum	1	Q6UWE0
Avasorum	1	Q6UWH4
Avasorum	1	Q6UWP7
Avasorum	1	Q6UWZ7
Avasorum	1	Q6UX04
Avasorum	1	Q6UXH1
Avasorum	1	Q6UXN9
Avasorum	1	Q6UXV4
Avasorum	1	Q6V0I7
Avasorum	1	Q6V1X1
Avasorum	1	Q6VMQ6
Avasorum	1	Q6VN20
Avasorum	1	Q6VY07
Avasorum	1	Q6WCQ1
Avasorum	1	Q6XE24
Avasorum	1	Q6XQN6
Avasorum	1	Q6XZF7
Avasorum	1	Q6Y1H2
Avasorum	1	Q6Y288
Avasorum	1	Q6Y7W6
Avasorum	1	Q6YHK3
Avasorum	1	Q6YHU6
Avasorum	1	Q6YN16
Avasorum	1	Q6YP21
Avasorum	1	Q6ZMI0
Avasorum	1	Q6ZMP0
Avasorum	1	Q6ZMZ3

Avasorum	1	Q6ZN55
Avasorum	1	Q6ZNB6
Avasorum	1	Q6ZNJ1
Avasorum	1	Q6ZNL6
Avasorum	1	Q6ZRP7
Avasorum	1	Q6ZRS2
Avasorum	1	Q6ZS17
Avasorum	1	Q6ZSR9
Avasorum	1	Q6ZSZ5
Avasorum	1	Q6ZT07
Avasorum	1	Q6ZT12
Avasorum	1	Q6ZT21
Avasorum	1	Q6ZU35
Avasorum	1	Q6ZUT6
Avasorum	1	Q6ZVK8
Avasorum	1	Q6ZW31
Avasorum	1	Q6ZW49
Avasorum	1	Q6ZWJ1
Avasorum	1	Q6ZXV5
Avasorum	1	Q709C8
Avasorum	1	Q70CQ2
Avasorum	1	Q70E73
Avasorum	1	Q70IA6
Avasorum	1	Q70UQ0
Avasorum	1	Q70Z35
Avasorum	1	Q70Z53
Avasorum	1	Q712K3
Avasorum	1	Q71F56
Avasorum	1	Q71RC2
Avasorum	1	Q71SY5
Avasorum	1	Q71UM5
Avasorum	1	Q75N03
Avasorum	1	Q75QN2
Avasorum	1	Q7KYR7
Avasorum	1	Q7KZ85
Avasorum	1	Q7KZF4
Avasorum	1	Q7KZI7
Avasorum	1	Q7KZN9
Avasorum	1	Q7L014
Avasorum	1	Q7L099
Avasorum	1	Q7L0Y3
Avasorum	1	Q7L1Q6
Avasorum	1	Q7L1V2
Avasorum	1	Q7L266
Avasorum	1	Q7L273
Avasorum	1	Q7L2E3
Avasorum	1	Q7L2H7
Avasorum	1	Q7L2J0
Avasorum	1	Q7L311
Avasorum	1	Q7L3T8

Avasorum	1	Q7L4I2
Avasorum	1	Q7L523
Avasorum	1	Q7L576
Avasorum	1	Q7L592
Avasorum	1	Q7L5D6
Avasorum	1	Q7L5N1
Avasorum	1	Q7L5N7
Avasorum	1	Q7L5Y1
Avasorum	1	Q7L5Y9
Avasorum	1	Q7L775
Avasorum	1	Q7L7V1
Avasorum	1	Q7L7X3
Avasorum	1	Q7L8L6
Avasorum	1	Q7L9L4
Avasorum	1	Q7LBC6
Avasorum	1	Q7LBR1
Avasorum	1	Q7LG56
Avasorum	1	Q7LGA3
Avasorum	1	Q7RTN6
Avasorum	1	Q7RTP6
Avasorum	1	Q7RTS9
Avasorum	1	Q7RTV0
Avasorum	1	Q7RTV5
Avasorum	1	Q7Z2E3
Avasorum	1	Q7Z2K6
Avasorum	1	Q7Z2K8
Avasorum	1	Q7Z2T5
Avasorum	1	Q7Z2W4
Avasorum	1	Q7Z2W9
Avasorum	1	Q7Z2Z2
Avasorum	1	Q7Z333
Avasorum	1	Q7Z392
Avasorum	1	Q7Z3B3
Avasorum	1	Q7Z3B4
Avasorum	1	Q7Z3C6
Avasorum	1	Q7Z3D6
Avasorum	1	Q7Z3E2
Avasorum	1	Q7Z3E5
Avasorum	1	Q7Z3J2
Avasorum	1	Q7Z3K3
Avasorum	1	Q7Z3T8
Avasorum	1	Q7Z3U7
Avasorum	1	Q7Z401
Avasorum	1	Q7Z406
Avasorum	1	Q7Z417
Avasorum	1	Q7Z422
Avasorum	1	Q7Z434
Avasorum	1	Q7Z460
Avasorum	1	Q7Z478
Avasorum	1	Q7Z494

Avasorum	1	Q7Z4F1
Avasorum	1	Q7Z4H7
Avasorum	1	Q7Z4H8
Avasorum	1	Q7Z4L5
Avasorum	1	Q7Z4Q2
Avasorum	1	Q7Z4S6
Avasorum	1	Q7Z4V5
Avasorum	1	Q7Z4W1
Avasorum	1	Q7Z569
Avasorum	1	Q7Z589
Avasorum	1	Q7Z5G4
Avasorum	1	Q7Z5H3
Avasorum	1	Q7Z5K2
Avasorum	1	Q7Z5L9
Avasorum	1	Q7Z6E9
Avasorum	1	Q7Z6J0
Avasorum	1	Q7Z6J2
Avasorum	1	Q7Z6J6
Avasorum	1	Q7Z6J9
Avasorum	1	Q7Z6K3
Avasorum	1	Q7Z6K5
Avasorum	1	Q7Z6L1
Avasorum	1	Q7Z6M1
Avasorum	1	Q7Z6M4
Avasorum	1	Q7Z6Z7
Avasorum	1	Q7Z739
Avasorum	1	Q7Z7A3
Avasorum	1	Q7Z7C8
Avasorum	1	Q7Z7E8
Avasorum	1	Q7Z7F7
Avasorum	1	Q7Z7H5
Avasorum	1	Q7Z7H8
Avasorum	1	Q7Z7K0
Avasorum	1	Q7Z7K6
Avasorum	1	Q7Z7L1
Avasorum	1	Q7Z7M0
Avasorum	1	Q7Z7N9
Avasorum	1	Q86SF2
Avasorum	1	Q86SK9
Avasorum	1	Q86SQ0
Avasorum	1	Q86SQ4
Avasorum	1	Q86SQ9
Avasorum	1	Q86SR1
Avasorum	1	Q86SZ2
Avasorum	1	Q86T03
Avasorum	1	Q86T13
Avasorum	1	Q86T24
Avasorum	1	Q86TB9
Avasorum	1	Q86TI2
Avasorum	1	Q86TM6

Avasorum	1	Q86TN4
Avasorum	1	Q86TU7
Avasorum	1	Q86TV6
Avasorum	1	Q86TX2
Avasorum	1	Q86U38
Avasorum	1	Q86U42
Avasorum	1	Q86U44
Avasorum	1	Q86U86
Avasorum	1	Q86U90
Avasorum	1	Q86UA1
Avasorum	1	Q86UE4
Avasorum	1	Q86UK7
Avasorum	1	Q86UL3
Avasorum	1	Q86UP2
Avasorum	1	Q86US8
Avasorum	1	Q86UT6
Avasorum	1	Q86UU0
Avasorum	1	Q86UU1
Avasorum	1	Q86UV5
Avasorum	1	Q86UW7
Avasorum	1	Q86UX7
Avasorum	1	Q86UY6
Avasorum	1	Q86V21
Avasorum	1	Q86V48
Avasorum	1	Q86V81
Avasorum	1	Q86V85
Avasorum	1	Q86V87
Avasorum	1	Q86VI3
Avasorum	1	Q86VM9
Avasorum	1	Q86VN1
Avasorum	1	Q86VP6
Avasorum	1	Q86VR2
Avasorum	1	Q86VS8
Avasorum	1	Q86VW0
Avasorum	1	Q86VX2
Avasorum	1	Q86VX9
Avasorum	1	Q86W34
Avasorum	1	Q86W42
Avasorum	1	Q86W50
Avasorum	1	Q86W56
Avasorum	1	Q86W92
Avasorum	1	Q86WA8
Avasorum	1	Q86WB0
Avasorum	1	Q86WJ1
Avasorum	1	Q86WN1
Avasorum	1	Q86WR0
Avasorum	1	Q86WR7
Avasorum	1	Q86WV6
Avasorum	1	Q86WX3
Avasorum	1	Q86X02

Avasorum	1	Q86X10
Avasorum	1	Q86X27
Avasorum	1	Q86X55
Avasorum	1	Q86X76
Avasorum	1	Q86X83
Avasorum	1	Q86XA9
Avasorum	1	Q86XI2
Avasorum	1	Q86XI6
Avasorum	1	Q86XK2
Avasorum	1	Q86XL3
Avasorum	1	Q86XN8
Avasorum	1	Q86XP1
Avasorum	1	Q86XP3
Avasorum	1	Q86XZ4
Avasorum	1	Q86Y07
Avasorum	1	Q86Y37
Avasorum	1	Q86Y39
Avasorum	1	Q86Y56
Avasorum	1	Q86Y79
Avasorum	1	Q86Y82
Avasorum	1	Q86YP4
Avasorum	1	Q86YQ8
Avasorum	1	Q86YR5
Avasorum	1	Q86YS6
Avasorum	1	Q86YS7
Avasorum	1	Q86YT6
Avasorum	1	Q86YV5
Avasorum	1	Q86YV9
Avasorum	1	Q8IU81
Avasorum	1	Q8IUD2
Avasorum	1	Q8IUF8
Avasorum	1	Q8IUH4
Avasorum	1	Q8IUI8
Avasorum	1	Q8IUR0
Avasorum	1	Q8IUR7
Avasorum	1	Q8IUW5
Avasorum	1	Q8IUX1
Avasorum	1	Q8IV08
Avasorum	1	Q8IV36
Avasorum	1	Q8IV38
Avasorum	1	Q8IV48
Avasorum	1	Q8IV63
Avasorum	1	Q8IVB5
Avasorum	1	Q8IVD9
Avasorum	1	Q8IVF2
Avasorum	1	Q8IVF7
Avasorum	1	Q8IVH4
Avasorum	1	Q8IVH8
Avasorum	1	Q8IVL0
Avasorum	1	Q8IVL6

Avasorum	1	Q8IVM0
Avasorum	1	Q8IVS2
Avasorum	1	Q8IVT5
Avasorum	1	Q8IW35
Avasorum	1	Q8IW45
Avasorum	1	Q8IWA0
Avasorum	1	Q8IWA4
Avasorum	1	Q8IWA5
Avasorum	1	Q8IWB1
Avasorum	1	Q8IWB7
Avasorum	1	Q8IWC1
Avasorum	1	Q8IWD4
Avasorum	1	Q8IWE2
Avasorum	1	Q8IWE4
Avasorum	1	Q8IWF6
Avasorum	1	Q8IWI9
Avasorum	1	Q8IWJ2
Avasorum	1	Q8IWR0
Avasorum	1	Q8IWS0
Avasorum	1	Q8IWT0
Avasorum	1	Q8IWT6
Avasorum	1	Q8IWU5
Avasorum	1	Q8IWV7
Avasorum	1	Q8IWV8
Avasorum	1	Q8IWW6
Avasorum	1	Q8IWX8
Avasorum	1	Q8IWY9
Avasorum	1	Q8IWZ3
Avasorum	1	Q8IWZ8
Avasorum	1	Q8IX01
Avasorum	1	Q8IX04
Avasorum	1	Q8IX12
Avasorum	1	Q8IX18
Avasorum	1	Q8IXB1
Avasorum	1	Q8IXH7
Avasorum	1	Q8IXI1
Avasorum	1	Q8IXI2
Avasorum	1	Q8IXJ6
Avasorum	1	Q8IXK0
Avasorum	1	Q8IXM2
Avasorum	1	Q8IXM3
Avasorum	1	Q8IXM6
Avasorum	1	Q8IXQ4
Avasorum	1	Q8IXQ6
Avasorum	1	Q8IXT5
Avasorum	1	Q8IXU6
Avasorum	1	Q8IXW5
Avasorum	1	Q8IY17
Avasorum	1	Q8IY18
Avasorum	1	Q8IY21

Avasorum	1	Q8IY22
Avasorum	1	Q8IY33
Avasorum	1	Q8IY37
Avasorum	1	Q8IY47
Avasorum	1	Q8IY67
Avasorum	1	Q8IY81
Avasorum	1	Q8IY95
Avasorum	1	Q8IYA6
Avasorum	1	Q8IYB1
Avasorum	1	Q8IYB3
Avasorum	1	Q8IYB5
Avasorum	1	Q8IYB7
Avasorum	1	Q8IYB8
Avasorum	1	Q8IYD1
Avasorum	1	Q8IYH5
Avasorum	1	Q8IYI6
Avasorum	1	Q8IYJ2
Avasorum	1	Q8IYM9
Avasorum	1	Q8IYQ7
Avasorum	1	Q8IYS2
Avasorum	1	Q8IYU8
Avasorum	1	Q8IZ07
Avasorum	1	Q8IZ21
Avasorum	1	Q8IZ69
Avasorum	1	Q8IZ73
Avasorum	1	Q8IZ81
Avasorum	1	Q8IZ83
Avasorum	1	Q8IZA0
Avasorum	1	Q8IZF2
Avasorum	1	Q8IZH2
Avasorum	1	Q8IZL8
Avasorum	1	Q8IZP0
Avasorum	1	Q8IZQ1
Avasorum	1	Q8IZQ5
Avasorum	1	Q8N0U8
Avasorum	1	Q8N0X7
Avasorum	1	Q8N0Z6
Avasorum	1	Q8N108
Avasorum	1	Q8N122
Avasorum	1	Q8N138
Avasorum	1	Q8N163
Avasorum	1	Q8N183
Avasorum	1	Q8N1B4
Avasorum	1	Q8N1F7
Avasorum	1	Q8N1F8
Avasorum	1	Q8N1G0
Avasorum	1	Q8N1G2
Avasorum	1	Q8N1G4
Avasorum	1	Q8N1I0
Avasorum	1	Q8N1Q1

Avasorum	1	Q8N1W1
Avasorum	1	Q8N201
Avasorum	1	Q8N257
Avasorum	1	Q8N264
Avasorum	1	Q8N2F6
Avasorum	1	Q8N2K0
Avasorum	1	Q8N2M8
Avasorum	1	Q8N2R8
Avasorum	1	Q8N2U0
Avasorum	1	Q8N2W9
Avasorum	1	Q8N335
Avasorum	1	Q8N357
Avasorum	1	Q8N392
Avasorum	1	Q8N3C0
Avasorum	1	Q8N3F8
Avasorum	1	Q8N3P4
Avasorum	1	Q8N3R9
Avasorum	1	Q8N3U4
Avasorum	1	Q8N3V7
Avasorum	1	Q8N3X1
Avasorum	1	Q8N442
Avasorum	1	Q8N488
Avasorum	1	Q8N4A0
Avasorum	1	Q8N4C8
Avasorum	1	Q8N4P3
Avasorum	1	Q8N4Q0
Avasorum	1	Q8N4T8
Avasorum	1	Q8N4V1
Avasorum	1	Q8N511
Avasorum	1	Q8N531
Avasorum	1	Q8N543
Avasorum	1	Q8N556
Avasorum	1	Q8N567
Avasorum	1	Q8N573
Avasorum	1	Q8N5A5
Avasorum	1	Q8N5C1
Avasorum	1	Q8N5C6
Avasorum	1	Q8N5D0
Avasorum	1	Q8N5F7
Avasorum	1	Q8N5G2
Avasorum	1	Q8N5H7
Avasorum	1	Q8N5I2
Avasorum	1	Q8N5I4
Avasorum	1	Q8N5K1
Avasorum	1	Q8N5L8
Avasorum	1	Q8N5M1
Avasorum	1	Q8N5M4
Avasorum	1	Q8N5M9
Avasorum	1	Q8N5W9
Avasorum	1	Q8N653

Avasorum	1	Q8N668
Avasorum	1	Q8N684
Avasorum	1	Q8N697
Avasorum	1	Q8N699
Avasorum	1	Q8N6G6
Avasorum	1	Q8N6H7
Avasorum	1	Q8N6M0
Avasorum	1	Q8N6M3
Avasorum	1	Q8N6R0
Avasorum	1	Q8N6S5
Avasorum	1	Q8N6T3
Avasorum	1	Q8N6T7
Avasorum	1	Q8N755
Avasorum	1	Q8N766
Avasorum	1	Q8N7H5
Avasorum	1	Q8N7R7
Avasorum	1	Q8N806
Avasorum	1	Q8N8A6
Avasorum	1	Q8N8J7
Avasorum	1	Q8N8R3
Avasorum	1	Q8N8S7
Avasorum	1	Q8N954
Avasorum	1	Q8N983
Avasorum	1	Q8N999
Avasorum	1	Q8N9F7
Avasorum	1	Q8N9M1
Avasorum	1	Q8N9N2
Avasorum	1	Q8N9N7
Avasorum	1	Q8N9N8
Avasorum	1	Q8N9T8
Avasorum	1	Q8N9Z2
Avasorum	1	Q8NAF0
Avasorum	1	Q8NAV1
Avasorum	1	Q8NB16
Avasorum	1	Q8NB37
Avasorum	1	Q8NB46
Avasorum	1	Q8NB49
Avasorum	1	Q8NB90
Avasorum	1	Q8NBF2
Avasorum	1	Q8NBF6
Avasorum	1	Q8NBI5
Avasorum	1	Q8NBI6
Avasorum	1	Q8NBJ4
Avasorum	1	Q8NBJ5
Avasorum	1	Q8NBJ7
Avasorum	1	Q8NBJ9
Avasorum	1	Q8NBK3
Avasorum	1	Q8NBL1
Avasorum	1	Q8NBM4
Avasorum	1	Q8NBM8

Avasorum	1	Q8NBN3
Avasorum	1	Q8NBN7
Avasorum	1	Q8NBP0
Avasorum	1	Q8NBQ5
Avasorum	1	Q8NBS9
Avasorum	1	Q8NBT2
Avasorum	1	Q8NBU5
Avasorum	1	Q8NBX0
Avasorum	1	Q8NBZ7
Avasorum	1	Q8NC42
Avasorum	1	Q8NC44
Avasorum	1	Q8NC51
Avasorum	1	Q8NC56
Avasorum	1	Q8NC60
Avasorum	1	Q8NC96
Avasorum	1	Q8NCA5
Avasorum	1	Q8NCC3
Avasorum	1	Q8NCE2
Avasorum	1	Q8NCF5
Avasorum	1	Q8NCG7
Avasorum	1	Q8NCH0
Avasorum	1	Q8NCL4
Avasorum	1	Q8NCN4
Avasorum	1	Q8NCN5
Avasorum	1	Q8NCW5
Avasorum	1	Q8ND04
Avasorum	1	Q8ND24
Avasorum	1	Q8ND56
Avasorum	1	Q8ND71
Avasorum	1	Q8ND76
Avasorum	1	Q8NDA8
Avasorum	1	Q8NDF8
Avasorum	1	Q8NDH3
Avasorum	1	Q8NDI1
Avasorum	1	Q8NDT2
Avasorum	1	Q8NDV1
Avasorum	1	Q8NDV7
Avasorum	1	Q8NDX5
Avasorum	1	Q8NDZ4
Avasorum	1	Q8NE01
Avasorum	1	Q8NE71
Avasorum	1	Q8NE86
Avasorum	1	Q8NEB9
Avasorum	1	Q8NEC7
Avasorum	1	Q8NEF9
Avasorum	1	Q8NEJ9
Avasorum	1	Q8NEM2
Avasorum	1	Q8NEN9
Avasorum	1	Q8NEU8
Avasorum	1	Q8NEW0

Avasorum	1	Q8NEY1
Avasorum	1	Q8NEY8
Avasorum	1	Q8NEZ3
Avasorum	1	Q8NEZ5
Avasorum	1	Q8NF37
Avasorum	1	Q8NF64
Avasorum	1	Q8NF91
Avasorum	1	Q8NFA0
Avasorum	1	Q8NFC6
Avasorum	1	Q8NFD5
Avasorum	1	Q8NFF5
Avasorum	1	Q8NFG4
Avasorum	1	Q8NFH3
Avasorum	1	Q8NFH4
Avasorum	1	Q8NFH5
Avasorum	1	Q8NFI3
Avasorum	1	Q8NFQ8
Avasorum	1	Q8NFV4
Avasorum	1	Q8NFW8
Avasorum	1	Q8NFZ0
Avasorum	1	Q8NG11
Avasorum	1	Q8NG68
Avasorum	1	Q8NHG7
Avasorum	1	Q8NHH9
Avasorum	1	Q8NHP6
Avasorum	1	Q8NHP8
Avasorum	1	Q8NHQ8
Avasorum	1	Q8NHQ9
Avasorum	1	Q8NHS3
Avasorum	1	Q8NHU6
Avasorum	1	Q8NHV1
Avasorum	1	Q8NHV4
Avasorum	1	Q8NI08
Avasorum	1	Q8NI27
Avasorum	1	Q8NI36
Avasorum	1	Q8NI37
Avasorum	1	Q8TA86
Avasorum	1	Q8TAA9
Avasorum	1	Q8TAD8
Avasorum	1	Q8TAE8
Avasorum	1	Q8TAF3
Avasorum	1	Q8TAG9
Avasorum	1	Q8TAQ2
Avasorum	1	Q8TAT6
Avasorum	1	Q8TB03
Avasorum	1	Q8TB22
Avasorum	1	Q8TB40
Avasorum	1	Q8TB52
Avasorum	1	Q8TB61
Avasorum	1	Q8TB72

Avasorum	1	Q8TB96
Avasorum	1	Q8TBA6
Avasorum	1	Q8TBB5
Avasorum	1	Q8TBC3
Avasorum	1	Q8TBC4
Avasorum	1	Q8TBF2
Avasorum	1	Q8TBM8
Avasorum	1	Q8TBQ9
Avasorum	1	Q8TBX8
Avasorum	1	Q8TBZ3
Avasorum	1	Q8TC07
Avasorum	1	Q8TC12
Avasorum	1	Q8TCA0
Avasorum	1	Q8TCC3
Avasorum	1	Q8TCF1
Avasorum	1	Q8TCG1
Avasorum	1	Q8TCG2
Avasorum	1	Q8TCJ2
Avasorum	1	Q8TCS8
Avasorum	1	Q8TCT7
Avasorum	1	Q8TCT9
Avasorum	1	Q8TCU6
Avasorum	1	Q8TCY9
Avasorum	1	Q8TD16
Avasorum	1	Q8TD19
Avasorum	1	Q8TD55
Avasorum	1	Q8TDB6
Avasorum	1	Q8TDD1
Avasorum	1	Q8TDM6
Avasorum	1	Q8TDN6
Avasorum	1	Q8TDQ7
Avasorum	1	Q8TDW0
Avasorum	1	Q8TDX7
Avasorum	1	Q8TDY2
Avasorum	1	Q8TDZ2
Avasorum	1	Q8TE02
Avasorum	1	Q8TE77
Avasorum	1	Q8TE82
Avasorum	1	Q8TEA7
Avasorum	1	Q8TEA8
Avasorum	1	Q8TEB1
Avasorum	1	Q8TED0
Avasorum	1	Q8TED1
Avasorum	1	Q8TED9
Avasorum	1	Q8TEJ3
Avasorum	1	Q8TEL6
Avasorum	1	Q8TEP8
Avasorum	1	Q8TEQ6
Avasorum	1	Q8TEQ8
Avasorum	1	Q8TEU7

Avasorum	1	Q8TEV9
Avasorum	1	Q8TEW0
Avasorum	1	Q8TEX9
Avasorum	1	Q8TEY7
Avasorum	1	Q8TF01
Avasorum	1	Q8TF05
Avasorum	1	Q8TF42
Avasorum	1	Q8TF65
Avasorum	1	Q8TF74
Avasorum	1	Q8WTS1
Avasorum	1	Q8WTS6
Avasorum	1	Q8WTT2
Avasorum	1	Q8WTV0
Avasorum	1	Q8WTW3
Avasorum	1	Q8WU76
Avasorum	1	Q8WU79
Avasorum	1	Q8WU90
Avasorum	1	Q8WUA2
Avasorum	1	Q8WUA4
Avasorum	1	Q8WUA7
Avasorum	1	Q8WUB8
Avasorum	1	Q8WUD1
Avasorum	1	Q8WUD4
Avasorum	1	Q8WUF5
Avasorum	1	Q8WUF8
Avasorum	1	Q8WUH1
Avasorum	1	Q8WUH2
Avasorum	1	Q8WUH6
Avasorum	1	Q8WUI4
Avasorum	1	Q8WUJ0
Avasorum	1	Q8WUK0
Avasorum	1	Q8WUM0
Avasorum	1	Q8WUM4
Avasorum	1	Q8WUM9
Avasorum	1	Q8WUP2
Avasorum	1	Q8WUQ7
Avasorum	1	Q8WUX2
Avasorum	1	Q8WUX9
Avasorum	1	Q8WUY1
Avasorum	1	Q8WUY8
Avasorum	1	Q8WUZ0
Avasorum	1	Q8WV22
Avasorum	1	Q8WV92
Avasorum	1	Q8WVB6
Avasorum	1	Q8WVC0
Avasorum	1	Q8WVC6
Avasorum	1	Q8WVJ2
Avasorum	1	Q8WVK2
Avasorum	1	Q8WVM0
Avasorum	1	Q8WVM7

Avasorum	1	Q8WVM8
Avasorum	1	Q8WVP5
Avasorum	1	Q8WVQ1
Avasorum	1	Q8WVT3
Avasorum	1	Q8WVV9
Avasorum	1	Q8WVX9
Avasorum	1	Q8WVY7
Avasorum	1	Q8WW01
Avasorum	1	Q8WW12
Avasorum	1	Q8WW22
Avasorum	1	Q8WW59
Avasorum	1	Q8WWC4
Avasorum	1	Q8WWH5
Avasorum	1	Q8WWI1
Avasorum	1	Q8WWI5
Avasorum	1	Q8WWK9
Avasorum	1	Q8WWM7
Avasorum	1	Q8WWN8
Avasorum	1	Q8WWP7
Avasorum	1	Q8WWQ0
Avasorum	1	Q8WWY3
Avasorum	1	Q8WX92
Avasorum	1	Q8WX93
Avasorum	1	Q8WXA3
Avasorum	1	Q8WXA9
Avasorum	1	Q8WXD5
Avasorum	1	Q8WXE0
Avasorum	1	Q8WXF1
Avasorum	1	Q8WXG6
Avasorum	1	Q8WXH0
Avasorum	1	Q8WXI9
Avasorum	1	Q8WXX5
Avasorum	1	Q8WY22
Avasorum	1	Q8WYA6
Avasorum	1	Q8WYN0
Avasorum	1	Q8WYP3
Avasorum	1	Q8WYP5
Avasorum	1	Q8WYQ5
Avasorum	1	Q8WZ75
Avasorum	1	Q8WZ82
Avasorum	1	Q8WZA9
Avasorum	1	Q92466
Avasorum	1	Q92478
Avasorum	1	Q92499
Avasorum	1	Q92503
Avasorum	1	Q92508
Avasorum	1	Q92520
Avasorum	1	Q92522
Avasorum	1	Q92530
Avasorum	1	Q92538

Avasorum	1	Q92541
Avasorum	1	Q92542
Avasorum	1	Q92543
Avasorum	1	Q92544
Avasorum	1	Q92545
Avasorum	1	Q92552
Avasorum	1	Q92556
Avasorum	1	Q92562
Avasorum	1	Q92567
Avasorum	1	Q92572
Avasorum	1	Q92574
Avasorum	1	Q92575
Avasorum	1	Q92576
Avasorum	1	Q92597
Avasorum	1	Q92598
Avasorum	1	Q92599
Avasorum	1	Q92600
Avasorum	1	Q92604
Avasorum	1	Q92609
Avasorum	1	Q92610
Avasorum	1	Q92613
Avasorum	1	Q92614
Avasorum	1	Q92615
Avasorum	1	Q92616
Avasorum	1	Q92619
Avasorum	1	Q92620
Avasorum	1	Q92621
Avasorum	1	Q92625
Avasorum	1	Q92626
Avasorum	1	Q92636
Avasorum	1	Q92643
Avasorum	1	Q92665
Avasorum	1	Q92667
Avasorum	1	Q92685
Avasorum	1	Q92686
Avasorum	1	Q92688
Avasorum	1	Q92692
Avasorum	1	Q92696
Avasorum	1	Q92734
Avasorum	1	Q92743
Avasorum	1	Q92747
Avasorum	1	Q92759
Avasorum	1	Q92766
Avasorum	1	Q92769
Avasorum	1	Q92783
Avasorum	1	Q92785
Avasorum	1	Q92791
Avasorum	1	Q92793
Avasorum	1	Q92797
Avasorum	1	Q92804

Avasorum	1	Q92805
Avasorum	1	Q92820
Avasorum	1	Q92823
Avasorum	1	Q92834
Avasorum	1	Q92835
Avasorum	1	Q92841
Avasorum	1	Q92845
Avasorum	1	Q92851
Avasorum	1	Q92870
Avasorum	1	Q92878
Avasorum	1	Q92879
Avasorum	1	Q92882
Avasorum	1	Q92888
Avasorum	1	Q92889
Avasorum	1	Q92890
Avasorum	1	Q92896
Avasorum	1	Q92900
Avasorum	1	Q92905
Avasorum	1	Q92917
Avasorum	1	Q92922
Avasorum	1	Q92925
Avasorum	1	Q92930
Avasorum	1	Q92934
Avasorum	1	Q92945
Avasorum	1	Q92947
Avasorum	1	Q92963
Avasorum	1	Q92968
Avasorum	1	Q92973
Avasorum	1	Q92974
Avasorum	1	Q92979
Avasorum	1	Q92989
Avasorum	1	Q92990
Avasorum	1	Q92995
Avasorum	1	Q92997
Avasorum	1	Q93008
Avasorum	1	Q93009
Avasorum	1	Q93034
Avasorum	1	Q93045
Avasorum	1	Q93050
Avasorum	1	Q93052
Avasorum	1	Q93062
Avasorum	1	Q93063
Avasorum	1	Q93074
Avasorum	1	Q93096
Avasorum	1	Q93100
Avasorum	1	Q969E2
Avasorum	1	Q969F9
Avasorum	1	Q969G3
Avasorum	1	Q969G5
Avasorum	1	Q969G6

Avasorum	1	Q969H8
Avasorum	1	Q969M1
Avasorum	1	Q969M3
Avasorum	1	Q969N2
Avasorum	1	Q969P0
Avasorum	1	Q969Q0
Avasorum	1	Q969Q5
Avasorum	1	Q969R2
Avasorum	1	Q969S3
Avasorum	1	Q969S9
Avasorum	1	Q969T4
Avasorum	1	Q969T7
Avasorum	1	Q969T9
Avasorum	1	Q969U7
Avasorum	1	Q969V3
Avasorum	1	Q969V5
Avasorum	1	Q969V6
Avasorum	1	Q969X1
Avasorum	1	Q969X5
Avasorum	1	Q969X6
Avasorum	1	Q969Z0
Avasorum	1	Q969Z3
Avasorum	1	Q96A26
Avasorum	1	Q96A33
Avasorum	1	Q96A35
Avasorum	1	Q96A65
Avasorum	1	Q96A72
Avasorum	1	Q96A73
Avasorum	1	Q96AA3
Avasorum	1	Q96AB3
Avasorum	1	Q96AB6
Avasorum	1	Q96AC1
Avasorum	1	Q96AE4
Avasorum	1	Q96AE7
Avasorum	1	Q96AG4
Avasorum	1	Q96AJ9
Avasorum	1	Q96AP7
Avasorum	1	Q96AQ6
Avasorum	1	Q96AQ8
Avasorum	1	Q96AT1
Avasorum	1	Q96AX1
Avasorum	1	Q96AY3
Avasorum	1	Q96AY4
Avasorum	1	Q96B26
Avasorum	1	Q96B54
Avasorum	1	Q96B97
Avasorum	1	Q96BD8
Avasorum	1	Q96BF6
Avasorum	1	Q96BH1
Avasorum	1	Q96BJ3

Avasorum	1	Q96BK5
Avasorum	1	Q96BM9
Avasorum	1	Q96BN8
Avasorum	1	Q96BP2
Avasorum	1	Q96BP3
Avasorum	1	Q96BQ5
Avasorum	1	Q96BR5
Avasorum	1	Q96BW1
Avasorum	1	Q96BW9
Avasorum	1	Q96BX8
Avasorum	1	Q96BY6
Avasorum	1	Q96BY7
Avasorum	1	Q96BZ8
Avasorum	1	Q96BZ9
Avasorum	1	Q96C19
Avasorum	1	Q96C23
Avasorum	1	Q96C24
Avasorum	1	Q96C36
Avasorum	1	Q96C57
Avasorum	1	Q96C86
Avasorum	1	Q96C90
Avasorum	1	Q96CB8
Avasorum	1	Q96CB9
Avasorum	1	Q96CC6
Avasorum	1	Q96CG8
Avasorum	1	Q96CM8
Avasorum	1	Q96CN7
Avasorum	1	Q96CN9
Avasorum	1	Q96CP2
Avasorum	1	Q96CP6
Avasorum	1	Q96CS2
Avasorum	1	Q96CS3
Avasorum	1	Q96CT7
Avasorum	1	Q96CU9
Avasorum	1	Q96CV9
Avasorum	1	Q96CW1
Avasorum	1	Q96CW5
Avasorum	1	Q96CW6
Avasorum	1	Q96CX2
Avasorum	1	Q96D15
Avasorum	1	Q96D46
Avasorum	1	Q96D71
Avasorum	1	Q96DA6
Avasorum	1	Q96DB5
Avasorum	1	Q96DE0
Avasorum	1	Q96DF8
Avasorum	1	Q96DG6
Avasorum	1	Q96DH6
Avasorum	1	Q96DI7
Avasorum	1	Q96DM3

Avasorum	1	Q96DV4
Avasorum	1	Q96DX4
Avasorum	1	Q96DZ1
Avasorum	1	Q96E11
Avasorum	1	Q96E29
Avasorum	1	Q96EA4
Avasorum	1	Q96EB1
Avasorum	1	Q96EB6
Avasorum	1	Q96EC8
Avasorum	1	Q96EE3
Avasorum	1	Q96EI5
Avasorum	1	Q96EK5
Avasorum	1	Q96EK6
Avasorum	1	Q96EK7
Avasorum	1	Q96EK9
Avasorum	1	Q96EL2
Avasorum	1	Q96EL3
Avasorum	1	Q96EM0
Avasorum	1	Q96EP0
Avasorum	1	Q96EP5
Avasorum	1	Q96EQ0
Avasorum	1	Q96ER3
Avasorum	1	Q96ER9
Avasorum	1	Q96ES7
Avasorum	1	Q96EU6
Avasorum	1	Q96EU7
Avasorum	1	Q96EV2
Avasorum	1	Q96EV8
Avasorum	1	Q96EY1
Avasorum	1	Q96EY4
Avasorum	1	Q96EY5
Avasorum	1	Q96EY7
Avasorum	1	Q96EY8
Avasorum	1	Q96F07
Avasorum	1	Q96F15
Avasorum	1	Q96F44
Avasorum	1	Q96F63
Avasorum	1	Q96F85
Avasorum	1	Q96F86
Avasorum	1	Q96FJ2
Avasorum	1	Q96FN4
Avasorum	1	Q96FQ6
Avasorum	1	Q96FS4
Avasorum	1	Q96FV2
Avasorum	1	Q96FV9
Avasorum	1	Q96FW1
Avasorum	1	Q96FX7
Avasorum	1	Q96FZ2
Avasorum	1	Q96FZ7
Avasorum	1	Q96G01

Avasorum	1	Q96G03
Avasorum	1	Q96G21
Avasorum	1	Q96G23
Avasorum	1	Q96G46
Avasorum	1	Q96GA3
Avasorum	1	Q96GA7
Avasorum	1	Q96GC5
Avasorum	1	Q96GD0
Avasorum	1	Q96GD4
Avasorum	1	Q96GG9
Avasorum	1	Q96GK7
Avasorum	1	Q96GM5
Avasorum	1	Q96GM8
Avasorum	1	Q96GN5
Avasorum	1	Q96GQ5
Avasorum	1	Q96GQ7
Avasorum	1	Q96GS4
Avasorum	1	Q96GW9
Avasorum	1	Q96GX2
Avasorum	1	Q96GX5
Avasorum	1	Q96GX9
Avasorum	1	Q96GY0
Avasorum	1	Q96GY3
Avasorum	1	Q96GZ6
Avasorum	1	Q96H20
Avasorum	1	Q96H79
Avasorum	1	Q96HA1
Avasorum	1	Q96HA7
Avasorum	1	Q96HC4
Avasorum	1	Q96HD1
Avasorum	1	Q96HE7
Avasorum	1	Q96HE9
Avasorum	1	Q96HJ9
Avasorum	1	Q96HN2
Avasorum	1	Q96HP0
Avasorum	1	Q96HR3
Avasorum	1	Q96HS1
Avasorum	1	Q96HW7
Avasorum	1	Q96HY6
Avasorum	1	Q96HY7
Avasorum	1	Q96I15
Avasorum	1	Q96I24
Avasorum	1	Q96I25
Avasorum	1	Q96I51
Avasorum	1	Q96I59
Avasorum	1	Q96I99
Avasorum	1	Q96II8
Avasorum	1	Q96IJ6
Avasorum	1	Q96IR7
Avasorum	1	Q96IU4

Avasorum	1	Q96IV0
Avasorum	1	Q96IW7
Avasorum	1	Q96IX5
Avasorum	1	Q96IY1
Avasorum	1	Q96IZ0
Avasorum	1	Q96IZ6
Avasorum	1	Q96J01
Avasorum	1	Q96J02
Avasorum	1	Q96J42
Avasorum	1	Q96J84
Avasorum	1	Q96JB2
Avasorum	1	Q96JB5
Avasorum	1	Q96JC1
Avasorum	1	Q96JG6
Avasorum	1	Q96JH7
Avasorum	1	Q96JI7
Avasorum	1	Q96JJ3
Avasorum	1	Q96JJ7
Avasorum	1	Q96JK2
Avasorum	1	Q96JM3
Avasorum	1	Q96JM7
Avasorum	1	Q96JP5
Avasorum	1	Q96JQ0
Avasorum	1	Q96JQ2
Avasorum	1	Q96JY6
Avasorum	1	Q96K12
Avasorum	1	Q96K17
Avasorum	1	Q96K19
Avasorum	1	Q96K21
Avasorum	1	Q96K37
Avasorum	1	Q96K76
Avasorum	1	Q96K83
Avasorum	1	Q96KA5
Avasorum	1	Q96KB5
Avasorum	1	Q96KC2
Avasorum	1	Q96KC8
Avasorum	1	Q96KG9
Avasorum	1	Q96KM6
Avasorum	1	Q96KN1
Avasorum	1	Q96KP1
Avasorum	1	Q96KP4
Avasorum	1	Q96KQ7
Avasorum	1	Q96KR1
Avasorum	1	Q96KR6
Avasorum	1	Q96L58
Avasorum	1	Q96L91
Avasorum	1	Q96L92
Avasorum	1	Q96L93
Avasorum	1	Q96LB3
Avasorum	1	Q96LD4

Avasorum	1	Q96LI5
Avasorum	1	Q96LJ7
Avasorum	1	Q96LL9
Avasorum	1	Q96LR5
Avasorum	1	Q96LW7
Avasorum	1	Q96M27
Avasorum	1	Q96M96
Avasorum	1	Q96ME1
Avasorum	1	Q96ME7
Avasorum	1	Q96MF7
Avasorum	1	Q96MG7
Avasorum	1	Q96MG8
Avasorum	1	Q96MM6
Avasorum	1	Q96MU7
Avasorum	1	Q96MW5
Avasorum	1	Q96MX0
Avasorum	1	Q96MX6
Avasorum	1	Q96MY1
Avasorum	1	Q96N66
Avasorum	1	Q96N67
Avasorum	1	Q96NB2
Avasorum	1	Q96ND0
Avasorum	1	Q96NE9
Avasorum	1	Q96P11
Avasorum	1	Q96P16
Avasorum	1	Q96P47
Avasorum	1	Q96P48
Avasorum	1	Q96P70
Avasorum	1	Q96PC5
Avasorum	1	Q96PD2
Avasorum	1	Q96PE2
Avasorum	1	Q96PE3
Avasorum	1	Q96PK6
Avasorum	1	Q96PM5
Avasorum	1	Q96PQ7
Avasorum	1	Q96PU4
Avasorum	1	Q96PU5
Avasorum	1	Q96PU8
Avasorum	1	Q96PV6
Avasorum	1	Q96PY5
Avasorum	1	Q96PZ0
Avasorum	1	Q96PZ2
Avasorum	1	Q96Q05
Avasorum	1	Q96Q11
Avasorum	1	Q96Q15
Avasorum	1	Q96Q42
Avasorum	1	Q96Q45
Avasorum	1	Q96Q83
Avasorum	1	Q96QB1
Avasorum	1	Q96QC0

Avasorum	1	Q96QD8
Avasorum	1	Q96QD9
Avasorum	1	Q96QG7
Avasorum	1	Q96QK1
Avasorum	1	Q96QR8
Avasorum	1	Q96QU8
Avasorum	1	Q96QV1
Avasorum	1	Q96QZ7
Avasorum	1	Q96R06
Avasorum	1	Q96RE7
Avasorum	1	Q96RF0
Avasorum	1	Q96RG2
Avasorum	1	Q96RK0
Avasorum	1	Q96RL1
Avasorum	1	Q96RL7
Avasorum	1	Q96RN5
Avasorum	1	Q96RP9
Avasorum	1	Q96RQ1
Avasorum	1	Q96RQ3
Avasorum	1	Q96RR4
Avasorum	1	Q96RS0
Avasorum	1	Q96RS6
Avasorum	1	Q96RT1
Avasorum	1	Q96RT7
Avasorum	1	Q96RT8
Avasorum	1	Q96RU2
Avasorum	1	Q96RU3
Avasorum	1	Q96S44
Avasorum	1	Q96S52
Avasorum	1	Q96S55
Avasorum	1	Q96S59
Avasorum	1	Q96S66
Avasorum	1	Q96S97
Avasorum	1	Q96SB3
Avasorum	1	Q96SB4
Avasorum	1	Q96SB8
Avasorum	1	Q96SI1
Avasorum	1	Q96SI9
Avasorum	1	Q96SK2
Avasorum	1	Q96SL4
Avasorum	1	Q96SN8
Avasorum	1	Q96SQ9
Avasorum	1	Q96ST2
Avasorum	1	Q96ST3
Avasorum	1	Q96SU4
Avasorum	1	Q96SW2
Avasorum	1	Q96SY0
Avasorum	1	Q96SZ5
Avasorum	1	Q96SZ6
Avasorum	1	Q96T23

Avasorum	1	Q96T37
Avasorum	1	Q96T51
Avasorum	1	Q96T58
Avasorum	1	Q96T60
Avasorum	1	Q96T76
Avasorum	1	Q96T88
Avasorum	1	Q96TA1
Avasorum	1	Q96TA2
Avasorum	1	Q96TC7
Avasorum	1	Q99081
Avasorum	1	Q99418
Avasorum	1	Q99426
Avasorum	1	Q99436
Avasorum	1	Q99439
Avasorum	1	Q99442
Avasorum	1	Q99447
Avasorum	1	Q99459
Avasorum	1	Q99460
Avasorum	1	Q99470
Avasorum	1	Q99471
Avasorum	1	Q99496
Avasorum	1	Q99497
Avasorum	1	Q99500
Avasorum	1	Q99501
Avasorum	1	Q99504
Avasorum	1	Q99519
Avasorum	1	Q99523
Avasorum	1	Q99536
Avasorum	1	Q99538
Avasorum	1	Q99541
Avasorum	1	Q99543
Avasorum	1	Q99549
Avasorum	1	Q99567
Avasorum	1	Q99569
Avasorum	1	Q99570
Avasorum	1	Q99571
Avasorum	1	Q99575
Avasorum	1	Q99584
Avasorum	1	Q99590
Avasorum	1	Q99598
Avasorum	1	Q99611
Avasorum	1	Q99613
Avasorum	1	Q99614
Avasorum	1	Q99615
Avasorum	1	Q99623
Avasorum	1	Q99627
Avasorum	1	Q99653
Avasorum	1	Q99661
Avasorum	1	Q99666
Avasorum	1	Q99685

Avasorum	1	Q99700
Avasorum	1	Q99704
Avasorum	1	Q99707
Avasorum	1	Q99714
Avasorum	1	Q99715
Avasorum	1	Q99717
Avasorum	1	Q99720
Avasorum	1	Q99729
Avasorum	1	Q99733
Avasorum	1	Q99735
Avasorum	1	Q99747
Avasorum	1	Q99755
Avasorum	1	Q99758
Avasorum	1	Q99759
Avasorum	1	Q99766
Avasorum	1	Q99797
Avasorum	1	Q99798
Avasorum	1	Q99805
Avasorum	1	Q99808
Avasorum	1	Q99816
Avasorum	1	Q99829
Avasorum	1	Q99832
Avasorum	1	Q99836
Avasorum	1	Q99848
Avasorum	1	Q99873
Avasorum	1	Q99933
Avasorum	1	Q99941
Avasorum	1	Q99958
Avasorum	1	Q99961
Avasorum	1	Q99973
Avasorum	1	Q99986
Avasorum	1	Q99988
Avasorum	1	Q99996
Avasorum	1	Q9BPW8
Avasorum	1	Q9BPX3
Avasorum	1	Q9BPX5
Avasorum	1	Q9BPX6
Avasorum	1	Q9BPX7
Avasorum	1	Q9BPY3
Avasorum	1	Q9BPZ7
Avasorum	1	Q9BQ04
Avasorum	1	Q9BQ24
Avasorum	1	Q9BQ39
Avasorum	1	Q9BQ48
Avasorum	1	Q9BQ52
Avasorum	1	Q9BQ61
Avasorum	1	Q9BQ67
Avasorum	1	Q9BQ69
Avasorum	1	Q9BQ70
Avasorum	1	Q9BQ75

Avasorum	1	Q9BQ90
Avasorum	1	Q9BQ95
Avasorum	1	Q9BQA1
Avasorum	1	Q9BQA9
Avasorum	1	Q9BQB6
Avasorum	1	Q9BQC3
Avasorum	1	Q9BQE3
Avasorum	1	Q9BQE4
Avasorum	1	Q9BQE5
Avasorum	1	Q9BQG0
Avasorum	1	Q9BQP7
Avasorum	1	Q9BQQ3
Avasorum	1	Q9BQS8
Avasorum	1	Q9BR61
Avasorum	1	Q9BR76
Avasorum	1	Q9BRA2
Avasorum	1	Q9BRD0
Avasorum	1	Q9BRF8
Avasorum	1	Q9BRG1
Avasorum	1	Q9BRJ2
Avasorum	1	Q9BRJ6
Avasorum	1	Q9BRJ7
Avasorum	1	Q9BRK5
Avasorum	1	Q9BRP1
Avasorum	1	Q9BRP4
Avasorum	1	Q9BRP8
Avasorum	1	Q9BRQ6
Avasorum	1	Q9BRR6
Avasorum	1	Q9BRR8
Avasorum	1	Q9BRS2
Avasorum	1	Q9BRT6
Avasorum	1	Q9BRT9
Avasorum	1	Q9BRU9
Avasorum	1	Q9BRX2
Avasorum	1	Q9BRX8
Avasorum	1	Q9BRZ2
Avasorum	1	Q9BS26
Avasorum	1	Q9BS40
Avasorum	1	Q9BSC4
Avasorum	1	Q9BSD7
Avasorum	1	Q9BSF4
Avasorum	1	Q9BSH4
Avasorum	1	Q9BSJ2
Avasorum	1	Q9BSJ8
Avasorum	1	Q9BSL1
Avasorum	1	Q9BSR8
Avasorum	1	Q9BSU1
Avasorum	1	Q9BSV6
Avasorum	1	Q9BT09
Avasorum	1	Q9BT17

Avasorum	1	Q9BT22
Avasorum	1	Q9BT23
Avasorum	1	Q9BT25
Avasorum	1	Q9BT40
Avasorum	1	Q9BT73
Avasorum	1	Q9BT78
Avasorum	1	Q9BT88
Avasorum	1	Q9BTA9
Avasorum	1	Q9BTC0
Avasorum	1	Q9BTC8
Avasorum	1	Q9BTD8
Avasorum	1	Q9BTE1
Avasorum	1	Q9BTE3
Avasorum	1	Q9BTE6
Avasorum	1	Q9BTE7
Avasorum	1	Q9BTT0
Avasorum	1	Q9BTT6
Avasorum	1	Q9BTU6
Avasorum	1	Q9BTV4
Avasorum	1	Q9BTV5
Avasorum	1	Q9BTW9
Avasorum	1	Q9BTX1
Avasorum	1	Q9BTY7
Avasorum	1	Q9BTZ2
Avasorum	1	Q9BU23
Avasorum	1	Q9BU61
Avasorum	1	Q9BU76
Avasorum	1	Q9BUB7
Avasorum	1	Q9BUE0
Avasorum	1	Q9BUE6
Avasorum	1	Q9BUF5
Avasorum	1	Q9BUH6
Avasorum	1	Q9BUI4
Avasorum	1	Q9BUJ2
Avasorum	1	Q9BUK6
Avasorum	1	Q9BUL5
Avasorum	1	Q9BUL8
Avasorum	1	Q9BUL9
Avasorum	1	Q9BUN8
Avasorum	1	Q9BUP3
Avasorum	1	Q9BUQ8
Avasorum	1	Q9BUR5
Avasorum	1	Q9BUT1
Avasorum	1	Q9BV20
Avasorum	1	Q9BV38
Avasorum	1	Q9BV40
Avasorum	1	Q9BV44
Avasorum	1	Q9BV57
Avasorum	1	Q9BV79
Avasorum	1	Q9BV81

Avasorum	1	Q9BV86
Avasorum	1	Q9BV94
Avasorum	1	Q9BVA0
Avasorum	1	Q9BVC4
Avasorum	1	Q9BVC5
Avasorum	1	Q9BVC6
Avasorum	1	Q9BVG4
Avasorum	1	Q9BVG9
Avasorum	1	Q9BVI4
Avasorum	1	Q9BVJ6
Avasorum	1	Q9BVJ7
Avasorum	1	Q9BVK6
Avasorum	1	Q9BVK8
Avasorum	1	Q9BVL2
Avasorum	1	Q9BVL4
Avasorum	1	Q9BVM2
Avasorum	1	Q9BVP2
Avasorum	1	Q9BVQ7
Avasorum	1	Q9BVS4
Avasorum	1	Q9BVS5
Avasorum	1	Q9BVT8
Avasorum	1	Q9BVV7
Avasorum	1	Q9BW19
Avasorum	1	Q9BW27
Avasorum	1	Q9BW60
Avasorum	1	Q9BW62
Avasorum	1	Q9BW71
Avasorum	1	Q9BW83
Avasorum	1	Q9BW85
Avasorum	1	Q9BW91
Avasorum	1	Q9BW92
Avasorum	1	Q9BWD1
Avasorum	1	Q9BWE0
Avasorum	1	Q9BWF3
Avasorum	1	Q9BWH2
Avasorum	1	Q9BWH6
Avasorum	1	Q9BWJ5
Avasorum	1	Q9BWM7
Avasorum	1	Q9BWN1
Avasorum	1	Q9BWS9
Avasorum	1	Q9BWT3
Avasorum	1	Q9BWT7
Avasorum	1	Q9BWU0
Avasorum	1	Q9BWU1
Avasorum	1	Q9BX40
Avasorum	1	Q9BX59
Avasorum	1	Q9BX67
Avasorum	1	Q9BX69
Avasorum	1	Q9BX70
Avasorum	1	Q9BXB4

Avasorum	1	Q9BXB5
Avasorum	1	Q9BXF6
Avasorum	1	Q9BXI6
Avasorum	1	Q9BXJ0
Avasorum	1	Q9BXJ9
Avasorum	1	Q9BXK5
Avasorum	1	Q9BXL7
Avasorum	1	Q9BXP2
Avasorum	1	Q9BXP5
Avasorum	1	Q9BXR0
Avasorum	1	Q9BXS5
Avasorum	1	Q9BXS6
Avasorum	1	Q9BXW7
Avasorum	1	Q9BXW9
Avasorum	1	Q9BXY0
Avasorum	1	Q9BY41
Avasorum	1	Q9BY42
Avasorum	1	Q9BY43
Avasorum	1	Q9BY44
Avasorum	1	Q9BY77
Avasorum	1	Q9BY89
Avasorum	1	Q9BYB0
Avasorum	1	Q9BYB4
Avasorum	1	Q9BYC5
Avasorum	1	Q9BYC8
Avasorum	1	Q9BYC9
Avasorum	1	Q9BYD1
Avasorum	1	Q9BYD2
Avasorum	1	Q9BYD3
Avasorum	1	Q9BYD6
Avasorum	1	Q9BYG3
Avasorum	1	Q9BYI3
Avasorum	1	Q9BYK8
Avasorum	1	Q9BYM8
Avasorum	1	Q9BYN8
Avasorum	1	Q9BYT8
Avasorum	1	Q9BYV8
Avasorum	1	Q9BYW2
Avasorum	1	Q9BZ23
Avasorum	1	Q9BZ29
Avasorum	1	Q9BZ67
Avasorum	1	Q9BZ76
Avasorum	1	Q9BZ95
Avasorum	1	Q9BZD4
Avasorum	1	Q9BZE1
Avasorum	1	Q9BZE4
Avasorum	1	Q9BZE9
Avasorum	1	Q9BZF1
Avasorum	1	Q9BZF9
Avasorum	1	Q9BZG1

Avasorum	1	Q9BZG8
Avasorum	1	Q9BZH6
Avasorum	1	Q9BZI7
Avasorum	1	Q9BZJ0
Avasorum	1	Q9BZK7
Avasorum	1	Q9BZL1
Avasorum	1	Q9BZL4
Avasorum	1	Q9BZL6
Avasorum	1	Q9BZQ6
Avasorum	1	Q9BZQ8
Avasorum	1	Q9BZV1
Avasorum	1	Q9BZW5
Avasorum	1	Q9BZX2
Avasorum	1	Q9BZZ2
Avasorum	1	Q9BZZ5
Avasorum	1	Q9C000
Avasorum	1	Q9C035
Avasorum	1	Q9C037
Avasorum	1	Q9C0B1
Avasorum	1	Q9C0B5
Avasorum	1	Q9C0B7
Avasorum	1	Q9C0C2
Avasorum	1	Q9C0C9
Avasorum	1	Q9C0D3
Avasorum	1	Q9C0D5
Avasorum	1	Q9C0D9
Avasorum	1	Q9C0E2
Avasorum	1	Q9C0E8
Avasorum	1	Q9C0F1
Avasorum	1	Q9C0H2
Avasorum	1	Q9C0H6
Avasorum	1	Q9C0I1
Avasorum	1	Q9C0J8
Avasorum	1	Q9GZL7
Avasorum	1	Q9GZM5
Avasorum	1	Q9GZM7
Avasorum	1	Q9GZN8
Avasorum	1	Q9GZP4
Avasorum	1	Q9GZP9
Avasorum	1	Q9GZQ3
Avasorum	1	Q9GZR1
Avasorum	1	Q9GZR2
Avasorum	1	Q9GZR7
Avasorum	1	Q9GZS1
Avasorum	1	Q9GZS3
Avasorum	1	Q9GZT3
Avasorum	1	Q9GZT4
Avasorum	1	Q9GZT8
Avasorum	1	Q9GZT9
Avasorum	1	Q9GZU1

Avasorum	1	Q9GZU8
Avasorum	1	Q9GZY8
Avasorum	1	Q9GZZ1
Avasorum	1	Q9GZZ9
Avasorum	1	Q9H000
Avasorum	1	Q9H061
Avasorum	1	Q9H074
Avasorum	1	Q9H078
Avasorum	1	Q9H082
Avasorum	1	Q9H089
Avasorum	1	Q9H098
Avasorum	1	Q9H0A0
Avasorum	1	Q9H0A8
Avasorum	1	Q9H0B6
Avasorum	1	Q9H0C8
Avasorum	1	Q9H0D6
Avasorum	1	Q9H0E2
Avasorum	1	Q9H0E3
Avasorum	1	Q9H0E9
Avasorum	1	Q9H0G5
Avasorum	1	Q9H0H0
Avasorum	1	Q9H0H5
Avasorum	1	Q9H0J9
Avasorum	1	Q9H0L4
Avasorum	1	Q9H0P0
Avasorum	1	Q9H0Q0
Avasorum	1	Q9H0R3
Avasorum	1	Q9H0R4
Avasorum	1	Q9H0R6
Avasorum	1	Q9H0S4
Avasorum	1	Q9H0U3
Avasorum	1	Q9H0U4
Avasorum	1	Q9H0U6
Avasorum	1	Q9H0U9
Avasorum	1	Q9H0V1
Avasorum	1	Q9H0V9
Avasorum	1	Q9H0W8
Avasorum	1	Q9H0X4
Avasorum	1	Q9H0X9
Avasorum	1	Q9H147
Avasorum	1	Q9H173
Avasorum	1	Q9H1A3
Avasorum	1	Q9H1A4
Avasorum	1	Q9H1B7
Avasorum	1	Q9H1C7
Avasorum	1	Q9H1D9
Avasorum	1	Q9H1E3
Avasorum	1	Q9H1E5
Avasorum	1	Q9H1H9
Avasorum	1	Q9H1I8

Avasorum	1	Q9H1K0
Avasorum	1	Q9H1K1
Avasorum	1	Q9H1P3
Avasorum	1	Q9H1Y0
Avasorum	1	Q9H1Z4
Avasorum	1	Q9H201
Avasorum	1	Q9H223
Avasorum	1	Q9H267
Avasorum	1	Q9H269
Avasorum	1	Q9H270
Avasorum	1	Q9H299
Avasorum	1	Q9H2C0
Avasorum	1	Q9H2D1
Avasorum	1	Q9H2D6
Avasorum	1	Q9H2G2
Avasorum	1	Q9H2H8
Avasorum	1	Q9H2H9
Avasorum	1	Q9H2J4
Avasorum	1	Q9H2K8
Avasorum	1	Q9H2M9
Avasorum	1	Q9H2P0
Avasorum	1	Q9H2P9
Avasorum	1	Q9H2U1
Avasorum	1	Q9H2U2
Avasorum	1	Q9H2V7
Avasorum	1	Q9H2W6
Avasorum	1	Q9H2X9
Avasorum	1	Q9H300
Avasorum	1	Q9H307
Avasorum	1	Q9H330
Avasorum	1	Q9H334
Avasorum	1	Q9H3F6
Avasorum	1	Q9H3H1
Avasorum	1	Q9H3H3
Avasorum	1	Q9H3K2
Avasorum	1	Q9H3K6
Avasorum	1	Q9H3L0
Avasorum	1	Q9H3M7
Avasorum	1	Q9H3N1
Avasorum	1	Q9H3P2
Avasorum	1	Q9H3P7
Avasorum	1	Q9H3Q1
Avasorum	1	Q9H3S7
Avasorum	1	Q9H3T3
Avasorum	1	Q9H3U1
Avasorum	1	Q9H3U5
Avasorum	1	Q9H3Z4
Avasorum	1	Q9H410
Avasorum	1	Q9H444
Avasorum	1	Q9H446

Avasorum	1	Q9H467
Avasorum	1	Q9H469
Avasorum	1	Q9H479
Avasorum	1	Q9H488
Avasorum	1	Q9H490
Avasorum	1	Q9H497
Avasorum	1	Q9H4A3
Avasorum	1	Q9H4A4
Avasorum	1	Q9H4A5
Avasorum	1	Q9H4A6
Avasorum	1	Q9H4B0
Avasorum	1	Q9H4F1
Avasorum	1	Q9H4G4
Avasorum	1	Q9H4I3
Avasorum	1	Q9H4K7
Avasorum	1	Q9H4L4
Avasorum	1	Q9H4L5
Avasorum	1	Q9H4L7
Avasorum	1	Q9H4M9
Avasorum	1	Q9H4Z3
Avasorum	1	Q9H501
Avasorum	1	Q9H553
Avasorum	1	Q9H583
Avasorum	1	Q9H5K3
Avasorum	1	Q9H5N1
Avasorum	1	Q9H5Q4
Avasorum	1	Q9H5U6
Avasorum	1	Q9H5V9
Avasorum	1	Q9H5X1
Avasorum	1	Q9H5Z1
Avasorum	1	Q9H5Z6
Avasorum	1	Q9H6D7
Avasorum	1	Q9H6E4
Avasorum	1	Q9H6F5
Avasorum	1	Q9H6H4
Avasorum	1	Q9H6I2
Avasorum	1	Q9H6K4
Avasorum	1	Q9H6Q4
Avasorum	1	Q9H6R0
Avasorum	1	Q9H6R4
Avasorum	1	Q9H6R7
Avasorum	1	Q9H6S0
Avasorum	1	Q9H6S1
Avasorum	1	Q9H6T3
Avasorum	1	Q9H6U6
Avasorum	1	Q9H6U8
Avasorum	1	Q9H6V9
Avasorum	1	Q9H6W3
Avasorum	1	Q9H6Y2
Avasorum	1	Q9H6Z4

Avasorum	1	Q9H773
Avasorum	1	Q9H792
Avasorum	1	Q9H7B2
Avasorum	1	Q9H7B4
Avasorum	1	Q9H7C9
Avasorum	1	Q9H7D0
Avasorum	1	Q9H7D7
Avasorum	1	Q9H7E2
Avasorum	1	Q9H7E9
Avasorum	1	Q9H7F0
Avasorum	1	Q9H7H0
Avasorum	1	Q9H7N4
Avasorum	1	Q9H7Z7
Avasorum	1	Q9H814
Avasorum	1	Q9H832
Avasorum	1	Q9H845
Avasorum	1	Q9H857
Avasorum	1	Q9H875
Avasorum	1	Q9H8G2
Avasorum	1	Q9H8H0
Avasorum	1	Q9H8H2
Avasorum	1	Q9H8H3
Avasorum	1	Q9H8K7
Avasorum	1	Q9H8L6
Avasorum	1	Q9H8M7
Avasorum	1	Q9H8M9
Avasorum	1	Q9H8T0
Avasorum	1	Q9H8W4
Avasorum	1	Q9H8Y5
Avasorum	1	Q9H8Y8
Avasorum	1	Q9H900
Avasorum	1	Q9H910
Avasorum	1	Q9H936
Avasorum	1	Q9H939
Avasorum	1	Q9H944
Avasorum	1	Q9H974
Avasorum	1	Q9H981
Avasorum	1	Q9H993
Avasorum	1	Q9H9A5
Avasorum	1	Q9H9A6
Avasorum	1	Q9H9B1
Avasorum	1	Q9H9B4
Avasorum	1	Q9H9C1
Avasorum	1	Q9H9E3
Avasorum	1	Q9H9F9
Avasorum	1	Q9H9H4
Avasorum	1	Q9H9J2
Avasorum	1	Q9H9P8
Avasorum	1	Q9H9Q2
Avasorum	1	Q9H9Q4

Avasorum	1	Q9H9S3
Avasorum	1	Q9H9S5
Avasorum	1	Q9H9T3
Avasorum	1	Q9H9Y2
Avasorum	1	Q9H9Y6
Avasorum	1	Q9HA38
Avasorum	1	Q9HA47
Avasorum	1	Q9HA64
Avasorum	1	Q9HA65
Avasorum	1	Q9HA77
Avasorum	1	Q9HAB3
Avasorum	1	Q9HAB8
Avasorum	1	Q9HAC8
Avasorum	1	Q9HAD4
Avasorum	1	Q9HAN9
Avasorum	1	Q9HAS0
Avasorum	1	Q9HAT2
Avasorum	1	Q9HAU0
Avasorum	1	Q9HAU4
Avasorum	1	Q9HAU5
Avasorum	1	Q9HAV0
Avasorum	1	Q9HAV4
Avasorum	1	Q9HAV7
Avasorum	1	Q9HB07
Avasorum	1	Q9HB20
Avasorum	1	Q9HB21
Avasorum	1	Q9HB40
Avasorum	1	Q9HB63
Avasorum	1	Q9HB71
Avasorum	1	Q9HB90
Avasorum	1	Q9HBF4
Avasorum	1	Q9HBG6
Avasorum	1	Q9HBH5
Avasorum	1	Q9HBI1
Avasorum	1	Q9HBL0
Avasorum	1	Q9HBL7
Avasorum	1	Q9HBL8
Avasorum	1	Q9HBM0
Avasorum	1	Q9HBM1
Avasorum	1	Q9HBM6
Avasorum	1	Q9HBR0
Avasorum	1	Q9HBU6
Avasorum	1	Q9HBW9
Avasorum	1	Q9HC07
Avasorum	1	Q9HC21
Avasorum	1	Q9HC35
Avasorum	1	Q9HC36
Avasorum	1	Q9HC38
Avasorum	1	Q9HC98
Avasorum	1	Q9HCC0

Avasorum	1	Q9HCD5
Avasorum	1	Q9HCE0
Avasorum	1	Q9HCE1
Avasorum	1	Q9HCE5
Avasorum	1	Q9HCG7
Avasorum	1	Q9HCG8
Avasorum	1	Q9HCJ3
Avasorum	1	Q9HCK8
Avasorum	1	Q9HCM4
Avasorum	1	Q9HCN4
Avasorum	1	Q9HCN8
Avasorum	1	Q9HCP0
Avasorum	1	Q9HCS7
Avasorum	1	Q9HCU5
Avasorum	1	Q9HD15
Avasorum	1	Q9HD20
Avasorum	1	Q9HD26
Avasorum	1	Q9HD33
Avasorum	1	Q9HD34
Avasorum	1	Q9HD42
Avasorum	1	Q9HD45
Avasorum	1	Q9HD67
Avasorum	1	Q9HDC9
Avasorum	1	Q9NNW5
Avasorum	1	Q9NNW7
Avasorum	1	Q9NP58
Avasorum	1	Q9NP61
Avasorum	1	Q9NP64
Avasorum	1	Q9NP72
Avasorum	1	Q9NP74
Avasorum	1	Q9NP77
Avasorum	1	Q9NP79
Avasorum	1	Q9NP81
Avasorum	1	Q9NP92
Avasorum	1	Q9NP97
Avasorum	1	Q9NPA0
Avasorum	1	Q9NPA8
Avasorum	1	Q9NPD3
Avasorum	1	Q9NPD8
Avasorum	1	Q9NPE3
Avasorum	1	Q9NPF4
Avasorum	1	Q9NPF5
Avasorum	1	Q9NPG4
Avasorum	1	Q9NPH2
Avasorum	1	Q9NPI1
Avasorum	1	Q9NPI6
Avasorum	1	Q9NPJ3
Avasorum	1	Q9NPJ6
Avasorum	1	Q9NPL8
Avasorum	1	Q9NPQ8

Avasorum	1	Q9NPY3
Avasorum	1	Q9NQ29
Avasorum	1	Q9NQ30
Avasorum	1	Q9NQ48
Avasorum	1	Q9NQ50
Avasorum	1	Q9NQ55
Avasorum	1	Q9NQ66
Avasorum	1	Q9NQ88
Avasorum	1	Q9NQ89
Avasorum	1	Q9NQC3
Avasorum	1	Q9NQG5
Avasorum	1	Q9NQH7
Avasorum	1	Q9NQR4
Avasorum	1	Q9NQS1
Avasorum	1	Q9NQT4
Avasorum	1	Q9NQT5
Avasorum	1	Q9NQT8
Avasorum	1	Q9NQW6
Avasorum	1	Q9NQW7
Avasorum	1	Q9NQX3
Avasorum	1	Q9NQY0
Avasorum	1	Q9NQZ2
Avasorum	1	Q9NQZ5
Avasorum	1	Q9NR09
Avasorum	1	Q9NR12
Avasorum	1	Q9NR19
Avasorum	1	Q9NR28
Avasorum	1	Q9NR30
Avasorum	1	Q9NR31
Avasorum	1	Q9NR45
Avasorum	1	Q9NR46
Avasorum	1	Q9NR48
Avasorum	1	Q9NR50
Avasorum	1	Q9NR56
Avasorum	1	Q9NRA2
Avasorum	1	Q9NRA8
Avasorum	1	Q9NRB3
Avasorum	1	Q9NRC1
Avasorum	1	Q9NRD5
Avasorum	1	Q9NRF2
Avasorum	1	Q9NRF8
Avasorum	1	Q9NRF9
Avasorum	1	Q9NRG0
Avasorum	1	Q9NRG9
Avasorum	1	Q9NRH2
Avasorum	1	Q9NRK6
Avasorum	1	Q9NRL2
Avasorum	1	Q9NRL3
Avasorum	1	Q9NRN7
Avasorum	1	Q9NRN9

Avasorum	1	Q9NRP0
Avasorum	1	Q9NRQ2
Avasorum	1	Q9NRR4
Avasorum	1	Q9NRS6
Avasorum	1	Q9NRV9
Avasorum	1	Q9NRW3
Avasorum	1	Q9NRW7
Avasorum	1	Q9NRX1
Avasorum	1	Q9NRX2
Avasorum	1	Q9NRX4
Avasorum	1	Q9NRX5
Avasorum	1	Q9NRY4
Avasorum	1	Q9NRY5
Avasorum	1	Q9NRY6
Avasorum	1	Q9NRZ5
Avasorum	1	Q9NRZ7
Avasorum	1	Q9NRZ9
Avasorum	1	Q9NS00
Avasorum	1	Q9NS15
Avasorum	1	Q9NS69
Avasorum	1	Q9NS86
Avasorum	1	Q9NS87
Avasorum	1	Q9NS93
Avasorum	1	Q9NSC5
Avasorum	1	Q9NSD9
Avasorum	1	Q9NSE4
Avasorum	1	Q9NSI2
Avasorum	1	Q9NSI8
Avasorum	1	Q9NSK0
Avasorum	1	Q9NSV4
Avasorum	1	Q9NSY1
Avasorum	1	Q9NT62
Avasorum	1	Q9NTI5
Avasorum	1	Q9NTJ3
Avasorum	1	Q9NTJ4
Avasorum	1	Q9NTJ5
Avasorum	1	Q9NTK1
Avasorum	1	Q9NTK5
Avasorum	1	Q9NTM9
Avasorum	1	Q9NTW7
Avasorum	1	Q9NTX5
Avasorum	1	Q9NTZ6
Avasorum	1	Q9NU19
Avasorum	1	Q9NU22
Avasorum	1	Q9NUB1
Avasorum	1	Q9NUD5
Avasorum	1	Q9NUI1
Avasorum	1	Q9NUJ1
Avasorum	1	Q9NUJ3
Avasorum	1	Q9NUL3

Avasorum	1	Q9NUL5
Avasorum	1	Q9NUL7
Avasorum	1	Q9NUM4
Avasorum	1	Q9NUP9
Avasorum	1	Q9NUQ2
Avasorum	1	Q9NUQ3
Avasorum	1	Q9NUQ6
Avasorum	1	Q9NUQ7
Avasorum	1	Q9NUQ8
Avasorum	1	Q9NUQ9
Avasorum	1	Q9NUT2
Avasorum	1	Q9NUU7
Avasorum	1	Q9NUV9
Avasorum	1	Q9NUW8
Avasorum	1	Q9NUX5
Avasorum	1	Q9NUY8
Avasorum	1	Q9NV06
Avasorum	1	Q9NV31
Avasorum	1	Q9NV56
Avasorum	1	Q9NV66
Avasorum	1	Q9NV70
Avasorum	1	Q9NV79
Avasorum	1	Q9NV88
Avasorum	1	Q9NV96
Avasorum	1	Q9NVA1
Avasorum	1	Q9NVA2
Avasorum	1	Q9NVC3
Avasorum	1	Q9NVC6
Avasorum	1	Q9NVD7
Avasorum	1	Q9NVE5
Avasorum	1	Q9NVE7
Avasorum	1	Q9NVF7
Avasorum	1	Q9NVG8
Avasorum	1	Q9NVH0
Avasorum	1	Q9NVH1
Avasorum	1	Q9NVH2
Avasorum	1	Q9NVI1
Avasorum	1	Q9NVI7
Avasorum	1	Q9NVJ2
Avasorum	1	Q9NVM6
Avasorum	1	Q9NVM9
Avasorum	1	Q9NVN3
Avasorum	1	Q9NVN8
Avasorum	1	Q9NVP1
Avasorum	1	Q9NVP2
Avasorum	1	Q9NVR0
Avasorum	1	Q9NVR2
Avasorum	1	Q9NVS2
Avasorum	1	Q9NVS9
Avasorum	1	Q9NVT9

Avasorum	1	Q9NVU0
Avasorum	1	Q9NVU7
Avasorum	1	Q9NVV4
Avasorum	1	Q9NVX2
Avasorum	1	Q9NVZ3
Avasorum	1	Q9NW08
Avasorum	1	Q9NW13
Avasorum	1	Q9NW15
Avasorum	1	Q9NW64
Avasorum	1	Q9NW81
Avasorum	1	Q9NW82
Avasorum	1	Q9NW97
Avasorum	1	Q9NWB6
Avasorum	1	Q9NWB7
Avasorum	1	Q9NWH9
Avasorum	1	Q9NWM8
Avasorum	1	Q9NWS8
Avasorum	1	Q9NWT1
Avasorum	1	Q9NWT6
Avasorum	1	Q9NWU1
Avasorum	1	Q9NWU5
Avasorum	1	Q9NWV4
Avasorum	1	Q9NWV8
Avasorum	1	Q9NWW5
Avasorum	1	Q9NWX6
Avasorum	1	Q9NWY4
Avasorum	1	Q9NWZ3
Avasorum	1	Q9NWZ5
Avasorum	1	Q9NX00
Avasorum	1	Q9NX02
Avasorum	1	Q9NX05
Avasorum	1	Q9NX07
Avasorum	1	Q9NX14
Avasorum	1	Q9NX20
Avasorum	1	Q9NX24
Avasorum	1	Q9NX40
Avasorum	1	Q9NX46
Avasorum	1	Q9NX47
Avasorum	1	Q9NX58
Avasorum	1	Q9NX61
Avasorum	1	Q9NX62
Avasorum	1	Q9NX63
Avasorum	1	Q9NX74
Avasorum	1	Q9NX76
Avasorum	1	Q9NXA8
Avasorum	1	Q9NXC5
Avasorum	1	Q9NXD2
Avasorum	1	Q9NXE4
Avasorum	1	Q9NXE8
Avasorum	1	Q9NXF1

Avasorum	1	Q9NXF7
Avasorum	1	Q9NXG2
Avasorum	1	Q9NXH8
Avasorum	1	Q9NXH9
Avasorum	1	Q9NXN4
Avasorum	1	Q9NXR1
Avasorum	1	Q9NXR7
Avasorum	1	Q9NXS2
Avasorum	1	Q9NXU5
Avasorum	1	Q9NXV2
Avasorum	1	Q9NXV6
Avasorum	1	Q9NXW2
Avasorum	1	Q9NXW9
Avasorum	1	Q9NXX6
Avasorum	1	Q9NY12
Avasorum	1	Q9NY15
Avasorum	1	Q9NY27
Avasorum	1	Q9NY33
Avasorum	1	Q9NY61
Avasorum	1	Q9NY65
Avasorum	1	Q9NY93
Avasorum	1	Q9NYB0
Avasorum	1	Q9NYB9
Avasorum	1	Q9NYF8
Avasorum	1	Q9NYH9
Avasorum	1	Q9NYJ8
Avasorum	1	Q9NYK5
Avasorum	1	Q9NYL2
Avasorum	1	Q9NYL4
Avasorum	1	Q9NYL9
Avasorum	1	Q9NYM9
Avasorum	1	Q9NYP7
Avasorum	1	Q9NYR9
Avasorum	1	Q9NYU1
Avasorum	1	Q9NYU2
Avasorum	1	Q9NYV4
Avasorum	1	Q9NYY8
Avasorum	1	Q9NZ01
Avasorum	1	Q9NZ08
Avasorum	1	Q9NZ32
Avasorum	1	Q9NZ43
Avasorum	1	Q9NZ45
Avasorum	1	Q9NZ52
Avasorum	1	Q9NZ63
Avasorum	1	Q9NZB2
Avasorum	1	Q9NZC3
Avasorum	1	Q9NZC9
Avasorum	1	Q9NZD2
Avasorum	1	Q9NZD8
Avasorum	1	Q9NZE8

Avasorum	1	Q9NZI7
Avasorum	1	Q9NZI8
Avasorum	1	Q9NZJ4
Avasorum	1	Q9NZJ7
Avasorum	1	Q9NZJ9
Avasorum	1	Q9NZL4
Avasorum	1	Q9NZL9
Avasorum	1	Q9NZM1
Avasorum	1	Q9NZM3
Avasorum	1	Q9NZM5
Avasorum	1	Q9NZN3
Avasorum	1	Q9NZN4
Avasorum	1	Q9NZN5
Avasorum	1	Q9NZN8
Avasorum	1	Q9NZQ3
Avasorum	1	Q9NZQ7
Avasorum	1	Q9NZT2
Avasorum	1	Q9NZU5
Avasorum	1	Q9NZV1
Avasorum	1	Q9NZV5
Avasorum	1	Q9NZW5
Avasorum	1	Q9NZZ3
Avasorum	1	Q9P013
Avasorum	1	Q9P015
Avasorum	1	Q9P016
Avasorum	1	Q9P031
Avasorum	1	Q9P032
Avasorum	1	Q9P035
Avasorum	1	Q9P0I2
Avasorum	1	Q9P0J0
Avasorum	1	Q9P0J1
Avasorum	1	Q9P0J7
Avasorum	1	Q9P0K7
Avasorum	1	Q9P0L0
Avasorum	1	Q9P0M9
Avasorum	1	Q9P0T7
Avasorum	1	Q9P0U3
Avasorum	1	Q9P0U4
Avasorum	1	Q9P0V3
Avasorum	1	Q9P0V9
Avasorum	1	Q9P107
Avasorum	1	Q9P1Y6
Avasorum	1	Q9P215
Avasorum	1	Q9P219
Avasorum	1	Q9P227
Avasorum	1	Q9P253
Avasorum	1	Q9P258
Avasorum	1	Q9P260
Avasorum	1	Q9P265
Avasorum	1	Q9P266

Avasorum	1	Q9P270
Avasorum	1	Q9P273
Avasorum	1	Q9P275
Avasorum	1	Q9P287
Avasorum	1	Q9P289
Avasorum	1	Q9P291
Avasorum	1	Q9P2A4
Avasorum	1	Q9P2B2
Avasorum	1	Q9P2B4
Avasorum	1	Q9P2C4
Avasorum	1	Q9P2D0
Avasorum	1	Q9P2D3
Avasorum	1	Q9P2E3
Avasorum	1	Q9P2E5
Avasorum	1	Q9P2E7
Avasorum	1	Q9P2E9
Avasorum	1	Q9P2I0
Avasorum	1	Q9P2J3
Avasorum	1	Q9P2J5
Avasorum	1	Q9P2K5
Avasorum	1	Q9P2K6
Avasorum	1	Q9P2K8
Avasorum	1	Q9P2L0
Avasorum	1	Q9P2N5
Avasorum	1	Q9P2N6
Avasorum	1	Q9P2P5
Avasorum	1	Q9P2Q2
Avasorum	1	Q9P2R3
Avasorum	1	Q9P2R6
Avasorum	1	Q9P2R7
Avasorum	1	Q9P2T1
Avasorum	1	Q9P2W9
Avasorum	1	Q9P2X0
Avasorum	1	Q9P2X3
Avasorum	1	Q9P2Y5
Avasorum	1	Q9UBB4
Avasorum	1	Q9UBB5
Avasorum	1	Q9UBB6
Avasorum	1	Q9UBB9
Avasorum	1	Q9UBC2
Avasorum	1	Q9UBC5
Avasorum	1	Q9UBD5
Avasorum	1	Q9UBE0
Avasorum	1	Q9UBF2
Avasorum	1	Q9UBF8
Avasorum	1	Q9UBG0
Avasorum	1	Q9UBI1
Avasorum	1	Q9UBI6
Avasorum	1	Q9UBK7
Avasorum	1	Q9UBK8

Avasorum	1	Q9UBK9
Avasorum	1	Q9UBL3
Avasorum	1	Q9UBM7
Avasorum	1	Q9UBN6
Avasorum	1	Q9UBN7
Avasorum	1	Q9UBP0
Avasorum	1	Q9UBP4
Avasorum	1	Q9UBP6
Avasorum	1	Q9UBP9
Avasorum	1	Q9UBQ0
Avasorum	1	Q9UBQ5
Avasorum	1	Q9UBQ6
Avasorum	1	Q9UBQ7
Avasorum	1	Q9UBR2
Avasorum	1	Q9UBS0
Avasorum	1	Q9UBS4
Avasorum	1	Q9UBS8
Avasorum	1	Q9UBT2
Avasorum	1	Q9UBU8
Avasorum	1	Q9UBU9
Avasorum	1	Q9UBV2
Avasorum	1	Q9UBV7
Avasorum	1	Q9UBV8
Avasorum	1	Q9UBW7
Avasorum	1	Q9UBW8
Avasorum	1	Q9UBX3
Avasorum	1	Q9UDR5
Avasorum	1	Q9UDT6
Avasorum	1	Q9UDW1
Avasorum	1	Q9UDX5
Avasorum	1	Q9UDY2
Avasorum	1	Q9UDY4
Avasorum	1	Q9UEE5
Avasorum	1	Q9UEE9
Avasorum	1	Q9UER7
Avasorum	1	Q9UET6
Avasorum	1	Q9UEU0
Avasorum	1	Q9UEW8
Avasorum	1	Q9UEY8
Avasorum	1	Q9UFC0
Avasorum	1	Q9UFN0
Avasorum	1	Q9UFW8
Avasorum	1	Q9UG56
Avasorum	1	Q9UG63
Avasorum	1	Q9UGI8
Avasorum	1	Q9UGJ0
Avasorum	1	Q9UGJ1
Avasorum	1	Q9UGL1
Avasorum	1	Q9UGM6
Avasorum	1	Q9UGP4

Avasorum	1	Q9UGP8
Avasorum	1	Q9UGQ3
Avasorum	1	Q9UGR2
Avasorum	1	Q9UGV2
Avasorum	1	Q9UH62
Avasorum	1	Q9UH65
Avasorum	1	Q9UH99
Avasorum	1	Q9UHA3
Avasorum	1	Q9UHA4
Avasorum	1	Q9UHB6
Avasorum	1	Q9UHB7
Avasorum	1	Q9UHB9
Avasorum	1	Q9UHD1
Avasorum	1	Q9UHD2
Avasorum	1	Q9UHD8
Avasorum	1	Q9UHG3
Avasorum	1	Q9UHI6
Avasorum	1	Q9UHJ6
Avasorum	1	Q9UHL4
Avasorum	1	Q9UHN6
Avasorum	1	Q9UHP3
Avasorum	1	Q9UHQ4
Avasorum	1	Q9UHQ9
Avasorum	1	Q9UHR5
Avasorum	1	Q9UHR6
Avasorum	1	Q9UHV9
Avasorum	1	Q9UHW5
Avasorum	1	Q9UHX1
Avasorum	1	Q9UHY1
Avasorum	1	Q9UHY7
Avasorum	1	Q9UHY8
Avasorum	1	Q9UI09
Avasorum	1	Q9UI10
Avasorum	1	Q9UI12
Avasorum	1	Q9UI14
Avasorum	1	Q9UI26
Avasorum	1	Q9UI30
Avasorum	1	Q9UI36
Avasorum	1	Q9UIA9
Avasorum	1	Q9UIC8
Avasorum	1	Q9UID3
Avasorum	1	Q9UIF9
Avasorum	1	Q9UIG0
Avasorum	1	Q9UII2
Avasorum	1	Q9UIJ7
Avasorum	1	Q9UIQ6
Avasorum	1	Q9UIV1
Avasorum	1	Q9UJ41
Avasorum	1	Q9UJ68
Avasorum	1	Q9UJ70

Avasorum	1	Q9UJ83
Avasorum	1	Q9UJA5
Avasorum	1	Q9UJC5
Avasorum	1	Q9UJF2
Avasorum	1	Q9UJK0
Avasorum	1	Q9UJS0
Avasorum	1	Q9UJU6
Avasorum	1	Q9UJV9
Avasorum	1	Q9UJW0
Avasorum	1	Q9UJX2
Avasorum	1	Q9UJX3
Avasorum	1	Q9UJX4
Avasorum	1	Q9UJX5
Avasorum	1	Q9UJX6
Avasorum	1	Q9UJY4
Avasorum	1	Q9UJY5
Avasorum	1	Q9UJZ1
Avasorum	1	Q9UK23
Avasorum	1	Q9UK41
Avasorum	1	Q9UK45
Avasorum	1	Q9UK59
Avasorum	1	Q9UK61
Avasorum	1	Q9UK76
Avasorum	1	Q9UK99
Avasorum	1	Q9UKA4
Avasorum	1	Q9UKB1
Avasorum	1	Q9UKD1
Avasorum	1	Q9UKD2
Avasorum	1	Q9UKE5
Avasorum	1	Q9UKF6
Avasorum	1	Q9UKG1
Avasorum	1	Q9UKI2
Avasorum	1	Q9UKI8
Avasorum	1	Q9UKJ3
Avasorum	1	Q9UKK3
Avasorum	1	Q9UKK6
Avasorum	1	Q9UKK9
Avasorum	1	Q9UKL0
Avasorum	1	Q9UKM7
Avasorum	1	Q9UKM9
Avasorum	1	Q9UKN8
Avasorum	1	Q9UKR5
Avasorum	1	Q9UKS6
Avasorum	1	Q9UKU7
Avasorum	1	Q9UKU9
Avasorum	1	Q9UKV3
Avasorum	1	Q9UKV5
Avasorum	1	Q9UKV8
Avasorum	1	Q9UKX7
Avasorum	1	Q9UKY7

Avasorum	1	Q9UKZ1
Avasorum	1	Q9UL03
Avasorum	1	Q9UL15
Avasorum	1	Q9UL18
Avasorum	1	Q9UL25
Avasorum	1	Q9UL26
Avasorum	1	Q9UL33
Avasorum	1	Q9UL40
Avasorum	1	Q9UL46
Avasorum	1	Q9UL54
Avasorum	1	Q9UL63
Avasorum	1	Q9ULA0
Avasorum	1	Q9ULC3
Avasorum	1	Q9ULC4
Avasorum	1	Q9ULD2
Avasorum	1	Q9ULE0
Avasorum	1	Q9ULE6
Avasorum	1	Q9ULF5
Avasorum	1	Q9ULG1
Avasorum	1	Q9ULG6
Avasorum	1	Q9ULH0
Avasorum	1	Q9ULH1
Avasorum	1	Q9ULH7
Avasorum	1	Q9ULJ7
Avasorum	1	Q9ULK4
Avasorum	1	Q9ULL8
Avasorum	1	Q9ULM3
Avasorum	1	Q9ULP0
Avasorum	1	Q9ULP9
Avasorum	1	Q9ULR0
Avasorum	1	Q9ULS5
Avasorum	1	Q9ULT8
Avasorum	1	Q9ULU4
Avasorum	1	Q9ULV3
Avasorum	1	Q9ULV4
Avasorum	1	Q9ULW0
Avasorum	1	Q9ULW3
Avasorum	1	Q9ULX3
Avasorum	1	Q9ULX6
Avasorum	1	Q9ULX9
Avasorum	1	Q9ULZ3
Avasorum	1	Q9UM00
Avasorum	1	Q9UM11
Avasorum	1	Q9UM13
Avasorum	1	Q9UM54
Avasorum	1	Q9UMR2
Avasorum	1	Q9UMR5
Avasorum	1	Q9UMS4
Avasorum	1	Q9UMX1
Avasorum	1	Q9UMX3

Avasorum	1	Q9UMY1
Avasorum	1	Q9UMY4
Avasorum	1	Q9UMZ2
Avasorum	1	Q9UN37
Avasorum	1	Q9UN86
Avasorum	1	Q9UNE7
Avasorum	1	Q9UNF0
Avasorum	1	Q9UNF1
Avasorum	1	Q9UNH6
Avasorum	1	Q9UNH7
Avasorum	1	Q9UNI6
Avasorum	1	Q9UNK0
Avasorum	1	Q9UNL2
Avasorum	1	Q9UNM6
Avasorum	1	Q9UNN5
Avasorum	1	Q9UNN8
Avasorum	1	Q9UNP9
Avasorum	1	Q9UNQ2
Avasorum	1	Q9UNS1
Avasorum	1	Q9UNS2
Avasorum	1	Q9UNW1
Avasorum	1	Q9UNW9
Avasorum	1	Q9UNX4
Avasorum	1	Q9UNY4
Avasorum	1	Q9UNZ2
Avasorum	1	Q9UP83
Avasorum	1	Q9UP95
Avasorum	1	Q9UPM8
Avasorum	1	Q9UPN3
Avasorum	1	Q9UPN4
Avasorum	1	Q9UPN6
Avasorum	1	Q9UPN7
Avasorum	1	Q9UPN9
Avasorum	1	Q9UPP1
Avasorum	1	Q9UPQ9
Avasorum	1	Q9UPR0
Avasorum	1	Q9UPR3
Avasorum	1	Q9UPT5
Avasorum	1	Q9UPT8
Avasorum	1	Q9UPU5
Avasorum	1	Q9UPU7
Avasorum	1	Q9UPU9
Avasorum	1	Q9UPW0
Avasorum	1	Q9UPW5
Avasorum	1	Q9UPY3
Avasorum	1	Q9UPY5
Avasorum	1	Q9UPY8
Avasorum	1	Q9UQ13
Avasorum	1	Q9UQ35
Avasorum	1	Q9UQ80

Avasorum	1	Q9UQ90
Avasorum	1	Q9UQB8
Avasorum	1	Q9UQC2
Avasorum	1	Q9UQE7
Avasorum	1	Q9UQN3
Avasorum	1	Q9UQQ2
Avasorum	1	Q9UQR1
Avasorum	1	Q9Y217
Avasorum	1	Q9Y219
Avasorum	1	Q9Y221
Avasorum	1	Q9Y223
Avasorum	1	Q9Y224
Avasorum	1	Q9Y230
Avasorum	1	Q9Y232
Avasorum	1	Q9Y237
Avasorum	1	Q9Y243
Avasorum	1	Q9Y259
Avasorum	1	Q9Y262
Avasorum	1	Q9Y263
Avasorum	1	Q9Y265
Avasorum	1	Q9Y266
Avasorum	1	Q9Y276
Avasorum	1	Q9Y277
Avasorum	1	Q9Y281
Avasorum	1	Q9Y282
Avasorum	1	Q9Y285
Avasorum	1	Q9Y289
Avasorum	1	Q9Y291
Avasorum	1	Q9Y294
Avasorum	1	Q9Y295
Avasorum	1	Q9Y296
Avasorum	1	Q9Y2A7
Avasorum	1	Q9Y2B0
Avasorum	1	Q9Y2C4
Avasorum	1	Q9Y2D4
Avasorum	1	Q9Y2D5
Avasorum	1	Q9Y2G2
Avasorum	1	Q9Y2G3
Avasorum	1	Q9Y2G5
Avasorum	1	Q9Y2G8
Avasorum	1	Q9Y2H0
Avasorum	1	Q9Y2H1
Avasorum	1	Q9Y2H2
Avasorum	1	Q9Y2H6
Avasorum	1	Q9Y2I1
Avasorum	1	Q9Y2I7
Avasorum	1	Q9Y2I8
Avasorum	1	Q9Y2J2
Avasorum	1	Q9Y2J4
Avasorum	1	Q9Y2K2

Avasorum	1	Q9Y2K7
Avasorum	1	Q9Y2L1
Avasorum	1	Q9Y2L5
Avasorum	1	Q9Y2L9
Avasorum	1	Q9Y2P8
Avasorum	1	Q9Y2Q3
Avasorum	1	Q9Y2Q5
Avasorum	1	Q9Y2Q9
Avasorum	1	Q9Y2R0
Avasorum	1	Q9Y2R4
Avasorum	1	Q9Y2R5
Avasorum	1	Q9Y2R9
Avasorum	1	Q9Y2S2
Avasorum	1	Q9Y2S6
Avasorum	1	Q9Y2S7
Avasorum	1	Q9Y2T2
Avasorum	1	Q9Y2U8
Avasorum	1	Q9Y2V2
Avasorum	1	Q9Y2V7
Avasorum	1	Q9Y2W1
Avasorum	1	Q9Y2W2
Avasorum	1	Q9Y2W6
Avasorum	1	Q9Y2X0
Avasorum	1	Q9Y2X3
Avasorum	1	Q9Y2X7
Avasorum	1	Q9Y2X9
Avasorum	1	Q9Y2Y0
Avasorum	1	Q9Y2Y6
Avasorum	1	Q9Y2Z0
Avasorum	1	Q9Y2Z4
Avasorum	1	Q9Y303
Avasorum	1	Q9Y305
Avasorum	1	Q9Y312
Avasorum	1	Q9Y314
Avasorum	1	Q9Y315
Avasorum	1	Q9Y316
Avasorum	1	Q9Y320
Avasorum	1	Q9Y324
Avasorum	1	Q9Y333
Avasorum	1	Q9Y371
Avasorum	1	Q9Y375
Avasorum	1	Q9Y376
Avasorum	1	Q9Y383
Avasorum	1	Q9Y385
Avasorum	1	Q9Y388
Avasorum	1	Q9Y394
Avasorum	1	Q9Y399
Avasorum	1	Q9Y3A2
Avasorum	1	Q9Y3A3
Avasorum	1	Q9Y3A4

Avasorum	1	Q9Y3A5
Avasorum	1	Q9Y3A6
Avasorum	1	Q9Y3B2
Avasorum	1	Q9Y3B3
Avasorum	1	Q9Y3B4
Avasorum	1	Q9Y3B7
Avasorum	1	Q9Y3B8
Avasorum	1	Q9Y3B9
Avasorum	1	Q9Y3C0
Avasorum	1	Q9Y3C1
Avasorum	1	Q9Y3C4
Avasorum	1	Q9Y3C6
Avasorum	1	Q9Y3C8
Avasorum	1	Q9Y3D0
Avasorum	1	Q9Y3D3
Avasorum	1	Q9Y3D5
Avasorum	1	Q9Y3D6
Avasorum	1	Q9Y3D7
Avasorum	1	Q9Y3D8
Avasorum	1	Q9Y3D9
Avasorum	1	Q9Y3E0
Avasorum	1	Q9Y3E1
Avasorum	1	Q9Y3E5
Avasorum	1	Q9Y3E7
Avasorum	1	Q9Y3F4
Avasorum	1	Q9Y3I0
Avasorum	1	Q9Y3I1
Avasorum	1	Q9Y3L5
Avasorum	1	Q9Y3M8
Avasorum	1	Q9Y3P9
Avasorum	1	Q9Y3Q3
Avasorum	1	Q9Y3Q8
Avasorum	1	Q9Y3R5
Avasorum	1	Q9Y3S2
Avasorum	1	Q9Y3T6
Avasorum	1	Q9Y3T9
Avasorum	1	Q9Y3U8
Avasorum	1	Q9Y3X0
Avasorum	1	Q9Y3Y2
Avasorum	1	Q9Y3Z3
Avasorum	1	Q9Y448
Avasorum	1	Q9Y450
Avasorum	1	Q9Y478
Avasorum	1	Q9Y485
Avasorum	1	Q9Y487
Avasorum	1	Q9Y490
Avasorum	1	Q9Y496
Avasorum	1	Q9Y4A5
Avasorum	1	Q9Y4B5
Avasorum	1	Q9Y4B6

Avasorum	1	Q9Y4C2
Avasorum	1	Q9Y4C8
Avasorum	1	Q9Y4D1
Avasorum	1	Q9Y4D7
Avasorum	1	Q9Y4D8
Avasorum	1	Q9Y4E1
Avasorum	1	Q9Y4E6
Avasorum	1	Q9Y4E8
Avasorum	1	Q9Y4F1
Avasorum	1	Q9Y4F3
Avasorum	1	Q9Y4F5
Avasorum	1	Q9Y4G8
Avasorum	1	Q9Y4I1
Avasorum	1	Q9Y4J8
Avasorum	1	Q9Y4K0
Avasorum	1	Q9Y4K1
Avasorum	1	Q9Y4K3
Avasorum	1	Q9Y4K4
Avasorum	1	Q9Y4L1
Avasorum	1	Q9Y4P1
Avasorum	1	Q9Y4P3
Avasorum	1	Q9Y4P8
Avasorum	1	Q9Y4R8
Avasorum	1	Q9Y4W2
Avasorum	1	Q9Y4W6
Avasorum	1	Q9Y4X4
Avasorum	1	Q9Y4X5
Avasorum	1	Q9Y4Z0
Avasorum	1	Q9Y508
Avasorum	1	Q9Y512
Avasorum	1	Q9Y520
Avasorum	1	Q9Y530
Avasorum	1	Q9Y547
Avasorum	1	Q9Y570
Avasorum	1	Q9Y580
Avasorum	1	Q9Y584
Avasorum	1	Q9Y5A7
Avasorum	1	Q9Y5A9
Avasorum	1	Q9Y5B0
Avasorum	1	Q9Y5B6
Avasorum	1	Q9Y5B8
Avasorum	1	Q9Y5B9
Avasorum	1	Q9Y5J1
Avasorum	1	Q9Y5K5
Avasorum	1	Q9Y5K6
Avasorum	1	Q9Y5K8
Avasorum	1	Q9Y5L0
Avasorum	1	Q9Y5M8
Avasorum	1	Q9Y5P4
Avasorum	1	Q9Y5P6

Avasorum	1	Q9Y5Q0
Avasorum	1	Q9Y5Q8
Avasorum	1	Q9Y5Q9
Avasorum	1	Q9Y5R8
Avasorum	1	Q9Y5S1
Avasorum	1	Q9Y5S2
Avasorum	1	Q9Y5S9
Avasorum	1	Q9Y5T4
Avasorum	1	Q9Y5T5
Avasorum	1	Q9Y5U2
Avasorum	1	Q9Y5V0
Avasorum	1	Q9Y5V3
Avasorum	1	Q9Y5W7
Avasorum	1	Q9Y5W9
Avasorum	1	Q9Y5X1
Avasorum	1	Q9Y5X2
Avasorum	1	Q9Y5X3
Avasorum	1	Q9Y5X9
Avasorum	1	Q9Y5Y2
Avasorum	1	Q9Y5Y5
Avasorum	1	Q9Y5Y7
Avasorum	1	Q9Y5Z0
Avasorum	1	Q9Y5Z4
Avasorum	1	Q9Y5Z7
Avasorum	1	Q9Y5Z9
Avasorum	1	Q9Y606
Avasorum	1	Q9Y608
Avasorum	1	Q9Y613
Avasorum	1	Q9Y617
Avasorum	1	Q9Y618
Avasorum	1	Q9Y619
Avasorum	1	Q9Y624
Avasorum	1	Q9Y639
Avasorum	1	Q9Y653
Avasorum	1	Q9Y657
Avasorum	1	Q9Y666
Avasorum	1	Q9Y672
Avasorum	1	Q9Y673
Avasorum	1	Q9Y676
Avasorum	1	Q9Y678
Avasorum	1	Q9Y679
Avasorum	1	Q9Y680
Avasorum	1	Q9Y696
Avasorum	1	Q9Y697
Avasorum	1	Q9Y6A4
Avasorum	1	Q9Y6A9
Avasorum	1	Q9Y6B6
Avasorum	1	Q9Y6B7
Avasorum	1	Q9Y6C9
Avasorum	1	Q9Y6D0

Avasorum	1	Q9Y6D5
Avasorum	1	Q9Y6D6
Avasorum	1	Q9Y6D9
Avasorum	1	Q9Y6E0
Avasorum	1	Q9Y6E2
Avasorum	1	Q9Y6G5
Avasorum	1	Q9Y6G9
Avasorum	1	Q9Y6I3
Avasorum	1	Q9Y6I4
Avasorum	1	Q9Y6I9
Avasorum	1	Q9Y6J0
Avasorum	1	Q9Y6K0
Avasorum	1	Q9Y6K1
Avasorum	1	Q9Y6K5
Avasorum	1	Q9Y6K8
Avasorum	1	Q9Y6K9
Avasorum	1	Q9Y6M1
Avasorum	1	Q9Y6M5
Avasorum	1	Q9Y6M7
Avasorum	1	Q9Y6M9
Avasorum	1	Q9Y6N5
Avasorum	1	Q9Y6N7
Avasorum	1	Q9Y6Q2
Avasorum	1	Q9Y6Q9
Avasorum	1	Q9Y6R0
Avasorum	1	Q9Y6R4
Avasorum	1	Q9Y6V7
Avasorum	1	Q9Y6W3
Avasorum	1	Q9Y6W5
Avasorum	1	Q9Y6X2
Avasorum	1	Q9Y6X3
Avasorum	1	Q9Y6X9
Avasorum	1	Q9Y6Y0
Avasorum	1	Q9Y6Y8
Avasorum	2	A0A0B4J2D5
Avasorum	2	A0AVF1
Avasorum	2	A0AVT1
Avasorum	2	A0FGR8
Avasorum	2	A0JNW5
Avasorum	2	A0MZ66
Avasorum	2	A0PJW6
Avasorum	2	A1A4S6
Avasorum	2	A1L0T0
Avasorum	2	A1X283
Avasorum	2	A2RRP1
Avasorum	2	A2RUC4
Avasorum	2	A2RUS2
Avasorum	2	A3KMH1
Avasorum	2	A3KN83
Avasorum	2	A4D1E9

Avasorum	2	A4D1P6
Avasorum	2	A4D1U4
Avasorum	2	A5PLN9
Avasorum	2	A5YKK6
Avasorum	2	A6NCE7
Avasorum	2	A6NDG6
Avasorum	2	A6NDU8
Avasorum	2	A6NED2
Avasorum	2	A6NFQ2
Avasorum	2	A6NHR9
Avasorum	2	A6NHX0
Avasorum	2	A6NIH7
Avasorum	2	A6NJ78
Avasorum	2	A6NKD9
Avasorum	2	A6ZKI3
Avasorum	2	A7E2V4
Avasorum	2	A8CG34
Avasorum	2	A8MW92
Avasorum	2	A8MWD9
Avasorum	2	A8MWY0
Avasorum	2	A8MXV4
Avasorum	2	A9UHW6
Avasorum	2	P0DP23
Avasorum	2	B2RTY4
Avasorum	2	B7ZAQ6
Avasorum	2	C4AMC7
Avasorum	2	C9JLW8
Avasorum	2	E9PAV3
Avasorum	2	E9PRG8
Avasorum	2	L0R819
Avasorum	2	L0R8F8
Avasorum	2	O00115
Avasorum	2	O00116
Avasorum	2	O00139
Avasorum	2	O00148
Avasorum	2	O00151
Avasorum	2	O00154
Avasorum	2	O00159
Avasorum	2	O00161
Avasorum	2	O00165
Avasorum	2	O00170
Avasorum	2	O00178
Avasorum	2	O00182
Avasorum	2	O00186
Avasorum	2	O00189
Avasorum	2	O00192
Avasorum	2	O00193
Avasorum	2	O00203
Avasorum	2	O00213
Avasorum	2	O00214

Avasorum	2	O00217
Avasorum	2	O00221
Avasorum	2	O00231
Avasorum	2	O00232
Avasorum	2	O00233
Avasorum	2	O00255
Avasorum	2	O00258
Avasorum	2	O00264
Avasorum	2	O00267
Avasorum	2	O00268
Avasorum	2	O00273
Avasorum	2	O00291
Avasorum	2	O00299
Avasorum	2	O00303
Avasorum	2	O00308
Avasorum	2	O00330
Avasorum	2	O00391
Avasorum	2	O00399
Avasorum	2	O00400
Avasorum	2	O00401
Avasorum	2	O00410
Avasorum	2	O00411
Avasorum	2	O00418
Avasorum	2	O00422
Avasorum	2	O00423
Avasorum	2	O00425
Avasorum	2	O00429
Avasorum	2	O00442
Avasorum	2	O00443
Avasorum	2	O00461
Avasorum	2	O00468
Avasorum	2	O00469
Avasorum	2	O00471
Avasorum	2	O00481
Avasorum	2	O00483
Avasorum	2	O00487
Avasorum	2	O00499
Avasorum	2	O00501
Avasorum	2	O00505
Avasorum	2	O00506
Avasorum	2	O00522
Avasorum	2	O00534
Avasorum	2	O00541
Avasorum	2	O00560
Avasorum	2	O00562
Avasorum	2	O00566
Avasorum	2	O00567
Avasorum	2	O00571
Avasorum	2	O00587
Avasorum	2	O00592

Avasorum	2	O00622
Avasorum	2	O00625
Avasorum	2	O00629
Avasorum	2	O00635
Avasorum	2	O00712
Avasorum	2	O00743
Avasorum	2	O00746
Avasorum	2	O00750
Avasorum	2	O00754
Avasorum	2	O00764
Avasorum	2	O00767
Avasorum	2	O14497
Avasorum	2	O14524
Avasorum	2	O14530
Avasorum	2	O14531
Avasorum	2	O14545
Avasorum	2	O14548
Avasorum	2	O14562
Avasorum	2	O14578
Avasorum	2	O14579
Avasorum	2	O14613
Avasorum	2	O14617
Avasorum	2	O14618
Avasorum	2	O14639
Avasorum	2	O14640
Avasorum	2	O14641
Avasorum	2	O14646
Avasorum	2	O14647
Avasorum	2	O14653
Avasorum	2	O14656
Avasorum	2	O14657
Avasorum	2	O14662
Avasorum	2	O14672
Avasorum	2	O14678
Avasorum	2	O14681
Avasorum	2	O14683
Avasorum	2	O14686
Avasorum	2	O14727
Avasorum	2	O14730
Avasorum	2	O14733
Avasorum	2	O14734
Avasorum	2	O14735
Avasorum	2	O14737
Avasorum	2	O14744
Avasorum	2	O14745
Avasorum	2	O14757
Avasorum	2	O14763
Avasorum	2	O14773
Avasorum	2	O14776
Avasorum	2	O14777

Avasorum	2	O14782
Avasorum	2	O14786
Avasorum	2	O14787
Avasorum	2	O14795
Avasorum	2	O14798
Avasorum	2	O14802
Avasorum	2	O14817
Avasorum	2	O14818
Avasorum	2	O14828
Avasorum	2	O14874
Avasorum	2	O14880
Avasorum	2	O14907
Avasorum	2	O14908
Avasorum	2	O14920
Avasorum	2	O14924
Avasorum	2	O14925
Avasorum	2	O14929
Avasorum	2	O14936
Avasorum	2	O14939
Avasorum	2	O14949
Avasorum	2	O14950
Avasorum	2	O14964
Avasorum	2	O14966
Avasorum	2	O14972
Avasorum	2	O14974
Avasorum	2	O14976
Avasorum	2	O14979
Avasorum	2	O14980
Avasorum	2	O14981
Avasorum	2	O15013
Avasorum	2	O15014
Avasorum	2	O15020
Avasorum	2	O15021
Avasorum	2	O15027
Avasorum	2	O15031
Avasorum	2	O15037
Avasorum	2	O15040
Avasorum	2	O15042
Avasorum	2	O15047
Avasorum	2	O15049
Avasorum	2	O15056
Avasorum	2	O15061
Avasorum	2	O15063
Avasorum	2	O15066
Avasorum	2	O15067
Avasorum	2	O15084
Avasorum	2	O15085
Avasorum	2	O15091
Avasorum	2	O15111
Avasorum	2	O15118

Avasorum	2	O15121
Avasorum	2	O15123
Avasorum	2	O15126
Avasorum	2	O15127
Avasorum	2	O15143
Avasorum	2	O15144
Avasorum	2	O15145
Avasorum	2	O15160
Avasorum	2	O15162
Avasorum	2	O15164
Avasorum	2	O15173
Avasorum	2	O15211
Avasorum	2	O15213
Avasorum	2	O15226
Avasorum	2	O15228
Avasorum	2	O15230
Avasorum	2	O15231
Avasorum	2	O15234
Avasorum	2	O15235
Avasorum	2	O15254
Avasorum	2	O15258
Avasorum	2	O15260
Avasorum	2	O15264
Avasorum	2	O15269
Avasorum	2	O15270
Avasorum	2	O15294
Avasorum	2	O15305
Avasorum	2	O15321
Avasorum	2	O15344
Avasorum	2	O15347
Avasorum	2	O15355
Avasorum	2	O15357
Avasorum	2	O15371
Avasorum	2	O15372
Avasorum	2	O15379
Avasorum	2	O15381
Avasorum	2	O15382
Avasorum	2	O15397
Avasorum	2	O15400
Avasorum	2	O15427
Avasorum	2	O15439
Avasorum	2	O15446
Avasorum	2	O15460
Avasorum	2	O15484
Avasorum	2	O15498
Avasorum	2	O15504
Avasorum	2	O15511
Avasorum	2	O15519
Avasorum	2	O15525
Avasorum	2	O15530

Avasorum	2	O15533
Avasorum	2	O15541
Avasorum	2	O15550
Avasorum	2	O43143
Avasorum	2	O43148
Avasorum	2	O43149
Avasorum	2	O43150
Avasorum	2	O43155
Avasorum	2	O43156
Avasorum	2	O43159
Avasorum	2	O43164
Avasorum	2	O43169
Avasorum	2	O43172
Avasorum	2	O43175
Avasorum	2	O43181
Avasorum	2	O43237
Avasorum	2	O43242
Avasorum	2	O43251
Avasorum	2	O43252
Avasorum	2	O43264
Avasorum	2	O43286
Avasorum	2	O43290
Avasorum	2	O43292
Avasorum	2	O43293
Avasorum	2	O43294
Avasorum	2	O43299
Avasorum	2	O43310
Avasorum	2	O43312
Avasorum	2	O43314
Avasorum	2	O43318
Avasorum	2	O43324
Avasorum	2	O43353
Avasorum	2	O43390
Avasorum	2	O43395
Avasorum	2	O43396
Avasorum	2	O43399
Avasorum	2	O43402
Avasorum	2	O43414
Avasorum	2	O43426
Avasorum	2	O43427
Avasorum	2	O43432
Avasorum	2	O43447
Avasorum	2	O43464
Avasorum	2	O43482
Avasorum	2	O43488
Avasorum	2	O43491
Avasorum	2	O43493
Avasorum	2	O43505
Avasorum	2	O43516
Avasorum	2	O43520

Avasorum	2	O43572
Avasorum	2	O43583
Avasorum	2	O43592
Avasorum	2	O43598
Avasorum	2	O43615
Avasorum	2	O43617
Avasorum	2	O43633
Avasorum	2	O43639
Avasorum	2	O43657
Avasorum	2	O43660
Avasorum	2	O43663
Avasorum	2	O43665
Avasorum	2	O43670
Avasorum	2	O43674
Avasorum	2	O43676
Avasorum	2	O43678
Avasorum	2	O43679
Avasorum	2	O43681
Avasorum	2	O43684
Avasorum	2	O43704
Avasorum	2	O43707
Avasorum	2	O43709
Avasorum	2	O43719
Avasorum	2	O43731
Avasorum	2	O43747
Avasorum	2	O43752
Avasorum	2	O43760
Avasorum	2	O43765
Avasorum	2	O43768
Avasorum	2	O43772
Avasorum	2	O43776
Avasorum	2	O43795
Avasorum	2	O43808
Avasorum	2	O43809
Avasorum	2	O43813
Avasorum	2	O43815
Avasorum	2	O43818
Avasorum	2	O43819
Avasorum	2	O43822
Avasorum	2	O43823
Avasorum	2	O43824
Avasorum	2	O43826
Avasorum	2	O43837
Avasorum	2	O43847
Avasorum	2	O43852
Avasorum	2	O43854
Avasorum	2	O43861
Avasorum	2	O43865
Avasorum	2	O43896
Avasorum	2	O43909

Avasorum	2	O43913
Avasorum	2	O43920
Avasorum	2	O43924
Avasorum	2	O43929
Avasorum	2	O43933
Avasorum	2	O60216
Avasorum	2	O60231
Avasorum	2	O60234
Avasorum	2	O60239
Avasorum	2	O60242
Avasorum	2	O60244
Avasorum	2	O60245
Avasorum	2	O60256
Avasorum	2	O60264
Avasorum	2	O60271
Avasorum	2	O60285
Avasorum	2	O60287
Avasorum	2	O60292
Avasorum	2	O60293
Avasorum	2	O60306
Avasorum	2	O60313
Avasorum	2	O60315
Avasorum	2	O60318
Avasorum	2	O60330
Avasorum	2	O60331
Avasorum	2	O60333
Avasorum	2	O60336
Avasorum	2	O60341
Avasorum	2	O60343
Avasorum	2	O60427
Avasorum	2	O60443
Avasorum	2	O60447
Avasorum	2	O60462
Avasorum	2	O60476
Avasorum	2	O60487
Avasorum	2	O60488
Avasorum	2	O60493
Avasorum	2	O60502
Avasorum	2	O60504
Avasorum	2	O60506
Avasorum	2	O60508
Avasorum	2	O60518
Avasorum	2	O60524
Avasorum	2	O60547
Avasorum	2	O60551
Avasorum	2	O60563
Avasorum	2	O60566
Avasorum	2	O60568
Avasorum	2	O60573
Avasorum	2	O60610

Avasorum	2	O60637
Avasorum	2	O60645
Avasorum	2	O60664
Avasorum	2	O60678
Avasorum	2	O60684
Avasorum	2	O60701
Avasorum	2	O60704
Avasorum	2	O60711
Avasorum	2	O60716
Avasorum	2	O60725
Avasorum	2	O60739
Avasorum	2	O60749
Avasorum	2	O60762
Avasorum	2	O60763
Avasorum	2	O60783
Avasorum	2	O60784
Avasorum	2	O60814
Avasorum	2	O60826
Avasorum	2	O60828
Avasorum	2	O60830
Avasorum	2	O60831
Avasorum	2	O60832
Avasorum	2	O60841
Avasorum	2	O60869
Avasorum	2	O60870
Avasorum	2	O60879
Avasorum	2	O60884
Avasorum	2	O60885
Avasorum	2	O60921
Avasorum	2	O60925
Avasorum	2	O60927
Avasorum	2	O60934
Avasorum	2	O60942
Avasorum	2	O75027
Avasorum	2	O75044
Avasorum	2	O75051
Avasorum	2	O75063
Avasorum	2	O75083
Avasorum	2	O75110
Avasorum	2	O75113
Avasorum	2	O75116
Avasorum	2	O75122
Avasorum	2	O75127
Avasorum	2	O75131
Avasorum	2	O75143
Avasorum	2	O75146
Avasorum	2	O75150
Avasorum	2	O75151
Avasorum	2	O75152
Avasorum	2	O75153

Avasorum	2	O75155
Avasorum	2	O75157
Avasorum	2	O75165
Avasorum	2	O75167
Avasorum	2	O75170
Avasorum	2	O75173
Avasorum	2	O75175
Avasorum	2	O75179
Avasorum	2	O75182
Avasorum	2	O75190
Avasorum	2	O75208
Avasorum	2	O75223
Avasorum	2	O75251
Avasorum	2	O75306
Avasorum	2	O75312
Avasorum	2	O75319
Avasorum	2	O75323
Avasorum	2	O75330
Avasorum	2	O75340
Avasorum	2	O75347
Avasorum	2	O75348
Avasorum	2	O75351
Avasorum	2	O75352
Avasorum	2	O75367
Avasorum	2	O75368
Avasorum	2	O75369
Avasorum	2	O75376
Avasorum	2	O75381
Avasorum	2	O75382
Avasorum	2	O75390
Avasorum	2	O75391
Avasorum	2	O75396
Avasorum	2	O75400
Avasorum	2	O75410
Avasorum	2	O75414
Avasorum	2	O75419
Avasorum	2	O75427
Avasorum	2	O75431
Avasorum	2	O75436
Avasorum	2	O75439
Avasorum	2	O75448
Avasorum	2	O75449
Avasorum	2	O75475
Avasorum	2	O75477
Avasorum	2	O75489
Avasorum	2	O75494
Avasorum	2	O75503
Avasorum	2	O75508
Avasorum	2	O75521
Avasorum	2	O75525

Avasorum	2	O75530
Avasorum	2	O75531
Avasorum	2	O75533
Avasorum	2	O75534
Avasorum	2	O75554
Avasorum	2	O75569
Avasorum	2	O75578
Avasorum	2	O75582
Avasorum	2	O75586
Avasorum	2	O75592
Avasorum	2	O75600
Avasorum	2	O75607
Avasorum	2	O75608
Avasorum	2	O75616
Avasorum	2	O75643
Avasorum	2	O75648
Avasorum	2	O75663
Avasorum	2	O75665
Avasorum	2	O75676
Avasorum	2	O75683
Avasorum	2	O75688
Avasorum	2	O75691
Avasorum	2	O75694
Avasorum	2	O75695
Avasorum	2	O75717
Avasorum	2	O75718
Avasorum	2	O75746
Avasorum	2	O75787
Avasorum	2	O75792
Avasorum	2	O75794
Avasorum	2	O75815
Avasorum	2	O75817
Avasorum	2	O75818
Avasorum	2	O75821
Avasorum	2	O75822
Avasorum	2	O75828
Avasorum	2	O75832
Avasorum	2	O75843
Avasorum	2	O75844
Avasorum	2	O75874
Avasorum	2	O75879
Avasorum	2	O75880
Avasorum	2	O75882
Avasorum	2	O75884
Avasorum	2	O75886
Avasorum	2	O75907
Avasorum	2	O75909
Avasorum	2	O75915
Avasorum	2	O75923
Avasorum	2	O75925

Avasorum	2	O75934
Avasorum	2	O75935
Avasorum	2	O75937
Avasorum	2	O75940
Avasorum	2	O75943
Avasorum	2	O75947
Avasorum	2	O75954
Avasorum	2	O75955
Avasorum	2	O75962
Avasorum	2	O75964
Avasorum	2	O75970
Avasorum	2	O75976
Avasorum	2	O76003
Avasorum	2	O76021
Avasorum	2	O76024
Avasorum	2	O76031
Avasorum	2	O76054
Avasorum	2	O76070
Avasorum	2	O76071
Avasorum	2	O76094
Avasorum	2	O94760
Avasorum	2	O94762
Avasorum	2	O94763
Avasorum	2	O94766
Avasorum	2	O94776
Avasorum	2	O94788
Avasorum	2	O94804
Avasorum	2	O94806
Avasorum	2	O94808
Avasorum	2	O94813
Avasorum	2	O94822
Avasorum	2	O94826
Avasorum	2	O94827
Avasorum	2	O94829
Avasorum	2	O94830
Avasorum	2	O94832
Avasorum	2	O94842
Avasorum	2	O94851
Avasorum	2	O94855
Avasorum	2	O94868
Avasorum	2	O94874
Avasorum	2	O94875
Avasorum	2	O94885
Avasorum	2	O94888
Avasorum	2	O94889
Avasorum	2	O94901
Avasorum	2	O94903
Avasorum	2	O94905
Avasorum	2	O94906
Avasorum	2	O94913

Avasorum	2	O94915
Avasorum	2	O94916
Avasorum	2	O94919
Avasorum	2	O94923
Avasorum	2	O94925
Avasorum	2	O94927
Avasorum	2	O94929
Avasorum	2	O94952
Avasorum	2	O94953
Avasorum	2	O94964
Avasorum	2	O94966
Avasorum	2	O94967
Avasorum	2	O94973
Avasorum	2	O94979
Avasorum	2	O94989
Avasorum	2	O94992
Avasorum	2	O95059
Avasorum	2	O95067
Avasorum	2	O95070
Avasorum	2	O95071
Avasorum	2	O95081
Avasorum	2	O95084
Avasorum	2	O95104
Avasorum	2	O95139
Avasorum	2	O95140
Avasorum	2	O95149
Avasorum	2	O95155
Avasorum	2	O95159
Avasorum	2	O95163
Avasorum	2	O95168
Avasorum	2	O95169
Avasorum	2	O95182
Avasorum	2	O95183
Avasorum	2	O95197
Avasorum	2	O95202
Avasorum	2	O95208
Avasorum	2	O95218
Avasorum	2	O95219
Avasorum	2	O95232
Avasorum	2	O95235
Avasorum	2	O95236
Avasorum	2	O95239
Avasorum	2	O95243
Avasorum	2	O95248
Avasorum	2	O95249
Avasorum	2	O95251
Avasorum	2	O95260
Avasorum	2	O95292
Avasorum	2	O95295
Avasorum	2	O95297

Avasorum	2	O95298
Avasorum	2	O95299
Avasorum	2	O95302
Avasorum	2	O95319
Avasorum	2	O95336
Avasorum	2	O95340
Avasorum	2	O95347
Avasorum	2	O95352
Avasorum	2	O95359
Avasorum	2	O95361
Avasorum	2	O95363
Avasorum	2	O95372
Avasorum	2	O95373
Avasorum	2	O95376
Avasorum	2	O95391
Avasorum	2	O95394
Avasorum	2	O95396
Avasorum	2	O95400
Avasorum	2	O95425
Avasorum	2	O95433
Avasorum	2	O95453
Avasorum	2	O95456
Avasorum	2	O95470
Avasorum	2	O95476
Avasorum	2	O95478
Avasorum	2	O95479
Avasorum	2	O95486
Avasorum	2	O95487
Avasorum	2	O95490
Avasorum	2	O95544
Avasorum	2	O95551
Avasorum	2	O95563
Avasorum	2	O95571
Avasorum	2	O95573
Avasorum	2	O95602
Avasorum	2	O95619
Avasorum	2	O95628
Avasorum	2	O95630
Avasorum	2	O95639
Avasorum	2	O95644
Avasorum	2	O95671
Avasorum	2	O95674
Avasorum	2	O95685
Avasorum	2	O95696
Avasorum	2	O95707
Avasorum	2	O95714
Avasorum	2	O95716
Avasorum	2	O95721
Avasorum	2	O95747
Avasorum	2	O95749

Avasorum	2	O95757
Avasorum	2	O95758
Avasorum	2	O95759
Avasorum	2	O95760
Avasorum	2	O95772
Avasorum	2	O95777
Avasorum	2	O95782
Avasorum	2	O95785
Avasorum	2	O95786
Avasorum	2	O95793
Avasorum	2	O95801
Avasorum	2	O95807
Avasorum	2	O95810
Avasorum	2	O95816
Avasorum	2	O95817
Avasorum	2	O95819
Avasorum	2	O95822
Avasorum	2	O95825
Avasorum	2	O95831
Avasorum	2	O95834
Avasorum	2	O95835
Avasorum	2	O95848
Avasorum	2	O95858
Avasorum	2	O95861
Avasorum	2	O95864
Avasorum	2	O95865
Avasorum	2	O95870
Avasorum	2	O95881
Avasorum	2	O95905
Avasorum	2	O95980
Avasorum	2	O95983
Avasorum	2	O95985
Avasorum	2	O95989
Avasorum	2	O95999
Avasorum	2	O96000
Avasorum	2	O96005
Avasorum	2	O96006
Avasorum	2	O96007
Avasorum	2	O96008
Avasorum	2	O96011
Avasorum	2	O96013
Avasorum	2	O96018
Avasorum	2	O96019
Avasorum	2	O96028
Avasorum	2	P00338
Avasorum	2	P00352
Avasorum	2	P00367
Avasorum	2	P00374
Avasorum	2	P00387
Avasorum	2	P00390

Avasorum	2	P00395
Avasorum	2	P00403
Avasorum	2	P00491
Avasorum	2	P00492
Avasorum	2	P00505
Avasorum	2	P00519
Avasorum	2	P00558
Avasorum	2	P00568
Avasorum	2	P00749
Avasorum	2	P00750
Avasorum	2	P00813
Avasorum	2	P01034
Avasorum	2	P01111
Avasorum	2	P01112
Avasorum	2	P01116
Avasorum	2	P01127
Avasorum	2	P01130
Avasorum	2	P01137
Avasorum	2	P01889
Avasorum	2	P01893
Avasorum	2	P02452
Avasorum	2	P02462
Avasorum	2	P02545
Avasorum	2	P02647
Avasorum	2	P02751
Avasorum	2	P02765
Avasorum	2	P02768
Avasorum	2	P02786
Avasorum	2	P02792
Avasorum	2	P02794
Avasorum	2	P02795
Avasorum	2	P03886
Avasorum	2	P03905
Avasorum	2	P03915
Avasorum	2	P03956
Avasorum	2	P04035
Avasorum	2	P04040
Avasorum	2	P04049
Avasorum	2	P04062
Avasorum	2	P04075
Avasorum	2	P04080
Avasorum	2	P04083
Avasorum	2	P04114
Avasorum	2	P04156
Avasorum	2	P04179
Avasorum	2	P04181
Avasorum	2	P04183
Avasorum	2	P04275
Avasorum	2	P04350
Avasorum	2	P04406

Avasorum	2	P04424
Avasorum	2	P04439
Avasorum	2	P04632
Avasorum	2	P04732
Avasorum	2	P04792
Avasorum	2	P04818
Avasorum	2	P04843
Avasorum	2	P04844
Avasorum	2	P04899
Avasorum	2	P04908
Avasorum	2	P05023
Avasorum	2	P05026
Avasorum	2	P05067
Avasorum	2	P05091
Avasorum	2	P05106
Avasorum	2	P05109
Avasorum	2	P05114
Avasorum	2	P05120
Avasorum	2	P05121
Avasorum	2	P05141
Avasorum	2	P05161
Avasorum	2	P05165
Avasorum	2	P05166
Avasorum	2	P05198
Avasorum	2	P05204
Avasorum	2	P05362
Avasorum	2	P05387
Avasorum	2	P05388
Avasorum	2	P05412
Avasorum	2	P05423
Avasorum	2	P05455
Avasorum	2	P05556
Avasorum	2	P05783
Avasorum	2	P05997
Avasorum	2	P06132
Avasorum	2	P06241
Avasorum	2	P06280
Avasorum	2	P06396
Avasorum	2	P06400
Avasorum	2	P06454
Avasorum	2	P06493
Avasorum	2	P06576
Avasorum	2	P06703
Avasorum	2	P06730
Avasorum	2	P06733
Avasorum	2	P06737
Avasorum	2	P06744
Avasorum	2	P06746
Avasorum	2	P06748
Avasorum	2	P06753

Avasorum	2	P06756
Avasorum	2	P06865
Avasorum	2	P07099
Avasorum	2	P07195
Avasorum	2	P07199
Avasorum	2	P07203
Avasorum	2	P07237
Avasorum	2	P07305
Avasorum	2	P07311
Avasorum	2	P07332
Avasorum	2	P07339
Avasorum	2	P07355
Avasorum	2	P07384
Avasorum	2	P07437
Avasorum	2	P07602
Avasorum	2	P07686
Avasorum	2	P07711
Avasorum	2	P07737
Avasorum	2	P07738
Avasorum	2	P07741
Avasorum	2	P07814
Avasorum	2	P07858
Avasorum	2	P07900
Avasorum	2	P07902
Avasorum	2	P07910
Avasorum	2	P07942
Avasorum	2	P07947
Avasorum	2	P07948
Avasorum	2	P07954
Avasorum	2	P07992
Avasorum	2	P07996
Avasorum	2	P08047
Avasorum	2	P08069
Avasorum	2	P08123
Avasorum	2	P08133
Avasorum	2	P08134
Avasorum	2	P08174
Avasorum	2	P08195
Avasorum	2	P08236
Avasorum	2	P08237
Avasorum	2	P08238
Avasorum	2	P08240
Avasorum	2	P08243
Avasorum	2	P08253
Avasorum	2	P08397
Avasorum	2	P08473
Avasorum	2	P08493
Avasorum	2	P08559
Avasorum	2	P08572
Avasorum	2	P08574

Avasorum	2	P08579
Avasorum	2	P08581
Avasorum	2	P08621
Avasorum	2	P08648
Avasorum	2	P08651
Avasorum	2	P08670
Avasorum	2	P08708
Avasorum	2	P08754
Avasorum	2	P08758
Avasorum	2	P08865
Avasorum	2	P08962
Avasorum	2	P09001
Avasorum	2	P09012
Avasorum	2	P09104
Avasorum	2	P09110
Avasorum	2	P09132
Avasorum	2	P09211
Avasorum	2	P09234
Avasorum	2	P09382
Avasorum	2	P09417
Avasorum	2	P09429
Avasorum	2	P09486
Avasorum	2	P09493
Avasorum	2	P09496
Avasorum	2	P09497
Avasorum	2	P09525
Avasorum	2	P09543
Avasorum	2	P09601
Avasorum	2	P09622
Avasorum	2	P09651
Avasorum	2	P09661
Avasorum	2	P09669
Avasorum	2	P09758
Avasorum	2	P09874
Avasorum	2	P09884
Avasorum	2	P09936
Avasorum	2	P09960
Avasorum	2	P09972
Avasorum	2	P0C0S5
Avasorum	2	P0C0S8
Avasorum	2	P0CAP1
Avasorum	2	P0CAP2
Avasorum	2	P0CG29
Avasorum	2	P0CG39
Avasorum	2	P0CW19
Avasorum	2	P0DMV8
Avasorum	2	P0DP91
Avasorum	2	P0DPH7
Avasorum	2	P0DTL6
Avasorum	2	P10114

Avasorum	2	P10124
Avasorum	2	P10155
Avasorum	2	P10253
Avasorum	2	P10301
Avasorum	2	P10321
Avasorum	2	P10398
Avasorum	2	P10412
Avasorum	2	P10515
Avasorum	2	P10586
Avasorum	2	P10589
Avasorum	2	P10599
Avasorum	2	P10606
Avasorum	2	P10619
Avasorum	2	P10620
Avasorum	2	P10644
Avasorum	2	P10646
Avasorum	2	P10768
Avasorum	2	P10809
Avasorum	2	P10909
Avasorum	2	P11021
Avasorum	2	P11047
Avasorum	2	P11117
Avasorum	2	P11137
Avasorum	2	P11142
Avasorum	2	P11166
Avasorum	2	P11169
Avasorum	2	P11171
Avasorum	2	P11172
Avasorum	2	P11177
Avasorum	2	P11182
Avasorum	2	P11216
Avasorum	2	P11217
Avasorum	2	P11233
Avasorum	2	P11234
Avasorum	2	P11274
Avasorum	2	P11279
Avasorum	2	P11308
Avasorum	2	P11310
Avasorum	2	P11387
Avasorum	2	P11388
Avasorum	2	P11413
Avasorum	2	P11441
Avasorum	2	P11498
Avasorum	2	P11586
Avasorum	2	P11717
Avasorum	2	P11766
Avasorum	2	P11802
Avasorum	2	P11908
Avasorum	2	P11940
Avasorum	2	P12004

Avasorum	2	P12081
Avasorum	2	P12109
Avasorum	2	P12235
Avasorum	2	P12236
Avasorum	2	P12268
Avasorum	2	P12270
Avasorum	2	P12429
Avasorum	2	P12694
Avasorum	2	P12814
Avasorum	2	P12821
Avasorum	2	P12931
Avasorum	2	P12955
Avasorum	2	P12956
Avasorum	2	P13010
Avasorum	2	P13051
Avasorum	2	P13073
Avasorum	2	P13196
Avasorum	2	P13473
Avasorum	2	P13489
Avasorum	2	P13598
Avasorum	2	P13639
Avasorum	2	P13667
Avasorum	2	P13674
Avasorum	2	P13693
Avasorum	2	P13716
Avasorum	2	P13747
Avasorum	2	P13797
Avasorum	2	P13798
Avasorum	2	P13804
Avasorum	2	P13807
Avasorum	2	P13861
Avasorum	2	P13984
Avasorum	2	P13987
Avasorum	2	P13995
Avasorum	2	P14174
Avasorum	2	P14209
Avasorum	2	P14314
Avasorum	2	P14317
Avasorum	2	P14324
Avasorum	2	P14406
Avasorum	2	P14543
Avasorum	2	P14550
Avasorum	2	P14618
Avasorum	2	P14625
Avasorum	2	P14635
Avasorum	2	P14649
Avasorum	2	P14678
Avasorum	2	P14735
Avasorum	2	P14859
Avasorum	2	P14866

Avasorum	2	P14868
Avasorum	2	P14921
Avasorum	2	P14923
Avasorum	2	P14927
Avasorum	2	P15056
Avasorum	2	P15090
Avasorum	2	P15104
Avasorum	2	P15121
Avasorum	2	P15144
Avasorum	2	P15151
Avasorum	2	P15153
Avasorum	2	P15170
Avasorum	2	P15289
Avasorum	2	P15291
Avasorum	2	P15311
Avasorum	2	P15374
Avasorum	2	P15407
Avasorum	2	P15408
Avasorum	2	P15529
Avasorum	2	P15531
Avasorum	2	P15559
Avasorum	2	P15586
Avasorum	2	P15735
Avasorum	2	P15848
Avasorum	2	P15880
Avasorum	2	P15884
Avasorum	2	P15924
Avasorum	2	P15927
Avasorum	2	P16035
Avasorum	2	P16070
Avasorum	2	P16152
Avasorum	2	P16219
Avasorum	2	P16220
Avasorum	2	P16278
Avasorum	2	P16284
Avasorum	2	P16298
Avasorum	2	P16333
Avasorum	2	P16383
Avasorum	2	P16401
Avasorum	2	P16402
Avasorum	2	P16403
Avasorum	2	P16435
Avasorum	2	P16591
Avasorum	2	P16615
Avasorum	2	P16885
Avasorum	2	P16930
Avasorum	2	P16949
Avasorum	2	P16989
Avasorum	2	P17026
Avasorum	2	P17050

Avasorum	2	P17096
Avasorum	2	P17152
Avasorum	2	P17174
Avasorum	2	P17252
Avasorum	2	P17301
Avasorum	2	P17302
Avasorum	2	P17405
Avasorum	2	P17480
Avasorum	2	P17535
Avasorum	2	P17542
Avasorum	2	P17544
Avasorum	2	P17568
Avasorum	2	P17612
Avasorum	2	P17655
Avasorum	2	P17706
Avasorum	2	P17812
Avasorum	2	P17813
Avasorum	2	P17844
Avasorum	2	P17858
Avasorum	2	P17948
Avasorum	2	P17980
Avasorum	2	P17987
Avasorum	2	P18031
Avasorum	2	P18074
Avasorum	2	P18077
Avasorum	2	P18084
Avasorum	2	P18085
Avasorum	2	P18124
Avasorum	2	P18206
Avasorum	2	P18433
Avasorum	2	P18440
Avasorum	2	P18583
Avasorum	2	P18615
Avasorum	2	P18621
Avasorum	2	P18669
Avasorum	2	P18754
Avasorum	2	P18846
Avasorum	2	P18850
Avasorum	2	P18858
Avasorum	2	P18887
Avasorum	2	P19021
Avasorum	2	P19022
Avasorum	2	P19174
Avasorum	2	P19338
Avasorum	2	P19367
Avasorum	2	P19387
Avasorum	2	P19388
Avasorum	2	P19404
Avasorum	2	P19447
Avasorum	2	P19474

Avasorum	2	P19525
Avasorum	2	P19623
Avasorum	2	P19634
Avasorum	2	P19784
Avasorum	2	P19838
Avasorum	2	P20020
Avasorum	2	P20042
Avasorum	2	P20073
Avasorum	2	P20226
Avasorum	2	P20248
Avasorum	2	P20290
Avasorum	2	P20337
Avasorum	2	P20338
Avasorum	2	P20339
Avasorum	2	P20340
Avasorum	2	P20585
Avasorum	2	P20594
Avasorum	2	P20618
Avasorum	2	P20645
Avasorum	2	P20674
Avasorum	2	P20700
Avasorum	2	P20810
Avasorum	2	P20839
Avasorum	2	P20908
Avasorum	2	P20929
Avasorum	2	P20933
Avasorum	2	P20936
Avasorum	2	P20962
Avasorum	2	P21127
Avasorum	2	P21266
Avasorum	2	P21281
Avasorum	2	P21283
Avasorum	2	P21291
Avasorum	2	P21333
Avasorum	2	P21359
Avasorum	2	P21397
Avasorum	2	P21399
Avasorum	2	P21453
Avasorum	2	P21579
Avasorum	2	P21589
Avasorum	2	P21741
Avasorum	2	P21796
Avasorum	2	P21810
Avasorum	2	P21912
Avasorum	2	P21926
Avasorum	2	P21964
Avasorum	2	P21980
Avasorum	2	P22033
Avasorum	2	P22059
Avasorum	2	P22061

Avasorum	2	P22087
Avasorum	2	P22102
Avasorum	2	P22234
Avasorum	2	P22307
Avasorum	2	P22314
Avasorum	2	P22392
Avasorum	2	P22570
Avasorum	2	P22626
Avasorum	2	P22670
Avasorum	2	P22681
Avasorum	2	P22692
Avasorum	2	P22694
Avasorum	2	P22695
Avasorum	2	P22830
Avasorum	2	P23025
Avasorum	2	P23193
Avasorum	2	P23219
Avasorum	2	P23229
Avasorum	2	P23246
Avasorum	2	P23258
Avasorum	2	P23284
Avasorum	2	P23368
Avasorum	2	P23381
Avasorum	2	P23396
Avasorum	2	P23443
Avasorum	2	P23458
Avasorum	2	P23467
Avasorum	2	P23469
Avasorum	2	P23470
Avasorum	2	P23497
Avasorum	2	P23508
Avasorum	2	P23510
Avasorum	2	P23526
Avasorum	2	P23528
Avasorum	2	P23588
Avasorum	2	P23610
Avasorum	2	P23634
Avasorum	2	P23743
Avasorum	2	P23786
Avasorum	2	P23919
Avasorum	2	P23921
Avasorum	2	P24385
Avasorum	2	P24386
Avasorum	2	P24390
Avasorum	2	P24468
Avasorum	2	P24534
Avasorum	2	P24539
Avasorum	2	P24666
Avasorum	2	P24723
Avasorum	2	P24752

Avasorum	2	P24844
Avasorum	2	P24928
Avasorum	2	P24941
Avasorum	2	P25098
Avasorum	2	P25116
Avasorum	2	P25205
Avasorum	2	P25325
Avasorum	2	P25398
Avasorum	2	P25440
Avasorum	2	P25445
Avasorum	2	P25490
Avasorum	2	P25685
Avasorum	2	P25686
Avasorum	2	P25705
Avasorum	2	P25774
Avasorum	2	P25786
Avasorum	2	P25787
Avasorum	2	P25788
Avasorum	2	P25789
Avasorum	2	P25942
Avasorum	2	P26006
Avasorum	2	P26022
Avasorum	2	P26038
Avasorum	2	P26196
Avasorum	2	P26358
Avasorum	2	P26368
Avasorum	2	P26373
Avasorum	2	P26374
Avasorum	2	P26440
Avasorum	2	P26572
Avasorum	2	P26583
Avasorum	2	P26599
Avasorum	2	P26639
Avasorum	2	P26640
Avasorum	2	P26641
Avasorum	2	P26885
Avasorum	2	P26951
Avasorum	2	P27105
Avasorum	2	P27144
Avasorum	2	P27348
Avasorum	2	P27361
Avasorum	2	P27448
Avasorum	2	P27635
Avasorum	2	P27658
Avasorum	2	P27694
Avasorum	2	P27695
Avasorum	2	P27701
Avasorum	2	P27707
Avasorum	2	P27708
Avasorum	2	P27797

Avasorum	2	P27816
Avasorum	2	P27824
Avasorum	2	P27986
Avasorum	2	P27987
Avasorum	2	P28062
Avasorum	2	P28065
Avasorum	2	P28066
Avasorum	2	P28070
Avasorum	2	P28072
Avasorum	2	P28074
Avasorum	2	P28161
Avasorum	2	P28288
Avasorum	2	P28290
Avasorum	2	P28300
Avasorum	2	P28331
Avasorum	2	P28340
Avasorum	2	P28370
Avasorum	2	P28482
Avasorum	2	P28702
Avasorum	2	P28715
Avasorum	2	P28799
Avasorum	2	P28827
Avasorum	2	P28838
Avasorum	2	P28906
Avasorum	2	P29083
Avasorum	2	P29084
Avasorum	2	P29144
Avasorum	2	P29218
Avasorum	2	P29279
Avasorum	2	P29317
Avasorum	2	P29323
Avasorum	2	P29353
Avasorum	2	P29372
Avasorum	2	P29401
Avasorum	2	P29466
Avasorum	2	P29474
Avasorum	2	P29558
Avasorum	2	P29590
Avasorum	2	P29597
Avasorum	2	P29692
Avasorum	2	P29966
Avasorum	2	P29992
Avasorum	2	P30038
Avasorum	2	P30040
Avasorum	2	P30041
Avasorum	2	P30043
Avasorum	2	P30044
Avasorum	2	P30048
Avasorum	2	P30050
Avasorum	2	P30084

Avasorum	2	P30085
Avasorum	2	P30086
Avasorum	2	P30101
Avasorum	2	P30153
Avasorum	2	P30154
Avasorum	2	P30260
Avasorum	2	P30405
Avasorum	2	P30414
Avasorum	2	P30419
Avasorum	2	P30519
Avasorum	2	P30520
Avasorum	2	P30530
Avasorum	2	P30533
Avasorum	2	P30536
Avasorum	2	P30566
Avasorum	2	P30622
Avasorum	2	P30626
Avasorum	2	P30740
Avasorum	2	P30825
Avasorum	2	P30837
Avasorum	2	P30876
Avasorum	2	P31040
Avasorum	2	P31146
Avasorum	2	P31150
Avasorum	2	P31151
Avasorum	2	P31153
Avasorum	2	P31323
Avasorum	2	P31350
Avasorum	2	P31431
Avasorum	2	P31483
Avasorum	2	P31641
Avasorum	2	P31689
Avasorum	2	P31749
Avasorum	2	P31930
Avasorum	2	P31937
Avasorum	2	P31939
Avasorum	2	P31942
Avasorum	2	P31943
Avasorum	2	P31944
Avasorum	2	P31946
Avasorum	2	P31948
Avasorum	2	P31949
Avasorum	2	P32119
Avasorum	2	P32121
Avasorum	2	P32189
Avasorum	2	P32321
Avasorum	2	P32322
Avasorum	2	P32455
Avasorum	2	P32456
Avasorum	2	P32519

Avasorum	2	P32780
Avasorum	2	P32856
Avasorum	2	P32969
Avasorum	2	P33121
Avasorum	2	P33151
Avasorum	2	P33176
Avasorum	2	P33316
Avasorum	2	P33527
Avasorum	2	P33897
Avasorum	2	P33981
Avasorum	2	P33991
Avasorum	2	P33992
Avasorum	2	P33993
Avasorum	2	P34059
Avasorum	2	P34896
Avasorum	2	P34897
Avasorum	2	P34931
Avasorum	2	P34932
Avasorum	2	P34947
Avasorum	2	P34949
Avasorum	2	P35052
Avasorum	2	P35080
Avasorum	2	P35221
Avasorum	2	P35222
Avasorum	2	P35232
Avasorum	2	P35237
Avasorum	2	P35240
Avasorum	2	P35241
Avasorum	2	P35244
Avasorum	2	P35249
Avasorum	2	P35250
Avasorum	2	P35251
Avasorum	2	P35268
Avasorum	2	P35269
Avasorum	2	P35270
Avasorum	2	P35367
Avasorum	2	P35520
Avasorum	2	P35555
Avasorum	2	P35556
Avasorum	2	P35573
Avasorum	2	P35579
Avasorum	2	P35580
Avasorum	2	P35590
Avasorum	2	P35606
Avasorum	2	P35610
Avasorum	2	P35611
Avasorum	2	P35613
Avasorum	2	P35637
Avasorum	2	P35658
Avasorum	2	P35659

Avasorum	2	P35713
Avasorum	2	P35749
Avasorum	2	P35813
Avasorum	2	P35914
Avasorum	2	P35916
Avasorum	2	P35968
Avasorum	2	P35998
Avasorum	2	P36269
Avasorum	2	P36404
Avasorum	2	P36405
Avasorum	2	P36507
Avasorum	2	P36542
Avasorum	2	P36543
Avasorum	2	P36551
Avasorum	2	P36578
Avasorum	2	P36639
Avasorum	2	P36776
Avasorum	2	P36871
Avasorum	2	P36873
Avasorum	2	P36915
Avasorum	2	P36957
Avasorum	2	P36959
Avasorum	2	P36969
Avasorum	2	P37023
Avasorum	2	P37059
Avasorum	2	P37108
Avasorum	2	P37173
Avasorum	2	P37198
Avasorum	2	P37235
Avasorum	2	P37268
Avasorum	2	P37275
Avasorum	2	P37802
Avasorum	2	P37837
Avasorum	2	P37840
Avasorum	2	P38117
Avasorum	2	P38159
Avasorum	2	P38432
Avasorum	2	P38435
Avasorum	2	P38571
Avasorum	2	P38606
Avasorum	2	P38646
Avasorum	2	P38919
Avasorum	2	P38935
Avasorum	2	P38936
Avasorum	2	P39019
Avasorum	2	P39023
Avasorum	2	P39060
Avasorum	2	P39656
Avasorum	2	P39687
Avasorum	2	P39748

Avasorum	2	P39880
Avasorum	2	P40121
Avasorum	2	P40123
Avasorum	2	P40189
Avasorum	2	P40222
Avasorum	2	P40227
Avasorum	2	P40261
Avasorum	2	P40306
Avasorum	2	P40425
Avasorum	2	P40429
Avasorum	2	P40616
Avasorum	2	P40692
Avasorum	2	P40763
Avasorum	2	P40818
Avasorum	2	P40925
Avasorum	2	P40926
Avasorum	2	P40937
Avasorum	2	P40938
Avasorum	2	P40939
Avasorum	2	P41091
Avasorum	2	P41162
Avasorum	2	P41208
Avasorum	2	P41212
Avasorum	2	P41214
Avasorum	2	P41223
Avasorum	2	P41226
Avasorum	2	P41227
Avasorum	2	P41229
Avasorum	2	P41240
Avasorum	2	P41250
Avasorum	2	P41252
Avasorum	2	P41440
Avasorum	2	P41567
Avasorum	2	P41743
Avasorum	2	P41970
Avasorum	2	P42025
Avasorum	2	P42126
Avasorum	2	P42166
Avasorum	2	P42167
Avasorum	2	P42224
Avasorum	2	P42226
Avasorum	2	P42229
Avasorum	2	P42285
Avasorum	2	P42330
Avasorum	2	P42336
Avasorum	2	P42338
Avasorum	2	P42345
Avasorum	2	P42356
Avasorum	2	P42566
Avasorum	2	P42574

Avasorum	2	P42575
Avasorum	2	P42677
Avasorum	2	P42684
Avasorum	2	P42694
Avasorum	2	P42695
Avasorum	2	P42696
Avasorum	2	P42704
Avasorum	2	P42765
Avasorum	2	P42766
Avasorum	2	P42785
Avasorum	2	P42858
Avasorum	2	P42892
Avasorum	2	P42898
Avasorum	2	P43007
Avasorum	2	P43034
Avasorum	2	P43121
Avasorum	2	P43155
Avasorum	2	P43243
Avasorum	2	P43246
Avasorum	2	P43250
Avasorum	2	P43304
Avasorum	2	P43307
Avasorum	2	P43378
Avasorum	2	P43487
Avasorum	2	P43490
Avasorum	2	P43686
Avasorum	2	P43897
Avasorum	2	P45877
Avasorum	2	P45880
Avasorum	2	P45954
Avasorum	2	P45973
Avasorum	2	P45974
Avasorum	2	P45983
Avasorum	2	P45985
Avasorum	2	P46013
Avasorum	2	P46020
Avasorum	2	P46060
Avasorum	2	P46063
Avasorum	2	P46087
Avasorum	2	P46100
Avasorum	2	P46108
Avasorum	2	P46109
Avasorum	2	P46199
Avasorum	2	P46379
Avasorum	2	P46459
Avasorum	2	P46531
Avasorum	2	P46734
Avasorum	2	P46736
Avasorum	2	P46776
Avasorum	2	P46777

Avasorum	2	P46778
Avasorum	2	P46779
Avasorum	2	P46781
Avasorum	2	P46782
Avasorum	2	P46783
Avasorum	2	P46821
Avasorum	2	P46926
Avasorum	2	P46934
Avasorum	2	P46939
Avasorum	2	P46940
Avasorum	2	P46976
Avasorum	2	P46977
Avasorum	2	P47712
Avasorum	2	P47755
Avasorum	2	P47756
Avasorum	2	P47813
Avasorum	2	P47895
Avasorum	2	P47897
Avasorum	2	P47914
Avasorum	2	P47974
Avasorum	2	P47985
Avasorum	2	P48047
Avasorum	2	P48059
Avasorum	2	P48060
Avasorum	2	P48147
Avasorum	2	P48163
Avasorum	2	P48200
Avasorum	2	P48307
Avasorum	2	P48382
Avasorum	2	P48426
Avasorum	2	P48444
Avasorum	2	P48449
Avasorum	2	P48454
Avasorum	2	P48506
Avasorum	2	P48507
Avasorum	2	P48509
Avasorum	2	P48553
Avasorum	2	P48556
Avasorum	2	P48634
Avasorum	2	P48637
Avasorum	2	P48643
Avasorum	2	P48651
Avasorum	2	P48681
Avasorum	2	P48723
Avasorum	2	P48729
Avasorum	2	P48730
Avasorum	2	P48735
Avasorum	2	P48736
Avasorum	2	P48739
Avasorum	2	P49005

Avasorum	2	P49006
Avasorum	2	P49023
Avasorum	2	P49069
Avasorum	2	P49116
Avasorum	2	P49137
Avasorum	2	P49184
Avasorum	2	P49189
Avasorum	2	P49207
Avasorum	2	P49247
Avasorum	2	P49257
Avasorum	2	P49281
Avasorum	2	P49321
Avasorum	2	P49327
Avasorum	2	P49354
Avasorum	2	P49356
Avasorum	2	P49366
Avasorum	2	P49368
Avasorum	2	P49406
Avasorum	2	P49407
Avasorum	2	P49411
Avasorum	2	P49419
Avasorum	2	P49427
Avasorum	2	P49441
Avasorum	2	P49454
Avasorum	2	P49458
Avasorum	2	P49585
Avasorum	2	P49588
Avasorum	2	P49589
Avasorum	2	P49590
Avasorum	2	P49591
Avasorum	2	P49593
Avasorum	2	P49641
Avasorum	2	P49642
Avasorum	2	P49643
Avasorum	2	P49662
Avasorum	2	P49674
Avasorum	2	P49720
Avasorum	2	P49721
Avasorum	2	P49736
Avasorum	2	P49748
Avasorum	2	P49750
Avasorum	2	P49754
Avasorum	2	P49755
Avasorum	2	P49756
Avasorum	2	P49757
Avasorum	2	P49761
Avasorum	2	P49768
Avasorum	2	P49770
Avasorum	2	P49773
Avasorum	2	P49790

Avasorum	2	P49792
Avasorum	2	P49795
Avasorum	2	P49796
Avasorum	2	P49810
Avasorum	2	P49815
Avasorum	2	P49821
Avasorum	2	P49840
Avasorum	2	P49841
Avasorum	2	P49848
Avasorum	2	P49902
Avasorum	2	P49903
Avasorum	2	P49914
Avasorum	2	P49915
Avasorum	2	P49916
Avasorum	2	P49917
Avasorum	2	P49959
Avasorum	2	P49961
Avasorum	2	P50148
Avasorum	2	P50213
Avasorum	2	P50225
Avasorum	2	P50281
Avasorum	2	P50336
Avasorum	2	P50395
Avasorum	2	P50402
Avasorum	2	P50416
Avasorum	2	P50443
Avasorum	2	P50452
Avasorum	2	P50453
Avasorum	2	P50454
Avasorum	2	P50479
Avasorum	2	P50502
Avasorum	2	P50542
Avasorum	2	P50548
Avasorum	2	P50552
Avasorum	2	P50570
Avasorum	2	P50579
Avasorum	2	P50583
Avasorum	2	P50613
Avasorum	2	P50748
Avasorum	2	P50749
Avasorum	2	P50750
Avasorum	2	P50851
Avasorum	2	P50895
Avasorum	2	P50897
Avasorum	2	P50914
Avasorum	2	P50990
Avasorum	2	P50991
Avasorum	2	P50995
Avasorum	2	P51003
Avasorum	2	P51114

Avasorum	2	P51116
Avasorum	2	P51148
Avasorum	2	P51149
Avasorum	2	P51151
Avasorum	2	P51153
Avasorum	2	P51159
Avasorum	2	P51178
Avasorum	2	P51398
Avasorum	2	P51452
Avasorum	2	P51531
Avasorum	2	P51532
Avasorum	2	P51553
Avasorum	2	P51570
Avasorum	2	P51571
Avasorum	2	P51572
Avasorum	2	P51580
Avasorum	2	P51608
Avasorum	2	P51610
Avasorum	2	P51617
Avasorum	2	P51636
Avasorum	2	P51648
Avasorum	2	P51659
Avasorum	2	P51665
Avasorum	2	P51687
Avasorum	2	P51688
Avasorum	2	P51692
Avasorum	2	P51784
Avasorum	2	P51798
Avasorum	2	P51809
Avasorum	2	P51812
Avasorum	2	P51813
Avasorum	2	P51825
Avasorum	2	P51858
Avasorum	2	P51946
Avasorum	2	P51948
Avasorum	2	P51956
Avasorum	2	P51965
Avasorum	2	P51970
Avasorum	2	P51991
Avasorum	2	P52209
Avasorum	2	P52272
Avasorum	2	P52292
Avasorum	2	P52294
Avasorum	2	P52298
Avasorum	2	P52306
Avasorum	2	P52434
Avasorum	2	P52564
Avasorum	2	P52565
Avasorum	2	P52566
Avasorum	2	P52594

Avasorum	2	P52597
Avasorum	2	P52630
Avasorum	2	P52701
Avasorum	2	P52732
Avasorum	2	P52735
Avasorum	2	P52747
Avasorum	2	P52756
Avasorum	2	P52758
Avasorum	2	P52788
Avasorum	2	P52789
Avasorum	2	P52799
Avasorum	2	P52815
Avasorum	2	P52824
Avasorum	2	P52848
Avasorum	2	P52888
Avasorum	2	P52907
Avasorum	2	P52926
Avasorum	2	P52943
Avasorum	2	P52948
Avasorum	2	P53004
Avasorum	2	P53007
Avasorum	2	P53041
Avasorum	2	P53350
Avasorum	2	P53355
Avasorum	2	P53365
Avasorum	2	P53367
Avasorum	2	P53384
Avasorum	2	P53396
Avasorum	2	P53582
Avasorum	2	P53597
Avasorum	2	P53602
Avasorum	2	P53609
Avasorum	2	P53611
Avasorum	2	P53618
Avasorum	2	P53621
Avasorum	2	P53634
Avasorum	2	P53667
Avasorum	2	P53671
Avasorum	2	P53675
Avasorum	2	P53677
Avasorum	2	P53680
Avasorum	2	P53701
Avasorum	2	P53778
Avasorum	2	P53801
Avasorum	2	P53814
Avasorum	2	P53985
Avasorum	2	P53990
Avasorum	2	P53992
Avasorum	2	P53999
Avasorum	2	P54098

Avasorum	2	P54105
Avasorum	2	P54136
Avasorum	2	P54198
Avasorum	2	P54252
Avasorum	2	P54259
Avasorum	2	P54277
Avasorum	2	P54278
Avasorum	2	P54289
Avasorum	2	P54577
Avasorum	2	P54578
Avasorum	2	P54619
Avasorum	2	P54652
Avasorum	2	P54687
Avasorum	2	P54709
Avasorum	2	P54727
Avasorum	2	P54760
Avasorum	2	P54802
Avasorum	2	P54819
Avasorum	2	P54886
Avasorum	2	P54920
Avasorum	2	P55010
Avasorum	2	P55011
Avasorum	2	P55036
Avasorum	2	P55039
Avasorum	2	P55060
Avasorum	2	P55072
Avasorum	2	P55081
Avasorum	2	P55084
Avasorum	2	P55145
Avasorum	2	P55196
Avasorum	2	P55199
Avasorum	2	P55201
Avasorum	2	P55209
Avasorum	2	P55210
Avasorum	2	P55212
Avasorum	2	P55263
Avasorum	2	P55265
Avasorum	2	P55268
Avasorum	2	P55290
Avasorum	2	P55735
Avasorum	2	P55769
Avasorum	2	P55786
Avasorum	2	P55795
Avasorum	2	P55809
Avasorum	2	P55884
Avasorum	2	P55899
Avasorum	2	P55957
Avasorum	2	P56134
Avasorum	2	P56182
Avasorum	2	P56192

Avasorum	2	P56211
Avasorum	2	P56377
Avasorum	2	P56378
Avasorum	2	P56381
Avasorum	2	P56385
Avasorum	2	P56524
Avasorum	2	P56537
Avasorum	2	P56545
Avasorum	2	P56556
Avasorum	2	P56589
Avasorum	2	P56937
Avasorum	2	P56945
Avasorum	2	P56962
Avasorum	2	P57076
Avasorum	2	P57081
Avasorum	2	P57088
Avasorum	2	P57678
Avasorum	2	P57737
Avasorum	2	P57740
Avasorum	2	P57764
Avasorum	2	P57772
Avasorum	2	P58004
Avasorum	2	P58335
Avasorum	2	P58546
Avasorum	2	P59998
Avasorum	2	P60033
Avasorum	2	P60059
Avasorum	2	P60174
Avasorum	2	P60228
Avasorum	2	P60468
Avasorum	2	P60484
Avasorum	2	P60510
Avasorum	2	P60520
Avasorum	2	P60602
Avasorum	2	P60660
Avasorum	2	P60709
Avasorum	2	P60842
Avasorum	2	P60866
Avasorum	2	P60891
Avasorum	2	P60900
Avasorum	2	P60903
Avasorum	2	P60953
Avasorum	2	P60981
Avasorum	2	P60983
Avasorum	2	P61006
Avasorum	2	P61009
Avasorum	2	P61011
Avasorum	2	P61018
Avasorum	2	P61019
Avasorum	2	P61020

Avasorum	2	P61026
Avasorum	2	P61077
Avasorum	2	P61081
Avasorum	2	P61086
Avasorum	2	P61088
Avasorum	2	P61106
Avasorum	2	P61158
Avasorum	2	P61160
Avasorum	2	P61163
Avasorum	2	P61201
Avasorum	2	P61221
Avasorum	2	P61224
Avasorum	2	P61225
Avasorum	2	P61244
Avasorum	2	P61247
Avasorum	2	P61254
Avasorum	2	P61289
Avasorum	2	P61313
Avasorum	2	P61353
Avasorum	2	P61421
Avasorum	2	P61513
Avasorum	2	P61586
Avasorum	2	P61587
Avasorum	2	P61599
Avasorum	2	P61604
Avasorum	2	P61619
Avasorum	2	P61758
Avasorum	2	P61764
Avasorum	2	P61769
Avasorum	2	P61803
Avasorum	2	P61916
Avasorum	2	P61923
Avasorum	2	P61927
Avasorum	2	P61956
Avasorum	2	P61962
Avasorum	2	P61964
Avasorum	2	P61966
Avasorum	2	P61970
Avasorum	2	P61978
Avasorum	2	P61981
Avasorum	2	P62068
Avasorum	2	P62070
Avasorum	2	P62081
Avasorum	2	P62136
Avasorum	2	P62140
Avasorum	2	P62191
Avasorum	2	P62195
Avasorum	2	P62241
Avasorum	2	P62244
Avasorum	2	P62249

Avasorum	2	P62253
Avasorum	2	P62256
Avasorum	2	P62258
Avasorum	2	P62263
Avasorum	2	P62266
Avasorum	2	P62269
Avasorum	2	P62273
Avasorum	2	P62277
Avasorum	2	P62280
Avasorum	2	P62304
Avasorum	2	P62306
Avasorum	2	P62310
Avasorum	2	P62312
Avasorum	2	P62314
Avasorum	2	P62316
Avasorum	2	P62318
Avasorum	2	P62328
Avasorum	2	P62330
Avasorum	2	P62333
Avasorum	2	P62341
Avasorum	2	P62380
Avasorum	2	P62424
Avasorum	2	P62487
Avasorum	2	P62491
Avasorum	2	P62495
Avasorum	2	P62633
Avasorum	2	P62699
Avasorum	2	P62701
Avasorum	2	P62714
Avasorum	2	P62736
Avasorum	2	P62745
Avasorum	2	P62750
Avasorum	2	P62753
Avasorum	2	P62805
Avasorum	2	P62820
Avasorum	2	P62826
Avasorum	2	P62829
Avasorum	2	P62834
Avasorum	2	P62841
Avasorum	2	P62847
Avasorum	2	P62851
Avasorum	2	P62854
Avasorum	2	P62857
Avasorum	2	P62861
Avasorum	2	P62873
Avasorum	2	P62877
Avasorum	2	P62879
Avasorum	2	P62888
Avasorum	2	P62891
Avasorum	2	P62899

Avasorum	2	P62906
Avasorum	2	P62910
Avasorum	2	P62913
Avasorum	2	P62917
Avasorum	2	P62937
Avasorum	2	P62942
Avasorum	2	P62979
Avasorum	2	P62993
Avasorum	2	P62995
Avasorum	2	P63000
Avasorum	2	P63010
Avasorum	2	P63027
Avasorum	2	P63092
Avasorum	2	P63096
Avasorum	2	P63104
Avasorum	2	P63151
Avasorum	2	P63165
Avasorum	2	P63167
Avasorum	2	P63172
Avasorum	2	P63173
Avasorum	2	P63208
Avasorum	2	P63218
Avasorum	2	P63220
Avasorum	2	P63241
Avasorum	2	P63244
Avasorum	2	P63272
Avasorum	2	P63279
Avasorum	2	P63313
Avasorum	2	P67775
Avasorum	2	P67809
Avasorum	2	P67812
Avasorum	2	P67870
Avasorum	2	P67936
Avasorum	2	P68036
Avasorum	2	P68104
Avasorum	2	P68363
Avasorum	2	P68366
Avasorum	2	P68371
Avasorum	2	P68400
Avasorum	2	P68402
Avasorum	2	P68431
Avasorum	2	P68871
Avasorum	2	P69905
Avasorum	2	P78310
Avasorum	2	P78312
Avasorum	2	P78316
Avasorum	2	P78318
Avasorum	2	P78324
Avasorum	2	P78330
Avasorum	2	P78332

Avasorum	2	P78344
Avasorum	2	P78345
Avasorum	2	P78346
Avasorum	2	P78347
Avasorum	2	P78356
Avasorum	2	P78357
Avasorum	2	P78362
Avasorum	2	P78371
Avasorum	2	P78381
Avasorum	2	P78406
Avasorum	2	P78417
Avasorum	2	P78527
Avasorum	2	P78536
Avasorum	2	P78539
Avasorum	2	P78540
Avasorum	2	P78549
Avasorum	2	P80217
Avasorum	2	P80303
Avasorum	2	P80723
Avasorum	2	P81605
Avasorum	2	P82094
Avasorum	2	P82650
Avasorum	2	P82663
Avasorum	2	P82664
Avasorum	2	P82673
Avasorum	2	P82675
Avasorum	2	P82909
Avasorum	2	P82912
Avasorum	2	P82914
Avasorum	2	P82921
Avasorum	2	P82930
Avasorum	2	P82932
Avasorum	2	P82933
Avasorum	2	P82979
Avasorum	2	P83111
Avasorum	2	P83436
Avasorum	2	P83731
Avasorum	2	P83876
Avasorum	2	P83881
Avasorum	2	P83916
Avasorum	2	P84022
Avasorum	2	P84077
Avasorum	2	P84085
Avasorum	2	P84090
Avasorum	2	P84095
Avasorum	2	P84098
Avasorum	2	P84101
Avasorum	2	P84103
Avasorum	2	P85037
Avasorum	2	P86790

Avasorum	2	P98082
Avasorum	2	P98160
Avasorum	2	P98170
Avasorum	2	P98172
Avasorum	2	P98175
Avasorum	2	P98179
Avasorum	2	P98194
Avasorum	2	P98196
Avasorum	2	P99999
Avasorum	2	Q00013
Avasorum	2	Q00059
Avasorum	2	Q00169
Avasorum	2	Q00325
Avasorum	2	Q00341
Avasorum	2	Q00403
Avasorum	2	Q00534
Avasorum	2	Q00535
Avasorum	2	Q00537
Avasorum	2	Q00577
Avasorum	2	Q00587
Avasorum	2	Q00610
Avasorum	2	Q00613
Avasorum	2	Q00653
Avasorum	2	Q00688
Avasorum	2	Q00765
Avasorum	2	Q00796
Avasorum	2	Q00839
Avasorum	2	Q01081
Avasorum	2	Q01082
Avasorum	2	Q01085
Avasorum	2	Q01105
Avasorum	2	Q01130
Avasorum	2	Q01167
Avasorum	2	Q01201
Avasorum	2	Q01415
Avasorum	2	Q01433
Avasorum	2	Q01469
Avasorum	2	Q01518
Avasorum	2	Q01543
Avasorum	2	Q01581
Avasorum	2	Q01628
Avasorum	2	Q01650
Avasorum	2	Q01658
Avasorum	2	Q01780
Avasorum	2	Q01804
Avasorum	2	Q01813
Avasorum	2	Q01831
Avasorum	2	Q01844
Avasorum	2	Q01850
Avasorum	2	Q01968

Avasorum	2	Q01970
Avasorum	2	Q01995
Avasorum	2	Q02040
Avasorum	2	Q02127
Avasorum	2	Q02218
Avasorum	2	Q02241
Avasorum	2	Q02252
Avasorum	2	Q02297
Avasorum	2	Q02318
Avasorum	2	Q02447
Avasorum	2	Q02539
Avasorum	2	Q02543
Avasorum	2	Q02750
Avasorum	2	Q02763
Avasorum	2	Q02790
Avasorum	2	Q02809
Avasorum	2	Q02818
Avasorum	2	Q02878
Avasorum	2	Q02880
Avasorum	2	Q02952
Avasorum	2	Q02978
Avasorum	2	Q03001
Avasorum	2	Q03111
Avasorum	2	Q03112
Avasorum	2	Q03113
Avasorum	2	Q03135
Avasorum	2	Q03154
Avasorum	2	Q03169
Avasorum	2	Q03252
Avasorum	2	Q03393
Avasorum	2	Q03405
Avasorum	2	Q03426
Avasorum	2	Q03468
Avasorum	2	Q03518
Avasorum	2	Q03519
Avasorum	2	Q03701
Avasorum	2	Q03936
Avasorum	2	Q04206
Avasorum	2	Q04323
Avasorum	2	Q04446
Avasorum	2	Q04637
Avasorum	2	Q04656
Avasorum	2	Q04721
Avasorum	2	Q04724
Avasorum	2	Q04726
Avasorum	2	Q04760
Avasorum	2	Q04771
Avasorum	2	Q04837
Avasorum	2	Q04864
Avasorum	2	Q04917

Avasorum	2	Q04941
Avasorum	2	Q05048
Avasorum	2	Q05086
Avasorum	2	Q05193
Avasorum	2	Q05209
Avasorum	2	Q05397
Avasorum	2	Q05519
Avasorum	2	Q05655
Avasorum	2	Q05682
Avasorum	2	Q05932
Avasorum	2	Q05D32
Avasorum	2	Q06124
Avasorum	2	Q06136
Avasorum	2	Q06203
Avasorum	2	Q06210
Avasorum	2	Q06265
Avasorum	2	Q06323
Avasorum	2	Q06330
Avasorum	2	Q06413
Avasorum	2	Q06481
Avasorum	2	Q06546
Avasorum	2	Q06587
Avasorum	2	Q06787
Avasorum	2	Q06830
Avasorum	2	Q07020
Avasorum	2	Q07021
Avasorum	2	Q07065
Avasorum	2	Q07157
Avasorum	2	Q07352
Avasorum	2	Q07617
Avasorum	2	Q07666
Avasorum	2	Q07812
Avasorum	2	Q07817
Avasorum	2	Q07820
Avasorum	2	Q07864
Avasorum	2	Q07866
Avasorum	2	Q07889
Avasorum	2	Q07955
Avasorum	2	Q07960
Avasorum	2	Q08117
Avasorum	2	Q08170
Avasorum	2	Q08174
Avasorum	2	Q08209
Avasorum	2	Q08211
Avasorum	2	Q08257
Avasorum	2	Q08378
Avasorum	2	Q08379
Avasorum	2	Q08426
Avasorum	2	Q08431
Avasorum	2	Q08499

Avasorum	2	Q08554
Avasorum	2	Q08623
Avasorum	2	Q08629
Avasorum	2	Q08722
Avasorum	2	Q08752
Avasorum	2	Q08945
Avasorum	2	Q08AD1
Avasorum	2	Q08AE8
Avasorum	2	Q08AF3
Avasorum	2	Q08AM6
Avasorum	2	Q08J23
Avasorum	2	Q09019
Avasorum	2	Q09028
Avasorum	2	Q09161
Avasorum	2	Q09328
Avasorum	2	Q09472
Avasorum	2	Q09666
Avasorum	2	Q0JRZ9
Avasorum	2	Q0PNE2
Avasorum	2	Q0VDF9
Avasorum	2	Q0VDG4
Avasorum	2	Q0VF96
Avasorum	2	Q0VGL1
Avasorum	2	Q0ZGT2
Avasorum	2	Q10469
Avasorum	2	Q10471
Avasorum	2	Q10472
Avasorum	2	Q10567
Avasorum	2	Q10570
Avasorum	2	Q10589
Avasorum	2	Q10713
Avasorum	2	Q11201
Avasorum	2	Q12765
Avasorum	2	Q12768
Avasorum	2	Q12769
Avasorum	2	Q12770
Avasorum	2	Q12778
Avasorum	2	Q12788
Avasorum	2	Q12789
Avasorum	2	Q12792
Avasorum	2	Q12797
Avasorum	2	Q12800
Avasorum	2	Q12802
Avasorum	2	Q12805
Avasorum	2	Q12824
Avasorum	2	Q12830
Avasorum	2	Q12834
Avasorum	2	Q12841
Avasorum	2	Q12846
Avasorum	2	Q12849

Avasorum	2	Q12851
Avasorum	2	Q12866
Avasorum	2	Q12872
Avasorum	2	Q12873
Avasorum	2	Q12874
Avasorum	2	Q12882
Avasorum	2	Q12888
Avasorum	2	Q12893
Avasorum	2	Q12894
Avasorum	2	Q12899
Avasorum	2	Q12904
Avasorum	2	Q12905
Avasorum	2	Q12906
Avasorum	2	Q12907
Avasorum	2	Q12931
Avasorum	2	Q12933
Avasorum	2	Q12959
Avasorum	2	Q12965
Avasorum	2	Q12972
Avasorum	2	Q12974
Avasorum	2	Q12979
Avasorum	2	Q12980
Avasorum	2	Q12981
Avasorum	2	Q12982
Avasorum	2	Q12986
Avasorum	2	Q12996
Avasorum	2	Q12999
Avasorum	2	Q13011
Avasorum	2	Q13017
Avasorum	2	Q13033
Avasorum	2	Q13042
Avasorum	2	Q13043
Avasorum	2	Q13045
Avasorum	2	Q13049
Avasorum	2	Q13057
Avasorum	2	Q13084
Avasorum	2	Q13085
Avasorum	2	Q13098
Avasorum	2	Q13107
Avasorum	2	Q13111
Avasorum	2	Q13112
Avasorum	2	Q13123
Avasorum	2	Q13126
Avasorum	2	Q13131
Avasorum	2	Q13136
Avasorum	2	Q13144
Avasorum	2	Q13148
Avasorum	2	Q13151
Avasorum	2	Q13155
Avasorum	2	Q13158

Avasorum	2	Q13162
Avasorum	2	Q13163
Avasorum	2	Q13164
Avasorum	2	Q13177
Avasorum	2	Q13185
Avasorum	2	Q13188
Avasorum	2	Q13190
Avasorum	2	Q13200
Avasorum	2	Q13201
Avasorum	2	Q13206
Avasorum	2	Q13217
Avasorum	2	Q13228
Avasorum	2	Q13232
Avasorum	2	Q13242
Avasorum	2	Q13243
Avasorum	2	Q13247
Avasorum	2	Q13257
Avasorum	2	Q13263
Avasorum	2	Q13275
Avasorum	2	Q13277
Avasorum	2	Q13283
Avasorum	2	Q13287
Avasorum	2	Q13308
Avasorum	2	Q13310
Avasorum	2	Q13315
Avasorum	2	Q13322
Avasorum	2	Q13325
Avasorum	2	Q13330
Avasorum	2	Q13347
Avasorum	2	Q13356
Avasorum	2	Q13362
Avasorum	2	Q13363
Avasorum	2	Q13371
Avasorum	2	Q13393
Avasorum	2	Q13395
Avasorum	2	Q13404
Avasorum	2	Q13405
Avasorum	2	Q13409
Avasorum	2	Q13416
Avasorum	2	Q13418
Avasorum	2	Q13423
Avasorum	2	Q13425
Avasorum	2	Q13426
Avasorum	2	Q13427
Avasorum	2	Q13428
Avasorum	2	Q13432
Avasorum	2	Q13435
Avasorum	2	Q13438
Avasorum	2	Q13439
Avasorum	2	Q13442

Avasorum	2	Q13443
Avasorum	2	Q13444
Avasorum	2	Q13445
Avasorum	2	Q13451
Avasorum	2	Q13459
Avasorum	2	Q13464
Avasorum	2	Q13469
Avasorum	2	Q13472
Avasorum	2	Q13485
Avasorum	2	Q13488
Avasorum	2	Q13490
Avasorum	2	Q13492
Avasorum	2	Q13496
Avasorum	2	Q13501
Avasorum	2	Q13505
Avasorum	2	Q13509
Avasorum	2	Q13523
Avasorum	2	Q13526
Avasorum	2	Q13535
Avasorum	2	Q13541
Avasorum	2	Q13546
Avasorum	2	Q13547
Avasorum	2	Q13555
Avasorum	2	Q13557
Avasorum	2	Q13561
Avasorum	2	Q13563
Avasorum	2	Q13564
Avasorum	2	Q13572
Avasorum	2	Q13573
Avasorum	2	Q13574
Avasorum	2	Q13586
Avasorum	2	Q13588
Avasorum	2	Q13595
Avasorum	2	Q13596
Avasorum	2	Q13601
Avasorum	2	Q13610
Avasorum	2	Q13613
Avasorum	2	Q13614
Avasorum	2	Q13616
Avasorum	2	Q13617
Avasorum	2	Q13618
Avasorum	2	Q13619
Avasorum	2	Q13620
Avasorum	2	Q13625
Avasorum	2	Q13627
Avasorum	2	Q13630
Avasorum	2	Q13636
Avasorum	2	Q13637
Avasorum	2	Q13641
Avasorum	2	Q13642

Avasorum	2	Q13643
Avasorum	2	Q13671
Avasorum	2	Q13685
Avasorum	2	Q13724
Avasorum	2	Q13740
Avasorum	2	Q13769
Avasorum	2	Q13796
Avasorum	2	Q13813
Avasorum	2	Q13823
Avasorum	2	Q13825
Avasorum	2	Q13838
Avasorum	2	Q13867
Avasorum	2	Q13868
Avasorum	2	Q13873
Avasorum	2	Q13885
Avasorum	2	Q13888
Avasorum	2	Q13889
Avasorum	2	Q13895
Avasorum	2	Q13905
Avasorum	2	Q13907
Avasorum	2	Q13951
Avasorum	2	Q13952
Avasorum	2	Q14004
Avasorum	2	Q14008
Avasorum	2	Q14011
Avasorum	2	Q14012
Avasorum	2	Q14019
Avasorum	2	Q14103
Avasorum	2	Q14108
Avasorum	2	Q14112
Avasorum	2	Q14114
Avasorum	2	Q14118
Avasorum	2	Q14137
Avasorum	2	Q14139
Avasorum	2	Q14141
Avasorum	2	Q14145
Avasorum	2	Q14146
Avasorum	2	Q14147
Avasorum	2	Q14149
Avasorum	2	Q14151
Avasorum	2	Q14152
Avasorum	2	Q14155
Avasorum	2	Q14156
Avasorum	2	Q14157
Avasorum	2	Q14160
Avasorum	2	Q14161
Avasorum	2	Q14162
Avasorum	2	Q14165
Avasorum	2	Q14166
Avasorum	2	Q14181

Avasorum	2	Q14185
Avasorum	2	Q14186
Avasorum	2	Q14192
Avasorum	2	Q14195
Avasorum	2	Q14197
Avasorum	2	Q14202
Avasorum	2	Q14203
Avasorum	2	Q14204
Avasorum	2	Q14232
Avasorum	2	Q14240
Avasorum	2	Q14241
Avasorum	2	Q14247
Avasorum	2	Q14249
Avasorum	2	Q14254
Avasorum	2	Q14257
Avasorum	2	Q14258
Avasorum	2	Q14315
Avasorum	2	Q14318
Avasorum	2	Q14320
Avasorum	2	Q14331
Avasorum	2	Q14344
Avasorum	2	Q14353
Avasorum	2	Q14376
Avasorum	2	Q14392
Avasorum	2	Q14444
Avasorum	2	Q14457
Avasorum	2	Q14498
Avasorum	2	Q14511
Avasorum	2	Q14527
Avasorum	2	Q14534
Avasorum	2	Q14554
Avasorum	2	Q14558
Avasorum	2	Q14562
Avasorum	2	Q14566
Avasorum	2	Q14571
Avasorum	2	Q14573
Avasorum	2	Q14643
Avasorum	2	Q14644
Avasorum	2	Q14651
Avasorum	2	Q14653
Avasorum	2	Q14657
Avasorum	2	Q14667
Avasorum	2	Q14669
Avasorum	2	Q14671
Avasorum	2	Q14676
Avasorum	2	Q14677
Avasorum	2	Q14678
Avasorum	2	Q14683
Avasorum	2	Q14684
Avasorum	2	Q14689

Avasorum	2	Q14690
Avasorum	2	Q14691
Avasorum	2	Q14692
Avasorum	2	Q14693
Avasorum	2	Q14694
Avasorum	2	Q14696
Avasorum	2	Q14697
Avasorum	2	Q14699
Avasorum	2	Q14728
Avasorum	2	Q14738
Avasorum	2	Q14739
Avasorum	2	Q14746
Avasorum	2	Q14764
Avasorum	2	Q14766
Avasorum	2	Q14767
Avasorum	2	Q14781
Avasorum	2	Q14789
Avasorum	2	Q14790
Avasorum	2	Q147X3
Avasorum	2	Q14807
Avasorum	2	Q14814
Avasorum	2	Q14839
Avasorum	2	Q14847
Avasorum	2	Q14914
Avasorum	2	Q14919
Avasorum	2	Q14934
Avasorum	2	Q14966
Avasorum	2	Q14974
Avasorum	2	Q14978
Avasorum	2	Q14980
Avasorum	2	Q14997
Avasorum	2	Q14999
Avasorum	2	Q14BN4
Avasorum	2	Q14C86
Avasorum	2	Q14CX7
Avasorum	2	Q14D04
Avasorum	2	Q15003
Avasorum	2	Q15004
Avasorum	2	Q15005
Avasorum	2	Q15006
Avasorum	2	Q15007
Avasorum	2	Q15008
Avasorum	2	Q15011
Avasorum	2	Q15013
Avasorum	2	Q15014
Avasorum	2	Q15018
Avasorum	2	Q15019
Avasorum	2	Q15020
Avasorum	2	Q15021
Avasorum	2	Q15022

Avasorum	2	Q15024
Avasorum	2	Q15025
Avasorum	2	Q15029
Avasorum	2	Q15031
Avasorum	2	Q15032
Avasorum	2	Q15035
Avasorum	2	Q15036
Avasorum	2	Q15041
Avasorum	2	Q15042
Avasorum	2	Q15043
Avasorum	2	Q15046
Avasorum	2	Q15047
Avasorum	2	Q15048
Avasorum	2	Q15050
Avasorum	2	Q15054
Avasorum	2	Q15056
Avasorum	2	Q15057
Avasorum	2	Q15058
Avasorum	2	Q15059
Avasorum	2	Q15061
Avasorum	2	Q15067
Avasorum	2	Q15070
Avasorum	2	Q15075
Avasorum	2	Q15084
Avasorum	2	Q15102
Avasorum	2	Q15120
Avasorum	2	Q15121
Avasorum	2	Q15125
Avasorum	2	Q15126
Avasorum	2	Q15139
Avasorum	2	Q15149
Avasorum	2	Q15154
Avasorum	2	Q15155
Avasorum	2	Q15165
Avasorum	2	Q15172
Avasorum	2	Q15181
Avasorum	2	Q15185
Avasorum	2	Q15208
Avasorum	2	Q15233
Avasorum	2	Q15257
Avasorum	2	Q15262
Avasorum	2	Q15269
Avasorum	2	Q15276
Avasorum	2	Q15286
Avasorum	2	Q15287
Avasorum	2	Q15291
Avasorum	2	Q15293
Avasorum	2	Q15311
Avasorum	2	Q15334
Avasorum	2	Q15345

Avasorum	2	Q15361
Avasorum	2	Q15363
Avasorum	2	Q15365
Avasorum	2	Q15366
Avasorum	2	Q15369
Avasorum	2	Q15370
Avasorum	2	Q15382
Avasorum	2	Q15386
Avasorum	2	Q15388
Avasorum	2	Q15390
Avasorum	2	Q15392
Avasorum	2	Q15393
Avasorum	2	Q15397
Avasorum	2	Q15398
Avasorum	2	Q15404
Avasorum	2	Q15417
Avasorum	2	Q15418
Avasorum	2	Q15424
Avasorum	2	Q15428
Avasorum	2	Q15434
Avasorum	2	Q15435
Avasorum	2	Q15436
Avasorum	2	Q15437
Avasorum	2	Q15438
Avasorum	2	Q15459
Avasorum	2	Q15477
Avasorum	2	Q15526
Avasorum	2	Q15542
Avasorum	2	Q15545
Avasorum	2	Q15554
Avasorum	2	Q15555
Avasorum	2	Q15599
Avasorum	2	Q15628
Avasorum	2	Q15629
Avasorum	2	Q15631
Avasorum	2	Q15633
Avasorum	2	Q15637
Avasorum	2	Q15642
Avasorum	2	Q15643
Avasorum	2	Q15645
Avasorum	2	Q15648
Avasorum	2	Q15650
Avasorum	2	Q15651
Avasorum	2	Q15652
Avasorum	2	Q15653
Avasorum	2	Q15654
Avasorum	2	Q15678
Avasorum	2	Q15691
Avasorum	2	Q15717
Avasorum	2	Q15738

Avasorum	2	Q15746
Avasorum	2	Q15750
Avasorum	2	Q15751
Avasorum	2	Q15758
Avasorum	2	Q15773
Avasorum	2	Q15785
Avasorum	2	Q15796
Avasorum	2	Q15797
Avasorum	2	Q15800
Avasorum	2	Q15811
Avasorum	2	Q15813
Avasorum	2	Q15814
Avasorum	2	Q15819
Avasorum	2	Q15831
Avasorum	2	Q15833
Avasorum	2	Q15836
Avasorum	2	Q15843
Avasorum	2	Q15904
Avasorum	2	Q15906
Avasorum	2	Q15907
Avasorum	2	Q15942
Avasorum	2	Q16134
Avasorum	2	Q16181
Avasorum	2	Q16186
Avasorum	2	Q16204
Avasorum	2	Q16222
Avasorum	2	Q16254
Avasorum	2	Q16270
Avasorum	2	Q16342
Avasorum	2	Q16363
Avasorum	2	Q16401
Avasorum	2	Q16512
Avasorum	2	Q16513
Avasorum	2	Q16514
Avasorum	2	Q16527
Avasorum	2	Q16531
Avasorum	2	Q16537
Avasorum	2	Q16539
Avasorum	2	Q16540
Avasorum	2	Q16543
Avasorum	2	Q16555
Avasorum	2	Q16563
Avasorum	2	Q16576
Avasorum	2	Q16584
Avasorum	2	Q16594
Avasorum	2	Q16602
Avasorum	2	Q16611
Avasorum	2	Q16629
Avasorum	2	Q16630
Avasorum	2	Q16637

Avasorum	2	Q16643
Avasorum	2	Q16656
Avasorum	2	Q16658
Avasorum	2	Q16666
Avasorum	2	Q16698
Avasorum	2	Q16706
Avasorum	2	Q16718
Avasorum	2	Q16739
Avasorum	2	Q16740
Avasorum	2	Q16762
Avasorum	2	Q16763
Avasorum	2	Q16773
Avasorum	2	Q16774
Avasorum	2	Q16775
Avasorum	2	Q16795
Avasorum	2	Q16798
Avasorum	2	Q16822
Avasorum	2	Q16831
Avasorum	2	Q16832
Avasorum	2	Q16836
Avasorum	2	Q16850
Avasorum	2	Q16851
Avasorum	2	Q16875
Avasorum	2	Q16881
Avasorum	2	Q16891
Avasorum	2	Q19T08
Avasorum	2	Q1ED39
Avasorum	2	Q1KMD3
Avasorum	2	Q24JP5
Avasorum	2	Q27J81
Avasorum	2	Q29RF7
Avasorum	2	Q2KHT3
Avasorum	2	Q2M1P5
Avasorum	2	Q2M1Z3
Avasorum	2	Q2M296
Avasorum	2	Q2M2I8
Avasorum	2	Q2M389
Avasorum	2	Q2NKX8
Avasorum	2	Q2NL82
Avasorum	2	Q2PPJ7
Avasorum	2	Q2PZI1
Avasorum	2	Q2TAA2
Avasorum	2	Q2TAA5
Avasorum	2	Q2TAL8
Avasorum	2	Q2TAY7
Avasorum	2	Q2VPK5
Avasorum	2	Q32M88
Avasorum	2	Q32MZ4
Avasorum	2	Q32NB8
Avasorum	2	Q32NC0

Avasorum	2	Q32P28
Avasorum	2	Q32P41
Avasorum	2	Q32P44
Avasorum	2	Q3B726
Avasorum	2	Q3KQU3
Avasorum	2	Q3KQV9
Avasorum	2	Q3KQZ1
Avasorum	2	Q3L8U1
Avasorum	2	Q3LXA3
Avasorum	2	Q3MHD2
Avasorum	2	Q3SXM5
Avasorum	2	Q3SY69
Avasorum	2	Q3T906
Avasorum	2	Q3V6T2
Avasorum	2	Q3YEC7
Avasorum	2	Q3ZCQ8
Avasorum	2	Q460N5
Avasorum	2	Q495W5
Avasorum	2	Q49A26
Avasorum	2	Q49AR2
Avasorum	2	Q4G0F5
Avasorum	2	Q4G0J3
Avasorum	2	Q4G0N4
Avasorum	2	Q4G0X4
Avasorum	2	Q4G148
Avasorum	2	Q4G176
Avasorum	2	Q4J6C6
Avasorum	2	Q4KMP7
Avasorum	2	Q4KMQ1
Avasorum	2	Q4KMQ2
Avasorum	2	Q4L180
Avasorum	2	Q4V328
Avasorum	2	Q4V339
Avasorum	2	Q4VC31
Avasorum	2	Q4ZIN3
Avasorum	2	Q52LJ0
Avasorum	2	Q52LW3
Avasorum	2	Q53EL6
Avasorum	2	Q53EP0
Avasorum	2	Q53ET0
Avasorum	2	Q53EU6
Avasorum	2	Q53EZ4
Avasorum	2	Q53F19
Avasorum	2	Q53FA7
Avasorum	2	Q53FP2
Avasorum	2	Q53GA4
Avasorum	2	Q53GG5
Avasorum	2	Q53GL7
Avasorum	2	Q53GQ0
Avasorum	2	Q53GS7

Avasorum	2	Q53GS9
Avasorum	2	Q53GT1
Avasorum	2	Q53H12
Avasorum	2	Q53H47
Avasorum	2	Q53H82
Avasorum	2	Q53H96
Avasorum	2	Q53HC9
Avasorum	2	Q53HL2
Avasorum	2	Q53SF7
Avasorum	2	Q53T59
Avasorum	2	Q53TN4
Avasorum	2	Q562E7
Avasorum	2	Q562R1
Avasorum	2	Q567U6
Avasorum	2	Q56VL3
Avasorum	2	Q58EX7
Avasorum	2	Q58FG1
Avasorum	2	Q58WW2
Avasorum	2	Q5BJD5
Avasorum	2	Q5BJF2
Avasorum	2	Q5BJH7
Avasorum	2	Q5BKZ1
Avasorum	2	Q5C9Z4
Avasorum	2	Q5EBL4
Avasorum	2	Q5F1R6
Avasorum	2	Q5GJ75
Avasorum	2	Q5GLZ8
Avasorum	2	Q5H8A4
Avasorum	2	Q5H9R7
Avasorum	2	Q5H9U9
Avasorum	2	Q5HYI8
Avasorum	2	Q5HYJ3
Avasorum	2	Q5HYK3
Avasorum	2	Q5HYK7
Avasorum	2	Q5HYW2
Avasorum	2	Q5J8M3
Avasorum	2	Q5JPH6
Avasorum	2	Q5JPI3
Avasorum	2	Q5JPI9
Avasorum	2	Q5JRA6
Avasorum	2	Q5JRX3
Avasorum	2	Q5JSH3
Avasorum	2	Q5JSZ5
Avasorum	2	Q5JTD0
Avasorum	2	Q5JTH9
Avasorum	2	Q5JTJ3
Avasorum	2	Q5JTV8
Avasorum	2	Q5JTW2
Avasorum	2	Q5JTZ9
Avasorum	2	Q5JU69

Avasorum	2	Q5JVF3
Avasorum	2	Q5JVS0
Avasorum	2	Q5K4L6
Avasorum	2	Q5K651
Avasorum	2	Q5MIZ7
Avasorum	2	Q5MNZ6
Avasorum	2	Q5MNZ9
Avasorum	2	Q5NDL2
Avasorum	2	Q5PRF9
Avasorum	2	Q5QJ74
Avasorum	2	Q5QJE6
Avasorum	2	Q5R372
Avasorum	2	Q5R3I4
Avasorum	2	Q5RI15
Avasorum	2	Q5RKV6
Avasorum	2	Q5SNT2
Avasorum	2	Q5SQI0
Avasorum	2	Q5SQN1
Avasorum	2	Q5SRE5
Avasorum	2	Q5SSJ5
Avasorum	2	Q5ST30
Avasorum	2	Q5SW79
Avasorum	2	Q5SW96
Avasorum	2	Q5SWX8
Avasorum	2	Q5SY16
Avasorum	2	Q5SYE7
Avasorum	2	Q5T013
Avasorum	2	Q5T0D9
Avasorum	2	Q5T0F9
Avasorum	2	Q5T0N5
Avasorum	2	Q5T160
Avasorum	2	Q5T1C6
Avasorum	2	Q5T1J5
Avasorum	2	Q5T1M5
Avasorum	2	Q5T200
Avasorum	2	Q5T280
Avasorum	2	Q5T2E6
Avasorum	2	Q5T3F8
Avasorum	2	Q5T3I0
Avasorum	2	Q5T440
Avasorum	2	Q5T447
Avasorum	2	Q5T4B2
Avasorum	2	Q5T4S7
Avasorum	2	Q5T5C0
Avasorum	2	Q5T5U3
Avasorum	2	Q5T5Y3
Avasorum	2	Q5T653
Avasorum	2	Q5T6F2
Avasorum	2	Q5T6V5
Avasorum	2	Q5T749

Avasorum	2	Q5T7W0
Avasorum	2	Q5T8D3
Avasorum	2	Q5T8P6
Avasorum	2	Q5T9A4
Avasorum	2	Q5T9L3
Avasorum	2	Q5TA45
Avasorum	2	Q5TA50
Avasorum	2	Q5TAQ9
Avasorum	2	Q5TAT6
Avasorum	2	Q5TAX3
Avasorum	2	Q5TBA9
Avasorum	2	Q5TBB1
Avasorum	2	Q5TC12
Avasorum	2	Q5TC82
Avasorum	2	Q5TDH0
Avasorum	2	Q5TEJ8
Avasorum	2	Q5TEU4
Avasorum	2	Q5TFE4
Avasorum	2	Q5TH69
Avasorum	2	Q5THJ4
Avasorum	2	Q5TZA2
Avasorum	2	Q5U5X0
Avasorum	2	Q5U651
Avasorum	2	Q5UIP0
Avasorum	2	Q5VIR6
Avasorum	2	Q5VSL9
Avasorum	2	Q5VT25
Avasorum	2	Q5VT52
Avasorum	2	Q5VTB9
Avasorum	2	Q5VTL8
Avasorum	2	Q5VTR2
Avasorum	2	Q5VU43
Avasorum	2	Q5VUA4
Avasorum	2	Q5VUB5
Avasorum	2	Q5VUD6
Avasorum	2	Q5VUJ6
Avasorum	2	Q5VV42
Avasorum	2	Q5VW32
Avasorum	2	Q5VW36
Avasorum	2	Q5VWQ0
Avasorum	2	Q5VWQ8
Avasorum	2	Q5VWZ2
Avasorum	2	Q5VY43
Avasorum	2	Q5VYK3
Avasorum	2	Q5VYS8
Avasorum	2	Q5VZ18
Avasorum	2	Q5VZ89
Avasorum	2	Q5VZE5
Avasorum	2	Q5VZL5
Avasorum	2	Q5W0V3

Avasorum	2	Q5W0Z9
Avasorum	2	Q5W111
Avasorum	2	Q5XUX1
Avasorum	2	Q5ZPR3
Avasorum	2	Q63HN8
Avasorum	2	Q63HR2
Avasorum	2	Q63ZY3
Avasorum	2	Q641Q2
Avasorum	2	Q643R3
Avasorum	2	Q658P3
Avasorum	2	Q658Y4
Avasorum	2	Q66K14
Avasorum	2	Q66K74
Avasorum	2	Q66LE6
Avasorum	2	Q66PJ3
Avasorum	2	Q676U5
Avasorum	2	Q68CP4
Avasorum	2	Q68CP9
Avasorum	2	Q68CQ4
Avasorum	2	Q68CQ7
Avasorum	2	Q68CR1
Avasorum	2	Q68CZ2
Avasorum	2	Q68CZ6
Avasorum	2	Q68D10
Avasorum	2	Q68D91
Avasorum	2	Q68DQ2
Avasorum	2	Q68E01
Avasorum	2	Q68EM7
Avasorum	2	Q69YN2
Avasorum	2	Q69YN4
Avasorum	2	Q69YQ0
Avasorum	2	Q6AI08
Avasorum	2	Q6AI12
Avasorum	2	Q6AWC2
Avasorum	2	Q6AZY7
Avasorum	2	Q6DD88
Avasorum	2	Q6DKI1
Avasorum	2	Q6DKJ4
Avasorum	2	Q6DKK2
Avasorum	2	Q6DN90
Avasorum	2	Q6FI81
Avasorum	2	Q6FIF0
Avasorum	2	Q6GMV2
Avasorum	2	Q6GQQ9
Avasorum	2	Q6I9Y2
Avasorum	2	Q6IA69
Avasorum	2	Q6IA86
Avasorum	2	Q6IAA8
Avasorum	2	Q6IAN0
Avasorum	2	Q6IBS0

Avasorum	2	Q6IBW4
Avasorum	2	Q6ICG6
Avasorum	2	Q6ICL3
Avasorum	2	Q6IN85
Avasorum	2	Q6IQ22
Avasorum	2	Q6IQ26
Avasorum	2	Q6IQ49
Avasorum	2	Q6KB66
Avasorum	2	Q6KC79
Avasorum	2	Q6KCM7
Avasorum	2	Q6L8Q7
Avasorum	2	Q6N069
Avasorum	2	Q6NSW5
Avasorum	2	Q6NTF9
Avasorum	2	Q6NUK1
Avasorum	2	Q6NUK4
Avasorum	2	Q6NUM9
Avasorum	2	Q6NUQ1
Avasorum	2	Q6NUQ4
Avasorum	2	Q6NVY1
Avasorum	2	Q6NW29
Avasorum	2	Q6NW34
Avasorum	2	Q6NXE6
Avasorum	2	Q6NXR4
Avasorum	2	Q6NXT1
Avasorum	2	Q6NXT4
Avasorum	2	Q6NY19
Avasorum	2	Q6NYC1
Avasorum	2	Q6NYC8
Avasorum	2	Q6NZI2
Avasorum	2	Q6NZY4
Avasorum	2	Q6P158
Avasorum	2	Q6P179
Avasorum	2	Q6P1A2
Avasorum	2	Q6P1J9
Avasorum	2	Q6P1L8
Avasorum	2	Q6P1M0
Avasorum	2	Q6P1N0
Avasorum	2	Q6P1N9
Avasorum	2	Q6P1Q9
Avasorum	2	Q6P1R3
Avasorum	2	Q6P1X5
Avasorum	2	Q6P1X6
Avasorum	2	Q6P2C8
Avasorum	2	Q6P2E9
Avasorum	2	Q6P2P2
Avasorum	2	Q6P2Q9
Avasorum	2	Q6P3S6
Avasorum	2	Q6P3W7
Avasorum	2	Q6P3X3

Avasorum	2	Q6P4A7
Avasorum	2	Q6P4E1
Avasorum	2	Q6P4R8
Avasorum	2	Q6P587
Avasorum	2	Q6P5R6
Avasorum	2	Q6P5Z2
Avasorum	2	Q6P6C2
Avasorum	2	Q6P996
Avasorum	2	Q6P9B6
Avasorum	2	Q6P9B9
Avasorum	2	Q6P9H5
Avasorum	2	Q6PCB7
Avasorum	2	Q6PCE3
Avasorum	2	Q6PD62
Avasorum	2	Q6PD74
Avasorum	2	Q6PEY1
Avasorum	2	Q6PGN9
Avasorum	2	Q6PGP7
Avasorum	2	Q6PHR2
Avasorum	2	Q6PI48
Avasorum	2	Q6PI78
Avasorum	2	Q6PI98
Avasorum	2	Q6PIJ6
Avasorum	2	Q6PIU2
Avasorum	2	Q6PIW4
Avasorum	2	Q6PJ69
Avasorum	2	Q6PJF5
Avasorum	2	Q6PJG2
Avasorum	2	Q6PJG6
Avasorum	2	Q6PJI9
Avasorum	2	Q6PJT7
Avasorum	2	Q6PK04
Avasorum	2	Q6PKC3
Avasorum	2	Q6PKG0
Avasorum	2	Q6PL18
Avasorum	2	Q6PL24
Avasorum	2	Q6PML9
Avasorum	2	Q6Q0C0
Avasorum	2	Q6R327
Avasorum	2	Q6RFH5
Avasorum	2	Q6RW13
Avasorum	2	Q6STE5
Avasorum	2	Q6UB35
Avasorum	2	Q6ULP2
Avasorum	2	Q6UN15
Avasorum	2	Q6UUV7
Avasorum	2	Q6UUV9
Avasorum	2	Q6UW02
Avasorum	2	Q6UW63
Avasorum	2	Q6UW68

Avasorum	2	Q6UWE0
Avasorum	2	Q6UWH4
Avasorum	2	Q6UWP7
Avasorum	2	Q6UWZ7
Avasorum	2	Q6UX04
Avasorum	2	Q6UXH1
Avasorum	2	Q6UXN9
Avasorum	2	Q6UXV4
Avasorum	2	Q6V0I7
Avasorum	2	Q6V1X1
Avasorum	2	Q6VMQ6
Avasorum	2	Q6VN20
Avasorum	2	Q6VY07
Avasorum	2	Q6WCQ1
Avasorum	2	Q6XE24
Avasorum	2	Q6XQN6
Avasorum	2	Q6XZF7
Avasorum	2	Q6Y1H2
Avasorum	2	Q6Y288
Avasorum	2	Q6Y7W6
Avasorum	2	Q6YHK3
Avasorum	2	Q6YHU6
Avasorum	2	Q6YN16
Avasorum	2	Q6YP21
Avasorum	2	Q6ZMI0
Avasorum	2	Q6ZMP0
Avasorum	2	Q6ZMZ3
Avasorum	2	Q6ZN55
Avasorum	2	Q6ZNB6
Avasorum	2	Q6ZNJ1
Avasorum	2	Q6ZNL6
Avasorum	2	Q6ZRP7
Avasorum	2	Q6ZRS2
Avasorum	2	Q6ZS17
Avasorum	2	Q6ZSR9
Avasorum	2	Q6ZSZ5
Avasorum	2	Q6ZT07
Avasorum	2	Q6ZT12
Avasorum	2	Q6ZT21
Avasorum	2	Q6ZU35
Avasorum	2	Q6ZUT6
Avasorum	2	Q6ZVK8
Avasorum	2	Q6ZW31
Avasorum	2	Q6ZW49
Avasorum	2	Q6ZXV5
Avasorum	2	Q709C8
Avasorum	2	Q70CQ2
Avasorum	2	Q70E73
Avasorum	2	Q70IA6
Avasorum	2	Q70UQ0

Avasorum	2	Q70Z35
Avasorum	2	Q70Z53
Avasorum	2	Q712K3
Avasorum	2	Q71F56
Avasorum	2	Q71RC2
Avasorum	2	Q71SY5
Avasorum	2	Q71UM5
Avasorum	2	Q75N03
Avasorum	2	Q75QN2
Avasorum	2	Q7KYR7
Avasorum	2	Q7KZ85
Avasorum	2	Q7KZF4
Avasorum	2	Q7KZI7
Avasorum	2	Q7KZN9
Avasorum	2	Q7L014
Avasorum	2	Q7L099
Avasorum	2	Q7L0Y3
Avasorum	2	Q7L1Q6
Avasorum	2	Q7L1V2
Avasorum	2	Q7L1W4
Avasorum	2	Q7L266
Avasorum	2	Q7L273
Avasorum	2	Q7L2E3
Avasorum	2	Q7L2H7
Avasorum	2	Q7L2J0
Avasorum	2	Q7L311
Avasorum	2	Q7L3T8
Avasorum	2	Q7L4I2
Avasorum	2	Q7L523
Avasorum	2	Q7L576
Avasorum	2	Q7L592
Avasorum	2	Q7L5D6
Avasorum	2	Q7L5N1
Avasorum	2	Q7L5N7
Avasorum	2	Q7L5Y1
Avasorum	2	Q7L5Y9
Avasorum	2	Q7L775
Avasorum	2	Q7L7V1
Avasorum	2	Q7L7X3
Avasorum	2	Q7L8L6
Avasorum	2	Q7L9L4
Avasorum	2	Q7LBC6
Avasorum	2	Q7LBR1
Avasorum	2	Q7LG56
Avasorum	2	Q7LGA3
Avasorum	2	Q7RTN6
Avasorum	2	Q7RTP6
Avasorum	2	Q7RTS9
Avasorum	2	Q7RTV0
Avasorum	2	Q7RTV5

Avasorum	2	Q7Z2E3
Avasorum	2	Q7Z2K6
Avasorum	2	Q7Z2K8
Avasorum	2	Q7Z2T5
Avasorum	2	Q7Z2W4
Avasorum	2	Q7Z2W9
Avasorum	2	Q7Z2Z2
Avasorum	2	Q7Z333
Avasorum	2	Q7Z392
Avasorum	2	Q7Z3B3
Avasorum	2	Q7Z3B4
Avasorum	2	Q7Z3C6
Avasorum	2	Q7Z3D6
Avasorum	2	Q7Z3E2
Avasorum	2	Q7Z3E5
Avasorum	2	Q7Z3J2
Avasorum	2	Q7Z3K3
Avasorum	2	Q7Z3T8
Avasorum	2	Q7Z3U7
Avasorum	2	Q7Z401
Avasorum	2	Q7Z406
Avasorum	2	Q7Z417
Avasorum	2	Q7Z422
Avasorum	2	Q7Z434
Avasorum	2	Q7Z460
Avasorum	2	Q7Z478
Avasorum	2	Q7Z494
Avasorum	2	Q7Z4F1
Avasorum	2	Q7Z4H7
Avasorum	2	Q7Z4H8
Avasorum	2	Q7Z4L5
Avasorum	2	Q7Z4Q2
Avasorum	2	Q7Z4S6
Avasorum	2	Q7Z4V5
Avasorum	2	Q7Z4W1
Avasorum	2	Q7Z569
Avasorum	2	Q7Z589
Avasorum	2	Q7Z5G4
Avasorum	2	Q7Z5H3
Avasorum	2	Q7Z5K2
Avasorum	2	Q7Z5L9
Avasorum	2	Q7Z6E9
Avasorum	2	Q7Z6J0
Avasorum	2	Q7Z6J2
Avasorum	2	Q7Z6J6
Avasorum	2	Q7Z6J9
Avasorum	2	Q7Z6K3
Avasorum	2	Q7Z6K5
Avasorum	2	Q7Z6L1
Avasorum	2	Q7Z6M1

Avasorum	2	Q7Z6M4
Avasorum	2	Q7Z6Z7
Avasorum	2	Q7Z739
Avasorum	2	Q7Z7A3
Avasorum	2	Q7Z7C8
Avasorum	2	Q7Z7E8
Avasorum	2	Q7Z7F7
Avasorum	2	Q7Z7H5
Avasorum	2	Q7Z7H8
Avasorum	2	Q7Z7K0
Avasorum	2	Q7Z7K6
Avasorum	2	Q7Z7L1
Avasorum	2	Q7Z7M0
Avasorum	2	Q7Z7N9
Avasorum	2	Q86SF2
Avasorum	2	Q86SK9
Avasorum	2	Q86SQ0
Avasorum	2	Q86SQ4
Avasorum	2	Q86SQ9
Avasorum	2	Q86SR1
Avasorum	2	Q86SZ2
Avasorum	2	Q86T03
Avasorum	2	Q86T13
Avasorum	2	Q86TB9
Avasorum	2	Q86TI2
Avasorum	2	Q86TM6
Avasorum	2	Q86TN4
Avasorum	2	Q86TU7
Avasorum	2	Q86TV6
Avasorum	2	Q86TX2
Avasorum	2	Q86U38
Avasorum	2	Q86U42
Avasorum	2	Q86U44
Avasorum	2	Q86U86
Avasorum	2	Q86U90
Avasorum	2	Q86UA1
Avasorum	2	Q86UE4
Avasorum	2	Q86UK7
Avasorum	2	Q86UL3
Avasorum	2	Q86UP2
Avasorum	2	Q86US8
Avasorum	2	Q86UT6
Avasorum	2	Q86UU0
Avasorum	2	Q86UU1
Avasorum	2	Q86UV5
Avasorum	2	Q86UW7
Avasorum	2	Q86UX7
Avasorum	2	Q86UY6
Avasorum	2	Q86V21
Avasorum	2	Q86V48

Avasorum	2	Q86V81
Avasorum	2	Q86V85
Avasorum	2	Q86V87
Avasorum	2	Q86VI3
Avasorum	2	Q86VM9
Avasorum	2	Q86VN1
Avasorum	2	Q86VP6
Avasorum	2	Q86VR2
Avasorum	2	Q86VS8
Avasorum	2	Q86VW0
Avasorum	2	Q86VX2
Avasorum	2	Q86VX9
Avasorum	2	Q86W34
Avasorum	2	Q86W42
Avasorum	2	Q86W50
Avasorum	2	Q86W56
Avasorum	2	Q86W92
Avasorum	2	Q86WA8
Avasorum	2	Q86WB0
Avasorum	2	Q86WJ1
Avasorum	2	Q86WN1
Avasorum	2	Q86WR0
Avasorum	2	Q86WR7
Avasorum	2	Q86WV6
Avasorum	2	Q86WX3
Avasorum	2	Q86X02
Avasorum	2	Q86X10
Avasorum	2	Q86X27
Avasorum	2	Q86X55
Avasorum	2	Q86X76
Avasorum	2	Q86X83
Avasorum	2	Q86XA9
Avasorum	2	Q86XI2
Avasorum	2	Q86XI6
Avasorum	2	Q86XK2
Avasorum	2	Q86XL3
Avasorum	2	Q86XN8
Avasorum	2	Q86XP3
Avasorum	2	Q86XZ4
Avasorum	2	Q86Y07
Avasorum	2	Q86Y37
Avasorum	2	Q86Y39
Avasorum	2	Q86Y56
Avasorum	2	Q86Y79
Avasorum	2	Q86Y82
Avasorum	2	Q86YP4
Avasorum	2	Q86YQ8
Avasorum	2	Q86YR5
Avasorum	2	Q86YS6
Avasorum	2	Q86YS7

Avasorum	2	Q86YT6
Avasorum	2	Q86YV5
Avasorum	2	Q86YV9
Avasorum	2	Q8IU81
Avasorum	2	Q8IUD2
Avasorum	2	Q8IUF8
Avasorum	2	Q8IUH4
Avasorum	2	Q8IUH5
Avasorum	2	Q8IUI8
Avasorum	2	Q8IUR0
Avasorum	2	Q8IUR7
Avasorum	2	Q8IUW5
Avasorum	2	Q8IUX1
Avasorum	2	Q8IV08
Avasorum	2	Q8IV36
Avasorum	2	Q8IV38
Avasorum	2	Q8IV48
Avasorum	2	Q8IV63
Avasorum	2	Q8IVB5
Avasorum	2	Q8IVD9
Avasorum	2	Q8IVF2
Avasorum	2	Q8IVF7
Avasorum	2	Q8IVH4
Avasorum	2	Q8IVH8
Avasorum	2	Q8IVL0
Avasorum	2	Q8IVL6
Avasorum	2	Q8IVM0
Avasorum	2	Q8IVS2
Avasorum	2	Q8IVT5
Avasorum	2	Q8IW35
Avasorum	2	Q8IW45
Avasorum	2	Q8IWA0
Avasorum	2	Q8IWA4
Avasorum	2	Q8IWA5
Avasorum	2	Q8IWB1
Avasorum	2	Q8IWB7
Avasorum	2	Q8IWC1
Avasorum	2	Q8IWD4
Avasorum	2	Q8IWE2
Avasorum	2	Q8IWE4
Avasorum	2	Q8IWF6
Avasorum	2	Q8IWI9
Avasorum	2	Q8IWJ2
Avasorum	2	Q8IWR0
Avasorum	2	Q8IWS0
Avasorum	2	Q8IWT0
Avasorum	2	Q8IWT6
Avasorum	2	Q8IWU5
Avasorum	2	Q8IWV7
Avasorum	2	Q8IWV8

Avasorum	2	Q8IWW6
Avasorum	2	Q8IWX8
Avasorum	2	Q8IWY9
Avasorum	2	Q8IWZ3
Avasorum	2	Q8IWZ8
Avasorum	2	Q8IX01
Avasorum	2	Q8IX04
Avasorum	2	Q8IX12
Avasorum	2	Q8IX18
Avasorum	2	Q8IXB1
Avasorum	2	Q8IXH7
Avasorum	2	Q8IXI1
Avasorum	2	Q8IXI2
Avasorum	2	Q8IXJ6
Avasorum	2	Q8IXK0
Avasorum	2	Q8IXM2
Avasorum	2	Q8IXM3
Avasorum	2	Q8IXM6
Avasorum	2	Q8IXQ4
Avasorum	2	Q8IXQ6
Avasorum	2	Q8IXT5
Avasorum	2	Q8IXU6
Avasorum	2	Q8IY17
Avasorum	2	Q8IY18
Avasorum	2	Q8IY21
Avasorum	2	Q8IY22
Avasorum	2	Q8IY33
Avasorum	2	Q8IY37
Avasorum	2	Q8IY47
Avasorum	2	Q8IY67
Avasorum	2	Q8IY81
Avasorum	2	Q8IY95
Avasorum	2	Q8IYA6
Avasorum	2	Q8IYB1
Avasorum	2	Q8IYB3
Avasorum	2	Q8IYB5
Avasorum	2	Q8IYB7
Avasorum	2	Q8IYB8
Avasorum	2	Q8IYD1
Avasorum	2	Q8IYH5
Avasorum	2	Q8IYI6
Avasorum	2	Q8IYJ2
Avasorum	2	Q8IYM9
Avasorum	2	Q8IYQ7
Avasorum	2	Q8IYS2
Avasorum	2	Q8IYU8
Avasorum	2	Q8IZ07
Avasorum	2	Q8IZ21
Avasorum	2	Q8IZ69
Avasorum	2	Q8IZ73

Avasorum	2	Q8IZ81
Avasorum	2	Q8IZ83
Avasorum	2	Q8IZA0
Avasorum	2	Q8IZF2
Avasorum	2	Q8IZH2
Avasorum	2	Q8IZL8
Avasorum	2	Q8IZP0
Avasorum	2	Q8IZQ1
Avasorum	2	Q8IZQ5
Avasorum	2	Q8N0U8
Avasorum	2	Q8N0X7
Avasorum	2	Q8N0Z6
Avasorum	2	Q8N108
Avasorum	2	Q8N122
Avasorum	2	Q8N138
Avasorum	2	Q8N163
Avasorum	2	Q8N183
Avasorum	2	Q8N1B4
Avasorum	2	Q8N1F7
Avasorum	2	Q8N1F8
Avasorum	2	Q8N1G0
Avasorum	2	Q8N1G2
Avasorum	2	Q8N1G4
Avasorum	2	Q8N1I0
Avasorum	2	Q8N1Q1
Avasorum	2	Q8N1W1
Avasorum	2	Q8N201
Avasorum	2	Q8N257
Avasorum	2	Q8N264
Avasorum	2	Q8N2F6
Avasorum	2	Q8N2K0
Avasorum	2	Q8N2M8
Avasorum	2	Q8N2R8
Avasorum	2	Q8N2U0
Avasorum	2	Q8N2W9
Avasorum	2	Q8N335
Avasorum	2	Q8N357
Avasorum	2	Q8N392
Avasorum	2	Q8N3C0
Avasorum	2	Q8N3F8
Avasorum	2	Q8N3P4
Avasorum	2	Q8N3R9
Avasorum	2	Q8N3U4
Avasorum	2	Q8N3V7
Avasorum	2	Q8N3X1
Avasorum	2	Q8N442
Avasorum	2	Q8N488
Avasorum	2	Q8N4A0
Avasorum	2	Q8N4C8
Avasorum	2	Q8N4P3

Avasorum	2	Q8N4Q0
Avasorum	2	Q8N4T8
Avasorum	2	Q8N4V1
Avasorum	2	Q8N511
Avasorum	2	Q8N531
Avasorum	2	Q8N543
Avasorum	2	Q8N556
Avasorum	2	Q8N567
Avasorum	2	Q8N573
Avasorum	2	Q8N5A5
Avasorum	2	Q8N5C1
Avasorum	2	Q8N5C6
Avasorum	2	Q8N5D0
Avasorum	2	Q8N5F7
Avasorum	2	Q8N5G2
Avasorum	2	Q8N5H7
Avasorum	2	Q8N5I2
Avasorum	2	Q8N5I4
Avasorum	2	Q8N5K1
Avasorum	2	Q8N5L8
Avasorum	2	Q8N5M1
Avasorum	2	Q8N5M4
Avasorum	2	Q8N5M9
Avasorum	2	Q8N5U6
Avasorum	2	Q8N5W9
Avasorum	2	Q8N653
Avasorum	2	Q8N668
Avasorum	2	Q8N684
Avasorum	2	Q8N697
Avasorum	2	Q8N699
Avasorum	2	Q8N6G6
Avasorum	2	Q8N6H7
Avasorum	2	Q8N6M0
Avasorum	2	Q8N6M3
Avasorum	2	Q8N6N3
Avasorum	2	Q8N6R0
Avasorum	2	Q8N6S5
Avasorum	2	Q8N6T3
Avasorum	2	Q8N6T7
Avasorum	2	Q8N755
Avasorum	2	Q8N766
Avasorum	2	Q8N7H5
Avasorum	2	Q8N7R7
Avasorum	2	Q8N806
Avasorum	2	Q8N8A6
Avasorum	2	Q8N8J7
Avasorum	2	Q8N8R3
Avasorum	2	Q8N8S7
Avasorum	2	Q8N8Z6
Avasorum	2	Q8N954

Avasorum	2	Q8N983
Avasorum	2	Q8N999
Avasorum	2	Q8N9F7
Avasorum	2	Q8N9M1
Avasorum	2	Q8N9N2
Avasorum	2	Q8N9N7
Avasorum	2	Q8N9N8
Avasorum	2	Q8N9T8
Avasorum	2	Q8N9Z2
Avasorum	2	Q8NAF0
Avasorum	2	Q8NAV1
Avasorum	2	Q8NB16
Avasorum	2	Q8NB37
Avasorum	2	Q8NB46
Avasorum	2	Q8NB49
Avasorum	2	Q8NB90
Avasorum	2	Q8NBF2
Avasorum	2	Q8NBF6
Avasorum	2	Q8NBI5
Avasorum	2	Q8NBI6
Avasorum	2	Q8NBJ4
Avasorum	2	Q8NBJ5
Avasorum	2	Q8NBJ7
Avasorum	2	Q8NBJ9
Avasorum	2	Q8NBK3
Avasorum	2	Q8NBL1
Avasorum	2	Q8NBM4
Avasorum	2	Q8NBM8
Avasorum	2	Q8NBN3
Avasorum	2	Q8NBN7
Avasorum	2	Q8NBP0
Avasorum	2	Q8NBQ5
Avasorum	2	Q8NBS9
Avasorum	2	Q8NBT2
Avasorum	2	Q8NBU5
Avasorum	2	Q8NBX0
Avasorum	2	Q8NBZ7
Avasorum	2	Q8NC42
Avasorum	2	Q8NC44
Avasorum	2	Q8NC51
Avasorum	2	Q8NC56
Avasorum	2	Q8NC60
Avasorum	2	Q8NC96
Avasorum	2	Q8NCA5
Avasorum	2	Q8NCC3
Avasorum	2	Q8NCE2
Avasorum	2	Q8NCF5
Avasorum	2	Q8NCG7
Avasorum	2	Q8NCH0
Avasorum	2	Q8NCL4

Avasorum	2	Q8NCN4
Avasorum	2	Q8NCN5
Avasorum	2	Q8NCW5
Avasorum	2	Q8ND04
Avasorum	2	Q8ND24
Avasorum	2	Q8ND56
Avasorum	2	Q8ND71
Avasorum	2	Q8ND76
Avasorum	2	Q8NDA8
Avasorum	2	Q8NDF8
Avasorum	2	Q8NDH3
Avasorum	2	Q8NDI1
Avasorum	2	Q8NDT2
Avasorum	2	Q8NDV1
Avasorum	2	Q8NDV7
Avasorum	2	Q8NDX5
Avasorum	2	Q8NDZ4
Avasorum	2	Q8NE01
Avasorum	2	Q8NE71
Avasorum	2	Q8NE86
Avasorum	2	Q8NEB9
Avasorum	2	Q8NEC7
Avasorum	2	Q8NEF9
Avasorum	2	Q8NEJ9
Avasorum	2	Q8NEM2
Avasorum	2	Q8NEN9
Avasorum	2	Q8NEU8
Avasorum	2	Q8NEW0
Avasorum	2	Q8NEY1
Avasorum	2	Q8NEY8
Avasorum	2	Q8NEZ3
Avasorum	2	Q8NEZ5
Avasorum	2	Q8NF37
Avasorum	2	Q8NF64
Avasorum	2	Q8NF91
Avasorum	2	Q8NFA0
Avasorum	2	Q8NFC6
Avasorum	2	Q8NFD5
Avasorum	2	Q8NFF5
Avasorum	2	Q8NFG4
Avasorum	2	Q8NFH3
Avasorum	2	Q8NFH4
Avasorum	2	Q8NFH5
Avasorum	2	Q8NFI3
Avasorum	2	Q8NFJ5
Avasorum	2	Q8NFQ8
Avasorum	2	Q8NFV4
Avasorum	2	Q8NFW8
Avasorum	2	Q8NFZ0
Avasorum	2	Q8NG11

Avasorum	2	Q8NG68
Avasorum	2	Q8NHG7
Avasorum	2	Q8NHH9
Avasorum	2	Q8NHP6
Avasorum	2	Q8NHP8
Avasorum	2	Q8NHQ8
Avasorum	2	Q8NHQ9
Avasorum	2	Q8NHS3
Avasorum	2	Q8NHU6
Avasorum	2	Q8NHV1
Avasorum	2	Q8NHV4
Avasorum	2	Q8NI08
Avasorum	2	Q8NI27
Avasorum	2	Q8NI36
Avasorum	2	Q8NI37
Avasorum	2	Q8TA86
Avasorum	2	Q8TAA9
Avasorum	2	Q8TAD8
Avasorum	2	Q8TAE8
Avasorum	2	Q8TAF3
Avasorum	2	Q8TAG9
Avasorum	2	Q8TAQ2
Avasorum	2	Q8TAT6
Avasorum	2	Q8TB03
Avasorum	2	Q8TB22
Avasorum	2	Q8TB37
Avasorum	2	Q8TB40
Avasorum	2	Q8TB52
Avasorum	2	Q8TB61
Avasorum	2	Q8TB72
Avasorum	2	Q8TB96
Avasorum	2	Q8TBA6
Avasorum	2	Q8TBB5
Avasorum	2	Q8TBC3
Avasorum	2	Q8TBC4
Avasorum	2	Q8TBF2
Avasorum	2	Q8TBM8
Avasorum	2	Q8TBQ9
Avasorum	2	Q8TBX8
Avasorum	2	Q8TBZ3
Avasorum	2	Q8TC07
Avasorum	2	Q8TC12
Avasorum	2	Q8TCA0
Avasorum	2	Q8TCB0
Avasorum	2	Q8TCC3
Avasorum	2	Q8TCF1
Avasorum	2	Q8TCG1
Avasorum	2	Q8TCG2
Avasorum	2	Q8TCJ2
Avasorum	2	Q8TCS8

Avasorum	2	Q8TCT7
Avasorum	2	Q8TCT9
Avasorum	2	Q8TCU6
Avasorum	2	Q8TCY9
Avasorum	2	Q8TD16
Avasorum	2	Q8TD19
Avasorum	2	Q8TD55
Avasorum	2	Q8TDB6
Avasorum	2	Q8TDD1
Avasorum	2	Q8TDM6
Avasorum	2	Q8TDN6
Avasorum	2	Q8TDQ7
Avasorum	2	Q8TDW0
Avasorum	2	Q8TDX7
Avasorum	2	Q8TDY2
Avasorum	2	Q8TDZ2
Avasorum	2	Q8TE02
Avasorum	2	Q8TE77
Avasorum	2	Q8TE82
Avasorum	2	Q8TEA7
Avasorum	2	Q8TEA8
Avasorum	2	Q8TEB1
Avasorum	2	Q8TED0
Avasorum	2	Q8TED1
Avasorum	2	Q8TED9
Avasorum	2	Q8TEJ3
Avasorum	2	Q8TEL6
Avasorum	2	Q8TEP8
Avasorum	2	Q8TEQ6
Avasorum	2	Q8TEQ8
Avasorum	2	Q8TEU7
Avasorum	2	Q8TEW0
Avasorum	2	Q8TEX9
Avasorum	2	Q8TEY7
Avasorum	2	Q8TF01
Avasorum	2	Q8TF05
Avasorum	2	Q8TF42
Avasorum	2	Q8TF65
Avasorum	2	Q8TF74
Avasorum	2	Q8WTS1
Avasorum	2	Q8WTS6
Avasorum	2	Q8WTT2
Avasorum	2	Q8WTV0
Avasorum	2	Q8WTW3
Avasorum	2	Q8WU76
Avasorum	2	Q8WU79
Avasorum	2	Q8WU90
Avasorum	2	Q8WUA2
Avasorum	2	Q8WUA4
Avasorum	2	Q8WUA7

Avasorum	2	Q8WUB8
Avasorum	2	Q8WUD1
Avasorum	2	Q8WUD4
Avasorum	2	Q8WUF5
Avasorum	2	Q8WUF8
Avasorum	2	Q8WUH1
Avasorum	2	Q8WUH2
Avasorum	2	Q8WUH6
Avasorum	2	Q8WUI4
Avasorum	2	Q8WUJ0
Avasorum	2	Q8WUK0
Avasorum	2	Q8WUM0
Avasorum	2	Q8WUM4
Avasorum	2	Q8WUM9
Avasorum	2	Q8WUP2
Avasorum	2	Q8WUQ7
Avasorum	2	Q8WUX2
Avasorum	2	Q8WUX9
Avasorum	2	Q8WUY1
Avasorum	2	Q8WUY8
Avasorum	2	Q8WUZ0
Avasorum	2	Q8WV22
Avasorum	2	Q8WV92
Avasorum	2	Q8WVB6
Avasorum	2	Q8WVC0
Avasorum	2	Q8WVC6
Avasorum	2	Q8WVJ2
Avasorum	2	Q8WVK2
Avasorum	2	Q8WVM0
Avasorum	2	Q8WVM7
Avasorum	2	Q8WVM8
Avasorum	2	Q8WVP5
Avasorum	2	Q8WVQ1
Avasorum	2	Q8WVT3
Avasorum	2	Q8WVV9
Avasorum	2	Q8WVX9
Avasorum	2	Q8WVY7
Avasorum	2	Q8WW01
Avasorum	2	Q8WW12
Avasorum	2	Q8WW22
Avasorum	2	Q8WW59
Avasorum	2	Q8WWC4
Avasorum	2	Q8WWH5
Avasorum	2	Q8WWI1
Avasorum	2	Q8WWI5
Avasorum	2	Q8WWK9
Avasorum	2	Q8WWM7
Avasorum	2	Q8WWN8
Avasorum	2	Q8WWP7
Avasorum	2	Q8WWQ0

Avasorum	2	Q8WWY3
Avasorum	2	Q8WX92
Avasorum	2	Q8WX93
Avasorum	2	Q8WXA3
Avasorum	2	Q8WXA9
Avasorum	2	Q8WXD5
Avasorum	2	Q8WXE0
Avasorum	2	Q8WXF1
Avasorum	2	Q8WXG6
Avasorum	2	Q8WXH0
Avasorum	2	Q8WXI9
Avasorum	2	Q8WXX5
Avasorum	2	Q8WY22
Avasorum	2	Q8WYA6
Avasorum	2	Q8WYN0
Avasorum	2	Q8WYP3
Avasorum	2	Q8WYP5
Avasorum	2	Q8WYQ5
Avasorum	2	Q8WZ42
Avasorum	2	Q8WZ75
Avasorum	2	Q8WZ82
Avasorum	2	Q8WZA9
Avasorum	2	Q92466
Avasorum	2	Q92478
Avasorum	2	Q92499
Avasorum	2	Q92503
Avasorum	2	Q92508
Avasorum	2	Q92520
Avasorum	2	Q92522
Avasorum	2	Q92530
Avasorum	2	Q92538
Avasorum	2	Q92540
Avasorum	2	Q92541
Avasorum	2	Q92542
Avasorum	2	Q92543
Avasorum	2	Q92544
Avasorum	2	Q92545
Avasorum	2	Q92551
Avasorum	2	Q92552
Avasorum	2	Q92556
Avasorum	2	Q92562
Avasorum	2	Q92567
Avasorum	2	Q92572
Avasorum	2	Q92574
Avasorum	2	Q92575
Avasorum	2	Q92576
Avasorum	2	Q92597
Avasorum	2	Q92598
Avasorum	2	Q92599
Avasorum	2	Q92600

Avasorum	2	Q92604
Avasorum	2	Q92609
Avasorum	2	Q92610
Avasorum	2	Q92613
Avasorum	2	Q92614
Avasorum	2	Q92615
Avasorum	2	Q92616
Avasorum	2	Q92619
Avasorum	2	Q92620
Avasorum	2	Q92621
Avasorum	2	Q92625
Avasorum	2	Q92626
Avasorum	2	Q92636
Avasorum	2	Q92643
Avasorum	2	Q92665
Avasorum	2	Q92667
Avasorum	2	Q92685
Avasorum	2	Q92686
Avasorum	2	Q92688
Avasorum	2	Q92692
Avasorum	2	Q92696
Avasorum	2	Q92734
Avasorum	2	Q92743
Avasorum	2	Q92747
Avasorum	2	Q92759
Avasorum	2	Q92766
Avasorum	2	Q92769
Avasorum	2	Q92783
Avasorum	2	Q92785
Avasorum	2	Q92791
Avasorum	2	Q92793
Avasorum	2	Q92794
Avasorum	2	Q92797
Avasorum	2	Q92804
Avasorum	2	Q92805
Avasorum	2	Q92820
Avasorum	2	Q92823
Avasorum	2	Q92834
Avasorum	2	Q92835
Avasorum	2	Q92841
Avasorum	2	Q92845
Avasorum	2	Q92851
Avasorum	2	Q92870
Avasorum	2	Q92878
Avasorum	2	Q92879
Avasorum	2	Q92882
Avasorum	2	Q92888
Avasorum	2	Q92889
Avasorum	2	Q92890
Avasorum	2	Q92896

Avasorum	2	Q92900
Avasorum	2	Q92905
Avasorum	2	Q92917
Avasorum	2	Q92922
Avasorum	2	Q92925
Avasorum	2	Q92930
Avasorum	2	Q92934
Avasorum	2	Q92945
Avasorum	2	Q92947
Avasorum	2	Q92963
Avasorum	2	Q92968
Avasorum	2	Q92973
Avasorum	2	Q92974
Avasorum	2	Q92979
Avasorum	2	Q92989
Avasorum	2	Q92990
Avasorum	2	Q92995
Avasorum	2	Q92997
Avasorum	2	Q93008
Avasorum	2	Q93009
Avasorum	2	Q93034
Avasorum	2	Q93045
Avasorum	2	Q93050
Avasorum	2	Q93052
Avasorum	2	Q93062
Avasorum	2	Q93063
Avasorum	2	Q93074
Avasorum	2	Q93096
Avasorum	2	Q93100
Avasorum	2	Q969E2
Avasorum	2	Q969F9
Avasorum	2	Q969G3
Avasorum	2	Q969G5
Avasorum	2	Q969G6
Avasorum	2	Q969H8
Avasorum	2	Q969M1
Avasorum	2	Q969M3
Avasorum	2	Q969N2
Avasorum	2	Q969P0
Avasorum	2	Q969Q0
Avasorum	2	Q969Q5
Avasorum	2	Q969R2
Avasorum	2	Q969S3
Avasorum	2	Q969S9
Avasorum	2	Q969T4
Avasorum	2	Q969T7
Avasorum	2	Q969T9
Avasorum	2	Q969U7
Avasorum	2	Q969V3
Avasorum	2	Q969V5

Avasorum	2	Q969V6
Avasorum	2	Q969X1
Avasorum	2	Q969X5
Avasorum	2	Q969X6
Avasorum	2	Q969Z0
Avasorum	2	Q969Z3
Avasorum	2	Q96A26
Avasorum	2	Q96A33
Avasorum	2	Q96A35
Avasorum	2	Q96A65
Avasorum	2	Q96A72
Avasorum	2	Q96A73
Avasorum	2	Q96AA3
Avasorum	2	Q96AB3
Avasorum	2	Q96AB6
Avasorum	2	Q96AC1
Avasorum	2	Q96AE4
Avasorum	2	Q96AE7
Avasorum	2	Q96AG4
Avasorum	2	Q96AJ9
Avasorum	2	Q96AP7
Avasorum	2	Q96AQ6
Avasorum	2	Q96AQ8
Avasorum	2	Q96AT1
Avasorum	2	Q96AX1
Avasorum	2	Q96AY3
Avasorum	2	Q96AY4
Avasorum	2	Q96B26
Avasorum	2	Q96B54
Avasorum	2	Q96B97
Avasorum	2	Q96BD8
Avasorum	2	Q96BH1
Avasorum	2	Q96BJ3
Avasorum	2	Q96BK5
Avasorum	2	Q96BM9
Avasorum	2	Q96BN8
Avasorum	2	Q96BP2
Avasorum	2	Q96BP3
Avasorum	2	Q96BQ5
Avasorum	2	Q96BW1
Avasorum	2	Q96BW9
Avasorum	2	Q96BX8
Avasorum	2	Q96BY6
Avasorum	2	Q96BY7
Avasorum	2	Q96BZ8
Avasorum	2	Q96BZ9
Avasorum	2	Q96C19
Avasorum	2	Q96C23
Avasorum	2	Q96C24
Avasorum	2	Q96C36

Avasorum	2	Q96C57
Avasorum	2	Q96C86
Avasorum	2	Q96C90
Avasorum	2	Q96CB8
Avasorum	2	Q96CB9
Avasorum	2	Q96CC6
Avasorum	2	Q96CG8
Avasorum	2	Q96CM8
Avasorum	2	Q96CN7
Avasorum	2	Q96CN9
Avasorum	2	Q96CP2
Avasorum	2	Q96CP6
Avasorum	2	Q96CS2
Avasorum	2	Q96CS3
Avasorum	2	Q96CT7
Avasorum	2	Q96CU9
Avasorum	2	Q96CV9
Avasorum	2	Q96CW1
Avasorum	2	Q96CW5
Avasorum	2	Q96CW6
Avasorum	2	Q96CX2
Avasorum	2	Q96D15
Avasorum	2	Q96D46
Avasorum	2	Q96D71
Avasorum	2	Q96DA6
Avasorum	2	Q96DB5
Avasorum	2	Q96DE0
Avasorum	2	Q96DF8
Avasorum	2	Q96DG6
Avasorum	2	Q96DH6
Avasorum	2	Q96DI7
Avasorum	2	Q96DM3
Avasorum	2	Q96DV4
Avasorum	2	Q96DX4
Avasorum	2	Q96DZ1
Avasorum	2	Q96E11
Avasorum	2	Q96EA4
Avasorum	2	Q96EB1
Avasorum	2	Q96EB6
Avasorum	2	Q96EC8
Avasorum	2	Q96EE3
Avasorum	2	Q96EI5
Avasorum	2	Q96EK5
Avasorum	2	Q96EK6
Avasorum	2	Q96EK7
Avasorum	2	Q96EK9
Avasorum	2	Q96EL2
Avasorum	2	Q96EL3
Avasorum	2	Q96EM0
Avasorum	2	Q96EP0

Avasorum	2	Q96EP5
Avasorum	2	Q96EQ0
Avasorum	2	Q96ER3
Avasorum	2	Q96ER9
Avasorum	2	Q96ES7
Avasorum	2	Q96EU6
Avasorum	2	Q96EU7
Avasorum	2	Q96EV2
Avasorum	2	Q96EV8
Avasorum	2	Q96EY1
Avasorum	2	Q96EY4
Avasorum	2	Q96EY5
Avasorum	2	Q96EY7
Avasorum	2	Q96EY8
Avasorum	2	Q96F07
Avasorum	2	Q96F15
Avasorum	2	Q96F44
Avasorum	2	Q96F63
Avasorum	2	Q96F85
Avasorum	2	Q96F86
Avasorum	2	Q96FJ2
Avasorum	2	Q96FN4
Avasorum	2	Q96FQ6
Avasorum	2	Q96FS4
Avasorum	2	Q96FV2
Avasorum	2	Q96FV9
Avasorum	2	Q96FW1
Avasorum	2	Q96FX7
Avasorum	2	Q96FZ2
Avasorum	2	Q96FZ7
Avasorum	2	Q96G01
Avasorum	2	Q96G03
Avasorum	2	Q96G21
Avasorum	2	Q96G23
Avasorum	2	Q96G46
Avasorum	2	Q96GA3
Avasorum	2	Q96GA7
Avasorum	2	Q96GC5
Avasorum	2	Q96GD0
Avasorum	2	Q96GD4
Avasorum	2	Q96GG9
Avasorum	2	Q96GK7
Avasorum	2	Q96GM5
Avasorum	2	Q96GM8
Avasorum	2	Q96GN5
Avasorum	2	Q96GQ5
Avasorum	2	Q96GQ7
Avasorum	2	Q96GS4
Avasorum	2	Q96GW9
Avasorum	2	Q96GX2

Avasorum	2	Q96GX9
Avasorum	2	Q96GY0
Avasorum	2	Q96GY3
Avasorum	2	Q96GZ6
Avasorum	2	Q96H20
Avasorum	2	Q96H79
Avasorum	2	Q96HA1
Avasorum	2	Q96HA7
Avasorum	2	Q96HC4
Avasorum	2	Q96HD1
Avasorum	2	Q96HE7
Avasorum	2	Q96HE9
Avasorum	2	Q96HJ9
Avasorum	2	Q96HN2
Avasorum	2	Q96HP0
Avasorum	2	Q96HR3
Avasorum	2	Q96HS1
Avasorum	2	Q96HW7
Avasorum	2	Q96HY6
Avasorum	2	Q96HY7
Avasorum	2	Q96I15
Avasorum	2	Q96I24
Avasorum	2	Q96I25
Avasorum	2	Q96I51
Avasorum	2	Q96I59
Avasorum	2	Q96I99
Avasorum	2	Q96II8
Avasorum	2	Q96IJ6
Avasorum	2	Q96IR7
Avasorum	2	Q96IU4
Avasorum	2	Q96IV0
Avasorum	2	Q96IW7
Avasorum	2	Q96IX5
Avasorum	2	Q96IY1
Avasorum	2	Q96IZ0
Avasorum	2	Q96IZ6
Avasorum	2	Q96J01
Avasorum	2	Q96J02
Avasorum	2	Q96J42
Avasorum	2	Q96J84
Avasorum	2	Q96JA1
Avasorum	2	Q96JB2
Avasorum	2	Q96JB5
Avasorum	2	Q96JC1
Avasorum	2	Q96JG6
Avasorum	2	Q96JH7
Avasorum	2	Q96JI7
Avasorum	2	Q96JJ3
Avasorum	2	Q96JJ7
Avasorum	2	Q96JK2

Avasorum	2	Q96JM3
Avasorum	2	Q96JM7
Avasorum	2	Q96JP5
Avasorum	2	Q96JQ0
Avasorum	2	Q96JQ2
Avasorum	2	Q96JY6
Avasorum	2	Q96K12
Avasorum	2	Q96K17
Avasorum	2	Q96K19
Avasorum	2	Q96K21
Avasorum	2	Q96K37
Avasorum	2	Q96K76
Avasorum	2	Q96K83
Avasorum	2	Q96KA5
Avasorum	2	Q96KB5
Avasorum	2	Q96KC2
Avasorum	2	Q96KC8
Avasorum	2	Q96KG9
Avasorum	2	Q96KM6
Avasorum	2	Q96KN1
Avasorum	2	Q96KP1
Avasorum	2	Q96KP4
Avasorum	2	Q96KQ7
Avasorum	2	Q96KR1
Avasorum	2	Q96KR6
Avasorum	2	Q96L58
Avasorum	2	Q96L91
Avasorum	2	Q96L92
Avasorum	2	Q96L93
Avasorum	2	Q96LB3
Avasorum	2	Q96LD4
Avasorum	2	Q96LI5
Avasorum	2	Q96LJ7
Avasorum	2	Q96LL9
Avasorum	2	Q96LR5
Avasorum	2	Q96LW7
Avasorum	2	Q96M27
Avasorum	2	Q96M96
Avasorum	2	Q96ME1
Avasorum	2	Q96ME7
Avasorum	2	Q96MF7
Avasorum	2	Q96MG7
Avasorum	2	Q96MM6
Avasorum	2	Q96MU7
Avasorum	2	Q96MW1
Avasorum	2	Q96MW5
Avasorum	2	Q96MX0
Avasorum	2	Q96MX6
Avasorum	2	Q96MY1
Avasorum	2	Q96N66

Avasorum	2	Q96N67
Avasorum	2	Q96NB2
Avasorum	2	Q96NB3
Avasorum	2	Q96ND0
Avasorum	2	Q96NE9
Avasorum	2	Q96P11
Avasorum	2	Q96P16
Avasorum	2	Q96P47
Avasorum	2	Q96P48
Avasorum	2	Q96P70
Avasorum	2	Q96PC5
Avasorum	2	Q96PD2
Avasorum	2	Q96PE2
Avasorum	2	Q96PE3
Avasorum	2	Q96PK6
Avasorum	2	Q96PM5
Avasorum	2	Q96PQ7
Avasorum	2	Q96PU4
Avasorum	2	Q96PU5
Avasorum	2	Q96PU8
Avasorum	2	Q96PV6
Avasorum	2	Q96PY5
Avasorum	2	Q96PZ0
Avasorum	2	Q96PZ2
Avasorum	2	Q96Q05
Avasorum	2	Q96Q11
Avasorum	2	Q96Q15
Avasorum	2	Q96Q42
Avasorum	2	Q96Q45
Avasorum	2	Q96Q83
Avasorum	2	Q96QB1
Avasorum	2	Q96QC0
Avasorum	2	Q96QD8
Avasorum	2	Q96QD9
Avasorum	2	Q96QG7
Avasorum	2	Q96QK1
Avasorum	2	Q96QR8
Avasorum	2	Q96QU8
Avasorum	2	Q96QV1
Avasorum	2	Q96QZ7
Avasorum	2	Q96R06
Avasorum	2	Q96RE7
Avasorum	2	Q96RF0
Avasorum	2	Q96RG2
Avasorum	2	Q96RK0
Avasorum	2	Q96RL1
Avasorum	2	Q96RL7
Avasorum	2	Q96RN5
Avasorum	2	Q96RP9
Avasorum	2	Q96RQ1

Avasorum	2	Q96RQ3
Avasorum	2	Q96RR4
Avasorum	2	Q96RS0
Avasorum	2	Q96RS6
Avasorum	2	Q96RT1
Avasorum	2	Q96RT7
Avasorum	2	Q96RT8
Avasorum	2	Q96RU2
Avasorum	2	Q96RU3
Avasorum	2	Q96S44
Avasorum	2	Q96S52
Avasorum	2	Q96S55
Avasorum	2	Q96S59
Avasorum	2	Q96S66
Avasorum	2	Q96S97
Avasorum	2	Q96SB3
Avasorum	2	Q96SB4
Avasorum	2	Q96SB8
Avasorum	2	Q96SI1
Avasorum	2	Q96SI9
Avasorum	2	Q96SK2
Avasorum	2	Q96SL4
Avasorum	2	Q96SN8
Avasorum	2	Q96SQ9
Avasorum	2	Q96ST2
Avasorum	2	Q96ST3
Avasorum	2	Q96SU4
Avasorum	2	Q96SW2
Avasorum	2	Q96SY0
Avasorum	2	Q96SZ5
Avasorum	2	Q96SZ6
Avasorum	2	Q96T23
Avasorum	2	Q96T37
Avasorum	2	Q96T49
Avasorum	2	Q96T51
Avasorum	2	Q96T58
Avasorum	2	Q96T60
Avasorum	2	Q96T76
Avasorum	2	Q96T88
Avasorum	2	Q96TA1
Avasorum	2	Q96TA2
Avasorum	2	Q96TC7
Avasorum	2	Q99081
Avasorum	2	Q99418
Avasorum	2	Q99426
Avasorum	2	Q99436
Avasorum	2	Q99439
Avasorum	2	Q99442
Avasorum	2	Q99447
Avasorum	2	Q99459

Avasorum	2	Q99460
Avasorum	2	Q99470
Avasorum	2	Q99471
Avasorum	2	Q99496
Avasorum	2	Q99497
Avasorum	2	Q99500
Avasorum	2	Q99501
Avasorum	2	Q99504
Avasorum	2	Q99519
Avasorum	2	Q99523
Avasorum	2	Q99536
Avasorum	2	Q99538
Avasorum	2	Q99541
Avasorum	2	Q99543
Avasorum	2	Q99549
Avasorum	2	Q99567
Avasorum	2	Q99569
Avasorum	2	Q99570
Avasorum	2	Q99571
Avasorum	2	Q99575
Avasorum	2	Q99584
Avasorum	2	Q99590
Avasorum	2	Q99598
Avasorum	2	Q99611
Avasorum	2	Q99613
Avasorum	2	Q99614
Avasorum	2	Q99615
Avasorum	2	Q99623
Avasorum	2	Q99627
Avasorum	2	Q99633
Avasorum	2	Q99653
Avasorum	2	Q99661
Avasorum	2	Q99666
Avasorum	2	Q99685
Avasorum	2	Q99700
Avasorum	2	Q99704
Avasorum	2	Q99707
Avasorum	2	Q99714
Avasorum	2	Q99715
Avasorum	2	Q99717
Avasorum	2	Q99720
Avasorum	2	Q99729
Avasorum	2	Q99733
Avasorum	2	Q99735
Avasorum	2	Q99747
Avasorum	2	Q99755
Avasorum	2	Q99758
Avasorum	2	Q99759
Avasorum	2	Q99766
Avasorum	2	Q99797

Avasorum	2	Q99798
Avasorum	2	Q99805
Avasorum	2	Q99808
Avasorum	2	Q99816
Avasorum	2	Q99829
Avasorum	2	Q99832
Avasorum	2	Q99836
Avasorum	2	Q99848
Avasorum	2	Q99873
Avasorum	2	Q99933
Avasorum	2	Q99941
Avasorum	2	Q99961
Avasorum	2	Q99973
Avasorum	2	Q99986
Avasorum	2	Q99988
Avasorum	2	Q99996
Avasorum	2	Q9BPW8
Avasorum	2	Q9BPX3
Avasorum	2	Q9BPX5
Avasorum	2	Q9BPX6
Avasorum	2	Q9BPX7
Avasorum	2	Q9BPY3
Avasorum	2	Q9BPZ7
Avasorum	2	Q9BQ04
Avasorum	2	Q9BQ24
Avasorum	2	Q9BQ39
Avasorum	2	Q9BQ48
Avasorum	2	Q9BQ52
Avasorum	2	Q9BQ61
Avasorum	2	Q9BQ67
Avasorum	2	Q9BQ69
Avasorum	2	Q9BQ70
Avasorum	2	Q9BQ75
Avasorum	2	Q9BQ90
Avasorum	2	Q9BQ95
Avasorum	2	Q9BQA1
Avasorum	2	Q9BQA9
Avasorum	2	Q9BQB6
Avasorum	2	Q9BQC3
Avasorum	2	Q9BQE3
Avasorum	2	Q9BQE4
Avasorum	2	Q9BQE5
Avasorum	2	Q9BQG0
Avasorum	2	Q9BQL6
Avasorum	2	Q9BQP7
Avasorum	2	Q9BQQ3
Avasorum	2	Q9BQS8
Avasorum	2	Q9BR61
Avasorum	2	Q9BR76
Avasorum	2	Q9BRA2

Avasorum	2	Q9BRD0
Avasorum	2	Q9BRF8
Avasorum	2	Q9BRG1
Avasorum	2	Q9BRJ2
Avasorum	2	Q9BRJ6
Avasorum	2	Q9BRJ7
Avasorum	2	Q9BRK5
Avasorum	2	Q9BRP1
Avasorum	2	Q9BRP4
Avasorum	2	Q9BRP8
Avasorum	2	Q9BRQ6
Avasorum	2	Q9BRR6
Avasorum	2	Q9BRR8
Avasorum	2	Q9BRS2
Avasorum	2	Q9BRT6
Avasorum	2	Q9BRT9
Avasorum	2	Q9BRU9
Avasorum	2	Q9BRX2
Avasorum	2	Q9BRX8
Avasorum	2	Q9BRZ2
Avasorum	2	Q9BS26
Avasorum	2	Q9BS40
Avasorum	2	Q9BSC4
Avasorum	2	Q9BSD7
Avasorum	2	Q9BSF4
Avasorum	2	Q9BSH4
Avasorum	2	Q9BSJ2
Avasorum	2	Q9BSJ8
Avasorum	2	Q9BSL1
Avasorum	2	Q9BSR8
Avasorum	2	Q9BSU1
Avasorum	2	Q9BSV6
Avasorum	2	Q9BT09
Avasorum	2	Q9BT17
Avasorum	2	Q9BT22
Avasorum	2	Q9BT23
Avasorum	2	Q9BT25
Avasorum	2	Q9BT40
Avasorum	2	Q9BT73
Avasorum	2	Q9BT78
Avasorum	2	Q9BT88
Avasorum	2	Q9BTA9
Avasorum	2	Q9BTC0
Avasorum	2	Q9BTC8
Avasorum	2	Q9BTD8
Avasorum	2	Q9BTE1
Avasorum	2	Q9BTE3
Avasorum	2	Q9BTE6
Avasorum	2	Q9BTE7
Avasorum	2	Q9BTT0

Avasorum	2	Q9BTT6
Avasorum	2	Q9BTU6
Avasorum	2	Q9BTV4
Avasorum	2	Q9BTV5
Avasorum	2	Q9BTW9
Avasorum	2	Q9BTX1
Avasorum	2	Q9BTY7
Avasorum	2	Q9BTZ2
Avasorum	2	Q9BU23
Avasorum	2	Q9BU61
Avasorum	2	Q9BU76
Avasorum	2	Q9BUB7
Avasorum	2	Q9BUE0
Avasorum	2	Q9BUE6
Avasorum	2	Q9BUF5
Avasorum	2	Q9BUH6
Avasorum	2	Q9BUI4
Avasorum	2	Q9BUJ2
Avasorum	2	Q9BUK6
Avasorum	2	Q9BUL5
Avasorum	2	Q9BUL8
Avasorum	2	Q9BUL9
Avasorum	2	Q9BUN8
Avasorum	2	Q9BUP3
Avasorum	2	Q9BUQ8
Avasorum	2	Q9BUR4
Avasorum	2	Q9BUR5
Avasorum	2	Q9BUT1
Avasorum	2	Q9BV20
Avasorum	2	Q9BV38
Avasorum	2	Q9BV40
Avasorum	2	Q9BV44
Avasorum	2	Q9BV57
Avasorum	2	Q9BV79
Avasorum	2	Q9BV81
Avasorum	2	Q9BV86
Avasorum	2	Q9BVA0
Avasorum	2	Q9BVC4
Avasorum	2	Q9BVC5
Avasorum	2	Q9BVC6
Avasorum	2	Q9BVG4
Avasorum	2	Q9BVG9
Avasorum	2	Q9BVI4
Avasorum	2	Q9BVJ6
Avasorum	2	Q9BVJ7
Avasorum	2	Q9BVK6
Avasorum	2	Q9BVK8
Avasorum	2	Q9BVL2
Avasorum	2	Q9BVL4
Avasorum	2	Q9BVM2

Avasorum	2	Q9BVP2
Avasorum	2	Q9BVQ7
Avasorum	2	Q9BVS4
Avasorum	2	Q9BVS5
Avasorum	2	Q9BVT8
Avasorum	2	Q9BVV7
Avasorum	2	Q9BW19
Avasorum	2	Q9BW27
Avasorum	2	Q9BW60
Avasorum	2	Q9BW62
Avasorum	2	Q9BW71
Avasorum	2	Q9BW83
Avasorum	2	Q9BW85
Avasorum	2	Q9BW91
Avasorum	2	Q9BW92
Avasorum	2	Q9BWD1
Avasorum	2	Q9BWE0
Avasorum	2	Q9BWF3
Avasorum	2	Q9BWH2
Avasorum	2	Q9BWH6
Avasorum	2	Q9BWJ5
Avasorum	2	Q9BWM7
Avasorum	2	Q9BWS9
Avasorum	2	Q9BWT3
Avasorum	2	Q9BWT7
Avasorum	2	Q9BWU0
Avasorum	2	Q9BWU1
Avasorum	2	Q9BX40
Avasorum	2	Q9BX59
Avasorum	2	Q9BX67
Avasorum	2	Q9BX69
Avasorum	2	Q9BX70
Avasorum	2	Q9BXB4
Avasorum	2	Q9BXB5
Avasorum	2	Q9BXF6
Avasorum	2	Q9BXI6
Avasorum	2	Q9BXJ0
Avasorum	2	Q9BXJ9
Avasorum	2	Q9BXK5
Avasorum	2	Q9BXL7
Avasorum	2	Q9BXP2
Avasorum	2	Q9BXP5
Avasorum	2	Q9BXR0
Avasorum	2	Q9BXS5
Avasorum	2	Q9BXS6
Avasorum	2	Q9BXW7
Avasorum	2	Q9BXW9
Avasorum	2	Q9BXY0
Avasorum	2	Q9BY41
Avasorum	2	Q9BY42

Avasorum	2	Q9BY43
Avasorum	2	Q9BY44
Avasorum	2	Q9BY77
Avasorum	2	Q9BY89
Avasorum	2	Q9BYB0
Avasorum	2	Q9BYB4
Avasorum	2	Q9BYC5
Avasorum	2	Q9BYC8
Avasorum	2	Q9BYC9
Avasorum	2	Q9BYD1
Avasorum	2	Q9BYD2
Avasorum	2	Q9BYD3
Avasorum	2	Q9BYD6
Avasorum	2	Q9BYG3
Avasorum	2	Q9BYI3
Avasorum	2	Q9BYJ9
Avasorum	2	Q9BYK8
Avasorum	2	Q9BYM8
Avasorum	2	Q9BYN8
Avasorum	2	Q9BYT8
Avasorum	2	Q9BYV8
Avasorum	2	Q9BYW2
Avasorum	2	Q9BZ23
Avasorum	2	Q9BZ29
Avasorum	2	Q9BZ67
Avasorum	2	Q9BZ76
Avasorum	2	Q9BZ95
Avasorum	2	Q9BZD4
Avasorum	2	Q9BZE1
Avasorum	2	Q9BZE4
Avasorum	2	Q9BZE9
Avasorum	2	Q9BZF1
Avasorum	2	Q9BZF9
Avasorum	2	Q9BZG1
Avasorum	2	Q9BZG8
Avasorum	2	Q9BZH6
Avasorum	2	Q9BZI7
Avasorum	2	Q9BZJ0
Avasorum	2	Q9BZK7
Avasorum	2	Q9BZL1
Avasorum	2	Q9BZL4
Avasorum	2	Q9BZL6
Avasorum	2	Q9BZQ6
Avasorum	2	Q9BZQ8
Avasorum	2	Q9BZV1
Avasorum	2	Q9BZW5
Avasorum	2	Q9BZX2
Avasorum	2	Q9BZZ2
Avasorum	2	Q9BZZ5
Avasorum	2	Q9C000

Avasorum	2	Q9C035
Avasorum	2	Q9C037
Avasorum	2	Q9C0B1
Avasorum	2	Q9C0B5
Avasorum	2	Q9C0B7
Avasorum	2	Q9C0C2
Avasorum	2	Q9C0C9
Avasorum	2	Q9C0D3
Avasorum	2	Q9C0D5
Avasorum	2	Q9C0D9
Avasorum	2	Q9C0E2
Avasorum	2	Q9C0E8
Avasorum	2	Q9C0F1
Avasorum	2	Q9C0H2
Avasorum	2	Q9C0H6
Avasorum	2	Q9C0I1
Avasorum	2	Q9C0J8
Avasorum	2	Q9GZL7
Avasorum	2	Q9GZM5
Avasorum	2	Q9GZM7
Avasorum	2	Q9GZN8
Avasorum	2	Q9GZP4
Avasorum	2	Q9GZP9
Avasorum	2	Q9GZQ3
Avasorum	2	Q9GZR1
Avasorum	2	Q9GZR2
Avasorum	2	Q9GZR7
Avasorum	2	Q9GZS1
Avasorum	2	Q9GZS3
Avasorum	2	Q9GZT3
Avasorum	2	Q9GZT4
Avasorum	2	Q9GZT8
Avasorum	2	Q9GZT9
Avasorum	2	Q9GZU1
Avasorum	2	Q9GZU8
Avasorum	2	Q9GZY8
Avasorum	2	Q9GZZ1
Avasorum	2	Q9GZZ9
Avasorum	2	Q9H000
Avasorum	2	Q9H061
Avasorum	2	Q9H074
Avasorum	2	Q9H078
Avasorum	2	Q9H082
Avasorum	2	Q9H089
Avasorum	2	Q9H098
Avasorum	2	Q9H0A0
Avasorum	2	Q9H0A8
Avasorum	2	Q9H0B6
Avasorum	2	Q9H0C8
Avasorum	2	Q9H0D6

Avasorum	2	Q9H0E2
Avasorum	2	Q9H0E3
Avasorum	2	Q9H0G5
Avasorum	2	Q9H0H0
Avasorum	2	Q9H0H5
Avasorum	2	Q9H0J9
Avasorum	2	Q9H0L4
Avasorum	2	Q9H0P0
Avasorum	2	Q9H0Q0
Avasorum	2	Q9H0R3
Avasorum	2	Q9H0R4
Avasorum	2	Q9H0R6
Avasorum	2	Q9H0S4
Avasorum	2	Q9H0U3
Avasorum	2	Q9H0U4
Avasorum	2	Q9H0U6
Avasorum	2	Q9H0U9
Avasorum	2	Q9H0V1
Avasorum	2	Q9H0V9
Avasorum	2	Q9H0W8
Avasorum	2	Q9H0X4
Avasorum	2	Q9H0X9
Avasorum	2	Q9H147
Avasorum	2	Q9H173
Avasorum	2	Q9H1A3
Avasorum	2	Q9H1A4
Avasorum	2	Q9H1B7
Avasorum	2	Q9H1C7
Avasorum	2	Q9H1D9
Avasorum	2	Q9H1E3
Avasorum	2	Q9H1E5
Avasorum	2	Q9H1H9
Avasorum	2	Q9H1I8
Avasorum	2	Q9H1K0
Avasorum	2	Q9H1K1
Avasorum	2	Q9H1P3
Avasorum	2	Q9H1Y0
Avasorum	2	Q9H1Z4
Avasorum	2	Q9H201
Avasorum	2	Q9H223
Avasorum	2	Q9H267
Avasorum	2	Q9H269
Avasorum	2	Q9H270
Avasorum	2	Q9H299
Avasorum	2	Q9H2C0
Avasorum	2	Q9H2D1
Avasorum	2	Q9H2D6
Avasorum	2	Q9H2F5
Avasorum	2	Q9H2G2
Avasorum	2	Q9H2H8

Avasorum	2	Q9H2H9
Avasorum	2	Q9H2J4
Avasorum	2	Q9H2K8
Avasorum	2	Q9H2M9
Avasorum	2	Q9H2P0
Avasorum	2	Q9H2P9
Avasorum	2	Q9H2U1
Avasorum	2	Q9H2U2
Avasorum	2	Q9H2V7
Avasorum	2	Q9H2W6
Avasorum	2	Q9H2X9
Avasorum	2	Q9H300
Avasorum	2	Q9H307
Avasorum	2	Q9H330
Avasorum	2	Q9H334
Avasorum	2	Q9H3F6
Avasorum	2	Q9H3H1
Avasorum	2	Q9H3H3
Avasorum	2	Q9H3K2
Avasorum	2	Q9H3K6
Avasorum	2	Q9H3M7
Avasorum	2	Q9H3N1
Avasorum	2	Q9H3P2
Avasorum	2	Q9H3P7
Avasorum	2	Q9H3Q1
Avasorum	2	Q9H3S7
Avasorum	2	Q9H3T3
Avasorum	2	Q9H3U1
Avasorum	2	Q9H3U5
Avasorum	2	Q9H3Z4
Avasorum	2	Q9H410
Avasorum	2	Q9H444
Avasorum	2	Q9H446
Avasorum	2	Q9H467
Avasorum	2	Q9H469
Avasorum	2	Q9H479
Avasorum	2	Q9H488
Avasorum	2	Q9H490
Avasorum	2	Q9H497
Avasorum	2	Q9H4A3
Avasorum	2	Q9H4A4
Avasorum	2	Q9H4A5
Avasorum	2	Q9H4A6
Avasorum	2	Q9H4B0
Avasorum	2	Q9H4G4
Avasorum	2	Q9H4I3
Avasorum	2	Q9H4K7
Avasorum	2	Q9H4L4
Avasorum	2	Q9H4L5
Avasorum	2	Q9H4L7

Avasorum	2	Q9H4M9
Avasorum	2	Q9H4Z3
Avasorum	2	Q9H501
Avasorum	2	Q9H553
Avasorum	2	Q9H583
Avasorum	2	Q9H5K3
Avasorum	2	Q9H5N1
Avasorum	2	Q9H5Q4
Avasorum	2	Q9H5U6
Avasorum	2	Q9H5V9
Avasorum	2	Q9H5X1
Avasorum	2	Q9H5Z1
Avasorum	2	Q9H5Z6
Avasorum	2	Q9H6D7
Avasorum	2	Q9H6E4
Avasorum	2	Q9H6F5
Avasorum	2	Q9H6H4
Avasorum	2	Q9H6I2
Avasorum	2	Q9H6K4
Avasorum	2	Q9H6Q4
Avasorum	2	Q9H6R0
Avasorum	2	Q9H6R4
Avasorum	2	Q9H6R7
Avasorum	2	Q9H6S0
Avasorum	2	Q9H6S1
Avasorum	2	Q9H6T3
Avasorum	2	Q9H6U6
Avasorum	2	Q9H6U8
Avasorum	2	Q9H6V9
Avasorum	2	Q9H6W3
Avasorum	2	Q9H6Y2
Avasorum	2	Q9H6Z4
Avasorum	2	Q9H773
Avasorum	2	Q9H792
Avasorum	2	Q9H7B2
Avasorum	2	Q9H7B4
Avasorum	2	Q9H7C9
Avasorum	2	Q9H7D0
Avasorum	2	Q9H7D7
Avasorum	2	Q9H7E2
Avasorum	2	Q9H7E9
Avasorum	2	Q9H7F0
Avasorum	2	Q9H7H0
Avasorum	2	Q9H7N4
Avasorum	2	Q9H7Z3
Avasorum	2	Q9H7Z7
Avasorum	2	Q9H814
Avasorum	2	Q9H832
Avasorum	2	Q9H845
Avasorum	2	Q9H857

Avasorum	2	Q9H875
Avasorum	2	Q9H8G2
Avasorum	2	Q9H8H0
Avasorum	2	Q9H8H2
Avasorum	2	Q9H8H3
Avasorum	2	Q9H8K7
Avasorum	2	Q9H8L6
Avasorum	2	Q9H8M7
Avasorum	2	Q9H8M9
Avasorum	2	Q9H8T0
Avasorum	2	Q9H8W4
Avasorum	2	Q9H8Y5
Avasorum	2	Q9H8Y8
Avasorum	2	Q9H900
Avasorum	2	Q9H910
Avasorum	2	Q9H936
Avasorum	2	Q9H939
Avasorum	2	Q9H944
Avasorum	2	Q9H967
Avasorum	2	Q9H974
Avasorum	2	Q9H981
Avasorum	2	Q9H993
Avasorum	2	Q9H999
Avasorum	2	Q9H9A5
Avasorum	2	Q9H9A6
Avasorum	2	Q9H9B1
Avasorum	2	Q9H9B4
Avasorum	2	Q9H9C1
Avasorum	2	Q9H9E3
Avasorum	2	Q9H9F9
Avasorum	2	Q9H9H4
Avasorum	2	Q9H9J2
Avasorum	2	Q9H9P8
Avasorum	2	Q9H9Q2
Avasorum	2	Q9H9Q4
Avasorum	2	Q9H9S3
Avasorum	2	Q9H9S5
Avasorum	2	Q9H9T3
Avasorum	2	Q9H9Y2
Avasorum	2	Q9H9Y6
Avasorum	2	Q9HA38
Avasorum	2	Q9HA47
Avasorum	2	Q9HA64
Avasorum	2	Q9HA65
Avasorum	2	Q9HA77
Avasorum	2	Q9HAB3
Avasorum	2	Q9HAB8
Avasorum	2	Q9HAC8
Avasorum	2	Q9HAD4
Avasorum	2	Q9HAH7

Avasorum	2	Q9HAN9
Avasorum	2	Q9HAS0
Avasorum	2	Q9HAT2
Avasorum	2	Q9HAU0
Avasorum	2	Q9HAU4
Avasorum	2	Q9HAU5
Avasorum	2	Q9HAV0
Avasorum	2	Q9HAV4
Avasorum	2	Q9HAV7
Avasorum	2	Q9HB07
Avasorum	2	Q9HB20
Avasorum	2	Q9HB21
Avasorum	2	Q9HB40
Avasorum	2	Q9HB63
Avasorum	2	Q9HB71
Avasorum	2	Q9HB90
Avasorum	2	Q9HBF4
Avasorum	2	Q9HBG6
Avasorum	2	Q9HBH5
Avasorum	2	Q9HBI1
Avasorum	2	Q9HBL0
Avasorum	2	Q9HBL7
Avasorum	2	Q9HBL8
Avasorum	2	Q9HBM0
Avasorum	2	Q9HBM1
Avasorum	2	Q9HBM6
Avasorum	2	Q9HBR0
Avasorum	2	Q9HBU6
Avasorum	2	Q9HBW9
Avasorum	2	Q9HC07
Avasorum	2	Q9HC21
Avasorum	2	Q9HC35
Avasorum	2	Q9HC36
Avasorum	2	Q9HC38
Avasorum	2	Q9HC98
Avasorum	2	Q9HCC0
Avasorum	2	Q9HCD5
Avasorum	2	Q9HCE0
Avasorum	2	Q9HCE1
Avasorum	2	Q9HCE5
Avasorum	2	Q9HCG7
Avasorum	2	Q9HCG8
Avasorum	2	Q9HCJ3
Avasorum	2	Q9HCK8
Avasorum	2	Q9HCM4
Avasorum	2	Q9HCN4
Avasorum	2	Q9HCN8
Avasorum	2	Q9HCP0
Avasorum	2	Q9HCS7
Avasorum	2	Q9HCU5

Avasorum	2	Q9HD15
Avasorum	2	Q9HD20
Avasorum	2	Q9HD26
Avasorum	2	Q9HD33
Avasorum	2	Q9HD34
Avasorum	2	Q9HD40
Avasorum	2	Q9HD42
Avasorum	2	Q9HD45
Avasorum	2	Q9HD67
Avasorum	2	Q9HDC9
Avasorum	2	Q9NNW5
Avasorum	2	Q9NNW7
Avasorum	2	Q9NP58
Avasorum	2	Q9NP61
Avasorum	2	Q9NP64
Avasorum	2	Q9NP72
Avasorum	2	Q9NP74
Avasorum	2	Q9NP77
Avasorum	2	Q9NP79
Avasorum	2	Q9NP81
Avasorum	2	Q9NP92
Avasorum	2	Q9NP97
Avasorum	2	Q9NPA0
Avasorum	2	Q9NPA8
Avasorum	2	Q9NPD3
Avasorum	2	Q9NPD8
Avasorum	2	Q9NPE3
Avasorum	2	Q9NPF4
Avasorum	2	Q9NPF5
Avasorum	2	Q9NPG4
Avasorum	2	Q9NPH2
Avasorum	2	Q9NPI1
Avasorum	2	Q9NPI6
Avasorum	2	Q9NPJ3
Avasorum	2	Q9NPJ6
Avasorum	2	Q9NPL8
Avasorum	2	Q9NPQ8
Avasorum	2	Q9NPY3
Avasorum	2	Q9NQ29
Avasorum	2	Q9NQ30
Avasorum	2	Q9NQ48
Avasorum	2	Q9NQ50
Avasorum	2	Q9NQ55
Avasorum	2	Q9NQ66
Avasorum	2	Q9NQ88
Avasorum	2	Q9NQ89
Avasorum	2	Q9NQC3
Avasorum	2	Q9NQG5
Avasorum	2	Q9NQH7
Avasorum	2	Q9NQR4

Avasorum	2	Q9NQS1
Avasorum	2	Q9NQS7
Avasorum	2	Q9NQT4
Avasorum	2	Q9NQT5
Avasorum	2	Q9NQT8
Avasorum	2	Q9NQW6
Avasorum	2	Q9NQW7
Avasorum	2	Q9NQX3
Avasorum	2	Q9NQY0
Avasorum	2	Q9NQZ2
Avasorum	2	Q9NQZ5
Avasorum	2	Q9NR09
Avasorum	2	Q9NR12
Avasorum	2	Q9NR19
Avasorum	2	Q9NR28
Avasorum	2	Q9NR30
Avasorum	2	Q9NR31
Avasorum	2	Q9NR45
Avasorum	2	Q9NR46
Avasorum	2	Q9NR48
Avasorum	2	Q9NR50
Avasorum	2	Q9NR56
Avasorum	2	Q9NRA2
Avasorum	2	Q9NRA8
Avasorum	2	Q9NRB3
Avasorum	2	Q9NRC1
Avasorum	2	Q9NRD5
Avasorum	2	Q9NRF2
Avasorum	2	Q9NRF8
Avasorum	2	Q9NRF9
Avasorum	2	Q9NRG0
Avasorum	2	Q9NRG9
Avasorum	2	Q9NRH2
Avasorum	2	Q9NRK6
Avasorum	2	Q9NRL2
Avasorum	2	Q9NRL3
Avasorum	2	Q9NRN7
Avasorum	2	Q9NRN9
Avasorum	2	Q9NRP0
Avasorum	2	Q9NRP2
Avasorum	2	Q9NRQ2
Avasorum	2	Q9NRR4
Avasorum	2	Q9NRS6
Avasorum	2	Q9NRV9
Avasorum	2	Q9NRW3
Avasorum	2	Q9NRW7
Avasorum	2	Q9NRX1
Avasorum	2	Q9NRX2
Avasorum	2	Q9NRX4
Avasorum	2	Q9NRX5

Avasorum	2	Q9NRY4
Avasorum	2	Q9NRY5
Avasorum	2	Q9NRY6
Avasorum	2	Q9NRZ7
Avasorum	2	Q9NRZ9
Avasorum	2	Q9NS00
Avasorum	2	Q9NS15
Avasorum	2	Q9NS69
Avasorum	2	Q9NS86
Avasorum	2	Q9NS87
Avasorum	2	Q9NS93
Avasorum	2	Q9NSC5
Avasorum	2	Q9NSD9
Avasorum	2	Q9NSE4
Avasorum	2	Q9NSI2
Avasorum	2	Q9NSK0
Avasorum	2	Q9NSV4
Avasorum	2	Q9NSY1
Avasorum	2	Q9NT62
Avasorum	2	Q9NTG7
Avasorum	2	Q9NTI5
Avasorum	2	Q9NTJ3
Avasorum	2	Q9NTJ4
Avasorum	2	Q9NTJ5
Avasorum	2	Q9NTK1
Avasorum	2	Q9NTK5
Avasorum	2	Q9NTM9
Avasorum	2	Q9NTW7
Avasorum	2	Q9NTX5
Avasorum	2	Q9NTZ6
Avasorum	2	Q9NU19
Avasorum	2	Q9NU22
Avasorum	2	Q9NUB1
Avasorum	2	Q9NUD5
Avasorum	2	Q9NUI1
Avasorum	2	Q9NUJ1
Avasorum	2	Q9NUJ3
Avasorum	2	Q9NUL3
Avasorum	2	Q9NUL5
Avasorum	2	Q9NUL7
Avasorum	2	Q9NUM4
Avasorum	2	Q9NUP9
Avasorum	2	Q9NUQ2
Avasorum	2	Q9NUQ3
Avasorum	2	Q9NUQ6
Avasorum	2	Q9NUQ7
Avasorum	2	Q9NUQ8
Avasorum	2	Q9NUQ9
Avasorum	2	Q9NUT2
Avasorum	2	Q9NUU7

Avasorum	2	Q9NUV9
Avasorum	2	Q9NUW8
Avasorum	2	Q9NUX5
Avasorum	2	Q9NUY8
Avasorum	2	Q9NV06
Avasorum	2	Q9NV31
Avasorum	2	Q9NV56
Avasorum	2	Q9NV66
Avasorum	2	Q9NV70
Avasorum	2	Q9NV79
Avasorum	2	Q9NV88
Avasorum	2	Q9NV96
Avasorum	2	Q9NVA1
Avasorum	2	Q9NVA2
Avasorum	2	Q9NVC3
Avasorum	2	Q9NVC6
Avasorum	2	Q9NVD7
Avasorum	2	Q9NVE5
Avasorum	2	Q9NVE7
Avasorum	2	Q9NVF7
Avasorum	2	Q9NVG8
Avasorum	2	Q9NVH0
Avasorum	2	Q9NVH1
Avasorum	2	Q9NVH2
Avasorum	2	Q9NVI1
Avasorum	2	Q9NVI7
Avasorum	2	Q9NVJ2
Avasorum	2	Q9NVM6
Avasorum	2	Q9NVM9
Avasorum	2	Q9NVN3
Avasorum	2	Q9NVN8
Avasorum	2	Q9NVP1
Avasorum	2	Q9NVP2
Avasorum	2	Q9NVR0
Avasorum	2	Q9NVR2
Avasorum	2	Q9NVS2
Avasorum	2	Q9NVS9
Avasorum	2	Q9NVT9
Avasorum	2	Q9NVU0
Avasorum	2	Q9NVU7
Avasorum	2	Q9NVV4
Avasorum	2	Q9NVX2
Avasorum	2	Q9NVZ3
Avasorum	2	Q9NW08
Avasorum	2	Q9NW13
Avasorum	2	Q9NW15
Avasorum	2	Q9NW64
Avasorum	2	Q9NW81
Avasorum	2	Q9NW82
Avasorum	2	Q9NW97

Avasorum	2	Q9NWB6
Avasorum	2	Q9NWH9
Avasorum	2	Q9NWM8
Avasorum	2	Q9NWS8
Avasorum	2	Q9NWT1
Avasorum	2	Q9NWT6
Avasorum	2	Q9NWU1
Avasorum	2	Q9NWU5
Avasorum	2	Q9NWV4
Avasorum	2	Q9NWV8
Avasorum	2	Q9NWW5
Avasorum	2	Q9NWX5
Avasorum	2	Q9NWX6
Avasorum	2	Q9NWY4
Avasorum	2	Q9NWZ3
Avasorum	2	Q9NWZ5
Avasorum	2	Q9NX00
Avasorum	2	Q9NX02
Avasorum	2	Q9NX05
Avasorum	2	Q9NX07
Avasorum	2	Q9NX14
Avasorum	2	Q9NX20
Avasorum	2	Q9NX24
Avasorum	2	Q9NX40
Avasorum	2	Q9NX46
Avasorum	2	Q9NX47
Avasorum	2	Q9NX58
Avasorum	2	Q9NX61
Avasorum	2	Q9NX62
Avasorum	2	Q9NX63
Avasorum	2	Q9NX74
Avasorum	2	Q9NX76
Avasorum	2	Q9NXA8
Avasorum	2	Q9NXC5
Avasorum	2	Q9NXD2
Avasorum	2	Q9NXE4
Avasorum	2	Q9NXE8
Avasorum	2	Q9NXF1
Avasorum	2	Q9NXF7
Avasorum	2	Q9NXG2
Avasorum	2	Q9NXG6
Avasorum	2	Q9NXH8
Avasorum	2	Q9NXH9
Avasorum	2	Q9NXN4
Avasorum	2	Q9NXR1
Avasorum	2	Q9NXR7
Avasorum	2	Q9NXS2
Avasorum	2	Q9NXU5
Avasorum	2	Q9NXV2
Avasorum	2	Q9NXV6

Avasorum	2	Q9NXW2
Avasorum	2	Q9NXX6
Avasorum	2	Q9NY12
Avasorum	2	Q9NY15
Avasorum	2	Q9NY27
Avasorum	2	Q9NY33
Avasorum	2	Q9NY61
Avasorum	2	Q9NY65
Avasorum	2	Q9NY93
Avasorum	2	Q9NYA1
Avasorum	2	Q9NYB0
Avasorum	2	Q9NYB9
Avasorum	2	Q9NYF8
Avasorum	2	Q9NYH9
Avasorum	2	Q9NYJ8
Avasorum	2	Q9NYK5
Avasorum	2	Q9NYL2
Avasorum	2	Q9NYL4
Avasorum	2	Q9NYL9
Avasorum	2	Q9NYM9
Avasorum	2	Q9NYP7
Avasorum	2	Q9NYR9
Avasorum	2	Q9NYU1
Avasorum	2	Q9NYU2
Avasorum	2	Q9NYV4
Avasorum	2	Q9NYY8
Avasorum	2	Q9NZ01
Avasorum	2	Q9NZ08
Avasorum	2	Q9NZ32
Avasorum	2	Q9NZ43
Avasorum	2	Q9NZ45
Avasorum	2	Q9NZ52
Avasorum	2	Q9NZ63
Avasorum	2	Q9NZB2
Avasorum	2	Q9NZC3
Avasorum	2	Q9NZC9
Avasorum	2	Q9NZD2
Avasorum	2	Q9NZD8
Avasorum	2	Q9NZE8
Avasorum	2	Q9NZI7
Avasorum	2	Q9NZI8
Avasorum	2	Q9NZJ4
Avasorum	2	Q9NZJ7
Avasorum	2	Q9NZJ9
Avasorum	2	Q9NZL4
Avasorum	2	Q9NZL9
Avasorum	2	Q9NZM1
Avasorum	2	Q9NZM3
Avasorum	2	Q9NZM5
Avasorum	2	Q9NZN3

Avasorum	2	Q9NZN4
Avasorum	2	Q9NZN5
Avasorum	2	Q9NZN8
Avasorum	2	Q9NZQ3
Avasorum	2	Q9NZQ7
Avasorum	2	Q9NZT2
Avasorum	2	Q9NZU5
Avasorum	2	Q9NZV1
Avasorum	2	Q9NZV5
Avasorum	2	Q9NZW5
Avasorum	2	Q9NZZ3
Avasorum	2	Q9P013
Avasorum	2	Q9P015
Avasorum	2	Q9P016
Avasorum	2	Q9P031
Avasorum	2	Q9P032
Avasorum	2	Q9P035
Avasorum	2	Q9P0I2
Avasorum	2	Q9P0J0
Avasorum	2	Q9P0J1
Avasorum	2	Q9P0J7
Avasorum	2	Q9P0K7
Avasorum	2	Q9P0L0
Avasorum	2	Q9P0M9
Avasorum	2	Q9P0T7
Avasorum	2	Q9P0U3
Avasorum	2	Q9P0U4
Avasorum	2	Q9P0V3
Avasorum	2	Q9P0V9
Avasorum	2	Q9P107
Avasorum	2	Q9P1Y6
Avasorum	2	Q9P215
Avasorum	2	Q9P219
Avasorum	2	Q9P227
Avasorum	2	Q9P253
Avasorum	2	Q9P258
Avasorum	2	Q9P260
Avasorum	2	Q9P265
Avasorum	2	Q9P266
Avasorum	2	Q9P270
Avasorum	2	Q9P273
Avasorum	2	Q9P275
Avasorum	2	Q9P287
Avasorum	2	Q9P289
Avasorum	2	Q9P291
Avasorum	2	Q9P2A4
Avasorum	2	Q9P2B2
Avasorum	2	Q9P2B4
Avasorum	2	Q9P2C4
Avasorum	2	Q9P2D0

Avasorum	2	Q9P2D3
Avasorum	2	Q9P2E3
Avasorum	2	Q9P2E5
Avasorum	2	Q9P2E7
Avasorum	2	Q9P2E9
Avasorum	2	Q9P2I0
Avasorum	2	Q9P2J3
Avasorum	2	Q9P2J5
Avasorum	2	Q9P2K5
Avasorum	2	Q9P2K6
Avasorum	2	Q9P2K8
Avasorum	2	Q9P2L0
Avasorum	2	Q9P2N5
Avasorum	2	Q9P2N6
Avasorum	2	Q9P2P5
Avasorum	2	Q9P2Q2
Avasorum	2	Q9P2R3
Avasorum	2	Q9P2R7
Avasorum	2	Q9P2T1
Avasorum	2	Q9P2W9
Avasorum	2	Q9P2X0
Avasorum	2	Q9P2X3
Avasorum	2	Q9P2Y5
Avasorum	2	Q9UBB4
Avasorum	2	Q9UBB5
Avasorum	2	Q9UBB6
Avasorum	2	Q9UBB9
Avasorum	2	Q9UBC2
Avasorum	2	Q9UBC5
Avasorum	2	Q9UBD5
Avasorum	2	Q9UBE0
Avasorum	2	Q9UBF2
Avasorum	2	Q9UBF8
Avasorum	2	Q9UBG0
Avasorum	2	Q9UBI1
Avasorum	2	Q9UBI6
Avasorum	2	Q9UBK7
Avasorum	2	Q9UBK8
Avasorum	2	Q9UBK9
Avasorum	2	Q9UBL3
Avasorum	2	Q9UBM7
Avasorum	2	Q9UBN6
Avasorum	2	Q9UBN7
Avasorum	2	Q9UBP0
Avasorum	2	Q9UBP4
Avasorum	2	Q9UBP6
Avasorum	2	Q9UBP9
Avasorum	2	Q9UBQ0
Avasorum	2	Q9UBQ5
Avasorum	2	Q9UBQ6

Avasorum	2	Q9UBQ7
Avasorum	2	Q9UBR2
Avasorum	2	Q9UBS0
Avasorum	2	Q9UBS4
Avasorum	2	Q9UBS8
Avasorum	2	Q9UBT2
Avasorum	2	Q9UBU8
Avasorum	2	Q9UBU9
Avasorum	2	Q9UBV2
Avasorum	2	Q9UBV7
Avasorum	2	Q9UBV8
Avasorum	2	Q9UBW7
Avasorum	2	Q9UBW8
Avasorum	2	Q9UBX3
Avasorum	2	Q9UDR5
Avasorum	2	Q9UDT6
Avasorum	2	Q9UDW1
Avasorum	2	Q9UDX5
Avasorum	2	Q9UDY2
Avasorum	2	Q9UDY4
Avasorum	2	Q9UEE5
Avasorum	2	Q9UEE9
Avasorum	2	Q9UER7
Avasorum	2	Q9UET6
Avasorum	2	Q9UEU0
Avasorum	2	Q9UEW8
Avasorum	2	Q9UEY8
Avasorum	2	Q9UFC0
Avasorum	2	Q9UFN0
Avasorum	2	Q9UFW8
Avasorum	2	Q9UG56
Avasorum	2	Q9UG63
Avasorum	2	Q9UGI8
Avasorum	2	Q9UGJ0
Avasorum	2	Q9UGJ1
Avasorum	2	Q9UGM6
Avasorum	2	Q9UGP4
Avasorum	2	Q9UGP8
Avasorum	2	Q9UGQ3
Avasorum	2	Q9UGR2
Avasorum	2	Q9UGV2
Avasorum	2	Q9UH62
Avasorum	2	Q9UH65
Avasorum	2	Q9UH99
Avasorum	2	Q9UHA3
Avasorum	2	Q9UHA4
Avasorum	2	Q9UHB6
Avasorum	2	Q9UHB7
Avasorum	2	Q9UHB9
Avasorum	2	Q9UHD1

Avasorum	2	Q9UHD2
Avasorum	2	Q9UHD8
Avasorum	2	Q9UHG3
Avasorum	2	Q9UHI6
Avasorum	2	Q9UHJ6
Avasorum	2	Q9UHL4
Avasorum	2	Q9UHN6
Avasorum	2	Q9UHP3
Avasorum	2	Q9UHQ4
Avasorum	2	Q9UHQ9
Avasorum	2	Q9UHR5
Avasorum	2	Q9UHR6
Avasorum	2	Q9UHV9
Avasorum	2	Q9UHW5
Avasorum	2	Q9UHX1
Avasorum	2	Q9UHY1
Avasorum	2	Q9UHY7
Avasorum	2	Q9UHY8
Avasorum	2	Q9UI09
Avasorum	2	Q9UI10
Avasorum	2	Q9UI12
Avasorum	2	Q9UI14
Avasorum	2	Q9UI26
Avasorum	2	Q9UI30
Avasorum	2	Q9UI36
Avasorum	2	Q9UIA9
Avasorum	2	Q9UIC8
Avasorum	2	Q9UID3
Avasorum	2	Q9UIF9
Avasorum	2	Q9UIG0
Avasorum	2	Q9UII2
Avasorum	2	Q9UIJ7
Avasorum	2	Q9UIQ6
Avasorum	2	Q9UIS9
Avasorum	2	Q9UIV1
Avasorum	2	Q9UJ41
Avasorum	2	Q9UJ68
Avasorum	2	Q9UJ70
Avasorum	2	Q9UJ83
Avasorum	2	Q9UJA5
Avasorum	2	Q9UJC5
Avasorum	2	Q9UJF2
Avasorum	2	Q9UJK0
Avasorum	2	Q9UJS0
Avasorum	2	Q9UJU6
Avasorum	2	Q9UJV9
Avasorum	2	Q9UJW0
Avasorum	2	Q9UJX2
Avasorum	2	Q9UJX3
Avasorum	2	Q9UJX4

Avasorum	2	Q9UJX5
Avasorum	2	Q9UJX6
Avasorum	2	Q9UJY4
Avasorum	2	Q9UJY5
Avasorum	2	Q9UJZ1
Avasorum	2	Q9UK23
Avasorum	2	Q9UK41
Avasorum	2	Q9UK45
Avasorum	2	Q9UK59
Avasorum	2	Q9UK61
Avasorum	2	Q9UK76
Avasorum	2	Q9UK99
Avasorum	2	Q9UKA4
Avasorum	2	Q9UKB1
Avasorum	2	Q9UKD1
Avasorum	2	Q9UKD2
Avasorum	2	Q9UKE5
Avasorum	2	Q9UKF6
Avasorum	2	Q9UKG1
Avasorum	2	Q9UKI2
Avasorum	2	Q9UKI8
Avasorum	2	Q9UKJ3
Avasorum	2	Q9UKK3
Avasorum	2	Q9UKK6
Avasorum	2	Q9UKK9
Avasorum	2	Q9UKL0
Avasorum	2	Q9UKL6
Avasorum	2	Q9UKM7
Avasorum	2	Q9UKM9
Avasorum	2	Q9UKN8
Avasorum	2	Q9UKR5
Avasorum	2	Q9UKS6
Avasorum	2	Q9UKU7
Avasorum	2	Q9UKU9
Avasorum	2	Q9UKV3
Avasorum	2	Q9UKV5
Avasorum	2	Q9UKV8
Avasorum	2	Q9UKX7
Avasorum	2	Q9UKY7
Avasorum	2	Q9UKZ1
Avasorum	2	Q9UL03
Avasorum	2	Q9UL15
Avasorum	2	Q9UL18
Avasorum	2	Q9UL25
Avasorum	2	Q9UL26
Avasorum	2	Q9UL33
Avasorum	2	Q9UL40
Avasorum	2	Q9UL46
Avasorum	2	Q9UL54
Avasorum	2	Q9UL63

Avasorum	2	Q9ULA0
Avasorum	2	Q9ULC3
Avasorum	2	Q9ULC4
Avasorum	2	Q9ULD2
Avasorum	2	Q9ULE0
Avasorum	2	Q9ULE6
Avasorum	2	Q9ULF5
Avasorum	2	Q9ULG1
Avasorum	2	Q9ULG6
Avasorum	2	Q9ULH0
Avasorum	2	Q9ULH1
Avasorum	2	Q9ULH7
Avasorum	2	Q9ULJ7
Avasorum	2	Q9ULK4
Avasorum	2	Q9ULL8
Avasorum	2	Q9ULM3
Avasorum	2	Q9ULP0
Avasorum	2	Q9ULP9
Avasorum	2	Q9ULR0
Avasorum	2	Q9ULS5
Avasorum	2	Q9ULT8
Avasorum	2	Q9ULU4
Avasorum	2	Q9ULV3
Avasorum	2	Q9ULV4
Avasorum	2	Q9ULW0
Avasorum	2	Q9ULW3
Avasorum	2	Q9ULX3
Avasorum	2	Q9ULX6
Avasorum	2	Q9ULX9
Avasorum	2	Q9ULZ3
Avasorum	2	Q9UM00
Avasorum	2	Q9UM11
Avasorum	2	Q9UM13
Avasorum	2	Q9UM54
Avasorum	2	Q9UMR2
Avasorum	2	Q9UMR5
Avasorum	2	Q9UMS4
Avasorum	2	Q9UMX1
Avasorum	2	Q9UMX3
Avasorum	2	Q9UMY1
Avasorum	2	Q9UMY4
Avasorum	2	Q9UMZ2
Avasorum	2	Q9UN37
Avasorum	2	Q9UN86
Avasorum	2	Q9UNE7
Avasorum	2	Q9UNF0
Avasorum	2	Q9UNF1
Avasorum	2	Q9UNH6
Avasorum	2	Q9UNH7
Avasorum	2	Q9UNI6

Avasorum	2	Q9UNK0
Avasorum	2	Q9UNL2
Avasorum	2	Q9UNM6
Avasorum	2	Q9UNN5
Avasorum	2	Q9UNN8
Avasorum	2	Q9UNP9
Avasorum	2	Q9UNQ2
Avasorum	2	Q9UNS1
Avasorum	2	Q9UNS2
Avasorum	2	Q9UNW1
Avasorum	2	Q9UNW9
Avasorum	2	Q9UNX4
Avasorum	2	Q9UNY4
Avasorum	2	Q9UNZ2
Avasorum	2	Q9UP83
Avasorum	2	Q9UP95
Avasorum	2	Q9UPM8
Avasorum	2	Q9UPN3
Avasorum	2	Q9UPN4
Avasorum	2	Q9UPN6
Avasorum	2	Q9UPN7
Avasorum	2	Q9UPN9
Avasorum	2	Q9UPP1
Avasorum	2	Q9UPQ9
Avasorum	2	Q9UPR0
Avasorum	2	Q9UPR3
Avasorum	2	Q9UPT5
Avasorum	2	Q9UPT8
Avasorum	2	Q9UPU5
Avasorum	2	Q9UPU7
Avasorum	2	Q9UPU9
Avasorum	2	Q9UPW0
Avasorum	2	Q9UPW5
Avasorum	2	Q9UPY3
Avasorum	2	Q9UPY5
Avasorum	2	Q9UPY8
Avasorum	2	Q9UQ13
Avasorum	2	Q9UQ35
Avasorum	2	Q9UQ80
Avasorum	2	Q9UQ90
Avasorum	2	Q9UQB8
Avasorum	2	Q9UQC2
Avasorum	2	Q9UQE7
Avasorum	2	Q9UQN3
Avasorum	2	Q9UQQ2
Avasorum	2	Q9UQR1
Avasorum	2	Q9Y217
Avasorum	2	Q9Y219
Avasorum	2	Q9Y221
Avasorum	2	Q9Y223

Avasorum	2	Q9Y224
Avasorum	2	Q9Y230
Avasorum	2	Q9Y232
Avasorum	2	Q9Y237
Avasorum	2	Q9Y243
Avasorum	2	Q9Y256
Avasorum	2	Q9Y259
Avasorum	2	Q9Y262
Avasorum	2	Q9Y263
Avasorum	2	Q9Y265
Avasorum	2	Q9Y266
Avasorum	2	Q9Y276
Avasorum	2	Q9Y277
Avasorum	2	Q9Y281
Avasorum	2	Q9Y282
Avasorum	2	Q9Y285
Avasorum	2	Q9Y289
Avasorum	2	Q9Y291
Avasorum	2	Q9Y294
Avasorum	2	Q9Y295
Avasorum	2	Q9Y296
Avasorum	2	Q9Y2A7
Avasorum	2	Q9Y2B0
Avasorum	2	Q9Y2C4
Avasorum	2	Q9Y2D4
Avasorum	2	Q9Y2D5
Avasorum	2	Q9Y2G2
Avasorum	2	Q9Y2G3
Avasorum	2	Q9Y2G5
Avasorum	2	Q9Y2H0
Avasorum	2	Q9Y2H1
Avasorum	2	Q9Y2H2
Avasorum	2	Q9Y2H6
Avasorum	2	Q9Y2I1
Avasorum	2	Q9Y2I7
Avasorum	2	Q9Y2I8
Avasorum	2	Q9Y2J2
Avasorum	2	Q9Y2J4
Avasorum	2	Q9Y2K2
Avasorum	2	Q9Y2K7
Avasorum	2	Q9Y2L1
Avasorum	2	Q9Y2L5
Avasorum	2	Q9Y2L9
Avasorum	2	Q9Y2P8
Avasorum	2	Q9Y2Q3
Avasorum	2	Q9Y2Q5
Avasorum	2	Q9Y2Q9
Avasorum	2	Q9Y2R0
Avasorum	2	Q9Y2R4
Avasorum	2	Q9Y2R5

Avasorum	2	Q9Y2R9
Avasorum	2	Q9Y2S2
Avasorum	2	Q9Y2S6
Avasorum	2	Q9Y2S7
Avasorum	2	Q9Y2T2
Avasorum	2	Q9Y2U8
Avasorum	2	Q9Y2V2
Avasorum	2	Q9Y2V7
Avasorum	2	Q9Y2W1
Avasorum	2	Q9Y2W2
Avasorum	2	Q9Y2W6
Avasorum	2	Q9Y2X0
Avasorum	2	Q9Y2X3
Avasorum	2	Q9Y2X7
Avasorum	2	Q9Y2X9
Avasorum	2	Q9Y2Y0
Avasorum	2	Q9Y2Y6
Avasorum	2	Q9Y2Z0
Avasorum	2	Q9Y2Z4
Avasorum	2	Q9Y303
Avasorum	2	Q9Y305
Avasorum	2	Q9Y312
Avasorum	2	Q9Y314
Avasorum	2	Q9Y315
Avasorum	2	Q9Y316
Avasorum	2	Q9Y320
Avasorum	2	Q9Y324
Avasorum	2	Q9Y333
Avasorum	2	Q9Y371
Avasorum	2	Q9Y375
Avasorum	2	Q9Y376
Avasorum	2	Q9Y383
Avasorum	2	Q9Y385
Avasorum	2	Q9Y388
Avasorum	2	Q9Y394
Avasorum	2	Q9Y399
Avasorum	2	Q9Y3A2
Avasorum	2	Q9Y3A3
Avasorum	2	Q9Y3A4
Avasorum	2	Q9Y3A5
Avasorum	2	Q9Y3A6
Avasorum	2	Q9Y3B2
Avasorum	2	Q9Y3B3
Avasorum	2	Q9Y3B4
Avasorum	2	Q9Y3B7
Avasorum	2	Q9Y3B8
Avasorum	2	Q9Y3B9
Avasorum	2	Q9Y3C0
Avasorum	2	Q9Y3C1
Avasorum	2	Q9Y3C4

Avasorum	2	Q9Y3C6
Avasorum	2	Q9Y3C8
Avasorum	2	Q9Y3D0
Avasorum	2	Q9Y3D3
Avasorum	2	Q9Y3D5
Avasorum	2	Q9Y3D6
Avasorum	2	Q9Y3D7
Avasorum	2	Q9Y3D8
Avasorum	2	Q9Y3D9
Avasorum	2	Q9Y3E0
Avasorum	2	Q9Y3E1
Avasorum	2	Q9Y3E5
Avasorum	2	Q9Y3E7
Avasorum	2	Q9Y3F4
Avasorum	2	Q9Y3I0
Avasorum	2	Q9Y3I1
Avasorum	2	Q9Y3L5
Avasorum	2	Q9Y3M8
Avasorum	2	Q9Y3P9
Avasorum	2	Q9Y3Q3
Avasorum	2	Q9Y3Q8
Avasorum	2	Q9Y3R5
Avasorum	2	Q9Y3S2
Avasorum	2	Q9Y3T6
Avasorum	2	Q9Y3T9
Avasorum	2	Q9Y3U8
Avasorum	2	Q9Y3X0
Avasorum	2	Q9Y3Y2
Avasorum	2	Q9Y3Z3
Avasorum	2	Q9Y448
Avasorum	2	Q9Y450
Avasorum	2	Q9Y478
Avasorum	2	Q9Y484
Avasorum	2	Q9Y485
Avasorum	2	Q9Y487
Avasorum	2	Q9Y490
Avasorum	2	Q9Y496
Avasorum	2	Q9Y4A5
Avasorum	2	Q9Y4B5
Avasorum	2	Q9Y4B6
Avasorum	2	Q9Y4C2
Avasorum	2	Q9Y4C8
Avasorum	2	Q9Y4D1
Avasorum	2	Q9Y4D7
Avasorum	2	Q9Y4D8
Avasorum	2	Q9Y4E1
Avasorum	2	Q9Y4E8
Avasorum	2	Q9Y4F1
Avasorum	2	Q9Y4F3
Avasorum	2	Q9Y4F5

Avasorum	2	Q9Y4G8
Avasorum	2	Q9Y4I1
Avasorum	2	Q9Y4J8
Avasorum	2	Q9Y4K0
Avasorum	2	Q9Y4K1
Avasorum	2	Q9Y4K3
Avasorum	2	Q9Y4K4
Avasorum	2	Q9Y4L1
Avasorum	2	Q9Y4P1
Avasorum	2	Q9Y4P3
Avasorum	2	Q9Y4P8
Avasorum	2	Q9Y4R8
Avasorum	2	Q9Y4W2
Avasorum	2	Q9Y4W6
Avasorum	2	Q9Y4X4
Avasorum	2	Q9Y4X5
Avasorum	2	Q9Y4Z0
Avasorum	2	Q9Y508
Avasorum	2	Q9Y512
Avasorum	2	Q9Y520
Avasorum	2	Q9Y530
Avasorum	2	Q9Y547
Avasorum	2	Q9Y570
Avasorum	2	Q9Y580
Avasorum	2	Q9Y584
Avasorum	2	Q9Y5A7
Avasorum	2	Q9Y5A9
Avasorum	2	Q9Y5B0
Avasorum	2	Q9Y5B6
Avasorum	2	Q9Y5B8
Avasorum	2	Q9Y5B9
Avasorum	2	Q9Y5J1
Avasorum	2	Q9Y5K5
Avasorum	2	Q9Y5K6
Avasorum	2	Q9Y5K8
Avasorum	2	Q9Y5L0
Avasorum	2	Q9Y5M8
Avasorum	2	Q9Y5P4
Avasorum	2	Q9Y5P6
Avasorum	2	Q9Y5Q0
Avasorum	2	Q9Y5Q8
Avasorum	2	Q9Y5Q9
Avasorum	2	Q9Y5R8
Avasorum	2	Q9Y5S1
Avasorum	2	Q9Y5S2
Avasorum	2	Q9Y5S9
Avasorum	2	Q9Y5T4
Avasorum	2	Q9Y5T5
Avasorum	2	Q9Y5U2
Avasorum	2	Q9Y5V0

Avasorum	2	Q9Y5V3
Avasorum	2	Q9Y5W7
Avasorum	2	Q9Y5W9
Avasorum	2	Q9Y5X1
Avasorum	2	Q9Y5X2
Avasorum	2	Q9Y5X3
Avasorum	2	Q9Y5X9
Avasorum	2	Q9Y5Y2
Avasorum	2	Q9Y5Y5
Avasorum	2	Q9Y5Y7
Avasorum	2	Q9Y5Z0
Avasorum	2	Q9Y5Z4
Avasorum	2	Q9Y5Z7
Avasorum	2	Q9Y5Z9
Avasorum	2	Q9Y606
Avasorum	2	Q9Y608
Avasorum	2	Q9Y613
Avasorum	2	Q9Y617
Avasorum	2	Q9Y618
Avasorum	2	Q9Y619
Avasorum	2	Q9Y624
Avasorum	2	Q9Y639
Avasorum	2	Q9Y653
Avasorum	2	Q9Y657
Avasorum	2	Q9Y666
Avasorum	2	Q9Y672
Avasorum	2	Q9Y673
Avasorum	2	Q9Y676
Avasorum	2	Q9Y678
Avasorum	2	Q9Y679
Avasorum	2	Q9Y680
Avasorum	2	Q9Y696
Avasorum	2	Q9Y697
Avasorum	2	Q9Y6A4
Avasorum	2	Q9Y6A5
Avasorum	2	Q9Y6A9
Avasorum	2	Q9Y6B6
Avasorum	2	Q9Y6B7
Avasorum	2	Q9Y6C9
Avasorum	2	Q9Y6D0
Avasorum	2	Q9Y6D5
Avasorum	2	Q9Y6D6
Avasorum	2	Q9Y6D9
Avasorum	2	Q9Y6E0
Avasorum	2	Q9Y6E2
Avasorum	2	Q9Y6G5
Avasorum	2	Q9Y6G9
Avasorum	2	Q9Y6I3
Avasorum	2	Q9Y6I4
Avasorum	2	Q9Y6I9

Avasorum	2	Q9Y6J0
Avasorum	2	Q9Y6K0
Avasorum	2	Q9Y6K1
Avasorum	2	Q9Y6K5
Avasorum	2	Q9Y6K8
Avasorum	2	Q9Y6K9
Avasorum	2	Q9Y6M1
Avasorum	2	Q9Y6M5
Avasorum	2	Q9Y6M7
Avasorum	2	Q9Y6M9
Avasorum	2	Q9Y6N5
Avasorum	2	Q9Y6N7
Avasorum	2	Q9Y6Q2
Avasorum	2	Q9Y6Q9
Avasorum	2	Q9Y6R0
Avasorum	2	Q9Y6R4
Avasorum	2	Q9Y6V7
Avasorum	2	Q9Y6W3
Avasorum	2	Q9Y6W5
Avasorum	2	Q9Y6X3
Avasorum	2	Q9Y6X9
Avasorum	2	Q9Y6Y0
Avasorum	2	Q9Y6Y8
Avasorum	3	A0A0B4J2D5
Avasorum	3	A0AVF1
Avasorum	3	A0AVT1
Avasorum	3	A0FGR8
Avasorum	3	A0MZ66
Avasorum	3	A0PJW6
Avasorum	3	A1A4S6
Avasorum	3	A1L0T0
Avasorum	3	A1X283
Avasorum	3	A2RRP1
Avasorum	3	A2RUC4
Avasorum	3	A2RUS2
Avasorum	3	A3KMH1
Avasorum	3	A3KN83
Avasorum	3	A4D1E9
Avasorum	3	A4D1P6
Avasorum	3	A4D1U4
Avasorum	3	A5PLL7
Avasorum	3	A5PLN9
Avasorum	3	A5YKK6
Avasorum	3	A6NCE7
Avasorum	3	A6NDG6
Avasorum	3	A6NDU8
Avasorum	3	A6NFQ2
Avasorum	3	A6NHR9
Avasorum	3	A6NHX0
Avasorum	3	A6NIH7

Avasorum	3	A6NJ78
Avasorum	3	A6NKD9
Avasorum	3	A6ZKI3
Avasorum	3	A7E2V4
Avasorum	3	A8CG34
Avasorum	3	A8MW92
Avasorum	3	A8MWD9
Avasorum	3	A8MWY0
Avasorum	3	A8MXV4
Avasorum	3	A9UHW6
Avasorum	3	P0DP23
Avasorum	3	B2RTY4
Avasorum	3	B7ZAQ6
Avasorum	3	C4AMC7
Avasorum	3	C9JLW8
Avasorum	3	E9PAV3
Avasorum	3	E9PRG8
Avasorum	3	L0R819
Avasorum	3	L0R8F8
Avasorum	3	O00115
Avasorum	3	O00116
Avasorum	3	O00139
Avasorum	3	O00148
Avasorum	3	O00151
Avasorum	3	O00154
Avasorum	3	O00159
Avasorum	3	O00161
Avasorum	3	O00165
Avasorum	3	O00170
Avasorum	3	O00178
Avasorum	3	O00182
Avasorum	3	O00186
Avasorum	3	O00189
Avasorum	3	O00192
Avasorum	3	O00193
Avasorum	3	O00203
Avasorum	3	O00213
Avasorum	3	O00214
Avasorum	3	O00217
Avasorum	3	O00221
Avasorum	3	O00231
Avasorum	3	O00232
Avasorum	3	O00233
Avasorum	3	O00255
Avasorum	3	O00258
Avasorum	3	O00264
Avasorum	3	O00267
Avasorum	3	O00268
Avasorum	3	O00273
Avasorum	3	O00291

Avasorum	3	O00299
Avasorum	3	O00303
Avasorum	3	O00308
Avasorum	3	O00330
Avasorum	3	O00391
Avasorum	3	O00399
Avasorum	3	O00400
Avasorum	3	O00401
Avasorum	3	O00410
Avasorum	3	O00411
Avasorum	3	O00418
Avasorum	3	O00422
Avasorum	3	O00423
Avasorum	3	O00425
Avasorum	3	O00429
Avasorum	3	O00442
Avasorum	3	O00443
Avasorum	3	O00461
Avasorum	3	O00468
Avasorum	3	O00469
Avasorum	3	O00471
Avasorum	3	O00481
Avasorum	3	O00483
Avasorum	3	O00487
Avasorum	3	O00499
Avasorum	3	O00501
Avasorum	3	O00505
Avasorum	3	O00506
Avasorum	3	O00522
Avasorum	3	O00534
Avasorum	3	O00541
Avasorum	3	O00560
Avasorum	3	O00562
Avasorum	3	O00566
Avasorum	3	O00567
Avasorum	3	O00571
Avasorum	3	O00587
Avasorum	3	O00592
Avasorum	3	O00622
Avasorum	3	O00625
Avasorum	3	O00629
Avasorum	3	O00635
Avasorum	3	O00712
Avasorum	3	O00743
Avasorum	3	O00746
Avasorum	3	O00750
Avasorum	3	O00754
Avasorum	3	O00764
Avasorum	3	O00767
Avasorum	3	O14497

Avasorum	3	O14524
Avasorum	3	O14530
Avasorum	3	O14531
Avasorum	3	O14545
Avasorum	3	O14548
Avasorum	3	O14562
Avasorum	3	O14578
Avasorum	3	O14579
Avasorum	3	O14613
Avasorum	3	O14617
Avasorum	3	O14618
Avasorum	3	O14639
Avasorum	3	O14641
Avasorum	3	O14646
Avasorum	3	O14647
Avasorum	3	O14653
Avasorum	3	O14656
Avasorum	3	O14657
Avasorum	3	O14662
Avasorum	3	O14672
Avasorum	3	O14678
Avasorum	3	O14681
Avasorum	3	O14683
Avasorum	3	O14686
Avasorum	3	O14727
Avasorum	3	O14730
Avasorum	3	O14733
Avasorum	3	O14734
Avasorum	3	O14735
Avasorum	3	O14737
Avasorum	3	O14744
Avasorum	3	O14745
Avasorum	3	O14757
Avasorum	3	O14763
Avasorum	3	O14773
Avasorum	3	O14776
Avasorum	3	O14777
Avasorum	3	O14782
Avasorum	3	O14786
Avasorum	3	O14787
Avasorum	3	O14795
Avasorum	3	O14798
Avasorum	3	O14802
Avasorum	3	O14817
Avasorum	3	O14818
Avasorum	3	O14828
Avasorum	3	O14874
Avasorum	3	O14880
Avasorum	3	O14907
Avasorum	3	O14908

Avasorum	3	O14920
Avasorum	3	O14924
Avasorum	3	O14925
Avasorum	3	O14929
Avasorum	3	O14936
Avasorum	3	O14939
Avasorum	3	O14949
Avasorum	3	O14950
Avasorum	3	O14964
Avasorum	3	O14966
Avasorum	3	O14972
Avasorum	3	O14974
Avasorum	3	O14976
Avasorum	3	O14979
Avasorum	3	O14980
Avasorum	3	O14981
Avasorum	3	O15013
Avasorum	3	O15014
Avasorum	3	O15020
Avasorum	3	O15021
Avasorum	3	O15027
Avasorum	3	O15031
Avasorum	3	O15037
Avasorum	3	O15040
Avasorum	3	O15042
Avasorum	3	O15047
Avasorum	3	O15049
Avasorum	3	O15056
Avasorum	3	O15061
Avasorum	3	O15063
Avasorum	3	O15066
Avasorum	3	O15067
Avasorum	3	O15084
Avasorum	3	O15085
Avasorum	3	O15091
Avasorum	3	O15111
Avasorum	3	O15118
Avasorum	3	O15121
Avasorum	3	O15123
Avasorum	3	O15126
Avasorum	3	O15127
Avasorum	3	O15143
Avasorum	3	O15144
Avasorum	3	O15145
Avasorum	3	O15160
Avasorum	3	O15162
Avasorum	3	O15164
Avasorum	3	O15173
Avasorum	3	O15211
Avasorum	3	O15213

Avasorum	3	O15226
Avasorum	3	O15228
Avasorum	3	O15230
Avasorum	3	O15231
Avasorum	3	O15234
Avasorum	3	O15235
Avasorum	3	O15254
Avasorum	3	O15258
Avasorum	3	O15260
Avasorum	3	O15264
Avasorum	3	O15269
Avasorum	3	O15270
Avasorum	3	O15294
Avasorum	3	O15305
Avasorum	3	O15321
Avasorum	3	O15344
Avasorum	3	O15347
Avasorum	3	O15355
Avasorum	3	O15357
Avasorum	3	O15371
Avasorum	3	O15372
Avasorum	3	O15379
Avasorum	3	O15381
Avasorum	3	O15382
Avasorum	3	O15397
Avasorum	3	O15400
Avasorum	3	O15427
Avasorum	3	O15439
Avasorum	3	O15446
Avasorum	3	O15460
Avasorum	3	O15484
Avasorum	3	O15498
Avasorum	3	O15504
Avasorum	3	O15511
Avasorum	3	O15525
Avasorum	3	O15530
Avasorum	3	O15533
Avasorum	3	O15541
Avasorum	3	O15550
Avasorum	3	O43143
Avasorum	3	O43148
Avasorum	3	O43149
Avasorum	3	O43150
Avasorum	3	O43155
Avasorum	3	O43156
Avasorum	3	O43159
Avasorum	3	O43164
Avasorum	3	O43169
Avasorum	3	O43172
Avasorum	3	O43175

Avasorum	3	O43181
Avasorum	3	O43237
Avasorum	3	O43242
Avasorum	3	O43251
Avasorum	3	O43252
Avasorum	3	O43264
Avasorum	3	O43286
Avasorum	3	O43290
Avasorum	3	O43292
Avasorum	3	O43293
Avasorum	3	O43294
Avasorum	3	O43299
Avasorum	3	O43310
Avasorum	3	O43312
Avasorum	3	O43314
Avasorum	3	O43318
Avasorum	3	O43324
Avasorum	3	O43353
Avasorum	3	O43390
Avasorum	3	O43395
Avasorum	3	O43396
Avasorum	3	O43399
Avasorum	3	O43402
Avasorum	3	O43414
Avasorum	3	O43426
Avasorum	3	O43427
Avasorum	3	O43432
Avasorum	3	O43447
Avasorum	3	O43464
Avasorum	3	O43482
Avasorum	3	O43488
Avasorum	3	O43491
Avasorum	3	O43493
Avasorum	3	O43505
Avasorum	3	O43516
Avasorum	3	O43520
Avasorum	3	O43572
Avasorum	3	O43583
Avasorum	3	O43592
Avasorum	3	O43598
Avasorum	3	O43615
Avasorum	3	O43617
Avasorum	3	O43633
Avasorum	3	O43639
Avasorum	3	O43657
Avasorum	3	O43660
Avasorum	3	O43663
Avasorum	3	O43665
Avasorum	3	O43670
Avasorum	3	O43674

Avasorum	3	O43676
Avasorum	3	O43678
Avasorum	3	O43679
Avasorum	3	O43681
Avasorum	3	O43683
Avasorum	3	O43684
Avasorum	3	O43704
Avasorum	3	O43707
Avasorum	3	O43709
Avasorum	3	O43719
Avasorum	3	O43731
Avasorum	3	O43747
Avasorum	3	O43752
Avasorum	3	O43760
Avasorum	3	O43765
Avasorum	3	O43768
Avasorum	3	O43772
Avasorum	3	O43776
Avasorum	3	O43795
Avasorum	3	O43808
Avasorum	3	O43809
Avasorum	3	O43813
Avasorum	3	O43815
Avasorum	3	O43818
Avasorum	3	O43819
Avasorum	3	O43822
Avasorum	3	O43823
Avasorum	3	O43824
Avasorum	3	O43826
Avasorum	3	O43837
Avasorum	3	O43847
Avasorum	3	O43852
Avasorum	3	O43854
Avasorum	3	O43861
Avasorum	3	O43865
Avasorum	3	O43896
Avasorum	3	O43909
Avasorum	3	O43913
Avasorum	3	O43920
Avasorum	3	O43924
Avasorum	3	O43929
Avasorum	3	O43933
Avasorum	3	O60216
Avasorum	3	O60231
Avasorum	3	O60234
Avasorum	3	O60239
Avasorum	3	O60242
Avasorum	3	O60244
Avasorum	3	O60256
Avasorum	3	O60264

Avasorum	3	O60271
Avasorum	3	O60285
Avasorum	3	O60287
Avasorum	3	O60292
Avasorum	3	O60293
Avasorum	3	O60306
Avasorum	3	O60313
Avasorum	3	O60315
Avasorum	3	O60318
Avasorum	3	O60330
Avasorum	3	O60331
Avasorum	3	O60333
Avasorum	3	O60336
Avasorum	3	O60341
Avasorum	3	O60343
Avasorum	3	O60427
Avasorum	3	O60443
Avasorum	3	O60447
Avasorum	3	O60462
Avasorum	3	O60476
Avasorum	3	O60487
Avasorum	3	O60488
Avasorum	3	O60493
Avasorum	3	O60502
Avasorum	3	O60504
Avasorum	3	O60506
Avasorum	3	O60508
Avasorum	3	O60518
Avasorum	3	O60524
Avasorum	3	O60547
Avasorum	3	O60551
Avasorum	3	O60563
Avasorum	3	O60566
Avasorum	3	O60568
Avasorum	3	O60573
Avasorum	3	O60610
Avasorum	3	O60637
Avasorum	3	O60645
Avasorum	3	O60664
Avasorum	3	O60678
Avasorum	3	O60684
Avasorum	3	O60701
Avasorum	3	O60704
Avasorum	3	O60711
Avasorum	3	O60716
Avasorum	3	O60725
Avasorum	3	O60739
Avasorum	3	O60749
Avasorum	3	O60762
Avasorum	3	O60763

Avasorum	3	O60783
Avasorum	3	O60784
Avasorum	3	O60814
Avasorum	3	O60826
Avasorum	3	O60828
Avasorum	3	O60830
Avasorum	3	O60831
Avasorum	3	O60832
Avasorum	3	O60841
Avasorum	3	O60869
Avasorum	3	O60870
Avasorum	3	O60879
Avasorum	3	O60884
Avasorum	3	O60885
Avasorum	3	O60921
Avasorum	3	O60925
Avasorum	3	O60927
Avasorum	3	O60934
Avasorum	3	O60942
Avasorum	3	O75027
Avasorum	3	O75044
Avasorum	3	O75051
Avasorum	3	O75063
Avasorum	3	O75083
Avasorum	3	O75110
Avasorum	3	O75113
Avasorum	3	O75116
Avasorum	3	O75122
Avasorum	3	O75127
Avasorum	3	O75131
Avasorum	3	O75143
Avasorum	3	O75146
Avasorum	3	O75150
Avasorum	3	O75152
Avasorum	3	O75153
Avasorum	3	O75155
Avasorum	3	O75157
Avasorum	3	O75165
Avasorum	3	O75167
Avasorum	3	O75170
Avasorum	3	O75173
Avasorum	3	O75175
Avasorum	3	O75179
Avasorum	3	O75182
Avasorum	3	O75190
Avasorum	3	O75208
Avasorum	3	O75223
Avasorum	3	O75251
Avasorum	3	O75306
Avasorum	3	O75312

Avasorum	3	O75319
Avasorum	3	O75323
Avasorum	3	O75330
Avasorum	3	O75340
Avasorum	3	O75347
Avasorum	3	O75348
Avasorum	3	O75351
Avasorum	3	O75352
Avasorum	3	O75367
Avasorum	3	O75368
Avasorum	3	O75369
Avasorum	3	O75376
Avasorum	3	O75381
Avasorum	3	O75382
Avasorum	3	O75390
Avasorum	3	O75391
Avasorum	3	O75396
Avasorum	3	O75400
Avasorum	3	O75410
Avasorum	3	O75414
Avasorum	3	O75419
Avasorum	3	O75427
Avasorum	3	O75431
Avasorum	3	O75436
Avasorum	3	O75439
Avasorum	3	O75448
Avasorum	3	O75449
Avasorum	3	O75475
Avasorum	3	O75477
Avasorum	3	O75489
Avasorum	3	O75494
Avasorum	3	O75503
Avasorum	3	O75508
Avasorum	3	O75521
Avasorum	3	O75525
Avasorum	3	O75530
Avasorum	3	O75531
Avasorum	3	O75533
Avasorum	3	O75534
Avasorum	3	O75554
Avasorum	3	O75569
Avasorum	3	O75578
Avasorum	3	O75582
Avasorum	3	O75586
Avasorum	3	O75592
Avasorum	3	O75600
Avasorum	3	O75607
Avasorum	3	O75608
Avasorum	3	O75616
Avasorum	3	O75643

Avasorum	3	O75648
Avasorum	3	O75663
Avasorum	3	O75665
Avasorum	3	O75676
Avasorum	3	O75683
Avasorum	3	O75688
Avasorum	3	O75691
Avasorum	3	O75694
Avasorum	3	O75695
Avasorum	3	O75717
Avasorum	3	O75718
Avasorum	3	O75746
Avasorum	3	O75787
Avasorum	3	O75792
Avasorum	3	O75794
Avasorum	3	O75815
Avasorum	3	O75817
Avasorum	3	O75818
Avasorum	3	O75821
Avasorum	3	O75822
Avasorum	3	O75828
Avasorum	3	O75832
Avasorum	3	O75843
Avasorum	3	O75844
Avasorum	3	O75874
Avasorum	3	O75879
Avasorum	3	O75880
Avasorum	3	O75882
Avasorum	3	O75884
Avasorum	3	O75886
Avasorum	3	O75907
Avasorum	3	O75909
Avasorum	3	O75915
Avasorum	3	O75923
Avasorum	3	O75925
Avasorum	3	O75934
Avasorum	3	O75935
Avasorum	3	O75937
Avasorum	3	O75940
Avasorum	3	O75943
Avasorum	3	O75947
Avasorum	3	O75954
Avasorum	3	O75955
Avasorum	3	O75962
Avasorum	3	O75964
Avasorum	3	O75970
Avasorum	3	O75976
Avasorum	3	O76003
Avasorum	3	O76021
Avasorum	3	O76024

Avasorum	3	O76031
Avasorum	3	O76054
Avasorum	3	O76070
Avasorum	3	O76071
Avasorum	3	O76094
Avasorum	3	O94760
Avasorum	3	O94762
Avasorum	3	O94763
Avasorum	3	O94766
Avasorum	3	O94776
Avasorum	3	O94788
Avasorum	3	O94804
Avasorum	3	O94806
Avasorum	3	O94808
Avasorum	3	O94813
Avasorum	3	O94822
Avasorum	3	O94826
Avasorum	3	O94827
Avasorum	3	O94829
Avasorum	3	O94830
Avasorum	3	O94832
Avasorum	3	O94842
Avasorum	3	O94851
Avasorum	3	O94855
Avasorum	3	O94868
Avasorum	3	O94874
Avasorum	3	O94875
Avasorum	3	O94885
Avasorum	3	O94888
Avasorum	3	O94889
Avasorum	3	O94901
Avasorum	3	O94903
Avasorum	3	O94905
Avasorum	3	O94906
Avasorum	3	O94913
Avasorum	3	O94915
Avasorum	3	O94916
Avasorum	3	O94919
Avasorum	3	O94923
Avasorum	3	O94925
Avasorum	3	O94927
Avasorum	3	O94929
Avasorum	3	O94952
Avasorum	3	O94964
Avasorum	3	O94966
Avasorum	3	O94967
Avasorum	3	O94973
Avasorum	3	O94979
Avasorum	3	O94989
Avasorum	3	O94992

Avasorum	3	O95059
Avasorum	3	O95067
Avasorum	3	O95070
Avasorum	3	O95071
Avasorum	3	O95081
Avasorum	3	O95084
Avasorum	3	O95104
Avasorum	3	O95139
Avasorum	3	O95140
Avasorum	3	O95149
Avasorum	3	O95155
Avasorum	3	O95159
Avasorum	3	O95163
Avasorum	3	O95168
Avasorum	3	O95169
Avasorum	3	O95182
Avasorum	3	O95183
Avasorum	3	O95197
Avasorum	3	O95202
Avasorum	3	O95208
Avasorum	3	O95218
Avasorum	3	O95219
Avasorum	3	O95232
Avasorum	3	O95235
Avasorum	3	O95236
Avasorum	3	O95239
Avasorum	3	O95248
Avasorum	3	O95249
Avasorum	3	O95251
Avasorum	3	O95260
Avasorum	3	O95292
Avasorum	3	O95295
Avasorum	3	O95297
Avasorum	3	O95298
Avasorum	3	O95299
Avasorum	3	O95302
Avasorum	3	O95319
Avasorum	3	O95336
Avasorum	3	O95340
Avasorum	3	O95347
Avasorum	3	O95352
Avasorum	3	O95359
Avasorum	3	O95361
Avasorum	3	O95363
Avasorum	3	O95365
Avasorum	3	O95372
Avasorum	3	O95373
Avasorum	3	O95376
Avasorum	3	O95391
Avasorum	3	O95394

Avasorum	3	O95396
Avasorum	3	O95400
Avasorum	3	O95425
Avasorum	3	O95433
Avasorum	3	O95453
Avasorum	3	O95456
Avasorum	3	O95470
Avasorum	3	O95476
Avasorum	3	O95478
Avasorum	3	O95479
Avasorum	3	O95486
Avasorum	3	O95487
Avasorum	3	O95490
Avasorum	3	O95544
Avasorum	3	O95551
Avasorum	3	O95563
Avasorum	3	O95571
Avasorum	3	O95573
Avasorum	3	O95602
Avasorum	3	O95619
Avasorum	3	O95625
Avasorum	3	O95628
Avasorum	3	O95630
Avasorum	3	O95639
Avasorum	3	O95644
Avasorum	3	O95671
Avasorum	3	O95674
Avasorum	3	O95685
Avasorum	3	O95696
Avasorum	3	O95707
Avasorum	3	O95714
Avasorum	3	O95716
Avasorum	3	O95721
Avasorum	3	O95747
Avasorum	3	O95749
Avasorum	3	O95757
Avasorum	3	O95758
Avasorum	3	O95759
Avasorum	3	O95760
Avasorum	3	O95772
Avasorum	3	O95777
Avasorum	3	O95782
Avasorum	3	O95785
Avasorum	3	O95786
Avasorum	3	O95793
Avasorum	3	O95801
Avasorum	3	O95807
Avasorum	3	O95810
Avasorum	3	O95816
Avasorum	3	O95817

Avasorum	3	O95819
Avasorum	3	O95822
Avasorum	3	O95825
Avasorum	3	O95831
Avasorum	3	O95834
Avasorum	3	O95835
Avasorum	3	O95848
Avasorum	3	O95858
Avasorum	3	O95861
Avasorum	3	O95864
Avasorum	3	O95865
Avasorum	3	O95870
Avasorum	3	O95881
Avasorum	3	O95905
Avasorum	3	O95980
Avasorum	3	O95983
Avasorum	3	O95985
Avasorum	3	O95989
Avasorum	3	O95999
Avasorum	3	O96000
Avasorum	3	O96005
Avasorum	3	O96006
Avasorum	3	O96007
Avasorum	3	O96008
Avasorum	3	O96011
Avasorum	3	O96013
Avasorum	3	O96018
Avasorum	3	O96019
Avasorum	3	O96028
Avasorum	3	P00338
Avasorum	3	P00352
Avasorum	3	P00367
Avasorum	3	P00374
Avasorum	3	P00387
Avasorum	3	P00390
Avasorum	3	P00395
Avasorum	3	P00403
Avasorum	3	P00491
Avasorum	3	P00492
Avasorum	3	P00505
Avasorum	3	P00519
Avasorum	3	P00558
Avasorum	3	P00568
Avasorum	3	P00749
Avasorum	3	P00750
Avasorum	3	P00813
Avasorum	3	P01009
Avasorum	3	P01023
Avasorum	3	P01034
Avasorum	3	P01111

Avasorum	3	P01112
Avasorum	3	P01116
Avasorum	3	P01127
Avasorum	3	P01130
Avasorum	3	P01137
Avasorum	3	P01889
Avasorum	3	P01893
Avasorum	3	P02452
Avasorum	3	P02462
Avasorum	3	P02545
Avasorum	3	P02647
Avasorum	3	P02751
Avasorum	3	P02765
Avasorum	3	P02768
Avasorum	3	P02786
Avasorum	3	P02792
Avasorum	3	P02794
Avasorum	3	P02795
Avasorum	3	P03886
Avasorum	3	P03905
Avasorum	3	P03915
Avasorum	3	P03956
Avasorum	3	P04035
Avasorum	3	P04040
Avasorum	3	P04049
Avasorum	3	P04062
Avasorum	3	P04075
Avasorum	3	P04080
Avasorum	3	P04083
Avasorum	3	P04114
Avasorum	3	P04156
Avasorum	3	P04179
Avasorum	3	P04181
Avasorum	3	P04183
Avasorum	3	P04275
Avasorum	3	P04350
Avasorum	3	P04406
Avasorum	3	P04424
Avasorum	3	P04439
Avasorum	3	P04632
Avasorum	3	P04732
Avasorum	3	P04792
Avasorum	3	P04818
Avasorum	3	P04843
Avasorum	3	P04844
Avasorum	3	P04899
Avasorum	3	P04908
Avasorum	3	P04920
Avasorum	3	P05023
Avasorum	3	P05026

Avasorum	3	P05067
Avasorum	3	P05091
Avasorum	3	P05106
Avasorum	3	P05114
Avasorum	3	P05120
Avasorum	3	P05121
Avasorum	3	P05141
Avasorum	3	P05161
Avasorum	3	P05165
Avasorum	3	P05166
Avasorum	3	P05198
Avasorum	3	P05204
Avasorum	3	P05362
Avasorum	3	P05387
Avasorum	3	P05388
Avasorum	3	P05412
Avasorum	3	P05423
Avasorum	3	P05455
Avasorum	3	P05556
Avasorum	3	P05783
Avasorum	3	P05997
Avasorum	3	P06132
Avasorum	3	P06241
Avasorum	3	P06280
Avasorum	3	P06396
Avasorum	3	P06400
Avasorum	3	P06454
Avasorum	3	P06493
Avasorum	3	P06576
Avasorum	3	P06702
Avasorum	3	P06703
Avasorum	3	P06730
Avasorum	3	P06733
Avasorum	3	P06737
Avasorum	3	P06744
Avasorum	3	P06746
Avasorum	3	P06748
Avasorum	3	P06753
Avasorum	3	P06756
Avasorum	3	P06865
Avasorum	3	P07099
Avasorum	3	P07195
Avasorum	3	P07199
Avasorum	3	P07203
Avasorum	3	P07237
Avasorum	3	P07305
Avasorum	3	P07311
Avasorum	3	P07332
Avasorum	3	P07339
Avasorum	3	P07355

Avasorum	3	P07384
Avasorum	3	P07437
Avasorum	3	P07602
Avasorum	3	P07686
Avasorum	3	P07711
Avasorum	3	P07737
Avasorum	3	P07738
Avasorum	3	P07741
Avasorum	3	P07814
Avasorum	3	P07858
Avasorum	3	P07900
Avasorum	3	P07902
Avasorum	3	P07910
Avasorum	3	P07942
Avasorum	3	P07947
Avasorum	3	P07948
Avasorum	3	P07954
Avasorum	3	P07992
Avasorum	3	P07996
Avasorum	3	P08047
Avasorum	3	P08069
Avasorum	3	P08123
Avasorum	3	P08133
Avasorum	3	P08134
Avasorum	3	P08174
Avasorum	3	P08195
Avasorum	3	P08236
Avasorum	3	P08237
Avasorum	3	P08238
Avasorum	3	P08240
Avasorum	3	P08243
Avasorum	3	P08253
Avasorum	3	P08397
Avasorum	3	P08473
Avasorum	3	P08493
Avasorum	3	P08559
Avasorum	3	P08572
Avasorum	3	P08574
Avasorum	3	P08579
Avasorum	3	P08581
Avasorum	3	P08621
Avasorum	3	P08648
Avasorum	3	P08651
Avasorum	3	P08670
Avasorum	3	P08708
Avasorum	3	P08754
Avasorum	3	P08758
Avasorum	3	P08865
Avasorum	3	P08962
Avasorum	3	P09001

Avasorum	3	P09012
Avasorum	3	P09104
Avasorum	3	P09110
Avasorum	3	P09132
Avasorum	3	P09211
Avasorum	3	P09234
Avasorum	3	P09382
Avasorum	3	P09417
Avasorum	3	P09429
Avasorum	3	P09486
Avasorum	3	P09493
Avasorum	3	P09496
Avasorum	3	P09497
Avasorum	3	P09525
Avasorum	3	P09543
Avasorum	3	P09601
Avasorum	3	P09622
Avasorum	3	P09651
Avasorum	3	P09661
Avasorum	3	P09669
Avasorum	3	P09758
Avasorum	3	P09874
Avasorum	3	P09884
Avasorum	3	P09936
Avasorum	3	P09960
Avasorum	3	P09972
Avasorum	3	P0C0S5
Avasorum	3	P0C0S8
Avasorum	3	P0CAP1
Avasorum	3	P0CAP2
Avasorum	3	P0CG29
Avasorum	3	P0CG39
Avasorum	3	P0CW19
Avasorum	3	P0DMV8
Avasorum	3	P0DP91
Avasorum	3	P0DPH7
Avasorum	3	P0DTL6
Avasorum	3	P10114
Avasorum	3	P10124
Avasorum	3	P10155
Avasorum	3	P10253
Avasorum	3	P10301
Avasorum	3	P10321
Avasorum	3	P10398
Avasorum	3	P10412
Avasorum	3	P10515
Avasorum	3	P10586
Avasorum	3	P10589
Avasorum	3	P10599
Avasorum	3	P10606

Avasorum	3	P10619
Avasorum	3	P10620
Avasorum	3	P10644
Avasorum	3	P10646
Avasorum	3	P10768
Avasorum	3	P10809
Avasorum	3	P10909
Avasorum	3	P11021
Avasorum	3	P11047
Avasorum	3	P11117
Avasorum	3	P11137
Avasorum	3	P11142
Avasorum	3	P11166
Avasorum	3	P11169
Avasorum	3	P11171
Avasorum	3	P11172
Avasorum	3	P11177
Avasorum	3	P11182
Avasorum	3	P11216
Avasorum	3	P11217
Avasorum	3	P11233
Avasorum	3	P11234
Avasorum	3	P11274
Avasorum	3	P11279
Avasorum	3	P11308
Avasorum	3	P11310
Avasorum	3	P11387
Avasorum	3	P11388
Avasorum	3	P11413
Avasorum	3	P11441
Avasorum	3	P11498
Avasorum	3	P11586
Avasorum	3	P11717
Avasorum	3	P11766
Avasorum	3	P11802
Avasorum	3	P11908
Avasorum	3	P11940
Avasorum	3	P12004
Avasorum	3	P12081
Avasorum	3	P12109
Avasorum	3	P12235
Avasorum	3	P12236
Avasorum	3	P12268
Avasorum	3	P12270
Avasorum	3	P12429
Avasorum	3	P12694
Avasorum	3	P12814
Avasorum	3	P12821
Avasorum	3	P12931
Avasorum	3	P12955

Avasorum	3	P12956
Avasorum	3	P13010
Avasorum	3	P13051
Avasorum	3	P13073
Avasorum	3	P13196
Avasorum	3	P13473
Avasorum	3	P13489
Avasorum	3	P13598
Avasorum	3	P13639
Avasorum	3	P13667
Avasorum	3	P13674
Avasorum	3	P13693
Avasorum	3	P13716
Avasorum	3	P13747
Avasorum	3	P13797
Avasorum	3	P13798
Avasorum	3	P13804
Avasorum	3	P13807
Avasorum	3	P13861
Avasorum	3	P13984
Avasorum	3	P13987
Avasorum	3	P13995
Avasorum	3	P14174
Avasorum	3	P14209
Avasorum	3	P14314
Avasorum	3	P14317
Avasorum	3	P14324
Avasorum	3	P14406
Avasorum	3	P14543
Avasorum	3	P14550
Avasorum	3	P14618
Avasorum	3	P14625
Avasorum	3	P14635
Avasorum	3	P14649
Avasorum	3	P14678
Avasorum	3	P14735
Avasorum	3	P14859
Avasorum	3	P14866
Avasorum	3	P14868
Avasorum	3	P14921
Avasorum	3	P14923
Avasorum	3	P14927
Avasorum	3	P15056
Avasorum	3	P15090
Avasorum	3	P15104
Avasorum	3	P15121
Avasorum	3	P15144
Avasorum	3	P15151
Avasorum	3	P15153
Avasorum	3	P15170

Avasorum	3	P15289
Avasorum	3	P15291
Avasorum	3	P15311
Avasorum	3	P15374
Avasorum	3	P15407
Avasorum	3	P15408
Avasorum	3	P15529
Avasorum	3	P15531
Avasorum	3	P15559
Avasorum	3	P15586
Avasorum	3	P15735
Avasorum	3	P15848
Avasorum	3	P15880
Avasorum	3	P15884
Avasorum	3	P15924
Avasorum	3	P15927
Avasorum	3	P16035
Avasorum	3	P16070
Avasorum	3	P16152
Avasorum	3	P16219
Avasorum	3	P16220
Avasorum	3	P16278
Avasorum	3	P16284
Avasorum	3	P16298
Avasorum	3	P16333
Avasorum	3	P16383
Avasorum	3	P16401
Avasorum	3	P16402
Avasorum	3	P16403
Avasorum	3	P16435
Avasorum	3	P16591
Avasorum	3	P16615
Avasorum	3	P16885
Avasorum	3	P16930
Avasorum	3	P16949
Avasorum	3	P16989
Avasorum	3	P17026
Avasorum	3	P17028
Avasorum	3	P17050
Avasorum	3	P17096
Avasorum	3	P17152
Avasorum	3	P17174
Avasorum	3	P17252
Avasorum	3	P17301
Avasorum	3	P17302
Avasorum	3	P17405
Avasorum	3	P17480
Avasorum	3	P17535
Avasorum	3	P17542
Avasorum	3	P17544

Avasorum	3	P17568
Avasorum	3	P17612
Avasorum	3	P17655
Avasorum	3	P17706
Avasorum	3	P17812
Avasorum	3	P17813
Avasorum	3	P17844
Avasorum	3	P17858
Avasorum	3	P17948
Avasorum	3	P17980
Avasorum	3	P17987
Avasorum	3	P18031
Avasorum	3	P18074
Avasorum	3	P18077
Avasorum	3	P18084
Avasorum	3	P18085
Avasorum	3	P18124
Avasorum	3	P18206
Avasorum	3	P18433
Avasorum	3	P18440
Avasorum	3	P18583
Avasorum	3	P18615
Avasorum	3	P18621
Avasorum	3	P18669
Avasorum	3	P18754
Avasorum	3	P18846
Avasorum	3	P18850
Avasorum	3	P18858
Avasorum	3	P18887
Avasorum	3	P19021
Avasorum	3	P19022
Avasorum	3	P19174
Avasorum	3	P19338
Avasorum	3	P19367
Avasorum	3	P19387
Avasorum	3	P19388
Avasorum	3	P19404
Avasorum	3	P19447
Avasorum	3	P19474
Avasorum	3	P19525
Avasorum	3	P19623
Avasorum	3	P19634
Avasorum	3	P19784
Avasorum	3	P19838
Avasorum	3	P20020
Avasorum	3	P20042
Avasorum	3	P20073
Avasorum	3	P20226
Avasorum	3	P20290
Avasorum	3	P20337

Avasorum	3	P20338
Avasorum	3	P20339
Avasorum	3	P20340
Avasorum	3	P20585
Avasorum	3	P20594
Avasorum	3	P20618
Avasorum	3	P20645
Avasorum	3	P20700
Avasorum	3	P20810
Avasorum	3	P20839
Avasorum	3	P20908
Avasorum	3	P20929
Avasorum	3	P20933
Avasorum	3	P20936
Avasorum	3	P20962
Avasorum	3	P21127
Avasorum	3	P21266
Avasorum	3	P21281
Avasorum	3	P21283
Avasorum	3	P21291
Avasorum	3	P21333
Avasorum	3	P21359
Avasorum	3	P21397
Avasorum	3	P21399
Avasorum	3	P21453
Avasorum	3	P21579
Avasorum	3	P21589
Avasorum	3	P21675
Avasorum	3	P21741
Avasorum	3	P21796
Avasorum	3	P21810
Avasorum	3	P21912
Avasorum	3	P21926
Avasorum	3	P21964
Avasorum	3	P21980
Avasorum	3	P22033
Avasorum	3	P22059
Avasorum	3	P22061
Avasorum	3	P22087
Avasorum	3	P22102
Avasorum	3	P22234
Avasorum	3	P22307
Avasorum	3	P22314
Avasorum	3	P22392
Avasorum	3	P22570
Avasorum	3	P22626
Avasorum	3	P22670
Avasorum	3	P22681
Avasorum	3	P22692
Avasorum	3	P22694

Avasorum	3	P22695
Avasorum	3	P22830
Avasorum	3	P23025
Avasorum	3	P23193
Avasorum	3	P23219
Avasorum	3	P23229
Avasorum	3	P23246
Avasorum	3	P23258
Avasorum	3	P23284
Avasorum	3	P23368
Avasorum	3	P23381
Avasorum	3	P23396
Avasorum	3	P23443
Avasorum	3	P23458
Avasorum	3	P23467
Avasorum	3	P23469
Avasorum	3	P23470
Avasorum	3	P23497
Avasorum	3	P23508
Avasorum	3	P23510
Avasorum	3	P23526
Avasorum	3	P23528
Avasorum	3	P23588
Avasorum	3	P23610
Avasorum	3	P23634
Avasorum	3	P23743
Avasorum	3	P23786
Avasorum	3	P23919
Avasorum	3	P23921
Avasorum	3	P24385
Avasorum	3	P24386
Avasorum	3	P24390
Avasorum	3	P24468
Avasorum	3	P24534
Avasorum	3	P24539
Avasorum	3	P24666
Avasorum	3	P24723
Avasorum	3	P24752
Avasorum	3	P24844
Avasorum	3	P24928
Avasorum	3	P24941
Avasorum	3	P25098
Avasorum	3	P25116
Avasorum	3	P25205
Avasorum	3	P25325
Avasorum	3	P25398
Avasorum	3	P25440
Avasorum	3	P25445
Avasorum	3	P25490
Avasorum	3	P25685

Avasorum	3	P25686
Avasorum	3	P25705
Avasorum	3	P25786
Avasorum	3	P25787
Avasorum	3	P25788
Avasorum	3	P25789
Avasorum	3	P25942
Avasorum	3	P26006
Avasorum	3	P26022
Avasorum	3	P26038
Avasorum	3	P26196
Avasorum	3	P26358
Avasorum	3	P26368
Avasorum	3	P26373
Avasorum	3	P26374
Avasorum	3	P26440
Avasorum	3	P26572
Avasorum	3	P26583
Avasorum	3	P26599
Avasorum	3	P26639
Avasorum	3	P26640
Avasorum	3	P26641
Avasorum	3	P26885
Avasorum	3	P26951
Avasorum	3	P27105
Avasorum	3	P27144
Avasorum	3	P27348
Avasorum	3	P27361
Avasorum	3	P27448
Avasorum	3	P27635
Avasorum	3	P27658
Avasorum	3	P27694
Avasorum	3	P27695
Avasorum	3	P27701
Avasorum	3	P27707
Avasorum	3	P27708
Avasorum	3	P27797
Avasorum	3	P27816
Avasorum	3	P27824
Avasorum	3	P27986
Avasorum	3	P27987
Avasorum	3	P28062
Avasorum	3	P28065
Avasorum	3	P28066
Avasorum	3	P28070
Avasorum	3	P28072
Avasorum	3	P28074
Avasorum	3	P28161
Avasorum	3	P28288
Avasorum	3	P28290

Avasorum	3	P28300
Avasorum	3	P28331
Avasorum	3	P28340
Avasorum	3	P28370
Avasorum	3	P28482
Avasorum	3	P28702
Avasorum	3	P28715
Avasorum	3	P28799
Avasorum	3	P28827
Avasorum	3	P28838
Avasorum	3	P28906
Avasorum	3	P29083
Avasorum	3	P29084
Avasorum	3	P29144
Avasorum	3	P29218
Avasorum	3	P29279
Avasorum	3	P29317
Avasorum	3	P29323
Avasorum	3	P29353
Avasorum	3	P29372
Avasorum	3	P29401
Avasorum	3	P29466
Avasorum	3	P29474
Avasorum	3	P29558
Avasorum	3	P29590
Avasorum	3	P29597
Avasorum	3	P29692
Avasorum	3	P29966
Avasorum	3	P29992
Avasorum	3	P30038
Avasorum	3	P30040
Avasorum	3	P30041
Avasorum	3	P30043
Avasorum	3	P30044
Avasorum	3	P30048
Avasorum	3	P30050
Avasorum	3	P30084
Avasorum	3	P30085
Avasorum	3	P30086
Avasorum	3	P30101
Avasorum	3	P30153
Avasorum	3	P30154
Avasorum	3	P30260
Avasorum	3	P30405
Avasorum	3	P30414
Avasorum	3	P30419
Avasorum	3	P30519
Avasorum	3	P30520
Avasorum	3	P30530
Avasorum	3	P30533

Avasorum	3	P30536
Avasorum	3	P30566
Avasorum	3	P30622
Avasorum	3	P30626
Avasorum	3	P30740
Avasorum	3	P30825
Avasorum	3	P30837
Avasorum	3	P30876
Avasorum	3	P31040
Avasorum	3	P31146
Avasorum	3	P31150
Avasorum	3	P31153
Avasorum	3	P31323
Avasorum	3	P31350
Avasorum	3	P31483
Avasorum	3	P31641
Avasorum	3	P31689
Avasorum	3	P31749
Avasorum	3	P31930
Avasorum	3	P31937
Avasorum	3	P31939
Avasorum	3	P31942
Avasorum	3	P31943
Avasorum	3	P31946
Avasorum	3	P31948
Avasorum	3	P31949
Avasorum	3	P32119
Avasorum	3	P32121
Avasorum	3	P32189
Avasorum	3	P32321
Avasorum	3	P32322
Avasorum	3	P32455
Avasorum	3	P32456
Avasorum	3	P32519
Avasorum	3	P32780
Avasorum	3	P32856
Avasorum	3	P32969
Avasorum	3	P33121
Avasorum	3	P33151
Avasorum	3	P33176
Avasorum	3	P33316
Avasorum	3	P33527
Avasorum	3	P33897
Avasorum	3	P33981
Avasorum	3	P33991
Avasorum	3	P33992
Avasorum	3	P33993
Avasorum	3	P34059
Avasorum	3	P34896
Avasorum	3	P34897

Avasorum	3	P34931
Avasorum	3	P34932
Avasorum	3	P34947
Avasorum	3	P34949
Avasorum	3	P35052
Avasorum	3	P35080
Avasorum	3	P35221
Avasorum	3	P35222
Avasorum	3	P35232
Avasorum	3	P35237
Avasorum	3	P35240
Avasorum	3	P35241
Avasorum	3	P35244
Avasorum	3	P35249
Avasorum	3	P35250
Avasorum	3	P35251
Avasorum	3	P35268
Avasorum	3	P35269
Avasorum	3	P35270
Avasorum	3	P35367
Avasorum	3	P35520
Avasorum	3	P35555
Avasorum	3	P35556
Avasorum	3	P35573
Avasorum	3	P35579
Avasorum	3	P35580
Avasorum	3	P35590
Avasorum	3	P35606
Avasorum	3	P35610
Avasorum	3	P35611
Avasorum	3	P35613
Avasorum	3	P35637
Avasorum	3	P35658
Avasorum	3	P35659
Avasorum	3	P35713
Avasorum	3	P35749
Avasorum	3	P35813
Avasorum	3	P35914
Avasorum	3	P35916
Avasorum	3	P35968
Avasorum	3	P35998
Avasorum	3	P36269
Avasorum	3	P36404
Avasorum	3	P36405
Avasorum	3	P36507
Avasorum	3	P36542
Avasorum	3	P36543
Avasorum	3	P36551
Avasorum	3	P36578
Avasorum	3	P36639

Avasorum	3	P36776
Avasorum	3	P36871
Avasorum	3	P36873
Avasorum	3	P36915
Avasorum	3	P36957
Avasorum	3	P36959
Avasorum	3	P36969
Avasorum	3	P37023
Avasorum	3	P37059
Avasorum	3	P37108
Avasorum	3	P37173
Avasorum	3	P37198
Avasorum	3	P37235
Avasorum	3	P37268
Avasorum	3	P37275
Avasorum	3	P37802
Avasorum	3	P37837
Avasorum	3	P37840
Avasorum	3	P38117
Avasorum	3	P38159
Avasorum	3	P38432
Avasorum	3	P38435
Avasorum	3	P38571
Avasorum	3	P38606
Avasorum	3	P38646
Avasorum	3	P38919
Avasorum	3	P38935
Avasorum	3	P38936
Avasorum	3	P39019
Avasorum	3	P39023
Avasorum	3	P39060
Avasorum	3	P39656
Avasorum	3	P39687
Avasorum	3	P39748
Avasorum	3	P39880
Avasorum	3	P40121
Avasorum	3	P40123
Avasorum	3	P40189
Avasorum	3	P40222
Avasorum	3	P40227
Avasorum	3	P40261
Avasorum	3	P40306
Avasorum	3	P40425
Avasorum	3	P40429
Avasorum	3	P40616
Avasorum	3	P40692
Avasorum	3	P40763
Avasorum	3	P40818
Avasorum	3	P40925
Avasorum	3	P40926

Avasorum	3	P40937
Avasorum	3	P40938
Avasorum	3	P40939
Avasorum	3	P41091
Avasorum	3	P41162
Avasorum	3	P41208
Avasorum	3	P41212
Avasorum	3	P41214
Avasorum	3	P41223
Avasorum	3	P41226
Avasorum	3	P41227
Avasorum	3	P41229
Avasorum	3	P41240
Avasorum	3	P41250
Avasorum	3	P41252
Avasorum	3	P41440
Avasorum	3	P41567
Avasorum	3	P41743
Avasorum	3	P41970
Avasorum	3	P42025
Avasorum	3	P42126
Avasorum	3	P42166
Avasorum	3	P42167
Avasorum	3	P42224
Avasorum	3	P42226
Avasorum	3	P42229
Avasorum	3	P42285
Avasorum	3	P42330
Avasorum	3	P42336
Avasorum	3	P42338
Avasorum	3	P42345
Avasorum	3	P42356
Avasorum	3	P42566
Avasorum	3	P42574
Avasorum	3	P42575
Avasorum	3	P42677
Avasorum	3	P42684
Avasorum	3	P42694
Avasorum	3	P42695
Avasorum	3	P42696
Avasorum	3	P42704
Avasorum	3	P42765
Avasorum	3	P42766
Avasorum	3	P42785
Avasorum	3	P42858
Avasorum	3	P42892
Avasorum	3	P42898
Avasorum	3	P43007
Avasorum	3	P43034
Avasorum	3	P43121

Avasorum	3	P43155
Avasorum	3	P43243
Avasorum	3	P43246
Avasorum	3	P43250
Avasorum	3	P43304
Avasorum	3	P43307
Avasorum	3	P43378
Avasorum	3	P43487
Avasorum	3	P43490
Avasorum	3	P43686
Avasorum	3	P43897
Avasorum	3	P45877
Avasorum	3	P45880
Avasorum	3	P45954
Avasorum	3	P45973
Avasorum	3	P45974
Avasorum	3	P45983
Avasorum	3	P45985
Avasorum	3	P46013
Avasorum	3	P46019
Avasorum	3	P46020
Avasorum	3	P46060
Avasorum	3	P46063
Avasorum	3	P46087
Avasorum	3	P46100
Avasorum	3	P46108
Avasorum	3	P46109
Avasorum	3	P46199
Avasorum	3	P46379
Avasorum	3	P46459
Avasorum	3	P46531
Avasorum	3	P46734
Avasorum	3	P46736
Avasorum	3	P46776
Avasorum	3	P46777
Avasorum	3	P46778
Avasorum	3	P46779
Avasorum	3	P46781
Avasorum	3	P46782
Avasorum	3	P46783
Avasorum	3	P46821
Avasorum	3	P46926
Avasorum	3	P46934
Avasorum	3	P46939
Avasorum	3	P46940
Avasorum	3	P46976
Avasorum	3	P46977
Avasorum	3	P47712
Avasorum	3	P47755
Avasorum	3	P47756

Avasorum	3	P47813
Avasorum	3	P47895
Avasorum	3	P47897
Avasorum	3	P47914
Avasorum	3	P47929
Avasorum	3	P47974
Avasorum	3	P47985
Avasorum	3	P48047
Avasorum	3	P48059
Avasorum	3	P48060
Avasorum	3	P48147
Avasorum	3	P48163
Avasorum	3	P48200
Avasorum	3	P48307
Avasorum	3	P48382
Avasorum	3	P48426
Avasorum	3	P48444
Avasorum	3	P48449
Avasorum	3	P48454
Avasorum	3	P48506
Avasorum	3	P48507
Avasorum	3	P48509
Avasorum	3	P48553
Avasorum	3	P48556
Avasorum	3	P48634
Avasorum	3	P48637
Avasorum	3	P48643
Avasorum	3	P48651
Avasorum	3	P48681
Avasorum	3	P48723
Avasorum	3	P48729
Avasorum	3	P48730
Avasorum	3	P48735
Avasorum	3	P48736
Avasorum	3	P48739
Avasorum	3	P49005
Avasorum	3	P49006
Avasorum	3	P49023
Avasorum	3	P49069
Avasorum	3	P49116
Avasorum	3	P49137
Avasorum	3	P49184
Avasorum	3	P49189
Avasorum	3	P49207
Avasorum	3	P49247
Avasorum	3	P49257
Avasorum	3	P49281
Avasorum	3	P49321
Avasorum	3	P49327
Avasorum	3	P49354

Avasorum	3	P49356
Avasorum	3	P49366
Avasorum	3	P49368
Avasorum	3	P49406
Avasorum	3	P49407
Avasorum	3	P49411
Avasorum	3	P49419
Avasorum	3	P49427
Avasorum	3	P49441
Avasorum	3	P49454
Avasorum	3	P49458
Avasorum	3	P49585
Avasorum	3	P49588
Avasorum	3	P49589
Avasorum	3	P49590
Avasorum	3	P49591
Avasorum	3	P49593
Avasorum	3	P49641
Avasorum	3	P49642
Avasorum	3	P49643
Avasorum	3	P49662
Avasorum	3	P49674
Avasorum	3	P49720
Avasorum	3	P49721
Avasorum	3	P49736
Avasorum	3	P49748
Avasorum	3	P49750
Avasorum	3	P49754
Avasorum	3	P49755
Avasorum	3	P49756
Avasorum	3	P49757
Avasorum	3	P49761
Avasorum	3	P49768
Avasorum	3	P49770
Avasorum	3	P49773
Avasorum	3	P49790
Avasorum	3	P49792
Avasorum	3	P49795
Avasorum	3	P49796
Avasorum	3	P49815
Avasorum	3	P49821
Avasorum	3	P49840
Avasorum	3	P49841
Avasorum	3	P49848
Avasorum	3	P49902
Avasorum	3	P49903
Avasorum	3	P49914
Avasorum	3	P49915
Avasorum	3	P49916
Avasorum	3	P49917

Avasorum	3	P49959
Avasorum	3	P49961
Avasorum	3	P50148
Avasorum	3	P50213
Avasorum	3	P50225
Avasorum	3	P50281
Avasorum	3	P50336
Avasorum	3	P50395
Avasorum	3	P50402
Avasorum	3	P50416
Avasorum	3	P50443
Avasorum	3	P50452
Avasorum	3	P50453
Avasorum	3	P50454
Avasorum	3	P50479
Avasorum	3	P50502
Avasorum	3	P50542
Avasorum	3	P50548
Avasorum	3	P50552
Avasorum	3	P50570
Avasorum	3	P50579
Avasorum	3	P50583
Avasorum	3	P50613
Avasorum	3	P50748
Avasorum	3	P50749
Avasorum	3	P50750
Avasorum	3	P50851
Avasorum	3	P50895
Avasorum	3	P50897
Avasorum	3	P50914
Avasorum	3	P50990
Avasorum	3	P50991
Avasorum	3	P50995
Avasorum	3	P51003
Avasorum	3	P51114
Avasorum	3	P51116
Avasorum	3	P51148
Avasorum	3	P51149
Avasorum	3	P51151
Avasorum	3	P51153
Avasorum	3	P51159
Avasorum	3	P51178
Avasorum	3	P51398
Avasorum	3	P51452
Avasorum	3	P51531
Avasorum	3	P51532
Avasorum	3	P51553
Avasorum	3	P51570
Avasorum	3	P51571
Avasorum	3	P51572

Avasorum	3	P51580
Avasorum	3	P51608
Avasorum	3	P51610
Avasorum	3	P51617
Avasorum	3	P51636
Avasorum	3	P51648
Avasorum	3	P51659
Avasorum	3	P51665
Avasorum	3	P51687
Avasorum	3	P51688
Avasorum	3	P51692
Avasorum	3	P51784
Avasorum	3	P51798
Avasorum	3	P51809
Avasorum	3	P51812
Avasorum	3	P51813
Avasorum	3	P51825
Avasorum	3	P51858
Avasorum	3	P51946
Avasorum	3	P51948
Avasorum	3	P51956
Avasorum	3	P51965
Avasorum	3	P51970
Avasorum	3	P51991
Avasorum	3	P52209
Avasorum	3	P52272
Avasorum	3	P52292
Avasorum	3	P52294
Avasorum	3	P52298
Avasorum	3	P52306
Avasorum	3	P52434
Avasorum	3	P52564
Avasorum	3	P52565
Avasorum	3	P52566
Avasorum	3	P52594
Avasorum	3	P52597
Avasorum	3	P52630
Avasorum	3	P52701
Avasorum	3	P52732
Avasorum	3	P52735
Avasorum	3	P52747
Avasorum	3	P52756
Avasorum	3	P52758
Avasorum	3	P52788
Avasorum	3	P52789
Avasorum	3	P52799
Avasorum	3	P52815
Avasorum	3	P52824
Avasorum	3	P52848
Avasorum	3	P52888

Avasorum	3	P52907
Avasorum	3	P52926
Avasorum	3	P52943
Avasorum	3	P52948
Avasorum	3	P53004
Avasorum	3	P53007
Avasorum	3	P53041
Avasorum	3	P53350
Avasorum	3	P53355
Avasorum	3	P53365
Avasorum	3	P53367
Avasorum	3	P53384
Avasorum	3	P53396
Avasorum	3	P53582
Avasorum	3	P53597
Avasorum	3	P53602
Avasorum	3	P53609
Avasorum	3	P53611
Avasorum	3	P53618
Avasorum	3	P53621
Avasorum	3	P53634
Avasorum	3	P53667
Avasorum	3	P53671
Avasorum	3	P53675
Avasorum	3	P53677
Avasorum	3	P53680
Avasorum	3	P53701
Avasorum	3	P53778
Avasorum	3	P53801
Avasorum	3	P53814
Avasorum	3	P53985
Avasorum	3	P53990
Avasorum	3	P53992
Avasorum	3	P53999
Avasorum	3	P54098
Avasorum	3	P54105
Avasorum	3	P54136
Avasorum	3	P54198
Avasorum	3	P54252
Avasorum	3	P54259
Avasorum	3	P54277
Avasorum	3	P54278
Avasorum	3	P54289
Avasorum	3	P54577
Avasorum	3	P54578
Avasorum	3	P54619
Avasorum	3	P54652
Avasorum	3	P54687
Avasorum	3	P54709
Avasorum	3	P54727

Avasorum	3	P54760
Avasorum	3	P54802
Avasorum	3	P54819
Avasorum	3	P54886
Avasorum	3	P54920
Avasorum	3	P55010
Avasorum	3	P55011
Avasorum	3	P55036
Avasorum	3	P55039
Avasorum	3	P55060
Avasorum	3	P55072
Avasorum	3	P55081
Avasorum	3	P55084
Avasorum	3	P55145
Avasorum	3	P55196
Avasorum	3	P55199
Avasorum	3	P55201
Avasorum	3	P55209
Avasorum	3	P55210
Avasorum	3	P55212
Avasorum	3	P55263
Avasorum	3	P55265
Avasorum	3	P55268
Avasorum	3	P55290
Avasorum	3	P55327
Avasorum	3	P55735
Avasorum	3	P55769
Avasorum	3	P55786
Avasorum	3	P55789
Avasorum	3	P55795
Avasorum	3	P55809
Avasorum	3	P55884
Avasorum	3	P55899
Avasorum	3	P55957
Avasorum	3	P56134
Avasorum	3	P56182
Avasorum	3	P56192
Avasorum	3	P56211
Avasorum	3	P56377
Avasorum	3	P56378
Avasorum	3	P56381
Avasorum	3	P56385
Avasorum	3	P56524
Avasorum	3	P56537
Avasorum	3	P56545
Avasorum	3	P56556
Avasorum	3	P56589
Avasorum	3	P56937
Avasorum	3	P56945
Avasorum	3	P56962

Avasorum	3	P57076
Avasorum	3	P57081
Avasorum	3	P57088
Avasorum	3	P57678
Avasorum	3	P57737
Avasorum	3	P57740
Avasorum	3	P57764
Avasorum	3	P57772
Avasorum	3	P58004
Avasorum	3	P58335
Avasorum	3	P58546
Avasorum	3	P59998
Avasorum	3	P60033
Avasorum	3	P60059
Avasorum	3	P60174
Avasorum	3	P60228
Avasorum	3	P60468
Avasorum	3	P60484
Avasorum	3	P60510
Avasorum	3	P60520
Avasorum	3	P60602
Avasorum	3	P60660
Avasorum	3	P60709
Avasorum	3	P60842
Avasorum	3	P60866
Avasorum	3	P60891
Avasorum	3	P60900
Avasorum	3	P60903
Avasorum	3	P60953
Avasorum	3	P60981
Avasorum	3	P60983
Avasorum	3	P61006
Avasorum	3	P61009
Avasorum	3	P61011
Avasorum	3	P61018
Avasorum	3	P61019
Avasorum	3	P61020
Avasorum	3	P61026
Avasorum	3	P61077
Avasorum	3	P61081
Avasorum	3	P61086
Avasorum	3	P61088
Avasorum	3	P61106
Avasorum	3	P61158
Avasorum	3	P61160
Avasorum	3	P61163
Avasorum	3	P61201
Avasorum	3	P61221
Avasorum	3	P61224
Avasorum	3	P61225

Avasorum	3	P61244
Avasorum	3	P61247
Avasorum	3	P61254
Avasorum	3	P61289
Avasorum	3	P61313
Avasorum	3	P61353
Avasorum	3	P61421
Avasorum	3	P61513
Avasorum	3	P61586
Avasorum	3	P61587
Avasorum	3	P61599
Avasorum	3	P61604
Avasorum	3	P61619
Avasorum	3	P61758
Avasorum	3	P61764
Avasorum	3	P61769
Avasorum	3	P61803
Avasorum	3	P61916
Avasorum	3	P61923
Avasorum	3	P61927
Avasorum	3	P61956
Avasorum	3	P61962
Avasorum	3	P61964
Avasorum	3	P61966
Avasorum	3	P61970
Avasorum	3	P61978
Avasorum	3	P61981
Avasorum	3	P62068
Avasorum	3	P62070
Avasorum	3	P62081
Avasorum	3	P62136
Avasorum	3	P62140
Avasorum	3	P62191
Avasorum	3	P62195
Avasorum	3	P62241
Avasorum	3	P62244
Avasorum	3	P62249
Avasorum	3	P62253
Avasorum	3	P62256
Avasorum	3	P62258
Avasorum	3	P62263
Avasorum	3	P62266
Avasorum	3	P62269
Avasorum	3	P62273
Avasorum	3	P62277
Avasorum	3	P62280
Avasorum	3	P62304
Avasorum	3	P62306
Avasorum	3	P62310
Avasorum	3	P62312

Avasorum	3	P62314
Avasorum	3	P62316
Avasorum	3	P62318
Avasorum	3	P62328
Avasorum	3	P62330
Avasorum	3	P62333
Avasorum	3	P62341
Avasorum	3	P62380
Avasorum	3	P62424
Avasorum	3	P62487
Avasorum	3	P62491
Avasorum	3	P62495
Avasorum	3	P62633
Avasorum	3	P62699
Avasorum	3	P62701
Avasorum	3	P62714
Avasorum	3	P62736
Avasorum	3	P62745
Avasorum	3	P62750
Avasorum	3	P62753
Avasorum	3	P62805
Avasorum	3	P62820
Avasorum	3	P62826
Avasorum	3	P62829
Avasorum	3	P62834
Avasorum	3	P62841
Avasorum	3	P62847
Avasorum	3	P62851
Avasorum	3	P62854
Avasorum	3	P62857
Avasorum	3	P62861
Avasorum	3	P62873
Avasorum	3	P62877
Avasorum	3	P62879
Avasorum	3	P62888
Avasorum	3	P62891
Avasorum	3	P62899
Avasorum	3	P62906
Avasorum	3	P62910
Avasorum	3	P62913
Avasorum	3	P62917
Avasorum	3	P62937
Avasorum	3	P62942
Avasorum	3	P62979
Avasorum	3	P62993
Avasorum	3	P62995
Avasorum	3	P63000
Avasorum	3	P63010
Avasorum	3	P63027
Avasorum	3	P63092

Avasorum	3	P63096
Avasorum	3	P63104
Avasorum	3	P63151
Avasorum	3	P63165
Avasorum	3	P63167
Avasorum	3	P63172
Avasorum	3	P63173
Avasorum	3	P63208
Avasorum	3	P63218
Avasorum	3	P63220
Avasorum	3	P63241
Avasorum	3	P63244
Avasorum	3	P63272
Avasorum	3	P63279
Avasorum	3	P63313
Avasorum	3	P67775
Avasorum	3	P67809
Avasorum	3	P67812
Avasorum	3	P67870
Avasorum	3	P67936
Avasorum	3	P68036
Avasorum	3	P68104
Avasorum	3	P68363
Avasorum	3	P68366
Avasorum	3	P68371
Avasorum	3	P68400
Avasorum	3	P68402
Avasorum	3	P68431
Avasorum	3	P68871
Avasorum	3	P69905
Avasorum	3	P78310
Avasorum	3	P78312
Avasorum	3	P78316
Avasorum	3	P78318
Avasorum	3	P78324
Avasorum	3	P78330
Avasorum	3	P78332
Avasorum	3	P78344
Avasorum	3	P78345
Avasorum	3	P78346
Avasorum	3	P78347
Avasorum	3	P78356
Avasorum	3	P78357
Avasorum	3	P78362
Avasorum	3	P78371
Avasorum	3	P78381
Avasorum	3	P78406
Avasorum	3	P78417
Avasorum	3	P78527
Avasorum	3	P78536

Avasorum	3	P78537
Avasorum	3	P78539
Avasorum	3	P78540
Avasorum	3	P78549
Avasorum	3	P80217
Avasorum	3	P80303
Avasorum	3	P80723
Avasorum	3	P81605
Avasorum	3	P82094
Avasorum	3	P82650
Avasorum	3	P82663
Avasorum	3	P82664
Avasorum	3	P82673
Avasorum	3	P82675
Avasorum	3	P82909
Avasorum	3	P82912
Avasorum	3	P82914
Avasorum	3	P82921
Avasorum	3	P82930
Avasorum	3	P82932
Avasorum	3	P82933
Avasorum	3	P82979
Avasorum	3	P83111
Avasorum	3	P83436
Avasorum	3	P83731
Avasorum	3	P83876
Avasorum	3	P83881
Avasorum	3	P83916
Avasorum	3	P84022
Avasorum	3	P84077
Avasorum	3	P84085
Avasorum	3	P84090
Avasorum	3	P84095
Avasorum	3	P84098
Avasorum	3	P84101
Avasorum	3	P84103
Avasorum	3	P85037
Avasorum	3	P86790
Avasorum	3	P98082
Avasorum	3	P98160
Avasorum	3	P98170
Avasorum	3	P98172
Avasorum	3	P98175
Avasorum	3	P98179
Avasorum	3	P98194
Avasorum	3	P98196
Avasorum	3	P99999
Avasorum	3	Q00013
Avasorum	3	Q00059
Avasorum	3	Q00169

Avasorum	3	Q00325
Avasorum	3	Q00341
Avasorum	3	Q00403
Avasorum	3	Q00534
Avasorum	3	Q00535
Avasorum	3	Q00537
Avasorum	3	Q00577
Avasorum	3	Q00587
Avasorum	3	Q00610
Avasorum	3	Q00613
Avasorum	3	Q00653
Avasorum	3	Q00688
Avasorum	3	Q00765
Avasorum	3	Q00796
Avasorum	3	Q00839
Avasorum	3	Q01081
Avasorum	3	Q01082
Avasorum	3	Q01085
Avasorum	3	Q01105
Avasorum	3	Q01130
Avasorum	3	Q01167
Avasorum	3	Q01201
Avasorum	3	Q01415
Avasorum	3	Q01433
Avasorum	3	Q01469
Avasorum	3	Q01518
Avasorum	3	Q01543
Avasorum	3	Q01581
Avasorum	3	Q01628
Avasorum	3	Q01650
Avasorum	3	Q01658
Avasorum	3	Q01780
Avasorum	3	Q01804
Avasorum	3	Q01813
Avasorum	3	Q01831
Avasorum	3	Q01844
Avasorum	3	Q01850
Avasorum	3	Q01968
Avasorum	3	Q01970
Avasorum	3	Q01995
Avasorum	3	Q02040
Avasorum	3	Q02127
Avasorum	3	Q02218
Avasorum	3	Q02224
Avasorum	3	Q02241
Avasorum	3	Q02252
Avasorum	3	Q02297
Avasorum	3	Q02318
Avasorum	3	Q02413
Avasorum	3	Q02447

Avasorum	3	Q02539
Avasorum	3	Q02543
Avasorum	3	Q02750
Avasorum	3	Q02763
Avasorum	3	Q02790
Avasorum	3	Q02809
Avasorum	3	Q02818
Avasorum	3	Q02878
Avasorum	3	Q02880
Avasorum	3	Q02952
Avasorum	3	Q02978
Avasorum	3	Q03001
Avasorum	3	Q03111
Avasorum	3	Q03112
Avasorum	3	Q03113
Avasorum	3	Q03135
Avasorum	3	Q03154
Avasorum	3	Q03169
Avasorum	3	Q03252
Avasorum	3	Q03393
Avasorum	3	Q03405
Avasorum	3	Q03426
Avasorum	3	Q03468
Avasorum	3	Q03518
Avasorum	3	Q03519
Avasorum	3	Q03701
Avasorum	3	Q03936
Avasorum	3	Q04206
Avasorum	3	Q04323
Avasorum	3	Q04446
Avasorum	3	Q04637
Avasorum	3	Q04721
Avasorum	3	Q04724
Avasorum	3	Q04726
Avasorum	3	Q04760
Avasorum	3	Q04771
Avasorum	3	Q04837
Avasorum	3	Q04917
Avasorum	3	Q04941
Avasorum	3	Q05048
Avasorum	3	Q05086
Avasorum	3	Q05193
Avasorum	3	Q05209
Avasorum	3	Q05397
Avasorum	3	Q05519
Avasorum	3	Q05655
Avasorum	3	Q05682
Avasorum	3	Q05932
Avasorum	3	Q05D32
Avasorum	3	Q06124

Avasorum	3	Q06136
Avasorum	3	Q06203
Avasorum	3	Q06210
Avasorum	3	Q06265
Avasorum	3	Q06323
Avasorum	3	Q06330
Avasorum	3	Q06413
Avasorum	3	Q06481
Avasorum	3	Q06546
Avasorum	3	Q06587
Avasorum	3	Q06787
Avasorum	3	Q06830
Avasorum	3	Q07020
Avasorum	3	Q07021
Avasorum	3	Q07065
Avasorum	3	Q07157
Avasorum	3	Q07352
Avasorum	3	Q07617
Avasorum	3	Q07666
Avasorum	3	Q07812
Avasorum	3	Q07817
Avasorum	3	Q07820
Avasorum	3	Q07864
Avasorum	3	Q07866
Avasorum	3	Q07889
Avasorum	3	Q07955
Avasorum	3	Q07960
Avasorum	3	Q08117
Avasorum	3	Q08170
Avasorum	3	Q08174
Avasorum	3	Q08188
Avasorum	3	Q08209
Avasorum	3	Q08211
Avasorum	3	Q08257
Avasorum	3	Q08378
Avasorum	3	Q08379
Avasorum	3	Q08426
Avasorum	3	Q08431
Avasorum	3	Q08499
Avasorum	3	Q08554
Avasorum	3	Q08623
Avasorum	3	Q08629
Avasorum	3	Q08722
Avasorum	3	Q08752
Avasorum	3	Q08945
Avasorum	3	Q08AD1
Avasorum	3	Q08AE8
Avasorum	3	Q08AF3
Avasorum	3	Q08AM6
Avasorum	3	Q08J23

Avasorum	3	Q09019
Avasorum	3	Q09028
Avasorum	3	Q09161
Avasorum	3	Q09328
Avasorum	3	Q09472
Avasorum	3	Q09666
Avasorum	3	Q0JRZ9
Avasorum	3	Q0PNE2
Avasorum	3	Q0VDF9
Avasorum	3	Q0VDG4
Avasorum	3	Q0VF96
Avasorum	3	Q0VGL1
Avasorum	3	Q0ZGT2
Avasorum	3	Q10469
Avasorum	3	Q10471
Avasorum	3	Q10472
Avasorum	3	Q10567
Avasorum	3	Q10570
Avasorum	3	Q10589
Avasorum	3	Q10713
Avasorum	3	Q11201
Avasorum	3	Q12765
Avasorum	3	Q12768
Avasorum	3	Q12769
Avasorum	3	Q12770
Avasorum	3	Q12778
Avasorum	3	Q12788
Avasorum	3	Q12789
Avasorum	3	Q12792
Avasorum	3	Q12797
Avasorum	3	Q12800
Avasorum	3	Q12802
Avasorum	3	Q12805
Avasorum	3	Q12824
Avasorum	3	Q12830
Avasorum	3	Q12834
Avasorum	3	Q12841
Avasorum	3	Q12846
Avasorum	3	Q12849
Avasorum	3	Q12851
Avasorum	3	Q12866
Avasorum	3	Q12872
Avasorum	3	Q12873
Avasorum	3	Q12874
Avasorum	3	Q12882
Avasorum	3	Q12888
Avasorum	3	Q12893
Avasorum	3	Q12894
Avasorum	3	Q12899
Avasorum	3	Q12904

Avasorum	3	Q12905
Avasorum	3	Q12906
Avasorum	3	Q12907
Avasorum	3	Q12931
Avasorum	3	Q12933
Avasorum	3	Q12959
Avasorum	3	Q12965
Avasorum	3	Q12972
Avasorum	3	Q12974
Avasorum	3	Q12979
Avasorum	3	Q12980
Avasorum	3	Q12981
Avasorum	3	Q12982
Avasorum	3	Q12986
Avasorum	3	Q12996
Avasorum	3	Q12999
Avasorum	3	Q13011
Avasorum	3	Q13017
Avasorum	3	Q13033
Avasorum	3	Q13042
Avasorum	3	Q13043
Avasorum	3	Q13045
Avasorum	3	Q13049
Avasorum	3	Q13057
Avasorum	3	Q13084
Avasorum	3	Q13085
Avasorum	3	Q13098
Avasorum	3	Q13107
Avasorum	3	Q13111
Avasorum	3	Q13112
Avasorum	3	Q13123
Avasorum	3	Q13126
Avasorum	3	Q13131
Avasorum	3	Q13136
Avasorum	3	Q13137
Avasorum	3	Q13144
Avasorum	3	Q13148
Avasorum	3	Q13151
Avasorum	3	Q13155
Avasorum	3	Q13158
Avasorum	3	Q13162
Avasorum	3	Q13163
Avasorum	3	Q13164
Avasorum	3	Q13177
Avasorum	3	Q13185
Avasorum	3	Q13188
Avasorum	3	Q13190
Avasorum	3	Q13200
Avasorum	3	Q13201
Avasorum	3	Q13206

Avasorum	3	Q13217
Avasorum	3	Q13228
Avasorum	3	Q13232
Avasorum	3	Q13242
Avasorum	3	Q13243
Avasorum	3	Q13247
Avasorum	3	Q13257
Avasorum	3	Q13263
Avasorum	3	Q13275
Avasorum	3	Q13277
Avasorum	3	Q13283
Avasorum	3	Q13287
Avasorum	3	Q13308
Avasorum	3	Q13310
Avasorum	3	Q13315
Avasorum	3	Q13322
Avasorum	3	Q13330
Avasorum	3	Q13347
Avasorum	3	Q13356
Avasorum	3	Q13362
Avasorum	3	Q13363
Avasorum	3	Q13371
Avasorum	3	Q13393
Avasorum	3	Q13395
Avasorum	3	Q13404
Avasorum	3	Q13405
Avasorum	3	Q13409
Avasorum	3	Q13416
Avasorum	3	Q13418
Avasorum	3	Q13423
Avasorum	3	Q13425
Avasorum	3	Q13426
Avasorum	3	Q13427
Avasorum	3	Q13428
Avasorum	3	Q13432
Avasorum	3	Q13435
Avasorum	3	Q13438
Avasorum	3	Q13439
Avasorum	3	Q13442
Avasorum	3	Q13443
Avasorum	3	Q13444
Avasorum	3	Q13445
Avasorum	3	Q13451
Avasorum	3	Q13459
Avasorum	3	Q13464
Avasorum	3	Q13469
Avasorum	3	Q13472
Avasorum	3	Q13485
Avasorum	3	Q13488
Avasorum	3	Q13490

Avasorum	3	Q13492
Avasorum	3	Q13496
Avasorum	3	Q13501
Avasorum	3	Q13505
Avasorum	3	Q13509
Avasorum	3	Q13510
Avasorum	3	Q13523
Avasorum	3	Q13526
Avasorum	3	Q13535
Avasorum	3	Q13541
Avasorum	3	Q13546
Avasorum	3	Q13547
Avasorum	3	Q13555
Avasorum	3	Q13557
Avasorum	3	Q13561
Avasorum	3	Q13563
Avasorum	3	Q13564
Avasorum	3	Q13572
Avasorum	3	Q13573
Avasorum	3	Q13574
Avasorum	3	Q13586
Avasorum	3	Q13588
Avasorum	3	Q13595
Avasorum	3	Q13596
Avasorum	3	Q13601
Avasorum	3	Q13610
Avasorum	3	Q13613
Avasorum	3	Q13614
Avasorum	3	Q13616
Avasorum	3	Q13617
Avasorum	3	Q13618
Avasorum	3	Q13619
Avasorum	3	Q13620
Avasorum	3	Q13625
Avasorum	3	Q13627
Avasorum	3	Q13630
Avasorum	3	Q13636
Avasorum	3	Q13637
Avasorum	3	Q13642
Avasorum	3	Q13643
Avasorum	3	Q13671
Avasorum	3	Q13685
Avasorum	3	Q13724
Avasorum	3	Q13740
Avasorum	3	Q13769
Avasorum	3	Q13796
Avasorum	3	Q13813
Avasorum	3	Q13823
Avasorum	3	Q13825
Avasorum	3	Q13838

Avasorum	3	Q13867
Avasorum	3	Q13868
Avasorum	3	Q13873
Avasorum	3	Q13885
Avasorum	3	Q13888
Avasorum	3	Q13889
Avasorum	3	Q13895
Avasorum	3	Q13905
Avasorum	3	Q13907
Avasorum	3	Q13951
Avasorum	3	Q13952
Avasorum	3	Q14004
Avasorum	3	Q14008
Avasorum	3	Q14011
Avasorum	3	Q14012
Avasorum	3	Q14019
Avasorum	3	Q14103
Avasorum	3	Q14108
Avasorum	3	Q14112
Avasorum	3	Q14114
Avasorum	3	Q14118
Avasorum	3	Q14119
Avasorum	3	Q14137
Avasorum	3	Q14139
Avasorum	3	Q14141
Avasorum	3	Q14145
Avasorum	3	Q14146
Avasorum	3	Q14147
Avasorum	3	Q14149
Avasorum	3	Q14151
Avasorum	3	Q14152
Avasorum	3	Q14155
Avasorum	3	Q14156
Avasorum	3	Q14157
Avasorum	3	Q14160
Avasorum	3	Q14161
Avasorum	3	Q14162
Avasorum	3	Q14165
Avasorum	3	Q14166
Avasorum	3	Q14181
Avasorum	3	Q14185
Avasorum	3	Q14186
Avasorum	3	Q14192
Avasorum	3	Q14195
Avasorum	3	Q14197
Avasorum	3	Q14202
Avasorum	3	Q14203
Avasorum	3	Q14204
Avasorum	3	Q14232
Avasorum	3	Q14240

Avasorum	3	Q14241
Avasorum	3	Q14247
Avasorum	3	Q14249
Avasorum	3	Q14254
Avasorum	3	Q14257
Avasorum	3	Q14258
Avasorum	3	Q14315
Avasorum	3	Q14318
Avasorum	3	Q14320
Avasorum	3	Q14331
Avasorum	3	Q14344
Avasorum	3	Q14353
Avasorum	3	Q14376
Avasorum	3	Q14392
Avasorum	3	Q14444
Avasorum	3	Q14457
Avasorum	3	Q14498
Avasorum	3	Q14527
Avasorum	3	Q14534
Avasorum	3	Q14554
Avasorum	3	Q14558
Avasorum	3	Q14562
Avasorum	3	Q14566
Avasorum	3	Q14571
Avasorum	3	Q14573
Avasorum	3	Q14643
Avasorum	3	Q14644
Avasorum	3	Q14651
Avasorum	3	Q14653
Avasorum	3	Q14657
Avasorum	3	Q14667
Avasorum	3	Q14669
Avasorum	3	Q14671
Avasorum	3	Q14676
Avasorum	3	Q14677
Avasorum	3	Q14678
Avasorum	3	Q14683
Avasorum	3	Q14684
Avasorum	3	Q14689
Avasorum	3	Q14690
Avasorum	3	Q14691
Avasorum	3	Q14692
Avasorum	3	Q14693
Avasorum	3	Q14694
Avasorum	3	Q14696
Avasorum	3	Q14697
Avasorum	3	Q14699
Avasorum	3	Q14728
Avasorum	3	Q14738
Avasorum	3	Q14739

Avasorum	3	Q14746
Avasorum	3	Q14764
Avasorum	3	Q14766
Avasorum	3	Q14767
Avasorum	3	Q14781
Avasorum	3	Q14789
Avasorum	3	Q14790
Avasorum	3	Q147X3
Avasorum	3	Q14807
Avasorum	3	Q14814
Avasorum	3	Q14839
Avasorum	3	Q14847
Avasorum	3	Q14914
Avasorum	3	Q14919
Avasorum	3	Q14934
Avasorum	3	Q14966
Avasorum	3	Q14974
Avasorum	3	Q14978
Avasorum	3	Q14980
Avasorum	3	Q14997
Avasorum	3	Q14999
Avasorum	3	Q14BN4
Avasorum	3	Q14C86
Avasorum	3	Q14CX7
Avasorum	3	Q14D04
Avasorum	3	Q15003
Avasorum	3	Q15004
Avasorum	3	Q15005
Avasorum	3	Q15006
Avasorum	3	Q15007
Avasorum	3	Q15008
Avasorum	3	Q15011
Avasorum	3	Q15013
Avasorum	3	Q15014
Avasorum	3	Q15018
Avasorum	3	Q15019
Avasorum	3	Q15020
Avasorum	3	Q15021
Avasorum	3	Q15022
Avasorum	3	Q15024
Avasorum	3	Q15025
Avasorum	3	Q15029
Avasorum	3	Q15031
Avasorum	3	Q15032
Avasorum	3	Q15035
Avasorum	3	Q15036
Avasorum	3	Q15041
Avasorum	3	Q15042
Avasorum	3	Q15043
Avasorum	3	Q15046

Avasorum	3	Q15047
Avasorum	3	Q15048
Avasorum	3	Q15050
Avasorum	3	Q15054
Avasorum	3	Q15056
Avasorum	3	Q15057
Avasorum	3	Q15058
Avasorum	3	Q15059
Avasorum	3	Q15061
Avasorum	3	Q15067
Avasorum	3	Q15070
Avasorum	3	Q15075
Avasorum	3	Q15084
Avasorum	3	Q15102
Avasorum	3	Q15120
Avasorum	3	Q15121
Avasorum	3	Q15125
Avasorum	3	Q15126
Avasorum	3	Q15139
Avasorum	3	Q15149
Avasorum	3	Q15154
Avasorum	3	Q15155
Avasorum	3	Q15165
Avasorum	3	Q15172
Avasorum	3	Q15181
Avasorum	3	Q15185
Avasorum	3	Q15208
Avasorum	3	Q15233
Avasorum	3	Q15257
Avasorum	3	Q15262
Avasorum	3	Q15269
Avasorum	3	Q15276
Avasorum	3	Q15286
Avasorum	3	Q15287
Avasorum	3	Q15291
Avasorum	3	Q15293
Avasorum	3	Q15311
Avasorum	3	Q15334
Avasorum	3	Q15361
Avasorum	3	Q15363
Avasorum	3	Q15365
Avasorum	3	Q15366
Avasorum	3	Q15369
Avasorum	3	Q15370
Avasorum	3	Q15382
Avasorum	3	Q15386
Avasorum	3	Q15388
Avasorum	3	Q15390
Avasorum	3	Q15392
Avasorum	3	Q15393

Avasorum	3	Q15397
Avasorum	3	Q15398
Avasorum	3	Q15404
Avasorum	3	Q15417
Avasorum	3	Q15418
Avasorum	3	Q15424
Avasorum	3	Q15428
Avasorum	3	Q15434
Avasorum	3	Q15435
Avasorum	3	Q15436
Avasorum	3	Q15437
Avasorum	3	Q15438
Avasorum	3	Q15459
Avasorum	3	Q15477
Avasorum	3	Q15526
Avasorum	3	Q15545
Avasorum	3	Q15554
Avasorum	3	Q15555
Avasorum	3	Q15583
Avasorum	3	Q15599
Avasorum	3	Q15628
Avasorum	3	Q15629
Avasorum	3	Q15631
Avasorum	3	Q15633
Avasorum	3	Q15637
Avasorum	3	Q15642
Avasorum	3	Q15643
Avasorum	3	Q15645
Avasorum	3	Q15648
Avasorum	3	Q15650
Avasorum	3	Q15652
Avasorum	3	Q15653
Avasorum	3	Q15654
Avasorum	3	Q15678
Avasorum	3	Q15691
Avasorum	3	Q15717
Avasorum	3	Q15738
Avasorum	3	Q15746
Avasorum	3	Q15750
Avasorum	3	Q15751
Avasorum	3	Q15758
Avasorum	3	Q15773
Avasorum	3	Q15785
Avasorum	3	Q15796
Avasorum	3	Q15797
Avasorum	3	Q15800
Avasorum	3	Q15811
Avasorum	3	Q15813
Avasorum	3	Q15814
Avasorum	3	Q15819

Avasorum	3	Q15831
Avasorum	3	Q15833
Avasorum	3	Q15836
Avasorum	3	Q15843
Avasorum	3	Q15904
Avasorum	3	Q15906
Avasorum	3	Q15907
Avasorum	3	Q15942
Avasorum	3	Q16134
Avasorum	3	Q16181
Avasorum	3	Q16186
Avasorum	3	Q16204
Avasorum	3	Q16222
Avasorum	3	Q16254
Avasorum	3	Q16270
Avasorum	3	Q16342
Avasorum	3	Q16363
Avasorum	3	Q16401
Avasorum	3	Q16512
Avasorum	3	Q16513
Avasorum	3	Q16514
Avasorum	3	Q16527
Avasorum	3	Q16531
Avasorum	3	Q16537
Avasorum	3	Q16539
Avasorum	3	Q16540
Avasorum	3	Q16543
Avasorum	3	Q16555
Avasorum	3	Q16563
Avasorum	3	Q16576
Avasorum	3	Q16584
Avasorum	3	Q16594
Avasorum	3	Q16602
Avasorum	3	Q16611
Avasorum	3	Q16629
Avasorum	3	Q16630
Avasorum	3	Q16637
Avasorum	3	Q16643
Avasorum	3	Q16656
Avasorum	3	Q16658
Avasorum	3	Q16666
Avasorum	3	Q16698
Avasorum	3	Q16706
Avasorum	3	Q16718
Avasorum	3	Q16739
Avasorum	3	Q16740
Avasorum	3	Q16762
Avasorum	3	Q16763
Avasorum	3	Q16773
Avasorum	3	Q16774

Avasorum	3	Q16775
Avasorum	3	Q16795
Avasorum	3	Q16798
Avasorum	3	Q16822
Avasorum	3	Q16831
Avasorum	3	Q16832
Avasorum	3	Q16836
Avasorum	3	Q16850
Avasorum	3	Q16851
Avasorum	3	Q16875
Avasorum	3	Q16881
Avasorum	3	Q16891
Avasorum	3	Q19T08
Avasorum	3	Q1ED39
Avasorum	3	Q1KMD3
Avasorum	3	Q24JP5
Avasorum	3	Q27J81
Avasorum	3	Q29RF7
Avasorum	3	Q2KHT3
Avasorum	3	Q2M1P5
Avasorum	3	Q2M1Z3
Avasorum	3	Q2M296
Avasorum	3	Q2M2I8
Avasorum	3	Q2M389
Avasorum	3	Q2NKX8
Avasorum	3	Q2NL82
Avasorum	3	Q2PPJ7
Avasorum	3	Q2PZI1
Avasorum	3	Q2TAA2
Avasorum	3	Q2TAA5
Avasorum	3	Q2TAL8
Avasorum	3	Q2TAY7
Avasorum	3	Q2VPK5
Avasorum	3	Q32M88
Avasorum	3	Q32MZ4
Avasorum	3	Q32NB8
Avasorum	3	Q32NC0
Avasorum	3	Q32P28
Avasorum	3	Q32P41
Avasorum	3	Q32P44
Avasorum	3	Q3B726
Avasorum	3	Q3KQU3
Avasorum	3	Q3KQV9
Avasorum	3	Q3KQZ1
Avasorum	3	Q3L8U1
Avasorum	3	Q3LXA3
Avasorum	3	Q3MHD2
Avasorum	3	Q3SXM5
Avasorum	3	Q3SY69
Avasorum	3	Q3T906

Avasorum	3	Q3V6T2
Avasorum	3	Q3YEC7
Avasorum	3	Q3ZCQ8
Avasorum	3	Q460N5
Avasorum	3	Q495W5
Avasorum	3	Q49A26
Avasorum	3	Q49AR2
Avasorum	3	Q4AC94
Avasorum	3	Q4G0F5
Avasorum	3	Q4G0J3
Avasorum	3	Q4G0N4
Avasorum	3	Q4G0X4
Avasorum	3	Q4G148
Avasorum	3	Q4G176
Avasorum	3	Q4J6C6
Avasorum	3	Q4KMP7
Avasorum	3	Q4KMQ1
Avasorum	3	Q4KMQ2
Avasorum	3	Q4L180
Avasorum	3	Q4V328
Avasorum	3	Q4V339
Avasorum	3	Q4VC31
Avasorum	3	Q4ZIN3
Avasorum	3	Q52LJ0
Avasorum	3	Q52LW3
Avasorum	3	Q53EL6
Avasorum	3	Q53EP0
Avasorum	3	Q53ET0
Avasorum	3	Q53EU6
Avasorum	3	Q53EZ4
Avasorum	3	Q53F19
Avasorum	3	Q53FA7
Avasorum	3	Q53FP2
Avasorum	3	Q53GA4
Avasorum	3	Q53GG5
Avasorum	3	Q53GL7
Avasorum	3	Q53GQ0
Avasorum	3	Q53GS7
Avasorum	3	Q53GS9
Avasorum	3	Q53GT1
Avasorum	3	Q53H12
Avasorum	3	Q53H47
Avasorum	3	Q53H82
Avasorum	3	Q53H96
Avasorum	3	Q53HC9
Avasorum	3	Q53HL2
Avasorum	3	Q53SF7
Avasorum	3	Q53T59
Avasorum	3	Q53TN4
Avasorum	3	Q562E7

Avasorum	3	Q562R1
Avasorum	3	Q567U6
Avasorum	3	Q56VL3
Avasorum	3	Q58EX7
Avasorum	3	Q58FG1
Avasorum	3	Q58WW2
Avasorum	3	Q5BJD5
Avasorum	3	Q5BJF2
Avasorum	3	Q5BJH7
Avasorum	3	Q5BKZ1
Avasorum	3	Q5C9Z4
Avasorum	3	Q5EBL4
Avasorum	3	Q5F1R6
Avasorum	3	Q5GJ75
Avasorum	3	Q5GLZ8
Avasorum	3	Q5H8A4
Avasorum	3	Q5H9R7
Avasorum	3	Q5H9U9
Avasorum	3	Q5HYI8
Avasorum	3	Q5HYJ3
Avasorum	3	Q5HYK3
Avasorum	3	Q5HYK7
Avasorum	3	Q5HYW2
Avasorum	3	Q5J8M3
Avasorum	3	Q5JPH6
Avasorum	3	Q5JPI3
Avasorum	3	Q5JPI9
Avasorum	3	Q5JRA6
Avasorum	3	Q5JRX3
Avasorum	3	Q5JSH3
Avasorum	3	Q5JSZ5
Avasorum	3	Q5JTD0
Avasorum	3	Q5JTH9
Avasorum	3	Q5JTJ3
Avasorum	3	Q5JTV8
Avasorum	3	Q5JTW2
Avasorum	3	Q5JTZ9
Avasorum	3	Q5JU69
Avasorum	3	Q5JVF3
Avasorum	3	Q5JVS0
Avasorum	3	Q5K4L6
Avasorum	3	Q5K651
Avasorum	3	Q5MIZ7
Avasorum	3	Q5MNZ6
Avasorum	3	Q5MNZ9
Avasorum	3	Q5NDL2
Avasorum	3	Q5PRF9
Avasorum	3	Q5QJ74
Avasorum	3	Q5QJE6
Avasorum	3	Q5R372

Avasorum	3	Q5R3I4
Avasorum	3	Q5RI15
Avasorum	3	Q5RKV6
Avasorum	3	Q5SNT2
Avasorum	3	Q5SQI0
Avasorum	3	Q5SQN1
Avasorum	3	Q5SRE5
Avasorum	3	Q5SSJ5
Avasorum	3	Q5ST30
Avasorum	3	Q5SW79
Avasorum	3	Q5SW96
Avasorum	3	Q5SWX8
Avasorum	3	Q5SY16
Avasorum	3	Q5SYE7
Avasorum	3	Q5T013
Avasorum	3	Q5T0D9
Avasorum	3	Q5T0F9
Avasorum	3	Q5T0N5
Avasorum	3	Q5T160
Avasorum	3	Q5T1C6
Avasorum	3	Q5T1J5
Avasorum	3	Q5T1M5
Avasorum	3	Q5T200
Avasorum	3	Q5T280
Avasorum	3	Q5T2E6
Avasorum	3	Q5T3F8
Avasorum	3	Q5T3I0
Avasorum	3	Q5T440
Avasorum	3	Q5T447
Avasorum	3	Q5T4B2
Avasorum	3	Q5T4S7
Avasorum	3	Q5T5C0
Avasorum	3	Q5T5U3
Avasorum	3	Q5T5Y3
Avasorum	3	Q5T653
Avasorum	3	Q5T6F2
Avasorum	3	Q5T6V5
Avasorum	3	Q5T749
Avasorum	3	Q5T7W0
Avasorum	3	Q5T8D3
Avasorum	3	Q5T8P6
Avasorum	3	Q5T9A4
Avasorum	3	Q5T9L3
Avasorum	3	Q5TA45
Avasorum	3	Q5TA50
Avasorum	3	Q5TAQ9
Avasorum	3	Q5TAT6
Avasorum	3	Q5TAX3
Avasorum	3	Q5TBA9
Avasorum	3	Q5TBB1

Avasorum	3	Q5TC12
Avasorum	3	Q5TC82
Avasorum	3	Q5TDH0
Avasorum	3	Q5TEJ8
Avasorum	3	Q5TEU4
Avasorum	3	Q5TFE4
Avasorum	3	Q5TH69
Avasorum	3	Q5THJ4
Avasorum	3	Q5TZA2
Avasorum	3	Q5U5X0
Avasorum	3	Q5U651
Avasorum	3	Q5UIP0
Avasorum	3	Q5VIR6
Avasorum	3	Q5VSL9
Avasorum	3	Q5VT25
Avasorum	3	Q5VT52
Avasorum	3	Q5VTB9
Avasorum	3	Q5VTL8
Avasorum	3	Q5VTR2
Avasorum	3	Q5VU43
Avasorum	3	Q5VUA4
Avasorum	3	Q5VUB5
Avasorum	3	Q5VUD6
Avasorum	3	Q5VUJ6
Avasorum	3	Q5VV42
Avasorum	3	Q5VW32
Avasorum	3	Q5VW36
Avasorum	3	Q5VWQ0
Avasorum	3	Q5VWQ8
Avasorum	3	Q5VWZ2
Avasorum	3	Q5VY43
Avasorum	3	Q5VYK3
Avasorum	3	Q5VYS8
Avasorum	3	Q5VZ18
Avasorum	3	Q5VZ89
Avasorum	3	Q5VZE5
Avasorum	3	Q5VZL5
Avasorum	3	Q5W0V3
Avasorum	3	Q5W0Z9
Avasorum	3	Q5W111
Avasorum	3	Q5XUX1
Avasorum	3	Q5ZPR3
Avasorum	3	Q63HN8
Avasorum	3	Q63HR2
Avasorum	3	Q63ZY3
Avasorum	3	Q641Q2
Avasorum	3	Q643R3
Avasorum	3	Q658P3
Avasorum	3	Q658Y4
Avasorum	3	Q66K14

Avasorum	3	Q66K74
Avasorum	3	Q66LE6
Avasorum	3	Q66PJ3
Avasorum	3	Q676U5
Avasorum	3	Q68CP4
Avasorum	3	Q68CP9
Avasorum	3	Q68CQ4
Avasorum	3	Q68CQ7
Avasorum	3	Q68CR1
Avasorum	3	Q68CZ2
Avasorum	3	Q68CZ6
Avasorum	3	Q68D10
Avasorum	3	Q68D91
Avasorum	3	Q68DQ2
Avasorum	3	Q68E01
Avasorum	3	Q68EM7
Avasorum	3	Q69YL0
Avasorum	3	Q69YN2
Avasorum	3	Q69YN4
Avasorum	3	Q69YQ0
Avasorum	3	Q6AI08
Avasorum	3	Q6AI12
Avasorum	3	Q6AWC2
Avasorum	3	Q6AZY7
Avasorum	3	Q6DD88
Avasorum	3	Q6DKI1
Avasorum	3	Q6DKJ4
Avasorum	3	Q6DKK2
Avasorum	3	Q6DN90
Avasorum	3	Q6FI81
Avasorum	3	Q6FIF0
Avasorum	3	Q6GMV2
Avasorum	3	Q6GQQ9
Avasorum	3	Q6I9Y2
Avasorum	3	Q6IA69
Avasorum	3	Q6IA86
Avasorum	3	Q6IAA8
Avasorum	3	Q6IAN0
Avasorum	3	Q6IBS0
Avasorum	3	Q6IBW4
Avasorum	3	Q6ICG6
Avasorum	3	Q6ICL3
Avasorum	3	Q6IN85
Avasorum	3	Q6IQ22
Avasorum	3	Q6IQ26
Avasorum	3	Q6IQ49
Avasorum	3	Q6KB66
Avasorum	3	Q6KC79
Avasorum	3	Q6KCM7
Avasorum	3	Q6L8Q7

Avasorum	3	Q6N069
Avasorum	3	Q6NSW5
Avasorum	3	Q6NTF9
Avasorum	3	Q6NUK1
Avasorum	3	Q6NUK4
Avasorum	3	Q6NUM9
Avasorum	3	Q6NUQ1
Avasorum	3	Q6NUQ4
Avasorum	3	Q6NVY1
Avasorum	3	Q6NW29
Avasorum	3	Q6NW34
Avasorum	3	Q6NXE6
Avasorum	3	Q6NXR4
Avasorum	3	Q6NXT1
Avasorum	3	Q6NXT4
Avasorum	3	Q6NXT6
Avasorum	3	Q6NY19
Avasorum	3	Q6NYC1
Avasorum	3	Q6NYC8
Avasorum	3	Q6NZI2
Avasorum	3	Q6NZY4
Avasorum	3	Q6P158
Avasorum	3	Q6P179
Avasorum	3	Q6P1A2
Avasorum	3	Q6P1J9
Avasorum	3	Q6P1L8
Avasorum	3	Q6P1M0
Avasorum	3	Q6P1N0
Avasorum	3	Q6P1N9
Avasorum	3	Q6P1Q9
Avasorum	3	Q6P1R3
Avasorum	3	Q6P1X5
Avasorum	3	Q6P1X6
Avasorum	3	Q6P2C8
Avasorum	3	Q6P2E9
Avasorum	3	Q6P2H3
Avasorum	3	Q6P2P2
Avasorum	3	Q6P2Q9
Avasorum	3	Q6P3S6
Avasorum	3	Q6P3W7
Avasorum	3	Q6P3X3
Avasorum	3	Q6P4A7
Avasorum	3	Q6P4E1
Avasorum	3	Q6P4R8
Avasorum	3	Q6P587
Avasorum	3	Q6P5R6
Avasorum	3	Q6P5Z2
Avasorum	3	Q6P6C2
Avasorum	3	Q6P996
Avasorum	3	Q6P9B6

Avasorum	3	Q6P9B9
Avasorum	3	Q6P9H5
Avasorum	3	Q6PCB7
Avasorum	3	Q6PCE3
Avasorum	3	Q6PD62
Avasorum	3	Q6PD74
Avasorum	3	Q6PEY1
Avasorum	3	Q6PGN9
Avasorum	3	Q6PGP7
Avasorum	3	Q6PHR2
Avasorum	3	Q6PI48
Avasorum	3	Q6PI78
Avasorum	3	Q6PI98
Avasorum	3	Q6PIJ6
Avasorum	3	Q6PIU2
Avasorum	3	Q6PIW4
Avasorum	3	Q6PJ69
Avasorum	3	Q6PJF5
Avasorum	3	Q6PJG2
Avasorum	3	Q6PJG6
Avasorum	3	Q6PJI9
Avasorum	3	Q6PJT7
Avasorum	3	Q6PK04
Avasorum	3	Q6PK18
Avasorum	3	Q6PKC3
Avasorum	3	Q6PKG0
Avasorum	3	Q6PL24
Avasorum	3	Q6PML9
Avasorum	3	Q6Q0C0
Avasorum	3	Q6R327
Avasorum	3	Q6RFH5
Avasorum	3	Q6RW13
Avasorum	3	Q6STE5
Avasorum	3	Q6UB35
Avasorum	3	Q6ULP2
Avasorum	3	Q6UN15
Avasorum	3	Q6UUV7
Avasorum	3	Q6UUV9
Avasorum	3	Q6UW02
Avasorum	3	Q6UW63
Avasorum	3	Q6UW68
Avasorum	3	Q6UWE0
Avasorum	3	Q6UWH4
Avasorum	3	Q6UWP7
Avasorum	3	Q6UWZ7
Avasorum	3	Q6UX04
Avasorum	3	Q6UXH1
Avasorum	3	Q6UXN9
Avasorum	3	Q6UXV4
Avasorum	3	Q6V0I7

Avasorum	3	Q6V1X1
Avasorum	3	Q6VMQ6
Avasorum	3	Q6VN20
Avasorum	3	Q6VY07
Avasorum	3	Q6WCQ1
Avasorum	3	Q6XE24
Avasorum	3	Q6XQN6
Avasorum	3	Q6XZF7
Avasorum	3	Q6Y1H2
Avasorum	3	Q6Y288
Avasorum	3	Q6Y7W6
Avasorum	3	Q6YHK3
Avasorum	3	Q6YHU6
Avasorum	3	Q6YN16
Avasorum	3	Q6YP21
Avasorum	3	Q6ZMI0
Avasorum	3	Q6ZMP0
Avasorum	3	Q6ZMZ3
Avasorum	3	Q6ZN55
Avasorum	3	Q6ZNB6
Avasorum	3	Q6ZNJ1
Avasorum	3	Q6ZNL6
Avasorum	3	Q6ZRP7
Avasorum	3	Q6ZRS2
Avasorum	3	Q6ZS17
Avasorum	3	Q6ZSR9
Avasorum	3	Q6ZSZ5
Avasorum	3	Q6ZT07
Avasorum	3	Q6ZT12
Avasorum	3	Q6ZT21
Avasorum	3	Q6ZU35
Avasorum	3	Q6ZUT6
Avasorum	3	Q6ZVK8
Avasorum	3	Q6ZW49
Avasorum	3	Q6ZWJ1
Avasorum	3	Q6ZXV5
Avasorum	3	Q709C8
Avasorum	3	Q70CQ2
Avasorum	3	Q70E73
Avasorum	3	Q70IA6
Avasorum	3	Q70UQ0
Avasorum	3	Q70Z35
Avasorum	3	Q70Z53
Avasorum	3	Q712K3
Avasorum	3	Q71F56
Avasorum	3	Q71RC2
Avasorum	3	Q71SY5
Avasorum	3	Q71UM5
Avasorum	3	Q75N03
Avasorum	3	Q75QN2

Avasorum	3	Q7KYR7
Avasorum	3	Q7KZ85
Avasorum	3	Q7KZF4
Avasorum	3	Q7KZI7
Avasorum	3	Q7KZN9
Avasorum	3	Q7L014
Avasorum	3	Q7L099
Avasorum	3	Q7L0Y3
Avasorum	3	Q7L1Q6
Avasorum	3	Q7L1V2
Avasorum	3	Q7L1W4
Avasorum	3	Q7L266
Avasorum	3	Q7L273
Avasorum	3	Q7L2E3
Avasorum	3	Q7L2H7
Avasorum	3	Q7L2J0
Avasorum	3	Q7L311
Avasorum	3	Q7L3T8
Avasorum	3	Q7L4I2
Avasorum	3	Q7L523
Avasorum	3	Q7L576
Avasorum	3	Q7L592
Avasorum	3	Q7L5D6
Avasorum	3	Q7L5N1
Avasorum	3	Q7L5N7
Avasorum	3	Q7L5Y1
Avasorum	3	Q7L5Y9
Avasorum	3	Q7L775
Avasorum	3	Q7L7V1
Avasorum	3	Q7L7X3
Avasorum	3	Q7L8L6
Avasorum	3	Q7L9L4
Avasorum	3	Q7LBC6
Avasorum	3	Q7LBR1
Avasorum	3	Q7LG56
Avasorum	3	Q7LGA3
Avasorum	3	Q7RTN6
Avasorum	3	Q7RTP6
Avasorum	3	Q7RTS9
Avasorum	3	Q7RTV0
Avasorum	3	Q7RTV5
Avasorum	3	Q7Z2E3
Avasorum	3	Q7Z2K6
Avasorum	3	Q7Z2K8
Avasorum	3	Q7Z2T5
Avasorum	3	Q7Z2W4
Avasorum	3	Q7Z2W9
Avasorum	3	Q7Z2Z2
Avasorum	3	Q7Z333
Avasorum	3	Q7Z392

Avasorum	3	Q7Z3B3
Avasorum	3	Q7Z3B4
Avasorum	3	Q7Z3C6
Avasorum	3	Q7Z3D6
Avasorum	3	Q7Z3E2
Avasorum	3	Q7Z3E5
Avasorum	3	Q7Z3J2
Avasorum	3	Q7Z3K3
Avasorum	3	Q7Z3T8
Avasorum	3	Q7Z3U7
Avasorum	3	Q7Z401
Avasorum	3	Q7Z406
Avasorum	3	Q7Z417
Avasorum	3	Q7Z422
Avasorum	3	Q7Z434
Avasorum	3	Q7Z460
Avasorum	3	Q7Z478
Avasorum	3	Q7Z494
Avasorum	3	Q7Z4F1
Avasorum	3	Q7Z4H7
Avasorum	3	Q7Z4H8
Avasorum	3	Q7Z4L5
Avasorum	3	Q7Z4Q2
Avasorum	3	Q7Z4S6
Avasorum	3	Q7Z4V5
Avasorum	3	Q7Z4W1
Avasorum	3	Q7Z569
Avasorum	3	Q7Z589
Avasorum	3	Q7Z5G4
Avasorum	3	Q7Z5H3
Avasorum	3	Q7Z5K2
Avasorum	3	Q7Z5L9
Avasorum	3	Q7Z6E9
Avasorum	3	Q7Z6J0
Avasorum	3	Q7Z6J2
Avasorum	3	Q7Z6J6
Avasorum	3	Q7Z6J9
Avasorum	3	Q7Z6K3
Avasorum	3	Q7Z6K5
Avasorum	3	Q7Z6L1
Avasorum	3	Q7Z6M1
Avasorum	3	Q7Z6M4
Avasorum	3	Q7Z6Z7
Avasorum	3	Q7Z739
Avasorum	3	Q7Z7A3
Avasorum	3	Q7Z7E8
Avasorum	3	Q7Z7F7
Avasorum	3	Q7Z7H5
Avasorum	3	Q7Z7H8
Avasorum	3	Q7Z7K0

Avasorum	3	Q7Z7K6
Avasorum	3	Q7Z7L1
Avasorum	3	Q7Z7M0
Avasorum	3	Q7Z7N9
Avasorum	3	Q86SF2
Avasorum	3	Q86SK9
Avasorum	3	Q86SQ0
Avasorum	3	Q86SQ4
Avasorum	3	Q86SQ9
Avasorum	3	Q86SR1
Avasorum	3	Q86SZ2
Avasorum	3	Q86T03
Avasorum	3	Q86T13
Avasorum	3	Q86TB9
Avasorum	3	Q86TI2
Avasorum	3	Q86TM6
Avasorum	3	Q86TN4
Avasorum	3	Q86TU7
Avasorum	3	Q86TV6
Avasorum	3	Q86TX2
Avasorum	3	Q86U38
Avasorum	3	Q86U42
Avasorum	3	Q86U44
Avasorum	3	Q86U86
Avasorum	3	Q86U90
Avasorum	3	Q86UA1
Avasorum	3	Q86UE4
Avasorum	3	Q86UK7
Avasorum	3	Q86UL3
Avasorum	3	Q86UP2
Avasorum	3	Q86US8
Avasorum	3	Q86UT6
Avasorum	3	Q86UU0
Avasorum	3	Q86UU1
Avasorum	3	Q86UV5
Avasorum	3	Q86UW7
Avasorum	3	Q86UX7
Avasorum	3	Q86UY6
Avasorum	3	Q86UY8
Avasorum	3	Q86V21
Avasorum	3	Q86V48
Avasorum	3	Q86V81
Avasorum	3	Q86V85
Avasorum	3	Q86V87
Avasorum	3	Q86VI3
Avasorum	3	Q86VM9
Avasorum	3	Q86VN1
Avasorum	3	Q86VP6
Avasorum	3	Q86VR2
Avasorum	3	Q86VS8

Avasorum	3	Q86VW0
Avasorum	3	Q86VX2
Avasorum	3	Q86VX9
Avasorum	3	Q86W34
Avasorum	3	Q86W42
Avasorum	3	Q86W50
Avasorum	3	Q86W56
Avasorum	3	Q86W92
Avasorum	3	Q86WA8
Avasorum	3	Q86WB0
Avasorum	3	Q86WJ1
Avasorum	3	Q86WN1
Avasorum	3	Q86WR0
Avasorum	3	Q86WR7
Avasorum	3	Q86WV6
Avasorum	3	Q86WX3
Avasorum	3	Q86X02
Avasorum	3	Q86X10
Avasorum	3	Q86X27
Avasorum	3	Q86X55
Avasorum	3	Q86X76
Avasorum	3	Q86X83
Avasorum	3	Q86XA9
Avasorum	3	Q86XI2
Avasorum	3	Q86XI6
Avasorum	3	Q86XK2
Avasorum	3	Q86XL3
Avasorum	3	Q86XN8
Avasorum	3	Q86XP1
Avasorum	3	Q86XP3
Avasorum	3	Q86XZ4
Avasorum	3	Q86Y07
Avasorum	3	Q86Y37
Avasorum	3	Q86Y39
Avasorum	3	Q86Y56
Avasorum	3	Q86Y79
Avasorum	3	Q86Y82
Avasorum	3	Q86YP4
Avasorum	3	Q86YQ8
Avasorum	3	Q86YR5
Avasorum	3	Q86YS6
Avasorum	3	Q86YS7
Avasorum	3	Q86YT6
Avasorum	3	Q86YV5
Avasorum	3	Q86YV9
Avasorum	3	Q8IU81
Avasorum	3	Q8IUD2
Avasorum	3	Q8IUF8
Avasorum	3	Q8IUH4
Avasorum	3	Q8IUH5

Avasorum	3	Q8IUI8
Avasorum	3	Q8IUR0
Avasorum	3	Q8IUR7
Avasorum	3	Q8IUW5
Avasorum	3	Q8IUX1
Avasorum	3	Q8IV08
Avasorum	3	Q8IV36
Avasorum	3	Q8IV38
Avasorum	3	Q8IV48
Avasorum	3	Q8IV63
Avasorum	3	Q8IVB5
Avasorum	3	Q8IVD9
Avasorum	3	Q8IVF2
Avasorum	3	Q8IVF7
Avasorum	3	Q8IVH4
Avasorum	3	Q8IVH8
Avasorum	3	Q8IVL0
Avasorum	3	Q8IVL6
Avasorum	3	Q8IVM0
Avasorum	3	Q8IVS2
Avasorum	3	Q8IVT5
Avasorum	3	Q8IW35
Avasorum	3	Q8IW45
Avasorum	3	Q8IWA0
Avasorum	3	Q8IWA4
Avasorum	3	Q8IWA5
Avasorum	3	Q8IWB1
Avasorum	3	Q8IWB7
Avasorum	3	Q8IWC1
Avasorum	3	Q8IWD4
Avasorum	3	Q8IWE2
Avasorum	3	Q8IWE4
Avasorum	3	Q8IWF6
Avasorum	3	Q8IWI9
Avasorum	3	Q8IWJ2
Avasorum	3	Q8IWR0
Avasorum	3	Q8IWS0
Avasorum	3	Q8IWT0
Avasorum	3	Q8IWT6
Avasorum	3	Q8IWU5
Avasorum	3	Q8IWV7
Avasorum	3	Q8IWV8
Avasorum	3	Q8IWW6
Avasorum	3	Q8IWX8
Avasorum	3	Q8IWY9
Avasorum	3	Q8IWZ3
Avasorum	3	Q8IWZ8
Avasorum	3	Q8IX01
Avasorum	3	Q8IX04
Avasorum	3	Q8IX12

Avasorum	3	Q8IX18
Avasorum	3	Q8IXB1
Avasorum	3	Q8IXH7
Avasorum	3	Q8IXI1
Avasorum	3	Q8IXI2
Avasorum	3	Q8IXJ6
Avasorum	3	Q8IXK0
Avasorum	3	Q8IXM2
Avasorum	3	Q8IXM3
Avasorum	3	Q8IXM6
Avasorum	3	Q8IXQ4
Avasorum	3	Q8IXQ6
Avasorum	3	Q8IXT5
Avasorum	3	Q8IXU6
Avasorum	3	Q8IXW5
Avasorum	3	Q8IY17
Avasorum	3	Q8IY18
Avasorum	3	Q8IY21
Avasorum	3	Q8IY22
Avasorum	3	Q8IY33
Avasorum	3	Q8IY37
Avasorum	3	Q8IY47
Avasorum	3	Q8IY67
Avasorum	3	Q8IY81
Avasorum	3	Q8IY95
Avasorum	3	Q8IYB1
Avasorum	3	Q8IYB3
Avasorum	3	Q8IYB5
Avasorum	3	Q8IYB7
Avasorum	3	Q8IYB8
Avasorum	3	Q8IYD1
Avasorum	3	Q8IYH5
Avasorum	3	Q8IYI6
Avasorum	3	Q8IYJ2
Avasorum	3	Q8IYM9
Avasorum	3	Q8IYQ7
Avasorum	3	Q8IYS2
Avasorum	3	Q8IYU8
Avasorum	3	Q8IZ07
Avasorum	3	Q8IZ21
Avasorum	3	Q8IZ69
Avasorum	3	Q8IZ73
Avasorum	3	Q8IZ81
Avasorum	3	Q8IZ83
Avasorum	3	Q8IZA0
Avasorum	3	Q8IZF2
Avasorum	3	Q8IZH2
Avasorum	3	Q8IZL8
Avasorum	3	Q8IZP0
Avasorum	3	Q8IZQ1

Avasorum	3	Q8IZQ5
Avasorum	3	Q8N0U8
Avasorum	3	Q8N0X7
Avasorum	3	Q8N0Z6
Avasorum	3	Q8N108
Avasorum	3	Q8N122
Avasorum	3	Q8N138
Avasorum	3	Q8N163
Avasorum	3	Q8N183
Avasorum	3	Q8N1B4
Avasorum	3	Q8N1F7
Avasorum	3	Q8N1F8
Avasorum	3	Q8N1G0
Avasorum	3	Q8N1G2
Avasorum	3	Q8N1G4
Avasorum	3	Q8N1I0
Avasorum	3	Q8N1Q1
Avasorum	3	Q8N1W1
Avasorum	3	Q8N201
Avasorum	3	Q8N257
Avasorum	3	Q8N264
Avasorum	3	Q8N2F6
Avasorum	3	Q8N2K0
Avasorum	3	Q8N2M8
Avasorum	3	Q8N2R8
Avasorum	3	Q8N2U0
Avasorum	3	Q8N2W9
Avasorum	3	Q8N335
Avasorum	3	Q8N357
Avasorum	3	Q8N392
Avasorum	3	Q8N3C0
Avasorum	3	Q8N3F8
Avasorum	3	Q8N3P4
Avasorum	3	Q8N3R9
Avasorum	3	Q8N3U4
Avasorum	3	Q8N3V7
Avasorum	3	Q8N3X1
Avasorum	3	Q8N442
Avasorum	3	Q8N488
Avasorum	3	Q8N4A0
Avasorum	3	Q8N4C8
Avasorum	3	Q8N4P3
Avasorum	3	Q8N4Q0
Avasorum	3	Q8N4T8
Avasorum	3	Q8N4V1
Avasorum	3	Q8N511
Avasorum	3	Q8N531
Avasorum	3	Q8N543
Avasorum	3	Q8N556
Avasorum	3	Q8N573

Avasorum	3	Q8N5A5
Avasorum	3	Q8N5C1
Avasorum	3	Q8N5C6
Avasorum	3	Q8N5D0
Avasorum	3	Q8N5F7
Avasorum	3	Q8N5G2
Avasorum	3	Q8N5H7
Avasorum	3	Q8N5I2
Avasorum	3	Q8N5I4
Avasorum	3	Q8N5K1
Avasorum	3	Q8N5L8
Avasorum	3	Q8N5M1
Avasorum	3	Q8N5M4
Avasorum	3	Q8N5M9
Avasorum	3	Q8N5U6
Avasorum	3	Q8N5W9
Avasorum	3	Q8N653
Avasorum	3	Q8N668
Avasorum	3	Q8N684
Avasorum	3	Q8N697
Avasorum	3	Q8N699
Avasorum	3	Q8N6G6
Avasorum	3	Q8N6H7
Avasorum	3	Q8N6M0
Avasorum	3	Q8N6M3
Avasorum	3	Q8N6N3
Avasorum	3	Q8N6R0
Avasorum	3	Q8N6S5
Avasorum	3	Q8N6T3
Avasorum	3	Q8N6T7
Avasorum	3	Q8N766
Avasorum	3	Q8N7H5
Avasorum	3	Q8N7R7
Avasorum	3	Q8N806
Avasorum	3	Q8N8A6
Avasorum	3	Q8N8J7
Avasorum	3	Q8N8R3
Avasorum	3	Q8N8S7
Avasorum	3	Q8N8Z6
Avasorum	3	Q8N954
Avasorum	3	Q8N983
Avasorum	3	Q8N999
Avasorum	3	Q8N9M1
Avasorum	3	Q8N9N2
Avasorum	3	Q8N9N7
Avasorum	3	Q8N9N8
Avasorum	3	Q8N9T8
Avasorum	3	Q8N9Z2
Avasorum	3	Q8NAF0
Avasorum	3	Q8NAV1

Avasorum	3	Q8NB16
Avasorum	3	Q8NB37
Avasorum	3	Q8NB46
Avasorum	3	Q8NB49
Avasorum	3	Q8NB90
Avasorum	3	Q8NBF2
Avasorum	3	Q8NBF6
Avasorum	3	Q8NBI5
Avasorum	3	Q8NBI6
Avasorum	3	Q8NBJ4
Avasorum	3	Q8NBJ5
Avasorum	3	Q8NBJ7
Avasorum	3	Q8NBJ9
Avasorum	3	Q8NBK3
Avasorum	3	Q8NBL1
Avasorum	3	Q8NBM4
Avasorum	3	Q8NBM8
Avasorum	3	Q8NBN3
Avasorum	3	Q8NBN7
Avasorum	3	Q8NBP0
Avasorum	3	Q8NBQ5
Avasorum	3	Q8NBS9
Avasorum	3	Q8NBT2
Avasorum	3	Q8NBU5
Avasorum	3	Q8NBX0
Avasorum	3	Q8NBZ7
Avasorum	3	Q8NC42
Avasorum	3	Q8NC44
Avasorum	3	Q8NC51
Avasorum	3	Q8NC56
Avasorum	3	Q8NC60
Avasorum	3	Q8NC96
Avasorum	3	Q8NCA5
Avasorum	3	Q8NCC3
Avasorum	3	Q8NCE2
Avasorum	3	Q8NCF5
Avasorum	3	Q8NCG7
Avasorum	3	Q8NCH0
Avasorum	3	Q8NCL4
Avasorum	3	Q8NCN4
Avasorum	3	Q8NCN5
Avasorum	3	Q8NCW5
Avasorum	3	Q8ND04
Avasorum	3	Q8ND24
Avasorum	3	Q8ND56
Avasorum	3	Q8ND71
Avasorum	3	Q8ND76
Avasorum	3	Q8NDA8
Avasorum	3	Q8NDF8
Avasorum	3	Q8NDH3

Avasorum	3	Q8NDI1
Avasorum	3	Q8NDT2
Avasorum	3	Q8NDV1
Avasorum	3	Q8NDV7
Avasorum	3	Q8NDX5
Avasorum	3	Q8NDZ4
Avasorum	3	Q8NE01
Avasorum	3	Q8NE71
Avasorum	3	Q8NE86
Avasorum	3	Q8NEB9
Avasorum	3	Q8NEC7
Avasorum	3	Q8NEF9
Avasorum	3	Q8NEJ9
Avasorum	3	Q8NEM2
Avasorum	3	Q8NEN9
Avasorum	3	Q8NEU8
Avasorum	3	Q8NEW0
Avasorum	3	Q8NEY1
Avasorum	3	Q8NEY8
Avasorum	3	Q8NEZ3
Avasorum	3	Q8NEZ5
Avasorum	3	Q8NF37
Avasorum	3	Q8NF64
Avasorum	3	Q8NF91
Avasorum	3	Q8NFA0
Avasorum	3	Q8NFC6
Avasorum	3	Q8NFD5
Avasorum	3	Q8NFF5
Avasorum	3	Q8NFG4
Avasorum	3	Q8NFH3
Avasorum	3	Q8NFH4
Avasorum	3	Q8NFH5
Avasorum	3	Q8NFI3
Avasorum	3	Q8NFJ5
Avasorum	3	Q8NFQ8
Avasorum	3	Q8NFV4
Avasorum	3	Q8NFW8
Avasorum	3	Q8NG11
Avasorum	3	Q8NG68
Avasorum	3	Q8NHG7
Avasorum	3	Q8NHH9
Avasorum	3	Q8NHP6
Avasorum	3	Q8NHP8
Avasorum	3	Q8NHQ8
Avasorum	3	Q8NHQ9
Avasorum	3	Q8NHS3
Avasorum	3	Q8NHU6
Avasorum	3	Q8NHV1
Avasorum	3	Q8NHV4
Avasorum	3	Q8NI08

Avasorum	3	Q8NI27
Avasorum	3	Q8NI36
Avasorum	3	Q8NI37
Avasorum	3	Q8TA86
Avasorum	3	Q8TAA9
Avasorum	3	Q8TAD8
Avasorum	3	Q8TAE8
Avasorum	3	Q8TAF3
Avasorum	3	Q8TAG9
Avasorum	3	Q8TAQ2
Avasorum	3	Q8TAT6
Avasorum	3	Q8TB03
Avasorum	3	Q8TB22
Avasorum	3	Q8TB37
Avasorum	3	Q8TB40
Avasorum	3	Q8TB52
Avasorum	3	Q8TB61
Avasorum	3	Q8TB72
Avasorum	3	Q8TB96
Avasorum	3	Q8TBA6
Avasorum	3	Q8TBB5
Avasorum	3	Q8TBC3
Avasorum	3	Q8TBC4
Avasorum	3	Q8TBF2
Avasorum	3	Q8TBM8
Avasorum	3	Q8TBQ9
Avasorum	3	Q8TBX8
Avasorum	3	Q8TBZ3
Avasorum	3	Q8TC07
Avasorum	3	Q8TC12
Avasorum	3	Q8TCA0
Avasorum	3	Q8TCB0
Avasorum	3	Q8TCC3
Avasorum	3	Q8TCF1
Avasorum	3	Q8TCG1
Avasorum	3	Q8TCG2
Avasorum	3	Q8TCJ2
Avasorum	3	Q8TCS8
Avasorum	3	Q8TCT7
Avasorum	3	Q8TCT9
Avasorum	3	Q8TCU6
Avasorum	3	Q8TCY9
Avasorum	3	Q8TD16
Avasorum	3	Q8TD19
Avasorum	3	Q8TD55
Avasorum	3	Q8TDB6
Avasorum	3	Q8TDD1
Avasorum	3	Q8TDM6
Avasorum	3	Q8TDN6
Avasorum	3	Q8TDQ7

Avasorum	3	Q8TDW0
Avasorum	3	Q8TDX7
Avasorum	3	Q8TDY2
Avasorum	3	Q8TDZ2
Avasorum	3	Q8TE02
Avasorum	3	Q8TE77
Avasorum	3	Q8TE82
Avasorum	3	Q8TEA7
Avasorum	3	Q8TEA8
Avasorum	3	Q8TEB1
Avasorum	3	Q8TED0
Avasorum	3	Q8TED1
Avasorum	3	Q8TED9
Avasorum	3	Q8TEJ3
Avasorum	3	Q8TEL6
Avasorum	3	Q8TEP8
Avasorum	3	Q8TEQ6
Avasorum	3	Q8TEQ8
Avasorum	3	Q8TEU7
Avasorum	3	Q8TEV9
Avasorum	3	Q8TEW0
Avasorum	3	Q8TEX9
Avasorum	3	Q8TEY7
Avasorum	3	Q8TF01
Avasorum	3	Q8TF05
Avasorum	3	Q8TF42
Avasorum	3	Q8TF65
Avasorum	3	Q8TF74
Avasorum	3	Q8WTS1
Avasorum	3	Q8WTS6
Avasorum	3	Q8WTT2
Avasorum	3	Q8WTV0
Avasorum	3	Q8WTW3
Avasorum	3	Q8WU76
Avasorum	3	Q8WU79
Avasorum	3	Q8WU90
Avasorum	3	Q8WUA2
Avasorum	3	Q8WUA4
Avasorum	3	Q8WUA7
Avasorum	3	Q8WUB8
Avasorum	3	Q8WUD1
Avasorum	3	Q8WUD4
Avasorum	3	Q8WUF5
Avasorum	3	Q8WUF8
Avasorum	3	Q8WUH1
Avasorum	3	Q8WUH2
Avasorum	3	Q8WUH6
Avasorum	3	Q8WUI4
Avasorum	3	Q8WUJ0
Avasorum	3	Q8WUK0

Avasorum	3	Q8WUM0
Avasorum	3	Q8WUM4
Avasorum	3	Q8WUP2
Avasorum	3	Q8WUQ7
Avasorum	3	Q8WUX2
Avasorum	3	Q8WUX9
Avasorum	3	Q8WUY1
Avasorum	3	Q8WUY8
Avasorum	3	Q8WUZ0
Avasorum	3	Q8WV92
Avasorum	3	Q8WVB6
Avasorum	3	Q8WVC0
Avasorum	3	Q8WVC6
Avasorum	3	Q8WVJ2
Avasorum	3	Q8WVK2
Avasorum	3	Q8WVM0
Avasorum	3	Q8WVM7
Avasorum	3	Q8WVM8
Avasorum	3	Q8WVP5
Avasorum	3	Q8WVQ1
Avasorum	3	Q8WVT3
Avasorum	3	Q8WVV9
Avasorum	3	Q8WVX9
Avasorum	3	Q8WVY7
Avasorum	3	Q8WW01
Avasorum	3	Q8WW12
Avasorum	3	Q8WW22
Avasorum	3	Q8WW59
Avasorum	3	Q8WWC4
Avasorum	3	Q8WWH5
Avasorum	3	Q8WWI1
Avasorum	3	Q8WWI5
Avasorum	3	Q8WWK9
Avasorum	3	Q8WWM7
Avasorum	3	Q8WWN8
Avasorum	3	Q8WWP7
Avasorum	3	Q8WWY3
Avasorum	3	Q8WX92
Avasorum	3	Q8WX93
Avasorum	3	Q8WXA3
Avasorum	3	Q8WXA9
Avasorum	3	Q8WXD5
Avasorum	3	Q8WXE0
Avasorum	3	Q8WXE1
Avasorum	3	Q8WXF1
Avasorum	3	Q8WXG6
Avasorum	3	Q8WXH0
Avasorum	3	Q8WXI9
Avasorum	3	Q8WXX5
Avasorum	3	Q8WY22

Avasorum	3	Q8WYA6
Avasorum	3	Q8WYN0
Avasorum	3	Q8WYP3
Avasorum	3	Q8WYP5
Avasorum	3	Q8WYQ5
Avasorum	3	Q8WZ75
Avasorum	3	Q8WZ82
Avasorum	3	Q8WZA9
Avasorum	3	Q92466
Avasorum	3	Q92478
Avasorum	3	Q92499
Avasorum	3	Q92503
Avasorum	3	Q92508
Avasorum	3	Q92520
Avasorum	3	Q92522
Avasorum	3	Q92530
Avasorum	3	Q92538
Avasorum	3	Q92540
Avasorum	3	Q92541
Avasorum	3	Q92542
Avasorum	3	Q92543
Avasorum	3	Q92544
Avasorum	3	Q92545
Avasorum	3	Q92551
Avasorum	3	Q92552
Avasorum	3	Q92556
Avasorum	3	Q92562
Avasorum	3	Q92572
Avasorum	3	Q92574
Avasorum	3	Q92575
Avasorum	3	Q92576
Avasorum	3	Q92597
Avasorum	3	Q92598
Avasorum	3	Q92599
Avasorum	3	Q92600
Avasorum	3	Q92604
Avasorum	3	Q92609
Avasorum	3	Q92610
Avasorum	3	Q92613
Avasorum	3	Q92614
Avasorum	3	Q92615
Avasorum	3	Q92616
Avasorum	3	Q92619
Avasorum	3	Q92620
Avasorum	3	Q92621
Avasorum	3	Q92625
Avasorum	3	Q92626
Avasorum	3	Q92636
Avasorum	3	Q92643
Avasorum	3	Q92665

Avasorum	3	Q92667
Avasorum	3	Q92685
Avasorum	3	Q92686
Avasorum	3	Q92688
Avasorum	3	Q92692
Avasorum	3	Q92696
Avasorum	3	Q92734
Avasorum	3	Q92743
Avasorum	3	Q92747
Avasorum	3	Q92759
Avasorum	3	Q92766
Avasorum	3	Q92769
Avasorum	3	Q92783
Avasorum	3	Q92785
Avasorum	3	Q92791
Avasorum	3	Q92793
Avasorum	3	Q92794
Avasorum	3	Q92797
Avasorum	3	Q92804
Avasorum	3	Q92805
Avasorum	3	Q92820
Avasorum	3	Q92823
Avasorum	3	Q92834
Avasorum	3	Q92835
Avasorum	3	Q92841
Avasorum	3	Q92845
Avasorum	3	Q92851
Avasorum	3	Q92870
Avasorum	3	Q92878
Avasorum	3	Q92879
Avasorum	3	Q92882
Avasorum	3	Q92888
Avasorum	3	Q92889
Avasorum	3	Q92890
Avasorum	3	Q92896
Avasorum	3	Q92900
Avasorum	3	Q92905
Avasorum	3	Q92917
Avasorum	3	Q92922
Avasorum	3	Q92925
Avasorum	3	Q92930
Avasorum	3	Q92934
Avasorum	3	Q92945
Avasorum	3	Q92947
Avasorum	3	Q92963
Avasorum	3	Q92968
Avasorum	3	Q92973
Avasorum	3	Q92974
Avasorum	3	Q92979
Avasorum	3	Q92989

Avasorum	3	Q92990
Avasorum	3	Q92995
Avasorum	3	Q92997
Avasorum	3	Q93008
Avasorum	3	Q93009
Avasorum	3	Q93034
Avasorum	3	Q93045
Avasorum	3	Q93050
Avasorum	3	Q93052
Avasorum	3	Q93062
Avasorum	3	Q93063
Avasorum	3	Q93074
Avasorum	3	Q93096
Avasorum	3	Q93100
Avasorum	3	Q969E2
Avasorum	3	Q969F9
Avasorum	3	Q969G3
Avasorum	3	Q969G5
Avasorum	3	Q969H8
Avasorum	3	Q969M1
Avasorum	3	Q969M3
Avasorum	3	Q969N2
Avasorum	3	Q969P0
Avasorum	3	Q969Q0
Avasorum	3	Q969Q5
Avasorum	3	Q969R2
Avasorum	3	Q969S3
Avasorum	3	Q969S9
Avasorum	3	Q969T4
Avasorum	3	Q969T7
Avasorum	3	Q969T9
Avasorum	3	Q969U7
Avasorum	3	Q969V3
Avasorum	3	Q969V5
Avasorum	3	Q969V6
Avasorum	3	Q969X1
Avasorum	3	Q969X5
Avasorum	3	Q969X6
Avasorum	3	Q969Z0
Avasorum	3	Q969Z3
Avasorum	3	Q96A26
Avasorum	3	Q96A33
Avasorum	3	Q96A35
Avasorum	3	Q96A65
Avasorum	3	Q96A72
Avasorum	3	Q96A73
Avasorum	3	Q96AA3
Avasorum	3	Q96AB3
Avasorum	3	Q96AB6
Avasorum	3	Q96AC1

Avasorum	3	Q96AE4
Avasorum	3	Q96AE7
Avasorum	3	Q96AG4
Avasorum	3	Q96AJ9
Avasorum	3	Q96AP7
Avasorum	3	Q96AQ6
Avasorum	3	Q96AQ8
Avasorum	3	Q96AT1
Avasorum	3	Q96AX1
Avasorum	3	Q96AY3
Avasorum	3	Q96AY4
Avasorum	3	Q96B26
Avasorum	3	Q96B54
Avasorum	3	Q96B97
Avasorum	3	Q96BD8
Avasorum	3	Q96BH1
Avasorum	3	Q96BJ3
Avasorum	3	Q96BK5
Avasorum	3	Q96BM9
Avasorum	3	Q96BN8
Avasorum	3	Q96BP2
Avasorum	3	Q96BP3
Avasorum	3	Q96BW1
Avasorum	3	Q96BW9
Avasorum	3	Q96BX8
Avasorum	3	Q96BY6
Avasorum	3	Q96BY7
Avasorum	3	Q96BZ8
Avasorum	3	Q96BZ9
Avasorum	3	Q96C19
Avasorum	3	Q96C23
Avasorum	3	Q96C24
Avasorum	3	Q96C36
Avasorum	3	Q96C57
Avasorum	3	Q96C86
Avasorum	3	Q96C90
Avasorum	3	Q96CB8
Avasorum	3	Q96CB9
Avasorum	3	Q96CC6
Avasorum	3	Q96CG8
Avasorum	3	Q96CM8
Avasorum	3	Q96CN7
Avasorum	3	Q96CN9
Avasorum	3	Q96CP2
Avasorum	3	Q96CP6
Avasorum	3	Q96CS2
Avasorum	3	Q96CS3
Avasorum	3	Q96CT7
Avasorum	3	Q96CU9
Avasorum	3	Q96CV9

Avasorum	3	Q96CW1
Avasorum	3	Q96CW5
Avasorum	3	Q96CW6
Avasorum	3	Q96CX2
Avasorum	3	Q96D15
Avasorum	3	Q96D46
Avasorum	3	Q96D71
Avasorum	3	Q96DA6
Avasorum	3	Q96DB5
Avasorum	3	Q96DE0
Avasorum	3	Q96DF8
Avasorum	3	Q96DG6
Avasorum	3	Q96DH6
Avasorum	3	Q96DI7
Avasorum	3	Q96DM3
Avasorum	3	Q96DV4
Avasorum	3	Q96DX4
Avasorum	3	Q96DZ1
Avasorum	3	Q96E11
Avasorum	3	Q96EA4
Avasorum	3	Q96EB1
Avasorum	3	Q96EB6
Avasorum	3	Q96EC8
Avasorum	3	Q96EE3
Avasorum	3	Q96EI5
Avasorum	3	Q96EK5
Avasorum	3	Q96EK6
Avasorum	3	Q96EK7
Avasorum	3	Q96EK9
Avasorum	3	Q96EL2
Avasorum	3	Q96EL3
Avasorum	3	Q96EM0
Avasorum	3	Q96EP0
Avasorum	3	Q96EP5
Avasorum	3	Q96EQ0
Avasorum	3	Q96ER3
Avasorum	3	Q96ER9
Avasorum	3	Q96ES7
Avasorum	3	Q96EU6
Avasorum	3	Q96EU7
Avasorum	3	Q96EV2
Avasorum	3	Q96EV8
Avasorum	3	Q96EY1
Avasorum	3	Q96EY4
Avasorum	3	Q96EY5
Avasorum	3	Q96EY7
Avasorum	3	Q96EY8
Avasorum	3	Q96F07
Avasorum	3	Q96F15
Avasorum	3	Q96F44

Avasorum	3	Q96F63
Avasorum	3	Q96F85
Avasorum	3	Q96F86
Avasorum	3	Q96FJ2
Avasorum	3	Q96FN4
Avasorum	3	Q96FQ6
Avasorum	3	Q96FS4
Avasorum	3	Q96FV2
Avasorum	3	Q96FV9
Avasorum	3	Q96FW1
Avasorum	3	Q96FX7
Avasorum	3	Q96FZ2
Avasorum	3	Q96FZ7
Avasorum	3	Q96G01
Avasorum	3	Q96G03
Avasorum	3	Q96G21
Avasorum	3	Q96G23
Avasorum	3	Q96G46
Avasorum	3	Q96GA3
Avasorum	3	Q96GA7
Avasorum	3	Q96GC5
Avasorum	3	Q96GD0
Avasorum	3	Q96GD4
Avasorum	3	Q96GG9
Avasorum	3	Q96GK7
Avasorum	3	Q96GM5
Avasorum	3	Q96GM8
Avasorum	3	Q96GQ5
Avasorum	3	Q96GQ7
Avasorum	3	Q96GS4
Avasorum	3	Q96GW9
Avasorum	3	Q96GX2
Avasorum	3	Q96GX5
Avasorum	3	Q96GX9
Avasorum	3	Q96GY0
Avasorum	3	Q96GY3
Avasorum	3	Q96GZ6
Avasorum	3	Q96H20
Avasorum	3	Q96H79
Avasorum	3	Q96HA1
Avasorum	3	Q96HA7
Avasorum	3	Q96HC4
Avasorum	3	Q96HD1
Avasorum	3	Q96HE7
Avasorum	3	Q96HE9
Avasorum	3	Q96HJ9
Avasorum	3	Q96HN2
Avasorum	3	Q96HP0
Avasorum	3	Q96HR3
Avasorum	3	Q96HS1

Avasorum	3	Q96HW7
Avasorum	3	Q96HY6
Avasorum	3	Q96HY7
Avasorum	3	Q96I15
Avasorum	3	Q96I24
Avasorum	3	Q96I25
Avasorum	3	Q96I51
Avasorum	3	Q96I59
Avasorum	3	Q96I99
Avasorum	3	Q96II8
Avasorum	3	Q96IJ6
Avasorum	3	Q96IR7
Avasorum	3	Q96IU4
Avasorum	3	Q96IV0
Avasorum	3	Q96IW7
Avasorum	3	Q96IX5
Avasorum	3	Q96IY1
Avasorum	3	Q96IZ0
Avasorum	3	Q96IZ6
Avasorum	3	Q96J01
Avasorum	3	Q96J02
Avasorum	3	Q96J42
Avasorum	3	Q96J84
Avasorum	3	Q96JA1
Avasorum	3	Q96JB2
Avasorum	3	Q96JB5
Avasorum	3	Q96JC1
Avasorum	3	Q96JG6
Avasorum	3	Q96JH7
Avasorum	3	Q96JI7
Avasorum	3	Q96JJ3
Avasorum	3	Q96JJ7
Avasorum	3	Q96JK2
Avasorum	3	Q96JM3
Avasorum	3	Q96JM7
Avasorum	3	Q96JP5
Avasorum	3	Q96JQ0
Avasorum	3	Q96JQ2
Avasorum	3	Q96JY6
Avasorum	3	Q96K12
Avasorum	3	Q96K17
Avasorum	3	Q96K19
Avasorum	3	Q96K21
Avasorum	3	Q96K37
Avasorum	3	Q96K76
Avasorum	3	Q96K83
Avasorum	3	Q96KA5
Avasorum	3	Q96KB5
Avasorum	3	Q96KC2
Avasorum	3	Q96KC8

Avasorum	3	Q96KG9
Avasorum	3	Q96KM6
Avasorum	3	Q96KN1
Avasorum	3	Q96KP1
Avasorum	3	Q96KP4
Avasorum	3	Q96KQ7
Avasorum	3	Q96KR1
Avasorum	3	Q96KR6
Avasorum	3	Q96L58
Avasorum	3	Q96L91
Avasorum	3	Q96L92
Avasorum	3	Q96L93
Avasorum	3	Q96LB3
Avasorum	3	Q96LD4
Avasorum	3	Q96LI5
Avasorum	3	Q96LJ7
Avasorum	3	Q96LL9
Avasorum	3	Q96LR5
Avasorum	3	Q96LW7
Avasorum	3	Q96M27
Avasorum	3	Q96M96
Avasorum	3	Q96ME1
Avasorum	3	Q96ME7
Avasorum	3	Q96MF7
Avasorum	3	Q96MG7
Avasorum	3	Q96MG8
Avasorum	3	Q96MM6
Avasorum	3	Q96MU7
Avasorum	3	Q96MW1
Avasorum	3	Q96MW5
Avasorum	3	Q96MX0
Avasorum	3	Q96MX6
Avasorum	3	Q96MY1
Avasorum	3	Q96N66
Avasorum	3	Q96N67
Avasorum	3	Q96NB3
Avasorum	3	Q96ND0
Avasorum	3	Q96NE9
Avasorum	3	Q96P11
Avasorum	3	Q96P16
Avasorum	3	Q96P47
Avasorum	3	Q96P48
Avasorum	3	Q96P70
Avasorum	3	Q96PC5
Avasorum	3	Q96PD2
Avasorum	3	Q96PE2
Avasorum	3	Q96PE3
Avasorum	3	Q96PK6
Avasorum	3	Q96PM5
Avasorum	3	Q96PQ7

Avasorum	3	Q96PU4
Avasorum	3	Q96PU5
Avasorum	3	Q96PU8
Avasorum	3	Q96PV6
Avasorum	3	Q96PY5
Avasorum	3	Q96PZ0
Avasorum	3	Q96PZ2
Avasorum	3	Q96Q05
Avasorum	3	Q96Q11
Avasorum	3	Q96Q15
Avasorum	3	Q96Q42
Avasorum	3	Q96Q45
Avasorum	3	Q96Q83
Avasorum	3	Q96QB1
Avasorum	3	Q96QC0
Avasorum	3	Q96QD8
Avasorum	3	Q96QD9
Avasorum	3	Q96QG7
Avasorum	3	Q96QK1
Avasorum	3	Q96QR8
Avasorum	3	Q96QU8
Avasorum	3	Q96QV1
Avasorum	3	Q96QZ7
Avasorum	3	Q96R06
Avasorum	3	Q96RE7
Avasorum	3	Q96RF0
Avasorum	3	Q96RG2
Avasorum	3	Q96RK0
Avasorum	3	Q96RL1
Avasorum	3	Q96RL7
Avasorum	3	Q96RN5
Avasorum	3	Q96RP9
Avasorum	3	Q96RQ1
Avasorum	3	Q96RQ3
Avasorum	3	Q96RR4
Avasorum	3	Q96RS0
Avasorum	3	Q96RS6
Avasorum	3	Q96RT1
Avasorum	3	Q96RT7
Avasorum	3	Q96RT8
Avasorum	3	Q96RU2
Avasorum	3	Q96RU3
Avasorum	3	Q96S44
Avasorum	3	Q96S52
Avasorum	3	Q96S55
Avasorum	3	Q96S59
Avasorum	3	Q96S66
Avasorum	3	Q96S97
Avasorum	3	Q96SB3
Avasorum	3	Q96SB4

Avasorum	3	Q96SB8
Avasorum	3	Q96SI1
Avasorum	3	Q96SI9
Avasorum	3	Q96SK2
Avasorum	3	Q96SL4
Avasorum	3	Q96SQ9
Avasorum	3	Q96ST2
Avasorum	3	Q96ST3
Avasorum	3	Q96SU4
Avasorum	3	Q96SW2
Avasorum	3	Q96SY0
Avasorum	3	Q96SZ5
Avasorum	3	Q96SZ6
Avasorum	3	Q96T23
Avasorum	3	Q96T37
Avasorum	3	Q96T49
Avasorum	3	Q96T51
Avasorum	3	Q96T58
Avasorum	3	Q96T60
Avasorum	3	Q96T76
Avasorum	3	Q96T88
Avasorum	3	Q96TA1
Avasorum	3	Q96TA2
Avasorum	3	Q96TC7
Avasorum	3	Q99081
Avasorum	3	Q99418
Avasorum	3	Q99426
Avasorum	3	Q99436
Avasorum	3	Q99439
Avasorum	3	Q99442
Avasorum	3	Q99447
Avasorum	3	Q99459
Avasorum	3	Q99460
Avasorum	3	Q99470
Avasorum	3	Q99471
Avasorum	3	Q99496
Avasorum	3	Q99497
Avasorum	3	Q99500
Avasorum	3	Q99501
Avasorum	3	Q99504
Avasorum	3	Q99519
Avasorum	3	Q99523
Avasorum	3	Q99536
Avasorum	3	Q99538
Avasorum	3	Q99541
Avasorum	3	Q99543
Avasorum	3	Q99549
Avasorum	3	Q99567
Avasorum	3	Q99569
Avasorum	3	Q99570

Avasorum	3	Q99571
Avasorum	3	Q99575
Avasorum	3	Q99584
Avasorum	3	Q99590
Avasorum	3	Q99598
Avasorum	3	Q99611
Avasorum	3	Q99613
Avasorum	3	Q99614
Avasorum	3	Q99615
Avasorum	3	Q99623
Avasorum	3	Q99627
Avasorum	3	Q99633
Avasorum	3	Q99653
Avasorum	3	Q99661
Avasorum	3	Q99666
Avasorum	3	Q99685
Avasorum	3	Q99700
Avasorum	3	Q99704
Avasorum	3	Q99707
Avasorum	3	Q99714
Avasorum	3	Q99715
Avasorum	3	Q99717
Avasorum	3	Q99720
Avasorum	3	Q99729
Avasorum	3	Q99733
Avasorum	3	Q99735
Avasorum	3	Q99747
Avasorum	3	Q99755
Avasorum	3	Q99758
Avasorum	3	Q99759
Avasorum	3	Q99766
Avasorum	3	Q99797
Avasorum	3	Q99798
Avasorum	3	Q99805
Avasorum	3	Q99808
Avasorum	3	Q99816
Avasorum	3	Q99829
Avasorum	3	Q99832
Avasorum	3	Q99836
Avasorum	3	Q99848
Avasorum	3	Q99873
Avasorum	3	Q99933
Avasorum	3	Q99941
Avasorum	3	Q99961
Avasorum	3	Q99973
Avasorum	3	Q99986
Avasorum	3	Q99988
Avasorum	3	Q99996
Avasorum	3	Q9BPW8
Avasorum	3	Q9BPX3

Avasorum	3	Q9BPX5
Avasorum	3	Q9BPX6
Avasorum	3	Q9BPX7
Avasorum	3	Q9BPY3
Avasorum	3	Q9BPZ7
Avasorum	3	Q9BQ04
Avasorum	3	Q9BQ39
Avasorum	3	Q9BQ48
Avasorum	3	Q9BQ52
Avasorum	3	Q9BQ61
Avasorum	3	Q9BQ67
Avasorum	3	Q9BQ69
Avasorum	3	Q9BQ70
Avasorum	3	Q9BQ75
Avasorum	3	Q9BQ90
Avasorum	3	Q9BQ95
Avasorum	3	Q9BQA1
Avasorum	3	Q9BQA9
Avasorum	3	Q9BQB6
Avasorum	3	Q9BQC3
Avasorum	3	Q9BQE3
Avasorum	3	Q9BQE4
Avasorum	3	Q9BQE5
Avasorum	3	Q9BQG0
Avasorum	3	Q9BQL6
Avasorum	3	Q9BQP7
Avasorum	3	Q9BQQ3
Avasorum	3	Q9BQS8
Avasorum	3	Q9BR61
Avasorum	3	Q9BR76
Avasorum	3	Q9BRA2
Avasorum	3	Q9BRD0
Avasorum	3	Q9BRF8
Avasorum	3	Q9BRG1
Avasorum	3	Q9BRJ2
Avasorum	3	Q9BRJ6
Avasorum	3	Q9BRJ7
Avasorum	3	Q9BRK5
Avasorum	3	Q9BRP1
Avasorum	3	Q9BRP4
Avasorum	3	Q9BRP8
Avasorum	3	Q9BRQ6
Avasorum	3	Q9BRR6
Avasorum	3	Q9BRR8
Avasorum	3	Q9BRS2
Avasorum	3	Q9BRT6
Avasorum	3	Q9BRT9
Avasorum	3	Q9BRU9
Avasorum	3	Q9BRX2
Avasorum	3	Q9BRX8

Avasorum	3	Q9BRZ2
Avasorum	3	Q9BS26
Avasorum	3	Q9BS40
Avasorum	3	Q9BSC4
Avasorum	3	Q9BSD7
Avasorum	3	Q9BSF4
Avasorum	3	Q9BSH4
Avasorum	3	Q9BSJ2
Avasorum	3	Q9BSJ8
Avasorum	3	Q9BSL1
Avasorum	3	Q9BSR8
Avasorum	3	Q9BSU1
Avasorum	3	Q9BSV6
Avasorum	3	Q9BT09
Avasorum	3	Q9BT17
Avasorum	3	Q9BT22
Avasorum	3	Q9BT23
Avasorum	3	Q9BT25
Avasorum	3	Q9BT40
Avasorum	3	Q9BT73
Avasorum	3	Q9BT78
Avasorum	3	Q9BT88
Avasorum	3	Q9BTA9
Avasorum	3	Q9BTC0
Avasorum	3	Q9BTC8
Avasorum	3	Q9BTD8
Avasorum	3	Q9BTE1
Avasorum	3	Q9BTE3
Avasorum	3	Q9BTE6
Avasorum	3	Q9BTE7
Avasorum	3	Q9BTT0
Avasorum	3	Q9BTT6
Avasorum	3	Q9BTU6
Avasorum	3	Q9BTV4
Avasorum	3	Q9BTV5
Avasorum	3	Q9BTW9
Avasorum	3	Q9BTX1
Avasorum	3	Q9BTZ2
Avasorum	3	Q9BU23
Avasorum	3	Q9BU61
Avasorum	3	Q9BU76
Avasorum	3	Q9BUB7
Avasorum	3	Q9BUE0
Avasorum	3	Q9BUE6
Avasorum	3	Q9BUF5
Avasorum	3	Q9BUH6
Avasorum	3	Q9BUI4
Avasorum	3	Q9BUJ2
Avasorum	3	Q9BUK6
Avasorum	3	Q9BUL5

Avasorum	3	Q9BUL8
Avasorum	3	Q9BUL9
Avasorum	3	Q9BUN8
Avasorum	3	Q9BUP3
Avasorum	3	Q9BUQ8
Avasorum	3	Q9BUR4
Avasorum	3	Q9BUR5
Avasorum	3	Q9BUT1
Avasorum	3	Q9BV20
Avasorum	3	Q9BV38
Avasorum	3	Q9BV40
Avasorum	3	Q9BV44
Avasorum	3	Q9BV57
Avasorum	3	Q9BV79
Avasorum	3	Q9BV81
Avasorum	3	Q9BV86
Avasorum	3	Q9BVA0
Avasorum	3	Q9BVC4
Avasorum	3	Q9BVC5
Avasorum	3	Q9BVC6
Avasorum	3	Q9BVG4
Avasorum	3	Q9BVG9
Avasorum	3	Q9BVI4
Avasorum	3	Q9BVJ6
Avasorum	3	Q9BVJ7
Avasorum	3	Q9BVK6
Avasorum	3	Q9BVK8
Avasorum	3	Q9BVL2
Avasorum	3	Q9BVL4
Avasorum	3	Q9BVM2
Avasorum	3	Q9BVP2
Avasorum	3	Q9BVQ7
Avasorum	3	Q9BVS4
Avasorum	3	Q9BVS5
Avasorum	3	Q9BVT8
Avasorum	3	Q9BVV7
Avasorum	3	Q9BW19
Avasorum	3	Q9BW27
Avasorum	3	Q9BW60
Avasorum	3	Q9BW62
Avasorum	3	Q9BW71
Avasorum	3	Q9BW83
Avasorum	3	Q9BW85
Avasorum	3	Q9BW91
Avasorum	3	Q9BW92
Avasorum	3	Q9BWD1
Avasorum	3	Q9BWE0
Avasorum	3	Q9BWF3
Avasorum	3	Q9BWH2
Avasorum	3	Q9BWH6

Avasorum	3	Q9BWJ5
Avasorum	3	Q9BWM7
Avasorum	3	Q9BWS9
Avasorum	3	Q9BWT3
Avasorum	3	Q9BWT7
Avasorum	3	Q9BWU0
Avasorum	3	Q9BWU1
Avasorum	3	Q9BX40
Avasorum	3	Q9BX59
Avasorum	3	Q9BX67
Avasorum	3	Q9BX69
Avasorum	3	Q9BX70
Avasorum	3	Q9BX95
Avasorum	3	Q9BXB4
Avasorum	3	Q9BXB5
Avasorum	3	Q9BXF6
Avasorum	3	Q9BXI6
Avasorum	3	Q9BXJ0
Avasorum	3	Q9BXJ9
Avasorum	3	Q9BXK1
Avasorum	3	Q9BXK5
Avasorum	3	Q9BXL7
Avasorum	3	Q9BXP2
Avasorum	3	Q9BXP5
Avasorum	3	Q9BXR0
Avasorum	3	Q9BXS5
Avasorum	3	Q9BXS6
Avasorum	3	Q9BXW7
Avasorum	3	Q9BXW9
Avasorum	3	Q9BXY0
Avasorum	3	Q9BY41
Avasorum	3	Q9BY42
Avasorum	3	Q9BY43
Avasorum	3	Q9BY44
Avasorum	3	Q9BY77
Avasorum	3	Q9BY89
Avasorum	3	Q9BYB0
Avasorum	3	Q9BYB4
Avasorum	3	Q9BYC5
Avasorum	3	Q9BYC8
Avasorum	3	Q9BYC9
Avasorum	3	Q9BYD1
Avasorum	3	Q9BYD2
Avasorum	3	Q9BYD3
Avasorum	3	Q9BYD6
Avasorum	3	Q9BYG3
Avasorum	3	Q9BYI3
Avasorum	3	Q9BYJ9
Avasorum	3	Q9BYK8
Avasorum	3	Q9BYM8

Avasorum	3	Q9BYN8
Avasorum	3	Q9BYT8
Avasorum	3	Q9BYV8
Avasorum	3	Q9BYW2
Avasorum	3	Q9BZ23
Avasorum	3	Q9BZ29
Avasorum	3	Q9BZ67
Avasorum	3	Q9BZ76
Avasorum	3	Q9BZ95
Avasorum	3	Q9BZD4
Avasorum	3	Q9BZE1
Avasorum	3	Q9BZE4
Avasorum	3	Q9BZE9
Avasorum	3	Q9BZF1
Avasorum	3	Q9BZF9
Avasorum	3	Q9BZG1
Avasorum	3	Q9BZG8
Avasorum	3	Q9BZH6
Avasorum	3	Q9BZI7
Avasorum	3	Q9BZJ0
Avasorum	3	Q9BZK7
Avasorum	3	Q9BZL1
Avasorum	3	Q9BZL4
Avasorum	3	Q9BZL6
Avasorum	3	Q9BZQ6
Avasorum	3	Q9BZQ8
Avasorum	3	Q9BZV1
Avasorum	3	Q9BZW5
Avasorum	3	Q9BZX2
Avasorum	3	Q9BZZ2
Avasorum	3	Q9BZZ5
Avasorum	3	Q9C000
Avasorum	3	Q9C035
Avasorum	3	Q9C037
Avasorum	3	Q9C0B1
Avasorum	3	Q9C0B5
Avasorum	3	Q9C0B7
Avasorum	3	Q9C0C2
Avasorum	3	Q9C0C9
Avasorum	3	Q9C0D3
Avasorum	3	Q9C0D5
Avasorum	3	Q9C0D9
Avasorum	3	Q9C0E2
Avasorum	3	Q9C0E8
Avasorum	3	Q9C0F1
Avasorum	3	Q9C0H2
Avasorum	3	Q9C0H6
Avasorum	3	Q9C0I1
Avasorum	3	Q9C0J8
Avasorum	3	Q9GZL7

Avasorum	3	Q9GZM5
Avasorum	3	Q9GZM7
Avasorum	3	Q9GZN8
Avasorum	3	Q9GZP4
Avasorum	3	Q9GZP9
Avasorum	3	Q9GZQ3
Avasorum	3	Q9GZR1
Avasorum	3	Q9GZR2
Avasorum	3	Q9GZR7
Avasorum	3	Q9GZS1
Avasorum	3	Q9GZS3
Avasorum	3	Q9GZT3
Avasorum	3	Q9GZT4
Avasorum	3	Q9GZT8
Avasorum	3	Q9GZT9
Avasorum	3	Q9GZU1
Avasorum	3	Q9GZU8
Avasorum	3	Q9GZY8
Avasorum	3	Q9GZZ1
Avasorum	3	Q9GZZ9
Avasorum	3	Q9H000
Avasorum	3	Q9H061
Avasorum	3	Q9H074
Avasorum	3	Q9H078
Avasorum	3	Q9H082
Avasorum	3	Q9H089
Avasorum	3	Q9H098
Avasorum	3	Q9H0A0
Avasorum	3	Q9H0A8
Avasorum	3	Q9H0B6
Avasorum	3	Q9H0C8
Avasorum	3	Q9H0D6
Avasorum	3	Q9H0E2
Avasorum	3	Q9H0E3
Avasorum	3	Q9H0E9
Avasorum	3	Q9H0G5
Avasorum	3	Q9H0H0
Avasorum	3	Q9H0H5
Avasorum	3	Q9H0J9
Avasorum	3	Q9H0L4
Avasorum	3	Q9H0P0
Avasorum	3	Q9H0Q0
Avasorum	3	Q9H0R3
Avasorum	3	Q9H0R4
Avasorum	3	Q9H0R6
Avasorum	3	Q9H0S4
Avasorum	3	Q9H0U3
Avasorum	3	Q9H0U4
Avasorum	3	Q9H0U6
Avasorum	3	Q9H0U9

Avasorum	3	Q9H0V1
Avasorum	3	Q9H0V9
Avasorum	3	Q9H0W8
Avasorum	3	Q9H0X4
Avasorum	3	Q9H0X9
Avasorum	3	Q9H147
Avasorum	3	Q9H173
Avasorum	3	Q9H1A3
Avasorum	3	Q9H1A4
Avasorum	3	Q9H1C7
Avasorum	3	Q9H1D9
Avasorum	3	Q9H1E3
Avasorum	3	Q9H1E5
Avasorum	3	Q9H1H9
Avasorum	3	Q9H1I8
Avasorum	3	Q9H1K0
Avasorum	3	Q9H1K1
Avasorum	3	Q9H1P3
Avasorum	3	Q9H1Y0
Avasorum	3	Q9H1Z4
Avasorum	3	Q9H201
Avasorum	3	Q9H223
Avasorum	3	Q9H267
Avasorum	3	Q9H269
Avasorum	3	Q9H270
Avasorum	3	Q9H299
Avasorum	3	Q9H2C0
Avasorum	3	Q9H2D1
Avasorum	3	Q9H2D6
Avasorum	3	Q9H2G2
Avasorum	3	Q9H2H8
Avasorum	3	Q9H2H9
Avasorum	3	Q9H2J4
Avasorum	3	Q9H2K8
Avasorum	3	Q9H2M9
Avasorum	3	Q9H2P0
Avasorum	3	Q9H2P9
Avasorum	3	Q9H2U1
Avasorum	3	Q9H2U2
Avasorum	3	Q9H2V7
Avasorum	3	Q9H2W6
Avasorum	3	Q9H2X9
Avasorum	3	Q9H300
Avasorum	3	Q9H307
Avasorum	3	Q9H330
Avasorum	3	Q9H334
Avasorum	3	Q9H3F6
Avasorum	3	Q9H3H1
Avasorum	3	Q9H3H3
Avasorum	3	Q9H3K2

Avasorum	3	Q9H3K6
Avasorum	3	Q9H3L0
Avasorum	3	Q9H3M7
Avasorum	3	Q9H3N1
Avasorum	3	Q9H3P2
Avasorum	3	Q9H3P7
Avasorum	3	Q9H3Q1
Avasorum	3	Q9H3S7
Avasorum	3	Q9H3T3
Avasorum	3	Q9H3U1
Avasorum	3	Q9H3U5
Avasorum	3	Q9H3Z4
Avasorum	3	Q9H410
Avasorum	3	Q9H444
Avasorum	3	Q9H446
Avasorum	3	Q9H467
Avasorum	3	Q9H469
Avasorum	3	Q9H479
Avasorum	3	Q9H488
Avasorum	3	Q9H490
Avasorum	3	Q9H497
Avasorum	3	Q9H4A3
Avasorum	3	Q9H4A4
Avasorum	3	Q9H4A5
Avasorum	3	Q9H4A6
Avasorum	3	Q9H4B0
Avasorum	3	Q9H4F1
Avasorum	3	Q9H4G4
Avasorum	3	Q9H4I3
Avasorum	3	Q9H4K7
Avasorum	3	Q9H4L4
Avasorum	3	Q9H4L5
Avasorum	3	Q9H4L7
Avasorum	3	Q9H4M9
Avasorum	3	Q9H4Z3
Avasorum	3	Q9H501
Avasorum	3	Q9H553
Avasorum	3	Q9H583
Avasorum	3	Q9H5K3
Avasorum	3	Q9H5N1
Avasorum	3	Q9H5Q4
Avasorum	3	Q9H5U6
Avasorum	3	Q9H5V9
Avasorum	3	Q9H5X1
Avasorum	3	Q9H5Z1
Avasorum	3	Q9H5Z6
Avasorum	3	Q9H6D7
Avasorum	3	Q9H6E4
Avasorum	3	Q9H6F5
Avasorum	3	Q9H6H4

Avasorum	3	Q9H6I2
Avasorum	3	Q9H6K4
Avasorum	3	Q9H6Q4
Avasorum	3	Q9H6R0
Avasorum	3	Q9H6R4
Avasorum	3	Q9H6R7
Avasorum	3	Q9H6S0
Avasorum	3	Q9H6S1
Avasorum	3	Q9H6T3
Avasorum	3	Q9H6U6
Avasorum	3	Q9H6U8
Avasorum	3	Q9H6V9
Avasorum	3	Q9H6W3
Avasorum	3	Q9H6Y2
Avasorum	3	Q9H6Z4
Avasorum	3	Q9H792
Avasorum	3	Q9H7B2
Avasorum	3	Q9H7B4
Avasorum	3	Q9H7C9
Avasorum	3	Q9H7D0
Avasorum	3	Q9H7D7
Avasorum	3	Q9H7E2
Avasorum	3	Q9H7E9
Avasorum	3	Q9H7H0
Avasorum	3	Q9H7N4
Avasorum	3	Q9H7Z3
Avasorum	3	Q9H7Z7
Avasorum	3	Q9H814
Avasorum	3	Q9H832
Avasorum	3	Q9H845
Avasorum	3	Q9H857
Avasorum	3	Q9H875
Avasorum	3	Q9H8G2
Avasorum	3	Q9H8H0
Avasorum	3	Q9H8H2
Avasorum	3	Q9H8H3
Avasorum	3	Q9H8K7
Avasorum	3	Q9H8L6
Avasorum	3	Q9H8M7
Avasorum	3	Q9H8M9
Avasorum	3	Q9H8T0
Avasorum	3	Q9H8W4
Avasorum	3	Q9H8Y5
Avasorum	3	Q9H8Y8
Avasorum	3	Q9H900
Avasorum	3	Q9H910
Avasorum	3	Q9H936
Avasorum	3	Q9H939
Avasorum	3	Q9H944
Avasorum	3	Q9H967

Avasorum	3	Q9H974
Avasorum	3	Q9H981
Avasorum	3	Q9H993
Avasorum	3	Q9H999
Avasorum	3	Q9H9A5
Avasorum	3	Q9H9A6
Avasorum	3	Q9H9B1
Avasorum	3	Q9H9B4
Avasorum	3	Q9H9C1
Avasorum	3	Q9H9E3
Avasorum	3	Q9H9F9
Avasorum	3	Q9H9H4
Avasorum	3	Q9H9J2
Avasorum	3	Q9H9P8
Avasorum	3	Q9H9Q2
Avasorum	3	Q9H9Q4
Avasorum	3	Q9H9S3
Avasorum	3	Q9H9S5
Avasorum	3	Q9H9T3
Avasorum	3	Q9H9Y2
Avasorum	3	Q9H9Y6
Avasorum	3	Q9HA47
Avasorum	3	Q9HA64
Avasorum	3	Q9HA65
Avasorum	3	Q9HA77
Avasorum	3	Q9HAB3
Avasorum	3	Q9HAB8
Avasorum	3	Q9HAC8
Avasorum	3	Q9HAD4
Avasorum	3	Q9HAH7
Avasorum	3	Q9HAN9
Avasorum	3	Q9HAS0
Avasorum	3	Q9HAT2
Avasorum	3	Q9HAU0
Avasorum	3	Q9HAU4
Avasorum	3	Q9HAU5
Avasorum	3	Q9HAV0
Avasorum	3	Q9HAV4
Avasorum	3	Q9HAV7
Avasorum	3	Q9HB07
Avasorum	3	Q9HB20
Avasorum	3	Q9HB21
Avasorum	3	Q9HB40
Avasorum	3	Q9HB63
Avasorum	3	Q9HB71
Avasorum	3	Q9HB90
Avasorum	3	Q9HBF4
Avasorum	3	Q9HBG6
Avasorum	3	Q9HBH5
Avasorum	3	Q9HBI1

Avasorum	3	Q9HBL0
Avasorum	3	Q9HBL7
Avasorum	3	Q9HBL8
Avasorum	3	Q9HBM0
Avasorum	3	Q9HBM1
Avasorum	3	Q9HBM6
Avasorum	3	Q9HBR0
Avasorum	3	Q9HBU6
Avasorum	3	Q9HBW9
Avasorum	3	Q9HC07
Avasorum	3	Q9HC21
Avasorum	3	Q9HC35
Avasorum	3	Q9HC36
Avasorum	3	Q9HC38
Avasorum	3	Q9HC98
Avasorum	3	Q9HCC0
Avasorum	3	Q9HCD5
Avasorum	3	Q9HCE0
Avasorum	3	Q9HCE1
Avasorum	3	Q9HCE5
Avasorum	3	Q9HCG7
Avasorum	3	Q9HCG8
Avasorum	3	Q9HCJ3
Avasorum	3	Q9HCK8
Avasorum	3	Q9HCM4
Avasorum	3	Q9HCN4
Avasorum	3	Q9HCN8
Avasorum	3	Q9HCP0
Avasorum	3	Q9HCS7
Avasorum	3	Q9HCU5
Avasorum	3	Q9HD15
Avasorum	3	Q9HD20
Avasorum	3	Q9HD26
Avasorum	3	Q9HD33
Avasorum	3	Q9HD34
Avasorum	3	Q9HD40
Avasorum	3	Q9HD42
Avasorum	3	Q9HD45
Avasorum	3	Q9HD67
Avasorum	3	Q9HDC9
Avasorum	3	Q9NNW5
Avasorum	3	Q9NNW7
Avasorum	3	Q9NP58
Avasorum	3	Q9NP61
Avasorum	3	Q9NP64
Avasorum	3	Q9NP72
Avasorum	3	Q9NP74
Avasorum	3	Q9NP77
Avasorum	3	Q9NP79
Avasorum	3	Q9NP81

Avasorum	3	Q9NP92
Avasorum	3	Q9NP97
Avasorum	3	Q9NPA0
Avasorum	3	Q9NPA8
Avasorum	3	Q9NPD3
Avasorum	3	Q9NPD8
Avasorum	3	Q9NPE3
Avasorum	3	Q9NPF4
Avasorum	3	Q9NPF5
Avasorum	3	Q9NPG4
Avasorum	3	Q9NPH2
Avasorum	3	Q9NPI1
Avasorum	3	Q9NPI6
Avasorum	3	Q9NPJ3
Avasorum	3	Q9NPJ6
Avasorum	3	Q9NPL8
Avasorum	3	Q9NPQ8
Avasorum	3	Q9NPY3
Avasorum	3	Q9NQ29
Avasorum	3	Q9NQ30
Avasorum	3	Q9NQ48
Avasorum	3	Q9NQ50
Avasorum	3	Q9NQ55
Avasorum	3	Q9NQ66
Avasorum	3	Q9NQ88
Avasorum	3	Q9NQ89
Avasorum	3	Q9NQC3
Avasorum	3	Q9NQG5
Avasorum	3	Q9NQH7
Avasorum	3	Q9NQR4
Avasorum	3	Q9NQS1
Avasorum	3	Q9NQS7
Avasorum	3	Q9NQT4
Avasorum	3	Q9NQT5
Avasorum	3	Q9NQT8
Avasorum	3	Q9NQW6
Avasorum	3	Q9NQW7
Avasorum	3	Q9NQX3
Avasorum	3	Q9NQY0
Avasorum	3	Q9NQZ2
Avasorum	3	Q9NQZ5
Avasorum	3	Q9NR09
Avasorum	3	Q9NR12
Avasorum	3	Q9NR19
Avasorum	3	Q9NR28
Avasorum	3	Q9NR30
Avasorum	3	Q9NR31
Avasorum	3	Q9NR45
Avasorum	3	Q9NR46
Avasorum	3	Q9NR48

Avasorum	3	Q9NR50
Avasorum	3	Q9NR56
Avasorum	3	Q9NRA2
Avasorum	3	Q9NRA8
Avasorum	3	Q9NRB3
Avasorum	3	Q9NRC1
Avasorum	3	Q9NRD5
Avasorum	3	Q9NRF2
Avasorum	3	Q9NRF8
Avasorum	3	Q9NRF9
Avasorum	3	Q9NRG0
Avasorum	3	Q9NRG9
Avasorum	3	Q9NRH2
Avasorum	3	Q9NRK6
Avasorum	3	Q9NRL2
Avasorum	3	Q9NRL3
Avasorum	3	Q9NRN7
Avasorum	3	Q9NRN9
Avasorum	3	Q9NRP0
Avasorum	3	Q9NRP2
Avasorum	3	Q9NRQ2
Avasorum	3	Q9NRR4
Avasorum	3	Q9NRS6
Avasorum	3	Q9NRV9
Avasorum	3	Q9NRW3
Avasorum	3	Q9NRW7
Avasorum	3	Q9NRX1
Avasorum	3	Q9NRX2
Avasorum	3	Q9NRX4
Avasorum	3	Q9NRX5
Avasorum	3	Q9NRY4
Avasorum	3	Q9NRY5
Avasorum	3	Q9NRY6
Avasorum	3	Q9NRZ5
Avasorum	3	Q9NRZ7
Avasorum	3	Q9NRZ9
Avasorum	3	Q9NS00
Avasorum	3	Q9NS15
Avasorum	3	Q9NS69
Avasorum	3	Q9NS86
Avasorum	3	Q9NS87
Avasorum	3	Q9NS93
Avasorum	3	Q9NSC5
Avasorum	3	Q9NSD9
Avasorum	3	Q9NSE4
Avasorum	3	Q9NSI2
Avasorum	3	Q9NSI8
Avasorum	3	Q9NSK0
Avasorum	3	Q9NSV4
Avasorum	3	Q9NSY1

Avasorum	3	Q9NT62
Avasorum	3	Q9NTG7
Avasorum	3	Q9NTI5
Avasorum	3	Q9NTJ3
Avasorum	3	Q9NTJ4
Avasorum	3	Q9NTJ5
Avasorum	3	Q9NTK1
Avasorum	3	Q9NTK5
Avasorum	3	Q9NTM9
Avasorum	3	Q9NTW7
Avasorum	3	Q9NTX5
Avasorum	3	Q9NTZ6
Avasorum	3	Q9NU19
Avasorum	3	Q9NU22
Avasorum	3	Q9NUB1
Avasorum	3	Q9NUD5
Avasorum	3	Q9NUI1
Avasorum	3	Q9NUJ1
Avasorum	3	Q9NUJ3
Avasorum	3	Q9NUL3
Avasorum	3	Q9NUL5
Avasorum	3	Q9NUL7
Avasorum	3	Q9NUM4
Avasorum	3	Q9NUP9
Avasorum	3	Q9NUQ2
Avasorum	3	Q9NUQ3
Avasorum	3	Q9NUQ6
Avasorum	3	Q9NUQ7
Avasorum	3	Q9NUQ8
Avasorum	3	Q9NUQ9
Avasorum	3	Q9NUT2
Avasorum	3	Q9NUU7
Avasorum	3	Q9NUV9
Avasorum	3	Q9NUW8
Avasorum	3	Q9NUX5
Avasorum	3	Q9NUY8
Avasorum	3	Q9NV06
Avasorum	3	Q9NV31
Avasorum	3	Q9NV56
Avasorum	3	Q9NV66
Avasorum	3	Q9NV70
Avasorum	3	Q9NV79
Avasorum	3	Q9NV88
Avasorum	3	Q9NV96
Avasorum	3	Q9NVA1
Avasorum	3	Q9NVA2
Avasorum	3	Q9NVC3
Avasorum	3	Q9NVC6
Avasorum	3	Q9NVD7
Avasorum	3	Q9NVE5

Avasorum	3	Q9NVE7
Avasorum	3	Q9NVF7
Avasorum	3	Q9NVG8
Avasorum	3	Q9NVH0
Avasorum	3	Q9NVH1
Avasorum	3	Q9NVH2
Avasorum	3	Q9NVI1
Avasorum	3	Q9NVI7
Avasorum	3	Q9NVJ2
Avasorum	3	Q9NVM6
Avasorum	3	Q9NVM9
Avasorum	3	Q9NVN3
Avasorum	3	Q9NVN8
Avasorum	3	Q9NVP1
Avasorum	3	Q9NVP2
Avasorum	3	Q9NVR0
Avasorum	3	Q9NVR2
Avasorum	3	Q9NVS2
Avasorum	3	Q9NVS9
Avasorum	3	Q9NVT9
Avasorum	3	Q9NVU0
Avasorum	3	Q9NVU7
Avasorum	3	Q9NVV4
Avasorum	3	Q9NVX2
Avasorum	3	Q9NVZ3
Avasorum	3	Q9NW08
Avasorum	3	Q9NW13
Avasorum	3	Q9NW15
Avasorum	3	Q9NW64
Avasorum	3	Q9NW81
Avasorum	3	Q9NW82
Avasorum	3	Q9NW97
Avasorum	3	Q9NWB6
Avasorum	3	Q9NWB7
Avasorum	3	Q9NWH9
Avasorum	3	Q9NWM8
Avasorum	3	Q9NWS8
Avasorum	3	Q9NWT1
Avasorum	3	Q9NWT6
Avasorum	3	Q9NWU1
Avasorum	3	Q9NWU5
Avasorum	3	Q9NWV4
Avasorum	3	Q9NWV8
Avasorum	3	Q9NWW5
Avasorum	3	Q9NWX5
Avasorum	3	Q9NWX6
Avasorum	3	Q9NWY4
Avasorum	3	Q9NWZ3
Avasorum	3	Q9NWZ5
Avasorum	3	Q9NX00

Avasorum	3	Q9NX02
Avasorum	3	Q9NX05
Avasorum	3	Q9NX07
Avasorum	3	Q9NX14
Avasorum	3	Q9NX20
Avasorum	3	Q9NX24
Avasorum	3	Q9NX40
Avasorum	3	Q9NX46
Avasorum	3	Q9NX47
Avasorum	3	Q9NX58
Avasorum	3	Q9NX61
Avasorum	3	Q9NX62
Avasorum	3	Q9NX63
Avasorum	3	Q9NX74
Avasorum	3	Q9NX76
Avasorum	3	Q9NXA8
Avasorum	3	Q9NXC5
Avasorum	3	Q9NXD2
Avasorum	3	Q9NXE4
Avasorum	3	Q9NXE8
Avasorum	3	Q9NXF1
Avasorum	3	Q9NXF7
Avasorum	3	Q9NXG2
Avasorum	3	Q9NXG6
Avasorum	3	Q9NXH8
Avasorum	3	Q9NXH9
Avasorum	3	Q9NXN4
Avasorum	3	Q9NXR1
Avasorum	3	Q9NXR7
Avasorum	3	Q9NXS2
Avasorum	3	Q9NXU5
Avasorum	3	Q9NXV2
Avasorum	3	Q9NXV6
Avasorum	3	Q9NXW2
Avasorum	3	Q9NXW9
Avasorum	3	Q9NXX6
Avasorum	3	Q9NY12
Avasorum	3	Q9NY15
Avasorum	3	Q9NY27
Avasorum	3	Q9NY33
Avasorum	3	Q9NY61
Avasorum	3	Q9NY65
Avasorum	3	Q9NY93
Avasorum	3	Q9NYA1
Avasorum	3	Q9NYB0
Avasorum	3	Q9NYB9
Avasorum	3	Q9NYF8
Avasorum	3	Q9NYH9
Avasorum	3	Q9NYJ8
Avasorum	3	Q9NYK5

Avasorum	3	Q9NYL2
Avasorum	3	Q9NYL4
Avasorum	3	Q9NYL9
Avasorum	3	Q9NYM9
Avasorum	3	Q9NYP7
Avasorum	3	Q9NYR9
Avasorum	3	Q9NYU1
Avasorum	3	Q9NYU2
Avasorum	3	Q9NYV4
Avasorum	3	Q9NYY8
Avasorum	3	Q9NZ01
Avasorum	3	Q9NZ08
Avasorum	3	Q9NZ32
Avasorum	3	Q9NZ43
Avasorum	3	Q9NZ45
Avasorum	3	Q9NZ52
Avasorum	3	Q9NZ63
Avasorum	3	Q9NZB2
Avasorum	3	Q9NZC9
Avasorum	3	Q9NZD2
Avasorum	3	Q9NZD8
Avasorum	3	Q9NZE8
Avasorum	3	Q9NZI7
Avasorum	3	Q9NZI8
Avasorum	3	Q9NZJ4
Avasorum	3	Q9NZJ7
Avasorum	3	Q9NZJ9
Avasorum	3	Q9NZL4
Avasorum	3	Q9NZL9
Avasorum	3	Q9NZM1
Avasorum	3	Q9NZM3
Avasorum	3	Q9NZM5
Avasorum	3	Q9NZN3
Avasorum	3	Q9NZN4
Avasorum	3	Q9NZN5
Avasorum	3	Q9NZN8
Avasorum	3	Q9NZQ3
Avasorum	3	Q9NZQ7
Avasorum	3	Q9NZT1
Avasorum	3	Q9NZT2
Avasorum	3	Q9NZU5
Avasorum	3	Q9NZV1
Avasorum	3	Q9NZV5
Avasorum	3	Q9NZW5
Avasorum	3	Q9NZZ3
Avasorum	3	Q9P013
Avasorum	3	Q9P015
Avasorum	3	Q9P016
Avasorum	3	Q9P031
Avasorum	3	Q9P032

Avasorum	3	Q9P035
Avasorum	3	Q9P0I2
Avasorum	3	Q9P0J0
Avasorum	3	Q9P0J1
Avasorum	3	Q9P0J7
Avasorum	3	Q9P0K7
Avasorum	3	Q9P0L0
Avasorum	3	Q9P0M9
Avasorum	3	Q9P0T7
Avasorum	3	Q9P0U3
Avasorum	3	Q9P0U4
Avasorum	3	Q9P0V3
Avasorum	3	Q9P0V9
Avasorum	3	Q9P107
Avasorum	3	Q9P1Y6
Avasorum	3	Q9P215
Avasorum	3	Q9P219
Avasorum	3	Q9P227
Avasorum	3	Q9P253
Avasorum	3	Q9P258
Avasorum	3	Q9P260
Avasorum	3	Q9P265
Avasorum	3	Q9P266
Avasorum	3	Q9P270
Avasorum	3	Q9P273
Avasorum	3	Q9P275
Avasorum	3	Q9P287
Avasorum	3	Q9P289
Avasorum	3	Q9P291
Avasorum	3	Q9P2A4
Avasorum	3	Q9P2B2
Avasorum	3	Q9P2B4
Avasorum	3	Q9P2C4
Avasorum	3	Q9P2D0
Avasorum	3	Q9P2D3
Avasorum	3	Q9P2E3
Avasorum	3	Q9P2E5
Avasorum	3	Q9P2E7
Avasorum	3	Q9P2E9
Avasorum	3	Q9P2I0
Avasorum	3	Q9P2J3
Avasorum	3	Q9P2J5
Avasorum	3	Q9P2K5
Avasorum	3	Q9P2K6
Avasorum	3	Q9P2K8
Avasorum	3	Q9P2N5
Avasorum	3	Q9P2P5
Avasorum	3	Q9P2Q2
Avasorum	3	Q9P2R3
Avasorum	3	Q9P2R6

Avasorum	3	Q9P2R7
Avasorum	3	Q9P2T1
Avasorum	3	Q9P2W9
Avasorum	3	Q9P2X0
Avasorum	3	Q9P2X3
Avasorum	3	Q9P2Y5
Avasorum	3	Q9UBB4
Avasorum	3	Q9UBB5
Avasorum	3	Q9UBB6
Avasorum	3	Q9UBB9
Avasorum	3	Q9UBC2
Avasorum	3	Q9UBC5
Avasorum	3	Q9UBD5
Avasorum	3	Q9UBE0
Avasorum	3	Q9UBF2
Avasorum	3	Q9UBF8
Avasorum	3	Q9UBG0
Avasorum	3	Q9UBI1
Avasorum	3	Q9UBI6
Avasorum	3	Q9UBK7
Avasorum	3	Q9UBK8
Avasorum	3	Q9UBK9
Avasorum	3	Q9UBL3
Avasorum	3	Q9UBM7
Avasorum	3	Q9UBN6
Avasorum	3	Q9UBN7
Avasorum	3	Q9UBP0
Avasorum	3	Q9UBP4
Avasorum	3	Q9UBP6
Avasorum	3	Q9UBP9
Avasorum	3	Q9UBQ0
Avasorum	3	Q9UBQ5
Avasorum	3	Q9UBQ6
Avasorum	3	Q9UBQ7
Avasorum	3	Q9UBR2
Avasorum	3	Q9UBS0
Avasorum	3	Q9UBS4
Avasorum	3	Q9UBS8
Avasorum	3	Q9UBT2
Avasorum	3	Q9UBU8
Avasorum	3	Q9UBU9
Avasorum	3	Q9UBV2
Avasorum	3	Q9UBV7
Avasorum	3	Q9UBV8
Avasorum	3	Q9UBW7
Avasorum	3	Q9UBW8
Avasorum	3	Q9UBX3
Avasorum	3	Q9UDR5
Avasorum	3	Q9UDT6
Avasorum	3	Q9UDW1

Avasorum	3	Q9UDX5
Avasorum	3	Q9UDY2
Avasorum	3	Q9UDY4
Avasorum	3	Q9UEE5
Avasorum	3	Q9UEE9
Avasorum	3	Q9UER7
Avasorum	3	Q9UET6
Avasorum	3	Q9UEU0
Avasorum	3	Q9UEW8
Avasorum	3	Q9UEY8
Avasorum	3	Q9UFC0
Avasorum	3	Q9UFN0
Avasorum	3	Q9UFW8
Avasorum	3	Q9UG56
Avasorum	3	Q9UG63
Avasorum	3	Q9UGI8
Avasorum	3	Q9UGJ0
Avasorum	3	Q9UGJ1
Avasorum	3	Q9UGL1
Avasorum	3	Q9UGM6
Avasorum	3	Q9UGP4
Avasorum	3	Q9UGP8
Avasorum	3	Q9UGQ3
Avasorum	3	Q9UGR2
Avasorum	3	Q9UGV2
Avasorum	3	Q9UH62
Avasorum	3	Q9UH65
Avasorum	3	Q9UH99
Avasorum	3	Q9UHA3
Avasorum	3	Q9UHA4
Avasorum	3	Q9UHB6
Avasorum	3	Q9UHB7
Avasorum	3	Q9UHB9
Avasorum	3	Q9UHD1
Avasorum	3	Q9UHD2
Avasorum	3	Q9UHD8
Avasorum	3	Q9UHG3
Avasorum	3	Q9UHI6
Avasorum	3	Q9UHJ6
Avasorum	3	Q9UHL4
Avasorum	3	Q9UHN6
Avasorum	3	Q9UHP3
Avasorum	3	Q9UHQ4
Avasorum	3	Q9UHQ9
Avasorum	3	Q9UHR5
Avasorum	3	Q9UHR6
Avasorum	3	Q9UHV9
Avasorum	3	Q9UHW5
Avasorum	3	Q9UHX1
Avasorum	3	Q9UHY1

Avasorum	3	Q9UHY7
Avasorum	3	Q9UHY8
Avasorum	3	Q9UI09
Avasorum	3	Q9UI10
Avasorum	3	Q9UI12
Avasorum	3	Q9UI14
Avasorum	3	Q9UI26
Avasorum	3	Q9UI30
Avasorum	3	Q9UI36
Avasorum	3	Q9UIA9
Avasorum	3	Q9UIC8
Avasorum	3	Q9UID3
Avasorum	3	Q9UIF9
Avasorum	3	Q9UIG0
Avasorum	3	Q9UII2
Avasorum	3	Q9UIJ7
Avasorum	3	Q9UIQ6
Avasorum	3	Q9UIS9
Avasorum	3	Q9UIV1
Avasorum	3	Q9UJ41
Avasorum	3	Q9UJ68
Avasorum	3	Q9UJ70
Avasorum	3	Q9UJ83
Avasorum	3	Q9UJA5
Avasorum	3	Q9UJC5
Avasorum	3	Q9UJF2
Avasorum	3	Q9UJK0
Avasorum	3	Q9UJS0
Avasorum	3	Q9UJU6
Avasorum	3	Q9UJV9
Avasorum	3	Q9UJW0
Avasorum	3	Q9UJX2
Avasorum	3	Q9UJX3
Avasorum	3	Q9UJX4
Avasorum	3	Q9UJX5
Avasorum	3	Q9UJX6
Avasorum	3	Q9UJY4
Avasorum	3	Q9UJY5
Avasorum	3	Q9UJZ1
Avasorum	3	Q9UK23
Avasorum	3	Q9UK41
Avasorum	3	Q9UK45
Avasorum	3	Q9UK59
Avasorum	3	Q9UK61
Avasorum	3	Q9UK76
Avasorum	3	Q9UK99
Avasorum	3	Q9UKA4
Avasorum	3	Q9UKB1
Avasorum	3	Q9UKD1
Avasorum	3	Q9UKD2

Avasorum	3	Q9UKE5
Avasorum	3	Q9UKF6
Avasorum	3	Q9UKG1
Avasorum	3	Q9UKI2
Avasorum	3	Q9UKI8
Avasorum	3	Q9UKJ3
Avasorum	3	Q9UKK3
Avasorum	3	Q9UKK6
Avasorum	3	Q9UKK9
Avasorum	3	Q9UKL0
Avasorum	3	Q9UKL6
Avasorum	3	Q9UKM7
Avasorum	3	Q9UKM9
Avasorum	3	Q9UKN8
Avasorum	3	Q9UKR5
Avasorum	3	Q9UKS6
Avasorum	3	Q9UKU7
Avasorum	3	Q9UKU9
Avasorum	3	Q9UKV3
Avasorum	3	Q9UKV5
Avasorum	3	Q9UKV8
Avasorum	3	Q9UKX7
Avasorum	3	Q9UKY7
Avasorum	3	Q9UKZ1
Avasorum	3	Q9UL03
Avasorum	3	Q9UL15
Avasorum	3	Q9UL18
Avasorum	3	Q9UL25
Avasorum	3	Q9UL26
Avasorum	3	Q9UL33
Avasorum	3	Q9UL40
Avasorum	3	Q9UL46
Avasorum	3	Q9UL54
Avasorum	3	Q9UL63
Avasorum	3	Q9ULA0
Avasorum	3	Q9ULC3
Avasorum	3	Q9ULC4
Avasorum	3	Q9ULD2
Avasorum	3	Q9ULE0
Avasorum	3	Q9ULE6
Avasorum	3	Q9ULF5
Avasorum	3	Q9ULG1
Avasorum	3	Q9ULG6
Avasorum	3	Q9ULH0
Avasorum	3	Q9ULH1
Avasorum	3	Q9ULH7
Avasorum	3	Q9ULJ7
Avasorum	3	Q9ULK4
Avasorum	3	Q9ULL8
Avasorum	3	Q9ULM3

Avasorum	3	Q9ULP0
Avasorum	3	Q9ULP9
Avasorum	3	Q9ULR0
Avasorum	3	Q9ULS5
Avasorum	3	Q9ULT8
Avasorum	3	Q9ULU4
Avasorum	3	Q9ULV3
Avasorum	3	Q9ULV4
Avasorum	3	Q9ULW0
Avasorum	3	Q9ULW3
Avasorum	3	Q9ULX3
Avasorum	3	Q9ULX6
Avasorum	3	Q9ULX9
Avasorum	3	Q9ULZ3
Avasorum	3	Q9UM00
Avasorum	3	Q9UM11
Avasorum	3	Q9UM13
Avasorum	3	Q9UM54
Avasorum	3	Q9UMR2
Avasorum	3	Q9UMR5
Avasorum	3	Q9UMS4
Avasorum	3	Q9UMX1
Avasorum	3	Q9UMY1
Avasorum	3	Q9UMY4
Avasorum	3	Q9UMZ2
Avasorum	3	Q9UN37
Avasorum	3	Q9UN86
Avasorum	3	Q9UNE7
Avasorum	3	Q9UNF0
Avasorum	3	Q9UNF1
Avasorum	3	Q9UNH6
Avasorum	3	Q9UNH7
Avasorum	3	Q9UNI6
Avasorum	3	Q9UNK0
Avasorum	3	Q9UNL2
Avasorum	3	Q9UNM6
Avasorum	3	Q9UNN5
Avasorum	3	Q9UNN8
Avasorum	3	Q9UNP9
Avasorum	3	Q9UNQ2
Avasorum	3	Q9UNS2
Avasorum	3	Q9UNW1
Avasorum	3	Q9UNW9
Avasorum	3	Q9UNX4
Avasorum	3	Q9UNY4
Avasorum	3	Q9UNZ2
Avasorum	3	Q9UP83
Avasorum	3	Q9UP95
Avasorum	3	Q9UPM8
Avasorum	3	Q9UPN3

Avasorum	3	Q9UPN4
Avasorum	3	Q9UPN6
Avasorum	3	Q9UPN7
Avasorum	3	Q9UPN9
Avasorum	3	Q9UPP1
Avasorum	3	Q9UPQ9
Avasorum	3	Q9UPR0
Avasorum	3	Q9UPR3
Avasorum	3	Q9UPT5
Avasorum	3	Q9UPT8
Avasorum	3	Q9UPU5
Avasorum	3	Q9UPU7
Avasorum	3	Q9UPU9
Avasorum	3	Q9UPW0
Avasorum	3	Q9UPW5
Avasorum	3	Q9UPY3
Avasorum	3	Q9UPY5
Avasorum	3	Q9UPY8
Avasorum	3	Q9UQ13
Avasorum	3	Q9UQ35
Avasorum	3	Q9UQ53
Avasorum	3	Q9UQ80
Avasorum	3	Q9UQ90
Avasorum	3	Q9UQB8
Avasorum	3	Q9UQC2
Avasorum	3	Q9UQE7
Avasorum	3	Q9UQN3
Avasorum	3	Q9UQQ2
Avasorum	3	Q9UQR1
Avasorum	3	Q9Y217
Avasorum	3	Q9Y219
Avasorum	3	Q9Y221
Avasorum	3	Q9Y223
Avasorum	3	Q9Y224
Avasorum	3	Q9Y230
Avasorum	3	Q9Y232
Avasorum	3	Q9Y237
Avasorum	3	Q9Y243
Avasorum	3	Q9Y256
Avasorum	3	Q9Y259
Avasorum	3	Q9Y262
Avasorum	3	Q9Y263
Avasorum	3	Q9Y265
Avasorum	3	Q9Y266
Avasorum	3	Q9Y276
Avasorum	3	Q9Y277
Avasorum	3	Q9Y281
Avasorum	3	Q9Y282
Avasorum	3	Q9Y285
Avasorum	3	Q9Y289

Avasorum	3	Q9Y291
Avasorum	3	Q9Y294
Avasorum	3	Q9Y295
Avasorum	3	Q9Y296
Avasorum	3	Q9Y2A7
Avasorum	3	Q9Y2B0
Avasorum	3	Q9Y2C4
Avasorum	3	Q9Y2D4
Avasorum	3	Q9Y2D5
Avasorum	3	Q9Y2G2
Avasorum	3	Q9Y2G3
Avasorum	3	Q9Y2G5
Avasorum	3	Q9Y2G8
Avasorum	3	Q9Y2H0
Avasorum	3	Q9Y2H1
Avasorum	3	Q9Y2H2
Avasorum	3	Q9Y2H6
Avasorum	3	Q9Y2I1
Avasorum	3	Q9Y2I7
Avasorum	3	Q9Y2I8
Avasorum	3	Q9Y2J2
Avasorum	3	Q9Y2J4
Avasorum	3	Q9Y2K2
Avasorum	3	Q9Y2K7
Avasorum	3	Q9Y2L1
Avasorum	3	Q9Y2L5
Avasorum	3	Q9Y2L9
Avasorum	3	Q9Y2P8
Avasorum	3	Q9Y2Q3
Avasorum	3	Q9Y2Q5
Avasorum	3	Q9Y2Q9
Avasorum	3	Q9Y2R0
Avasorum	3	Q9Y2R4
Avasorum	3	Q9Y2R5
Avasorum	3	Q9Y2R9
Avasorum	3	Q9Y2S2
Avasorum	3	Q9Y2S6
Avasorum	3	Q9Y2S7
Avasorum	3	Q9Y2T2
Avasorum	3	Q9Y2U8
Avasorum	3	Q9Y2V2
Avasorum	3	Q9Y2V7
Avasorum	3	Q9Y2W1
Avasorum	3	Q9Y2W2
Avasorum	3	Q9Y2W6
Avasorum	3	Q9Y2X0
Avasorum	3	Q9Y2X3
Avasorum	3	Q9Y2X7
Avasorum	3	Q9Y2X9
Avasorum	3	Q9Y2Y0

Avasorum	3	Q9Y2Y6
Avasorum	3	Q9Y2Z0
Avasorum	3	Q9Y2Z4
Avasorum	3	Q9Y303
Avasorum	3	Q9Y305
Avasorum	3	Q9Y312
Avasorum	3	Q9Y314
Avasorum	3	Q9Y315
Avasorum	3	Q9Y316
Avasorum	3	Q9Y320
Avasorum	3	Q9Y324
Avasorum	3	Q9Y333
Avasorum	3	Q9Y371
Avasorum	3	Q9Y375
Avasorum	3	Q9Y376
Avasorum	3	Q9Y383
Avasorum	3	Q9Y385
Avasorum	3	Q9Y388
Avasorum	3	Q9Y394
Avasorum	3	Q9Y399
Avasorum	3	Q9Y3A2
Avasorum	3	Q9Y3A3
Avasorum	3	Q9Y3A4
Avasorum	3	Q9Y3A5
Avasorum	3	Q9Y3A6
Avasorum	3	Q9Y3B2
Avasorum	3	Q9Y3B3
Avasorum	3	Q9Y3B4
Avasorum	3	Q9Y3B7
Avasorum	3	Q9Y3B8
Avasorum	3	Q9Y3B9
Avasorum	3	Q9Y3C0
Avasorum	3	Q9Y3C1
Avasorum	3	Q9Y3C4
Avasorum	3	Q9Y3C6
Avasorum	3	Q9Y3C8
Avasorum	3	Q9Y3D0
Avasorum	3	Q9Y3D3
Avasorum	3	Q9Y3D5
Avasorum	3	Q9Y3D6
Avasorum	3	Q9Y3D7
Avasorum	3	Q9Y3D8
Avasorum	3	Q9Y3D9
Avasorum	3	Q9Y3E0
Avasorum	3	Q9Y3E1
Avasorum	3	Q9Y3E5
Avasorum	3	Q9Y3E7
Avasorum	3	Q9Y3F4
Avasorum	3	Q9Y3I0
Avasorum	3	Q9Y3I1

Avasorum	3	Q9Y3L5
Avasorum	3	Q9Y3M8
Avasorum	3	Q9Y3P9
Avasorum	3	Q9Y3Q3
Avasorum	3	Q9Y3Q8
Avasorum	3	Q9Y3R5
Avasorum	3	Q9Y3S2
Avasorum	3	Q9Y3T6
Avasorum	3	Q9Y3T9
Avasorum	3	Q9Y3U8
Avasorum	3	Q9Y3X0
Avasorum	3	Q9Y3Y2
Avasorum	3	Q9Y3Z3
Avasorum	3	Q9Y448
Avasorum	3	Q9Y450
Avasorum	3	Q9Y478
Avasorum	3	Q9Y484
Avasorum	3	Q9Y485
Avasorum	3	Q9Y487
Avasorum	3	Q9Y490
Avasorum	3	Q9Y496
Avasorum	3	Q9Y4A5
Avasorum	3	Q9Y4B5
Avasorum	3	Q9Y4B6
Avasorum	3	Q9Y4C2
Avasorum	3	Q9Y4C8
Avasorum	3	Q9Y4D1
Avasorum	3	Q9Y4D7
Avasorum	3	Q9Y4D8
Avasorum	3	Q9Y4E1
Avasorum	3	Q9Y4E8
Avasorum	3	Q9Y4F1
Avasorum	3	Q9Y4F3
Avasorum	3	Q9Y4F5
Avasorum	3	Q9Y4G8
Avasorum	3	Q9Y4I1
Avasorum	3	Q9Y4J8
Avasorum	3	Q9Y4K0
Avasorum	3	Q9Y4K1
Avasorum	3	Q9Y4K3
Avasorum	3	Q9Y4K4
Avasorum	3	Q9Y4L1
Avasorum	3	Q9Y4P1
Avasorum	3	Q9Y4P3
Avasorum	3	Q9Y4P8
Avasorum	3	Q9Y4R8
Avasorum	3	Q9Y4W2
Avasorum	3	Q9Y4W6
Avasorum	3	Q9Y4X4
Avasorum	3	Q9Y4X5

Avasorum	3	Q9Y4Z0
Avasorum	3	Q9Y508
Avasorum	3	Q9Y512
Avasorum	3	Q9Y520
Avasorum	3	Q9Y530
Avasorum	3	Q9Y547
Avasorum	3	Q9Y570
Avasorum	3	Q9Y580
Avasorum	3	Q9Y584
Avasorum	3	Q9Y5A7
Avasorum	3	Q9Y5A9
Avasorum	3	Q9Y5B0
Avasorum	3	Q9Y5B6
Avasorum	3	Q9Y5B8
Avasorum	3	Q9Y5B9
Avasorum	3	Q9Y5J1
Avasorum	3	Q9Y5K5
Avasorum	3	Q9Y5K6
Avasorum	3	Q9Y5K8
Avasorum	3	Q9Y5L0
Avasorum	3	Q9Y5M8
Avasorum	3	Q9Y5N6
Avasorum	3	Q9Y5P4
Avasorum	3	Q9Y5P6
Avasorum	3	Q9Y5Q0
Avasorum	3	Q9Y5Q8
Avasorum	3	Q9Y5Q9
Avasorum	3	Q9Y5R8
Avasorum	3	Q9Y5S1
Avasorum	3	Q9Y5S2
Avasorum	3	Q9Y5S9
Avasorum	3	Q9Y5T4
Avasorum	3	Q9Y5T5
Avasorum	3	Q9Y5U2
Avasorum	3	Q9Y5V0
Avasorum	3	Q9Y5V3
Avasorum	3	Q9Y5W7
Avasorum	3	Q9Y5W9
Avasorum	3	Q9Y5X1
Avasorum	3	Q9Y5X2
Avasorum	3	Q9Y5X3
Avasorum	3	Q9Y5X9
Avasorum	3	Q9Y5Y2
Avasorum	3	Q9Y5Y5
Avasorum	3	Q9Y5Y7
Avasorum	3	Q9Y5Z0
Avasorum	3	Q9Y5Z4
Avasorum	3	Q9Y5Z7
Avasorum	3	Q9Y5Z9
Avasorum	3	Q9Y606

Avasorum	3	Q9Y608
Avasorum	3	Q9Y613
Avasorum	3	Q9Y617
Avasorum	3	Q9Y618
Avasorum	3	Q9Y619
Avasorum	3	Q9Y624
Avasorum	3	Q9Y639
Avasorum	3	Q9Y653
Avasorum	3	Q9Y657
Avasorum	3	Q9Y666
Avasorum	3	Q9Y672
Avasorum	3	Q9Y673
Avasorum	3	Q9Y676
Avasorum	3	Q9Y678
Avasorum	3	Q9Y679
Avasorum	3	Q9Y680
Avasorum	3	Q9Y696
Avasorum	3	Q9Y697
Avasorum	3	Q9Y6A4
Avasorum	3	Q9Y6A5
Avasorum	3	Q9Y6A9
Avasorum	3	Q9Y6B6
Avasorum	3	Q9Y6B7
Avasorum	3	Q9Y6C9
Avasorum	3	Q9Y6D0
Avasorum	3	Q9Y6D5
Avasorum	3	Q9Y6D6
Avasorum	3	Q9Y6D9
Avasorum	3	Q9Y6E0
Avasorum	3	Q9Y6E2
Avasorum	3	Q9Y6G5
Avasorum	3	Q9Y6G9
Avasorum	3	Q9Y6I3
Avasorum	3	Q9Y6I4
Avasorum	3	Q9Y6I9
Avasorum	3	Q9Y6K0
Avasorum	3	Q9Y6K1
Avasorum	3	Q9Y6K5
Avasorum	3	Q9Y6K8
Avasorum	3	Q9Y6K9
Avasorum	3	Q9Y6M1
Avasorum	3	Q9Y6M5
Avasorum	3	Q9Y6M7
Avasorum	3	Q9Y6M9
Avasorum	3	Q9Y6N5
Avasorum	3	Q9Y6N7
Avasorum	3	Q9Y6Q2
Avasorum	3	Q9Y6Q9
Avasorum	3	Q9Y6R0
Avasorum	3	Q9Y6R4

Avasorum	3	Q9Y6V7
Avasorum	3	Q9Y6W3
Avasorum	3	Q9Y6W5
Avasorum	3	Q9Y6X3
Avasorum	3	Q9Y6X9
Avasorum	3	Q9Y6Y0
Avasorum	3	Q9Y6Y8

d in the lysates of the three replicates of cells treated with *A.vasorum* somatic antigen.

**PG.ProteinDescriptions**
Putative glutamine amidotransferase-like class 1 domain-containing protein 3B, mitochondria
Intraflagellar transport protein 56
Ubiquitin-like modifier-activating enzyme 6
Extended synaptotagmin-2
Bridge-like lipid transfer protein family member 3B
Shootin-1
Transmembrane protein 223
Rho GTPase-activating protein 10
2-hydroxyacyl-CoA lyase 2
SH3 and PX domain-containing protein 2B
NBAS subunit of NRZ tethering complex
tRNA wybutosine-synthesizing protein 5
DENN domain-containing protein 3
von Willebrand factor A domain-containing protein 8
Protein strawberry notch homolog 1
GTP-binding protein 10
WD repeat-containing protein 91
DENN domain-containing protein 11
Plasmanylethanolamine desaturase 1
Trafficking protein particle complex subunit 13
CCR4-NOT transcription complex subunit 1
Microtubule-associated proteins 1A/1B light chain 3 beta 2
Glycerol-3-phosphate phosphatase
RAB7A-interacting MON1-CCZ1 complex subunit 1
RCC1 domain-containing protein 1
TRPM8 channel-associated factor 2
Structural maintenance of chromosomes flexible hinge domain-containing protein 1
Cytosolic arginine sensor for mTORC1 subunit 2
Protein unc-119 homolog B
12S rRNA N4-methylcytidine (m4C) methyltransferase
Coiled-coil domain-containing protein 85C
Retrotransposon Gag-like protein 8C
Zinc finger SWIM domain-containing protein 8
Nuclear envelope pore membrane protein POM 121C
PHD finger protein 20-like protein 1
Putative small nuclear ribonucleoprotein G-like protein 15
Endosome/lysosome-associated apoptosis and autophagy regulator family member 2
Acyl-coenzyme A diphosphatase NUDT19
MIF4G domain-containing protein
Calmodulin-1
Unconventional myosin-IXa
Golgi pH regulator A
Putative WAS protein family homolog 3
Mapk-regulated corepressor-interacting protein 1
Nascent polypeptide-associated complex subunit alpha, muscle-specific form
Uncharacterized protein C11orf98
ASNSD1 upstream open reading frame protein
Mitochondrial ribosome and complex I assembly factor AltMIEF1

Deoxyribonuclease-2-alpha
Alkyldihydroxyacetonephosphate synthase, peroxisomal
Kinesin-like protein KIF2A
ATP-dependent RNA helicase DDX39A
PDZ and LIM domain protein 1
Cytosolic acyl coenzyme A thioester hydrolase
Unconventional myosin-Ic
Synaptosomal-associated protein 23
HCLS1-associated protein X-1
AH receptor-interacting protein
GTP-binding protein 1
Galectin-9
Syntaxin-binding protein 3
AP-4 complex subunit mu-1
Splicing regulator ARVCF
Small acidic protein
AP-3 complex subunit beta-1
Amyloid beta precursor protein binding family B member 1
Galectin-8
NADH dehydrogenase [ubiquinone] iron-sulfur protein 8, mitochondrial
NF-kappa-B inhibitor epsilon
26S proteasome non-ATPase regulatory subunit 11
26S proteasome non-ATPase regulatory subunit 12
26S proteasome non-ATPase regulatory subunit 9
Menin
Guided entry of tail-anchored proteins factor 1
Membrane-associated progesterone receptor component 1
Transcription elongation factor SPT5
Transcription initiation factor TFIID subunit 4
DNA fragmentation factor subunit alpha
Huntingtin-interacting protein 1
Chloride intracellular channel protein 1
Eukaryotic translation initiation factor 3 subunit F
NEDD4-like E3 ubiquitin-protein ligase WWP2
Pyruvate dehydrogenase protein X component, mitochondrial
Sulfhydryl oxidase 1
Dynactin subunit 6
Acetyl-coenzyme A transporter 1
Actin nucleation-promoting factor WASL
Importin-5
DNA-directed RNA polymerase, mitochondrial
Eukaryotic elongation factor 2 kinase
Histone deacetylase complex subunit SAP18
Echinoderm microtubule-associated protein-like 1
Insulin-like growth factor 2 mRNA-binding protein 3
Dynamin-1-like protein
RNA 3'-terminal phosphate cyclase
Phosphatidylinositol 4-phosphate 3-kinase C2 domain-containing subunit alpha
Golgi integral membrane protein 4
Agrin

Procollagen-lysine,2-oxoglutarate 5-dioxygenase 2
Exocyst complex component 5
Butyrophilin subfamily 3 member A1
Cytochrome c oxidase subunit NDUFA4
26S proteasome non-ATPase regulatory subunit 14
Myc box-dependent-interacting protein 1
Claudin-5
Importin subunit alpha-4
Serine/threonine-protein kinase 25
Krev interaction trapped protein 1
von Willebrand factor A domain-containing protein 5A
Pescadillo homolog
Syntenin-1
Membrane-associated phosphatidylinositol transfer protein 1
U3 small nucleolar ribonucleoprotein protein MPP10
Nucleolar protein 56
ATP-dependent RNA helicase DDX3X
Beta-1,3-N-acetylglucosaminyltransferase manic fringe
Podocalyxin
CCN family member 1
Pirin
Importin subunit alpha-3
E3 ubiquitin-protein ligase TRIM38
Nuclear factor 1 B-type
Serine/threonine-protein phosphatase 6 catalytic subunit
Nucleoside diphosphate kinase, mitochondrial
Phosphatidylinositol 4-phosphate 3-kinase C2 domain-containing subunit beta
Lysosomal alpha-mannosidase
Pyridoxal kinase
Stearoyl-CoA desaturase
AT-rich interactive domain-containing protein 1A
Nuclear envelope integral membrane protein 1
Thioredoxin domain-containing protein 9
Dihydropyrimidinase-related protein 4
TRAF-type zinc finger domain-containing protein 1
Cytochrome c oxidase subunit 7A-related protein, mitochondrial
Ubiquitin domain-containing protein UBFD1
Citron Rho-interacting kinase
Coatomer subunit epsilon
Cdc42 effector protein 2
AP-3 complex subunit delta-1
Copper chaperone for superoxide dismutase
Actin-binding LIM protein 1
Segment polarity protein dishevelled homolog DVL-2
Chromodomain-helicase-DNA-binding protein 1
Chromodomain-helicase-DNA-binding protein 2
Golgi SNAP receptor complex member 2
Torsin-1A
Torsin-1B
Syntaxin-16

Disintegrin and metalloproteinase domain-containing protein 10
Lysosomal cobalamin transporter ABCD4
Etoposide-induced protein 2.4 homolog
Tumor protein p53-inducible protein 11
Histone-lysine N-methyltransferase 2D
Apoptotic protease-activating factor 1
Serine/threonine-protein kinase RIO3
Dual specificity mitogen-activated protein kinase kinase 7
Acyl-coenzyme A thioesterase 8
CDP-diacylglycerol--inositol 3-phosphatidyltransferase
Programmed cell death protein 5
Protein arginine N-methyltransferase 5
Na(+)/H(+) exchange regulatory cofactor NHE-RF1
Serine/threonine-protein kinase Chk1
Tumor necrosis factor receptor superfamily member 10B
Tripeptidyl-peptidase 1
Transcription elongation regulator 1
Kinetochore protein NDC80 homolog
Kinesin-like protein KIF3C
Neuropilin-1
Transportin-2
Protein unc-13 homolog B
Tumor necrosis factor receptor superfamily member 10C
DNA-directed RNA polymerase III subunit RPC1
Tetraspanin-4
Proteasome subunit alpha type-7
Secretory carrier-associated membrane protein 3
Branched-chain alpha-ketoacid dehydrogenase kinase
Glutathione S-transferase 3, mitochondrial
Tax1-binding protein 3
PDZ domain-containing protein GIPC1
Inhibitor of nuclear factor kappa-B kinase subunit beta
Regulator of G-protein signaling 12
Mitochondrial import inner membrane translocase subunit Tim23
Histone acetyltransferase type B catalytic subunit
Peripheral plasma membrane protein CASK
Phospholipase D2
Cytochrome b-c1 complex subunit 8
Myosin regulatory light chain 12B
Hepatocyte growth factor-regulated tyrosine kinase substrate
Ras-related protein Rab-7L1
Vacuolar protein sorting-associated protein 26C
Protein phosphatase 1 regulatory subunit 12A
Cyclin-G-associated kinase
Heterogeneous nuclear ribonucleoprotein D-like
Exportin-1
TATA-binding protein-associated factor 172
Rho guanine nucleotide exchange factor 10
Zinc finger protein 609
Spectrin beta chain, non-erythrocytic 2

Microtubule-associated serine/threonine-protein kinase 4
Protein transport protein Sec16A
Plexin-B2
Protein KHNYN
Tectonin beta-propeller repeat-containing protein 2
U2 snRNP-associated SURP motif-containing protein
Histone-lysine N-methyltransferase SETD1A
NEDD4-binding protein 3
Synaptojanin-2
Synemin
Granule associated Rac and RHOG effector protein 1
Kinesin-like protein KIF3B
Phosphoribosylformylglycinamidine synthase
Serine/threonine-protein phosphatase 6 regulatory ankyrin repeat subunit A
Rho guanine nucleotide exchange factor 11
Mitochondrial ribonuclease P catalytic subunit
Inhibitor of nuclear factor kappa-B kinase subunit alpha
NPC intracellular cholesterol transporter 1
Sphingolipid delta(4)-desaturase DES1
Angiopoietin-2
Secretory carrier-associated membrane protein 1
Secretory carrier-associated membrane protein 2
Actin-related protein 2/3 complex subunit 1B
Actin-related protein 2/3 complex subunit 2
Actin-related protein 2/3 complex subunit 3
DNA-directed RNA polymerases I and III subunit RPAC1
Phospholipid scramblase 1
Transcription intermediary factor 1-alpha
Membrane-associated progesterone receptor component 2
Ral guanine nucleotide dissociation stimulator-like 2
WD repeat-containing protein 46
NF-kappa-B-repressing factor
Dihydroxyacetone phosphate acyltransferase
Laminin subunit alpha-5
Zinc finger protein 185
Protein CASC3
Small ribosomal subunit protein uS12m
Peroxisomal acyl-coenzyme A oxidase 3
Protein RER1
Surfeit locus protein 4
Mitogen-activated protein kinase 13
Serine palmitoyltransferase 1
Serine palmitoyltransferase 2
UDP-N-acetylglucosamine--peptide N-acetylglucosaminyltransferase 110 kDa subunit
Phosphomannomutase 2
Transmembrane 9 superfamily member 1
High mobility group protein B3
Protein phosphatase 1G
Phosphatidylinositol 3,4,5-trisphosphate 5-phosphatase 2
Eukaryotic translation initiation factor 3 subunit D

Eukaryotic translation initiation factor 3 subunit H
Histone deacetylase 3
Nuclear valosin-containing protein-like
Branched-chain-amino-acid aminotransferase, mitochondrial
Importin-8
Syntaxin-7
Monocarboxylate transporter 4
ATP-binding cassette sub-family C member 4
DNA-directed RNA polymerase I subunit RPA34
Prolyl 4-hydroxylase subunit alpha-2
Calpain-5
Synaptobrevin homolog YKT6
Nucleoporin NUP42
Actin-related protein 2/3 complex subunit 5
CASP8 and FADD-like apoptosis regulator
3-phosphoinositide-dependent protein kinase 1
Tapasin
E3 ubiquitin-protein ligase RNF113A
Lysine-specific demethylase 6A
ATP-dependent RNA helicase DHX15
mRNA cap guanine-N7 methyltransferase
Zinc finger ZZ-type and EF-hand domain-containing protein 1
Arf-GAP with SH3 domain, ANK repeat and PH domain-containing protein 2
Leucine-rich repeat transmembrane protein FLRT2
TELO2-interacting protein 1 homolog
Ribosomal RNA-processing protein 8
E3 ubiquitin-protein ligase Praja-2
Cytochrome b5 type B
U4/U6 small nuclear ribonucleoprotein Prp4
D-3-phosphoglycerate dehydrogenase
NADH dehydrogenase [ubiquinone] iron-sulfur protein 4, mitochondrial
Cytoplasmic dynein 1 light intermediate chain 2
26S proteasome non-ATPase regulatory subunit 3
RNA binding protein fox-1 homolog 2
Bifunctional 3'-phosphoadenosine 5'-phosphosulfate synthase 1
Centromere/kinetochore protein zw10 homolog
Beta-1,4-galactosyltransferase 5
U4/U6.U5 tri-snRNP-associated protein 1
Glycosylphosphatidylinositol anchor attachment 1 protein
Death-associated protein kinase 3
Transforming growth factor beta-1-induced transcript 1 protein
AP-5 complex subunit zeta-1
CBP80/20-dependent translation initiation factor
Protein MTSS 1
Inositol hexakisphosphate and diphosphoinositol-pentakisphosphate kinase 2
Mitogen-activated protein kinase kinase kinase 7
Eukaryotic translation elongation factor 1 epsilon-1
Receptor-interacting serine/threonine-protein kinase 2
Heterogeneous nuclear ribonucleoprotein R
U4/U6 small nuclear ribonucleoprotein Prp3

Thioredoxin-like protein 1
Tumor protein D54
ER membrane protein complex subunit 8
ERI1 exoribonuclease 3
Synaptojanin-1
Acidic fibroblast growth factor intracellular-binding protein
Eukaryotic translation initiation factor 4 gamma 3
Peptidyl-prolyl cis-trans isomerase H
Serine protease HTRA2, mitochondrial
Protein Mis18-beta
Aflatoxin B1 aldehyde reductase member 2
Band 4.1-like protein 2
Trans-Golgi network integral membrane protein 2
Beta-1,4-glucuronyltransferase 1
WAS/WASL-interacting protein family member 1
Phospholipid-transporting ATPase IC
A-kinase anchor protein 10, mitochondrial
Density-regulated protein
Exportin-T
5-hydroxymethyl-dUMP N-hydrolase
Mitochondrial import inner membrane translocase subunit TIM44
Trafficking protein particle complex subunit 3
Charged multivesicular body protein 2a
Cytoplasmic protein NCK2
Tetraspanin-6
Pleiotropic regulator 1
Protein regulator of cytokinesis 1
Regulator of G-protein signaling 10
BUB3-interacting and GLEBS motif-containing protein ZNF207
NADH dehydrogenase [ubiquinone] 1 beta subcomplex subunit 5, mitochondrial
NADH dehydrogenase [ubiquinone] 1 beta subcomplex subunit 3
NADH dehydrogenase [ubiquinone] 1 alpha subcomplex subunit 2
LIM domain-binding protein 2
ATPase GET3
Mitotic checkpoint serine/threonine-protein kinase BUB1
Mitotic checkpoint protein BUB3
Sulfotransferase 1B1
Alpha-actinin-4
Probable 18S rRNA (guanine-N(7))-methyltransferase
17S U2 SnRNP complex component HTATSF1
ER lumen protein-retaining receptor 3
AP-1 complex subunit gamma-1
Syntaxin-6
Synaptogyrin-2
Small glutamine-rich tetratricopeptide repeat-containing protein alpha
Alpha-endosulfine
Mitochondrial carnitine/acylcarnitine carrier protein
Asparagine--tRNA ligase, cytoplasmic
Unconventional myosin-Ib
Peroxisomal membrane protein PMP34

Cleavage and polyadenylation specificity factor subunit 5
Glutathione S-transferase LANCL1
Striatin
U3 small nucleolar RNA-interacting protein 2
Protein SCO2 homolog, mitochondrial
Cilia- and flagella-associated protein 410
A-kinase anchor protein 8
Putative GTP-binding protein 6
Glucose-6-phosphate exchanger SLC37A4
Isocitrate dehydrogenase [NAD] subunit beta, mitochondrial
Nardilysin
Calumenin
EGF-like repeat and discoidin I-like domain-containing protein 3
S-adenosylhomocysteine hydrolase-like protein 1
Kinesin-like protein KIF1C
Exostosin-like 3
Origin recognition complex subunit 5
NADH dehydrogenase [ubiquinone] iron-sulfur protein 5
Retinal rod rhodopsin-sensitive cGMP 3',5'-cyclic phosphodiesterase subunit delta
Origin recognition complex subunit 4
Peroxisomal ATPase PEX1
Double-strand-break repair protein rad21 homolog
Pre-mRNA-splicing factor ATP-dependent RNA helicase DHX16
Glia maturation factor gamma
SH3 domain-binding protein 5
Adhesion G protein-coupled receptor B3
Mediator of RNA polymerase II transcription subunit 14
Protocadherin-7
Phosphoribosyl pyrophosphate synthase-associated protein 2
SWI/SNF-related matrix-associated actin-dependent regulator of chromatin subfamily A membe
C-Jun-amino-terminal kinase-interacting protein 4
NUAK family SNF1-like kinase 1
Nucleolar pre-ribosomal-associated protein 1
Signal-induced proliferation-associated 1-like protein 3
Zinc finger C3H1 domain-containing protein
RNA helicase aquarius
Dynamin-like 120 kDa protein, mitochondrial
Zinc finger E-box-binding homeobox 2
Germinal-center associated nuclear protein
Protocadherin gamma-A12
Phosphatidylinositol 4-phosphate 5-kinase type-1 gamma
Kinesin-like protein KIF1B
Mitogen-activated protein kinase-binding protein 1
Lysine-specific histone demethylase 1A
TBC1 domain family member 4
Acyl-CoA (8-3)-desaturase
Gasdermin-E
Ecotropic viral integration site 5 protein homolog
Neuropilin-2
Mannosyl-oligosaccharide 1,2-alpha-mannosidase IB

Myelin protein zero-like protein 2
Long-chain-fatty-acid--CoA ligase 4
Sorting nexin-3
Protein O-GlcNAcase
Vinexin
Heterogeneous nuclear ribonucleoprotein Q
Pre-mRNA-processing factor 17
Ran-binding protein 6
Ribosome quality control complex subunit NEMF
GDP-mannose 4,6 dehydratase
Glycylpeptide N-tetradecanoyltransferase 2
Cyclin-T1
Mitotic checkpoint serine/threonine-protein kinase BUB1 beta
Multifunctional procollagen lysine hydroxylase and glycosyltransferase LH3
Eukaryotic translation initiation factor 4E type 2
Protein diaphanous homolog 1
Tetraspanin-3
Exocyst complex component 3
Perilipin-3
Protein arginine N-methyltransferase 3
Importin subunit alpha-7
UDP-glucose 6-dehydrogenase
Protein-tyrosine sulfotransferase 2
Leupaxin
Catenin delta-1
Protein-S-isoprenylcysteine O-methyltransferase
Eukaryotic translation initiation factor 1b
Sorting nexin-2
Dolichol-phosphate mannosyltransferase subunit 1
General vesicular transport factor p115
Small ribosomal subunit protein uS14m
Target of Myb1 membrane trafficking protein
Histone H2B type 1-K
Coiled-coil domain-containing protein 22
Polyglutamine-binding protein 1
Mitochondrial import inner membrane translocase subunit Tim17-B
PRA1 family protein 2
H/ACA ribonucleoprotein complex subunit DKC1
Eukaryotic translation initiation factor 5B
Endothelial differentiation-related factor 1
DNA/RNA-binding protein KIN17
Protein diaphanous homolog 2
DnaJ homolog subfamily A member 2
Bromodomain-containing protein 4
Checkpoint protein HUS1
Prefoldin subunit 1
E3 ubiquitin-protein ligase PPP1R11
Nibrin
mRNA-capping enzyme
Iron-sulfur clusters transporter ABCB7, mitochondrial

SLIT-ROBO Rho GTPase-activating protein 2
Plexin-A2
Glycosaminoglycan xylosylkinase
WD repeat-containing protein 1
Probable phospholipid-transporting ATPase IIA
NEDD4-binding protein 1
Rho-associated protein kinase 2
CLIP-associating protein 2
Pentatricopeptide repeat-containing protein 1, mitochondrial
Copine-3
Autophagy-related protein 13
Huntingtin-interacting protein 1-related protein
E3 ubiquitin-protein ligase BRE1B
Lysine-specific demethylase PHF2
Zinc finger CCCH domain-containing protein 11A
Clustered mitochondria protein homolog
Cullin-associated NEDD8-dissociated protein 2
TSC22 domain family protein 2
DnaJ homolog subfamily C member 13
Phosphatase and actin regulator 2
Serine/threonine-protein phosphatase 6 regulatory subunit 2
A disintegrin and metalloproteinase with thrombospondin motifs 4
CCR4-NOT transcription complex subunit 3
Ankyrin repeat domain-containing protein 17
Paired amphipathic helix protein Sin3b
DnaJ homolog subfamily B member 6
Ubiquinone biosynthesis protein COQ9, mitochondrial
Gamma-glutamylcyclotransferase
NADH dehydrogenase [ubiquinone] iron-sulfur protein 7, mitochondrial
NADH dehydrogenase [ubiquinone] iron-sulfur protein 2, mitochondrial
Zinc finger protein ZPR1
RNA/RNP complex-1-interacting phosphatase
Protein NipSnap homolog 2
Hyaluronan mediated motility receptor
Programmed cell death protein 6
Tubulin-specific chaperone A
V-type proton ATPase subunit G 1
Vacuolar protein sorting-associated protein 4B
Mannose-P-dolichol utilization defect 1 protein
Core histone macro-H2A.1
Adapter SH3BGRL
Filamin-B
Nuclear receptor corepressor 1
Peroxisomal membrane protein PEX14
Tripartite motif-containing protein 3
Citrate synthase, mitochondrial
Sperm-associated antigen 7
Vesicle-trafficking protein SEC22b
Pre-mRNA-processing factor 40 homolog A
Transforming acidic coiled-coil-containing protein 1

Nucleoside diphosphate kinase 6
Cell division control protein 45 homolog
Leucine-rich repeat and calponin homology domain-containing protein 4
Metaxin-2
Vacuolar protein sorting-associated protein 26A
Mitochondrial-processing peptidase subunit beta
Mediator of RNA polymerase II transcription subunit 24
Katanin p60 ATPase-containing subunit A1
PC4 and SFRS1-interacting protein
Erlin-1
NADH dehydrogenase [ubiquinone] iron-sulfur protein 3, mitochondrial
Serine/arginine-rich splicing factor 10
Ceroid-lipofuscinosis neuronal protein 5
Claudin-11
Enoyl-CoA delta isomerase 2
KH domain-containing, RNA-binding, signal transduction-associated protein 3
Polycomb protein EED
Barrier-to-autointegration factor
Splicing factor 3B subunit 1
Cold shock domain-containing protein E1
WW domain-binding protein 4
Interferon-inducible double-stranded RNA-dependent protein kinase activator A
Integrin alpha-10
Ribosomal protein S6 kinase alpha-5
Mediator of RNA polymerase II transcription subunit 6
E3 ubiquitin-protein ligase MYCBP2
2-amino-3-ketobutyrate coenzyme A ligase, mitochondrial
Nucleoplasmin-3
Acyl-protein thioesterase 1
GTPase Era, mitochondrial
U5 small nuclear ribonucleoprotein 200 kDa helicase
Mitochondrial tRNA-specific 2-thiouridylase 1
TIP41-like protein
Centriole and centriolar satellite protein OFD1
Ribosomal protein S6 kinase alpha-4
Surfeit locus protein 6
Protein phosphatase 1B
Small subunit processome component 20 homolog
Nuclear pore complex protein Nup155
Protein XRP2
WD repeat and HMG-box DNA-binding protein 1
Cartilage-associated protein
Electrogenic aspartate/glutamate antiporter SLC25A12, mitochondrial
Renin receptor
Ribonuclease H2 subunit A
Translation initiation factor eIF2 assembly protein
Breast cancer anti-estrogen resistance protein 3
Ribonuclease P protein subunit p20
Ribonuclease P protein subunit p40
Eukaryotic translation initiation factor 3 subunit G

Eukaryotic translation initiation factor 3 subunit J
Carbonyl reductase [NADPH] 3
26S proteasome non-ATPase regulatory subunit 10
AP-1 complex subunit gamma-like 2
CAAX prenyl protease 1 homolog
Isocitrate dehydrogenase [NADP] cytoplasmic
Glutamyl-tRNA(Gln) amidotransferase subunit B, mitochondrial
Protein SCO1 homolog, mitochondrial
Attractin
Serine hydrolase RBBP9
Signal transducing adapter molecule 2
Cyclin-K
PRA1 family protein 3
Dysferlin
E3 SUMO-protein ligase PIAS1
Pre-mRNA-splicing factor SPF27
Dynactin subunit 3
DnaJ homolog subfamily C member 8
Survival of motor neuron-related-splicing factor 30
Cell cycle checkpoint protein RAD17
ATP synthase subunit d, mitochondrial
Tetraspanin-9
Flotillin-1
Triple functional domain protein
ATP synthase subunit g, mitochondrial
Multiple PDZ domain protein
Carboxypeptidase D
Glutaredoxin-3
Ribosomal L1 domain-containing protein 1
Wolframin
ATP-dependent Clp protease ATP-binding subunit clpX-like, mitochondrial
SEC14-like protein 2
Gamma-synuclein
Probable cytosolic iron-sulfur protein assembly protein CIAO1
Signal recognition particle subunit SRP72
N(G),N(G)-dimethylarginine dimethylaminohydrolase 1
ATP-dependent DNA helicase Q5
Unconventional prefoldin RPB5 interactor 1
Galactosylgalactosylxylosylprotein 3-beta-glucuronosyltransferase 3
Metastasis-associated protein MTA2
Retinal dehydrogenase 2
Serine/threonine-protein kinase 10
Serine/threonine-protein kinase D3
Glutamine--fructose-6-phosphate aminotransferase [isomerizing] 2
Slit homolog 2 protein
E3 ubiquitin-protein ligase listerin
Mitochondrial import receptor subunit TOM70
Pleckstrin homology domain-containing family G member 5
Importin-13
Phospholipase DDHD2

Unconventional myosin-Id
TOX high mobility group box family member 4
[F-actin]-monooxygenase MICAL2
Protein transport protein Sec24D
F-BAR and double SH3 domains protein 2
E3 UFM1-protein ligase 1
Sorbin and SH3 domain-containing protein 2
SAM and SH3 domain-containing protein 1
UBX domain-containing protein 7
Kelch-like protein 18
SUN domain-containing protein 1
Pyridoxal phosphate homeostasis protein
Erlin-2
Pre-mRNA-processing factor 6
Pre-mRNA cleavage complex 2 protein Pcf11
Protein furry homolog-like
Nuclear factor of activated T-cells 5
Endonuclease domain-containing 1 protein
D-glucuronyl C5-epimerase
Glutaminase kidney isoform, mitochondrial
HAUS augmin-like complex subunit 5
Actin-binding LIM protein 3
F-box only protein 21
Microtubule cross-linking factor 2
Ubiquitin carboxyl-terminal hydrolase 19
WD repeat-containing protein 47
AP-2 complex subunit alpha-2
Protein transport protein Sec31A
Rho guanine nucleotide exchange factor 15
Protein HEXIM1
Ribonuclease P protein subunit p14
G2/mitotic-specific cyclin-B2
Protein YIF1A
E3 ubiquitin-protein ligase UBR5
Arf-GAP domain and FG repeat-containing protein 2
Serine protease 23
SR-related and CTD-associated factor 4
NADH dehydrogenase [ubiquinone] 1 beta subcomplex subunit 6
Mitofusin-2
Snurportin-1
Ubiquitin conjugation factor E4 B
Zinc finger protein-like 1
Elongator complex protein 1
NADH dehydrogenase [ubiquinone] 1 beta subcomplex subunit 4
NADH dehydrogenase [ubiquinone] 1 beta subcomplex subunit 8, mitochondrial
NADH dehydrogenase [ubiquinone] 1 alpha subcomplex subunit 7
Vesicle-associated membrane protein 5
Reticulon-3
Mitochondrial proton/calcium exchanger protein
Epsin-2

Zinc finger Ran-binding domain-containing protein 2
Sorting nexin-4
Luc7-like protein 3
Kinesin-like protein KIF20A
Apolipoprotein L3
Chromosome-associated kinesin KIF4A
Methyl-CpG-binding domain protein 4
Myotubularin-related protein 5
Golgi SNAP receptor complex member 1
Histone acetyltransferase KAT7
Arginyl-tRNA--protein transferase 1
Vesicle-associated membrane protein-associated protein B/C
SNARE-associated protein Snapin
Myelin protein zero-like protein 1
NADH dehydrogenase [ubiquinone] 1 subunit C2
NADH dehydrogenase [ubiquinone] 1 alpha subcomplex subunit 10, mitochondrial
Peptidyl-prolyl cis-trans isomerase FKBP9
CUGBP Elav-like family member 2
6-phosphogluconolactonase
Bifunctional 3'-phosphoadenosine 5'-phosphosulfate synthase 2
Structural maintenance of chromosomes protein 2
Ubiquitin-like modifier-activating enzyme ATG7
Transforming acidic coiled-coil-containing protein 2
Tripartite motif-containing protein 16
Phenylalanine--tRNA ligase, mitochondrial
Zinc finger and BTB domain-containing protein 7A
Acyl-protein thioesterase 2
Importin-7
E3 ubiquitin-protein ligase ARIH2
Pre-mRNA-splicing factor SLU7
Phosphoacetylglucosamine mutase
Adenylyltransferase and sulfurtransferase MOCS3
CD2 antigen cytoplasmic tail-binding protein 2
Supervillin
Activator of 90 kDa heat shock protein ATPase homolog 1
Poly(A)-specific ribonuclease PARN
Proteasome assembly chaperone 1
Sphingosine-1-phosphate lyase 1
CTD nuclear envelope phosphatase 1
Ribosome biogenesis protein NSA2 homolog
GDH/6PGL endoplasmic bifunctional protein
Protein transport protein Sec24A
Protein transport protein Sec24B
Adhesion G protein-coupled receptor L2
NAD kinase
Tyrosyl-DNA phosphodiesterase 2
Mitochondrial pyruvate carrier 2
Persulfide dioxygenase ETHE1, mitochondrial
Fatty acid CoA ligase Acsl3
DNA-directed RNA polymerase I subunit RPA1

YEATS domain-containing protein 4
CCR4-NOT transcription complex subunit 4
STAM-binding protein
Cleavage and polyadenylation specificity factor subunit 4
Nuclear factor of activated T-cells, cytoplasmic 1
Probable bifunctional dTTP/UTP pyrophosphatase/methyltransferase protein
Phosphatidate cytidylyltransferase 2
Protein phosphatase 1 regulatory subunit 3D
Bromodomain-containing protein 1
Ribonuclease P protein subunit p29
E3 ubiquitin-protein ligase HERC2
Ras-related protein Rab-3D
Synaptosomal-associated protein 29
Serine/threonine-protein kinase OSR1
Geranylgeranyl pyrophosphate synthase
Heat shock 70 kDa protein 4L
Polypyrimidine tract-binding protein 3
TBC1 domain family member 8
Interleukin-33
STARD3 N-terminal-like protein
U6 snRNA-associated Sm-like protein LSm8
AP-2 complex subunit alpha-1
Protein Wiz
Antiviral innate immune response receptor RIG-I
Double-stranded RNA-binding protein Staufen homolog 1
Tetratricopeptide repeat protein 4
Transmembrane protein 50A
Caveolae-associated protein 2
BAG family molecular chaperone regulator 2
BAG family molecular chaperone regulator 3
Mitogen-activated protein kinase kinase kinase kinase 4
Malonyl-CoA decarboxylase, mitochondrial
Quinone oxidoreductase-like protein 1
Apoptosis-inducing factor 1, mitochondrial
Echinoderm microtubule-associated protein-like 2
Serine/threonine-protein kinase LATS1
Uridine diphosphate glucose pyrophosphatase NUDT14
Tetraspanin-15
3'(2'),5'-bisphosphate nucleotidase 1
Acyl-CoA 6-desaturase
Putative hydrolase DDAH2
Phosphatidylserine lipase ABHD16A
Thioredoxin domain-containing protein 12
Protein ecdysoneless homolog
Reversion-inducing cysteine-rich protein with Kazal motifs
Methyl-CpG-binding domain protein 3
DNA topoisomerase 3-beta-1
Diphosphoinositol polyphosphate phosphohydrolase 1
B-cell lymphoma/leukemia 10
NADH dehydrogenase [ubiquinone] 1 beta subcomplex subunit 10

Putative lipid scramblase CLPTM1
E3 SUMO-protein ligase ZBED1
Molybdopterin synthase catalytic subunit
Mitochondrial import receptor subunit TOM40 homolog
Peroxisomal membrane protein 11B
Serine/threonine-protein kinase PAK 4
Amyloid-beta A4 precursor protein-binding family A member 3
Actin-like protein 6A
Histone-lysine N-methyltransferase NSD2
L-lactate dehydrogenase A chain
Aldehyde dehydrogenase 1A1
Glutamate dehydrogenase 1, mitochondrial
Dihydrofolate reductase
NADH-cytochrome b5 reductase 3
Glutathione reductase, mitochondrial
Cytochrome c oxidase subunit 1
Cytochrome c oxidase subunit 2
Purine nucleoside phosphorylase
Hypoxanthine-guanine phosphoribosyltransferase
Aspartate aminotransferase, mitochondrial
Tyrosine-protein kinase ABL1
Phosphoglycerate kinase 1
Adenylate kinase isoenzyme 1
Urokinase-type plasminogen activator
Tissue-type plasminogen activator
Adenosine deaminase
Alpha-1-antitrypsin
Alpha-2-macroglobulin
Cystatin-C
GTPase NRas
GTPase HRas
GTPase KRas
Platelet-derived growth factor subunit B
Low-density lipoprotein receptor
Transforming growth factor beta-1 proprotein
HLA class I histocompatibility antigen, B alpha chain
Putative HLA class I histocompatibility antigen, alpha chain H
Collagen alpha-1(I) chain
Collagen alpha-1(IV) chain
Prelamin-A/C
Apolipoprotein A-I
Fibronectin
Alpha-2-HS-glycoprotein
Albumin
Transferrin receptor protein 1
Ferritin light chain
Ferritin heavy chain
Metallothionein-2
NADH-ubiquinone oxidoreductase chain 1
NADH-ubiquinone oxidoreductase chain 4

NADH-ubiquinone oxidoreductase chain 5
Interstitial collagenase
3-hydroxy-3-methylglutaryl-coenzyme A reductase
Catalase
RAF proto-oncogene serine/threonine-protein kinase
Lysosomal acid glucosylceramidase
Fructose-bisphosphate aldolase A
Cystatin-B
Annexin A1
Apolipoprotein B-100
Major prion protein
Superoxide dismutase [Mn], mitochondrial
Ornithine aminotransferase, mitochondrial
Thymidine kinase, cytosolic
von Willebrand factor
Tubulin beta-4A chain
Glyceraldehyde-3-phosphate dehydrogenase
Argininosuccinate lyase
HLA class I histocompatibility antigen, A alpha chain
Calpain small subunit 1
Metallothionein-1E
Heat shock protein beta-1
Thymidylate synthase
Dolichyl-diphosphooligosaccharide--protein glycosyltransferase subunit 1
Dolichyl-diphosphooligosaccharide--protein glycosyltransferase subunit 2
Guanine nucleotide-binding protein G(i) subunit alpha-2
Histone H2A type 1-B/E
Sodium/potassium-transporting ATPase subunit alpha-1
Sodium/potassium-transporting ATPase subunit beta-1
Amyloid-beta precursor protein
Aldehyde dehydrogenase, mitochondrial
Integrin beta-3
Protein S100-A8
Non-histone chromosomal protein HMG-14
Plasminogen activator inhibitor 2
Plasminogen activator inhibitor 1
ADP/ATP translocase 2
Ubiquitin-like protein ISG15
Propionyl-CoA carboxylase alpha chain, mitochondrial
Propionyl-CoA carboxylase beta chain, mitochondrial
Eukaryotic translation initiation factor 2 subunit 1
Non-histone chromosomal protein HMG-17
Intercellular adhesion molecule 1
Large ribosomal subunit protein P2
Large ribosomal subunit protein uL10
Transcription factor Jun
DNA-directed RNA polymerase III subunit RPC4
Lupus La protein
Integrin beta-1
Keratin, type I cytoskeletal 18

Collagen alpha-2(V) chain
Uroporphyrinogen decarboxylase
Tyrosine-protein kinase Fyn
Alpha-galactosidase A
Gelsolin
Retinoblastoma-associated protein
Prothymosin alpha
Cyclin-dependent kinase 1
ATP synthase subunit beta, mitochondrial
Protein S100-A6
Eukaryotic translation initiation factor 4E
Alpha-enolase
Glycogen phosphorylase, liver form
Glucose-6-phosphate isomerase
DNA polymerase beta
Nucleophosmin
Tropomyosin alpha-3 chain
Integrin alpha-V
Beta-hexosaminidase subunit alpha
Epoxide hydrolase 1
L-lactate dehydrogenase B chain
Major centromere autoantigen B
Glutathione peroxidase 1
Protein disulfide-isomerase
Histone H1.0
Acylphosphatase-1
Tyrosine-protein kinase Fes/Fps
Cathepsin D
Annexin A2
Calpain-1 catalytic subunit
Tubulin beta chain
Prosaposin
Beta-hexosaminidase subunit beta
Procathepsin L
Profilin-1
Bisphosphoglycerate mutase
Adenine phosphoribosyltransferase
Bifunctional glutamate/proline--tRNA ligase
Cathepsin B
Heat shock protein HSP 90-alpha
Galactose-1-phosphate uridylyltransferase
Heterogeneous nuclear ribonucleoproteins C1/C2
Laminin subunit beta-1
Tyrosine-protein kinase Yes
Tyrosine-protein kinase Lyn
Fumarate hydratase, mitochondrial
DNA excision repair protein ERCC-1
Thrombospondin-1
Transcription factor Sp1
Insulin-like growth factor 1 receptor

Collagen alpha-2(I) chain
Annexin A6
Rho-related GTP-binding protein RhoC
Complement decay-accelerating factor
Amino acid transporter heavy chain SLC3A2
Beta-glucuronidase
ATP-dependent 6-phosphofructokinase, muscle type
Heat shock protein HSP 90-beta
Signal recognition particle receptor subunit alpha
Asparagine synthetase [glutamine-hydrolyzing]
72 kDa type IV collagenase
Porphobilinogen deaminase
Neprilysin
Matrix Gla protein
Pyruvate dehydrogenase E1 component subunit alpha, somatic form, mitochondrial
Collagen alpha-2(IV) chain
Cytochrome c1, heme protein, mitochondrial
U2 small nuclear ribonucleoprotein B''
Hepatocyte growth factor receptor
U1 small nuclear ribonucleoprotein 70 kDa
Integrin alpha-5
Nuclear factor 1 C-type
Vimentin
Small ribosomal subunit protein eS17
Guanine nucleotide-binding protein G(i) subunit alpha-3
Annexin A5
Small ribosomal subunit protein uS2
CD63 antigen
Large ribosomal subunit protein uL3m
U1 small nuclear ribonucleoprotein A
Gamma-enolase
3-ketoacyl-CoA thiolase, peroxisomal
Signal recognition particle 19 kDa protein
Glutathione S-transferase P
U1 small nuclear ribonucleoprotein C
Galectin-1
Dihydropteridine reductase
High mobility group protein B1
SPARC
Tropomyosin alpha-1 chain
Clathrin light chain A
Clathrin light chain B
Annexin A4
2',3'-cyclic-nucleotide 3'-phosphodiesterase
Heme oxygenase 1
Dihydrolipoyl dehydrogenase, mitochondrial
Heterogeneous nuclear ribonucleoprotein A1
U2 small nuclear ribonucleoprotein A'
Cytochrome c oxidase subunit 6C
Tumor-associated calcium signal transducer 2

Poly [ADP-ribose] polymerase 1
DNA polymerase alpha catalytic subunit
Ubiquitin carboxyl-terminal hydrolase isozyme L1
Leukotriene A-4 hydrolase
Fructose-bisphosphate aldolase C
Histone H2A.Z
Histone H2A type 1
Myocardial zonula adherens protein
DNA-directed RNA polymerase II subunit GRINL1A
Glutathione S-transferase theta-2
POTE ankyrin domain family member J
LIM and senescent cell antigen-like-containing domain protein 3
Heat shock 70 kDa protein 1A
Chimeric ERCC6-PGBD3 protein
Tubulin alpha-3C chain
Zinc finger TRAF-type-containing protein 1
Ras-related protein Rap-2a
Serglycin
RNA-binding protein RO60
Lysosomal alpha-glucosidase
Ras-related protein R-Ras
HLA class I histocompatibility antigen, C alpha chain
Serine/threonine-protein kinase A-Raf
Histone H1.4
Dihydrolipoyllysine-residue acetyltransferase component of pyruvate dehydrogenase complex, [illegible]
Receptor-type tyrosine-protein phosphatase F
COUP transcription factor 1
Thioredoxin
Cytochrome c oxidase subunit 5B, mitochondrial
Lysosomal protective protein
Microsomal glutathione S-transferase 1
cAMP-dependent protein kinase type I-alpha regulatory subunit
Tissue factor pathway inhibitor
S-formylglutathione hydrolase
60 kDa heat shock protein, mitochondrial
Clusterin
Endoplasmic reticulum chaperone BiP
Laminin subunit gamma-1
Lysosomal acid phosphatase
Microtubule-associated protein 2
Heat shock cognate 71 kDa protein
Solute carrier family 2, facilitated glucose transporter member 1
Solute carrier family 2, facilitated glucose transporter member 3
Protein 4.1
Uridine 5'-monophosphate synthase
Pyruvate dehydrogenase E1 component subunit beta, mitochondrial
Lipoamide acyltransferase component of branched-chain alpha-keto acid dehydrogenase compl[illegible]
Glycogen phosphorylase, brain form
Glycogen phosphorylase, muscle form
Ras-related protein Ral-A

Ras-related protein Ral-B
Breakpoint cluster region protein
Lysosome-associated membrane glycoprotein 1
Transcriptional regulator ERG
Medium-chain specific acyl-CoA dehydrogenase, mitochondrial
DNA topoisomerase 1
DNA topoisomerase 2-alpha
Glucose-6-phosphate 1-dehydrogenase
Ubiquitin-like protein 4A
Pyruvate carboxylase, mitochondrial
C-1-tetrahydrofolate synthase, cytoplasmic
Cation-independent mannose-6-phosphate receptor
Alcohol dehydrogenase class-3
Cyclin-dependent kinase 4
Ribose-phosphate pyrophosphokinase 2
Polyadenylate-binding protein 1
Proliferating cell nuclear antigen
Histidine--tRNA ligase, cytoplasmic
Collagen alpha-1(VI) chain
ADP/ATP translocase 1
ADP/ATP translocase 3
Inosine-5'-monophosphate dehydrogenase 2
Nucleoprotein TPR
Annexin A3
2-oxoisovalerate dehydrogenase subunit alpha, mitochondrial
Alpha-actinin-1
Angiotensin-converting enzyme
Proto-oncogene tyrosine-protein kinase Src
Xaa-Pro dipeptidase
X-ray repair cross-complementing protein 6
X-ray repair cross-complementing protein 5
Uracil-DNA glycosylase
Cytochrome c oxidase subunit 4 isoform 1, mitochondrial
5-aminolevulinate synthase, non-specific, mitochondrial
Lysosome-associated membrane glycoprotein 2
Ribonuclease inhibitor
Intercellular adhesion molecule 2
Elongation factor 2
Protein disulfide-isomerase A4
Prolyl 4-hydroxylase subunit alpha-1
Translationally-controlled tumor protein
Delta-aminolevulinic acid dehydratase
HLA class I histocompatibility antigen, alpha chain E
Plastin-3
Acylamino-acid-releasing enzyme
Electron transfer flavoprotein subunit alpha, mitochondrial
Glycogen [starch] synthase, muscle
cAMP-dependent protein kinase type II-alpha regulatory subunit
General transcription factor IIF subunit 2
CD59 glycoprotein

Bifunctional methylenetetrahydrofolate dehydrogenase/cyclohydrolase, mitochondrial
Macrophage migration inhibitory factor
CD99 antigen
Glucosidase 2 subunit beta
Hematopoietic lineage cell-specific protein
Farnesyl pyrophosphate synthase
Cytochrome c oxidase subunit 7A2, mitochondrial
Nidogen-1
Aldo-keto reductase family 1 member A1
Pyruvate kinase PKM
Endoplasmin
G2/mitotic-specific cyclin-B1
Myosin light chain 6B
Small nuclear ribonucleoprotein-associated proteins B and B'
Insulin-degrading enzyme
POU domain, class 2, transcription factor 1
Heterogeneous nuclear ribonucleoprotein L
Aspartate--tRNA ligase, cytoplasmic
Protein C-ets-1
Junction plakoglobin
Cytochrome b-c1 complex subunit 7
Serine/threonine-protein kinase B-raf
Fatty acid-binding protein, adipocyte
Glutamine synthetase
Aldo-keto reductase family 1 member B1
Aminopeptidase N
Poliovirus receptor
Ras-related C3 botulinum toxin substrate 2
Eukaryotic peptide chain release factor GTP-binding subunit ERF3A
Arylsulfatase A
Beta-1,4-galactosyltransferase 1
Ezrin
Ubiquitin carboxyl-terminal hydrolase isozyme L3
Fos-related antigen 1
Fos-related antigen 2
Membrane cofactor protein
Nucleoside diphosphate kinase A
NAD(P)H dehydrogenase [quinone] 1
N-acetylglucosamine-6-sulfatase
Phosphorylase b kinase gamma catalytic chain, liver/testis isoform
Arylsulfatase B
Small ribosomal subunit protein uS5
Transcription factor 4
Desmoplakin
Replication protein A 32 kDa subunit
Metalloproteinase inhibitor 2
CD44 antigen
Carbonyl reductase [NADPH] 1
Short-chain specific acyl-CoA dehydrogenase, mitochondrial
Cyclic AMP-responsive element-binding protein 1

Beta-galactosidase
Platelet endothelial cell adhesion molecule
Serine/threonine-protein phosphatase 2B catalytic subunit beta isoform
SH2/SH3 adapter protein NCK1
Intron Large complex component GCFC2
Histone H1.5
Histone H1.3
Histone H1.2
NADPH--cytochrome P450 reductase
Tyrosine-protein kinase Fer
Sarcoplasmic/endoplasmic reticulum calcium ATPase 2
1-phosphatidylinositol 4,5-bisphosphate phosphodiesterase gamma-2
Fumarylacetoacetase
Stathmin
Y-box-binding protein 3
Zinc finger protein 22
Alpha-N-acetylgalactosaminidase
High mobility group protein HMG-I/HMG-Y
Transmembrane protein 11, mitochondrial
Aspartate aminotransferase, cytoplasmic
Protein kinase C alpha type
Integrin alpha-2
Gap junction alpha-1 protein
Sphingomyelin phosphodiesterase
Nucleolar transcription factor 1
Transcription factor JunD
T-cell acute lymphocytic leukemia protein 1
Cyclic AMP-dependent transcription factor ATF-7
NADH dehydrogenase [ubiquinone] 1 beta subcomplex subunit 7
cAMP-dependent protein kinase catalytic subunit alpha
Calpain-2 catalytic subunit
Tyrosine-protein phosphatase non-receptor type 2
CTP synthase 1
Endoglin
Probable ATP-dependent RNA helicase DDX5
ATP-dependent 6-phosphofructokinase, liver type
Vascular endothelial growth factor receptor 1
26S proteasome regulatory subunit 6A
T-complex protein 1 subunit alpha
Tyrosine-protein phosphatase non-receptor type 1
General transcription and DNA repair factor IIH helicase subunit XPD
Large ribosomal subunit protein eL33
Integrin beta-5
ADP-ribosylation factor 4
Large ribosomal subunit protein uL30
Vinculin
Receptor-type tyrosine-protein phosphatase alpha
Arylamine N-acetyltransferase 1
Protein SON
Negative elongation factor E

Large ribosomal subunit protein uL22
Phosphoglycerate mutase 1
Regulator of chromosome condensation
Cyclic AMP-dependent transcription factor ATF-1
Cyclic AMP-dependent transcription factor ATF-6 alpha
DNA ligase 1
DNA repair protein XRCC1
Peptidyl-glycine alpha-amidating monooxygenase
Cadherin-2
1-phosphatidylinositol 4,5-bisphosphate phosphodiesterase gamma-1
Nucleolin
Hexokinase-1
DNA-directed RNA polymerase II subunit RPB3
DNA-directed RNA polymerases I, II, and III subunit RPABC1
NADH dehydrogenase [ubiquinone] flavoprotein 2, mitochondrial
General transcription and DNA repair factor IIH helicase subunit XPB
E3 ubiquitin-protein ligase TRIM21
Interferon-induced, double-stranded RNA-activated protein kinase
Spermidine synthase
Sodium/hydrogen exchanger 1
Casein kinase II subunit alpha'
Nuclear factor NF-kappa-B p105 subunit
Plasma membrane calcium-transporting ATPase 1
Eukaryotic translation initiation factor 2 subunit 2
Annexin A7
TATA-box-binding protein
Cyclin-A2
Transcription factor BTF3
Ras-related protein Rab-3B
Ras-related protein Rab-4A
Ras-related protein Rab-5A
Ras-related protein Rab-6A
DNA mismatch repair protein Msh3
Atrial natriuretic peptide receptor 2
Proteasome subunit beta type-1
Cation-dependent mannose-6-phosphate receptor
Cytochrome c oxidase subunit 5A, mitochondrial
Lamin-B1
Calpastatin
Inosine-5'-monophosphate dehydrogenase 1
Collagen alpha-1(V) chain
Nebulin
N(4)-(beta-N-acetylglucosaminyl)-L-asparaginase
Ras GTPase-activating protein 1
Parathymosin
Cyclin-dependent kinase 11B
Glutathione S-transferase Mu 3
V-type proton ATPase subunit B, brain isoform
V-type proton ATPase subunit C 1
Cysteine and glycine-rich protein 1

Filamin-A
Neurofibromin
Amine oxidase [flavin-containing] A
Cytoplasmic aconitate hydratase
Sphingosine 1-phosphate receptor 1
Synaptotagmin-1
5'-nucleotidase
Transcription initiation factor TFIID subunit 1
Midkine
Voltage-dependent anion-selective channel protein 1
Biglycan
Succinate dehydrogenase [ubiquinone] iron-sulfur subunit, mitochondrial
CD9 antigen
Catechol O-methyltransferase
Protein-glutamine gamma-glutamyltransferase 2
Methylmalonyl-CoA mutase, mitochondrial
Oxysterol-binding protein 1
Protein-L-isoaspartate(D-aspartate) O-methyltransferase
rRNA 2'-O-methyltransferase fibrillarin
Trifunctional purine biosynthetic protein adenosine-3
Bifunctional phosphoribosylaminoimidazole carboxylase/phosphoribosylaminoimidazole succino
Sterol carrier protein 2
Ubiquitin-like modifier-activating enzyme 1
Nucleoside diphosphate kinase B
NADPH:adrenodoxin oxidoreductase, mitochondrial
Heterogeneous nuclear ribonucleoproteins A2/B1
MHC class II regulatory factor RFX1
E3 ubiquitin-protein ligase CBL
Insulin-like growth factor-binding protein 4
cAMP-dependent protein kinase catalytic subunit beta
Cytochrome b-c1 complex subunit 2, mitochondrial
Ferrochelatase, mitochondrial
DNA repair protein complementing XP-A cells
Transcription elongation factor A protein 1
Prostaglandin G/H synthase 1
Integrin alpha-6
Splicing factor, proline- and glutamine-rich
Tubulin gamma-1 chain
Peptidyl-prolyl cis-trans isomerase B
NAD-dependent malic enzyme, mitochondrial
Tryptophan--tRNA ligase, cytoplasmic
Small ribosomal subunit protein uS3
Ribosomal protein S6 kinase beta-1
Tyrosine-protein kinase JAK1
Receptor-type tyrosine-protein phosphatase beta
Receptor-type tyrosine-protein phosphatase epsilon
Receptor-type tyrosine-protein phosphatase gamma
Nuclear autoantigen Sp-100
Colorectal mutant cancer protein
Tumor necrosis factor ligand superfamily member 4

Adenosylhomocysteinase
Cofilin-1
Eukaryotic translation initiation factor 4B
40-kDa huntingtin-associated protein
Plasma membrane calcium-transporting ATPase 4
Diacylglycerol kinase alpha
Carnitine O-palmitoyltransferase 2, mitochondrial
Thymidylate kinase
Ribonucleoside-diphosphate reductase large subunit
G1/S-specific cyclin-D1
Rab proteins geranylgeranyltransferase component A 1
ER lumen protein-retaining receptor 1
COUP transcription factor 2
Elongation factor 1-beta
ATP synthase F(0) complex subunit B1, mitochondrial
Low molecular weight phosphotyrosine protein phosphatase
Protein kinase C eta type
Acetyl-CoA acetyltransferase, mitochondrial
Myosin regulatory light polypeptide 9
DNA-directed RNA polymerase II subunit RPB1
Cyclin-dependent kinase 2
Beta-adrenergic receptor kinase 1
Proteinase-activated receptor 1
DNA replication licensing factor MCM3
3-mercaptopyruvate sulfurtransferase
Small ribosomal subunit protein eS12
Bromodomain-containing protein 2
Tumor necrosis factor receptor superfamily member 6
Transcriptional repressor protein YY1
DnaJ homolog subfamily B member 1
DnaJ homolog subfamily B member 2
ATP synthase subunit alpha, mitochondrial
Cathepsin S
Proteasome subunit alpha type-1
Proteasome subunit alpha type-2
Proteasome subunit alpha type-3
Proteasome subunit alpha type-4
Tumor necrosis factor receptor superfamily member 5
Integrin alpha-3
Pentraxin-related protein PTX3
Moesin
Probable ATP-dependent RNA helicase DDX6
DNA (cytosine-5)-methyltransferase 1
Splicing factor U2AF 65 kDa subunit
Large ribosomal subunit protein eL13
Rab proteins geranylgeranyltransferase component A 2
Isovaleryl-CoA dehydrogenase, mitochondrial
Alpha-1,3-mannosyl-glycoprotein 2-beta-N-acetylglucosaminyltransferase
High mobility group protein B2
Polypyrimidine tract-binding protein 1

Threonine--tRNA ligase 1, cytoplasmic
Valine--tRNA ligase
Elongation factor 1-gamma
Peptidyl-prolyl cis-trans isomerase FKBP2
Interleukin-3 receptor subunit alpha
Stomatin
Adenylate kinase 4, mitochondrial
14-3-3 protein theta
Mitogen-activated protein kinase 3
MAP/microtubule affinity-regulating kinase 3
Large ribosomal subunit protein uL16
Collagen alpha-1(VIII) chain
Replication protein A 70 kDa DNA-binding subunit
DNA-(apurinic or apyrimidinic site) endonuclease
CD82 antigen
Deoxycytidine kinase
Multifunctional protein CAD
Calreticulin
Microtubule-associated protein 4
Calnexin
Phosphatidylinositol 3-kinase regulatory subunit alpha
Inositol-trisphosphate 3-kinase B
Proteasome subunit beta type-8
Proteasome subunit beta type-9
Proteasome subunit alpha type-5
Proteasome subunit beta type-4
Proteasome subunit beta type-6
Proteasome subunit beta type-5
Glutathione S-transferase Mu 2
ATP-binding cassette sub-family D member 3
Protein ITPRID2
Protein-lysine 6-oxidase
NADH-ubiquinone oxidoreductase 75 kDa subunit, mitochondrial
DNA polymerase delta catalytic subunit
Probable global transcription activator SNF2L1
Mitogen-activated protein kinase 1
Retinoic acid receptor RXR-beta
DNA excision repair protein ERCC-5
Progranulin
Receptor-type tyrosine-protein phosphatase mu
Cytosol aminopeptidase
Hematopoietic progenitor cell antigen CD34
General transcription factor IIE subunit 1
Transcription initiation factor IIE subunit beta
Tripeptidyl-peptidase 2
Inositol monophosphatase 1
CCN family member 2
Ephrin type-A receptor 2
Ephrin type-B receptor 2
SHC-transforming protein 1

DNA-3-methyladenine glycosylase
Transketolase
Caspase-1
Nitric oxide synthase 3
RNA-binding motif, single-stranded-interacting protein 1
Protein PML
Non-receptor tyrosine-protein kinase TYK2
Elongation factor 1-delta
Myristoylated alanine-rich C-kinase substrate
Guanine nucleotide-binding protein subunit alpha-11
Delta-1-pyrroline-5-carboxylate dehydrogenase, mitochondrial
Endoplasmic reticulum resident protein 29
Peroxiredoxin-6
Flavin reductase (NADPH)
Peroxiredoxin-5, mitochondrial
Thioredoxin-dependent peroxide reductase, mitochondrial
Large ribosomal subunit protein uL11
Enoyl-CoA hydratase, mitochondrial
UMP-CMP kinase
Phosphatidylethanolamine-binding protein 1
Protein disulfide-isomerase A3
Serine/threonine-protein phosphatase 2A 65 kDa regulatory subunit A alpha isoform
Serine/threonine-protein phosphatase 2A 65 kDa regulatory subunit A beta isoform
Cell division cycle protein 27 homolog
Peptidyl-prolyl cis-trans isomerase F, mitochondrial
NK-tumor recognition protein
Glycylpeptide N-tetradecanoyltransferase 1
Heme oxygenase 2
Adenylosuccinate synthetase isozyme 2
Tyrosine-protein kinase receptor UFO
Alpha-2-macroglobulin receptor-associated protein
Translocator protein
Adenylosuccinate lyase
CAP-Gly domain-containing linker protein 1
Sorcin
Leukocyte elastase inhibitor
High affinity cationic amino acid transporter 1
Aldehyde dehydrogenase X, mitochondrial
DNA-directed RNA polymerase II subunit RPB2
Succinate dehydrogenase [ubiquinone] flavoprotein subunit, mitochondrial
Coronin-1A
Rab GDP dissociation inhibitor alpha
S-adenosylmethionine synthase isoform type-2
cAMP-dependent protein kinase type II-beta regulatory subunit
Ribonucleoside-diphosphate reductase subunit M2
Syndecan-4
Cytotoxic granule associated RNA binding protein TIA1
Sodium- and chloride-dependent taurine transporter
DnaJ homolog subfamily A member 1
RAC-alpha serine/threonine-protein kinase

Cytochrome b-c1 complex subunit 1, mitochondrial
3-hydroxyisobutyrate dehydrogenase, mitochondrial
Bifunctional purine biosynthesis protein ATIC
Heterogeneous nuclear ribonucleoprotein H3
Heterogeneous nuclear ribonucleoprotein H
14-3-3 protein beta/alpha
Stress-induced-phosphoprotein 1
Protein S100-A11
Peroxiredoxin-2
Beta-arrestin-2
Glycerol kinase
Deoxycytidylate deaminase
Pyrroline-5-carboxylate reductase 1, mitochondrial
Guanylate-binding protein 1
Guanylate-binding protein 2
ETS-related transcription factor Elf-1
General transcription factor IIH subunit 1
Syntaxin-2
Large ribosomal subunit protein uL6
Long-chain-fatty-acid--CoA ligase 1
Cadherin-5
Kinesin-1 heavy chain
Deoxyuridine 5'-triphosphate nucleotidohydrolase, mitochondrial
Multidrug resistance-associated protein 1
ATP-binding cassette sub-family D member 1
Dual specificity protein kinase TTK
DNA replication licensing factor MCM4
DNA replication licensing factor MCM5
DNA replication licensing factor MCM7
Serine hydroxymethyltransferase, cytosolic
Serine hydroxymethyltransferase, mitochondrial
Heat shock 70 kDa protein 1-like
Heat shock 70 kDa protein 4
G protein-coupled receptor kinase 5
Mannose-6-phosphate isomerase
Glypican-1
Profilin-2
Catenin alpha-1
Catenin beta-1
Prohibitin 1
Serpin B6
Merlin
Radixin
Replication protein A 14 kDa subunit
Replication factor C subunit 4
Replication factor C subunit 2
Replication factor C subunit 1
Large ribosomal subunit protein eL22
General transcription factor IIF subunit 1
Sepiapterin reductase

Histamine H1 receptor
Cystathionine beta-synthase
Fibrillin-1
Fibrillin-2
Glycogen debranching enzyme
Myosin-9
Myosin-10
Tyrosine-protein kinase receptor Tie-1
Coatomer subunit beta'
Sterol O-acyltransferase 1
Alpha-adducin
Basigin
RNA-binding protein FUS
Nuclear pore complex protein Nup214
Protein DEK
Transcription factor SOX-18
Myosin-11
Protein phosphatase 1A
Hydroxymethylglutaryl-CoA lyase, mitochondrial
Vascular endothelial growth factor receptor 3
Vascular endothelial growth factor receptor 2
26S proteasome regulatory subunit 7
Glutathione hydrolase 5 proenzyme
ADP-ribosylation factor-like protein 2
ADP-ribosylation factor-like protein 3
Dual specificity mitogen-activated protein kinase kinase 2
ATP synthase subunit gamma, mitochondrial
V-type proton ATPase subunit E 1
Oxygen-dependent coproporphyrinogen-III oxidase, mitochondrial
Large ribosomal subunit protein uL4
Oxidized purine nucleoside triphosphate hydrolase
Lon protease homolog, mitochondrial
Phosphoglucomutase-1
Serine/threonine-protein phosphatase PP1-gamma catalytic subunit
Guanine nucleotide-binding protein-like 1
Dihydrolipoyllysine-residue succinyltransferase component of 2-oxoglutarate dehydrogenase co
GMP reductase 1
Phospholipid hydroperoxide glutathione peroxidase GPX4
Serine/threonine-protein kinase receptor R3
17-beta-hydroxysteroid dehydrogenase type 2
Signal recognition particle 14 kDa protein
TGF-beta receptor type-2
Nuclear pore glycoprotein p62
Hippocalcin-like protein 1
Squalene synthase
Zinc finger E-box-binding homeobox 1
Transgelin-2
Transaldolase
Alpha-synuclein
Electron transfer flavoprotein subunit beta

RNA-binding motif protein, X chromosome
Coilin
Vitamin K-dependent gamma-carboxylase
Lysosomal acid lipase/cholesteryl ester hydrolase
V-type proton ATPase catalytic subunit A
Stress-70 protein, mitochondrial
Eukaryotic initiation factor 4A-III
DNA-binding protein SMUBP-2
Cyclin-dependent kinase inhibitor 1
Small ribosomal subunit protein eS19
Large ribosomal subunit protein uL3
Collagen alpha-1(XVIII) chain
Dolichyl-diphosphooligosaccharide--protein glycosyltransferase 48 kDa subunit
Acidic leucine-rich nuclear phosphoprotein 32 family member A
Flap endonuclease 1
Homeobox protein cut-like 1
Macrophage-capping protein
Adenylyl cyclase-associated protein 2
Interleukin-6 receptor subunit beta
Alpha-taxilin
T-complex protein 1 subunit zeta
Nicotinamide N-methyltransferase
Proteasome subunit beta type-10
Pre-B-cell leukemia transcription factor 2
Large ribosomal subunit protein uL13
ADP-ribosylation factor-like protein 1
DNA mismatch repair protein Mlh1
Signal transducer and activator of transcription 3
Ubiquitin carboxyl-terminal hydrolase 8
Malate dehydrogenase, cytoplasmic
Malate dehydrogenase, mitochondrial
Replication factor C subunit 5
Replication factor C subunit 3
Trifunctional enzyme subunit alpha, mitochondrial
Eukaryotic translation initiation factor 2 subunit 3
Centrin-2
Transcription factor ETV6
Eukaryotic translation initiation factor 2D
Protein BUD31 homolog
Ubiquitin-like modifier-activating enzyme 7
N-alpha-acetyltransferase 10
Lysine-specific demethylase 5C
Tyrosine-protein kinase CSK
Glycine--tRNA ligase
Isoleucine--tRNA ligase, cytoplasmic
Reduced folate transporter
Eukaryotic translation initiation factor 1
Protein kinase C iota type
ETS domain-containing protein Elk-3
Beta-centractin

Enoyl-CoA delta isomerase 1, mitochondrial
Lamina-associated polypeptide 2, isoform alpha
Lamina-associated polypeptide 2, isoforms beta/gamma
Signal transducer and activator of transcription 1-alpha/beta
Signal transducer and activator of transcription 6
Signal transducer and activator of transcription 5A
Exosome RNA helicase MTR4
Aldo-keto reductase family 1 member C3
Phosphatidylinositol 4,5-bisphosphate 3-kinase catalytic subunit alpha isoform
Phosphatidylinositol 4,5-bisphosphate 3-kinase catalytic subunit beta isoform
Serine/threonine-protein kinase mTOR
Phosphatidylinositol 4-kinase alpha
Epidermal growth factor receptor substrate 15
Caspase-3
Caspase-2
Small ribosomal subunit protein eS27
Tyrosine-protein kinase ABL2
Probable helicase with zinc finger domain
Condensin-2 complex subunit D3
RNA-binding protein 34
Leucine-rich PPR motif-containing protein, mitochondrial
3-ketoacyl-CoA thiolase, mitochondrial
Large ribosomal subunit protein uL29
Lysosomal Pro-X carboxypeptidase
Huntingtin
Endothelin-converting enzyme 1
Methylenetetrahydrofolate reductase (NADPH)
Neutral amino acid transporter A
Platelet-activating factor acetylhydrolase IB subunit beta
Cell surface glycoprotein MUC18
Carnitine O-acetyltransferase
Matrin-3
DNA mismatch repair protein Msh2
G protein-coupled receptor kinase 6
Glycerol-3-phosphate dehydrogenase, mitochondrial
Translocon-associated protein subunit alpha
Tyrosine-protein phosphatase non-receptor type 9
Ran-specific GTPase-activating protein
Nicotinamide phosphoribosyltransferase
26S proteasome regulatory subunit 6B
Elongation factor Ts, mitochondrial
Peptidyl-prolyl cis-trans isomerase C
Voltage-dependent anion-selective channel protein 2
Short/branched chain specific acyl-CoA dehydrogenase, mitochondrial
Chromobox protein homolog 5
Ubiquitin carboxyl-terminal hydrolase 5
Mitogen-activated protein kinase 8
Dual specificity mitogen-activated protein kinase kinase 4
Proliferation marker protein Ki-67
Phosphorylase b kinase regulatory subunit alpha, liver isoform

Phosphorylase b kinase regulatory subunit alpha, skeletal muscle isoform
Ran GTPase-activating protein 1
ATP-dependent DNA helicase Q1
Probable 28S rRNA (cytosine(4447)-C(5))-methyltransferase
Transcriptional regulator ATRX
Adapter molecule crk
Crk-like protein
Translation initiation factor IF-2, mitochondrial
Large proline-rich protein BAG6
Vesicle-fusing ATPase
Neurogenic locus notch homolog protein 1
Dual specificity mitogen-activated protein kinase kinase 3
Lys-63-specific deubiquitinase BRCC36
Large ribosomal subunit protein uL15
Large ribosomal subunit protein uL18
Large ribosomal subunit protein eL21
Large ribosomal subunit protein eL28
Small ribosomal subunit protein uS4
Small ribosomal subunit protein uS7
Small ribosomal subunit protein eS10
Microtubule-associated protein 1B
Glucosamine-6-phosphate isomerase 1
E3 ubiquitin-protein ligase NEDD4
Utrophin
Ras GTPase-activating-like protein IQGAP1
Glycogenin-1
Dolichyl-diphosphooligosaccharide--protein glycosyltransferase subunit STT3A
Cytosolic phospholipase A2
F-actin-capping protein subunit alpha-2
F-actin-capping protein subunit beta
Eukaryotic translation initiation factor 1A, X-chromosomal
Retinaldehyde dehydrogenase 3
Glutamine--tRNA ligase
Large ribosomal subunit protein eL29
mRNA decay activator protein ZFP36L2
Cytochrome b-c1 complex subunit Rieske, mitochondrial
ATP synthase subunit O, mitochondrial
LIM and senescent cell antigen-like-containing domain protein 1
Glioma pathogenesis-related protein 1
Prolyl endopeptidase
NADP-dependent malic enzyme
Iron-responsive element-binding protein 2
Tissue factor pathway inhibitor 2
DNA-binding protein RFX5
Phosphatidylinositol 5-phosphate 4-kinase type-2 alpha
Coatomer subunit delta
Lanosterol synthase
Serine/threonine-protein phosphatase 2B catalytic subunit gamma isoform
Glutamate--cysteine ligase catalytic subunit
Glutamate--cysteine ligase regulatory subunit

CD151 antigen
Trafficking protein particle complex subunit 10
26S proteasome non-ATPase regulatory subunit 8
Protein PRRC2A
Glutathione synthetase
T-complex protein 1 subunit epsilon
Phosphatidylserine synthase 1
Nestin
Heat shock 70 kDa protein 13
Casein kinase I isoform alpha
Casein kinase I isoform delta
Isocitrate dehydrogenase [NADP], mitochondrial
Phosphatidylinositol 4,5-bisphosphate 3-kinase catalytic subunit gamma isoform
Phosphatidylinositol transfer protein beta isoform
DNA polymerase delta subunit 2
MARCKS-related protein
Paxillin
Guided entry of tail-anchored proteins factor CAMLG
Nuclear receptor subfamily 2 group C member 2
MAP kinase-activated protein kinase 2
Deoxyribonuclease-1-like 1
4-trimethylaminobutyraldehyde dehydrogenase
Large ribosomal subunit protein eL34
Ribose-5-phosphate isomerase
Protein ERGIC-53
Natural resistance-associated macrophage protein 2
Nuclear autoantigenic sperm protein
Fatty acid synthase
Protein farnesyltransferase/geranylgeranyltransferase type-1 subunit alpha
Protein farnesyltransferase subunit beta
Deoxyhypusine synthase
T-complex protein 1 subunit gamma
Large ribosomal subunit protein bL19m
Beta-arrestin-1
Elongation factor Tu, mitochondrial
Alpha-aminoadipic semialdehyde dehydrogenase
Ubiquitin-conjugating enzyme E2 R1
Inositol polyphosphate 1-phosphatase
Signal recognition particle 9 kDa protein
Choline-phosphate cytidylyltransferase A
Alanine--tRNA ligase, cytoplasmic
Cysteine--tRNA ligase, cytoplasmic
Histidine--tRNA ligase, mitochondrial
Serine--tRNA ligase, cytoplasmic
Protein phosphatase 1F
Alpha-mannosidase 2x
DNA primase small subunit
DNA primase large subunit
Caspase-4
Casein kinase I isoform epsilon

Proteasome subunit beta type-3
Proteasome subunit beta type-2
DNA replication licensing factor MCM2
Very long-chain specific acyl-CoA dehydrogenase, mitochondrial
YLP motif-containing protein 1
Vacuolar protein sorting-associated protein 41 homolog
Transmembrane emp24 domain-containing protein 10
RNA-binding protein 25
Protein numb homolog
Dual specificity protein kinase CLK3
Presenilin-1
Translation initiation factor eIF2B subunit beta
Adenosine 5'-monophosphoramidase HINT1
Nuclear pore complex protein Nup153
E3 SUMO-protein ligase RanBP2
Regulator of G-protein signaling 19
Regulator of G-protein signaling 3
Presenilin-2
Tuberin
NADH dehydrogenase [ubiquinone] flavoprotein 1, mitochondrial
Glycogen synthase kinase-3 alpha
Glycogen synthase kinase-3 beta
Transcription initiation factor TFIID subunit 6
Cytosolic purine 5'-nucleotidase
Selenide, water dikinase 1
5-formyltetrahydrofolate cyclo-ligase
GMP synthase [glutamine-hydrolyzing]
DNA ligase 3
DNA ligase 4
Double-strand break repair protein MRE11
Ectonucleoside triphosphate diphosphohydrolase 1
Guanine nucleotide-binding protein G(q) subunit alpha
Isocitrate dehydrogenase [NAD] subunit alpha, mitochondrial
Sulfotransferase 1A1
Matrix metalloproteinase-14
Protoporphyrinogen oxidase
Rab GDP dissociation inhibitor beta
Emerin
Carnitine O-palmitoyltransferase 1, liver isoform
Sulfate transporter
Serpin B8
Serpin B9
Serpin H1
PDZ and LIM domain protein 4
Hsc70-interacting protein
Peroxisomal targeting signal 1 receptor
ETS domain-containing transcription factor ERF
Vasodilator-stimulated phosphoprotein
Dynamin-2
Methionine aminopeptidase 2

Bis(5'-nucleosyl)-tetraphosphatase [asymmetrical]
Cyclin-dependent kinase 7
Kinetochore-associated protein 1
Ras association domain-containing protein 2
Cyclin-dependent kinase 9
Lipopolysaccharide-responsive and beige-like anchor protein
Basal cell adhesion molecule
Palmitoyl-protein thioesterase 1
Large ribosomal subunit protein eL14
T-complex protein 1 subunit theta
T-complex protein 1 subunit delta
Annexin A11
Poly(A) polymerase alpha
RNA-binding protein FXR1
RNA-binding protein FXR2
Ras-related protein Rab-5C
Ras-related protein Rab-7a
Ras-related protein Rab-9A
Ras-related protein Rab-13
Ras-related protein Rab-27A
1-phosphatidylinositol 4,5-bisphosphate phosphodiesterase delta-1
Small ribosomal subunit protein mS29
Dual specificity protein phosphatase 3
Transcription activator BRG1
Isocitrate dehydrogenase [NAD] subunit gamma, mitochondrial
Galactokinase
Translocon-associated protein subunit delta
B-cell receptor-associated protein 31
Thiopurine S-methyltransferase
Methyl-CpG-binding protein 2
Host cell factor 1
Interleukin-1 receptor-associated kinase 1
Caveolin-2
Aldehyde dehydrogenase family 3 member A2
Peroxisomal multifunctional enzyme type 2
26S proteasome non-ATPase regulatory subunit 7
Sulfite oxidase, mitochondrial
N-sulphoglucosamine sulphohydrolase
Signal transducer and activator of transcription 5B
Ubiquitin carboxyl-terminal hydrolase 11
H(+)/Cl(-) exchange transporter 7
Vesicle-associated membrane protein 7
Ribosomal protein S6 kinase alpha-3
Cytoplasmic tyrosine-protein kinase BMX
AF4/FMR2 family member 1
Hepatoma-derived growth factor
Cyclin-H
CDK-activating kinase assembly factor MAT1
Serine/threonine-protein kinase Nek3
Ubiquitin-conjugating enzyme E2 E1

NADH dehydrogenase [ubiquinone] 1 alpha subcomplex subunit 8
Heterogeneous nuclear ribonucleoprotein A3
6-phosphogluconate dehydrogenase, decarboxylating
Heterogeneous nuclear ribonucleoprotein M
Importin subunit alpha-1
Importin subunit alpha-5
Nuclear cap-binding protein subunit 2
Rap1 GTPase-GDP dissociation stimulator 1
DNA-directed RNA polymerases I, II, and III subunit RPABC3
Dual specificity mitogen-activated protein kinase kinase 6
Rho GDP-dissociation inhibitor 1
Rho GDP-dissociation inhibitor 2
Arf-GAP domain and FG repeat-containing protein 1
Heterogeneous nuclear ribonucleoprotein F
Signal transducer and activator of transcription 2
DNA mismatch repair protein Msh6
Kinesin-like protein KIF11
Guanine nucleotide exchange factor VAV2
Zinc finger protein 143
RNA-binding protein 5
2-iminobutanoate/2-iminopropanoate deaminase
Spermine synthase
Hexokinase-2
Ephrin-B2
Large ribosomal subunit protein bL12m
Diacylglycerol kinase theta
Bifunctional heparan sulfate N-deacetylase/N-sulfotransferase 1
Thimet oligopeptidase
F-actin-capping protein subunit alpha-1
High mobility group protein HMGI-C
Cysteine-rich protein 2
Nuclear pore complex protein Nup98-Nup96
Biliverdin reductase A
Tricarboxylate transport protein, mitochondrial
Serine/threonine-protein phosphatase 5
Serine/threonine-protein kinase PLK1
Death-associated protein kinase 1
Arfaptin-2
Arfaptin-1
Cytosolic Fe-S cluster assembly factor NUBP1
ATP-citrate synthase
Methionine aminopeptidase 1
Succinate--CoA ligase [ADP/GDP-forming] subunit alpha, mitochondrial
Diphosphomevalonate decarboxylase
Geranylgeranyl transferase type-1 subunit beta
Geranylgeranyl transferase type-2 subunit beta
Coatomer subunit beta
Coatomer subunit alpha
Dipeptidyl peptidase 1
LIM domain kinase 1

LIM domain kinase 2
Clathrin heavy chain 2
AP-3 complex subunit mu-2
AP-2 complex subunit sigma
Holocytochrome c-type synthase
Mitogen-activated protein kinase 12
Pituitary tumor-transforming gene 1 protein-interacting protein
Smoothelin
Monocarboxylate transporter 1
IST1 homolog
Protein transport protein Sec24C
Activated RNA polymerase II transcriptional coactivator p15
DNA polymerase subunit gamma-1
Methylosome subunit pICln
Arginine--tRNA ligase, cytoplasmic
Protein HIRA
Ataxin-3
Atrophin-1
PMS1 protein homolog 1
Mismatch repair endonuclease PMS2
Voltage-dependent calcium channel subunit alpha-2/delta-1
Tyrosine--tRNA ligase, cytoplasmic
Ubiquitin carboxyl-terminal hydrolase 14
5'-AMP-activated protein kinase subunit gamma-1
Heat shock-related 70 kDa protein 2
Branched-chain-amino-acid aminotransferase, cytosolic
Sodium/potassium-transporting ATPase subunit beta-3
UV excision repair protein RAD23 homolog B
Ephrin type-B receptor 4
Alpha-N-acetylglucosaminidase
Adenylate kinase 2, mitochondrial
Delta-1-pyrroline-5-carboxylate synthase
Alpha-soluble NSF attachment protein
Eukaryotic translation initiation factor 5
Solute carrier family 12 member 2
26S proteasome non-ATPase regulatory subunit 4
Developmentally-regulated GTP-binding protein 2
Exportin-2
Transitional endoplasmic reticulum ATPase
Microfibrillar-associated protein 1
Trifunctional enzyme subunit beta, mitochondrial
Mesencephalic astrocyte-derived neurotrophic factor
Afadin
RNA polymerase II elongation factor ELL
Peregrin
Nucleosome assembly protein 1-like 1
Caspase-7
Caspase-6
Adenosine kinase
Double-stranded RNA-specific adenosine deaminase

Laminin subunit beta-2
Cadherin-13
Tumor protein D52
Protein SEC13 homolog
NHP2-like protein 1
Puromycin-sensitive aminopeptidase
FAD-linked sulfhydryl oxidase ALR
Heterogeneous nuclear ribonucleoprotein H2
Succinyl-CoA:3-ketoacid coenzyme A transferase 1, mitochondrial
Eukaryotic translation initiation factor 3 subunit B
IgG receptor FcRn large subunit p51
BH3-interacting domain death agonist
ATP synthase subunit f, mitochondrial
Ribosomal RNA processing protein 1 homolog A
Methionine--tRNA ligase, cytoplasmic
cAMP-regulated phosphoprotein 19
AP-1 complex subunit sigma-2
ATP synthase subunit ATP5MJ, mitochondrial
ATP synthase subunit epsilon, mitochondrial
ATP synthase subunit e, mitochondrial
Histone deacetylase 4
Eukaryotic translation initiation factor 6
C-terminal-binding protein 2
NADH dehydrogenase [ubiquinone] 1 alpha subcomplex subunit 6
Peroxisomal biogenesis factor 3
3-keto-steroid reductase/17-beta-hydroxysteroid dehydrogenase 7
Breast cancer anti-estrogen resistance protein 1
Syntaxin-17
Cilia- and flagella-associated protein 298
tRNA (guanine-N(7)-)-methyltransferase non-catalytic subunit WDR4
Transmembrane protein 33
Gem-associated protein 4
Coronin-7
Nuclear pore complex protein Nup107
Gasdermin-D
Selenocysteine-specific elongation factor
Sestrin-2
Anthrax toxin receptor 2
Myotrophin
Actin-related protein 2/3 complex subunit 4
CD81 antigen
Protein transport protein Sec61 subunit gamma
Triosephosphate isomerase
Eukaryotic translation initiation factor 3 subunit E
Protein transport protein Sec61 subunit beta
Phosphatidylinositol 3,4,5-trisphosphate 3-phosphatase and dual-specificity protein phosphatas
Serine/threonine-protein phosphatase 4 catalytic subunit
Gamma-aminobutyric acid receptor-associated protein-like 2
Reactive oxygen species modulator 1
Myosin light polypeptide 6

Actin, cytoplasmic 1
Eukaryotic initiation factor 4A-I
Small ribosomal subunit protein uS10
Ribose-phosphate pyrophosphokinase 1
Proteasome subunit alpha type-6
Protein S100-A10
Cell division control protein 42 homolog
Destrin
Glia maturation factor beta
Ras-related protein Rab-8A
Signal peptidase complex subunit 3
Signal recognition particle subunit SRP54
Ras-related protein Rab-4B
Ras-related protein Rab-2A
Ras-related protein Rab-5B
Ras-related protein Rab-10
Ubiquitin-conjugating enzyme E2 D3
NEDD8-conjugating enzyme Ubc12
Ubiquitin-conjugating enzyme E2 K
Ubiquitin-conjugating enzyme E2 N
Ras-related protein Rab-14
Actin-related protein 3
Actin-related protein 2
Alpha-centractin
COP9 signalosome complex subunit 2
ATP-binding cassette sub-family E member 1
Ras-related protein Rap-1b
Ras-related protein Rap-2b
Protein max
Small ribosomal subunit protein eS1
Large ribosomal subunit protein uL24
Proteasome activator complex subunit 3
Large ribosomal subunit protein eL15
Large ribosomal subunit protein eL27
V-type proton ATPase subunit d 1
Large ribosomal subunit protein eL43
Transforming protein RhoA
Rho-related GTP-binding protein RhoE
N-alpha-acetyltransferase 20
10 kDa heat shock protein, mitochondrial
Protein transport protein Sec61 subunit alpha isoform 1
Prefoldin subunit 3
Syntaxin-binding protein 1
Beta-2-microglobulin
Dolichyl-diphosphooligosaccharide--protein glycosyltransferase subunit DAD1
NPC intracellular cholesterol transporter 2
Coatomer subunit zeta-1
Large ribosomal subunit protein eL37
Small ubiquitin-related modifier 2
DDB1- and CUL4-associated factor 7

WD repeat-containing protein 5
AP-1 complex subunit sigma-1A
Nuclear transport factor 2
Heterogeneous nuclear ribonucleoprotein K
14-3-3 protein gamma
Ubiquitin carboxyl-terminal hydrolase 46
Ras-related protein R-Ras2
Small ribosomal subunit protein eS7
Serine/threonine-protein phosphatase PP1-alpha catalytic subunit
Serine/threonine-protein phosphatase PP1-beta catalytic subunit
26S proteasome regulatory subunit 4
26S proteasome regulatory subunit 8
Small ribosomal subunit protein eS8
Small ribosomal subunit protein uS8
Small ribosomal subunit protein uS9
Ubiquitin-conjugating enzyme E2 G1
Ubiquitin-conjugating enzyme E2 H
14-3-3 protein epsilon
Small ribosomal subunit protein uS11
Small ribosomal subunit protein uS12
Small ribosomal subunit protein uS13
Small ribosomal subunit protein uS14
Small ribosomal subunit protein uS15
Small ribosomal subunit protein uS17
Small nuclear ribonucleoprotein E
Small nuclear ribonucleoprotein F
U6 snRNA-associated Sm-like protein LSm3
U6 snRNA-associated Sm-like protein LSm6
Small nuclear ribonucleoprotein Sm D1
Small nuclear ribonucleoprotein Sm D2
Small nuclear ribonucleoprotein Sm D3
Thymosin beta-4
ADP-ribosylation factor 6
26S proteasome regulatory subunit 10B
Thioredoxin reductase-like selenoprotein T
TATA box-binding protein-like 1
Large ribosomal subunit protein eL8
DNA-directed RNA polymerase II subunit RPB7
Ras-related protein Rab-11A
Eukaryotic peptide chain release factor subunit 1
CCHC-type zinc finger nucleic acid binding protein
Protein yippee-like 5
Small ribosomal subunit protein eS4, X isoform
Serine/threonine-protein phosphatase 2A catalytic subunit beta isoform
Actin, aortic smooth muscle
Rho-related GTP-binding protein RhoB
Large ribosomal subunit protein uL23
Small ribosomal subunit protein eS6
Histone H4
Ras-related protein Rab-1A

GTP-binding nuclear protein Ran
Large ribosomal subunit protein uL14
Ras-related protein Rap-1A
Small ribosomal subunit protein uS19
Small ribosomal subunit protein eS24
Small ribosomal subunit protein eS25
Small ribosomal subunit protein eS26
Small ribosomal subunit protein eS28
Ubiquitin-like FUBI-ribosomal protein eS30 fusion protein
Guanine nucleotide-binding protein G(I)/G(S)/G(T) subunit beta-1
E3 ubiquitin-protein ligase RBX1
Guanine nucleotide-binding protein G(I)/G(S)/G(T) subunit beta-2
Large ribosomal subunit protein eL30
Large ribosomal subunit protein eL39
Large ribosomal subunit protein eL31
Large ribosomal subunit protein uL1
Large ribosomal subunit protein eL32
Large ribosomal subunit protein uL5
Large ribosomal subunit protein uL2
Peptidyl-prolyl cis-trans isomerase A
Peptidyl-prolyl cis-trans isomerase FKBP1A
Ubiquitin-ribosomal protein eS31 fusion protein
Growth factor receptor-bound protein 2
Transformer-2 protein homolog beta
Ras-related C3 botulinum toxin substrate 1
AP-2 complex subunit beta
Vesicle-associated membrane protein 2
Guanine nucleotide-binding protein G(s) subunit alpha isoforms short
Guanine nucleotide-binding protein G(i) subunit alpha-1
14-3-3 protein zeta/delta
Serine/threonine-protein phosphatase 2A 55 kDa regulatory subunit B alpha isoform
Small ubiquitin-related modifier 1
Dynein light chain 1, cytoplasmic
Dynein light chain Tctex-type 1
Large ribosomal subunit protein eL38
S-phase kinase-associated protein 1
Guanine nucleotide-binding protein G(I)/G(S)/G(O) subunit gamma-5
Small ribosomal subunit protein eS21
Eukaryotic translation initiation factor 5A-1
Small ribosomal subunit protein RACK1
Transcription elongation factor SPT4
SUMO-conjugating enzyme UBC9
Thymosin beta-10
Serine/threonine-protein phosphatase 2A catalytic subunit alpha isoform
Y-box-binding protein 1
Signal peptidase complex catalytic subunit SEC11A
Casein kinase II subunit beta
Tropomyosin alpha-4 chain
Ubiquitin-conjugating enzyme E2 L3
Elongation factor 1-alpha 1

Tubulin alpha-1B chain
Tubulin alpha-4A chain
Tubulin beta-4B chain
Casein kinase II subunit alpha
Platelet-activating factor acetylhydrolase IB subunit alpha2
Histone H3.1
Hemoglobin subunit beta
Hemoglobin subunit alpha
Coxsackievirus and adenovirus receptor
Protein FAM193A
Nucleolar protein 14
Immunoglobulin-binding protein 1
Tyrosine-protein phosphatase non-receptor type substrate 1
Phosphoserine phosphatase
RNA-binding protein 6
Eukaryotic translation initiation factor 4 gamma 2
Ribonuclease P protein subunit p38
Ribonuclease P protein subunit p30
General transcription factor II-I
Phosphatidylinositol 5-phosphate 4-kinase type-2 beta
Contactin-associated protein 1
SRSF protein kinase 2
T-complex protein 1 subunit beta
UDP-galactose translocator
mRNA export factor RAE1
Glutathione S-transferase omega-1
DNA-dependent protein kinase catalytic subunit
Disintegrin and metalloproteinase domain-containing protein 17
Biogenesis of lysosome-related organelles complex 1 subunit 1
Sushi repeat-containing protein SRPX
Arginase-2, mitochondrial
Endonuclease III-like protein 1
Interferon-induced 35 kDa protein
Nucleobindin-2
Brain acid soluble protein 1
Dermcidin
TATA element modulatory factor
Small ribosomal subunit protein mS22
Small ribosomal subunit protein mS25
Small ribosomal subunit protein uS10m
Small ribosomal subunit protein mS35
Small ribosomal subunit protein uS5m
Alpha-ketoglutarate dehydrogenase component 4
Small ribosomal subunit protein uS11m
Small ribosomal subunit protein uS15m
Small ribosomal subunit protein bS21m
Small ribosomal subunit protein mS34
Small ribosomal subunit protein bS6m
Small ribosomal subunit protein uS9m
SAP domain-containing ribonucleoprotein

Serine beta-lactamase-like protein LACTB, mitochondrial
Conserved oligomeric Golgi complex subunit 7
Large ribosomal subunit protein eL24
Thioredoxin-like protein 4A
Large ribosomal subunit protein eL42
Chromobox protein homolog 1
Mothers against decapentaplegic homolog 3
ADP-ribosylation factor 1
ADP-ribosylation factor 5
Enhancer of rudimentary homolog
Rho-related GTP-binding protein RhoG
Large ribosomal subunit protein eL19
Small EDRK-rich factor 2
Serine/arginine-rich splicing factor 3
Forkhead box protein K1
Vacuolar fusion protein CCZ1 homolog B
Disabled homolog 2
Basement membrane-specific heparan sulfate proteoglycan core protein
E3 ubiquitin-protein ligase XIAP
Ephrin-B1
RNA-binding protein 10
RNA-binding protein 3
Calcium-transporting ATPase type 2C member 1
Phospholipid-transporting ATPase IH
Cytochrome c
55 kDa erythrocyte membrane protein
Transcription factor A, mitochondrial
Phosphatidylinositol transfer protein alpha isoform
Solute carrier family 25 member 3
Vigilin
Transcription initiation factor IIB
Cyclin-dependent kinase 6
Cyclin-dependent kinase 5
Cyclin-dependent kinase 17
Transcriptional activator protein Pur-alpha
Cdc42 effector protein 1
Clathrin heavy chain 1
Heat shock factor protein 1
Nuclear factor NF-kappa-B p100 subunit
Peptidyl-prolyl cis-trans isomerase FKBP3
Receptor expression-enhancing protein 5
Sorbitol dehydrogenase
Heterogeneous nuclear ribonucleoprotein U
Splicing factor U2AF 35 kDa subunit
Spectrin beta chain, non-erythrocytic 1
Nucleolysin TIAR
Protein SET
Serine/arginine-rich splicing factor 2
Forkhead box protein K2
Transcription factor RelB

N-acetylgalactosamine kinase
AMP deaminase 2
Fatty acid-binding protein 5
Adenylyl cyclase-associated protein 1
Friend leukemia integration 1 transcription factor
Hydroxymethylglutaryl-CoA synthase, cytoplasmic
Interferon-induced transmembrane protein 3
Large neutral amino acids transporter small subunit 1
Protein Dr1
Exosome complex component 10
OTU domain-containing protein 4
ATP-dependent 6-phosphofructokinase, platelet type
DNA repair protein complementing XP-C cells
RNA-binding protein EWS
Cerebellar degeneration-related protein 2
Inositol polyphosphate 5-phosphatase OCRL
1-phosphatidylinositol 4,5-bisphosphate phosphodiesterase beta-3
Transgelin
A-kinase anchor protein 17A
Dihydroorotate dehydrogenase (quinone), mitochondrial
2-oxoglutarate dehydrogenase complex component E1
Kinesin-like protein KIF23
Methylmalonate-semialdehyde/malonate-semialdehyde dehydrogenase [acylating], mitochondr
Pro-neuregulin-1, membrane-bound isoform
Sterol 26-hydroxylase, mitochondrial
Desmoglein-1
Transcription factor Sp3
Histone H1.1
Large ribosomal subunit protein eL20
Dual specificity mitogen-activated protein kinase kinase 1
Angiopoietin-1 receptor
Peptidyl-prolyl cis-trans isomerase FKBP4
Procollagen-lysine,2-oxoglutarate 5-dioxygenase 1
Nucleobindin-1
Large ribosomal subunit protein eL6
DNA topoisomerase 2-beta
A-kinase anchor protein 12
Mitochondrial 2-oxoglutarate/malate carrier protein
Dystonin
Protein ENL
Histone-lysine N-methyltransferase MECOM
Guanine nucleotide-binding protein subunit alpha-12
Caveolin-1
Aminoacylase-1
Tumor necrosis factor alpha-induced protein 2
Lamin-B2
6-pyruvoyl tetrahydrobiopterin synthase
Urokinase plasminogen activator surface receptor
Mevalonate kinase
DNA excision repair protein ERCC-6

Antigen peptide transporter 1
Antigen peptide transporter 2
CCAAT/enhancer-binding protein zeta
Zinc finger protein 92
Transcription factor p65
UBX domain-containing protein 1
1,4-alpha-glucan-branching enzyme
Eukaryotic translation initiation factor 4 gamma 1
Neurogenic locus notch homolog protein 2
Transducin-like enhancer protein 1
Transducin-like enhancer protein 3
Lactoylglutathione lyase
Activin receptor type-1
Single-stranded DNA-binding protein, mitochondrial
Proto-oncogene c-Rel
14-3-3 protein eta
Proteolipid protein 2
Cleavage stimulation factor subunit 1
Ubiquitin-protein ligase E3A
Dynamin-1
Tyrosine-protein phosphatase non-receptor type 12
Focal adhesion kinase 1
Serine/arginine-rich splicing factor 11
Protein kinase C delta type
Caldesmon
Folylpolyglutamate synthase, mitochondrial
CTD small phosphatase-like protein 2
Tyrosine-protein phosphatase non-receptor type 11
3-ketodihydrosphingosine reductase
Amidophosphoribosyltransferase
Glutamine--fructose-6-phosphate aminotransferase [isomerizing] 1
Exosome complex component RRP45
Proteasome activator complex subunit 1
Recombining binding protein suppressor of hairless
Myocyte-specific enhancer factor 2C
Amyloid beta precursor like protein 2
GA-binding protein alpha chain
E3 ubiquitin-protein ligase RING1
Fragile X messenger ribonucleoprotein 1
Peroxiredoxin-1
Large ribosomal subunit protein eL18
Complement component 1 Q subcomponent-binding protein, mitochondrial
Cytoskeleton-associated protein 4
Tight junction protein ZO-1
mRNA decay activator protein ZFP36L1
Sperm-associated antigen 1
KH domain-containing, RNA-binding, signal transduction-associated protein 1
Apoptosis regulator BAX
Bcl-2-like protein 1
Induced myeloid leukemia cell differentiation protein Mcl-1

DNA polymerase epsilon catalytic subunit A
Kinesin light chain 1
Son of sevenless homolog 1
Serine/arginine-rich splicing factor 1
Rho GTPase-activating protein 1
TLE family member 5
Serine/arginine-rich splicing factor 4
Protocadherin-1
Protein phosphatase 3 catalytic subunit alpha
ATP-dependent RNA helicase A
Quinone oxidoreductase
Golgin subfamily A member 3
Golgin subfamily A member 2
Peroxisomal bifunctional enzyme
Lactadherin
3',5'-cyclic-AMP phosphodiesterase 4D
Desmocollin-1
Pseudouridine-5'-phosphatase
Testican-1
Leukocyte surface antigen CD47
Peptidyl-prolyl cis-trans isomerase D
FACT complex subunit SSRP1
Calmodulin-regulated spectrin-associated protein 2
Protein spire homolog 1
Schlafen family member 5
Protein VAC14 homolog
RNA cytosine C(5)-methyltransferase NSUN2
Dystrophia myotonica WD repeat-containing protein
Histone-binding protein RBBP4
Nuclear cap-binding protein subunit 1
Alpha-1,6-mannosylglycoprotein 6-beta-N-acetylglucosaminyltransferase A
Histone acetyltransferase p300
Neuroblast differentiation-associated protein AHNAK
F-BAR domain only protein 2
Elongator complex protein 6
Heat shock 70 kDa protein 14
Secernin-3
Cingulin-like protein 1
Ragulator complex protein LAMTOR4
Nexilin
Alpha-1,6-mannosyl-glycoprotein 2-beta-N-acetylglucosaminyltransferase
Polypeptide N-acetylgalactosaminyltransferase 2
Polypeptide N-acetylgalactosaminyltransferase 1
AP-1 complex subunit beta-1
Cleavage and polyadenylation specificity factor subunit 1
Bone marrow stromal antigen 2
Mitochondrial-processing peptidase subunit alpha
CMP-N-acetylneuraminate-beta-galactosamide-alpha-2,3-sialyltransferase 1
Secernin-1
WASH complex subunit 5

Nuclear pore complex protein Nup160
Sterol regulatory element-binding protein cleavage-activating protein
Forkhead box protein O1
Transducin beta-like protein 3
General transcription factor 3C polypeptide 1
Twinfilin-1
Aspartyl/asparaginyl beta-hydroxylase
Alpha-globin transcription factor CP2
A-kinase anchor protein 13
EGF-containing fibulin-like extracellular matrix protein 1
SWI/SNF-related matrix-associated actin-dependent regulator of chromatin subfamily B membe
Nucleosome-remodeling factor subunit BPTF
Cell division cycle protein 20 homolog
Follistatin-related protein 1
Syntaxin-4
G-rich sequence factor 1
Mitogen-activated protein kinase kinase kinase kinase 2
Tyrosine-protein kinase Mer
Splicing factor, suppressor of white-apricot homolog
Chromodomain-helicase-DNA-binding protein 3
Splicing factor 3A subunit 3
Dihydropyrimidine dehydrogenase [NADP(+)]
TP53-binding protein 1
Transmembrane protein 115
Interferon-related developmental regulator 2
Tripartite motif-containing protein 26
Aminoacyl tRNA synthase complex-interacting multifunctional protein 1
Interleukin enhancer-binding factor 2
Interleukin enhancer-binding factor 3
Vesicular integral-membrane protein VIP36
Heat shock protein 75 kDa, mitochondrial
TNF receptor-associated factor 2
Disks large homolog 1
Unconventional myosin-Ie
Nuclear inhibitor of protein phosphatase 1
Protein tyrosine phosphatase type IVA 2
Active breakpoint cluster region-related protein
GATOR1 complex protein NPRL3
Vesicle transport protein SEC20
BCL2/adenovirus E1B 19 kDa protein-interacting protein 2
Transcriptional repressor NF-X1
Cleavage stimulation factor subunit 3
Tetraspanin-31
Delta(3,5)-Delta(2,4)-dienoyl-CoA isomerase, mitochondrial
Rho GTPase-activating protein 5
Striatin-3
Cell division cycle protein 16 homolog
Serine/threonine-protein kinase 4
Protein flightless-1 homolog
E3 ubiquitin-protein ligase TRIM32

Bifunctional coenzyme A synthase
Large ribosomal subunit protein bL28m
Acetyl-CoA carboxylase 1
COP9 signalosome complex subunit 1
Ubiquitin carboxyl-terminal hydrolase 4
Chromatin assembly factor 1 subunit A
Chromatin assembly factor 1 subunit B
Protein Red
S-methyl-5'-thioadenosine phosphorylase
5'-AMP-activated protein kinase catalytic subunit alpha-1
Liprin-alpha-1
Calcium-binding and coiled-coil domain-containing protein 2
Translation initiation factor eIF2B subunit epsilon
TAR DNA-binding protein 43
Heterogeneous nuclear ribonucleoprotein A0
Aminoacyl tRNA synthase complex-interacting multifunctional protein 2
FAS-associated death domain protein
Peroxiredoxin-4
Dual specificity mitogen-activated protein kinase kinase 5
Mitogen-activated protein kinase 7
Serine/threonine-protein kinase PAK 2
Chromobox protein homolog 3
Serine/threonine-protein kinase 3
Syntaxin-5
26S proteasome non-ATPase regulatory subunit 2
Multimerin-1
Probable ATP-dependent RNA helicase DDX10
DnaJ homolog subfamily C member 3
Methanethiol oxidase
Nucleoside diphosphate kinase 3
Serine/arginine-rich splicing factor 9
Serine/arginine-rich splicing factor 5
Serine/arginine-rich splicing factor 6
Mitotic spindle assembly checkpoint protein MAD2A
Transcription intermediary factor 1-beta
Semaphorin-3F
Syntaxin-3
Ras GTPase-activating protein-binding protein 1
N-myc-interactor
Inactive tyrosine-protein kinase 7
Polyadenylate-binding protein 4
Serine-protein kinase ATM
Growth factor receptor-bound protein 10
Interferon-induced protein with tetratricopeptide repeats 5
Metastasis-associated protein MTA1
Eukaryotic translation initiation factor 3 subunit I
RING-type E3 ubiquitin-protein ligase PPIL2
Serine/threonine-protein phosphatase 2A 56 kDa regulatory subunit gamma isoform
C-terminal-binding protein 1
Phosducin-like protein

Phospholipase D1
Probable methyltransferase TARBP1
Ubiquitin-conjugating enzyme E2 variant 1
Large ribosomal subunit protein mL49
Cytoplasmic dynein 1 intermediate chain 2
Origin recognition complex subunit 2
Integrin-linked protein kinase
NAD(P) transhydrogenase, mitochondrial
Beta-2-syntrophin
DNA repair protein XRCC4
Peptidyl-prolyl cis-trans isomerase G
Treacle protein
Protein unc-119 homolog A
Splicing factor 3B subunit 2
Protein OS-9
Golgin subfamily A member 4
28 kDa heat- and acid-stable phosphoprotein
Disintegrin and metalloproteinase domain-containing protein 9
Disintegrin and metalloproteinase domain-containing protein 15
Transmembrane emp24 domain-containing protein 1
Peptidyl-prolyl cis-trans isomerase FKBP5
Unconventional myosin-IXb
Rho-associated protein kinase 1
Nuclear factor of activated T-cells, cytoplasmic 2
DNA topoisomerase 3-alpha
Mothers against decapentaplegic homolog 4
V-type proton ATPase 116 kDa subunit a 3
Baculoviral IAP repeat-containing protein 2
Phosphatidylinositol-binding clathrin assembly protein
Myotubularin
Sequestosome-1
Metaxin-1
Tubulin beta-3 chain
Acid ceramidase
Serine/threonine-protein kinase PRP4 homolog
Peptidyl-prolyl cis-trans isomerase NIMA-interacting 1
Serine/threonine-protein kinase ATR
Eukaryotic translation initiation factor 4E-binding protein 1
Receptor-interacting serine/threonine-protein kinase 1
Histone deacetylase 1
Calcium/calmodulin-dependent protein kinase type II subunit gamma
Calcium/calmodulin-dependent protein kinase type II subunit delta
Dynactin subunit 2
Polycystin-2
NEDD8-activating enzyme E1 regulatory subunit
Inositol-tetrakisphosphate 1-kinase
SNW domain-containing protein 1
Diacylglycerol kinase zeta
Stromal interaction molecule 1
GRB2-related adapter protein

Transformer-2 protein homolog alpha
Sorting nexin-1
KRR1 small subunit processome component homolog
Periodic tryptophan protein 1 homolog
Myotubularin-related protein 1
Myotubularin-related protein 2
Cullin-1
Cullin-2
Cullin-3
Cullin-4A
Cullin-4B
Apoptosis-stimulating of p53 protein 2
GDP-L-fucose synthase
Ras-related protein Rab-31
Ras-related protein Rab-32
Trophoblast glycoprotein
Four and a half LIM domains protein 1
Four and a half LIM domains protein 3
Ras and Rab interactor 1
Angio-associated migratory cell protein
Mannosyl-oligosaccharide glucosidase
CD166 antigen
THO complex subunit 5 homolog
Protein Shroom2
Spectrin alpha chain, non-erythrocytic 1
Nucleolar GTP-binding protein 2
Methylglutaconyl-CoA hydratase, mitochondrial
Spliceosome RNA helicase DDX39B
Bleomycin hydrolase
Exosome complex component RRP4
Bone morphogenetic protein receptor type-2
Tubulin beta-2A chain
General transcription factor IIH subunit 2
General transcription factor IIH subunit 3
Bystin
Rap guanine nucleotide exchange factor 1
Isopentenyl-diphosphate Delta-isomerase 1
Core-binding factor subunit beta
Nuclear transcription factor Y subunit gamma
Cyclin-dependent kinase 13
Cytoskeleton-associated protein 5
Cold-inducible RNA-binding protein
Calcium/calmodulin-dependent protein kinase type 1
Coactosin-like protein
Heterogeneous nuclear ribonucleoprotein D0
Lysosome membrane protein 2
Nidogen-2
Low-density lipoprotein receptor-related protein 8
Dystroglycan 1
Vascular endothelial zinc finger 1

Ribosome biogenesis protein BOP1
Ubiquitin conjugation factor E4 A
Septin-6
Kelch-like ECH-associated protein 1
Unhealthy ribosome biogenesis protein 2 homolog
Probable ATP-dependent RNA helicase DHX34
MORC family CW-type zinc finger protein 3
Scaffold attachment factor B2
Eukaryotic translation initiation factor 3 subunit A
Rho guanine nucleotide exchange factor 7
Protein EFR3 homolog A
Ubiquitin-associated protein 2-like
Protein scribble homolog
ARF GTPase-activating protein GIT2
Scavenger receptor class F member 1
Malectin
Tubulin--tyrosine ligase-like protein 12
DNA polymerase alpha subunit B
Dedicator of cytokinesis protein 1
Transcription factor Dp-1
Four and a half LIM domains protein 2
Dihydropyrimidinase-related protein 3
Large ribosomal subunit protein mL62
Zinc finger MYM-type protein 3
Dynactin subunit 1
Cytoplasmic dynein 1 heavy chain 1
Translation initiation factor eIF2B subunit alpha
Eukaryotic initiation factor 4A-II
Elongin-A
Src substrate cortactin
Endonuclease G, mitochondrial
Flotillin-2
Reticulocalbin-2
E3 ubiquitin/ISG15 ligase TRIM25
Filamin-C
Peptidyl-prolyl cis-trans isomerase FKBP8
Protein FAM50A
Protein FRG1
Guanine nucleotide-binding protein subunit alpha-13
Guanidinoacetate N-methyltransferase
UDP-glucose 4-epimerase
Transforming growth factor beta activator LRRC32
Caprin-1
Beclin-1
RNA-binding protein 39
Enhancer of filamentation 1
Helicase-like transcription factor
Squalene monooxygenase
Protein disulfide-isomerase A5
Phosphoribosyl pyrophosphate synthase-associated protein 1

ATP-dependent RNA helicase DHX8
DNA replication licensing factor MCM6
Inositol 1,4,5-trisphosphate receptor type 2
Inositol 1,4,5-trisphosphate receptor type 3
Inositol 1,4,5-trisphosphate receptor type 1
Ras GTPase-activating protein 3
Plastin-1
Interferon regulatory factor 3
EKC/KEOPS complex subunit LAGE3
Bridge-like lipid transfer protein family member 2
E3 ubiquitin-protein ligase TRIP12
Pumilio homolog 1
Mediator of DNA damage checkpoint protein 1
Clathrin interactor 1
KN motif and ankyrin repeat domain-containing protein 1
Structural maintenance of chromosomes protein 1A
Ribosomal RNA processing protein 1 homolog B
Disco-interacting protein 2 homolog A
Protein RRP5 homolog
DNA replication complex GINS protein PSF1
Ribosome biogenesis protein BMS1 homolog
Phosphatidate phosphatase LPIN1
Ubiquitin carboxyl-terminal hydrolase 10
LRP chaperone MESD
Neutral alpha-glucosidase AB
Raftlin
Major facilitator superfamily domain-containing protein 10
Serine/threonine-protein phosphatase 2A 56 kDa regulatory subunit delta isoform
Delta(14)-sterol reductase LBR
Conserved oligomeric Golgi complex subunit 2
Major vault protein
Latent-transforming growth factor beta-binding protein 1
Latent-transforming growth factor beta-binding protein 2
Chromobox protein homolog 2
Golgin subfamily B member 1
Caspase-8
N-alpha-acetyltransferase 30
Kinesin-like protein KIF22
Myocyte-specific enhancer factor 2D
Chromodomain-helicase-DNA-binding protein 4
LIM and SH3 domain protein 1
Prostaglandin reductase 1
Dr1-associated corepressor
Nuclear factor of activated T-cells, cytoplasmic 4
Zinc finger protein 638
Importin subunit beta-1
Nucleolar and coiled-body phosphoprotein 1
Nuclear mitotic apparatus protein 1
Proteasome activator complex subunit 4
Cullin-7

Sarcolemmal membrane-associated protein
GTPase-activating protein and VPS9 domain-containing protein 1
N-alpha-acetyltransferase 25, NatB auxiliary subunit
Ventricular zone-expressed PH domain-containing protein homolog 1
Condensin complex subunit 2
PCNA-associated factor
Signal peptidase complex subunit 2
ER membrane protein complex subunit 2
Pre-mRNA-splicing regulator WTAP
26S proteasome non-ATPase regulatory subunit 6
Homocysteine-responsive endoplasmic reticulum-resident ubiquitin-like domain member 1 prot
MAD2L1-binding protein
Mortality factor 4-like protein 2
BRISC complex subunit Abraxas 2
Septin-2
Squamous cell carcinoma antigen recognized by T-cells 3
Condensin complex subunit 1
Polycomb protein SUZ12
Exosome complex component RRP42
TNFAIP3-interacting protein 1
116 kDa U5 small nuclear ribonucleoprotein component
Leucine--tRNA ligase, mitochondrial
R3H domain-containing protein 1
Translocating chain-associated membrane protein 2
Sorting nexin-17
ADP-ribosylation factor-like protein 6-interacting protein 1
Rab3 GTPase-activating protein catalytic subunit
Metal cation symporter ZIP14
Lysine--tRNA ligase
Histone-lysine N-methyltransferase SETDB1
Leucine-rich repeat-containing protein 14
Ribosome biogenesis regulatory protein homolog
DNA polymerase delta subunit 3
Eukaryotic translation initiation factor 4H
Arf-GAP with coiled-coil, ANK repeat and PH domain-containing protein 2
Kinesin-like protein KIF14
Bromodomain-containing protein 3
WD repeat-containing protein 43
Peroxisomal acyl-coenzyme A oxidase 1
Mitochondrial inner membrane protein OXA1L
Early endosome antigen 1
Protein disulfide-isomerase A6
Platelet-activating factor acetylhydrolase IB subunit alpha1
[Pyruvate dehydrogenase (acetyl-transferring)] kinase isozyme 3, mitochondrial
Astrocytic phosphoprotein PEA-15
3-beta-hydroxysteroid-Delta(8),Delta(7)-isomerase
Phosphomevalonate kinase
Serine/threonine-protein kinase D1
Plectin
Pericentriolar material 1 protein

BOS complex subunit NOMO1
Serum paraoxonase/arylesterase 2
Serine/threonine-protein phosphatase 2A 56 kDa regulatory subunit alpha isoform
Inorganic pyrophosphatase
Prostaglandin E synthase 3
Serine/threonine-protein kinase 38
Non-POU domain-containing octamer-binding protein
Serine/threonine-protein phosphatase 2A activator
Receptor-type tyrosine-protein phosphatase kappa
Periodic tryptophan protein 2 homolog
Rab GTPase-binding effector protein 1
Ras-related protein Rab-35
RNA-binding protein with serine-rich domain 1
Retinoblastoma-binding protein 5
Reticulocalbin-1
RalA-binding protein 1
Lethal(2) giant larvae protein homolog 1
Leucine-rich repeat-containing protein 41
Transcription termination factor 1
Transmembrane emp24 domain-containing protein 2
Poly(rC)-binding protein 1
Poly(rC)-binding protein 2
Elongin-C
Elongin-B
GTP-binding protein Rheb
Ubiquitin-protein ligase E3C
Mitochondrial import receptor subunit TOM20 homolog
Mitochondrial fission regulator 1
Delta(24)-sterol reductase
Splicing factor 3B subunit 3
Pumilio homolog 3
Disks large-associated protein 5
Ras suppressor protein 1
Calponin-3
Ribosomal protein S6 kinase alpha-1
Scaffold attachment factor B1
Splicing factor 3A subunit 2
RNA-binding motif, single-stranded-interacting protein 2
Protein phosphatase 1 regulatory subunit 7
Protein transport protein Sec23A
Protein transport protein Sec23B
Cytohesin-1
Splicing factor 3A subunit 1
Superkiller complex protein 2
Corneodesmosin
Surfeit locus protein 1
Transcription initiation factor TFIID subunit 5
Transcription initiation factor TFIID subunit 7
Telomeric repeat-binding factor 2
Microtubule-associated protein RP/EB family member 2

Homeobox protein TGIF1
Na(+)/H(+) exchange regulatory cofactor NHE-RF2
Tumor necrosis factor receptor type 1-associated DEATH domain protein
Translocating chain-associated membrane protein 1
Translin
RISC-loading complex subunit TARBP2
Splicing factor 1
Cdc42-interacting protein 4
Thyroid receptor-interacting protein 11
Pachytene checkpoint protein 2 homolog
Mediator of RNA polymerase II transcription subunit 1
Activating signal cointegrator 1
Probable JmjC domain-containing histone demethylation protein 2C
NF-kappa-B inhibitor beta
Thyroid receptor-interacting protein 6
Tyrosine-protein phosphatase non-receptor type 14
Microtubule-associated protein RP/EB family member 1
ELAV-like protein 1
Sterol-4-alpha-carboxylate 3-dehydrogenase, decarboxylating
NGFI-A-binding protein 2
Myosin light chain kinase, smooth muscle
TGF-beta-activated kinase 1 and MAP3K7-binding protein 1
Probable E3 ubiquitin-protein ligase HERC1
Neutral amino acid transporter B(0)
Myeloid leukemia factor 2
Mitochondrial import receptor subunit TOM34
Mothers against decapentaplegic homolog 2
Mothers against decapentaplegic homolog 1
Methylsterol monooxygenase 1
Intersectin-1
Tubulin-specific chaperone E
Tubulin-specific chaperone C
Ubiquitin-conjugating enzyme E2 variant 2
Serine/threonine-protein kinase STK11
Syntaxin-binding protein 2
Vesicle-associated membrane protein 3
NEDD8
V-type proton ATPase subunit S1
Vacuolar protein sorting-associated protein 72 homolog
Ras-related protein Rab-11B
Zyxin
Electron transfer flavoprotein-ubiquinone oxidoreductase, mitochondrial
Septin-7
Proteasomal ubiquitin receptor ADRM1
Coiled-coil domain-containing protein 6
UDP-N-acetylhexosamine pyrophosphorylase
Transcription factor E2F4
Insulin-like growth factor-binding protein 7
Programmed cell death protein 2
Laminin subunit alpha-4

26S proteasome non-ATPase regulatory subunit 5
Serine/threonine-protein kinase N1
Serine/threonine-protein kinase N2
Transcription initiation factor TFIID subunit 12
Cysteine and glycine-rich protein 2
DNA damage-binding protein 1
Serine/threonine-protein phosphatase 2A 56 kDa regulatory subunit epsilon isoform
Mitogen-activated protein kinase 14
Large ribosomal subunit protein uL23m
Hsp90 co-chaperone Cdc37
Dihydropyrimidinase-related protein 2
Synaptophysin-like protein 1
Histone-binding protein RBBP7
Mitogen-activated protein kinase kinase kinase 11
Transcription initiation factor TFIID subunit 9
Calcitonin gene-related peptide type 1 receptor
Bcl-2 homologous antagonist/killer
Serine/arginine-rich splicing factor 7
Cleavage and polyadenylation specificity factor subunit 6
Survival motor neuron protein
Drebrin
Nuclear respiratory factor 1
Fascin
Gamma-interferon-inducible protein 16
2,4-dienoyl-CoA reductase [(3E)-enoyl-CoA-producing], mitochondrial
Alpha-mannosidase 2
NADH dehydrogenase [ubiquinone] 1 alpha subcomplex subunit 5
Ceramide glucosyltransferase
ATP-dependent Clp protease proteolytic subunit, mitochondrial
Thiosulfate sulfurtransferase
Ubiquitin-conjugating enzyme E2 S
Kynurenine--oxoglutarate transaminase 1
Guanylate kinase
Hydroxyacylglutathione hydrolase, mitochondrial
NADH dehydrogenase [ubiquinone] 1 alpha subcomplex subunit 9, mitochondrial
NADP-dependent malic enzyme, mitochondrial
Phosphoenolpyruvate carboxykinase [GTP], mitochondrial
Uridine phosphorylase 1
Discoidin domain-containing receptor 2
Hydroxyacyl-coenzyme A dehydrogenase, mitochondrial
Lanosterol 14-alpha demethylase
UTP--glucose-1-phosphate uridylyltransferase
6-phosphofructo-2-kinase/fructose-2,6-bisphosphatase 3
Thioredoxin reductase 1, cytoplasmic
MICOS complex subunit MIC60
Endothelial cell-specific chemotaxis regulator
Lysine-rich nucleolar protein 1
Heterogeneous nuclear ribonucleoprotein U-like protein 2
Transmembrane protein 132A
Inverted formin-2

Sister chromatid cohesion protein PDS5 homolog A
Protein CLEC16A
Kinesin-like protein KIF7
Rho GTPase-activating protein 31
Methenyltetrahydrofolate synthase domain-containing protein
AP2-associated protein kinase 1
WASH complex subunit 4
DNA excision repair protein ERCC-6-like
Pre-rRNA-processing protein TSR1 homolog
Ral GTPase-activating protein subunit alpha-2
Protein C-mannosyl-transferase DPY19L1
Isoamyl acetate-hydrolyzing esterase 1 homolog
GDP-Man:Man(3)GlcNAc(2)-PP-Dol alpha-1,2-mannosyltransferase
Transcriptional regulator QRICH1
WD40 repeat-containing protein SMU1
Cytoplasmic tRNA 2-thiolation protein 2
Protein-glucosylgalactosylhydroxylysine glucosidase
Leucine-rich repeat flightless-interacting protein 1
CDP-diacylglycerol--glycerol-3-phosphate 3-phosphatidyltransferase, mitochondrial
UPF0711 protein C18orf21
Prolyl 3-hydroxylase 1
tRNA (guanine(37)-N1)-methyltransferase
Echinoderm microtubule-associated protein-like 3
DNA-directed RNA polymerase I subunit RPA43
MAP7 domain-containing protein 1
UDP-N-acetylhexosamine pyrophosphorylase-like protein 1
Solute carrier family 25 member 35
Chromodomain-helicase-DNA-binding protein 9
Triokinase/FMN cyclase
Protein LSM12
Inactive hydroxysteroid dehydrogenase-like protein 1
Mitochondrial 10-formyltetrahydrofolate dehydrogenase
N-acetylglucosamine-1-phosphotransferase subunits alpha/beta
Girdin
Rab-like protein 6
Mitochondrial import inner membrane translocase subunit TIM50
Protein mono-ADP-ribosyltransferase PARP14
Alpha-(1,3)-fucosyltransferase 11
Cytokine-like nuclear factor N-PAC
UPF0489 protein C5orf22
C2 domain-containing protein 3
Vacuolar protein sorting-associated protein 26B
La-related protein 7
NAD kinase 2, mitochondrial
BTB/POZ domain-containing protein KCTD21
Glucoside xylosyltransferase 1
Malonate--CoA ligase ACSF3, mitochondrial
Prolyl endopeptidase-like
TBC1 domain family member 10B
Taperin

Anoctamin-6
Filamin A-interacting protein 1-like
GRIP1-associated protein 1
Zinc-regulated GTPase metalloprotein activator 1F
Protein MIX23
Membralin
Protein FAM98B
Rho GTPase-activating protein 29
Programmed cell death protein 4
Fibronectin type III domain-containing protein 3B
CREB-regulated transcription coactivator 2
Glycerol-3-phosphate acyltransferase 3
Centrosomal protein of 55 kDa
Nuclear cap-binding protein subunit 3
Quinone oxidoreductase PIG3
Novel acetylcholine receptor chaperone
Pleckstrin homology-like domain family A member 2
PDZ and LIM domain protein 3
Protein mono-ADP-ribosyltransferase PARP10
Very-long-chain 3-oxoacyl-CoA reductase
mRNA export factor GLE1
Ubiquitin carboxyl-terminal hydrolase 39
Kelch-like protein 22
Acylglycerol kinase, mitochondrial
Histone-lysine N-methyltransferase SETMAR
Endoribonuclease LACTB2
Pyrroline-5-carboxylate reductase 3
EARP and GARP complex-interacting protein 1
Borealin
Cordon-bleu protein-like 1
HCLS1-binding protein 3
Plasma membrane ascorbate-dependent reductase CYBRD1
WD repeat-containing protein 81
Beta-actin-like protein 2
Coiled-coil domain-containing protein 93
OCIA domain-containing protein 2
Puratrophin-1
Putative heat shock protein HSP 90-alpha A4
DDB1- and CUL4-associated factor 6
Transmembrane protein 41B
Sigma intracellular receptor 2
Protein YIF1B
DBIRD complex subunit ZNF326
Nucleolar MIF4G domain-containing protein 1
RILP-like protein 1
DnaJ homolog subfamily C member 21
Tumor necrosis factor alpha-induced protein 8-like protein 3
Probable E3 ubiquitin-protein ligase HERC4
GPI ethanolamine phosphate transferase 2
Serine/threonine-protein phosphatase 6 regulatory subunit 3

Probable ATP-dependent RNA helicase DDX60-like
Rab-like protein 3
Protein FAM76B
2-methoxy-6-polyprenyl-1,4-benzoquinol methylase, mitochondrial
SH3 domain-containing protein 19
NHS-like protein 2
ER membrane protein complex subunit 4
Nondiscriminating glutamyl-tRNA synthetase EARS2, mitochondrial
Uncharacterized protein C3orf38
EEF1A lysine methyltransferase 2
Transport and Golgi organization protein 1 homolog
Presequence protease, mitochondrial
WD repeat-containing protein 44
Protein PRRC2B
Tight junction-associated protein 1
RRP12-like protein
Cytochrome c oxidase assembly factor 6 homolog
Torsin-1A-interacting protein 1
Centrosomal protein of 78 kDa
Alanine--tRNA ligase, mitochondrial
Torsin-2A
PCI domain-containing protein 2
Intracellular hyaluronan-binding protein 4
Long-chain fatty acid transport protein 3
Sterile alpha motif domain-containing protein 9
Serine/threonine-protein phosphatase 4 regulatory subunit 3B
WD repeat domain phosphoinositide-interacting protein 3
WD repeat domain phosphoinositide-interacting protein 1
EGF domain-specific O-linked N-acetylglucosamine transferase
Protein Smaug homolog 2
Tubulin-specific chaperone cofactor E-like protein
Deoxynucleotidyltransferase terminal-interacting protein 2
Rab GTPase-activating protein 1-like
Tetratricopeptide repeat protein 38
Cytochrome c oxidase assembly protein COX20, mitochondrial
Exosome complex component MTR3
Transmembrane protein 201
Alpha-tubulin N-acetyltransferase 1
Synaptosomal-associated protein 47
Nucleoporin NUP188
Heterochromatin protein 1-binding protein 3
Valine--tRNA ligase, mitochondrial
Centrosomal protein of 170 kDa
Low density lipoprotein receptor adapter protein 1
Protein odr-4 homolog
Polynucleotide 5'-hydroxyl-kinase NOL9
NHS-like protein 1
Putative hydroxypyruvate isomerase
Tumor protein p63-regulated gene 1-like protein
Coiled-coil and C2 domain-containing protein 1B

Formin-binding protein 1-like
Probable arginine--tRNA ligase, mitochondrial
Acyl-coenzyme A thioesterase THEM4
Putative coiled-coil-helix-coiled-coil-helix domain-containing protein CHCHD2P9, mitochondrial
FK506-binding protein 15
Zinc finger CCCH domain-containing protein 13
Putative methyltransferase C9orf114
Armadillo-like helical domain-containing protein 3
CSC1-like protein 2
G patch domain-containing protein 4
Putative transferase CAF17, mitochondrial
E3 ubiquitin-protein ligase HECTD3
Inactive glycosyltransferase 25 family member 3
E3 ubiquitin-protein ligase UBR4
Syntaxin-binding protein 5
Rho GTPase-activating protein 21
Calmodulin-regulated spectrin-associated protein 1
Large ribosomal subunit protein uL2m
Ubiquitin-associated protein 2
Queuosine 5'-phosphate N-glycosylase/hydrolase
Keratinocyte proline-rich protein
Zinc finger protein 618
Acyl-CoA-binding domain-containing protein 5
RNA-binding protein 26
ATPase family AAA domain-containing protein 3B
Protein wntless homolog
Integrator complex subunit 11
Ceramide-1-phosphate transfer protein
DDB1- and CUL4-associated factor 8
Collagen alpha-1(XIII) chain
Terminal uridylyltransferase 4
Protein furry homolog
Ribonuclease H2 subunit B
ATP synthase mitochondrial F1 complex assembly factor 1
Roquin-1
Protein DDI1 homolog 2
Protein THEMIS2
Arginine-hydroxylase NDUFAF5, mitochondrial
5'-nucleotidase domain-containing protein 1
Brefeldin A-inhibited guanine nucleotide-exchange protein 3
Intermembrane lipid transfer protein VPS13D
Rootletin
Complex III assembly factor LYRM7
Ras-interacting protein 1
Telomere-associated protein RIF1
Vacuolar protein sorting-associated protein 53 homolog
Striatin-interacting protein 1
Serine/threonine-protein kinase MRCK alpha
Regulation of nuclear pre-mRNA domain-containing protein 2
E3 ubiquitin-protein ligase RNF220

Pre-mRNA-splicing factor 38B
E3 ubiquitin-protein ligase BRE1A
Myomegalin
Zinc finger protein 318
Protein FAM171A1
Divergent protein kinase domain 1B
Leucine-rich repeat and calponin homology domain-containing protein 2
Threonylcarbamoyladenosine tRNA methylthiotransferase
BRO1 domain-containing protein BROX
Focadhesin
Lysine-specific demethylase 9
Disabled homolog 2-interacting protein
Lysophospholipase-like protein 1
Platelet endothelial aggregation receptor 1
Proteasome adapter and scaffold protein ECM29
Terminal uridylyltransferase 7
SH2 domain-containing adapter protein E
DENN domain-containing protein 4C
N-alpha-acetyltransferase 35, NatC auxiliary subunit
Zinc finger MYM-type protein 4
FHF complex subunit HOOK interacting protein 2A
Palmitoyltransferase ZDHHC20
SPRY domain-containing protein 7
F-box/WD repeat-containing protein 9
CD276 antigen
E3 ubiquitin-protein ligase RNF213
Tensin-2
KN motif and ankyrin repeat domain-containing protein 2
WASH complex subunit 2A
Lysophospholipid acyltransferase LPCAT4
Metalloreductase STEAP3
Protein FAM91A1
TBC1 domain family member 9B
Microtubule-associated protein 1S
Serine/threonine-protein phosphatase 2A 55 kDa regulatory subunit B delta isoform
ADP-ribosylation factor-like protein 6-interacting protein 4
Autophagy-related protein 16-1
Heparan-alpha-glucosaminide N-acetyltransferase
AT-rich interactive domain-containing protein 2
U3 small nucleolar RNA-associated protein 25 homolog
Glycosyltransferase 8 domain-containing protein 1
Protein sel-1 homolog 3
Tensin-3
HAUS augmin-like complex subunit 3
Protein SPT2 homolog
Acyl-coenzyme A thioesterase MBLAC2
Very large A-kinase anchor protein
Integrator complex subunit 3
Rho GTPase-activating protein 17
CWF19-like protein 1

Protein virilizer homolog
Cytospin-A
HEAT repeat-containing protein 6
Ankyrin repeat domain-containing protein 40
Protein WWC2
Scavenger receptor class A member 3
Atlastin-3
Ribosomal protein uL30-like
Nucleoredoxin
Tetratricopeptide repeat protein 19, mitochondrial
IQ motif and SEC7 domain-containing protein 1
Anamorsin
AN1-type zinc finger protein 6
Histone-lysine N-trimethyltransferase SMYD5
OTU domain-containing protein 7B
THO complex subunit 7 homolog
Glutamine-dependent NAD(+) synthetase
Elongator complex protein 2
Ragulator complex protein LAMTOR1
Dehydrogenase/reductase SDR family member 7B
Twinfilin-2
Condensin-2 complex subunit H2
Uncharacterized protein KIAA0930
Transport and Golgi organization protein 2 homolog
Serine/threonine-protein phosphatase 4 regulatory subunit 3A
Ras-related protein Rab-12
DENN domain-containing protein 5A
Splicing regulator SDE2
Keratin, type II cytoskeletal 80
Nipped-B-like protein
Mitochondrial adenyl nucleotide antiporter SLC25A25
2',5'-phosphodiesterase 12
N-alpha-acetyltransferase 16, NatA auxiliary subunit
Putative DENN domain-containing protein 10 B
Rhomboid domain-containing protein 2
Mitochondrial adenyl nucleotide antiporter SLC25A24
Receptor expression-enhancing protein 3
All-trans-retinol 13,14-reductase
RAD50-interacting protein 1
Transmembrane protein 214
3-hydroxyisobutyryl-CoA hydrolase, mitochondrial
RWD domain-containing protein 4
Nucleolus and neural progenitor protein
Armadillo repeat-containing protein 6
TELO2-interacting protein 2
Ankyrin repeat domain-containing protein 54
Zinc transporter 6
Transmembrane anterior posterior transformation protein 1 homolog
KN motif and ankyrin repeat domain-containing protein 3
Bifunctional arginine demethylase and lysyl-hydroxylase JMJD6

Phostensin
Caveolae-associated protein 1
Zinc finger CCHC domain-containing protein 8
Putative ATP-dependent RNA helicase DHX57
Endoplasmic reticulum aminopeptidase 2
Lysophospholipid acyltransferase 5
Parafibromin
Large ribosomal subunit protein uL14m
Long-chain fatty acid transport protein 4
Coiled-coil and C2 domain-containing protein 1A
Deoxyribonuclease TATDN1
tRNA N(3)-methylcytidine methyltransferase METTL2B
Myb/SANT-like DNA-binding domain-containing protein 2
Transcription initiation factor TFIID subunit 2
UPF0598 protein C8orf82
Mediator of RNA polymerase II transcription subunit 27
Enhancer of mRNA-decapping protein 4
Protein arginine N-methyltransferase 9
Pre-mRNA-processing-splicing factor 8
F-box only protein 42
SCY1-like protein 2
Tetratricopeptide repeat protein 27
Sideroflexin-4
Protein GOLM2
Nuclear factor related to kappa-B-binding protein
Acylpyruvase FAHD1, mitochondrial
Ribosomal protein eL22-like
Serine/threonine-protein kinase N3
RNA demethylase ALKBH5
Pyridoxal-dependent decarboxylase domain-containing protein 1
MTOR-associated protein MEAK7
Integrator complex subunit 5
GTPase IMAP family member 6
Long-chain fatty acid transport protein 1
Glucose 1,6-bisphosphate synthase
RNA polymerase-associated protein CTR9 homolog
Alpha- and gamma-adaptin-binding protein p34
Transmembrane protein 88
Proline/serine-rich coiled-coil protein 1
Superkiller complex protein 3
Serine/threonine-protein kinase ULK3
Aspartate--tRNA ligase, mitochondrial
Transmembrane protein 65
INO80 complex subunit C
F-box only protein 38
Neutral cholesterol ester hydrolase 1
Fidgetin-like protein 1
E3 ubiquitin-protein ligase TRIM65
Inactive rhomboid protein 2
Mitotic deacetylase-associated SANT domain protein

BRCA1-associated ATM activator 1
GATOR2 complex protein WDR59
Zinc finger CCCH domain-containing protein 14
Coiled-coil domain-containing protein 137
2-oxoglutarate and iron-dependent oxygenase domain-containing protein 3
Thioredoxin domain-containing protein 11
La-related protein 1
ATPase family AAA domain-containing protein 2
Protein TMED8
Proton-coupled zinc antiporter SLC30A9, mitochondrial
E3 ubiquitin-protein ligase TRAF7
Rapamycin-insensitive companion of mTOR
WD repeat-containing protein 74
Type-1 angiotensin II receptor-associated protein
SWI/SNF-related matrix-associated actin-dependent regulator of chromatin subfamily D membe
Monofunctional C1-tetrahydrofolate synthase, mitochondrial
Aftiphilin
Pre-mRNA 3'-end-processing factor FIP1
CREB-regulated transcription coactivator 3
CREB-regulated transcription coactivator 1
Cytochrome P450 20A1
Protein O-glucosyltransferase 2
Transmembrane protein 205
E3 ubiquitin-protein ligase LRSAM1
Golgi-associated kinase 1B
Lysocardiolipin acyltransferase 1
BRCA1-A complex subunit Abraxas 1
Spliceosome-associated protein CWC27 homolog
Protein disulfide isomerase CRELD2
WD repeat-containing protein 82
MICOS complex subunit MIC27
Protocadherin Fat 4
Dipeptidyl peptidase 8
Activating transcription factor 7-interacting protein 1
Ran-binding protein 10
Phosphofurin acidic cluster sorting protein 1
Myosin phosphatase Rho-interacting protein
RNA-binding motif, single-stranded-interacting protein 3
Nicotinate phosphoribosyltransferase
Dynamin-binding protein
Very-long-chain (3R)-3-hydroxyacyl-CoA dehydratase 2
Beta-1,3-glucosyltransferase
GRB10-interacting GYF protein 2
CD109 antigen
tRNA (32-2'-O)-methyltransferase regulator THADA
Hydroxysteroid dehydrogenase-like protein 2
Kynurenine--oxoglutarate transaminase 3
Protein phosphatase 1 regulatory subunit 21
Thrombospondin type-1 domain-containing protein 4
Nesprin-3

Zinc finger protein 574
NF-X1-type zinc finger protein NFXL1
Neurobeachin-like protein 2
FYVE, RhoGEF and PH domain-containing protein 5
Sulfhydryl oxidase 2
Helicase SRCAP
Rho family-interacting cell polarization regulator 1
Uncharacterized protein FLJ45252
Rho guanine nucleotide exchange factor 18
TBC1 domain family member 9
E3 ubiquitin-protein ligase UBR3
Transmembrane protein with metallophosphoesterase domain
Capping protein-inhibiting regulator of actin dynamics
Coiled-coil domain-containing protein 9B
8-oxo-dGDP phosphatase NUDT18
Rho GTPase-activating protein SYDE1
PAX-interacting protein 1
Syntaxin-binding protein 4
Protein O-mannosyl-transferase TMTC3
Intermembrane lipid transfer protein VPS13C
Ubiquitin carboxyl-terminal hydrolase 34
Ras-associated and pleckstrin homology domains-containing protein 1
MOB kinase activator 2
Inhibitor of nuclear factor kappa-B kinase-interacting protein
Phosphatidylinositol 3,4,5-trisphosphate-dependent Rac exchanger 2 protein
Protein FRA10AC1
Ubiquitin-conjugating enzyme E2 R2
Mediator of RNA polymerase II transcription subunit 13-like
La-related protein 4
Mediator of RNA polymerase II transcription subunit 25
Ribosomal protein eS27-like
E3 ubiquitin-protein ligase Hakai
Integrator complex subunit 8
Butyrophilin subfamily 2 member A1
Transcription elongation factor SPT6
Staphylococcal nuclease domain-containing protein 1
Serine/threonine-protein kinase MARK2
Cytochrome c oxidase assembly protein COX15 homolog
Probable ATP-dependent RNA helicase DDX46
Protein RUFY3
tRNA methyltransferase 10 homolog C
eIF5-mimic protein 2
Vacuolar fusion protein MON1 homolog B
Isoaspartyl peptidase/L-asparaginase
BTB/POZ domain-containing protein KCTD9
ATP-dependent RNA helicase DHX30
Eukaryotic translation initiation factor 3 subunit M
7SK snRNA methylphosphate capping enzyme
Armadillo repeat-containing X-linked protein 2
Probable proline--tRNA ligase, mitochondrial

Arginine/serine-rich coiled-coil protein 2
Ras-related GTP-binding protein A
Cytoplasmic FMR1-interacting protein 1
Protein arginine methyltransferase NDUFAF7, mitochondrial
Golgi to ER traffic protein 4 homolog
COP9 signalosome complex subunit 6
Lysophosphatidylcholine acyltransferase 2
Mitochondrial enolase superfamily member 1
E3 ubiquitin-protein transferase MAEA
EPM2A-interacting protein 1
Putative pre-mRNA-splicing factor ATP-dependent RNA helicase DHX32
Serine/threonine-protein kinase TAO1
FAST kinase domain-containing protein 5, mitochondrial
MOB kinase activator 1B
Lysine-specific demethylase 3B
Charged multivesicular body protein 1b
Ribonucleoside-diphosphate reductase subunit M2 B
Heparan sulfate 2-O-sulfotransferase 1
STE20-related kinase adapter protein alpha
[F-actin]-monooxygenase MICAL3
Dymeclin
PHD finger-like domain-containing protein 5A
Peroxiredoxin-like 2C
Aprataxin
Endoplasmic reticulum metallopeptidase 1
G protein-regulated inducer of neurite outgrowth 1
TRMT1-like protein
Zinc finger CCCH-type antiviral protein 1
Large ribosomal subunit protein bL21m
Elongation factor-like GTPase 1
Probable helicase senataxin
Trafficking protein particle complex subunit 11
KAT8 regulatory NSL complex subunit 1
Nucleoporin p54
Autophagy-related protein 9A
D-glutamate cyclase, mitochondrial
Coiled-coil domain-containing protein 186
LisH domain-containing protein ARMC9
VPS35 endosomal protein-sorting factor-like
Pogo transposable element with ZNF domain
Zinc finger FYVE domain-containing protein 16
Protein MON2 homolog
C-myc promoter-binding protein
Myosin-14
FMR1-interacting protein NUFIP2
SUZ domain-containing protein 1
Mitochondrial antiviral-signaling protein
CLIP-associating protein 1
ATP-dependent RNA helicase DHX29
Nephrocystin-3

Low-density lipoprotein receptor-related protein 10
HAUS augmin-like complex subunit 6
Protein O-glucosyltransferase 3
Tetratricopeptide repeat protein 21B
HEAT repeat-containing protein 3
Kinesin-like protein KIF21A
Hepatoma-derived growth factor-related protein 2
L-xylulose reductase
BRCA1-associated protein
BRCA2-interacting transcriptional repressor EMSY
Golgin subfamily A member 7
Rho GTPase-activating protein 22
Wings apart-like protein homolog
Interferon regulatory factor 2-binding protein 2
E3 ubiquitin-protein ligase RBBP6
E3 ubiquitin-protein ligase SH3RF1
Protein TAMALIN
FERM domain-containing protein 5
tRNA-splicing endonuclease subunit Sen54
Protein prenyltransferase alpha subunit repeat-containing protein 1
Arpin
Tectonin beta-propeller repeat-containing protein 1
Rab9 effector protein with kelch motifs
Transcription termination factor 4, mitochondrial
E3 ubiquitin-protein ligase HUWE1
YTH domain-containing family protein 3
Cytoplasmic tRNA 2-thiolation protein 1
Transcription initiation factor TFIID subunit 8
Ubiquitin-conjugating enzyme E2 Q1
Large ribosomal subunit protein mL55
Transmembrane emp24 domain-containing protein 4
Large ribosomal subunit protein uL10m
COX assembly mitochondrial protein homolog
Centromere protein V
Schlafen family member 11
Multiple epidermal growth factor-like domains protein 8
Transmembrane protein 179B
N-acetylgalactosaminyltransferase 7
Stearoyl-CoA desaturase 5
Pleckstrin homology-like domain family B member 2
Adhesion G-protein coupled receptor G6
Dehydrodolichyl diphosphate synthase complex subunit DHDDS
Polypeptide N-acetylgalactosaminyltransferase 10
Trafficking protein particle complex subunit 6B
Type 1 phosphatidylinositol 4,5-bisphosphate 4-phosphatase
C-type lectin domain family 14 member A
Transcriptional regulator Kaiso
Protein PAT1 homolog 1
Dipeptidyl peptidase 9
E3 ubiquitin-protein ligase synoviolin

tRNA 2'-phosphotransferase 1
Actin-histidine N-methyltransferase
Tetratricopeptide repeat protein 7B
Acyl-coenzyme A thioesterase 1
Nucleolar protein 9
Polyadenylate-binding protein 2
N6-adenosine-methyltransferase catalytic subunit
Protein polybromo-1
Threonylcarbamoyl-AMP synthase
Pre-mRNA-processing factor 39
Protein LYRIC
E3 ubiquitin-protein ligase ZNF598
Glycerol-3-phosphate acyltransferase 4
Kinectin
Telomerase-binding protein EST1A
NLR family member X1
B-cell CLL/lymphoma 9-like protein
Pleckstrin homology-like domain family B member 1
Ubiquitin carboxyl-terminal hydrolase 48
Calcium-dependent secretion activator 2
Fermitin family homolog 3
N-alpha-acetyltransferase 40
Acetoacetyl-CoA synthetase
Leucine zipper protein 1
THO complex subunit 4
Integral membrane protein GPR180
FHF complex subunit HOOK-interacting protein 2B
Ras GTPase-activating-like protein IQGAP3
Zinc finger CCCH domain-containing protein 18
Vacuolar protein-sorting-associated protein 36
Cullin-associated NEDD8-dissociated protein 1
Reticulophagy regulator 3
Protein Hook homolog 3
SEC14 domain and spectrin repeat-containing protein 1
COMM domain-containing protein 7
Vacuolar fusion protein MON1 homolog A
Archaemetzincin-2
THO complex subunit 6 homolog
RNA N6-adenosine-methyltransferase METTL16
Poly(ADP-ribose) glycohydrolase
Liprin-beta-1
Lon protease homolog 2, peroxisomal
Zinc finger C3HC-type protein 1
Chromodomain-helicase-DNA-binding protein 1-like
F-BAR and double SH3 domains protein 1
Coiled-coil domain-containing protein 25
Proline and serine-rich protein 2
Stimulator of interferon genes protein
Active regulator of SIRT1
Cerebellar degeneration-related protein 2-like

Ral GTPase-activating protein subunit beta
Ras-specific guanine nucleotide-releasing factor RalGPS2
Histone-arginine methyltransferase CARM1
Deaminated glutathione amidase
COMM domain-containing protein 2
HEAT repeat-containing protein 5A
Condensin-2 complex subunit G2
Protein phosphatase 1 regulatory subunit 3B
F-box only protein 11
Ankyrin repeat and LEM domain-containing protein 2
RNA-binding protein MEX3D
Diacylglycerol kinase eta
ATP-dependent RNA helicase DDX42
Spermatogenesis-associated serine-rich protein 2
Serine/threonine-protein kinase VRK2
CDK2-associated and cullin domain-containing protein 1
NADH dehydrogenase [ubiquinone] 1 alpha subcomplex subunit 11
Dynein axonemal assembly factor 5
Peptidyl-tRNA hydrolase
Syntaxin-12
Transcriptional repressor p66-alpha
Copine-8
G-protein-signaling modulator 1
Ras-related protein Rab-43
C2 domain-containing protein 5
E3 ubiquitin-protein ligase MIB1
Inactive tyrosine-protein kinase PRAG1
BLOC-2 complex member HPS6
Interferon regulatory factor 2-binding protein 1
ELKS/Rab6-interacting/CAST family member 1
Ribosomal oxygenase 2
Palmitoyltransferase ZDHHC13
Cytokine receptor-like factor 3
Trafficking protein particle complex subunit 5
Armadillo repeat-containing protein 8
RELT-like protein 1
Complex I assembly factor TMEM126B, mitochondrial
5'-3' exonuclease PLD3
Protein HID1
Ankyrin repeat and MYND domain-containing protein 2
3'-5' exoribonuclease 1
Inactive serine/threonine-protein kinase VRK3
LIX1-like protein
NudC domain-containing protein 3
Protein AHNAK2
Formin-like protein 3
Methylmalonic aciduria type A protein, mitochondrial
Mitogen-activated protein kinase kinase kinase kinase 3
Neuron navigator 3
Prolyl 3-hydroxylase 3

Coiled-coil domain-containing protein 50
Malonyl-CoA-acyl carrier protein transacylase, mitochondrial
Kinase suppressor of Ras 1
Centrosomal protein of 97 kDa
ATP-dependent (S)-NAD(P)H-hydrate dehydratase
WD repeat-containing protein 75
Mitofusin-1
Choline transporter-like protein 2
Inositol 1,4,5-trisphosphate receptor-interacting protein
WD repeat and FYVE domain-containing protein 1
MAP7 domain-containing protein 3
Coiled-coil domain-containing protein 117
Protein NOXP20
DCN1-like protein 3
Protein DENND6A
MAX gene-associated protein
GRIP and coiled-coil domain-containing protein 2
Zinc finger CCCH domain-containing protein 7A
PHD finger protein 6
Protein archease
Volume-regulated anion channel subunit LRRC8A
Extracellular sulfatase Sulf-2
E3 ubiquitin-protein ligase UBR1
E3 ubiquitin-protein ligase UBR2
Rho GTPase-activating protein 12
Calcium homeostasis endoplasmic reticulum protein
Codanin-1
Ankyrin repeat and KH domain-containing protein 1
SURP and G-patch domain-containing protein 1
SURP and G-patch domain-containing protein 2
Ubiquitin-conjugating enzyme E2 variant 3
Cell division cycle and apoptosis regulator protein 1
Probable ATP-dependent RNA helicase DHX40
DnaJ homolog subfamily C member 10
Negative elongation factor C/D
Mitochondrial Rho GTPase 2
Mitochondrial Rho GTPase 1
NAD-dependent protein deacetylase sirtuin-2
Polyhomeotic-like protein 2
Chromatin complexes subunit BAP18
Large ribosomal subunit protein mL41
Nurim
GPALPP motifs-containing protein 1
Protein mono-ADP-ribosyltransferase PARP9
RNA-binding protein 12B
Solute carrier family 35 member F2
Putative RNA polymerase II subunit B1 CTD phosphatase RPAP2
Patatin-like phospholipase domain-containing protein 6
Structural maintenance of chromosomes protein 5
Probable ATP-dependent RNA helicase DDX60

C-Maf-inducing protein
MICAL-like protein 2
Probable ATP-dependent RNA helicase DHX37
Kelch repeat and BTB domain-containing protein 2
Ribonucleoprotein PTB-binding 1
pre-rRNA 2'-O-ribose RNA methyltransferase FTSJ3
Transmembrane protein 192
Cytoskeleton-associated protein 2-like
Nucleotidyltransferase MB21D2
Serine/arginine repetitive matrix protein 1
Stromal membrane-associated protein 1
DIS3-like exonuclease 2
ATP-dependent RNA helicase SUPV3L1, mitochondrial
Eukaryotic peptide chain release factor GTP-binding subunit ERF3B
ZZ-type zinc finger-containing protein 3
Exocyst complex component 8
Uncharacterized protein C10orf67, mitochondrial
E3 ubiquitin-protein ligase TRIM22
Threonine synthase-like 1
Uncharacterized protein KIAA2013
Calcium uptake protein 2, mitochondrial
Ankyrin repeat domain-containing protein 13A
Phosphatase and actin regulator 4
tRNA (uracil-5-)-methyltransferase homolog A
Pseudouridylate synthase RPUSD2
ELMO domain-containing protein 2
Aldehyde dehydrogenase family 16 member A1
Dyslexia-associated protein KIAA0319-like protein
Adhesion G protein-coupled receptor F5
5'-3' exoribonuclease 1
Proline-, glutamic acid- and leucine-rich protein 1
Abl interactor 1
WD repeat and FYVE domain-containing protein 3
Selenoprotein H
Vitamin K epoxide reductase complex subunit 1-like protein 1
Spartin
Tetratricopeptide repeat protein 5
Mesoderm induction early response protein 1
Regulatory-associated protein of mTOR
ORM1-like protein 3
Cell cycle and apoptosis regulator protein 2
NADH dehydrogenase [ubiquinone] 1 alpha subcomplex assembly factor 2
Vacuolar protein sorting-associated protein 52 homolog
Nuclear pore complex protein Nup93
Serine/threonine-protein kinase 11-interacting protein
Zinc finger protein 687
Cap-specific mRNA (nucleoside-2'-O-)-methyltransferase 1
Leucine-rich repeat-containing protein 47
Dedicator of cytokinesis protein 4
Carbonic anhydrase 13

Rho guanine nucleotide exchange factor 28
Integrator complex subunit 1
Histone H2B type 3-B
Rho GTPase-activating protein 24
Armadillo repeat-containing protein 10
Lysophosphatidylserine lipase ABHD12
CLK4-associating serine/arginine rich protein
Protein FAM43A
Transmembrane protein 256
E3 SUMO-protein ligase PIAS4
Glycerol-3-phosphate dehydrogenase 1-like protein
Solute carrier family 35 member F6
Rho GTPase-activating protein 18
Activating signal cointegrator 1 complex subunit 3
MICAL-like protein 1
Vacuolar protein sorting-associated protein 8 homolog
Protein PALS1
Cohesin subunit SA-2
Synaptopodin
Formin-binding protein 4
Translation factor GUF1, mitochondrial
RING1 and YY1-binding protein
Polypeptide N-acetylgalactosaminyltransferase 4
Misshapen-like kinase 1
Guanosine-3',5'-bis(diphosphate) 3'-pyrophosphohydrolase MESH1
Prostaglandin reductase 3
3-oxoacyl-[acyl-carrier-protein] reductase
ER membrane protein complex subunit 5
Transmembrane protein 199
F-box/LRR-repeat protein 6
Prolyl 3-hydroxylase OGFOD1
Actin filament-associated protein 1
Zinc finger CCHC domain-containing protein 9
Oxidation resistance protein 1
Zinc finger CCCH-type with G patch domain-containing protein
Calcium homeostasis modulator protein 5
S1 RNA-binding domain-containing protein 1
WD and tetratricopeptide repeats protein 1
NF-kappa-B-activating protein
Macoilin
SH2 domain-containing protein 3C
Arrestin domain-containing protein 1
Dehydrogenase/reductase SDR family member on chromosome X
CDGSH iron-sulfur domain-containing protein 2
Ribonuclease P protein subunit p25-like protein
ATP synthase mitochondrial F1 complex assembly factor 2
Tetratricopeptide repeat protein 9C
Protein jagunal homolog 1
Refilin-B
Leucine-zipper-like transcriptional regulator 1

COMM domain-containing protein 1
Cleavage and polyadenylation specificity factor subunit 7
Solute carrier family 15 member 4
Myc target protein 1
ADAMTS-like protein 1
ADP-ribosylation factor GTPase-activating protein 2
Deubiquitinase OTUD6B
Acyl-coenzyme A diphosphatase FITM2
eEF1A lysine and N-terminal methyltransferase
ADP-ribosylation factor-like protein 6-interacting protein 6
ADP-ribosylation factor GTPase-activating protein 1
NAD-dependent protein deacylase sirtuin-6
Solute carrier family 66 member 3
ER membrane protein complex subunit 1
RNA polymerase II-associated factor 1 homolog
Cyclin-Y-like protein 1
Putative E3 ubiquitin-protein ligase UBR7
ATP-dependent RNA helicase DDX51
Uncharacterized protein FAM241A
Mitochondrial basic amino acids transporter
Protein enabled homolog
G patch domain-containing protein 11
Large ribosomal subunit protein mL43
RNA ligase 1
Lysophospholipase D GDPD1
Uncharacterized protein C19orf47
Activating signal cointegrator 1 complex subunit 1
Leucine-rich repeat-containing protein 57
Probable RNA-binding protein EIF1AD
Protein KRI1 homolog
Coiled-coil domain-containing protein 71L
Zinc finger protein 579
Pre-mRNA-splicing factor 38A
Mixed lineage kinase domain-like protein
Glutamine amidotransferase-like class 1 domain-containing protein 1
Serine/threonine-protein phosphatase 6 regulatory ankyrin repeat subunit C
Phospholipid-transporting ATPase IG
ATPase family gene 2 protein homolog A
NHL repeat-containing protein 2
Late secretory pathway protein AVL9 homolog
Equilibrative nucleobase transporter 1
Xyloside xylosyltransferase 1
Golgi membrane protein 1
Procollagen galactosyltransferase 1
Inactive C-alpha-formylglycine-generating enzyme 2
SID1 transmembrane family member 2
Formylglycine-generating enzyme
Protein O-glucosyltransferase 1
Ubiquitin-associated domain-containing protein 2
Prenylcysteine oxidase-like

Transmembrane protein 87A
Retinol dehydrogenase 13
Tetratricopeptide repeat protein 13
Estradiol 17-beta-dehydrogenase 11
Thioredoxin domain-containing protein 5
Kinetochore protein Spc24
Outer mitochondrial transmembrane helix translocase
Saccharopine dehydrogenase-like oxidoreductase
UDP-glucuronic acid decarboxylase 1
E3 ubiquitin-protein ligase RNF149
Reticulophagy regulator 2
SERPINE1 mRNA-binding protein 1
LEM domain-containing protein 2
Nitric oxide-associated protein 1
Adaptin ear-binding coat-associated protein 1
Protein FAM98A
Phospholipase A2 group XV
Myotubularin-related protein 14
NFATC2-interacting protein
Diacylglycerol lipase-beta
Carbohydrate sulfotransferase 14
Polypeptide N-acetylgalactosaminyltransferase 6
E3 ubiquitin-protein ligase RNF169
Pyruvate dehydrogenase phosphatase regulatory subunit, mitochondrial
NAD(P)H-hydrate epimerase
Nonsense-mediated mRNA decay factor SMG8
RING finger protein 214
Protein LSM14 homolog A
GTPase IMAP family member 8
Cyclin-Y
Maestro heat-like repeat-containing protein family member 1
Terminal nucleotidyltransferase 4B
Probable aminopeptidase NPEPL1
EH domain-binding protein 1
Putative RNA-binding protein 15B
Alpha-N-acetylgalactosaminide alpha-2,6-sialyltransferase 3
Trinucleotide repeat-containing gene 6A protein
Polyhomeotic-like protein 3
Divergent protein kinase domain 2A
Metal transporter CNNM3
ATP-binding cassette sub-family F member 1
Calcium uniporter protein, mitochondrial
Phosphatidylinositol 3-kinase catalytic subunit type 3
Glutathione S-transferase C-terminal domain-containing protein
Serum response factor-binding protein 1
Neuroguidin
SHC SH2 domain-binding protein 1
PDZ domain-containing protein 8
DCC-interacting protein 13-beta
Zinc transporter 7

Neuron navigator 1
Periphilin-1
WD repeat-containing protein 19
F-box only protein 22
Lysophosphatidylcholine acyltransferase 1
Zinc finger MIZ domain-containing protein 2
Nesprin-1
Ubiquitin carboxyl-terminal hydrolase 32
Biorientation of chromosomes in cell division protein 1-like 1
AT-rich interactive domain-containing protein 1B
FAD synthase
Folliculin
Nucleoporin Nup43
Nucleoporin Nup37
Nucleoporin NUP35
Cytosolic endo-beta-N-acetylglucosaminidase
Torsin-1A-interacting protein 2
sn-1-specific diacylglycerol lipase ABHD11
N-acylneuraminate cytidylyltransferase
F-box DNA helicase 1
Tetraspanin-14
Tubulin--tyrosine ligase
Small VCP/p97-interacting protein
Atlastin-2
Motile sperm domain-containing protein 2
Putative phospholipase B-like 2
Ras association domain-containing protein 8
ATP-dependent RNA helicase DDX55
Major facilitator superfamily domain-containing protein 8
Tudor domain-containing protein 7
GTPase IMAP family member 7
Protein NEDD1
Nuclear receptor coactivator 7
THO complex subunit 2
WD repeat-containing protein 36
Protein phosphatase PTC7 homolog
Retinitis pigmentosa 9 protein
Vang-like protein 1
Smad nuclear-interacting protein 1
Large ribosomal subunit protein mL64
WD repeat-containing protein 48
Exocyst complex component 6
SWI/SNF complex subunit SMARCC2
Nuclear protein localization protein 4 homolog
Uncharacterized protein CXorf38
Spermatogenesis-associated protein 20
(Lyso)-N-acylphosphatidylethanolamine lipase
F-box only protein 30
Adenosine 3'-phospho 5'-phosphosulfate transporter 1
Pumilio homolog 2

T-cell immunomodulatory protein
Golgin subfamily A member 5
Kelch domain-containing protein 4
SH3KBP1-binding protein 1
NEDD8-activating enzyme E1 catalytic subunit
Prostamide/prostaglandin F synthase
DnaJ homolog subfamily B member 14
Protein kish-A
Phosphatidylinositol 5-phosphate 4-kinase type-2 gamma
WD repeat-containing protein 20
TBC1 domain family member 15
Retinol dehydrogenase 11
Leucine-rich repeat-containing protein 20
Large ribosomal subunit protein uL30m
AN1-type zinc finger protein 1
Protein CIP2A
Phosphatidylinositol 4-kinase type 2-beta
Dolichyl-diphosphooligosaccharide--protein glycosyltransferase subunit STT3B
Polyribonucleotide nucleotidyltransferase 1, mitochondrial
Signal peptide peptidase-like 2B
Minor histocompatibility antigen H13
Phosphatidylinositol 3,4,5-trisphosphate-dependent Rac exchanger 1 protein
Up-regulator of cell proliferation
Protein bicaudal D homolog 2
Serine/threonine-protein kinase Nek9
Pleckstrin homology domain-containing family O member 2
E3 ubiquitin-protein ligase DTX3L
ATP-dependent RNA helicase DDX54
Disks large homolog 5
Ribosome biogenesis protein BRX1 homolog
Glucosamine-6-phosphate isomerase 2
Volume-regulated anion channel subunit LRRC8C
Serine/threonine-protein kinase Nek7
RB1-inducible coiled-coil protein 1
[F-actin]-monooxygenase MICAL1
Elongator complex protein 5
Protein phosphatase Slingshot homolog 3
SH3 domain and tetratricopeptide repeat-containing protein 1
TBC domain-containing protein kinase-like protein
D-aminoacyl-tRNA deacylase 1
DDB1- and CUL4-associated factor 11
U3 small nucleolar RNA-associated protein 15 homolog
Probable glutathione peroxidase 8
Actin filament-associated protein 1-like 1
E3 ubiquitin-protein ligase SH3RF3
Short transient receptor potential channel 4-associated protein
Centrosomal protein of 192 kDa
Gem-associated protein 5
GPI ethanolamine phosphate transferase 3
Rap guanine nucleotide exchange factor 6

Guanine nucleotide exchange protein SMCR8
Partitioning defective 3 homolog
Importin-4
Ubiquitin carboxyl-terminal hydrolase 33
Arginine/serine-rich protein PNISR
Serine/threonine-protein phosphatase 4 regulatory subunit 1
Ubiquitin-associated and SH3 domain-containing protein B
PDZ domain-containing protein GIPC2
WAS/WASL-interacting protein family member 2
1-acylglycerol-3-phosphate O-acyltransferase ABHD5
Histone-lysine N-methyltransferase SETD7
Nucleolar complex protein 3 homolog
Scavenger receptor class B member 1
Conserved oligomeric Golgi complex subunit 1
Sec1 family domain-containing protein 2
Stromal membrane-associated protein 2
Zinc finger CCCH domain-containing protein 15
Peptidyl-prolyl cis-trans isomerase-like 4
General transcription factor 3C polypeptide 2
TBC1 domain family member 22A
PHD finger protein 10
Ras-related protein Rab-2B
Coiled-coil domain-containing protein 12
RelA-associated inhibitor
Cotranscriptional regulator ARB2A
Protein Churchill
Transforming growth factor-beta receptor-associated protein 1
Transmembrane protein 263
Histone deacetylase 7
Serine/threonine/tyrosine-interacting protein
Phosphatidylglycerophosphatase and protein-tyrosine phosphatase 1
Nuclear pore complex protein Nup133
Programmed cell death 6-interacting protein
Sodium-dependent phosphate transporter 1
Filamin-binding LIM protein 1
Splicing factor Cactin
Glutathione-specific gamma-glutamylcyclotransferase 2
Charged multivesicular body protein 7
Protein THEM6
Probable N-acetyltransferase 14
B-cell CLL/lymphoma 7 protein family member C
Non-structural maintenance of chromosomes element 1 homolog
MIT domain-containing protein 1
Chromosome transmission fidelity protein 18 homolog
RNA polymerase-associated protein LEO1
Dephospho-CoA kinase domain-containing protein
NudC domain-containing protein 2
U4/U6.U5 small nuclear ribonucleoprotein 27 kDa protein
Dimethyladenosine transferase 1, mitochondrial
Cohesin subunit SA-1

Sec1 family domain-containing protein 1
Tumor necrosis factor alpha-induced protein 8-like protein 1
Soluble calcium-activated nucleotidase 1
Trafficking protein particle complex subunit 12
Heterogeneous nuclear ribonucleoprotein L-like
Fatty acyl-CoA reductase 1
Ubiquitin-like domain-containing CTD phosphatase 1
tRNA-splicing endonuclease subunit Sen15
PEST proteolytic signal-containing nuclear protein
DnaJ homolog subfamily A member 4
SPRY domain-containing protein 4
m-AAA protease-interacting protein 1, mitochondrial
Pseudouridylate synthase TRUB1
LIM domain only protein 7
Choline transporter-like protein 1
Cytoskeleton-associated protein 2
Ataxin-2-like protein
Arf-GAP with Rho-GAP domain, ANK repeat and PH domain-containing protein 3
GTPase IMAP family member 1
PH-interacting protein
U4/U6 small nuclear ribonucleoprotein Prp31
Negative elongation factor B
Palladin
RUN and FYVE domain-containing protein 2
Splicing regulatory glutamine/lysine-rich protein 1
Gem-associated protein 6
Caskin-2
Paraspeckle component 1
MAP kinase-activating death domain protein
Nesprin-2
Transcriptional repressor p66-beta
DnaJ homolog subfamily C member 9
BRI3-binding protein
Beta-catenin-like protein 1
Cysteine protease ATG4A
Ras and Rab interactor 2
Protein ELYS
Microprocessor complex subunit DGCR8
Roundabout homolog 4
Esterase OVCA2
Immunity-related GTPase family Q protein
DNA damage-binding protein 2
C-type lectin domain family 2 member B
ATP-dependent RNA helicase DDX1
SEC14-like protein 1
Piezo-type mechanosensitive ion channel component 1
Protein FAM3C
Histone H1.10
Proteasome inhibitor PI31 subunit
Golgi-specific brefeldin A-resistance guanine nucleotide exchange factor 1

RNA polymerase-associated protein RTF1 homolog
Nicastrin
Sorting nexin-19
Transmembrane 9 superfamily member 4
Transmembrane protein 131
Small ribosomal subunit protein mS27
Engulfment and cell motility protein 1
Polyphosphoinositide phosphatase
Protein FAM168A
AP-3 complex subunit sigma-1
Hamartin
UBX domain-containing protein 4
PHD finger protein 3
Protein NDRG1
Heat shock protein 105 kDa
Septin-8
CCR4-NOT transcription complex subunit 9
Acyl-CoA:lysophosphatidylglycerol acyltransferase 1
TBC1 domain family member 5
Zinc finger protein 592
Protein Jade-3
Unconventional myosin-XVIIIa
La-related protein 4B
Stalled ribosome sensor GCN1
Rho GTPase-activating protein 45
Pre-mRNA-splicing factor ATP-dependent RNA helicase PRP16
Nuclear pore complex protein Nup205
Ankyrin repeat and SAM domain-containing protein 1A
Peroxidasin homolog
Protein FAN
GPI-anchor transamidase
Small ribosomal subunit protein mS31
A-kinase anchor protein 1, mitochondrial
Dol-P-Man:Man(5)GlcNAc(2)-PP-Dol alpha-1,3-mannosyltransferase
Neurogranin
Acidic leucine-rich nuclear phosphoprotein 32 family member B
Nectin-2
Geranylgeranyl transferase type-2 subunit alpha
Protein TFG
Serine protease HTRA1
Actin-related protein 2/3 complex subunit 1A
General transcription factor IIH subunit 4
Ras-responsive element-binding protein 1
Histone deacetylase 2
Signal transducing adapter molecule 1
Zinc finger protein ubi-d4
Endoplasmic reticulum protein SC65
CREB-binding protein
Symplekin
TATA-binding protein-associated factor 2N

Golgin subfamily A member 1
Gamma-glutamyl hydrolase
Neuronal cell adhesion molecule
X-linked retinitis pigmentosa GTPase regulator
Phosphatidylinositol 3,4,5-trisphosphate 5-phosphatase 1
Probable ATP-dependent RNA helicase DDX17
Kinesin-associated protein 3
Caspase-10
Amyloid beta precursor protein binding family B member 2
DNA repair protein RAD50
CUGBP Elav-like family member 1
Osteoclast-stimulating factor 1
Rho guanine nucleotide exchange factor 1
DNA repair endonuclease XPF
Ubiquitin recognition factor in ER-associated degradation protein 1
Golgi apparatus protein 1
Regulator of nonsense transcripts 1
COP9 signalosome complex subunit 5
G-patch domain and KOW motifs-containing protein
SWI/SNF complex subunit SMARCC1
SWI/SNF-related matrix-associated actin-dependent regulator of chromatin subfamily D membe
Ras-related protein Rab-8B
Bcl2-associated agonist of cell death
Far upstream element-binding protein 2
Glutaryl-CoA dehydrogenase, mitochondrial
GTP-binding protein Rit1
Peroxisomal membrane protein PEX13
Transportin-1
Rho guanine nucleotide exchange factor 2
Ribosomal RNA small subunit methyltransferase NEP1
Polyribonucleotide 5'-hydroxyl-kinase Clp1
Glomulin
Ubiquitin carboxyl-terminal hydrolase 13
Segment polarity protein dishevelled homolog DVL-3
Probable ubiquitin carboxyl-terminal hydrolase FAF-X
Ubiquitin carboxyl-terminal hydrolase 7
Cullin-5
Stathmin-2
V-type proton ATPase 116 kDa subunit a 1
Lipoma-preferred partner
RNA-binding protein with multiple splicing
Exostosin-2
Mediator of RNA polymerase II transcription subunit 12
Protein tyrosine phosphatase type IVA 1
Phosphorylase b kinase regulatory subunit beta
Secretory carrier-associated membrane protein 4
BLOC-2 complex member HPS3
SWI/SNF-related matrix-associated actin-dependent regulator of chromatin subfamily E membe
Caveolae-associated protein 3
Riboflavin kinase

Myeloid-derived growth factor
Mitochondrial import receptor subunit TOM40B
Protein YIPF5
GPI transamidase component PIG-T
Immunoglobulin superfamily member 8
Ribosomal protein eL42-like
Ras-related protein Rab-24
Oxysterol-binding protein 2
Cytoplasmic 60S subunit biogenesis factor ZNF622
Ribosome-releasing factor 2, mitochondrial
Ubiquitin-conjugating enzyme E2 E3
7-methylguanosine phosphate-specific 5'-nucleotidase
WW domain-binding protein 2
Proteasome assembly chaperone 2
BOS complex subunit NCLN
Mitochondrial ubiquitin ligase activator of NFKB 1
Myocardin-related transcription factor A
Protein lifeguard 3
Endoplasmic reticulum-Golgi intermediate compartment protein 1
U3 small nucleolar RNA-associated protein 4 homolog
FAST kinase domain-containing protein 4
Mitochondrial amidoxime reducing component 2
Protein FAM162A
PAT complex subunit CCDC47
Large ribosomal subunit protein uL24m
Exocyst complex component 4
Protein mago nashi homolog 2
Putative monooxygenase p33MONOX
Protein RFT1 homolog
Isochorismatase domain-containing protein 2
Protein N-terminal asparagine amidohydrolase
Fermitin family homolog 2
Far upstream element-binding protein 1
Tetratricopeptide repeat protein 17
Leucine-rich repeat-containing protein 59
Vesicle transport through interaction with t-SNAREs homolog 1A
Endothelial cell-selective adhesion molecule
Pre-B-cell leukemia transcription factor-interacting protein 1
Mitochondrial calcium uniporter regulator 1
Uncharacterized protein KIAA1143
Vacuolar protein sorting-associated protein 33A
Peptidyl-prolyl cis-trans isomerase FKBP10
Tetratricopeptide repeat protein 28
Exosome complex component RRP43
Zinc finger protein 428
SH3 domain-containing kinase-binding protein 1
Spindle and kinetochore-associated protein 1
Nucleus accumbens-associated protein 2
E3 ubiquitin-protein ligase RNF25
Axin interactor, dorsalization-associated protein

PIN2/TERF1-interacting telomerase inhibitor 1
ADP-ribosylation factor-like protein 8A
Ubiquitin thioesterase otulin
Small ribosomal subunit protein mS37
Peptidylprolyl isomerase domain and WD repeat-containing protein 1
Coiled-coil domain-containing protein 127
Cytochrome c oxidase assembly factor 7
Uracil phosphoribosyltransferase homolog
Phosphatidate cytidylyltransferase, mitochondrial
MOB kinase activator 3A
Dedicator of cytokinesis protein 10
Autophagy-related protein 2 homolog B
Leukocyte receptor cluster member 1
TBC1 domain family member 20
EF-hand domain-containing protein D2
Galactose mutarotase
Synaptotagmin-like protein 4
Pyrroline-5-carboxylate reductase 2
Protein CUSTOS
m7GpppX diphosphatase
Protein phosphatase 1 regulatory subunit 14B
Integrator complex subunit 12
5-methylcytosine rRNA methyltransferase NSUN4
Inactive rhomboid protein 1
Collagen triple helix repeat-containing protein 1
Medium-chain acyl-CoA ligase ACSF2, mitochondrial
Isochorismatase domain-containing protein 1
GRIP and coiled-coil domain-containing protein 1
FLYWCH family member 2
Protein Aster-A
HAUS augmin-like complex subunit 1
FAS-associated factor 2
Coiled-coil domain-containing protein 124
FAD-dependent oxidoreductase domain-containing protein 1
Optineurin
AP-2 complex subunit mu
Gamma-tubulin complex component 3
Probable RNA polymerase II nuclear localization protein SLC7A6OS
BTB/POZ domain-containing protein KCTD12
Reticulocalbin-3
60S ribosomal export protein NMD3
RalBP1-associated Eps domain-containing protein 1
Mitochondrial import inner membrane translocase subunit TIM14
Regulator of microtubule dynamics protein 1
U8 snoRNA-decapping enzyme
Splicing factor ESS-2 homolog
Carboxymethylenebutenolidase homolog
RNA-binding protein Musashi homolog 2
U5 small nuclear ribonucleoprotein 40 kDa protein
Regulator of MON1-CCZ1 complex

Large ribosomal subunit protein mL38
RING finger and SPRY domain-containing protein 1
Endoplasmic reticulum lectin 1
Ribosome-recycling factor, mitochondrial
Transcription termination factor 3, mitochondrial
Protein Spindly
Elongator complex protein 4
NAD-dependent protein deacetylase sirtuin-1
Protein YIPF6
Nucleoporin SEH1
Transcription elongation factor A protein-like 4
KIF-binding protein
Glucosamine 6-phosphate N-acetyltransferase
Constitutive coactivator of peroxisome proliferator-activated receptor gamma
Protein KTI12 homolog
Small ribosomal subunit protein uS3m
Large ribosomal subunit protein mL53
Trans-3-hydroxy-L-proline dehydratase
E3 ubiquitin-protein ligase RNF31
DAZ-associated protein 1
Small glutamine-rich tetratricopeptide repeat-containing protein beta
Protein SAAL1
Mitochondrial potassium channel
SAGA-associated factor 29
Ribosomal RNA processing protein 36 homolog
C1GALT1-specific chaperone 1
RNA-binding protein 33
Dysbindin
DnaJ homolog subfamily A member 3, mitochondrial
Translation machinery-associated protein 16
Multivesicular body subunit 12A
Small ribosomal subunit protein mS39
Corrinoid adenosyltransferase MMAB
Cytoplasmic FMR1-interacting protein 2
GTPase IMAP family member 5
E3 ubiquitin-protein ligase TRIM11
Coiled-coil domain-containing protein 97
CB1 cannabinoid receptor-interacting protein 1
Enhancer of mRNA-decapping protein 3
Dynein light chain 2, cytoplasmic
Copine-2
Protein S100-A16
Signal-induced proliferation-associated protein 1
Secernin-2
THO complex subunit 1
Ubiquitin thioesterase OTUB1
tRNA (adenine(58)-N(1))-methyltransferase catalytic subunit TRMT61A
Abasic site processing protein HMCES
Charged multivesicular body protein 6
Protein bicaudal D homolog 1

Phosphopentomutase
U3 small nucleolar ribonucleoprotein protein IMP4
Ceramide synthase 2
tRNA-dihydrouridine(47) synthase [NAD(P)(+)]-like
Protein LTV1 homolog
Serine dehydratase-like
Large ribosomal subunit protein mL48
Chronophin
Aurora kinase B
DCN1-like protein 1
Fumarylacetoacetate hydrolase domain-containing protein 2A
SWI/SNF-related matrix-associated actin-dependent regulator of chromatin subfamily D membe
Target of EGR1 protein 1
Cell division cycle-associated 7-like protein
RUS family member 1
Probable ATP-dependent RNA helicase DDX27
BLOC-1-related complex subunit 6
Methionine--tRNA ligase, mitochondrial
Ataxin-7-like protein 3B
Serine/threonine-protein kinase greatwall
Methylthioribulose-1-phosphate dehydratase
Zinc finger C2HC domain-containing protein 1A
Protein lin-37 homolog
Solute carrier family 41 member 3
Vacuolar-sorting protein SNF8
Zinc finger CCCH-type antiviral protein 1-like
Nuclear envelope pore membrane protein POM 121
Tonsoku-like protein
PDZ and LIM domain protein 5
Protein disulfide isomerase CRELD1
ERO1-like protein alpha
Proline-rich protein 11
Protein FMC1 homolog
Adenosylhomocysteinase 3
Dedicator of cytokinesis protein 6
Mediator of RNA polymerase II transcription subunit 30
Serine/threonine-protein phosphatase PGAM5, mitochondrial
Integrator complex subunit 4
DDRGK domain-containing protein 1
2-oxoadipate dehydrogenase complex component E1
Selenocysteine lyase
Far upstream element-binding protein 3
Splicing factor 45
RCC1-like G exchanging factor-like protein
Asparaginyl-tRNA synthetase
Succinate--CoA ligase [GDP-forming] subunit beta, mitochondrial
DISP complex protein LRCH3
Mannose-1-phosphate guanyltransferase alpha
4-hydroxyphenylpyruvate dioxygenase-like protein
Putative protein-lysine deacylase ABHD14B

Peptide-N(4)-(N-acetyl-beta-glucosaminyl)asparagine amidase
Vesicle-trafficking protein SEC22a
ATP synthase membrane subunit K, mitochondrial
Kinetochore-associated protein NSL1 homolog
PRKC apoptosis WT1 regulator protein
tRNA N(3)-methylcytidine methyltransferase METTL2A
THO complex subunit 3
E3 ubiquitin-protein ligase Itchy homolog
Thioredoxin domain-containing protein 15
Kin of IRRE-like protein 1
Conserved oligomeric Golgi complex subunit 3
CDK5 regulatory subunit-associated protein 3
Vam6/Vps39-like protein
Syndetin
Deubiquitinating protein VCPIP1
Spatacsin
Engulfment and cell motility protein 2
Protein disulfide-isomerase TMX3
DDB1- and CUL4-associated factor 5
Chromosome alignment-maintaining phosphoprotein 1
Lethal(3)malignant brain tumor-like protein 3
E3 ubiquitin-protein ligase ZFP91
Protocadherin-16
Calmin
PDZ and LIM domain protein 2
Fatty acyl-CoA reductase 2
Transcription factor BTF3 homolog 4
E3 ubiquitin-protein ligase RNF170
Abscission/NoCut checkpoint regulator
Solute carrier family 35 member E1
Ubiquitin carboxyl-terminal hydrolase 47
Zinc finger protein 521
Lipid scramblase CLPTM1L
Lymphokine-activated killer T-cell-originated protein kinase
ADP-ribosylation factor-like protein 5B
DnaJ homolog subfamily C member 1
N-terminal kinase-like protein
Zinc finger protein 512B
Protein LRATD2
Exocyst complex component 2
Cytosolic non-specific dipeptidase
Histone-lysine N-methyltransferase EHMT2
Zinc finger RNA-binding protein
Protein FAM210B, mitochondrial
Beta-1,3-galactosyltransferase 6
E1A-binding protein p400
Sorting nexin-27
Kinesin-like protein KIF16B
Intraflagellar transport protein 74 homolog
E3 ubiquitin-protein ligase TRIM47

CCR4-NOT transcription complex subunit 6-like
Dehydrogenase/reductase SDR family member 1
DnaJ homolog subfamily C member 30, mitochondrial
Ubiquitin-conjugating enzyme E2 E2
Caspase recruitment domain-containing protein 19
Protein PRRC1
FYVE, RhoGEF and PH domain-containing protein 4
F-box/LRR-repeat protein 18
Zinc finger protein 512
E3 SUMO-protein ligase NSE2
Non-structural maintenance of chromosomes element 3 homolog
Protein-L-isoaspartate O-methyltransferase domain-containing protein 1
Heat shock 70 kDa protein 12B
YTH domain-containing protein 1
Conserved oligomeric Golgi complex subunit 8
CKLF-like MARVEL transmembrane domain-containing protein 3
Dynein axonemal assembly factor 10
Nucleolar protein 4-like
Lysophospholipid acyltransferase 7
Dedicator of cytokinesis protein 7
Sideroflexin-2
Protein FAM210A
FERM domain-containing protein 6
28S rRNA (cytosine-C(5))-methyltransferase
Regulation of nuclear pre-mRNA domain-containing protein 1A
Arf-GAP with GTPase, ANK repeat and PH domain-containing protein 3
Arf-GAP with Rho-GAP domain, ANK repeat and PH domain-containing protein 1
Importin-9
Melanoma inhibitory activity protein 2
Discoidin, CUB and LCCL domain-containing protein 2
Rho guanine nucleotide exchange factor 17
Inositol polyphosphate-4-phosphatase type I A
RNA-binding protein 14
RING finger and CHY zinc finger domain-containing protein 1
Kelch-like protein 5
E3 ubiquitin-protein ligase UHRF2
E3 ubiquitin-protein ligase NEDD4-like
KH domain-containing RNA-binding protein QKI
Leukocyte receptor cluster member 8
Formin-like protein 2
Pseudouridylate synthase 7 homolog
Serine protease FAM111A
Trafficking protein particle complex subunit 9
CCA tRNA nucleotidyltransferase 1, mitochondrial
Serine/threonine-protein kinase SMG1
Alsin
Transmembrane protein 237
Alpha-ketoglutarate-dependent dioxygenase alkB homolog 3
Rho GTPase-activating protein 7
Serine/threonine-protein phosphatase 1 regulatory subunit 10

Sodium-coupled neutral amino acid symporter 2
UAP56-interacting factor
Myotubularin-related protein 9
Vacuolar protein sorting-associated protein 35
Transcriptional activator protein Pur-beta
Exportin-6
Hedgehog-interacting protein
Membrane-associated guanylate kinase, WW and PDZ domain-containing protein 1
Sperm-associated antigen 5
Nucleus accumbens-associated protein 1
Sorting nexin-18
PAS domain-containing serine/threonine-protein kinase
Protein capicua homolog
BRCA1-A complex subunit RAP80
Intermembrane lipid transfer protein VPS13A
Mediator of RNA polymerase II transcription subunit 15
Elongation factor G, mitochondrial
Endoplasmic reticulum-Golgi intermediate compartment protein 2
Methylcrotonoyl-CoA carboxylase subunit alpha, mitochondrial
Calcium/calmodulin-dependent protein kinase kinase 2
Trimethylguanosine synthase
NudC domain-containing protein 1
Erbin
Gamma-tubulin complex component 6
Gamma-tubulin complex component 5
Ubiquitin carboxyl-terminal hydrolase 28
Formin-binding protein 1
EKC/KEOPS complex subunit TP53RK
GPI transamidase component PIG-S
ATPase WRNIP1
Ran-binding protein 9
Chloride channel CLIC-like protein 1
Myeloid-associated differentiation marker
Neurabin-2
SRSF protein kinase 1
Structural maintenance of chromosomes protein 6
BTB/POZ domain-containing protein KCTD15
Spermatid perinuclear RNA-binding protein
Transmembrane protein 209
Glutathione peroxidase 7
CDK5 regulatory subunit-associated protein 2
Cytochrome P450 2S1
Protein IWS1 homolog
Paired amphipathic helix protein Sin3a
Oxysterol-binding protein-related protein 9
Protein cereblon
Integrator complex subunit 14
2-aminoethanethiol dioxygenase
Mitochondrial tRNA methylthiotransferase CDK5RAP1
Remodeling and spacing factor 1

RNA-binding protein 15
RUN and FYVE domain-containing protein 1
Msx2-interacting protein
Bifunctional polynucleotide phosphatase/kinase
MMS19 nucleotide excision repair protein homolog
E3 ubiquitin-protein ligase UHRF1
Protein Niban 2
ATP-dependent zinc metalloprotease YME1L1
Regulator of microtubule dynamics protein 3
Transcription factor 12
Cytohesin-2
Tubulin-folding cofactor B
Proteasome subunit beta type-7
Calponin-2
Translocation protein SEC62
Ethanolamine-phosphate cytidylyltransferase
Cell division cycle 5-like protein
26S proteasome non-ATPase regulatory subunit 1
Stromal cell-derived factor 2
Prefoldin subunit 5
E3 ubiquitin-protein ligase RING2
Parkinson disease protein 7
Sphingosine 1-phosphate receptor 3
GAS2-like protein 1
Eyes absent homolog 3
Sialidase-1
Sortilin
Synaptic vesicle membrane protein VAT-1 homolog
Legumain
Perilipin-2
DnaJ homolog subfamily C member 2
M-phase phosphoprotein 8
Nuclear pore complex protein Nup88
Plakophilin-4
Phosphoinositide 3-kinase regulatory subunit 4
P2X purinoceptor 4
Ribonucleases P/MRP protein subunit POP1
Protein S100-A13
Protein SCAF11
Translin-associated protein X
Selenide, water dikinase 2
Eukaryotic translation initiation factor 3 subunit C
Tetratricopeptide repeat protein 1
DnaJ homolog subfamily C member 7
Prohibitin-2
COP9 signalosome complex subunit 8
Calcineurin B homologous protein 1
Kinesin-like protein KIF2C
RANBP2-like and GRIP domain-containing protein 5/6
Monoglyceride lipase

Ataxin-2
Docking protein 1
Methionine synthase
3-hydroxyacyl-CoA dehydrogenase type-2
Collagen alpha-1(XII) chain
Mothers against decapentaplegic homolog 5
Sigma non-opioid intracellular receptor 1
Heterogeneous nuclear ribonucleoprotein A/B
Nucleosome assembly protein 1-like 4
Microsomal glutathione S-transferase 2
Gamma-soluble NSF attachment protein
Phosphatidylinositol 4-phosphate 5-kinase type-1 alpha
Phospholipid-transporting ATPase ABCA3
Mitogen-activated protein kinase kinase kinase 3
ATP synthase subunit s, mitochondrial
Mitochondrial intermediate peptidase
Aconitate hydratase, mitochondrial
Transmembrane 9 superfamily member 2
Equilibrative nucleoside transporter 1
Tumor susceptibility gene 101 protein
Copine-1
T-complex protein 1 subunit eta
Myeloid differentiation primary response protein MyD88
Probable rRNA-processing protein EBP2
Protein arginine N-methyltransferase 1
BAG family molecular chaperone regulator 1
Cyclic AMP-dependent transcription factor ATF-6 beta
Forkhead box protein C2
Endophilin-A2
Telomerase protein component 1
Serine/threonine-protein kinase VRK1
Growth/differentiation factor 15
A-kinase anchor protein 9
Protein NipSnap homolog 1
Condensin complex subunit 3
Actin-related protein 2/3 complex subunit 5-like protein
Calcium uptake protein 1, mitochondrial
UPF0415 protein C7orf25
Protein FAM118B
Target of rapamycin complex 2 subunit MAPKAP1
RNA-binding protein 4B
Zinc finger FYVE domain-containing protein 21
ATP-dependent RNA helicase DDX50
Large ribosomal subunit protein bL34m
Zinc phosphodiesterase ELAC protein 2
Telomerase RNA component interacting RNase
Glutamate-rich WD repeat-containing protein 1
ADP-ribose glycohydrolase MACROD1
Ribosome quality control complex subunit TCF25
Protein CMSS1

Kelch domain-containing protein 3
Evolutionarily conserved signaling intermediate in Toll pathway, mitochondrial
Methylosome protein WDR77
Cytochrome b-245 chaperone 1
Vitamin K epoxide reductase complex subunit 1
2-(3-amino-3-carboxypropyl)histidine synthase subunit 2
Tubulin alpha-1C chain
Selenoprotein S
Apolipoprotein L2
Myb-binding protein 1A
Mitochondrial genome maintenance exonuclease 1
Golgi reassembly-stacking protein 1
FYVE and coiled-coil domain-containing protein 1
Acyl-CoA-binding domain-containing protein 6
Coronin-1B
Thioredoxin domain-containing protein 17
BUD13 homolog
Serine/threonine-protein phosphatase CPPED1
Vacuolar protein-sorting-associated protein 25
Large ribosomal subunit protein mL45
Uncharacterized protein C7orf50
Tudor-interacting repair regulator protein
45 kDa calcium-binding protein
Programmed cell death protein 2-like
Proteasomal ATPase-associated factor 1
Partner of Y14 and mago
MICOS complex subunit MIC25
ADP-dependent glucokinase
G patch domain-containing protein 1
Serine/threonine-protein kinase RIO1
Protein LLP homolog
DNA replication complex GINS protein SLD5
rRNA-processing protein UTP23 homolog
Protein pelota homolog
Peroxiredoxin-like 2A
E3 ubiquitin-protein ligase TRIM56
Endoplasmic reticulum resident protein 44
Latexin
Nucleolar protein 10
Cancer-related nucleoside-triphosphatase
Mitochondrial import inner membrane translocase subunit Tim29
Translational activator of cytochrome c oxidase 1
Gamma-tubulin complex component 2
Extended synaptotagmin-1
Ubiquitin-associated domain-containing protein 1
Protein YIPF4
Phagosome assembly factor 1
tRNA-splicing endonuclease subunit Sen34
Protein canopy homolog 3
Mitochondrial ribosome-associated GTPase 1

Chitobiosyldiphosphodolichol beta-mannosyltransferase
LIM domain-containing protein 2
HAUS augmin-like complex subunit 8
Inositol polyphosphate 5-phosphatase K
Proteasome assembly chaperone 3
COP9 signalosome complex subunit 4
Synaptotagmin-11
WW domain-containing adapter protein with coiled-coil
Death-inducer obliterator 1
Metastasis-associated protein MTA3
RNA-binding protein 42
Dynactin subunit 5
Mini-chromosome maintenance complex-binding protein
Alanyl-tRNA editing protein Aarsd1
DCN1-like protein 5
Acidic leucine-rich nuclear phosphoprotein 32 family member E
Leucine-rich repeat-containing protein 1
Phosphatidylinositol 4-kinase type 2-alpha
Transmembrane protein 43
Fibronectin type III and SPRY domain-containing protein 1
Tubulin-specific chaperone D
Nucleoporin NDC1
Protein HGH1 homolog
Dehydrogenase/reductase SDR family member 4
Lipase maturation factor 2
NADH dehydrogenase [ubiquinone] 1 alpha subcomplex assembly factor 3
Multiple myeloma tumor-associated protein 2
Transmembrane protein 70, mitochondrial
Mediator of RNA polymerase II transcription subunit 18
Iron-sulfur cluster assembly 1 homolog, mitochondrial
Tubulin beta-6 chain
Protein PAXX
DNA-directed RNA polymerase III subunit RPC3
Heterogeneous nuclear ribonucleoprotein U-like protein 1
Protein misato homolog 1
PHD finger protein 23
Programmed cell death protein 10
Ribonuclease P protein subunit p25
Derlin-1
Oxidoreductase HTATIP2
Probable ATP-dependent RNA helicase DDX23
MICOS complex subunit MIC26
Dehydrogenase/reductase SDR family member 6
Methylthioribose-1-phosphate isomerase
WD repeat-containing protein 18
Vesicle-associated membrane protein 8
tRNA (guanine(6)-N2)-methyltransferase THUMP3
Acireductone dioxygenase
Enoyl-[acyl-carrier-protein] reductase, mitochondrial
ER membrane protein complex subunit 6

N-terminal Xaa-Pro-Lys N-methyltransferase 1
ER degradation-enhancing alpha-mannosidase-like protein 2
Katanin p80 WD40 repeat-containing subunit B1
Target of rapamycin complex subunit LST8
Ashwin
Voltage-gated monoatomic cation channel TMEM109
Protein PBDC1
Phosphatidylserine synthase 2
Nucleolar complex protein 4 homolog
U3 small nucleolar RNA-associated protein 14 homolog A
Dual specificity protein phosphatase 23
Transmembrane emp24 domain-containing protein 9
BOS complex subunit TMEM147
Nucleoporin p58/p45
Protein adenylyltransferase SelO, mitochondrial
Protein DPCD
Guanine nucleotide-binding protein-like 3
ATPase family gene 2 protein homolog B
Serine/threonine-protein kinase RIO2
tRNA (adenine(58)-N(1))-methyltransferase, mitochondrial
Transmembrane and ubiquitin-like domain-containing protein 1
Mitochondrial import inner membrane translocase subunit Tim21
Kinesin-like protein KIFC1
Nuclear pore complex protein Nup85
Very long chain fatty acid elongase 1
Katanin p60 ATPase-containing subunit A-like 1
HIRA-interacting protein 3
Intraflagellar transport protein 27 homolog
Splicing factor YJU2
ADP-ribose pyrophosphatase, mitochondrial
Threonine--tRNA ligase, mitochondrial
Acetyl-CoA acetyltransferase, cytosolic
Replication initiator 1
RNA-binding protein 4
FUN14 domain-containing protein 2
RNA polymerase II-associated protein 1
Splicing factor 3B subunit 5
Sideroflexin-3
Proline-rich protein 14
Chitinase domain-containing protein 1
Poly(A) polymerase gamma
Caspase recruitment domain-containing protein 10
Kanadaptin
Cyclin-dependent kinase 19
Protein LSM14 homolog B
Tapasin-related protein
Junctional adhesion molecule C
Caspase recruitment domain-containing protein 6
BTB/POZ domain-containing protein 2
Oxysterol-binding protein-related protein 11

Oxysterol-binding protein-related protein 10
Rab11 family-interacting protein 5
TBC1 domain family member 10A
Complement C1q tumor necrosis factor-related protein 5
N-alpha-acetyltransferase 15, NatA auxiliary subunit
Bcl-2-like protein 13
Caspase recruitment domain-containing protein 11
Solute carrier family 12 member 9
Serrate RNA effector molecule homolog
Queuine tRNA-ribosyltransferase catalytic subunit 1
AP-1 complex subunit mu-1
Nucleolar and spindle-associated protein 1
Haloacid dehalogenase-like hydrolase domain-containing 5
Fanconi anemia group D2 protein
Protein MAK16 homolog
Histone deacetylase 8
Replication termination factor 2
Charged multivesicular body protein 4a
Eukaryotic translation initiation factor 2A
Polymerase delta-interacting protein 3
Uncharacterized protein KIAA1671
SH3 and multiple ankyrin repeat domains protein 3
Guanine nucleotide-binding protein subunit beta-like protein 1
Alpha-(1,6)-fucosyltransferase
Large ribosomal subunit protein bL32m
Large ribosomal subunit protein bL20m
Large ribosomal subunit protein uL13m
Large ribosomal subunit protein bL9m
Large ribosomal subunit protein uL4m
Large ribosomal subunit protein uL1m
MKI67 FHA domain-interacting nucleolar phosphoprotein
Hyccin
3'-5' exoribonuclease HELZ2
RanBP-type and C3HC4-type zinc finger-containing protein 1
Small ribosomal subunit protein mS26
Neurolysin, mitochondrial
Centrosomal protein of 41 kDa
Histone-lysine N-methyltransferase SETD2
Pantothenate kinase 2, mitochondrial
Dedicator of cytokinesis protein 9
FERM domain-containing protein 8
Contactin-associated protein-like 3
Histone-lysine N-methyltransferase NSD3
Kinetochore protein Nuf2
Large ribosomal subunit protein mL37
GTP-binding protein 4
Tether containing UBX domain for GLUT4
Oxysterol-binding protein-related protein 8
Uveal autoantigen with coiled-coil domains and ankyrin repeats
Ras-related protein Rab-34

2-(3-amino-3-carboxypropyl)histidine synthase subunit 1
WD repeat-containing protein 11
Regulator of nonsense transcripts 3B
Crooked neck-like protein 1
F-box-like/WD repeat-containing protein TBL1XR1
Ubiquitin-like protein 5
Protein phosphatase 1 regulatory subunit 12C
Serine/threonine-protein kinase D2
ER degradation-enhancing alpha-mannosidase-like protein 3
Protein Niban 1
UBX domain-containing protein 6
Transmembrane 6 superfamily member 1
Uridine-cytidine kinase 2
Sialoadhesin
Apoptosis inhibitor 5
NACHT, LRR and PYD domains-containing protein 1
Tripartite motif-containing protein 5
E3 ubiquitin-protein ligase TRIM4
Alpha-ketoglutarate-dependent dioxygenase FTO
Palmitoyltransferase ZDHHC5
Transport and Golgi organization protein 6 homolog
182 kDa tankyrase-1-binding protein
(E3-independent) E2 ubiquitin-conjugating enzyme
Protein zyg-11 homolog B
Protein TANC1
Ethanolaminephosphotransferase 1
Exportin-4
Endoplasmic reticulum junction formation protein lunapark
Centrosomal protein of 44 kDa
Protein tweety homolog 3
Kelch-like protein 4
Myotubularin-related protein 12
pre-mRNA 3' end processing protein WDR33
Ribosome biogenesis protein WDR12
Protein YIPF3
Tubulointerstitial nephritis antigen-like
Adipose-secreted signaling protein
PITH domain-containing protein 1
Derlin-2
COMM domain-containing protein 5
Sentrin-specific protease 6
RNA exonuclease 4
ATP-dependent RNA helicase DDX24
DNA-directed RNA polymerase I subunit RPA49
Superkiller complex protein 8
SRA stem-loop-interacting RNA-binding protein, mitochondrial
Serine racemase
NIF3-like protein 1
Egl nine homolog 1
Mucolipin-1

PSME3-interacting protein
Mitochondrial fission factor
N-alpha-acetyltransferase 50
Ubiquitin-like modifier-activating enzyme 5
E3 ubiquitin-protein ligase makorin-2
Transmembrane protein 126A
Polyadenylate-binding protein-interacting protein 1
Mitochondrial disaggregase
Ras-related protein Rab-33B
Large subunit GTPase 1 homolog
Protein FAM107B
RNA cytidine acetyltransferase
COMM domain-containing protein 4
Kinesin light chain 2
Integrin-linked kinase-associated serine/threonine phosphatase 2C
5'-3' exoribonuclease 2
Toll-interacting protein
Histone deacetylase complex subunit SAP130
Bromodomain-containing protein 8
Nuclear speckle splicing regulatory protein 1
Integrator complex subunit 2
Rac GTPase-activating protein 1
Protein mono-ADP-ribosyltransferase PARP12
Cleavage stimulation factor subunit 2 tau variant
Cytosolic 5'-nucleotidase 3A
CYFIP-related Rac1 interactor A
Transmembrane protein 222
Haloacid dehalogenase-like hydrolase domain-containing protein 2
Glutamyl-tRNA(Gln) amidotransferase subunit A, mitochondrial
Probable ATP-dependent RNA helicase DDX47
Magnesium transporter protein 1
Ras-related protein Rab-1B
Large ribosomal subunit protein uL18m
Testis-specific Y-encoded-like protein 1
Transmembrane protein 168
VIP36-like protein
Nonsense-mediated mRNA decay factor SMG9
Protein FAM234A
Oxysterol-binding protein-related protein 5
Deoxynucleotidyltransferase terminal-interacting protein 1
Nucleotide exchange factor SIL1
Protein-L-histidine N-pros-methyltransferase
Anaphase-promoting complex subunit 1
Probable E3 ubiquitin-protein ligase IRF2BPL
Cysteine-rich and transmembrane domain-containing protein 1
DNA-directed RNA polymerase III subunit RPC6
Nuclear ubiquitous casein and cyclin-dependent kinase substrate 1
Thioredoxin-related transmembrane protein 4
Kinesin-like protein KIF13A
Activating signal cointegrator 1 complex subunit 2

Rabenosyn-5
Iron-sulfur cluster assembly enzyme ISCU
Oxysterol-binding protein-related protein 2
Autophagy protein 5
WD repeat-containing protein 13
Epsin-3
EH domain-containing protein 4
Vacuolar protein sorting-associated protein 33B
Vacuolar protein sorting-associated protein 16 homolog
Vacuolar protein sorting-associated protein 11 homolog
SH3 domain-binding glutamic acid-rich-like protein 3
Gigaxonin
Solute carrier family 25 member 32
TRIO and F-actin-binding protein
STE20-like serine/threonine-protein kinase
Peptidyl-prolyl cis-trans isomerase-like 3
Sodium-coupled neutral amino acid symporter 1
Phosducin-like protein 3
Serine/threonine-protein kinase TAO3
Rab3 GTPase-activating protein non-catalytic subunit
Activity-dependent neuroprotector homeobox protein
Diphthine methyl ester synthase
ATP-dependent DNA/RNA helicase DHX36
Inorganic pyrophosphatase 2, mitochondrial
Protein spinster homolog 1
Large ribosomal subunit protein mL46
Solute carrier family 12 member 5
Presenilin-associated rhomboid-like protein, mitochondrial
Pinin
Transmembrane protein 245
Forkhead box protein P1
BTB/POZ domain-containing adapter for CUL3-mediated RhoA degradation protein 3
tRNA dimethylallyltransferase
UPF0696 protein C11orf68
Growth hormone-inducible transmembrane protein
BolA-like protein 2
Cobalamin trafficking protein CblD
Thioredoxin-interacting protein
Thioredoxin-related transmembrane protein 1
Negative elongation factor A
Golgi resident protein GCP60
Cdc42 effector protein 4
Tyrosine-protein phosphatase non-receptor type 23
Semaphorin-6B
Protein unc-45 homolog A
Major facilitator superfamily domain-containing protein 1
DnaJ homolog subfamily C member 5
Kinetochore-associated protein DSN1 homolog
Charged multivesicular body protein 4b
RWD domain-containing protein 1

CUE domain-containing protein 2
F-box/LRR-repeat protein 15
Fructosamine-3-kinase
GDP-fucose protein O-fucosyltransferase 1
Phosphatidylinositol glycan anchor biosynthesis class U protein
Torsin-3A
Serine/threonine-protein kinase WNK1
Aminopeptidase B
Golgi phosphoprotein 3-like
Golgi phosphoprotein 3
tRNA N6-adenosine threonylcarbamoyltransferase, mitochondrial
Alpha-N-acetyl-neuraminyl-2,3-beta-galactosyl-1,3-N-acetyl-galactosaminide alpha-2,6-sialyltran
Golgi-associated plant pathogenesis-related protein 1
TraB domain-containing protein
Mitochondrial ribosome-associated GTPase 2
Sentrin-specific protease 3
Oxysterol-binding protein-related protein 3
SWI/SNF-related matrix-associated actin-dependent regulator of chromatin subfamily A contain
EH domain-containing protein 1
mRNA (2'-O-methyladenosine-N(6)-)-methyltransferase
ESF1 homolog
Alpha-1,3/1,6-mannosyltransferase ALG2
HEAT repeat-containing protein 1
Protein O-mannose kinase
Rab GTPase-binding effector protein 2
Dimethyladenosine transferase 2, mitochondrial
rRNA N6-adenosine-methyltransferase ZCCHC4
STING ER exit protein
Cytosolic iron-sulfur assembly component 2A
Probable ATP-dependent RNA helicase DHX35
Protein FAM124B
HAUS augmin-like complex subunit 4
Coiled-coil domain-containing protein 134
Coiled-coil domain-containing protein 86
Receptor expression-enhancing protein 4
Transcription factor SOX-17
Optic atrophy 3 protein
Cytosolic iron-sulfur assembly component 3
ATP-dependent RNA helicase DHX33
Nucleolar protein 6
WD repeat and coiled-coil-containing protein
3'-5' RNA helicase YTHDC2
5-azacytidine-induced protein 2
RNA polymerase II-associated protein 3
BCAS3 microtubule associated cell migration factor
Alpha-1,2-mannosyltransferase ALG9
Lipid droplet-associated hydrolase
Ribosomal oxygenase 1
WD repeat-containing protein 55
Ran-binding protein 3

dCTP pyrophosphatase 1
Inactive tyrosine-protein kinase PEAK1
Ribosome production factor 2 homolog
Histone-lysine N-methyltransferase SMYD3
Mth938 domain-containing protein
Dedicator of cytokinesis protein 5
WD repeat-containing protein 26
Tudor domain-containing protein 3
UPF0488 protein C8orf33
Polyamine-transporting ATPase 13A3
Methyltransferase-like protein 17, mitochondrial
Splicing factor, arginine/serine-rich 19
Prostaglandin E synthase 2
Phosphorylated adapter RNA export protein
Ubiquitin-conjugating enzyme E2 Z
Complex I assembly factor ACAD9, mitochondrial
5'-nucleotidase domain-containing protein 2
PRKR-interacting protein 1
Caspase activity and apoptosis inhibitor 1
Nucleolar protein 11
Probable ATP-dependent RNA helicase DDX31
Thiol S-methyltransferase TMT1A
ATPase PAAT
Multimerin-2
Ubiquitin carboxyl-terminal hydrolase MINDY-3
Protein eva-1 homolog A
AKT-interacting protein
Pleckstrin homology domain-containing family F member 2
tRNA endonuclease ANKZF1
Golgi reassembly-stacking protein 2
Protein zwilch homolog
Jupiter microtubule associated homolog 2
Mitochondrial glutamate carrier 1
Proline-serine-threonine phosphatase-interacting protein 2
Mediator of RNA polymerase II transcription subunit 20
Queuine tRNA-ribosyltransferase accessory subunit 2
Actin-related protein 8
Damage-control phosphatase ARMT1
CCR4-NOT transcription complex subunit 10
Leucine-rich repeat-containing protein 40
Histone-lysine N-methyltransferase EHMT1
Sideroflexin-1
Spermatogenesis-defective protein 39 homolog
Conserved oligomeric Golgi complex subunit 4
Actin-related protein 5
Vacuolar protein sorting-associated protein 37B
Large ribosomal subunit protein mL44
L-2-hydroxyglutarate dehydrogenase, mitochondrial
COP9 signalosome complex subunit 7b
Non-homologous end-joining factor 1

Protein transport protein Sec61 subunit alpha isoform 2
Ribitol 5-phosphate transferase FKRP
Elongator complex protein 3
Ribosome production factor 1
DNA-directed RNA polymerase I subunit RPA2
Zinc finger matrin-type protein 3
Uridine-cytidine kinase 1
Ketosamine-3-kinase
TBC1 domain family member 17
Probable cysteine--tRNA ligase, mitochondrial
Solute carrier family 52, riboflavin transporter, member 2
Phosphopantothenate--cysteine ligase
Ubiquitin domain-containing protein 1
WD repeat-containing protein 41
Nicotinamide/nicotinic acid mononucleotide adenylyltransferase 1
Protein Njmu-R1
Sialate O-acetylesterase
Pleckstrin homology domain-containing family A member 5
E3 ubiquitin-protein ligase SMURF2
Regulator of nonsense transcripts 2
Guanine nucleotide-binding protein subunit beta-4
Exportin-5
GrpE protein homolog 1, mitochondrial
MYG1 exonuclease
Pleckstrin homology domain-containing family A member 3
Pleckstrin homology domain-containing family A member 1
Retinoid-inducible serine carboxypeptidase
Netrin-4
Calcyclin-binding protein
Ras-related GTP-binding protein C
Zinc finger FYVE domain-containing protein 1
Intraflagellar transport protein 122 homolog
Retinol dehydrogenase 14
Beta-parvin
Tensin-1
Plasminogen receptor (KT)
NmrA-like family domain-containing protein 1
Vezatin
Kinetochore protein Spc25
Transcription initiation factor TFIID subunit 9B
Solute carrier family 38 member 10
Ethanolamine kinase 1
Adhesion G protein-coupled receptor L4
Putative divalent cation/proton antiporter TMEM165
Mitochondrial thiamine pyrophosphate carrier
Echinoderm microtubule-associated protein-like 4
rRNA methyltransferase 3, mitochondrial
Glyoxalase domain-containing protein 4
Serine/threonine-protein kinase Nek6
Methylcrotonoyl-CoA carboxylase beta chain, mitochondrial

Nuclear receptor coactivator 5
Ectopic P granules protein 5 homolog
Helicase MOV-10
N6-adenosine-methyltransferase non-catalytic subunit
Non-lysosomal glucosylceramidase
Pre-mRNA-splicing factor CWC22 homolog
Ribonucleoprotein PTB-binding 2
Chromodomain-helicase-DNA-binding protein 8
Band 4.1-like protein 5
GPN-loop GTPase 1
Stromal cell-derived factor 2-like protein 1
Casein kinase I isoform gamma-1
Pre-mRNA-splicing factor SYF1
Prolactin regulatory element-binding protein
Steroid receptor RNA activator 1
Endoplasmic reticulum transmembrane helix translocase
Golgi-associated PDZ and coiled-coil motif-containing protein
Large ribosomal subunit protein uL29m
LYR motif-containing protein 4
Charged multivesicular body protein 1a
Transmembrane 9 superfamily member 3
Unconventional myosin-X
Adipocyte plasma membrane-associated protein
tRNA (34-2'-O)-methyltransferase regulator WDR6
Thioredoxin reductase 2, mitochondrial
ATP-binding cassette sub-family B member 6
ADP-ribosylation factor GTPase-activating protein 3
Zinc finger CCHC domain-containing protein 17
Ras-related protein Rab-18
Palmdelphin
RNA polymerase II subunit A C-terminal domain phosphatase SSU72
Vacuolar protein sorting-associated protein VTA1 homolog
Serine--tRNA ligase, mitochondrial
Large ribosomal subunit protein mL65
Dynein light chain roadblock-type 1
ER membrane protein complex subunit 7
Transcription and mRNA export factor ENY2
Exosome complex component RRP41
Ubiquitin-conjugating enzyme E2 T
H/ACA ribonucleoprotein complex subunit 3
tRNA N6-adenosine threonylcarbamoyltransferase
DNA methyltransferase 1-associated protein 1
Protocadherin-12
Inositol-3-phosphate synthase 1
Bromodomain-containing protein 7
mRNA-decapping enzyme 1A
Acyl-coenzyme A thioesterase 13
Mediator of RNA polymerase II transcription subunit 4
Complex I assembly factor TIMMDC1, mitochondrial
Synembryn-A

Complement component C1q receptor
Putative RNA-binding protein Luc7-like 1
Endothelial cell-specific molecule 1
Leucine zipper transcription factor-like protein 1
Large ribosomal subunit protein mL40
Suppressor of SWI4 1 homolog
1-phosphatidylinositol 4,5-bisphosphate phosphodiesterase beta-1
Fructose-2,6-bisphosphatase TIGAR
Protein C12orf4
Reticulon-4
Regulation of nuclear pre-mRNA domain-containing protein 1B
Xaa-Pro aminopeptidase 3
Omega-amidase NIT2
Cell death regulator Aven
Exosome complex component RRP46
Exosome complex component RRP40
Kinesin-like protein KIF13B
Anillin
Xaa-Pro aminopeptidase 1
Gephyrin
Bridging integrator 3
Something about silencing protein 10
StAR-related lipid transfer protein 7, mitochondrial
Baculoviral IAP repeat-containing protein 6
PDZ and LIM domain protein 7
Acetyl-coenzyme A synthetase, cytoplasmic
Diablo IAP-binding mitochondrial protein
Nucleolar RNA helicase 2
GTP-binding protein SAR1a
Sialic acid synthase
Endophilin-B2
Histone-lysine N-methyltransferase ASH1L
Translation initiation factor eIF2B subunit gamma
Muscleblind-like protein 1
Sialin
Eukaryotic translation initiation factor 4E transporter
Carbohydrate sulfotransferase 12
Suppressor of tumorigenicity 7 protein
PRKCA-binding protein
SH2B adapter protein 1
CTP synthase 2
DNA polymerase epsilon subunit 3
Chromatin accessibility complex protein 1
Aladin
SNF-related serine/threonine-protein kinase
ATP-binding cassette sub-family B member 10, mitochondrial
Bromodomain adjacent to zinc finger domain protein 1A
Striatin-4
L-aminoadipate-semialdehyde dehydrogenase-phosphopantetheinyl transferase
rRNA N6-adenosine-methyltransferase METTL5

Oligosaccharyltransferase complex subunit OSTC
Phospholipid scramblase 4
Ribonuclease 3
Sorting nexin-15
Heme-binding protein 1
DNA dC->dU-editing enzyme APOBEC-3C
Vacuolar protein sorting-associated protein 45
RNA-binding protein PNO1
Large ribosomal subunit protein bL17m
14 kDa phosphohistidine phosphatase
Serine incorporator 1
Rho GTPase-activating protein 35
Protein FAM114A2
Phospholipid scramblase 3
1-acyl-sn-glycerol-3-phosphate acyltransferase delta
1-acyl-sn-glycerol-3-phosphate acyltransferase gamma
Lymphoid-specific helicase
Glycoprotein-N-acetylgalactosamine 3-beta-galactosyltransferase 1
Latent-transforming growth factor beta-binding protein 3
Mitochondrial import receptor subunit TOM22 homolog
LanC-like protein 2
Kinesin-like protein KIF15
Transmembrane 7 superfamily member 3
Homer protein homolog 3
Phenylalanine--tRNA ligase beta subunit
Isoleucine--tRNA ligase, mitochondrial
Ribosome biogenesis protein SLX9 homolog
SAM domain-containing protein SAMSN-1
Kinesin light chain 4
Protein diaphanous homolog 3
BMP-2-inducible protein kinase
Ubiquitin-like-conjugating enzyme ATG3
Sister chromatid cohesion protein PDS5 homolog B
Structural maintenance of chromosomes protein 4
Alpha-mannosidase 2C1
Phosphatidylinositol-3-phosphatase SAC1
Protein DEPP1
Obg-like ATPase 1
Copper homeostasis protein cutC homolog
Zinc finger protein 64
Ethylmalonyl-CoA decarboxylase
RNA-binding protein 12
TBC1 domain family member 22B
Midasin
Acetyl-coenzyme A synthetase 2-like, mitochondrial
Zinc finger CCHC domain-containing protein 3
Peroxisomal 2,4-dienoyl-CoA reductase [(3E)-enoyl-CoA-producing]
Palmitoyl-protein thioesterase ABHD10, mitochondrial
T-complex protein 11-like protein 1
Double-stranded RNA-binding protein Staufen homolog 2

Shiftless antiviral inhibitor of ribosomal frameshifting protein
Probable ATP-dependent RNA helicase DDX28
Transmembrane protein 106B
Protein lin-7 homolog C
1-acyl-sn-glycerol-3-phosphate acyltransferase epsilon
Gamma-taxilin
SPATS2-like protein
Ufm1-specific protease 2
ATP-binding cassette sub-family F member 3
CYFIP-related Rac1 interactor B
Mitochondrial potassium channel ATP-binding subunit
ATP-dependent RNA helicase DDX19A
GTPase IMAP family member 4
Tyrosyl-DNA phosphodiesterase 1
Protection of telomeres protein 1
TBC1 domain family member 23
DDB1- and CUL4-associated factor 13
U3 small nucleolar ribonucleoprotein protein IMP3
MRG/MORF4L-binding protein
S-adenosyl-L-methionine-dependent tRNA 4-demethylwyosine synthase TYW1
Exocyst complex component 1
Protein-L-isoaspartate O-methyltransferase domain-containing protein 2
Integrator complex subunit 9
Cell cycle control protein 50A
Ubiquinol-cytochrome c reductase complex assembly factor 1
Septin-11
Sodium-coupled neutral amino acid transporter 7
Mediator of RNA polymerase II transcription subunit 17
Alpha-parvin
Ubiquitin carboxyl-terminal hydrolase 40
4'-phosphopantetheine phosphatase
F-box only protein 28
TBC1 domain family member 13
Exonuclease 3'-5' domain-containing protein 2
DnaJ homolog subfamily C member 11
Integrator complex subunit 7
Fanconi anemia group I protein
ATPase family AAA domain-containing protein 3A
ADP-ribosylation factor-like protein 8B
DnaJ homolog subfamily C member 17
Integrator complex subunit 13
Synembryn-B
Guanine nucleotide-binding protein-like 3-like protein
ATP-dependent RNA helicase DDX18
Histone chaperone ASF1B
Kelch-like protein 11
Integrator complex subunit 10
Large ribosomal subunit protein mL66
Pyridoxine-5'-phosphate oxidase
Armadillo repeat-containing protein 1

DNA-directed RNA polymerase III subunit RPC5
Protein SDA1 homolog
Poly(A) RNA polymerase, mitochondrial
Notchless protein homolog 1
Adaptin ear-binding coat-associated protein 2
DNA-directed RNA polymerase III subunit RPC2
RNA-binding protein 28
Anoctamin-10
Pre-mRNA-splicing factor RBM22
Distal membrane-arm assembly complex protein 2
WD repeat-containing protein 70
Transmembrane protein 51
Arginine and glutamate-rich protein 1
Intraflagellar transport protein 57 homolog
SAFB-like transcription modulator
Peptidyl-prolyl cis-trans isomerase FKBP14
Required for meiotic nuclear division protein 1 homolog
p21-activated protein kinase-interacting protein 1
Hypoxia-inducible factor 1-alpha inhibitor
3-oxoacyl-[acyl-carrier-protein] synthase, mitochondrial
Large ribosomal subunit protein uL22m
CXXC motif containing zinc binding protein
BRISC and BRCA1-A complex member 1
Ceroid-lipofuscinosis neuronal protein 6
Probable tRNA(His) guanylyltransferase
Histone PARylation factor 1
Interleukin-1 receptor-associated kinase 4
Uridine-cytidine kinase-like 1
Transmembrane protein 160
NACHT, LRR and PYD domains-containing protein 2
Constitutive coactivator of PPAR-gamma-like protein 2
tRNA selenocysteine 1-associated protein 1
NADH dehydrogenase [ubiquinone] 1 beta subcomplex subunit 11, mitochondrial
Large ribosomal subunit protein uL16m
H/ACA ribonucleoprotein complex subunit 2
OCIA domain-containing protein 1
ADP-ribosylhydrolase ARH3
E3 ubiquitin-protein ligase MARCHF5
Cell growth-regulating nucleolar protein
Transmembrane protein 161A
Golgi-resident adenosine 3',5'-bisphosphate 3'-phosphatase
MICOS complex subunit MIC19
tRNA-dihydrouridine(20) synthase [NAD(P)+]-like
CKLF-like MARVEL transmembrane domain-containing protein 6
NAD-dependent protein deacylase sirtuin-5, mitochondrial
GATOR2 complex protein MIOS
Myotubularin-related protein 10
Sphingomyelin phosphodiesterase 4
Pre-mRNA-splicing factor CWC25 homolog
Testis-expressed protein 10

DDB1- and CUL4-associated factor 16
THUMP domain-containing protein 1
Torsin-4A
tRNA (guanine(26)-N(2))-dimethyltransferase
Ganglioside-induced differentiation-associated protein 2
Nuclear distribution protein nudE homolog 1
BRISC and BRCA1-A complex member 2
Glutaminyl-peptide cyclotransferase-like protein
ADP-ribosylation factor-like protein 15
BTB/POZ domain-containing protein KCTD5
CDKN2A-interacting protein
DnaJ homolog subfamily B member 12
Alpha-ketoglutarate-dependent dioxygenase alkB homolog 4
Non-structural maintenance of chromosomes element 4 homolog A
H/ACA ribonucleoprotein complex subunit 1
Stabilin-1
Serine/threonine-protein phosphatase 4 regulatory subunit 2
Dipeptidyl peptidase 3
Protein AATF
Tubulin alpha-8 chain
Probable ATP-dependent RNA helicase DDX56
Telomeric repeat-binding factor 2-interacting protein 1
Abl interactor 2
Bcl-2-associated transcription factor 1
U3 small nucleolar RNA-associated protein 6 homolog
TGF-beta-activated kinase 1 and MAP3K7-binding protein 2
Large ribosomal subunit protein mL39
Mitogen-activated protein kinase kinase kinase 20
Peptidyl-prolyl cis-trans isomerase FKBP11
Tropomodulin-3
BET1-like protein
Very long chain fatty acid elongase 5
NF-kappa-B inhibitor-interacting Ras-like protein 2
UDP-glucose:glycoprotein glucosyltransferase 2
UDP-glucose:glycoprotein glucosyltransferase 1
Cyclin-dependent kinase 12
FAST kinase domain-containing protein 2, mitochondrial
Very-long-chain enoyl-CoA reductase
Endoplasmic reticulum aminopeptidase 1
Actin-related protein 10
Vesicle transport protein USE1
CDGSH iron-sulfur domain-containing protein 1
ADP-ribosylation factor-binding protein GGA3
Splicing factor C9orf78
Constitutive coactivator of PPAR-gamma-like protein 1
Glycerophosphodiester phosphodiesterase 1
SWI/SNF-related matrix-associated actin-dependent regulator of chromatin subfamily A-like pro
Glycolipid transfer protein
Maspardin
Large ribosomal subunit protein bL35m

Upstream-binding protein 1
Insulin-like growth factor 2 mRNA-binding protein 1
Sacsin
Mitochondrial carrier homolog 1
Diphosphoinositol polyphosphate phosphohydrolase 2
Hsp70-binding protein 1
Methionine adenosyltransferase 2 subunit beta
Myoferlin
Intersectin-2
Ribosome biogenesis protein NOP53
EH domain-containing protein 3
EH domain-containing protein 2
Rho guanine nucleotide exchange factor 12
CCR4-NOT transcription complex subunit 2
NCK-interacting protein with SH3 domain
Programmed cell death 1 ligand 1
Opioid growth factor receptor
LIM and cysteine-rich domains protein 1
Cysteine-rich motor neuron 1 protein
Selenoprotein N
Protein PALS2
Charged multivesicular body protein 5
Spliceosome-associated protein CWC15 homolog
Large ribosomal subunit protein uL15m
Thymocyte nuclear protein 1
Thyroid transcription factor 1-associated protein 26
NADH dehydrogenase [ubiquinone] 1 alpha subcomplex assembly factor 4
Very-long-chain (3R)-3-hydroxyacyl-CoA dehydratase 3
ER membrane protein complex subunit 3
NADH dehydrogenase [ubiquinone] 1 alpha subcomplex subunit 13
[Pyruvate dehydrogenase [acetyl-transferring]]-phosphatase 1, mitochondrial
E3 ubiquitin-protein ligase KCMF1
Ankycorbin
Vesicle-associated membrane protein-associated protein A
Large ribosomal subunit protein bL27m
Proton-transporting V-type ATPase complex assembly regulator TMEM9
Sentrin-specific protease 1
CXXC-type zinc finger protein 1
SH3 domain-binding protein 4
Septin-10
GEM-interacting protein
PHD and RING finger domain-containing protein 1
Pogo transposable element with KRAB domain
Protein Daple
Rho GTPase-activating protein 23
Vacuolar protein sorting-associated protein 18 homolog
Protein RCC2
RAB11-binding protein RELCH
Disco-interacting protein 2 homolog B
Junctional cadherin 5-associated protein

SLAIN motif-containing protein 2
Teneurin-3
Ubiquitin carboxyl-terminal hydrolase 36
BRCA2 and CDKN1A-interacting protein
Serine/threonine-protein kinase 26
Armadillo repeat-containing X-linked protein 1
ABI gene family member 3
Prostaglandin F2 receptor negative regulator
CTTNBP2 N-terminal-like protein
Transmembrane protein 181
Inhibitor of Bruton tyrosine kinase
HEAT repeat-containing protein 5B
NFX1-type zinc finger-containing protein 1
Chondroitin sulfate glucuronyltransferase
Protocadherin-10
Ribosome-binding protein 1
Cleavage and polyadenylation specificity factor subunit 2
Kelch-like protein 9
Leucine--tRNA ligase, cytoplasmic
Myelin expression factor 2
Kelch-like protein 42
eIF-2-alpha kinase GCN2
WD repeat-containing protein 35
RNA-binding protein 27
KAT8 regulatory NSL complex subunit 3
E3 ubiquitin-protein ligase HECW2
FERM domain-containing protein 4A
Rabankyrin-5
Arginine-glutamic acid dipeptide repeats protein
Succinate--CoA ligase [ADP-forming] subunit beta, mitochondrial
GMP reductase 2
Syntaxin-18
Dolichol-phosphate mannosyltransferase subunit 3
Protein IMPACT
UV radiation resistance-associated gene protein
Ataxin-10
Methyl-CpG-binding domain protein 2
Neurochondrin
Tuftelin-interacting protein 11
Epidermal growth factor receptor substrate 15-like 1
Unconventional myosin-Ia
Origin recognition complex subunit 3
SUMO-activating enzyme subunit 1
Coatomer subunit gamma-2
Phosphatidylinositol 4-kinase beta
C-type mannose receptor 2
COMM domain-containing protein 3
Guanine nucleotide-binding protein G(I)/G(S)/G(O) subunit gamma-12
Rab-like protein 2A
Methionine synthase reductase

Protein UXT
Set1/Ash2 histone methyltransferase complex subunit ASH2
7-dehydrocholesterol reductase
Tumor necrosis factor receptor superfamily member 10D
Histone deacetylase 6
Spastin
Dickkopf-related protein 3
tRNA (guanine-N(7)-)-methyltransferase
PTB domain-containing engulfment adapter protein 1
Vacuolar protein sorting-associated protein 29
Eukaryotic translation initiation factor 3 subunit K
Exostosin-like 2
Glyoxylate reductase/hydroxypyruvate reductase
Cathepsin Z
Ribosomal protein S6 kinase beta-2
DnaJ homolog subfamily B member 11
E3 ubiquitin-protein ligase RNF14
SUMO-activating enzyme subunit 2
Mortality factor 4-like protein 1
Nuclear RNA export factor 1
Protein sel-1 homolog 1
Beta-1,4-galactosyltransferase 7
Peflin
Zinc finger MYM-type protein 2
COP9 signalosome complex subunit 7a
Mitochondrial dicarboxylate carrier
Alpha-aminoadipic semialdehyde synthase, mitochondrial
CAP-Gly domain-containing linker protein 2
Cytochrome b-c1 complex subunit 9
Mitochondrial fission process protein 1
Tight junction protein ZO-2
DnaJ homolog subfamily B member 4
Serine/threonine-protein kinase 17A
Craniofacial development protein 1
Death domain-associated protein 6
tRNA (cytidine(32)/guanosine(34)-2'-O)-methyltransferase
Vesicle transport through interaction with t-SNAREs homolog 1B
STE20/SPS1-related proline-alanine-rich protein kinase
Gamma-adducin
Leucine-rich repeat and WD repeat-containing protein 1
Protein NipSnap homolog 3A
CGG triplet repeat-binding protein 1
Phosphatidylserine decarboxylase proenzyme, mitochondrial
ATP-binding cassette sub-family F member 2
Testin
5'-AMP-activated protein kinase subunit gamma-2
Gamma-tubulin complex component 4
Lysine-specific demethylase 5B
Tryptophan--tRNA ligase, mitochondrial
LIM domain-containing protein 1

Translocation protein SEC63 homolog
Solute carrier family 2, facilitated glucose transporter member 6
Zinc finger CCCH domain-containing protein 7B
Protein NDRG3
Armadillo repeat-containing X-linked protein 3
Switch-associated protein 70
SUN domain-containing protein 2
Probable ribosome biogenesis protein RLP24
Ragulator complex protein LAMTOR3
LIM domain and actin-binding protein 1
AF4/FMR2 family member 4
Signal recognition particle subunit SRP68
Cysteine and histidine-rich domain-containing protein 1
Serine/threonine-protein kinase TBK1
Septin-9
Prenylcysteine oxidase 1
Probable ATP-dependent RNA helicase DDX20
Sedoheptulokinase
Dipeptidyl peptidase 2
Inactive cell surface hyaluronidase CEMIP2
Ubiquitin carboxyl-terminal hydrolase 25
B-cell receptor-associated protein 29
NADH-cytochrome b5 reductase 1
SAP30-binding protein
Zinc finger HIT domain-containing protein 2
Prefoldin subunit 2
GPN-loop GTPase 3
Poly(U)-binding-splicing factor PUF60
Nuclear receptor-binding protein
Enolase-phosphatase E1
Fasciculation and elongation protein zeta-2
NADH dehydrogenase [ubiquinone] 1 alpha subcomplex subunit 12
Translation initiation factor eIF2B subunit delta
V-type proton ATPase subunit H
Prenylated Rab acceptor protein 1
Importin-11
Multifunctional methyltransferase subunit TRM112-like protein
Dachshund homolog 1
Exportin-7
Leucine carboxyl methyltransferase 1
Vacuolar protein sorting-associated protein 51 homolog
Bromodomain adjacent to zinc finger domain protein 2A
Tyrosine-protein kinase BAZ1B
ATPase inhibitor, mitochondrial
GTP:AMP phosphotransferase AK3, mitochondrial
Leucyl-cystinyl aminopeptidase
CCR4-NOT transcription complex subunit 7
Rab5 GDP/GTP exchange factor
Mitochondrial peptide methionine sulfoxide reductase
N-acetyl-D-glucosamine kinase

2-hydroxyacyl-CoA lyase 1
tRNA (adenine(58)-N(1))-methyltransferase non-catalytic subunit TRM6
SH3 domain-binding glutamic acid-rich-like protein 2
Ras GTPase-activating protein nGAP
18S rRNA aminocarboxypropyltransferase
Electrogenic aspartate/glutamate antiporter SLC25A13, mitochondrial
Drebrin-like protein
Probable ATP-dependent RNA helicase DDX41
Dynactin subunit 4
Cell division cycle protein 23 homolog
Anaphase-promoting complex subunit 7
Anaphase-promoting complex subunit 5
Anaphase-promoting complex subunit 4
Anaphase-promoting complex subunit 2
ADP-ribosylation factor-binding protein GGA2
ADP-ribosylation factor-binding protein GGA1
Stomatin-like protein 2, mitochondrial
N-acetylglucosamine-1-phosphodiester alpha-N-acetylglucosaminidase
Vacuolar protein sorting-associated protein 28 homolog
U6 snRNA-associated Sm-like protein LSm7
Lariat debranching enzyme
Protein TASOR
Jupiter microtubule associated homolog 1
F-box only protein 3
A-kinase anchor protein 11
F-box/WD repeat-containing protein 11
Glucocorticoid modulatory element-binding protein 2
mRNA turnover protein 4 homolog
TRAF2 and NCK-interacting protein kinase
Cleavage and polyadenylation specificity factor subunit 3
DCC-interacting protein 13-alpha
Cdc42 effector protein 3
Serine/threonine-protein kinase tousled-like 1
G patch domain-containing protein 8
Protein mono-ADP-ribosyltransferase PARP4
NTF2-related export protein 1
ADP-sugar pyrophosphatase
REST corepressor 1
Endoplasmic reticulum mannosyl-oligosaccharide 1,2-alpha-mannosidase
RNA-binding protein Raly
General transcription factor 3C polypeptide 4
Ergosterol biosynthetic protein 28 homolog
Protein kinase C and casein kinase substrate in neurons protein 3
Isobutyryl-CoA dehydrogenase, mitochondrial
Angiopoietin-related protein 2
Apoptotic chromatin condensation inducer in the nucleus
E3 ubiquitin-protein ligase AMFR
Protein argonaute-2
Nuclear pore complex protein Nup50
Protein CDV3 homolog

CCR4-NOT transcription complex subunit 11
Integrator complex subunit 6
BAG family molecular chaperone regulator 5
Protein argonaute-1
Ras-related protein Rab-21
Ras-related protein Rab-22A
Trafficking protein particle complex subunit 2-like protein
Zinc finger protein 346
Proteasome activator complex subunit 2
Serine/threonine-protein kinase TAO2
Muskelin
Aspartyl aminopeptidase
Ras-related protein Rab-23
Malignant T-cell-amplified sequence 1
Microtubule-associated tumor suppressor 1
Protein WWC3
Paladin
Zinc transporter ZIP10
Chromatin-remodeling ATPase INO80
Cell cycle progression protein 1
Kinase D-interacting substrate of 220 kDa
Arf-GAP with SH3 domain, ANK repeat and PH domain-containing protein 1
Myocardin-related transcription factor B
Ankyrin repeat domain-containing protein 50
Mediator of RNA polymerase II transcription subunit 23
Protein Shroom4
YEATS domain-containing protein 2
Protein NDRG4
TBC1 domain family member 24
Pre-mRNA-splicing factor ISY1 homolog
Transmembrane and coiled-coil domain protein 3
E3 ubiquitin-protein ligase HECTD1
MYND-type zinc finger-containing chromatin reader ZMYND8
Cip1-interacting zinc finger protein
Coronin-1C
Targeting protein for Xklp2
Activator of basal transcription 1
RNA-binding protein NOB1
A-kinase anchor protein 8-like
Transcription factor MafF
Apoptosis-associated speck-like protein containing a CARD
Calcium load-activated calcium channel
Fizzy-related protein homolog
Anaphase-promoting complex subunit 10
Unconventional myosin-VI
ATP-dependent RNA helicase DDX19B
Lysosomal thioesterase PPT2
Pre-mRNA-processing factor 19
Suppressor of fused homolog
Bcl-2-related ovarian killer protein

Nucleolar protein 7
Sorting nexin-12
Synergin gamma
Vacuolar protein sorting-associated protein 4A
Ras GTPase-activating protein-binding protein 2
E3 ubiquitin-protein ligase CHIP
Protein kinase C and casein kinase substrate in neurons protein 2
Melanoma-associated antigen D2
Sorting nexin-7
Sorting nexin-6
Dual specificity protein phosphatase 12
Syntaxin-8
Translocon-associated protein subunit gamma
26S proteasome non-ATPase regulatory subunit 13
FAS-associated factor 1
Endothelial protein C receptor
Peptidyl-prolyl cis-trans isomerase E
Probable dimethyladenosine transferase
Protein timeless homolog
COP9 signalosome complex subunit 3
Multiple inositol polyphosphate phosphatase 1
RNA-binding protein Nova-2
WD repeat-containing protein 3
Transcription termination factor 2
NSFL1 cofactor p47
Conserved oligomeric Golgi complex subunit 5
Solute carrier family 12 member 4
AP-4 complex subunit epsilon-1
Microtubule-actin cross-linking factor 1, isoforms 1/2/3/4/5
Centrosomal protein of 131 kDa
SR-related and CTD-associated factor 8
Serine/threonine-protein phosphatase 6 regulatory subunit 1
E3 ubiquitin-protein ligase TRIM33
Histone lysine demethylase PHF8
Trinucleotide repeat-containing gene 6B protein
Inactive phospholipase C-like protein 2
Nonsense-mediated mRNA decay factor SMG5
Exocyst complex component 7
Zinc finger CCCH domain-containing protein 4
Ubiquitin carboxyl-terminal hydrolase 24
TBC1 domain family member 2B
Protein Smaug homolog 1
Forkhead box protein J3
Cytosolic carboxypeptidase 1
Endoribonuclease Dicer
Cystine/glutamate transporter
Microtubule-associated protein RP/EB family member 3
Leucine-rich repeat protein SHOC-2
Serine/arginine repetitive matrix protein 2
Proliferation-associated protein 2G4

Paraplegin
Brain-specific angiogenesis inhibitor 1-associated protein 2
GRB2-associated-binding protein 2
Structural maintenance of chromosomes protein 3
Charged multivesicular body protein 2b
SH2B adapter protein 3
Zinc finger protein 148
Myotubularin-related protein 6
Protein jagged-2
60S ribosome subunit biogenesis protein NIP7 homolog
Bifunctional UDP-N-acetylglucosamine 2-epimerase/N-acetylmannosamine kinase
RNA transcription, translation and transport factor protein
RuvB-like 2
Chromodomain Y-like protein
Peptidyl-prolyl cis-trans isomerase NIMA-interacting 4
RAC-gamma serine/threonine-protein kinase
Choline/ethanolamine kinase
Eukaryotic translation initiation factor 3 subunit L
Phospholipase A-2-activating protein
RuvB-like 1
Nuclear migration protein nudC
Mitochondrial chaperone BCS1
Voltage-dependent anion-selective channel protein 3
Cofilin-2
Endoplasmic reticulum-Golgi intermediate compartment protein 3
Phenylalanine--tRNA ligase alpha subunit
Sodium-dependent multivitamin transporter
Small ribosomal subunit protein mS33
Histone chaperone ASF1A
Developmentally-regulated GTP-binding protein 1
Trafficking protein particle complex subunit 4
Nck-associated protein 1
Protein canopy homolog 2
Nuclease EXOG, mitochondrial
Exocyst complex component 6B
PALM2-AKAP2 fusion protein
Caspase recruitment domain-containing protein 8
Phospholipid-transporting ATPase IF
GDP-fucose protein O-fucosyltransferase 2
DnaJ homolog subfamily C member 16
Disks large-associated protein 4
Serine/threonine-protein kinase 38-like
Phosphatidylinositide phosphatase SAC2
Fibronectin type-III domain-containing protein 3A
Nischarin
1-phosphatidylinositol 3-phosphate 5-kinase
WD repeat-containing protein 37
Band 4.1-like protein 3
Angiomotin-like protein 2
Serine/threonine-protein kinase SIK3

Lysine-specific demethylase 2A
Exosome complex exonuclease RRP44
Trafficking protein particle complex subunit 8
Leucine-rich repeat and calponin homology domain-containing protein 1
RNA 3'-terminal phosphate cyclase-like protein
Glutathione S-transferase kappa 1
Ragulator complex protein LAMTOR2
Small ribosomal subunit protein bS1m
Cytochrome c oxidase assembly factor 3 homolog, mitochondrial
Probable ATP-dependent RNA helicase DDX52
Small ribosomal subunit protein uS17m
Small ribosomal subunit protein uS7m
Lambda-crystallin homolog
Translation machinery-associated protein 7
Polymerase delta-interacting protein 2
AP-3 complex subunit mu-1
Inner nuclear membrane protein Man1
Calcium-regulated heat-stable protein 1
Conserved oligomeric Golgi complex subunit 6
Thyroid hormone receptor-associated protein 3
WW domain-binding protein 11
Tudor and KH domain-containing protein
Mediator of RNA polymerase II transcription subunit 16
Nucleolar protein 58
ARF GTPase-activating protein GIT1
Zinc finger protein 281
ADP-ribosylation factor-like protein 2-binding protein
Transmembrane protein 98
Protein SGT1 homolog
Tyrosine--tRNA ligase, mitochondrial
N-acetylglucosamine-6-phosphate deacetylase
Acyl-coenzyme A thioesterase 9, mitochondrial
Protein AAR2 homolog
Nitric oxide synthase-interacting protein
Deoxyribose-phosphate aldolase
Protein MEMO1
Thioredoxin-related transmembrane protein 2
rRNA-processing protein FCF1 homolog
U6 snRNA-associated Sm-like protein LSm2
Endophilin-B1
Complex I intermediate-associated protein 30, mitochondrial
Calcium-binding protein 39
Putative RNA-binding protein Luc7-like 2
Ubiquitin-conjugating enzyme E2 J1
RNA-binding motif protein, X-linked 2
Dehydrogenase/reductase SDR family member 7
Small ribosomal subunit protein uS2m
Probable U3 small nucleolar RNA-associated protein 11
MOB-like protein phocein
Ribosomal RNA-processing protein 7 homolog A

Ribosome maturation protein SBDS
Transmembrane emp24 domain-containing protein 5
Exosome complex component CSL4
Transmembrane emp24 domain-containing protein 7
Splicing factor 3B subunit 6
Large ribosomal subunit protein uL11m
Oligoribonuclease, mitochondrial
RRP15-like protein
WASH complex subunit 3
Nucleolar protein 16
EKC/KEOPS complex subunit TPRKB
Peptidyl-prolyl cis-trans isomerase-like 1
Ubiquitin-fold modifier-conjugating enzyme 1
Cytosolic iron-sulfur assembly component 2B
Small ribosomal subunit protein bS16m
Small ribosomal subunit protein bS18m
Mitochondrial fission 1 protein
Mitochondrial import inner membrane translocase subunit TIM16
Adenylate kinase isoenzyme 6
Small ribosomal subunit protein mS23
Vesicle transport protein GOT1B
Hepatoma-derived growth factor-related protein 3
Peptidyl-tRNA hydrolase 2, mitochondrial
Charged multivesicular body protein 3
Serine-threonine kinase receptor-associated protein
RNA-splicing ligase RtcB homolog
F-box only protein 7
Ras-related protein Rap-2c
StAR-related lipid transfer protein 13
Rab GTPase-activating protein 1
Transmembrane emp24 domain-containing protein 3
TSC22 domain family protein 4
Protein dopey-2
Zinc finger protein 330
R3H and coiled-coil domain-containing protein 1
Nucleolar complex protein 2 homolog
Large ribosomal subunit protein eL36
Coiled-coil domain-containing protein 9
Chromatin target of PRMT1 protein
Deoxynucleoside triphosphate triphosphohydrolase SAMHD1
Small kinetochore-associated protein
HBS1-like protein
5'-AMP-activated protein kinase subunit beta-1
DmX-like protein 1
V-type proton ATPase 116 kDa subunit a 2
Talin-1
Kinesin-like protein KIF3A
Transformation/transcription domain-associated protein
Microtubule cross-linking factor 1
DDB1- and CUL4-associated factor 1

TRPM8 channel-associated factor 1
Probable RNA-binding protein 19
Disheveled-associated activator of morphogenesis 1
Plexin-D1
Probable E3 ubiquitin-protein ligase HECTD4
WASH complex subunit 2C
WD repeat-containing protein 7
Ubiquitin carboxyl-terminal hydrolase 15
FERM, ARHGEF and pleckstrin domain-containing protein 1
Meiosis regulator and mRNA stability factor 1
Centrosomal protein of 170 kDa protein B
Rap guanine nucleotide exchange factor 2
Unconventional myosin-Va
Dystrobrevin alpha
Lysyl oxidase homolog 2
Beta/gamma crystallin domain-containing protein 1
TNF receptor-associated factor 6
Mitogen-activated protein kinase kinase kinase kinase 5
Hypoxia up-regulated protein 1
Cysteine protease ATG4B
Transducin beta-like protein 2
WD repeat domain phosphoinositide-interacting protein 2
Telomere length regulation protein TEL2 homolog
Ribosomal biogenesis protein LAS1L
AFG3-like protein 2
Krueppel-like factor 12
E3 ubiquitin-protein ligase ARIH1
U6 snRNA-associated Sm-like protein LSm4
E3 ubiquitin-protein ligase RNF114
Sorting and assembly machinery component 50 homolog
Protein PRRC2C
ADP-ribose glycohydrolase OARD1
Intraflagellar transport protein 25 homolog
Protein phosphatase methylesterase 1
RNA-binding protein 7
Mitochondrial import inner membrane translocase subunit Tim22
NEDD8 ultimate buster 1
YTH domain-containing family protein 2
RNA polymerase II subunit A C-terminal domain phosphatase
PAX3- and PAX7-binding protein 1
Nucleoside diphosphate kinase 7
FACT complex subunit SPT16
U3 small nucleolar RNA-associated protein 18 homolog
Ubiquitin carboxyl-terminal hydrolase isozyme L5
CD2-associated protein
V-type proton ATPase subunit D
Transportin-3
Signal recognition particle receptor subunit beta
Ceramide transfer protein
Mannose-1-phosphate guanyltransferase beta

Fatty acid desaturase 3
General transcription factor 3C polypeptide 5
General transcription factor 3C polypeptide 3
Trafficking protein particle complex subunit 1
Transient receptor potential cation channel subfamily V member 2
Serine/threonine-protein kinase MRCK beta
RNA-binding protein 8A
DnaJ homolog subfamily C member 15
Ubiquitin carboxyl-terminal hydrolase 16
U5 small nuclear ribonucleoprotein TSSC4
Zinc finger protein 706
Melanoma-associated antigen D1
Sorting nexin-14
Sorting nexin-11
Sorting nexin-9
Sorting nexin-8
Sorting nexin-5
Endothelial lipase
Cytosolic Fe-S cluster assembly factor NUBP2
Peroxisomal membrane protein PEX16
Lymphatic vessel endothelial hyaluronic acid receptor 1
Beta-secretase 2
Heme-binding protein 2
Host cell factor 2
UbiA prenyltransferase domain-containing protein 1
Pseudouridylate synthase 1 homolog
Leucine-rich repeat flightless-interacting protein 2
FH1/FH2 domain-containing protein 1
Phosphoserine aminotransferase
Nuclear receptor corepressor 2
Mitochondrial ornithine transporter 1
Junctional adhesion molecule A
Neuroplastin
Adhesion G-protein coupled receptor G1
Spindlin-1
Solute carrier family 12 member 7
Dolichyl pyrophosphate Man9GlcNAc2 alpha-1,3-glucosyltransferase
Dolichyl-phosphate beta-glucosyltransferase
Small ribosomal subunit protein mS40
Coatomer subunit gamma-1
Lipid droplet-regulating VLDL assembly factor AUP1
Peptidyl-prolyl cis-trans isomerase FKBP7
Chloride intracellular channel protein 4
Cysteine desulfurase
Cilia- and flagella-associated protein 20
Signal peptidase complex subunit 1
GTP-binding protein SAR1b
AP-4 complex subunit beta-1
Mitochondrial carrier homolog 2
Selenoprotein K

Brefeldin A-inhibited guanine nucleotide-exchange protein 2
Brefeldin A-inhibited guanine nucleotide-exchange protein 1
Mitotic spindle assembly checkpoint protein MAD1
Serine/threonine-protein kinase 24
eIF5-mimic protein 1
COMM domain-containing protein 10
Cytoplasmic dynein 1 light intermediate chain 1
Epsin-1
Ubiquitin carboxyl-terminal hydrolase 3
Testis-expressed protein 264
Calcineurin-binding protein cabin-1
Choline/ethanolaminephosphotransferase 1
DNA (cytosine-5)-methyltransferase 3A
2'-5'-oligoadenylate synthase 3
Adenylate kinase isoenzyme 5
NF-kappa-B essential modulator
Insulin-like growth factor 2 mRNA-binding protein 2
Proton-coupled zinc antiporter SLC30A1
Sodium bicarbonate cotransporter 3
NADH dehydrogenase [ubiquinone] 1 beta subcomplex subunit 9
Sulfide:quinone oxidoreductase, mitochondrial
Roundabout homolog 1
Stonin-1
Nuclear receptor coactivator 3
Numb-like protein
Mitogen-activated protein kinase kinase kinase 4
Probable ATP-dependent RNA helicase DDX49
Calpain-7
Actin-binding protein WASF2
E3 SUMO-protein ligase PIAS3
MAU2 chromatid cohesion factor homolog
ATPase MORC2
Influenza virus NS1A-binding protein
SEC23-interacting protein
Putative glutamine amidotransferase-like class 1 domain-containing protein 3B, mitochondrial
Intraflagellar transport protein 56
Ubiquitin-like modifier-activating enzyme 6
Extended synaptotagmin-2
Bridge-like lipid transfer protein family member 3B
Shootin-1
Transmembrane protein 223
Rho GTPase-activating protein 10
2-hydroxyacyl-CoA lyase 2
SH3 and PX domain-containing protein 2B
NBAS subunit of NRZ tethering complex
tRNA wybutosine-synthesizing protein 5
DENN domain-containing protein 3
von Willebrand factor A domain-containing protein 8
Protein strawberry notch homolog 1
GTP-binding protein 10

WD repeat-containing protein 91
DENN domain-containing protein 11
Trafficking protein particle complex subunit 13
CCR4-NOT transcription complex subunit 1
Microtubule-associated proteins 1A/1B light chain 3 beta 2
Glycerol-3-phosphate phosphatase
RAB7A-interacting MON1-CCZ1 complex subunit 1
RCC1 domain-containing protein 1
TRPM8 channel-associated factor 2
Structural maintenance of chromosomes flexible hinge domain-containing protein 1
Cytosolic arginine sensor for mTORC1 subunit 2
Protein unc-119 homolog B
12S rRNA N4-methylcytidine (m4C) methyltransferase
Coiled-coil domain-containing protein 85C
Retrotransposon Gag-like protein 8C
Zinc finger SWIM domain-containing protein 8
Nuclear envelope pore membrane protein POM 121C
PHD finger protein 20-like protein 1
Putative small nuclear ribonucleoprotein G-like protein 15
Endosome/lysosome-associated apoptosis and autophagy regulator family member 2
Acyl-coenzyme A diphosphatase NUDT19
MIF4G domain-containing protein
Calmodulin-1
Unconventional myosin-IXa
Golgi pH regulator A
Putative WAS protein family homolog 3
Mapk-regulated corepressor-interacting protein 1
Nascent polypeptide-associated complex subunit alpha, muscle-specific form
Uncharacterized protein C11orf98
ASNSD1 upstream open reading frame protein
Mitochondrial ribosome and complex I assembly factor AltMIEF1
Deoxyribonuclease-2-alpha
Alkyldihydroxyacetonephosphate synthase, peroxisomal
Kinesin-like protein KIF2A
ATP-dependent RNA helicase DDX39A
PDZ and LIM domain protein 1
Cytosolic acyl coenzyme A thioester hydrolase
Unconventional myosin-Ic
Synaptosomal-associated protein 23
HCLS1-associated protein X-1
AH receptor-interacting protein
GTP-binding protein 1
Galectin-9
Syntaxin-binding protein 3
AP-4 complex subunit mu-1
Splicing regulator ARVCF
Small acidic protein
AP-3 complex subunit beta-1
Amyloid beta precursor protein binding family B member 1
Galectin-8

NADH dehydrogenase [ubiquinone] iron-sulfur protein 8, mitochondrial
NF-kappa-B inhibitor epsilon
26S proteasome non-ATPase regulatory subunit 11
26S proteasome non-ATPase regulatory subunit 12
26S proteasome non-ATPase regulatory subunit 9
Menin
Guided entry of tail-anchored proteins factor 1
Membrane-associated progesterone receptor component 1
Transcription elongation factor SPT5
Transcription initiation factor TFIID subunit 4
DNA fragmentation factor subunit alpha
Huntingtin-interacting protein 1
Chloride intracellular channel protein 1
Eukaryotic translation initiation factor 3 subunit F
NEDD4-like E3 ubiquitin-protein ligase WWP2
Pyruvate dehydrogenase protein X component, mitochondrial
Sulfhydryl oxidase 1
Dynactin subunit 6
Acetyl-coenzyme A transporter 1
Actin nucleation-promoting factor WASL
Importin-5
DNA-directed RNA polymerase, mitochondrial
Eukaryotic elongation factor 2 kinase
Histone deacetylase complex subunit SAP18
Echinoderm microtubule-associated protein-like 1
Insulin-like growth factor 2 mRNA-binding protein 3
Dynamin-1-like protein
RNA 3'-terminal phosphate cyclase
Phosphatidylinositol 4-phosphate 3-kinase C2 domain-containing subunit alpha
Golgi integral membrane protein 4
Agrin
Procollagen-lysine,2-oxoglutarate 5-dioxygenase 2
Exocyst complex component 5
Butyrophilin subfamily 3 member A1
Cytochrome c oxidase subunit NDUFA4
26S proteasome non-ATPase regulatory subunit 14
Myc box-dependent-interacting protein 1
Claudin-5
Importin subunit alpha-4
Serine/threonine-protein kinase 25
Krev interaction trapped protein 1
von Willebrand factor A domain-containing protein 5A
Pescadillo homolog
Syntenin-1
Membrane-associated phosphatidylinositol transfer protein 1
U3 small nucleolar ribonucleoprotein protein MPP10
Nucleolar protein 56
ATP-dependent RNA helicase DDX3X
Beta-1,3-N-acetylglucosaminyltransferase manic fringe
Podocalyxin

CCN family member 1
Pirin
Importin subunit alpha-3
E3 ubiquitin-protein ligase TRIM38
Nuclear factor 1 B-type
Serine/threonine-protein phosphatase 6 catalytic subunit
Nucleoside diphosphate kinase, mitochondrial
Phosphatidylinositol 4-phosphate 3-kinase C2 domain-containing subunit beta
Lysosomal alpha-mannosidase
Pyridoxal kinase
Stearoyl-CoA desaturase
AT-rich interactive domain-containing protein 1A
Nuclear envelope integral membrane protein 1
Thioredoxin domain-containing protein 9
Dihydropyrimidinase-related protein 4
TRAF-type zinc finger domain-containing protein 1
Cytochrome c oxidase subunit 7A-related protein, mitochondrial
Ubiquitin domain-containing protein UBFD1
Citron Rho-interacting kinase
Coatomer subunit epsilon
Cdc42 effector protein 2
AP-3 complex subunit delta-1
Copper chaperone for superoxide dismutase
Actin-binding LIM protein 1
Segment polarity protein dishevelled homolog DVL-1
Segment polarity protein dishevelled homolog DVL-2
Chromodomain-helicase-DNA-binding protein 1
Chromodomain-helicase-DNA-binding protein 2
Golgi SNAP receptor complex member 2
Torsin-1A
Torsin-1B
Syntaxin-16
Disintegrin and metalloproteinase domain-containing protein 10
Lysosomal cobalamin transporter ABCD4
Etoposide-induced protein 2.4 homolog
Tumor protein p53-inducible protein 11
Histone-lysine N-methyltransferase 2D
Apoptotic protease-activating factor 1
Serine/threonine-protein kinase RIO3
Dual specificity mitogen-activated protein kinase kinase 7
Acyl-coenzyme A thioesterase 8
CDP-diacylglycerol--inositol 3-phosphatidyltransferase
Programmed cell death protein 5
Protein arginine N-methyltransferase 5
Na(+)/H(+) exchange regulatory cofactor NHE-RF1
Serine/threonine-protein kinase Chk1
Tumor necrosis factor receptor superfamily member 10B
Tripeptidyl-peptidase 1
Transcription elongation regulator 1
Kinetochore protein NDC80 homolog

Kinesin-like protein KIF3C
Neuropilin-1
Transportin-2
Protein unc-13 homolog B
Tumor necrosis factor receptor superfamily member 10C
DNA-directed RNA polymerase III subunit RPC1
Tetraspanin-4
Proteasome subunit alpha type-7
Secretory carrier-associated membrane protein 3
Branched-chain alpha-ketoacid dehydrogenase kinase
Glutathione S-transferase 3, mitochondrial
Tax1-binding protein 3
PDZ domain-containing protein GIPC1
Inhibitor of nuclear factor kappa-B kinase subunit beta
Regulator of G-protein signaling 12
Mitochondrial import inner membrane translocase subunit Tim23
Histone acetyltransferase type B catalytic subunit
Peripheral plasma membrane protein CASK
Phospholipase D2
Cytochrome b-c1 complex subunit 8
Myosin regulatory light chain 12B
Hepatocyte growth factor-regulated tyrosine kinase substrate
Ras-related protein Rab-7L1
Vacuolar protein sorting-associated protein 26C
Protein phosphatase 1 regulatory subunit 12A
Cyclin-G-associated kinase
Heterogeneous nuclear ribonucleoprotein D-like
Exportin-1
TATA-binding protein-associated factor 172
Rho guanine nucleotide exchange factor 10
Zinc finger protein 609
Spectrin beta chain, non-erythrocytic 2
Microtubule-associated serine/threonine-protein kinase 4
Protein transport protein Sec16A
Plexin-B2
Protein KHNYN
Tectonin beta-propeller repeat-containing protein 2
U2 snRNP-associated SURP motif-containing protein
Histone-lysine N-methyltransferase SETD1A
NEDD4-binding protein 3
Synaptojanin-2
Synemin
Granule associated Rac and RHOG effector protein 1
Kinesin-like protein KIF3B
Phosphoribosylformylglycinamidine synthase
Serine/threonine-protein phosphatase 6 regulatory ankyrin repeat subunit A
Rho guanine nucleotide exchange factor 11
Mitochondrial ribonuclease P catalytic subunit
Inhibitor of nuclear factor kappa-B kinase subunit alpha
NPC intracellular cholesterol transporter 1

Sphingolipid delta(4)-desaturase DES1
Angiopoietin-2
Secretory carrier-associated membrane protein 1
Secretory carrier-associated membrane protein 2
Actin-related protein 2/3 complex subunit 1B
Actin-related protein 2/3 complex subunit 2
Actin-related protein 2/3 complex subunit 3
DNA-directed RNA polymerases I and III subunit RPAC1
Phospholipid scramblase 1
Transcription intermediary factor 1-alpha
Membrane-associated progesterone receptor component 2
Ral guanine nucleotide dissociation stimulator-like 2
WD repeat-containing protein 46
NF-kappa-B-repressing factor
Dihydroxyacetone phosphate acyltransferase
Laminin subunit alpha-5
Zinc finger protein 185
Protein CASC3
Small ribosomal subunit protein uS12m
Peroxisomal acyl-coenzyme A oxidase 3
Protein RER1
Surfeit locus protein 4
Mitogen-activated protein kinase 13
Serine palmitoyltransferase 1
Serine palmitoyltransferase 2
UDP-N-acetylglucosamine--peptide N-acetylglucosaminyltransferase 110 kDa subunit
Phosphomannomutase 2
Transmembrane 9 superfamily member 1
E3 ubiquitin-protein ligase Midline-1
High mobility group protein B3
Protein phosphatase 1G
Phosphatidylinositol 3,4,5-trisphosphate 5-phosphatase 2
Eukaryotic translation initiation factor 3 subunit D
Eukaryotic translation initiation factor 3 subunit H
Histone deacetylase 3
Nuclear valosin-containing protein-like
Branched-chain-amino-acid aminotransferase, mitochondrial
Importin-8
Syntaxin-7
Monocarboxylate transporter 4
ATP-binding cassette sub-family C member 4
DNA-directed RNA polymerase I subunit RPA34
Prolyl 4-hydroxylase subunit alpha-2
Calpain-5
Synaptobrevin homolog YKT6
Nucleoporin NUP42
Actin-related protein 2/3 complex subunit 5
CASP8 and FADD-like apoptosis regulator
Transcription factor MafG
3-phosphoinositide-dependent protein kinase 1

Tapasin
E3 ubiquitin-protein ligase RNF113A
Lysine-specific demethylase 6A
ATP-dependent RNA helicase DHX15
mRNA cap guanine-N7 methyltransferase
Zinc finger ZZ-type and EF-hand domain-containing protein 1
Arf-GAP with SH3 domain, ANK repeat and PH domain-containing protein 2
Leucine-rich repeat transmembrane protein FLRT2
TELO2-interacting protein 1 homolog
Ribosomal RNA-processing protein 8
E3 ubiquitin-protein ligase Praja-2
Cytochrome b5 type B
U4/U6 small nuclear ribonucleoprotein Prp4
D-3-phosphoglycerate dehydrogenase
NADH dehydrogenase [ubiquinone] iron-sulfur protein 4, mitochondrial
Cytoplasmic dynein 1 light intermediate chain 2
26S proteasome non-ATPase regulatory subunit 3
RNA binding protein fox-1 homolog 2
Bifunctional 3'-phosphoadenosine 5'-phosphosulfate synthase 1
Centromere/kinetochore protein zw10 homolog
Beta-1,4-galactosyltransferase 5
U4/U6.U5 tri-snRNP-associated protein 1
Glycosylphosphatidylinositol anchor attachment 1 protein
Death-associated protein kinase 3
Transforming growth factor beta-1-induced transcript 1 protein
AP-5 complex subunit zeta-1
CBP80/20-dependent translation initiation factor
Protein MTSS 1
Inositol hexakisphosphate and diphosphoinositol-pentakisphosphate kinase 2
Mitogen-activated protein kinase kinase kinase 7
Eukaryotic translation elongation factor 1 epsilon-1
Receptor-interacting serine/threonine-protein kinase 2
Heterogeneous nuclear ribonucleoprotein R
U4/U6 small nuclear ribonucleoprotein Prp3
Thioredoxin-like protein 1
Tumor protein D54
ER membrane protein complex subunit 8
ERI1 exoribonuclease 3
Synaptojanin-1
Acidic fibroblast growth factor intracellular-binding protein
Eukaryotic translation initiation factor 4 gamma 3
Peptidyl-prolyl cis-trans isomerase H
Serine protease HTRA2, mitochondrial
Protein Mis18-beta
Aflatoxin B1 aldehyde reductase member 2
Band 4.1-like protein 2
Trans-Golgi network integral membrane protein 2
Beta-1,4-glucuronyltransferase 1
WAS/WASL-interacting protein family member 1
Phospholipid-transporting ATPase IC

A-kinase anchor protein 10, mitochondrial
Density-regulated protein
Exportin-T
5-hydroxymethyl-dUMP N-hydrolase
Mitochondrial import inner membrane translocase subunit TIM44
Trafficking protein particle complex subunit 3
Charged multivesicular body protein 2a
Cytoplasmic protein NCK2
Tetraspanin-6
Pleiotropic regulator 1
Protein regulator of cytokinesis 1
Regulator of G-protein signaling 10
BUB3-interacting and GLEBS motif-containing protein ZNF207
NADH dehydrogenase [ubiquinone] 1 beta subcomplex subunit 5, mitochondrial
NADH dehydrogenase [ubiquinone] 1 beta subcomplex subunit 3
NADH dehydrogenase [ubiquinone] 1 alpha subcomplex subunit 2
LIM domain-binding protein 2
ATPase GET3
Mitotic checkpoint protein BUB3
Sulfotransferase 1B1
Alpha-actinin-4
Probable 18S rRNA (guanine-N(7))-methyltransferase
17S U2 SnRNP complex component HTATSF1
ER lumen protein-retaining receptor 3
AP-1 complex subunit gamma-1
Syntaxin-6
Synaptogyrin-2
Small glutamine-rich tetratricopeptide repeat-containing protein alpha
Alpha-endosulfine
Mitochondrial carnitine/acylcarnitine carrier protein
Asparagine--tRNA ligase, cytoplasmic
Unconventional myosin-Ib
Peroxisomal membrane protein PMP34
Cleavage and polyadenylation specificity factor subunit 5
Glutathione S-transferase LANCL1
Striatin
U3 small nucleolar RNA-interacting protein 2
Protein SCO2 homolog, mitochondrial
Cilia- and flagella-associated protein 410
A-kinase anchor protein 8
Putative GTP-binding protein 6
Glucose-6-phosphate exchanger SLC37A4
Isocitrate dehydrogenase [NAD] subunit beta, mitochondrial
Nardilysin
Calumenin
EGF-like repeat and discoidin I-like domain-containing protein 3
Probable phospholipid-transporting ATPase IIB
S-adenosylhomocysteine hydrolase-like protein 1
Kinesin-like protein KIF1C
Exostosin-like 3

Origin recognition complex subunit 5
NADH dehydrogenase [ubiquinone] iron-sulfur protein 5
Retinal rod rhodopsin-sensitive cGMP 3',5'-cyclic phosphodiesterase subunit delta
Origin recognition complex subunit 4
Peroxisomal ATPase PEX1
Double-strand-break repair protein rad21 homolog
Pre-mRNA-splicing factor ATP-dependent RNA helicase DHX16
Glia maturation factor gamma
SH3 domain-binding protein 5
Adhesion G protein-coupled receptor B3
Mediator of RNA polymerase II transcription subunit 14
Protocadherin-7
Phosphoribosyl pyrophosphate synthase-associated protein 2
SWI/SNF-related matrix-associated actin-dependent regulator of chromatin subfamily A membe
C-Jun-amino-terminal kinase-interacting protein 4
NUAK family SNF1-like kinase 1
Nucleolar pre-ribosomal-associated protein 1
Signal-induced proliferation-associated 1-like protein 3
Zinc finger C3H1 domain-containing protein
RNA helicase aquarius
Dynamin-like 120 kDa protein, mitochondrial
Zinc finger E-box-binding homeobox 2
Germinal-center associated nuclear protein
Protocadherin gamma-A12
Phosphatidylinositol 4-phosphate 5-kinase type-1 gamma
Kinesin-like protein KIF1B
Mitogen-activated protein kinase-binding protein 1
Lysine-specific histone demethylase 1A
TBC1 domain family member 4
Acyl-CoA (8-3)-desaturase
Gasdermin-E
Ecotropic viral integration site 5 protein homolog
Neuropilin-2
Mannosyl-oligosaccharide 1,2-alpha-mannosidase IB
Myelin protein zero-like protein 2
Long-chain-fatty-acid--CoA ligase 4
Sorting nexin-3
Protein O-GlcNAcase
Vinexin
Heterogeneous nuclear ribonucleoprotein Q
Pre-mRNA-processing factor 17
Ran-binding protein 6
Ribosome quality control complex subunit NEMF
GDP-mannose 4,6 dehydratase
Glycylpeptide N-tetradecanoyltransferase 2
Cyclin-T1
Mitotic checkpoint serine/threonine-protein kinase BUB1 beta
Multifunctional procollagen lysine hydroxylase and glycosyltransferase LH3
Eukaryotic translation initiation factor 4E type 2
Protein diaphanous homolog 1

Tetraspanin-3
Exocyst complex component 3
Perilipin-3
Protein arginine N-methyltransferase 3
Importin subunit alpha-7
UDP-glucose 6-dehydrogenase
Protein-tyrosine sulfotransferase 2
Leupaxin
Catenin delta-1
Protein-S-isoprenylcysteine O-methyltransferase
Eukaryotic translation initiation factor 1b
Sorting nexin-2
Dolichol-phosphate mannosyltransferase subunit 1
General vesicular transport factor p115
Small ribosomal subunit protein uS14m
Target of Myb1 membrane trafficking protein
Histone H2B type 1-K
Coiled-coil domain-containing protein 22
Polyglutamine-binding protein 1
Mitochondrial import inner membrane translocase subunit Tim17-B
PRA1 family protein 2
H/ACA ribonucleoprotein complex subunit DKC1
Eukaryotic translation initiation factor 5B
Endothelial differentiation-related factor 1
DNA/RNA-binding protein KIN17
Protein diaphanous homolog 2
DnaJ homolog subfamily A member 2
Bromodomain-containing protein 4
Checkpoint protein HUS1
Prefoldin subunit 1
E3 ubiquitin-protein ligase PPP1R11
Nibrin
mRNA-capping enzyme
Iron-sulfur clusters transporter ABCB7, mitochondrial
SLIT-ROBO Rho GTPase-activating protein 2
Plexin-A2
Glycosaminoglycan xylosylkinase
WD repeat-containing protein 1
Probable phospholipid-transporting ATPase IIA
NEDD4-binding protein 1
Rho-associated protein kinase 2
CLIP-associating protein 2
Pentatricopeptide repeat-containing protein 1, mitochondrial
Copine-3
Autophagy-related protein 13
Huntingtin-interacting protein 1-related protein
E3 ubiquitin-protein ligase BRE1B
Lysine-specific demethylase PHF2
Zinc finger CCCH domain-containing protein 11A
Clustered mitochondria protein homolog

Cullin-associated NEDD8-dissociated protein 2
TSC22 domain family protein 2
DnaJ homolog subfamily C member 13
Phosphatase and actin regulator 2
Serine/threonine-protein phosphatase 6 regulatory subunit 2
A disintegrin and metalloproteinase with thrombospondin motifs 4
CCR4-NOT transcription complex subunit 3
Ankyrin repeat domain-containing protein 17
Paired amphipathic helix protein Sin3b
DnaJ homolog subfamily B member 6
Ubiquinone biosynthesis protein COQ9, mitochondrial
Gamma-glutamylcyclotransferase
NADH dehydrogenase [ubiquinone] iron-sulfur protein 7, mitochondrial
NADH dehydrogenase [ubiquinone] iron-sulfur protein 2, mitochondrial
Zinc finger protein ZPR1
RNA/RNP complex-1-interacting phosphatase
Protein NipSnap homolog 2
Hyaluronan mediated motility receptor
Programmed cell death protein 6
Tubulin-specific chaperone A
V-type proton ATPase subunit G 1
Vacuolar protein sorting-associated protein 4B
Mannose-P-dolichol utilization defect 1 protein
Core histone macro-H2A.1
Adapter SH3BGRL
Filamin-B
Nuclear receptor corepressor 1
Peroxisomal membrane protein PEX14
Tripartite motif-containing protein 3
Citrate synthase, mitochondrial
Sperm-associated antigen 7
Vesicle-trafficking protein SEC22b
Pre-mRNA-processing factor 40 homolog A
Transforming acidic coiled-coil-containing protein 1
Nucleoside diphosphate kinase 6
Cell division control protein 45 homolog
Leucine-rich repeat and calponin homology domain-containing protein 4
Metaxin-2
Vacuolar protein sorting-associated protein 26A
Mitochondrial-processing peptidase subunit beta
Mediator of RNA polymerase II transcription subunit 24
Katanin p60 ATPase-containing subunit A1
PC4 and SFRS1-interacting protein
Erlin-1
NADH dehydrogenase [ubiquinone] iron-sulfur protein 3, mitochondrial
Serine/arginine-rich splicing factor 10
Ceroid-lipofuscinosis neuronal protein 5
Claudin-11
Enoyl-CoA delta isomerase 2
KH domain-containing, RNA-binding, signal transduction-associated protein 3

Polycomb protein EED
Barrier-to-autointegration factor
Splicing factor 3B subunit 1
Cold shock domain-containing protein E1
WW domain-binding protein 4
Interferon-inducible double-stranded RNA-dependent protein kinase activator A
Integrin alpha-10
Ribosomal protein S6 kinase alpha-5
Mediator of RNA polymerase II transcription subunit 6
E3 ubiquitin-protein ligase MYCBP2
2-amino-3-ketobutyrate coenzyme A ligase, mitochondrial
Nucleoplasmin-3
Acyl-protein thioesterase 1
GTPase Era, mitochondrial
U5 small nuclear ribonucleoprotein 200 kDa helicase
Mitochondrial tRNA-specific 2-thiouridylase 1
TIP41-like protein
Centriole and centriolar satellite protein OFD1
Ribosomal protein S6 kinase alpha-4
Surfeit locus protein 6
Protein phosphatase 1B
Small subunit processome component 20 homolog
Nuclear pore complex protein Nup155
Protein XRP2
WD repeat and HMG-box DNA-binding protein 1
Cartilage-associated protein
Electrogenic aspartate/glutamate antiporter SLC25A12, mitochondrial
Renin receptor
Ribonuclease H2 subunit A
Translation initiation factor eIF2 assembly protein
Breast cancer anti-estrogen resistance protein 3
Ribonuclease P protein subunit p20
Ribonuclease P protein subunit p40
Eukaryotic translation initiation factor 3 subunit G
Eukaryotic translation initiation factor 3 subunit J
Carbonyl reductase [NADPH] 3
26S proteasome non-ATPase regulatory subunit 10
AP-1 complex subunit gamma-like 2
CAAX prenyl protease 1 homolog
Isocitrate dehydrogenase [NADP] cytoplasmic
Glutamyl-tRNA(Gln) amidotransferase subunit B, mitochondrial
Protein SCO1 homolog, mitochondrial
Attractin
Serine hydrolase RBBP9
Signal transducing adapter molecule 2
Diacylglycerol O-acyltransferase 1
Cyclin-K
PRA1 family protein 3
Dysferlin
E3 SUMO-protein ligase PIAS1

Pre-mRNA-splicing factor SPF27
Dynactin subunit 3
DnaJ homolog subfamily C member 8
Survival of motor neuron-related-splicing factor 30
Cell cycle checkpoint protein RAD17
ATP synthase subunit d, mitochondrial
Tetraspanin-9
Flotillin-1
Triple functional domain protein
ATP synthase subunit g, mitochondrial
Multiple PDZ domain protein
Carboxypeptidase D
Glutaredoxin-3
Ribosomal L1 domain-containing protein 1
Wolframin
ATP-dependent Clp protease ATP-binding subunit clpX-like, mitochondrial
SEC14-like protein 2
Gamma-synuclein
Probable cytosolic iron-sulfur protein assembly protein CIAO1
Signal recognition particle subunit SRP72
N(G),N(G)-dimethylarginine dimethylaminohydrolase 1
ATP-dependent DNA helicase Q5
Unconventional prefoldin RPB5 interactor 1
Galactosylgalactosylxylosylprotein 3-beta-glucuronosyltransferase 3
Metastasis-associated protein MTA2
Retinal dehydrogenase 2
Serine/threonine-protein kinase 10
Serine/threonine-protein kinase D3
Glutamine--fructose-6-phosphate aminotransferase [isomerizing] 2
Slit homolog 2 protein
E3 ubiquitin-protein ligase listerin
Mitochondrial import receptor subunit TOM70
Pleckstrin homology domain-containing family G member 5
Importin-13
Phospholipase DDHD2
Unconventional myosin-Id
TOX high mobility group box family member 4
[F-actin]-monooxygenase MICAL2
Protein transport protein Sec24D
F-BAR and double SH3 domains protein 2
E3 UFM1-protein ligase 1
Sorbin and SH3 domain-containing protein 2
SAM and SH3 domain-containing protein 1
UBX domain-containing protein 7
Kelch-like protein 18
SUN domain-containing protein 1
Pyridoxal phosphate homeostasis protein
Erlin-2
Pre-mRNA-processing factor 6
Pre-mRNA cleavage complex 2 protein Pcf11

Protein furry homolog-like
Nuclear factor of activated T-cells 5
Endonuclease domain-containing 1 protein
D-glucuronyl C5-epimerase
Glutaminase kidney isoform, mitochondrial
HAUS augmin-like complex subunit 5
Actin-binding LIM protein 3
F-box only protein 21
Lysine-specific demethylase 4B
Microtubule cross-linking factor 2
Ubiquitin carboxyl-terminal hydrolase 19
WD repeat-containing protein 47
AP-2 complex subunit alpha-2
Protein transport protein Sec31A
Rho guanine nucleotide exchange factor 15
Protein HEXIM1
Ribonuclease P protein subunit p14
G2/mitotic-specific cyclin-B2
Protein YIF1A
E3 ubiquitin-protein ligase UBR5
Arf-GAP domain and FG repeat-containing protein 2
Serine protease 23
SR-related and CTD-associated factor 4
NADH dehydrogenase [ubiquinone] 1 beta subcomplex subunit 6
Mitofusin-2
Snurportin-1
Ubiquitin conjugation factor E4 B
Zinc finger protein-like 1
Elongator complex protein 1
NADH dehydrogenase [ubiquinone] 1 beta subcomplex subunit 4
NADH dehydrogenase [ubiquinone] 1 beta subcomplex subunit 8, mitochondrial
NADH dehydrogenase [ubiquinone] 1 alpha subcomplex subunit 7
Vesicle-associated membrane protein 5
Reticulon-3
Mitochondrial proton/calcium exchanger protein
Epsin-2
Zinc finger Ran-binding domain-containing protein 2
Sorting nexin-4
Luc7-like protein 3
Kinesin-like protein KIF20A
Apolipoprotein L3
Chromosome-associated kinesin KIF4A
Methyl-CpG-binding domain protein 4
Myotubularin-related protein 5
Golgi SNAP receptor complex member 1
Histone acetyltransferase KAT7
Arginyl-tRNA--protein transferase 1
Vesicle-associated membrane protein-associated protein B/C
SNARE-associated protein Snapin
Myelin protein zero-like protein 1

NADH dehydrogenase [ubiquinone] 1 subunit C2
NADH dehydrogenase [ubiquinone] 1 alpha subcomplex subunit 10, mitochondrial
Peptidyl-prolyl cis-trans isomerase FKBP9
CUGBP Elav-like family member 2
6-phosphogluconolactonase
Bifunctional 3'-phosphoadenosine 5'-phosphosulfate synthase 2
Structural maintenance of chromosomes protein 2
Ubiquitin-like modifier-activating enzyme ATG7
Transforming acidic coiled-coil-containing protein 2
Tripartite motif-containing protein 16
Phenylalanine--tRNA ligase, mitochondrial
Acyl-protein thioesterase 2
Importin-7
E3 ubiquitin-protein ligase ARIH2
Pre-mRNA-splicing factor SLU7
Phosphoacetylglucosamine mutase
Adenylyltransferase and sulfurtransferase MOCS3
CD2 antigen cytoplasmic tail-binding protein 2
Supervillin
Activator of 90 kDa heat shock protein ATPase homolog 1
Poly(A)-specific ribonuclease PARN
Proteasome assembly chaperone 1
Sphingosine-1-phosphate lyase 1
CTD nuclear envelope phosphatase 1
Ribosome biogenesis protein NSA2 homolog
GDH/6PGL endoplasmic bifunctional protein
Protein transport protein Sec24A
Protein transport protein Sec24B
Adhesion G protein-coupled receptor L2
NAD kinase
Tyrosyl-DNA phosphodiesterase 2
Mitochondrial pyruvate carrier 2
Persulfide dioxygenase ETHE1, mitochondrial
Fatty acid CoA ligase Acsl3
DNA-directed RNA polymerase I subunit RPA1
YEATS domain-containing protein 4
CCR4-NOT transcription complex subunit 4
STAM-binding protein
Cleavage and polyadenylation specificity factor subunit 4
Nuclear factor of activated T-cells, cytoplasmic 1
Probable bifunctional dTTP/UTP pyrophosphatase/methyltransferase protein
Phosphatidate cytidylyltransferase 2
Protein phosphatase 1 regulatory subunit 3D
Bromodomain-containing protein 1
Ribonuclease P protein subunit p29
E3 ubiquitin-protein ligase HERC2
Ras-related protein Rab-3D
Synaptosomal-associated protein 29
Serine/threonine-protein kinase OSR1
Geranylgeranyl pyrophosphate synthase

Heat shock 70 kDa protein 4L
Polypyrimidine tract-binding protein 3
TBC1 domain family member 8
Interleukin-33
STARD3 N-terminal-like protein
U6 snRNA-associated Sm-like protein LSm8
AP-2 complex subunit alpha-1
Protein Wiz
Antiviral innate immune response receptor RIG-I
Double-stranded RNA-binding protein Staufen homolog 1
Tetratricopeptide repeat protein 4
Transmembrane protein 50A
Caveolae-associated protein 2
BAG family molecular chaperone regulator 2
BAG family molecular chaperone regulator 3
Mitogen-activated protein kinase kinase kinase kinase 4
Malonyl-CoA decarboxylase, mitochondrial
Quinone oxidoreductase-like protein 1
Apoptosis-inducing factor 1, mitochondrial
Echinoderm microtubule-associated protein-like 2
Serine/threonine-protein kinase LATS1
Uridine diphosphate glucose pyrophosphatase NUDT14
Tetraspanin-15
3'(2'),5'-bisphosphate nucleotidase 1
Acyl-CoA 6-desaturase
Putative hydrolase DDAH2
Phosphatidylserine lipase ABHD16A
Thioredoxin domain-containing protein 12
Protein ecdysoneless homolog
Reversion-inducing cysteine-rich protein with Kazal motifs
Methyl-CpG-binding domain protein 3
DNA topoisomerase 3-beta-1
Diphosphoinositol polyphosphate phosphohydrolase 1
B-cell lymphoma/leukemia 10
NADH dehydrogenase [ubiquinone] 1 beta subcomplex subunit 10
Putative lipid scramblase CLPTM1
E3 SUMO-protein ligase ZBED1
Molybdopterin synthase catalytic subunit
Mitochondrial import receptor subunit TOM40 homolog
Peroxisomal membrane protein 11B
Serine/threonine-protein kinase PAK 4
Amyloid-beta A4 precursor protein-binding family A member 3
Actin-like protein 6A
Histone-lysine N-methyltransferase NSD2
L-lactate dehydrogenase A chain
Aldehyde dehydrogenase 1A1
Glutamate dehydrogenase 1, mitochondrial
Dihydrofolate reductase
NADH-cytochrome b5 reductase 3
Glutathione reductase, mitochondrial

Cytochrome c oxidase subunit 1
Cytochrome c oxidase subunit 2
Purine nucleoside phosphorylase
Hypoxanthine-guanine phosphoribosyltransferase
Aspartate aminotransferase, mitochondrial
Tyrosine-protein kinase ABL1
Phosphoglycerate kinase 1
Adenylate kinase isoenzyme 1
Urokinase-type plasminogen activator
Tissue-type plasminogen activator
Adenosine deaminase
Cystatin-C
GTPase NRas
GTPase HRas
GTPase KRas
Platelet-derived growth factor subunit B
Low-density lipoprotein receptor
Transforming growth factor beta-1 proprotein
HLA class I histocompatibility antigen, B alpha chain
Putative HLA class I histocompatibility antigen, alpha chain H
Collagen alpha-1(I) chain
Collagen alpha-1(IV) chain
Prelamin-A/C
Apolipoprotein A-I
Fibronectin
Alpha-2-HS-glycoprotein
Albumin
Transferrin receptor protein 1
Ferritin light chain
Ferritin heavy chain
Metallothionein-2
NADH-ubiquinone oxidoreductase chain 1
NADH-ubiquinone oxidoreductase chain 4
NADH-ubiquinone oxidoreductase chain 5
Interstitial collagenase
3-hydroxy-3-methylglutaryl-coenzyme A reductase
Catalase
RAF proto-oncogene serine/threonine-protein kinase
Lysosomal acid glucosylceramidase
Fructose-bisphosphate aldolase A
Cystatin-B
Annexin A1
Apolipoprotein B-100
Major prion protein
Superoxide dismutase [Mn], mitochondrial
Ornithine aminotransferase, mitochondrial
Thymidine kinase, cytosolic
von Willebrand factor
Tubulin beta-4A chain
Glyceraldehyde-3-phosphate dehydrogenase

Argininosuccinate lyase
HLA class I histocompatibility antigen, A alpha chain
Calpain small subunit 1
Metallothionein-1E
Heat shock protein beta-1
Thymidylate synthase
Dolichyl-diphosphooligosaccharide--protein glycosyltransferase subunit 1
Dolichyl-diphosphooligosaccharide--protein glycosyltransferase subunit 2
Guanine nucleotide-binding protein G(i) subunit alpha-2
Histone H2A type 1-B/E
Sodium/potassium-transporting ATPase subunit alpha-1
Sodium/potassium-transporting ATPase subunit beta-1
Amyloid-beta precursor protein
Aldehyde dehydrogenase, mitochondrial
Integrin beta-3
Protein S100-A8
Non-histone chromosomal protein HMG-14
Plasminogen activator inhibitor 2
Plasminogen activator inhibitor 1
ADP/ATP translocase 2
Ubiquitin-like protein ISG15
Propionyl-CoA carboxylase alpha chain, mitochondrial
Propionyl-CoA carboxylase beta chain, mitochondrial
Eukaryotic translation initiation factor 2 subunit 1
Non-histone chromosomal protein HMG-17
Intercellular adhesion molecule 1
Large ribosomal subunit protein P2
Large ribosomal subunit protein uL10
Transcription factor Jun
DNA-directed RNA polymerase III subunit RPC4
Lupus La protein
Integrin beta-1
Keratin, type I cytoskeletal 18
Collagen alpha-2(V) chain
Uroporphyrinogen decarboxylase
Tyrosine-protein kinase Fyn
Alpha-galactosidase A
Gelsolin
Retinoblastoma-associated protein
Prothymosin alpha
Cyclin-dependent kinase 1
ATP synthase subunit beta, mitochondrial
Protein S100-A6
Eukaryotic translation initiation factor 4E
Alpha-enolase
Glycogen phosphorylase, liver form
Glucose-6-phosphate isomerase
DNA polymerase beta
Nucleophosmin
Tropomyosin alpha-3 chain

Integrin alpha-V
Beta-hexosaminidase subunit alpha
Epoxide hydrolase 1
L-lactate dehydrogenase B chain
Major centromere autoantigen B
Glutathione peroxidase 1
Protein disulfide-isomerase
Histone H1.0
Acylphosphatase-1
Tyrosine-protein kinase Fes/Fps
Cathepsin D
Annexin A2
Calpain-1 catalytic subunit
Tubulin beta chain
Prosaposin
Beta-hexosaminidase subunit beta
Procathepsin L
Profilin-1
Bisphosphoglycerate mutase
Adenine phosphoribosyltransferase
Bifunctional glutamate/proline--tRNA ligase
Cathepsin B
Heat shock protein HSP 90-alpha
Galactose-1-phosphate uridylyltransferase
Heterogeneous nuclear ribonucleoproteins C1/C2
Laminin subunit beta-1
Tyrosine-protein kinase Yes
Tyrosine-protein kinase Lyn
Fumarate hydratase, mitochondrial
DNA excision repair protein ERCC-1
Thrombospondin-1
Transcription factor Sp1
Insulin-like growth factor 1 receptor
Collagen alpha-2(I) chain
Annexin A6
Rho-related GTP-binding protein RhoC
Complement decay-accelerating factor
Amino acid transporter heavy chain SLC3A2
Beta-glucuronidase
ATP-dependent 6-phosphofructokinase, muscle type
Heat shock protein HSP 90-beta
Signal recognition particle receptor subunit alpha
Asparagine synthetase [glutamine-hydrolyzing]
72 kDa type IV collagenase
Porphobilinogen deaminase
Neprilysin
Matrix Gla protein
Pyruvate dehydrogenase E1 component subunit alpha, somatic form, mitochondrial
Collagen alpha-2(IV) chain
Cytochrome c1, heme protein, mitochondrial

U2 small nuclear ribonucleoprotein B''
Hepatocyte growth factor receptor
U1 small nuclear ribonucleoprotein 70 kDa
Integrin alpha-5
Nuclear factor 1 C-type
Vimentin
Small ribosomal subunit protein eS17
Guanine nucleotide-binding protein G(i) subunit alpha-3
Annexin A5
Small ribosomal subunit protein uS2
CD63 antigen
Large ribosomal subunit protein uL3m
U1 small nuclear ribonucleoprotein A
Gamma-enolase
3-ketoacyl-CoA thiolase, peroxisomal
Signal recognition particle 19 kDa protein
Glutathione S-transferase P
U1 small nuclear ribonucleoprotein C
Galectin-1
Dihydropteridine reductase
High mobility group protein B1
SPARC
Tropomyosin alpha-1 chain
Clathrin light chain A
Clathrin light chain B
Annexin A4
2',3'-cyclic-nucleotide 3'-phosphodiesterase
Heme oxygenase 1
Dihydrolipoyl dehydrogenase, mitochondrial
Heterogeneous nuclear ribonucleoprotein A1
U2 small nuclear ribonucleoprotein A'
Cytochrome c oxidase subunit 6C
Tumor-associated calcium signal transducer 2
Poly [ADP-ribose] polymerase 1
DNA polymerase alpha catalytic subunit
Ubiquitin carboxyl-terminal hydrolase isozyme L1
Leukotriene A-4 hydrolase
Fructose-bisphosphate aldolase C
Histone H2A.Z
Histone H2A type 1
Myocardial zonula adherens protein
DNA-directed RNA polymerase II subunit GRINL1A
Glutathione S-transferase theta-2
POTE ankyrin domain family member J
LIM and senescent cell antigen-like-containing domain protein 3
Heat shock 70 kDa protein 1A
Chimeric ERCC6-PGBD3 protein
Tubulin alpha-3C chain
Zinc finger TRAF-type-containing protein 1
Ras-related protein Rap-2a

Serglycin
RNA-binding protein RO60
Lysosomal alpha-glucosidase
Ras-related protein R-Ras
HLA class I histocompatibility antigen, C alpha chain
Serine/threonine-protein kinase A-Raf
Histone H1.4
Dihydrolipoyllysine-residue acetyltransferase component of pyruvate dehydrogenase complex, [illegible]
Receptor-type tyrosine-protein phosphatase F
COUP transcription factor 1
Thioredoxin
Cytochrome c oxidase subunit 5B, mitochondrial
Lysosomal protective protein
Microsomal glutathione S-transferase 1
cAMP-dependent protein kinase type I-alpha regulatory subunit
Tissue factor pathway inhibitor
S-formylglutathione hydrolase
60 kDa heat shock protein, mitochondrial
Clusterin
Endoplasmic reticulum chaperone BiP
Laminin subunit gamma-1
Lysosomal acid phosphatase
Microtubule-associated protein 2
Heat shock cognate 71 kDa protein
Solute carrier family 2, facilitated glucose transporter member 1
Solute carrier family 2, facilitated glucose transporter member 3
Protein 4.1
Uridine 5'-monophosphate synthase
Pyruvate dehydrogenase E1 component subunit beta, mitochondrial
Lipoamide acyltransferase component of branched-chain alpha-keto acid dehydrogenase compl[illegible]
Glycogen phosphorylase, brain form
Glycogen phosphorylase, muscle form
Ras-related protein Ral-A
Ras-related protein Ral-B
Breakpoint cluster region protein
Lysosome-associated membrane glycoprotein 1
Transcriptional regulator ERG
Medium-chain specific acyl-CoA dehydrogenase, mitochondrial
DNA topoisomerase 1
DNA topoisomerase 2-alpha
Glucose-6-phosphate 1-dehydrogenase
Ubiquitin-like protein 4A
Pyruvate carboxylase, mitochondrial
C-1-tetrahydrofolate synthase, cytoplasmic
Cation-independent mannose-6-phosphate receptor
Alcohol dehydrogenase class-3
Cyclin-dependent kinase 4
Ribose-phosphate pyrophosphokinase 2
Polyadenylate-binding protein 1
Proliferating cell nuclear antigen

Histidine--tRNA ligase, cytoplasmic
Collagen alpha-1(VI) chain
ADP/ATP translocase 1
ADP/ATP translocase 3
Inosine-5'-monophosphate dehydrogenase 2
Nucleoprotein TPR
Annexin A3
2-oxoisovalerate dehydrogenase subunit alpha, mitochondrial
Alpha-actinin-1
Angiotensin-converting enzyme
Proto-oncogene tyrosine-protein kinase Src
Xaa-Pro dipeptidase
X-ray repair cross-complementing protein 6
X-ray repair cross-complementing protein 5
Uracil-DNA glycosylase
Cytochrome c oxidase subunit 4 isoform 1, mitochondrial
5-aminolevulinate synthase, non-specific, mitochondrial
Lysosome-associated membrane glycoprotein 2
Ribonuclease inhibitor
Intercellular adhesion molecule 2
Elongation factor 2
Protein disulfide-isomerase A4
Prolyl 4-hydroxylase subunit alpha-1
Translationally-controlled tumor protein
Delta-aminolevulinic acid dehydratase
HLA class I histocompatibility antigen, alpha chain E
Plastin-3
Acylamino-acid-releasing enzyme
Electron transfer flavoprotein subunit alpha, mitochondrial
Glycogen [starch] synthase, muscle
cAMP-dependent protein kinase type II-alpha regulatory subunit
General transcription factor IIF subunit 2
CD59 glycoprotein
Bifunctional methylenetetrahydrofolate dehydrogenase/cyclohydrolase, mitochondrial
Macrophage migration inhibitory factor
CD99 antigen
Glucosidase 2 subunit beta
Hematopoietic lineage cell-specific protein
Farnesyl pyrophosphate synthase
Cytochrome c oxidase subunit 7A2, mitochondrial
Nidogen-1
Aldo-keto reductase family 1 member A1
Pyruvate kinase PKM
Endoplasmin
G2/mitotic-specific cyclin-B1
Myosin light chain 6B
Small nuclear ribonucleoprotein-associated proteins B and B'
Insulin-degrading enzyme
POU domain, class 2, transcription factor 1
Heterogeneous nuclear ribonucleoprotein L

Aspartate--tRNA ligase, cytoplasmic
Protein C-ets-1
Junction plakoglobin
Cytochrome b-c1 complex subunit 7
Serine/threonine-protein kinase B-raf
Fatty acid-binding protein, adipocyte
Glutamine synthetase
Aldo-keto reductase family 1 member B1
Aminopeptidase N
Poliovirus receptor
Ras-related C3 botulinum toxin substrate 2
Eukaryotic peptide chain release factor GTP-binding subunit ERF3A
Arylsulfatase A
Beta-1,4-galactosyltransferase 1
Ezrin
Ubiquitin carboxyl-terminal hydrolase isozyme L3
Fos-related antigen 1
Fos-related antigen 2
Membrane cofactor protein
Nucleoside diphosphate kinase A
NAD(P)H dehydrogenase [quinone] 1
N-acetylglucosamine-6-sulfatase
Phosphorylase b kinase gamma catalytic chain, liver/testis isoform
Arylsulfatase B
Small ribosomal subunit protein uS5
Transcription factor 4
Desmoplakin
Replication protein A 32 kDa subunit
Metalloproteinase inhibitor 2
CD44 antigen
Carbonyl reductase [NADPH] 1
Short-chain specific acyl-CoA dehydrogenase, mitochondrial
Cyclic AMP-responsive element-binding protein 1
Beta-galactosidase
Platelet endothelial cell adhesion molecule
Serine/threonine-protein phosphatase 2B catalytic subunit beta isoform
SH2/SH3 adapter protein NCK1
Intron Large complex component GCFC2
Histone H1.5
Histone H1.3
Histone H1.2
NADPH--cytochrome P450 reductase
Tyrosine-protein kinase Fer
Sarcoplasmic/endoplasmic reticulum calcium ATPase 2
1-phosphatidylinositol 4,5-bisphosphate phosphodiesterase gamma-2
Fumarylacetoacetase
Stathmin
Y-box-binding protein 3
Zinc finger protein 22
Alpha-N-acetylgalactosaminidase

High mobility group protein HMG-I/HMG-Y
Transmembrane protein 11, mitochondrial
Aspartate aminotransferase, cytoplasmic
Protein kinase C alpha type
Integrin alpha-2
Gap junction alpha-1 protein
Sphingomyelin phosphodiesterase
Nucleolar transcription factor 1
Transcription factor JunD
T-cell acute lymphocytic leukemia protein 1
Cyclic AMP-dependent transcription factor ATF-7
NADH dehydrogenase [ubiquinone] 1 beta subcomplex subunit 7
cAMP-dependent protein kinase catalytic subunit alpha
Calpain-2 catalytic subunit
Tyrosine-protein phosphatase non-receptor type 2
CTP synthase 1
Endoglin
Probable ATP-dependent RNA helicase DDX5
ATP-dependent 6-phosphofructokinase, liver type
Vascular endothelial growth factor receptor 1
26S proteasome regulatory subunit 6A
T-complex protein 1 subunit alpha
Tyrosine-protein phosphatase non-receptor type 1
General transcription and DNA repair factor IIH helicase subunit XPD
Large ribosomal subunit protein eL33
Integrin beta-5
ADP-ribosylation factor 4
Large ribosomal subunit protein uL30
Vinculin
Receptor-type tyrosine-protein phosphatase alpha
Arylamine N-acetyltransferase 1
Protein SON
Negative elongation factor E
Large ribosomal subunit protein uL22
Phosphoglycerate mutase 1
Regulator of chromosome condensation
Cyclic AMP-dependent transcription factor ATF-1
Cyclic AMP-dependent transcription factor ATF-6 alpha
DNA ligase 1
DNA repair protein XRCC1
Peptidyl-glycine alpha-amidating monooxygenase
Cadherin-2
1-phosphatidylinositol 4,5-bisphosphate phosphodiesterase gamma-1
Nucleolin
Hexokinase-1
DNA-directed RNA polymerase II subunit RPB3
DNA-directed RNA polymerases I, II, and III subunit RPABC1
NADH dehydrogenase [ubiquinone] flavoprotein 2, mitochondrial
General transcription and DNA repair factor IIH helicase subunit XPB
E3 ubiquitin-protein ligase TRIM21

Interferon-induced, double-stranded RNA-activated protein kinase
Spermidine synthase
Sodium/hydrogen exchanger 1
Casein kinase II subunit alpha'
Nuclear factor NF-kappa-B p105 subunit
Plasma membrane calcium-transporting ATPase 1
Eukaryotic translation initiation factor 2 subunit 2
Annexin A7
TATA-box-binding protein
Cyclin-A2
Transcription factor BTF3
Ras-related protein Rab-3B
Ras-related protein Rab-4A
Ras-related protein Rab-5A
Ras-related protein Rab-6A
DNA mismatch repair protein Msh3
Atrial natriuretic peptide receptor 2
Proteasome subunit beta type-1
Cation-dependent mannose-6-phosphate receptor
Cytochrome c oxidase subunit 5A, mitochondrial
Lamin-B1
Calpastatin
Inosine-5'-monophosphate dehydrogenase 1
Collagen alpha-1(V) chain
Nebulin
N(4)-(beta-N-acetylglucosaminyl)-L-asparaginase
Ras GTPase-activating protein 1
Parathymosin
Cyclin-dependent kinase 11B
Glutathione S-transferase Mu 3
V-type proton ATPase subunit B, brain isoform
V-type proton ATPase subunit C 1
Cysteine and glycine-rich protein 1
Filamin-A
Neurofibromin
Amine oxidase [flavin-containing] A
Cytoplasmic aconitate hydratase
Sphingosine 1-phosphate receptor 1
Synaptotagmin-1
5'-nucleotidase
Midkine
Voltage-dependent anion-selective channel protein 1
Biglycan
Succinate dehydrogenase [ubiquinone] iron-sulfur subunit, mitochondrial
CD9 antigen
Catechol O-methyltransferase
Protein-glutamine gamma-glutamyltransferase 2
Methylmalonyl-CoA mutase, mitochondrial
Oxysterol-binding protein 1
Protein-L-isoaspartate(D-aspartate) O-methyltransferase

rRNA 2'-O-methyltransferase fibrillarin
Trifunctional purine biosynthetic protein adenosine-3
Bifunctional phosphoribosylaminoimidazole carboxylase/phosphoribosylaminoimidazole succin
Sterol carrier protein 2
Ubiquitin-like modifier-activating enzyme 1
Nucleoside diphosphate kinase B
NADPH:adrenodoxin oxidoreductase, mitochondrial
Heterogeneous nuclear ribonucleoproteins A2/B1
MHC class II regulatory factor RFX1
E3 ubiquitin-protein ligase CBL
Insulin-like growth factor-binding protein 4
cAMP-dependent protein kinase catalytic subunit beta
Cytochrome b-c1 complex subunit 2, mitochondrial
Ferrochelatase, mitochondrial
DNA repair protein complementing XP-A cells
Transcription elongation factor A protein 1
Prostaglandin G/H synthase 1
Integrin alpha-6
Splicing factor, proline- and glutamine-rich
Tubulin gamma-1 chain
Peptidyl-prolyl cis-trans isomerase B
NAD-dependent malic enzyme, mitochondrial
Tryptophan--tRNA ligase, cytoplasmic
Small ribosomal subunit protein uS3
Ribosomal protein S6 kinase beta-1
Tyrosine-protein kinase JAK1
Receptor-type tyrosine-protein phosphatase beta
Receptor-type tyrosine-protein phosphatase epsilon
Receptor-type tyrosine-protein phosphatase gamma
Nuclear autoantigen Sp-100
Colorectal mutant cancer protein
Tumor necrosis factor ligand superfamily member 4
Adenosylhomocysteinase
Cofilin-1
Eukaryotic translation initiation factor 4B
40-kDa huntingtin-associated protein
Plasma membrane calcium-transporting ATPase 4
Diacylglycerol kinase alpha
Carnitine O-palmitoyltransferase 2, mitochondrial
Thymidylate kinase
Ribonucleoside-diphosphate reductase large subunit
G1/S-specific cyclin-D1
Rab proteins geranylgeranyltransferase component A 1
ER lumen protein-retaining receptor 1
COUP transcription factor 2
Elongation factor 1-beta
ATP synthase F(0) complex subunit B1, mitochondrial
Low molecular weight phosphotyrosine protein phosphatase
Protein kinase C eta type
Acetyl-CoA acetyltransferase, mitochondrial

Myosin regulatory light polypeptide 9
DNA-directed RNA polymerase II subunit RPB1
Cyclin-dependent kinase 2
Beta-adrenergic receptor kinase 1
Proteinase-activated receptor 1
DNA replication licensing factor MCM3
3-mercaptopyruvate sulfurtransferase
Small ribosomal subunit protein eS12
Bromodomain-containing protein 2
Tumor necrosis factor receptor superfamily member 6
Transcriptional repressor protein YY1
DnaJ homolog subfamily B member 1
DnaJ homolog subfamily B member 2
ATP synthase subunit alpha, mitochondrial
Cathepsin S
Proteasome subunit alpha type-1
Proteasome subunit alpha type-2
Proteasome subunit alpha type-3
Proteasome subunit alpha type-4
Tumor necrosis factor receptor superfamily member 5
Integrin alpha-3
Pentraxin-related protein PTX3
Moesin
Probable ATP-dependent RNA helicase DDX6
DNA (cytosine-5)-methyltransferase 1
Splicing factor U2AF 65 kDa subunit
Large ribosomal subunit protein eL13
Rab proteins geranylgeranyltransferase component A 2
Isovaleryl-CoA dehydrogenase, mitochondrial
Alpha-1,3-mannosyl-glycoprotein 2-beta-N-acetylglucosaminyltransferase
High mobility group protein B2
Polypyrimidine tract-binding protein 1
Threonine--tRNA ligase 1, cytoplasmic
Valine--tRNA ligase
Elongation factor 1-gamma
Peptidyl-prolyl cis-trans isomerase FKBP2
Interleukin-3 receptor subunit alpha
Stomatin
Adenylate kinase 4, mitochondrial
14-3-3 protein theta
Mitogen-activated protein kinase 3
MAP/microtubule affinity-regulating kinase 3
Large ribosomal subunit protein uL16
Collagen alpha-1(VIII) chain
Replication protein A 70 kDa DNA-binding subunit
DNA-(apurinic or apyrimidinic site) endonuclease
CD82 antigen
Deoxycytidine kinase
Multifunctional protein CAD
Calreticulin

Microtubule-associated protein 4
Calnexin
Phosphatidylinositol 3-kinase regulatory subunit alpha
Inositol-trisphosphate 3-kinase B
Proteasome subunit beta type-8
Proteasome subunit beta type-9
Proteasome subunit alpha type-5
Proteasome subunit beta type-4
Proteasome subunit beta type-6
Proteasome subunit beta type-5
Glutathione S-transferase Mu 2
ATP-binding cassette sub-family D member 3
Protein ITPRID2
Protein-lysine 6-oxidase
NADH-ubiquinone oxidoreductase 75 kDa subunit, mitochondrial
DNA polymerase delta catalytic subunit
Probable global transcription activator SNF2L1
Mitogen-activated protein kinase 1
Retinoic acid receptor RXR-beta
DNA excision repair protein ERCC-5
Progranulin
Receptor-type tyrosine-protein phosphatase mu
Cytosol aminopeptidase
Hematopoietic progenitor cell antigen CD34
General transcription factor IIE subunit 1
Transcription initiation factor IIE subunit beta
Tripeptidyl-peptidase 2
Inositol monophosphatase 1
CCN family member 2
Ephrin type-A receptor 2
Ephrin type-B receptor 2
SHC-transforming protein 1
DNA-3-methyladenine glycosylase
Transketolase
Caspase-1
Nitric oxide synthase 3
RNA-binding motif, single-stranded-interacting protein 1
Protein PML
Non-receptor tyrosine-protein kinase TYK2
Elongation factor 1-delta
Myristoylated alanine-rich C-kinase substrate
Guanine nucleotide-binding protein subunit alpha-11
Delta-1-pyrroline-5-carboxylate dehydrogenase, mitochondrial
Endoplasmic reticulum resident protein 29
Peroxiredoxin-6
Flavin reductase (NADPH)
Peroxiredoxin-5, mitochondrial
Thioredoxin-dependent peroxide reductase, mitochondrial
Large ribosomal subunit protein uL11
Enoyl-CoA hydratase, mitochondrial

UMP-CMP kinase
Phosphatidylethanolamine-binding protein 1
Protein disulfide-isomerase A3
Serine/threonine-protein phosphatase 2A 65 kDa regulatory subunit A alpha isoform
Serine/threonine-protein phosphatase 2A 65 kDa regulatory subunit A beta isoform
Cell division cycle protein 27 homolog
Peptidyl-prolyl cis-trans isomerase F, mitochondrial
NK-tumor recognition protein
Glycylpeptide N-tetradecanoyltransferase 1
Heme oxygenase 2
Adenylosuccinate synthetase isozyme 2
Tyrosine-protein kinase receptor UFO
Alpha-2-macroglobulin receptor-associated protein
Translocator protein
Adenylosuccinate lyase
CAP-Gly domain-containing linker protein 1
Sorcin
Leukocyte elastase inhibitor
High affinity cationic amino acid transporter 1
Aldehyde dehydrogenase X, mitochondrial
DNA-directed RNA polymerase II subunit RPB2
Succinate dehydrogenase [ubiquinone] flavoprotein subunit, mitochondrial
Coronin-1A
Rab GDP dissociation inhibitor alpha
Protein S100-A7
S-adenosylmethionine synthase isoform type-2
cAMP-dependent protein kinase type II-beta regulatory subunit
Ribonucleoside-diphosphate reductase subunit M2
Syndecan-4
Cytotoxic granule associated RNA binding protein TIA1
Sodium- and chloride-dependent taurine transporter
DnaJ homolog subfamily A member 1
RAC-alpha serine/threonine-protein kinase
Cytochrome b-c1 complex subunit 1, mitochondrial
3-hydroxyisobutyrate dehydrogenase, mitochondrial
Bifunctional purine biosynthesis protein ATIC
Heterogeneous nuclear ribonucleoprotein H3
Heterogeneous nuclear ribonucleoprotein H
Caspase-14
14-3-3 protein beta/alpha
Stress-induced-phosphoprotein 1
Protein S100-A11
Peroxiredoxin-2
Beta-arrestin-2
Glycerol kinase
Deoxycytidylate deaminase
Pyrroline-5-carboxylate reductase 1, mitochondrial
Guanylate-binding protein 1
Guanylate-binding protein 2
ETS-related transcription factor Elf-1

General transcription factor IIH subunit 1
Syntaxin-2
Large ribosomal subunit protein uL6
Long-chain-fatty-acid--CoA ligase 1
Cadherin-5
Kinesin-1 heavy chain
Deoxyuridine 5'-triphosphate nucleotidohydrolase, mitochondrial
Multidrug resistance-associated protein 1
ATP-binding cassette sub-family D member 1
Dual specificity protein kinase TTK
DNA replication licensing factor MCM4
DNA replication licensing factor MCM5
DNA replication licensing factor MCM7
N-acetylgalactosamine-6-sulfatase
Serine hydroxymethyltransferase, cytosolic
Serine hydroxymethyltransferase, mitochondrial
Heat shock 70 kDa protein 1-like
Heat shock 70 kDa protein 4
G protein-coupled receptor kinase 5
Mannose-6-phosphate isomerase
Glypican-1
Profilin-2
Catenin alpha-1
Catenin beta-1
Prohibitin 1
Serpin B6
Merlin
Radixin
Replication protein A 14 kDa subunit
Replication factor C subunit 4
Replication factor C subunit 2
Replication factor C subunit 1
Large ribosomal subunit protein eL22
General transcription factor IIF subunit 1
Sepiapterin reductase
Histamine H1 receptor
Cystathionine beta-synthase
Fibrillin-1
Fibrillin-2
Glycogen debranching enzyme
Myosin-9
Myosin-10
Tyrosine-protein kinase receptor Tie-1
Coatomer subunit beta'
Sterol O-acyltransferase 1
Alpha-adducin
Basigin
RNA-binding protein FUS
Nuclear pore complex protein Nup214
Protein DEK

Transcription factor SOX-18
Myosin-11
Protein phosphatase 1A
Hydroxymethylglutaryl-CoA lyase, mitochondrial
Vascular endothelial growth factor receptor 3
Vascular endothelial growth factor receptor 2
26S proteasome regulatory subunit 7
Glutathione hydrolase 5 proenzyme
ADP-ribosylation factor-like protein 2
ADP-ribosylation factor-like protein 3
Dual specificity mitogen-activated protein kinase kinase 2
ATP synthase subunit gamma, mitochondrial
V-type proton ATPase subunit E 1
Oxygen-dependent coproporphyrinogen-III oxidase, mitochondrial
Large ribosomal subunit protein uL4
Oxidized purine nucleoside triphosphate hydrolase
Lon protease homolog, mitochondrial
Phosphoglucomutase-1
Serine/threonine-protein phosphatase PP1-gamma catalytic subunit
Guanine nucleotide-binding protein-like 1
Dihydrolipoyllysine-residue succinyltransferase component of 2-oxoglutarate dehydrogenase co
GMP reductase 1
Phospholipid hydroperoxide glutathione peroxidase GPX4
Serine/threonine-protein kinase receptor R3
17-beta-hydroxysteroid dehydrogenase type 2
Signal recognition particle 14 kDa protein
TGF-beta receptor type-2
Nuclear pore glycoprotein p62
Hippocalcin-like protein 1
Squalene synthase
Zinc finger E-box-binding homeobox 1
Transgelin-2
Transaldolase
Alpha-synuclein
Electron transfer flavoprotein subunit beta
RNA-binding motif protein, X chromosome
Coilin
Vitamin K-dependent gamma-carboxylase
Lysosomal acid lipase/cholesteryl ester hydrolase
V-type proton ATPase catalytic subunit A
Stress-70 protein, mitochondrial
Eukaryotic initiation factor 4A-III
DNA-binding protein SMUBP-2
Cyclin-dependent kinase inhibitor 1
Small ribosomal subunit protein eS19
Large ribosomal subunit protein uL3
Collagen alpha-1(XVIII) chain
Dolichyl-diphosphooligosaccharide--protein glycosyltransferase 48 kDa subunit
Acidic leucine-rich nuclear phosphoprotein 32 family member A
Flap endonuclease 1

Homeobox protein cut-like 1
Macrophage-capping protein
Adenylyl cyclase-associated protein 2
Interleukin-6 receptor subunit beta
Alpha-taxilin
T-complex protein 1 subunit zeta
Nicotinamide N-methyltransferase
Proteasome subunit beta type-10
Pre-B-cell leukemia transcription factor 2
Large ribosomal subunit protein uL13
ADP-ribosylation factor-like protein 1
DNA mismatch repair protein Mlh1
Signal transducer and activator of transcription 3
Ubiquitin carboxyl-terminal hydrolase 8
Malate dehydrogenase, cytoplasmic
Malate dehydrogenase, mitochondrial
Replication factor C subunit 5
Replication factor C subunit 3
Trifunctional enzyme subunit alpha, mitochondrial
Eukaryotic translation initiation factor 2 subunit 3
ETS translocation variant 3
Centrin-2
Transcription factor ETV6
Eukaryotic translation initiation factor 2D
Protein BUD31 homolog
Ubiquitin-like modifier-activating enzyme 7
N-alpha-acetyltransferase 10
Lysine-specific demethylase 5C
Tyrosine-protein kinase CSK
Glycine--tRNA ligase
Isoleucine--tRNA ligase, cytoplasmic
Reduced folate transporter
Eukaryotic translation initiation factor 1
Protein kinase C iota type
ETS domain-containing protein Elk-3
Beta-centractin
Enoyl-CoA delta isomerase 1, mitochondrial
Lamina-associated polypeptide 2, isoform alpha
Lamina-associated polypeptide 2, isoforms beta/gamma
Signal transducer and activator of transcription 1-alpha/beta
Signal transducer and activator of transcription 6
Signal transducer and activator of transcription 5A
Exosome RNA helicase MTR4
Aldo-keto reductase family 1 member C3
Phosphatidylinositol 4,5-bisphosphate 3-kinase catalytic subunit alpha isoform
Phosphatidylinositol 4,5-bisphosphate 3-kinase catalytic subunit beta isoform
Serine/threonine-protein kinase mTOR
Phosphatidylinositol 4-kinase alpha
Epidermal growth factor receptor substrate 15
Caspase-3

Caspase-2
Small ribosomal subunit protein eS27
Tyrosine-protein kinase ABL2
Probable helicase with zinc finger domain
Condensin-2 complex subunit D3
RNA-binding protein 34
Leucine-rich PPR motif-containing protein, mitochondrial
3-ketoacyl-CoA thiolase, mitochondrial
Large ribosomal subunit protein uL29
Lysosomal Pro-X carboxypeptidase
Huntingtin
Endothelin-converting enzyme 1
Methylenetetrahydrofolate reductase (NADPH)
Neutral amino acid transporter A
Platelet-activating factor acetylhydrolase IB subunit beta
Cell surface glycoprotein MUC18
Carnitine O-acetyltransferase
Matrin-3
DNA mismatch repair protein Msh2
G protein-coupled receptor kinase 6
Glycerol-3-phosphate dehydrogenase, mitochondrial
Translocon-associated protein subunit alpha
Tyrosine-protein phosphatase non-receptor type 9
Ran-specific GTPase-activating protein
Nicotinamide phosphoribosyltransferase
26S proteasome regulatory subunit 6B
Elongation factor Ts, mitochondrial
Peptidyl-prolyl cis-trans isomerase C
Voltage-dependent anion-selective channel protein 2
Short/branched chain specific acyl-CoA dehydrogenase, mitochondrial
Chromobox protein homolog 5
Ubiquitin carboxyl-terminal hydrolase 5
Mitogen-activated protein kinase 8
Dual specificity mitogen-activated protein kinase kinase 4
Proliferation marker protein Ki-67
Phosphorylase b kinase regulatory subunit alpha, skeletal muscle isoform
Ran GTPase-activating protein 1
ATP-dependent DNA helicase Q1
Probable 28S rRNA (cytosine(4447)-C(5))-methyltransferase
Transcriptional regulator ATRX
Adapter molecule crk
Crk-like protein
Translation initiation factor IF-2, mitochondrial
Large proline-rich protein BAG6
Vesicle-fusing ATPase
Neurogenic locus notch homolog protein 1
Dual specificity mitogen-activated protein kinase kinase 3
Lys-63-specific deubiquitinase BRCC36
Large ribosomal subunit protein uL15
Large ribosomal subunit protein uL18

Large ribosomal subunit protein eL21
Large ribosomal subunit protein eL28
Small ribosomal subunit protein uS4
Small ribosomal subunit protein uS7
Small ribosomal subunit protein eS10
Microtubule-associated protein 1B
Glucosamine-6-phosphate isomerase 1
E3 ubiquitin-protein ligase NEDD4
Utrophin
Ras GTPase-activating-like protein IQGAP1
Glycogenin-1
Dolichyl-diphosphooligosaccharide--protein glycosyltransferase subunit STT3A
Cytosolic phospholipase A2
F-actin-capping protein subunit alpha-2
F-actin-capping protein subunit beta
Eukaryotic translation initiation factor 1A, X-chromosomal
Retinaldehyde dehydrogenase 3
Glutamine--tRNA ligase
Large ribosomal subunit protein eL29
mRNA decay activator protein ZFP36L2
Cytochrome b-c1 complex subunit Rieske, mitochondrial
ATP synthase subunit O, mitochondrial
LIM and senescent cell antigen-like-containing domain protein 1
Glioma pathogenesis-related protein 1
Prolyl endopeptidase
NADP-dependent malic enzyme
Iron-responsive element-binding protein 2
Tissue factor pathway inhibitor 2
DNA-binding protein RFX5
Phosphatidylinositol 5-phosphate 4-kinase type-2 alpha
Coatomer subunit delta
Lanosterol synthase
Serine/threonine-protein phosphatase 2B catalytic subunit gamma isoform
Glutamate--cysteine ligase catalytic subunit
Glutamate--cysteine ligase regulatory subunit
CD151 antigen
Trafficking protein particle complex subunit 10
26S proteasome non-ATPase regulatory subunit 8
Protein PRRC2A
Glutathione synthetase
T-complex protein 1 subunit epsilon
Phosphatidylserine synthase 1
Nestin
Heat shock 70 kDa protein 13
Casein kinase I isoform alpha
Casein kinase I isoform delta
Isocitrate dehydrogenase [NADP], mitochondrial
Phosphatidylinositol 4,5-bisphosphate 3-kinase catalytic subunit gamma isoform
Phosphatidylinositol transfer protein beta isoform
DNA polymerase delta subunit 2

MARCKS-related protein
Paxillin
Guided entry of tail-anchored proteins factor CAMLG
Nuclear receptor subfamily 2 group C member 2
MAP kinase-activated protein kinase 2
Deoxyribonuclease-1-like 1
4-trimethylaminobutyraldehyde dehydrogenase
Large ribosomal subunit protein eL34
Ribose-5-phosphate isomerase
Protein ERGIC-53
Natural resistance-associated macrophage protein 2
Nuclear autoantigenic sperm protein
Fatty acid synthase
Protein farnesyltransferase/geranylgeranyltransferase type-1 subunit alpha
Protein farnesyltransferase subunit beta
Deoxyhypusine synthase
T-complex protein 1 subunit gamma
Large ribosomal subunit protein bL19m
Beta-arrestin-1
Elongation factor Tu, mitochondrial
Alpha-aminoadipic semialdehyde dehydrogenase
Ubiquitin-conjugating enzyme E2 R1
Inositol polyphosphate 1-phosphatase
Centromere protein F
Signal recognition particle 9 kDa protein
Choline-phosphate cytidylyltransferase A
Alanine--tRNA ligase, cytoplasmic
Cysteine--tRNA ligase, cytoplasmic
Histidine--tRNA ligase, mitochondrial
Serine--tRNA ligase, cytoplasmic
Protein phosphatase 1F
Alpha-mannosidase 2x
DNA primase small subunit
DNA primase large subunit
Caspase-4
Casein kinase I isoform epsilon
Proteasome subunit beta type-3
Proteasome subunit beta type-2
DNA replication licensing factor MCM2
Very long-chain specific acyl-CoA dehydrogenase, mitochondrial
YLP motif-containing protein 1
Vacuolar protein sorting-associated protein 41 homolog
Transmembrane emp24 domain-containing protein 10
RNA-binding protein 25
Protein numb homolog
Dual specificity protein kinase CLK3
Presenilin-1
Translation initiation factor eIF2B subunit beta
Adenosine 5'-monophosphoramidase HINT1
Nuclear pore complex protein Nup153

E3 SUMO-protein ligase RanBP2
Regulator of G-protein signaling 19
Regulator of G-protein signaling 3
Presenilin-2
Tuberin
NADH dehydrogenase [ubiquinone] flavoprotein 1, mitochondrial
Glycogen synthase kinase-3 alpha
Glycogen synthase kinase-3 beta
Transcription initiation factor TFIID subunit 6
Cytosolic purine 5'-nucleotidase
Selenide, water dikinase 1
5-formyltetrahydrofolate cyclo-ligase
GMP synthase [glutamine-hydrolyzing]
DNA ligase 3
DNA ligase 4
Double-strand break repair protein MRE11
Ectonucleoside triphosphate diphosphohydrolase 1
Guanine nucleotide-binding protein G(q) subunit alpha
Isocitrate dehydrogenase [NAD] subunit alpha, mitochondrial
Sulfotransferase 1A1
Matrix metalloproteinase-14
Protoporphyrinogen oxidase
Rab GDP dissociation inhibitor beta
Emerin
Carnitine O-palmitoyltransferase 1, liver isoform
Sulfate transporter
Serpin B8
Serpin B9
Serpin H1
PDZ and LIM domain protein 4
Hsc70-interacting protein
Peroxisomal targeting signal 1 receptor
ETS domain-containing transcription factor ERF
Vasodilator-stimulated phosphoprotein
Dynamin-2
Methionine aminopeptidase 2
Bis(5'-nucleosyl)-tetraphosphatase [asymmetrical]
Cyclin-dependent kinase 7
Kinetochore-associated protein 1
Ras association domain-containing protein 2
Cyclin-dependent kinase 9
Lipopolysaccharide-responsive and beige-like anchor protein
Basal cell adhesion molecule
Palmitoyl-protein thioesterase 1
Large ribosomal subunit protein eL14
T-complex protein 1 subunit theta
T-complex protein 1 subunit delta
Annexin A11
Poly(A) polymerase alpha
RNA-binding protein FXR1

RNA-binding protein FXR2
Ras-related protein Rab-5C
Ras-related protein Rab-7a
Ras-related protein Rab-9A
Ras-related protein Rab-13
Ras-related protein Rab-27A
1-phosphatidylinositol 4,5-bisphosphate phosphodiesterase delta-1
Small ribosomal subunit protein mS29
Dual specificity protein phosphatase 3
Probable global transcription activator SNF2L2
Transcription activator BRG1
Isocitrate dehydrogenase [NAD] subunit gamma, mitochondrial
Galactokinase
Translocon-associated protein subunit delta
B-cell receptor-associated protein 31
Thiopurine S-methyltransferase
Methyl-CpG-binding protein 2
Host cell factor 1
Interleukin-1 receptor-associated kinase 1
Caveolin-2
Aldehyde dehydrogenase family 3 member A2
Peroxisomal multifunctional enzyme type 2
26S proteasome non-ATPase regulatory subunit 7
Sulfite oxidase, mitochondrial
N-sulphoglucosamine sulphohydrolase
Signal transducer and activator of transcription 5B
Ubiquitin carboxyl-terminal hydrolase 11
H(+)/Cl(-) exchange transporter 7
Vesicle-associated membrane protein 7
Ribosomal protein S6 kinase alpha-3
Cytoplasmic tyrosine-protein kinase BMX
AF4/FMR2 family member 1
Hepatoma-derived growth factor
Cyclin-H
CDK-activating kinase assembly factor MAT1
Serine/threonine-protein kinase Nek3
Ubiquitin-conjugating enzyme E2 E1
NADH dehydrogenase [ubiquinone] 1 alpha subcomplex subunit 8
Heterogeneous nuclear ribonucleoprotein A3
6-phosphogluconate dehydrogenase, decarboxylating
Heterogeneous nuclear ribonucleoprotein M
Importin subunit alpha-1
Importin subunit alpha-5
Nuclear cap-binding protein subunit 2
Rap1 GTPase-GDP dissociation stimulator 1
DNA-directed RNA polymerases I, II, and III subunit RPABC3
Dual specificity mitogen-activated protein kinase kinase 6
Rho GDP-dissociation inhibitor 1
Rho GDP-dissociation inhibitor 2
Arf-GAP domain and FG repeat-containing protein 1

Heterogeneous nuclear ribonucleoprotein F
Signal transducer and activator of transcription 2
DNA mismatch repair protein Msh6
Kinesin-like protein KIF11
Guanine nucleotide exchange factor VAV2
Zinc finger protein 143
RNA-binding protein 5
2-iminobutanoate/2-iminopropanoate deaminase
Spermine synthase
Hexokinase-2
Ephrin-B2
Large ribosomal subunit protein bL12m
Diacylglycerol kinase theta
Bifunctional heparan sulfate N-deacetylase/N-sulfotransferase 1
Thimet oligopeptidase
F-actin-capping protein subunit alpha-1
High mobility group protein HMGI-C
Cysteine-rich protein 2
Nuclear pore complex protein Nup98-Nup96
Biliverdin reductase A
Tricarboxylate transport protein, mitochondrial
Serine/threonine-protein phosphatase 5
Serine/threonine-protein kinase PLK1
Death-associated protein kinase 1
Arfaptin-2
Arfaptin-1
Cytosolic Fe-S cluster assembly factor NUBP1
ATP-citrate synthase
Methionine aminopeptidase 1
Succinate--CoA ligase [ADP/GDP-forming] subunit alpha, mitochondrial
Diphosphomevalonate decarboxylase
Geranylgeranyl transferase type-1 subunit beta
Geranylgeranyl transferase type-2 subunit beta
Coatomer subunit beta
Coatomer subunit alpha
Dipeptidyl peptidase 1
LIM domain kinase 1
LIM domain kinase 2
Clathrin heavy chain 2
AP-3 complex subunit mu-2
AP-2 complex subunit sigma
Holocytochrome c-type synthase
Mitogen-activated protein kinase 12
Pituitary tumor-transforming gene 1 protein-interacting protein
Smoothelin
Monocarboxylate transporter 1
IST1 homolog
Protein transport protein Sec24C
Activated RNA polymerase II transcriptional coactivator p15
DNA polymerase subunit gamma-1

Methylosome subunit pICln
Arginine--tRNA ligase, cytoplasmic
Protein HIRA
Ataxin-3
Atrophin-1
PMS1 protein homolog 1
Mismatch repair endonuclease PMS2
Voltage-dependent calcium channel subunit alpha-2/delta-1
Tyrosine--tRNA ligase, cytoplasmic
Ubiquitin carboxyl-terminal hydrolase 14
5'-AMP-activated protein kinase subunit gamma-1
Heat shock-related 70 kDa protein 2
Branched-chain-amino-acid aminotransferase, cytosolic
Sodium/potassium-transporting ATPase subunit beta-3
UV excision repair protein RAD23 homolog B
Ephrin type-B receptor 4
Alpha-N-acetylglucosaminidase
Adenylate kinase 2, mitochondrial
Delta-1-pyrroline-5-carboxylate synthase
Alpha-soluble NSF attachment protein
Eukaryotic translation initiation factor 5
Solute carrier family 12 member 2
26S proteasome non-ATPase regulatory subunit 4
Developmentally-regulated GTP-binding protein 2
Exportin-2
Transitional endoplasmic reticulum ATPase
Microfibrillar-associated protein 1
Trifunctional enzyme subunit beta, mitochondrial
Mesencephalic astrocyte-derived neurotrophic factor
Afadin
RNA polymerase II elongation factor ELL
Peregrin
Nucleosome assembly protein 1-like 1
Caspase-7
Caspase-6
Adenosine kinase
Double-stranded RNA-specific adenosine deaminase
Laminin subunit beta-2
Cadherin-13
Protein SEC13 homolog
NHP2-like protein 1
Puromycin-sensitive aminopeptidase
Heterogeneous nuclear ribonucleoprotein H2
Succinyl-CoA:3-ketoacid coenzyme A transferase 1, mitochondrial
Eukaryotic translation initiation factor 3 subunit B
IgG receptor FcRn large subunit p51
BH3-interacting domain death agonist
ATP synthase subunit f, mitochondrial
Ribosomal RNA processing protein 1 homolog A
Methionine--tRNA ligase, cytoplasmic

cAMP-regulated phosphoprotein 19
AP-1 complex subunit sigma-2
ATP synthase subunit ATP5MJ, mitochondrial
ATP synthase subunit epsilon, mitochondrial
ATP synthase subunit e, mitochondrial
Histone deacetylase 4
Eukaryotic translation initiation factor 6
C-terminal-binding protein 2
NADH dehydrogenase [ubiquinone] 1 alpha subcomplex subunit 6
Peroxisomal biogenesis factor 3
3-keto-steroid reductase/17-beta-hydroxysteroid dehydrogenase 7
Breast cancer anti-estrogen resistance protein 1
Syntaxin-17
Cilia- and flagella-associated protein 298
tRNA (guanine-N(7)-)-methyltransferase non-catalytic subunit WDR4
Transmembrane protein 33
Gem-associated protein 4
Coronin-7
Nuclear pore complex protein Nup107
Gasdermin-D
Selenocysteine-specific elongation factor
Sestrin-2
Anthrax toxin receptor 2
Myotrophin
Actin-related protein 2/3 complex subunit 4
CD81 antigen
Protein transport protein Sec61 subunit gamma
Triosephosphate isomerase
Eukaryotic translation initiation factor 3 subunit E
Protein transport protein Sec61 subunit beta
Phosphatidylinositol 3,4,5-trisphosphate 3-phosphatase and dual-specificity protein phosphatas
Serine/threonine-protein phosphatase 4 catalytic subunit
Gamma-aminobutyric acid receptor-associated protein-like 2
Reactive oxygen species modulator 1
Myosin light polypeptide 6
Actin, cytoplasmic 1
Eukaryotic initiation factor 4A-I
Small ribosomal subunit protein uS10
Ribose-phosphate pyrophosphokinase 1
Proteasome subunit alpha type-6
Protein S100-A10
Cell division control protein 42 homolog
Destrin
Glia maturation factor beta
Ras-related protein Rab-8A
Signal peptidase complex subunit 3
Signal recognition particle subunit SRP54
Ras-related protein Rab-4B
Ras-related protein Rab-2A
Ras-related protein Rab-5B

Ras-related protein Rab-10
Ubiquitin-conjugating enzyme E2 D3
NEDD8-conjugating enzyme Ubc12
Ubiquitin-conjugating enzyme E2 K
Ubiquitin-conjugating enzyme E2 N
Ras-related protein Rab-14
Actin-related protein 3
Actin-related protein 2
Alpha-centractin
COP9 signalosome complex subunit 2
ATP-binding cassette sub-family E member 1
Ras-related protein Rap-1b
Ras-related protein Rap-2b
Protein max
Small ribosomal subunit protein eS1
Large ribosomal subunit protein uL24
Proteasome activator complex subunit 3
Large ribosomal subunit protein eL15
Large ribosomal subunit protein eL27
V-type proton ATPase subunit d 1
Large ribosomal subunit protein eL43
Transforming protein RhoA
Rho-related GTP-binding protein RhoE
N-alpha-acetyltransferase 20
10 kDa heat shock protein, mitochondrial
Protein transport protein Sec61 subunit alpha isoform 1
Prefoldin subunit 3
Syntaxin-binding protein 1
Beta-2-microglobulin
Dolichyl-diphosphooligosaccharide--protein glycosyltransferase subunit DAD1
NPC intracellular cholesterol transporter 2
Coatomer subunit zeta-1
Large ribosomal subunit protein eL37
Small ubiquitin-related modifier 2
DDB1- and CUL4-associated factor 7
WD repeat-containing protein 5
AP-1 complex subunit sigma-1A
Nuclear transport factor 2
Heterogeneous nuclear ribonucleoprotein K
14-3-3 protein gamma
Ubiquitin carboxyl-terminal hydrolase 46
Ras-related protein R-Ras2
Small ribosomal subunit protein eS7
Serine/threonine-protein phosphatase PP1-alpha catalytic subunit
Serine/threonine-protein phosphatase PP1-beta catalytic subunit
26S proteasome regulatory subunit 4
26S proteasome regulatory subunit 8
Small ribosomal subunit protein eS8
Small ribosomal subunit protein uS8
Small ribosomal subunit protein uS9

Ubiquitin-conjugating enzyme E2 G1
Ubiquitin-conjugating enzyme E2 H
14-3-3 protein epsilon
Small ribosomal subunit protein uS11
Small ribosomal subunit protein uS12
Small ribosomal subunit protein uS13
Small ribosomal subunit protein uS14
Small ribosomal subunit protein uS15
Small ribosomal subunit protein uS17
Small nuclear ribonucleoprotein E
Small nuclear ribonucleoprotein F
U6 snRNA-associated Sm-like protein LSm3
U6 snRNA-associated Sm-like protein LSm6
Small nuclear ribonucleoprotein Sm D1
Small nuclear ribonucleoprotein Sm D2
Small nuclear ribonucleoprotein Sm D3
Thymosin beta-4
ADP-ribosylation factor 6
26S proteasome regulatory subunit 10B
Thioredoxin reductase-like selenoprotein T
TATA box-binding protein-like 1
Large ribosomal subunit protein eL8
DNA-directed RNA polymerase II subunit RPB7
Ras-related protein Rab-11A
Eukaryotic peptide chain release factor subunit 1
CCHC-type zinc finger nucleic acid binding protein
Protein yippee-like 5
Small ribosomal subunit protein eS4, X isoform
Serine/threonine-protein phosphatase 2A catalytic subunit beta isoform
Actin, aortic smooth muscle
Rho-related GTP-binding protein RhoB
Large ribosomal subunit protein uL23
Small ribosomal subunit protein eS6
Histone H4
Ras-related protein Rab-1A
GTP-binding nuclear protein Ran
Large ribosomal subunit protein uL14
Ras-related protein Rap-1A
Small ribosomal subunit protein uS19
Small ribosomal subunit protein eS24
Small ribosomal subunit protein eS25
Small ribosomal subunit protein eS26
Small ribosomal subunit protein eS28
Ubiquitin-like FUBI-ribosomal protein eS30 fusion protein
Guanine nucleotide-binding protein G(I)/G(S)/G(T) subunit beta-1
E3 ubiquitin-protein ligase RBX1
Guanine nucleotide-binding protein G(I)/G(S)/G(T) subunit beta-2
Large ribosomal subunit protein eL30
Large ribosomal subunit protein eL39
Large ribosomal subunit protein eL31

Large ribosomal subunit protein uL1
Large ribosomal subunit protein eL32
Large ribosomal subunit protein uL5
Large ribosomal subunit protein uL2
Peptidyl-prolyl cis-trans isomerase A
Peptidyl-prolyl cis-trans isomerase FKBP1A
Ubiquitin-ribosomal protein eS31 fusion protein
Growth factor receptor-bound protein 2
Transformer-2 protein homolog beta
Ras-related C3 botulinum toxin substrate 1
AP-2 complex subunit beta
Vesicle-associated membrane protein 2
Guanine nucleotide-binding protein G(s) subunit alpha isoforms short
Guanine nucleotide-binding protein G(i) subunit alpha-1
14-3-3 protein zeta/delta
Serine/threonine-protein phosphatase 2A 55 kDa regulatory subunit B alpha isoform
Small ubiquitin-related modifier 1
Dynein light chain 1, cytoplasmic
Dynein light chain Tctex-type 1
Large ribosomal subunit protein eL38
S-phase kinase-associated protein 1
Guanine nucleotide-binding protein G(I)/G(S)/G(O) subunit gamma-5
Small ribosomal subunit protein eS21
Eukaryotic translation initiation factor 5A-1
Small ribosomal subunit protein RACK1
Transcription elongation factor SPT4
SUMO-conjugating enzyme UBC9
Thymosin beta-10
Serine/threonine-protein phosphatase 2A catalytic subunit alpha isoform
Y-box-binding protein 1
Signal peptidase complex catalytic subunit SEC11A
Casein kinase II subunit beta
Tropomyosin alpha-4 chain
Ubiquitin-conjugating enzyme E2 L3
Elongation factor 1-alpha 1
Tubulin alpha-1B chain
Tubulin alpha-4A chain
Tubulin beta-4B chain
Casein kinase II subunit alpha
Platelet-activating factor acetylhydrolase IB subunit alpha2
Histone H3.1
Hemoglobin subunit beta
Hemoglobin subunit alpha
Coxsackievirus and adenovirus receptor
Protein FAM193A
Nucleolar protein 14
Immunoglobulin-binding protein 1
Tyrosine-protein phosphatase non-receptor type substrate 1
Phosphoserine phosphatase
RNA-binding protein 6

Eukaryotic translation initiation factor 4 gamma 2
Ribonuclease P protein subunit p38
Ribonuclease P protein subunit p30
General transcription factor II-I
Phosphatidylinositol 5-phosphate 4-kinase type-2 beta
Contactin-associated protein 1
SRSF protein kinase 2
T-complex protein 1 subunit beta
UDP-galactose translocator
mRNA export factor RAE1
Glutathione S-transferase omega-1
DNA-dependent protein kinase catalytic subunit
Disintegrin and metalloproteinase domain-containing protein 17
Sushi repeat-containing protein SRPX
Arginase-2, mitochondrial
Endonuclease III-like protein 1
Interferon-induced 35 kDa protein
Nucleobindin-2
Brain acid soluble protein 1
Dermcidin
TATA element modulatory factor
Small ribosomal subunit protein mS22
Small ribosomal subunit protein mS25
Small ribosomal subunit protein uS10m
Small ribosomal subunit protein mS35
Small ribosomal subunit protein uS5m
Alpha-ketoglutarate dehydrogenase component 4
Small ribosomal subunit protein uS11m
Small ribosomal subunit protein uS15m
Small ribosomal subunit protein bS21m
Small ribosomal subunit protein mS34
Small ribosomal subunit protein bS6m
Small ribosomal subunit protein uS9m
SAP domain-containing ribonucleoprotein
Serine beta-lactamase-like protein LACTB, mitochondrial
Conserved oligomeric Golgi complex subunit 7
Large ribosomal subunit protein eL24
Thioredoxin-like protein 4A
Large ribosomal subunit protein eL42
Chromobox protein homolog 1
Mothers against decapentaplegic homolog 3
ADP-ribosylation factor 1
ADP-ribosylation factor 5
Enhancer of rudimentary homolog
Rho-related GTP-binding protein RhoG
Large ribosomal subunit protein eL19
Small EDRK-rich factor 2
Serine/arginine-rich splicing factor 3
Forkhead box protein K1
Vacuolar fusion protein CCZ1 homolog B

Disabled homolog 2
Basement membrane-specific heparan sulfate proteoglycan core protein
E3 ubiquitin-protein ligase XIAP
Ephrin-B1
RNA-binding protein 10
RNA-binding protein 3
Calcium-transporting ATPase type 2C member 1
Phospholipid-transporting ATPase IH
Cytochrome c
55 kDa erythrocyte membrane protein
Transcription factor A, mitochondrial
Phosphatidylinositol transfer protein alpha isoform
Solute carrier family 25 member 3
Vigilin
Transcription initiation factor IIB
Cyclin-dependent kinase 6
Cyclin-dependent kinase 5
Cyclin-dependent kinase 17
Transcriptional activator protein Pur-alpha
Cdc42 effector protein 1
Clathrin heavy chain 1
Heat shock factor protein 1
Nuclear factor NF-kappa-B p100 subunit
Peptidyl-prolyl cis-trans isomerase FKBP3
Receptor expression-enhancing protein 5
Sorbitol dehydrogenase
Heterogeneous nuclear ribonucleoprotein U
Splicing factor U2AF 35 kDa subunit
Spectrin beta chain, non-erythrocytic 1
Nucleolysin TIAR
Protein SET
Serine/arginine-rich splicing factor 2
Forkhead box protein K2
Transcription factor RelB
N-acetylgalactosamine kinase
AMP deaminase 2
Fatty acid-binding protein 5
Adenylyl cyclase-associated protein 1
Friend leukemia integration 1 transcription factor
Hydroxymethylglutaryl-CoA synthase, cytoplasmic
Interferon-induced transmembrane protein 3
Large neutral amino acids transporter small subunit 1
Protein Dr1
Exosome complex component 10
OTU domain-containing protein 4
ATP-dependent 6-phosphofructokinase, platelet type
DNA repair protein complementing XP-C cells
RNA-binding protein EWS
Cerebellar degeneration-related protein 2
Inositol polyphosphate 5-phosphatase OCRL

1-phosphatidylinositol 4,5-bisphosphate phosphodiesterase beta-3
Transgelin
A-kinase anchor protein 17A
Dihydroorotate dehydrogenase (quinone), mitochondrial
2-oxoglutarate dehydrogenase complex component E1
Kinesin-like protein KIF23
Methylmalonate-semialdehyde/malonate-semialdehyde dehydrogenase [acylating], mitochondr
Pro-neuregulin-1, membrane-bound isoform
Sterol 26-hydroxylase, mitochondrial
Transcription factor Sp3
Histone H1.1
Large ribosomal subunit protein eL20
Dual specificity mitogen-activated protein kinase kinase 1
Angiopoietin-1 receptor
Peptidyl-prolyl cis-trans isomerase FKBP4
Procollagen-lysine,2-oxoglutarate 5-dioxygenase 1
Nucleobindin-1
Large ribosomal subunit protein eL6
DNA topoisomerase 2-beta
A-kinase anchor protein 12
Mitochondrial 2-oxoglutarate/malate carrier protein
Dystonin
Protein ENL
Histone-lysine N-methyltransferase MECOM
Guanine nucleotide-binding protein subunit alpha-12
Caveolin-1
Aminoacylase-1
Tumor necrosis factor alpha-induced protein 2
Lamin-B2
6-pyruvoyl tetrahydrobiopterin synthase
Urokinase plasminogen activator surface receptor
Mevalonate kinase
DNA excision repair protein ERCC-6
Antigen peptide transporter 1
Antigen peptide transporter 2
CCAAT/enhancer-binding protein zeta
Zinc finger protein 92
Transcription factor p65
UBX domain-containing protein 1
1,4-alpha-glucan-branching enzyme
Eukaryotic translation initiation factor 4 gamma 1
Copper-transporting ATPase 1
Neurogenic locus notch homolog protein 2
Transducin-like enhancer protein 1
Transducin-like enhancer protein 3
Lactoylglutathione lyase
Activin receptor type-1
Single-stranded DNA-binding protein, mitochondrial
Proto-oncogene c-Rel
14-3-3 protein eta

Proteolipid protein 2
Cleavage stimulation factor subunit 1
Ubiquitin-protein ligase E3A
Dynamin-1
Tyrosine-protein phosphatase non-receptor type 12
Focal adhesion kinase 1
Serine/arginine-rich splicing factor 11
Protein kinase C delta type
Caldesmon
Folylpolyglutamate synthase, mitochondrial
CTD small phosphatase-like protein 2
Tyrosine-protein phosphatase non-receptor type 11
3-ketodihydrosphingosine reductase
Amidophosphoribosyltransferase
Glutamine--fructose-6-phosphate aminotransferase [isomerizing] 1
Exosome complex component RRP45
Proteasome activator complex subunit 1
Recombining binding protein suppressor of hairless
Myocyte-specific enhancer factor 2C
Amyloid beta precursor like protein 2
GA-binding protein alpha chain
E3 ubiquitin-protein ligase RING1
Fragile X messenger ribonucleoprotein 1
Peroxiredoxin-1
Large ribosomal subunit protein eL18
Complement component 1 Q subcomponent-binding protein, mitochondrial
Cytoskeleton-associated protein 4
Tight junction protein ZO-1
mRNA decay activator protein ZFP36L1
Sperm-associated antigen 1
KH domain-containing, RNA-binding, signal transduction-associated protein 1
Apoptosis regulator BAX
Bcl-2-like protein 1
Induced myeloid leukemia cell differentiation protein Mcl-1
DNA polymerase epsilon catalytic subunit A
Kinesin light chain 1
Son of sevenless homolog 1
Serine/arginine-rich splicing factor 1
Rho GTPase-activating protein 1
TLE family member 5
Serine/arginine-rich splicing factor 4
Protocadherin-1
Protein phosphatase 3 catalytic subunit alpha
ATP-dependent RNA helicase A
Quinone oxidoreductase
Golgin subfamily A member 3
Golgin subfamily A member 2
Peroxisomal bifunctional enzyme
Lactadherin
3',5'-cyclic-AMP phosphodiesterase 4D

Desmocollin-1
Pseudouridine-5'-phosphatase
Testican-1
Leukocyte surface antigen CD47
Peptidyl-prolyl cis-trans isomerase D
FACT complex subunit SSRP1
Calmodulin-regulated spectrin-associated protein 2
Protein spire homolog 1
Schlafen family member 5
Protein VAC14 homolog
RNA cytosine C(5)-methyltransferase NSUN2
Dystrophia myotonica WD repeat-containing protein
Histone-binding protein RBBP4
Nuclear cap-binding protein subunit 1
Alpha-1,6-mannosylglycoprotein 6-beta-N-acetylglucosaminyltransferase A
Histone acetyltransferase p300
Neuroblast differentiation-associated protein AHNAK
F-BAR domain only protein 2
Elongator complex protein 6
Heat shock 70 kDa protein 14
Secernin-3
Cingulin-like protein 1
Ragulator complex protein LAMTOR4
Nexilin
Alpha-1,6-mannosyl-glycoprotein 2-beta-N-acetylglucosaminyltransferase
Polypeptide N-acetylgalactosaminyltransferase 2
Polypeptide N-acetylgalactosaminyltransferase 1
AP-1 complex subunit beta-1
Cleavage and polyadenylation specificity factor subunit 1
Bone marrow stromal antigen 2
Mitochondrial-processing peptidase subunit alpha
CMP-N-acetylneuraminate-beta-galactosamide-alpha-2,3-sialyltransferase 1
Secernin-1
WASH complex subunit 5
Nuclear pore complex protein Nup160
Sterol regulatory element-binding protein cleavage-activating protein
Forkhead box protein O1
Transducin beta-like protein 3
General transcription factor 3C polypeptide 1
Twinfilin-1
Aspartyl/asparaginyl beta-hydroxylase
Alpha-globin transcription factor CP2
A-kinase anchor protein 13
EGF-containing fibulin-like extracellular matrix protein 1
SWI/SNF-related matrix-associated actin-dependent regulator of chromatin subfamily B membe
Nucleosome-remodeling factor subunit BPTF
Cell division cycle protein 20 homolog
Follistatin-related protein 1
Syntaxin-4
G-rich sequence factor 1

Mitogen-activated protein kinase kinase kinase kinase 2
Tyrosine-protein kinase Mer
Splicing factor, suppressor of white-apricot homolog
Chromodomain-helicase-DNA-binding protein 3
Splicing factor 3A subunit 3
Dihydropyrimidine dehydrogenase [NADP(+)]
TP53-binding protein 1
Transmembrane protein 115
Interferon-related developmental regulator 2
Tripartite motif-containing protein 26
Aminoacyl tRNA synthase complex-interacting multifunctional protein 1
Interleukin enhancer-binding factor 2
Interleukin enhancer-binding factor 3
Vesicular integral-membrane protein VIP36
Heat shock protein 75 kDa, mitochondrial
TNF receptor-associated factor 2
Disks large homolog 1
Unconventional myosin-Ie
Nuclear inhibitor of protein phosphatase 1
Protein tyrosine phosphatase type IVA 2
Active breakpoint cluster region-related protein
GATOR1 complex protein NPRL3
Vesicle transport protein SEC20
BCL2/adenovirus E1B 19 kDa protein-interacting protein 2
Transcriptional repressor NF-X1
Cleavage stimulation factor subunit 3
Tetraspanin-31
Delta(3,5)-Delta(2,4)-dienoyl-CoA isomerase, mitochondrial
Rho GTPase-activating protein 5
Striatin-3
Cell division cycle protein 16 homolog
Serine/threonine-protein kinase 4
Protein flightless-1 homolog
E3 ubiquitin-protein ligase TRIM32
Bifunctional coenzyme A synthase
Large ribosomal subunit protein bL28m
Acetyl-CoA carboxylase 1
COP9 signalosome complex subunit 1
Ubiquitin carboxyl-terminal hydrolase 4
Chromatin assembly factor 1 subunit A
Chromatin assembly factor 1 subunit B
Protein Red
S-methyl-5'-thioadenosine phosphorylase
5'-AMP-activated protein kinase catalytic subunit alpha-1
Liprin-alpha-1
Translation initiation factor eIF2B subunit epsilon
TAR DNA-binding protein 43
Heterogeneous nuclear ribonucleoprotein A0
Aminoacyl tRNA synthase complex-interacting multifunctional protein 2
FAS-associated death domain protein

Peroxiredoxin-4
Dual specificity mitogen-activated protein kinase kinase 5
Mitogen-activated protein kinase 7
Serine/threonine-protein kinase PAK 2
Chromobox protein homolog 3
Serine/threonine-protein kinase 3
Syntaxin-5
26S proteasome non-ATPase regulatory subunit 2
Multimerin-1
Probable ATP-dependent RNA helicase DDX10
DnaJ homolog subfamily C member 3
Methanethiol oxidase
Nucleoside diphosphate kinase 3
Serine/arginine-rich splicing factor 9
Serine/arginine-rich splicing factor 5
Serine/arginine-rich splicing factor 6
Mitotic spindle assembly checkpoint protein MAD2A
Transcription intermediary factor 1-beta
Semaphorin-3F
Syntaxin-3
Ras GTPase-activating protein-binding protein 1
N-myc-interactor
Inactive tyrosine-protein kinase 7
Polyadenylate-binding protein 4
Serine-protein kinase ATM
Growth factor receptor-bound protein 10
Interferon-induced protein with tetratricopeptide repeats 5
Metastasis-associated protein MTA1
Eukaryotic translation initiation factor 3 subunit I
RING-type E3 ubiquitin-protein ligase PPIL2
Serine/threonine-protein phosphatase 2A 56 kDa regulatory subunit gamma isoform
C-terminal-binding protein 1
Phosducin-like protein
Phospholipase D1
Probable methyltransferase TARBP1
Ubiquitin-conjugating enzyme E2 variant 1
Large ribosomal subunit protein mL49
Cytoplasmic dynein 1 intermediate chain 2
Origin recognition complex subunit 2
Integrin-linked protein kinase
NAD(P) transhydrogenase, mitochondrial
Beta-2-syntrophin
DNA repair protein XRCC4
Peptidyl-prolyl cis-trans isomerase G
Treacle protein
Protein unc-119 homolog A
Splicing factor 3B subunit 2
Protein OS-9
Golgin subfamily A member 4
28 kDa heat- and acid-stable phosphoprotein

Disintegrin and metalloproteinase domain-containing protein 9
Disintegrin and metalloproteinase domain-containing protein 15
Transmembrane emp24 domain-containing protein 1
Peptidyl-prolyl cis-trans isomerase FKBP5
Unconventional myosin-IXb
Rho-associated protein kinase 1
Nuclear factor of activated T-cells, cytoplasmic 2
DNA topoisomerase 3-alpha
Mothers against decapentaplegic homolog 4
V-type proton ATPase 116 kDa subunit a 3
Baculoviral IAP repeat-containing protein 2
Phosphatidylinositol-binding clathrin assembly protein
Myotubularin
Sequestosome-1
Metaxin-1
Tubulin beta-3 chain
Serine/threonine-protein kinase PRP4 homolog
Peptidyl-prolyl cis-trans isomerase NIMA-interacting 1
Serine/threonine-protein kinase ATR
Eukaryotic translation initiation factor 4E-binding protein 1
Receptor-interacting serine/threonine-protein kinase 1
Histone deacetylase 1
Calcium/calmodulin-dependent protein kinase type II subunit gamma
Calcium/calmodulin-dependent protein kinase type II subunit delta
Dynactin subunit 2
Polycystin-2
NEDD8-activating enzyme E1 regulatory subunit
Inositol-tetrakisphosphate 1-kinase
SNW domain-containing protein 1
Diacylglycerol kinase zeta
Stromal interaction molecule 1
GRB2-related adapter protein
Transformer-2 protein homolog alpha
Sorting nexin-1
KRR1 small subunit processome component homolog
Periodic tryptophan protein 1 homolog
Myotubularin-related protein 1
Myotubularin-related protein 2
Cullin-1
Cullin-2
Cullin-3
Cullin-4A
Cullin-4B
Apoptosis-stimulating of p53 protein 2
Dual specificity tyrosine-phosphorylation-regulated kinase 1A
GDP-L-fucose synthase
Ras-related protein Rab-31
Ras-related protein Rab-32
Trophoblast glycoprotein
Four and a half LIM domains protein 1

Four and a half LIM domains protein 3
Ras and Rab interactor 1
Angio-associated migratory cell protein
Mannosyl-oligosaccharide glucosidase
CD166 antigen
THO complex subunit 5 homolog
Protein Shroom2
Spectrin alpha chain, non-erythrocytic 1
Nucleolar GTP-binding protein 2
Methylglutaconyl-CoA hydratase, mitochondrial
Spliceosome RNA helicase DDX39B
Bleomycin hydrolase
Exosome complex component RRP4
Bone morphogenetic protein receptor type-2
Tubulin beta-2A chain
General transcription factor IIH subunit 2
General transcription factor IIH subunit 3
Bystin
Rap guanine nucleotide exchange factor 1
Isopentenyl-diphosphate Delta-isomerase 1
Core-binding factor subunit beta
Nuclear transcription factor Y subunit gamma
Cyclin-dependent kinase 13
Cytoskeleton-associated protein 5
Cold-inducible RNA-binding protein
Calcium/calmodulin-dependent protein kinase type 1
Coactosin-like protein
Heterogeneous nuclear ribonucleoprotein D0
Lysosome membrane protein 2
Nidogen-2
Low-density lipoprotein receptor-related protein 8
Dystroglycan 1
Ribosome biogenesis protein BOP1
Ubiquitin conjugation factor E4 A
Septin-6
Kelch-like ECH-associated protein 1
Unhealthy ribosome biogenesis protein 2 homolog
Probable ATP-dependent RNA helicase DHX34
MORC family CW-type zinc finger protein 3
Scaffold attachment factor B2
Eukaryotic translation initiation factor 3 subunit A
Rho guanine nucleotide exchange factor 7
Protein EFR3 homolog A
Ubiquitin-associated protein 2-like
Protein scribble homolog
ARF GTPase-activating protein GIT2
Scavenger receptor class F member 1
Malectin
Tubulin--tyrosine ligase-like protein 12
DNA polymerase alpha subunit B

Dedicator of cytokinesis protein 1
Transcription factor Dp-1
Four and a half LIM domains protein 2
Dihydropyrimidinase-related protein 3
Large ribosomal subunit protein mL62
Zinc finger MYM-type protein 3
Dynactin subunit 1
Cytoplasmic dynein 1 heavy chain 1
Translation initiation factor eIF2B subunit alpha
Eukaryotic initiation factor 4A-II
Elongin-A
Src substrate cortactin
Endonuclease G, mitochondrial
Flotillin-2
Reticulocalbin-2
E3 ubiquitin/ISG15 ligase TRIM25
Filamin-C
Peptidyl-prolyl cis-trans isomerase FKBP8
Protein FAM50A
Protein FRG1
Guanine nucleotide-binding protein subunit alpha-13
Guanidinoacetate N-methyltransferase
UDP-glucose 4-epimerase
Transforming growth factor beta activator LRRC32
Caprin-1
Beclin-1
RNA-binding protein 39
Enhancer of filamentation 1
Helicase-like transcription factor
Squalene monooxygenase
Protein disulfide-isomerase A5
Phosphoribosyl pyrophosphate synthase-associated protein 1
ATP-dependent RNA helicase DHX8
DNA replication licensing factor MCM6
Inositol 1,4,5-trisphosphate receptor type 2
Inositol 1,4,5-trisphosphate receptor type 3
Inositol 1,4,5-trisphosphate receptor type 1
Ras GTPase-activating protein 3
Plastin-1
Interferon regulatory factor 3
EKC/KEOPS complex subunit LAGE3
Bridge-like lipid transfer protein family member 2
E3 ubiquitin-protein ligase TRIP12
Pumilio homolog 1
Mediator of DNA damage checkpoint protein 1
Clathrin interactor 1
KN motif and ankyrin repeat domain-containing protein 1
Structural maintenance of chromosomes protein 1A
Ribosomal RNA processing protein 1 homolog B
Disco-interacting protein 2 homolog A

Protein RRP5 homolog
DNA replication complex GINS protein PSF1
Ribosome biogenesis protein BMS1 homolog
Phosphatidate phosphatase LPIN1
Ubiquitin carboxyl-terminal hydrolase 10
LRP chaperone MESD
Neutral alpha-glucosidase AB
Raftlin
Major facilitator superfamily domain-containing protein 10
Serine/threonine-protein phosphatase 2A 56 kDa regulatory subunit delta isoform
Delta(14)-sterol reductase LBR
Conserved oligomeric Golgi complex subunit 2
Major vault protein
Latent-transforming growth factor beta-binding protein 1
Latent-transforming growth factor beta-binding protein 2
Chromobox protein homolog 2
Golgin subfamily B member 1
Caspase-8
N-alpha-acetyltransferase 30
Kinesin-like protein KIF22
Myocyte-specific enhancer factor 2D
Chromodomain-helicase-DNA-binding protein 4
LIM and SH3 domain protein 1
Prostaglandin reductase 1
Dr1-associated corepressor
Nuclear factor of activated T-cells, cytoplasmic 4
Zinc finger protein 638
Importin subunit beta-1
Nucleolar and coiled-body phosphoprotein 1
Nuclear mitotic apparatus protein 1
Proteasome activator complex subunit 4
Cullin-7
Sarcolemmal membrane-associated protein
GTPase-activating protein and VPS9 domain-containing protein 1
N-alpha-acetyltransferase 25, NatB auxiliary subunit
Ventricular zone-expressed PH domain-containing protein homolog 1
Condensin complex subunit 2
PCNA-associated factor
Signal peptidase complex subunit 2
ER membrane protein complex subunit 2
Pre-mRNA-splicing regulator WTAP
26S proteasome non-ATPase regulatory subunit 6
Homocysteine-responsive endoplasmic reticulum-resident ubiquitin-like domain member 1 prot
MAD2L1-binding protein
Mortality factor 4-like protein 2
BRISC complex subunit Abraxas 2
Septin-2
Squamous cell carcinoma antigen recognized by T-cells 3
Condensin complex subunit 1
Polycomb protein SUZ12

Exosome complex component RRP42
TNFAIP3-interacting protein 1
116 kDa U5 small nuclear ribonucleoprotein component
Leucine--tRNA ligase, mitochondrial
R3H domain-containing protein 1
Translocating chain-associated membrane protein 2
Sorting nexin-17
ADP-ribosylation factor-like protein 6-interacting protein 1
Rab3 GTPase-activating protein catalytic subunit
Metal cation symporter ZIP14
Lysine--tRNA ligase
Histone-lysine N-methyltransferase SETDB1
Leucine-rich repeat-containing protein 14
Ribosome biogenesis regulatory protein homolog
DNA polymerase delta subunit 3
Eukaryotic translation initiation factor 4H
Arf-GAP with coiled-coil, ANK repeat and PH domain-containing protein 2
Kinesin-like protein KIF14
Bromodomain-containing protein 3
WD repeat-containing protein 43
Peroxisomal acyl-coenzyme A oxidase 1
Mitochondrial inner membrane protein OXA1L
Early endosome antigen 1
Protein disulfide-isomerase A6
Platelet-activating factor acetylhydrolase IB subunit alpha1
[Pyruvate dehydrogenase (acetyl-transferring)] kinase isozyme 3, mitochondrial
Astrocytic phosphoprotein PEA-15
3-beta-hydroxysteroid-Delta(8),Delta(7)-isomerase
Phosphomevalonate kinase
Serine/threonine-protein kinase D1
Plectin
Pericentriolar material 1 protein
BOS complex subunit NOMO1
Serum paraoxonase/arylesterase 2
Serine/threonine-protein phosphatase 2A 56 kDa regulatory subunit alpha isoform
Inorganic pyrophosphatase
Prostaglandin E synthase 3
Serine/threonine-protein kinase 38
Non-POU domain-containing octamer-binding protein
Serine/threonine-protein phosphatase 2A activator
Receptor-type tyrosine-protein phosphatase kappa
Periodic tryptophan protein 2 homolog
Rab GTPase-binding effector protein 1
Ras-related protein Rab-35
RNA-binding protein with serine-rich domain 1
Retinoblastoma-binding protein 5
Reticulocalbin-1
RalA-binding protein 1
Lethal(2) giant larvae protein homolog 1
Leucine-rich repeat-containing protein 41

Transcription termination factor 1
Transmembrane emp24 domain-containing protein 2
Poly(rC)-binding protein 1
Poly(rC)-binding protein 2
Elongin-C
Elongin-B
GTP-binding protein Rheb
Ubiquitin-protein ligase E3C
Mitochondrial import receptor subunit TOM20 homolog
Mitochondrial fission regulator 1
Delta(24)-sterol reductase
Splicing factor 3B subunit 3
Pumilio homolog 3
Disks large-associated protein 5
Ras suppressor protein 1
Calponin-3
Ribosomal protein S6 kinase alpha-1
Scaffold attachment factor B1
Splicing factor 3A subunit 2
RNA-binding motif, single-stranded-interacting protein 2
Protein phosphatase 1 regulatory subunit 7
Protein transport protein Sec23A
Protein transport protein Sec23B
Cytohesin-1
Splicing factor 3A subunit 1
Superkiller complex protein 2
Surfeit locus protein 1
Transcription initiation factor TFIID subunit 5
Transcription initiation factor TFIID subunit 7
Telomeric repeat-binding factor 2
Microtubule-associated protein RP/EB family member 2
Na(+)/H(+) exchange regulatory cofactor NHE-RF2
Tumor necrosis factor receptor type 1-associated DEATH domain protein
Translocating chain-associated membrane protein 1
Translin
RISC-loading complex subunit TARBP2
Splicing factor 1
Cdc42-interacting protein 4
Thyroid receptor-interacting protein 11
Pachytene checkpoint protein 2 homolog
Mediator of RNA polymerase II transcription subunit 1
Activating signal cointegrator 1
High mobility group nucleosome-binding domain-containing protein 3
Probable JmjC domain-containing histone demethylation protein 2C
NF-kappa-B inhibitor beta
Thyroid receptor-interacting protein 6
Tyrosine-protein phosphatase non-receptor type 14
Microtubule-associated protein RP/EB family member 1
ELAV-like protein 1
Sterol-4-alpha-carboxylate 3-dehydrogenase, decarboxylating

Myosin light chain kinase, smooth muscle
TGF-beta-activated kinase 1 and MAP3K7-binding protein 1
Probable E3 ubiquitin-protein ligase HERC1
Neutral amino acid transporter B(0)
Myeloid leukemia factor 2
Mitochondrial import receptor subunit TOM34
Mothers against decapentaplegic homolog 2
Mothers against decapentaplegic homolog 1
Methylsterol monooxygenase 1
Intersectin-1
Tubulin-specific chaperone E
Tubulin-specific chaperone C
Ubiquitin-conjugating enzyme E2 variant 2
Serine/threonine-protein kinase STK11
Syntaxin-binding protein 2
Vesicle-associated membrane protein 3
NEDD8
V-type proton ATPase subunit S1
Vacuolar protein sorting-associated protein 72 homolog
Ras-related protein Rab-11B
Zyxin
Electron transfer flavoprotein-ubiquinone oxidoreductase, mitochondrial
Septin-7
Proteasomal ubiquitin receptor ADRM1
Coiled-coil domain-containing protein 6
UDP-N-acetylhexosamine pyrophosphorylase
Transcription factor E2F4
Insulin-like growth factor-binding protein 7
Programmed cell death protein 2
Laminin subunit alpha-4
26S proteasome non-ATPase regulatory subunit 5
Serine/threonine-protein kinase N1
Serine/threonine-protein kinase N2
Transcription initiation factor TFIID subunit 12
Cysteine and glycine-rich protein 2
DNA damage-binding protein 1
Serine/threonine-protein phosphatase 2A 56 kDa regulatory subunit epsilon isoform
Mitogen-activated protein kinase 14
Large ribosomal subunit protein uL23m
Hsp90 co-chaperone Cdc37
Dihydropyrimidinase-related protein 2
Synaptophysin-like protein 1
Histone-binding protein RBBP7
Mitogen-activated protein kinase kinase kinase 11
Transcription initiation factor TFIID subunit 9
Calcitonin gene-related peptide type 1 receptor
Bcl-2 homologous antagonist/killer
Serine/arginine-rich splicing factor 7
Cleavage and polyadenylation specificity factor subunit 6
Survival motor neuron protein

Drebrin
Nuclear respiratory factor 1
Fascin
Gamma-interferon-inducible protein 16
2,4-dienoyl-CoA reductase [(3E)-enoyl-CoA-producing], mitochondrial
Alpha-mannosidase 2
NADH dehydrogenase [ubiquinone] 1 alpha subcomplex subunit 5
Ceramide glucosyltransferase
ATP-dependent Clp protease proteolytic subunit, mitochondrial
Thiosulfate sulfurtransferase
Ubiquitin-conjugating enzyme E2 S
Kynurenine--oxoglutarate transaminase 1
Guanylate kinase
Hydroxyacylglutathione hydrolase, mitochondrial
NADH dehydrogenase [ubiquinone] 1 alpha subcomplex subunit 9, mitochondrial
NADP-dependent malic enzyme, mitochondrial
Phosphoenolpyruvate carboxykinase [GTP], mitochondrial
Uridine phosphorylase 1
Discoidin domain-containing receptor 2
Hydroxyacyl-coenzyme A dehydrogenase, mitochondrial
Lanosterol 14-alpha demethylase
UTP--glucose-1-phosphate uridylyltransferase
6-phosphofructo-2-kinase/fructose-2,6-bisphosphatase 3
Thioredoxin reductase 1, cytoplasmic
MICOS complex subunit MIC60
Endothelial cell-specific chemotaxis regulator
Lysine-rich nucleolar protein 1
Heterogeneous nuclear ribonucleoprotein U-like protein 2
Transmembrane protein 132A
Inverted formin-2
Sister chromatid cohesion protein PDS5 homolog A
Protein CLEC16A
Kinesin-like protein KIF7
Rho GTPase-activating protein 31
Methenyltetrahydrofolate synthase domain-containing protein
AP2-associated protein kinase 1
WASH complex subunit 4
DNA excision repair protein ERCC-6-like
Pre-rRNA-processing protein TSR1 homolog
Ral GTPase-activating protein subunit alpha-2
Protein C-mannosyl-transferase DPY19L1
Isoamyl acetate-hydrolyzing esterase 1 homolog
GDP-Man:Man(3)GlcNAc(2)-PP-Dol alpha-1,2-mannosyltransferase
Transcriptional regulator QRICH1
WD40 repeat-containing protein SMU1
Cytoplasmic tRNA 2-thiolation protein 2
Protein-glucosylgalactosylhydroxylysine glucosidase
Leucine-rich repeat flightless-interacting protein 1
CDP-diacylglycerol--glycerol-3-phosphate 3-phosphatidyltransferase, mitochondrial
UPF0711 protein C18orf21

Prolyl 3-hydroxylase 1
tRNA (guanine(37)-N1)-methyltransferase
Echinoderm microtubule-associated protein-like 3
DNA-directed RNA polymerase I subunit RPA43
MAP7 domain-containing protein 1
UDP-N-acetylhexosamine pyrophosphorylase-like protein 1
Solute carrier family 25 member 35
Chromodomain-helicase-DNA-binding protein 9
Triokinase/FMN cyclase
Protein LSM12
Inactive hydroxysteroid dehydrogenase-like protein 1
Mitochondrial 10-formyltetrahydrofolate dehydrogenase
N-acetylglucosamine-1-phosphotransferase subunits alpha/beta
Girdin
Rab-like protein 6
Mitochondrial import inner membrane translocase subunit TIM50
Protein mono-ADP-ribosyltransferase PARP14
Alpha-(1,3)-fucosyltransferase 11
Cytokine-like nuclear factor N-PAC
UPF0489 protein C5orf22
Vacuolar protein sorting-associated protein 26B
La-related protein 7
NAD kinase 2, mitochondrial
BTB/POZ domain-containing protein KCTD21
Glucoside xylosyltransferase 1
Malonate--CoA ligase ACSF3, mitochondrial
Prolyl endopeptidase-like
TBC1 domain family member 10B
Taperin
Anoctamin-6
Filamin A-interacting protein 1-like
GRIP1-associated protein 1
Zinc-regulated GTPase metalloprotein activator 1F
Protein MIX23
Membralin
Protein FAM98B
Rho GTPase-activating protein 29
Programmed cell death protein 4
Fibronectin type III domain-containing protein 3B
CREB-regulated transcription coactivator 2
Glycerol-3-phosphate acyltransferase 3
Centrosomal protein of 55 kDa
Nuclear cap-binding protein subunit 3
Quinone oxidoreductase PIG3
Novel acetylcholine receptor chaperone
Pleckstrin homology-like domain family A member 2
PDZ and LIM domain protein 3
Protein mono-ADP-ribosyltransferase PARP10
Very-long-chain 3-oxoacyl-CoA reductase
mRNA export factor GLE1

Ubiquitin carboxyl-terminal hydrolase 39
Kelch-like protein 22
Acylglycerol kinase, mitochondrial
Histone-lysine N-methyltransferase SETMAR
Endoribonuclease LACTB2
Pyrroline-5-carboxylate reductase 3
EARP and GARP complex-interacting protein 1
Borealin
Cordon-bleu protein-like 1
HCLS1-binding protein 3
Plasma membrane ascorbate-dependent reductase CYBRD1
WD repeat-containing protein 81
Beta-actin-like protein 2
Coiled-coil domain-containing protein 93
OCIA domain-containing protein 2
Puratrophin-1
Putative heat shock protein HSP 90-alpha A4
DDB1- and CUL4-associated factor 6
Transmembrane protein 41B
Sigma intracellular receptor 2
Protein YIF1B
DBIRD complex subunit ZNF326
Nucleolar MIF4G domain-containing protein 1
RILP-like protein 1
DnaJ homolog subfamily C member 21
Tumor necrosis factor alpha-induced protein 8-like protein 3
Probable E3 ubiquitin-protein ligase HERC4
GPI ethanolamine phosphate transferase 2
Serine/threonine-protein phosphatase 6 regulatory subunit 3
Probable ATP-dependent RNA helicase DDX60-like
Rab-like protein 3
Protein FAM76B
2-methoxy-6-polyprenyl-1,4-benzoquinol methylase, mitochondrial
SH3 domain-containing protein 19
NHS-like protein 2
ER membrane protein complex subunit 4
Nondiscriminating glutamyl-tRNA synthetase EARS2, mitochondrial
Uncharacterized protein C3orf38
EEF1A lysine methyltransferase 2
Transport and Golgi organization protein 1 homolog
Presequence protease, mitochondrial
WD repeat-containing protein 44
Protein PRRC2B
Tight junction-associated protein 1
RRP12-like protein
Cytochrome c oxidase assembly factor 6 homolog
Torsin-1A-interacting protein 1
Centrosomal protein of 78 kDa
Alanine--tRNA ligase, mitochondrial
Torsin-2A

PCI domain-containing protein 2
Intracellular hyaluronan-binding protein 4
Long-chain fatty acid transport protein 3
Sterile alpha motif domain-containing protein 9
Serine/threonine-protein phosphatase 4 regulatory subunit 3B
WD repeat domain phosphoinositide-interacting protein 3
WD repeat domain phosphoinositide-interacting protein 1
EGF domain-specific O-linked N-acetylglucosamine transferase
Protein Smaug homolog 2
Tubulin-specific chaperone cofactor E-like protein
Deoxynucleotidyltransferase terminal-interacting protein 2
Rab GTPase-activating protein 1-like
Tetratricopeptide repeat protein 38
Cytochrome c oxidase assembly protein COX20, mitochondrial
Exosome complex component MTR3
Transmembrane protein 201
Alpha-tubulin N-acetyltransferase 1
Synaptosomal-associated protein 47
Nucleoporin NUP188
Heterochromatin protein 1-binding protein 3
Valine--tRNA ligase, mitochondrial
Centrosomal protein of 170 kDa
Low density lipoprotein receptor adapter protein 1
Protein odr-4 homolog
Polynucleotide 5'-hydroxyl-kinase NOL9
NHS-like protein 1
Putative hydroxypyruvate isomerase
Tumor protein p63-regulated gene 1-like protein
Coiled-coil and C2 domain-containing protein 1B
Formin-binding protein 1-like
Probable arginine--tRNA ligase, mitochondrial
Acyl-coenzyme A thioesterase THEM4
Putative coiled-coil-helix-coiled-coil-helix domain-containing protein CHCHD2P9, mitochondrial
FK506-binding protein 15
Zinc finger CCCH domain-containing protein 13
Putative methyltransferase C9orf114
Armadillo-like helical domain-containing protein 3
CSC1-like protein 2
G patch domain-containing protein 4
Putative transferase CAF17, mitochondrial
E3 ubiquitin-protein ligase HECTD3
Inactive glycosyltransferase 25 family member 3
E3 ubiquitin-protein ligase UBR4
Syntaxin-binding protein 5
Rho GTPase-activating protein 21
Calmodulin-regulated spectrin-associated protein 1
Large ribosomal subunit protein uL2m
Ubiquitin-associated protein 2
Queuosine 5'-phosphate N-glycosylase/hydrolase
Keratinocyte proline-rich protein

Zinc finger protein 618
Acyl-CoA-binding domain-containing protein 5
RNA-binding protein 26
ATPase family AAA domain-containing protein 3B
Protein wntless homolog
Integrator complex subunit 11
Ceramide-1-phosphate transfer protein
DDB1- and CUL4-associated factor 8
Collagen alpha-1(XIII) chain
Terminal uridylyltransferase 4
Protein furry homolog
Ribonuclease H2 subunit B
ATP synthase mitochondrial F1 complex assembly factor 1
Roquin-1
Protein DDI1 homolog 2
Protein THEMIS2
Arginine-hydroxylase NDUFAF5, mitochondrial
5'-nucleotidase domain-containing protein 1
Brefeldin A-inhibited guanine nucleotide-exchange protein 3
Intermembrane lipid transfer protein VPS13D
Rootletin
Complex III assembly factor LYRM7
Ras-interacting protein 1
Telomere-associated protein RIF1
Vacuolar protein sorting-associated protein 53 homolog
Striatin-interacting protein 1
Serine/threonine-protein kinase MRCK alpha
Regulation of nuclear pre-mRNA domain-containing protein 2
E3 ubiquitin-protein ligase RNF220
Pre-mRNA-splicing factor 38B
E3 ubiquitin-protein ligase BRE1A
Myomegalin
Zinc finger protein 318
Protein FAM171A1
Divergent protein kinase domain 1B
Leucine-rich repeat and calponin homology domain-containing protein 2
Threonylcarbamoyladenosine tRNA methylthiotransferase
BRO1 domain-containing protein BROX
Focadhesin
Lysine-specific demethylase 9
Disabled homolog 2-interacting protein
Lysophospholipase-like protein 1
Platelet endothelial aggregation receptor 1
Proteasome adapter and scaffold protein ECM29
Terminal uridylyltransferase 7
SH2 domain-containing adapter protein E
DENN domain-containing protein 4C
N-alpha-acetyltransferase 35, NatC auxiliary subunit
Zinc finger MYM-type protein 4
FHF complex subunit HOOK interacting protein 2A

Palmitoyltransferase ZDHHC20
SPRY domain-containing protein 7
F-box/WD repeat-containing protein 9
CD276 antigen
E3 ubiquitin-protein ligase RNF213
Tensin-2
KN motif and ankyrin repeat domain-containing protein 2
WASH complex subunit 2A
Lysophospholipid acyltransferase LPCAT4
Metalloreductase STEAP3
Protein FAM91A1
TBC1 domain family member 9B
Microtubule-associated protein 1S
Serine/threonine-protein phosphatase 2A 55 kDa regulatory subunit B delta isoform
ADP-ribosylation factor-like protein 6-interacting protein 4
Autophagy-related protein 16-1
Heparan-alpha-glucosaminide N-acetyltransferase
AT-rich interactive domain-containing protein 2
U3 small nucleolar RNA-associated protein 25 homolog
Glycosyltransferase 8 domain-containing protein 1
Protein sel-1 homolog 3
Tensin-3
HAUS augmin-like complex subunit 3
Protein SPT2 homolog
Acyl-coenzyme A thioesterase MBLAC2
Very large A-kinase anchor protein
Integrator complex subunit 3
Rho GTPase-activating protein 17
CWF19-like protein 1
Protein virilizer homolog
Cytospin-A
HEAT repeat-containing protein 6
Ankyrin repeat domain-containing protein 40
Protein WWC2
Scavenger receptor class A member 3
Atlastin-3
Ribosomal protein uL30-like
Nucleoredoxin
Tetratricopeptide repeat protein 19, mitochondrial
IQ motif and SEC7 domain-containing protein 1
Anamorsin
AN1-type zinc finger protein 6
Histone-lysine N-trimethyltransferase SMYD5
OTU domain-containing protein 7B
THO complex subunit 7 homolog
Glutamine-dependent NAD(+) synthetase
Elongator complex protein 2
Ragulator complex protein LAMTOR1
Dehydrogenase/reductase SDR family member 7B
Twinfilin-2

Condensin-2 complex subunit H2
Uncharacterized protein KIAA0930
Transport and Golgi organization protein 2 homolog
Serine/threonine-protein phosphatase 4 regulatory subunit 3A
Ras-related protein Rab-12
DENN domain-containing protein 5A
Splicing regulator SDE2
Keratin, type II cytoskeletal 80
Nipped-B-like protein
Mitochondrial adenyl nucleotide antiporter SLC25A25
2',5'-phosphodiesterase 12
N-alpha-acetyltransferase 16, NatA auxiliary subunit
Putative DENN domain-containing protein 10 B
Rhomboid domain-containing protein 2
Mitochondrial adenyl nucleotide antiporter SLC25A24
Receptor expression-enhancing protein 3
All-trans-retinol 13,14-reductase
RAD50-interacting protein 1
Transmembrane protein 214
3-hydroxyisobutyryl-CoA hydrolase, mitochondrial
RWD domain-containing protein 4
Nucleolus and neural progenitor protein
Armadillo repeat-containing protein 6
TELO2-interacting protein 2
Ankyrin repeat domain-containing protein 54
Zinc transporter 6
KN motif and ankyrin repeat domain-containing protein 3
Bifunctional arginine demethylase and lysyl-hydroxylase JMJD6
Phostensin
Caveolae-associated protein 1
Zinc finger CCHC domain-containing protein 8
Putative ATP-dependent RNA helicase DHX57
Endoplasmic reticulum aminopeptidase 2
Lysophospholipid acyltransferase 5
Parafibromin
Large ribosomal subunit protein uL14m
Long-chain fatty acid transport protein 4
Coiled-coil and C2 domain-containing protein 1A
Deoxyribonuclease TATDN1
tRNA N(3)-methylcytidine methyltransferase METTL2B
Myb/SANT-like DNA-binding domain-containing protein 2
Transcription initiation factor TFIID subunit 2
UPF0598 protein C8orf82
Mediator of RNA polymerase II transcription subunit 27
Enhancer of mRNA-decapping protein 4
Protein arginine N-methyltransferase 9
Pre-mRNA-processing-splicing factor 8
F-box only protein 42
SCY1-like protein 2
Tetratricopeptide repeat protein 27

Sideroflexin-4
Protein GOLM2
Nuclear factor related to kappa-B-binding protein
Acylpyruvase FAHD1, mitochondrial
Ribosomal protein eL22-like
Serine/threonine-protein kinase N3
RNA demethylase ALKBH5
Pyridoxal-dependent decarboxylase domain-containing protein 1
MTOR-associated protein MEAK7
Integrator complex subunit 5
GTPase IMAP family member 6
Long-chain fatty acid transport protein 1
Glucose 1,6-bisphosphate synthase
RNA polymerase-associated protein CTR9 homolog
Alpha- and gamma-adaptin-binding protein p34
Transmembrane protein 88
Proline/serine-rich coiled-coil protein 1
Superkiller complex protein 3
Serine/threonine-protein kinase ULK3
Aspartate--tRNA ligase, mitochondrial
Transmembrane protein 65
INO80 complex subunit C
F-box only protein 38
Neutral cholesterol ester hydrolase 1
Fidgetin-like protein 1
E3 ubiquitin-protein ligase TRIM65
Inactive rhomboid protein 2
Mitotic deacetylase-associated SANT domain protein
BRCA1-associated ATM activator 1
GATOR2 complex protein WDR59
Zinc finger CCCH domain-containing protein 14
Coiled-coil domain-containing protein 137
Thioredoxin domain-containing protein 11
La-related protein 1
ATPase family AAA domain-containing protein 2
Protein TMED8
Proton-coupled zinc antiporter SLC30A9, mitochondrial
E3 ubiquitin-protein ligase TRAF7
Rapamycin-insensitive companion of mTOR
WD repeat-containing protein 74
Type-1 angiotensin II receptor-associated protein
SWI/SNF-related matrix-associated actin-dependent regulator of chromatin subfamily D membe
Monofunctional C1-tetrahydrofolate synthase, mitochondrial
Aftiphilin
Pre-mRNA 3'-end-processing factor FIP1
CREB-regulated transcription coactivator 3
CREB-regulated transcription coactivator 1
Cytochrome P450 20A1
Protein O-glucosyltransferase 2
Transmembrane protein 205

E3 ubiquitin-protein ligase LRSAM1
Golgi-associated kinase 1B
Lysocardiolipin acyltransferase 1
BRCA1-A complex subunit Abraxas 1
Spliceosome-associated protein CWC27 homolog
Protein disulfide isomerase CRELD2
WD repeat-containing protein 82
MICOS complex subunit MIC27
Protocadherin Fat 4
Dipeptidyl peptidase 8
Activating transcription factor 7-interacting protein 1
Ran-binding protein 10
Phosphofurin acidic cluster sorting protein 1
Myosin phosphatase Rho-interacting protein
RNA-binding motif, single-stranded-interacting protein 3
Nicotinate phosphoribosyltransferase
Dynamin-binding protein
Very-long-chain (3R)-3-hydroxyacyl-CoA dehydratase 2
Beta-1,3-glucosyltransferase
GRB10-interacting GYF protein 2
CD109 antigen
tRNA (32-2'-O)-methyltransferase regulator THADA
Hydroxysteroid dehydrogenase-like protein 2
Kynurenine--oxoglutarate transaminase 3
Protein phosphatase 1 regulatory subunit 21
Thrombospondin type-1 domain-containing protein 4
Nesprin-3
Zinc finger protein 574
NF-X1-type zinc finger protein NFXL1
Neurobeachin-like protein 2
FYVE, RhoGEF and PH domain-containing protein 5
Sulfhydryl oxidase 2
Helicase SRCAP
Rho family-interacting cell polarization regulator 1
Uncharacterized protein FLJ45252
Rho guanine nucleotide exchange factor 18
TBC1 domain family member 9
E3 ubiquitin-protein ligase UBR3
Transmembrane protein with metallophosphoesterase domain
Capping protein-inhibiting regulator of actin dynamics
Coiled-coil domain-containing protein 9B
8-oxo-dGDP phosphatase NUDT18
Rho GTPase-activating protein SYDE1
PAX-interacting protein 1
Protein O-mannosyl-transferase TMTC3
Intermembrane lipid transfer protein VPS13C
Ubiquitin carboxyl-terminal hydrolase 34
Ras-associated and pleckstrin homology domains-containing protein 1
MOB kinase activator 2
Inhibitor of nuclear factor kappa-B kinase-interacting protein

Phosphatidylinositol 3,4,5-trisphosphate-dependent Rac exchanger 2 protein
Protein FRA10AC1
Ubiquitin-conjugating enzyme E2 R2
Mediator of RNA polymerase II transcription subunit 13-like
La-related protein 4
Mediator of RNA polymerase II transcription subunit 25
Ribosomal protein eS27-like
E3 ubiquitin-protein ligase Hakai
Integrator complex subunit 8
Butyrophilin subfamily 2 member A1
Transcription elongation factor SPT6
Staphylococcal nuclease domain-containing protein 1
Serine/threonine-protein kinase MARK2
Cytochrome c oxidase assembly protein COX15 homolog
Probable ATP-dependent RNA helicase DDX46
Protein RUFY3
tRNA methyltransferase 10 homolog C
eIF5-mimic protein 2
Vacuolar fusion protein MON1 homolog B
Volume-regulated anion channel subunit LRRC8D
Isoaspartyl peptidase/L-asparaginase
BTB/POZ domain-containing protein KCTD9
ATP-dependent RNA helicase DHX30
Eukaryotic translation initiation factor 3 subunit M
7SK snRNA methylphosphate capping enzyme
Armadillo repeat-containing X-linked protein 2
Probable proline--tRNA ligase, mitochondrial
Arginine/serine-rich coiled-coil protein 2
Ras-related GTP-binding protein A
Cytoplasmic FMR1-interacting protein 1
Protein arginine methyltransferase NDUFAF7, mitochondrial
Golgi to ER traffic protein 4 homolog
COP9 signalosome complex subunit 6
Lysophosphatidylcholine acyltransferase 2
Mitochondrial enolase superfamily member 1
E3 ubiquitin-protein transferase MAEA
EPM2A-interacting protein 1
Putative pre-mRNA-splicing factor ATP-dependent RNA helicase DHX32
Serine/threonine-protein kinase TAO1
FAST kinase domain-containing protein 5, mitochondrial
MOB kinase activator 1B
Lysine-specific demethylase 3B
Charged multivesicular body protein 1b
Ribonucleoside-diphosphate reductase subunit M2 B
Heparan sulfate 2-O-sulfotransferase 1
STE20-related kinase adapter protein alpha
[F-actin]-monooxygenase MICAL3
Dymeclin
PHD finger-like domain-containing protein 5A
Peroxiredoxin-like 2C

Aprataxin
Endoplasmic reticulum metallopeptidase 1
G protein-regulated inducer of neurite outgrowth 1
TRMT1-like protein
Zinc finger CCCH-type antiviral protein 1
Large ribosomal subunit protein bL21m
Elongation factor-like GTPase 1
Probable helicase senataxin
Trafficking protein particle complex subunit 11
KAT8 regulatory NSL complex subunit 1
Nucleoporin p54
Autophagy-related protein 9A
D-glutamate cyclase, mitochondrial
Coiled-coil domain-containing protein 186
LisH domain-containing protein ARMC9
VPS35 endosomal protein-sorting factor-like
Pogo transposable element with ZNF domain
Zinc finger FYVE domain-containing protein 16
Protein MON2 homolog
C-myc promoter-binding protein
Myosin-14
FMR1-interacting protein NUFIP2
SUZ domain-containing protein 1
Mitochondrial antiviral-signaling protein
CLIP-associating protein 1
ATP-dependent RNA helicase DHX29
Nephrocystin-3
Low-density lipoprotein receptor-related protein 10
HAUS augmin-like complex subunit 6
Protein O-glucosyltransferase 3
Tetratricopeptide repeat protein 21B
HEAT repeat-containing protein 3
Kinesin-like protein KIF21A
Hepatoma-derived growth factor-related protein 2
L-xylulose reductase
BRCA1-associated protein
BRCA2-interacting transcriptional repressor EMSY
Golgin subfamily A member 7
Rho GTPase-activating protein 22
Wings apart-like protein homolog
Interferon regulatory factor 2-binding protein 2
E3 ubiquitin-protein ligase RBBP6
E3 ubiquitin-protein ligase SH3RF1
Protein TAMALIN
FERM domain-containing protein 5
tRNA-splicing endonuclease subunit Sen54
Protein prenyltransferase alpha subunit repeat-containing protein 1
Arpin
Tectonin beta-propeller repeat-containing protein 1
Rab9 effector protein with kelch motifs

Transcription termination factor 4, mitochondrial
E3 ubiquitin-protein ligase HUWE1
YTH domain-containing family protein 3
Cytoplasmic tRNA 2-thiolation protein 1
Transcription initiation factor TFIID subunit 8
Ubiquitin-conjugating enzyme E2 Q1
Large ribosomal subunit protein mL55
Transmembrane emp24 domain-containing protein 4
Large ribosomal subunit protein uL10m
COX assembly mitochondrial protein homolog
Centromere protein V
Schlafen family member 11
Multiple epidermal growth factor-like domains protein 8
Transmembrane protein 179B
N-acetylgalactosaminyltransferase 7
Stearoyl-CoA desaturase 5
Pleckstrin homology-like domain family B member 2
Adhesion G-protein coupled receptor G6
Dehydrodolichyl diphosphate synthase complex subunit DHDDS
Polypeptide N-acetylgalactosaminyltransferase 10
Trafficking protein particle complex subunit 6B
Type 1 phosphatidylinositol 4,5-bisphosphate 4-phosphatase
C-type lectin domain family 14 member A
Protein PAT1 homolog 1
Dipeptidyl peptidase 9
E3 ubiquitin-protein ligase synoviolin
tRNA 2'-phosphotransferase 1
Actin-histidine N-methyltransferase
Tetratricopeptide repeat protein 7B
Acyl-coenzyme A thioesterase 1
Nucleolar protein 9
Polyadenylate-binding protein 2
N6-adenosine-methyltransferase catalytic subunit
Protein polybromo-1
Threonylcarbamoyl-AMP synthase
Pre-mRNA-processing factor 39
Protein LYRIC
E3 ubiquitin-protein ligase ZNF598
Glycerol-3-phosphate acyltransferase 4
Kinectin
Telomerase-binding protein EST1A
NLR family member X1
B-cell CLL/lymphoma 9-like protein
Pleckstrin homology-like domain family B member 1
Ubiquitin carboxyl-terminal hydrolase 48
Calcium-dependent secretion activator 2
Fermitin family homolog 3
N-alpha-acetyltransferase 40
Acetoacetyl-CoA synthetase
Leucine zipper protein 1

THO complex subunit 4
Integral membrane protein GPR180
FHF complex subunit HOOK-interacting protein 2B
Ras GTPase-activating-like protein IQGAP3
Zinc finger CCCH domain-containing protein 18
Vacuolar protein-sorting-associated protein 36
Cullin-associated NEDD8-dissociated protein 1
Reticulophagy regulator 3
Protein Hook homolog 3
SEC14 domain and spectrin repeat-containing protein 1
COMM domain-containing protein 7
Vacuolar fusion protein MON1 homolog A
Archaemetzincin-2
THO complex subunit 6 homolog
RNA N6-adenosine-methyltransferase METTL16
Poly(ADP-ribose) glycohydrolase
Liprin-beta-1
Lon protease homolog 2, peroxisomal
Zinc finger C3HC-type protein 1
Chromodomain-helicase-DNA-binding protein 1-like
F-BAR and double SH3 domains protein 1
Coiled-coil domain-containing protein 25
Proline and serine-rich protein 2
Stimulator of interferon genes protein
Active regulator of SIRT1
Cerebellar degeneration-related protein 2-like
Ral GTPase-activating protein subunit beta
Ras-specific guanine nucleotide-releasing factor RalGPS2
Histone-arginine methyltransferase CARM1
Deaminated glutathione amidase
COMM domain-containing protein 2
HEAT repeat-containing protein 5A
Condensin-2 complex subunit G2
Protein phosphatase 1 regulatory subunit 3B
F-box only protein 11
Ankyrin repeat and LEM domain-containing protein 2
RNA-binding protein MEX3D
ATP-dependent RNA helicase DDX42
Spermatogenesis-associated serine-rich protein 2
Serine/threonine-protein kinase VRK2
CDK2-associated and cullin domain-containing protein 1
NADH dehydrogenase [ubiquinone] 1 alpha subcomplex subunit 11
Dynein axonemal assembly factor 5
Peptidyl-tRNA hydrolase
Syntaxin-12
Transcriptional repressor p66-alpha
Copine-8
G-protein-signaling modulator 1
Ras-related protein Rab-43
C2 domain-containing protein 5

E3 ubiquitin-protein ligase MIB1
Inactive tyrosine-protein kinase PRAG1
BLOC-2 complex member HPS6
Interferon regulatory factor 2-binding protein 1
ELKS/Rab6-interacting/CAST family member 1
Ribosomal oxygenase 2
Palmitoyltransferase ZDHHC13
Palmitoyltransferase ZDHHC17
Cytokine receptor-like factor 3
Trafficking protein particle complex subunit 5
Armadillo repeat-containing protein 8
RELT-like protein 1
Complex I assembly factor TMEM126B, mitochondrial
5'-3' exonuclease PLD3
Protein HID1
Ankyrin repeat and MYND domain-containing protein 2
3'-5' exoribonuclease 1
Inactive serine/threonine-protein kinase VRK3
LIX1-like protein
NudC domain-containing protein 3
Protein AHNAK2
Formin-like protein 3
Methylmalonic aciduria type A protein, mitochondrial
Mitogen-activated protein kinase kinase kinase kinase 3
Neuron navigator 3
Prolyl 3-hydroxylase 3
Coiled-coil domain-containing protein 50
Malonyl-CoA-acyl carrier protein transacylase, mitochondrial
Kinase suppressor of Ras 1
Centrosomal protein of 97 kDa
ATP-dependent (S)-NAD(P)H-hydrate dehydratase
WD repeat-containing protein 75
Mitofusin-1
Choline transporter-like protein 2
Inositol 1,4,5-trisphosphate receptor-interacting protein
WD repeat and FYVE domain-containing protein 1
MAP7 domain-containing protein 3
Coiled-coil domain-containing protein 117
Protein NOXP20
DCN1-like protein 3
Protein DENND6A
MAX gene-associated protein
GRIP and coiled-coil domain-containing protein 2
Zinc finger CCCH domain-containing protein 7A
PHD finger protein 6
Protein archease
Volume-regulated anion channel subunit LRRC8A
Extracellular sulfatase Sulf-2
E3 ubiquitin-protein ligase UBR1
E3 ubiquitin-protein ligase UBR2

Rho GTPase-activating protein 12
Calcium homeostasis endoplasmic reticulum protein
Codanin-1
Ankyrin repeat and KH domain-containing protein 1
SURP and G-patch domain-containing protein 1
SURP and G-patch domain-containing protein 2
Ubiquitin-conjugating enzyme E2 variant 3
Cell division cycle and apoptosis regulator protein 1
Probable ATP-dependent RNA helicase DHX40
DnaJ homolog subfamily C member 10
Negative elongation factor C/D
Mitochondrial Rho GTPase 2
Mitochondrial Rho GTPase 1
NAD-dependent protein deacetylase sirtuin-2
Polyhomeotic-like protein 2
Chromatin complexes subunit BAP18
Large ribosomal subunit protein mL41
Nurim
GPALPP motifs-containing protein 1
Protein mono-ADP-ribosyltransferase PARP9
RNA-binding protein 12B
Solute carrier family 35 member F2
Patatin-like phospholipase domain-containing protein 6
Structural maintenance of chromosomes protein 5
Probable ATP-dependent RNA helicase DDX60
C-Maf-inducing protein
MICAL-like protein 2
Probable ATP-dependent RNA helicase DHX37
Kelch repeat and BTB domain-containing protein 2
Ribonucleoprotein PTB-binding 1
pre-rRNA 2'-O-ribose RNA methyltransferase FTSJ3
Transmembrane protein 192
Cytoskeleton-associated protein 2-like
Nucleotidyltransferase MB21D2
Serine/arginine repetitive matrix protein 1
Stromal membrane-associated protein 1
DIS3-like exonuclease 2
ATP-dependent RNA helicase SUPV3L1, mitochondrial
Eukaryotic peptide chain release factor GTP-binding subunit ERF3B
ZZ-type zinc finger-containing protein 3
Exocyst complex component 8
Uncharacterized protein C10orf67, mitochondrial
E3 ubiquitin-protein ligase TRIM22
Threonine synthase-like 1
Uncharacterized protein KIAA2013
Calcium uptake protein 2, mitochondrial
Ankyrin repeat domain-containing protein 13A
Phosphatase and actin regulator 4
tRNA (uracil-5-)-methyltransferase homolog A
Pseudouridylate synthase RPUSD2

ELMO domain-containing protein 2
Aldehyde dehydrogenase family 16 member A1
Dyslexia-associated protein KIAA0319-like protein
Adhesion G protein-coupled receptor F5
5'-3' exoribonuclease 1
Proline-, glutamic acid- and leucine-rich protein 1
Abl interactor 1
WD repeat and FYVE domain-containing protein 3
Selenoprotein H
Vitamin K epoxide reductase complex subunit 1-like protein 1
Spartin
Tetratricopeptide repeat protein 5
Mesoderm induction early response protein 1
Regulatory-associated protein of mTOR
ORM1-like protein 3
Cell cycle and apoptosis regulator protein 2
NADH dehydrogenase [ubiquinone] 1 alpha subcomplex assembly factor 2
Vacuolar protein sorting-associated protein 52 homolog
Nuclear pore complex protein Nup93
Serine/threonine-protein kinase 11-interacting protein
Zinc finger protein 687
Cap-specific mRNA (nucleoside-2'-O-)-methyltransferase 1
Leucine-rich repeat-containing protein 47
Dedicator of cytokinesis protein 4
Carbonic anhydrase 13
Rho guanine nucleotide exchange factor 28
Integrator complex subunit 1
Histone H2B type 3-B
Rho GTPase-activating protein 24
Armadillo repeat-containing protein 10
Lysophosphatidylserine lipase ABHD12
CLK4-associating serine/arginine rich protein
Protein FAM43A
Transmembrane protein 256
E3 SUMO-protein ligase PIAS4
Glycerol-3-phosphate dehydrogenase 1-like protein
Solute carrier family 35 member F6
Rho GTPase-activating protein 18
Activating signal cointegrator 1 complex subunit 3
MICAL-like protein 1
Vacuolar protein sorting-associated protein 8 homolog
Protein PALS1
Cohesin subunit SA-2
Synaptopodin
Formin-binding protein 4
Translation factor GUF1, mitochondrial
RING1 and YY1-binding protein
Polypeptide N-acetylgalactosaminyltransferase 4
Misshapen-like kinase 1
Guanosine-3',5'-bis(diphosphate) 3'-pyrophosphohydrolase MESH1

Prostaglandin reductase 3
3-oxoacyl-[acyl-carrier-protein] reductase
ER membrane protein complex subunit 5
Transmembrane protein 199
F-box/LRR-repeat protein 6
Prolyl 3-hydroxylase OGFOD1
Actin filament-associated protein 1
Zinc finger CCHC domain-containing protein 9
Oxidation resistance protein 1
Zinc finger CCCH-type with G patch domain-containing protein
Calcium homeostasis modulator protein 5
S1 RNA-binding domain-containing protein 1
WD and tetratricopeptide repeats protein 1
NF-kappa-B-activating protein
Macoilin
SH2 domain-containing protein 3C
Arrestin domain-containing protein 1
Dehydrogenase/reductase SDR family member on chromosome X
CDGSH iron-sulfur domain-containing protein 2
Ribonuclease P protein subunit p25-like protein
ATP synthase mitochondrial F1 complex assembly factor 2
Tetratricopeptide repeat protein 9C
Protein jagunal homolog 1
E3 ubiquitin-protein ligase RNF10
Refilin-B
Leucine-zipper-like transcriptional regulator 1
COMM domain-containing protein 1
Cleavage and polyadenylation specificity factor subunit 7
Solute carrier family 15 member 4
Myc target protein 1
ADAMTS-like protein 1
ADP-ribosylation factor GTPase-activating protein 2
Deubiquitinase OTUD6B
Acyl-coenzyme A diphosphatase FITM2
UPF0690 protein C1orf52
eEF1A lysine and N-terminal methyltransferase
ADP-ribosylation factor-like protein 6-interacting protein 6
ADP-ribosylation factor GTPase-activating protein 1
NAD-dependent protein deacylase sirtuin-6
Solute carrier family 66 member 3
ER membrane protein complex subunit 1
RNA polymerase II-associated factor 1 homolog
Cyclin-Y-like protein 1
Putative E3 ubiquitin-protein ligase UBR7
ATP-dependent RNA helicase DDX51
Uncharacterized protein FAM241A
Mitochondrial basic amino acids transporter
Protein enabled homolog
Discoidin, CUB and LCCL domain-containing protein 1
G patch domain-containing protein 11

Large ribosomal subunit protein mL43
RNA ligase 1
Lysophospholipase D GDPD1
Uncharacterized protein C19orf47
Activating signal cointegrator 1 complex subunit 1
Leucine-rich repeat-containing protein 57
Probable RNA-binding protein EIF1AD
Protein KRI1 homolog
Coiled-coil domain-containing protein 71L
Zinc finger protein 579
Pre-mRNA-splicing factor 38A
Mixed lineage kinase domain-like protein
Glutamine amidotransferase-like class 1 domain-containing protein 1
Serine/threonine-protein phosphatase 6 regulatory ankyrin repeat subunit C
Phospholipid-transporting ATPase IG
ATPase family gene 2 protein homolog A
NHL repeat-containing protein 2
Late secretory pathway protein AVL9 homolog
Equilibrative nucleobase transporter 1
Xyloside xylosyltransferase 1
Golgi membrane protein 1
Procollagen galactosyltransferase 1
Inactive C-alpha-formylglycine-generating enzyme 2
SID1 transmembrane family member 2
Formylglycine-generating enzyme
Protein O-glucosyltransferase 1
Ubiquitin-associated domain-containing protein 2
Prenylcysteine oxidase-like
Transmembrane protein 87A
Retinol dehydrogenase 13
Tetratricopeptide repeat protein 13
Estradiol 17-beta-dehydrogenase 11
Thioredoxin domain-containing protein 5
Kinetochore protein Spc24
Outer mitochondrial transmembrane helix translocase
Saccharopine dehydrogenase-like oxidoreductase
UDP-glucuronic acid decarboxylase 1
E3 ubiquitin-protein ligase RNF149
Reticulophagy regulator 2
SERPINE1 mRNA-binding protein 1
LEM domain-containing protein 2
Nitric oxide-associated protein 1
Adaptin ear-binding coat-associated protein 1
Protein FAM98A
Phospholipase A2 group XV
Myotubularin-related protein 14
NFATC2-interacting protein
Diacylglycerol lipase-beta
Carbohydrate sulfotransferase 14
Polypeptide N-acetylgalactosaminyltransferase 6

E3 ubiquitin-protein ligase RNF169
Pyruvate dehydrogenase phosphatase regulatory subunit, mitochondrial
NAD(P)H-hydrate epimerase
Nonsense-mediated mRNA decay factor SMG8
RING finger protein 214
Protein LSM14 homolog A
GTPase IMAP family member 8
Cyclin-Y
Maestro heat-like repeat-containing protein family member 1
Terminal nucleotidyltransferase 4B
Probable aminopeptidase NPEPL1
EH domain-binding protein 1
Putative RNA-binding protein 15B
Alpha-N-acetylgalactosaminide alpha-2,6-sialyltransferase 3
Trinucleotide repeat-containing gene 6A protein
Polyhomeotic-like protein 3
Divergent protein kinase domain 2A
Metal transporter CNNM3
ATP-binding cassette sub-family F member 1
Calcium uniporter protein, mitochondrial
Phosphatidylinositol 3-kinase catalytic subunit type 3
Glutathione S-transferase C-terminal domain-containing protein
Serum response factor-binding protein 1
Neuroguidin
SHC SH2 domain-binding protein 1
PDZ domain-containing protein 8
DCC-interacting protein 13-beta
Zinc transporter 7
Neuron navigator 1
Periphilin-1
WD repeat-containing protein 19
F-box only protein 22
Lysophosphatidylcholine acyltransferase 1
Zinc finger MIZ domain-containing protein 2
Nesprin-1
Ubiquitin carboxyl-terminal hydrolase 32
Biorientation of chromosomes in cell division protein 1-like 1
AT-rich interactive domain-containing protein 1B
FAD synthase
Folliculin
Nucleoporin Nup43
Nucleoporin Nup37
Nucleoporin NUP35
Cytosolic endo-beta-N-acetylglucosaminidase
Retinoic acid-induced protein 3
Torsin-1A-interacting protein 2
sn-1-specific diacylglycerol lipase ABHD11
N-acylneuraminate cytidylyltransferase
F-box DNA helicase 1
Tetraspanin-14

Tubulin--tyrosine ligase
Small VCP/p97-interacting protein
Atlastin-2
Motile sperm domain-containing protein 2
Putative phospholipase B-like 2
Ras association domain-containing protein 8
ATP-dependent RNA helicase DDX55
Major facilitator superfamily domain-containing protein 8
Tudor domain-containing protein 7
GTPase IMAP family member 7
Protein NEDD1
Nuclear receptor coactivator 7
THO complex subunit 2
WD repeat-containing protein 36
Protein phosphatase PTC7 homolog
Retinitis pigmentosa 9 protein
Vang-like protein 1
Smad nuclear-interacting protein 1
Large ribosomal subunit protein mL64
WD repeat-containing protein 48
Exocyst complex component 6
SWI/SNF complex subunit SMARCC2
Nuclear protein localization protein 4 homolog
Uncharacterized protein CXorf38
Spermatogenesis-associated protein 20
Iron-sulfur cluster transfer protein NUBPL
(Lyso)-N-acylphosphatidylethanolamine lipase
F-box only protein 30
Adenosine 3'-phospho 5'-phosphosulfate transporter 1
Pumilio homolog 2
T-cell immunomodulatory protein
Golgin subfamily A member 5
Kelch domain-containing protein 4
SH3KBP1-binding protein 1
NEDD8-activating enzyme E1 catalytic subunit
Prostamide/prostaglandin F synthase
DnaJ homolog subfamily B member 14
Protein kish-A
Phosphatidylinositol 5-phosphate 4-kinase type-2 gamma
WD repeat-containing protein 20
TBC1 domain family member 15
Retinol dehydrogenase 11
Leucine-rich repeat-containing protein 20
Interferon-induced protein 44
Large ribosomal subunit protein uL30m
AN1-type zinc finger protein 1
Protein CIP2A
Phosphatidylinositol 4-kinase type 2-beta
Dolichyl-diphosphooligosaccharide--protein glycosyltransferase subunit STT3B
Polyribonucleotide nucleotidyltransferase 1, mitochondrial

Signal peptide peptidase-like 2B
Minor histocompatibility antigen H13
Phosphatidylinositol 3,4,5-trisphosphate-dependent Rac exchanger 1 protein
Up-regulator of cell proliferation
Protein bicaudal D homolog 2
Serine/threonine-protein kinase Nek9
Pleckstrin homology domain-containing family O member 2
E3 ubiquitin-protein ligase DTX3L
ATP-dependent RNA helicase DDX54
Disks large homolog 5
Ribosome biogenesis protein BRX1 homolog
Glucosamine-6-phosphate isomerase 2
Volume-regulated anion channel subunit LRRC8C
Serine/threonine-protein kinase Nek7
RB1-inducible coiled-coil protein 1
[F-actin]-monooxygenase MICAL1
Elongator complex protein 5
Protein phosphatase Slingshot homolog 3
SH3 domain and tetratricopeptide repeat-containing protein 1
TBC domain-containing protein kinase-like protein
D-aminoacyl-tRNA deacylase 1
DDB1- and CUL4-associated factor 11
U3 small nucleolar RNA-associated protein 15 homolog
Probable glutathione peroxidase 8
Actin filament-associated protein 1-like 1
E3 ubiquitin-protein ligase SH3RF3
Short transient receptor potential channel 4-associated protein
Centrosomal protein of 192 kDa
Gem-associated protein 5
GPI ethanolamine phosphate transferase 3
Rap guanine nucleotide exchange factor 6
Partitioning defective 3 homolog
Importin-4
Ubiquitin carboxyl-terminal hydrolase 33
Arginine/serine-rich protein PNISR
Serine/threonine-protein phosphatase 4 regulatory subunit 1
Ubiquitin-associated and SH3 domain-containing protein B
PDZ domain-containing protein GIPC2
WAS/WASL-interacting protein family member 2
1-acylglycerol-3-phosphate O-acyltransferase ABHD5
Histone-lysine N-methyltransferase SETD7
Nucleolar complex protein 3 homolog
Scavenger receptor class B member 1
Conserved oligomeric Golgi complex subunit 1
Sec1 family domain-containing protein 2
Stromal membrane-associated protein 2
Zinc finger CCCH domain-containing protein 15
Peptidyl-prolyl cis-trans isomerase-like 4
General transcription factor 3C polypeptide 2
TBC1 domain family member 22A

PHD finger protein 10
Ras-related protein Rab-2B
Coiled-coil domain-containing protein 12
RelA-associated inhibitor
Cotranscriptional regulator ARB2A
Protein Churchill
Transforming growth factor-beta receptor-associated protein 1
Transmembrane protein 263
Histone deacetylase 7
Serine/threonine/tyrosine-interacting protein
Phosphatidylglycerophosphatase and protein-tyrosine phosphatase 1
Nuclear pore complex protein Nup133
Programmed cell death 6-interacting protein
Sodium-dependent phosphate transporter 1
Filamin-binding LIM protein 1
Splicing factor Cactin
Glutathione-specific gamma-glutamylcyclotransferase 2
Charged multivesicular body protein 7
Protein THEM6
Probable N-acetyltransferase 14
B-cell CLL/lymphoma 7 protein family member C
Non-structural maintenance of chromosomes element 1 homolog
MIT domain-containing protein 1
Chromosome transmission fidelity protein 18 homolog
RNA polymerase-associated protein LEO1
Dephospho-CoA kinase domain-containing protein
NudC domain-containing protein 2
U4/U6.U5 small nuclear ribonucleoprotein 27 kDa protein
Dimethyladenosine transferase 1, mitochondrial
Cohesin subunit SA-1
Sec1 family domain-containing protein 1
Tumor necrosis factor alpha-induced protein 8-like protein 1
Soluble calcium-activated nucleotidase 1
Trafficking protein particle complex subunit 12
Heterogeneous nuclear ribonucleoprotein L-like
Fatty acyl-CoA reductase 1
Ubiquitin-like domain-containing CTD phosphatase 1
tRNA-splicing endonuclease subunit Sen15
PEST proteolytic signal-containing nuclear protein
DnaJ homolog subfamily A member 4
SPRY domain-containing protein 4
m-AAA protease-interacting protein 1, mitochondrial
Pseudouridylate synthase TRUB1
LIM domain only protein 7
Choline transporter-like protein 1
Cytoskeleton-associated protein 2
Ataxin-2-like protein
Arf-GAP with Rho-GAP domain, ANK repeat and PH domain-containing protein 3
GTPase IMAP family member 1
PH-interacting protein

U4/U6 small nuclear ribonucleoprotein Prp31
Negative elongation factor B
Palladin
RUN and FYVE domain-containing protein 2
Splicing regulatory glutamine/lysine-rich protein 1
Gem-associated protein 6
Caskin-2
Paraspeckle component 1
MAP kinase-activating death domain protein
Nesprin-2
Transcriptional repressor p66-beta
DnaJ homolog subfamily C member 9
BRI3-binding protein
Beta-catenin-like protein 1
Cysteine protease ATG4A
Ras and Rab interactor 2
Protein ELYS
Microprocessor complex subunit DGCR8
Titin
Roundabout homolog 4
Esterase OVCA2
Immunity-related GTPase family Q protein
DNA damage-binding protein 2
C-type lectin domain family 2 member B
ATP-dependent RNA helicase DDX1
SEC14-like protein 1
Piezo-type mechanosensitive ion channel component 1
Protein FAM3C
Histone H1.10
Proteasome inhibitor PI31 subunit
Golgi-specific brefeldin A-resistance guanine nucleotide exchange factor 1
Nonsense-mediated mRNA decay factor SMG7
RNA polymerase-associated protein RTF1 homolog
Nicastrin
Sorting nexin-19
Transmembrane 9 superfamily member 4
Transmembrane protein 131
Inositol hexakisphosphate kinase 1
Small ribosomal subunit protein mS27
Engulfment and cell motility protein 1
Polyphosphoinositide phosphatase
Protein FAM168A
AP-3 complex subunit sigma-1
Hamartin
UBX domain-containing protein 4
PHD finger protein 3
Protein NDRG1
Heat shock protein 105 kDa
Septin-8
CCR4-NOT transcription complex subunit 9

Acyl-CoA:lysophosphatidylglycerol acyltransferase 1
TBC1 domain family member 5
Zinc finger protein 592
Protein Jade-3
Unconventional myosin-XVIIIa
La-related protein 4B
Stalled ribosome sensor GCN1
Rho GTPase-activating protein 45
Pre-mRNA-splicing factor ATP-dependent RNA helicase PRP16
Nuclear pore complex protein Nup205
Ankyrin repeat and SAM domain-containing protein 1A
Peroxidasin homolog
Protein FAN
GPI-anchor transamidase
Small ribosomal subunit protein mS31
A-kinase anchor protein 1, mitochondrial
Dol-P-Man:Man(5)GlcNAc(2)-PP-Dol alpha-1,3-mannosyltransferase
Neurogranin
Acidic leucine-rich nuclear phosphoprotein 32 family member B
Nectin-2
Geranylgeranyl transferase type-2 subunit alpha
Protein TFG
Serine protease HTRA1
Actin-related protein 2/3 complex subunit 1A
General transcription factor IIH subunit 4
Ras-responsive element-binding protein 1
Histone deacetylase 2
Signal transducing adapter molecule 1
Zinc finger protein ubi-d4
Endoplasmic reticulum protein SC65
CREB-binding protein
Histone acetyltransferase KAT6A
Symplekin
TATA-binding protein-associated factor 2N
Golgin subfamily A member 1
Gamma-glutamyl hydrolase
Neuronal cell adhesion molecule
X-linked retinitis pigmentosa GTPase regulator
Phosphatidylinositol 3,4,5-trisphosphate 5-phosphatase 1
Probable ATP-dependent RNA helicase DDX17
Kinesin-associated protein 3
Caspase-10
Amyloid beta precursor protein binding family B member 2
DNA repair protein RAD50
CUGBP Elav-like family member 1
Osteoclast-stimulating factor 1
Rho guanine nucleotide exchange factor 1
DNA repair endonuclease XPF
Ubiquitin recognition factor in ER-associated degradation protein 1
Golgi apparatus protein 1

Regulator of nonsense transcripts 1
COP9 signalosome complex subunit 5
G-patch domain and KOW motifs-containing protein
SWI/SNF complex subunit SMARCC1
SWI/SNF-related matrix-associated actin-dependent regulator of chromatin subfamily D membe
Ras-related protein Rab-8B
Bcl2-associated agonist of cell death
Far upstream element-binding protein 2
Glutaryl-CoA dehydrogenase, mitochondrial
GTP-binding protein Rit1
Peroxisomal membrane protein PEX13
Transportin-1
Rho guanine nucleotide exchange factor 2
Ribosomal RNA small subunit methyltransferase NEP1
Polyribonucleotide 5'-hydroxyl-kinase Clp1
Glomulin
Ubiquitin carboxyl-terminal hydrolase 13
Segment polarity protein dishevelled homolog DVL-3
Probable ubiquitin carboxyl-terminal hydrolase FAF-X
Ubiquitin carboxyl-terminal hydrolase 7
Cullin-5
Stathmin-2
V-type proton ATPase 116 kDa subunit a 1
Lipoma-preferred partner
RNA-binding protein with multiple splicing
Exostosin-2
Mediator of RNA polymerase II transcription subunit 12
Protein tyrosine phosphatase type IVA 1
Phosphorylase b kinase regulatory subunit beta
Secretory carrier-associated membrane protein 4
BLOC-2 complex member HPS3
SWI/SNF-related matrix-associated actin-dependent regulator of chromatin subfamily E membe
Caveolae-associated protein 3
Riboflavin kinase
Myeloid-derived growth factor
Mitochondrial import receptor subunit TOM40B
Protein YIPF5
GPI transamidase component PIG-T
Immunoglobulin superfamily member 8
Ribosomal protein eL42-like
Ras-related protein Rab-24
Oxysterol-binding protein 2
Cytoplasmic 60S subunit biogenesis factor ZNF622
Ribosome-releasing factor 2, mitochondrial
Ubiquitin-conjugating enzyme E2 E3
7-methylguanosine phosphate-specific 5'-nucleotidase
WW domain-binding protein 2
Proteasome assembly chaperone 2
BOS complex subunit NCLN
Mitochondrial ubiquitin ligase activator of NFKB 1

Myocardin-related transcription factor A
Protein lifeguard 3
Endoplasmic reticulum-Golgi intermediate compartment protein 1
U3 small nucleolar RNA-associated protein 4 homolog
FAST kinase domain-containing protein 4
Mitochondrial amidoxime reducing component 2
Protein FAM162A
PAT complex subunit CCDC47
Large ribosomal subunit protein uL24m
Exocyst complex component 4
Protein mago nashi homolog 2
Putative monooxygenase p33MONOX
Protein RFT1 homolog
Isochorismatase domain-containing protein 2
Protein N-terminal asparagine amidohydrolase
Fermitin family homolog 2
Far upstream element-binding protein 1
Tetratricopeptide repeat protein 17
Leucine-rich repeat-containing protein 59
Vesicle transport through interaction with t-SNAREs homolog 1A
Endothelial cell-selective adhesion molecule
Pre-B-cell leukemia transcription factor-interacting protein 1
Mitochondrial calcium uniporter regulator 1
Uncharacterized protein KIAA1143
Vacuolar protein sorting-associated protein 33A
Peptidyl-prolyl cis-trans isomerase FKBP10
Tetratricopeptide repeat protein 28
Exosome complex component RRP43
Zinc finger protein 428
SH3 domain-containing kinase-binding protein 1
Spindle and kinetochore-associated protein 1
E3 ubiquitin-protein ligase RNF25
Axin interactor, dorsalization-associated protein
PIN2/TERF1-interacting telomerase inhibitor 1
ADP-ribosylation factor-like protein 8A
Ubiquitin thioesterase otulin
Small ribosomal subunit protein mS37
Peptidylprolyl isomerase domain and WD repeat-containing protein 1
Coiled-coil domain-containing protein 127
Uracil phosphoribosyltransferase homolog
Phosphatidate cytidylyltransferase, mitochondrial
MOB kinase activator 3A
Dedicator of cytokinesis protein 10
Autophagy-related protein 2 homolog B
Leukocyte receptor cluster member 1
TBC1 domain family member 20
EF-hand domain-containing protein D2
Galactose mutarotase
Synaptotagmin-like protein 4
Pyrroline-5-carboxylate reductase 2

Protein CUSTOS
m7GpppX diphosphatase
Protein phosphatase 1 regulatory subunit 14B
Integrator complex subunit 12
5-methylcytosine rRNA methyltransferase NSUN4
Inactive rhomboid protein 1
Collagen triple helix repeat-containing protein 1
Medium-chain acyl-CoA ligase ACSF2, mitochondrial
Isochorismatase domain-containing protein 1
GRIP and coiled-coil domain-containing protein 1
FLYWCH family member 2
Protein Aster-A
HAUS augmin-like complex subunit 1
FAS-associated factor 2
Coiled-coil domain-containing protein 124
FAD-dependent oxidoreductase domain-containing protein 1
Optineurin
AP-2 complex subunit mu
Gamma-tubulin complex component 3
Probable RNA polymerase II nuclear localization protein SLC7A6OS
BTB/POZ domain-containing protein KCTD12
Reticulocalbin-3
60S ribosomal export protein NMD3
RalBP1-associated Eps domain-containing protein 1
Mitochondrial import inner membrane translocase subunit TIM14
Regulator of microtubule dynamics protein 1
U8 snoRNA-decapping enzyme
Splicing factor ESS-2 homolog
Carboxymethylenebutenolidase homolog
RNA-binding protein Musashi homolog 2
U5 small nuclear ribonucleoprotein 40 kDa protein
Regulator of MON1-CCZ1 complex
Large ribosomal subunit protein mL38
RING finger and SPRY domain-containing protein 1
Endoplasmic reticulum lectin 1
Ribosome-recycling factor, mitochondrial
Protein Spindly
Elongator complex protein 4
NAD-dependent protein deacetylase sirtuin-1
Protein YIPF6
Nucleoporin SEH1
Transcription elongation factor A protein-like 4
KIF-binding protein
Glucosamine 6-phosphate N-acetyltransferase
Constitutive coactivator of peroxisome proliferator-activated receptor gamma
Protein KTI12 homolog
Small ribosomal subunit protein uS3m
Large ribosomal subunit protein mL53
Trans-3-hydroxy-L-proline dehydratase
E3 ubiquitin-protein ligase RNF31

DAZ-associated protein 1
Small glutamine-rich tetratricopeptide repeat-containing protein beta
Protein SAAL1
Mitochondrial potassium channel
SAGA-associated factor 29
Ribosomal RNA processing protein 36 homolog
C1GALT1-specific chaperone 1
RNA-binding protein 33
Dysbindin
DnaJ homolog subfamily A member 3, mitochondrial
Translation machinery-associated protein 16
Multivesicular body subunit 12A
Small ribosomal subunit protein mS39
Corrinoid adenosyltransferase MMAB
Cytoplasmic FMR1-interacting protein 2
GTPase IMAP family member 5
E3 ubiquitin-protein ligase TRIM11
Coiled-coil domain-containing protein 97
CB1 cannabinoid receptor-interacting protein 1
Enhancer of mRNA-decapping protein 3
Dynein light chain 2, cytoplasmic
Copine-2
Protein S100-A16
Signal-induced proliferation-associated protein 1
Secernin-2
THO complex subunit 1
Ubiquitin thioesterase OTUB1
tRNA (adenine(58)-N(1))-methyltransferase catalytic subunit TRMT61A
Abasic site processing protein HMCES
Charged multivesicular body protein 6
Protein bicaudal D homolog 1
Phosphopentomutase
U3 small nucleolar ribonucleoprotein protein IMP4
Ceramide synthase 2
tRNA-dihydrouridine(47) synthase [NAD(P)(+)]-like
Protein LTV1 homolog
Serine dehydratase-like
Large ribosomal subunit protein mL48
Chronophin
Aurora kinase B
DCN1-like protein 1
Fumarylacetoacetate hydrolase domain-containing protein 2A
SWI/SNF-related matrix-associated actin-dependent regulator of chromatin subfamily D membe
Target of EGR1 protein 1
Cell division cycle-associated 7-like protein
RUS family member 1
Probable ATP-dependent RNA helicase DDX27
BLOC-1-related complex subunit 6
Methionine--tRNA ligase, mitochondrial
Ataxin-7-like protein 3B

Methylthioribulose-1-phosphate dehydratase
Zinc finger C2HC domain-containing protein 1A
Protein lin-37 homolog
Solute carrier family 41 member 3
Vacuolar-sorting protein SNF8
Zinc finger CCCH-type antiviral protein 1-like
Nuclear envelope pore membrane protein POM 121
Tonsoku-like protein
PDZ and LIM domain protein 5
Protein disulfide isomerase CRELD1
ERO1-like protein alpha
Proline-rich protein 11
Protein FMC1 homolog
Adenosylhomocysteinase 3
Dedicator of cytokinesis protein 6
Mediator of RNA polymerase II transcription subunit 30
Serine/threonine-protein phosphatase PGAM5, mitochondrial
Integrator complex subunit 4
DDRGK domain-containing protein 1
2-oxoadipate dehydrogenase complex component E1
Selenocysteine lyase
Far upstream element-binding protein 3
Splicing factor 45
RCC1-like G exchanging factor-like protein
Asparaginyl-tRNA synthetase
Succinate--CoA ligase [GDP-forming] subunit beta, mitochondrial
DISP complex protein LRCH3
Mannose-1-phosphate guanyltransferase alpha
4-hydroxyphenylpyruvate dioxygenase-like protein
Putative protein-lysine deacylase ABHD14B
Peptide-N(4)-(N-acetyl-beta-glucosaminyl)asparagine amidase
Vesicle-trafficking protein SEC22a
ATP synthase membrane subunit K, mitochondrial
Kinetochore-associated protein NSL1 homolog
PRKC apoptosis WT1 regulator protein
tRNA N(3)-methylcytidine methyltransferase METTL2A
THO complex subunit 3
E3 ubiquitin-protein ligase Itchy homolog
Thioredoxin domain-containing protein 15
Kin of IRRE-like protein 1
Leucine-rich repeats and immunoglobulin-like domains protein 1
Conserved oligomeric Golgi complex subunit 3
CDK5 regulatory subunit-associated protein 3
Vam6/Vps39-like protein
Syndetin
Deubiquitinating protein VCPIP1
Spatacsin
Engulfment and cell motility protein 2
Protein disulfide-isomerase TMX3
DDB1- and CUL4-associated factor 5

Chromosome alignment-maintaining phosphoprotein 1
Lethal(3)malignant brain tumor-like protein 3
E3 ubiquitin-protein ligase ZFP91
Protocadherin-16
Calmin
PDZ and LIM domain protein 2
Fatty acyl-CoA reductase 2
Transcription factor BTF3 homolog 4
E3 ubiquitin-protein ligase RNF170
Abscission/NoCut checkpoint regulator
Solute carrier family 35 member E1
Ubiquitin carboxyl-terminal hydrolase 47
Zinc finger protein 521
Lipid scramblase CLPTM1L
Lymphokine-activated killer T-cell-originated protein kinase
ADP-ribosylation factor-like protein 5B
DnaJ homolog subfamily C member 1
N-terminal kinase-like protein
Zinc finger protein 512B
Protein LRATD2
Exocyst complex component 2
Cytosolic non-specific dipeptidase
Histone-lysine N-methyltransferase EHMT2
Zinc finger RNA-binding protein
Protein FAM210B, mitochondrial
Beta-1,3-galactosyltransferase 6
E1A-binding protein p400
Sorting nexin-27
Kinesin-like protein KIF16B
Intraflagellar transport protein 74 homolog
E3 ubiquitin-protein ligase TRIM47
CCR4-NOT transcription complex subunit 6-like
Dehydrogenase/reductase SDR family member 1
DnaJ homolog subfamily C member 30, mitochondrial
Ubiquitin-conjugating enzyme E2 E2
Caspase recruitment domain-containing protein 19
Protein PRRC1
FYVE, RhoGEF and PH domain-containing protein 4
F-box/LRR-repeat protein 18
Zinc finger protein 512
E3 SUMO-protein ligase NSE2
Non-structural maintenance of chromosomes element 3 homolog
Heat shock 70 kDa protein 12B
YTH domain-containing protein 1
Coiled-coil domain-containing protein 43
Conserved oligomeric Golgi complex subunit 8
CKLF-like MARVEL transmembrane domain-containing protein 3
Dynein axonemal assembly factor 10
Nucleolar protein 4-like
Lysophospholipid acyltransferase 7

Dedicator of cytokinesis protein 7
Sideroflexin-2
Zinc finger protein 830
Protein FAM210A
FERM domain-containing protein 6
28S rRNA (cytosine-C(5))-methyltransferase
Regulation of nuclear pre-mRNA domain-containing protein 1A
Arf-GAP with GTPase, ANK repeat and PH domain-containing protein 3
Arf-GAP with Rho-GAP domain, ANK repeat and PH domain-containing protein 1
Importin-9
Melanoma inhibitory activity protein 2
Discoidin, CUB and LCCL domain-containing protein 2
Rho guanine nucleotide exchange factor 17
Inositol polyphosphate-4-phosphatase type I A
RNA-binding protein 14
RING finger and CHY zinc finger domain-containing protein 1
Kelch-like protein 5
E3 ubiquitin-protein ligase UHRF2
E3 ubiquitin-protein ligase NEDD4-like
KH domain-containing RNA-binding protein QKI
Leukocyte receptor cluster member 8
Formin-like protein 2
Pseudouridylate synthase 7 homolog
Serine protease FAM111A
Trafficking protein particle complex subunit 9
CCA tRNA nucleotidyltransferase 1, mitochondrial
Serine/threonine-protein kinase SMG1
Alsin
Transmembrane protein 237
Alpha-ketoglutarate-dependent dioxygenase alkB homolog 3
Rho GTPase-activating protein 7
Serine/threonine-protein phosphatase 1 regulatory subunit 10
Sodium-coupled neutral amino acid symporter 2
UAP56-interacting factor
Myotubularin-related protein 9
Vacuolar protein sorting-associated protein 35
Transcriptional activator protein Pur-beta
Exportin-6
Hedgehog-interacting protein
Membrane-associated guanylate kinase, WW and PDZ domain-containing protein 1
Sperm-associated antigen 5
Nucleus accumbens-associated protein 1
Sorting nexin-18
PAS domain-containing serine/threonine-protein kinase
Protein capicua homolog
BRCA1-A complex subunit RAP80
Intermembrane lipid transfer protein VPS13A
Mediator of RNA polymerase II transcription subunit 15
Elongation factor G, mitochondrial
Endoplasmic reticulum-Golgi intermediate compartment protein 2

Methylcrotonoyl-CoA carboxylase subunit alpha, mitochondrial
Calcium/calmodulin-dependent protein kinase kinase 2
Trimethylguanosine synthase
NudC domain-containing protein 1
Erbin
Gamma-tubulin complex component 6
Gamma-tubulin complex component 5
Ubiquitin carboxyl-terminal hydrolase 28
Formin-binding protein 1
EKC/KEOPS complex subunit TP53RK
GPI transamidase component PIG-S
ATPase WRNIP1
Ran-binding protein 9
Chloride channel CLIC-like protein 1
Myeloid-associated differentiation marker
Neurabin-2
SRSF protein kinase 1
Structural maintenance of chromosomes protein 6
BTB/POZ domain-containing protein KCTD15
Spermatid perinuclear RNA-binding protein
Transmembrane protein 209
Glutathione peroxidase 7
CDK5 regulatory subunit-associated protein 2
Cytochrome P450 2S1
Protein IWS1 homolog
Paired amphipathic helix protein Sin3a
Oxysterol-binding protein-related protein 9
Protein cereblon
Integrator complex subunit 14
2-aminoethanethiol dioxygenase
Mitochondrial tRNA methylthiotransferase CDK5RAP1
Remodeling and spacing factor 1
RNA-binding protein 15
Protein phosphatase 1 regulatory inhibitor subunit 16B
RUN and FYVE domain-containing protein 1
Msx2-interacting protein
Bifunctional polynucleotide phosphatase/kinase
MMS19 nucleotide excision repair protein homolog
E3 ubiquitin-protein ligase UHRF1
Protein Niban 2
ATP-dependent zinc metalloprotease YME1L1
Regulator of microtubule dynamics protein 3
Transcription factor 12
Cytohesin-2
Tubulin-folding cofactor B
Proteasome subunit beta type-7
Calponin-2
Translocation protein SEC62
Ethanolamine-phosphate cytidylyltransferase
Cell division cycle 5-like protein

26S proteasome non-ATPase regulatory subunit 1
Stromal cell-derived factor 2
Prefoldin subunit 5
E3 ubiquitin-protein ligase RING2
Parkinson disease protein 7
Sphingosine 1-phosphate receptor 3
GAS2-like protein 1
Eyes absent homolog 3
Sialidase-1
Sortilin
Synaptic vesicle membrane protein VAT-1 homolog
Legumain
Perilipin-2
DnaJ homolog subfamily C member 2
M-phase phosphoprotein 8
Nuclear pore complex protein Nup88
Plakophilin-4
Phosphoinositide 3-kinase regulatory subunit 4
P2X purinoceptor 4
Ribonucleases P/MRP protein subunit POP1
Protein S100-A13
Protein SCAF11
Translin-associated protein X
Selenide, water dikinase 2
Eukaryotic translation initiation factor 3 subunit C
Tetratricopeptide repeat protein 1
DnaJ homolog subfamily C member 7
Prohibitin-2
COP9 signalosome complex subunit 8
Pre-mRNA-splicing factor 18
Calcineurin B homologous protein 1
Kinesin-like protein KIF2C
RANBP2-like and GRIP domain-containing protein 5/6
Monoglyceride lipase
Ataxin-2
Docking protein 1
Methionine synthase
3-hydroxyacyl-CoA dehydrogenase type-2
Collagen alpha-1(XII) chain
Mothers against decapentaplegic homolog 5
Sigma non-opioid intracellular receptor 1
Heterogeneous nuclear ribonucleoprotein A/B
Nucleosome assembly protein 1-like 4
Microsomal glutathione S-transferase 2
Gamma-soluble NSF attachment protein
Phosphatidylinositol 4-phosphate 5-kinase type-1 alpha
Phospholipid-transporting ATPase ABCA3
Mitogen-activated protein kinase kinase kinase 3
ATP synthase subunit s, mitochondrial
Mitochondrial intermediate peptidase

Aconitate hydratase, mitochondrial
Transmembrane 9 superfamily member 2
Equilibrative nucleoside transporter 1
Tumor susceptibility gene 101 protein
Copine-1
T-complex protein 1 subunit eta
Myeloid differentiation primary response protein MyD88
Probable rRNA-processing protein EBP2
Protein arginine N-methyltransferase 1
BAG family molecular chaperone regulator 1
Cyclic AMP-dependent transcription factor ATF-6 beta
Endophilin-A2
Telomerase protein component 1
Serine/threonine-protein kinase VRK1
Growth/differentiation factor 15
A-kinase anchor protein 9
Protein NipSnap homolog 1
Condensin complex subunit 3
Actin-related protein 2/3 complex subunit 5-like protein
Calcium uptake protein 1, mitochondrial
UPF0415 protein C7orf25
Protein FAM118B
Target of rapamycin complex 2 subunit MAPKAP1
RNA-binding protein 4B
Zinc finger FYVE domain-containing protein 21
ATP-dependent RNA helicase DDX50
Large ribosomal subunit protein bL34m
Zinc phosphodiesterase ELAC protein 2
Telomerase RNA component interacting RNase
Glutamate-rich WD repeat-containing protein 1
ADP-ribose glycohydrolase MACROD1
Ribosome quality control complex subunit TCF25
Protein CMSS1
Kelch domain-containing protein 3
Evolutionarily conserved signaling intermediate in Toll pathway, mitochondrial
Methylosome protein WDR77
Cytochrome b-245 chaperone 1
Vitamin K epoxide reductase complex subunit 1
2-(3-amino-3-carboxypropyl)histidine synthase subunit 2
Tubulin alpha-1C chain
Selenoprotein S
Apolipoprotein L2
Myb-binding protein 1A
Fermitin family homolog 1
Mitochondrial genome maintenance exonuclease 1
Golgi reassembly-stacking protein 1
FYVE and coiled-coil domain-containing protein 1
Acyl-CoA-binding domain-containing protein 6
Coronin-1B
Thioredoxin domain-containing protein 17

BUD13 homolog
Serine/threonine-protein phosphatase CPPED1
Vacuolar protein-sorting-associated protein 25
Large ribosomal subunit protein mL45
Uncharacterized protein C7orf50
Tudor-interacting repair regulator protein
45 kDa calcium-binding protein
Programmed cell death protein 2-like
Proteasomal ATPase-associated factor 1
Partner of Y14 and mago
MICOS complex subunit MIC25
ADP-dependent glucokinase
G patch domain-containing protein 1
Serine/threonine-protein kinase RIO1
Protein LLP homolog
DNA replication complex GINS protein SLD5
rRNA-processing protein UTP23 homolog
Protein pelota homolog
Peroxiredoxin-like 2A
E3 ubiquitin-protein ligase TRIM56
Endoplasmic reticulum resident protein 44
Latexin
Nucleolar protein 10
Cancer-related nucleoside-triphosphatase
Mitochondrial import inner membrane translocase subunit Tim29
Translational activator of cytochrome c oxidase 1
Gamma-tubulin complex component 2
Extended synaptotagmin-1
Ubiquitin-associated domain-containing protein 1
Protein YIPF4
Phagosome assembly factor 1
tRNA-splicing endonuclease subunit Sen34
Protein canopy homolog 3
Mitochondrial ribosome-associated GTPase 1
Chitobiosyldiphosphodolichol beta-mannosyltransferase
LIM domain-containing protein 2
HAUS augmin-like complex subunit 8
Inositol polyphosphate 5-phosphatase K
Proteasome assembly chaperone 3
COP9 signalosome complex subunit 4
Synaptotagmin-11
WW domain-containing adapter protein with coiled-coil
Death-inducer obliterator 1
Metastasis-associated protein MTA3
RNA-binding protein 42
Dynactin subunit 5
Mini-chromosome maintenance complex-binding protein
Alanyl-tRNA editing protein Aarsd1
DCN1-like protein 5
Acidic leucine-rich nuclear phosphoprotein 32 family member E

Leucine-rich repeat-containing protein 1
Phosphatidylinositol 4-kinase type 2-alpha
Transmembrane protein 43
Fibronectin type III and SPRY domain-containing protein 1
Tubulin-specific chaperone D
Nucleoporin NDC1
Protein HGH1 homolog
Dehydrogenase/reductase SDR family member 4
Lipase maturation factor 2
NADH dehydrogenase [ubiquinone] 1 alpha subcomplex assembly factor 3
Multiple myeloma tumor-associated protein 2
Transmembrane protein 70, mitochondrial
Mediator of RNA polymerase II transcription subunit 18
Iron-sulfur cluster assembly 1 homolog, mitochondrial
Tubulin beta-6 chain
Protein PAXX
DNA-directed RNA polymerase III subunit RPC3
Heterogeneous nuclear ribonucleoprotein U-like protein 1
Protein misato homolog 1
PHD finger protein 23
Programmed cell death protein 10
Ribonuclease P protein subunit p25
Derlin-1
Oxidoreductase HTATIP2
Probable ATP-dependent RNA helicase DDX23
Telomerase Cajal body protein 1
MICOS complex subunit MIC26
Dehydrogenase/reductase SDR family member 6
Methylthioribose-1-phosphate isomerase
WD repeat-containing protein 18
Vesicle-associated membrane protein 8
tRNA (guanine(6)-N2)-methyltransferase THUMP3
Acireductone dioxygenase
Enoyl-[acyl-carrier-protein] reductase, mitochondrial
ER membrane protein complex subunit 6
N-terminal Xaa-Pro-Lys N-methyltransferase 1
Katanin p80 WD40 repeat-containing subunit B1
Target of rapamycin complex subunit LST8
Ashwin
Voltage-gated monoatomic cation channel TMEM109
Protein PBDC1
Phosphatidylserine synthase 2
Nucleolar complex protein 4 homolog
U3 small nucleolar RNA-associated protein 14 homolog A
Dual specificity protein phosphatase 23
Transmembrane emp24 domain-containing protein 9
BOS complex subunit TMEM147
Nucleoporin p58/p45
Protein adenylyltransferase SelO, mitochondrial
Protein DPCD

Guanine nucleotide-binding protein-like 3
ATPase family gene 2 protein homolog B
Serine/threonine-protein kinase RIO2
tRNA (adenine(58)-N(1))-methyltransferase, mitochondrial
Transmembrane and ubiquitin-like domain-containing protein 1
Mitochondrial import inner membrane translocase subunit Tim21
Kinesin-like protein KIFC1
Nuclear pore complex protein Nup85
Very long chain fatty acid elongase 1
Katanin p60 ATPase-containing subunit A-like 1
HIRA-interacting protein 3
Intraflagellar transport protein 27 homolog
Splicing factor YJU2
ADP-ribose pyrophosphatase, mitochondrial
Threonine--tRNA ligase, mitochondrial
Acetyl-CoA acetyltransferase, cytosolic
Replication initiator 1
RNA-binding protein 4
FUN14 domain-containing protein 2
RNA polymerase II-associated protein 1
Splicing factor 3B subunit 5
Sideroflexin-3
Chitinase domain-containing protein 1
Poly(A) polymerase gamma
Caspase recruitment domain-containing protein 10
Kanadaptin
Cyclin-dependent kinase 19
Protein LSM14 homolog B
Tapasin-related protein
Junctional adhesion molecule C
Caspase recruitment domain-containing protein 6
BTB/POZ domain-containing protein 2
Oxysterol-binding protein-related protein 11
Oxysterol-binding protein-related protein 10
Rab11 family-interacting protein 5
TBC1 domain family member 10A
Complement C1q tumor necrosis factor-related protein 5
N-alpha-acetyltransferase 15, NatA auxiliary subunit
Bcl-2-like protein 13
Caspase recruitment domain-containing protein 11
Solute carrier family 12 member 9
Serrate RNA effector molecule homolog
Queuine tRNA-ribosyltransferase catalytic subunit 1
AP-1 complex subunit mu-1
Nucleolar and spindle-associated protein 1
Haloacid dehalogenase-like hydrolase domain-containing 5
Fanconi anemia group D2 protein
Protein MAK16 homolog
Histone deacetylase 8
Replication termination factor 2

Charged multivesicular body protein 4a
Eukaryotic translation initiation factor 2A
Polymerase delta-interacting protein 3
Uncharacterized protein KIAA1671
SH3 and multiple ankyrin repeat domains protein 3
Guanine nucleotide-binding protein subunit beta-like protein 1
Alpha-(1,6)-fucosyltransferase
Large ribosomal subunit protein bL32m
Large ribosomal subunit protein bL20m
Large ribosomal subunit protein uL13m
Large ribosomal subunit protein bL9m
Large ribosomal subunit protein uL4m
Large ribosomal subunit protein uL1m
MKI67 FHA domain-interacting nucleolar phosphoprotein
Hyccin
YTH domain-containing family protein 1
3'-5' exoribonuclease HELZ2
RanBP-type and C3HC4-type zinc finger-containing protein 1
Small ribosomal subunit protein mS26
Neurolysin, mitochondrial
Centrosomal protein of 41 kDa
Histone-lysine N-methyltransferase SETD2
Pantothenate kinase 2, mitochondrial
Dedicator of cytokinesis protein 9
FERM domain-containing protein 8
Contactin-associated protein-like 3
Histone-lysine N-methyltransferase NSD3
Kinetochore protein Nuf2
Large ribosomal subunit protein mL37
GTP-binding protein 4
Tether containing UBX domain for GLUT4
Oxysterol-binding protein-related protein 8
Uveal autoantigen with coiled-coil domains and ankyrin repeats
Ras-related protein Rab-34
2-(3-amino-3-carboxypropyl)histidine synthase subunit 1
WD repeat-containing protein 11
Regulator of nonsense transcripts 3B
Crooked neck-like protein 1
F-box-like/WD repeat-containing protein TBL1XR1
Ubiquitin-like protein 5
Protein phosphatase 1 regulatory subunit 12C
Serine/threonine-protein kinase D2
ER degradation-enhancing alpha-mannosidase-like protein 3
Protein Niban 1
UBX domain-containing protein 6
Transmembrane 6 superfamily member 1
Uridine-cytidine kinase 2
Sialoadhesin
Apoptosis inhibitor 5
NACHT, LRR and PYD domains-containing protein 1

Tripartite motif-containing protein 5
E3 ubiquitin-protein ligase TRIM4
Alpha-ketoglutarate-dependent dioxygenase FTO
Palmitoyltransferase ZDHHC5
Transport and Golgi organization protein 6 homolog
182 kDa tankyrase-1-binding protein
(E3-independent) E2 ubiquitin-conjugating enzyme
Protein zyg-11 homolog B
Protein TANC1
Ethanolaminephosphotransferase 1
Exportin-4
Endoplasmic reticulum junction formation protein lunapark
Centrosomal protein of 44 kDa
Protein tweety homolog 3
Kelch-like protein 4
Myotubularin-related protein 12
pre-mRNA 3' end processing protein WDR33
Ribosome biogenesis protein WDR12
Protein YIPF3
Tubulointerstitial nephritis antigen-like
Adipose-secreted signaling protein
PITH domain-containing protein 1
Derlin-2
COMM domain-containing protein 5
Sentrin-specific protease 6
RNA exonuclease 4
ATP-dependent RNA helicase DDX24
DNA-directed RNA polymerase I subunit RPA49
Superkiller complex protein 8
SRA stem-loop-interacting RNA-binding protein, mitochondrial
Serine racemase
NIF3-like protein 1
Egl nine homolog 1
Mucolipin-1
PSME3-interacting protein
Mitochondrial fission factor
N-alpha-acetyltransferase 50
Ubiquitin-like modifier-activating enzyme 5
E3 ubiquitin-protein ligase makorin-2
Transmembrane protein 126A
Polyadenylate-binding protein-interacting protein 1
Mitochondrial disaggregase
Ras-related protein Rab-33B
Large subunit GTPase 1 homolog
Protein FAM107B
RNA cytidine acetyltransferase
COMM domain-containing protein 4
Kinesin light chain 2
Integrin-linked kinase-associated serine/threonine phosphatase 2C
5'-3' exoribonuclease 2

Toll-interacting protein
Histone deacetylase complex subunit SAP130
Nuclear speckle splicing regulatory protein 1
Integrator complex subunit 2
Rac GTPase-activating protein 1
Protein mono-ADP-ribosyltransferase PARP12
Cleavage stimulation factor subunit 2 tau variant
Cytosolic 5'-nucleotidase 3A
CYFIP-related Rac1 interactor A
Transmembrane protein 222
Haloacid dehalogenase-like hydrolase domain-containing protein 2
Glutamyl-tRNA(Gln) amidotransferase subunit A, mitochondrial
Probable ATP-dependent RNA helicase DDX47
Magnesium transporter protein 1
Ras-related protein Rab-1B
Large ribosomal subunit protein uL18m
Testis-specific Y-encoded-like protein 1
Transmembrane protein 168
VIP36-like protein
Nonsense-mediated mRNA decay factor SMG9
Protein FAM234A
Oxysterol-binding protein-related protein 5
Deoxynucleotidyltransferase terminal-interacting protein 1
Nucleotide exchange factor SIL1
Protein-L-histidine N-pros-methyltransferase
Anaphase-promoting complex subunit 1
Probable E3 ubiquitin-protein ligase IRF2BPL
Cysteine-rich and transmembrane domain-containing protein 1
DNA-directed RNA polymerase III subunit RPC6
Nuclear ubiquitous casein and cyclin-dependent kinase substrate 1
Thioredoxin-related transmembrane protein 4
Kinesin-like protein KIF13A
Activating signal cointegrator 1 complex subunit 2
Rabenosyn-5
Iron-sulfur cluster assembly enzyme ISCU
Oxysterol-binding protein-related protein 2
Autophagy protein 5
WD repeat-containing protein 13
Epsin-3
EH domain-containing protein 4
Vacuolar protein sorting-associated protein 33B
Vacuolar protein sorting-associated protein 16 homolog
Vacuolar protein sorting-associated protein 11 homolog
SH3 domain-binding glutamic acid-rich-like protein 3
Gigaxonin
Solute carrier family 25 member 32
TRIO and F-actin-binding protein
Enhancer of polycomb homolog 1
STE20-like serine/threonine-protein kinase
Peptidyl-prolyl cis-trans isomerase-like 3

Sodium-coupled neutral amino acid symporter 1
Phosducin-like protein 3
Serine/threonine-protein kinase TAO3
Rab3 GTPase-activating protein non-catalytic subunit
Activity-dependent neuroprotector homeobox protein
Diphthine methyl ester synthase
ATP-dependent DNA/RNA helicase DHX36
Inorganic pyrophosphatase 2, mitochondrial
Protein spinster homolog 1
Large ribosomal subunit protein mL46
Solute carrier family 12 member 5
Presenilin-associated rhomboid-like protein, mitochondrial
Pinin
Transmembrane protein 245
Forkhead box protein P1
BTB/POZ domain-containing adapter for CUL3-mediated RhoA degradation protein 3
tRNA dimethylallyltransferase
UPF0696 protein C11orf68
Growth hormone-inducible transmembrane protein
BolA-like protein 2
Thioredoxin-interacting protein
Thioredoxin-related transmembrane protein 1
Negative elongation factor A
Golgi resident protein GCP60
Cdc42 effector protein 4
Tyrosine-protein phosphatase non-receptor type 23
Semaphorin-6B
Protein unc-45 homolog A
Major facilitator superfamily domain-containing protein 1
DnaJ homolog subfamily C member 5
Kinetochore-associated protein DSN1 homolog
Charged multivesicular body protein 4b
RWD domain-containing protein 1
CUE domain-containing protein 2
F-box/LRR-repeat protein 15
Fructosamine-3-kinase
GDP-fucose protein O-fucosyltransferase 1
Phosphatidylinositol glycan anchor biosynthesis class U protein
Torsin-3A
Serine/threonine-protein kinase WNK1
Aminopeptidase B
Golgi phosphoprotein 3-like
Golgi phosphoprotein 3
tRNA N6-adenosine threonylcarbamoyltransferase, mitochondrial
Golgi-associated plant pathogenesis-related protein 1
TraB domain-containing protein
Mitochondrial ribosome-associated GTPase 2
Sentrin-specific protease 3
Oxysterol-binding protein-related protein 3
SWI/SNF-related matrix-associated actin-dependent regulator of chromatin subfamily A contain

EH domain-containing protein 1
mRNA (2'-O-methyladenosine-N(6)-)-methyltransferase
ESF1 homolog
Alpha-1,3/1,6-mannosyltransferase ALG2
HEAT repeat-containing protein 1
Protein O-mannose kinase
Rab GTPase-binding effector protein 2
Dimethyladenosine transferase 2, mitochondrial
rRNA N6-adenosine-methyltransferase ZCCHC4
STING ER exit protein
Cytosolic iron-sulfur assembly component 2A
Probable ATP-dependent RNA helicase DHX35
Protein FAM124B
HAUS augmin-like complex subunit 4
Coiled-coil domain-containing protein 134
Coiled-coil domain-containing protein 86
Receptor expression-enhancing protein 4
Transcription factor SOX-17
Optic atrophy 3 protein
Cytosolic iron-sulfur assembly component 3
ATP-dependent RNA helicase DHX33
Nucleolar protein 6
WD repeat and coiled-coil-containing protein
3'-5' RNA helicase YTHDC2
5-azacytidine-induced protein 2
RNA polymerase II-associated protein 3
BCAS3 microtubule associated cell migration factor
Alpha-1,2-mannosyltransferase ALG9
Lipid droplet-associated hydrolase
Ribosomal oxygenase 1
WD repeat-containing protein 55
Ran-binding protein 3
dCTP pyrophosphatase 1
Inactive tyrosine-protein kinase PEAK1
Ribosome production factor 2 homolog
Histone-lysine N-methyltransferase SMYD3
Mth938 domain-containing protein
Dedicator of cytokinesis protein 5
WD repeat-containing protein 26
Tudor domain-containing protein 3
UPF0488 protein C8orf33
Polyamine-transporting ATPase 13A3
Methyltransferase-like protein 17, mitochondrial
Splicing factor, arginine/serine-rich 19
Nuclear exosome regulator NRDE2
Prostaglandin E synthase 2
Phosphorylated adapter RNA export protein
Ubiquitin-conjugating enzyme E2 Z
Complex I assembly factor ACAD9, mitochondrial
5'-nucleotidase domain-containing protein 2

PRKR-interacting protein 1
Caspase activity and apoptosis inhibitor 1
Nucleolar protein 11
Probable ATP-dependent RNA helicase DDX31
Thiol S-methyltransferase TMT1A
ATPase PAAT
Multimerin-2
Ubiquitin carboxyl-terminal hydrolase MINDY-3
Protein eva-1 homolog A
AKT-interacting protein
Pleckstrin homology domain-containing family F member 2
tRNA endonuclease ANKZF1
Golgi reassembly-stacking protein 2
Protein zwilch homolog
Jupiter microtubule associated homolog 2
Mitochondrial glutamate carrier 1
Proline-serine-threonine phosphatase-interacting protein 2
Mediator of RNA polymerase II transcription subunit 20
WD repeat-containing protein 76
Queuine tRNA-ribosyltransferase accessory subunit 2
Actin-related protein 8
Damage-control phosphatase ARMT1
Pantothenate kinase 3
CCR4-NOT transcription complex subunit 10
Leucine-rich repeat-containing protein 40
Histone-lysine N-methyltransferase EHMT1
Sideroflexin-1
Spermatogenesis-defective protein 39 homolog
Conserved oligomeric Golgi complex subunit 4
Actin-related protein 5
Vacuolar protein sorting-associated protein 37B
Large ribosomal subunit protein mL44
L-2-hydroxyglutarate dehydrogenase, mitochondrial
COP9 signalosome complex subunit 7b
Non-homologous end-joining factor 1
Protein transport protein Sec61 subunit alpha isoform 2
Ribitol 5-phosphate transferase FKRP
Elongator complex protein 3
Ribosome production factor 1
DNA-directed RNA polymerase I subunit RPA2
Zinc finger matrin-type protein 3
Uridine-cytidine kinase 1
Ketosamine-3-kinase
TBC1 domain family member 17
Probable cysteine--tRNA ligase, mitochondrial
Solute carrier family 52, riboflavin transporter, member 2
Phosphopantothenate--cysteine ligase
Ubiquitin domain-containing protein 1
WD repeat-containing protein 41
Probable fibrosin-1

Nicotinamide/nicotinic acid mononucleotide adenylyltransferase 1
Protein Njmu-R1
Sialate O-acetylesterase
Pleckstrin homology domain-containing family A member 5
E3 ubiquitin-protein ligase SMURF2
Regulator of nonsense transcripts 2
Guanine nucleotide-binding protein subunit beta-4
Exportin-5
GrpE protein homolog 1, mitochondrial
MYG1 exonuclease
Pleckstrin homology domain-containing family A member 3
Pleckstrin homology domain-containing family A member 1
Retinoid-inducible serine carboxypeptidase
Netrin-4
Calcyclin-binding protein
Ras-related GTP-binding protein C
Zinc finger FYVE domain-containing protein 1
Intraflagellar transport protein 122 homolog
Retinol dehydrogenase 14
Beta-parvin
Tensin-1
Plasminogen receptor (KT)
NmrA-like family domain-containing protein 1
Vezatin
Kinetochore protein Spc25
Transcription initiation factor TFIID subunit 9B
Solute carrier family 38 member 10
Ethanolamine kinase 1
Adhesion G protein-coupled receptor L4
Putative divalent cation/proton antiporter TMEM165
Mitochondrial thiamine pyrophosphate carrier
Echinoderm microtubule-associated protein-like 4
rRNA methyltransferase 3, mitochondrial
Glyoxalase domain-containing protein 4
Serine/threonine-protein kinase Nek6
Methylcrotonoyl-CoA carboxylase beta chain, mitochondrial
Nuclear receptor coactivator 5
Ectopic P granules protein 5 homolog
Helicase MOV-10
N6-adenosine-methyltransferase non-catalytic subunit
Non-lysosomal glucosylceramidase
Pre-mRNA-splicing factor CWC22 homolog
Ribonucleoprotein PTB-binding 2
Chromodomain-helicase-DNA-binding protein 8
Band 4.1-like protein 5
GPN-loop GTPase 1
Stromal cell-derived factor 2-like protein 1
Casein kinase I isoform gamma-1
Pre-mRNA-splicing factor SYF1
Prolactin regulatory element-binding protein

Steroid receptor RNA activator 1
Endoplasmic reticulum transmembrane helix translocase
Golgi-associated PDZ and coiled-coil motif-containing protein
Large ribosomal subunit protein uL29m
LYR motif-containing protein 4
O-phosphoseryl-tRNA(Sec) selenium transferase
Charged multivesicular body protein 1a
Transmembrane 9 superfamily member 3
Unconventional myosin-X
Adipocyte plasma membrane-associated protein
tRNA (34-2'-O)-methyltransferase regulator WDR6
Thioredoxin reductase 2, mitochondrial
ATP-binding cassette sub-family B member 6
ADP-ribosylation factor GTPase-activating protein 3
Zinc finger CCHC domain-containing protein 17
Ras-related protein Rab-18
Palmdelphin
RNA polymerase II subunit A C-terminal domain phosphatase SSU72
Vacuolar protein sorting-associated protein VTA1 homolog
Serine--tRNA ligase, mitochondrial
Large ribosomal subunit protein mL65
Dynein light chain roadblock-type 1
ER membrane protein complex subunit 7
Transcription and mRNA export factor ENY2
Exosome complex component RRP41
Ubiquitin-conjugating enzyme E2 T
H/ACA ribonucleoprotein complex subunit 3
tRNA N6-adenosine threonylcarbamoyltransferase
DNA methyltransferase 1-associated protein 1
Protocadherin-12
Inositol-3-phosphate synthase 1
Bromodomain-containing protein 7
mRNA-decapping enzyme 1A
Acyl-coenzyme A thioesterase 13
Mediator of RNA polymerase II transcription subunit 4
Complex I assembly factor TIMMDC1, mitochondrial
Synembryn-A
Complement component C1q receptor
Putative RNA-binding protein Luc7-like 1
Endothelial cell-specific molecule 1
Leucine zipper transcription factor-like protein 1
Large ribosomal subunit protein mL40
Suppressor of SWI4 1 homolog
1-phosphatidylinositol 4,5-bisphosphate phosphodiesterase beta-1
Fructose-2,6-bisphosphatase TIGAR
Protein C12orf4
Reticulon-4
Regulation of nuclear pre-mRNA domain-containing protein 1B
Xaa-Pro aminopeptidase 3
Omega-amidase NIT2

Cell death regulator Aven
Inner centromere protein
Exosome complex component RRP46
Exosome complex component RRP40
Kinesin-like protein KIF13B
Anillin
Xaa-Pro aminopeptidase 1
Gephyrin
Bridging integrator 3
Something about silencing protein 10
StAR-related lipid transfer protein 7, mitochondrial
Baculoviral IAP repeat-containing protein 6
PDZ and LIM domain protein 7
Acetyl-coenzyme A synthetase, cytoplasmic
Diablo IAP-binding mitochondrial protein
Nucleolar RNA helicase 2
GTP-binding protein SAR1a
Sialic acid synthase
Endophilin-B2
Histone-lysine N-methyltransferase ASH1L
Translation initiation factor eIF2B subunit gamma
Muscleblind-like protein 1
Sialin
Eukaryotic translation initiation factor 4E transporter
Carbohydrate sulfotransferase 12
Suppressor of tumorigenicity 7 protein
PRKCA-binding protein
SH2B adapter protein 1
CTP synthase 2
DNA polymerase epsilon subunit 3
Chromatin accessibility complex protein 1
Aladin
SNF-related serine/threonine-protein kinase
ATP-binding cassette sub-family B member 10, mitochondrial
Bromodomain adjacent to zinc finger domain protein 1A
Striatin-4
L-aminoadipate-semialdehyde dehydrogenase-phosphopantetheinyl transferase
rRNA N6-adenosine-methyltransferase METTL5
Oligosaccharyltransferase complex subunit OSTC
COX assembly mitochondrial protein 2 homolog
Phospholipid scramblase 4
Ribonuclease 3
Sorting nexin-15
Heme-binding protein 1
DNA dC->dU-editing enzyme APOBEC-3C
Vacuolar protein sorting-associated protein 45
RNA-binding protein PNO1
Large ribosomal subunit protein bL17m
14 kDa phosphohistidine phosphatase
Serine incorporator 1

Rho GTPase-activating protein 35
Protein FAM114A2
Phospholipid scramblase 3
1-acyl-sn-glycerol-3-phosphate acyltransferase gamma
Lymphoid-specific helicase
Glycoprotein-N-acetylgalactosamine 3-beta-galactosyltransferase 1
Latent-transforming growth factor beta-binding protein 3
Mitochondrial import receptor subunit TOM22 homolog
LanC-like protein 2
Kinesin-like protein KIF15
Transmembrane 7 superfamily member 3
Homer protein homolog 3
Phenylalanine--tRNA ligase beta subunit
Isoleucine--tRNA ligase, mitochondrial
Ribosome biogenesis protein SLX9 homolog
Kinesin light chain 4
Protein diaphanous homolog 3
BMP-2-inducible protein kinase
Ubiquitin-like-conjugating enzyme ATG3
NAD-dependent protein deacetylase sirtuin-3, mitochondrial
Sister chromatid cohesion protein PDS5 homolog B
Structural maintenance of chromosomes protein 4
Alpha-mannosidase 2C1
Phosphatidylinositol-3-phosphatase SAC1
Protein DEPP1
Obg-like ATPase 1
Copper homeostasis protein cutC homolog
Zinc finger protein 64
Ethylmalonyl-CoA decarboxylase
RNA-binding protein 12
TBC1 domain family member 22B
Midasin
Acetyl-coenzyme A synthetase 2-like, mitochondrial
Zinc finger CCHC domain-containing protein 3
Peroxisomal 2,4-dienoyl-CoA reductase [(3E)-enoyl-CoA-producing]
Palmitoyl-protein thioesterase ABHD10, mitochondrial
T-complex protein 11-like protein 1
Double-stranded RNA-binding protein Staufen homolog 2
Shiftless antiviral inhibitor of ribosomal frameshifting protein
Probable ATP-dependent RNA helicase DDX28
Transmembrane protein 106B
Protein lin-7 homolog C
1-acyl-sn-glycerol-3-phosphate acyltransferase epsilon
Gamma-taxilin
SPATS2-like protein
Ufm1-specific protease 2
ATP-binding cassette sub-family F member 3
CYFIP-related Rac1 interactor B
Mitochondrial potassium channel ATP-binding subunit
ATP-dependent RNA helicase DDX19A

GTPase IMAP family member 4
Tyrosyl-DNA phosphodiesterase 1
Protection of telomeres protein 1
TBC1 domain family member 23
DDB1- and CUL4-associated factor 13
U3 small nucleolar ribonucleoprotein protein IMP3
MRG/MORF4L-binding protein
S-adenosyl-L-methionine-dependent tRNA 4-demethylwyosine synthase TYW1
Exocyst complex component 1
Protein-L-isoaspartate O-methyltransferase domain-containing protein 2
Integrator complex subunit 9
Cell cycle control protein 50A
Ubiquinol-cytochrome c reductase complex assembly factor 1
Septin-11
Sodium-coupled neutral amino acid transporter 7
Mediator of RNA polymerase II transcription subunit 17
Alpha-parvin
Ubiquitin carboxyl-terminal hydrolase 40
4'-phosphopantetheine phosphatase
F-box only protein 28
TBC1 domain family member 13
Exonuclease 3'-5' domain-containing protein 2
DnaJ homolog subfamily C member 11
Integrator complex subunit 7
Fanconi anemia group I protein
ATPase family AAA domain-containing protein 3A
ADP-ribosylation factor-like protein 8B
DnaJ homolog subfamily C member 17
Integrator complex subunit 13
Synembryn-B
Guanine nucleotide-binding protein-like 3-like protein
ATP-dependent RNA helicase DDX18
Histone chaperone ASF1B
Kelch-like protein 11
Integrator complex subunit 10
Large ribosomal subunit protein mL66
Pyridoxine-5'-phosphate oxidase
Armadillo repeat-containing protein 1
DNA-directed RNA polymerase III subunit RPC5
Protein SDA1 homolog
Poly(A) RNA polymerase, mitochondrial
Notchless protein homolog 1
Adaptin ear-binding coat-associated protein 2
DNA-directed RNA polymerase III subunit RPC2
RNA-binding protein 28
Anoctamin-10
Pre-mRNA-splicing factor RBM22
Distal membrane-arm assembly complex protein 2
WD repeat-containing protein 70
Transmembrane protein 51

Arginine and glutamate-rich protein 1
SAFB-like transcription modulator
Peptidyl-prolyl cis-trans isomerase FKBP14
Required for meiotic nuclear division protein 1 homolog
p21-activated protein kinase-interacting protein 1
Hypoxia-inducible factor 1-alpha inhibitor
3-oxoacyl-[acyl-carrier-protein] synthase, mitochondrial
Large ribosomal subunit protein uL22m
CXXC motif containing zinc binding protein
BRISC and BRCA1-A complex member 1
Ceroid-lipofuscinosis neuronal protein 6
Ankyrin repeat and SOCS box protein 6
Probable tRNA(His) guanylyltransferase
Histone PARylation factor 1
Interleukin-1 receptor-associated kinase 4
Uridine-cytidine kinase-like 1
Transmembrane protein 160
NACHT, LRR and PYD domains-containing protein 2
Constitutive coactivator of PPAR-gamma-like protein 2
tRNA selenocysteine 1-associated protein 1
NADH dehydrogenase [ubiquinone] 1 beta subcomplex subunit 11, mitochondrial
Large ribosomal subunit protein uL16m
H/ACA ribonucleoprotein complex subunit 2
OCIA domain-containing protein 1
ADP-ribosylhydrolase ARH3
E3 ubiquitin-protein ligase MARCHF5
Cell growth-regulating nucleolar protein
Transmembrane protein 161A
Golgi-resident adenosine 3',5'-bisphosphate 3'-phosphatase
MICOS complex subunit MIC19
tRNA-dihydrouridine(20) synthase [NAD(P)+]-like
CKLF-like MARVEL transmembrane domain-containing protein 6
NAD-dependent protein deacylase sirtuin-5, mitochondrial
GATOR2 complex protein MIOS
Myotubularin-related protein 10
Sphingomyelin phosphodiesterase 4
Pre-mRNA-splicing factor CWC25 homolog
Testis-expressed protein 10
DDB1- and CUL4-associated factor 16
THUMP domain-containing protein 1
Transmembrane prolyl 4-hydroxylase
Torsin-4A
tRNA (guanine(26)-N(2))-dimethyltransferase
Ganglioside-induced differentiation-associated protein 2
Nuclear distribution protein nudE homolog 1
BRISC and BRCA1-A complex member 2
Glutaminyl-peptide cyclotransferase-like protein
ADP-ribosylation factor-like protein 15
BTB/POZ domain-containing protein KCTD5
CDKN2A-interacting protein

DnaJ homolog subfamily B member 12
Non-structural maintenance of chromosomes element 4 homolog A
H/ACA ribonucleoprotein complex subunit 1
Stabilin-1
Serine/threonine-protein phosphatase 4 regulatory subunit 2
Dipeptidyl peptidase 3
Protein AATF
Tubulin alpha-8 chain
Probable ATP-dependent RNA helicase DDX56
Sphingosine kinase 1
Telomeric repeat-binding factor 2-interacting protein 1
Abl interactor 2
Bcl-2-associated transcription factor 1
U3 small nucleolar RNA-associated protein 6 homolog
TGF-beta-activated kinase 1 and MAP3K7-binding protein 2
Large ribosomal subunit protein mL39
Mitogen-activated protein kinase kinase kinase 20
Peptidyl-prolyl cis-trans isomerase FKBP11
Tropomodulin-3
BET1-like protein
Very long chain fatty acid elongase 5
NF-kappa-B inhibitor-interacting Ras-like protein 2
UDP-glucose:glycoprotein glucosyltransferase 2
UDP-glucose:glycoprotein glucosyltransferase 1
Cyclin-dependent kinase 12
FAST kinase domain-containing protein 2, mitochondrial
Very-long-chain enoyl-CoA reductase
Endoplasmic reticulum aminopeptidase 1
Actin-related protein 10
Vesicle transport protein USE1
CDGSH iron-sulfur domain-containing protein 1
ADP-ribosylation factor-binding protein GGA3
Splicing factor C9orf78
Constitutive coactivator of PPAR-gamma-like protein 1
Glycerophosphodiester phosphodiesterase 1
SWI/SNF-related matrix-associated actin-dependent regulator of chromatin subfamily A-like pro
Glycolipid transfer protein
Maspardin
Large ribosomal subunit protein bL35m
Upstream-binding protein 1
Insulin-like growth factor 2 mRNA-binding protein 1
Sacsin
Mitochondrial carrier homolog 1
Diphosphoinositol polyphosphate phosphohydrolase 2
Hsp70-binding protein 1
Methionine adenosyltransferase 2 subunit beta
Myoferlin
Intersectin-2
Ribosome biogenesis protein NOP53
EH domain-containing protein 3

EH domain-containing protein 2
Rho guanine nucleotide exchange factor 12
CCR4-NOT transcription complex subunit 2
NCK-interacting protein with SH3 domain
Programmed cell death 1 ligand 1
Opioid growth factor receptor
LIM and cysteine-rich domains protein 1
Cysteine-rich motor neuron 1 protein
Selenoprotein N
Protein PALS2
Charged multivesicular body protein 5
Spliceosome-associated protein CWC15 homolog
Large ribosomal subunit protein uL15m
Thymocyte nuclear protein 1
Thyroid transcription factor 1-associated protein 26
NADH dehydrogenase [ubiquinone] 1 alpha subcomplex assembly factor 4
Very-long-chain (3R)-3-hydroxyacyl-CoA dehydratase 3
ER membrane protein complex subunit 3
NADH dehydrogenase [ubiquinone] 1 alpha subcomplex subunit 13
[Pyruvate dehydrogenase [acetyl-transferring]]-phosphatase 1, mitochondrial
E3 ubiquitin-protein ligase KCMF1
Ankycorbin
Vesicle-associated membrane protein-associated protein A
Large ribosomal subunit protein bL27m
Proton-transporting V-type ATPase complex assembly regulator TMEM9
Sentrin-specific protease 1
CXXC-type zinc finger protein 1
SH3 domain-binding protein 4
Septin-10
GEM-interacting protein
PHD and RING finger domain-containing protein 1
Pogo transposable element with KRAB domain
Protein Daple
Rho GTPase-activating protein 23
Vacuolar protein sorting-associated protein 18 homolog
Protein RCC2
RAB11-binding protein RELCH
Disco-interacting protein 2 homolog B
Junctional cadherin 5-associated protein
SLAIN motif-containing protein 2
Teneurin-3
Ubiquitin carboxyl-terminal hydrolase 36
BRCA2 and CDKN1A-interacting protein
Serine/threonine-protein kinase 26
Armadillo repeat-containing X-linked protein 1
ABI gene family member 3
Prostaglandin F2 receptor negative regulator
CTTNBP2 N-terminal-like protein
Transmembrane protein 181
Inhibitor of Bruton tyrosine kinase

HEAT repeat-containing protein 5B
NFX1-type zinc finger-containing protein 1
Chondroitin sulfate glucuronyltransferase
Protocadherin-10
Ribosome-binding protein 1
Cleavage and polyadenylation specificity factor subunit 2
Kelch-like protein 9
Leucine--tRNA ligase, cytoplasmic
Myelin expression factor 2
Kelch-like protein 42
eIF-2-alpha kinase GCN2
WD repeat-containing protein 35
RNA-binding protein 27
KAT8 regulatory NSL complex subunit 3
E3 ubiquitin-protein ligase HECW2
FERM domain-containing protein 4A
Rabankyrin-5
Succinate--CoA ligase [ADP-forming] subunit beta, mitochondrial
GMP reductase 2
Syntaxin-18
Dolichol-phosphate mannosyltransferase subunit 3
Protein IMPACT
UV radiation resistance-associated gene protein
Ataxin-10
Methyl-CpG-binding domain protein 2
Neurochondrin
Tuftelin-interacting protein 11
Epidermal growth factor receptor substrate 15-like 1
Unconventional myosin-Ia
Origin recognition complex subunit 3
SUMO-activating enzyme subunit 1
Coatomer subunit gamma-2
Phosphatidylinositol 4-kinase beta
C-type mannose receptor 2
COMM domain-containing protein 3
Guanine nucleotide-binding protein G(I)/G(S)/G(O) subunit gamma-12
Rab-like protein 2A
Methionine synthase reductase
Protein UXT
Set1/Ash2 histone methyltransferase complex subunit ASH2
7-dehydrocholesterol reductase
Tumor necrosis factor receptor superfamily member 10D
Histone deacetylase 6
Spastin
Dickkopf-related protein 3
tRNA (guanine-N(7)-)-methyltransferase
PTB domain-containing engulfment adapter protein 1
Vacuolar protein sorting-associated protein 29
Eukaryotic translation initiation factor 3 subunit K
Exostosin-like 2

Glyoxylate reductase/hydroxypyruvate reductase
Cathepsin Z
Ribosomal protein S6 kinase beta-2
DnaJ homolog subfamily B member 11
E3 ubiquitin-protein ligase RNF14
SUMO-activating enzyme subunit 2
Mortality factor 4-like protein 1
Nuclear RNA export factor 1
Protein sel-1 homolog 1
Beta-1,4-galactosyltransferase 7
Peflin
Zinc finger MYM-type protein 2
COP9 signalosome complex subunit 7a
Mitochondrial dicarboxylate carrier
Alpha-aminoadipic semialdehyde synthase, mitochondrial
CAP-Gly domain-containing linker protein 2
Cytochrome b-c1 complex subunit 9
Mitochondrial fission process protein 1
Tight junction protein ZO-2
DnaJ homolog subfamily B member 4
Serine/threonine-protein kinase 17A
Craniofacial development protein 1
Death domain-associated protein 6
tRNA (cytidine(32)/guanosine(34)-2'-O)-methyltransferase
Vesicle transport through interaction with t-SNAREs homolog 1B
STE20/SPS1-related proline-alanine-rich protein kinase
Gamma-adducin
Leucine-rich repeat and WD repeat-containing protein 1
Protein NipSnap homolog 3A
CGG triplet repeat-binding protein 1
Phosphatidylserine decarboxylase proenzyme, mitochondrial
ATP-binding cassette sub-family F member 2
Testin
5'-AMP-activated protein kinase subunit gamma-2
Gamma-tubulin complex component 4
Tryptophan--tRNA ligase, mitochondrial
LIM domain-containing protein 1
Translocation protein SEC63 homolog
Solute carrier family 2, facilitated glucose transporter member 6
Zinc finger CCCH domain-containing protein 7B
Protein NDRG3
Armadillo repeat-containing X-linked protein 3
Switch-associated protein 70
SUN domain-containing protein 2
Probable ribosome biogenesis protein RLP24
Ragulator complex protein LAMTOR3
LIM domain and actin-binding protein 1
AF4/FMR2 family member 4
Signal recognition particle subunit SRP68
Cysteine and histidine-rich domain-containing protein 1

Serine/threonine-protein kinase TBK1
Septin-9
Prenylcysteine oxidase 1
Probable ATP-dependent RNA helicase DDX20
Sedoheptulokinase
Dipeptidyl peptidase 2
Inactive cell surface hyaluronidase CEMIP2
Ubiquitin carboxyl-terminal hydrolase 25
B-cell receptor-associated protein 29
NADH-cytochrome b5 reductase 1
SAP30-binding protein
Zinc finger HIT domain-containing protein 2
Prefoldin subunit 2
GPN-loop GTPase 3
Poly(U)-binding-splicing factor PUF60
Nuclear receptor-binding protein
Enolase-phosphatase E1
Fasciculation and elongation protein zeta-2
NADH dehydrogenase [ubiquinone] 1 alpha subcomplex subunit 12
Translation initiation factor eIF2B subunit delta
V-type proton ATPase subunit H
Prenylated Rab acceptor protein 1
Importin-11
Multifunctional methyltransferase subunit TRM112-like protein
Dachshund homolog 1
Exportin-7
Leucine carboxyl methyltransferase 1
Vacuolar protein sorting-associated protein 51 homolog
Bromodomain adjacent to zinc finger domain protein 2A
Tyrosine-protein kinase BAZ1B
ATPase inhibitor, mitochondrial
GTP:AMP phosphotransferase AK3, mitochondrial
Leucyl-cystinyl aminopeptidase
Methyl-CpG-binding domain protein 1
CCR4-NOT transcription complex subunit 7
Rab5 GDP/GTP exchange factor
Mitochondrial peptide methionine sulfoxide reductase
N-acetyl-D-glucosamine kinase
2-hydroxyacyl-CoA lyase 1
tRNA (adenine(58)-N(1))-methyltransferase non-catalytic subunit TRM6
SH3 domain-binding glutamic acid-rich-like protein 2
Ras GTPase-activating protein nGAP
18S rRNA aminocarboxypropyltransferase
Electrogenic aspartate/glutamate antiporter SLC25A13, mitochondrial
Drebrin-like protein
Probable ATP-dependent RNA helicase DDX41
Dynactin subunit 4
Cell division cycle protein 23 homolog
Anaphase-promoting complex subunit 7
Anaphase-promoting complex subunit 5

Anaphase-promoting complex subunit 4
Anaphase-promoting complex subunit 2
ADP-ribosylation factor-binding protein GGA2
ADP-ribosylation factor-binding protein GGA1
Stomatin-like protein 2, mitochondrial
N-acetylglucosamine-1-phosphodiester alpha-N-acetylglucosaminidase
Vacuolar protein sorting-associated protein 28 homolog
U6 snRNA-associated Sm-like protein LSm7
Lariat debranching enzyme
Protein TASOR
Jupiter microtubule associated homolog 1
F-box only protein 3
A-kinase anchor protein 11
F-box/WD repeat-containing protein 11
Glucocorticoid modulatory element-binding protein 2
mRNA turnover protein 4 homolog
TRAF2 and NCK-interacting protein kinase
Cleavage and polyadenylation specificity factor subunit 3
DCC-interacting protein 13-alpha
Cdc42 effector protein 3
Serine/threonine-protein kinase tousled-like 1
G patch domain-containing protein 8
Protein mono-ADP-ribosyltransferase PARP4
NTF2-related export protein 1
ADP-sugar pyrophosphatase
REST corepressor 1
Phosphatidylcholine transfer protein
Endoplasmic reticulum mannosyl-oligosaccharide 1,2-alpha-mannosidase
RNA-binding protein Raly
General transcription factor 3C polypeptide 4
Ergosterol biosynthetic protein 28 homolog
Protein kinase C and casein kinase substrate in neurons protein 3
Isobutyryl-CoA dehydrogenase, mitochondrial
Angiopoietin-related protein 2
Apoptotic chromatin condensation inducer in the nucleus
E3 ubiquitin-protein ligase AMFR
Protein argonaute-2
Nuclear pore complex protein Nup50
Protein CDV3 homolog
CCR4-NOT transcription complex subunit 11
Integrator complex subunit 6
BAG family molecular chaperone regulator 5
Protein argonaute-1
Ras-related protein Rab-21
Ras-related protein Rab-22A
Trafficking protein particle complex subunit 2-like protein
Zinc finger protein 346
Proteasome activator complex subunit 2
Serine/threonine-protein kinase TAO2
Muskelin

Aspartyl aminopeptidase
Ras-related protein Rab-23
Malignant T-cell-amplified sequence 1
Microtubule-associated tumor suppressor 1
Protein WWC3
Paladin
Zinc transporter ZIP10
Chromatin-remodeling ATPase INO80
Cell cycle progression protein 1
Kinase D-interacting substrate of 220 kDa
Arf-GAP with SH3 domain, ANK repeat and PH domain-containing protein 1
Myocardin-related transcription factor B
Ankyrin repeat domain-containing protein 50
Mediator of RNA polymerase II transcription subunit 23
Protein Shroom4
YEATS domain-containing protein 2
Protein NDRG4
TBC1 domain family member 24
Pre-mRNA-splicing factor ISY1 homolog
Transmembrane and coiled-coil domain protein 3
E3 ubiquitin-protein ligase HECTD1
MYND-type zinc finger-containing chromatin reader ZMYND8
Cip1-interacting zinc finger protein
Coronin-1C
Targeting protein for Xklp2
Activator of basal transcription 1
RNA-binding protein NOB1
A-kinase anchor protein 8-like
Transcription factor MafF
Apoptosis-associated speck-like protein containing a CARD
Calcium load-activated calcium channel
Fizzy-related protein homolog
Anaphase-promoting complex subunit 10
Unconventional myosin-VI
ATP-dependent RNA helicase DDX19B
Lysosomal thioesterase PPT2
Pre-mRNA-processing factor 19
Suppressor of fused homolog
Bcl-2-related ovarian killer protein
Nucleolar protein 7
Sorting nexin-12
Synergin gamma
Vacuolar protein sorting-associated protein 4A
Ras GTPase-activating protein-binding protein 2
E3 ubiquitin-protein ligase CHIP
Protein kinase C and casein kinase substrate in neurons protein 2
Melanoma-associated antigen D2
Sorting nexin-7
Sorting nexin-6
Dual specificity protein phosphatase 12

Syntaxin-8
Translocon-associated protein subunit gamma
26S proteasome non-ATPase regulatory subunit 13
FAS-associated factor 1
Endothelial protein C receptor
Peptidyl-prolyl cis-trans isomerase E
Probable dimethyladenosine transferase
Protein timeless homolog
COP9 signalosome complex subunit 3
Multiple inositol polyphosphate phosphatase 1
RNA-binding protein Nova-2
WD repeat-containing protein 3
Transcription termination factor 2
NSFL1 cofactor p47
Conserved oligomeric Golgi complex subunit 5
Solute carrier family 12 member 4
AP-4 complex subunit epsilon-1
Microtubule-actin cross-linking factor 1, isoforms 1/2/3/4/5
Centrosomal protein of 131 kDa
SR-related and CTD-associated factor 8
Serine/threonine-protein phosphatase 6 regulatory subunit 1
E3 ubiquitin-protein ligase TRIM33
Histone lysine demethylase PHF8
Trinucleotide repeat-containing gene 6B protein
Inactive phospholipase C-like protein 2
Nonsense-mediated mRNA decay factor SMG5
Exocyst complex component 7
Zinc finger CCCH domain-containing protein 4
Ubiquitin carboxyl-terminal hydrolase 24
TBC1 domain family member 2B
Protein Smaug homolog 1
Forkhead box protein J3
Cytosolic carboxypeptidase 1
Endoribonuclease Dicer
Cystine/glutamate transporter
Microtubule-associated protein RP/EB family member 3
Leucine-rich repeat protein SHOC-2
Serine/arginine repetitive matrix protein 2
Proliferation-associated protein 2G4
Paraplegin
Brain-specific angiogenesis inhibitor 1-associated protein 2
GRB2-associated-binding protein 2
Structural maintenance of chromosomes protein 3
Charged multivesicular body protein 2b
SH2B adapter protein 3
Zinc finger protein 148
Myotubularin-related protein 6
Protein jagged-2
60S ribosome subunit biogenesis protein NIP7 homolog
Bifunctional UDP-N-acetylglucosamine 2-epimerase/N-acetylmannosamine kinase

RNA transcription, translation and transport factor protein
RuvB-like 2
Chromodomain Y-like protein
Peptidyl-prolyl cis-trans isomerase NIMA-interacting 4
RAC-gamma serine/threonine-protein kinase
CAAX prenyl protease 2
Choline/ethanolamine kinase
Eukaryotic translation initiation factor 3 subunit L
Phospholipase A-2-activating protein
RuvB-like 1
Nuclear migration protein nudC
Mitochondrial chaperone BCS1
Voltage-dependent anion-selective channel protein 3
Cofilin-2
Endoplasmic reticulum-Golgi intermediate compartment protein 3
Phenylalanine--tRNA ligase alpha subunit
Sodium-dependent multivitamin transporter
Small ribosomal subunit protein mS33
Histone chaperone ASF1A
Developmentally-regulated GTP-binding protein 1
Trafficking protein particle complex subunit 4
Nck-associated protein 1
Protein canopy homolog 2
Nuclease EXOG, mitochondrial
Exocyst complex component 6B
PALM2-AKAP2 fusion protein
Caspase recruitment domain-containing protein 8
Phospholipid-transporting ATPase IF
GDP-fucose protein O-fucosyltransferase 2
Disks large-associated protein 4
Serine/threonine-protein kinase 38-like
Phosphatidylinositide phosphatase SAC2
Fibronectin type-III domain-containing protein 3A
Nischarin
1-phosphatidylinositol 3-phosphate 5-kinase
WD repeat-containing protein 37
Band 4.1-like protein 3
Angiomotin-like protein 2
Serine/threonine-protein kinase SIK3
Lysine-specific demethylase 2A
Exosome complex exonuclease RRP44
Trafficking protein particle complex subunit 8
Leucine-rich repeat and calponin homology domain-containing protein 1
RNA 3'-terminal phosphate cyclase-like protein
Glutathione S-transferase kappa 1
Ragulator complex protein LAMTOR2
Small ribosomal subunit protein bS1m
Cytochrome c oxidase assembly factor 3 homolog, mitochondrial
Probable ATP-dependent RNA helicase DDX52
Small ribosomal subunit protein uS17m

Small ribosomal subunit protein uS7m
Lambda-crystallin homolog
Translation machinery-associated protein 7
Polymerase delta-interacting protein 2
AP-3 complex subunit mu-1
Inner nuclear membrane protein Man1
Calcium-regulated heat-stable protein 1
Conserved oligomeric Golgi complex subunit 6
Thyroid hormone receptor-associated protein 3
WW domain-binding protein 11
Tudor and KH domain-containing protein
Mediator of RNA polymerase II transcription subunit 16
Nucleolar protein 58
ARF GTPase-activating protein GIT1
Zinc finger protein 281
ADP-ribosylation factor-like protein 2-binding protein
Transmembrane protein 98
Protein SGT1 homolog
Tyrosine--tRNA ligase, mitochondrial
N-acetylglucosamine-6-phosphate deacetylase
Acyl-coenzyme A thioesterase 9, mitochondrial
Protein AAR2 homolog
Nitric oxide synthase-interacting protein
Deoxyribose-phosphate aldolase
Protein MEMO1
Thioredoxin-related transmembrane protein 2
rRNA-processing protein FCF1 homolog
U6 snRNA-associated Sm-like protein LSm2
Endophilin-B1
Complex I intermediate-associated protein 30, mitochondrial
Calcium-binding protein 39
Putative RNA-binding protein Luc7-like 2
Ubiquitin-conjugating enzyme E2 J1
RNA-binding motif protein, X-linked 2
Dehydrogenase/reductase SDR family member 7
Small ribosomal subunit protein uS2m
Probable U3 small nucleolar RNA-associated protein 11
MOB-like protein phocein
Ribosomal RNA-processing protein 7 homolog A
Ribosome maturation protein SBDS
Transmembrane emp24 domain-containing protein 5
Exosome complex component CSL4
Transmembrane emp24 domain-containing protein 7
Splicing factor 3B subunit 6
Large ribosomal subunit protein uL11m
Oligoribonuclease, mitochondrial
RRP15-like protein
WASH complex subunit 3
Nucleolar protein 16
EKC/KEOPS complex subunit TPRKB

Peptidyl-prolyl cis-trans isomerase-like 1
Ubiquitin-fold modifier-conjugating enzyme 1
Cytosolic iron-sulfur assembly component 2B
Small ribosomal subunit protein bS16m
Small ribosomal subunit protein bS18m
Mitochondrial fission 1 protein
Mitochondrial import inner membrane translocase subunit TIM16
Adenylate kinase isoenzyme 6
Small ribosomal subunit protein mS23
Vesicle transport protein GOT1B
Hepatoma-derived growth factor-related protein 3
Peptidyl-tRNA hydrolase 2, mitochondrial
Charged multivesicular body protein 3
Serine-threonine kinase receptor-associated protein
RNA-splicing ligase RtcB homolog
F-box only protein 7
Ras-related protein Rap-2c
StAR-related lipid transfer protein 13
Rab GTPase-activating protein 1
Transmembrane emp24 domain-containing protein 3
TSC22 domain family protein 4
Protein dopey-2
Zinc finger protein 330
R3H and coiled-coil domain-containing protein 1
Nucleolar complex protein 2 homolog
Large ribosomal subunit protein eL36
Coiled-coil domain-containing protein 9
Chromatin target of PRMT1 protein
Deoxynucleoside triphosphate triphosphohydrolase SAMHD1
Small kinetochore-associated protein
HBS1-like protein
5'-AMP-activated protein kinase subunit beta-1
WD repeat domain phosphoinositide-interacting protein 4
DmX-like protein 1
V-type proton ATPase 116 kDa subunit a 2
Talin-1
Kinesin-like protein KIF3A
Transformation/transcription domain-associated protein
Microtubule cross-linking factor 1
DDB1- and CUL4-associated factor 1
TRPM8 channel-associated factor 1
Probable RNA-binding protein 19
Disheveled-associated activator of morphogenesis 1
Plexin-D1
Probable E3 ubiquitin-protein ligase HECTD4
WASH complex subunit 2C
Ubiquitin carboxyl-terminal hydrolase 15
FERM, ARHGEF and pleckstrin domain-containing protein 1
Meiosis regulator and mRNA stability factor 1
Centrosomal protein of 170 kDa protein B

Rap guanine nucleotide exchange factor 2
Unconventional myosin-Va
Dystrobrevin alpha
Lysyl oxidase homolog 2
Beta/gamma crystallin domain-containing protein 1
TNF receptor-associated factor 6
Mitogen-activated protein kinase kinase kinase kinase 5
Hypoxia up-regulated protein 1
Cysteine protease ATG4B
Transducin beta-like protein 2
WD repeat domain phosphoinositide-interacting protein 2
Telomere length regulation protein TEL2 homolog
Ribosomal biogenesis protein LAS1L
AFG3-like protein 2
Krueppel-like factor 12
E3 ubiquitin-protein ligase ARIH1
U6 snRNA-associated Sm-like protein LSm4
E3 ubiquitin-protein ligase RNF114
Sorting and assembly machinery component 50 homolog
Protein PRRC2C
ADP-ribose glycohydrolase OARD1
Intraflagellar transport protein 25 homolog
Protein phosphatase methylesterase 1
RNA-binding protein 7
Mitochondrial import inner membrane translocase subunit Tim22
NEDD8 ultimate buster 1
YTH domain-containing family protein 2
RNA polymerase II subunit A C-terminal domain phosphatase
PAX3- and PAX7-binding protein 1
Nucleoside diphosphate kinase 7
FACT complex subunit SPT16
U3 small nucleolar RNA-associated protein 18 homolog
Ubiquitin carboxyl-terminal hydrolase isozyme L5
CD2-associated protein
V-type proton ATPase subunit D
Transportin-3
Signal recognition particle receptor subunit beta
Ceramide transfer protein
Mannose-1-phosphate guanyltransferase beta
Fatty acid desaturase 3
General transcription factor 3C polypeptide 5
General transcription factor 3C polypeptide 3
Trafficking protein particle complex subunit 1
Transient receptor potential cation channel subfamily V member 2
Serine/threonine-protein kinase MRCK beta
RNA-binding protein 8A
DnaJ homolog subfamily C member 15
Ubiquitin carboxyl-terminal hydrolase 16
U5 small nuclear ribonucleoprotein TSSC4
Zinc finger protein 706

Melanoma-associated antigen D1
Sorting nexin-14
Sorting nexin-11
Sorting nexin-9
Sorting nexin-8
Sorting nexin-5
Endothelial lipase
Cytosolic Fe-S cluster assembly factor NUBP2
Peroxisomal membrane protein PEX16
Lymphatic vessel endothelial hyaluronic acid receptor 1
Beta-secretase 2
Heme-binding protein 2
Host cell factor 2
UbiA prenyltransferase domain-containing protein 1
Pseudouridylate synthase 1 homolog
Leucine-rich repeat flightless-interacting protein 2
FH1/FH2 domain-containing protein 1
Phosphoserine aminotransferase
Nuclear receptor corepressor 2
Mitochondrial ornithine transporter 1
Junctional adhesion molecule A
Neuroplastin
Adhesion G-protein coupled receptor G1
Spindlin-1
Solute carrier family 12 member 7
Dolichyl pyrophosphate Man9GlcNAc2 alpha-1,3-glucosyltransferase
Dolichyl-phosphate beta-glucosyltransferase
Small ribosomal subunit protein mS40
Coatomer subunit gamma-1
Lipid droplet-regulating VLDL assembly factor AUP1
Peptidyl-prolyl cis-trans isomerase FKBP7
Chloride intracellular channel protein 4
Cysteine desulfurase
Cilia- and flagella-associated protein 20
Transforming acidic coiled-coil-containing protein 3
Signal peptidase complex subunit 1
GTP-binding protein SAR1b
AP-4 complex subunit beta-1
Mitochondrial carrier homolog 2
Selenoprotein K
Brefeldin A-inhibited guanine nucleotide-exchange protein 2
Brefeldin A-inhibited guanine nucleotide-exchange protein 1
Mitotic spindle assembly checkpoint protein MAD1
Serine/threonine-protein kinase 24
eIF5-mimic protein 1
COMM domain-containing protein 10
Cytoplasmic dynein 1 light intermediate chain 1
Epsin-1
Ubiquitin carboxyl-terminal hydrolase 3
Testis-expressed protein 264

Calcineurin-binding protein cabin-1
Choline/ethanolaminephosphotransferase 1
DNA (cytosine-5)-methyltransferase 3A
2'-5'-oligoadenylate synthase 3
Adenylate kinase isoenzyme 5
NF-kappa-B essential modulator
Insulin-like growth factor 2 mRNA-binding protein 2
Proton-coupled zinc antiporter SLC30A1
Sodium bicarbonate cotransporter 3
NADH dehydrogenase [ubiquinone] 1 beta subcomplex subunit 9
Sulfide:quinone oxidoreductase, mitochondrial
Roundabout homolog 1
Stonin-1
Nuclear receptor coactivator 3
Numb-like protein
Mitogen-activated protein kinase kinase kinase 4
Probable ATP-dependent RNA helicase DDX49
Calpain-7
Actin-binding protein WASF2
MAU2 chromatid cohesion factor homolog
ATPase MORC2
Influenza virus NS1A-binding protein
SEC23-interacting protein
Putative glutamine amidotransferase-like class 1 domain-containing protein 3B, mitochondrial
Intraflagellar transport protein 56
Ubiquitin-like modifier-activating enzyme 6
Extended synaptotagmin-2
Shootin-1
Transmembrane protein 223
Rho GTPase-activating protein 10
2-hydroxyacyl-CoA lyase 2
SH3 and PX domain-containing protein 2B
NBAS subunit of NRZ tethering complex
tRNA wybutosine-synthesizing protein 5
DENN domain-containing protein 3
von Willebrand factor A domain-containing protein 8
Protein strawberry notch homolog 1
GTP-binding protein 10
WD repeat-containing protein 91
DENN domain-containing protein 11
Plasmanylethanolamine desaturase 1
Trafficking protein particle complex subunit 13
CCR4-NOT transcription complex subunit 1
Microtubule-associated proteins 1A/1B light chain 3 beta 2
Glycerol-3-phosphate phosphatase
RAB7A-interacting MON1-CCZ1 complex subunit 1
TRPM8 channel-associated factor 2
Structural maintenance of chromosomes flexible hinge domain-containing protein 1
Cytosolic arginine sensor for mTORC1 subunit 2
Protein unc-119 homolog B

12S rRNA N4-methylcytidine (m4C) methyltransferase
Coiled-coil domain-containing protein 85C
Retrotransposon Gag-like protein 8C
Zinc finger SWIM domain-containing protein 8
Nuclear envelope pore membrane protein POM 121C
PHD finger protein 20-like protein 1
Putative small nuclear ribonucleoprotein G-like protein 15
Endosome/lysosome-associated apoptosis and autophagy regulator family member 2
Acyl-coenzyme A diphosphatase NUDT19
MIF4G domain-containing protein
Calmodulin-1
Unconventional myosin-IXa
Golgi pH regulator A
Putative WAS protein family homolog 3
Mapk-regulated corepressor-interacting protein 1
Nascent polypeptide-associated complex subunit alpha, muscle-specific form
Uncharacterized protein C11orf98
ASNSD1 upstream open reading frame protein
Mitochondrial ribosome and complex I assembly factor AltMIEF1
Deoxyribonuclease-2-alpha
Alkyldihydroxyacetonephosphate synthase, peroxisomal
Kinesin-like protein KIF2A
ATP-dependent RNA helicase DDX39A
PDZ and LIM domain protein 1
Cytosolic acyl coenzyme A thioester hydrolase
Unconventional myosin-Ic
Synaptosomal-associated protein 23
HCLS1-associated protein X-1
AH receptor-interacting protein
GTP-binding protein 1
Galectin-9
Syntaxin-binding protein 3
AP-4 complex subunit mu-1
Splicing regulator ARVCF
Small acidic protein
AP-3 complex subunit beta-1
Amyloid beta precursor protein binding family B member 1
Galectin-8
NADH dehydrogenase [ubiquinone] iron-sulfur protein 8, mitochondrial
NF-kappa-B inhibitor epsilon
26S proteasome non-ATPase regulatory subunit 11
26S proteasome non-ATPase regulatory subunit 12
26S proteasome non-ATPase regulatory subunit 9
Menin
Guided entry of tail-anchored proteins factor 1
Membrane-associated progesterone receptor component 1
Transcription elongation factor SPT5
Transcription initiation factor TFIID subunit 4
DNA fragmentation factor subunit alpha
Huntingtin-interacting protein 1

Chloride intracellular channel protein 1
Eukaryotic translation initiation factor 3 subunit F
NEDD4-like E3 ubiquitin-protein ligase WWP2
Pyruvate dehydrogenase protein X component, mitochondrial
Sulfhydryl oxidase 1
Dynactin subunit 6
Acetyl-coenzyme A transporter 1
Actin nucleation-promoting factor WASL
Importin-5
DNA-directed RNA polymerase, mitochondrial
Eukaryotic elongation factor 2 kinase
Histone deacetylase complex subunit SAP18
Echinoderm microtubule-associated protein-like 1
Insulin-like growth factor 2 mRNA-binding protein 3
Dynamin-1-like protein
RNA 3'-terminal phosphate cyclase
Phosphatidylinositol 4-phosphate 3-kinase C2 domain-containing subunit alpha
Golgi integral membrane protein 4
Agrin
Procollagen-lysine,2-oxoglutarate 5-dioxygenase 2
Exocyst complex component 5
Butyrophilin subfamily 3 member A1
Cytochrome c oxidase subunit NDUFA4
26S proteasome non-ATPase regulatory subunit 14
Myc box-dependent-interacting protein 1
Claudin-5
Importin subunit alpha-4
Serine/threonine-protein kinase 25
Krev interaction trapped protein 1
von Willebrand factor A domain-containing protein 5A
Pescadillo homolog
Syntenin-1
Membrane-associated phosphatidylinositol transfer protein 1
U3 small nucleolar ribonucleoprotein protein MPP10
Nucleolar protein 56
ATP-dependent RNA helicase DDX3X
Beta-1,3-N-acetylglucosaminyltransferase manic fringe
Podocalyxin
CCN family member 1
Pirin
Importin subunit alpha-3
E3 ubiquitin-protein ligase TRIM38
Nuclear factor 1 B-type
Serine/threonine-protein phosphatase 6 catalytic subunit
Nucleoside diphosphate kinase, mitochondrial
Phosphatidylinositol 4-phosphate 3-kinase C2 domain-containing subunit beta
Lysosomal alpha-mannosidase
Pyridoxal kinase
Stearoyl-CoA desaturase
AT-rich interactive domain-containing protein 1A

Nuclear envelope integral membrane protein 1
Thioredoxin domain-containing protein 9
Dihydropyrimidinase-related protein 4
TRAF-type zinc finger domain-containing protein 1
Cytochrome c oxidase subunit 7A-related protein, mitochondrial
Ubiquitin domain-containing protein UBFD1
Citron Rho-interacting kinase
Coatomer subunit epsilon
Cdc42 effector protein 2
AP-3 complex subunit delta-1
Copper chaperone for superoxide dismutase
Actin-binding LIM protein 1
Segment polarity protein dishevelled homolog DVL-2
Chromodomain-helicase-DNA-binding protein 1
Chromodomain-helicase-DNA-binding protein 2
Golgi SNAP receptor complex member 2
Torsin-1A
Torsin-1B
Syntaxin-16
Disintegrin and metalloproteinase domain-containing protein 10
Lysosomal cobalamin transporter ABCD4
Etoposide-induced protein 2.4 homolog
Tumor protein p53-inducible protein 11
Histone-lysine N-methyltransferase 2D
Apoptotic protease-activating factor 1
Serine/threonine-protein kinase RIO3
Dual specificity mitogen-activated protein kinase kinase 7
Acyl-coenzyme A thioesterase 8
CDP-diacylglycerol--inositol 3-phosphatidyltransferase
Programmed cell death protein 5
Protein arginine N-methyltransferase 5
Na(+)/H(+) exchange regulatory cofactor NHE-RF1
Serine/threonine-protein kinase Chk1
Tumor necrosis factor receptor superfamily member 10B
Tripeptidyl-peptidase 1
Transcription elongation regulator 1
Kinetochore protein NDC80 homolog
Kinesin-like protein KIF3C
Neuropilin-1
Transportin-2
Protein unc-13 homolog B
Tumor necrosis factor receptor superfamily member 10C
DNA-directed RNA polymerase III subunit RPC1
Tetraspanin-4
Proteasome subunit alpha type-7
Secretory carrier-associated membrane protein 3
Branched-chain alpha-ketoacid dehydrogenase kinase
Glutathione S-transferase 3, mitochondrial
Tax1-binding protein 3
PDZ domain-containing protein GIPC1

Inhibitor of nuclear factor kappa-B kinase subunit beta
Regulator of G-protein signaling 12
Mitochondrial import inner membrane translocase subunit Tim23
Histone acetyltransferase type B catalytic subunit
Peripheral plasma membrane protein CASK
Phospholipase D2
Cytochrome b-c1 complex subunit 8
Myosin regulatory light chain 12B
Hepatocyte growth factor-regulated tyrosine kinase substrate
Ras-related protein Rab-7L1
Vacuolar protein sorting-associated protein 26C
Protein phosphatase 1 regulatory subunit 12A
Cyclin-G-associated kinase
Heterogeneous nuclear ribonucleoprotein D-like
Exportin-1
TATA-binding protein-associated factor 172
Rho guanine nucleotide exchange factor 10
Zinc finger protein 609
Spectrin beta chain, non-erythrocytic 2
Microtubule-associated serine/threonine-protein kinase 4
Protein transport protein Sec16A
Plexin-B2
Protein KHNYN
Tectonin beta-propeller repeat-containing protein 2
U2 snRNP-associated SURP motif-containing protein
Histone-lysine N-methyltransferase SETD1A
NEDD4-binding protein 3
Synaptojanin-2
Synemin
Granule associated Rac and RHOG effector protein 1
Kinesin-like protein KIF3B
Phosphoribosylformylglycinamidine synthase
Serine/threonine-protein phosphatase 6 regulatory ankyrin repeat subunit A
Rho guanine nucleotide exchange factor 11
Mitochondrial ribonuclease P catalytic subunit
Inhibitor of nuclear factor kappa-B kinase subunit alpha
NPC intracellular cholesterol transporter 1
Sphingolipid delta(4)-desaturase DES1
Angiopoietin-2
Secretory carrier-associated membrane protein 1
Secretory carrier-associated membrane protein 2
Actin-related protein 2/3 complex subunit 1B
Actin-related protein 2/3 complex subunit 2
Actin-related protein 2/3 complex subunit 3
DNA-directed RNA polymerases I and III subunit RPAC1
Phospholipid scramblase 1
Transcription intermediary factor 1-alpha
Membrane-associated progesterone receptor component 2
Ral guanine nucleotide dissociation stimulator-like 2
WD repeat-containing protein 46

NF-kappa-B-repressing factor
Dihydroxyacetone phosphate acyltransferase
Laminin subunit alpha-5
Zinc finger protein 185
Protein CASC3
Small ribosomal subunit protein uS12m
Peroxisomal acyl-coenzyme A oxidase 3
Protein RER1
Surfeit locus protein 4
Mitogen-activated protein kinase 13
Serine palmitoyltransferase 1
Serine palmitoyltransferase 2
UDP-N-acetylglucosamine--peptide N-acetylglucosaminyltransferase 110 kDa subunit
Phosphomannomutase 2
Transmembrane 9 superfamily member 1
E3 ubiquitin-protein ligase Midline-1
High mobility group protein B3
Protein phosphatase 1G
Phosphatidylinositol 3,4,5-trisphosphate 5-phosphatase 2
Eukaryotic translation initiation factor 3 subunit D
Eukaryotic translation initiation factor 3 subunit H
Histone deacetylase 3
Nuclear valosin-containing protein-like
Branched-chain-amino-acid aminotransferase, mitochondrial
Importin-8
Syntaxin-7
Monocarboxylate transporter 4
ATP-binding cassette sub-family C member 4
DNA-directed RNA polymerase I subunit RPA34
Prolyl 4-hydroxylase subunit alpha-2
Calpain-5
Synaptobrevin homolog YKT6
Nucleoporin NUP42
Actin-related protein 2/3 complex subunit 5
Transcription factor MafG
3-phosphoinositide-dependent protein kinase 1
Tapasin
E3 ubiquitin-protein ligase RNF113A
Lysine-specific demethylase 6A
ATP-dependent RNA helicase DHX15
mRNA cap guanine-N7 methyltransferase
Zinc finger ZZ-type and EF-hand domain-containing protein 1
Arf-GAP with SH3 domain, ANK repeat and PH domain-containing protein 2
Leucine-rich repeat transmembrane protein FLRT2
TELO2-interacting protein 1 homolog
Ribosomal RNA-processing protein 8
E3 ubiquitin-protein ligase Praja-2
Cytochrome b5 type B
U4/U6 small nuclear ribonucleoprotein Prp4
D-3-phosphoglycerate dehydrogenase

NADH dehydrogenase [ubiquinone] iron-sulfur protein 4, mitochondrial
Cytoplasmic dynein 1 light intermediate chain 2
26S proteasome non-ATPase regulatory subunit 3
RNA binding protein fox-1 homolog 2
Bifunctional 3'-phosphoadenosine 5'-phosphosulfate synthase 1
Centromere/kinetochore protein zw10 homolog
Beta-1,4-galactosyltransferase 5
U4/U6.U5 tri-snRNP-associated protein 1
Glycosylphosphatidylinositol anchor attachment 1 protein
Death-associated protein kinase 3
Transforming growth factor beta-1-induced transcript 1 protein
AP-5 complex subunit zeta-1
CBP80/20-dependent translation initiation factor
Protein MTSS 1
Inositol hexakisphosphate and diphosphoinositol-pentakisphosphate kinase 2
Mitogen-activated protein kinase kinase kinase 7
Eukaryotic translation elongation factor 1 epsilon-1
Receptor-interacting serine/threonine-protein kinase 2
Heterogeneous nuclear ribonucleoprotein R
U4/U6 small nuclear ribonucleoprotein Prp3
Thioredoxin-like protein 1
Tumor protein D54
ER membrane protein complex subunit 8
ERI1 exoribonuclease 3
Synaptojanin-1
Acidic fibroblast growth factor intracellular-binding protein
Eukaryotic translation initiation factor 4 gamma 3
Peptidyl-prolyl cis-trans isomerase H
Serine protease HTRA2, mitochondrial
Protein Mis18-beta
Aflatoxin B1 aldehyde reductase member 2
Band 4.1-like protein 2
Trans-Golgi network integral membrane protein 2
Beta-1,4-glucuronyltransferase 1
WAS/WASL-interacting protein family member 1
Phospholipid-transporting ATPase IC
A-kinase anchor protein 10, mitochondrial
Density-regulated protein
Exportin-T
5-hydroxymethyl-dUMP N-hydrolase
Mitochondrial import inner membrane translocase subunit TIM44
Trafficking protein particle complex subunit 3
Charged multivesicular body protein 2a
Cytoplasmic protein NCK2
Tetraspanin-6
Pleiotropic regulator 1
Protein regulator of cytokinesis 1
Regulator of G-protein signaling 10
BUB3-interacting and GLEBS motif-containing protein ZNF207
NADH dehydrogenase [ubiquinone] 1 beta subcomplex subunit 5, mitochondrial

NADH dehydrogenase [ubiquinone] 1 beta subcomplex subunit 3
NADH dehydrogenase [ubiquinone] 1 alpha subcomplex subunit 2
LIM domain-binding protein 2
ATPase GET3
Mitotic checkpoint serine/threonine-protein kinase BUB1
Mitotic checkpoint protein BUB3
Sulfotransferase 1B1
Alpha-actinin-4
Probable 18S rRNA (guanine-N(7))-methyltransferase
17S U2 SnRNP complex component HTATSF1
ER lumen protein-retaining receptor 3
AP-1 complex subunit gamma-1
Syntaxin-6
Synaptogyrin-2
Small glutamine-rich tetratricopeptide repeat-containing protein alpha
Alpha-endosulfine
Mitochondrial carnitine/acylcarnitine carrier protein
Asparagine--tRNA ligase, cytoplasmic
Unconventional myosin-Ib
Peroxisomal membrane protein PMP34
Cleavage and polyadenylation specificity factor subunit 5
Glutathione S-transferase LANCL1
Striatin
U3 small nucleolar RNA-interacting protein 2
Protein SCO2 homolog, mitochondrial
Cilia- and flagella-associated protein 410
A-kinase anchor protein 8
Putative GTP-binding protein 6
Glucose-6-phosphate exchanger SLC37A4
Isocitrate dehydrogenase [NAD] subunit beta, mitochondrial
Nardilysin
Calumenin
EGF-like repeat and discoidin I-like domain-containing protein 3
Probable phospholipid-transporting ATPase IIB
S-adenosylhomocysteine hydrolase-like protein 1
Kinesin-like protein KIF1C
Exostosin-like 3
Origin recognition complex subunit 5
NADH dehydrogenase [ubiquinone] iron-sulfur protein 5
Retinal rod rhodopsin-sensitive cGMP 3',5'-cyclic phosphodiesterase subunit delta
Origin recognition complex subunit 4
Peroxisomal ATPase PEX1
Double-strand-break repair protein rad21 homolog
Pre-mRNA-splicing factor ATP-dependent RNA helicase DHX16
Glia maturation factor gamma
SH3 domain-binding protein 5
Adhesion G protein-coupled receptor B3
Mediator of RNA polymerase II transcription subunit 14
Phosphoribosyl pyrophosphate synthase-associated protein 2
SWI/SNF-related matrix-associated actin-dependent regulator of chromatin subfamily A membe

C-Jun-amino-terminal kinase-interacting protein 4
NUAK family SNF1-like kinase 1
Nucleolar pre-ribosomal-associated protein 1
Signal-induced proliferation-associated 1-like protein 3
Zinc finger C3H1 domain-containing protein
RNA helicase aquarius
Dynamin-like 120 kDa protein, mitochondrial
Zinc finger E-box-binding homeobox 2
Germinal-center associated nuclear protein
Protocadherin gamma-A12
Phosphatidylinositol 4-phosphate 5-kinase type-1 gamma
Kinesin-like protein KIF1B
Mitogen-activated protein kinase-binding protein 1
Lysine-specific histone demethylase 1A
TBC1 domain family member 4
Acyl-CoA (8-3)-desaturase
Gasdermin-E
Ecotropic viral integration site 5 protein homolog
Neuropilin-2
Mannosyl-oligosaccharide 1,2-alpha-mannosidase IB
Myelin protein zero-like protein 2
Long-chain-fatty-acid--CoA ligase 4
Sorting nexin-3
Protein O-GlcNAcase
Vinexin
Heterogeneous nuclear ribonucleoprotein Q
Pre-mRNA-processing factor 17
Ran-binding protein 6
Ribosome quality control complex subunit NEMF
GDP-mannose 4,6 dehydratase
Glycylpeptide N-tetradecanoyltransferase 2
Cyclin-T1
Mitotic checkpoint serine/threonine-protein kinase BUB1 beta
Multifunctional procollagen lysine hydroxylase and glycosyltransferase LH3
Eukaryotic translation initiation factor 4E type 2
Protein diaphanous homolog 1
Tetraspanin-3
Exocyst complex component 3
Perilipin-3
Protein arginine N-methyltransferase 3
Importin subunit alpha-7
UDP-glucose 6-dehydrogenase
Protein-tyrosine sulfotransferase 2
Leupaxin
Catenin delta-1
Protein-S-isoprenylcysteine O-methyltransferase
Eukaryotic translation initiation factor 1b
Sorting nexin-2
Dolichol-phosphate mannosyltransferase subunit 1
General vesicular transport factor p115

Small ribosomal subunit protein uS14m
Target of Myb1 membrane trafficking protein
Histone H2B type 1-K
Coiled-coil domain-containing protein 22
Polyglutamine-binding protein 1
Mitochondrial import inner membrane translocase subunit Tim17-B
PRA1 family protein 2
H/ACA ribonucleoprotein complex subunit DKC1
Eukaryotic translation initiation factor 5B
Endothelial differentiation-related factor 1
DNA/RNA-binding protein KIN17
Protein diaphanous homolog 2
DnaJ homolog subfamily A member 2
Bromodomain-containing protein 4
Checkpoint protein HUS1
Prefoldin subunit 1
E3 ubiquitin-protein ligase PPP1R11
Nibrin
mRNA-capping enzyme
Iron-sulfur clusters transporter ABCB7, mitochondrial
SLIT-ROBO Rho GTPase-activating protein 2
Plexin-A2
Glycosaminoglycan xylosylkinase
WD repeat-containing protein 1
Probable phospholipid-transporting ATPase IIA
NEDD4-binding protein 1
Rho-associated protein kinase 2
CLIP-associating protein 2
Pentatricopeptide repeat-containing protein 1, mitochondrial
Copine-3
Autophagy-related protein 13
Huntingtin-interacting protein 1-related protein
E3 ubiquitin-protein ligase BRE1B
Zinc finger CCCH domain-containing protein 11A
Clustered mitochondria protein homolog
Cullin-associated NEDD8-dissociated protein 2
TSC22 domain family protein 2
DnaJ homolog subfamily C member 13
Phosphatase and actin regulator 2
Serine/threonine-protein phosphatase 6 regulatory subunit 2
A disintegrin and metalloproteinase with thrombospondin motifs 4
CCR4-NOT transcription complex subunit 3
Ankyrin repeat domain-containing protein 17
Paired amphipathic helix protein Sin3b
DnaJ homolog subfamily B member 6
Ubiquinone biosynthesis protein COQ9, mitochondrial
Gamma-glutamylcyclotransferase
NADH dehydrogenase [ubiquinone] iron-sulfur protein 7, mitochondrial
NADH dehydrogenase [ubiquinone] iron-sulfur protein 2, mitochondrial
Zinc finger protein ZPR1

RNA/RNP complex-1-interacting phosphatase
Protein NipSnap homolog 2
Hyaluronan mediated motility receptor
Programmed cell death protein 6
Tubulin-specific chaperone A
V-type proton ATPase subunit G 1
Vacuolar protein sorting-associated protein 4B
Mannose-P-dolichol utilization defect 1 protein
Core histone macro-H2A.1
Adapter SH3BGRL
Filamin-B
Nuclear receptor corepressor 1
Peroxisomal membrane protein PEX14
Tripartite motif-containing protein 3
Citrate synthase, mitochondrial
Sperm-associated antigen 7
Vesicle-trafficking protein SEC22b
Pre-mRNA-processing factor 40 homolog A
Transforming acidic coiled-coil-containing protein 1
Nucleoside diphosphate kinase 6
Cell division control protein 45 homolog
Leucine-rich repeat and calponin homology domain-containing protein 4
Metaxin-2
Vacuolar protein sorting-associated protein 26A
Mitochondrial-processing peptidase subunit beta
Mediator of RNA polymerase II transcription subunit 24
Katanin p60 ATPase-containing subunit A1
PC4 and SFRS1-interacting protein
Erlin-1
NADH dehydrogenase [ubiquinone] iron-sulfur protein 3, mitochondrial
Serine/arginine-rich splicing factor 10
Ceroid-lipofuscinosis neuronal protein 5
Claudin-11
Enoyl-CoA delta isomerase 2
KH domain-containing, RNA-binding, signal transduction-associated protein 3
Polycomb protein EED
Barrier-to-autointegration factor
Splicing factor 3B subunit 1
Cold shock domain-containing protein E1
WW domain-binding protein 4
Interferon-inducible double-stranded RNA-dependent protein kinase activator A
Integrin alpha-10
Ribosomal protein S6 kinase alpha-5
Mediator of RNA polymerase II transcription subunit 6
E3 ubiquitin-protein ligase MYCBP2
2-amino-3-ketobutyrate coenzyme A ligase, mitochondrial
Nucleoplasmin-3
Acyl-protein thioesterase 1
GTPase Era, mitochondrial
U5 small nuclear ribonucleoprotein 200 kDa helicase

Mitochondrial tRNA-specific 2-thiouridylase 1
TIP41-like protein
Centriole and centriolar satellite protein OFD1
Ribosomal protein S6 kinase alpha-4
Surfeit locus protein 6
Protein phosphatase 1B
Small subunit processome component 20 homolog
Nuclear pore complex protein Nup155
Protein XRP2
WD repeat and HMG-box DNA-binding protein 1
Cartilage-associated protein
Electrogenic aspartate/glutamate antiporter SLC25A12, mitochondrial
Renin receptor
Ribonuclease H2 subunit A
Translation initiation factor eIF2 assembly protein
Breast cancer anti-estrogen resistance protein 3
Ribonuclease P protein subunit p20
Ribonuclease P protein subunit p40
Eukaryotic translation initiation factor 3 subunit G
Eukaryotic translation initiation factor 3 subunit J
Carbonyl reductase [NADPH] 3
26S proteasome non-ATPase regulatory subunit 10
AP-1 complex subunit gamma-like 2
CAAX prenyl protease 1 homolog
Isocitrate dehydrogenase [NADP] cytoplasmic
Glutamyl-tRNA(Gln) amidotransferase subunit B, mitochondrial
Protein SCO1 homolog, mitochondrial
Attractin
Serine hydrolase RBBP9
Signal transducing adapter molecule 2
Diacylglycerol O-acyltransferase 1
Cyclin-K
PRA1 family protein 3
Dysferlin
E3 SUMO-protein ligase PIAS1
Pre-mRNA-splicing factor SPF27
Dynactin subunit 3
DnaJ homolog subfamily C member 8
Survival of motor neuron-related-splicing factor 30
Cell cycle checkpoint protein RAD17
ATP synthase subunit d, mitochondrial
Tetraspanin-9
Flotillin-1
Triple functional domain protein
ATP synthase subunit g, mitochondrial
Multiple PDZ domain protein
Carboxypeptidase D
Glutaredoxin-3
Ribosomal L1 domain-containing protein 1
Wolframin

ATP-dependent Clp protease ATP-binding subunit clpX-like, mitochondrial
SEC14-like protein 2
Gamma-synuclein
Probable cytosolic iron-sulfur protein assembly protein CIAO1
Signal recognition particle subunit SRP72
N(G),N(G)-dimethylarginine dimethylaminohydrolase 1
ATP-dependent DNA helicase Q5
Unconventional prefoldin RPB5 interactor 1
Galactosylgalactosylxylosylprotein 3-beta-glucuronosyltransferase 3
Metastasis-associated protein MTA2
Retinal dehydrogenase 2
Serine/threonine-protein kinase 10
Serine/threonine-protein kinase D3
Glutamine--fructose-6-phosphate aminotransferase [isomerizing] 2
Slit homolog 2 protein
E3 ubiquitin-protein ligase listerin
Mitochondrial import receptor subunit TOM70
Pleckstrin homology domain-containing family G member 5
Importin-13
Phospholipase DDHD2
Unconventional myosin-Id
TOX high mobility group box family member 4
[F-actin]-monooxygenase MICAL2
Protein transport protein Sec24D
F-BAR and double SH3 domains protein 2
E3 UFM1-protein ligase 1
Sorbin and SH3 domain-containing protein 2
SAM and SH3 domain-containing protein 1
UBX domain-containing protein 7
Kelch-like protein 18
SUN domain-containing protein 1
Pyridoxal phosphate homeostasis protein
Erlin-2
Pre-mRNA-processing factor 6
Pre-mRNA cleavage complex 2 protein Pcf11
Protein furry homolog-like
Nuclear factor of activated T-cells 5
Endonuclease domain-containing 1 protein
D-glucuronyl C5-epimerase
Glutaminase kidney isoform, mitochondrial
HAUS augmin-like complex subunit 5
Actin-binding LIM protein 3
F-box only protein 21
Microtubule cross-linking factor 2
Ubiquitin carboxyl-terminal hydrolase 19
WD repeat-containing protein 47
AP-2 complex subunit alpha-2
Protein transport protein Sec31A
Rho guanine nucleotide exchange factor 15
Protein HEXIM1

Ribonuclease P protein subunit p14
G2/mitotic-specific cyclin-B2
Protein YIF1A
E3 ubiquitin-protein ligase UBR5
Arf-GAP domain and FG repeat-containing protein 2
Serine protease 23
SR-related and CTD-associated factor 4
NADH dehydrogenase [ubiquinone] 1 beta subcomplex subunit 6
Mitofusin-2
Snurportin-1
Ubiquitin conjugation factor E4 B
Zinc finger protein-like 1
Elongator complex protein 1
NADH dehydrogenase [ubiquinone] 1 beta subcomplex subunit 4
NADH dehydrogenase [ubiquinone] 1 beta subcomplex subunit 8, mitochondrial
NADH dehydrogenase [ubiquinone] 1 alpha subcomplex subunit 7
Vesicle-associated membrane protein 5
Reticulon-3
Mitochondrial proton/calcium exchanger protein
Epsin-2
Zinc finger Ran-binding domain-containing protein 2
Sorting nexin-4
Luc7-like protein 3
Kinesin-like protein KIF20A
Apolipoprotein L3
Chromosome-associated kinesin KIF4A
Myotubularin-related protein 5
Golgi SNAP receptor complex member 1
Histone acetyltransferase KAT7
Arginyl-tRNA--protein transferase 1
Vesicle-associated membrane protein-associated protein B/C
SNARE-associated protein Snapin
Myelin protein zero-like protein 1
NADH dehydrogenase [ubiquinone] 1 subunit C2
NADH dehydrogenase [ubiquinone] 1 alpha subcomplex subunit 10, mitochondrial
Peptidyl-prolyl cis-trans isomerase FKBP9
CUGBP Elav-like family member 2
6-phosphogluconolactonase
Bifunctional 3'-phosphoadenosine 5'-phosphosulfate synthase 2
Structural maintenance of chromosomes protein 2
Ubiquitin-like modifier-activating enzyme ATG7
Transforming acidic coiled-coil-containing protein 2
Tripartite motif-containing protein 16
Phenylalanine--tRNA ligase, mitochondrial
Zinc finger and BTB domain-containing protein 7A
Acyl-protein thioesterase 2
Importin-7
E3 ubiquitin-protein ligase ARIH2
Pre-mRNA-splicing factor SLU7
Phosphoacetylglucosamine mutase

Adenylyltransferase and sulfurtransferase MOCS3
CD2 antigen cytoplasmic tail-binding protein 2
Supervillin
Activator of 90 kDa heat shock protein ATPase homolog 1
Poly(A)-specific ribonuclease PARN
Proteasome assembly chaperone 1
Sphingosine-1-phosphate lyase 1
CTD nuclear envelope phosphatase 1
Ribosome biogenesis protein NSA2 homolog
GDH/6PGL endoplasmic bifunctional protein
Protein transport protein Sec24A
Protein transport protein Sec24B
Adhesion G protein-coupled receptor L2
NAD kinase
Tyrosyl-DNA phosphodiesterase 2
Mitochondrial pyruvate carrier 2
Persulfide dioxygenase ETHE1, mitochondrial
Fatty acid CoA ligase Acsl3
DNA-directed RNA polymerase I subunit RPA1
YEATS domain-containing protein 4
Zinc finger and BTB domain-containing protein 11
CCR4-NOT transcription complex subunit 4
STAM-binding protein
Cleavage and polyadenylation specificity factor subunit 4
Nuclear factor of activated T-cells, cytoplasmic 1
Probable bifunctional dTTP/UTP pyrophosphatase/methyltransferase protein
Phosphatidate cytidylyltransferase 2
Protein phosphatase 1 regulatory subunit 3D
Bromodomain-containing protein 1
Ribonuclease P protein subunit p29
E3 ubiquitin-protein ligase HERC2
Ras-related protein Rab-3D
Synaptosomal-associated protein 29
Serine/threonine-protein kinase OSR1
Geranylgeranyl pyrophosphate synthase
Heat shock 70 kDa protein 4L
Polypyrimidine tract-binding protein 3
TBC1 domain family member 8
Interleukin-33
STARD3 N-terminal-like protein
U6 snRNA-associated Sm-like protein LSm8
AP-2 complex subunit alpha-1
Protein Wiz
Antiviral innate immune response receptor RIG-I
Double-stranded RNA-binding protein Staufen homolog 1
Tetratricopeptide repeat protein 4
Transmembrane protein 50A
Caveolae-associated protein 2
BAG family molecular chaperone regulator 2
BAG family molecular chaperone regulator 3

Mitogen-activated protein kinase kinase kinase kinase 4
Malonyl-CoA decarboxylase, mitochondrial
Quinone oxidoreductase-like protein 1
Apoptosis-inducing factor 1, mitochondrial
Echinoderm microtubule-associated protein-like 2
Serine/threonine-protein kinase LATS1
Uridine diphosphate glucose pyrophosphatase NUDT14
Tetraspanin-15
3'(2'),5'-bisphosphate nucleotidase 1
Acyl-CoA 6-desaturase
Putative hydrolase DDAH2
Phosphatidylserine lipase ABHD16A
Thioredoxin domain-containing protein 12
Protein ecdysoneless homolog
Reversion-inducing cysteine-rich protein with Kazal motifs
Methyl-CpG-binding domain protein 3
DNA topoisomerase 3-beta-1
Diphosphoinositol polyphosphate phosphohydrolase 1
B-cell lymphoma/leukemia 10
NADH dehydrogenase [ubiquinone] 1 beta subcomplex subunit 10
Putative lipid scramblase CLPTM1
E3 SUMO-protein ligase ZBED1
Molybdopterin synthase catalytic subunit
Mitochondrial import receptor subunit TOM40 homolog
Peroxisomal membrane protein 11B
Serine/threonine-protein kinase PAK 4
Amyloid-beta A4 precursor protein-binding family A member 3
Actin-like protein 6A
Histone-lysine N-methyltransferase NSD2
L-lactate dehydrogenase A chain
Aldehyde dehydrogenase 1A1
Glutamate dehydrogenase 1, mitochondrial
Dihydrofolate reductase
NADH-cytochrome b5 reductase 3
Glutathione reductase, mitochondrial
Cytochrome c oxidase subunit 1
Cytochrome c oxidase subunit 2
Purine nucleoside phosphorylase
Hypoxanthine-guanine phosphoribosyltransferase
Aspartate aminotransferase, mitochondrial
Tyrosine-protein kinase ABL1
Phosphoglycerate kinase 1
Adenylate kinase isoenzyme 1
Urokinase-type plasminogen activator
Tissue-type plasminogen activator
Adenosine deaminase
Alpha-1-antitrypsin
Alpha-2-macroglobulin
Cystatin-C
GTPase NRas

GTPase HRas
GTPase KRas
Platelet-derived growth factor subunit B
Low-density lipoprotein receptor
Transforming growth factor beta-1 proprotein
HLA class I histocompatibility antigen, B alpha chain
Putative HLA class I histocompatibility antigen, alpha chain H
Collagen alpha-1(I) chain
Collagen alpha-1(IV) chain
Prelamin-A/C
Apolipoprotein A-I
Fibronectin
Alpha-2-HS-glycoprotein
Albumin
Transferrin receptor protein 1
Ferritin light chain
Ferritin heavy chain
Metallothionein-2
NADH-ubiquinone oxidoreductase chain 1
NADH-ubiquinone oxidoreductase chain 4
NADH-ubiquinone oxidoreductase chain 5
Interstitial collagenase
3-hydroxy-3-methylglutaryl-coenzyme A reductase
Catalase
RAF proto-oncogene serine/threonine-protein kinase
Lysosomal acid glucosylceramidase
Fructose-bisphosphate aldolase A
Cystatin-B
Annexin A1
Apolipoprotein B-100
Major prion protein
Superoxide dismutase [Mn], mitochondrial
Ornithine aminotransferase, mitochondrial
Thymidine kinase, cytosolic
von Willebrand factor
Tubulin beta-4A chain
Glyceraldehyde-3-phosphate dehydrogenase
Argininosuccinate lyase
HLA class I histocompatibility antigen, A alpha chain
Calpain small subunit 1
Metallothionein-1E
Heat shock protein beta-1
Thymidylate synthase
Dolichyl-diphosphooligosaccharide--protein glycosyltransferase subunit 1
Dolichyl-diphosphooligosaccharide--protein glycosyltransferase subunit 2
Guanine nucleotide-binding protein G(i) subunit alpha-2
Histone H2A type 1-B/E
Anion exchange protein 2
Sodium/potassium-transporting ATPase subunit alpha-1
Sodium/potassium-transporting ATPase subunit beta-1

Amyloid-beta precursor protein
Aldehyde dehydrogenase, mitochondrial
Integrin beta-3
Non-histone chromosomal protein HMG-14
Plasminogen activator inhibitor 2
Plasminogen activator inhibitor 1
ADP/ATP translocase 2
Ubiquitin-like protein ISG15
Propionyl-CoA carboxylase alpha chain, mitochondrial
Propionyl-CoA carboxylase beta chain, mitochondrial
Eukaryotic translation initiation factor 2 subunit 1
Non-histone chromosomal protein HMG-17
Intercellular adhesion molecule 1
Large ribosomal subunit protein P2
Large ribosomal subunit protein uL10
Transcription factor Jun
DNA-directed RNA polymerase III subunit RPC4
Lupus La protein
Integrin beta-1
Keratin, type I cytoskeletal 18
Collagen alpha-2(V) chain
Uroporphyrinogen decarboxylase
Tyrosine-protein kinase Fyn
Alpha-galactosidase A
Gelsolin
Retinoblastoma-associated protein
Prothymosin alpha
Cyclin-dependent kinase 1
ATP synthase subunit beta, mitochondrial
Protein S100-A9
Protein S100-A6
Eukaryotic translation initiation factor 4E
Alpha-enolase
Glycogen phosphorylase, liver form
Glucose-6-phosphate isomerase
DNA polymerase beta
Nucleophosmin
Tropomyosin alpha-3 chain
Integrin alpha-V
Beta-hexosaminidase subunit alpha
Epoxide hydrolase 1
L-lactate dehydrogenase B chain
Major centromere autoantigen B
Glutathione peroxidase 1
Protein disulfide-isomerase
Histone H1.0
Acylphosphatase-1
Tyrosine-protein kinase Fes/Fps
Cathepsin D
Annexin A2

Calpain-1 catalytic subunit
Tubulin beta chain
Prosaposin
Beta-hexosaminidase subunit beta
Procathepsin L
Profilin-1
Bisphosphoglycerate mutase
Adenine phosphoribosyltransferase
Bifunctional glutamate/proline--tRNA ligase
Cathepsin B
Heat shock protein HSP 90-alpha
Galactose-1-phosphate uridylyltransferase
Heterogeneous nuclear ribonucleoproteins C1/C2
Laminin subunit beta-1
Tyrosine-protein kinase Yes
Tyrosine-protein kinase Lyn
Fumarate hydratase, mitochondrial
DNA excision repair protein ERCC-1
Thrombospondin-1
Transcription factor Sp1
Insulin-like growth factor 1 receptor
Collagen alpha-2(I) chain
Annexin A6
Rho-related GTP-binding protein RhoC
Complement decay-accelerating factor
Amino acid transporter heavy chain SLC3A2
Beta-glucuronidase
ATP-dependent 6-phosphofructokinase, muscle type
Heat shock protein HSP 90-beta
Signal recognition particle receptor subunit alpha
Asparagine synthetase [glutamine-hydrolyzing]
72 kDa type IV collagenase
Porphobilinogen deaminase
Neprilysin
Matrix Gla protein
Pyruvate dehydrogenase E1 component subunit alpha, somatic form, mitochondrial
Collagen alpha-2(IV) chain
Cytochrome c1, heme protein, mitochondrial
U2 small nuclear ribonucleoprotein B''
Hepatocyte growth factor receptor
U1 small nuclear ribonucleoprotein 70 kDa
Integrin alpha-5
Nuclear factor 1 C-type
Vimentin
Small ribosomal subunit protein eS17
Guanine nucleotide-binding protein G(i) subunit alpha-3
Annexin A5
Small ribosomal subunit protein uS2
CD63 antigen
Large ribosomal subunit protein uL3m

U1 small nuclear ribonucleoprotein A
Gamma-enolase
3-ketoacyl-CoA thiolase, peroxisomal
Signal recognition particle 19 kDa protein
Glutathione S-transferase P
U1 small nuclear ribonucleoprotein C
Galectin-1
Dihydropteridine reductase
High mobility group protein B1
SPARC
Tropomyosin alpha-1 chain
Clathrin light chain A
Clathrin light chain B
Annexin A4
2',3'-cyclic-nucleotide 3'-phosphodiesterase
Heme oxygenase 1
Dihydrolipoyl dehydrogenase, mitochondrial
Heterogeneous nuclear ribonucleoprotein A1
U2 small nuclear ribonucleoprotein A'
Cytochrome c oxidase subunit 6C
Tumor-associated calcium signal transducer 2
Poly [ADP-ribose] polymerase 1
DNA polymerase alpha catalytic subunit
Ubiquitin carboxyl-terminal hydrolase isozyme L1
Leukotriene A-4 hydrolase
Fructose-bisphosphate aldolase C
Histone H2A.Z
Histone H2A type 1
Myocardial zonula adherens protein
DNA-directed RNA polymerase II subunit GRINL1A
Glutathione S-transferase theta-2
POTE ankyrin domain family member J
LIM and senescent cell antigen-like-containing domain protein 3
Heat shock 70 kDa protein 1A
Chimeric ERCC6-PGBD3 protein
Tubulin alpha-3C chain
Zinc finger TRAF-type-containing protein 1
Ras-related protein Rap-2a
Serglycin
RNA-binding protein RO60
Lysosomal alpha-glucosidase
Ras-related protein R-Ras
HLA class I histocompatibility antigen, C alpha chain
Serine/threonine-protein kinase A-Raf
Histone H1.4
Dihydrolipoyllysine-residue acetyltransferase component of pyruvate dehydrogenase complex, [illegible]
Receptor-type tyrosine-protein phosphatase F
COUP transcription factor 1
Thioredoxin
Cytochrome c oxidase subunit 5B, mitochondrial

Lysosomal protective protein
Microsomal glutathione S-transferase 1
cAMP-dependent protein kinase type I-alpha regulatory subunit
Tissue factor pathway inhibitor
S-formylglutathione hydrolase
60 kDa heat shock protein, mitochondrial
Clusterin
Endoplasmic reticulum chaperone BiP
Laminin subunit gamma-1
Lysosomal acid phosphatase
Microtubule-associated protein 2
Heat shock cognate 71 kDa protein
Solute carrier family 2, facilitated glucose transporter member 1
Solute carrier family 2, facilitated glucose transporter member 3
Protein 4.1
Uridine 5'-monophosphate synthase
Pyruvate dehydrogenase E1 component subunit beta, mitochondrial
Lipoamide acyltransferase component of branched-chain alpha-keto acid dehydrogenase compl
Glycogen phosphorylase, brain form
Glycogen phosphorylase, muscle form
Ras-related protein Ral-A
Ras-related protein Ral-B
Breakpoint cluster region protein
Lysosome-associated membrane glycoprotein 1
Transcriptional regulator ERG
Medium-chain specific acyl-CoA dehydrogenase, mitochondrial
DNA topoisomerase 1
DNA topoisomerase 2-alpha
Glucose-6-phosphate 1-dehydrogenase
Ubiquitin-like protein 4A
Pyruvate carboxylase, mitochondrial
C-1-tetrahydrofolate synthase, cytoplasmic
Cation-independent mannose-6-phosphate receptor
Alcohol dehydrogenase class-3
Cyclin-dependent kinase 4
Ribose-phosphate pyrophosphokinase 2
Polyadenylate-binding protein 1
Proliferating cell nuclear antigen
Histidine--tRNA ligase, cytoplasmic
Collagen alpha-1(VI) chain
ADP/ATP translocase 1
ADP/ATP translocase 3
Inosine-5'-monophosphate dehydrogenase 2
Nucleoprotein TPR
Annexin A3
2-oxoisovalerate dehydrogenase subunit alpha, mitochondrial
Alpha-actinin-1
Angiotensin-converting enzyme
Proto-oncogene tyrosine-protein kinase Src
Xaa-Pro dipeptidase

X-ray repair cross-complementing protein 6
X-ray repair cross-complementing protein 5
Uracil-DNA glycosylase
Cytochrome c oxidase subunit 4 isoform 1, mitochondrial
5-aminolevulinate synthase, non-specific, mitochondrial
Lysosome-associated membrane glycoprotein 2
Ribonuclease inhibitor
Intercellular adhesion molecule 2
Elongation factor 2
Protein disulfide-isomerase A4
Prolyl 4-hydroxylase subunit alpha-1
Translationally-controlled tumor protein
Delta-aminolevulinic acid dehydratase
HLA class I histocompatibility antigen, alpha chain E
Plastin-3
Acylamino-acid-releasing enzyme
Electron transfer flavoprotein subunit alpha, mitochondrial
Glycogen [starch] synthase, muscle
cAMP-dependent protein kinase type II-alpha regulatory subunit
General transcription factor IIF subunit 2
CD59 glycoprotein
Bifunctional methylenetetrahydrofolate dehydrogenase/cyclohydrolase, mitochondrial
Macrophage migration inhibitory factor
CD99 antigen
Glucosidase 2 subunit beta
Hematopoietic lineage cell-specific protein
Farnesyl pyrophosphate synthase
Cytochrome c oxidase subunit 7A2, mitochondrial
Nidogen-1
Aldo-keto reductase family 1 member A1
Pyruvate kinase PKM
Endoplasmin
G2/mitotic-specific cyclin-B1
Myosin light chain 6B
Small nuclear ribonucleoprotein-associated proteins B and B'
Insulin-degrading enzyme
POU domain, class 2, transcription factor 1
Heterogeneous nuclear ribonucleoprotein L
Aspartate--tRNA ligase, cytoplasmic
Protein C-ets-1
Junction plakoglobin
Cytochrome b-c1 complex subunit 7
Serine/threonine-protein kinase B-raf
Fatty acid-binding protein, adipocyte
Glutamine synthetase
Aldo-keto reductase family 1 member B1
Aminopeptidase N
Poliovirus receptor
Ras-related C3 botulinum toxin substrate 2
Eukaryotic peptide chain release factor GTP-binding subunit ERF3A

Arylsulfatase A
Beta-1,4-galactosyltransferase 1
Ezrin
Ubiquitin carboxyl-terminal hydrolase isozyme L3
Fos-related antigen 1
Fos-related antigen 2
Membrane cofactor protein
Nucleoside diphosphate kinase A
NAD(P)H dehydrogenase [quinone] 1
N-acetylglucosamine-6-sulfatase
Phosphorylase b kinase gamma catalytic chain, liver/testis isoform
Arylsulfatase B
Small ribosomal subunit protein uS5
Transcription factor 4
Desmoplakin
Replication protein A 32 kDa subunit
Metalloproteinase inhibitor 2
CD44 antigen
Carbonyl reductase [NADPH] 1
Short-chain specific acyl-CoA dehydrogenase, mitochondrial
Cyclic AMP-responsive element-binding protein 1
Beta-galactosidase
Platelet endothelial cell adhesion molecule
Serine/threonine-protein phosphatase 2B catalytic subunit beta isoform
SH2/SH3 adapter protein NCK1
Intron Large complex component GCFC2
Histone H1.5
Histone H1.3
Histone H1.2
NADPH--cytochrome P450 reductase
Tyrosine-protein kinase Fer
Sarcoplasmic/endoplasmic reticulum calcium ATPase 2
1-phosphatidylinositol 4,5-bisphosphate phosphodiesterase gamma-2
Fumarylacetoacetase
Stathmin
Y-box-binding protein 3
Zinc finger protein 22
Zinc finger protein 24
Alpha-N-acetylgalactosaminidase
High mobility group protein HMG-I/HMG-Y
Transmembrane protein 11, mitochondrial
Aspartate aminotransferase, cytoplasmic
Protein kinase C alpha type
Integrin alpha-2
Gap junction alpha-1 protein
Sphingomyelin phosphodiesterase
Nucleolar transcription factor 1
Transcription factor JunD
T-cell acute lymphocytic leukemia protein 1
Cyclic AMP-dependent transcription factor ATF-7

NADH dehydrogenase [ubiquinone] 1 beta subcomplex subunit 7
cAMP-dependent protein kinase catalytic subunit alpha
Calpain-2 catalytic subunit
Tyrosine-protein phosphatase non-receptor type 2
CTP synthase 1
Endoglin
Probable ATP-dependent RNA helicase DDX5
ATP-dependent 6-phosphofructokinase, liver type
Vascular endothelial growth factor receptor 1
26S proteasome regulatory subunit 6A
T-complex protein 1 subunit alpha
Tyrosine-protein phosphatase non-receptor type 1
General transcription and DNA repair factor IIH helicase subunit XPD
Large ribosomal subunit protein eL33
Integrin beta-5
ADP-ribosylation factor 4
Large ribosomal subunit protein uL30
Vinculin
Receptor-type tyrosine-protein phosphatase alpha
Arylamine N-acetyltransferase 1
Protein SON
Negative elongation factor E
Large ribosomal subunit protein uL22
Phosphoglycerate mutase 1
Regulator of chromosome condensation
Cyclic AMP-dependent transcription factor ATF-1
Cyclic AMP-dependent transcription factor ATF-6 alpha
DNA ligase 1
DNA repair protein XRCC1
Peptidyl-glycine alpha-amidating monooxygenase
Cadherin-2
1-phosphatidylinositol 4,5-bisphosphate phosphodiesterase gamma-1
Nucleolin
Hexokinase-1
DNA-directed RNA polymerase II subunit RPB3
DNA-directed RNA polymerases I, II, and III subunit RPABC1
NADH dehydrogenase [ubiquinone] flavoprotein 2, mitochondrial
General transcription and DNA repair factor IIH helicase subunit XPB
E3 ubiquitin-protein ligase TRIM21
Interferon-induced, double-stranded RNA-activated protein kinase
Spermidine synthase
Sodium/hydrogen exchanger 1
Casein kinase II subunit alpha'
Nuclear factor NF-kappa-B p105 subunit
Plasma membrane calcium-transporting ATPase 1
Eukaryotic translation initiation factor 2 subunit 2
Annexin A7
TATA-box-binding protein
Transcription factor BTF3
Ras-related protein Rab-3B

Ras-related protein Rab-4A
Ras-related protein Rab-5A
Ras-related protein Rab-6A
DNA mismatch repair protein Msh3
Atrial natriuretic peptide receptor 2
Proteasome subunit beta type-1
Cation-dependent mannose-6-phosphate receptor
Lamin-B1
Calpastatin
Inosine-5'-monophosphate dehydrogenase 1
Collagen alpha-1(V) chain
Nebulin
N(4)-(beta-N-acetylglucosaminyl)-L-asparaginase
Ras GTPase-activating protein 1
Parathymosin
Cyclin-dependent kinase 11B
Glutathione S-transferase Mu 3
V-type proton ATPase subunit B, brain isoform
V-type proton ATPase subunit C 1
Cysteine and glycine-rich protein 1
Filamin-A
Neurofibromin
Amine oxidase [flavin-containing] A
Cytoplasmic aconitate hydratase
Sphingosine 1-phosphate receptor 1
Synaptotagmin-1
5'-nucleotidase
Transcription initiation factor TFIID subunit 1
Midkine
Voltage-dependent anion-selective channel protein 1
Biglycan
Succinate dehydrogenase [ubiquinone] iron-sulfur subunit, mitochondrial
CD9 antigen
Catechol O-methyltransferase
Protein-glutamine gamma-glutamyltransferase 2
Methylmalonyl-CoA mutase, mitochondrial
Oxysterol-binding protein 1
Protein-L-isoaspartate(D-aspartate) O-methyltransferase
rRNA 2'-O-methyltransferase fibrillarin
Trifunctional purine biosynthetic protein adenosine-3
Bifunctional phosphoribosylaminoimidazole carboxylase/phosphoribosylaminoimidazole succin
Sterol carrier protein 2
Ubiquitin-like modifier-activating enzyme 1
Nucleoside diphosphate kinase B
NADPH:adrenodoxin oxidoreductase, mitochondrial
Heterogeneous nuclear ribonucleoproteins A2/B1
MHC class II regulatory factor RFX1
E3 ubiquitin-protein ligase CBL
Insulin-like growth factor-binding protein 4
cAMP-dependent protein kinase catalytic subunit beta

Cytochrome b-c1 complex subunit 2, mitochondrial
Ferrochelatase, mitochondrial
DNA repair protein complementing XP-A cells
Transcription elongation factor A protein 1
Prostaglandin G/H synthase 1
Integrin alpha-6
Splicing factor, proline- and glutamine-rich
Tubulin gamma-1 chain
Peptidyl-prolyl cis-trans isomerase B
NAD-dependent malic enzyme, mitochondrial
Tryptophan--tRNA ligase, cytoplasmic
Small ribosomal subunit protein uS3
Ribosomal protein S6 kinase beta-1
Tyrosine-protein kinase JAK1
Receptor-type tyrosine-protein phosphatase beta
Receptor-type tyrosine-protein phosphatase epsilon
Receptor-type tyrosine-protein phosphatase gamma
Nuclear autoantigen Sp-100
Colorectal mutant cancer protein
Tumor necrosis factor ligand superfamily member 4
Adenosylhomocysteinase
Cofilin-1
Eukaryotic translation initiation factor 4B
40-kDa huntingtin-associated protein
Plasma membrane calcium-transporting ATPase 4
Diacylglycerol kinase alpha
Carnitine O-palmitoyltransferase 2, mitochondrial
Thymidylate kinase
Ribonucleoside-diphosphate reductase large subunit
G1/S-specific cyclin-D1
Rab proteins geranylgeranyltransferase component A 1
ER lumen protein-retaining receptor 1
COUP transcription factor 2
Elongation factor 1-beta
ATP synthase F(0) complex subunit B1, mitochondrial
Low molecular weight phosphotyrosine protein phosphatase
Protein kinase C eta type
Acetyl-CoA acetyltransferase, mitochondrial
Myosin regulatory light polypeptide 9
DNA-directed RNA polymerase II subunit RPB1
Cyclin-dependent kinase 2
Beta-adrenergic receptor kinase 1
Proteinase-activated receptor 1
DNA replication licensing factor MCM3
3-mercaptopyruvate sulfurtransferase
Small ribosomal subunit protein eS12
Bromodomain-containing protein 2
Tumor necrosis factor receptor superfamily member 6
Transcriptional repressor protein YY1
DnaJ homolog subfamily B member 1

DnaJ homolog subfamily B member 2
ATP synthase subunit alpha, mitochondrial
Proteasome subunit alpha type-1
Proteasome subunit alpha type-2
Proteasome subunit alpha type-3
Proteasome subunit alpha type-4
Tumor necrosis factor receptor superfamily member 5
Integrin alpha-3
Pentraxin-related protein PTX3
Moesin
Probable ATP-dependent RNA helicase DDX6
DNA (cytosine-5)-methyltransferase 1
Splicing factor U2AF 65 kDa subunit
Large ribosomal subunit protein eL13
Rab proteins geranylgeranyltransferase component A 2
Isovaleryl-CoA dehydrogenase, mitochondrial
Alpha-1,3-mannosyl-glycoprotein 2-beta-N-acetylglucosaminyltransferase
High mobility group protein B2
Polypyrimidine tract-binding protein 1
Threonine--tRNA ligase 1, cytoplasmic
Valine--tRNA ligase
Elongation factor 1-gamma
Peptidyl-prolyl cis-trans isomerase FKBP2
Interleukin-3 receptor subunit alpha
Stomatin
Adenylate kinase 4, mitochondrial
14-3-3 protein theta
Mitogen-activated protein kinase 3
MAP/microtubule affinity-regulating kinase 3
Large ribosomal subunit protein uL16
Collagen alpha-1(VIII) chain
Replication protein A 70 kDa DNA-binding subunit
DNA-(apurinic or apyrimidinic site) endonuclease
CD82 antigen
Deoxycytidine kinase
Multifunctional protein CAD
Calreticulin
Microtubule-associated protein 4
Calnexin
Phosphatidylinositol 3-kinase regulatory subunit alpha
Inositol-trisphosphate 3-kinase B
Proteasome subunit beta type-8
Proteasome subunit beta type-9
Proteasome subunit alpha type-5
Proteasome subunit beta type-4
Proteasome subunit beta type-6
Proteasome subunit beta type-5
Glutathione S-transferase Mu 2
ATP-binding cassette sub-family D member 3
Protein ITPRID2

Protein-lysine 6-oxidase
NADH-ubiquinone oxidoreductase 75 kDa subunit, mitochondrial
DNA polymerase delta catalytic subunit
Probable global transcription activator SNF2L1
Mitogen-activated protein kinase 1
Retinoic acid receptor RXR-beta
DNA excision repair protein ERCC-5
Progranulin
Receptor-type tyrosine-protein phosphatase mu
Cytosol aminopeptidase
Hematopoietic progenitor cell antigen CD34
General transcription factor IIE subunit 1
Transcription initiation factor IIE subunit beta
Tripeptidyl-peptidase 2
Inositol monophosphatase 1
CCN family member 2
Ephrin type-A receptor 2
Ephrin type-B receptor 2
SHC-transforming protein 1
DNA-3-methyladenine glycosylase
Transketolase
Caspase-1
Nitric oxide synthase 3
RNA-binding motif, single-stranded-interacting protein 1
Protein PML
Non-receptor tyrosine-protein kinase TYK2
Elongation factor 1-delta
Myristoylated alanine-rich C-kinase substrate
Guanine nucleotide-binding protein subunit alpha-11
Delta-1-pyrroline-5-carboxylate dehydrogenase, mitochondrial
Endoplasmic reticulum resident protein 29
Peroxiredoxin-6
Flavin reductase (NADPH)
Peroxiredoxin-5, mitochondrial
Thioredoxin-dependent peroxide reductase, mitochondrial
Large ribosomal subunit protein uL11
Enoyl-CoA hydratase, mitochondrial
UMP-CMP kinase
Phosphatidylethanolamine-binding protein 1
Protein disulfide-isomerase A3
Serine/threonine-protein phosphatase 2A 65 kDa regulatory subunit A alpha isoform
Serine/threonine-protein phosphatase 2A 65 kDa regulatory subunit A beta isoform
Cell division cycle protein 27 homolog
Peptidyl-prolyl cis-trans isomerase F, mitochondrial
NK-tumor recognition protein
Glycylpeptide N-tetradecanoyltransferase 1
Heme oxygenase 2
Adenylosuccinate synthetase isozyme 2
Tyrosine-protein kinase receptor UFO
Alpha-2-macroglobulin receptor-associated protein

Translocator protein
Adenylosuccinate lyase
CAP-Gly domain-containing linker protein 1
Sorcin
Leukocyte elastase inhibitor
High affinity cationic amino acid transporter 1
Aldehyde dehydrogenase X, mitochondrial
DNA-directed RNA polymerase II subunit RPB2
Succinate dehydrogenase [ubiquinone] flavoprotein subunit, mitochondrial
Coronin-1A
Rab GDP dissociation inhibitor alpha
S-adenosylmethionine synthase isoform type-2
cAMP-dependent protein kinase type II-beta regulatory subunit
Ribonucleoside-diphosphate reductase subunit M2
Cytotoxic granule associated RNA binding protein TIA1
Sodium- and chloride-dependent taurine transporter
DnaJ homolog subfamily A member 1
RAC-alpha serine/threonine-protein kinase
Cytochrome b-c1 complex subunit 1, mitochondrial
3-hydroxyisobutyrate dehydrogenase, mitochondrial
Bifunctional purine biosynthesis protein ATIC
Heterogeneous nuclear ribonucleoprotein H3
Heterogeneous nuclear ribonucleoprotein H
14-3-3 protein beta/alpha
Stress-induced-phosphoprotein 1
Protein S100-A11
Peroxiredoxin-2
Beta-arrestin-2
Glycerol kinase
Deoxycytidylate deaminase
Pyrroline-5-carboxylate reductase 1, mitochondrial
Guanylate-binding protein 1
Guanylate-binding protein 2
ETS-related transcription factor Elf-1
General transcription factor IIH subunit 1
Syntaxin-2
Large ribosomal subunit protein uL6
Long-chain-fatty-acid--CoA ligase 1
Cadherin-5
Kinesin-1 heavy chain
Deoxyuridine 5'-triphosphate nucleotidohydrolase, mitochondrial
Multidrug resistance-associated protein 1
ATP-binding cassette sub-family D member 1
Dual specificity protein kinase TTK
DNA replication licensing factor MCM4
DNA replication licensing factor MCM5
DNA replication licensing factor MCM7
N-acetylgalactosamine-6-sulfatase
Serine hydroxymethyltransferase, cytosolic
Serine hydroxymethyltransferase, mitochondrial

Heat shock 70 kDa protein 1-like
Heat shock 70 kDa protein 4
G protein-coupled receptor kinase 5
Mannose-6-phosphate isomerase
Glypican-1
Profilin-2
Catenin alpha-1
Catenin beta-1
Prohibitin 1
Serpin B6
Merlin
Radixin
Replication protein A 14 kDa subunit
Replication factor C subunit 4
Replication factor C subunit 2
Replication factor C subunit 1
Large ribosomal subunit protein eL22
General transcription factor IIF subunit 1
Sepiapterin reductase
Histamine H1 receptor
Cystathionine beta-synthase
Fibrillin-1
Fibrillin-2
Glycogen debranching enzyme
Myosin-9
Myosin-10
Tyrosine-protein kinase receptor Tie-1
Coatomer subunit beta'
Sterol O-acyltransferase 1
Alpha-adducin
Basigin
RNA-binding protein FUS
Nuclear pore complex protein Nup214
Protein DEK
Transcription factor SOX-18
Myosin-11
Protein phosphatase 1A
Hydroxymethylglutaryl-CoA lyase, mitochondrial
Vascular endothelial growth factor receptor 3
Vascular endothelial growth factor receptor 2
26S proteasome regulatory subunit 7
Glutathione hydrolase 5 proenzyme
ADP-ribosylation factor-like protein 2
ADP-ribosylation factor-like protein 3
Dual specificity mitogen-activated protein kinase kinase 2
ATP synthase subunit gamma, mitochondrial
V-type proton ATPase subunit E 1
Oxygen-dependent coproporphyrinogen-III oxidase, mitochondrial
Large ribosomal subunit protein uL4
Oxidized purine nucleoside triphosphate hydrolase

Lon protease homolog, mitochondrial
Phosphoglucomutase-1
Serine/threonine-protein phosphatase PP1-gamma catalytic subunit
Guanine nucleotide-binding protein-like 1
Dihydrolipoyllysine-residue succinyltransferase component of 2-oxoglutarate dehydrogenase co
GMP reductase 1
Phospholipid hydroperoxide glutathione peroxidase GPX4
Serine/threonine-protein kinase receptor R3
17-beta-hydroxysteroid dehydrogenase type 2
Signal recognition particle 14 kDa protein
TGF-beta receptor type-2
Nuclear pore glycoprotein p62
Hippocalcin-like protein 1
Squalene synthase
Zinc finger E-box-binding homeobox 1
Transgelin-2
Transaldolase
Alpha-synuclein
Electron transfer flavoprotein subunit beta
RNA-binding motif protein, X chromosome
Coilin
Vitamin K-dependent gamma-carboxylase
Lysosomal acid lipase/cholesteryl ester hydrolase
V-type proton ATPase catalytic subunit A
Stress-70 protein, mitochondrial
Eukaryotic initiation factor 4A-III
DNA-binding protein SMUBP-2
Cyclin-dependent kinase inhibitor 1
Small ribosomal subunit protein eS19
Large ribosomal subunit protein uL3
Collagen alpha-1(XVIII) chain
Dolichyl-diphosphooligosaccharide--protein glycosyltransferase 48 kDa subunit
Acidic leucine-rich nuclear phosphoprotein 32 family member A
Flap endonuclease 1
Homeobox protein cut-like 1
Macrophage-capping protein
Adenylyl cyclase-associated protein 2
Interleukin-6 receptor subunit beta
Alpha-taxilin
T-complex protein 1 subunit zeta
Nicotinamide N-methyltransferase
Proteasome subunit beta type-10
Pre-B-cell leukemia transcription factor 2
Large ribosomal subunit protein uL13
ADP-ribosylation factor-like protein 1
DNA mismatch repair protein Mlh1
Signal transducer and activator of transcription 3
Ubiquitin carboxyl-terminal hydrolase 8
Malate dehydrogenase, cytoplasmic
Malate dehydrogenase, mitochondrial

Replication factor C subunit 5
Replication factor C subunit 3
Trifunctional enzyme subunit alpha, mitochondrial
Eukaryotic translation initiation factor 2 subunit 3
ETS translocation variant 3
Centrin-2
Transcription factor ETV6
Eukaryotic translation initiation factor 2D
Protein BUD31 homolog
Ubiquitin-like modifier-activating enzyme 7
N-alpha-acetyltransferase 10
Lysine-specific demethylase 5C
Tyrosine-protein kinase CSK
Glycine--tRNA ligase
Isoleucine--tRNA ligase, cytoplasmic
Reduced folate transporter
Eukaryotic translation initiation factor 1
Protein kinase C iota type
ETS domain-containing protein Elk-3
Beta-centractin
Enoyl-CoA delta isomerase 1, mitochondrial
Lamina-associated polypeptide 2, isoform alpha
Lamina-associated polypeptide 2, isoforms beta/gamma
Signal transducer and activator of transcription 1-alpha/beta
Signal transducer and activator of transcription 6
Signal transducer and activator of transcription 5A
Exosome RNA helicase MTR4
Aldo-keto reductase family 1 member C3
Phosphatidylinositol 4,5-bisphosphate 3-kinase catalytic subunit alpha isoform
Phosphatidylinositol 4,5-bisphosphate 3-kinase catalytic subunit beta isoform
Serine/threonine-protein kinase mTOR
Phosphatidylinositol 4-kinase alpha
Epidermal growth factor receptor substrate 15
Caspase-3
Caspase-2
Small ribosomal subunit protein eS27
Tyrosine-protein kinase ABL2
Probable helicase with zinc finger domain
Condensin-2 complex subunit D3
RNA-binding protein 34
Leucine-rich PPR motif-containing protein, mitochondrial
3-ketoacyl-CoA thiolase, mitochondrial
Large ribosomal subunit protein uL29
Lysosomal Pro-X carboxypeptidase
Huntingtin
Endothelin-converting enzyme 1
Methylenetetrahydrofolate reductase (NADPH)
Neutral amino acid transporter A
Platelet-activating factor acetylhydrolase IB subunit beta
Cell surface glycoprotein MUC18

Carnitine O-acetyltransferase
Matrin-3
DNA mismatch repair protein Msh2
G protein-coupled receptor kinase 6
Glycerol-3-phosphate dehydrogenase, mitochondrial
Translocon-associated protein subunit alpha
Tyrosine-protein phosphatase non-receptor type 9
Ran-specific GTPase-activating protein
Nicotinamide phosphoribosyltransferase
26S proteasome regulatory subunit 6B
Elongation factor Ts, mitochondrial
Peptidyl-prolyl cis-trans isomerase C
Voltage-dependent anion-selective channel protein 2
Short/branched chain specific acyl-CoA dehydrogenase, mitochondrial
Chromobox protein homolog 5
Ubiquitin carboxyl-terminal hydrolase 5
Mitogen-activated protein kinase 8
Dual specificity mitogen-activated protein kinase kinase 4
Proliferation marker protein Ki-67
Phosphorylase b kinase regulatory subunit alpha, liver isoform
Phosphorylase b kinase regulatory subunit alpha, skeletal muscle isoform
Ran GTPase-activating protein 1
ATP-dependent DNA helicase Q1
Probable 28S rRNA (cytosine(4447)-C(5))-methyltransferase
Transcriptional regulator ATRX
Adapter molecule crk
Crk-like protein
Translation initiation factor IF-2, mitochondrial
Large proline-rich protein BAG6
Vesicle-fusing ATPase
Neurogenic locus notch homolog protein 1
Dual specificity mitogen-activated protein kinase kinase 3
Lys-63-specific deubiquitinase BRCC36
Large ribosomal subunit protein uL15
Large ribosomal subunit protein uL18
Large ribosomal subunit protein eL21
Large ribosomal subunit protein eL28
Small ribosomal subunit protein uS4
Small ribosomal subunit protein uS7
Small ribosomal subunit protein eS10
Microtubule-associated protein 1B
Glucosamine-6-phosphate isomerase 1
E3 ubiquitin-protein ligase NEDD4
Utrophin
Ras GTPase-activating-like protein IQGAP1
Glycogenin-1
Dolichyl-diphosphooligosaccharide--protein glycosyltransferase subunit STT3A
Cytosolic phospholipase A2
F-actin-capping protein subunit alpha-2
F-actin-capping protein subunit beta

Eukaryotic translation initiation factor 1A, X-chromosomal
Retinaldehyde dehydrogenase 3
Glutamine--tRNA ligase
Large ribosomal subunit protein eL29
Galectin-7
mRNA decay activator protein ZFP36L2
Cytochrome b-c1 complex subunit Rieske, mitochondrial
ATP synthase subunit O, mitochondrial
LIM and senescent cell antigen-like-containing domain protein 1
Glioma pathogenesis-related protein 1
Prolyl endopeptidase
NADP-dependent malic enzyme
Iron-responsive element-binding protein 2
Tissue factor pathway inhibitor 2
DNA-binding protein RFX5
Phosphatidylinositol 5-phosphate 4-kinase type-2 alpha
Coatomer subunit delta
Lanosterol synthase
Serine/threonine-protein phosphatase 2B catalytic subunit gamma isoform
Glutamate--cysteine ligase catalytic subunit
Glutamate--cysteine ligase regulatory subunit
CD151 antigen
Trafficking protein particle complex subunit 10
26S proteasome non-ATPase regulatory subunit 8
Protein PRRC2A
Glutathione synthetase
T-complex protein 1 subunit epsilon
Phosphatidylserine synthase 1
Nestin
Heat shock 70 kDa protein 13
Casein kinase I isoform alpha
Casein kinase I isoform delta
Isocitrate dehydrogenase [NADP], mitochondrial
Phosphatidylinositol 4,5-bisphosphate 3-kinase catalytic subunit gamma isoform
Phosphatidylinositol transfer protein beta isoform
DNA polymerase delta subunit 2
MARCKS-related protein
Paxillin
Guided entry of tail-anchored proteins factor CAMLG
Nuclear receptor subfamily 2 group C member 2
MAP kinase-activated protein kinase 2
Deoxyribonuclease-1-like 1
4-trimethylaminobutyraldehyde dehydrogenase
Large ribosomal subunit protein eL34
Ribose-5-phosphate isomerase
Protein ERGIC-53
Natural resistance-associated macrophage protein 2
Nuclear autoantigenic sperm protein
Fatty acid synthase
Protein farnesyltransferase/geranylgeranyltransferase type-1 subunit alpha

Protein farnesyltransferase subunit beta
Deoxyhypusine synthase
T-complex protein 1 subunit gamma
Large ribosomal subunit protein bL19m
Beta-arrestin-1
Elongation factor Tu, mitochondrial
Alpha-aminoadipic semialdehyde dehydrogenase
Ubiquitin-conjugating enzyme E2 R1
Inositol polyphosphate 1-phosphatase
Centromere protein F
Signal recognition particle 9 kDa protein
Choline-phosphate cytidylyltransferase A
Alanine--tRNA ligase, cytoplasmic
Cysteine--tRNA ligase, cytoplasmic
Histidine--tRNA ligase, mitochondrial
Serine--tRNA ligase, cytoplasmic
Protein phosphatase 1F
Alpha-mannosidase 2x
DNA primase small subunit
DNA primase large subunit
Caspase-4
Casein kinase I isoform epsilon
Proteasome subunit beta type-3
Proteasome subunit beta type-2
DNA replication licensing factor MCM2
Very long-chain specific acyl-CoA dehydrogenase, mitochondrial
YLP motif-containing protein 1
Vacuolar protein sorting-associated protein 41 homolog
Transmembrane emp24 domain-containing protein 10
RNA-binding protein 25
Protein numb homolog
Dual specificity protein kinase CLK3
Presenilin-1
Translation initiation factor eIF2B subunit beta
Adenosine 5'-monophosphoramidase HINT1
Nuclear pore complex protein Nup153
E3 SUMO-protein ligase RanBP2
Regulator of G-protein signaling 19
Regulator of G-protein signaling 3
Tuberin
NADH dehydrogenase [ubiquinone] flavoprotein 1, mitochondrial
Glycogen synthase kinase-3 alpha
Glycogen synthase kinase-3 beta
Transcription initiation factor TFIID subunit 6
Cytosolic purine 5'-nucleotidase
Selenide, water dikinase 1
5-formyltetrahydrofolate cyclo-ligase
GMP synthase [glutamine-hydrolyzing]
DNA ligase 3
DNA ligase 4

Double-strand break repair protein MRE11
Ectonucleoside triphosphate diphosphohydrolase 1
Guanine nucleotide-binding protein G(q) subunit alpha
Isocitrate dehydrogenase [NAD] subunit alpha, mitochondrial
Sulfotransferase 1A1
Matrix metalloproteinase-14
Protoporphyrinogen oxidase
Rab GDP dissociation inhibitor beta
Emerin
Carnitine O-palmitoyltransferase 1, liver isoform
Sulfate transporter
Serpin B8
Serpin B9
Serpin H1
PDZ and LIM domain protein 4
Hsc70-interacting protein
Peroxisomal targeting signal 1 receptor
ETS domain-containing transcription factor ERF
Vasodilator-stimulated phosphoprotein
Dynamin-2
Methionine aminopeptidase 2
Bis(5'-nucleosyl)-tetraphosphatase [asymmetrical]
Cyclin-dependent kinase 7
Kinetochore-associated protein 1
Ras association domain-containing protein 2
Cyclin-dependent kinase 9
Lipopolysaccharide-responsive and beige-like anchor protein
Basal cell adhesion molecule
Palmitoyl-protein thioesterase 1
Large ribosomal subunit protein eL14
T-complex protein 1 subunit theta
T-complex protein 1 subunit delta
Annexin A11
Poly(A) polymerase alpha
RNA-binding protein FXR1
RNA-binding protein FXR2
Ras-related protein Rab-5C
Ras-related protein Rab-7a
Ras-related protein Rab-9A
Ras-related protein Rab-13
Ras-related protein Rab-27A
1-phosphatidylinositol 4,5-bisphosphate phosphodiesterase delta-1
Small ribosomal subunit protein mS29
Dual specificity protein phosphatase 3
Probable global transcription activator SNF2L2
Transcription activator BRG1
Isocitrate dehydrogenase [NAD] subunit gamma, mitochondrial
Galactokinase
Translocon-associated protein subunit delta
B-cell receptor-associated protein 31

Thiopurine S-methyltransferase
Methyl-CpG-binding protein 2
Host cell factor 1
Interleukin-1 receptor-associated kinase 1
Caveolin-2
Aldehyde dehydrogenase family 3 member A2
Peroxisomal multifunctional enzyme type 2
26S proteasome non-ATPase regulatory subunit 7
Sulfite oxidase, mitochondrial
N-sulphoglucosamine sulphohydrolase
Signal transducer and activator of transcription 5B
Ubiquitin carboxyl-terminal hydrolase 11
H(+)/Cl(-) exchange transporter 7
Vesicle-associated membrane protein 7
Ribosomal protein S6 kinase alpha-3
Cytoplasmic tyrosine-protein kinase BMX
AF4/FMR2 family member 1
Hepatoma-derived growth factor
Cyclin-H
CDK-activating kinase assembly factor MAT1
Serine/threonine-protein kinase Nek3
Ubiquitin-conjugating enzyme E2 E1
NADH dehydrogenase [ubiquinone] 1 alpha subcomplex subunit 8
Heterogeneous nuclear ribonucleoprotein A3
6-phosphogluconate dehydrogenase, decarboxylating
Heterogeneous nuclear ribonucleoprotein M
Importin subunit alpha-1
Importin subunit alpha-5
Nuclear cap-binding protein subunit 2
Rap1 GTPase-GDP dissociation stimulator 1
DNA-directed RNA polymerases I, II, and III subunit RPABC3
Dual specificity mitogen-activated protein kinase kinase 6
Rho GDP-dissociation inhibitor 1
Rho GDP-dissociation inhibitor 2
Arf-GAP domain and FG repeat-containing protein 1
Heterogeneous nuclear ribonucleoprotein F
Signal transducer and activator of transcription 2
DNA mismatch repair protein Msh6
Kinesin-like protein KIF11
Guanine nucleotide exchange factor VAV2
Zinc finger protein 143
RNA-binding protein 5
2-iminobutanoate/2-iminopropanoate deaminase
Spermine synthase
Hexokinase-2
Ephrin-B2
Large ribosomal subunit protein bL12m
Diacylglycerol kinase theta
Bifunctional heparan sulfate N-deacetylase/N-sulfotransferase 1
Thimet oligopeptidase

F-actin-capping protein subunit alpha-1
High mobility group protein HMGI-C
Cysteine-rich protein 2
Nuclear pore complex protein Nup98-Nup96
Biliverdin reductase A
Tricarboxylate transport protein, mitochondrial
Serine/threonine-protein phosphatase 5
Serine/threonine-protein kinase PLK1
Death-associated protein kinase 1
Arfaptin-2
Arfaptin-1
Cytosolic Fe-S cluster assembly factor NUBP1
ATP-citrate synthase
Methionine aminopeptidase 1
Succinate--CoA ligase [ADP/GDP-forming] subunit alpha, mitochondrial
Diphosphomevalonate decarboxylase
Geranylgeranyl transferase type-1 subunit beta
Geranylgeranyl transferase type-2 subunit beta
Coatomer subunit beta
Coatomer subunit alpha
Dipeptidyl peptidase 1
LIM domain kinase 1
LIM domain kinase 2
Clathrin heavy chain 2
AP-3 complex subunit mu-2
AP-2 complex subunit sigma
Holocytochrome c-type synthase
Mitogen-activated protein kinase 12
Pituitary tumor-transforming gene 1 protein-interacting protein
Smoothelin
Monocarboxylate transporter 1
IST1 homolog
Protein transport protein Sec24C
Activated RNA polymerase II transcriptional coactivator p15
DNA polymerase subunit gamma-1
Methylosome subunit pICln
Arginine--tRNA ligase, cytoplasmic
Protein HIRA
Ataxin-3
Atrophin-1
PMS1 protein homolog 1
Mismatch repair endonuclease PMS2
Voltage-dependent calcium channel subunit alpha-2/delta-1
Tyrosine--tRNA ligase, cytoplasmic
Ubiquitin carboxyl-terminal hydrolase 14
5'-AMP-activated protein kinase subunit gamma-1
Heat shock-related 70 kDa protein 2
Branched-chain-amino-acid aminotransferase, cytosolic
Sodium/potassium-transporting ATPase subunit beta-3
UV excision repair protein RAD23 homolog B

Ephrin type-B receptor 4
Alpha-N-acetylglucosaminidase
Adenylate kinase 2, mitochondrial
Delta-1-pyrroline-5-carboxylate synthase
Alpha-soluble NSF attachment protein
Eukaryotic translation initiation factor 5
Solute carrier family 12 member 2
26S proteasome non-ATPase regulatory subunit 4
Developmentally-regulated GTP-binding protein 2
Exportin-2
Transitional endoplasmic reticulum ATPase
Microfibrillar-associated protein 1
Trifunctional enzyme subunit beta, mitochondrial
Mesencephalic astrocyte-derived neurotrophic factor
Afadin
RNA polymerase II elongation factor ELL
Peregrin
Nucleosome assembly protein 1-like 1
Caspase-7
Caspase-6
Adenosine kinase
Double-stranded RNA-specific adenosine deaminase
Laminin subunit beta-2
Cadherin-13
Tumor protein D52
Protein SEC13 homolog
NHP2-like protein 1
Puromycin-sensitive aminopeptidase
FAD-linked sulfhydryl oxidase ALR
Heterogeneous nuclear ribonucleoprotein H2
Succinyl-CoA:3-ketoacid coenzyme A transferase 1, mitochondrial
Eukaryotic translation initiation factor 3 subunit B
IgG receptor FcRn large subunit p51
BH3-interacting domain death agonist
ATP synthase subunit f, mitochondrial
Ribosomal RNA processing protein 1 homolog A
Methionine--tRNA ligase, cytoplasmic
cAMP-regulated phosphoprotein 19
AP-1 complex subunit sigma-2
ATP synthase subunit ATP5MJ, mitochondrial
ATP synthase subunit epsilon, mitochondrial
ATP synthase subunit e, mitochondrial
Histone deacetylase 4
Eukaryotic translation initiation factor 6
C-terminal-binding protein 2
NADH dehydrogenase [ubiquinone] 1 alpha subcomplex subunit 6
Peroxisomal biogenesis factor 3
3-keto-steroid reductase/17-beta-hydroxysteroid dehydrogenase 7
Breast cancer anti-estrogen resistance protein 1
Syntaxin-17

Cilia- and flagella-associated protein 298
tRNA (guanine-N(7)-)-methyltransferase non-catalytic subunit WDR4
Transmembrane protein 33
Gem-associated protein 4
Coronin-7
Nuclear pore complex protein Nup107
Gasdermin-D
Selenocysteine-specific elongation factor
Sestrin-2
Anthrax toxin receptor 2
Myotrophin
Actin-related protein 2/3 complex subunit 4
CD81 antigen
Protein transport protein Sec61 subunit gamma
Triosephosphate isomerase
Eukaryotic translation initiation factor 3 subunit E
Protein transport protein Sec61 subunit beta
Phosphatidylinositol 3,4,5-trisphosphate 3-phosphatase and dual-specificity protein phosphatas
Serine/threonine-protein phosphatase 4 catalytic subunit
Gamma-aminobutyric acid receptor-associated protein-like 2
Reactive oxygen species modulator 1
Myosin light polypeptide 6
Actin, cytoplasmic 1
Eukaryotic initiation factor 4A-I
Small ribosomal subunit protein uS10
Ribose-phosphate pyrophosphokinase 1
Proteasome subunit alpha type-6
Protein S100-A10
Cell division control protein 42 homolog
Destrin
Glia maturation factor beta
Ras-related protein Rab-8A
Signal peptidase complex subunit 3
Signal recognition particle subunit SRP54
Ras-related protein Rab-4B
Ras-related protein Rab-2A
Ras-related protein Rab-5B
Ras-related protein Rab-10
Ubiquitin-conjugating enzyme E2 D3
NEDD8-conjugating enzyme Ubc12
Ubiquitin-conjugating enzyme E2 K
Ubiquitin-conjugating enzyme E2 N
Ras-related protein Rab-14
Actin-related protein 3
Actin-related protein 2
Alpha-centractin
COP9 signalosome complex subunit 2
ATP-binding cassette sub-family E member 1
Ras-related protein Rap-1b
Ras-related protein Rap-2b

Protein max
Small ribosomal subunit protein eS1
Large ribosomal subunit protein uL24
Proteasome activator complex subunit 3
Large ribosomal subunit protein eL15
Large ribosomal subunit protein eL27
V-type proton ATPase subunit d 1
Large ribosomal subunit protein eL43
Transforming protein RhoA
Rho-related GTP-binding protein RhoE
N-alpha-acetyltransferase 20
10 kDa heat shock protein, mitochondrial
Protein transport protein Sec61 subunit alpha isoform 1
Prefoldin subunit 3
Syntaxin-binding protein 1
Beta-2-microglobulin
Dolichyl-diphosphooligosaccharide--protein glycosyltransferase subunit DAD1
NPC intracellular cholesterol transporter 2
Coatomer subunit zeta-1
Large ribosomal subunit protein eL37
Small ubiquitin-related modifier 2
DDB1- and CUL4-associated factor 7
WD repeat-containing protein 5
AP-1 complex subunit sigma-1A
Nuclear transport factor 2
Heterogeneous nuclear ribonucleoprotein K
14-3-3 protein gamma
Ubiquitin carboxyl-terminal hydrolase 46
Ras-related protein R-Ras2
Small ribosomal subunit protein eS7
Serine/threonine-protein phosphatase PP1-alpha catalytic subunit
Serine/threonine-protein phosphatase PP1-beta catalytic subunit
26S proteasome regulatory subunit 4
26S proteasome regulatory subunit 8
Small ribosomal subunit protein eS8
Small ribosomal subunit protein uS8
Small ribosomal subunit protein uS9
Ubiquitin-conjugating enzyme E2 G1
Ubiquitin-conjugating enzyme E2 H
14-3-3 protein epsilon
Small ribosomal subunit protein uS11
Small ribosomal subunit protein uS12
Small ribosomal subunit protein uS13
Small ribosomal subunit protein uS14
Small ribosomal subunit protein uS15
Small ribosomal subunit protein uS17
Small nuclear ribonucleoprotein E
Small nuclear ribonucleoprotein F
U6 snRNA-associated Sm-like protein LSm3
U6 snRNA-associated Sm-like protein LSm6

Small nuclear ribonucleoprotein Sm D1
Small nuclear ribonucleoprotein Sm D2
Small nuclear ribonucleoprotein Sm D3
Thymosin beta-4
ADP-ribosylation factor 6
26S proteasome regulatory subunit 10B
Thioredoxin reductase-like selenoprotein T
TATA box-binding protein-like 1
Large ribosomal subunit protein eL8
DNA-directed RNA polymerase II subunit RPB7
Ras-related protein Rab-11A
Eukaryotic peptide chain release factor subunit 1
CCHC-type zinc finger nucleic acid binding protein
Protein yippee-like 5
Small ribosomal subunit protein eS4, X isoform
Serine/threonine-protein phosphatase 2A catalytic subunit beta isoform
Actin, aortic smooth muscle
Rho-related GTP-binding protein RhoB
Large ribosomal subunit protein uL23
Small ribosomal subunit protein eS6
Histone H4
Ras-related protein Rab-1A
GTP-binding nuclear protein Ran
Large ribosomal subunit protein uL14
Ras-related protein Rap-1A
Small ribosomal subunit protein uS19
Small ribosomal subunit protein eS24
Small ribosomal subunit protein eS25
Small ribosomal subunit protein eS26
Small ribosomal subunit protein eS28
Ubiquitin-like FUBI-ribosomal protein eS30 fusion protein
Guanine nucleotide-binding protein G(I)/G(S)/G(T) subunit beta-1
E3 ubiquitin-protein ligase RBX1
Guanine nucleotide-binding protein G(I)/G(S)/G(T) subunit beta-2
Large ribosomal subunit protein eL30
Large ribosomal subunit protein eL39
Large ribosomal subunit protein eL31
Large ribosomal subunit protein uL1
Large ribosomal subunit protein eL32
Large ribosomal subunit protein uL5
Large ribosomal subunit protein uL2
Peptidyl-prolyl cis-trans isomerase A
Peptidyl-prolyl cis-trans isomerase FKBP1A
Ubiquitin-ribosomal protein eS31 fusion protein
Growth factor receptor-bound protein 2
Transformer-2 protein homolog beta
Ras-related C3 botulinum toxin substrate 1
AP-2 complex subunit beta
Vesicle-associated membrane protein 2
Guanine nucleotide-binding protein G(s) subunit alpha isoforms short

Guanine nucleotide-binding protein G(i) subunit alpha-1
14-3-3 protein zeta/delta
Serine/threonine-protein phosphatase 2A 55 kDa regulatory subunit B alpha isoform
Small ubiquitin-related modifier 1
Dynein light chain 1, cytoplasmic
Dynein light chain Tctex-type 1
Large ribosomal subunit protein eL38
S-phase kinase-associated protein 1
Guanine nucleotide-binding protein G(I)/G(S)/G(O) subunit gamma-5
Small ribosomal subunit protein eS21
Eukaryotic translation initiation factor 5A-1
Small ribosomal subunit protein RACK1
Transcription elongation factor SPT4
SUMO-conjugating enzyme UBC9
Thymosin beta-10
Serine/threonine-protein phosphatase 2A catalytic subunit alpha isoform
Y-box-binding protein 1
Signal peptidase complex catalytic subunit SEC11A
Casein kinase II subunit beta
Tropomyosin alpha-4 chain
Ubiquitin-conjugating enzyme E2 L3
Elongation factor 1-alpha 1
Tubulin alpha-1B chain
Tubulin alpha-4A chain
Tubulin beta-4B chain
Casein kinase II subunit alpha
Platelet-activating factor acetylhydrolase IB subunit alpha2
Histone H3.1
Hemoglobin subunit beta
Hemoglobin subunit alpha
Coxsackievirus and adenovirus receptor
Protein FAM193A
Nucleolar protein 14
Immunoglobulin-binding protein 1
Tyrosine-protein phosphatase non-receptor type substrate 1
Phosphoserine phosphatase
RNA-binding protein 6
Eukaryotic translation initiation factor 4 gamma 2
Ribonuclease P protein subunit p38
Ribonuclease P protein subunit p30
General transcription factor II-I
Phosphatidylinositol 5-phosphate 4-kinase type-2 beta
Contactin-associated protein 1
SRSF protein kinase 2
T-complex protein 1 subunit beta
UDP-galactose translocator
mRNA export factor RAE1
Glutathione S-transferase omega-1
DNA-dependent protein kinase catalytic subunit
Disintegrin and metalloproteinase domain-containing protein 17

Biogenesis of lysosome-related organelles complex 1 subunit 1
Sushi repeat-containing protein SRPX
Arginase-2, mitochondrial
Endonuclease III-like protein 1
Interferon-induced 35 kDa protein
Nucleobindin-2
Brain acid soluble protein 1
Dermcidin
TATA element modulatory factor
Small ribosomal subunit protein mS22
Small ribosomal subunit protein mS25
Small ribosomal subunit protein uS10m
Small ribosomal subunit protein mS35
Small ribosomal subunit protein uS5m
Alpha-ketoglutarate dehydrogenase component 4
Small ribosomal subunit protein uS11m
Small ribosomal subunit protein uS15m
Small ribosomal subunit protein bS21m
Small ribosomal subunit protein mS34
Small ribosomal subunit protein bS6m
Small ribosomal subunit protein uS9m
SAP domain-containing ribonucleoprotein
Serine beta-lactamase-like protein LACTB, mitochondrial
Conserved oligomeric Golgi complex subunit 7
Large ribosomal subunit protein eL24
Thioredoxin-like protein 4A
Large ribosomal subunit protein eL42
Chromobox protein homolog 1
Mothers against decapentaplegic homolog 3
ADP-ribosylation factor 1
ADP-ribosylation factor 5
Enhancer of rudimentary homolog
Rho-related GTP-binding protein RhoG
Large ribosomal subunit protein eL19
Small EDRK-rich factor 2
Serine/arginine-rich splicing factor 3
Forkhead box protein K1
Vacuolar fusion protein CCZ1 homolog B
Disabled homolog 2
Basement membrane-specific heparan sulfate proteoglycan core protein
E3 ubiquitin-protein ligase XIAP
Ephrin-B1
RNA-binding protein 10
RNA-binding protein 3
Calcium-transporting ATPase type 2C member 1
Phospholipid-transporting ATPase IH
Cytochrome c
55 kDa erythrocyte membrane protein
Transcription factor A, mitochondrial
Phosphatidylinositol transfer protein alpha isoform

Solute carrier family 25 member 3
Vigilin
Transcription initiation factor IIB
Cyclin-dependent kinase 6
Cyclin-dependent kinase 5
Cyclin-dependent kinase 17
Transcriptional activator protein Pur-alpha
Cdc42 effector protein 1
Clathrin heavy chain 1
Heat shock factor protein 1
Nuclear factor NF-kappa-B p100 subunit
Peptidyl-prolyl cis-trans isomerase FKBP3
Receptor expression-enhancing protein 5
Sorbitol dehydrogenase
Heterogeneous nuclear ribonucleoprotein U
Splicing factor U2AF 35 kDa subunit
Spectrin beta chain, non-erythrocytic 1
Nucleolysin TIAR
Protein SET
Serine/arginine-rich splicing factor 2
Forkhead box protein K2
Transcription factor RelB
N-acetylgalactosamine kinase
AMP deaminase 2
Fatty acid-binding protein 5
Adenylyl cyclase-associated protein 1
Friend leukemia integration 1 transcription factor
Hydroxymethylglutaryl-CoA synthase, cytoplasmic
Interferon-induced transmembrane protein 3
Large neutral amino acids transporter small subunit 1
Protein Dr1
Exosome complex component 10
OTU domain-containing protein 4
ATP-dependent 6-phosphofructokinase, platelet type
DNA repair protein complementing XP-C cells
RNA-binding protein EWS
Cerebellar degeneration-related protein 2
Inositol polyphosphate 5-phosphatase OCRL
1-phosphatidylinositol 4,5-bisphosphate phosphodiesterase beta-3
Transgelin
A-kinase anchor protein 17A
Dihydroorotate dehydrogenase (quinone), mitochondrial
2-oxoglutarate dehydrogenase complex component E1
Centromere-associated protein E
Kinesin-like protein KIF23
Methylmalonate-semialdehyde/malonate-semialdehyde dehydrogenase [acylating], mitochondr
Pro-neuregulin-1, membrane-bound isoform
Sterol 26-hydroxylase, mitochondrial
Desmoglein-1
Transcription factor Sp3

Histone H1.1
Large ribosomal subunit protein eL20
Dual specificity mitogen-activated protein kinase kinase 1
Angiopoietin-1 receptor
Peptidyl-prolyl cis-trans isomerase FKBP4
Procollagen-lysine,2-oxoglutarate 5-dioxygenase 1
Nucleobindin-1
Large ribosomal subunit protein eL6
DNA topoisomerase 2-beta
A-kinase anchor protein 12
Mitochondrial 2-oxoglutarate/malate carrier protein
Dystonin
Protein ENL
Histone-lysine N-methyltransferase MECOM
Guanine nucleotide-binding protein subunit alpha-12
Caveolin-1
Aminoacylase-1
Tumor necrosis factor alpha-induced protein 2
Lamin-B2
6-pyruvoyl tetrahydrobiopterin synthase
Urokinase plasminogen activator surface receptor
Mevalonate kinase
DNA excision repair protein ERCC-6
Antigen peptide transporter 1
Antigen peptide transporter 2
CCAAT/enhancer-binding protein zeta
Zinc finger protein 92
Transcription factor p65
UBX domain-containing protein 1
1,4-alpha-glucan-branching enzyme
Eukaryotic translation initiation factor 4 gamma 1
Neurogenic locus notch homolog protein 2
Transducin-like enhancer protein 1
Transducin-like enhancer protein 3
Lactoylglutathione lyase
Activin receptor type-1
Single-stranded DNA-binding protein, mitochondrial
14-3-3 protein eta
Proteolipid protein 2
Cleavage stimulation factor subunit 1
Ubiquitin-protein ligase E3A
Dynamin-1
Tyrosine-protein phosphatase non-receptor type 12
Focal adhesion kinase 1
Serine/arginine-rich splicing factor 11
Protein kinase C delta type
Caldesmon
Folylpolyglutamate synthase, mitochondrial
CTD small phosphatase-like protein 2
Tyrosine-protein phosphatase non-receptor type 11

3-ketodihydrosphingosine reductase
Amidophosphoribosyltransferase
Glutamine--fructose-6-phosphate aminotransferase [isomerizing] 1
Exosome complex component RRP45
Proteasome activator complex subunit 1
Recombining binding protein suppressor of hairless
Myocyte-specific enhancer factor 2C
Amyloid beta precursor like protein 2
GA-binding protein alpha chain
E3 ubiquitin-protein ligase RING1
Fragile X messenger ribonucleoprotein 1
Peroxiredoxin-1
Large ribosomal subunit protein eL18
Complement component 1 Q subcomponent-binding protein, mitochondrial
Cytoskeleton-associated protein 4
Tight junction protein ZO-1
mRNA decay activator protein ZFP36L1
Sperm-associated antigen 1
KH domain-containing, RNA-binding, signal transduction-associated protein 1
Apoptosis regulator BAX
Bcl-2-like protein 1
Induced myeloid leukemia cell differentiation protein Mcl-1
DNA polymerase epsilon catalytic subunit A
Kinesin light chain 1
Son of sevenless homolog 1
Serine/arginine-rich splicing factor 1
Rho GTPase-activating protein 1
TLE family member 5
Serine/arginine-rich splicing factor 4
Protocadherin-1
Protein-glutamine gamma-glutamyltransferase E
Protein phosphatase 3 catalytic subunit alpha
ATP-dependent RNA helicase A
Quinone oxidoreductase
Golgin subfamily A member 3
Golgin subfamily A member 2
Peroxisomal bifunctional enzyme
Lactadherin
3',5'-cyclic-AMP phosphodiesterase 4D
Desmocollin-1
Pseudouridine-5'-phosphatase
Testican-1
Leukocyte surface antigen CD47
Peptidyl-prolyl cis-trans isomerase D
FACT complex subunit SSRP1
Calmodulin-regulated spectrin-associated protein 2
Protein spire homolog 1
Schlafen family member 5
Protein VAC14 homolog
RNA cytosine C(5)-methyltransferase NSUN2

Dystrophia myotonica WD repeat-containing protein
Histone-binding protein RBBP4
Nuclear cap-binding protein subunit 1
Alpha-1,6-mannosylglycoprotein 6-beta-N-acetylglucosaminyltransferase A
Histone acetyltransferase p300
Neuroblast differentiation-associated protein AHNAK
F-BAR domain only protein 2
Elongator complex protein 6
Heat shock 70 kDa protein 14
Secernin-3
Cingulin-like protein 1
Ragulator complex protein LAMTOR4
Nexilin
Alpha-1,6-mannosyl-glycoprotein 2-beta-N-acetylglucosaminyltransferase
Polypeptide N-acetylgalactosaminyltransferase 2
Polypeptide N-acetylgalactosaminyltransferase 1
AP-1 complex subunit beta-1
Cleavage and polyadenylation specificity factor subunit 1
Bone marrow stromal antigen 2
Mitochondrial-processing peptidase subunit alpha
CMP-N-acetylneuraminate-beta-galactosamide-alpha-2,3-sialyltransferase 1
Secernin-1
WASH complex subunit 5
Nuclear pore complex protein Nup160
Sterol regulatory element-binding protein cleavage-activating protein
Forkhead box protein O1
Transducin beta-like protein 3
General transcription factor 3C polypeptide 1
Twinfilin-1
Aspartyl/asparaginyl beta-hydroxylase
Alpha-globin transcription factor CP2
A-kinase anchor protein 13
EGF-containing fibulin-like extracellular matrix protein 1
SWI/SNF-related matrix-associated actin-dependent regulator of chromatin subfamily B membe
Nucleosome-remodeling factor subunit BPTF
Cell division cycle protein 20 homolog
Follistatin-related protein 1
Syntaxin-4
G-rich sequence factor 1
Mitogen-activated protein kinase kinase kinase kinase 2
Tyrosine-protein kinase Mer
Splicing factor, suppressor of white-apricot homolog
Chromodomain-helicase-DNA-binding protein 3
Splicing factor 3A subunit 3
Dihydropyrimidine dehydrogenase [NADP(+)]
TP53-binding protein 1
Transmembrane protein 115
Interferon-related developmental regulator 2
Tripartite motif-containing protein 26
Aminoacyl tRNA synthase complex-interacting multifunctional protein 1

Interleukin enhancer-binding factor 2
Interleukin enhancer-binding factor 3
Vesicular integral-membrane protein VIP36
Heat shock protein 75 kDa, mitochondrial
TNF receptor-associated factor 2
Disks large homolog 1
Unconventional myosin-Ie
Nuclear inhibitor of protein phosphatase 1
Protein tyrosine phosphatase type IVA 2
Active breakpoint cluster region-related protein
GATOR1 complex protein NPRL3
Vesicle transport protein SEC20
BCL2/adenovirus E1B 19 kDa protein-interacting protein 2
Transcriptional repressor NF-X1
Cleavage stimulation factor subunit 3
Tetraspanin-31
Delta(3,5)-Delta(2,4)-dienoyl-CoA isomerase, mitochondrial
Rho GTPase-activating protein 5
Striatin-3
Cell division cycle protein 16 homolog
Serine/threonine-protein kinase 4
Protein flightless-1 homolog
E3 ubiquitin-protein ligase TRIM32
Bifunctional coenzyme A synthase
Large ribosomal subunit protein bL28m
Acetyl-CoA carboxylase 1
COP9 signalosome complex subunit 1
Ubiquitin carboxyl-terminal hydrolase 4
Chromatin assembly factor 1 subunit A
Chromatin assembly factor 1 subunit B
Protein Red
S-methyl-5'-thioadenosine phosphorylase
5'-AMP-activated protein kinase catalytic subunit alpha-1
Liprin-alpha-1
Calcium-binding and coiled-coil domain-containing protein 2
Translation initiation factor eIF2B subunit epsilon
TAR DNA-binding protein 43
Heterogeneous nuclear ribonucleoprotein A0
Aminoacyl tRNA synthase complex-interacting multifunctional protein 2
FAS-associated death domain protein
Peroxiredoxin-4
Dual specificity mitogen-activated protein kinase kinase 5
Mitogen-activated protein kinase 7
Serine/threonine-protein kinase PAK 2
Chromobox protein homolog 3
Serine/threonine-protein kinase 3
Syntaxin-5
26S proteasome non-ATPase regulatory subunit 2
Multimerin-1
Probable ATP-dependent RNA helicase DDX10

DnaJ homolog subfamily C member 3
Methanethiol oxidase
Nucleoside diphosphate kinase 3
Serine/arginine-rich splicing factor 9
Serine/arginine-rich splicing factor 5
Serine/arginine-rich splicing factor 6
Mitotic spindle assembly checkpoint protein MAD2A
Transcription intermediary factor 1-beta
Semaphorin-3F
Syntaxin-3
Ras GTPase-activating protein-binding protein 1
N-myc-interactor
Inactive tyrosine-protein kinase 7
Polyadenylate-binding protein 4
Serine-protein kinase ATM
Growth factor receptor-bound protein 10
Metastasis-associated protein MTA1
Eukaryotic translation initiation factor 3 subunit I
RING-type E3 ubiquitin-protein ligase PPIL2
Serine/threonine-protein phosphatase 2A 56 kDa regulatory subunit gamma isoform
C-terminal-binding protein 1
Phosducin-like protein
Phospholipase D1
Probable methyltransferase TARBP1
Ubiquitin-conjugating enzyme E2 variant 1
Large ribosomal subunit protein mL49
Cytoplasmic dynein 1 intermediate chain 2
Origin recognition complex subunit 2
Integrin-linked protein kinase
NAD(P) transhydrogenase, mitochondrial
Beta-2-syntrophin
DNA repair protein XRCC4
Peptidyl-prolyl cis-trans isomerase G
Treacle protein
Protein unc-119 homolog A
Splicing factor 3B subunit 2
Protein OS-9
Golgin subfamily A member 4
28 kDa heat- and acid-stable phosphoprotein
Disintegrin and metalloproteinase domain-containing protein 9
Disintegrin and metalloproteinase domain-containing protein 15
Transmembrane emp24 domain-containing protein 1
Peptidyl-prolyl cis-trans isomerase FKBP5
Unconventional myosin-IXb
Rho-associated protein kinase 1
Nuclear factor of activated T-cells, cytoplasmic 2
DNA topoisomerase 3-alpha
Mothers against decapentaplegic homolog 4
V-type proton ATPase 116 kDa subunit a 3
Baculoviral IAP repeat-containing protein 2

Phosphatidylinositol-binding clathrin assembly protein
Myotubularin
Sequestosome-1
Metaxin-1
Tubulin beta-3 chain
Acid ceramidase
Serine/threonine-protein kinase PRP4 homolog
Peptidyl-prolyl cis-trans isomerase NIMA-interacting 1
Serine/threonine-protein kinase ATR
Eukaryotic translation initiation factor 4E-binding protein 1
Receptor-interacting serine/threonine-protein kinase 1
Histone deacetylase 1
Calcium/calmodulin-dependent protein kinase type II subunit gamma
Calcium/calmodulin-dependent protein kinase type II subunit delta
Dynactin subunit 2
Polycystin-2
NEDD8-activating enzyme E1 regulatory subunit
Inositol-tetrakisphosphate 1-kinase
SNW domain-containing protein 1
Diacylglycerol kinase zeta
Stromal interaction molecule 1
GRB2-related adapter protein
Transformer-2 protein homolog alpha
Sorting nexin-1
KRR1 small subunit processome component homolog
Periodic tryptophan protein 1 homolog
Myotubularin-related protein 1
Myotubularin-related protein 2
Cullin-1
Cullin-2
Cullin-3
Cullin-4A
Cullin-4B
Apoptosis-stimulating of p53 protein 2
Dual specificity tyrosine-phosphorylation-regulated kinase 1A
GDP-L-fucose synthase
Ras-related protein Rab-31
Ras-related protein Rab-32
Four and a half LIM domains protein 1
Four and a half LIM domains protein 3
Ras and Rab interactor 1
Angio-associated migratory cell protein
Mannosyl-oligosaccharide glucosidase
CD166 antigen
THO complex subunit 5 homolog
Protein Shroom2
Spectrin alpha chain, non-erythrocytic 1
Nucleolar GTP-binding protein 2
Methylglutaconyl-CoA hydratase, mitochondrial
Spliceosome RNA helicase DDX39B

Bleomycin hydrolase
Exosome complex component RRP4
Bone morphogenetic protein receptor type-2
Tubulin beta-2A chain
General transcription factor IIH subunit 2
General transcription factor IIH subunit 3
Bystin
Rap guanine nucleotide exchange factor 1
Isopentenyl-diphosphate Delta-isomerase 1
Core-binding factor subunit beta
Nuclear transcription factor Y subunit gamma
Cyclin-dependent kinase 13
Cytoskeleton-associated protein 5
Cold-inducible RNA-binding protein
Calcium/calmodulin-dependent protein kinase type 1
Coactosin-like protein
Heterogeneous nuclear ribonucleoprotein D0
Lysosome membrane protein 2
Nidogen-2
Low-density lipoprotein receptor-related protein 8
Dystroglycan 1
Vascular endothelial zinc finger 1
Ribosome biogenesis protein BOP1
Ubiquitin conjugation factor E4 A
Septin-6
Kelch-like ECH-associated protein 1
Unhealthy ribosome biogenesis protein 2 homolog
Probable ATP-dependent RNA helicase DHX34
MORC family CW-type zinc finger protein 3
Scaffold attachment factor B2
Eukaryotic translation initiation factor 3 subunit A
Rho guanine nucleotide exchange factor 7
Protein EFR3 homolog A
Ubiquitin-associated protein 2-like
Protein scribble homolog
ARF GTPase-activating protein GIT2
Scavenger receptor class F member 1
Malectin
Tubulin--tyrosine ligase-like protein 12
DNA polymerase alpha subunit B
Dedicator of cytokinesis protein 1
Transcription factor Dp-1
Four and a half LIM domains protein 2
Dihydropyrimidinase-related protein 3
Large ribosomal subunit protein mL62
Zinc finger MYM-type protein 3
Dynactin subunit 1
Cytoplasmic dynein 1 heavy chain 1
Translation initiation factor eIF2B subunit alpha
Eukaryotic initiation factor 4A-II

Elongin-A
Src substrate cortactin
Endonuclease G, mitochondrial
Flotillin-2
Reticulocalbin-2
E3 ubiquitin/ISG15 ligase TRIM25
Filamin-C
Peptidyl-prolyl cis-trans isomerase FKBP8
Protein FAM50A
Protein FRG1
Guanine nucleotide-binding protein subunit alpha-13
Guanidinoacetate N-methyltransferase
UDP-glucose 4-epimerase
Transforming growth factor beta activator LRRC32
Caprin-1
Beclin-1
RNA-binding protein 39
Helicase-like transcription factor
Squalene monooxygenase
Protein disulfide-isomerase A5
Phosphoribosyl pyrophosphate synthase-associated protein 1
ATP-dependent RNA helicase DHX8
DNA replication licensing factor MCM6
Inositol 1,4,5-trisphosphate receptor type 2
Inositol 1,4,5-trisphosphate receptor type 3
Inositol 1,4,5-trisphosphate receptor type 1
Ras GTPase-activating protein 3
Plastin-1
Interferon regulatory factor 3
EKC/KEOPS complex subunit LAGE3
Bridge-like lipid transfer protein family member 2
E3 ubiquitin-protein ligase TRIP12
Pumilio homolog 1
Mediator of DNA damage checkpoint protein 1
Clathrin interactor 1
KN motif and ankyrin repeat domain-containing protein 1
Structural maintenance of chromosomes protein 1A
Ribosomal RNA processing protein 1 homolog B
Disco-interacting protein 2 homolog A
Protein RRP5 homolog
DNA replication complex GINS protein PSF1
Ribosome biogenesis protein BMS1 homolog
Phosphatidate phosphatase LPIN1
Ubiquitin carboxyl-terminal hydrolase 10
LRP chaperone MESD
Neutral alpha-glucosidase AB
Raftlin
Major facilitator superfamily domain-containing protein 10
Serine/threonine-protein phosphatase 2A 56 kDa regulatory subunit delta isoform
Delta(14)-sterol reductase LBR

Conserved oligomeric Golgi complex subunit 2
Major vault protein
Latent-transforming growth factor beta-binding protein 1
Latent-transforming growth factor beta-binding protein 2
Chromobox protein homolog 2
Golgin subfamily B member 1
Caspase-8
N-alpha-acetyltransferase 30
Kinesin-like protein KIF22
Myocyte-specific enhancer factor 2D
Chromodomain-helicase-DNA-binding protein 4
LIM and SH3 domain protein 1
Prostaglandin reductase 1
Dr1-associated corepressor
Nuclear factor of activated T-cells, cytoplasmic 4
Zinc finger protein 638
Importin subunit beta-1
Nucleolar and coiled-body phosphoprotein 1
Nuclear mitotic apparatus protein 1
Proteasome activator complex subunit 4
Cullin-7
Sarcolemmal membrane-associated protein
GTPase-activating protein and VPS9 domain-containing protein 1
N-alpha-acetyltransferase 25, NatB auxiliary subunit
Ventricular zone-expressed PH domain-containing protein homolog 1
Condensin complex subunit 2
PCNA-associated factor
Signal peptidase complex subunit 2
ER membrane protein complex subunit 2
Pre-mRNA-splicing regulator WTAP
26S proteasome non-ATPase regulatory subunit 6
Homocysteine-responsive endoplasmic reticulum-resident ubiquitin-like domain member 1 prot
MAD2L1-binding protein
Mortality factor 4-like protein 2
BRISC complex subunit Abraxas 2
Septin-2
Squamous cell carcinoma antigen recognized by T-cells 3
Condensin complex subunit 1
Polycomb protein SUZ12
Exosome complex component RRP42
TNFAIP3-interacting protein 1
116 kDa U5 small nuclear ribonucleoprotein component
Leucine--tRNA ligase, mitochondrial
R3H domain-containing protein 1
Translocating chain-associated membrane protein 2
Sorting nexin-17
ADP-ribosylation factor-like protein 6-interacting protein 1
Rab3 GTPase-activating protein catalytic subunit
Metal cation symporter ZIP14
Lysine--tRNA ligase

Histone-lysine N-methyltransferase SETDB1
Leucine-rich repeat-containing protein 14
Ribosome biogenesis regulatory protein homolog
DNA polymerase delta subunit 3
Eukaryotic translation initiation factor 4H
Arf-GAP with coiled-coil, ANK repeat and PH domain-containing protein 2
Kinesin-like protein KIF14
Bromodomain-containing protein 3
WD repeat-containing protein 43
Peroxisomal acyl-coenzyme A oxidase 1
Mitochondrial inner membrane protein OXA1L
Early endosome antigen 1
Protein disulfide-isomerase A6
Platelet-activating factor acetylhydrolase IB subunit alpha1
[Pyruvate dehydrogenase (acetyl-transferring)] kinase isozyme 3, mitochondrial
Astrocytic phosphoprotein PEA-15
3-beta-hydroxysteroid-Delta(8),Delta(7)-isomerase
Phosphomevalonate kinase
Serine/threonine-protein kinase D1
Plectin
Pericentriolar material 1 protein
BOS complex subunit NOMO1
Serum paraoxonase/arylesterase 2
Serine/threonine-protein phosphatase 2A 56 kDa regulatory subunit alpha isoform
Inorganic pyrophosphatase
Prostaglandin E synthase 3
Serine/threonine-protein kinase 38
Non-POU domain-containing octamer-binding protein
Serine/threonine-protein phosphatase 2A activator
Receptor-type tyrosine-protein phosphatase kappa
Periodic tryptophan protein 2 homolog
Rab GTPase-binding effector protein 1
Ras-related protein Rab-35
RNA-binding protein with serine-rich domain 1
Retinoblastoma-binding protein 5
Reticulocalbin-1
RalA-binding protein 1
Lethal(2) giant larvae protein homolog 1
Transcription termination factor 1
Transmembrane emp24 domain-containing protein 2
Poly(rC)-binding protein 1
Poly(rC)-binding protein 2
Elongin-C
Elongin-B
GTP-binding protein Rheb
Ubiquitin-protein ligase E3C
Mitochondrial import receptor subunit TOM20 homolog
Mitochondrial fission regulator 1
Delta(24)-sterol reductase
Splicing factor 3B subunit 3

Pumilio homolog 3
Disks large-associated protein 5
Ras suppressor protein 1
Calponin-3
Ribosomal protein S6 kinase alpha-1
Scaffold attachment factor B1
Splicing factor 3A subunit 2
RNA-binding motif, single-stranded-interacting protein 2
Protein phosphatase 1 regulatory subunit 7
Protein transport protein Sec23A
Protein transport protein Sec23B
Cytohesin-1
Splicing factor 3A subunit 1
Superkiller complex protein 2
Surfeit locus protein 1
Transcription initiation factor TFIID subunit 7
Telomeric repeat-binding factor 2
Microtubule-associated protein RP/EB family member 2
Homeobox protein TGIF1
Na(+)/H(+) exchange regulatory cofactor NHE-RF2
Tumor necrosis factor receptor type 1-associated DEATH domain protein
Translocating chain-associated membrane protein 1
Translin
RISC-loading complex subunit TARBP2
Splicing factor 1
Cdc42-interacting protein 4
Thyroid receptor-interacting protein 11
Pachytene checkpoint protein 2 homolog
Mediator of RNA polymerase II transcription subunit 1
Activating signal cointegrator 1
Probable JmjC domain-containing histone demethylation protein 2C
NF-kappa-B inhibitor beta
Thyroid receptor-interacting protein 6
Tyrosine-protein phosphatase non-receptor type 14
Microtubule-associated protein RP/EB family member 1
ELAV-like protein 1
Sterol-4-alpha-carboxylate 3-dehydrogenase, decarboxylating
Myosin light chain kinase, smooth muscle
TGF-beta-activated kinase 1 and MAP3K7-binding protein 1
Probable E3 ubiquitin-protein ligase HERC1
Neutral amino acid transporter B(0)
Myeloid leukemia factor 2
Mitochondrial import receptor subunit TOM34
Mothers against decapentaplegic homolog 2
Mothers against decapentaplegic homolog 1
Methylsterol monooxygenase 1
Intersectin-1
Tubulin-specific chaperone E
Tubulin-specific chaperone C
Ubiquitin-conjugating enzyme E2 variant 2

Serine/threonine-protein kinase STK11
Syntaxin-binding protein 2
Vesicle-associated membrane protein 3
NEDD8
V-type proton ATPase subunit S1
Vacuolar protein sorting-associated protein 72 homolog
Ras-related protein Rab-11B
Zyxin
Electron transfer flavoprotein-ubiquinone oxidoreductase, mitochondrial
Septin-7
Proteasomal ubiquitin receptor ADRM1
Coiled-coil domain-containing protein 6
UDP-N-acetylhexosamine pyrophosphorylase
Transcription factor E2F4
Insulin-like growth factor-binding protein 7
Programmed cell death protein 2
Laminin subunit alpha-4
26S proteasome non-ATPase regulatory subunit 5
Serine/threonine-protein kinase N1
Serine/threonine-protein kinase N2
Transcription initiation factor TFIID subunit 12
Cysteine and glycine-rich protein 2
DNA damage-binding protein 1
Serine/threonine-protein phosphatase 2A 56 kDa regulatory subunit epsilon isoform
Mitogen-activated protein kinase 14
Large ribosomal subunit protein uL23m
Hsp90 co-chaperone Cdc37
Dihydropyrimidinase-related protein 2
Synaptophysin-like protein 1
Histone-binding protein RBBP7
Mitogen-activated protein kinase kinase kinase 11
Transcription initiation factor TFIID subunit 9
Calcitonin gene-related peptide type 1 receptor
Bcl-2 homologous antagonist/killer
Serine/arginine-rich splicing factor 7
Cleavage and polyadenylation specificity factor subunit 6
Survival motor neuron protein
Drebrin
Nuclear respiratory factor 1
Fascin
Gamma-interferon-inducible protein 16
2,4-dienoyl-CoA reductase [(3E)-enoyl-CoA-producing], mitochondrial
Alpha-mannosidase 2
NADH dehydrogenase [ubiquinone] 1 alpha subcomplex subunit 5
Ceramide glucosyltransferase
ATP-dependent Clp protease proteolytic subunit, mitochondrial
Thiosulfate sulfurtransferase
Ubiquitin-conjugating enzyme E2 S
Kynurenine--oxoglutarate transaminase 1
Guanylate kinase

Hydroxyacylglutathione hydrolase, mitochondrial
NADH dehydrogenase [ubiquinone] 1 alpha subcomplex subunit 9, mitochondrial
NADP-dependent malic enzyme, mitochondrial
Phosphoenolpyruvate carboxykinase [GTP], mitochondrial
Uridine phosphorylase 1
Discoidin domain-containing receptor 2
Hydroxyacyl-coenzyme A dehydrogenase, mitochondrial
Lanosterol 14-alpha demethylase
UTP--glucose-1-phosphate uridylyltransferase
6-phosphofructo-2-kinase/fructose-2,6-bisphosphatase 3
Thioredoxin reductase 1, cytoplasmic
MICOS complex subunit MIC60
Endothelial cell-specific chemotaxis regulator
Lysine-rich nucleolar protein 1
Heterogeneous nuclear ribonucleoprotein U-like protein 2
Transmembrane protein 132A
Inverted formin-2
Sister chromatid cohesion protein PDS5 homolog A
Protein CLEC16A
Kinesin-like protein KIF7
Rho GTPase-activating protein 31
Methenyltetrahydrofolate synthase domain-containing protein
AP2-associated protein kinase 1
WASH complex subunit 4
DNA excision repair protein ERCC-6-like
Pre-rRNA-processing protein TSR1 homolog
Ral GTPase-activating protein subunit alpha-2
Protein C-mannosyl-transferase DPY19L1
Isoamyl acetate-hydrolyzing esterase 1 homolog
GDP-Man:Man(3)GlcNAc(2)-PP-Dol alpha-1,2-mannosyltransferase
Transcriptional regulator QRICH1
WD40 repeat-containing protein SMU1
Cytoplasmic tRNA 2-thiolation protein 2
Protein-glucosylgalactosylhydroxylysine glucosidase
Leucine-rich repeat flightless-interacting protein 1
CDP-diacylglycerol--glycerol-3-phosphate 3-phosphatidyltransferase, mitochondrial
UPF0711 protein C18orf21
Prolyl 3-hydroxylase 1
tRNA (guanine(37)-N1)-methyltransferase
Echinoderm microtubule-associated protein-like 3
DNA-directed RNA polymerase I subunit RPA43
MAP7 domain-containing protein 1
UDP-N-acetylhexosamine pyrophosphorylase-like protein 1
Solute carrier family 25 member 35
Chromodomain-helicase-DNA-binding protein 9
Triokinase/FMN cyclase
Protein LSM12
Inactive hydroxysteroid dehydrogenase-like protein 1
Mitochondrial 10-formyltetrahydrofolate dehydrogenase
N-acetylglucosamine-1-phosphotransferase subunits alpha/beta

Girdin
Rab-like protein 6
Mitochondrial import inner membrane translocase subunit TIM50
Protein mono-ADP-ribosyltransferase PARP14
Alpha-(1,3)-fucosyltransferase 11
Cytokine-like nuclear factor N-PAC
UPF0489 protein C5orf22
C2 domain-containing protein 3
Vacuolar protein sorting-associated protein 26B
La-related protein 7
NAD kinase 2, mitochondrial
BTB/POZ domain-containing protein KCTD21
Glucoside xylosyltransferase 1
Malonate--CoA ligase ACSF3, mitochondrial
Prolyl endopeptidase-like
TBC1 domain family member 10B
Taperin
Anoctamin-6
Filamin A-interacting protein 1-like
GRIP1-associated protein 1
Zinc-regulated GTPase metalloprotein activator 1F
Protein MIX23
Membralin
Protein FAM98B
Rho GTPase-activating protein 29
Programmed cell death protein 4
Fibronectin type III domain-containing protein 3B
CREB-regulated transcription coactivator 2
Glycerol-3-phosphate acyltransferase 3
Centrosomal protein of 55 kDa
Nuclear cap-binding protein subunit 3
Quinone oxidoreductase PIG3
Novel acetylcholine receptor chaperone
Pleckstrin homology-like domain family A member 2
PDZ and LIM domain protein 3
Protein mono-ADP-ribosyltransferase PARP10
Very-long-chain 3-oxoacyl-CoA reductase
mRNA export factor GLE1
Ubiquitin carboxyl-terminal hydrolase 39
Kelch-like protein 22
Acylglycerol kinase, mitochondrial
Histone-lysine N-methyltransferase SETMAR
Endoribonuclease LACTB2
Pyrroline-5-carboxylate reductase 3
EARP and GARP complex-interacting protein 1
Borealin
Cordon-bleu protein-like 1
HCLS1-binding protein 3
Plasma membrane ascorbate-dependent reductase CYBRD1
WD repeat-containing protein 81

Beta-actin-like protein 2
Coiled-coil domain-containing protein 93
OCIA domain-containing protein 2
Puratrophin-1
Putative heat shock protein HSP 90-alpha A4
DDB1- and CUL4-associated factor 6
Transmembrane protein 41B
Sigma intracellular receptor 2
Protein YIF1B
DBIRD complex subunit ZNF326
Nucleolar MIF4G domain-containing protein 1
RILP-like protein 1
DnaJ homolog subfamily C member 21
Tumor necrosis factor alpha-induced protein 8-like protein 3
Probable E3 ubiquitin-protein ligase HERC4
GPI ethanolamine phosphate transferase 2
Serine/threonine-protein phosphatase 6 regulatory subunit 3
Probable ATP-dependent RNA helicase DDX60-like
Rab-like protein 3
Protein FAM76B
2-methoxy-6-polyprenyl-1,4-benzoquinol methylase, mitochondrial
SH3 domain-containing protein 19
NHS-like protein 2
ER membrane protein complex subunit 4
Nondiscriminating glutamyl-tRNA synthetase EARS2, mitochondrial
Uncharacterized protein C3orf38
EEF1A lysine methyltransferase 2
Transport and Golgi organization protein 1 homolog
Presequence protease, mitochondrial
WD repeat-containing protein 44
Protein PRRC2B
Tight junction-associated protein 1
RRP12-like protein
Cytochrome c oxidase assembly factor 6 homolog
Torsin-1A-interacting protein 1
Centrosomal protein of 78 kDa
Alanine--tRNA ligase, mitochondrial
Torsin-2A
PCI domain-containing protein 2
Intracellular hyaluronan-binding protein 4
Long-chain fatty acid transport protein 3
Sterile alpha motif domain-containing protein 9
Serine/threonine-protein phosphatase 4 regulatory subunit 3B
WD repeat domain phosphoinositide-interacting protein 3
WD repeat domain phosphoinositide-interacting protein 1
EGF domain-specific O-linked N-acetylglucosamine transferase
Protein Smaug homolog 2
Tubulin-specific chaperone cofactor E-like protein
Deoxynucleotidyltransferase terminal-interacting protein 2
Rab GTPase-activating protein 1-like

Tetratricopeptide repeat protein 38
Cytochrome c oxidase assembly protein COX20, mitochondrial
Exosome complex component MTR3
Transmembrane protein 201
Alpha-tubulin N-acetyltransferase 1
Synaptosomal-associated protein 47
Nucleoporin NUP188
Heterochromatin protein 1-binding protein 3
Valine--tRNA ligase, mitochondrial
Centrosomal protein of 170 kDa
Low density lipoprotein receptor adapter protein 1
Protein odr-4 homolog
Polynucleotide 5'-hydroxyl-kinase NOL9
NHS-like protein 1
Putative hydroxypyruvate isomerase
Tumor protein p63-regulated gene 1-like protein
Coiled-coil and C2 domain-containing protein 1B
Formin-binding protein 1-like
Probable arginine--tRNA ligase, mitochondrial
Acyl-coenzyme A thioesterase THEM4
Putative coiled-coil-helix-coiled-coil-helix domain-containing protein CHCHD2P9, mitochondrial
FK506-binding protein 15
Zinc finger CCCH domain-containing protein 13
Putative methyltransferase C9orf114
Armadillo-like helical domain-containing protein 3
CSC1-like protein 2
G patch domain-containing protein 4
Putative transferase CAF17, mitochondrial
E3 ubiquitin-protein ligase HECTD3
Inactive glycosyltransferase 25 family member 3
E3 ubiquitin-protein ligase UBR4
Syntaxin-binding protein 5
Rho GTPase-activating protein 21
Calmodulin-regulated spectrin-associated protein 1
Large ribosomal subunit protein uL2m
Ubiquitin-associated protein 2
Queuosine 5'-phosphate N-glycosylase/hydrolase
Keratinocyte proline-rich protein
Zinc finger protein 618
Acyl-CoA-binding domain-containing protein 5
RNA-binding protein 26
ATPase family AAA domain-containing protein 3B
Protein wntless homolog
Integrator complex subunit 11
Ceramide-1-phosphate transfer protein
DDB1- and CUL4-associated factor 8
Collagen alpha-1(XIII) chain
Terminal uridylyltransferase 4
Protein furry homolog
Ribonuclease H2 subunit B

ATP synthase mitochondrial F1 complex assembly factor 1
Roquin-1
Protein DDI1 homolog 2
Protein THEMIS2
Arginine-hydroxylase NDUFAF5, mitochondrial
5'-nucleotidase domain-containing protein 1
Brefeldin A-inhibited guanine nucleotide-exchange protein 3
Intermembrane lipid transfer protein VPS13D
Rootletin
Complex III assembly factor LYRM7
Ras-interacting protein 1
Telomere-associated protein RIF1
Vacuolar protein sorting-associated protein 53 homolog
Striatin-interacting protein 1
Serine/threonine-protein kinase MRCK alpha
Regulation of nuclear pre-mRNA domain-containing protein 2
E3 ubiquitin-protein ligase RNF220
Pre-mRNA-splicing factor 38B
E3 ubiquitin-protein ligase BRE1A
Myomegalin
Zinc finger protein 318
Protein FAM171A1
Divergent protein kinase domain 1B
Leucine-rich repeat and calponin homology domain-containing protein 2
Threonylcarbamoyladenosine tRNA methylthiotransferase
BRO1 domain-containing protein BROX
Focadhesin
Lysine-specific demethylase 9
Disabled homolog 2-interacting protein
Lysophospholipase-like protein 1
Platelet endothelial aggregation receptor 1
Proteasome adapter and scaffold protein ECM29
Terminal uridylyltransferase 7
SH2 domain-containing adapter protein E
DENN domain-containing protein 4C
N-alpha-acetyltransferase 35, NatC auxiliary subunit
Zinc finger MYM-type protein 4
FHF complex subunit HOOK interacting protein 2A
Palmitoyltransferase ZDHHC20
SPRY domain-containing protein 7
F-box/WD repeat-containing protein 9
CD276 antigen
E3 ubiquitin-protein ligase RNF213
Tensin-2
KN motif and ankyrin repeat domain-containing protein 2
WASH complex subunit 2A
Lysophospholipid acyltransferase LPCAT4
Metalloreductase STEAP3
Protein FAM91A1
TBC1 domain family member 9B

Microtubule-associated protein 1S
Serine/threonine-protein phosphatase 2A 55 kDa regulatory subunit B delta isoform
ADP-ribosylation factor-like protein 6-interacting protein 4
Autophagy-related protein 16-1
Heparan-alpha-glucosaminide N-acetyltransferase
AT-rich interactive domain-containing protein 2
U3 small nucleolar RNA-associated protein 25 homolog
Glycosyltransferase 8 domain-containing protein 1
Protein sel-1 homolog 3
Tensin-3
HAUS augmin-like complex subunit 3
Protein SPT2 homolog
Acyl-coenzyme A thioesterase MBLAC2
Very large A-kinase anchor protein
Integrator complex subunit 3
Rho GTPase-activating protein 17
Protein NCBP2AS2
CWF19-like protein 1
Protein virilizer homolog
Cytospin-A
HEAT repeat-containing protein 6
Ankyrin repeat domain-containing protein 40
Protein WWC2
Scavenger receptor class A member 3
Atlastin-3
Ribosomal protein uL30-like
Nucleoredoxin
Tetratricopeptide repeat protein 19, mitochondrial
IQ motif and SEC7 domain-containing protein 1
Anamorsin
AN1-type zinc finger protein 6
Histone-lysine N-trimethyltransferase SMYD5
OTU domain-containing protein 7B
THO complex subunit 7 homolog
Glutamine-dependent NAD(+) synthetase
Elongator complex protein 2
Ragulator complex protein LAMTOR1
Dehydrogenase/reductase SDR family member 7B
Twinfilin-2
Condensin-2 complex subunit H2
Uncharacterized protein KIAA0930
Transport and Golgi organization protein 2 homolog
Serine/threonine-protein phosphatase 4 regulatory subunit 3A
Ras-related protein Rab-12
DENN domain-containing protein 5A
Splicing regulator SDE2
Keratin, type II cytoskeletal 80
Nipped-B-like protein
Mitochondrial adenyl nucleotide antiporter SLC25A25
2',5'-phosphodiesterase 12

N-alpha-acetyltransferase 16, NatA auxiliary subunit
Putative DENN domain-containing protein 10 B
Rhomboid domain-containing protein 2
Mitochondrial adenyl nucleotide antiporter SLC25A24
Receptor expression-enhancing protein 3
All-trans-retinol 13,14-reductase
RAD50-interacting protein 1
Transmembrane protein 214
3-hydroxyisobutyryl-CoA hydrolase, mitochondrial
RWD domain-containing protein 4
Nucleolus and neural progenitor protein
Armadillo repeat-containing protein 6
TELO2-interacting protein 2
Ankyrin repeat domain-containing protein 54
Zinc transporter 6
Transmembrane anterior posterior transformation protein 1 homolog
KN motif and ankyrin repeat domain-containing protein 3
Bifunctional arginine demethylase and lysyl-hydroxylase JMJD6
Phostensin
Caveolae-associated protein 1
Zinc finger CCHC domain-containing protein 8
Putative ATP-dependent RNA helicase DHX57
Endoplasmic reticulum aminopeptidase 2
Lysophospholipid acyltransferase 5
Parafibromin
Large ribosomal subunit protein uL14m
Long-chain fatty acid transport protein 4
Coiled-coil and C2 domain-containing protein 1A
Deoxyribonuclease TATDN1
tRNA N(3)-methylcytidine methyltransferase METTL2B
Myb/SANT-like DNA-binding domain-containing protein 2
Transcription initiation factor TFIID subunit 2
UPF0598 protein C8orf82
Mediator of RNA polymerase II transcription subunit 27
Enhancer of mRNA-decapping protein 4
Centrosomal protein of 85 kDa
Protein arginine N-methyltransferase 9
Pre-mRNA-processing-splicing factor 8
F-box only protein 42
SCY1-like protein 2
Tetratricopeptide repeat protein 27
Sideroflexin-4
Protein GOLM2
Nuclear factor related to kappa-B-binding protein
Acylpyruvase FAHD1, mitochondrial
Ribosomal protein eL22-like
Serine/threonine-protein kinase N3
RNA demethylase ALKBH5
Pyridoxal-dependent decarboxylase domain-containing protein 1
MTOR-associated protein MEAK7

Integrator complex subunit 5
GTPase IMAP family member 6
Long-chain fatty acid transport protein 1
Glucose 1,6-bisphosphate synthase
RNA polymerase-associated protein CTR9 homolog
Alpha- and gamma-adaptin-binding protein p34
Transmembrane protein 88
Proline/serine-rich coiled-coil protein 1
Superkiller complex protein 3
Serine/threonine-protein kinase ULK3
Aspartate--tRNA ligase, mitochondrial
Transmembrane protein 65
INO80 complex subunit C
F-box only protein 38
Neutral cholesterol ester hydrolase 1
Fidgetin-like protein 1
E3 ubiquitin-protein ligase TRIM65
Inactive rhomboid protein 2
Mitotic deacetylase-associated SANT domain protein
BRCA1-associated ATM activator 1
GATOR2 complex protein WDR59
Zinc finger CCCH domain-containing protein 14
Coiled-coil domain-containing protein 137
2-oxoglutarate and iron-dependent oxygenase domain-containing protein 3
Thioredoxin domain-containing protein 11
La-related protein 1
Protein TMED8
Proton-coupled zinc antiporter SLC30A9, mitochondrial
E3 ubiquitin-protein ligase TRAF7
Rapamycin-insensitive companion of mTOR
WD repeat-containing protein 74
Type-1 angiotensin II receptor-associated protein
SWI/SNF-related matrix-associated actin-dependent regulator of chromatin subfamily D membe
Monofunctional C1-tetrahydrofolate synthase, mitochondrial
Aftiphilin
Pre-mRNA 3'-end-processing factor FIP1
CREB-regulated transcription coactivator 3
CREB-regulated transcription coactivator 1
Cytochrome P450 20A1
Protein O-glucosyltransferase 2
Transmembrane protein 205
E3 ubiquitin-protein ligase LRSAM1
Golgi-associated kinase 1B
Lysocardiolipin acyltransferase 1
BRCA1-A complex subunit Abraxas 1
Spliceosome-associated protein CWC27 homolog
Protein disulfide isomerase CRELD2
WD repeat-containing protein 82
MICOS complex subunit MIC27
Protocadherin Fat 4

Dipeptidyl peptidase 8
Activating transcription factor 7-interacting protein 1
Ran-binding protein 10
Phosphofurin acidic cluster sorting protein 1
Myosin phosphatase Rho-interacting protein
RNA-binding motif, single-stranded-interacting protein 3
Nicotinate phosphoribosyltransferase
Dynamin-binding protein
Very-long-chain (3R)-3-hydroxyacyl-CoA dehydratase 2
Beta-1,3-glucosyltransferase
GRB10-interacting GYF protein 2
CD109 antigen
tRNA (32-2'-O)-methyltransferase regulator THADA
Hydroxysteroid dehydrogenase-like protein 2
Kynurenine--oxoglutarate transaminase 3
Protein phosphatase 1 regulatory subunit 21
Thrombospondin type-1 domain-containing protein 4
Nesprin-3
Zinc finger protein 574
NF-X1-type zinc finger protein NFXL1
Neurobeachin-like protein 2
FYVE, RhoGEF and PH domain-containing protein 5
Sulfhydryl oxidase 2
Helicase SRCAP
Rho family-interacting cell polarization regulator 1
Uncharacterized protein FLJ45252
Rho guanine nucleotide exchange factor 18
TBC1 domain family member 9
E3 ubiquitin-protein ligase UBR3
Transmembrane protein with metallophosphoesterase domain
Capping protein-inhibiting regulator of actin dynamics
Coiled-coil domain-containing protein 9B
8-oxo-dGDP phosphatase NUDT18
PAX-interacting protein 1
Syntaxin-binding protein 4
Protein O-mannosyl-transferase TMTC3
Intermembrane lipid transfer protein VPS13C
Ubiquitin carboxyl-terminal hydrolase 34
Ras-associated and pleckstrin homology domains-containing protein 1
MOB kinase activator 2
Inhibitor of nuclear factor kappa-B kinase-interacting protein
Phosphatidylinositol 3,4,5-trisphosphate-dependent Rac exchanger 2 protein
Protein FRA10AC1
Ubiquitin-conjugating enzyme E2 R2
Mediator of RNA polymerase II transcription subunit 13-like
La-related protein 4
Mediator of RNA polymerase II transcription subunit 25
Ribosomal protein eS27-like
E3 ubiquitin-protein ligase Hakai
Integrator complex subunit 8

Butyrophilin subfamily 2 member A1
Transcription elongation factor SPT6
Staphylococcal nuclease domain-containing protein 1
Serine/threonine-protein kinase MARK2
Cytochrome c oxidase assembly protein COX15 homolog
Probable ATP-dependent RNA helicase DDX46
Protein RUFY3
tRNA methyltransferase 10 homolog C
eIF5-mimic protein 2
Vacuolar fusion protein MON1 homolog B
Volume-regulated anion channel subunit LRRC8D
Isoaspartyl peptidase/L-asparaginase
BTB/POZ domain-containing protein KCTD9
ATP-dependent RNA helicase DHX30
Eukaryotic translation initiation factor 3 subunit M
7SK snRNA methylphosphate capping enzyme
Armadillo repeat-containing X-linked protein 2
Probable proline--tRNA ligase, mitochondrial
Arginine/serine-rich coiled-coil protein 2
Ras-related GTP-binding protein A
Cytoplasmic FMR1-interacting protein 1
Protein arginine methyltransferase NDUFAF7, mitochondrial
Golgi to ER traffic protein 4 homolog
COP9 signalosome complex subunit 6
Lysophosphatidylcholine acyltransferase 2
Mitochondrial enolase superfamily member 1
E3 ubiquitin-protein transferase MAEA
EPM2A-interacting protein 1
Putative pre-mRNA-splicing factor ATP-dependent RNA helicase DHX32
Serine/threonine-protein kinase TAO1
FAST kinase domain-containing protein 5, mitochondrial
MOB kinase activator 1B
Lysine-specific demethylase 3B
Charged multivesicular body protein 1b
Ribonucleoside-diphosphate reductase subunit M2 B
Heparan sulfate 2-O-sulfotransferase 1
STE20-related kinase adapter protein alpha
[F-actin]-monooxygenase MICAL3
Dymeclin
PHD finger-like domain-containing protein 5A
Peroxiredoxin-like 2C
Aprataxin
Endoplasmic reticulum metallopeptidase 1
G protein-regulated inducer of neurite outgrowth 1
TRMT1-like protein
Zinc finger CCCH-type antiviral protein 1
Large ribosomal subunit protein bL21m
Elongation factor-like GTPase 1
Probable helicase senataxin
Trafficking protein particle complex subunit 11

KAT8 regulatory NSL complex subunit 1
Nucleoporin p54
Autophagy-related protein 9A
D-glutamate cyclase, mitochondrial
Coiled-coil domain-containing protein 186
LisH domain-containing protein ARMC9
VPS35 endosomal protein-sorting factor-like
Pogo transposable element with ZNF domain
Zinc finger FYVE domain-containing protein 16
Protein MON2 homolog
C-myc promoter-binding protein
Myosin-14
FMR1-interacting protein NUFIP2
SUZ domain-containing protein 1
Mitochondrial antiviral-signaling protein
CLIP-associating protein 1
ATP-dependent RNA helicase DHX29
Nephrocystin-3
Low-density lipoprotein receptor-related protein 10
HAUS augmin-like complex subunit 6
Protein O-glucosyltransferase 3
Tetratricopeptide repeat protein 21B
HEAT repeat-containing protein 3
Kinesin-like protein KIF21A
Hepatoma-derived growth factor-related protein 2
L-xylulose reductase
BRCA1-associated protein
BRCA2-interacting transcriptional repressor EMSY
Golgin subfamily A member 7
Rho GTPase-activating protein 22
Wings apart-like protein homolog
Interferon regulatory factor 2-binding protein 2
E3 ubiquitin-protein ligase RBBP6
E3 ubiquitin-protein ligase SH3RF1
Protein TAMALIN
FERM domain-containing protein 5
tRNA-splicing endonuclease subunit Sen54
Protein prenyltransferase alpha subunit repeat-containing protein 1
Arpin
Tectonin beta-propeller repeat-containing protein 1
Rab9 effector protein with kelch motifs
Transcription termination factor 4, mitochondrial
E3 ubiquitin-protein ligase HUWE1
YTH domain-containing family protein 3
Cytoplasmic tRNA 2-thiolation protein 1
Ubiquitin-conjugating enzyme E2 Q1
Large ribosomal subunit protein mL55
Transmembrane emp24 domain-containing protein 4
Large ribosomal subunit protein uL10m
COX assembly mitochondrial protein homolog

Centromere protein V
Schlafen family member 11
Multiple epidermal growth factor-like domains protein 8
Transmembrane protein 179B
N-acetylgalactosaminyltransferase 7
Stearoyl-CoA desaturase 5
Pleckstrin homology-like domain family B member 2
Adhesion G-protein coupled receptor G6
Dehydrodolichyl diphosphate synthase complex subunit DHDDS
Polypeptide N-acetylgalactosaminyltransferase 10
Trafficking protein particle complex subunit 6B
Type 1 phosphatidylinositol 4,5-bisphosphate 4-phosphatase
C-type lectin domain family 14 member A
Protein PAT1 homolog 1
Dipeptidyl peptidase 9
E3 ubiquitin-protein ligase synoviolin
tRNA 2'-phosphotransferase 1
Actin-histidine N-methyltransferase
Tetratricopeptide repeat protein 7B
Acyl-coenzyme A thioesterase 1
Nucleolar protein 9
Polyadenylate-binding protein 2
N6-adenosine-methyltransferase catalytic subunit
Protein polybromo-1
Threonylcarbamoyl-AMP synthase
Pre-mRNA-processing factor 39
Protein LYRIC
E3 ubiquitin-protein ligase ZNF598
Glycerol-3-phosphate acyltransferase 4
Kinectin
Telomerase-binding protein EST1A
NLR family member X1
B-cell CLL/lymphoma 9-like protein
Pleckstrin homology-like domain family B member 1
Ubiquitin carboxyl-terminal hydrolase 48
Calcium-dependent secretion activator 2
Fermitin family homolog 3
N-alpha-acetyltransferase 40
5'-nucleotidase domain-containing protein 3
Acetoacetyl-CoA synthetase
Leucine zipper protein 1
THO complex subunit 4
Integral membrane protein GPR180
FHF complex subunit HOOK-interacting protein 2B
Ras GTPase-activating-like protein IQGAP3
Zinc finger CCCH domain-containing protein 18
Vacuolar protein-sorting-associated protein 36
Cullin-associated NEDD8-dissociated protein 1
Reticulophagy regulator 3
Protein Hook homolog 3

SEC14 domain and spectrin repeat-containing protein 1
COMM domain-containing protein 7
Vacuolar fusion protein MON1 homolog A
Archaemetzincin-2
THO complex subunit 6 homolog
RNA N6-adenosine-methyltransferase METTL16
Poly(ADP-ribose) glycohydrolase
Liprin-beta-1
Lon protease homolog 2, peroxisomal
Zinc finger C3HC-type protein 1
Chromodomain-helicase-DNA-binding protein 1-like
F-BAR and double SH3 domains protein 1
Coiled-coil domain-containing protein 25
Proline and serine-rich protein 2
Stimulator of interferon genes protein
Active regulator of SIRT1
Cerebellar degeneration-related protein 2-like
Ral GTPase-activating protein subunit beta
Ras-specific guanine nucleotide-releasing factor RalGPS2
Histone-arginine methyltransferase CARM1
Deaminated glutathione amidase
COMM domain-containing protein 2
HEAT repeat-containing protein 5A
Condensin-2 complex subunit G2
Protein phosphatase 1 regulatory subunit 3B
F-box only protein 11
Ankyrin repeat and LEM domain-containing protein 2
RNA-binding protein MEX3D
Diacylglycerol kinase eta
ATP-dependent RNA helicase DDX42
Spermatogenesis-associated serine-rich protein 2
Serine/threonine-protein kinase VRK2
CDK2-associated and cullin domain-containing protein 1
NADH dehydrogenase [ubiquinone] 1 alpha subcomplex subunit 11
Dynein axonemal assembly factor 5
Peptidyl-tRNA hydrolase
Syntaxin-12
Transcriptional repressor p66-alpha
Copine-8
G-protein-signaling modulator 1
Ras-related protein Rab-43
C2 domain-containing protein 5
E3 ubiquitin-protein ligase MIB1
Inactive tyrosine-protein kinase PRAG1
BLOC-2 complex member HPS6
Interferon regulatory factor 2-binding protein 1
ELKS/Rab6-interacting/CAST family member 1
Ribosomal oxygenase 2
Palmitoyltransferase ZDHHC13
Palmitoyltransferase ZDHHC17

Cytokine receptor-like factor 3
Trafficking protein particle complex subunit 5
Armadillo repeat-containing protein 8
RELT-like protein 1
Complex I assembly factor TMEM126B, mitochondrial
5'-3' exonuclease PLD3
Protein HID1
Ankyrin repeat and MYND domain-containing protein 2
3'-5' exoribonuclease 1
Inactive serine/threonine-protein kinase VRK3
LIX1-like protein
NudC domain-containing protein 3
Protein AHNAK2
Formin-like protein 3
Methylmalonic aciduria type A protein, mitochondrial
Mitogen-activated protein kinase kinase kinase kinase 3
Neuron navigator 3
Prolyl 3-hydroxylase 3
Coiled-coil domain-containing protein 50
Malonyl-CoA-acyl carrier protein transacylase, mitochondrial
Kinase suppressor of Ras 1
Centrosomal protein of 97 kDa
ATP-dependent (S)-NAD(P)H-hydrate dehydratase
WD repeat-containing protein 75
Mitofusin-1
Choline transporter-like protein 2
Inositol 1,4,5-trisphosphate receptor-interacting protein
WD repeat and FYVE domain-containing protein 1
MAP7 domain-containing protein 3
Coiled-coil domain-containing protein 117
Protein NOXP20
DCN1-like protein 3
Protein DENND6A
MAX gene-associated protein
GRIP and coiled-coil domain-containing protein 2
Zinc finger CCCH domain-containing protein 7A
PHD finger protein 6
Protein archease
Volume-regulated anion channel subunit LRRC8A
Extracellular sulfatase Sulf-2
E3 ubiquitin-protein ligase UBR1
E3 ubiquitin-protein ligase UBR2
Rho GTPase-activating protein 12
Calcium homeostasis endoplasmic reticulum protein
Codanin-1
Ankyrin repeat and KH domain-containing protein 1
SURP and G-patch domain-containing protein 1
SURP and G-patch domain-containing protein 2
Ubiquitin-conjugating enzyme E2 variant 3
Cell division cycle and apoptosis regulator protein 1

Probable ATP-dependent RNA helicase DHX40
DnaJ homolog subfamily C member 10
Negative elongation factor C/D
Mitochondrial Rho GTPase 2
Mitochondrial Rho GTPase 1
NAD-dependent protein deacetylase sirtuin-2
Polyhomeotic-like protein 2
Chromatin complexes subunit BAP18
Large ribosomal subunit protein mL41
Nurim
GPALPP motifs-containing protein 1
Protein mono-ADP-ribosyltransferase PARP9
RNA-binding protein 12B
Solute carrier family 35 member F2
Putative RNA polymerase II subunit B1 CTD phosphatase RPAP2
Patatin-like phospholipase domain-containing protein 6
Structural maintenance of chromosomes protein 5
Probable ATP-dependent RNA helicase DDX60
C-Maf-inducing protein
MICAL-like protein 2
Probable ATP-dependent RNA helicase DHX37
Kelch repeat and BTB domain-containing protein 2
Ribonucleoprotein PTB-binding 1
pre-rRNA 2'-O-ribose RNA methyltransferase FTSJ3
Transmembrane protein 192
Nucleotidyltransferase MB21D2
Serine/arginine repetitive matrix protein 1
Stromal membrane-associated protein 1
DIS3-like exonuclease 2
ATP-dependent RNA helicase SUPV3L1, mitochondrial
Eukaryotic peptide chain release factor GTP-binding subunit ERF3B
ZZ-type zinc finger-containing protein 3
Exocyst complex component 8
Uncharacterized protein C10orf67, mitochondrial
E3 ubiquitin-protein ligase TRIM22
Threonine synthase-like 1
Uncharacterized protein KIAA2013
Calcium uptake protein 2, mitochondrial
Ankyrin repeat domain-containing protein 13A
Phosphatase and actin regulator 4
tRNA (uracil-5-)-methyltransferase homolog A
Pseudouridylate synthase RPUSD2
ELMO domain-containing protein 2
Aldehyde dehydrogenase family 16 member A1
Dyslexia-associated protein KIAA0319-like protein
Adhesion G protein-coupled receptor F5
5'-3' exoribonuclease 1
Proline-, glutamic acid- and leucine-rich protein 1
Abl interactor 1
WD repeat and FYVE domain-containing protein 3

Selenoprotein H
Vitamin K epoxide reductase complex subunit 1-like protein 1
Spartin
Tetratricopeptide repeat protein 5
Mesoderm induction early response protein 1
Regulatory-associated protein of mTOR
ORM1-like protein 3
Cell cycle and apoptosis regulator protein 2
NADH dehydrogenase [ubiquinone] 1 alpha subcomplex assembly factor 2
Vacuolar protein sorting-associated protein 52 homolog
Nuclear pore complex protein Nup93
Serine/threonine-protein kinase 11-interacting protein
Zinc finger protein 687
Cap-specific mRNA (nucleoside-2'-O-)-methyltransferase 1
Leucine-rich repeat-containing protein 47
Dedicator of cytokinesis protein 4
Carbonic anhydrase 13
Rho guanine nucleotide exchange factor 28
Integrator complex subunit 1
Histone H2B type 3-B
Rho GTPase-activating protein 24
Armadillo repeat-containing protein 10
Lysophosphatidylserine lipase ABHD12
CLK4-associating serine/arginine rich protein
Protein FAM43A
Transmembrane protein 256
E3 SUMO-protein ligase PIAS4
Glycerol-3-phosphate dehydrogenase 1-like protein
Solute carrier family 35 member F6
Rho GTPase-activating protein 18
Activating signal cointegrator 1 complex subunit 3
MICAL-like protein 1
Vacuolar protein sorting-associated protein 8 homolog
Protein PALS1
Cohesin subunit SA-2
Synaptopodin
Formin-binding protein 4
Translation factor GUF1, mitochondrial
RING1 and YY1-binding protein
Polypeptide N-acetylgalactosaminyltransferase 4
Misshapen-like kinase 1
Guanosine-3',5'-bis(diphosphate) 3'-pyrophosphohydrolase MESH1
Prostaglandin reductase 3
3-oxoacyl-[acyl-carrier-protein] reductase
ER membrane protein complex subunit 5
Transmembrane protein 199
F-box/LRR-repeat protein 6
Prolyl 3-hydroxylase OGFOD1
Actin filament-associated protein 1
Oxidation resistance protein 1

Zinc finger CCCH-type with G patch domain-containing protein
Calcium homeostasis modulator protein 5
S1 RNA-binding domain-containing protein 1
WD and tetratricopeptide repeats protein 1
NF-kappa-B-activating protein
Macoilin
SH2 domain-containing protein 3C
Arrestin domain-containing protein 1
Dehydrogenase/reductase SDR family member on chromosome X
CDGSH iron-sulfur domain-containing protein 2
Ribonuclease P protein subunit p25-like protein
ATP synthase mitochondrial F1 complex assembly factor 2
Tetratricopeptide repeat protein 9C
Protein jagunal homolog 1
E3 ubiquitin-protein ligase RNF10
Refilin-B
Leucine-zipper-like transcriptional regulator 1
COMM domain-containing protein 1
Cleavage and polyadenylation specificity factor subunit 7
Solute carrier family 15 member 4
Myc target protein 1
ADAMTS-like protein 1
ADP-ribosylation factor GTPase-activating protein 2
Deubiquitinase OTUD6B
Acyl-coenzyme A diphosphatase FITM2
UPF0690 protein C1orf52
eEF1A lysine and N-terminal methyltransferase
ADP-ribosylation factor-like protein 6-interacting protein 6
ADP-ribosylation factor GTPase-activating protein 1
NAD-dependent protein deacylase sirtuin-6
ER membrane protein complex subunit 1
RNA polymerase II-associated factor 1 homolog
Cyclin-Y-like protein 1
Putative E3 ubiquitin-protein ligase UBR7
ATP-dependent RNA helicase DDX51
Uncharacterized protein FAM241A
Mitochondrial basic amino acids transporter
Protein enabled homolog
Discoidin, CUB and LCCL domain-containing protein 1
G patch domain-containing protein 11
Large ribosomal subunit protein mL43
RNA ligase 1
Uncharacterized protein C19orf47
Activating signal cointegrator 1 complex subunit 1
Leucine-rich repeat-containing protein 57
Probable RNA-binding protein EIF1AD
Protein KRI1 homolog
Coiled-coil domain-containing protein 71L
Zinc finger protein 579
Pre-mRNA-splicing factor 38A

Mixed lineage kinase domain-like protein
Glutamine amidotransferase-like class 1 domain-containing protein 1
Serine/threonine-protein phosphatase 6 regulatory ankyrin repeat subunit C
Phospholipid-transporting ATPase IG
ATPase family gene 2 protein homolog A
NHL repeat-containing protein 2
Late secretory pathway protein AVL9 homolog
Equilibrative nucleobase transporter 1
Xyloside xylosyltransferase 1
Golgi membrane protein 1
Procollagen galactosyltransferase 1
Inactive C-alpha-formylglycine-generating enzyme 2
SID1 transmembrane family member 2
Formylglycine-generating enzyme
Protein O-glucosyltransferase 1
Ubiquitin-associated domain-containing protein 2
Prenylcysteine oxidase-like
Transmembrane protein 87A
Retinol dehydrogenase 13
Tetratricopeptide repeat protein 13
Estradiol 17-beta-dehydrogenase 11
Thioredoxin domain-containing protein 5
Kinetochore protein Spc24
Outer mitochondrial transmembrane helix translocase
Saccharopine dehydrogenase-like oxidoreductase
UDP-glucuronic acid decarboxylase 1
E3 ubiquitin-protein ligase RNF149
Reticulophagy regulator 2
SERPINE1 mRNA-binding protein 1
LEM domain-containing protein 2
Nitric oxide-associated protein 1
Adaptin ear-binding coat-associated protein 1
Protein FAM98A
Phospholipase A2 group XV
Myotubularin-related protein 14
NFATC2-interacting protein
Diacylglycerol lipase-beta
Carbohydrate sulfotransferase 14
Polypeptide N-acetylgalactosaminyltransferase 6
E3 ubiquitin-protein ligase RNF169
Pyruvate dehydrogenase phosphatase regulatory subunit, mitochondrial
NAD(P)H-hydrate epimerase
Nonsense-mediated mRNA decay factor SMG8
RING finger protein 214
Protein LSM14 homolog A
GTPase IMAP family member 8
Cyclin-Y
Maestro heat-like repeat-containing protein family member 1
Terminal nucleotidyltransferase 4B
Probable aminopeptidase NPEPL1

EH domain-binding protein 1
Putative RNA-binding protein 15B
Alpha-N-acetylgalactosaminide alpha-2,6-sialyltransferase 3
Trinucleotide repeat-containing gene 6A protein
Polyhomeotic-like protein 3
Divergent protein kinase domain 2A
Metal transporter CNNM3
ATP-binding cassette sub-family F member 1
Calcium uniporter protein, mitochondrial
Phosphatidylinositol 3-kinase catalytic subunit type 3
Glutathione S-transferase C-terminal domain-containing protein
Serum response factor-binding protein 1
Neuroguidin
SHC SH2 domain-binding protein 1
PDZ domain-containing protein 8
DCC-interacting protein 13-beta
Zinc transporter 7
Neuron navigator 1
Periphilin-1
WD repeat-containing protein 19
F-box only protein 22
Lysophosphatidylcholine acyltransferase 1
Zinc finger MIZ domain-containing protein 2
Nesprin-1
Ubiquitin carboxyl-terminal hydrolase 32
Biorientation of chromosomes in cell division protein 1-like 1
AT-rich interactive domain-containing protein 1B
FAD synthase
Folliculin
Nucleoporin Nup43
Nucleoporin Nup37
Nucleoporin NUP35
Cytosolic endo-beta-N-acetylglucosaminidase
Retinoic acid-induced protein 3
Torsin-1A-interacting protein 2
sn-1-specific diacylglycerol lipase ABHD11
N-acylneuraminate cytidylyltransferase
Tetraspanin-14
Tubulin--tyrosine ligase
Small VCP/p97-interacting protein
Atlastin-2
Motile sperm domain-containing protein 2
Putative phospholipase B-like 2
Ras association domain-containing protein 8
ATP-dependent RNA helicase DDX55
Major facilitator superfamily domain-containing protein 8
Tudor domain-containing protein 7
GTPase IMAP family member 7
Protein NEDD1
Nuclear receptor coactivator 7

THO complex subunit 2
WD repeat-containing protein 36
Protein phosphatase PTC7 homolog
Retinitis pigmentosa 9 protein
Vang-like protein 1
Smad nuclear-interacting protein 1
Large ribosomal subunit protein mL64
WD repeat-containing protein 48
Exocyst complex component 6
SWI/SNF complex subunit SMARCC2
Nuclear protein localization protein 4 homolog
Uncharacterized protein CXorf38
Spermatogenesis-associated protein 20
Iron-sulfur cluster transfer protein NUBPL
(Lyso)-N-acylphosphatidylethanolamine lipase
F-box only protein 30
Adenosine 3'-phospho 5'-phosphosulfate transporter 1
Pumilio homolog 2
T-cell immunomodulatory protein
Golgin subfamily A member 5
Kelch domain-containing protein 4
SH3KBP1-binding protein 1
NEDD8-activating enzyme E1 catalytic subunit
Prostamide/prostaglandin F synthase
DnaJ homolog subfamily B member 14
Protein kish-A
Phosphatidylinositol 5-phosphate 4-kinase type-2 gamma
WD repeat-containing protein 20
TBC1 domain family member 15
Retinol dehydrogenase 11
Leucine-rich repeat-containing protein 20
Interferon-induced protein 44
Large ribosomal subunit protein uL30m
AN1-type zinc finger protein 1
Protein CIP2A
Phosphatidylinositol 4-kinase type 2-beta
Dolichyl-diphosphooligosaccharide--protein glycosyltransferase subunit STT3B
Polyribonucleotide nucleotidyltransferase 1, mitochondrial
Signal peptide peptidase-like 2B
Minor histocompatibility antigen H13
Phosphatidylinositol 3,4,5-trisphosphate-dependent Rac exchanger 1 protein
Up-regulator of cell proliferation
Protein bicaudal D homolog 2
Serine/threonine-protein kinase Nek9
Pleckstrin homology domain-containing family O member 2
E3 ubiquitin-protein ligase DTX3L
ATP-dependent RNA helicase DDX54
Disks large homolog 5
Ribosome biogenesis protein BRX1 homolog
Glucosamine-6-phosphate isomerase 2

Volume-regulated anion channel subunit LRRC8C
Serine/threonine-protein kinase Nek7
RB1-inducible coiled-coil protein 1
[F-actin]-monooxygenase MICAL1
Elongator complex protein 5
Protein phosphatase Slingshot homolog 3
SH3 domain and tetratricopeptide repeat-containing protein 1
TBC domain-containing protein kinase-like protein
D-aminoacyl-tRNA deacylase 1
DDB1- and CUL4-associated factor 11
U3 small nucleolar RNA-associated protein 15 homolog
Probable glutathione peroxidase 8
Actin filament-associated protein 1-like 1
E3 ubiquitin-protein ligase SH3RF3
Short transient receptor potential channel 4-associated protein
Centrosomal protein of 192 kDa
Gem-associated protein 5
GPI ethanolamine phosphate transferase 3
Rap guanine nucleotide exchange factor 6
Guanine nucleotide exchange protein SMCR8
Partitioning defective 3 homolog
Importin-4
Ubiquitin carboxyl-terminal hydrolase 33
Arginine/serine-rich protein PNISR
Serine/threonine-protein phosphatase 4 regulatory subunit 1
Ubiquitin-associated and SH3 domain-containing protein B
PDZ domain-containing protein GIPC2
WAS/WASL-interacting protein family member 2
1-acylglycerol-3-phosphate O-acyltransferase ABHD5
Histone-lysine N-methyltransferase SETD7
Nucleolar complex protein 3 homolog
Scavenger receptor class B member 1
Conserved oligomeric Golgi complex subunit 1
Sec1 family domain-containing protein 2
Stromal membrane-associated protein 2
Zinc finger CCCH domain-containing protein 15
Peptidyl-prolyl cis-trans isomerase-like 4
General transcription factor 3C polypeptide 2
TBC1 domain family member 22A
PHD finger protein 10
Ras-related protein Rab-2B
Coiled-coil domain-containing protein 12
RelA-associated inhibitor
Cotranscriptional regulator ARB2A
Protein Churchill
Transforming growth factor-beta receptor-associated protein 1
Transmembrane protein 263
Histone deacetylase 7
Serine/threonine/tyrosine-interacting protein
Phosphatidylglycerophosphatase and protein-tyrosine phosphatase 1

Nuclear pore complex protein Nup133
Programmed cell death 6-interacting protein
Filamin-binding LIM protein 1
Splicing factor Cactin
Glutathione-specific gamma-glutamylcyclotransferase 2
Charged multivesicular body protein 7
Protein THEM6
Probable N-acetyltransferase 14
B-cell CLL/lymphoma 7 protein family member C
MIT domain-containing protein 1
Chromosome transmission fidelity protein 18 homolog
RNA polymerase-associated protein LEO1
Dephospho-CoA kinase domain-containing protein
NudC domain-containing protein 2
U4/U6.U5 small nuclear ribonucleoprotein 27 kDa protein
Dimethyladenosine transferase 1, mitochondrial
Cohesin subunit SA-1
Sec1 family domain-containing protein 1
Tumor necrosis factor alpha-induced protein 8-like protein 1
Soluble calcium-activated nucleotidase 1
Trafficking protein particle complex subunit 12
Heterogeneous nuclear ribonucleoprotein L-like
Fatty acyl-CoA reductase 1
Ubiquitin-like domain-containing CTD phosphatase 1
tRNA-splicing endonuclease subunit Sen15
PEST proteolytic signal-containing nuclear protein
DnaJ homolog subfamily A member 4
SPRY domain-containing protein 4
m-AAA protease-interacting protein 1, mitochondrial
Pseudouridylate synthase TRUB1
LIM domain only protein 7
Choline transporter-like protein 1
Cytoskeleton-associated protein 2
Ataxin-2-like protein
Arf-GAP with Rho-GAP domain, ANK repeat and PH domain-containing protein 3
GTPase IMAP family member 1
U4/U6 small nuclear ribonucleoprotein Prp31
Negative elongation factor B
Palladin
RUN and FYVE domain-containing protein 2
Splicing regulatory glutamine/lysine-rich protein 1
Gem-associated protein 6
Caskin-2
ATR-interacting protein
Paraspeckle component 1
MAP kinase-activating death domain protein
Nesprin-2
Transcriptional repressor p66-beta
DnaJ homolog subfamily C member 9
BRI3-binding protein

Beta-catenin-like protein 1
Cysteine protease ATG4A
Ras and Rab interactor 2
Protein ELYS
Microprocessor complex subunit DGCR8
Roundabout homolog 4
Esterase OVCA2
Immunity-related GTPase family Q protein
DNA damage-binding protein 2
C-type lectin domain family 2 member B
ATP-dependent RNA helicase DDX1
SEC14-like protein 1
Piezo-type mechanosensitive ion channel component 1
Protein FAM3C
Histone H1.10
Proteasome inhibitor PI31 subunit
Golgi-specific brefeldin A-resistance guanine nucleotide exchange factor 1
Nonsense-mediated mRNA decay factor SMG7
RNA polymerase-associated protein RTF1 homolog
Nicastrin
Sorting nexin-19
Transmembrane 9 superfamily member 4
Transmembrane protein 131
Inositol hexakisphosphate kinase 1
Small ribosomal subunit protein mS27
Engulfment and cell motility protein 1
Polyphosphoinositide phosphatase
AP-3 complex subunit sigma-1
Hamartin
UBX domain-containing protein 4
PHD finger protein 3
Protein NDRG1
Heat shock protein 105 kDa
Septin-8
CCR4-NOT transcription complex subunit 9
Acyl-CoA:lysophosphatidylglycerol acyltransferase 1
TBC1 domain family member 5
Zinc finger protein 592
Protein Jade-3
Unconventional myosin-XVIIIa
La-related protein 4B
Stalled ribosome sensor GCN1
Rho GTPase-activating protein 45
Pre-mRNA-splicing factor ATP-dependent RNA helicase PRP16
Nuclear pore complex protein Nup205
Ankyrin repeat and SAM domain-containing protein 1A
Peroxidasin homolog
Protein FAN
GPI-anchor transamidase
Small ribosomal subunit protein mS31

A-kinase anchor protein 1, mitochondrial
Dol-P-Man:Man(5)GlcNAc(2)-PP-Dol alpha-1,3-mannosyltransferase
Neurogranin
Acidic leucine-rich nuclear phosphoprotein 32 family member B
Nectin-2
Geranylgeranyl transferase type-2 subunit alpha
Protein TFG
Serine protease HTRA1
Actin-related protein 2/3 complex subunit 1A
General transcription factor IIH subunit 4
Ras-responsive element-binding protein 1
Histone deacetylase 2
Signal transducing adapter molecule 1
Zinc finger protein ubi-d4
Endoplasmic reticulum protein SC65
CREB-binding protein
Histone acetyltransferase KAT6A
Symplekin
TATA-binding protein-associated factor 2N
Golgin subfamily A member 1
Gamma-glutamyl hydrolase
Neuronal cell adhesion molecule
X-linked retinitis pigmentosa GTPase regulator
Phosphatidylinositol 3,4,5-trisphosphate 5-phosphatase 1
Probable ATP-dependent RNA helicase DDX17
Kinesin-associated protein 3
Caspase-10
Amyloid beta precursor protein binding family B member 2
DNA repair protein RAD50
CUGBP Elav-like family member 1
Osteoclast-stimulating factor 1
Rho guanine nucleotide exchange factor 1
DNA repair endonuclease XPF
Ubiquitin recognition factor in ER-associated degradation protein 1
Golgi apparatus protein 1
Regulator of nonsense transcripts 1
COP9 signalosome complex subunit 5
G-patch domain and KOW motifs-containing protein
SWI/SNF complex subunit SMARCC1
SWI/SNF-related matrix-associated actin-dependent regulator of chromatin subfamily D membe
Ras-related protein Rab-8B
Bcl2-associated agonist of cell death
Far upstream element-binding protein 2
Glutaryl-CoA dehydrogenase, mitochondrial
GTP-binding protein Rit1
Peroxisomal membrane protein PEX13
Transportin-1
Rho guanine nucleotide exchange factor 2
Ribosomal RNA small subunit methyltransferase NEP1
Polyribonucleotide 5'-hydroxyl-kinase Clp1

Glomulin
Ubiquitin carboxyl-terminal hydrolase 13
Segment polarity protein dishevelled homolog DVL-3
Probable ubiquitin carboxyl-terminal hydrolase FAF-X
Ubiquitin carboxyl-terminal hydrolase 7
Cullin-5
Stathmin-2
V-type proton ATPase 116 kDa subunit a 1
Lipoma-preferred partner
RNA-binding protein with multiple splicing
Exostosin-2
Mediator of RNA polymerase II transcription subunit 12
Protein tyrosine phosphatase type IVA 1
Phosphorylase b kinase regulatory subunit beta
Secretory carrier-associated membrane protein 4
BLOC-2 complex member HPS3
SWI/SNF-related matrix-associated actin-dependent regulator of chromatin subfamily E membe
Caveolae-associated protein 3
Myeloid-derived growth factor
Mitochondrial import receptor subunit TOM40B
Protein YIPF5
GPI transamidase component PIG-T
Immunoglobulin superfamily member 8
Ribosomal protein eL42-like
Ras-related protein Rab-24
Oxysterol-binding protein 2
Cytoplasmic 60S subunit biogenesis factor ZNF622
Ribosome-releasing factor 2, mitochondrial
Ubiquitin-conjugating enzyme E2 E3
7-methylguanosine phosphate-specific 5'-nucleotidase
WW domain-binding protein 2
Proteasome assembly chaperone 2
BOS complex subunit NCLN
Mitochondrial ubiquitin ligase activator of NFKB 1
Myocardin-related transcription factor A
Protein lifeguard 3
Endoplasmic reticulum-Golgi intermediate compartment protein 1
U3 small nucleolar RNA-associated protein 4 homolog
FAST kinase domain-containing protein 4
Mitochondrial amidoxime reducing component 2
Protein FAM162A
PAT complex subunit CCDC47
Large ribosomal subunit protein uL24m
Exocyst complex component 4
Protein mago nashi homolog 2
Putative monooxygenase p33MONOX
Protein RFT1 homolog
Isochorismatase domain-containing protein 2
Protein N-terminal asparagine amidohydrolase
Fermitin family homolog 2

Far upstream element-binding protein 1
Tetratricopeptide repeat protein 17
Leucine-rich repeat-containing protein 59
Vesicle transport through interaction with t-SNAREs homolog 1A
Endothelial cell-selective adhesion molecule
Pre-B-cell leukemia transcription factor-interacting protein 1
Mitochondrial calcium uniporter regulator 1
Uncharacterized protein KIAA1143
Vacuolar protein sorting-associated protein 33A
Peptidyl-prolyl cis-trans isomerase FKBP10
Tetratricopeptide repeat protein 28
Exosome complex component RRP43
Zinc finger protein 428
SH3 domain-containing kinase-binding protein 1
Spindle and kinetochore-associated protein 1
E3 ubiquitin-protein ligase RNF25
Axin interactor, dorsalization-associated protein
PIN2/TERF1-interacting telomerase inhibitor 1
ADP-ribosylation factor-like protein 8A
Ubiquitin thioesterase otulin
Small ribosomal subunit protein mS37
Peptidylprolyl isomerase domain and WD repeat-containing protein 1
Uracil phosphoribosyltransferase homolog
Phosphatidate cytidylyltransferase, mitochondrial
MOB kinase activator 3A
Dedicator of cytokinesis protein 10
Autophagy-related protein 2 homolog B
Leukocyte receptor cluster member 1
TBC1 domain family member 20
EF-hand domain-containing protein D2
Galactose mutarotase
Synaptotagmin-like protein 4
Pyrroline-5-carboxylate reductase 2
Protein CUSTOS
m7GpppX diphosphatase
Protein phosphatase 1 regulatory subunit 14B
Integrator complex subunit 12
5-methylcytosine rRNA methyltransferase NSUN4
Inactive rhomboid protein 1
Collagen triple helix repeat-containing protein 1
Medium-chain acyl-CoA ligase ACSF2, mitochondrial
Isochorismatase domain-containing protein 1
GRIP and coiled-coil domain-containing protein 1
FLYWCH family member 2
Protein Aster-A
HAUS augmin-like complex subunit 1
FAS-associated factor 2
Coiled-coil domain-containing protein 124
FAD-dependent oxidoreductase domain-containing protein 1
Optineurin

AP-2 complex subunit mu
Gamma-tubulin complex component 3
Probable RNA polymerase II nuclear localization protein SLC7A6OS
BTB/POZ domain-containing protein KCTD12
Reticulocalbin-3
60S ribosomal export protein NMD3
RalBP1-associated Eps domain-containing protein 1
Mitochondrial import inner membrane translocase subunit TIM14
Regulator of microtubule dynamics protein 1
U8 snoRNA-decapping enzyme
Splicing factor ESS-2 homolog
Carboxymethylenebutenolidase homolog
RNA-binding protein Musashi homolog 2
U5 small nuclear ribonucleoprotein 40 kDa protein
Regulator of MON1-CCZ1 complex
Large ribosomal subunit protein mL38
RING finger and SPRY domain-containing protein 1
Endoplasmic reticulum lectin 1
Ribosome-recycling factor, mitochondrial
Protein Spindly
Elongator complex protein 4
NAD-dependent protein deacetylase sirtuin-1
Protein YIPF6
Nucleoporin SEH1
Transcription elongation factor A protein-like 4
KIF-binding protein
Glucosamine 6-phosphate N-acetyltransferase
Constitutive coactivator of peroxisome proliferator-activated receptor gamma
Protein KTI12 homolog
Small ribosomal subunit protein uS3m
Large ribosomal subunit protein mL53
Trans-3-hydroxy-L-proline dehydratase
E3 ubiquitin-protein ligase RNF31
DAZ-associated protein 1
Small glutamine-rich tetratricopeptide repeat-containing protein beta
Protein SAAL1
Mitochondrial potassium channel
SAGA-associated factor 29
Ribosomal RNA processing protein 36 homolog
C1GALT1-specific chaperone 1
RNA-binding protein 33
Dysbindin
DnaJ homolog subfamily A member 3, mitochondrial
Translation machinery-associated protein 16
Multivesicular body subunit 12A
Small ribosomal subunit protein mS39
Corrinoid adenosyltransferase MMAB
Cytoplasmic FMR1-interacting protein 2
GTPase IMAP family member 5
E3 ubiquitin-protein ligase TRIM11

Coiled-coil domain-containing protein 97
CB1 cannabinoid receptor-interacting protein 1
Enhancer of mRNA-decapping protein 3
Dynein light chain 2, cytoplasmic
Copine-2
Protein S100-A16
Signal-induced proliferation-associated protein 1
Secernin-2
THO complex subunit 1
Ubiquitin thioesterase OTUB1
tRNA (adenine(58)-N(1))-methyltransferase catalytic subunit TRMT61A
Abasic site processing protein HMCES
Charged multivesicular body protein 6
Protein bicaudal D homolog 1
Phosphopentomutase
U3 small nucleolar ribonucleoprotein protein IMP4
Ceramide synthase 2
tRNA-dihydrouridine(47) synthase [NAD(P)(+)]-like
Protein LTV1 homolog
Serine dehydratase-like
Large ribosomal subunit protein mL48
Chronophin
Aurora kinase B
DCN1-like protein 1
Fumarylacetoacetate hydrolase domain-containing protein 2A
SWI/SNF-related matrix-associated actin-dependent regulator of chromatin subfamily D membe
Target of EGR1 protein 1
RUS family member 1
Probable ATP-dependent RNA helicase DDX27
BLOC-1-related complex subunit 6
Methionine--tRNA ligase, mitochondrial
Ataxin-7-like protein 3B
Serine/threonine-protein kinase greatwall
Methylthioribulose-1-phosphate dehydratase
Zinc finger C2HC domain-containing protein 1A
Protein lin-37 homolog
Solute carrier family 41 member 3
Vacuolar-sorting protein SNF8
Zinc finger CCCH-type antiviral protein 1-like
Nuclear envelope pore membrane protein POM 121
Tonsoku-like protein
PDZ and LIM domain protein 5
Protein disulfide isomerase CRELD1
ERO1-like protein alpha
Proline-rich protein 11
Protein FMC1 homolog
Adenosylhomocysteinase 3
Dedicator of cytokinesis protein 6
Mediator of RNA polymerase II transcription subunit 30
Serine/threonine-protein phosphatase PGAM5, mitochondrial

Integrator complex subunit 4
DDRGK domain-containing protein 1
2-oxoadipate dehydrogenase complex component E1
Selenocysteine lyase
Far upstream element-binding protein 3
Splicing factor 45
RCC1-like G exchanging factor-like protein
Asparaginyl-tRNA synthetase
Succinate--CoA ligase [GDP-forming] subunit beta, mitochondrial
DISP complex protein LRCH3
Mannose-1-phosphate guanyltransferase alpha
4-hydroxyphenylpyruvate dioxygenase-like protein
Putative protein-lysine deacylase ABHD14B
Peptide-N(4)-(N-acetyl-beta-glucosaminyl)asparagine amidase
Vesicle-trafficking protein SEC22a
ATP synthase membrane subunit K, mitochondrial
Kinetochore-associated protein NSL1 homolog
PRKC apoptosis WT1 regulator protein
tRNA N(3)-methylcytidine methyltransferase METTL2A
THO complex subunit 3
E3 ubiquitin-protein ligase Itchy homolog
Thioredoxin domain-containing protein 15
Kin of IRRE-like protein 1
Leucine-rich repeats and immunoglobulin-like domains protein 1
Conserved oligomeric Golgi complex subunit 3
CDK5 regulatory subunit-associated protein 3
Vam6/Vps39-like protein
Syndetin
Deubiquitinating protein VCPIP1
Spatacsin
Engulfment and cell motility protein 2
Protein disulfide-isomerase TMX3
DDB1- and CUL4-associated factor 5
Chromosome alignment-maintaining phosphoprotein 1
Lethal(3)malignant brain tumor-like protein 3
E3 ubiquitin-protein ligase ZFP91
Protocadherin-16
Calmin
PDZ and LIM domain protein 2
Fatty acyl-CoA reductase 2
Transcription factor BTF3 homolog 4
E3 ubiquitin-protein ligase RNF170
Abscission/NoCut checkpoint regulator
Solute carrier family 35 member E1
Ubiquitin carboxyl-terminal hydrolase 47
Zinc finger protein 521
Lipid scramblase CLPTM1L
Lymphokine-activated killer T-cell-originated protein kinase
ADP-ribosylation factor-like protein 5B
DnaJ homolog subfamily C member 1

N-terminal kinase-like protein
Zinc finger protein 512B
Protein LRATD2
Exocyst complex component 2
Cytosolic non-specific dipeptidase
Histone-lysine N-methyltransferase EHMT2
Zinc finger RNA-binding protein
Protein FAM210B, mitochondrial
Beta-1,3-galactosyltransferase 6
E1A-binding protein p400
Sorting nexin-27
Kinesin-like protein KIF16B
Intraflagellar transport protein 74 homolog
E3 ubiquitin-protein ligase TRIM47
CCR4-NOT transcription complex subunit 6-like
Dehydrogenase/reductase SDR family member 1
DnaJ homolog subfamily C member 30, mitochondrial
Ubiquitin-conjugating enzyme E2 E2
Caspase recruitment domain-containing protein 19
Protein PRRC1
FYVE, RhoGEF and PH domain-containing protein 4
F-box/LRR-repeat protein 18
Zinc finger protein 512
E3 SUMO-protein ligase NSE2
Non-structural maintenance of chromosomes element 3 homolog
Protein-L-isoaspartate O-methyltransferase domain-containing protein 1
Heat shock 70 kDa protein 12B
YTH domain-containing protein 1
Coiled-coil domain-containing protein 43
Conserved oligomeric Golgi complex subunit 8
CKLF-like MARVEL transmembrane domain-containing protein 3
Dynein axonemal assembly factor 10
Nucleolar protein 4-like
Lysophospholipid acyltransferase 7
Dedicator of cytokinesis protein 7
Zinc finger protein 830
Protein FAM210A
FERM domain-containing protein 6
28S rRNA (cytosine-C(5))-methyltransferase
Regulation of nuclear pre-mRNA domain-containing protein 1A
Arf-GAP with GTPase, ANK repeat and PH domain-containing protein 3
Arf-GAP with Rho-GAP domain, ANK repeat and PH domain-containing protein 1
Importin-9
Melanoma inhibitory activity protein 2
Discoidin, CUB and LCCL domain-containing protein 2
Rho guanine nucleotide exchange factor 17
Inositol polyphosphate-4-phosphatase type I A
RNA-binding protein 14
RING finger and CHY zinc finger domain-containing protein 1
Kelch-like protein 5

E3 ubiquitin-protein ligase UHRF2
E3 ubiquitin-protein ligase NEDD4-like
KH domain-containing RNA-binding protein QKI
Leukocyte receptor cluster member 8
Formin-like protein 2
Pseudouridylate synthase 7 homolog
Serine protease FAM111A
Trafficking protein particle complex subunit 9
CCA tRNA nucleotidyltransferase 1, mitochondrial
Serine/threonine-protein kinase SMG1
Alsin
Transmembrane protein 237
Alpha-ketoglutarate-dependent dioxygenase alkB homolog 3
Rho GTPase-activating protein 7
Serine/threonine-protein phosphatase 1 regulatory subunit 10
Sodium-coupled neutral amino acid symporter 2
UAP56-interacting factor
Myotubularin-related protein 9
Vacuolar protein sorting-associated protein 35
Transcriptional activator protein Pur-beta
Exportin-6
Hedgehog-interacting protein
Membrane-associated guanylate kinase, WW and PDZ domain-containing protein 1
Sperm-associated antigen 5
Nucleus accumbens-associated protein 1
Sorting nexin-18
PAS domain-containing serine/threonine-protein kinase
Protein capicua homolog
BRCA1-A complex subunit RAP80
Intermembrane lipid transfer protein VPS13A
Mediator of RNA polymerase II transcription subunit 15
Elongation factor G, mitochondrial
Endoplasmic reticulum-Golgi intermediate compartment protein 2
Methylcrotonoyl-CoA carboxylase subunit alpha, mitochondrial
Calcium/calmodulin-dependent protein kinase kinase 2
Trimethylguanosine synthase
NudC domain-containing protein 1
Erbin
Gamma-tubulin complex component 6
Gamma-tubulin complex component 5
Ubiquitin carboxyl-terminal hydrolase 28
Formin-binding protein 1
EKC/KEOPS complex subunit TP53RK
GPI transamidase component PIG-S
ATPase WRNIP1
Ran-binding protein 9
Chloride channel CLIC-like protein 1
Myeloid-associated differentiation marker
Neurabin-2
SRSF protein kinase 1

Structural maintenance of chromosomes protein 6
BTB/POZ domain-containing protein KCTD15
Spermatid perinuclear RNA-binding protein
Transmembrane protein 209
Glutathione peroxidase 7
Cytochrome P450 2S1
Protein IWS1 homolog
Paired amphipathic helix protein Sin3a
Oxysterol-binding protein-related protein 9
Protein cereblon
Integrator complex subunit 14
2-aminoethanethiol dioxygenase
Mitochondrial tRNA methylthiotransferase CDK5RAP1
Remodeling and spacing factor 1
RNA-binding protein 15
Protein phosphatase 1 regulatory inhibitor subunit 16B
RUN and FYVE domain-containing protein 1
Msx2-interacting protein
Bifunctional polynucleotide phosphatase/kinase
MMS19 nucleotide excision repair protein homolog
E3 ubiquitin-protein ligase UHRF1
Protein Niban 2
ATP-dependent zinc metalloprotease YME1L1
Regulator of microtubule dynamics protein 3
Transcription factor 12
Cytohesin-2
Tubulin-folding cofactor B
Proteasome subunit beta type-7
Calponin-2
Translocation protein SEC62
Ethanolamine-phosphate cytidylyltransferase
Cell division cycle 5-like protein
26S proteasome non-ATPase regulatory subunit 1
Stromal cell-derived factor 2
Prefoldin subunit 5
E3 ubiquitin-protein ligase RING2
Parkinson disease protein 7
Sphingosine 1-phosphate receptor 3
GAS2-like protein 1
Eyes absent homolog 3
Sialidase-1
Sortilin
Synaptic vesicle membrane protein VAT-1 homolog
Legumain
Perilipin-2
DnaJ homolog subfamily C member 2
M-phase phosphoprotein 8
Nuclear pore complex protein Nup88
Plakophilin-4
Phosphoinositide 3-kinase regulatory subunit 4

P2X purinoceptor 4
Ribonucleases P/MRP protein subunit POP1
Protein S100-A13
Protein SCAF11
Translin-associated protein X
Selenide, water dikinase 2
Eukaryotic translation initiation factor 3 subunit C
Tetratricopeptide repeat protein 1
DnaJ homolog subfamily C member 7
Prohibitin-2
COP9 signalosome complex subunit 8
Pre-mRNA-splicing factor 18
Calcineurin B homologous protein 1
Kinesin-like protein KIF2C
RANBP2-like and GRIP domain-containing protein 5/6
Monoglyceride lipase
Ataxin-2
Docking protein 1
Methionine synthase
3-hydroxyacyl-CoA dehydrogenase type-2
Collagen alpha-1(XII) chain
Mothers against decapentaplegic homolog 5
Sigma non-opioid intracellular receptor 1
Heterogeneous nuclear ribonucleoprotein A/B
Nucleosome assembly protein 1-like 4
Microsomal glutathione S-transferase 2
Gamma-soluble NSF attachment protein
Phosphatidylinositol 4-phosphate 5-kinase type-1 alpha
Phospholipid-transporting ATPase ABCA3
Mitogen-activated protein kinase kinase kinase 3
ATP synthase subunit s, mitochondrial
Mitochondrial intermediate peptidase
Aconitate hydratase, mitochondrial
Transmembrane 9 superfamily member 2
Equilibrative nucleoside transporter 1
Tumor susceptibility gene 101 protein
Copine-1
T-complex protein 1 subunit eta
Myeloid differentiation primary response protein MyD88
Probable rRNA-processing protein EBP2
Protein arginine N-methyltransferase 1
BAG family molecular chaperone regulator 1
Cyclic AMP-dependent transcription factor ATF-6 beta
Endophilin-A2
Telomerase protein component 1
Serine/threonine-protein kinase VRK1
Growth/differentiation factor 15
A-kinase anchor protein 9
Protein NipSnap homolog 1
Condensin complex subunit 3

Actin-related protein 2/3 complex subunit 5-like protein
Calcium uptake protein 1, mitochondrial
UPF0415 protein C7orf25
Protein FAM118B
Target of rapamycin complex 2 subunit MAPKAP1
RNA-binding protein 4B
ATP-dependent RNA helicase DDX50
Large ribosomal subunit protein bL34m
Zinc phosphodiesterase ELAC protein 2
Telomerase RNA component interacting RNase
Glutamate-rich WD repeat-containing protein 1
ADP-ribose glycohydrolase MACROD1
Ribosome quality control complex subunit TCF25
Protein CMSS1
Kelch domain-containing protein 3
Evolutionarily conserved signaling intermediate in Toll pathway, mitochondrial
Methylosome protein WDR77
Cytochrome b-245 chaperone 1
Vitamin K epoxide reductase complex subunit 1
2-(3-amino-3-carboxypropyl)histidine synthase subunit 2
Tubulin alpha-1C chain
Selenoprotein S
Apolipoprotein L2
Myb-binding protein 1A
Fermitin family homolog 1
Mitochondrial genome maintenance exonuclease 1
Golgi reassembly-stacking protein 1
FYVE and coiled-coil domain-containing protein 1
Acyl-CoA-binding domain-containing protein 6
Coronin-1B
Thioredoxin domain-containing protein 17
BUD13 homolog
Serine/threonine-protein phosphatase CPPED1
Vacuolar protein-sorting-associated protein 25
Large ribosomal subunit protein mL45
Uncharacterized protein C7orf50
Tudor-interacting repair regulator protein
45 kDa calcium-binding protein
Programmed cell death protein 2-like
Proteasomal ATPase-associated factor 1
Partner of Y14 and mago
MICOS complex subunit MIC25
ADP-dependent glucokinase
G patch domain-containing protein 1
Serine/threonine-protein kinase RIO1
Protein LLP homolog
DNA replication complex GINS protein SLD5
rRNA-processing protein UTP23 homolog
Protein pelota homolog
Peroxiredoxin-like 2A

E3 ubiquitin-protein ligase TRIM56
Endoplasmic reticulum resident protein 44
Latexin
Nucleolar protein 10
Cancer-related nucleoside-triphosphatase
Mitochondrial import inner membrane translocase subunit Tim29
Translational activator of cytochrome c oxidase 1
Gamma-tubulin complex component 2
Extended synaptotagmin-1
Ubiquitin-associated domain-containing protein 1
Protein YIPF4
Phagosome assembly factor 1
tRNA-splicing endonuclease subunit Sen34
Protein canopy homolog 3
Mitochondrial ribosome-associated GTPase 1
Chitobiosyldiphosphodolichol beta-mannosyltransferase
LIM domain-containing protein 2
HAUS augmin-like complex subunit 8
Inositol polyphosphate 5-phosphatase K
Proteasome assembly chaperone 3
COP9 signalosome complex subunit 4
Synaptotagmin-11
WW domain-containing adapter protein with coiled-coil
Death-inducer obliterator 1
Metastasis-associated protein MTA3
RNA-binding protein 42
Dynactin subunit 5
Mini-chromosome maintenance complex-binding protein
Alanyl-tRNA editing protein Aarsd1
DCN1-like protein 5
Acidic leucine-rich nuclear phosphoprotein 32 family member E
Leucine-rich repeat-containing protein 1
Phosphatidylinositol 4-kinase type 2-alpha
Transmembrane protein 43
Fibronectin type III and SPRY domain-containing protein 1
Tubulin-specific chaperone D
Nucleoporin NDC1
Dehydrogenase/reductase SDR family member 4
Lipase maturation factor 2
NADH dehydrogenase [ubiquinone] 1 alpha subcomplex assembly factor 3
Multiple myeloma tumor-associated protein 2
Transmembrane protein 70, mitochondrial
Mediator of RNA polymerase II transcription subunit 18
Iron-sulfur cluster assembly 1 homolog, mitochondrial
Tubulin beta-6 chain
Protein PAXX
DNA-directed RNA polymerase III subunit RPC3
Heterogeneous nuclear ribonucleoprotein U-like protein 1
Protein misato homolog 1
PHD finger protein 23

Programmed cell death protein 10
Ribonuclease P protein subunit p25
Derlin-1
Oxidoreductase HTATIP2
Probable ATP-dependent RNA helicase DDX23
Telomerase Cajal body protein 1
MICOS complex subunit MIC26
Dehydrogenase/reductase SDR family member 6
Methylthioribose-1-phosphate isomerase
WD repeat-containing protein 18
Vesicle-associated membrane protein 8
tRNA (guanine(6)-N2)-methyltransferase THUMP3
Acireductone dioxygenase
Enoyl-[acyl-carrier-protein] reductase, mitochondrial
ER membrane protein complex subunit 6
N-terminal Xaa-Pro-Lys N-methyltransferase 1
Katanin p80 WD40 repeat-containing subunit B1
Target of rapamycin complex subunit LST8
Ashwin
Voltage-gated monoatomic cation channel TMEM109
Protein PBDC1
Phosphatidylserine synthase 2
Nucleolar complex protein 4 homolog
U3 small nucleolar RNA-associated protein 14 homolog A
Dual specificity protein phosphatase 23
Transmembrane emp24 domain-containing protein 9
BOS complex subunit TMEM147
Nucleoporin p58/p45
Protein adenylyltransferase SelO, mitochondrial
Protein DPCD
Guanine nucleotide-binding protein-like 3
ATPase family gene 2 protein homolog B
Serine/threonine-protein kinase RIO2
tRNA (adenine(58)-N(1))-methyltransferase, mitochondrial
Transmembrane and ubiquitin-like domain-containing protein 1
Mitochondrial import inner membrane translocase subunit Tim21
Kinesin-like protein KIFC1
Nuclear pore complex protein Nup85
Very long chain fatty acid elongase 1
Katanin p60 ATPase-containing subunit A-like 1
HIRA-interacting protein 3
Intraflagellar transport protein 27 homolog
Splicing factor YJU2
ADP-ribose pyrophosphatase, mitochondrial
Threonine--tRNA ligase, mitochondrial
Acetyl-CoA acetyltransferase, cytosolic
Replication initiator 1
RNA-binding protein 4
FUN14 domain-containing protein 2
RNA polymerase II-associated protein 1

Splicing factor 3B subunit 5
Sideroflexin-3
Chitinase domain-containing protein 1
Poly(A) polymerase gamma
Caspase recruitment domain-containing protein 10
Kanadaptin
Cyclin-dependent kinase 19
Protein LSM14 homolog B
Tapasin-related protein
Junctional adhesion molecule C
Caspase recruitment domain-containing protein 6
BTB/POZ domain-containing protein 2
Sphingosine-1-phosphate phosphatase 1
Oxysterol-binding protein-related protein 11
Oxysterol-binding protein-related protein 10
Rab11 family-interacting protein 5
TBC1 domain family member 10A
Complement C1q tumor necrosis factor-related protein 5
N-alpha-acetyltransferase 15, NatA auxiliary subunit
Krueppel-like factor 16
Bcl-2-like protein 13
Caspase recruitment domain-containing protein 11
Solute carrier family 12 member 9
Serrate RNA effector molecule homolog
Queuine tRNA-ribosyltransferase catalytic subunit 1
AP-1 complex subunit mu-1
Nucleolar and spindle-associated protein 1
Haloacid dehalogenase-like hydrolase domain-containing 5
Fanconi anemia group D2 protein
Protein MAK16 homolog
Histone deacetylase 8
Replication termination factor 2
Charged multivesicular body protein 4a
Eukaryotic translation initiation factor 2A
Polymerase delta-interacting protein 3
Uncharacterized protein KIAA1671
SH3 and multiple ankyrin repeat domains protein 3
Guanine nucleotide-binding protein subunit beta-like protein 1
Alpha-(1,6)-fucosyltransferase
Large ribosomal subunit protein bL32m
Large ribosomal subunit protein bL20m
Large ribosomal subunit protein uL13m
Large ribosomal subunit protein bL9m
Large ribosomal subunit protein uL4m
Large ribosomal subunit protein uL1m
MKI67 FHA domain-interacting nucleolar phosphoprotein
Hyccin
YTH domain-containing family protein 1
3'-5' exoribonuclease HELZ2
RanBP-type and C3HC4-type zinc finger-containing protein 1

Small ribosomal subunit protein mS26
Neurolysin, mitochondrial
Centrosomal protein of 41 kDa
Histone-lysine N-methyltransferase SETD2
Pantothenate kinase 2, mitochondrial
Dedicator of cytokinesis protein 9
FERM domain-containing protein 8
Contactin-associated protein-like 3
Histone-lysine N-methyltransferase NSD3
Kinetochore protein Nuf2
Large ribosomal subunit protein mL37
GTP-binding protein 4
Tether containing UBX domain for GLUT4
Oxysterol-binding protein-related protein 8
Uveal autoantigen with coiled-coil domains and ankyrin repeats
Ras-related protein Rab-34
2-(3-amino-3-carboxypropyl)histidine synthase subunit 1
WD repeat-containing protein 11
Regulator of nonsense transcripts 3B
Crooked neck-like protein 1
F-box-like/WD repeat-containing protein TBL1XR1
Ubiquitin-like protein 5
Protein phosphatase 1 regulatory subunit 12C
Serine/threonine-protein kinase D2
ER degradation-enhancing alpha-mannosidase-like protein 3
Protein Niban 1
UBX domain-containing protein 6
Transmembrane 6 superfamily member 1
Uridine-cytidine kinase 2
Sialoadhesin
Apoptosis inhibitor 5
NACHT, LRR and PYD domains-containing protein 1
Tripartite motif-containing protein 5
E3 ubiquitin-protein ligase TRIM4
Alpha-ketoglutarate-dependent dioxygenase FTO
Palmitoyltransferase ZDHHC5
Transport and Golgi organization protein 6 homolog
182 kDa tankyrase-1-binding protein
(E3-independent) E2 ubiquitin-conjugating enzyme
Protein zyg-11 homolog B
Protein TANC1
Ethanolaminephosphotransferase 1
Exportin-4
Endoplasmic reticulum junction formation protein lunapark
Centrosomal protein of 44 kDa
Protein tweety homolog 3
Kelch-like protein 4
Myotubularin-related protein 12
pre-mRNA 3' end processing protein WDR33
Ribosome biogenesis protein WDR12

Protein YIPF3
Tubulointerstitial nephritis antigen-like
Adipose-secreted signaling protein
PITH domain-containing protein 1
Derlin-2
COMM domain-containing protein 5
Sentrin-specific protease 6
RNA exonuclease 4
ATP-dependent RNA helicase DDX24
DNA-directed RNA polymerase I subunit RPA49
Superkiller complex protein 8
SRA stem-loop-interacting RNA-binding protein, mitochondrial
Serine racemase
NIF3-like protein 1
Egl nine homolog 1
Mucolipin-1
PSME3-interacting protein
Mitochondrial fission factor
N-alpha-acetyltransferase 50
Ubiquitin-like modifier-activating enzyme 5
E3 ubiquitin-protein ligase makorin-2
Transmembrane protein 126A
Polyadenylate-binding protein-interacting protein 1
Mitochondrial disaggregase
Ras-related protein Rab-33B
Large subunit GTPase 1 homolog
Protein FAM107B
RNA cytidine acetyltransferase
COMM domain-containing protein 4
Kinesin light chain 2
Integrin-linked kinase-associated serine/threonine phosphatase 2C
5'-3' exoribonuclease 2
Toll-interacting protein
Histone deacetylase complex subunit SAP130
Bromodomain-containing protein 8
Nuclear speckle splicing regulatory protein 1
Integrator complex subunit 2
Rac GTPase-activating protein 1
Protein mono-ADP-ribosyltransferase PARP12
Cleavage stimulation factor subunit 2 tau variant
Cytosolic 5'-nucleotidase 3A
CYFIP-related Rac1 interactor A
Transmembrane protein 222
Haloacid dehalogenase-like hydrolase domain-containing protein 2
Glutamyl-tRNA(Gln) amidotransferase subunit A, mitochondrial
Probable ATP-dependent RNA helicase DDX47
Magnesium transporter protein 1
Ras-related protein Rab-1B
Large ribosomal subunit protein uL18m
Testis-specific Y-encoded-like protein 1

Transmembrane protein 168
VIP36-like protein
Nonsense-mediated mRNA decay factor SMG9
Protein FAM234A
Oxysterol-binding protein-related protein 5
Deoxynucleotidyltransferase terminal-interacting protein 1
Nucleotide exchange factor SIL1
Protein-L-histidine N-pros-methyltransferase
Anaphase-promoting complex subunit 1
Cysteine-rich and transmembrane domain-containing protein 1
DNA-directed RNA polymerase III subunit RPC6
Nuclear ubiquitous casein and cyclin-dependent kinase substrate 1
Thioredoxin-related transmembrane protein 4
Kinesin-like protein KIF13A
Activating signal cointegrator 1 complex subunit 2
Rabenosyn-5
Iron-sulfur cluster assembly enzyme ISCU
Oxysterol-binding protein-related protein 2
Autophagy protein 5
WD repeat-containing protein 13
Epsin-3
EH domain-containing protein 4
Vacuolar protein sorting-associated protein 33B
Vacuolar protein sorting-associated protein 16 homolog
Vacuolar protein sorting-associated protein 11 homolog
SH3 domain-binding glutamic acid-rich-like protein 3
Gigaxonin
Solute carrier family 25 member 32
TRIO and F-actin-binding protein
STE20-like serine/threonine-protein kinase
Peptidyl-prolyl cis-trans isomerase-like 3
Sodium-coupled neutral amino acid symporter 1
Phosducin-like protein 3
Serine/threonine-protein kinase TAO3
Rab3 GTPase-activating protein non-catalytic subunit
Activity-dependent neuroprotector homeobox protein
Diphthine methyl ester synthase
ATP-dependent DNA/RNA helicase DHX36
Inorganic pyrophosphatase 2, mitochondrial
Protein spinster homolog 1
Large ribosomal subunit protein mL46
Solute carrier family 12 member 5
Presenilin-associated rhomboid-like protein, mitochondrial
Pinin
Transmembrane protein 245
Forkhead box protein P1
BTB/POZ domain-containing adapter for CUL3-mediated RhoA degradation protein 3
tRNA dimethylallyltransferase
UPF0696 protein C11orf68
Growth hormone-inducible transmembrane protein

BolA-like protein 2
Cobalamin trafficking protein CblD
Thioredoxin-interacting protein
Thioredoxin-related transmembrane protein 1
Negative elongation factor A
Golgi resident protein GCP60
Cdc42 effector protein 4
Tyrosine-protein phosphatase non-receptor type 23
Semaphorin-6B
Protein unc-45 homolog A
Major facilitator superfamily domain-containing protein 1
DnaJ homolog subfamily C member 5
Kinetochore-associated protein DSN1 homolog
Charged multivesicular body protein 4b
RWD domain-containing protein 1
CUE domain-containing protein 2
F-box/LRR-repeat protein 15
Fructosamine-3-kinase
GDP-fucose protein O-fucosyltransferase 1
Phosphatidylinositol glycan anchor biosynthesis class U protein
Torsin-3A
Serine/threonine-protein kinase WNK1
Aminopeptidase B
Golgi phosphoprotein 3-like
Golgi phosphoprotein 3
tRNA N6-adenosine threonylcarbamoyltransferase, mitochondrial
Alpha-N-acetyl-neuraminyl-2,3-beta-galactosyl-1,3-N-acetyl-galactosaminide alpha-2,6-sialyltran
Golgi-associated plant pathogenesis-related protein 1
TraB domain-containing protein
Mitochondrial ribosome-associated GTPase 2
Sentrin-specific protease 3
Oxysterol-binding protein-related protein 3
SWI/SNF-related matrix-associated actin-dependent regulator of chromatin subfamily A contain
EH domain-containing protein 1
mRNA (2'-O-methyladenosine-N(6)-)-methyltransferase
ESF1 homolog
Alpha-1,3/1,6-mannosyltransferase ALG2
HEAT repeat-containing protein 1
Protein O-mannose kinase
Rab GTPase-binding effector protein 2
Dimethyladenosine transferase 2, mitochondrial
rRNA N6-adenosine-methyltransferase ZCCHC4
STING ER exit protein
Cytosolic iron-sulfur assembly component 2A
Probable ATP-dependent RNA helicase DHX35
Protein FAM124B
HAUS augmin-like complex subunit 4
Coiled-coil domain-containing protein 134
Coiled-coil domain-containing protein 86
Receptor expression-enhancing protein 4

Transcription factor SOX-17
Optic atrophy 3 protein
Cytosolic iron-sulfur assembly component 3
ATP-dependent RNA helicase DHX33
Nucleolar protein 6
WD repeat and coiled-coil-containing protein
3'-5' RNA helicase YTHDC2
5-azacytidine-induced protein 2
RNA polymerase II-associated protein 3
BCAS3 microtubule associated cell migration factor
Alpha-1,2-mannosyltransferase ALG9
Lipid droplet-associated hydrolase
Ribosomal oxygenase 1
WD repeat-containing protein 55
Ran-binding protein 3
Inactive tyrosine-protein kinase PEAK1
Ribosome production factor 2 homolog
Histone-lysine N-methyltransferase SMYD3
Mth938 domain-containing protein
Dedicator of cytokinesis protein 5
WD repeat-containing protein 26
Tudor domain-containing protein 3
UPF0488 protein C8orf33
Methyltransferase-like protein 17, mitochondrial
Splicing factor, arginine/serine-rich 19
Nuclear exosome regulator NRDE2
Prostaglandin E synthase 2
Phosphorylated adapter RNA export protein
Ubiquitin-conjugating enzyme E2 Z
Complex I assembly factor ACAD9, mitochondrial
5'-nucleotidase domain-containing protein 2
PRKR-interacting protein 1
Caspase activity and apoptosis inhibitor 1
Nucleolar protein 11
Probable ATP-dependent RNA helicase DDX31
Thiol S-methyltransferase TMT1A
ATPase PAAT
Multimerin-2
Ubiquitin carboxyl-terminal hydrolase MINDY-3
Protein eva-1 homolog A
AKT-interacting protein
Pleckstrin homology domain-containing family F member 2
tRNA endonuclease ANKZF1
Golgi reassembly-stacking protein 2
Protein zwilch homolog
Jupiter microtubule associated homolog 2
Mitochondrial glutamate carrier 1
Proline-serine-threonine phosphatase-interacting protein 2
Mediator of RNA polymerase II transcription subunit 20
WD repeat-containing protein 76

Queuine tRNA-ribosyltransferase accessory subunit 2
Actin-related protein 8
Damage-control phosphatase ARMT1
Pantothenate kinase 3
CCR4-NOT transcription complex subunit 10
Leucine-rich repeat-containing protein 40
Histone-lysine N-methyltransferase EHMT1
Sideroflexin-1
Spermatogenesis-defective protein 39 homolog
Conserved oligomeric Golgi complex subunit 4
Actin-related protein 5
Vacuolar protein sorting-associated protein 37B
Large ribosomal subunit protein mL44
L-2-hydroxyglutarate dehydrogenase, mitochondrial
COP9 signalosome complex subunit 7b
Non-homologous end-joining factor 1
Protein transport protein Sec61 subunit alpha isoform 2
Ribitol 5-phosphate transferase FKRP
Elongator complex protein 3
Ribosome production factor 1
DNA-directed RNA polymerase I subunit RPA2
Uridine-cytidine kinase 1
Ketosamine-3-kinase
TBC1 domain family member 17
Probable cysteine--tRNA ligase, mitochondrial
Solute carrier family 52, riboflavin transporter, member 2
Phosphopantothenate--cysteine ligase
Ubiquitin domain-containing protein 1
WD repeat-containing protein 41
Probable fibrosin-1
Nicotinamide/nicotinic acid mononucleotide adenylyltransferase 1
Protein Njmu-R1
Sialate O-acetylesterase
Pleckstrin homology domain-containing family A member 5
E3 ubiquitin-protein ligase SMURF2
Regulator of nonsense transcripts 2
Guanine nucleotide-binding protein subunit beta-4
Exportin-5
GrpE protein homolog 1, mitochondrial
MYG1 exonuclease
Pleckstrin homology domain-containing family A member 3
Pleckstrin homology domain-containing family A member 1
Retinoid-inducible serine carboxypeptidase
Netrin-4
Calcyclin-binding protein
Ras-related GTP-binding protein C
Zinc finger FYVE domain-containing protein 1
Intraflagellar transport protein 122 homolog
Retinol dehydrogenase 14
Beta-parvin

Tensin-1
Plasminogen receptor (KT)
NmrA-like family domain-containing protein 1
Vezatin
Kinetochore protein Spc25
Transcription initiation factor TFIID subunit 9B
Solute carrier family 38 member 10
Ethanolamine kinase 1
Adhesion G protein-coupled receptor L4
Putative divalent cation/proton antiporter TMEM165
Mitochondrial thiamine pyrophosphate carrier
Echinoderm microtubule-associated protein-like 4
rRNA methyltransferase 3, mitochondrial
Glyoxalase domain-containing protein 4
Serine/threonine-protein kinase Nek6
Methylcrotonoyl-CoA carboxylase beta chain, mitochondrial
Nuclear receptor coactivator 5
Ectopic P granules protein 5 homolog
Helicase MOV-10
N6-adenosine-methyltransferase non-catalytic subunit
Non-lysosomal glucosylceramidase
Pre-mRNA-splicing factor CWC22 homolog
Ribonucleoprotein PTB-binding 2
Chromodomain-helicase-DNA-binding protein 8
Band 4.1-like protein 5
GPN-loop GTPase 1
Stromal cell-derived factor 2-like protein 1
Casein kinase I isoform gamma-1
Pre-mRNA-splicing factor SYF1
Prolactin regulatory element-binding protein
Steroid receptor RNA activator 1
Endoplasmic reticulum transmembrane helix translocase
Golgi-associated PDZ and coiled-coil motif-containing protein
Large ribosomal subunit protein uL29m
LYR motif-containing protein 4
O-phosphoseryl-tRNA(Sec) selenium transferase
Charged multivesicular body protein 1a
Transmembrane 9 superfamily member 3
Unconventional myosin-X
Adipocyte plasma membrane-associated protein
tRNA (34-2'-O)-methyltransferase regulator WDR6
Thioredoxin reductase 2, mitochondrial
ATP-binding cassette sub-family B member 6
ADP-ribosylation factor GTPase-activating protein 3
Zinc finger CCHC domain-containing protein 17
Ras-related protein Rab-18
Palmdelphin
RNA polymerase II subunit A C-terminal domain phosphatase SSU72
Vacuolar protein sorting-associated protein VTA1 homolog
Serine--tRNA ligase, mitochondrial

Large ribosomal subunit protein mL65
Dynein light chain roadblock-type 1
ER membrane protein complex subunit 7
Transcription and mRNA export factor ENY2
Exosome complex component RRP41
Ubiquitin-conjugating enzyme E2 T
H/ACA ribonucleoprotein complex subunit 3
tRNA N6-adenosine threonylcarbamoyltransferase
DNA methyltransferase 1-associated protein 1
Protocadherin-12
Inositol-3-phosphate synthase 1
Bromodomain-containing protein 7
mRNA-decapping enzyme 1A
Acyl-coenzyme A thioesterase 13
Mediator of RNA polymerase II transcription subunit 4
Complex I assembly factor TIMMDC1, mitochondrial
Synembryn-A
Complement component C1q receptor
Putative RNA-binding protein Luc7-like 1
Endothelial cell-specific molecule 1
Leucine zipper transcription factor-like protein 1
Large ribosomal subunit protein mL40
Suppressor of SWI4 1 homolog
1-phosphatidylinositol 4,5-bisphosphate phosphodiesterase beta-1
Fructose-2,6-bisphosphatase TIGAR
Protein C12orf4
Reticulon-4
Regulation of nuclear pre-mRNA domain-containing protein 1B
Xaa-Pro aminopeptidase 3
Omega-amidase NIT2
Cell death regulator Aven
Inner centromere protein
Exosome complex component RRP46
Exosome complex component RRP40
Kinesin-like protein KIF13B
Anillin
Xaa-Pro aminopeptidase 1
Gephyrin
Bridging integrator 3
Something about silencing protein 10
StAR-related lipid transfer protein 7, mitochondrial
Baculoviral IAP repeat-containing protein 6
PDZ and LIM domain protein 7
Acetyl-coenzyme A synthetase, cytoplasmic
Diablo IAP-binding mitochondrial protein
Nucleolar RNA helicase 2
GTP-binding protein SAR1a
Sialic acid synthase
Endophilin-B2
Histone-lysine N-methyltransferase ASH1L

Translation initiation factor eIF2B subunit gamma
Muscleblind-like protein 1
Sialin
Eukaryotic translation initiation factor 4E transporter
Carbohydrate sulfotransferase 12
Suppressor of tumorigenicity 7 protein
PRKCA-binding protein
SH2B adapter protein 1
CTP synthase 2
DNA polymerase epsilon subunit 3
Chromatin accessibility complex protein 1
Aladin
SNF-related serine/threonine-protein kinase
ATP-binding cassette sub-family B member 10, mitochondrial
Bromodomain adjacent to zinc finger domain protein 1A
Striatin-4
L-aminoadipate-semialdehyde dehydrogenase-phosphopantetheinyl transferase
rRNA N6-adenosine-methyltransferase METTL5
Oligosaccharyltransferase complex subunit OSTC
COX assembly mitochondrial protein 2 homolog
Phospholipid scramblase 4
Ribonuclease 3
Sorting nexin-15
Heme-binding protein 1
DNA dC->dU-editing enzyme APOBEC-3C
Vacuolar protein sorting-associated protein 45
RNA-binding protein PNO1
Large ribosomal subunit protein bL17m
14 kDa phosphohistidine phosphatase
Serine incorporator 1
Rho GTPase-activating protein 35
Protein FAM114A2
Phospholipid scramblase 3
1-acyl-sn-glycerol-3-phosphate acyltransferase delta
1-acyl-sn-glycerol-3-phosphate acyltransferase gamma
Lymphoid-specific helicase
Glycoprotein-N-acetylgalactosamine 3-beta-galactosyltransferase 1
Latent-transforming growth factor beta-binding protein 3
Mitochondrial import receptor subunit TOM22 homolog
LanC-like protein 2
Kinesin-like protein KIF15
Transmembrane 7 superfamily member 3
Homer protein homolog 3
Phenylalanine--tRNA ligase beta subunit
Isoleucine--tRNA ligase, mitochondrial
Ribosome biogenesis protein SLX9 homolog
SAM domain-containing protein SAMSN-1
Kinesin light chain 4
Protein diaphanous homolog 3
BMP-2-inducible protein kinase

Ubiquitin-like-conjugating enzyme ATG3
NAD-dependent protein deacetylase sirtuin-3, mitochondrial
Sister chromatid cohesion protein PDS5 homolog B
Structural maintenance of chromosomes protein 4
Alpha-mannosidase 2C1
Phosphatidylinositol-3-phosphatase SAC1
Protein DEPP1
Obg-like ATPase 1
Copper homeostasis protein cutC homolog
Zinc finger protein 64
Ethylmalonyl-CoA decarboxylase
RNA-binding protein 12
TBC1 domain family member 22B
Midasin
Acetyl-coenzyme A synthetase 2-like, mitochondrial
Zinc finger CCHC domain-containing protein 3
Peroxisomal 2,4-dienoyl-CoA reductase [(3E)-enoyl-CoA-producing]
Palmitoyl-protein thioesterase ABHD10, mitochondrial
T-complex protein 11-like protein 1
Double-stranded RNA-binding protein Staufen homolog 2
Shiftless antiviral inhibitor of ribosomal frameshifting protein
Probable ATP-dependent RNA helicase DDX28
Transmembrane protein 106B
Protein lin-7 homolog C
1-acyl-sn-glycerol-3-phosphate acyltransferase epsilon
Gamma-taxilin
SPATS2-like protein
Ufm1-specific protease 2
ATP-binding cassette sub-family F member 3
CYFIP-related Rac1 interactor B
Mitochondrial potassium channel ATP-binding subunit
ATP-dependent RNA helicase DDX19A
GTPase IMAP family member 4
Tyrosyl-DNA phosphodiesterase 1
Protection of telomeres protein 1
TBC1 domain family member 23
DDB1- and CUL4-associated factor 13
U3 small nucleolar ribonucleoprotein protein IMP3
MRG/MORF4L-binding protein
S-adenosyl-L-methionine-dependent tRNA 4-demethylwyosine synthase TYW1
Exocyst complex component 1
Protein-L-isoaspartate O-methyltransferase domain-containing protein 2
Integrator complex subunit 9
Cell cycle control protein 50A
Ubiquinol-cytochrome c reductase complex assembly factor 1
Septin-11
Sodium-coupled neutral amino acid transporter 7
Mediator of RNA polymerase II transcription subunit 17
Alpha-parvin
Ubiquitin carboxyl-terminal hydrolase 40

4'-phosphopantetheine phosphatase
F-box only protein 28
TBC1 domain family member 13
Exonuclease 3'-5' domain-containing protein 2
DnaJ homolog subfamily C member 11
Integrator complex subunit 7
Fanconi anemia group I protein
ATPase family AAA domain-containing protein 3A
ADP-ribosylation factor-like protein 8B
DnaJ homolog subfamily C member 17
Integrator complex subunit 13
Synembryn-B
Guanine nucleotide-binding protein-like 3-like protein
ATP-dependent RNA helicase DDX18
Histone chaperone ASF1B
Kelch-like protein 11
Integrator complex subunit 10
Large ribosomal subunit protein mL66
Pyridoxine-5'-phosphate oxidase
Armadillo repeat-containing protein 1
DNA-directed RNA polymerase III subunit RPC5
Protein SDA1 homolog
Poly(A) RNA polymerase, mitochondrial
Notchless protein homolog 1
Adaptin ear-binding coat-associated protein 2
DNA-directed RNA polymerase III subunit RPC2
RNA-binding protein 28
Anoctamin-10
Pre-mRNA-splicing factor RBM22
Distal membrane-arm assembly complex protein 2
WD repeat-containing protein 70
Transmembrane protein 51
Arginine and glutamate-rich protein 1
Intraflagellar transport protein 57 homolog
SAFB-like transcription modulator
Peptidyl-prolyl cis-trans isomerase FKBP14
Required for meiotic nuclear division protein 1 homolog
p21-activated protein kinase-interacting protein 1
Hypoxia-inducible factor 1-alpha inhibitor
3-oxoacyl-[acyl-carrier-protein] synthase, mitochondrial
Large ribosomal subunit protein uL22m
CXXC motif containing zinc binding protein
BRISC and BRCA1-A complex member 1
Ceroid-lipofuscinosis neuronal protein 6
Ankyrin repeat and SOCS box protein 6
Probable tRNA(His) guanylyltransferase
Histone PARylation factor 1
Interleukin-1 receptor-associated kinase 4
Uridine-cytidine kinase-like 1
Transmembrane protein 160

NACHT, LRR and PYD domains-containing protein 2
Constitutive coactivator of PPAR-gamma-like protein 2
tRNA selenocysteine 1-associated protein 1
NADH dehydrogenase [ubiquinone] 1 beta subcomplex subunit 11, mitochondrial
Large ribosomal subunit protein uL16m
H/ACA ribonucleoprotein complex subunit 2
OCIA domain-containing protein 1
ADP-ribosylhydrolase ARH3
E3 ubiquitin-protein ligase MARCHF5
Cell growth-regulating nucleolar protein
Transmembrane protein 161A
Golgi-resident adenosine 3',5'-bisphosphate 3'-phosphatase
MICOS complex subunit MIC19
tRNA-dihydrouridine(20) synthase [NAD(P)+]-like
CKLF-like MARVEL transmembrane domain-containing protein 6
NAD-dependent protein deacylase sirtuin-5, mitochondrial
GATOR2 complex protein MIOS
Myotubularin-related protein 10
Sphingomyelin phosphodiesterase 4
Pre-mRNA-splicing factor CWC25 homolog
Testis-expressed protein 10
DDB1- and CUL4-associated factor 16
THUMP domain-containing protein 1
Transmembrane prolyl 4-hydroxylase
Torsin-4A
tRNA (guanine(26)-N(2))-dimethyltransferase
Ganglioside-induced differentiation-associated protein 2
Nuclear distribution protein nudE homolog 1
BRISC and BRCA1-A complex member 2
Glutaminyl-peptide cyclotransferase-like protein
ADP-ribosylation factor-like protein 15
BTB/POZ domain-containing protein KCTD5
CDKN2A-interacting protein
DnaJ homolog subfamily B member 12
Alpha-ketoglutarate-dependent dioxygenase alkB homolog 4
Non-structural maintenance of chromosomes element 4 homolog A
H/ACA ribonucleoprotein complex subunit 1
Stabilin-1
Serine/threonine-protein phosphatase 4 regulatory subunit 2
Dipeptidyl peptidase 3
Protein AATF
Tubulin alpha-8 chain
Probable ATP-dependent RNA helicase DDX56
Sphingosine kinase 1
Telomeric repeat-binding factor 2-interacting protein 1
Abl interactor 2
Bcl-2-associated transcription factor 1
U3 small nucleolar RNA-associated protein 6 homolog
TGF-beta-activated kinase 1 and MAP3K7-binding protein 2
Large ribosomal subunit protein mL39

Mitogen-activated protein kinase kinase kinase 20
Peptidyl-prolyl cis-trans isomerase FKBP11
Tropomodulin-3
BET1-like protein
Very long chain fatty acid elongase 5
NF-kappa-B inhibitor-interacting Ras-like protein 2
UDP-glucose:glycoprotein glucosyltransferase 2
UDP-glucose:glycoprotein glucosyltransferase 1
Cyclin-dependent kinase 12
FAST kinase domain-containing protein 2, mitochondrial
Very-long-chain enoyl-CoA reductase
Endoplasmic reticulum aminopeptidase 1
Actin-related protein 10
Vesicle transport protein USE1
CDGSH iron-sulfur domain-containing protein 1
ADP-ribosylation factor-binding protein GGA3
Splicing factor C9orf78
Constitutive coactivator of PPAR-gamma-like protein 1
SWI/SNF-related matrix-associated actin-dependent regulator of chromatin subfamily A-like pro
Glycolipid transfer protein
Maspardin
Large ribosomal subunit protein bL35m
Upstream-binding protein 1
Insulin-like growth factor 2 mRNA-binding protein 1
Sacsin
Mitochondrial carrier homolog 1
Diphosphoinositol polyphosphate phosphohydrolase 2
Hsp70-binding protein 1
Methionine adenosyltransferase 2 subunit beta
Myoferlin
Intersectin-2
Ribosome biogenesis protein NOP53
EH domain-containing protein 3
EH domain-containing protein 2
Rho guanine nucleotide exchange factor 12
CCR4-NOT transcription complex subunit 2
NCK-interacting protein with SH3 domain
Programmed cell death 1 ligand 1
Calmodulin-like protein 5
Opioid growth factor receptor
LIM and cysteine-rich domains protein 1
Cysteine-rich motor neuron 1 protein
Selenoprotein N
Protein PALS2
Charged multivesicular body protein 5
Spliceosome-associated protein CWC15 homolog
Large ribosomal subunit protein uL15m
Thymocyte nuclear protein 1
Thyroid transcription factor 1-associated protein 26
NADH dehydrogenase [ubiquinone] 1 alpha subcomplex assembly factor 4

Very-long-chain (3R)-3-hydroxyacyl-CoA dehydratase 3
ER membrane protein complex subunit 3
NADH dehydrogenase [ubiquinone] 1 alpha subcomplex subunit 13
[Pyruvate dehydrogenase [acetyl-transferring]]-phosphatase 1, mitochondrial
E3 ubiquitin-protein ligase KCMF1
Ankycorbin
Vesicle-associated membrane protein-associated protein A
Large ribosomal subunit protein bL27m
Proton-transporting V-type ATPase complex assembly regulator TMEM9
Sentrin-specific protease 1
CXXC-type zinc finger protein 1
SH3 domain-binding protein 4
Septin-10
GEM-interacting protein
PHD and RING finger domain-containing protein 1
Pogo transposable element with KRAB domain
Protein Daple
Rho GTPase-activating protein 23
Vacuolar protein sorting-associated protein 18 homolog
Protein RCC2
RAB11-binding protein RELCH
Disco-interacting protein 2 homolog B
Junctional cadherin 5-associated protein
SLAIN motif-containing protein 2
Teneurin-3
Ubiquitin carboxyl-terminal hydrolase 36
BRCA2 and CDKN1A-interacting protein
Serine/threonine-protein kinase 26
Armadillo repeat-containing X-linked protein 1
ABI gene family member 3
Prostaglandin F2 receptor negative regulator
CTTNBP2 N-terminal-like protein
Transmembrane protein 181
Inhibitor of Bruton tyrosine kinase
HEAT repeat-containing protein 5B
NFX1-type zinc finger-containing protein 1
Chondroitin sulfate glucuronyltransferase
Protocadherin-10
Ribosome-binding protein 1
Cleavage and polyadenylation specificity factor subunit 2
Kelch-like protein 9
Leucine--tRNA ligase, cytoplasmic
Myelin expression factor 2
Kelch-like protein 42
eIF-2-alpha kinase GCN2
RNA-binding protein 27
E3 ubiquitin-protein ligase HECW2
FERM domain-containing protein 4A
Rabankyrin-5
Arginine-glutamic acid dipeptide repeats protein

Succinate--CoA ligase [ADP-forming] subunit beta, mitochondrial
GMP reductase 2
Syntaxin-18
Dolichol-phosphate mannosyltransferase subunit 3
Protein IMPACT
UV radiation resistance-associated gene protein
Ataxin-10
Methyl-CpG-binding domain protein 2
Neurochondrin
Tuftelin-interacting protein 11
Epidermal growth factor receptor substrate 15-like 1
Unconventional myosin-Ia
Origin recognition complex subunit 3
SUMO-activating enzyme subunit 1
Coatomer subunit gamma-2
Phosphatidylinositol 4-kinase beta
C-type mannose receptor 2
COMM domain-containing protein 3
Guanine nucleotide-binding protein G(I)/G(S)/G(O) subunit gamma-12
Rab-like protein 2A
Methionine synthase reductase
Protein UXT
Set1/Ash2 histone methyltransferase complex subunit ASH2
7-dehydrocholesterol reductase
Tumor necrosis factor receptor superfamily member 10D
Histone deacetylase 6
Spastin
Dickkopf-related protein 3
tRNA (guanine-N(7)-)-methyltransferase
PTB domain-containing engulfment adapter protein 1
Vacuolar protein sorting-associated protein 29
Eukaryotic translation initiation factor 3 subunit K
Exostosin-like 2
Glyoxylate reductase/hydroxypyruvate reductase
Cathepsin Z
Ribosomal protein S6 kinase beta-2
DnaJ homolog subfamily B member 11
E3 ubiquitin-protein ligase RNF14
SUMO-activating enzyme subunit 2
Mortality factor 4-like protein 1
Nuclear RNA export factor 1
Protein sel-1 homolog 1
Beta-1,4-galactosyltransferase 7
Peflin
Zinc finger MYM-type protein 2
COP9 signalosome complex subunit 7a
Mitochondrial dicarboxylate carrier
Alpha-aminoadipic semialdehyde synthase, mitochondrial
CAP-Gly domain-containing linker protein 2
Cytochrome b-c1 complex subunit 9

Mitochondrial fission process protein 1
Tight junction protein ZO-2
DnaJ homolog subfamily B member 4
Serine/threonine-protein kinase 17A
Craniofacial development protein 1
Death domain-associated protein 6
tRNA (cytidine(32)/guanosine(34)-2'-O)-methyltransferase
Vesicle transport through interaction with t-SNAREs homolog 1B
STE20/SPS1-related proline-alanine-rich protein kinase
Gamma-adducin
Leucine-rich repeat and WD repeat-containing protein 1
Protein NipSnap homolog 3A
CGG triplet repeat-binding protein 1
Phosphatidylserine decarboxylase proenzyme, mitochondrial
ATP-binding cassette sub-family F member 2
Testin
5'-AMP-activated protein kinase subunit gamma-2
Gamma-tubulin complex component 4
Lysine-specific demethylase 5B
Tryptophan--tRNA ligase, mitochondrial
LIM domain-containing protein 1
Translocation protein SEC63 homolog
Solute carrier family 2, facilitated glucose transporter member 6
Zinc finger CCCH domain-containing protein 7B
Protein NDRG3
Armadillo repeat-containing X-linked protein 3
Switch-associated protein 70
SUN domain-containing protein 2
Probable ribosome biogenesis protein RLP24
Ragulator complex protein LAMTOR3
LIM domain and actin-binding protein 1
AF4/FMR2 family member 4
Signal recognition particle subunit SRP68
Cysteine and histidine-rich domain-containing protein 1
Serine/threonine-protein kinase TBK1
Septin-9
Prenylcysteine oxidase 1
Probable ATP-dependent RNA helicase DDX20
Sedoheptulokinase
Dipeptidyl peptidase 2
Inactive cell surface hyaluronidase CEMIP2
Ubiquitin carboxyl-terminal hydrolase 25
B-cell receptor-associated protein 29
NADH-cytochrome b5 reductase 1
SAP30-binding protein
Zinc finger HIT domain-containing protein 2
Prefoldin subunit 2
GPN-loop GTPase 3
Poly(U)-binding-splicing factor PUF60
Nuclear receptor-binding protein

Enolase-phosphatase E1
Fasciculation and elongation protein zeta-2
NADH dehydrogenase [ubiquinone] 1 alpha subcomplex subunit 12
Translation initiation factor eIF2B subunit delta
V-type proton ATPase subunit H
Prenylated Rab acceptor protein 1
Importin-11
Multifunctional methyltransferase subunit TRM112-like protein
Dachshund homolog 1
Exportin-7
Leucine carboxyl methyltransferase 1
Vacuolar protein sorting-associated protein 51 homolog
Bromodomain adjacent to zinc finger domain protein 2A
Tyrosine-protein kinase BAZ1B
ATPase inhibitor, mitochondrial
GTP:AMP phosphotransferase AK3, mitochondrial
Leucyl-cystinyl aminopeptidase
Methyl-CpG-binding domain protein 1
CCR4-NOT transcription complex subunit 7
Rab5 GDP/GTP exchange factor
Mitochondrial peptide methionine sulfoxide reductase
N-acetyl-D-glucosamine kinase
2-hydroxyacyl-CoA lyase 1
tRNA (adenine(58)-N(1))-methyltransferase non-catalytic subunit TRM6
SH3 domain-binding glutamic acid-rich-like protein 2
Ras GTPase-activating protein nGAP
18S rRNA aminocarboxypropyltransferase
Electrogenic aspartate/glutamate antiporter SLC25A13, mitochondrial
Drebrin-like protein
Probable ATP-dependent RNA helicase DDX41
Dynactin subunit 4
Cell division cycle protein 23 homolog
Anaphase-promoting complex subunit 7
Anaphase-promoting complex subunit 5
Anaphase-promoting complex subunit 4
Anaphase-promoting complex subunit 2
ADP-ribosylation factor-binding protein GGA2
ADP-ribosylation factor-binding protein GGA1
Stomatin-like protein 2, mitochondrial
N-acetylglucosamine-1-phosphodiester alpha-N-acetylglucosaminidase
Vacuolar protein sorting-associated protein 28 homolog
U6 snRNA-associated Sm-like protein LSm7
Lariat debranching enzyme
Protein TASOR
Jupiter microtubule associated homolog 1
F-box only protein 3
A-kinase anchor protein 11
F-box/WD repeat-containing protein 11
Glucocorticoid modulatory element-binding protein 2
mRNA turnover protein 4 homolog

TRAF2 and NCK-interacting protein kinase
Cleavage and polyadenylation specificity factor subunit 3
DCC-interacting protein 13-alpha
Cdc42 effector protein 3
Serine/threonine-protein kinase tousled-like 1
G patch domain-containing protein 8
Protein mono-ADP-ribosyltransferase PARP4
NTF2-related export protein 1
ADP-sugar pyrophosphatase
REST corepressor 1
Phosphatidylcholine transfer protein
Endoplasmic reticulum mannosyl-oligosaccharide 1,2-alpha-mannosidase
RNA-binding protein Raly
General transcription factor 3C polypeptide 4
Ergosterol biosynthetic protein 28 homolog
Protein kinase C and casein kinase substrate in neurons protein 3
Isobutyryl-CoA dehydrogenase, mitochondrial
Angiopoietin-related protein 2
Apoptotic chromatin condensation inducer in the nucleus
E3 ubiquitin-protein ligase AMFR
Protein argonaute-2
Nuclear pore complex protein Nup50
Protein CDV3 homolog
CCR4-NOT transcription complex subunit 11
Integrator complex subunit 6
BAG family molecular chaperone regulator 5
Protein argonaute-1
Ras-related protein Rab-21
Ras-related protein Rab-22A
Trafficking protein particle complex subunit 2-like protein
Zinc finger protein 346
Proteasome activator complex subunit 2
Serine/threonine-protein kinase TAO2
Muskelin
Aspartyl aminopeptidase
Ras-related protein Rab-23
Malignant T-cell-amplified sequence 1
Microtubule-associated tumor suppressor 1
Protein WWC3
Paladin
Zinc transporter ZIP10
Chromatin-remodeling ATPase INO80
Cell cycle progression protein 1
Kinase D-interacting substrate of 220 kDa
Arf-GAP with SH3 domain, ANK repeat and PH domain-containing protein 1
Myocardin-related transcription factor B
Ankyrin repeat domain-containing protein 50
Mediator of RNA polymerase II transcription subunit 23
Protein Shroom4
YEATS domain-containing protein 2

Protein NDRG4
TBC1 domain family member 24
Pre-mRNA-splicing factor ISY1 homolog
Transmembrane and coiled-coil domain protein 3
E3 ubiquitin-protein ligase HECTD1
MYND-type zinc finger-containing chromatin reader ZMYND8
Cip1-interacting zinc finger protein
Coronin-1C
Targeting protein for Xklp2
Activator of basal transcription 1
RNA-binding protein NOB1
A-kinase anchor protein 8-like
Transcription factor MafF
Apoptosis-associated speck-like protein containing a CARD
Calcium load-activated calcium channel
Fizzy-related protein homolog
Anaphase-promoting complex subunit 10
Unconventional myosin-VI
ATP-dependent RNA helicase DDX19B
Lysosomal thioesterase PPT2
Pre-mRNA-processing factor 19
Suppressor of fused homolog
Nucleolar protein 7
Sorting nexin-12
Synergin gamma
Vacuolar protein sorting-associated protein 4A
Ras GTPase-activating protein-binding protein 2
E3 ubiquitin-protein ligase CHIP
Protein kinase C and casein kinase substrate in neurons protein 2
Melanoma-associated antigen D2
Sorting nexin-7
Sorting nexin-6
Dual specificity protein phosphatase 12
Syntaxin-8
Translocon-associated protein subunit gamma
26S proteasome non-ATPase regulatory subunit 13
FAS-associated factor 1
Endothelial protein C receptor
Peptidyl-prolyl cis-trans isomerase E
Probable dimethyladenosine transferase
COP9 signalosome complex subunit 3
Multiple inositol polyphosphate phosphatase 1
RNA-binding protein Nova-2
WD repeat-containing protein 3
Transcription termination factor 2
NSFL1 cofactor p47
Conserved oligomeric Golgi complex subunit 5
Solute carrier family 12 member 4
AP-4 complex subunit epsilon-1
Microtubule-actin cross-linking factor 1, isoforms 1/2/3/4/5

Centrosomal protein of 131 kDa
SR-related and CTD-associated factor 8
Serine/threonine-protein phosphatase 6 regulatory subunit 1
E3 ubiquitin-protein ligase TRIM33
Histone lysine demethylase PHF8
Trinucleotide repeat-containing gene 6B protein
Inactive phospholipase C-like protein 2
Nonsense-mediated mRNA decay factor SMG5
Exocyst complex component 7
Zinc finger CCCH domain-containing protein 4
Ubiquitin carboxyl-terminal hydrolase 24
TBC1 domain family member 2B
Protein Smaug homolog 1
Forkhead box protein J3
Cytosolic carboxypeptidase 1
Endoribonuclease Dicer
Cystine/glutamate transporter
Microtubule-associated protein RP/EB family member 3
Leucine-rich repeat protein SHOC-2
Serine/arginine repetitive matrix protein 2
Alpha-1,3-mannosyl-glycoprotein 4-beta-N-acetylglucosaminyltransferase B
Proliferation-associated protein 2G4
Paraplegin
Brain-specific angiogenesis inhibitor 1-associated protein 2
GRB2-associated-binding protein 2
Structural maintenance of chromosomes protein 3
Charged multivesicular body protein 2b
SH2B adapter protein 3
Zinc finger protein 148
Myotubularin-related protein 6
Protein jagged-2
60S ribosome subunit biogenesis protein NIP7 homolog
Bifunctional UDP-N-acetylglucosamine 2-epimerase/N-acetylmannosamine kinase
RNA transcription, translation and transport factor protein
RuvB-like 2
Chromodomain Y-like protein
Peptidyl-prolyl cis-trans isomerase NIMA-interacting 4
RAC-gamma serine/threonine-protein kinase
CAAX prenyl protease 2
Choline/ethanolamine kinase
Eukaryotic translation initiation factor 3 subunit L
Phospholipase A-2-activating protein
RuvB-like 1
Nuclear migration protein nudC
Mitochondrial chaperone BCS1
Voltage-dependent anion-selective channel protein 3
Cofilin-2
Endoplasmic reticulum-Golgi intermediate compartment protein 3
Phenylalanine--tRNA ligase alpha subunit
Sodium-dependent multivitamin transporter

Small ribosomal subunit protein mS33
Histone chaperone ASF1A
Developmentally-regulated GTP-binding protein 1
Trafficking protein particle complex subunit 4
Nck-associated protein 1
Protein canopy homolog 2
Nuclease EXOG, mitochondrial
Exocyst complex component 6B
PALM2-AKAP2 fusion protein
Caspase recruitment domain-containing protein 8
Phospholipid-transporting ATPase IF
GDP-fucose protein O-fucosyltransferase 2
DnaJ homolog subfamily C member 16
Disks large-associated protein 4
Serine/threonine-protein kinase 38-like
Phosphatidylinositide phosphatase SAC2
Fibronectin type-III domain-containing protein 3A
Nischarin
1-phosphatidylinositol 3-phosphate 5-kinase
WD repeat-containing protein 37
Band 4.1-like protein 3
Angiomotin-like protein 2
Serine/threonine-protein kinase SIK3
Lysine-specific demethylase 2A
Exosome complex exonuclease RRP44
Trafficking protein particle complex subunit 8
Leucine-rich repeat and calponin homology domain-containing protein 1
RNA 3'-terminal phosphate cyclase-like protein
Glutathione S-transferase kappa 1
Ragulator complex protein LAMTOR2
Small ribosomal subunit protein bS1m
Cytochrome c oxidase assembly factor 3 homolog, mitochondrial
Probable ATP-dependent RNA helicase DDX52
Small ribosomal subunit protein uS17m
Small ribosomal subunit protein uS7m
Lambda-crystallin homolog
Translation machinery-associated protein 7
Polymerase delta-interacting protein 2
AP-3 complex subunit mu-1
Inner nuclear membrane protein Man1
Calcium-regulated heat-stable protein 1
Conserved oligomeric Golgi complex subunit 6
Thyroid hormone receptor-associated protein 3
WW domain-binding protein 11
Tudor and KH domain-containing protein
Mediator of RNA polymerase II transcription subunit 16
Nucleolar protein 58
ARF GTPase-activating protein GIT1
Zinc finger protein 281
ADP-ribosylation factor-like protein 2-binding protein

Transmembrane protein 98
Protein SGT1 homolog
Tyrosine--tRNA ligase, mitochondrial
N-acetylglucosamine-6-phosphate deacetylase
Acyl-coenzyme A thioesterase 9, mitochondrial
Protein AAR2 homolog
Nitric oxide synthase-interacting protein
Deoxyribose-phosphate aldolase
Protein MEMO1
Thioredoxin-related transmembrane protein 2
rRNA-processing protein FCF1 homolog
U6 snRNA-associated Sm-like protein LSm2
Endophilin-B1
Complex I intermediate-associated protein 30, mitochondrial
Calcium-binding protein 39
Putative RNA-binding protein Luc7-like 2
Ubiquitin-conjugating enzyme E2 J1
RNA-binding motif protein, X-linked 2
Dehydrogenase/reductase SDR family member 7
Small ribosomal subunit protein uS2m
Probable U3 small nucleolar RNA-associated protein 11
MOB-like protein phocein
Ribosomal RNA-processing protein 7 homolog A
Ribosome maturation protein SBDS
Transmembrane emp24 domain-containing protein 5
Exosome complex component CSL4
Transmembrane emp24 domain-containing protein 7
Splicing factor 3B subunit 6
Large ribosomal subunit protein uL11m
Oligoribonuclease, mitochondrial
RRP15-like protein
WASH complex subunit 3
Nucleolar protein 16
EKC/KEOPS complex subunit TPRKB
Peptidyl-prolyl cis-trans isomerase-like 1
Ubiquitin-fold modifier-conjugating enzyme 1
Cytosolic iron-sulfur assembly component 2B
Small ribosomal subunit protein bS16m
Small ribosomal subunit protein bS18m
Mitochondrial fission 1 protein
Mitochondrial import inner membrane translocase subunit TIM16
Adenylate kinase isoenzyme 6
Small ribosomal subunit protein mS23
Vesicle transport protein GOT1B
Hepatoma-derived growth factor-related protein 3
Peptidyl-tRNA hydrolase 2, mitochondrial
Charged multivesicular body protein 3
Serine-threonine kinase receptor-associated protein
RNA-splicing ligase RtcB homolog
F-box only protein 7

Ras-related protein Rap-2c
StAR-related lipid transfer protein 13
Rab GTPase-activating protein 1
Transmembrane emp24 domain-containing protein 3
TSC22 domain family protein 4
Protein dopey-2
Zinc finger protein 330
R3H and coiled-coil domain-containing protein 1
Nucleolar complex protein 2 homolog
Large ribosomal subunit protein eL36
Coiled-coil domain-containing protein 9
Chromatin target of PRMT1 protein
Deoxynucleoside triphosphate triphosphohydrolase SAMHD1
Small kinetochore-associated protein
HBS1-like protein
5'-AMP-activated protein kinase subunit beta-1
WD repeat domain phosphoinositide-interacting protein 4
DmX-like protein 1
V-type proton ATPase 116 kDa subunit a 2
Talin-1
Kinesin-like protein KIF3A
Transformation/transcription domain-associated protein
Microtubule cross-linking factor 1
DDB1- and CUL4-associated factor 1
TRPM8 channel-associated factor 1
Probable RNA-binding protein 19
Disheveled-associated activator of morphogenesis 1
Plexin-D1
Probable E3 ubiquitin-protein ligase HECTD4
WASH complex subunit 2C
Ubiquitin carboxyl-terminal hydrolase 15
FERM, ARHGEF and pleckstrin domain-containing protein 1
Meiosis regulator and mRNA stability factor 1
Centrosomal protein of 170 kDa protein B
Rap guanine nucleotide exchange factor 2
Unconventional myosin-Va
Dystrobrevin alpha
Lysyl oxidase homolog 2
Beta/gamma crystallin domain-containing protein 1
TNF receptor-associated factor 6
Mitogen-activated protein kinase kinase kinase kinase 5
Hypoxia up-regulated protein 1
Cysteine protease ATG4B
Transducin beta-like protein 2
WD repeat domain phosphoinositide-interacting protein 2
Telomere length regulation protein TEL2 homolog
Ribosomal biogenesis protein LAS1L
AFG3-like protein 2
Krueppel-like factor 12
E3 ubiquitin-protein ligase ARIH1

U6 snRNA-associated Sm-like protein LSm4
E3 ubiquitin-protein ligase RNF114
Sorting and assembly machinery component 50 homolog
Protein PRRC2C
ADP-ribose glycohydrolase OARD1
Intraflagellar transport protein 25 homolog
Protein phosphatase methylesterase 1
RNA-binding protein 7
Mitochondrial import inner membrane translocase subunit Tim22
NEDD8 ultimate buster 1
YTH domain-containing family protein 2
RNA polymerase II subunit A C-terminal domain phosphatase
PAX3- and PAX7-binding protein 1
Nucleoside diphosphate kinase 7
FACT complex subunit SPT16
U3 small nucleolar RNA-associated protein 18 homolog
Ubiquitin carboxyl-terminal hydrolase isozyme L5
CD2-associated protein
V-type proton ATPase subunit D
Transportin-3
Signal recognition particle receptor subunit beta
Origin recognition complex subunit 6
Ceramide transfer protein
Mannose-1-phosphate guanyltransferase beta
Fatty acid desaturase 3
General transcription factor 3C polypeptide 5
General transcription factor 3C polypeptide 3
Trafficking protein particle complex subunit 1
Transient receptor potential cation channel subfamily V member 2
Serine/threonine-protein kinase MRCK beta
RNA-binding protein 8A
DnaJ homolog subfamily C member 15
Ubiquitin carboxyl-terminal hydrolase 16
U5 small nuclear ribonucleoprotein TSSC4
Zinc finger protein 706
Melanoma-associated antigen D1
Sorting nexin-14
Sorting nexin-11
Sorting nexin-9
Sorting nexin-8
Sorting nexin-5
Endothelial lipase
Cytosolic Fe-S cluster assembly factor NUBP2
Peroxisomal membrane protein PEX16
Lymphatic vessel endothelial hyaluronic acid receptor 1
Beta-secretase 2
Heme-binding protein 2
Host cell factor 2
UbiA prenyltransferase domain-containing protein 1
Pseudouridylate synthase 1 homolog

Leucine-rich repeat flightless-interacting protein 2
FH1/FH2 domain-containing protein 1
Phosphoserine aminotransferase
Nuclear receptor corepressor 2
Mitochondrial ornithine transporter 1
Junctional adhesion molecule A
Neuroplastin
Adhesion G-protein coupled receptor G1
Spindlin-1
Solute carrier family 12 member 7
Dolichyl pyrophosphate Man9GlcNAc2 alpha-1,3-glucosyltransferase
Dolichyl-phosphate beta-glucosyltransferase
Small ribosomal subunit protein mS40
Coatomer subunit gamma-1
Lipid droplet-regulating VLDL assembly factor AUP1
Peptidyl-prolyl cis-trans isomerase FKBP7
Chloride intracellular channel protein 4
Cysteine desulfurase
Cilia- and flagella-associated protein 20
Transforming acidic coiled-coil-containing protein 3
Signal peptidase complex subunit 1
GTP-binding protein SAR1b
AP-4 complex subunit beta-1
Mitochondrial carrier homolog 2
Selenoprotein K
Brefeldin A-inhibited guanine nucleotide-exchange protein 2
Brefeldin A-inhibited guanine nucleotide-exchange protein 1
Mitotic spindle assembly checkpoint protein MAD1
Serine/threonine-protein kinase 24
eIF5-mimic protein 1
COMM domain-containing protein 10
Cytoplasmic dynein 1 light intermediate chain 1
Epsin-1
Ubiquitin carboxyl-terminal hydrolase 3
Testis-expressed protein 264
Choline/ethanolaminephosphotransferase 1
DNA (cytosine-5)-methyltransferase 3A
2'-5'-oligoadenylate synthase 3
Adenylate kinase isoenzyme 5
NF-kappa-B essential modulator
Insulin-like growth factor 2 mRNA-binding protein 2
Proton-coupled zinc antiporter SLC30A1
Sodium bicarbonate cotransporter 3
NADH dehydrogenase [ubiquinone] 1 beta subcomplex subunit 9
Sulfide:quinone oxidoreductase, mitochondrial
Roundabout homolog 1
Stonin-1
Nuclear receptor coactivator 3
Numb-like protein
Mitogen-activated protein kinase kinase kinase 4

Probable ATP-dependent RNA helicase DDX49
Calpain-7
Actin-binding protein WASF2
MAU2 chromatid cohesion factor homolog
ATPase MORC2
Influenza virus NS1A-binding protein
SEC23-interacting protein

PG.ProteinNames	PG.Quantity
GAL3B_HUMAN	4607.2578
IFT56_HUMAN	2939.9065
UBA6_HUMAN	17574.371
ESYT2_HUMAN	14033.298
BLT3B_HUMAN	115.18124
SHOT1_HUMAN	2779.2881
TM223_HUMAN	1853.8815
RHG10_HUMAN	3089.3096
HACL2_HUMAN	8229.7744
SPD2B_HUMAN	11885.471
NBAS_HUMAN	2608.7358
TYW5_HUMAN	1042.1343
DEND3_HUMAN	1413.9778
VWA8_HUMAN	2056.9082
SBNO1_HUMAN	2265.5083
GTPBA_HUMAN	1289.2816
WDR91_HUMAN	776.20886
DEN11_HUMAN	549.69196
PDES1_HUMAN	753.94226
TPC13_HUMAN	1638.2078
CNOT1_HUMAN	8936.0586
MP3B2_HUMAN	24111.191
PGP_HUMAN	9017.2266
RIMC1_HUMAN	5696.9653
RCCD1_HUMAN	587.39392
TCAF2_HUMAN	5979.3423
SMHD1_HUMAN	5029.5742
CAST2_HUMAN	1614.4594
U119B_HUMAN	2636.0566
MET15_HUMAN	685.66486
CC85C_HUMAN	685.86829
RTL8C_HUMAN	3138.0015
ZSWM8_HUMAN	773.08679
P121C_HUMAN	2302.8809
P20L1_HUMAN	403.03693
RUXGL_HUMAN	11860.107
ELAP2_HUMAN	1085.8342
NUD19_HUMAN	2546.332
MI4GD_HUMAN	857.97552
CALM1_HUMAN	5295.5044
MYO9A_HUMAN	463.05264
GPHRA_HUMAN	1639.0488
WASH3_HUMAN	7566.2192
MCRI1_HUMAN	1624.8534
NACAM_HUMAN	51638.375
CK098_HUMAN	4316.167
ASURF_HUMAN	742.18073
MIDUO_HUMAN	2480.8091

DNS2A_HUMAN	4224.8618
ADAS_HUMAN	15071.146
KIF2A_HUMAN	9454.165
DX39A_HUMAN	63033.723
PDLI1_HUMAN	8306.7354
BACH_HUMAN	43393.348
MYO1C_HUMAN	32658.77
SNP23_HUMAN	8560.3945
HAX1_HUMAN	1763.7581
AIP_HUMAN	7029.4565
GTPB1_HUMAN	5486.0103
LEG9_HUMAN	584.8974
STXB3_HUMAN	9298.4736
AP4M1_HUMAN	1921.1887
ARVC_HUMAN	6459.0103
SMAP_HUMAN	1295.0129
AP3B1_HUMAN	13775.758
APBB1_HUMAN	1630.7042
LEG8_HUMAN	1228.2517
NDUS8_HUMAN	1378.6488
IKBE_HUMAN	1441.9089
PSD11_HUMAN	36088.176
PSD12_HUMAN	28236.844
PSMD9_HUMAN	2616.7888
MEN1_HUMAN	697.63177
GET1_HUMAN	3176.647
PGRC1_HUMAN	1898.578
SPT5H_HUMAN	4323.5361
TAF4_HUMAN	216.02187
DFFA_HUMAN	2720.5642
HIP1_HUMAN	2506.0559
CLIC1_HUMAN	184287.36
EIF3F_HUMAN	23379.707
WWP2_HUMAN	838.18005
ODPX_HUMAN	4042.9033
QSOX1_HUMAN	3071.0569
DCTN6_HUMAN	1534.6525
ACATN_HUMAN	4216.1079
WASL_HUMAN	5670.9907
IPO5_HUMAN	39863.094
RPOM_HUMAN	576.60437
EF2K_HUMAN	3857.4949
SAP18_HUMAN	11285.985
EMAL1_HUMAN	2781.0364
IF2B3_HUMAN	36176.504
DNM1L_HUMAN	41134.352
RTCA_HUMAN	7257.6997
P3C2A_HUMAN	6559.7158
GOLI4_HUMAN	2141.9863
AGRIN_HUMAN	2698.6138

PLOD2_HUMAN	39433.719
EXOC5_HUMAN	10112.724
BT3A1_HUMAN	518.67633
NDUA4_HUMAN	7217.0532
PSDE_HUMAN	41741.379
BIN1_HUMAN	3485.4004
CLD5_HUMAN	4616.1338
IMA4_HUMAN	24847.041
STK25_HUMAN	2930.8059
KRIT1_HUMAN	463.94949
VMA5A_HUMAN	1107.1216
PESC_HUMAN	5877.625
SDCB1_HUMAN	38640.828
PITM1_HUMAN	425.21237
MPP10_HUMAN	2679.9658
NOP56_HUMAN	3174.3059
DDX3X_HUMAN	65408.227
MFNG_HUMAN	572.72595
PODXL_HUMAN	38172.18
CCN1_HUMAN	54432.422
PIR_HUMAN	37123.992
IMA3_HUMAN	5731.3457
TRI38_HUMAN	619.98395
NFIB_HUMAN	9963.0654
PPP6_HUMAN	8788.1504
NDKM_HUMAN	1680.845
P3C2B_HUMAN	1369.8872
MA2B1_HUMAN	3055.3318
PDXK_HUMAN	4209.9468
SCD_HUMAN	4859.9751
ARI1A_HUMAN	2568.075
NEMP1_HUMAN	858.25653
TXND9_HUMAN	3620.3833
DPYL4_HUMAN	29504.279
TRAD1_HUMAN	1391.6958
COX7R_HUMAN	924.46503
UBFD1_HUMAN	5377.3779
CTRO_HUMAN	1758.3224
COPE_HUMAN	16486.064
BORG1_HUMAN	2026.4128
AP3D1_HUMAN	11837.204
CCS_HUMAN	1329.2338
ABLM1_HUMAN	5320.1221
DVL2_HUMAN	916.41205
CHD1_HUMAN	3229.3442
CHD2_HUMAN	3011.7173
GOSR2_HUMAN	5014.334
TOR1A_HUMAN	5818.8672
TOR1B_HUMAN	3537.5037
STX16_HUMAN	2365.6521

ADA10_HUMAN	9425.793
ABCD4_HUMAN	1569.274
EI24_HUMAN	3124.8337
P5I11_HUMAN	3415.8364
KMT2D_HUMAN	230.51105
APAF_HUMAN	2352.177
RIOK3_HUMAN	774.6264
MP2K7_HUMAN	329.40704
ACOT8_HUMAN	2086.7849
CDIPT_HUMAN	42691.059
PDCD5_HUMAN	2801.7615
ANM5_HUMAN	17627.061
NHRF1_HUMAN	5250.043
CHK1_HUMAN	941.73145
TR10B_HUMAN	4584.1372
TPP1_HUMAN	16158.857
TCRG1_HUMAN	17567.85
NDC80_HUMAN	2252.6226
KIF3C_HUMAN	188.92723
NRP1_HUMAN	14247.114
TNPO2_HUMAN	3390.3325
UN13B_HUMAN	923.80176
TR10C_HUMAN	413.27771
RPC1_HUMAN	1821.7819
TSN4_HUMAN	468.07236
PSA7_HUMAN	90310.602
SCAM3_HUMAN	4933.1899
BCKD_HUMAN	2692.9653
MGST3_HUMAN	6631.2505
TX1B3_HUMAN	12173.94
GIPC1_HUMAN	6363.1304
IKKB_HUMAN	3073.6758
RGS12_HUMAN	1487.4718
TIM23_HUMAN	1897.4346
HAT1_HUMAN	11353.014
CSKP_HUMAN	2704.2302
PLD2_HUMAN	1391.0859
QCR8_HUMAN	15708.106
ML12B_HUMAN	70454.75
HGS_HUMAN	6478.6558
RAB7L_HUMAN	735.00574
VP26C_HUMAN	1793.0134
MYPT1_HUMAN	11599.238
GAK_HUMAN	5276.7144
HNRDL_HUMAN	18475.67
XPO1_HUMAN	30361.178
BTAF1_HUMAN	1475.6696
ARHGA_HUMAN	3247.8865
ZN609_HUMAN	1115.744
SPTN2_HUMAN	1020.9598

MAST4_HUMAN	1233.4601
SC16A_HUMAN	6539.4985
PLXB2_HUMAN	2152.4248
KHNYN_HUMAN	376.24118
TCPR2_HUMAN	920.82874
SR140_HUMAN	26218.666
SET1A_HUMAN	685.00067
N4BP3_HUMAN	518.37402
SYNJ2_HUMAN	3611.1819
SYNEM_HUMAN	7205.0068
GRRE1_HUMAN	1321.3539
KIF3B_HUMAN	2036.6974
PUR4_HUMAN	23499.371
ANR28_HUMAN	1999.16
ARHGB_HUMAN	408.26501
MRPP3_HUMAN	1388.4716
IKKA_HUMAN	1684.9236
NPC1_HUMAN	3941.0183
DEGS1_HUMAN	2804.7383
ANGP2_HUMAN	6393.6079
SCAM1_HUMAN	4423.7554
SCAM2_HUMAN	2193.4678
ARC1B_HUMAN	38858.32
ARPC2_HUMAN	39776.348
ARPC3_HUMAN	48000.742
RPAC1_HUMAN	8717.4238
PLS1_HUMAN	1020.3962
TIF1A_HUMAN	421.98151
PGRC2_HUMAN	10117.679
RGL2_HUMAN	1917.0358
WDR46_HUMAN	401.96149
NKRF_HUMAN	1883.3298
GNPAT_HUMAN	2987.511
LAMA5_HUMAN	1432.5406
ZN185_HUMAN	6919.0757
CASC3_HUMAN	585.87909
RT12_HUMAN	1770.3756
ACOX3_HUMAN	2330.2844
RER1_HUMAN	16009.524
SURF4_HUMAN	21467.711
MK13_HUMAN	1981.3113
SPTC1_HUMAN	6736.2207
SPTC2_HUMAN	9910.7383
OGT1_HUMAN	6457.2729
PMM2_HUMAN	14418.719
TM9S1_HUMAN	2560.0225
HMGB3_HUMAN	22708.18
PPM1G_HUMAN	10509.248
SHIP2_HUMAN	4193.8657
EIF3D_HUMAN	23696.639

EIF3H_HUMAN	31679.25
HDAC3_HUMAN	1664.1112
NVL_HUMAN	1600.5791
BCAT2_HUMAN	8037.1978
IPO8_HUMAN	5620.8936
STX7_HUMAN	6073.2803
MOT4_HUMAN	13298.029
MRP4_HUMAN	1945.9106
RPA34_HUMAN	1304.2117
P4HA2_HUMAN	6117.6455
CAN5_HUMAN	1026.0321
YKT6_HUMAN	46446.703
NUP42_HUMAN	1489.6946
ARPC5_HUMAN	43339.273
CFLAR_HUMAN	1714.5135
PDPK1_HUMAN	1255.4469
TPSN_HUMAN	12508.79
R113A_HUMAN	596.52496
KDM6A_HUMAN	2437.49
DHX15_HUMAN	34187
MCES_HUMAN	6607.2866
ZZEF1_HUMAN	3301.4717
ASAP2_HUMAN	1113.1455
FLRT2_HUMAN	907.58575
TTI1_HUMAN	1318.1067
RRP8_HUMAN	1828.4564
PJA2_HUMAN	460.58362
CYB5B_HUMAN	2039.3804
PRP4_HUMAN	6880.144
SERA_HUMAN	27188.082
NDUS4_HUMAN	3686.0742
DC1L2_HUMAN	11280.086
PSMD3_HUMAN	42702.375
RFOX2_HUMAN	2514.1887
PAPS1_HUMAN	15875.057
ZW10_HUMAN	6674.8027
B4GT5_HUMAN	296.85587
SNUT1_HUMAN	7474.9321
GPAA1_HUMAN	6538.3965
DAPK3_HUMAN	6017.9204
TGFI1_HUMAN	2232.6909
AP5Z1_HUMAN	680.84741
CTIF_HUMAN	3089.4524
MTSS1_HUMAN	784.48083
VIP2_HUMAN	1594.0527
M3K7_HUMAN	582.18103
MCA3_HUMAN	25564.064
RIPK2_HUMAN	2290.707
HNRPR_HUMAN	60051.734
PRPF3_HUMAN	8797.4707

TXNL1_HUMAN	50933.613
TPD54_HUMAN	21354.967
EMC8_HUMAN	4563.7939
ERI3_HUMAN	2433.7385
SYNJ1_HUMAN	1558.3306
FIBP_HUMAN	3938.3115
IF4G3_HUMAN	25328.281
PPIH_HUMAN	6116.1465
HTRA2_HUMAN	2905.1621
MS18B_HUMAN	181.68346
ARK72_HUMAN	13593.218
E41L2_HUMAN	11935.049
TGON2_HUMAN	950.26086
B4GA1_HUMAN	870.76154
WIPF1_HUMAN	2036.8867
AT8B1_HUMAN	1372.2239
AKA10_HUMAN	393.43457
DENR_HUMAN	15156.902
XPOT_HUMAN	15667.745
DNPH1_HUMAN	10130.524
TIM44_HUMAN	10212.239
TPPC3_HUMAN	17625.232
CHM2A_HUMAN	10300.358
NCK2_HUMAN	2673.7017
TSN6_HUMAN	2090.5193
PLRG1_HUMAN	5874.0752
PRC1_HUMAN	1753.3673
RGS10_HUMAN	2417.5342
ZN207_HUMAN	1944.2167
NDUB5_HUMAN	5197.8545
NDUB3_HUMAN	9687.7705
NDUA2_HUMAN	2623.4573
LDB2_HUMAN	1913.6422
GET3_HUMAN	24937.053
BUB1_HUMAN	148.06979
BUB3_HUMAN	30119.219
ST1B1_HUMAN	8514.8555
ACTN4_HUMAN	53160.133
BUD23_HUMAN	1104.4232
HTSF1_HUMAN	2945.0591
ERD23_HUMAN	1934.4031
AP1G1_HUMAN	12731.923
STX6_HUMAN	1828.8192
SNG2_HUMAN	4753.2705
SGTA_HUMAN	2977.8164
ENSA_HUMAN	6794.6147
MCAT_HUMAN	7743.6035
SYNC_HUMAN	37363.766
MYO1B_HUMAN	6855.9746
PM34_HUMAN	1031.2273

CPSF5_HUMAN	17942.445
LANC1_HUMAN	12088.503
STRN_HUMAN	6232.6953
U3IP2_HUMAN	2237.73
SCO2_HUMAN	964.7536
CF410_HUMAN	231.56837
AKAP8_HUMAN	2650.8752
GTPB6_HUMAN	852.24432
G6PT1_HUMAN	1264.8446
IDH3B_HUMAN	8692.6426
NRDC_HUMAN	12777.804
CALU_HUMAN	25284.477
EDIL3_HUMAN	5537.6558
SAHH2_HUMAN	1685.2629
KIF1C_HUMAN	3515.7866
EXTL3_HUMAN	648.41528
ORC5_HUMAN	1273.5474
NDUS5_HUMAN	12558.554
PDE6D_HUMAN	4338.2168
ORC4_HUMAN	1229.9624
PEX1_HUMAN	1315.9863
RAD21_HUMAN	5718.0796
DHX16_HUMAN	4414.8428
GMFG_HUMAN	3863.2803
3BP5_HUMAN	3004.6746
AGRB3_HUMAN	1339.1807
MED14_HUMAN	12879.651
PCDH7_HUMAN	268.13858
KPRB_HUMAN	6305.6201
SMCA5_HUMAN	5652.374
JIP4_HUMAN	87817.648
NUAK1_HUMAN	393.38507
NPA1P_HUMAN	687.28949
SI1L3_HUMAN	248.69081
ZC3H1_HUMAN	329.63431
AQR_HUMAN	7099.4707
OPA1_HUMAN	6420.6655
ZEB2_HUMAN	821.11774
GANP_HUMAN	2073.27
PCDGC_HUMAN	934.14453
PI51C_HUMAN	3678.8518
KIF1B_HUMAN	2644.1362
MABP1_HUMAN	1148.7953
KDM1A_HUMAN	5979.6592
TBCD4_HUMAN	9038.9395
FADS1_HUMAN	2133.21
GSDME_HUMAN	8538.584
EVI5_HUMAN	300.84146
NRP2_HUMAN	7812.1924
MA1A2_HUMAN	2936.3972

MPZL2_HUMAN	3890.2158
ACSL4_HUMAN	11014.753
SNX3_HUMAN	34029.145
OGA_HUMAN	8997.583
VINEX_HUMAN	4197.3999
HNRPQ_HUMAN	39571.793
PRP17_HUMAN	1289.7961
RNBP6_HUMAN	31684.162
NEMF_HUMAN	5896.7241
GMDS_HUMAN	6018.7808
NMT2_HUMAN	5434.4067
CCNT1_HUMAN	1215.2523
BUB1B_HUMAN	1673.1097
PLOD3_HUMAN	26778.24
IF4E2_HUMAN	5780.3467
DIAP1_HUMAN	70987.352
TSN3_HUMAN	10274.623
EXOC3_HUMAN	10447.775
PLIN3_HUMAN	22174.85
ANM3_HUMAN	1267.4075
IMA7_HUMAN	8784.25
UGDH_HUMAN	37808.047
TPST2_HUMAN	1922.1024
LPXN_HUMAN	952.01154
CTND1_HUMAN	44392.582
ICMT_HUMAN	4604.7622
EIF1B_HUMAN	1853.2843
SNX2_HUMAN	18067.9
DPM1_HUMAN	16100.035
USO1_HUMAN	21698.371
RT14_HUMAN	2698.7261
TOM1_HUMAN	3869.6025
H2B1K_HUMAN	125156.42
CCD22_HUMAN	4209.2979
PQBP1_HUMAN	3325.8315
TI17B_HUMAN	764.45618
PRAF2_HUMAN	10663.394
DKC1_HUMAN	7813.2773
IF2P_HUMAN	29797.049
EDF1_HUMAN	24847.535
KIN17_HUMAN	928.69427
DIAP2_HUMAN	6866.9893
DNJA2_HUMAN	8556.0332
BRD4_HUMAN	3465.3069
HUS1_HUMAN	682.23492
PFD1_HUMAN	2581.8696
PP1RB_HUMAN	1953.6675
NBN_HUMAN	12939.612
MCE1_HUMAN	2081.8953
ABCB7_HUMAN	3225.636

SRGP2_HUMAN	1904.2157
PLXA2_HUMAN	6760.9453
XYLK_HUMAN	1434.5341
WDR1_HUMAN	102391.47
ATP9A_HUMAN	2908.2913
N4BP1_HUMAN	1101.1779
ROCK2_HUMAN	8515.6553
CLAP2_HUMAN	3431.9192
PTCD1_HUMAN	415.4317
CPNE3_HUMAN	17560.656
ATG13_HUMAN	1310.1954
HIP1R_HUMAN	10490.57
BRE1B_HUMAN	5211.3062
PHF2_HUMAN	1142.7615
ZC11A_HUMAN	2779.5352
CLU_HUMAN	7550.6377
CAND2_HUMAN	2126.7324
T22D2_HUMAN	392.96921
DJC13_HUMAN	8280.1982
PHAR2_HUMAN	1009.7051
PP6R2_HUMAN	1492.3358
ATS4_HUMAN	1582.9523
CNOT3_HUMAN	6261.8599
ANR17_HUMAN	5470.5078
SIN3B_HUMAN	935.82257
DNJB6_HUMAN	3909.156
COQ9_HUMAN	1563.4987
GGCT_HUMAN	6822.4253
NDUS7_HUMAN	13780.915
NDUS2_HUMAN	10729.213
ZPR1_HUMAN	3805.8591
DUS11_HUMAN	650.90125
NIPS2_HUMAN	9805.7734
HMMR_HUMAN	301.27808
PDCD6_HUMAN	15012.813
TBCA_HUMAN	19784.961
VATG1_HUMAN	12298.601
VPS4B_HUMAN	64312.723
MPU1_HUMAN	18959.588
H2AY_HUMAN	1805.1283
SH3L1_HUMAN	1447.5225
FLNB_HUMAN	121563.22
NCOR1_HUMAN	8025.3877
PEX14_HUMAN	5914.4458
TRIM3_HUMAN	3767.4534
CISY_HUMAN	109839.44
SPAG7_HUMAN	10021.864
SC22B_HUMAN	39163.574
PR40A_HUMAN	11642.678
TACC1_HUMAN	8725.9355

NDK6_HUMAN	1610.2094
CDC45_HUMAN	1100.4008
LRCH4_HUMAN	567.83984
MTX2_HUMAN	1691.2499
VP26A_HUMAN	37963.008
MPPB_HUMAN	11148.064
MED24_HUMAN	1104.0177
KTNA1_HUMAN	2309.7424
PSIP1_HUMAN	1834.8553
ERLN1_HUMAN	6417.457
NDUS3_HUMAN	19305.734
SRS10_HUMAN	4351.1143
CLN5_HUMAN	2277.8574
CLD11_HUMAN	1357.4305
ECI2_HUMAN	13185.837
KHDR3_HUMAN	653.94458
EED_HUMAN	2280.4407
BAF_HUMAN	3970.0576
SF3B1_HUMAN	25037.824
CSDE1_HUMAN	18109.896
WBP4_HUMAN	1178.7509
PRKRA_HUMAN	4756.8374
ITA10_HUMAN	2066.188
KS6A5_HUMAN	2354.9358
MED6_HUMAN	437.04837
MYCB2_HUMAN	2343.4402
KBL_HUMAN	1492.6301
NPM3_HUMAN	4927.5122
LYPA1_HUMAN	14988.836
ERAL1_HUMAN	5232.8589
U520_HUMAN	16204.205
MTU1_HUMAN	1413.599
TIPRL_HUMAN	10163.08
OFD1_HUMAN	1637.8254
KS6A4_HUMAN	1673.637
SURF6_HUMAN	858.57666
PPM1B_HUMAN	2618.1084
UTP20_HUMAN	4469.7573
NU155_HUMAN	10883.761
XRP2_HUMAN	7507.5166
WDHD1_HUMAN	3747.748
CRTAP_HUMAN	18260.842
S2512_HUMAN	12170.195
RENR_HUMAN	2314.252
RNH2A_HUMAN	2195.2771
CD123_HUMAN	2925.522
BCAR3_HUMAN	470.89639
POP7_HUMAN	1314.0005
RPP40_HUMAN	1084.2534
EIF3G_HUMAN	21974.398

EIF3J_HUMAN	20409.492
CBR3_HUMAN	14478.234
PSD10_HUMAN	3618.7944
AP1G2_HUMAN	2037.3137
FACE1_HUMAN	14112.789
IDHC_HUMAN	51889.191
GATB_HUMAN	1368.7802
SCO1_HUMAN	7623.4316
ATRN_HUMAN	551.30835
RBBP9_HUMAN	8220.3516
STAM2_HUMAN	5339.6001
CCNK_HUMAN	4548.2881
PRAF3_HUMAN	66288.266
DYSF_HUMAN	26510.621
PIAS1_HUMAN	447.89459
SPF27_HUMAN	640.22394
DCTN3_HUMAN	14128.56
DNJC8_HUMAN	15058.977
SPF30_HUMAN	3664.791
RAD17_HUMAN	266.54922
ATP5H_HUMAN	4873.3691
TSN9_HUMAN	2672.4041
FLOT1_HUMAN	5734.4023
TRIO_HUMAN	4148.6045
ATP5L_HUMAN	39454.348
MPDZ_HUMAN	2082.3396
CBPD_HUMAN	9307.0723
GLRX3_HUMAN	47226.414
RL1D1_HUMAN	6216.0244
WFS1_HUMAN	7841.5249
CLPX_HUMAN	4570.9272
S14L2_HUMAN	4196.7241
SYUG_HUMAN	1269.8086
CIAO1_HUMAN	3123.897
SRP72_HUMAN	11243.395
DDAH1_HUMAN	10317.446
RECQ5_HUMAN	556.41235
RMP_HUMAN	1311.4686
B3GA3_HUMAN	1168.6575
MTA2_HUMAN	12199.007
AL1A2_HUMAN	6102.5249
STK10_HUMAN	7253.5938
KPCD3_HUMAN	2063.4973
GFPT2_HUMAN	1459.3057
SLIT2_HUMAN	2918.7959
LTN1_HUMAN	2524.593
TOM70_HUMAN	8223.7852
PKHG5_HUMAN	1223.178
IPO13_HUMAN	1139.3213
DDHD2_HUMAN	838.24945

MYO1D_HUMAN	9577.9756
TOX4_HUMAN	2812.3843
MICA2_HUMAN	3307.9788
SC24D_HUMAN	16889.611
FCSD2_HUMAN	431.15524
UFL1_HUMAN	10313.4
SRBS2_HUMAN	8077.8784
SASH1_HUMAN	4114.1899
UBXN7_HUMAN	5927.749
KLH18_HUMAN	334.77667
SUN1_HUMAN	807.0683
PLPHP_HUMAN	9047.0508
ERLN2_HUMAN	13354.083
PRP6_HUMAN	13862.417
PCF11_HUMAN	570.73273
FRYL_HUMAN	7501.6426
NFAT5_HUMAN	233.98552
ENDD1_HUMAN	1038.3538
GLCE_HUMAN	5907.1245
GLSK_HUMAN	21760.336
HAUS5_HUMAN	766.23077
ABLM3_HUMAN	1160.7325
FBX21_HUMAN	1074.0332
MTCL2_HUMAN	2138.0608
UBP19_HUMAN	1153.2373
WDR47_HUMAN	2313.5581
AP2A2_HUMAN	20665.904
SC31A_HUMAN	41657.961
ARHGF_HUMAN	9241.0186
HEXI1_HUMAN	1405.1522
RPP14_HUMAN	1618.5254
CCNB2_HUMAN	213.00652
YIF1A_HUMAN	4358.2422
UBR5_HUMAN	2067.9353
AGFG2_HUMAN	2576.1504
PRS23_HUMAN	3877.082
SCAF4_HUMAN	2255.4009
NDUB6_HUMAN	6396.1035
MFN2_HUMAN	2029.9502
SPN1_HUMAN	1494.8898
UBE4B_HUMAN	6144.915
ZFPL1_HUMAN	4506.5234
ELP1_HUMAN	5614.3306
NDUB4_HUMAN	5253.4116
NDUB8_HUMAN	6220.873
NDUA7_HUMAN	4507.8804
VAMP5_HUMAN	1570.5271
RTN3_HUMAN	9179.2998
LETM1_HUMAN	13155.353
EPN2_HUMAN	2999.8572

ZRAB2_HUMAN	8029.666
SNX4_HUMAN	4140.7129
LC7L3_HUMAN	4710.9175
KI20A_HUMAN	916.36176
APOL3_HUMAN	795.35742
KIF4A_HUMAN	4390.8184
MBD4_HUMAN	513.33197
MTMR5_HUMAN	1292.0415
GOSR1_HUMAN	7524.0166
KAT7_HUMAN	1063.7362
ATE1_HUMAN	1790.0116
VAPB_HUMAN	4578.9551
SNAPN_HUMAN	2498.1091
MPZL1_HUMAN	2172.2781
NDUC2_HUMAN	9872.8682
NDUAA_HUMAN	13236.466
FKBP9_HUMAN	12963.239
CELF2_HUMAN	1136.1169
6PGL_HUMAN	5985.5742
PAPS2_HUMAN	23977.223
SMC2_HUMAN	11033.066
ATG7_HUMAN	8015.4019
TACC2_HUMAN	1464.4755
TRI16_HUMAN	3517.259
SYFM_HUMAN	1452.8563
ZBT7A_HUMAN	320.60934
LYPA2_HUMAN	5387.792
IPO7_HUMAN	36968.672
ARI2_HUMAN	1200.0665
SLU7_HUMAN	921.90143
AGM1_HUMAN	16387.742
MOCS3_HUMAN	762.54028
CD2B2_HUMAN	3888.9326
SVIL_HUMAN	6073.0127
AHSA1_HUMAN	33640.176
PARN_HUMAN	2719.6897
PSMG1_HUMAN	10694.319
SGPL1_HUMAN	3815.969
CNEP1_HUMAN	1566.4778
NSA2_HUMAN	722.39905
G6PE_HUMAN	8228.4971
SC24A_HUMAN	8226.8203
SC24B_HUMAN	8179.6802
AGRL2_HUMAN	2090.9553
NADK_HUMAN	625.0014
TYDP2_HUMAN	1608.0618
MPC2_HUMAN	2041.4269
ETHE1_HUMAN	8625.877
ACSL3_HUMAN	13610.646
RPA1_HUMAN	3009.4597

YETS4_HUMAN	806.03345
CNOT4_HUMAN	1030.6752
STABP_HUMAN	1860.307
CPSF4_HUMAN	1509.41
NFAC1_HUMAN	827.20337
ASML_HUMAN	5661.7485
CDS2_HUMAN	749.64954
PPR3D_HUMAN	964.37384
BRD1_HUMAN	424.15311
RPP29_HUMAN	1008.2269
HERC2_HUMAN	964.86121
RAB3D_HUMAN	2143.1311
SNP29_HUMAN	4670.584
OXSR1_HUMAN	5619.5693
GGPPS_HUMAN	1654.8225
HS74L_HUMAN	6997.0122
PTBP3_HUMAN	11643.088
TBCD8_HUMAN	956.54016
IL33_HUMAN	496.16748
STR3N_HUMAN	2668.176
LSM8_HUMAN	1942.8087
AP2A1_HUMAN	28178.408
WIZ_HUMAN	1780.2909
RIGI_HUMAN	7037.2866
STAU1_HUMAN	15548.157
TTC4_HUMAN	4012.551
TM50A_HUMAN	2646.9277
CAVN2_HUMAN	42702.793
BAG2_HUMAN	11217.4
BAG3_HUMAN	1670.2972
M4K4_HUMAN	9999.9717
DCMC_HUMAN	356.00015
QORL1_HUMAN	1550.1981
AIFM1_HUMAN	17685.914
EMAL2_HUMAN	1408.5837
LATS1_HUMAN	1529.8136
NUD14_HUMAN	4186.2393
TSN15_HUMAN	1180.8739
BPNT1_HUMAN	6508.7842
FADS2_HUMAN	25902.973
DDAH2_HUMAN	649.14117
ABHGA_HUMAN	3276.6453
TXD12_HUMAN	1133.4692
ECD_HUMAN	4198.749
RECK_HUMAN	4468.9419
MBD3_HUMAN	2005.6136
TOP3B_HUMAN	506.36884
NUDT3_HUMAN	3989.7773
BCL10_HUMAN	4013.7476
NDUBA_HUMAN	12948.949

CLPT1_HUMAN	6527.5229
ZBED1_HUMAN	990.57379
MOC2B_HUMAN	776.31256
TOM40_HUMAN	17132.498
PX11B_HUMAN	2990.1177
PAK4_HUMAN	7165.6152
APBA3_HUMAN	1253.0605
ACL6A_HUMAN	9360.4648
NSD2_HUMAN	891.04132
LDHA_HUMAN	263280.25
AL1A1_HUMAN	19458.818
DHE3_HUMAN	40034.652
DYR_HUMAN	13120.98
NB5R3_HUMAN	57729.273
GSHR_HUMAN	17429.59
COX1_HUMAN	786.07361
COX2_HUMAN	75560.703
PNPH_HUMAN	61724.918
HPRT_HUMAN	27037.736
AATM_HUMAN	51756.188
ABL1_HUMAN	805.53192
PGK1_HUMAN	159299.17
KAD1_HUMAN	28986.121
UROK_HUMAN	959.75244
TPA_HUMAN	421.90668
ADA_HUMAN	3976.1572
A1AT_HUMAN	22161.445
A2MG_HUMAN	193.14351
CYTC_HUMAN	6092.3926
RASN_HUMAN	28531.303
RASH_HUMAN	3435.1726
RASK_HUMAN	2206.4797
PDGFB_HUMAN	1099.5779
LDLR_HUMAN	2982.7742
TGFB1_HUMAN	3990.7031
HLAB_HUMAN	6363.6826
HLAH_HUMAN	67332.914
CO1A1_HUMAN	1990.7223
CO4A1_HUMAN	844.90338
LMNA_HUMAN	144043.2
APOA1_HUMAN	2837.0276
FINC_HUMAN	34003.254
FETUA_HUMAN	1835.0754
ALBU_HUMAN	96916.063
TFR1_HUMAN	16769.178
FRIL_HUMAN	21051.516
FRIH_HUMAN	11291.479
MT2_HUMAN	8127.2632
NU1M_HUMAN	6333.2144
NU4M_HUMAN	1916.7135

NU5M_HUMAN	2564.5098
MMP1_HUMAN	6995.3091
HMDH_HUMAN	345.92361
CATA_HUMAN	7852.8511
RAF1_HUMAN	1736.2336
GBA1_HUMAN	14328.278
ALDOA_HUMAN	275568.69
CYTB_HUMAN	5789.5288
ANXA1_HUMAN	194580.59
APOB_HUMAN	1448.6038
PRIO_HUMAN	1209.2426
SODM_HUMAN	3271.8953
OAT_HUMAN	24822.156
KITH_HUMAN	2432.8037
VWF_HUMAN	79927.672
TBB4A_HUMAN	77703.344
G3P_HUMAN	415545.44
ARLY_HUMAN	7531.7554
HLAA_HUMAN	41723.145
CPNS1_HUMAN	27219.213
MT1E_HUMAN	95.924088
HSPB1_HUMAN	138444.25
TYSY_HUMAN	9639.7607
RPN1_HUMAN	73016.469
RPN2_HUMAN	49037.246
GNAI2_HUMAN	29433.945
H2A1B_HUMAN	85554.625
AT1A1_HUMAN	48129.289
AT1B1_HUMAN	9046.1807
A4_HUMAN	7745.3022
ALDH2_HUMAN	29920.398
ITB3_HUMAN	6769.5269
S10A8_HUMAN	298.76645
HMGN1_HUMAN	761.2627
PAI2_HUMAN	10232.235
PAI1_HUMAN	71380.391
ADT2_HUMAN	216465.91
ISG15_HUMAN	4590.3862
PCCA_HUMAN	3345.7083
PCCB_HUMAN	4951.7773
IF2A_HUMAN	52425.637
HMGN2_HUMAN	540.65796
ICAM1_HUMAN	6653.1768
RLA2_HUMAN	6208.7207
RLA0_HUMAN	171662.31
JUN_HUMAN	1088.9485
RPC4_HUMAN	2236.1689
LA_HUMAN	30879.189
ITB1_HUMAN	100482.35
K1C18_HUMAN	52165.16

CO5A2_HUMAN	1706.2546
DCUP_HUMAN	26783.756
FYN_HUMAN	902.57788
AGAL_HUMAN	1157.0168
GELS_HUMAN	12683.868
RB_HUMAN	2191.4534
PTMA_HUMAN	5561.5415
CDK1_HUMAN	28598.779
ATPB_HUMAN	176653.58
S10A6_HUMAN	12548.543
IF4E_HUMAN	18221.117
ENOA_HUMAN	630395.06
PYGL_HUMAN	8872.9385
G6PI_HUMAN	62848.324
DPOLB_HUMAN	357.71698
NPM_HUMAN	64228.316
TPM3_HUMAN	55022.211
ITAV_HUMAN	16837.254
HEXA_HUMAN	15681.031
HYEP_HUMAN	9638.7832
LDHB_HUMAN	192431.7
CENPB_HUMAN	1314.5409
GPX1_HUMAN	17698.475
PDIA1_HUMAN	172804
H10_HUMAN	14681.925
ACYP1_HUMAN	3118.8743
FES_HUMAN	1243.382
CATD_HUMAN	74227.336
ANXA2_HUMAN	400610.69
CAN1_HUMAN	29518.762
TBB5_HUMAN	502602.34
SAP_HUMAN	4951.8306
HEXB_HUMAN	18875.992
CATL1_HUMAN	1712.4242
PROF1_HUMAN	290447.53
PMGE_HUMAN	3717.5576
APT_HUMAN	65545.32
SYEP_HUMAN	21275.967
CATB_HUMAN	76501.438
HS90A_HUMAN	150869.2
GALT_HUMAN	710.97443
HNRPC_HUMAN	88537.852
LAMB1_HUMAN	26706.83
YES_HUMAN	13259.105
LYN_HUMAN	3172.9229
FUMH_HUMAN	29075.576
ERCC1_HUMAN	962.96826
TSP1_HUMAN	349352.75
SP1_HUMAN	893.90015
IGF1R_HUMAN	1366.6851

CO1A2_HUMAN	487.89114
ANXA6_HUMAN	103000.36
RHOC_HUMAN	87205.117
DAF_HUMAN	6845.4189
4F2_HUMAN	14690.1
BGLR_HUMAN	1656.538
PFKAM_HUMAN	10024.112
HS90B_HUMAN	743914.69
SRPRA_HUMAN	11507.057
ASNS_HUMAN	4731.1323
MMP2_HUMAN	5985.3418
HEM3_HUMAN	1885.8177
NEP_HUMAN	606.1181
MGP_HUMAN	5577.5503
ODPA_HUMAN	30523.574
CO4A2_HUMAN	6888.1372
CY1_HUMAN	13532.486
RU2B_HUMAN	6341.6963
MET_HUMAN	3681.8892
RU17_HUMAN	44156.059
ITA5_HUMAN	66567.531
NFIC_HUMAN	767.72284
VIME_HUMAN	1347928.4
RS17_HUMAN	35154.27
GNAI3_HUMAN	6644.0776
ANXA5_HUMAN	461196.28
RSSA_HUMAN	120367.8
CD63_HUMAN	4617.939
RM03_HUMAN	5637.2612
SNRPA_HUMAN	19704.896
ENOG_HUMAN	10944.089
THIK_HUMAN	6573.7729
SRP19_HUMAN	4584.0288
GSTP1_HUMAN	129917.26
RU1C_HUMAN	4420.7539
LEG1_HUMAN	34981.195
DHPR_HUMAN	8751.75
HMGB1_HUMAN	70438.773
SPRC_HUMAN	3710.5791
TPM1_HUMAN	57230.074
CLCA_HUMAN	1868.7552
CLCB_HUMAN	879.37543
ANXA4_HUMAN	16091.581
CN37_HUMAN	11987.707
HMOX1_HUMAN	4416.5454
DLDH_HUMAN	18216.258
ROA1_HUMAN	137717.23
RU2A_HUMAN	21978.484
COX6C_HUMAN	18884.313
TACD2_HUMAN	3373.3672

PARP1_HUMAN	36841.512
DPOLA_HUMAN	11725.144
UCHL1_HUMAN	9766.5518
LKHA4_HUMAN	35240.48
ALDOC_HUMAN	20493.627
H2AZ_HUMAN	3939.6565
H2A1_HUMAN	2318.6992
MYZAP_HUMAN	300.74734
GRL1A_HUMAN	530.74402
GST2_HUMAN	4032.459
POTEJ_HUMAN	10374.991
LIMS3_HUMAN	4348.5537
HS71A_HUMAN	92232.805
ERPG3_HUMAN	1508.5717
TBA3C_HUMAN	57505.355
ZTRF1_HUMAN	936.40588
RAP2A_HUMAN	8321.6797
SRGN_HUMAN	1278.4047
RO60_HUMAN	11055.324
LYAG_HUMAN	5451.3286
RRAS_HUMAN	11240.377
HLAC_HUMAN	1911.2469
ARAF_HUMAN	1694.7242
H14_HUMAN	356072.78
ODP2_HUMAN	22414.385
PTPRF_HUMAN	4789.6377
COT1_HUMAN	3906.4126
THIO_HUMAN	5092.9883
COX5B_HUMAN	1404.576
PPGB_HUMAN	8281.9063
MGST1_HUMAN	10594.896
KAP0_HUMAN	17785.799
TFPI1_HUMAN	2206.6604
ESTD_HUMAN	19093.182
CH60_HUMAN	145107.48
CLUS_HUMAN	6372.9834
BIP_HUMAN	267732.38
LAMC1_HUMAN	20418.467
PPAL_HUMAN	6128.0469
MTAP2_HUMAN	3528.3188
HSP7C_HUMAN	245935.73
GTR1_HUMAN	4604.1567
GTR3_HUMAN	3011.6101
EPB41_HUMAN	26065.867
UMPS_HUMAN	13549.529
ODPB_HUMAN	16013.97
ODB2_HUMAN	1724.0645
PYGB_HUMAN	14277.022
PYGM_HUMAN	29558.691
RALA_HUMAN	31821.16

RALB_HUMAN	59349.547
BCR_HUMAN	5019.9448
LAMP1_HUMAN	73920.055
ERG_HUMAN	6571.2227
ACADM_HUMAN	13872.923
TOP1_HUMAN	34472.547
TOP2A_HUMAN	345.30859
G6PD_HUMAN	69071.656
UBL4A_HUMAN	966.65155
PYC_HUMAN	3877.8293
C1TC_HUMAN	54864.781
MPRI_HUMAN	5636.3218
ADHX_HUMAN	34568.828
CDK4_HUMAN	2477.8169
PRPS2_HUMAN	18711.914
PABP1_HUMAN	119110.33
PCNA_HUMAN	26877.49
HARS1_HUMAN	38999.918
CO6A1_HUMAN	1395.1567
ADT1_HUMAN	228859.34
ADT3_HUMAN	81607.055
IMDH2_HUMAN	42018.402
TPR_HUMAN	14928.447
ANXA3_HUMAN	53412.699
ODBA_HUMAN	5933.3896
ACTN1_HUMAN	87195.648
ACE_HUMAN	17711.686
SRC_HUMAN	3879.1182
PEPD_HUMAN	7758.9531
XRCC6_HUMAN	87781.133
XRCC5_HUMAN	56250.238
UNG_HUMAN	813.69641
COX41_HUMAN	57287.367
HEM1_HUMAN	1500.0824
LAMP2_HUMAN	56089.625
RINI_HUMAN	43102.371
ICAM2_HUMAN	49936.496
EF2_HUMAN	184454.25
PDIA4_HUMAN	121896.94
P4HA1_HUMAN	8914.5566
TCTP_HUMAN	16337.844
HEM2_HUMAN	5075.4551
HLAE_HUMAN	1584.8523
PLST_HUMAN	144026.27
ACPH_HUMAN	15920.848
ETFA_HUMAN	26740.256
GYS1_HUMAN	4624.3018
KAP2_HUMAN	8342.8467
T2FB_HUMAN	4336.6797
CD59_HUMAN	15545.715

MTDC_HUMAN	4437.9102
MIF_HUMAN	22580.24
CD99_HUMAN	1227.3181
GLU2B_HUMAN	70292.945
HCLS1_HUMAN	6115.3926
FPPS_HUMAN	63220.773
CX7A2_HUMAN	20557.342
NID1_HUMAN	16875.102
AK1A1_HUMAN	27731.168
KPYM_HUMAN	434190.38
ENPL_HUMAN	355322.53
CCNB1_HUMAN	1753.3027
MYL6B_HUMAN	9949.46
RSMB_HUMAN	46895.078
IDE_HUMAN	20115.775
PO2F1_HUMAN	575.53284
HNRPL_HUMAN	15545.881
SYDC_HUMAN	37746.012
ETS1_HUMAN	1351.1639
PLAK_HUMAN	24495.266
QCR7_HUMAN	1074.2371
BRAF_HUMAN	4294.6445
FABP4_HUMAN	29439.484
GLNA_HUMAN	500.15845
ALDR_HUMAN	46633.715
AMPN_HUMAN	51414.453
PVR_HUMAN	18136.506
RAC2_HUMAN	20627.389
ERF3A_HUMAN	12485.81
ARSA_HUMAN	1654.7946
B4GT1_HUMAN	2070.6711
EZRI_HUMAN	39179.836
UCHL3_HUMAN	6373.7588
FOSL1_HUMAN	699.44073
FOSL2_HUMAN	295.16467
MCP_HUMAN	3781.5969
NDKA_HUMAN	29236.584
NQO1_HUMAN	24529.979
GNS_HUMAN	45518.094
PHKG2_HUMAN	2264.6597
ARSB_HUMAN	2245.6704
RS2_HUMAN	141758.16
ITF2_HUMAN	650.43085
DESP_HUMAN	1128.5747
RFA2_HUMAN	9102.5986
TIMP2_HUMAN	4822.2622
CD44_HUMAN	8929.1641
CBR1_HUMAN	22586.982
ACADS_HUMAN	2081.5513
CREB1_HUMAN	3179.4602

BGAL_HUMAN	10916.927
PECA1_HUMAN	69292.398
PP2BB_HUMAN	5597.7153
NCK1_HUMAN	9915.0869
GCFC2_HUMAN	964.54303
H15_HUMAN	204835.72
H13_HUMAN	40379.086
H12_HUMAN	46997.852
NCPR_HUMAN	11932.806
FER_HUMAN	591.42566
AT2A2_HUMAN	39642.336
PLCG2_HUMAN	6254.4971
FAAA_HUMAN	5388.3535
STMN1_HUMAN	69934.305
YBOX3_HUMAN	13746.435
ZNF22_HUMAN	1138.0157
NAGAB_HUMAN	4282.1099
HMGA1_HUMAN	36989.691
TMM11_HUMAN	3988.3274
AATC_HUMAN	21876.693
KPCA_HUMAN	5386.2065
ITA2_HUMAN	19508.947
CXA1_HUMAN	1254.7799
ASM_HUMAN	1093.0795
UBF1_HUMAN	9900.3223
JUND_HUMAN	693.104
TAL1_HUMAN	948.23108
ATF7_HUMAN	131.82446
NDUB7_HUMAN	5389.751
KAPCA_HUMAN	11972.967
CAN2_HUMAN	39623.012
PTN2_HUMAN	2108.314
PYRG1_HUMAN	15254.519
EGLN_HUMAN	47194.961
DDX5_HUMAN	88554.102
PFKAL_HUMAN	46996.945
VGFR1_HUMAN	11280.547
PRS6A_HUMAN	43280.492
TCPA_HUMAN	63797.664
PTN1_HUMAN	18671.795
ERCC2_HUMAN	3995.8813
RL35A_HUMAN	78347.469
ITB5_HUMAN	7417.7964
ARF4_HUMAN	111626.95
RL7_HUMAN	180785.47
VINC_HUMAN	79424.336
PTPRA_HUMAN	2105.9866
ARY1_HUMAN	1509.441
SON_HUMAN	8675.7432
NELFE_HUMAN	5026.1885

RL17_HUMAN	92665.758
PGAM1_HUMAN	75572.703
RCC1_HUMAN	22047.41
ATF1_HUMAN	916.49762
ATF6A_HUMAN	470.56329
DNLI1_HUMAN	7363.8931
XRCC1_HUMAN	4632.3032
AMD_HUMAN	986.24841
CADH2_HUMAN	12462.793
PLCG1_HUMAN	11349.414
NUCL_HUMAN	169929.2
HXK1_HUMAN	51682.582
RPB3_HUMAN	2025.1322
RPAB1_HUMAN	4653.4995
NDUV2_HUMAN	3543.5608
ERCC3_HUMAN	3752.6567
RO52_HUMAN	5444.876
E2AK2_HUMAN	10130.57
SPEE_HUMAN	46019.273
SL9A1_HUMAN	1501.9545
CSK22_HUMAN	7138.3638
NFKB1_HUMAN	4916.4028
AT2B1_HUMAN	5892.2778
IF2B_HUMAN	22675.963
ANXA7_HUMAN	23868.35
TBP_HUMAN	844.11737
CCNA2_HUMAN	800.95673
BTF3_HUMAN	6366.4092
RAB3B_HUMAN	2891.0369
RAB4A_HUMAN	3621.0222
RAB5A_HUMAN	61661.656
RAB6A_HUMAN	41112.563
MSH3_HUMAN	1998.3606
ANPRB_HUMAN	436.92941
PSB1_HUMAN	37049.082
MPRD_HUMAN	5067.7085
COX5A_HUMAN	1073.5159
LMNB1_HUMAN	65082.59
ICAL_HUMAN	14924.603
IMDH1_HUMAN	6097.77
CO5A1_HUMAN	2344.3472
NEBU_HUMAN	1633.1442
ASPG_HUMAN	3080.3882
RASA1_HUMAN	6103.8306
PTMS_HUMAN	790.47937
CD11B_HUMAN	9807.4473
GSTM3_HUMAN	1491.7838
VATB2_HUMAN	12329.401
VATC1_HUMAN	5500.853
CSRP1_HUMAN	60031.859

FLNA_HUMAN	234274.22
NF1_HUMAN	680.61023
AOFA_HUMAN	1586.6565
ACOHC_HUMAN	22892.232
S1PR1_HUMAN	6836.0762
SYT1_HUMAN	1406.0717
5NTD_HUMAN	16023.894
TAF1_HUMAN	342.918
MK_HUMAN	1907.4556
VDAC1_HUMAN	82646.266
PGS1_HUMAN	12845.084
SDHB_HUMAN	21031.559
CD9_HUMAN	18841.979
COMT_HUMAN	6507.4575
TGM2_HUMAN	347451.59
MUTA_HUMAN	9123.4473
OSBP1_HUMAN	6974.5015
PIMT_HUMAN	2391.8025
FBRL_HUMAN	11991.319
PUR2_HUMAN	23926.66
PUR6_HUMAN	46169.223
SCP2_HUMAN	23367.002
UBA1_HUMAN	69182.945
NDKB_HUMAN	123871.73
ADRO_HUMAN	12494.466
ROA2_HUMAN	137389.89
RFX1_HUMAN	796.68048
CBL_HUMAN	5162.605
IBP4_HUMAN	985.50256
KAPCB_HUMAN	1840.0356
QCR2_HUMAN	19776.75
HEMH_HUMAN	4047.5652
XPA_HUMAN	789.15607
TCEA1_HUMAN	13277.942
PGH1_HUMAN	651.77759
ITA6_HUMAN	20486.033
SFPQ_HUMAN	99802.516
TBG1_HUMAN	12227.11
PPIB_HUMAN	157730.25
MAOM_HUMAN	14352.313
SYWC_HUMAN	32134.473
RS3_HUMAN	209943.89
KS6B1_HUMAN	3883.6018
JAK1_HUMAN	5687.6816
PTPRB_HUMAN	12655.444
PTPRE_HUMAN	7820.3945
PTPRG_HUMAN	988.3844
SP100_HUMAN	1907.2966
CRCM_HUMAN	493.6897
TNFL4_HUMAN	5913.1084

SAHH_HUMAN	81168.719
COF1_HUMAN	560878.31
IF4B_HUMAN	20053.877
HAP40_HUMAN	1821.6024
AT2B4_HUMAN	8954.5674
DGKA_HUMAN	5413.2075
CPT2_HUMAN	3477.907
KTHY_HUMAN	18964.801
RIR1_HUMAN	15321.891
CCND1_HUMAN	1605.0339
RAE1_HUMAN	664.52167
ERD21_HUMAN	1353.7333
COT2_HUMAN	1256.4491
EF1B_HUMAN	18988.158
AT5F1_HUMAN	16870.445
PPAC_HUMAN	15848.614
KPCL_HUMAN	4624.4531
THIL_HUMAN	27519.9
MYL9_HUMAN	1590.1799
RPB1_HUMAN	36424.805
CDK2_HUMAN	11312.94
ARBK1_HUMAN	4698.7827
PAR1_HUMAN	1600.9375
MCM3_HUMAN	48320.707
THTM_HUMAN	8200.5186
RS12_HUMAN	23424.451
BRD2_HUMAN	1136.0365
TNR6_HUMAN	71.612846
TYY1_HUMAN	2497.1328
DNJB1_HUMAN	9610.5381
DNJB2_HUMAN	549.72339
ATPA_HUMAN	226277.77
CATS_HUMAN	1915.2017
PSA1_HUMAN	94463.695
PSA2_HUMAN	26446.385
PSA3_HUMAN	38851.344
PSA4_HUMAN	79270.711
TNR5_HUMAN	2064.9919
ITA3_HUMAN	29955.045
PTX3_HUMAN	2370.1614
MOES_HUMAN	146513.64
DDX6_HUMAN	17168.803
DNMT1_HUMAN	8194.5693
U2AF2_HUMAN	41037.77
RL13_HUMAN	124270.67
RAE2_HUMAN	719.32074
IVD_HUMAN	2044.7245
MGAT1_HUMAN	4582.4819
HMGB2_HUMAN	20843.043
PTBP1_HUMAN	66962.422

SYTC_HUMAN	33415.109
SYVC_HUMAN	22045.979
EF1G_HUMAN	130441.89
FKBP2_HUMAN	20934.957
IL3RA_HUMAN	1009.7776
STOM_HUMAN	31518.754
KAD4_HUMAN	674.08911
1433T_HUMAN	57497.668
MK03_HUMAN	16960.723
MARK3_HUMAN	659.11169
RL10_HUMAN	44995.754
CO8A1_HUMAN	1771.0068
RFA1_HUMAN	16459.178
APEX1_HUMAN	30435.969
CD82_HUMAN	965.00067
DCK_HUMAN	3082.3049
PYR1_HUMAN	36344.078
CALR_HUMAN	190105.42
MAP4_HUMAN	12846.351
CALX_HUMAN	120058.35
P85A_HUMAN	1522.6318
IP3KB_HUMAN	1449.5415
PSB8_HUMAN	3903.4832
PSB9_HUMAN	3926.7507
PSA5_HUMAN	26879.02
PSB4_HUMAN	18715.645
PSB6_HUMAN	44580.336
PSB5_HUMAN	36575.113
GSTM2_HUMAN	811.59082
ABCD3_HUMAN	7980.9106
ITPI2_HUMAN	4097.7793
LYOX_HUMAN	2791.2441
NDUS1_HUMAN	17729.863
DPOD1_HUMAN	11064.794
SMCA1_HUMAN	1108.1094
MK01_HUMAN	48721.387
RXRB_HUMAN	1180.3344
ERCC5_HUMAN	1048.475
GRN_HUMAN	4768.228
PTPRM_HUMAN	1281.8965
AMPL_HUMAN	37338.629
CD34_HUMAN	4790.3979
T2EA_HUMAN	3315.5706
T2EB_HUMAN	3155.9155
TPP2_HUMAN	13628.165
IMPA1_HUMAN	7557.1255
CCN2_HUMAN	31984.688
EPHA2_HUMAN	12922.856
EPHB2_HUMAN	13806.188
SHC1_HUMAN	5310.9575

3MG_HUMAN	6728.8398
TKT_HUMAN	131761.8
CASP1_HUMAN	715.95251
NOS3_HUMAN	4218.5835
RBMS1_HUMAN	4811.603
PML_HUMAN	11437.765
TYK2_HUMAN	1695.2499
EF1D_HUMAN	27198.523
MARCS_HUMAN	35950.758
GNA11_HUMAN	18255.527
AL4A1_HUMAN	4804.77
ERP29_HUMAN	30347.586
PRDX6_HUMAN	138866.19
BLVRB_HUMAN	8129.5649
PRDX5_HUMAN	29139.029
PRDX3_HUMAN	25959.271
RL12_HUMAN	110491.98
ECHM_HUMAN	14660.548
KCY_HUMAN	27612.066
PEBP1_HUMAN	25733.848
PDIA3_HUMAN	163236.28
2AAA_HUMAN	64857.914
2AAB_HUMAN	5169.0366
CDC27_HUMAN	5295.3774
PPIF_HUMAN	12038.874
NKTR_HUMAN	1146.705
NMT1_HUMAN	21198.285
HMOX2_HUMAN	17206.92
PURA2_HUMAN	64854.637
UFO_HUMAN	2821.8923
AMRP_HUMAN	5605.2183
TSPO_HUMAN	11275.421
PUR8_HUMAN	18443.176
CLIP1_HUMAN	5910.1069
SORCN_HUMAN	5135.9629
ILEU_HUMAN	7315.2441
CTR1_HUMAN	4440.667
AL1B1_HUMAN	6561.8071
RPB2_HUMAN	8539.0781
SDHA_HUMAN	10926.857
COR1A_HUMAN	952.96545
GDIA_HUMAN	22240.139
METK2_HUMAN	45303.387
KAP3_HUMAN	10655.016
RIR2_HUMAN	12010.132
SDC4_HUMAN	449.49142
TIA1_HUMAN	17743.215
SC6A6_HUMAN	425.96136
DNJA1_HUMAN	13266.916
AKT1_HUMAN	3677.1672

QCR1_HUMAN	88209.625
3HIDH_HUMAN	6164.0835
PUR9_HUMAN	33326.695
HNRH3_HUMAN	7665.3848
HNRH1_HUMAN	53704.176
1433B_HUMAN	171325.19
STIP1_HUMAN	126000.66
S10AB_HUMAN	19167.063
PRDX2_HUMAN	51761.141
ARRB2_HUMAN	402.82175
GLPK_HUMAN	948.87927
DCTD_HUMAN	6959.8013
P5CR1_HUMAN	5343.5063
GBP1_HUMAN	4409.2363
GBP2_HUMAN	4745.9126
ELF1_HUMAN	1248.7272
TF2H1_HUMAN	752.24292
STX2_HUMAN	332.52179
RL9_HUMAN	69590.078
ACSL1_HUMAN	4774.0811
CADH5_HUMAN	31538.055
KINH_HUMAN	35940.059
DUT_HUMAN	7893.5361
MRP1_HUMAN	4488.603
ABCD1_HUMAN	678.65106
TTK_HUMAN	794.8653
MCM4_HUMAN	12189.012
MCM5_HUMAN	30692.289
MCM7_HUMAN	22200.145
GLYC_HUMAN	4887.7197
GLYM_HUMAN	57414.117
HS71L_HUMAN	1655.7719
HSP74_HUMAN	34852.285
GRK5_HUMAN	187.39117
MPI_HUMAN	3590.2429
GPC1_HUMAN	588.39423
PROF2_HUMAN	20621.381
CTNA1_HUMAN	54780.762
CTNB1_HUMAN	50255.52
PHB1_HUMAN	120611.07
SPB6_HUMAN	26250.859
MERL_HUMAN	3083.5
RADI_HUMAN	452688.31
RFA3_HUMAN	3168.9814
RFC4_HUMAN	9001.3809
RFC2_HUMAN	8263.7393
RFC1_HUMAN	3950.3677
RL22_HUMAN	132464.86
T2FA_HUMAN	6723.5444
SPRE_HUMAN	6612.8716

HRH1_HUMAN	136.86844
CBS_HUMAN	579.21277
FBN1_HUMAN	3538.2546
FBN2_HUMAN	185.99892
GDE_HUMAN	4596.6772
MYH9_HUMAN	217315.06
MYH10_HUMAN	198290.22
TIE1_HUMAN	17724.949
COPB2_HUMAN	47896.918
SOAT1_HUMAN	2506.7815
ADDA_HUMAN	7447.5186
BASI_HUMAN	59151.156
FUS_HUMAN	64598.82
NU214_HUMAN	7180.6558
DEK_HUMAN	21468.023
SOX18_HUMAN	1429.7357
MYH11_HUMAN	59317.129
PPM1A_HUMAN	5668.522
HMGCL_HUMAN	4987.2261
VGFR3_HUMAN	7784.2529
VGFR2_HUMAN	4206.6621
PRS7_HUMAN	63094.68
GGT5_HUMAN	2160.0308
ARL2_HUMAN	13167.86
ARL3_HUMAN	24767.965
MP2K2_HUMAN	17850.107
ATPG_HUMAN	43328.785
VATE1_HUMAN	9408.8027
HEM6_HUMAN	12129.152
RL4_HUMAN	154555.95
8ODP_HUMAN	1389.2751
LONM_HUMAN	12458.542
PGM1_HUMAN	10587.21
PP1G_HUMAN	10809.115
GNL1_HUMAN	31170.896
ODO2_HUMAN	23093.295
GMPR1_HUMAN	4135.4121
GPX4_HUMAN	10956.099
ACVL1_HUMAN	3631.0405
DHB2_HUMAN	1749.9944
SRP14_HUMAN	23675.799
TGFR2_HUMAN	4739.2949
NUP62_HUMAN	813.67969
HPCL1_HUMAN	7474.6479
FDFT_HUMAN	11112.704
ZEB1_HUMAN	1968.5884
TAGL2_HUMAN	46514.918
TALDO_HUMAN	119847.58
SYUA_HUMAN	3937.2314
ETFB_HUMAN	19420.623

RBMX_HUMAN	17117.602
COIL_HUMAN	2423.7979
VKGC_HUMAN	2719.281
LICH_HUMAN	3257.7861
VATA_HUMAN	19651.096
GRP75_HUMAN	122187.07
IF4A3_HUMAN	29505.904
SMBP2_HUMAN	597.84399
CDN1A_HUMAN	1170.6616
RS19_HUMAN	103118.49
RL3_HUMAN	42154.613
COIA1_HUMAN	30206.619
OST48_HUMAN	97193.633
AN32A_HUMAN	35692.664
FEN1_HUMAN	35843.91
CUX1_HUMAN	523.9657
CAPG_HUMAN	23614.377
CAP2_HUMAN	3811.4001
IL6RB_HUMAN	6566.6572
TXLNA_HUMAN	9039.6758
TCPZ_HUMAN	105738.97
NNMT_HUMAN	42241.895
PSB10_HUMAN	2054.0842
PBX2_HUMAN	1523.0085
RL13A_HUMAN	137697.64
ARL1_HUMAN	20241.004
MLH1_HUMAN	11211.166
STAT3_HUMAN	9042.0977
UBP8_HUMAN	5899.9751
MDHC_HUMAN	78333.328
MDHM_HUMAN	124775.66
RFC5_HUMAN	9962.5732
RFC3_HUMAN	4783.0747
ECHA_HUMAN	38832.059
IF2G_HUMAN	36552.723
CETN2_HUMAN	1007.7457
ETV6_HUMAN	2813.0615
EIF2D_HUMAN	5031.3931
BUD31_HUMAN	3281.8677
UBA7_HUMAN	2752.0916
NAA10_HUMAN	17944.529
KDM5C_HUMAN	295.02756
CSK_HUMAN	10629.829
GARS_HUMAN	23745.688
SYIC_HUMAN	51666.457
S19A1_HUMAN	1242.0193
EIF1_HUMAN	4574.7002
KPCI_HUMAN	610.53357
ELK3_HUMAN	967.62378
ACTY_HUMAN	27953.477

ECI1_HUMAN	7518.3545
LAP2A_HUMAN	7832.3101
LAP2B_HUMAN	96543.344
STAT1_HUMAN	21638.145
STAT6_HUMAN	6627.29
STA5A_HUMAN	8190.3081
MTREX_HUMAN	11552.879
AK1C3_HUMAN	3984.1858
PK3CA_HUMAN	1502.8998
PK3CB_HUMAN	1481.5663
MTOR_HUMAN	11141.524
PI4KA_HUMAN	4484.3794
EPS15_HUMAN	7864.9907
CASP3_HUMAN	22154.256
CASP2_HUMAN	370.74652
RS27_HUMAN	10662.353
ABL2_HUMAN	480.40259
HELZ_HUMAN	1419.8743
CNDD3_HUMAN	1473.4126
RBM34_HUMAN	7327.9517
LPPRC_HUMAN	42629.922
THIM_HUMAN	12759.361
RL35_HUMAN	55778.59
PCP_HUMAN	25797.563
HD_HUMAN	4447.3003
ECE1_HUMAN	15039.452
MTHR_HUMAN	693.92365
SATT_HUMAN	965.32733
LIS1_HUMAN	20534.76
MUC18_HUMAN	103321
CACP_HUMAN	4942.0322
MATR3_HUMAN	27927.438
MSH2_HUMAN	13999.829
GRK6_HUMAN	1788.1428
GPDM_HUMAN	10400.199
SSRA_HUMAN	35563.215
PTN9_HUMAN	2415.9482
RANG_HUMAN	49911.258
NAMPT_HUMAN	93131.781
PRS6B_HUMAN	29696.123
EFTS_HUMAN	6996.4521
PPIC_HUMAN	9268.5723
VDAC2_HUMAN	83494.688
ACDSB_HUMAN	3537.5027
CBX5_HUMAN	2487.9771
UBP5_HUMAN	38056.648
MK08_HUMAN	1786.6564
MP2K4_HUMAN	5223.5078
KI67_HUMAN	2560.7349
KPB2_HUMAN	861.72839

KPB1_HUMAN	1584.7535
RAGP1_HUMAN	22077.295
RECQ1_HUMAN	25380.945
NOP2_HUMAN	8034.603
ATRX_HUMAN	1915.608
CRK_HUMAN	8364.2451
CRKL_HUMAN	6487.9849
IF2M_HUMAN	3892.6506
BAG6_HUMAN	4557.8086
NSF_HUMAN	21949.498
NOTC1_HUMAN	3403.3281
MP2K3_HUMAN	5995.4443
BRCC3_HUMAN	2413.3716
RL27A_HUMAN	217050.41
RL5_HUMAN	56284.934
RL21_HUMAN	44968.293
RL28_HUMAN	46970.703
RS9_HUMAN	211001.88
RS5_HUMAN	112582.59
RS10_HUMAN	75139.539
MAP1B_HUMAN	26413.447
GNPI1_HUMAN	14325.009
NEDD4_HUMAN	3178.6785
UTRN_HUMAN	11602.329
IQGA1_HUMAN	161302.39
GLYG_HUMAN	13987.969
STT3A_HUMAN	52649.082
PA24A_HUMAN	2234.561
CAZA2_HUMAN	29131.941
CAPZB_HUMAN	44189.859
IF1AX_HUMAN	9563.5674
AL1A3_HUMAN	584.45044
SYQ_HUMAN	32525.063
RL29_HUMAN	34376.508
TISD_HUMAN	983.28088
UCRI_HUMAN	17907.48
ATPO_HUMAN	57067.836
LIMS1_HUMAN	14715.433
GLIP1_HUMAN	501.12036
PPCE_HUMAN	21199.074
MAOX_HUMAN	18166.012
IREB2_HUMAN	616.09686
TFPI2_HUMAN	539.14905
RFX5_HUMAN	4195.3257
PI42A_HUMAN	6516.666
COPD_HUMAN	46132.715
LSS_HUMAN	25337.494
PP2BC_HUMAN	1147.7574
GSH1_HUMAN	2800.4348
GSH0_HUMAN	9443.0713

CD151_HUMAN	8054.2217
TPC10_HUMAN	1090.7346
PSMD8_HUMAN	41004.613
PRC2A_HUMAN	2656.1333
GSHB_HUMAN	17319.068
TCPE_HUMAN	96814.719
PTSS1_HUMAN	6866.2505
NEST_HUMAN	57576.262
HSP13_HUMAN	10088.375
KC1A_HUMAN	11132.366
KC1D_HUMAN	4141.1123
IDHP_HUMAN	78000.422
PK3CG_HUMAN	2212.7534
PIPNB_HUMAN	16301.907
DPOD2_HUMAN	4952.6401
MRP_HUMAN	16470.666
PAXI_HUMAN	4264.5957
CAMLG_HUMAN	629.72437
NR2C2_HUMAN	789.11353
MAPK2_HUMAN	2200.5767
DNSL1_HUMAN	987.94312
AL9A1_HUMAN	13604.293
RL34_HUMAN	58295.992
RPIA_HUMAN	3092.5999
LMAN1_HUMAN	21501.572
NRAM2_HUMAN	949.32764
NASP_HUMAN	29847.951
FAS_HUMAN	69162.852
FNTA_HUMAN	10723.553
FNTB_HUMAN	4966.248
DHYS_HUMAN	1886.423
TCPG_HUMAN	77900.07
RM19_HUMAN	9196.4463
ARRB1_HUMAN	8261.79
EFTU_HUMAN	82754.555
AL7A1_HUMAN	24073.861
UB2R1_HUMAN	4891.5566
INPP_HUMAN	4450.877
SRP09_HUMAN	56043.633
PCY1A_HUMAN	11132.997
SYAC_HUMAN	24840.352
SYCC_HUMAN	17199.266
SYHM_HUMAN	6186.8848
SYSC_HUMAN	28443.141
PPM1F_HUMAN	26688.574
MA2A2_HUMAN	1976.1152
PRI1_HUMAN	2082.8806
PRI2_HUMAN	1795.1942
CASP4_HUMAN	2378.0112
KC1E_HUMAN	544.25769

PSB3_HUMAN	33271.184
PSB2_HUMAN	33639.43
MCM2_HUMAN	18535.391
ACADV_HUMAN	12520.3
YLPM1_HUMAN	7186.7598
VPS41_HUMAN	1534.3363
TMEDA_HUMAN	41312.176
RBM25_HUMAN	10849.525
NUMB_HUMAN	6609.6694
CLK3_HUMAN	1009.2434
PSN1_HUMAN	1632.2241
EI2BB_HUMAN	5241.3413
HINT1_HUMAN	6835.4409
NU153_HUMAN	6987.3994
RBP2_HUMAN	12821.955
RGS19_HUMAN	1052.4719
RGS3_HUMAN	3337.2651
PSN2_HUMAN	564.58667
TSC2_HUMAN	3082.1572
NDUV1_HUMAN	12946.105
GSK3A_HUMAN	2227.2664
GSK3B_HUMAN	6429.0356
TAF6_HUMAN	980.23529
5NTC_HUMAN	3255.7549
SPS1_HUMAN	6662.5103
MTHFS_HUMAN	1789.1283
GUAA_HUMAN	20881.539
DNLI3_HUMAN	5248.1587
DNLI4_HUMAN	2090.4792
MRE11_HUMAN	6321.9663
ENTP1_HUMAN	1204.8344
GNAQ_HUMAN	9532.7656
IDH3A_HUMAN	11821.114
ST1A1_HUMAN	2272.6086
MMP14_HUMAN	23670.453
PPOX_HUMAN	2730.0166
GDIB_HUMAN	73283.203
EMD_HUMAN	8438.2686
CPT1A_HUMAN	9552.8535
S26A2_HUMAN	941.88879
SPB8_HUMAN	13324.65
SPB9_HUMAN	54581.918
SERPH_HUMAN	103962.5
PDLI4_HUMAN	7850.063
F10A1_HUMAN	91254.844
PEX5_HUMAN	1364.9526
ERF_HUMAN	1154.2745
VASP_HUMAN	7700.0054
DYN2_HUMAN	26795.51
MAP2_HUMAN	5441.9434

AP4A_HUMAN	3844.1445
CDK7_HUMAN	3840.8721
KNTC1_HUMAN	2621.8645
RASF2_HUMAN	3219.6987
CDK9_HUMAN	4672.6465
LRBA_HUMAN	4886.0718
BCAM_HUMAN	5643.4482
PPT1_HUMAN	4978.7456
RL14_HUMAN	133087.61
TCPQ_HUMAN	169807.19
TCPD_HUMAN	45651.309
ANX11_HUMAN	26444.209
PAPOA_HUMAN	8123.0078
FXR1_HUMAN	17156.877
FXR2_HUMAN	14816.573
RAB5C_HUMAN	27774.527
RAB7A_HUMAN	85546.141
RAB9A_HUMAN	4559.0625
RAB13_HUMAN	12334.636
RB27A_HUMAN	8543.8154
PLCD1_HUMAN	1443.4373
RT29_HUMAN	5447.6372
DUS3_HUMAN	7506.1152
SMCA4_HUMAN	7784.7319
IDH3G_HUMAN	4303.0938
GALK1_HUMAN	15355.961
SSRD_HUMAN	38958.316
BAP31_HUMAN	16724.746
TPMT_HUMAN	3214.501
MECP2_HUMAN	782.96533
HCFC1_HUMAN	15502.865
IRAK1_HUMAN	1040.9404
CAV2_HUMAN	1103.0354
AL3A2_HUMAN	4512.1836
DHB4_HUMAN	17965.973
PSMD7_HUMAN	20969.346
SUOX_HUMAN	2798.4561
SPHM_HUMAN	7119.4404
STA5B_HUMAN	1826.9604
UBP11_HUMAN	2528.8274
CLCN7_HUMAN	2208.5723
VAMP7_HUMAN	2584.9375
KS6A3_HUMAN	6642.1812
BMX_HUMAN	8854.7188
AFF1_HUMAN	172.5405
HDGF_HUMAN	18935.133
CCNH_HUMAN	1046.3474
MAT1_HUMAN	1110.4924
NEK3_HUMAN	853.06818
UB2E1_HUMAN	2630.8914

NDUA8_HUMAN	7029.7544
ROA3_HUMAN	29503.615
6PGD_HUMAN	114165.84
HNRPM_HUMAN	106485.76
IMA1_HUMAN	29865.857
IMA5_HUMAN	5312.2163
NCBP2_HUMAN	3565.4055
GDS1_HUMAN	13005.42
RPAB3_HUMAN	2700.2327
MP2K6_HUMAN	2040.9758
GDIR1_HUMAN	70119.367
GDIR2_HUMAN	77377
AGFG1_HUMAN	4067.4541
HNRPF_HUMAN	14941.549
STAT2_HUMAN	2815.2126
MSH6_HUMAN	7829.4487
KIF11_HUMAN	4080.7021
VAV2_HUMAN	3854.9705
ZN143_HUMAN	1040.8418
RBM5_HUMAN	4364.1899
RIDA_HUMAN	350.3226
SPSY_HUMAN	25166.695
HXK2_HUMAN	27907.621
EFNB2_HUMAN	518.72815
RM12_HUMAN	29082.467
DGKQ_HUMAN	134.32155
NDST1_HUMAN	1152.0187
THOP1_HUMAN	12936.317
CAZA1_HUMAN	90618.008
HMGA2_HUMAN	626.73248
CRIP2_HUMAN	42227.063
NUP98_HUMAN	8694.3105
BIEA_HUMAN	26529.984
TXTP_HUMAN	17679.092
PPP5_HUMAN	11345.825
PLK1_HUMAN	2285.5811
DAPK1_HUMAN	1655.9705
ARFP2_HUMAN	3752.5823
ARFP1_HUMAN	6209.543
NUBP1_HUMAN	5219.1055
ACLY_HUMAN	85466.875
MAP11_HUMAN	5669.3887
SUCA_HUMAN	10023.239
MVD1_HUMAN	4877.3325
PGTB1_HUMAN	2778.3838
PGTB2_HUMAN	6198.1704
COPB_HUMAN	30771.094
COPA_HUMAN	57390.551
CATC_HUMAN	10697.513
LIMK1_HUMAN	8513.0488

LIMK2_HUMAN	522.4726
CLH2_HUMAN	548.76843
AP3M2_HUMAN	1374.8934
AP2S1_HUMAN	11708.354
CCHL_HUMAN	4893.0391
MK12_HUMAN	3627.0027
PTTG_HUMAN	2599.6895
SMTN_HUMAN	2245.5105
MOT1_HUMAN	7394.8403
IST1_HUMAN	10810.538
SC24C_HUMAN	20238.555
TCP4_HUMAN	46681.391
DPOG1_HUMAN	1203.09
ICLN_HUMAN	13765.764
SYRC_HUMAN	37066.441
HIRA_HUMAN	1549.7128
ATX3_HUMAN	3174.3979
ATN1_HUMAN	1194.1503
PMS1_HUMAN	660.1925
PMS2_HUMAN	407.552
CA2D1_HUMAN	2137.8101
SYYC_HUMAN	37485.008
UBP14_HUMAN	22156.984
AAKG1_HUMAN	3973.8047
HSP72_HUMAN	15876.151
BCAT1_HUMAN	23506.277
AT1B3_HUMAN	26732.854
RD23B_HUMAN	1514.1
EPHB4_HUMAN	3505.3032
ANAG_HUMAN	2630.3433
KAD2_HUMAN	5883.04
P5CS_HUMAN	18233.252
SNAA_HUMAN	20385.932
IF5_HUMAN	20783.857
S12A2_HUMAN	5599.7861
PSMD4_HUMAN	22762.998
DRG2_HUMAN	7642.3667
XPO2_HUMAN	180059.86
TERA_HUMAN	173620.5
MFAP1_HUMAN	1249.6158
ECHB_HUMAN	61984.871
MANF_HUMAN	17011.236
AFAD_HUMAN	5472.5874
ELL_HUMAN	997.39563
BRPF1_HUMAN	1715.6017
NP1L1_HUMAN	51465.625
CASP7_HUMAN	6880.0308
CASP6_HUMAN	4846.0405
ADK_HUMAN	11189.154
DSRAD_HUMAN	13574.426

LAMB2_HUMAN	4774.6382
CAD13_HUMAN	4594.6313
TPD52_HUMAN	171.40871
SEC13_HUMAN	12112.771
NH2L1_HUMAN	7065.0303
PSA_HUMAN	32391.719
ALR_HUMAN	321.41794
HNRH2_HUMAN	11720.253
SCOT1_HUMAN	13804.877
EIF3B_HUMAN	21163.893
FCGRN_HUMAN	9622.085
BID_HUMAN	1861.7214
ATPK_HUMAN	21813.365
RRP1_HUMAN	3395.5688
SYMC_HUMAN	33023.051
ARP19_HUMAN	5553.9507
AP1S2_HUMAN	5138.1909
ATP68_HUMAN	7608.5361
ATP5E_HUMA	6167.6646
ATP5I_HUMAN	24281.863
HDAC4_HUMAN	1245.3824
IF6_HUMAN	31900.133
CTBP2_HUMAN	23440.197
NDUA6_HUMAN	10155.073
PEX3_HUMAN	1033.1561
DHB7_HUMAN	2349.8108
BCAR1_HUMAN	9613.6836
STX17_HUMAN	2367.5034
CF298_HUMAN	769.14319
WDR4_HUMAN	1411.3285
TMM33_HUMAN	32118.365
GEMI4_HUMAN	3623.5906
CORO7_HUMAN	6357.6172
NU107_HUMAN	9468.6543
GSDMD_HUMAN	9626.667
SELB_HUMAN	2871.2813
SESN2_HUMAN	576.59955
ANTR2_HUMAN	1564.1658
MTPN_HUMAN	1219.6245
ARPC4_HUMAN	116111.72
CD81_HUMAN	7829.3779
SC61G_HUMAN	32237.779
TPIS_HUMAN	139919.75
EIF3E_HUMAN	31564.098
SC61B_HUMAN	20372.465
PTEN_HUMAN	120.26122
PP4C_HUMAN	9147.6025
GBRL2_HUMAN	9064.8242
ROMO1_HUMAN	3181.0781
MYL6_HUMAN	7895.7715

ACTB_HUMAN	1654821.9
IF4A1_HUMAN	168548.58
RS20_HUMAN	100602.07
PRPS1_HUMAN	5933.7031
PSA6_HUMAN	46150.953
S10AA_HUMAN	45414.473
CDC42_HUMAN	20754.143
DEST_HUMAN	15259.865
GMFB_HUMAN	4442.6484
RAB8A_HUMAN	3974.7139
SPCS3_HUMAN	18976.74
SRP54_HUMAN	9986.4268
RAB4B_HUMAN	2979.1775
RAB2A_HUMAN	33382.379
RAB5B_HUMAN	6368.249
RAB10_HUMAN	7273.5259
UB2D3_HUMAN	18617.799
UBC12_HUMAN	17792.406
UBE2K_HUMAN	20382.93
UBE2N_HUMAN	26718.854
RAB14_HUMAN	23847.314
ARP3_HUMAN	73831.773
ARP2_HUMAN	57675.078
ACTZ_HUMAN	21661.973
CSN2_HUMAN	12790.006
ABCE1_HUMAN	25712.957
RAP1B_HUMAN	36704.723
RAP2B_HUMAN	3239.8599
MAX_HUMAN	1313.5157
RS3A_HUMAN	97801.656
RL26_HUMAN	126625.21
PSME3_HUMAN	11694.925
RL15_HUMAN	172478.84
RL27_HUMAN	175455.67
VA0D1_HUMAN	12618.671
RL37A_HUMAN	6535.9961
RHOA_HUMAN	11484.96
RND3_HUMAN	634.45673
NAA20_HUMAN	2487.4634
CH10_HUMAN	11509.077
S61A1_HUMAN	27298.553
PFD3_HUMAN	11930.111
STXB1_HUMAN	7061.5811
B2MG_HUMAN	1780.1019
DAD1_HUMAN	35445.531
NPC2_HUMAN	5366.396
COPZ1_HUMAN	27137.041
RL37_HUMAN	799.36646
SUMO2_HUMAN	11309.223
DCAF7_HUMAN	3541.6951

WDR5_HUMAN	3001.0566
AP1S1_HUMAN	8195.46
NTF2_HUMAN	5297.6812
HNRPK_HUMAN	200774.8
1433G_HUMAN	56374.992
UBP46_HUMAN	599.11749
RRAS2_HUMAN	14787.567
RS7_HUMAN	70785.133
PP1A_HUMAN	26503.516
PP1B_HUMAN	26600.34
PRS4_HUMAN	41452.945
PRS8_HUMAN	51391.703
RS8_HUMAN	138133.8
RS15A_HUMAN	115670.53
RS16_HUMAN	228366.67
UB2G1_HUMAN	2748.4019
UBE2H_HUMAN	4864.0215
1433E_HUMAN	95212.219
RS14_HUMAN	150765.73
RS23_HUMAN	51567.297
RS18_HUMAN	230132.64
RS29_HUMAN	29360.432
RS13_HUMAN	109648.47
RS11_HUMAN	42731.359
RUXE_HUMAN	18204.586
RUXF_HUMAN	8717.5645
LSM3_HUMAN	4047.6497
LSM6_HUMAN	10084.955
SMD1_HUMAN	11064.144
SMD2_HUMAN	17084.355
SMD3_HUMAN	51441.32
TYB4_HUMAN	6676.2979
ARF6_HUMAN	21823.256
PRS10_HUMAN	27477.564
SELT_HUMAN	4495.9844
TBPL1_HUMAN	844.80902
RL7A_HUMAN	105633.73
RPB7_HUMAN	2689.3538
RB11A_HUMAN	72992.18
ERF1_HUMAN	22084.869
CNBP_HUMAN	6182.7046
YPEL5_HUMAN	932.02911
RS4X_HUMAN	160793.63
PP2AB_HUMAN	36689.645
ACTA_HUMAN	309592.28
RHOB_HUMAN	6728.1563
RL23A_HUMAN	175542.38
RS6_HUMAN	212268.19
H4_HUMAN	216155.48
RAB1A_HUMAN	9379.7402

RAN_HUMAN	273743.97
RL23_HUMAN	97670.82
RAP1A_HUMAN	5037.1382
RS15_HUMAN	26459.119
RS24_HUMAN	45863.098
RS25_HUMAN	205184.64
RS26_HUMAN	60964.871
RS28_HUMAN	34867.465
RS30_HUMAN	72243.563
GBB1_HUMAN	12152.536
RBX1_HUMAN	15834.58
GBB2_HUMAN	48161.879
RL30_HUMAN	77002.617
RL39_HUMAN	32646.506
RL31_HUMAN	100576.59
RL10A_HUMAN	68785.414
RL32_HUMAN	35734.445
RL11_HUMAN	84890.297
RL8_HUMAN	97745.047
PPIA_HUMAN	135695.33
FKB1A_HUMAN	25747.066
RS27A_HUMAN	101102.9
GRB2_HUMAN	7814.5488
TRA2B_HUMAN	8798.71
RAC1_HUMAN	66048.711
AP2B1_HUMAN	30057.127
VAMP2_HUMAN	8591.8584
GNAS2_HUMAN	13769.368
GNAI1_HUMAN	971.46918
1433Z_HUMAN	233514.91
2ABA_HUMAN	32758.611
SUMO1_HUMAN	13395.087
DYL1_HUMAN	10261.795
DYLT1_HUMAN	5080.1392
RL38_HUMAN	45150.152
SKP1_HUMAN	3898.7371
GBG5_HUMAN	1087.2799
RS21_HUMAN	15075.449
IF5A1_HUMAN	68165.188
RACK1_HUMAN	168460.08
SPT4H_HUMAN	2288.7947
UBC9_HUMAN	14491.524
TYB10_HUMAN	4604.0986
PP2AA_HUMAN	1624.2003
YBOX1_HUMAN	36110.605
SC11A_HUMAN	48073.16
CSK2B_HUMAN	7431.5864
TPM4_HUMAN	86432.844
UB2L3_HUMAN	36505.008
EF1A1_HUMAN	1074900.3

TBA1B_HUMAN	920479.63
TBA4A_HUMAN	4846.3491
TBB4B_HUMAN	278878.38
CSK21_HUMAN	10770.755
PA1B2_HUMAN	9481.9873
H31_HUMAN	29896.857
HBB_HUMAN	1632.8333
HBA_HUMAN	2721.895
CXAR_HUMAN	1922.7777
F193A_HUMAN	3197.6721
NOP14_HUMAN	1783.9652
IGBP1_HUMAN	5750.561
SHPS1_HUMAN	1339.4425
SERB_HUMAN	9760.624
RBM6_HUMAN	4905.519
IF4G2_HUMAN	30171.045
RPP38_HUMAN	2719.7981
RPP30_HUMAN	5709.7705
GTF2I_HUMAN	30122.004
PI42B_HUMAN	2225.3489
CNTP1_HUMAN	3121.4097
SRPK2_HUMAN	3310.6504
TCPB_HUMAN	73870.648
S35A2_HUMAN	1486.5364
RAE1L_HUMAN	9630.6201
GSTO1_HUMAN	141174.36
PRKDC_HUMAN	51117.953
ADA17_HUMAN	2614.5898
BL1S1_HUMAN	1334.1541
SRPX_HUMAN	3068.8811
ARGI2_HUMAN	876.79041
NTH_HUMAN	1626.8757
IN35_HUMAN	1083.9956
NUCB2_HUMAN	3993.9031
BASP1_HUMAN	7329.9795
DCD_HUMAN	1898.7072
TMF1_HUMAN	1851.6997
RT22_HUMAN	9168.2949
RT25_HUMAN	2578.8704
RT10_HUMAN	3966.8086
RT35_HUMAN	2222.2959
RT05_HUMAN	3061.9583
KGD4_HUMAN	222.15424
RT11_HUMAN	5457.5361
RT15_HUMAN	7032.4805
RT21_HUMAN	3822.5737
RT34_HUMAN	20352.205
RT06_HUMAN	3561.5371
RT09_HUMAN	4811.5557
SARNP_HUMAN	24794.891

LACTB_HUMAN	2508.9863
COG7_HUMAN	4232.5444
RL24_HUMAN	107985.55
TXN4A_HUMAN	2355.6333
RL36A_HUMAN	16403.221
CBX1_HUMAN	3386.9326
SMAD3_HUMAN	4722.7334
ARF1_HUMAN	100701.4
ARF5_HUMAN	12994.623
ERH_HUMAN	2644.4519
RHOG_HUMAN	9225.7744
RL19_HUMAN	99394.141
SERF2_HUMAN	3145.2695
SRSF3_HUMAN	85689.438
FOXK1_HUMAN	5958.0503
CCZ1B_HUMAN	3440.2783
DAB2_HUMAN	14908.832
PGBM_HUMAN	71780.438
XIAP_HUMAN	1321.7502
EFNB1_HUMAN	4455.75
RBM10_HUMAN	5875.0552
RBM3_HUMAN	13048.833
AT2C1_HUMAN	2821.8911
AT11A_HUMAN	1181.8326
CYC_HUMAN	69709.094
EM55_HUMAN	3309.7153
TFAM_HUMAN	11846.937
PIPNA_HUMAN	8835.6133
S25A3_HUMAN	121711.54
VIGLN_HUMAN	35430.473
TF2B_HUMAN	2858.7244
CDK6_HUMAN	1655.2811
CDK5_HUMAN	5466.6304
CDK17_HUMAN	8294.915
PURA_HUMAN	4429.4302
BORG5_HUMAN	3990.9666
CLH1_HUMAN	88991.555
HSF1_HUMAN	1085.9664
NFKB2_HUMAN	2291.5088
FKBP3_HUMAN	44961.211
REEP5_HUMAN	14157.232
DHSO_HUMAN	6827.606
HNRPU_HUMAN	80333.203
U2AF1_HUMAN	11035.123
SPTB2_HUMAN	55543.422
TIAR_HUMAN	8912.3311
SET_HUMAN	102971.09
SRSF2_HUMAN	35322.324
FOXK2_HUMAN	1628.2706
RELB_HUMAN	407.76498

GALK2_HUMAN	4472.9844
AMPD2_HUMAN	9694.8799
FABP5_HUMAN	63504.516
CAP1_HUMAN	69254.914
FLI1_HUMAN	6698.2817
HMCS1_HUMAN	13001.895
IFM3_HUMAN	5935.5293
LAT1_HUMAN	1731.886
NC2B_HUMAN	3803.0068
EXOSX_HUMAN	4026.2246
OTUD4_HUMAN	610.59692
PFKAP_HUMAN	47139.516
XPC_HUMAN	1524.3271
EWS_HUMAN	35321.371
CDR2_HUMAN	1845.576
OCRL_HUMAN	2853.2607
PLCB3_HUMAN	10215.179
TAGL_HUMAN	5923.1938
AK17A_HUMAN	2711.7966
PYRD_HUMAN	668.91504
ODO1_HUMAN	18600.865
KIF23_HUMAN	789.86389
MMSA_HUMAN	6464.6665
NRG1_HUMAN	281.53024
CP27A_HUMAN	1206.6818
DSG1_HUMAN	724.01752
SP3_HUMAN	244.48688
H11_HUMAN	2143.823
RL18A_HUMAN	84704.797
MP2K1_HUMAN	15151.514
TIE2_HUMAN	2352.3074
FKBP4_HUMAN	21197.125
PLOD1_HUMAN	18570.631
NUCB1_HUMAN	8140.4131
RL6_HUMAN	236886.97
TOP2B_HUMAN	1341.278
AKA12_HUMAN	21617.949
M2OM_HUMAN	20199.412
DYST_HUMAN	3398.551
ENL_HUMAN	821.19623
MECOM_HUMAN	1654.5024
GNA12_HUMAN	1082.1313
CAV1_HUMAN	91327.703
ACY1_HUMAN	2549.7561
TNAP2_HUMAN	1897.4484
LMNB2_HUMAN	21653.582
PTPS_HUMAN	894.72595
UPAR_HUMAN	565.57471
KIME_HUMAN	6148.7192
ERCC6_HUMAN	243.92821

TAP1_HUMAN	3379.4504
TAP2_HUMAN	2027.4617
CEBPZ_HUMAN	3416.645
ZNF92_HUMAN	1134.7111
TF65_HUMAN	8819.5596
UBXN1_HUMAN	9474.9932
GLGB_HUMAN	23981.947
IF4G1_HUMAN	27177.943
NOTC2_HUMAN	804.53027
TLE1_HUMAN	2880.6597
TLE3_HUMAN	1462.0664
LGUL_HUMAN	31591.828
ACVR1_HUMAN	732.00073
SSBP_HUMAN	14581.753
REL_HUMAN	693.70898
1433F_HUMAN	55152.41
PLP2_HUMAN	15023.91
CSTF1_HUMAN	7523.2847
UBE3A_HUMAN	6851.6133
DYN1_HUMAN	1123.5869
PTN12_HUMAN	6912.9224
FAK1_HUMAN	11175.619
SRS11_HUMAN	119659.94
KPCD_HUMAN	2268.2644
CALD1_HUMAN	38767.664
FOLC_HUMAN	1275.4532
CTSL2_HUMAN	1019.7776
PTN11_HUMAN	8894.8818
KDSR_HUMAN	3842.7092
PUR1_HUMAN	8630.4863
GFPT1_HUMAN	18462.035
EXOS9_HUMAN	3336.5413
PSME1_HUMAN	41891.434
SUH_HUMAN	929.86578
MEF2C_HUMAN	501.18341
APLP2_HUMAN	6094.6196
GABPA_HUMAN	2780.5276
RING1_HUMAN	366.73917
FMR1_HUMAN	976.02087
PRDX1_HUMAN	191811.72
RL18_HUMAN	135609.91
C1QBP_HUMAN	22740.957
CKAP4_HUMAN	126268.19
ZO1_HUMAN	18800.482
TISB_HUMAN	1237.8396
SPAG1_HUMAN	1260.9659
KHDR1_HUMAN	31062.98
BAX_HUMAN	19363.359
B2CL1_HUMAN	5177.7041
MCL1_HUMAN	203.89662

DPOE1_HUMAN	5268.8394
KLC1_HUMAN	21555.516
SOS1_HUMAN	1611.8103
SRSF1_HUMAN	85975.867
RHG01_HUMAN	16441.52
TLE5_HUMAN	375.08978
SRSF4_HUMAN	7088.4985
PCDH1_HUMAN	9135.6367
PP2BA_HUMAN	6590.252
DHX9_HUMAN	51972.051
QOR_HUMAN	8867.6338
GOGA3_HUMAN	2879.2207
GOGA2_HUMAN	4112.6128
ECHP_HUMAN	2047.5822
MFGM_HUMAN	3172.022
PDE4D_HUMAN	979.20923
DSC1_HUMAN	1680.5297
HDHD1_HUMAN	3141.6777
TICN1_HUMAN	1073.7427
CD47_HUMAN	15769.748
PPID_HUMAN	16124.484
SSRP1_HUMAN	21676.688
CAMP2_HUMAN	3126.6401
SPIR1_HUMAN	675.3147
SLFN5_HUMAN	3371.4287
VAC14_HUMAN	6266.3896
NSUN2_HUMAN	18074.514
DMWD_HUMAN	539.99884
RBBP4_HUMAN	56044.816
NCBP1_HUMAN	14475.284
MGT5A_HUMAN	452.97415
EP300_HUMAN	3504.5444
AHNK_HUMAN	126574.93
FCHO2_HUMAN	1548.7306
ELP6_HUMAN	817.18817
HSP7E_HUMAN	6115.2417
SCRN3_HUMAN	1878.4067
CGNL1_HUMAN	6724.0264
LTOR4_HUMAN	4276.3047
NEXN_HUMAN	2826.2383
MGAT2_HUMAN	5605.4785
GALT2_HUMAN	8295.4717
GALT1_HUMAN	9097.0459
AP1B1_HUMAN	33870.371
CPSF1_HUMAN	7723.481
BST2_HUMAN	1574.4053
MPPA_HUMAN	7402.0386
SIA4A_HUMAN	1172.1716
SCRN1_HUMAN	9546.1611
WASC5_HUMAN	7457.7188

NU160_HUMAN	8472.1982
SCAP_HUMAN	2041.0123
FOXO1_HUMAN	784.98645
TBL3_HUMAN	1929.3734
TF3C1_HUMAN	2699.144
TWF1_HUMAN	16929.262
ASPH_HUMAN	14366.358
TFCP2_HUMAN	2997.7126
AKP13_HUMAN	2394.9448
FBLN3_HUMAN	29061.188
SNF5_HUMAN	4176.4409
BPTF_HUMAN	1054.6115
CDC20_HUMAN	1778.7034
FSTL1_HUMAN	7762.6387
STX4_HUMAN	2490.8215
GRSF1_HUMAN	6360.4141
M4K2_HUMAN	1831.011
MERTK_HUMAN	3588.2859
SFSWA_HUMAN	1381.2947
CHD3_HUMAN	990.6391
SF3A3_HUMAN	25433.85
DPYD_HUMAN	667.27826
TP53B_HUMAN	4762.041
TM115_HUMAN	1924.2234
IFRD2_HUMAN	2033.109
TRI26_HUMAN	1647.9814
AIMP1_HUMAN	15604.5
ILF2_HUMAN	71978.914
ILF3_HUMAN	88068.031
LMAN2_HUMAN	55308.031
TRAP1_HUMAN	377511.59
TRAF2_HUMAN	3576.0154
DLG1_HUMAN	3058.7756
MYO1E_HUMAN	10459
PP1R8_HUMAN	4151.8179
TP4A2_HUMAN	4468.3438
ABR_HUMAN	5105.7764
NPRL3_HUMAN	768.19421
SEC20_HUMAN	5404.2651
BNIP2_HUMAN	2448.292
NFX1_HUMAN	140.43129
CSTF3_HUMAN	6484.8325
TSN31_HUMAN	1727.787
ECH1_HUMAN	10936.242
RHG05_HUMAN	882.24573
STRN3_HUMAN	5785.4077
CDC16_HUMAN	3540.8311
STK4_HUMAN	4353.2319
FLII_HUMAN	27971.41
TRI32_HUMAN	1292.2351

COASY_HUMAN	5833.3359
RM28_HUMAN	6491.335
ACACA_HUMAN	14580.501
CSN1_HUMAN	14235.05
UBP4_HUMAN	3979.8103
CAF1A_HUMAN	429.63889
CAF1B_HUMAN	1204.9363
RED_HUMAN	8594.6006
MTAP_HUMAN	8786.4219
AAPK1_HUMAN	4973.3154
LIPA1_HUMAN	2000.0463
CACO2_HUMAN	194.65176
EI2BE_HUMAN	18410.107
TADBP_HUMAN	15969.914
ROA0_HUMAN	19030.393
AIMP2_HUMAN	12945.966
FADD_HUMAN	4031.6841
PRDX4_HUMAN	39865.895
MP2K5_HUMAN	595.47998
MK07_HUMAN	825.15601
PAK2_HUMAN	16000.903
CBX3_HUMAN	22520.148
STK3_HUMAN	1191.1119
STX5_HUMAN	3794.3569
PSMD2_HUMAN	33633.008
MMRN1_HUMAN	12808.173
DDX10_HUMAN	823.11389
DNJC3_HUMAN	23362.646
SBP1_HUMAN	7424.3364
NDK3_HUMAN	3839.1394
SRSF9_HUMAN	9936.0947
SRSF5_HUMAN	6729.8179
SRSF6_HUMAN	59913.453
MD2L1_HUMAN	8047.2979
TIF1B_HUMAN	32971.184
SEM3F_HUMAN	470.81659
STX3_HUMAN	1599.1757
G3BP1_HUMAN	34153.73
NMI_HUMAN	5385.606
PTK7_HUMAN	4345.9321
PABP4_HUMAN	28436.953
ATM_HUMAN	1900.6888
GRB10_HUMAN	2781.3855
IFIT5_HUMAN	523.21429
MTA1_HUMAN	2960.8594
EIF3I_HUMAN	20400.613
PPIL2_HUMAN	1130.9117
2A5G_HUMAN	2827.6628
CTBP1_HUMAN	5710.1484
PHLP_HUMAN	5891.3438

PLD1_HUMAN	4664.9893
TARB1_HUMAN	800.93054
UB2V1_HUMAN	35748.813
RM49_HUMAN	862.80072
DC1I2_HUMAN	24246.926
ORC2_HUMAN	1271.1072
ILK_HUMAN	32267.023
NNTM_HUMAN	22509.193
SNTB2_HUMAN	15004.891
XRCC4_HUMAN	4008.3931
PPIG_HUMAN	7905.0249
TCOF_HUMAN	5650.2212
U119A_HUMAN	928.63959
SF3B2_HUMAN	19045.975
OS9_HUMAN	1585.25
GOGA4_HUMAN	2349.824
HAP28_HUMAN	16906.885
ADAM9_HUMAN	10023.81
ADA15_HUMAN	4616.0425
TMED1_HUMAN	4756.7573
FKBP5_HUMAN	24317.615
MYO9B_HUMAN	5309.54
ROCK1_HUMAN	9738.2158
NFAC2_HUMAN	1148.7207
TOP3A_HUMAN	675.52411
SMAD4_HUMAN	3712.687
VPP3_HUMAN	5336.8042
BIRC2_HUMAN	832.91199
PICAL_HUMAN	10797.726
MTM1_HUMAN	2774.7832
SQSTM_HUMAN	6982.9429
MTX1_HUMAN	5121.4673
TBB3_HUMAN	10885.987
ASAH1_HUMAN	664.59528
PRP4B_HUMAN	1841.323
PIN1_HUMAN	6967.8813
ATR_HUMAN	2135.9685
4EBP1_HUMAN	757.80469
RIPK1_HUMAN	1823.0481
HDAC1_HUMAN	18963.57
KCC2G_HUMAN	2892.7188
KCC2D_HUMAN	5115.5317
DCTN2_HUMAN	5653.1509
PKD2_HUMAN	507.67389
ULA1_HUMAN	9541.4814
ITPK1_HUMAN	1793.4835
SNW1_HUMAN	7007.2344
DGKZ_HUMAN	3788.2505
STIM1_HUMAN	2934.594
GRAP_HUMAN	3957.0737

TRA2A_HUMAN	2596.7942
SNX1_HUMAN	13198.551
KRR1_HUMAN	3825.9246
PWP1_HUMAN	4572.1157
MTMR1_HUMAN	981.63739
MTMR2_HUMAN	5927.9341
CUL1_HUMAN	6142.7974
CUL2_HUMAN	11516.884
CUL3_HUMAN	8307.9033
CUL4A_HUMAN	5105.9302
CUL4B_HUMAN	13923.404
ASPP2_HUMAN	487.09207
FCL_HUMAN	17559.395
RAB31_HUMAN	134.48445
RAB32_HUMAN	9258.0303
TPBG_HUMAN	857.85199
FHL1_HUMAN	10877.905
FHL3_HUMAN	1832.8271
RIN1_HUMAN	689.30505
AAMP_HUMAN	2530.458
MOGS_HUMAN	34858.789
CD166_HUMAN	5664.1318
THOC5_HUMAN	2242.3682
SHRM2_HUMAN	1491.6418
SPTN1_HUMAN	34556.434
NOG2_HUMAN	3134.0537
AUHM_HUMAN	1037.0956
DX39B_HUMAN	10060.18
BLMH_HUMAN	26886.865
EXOS2_HUMAN	4128.1431
BMPR2_HUMAN	1858.2813
TBB2A_HUMAN	171592.72
TF2H2_HUMAN	1768.8582
TF2H3_HUMAN	2031.4404
BYST_HUMAN	7522.3452
RPGF1_HUMAN	3031.5049
IDI1_HUMAN	8909.873
PEBB_HUMAN	7295.2217
NFYC_HUMAN	1037.6649
CDK13_HUMAN	1525.9767
CKAP5_HUMAN	27848.309
CIRBP_HUMAN	9194.7979
KCC1A_HUMAN	1742.3441
COTL1_HUMAN	32763.646
HNRPD_HUMAN	134561.2
SCRB2_HUMAN	33273.773
NID2_HUMAN	1056.6304
LRP8_HUMAN	401.26764
DAG1_HUMAN	4143.0518
VEZF1_HUMAN	69.414658

BOP1_HUMAN	3342.8206
UBE4A_HUMAN	3850.6443
SEPT6_HUMAN	5086.7314
KEAP1_HUMAN	1898.6212
URB2_HUMAN	1732.8951
DHX34_HUMAN	1646.6248
MORC3_HUMAN	1047.9119
SAFB2_HUMAN	8117.5488
EIF3A_HUMAN	42766.027
ARHG7_HUMAN	8120.7754
EFR3A_HUMAN	5405.4233
UBP2L_HUMAN	16463.648
SCRIB_HUMAN	6415.894
GIT2_HUMAN	4136.6938
SREC_HUMAN	4429.9375
MLEC_HUMAN	27124.154
TTL12_HUMAN	10871.201
DPOA2_HUMAN	2457.0608
DOCK1_HUMAN	7556.6465
TFDP1_HUMAN	488.07635
FHL2_HUMAN	18841.916
DPYL3_HUMAN	58093.977
ICT1_HUMAN	6214.603
ZMYM3_HUMAN	710.90033
DCTN1_HUMAN	14712.906
DYHC1_HUMAN	76695.633
EI2BA_HUMAN	8836.4473
IF4A2_HUMAN	7409.5513
ELOA1_HUMAN	2042.8197
SRC8_HUMAN	21124.426
NUCG_HUMAN	3567.6628
FLOT2_HUMAN	4322.1968
RCN2_HUMAN	16721.488
TRI25_HUMAN	20488.768
FLNC_HUMAN	48517.734
FKBP8_HUMAN	5300.6812
FA50A_HUMAN	6128.605
FRG1_HUMAN	2310.4822
GNA13_HUMAN	54116.09
GAMT_HUMAN	1584.4115
GALE_HUMAN	9269.4688
LRC32_HUMAN	1031.7319
CAPR1_HUMAN	22754.713
BECN1_HUMAN	1030.6108
RBM39_HUMAN	18046.762
CASL_HUMAN	994.58276
HLTF_HUMAN	1650.7139
ERG1_HUMAN	2510.009
PDIA5_HUMAN	12955.884
KPRA_HUMAN	9367.499

DHX8_HUMAN	2044.6383
MCM6_HUMAN	17950.055
ITPR2_HUMAN	6215.9175
ITPR3_HUMAN	6377.1021
ITPR1_HUMAN	692.75519
RASA3_HUMAN	2995.1506
PLSI_HUMAN	3857.2007
IRF3_HUMAN	4321.3896
LAGE3_HUMAN	875.16571
BLTP2_HUMAN	682.36292
TRIPC_HUMAN	5426.2529
PUM1_HUMAN	4361.3203
MDC1_HUMAN	256.15894
EPN4_HUMAN	10685.605
KANK1_HUMAN	2668.3259
SMC1A_HUMAN	57247.625
RRP1B_HUMAN	5532.4639
DIP2A_HUMAN	7160.7026
RRP5_HUMAN	3649.8667
PSF1_HUMAN	2561.5923
BMS1_HUMAN	2084.886
LPIN1_HUMAN	1980.1121
UBP10_HUMAN	10882.325
MESD_HUMAN	11965.617
GANAB_HUMAN	61798.336
RFTN1_HUMAN	4681.5513
MFS10_HUMAN	2005.0022
2A5D_HUMAN	13167.682
LBR_HUMAN	18913.24
COG2_HUMAN	998.05963
MVP_HUMAN	79378.891
LTBP1_HUMAN	1341.9653
LTBP2_HUMAN	2214.5691
CBX2_HUMAN	412.69336
GOGB1_HUMAN	3822.4224
CASP8_HUMAN	3070.8777
NAA30_HUMAN	3145.5305
KIF22_HUMAN	487.0766
MEF2D_HUMAN	1829.3401
CHD4_HUMAN	17629.957
LASP1_HUMAN	28994.16
PTGR1_HUMAN	9269.8789
NC2A_HUMAN	4417.4033
NFAC4_HUMAN	629.94543
ZN638_HUMAN	4499.252
IMB1_HUMAN	51770.219
NOLC1_HUMAN	2140.2432
NUMA1_HUMAN	11594.357
PSME4_HUMAN	7526.2773
CUL7_HUMAN	2235.7598

SLMAP_HUMAN	781.58588
GAPD1_HUMAN	10668.638
NAA25_HUMAN	8528.5586
MELT_HUMAN	1669.7109
CND2_HUMAN	6371.2876
PAF15_HUMAN	520.6236
SPCS2_HUMAN	20882.053
EMC2_HUMAN	5142.4766
FL2D_HUMAN	1155.7933
PSMD6_HUMAN	16142.966
HERP1_HUMAN	190.15852
MD2BP_HUMAN	1395.2996
MO4L2_HUMAN	2708.1609
ABRX2_HUMAN	2044.4342
SEPT2_HUMAN	42310.77
SART3_HUMAN	15950.833
CND1_HUMAN	12190.58
SUZ12_HUMAN	704.85107
EXOS7_HUMAN	3726.803
TNIP1_HUMAN	828.38934
U5S1_HUMAN	64250.52
SYLM_HUMAN	2770.9014
R3HD1_HUMAN	1941.0024
TRAM2_HUMAN	1043.8091
SNX17_HUMAN	3974.3079
AR6P1_HUMAN	3156.6702
RB3GP_HUMAN	3767.543
S39AE_HUMAN	2156.967
SYK_HUMAN	40671.523
SETB1_HUMAN	1199.6183
LRC14_HUMAN	1165.5725
RRS1_HUMAN	4370.9292
DPOD3_HUMAN	2252.04
IF4H_HUMAN	36116.137
ACAP2_HUMAN	4012.0515
KIF14_HUMAN	351.19437
BRD3_HUMAN	955.23535
WDR43_HUMAN	1329.8915
ACOX1_HUMAN	8398.8721
OXA1L_HUMAN	6956.3867
EEA1_HUMAN	13623.56
PDIA6_HUMAN	137587.38
PA1B3_HUMAN	6543.6582
PDK3_HUMAN	834.35376
PEA15_HUMAN	2612.9648
EBP_HUMAN	13021.274
PMVK_HUMAN	6425.2993
KPCD1_HUMAN	716.2699
PLEC_HUMAN	67663.125
PCM1_HUMAN	2428.241

NOMO1_HUMAN	24814.744
PON2_HUMAN	9299.4844
2A5A_HUMAN	3853.0012
IPYR_HUMAN	46024.602
TEBP_HUMAN	19054.242
STK38_HUMAN	2816.0156
NONO_HUMAN	62421.688
PTPA_HUMAN	25184.549
PTPRK_HUMAN	3636.4634
PWP2_HUMAN	2278.3091
RABE1_HUMAN	2109.3071
RAB35_HUMAN	6937.854
RNPS1_HUMAN	5864.8306
RBBP5_HUMAN	2501.9363
RCN1_HUMAN	20877.674
RBP1_HUMAN	525.92145
L2GL1_HUMAN	2384.9937
LRC41_HUMAN	2245.9751
TTF1_HUMAN	8949.376
TMED2_HUMAN	7680.9458
PCBP1_HUMAN	162678.88
PCBP2_HUMAN	154234.53
ELOC_HUMAN	4692.2275
ELOB_HUMAN	2949.6824
RHEB_HUMAN	11958.076
UBE3C_HUMAN	4540.3491
TOM20_HUMAN	8252.6289
MTFR1_HUMAN	316.51996
DHC24_HUMAN	13774.754
SF3B3_HUMAN	24949.277
PUM3_HUMAN	2300.7646
DLGP5_HUMAN	3211.4102
RSU1_HUMAN	49815.508
CNN3_HUMAN	26222.162
KS6A1_HUMAN	986.10553
SAFB1_HUMAN	6977.6313
SF3A2_HUMAN	4346.2661
RBMS2_HUMAN	6391.0972
PP1R7_HUMAN	16283.123
SC23A_HUMAN	22160.615
SC23B_HUMAN	4013.875
CYH1_HUMAN	5602.248
SF3A1_HUMAN	11626.596
SKI2_HUMAN	9521.6855
CDSN_HUMAN	370.2757
SURF1_HUMAN	1641.7645
TAF5_HUMAN	316.67395
TAF7_HUMAN	1507.1364
TERF2_HUMAN	1847.076
MARE2_HUMAN	11804.129

TGIF1_HUMAN	604.13904
NHRF2_HUMAN	3690.9521
TRADD_HUMAN	2402.0447
TRAM1_HUMAN	10085.743
TSN_HUMAN	10603.425
TRBP2_HUMAN	1464.5144
SF01_HUMAN	16263.383
CIP4_HUMAN	6909.5552
TRIPB_HUMAN	3218.9253
PCH2_HUMAN	9349.7295
MED1_HUMAN	1939.8481
TRIP4_HUMAN	3872.1646
JHD2C_HUMAN	276.54312
IKBB_HUMAN	2355.3831
TRIP6_HUMAN	5414.0225
PTN14_HUMAN	2057.4153
MARE1_HUMAN	38451.074
ELAV1_HUMAN	46386.523
NSDHL_HUMAN	18557.096
NAB2_HUMAN	517.91956
MYLK_HUMAN	3624.7214
TAB1_HUMAN	2878.0896
HERC1_HUMAN	2211.6418
AAAT_HUMAN	9668.0508
MLF2_HUMAN	1313.0414
TOM34_HUMAN	6274.6885
SMAD2_HUMAN	4279.1172
SMAD1_HUMAN	21526.488
MSMO1_HUMAN	6711.9468
ITSN1_HUMAN	2260.8872
TBCE_HUMAN	4440.29
TBCC_HUMAN	4856.8462
UB2V2_HUMAN	2489.1624
STK11_HUMAN	623.59161
STXB2_HUMAN	5817.0952
VAMP3_HUMAN	4094.9597
NEDD8_HUMAN	3988.5671
VAS1_HUMAN	3983.1558
VPS72_HUMAN	673.35944
RB11B_HUMAN	15453.208
ZYX_HUMAN	4067.0911
ETFD_HUMAN	1575.4796
SEPT7_HUMAN	31837.109
ADRM1_HUMAN	17005.625
CCDC6_HUMAN	5137.8164
UAP1_HUMAN	18619.873
E2F4_HUMAN	306.13773
IBP7_HUMAN	9062.9463
PDCD2_HUMAN	657.38702
LAMA4_HUMAN	16030.646

PSMD5_HUMAN	25511.912
PKN1_HUMAN	8735.4844
PKN2_HUMAN	7735.8335
TAF12_HUMAN	574.38623
CSRP2_HUMAN	1938.4928
DDB1_HUMAN	39368.262
2A5E_HUMAN	4801.1895
MK14_HUMAN	5868.4956
RM23_HUMAN	5038.0049
CDC37_HUMAN	34359
DPYL2_HUMAN	71075.063
SYPL1_HUMAN	12019.199
RBBP7_HUMAN	8685.8799
M3K11_HUMAN	1061.005
TAF9_HUMAN	2494.3359
CALRL_HUMAN	8085.1367
BAK_HUMAN	778.62073
SRSF7_HUMAN	40992.715
CPSF6_HUMAN	17967.896
SMN_HUMAN	3267.8611
DREB_HUMAN	11036.722
NRF1_HUMAN	1731.3566
FSCN1_HUMAN	157851.86
IF16_HUMAN	31814.662
DECR_HUMAN	29131.209
MA2A1_HUMAN	7663.2397
NDUA5_HUMAN	2071.8354
CEGT_HUMAN	1016.3506
CLPP_HUMAN	3913.1982
THTR_HUMAN	15415.92
UBE2S_HUMAN	888.39038
KAT1_HUMAN	922.06738
KGUA_HUMAN	3584.6392
GLO2_HUMAN	7306.1865
NDUA9_HUMAN	12768.98
MAON_HUMAN	2072.1567
PCKGM_HUMAN	4163.8818
UPP1_HUMAN	1843.0563
DDR2_HUMAN	2553.7058
HCDH_HUMAN	12937.32
CP51A_HUMAN	17652.641
UGPA_HUMAN	28769.83
F263_HUMAN	1780.2703
TRXR1_HUMAN	40162.199
MIC60_HUMAN	17040.998
ECSCR_HUMAN	3024.0266
KNOP1_HUMAN	1508.3091
HNRL2_HUMAN	15673.651
T132A_HUMAN	230.39034
INF2_HUMAN	6562.5054

PDS5A_HUMAN	7208.292
CL16A_HUMAN	1515.5801
KIF7_HUMAN	596.3067
RHG31_HUMAN	3207.7864
MTHSD_HUMAN	4909.7412
AAK1_HUMAN	4364.0566
WASC4_HUMAN	5368.2271
ERC6L_HUMAN	2295.063
TSR1_HUMAN	8096.7632
RGPA2_HUMAN	1264.4347
D19L1_HUMAN	4180.3442
IAH1_HUMAN	1826.0204
ALG11_HUMAN	2128.5791
QRIC1_HUMAN	3842.5483
SMU1_HUMAN	10105.929
CTU2_HUMAN	9621.5283
PGGHG_HUMAN	568.73267
LRRF1_HUMAN	5270.4316
PGPS1_HUMAN	1581.4299
CR021_HUMAN	112.66887
P3H1_HUMAN	20111.363
TRM5_HUMAN	3366.019
EMAL3_HUMAN	4777.6694
RPA43_HUMAN	2107.467
MA7D1_HUMAN	2947.145
UAP1L_HUMAN	13988.634
S2535_HUMAN	236.45438
CHD9_HUMAN	3964.6545
TKFC_HUMAN	3958.6785
LSM12_HUMAN	6116.0786
HSDL1_HUMAN	2839.3223
AL1L2_HUMAN	326.62552
GNPTA_HUMAN	480.22479
GRDN_HUMAN	1696.0344
RABL6_HUMAN	6019.9868
TIM50_HUMAN	6815.2695
PAR14_HUMAN	3615.7249
FUT11_HUMAN	1156.9406
GLYR1_HUMAN	8266.4492
CE022_HUMAN	2511.3777
C2CD3_HUMAN	234.11421
VP26B_HUMAN	4146.5645
LARP7_HUMAN	3045.1792
NAKD2_HUMAN	1213.1556
KCD21_HUMAN	1886.1965
GXLT1_HUMAN	495.56696
ACSF3_HUMAN	1824.0435
PPCEL_HUMAN	2549.303
TB10B_HUMAN	3857.2727
TPRN_HUMAN	224.59407

ANO6_HUMAN	3799.7773
FIL1L_HUMAN	1674.9573
GRAP1_HUMAN	3173.1626
ZNG1F_HUMAN	1800.6047
MIX23_HUMAN	4236.9424
MBRL_HUMAN	952.36786
FA98B_HUMAN	6871.1787
RHG29_HUMAN	7354.9775
PDCD4_HUMAN	9827.9629
FND3B_HUMAN	6337.5103
CRTC2_HUMAN	369.97742
GPAT3_HUMAN	703.06781
CEP55_HUMAN	2587.4146
NCBP3_HUMAN	1883.7452
QORX_HUMAN	20330.193
NACHO_HUMAN	3100.6511
PHLA2_HUMAN	1661.5983
PDLI3_HUMAN	784.3714
PAR10_HUMAN	430.67233
DHB12_HUMAN	23093.574
GLE1_HUMAN	3635.3552
UBP39_HUMAN	7109.729
KLH22_HUMAN	1513.0144
AGK_HUMAN	12157.314
SETMR_HUMAN	335.77335
LACB2_HUMAN	1933.1891
P5CR3_HUMAN	3539.5364
EIPR1_HUMAN	5376.0713
BOREA_HUMAN	230.34413
COBL1_HUMAN	1263.7186
H1BP3_HUMAN	4183.8804
CYBR1_HUMAN	880.01855
WDR81_HUMAN	3935.7146
ACTBL_HUMAN	238710.95
CCD93_HUMAN	4123.2524
OCAD2_HUMAN	11620.322
PKHG4_HUMAN	1232.2996
HS904_HUMAN	935.20032
DCAF6_HUMAN	429.858
TM41B_HUMAN	951.35944
SGMR2_HUMAN	11047.585
YIF1B_HUMAN	2995.7102
ZN326_HUMAN	3421.6179
NOM1_HUMAN	1242.5308
RIPL1_HUMAN	2399.4456
DJC21_HUMAN	1375.595
TP8L3_HUMAN	2835.4771
HERC4_HUMAN	6874.0273
PIGG_HUMAN	1322.8787
PP6R3_HUMAN	5762.2988

DDX6L_HUMAN	289.0498
RABL3_HUMAN	26436.354
FA76B_HUMAN	227.6839
COQ5_HUMAN	468.65222
SH319_HUMAN	3585.7612
NHSL2_HUMAN	2080.8855
EMC4_HUMAN	4651.9165
SYEM_HUMAN	1943.0081
CC038_HUMAN	722.86755
EFMT2_HUMAN	736.29041
TGO1_HUMAN	1920.5061
PREP_HUMAN	12711.21
WDR44_HUMAN	3123.3708
PRC2B_HUMAN	3485.8081
TJAP1_HUMAN	1034.6362
RRP12_HUMAN	7560.2378
COA6_HUMAN	381.36603
TOIP1_HUMAN	12053.329
CEP78_HUMAN	451.58334
SYAM_HUMAN	4436.3354
TOR2A_HUMAN	2304.813
PCID2_HUMAN	3203.7285
HABP4_HUMAN	2249.4675
S27A3_HUMAN	1087.1848
SAMD9_HUMAN	1823.233
P4R3B_HUMAN	2003.1809
WIPI3_HUMAN	2066.9363
WIPI1_HUMAN	989.07001
EOGT_HUMAN	12299.548
SMAG2_HUMAN	595.86591
TBCEL_HUMAN	4077.6208
TDIF2_HUMAN	764.91364
RBG1L_HUMAN	2325.312
TTC38_HUMAN	1349.6173
COX20_HUMAN	3855.0708
EXOS6_HUMAN	4816.7173
TM201_HUMAN	2018.795
ATAT_HUMAN	421.12964
SNP47_HUMAN	717.78192
NU188_HUMAN	6223.6714
HP1B3_HUMAN	35878.867
SYVM_HUMAN	609.81177
CE170_HUMAN	5056.0649
ARH_HUMAN	1441.9255
ODR4_HUMAN	3144.3582
NOL9_HUMAN	3266.6646
NHSL1_HUMAN	1178.1454
HYI_HUMAN	411.24973
TPRGL_HUMAN	988.16339
C2D1B_HUMAN	1580.588

FBP1L_HUMAN	17850.162
SYRM_HUMAN	1040.9512
THEM4_HUMAN	1520.5985
CHCH9_HUMAN	188.83986
FKB15_HUMAN	12137.652
ZC3HD_HUMAN	1318.8655
CI114_HUMAN	1492.9971
ARMD3_HUMAN	637.46942
CSCL2_HUMAN	1281.6018
GPTC4_HUMAN	475.47958
CAF17_HUMAN	1858.7534
HECD3_HUMAN	5034.5811
GT253_HUMAN	11651.196
UBR4_HUMAN	11562.563
STXB5_HUMAN	1168.347
RHG21_HUMAN	1373.8298
CAMP1_HUMAN	1536.6254
RM02_HUMAN	2962.7119
UBAP2_HUMAN	4468.2334
QNG1_HUMAN	2684.4373
KPRP_HUMAN	506.38766
ZN618_HUMAN	2839.9834
ACBD5_HUMAN	6583.1587
RBM26_HUMAN	3385.9924
ATD3B_HUMAN	1671.0807
WLS_HUMAN	5847.0674
INT11_HUMAN	3150.5586
CPTP_HUMAN	801.53748
DCAF8_HUMAN	3832.269
CODA1_HUMAN	396.81067
TUT4_HUMAN	2679.1494
FRY_HUMAN	973.63501
RNH2B_HUMAN	1621.0397
ATPF1_HUMAN	1911.8069
RC3H1_HUMAN	725.99982
DDI2_HUMAN	3585.1108
THMS2_HUMAN	684.50165
NDUF5_HUMAN	1319.225
NT5D1_HUMAN	2661.5195
BIG3_HUMAN	1198.606
VP13D_HUMAN	461.48181
CROCC_HUMAN	1270.3112
LYRM7_HUMAN	1217.1449
RAIN_HUMAN	23658.746
RIF1_HUMAN	2005.3787
VPS53_HUMAN	4171.0444
STRP1_HUMAN	4799.1084
MRCKA_HUMAN	6253.397
RPRD2_HUMAN	934.66461
RN220_HUMAN	329.1199

PR38B_HUMAN	5316.3018
BRE1A_HUMAN	4046.2297
MYOME_HUMAN	610.06787
ZN318_HUMAN	768.77594
F1711_HUMAN	982.87604
DIK1B_HUMAN	3626.4539
LRCH2_HUMAN	1243.8535
CDKAL_HUMAN	5813.2231
BROX_HUMAN	6214.354
FOCAD_HUMAN	7614.0576
RSBN1_HUMAN	315.24713
DAB2P_HUMAN	834.97235
LYPL1_HUMAN	8169.3203
PEAR1_HUMAN	2206.4089
ECM29_HUMAN	16197.446
TUT7_HUMAN	1013.0455
SHE_HUMAN	775.24988
DEN4C_HUMAN	3257.2144
NAA35_HUMAN	4631.0474
ZMYM4_HUMAN	2105.8293
FHI2A_HUMAN	4363.8525
ZDH20_HUMAN	2714.1357
SPRY7_HUMAN	1144.1595
FBXW9_HUMAN	219.09679
CD276_HUMAN	2615.0005
RN213_HUMAN	6377.0781
TENS2_HUMAN	2209.7622
KANK2_HUMAN	653.21515
WAC2A_HUMAN	1002.6899
LPCT4_HUMAN	1468.2952
STEA3_HUMAN	1344.0511
F91A1_HUMAN	4632.9082
TBC9B_HUMAN	7029.9038
MAP1S_HUMAN	9465.1152
2ABD_HUMAN	2937.1438
AR6P4_HUMAN	4295.729
A16L1_HUMAN	2560.2844
HGNAT_HUMAN	2364.7813
ARID2_HUMAN	547.86328
UTP25_HUMAN	1501.411
GL8D1_HUMAN	1847.6383
SE1L3_HUMAN	737.08081
TENS3_HUMAN	3016.3594
HAUS3_HUMAN	956.91217
SPT2_HUMAN	783.30249
MBLC2_HUMAN	1984.7588
CRBG3_HUMAN	875.35931
INT3_HUMAN	4559.2446
RHG17_HUMAN	17529.438
C19L1_HUMAN	10413.446

VIR_HUMAN	3346.6238
CYTSA_HUMAN	4910.6646
HEAT6_HUMAN	733.81134
ANR40_HUMAN	536.33624
WWC2_HUMAN	2094.8027
SCAR3_HUMAN	1163.598
ATLA3_HUMAN	19617.373
RL7L_HUMAN	6667.2275
NXN_HUMAN	2574.3193
TTC19_HUMAN	893.11328
IQEC1_HUMAN	3211.3906
CPIN1_HUMAN	9334.8652
ZFAN6_HUMAN	1547.4886
SMYD5_HUMAN	2667.9526
OTU7B_HUMAN	387.59894
THOC7_HUMAN	1391.6846
NADE_HUMAN	3352.9307
ELP2_HUMAN	2720.0747
LTOR1_HUMAN	1460.4948
DRS7B_HUMAN	1707.2173
TWF2_HUMAN	15382.907
CNDH2_HUMAN	668.38196
K0930_HUMAN	1295.1067
TNG2_HUMAN	6799.4375
P4R3A_HUMAN	2840.1824
RAB12_HUMAN	21063.191
DEN5A_HUMAN	276.72424
SDE2_HUMAN	268.70914
K2C80_HUMAN	867.7395
NIPBL_HUMAN	539.66925
SCMC2_HUMAN	3853.9456
PDE12_HUMAN	6835.7183
NAA16_HUMAN	1938.0792
DE10B_HUMAN	4108.3623
RHBD2_HUMAN	2074.3743
SCMC1_HUMAN	30865.455
REEP3_HUMAN	965.39417
RETST_HUMAN	3226.1255
RINT1_HUMAN	1110.547
TM214_HUMAN	10722.488
HIBCH_HUMAN	8034.6455
RWDD4_HUMAN	1638.037
NEPRO_HUMAN	1338.9341
ARMC6_HUMAN	4255.9141
TTI2_HUMAN	2076.5154
ANR54_HUMAN	2726.927
ZNT6_HUMAN	1094.1078
TAPT1_HUMAN	21146.209
KANK3_HUMAN	2413.6025
JMJD6_HUMAN	4387.855

PPR18_HUMAN	8239.8242
CAVN1_HUMAN	119425.59
ZCHC8_HUMAN	3006.5127
DHX57_HUMAN	3751.2781
ERAP2_HUMAN	13654.661
MBOA5_HUMAN	3508.7246
CDC73_HUMAN	6526.2183
RM14_HUMAN	3886.6953
S27A4_HUMAN	7692.0894
C2D1A_HUMAN	4403.1411
TATD1_HUMAN	2876.9041
MET2B_HUMAN	1773.9663
MSD2_HUMAN	1037.4778
TAF2_HUMAN	1932.8783
CH082_HUMAN	1523.5419
MED27_HUMAN	977.02228
EDC4_HUMAN	17663.146
ANM9_HUMAN	378.30023
PRP8_HUMAN	12656.285
FBX42_HUMAN	998.06787
SCYL2_HUMAN	4071.2031
TTC27_HUMAN	4769.6255
SFXN4_HUMAN	859.97614
GOLM2_HUMAN	3316.0803
NFRKB_HUMAN	595.68536
FAHD1_HUMAN	11051.755
RL22L_HUMAN	10268.102
PKN3_HUMAN	1096.2662
ALKB5_HUMAN	947.59125
PDXD1_HUMAN	7161.2139
MEAK7_HUMAN	3350.5317
INT5_HUMAN	3435.4734
GIMA6_HUMAN	751.59314
S27A1_HUMAN	1226.4395
PGM2L_HUMAN	10513.31
CTR9_HUMAN	5022.6201
AAGAB_HUMAN	2098.8452
TMM88_HUMAN	546.35205
PSRC1_HUMAN	209.85886
SKI3_HUMAN	5442.6748
ULK3_HUMAN	1502.8611
SYDM_HUMAN	9222.8799
TMM65_HUMAN	3679.9368
IN80C_HUMAN	1767.7561
FBX38_HUMAN	1780.4979
NCEH1_HUMAN	9192.041
FIGL1_HUMAN	807.73053
TRI65_HUMAN	1679.3062
RHDF2_HUMAN	751.09186
MDEAS_HUMAN	1727.6001

BRAT1_HUMAN	3382.3469
WDR59_HUMAN	942.8797
ZC3HE_HUMAN	1570.1378
CC137_HUMAN	972.35406
OGFD3_HUMAN	1221.3263
TXD11_HUMAN	250.42703
LARP1_HUMAN	14592.792
ATAD2_HUMAN	391.54922
TMED8_HUMAN	2524.2437
ZNT9_HUMAN	869.01709
TRAF7_HUMAN	1325.7295
RICTR_HUMAN	3979.8967
WDR74_HUMAN	609.05206
ATRAP_HUMAN	2128.3796
SMRD3_HUMAN	1745.3055
C1TM_HUMAN	22994.938
AFTIN_HUMAN	472.2229
FIP1_HUMAN	5952.3413
CRTC3_HUMAN	366.29135
CRTC1_HUMAN	649.83398
CP20A_HUMAN	3041.5127
PLGT2_HUMAN	3734.0967
TM205_HUMAN	8201.252
LRSM1_HUMAN	2547.021
GAK1B_HUMAN	4371.9736
LCLT1_HUMAN	3066.3987
ABRX1_HUMAN	271.84259
CWC27_HUMAN	1705.2125
CREL2_HUMAN	2091.1677
WDR82_HUMAN	4154.7744
MIC27_HUMAN	1839.0144
FAT4_HUMAN	1051.1841
DPP8_HUMAN	580.95624
MCAF1_HUMAN	1958.9224
RBP10_HUMAN	1749.051
PACS1_HUMAN	7413.9077
MPRIP_HUMAN	7547.8594
RBMS3_HUMAN	2305.2202
PNCB_HUMAN	5586.5068
DNMBP_HUMAN	4933.4692
HACD2_HUMAN	1289.1107
B3GLT_HUMAN	1418.0438
GGYF2_HUMAN	8381.0381
CD109_HUMAN	10386.126
THADA_HUMAN	1767.0211
HSDL2_HUMAN	4992.689
KAT3_HUMAN	7898.0879
PPR21_HUMAN	1336.5894
THSD4_HUMAN	1104.2889
SYNE3_HUMAN	1084.5771

ZN574_HUMAN	469.12061
NFXL1_HUMAN	468.49554
NBEL2_HUMAN	3333.0308
FGD5_HUMAN	4834.1914
QSOX2_HUMAN	2776.626
SRCAP_HUMAN	562.8609
RIPR1_HUMAN	3966.3972
YJ005_HUMAN	7026.3198
ARHGI_HUMAN	1338.1449
TBCD9_HUMAN	626.79578
UBR3_HUMAN	407.07709
TMPPE_HUMAN	817.20911
CRACD_HUMAN	452.50748
CCD9B_HUMAN	526.90399
NUD18_HUMAN	643.17084
SYDE1_HUMAN	328.07755
PAXI1_HUMAN	499.49744
STXB4_HUMAN	264.60403
TMTC3_HUMAN	6338.4072
VP13C_HUMAN	10825.128
UBP34_HUMAN	812.73291
RAPH1_HUMAN	2975.5327
MOB2_HUMAN	6192.4878
IKIP_HUMAN	25007.227
PREX2_HUMAN	1565.2744
F10C1_HUMAN	236.88606
UB2R2_HUMAN	6688.4248
MD13L_HUMAN	105.94209
LARP4_HUMAN	7631.0669
MED25_HUMAN	1142.5684
RS27L_HUMAN	56318.586
HAKAI_HUMAN	1224.3268
INT8_HUMAN	4579.0327
BT2A1_HUMAN	166.32066
SPT6H_HUMAN	3426.6504
SND1_HUMAN	85216.266
MARK2_HUMAN	1695.172
COX15_HUMAN	1914.9675
DDX46_HUMAN	14204.254
RUFY3_HUMAN	673.05969
TM10C_HUMAN	8633.8623
5MP2_HUMAN	26629.762
MON1B_HUMAN	1234.8656
ASGL1_HUMAN	1314.67
KCTD9_HUMAN	8732.4404
DHX30_HUMAN	8776.1494
EIF3M_HUMAN	16229.618
MEPCE_HUMAN	3211.8728
ARMX2_HUMAN	1484.631
SYPM_HUMAN	571.88763

RSRC2_HUMAN	1713.9293
RRAGA_HUMAN	4451.1646
CYFP1_HUMAN	26322.211
NDUF7_HUMAN	2433.281
GET4_HUMAN	8948.4014
CSN6_HUMAN	18803.928
PCAT2_HUMAN	18280.67
ENOF1_HUMAN	1874.9384
MAEA_HUMAN	1934.0266
EPMIP_HUMAN	1986.4279
DHX32_HUMAN	479.03952
TAOK1_HUMAN	2859.7085
FAKD5_HUMAN	1249.1217
MOB1B_HUMAN	12930.98
KDM3B_HUMAN	4149.3838
CHM1B_HUMAN	5755.9946
RIR2B_HUMAN	3751.8413
HS2ST_HUMAN	5031.8286
STRAA_HUMAN	1566.8087
MICA3_HUMAN	1127.6678
DYM_HUMAN	2230.3289
PHF5A_HUMAN	2482.3271
PXL2C_HUMAN	2501.5952
APTX_HUMAN	2486.0098
ERMP1_HUMAN	4873.938
GRIN1_HUMAN	254.67105
TRM1L_HUMAN	2509.5674
ZCCHV_HUMAN	9637.2422
RM21_HUMAN	4943.0015
EFL1_HUMAN	3987.1951
SETX_HUMAN	713.60272
TPC11_HUMAN	2201.1265
KANL1_HUMAN	165.98898
NUP54_HUMAN	9132.9658
ATG9A_HUMAN	1387.067
GLUCM_HUMAN	5899.7007
CC186_HUMAN	754.40808
ARMC9_HUMAN	1940.3757
VP35L_HUMAN	6137.7109
POGZ_HUMAN	3548.2993
ZFY16_HUMAN	1095.0406
MON2_HUMAN	3250.2102
MYCPP_HUMAN	738.45154
MYH14_HUMAN	7664.4258
NUFP2_HUMAN	5225.0923
SZRD1_HUMAN	1443.8467
MAVS_HUMAN	1027.7219
CLAP1_HUMAN	7251.7642
DHX29_HUMAN	4858.3022
NPHP3_HUMAN	453.78094

LRP10_HUMAN	1728.6355
HAUS6_HUMAN	1655.8785
PLGT3_HUMAN	6306.2349
TT21B_HUMAN	2924.9893
HEAT3_HUMAN	6630.8701
KI21A_HUMAN	390.0293
HDGR2_HUMAN	9542.4883
DCXR_HUMAN	7174.7222
BRAP_HUMAN	2716.7517
EMSY_HUMAN	797.38654
GOGA7_HUMAN	2380.6963
RHG22_HUMAN	661.26947
WAPL_HUMAN	1783.4985
I2BP2_HUMAN	936.27789
RBBP6_HUMAN	1932.3412
SH3R1_HUMAN	917.71857
GRASP_HUMAN	1609.2561
FRMD5_HUMAN	535.56567
SEN54_HUMAN	485.71677
PTAR1_HUMAN	549.35992
ARPIN_HUMAN	3639.4114
TCPR1_HUMAN	788.63788
RABEK_HUMAN	1190.5643
MTEF4_HUMAN	597.47296
HUWE1_HUMAN	13683.134
YTHD3_HUMAN	11116.061
CTU1_HUMAN	3431.781
TAF8_HUMAN	323.32852
UB2Q1_HUMAN	6465.9551
RM55_HUMAN	10784.298
TMED4_HUMAN	3626.5466
RM10_HUMAN	1295.0336
COXM1_HUMAN	5772.8296
CENPV_HUMAN	1444.184
SLN11_HUMAN	4728.2056
MEGF8_HUMAN	492.5762
T179B_HUMAN	1333.3523
GALT7_HUMAN	7084.459
SCD5_HUMAN	2823.3813
PHLB2_HUMAN	3510.585
AGRG6_HUMAN	2480.9688
DHDDS_HUMAN	488.99979
GLT10_HUMAN	477.95322
TPC6B_HUMAN	1146.4209
PP4P1_HUMAN	1428.8771
CLC14_HUMAN	14274.663
KAISO_HUMAN	571.42249
PATL1_HUMAN	1040.0331
DPP9_HUMAN	7138.041
SYVN1_HUMAN	2396.6816

TRPT1_HUMAN	358.57135
SETD3_HUMAN	6654.5103
TTC7B_HUMAN	1492.6162
ACOT1_HUMAN	4389.4458
NOP9_HUMAN	2506.4302
PABP2_HUMAN	20757.91
MTA70_HUMAN	1715.5176
PB1_HUMAN	3794.6316
YRDC_HUMAN	1347.2018
PRP39_HUMAN	2430.2319
LYRIC_HUMAN	14411.583
ZN598_HUMAN	4440.3237
GPAT4_HUMAN	1472.4193
KTN1_HUMAN	8214.6729
EST1A_HUMAN	1606.9823
NLRX1_HUMAN	1869.0916
BCL9L_HUMAN	392.09952
PHLB1_HUMAN	7873.1152
UBP48_HUMAN	7155.4346
CAPS2_HUMAN	426.04037
URP2_HUMAN	22776.332
NAA40_HUMAN	4205.8672
AACS_HUMAN	8462.1465
LUZP1_HUMAN	6887.7798
THOC4_HUMAN	20793.719
GP180_HUMAN	598.25189
FHI2B_HUMAN	3018.7744
IQGA3_HUMAN	3409.6067
ZCH18_HUMAN	2101.5259
VPS36_HUMAN	1479.3436
CAND1_HUMAN	38802.355
RETR3_HUMAN	2964.4419
HOOK3_HUMAN	6992.0371
SESD1_HUMAN	782.54578
COMD7_HUMAN	1269.1215
MON1A_HUMAN	1032.8956
AMZ2_HUMAN	1072.2153
THOC6_HUMAN	2245.6677
MET16_HUMAN	923.55078
PARG_HUMAN	504.39117
LIPB1_HUMAN	8866.4736
LONP2_HUMAN	1169.6381
ZC3C1_HUMAN	3537.9048
CHD1L_HUMAN	7494.8398
FCSD1_HUMAN	143.45065
CCD25_HUMAN	8030.6089
PRSR2_HUMAN	1034.9045
STING_HUMAN	8604.3125
AROS_HUMAN	584.66541
CDR2L_HUMAN	126.9217

RLGPB_HUMAN	1125.2603
RGPS2_HUMAN	580.41296
CARM1_HUMAN	8794.5303
NIT1_HUMAN	4746.8545
COMD2_HUMAN	4136.918
HTR5A_HUMAN	1538.929
CNDG2_HUMAN	927.43622
PPR3B_HUMAN	192.07207
FBX11_HUMAN	208.24379
ANKL2_HUMAN	2129.3401
MEX3D_HUMAN	2080.7952
DGKH_HUMAN	230.72339
DDX42_HUMAN	15566.575
SPAS2_HUMAN	2946.9241
VRK2_HUMAN	2820.2468
CACL1_HUMAN	499.69641
NDUAB_HUMAN	8047.0742
DAAF5_HUMAN	8761.3672
PTH_HUMAN	1090.9071
STX12_HUMAN	12068.336
P66A_HUMAN	3167.3711
CPNE8_HUMAN	25601.396
GPSM1_HUMAN	1064.1428
RAB43_HUMAN	1255.5313
C2CD5_HUMAN	1553.2034
MIB1_HUMAN	675.81555
PRAG1_HUMAN	1079.2903
HPS6_HUMAN	1684.5388
I2BP1_HUMAN	435.16406
RB6I2_HUMAN	4850.5879
RIOX2_HUMAN	1948.8864
ZDH13_HUMAN	7581.3369
CRLF3_HUMAN	1652.9891
TPPC5_HUMAN	6105.5205
ARMC8_HUMAN	2923.0073
RELL1_HUMAN	1137.9642
T126B_HUMAN	878.40045
PLD3_HUMAN	17568.455
HID1_HUMAN	2229.9165
ANKY2_HUMAN	1404.1207
ERI1_HUMAN	1372.9749
VRK3_HUMAN	820.57294
LIX1L_HUMAN	2183.3389
NUDC3_HUMAN	4886.519
AHNK2_HUMAN	19442.004
FMNL3_HUMAN	6841.9614
MMAA_HUMAN	2416.4026
M4K3_HUMAN	1247.3655
NAV3_HUMAN	2350.8113
P3H3_HUMAN	12158.497

CCD50_HUMAN	9325.6777
FABD_HUMAN	1715.775
KSR1_HUMAN	1674.6392
CEP97_HUMAN	1228.8551
NNRD_HUMAN	1516.0243
WDR75_HUMAN	828.19916
MFN1_HUMAN	2607.8662
CTL2_HUMAN	6593.2134
IPRI_HUMAN	1971.5092
WDFY1_HUMAN	3915.5762
MA7D3_HUMAN	2364.114
CC117_HUMAN	171.84392
NXP20_HUMAN	7118.9189
DCNL3_HUMAN	377.88495
DEN6A_HUMAN	960.72577
MGAP_HUMAN	475.3287
GCC2_HUMAN	3433.731
Z3H7A_HUMAN	1604.5343
PHF6_HUMAN	7525.7495
ARCH_HUMAN	897.8457
LRC8A_HUMAN	6200.8027
SULF2_HUMAN	3725.7771
UBR1_HUMAN	1412.5823
UBR2_HUMAN	966.18536
RHG12_HUMAN	1053.3107
CHERP_HUMAN	9063.582
CDAN1_HUMAN	665.73199
ANKH1_HUMAN	2662.9917
SUGP1_HUMAN	2027.1761
SUGP2_HUMAN	25394.525
UEVLD_HUMAN	1948.2283
CCAR1_HUMAN	9511.8105
DHX40_HUMAN	515.45715
DJC10_HUMAN	9977.0049
NELFD_HUMAN	6271.7515
MIRO2_HUMAN	5007.6016
MIRO1_HUMAN	5519.6406
SIR2_HUMAN	3188.1709
PHC2_HUMAN	1133.9119
BAP18_HUMAN	5357.4492
RM41_HUMAN	9661.7295
NRM_HUMAN	4535.291
GPAM1_HUMAN	1088.9207
PARP9_HUMAN	1108.1503
RB12B_HUMAN	3327.9202
S35F2_HUMAN	1686.3033
RPAP2_HUMAN	317.68112
PLPL6_HUMAN	6550.3579
SMC5_HUMAN	1168.3855
DDX60_HUMAN	995.62091

CMIP_HUMAN	495.88336
MILK2_HUMAN	658.79504
DHX37_HUMAN	2360.209
KBTB2_HUMAN	3981.1914
RAVR1_HUMAN	5240.8843
SPB1_HUMAN	6000.873
TM192_HUMAN	1546.0859
CKP2L_HUMAN	232.54124
M21D2_HUMAN	1609.4407
SRRM1_HUMAN	4336.5874
SMAP1_HUMAN	4759.5239
DI3L2_HUMAN	4064.7073
SUV3_HUMAN	4982.2559
ERF3B_HUMAN	7038.9204
ZZZ3_HUMAN	451.60709
EXOC8_HUMAN	9221.9502
CJ067_HUMAN	12559.957
TRI22_HUMAN	5870.8276
THNS1_HUMAN	661.58258
K2013_HUMAN	3537.8403
MICU2_HUMAN	1281.1134
AN13A_HUMAN	2891.5645
PHAR4_HUMAN	1781.2194
TRM2A_HUMAN	3601.1208
RUSD2_HUMAN	904.9248
ELMD2_HUMAN	2667.7617
A16A1_HUMAN	6834.9097
K319L_HUMAN	2090.9668
AGRF5_HUMAN	2231.9824
XRN1_HUMAN	4604.124
PELP1_HUMAN	13312.224
ABI1_HUMAN	6624.5542
WDFY3_HUMAN	4088.8894
SELH_HUMAN	6966.2476
VKORL_HUMAN	2472.3135
SPART_HUMAN	8191.2349
TTC5_HUMAN	1185.363
MIER1_HUMAN	1552.6937
RPTOR_HUMAN	1802.2238
ORML3_HUMAN	5148.8101
CCAR2_HUMAN	51344.016
NDUF2_HUMAN	7709.4873
VPS52_HUMAN	6366.9297
NUP93_HUMAN	23824.523
S11IP_HUMAN	1248.387
ZN687_HUMAN	515.82092
CMTR1_HUMAN	4292.7319
LRC47_HUMAN	15304.93
DOCK4_HUMAN	8503.7178
CAH13_HUMAN	1504.4877

ARG28_HUMAN	9923.0361
INT1_HUMAN	37026.098
H2B3B_HUMAN	3908.9456
RHG24_HUMAN	2571.3943
ARM10_HUMAN	3184.3406
ABD12_HUMAN	3402.6675
CLASR_HUMAN	171.24217
FA43A_HUMAN	2773.6135
TM256_HUMAN	2422.5676
PIAS4_HUMAN	1617.0764
GPD1L_HUMAN	5168.9131
S35F6_HUMAN	2171.4521
RHG18_HUMAN	23117.119
ASCC3_HUMAN	9595.6494
MILK1_HUMAN	1225.8595
VPS8_HUMAN	3065.4421
PALS1_HUMAN	968.67017
STAG2_HUMAN	3055.8672
SYNPO_HUMAN	9011.7656
FNBP4_HUMAN	1315.6105
GUF1_HUMAN	237.21799
RYBP_HUMAN	468.10898
GALT4_HUMAN	2798.0408
MINK1_HUMAN	1449.531
MESH1_HUMAN	1191.4932
PTGR3_HUMAN	2653.2769
CBR4_HUMAN	731.09967
EMC5_HUMAN	2844.6726
TM199_HUMAN	2427.925
FBXL6_HUMAN	160.06342
OGFD1_HUMAN	1912.6528
AFAP1_HUMAN	3992.2476
ZCHC9_HUMAN	613.69421
OXR1_HUMAN	1258.1281
ZGPAT_HUMAN	1363.7124
CAHM5_HUMAN	1891.2097
SRBD1_HUMAN	942.85858
WDTC1_HUMAN	2991.8767
NKAP_HUMAN	976.34241
MACOI_HUMAN	744.12262
SH2D3_HUMAN	3468.6238
ARRD1_HUMAN	954.49023
DHRSX_HUMAN	1571.3257
CISD2_HUMAN	10432.782
RP25L_HUMAN	2035.2924
ATPF2_HUMAN	1112.7034
TTC9C_HUMAN	1698.2734
JAGN1_HUMAN	3085.342
RFLB_HUMAN	2389.8806
LZTR1_HUMAN	437.57617

COMD1_HUMAN	3391.5979
CPSF7_HUMAN	14298.764
S15A4_HUMAN	304.36322
MYCT1_HUMAN	13359.241
ATL1_HUMAN	338.5878
ARFG2_HUMAN	4500.0396
OTU6B_HUMAN	2825.7549
FITM2_HUMAN	3716.1343
EFNMT_HUMAN	2802.7297
AR6P6_HUMAN	1000.0926
ARFG1_HUMAN	1584.6586
SIR6_HUMAN	606.72638
S66A3_HUMAN	721.07147
EMC1_HUMAN	12911.776
PAF1_HUMAN	3048.8123
CCYL1_HUMAN	1476.7361
UBR7_HUMAN	3661.2117
DDX51_HUMAN	1558.5786
F241A_HUMAN	5972.7261
S2529_HUMAN	3197.8989
ENAH_HUMAN	15153.511
GPT11_HUMAN	904.15558
RM43_HUMAN	7040.8052
RLIG1_HUMAN	2657.2966
GDPD1_HUMAN	451.52286
CS047_HUMAN	951.46112
ASCC1_HUMAN	2932.6604
LRC57_HUMAN	7865.1587
EIF1A_HUMAN	1077.08
KRI1_HUMAN	1639.147
CC71L_HUMAN	1335.0739
ZN579_HUMAN	2086.2109
PR38A_HUMAN	2474.4382
MLKL_HUMAN	5229.5522
GALD1_HUMAN	3073.3308
ANR52_HUMAN	680.04358
AT11C_HUMAN	1232.47
AFG2A_HUMAN	4568.9292
NHLC2_HUMAN	7943.2085
AVL9_HUMAN	1795.9785
S43A3_HUMAN	7123.3003
XXLT1_HUMAN	7335.4302
GOLM1_HUMAN	578.12488
GT251_HUMAN	29388.252
SUMF2_HUMAN	4540.2793
SIDT2_HUMAN	939.19977
SUMF1_HUMAN	4575.8101
PGLT1_HUMAN	3323.7837
UBAC2_HUMAN	1975.4789
PCYXL_HUMAN	2197.0481

TM87A_HUMAN	2375.2063
RDH13_HUMAN	2883.2002
TTC13_HUMAN	1109.7335
DHB11_HUMAN	9312.4307
TXND5_HUMAN	281569.19
SPC24_HUMAN	1836.7032
ATAD1_HUMAN	2507.9165
SCPDL_HUMAN	19428.83
UXS1_HUMAN	690.41583
RN149_HUMAN	1701.3284
RETR2_HUMAN	1267.4176
SERB1_HUMAN	11815.215
LEMD2_HUMAN	6715.2007
NOA1_HUMAN	1628.3353
NECP1_HUMAN	2618.8833
FA98A_HUMAN	4560.9019
PAG15_HUMAN	1164.4072
MTMRE_HUMAN	1558.0438
NF2IP_HUMAN	3814.5439
DGLB_HUMAN	1557.7833
CHSTE_HUMAN	5033.0142
GALT6_HUMAN	1821.3645
RN169_HUMAN	637.68683
PDPR_HUMAN	4839.0029
NNRE_HUMAN	3765.5537
SMG8_HUMAN	1455.8309
RN214_HUMAN	2677.5537
LS14A_HUMAN	3683.7778
GIMA8_HUMAN	17112.328
CCNY_HUMAN	3163.7729
MROH1_HUMAN	4454.1665
PAPD5_HUMAN	1590.0266
PEPL1_HUMAN	3109.1548
EHBP1_HUMAN	1168.337
RB15B_HUMAN	1818.7781
SIA7C_HUMAN	1719.6437
TNR6A_HUMAN	1205.2556
PHC3_HUMAN	698.19843
DIK2A_HUMAN	3217.7441
CNNM3_HUMAN	864.37659
ABCF1_HUMAN	12402.945
MCU_HUMAN	2132.0212
PK3C3_HUMAN	7283.3677
GSTCD_HUMAN	329.61063
SRFB1_HUMAN	1252.3313
NGDN_HUMAN	1269.2191
SHCBP_HUMAN	1636.6431
PDZD8_HUMAN	238.21829
DP13B_HUMAN	2308.1348
ZNT7_HUMAN	8762.6211

NAV1_HUMAN	1655.371
PPHLN_HUMAN	332.39047
WDR19_HUMAN	873.90082
FBX22_HUMAN	4256.9995
PCAT1_HUMAN	4486.1279
ZMIZ2_HUMAN	1197.2551
SYNE1_HUMAN	25473.707
UBP32_HUMAN	604.67773
BD1L1_HUMAN	975.67932
ARI1B_HUMAN	1198.3669
FAD1_HUMAN	5309.8447
FLCN_HUMAN	1764.5283
NUP43_HUMAN	5895.3008
NUP37_HUMAN	7978.8164
NUP35_HUMAN	3743.3032
ENASE_HUMAN	568.38696
TOIP2_HUMAN	7637.5161
ABHDB_HUMAN	1804.5341
NEUA_HUMAN	6776.7642
FBH1_HUMAN	696.03986
TSN14_HUMAN	4399.771
TTL_HUMAN	716.20953
SVIP_HUMAN	1952.3083
ATLA2_HUMAN	1618.8386
MSPD2_HUMAN	1304.0725
PLBL2_HUMAN	13658.628
RASF8_HUMAN	758.93756
DDX55_HUMAN	530.94037
MFSD8_HUMAN	497.63696
TDRD7_HUMAN	949.23822
GIMA7_HUMAN	7082.5151
NEDD1_HUMAN	1668.3624
NCOA7_HUMAN	2485.9832
THOC2_HUMAN	4337.6816
WDR36_HUMAN	2993.5671
PPTC7_HUMAN	649.6098
RP9_HUMAN	950.26196
VANG1_HUMAN	842.95093
SNIP1_HUMAN	420.79312
G45IP_HUMAN	4432.8931
WDR48_HUMAN	3241.7302
EXOC6_HUMAN	6667.0879
SMRC2_HUMAN	4565.5469
NPL4_HUMAN	9397.7041
CX038_HUMAN	2450.6135
SPT20_HUMAN	2206.8235
ABHD4_HUMAN	711.73303
FBX30_HUMAN	1002.0272
S35B2_HUMAN	7303.7305
PUM2_HUMAN	2902.428

TIP_HUMAN	1814.1824
GOGA5_HUMAN	1541.9279
KLDC4_HUMAN	1183.9973
SHKB1_HUMAN	1883.3804
UBA3_HUMAN	29041.855
PXL2B_HUMAN	876.30475
DJB14_HUMAN	612.85345
KISHA_HUMAN	23255.842
PI42C_HUMAN	1635.2399
WDR20_HUMAN	421.95273
TBC15_HUMAN	8536.666
RDH11_HUMAN	14120.252
LRC20_HUMAN	1120.9797
RM30_HUMAN	723.96326
ZFAN1_HUMAN	888.34772
CIP2A_HUMAN	3314.0664
P4K2B_HUMAN	937.52295
STT3B_HUMAN	33654.543
PNPT1_HUMAN	10115.434
SPP2B_HUMAN	1249.0652
HM13_HUMAN	47355.844
PREX1_HUMAN	4876.3296
URGCP_HUMAN	1635.5094
BICD2_HUMAN	4160.6768
NEK9_HUMAN	5416.0054
PKHO2_HUMAN	3577.603
DTX3L_HUMAN	1040.0444
DDX54_HUMAN	3463.4604
DLG5_HUMAN	246.4344
BRX1_HUMAN	4724.9771
GNPI2_HUMAN	3120.6292
LRC8C_HUMAN	16170.977
NEK7_HUMAN	2793.7964
RBCC1_HUMAN	566.99243
MICA1_HUMAN	3658.865
ELP5_HUMAN	3414.4509
SSH3_HUMAN	1286.2676
S3TC1_HUMAN	399.17816
TBCK_HUMAN	620.51581
DTD1_HUMAN	4763.6001
DCA11_HUMAN	903.98499
UTP15_HUMAN	897.43854
GPX8_HUMAN	22910.504
AF1L1_HUMAN	750.64813
SH3R3_HUMAN	6441.541
TP4AP_HUMAN	791.7674
CE192_HUMAN	672.73743
GEMI5_HUMAN	5337.3579
PIGO_HUMAN	227.94225
RPGF6_HUMAN	436.84326

SMCR8_HUMAN	686.55945
PARD3_HUMAN	962.84729
IPO4_HUMAN	13536.444
UBP33_HUMAN	482.12943
PNISR_HUMAN	1507.5538
PP4R1_HUMAN	7373.4722
UBS3B_HUMAN	1078.7454
GIPC2_HUMAN	478.19171
WIPF2_HUMAN	1300.0426
ABHD5_HUMAN	1031.1382
SETD7_HUMAN	5582.1558
NOC3L_HUMAN	3916.7544
SCRB1_HUMAN	20261.832
COG1_HUMAN	2976.7676
SCFD2_HUMAN	2995.9709
SMAP2_HUMAN	1878.7666
ZC3HF_HUMAN	15550.173
PPIL4_HUMAN	4051.3396
TF3C2_HUMAN	1912.6881
TB22A_HUMAN	3836.6982
PHF10_HUMAN	1365.5839
RAB2B_HUMAN	3902.8821
CCD12_HUMAN	1281.8674
IASPP_HUMAN	1552.6321
ARB2A_HUMAN	2623.2419
CHUR_HUMAN	2941.9854
TGFA1_HUMAN	1714.0953
TM263_HUMAN	5456.8022
HDAC7_HUMAN	6024.5024
STYX_HUMAN	661.9408
PTPM1_HUMAN	668.66846
NU133_HUMAN	8714.3828
PDC6I_HUMAN	52058.664
S20A1_HUMAN	499.73416
FBLI1_HUMAN	15027.975
CATIN_HUMAN	848.43121
CHAC2_HUMAN	1855.7241
CHMP7_HUMAN	5854.4941
THEM6_HUMAN	31285.342
NAT14_HUMAN	4989.8618
BCL7C_HUMAN	5419.5757
NSE1_HUMAN	899.96008
MITD1_HUMAN	542.41907
CTF18_HUMAN	726.83496
LEO1_HUMAN	3325.6951
DCAKD_HUMAN	639.53302
NUDC2_HUMAN	4965.4023
SNR27_HUMAN	2575.083
TFB1M_HUMAN	6699.5781
STAG1_HUMAN	1223.2457

SCFD1_HUMAN	10500.633
TP8L1_HUMAN	3072.343
CANT1_HUMAN	3095.2139
TPC12_HUMAN	1791.2623
HNRLL_HUMAN	3937.9895
FACR1_HUMAN	14556.89
UBCP1_HUMAN	6624.9194
SEN15_HUMAN	1129.6716
PCNP_HUMAN	7092.104
DNJA4_HUMAN	1375.2301
SPRY4_HUMAN	6210.4121
MAIP1_HUMAN	3147.9585
TRUB1_HUMAN	1666.2415
LMO7_HUMAN	1899.4529
CTL1_HUMAN	1415.6661
CKAP2_HUMAN	817.57831
ATX2L_HUMAN	13081.421
ARAP3_HUMAN	11002.799
GIMA1_HUMAN	9735.8311
PHIP_HUMAN	223.13591
PRP31_HUMAN	10883.025
NELFB_HUMAN	4584.1934
PALLD_HUMAN	14276.696
RUFY2_HUMAN	1807.425
SREK1_HUMAN	3459.5503
GEMI6_HUMAN	375.79916
CSKI2_HUMAN	2204.2986
PSPC1_HUMAN	16016.429
MADD_HUMAN	397.78745
SYNE2_HUMAN	9332.0771
P66B_HUMAN	2734.4668
DNJC9_HUMAN	15966.499
BRI3B_HUMAN	1884.4327
CTBL1_HUMAN	14028.742
ATG4A_HUMAN	3422.5212
RIN2_HUMAN	1195.6049
ELYS_HUMAN	2509.4875
DGCR8_HUMAN	1900.5309
ROBO4_HUMAN	3029.0381
OVCA2_HUMAN	2360.7195
IRGQ_HUMAN	1527.4156
DDB2_HUMAN	10432.465
CLC2B_HUMAN	1722.2074
DDX1_HUMAN	25170.105
S14L1_HUMAN	4596.5024
PIEZ1_HUMAN	1528.8116
FAM3C_HUMAN	5159.1499
H1X_HUMAN	14393.705
PSMF1_HUMAN	7766.1284
GBF1_HUMAN	9229.7148

RTF1_HUMAN	7147.3364
NICA_HUMAN	15188.455
SNX19_HUMAN	1152.516
TM9S4_HUMAN	9608.8379
TM131_HUMAN	874.5885
RT27_HUMAN	16370.921
ELMO1_HUMAN	2646.0461
FIG4_HUMAN	635.83746
F168A_HUMAN	348.92062
AP3S1_HUMAN	13629.126
TSC1_HUMAN	4457.2432
UBXN4_HUMAN	5109.1099
PHF3_HUMAN	1259.7264
NDRG1_HUMAN	3764.6973
HS105_HUMAN	22637.055
SEPT8_HUMAN	7150.6606
CNOT9_HUMAN	4798.3647
LGAT1_HUMAN	619.64301
TBCD5_HUMAN	4035.0933
ZN592_HUMAN	754.61084
JADE3_HUMAN	871.90125
MY18A_HUMAN	1421.7538
LAR4B_HUMAN	3418.5659
GCN1_HUMAN	20454.814
HMHA1_HUMAN	109.50047
PRP16_HUMAN	3460.8105
NU205_HUMAN	72156.289
ANS1A_HUMAN	2350.9001
PXDN_HUMAN	18537.191
FAN_HUMAN	720.80255
GPI8_HUMAN	2277.2493
RT31_HUMAN	9414.3506
AKAP1_HUMAN	152.41447
ALG3_HUMAN	1067.6127
NEUG_HUMAN	455.16391
AN32B_HUMAN	70538.172
NECT2_HUMAN	11640.399
PGTA_HUMAN	10922.183
TFG_HUMAN	12961.312
HTRA1_HUMAN	19425.941
ARC1A_HUMAN	8443.9834
TF2H4_HUMAN	1168.0104
RREB1_HUMAN	565.46472
HDAC2_HUMAN	12315.593
STAM1_HUMAN	2217.853
REQU_HUMAN	2169.4473
SC65_HUMAN	9734.875
CBP_HUMAN	281.55536
SYMPK_HUMAN	5194.3887
RBP56_HUMAN	2747.5654

GOGA1_HUMAN	1037.5768
GGH_HUMAN	9043.2939
NRCAM_HUMAN	5313.7505
RPGR_HUMAN	572.29156
SHIP1_HUMAN	5291.877
DDX17_HUMAN	90863.008
KIFA3_HUMAN	2116.7241
CASPA_HUMAN	2173.5779
APBB2_HUMAN	1338.4742
RAD50_HUMAN	13834.711
CELF1_HUMAN	6924.8613
OSTF1_HUMAN	6050.5317
ARHG1_HUMAN	12455.044
XPF_HUMAN	704.89752
UFD1_HUMAN	26384.188
GSLG1_HUMAN	18689.326
RENT1_HUMAN	11186.342
CSN5_HUMAN	15778.352
GPKOW_HUMAN	6368.854
SMRC1_HUMAN	2526.3403
SMRD2_HUMAN	3449.6572
RAB8B_HUMAN	21615.57
BAD_HUMAN	487.76825
FUBP2_HUMAN	15363.282
GCDH_HUMAN	10915.095
RIT1_HUMAN	824.71332
PEX13_HUMAN	502.55289
TNPO1_HUMAN	34325.289
ARHG2_HUMAN	24716.111
NEP1_HUMAN	5673.5176
CLP1_HUMAN	3290.2783
GLMN_HUMAN	6224.1597
UBP13_HUMAN	2177.248
DVL3_HUMAN	930.18066
USP9X_HUMAN	10913.738
UBP7_HUMAN	11352.963
CUL5_HUMAN	5457.0313
STMN2_HUMAN	61288.879
VPP1_HUMAN	2781.9473
LPP_HUMAN	10904.425
RBPMS_HUMAN	300.67365
EXT2_HUMAN	909.21301
MED12_HUMAN	925.01831
TP4A1_HUMAN	187.99265
KPBB_HUMAN	3791.8416
SCAM4_HUMAN	4046.3142
HPS3_HUMAN	1040.4088
SMCE1_HUMAN	4468.4336
CAVN3_HUMAN	10371.891
RIFK_HUMAN	591.19751

MYDGF_HUMAN	33546.063
TM40L_HUMAN	1668.3793
YIPF5_HUMAN	2342.0491
PIGT_HUMAN	4856.061
IGSF8_HUMAN	413.36713
RL36L_HUMAN	6382.4663
RAB24_HUMAN	714.0885
OSBP2_HUMAN	1216.4751
ZN622_HUMAN	2772.075
RRF2M_HUMAN	2578.3635
UB2E3_HUMAN	12169.681
5NT3B_HUMAN	5221.8911
WBP2_HUMAN	2745.6016
PSMG2_HUMAN	15264.102
NCLN_HUMAN	16034.405
MUL1_HUMAN	708.47797
MRTFA_HUMAN	414.32352
LFG3_HUMAN	3869.0901
ERGI1_HUMAN	12434.542
UTP4_HUMAN	442.25296
FAKD4_HUMAN	4864.0703
MARC2_HUMAN	3352.7039
F162A_HUMAN	9209.9219
CCD47_HUMAN	7196.042
RM24_HUMAN	6746.2456
EXOC4_HUMAN	10691.883
MGN2_HUMAN	35488.797
P33MX_HUMAN	1165.5459
RFT1_HUMAN	3418.7993
ISOC2_HUMAN	2715.126
NTAN1_HUMAN	1656.5563
FERM2_HUMAN	14677.311
FUBP1_HUMAN	46401.746
TTC17_HUMAN	879.82446
LRC59_HUMAN	87518.406
VTI1A_HUMAN	472.20474
ESAM_HUMAN	7071.8008
PBIP1_HUMAN	5934.2534
MCUR1_HUMAN	833.19275
K1143_HUMAN	1263.4769
VP33A_HUMAN	3848.271
FKB10_HUMAN	17023.746
TTC28_HUMAN	2587.637
EXOS8_HUMAN	2147.7808
ZN428_HUMAN	474.19336
SH3K1_HUMAN	2852.4783
SKA1_HUMAN	571.03973
NACC2_HUMAN	354.79926
RNF25_HUMAN	1605.2815
AIDA_HUMAN	7375.541

PINX1_HUMAN	551.69061
ARL8A_HUMAN	9440.6367
OTUL_HUMAN	2279.6814
CHCH1_HUMAN	2165.1157
PPWD1_HUMAN	5169.7202
CC127_HUMAN	547.39459
COA7_HUMAN	1373.8634
UPP_HUMAN	713.52899
TAM41_HUMAN	2938.3203
MOB3A_HUMAN	2655.5625
DOC10_HUMAN	1626.4661
ATG2B_HUMAN	1223.8156
LENG1_HUMAN	1127.8273
TBC20_HUMAN	702.36658
EFHD2_HUMAN	28622.156
GALM_HUMAN	2872.5979
SYTL4_HUMAN	3209.5828
P5CR2_HUMAN	7531.6196
CSTOS_HUMAN	1866.1294
DCPS_HUMAN	15710.201
PP14B_HUMAN	2570.1582
INT12_HUMAN	986.29663
NSUN4_HUMAN	2127.6211
RHDF1_HUMAN	602.42242
CTHR1_HUMAN	4737.728
ACSF2_HUMAN	1734.2576
ISOC1_HUMAN	7048.9106
GCC1_HUMAN	590.46906
FWCH2_HUMAN	3578.2502
ASTRA_HUMAN	1622.9276
HAUS1_HUMAN	433.61475
FAF2_HUMAN	10510.708
CC124_HUMAN	5818.3247
FXRD1_HUMAN	5171.7466
OPTN_HUMAN	2955.374
AP2M1_HUMAN	48751.52
GCP3_HUMAN	3061.623
S7A6O_HUMAN	638.03607
KCD12_HUMAN	60936.227
RCN3_HUMAN	1738.2792
NMD3_HUMAN	5829.5596
REPS1_HUMAN	731.02405
TIM14_HUMAN	4156.3438
RMD1_HUMAN	6938.5063
NUD16_HUMAN	7796.8286
ESS2_HUMAN	1354.1943
CMBL_HUMAN	3782.9026
MSI2H_HUMAN	3176.604
SNR40_HUMAN	6901.6655
RMC1_HUMAN	1331.6339

RM38_HUMAN	8905.125
RSPRY_HUMAN	568.85791
ERLEC_HUMAN	4037.4336
RRFM_HUMAN	6190.9028
MTEF3_HUMAN	838.91998
SPDLY_HUMAN	1051.9885
ELP4_HUMAN	2734.342
SIR1_HUMAN	764.79852
YIPF6_HUMAN	1442.6316
SEH1_HUMAN	4082.689
TCAL4_HUMAN	2352.7761
KBP_HUMAN	13135.63
GNA1_HUMAN	10237.459
F120B_HUMAN	2208.9409
KTI12_HUMAN	934.45746
RT24_HUMAN	6153.832
RM53_HUMAN	2005.7386
T3HPD_HUMAN	6559.7114
RNF31_HUMAN	1796.0923
DAZP1_HUMAN	14681.577
SGTB_HUMAN	1683.1274
SAAL1_HUMAN	2284.437
MITOK_HUMAN	1338.6425
SGF29_HUMAN	485.19016
RRP36_HUMAN	585.42712
C1GLC_HUMAN	1332.2906
RBM33_HUMAN	2522.3538
DTBP1_HUMAN	4431.0317
DNJA3_HUMAN	5641.8296
TMA16_HUMAN	5602.2856
MB12A_HUMAN	3354.8064
PTCD3_HUMAN	7468.5303
MMAB_HUMAN	5406.2183
CYFP2_HUMAN	433.34543
GIMA5_HUMAN	870.14093
TRI11_HUMAN	1428.3068
CCD97_HUMAN	991.34503
CNRP1_HUMAN	12796.969
EDC3_HUMAN	4706.5156
DYL2_HUMAN	6061.9409
CPNE2_HUMAN	6866.6206
S10AG_HUMAN	4743.3994
SIPA1_HUMAN	4209.8315
SCRN2_HUMAN	1477.1216
THOC1_HUMAN	4251.438
OTUB1_HUMAN	24725.076
TRM61_HUMAN	842.04785
HMCES_HUMAN	256.92484
CHMP6_HUMAN	3740.1208
BICD1_HUMAN	541.3114

PGM2_HUMAN	28099.25
IMP4_HUMAN	2701.4622
CERS2_HUMAN	5638.7881
DUS3L_HUMAN	1146.8793
LTV1_HUMAN	3708.8264
SDSL_HUMAN	870.09381
RM48_HUMAN	3019.7727
PLPP_HUMAN	1957.1061
AURKB_HUMAN	538.66046
DCNL1_HUMAN	2680.1514
FAH2A_HUMAN	4477.9448
SMRD1_HUMAN	1798.2991
TOE1_HUMAN	2401.4219
CDA7L_HUMAN	1130.4408
RUSF1_HUMAN	1670.2729
DDX27_HUMAN	3448.2881
BORC6_HUMAN	1273.7205
SYMM_HUMAN	926.05328
A7L3B_HUMAN	3952.1572
GWL_HUMAN	312.54352
MTNB_HUMAN	2787.1389
ZC21A_HUMAN	966.79016
LIN37_HUMAN	283.90652
S41A3_HUMAN	167.60429
SNF8_HUMAN	2639.0505
ZCCHL_HUMAN	2368.0063
P121A_HUMAN	231.77473
TONSL_HUMAN	580.35883
PDLI5_HUMAN	30015.746
CREL1_HUMAN	2793.7056
ERO1A_HUMAN	25012.021
PRR11_HUMAN	1218.5386
FMC1_HUMAN	2924.6667
SAHH3_HUMAN	12602.136
DOCK6_HUMAN	7126.6665
MED30_HUMAN	1080.624
PGAM5_HUMAN	13835.058
INT4_HUMAN	2500.3591
DDRGK_HUMAN	5292.0039
DHTK1_HUMAN	1187.6793
SCLY_HUMAN	3215.7407
FUBP3_HUMAN	16944.148
SPF45_HUMAN	4662.5737
RCC1L_HUMAN	1215.5946
SYNM_HUMAN	1437.1847
SUCB2_HUMAN	12420.153
LRCH3_HUMAN	3777.6077
GMPPA_HUMAN	8172.3828
HPDL_HUMAN	904.50671
ABHEB_HUMAN	2642.4976

NGLY1_HUMAN	1103.3971
SC22A_HUMAN	4993.5649
ATPMK_HUMAN	12885.707
NSL1_HUMAN	1754.7295
PAWR_HUMAN	4502.5889
MET2A_HUMAN	375.82019
THOC3_HUMAN	4395.543
ITCH_HUMAN	2992.3989
TXD15_HUMAN	859.6297
KIRR1_HUMAN	2966.8481
COG3_HUMAN	1992.8845
CK5P3_HUMAN	8808.5293
VPS39_HUMAN	3523.9817
VPS50_HUMAN	2392.3691
VCIP1_HUMAN	3733.3472
SPTCS_HUMAN	430.18613
ELMO2_HUMAN	7836.0073
TMX3_HUMAN	11688.634
DCAF5_HUMAN	1914.2207
CHAP1_HUMAN	2307.0698
LMBL3_HUMAN	4011.3604
ZFP91_HUMAN	686.66608
PCD16_HUMAN	2473.6968
CLMN_HUMAN	9026.7754
PDLI2_HUMAN	368.93054
FACR2_HUMAN	5958.5073
BT3L4_HUMAN	8378.7314
RN170_HUMAN	817.57587
ANCHR_HUMAN	3318.4976
S35E1_HUMAN	1561.708
UBP47_HUMAN	5194.0967
ZN521_HUMAN	577.15204
CLP1L_HUMAN	3245.6885
TOPK_HUMAN	3811.3887
ARL5B_HUMAN	1003.7791
DNJC1_HUMAN	1638.3477
SCYL1_HUMAN	6548.8281
Z512B_HUMAN	795.12592
LRAT2_HUMAN	683.18311
EXOC2_HUMAN	7603.5366
CNDP2_HUMAN	47390.195
EHMT2_HUMAN	798.73218
ZFR_HUMAN	3163.5215
F210B_HUMAN	962.12061
B3GT6_HUMAN	2065.7849
EP400_HUMAN	979.64319
SNX27_HUMAN	4497.3345
KI16B_HUMAN	245.96568
IFT74_HUMAN	574.99072
TRI47_HUMAN	5213.7251

CNO6L_HUMAN	3427.2847
DHRS1_HUMAN	400.67511
DJC30_HUMAN	312.93997
UB2E2_HUMAN	145.54041
CAR19_HUMAN	4128.1714
PRRC1_HUMAN	7027.2017
FGD4_HUMAN	3590.7141
FXL18_HUMAN	4004.428
ZN512_HUMAN	2609.0779
NSE2_HUMAN	826.62817
NSE3_HUMAN	285.3186
PCMD1_HUMAN	151.1366
HS12B_HUMAN	5561.9453
YTDC1_HUMAN	1873.8835
COG8_HUMAN	2943.4016
CKLF3_HUMAN	4702.1924
DAA10_HUMAN	2910.2966
NOL4L_HUMAN	5508.98
MBOA7_HUMAN	11826.634
DOCK7_HUMAN	7985.0688
SFXN2_HUMAN	680.55121
F210A_HUMAN	1318.0885
FRMD6_HUMAN	845.35931
NSUN5_HUMAN	362.73788
RPR1A_HUMAN	4059.2458
AGAP3_HUMAN	1577.2505
ARAP1_HUMAN	3798.6885
IPO9_HUMAN	17623.801
MIA2_HUMAN	1505.5193
DCBD2_HUMAN	838.75836
ARHGH_HUMAN	2111.3833
INP4A_HUMAN	828.36041
RBM14_HUMAN	20918.949
ZN363_HUMAN	461.54971
KLHL5_HUMAN	1013.517
UHRF2_HUMAN	5306.8916
NED4L_HUMAN	3379.209
QKI_HUMAN	15780.726
LENG8_HUMAN	797.21838
FMNL2_HUMAN	1284.5621
PUS7_HUMAN	6490.5205
F111A_HUMAN	1093.4438
TPPC9_HUMAN	820.24902
TRNT1_HUMAN	4549.5459
SMG1_HUMAN	1133.3457
ALS2_HUMAN	1157.4449
TM237_HUMAN	900.32013
ALKB3_HUMAN	346.06979
RHG07_HUMAN	510.49222
PP1RA_HUMAN	4463.7891

S38A2_HUMAN	484.9198
UIF_HUMAN	1943.1163
MTMR9_HUMAN	2092.9595
VPS35_HUMAN	28640.158
PURB_HUMAN	3217.0627
XPO6_HUMAN	2233.3894
HHIP_HUMAN	40375.996
MAGI1_HUMAN	1117.2786
SPAG5_HUMAN	874.67206
NACC1_HUMAN	4488.8091
SNX18_HUMAN	1785.262
PASK_HUMAN	270.29938
CIC_HUMAN	705.60736
UIMC1_HUMAN	297.16782
VP13A_HUMAN	2977.6775
MED15_HUMAN	2272.3979
EFGM_HUMAN	9568.7871
ERGI2_HUMAN	2543.9775
MCCA_HUMAN	2047.2416
KKCC2_HUMAN	840.62628
TGS1_HUMAN	802.47876
NUDC1_HUMAN	12588.816
ERBIN_HUMAN	2059.9902
GCP6_HUMAN	2078.3589
GCP5_HUMAN	559.58221
UBP28_HUMAN	885.46967
FNBP1_HUMAN	4836.3506
PRPK_HUMAN	5287.395
PIGS_HUMAN	5754.8223
WRIP1_HUMAN	3782.791
RANB9_HUMAN	2840.9819
CLCC1_HUMAN	2030.0168
MYADM_HUMAN	5874.8594
NEB2_HUMAN	1735.9697
SRPK1_HUMAN	4717.9009
SMC6_HUMAN	1020.5928
KCD15_HUMAN	1374.0653
STRBP_HUMAN	19996.021
TM209_HUMAN	3029.4233
GPX7_HUMAN	2243.4832
CK5P2_HUMAN	350.32733
CP2S1_HUMAN	1309.1316
IWS1_HUMAN	7897.6343
SIN3A_HUMAN	1500.0798
OSBL9_HUMAN	6640.9316
CRBN_HUMAN	1181.6222
INT14_HUMAN	1248.6334
AEDO_HUMAN	2949.6882
CK5P1_HUMAN	606.19495
RSF1_HUMAN	165.00851

RBM15_HUMAN	3341.1274
RUFY1_HUMAN	4738.0957
MINT_HUMAN	326.19153
PNKP_HUMAN	5494.8013
MMS19_HUMAN	10614.33
UHRF1_HUMAN	4054.6646
NIBA2_HUMAN	32103.32
YMEL1_HUMAN	7310.9785
RMD3_HUMAN	4664.8169
HTF4_HUMAN	980.92401
CYH2_HUMAN	2026.7142
TBCB_HUMAN	15493.056
PSB7_HUMAN	24185.658
CNN2_HUMAN	15113.403
SEC62_HUMAN	5939.3428
PCY2_HUMAN	6007.4839
CDC5L_HUMAN	13593.192
PSMD1_HUMAN	27783.352
SDF2_HUMAN	1913.4021
PFD5_HUMAN	1872.5369
RING2_HUMAN	2696.9597
PARK7_HUMAN	14672.091
S1PR3_HUMAN	434.18713
GA2L1_HUMAN	267.46936
EYA3_HUMAN	771.79132
NEUR1_HUMAN	7878.2529
SORT_HUMAN	413.44714
VAT1_HUMAN	112873.4
LGMN_HUMAN	2801.6064
PLIN2_HUMAN	520.4422
DNJC2_HUMAN	4497.9287
MPP8_HUMAN	294.32147
NUP88_HUMAN	9766.4111
PKP4_HUMAN	354.91086
PI3R4_HUMAN	5102.457
P2RX4_HUMAN	2479.2803
POP1_HUMAN	5111.1763
S10AD_HUMAN	27645.861
SCAFB_HUMAN	1286.7355
TSNAX_HUMAN	6649.0557
SPS2_HUMAN	2347.0398
EIF3C_HUMAN	26167.551
TTC1_HUMAN	931.50702
DNJC7_HUMAN	10319.021
PHB2_HUMAN	88556.266
CSN8_HUMAN	2144.2739
CHP1_HUMAN	5643.7539
KIF2C_HUMAN	2115.7112
RGPD5_HUMAN	288.49777
MGLL_HUMAN	2993.7207

ATX2_HUMAN	2781.082
DOK1_HUMAN	1018.9167
METH_HUMAN	9548.8594
HCD2_HUMAN	33328.012
COCA1_HUMAN	4683.9087
SMAD5_HUMAN	1822.6851
SGMR1_HUMAN	4499.0366
ROAA_HUMAN	12382.76
NP1L4_HUMAN	21666.828
MGST2_HUMAN	45823.754
SNAG_HUMAN	5466.8408
PI51A_HUMAN	1084.4869
ABCA3_HUMAN	1394.0195
M3K3_HUMAN	1348.0126
ATP5S_HUMAN	2385.3765
MIPEP_HUMAN	3287.4502
ACON_HUMAN	21369.824
TM9S2_HUMAN	6969.9775
S29A1_HUMAN	8316.6289
TS101_HUMAN	8785.2178
CPNE1_HUMAN	21164.043
TCPH_HUMAN	44661.391
MYD88_HUMAN	6084.1138
EBP2_HUMAN	4521.6714
ANM1_HUMAN	24201.26
BAG1_HUMAN	1274.2102
ATF6B_HUMAN	1353.0736
FOXC2_HUMAN	399.42694
SH3G1_HUMAN	27935.588
TEP1_HUMAN	8695.3457
VRK1_HUMAN	10795.189
GDF15_HUMAN	7430.6157
AKAP9_HUMAN	11123.895
NIPS1_HUMAN	13979.099
CND3_HUMAN	6460.1953
ARP5L_HUMAN	13052.789
MICU1_HUMAN	947.37225
CG025_HUMAN	862.11597
F118B_HUMAN	2437.9023
SIN1_HUMAN	366.93045
RBM4B_HUMAN	14450.894
ZFY21_HUMAN	216.84895
DDX50_HUMAN	3416.9392
RM34_HUMAN	2070.9382
RNZ2_HUMAN	6898.0454
TRIR_HUMAN	2704.2036
GRWD1_HUMAN	5358.7759
MACD1_HUMAN	1675.4579
TCF25_HUMAN	3147.8411
CMS1_HUMAN	3070.04

KLDC3_HUMAN	698.21478
ECSIT_HUMAN	2256.0535
MEP50_HUMAN	16084.267
CYBC1_HUMAN	1233.0668
VKOR1_HUMAN	3282.3799
DPH2_HUMAN	1866.2406
TBA1C_HUMAN	11520.333
SELS_HUMAN	2199.2183
APOL2_HUMAN	4921.0488
MBB1A_HUMAN	13984.457
MGME1_HUMAN	677.93463
GORS1_HUMAN	1546.7156
FYCO1_HUMAN	1470.8242
ACBD6_HUMAN	2827.8267
COR1B_HUMAN	33672.5
TXD17_HUMAN	14408.164
BUD13_HUMAN	704.36163
CPPED_HUMAN	22265.057
VPS25_HUMAN	3168.7043
RM45_HUMAN	12722.909
CG050_HUMAN	8501.2646
TIRR_HUMAN	2133.0452
CAB45_HUMAN	8364.6162
PDD2L_HUMAN	896.05835
PAAF1_HUMAN	4988.7422
PYM1_HUMAN	3602.4556
MIC25_HUMAN	1028.3937
ADPGK_HUMAN	5245.167
GPTC1_HUMAN	234.21129
RIOK1_HUMAN	1474.6149
LLPH_HUMAN	2303.9229
SLD5_HUMAN	1872.8278
UTP23_HUMAN	318.68607
PELO_HUMAN	5822.2798
PXL2A_HUMAN	85301.859
TRI56_HUMAN	1084.7141
ERP44_HUMAN	55124.957
LXN_HUMAN	22841.736
NOL10_HUMAN	1084.9504
NTPCR_HUMAN	6481.5269
TIM29_HUMAN	5495.772
TACO1_HUMAN	2523.7971
GCP2_HUMAN	5424.6465
ESYT1_HUMAN	30676.619
UBAC1_HUMAN	1228.0145
YIPF4_HUMAN	1989.5897
PHAF1_HUMAN	1901.4115
SEN34_HUMAN	1048.0293
CNPY3_HUMAN	1130.4187
MTG1_HUMAN	2414.4487

ALG1_HUMAN	3282.3813
LIMD2_HUMAN	872.0722
HAUS8_HUMAN	915.21649
INP5K_HUMAN	2868.3611
PSMG3_HUMAN	3903.5085
CSN4_HUMAN	12402.947
SYT11_HUMAN	1128.5223
WAC_HUMAN	398.11743
DIDO1_HUMAN	3301.3025
MTA3_HUMAN	4158.645
RBM42_HUMAN	9160.8076
DCTN5_HUMAN	2688.4846
MCMBP_HUMAN	4773.5552
AASD1_HUMAN	3979.0208
DCNL5_HUMAN	6387.6401
AN32E_HUMAN	6660.999
LRRC1_HUMAN	880.54889
P4K2A_HUMAN	2747.2593
TMM43_HUMAN	24816.676
FSD1_HUMAN	1154.1807
TBCD_HUMAN	9484.582
NDC1_HUMAN	1476.5217
HGH1_HUMAN	1515.4528
DHRS4_HUMAN	3726.823
LMF2_HUMAN	5601.729
NDUF3_HUMAN	7269.8467
MMTA2_HUMAN	1855.4313
TMM70_HUMAN	4818.0894
MED18_HUMAN	1602.527
ISCA1_HUMAN	665.40973
TBB6_HUMAN	114370.09
PAXX_HUMAN	1261.3242
RPC3_HUMAN	1709.8108
HNRL1_HUMAN	17285.809
MSTO1_HUMAN	6345.2026
PHF23_HUMAN	1439.1996
PDC10_HUMAN	14346.025
RPP25_HUMAN	2277.4482
DERL1_HUMAN	17767.822
HTAI2_HUMAN	5719.3403
DDX23_HUMAN	10080.123
MIC26_HUMAN	5361.6641
DHRS6_HUMAN	7995.3267
MTNA_HUMAN	4502.2261
WDR18_HUMAN	6639.8926
VAMP8_HUMAN	1084.9437
THUM3_HUMAN	2226.1672
MTND_HUMAN	5996.9692
MECR_HUMAN	2912.1714
EMC6_HUMAN	5848.3018

NTM1A_HUMAN	2431.958
EDEM2_HUMAN	1367.5371
KTNB1_HUMAN	451.5166
LST8_HUMAN	571.16156
ASHWN_HUMAN	1471.3561
TM109_HUMAN	50731.508
PBDC1_HUMAN	3683.6194
PTSS2_HUMAN	3178.5991
NOC4L_HUMAN	2212.0735
UT14A_HUMAN	2626.2915
DUS23_HUMAN	5262.686
TMED9_HUMAN	40553.355
TM147_HUMAN	2941.5171
NUP58_HUMAN	4118.4526
SELO_HUMAN	2840.7068
DPCD_HUMAN	2541.3921
GNL3_HUMAN	10089.193
AFG2B_HUMAN	5016.6475
RIOK2_HUMAN	1210.8762
TR61B_HUMAN	207.44756
TMUB1_HUMAN	1744.4662
TIM21_HUMAN	2994.6001
KIFC1_HUMAN	592.02618
NUP85_HUMAN	7477.0815
ELOV1_HUMAN	1733.733
KATL1_HUMAN	1083.7968
HIRP3_HUMAN	749.03821
IFT27_HUMAN	2684.1895
YJU2_HUMAN	1420.2634
NUDT9_HUMAN	5286.9526
SYTM_HUMAN	3308.2654
THIC_HUMAN	23910.797
REPI1_HUMAN	468.59335
RBM4_HUMAN	3050.075
FUND2_HUMAN	1136.171
RPAP1_HUMAN	2559.7786
SF3B5_HUMAN	1533.1031
SFXN3_HUMAN	17360.502
PRR14_HUMAN	2391.3967
CHID1_HUMAN	2569.8018
PAPOG_HUMAN	1273.3427
CAR10_HUMAN	586.09662
NADAP_HUMAN	6370.874
CDK19_HUMAN	1194.5825
LS14B_HUMAN	1422.1648
TPSNR_HUMAN	1077.9817
JAM3_HUMAN	8759.668
CARD6_HUMAN	1439.1198
BTBD2_HUMAN	727.74078
OSB11_HUMAN	1927.1808

OSB10_HUMAN	4671.5674
RFIP5_HUMAN	2426.2813
TB10A_HUMAN	241.2027
C1QT5_HUMAN	3688.3826
NAA15_HUMAN	20355.813
B2L13_HUMAN	720.32959
CAR11_HUMAN	950.01788
S12A9_HUMAN	1544.9208
SRRT_HUMAN	21846.051
TGT_HUMAN	4707.5708
AP1M1_HUMAN	16962.365
NUSAP_HUMAN	457.46103
HDHD5_HUMAN	3744.9617
FACD2_HUMAN	490.03729
MAK16_HUMAN	2094.3843
HDAC8_HUMAN	444.20395
RTF2_HUMAN	1037.3007
CHM4A_HUMAN	3299.7776
EIF2A_HUMAN	16078.214
PDIP3_HUMAN	3676.9771
K1671_HUMAN	1405.2593
SHAN3_HUMAN	11432.583
GNB1L_HUMAN	1865.8922
FUT8_HUMAN	573.59711
RM32_HUMAN	1162.4147
RM20_HUMAN	4669.2183
RM13_HUMAN	9482.4219
RM09_HUMAN	9795.0977
RM04_HUMAN	5695.5
RM01_HUMAN	8988.75
MK67I_HUMAN	1552.7775
HYCCI_HUMAN	1934.8243
HELZ2_HUMAN	885.45367
HOIL1_HUMAN	2777.0637
RT26_HUMAN	5866.7046
NEUL_HUMAN	11425.114
CEP41_HUMAN	3702.8074
SETD2_HUMAN	663.18536
PANK2_HUMAN	650.59235
DOCK9_HUMAN	9661.291
FRMD8_HUMAN	5263.0415
CNTP3_HUMAN	2122.4988
NSD3_HUMAN	1551.3489
NUF2_HUMAN	3036.0151
RM37_HUMAN	10381.749
GTPB4_HUMAN	5079.4688
ASPC1_HUMAN	1603.4338
OSBL8_HUMAN	2430.3379
UACA_HUMAN	2913.1824
RAB34_HUMAN	5466.2979

DPH1_HUMAN	1028.2834
WDR11_HUMAN	4177.9023
REN3B_HUMAN	2143.7549
CRNL1_HUMAN	3729.3508
TBL1R_HUMAN	3719.4863
UBL5_HUMAN	3962.5068
PP12C_HUMAN	2574.8484
KPCD2_HUMAN	5402.1523
EDEM3_HUMAN	3278.6255
NIBA1_HUMAN	616.04755
UBXN6_HUMAN	4102.4785
TM6S1_HUMAN	735.48749
UCK2_HUMAN	5769.0889
SN_HUMAN	1884.7927
API5_HUMAN	24505.549
NLRP1_HUMAN	802.81268
TRIM5_HUMAN	1231.8641
TRIM4_HUMAN	53440.992
FTO_HUMAN	6747.2314
ZDHC5_HUMAN	476.21417
TNG6_HUMAN	1537.9915
TB182_HUMAN	5821.9541
UBE2O_HUMAN	6033.1113
ZY11B_HUMAN	2099.7041
TANC1_HUMAN	5440.1328
EPT1_HUMAN	2420.1211
XPO4_HUMAN	7623.2476
LNP_HUMAN	1158.4043
CEP44_HUMAN	433.48984
TTYH3_HUMAN	9403.9316
KLHL4_HUMAN	2000.2031
MTMRC_HUMAN	632.20831
WDR33_HUMAN	5797.041
WDR12_HUMAN	3303.0491
YIPF3_HUMAN	5628.0649
TINAL_HUMAN	2935.2744
ADSSP_HUMAN	6819.3164
PITH1_HUMAN	5471.6377
DERL2_HUMAN	9721.3008
COMD5_HUMAN	2057.1785
SENP6_HUMAN	307.76511
REXO4_HUMAN	1870.6571
DDX24_HUMAN	4196.8149
RPA49_HUMAN	2844.1853
SKI8_HUMAN	7804.3589
SLIRP_HUMAN	7898.5034
SRR_HUMAN	1897.12
NIF3L_HUMAN	3437.1814
EGLN1_HUMAN	6226.8457
MCLN1_HUMAN	1087.7379

PIP30_HUMAN	3475.5254
MFF_HUMAN	1212.6564
NAA50_HUMAN	8686.5225
UBA5_HUMAN	5633.936
MKRN2_HUMAN	2365.8806
T126A_HUMAN	4137.5308
PAIP1_HUMAN	11428.004
CLPB_HUMAN	4347.1284
RB33B_HUMAN	3984.1157
LSG1_HUMAN	6450.9717
F107B_HUMAN	6773.3628
NAT10_HUMAN	12466.191
COMD4_HUMAN	2023.2789
KLC2_HUMAN	2761.8735
ILKAP_HUMAN	5588.4614
XRN2_HUMAN	16428.486
TOLIP_HUMAN	4831.2949
SP130_HUMAN	742.19373
BRD8_HUMAN	650.57983
NSRP1_HUMAN	188.7607
INT2_HUMAN	857.93518
RGAP1_HUMAN	554.4873
PAR12_HUMAN	2854.3748
CSTFT_HUMAN	1169.8568
5NT3A_HUMAN	2755.1504
CYRIA_HUMAN	17032.49
TM222_HUMAN	471.7196
HDHD2_HUMAN	2606.0149
GATA_HUMAN	2345.5615
DDX47_HUMAN	6190.1621
MAGT1_HUMAN	6534.9233
RAB1B_HUMAN	60481.93
RM18_HUMAN	4965.6797
TSYL1_HUMAN	860.27258
TM168_HUMAN	1249.4547
LMA2L_HUMAN	7883.98
SMG9_HUMAN	1755.5696
F234A_HUMAN	189.95039
OSBL5_HUMAN	406.82809
TDIF1_HUMAN	1920.583
SIL1_HUMAN	1391.4199
METL9_HUMAN	958.73938
APC1_HUMAN	4745.0513
I2BPL_HUMAN	732.51062
CYTM1_HUMAN	966.45636
RPC6_HUMAN	2567.4456
NUCKS_HUMAN	3482.2212
TMX4_HUMAN	7371.3574
KI13A_HUMAN	24118.715
ASCC2_HUMAN	6308.6738

RBNS5_HUMAN	666.17651
ISCU_HUMAN	2874.7153
OSBL2_HUMAN	1283.6035
ATG5_HUMAN	4233.6162
WDR13_HUMAN	2722.6001
EPN3_HUMAN	2223.9382
EHD4_HUMAN	97309.266
VP33B_HUMAN	4025.2146
VPS16_HUMAN	9002.2402
VPS11_HUMAN	5127.4482
SH3L3_HUMAN	16451.314
GAN_HUMAN	381.75073
S2532_HUMAN	1586.1874
TARA_HUMAN	7095.269
SLK_HUMAN	10270.135
PPIL3_HUMAN	417.47006
S38A1_HUMAN	527.78394
PDCL3_HUMAN	5386.416
TAOK3_HUMAN	5652.7144
RBGPR_HUMAN	9111.9229
ADNP_HUMAN	2044.6398
DPH5_HUMAN	2298.7102
DHX36_HUMAN	6751.5132
IPYR2_HUMAN	7920.519
SPNS1_HUMAN	4377.8135
RM46_HUMAN	2362.0779
S12A5_HUMAN	672.43695
PARL_HUMAN	1258.1671
PININ_HUMAN	7181.8037
TM245_HUMAN	1271.741
FOXP1_HUMAN	1430.7991
BACD3_HUMAN	4367.8804
MOD5_HUMAN	973.21552
CK068_HUMAN	6126.4912
GHITM_HUMAN	5482.8379
BOLA2_HUMAN	6619.3838
MMAD_HUMAN	628.56995
TXNIP_HUMAN	1275.2883
TMX1_HUMAN	19733.072
NELFA_HUMAN	3364.3652
GCP60_HUMAN	8477.7539
BORG4_HUMAN	799.46478
PTN23_HUMAN	8839.7461
SEM6B_HUMAN	6630.6079
UN45A_HUMAN	17429.434
MFSD1_HUMAN	2352.1687
DNJC5_HUMAN	590.41901
DSN1_HUMAN	318.40225
CHM4B_HUMAN	19896.449
RWDD1_HUMAN	2700.7637

CUED2_HUMAN	497.88489
FXL15_HUMAN	570.60474
FN3K_HUMAN	2702.2756
OFUT1_HUMAN	7576.1577
PIGU_HUMAN	4868.396
TOR3A_HUMAN	4295.0005
WNK1_HUMAN	3563.4556
AMPB_HUMAN	21457.182
GLP3L_HUMAN	5345.9316
GOLP3_HUMAN	4051.9922
OSGL1_HUMAN	2439.3811
SIA7D_HUMAN	470.61908
GAPR1_HUMAN	3982.3818
TRABD_HUMAN	2004.5502
MTG2_HUMAN	150.93318
SENP3_HUMAN	4379.2612
OSBL3_HUMAN	4677.5996
SMRCD_HUMAN	2533.6091
EHD1_HUMAN	22155.328
CAPAM_HUMAN	881.6095
ESF1_HUMAN	808.25909
ALG2_HUMAN	4428.1724
HEAT1_HUMAN	5085.8877
SG196_HUMAN	699.87897
RABE2_HUMAN	1473.9131
TFB2M_HUMAN	3298.7788
ZCHC4_HUMAN	2233.7891
STEEP_HUMAN	191.20313
CIA2A_HUMAN	1946.2931
DHX35_HUMAN	1775.1892
F124B_HUMAN	4350.5981
HAUS4_HUMAN	3213.261
CC134_HUMAN	5047.2188
CCD86_HUMAN	2452.8423
REEP4_HUMAN	1714.3447
SOX17_HUMAN	801.82007
OPA3_HUMAN	2628.541
CIAO3_HUMAN	1956.3052
DHX33_HUMAN	1062.9554
NOL6_HUMAN	2651.5061
WDCP_HUMAN	668.99475
YTDC2_HUMAN	3288.5332
AZI2_HUMAN	229.14462
RPAP3_HUMAN	3898.4084
BCAS3_HUMAN	471.40353
ALG9_HUMAN	3047.3792
LDAH_HUMAN	1818.8182
RIOX1_HUMAN	3232.0513
WDR55_HUMAN	2848.8777
RANB3_HUMAN	6653.7012

DCTP1_HUMAN	559.68958
PEAK1_HUMAN	1681.9949
RPF2_HUMAN	1486.9155
SMYD3_HUMAN	3952.2581
AAMDC_HUMAN	2029.7678
DOCK5_HUMAN	1526.1273
WDR26_HUMAN	3236.4402
TDRD3_HUMAN	2459.3044
CH033_HUMAN	1827.4004
AT133_HUMAN	416.07428
MET17_HUMAN	665.35767
SFR19_HUMAN	1465.3445
PGES2_HUMAN	5717.3921
PHAX_HUMAN	2843.7683
UBE2Z_HUMAN	6075.6758
ACAD9_HUMAN	6314.5171
NT5D2_HUMAN	11243.635
PKRI1_HUMAN	861.2821
CAAP1_HUMAN	1393.5135
NOL11_HUMAN	528.0954
DDX31_HUMAN	567.87213
TMT1A_HUMAN	3114.0674
PAAT_HUMAN	2688.2297
MMRN2_HUMAN	14582.361
MINY3_HUMAN	2213.075
EVA1A_HUMAN	1643.9077
AKTIP_HUMAN	875.146
PKHF2_HUMAN	2206.1296
ANKZ1_HUMAN	2608.2036
GORS2_HUMAN	3651.0383
ZWILC_HUMAN	2452.8257
JUPI2_HUMAN	1790.7817
GHC1_HUMAN	6183.0269
PPIP2_HUMAN	2795.0979
MED20_HUMAN	2079.2649
QTRT2_HUMAN	1777.0479
ARP8_HUMAN	1239.5719
ARMT1_HUMAN	4782.9063
CNO10_HUMAN	1483.6447
LRC40_HUMAN	18845.141
EHMT1_HUMAN	2224.4734
SFXN1_HUMAN	26580.365
SPE39_HUMAN	1617.3351
COG4_HUMAN	7156.125
ARP5_HUMAN	990.71295
VP37B_HUMAN	792.52637
RM44_HUMAN	8454.3809
L2HDH_HUMAN	1181.8599
CSN7B_HUMAN	3274.927
NHEJ1_HUMAN	704.92432

S61A2_HUMAN	13498.162
FKRP_HUMAN	690.01483
ELP3_HUMAN	7570.4126
RPF1_HUMAN	469.78076
RPA2_HUMAN	1527.2705
ZMAT3_HUMAN	495.9856
UCK1_HUMAN	827.57135
KT3K_HUMAN	1757.8523
TBC17_HUMAN	2029.4755
SYCM_HUMAN	4335.9731
S52A2_HUMAN	2191.2537
PPCS_HUMAN	11765.602
UBTD1_HUMAN	2504.4133
WDR41_HUMAN	671.51941
NMNA1_HUMAN	1294.1257
NJMU_HUMAN	1759.491
SIAE_HUMAN	875.24097
PKHA5_HUMAN	644.45178
SMUF2_HUMAN	1911.9652
RENT2_HUMAN	4375.2979
GBB4_HUMAN	1578.7219
XPO5_HUMAN	7275.0449
GRPE1_HUMAN	14260.823
MYG1_HUMAN	12124.618
PKHA3_HUMAN	321.63727
PKHA1_HUMAN	1476.3635
RISC_HUMAN	4450.0356
NET4_HUMAN	8337.5293
CYBP_HUMAN	30737.961
RRAGC_HUMAN	9256.3975
ZFYV1_HUMAN	1427.0938
IF122_HUMAN	1215.8228
RDH14_HUMAN	4575.5972
PARVB_HUMAN	11394.55
TENS1_HUMAN	2966.3618
PLRKT_HUMAN	2907.4583
NMRL1_HUMAN	2132.8987
VEZA_HUMAN	789.58325
SPC25_HUMAN	1673.2972
TAF9B_HUMAN	631.56238
S38AA_HUMAN	2377.6287
EKI1_HUMAN	1824.3525
AGRL4_HUMAN	9156.9541
TM165_HUMAN	4383.7246
TPC_HUMAN	498.17377
EMAL4_HUMAN	5580.5957
MRM3_HUMAN	2647.4995
GLOD4_HUMAN	48132.016
NEK6_HUMAN	1670.8639
MCCB_HUMAN	3493.4216

NCOA5_HUMAN	35751.699
EPG5_HUMAN	1410.915
MOV10_HUMAN	8511.6885
MET14_HUMAN	2649.9282
GBA2_HUMAN	2955.6633
CWC22_HUMAN	2217.2588
RAVR2_HUMAN	913.49786
CHD8_HUMAN	976.54425
E41L5_HUMAN	1644.1364
GPN1_HUMAN	4637.7725
SDF2L_HUMAN	5002.5112
KC1G1_HUMAN	3418.9495
SYF1_HUMAN	4724.2466
PREB_HUMAN	5100.5391
SRA1_HUMAN	1426.9109
AT131_HUMAN	5421.165
GOPC_HUMAN	1930.0718
RM47_HUMAN	5558.0454
LYRM4_HUMAN	2728.8811
CHM1A_HUMAN	8417.459
TM9S3_HUMAN	11746.881
MYO10_HUMAN	6094.772
APMAP_HUMAN	48981.668
WDR6_HUMAN	4381.9238
TRXR2_HUMAN	11431.313
ABCB6_HUMAN	897.74762
ARFG3_HUMAN	4629.3174
ZCC17_HUMAN	867.64819
RAB18_HUMAN	15527.935
PALMD_HUMAN	10155.934
SSU72_HUMAN	2584.4441
VTA1_HUMAN	20181.297
SYSM_HUMAN	3794.5918
RT30_HUMAN	5809.418
DLRB1_HUMAN	5832.7905
EMC7_HUMAN	7568.0273
ENY2_HUMAN	2258.5415
EXOS4_HUMAN	4065.4397
UBE2T_HUMAN	2783.1021
NOP10_HUMAN	3564.356
OSGEP_HUMAN	5397.9951
DMAP1_HUMAN	804.50299
PCD12_HUMAN	609.82849
INO1_HUMAN	7244.0049
BRD7_HUMAN	529.63135
DCP1A_HUMAN	4069.8604
ACO13_HUMAN	8245.8896
MED4_HUMAN	663.83405
TIDC1_HUMAN	237.06386
RIC8A_HUMAN	20065.502

C1QR1_HUMAN	36292.352
LUC7L_HUMAN	8640.4131
ESM1_HUMAN	832.07166
LZTL1_HUMAN	1921.3103
RM40_HUMAN	5351.7036
SSF1_HUMAN	1425.9587
PLCB1_HUMAN	1576.3912
TIGAR_HUMAN	15107.265
CL004_HUMAN	1339.9353
RTN4_HUMAN	14575.717
RPR1B_HUMAN	6829.3721
XPP3_HUMAN	2523.1509
NIT2_HUMAN	7557.5664
AVEN_HUMAN	549.60333
EXOS5_HUMAN	1886.8546
EXOS3_HUMAN	4727.2129
KI13B_HUMAN	5292.3057
ANLN_HUMAN	2411.4021
XPP1_HUMAN	13284.514
GEPH_HUMAN	4720.4946
BIN3_HUMAN	3148.7771
SAS10_HUMAN	1217.9773
STAR7_HUMAN	1164.3801
BIRC6_HUMAN	4663.251
PDLI7_HUMAN	15479.789
ACSA_HUMAN	11252.093
DBLOH_HUMAN	3562.3367
DDX21_HUMAN	9645.5195
SAR1A_HUMAN	28185.711
SIAS_HUMAN	32160.141
SHLB2_HUMAN	3603.3765
ASH1L_HUMAN	12067.014
EI2BG_HUMAN	9488.2705
MBNL1_HUMAN	10442.765
S17A5_HUMAN	3553.0947
4ET_HUMAN	1049.854
CHSTC_HUMAN	920.15344
ST7_HUMAN	1571.2646
PICK1_HUMAN	1117.8124
SH2B1_HUMAN	2188.3376
PYRG2_HUMAN	6174.0288
DPOE3_HUMAN	3064.5251
CHRC1_HUMAN	3051.0991
AAAS_HUMAN	6674.0596
SNRK_HUMAN	3678.0898
ABCBA_HUMAN	2867.0632
BAZ1A_HUMAN	1275.3018
STRN4_HUMAN	4153.5371
ADPPT_HUMAN	9613.3545
METL5_HUMAN	1785.2998

OSTC_HUMAN	21159.107
PLS4_HUMAN	2493.9541
RNC_HUMAN	748.44415
SNX15_HUMAN	702.90625
HEBP1_HUMAN	2937.8232
ABC3C_HUMAN	2129.063
VPS45_HUMAN	3244.3711
PNO1_HUMAN	1848.0057
RM17_HUMAN	10656.521
PHP14_HUMAN	427.08127
SERC1_HUMAN	855.53693
RHG35_HUMAN	3102.6565
F1142_HUMAN	2409.4768
PLS3_HUMAN	3650.5217
PLCD_HUMAN	1640.1255
PLCC_HUMAN	2377.2429
HELLS_HUMAN	1509.6296
C1GLT_HUMAN	1922.9631
LTBP3_HUMAN	429.44681
TOM22_HUMAN	4333.5767
LANC2_HUMAN	3955.2461
KIF15_HUMAN	1148.2262
TM7S3_HUMAN	1518.603
HOME3_HUMAN	6553.2153
SYFB_HUMAN	25424.465
SYIM_HUMAN	13931.97
SLX9_HUMAN	2521.0701
SAMN1_HUMAN	575.05988
KLC4_HUMAN	2000.1981
DIAP3_HUMAN	1572.3285
BMP2K_HUMAN	856.00165
ATG3_HUMAN	5197.0298
PDS5B_HUMAN	4901.2031
SMC4_HUMAN	30998.588
MA2C1_HUMAN	7775.9575
SAC1_HUMAN	8717.1406
DEPP1_HUMAN	1359.1146
OLA1_HUMAN	31824.049
CUTC_HUMAN	860.78967
ZF64B_HUMAN	1261.7195
ECHD1_HUMAN	11299.994
RBM12_HUMAN	87332.648
TB22B_HUMAN	1968.7196
MDN1_HUMAN	8298.5713
ACS2L_HUMAN	1252.2306
ZCHC3_HUMAN	979.63934
DECR2_HUMAN	5421.5405
ABHDA_HUMAN	2626.9028
T11L1_HUMAN	1823.3977
STAU2_HUMAN	5604.8628

SHFL_HUMAN	3106.5271
DDX28_HUMAN	1580.0963
T106B_HUMAN	2081.9827
LIN7C_HUMAN	814.20239
PLCE_HUMAN	4687.2393
TXLNG_HUMAN	1091.7322
SPS2L_HUMAN	2146.1399
UFSP2_HUMAN	3026.7153
ABCF3_HUMAN	7392.8169
CYRIB_HUMAN	12080.017
MITOS_HUMAN	1976.2103
DD19A_HUMAN	18560.063
GIMA4_HUMAN	33556.418
TYDP1_HUMAN	2150.0176
POTE1_HUMAN	580.67963
TBC23_HUMAN	4099.3994
DCA13_HUMAN	1096.2461
IMP3_HUMAN	2831.0544
MRGBP_HUMAN	1282.545
TYW1_HUMAN	171.93297
EXOC1_HUMAN	10468.458
PCMD2_HUMAN	982.28668
INT9_HUMAN	2577.8933
CC50A_HUMAN	14571.114
UQCC1_HUMAN	2694.1501
SEP11_HUMAN	21977.762
S38A7_HUMAN	16977.455
MED17_HUMAN	12580.352
PARVA_HUMAN	9759.0234
UBP40_HUMAN	646.28235
PANK4_HUMAN	10299.357
FBX28_HUMAN	551.51581
TBC13_HUMAN	6152.0659
EXD2_HUMAN	2174.821
DJC11_HUMAN	6239.1636
INT7_HUMAN	14677.413
FANCI_HUMAN	1062.9532
ATD3A_HUMAN	36501.395
ARL8B_HUMAN	10816.941
DJC17_HUMAN	5445.7734
INT13_HUMAN	3246.3884
RIC8B_HUMAN	858.88489
GNL3L_HUMAN	1845.2355
DDX18_HUMAN	4995.0137
ASF1B_HUMAN	2220.8472
KLH11_HUMAN	1404.0629
INT10_HUMAN	2508.6558
RT18A_HUMAN	7300.7158
PNPO_HUMAN	738.84674
ARMC1_HUMAN	3677.6882

RPC5_HUMAN	998.88031
SDA1_HUMAN	4002.54
PAPD1_HUMAN	5065.5967
NLE1_HUMAN	2637.4312
NECP2_HUMAN	13577.233
RPC2_HUMAN	5066.7891
RBM28_HUMAN	2564.0657
ANO10_HUMAN	8880.7588
RBM22_HUMAN	3131.2446
DMAC2_HUMAN	278.34299
WDR70_HUMAN	1308.8884
TMM51_HUMAN	258.20703
ARGL1_HUMAN	4871.5928
IFT57_HUMAN	288.81601
SLTM_HUMAN	2753.2659
FKB14_HUMAN	1248.4666
RMND1_HUMAN	2757.1292
PK1IP_HUMAN	1401.6606
HIF1N_HUMAN	2399.5789
OXSM_HUMAN	7611.9624
RM22_HUMAN	15241.948
CZIB_HUMAN	14091.633
BABA1_HUMAN	3999.1121
CLN6_HUMAN	310.93475
THG1_HUMAN	1186.3624
HPF1_HUMAN	2921.176
IRAK4_HUMAN	2592.4258
UCKL1_HUMAN	2923.2251
TM160_HUMAN	3636.1616
NALP2_HUMAN	3848.2546
F120C_HUMAN	846.35364
TSAP1_HUMAN	686.72229
NDUBB_HUMAN	2969.3472
RM16_HUMAN	9708.7842
NHP2_HUMAN	4202.0347
OCAD1_HUMAN	6881.6665
ADPRS_HUMAN	11955.891
MARH5_HUMAN	4436.6826
LYAR_HUMAN	5212.541
T161A_HUMAN	475.35364
IMPA3_HUMAN	4231.9116
MIC19_HUMAN	10894.853
DUS2L_HUMAN	196.45901
CKLF6_HUMAN	1794.7986
SIR5_HUMAN	1623.2418
MIOS_HUMAN	1147.432
MTMRA_HUMAN	4771.1855
NSMA3_HUMAN	4550.0376
CWC25_HUMAN	1563.9423
TEX10_HUMAN	4428.041

DCA16_HUMAN	1495.3674
THUM1_HUMAN	4434.6475
TOR4A_HUMAN	3964.9888
TRM1_HUMAN	3983.8281
GDAP2_HUMAN	1026.6437
NDE1_HUMAN	1295.1058
BABA2_HUMAN	1669.2206
QPCTL_HUMAN	2371.7107
ARL15_HUMAN	2957.6318
KCTD5_HUMAN	1059.2753
CARF_HUMAN	2985.4802
DJB12_HUMAN	2355.0872
ALKB4_HUMAN	386.69678
NSE4A_HUMAN	633.9646
GAR1_HUMAN	4605.3862
STAB1_HUMAN	10380.966
PP4R2_HUMAN	1580.7354
DPP3_HUMAN	22916.4
AATF_HUMAN	623.26117
TBA8_HUMAN	4027.4639
DDX56_HUMAN	1868.8356
TE2IP_HUMAN	225.06543
ABI2_HUMAN	3198.5571
BCLF1_HUMAN	12284.845
UTP6_HUMAN	823.19403
TAB2_HUMAN	175.37654
RM39_HUMAN	7428.8428
M3K20_HUMAN	2542.604
FKB11_HUMAN	11781.766
TMOD3_HUMAN	11906.979
BET1L_HUMAN	2410.8545
ELOV5_HUMAN	2488.0547
KBRS2_HUMAN	1720.5557
UGGG2_HUMAN	534.45105
UGGG1_HUMAN	29854.01
CDK12_HUMAN	2034.3593
FAKD2_HUMAN	4614.4629
TECR_HUMAN	6958.8667
ERAP1_HUMAN	14679.632
ARP10_HUMAN	4306.0938
USE1_HUMAN	1772.5372
CISD1_HUMAN	2366.1353
GGA3_HUMAN	2120.6934
TLS1_HUMAN	624.39905
F120A_HUMAN	8570.5537
GDE1_HUMAN	577.31396
SMAL1_HUMAN	1257.6362
GLTP_HUMAN	2485.9221
SPG21_HUMAN	3437.0039
RM35_HUMAN	7425.3081

UBIP1_HUMAN	958.3739
IF2B1_HUMAN	17618.369
SACS_HUMAN	2379.7217
MTCH1_HUMAN	7850.3252
NUDT4_HUMAN	9744.1611
HPBP1_HUMAN	8054.916
MAT2B_HUMAN	29026.313
MYOF_HUMAN	45263.078
ITSN2_HUMAN	3199.9133
NOP53_HUMAN	1181.439
EHD3_HUMAN	49984.637
EHD2_HUMAN	91475.633
ARHGC_HUMAN	3249.9729
CNOT2_HUMAN	4194.3052
SPN90_HUMAN	2135.5767
PD1L1_HUMAN	1195.9139
OGFR_HUMAN	7036.2866
LMCD1_HUMAN	1664.2117
CRIM1_HUMAN	2423.8318
SELN_HUMAN	4369.7603
PALS2_HUMAN	3127.3848
CHMP5_HUMAN	7401.2036
CWC15_HUMAN	2271.7732
RM15_HUMAN	12135.61
THYN1_HUMAN	9463.7158
TAP26_HUMAN	280.8988
NDUF4_HUMAN	3405.7305
HACD3_HUMAN	12041.924
EMC3_HUMAN	3395.9453
NDUAD_HUMAN	8187.2202
PDP1_HUMAN	5781.1909
KCMF1_HUMAN	1130.8479
RAI14_HUMAN	9266.3535
VAPA_HUMAN	11957.906
RM27_HUMAN	3238.4543
TMEM9_HUMAN	1331.3992
SENP1_HUMAN	917.24591
CXXC1_HUMAN	405.90598
SH3B4_HUMAN	6712.2295
SEP10_HUMAN	12022.474
GMIP_HUMAN	1001.9015
PHRF1_HUMAN	2164.1252
POGK_HUMAN	1241.9884
DAPLE_HUMAN	298.96213
RHG23_HUMAN	1456.1577
VPS18_HUMAN	10156.393
RCC2_HUMAN	43742.266
RELCH_HUMAN	1275.6649
DIP2B_HUMAN	4985.6543
JCAD_HUMAN	2061.3772

SLAI2_HUMAN	488.14047
TEN3_HUMAN	681.46454
UBP36_HUMAN	582.93109
BCCIP_HUMAN	13095.067
STK26_HUMAN	1168.5245
ARMX1_HUMAN	3009.4194
ABI3_HUMAN	6884.9375
FPRP_HUMAN	17332.928
CT2NL_HUMAN	5915.1475
TM181_HUMAN	2691.9141
IBTK_HUMAN	1552.3445
HTR5B_HUMAN	1754.9672
ZNFX1_HUMAN	1409.9579
CHPF2_HUMAN	3191.8928
PCD10_HUMAN	477.6821
RRBP1_HUMAN	40067.664
CPSF2_HUMAN	5561.7202
KLHL9_HUMAN	2957.8889
SYLC_HUMAN	27829.852
MYEF2_HUMAN	12593.821
KLH42_HUMAN	523.82751
E2AK4_HUMAN	4991.9595
WDR35_HUMAN	1158.4889
RBM27_HUMAN	2804.7244
KANL3_HUMAN	677.69751
HECW2_HUMAN	2906.1658
FRM4A_HUMAN	1024.4865
ANFY1_HUMAN	5899.6182
RERE_HUMAN	840.87518
SUCB1_HUMAN	9064.582
GMPR2_HUMAN	7009.4868
STX18_HUMAN	928.23743
DPM3_HUMAN	557.38196
IMPCT_HUMAN	1544.9615
UVRAG_HUMAN	2667.4912
ATX10_HUMAN	10839.055
MBD2_HUMAN	2851.6421
NCDN_HUMAN	2142.4646
TFP11_HUMAN	1092.6687
EP15R_HUMAN	10739.488
MYO1A_HUMAN	6418.0425
ORC3_HUMAN	1896.4181
SAE1_HUMAN	11777.221
COPG2_HUMAN	9968.3477
PI4KB_HUMAN	984.93121
MRC2_HUMAN	1447.3247
COMD3_HUMAN	1830.2813
GBG12_HUMAN	2058.5176
RBL2A_HUMAN	2992.9377
MTRR_HUMAN	426.46747

UXT_HUMAN	1309.0894
ASH2L_HUMAN	2814.0491
DHCR7_HUMAN	23581.756
TR10D_HUMAN	755.83112
HDAC6_HUMAN	2279.4407
SPAST_HUMAN	3882.0706
DKK3_HUMAN	733.60675
TRMB_HUMAN	6969.9858
GULP1_HUMAN	1393.3907
VPS29_HUMAN	16332.198
EIF3K_HUMAN	19958.639
EXTL2_HUMAN	1197.7319
GRHPR_HUMAN	15446.578
CATZ_HUMAN	25188.953
KS6B2_HUMAN	1532.5732
DJB11_HUMAN	13025.327
RNF14_HUMAN	2182.0012
SAE2_HUMAN	17288.24
MO4L1_HUMAN	1590.8862
NXF1_HUMAN	7985.7568
SE1L1_HUMAN	1641.1774
B4GT7_HUMAN	3711.2332
PEF1_HUMAN	4450.0752
ZMYM2_HUMAN	1045.7457
CSN7A_HUMAN	8297.1289
DIC_HUMAN	5776.9214
AASS_HUMAN	8189.5176
CLIP2_HUMAN	1315.1963
QCR9_HUMAN	13238.94
MTFP1_HUMAN	2274.7759
ZO2_HUMAN	21858.641
DNJB4_HUMAN	18322.926
ST17A_HUMAN	855.25446
CFDP1_HUMAN	4241.4834
DAXX_HUMAN	642.24426
TRM7_HUMAN	2918.6794
VTI1B_HUMAN	3028.365
STK39_HUMAN	2361.0537
ADDG_HUMAN	3704.2334
LRWD1_HUMAN	2832.6423
NPS3A_HUMAN	7359.8311
CGBP1_HUMAN	2976.8325
PISD_HUMAN	483.15402
ABCF2_HUMAN	12844.412
TES_HUMAN	6528.5542
AAKG2_HUMAN	4471.02
GCP4_HUMAN	3090.7585
KDM5B_HUMAN	164.51974
SYWM_HUMAN	1030.7332
LIMD1_HUMAN	996.71313

SEC63_HUMAN	10630.768
GTR6_HUMAN	1065.1509
Z3H7B_HUMAN	5658.3794
NDRG3_HUMAN	9280.0488
ARMX3_HUMAN	5023.2056
SWP70_HUMAN	21950.307
SUN2_HUMAN	9169.7451
RLP24_HUMAN	831.65167
LTOR3_HUMAN	4857.6357
LIMA1_HUMAN	12163.524
AFF4_HUMAN	7070.167
SRP68_HUMAN	12382.207
CHRD1_HUMAN	12394.743
TBK1_HUMAN	4754.3169
SEPT9_HUMAN	14711.844
PCYOX_HUMAN	10854.116
DDX20_HUMAN	5092.4722
SHPK_HUMAN	4827.8184
DPP2_HUMAN	4517.3975
CEIP2_HUMAN	5020.1421
UBP25_HUMAN	3146.9946
BAP29_HUMAN	4789.9658
NB5R1_HUMAN	6749.9458
S30BP_HUMAN	3307.8723
ZNHI2_HUMAN	9093.7734
PFD2_HUMAN	12935.636
GPN3_HUMAN	902.01074
PUF60_HUMAN	19739.793
NRBP_HUMAN	5175.0552
ENOPH_HUMAN	7485.5513
FEZ2_HUMAN	1665.0917
NDUAC_HUMAN	7588.9443
EI2BD_HUMAN	11755.034
VATH_HUMAN	5480.3301
PRAF1_HUMAN	2673.5083
IPO11_HUMAN	1822.9908
TR112_HUMAN	427.19064
DACH1_HUMAN	1452.0554
XPO7_HUMAN	6778.6382
LCMT1_HUMAN	4101.8584
VPS51_HUMAN	3886.9419
BAZ2A_HUMAN	288.05441
BAZ1B_HUMAN	1887.4244
ATIF1_HUMAN	1651.3368
KAD3_HUMAN	22769.428
LCAP_HUMAN	9568.251
CNOT7_HUMAN	2436.1409
RABX5_HUMAN	3165.8545
MSRA_HUMAN	2777.4983
NAGK_HUMAN	31557.781

HACL1_HUMAN	4316.7256
TRM6_HUMAN	1576.0994
SH3L2_HUMAN	1487.9392
NGAP_HUMAN	692.46667
TSR3_HUMAN	1467.2225
S2513_HUMAN	17949.951
DBNL_HUMAN	10890.5
DDX41_HUMAN	5601.9248
DCTN4_HUMAN	7733.7749
CDC23_HUMAN	4160.0938
APC7_HUMAN	5180.8726
APC5_HUMAN	848.50635
APC4_HUMAN	2259.5791
ANC2_HUMAN	1949.6057
GGA2_HUMAN	4954.04
GGA1_HUMAN	2513.9204
STML2_HUMAN	14251.38
NAGPA_HUMAN	908.89374
VPS28_HUMAN	8163.3389
LSM7_HUMAN	30271.074
DBR1_HUMAN	3863.2896
TASOR_HUMAN	450.17548
JUPI1_HUMAN	317.5658
FBX3_HUMAN	857.84711
AKA11_HUMAN	583.96289
FBW1B_HUMAN	1369.4402
GMEB2_HUMAN	748.31213
MRT4_HUMAN	11039.576
TNIK_HUMAN	1189.7186
CPSF3_HUMAN	5785.6211
DP13A_HUMAN	6400.9941
BORG2_HUMAN	1495.8927
TLK1_HUMAN	908.98169
GPTC8_HUMAN	1745.3137
PARP4_HUMAN	8768.4385
NXT1_HUMAN	699.79449
NUDT5_HUMAN	13669.22
RCOR1_HUMAN	1405.3446
MA1B1_HUMAN	2016.5029
RALY_HUMAN	13176.29
TF3C4_HUMAN	5122.2373
ERG28_HUMAN	2941.5059
PACN3_HUMAN	1150.3259
ACAD8_HUMAN	1687.1345
ANGL2_HUMAN	4581.5547
ACINU_HUMAN	7518.9268
AMFR_HUMAN	4128.1055
AGO2_HUMAN	4625.3628
NUP50_HUMAN	8720.4951
CDV3_HUMAN	16482.465

CNO11_HUMAN	1562.7046
INT6_HUMAN	2370.7556
BAG5_HUMAN	2879.6548
AGO1_HUMAN	4650.1987
RAB21_HUMAN	8158.9185
RB22A_HUMAN	5609.2124
TPC2L_HUMAN	2369.9561
ZN346_HUMAN	2252.3005
PSME2_HUMAN	16844.383
TAOK2_HUMAN	2546.4839
MKLN1_HUMAN	3557.228
DNPEP_HUMAN	7832.6831
RAB23_HUMAN	3701.5835
MCTS1_HUMAN	14130.248
MTUS1_HUMAN	707.56238
WWC3_HUMAN	684.98578
PALD_HUMAN	7107.4644
S39AA_HUMAN	1859.1207
INO80_HUMAN	351.73782
CCPG1_HUMAN	449.81088
KDIS_HUMAN	741.01129
ASAP1_HUMAN	3699.3999
MRTFB_HUMAN	1768.4252
ANR50_HUMAN	3932.0471
MED23_HUMAN	2766.6377
SHRM4_HUMAN	3305.9771
YETS2_HUMAN	1100.3707
NDRG4_HUMAN	351.08847
TBC24_HUMAN	1794.4727
ISY1_HUMAN	1345.4374
TMCC3_HUMAN	3310.313
HECD1_HUMAN	4371.7949
ZMYD8_HUMAN	2114.7739
CIZ1_HUMAN	334.74777
COR1C_HUMAN	15300.261
TPX2_HUMAN	1184.3237
ABT1_HUMAN	1989.8309
NOB1_HUMAN	5639.6904
AKP8L_HUMAN	4163.5615
MAFF_HUMAN	1108.6946
ASC_HUMAN	5594.9063
TMCO1_HUMAN	10269.315
FZR1_HUMAN	344.41437
APC10_HUMAN	1146.8005
MYO6_HUMAN	7327.2236
DD19B_HUMAN	1192.6343
PPT2_HUMAN	2848.8779
PRP19_HUMAN	23899.508
SUFU_HUMAN	1717.1014
BOK_HUMAN	3550.6213

NOL7_HUMAN	1698.9197
SNX12_HUMAN	10378.535
SYNRG_HUMAN	861.14801
VPS4A_HUMAN	9390.3574
G3BP2_HUMAN	10837.581
CHIP_HUMAN	5342.0928
PACN2_HUMAN	11252.727
MAGD2_HUMAN	19656.846
SNX7_HUMAN	2138.1099
SNX6_HUMAN	8837.1387
DUS12_HUMAN	4830.3701
STX8_HUMAN	1765.4087
SSRG_HUMAN	6742.8984
PSD13_HUMAN	36411.383
FAF1_HUMAN	6661.4473
EPCR_HUMAN	21423.793
PPIE_HUMAN	2616.0798
DIM1_HUMAN	4297.4727
TIM_HUMAN	348.84836
CSN3_HUMAN	9816.9238
MINP1_HUMAN	3373.231
NOVA2_HUMAN	8237.542
WDR3_HUMAN	2424.5964
TTF2_HUMAN	727.9306
NSF1C_HUMAN	10605.569
COG5_HUMAN	3199.9236
S12A4_HUMAN	6356.8623
AP4E1_HUMAN	223.96642
MACF1_HUMAN	40289.027
CP131_HUMAN	940.90973
SCAF8_HUMAN	2616.7786
PP6R1_HUMAN	1856.4954
TRI33_HUMAN	2729.1375
PHF8_HUMAN	914.29504
TNR6B_HUMAN	900.38062
PLCL2_HUMAN	606.43732
SMG5_HUMAN	1514.9165
EXOC7_HUMAN	10965.63
ZC3H4_HUMAN	94.315544
UBP24_HUMAN	4146.6177
TBD2B_HUMAN	2018.1337
SMAG1_HUMAN	1098.5372
FOXJ3_HUMAN	1297.205
CBPC1_HUMAN	756.70508
DICER_HUMAN	1173.0389
XCT_HUMAN	882.19653
MARE3_HUMAN	1855.3165
SHOC2_HUMAN	4990.5894
SRRM2_HUMAN	8608.6143
PA2G4_HUMAN	77655.078

SPG7_HUMAN	1264.8295
BAIP2_HUMAN	455.39722
GAB2_HUMAN	831.56995
SMC3_HUMAN	6624.6104
CHM2B_HUMAN	11912.819
SH2B3_HUMAN	1458.9868
ZN148_HUMAN	161.73604
MTMR6_HUMAN	4471.6519
JAG2_HUMAN	1268.093
NIP7_HUMAN	1069.5214
GLCNE_HUMAN	2877.0286
RTRAF_HUMAN	11561.561
RUVB2_HUMAN	31783.424
CDYL_HUMAN	476.64893
PIN4_HUMAN	6735.2266
AKT3_HUMAN	1400.1218
CHKB_HUMAN	663.8822
EIF3L_HUMAN	54329.141
PLAP_HUMAN	12147.093
RUVB1_HUMAN	49770.418
NUDC_HUMAN	27635.773
BCS1_HUMAN	2881.8928
VDAC3_HUMAN	21589.559
COF2_HUMAN	19582.574
ERGI3_HUMAN	2372.1572
SYFA_HUMAN	28368.238
SC5A6_HUMAN	955.69824
RT33_HUMAN	5631.0845
ASF1A_HUMAN	2864.4272
DRG1_HUMAN	15742.845
TPPC4_HUMAN	4241.0122
NCKP1_HUMAN	12954.058
CNPY2_HUMAN	9565.5723
EXOG_HUMAN	970.06006
EXC6B_HUMAN	2682.5061
PLAK2_HUMAN	15187.337
CARD8_HUMAN	2518.1404
AT11B_HUMAN	207.22925
OFUT2_HUMAN	7234.2109
DJC16_HUMAN	1179.9463
DLGP4_HUMAN	2604.4358
ST38L_HUMAN	4344.9531
SAC2_HUMAN	2487.4114
FND3A_HUMAN	4487.0552
NISCH_HUMAN	2107.4712
FYV1_HUMAN	1004.4075
WDR37_HUMAN	2943.3281
E41L3_HUMAN	18183.719
AMOL2_HUMAN	81.984215
SIK3_HUMAN	226.59999

KDM2A_HUMAN	1041.5162
RRP44_HUMAN	6320.6909
TPPC8_HUMAN	2414.0459
LRCH1_HUMAN	4437.9824
RCL1_HUMAN	2334.9785
GSTK1_HUMAN	9454.8545
LTOR2_HUMAN	1990.2715
RT28_HUMAN	11439.213
COA3_HUMAN	4422.9199
DDX52_HUMAN	1544.5873
RT17_HUMAN	5473.9072
RT07_HUMAN	7949.8301
CRYL1_HUMAN	3312.9583
TMA7_HUMAN	6075.939
PDIP2_HUMAN	10826.757
AP3M1_HUMAN	14204.945
MAN1_HUMAN	4270.749
CHSP1_HUMAN	1866.3677
COG6_HUMAN	2735.5061
TR150_HUMAN	17284.6
WBP11_HUMAN	4045.3767
TDRKH_HUMAN	776.789
MED16_HUMAN	1329.5356
NOP58_HUMAN	6726.6826
GIT1_HUMAN	6902.9292
ZN281_HUMAN	542.49396
AR2BP_HUMAN	3382.2134
TMM98_HUMAN	539.13684
SGT1_HUMAN	26144.166
SYYM_HUMAN	7125.8906
NAGA_HUMAN	4075.6926
ACOT9_HUMAN	26364.543
AAR2_HUMAN	1522.8303
NOSIP_HUMAN	3968.0779
DEOC_HUMAN	5561.9468
MEMO1_HUMAN	5510.7783
TMX2_HUMAN	5262.6089
FCF1_HUMAN	670.80048
LSM2_HUMAN	4763.5767
SHLB1_HUMAN	12544.881
CIA30_HUMAN	2034.4354
CAB39_HUMAN	14895.101
LC7L2_HUMAN	13160.941
UB2J1_HUMAN	5701.3413
RBMX2_HUMAN	461.05661
DHRS7_HUMAN	4685.3755
RT02_HUMAN	10740.238
UTP11_HUMAN	358.18903
PHOCN_HUMAN	11982.003
RRP7A_HUMAN	946.01965

SBDS_HUMAN	13938.303
TMED5_HUMAN	8446.2646
EXOS1_HUMAN	3098.2732
TMED7_HUMAN	4801.751
SF3B6_HUMAN	10392.288
RM11_HUMAN	12521.547
ORN_HUMAN	18294.184
RRP15_HUMAN	1997.0383
WASC3_HUMAN	1915.2751
NOP16_HUMAN	4740.7061
TPRKB_HUMAN	4012.9468
PPIL1_HUMAN	3909.3115
UFC1_HUMAN	6751.3799
CIA2B_HUMAN	404.82489
RT16_HUMAN	4691.7744
RT18C_HUMAN	1696.103
FIS1_HUMAN	9682.0498
TIM16_HUMAN	982.39929
KAD6_HUMAN	3415.7114
RT23_HUMAN	11559.169
GOT1B_HUMAN	4207.0449
HDGR3_HUMAN	2649.177
PTH2_HUMAN	6756.5132
CHMP3_HUMAN	48305.953
STRAP_HUMAN	18347.021
RTCB_HUMAN	46229.285
FBX7_HUMAN	3796.6931
RAP2C_HUMAN	1676.1781
STA13_HUMAN	2895.0146
RBGP1_HUMAN	1818.1189
TMED3_HUMAN	1202.4198
T22D4_HUMAN	1268.115
DOP2_HUMAN	597.96875
ZN330_HUMAN	626.37268
R3HC1_HUMAN	970.18054
NOC2L_HUMAN	7655.5098
RL36_HUMAN	55414.492
CCDC9_HUMAN	2854.6167
CHTOP_HUMAN	2298.2959
SAMH1_HUMAN	8554.7617
SKAP_HUMAN	582.80652
HBS1L_HUMAN	4422.0234
AAKB1_HUMAN	1245.7666
DMXL1_HUMAN	862.75659
VPP2_HUMAN	3207.6609
TLN1_HUMAN	96371.633
KIF3A_HUMAN	2255.8555
TRRAP_HUMAN	2666.9346
MTCL1_HUMAN	667.19879
DCAF1_HUMAN	655.9682

TCAF1_HUMAN	7046.2725
RBM19_HUMAN	467.13684
DAAM1_HUMAN	548.53687
PLXD1_HUMAN	20156.953
HECD4_HUMAN	353.70667
WAC2C_HUMAN	227.26871
WDR7_HUMAN	196.71268
UBP15_HUMAN	6395.9009
FARP1_HUMAN	1704.8882
MARF1_HUMAN	711.2099
C170B_HUMAN	772.04126
RPGF2_HUMAN	11934.517
MYO5A_HUMAN	7071.7495
DTNA_HUMAN	712.97894
LOXL2_HUMAN	5699.6289
CRBG1_HUMAN	3504.8335
TRAF6_HUMAN	2048.2026
M4K5_HUMAN	3287.9807
HYOU1_HUMAN	31493.912
ATG4B_HUMAN	5235.8066
TBL2_HUMAN	3952.0764
WIPI2_HUMAN	3327.7517
TELO2_HUMAN	2590.1836
LAS1L_HUMAN	5686.7417
AFG32_HUMAN	9529.0801
KLF12_HUMAN	568.84265
ARI1_HUMAN	6346.1206
LSM4_HUMAN	20031.834
RN114_HUMAN	3204.0676
SAM50_HUMAN	14873.94
PRC2C_HUMAN	16521.805
OARD1_HUMAN	6666.126
IFT25_HUMAN	478.30457
PPME1_HUMAN	12429.803
RBM7_HUMAN	2390.9617
TIM22_HUMAN	1522.2101
NUB1_HUMAN	1775.8699
YTHD2_HUMAN	2761.45
CTDP1_HUMAN	3573.1655
PAXB1_HUMAN	1135.9882
NDK7_HUMAN	2524.1401
SP16H_HUMAN	12719.732
UTP18_HUMAN	1295.9396
UCHL5_HUMAN	17451.658
CD2AP_HUMAN	11529.139
VATD_HUMAN	5739.6733
TNPO3_HUMAN	6627.7305
SRPRB_HUMAN	11708.063
CERT_HUMAN	1849.9686
GMPPB_HUMAN	16776.303

FADS3_HUMAN	1038.3456
TF3C5_HUMAN	6313.7646
TF3C3_HUMAN	3399.407
TPPC1_HUMAN	5571.7349
TRPV2_HUMAN	1235.3059
MRCKB_HUMAN	10972.952
RBM8A_HUMAN	15288.914
DJC15_HUMAN	797.13837
UBP16_HUMAN	1044.6066
TSSC4_HUMAN	2060.2183
ZN706_HUMAN	4760.1431
MAGD1_HUMAN	2727.7532
SNX14_HUMAN	678.16968
SNX11_HUMAN	893.1803
SNX9_HUMAN	12883.876
SNX8_HUMAN	1351.9213
SNX5_HUMAN	6299.7729
LIPG_HUMAN	1965.4055
NUBP2_HUMAN	5589.52
PEX16_HUMAN	1174.8835
LYVE1_HUMAN	5838.46
BACE2_HUMAN	2138.2402
HEBP2_HUMAN	910.02423
HCFC2_HUMAN	601.8996
UBIA1_HUMAN	802.87634
PUS1_HUMAN	4849.4487
LRRF2_HUMAN	4056.1643
FHOD1_HUMAN	6586.7188
SERC_HUMAN	19738.732
NCOR2_HUMAN	1799.6128
ORNT1_HUMAN	1327.6661
JAM1_HUMAN	19234.736
NPTN_HUMAN	1486.4613
AGRG1_HUMAN	1019.867
SPIN1_HUMAN	2180.7773
S12A7_HUMAN	7052.0532
ALG6_HUMAN	1978.0764
ALG5_HUMAN	7932.9063
RT18B_HUMAN	4913.6304
COPG1_HUMAN	29802.521
AUP1_HUMAN	2635.1025
FKBP7_HUMAN	1798.1107
CLIC4_HUMAN	48462.371
NFS1_HUMAN	4300.0713
CFA20_HUMAN	11828.111
SPCS1_HUMAN	6080.9282
SAR1B_HUMAN	6387.4238
AP4B1_HUMAN	980.78906
MTCH2_HUMAN	22311.908
SELK_HUMAN	1980.7303

BIG2_HUMAN	3385.1995
BIG1_HUMAN	4527.3979
MD1L1_HUMAN	3574.533
STK24_HUMAN	13697.979
5MP1_HUMAN	46152.441
COMDA_HUMAN	3036.0508
DC1L1_HUMAN	18991.621
EPN1_HUMAN	3730.9348
UBP3_HUMAN	14691.473
TX264_HUMAN	5492.9243
CABIN_HUMAN	229.95782
CEPT1_HUMAN	9960.1426
DNM3A_HUMAN	471.3183
OAS3_HUMAN	2146.3088
KAD5_HUMAN	18179.99
NEMO_HUMAN	4568.6201
IF2B2_HUMAN	23775.318
ZNT1_HUMAN	2737.0078
S4A7_HUMAN	2684.0979
NDUB9_HUMAN	13700.972
SQOR_HUMAN	3473.3345
ROBO1_HUMAN	572.0708
STON1_HUMAN	1658.8457
NCOA3_HUMAN	651.27789
NUMBL_HUMAN	1918.6694
M3K4_HUMAN	1319.9114
DDX49_HUMAN	1055.8268
CAN7_HUMAN	1130.5941
WASF2_HUMAN	10443.336
PIAS3_HUMAN	373.69888
SCC4_HUMAN	1680.854
MORC2_HUMAN	1262.9768
NS1BP_HUMAN	1426.3048
S23IP_HUMAN	17151.406
GAL3B_HUMAN	3718.3848
IFT56_HUMAN	690.29224
UBA6_HUMAN	16527.322
ESYT2_HUMAN	14269.814
BLT3B_HUMAN	94.892097
SHOT1_HUMAN	3117.1333
TM223_HUMAN	1643.4132
RHG10_HUMAN	3200.1697
HACL2_HUMAN	7267.7285
SPD2B_HUMAN	10583.02
NBAS_HUMAN	2651.5703
TYW5_HUMAN	653.31305
DEND3_HUMAN	1705.538
VWA8_HUMAN	2041.5193
SBNO1_HUMAN	1930.5609
GTPBA_HUMAN	1293.2698

WDR91_HUMAN	848.85016
DEN11_HUMAN	297.70654
TPC13_HUMAN	1499.1747
CNOT1_HUMAN	8756.3613
MP3B2_HUMAN	25866.018
PGP_HUMAN	8940.958
RIMC1_HUMAN	5456.1929
RCCD1_HUMAN	1292.2961
TCAF2_HUMAN	7653.4575
SMHD1_HUMAN	5427.6035
CAST2_HUMAN	1157.0878
U119B_HUMAN	3247.7305
MET15_HUMAN	914.8631
CC85C_HUMAN	429.14749
RTL8C_HUMAN	3730.4895
ZSWM8_HUMAN	756.77252
P121C_HUMAN	2181.8611
P20L1_HUMAN	328.65985
RUXGL_HUMAN	14993.993
ELAP2_HUMAN	1117.8998
NUD19_HUMAN	2358.9106
MI4GD_HUMAN	821.15186
CALM1_HUMAN	6610.8735
MYO9A_HUMAN	418.4682
GPHRA_HUMAN	1920.0924
WASH3_HUMAN	8171.3364
MCRI1_HUMAN	481.86621
NACAM_HUMAN	57558.602
CK098_HUMAN	5244.9507
ASURF_HUMAN	656.27399
MIDUO_HUMAN	2261.6394
DNS2A_HUMAN	4061.5708
ADAS_HUMAN	15018.935
KIF2A_HUMAN	8808.2412
DX39A_HUMAN	88646.992
PDLI1_HUMAN	7939.7192
BACH_HUMAN	40908.789
MYO1C_HUMAN	34789.773
SNP23_HUMAN	9733.793
HAX1_HUMAN	1810.387
AIP_HUMAN	6964.6299
GTPB1_HUMAN	5109.8828
LEG9_HUMAN	473.35208
STXB3_HUMAN	8359.4551
AP4M1_HUMAN	1866.5413
ARVC_HUMAN	5815.7637
SMAP_HUMAN	1406.2465
AP3B1_HUMAN	12725.233
APBB1_HUMAN	1887.8503
LEG8_HUMAN	1168.2889

NDUS8_HUMAN	1187.4877
IKBE_HUMAN	1581.1135
PSD11_HUMAN	37087.41
PSD12_HUMAN	27877.941
PSMD9_HUMAN	2495.1208
MEN1_HUMAN	903.28583
GET1_HUMAN	2594.9861
PGRC1_HUMAN	1524.9022
SPT5H_HUMAN	5450.6563
TAF4_HUMAN	262.39813
DFFA_HUMAN	2957.6921
HIP1_HUMAN	2089.686
CLIC1_HUMAN	188374.64
EIF3F_HUMAN	23159.338
WWP2_HUMAN	699.76917
ODPX_HUMAN	3709.1624
QSOX1_HUMAN	3475.0129
DCTN6_HUMAN	1491.4235
ACATN_HUMAN	4282.6245
WASL_HUMAN	5288.3906
IPO5_HUMAN	39069.465
RPOM_HUMAN	587.34741
EF2K_HUMAN	4040.5608
SAP18_HUMAN	13766.092
EMAL1_HUMAN	2833.6057
IF2B3_HUMAN	35348.492
DNM1L_HUMAN	38699.992
RTCA_HUMAN	7196.021
P3C2A_HUMAN	6565.3462
GOLI4_HUMAN	1979.0944
AGRIN_HUMAN	2161.2529
PLOD2_HUMAN	38938.832
EXOC5_HUMAN	9026.4111
BT3A1_HUMAN	685.10175
NDUA4_HUMAN	6699.2197
PSDE_HUMAN	43491.168
BIN1_HUMAN	3988.4717
CLD5_HUMAN	3868.1304
IMA4_HUMAN	25180.764
STK25_HUMAN	3156.2466
KRIT1_HUMAN	561.64508
VMA5A_HUMAN	999.13739
PESC_HUMAN	7701.873
SDCB1_HUMAN	41456.891
PITM1_HUMAN	588.13855
MPP10_HUMAN	4392.0884
NOP56_HUMAN	6773.064
DDX3X_HUMAN	65092.242
MFNG_HUMAN	435.41626
PODXL_HUMAN	38764.719

CCN1_HUMAN	48904.094
PIR_HUMAN	39043.172
IMA3_HUMAN	6221.5903
TRI38_HUMAN	692.94916
NFIB_HUMAN	7988.1182
PPP6_HUMAN	8851.2959
NDKM_HUMAN	1811.0912
P3C2B_HUMAN	834.729
MA2B1_HUMAN	2934.3335
PDXK_HUMAN	3630.4229
SCD_HUMAN	5370.0815
ARI1A_HUMAN	2815.8037
NEMP1_HUMAN	791.10535
TXND9_HUMAN	3098.8853
DPYL4_HUMAN	25594.26
TRAD1_HUMAN	1382.8605
COX7R_HUMAN	716.03845
UBFD1_HUMAN	4991.1963
CTRO_HUMAN	1762.0493
COPE_HUMAN	16150.746
BORG1_HUMAN	2042.5431
AP3D1_HUMAN	11741.152
CCS_HUMAN	1182.4502
ABLM1_HUMAN	4693.0249
DVL1_HUMAN	242.89551
DVL2_HUMAN	1203.8979
CHD1_HUMAN	3969.3269
CHD2_HUMAN	3375.6812
GOSR2_HUMAN	4723.8545
TOR1A_HUMAN	5766.5991
TOR1B_HUMAN	3886.3286
STX16_HUMAN	2252.1079
ADA10_HUMAN	8403.0508
ABCD4_HUMAN	1229.9058
EI24_HUMAN	2652.4878
P5I11_HUMAN	2399.104
KMT2D_HUMAN	138.26268
APAF_HUMAN	1919.9238
RIOK3_HUMAN	543.46906
MP2K7_HUMAN	624.12231
ACOT8_HUMAN	1790.5673
CDIPT_HUMAN	36348.125
PDCD5_HUMAN	3150.5051
ANM5_HUMAN	18268.369
NHRF1_HUMAN	5387.0703
CHK1_HUMAN	997.02997
TR10B_HUMAN	5918.4121
TPP1_HUMAN	17103.436
TCRG1_HUMAN	17855.74
NDC80_HUMAN	2571.4382

KIF3C_HUMAN	181.62798
NRP1_HUMAN	12909.354
TNPO2_HUMAN	2884.8474
UN13B_HUMAN	856.17163
TR10C_HUMAN	455.97925
RPC1_HUMAN	1832.6423
TSN4_HUMAN	632.91071
PSA7_HUMAN	92597.023
SCAM3_HUMAN	4398.8281
BCKD_HUMAN	2734.3076
MGST3_HUMAN	5648.7681
TX1B3_HUMAN	11293.618
GIPC1_HUMAN	6282.6812
IKKB_HUMAN	2919.9045
RGS12_HUMAN	1359.5671
TIM23_HUMAN	2319.2717
HAT1_HUMAN	12489.814
CSKP_HUMAN	2813.3794
PLD2_HUMAN	946.68744
QCR8_HUMAN	14573.854
ML12B_HUMAN	91301.164
HGS_HUMAN	6887.8833
RAB7L_HUMAN	978.03247
VP26C_HUMAN	1909.1647
MYPT1_HUMAN	12059.596
GAK_HUMAN	5105.5322
HNRDL_HUMAN	29050.688
XPO1_HUMAN	29567.322
BTAF1_HUMAN	1495.7279
ARHGA_HUMAN	3449.134
ZN609_HUMAN	1429.4508
SPTN2_HUMAN	1066.7317
MAST4_HUMAN	1281.5721
SC16A_HUMAN	6850.79
PLXB2_HUMAN	2095.1978
KHNYN_HUMAN	348.17212
TCPR2_HUMAN	737.59851
SR140_HUMAN	24477.705
SET1A_HUMAN	960.84229
N4BP3_HUMAN	373.5708
SYNJ2_HUMAN	3979.1567
SYNEM_HUMAN	6289.4312
GRRE1_HUMAN	755.52563
KIF3B_HUMAN	1651.7554
PUR4_HUMAN	23171.291
ANR28_HUMAN	1822.4449
ARHGB_HUMAN	473.79791
MRPP3_HUMAN	924.10541
IKKA_HUMAN	1914.5973
NPC1_HUMAN	3748.2244

DEGS1_HUMAN	2588.6411
ANGP2_HUMAN	5226.4653
SCAM1_HUMAN	4647.2412
SCAM2_HUMAN	2133.5227
ARC1B_HUMAN	37201.258
ARPC2_HUMAN	44241.512
ARPC3_HUMAN	43572.973
RPAC1_HUMAN	9303.5977
PLS1_HUMAN	955.30157
TIF1A_HUMAN	376.92517
PGRC2_HUMAN	11362.132
RGL2_HUMAN	2042.4226
WDR46_HUMAN	742.0614
NKRF_HUMAN	1737.8889
GNPAT_HUMAN	2994.4026
LAMA5_HUMAN	1315.4474
ZN185_HUMAN	8865.7793
CASC3_HUMAN	669.88776
RT12_HUMAN	2168.8628
ACOX3_HUMAN	2049.1062
RER1_HUMAN	16464.586
SURF4_HUMAN	21220.521
MK13_HUMAN	2309.8005
SPTC1_HUMAN	5619.7939
SPTC2_HUMAN	8957.1309
OGT1_HUMAN	6039.0239
PMM2_HUMAN	13398.077
TM9S1_HUMAN	2045.9265
TRI18_HUMAN	926.23883
HMGB3_HUMAN	21115.764
PPM1G_HUMAN	14285.798
SHIP2_HUMAN	5444.7856
EIF3D_HUMAN	25739.387
EIF3H_HUMAN	30861.814
HDAC3_HUMAN	2000.7188
NVL_HUMAN	1534.7567
BCAT2_HUMAN	7596.2051
IPO8_HUMAN	4926.6143
STX7_HUMAN	4957.7959
MOT4_HUMAN	13245.576
MRP4_HUMAN	1974.3765
RPA34_HUMAN	1668.7119
P4HA2_HUMAN	5609.3633
CAN5_HUMAN	618.67792
YKT6_HUMAN	44274.008
NUP42_HUMAN	1254.9834
ARPC5_HUMAN	37063.793
CFLAR_HUMAN	609.15863
MAFG_HUMAN	354.76263
PDPK1_HUMAN	1149.2921

TPSN_HUMAN	12487.199
R113A_HUMAN	891.85931
KDM6A_HUMAN	1663.5179
DHX15_HUMAN	36732.035
MCES_HUMAN	6273.769
ZZEF1_HUMAN	3346.5713
ASAP2_HUMAN	1128.4991
FLRT2_HUMAN	368.24524
TTI1_HUMAN	979.90576
RRP8_HUMAN	2093.7932
PJA2_HUMAN	564.07056
CYB5B_HUMAN	1707.7247
PRP4_HUMAN	7329.2686
SERA_HUMAN	23955.002
NDUS4_HUMAN	4133.3667
DC1L2_HUMAN	11118.488
PSMD3_HUMAN	43312.227
RFOX2_HUMAN	3084.709
PAPS1_HUMAN	14207.58
ZW10_HUMAN	6570.583
B4GT5_HUMAN	150.61848
SNUT1_HUMAN	8046.3916
GPAA1_HUMAN	7849.2471
DAPK3_HUMAN	6214.0659
TGFI1_HUMAN	2028.8491
AP5Z1_HUMAN	774.92474
CTIF_HUMAN	3646.675
MTSS1_HUMAN	843.67596
VIP2_HUMAN	1374.6251
M3K7_HUMAN	534.19061
MCA3_HUMAN	29467.91
RIPK2_HUMAN	1197.2374
HNRPR_HUMAN	103713.83
PRPF3_HUMAN	9199.6807
TXNL1_HUMAN	49520.223
TPD54_HUMAN	18132.752
EMC8_HUMAN	4895.8423
ERI3_HUMAN	2130.5376
SYNJ1_HUMAN	2023.6365
FIBP_HUMAN	2243.8894
IF4G3_HUMAN	25785.502
PPIH_HUMAN	6273.958
HTRA2_HUMAN	2860.0566
MS18B_HUMAN	302.75659
ARK72_HUMAN	13578.684
E41L2_HUMAN	13466.019
TGON2_HUMAN	868.55756
B4GA1_HUMAN	892.08789
WIPF1_HUMAN	1920.7156
AT8B1_HUMAN	1338.5039

AKA10_HUMAN	328.98166
DENR_HUMAN	16070.686
XPOT_HUMAN	16359.234
DNPH1_HUMAN	10982.282
TIM44_HUMAN	9523.251
TPPC3_HUMAN	18992.045
CHM2A_HUMAN	9311.6621
NCK2_HUMAN	2218.5808
TSN6_HUMAN	1524.6046
PLRG1_HUMAN	6718.3677
PRC1_HUMAN	1684.3054
RGS10_HUMAN	3042.6709
ZN207_HUMAN	2408.8828
NDUB5_HUMAN	5453.541
NDUB3_HUMAN	9780.5742
NDUA2_HUMAN	3300.6802
LDB2_HUMAN	1686.0049
GET3_HUMAN	25987.516
BUB3_HUMAN	28703.379
ST1B1_HUMAN	9173.9297
ACTN4_HUMAN	68041.125
BUD23_HUMAN	1073.3215
HTSF1_HUMAN	3097.4097
ERD23_HUMAN	2367.8816
AP1G1_HUMAN	12492.196
STX6_HUMAN	1731.4874
SNG2_HUMAN	4660.4019
SGTA_HUMAN	2449.2363
ENSA_HUMAN	6337.2344
MCAT_HUMAN	6920.3164
SYNC_HUMAN	36249.176
MYO1B_HUMAN	7180.23
PM34_HUMAN	656.93634
CPSF5_HUMAN	17152.291
LANC1_HUMAN	12004.653
STRN_HUMAN	6151.832
U3IP2_HUMAN	6115.2158
SCO2_HUMAN	1015.705
CF410_HUMAN	228.30034
AKAP8_HUMAN	2638.7932
GTPB6_HUMAN	716.46448
G6PT1_HUMAN	2084.8281
IDH3B_HUMAN	9177.3877
NRDC_HUMAN	12385.799
CALU_HUMAN	22891.697
EDIL3_HUMAN	5346.3335
ATP9B_HUMAN	695.64844
SAHH2_HUMAN	2342.0955
KIF1C_HUMAN	3177.5659
EXTL3_HUMAN	769.47601

ORC5_HUMAN	1036.1978
NDUS5_HUMAN	11633.856
PDE6D_HUMAN	3780.6221
ORC4_HUMAN	916.81219
PEX1_HUMAN	1352.5953
RAD21_HUMAN	8250.4912
DHX16_HUMAN	4295.1021
GMFG_HUMAN	3730.0044
3BP5_HUMAN	4261.498
AGRB3_HUMAN	1716.6392
MED14_HUMAN	16564.459
PCDH7_HUMAN	193.31726
KPRB_HUMAN	6671.0518
SMCA5_HUMAN	7070.377
JIP4_HUMAN	92398.242
NUAK1_HUMAN	350.32822
NPA1P_HUMAN	1275.8453
SI1L3_HUMAN	151.41603
ZC3H1_HUMAN	249.09482
AQR_HUMAN	8175.5732
OPA1_HUMAN	6294.0752
ZEB2_HUMAN	1001.4113
GANP_HUMAN	2011.6769
PCDGC_HUMAN	1067.27
PI51C_HUMAN	4105.5864
KIF1B_HUMAN	2615.0654
MABP1_HUMAN	1333.8812
KDM1A_HUMAN	6194.8306
TBCD4_HUMAN	8410.0352
FADS1_HUMAN	2724.4553
GSDME_HUMAN	8321.2422
EVI5_HUMAN	211.2285
NRP2_HUMAN	6854.6479
MA1A2_HUMAN	2930.7437
MPZL2_HUMAN	3491.3528
ACSL4_HUMAN	10862.012
SNX3_HUMAN	33193.863
OGA_HUMAN	7940.3071
VINEX_HUMAN	3763.0762
HNRPQ_HUMAN	45056.188
PRP17_HUMAN	1579.4962
RNBP6_HUMAN	33225.508
NEMF_HUMAN	6064.4092
GMDS_HUMAN	3852.7227
NMT2_HUMAN	5777.5132
CCNT1_HUMAN	1669.8633
BUB1B_HUMAN	1784.3383
PLOD3_HUMAN	25621.979
IF4E2_HUMAN	4842.812
DIAP1_HUMAN	67888.578

TSN3_HUMAN	7511.7451
EXOC3_HUMAN	10988.267
PLIN3_HUMAN	21021.992
ANM3_HUMAN	1039.4591
IMA7_HUMAN	9954.9248
UGDH_HUMAN	37916.176
TPST2_HUMAN	1883.0293
LPXN_HUMAN	1442.7566
CTND1_HUMAN	42393.535
ICMT_HUMAN	4096.7661
EIF1B_HUMAN	2361.4822
SNX2_HUMAN	17237.664
DPM1_HUMAN	15542.25
USO1_HUMAN	18708.109
RT14_HUMAN	2693.3066
TOM1_HUMAN	4249.7134
H2B1K_HUMAN	981622.56
CCD22_HUMAN	3791.2566
PQBP1_HUMAN	6786.7935
TI17B_HUMAN	754.91357
PRAF2_HUMAN	10115.232
DKC1_HUMAN	13745.14
IF2P_HUMAN	29324.016
EDF1_HUMAN	25569.514
KIN17_HUMAN	692.47974
DIAP2_HUMAN	6933.4946
DNJA2_HUMAN	9361.3271
BRD4_HUMAN	3888.2642
HUS1_HUMAN	1009.3242
PFD1_HUMAN	2662.3506
PP1RB_HUMAN	2501.9907
NBN_HUMAN	11617.58
MCE1_HUMAN	1982.3351
ABCB7_HUMAN	2678.4702
SRGP2_HUMAN	1898.3442
PLXA2_HUMAN	6612.0356
XYLK_HUMAN	634.24915
WDR1_HUMAN	97402.633
ATP9A_HUMAN	1764.1682
N4BP1_HUMAN	926.92285
ROCK2_HUMAN	7655.1563
CLAP2_HUMAN	3382.5232
PTCD1_HUMAN	556.61462
CPNE3_HUMAN	17054.521
ATG13_HUMAN	853.55042
HIP1R_HUMAN	9444.0449
BRE1B_HUMAN	5026.3687
PHF2_HUMAN	1067.5747
ZC11A_HUMAN	3125.5469
CLU_HUMAN	8662.1611

CAND2_HUMAN	2097.7983
T22D2_HUMAN	485.59656
DJC13_HUMAN	7601.8066
PHAR2_HUMAN	1034.0667
PP6R2_HUMAN	1779.2831
ATS4_HUMAN	2071.1914
CNOT3_HUMAN	6152.0137
ANR17_HUMAN	5050.1816
SIN3B_HUMAN	745.72034
DNJB6_HUMAN	4155.2412
COQ9_HUMAN	1536.4044
GGCT_HUMAN	6391.1069
NDUS7_HUMAN	12954.116
NDUS2_HUMAN	10713.871
ZPR1_HUMAN	3541.5605
DUS11_HUMAN	1070.8414
NIPS2_HUMAN	8527.0195
HMMR_HUMAN	452.1868
PDCD6_HUMAN	15112.984
TBCA_HUMAN	17238.922
VATG1_HUMAN	13688.484
VPS4B_HUMAN	60268.031
MPU1_HUMAN	18966.564
H2AY_HUMAN	13856.604
SH3L1_HUMAN	1214.0736
FLNB_HUMAN	119464.3
NCOR1_HUMAN	6377.9521
PEX14_HUMAN	4859.2031
TRIM3_HUMAN	3217.3455
CISY_HUMAN	110625.38
SPAG7_HUMAN	9515.9365
SC22B_HUMAN	37540.211
PR40A_HUMAN	11605.538
TACC1_HUMAN	8660.4941
NDK6_HUMAN	1502.2778
CDC45_HUMAN	999.44147
LRCH4_HUMAN	493.14154
MTX2_HUMAN	1887.326
VP26A_HUMAN	33426.398
MPPB_HUMAN	11066.377
MED24_HUMAN	1201.5643
KTNA1_HUMAN	2343.4568
PSIP1_HUMAN	3708.6428
ERLN1_HUMAN	6460.4302
NDUS3_HUMAN	19609.502
SRS10_HUMAN	7596.002
CLN5_HUMAN	2888.0642
CLD11_HUMAN	1644.9447
ECI2_HUMAN	15057.538
KHDR3_HUMAN	930.77222

EED_HUMAN	2100.9402
BAF_HUMAN	2881.4133
SF3B1_HUMAN	31256.957
CSDE1_HUMAN	19253.047
WBP4_HUMAN	1398.6936
PRKRA_HUMAN	5363.8579
ITA10_HUMAN	1646.6857
KS6A5_HUMAN	3190.7732
MED6_HUMAN	250.68909
MYCB2_HUMAN	1800.7025
KBL_HUMAN	1325.8826
NPM3_HUMAN	3811.7639
LYPA1_HUMAN	12685.608
ERAL1_HUMAN	5305.3794
U520_HUMAN	18300.791
MTU1_HUMAN	1407.9648
TIPRL_HUMAN	9985.2314
OFD1_HUMAN	1440.0443
KS6A4_HUMAN	1283.8527
SURF6_HUMAN	1058.4063
PPM1B_HUMAN	2231.0054
UTP20_HUMAN	5431.9365
NU155_HUMAN	10767.567
XRP2_HUMAN	7586.9951
WDHD1_HUMAN	4013.6226
CRTAP_HUMAN	18047.883
S2512_HUMAN	11891.582
RENR_HUMAN	2528.1487
RNH2A_HUMAN	1937.3606
CD123_HUMAN	3482.0105
BCAR3_HUMAN	966.47522
POP7_HUMAN	2117.3489
RPP40_HUMAN	949.35748
EIF3G_HUMAN	22249.748
EIF3J_HUMAN	20525.793
CBR3_HUMAN	14235.185
PSD10_HUMAN	3712.1572
AP1G2_HUMAN	2102.458
FACE1_HUMAN	13089.82
IDHC_HUMAN	49588.211
GATB_HUMAN	1330.8202
SCO1_HUMAN	6156.4678
ATRN_HUMAN	561.19604
RBBP9_HUMAN	7673.811
STAM2_HUMAN	4678.0605
DGAT1_HUMAN	854.89789
CCNK_HUMAN	5092.188
PRAF3_HUMAN	52025.223
DYSF_HUMAN	24764.521
PIAS1_HUMAN	1416.2682

SPF27_HUMAN	709.11029
DCTN3_HUMAN	14298.685
DNJC8_HUMAN	13390.351
SPF30_HUMAN	4406.6675
RAD17_HUMAN	233.41809
ATP5H_HUMAN	5545.4346
TSN9_HUMAN	2835.1516
FLOT1_HUMAN	7052.2622
TRIO_HUMAN	4522.9443
ATP5L_HUMAN	35038.805
MPDZ_HUMAN	2308.4963
CBPD_HUMAN	8776.7021
GLRX3_HUMAN	47508.484
RL1D1_HUMAN	16000.604
WFS1_HUMAN	7156.7915
CLPX_HUMAN	5060.2813
S14L2_HUMAN	3817.6785
SYUG_HUMAN	1453.6389
CIAO1_HUMAN	2395.0737
SRP72_HUMAN	10678.357
DDAH1_HUMAN	10134.516
RECQ5_HUMAN	1560.6632
RMP_HUMAN	1312.281
B3GA3_HUMAN	1178.5992
MTA2_HUMAN	13213.016
AL1A2_HUMAN	5571.1958
STK10_HUMAN	6354.689
KPCD3_HUMAN	2026.1115
GFPT2_HUMAN	1339.6824
SLIT2_HUMAN	2860.7217
LTN1_HUMAN	2395.3076
TOM70_HUMAN	8266.8193
PKHG5_HUMAN	1425.6785
IPO13_HUMAN	1060.896
DDHD2_HUMAN	545.04047
MYO1D_HUMAN	8844.3252
TOX4_HUMAN	2626.0015
MICA2_HUMAN	2783.9678
SC24D_HUMAN	16530.225
FCSD2_HUMAN	593.56213
UFL1_HUMAN	10004.162
SRBS2_HUMAN	7364.7109
SASH1_HUMAN	4455.8052
UBXN7_HUMAN	5437.1431
KLH18_HUMAN	692.37701
SUN1_HUMAN	1367.4767
PLPHP_HUMAN	8181.4126
ERLN2_HUMAN	13572.516
PRP6_HUMAN	13207.848
PCF11_HUMAN	776.8299

FRYL_HUMAN	7169.4692
NFAT5_HUMAN	323.12991
ENDD1_HUMAN	1859.0192
GLCE_HUMAN	5847.9805
GLSK_HUMAN	21994.486
HAUS5_HUMAN	913.39972
ABLM3_HUMAN	1429.8298
FBX21_HUMAN	860.36121
KDM4B_HUMAN	390.42679
MTCL2_HUMAN	2422.8672
UBP19_HUMAN	1375.0118
WDR47_HUMAN	1095.6248
AP2A2_HUMAN	22163.318
SC31A_HUMAN	36813.555
ARHGF_HUMAN	8071.6328
HEXI1_HUMAN	1381.2036
RPP14_HUMAN	1386.238
CCNB2_HUMAN	91.510559
YIF1A_HUMAN	4532.4482
UBR5_HUMAN	2349.7515
AGFG2_HUMAN	2340.2195
PRS23_HUMAN	4563.9604
SCAF4_HUMAN	3155.8486
NDUB6_HUMAN	6341.8638
MFN2_HUMAN	2223.9695
SPN1_HUMAN	1364.0052
UBE4B_HUMAN	5935.6816
ZFPL1_HUMAN	4602.27
ELP1_HUMAN	5649.1421
NDUB4_HUMAN	4964.145
NDUB8_HUMAN	6590.6123
NDUA7_HUMAN	5318.8145
VAMP5_HUMAN	1423.7108
RTN3_HUMAN	8333.4111
LETM1_HUMAN	13990.61
EPN2_HUMAN	2535.8076
ZRAB2_HUMAN	8047.167
SNX4_HUMAN	3608.834
LC7L3_HUMAN	4600.4473
KI20A_HUMAN	981.92035
APOL3_HUMAN	533.26685
KIF4A_HUMAN	4380.6211
MBD4_HUMAN	313.49051
MTMR5_HUMAN	1010.5452
GOSR1_HUMAN	6278.2324
KAT7_HUMAN	2020.3462
ATE1_HUMAN	1757.877
VAPB_HUMAN	5088.2476
SNAPN_HUMAN	2355.002
MPZL1_HUMAN	2044.8242

NDUC2_HUMAN	7711.7397
NDUAA_HUMAN	11947.994
FKBP9_HUMAN	12977.796
CELF2_HUMAN	1030.9343
6PGL_HUMAN	9286.0605
PAPS2_HUMAN	25430.023
SMC2_HUMAN	10708.453
ATG7_HUMAN	7539.4365
TACC2_HUMAN	1051.8069
TRI16_HUMAN	3464.9739
SYFM_HUMAN	1382.7754
LYPA2_HUMAN	5307.9287
IPO7_HUMAN	37155.176
ARI2_HUMAN	1073.1625
SLU7_HUMAN	893.01672
AGM1_HUMAN	16428.24
MOCS3_HUMAN	894.17792
CD2B2_HUMAN	3646.4741
SVIL_HUMAN	5903.5908
AHSA1_HUMAN	37307.707
PARN_HUMAN	2663.9624
PSMG1_HUMAN	10766.815
SGPL1_HUMAN	3839.2876
CNEP1_HUMAN	1120.991
NSA2_HUMAN	1692.0853
G6PE_HUMAN	8339.9473
SC24A_HUMAN	7527.5381
SC24B_HUMAN	7850.0957
AGRL2_HUMAN	1923.3246
NADK_HUMAN	429.32727
TYDP2_HUMAN	1079.7106
MPC2_HUMAN	1725.1516
ETHE1_HUMAN	7305.2021
ACSL3_HUMAN	12727.531
RPA1_HUMAN	3463.3013
YETS4_HUMAN	961.42999
CNOT4_HUMAN	1538.2454
STABP_HUMAN	1784.8002
CPSF4_HUMAN	1851.2858
NFAC1_HUMAN	712.49548
ASML_HUMAN	5053.043
CDS2_HUMAN	650.79108
PPR3D_HUMAN	1253.1846
BRD1_HUMAN	642.65546
RPP29_HUMAN	1003.3682
HERC2_HUMAN	958.80676
RAB3D_HUMAN	2325.2866
SNP29_HUMAN	4871.8286
OXSR1_HUMAN	5915.2061
GGPPS_HUMAN	1403.8213

HS74L_HUMAN	7482.4253
PTBP3_HUMAN	13069.136
TBCD8_HUMAN	433.97836
IL33_HUMAN	549.23315
STR3N_HUMAN	2965.7568
LSM8_HUMAN	541.89795
AP2A1_HUMAN	27105.701
WIZ_HUMAN	1953.745
RIGI_HUMAN	6428.1235
STAU1_HUMAN	15479.124
TTC4_HUMAN	4346.4321
TM50A_HUMAN	3155.1594
CAVN2_HUMAN	37902.445
BAG2_HUMAN	11703.235
BAG3_HUMAN	2735.2612
M4K4_HUMAN	10763.049
DCMC_HUMAN	199.89494
QORL1_HUMAN	1786.657
AIFM1_HUMAN	17393.938
EMAL2_HUMAN	1375.4757
LATS1_HUMAN	2334.4121
NUD14_HUMAN	3798.2578
TSN15_HUMAN	1174.1949
BPNT1_HUMAN	6669.0073
FADS2_HUMAN	27577.188
DDAH2_HUMAN	1118.556
ABHGA_HUMAN	2801.1477
TXD12_HUMAN	1666.7388
ECD_HUMAN	4502.875
RECK_HUMAN	13772.258
MBD3_HUMAN	2831.323
TOP3B_HUMAN	733.64667
NUDT3_HUMAN	3879.3398
BCL10_HUMAN	4569.7549
NDUBA_HUMAN	12729.769
CLPT1_HUMAN	6673.1875
ZBED1_HUMAN	1228.4719
MOC2B_HUMAN	1059.8677
TOM40_HUMAN	20753.285
PX11B_HUMAN	3164.084
PAK4_HUMAN	6952.5347
APBA3_HUMAN	1099.3491
ACL6A_HUMAN	9935.2432
NSD2_HUMAN	1153.4081
LDHA_HUMAN	275557.84
AL1A1_HUMAN	14731.904
DHE3_HUMAN	39014.328
DYR_HUMAN	12425.32
NB5R3_HUMAN	52701.449
GSHR_HUMAN	16844.539

COX1_HUMAN	661.71545
COX2_HUMAN	69149.063
PNPH_HUMAN	64643.086
HPRT_HUMAN	25175.781
AATM_HUMAN	53351.055
ABL1_HUMAN	886.32721
PGK1_HUMAN	154227.56
KAD1_HUMAN	25405.66
UROK_HUMAN	1486.7609
TPA_HUMAN	485.77246
ADA_HUMAN	3560.6755
CYTC_HUMAN	5645.4429
RASN_HUMAN	28959.291
RASH_HUMAN	3347.2839
RASK_HUMAN	2551.5012
PDGFB_HUMAN	1384.7775
LDLR_HUMAN	4094.061
TGFB1_HUMAN	3948.4109
HLAB_HUMAN	5306.1777
HLAH_HUMAN	55918.574
CO1A1_HUMAN	1248.9855
CO4A1_HUMAN	794.70264
LMNA_HUMAN	177256.89
APOA1_HUMAN	2227.8809
FINC_HUMAN	25928.924
FETUA_HUMAN	1240.1598
ALBU_HUMAN	48668.672
TFR1_HUMAN	12565.633
FRIL_HUMAN	23137.564
FRIH_HUMAN	15531.816
MT2_HUMAN	7963.7378
NU1M_HUMAN	7853.9961
NU4M_HUMAN	2167.6741
NU5M_HUMAN	2287.325
MMP1_HUMAN	6013.1655
HMDH_HUMAN	287.23831
CATA_HUMAN	7337.3496
RAF1_HUMAN	2005.2793
GBA1_HUMAN	12662.519
ALDOA_HUMAN	264747.91
CYTB_HUMAN	5408.1685
ANXA1_HUMAN	190153.94
APOB_HUMAN	1013.3217
PRIO_HUMAN	1292.0864
SODM_HUMAN	3043.5713
OAT_HUMAN	26637.85
KITH_HUMAN	2717.9414
VWF_HUMAN	63305.695
TBB4A_HUMAN	70456.703
G3P_HUMAN	440119.03

ARLY_HUMAN	6953.7119
HLAA_HUMAN	37631.738
CPNS1_HUMAN	27897.563
MT1E_HUMAN	110.41581
HSPB1_HUMAN	145965.3
TYSY_HUMAN	11689.247
RPN1_HUMAN	70289.625
RPN2_HUMAN	50518.156
GNAI2_HUMAN	33624.418
H2A1B_HUMAN	590899.38
AT1A1_HUMAN	47366.172
AT1B1_HUMAN	9315.8291
A4_HUMAN	6978.1304
ALDH2_HUMAN	27471.273
ITB3_HUMAN	6833.2197
S10A8_HUMAN	223.09917
HMGN1_HUMAN	1256.5216
PAI2_HUMAN	11455.567
PAI1_HUMAN	92427.086
ADT2_HUMAN	205660
ISG15_HUMAN	5277.4155
PCCA_HUMAN	3299.1882
PCCB_HUMAN	4410.5117
IF2A_HUMAN	53435.332
HMGN2_HUMAN	884.49951
ICAM1_HUMAN	7144.0713
RLA2_HUMAN	5213.0205
RLA0_HUMAN	166602.47
JUN_HUMAN	1338.379
RPC4_HUMAN	1981.4332
LA_HUMAN	32099.951
ITB1_HUMAN	104757.91
K1C18_HUMAN	55942.875
CO5A2_HUMAN	1570.7977
DCUP_HUMAN	27389.773
FYN_HUMAN	755.70197
AGAL_HUMAN	1271.9077
GELS_HUMAN	11098.312
RB_HUMAN	2360.7854
PTMA_HUMAN	5277.439
CDK1_HUMAN	28048.393
ATPB_HUMAN	176076.78
S10A6_HUMAN	12169.572
IF4E_HUMAN	15457.212
ENOA_HUMAN	642441.94
PYGL_HUMAN	8503.9697
G6PI_HUMAN	61511.348
DPOLB_HUMAN	384.23724
NPM_HUMAN	86489.211
TPM3_HUMAN	54771.34

ITAV_HUMAN	15144.299
HEXA_HUMAN	14722.838
HYEP_HUMAN	9842.623
LDHB_HUMAN	189797.45
CENPB_HUMAN	1253.6147
GPX1_HUMAN	16042.062
PDIA1_HUMAN	156616.36
H10_HUMAN	21604.334
ACYP1_HUMAN	3719.4893
FES_HUMAN	1473.6715
CATD_HUMAN	68562.594
ANXA2_HUMAN	390741.75
CAN1_HUMAN	28057.041
TBB5_HUMAN	485436.53
SAP_HUMAN	5213.4932
HEXB_HUMAN	18623.613
CATL1_HUMAN	1596.0481
PROF1_HUMAN	279810
PMGE_HUMAN	2974.3567
APT_HUMAN	61823.805
SYEP_HUMAN	20992.67
CATB_HUMAN	68315.234
HS90A_HUMAN	151416.08
GALT_HUMAN	605.28845
HNRPC_HUMAN	195324.09
LAMB1_HUMAN	23983.971
YES_HUMAN	17217.324
LYN_HUMAN	3751.4011
FUMH_HUMAN	28281.539
ERCC1_HUMAN	796.49341
TSP1_HUMAN	251957.16
SP1_HUMAN	899.34229
IGF1R_HUMAN	1317.8713
CO1A2_HUMAN	636.72546
ANXA6_HUMAN	93690.625
RHOC_HUMAN	90069.938
DAF_HUMAN	8547.4697
4F2_HUMAN	13598.996
BGLR_HUMAN	1301.1301
PFKAM_HUMAN	9606.9814
HS90B_HUMAN	721613
SRPRA_HUMAN	11326.26
ASNS_HUMAN	4449.625
MMP2_HUMAN	6602.0029
HEM3_HUMAN	2115.7083
NEP_HUMAN	1105.3363
MGP_HUMAN	4313.1782
ODPA_HUMAN	29613.049
CO4A2_HUMAN	6746.877
CY1_HUMAN	14420.258

RU2B_HUMAN	7995.583
MET_HUMAN	3629.5796
RU17_HUMAN	53409.695
ITA5_HUMAN	72377.68
NFIC_HUMAN	1134.1299
VIME_HUMAN	1388349.4
RS17_HUMAN	36848.02
GNAI3_HUMAN	7595.0444
ANXA5_HUMAN	441824.03
RSSA_HUMAN	128682.12
CD63_HUMAN	5365.8652
RM03_HUMAN	5467.1255
SNRPA_HUMAN	23400.936
ENOG_HUMAN	12244.714
THIK_HUMAN	6433.1025
SRP19_HUMAN	5210.2734
GSTP1_HUMAN	138184.95
RU1C_HUMAN	4192.5718
LEG1_HUMAN	45149.395
DHPR_HUMAN	9309.0254
HMGB1_HUMAN	67672.406
SPRC_HUMAN	3036.0298
TPM1_HUMAN	54465.352
CLCA_HUMAN	2009.4417
CLCB_HUMAN	680.40912
ANXA4_HUMAN	14585.29
CN37_HUMAN	11504.49
HMOX1_HUMAN	5805.5454
DLDH_HUMAN	16375.921
ROA1_HUMAN	171751.78
RU2A_HUMAN	27577.107
COX6C_HUMAN	13943.409
TACD2_HUMAN	3187.936
PARP1_HUMAN	37026.609
DPOLA_HUMAN	12990.325
UCHL1_HUMAN	9472.958
LKHA4_HUMAN	32255.789
ALDOC_HUMAN	21279.406
H2AZ_HUMAN	23631.248
H2A1_HUMAN	10476.593
MYZAP_HUMAN	570.6795
GRL1A_HUMAN	704.02557
GST2_HUMAN	4425.9126
POTEJ_HUMAN	14637.713
LIMS3_HUMAN	3644.1201
HS71A_HUMAN	83097.164
ERPG3_HUMAN	1624.0913
TBA3C_HUMAN	42772.168
ZTRF1_HUMAN	712.70691
RAP2A_HUMAN	9064.6182

SRGN_HUMAN	2519.0435
RO60_HUMAN	10642.253
LYAG_HUMAN	4629.9468
RRAS_HUMAN	11307.004
HLAC_HUMAN	1573.484
ARAF_HUMAN	1983.2975
H14_HUMAN	390470.03
ODP2_HUMAN	21448.17
PTPRF_HUMAN	4489.2671
COT1_HUMAN	4873.9995
THIO_HUMAN	4960.9165
COX5B_HUMAN	965.72327
PPGB_HUMAN	7856.394
MGST1_HUMAN	10759.13
KAP0_HUMAN	18035.559
TFPI1_HUMAN	1618.5813
ESTD_HUMAN	18356.666
CH60_HUMAN	138095.72
CLUS_HUMAN	5387.8481
BIP_HUMAN	245254.72
LAMC1_HUMAN	18141.221
PPAL_HUMAN	6112.1616
MTAP2_HUMAN	4226.354
HSP7C_HUMAN	248394.86
GTR1_HUMAN	3665.9214
GTR3_HUMAN	2865.9966
EPB41_HUMAN	24181.846
UMPS_HUMAN	13853.173
ODPB_HUMAN	15936.25
ODB2_HUMAN	1521.0815
PYGB_HUMAN	14432.544
PYGM_HUMAN	31059.422
RALA_HUMAN	25432.539
RALB_HUMAN	50675.301
BCR_HUMAN	4747.8921
LAMP1_HUMAN	80632.891
ERG_HUMAN	6472.146
ACADM_HUMAN	12903.024
TOP1_HUMAN	44933.316
TOP2A_HUMAN	964.37463
G6PD_HUMAN	66134.438
UBL4A_HUMAN	891.93335
PYC_HUMAN	3665.8694
C1TC_HUMAN	51400.563
MPRI_HUMAN	5175.8252
ADHX_HUMAN	34603.664
CDK4_HUMAN	2238.5078
PRPS2_HUMAN	17839.209
PABP1_HUMAN	119450.48
PCNA_HUMAN	28279.068

HARS1_HUMAN	37496.461
CO6A1_HUMAN	756.77814
ADT1_HUMAN	224516.75
ADT3_HUMAN	78817.742
IMDH2_HUMAN	39441.313
TPR_HUMAN	14280.182
ANXA3_HUMAN	56861.496
ODBA_HUMAN	5122.4346
ACTN1_HUMAN	103101.07
ACE_HUMAN	13539.423
SRC_HUMAN	3591.3318
PEPD_HUMAN	6527.5151
XRCC6_HUMAN	91463.289
XRCC5_HUMAN	56686.172
UNG_HUMAN	752.41364
COX41_HUMAN	53285.539
HEM1_HUMAN	1581.5393
LAMP2_HUMAN	52624.836
RINI_HUMAN	42402.211
ICAM2_HUMAN	48229.078
EF2_HUMAN	186138.39
PDIA4_HUMAN	113119.51
P4HA1_HUMAN	9002.4961
TCTP_HUMAN	17478.795
HEM2_HUMAN	4642.4507
HLAE_HUMAN	1167.0828
PLST_HUMAN	139801.45
ACPH_HUMAN	17504.252
ETFA_HUMAN	26281.67
GYS1_HUMAN	4763.8032
KAP2_HUMAN	7427.772
T2FB_HUMAN	4958.1992
CD59_HUMAN	23570.176
MTDC_HUMAN	5261.1304
MIF_HUMAN	12439.284
CD99_HUMAN	1164.6412
GLU2B_HUMAN	74168.672
HCLS1_HUMAN	7778.083
FPPS_HUMAN	58405.875
CX7A2_HUMAN	18660.168
NID1_HUMAN	13906.959
AK1A1_HUMAN	26597.527
KPYM_HUMAN	446673.13
ENPL_HUMAN	327276.19
CCNB1_HUMAN	2108.6558
MYL6B_HUMAN	8356.0449
RSMB_HUMAN	51477.945
IDE_HUMAN	19676.283
PO2F1_HUMAN	379.87982
HNRPL_HUMAN	26175.99

SYDC_HUMAN	35372.785
ETS1_HUMAN	1267.9358
PLAK_HUMAN	20783.367
QCR7_HUMAN	1298.3495
BRAF_HUMAN	3890.8157
FABP4_HUMAN	24783.381
GLNA_HUMAN	433.65936
ALDR_HUMAN	45306.359
AMPN_HUMAN	84831.594
PVR_HUMAN	18197.393
RAC2_HUMAN	21136.465
ERF3A_HUMAN	13515.642
ARSA_HUMAN	1492.3826
B4GT1_HUMAN	1126.9962
EZRI_HUMAN	38926.684
UCHL3_HUMAN	7595.1411
FOSL1_HUMAN	408.13702
FOSL2_HUMAN	270.58145
MCP_HUMAN	4550.8276
NDKA_HUMAN	29485.514
NQO1_HUMAN	24473.688
GNS_HUMAN	43060.82
PHKG2_HUMAN	1781.8994
ARSB_HUMAN	1837.6902
RS2_HUMAN	140734.27
ITF2_HUMAN	1571.2968
DESP_HUMAN	1010.2308
RFA2_HUMAN	7467.0371
TIMP2_HUMAN	5498.1919
CD44_HUMAN	13336.809
CBR1_HUMAN	22834.277
ACADS_HUMAN	1879.6134
CREB1_HUMAN	2972.0122
BGAL_HUMAN	10028.282
PECA1_HUMAN	63478.125
PP2BB_HUMAN	5744.8193
NCK1_HUMAN	9741.6436
GCFC2_HUMAN	1134.8229
H15_HUMAN	319667.78
H13_HUMAN	48015.324
H12_HUMAN	64947.949
NCPR_HUMAN	11865.838
FER_HUMAN	530.48553
AT2A2_HUMAN	38242.094
PLCG2_HUMAN	6567.0854
FAAA_HUMAN	6027.8691
STMN1_HUMAN	61646.832
YBOX3_HUMAN	15100.214
ZNF22_HUMAN	1026.2256
NAGAB_HUMAN	3916.6853

HMGA1_HUMAN	70734.664
TMM11_HUMAN	4114.7378
AATC_HUMAN	21718.635
KPCA_HUMAN	6151.3081
ITA2_HUMAN	19336.584
CXA1_HUMAN	1719.6388
ASM_HUMAN	1606.8896
UBF1_HUMAN	10293.664
JUND_HUMAN	493.12262
TAL1_HUMAN	1423.0164
ATF7_HUMAN	106.95537
NDUB7_HUMAN	5563.5303
KAPCA_HUMAN	12064.475
CAN2_HUMAN	39491.867
PTN2_HUMAN	2309.6331
PYRG1_HUMAN	16511.426
EGLN_HUMAN	45708.77
DDX5_HUMAN	88279.813
PFKAL_HUMAN	47769.074
VGFR1_HUMAN	8680.6758
PRS6A_HUMAN	44260.043
TCPA_HUMAN	64076.91
PTN1_HUMAN	18152.971
ERCC2_HUMAN	4223.3613
RL35A_HUMAN	78041.031
ITB5_HUMAN	5998.9702
ARF4_HUMAN	112286.95
RL7_HUMAN	183140.23
VINC_HUMAN	75182.531
PTPRA_HUMAN	2201.0359
ARY1_HUMAN	1533.8724
SON_HUMAN	10059.953
NELFE_HUMAN	4638.9482
RL17_HUMAN	90826.352
PGAM1_HUMAN	74057
RCC1_HUMAN	23918.381
ATF1_HUMAN	976.27863
ATF6A_HUMAN	475.30371
DNLI1_HUMAN	7130.1099
XRCC1_HUMAN	3887.2517
AMD_HUMAN	818.34845
CADH2_HUMAN	12715.29
PLCG1_HUMAN	10960.701
NUCL_HUMAN	181147.89
HXK1_HUMAN	54837.207
RPB3_HUMAN	2069.5229
RPAB1_HUMAN	5947.042
NDUV2_HUMAN	3876.8103
ERCC3_HUMAN	2427.5583
RO52_HUMAN	5776.9282

E2AK2_HUMAN	10887.927
SPEE_HUMAN	50951.918
SL9A1_HUMAN	1466.6609
CSK22_HUMAN	7299.1064
NFKB1_HUMAN	4428.6196
AT2B1_HUMAN	5264.8271
IF2B_HUMAN	21806.168
ANXA7_HUMAN	21543.004
TBP_HUMAN	928.61523
CCNA2_HUMAN	504.28665
BTF3_HUMAN	6202.1841
RAB3B_HUMAN	2801.853
RAB4A_HUMAN	3254.5034
RAB5A_HUMAN	55901.539
RAB6A_HUMAN	38710.23
MSH3_HUMAN	2191.0061
ANPRB_HUMAN	577.15985
PSB1_HUMAN	35343.672
MPRD_HUMAN	4655.0669
COX5A_HUMAN	1097.6857
LMNB1_HUMAN	84516.617
ICAL_HUMAN	20892.584
IMDH1_HUMAN	5433.7388
CO5A1_HUMAN	2065.4629
NEBU_HUMAN	2741.7568
ASPG_HUMAN	2469.8433
RASA1_HUMAN	5925.3569
PTMS_HUMAN	852.31537
CD11B_HUMAN	9545.957
GSTM3_HUMAN	1713.6655
VATB2_HUMAN	13064.772
VATC1_HUMAN	4787.8442
CSRP1_HUMAN	55748.055
FLNA_HUMAN	233387.03
NF1_HUMAN	435.79025
AOFA_HUMAN	1653.8325
ACOHC_HUMAN	23017.1
S1PR1_HUMAN	6517.3809
SYT1_HUMAN	1424.2709
5NTD_HUMAN	20039.258
MK_HUMAN	1878.0823
VDAC1_HUMAN	95999.656
PGS1_HUMAN	9201.3594
SDHB_HUMAN	18949.287
CD9_HUMAN	20495.057
COMT_HUMAN	5753.1841
TGM2_HUMAN	331958.97
MUTA_HUMAN	8249.1523
OSBP1_HUMAN	6753.2021
PIMT_HUMAN	2736.7373

FBRL_HUMAN	26827.201
PUR2_HUMAN	24025.787
PUR6_HUMAN	47941.281
SCP2_HUMAN	21268.037
UBA1_HUMAN	66366.164
NDKB_HUMAN	123842.45
ADRO_HUMAN	12139.465
ROA2_HUMAN	212737.38
RFX1_HUMAN	761.84497
CBL_HUMAN	4742.291
IBP4_HUMAN	817.50531
KAPCB_HUMAN	1804.4446
QCR2_HUMAN	18389.58
HEMH_HUMAN	3881.2014
XPA_HUMAN	757.25006
TCEA1_HUMAN	13171.732
PGH1_HUMAN	760.99066
ITA6_HUMAN	20133.596
SFPQ_HUMAN	109415.82
TBG1_HUMAN	12675.969
PPIB_HUMAN	153854.67
MAOM_HUMAN	14042.364
SYWC_HUMAN	28001.896
RS3_HUMAN	216780.56
KS6B1_HUMAN	3859.8167
JAK1_HUMAN	5246.2656
PTPRB_HUMAN	11999.424
PTPRE_HUMAN	5656.2036
PTPRG_HUMAN	1132.0767
SP100_HUMAN	5481.1177
CRCM_HUMAN	547.67322
TNFL4_HUMAN	6037.5776
SAHH_HUMAN	81057.375
COF1_HUMAN	536712.5
IF4B_HUMAN	22082.457
HAP40_HUMAN	1342.1134
AT2B4_HUMAN	7823.6167
DGKA_HUMAN	4342.3262
CPT2_HUMAN	3208.2559
KTHY_HUMAN	18011.488
RIR1_HUMAN	14818.859
CCND1_HUMAN	1855.1235
RAE1_HUMAN	1476.5237
ERD21_HUMAN	1365.3625
COT2_HUMAN	2538.897
EF1B_HUMAN	16783.402
AT5F1_HUMAN	17307.184
PPAC_HUMAN	14345.838
KPCL_HUMAN	3868.5813
THIL_HUMAN	27264.322

MYL9_HUMAN	1738.7697
RPB1_HUMAN	42708.566
CDK2_HUMAN	11133.991
ARBK1_HUMAN	4750.5229
PAR1_HUMAN	1736.3473
MCM3_HUMAN	48571.313
THTM_HUMAN	6995.1738
RS12_HUMAN	26305.201
BRD2_HUMAN	1912.8043
TNR6_HUMAN	61.767864
TYY1_HUMAN	2379.7954
DNJB1_HUMAN	8602.9365
DNJB2_HUMAN	441.19019
ATPA_HUMAN	221695.77
CATS_HUMAN	1151.8412
PSA1_HUMAN	94459.445
PSA2_HUMAN	24686.529
PSA3_HUMAN	40288.648
PSA4_HUMAN	81124.023
TNR5_HUMAN	2024.085
ITA3_HUMAN	24840.213
PTX3_HUMAN	2691.1672
MOES_HUMAN	137666.27
DDX6_HUMAN	16931.156
DNMT1_HUMAN	8720.8018
U2AF2_HUMAN	46703.18
RL13_HUMAN	120078.05
RAE2_HUMAN	826.52277
IVD_HUMAN	1828.0369
MGAT1_HUMAN	4210.1665
HMGB2_HUMAN	18397.08
PTBP1_HUMAN	66828.313
SYTC_HUMAN	34927.941
SYVC_HUMAN	22211.662
EF1G_HUMAN	127074.71
FKBP2_HUMAN	19923.693
IL3RA_HUMAN	1544.8528
STOM_HUMAN	37971.605
KAD4_HUMAN	1828.3917
1433T_HUMAN	55639.797
MK03_HUMAN	15426.653
MARK3_HUMAN	628.92188
RL10_HUMAN	47629.219
CO8A1_HUMAN	1703.2028
RFA1_HUMAN	16935.578
APEX1_HUMAN	28779.426
CD82_HUMAN	1004.5089
DCK_HUMAN	3621.5952
PYR1_HUMAN	37223.012
CALR_HUMAN	177395.69

MAP4_HUMAN	31607.637
CALX_HUMAN	116075.12
P85A_HUMAN	1403.7621
IP3KB_HUMAN	1069.9669
PSB8_HUMAN	4068.9929
PSB9_HUMAN	6140.4717
PSA5_HUMAN	29894.316
PSB4_HUMAN	18477.293
PSB6_HUMAN	44726.781
PSB5_HUMAN	35512.852
GSTM2_HUMAN	933.77301
ABCD3_HUMAN	6595.4902
ITPI2_HUMAN	5485.5674
LYOX_HUMAN	2693.8176
NDUS1_HUMAN	17170.953
DPOD1_HUMAN	10599.139
SMCA1_HUMAN	1239.2435
MK01_HUMAN	47454.574
RXRB_HUMAN	1336.359
ERCC5_HUMAN	1248.797
GRN_HUMAN	4625.2051
PTPRM_HUMAN	791.2475
AMPL_HUMAN	32475.27
CD34_HUMAN	3150.7136
T2EA_HUMAN	3186.6216
T2EB_HUMAN	3423.5903
TPP2_HUMAN	12989.882
IMPA1_HUMAN	6662.8066
CCN2_HUMAN	27220.332
EPHA2_HUMAN	14697.719
EPHB2_HUMAN	13243.793
SHC1_HUMAN	4847.6328
3MG_HUMAN	6106.3623
TKT_HUMAN	127309.53
CASP1_HUMAN	637.33844
NOS3_HUMAN	3990.9565
RBMS1_HUMAN	4963.4385
PML_HUMAN	11163.266
TYK2_HUMAN	1102.0126
EF1D_HUMAN	43629.906
MARCS_HUMAN	36778.082
GNA11_HUMAN	17632.113
AL4A1_HUMAN	3703.8774
ERP29_HUMAN	29023.516
PRDX6_HUMAN	150366.67
BLVRB_HUMAN	8487.0166
PRDX5_HUMAN	30161.625
PRDX3_HUMAN	25485.873
RL12_HUMAN	110806.51
ECHM_HUMAN	14432.526

KCY_HUMAN	29401.527
PEBP1_HUMAN	19823.523
PDIA3_HUMAN	152792.77
2AAA_HUMAN	62486.895
2AAB_HUMAN	4957.6494
CDC27_HUMAN	4793.064
PPIF_HUMAN	11572.915
NKTR_HUMAN	1463.7725
NMT1_HUMAN	19470.57
HMOX2_HUMAN	17062.621
PURA2_HUMAN	63967.934
UFO_HUMAN	2712.4392
AMRP_HUMAN	5041.8457
TSPO_HUMAN	11753.928
PUR8_HUMAN	18641.207
CLIP1_HUMAN	5473.6411
SORCN_HUMAN	5050.0488
ILEU_HUMAN	6709.0552
CTR1_HUMAN	4349.397
AL1B1_HUMAN	6177.9082
RPB2_HUMAN	9485.5078
SDHA_HUMAN	10287.968
COR1A_HUMAN	1980.1571
GDIA_HUMAN	20494.766
S10A7_HUMAN	213.76382
METK2_HUMAN	45613.035
KAP3_HUMAN	10571.738
RIR2_HUMAN	14357.26
SDC4_HUMAN	545.28265
TIA1_HUMAN	20093.578
SC6A6_HUMAN	378.21817
DNJA1_HUMAN	14022.031
AKT1_HUMAN	3210.7544
QCR1_HUMAN	90010.844
3HIDH_HUMAN	5349.4619
PUR9_HUMAN	34543.926
HNRH3_HUMAN	9887.8809
HNRH1_HUMAN	64729.715
CASPE_HUMAN	1244.2343
1433B_HUMAN	166744.2
STIP1_HUMAN	120230.16
S10AB_HUMAN	9170.9141
PRDX2_HUMAN	48037.383
ARRB2_HUMAN	501.75555
GLPK_HUMAN	832.95258
DCTD_HUMAN	8084.9839
P5CR1_HUMAN	5427.6216
GBP1_HUMAN	3824.877
GBP2_HUMAN	4129.3936
ELF1_HUMAN	1099.3063

TF2H1_HUMAN	1086.1486
STX2_HUMAN	209.36131
RL9_HUMAN	76484.891
ACSL1_HUMAN	4540.3423
CADH5_HUMAN	28523.4
KINH_HUMAN	34964.465
DUT_HUMAN	6568.1704
MRP1_HUMAN	4362.1675
ABCD1_HUMAN	726.26801
TTK_HUMAN	793.35596
MCM4_HUMAN	12952.563
MCM5_HUMAN	32306.73
MCM7_HUMAN	21236.723
GALNS_HUMAN	643.85815
GLYC_HUMAN	4840.3032
GLYM_HUMAN	58849.84
HS71L_HUMAN	1261.4744
HSP74_HUMAN	36323.719
GRK5_HUMAN	132.84808
MPI_HUMAN	3957.3613
GPC1_HUMAN	575.29205
PROF2_HUMAN	19258.463
CTNA1_HUMAN	51467.508
CTNB1_HUMAN	47313.02
PHB1_HUMAN	117043.23
SPB6_HUMAN	26212.336
MERL_HUMAN	3554.5176
RADI_HUMAN	420844.63
RFA3_HUMAN	2445.1008
RFC4_HUMAN	7953.9331
RFC2_HUMAN	7441.9575
RFC1_HUMAN	4333.8887
RL22_HUMAN	139651.58
T2FA_HUMAN	7007.1724
SPRE_HUMAN	8041.5649
HRH1_HUMAN	290.84146
CBS_HUMAN	832.21948
FBN1_HUMAN	3055.4363
FBN2_HUMAN	305.98059
GDE_HUMAN	4063.8438
MYH9_HUMAN	159980.63
MYH10_HUMAN	107617.47
TIE1_HUMAN	15955.622
COPB2_HUMAN	46343.801
SOAT1_HUMAN	2847.9409
ADDA_HUMAN	7475.3125
BASI_HUMAN	58037.988
FUS_HUMAN	71021.016
NU214_HUMAN	7822.2729
DEK_HUMAN	22188.844

SOX18_HUMAN	1421.874
MYH11_HUMAN	51030.996
PPM1A_HUMAN	4645.6377
HMGCL_HUMAN	4102.334
VGFR3_HUMAN	5072.293
VGFR2_HUMAN	3959.2996
PRS7_HUMAN	62805.352
GGT5_HUMAN	1844.2313
ARL2_HUMAN	13050.154
ARL3_HUMAN	24764.436
MP2K2_HUMAN	15854.611
ATPG_HUMAN	42316.57
VATE1_HUMAN	10395.462
HEM6_HUMAN	11968.943
RL4_HUMAN	153362.44
8ODP_HUMAN	1451.9178
LONM_HUMAN	12698.887
PGM1_HUMAN	9868.458
PP1G_HUMAN	10756.982
GNL1_HUMAN	31148.549
ODO2_HUMAN	22132
GMPR1_HUMAN	3507.9275
GPX4_HUMAN	10943.321
ACVL1_HUMAN	2712.9124
DHB2_HUMAN	1046.4545
SRP14_HUMAN	23542.68
TGFR2_HUMAN	3940.6499
NUP62_HUMAN	1281.7678
HPCL1_HUMAN	8547.2607
FDFT_HUMAN	11130.141
ZEB1_HUMAN	2192.1406
TAGL2_HUMAN	45840.934
TALDO_HUMAN	117890.28
SYUA_HUMAN	4658.9058
ETFB_HUMAN	19063.541
RBMX_HUMAN	40453.168
COIL_HUMAN	3316.4983
VKGC_HUMAN	2893.2942
LICH_HUMAN	2767.4238
VATA_HUMAN	20931.242
GRP75_HUMAN	119864.86
IF4A3_HUMAN	36633.055
SMBP2_HUMAN	781.74487
CDN1A_HUMAN	1858.9651
RS19_HUMAN	104072.39
RL3_HUMAN	41690.957
COIA1_HUMAN	30932.348
OST48_HUMAN	100382.87
AN32A_HUMAN	34517.66
FEN1_HUMAN	34209.965

CUX1_HUMAN	545.27649
CAPG_HUMAN	21924.801
CAP2_HUMAN	4308.9189
IL6RB_HUMAN	5895.8613
TXLNA_HUMAN	9410.1865
TCPZ_HUMAN	104331.73
NNMT_HUMAN	42425.477
PSB10_HUMAN	1647.689
PBX2_HUMAN	1413.5909
RL13A_HUMAN	132753.7
ARL1_HUMAN	20669.648
MLH1_HUMAN	11997.488
STAT3_HUMAN	8387.6484
UBP8_HUMAN	6292.2627
MDHC_HUMAN	75736.43
MDHM_HUMAN	117155.27
RFC5_HUMAN	10642.811
RFC3_HUMAN	4363.4316
ECHA_HUMAN	35350.906
IF2G_HUMAN	37378.754
ETV3_HUMAN	482.47794
CETN2_HUMAN	2007.6624
ETV6_HUMAN	2683.3411
EIF2D_HUMAN	5646.4536
BUD31_HUMAN	4273.9907
UBA7_HUMAN	2310.0208
NAA10_HUMAN	17004.408
KDM5C_HUMAN	514.62756
CSK_HUMAN	9918.0654
GARS_HUMAN	24246.131
SYIC_HUMAN	49682.887
S19A1_HUMAN	1210.3065
EIF1_HUMAN	4291.0928
KPCI_HUMAN	760.19019
ELK3_HUMAN	1183.1339
ACTY_HUMAN	25330.852
ECI1_HUMAN	5423.1592
LAP2A_HUMAN	9902.8145
LAP2B_HUMAN	108843.46
STAT1_HUMAN	21224.461
STAT6_HUMAN	6398.3569
STA5A_HUMAN	7368
MTREX_HUMAN	12577.527
AK1C3_HUMAN	3318.4055
PK3CA_HUMAN	1530.8702
PK3CB_HUMAN	2262.5566
MTOR_HUMAN	9692.7441
PI4KA_HUMAN	4676.8755
EPS15_HUMAN	7898.2051
CASP3_HUMAN	19502.639

CASP2_HUMAN	523.42322
RS27_HUMAN	9538.2363
ABL2_HUMAN	1013.1625
HELZ_HUMAN	1168.7175
CNDD3_HUMAN	1000.0044
RBM34_HUMAN	9333.9355
LPPRC_HUMAN	43218.082
THIM_HUMAN	11157.603
RL35_HUMAN	50547.336
PCP_HUMAN	21159.338
HD_HUMAN	4360.5229
ECE1_HUMAN	14811.729
MTHR_HUMAN	551.43048
SATT_HUMAN	943.01227
LIS1_HUMAN	20180.463
MUC18_HUMAN	103247.54
CACP_HUMAN	5151.1069
MATR3_HUMAN	35959.563
MSH2_HUMAN	13117.505
GRK6_HUMAN	1472.6296
GPDM_HUMAN	10783.314
SSRA_HUMAN	37864.711
PTN9_HUMAN	1943.0906
RANG_HUMAN	48231.559
NAMPT_HUMAN	88250.781
PRS6B_HUMAN	29776.131
EFTS_HUMAN	6976.1416
PPIC_HUMAN	10138.261
VDAC2_HUMAN	90497.023
ACDSB_HUMAN	3037.7634
CBX5_HUMAN	3585.5298
UBP5_HUMAN	35718.832
MK08_HUMAN	1244.4592
MP2K4_HUMAN	5108.271
KI67_HUMAN	4471.5078
KPB1_HUMAN	1175.1664
RAGP1_HUMAN	23110.438
RECQ1_HUMAN	24160.684
NOP2_HUMAN	14953.551
ATRX_HUMAN	2614.6096
CRK_HUMAN	7192.1997
CRKL_HUMAN	6091.3032
IF2M_HUMAN	3360.8198
BAG6_HUMAN	6724.9458
NSF_HUMAN	20788.359
NOTC1_HUMAN	3044.968
MP2K3_HUMAN	5632.9526
BRCC3_HUMAN	2162.0564
RL27A_HUMAN	207081.86
RL5_HUMAN	54977.066

RL21_HUMAN	40389.219
RL28_HUMAN	42870.164
RS9_HUMAN	219340.66
RS5_HUMAN	112547.41
RS10_HUMAN	80360.93
MAP1B_HUMAN	31301.342
GNPI1_HUMAN	15556.055
NEDD4_HUMAN	2699.0994
UTRN_HUMAN	10616.989
IQGA1_HUMAN	161942.45
GLYG_HUMAN	12317.744
STT3A_HUMAN	48307.863
PA24A_HUMAN	2522.2883
CAZA2_HUMAN	27092.711
CAPZB_HUMAN	42106.922
IF1AX_HUMAN	9647.2422
AL1A3_HUMAN	691.10498
SYQ_HUMAN	31609.773
RL29_HUMAN	32737.264
TISD_HUMAN	887.65546
UCRI_HUMAN	18058.719
ATPO_HUMAN	52988.957
LIMS1_HUMAN	15271.984
GLIP1_HUMAN	858.02155
PPCE_HUMAN	20038.957
MAOX_HUMAN	17604.744
IREB2_HUMAN	497.90515
TFPI2_HUMAN	432.74396
RFX5_HUMAN	5336.79
PI42A_HUMAN	5227.9072
COPD_HUMAN	47524.367
LSS_HUMAN	22410.326
PP2BC_HUMAN	905.01233
GSH1_HUMAN	1834.1085
GSH0_HUMAN	9369.7881
CD151_HUMAN	7367.1411
TPC10_HUMAN	1083.9614
PSMD8_HUMAN	38133.137
PRC2A_HUMAN	2924.6125
GSHB_HUMAN	16946.555
TCPE_HUMAN	94183.25
PTSS1_HUMAN	5478.8486
NEST_HUMAN	124740.57
HSP13_HUMAN	8789.8135
KC1A_HUMAN	9957.7627
KC1D_HUMAN	4842.1484
IDHP_HUMAN	67957.961
PK3CG_HUMAN	2485.9141
PIPNB_HUMAN	16239.057
DPOD2_HUMAN	5776.7202

MRP_HUMAN	13175.789
PAXI_HUMAN	4689.124
CAMLG_HUMAN	651.11548
NR2C2_HUMAN	839.25677
MAPK2_HUMAN	2111.5586
DNSL1_HUMAN	1698.5238
AL9A1_HUMAN	12626.839
RL34_HUMAN	58825.188
RPIA_HUMAN	3335.2432
LMAN1_HUMAN	18891.635
NRAM2_HUMAN	652.00195
NASP_HUMAN	30645.08
FAS_HUMAN	69125.008
FNTA_HUMAN	10598.909
FNTB_HUMAN	4818.3335
DHYS_HUMAN	2230.2419
TCPG_HUMAN	83040.273
RM19_HUMAN	9539.7734
ARRB1_HUMAN	6720.2129
EFTU_HUMAN	82358.25
AL7A1_HUMAN	21772.691
UB2R1_HUMAN	3949.2092
INPP_HUMAN	4312.1255
CENPF_HUMAN	318.53247
SRP09_HUMAN	51061.648
PCY1A_HUMAN	10320.677
SYAC_HUMAN	24110.49
SYCC_HUMAN	16154.122
SYHM_HUMAN	5364.0244
SYSC_HUMAN	28724.035
PPM1F_HUMAN	24167.348
MA2A2_HUMAN	2203.3069
PRI1_HUMAN	2799.1321
PRI2_HUMAN	1870.3246
CASP4_HUMAN	2491.2131
KC1E_HUMAN	499.41168
PSB3_HUMAN	32578.156
PSB2_HUMAN	30932.666
MCM2_HUMAN	20099.947
ACADV_HUMAN	12091.761
YLPM1_HUMAN	7337.645
VPS41_HUMAN	1084.7178
TMEDA_HUMAN	39111.035
RBM25_HUMAN	10680.588
NUMB_HUMAN	6033.7598
CLK3_HUMAN	1256.2343
PSN1_HUMAN	1635.9735
EI2BB_HUMAN	5668.0005
HINT1_HUMAN	8708.752
NU153_HUMAN	7059.1016

RBP2_HUMAN	12771.432
RGS19_HUMAN	1273.9186
RGS3_HUMAN	2884.8003
PSN2_HUMAN	459.57156
TSC2_HUMAN	3046.1338
NDUV1_HUMAN	12216.529
GSK3A_HUMAN	1983.7921
GSK3B_HUMAN	6238.3154
TAF6_HUMAN	1129.8586
5NTC_HUMAN	2732.2314
SPS1_HUMAN	6560.5498
MTHFS_HUMAN	1927.7958
GUAA_HUMAN	21349.049
DNLI3_HUMAN	5982.4238
DNLI4_HUMAN	1955.1163
MRE11_HUMAN	7055.8682
ENTP1_HUMAN	841.99286
GNAQ_HUMAN	7646.7754
IDH3A_HUMAN	10814.66
ST1A1_HUMAN	1622.009
MMP14_HUMAN	24358.18
PPOX_HUMAN	2466.4988
GDIB_HUMAN	68182.156
EMD_HUMAN	10278.069
CPT1A_HUMAN	8552.4229
S26A2_HUMAN	833.60687
SPB8_HUMAN	11805.492
SPB9_HUMAN	55769.027
SERPH_HUMAN	96667.148
PDLI4_HUMAN	9075.9941
F10A1_HUMAN	91255.898
PEX5_HUMAN	1064.6127
ERF_HUMAN	1783.3865
VASP_HUMAN	8758.8926
DYN2_HUMAN	26833.645
MAP2_HUMAN	6209.8374
AP4A_HUMAN	4092.134
CDK7_HUMAN	3820.6243
KNTC1_HUMAN	2342.2021
RASF2_HUMAN	2851.6492
CDK9_HUMAN	4522.0527
LRBA_HUMAN	5039.9731
BCAM_HUMAN	4663.271
PPT1_HUMAN	5515.9688
RL14_HUMAN	134143.31
TCPQ_HUMAN	165964.36
TCPD_HUMAN	45476.945
ANX11_HUMAN	24461.596
PAPOA_HUMAN	8385.8438
FXR1_HUMAN	18329.289

FXR2_HUMAN	14385.855
RAB5C_HUMAN	26623.359
RAB7A_HUMAN	84688.484
RAB9A_HUMAN	4196.6777
RAB13_HUMAN	12379.865
RB27A_HUMAN	8501.5645
PLCD1_HUMAN	1445.7893
RT29_HUMAN	5273.479
DUS3_HUMAN	7500.7471
SMCA2_HUMAN	528.25293
SMCA4_HUMAN	7768.5176
IDH3G_HUMAN	3539.9983
GALK1_HUMAN	15053.243
SSRD_HUMAN	44397.523
BAP31_HUMAN	16281.012
TPMT_HUMAN	4171.2627
MECP2_HUMAN	1112.2062
HCFC1_HUMAN	15296.369
IRAK1_HUMAN	1249.783
CAV2_HUMAN	1174.7681
AL3A2_HUMAN	4309.1968
DHB4_HUMAN	16984.793
PSMD7_HUMAN	19555.945
SUOX_HUMAN	2759.2075
SPHM_HUMAN	7103.8003
STA5B_HUMAN	1728.3876
UBP11_HUMAN	1819.9047
CLCN7_HUMAN	2067.9788
VAMP7_HUMAN	2330.082
KS6A3_HUMAN	6799.5894
BMX_HUMAN	8561.2695
AFF1_HUMAN	327.06183
HDGF_HUMAN	19908.857
CCNH_HUMAN	904.40686
MAT1_HUMAN	1112.0419
NEK3_HUMAN	790.49902
UB2E1_HUMAN	2078.7034
NDUA8_HUMAN	6550.626
ROA3_HUMAN	47649.387
6PGD_HUMAN	110215.27
HNRPM_HUMAN	108065.16
IMA1_HUMAN	31619.342
IMA5_HUMAN	5234.1128
NCBP2_HUMAN	2788.2773
GDS1_HUMAN	12352.06
RPAB3_HUMAN	3456.9705
MP2K6_HUMAN	1967.5941
GDIR1_HUMAN	67906.109
GDIR2_HUMAN	78096.078
AGFG1_HUMAN	4126.6016

HNRPF_HUMAN	16777.777
STAT2_HUMAN	2984.4209
MSH6_HUMAN	8228.8164
KIF11_HUMAN	4112.8223
VAV2_HUMAN	3051.0049
ZN143_HUMAN	715.01129
RBM5_HUMAN	4205.291
RIDA_HUMAN	276.26001
SPSY_HUMAN	25478.008
HXK2_HUMAN	29711.762
EFNB2_HUMAN	690.26691
RM12_HUMAN	25861.924
DGKQ_HUMAN	216.12984
NDST1_HUMAN	1013.5566
THOP1_HUMAN	12380.185
CAZA1_HUMAN	94111.734
HMGA2_HUMAN	2175.061
CRIP2_HUMAN	45357.41
NUP98_HUMAN	8890.5371
BIEA_HUMAN	25998.012
TXTP_HUMAN	15046.694
PPP5_HUMAN	10687.993
PLK1_HUMAN	2340.2983
DAPK1_HUMAN	1180.8431
ARFP2_HUMAN	2775.3137
ARFP1_HUMAN	6477.3462
NUBP1_HUMAN	5348.4751
ACLY_HUMAN	85558.953
MAP11_HUMAN	6635.9785
SUCA_HUMAN	9254.0625
MVD1_HUMAN	4701.7627
PGTB1_HUMAN	2558.0479
PGTB2_HUMAN	6366.0112
COPB_HUMAN	29839.564
COPA_HUMAN	54635.941
CATC_HUMAN	8944.9395
LIMK1_HUMAN	8679.6094
LIMK2_HUMAN	429.58905
CLH2_HUMAN	381.85208
AP3M2_HUMAN	1254.8595
AP2S1_HUMAN	12125.808
CCHL_HUMAN	4332.9688
MK12_HUMAN	3072.1228
PTTG_HUMAN	3034.3254
SMTN_HUMAN	2593.925
MOT1_HUMAN	8422.9629
IST1_HUMAN	10217.523
SC24C_HUMAN	20048.947
TCP4_HUMAN	50548.348
DPOG1_HUMAN	1131.4087

ICLN_HUMAN	11787.96
SYRC_HUMAN	35919.219
HIRA_HUMAN	1480.7645
ATX3_HUMAN	3168.2783
ATN1_HUMAN	1250.3602
PMS1_HUMAN	786.58173
PMS2_HUMAN	354.87271
CA2D1_HUMAN	2553.7156
SYYC_HUMAN	38272.957
UBP14_HUMAN	21294.426
AAKG1_HUMAN	3841.1995
HSP72_HUMAN	11567.036
BCAT1_HUMAN	23339.055
AT1B3_HUMAN	26464.342
RD23B_HUMAN	1533.5835
EPHB4_HUMAN	3470.4724
ANAG_HUMAN	2352.9358
KAD2_HUMAN	4968.0562
P5CS_HUMAN	18200.281
SNAA_HUMAN	19107.924
IF5_HUMAN	20933.533
S12A2_HUMAN	5838.605
PSMD4_HUMAN	21321.879
DRG2_HUMAN	8010.1885
XPO2_HUMAN	173686
TERA_HUMAN	165250.56
MFAP1_HUMAN	2880.0032
ECHB_HUMAN	58385.457
MANF_HUMAN	15760.75
AFAD_HUMAN	5440.7798
ELL_HUMAN	983.45056
BRPF1_HUMAN	1601.308
NP1L1_HUMAN	50978.094
CASP7_HUMAN	6881.5698
CASP6_HUMAN	5249.4473
ADK_HUMAN	11359.54
DSRAD_HUMAN	13138.752
LAMB2_HUMAN	4626.1646
CAD13_HUMAN	6216.2168
SEC13_HUMAN	10590.143
NH2L1_HUMAN	12482.597
PSA_HUMAN	31475.389
HNRH2_HUMAN	9705.0059
SCOT1_HUMAN	12855.03
EIF3B_HUMAN	21953.648
FCGRN_HUMAN	9006.8008
BID_HUMAN	1798.532
ATPK_HUMAN	21896.311
RRP1_HUMAN	5707.9805
SYMC_HUMAN	33066.898

ARP19_HUMAN	5484.5356
AP1S2_HUMAN	4191.2544
ATP68_HUMAN	8642.5508
ATP5E_HUMAN	5942.0693
ATP5I_HUMAN	22368.979
HDAC4_HUMAN	1504.9565
IF6_HUMAN	33758.789
CTBP2_HUMAN	22020.979
NDUA6_HUMAN	10317.699
PEX3_HUMAN	1156.4492
DHB7_HUMAN	2027.3763
BCAR1_HUMAN	9871.9492
STX17_HUMAN	2621.9724
CF298_HUMAN	698.74365
WDR4_HUMAN	1403.9073
TMM33_HUMAN	32853.25
GEMI4_HUMAN	3418.4155
CORO7_HUMAN	6173.7266
NU107_HUMAN	10069.787
GSDMD_HUMAN	9936.7031
SELB_HUMAN	2880.3596
SESN2_HUMAN	687.34308
ANTR2_HUMAN	1631.2844
MTPN_HUMAN	319.60873
ARPC4_HUMAN	111975.07
CD81_HUMAN	7504.2236
SC61G_HUMAN	32699.17
TPIS_HUMAN	145641.3
EIF3E_HUMAN	32951.023
SC61B_HUMAN	26682.102
PTEN_HUMAN	526.37048
PP4C_HUMAN	8272.7744
GBRL2_HUMAN	10015.075
ROMO1_HUMAN	3455.2478
MYL6_HUMAN	7642.0684
ACTB_HUMAN	1885050.5
IF4A1_HUMAN	179542.55
RS20_HUMAN	100907.3
PRPS1_HUMAN	5523.9175
PSA6_HUMAN	47063.988
S10AA_HUMAN	37715.5
CDC42_HUMAN	18333.617
DEST_HUMAN	15526.569
GMFB_HUMAN	4726.0728
RAB8A_HUMAN	3683.948
SPCS3_HUMAN	19534.156
SRP54_HUMAN	10351.541
RAB4B_HUMAN	2424.3955
RAB2A_HUMAN	31619.834
RAB5B_HUMAN	6458.145

RAB10_HUMAN	6845.4443
UB2D3_HUMAN	20058.734
UBC12_HUMAN	17555.016
UBE2K_HUMAN	19968.234
UBE2N_HUMAN	27135.213
RAB14_HUMAN	22532.039
ARP3_HUMAN	68660.086
ARP2_HUMAN	58916.129
ACTZ_HUMAN	20797.664
CSN2_HUMAN	13989.15
ABCE1_HUMAN	26234.41
RAP1B_HUMAN	33380.211
RAP2B_HUMAN	3809.8079
MAX_HUMAN	1428.2419
RS3A_HUMAN	99132.516
RL26_HUMAN	134561.58
PSME3_HUMAN	11296.58
RL15_HUMAN	156927.95
RL27_HUMAN	193085.22
VA0D1_HUMAN	14433.941
RL37A_HUMAN	7660.7397
RHOA_HUMAN	11567.875
RND3_HUMAN	728.71313
NAA20_HUMAN	2989.3906
CH10_HUMAN	10924.382
S61A1_HUMAN	26120.408
PFD3_HUMAN	11657.521
STXB1_HUMAN	7722.2188
B2MG_HUMAN	1577.8513
DAD1_HUMAN	32577.715
NPC2_HUMAN	4652.8823
COPZ1_HUMAN	25628.176
RL37_HUMAN	847.3772
SUMO2_HUMAN	12619.706
DCAF7_HUMAN	2929.8547
WDR5_HUMAN	3612.8379
AP1S1_HUMAN	7819.1167
NTF2_HUMAN	8358.0117
HNRPK_HUMAN	236951.88
1433G_HUMAN	52360.258
UBP46_HUMAN	2171.7341
RRAS2_HUMAN	15938.246
RS7_HUMAN	68947.93
PP1A_HUMAN	24169.111
PP1B_HUMAN	27588.398
PRS4_HUMAN	40061.43
PRS8_HUMAN	50113.156
RS8_HUMAN	140072.42
RS15A_HUMAN	115808.22
RS16_HUMAN	215757.8

UB2G1_HUMAN	2485.4692
UBE2H_HUMAN	4496.3628
1433E_HUMAN	95569.273
RS14_HUMAN	157036.78
RS23_HUMAN	49202.508
RS18_HUMAN	227143.84
RS29_HUMAN	33121.594
RS13_HUMAN	111079.21
RS11_HUMAN	42057.516
RUXE_HUMAN	20957.322
RUXF_HUMAN	8074.085
LSM3_HUMAN	4491.7119
LSM6_HUMAN	10495.932
SMD1_HUMAN	14324.847
SMD2_HUMAN	21419.357
SMD3_HUMAN	57270.691
TYB4_HUMAN	6580.563
ARF6_HUMAN	22047.725
PRS10_HUMAN	28007.578
SELT_HUMAN	4260.9966
TBPL1_HUMAN	978.03186
RL7A_HUMAN	109334.67
RPB7_HUMAN	3386.5872
RB11A_HUMAN	68884.016
ERF1_HUMAN	21878.199
CNBP_HUMAN	6611.1826
YPEL5_HUMAN	699.57062
RS4X_HUMAN	159291.25
PP2AB_HUMAN	37528.137
ACTA_HUMAN	351769.09
RHOB_HUMAN	5653.6519
RL23A_HUMAN	171735.75
RS6_HUMAN	200510.03
H4_HUMAN	1755774.3
RAB1A_HUMAN	8880.9824
RAN_HUMAN	262201.41
RL23_HUMAN	97773.969
RAP1A_HUMAN	5283.8223
RS15_HUMAN	26112.438
RS24_HUMAN	45731.969
RS25_HUMAN	201069.2
RS26_HUMAN	61286.434
RS28_HUMAN	36636.734
RS30_HUMAN	53863.203
GBB1_HUMAN	9116.1836
RBX1_HUMAN	15164.071
GBB2_HUMAN	55180.625
RL30_HUMAN	79914.203
RL39_HUMAN	36569.77
RL31_HUMAN	101599.5

RL10A_HUMAN	71407.891
RL32_HUMAN	34114.09
RL11_HUMAN	82792.5
RL8_HUMAN	97554.359
PPIA_HUMAN	132909.58
FKB1A_HUMAN	23407.063
RS27A_HUMAN	126016.04
GRB2_HUMAN	7280.021
TRA2B_HUMAN	12995.807
RAC1_HUMAN	69904.828
AP2B1_HUMAN	28576.211
VAMP2_HUMAN	7382.8589
GNAS2_HUMAN	14498.008
GNAI1_HUMAN	1147.6748
1433Z_HUMAN	230599.41
2ABA_HUMAN	30814.395
SUMO1_HUMAN	15201.528
DYL1_HUMAN	10395.62
DYLT1_HUMAN	4020.0823
RL38_HUMAN	45615.258
SKP1_HUMAN	3652.4099
GBG5_HUMAN	1214.822
RS21_HUMAN	19020.961
IF5A1_HUMAN	71187.766
RACK1_HUMAN	171775.06
SPT4H_HUMAN	2629.0044
UBC9_HUMAN	14295.894
TYB10_HUMAN	5543.1274
PP2AA_HUMAN	1590.6804
YBOX1_HUMAN	37856.047
SC11A_HUMAN	42273.801
CSK2B_HUMAN	7857.124
TPM4_HUMAN	75953.695
UB2L3_HUMAN	35241.824
EF1A1_HUMAN	1047366.6
TBA1B_HUMAN	930991.75
TBA4A_HUMAN	4017.124
TBB4B_HUMAN	284974.72
CSK21_HUMAN	9717.3232
PA1B2_HUMAN	8066.4814
H31_HUMAN	177195.5
HBB_HUMAN	2243.1431
HBA_HUMAN	3422.4902
CXAR_HUMAN	1971.88
F193A_HUMAN	3593.937
NOP14_HUMAN	3114.7815
IGBP1_HUMAN	6791.4316
SHPS1_HUMAN	835.36597
SERB_HUMAN	9156.3125
RBM6_HUMAN	3880.2002

IF4G2_HUMAN	30944.428
RPP38_HUMAN	3109.7014
RPP30_HUMAN	4691.6289
GTF2I_HUMAN	29080.344
PI42B_HUMAN	1888.2466
CNTP1_HUMAN	4626.3271
SRPK2_HUMAN	3573.7832
TCPB_HUMAN	74890.07
S35A2_HUMAN	1301.9268
RAE1L_HUMAN	9146.9463
GSTO1_HUMAN	144301.73
PRKDC_HUMAN	49181.621
ADA17_HUMAN	2365.877
SRPX_HUMAN	3600.9302
ARGI2_HUMAN	829.31781
NTH_HUMAN	1524.3235
IN35_HUMAN	1079.9498
NUCB2_HUMAN	3575.6101
BASP1_HUMAN	8776.2148
DCD_HUMAN	1619.2728
TMF1_HUMAN	1927.5894
RT22_HUMAN	9755.6865
RT25_HUMAN	2241.0469
RT10_HUMAN	3415.4504
RT35_HUMAN	2348.8992
RT05_HUMAN	3376.4778
KGD4_HUMAN	246.96834
RT11_HUMAN	5736.2002
RT15_HUMAN	6982.3716
RT21_HUMAN	4392.9463
RT34_HUMAN	18541.307
RT06_HUMAN	3687.5366
RT09_HUMAN	4746.4824
SARNP_HUMAN	28540.32
LACTB_HUMAN	2818.2209
COG7_HUMAN	4136.3418
RL24_HUMAN	105813.39
TXN4A_HUMAN	2767.9812
RL36A_HUMAN	15967.615
CBX1_HUMAN	3903.2102
SMAD3_HUMAN	4601.9448
ARF1_HUMAN	106853.6
ARF5_HUMAN	13440.144
ERH_HUMAN	2643.0085
RHOG_HUMAN	9620.2979
RL19_HUMAN	102217.11
SERF2_HUMAN	3207.6902
SRSF3_HUMAN	98743.586
FOXK1_HUMAN	5890.6514
CCZ1B_HUMAN	3249.7483

DAB2_HUMAN	15643.971
PGBM_HUMAN	59814.391
XIAP_HUMAN	1330.9763
EFNB1_HUMAN	4698.9941
RBM10_HUMAN	5596.5103
RBM3_HUMAN	12853.202
AT2C1_HUMAN	2381.4507
AT11A_HUMAN	871.48578
CYC_HUMAN	66421.977
EM55_HUMAN	2926.0574
TFAM_HUMAN	10533.723
PIPNA_HUMAN	7931.4287
S25A3_HUMAN	121203.14
VIGLN_HUMAN	34908.789
TF2B_HUMAN	3067.2068
CDK6_HUMAN	1909.2327
CDK5_HUMAN	3820.999
CDK17_HUMAN	8753.9814
PURA_HUMAN	4498.71
BORG5_HUMAN	4891.4673
CLH1_HUMAN	86748.984
HSF1_HUMAN	1422.2823
NFKB2_HUMAN	2362.6436
FKBP3_HUMAN	43421.59
REEP5_HUMAN	13437.847
DHSO_HUMAN	7592.2207
HNRPU_HUMAN	83940.516
U2AF1_HUMAN	12289.648
SPTB2_HUMAN	61977.082
TIAR_HUMAN	8026.9888
SET_HUMAN	93116.711
SRSF2_HUMAN	35122.484
FOXK2_HUMAN	1191.4966
RELB_HUMAN	406.71219
GALK2_HUMAN	5796.2754
AMPD2_HUMAN	9893.7578
FABP5_HUMAN	69385.398
CAP1_HUMAN	69671.398
FLI1_HUMAN	5408.7983
HMCS1_HUMAN	13182.115
IFM3_HUMAN	9058.1768
LAT1_HUMAN	2715.0273
NC2B_HUMAN	2659.7561
EXOSX_HUMAN	4417.3589
OTUD4_HUMAN	798.01776
PFKAP_HUMAN	47362.449
XPC_HUMAN	1710.6456
EWS_HUMAN	34161.711
CDR2_HUMAN	3393.8188
OCRL_HUMAN	2803.4397

PLCB3_HUMAN	10297.065
TAGL_HUMAN	4879.6973
AK17A_HUMAN	2904.2649
PYRD_HUMAN	695.83752
ODO1_HUMAN	18403.121
KIF23_HUMAN	908.32904
MMSA_HUMAN	5644.8955
NRG1_HUMAN	396.56393
CP27A_HUMAN	1330.5729
SP3_HUMAN	357.5249
H11_HUMAN	1880.6249
RL18A_HUMAN	71488.781
MP2K1_HUMAN	13240.714
TIE2_HUMAN	2179.3594
FKBP4_HUMAN	22457.865
PLOD1_HUMAN	17488.172
NUCB1_HUMAN	7628.6699
RL6_HUMAN	245425.5
TOP2B_HUMAN	5607.5059
AKA12_HUMAN	29070.211
M2OM_HUMAN	21477.156
DYST_HUMAN	3321.7627
ENL_HUMAN	1079.6259
MECOM_HUMAN	1339.1251
GNA12_HUMAN	374.54684
CAV1_HUMAN	86211.023
ACY1_HUMAN	2125.9795
TNAP2_HUMAN	2114.0959
LMNB2_HUMAN	28784.031
PTPS_HUMAN	872.78784
UPAR_HUMAN	874.21405
KIME_HUMAN	5861.541
ERCC6_HUMAN	172.98421
TAP1_HUMAN	3626.76
TAP2_HUMAN	1839.8276
CEBPZ_HUMAN	4211.0386
ZNF92_HUMAN	707.5542
TF65_HUMAN	8547.4277
UBXN1_HUMAN	9774.6475
GLGB_HUMAN	21498.871
IF4G1_HUMAN	28857.898
ATP7A_HUMAN	830.49329
NOTC2_HUMAN	1226.9801
TLE1_HUMAN	2302.0632
TLE3_HUMAN	1657.8695
LGUL_HUMAN	29440.357
ACVR1_HUMAN	551.99493
SSBP_HUMAN	14144.175
REL_HUMAN	427.44919
1433F_HUMAN	52962.969

PLP2_HUMAN	18925.488
CSTF1_HUMAN	6966.9053
UBE3A_HUMAN	6283.834
DYN1_HUMAN	444.26999
PTN12_HUMAN	7288.3848
FAK1_HUMAN	10898.487
SRS11_HUMAN	124245.88
KPCD_HUMAN	2489.0044
CALD1_HUMAN	34493.563
FOLC_HUMAN	1502.5139
CTSL2_HUMAN	1552.5337
PTN11_HUMAN	8643.9248
KDSR_HUMAN	3959.5938
PUR1_HUMAN	9374.5029
GFPT1_HUMAN	18780.449
EXOS9_HUMAN	3161.4282
PSME1_HUMAN	44941.793
SUH_HUMAN	567.34625
MEF2C_HUMAN	619.33801
APLP2_HUMAN	6159.8467
GABPA_HUMAN	2567.9617
RING1_HUMAN	316.42801
FMR1_HUMAN	857.68823
PRDX1_HUMAN	178356.03
RL18_HUMAN	138183.39
C1QBP_HUMAN	26951.846
CKAP4_HUMAN	118685.02
ZO1_HUMAN	18691.57
TISB_HUMAN	1962.6881
SPAG1_HUMAN	1222.3092
KHDR1_HUMAN	34642.914
BAX_HUMAN	18682.594
B2CL1_HUMAN	5486.9268
MCL1_HUMAN	515.9256
DPOE1_HUMAN	5738.916
KLC1_HUMAN	21080.779
SOS1_HUMAN	1496.8798
SRSF1_HUMAN	97784.18
RHG01_HUMAN	15618.853
TLE5_HUMAN	1390.2872
SRSF4_HUMAN	9521.2607
PCDH1_HUMAN	8103.7568
PP2BA_HUMAN	5507.5186
DHX9_HUMAN	60541.113
QOR_HUMAN	8427.3467
GOGA3_HUMAN	2952.6326
GOGA2_HUMAN	3714.9587
ECHP_HUMAN	1819.0227
MFGM_HUMAN	2828.5991
PDE4D_HUMAN	708.1651

DSC1_HUMAN	1575.1815
HDHD1_HUMAN	2804.2324
TICN1_HUMAN	1263.1135
CD47_HUMAN	16373.981
PPID_HUMAN	14495.602
SSRP1_HUMAN	28485.09
CAMP2_HUMAN	2929.2129
SPIR1_HUMAN	743.97833
SLFN5_HUMAN	3215.71
VAC14_HUMAN	6063.978
NSUN2_HUMAN	18188.512
DMWD_HUMAN	272.21622
RBBP4_HUMAN	60907.98
NCBP1_HUMAN	14997.548
MGT5A_HUMAN	620.95941
EP300_HUMAN	3260.8899
AHNK_HUMAN	141028.05
FCHO2_HUMAN	1524.3732
ELP6_HUMAN	1270.2266
HSP7E_HUMAN	6456.7188
SCRN3_HUMAN	2189.2742
CGNL1_HUMAN	5433.5581
LTOR4_HUMAN	4779.8032
NEXN_HUMAN	4387.7964
MGAT2_HUMAN	5712.4644
GALT2_HUMAN	7519.1006
GALT1_HUMAN	9066.1729
AP1B1_HUMAN	32771
CPSF1_HUMAN	8537.2783
BST2_HUMAN	1595.7039
MPPA_HUMAN	7347.9561
SIA4A_HUMAN	1064.1387
SCRN1_HUMAN	9526.6348
WASC5_HUMAN	7549.166
NU160_HUMAN	8572.2549
SCAP_HUMAN	2398.9858
FOXO1_HUMAN	720.60052
TBL3_HUMAN	3123.8994
TF3C1_HUMAN	2858.0244
TWF1_HUMAN	16421.73
ASPH_HUMAN	12632.496
TFCP2_HUMAN	2188.918
AKP13_HUMAN	2713.3706
FBLN3_HUMAN	25496.063
SNF5_HUMAN	4431.1343
BPTF_HUMAN	962.2254
CDC20_HUMAN	1726.1588
FSTL1_HUMAN	6371.2417
STX4_HUMAN	2201.9307
GRSF1_HUMAN	6422.6455

M4K2_HUMAN	1886.7686
MERTK_HUMAN	2863.6458
SFSWA_HUMAN	1475.7297
CHD3_HUMAN	747.89429
SF3A3_HUMAN	27844.742
DPYD_HUMAN	938.54492
TP53B_HUMAN	7519.6045
TM115_HUMAN	1561.8789
IFRD2_HUMAN	2603.5132
TRI26_HUMAN	1400.4622
AIMP1_HUMAN	15069.293
ILF2_HUMAN	87749.539
ILF3_HUMAN	100773.09
LMAN2_HUMAN	46596.695
TRAP1_HUMAN	366065.53
TRAF2_HUMAN	3595.386
DLG1_HUMAN	3253.1921
MYO1E_HUMAN	9197.8086
PP1R8_HUMAN	4220.0771
TP4A2_HUMAN	3931.5601
ABR_HUMAN	4479.7183
NPRL3_HUMAN	839.90271
SEC20_HUMAN	5501.4932
BNIP2_HUMAN	777.12622
NFX1_HUMAN	274.224
CSTF3_HUMAN	7035.2251
TSN31_HUMAN	1862.1047
ECH1_HUMAN	10446.991
RHG05_HUMAN	729.12799
STRN3_HUMAN	5189.7407
CDC16_HUMAN	3427.8176
STK4_HUMAN	3876.3513
FLII_HUMAN	25874.508
TRI32_HUMAN	1159.1167
COASY_HUMAN	5889.1714
RM28_HUMAN	6789.8081
ACACA_HUMAN	13644.287
CSN1_HUMAN	14096.371
UBP4_HUMAN	3173.5442
CAF1A_HUMAN	461.53223
CAF1B_HUMAN	1323.4954
RED_HUMAN	12470.908
MTAP_HUMAN	9559.9004
AAPK1_HUMAN	4566.5918
LIPA1_HUMAN	2158.6602
EI2BE_HUMAN	17468.648
TADBP_HUMAN	16136.618
ROA0_HUMAN	20556.574
AIMP2_HUMAN	12307.869
FADD_HUMAN	4013.9294

PRDX4_HUMAN	37321.18
MP2K5_HUMAN	471.30078
MK07_HUMAN	546.97107
PAK2_HUMAN	15944.33
CBX3_HUMAN	23249.521
STK3_HUMAN	976.00311
STX5_HUMAN	3256.7852
PSMD2_HUMAN	32888.566
MMRN1_HUMAN	10421.481
DDX10_HUMAN	998.32538
DNJC3_HUMAN	19757.949
SBP1_HUMAN	6578.7471
NDK3_HUMAN	4040.6973
SRSF9_HUMAN	17649.539
SRSF5_HUMAN	6793.9746
SRSF6_HUMAN	62803.629
MD2L1_HUMAN	8100.6641
TIF1B_HUMAN	33710.316
SEM3F_HUMAN	402.9855
STX3_HUMAN	1659.8915
G3BP1_HUMAN	36370.551
NMI_HUMAN	4228.7734
PTK7_HUMAN	3774.5413
PABP4_HUMAN	27690.547
ATM_HUMAN	2136.8293
GRB10_HUMAN	2701.0376
IFIT5_HUMAN	598.33533
MTA1_HUMAN	3567.7639
EIF3I_HUMAN	20155.707
PPIL2_HUMAN	1101.6132
2A5G_HUMAN	2892.9243
CTBP1_HUMAN	5235.2983
PHLP_HUMAN	5524.6787
PLD1_HUMAN	4192.3984
TARB1_HUMAN	294.7576
UB2V1_HUMAN	31537.471
RM49_HUMAN	640.53723
DC1I2_HUMAN	23772.504
ORC2_HUMAN	1591.0471
ILK_HUMAN	31588.93
NNTM_HUMAN	21179.338
SNTB2_HUMAN	14179.548
XRCC4_HUMAN	4046.2581
PPIG_HUMAN	7644.9194
TCOF_HUMAN	10474.123
U119A_HUMAN	1046.0631
SF3B2_HUMAN	25343.781
OS9_HUMAN	2037.9984
GOGA4_HUMAN	2813.5518
HAP28_HUMAN	20859.434

ADAM9_HUMAN	11685.061
ADA15_HUMAN	4699.7153
TMED1_HUMAN	4425.354
FKBP5_HUMAN	23219.768
MYO9B_HUMAN	5348.7686
ROCK1_HUMAN	8403.9385
NFAC2_HUMAN	970.56964
TOP3A_HUMAN	700.56464
SMAD4_HUMAN	3747.5808
VPP3_HUMAN	5817.7988
BIRC2_HUMAN	938.32965
PICAL_HUMAN	9787.7783
MTM1_HUMAN	2165.4712
SQSTM_HUMAN	8162.9854
MTX1_HUMAN	4923.4941
TBB3_HUMAN	11392.955
PRP4B_HUMAN	2844.5173
PIN1_HUMAN	5717.6152
ATR_HUMAN	1713.1561
4EBP1_HUMAN	851.72021
RIPK1_HUMAN	1695.1002
HDAC1_HUMAN	19762.461
KCC2G_HUMAN	3963.9763
KCC2D_HUMAN	5058.6758
DCTN2_HUMAN	6089.7817
PKD2_HUMAN	475.41455
ULA1_HUMAN	8721.4297
ITPK1_HUMAN	1862.4261
SNW1_HUMAN	10457.042
DGKZ_HUMAN	4467.4287
STIM1_HUMAN	2924.908
GRAP_HUMAN	3911.9702
TRA2A_HUMAN	3928.5483
SNX1_HUMAN	12795.362
KRR1_HUMAN	4843.2109
PWP1_HUMAN	4800.7603
MTMR1_HUMAN	1460.2366
MTMR2_HUMAN	5420.6724
CUL1_HUMAN	5902.5068
CUL2_HUMAN	11852.603
CUL3_HUMAN	7786.6538
CUL4A_HUMAN	5037.875
CUL4B_HUMAN	14679.107
ASPP2_HUMAN	546.38702
DYR1A_HUMAN	650.07031
FCL_HUMAN	15914.527
RAB31_HUMAN	156.56825
RAB32_HUMAN	9424.5068
TPBG_HUMAN	1512.8843
FHL1_HUMAN	11184.595

FHL3_HUMAN	1939.6124
RIN1_HUMAN	538.00446
AAMP_HUMAN	2752.9468
MOGS_HUMAN	32102.896
CD166_HUMAN	5851.9878
THOC5_HUMAN	4285.1548
SHRM2_HUMAN	1423.0028
SPTN1_HUMAN	41153.902
NOG2_HUMAN	4239.7642
AUHM_HUMAN	794.8183
DX39B_HUMAN	12703.392
BLMH_HUMAN	26336.25
EXOS2_HUMAN	4333.5742
BMPR2_HUMAN	1737.5062
TBB2A_HUMAN	171318.23
TF2H2_HUMAN	1473.2533
TF2H3_HUMAN	1905.4097
BYST_HUMAN	9602.6992
RPGF1_HUMAN	2923.4143
IDI1_HUMAN	9006.1064
PEBB_HUMAN	7197.8047
NFYC_HUMAN	1548.6885
CDK13_HUMAN	1893.9941
CKAP5_HUMAN	28445.75
CIRBP_HUMAN	7618.8418
KCC1A_HUMAN	2297.7371
COTL1_HUMAN	31269.67
HNRPD_HUMAN	139775.47
SCRB2_HUMAN	30001.256
NID2_HUMAN	814.20728
LRP8_HUMAN	157.57707
DAG1_HUMAN	4427.0776
BOP1_HUMAN	5868.5894
UBE4A_HUMAN	3986.8606
SEPT6_HUMAN	4819.9521
KEAP1_HUMAN	1572.5948
URB2_HUMAN	1745.1884
DHX34_HUMAN	397.6022
MORC3_HUMAN	882.6936
SAFB2_HUMAN	11884.886
EIF3A_HUMAN	43713.547
ARHG7_HUMAN	7500.2881
EFR3A_HUMAN	5415.5811
UBP2L_HUMAN	20537.881
SCRIB_HUMAN	6200.8647
GIT2_HUMAN	4152.4233
SREC_HUMAN	3721.9556
MLEC_HUMAN	25207.408
TTL12_HUMAN	10191.83
DPOA2_HUMAN	1653.3784

DOCK1_HUMAN	5901.5781
TFDP1_HUMAN	2060.6777
FHL2_HUMAN	20206.303
DPYL3_HUMAN	52886.949
ICT1_HUMAN	5713.627
ZMYM3_HUMAN	597.25964
DCTN1_HUMAN	13919.828
DYHC1_HUMAN	74462.445
EI2BA_HUMAN	8632.8018
IF4A2_HUMAN	7395.7964
ELOA1_HUMAN	2020.9155
SRC8_HUMAN	21510.453
NUCG_HUMAN	2924.624
FLOT2_HUMAN	5316.9766
RCN2_HUMAN	14213.416
TRI25_HUMAN	19023.559
FLNC_HUMAN	48151.695
FKBP8_HUMAN	4743.0283
FA50A_HUMAN	5982.937
FRG1_HUMAN	2491.3323
GNA13_HUMAN	55504.191
GAMT_HUMAN	643.68256
GALE_HUMAN	9753.3721
LRC32_HUMAN	888.79675
CAPR1_HUMAN	23268.914
BECN1_HUMAN	1025.2391
RBM39_HUMAN	16132.391
CASL_HUMAN	383.43097
HLTF_HUMAN	2261.4956
ERG1_HUMAN	2903.731
PDIA5_HUMAN	12244.646
KPRA_HUMAN	8928.375
DHX8_HUMAN	2209.8965
MCM6_HUMAN	19328.213
ITPR2_HUMAN	5781.7949
ITPR3_HUMAN	6797.9385
ITPR1_HUMAN	694.46497
RASA3_HUMAN	3548.0024
PLSI_HUMAN	4433.4844
IRF3_HUMAN	3699.2263
LAGE3_HUMAN	674.22424
BLTP2_HUMAN	627.73236
TRIPC_HUMAN	6189.71
PUM1_HUMAN	4531.1646
MDC1_HUMAN	566.37775
EPN4_HUMAN	10857.326
KANK1_HUMAN	2764.1506
SMC1A_HUMAN	61663.93
RRP1B_HUMAN	5770.4683
DIP2A_HUMAN	6482.4497

RRP5_HUMAN	5540.4014
PSF1_HUMAN	2696.0337
BMS1_HUMAN	3740.4548
LPIN1_HUMAN	2519.7991
UBP10_HUMAN	10488.741
MESD_HUMAN	11325.143
GANAB_HUMAN	59881.773
RFTN1_HUMAN	4307.1543
MFS10_HUMAN	2485.9719
2A5D_HUMAN	12388.923
LBR_HUMAN	19270.215
COG2_HUMAN	723.68872
MVP_HUMAN	83510.977
LTBP1_HUMAN	1223.0631
LTBP2_HUMAN	1926.8607
CBX2_HUMAN	331.12454
GOGB1_HUMAN	3660.0256
CASP8_HUMAN	1881.8328
NAA30_HUMAN	3312.4724
KIF22_HUMAN	665.0835
MEF2D_HUMAN	1954.7062
CHD4_HUMAN	19161.58
LASP1_HUMAN	45212.137
PTGR1_HUMAN	8444.7305
NC2A_HUMAN	5666.0474
NFAC4_HUMAN	471.24731
ZN638_HUMAN	4505.8242
IMB1_HUMAN	53791.523
NOLC1_HUMAN	4460.7695
NUMA1_HUMAN	11626.227
PSME4_HUMAN	7154.3999
CUL7_HUMAN	2164.6565
SLMAP_HUMAN	1203.1946
GAPD1_HUMAN	11015.882
NAA25_HUMAN	9184.459
MELT_HUMAN	1483.0546
CND2_HUMAN	6458.6138
PAF15_HUMAN	627.65997
SPCS2_HUMAN	20871.193
EMC2_HUMAN	5488.5444
FL2D_HUMAN	1397.1699
PSMD6_HUMAN	16190.938
HERP1_HUMAN	150.26508
MD2BP_HUMAN	1445.74
MO4L2_HUMAN	3718.4709
ABRX2_HUMAN	2399.4648
SEPT2_HUMAN	40021.246
SART3_HUMAN	14957.604
CND1_HUMAN	12136.175
SUZ12_HUMAN	1061.2361

EXOS7_HUMAN	3713.1865
TNIP1_HUMAN	823.49982
U5S1_HUMAN	72637.648
SYLM_HUMAN	2844.6101
R3HD1_HUMAN	1538.5394
TRAM2_HUMAN	1479.7554
SNX17_HUMAN	3725.1011
AR6P1_HUMAN	3538.6282
RB3GP_HUMAN	3735.572
S39AE_HUMAN	2722.5713
SYK_HUMAN	36947.73
SETB1_HUMAN	1266.9045
LRC14_HUMAN	1214.502
RRS1_HUMAN	6922.6016
DPOD3_HUMAN	1886.4482
IF4H_HUMAN	34779.293
ACAP2_HUMAN	4359.0239
KIF14_HUMAN	507.6796
BRD3_HUMAN	1201.007
WDR43_HUMAN	3206.8479
ACOX1_HUMAN	6900.6714
OXA1L_HUMAN	7195.7627
EEA1_HUMAN	13348.125
PDIA6_HUMAN	124375.4
PA1B3_HUMAN	6089.3643
PDK3_HUMAN	650.69818
PEA15_HUMAN	2988.4238
EBP_HUMAN	11848.166
PMVK_HUMAN	7285.9312
KPCD1_HUMAN	677.85297
PLEC_HUMAN	66041.984
PCM1_HUMAN	2535.1392
NOMO1_HUMAN	26111.842
PON2_HUMAN	8153.8535
2A5A_HUMAN	3982.7649
IPYR_HUMAN	47108.191
TEBP_HUMAN	18808.277
STK38_HUMAN	2663.0808
NONO_HUMAN	66008.617
PTPA_HUMAN	22825.891
PTPRK_HUMAN	3397.7041
PWP2_HUMAN	4025.3699
RABE1_HUMAN	1701.5256
RAB35_HUMAN	6462.5591
RNPS1_HUMAN	7945.853
RBBP5_HUMAN	2976.1509
RCN1_HUMAN	23327.391
RBP1_HUMAN	456.40314
L2GL1_HUMAN	2302.8152
LRC41_HUMAN	795.82056

TTF1_HUMAN	6551.9805
TMED2_HUMAN	6300.4829
PCBP1_HUMAN	162788.38
PCBP2_HUMAN	150176.36
ELOC_HUMAN	5602.2417
ELOB_HUMAN	3386.0251
RHEB_HUMAN	11900.052
UBE3C_HUMAN	5522.8228
TOM20_HUMAN	8978.1797
MTFR1_HUMAN	2660.4226
DHC24_HUMAN	12986.087
SF3B3_HUMAN	29065.295
PUM3_HUMAN	4101.8818
DLGP5_HUMAN	3645.9067
RSU1_HUMAN	47237.355
CNN3_HUMAN	24592.686
KS6A1_HUMAN	1102.0645
SAFB1_HUMAN	9767.3379
SF3A2_HUMAN	5110.793
RBMS2_HUMAN	7128.2744
PP1R7_HUMAN	15952.393
SC23A_HUMAN	21159.428
SC23B_HUMAN	4128.689
CYH1_HUMAN	5175.3569
SF3A1_HUMAN	15020.902
SKI2_HUMAN	8783.5811
SURF1_HUMAN	1858.1205
TAF5_HUMAN	336.12085
TAF7_HUMAN	1596.6174
TERF2_HUMAN	1628.5896
MARE2_HUMAN	14179.578
NHRF2_HUMAN	4174.8364
TRADD_HUMAN	2409.3787
TRAM1_HUMAN	10102.525
TSN_HUMAN	10638.326
TRBP2_HUMAN	1395.6355
SF01_HUMAN	17735.982
CIP4_HUMAN	6653.4434
TRIPB_HUMAN	2884.1833
PCH2_HUMAN	9145.9004
MED1_HUMAN	1519.1455
TRIP4_HUMAN	3744.6584
HMGN3_HUMAN	214.55618
JHD2C_HUMAN	335.09512
IKBB_HUMAN	2379.3342
TRIP6_HUMAN	4744.8247
PTN14_HUMAN	2597.4993
MARE1_HUMAN	54921.344
ELAV1_HUMAN	49898.539
NSDHL_HUMAN	16528.754

MYLK_HUMAN	2962.54
TAB1_HUMAN	3262.3706
HERC1_HUMAN	2157.9309
AAAT_HUMAN	10092.325
MLF2_HUMAN	1551.176
TOM34_HUMAN	8467.0557
SMAD2_HUMAN	4234.2168
SMAD1_HUMAN	17828.377
MSMO1_HUMAN	5640.124
ITSN1_HUMAN	2274.0056
TBCE_HUMAN	4509.2725
TBCC_HUMAN	5249.2422
UB2V2_HUMAN	2039.0786
STK11_HUMAN	441.94836
STXB2_HUMAN	5496.5874
VAMP3_HUMAN	3359.4365
NEDD8_HUMAN	4648.0024
VAS1_HUMAN	4694.4214
VPS72_HUMAN	1395.5776
RB11B_HUMAN	15201.065
ZYX_HUMAN	7266.3276
ETFD_HUMAN	1402.5703
SEPT7_HUMAN	30858.482
ADRM1_HUMAN	17127.771
CCDC6_HUMAN	5741.8154
UAP1_HUMAN	19894.301
E2F4_HUMAN	523.51526
IBP7_HUMAN	10483.008
PDCD2_HUMAN	739.78302
LAMA4_HUMAN	13876.938
PSMD5_HUMAN	26862.473
PKN1_HUMAN	8112.8164
PKN2_HUMAN	8037.833
TAF12_HUMAN	800.37402
CSRP2_HUMAN	1632.7697
DDB1_HUMAN	40176.547
2A5E_HUMAN	4684.6924
MK14_HUMAN	5494.9995
RM23_HUMAN	5383.1382
CDC37_HUMAN	34816.496
DPYL2_HUMAN	61953.313
SYPL1_HUMAN	12385.283
RBBP7_HUMAN	9273.3154
M3K11_HUMAN	1080.452
TAF9_HUMAN	2713.7542
CALRL_HUMAN	7468.2876
BAK_HUMAN	744.51733
SRSF7_HUMAN	43706.059
CPSF6_HUMAN	16566.641
SMN_HUMAN	2314.4346

DREB_HUMAN	16259.781
NRF1_HUMAN	1769.4933
FSCN1_HUMAN	137215.17
IF16_HUMAN	30725.531
DECR_HUMAN	26809.715
MA2A1_HUMAN	7167.8188
NDUA5_HUMAN	2105.8191
CEGT_HUMAN	412.38721
CLPP_HUMAN	4132.2563
THTR_HUMAN	14893.616
UBE2S_HUMAN	919.16809
KAT1_HUMAN	466.55469
KGUA_HUMAN	3677.3662
GLO2_HUMAN	8280.5615
NDUA9_HUMAN	12179.839
MAON_HUMAN	2166.2808
PCKGM_HUMAN	3806.625
UPP1_HUMAN	1623.6279
DDR2_HUMAN	2582.7207
HCDH_HUMAN	12589.1
CP51A_HUMAN	17521.711
UGPA_HUMAN	26907.063
F263_HUMAN	1599.5875
TRXR1_HUMAN	40881.555
MIC60_HUMAN	17820.006
ECSCR_HUMAN	3589.3269
KNOP1_HUMAN	1514.1333
HNRL2_HUMAN	17202.547
T132A_HUMAN	387.83563
INF2_HUMAN	6708.2627
PDS5A_HUMAN	7368.5269
CL16A_HUMAN	1592.037
KIF7_HUMAN	525.09174
RHG31_HUMAN	3314.5962
MTHSD_HUMAN	3547.5173
AAK1_HUMAN	4838.5845
WASC4_HUMAN	4930.0327
ERC6L_HUMAN	2004.4503
TSR1_HUMAN	7724.8599
RGPA2_HUMAN	925.86157
D19L1_HUMAN	4968.9111
IAH1_HUMAN	1956.2349
ALG11_HUMAN	2589.6387
QRIC1_HUMAN	4128.083
SMU1_HUMAN	14694.017
CTU2_HUMAN	10306.432
PGGHG_HUMAN	1081.3091
LRRF1_HUMAN	5261.8066
PGPS1_HUMAN	775.47034
CR021_HUMAN	154.93867

P3H1_HUMAN	19846.277
TRM5_HUMAN	3759.7097
EMAL3_HUMAN	4123.1382
RPA43_HUMAN	2363.1563
MA7D1_HUMAN	3703.2068
UAP1L_HUMAN	13173.395
S2535_HUMAN	174.32523
CHD9_HUMAN	4334.8652
TKFC_HUMAN	3444.6877
LSM12_HUMAN	5906.1841
HSDL1_HUMAN	3394.0056
AL1L2_HUMAN	1010.7501
GNPTA_HUMAN	551.42603
GRDN_HUMAN	1876.9983
RABL6_HUMAN	6877.7632
TIM50_HUMAN	6555.8511
PAR14_HUMAN	3465.1433
FUT11_HUMAN	948.63312
GLYR1_HUMAN	7584.1758
CE022_HUMAN	2437.0024
VP26B_HUMAN	3535.0381
LARP7_HUMAN	3292.0889
NAKD2_HUMAN	488.58917
KCD21_HUMAN	1874.4806
GXLT1_HUMAN	438.39331
ACSF3_HUMAN	1387.1583
PPCEL_HUMAN	1749.3936
TB10B_HUMAN	3678.3296
TPRN_HUMAN	242.02618
ANO6_HUMAN	3601.979
FIL1L_HUMAN	1867.5061
GRAP1_HUMAN	3146.9863
ZNG1F_HUMAN	1925.7505
MIX23_HUMAN	3523.0667
MBRL_HUMAN	1594.4642
FA98B_HUMAN	6955.5459
RHG29_HUMAN	7050.5635
PDCD4_HUMAN	8421.3525
FND3B_HUMAN	5982.7588
CRTC2_HUMAN	549.42365
GPAT3_HUMAN	470.20453
CEP55_HUMAN	3198.7151
NCBP3_HUMAN	2649.1333
QORX_HUMAN	20219.797
NACHO_HUMAN	2905.5957
PHLA2_HUMAN	673.2464
PDLI3_HUMAN	435.43668
PAR10_HUMAN	501.74094
DHB12_HUMAN	23082.498
GLE1_HUMAN	3107.05

UBP39_HUMAN	6720.0254
KLH22_HUMAN	1134.3196
AGK_HUMAN	11463.659
SETMR_HUMAN	204.38504
LACB2_HUMAN	1989.5619
P5CR3_HUMAN	3364.7942
EIPR1_HUMAN	4814.3022
BOREA_HUMAN	456.00137
COBL1_HUMAN	1136.9194
H1BP3_HUMAN	3840.6484
CYBR1_HUMAN	1351.9832
WDR81_HUMAN	3882.1233
ACTBL_HUMAN	237318.31
CCD93_HUMAN	3813.7617
OCAD2_HUMAN	10680.408
PKHG4_HUMAN	487.50143
HS904_HUMAN	957.32971
DCAF6_HUMAN	239.99196
TM41B_HUMAN	1058.7533
SGMR2_HUMAN	10665.965
YIF1B_HUMAN	3354.5928
ZN326_HUMAN	3912.4153
NOM1_HUMAN	1408.4132
RIPL1_HUMAN	2133.2844
DJC21_HUMAN	1425.0627
TP8L3_HUMAN	3784.0781
HERC4_HUMAN	6898.8232
PIGG_HUMAN	1254.1689
PP6R3_HUMAN	6265.3452
DDX6L_HUMAN	238.86526
RABL3_HUMAN	24957.598
FA76B_HUMAN	384.03958
COQ5_HUMAN	608.68164
SH319_HUMAN	3661.2791
NHSL2_HUMAN	2297.3909
EMC4_HUMAN	4137.1079
SYEM_HUMAN	1755.5854
CC038_HUMAN	788.03113
EFMT2_HUMAN	597.21655
TGO1_HUMAN	2287.0894
PREP_HUMAN	12160.955
WDR44_HUMAN	3072.6982
PRC2B_HUMAN	4059.0334
TJAP1_HUMAN	1039.8866
RRP12_HUMAN	9085.7666
COA6_HUMAN	308.0307
TOIP1_HUMAN	16119.695
CEP78_HUMAN	563.67169
SYAM_HUMAN	4340.687
TOR2A_HUMAN	2404.8479

PCID2_HUMAN	3531.7798
HABP4_HUMAN	2179.3694
S27A3_HUMAN	1397.213
SAMD9_HUMAN	1811.1112
P4R3B_HUMAN	2090.5115
WIPI3_HUMAN	1993.7386
WIPI1_HUMAN	928.18188
EOGT_HUMAN	11045.263
SMAG2_HUMAN	383.49112
TBCEL_HUMAN	2986.6487
TDIF2_HUMAN	1543.373
RBG1L_HUMAN	1432.2815
TTC38_HUMAN	1421.2953
COX20_HUMAN	4014.6223
EXOS6_HUMAN	5364.4399
TM201_HUMAN	2978.2327
ATAT_HUMAN	411.84537
SNP47_HUMAN	925.4425
NU188_HUMAN	6118.6372
HP1B3_HUMAN	37821.406
SYVM_HUMAN	869.14172
CE170_HUMAN	6880.9229
ARH_HUMAN	1326.3352
ODR4_HUMAN	2476.8037
NOL9_HUMAN	3172.3218
NHSL1_HUMAN	1489.897
HYI_HUMAN	808.31079
TPRGL_HUMAN	805.1131
C2D1B_HUMAN	1760.2711
FBP1L_HUMAN	12609.313
SYRM_HUMAN	760.90894
THEM4_HUMAN	1460.6957
CHCH9_HUMAN	1382.093
FKB15_HUMAN	11181.16
ZC3HD_HUMAN	2344.6348
CI114_HUMAN	1531.995
ARMD3_HUMAN	758.69714
CSCL2_HUMAN	1211.8843
GPTC4_HUMAN	374.02921
CAF17_HUMAN	1472.8008
HECD3_HUMAN	4307.7285
GT253_HUMAN	10558.213
UBR4_HUMAN	10745.628
STXB5_HUMAN	1177.7274
RHG21_HUMAN	1235.23
CAMP1_HUMAN	1208.0985
RM02_HUMAN	3670.6953
UBAP2_HUMAN	4802.1167
QNG1_HUMAN	2264.3376
KPRP_HUMAN	856.73633

ZN618_HUMAN	2370.0828
ACBD5_HUMAN	8741.8848
RBM26_HUMAN	3650.0464
ATD3B_HUMAN	1953.8005
WLS_HUMAN	5792.7686
INT11_HUMAN	3684.8301
CPTP_HUMAN	635.9007
DCAF8_HUMAN	4058.5586
CODA1_HUMAN	308.13214
TUT4_HUMAN	15915.613
FRY_HUMAN	1647.5162
RNH2B_HUMAN	1329.1775
ATPF1_HUMAN	1460.5806
RC3H1_HUMAN	680.8277
DDI2_HUMAN	4036.0793
THMS2_HUMAN	362.21182
NDUF5_HUMAN	1165.1338
NT5D1_HUMAN	3175.822
BIG3_HUMAN	1135.613
VP13D_HUMAN	567.47748
CROCC_HUMAN	1005.1442
LYRM7_HUMAN	1488.6066
RAIN_HUMAN	23096.678
RIF1_HUMAN	2322.2729
VPS53_HUMAN	4152.8901
STRP1_HUMAN	4552.4795
MRCKA_HUMAN	7218.7983
RPRD2_HUMAN	1109.4265
RN220_HUMAN	389.68707
PR38B_HUMAN	4508.5557
BRE1A_HUMAN	4190.0308
MYOME_HUMAN	829.26715
ZN318_HUMAN	989.40662
F1711_HUMAN	1318.407
DIK1B_HUMAN	2978.2148
LRCH2_HUMAN	1579.2517
CDKAL_HUMAN	6424.8867
BROX_HUMAN	5434.1089
FOCAD_HUMAN	7462.145
RSBN1_HUMAN	248.174
DAB2P_HUMAN	647.93115
LYPL1_HUMAN	7494.7046
PEAR1_HUMAN	1177.1116
ECM29_HUMAN	15052.533
TUT7_HUMAN	925.60291
SHE_HUMAN	598.20636
DEN4C_HUMAN	3121.5103
NAA35_HUMAN	4696.9316
ZMYM4_HUMAN	2135.8843
FHI2A_HUMAN	4801.8989

ZDH20_HUMAN	2438.3208
SPRY7_HUMAN	1785.8959
FBXW9_HUMAN	154.90422
CD276_HUMAN	2825.7078
RN213_HUMAN	6041.978
TENS2_HUMAN	1985.9739
KANK2_HUMAN	702.27051
WAC2A_HUMAN	2106.2402
LPCT4_HUMAN	1534.6356
STEA3_HUMAN	1568.8038
F91A1_HUMAN	4056.6057
TBC9B_HUMAN	6866.3535
MAP1S_HUMAN	9299.6367
2ABD_HUMAN	3254.4985
AR6P4_HUMAN	4621.3457
A16L1_HUMAN	2397.895
HGNAT_HUMAN	2234.291
ARID2_HUMAN	281.04901
UTP25_HUMAN	1632.0413
GL8D1_HUMAN	1365.8674
SE1L3_HUMAN	840.20105
TENS3_HUMAN	3549.0601
HAUS3_HUMAN	838.99316
SPT2_HUMAN	627.00238
MBLC2_HUMAN	1743.0948
CRBG3_HUMAN	800.11877
INT3_HUMAN	4722.2109
RHG17_HUMAN	18264.719
C19L1_HUMAN	10111.567
VIR_HUMAN	3805.4153
CYTSA_HUMAN	4846.6011
HEAT6_HUMAN	948.93689
ANR40_HUMAN	491.97592
WWC2_HUMAN	1344.6022
SCAR3_HUMAN	1117.014
ATLA3_HUMAN	19586.059
RL7L_HUMAN	12118.142
NXN_HUMAN	2212.5779
TTC19_HUMAN	939.9353
IQEC1_HUMAN	3399.0547
CPIN1_HUMAN	10434.24
ZFAN6_HUMAN	1765.0164
SMYD5_HUMAN	2511.9414
OTU7B_HUMAN	704.1488
THOC7_HUMAN	1822.6058
NADE_HUMAN	2849.8835
ELP2_HUMAN	2798.0059
LTOR1_HUMAN	2625.5212
DRS7B_HUMAN	1994.0212
TWF2_HUMAN	15548.162

CNDH2_HUMAN	385.90149
K0930_HUMAN	897.66705
TNG2_HUMAN	9687.3789
P4R3A_HUMAN	3675.1743
RAB12_HUMAN	22578.348
DEN5A_HUMAN	172.79906
SDE2_HUMAN	261.66281
K2C80_HUMAN	964.89386
NIPBL_HUMAN	717.14148
SCMC2_HUMAN	2369.7761
PDE12_HUMAN	7025.2207
NAA16_HUMAN	1798.5396
DE10B_HUMAN	4006.6855
RHBD2_HUMAN	1736.3187
SCMC1_HUMAN	28050.225
REEP3_HUMAN	1095.9215
RETST_HUMAN	2813.0356
RINT1_HUMAN	1467.2733
TM214_HUMAN	10580.857
HIBCH_HUMAN	7929.208
RWDD4_HUMAN	2726.1133
NEPRO_HUMAN	1403.0756
ARMC6_HUMAN	4285.2729
TTI2_HUMAN	1870.6433
ANR54_HUMAN	1987.0107
ZNT6_HUMAN	1109.2881
KANK3_HUMAN	2244.8596
JMJD6_HUMAN	5209.6499
PPR18_HUMAN	8631.9326
CAVN1_HUMAN	106997.59
ZCHC8_HUMAN	3410.688
DHX57_HUMAN	3507.8674
ERAP2_HUMAN	13563.564
MBOA5_HUMAN	3071.9106
CDC73_HUMAN	6727.7505
RM14_HUMAN	4216.668
S27A4_HUMAN	6624.5986
C2D1A_HUMAN	4321.3037
TATD1_HUMAN	3937.5156
MET2B_HUMAN	1741.2261
MSD2_HUMAN	952.04968
TAF2_HUMAN	1577.0303
CH082_HUMAN	1236.4932
MED27_HUMAN	1077.7655
EDC4_HUMAN	18168.826
ANM9_HUMAN	547.34241
PRP8_HUMAN	14954.301
FBX42_HUMAN	878.14294
SCYL2_HUMAN	3619.6025
TTC27_HUMAN	4438.3252

SFXN4_HUMAN	1174.7388
GOLM2_HUMAN	3303.1257
NFRKB_HUMAN	702.30298
FAHD1_HUMAN	11133.189
RL22L_HUMAN	10828.112
PKN3_HUMAN	1288.2209
ALKB5_HUMAN	1233.9727
PDXD1_HUMAN	7157.585
MEAK7_HUMAN	3558.0298
INT5_HUMAN	2793.0037
GIMA6_HUMAN	886.30231
S27A1_HUMAN	1174.0918
PGM2L_HUMAN	9681.2891
CTR9_HUMAN	4977.6377
AAGAB_HUMAN	2142.1243
TMM88_HUMAN	627.8197
PSRC1_HUMAN	336.11963
SKI3_HUMAN	4960.9297
ULK3_HUMAN	1392.1703
SYDM_HUMAN	9045.6523
TMM65_HUMAN	3958.1091
IN80C_HUMAN	2037.3066
FBX38_HUMAN	1743.4811
NCEH1_HUMAN	9312.457
FIGL1_HUMAN	545.10028
TRI65_HUMAN	1552.1343
RHDF2_HUMAN	609.51105
MDEAS_HUMAN	1861.2144
BRAT1_HUMAN	2958.3511
WDR59_HUMAN	353.66074
ZC3HE_HUMAN	2390.6011
CC137_HUMAN	1607.9149
TXD11_HUMAN	297.01233
LARP1_HUMAN	16823.934
ATAD2_HUMAN	298.31958
TMED8_HUMAN	2399.0444
ZNT9_HUMAN	691.68829
TRAF7_HUMAN	1320.5828
RICTR_HUMAN	3711.9541
WDR74_HUMAN	1330.9207
ATRAP_HUMAN	2936.5747
SMRD3_HUMAN	2192.2734
C1TM_HUMAN	21468.643
AFTIN_HUMAN	648.18549
FIP1_HUMAN	5831.1348
CRTC3_HUMAN	637.7262
CRTC1_HUMAN	404.33804
CP20A_HUMAN	3845.3716
PLGT2_HUMAN	3672.9429
TM205_HUMAN	7586.6367

LRSM1_HUMAN	2624.5215
GAK1B_HUMAN	3337.8149
LCLT1_HUMAN	2523.7312
ABRX1_HUMAN	166.84572
CWC27_HUMAN	1966.8538
CREL2_HUMAN	1882.3917
WDR82_HUMAN	3683.6033
MIC27_HUMAN	1872.3385
FAT4_HUMAN	1123.6759
DPP8_HUMAN	1037.5923
MCAF1_HUMAN	2386.0776
RBP10_HUMAN	1903.1287
PACS1_HUMAN	7149.0703
MPRIP_HUMAN	6754.9937
RBMS3_HUMAN	2833.9778
PNCB_HUMAN	5404.6362
DNMBP_HUMAN	5096.5625
HACD2_HUMAN	1342.2112
B3GLT_HUMAN	1688.8821
GGYF2_HUMAN	8536.5898
CD109_HUMAN	13775.657
THADA_HUMAN	1918.0876
HSDL2_HUMAN	4852.0894
KAT3_HUMAN	7001.7925
PPR21_HUMAN	1338.2601
THSD4_HUMAN	1058.3854
SYNE3_HUMAN	1548.3041
ZN574_HUMAN	466.04333
NFXL1_HUMAN	384.12119
NBEL2_HUMAN	3331.6987
FGD5_HUMAN	4789.0903
QSOX2_HUMAN	3082.6636
SRCAP_HUMAN	642.87158
RIPR1_HUMAN	4584.5142
YJ005_HUMAN	7074.3442
ARHGI_HUMAN	1325.5443
TBCD9_HUMAN	950.83716
UBR3_HUMAN	708.07428
TMPPE_HUMAN	463.95761
CRACD_HUMAN	618.57513
CCD9B_HUMAN	521.45544
NUD18_HUMAN	494.93826
SYDE1_HUMAN	178.22417
PAXI1_HUMAN	693.58136
TMTC3_HUMAN	6406.8916
VP13C_HUMAN	11179.928
UBP34_HUMAN	804.17511
RAPH1_HUMAN	2666.5359
MOB2_HUMAN	5587.1577
IKIP_HUMAN	25865.885

PREX2_HUMAN	927.45319
F10C1_HUMAN	504.79135
UB2R2_HUMAN	6268.6206
MD13L_HUMAN	448.31967
LARP4_HUMAN	8511.7607
MED25_HUMAN	1883.3082
RS27L_HUMAN	61475.418
HAKAI_HUMAN	1472.0841
INT8_HUMAN	3916.0808
BT2A1_HUMAN	383.15805
SPT6H_HUMAN	6005.9473
SND1_HUMAN	80190.563
MARK2_HUMAN	1909.0769
COX15_HUMAN	1349.1035
DDX46_HUMAN	13188.601
RUFY3_HUMAN	737.45343
TM10C_HUMAN	8816.1553
5MP2_HUMAN	26319.088
MON1B_HUMAN	1778.061
LRC8D_HUMAN	506.63138
ASGL1_HUMAN	1361.0276
KCTD9_HUMAN	3413.761
DHX30_HUMAN	9365.5527
EIF3M_HUMAN	16448.26
MEPCE_HUMAN	3333.6802
ARMX2_HUMAN	1540.5972
SYPM_HUMAN	450.64554
RSRC2_HUMAN	2022.6938
RRAGA_HUMAN	5256.9448
CYFP1_HUMAN	26289.582
NDUF7_HUMAN	2730.5928
GET4_HUMAN	10853.298
CSN6_HUMAN	20031.848
PCAT2_HUMAN	15974.952
ENOF1_HUMAN	2222.8923
MAEA_HUMAN	1769.2363
EPMIP_HUMAN	1388.0168
DHX32_HUMAN	493.70016
TAOK1_HUMAN	3103.6399
FAKD5_HUMAN	2598.3975
MOB1B_HUMAN	14807.038
KDM3B_HUMAN	4074.2356
CHM1B_HUMAN	5079.5117
RIR2B_HUMAN	3770.646
HS2ST_HUMAN	4676.7749
STRAA_HUMAN	1537.865
MICA3_HUMAN	1595.5503
DYM_HUMAN	1642.8984
PHF5A_HUMAN	2771.2378
PXL2C_HUMAN	3040.3596

APTX_HUMAN	2329.4155
ERMP1_HUMAN	4954.4023
GRIN1_HUMAN	257.39328
TRM1L_HUMAN	2708.8865
ZCCHV_HUMAN	9980.6533
RM21_HUMAN	4846.5264
EFL1_HUMAN	4045.134
SETX_HUMAN	473.58704
TPC11_HUMAN	2335.7556
KANL1_HUMAN	349.31387
NUP54_HUMAN	8975.9248
ATG9A_HUMAN	1452.7214
GLUCM_HUMAN	4855.8735
CC186_HUMAN	690.60315
ARMC9_HUMAN	2146.1792
VP35L_HUMAN	5562.9224
POGZ_HUMAN	3664.1096
ZFY16_HUMAN	985.07935
MON2_HUMAN	3431.6064
MYCPP_HUMAN	1021.2811
MYH14_HUMAN	4462.8296
NUFP2_HUMAN	5500.8467
SZRD1_HUMAN	1779.9615
MAVS_HUMAN	1650.0199
CLAP1_HUMAN	6806.1416
DHX29_HUMAN	4266.437
NPHP3_HUMAN	213.89525
LRP10_HUMAN	2033.5431
HAUS6_HUMAN	1677.7079
PLGT3_HUMAN	6562.3252
TT21B_HUMAN	962.14539
HEAT3_HUMAN	6877.8135
KI21A_HUMAN	551.92529
HDGR2_HUMAN	10756.521
DCXR_HUMAN	6567.9609
BRAP_HUMAN	2228.9453
EMSY_HUMAN	818.7077
GOGA7_HUMAN	2286.5969
RHG22_HUMAN	725.88226
WAPL_HUMAN	1732.8241
I2BP2_HUMAN	1814.2461
RBBP6_HUMAN	2250.5508
SH3R1_HUMAN	816.94543
GRASP_HUMAN	1539.5651
FRMD5_HUMAN	690.99298
SEN54_HUMAN	451.43573
PTAR1_HUMAN	889.7196
ARPIN_HUMAN	3201.6792
TCPR1_HUMAN	733.87024
RABEK_HUMAN	1087.8573

MTEF4_HUMAN	493.19235
HUWE1_HUMAN	13209.301
YTHD3_HUMAN	10127.189
CTU1_HUMAN	3208.7827
TAF8_HUMAN	254.60356
UB2Q1_HUMAN	6315.8174
RM55_HUMAN	10970.907
TMED4_HUMAN	4449.8267
RM10_HUMAN	1178.1577
COXM1_HUMAN	5265.1763
CENPV_HUMAN	2228.4729
SLN11_HUMAN	4782.2217
MEGF8_HUMAN	425.27026
T179B_HUMAN	958.88385
GALT7_HUMAN	6495.2266
SCD5_HUMAN	2600.0784
PHLB2_HUMAN	3190.5759
AGRG6_HUMAN	2842.6797
DHDDS_HUMAN	1141.7753
GLT10_HUMAN	418.28656
TPC6B_HUMAN	1275.8247
PP4P1_HUMAN	2055.5713
CLC14_HUMAN	13345.506
PATL1_HUMAN	1190.094
DPP9_HUMAN	6768.1484
SYVN1_HUMAN	1742.0548
TRPT1_HUMAN	1079.9037
SETD3_HUMAN	6193.0337
TTC7B_HUMAN	1285.3173
ACOT1_HUMAN	4422.8682
NOP9_HUMAN	2665.1841
PABP2_HUMAN	20944.348
MTA70_HUMAN	2009.0383
PB1_HUMAN	3704.917
YRDC_HUMAN	1865.8165
PRP39_HUMAN	1871.2961
LYRIC_HUMAN	15826.954
ZN598_HUMAN	4488.2539
GPAT4_HUMAN	1619.7145
KTN1_HUMAN	7499.1538
EST1A_HUMAN	1533.4701
NLRX1_HUMAN	819.32721
BCL9L_HUMAN	568.70483
PHLB1_HUMAN	8669.5977
UBP48_HUMAN	6580.0933
CAPS2_HUMAN	411.36942
URP2_HUMAN	22877.416
NAA40_HUMAN	3980.1069
AACS_HUMAN	7583.4736
LUZP1_HUMAN	6616.8516

THOC4_HUMAN	31884.186
GP180_HUMAN	1869.8938
FHI2B_HUMAN	2884.0344
IQGA3_HUMAN	3540.4849
ZCH18_HUMAN	2002.6754
VPS36_HUMAN	1883.7627
CAND1_HUMAN	36137.254
RETR3_HUMAN	3453.2068
HOOK3_HUMAN	6745.5854
SESD1_HUMAN	850.19269
COMD7_HUMAN	1416.8098
MON1A_HUMAN	1001.4216
AMZ2_HUMAN	1068.748
THOC6_HUMAN	2770.7825
MET16_HUMAN	1210.1208
PARG_HUMAN	378.79532
LIPB1_HUMAN	9109.4355
LONP2_HUMAN	1562.7622
ZC3C1_HUMAN	3462.5752
CHD1L_HUMAN	7815.4111
FCSD1_HUMAN	255.54085
CCD25_HUMAN	7386.0757
PRSR2_HUMAN	1344.7623
STING_HUMAN	7575.5342
AROS_HUMAN	638.98364
CDR2L_HUMAN	124.03345
RLGPB_HUMAN	1213.7605
RGPS2_HUMAN	501.65939
CARM1_HUMAN	7961.666
NIT1_HUMAN	5059.3076
COMD2_HUMAN	3704.8843
HTR5A_HUMAN	1256.9547
CNDG2_HUMAN	850.45404
PPR3B_HUMAN	132.61484
FBX11_HUMAN	227.08487
ANKL2_HUMAN	2076.5662
MEX3D_HUMAN	1739.092
DDX42_HUMAN	15816.787
SPAS2_HUMAN	3286.9907
VRK2_HUMAN	2944.5879
CACL1_HUMAN	299.4404
NDUAB_HUMAN	7181.5186
DAAF5_HUMAN	8595.8262
PTH_HUMAN	1057.4944
STX12_HUMAN	11916.393
P66A_HUMAN	3695.7178
CPNE8_HUMAN	20946.256
GPSM1_HUMAN	744.84027
RAB43_HUMAN	1784.4933
C2CD5_HUMAN	1653.5276

MIB1_HUMAN	540.56757
PRAG1_HUMAN	1439.9983
HPS6_HUMAN	1959.8444
I2BP1_HUMAN	1496.9615
RB6I2_HUMAN	4693.9219
RIOX2_HUMAN	2309.6689
ZDH13_HUMAN	8101.8374
ZDH17_HUMAN	670.27216
CRLF3_HUMAN	1509.0155
TPPC5_HUMAN	5156.0649
ARMC8_HUMAN	2648.8262
RELL1_HUMAN	1076.4126
T126B_HUMAN	987.59552
PLD3_HUMAN	16956.41
HID1_HUMAN	1519.2
ANKY2_HUMAN	1024.925
ERI1_HUMAN	1079.6394
VRK3_HUMAN	583.43103
LIX1L_HUMAN	1954.9253
NUDC3_HUMAN	5237.9727
AHNK2_HUMAN	21748.051
FMNL3_HUMAN	6923.8628
MMAA_HUMAN	1441.6261
M4K3_HUMAN	1207.6937
NAV3_HUMAN	1882.8818
P3H3_HUMAN	11017.047
CCD50_HUMAN	10246.664
FABD_HUMAN	1975.3998
KSR1_HUMAN	1475.1031
CEP97_HUMAN	1116.941
NNRD_HUMAN	1630.3456
WDR75_HUMAN	2053.3511
MFN1_HUMAN	2499.4229
CTL2_HUMAN	4576.5547
IPRI_HUMAN	1808.1628
WDFY1_HUMAN	4555.521
MA7D3_HUMAN	2683.4485
CC117_HUMAN	410.50562
NXP20_HUMAN	7018.2578
DCNL3_HUMAN	561.75385
DEN6A_HUMAN	795.47614
MGAP_HUMAN	629.42932
GCC2_HUMAN	2962.1396
Z3H7A_HUMAN	1787.2675
PHF6_HUMAN	7658.1538
ARCH_HUMAN	526.3761
LRC8A_HUMAN	5617.4761
SULF2_HUMAN	3828.093
UBR1_HUMAN	1729.4775
UBR2_HUMAN	1431.9486

RHG12_HUMAN	1131.4288
CHERP_HUMAN	10169.851
CDAN1_HUMAN	733.4303
ANKH1_HUMAN	2751.2314
SUGP1_HUMAN	2077.4944
SUGP2_HUMAN	25226.74
UEVLD_HUMAN	1629.0416
CCAR1_HUMAN	10004.426
DHX40_HUMAN	902.98834
DJC10_HUMAN	10490.26
NELFD_HUMAN	5750.041
MIRO2_HUMAN	4510.3818
MIRO1_HUMAN	4927.9126
SIR2_HUMAN	3117.2649
PHC2_HUMAN	1569.2172
BAP18_HUMAN	5443.6313
RM41_HUMAN	9773.4766
NRM_HUMAN	6201.1655
GPAM1_HUMAN	1180.0853
PARP9_HUMAN	1056.7992
RB12B_HUMAN	3750.5278
S35F2_HUMAN	1522.2012
PLPL6_HUMAN	6655.3335
SMC5_HUMAN	1172.4242
DDX60_HUMAN	623.2337
CMIP_HUMAN	558.85693
MILK2_HUMAN	441.67075
DHX37_HUMAN	2507.5083
KBTB2_HUMAN	3259.3411
RAVR1_HUMAN	5042.2524
SPB1_HUMAN	7273.0659
TM192_HUMAN	1145.2274
CKP2L_HUMAN	301.93143
M21D2_HUMAN	1859.9911
SRRM1_HUMAN	5469.2568
SMAP1_HUMAN	3616.4622
DI3L2_HUMAN	3883.2756
SUV3_HUMAN	4705.1514
ERF3B_HUMAN	7354.3555
ZZZ3_HUMAN	478.32367
EXOC8_HUMAN	8116.4917
CJ067_HUMAN	11854.897
TRI22_HUMAN	5590.8359
THNS1_HUMAN	477.48929
K2013_HUMAN	3558.0703
MICU2_HUMAN	1073.5989
AN13A_HUMAN	3003.4282
PHAR4_HUMAN	1962.8619
TRM2A_HUMAN	2956.728
RUSD2_HUMAN	943.72095

ELMD2_HUMAN	2257.2144
A16A1_HUMAN	6022.4863
K319L_HUMAN	1455.6606
AGRF5_HUMAN	1909.3977
XRN1_HUMAN	4605.3477
PELP1_HUMAN	13543.129
ABI1_HUMAN	5817.4995
WDFY3_HUMAN	3901.0757
SELH_HUMAN	7088.4336
VKORL_HUMAN	2225.967
SPART_HUMAN	8944.5098
TTC5_HUMAN	1306.5276
MIER1_HUMAN	1098.0419
RPTOR_HUMAN	1795.5001
ORML3_HUMAN	4762.8696
CCAR2_HUMAN	50937.719
NDUF2_HUMAN	7796.998
VPS52_HUMAN	5547.0811
NUP93_HUMAN	23313.533
S11IP_HUMAN	2542.5989
ZN687_HUMAN	610.80249
CMTR1_HUMAN	3918.7947
LRC47_HUMAN	15109.565
DOCK4_HUMAN	7447.6211
CAH13_HUMAN	1413.8816
ARG28_HUMAN	9065.3037
INT1_HUMAN	34471.418
H2B3B_HUMAN	42983.816
RHG24_HUMAN	2863.2634
ARM10_HUMAN	2909.0627
ABD12_HUMAN	2985.6736
CLASR_HUMAN	403.6571
FA43A_HUMAN	2421.6091
TM256_HUMAN	3001.6318
PIAS4_HUMAN	674.37354
GPD1L_HUMAN	4632.9624
S35F6_HUMAN	2513.9343
RHG18_HUMAN	21011.84
ASCC3_HUMAN	9113.6787
MILK1_HUMAN	2000.8848
VPS8_HUMAN	3046.4766
PALS1_HUMAN	933.24921
STAG2_HUMAN	3857.1365
SYNPO_HUMAN	9381.0977
FNBP4_HUMAN	1528.8854
GUF1_HUMAN	351.62717
RYBP_HUMAN	668.69733
GALT4_HUMAN	3074.3682
MINK1_HUMAN	1305.5908
MESH1_HUMAN	1368.1038

PTGR3_HUMAN	2393.1758
CBR4_HUMAN	663.77704
EMC5_HUMAN	3465.3723
TM199_HUMAN	2766.7715
FBXL6_HUMAN	678.38995
OGFD1_HUMAN	1659.8433
AFAP1_HUMAN	4226.645
ZCHC9_HUMAN	762.82745
OXR1_HUMAN	1077.6191
ZGPAT_HUMAN	1228.6338
CAHM5_HUMAN	1768.9789
SRBD1_HUMAN	1156.3888
WDTC1_HUMAN	3557.2883
NKAP_HUMAN	927.81201
MACOI_HUMAN	551.83807
SH2D3_HUMAN	3526.8196
ARRD1_HUMAN	539.84515
DHRSX_HUMAN	1273.0297
CISD2_HUMAN	11157.429
RP25L_HUMAN	1976.2224
ATPF2_HUMAN	1134.9956
TTC9C_HUMAN	1982.9972
JAGN1_HUMAN	2738.2239
RNF10_HUMAN	824.52679
RFLB_HUMAN	2722.3193
LZTR1_HUMAN	253.86598
COMD1_HUMAN	3328.5505
CPSF7_HUMAN	14296.199
S15A4_HUMAN	660.44135
MYCT1_HUMAN	12314.742
ATL1_HUMAN	391.26794
ARFG2_HUMAN	6344.1943
OTU6B_HUMAN	2786.7307
FITM2_HUMAN	1875.1671
CA052_HUMAN	1053.3896
EFNMT_HUMAN	2421.8655
AR6P6_HUMAN	374.25177
ARFG1_HUMAN	2728.2581
SIR6_HUMAN	664.81372
S66A3_HUMAN	1567.3595
EMC1_HUMAN	12710.787
PAF1_HUMAN	3255.1423
CCYL1_HUMAN	1715.0353
UBR7_HUMAN	3652.1121
DDX51_HUMAN	2068.9563
F241A_HUMAN	6134.2422
S2529_HUMAN	2983.9146
ENAH_HUMAN	14217.62
DCBD1_HUMAN	1054.3633
GPT11_HUMAN	1299.8961

RM43_HUMAN	5857.5381
RLIG1_HUMAN	2859.4343
GDPD1_HUMAN	768.12518
CS047_HUMAN	991.58459
ASCC1_HUMAN	3395.2363
LRC57_HUMAN	7435.4131
EIF1A_HUMAN	765.97827
KRI1_HUMAN	1919.5348
CC71L_HUMAN	1679.2632
ZN579_HUMAN	1700.5277
PR38A_HUMAN	3278.1497
MLKL_HUMAN	5378.4995
GALD1_HUMAN	4028.1924
ANR52_HUMAN	682.10217
AT11C_HUMAN	1313.7708
AFG2A_HUMAN	4001.2793
NHLC2_HUMAN	7030.9043
AVL9_HUMAN	1839.3704
S43A3_HUMAN	5299.7466
XXLT1_HUMAN	7562.2051
GOLM1_HUMAN	889.45831
GT251_HUMAN	28048.938
SUMF2_HUMAN	4123.459
SIDT2_HUMAN	1170.1515
SUMF1_HUMAN	4323.9722
PGLT1_HUMAN	2757.0229
UBAC2_HUMAN	1877.8815
PCYXL_HUMAN	1796.4458
TM87A_HUMAN	2155.2832
RDH13_HUMAN	2699.7476
TTC13_HUMAN	1256.4333
DHB11_HUMAN	9572.1582
TXND5_HUMAN	249049.33
SPC24_HUMAN	1870.7751
ATAD1_HUMAN	2158.8435
SCPDL_HUMAN	18694.229
UXS1_HUMAN	107.40096
RN149_HUMAN	1834.5973
RETR2_HUMAN	996.12714
SERB1_HUMAN	18256.416
LEMD2_HUMAN	6645.4146
NOA1_HUMAN	1943.589
NECP1_HUMAN	3060.7239
FA98A_HUMAN	4319.7017
PAG15_HUMAN	1334.5801
MTMRE_HUMAN	1412.2764
NF2IP_HUMAN	3988.4607
DGLB_HUMAN	1371.6685
CHSTE_HUMAN	4343.103
GALT6_HUMAN	2134.0459

RN169_HUMAN	705.03351
PDPR_HUMAN	4273.5898
NNRE_HUMAN	3794.0498
SMG8_HUMAN	1238.0325
RN214_HUMAN	2612.8979
LS14A_HUMAN	4146.9746
GIMA8_HUMAN	12318.667
CCNY_HUMAN	4256.3071
MROH1_HUMAN	3897.0713
PAPD5_HUMAN	1494.0897
PEPL1_HUMAN	2652.6313
EHBP1_HUMAN	1160.0393
RB15B_HUMAN	2611.5002
SIA7C_HUMAN	1392.6838
TNR6A_HUMAN	1409.4258
PHC3_HUMAN	618.69385
DIK2A_HUMAN	3895.6814
CNNM3_HUMAN	754.96509
ABCF1_HUMAN	13168.924
MCU_HUMAN	1969.1115
PK3C3_HUMAN	6650.6284
GSTCD_HUMAN	342.0181
SRFB1_HUMAN	1835.1792
NGDN_HUMAN	1699.8826
SHCBP_HUMAN	1509.4508
PDZD8_HUMAN	576.33368
DP13B_HUMAN	2327.4229
ZNT7_HUMAN	8607.9395
NAV1_HUMAN	1780.2131
PPHLN_HUMAN	926.25317
WDR19_HUMAN	431.85422
FBX22_HUMAN	4821.1147
PCAT1_HUMAN	4627.9785
ZMIZ2_HUMAN	1093.7903
SYNE1_HUMAN	30177.068
UBP32_HUMAN	1005.9272
BD1L1_HUMAN	1256.7538
ARI1B_HUMAN	1134.1271
FAD1_HUMAN	4906.4102
FLCN_HUMAN	1753.5504
NUP43_HUMAN	5433.6201
NUP37_HUMAN	8372.2363
NUP35_HUMAN	3730.9294
ENASE_HUMAN	498.79309
RAI3_HUMAN	1007.2626
TOIP2_HUMAN	6991.6831
ABHDB_HUMAN	2983.1199
NEUA_HUMAN	7227.752
FBH1_HUMAN	683.75049
TSN14_HUMAN	3982.0493

TTL_HUMAN	454.12769
SVIP_HUMAN	1664.5842
ATLA2_HUMAN	1605.3357
MSPD2_HUMAN	1263.0518
PLBL2_HUMAN	12161.213
RASF8_HUMAN	724.43591
DDX55_HUMAN	612.20551
MFSD8_HUMAN	319.75568
TDRD7_HUMAN	1001.7558
GIMA7_HUMAN	5762.4849
NEDD1_HUMAN	1922.4934
NCOA7_HUMAN	2443.5146
THOC2_HUMAN	5477.3618
WDR36_HUMAN	5484.4482
PPTC7_HUMAN	866.18042
RP9_HUMAN	487.61172
VANG1_HUMAN	1012.4958
SNIP1_HUMAN	568.19641
G45IP_HUMAN	4158.7783
WDR48_HUMAN	3166.2505
EXOC6_HUMAN	5924.5327
SMRC2_HUMAN	5547.8296
NPL4_HUMAN	7860.4653
CX038_HUMAN	2576.0876
SPT20_HUMAN	1997.9388
NUBPL_HUMAN	320.07953
ABHD4_HUMAN	897.40442
FBX30_HUMAN	1003.9049
S35B2_HUMAN	7178.2959
PUM2_HUMAN	2821.2419
TIP_HUMAN	1828.1287
GOGA5_HUMAN	1236.5946
KLDC4_HUMAN	749.12335
SHKB1_HUMAN	2777.5669
UBA3_HUMAN	30139.045
PXL2B_HUMAN	906.44635
DJB14_HUMAN	653.36151
KISHA_HUMAN	15554.07
PI42C_HUMAN	1578.7814
WDR20_HUMAN	363.42819
TBC15_HUMAN	8322.8027
RDH11_HUMAN	13693.238
LRC20_HUMAN	1387.8119
IFI44_HUMAN	304.98654
RM30_HUMAN	789.19971
ZFAN1_HUMAN	804.92926
CIP2A_HUMAN	3516.9736
P4K2B_HUMAN	840.59851
STT3B_HUMAN	34754.23
PNPT1_HUMAN	9671.8369

SPP2B_HUMAN	1307.2727
HM13_HUMAN	45151.223
PREX1_HUMAN	3693.1709
URGCP_HUMAN	1584.1005
BICD2_HUMAN	4072.821
NEK9_HUMAN	5426.1646
PKHO2_HUMAN	3457.5925
DTX3L_HUMAN	1073.2374
DDX54_HUMAN	4484.002
DLG5_HUMAN	428.94354
BRX1_HUMAN	7595.7661
GNPI2_HUMAN	2335.7659
LRC8C_HUMAN	15041.915
NEK7_HUMAN	2976.6584
RBCC1_HUMAN	335.29831
MICA1_HUMAN	3910.3457
ELP5_HUMAN	2953.4956
SSH3_HUMAN	1356.0161
S3TC1_HUMAN	460.58209
TBCK_HUMAN	1149.6676
DTD1_HUMAN	4762.9761
DCA11_HUMAN	896.83167
UTP15_HUMAN	2093.0706
GPX8_HUMAN	21937.719
AF1L1_HUMAN	762.61554
SH3R3_HUMAN	3818.4907
TP4AP_HUMAN	606.94995
CE192_HUMAN	769.03088
GEMI5_HUMAN	5595.4473
PIGO_HUMAN	231.51155
RPGF6_HUMAN	632.82965
PARD3_HUMAN	1323.0662
IPO4_HUMAN	12447.495
UBP33_HUMAN	567.87842
PNISR_HUMAN	1655.5808
PP4R1_HUMAN	6823.3804
UBS3B_HUMAN	1183.618
GIPC2_HUMAN	440.5723
WIPF2_HUMAN	1128.474
ABHD5_HUMAN	1134.9817
SETD7_HUMAN	5008.9551
NOC3L_HUMAN	4765.0601
SCRB1_HUMAN	20386.125
COG1_HUMAN	2426.1064
SCFD2_HUMAN	2847.3003
SMAP2_HUMAN	1648.9451
ZC3HF_HUMAN	15531.28
PPIL4_HUMAN	4432.0034
TF3C2_HUMAN	2410.3616
TB22A_HUMAN	2995.1204

PHF10_HUMAN	1618.4425
RAB2B_HUMAN	4198.5757
CCD12_HUMAN	1270.7522
IASPP_HUMAN	1482.8674
ARB2A_HUMAN	1864.1061
CHUR_HUMAN	3843.6836
TGFA1_HUMAN	940.97766
TM263_HUMAN	5741.6685
HDAC7_HUMAN	5651.7476
STYX_HUMAN	1400.5022
PTPM1_HUMAN	365.55289
NU133_HUMAN	8289.6611
PDC6I_HUMAN	51288.676
S20A1_HUMAN	309.26801
FBLI1_HUMAN	15757.617
CATIN_HUMAN	1362.3959
CHAC2_HUMAN	2126.0493
CHMP7_HUMAN	5312.7612
THEM6_HUMAN	24687.902
NAT14_HUMAN	4410.9351
BCL7C_HUMAN	4608.9858
NSE1_HUMAN	709.58258
MITD1_HUMAN	586.4292
CTF18_HUMAN	623.36627
LEO1_HUMAN	3530.4514
DCAKD_HUMAN	1239.4686
NUDC2_HUMAN	4871.7646
SNR27_HUMAN	1846.8115
TFB1M_HUMAN	7818.605
STAG1_HUMAN	1333.9548
SCFD1_HUMAN	9312.4961
TP8L1_HUMAN	3467.7559
CANT1_HUMAN	3378.9307
TPC12_HUMAN	1942.5612
HNRLL_HUMAN	4067.8916
FACR1_HUMAN	14537.625
UBCP1_HUMAN	6244.3208
SEN15_HUMAN	981.81763
PCNP_HUMAN	9452.4736
DNJA4_HUMAN	1609.22
SPRY4_HUMAN	5670.564
MAIP1_HUMAN	3084.3809
TRUB1_HUMAN	1877.0616
LMO7_HUMAN	1934.9764
CTL1_HUMAN	1664.5265
CKAP2_HUMAN	919.60797
ATX2L_HUMAN	13601.436
ARAP3_HUMAN	9878.2578
GIMA1_HUMAN	7307.1792
PHIP_HUMAN	545.95441

PRP31_HUMAN	10667.533
NELFB_HUMAN	4444.3613
PALLD_HUMAN	14896.104
RUFY2_HUMAN	1703.3065
SREK1_HUMAN	2998.7852
GEMI6_HUMAN	1849.0359
CSKI2_HUMAN	2973.561
PSPC1_HUMAN	17938.936
MADD_HUMAN	443.76691
SYNE2_HUMAN	8914.5869
P66B_HUMAN	3419.1658
DNJC9_HUMAN	16304.466
BRI3B_HUMAN	1718.8027
CTBL1_HUMAN	14095.97
ATG4A_HUMAN	2168.1411
RIN2_HUMAN	1039.7682
ELYS_HUMAN	2760.4331
DGCR8_HUMAN	515.03748
TITIN_HUMAN	251.65514
ROBO4_HUMAN	2786.7422
OVCA2_HUMAN	1989.2372
IRGQ_HUMAN	2741.0469
DDB2_HUMAN	10061.775
CLC2B_HUMAN	1698.5908
DDX1_HUMAN	23295.184
S14L1_HUMAN	5575.7773
PIEZ1_HUMAN	1206.6959
FAM3C_HUMAN	5819.0605
H1X_HUMAN	16528.695
PSMF1_HUMAN	6991.644
GBF1_HUMAN	9112.4854
SMG7_HUMAN	98.78788
RTF1_HUMAN	6372.2866
NICA_HUMAN	14231.296
SNX19_HUMAN	542.2077
TM9S4_HUMAN	9795.9854
TM131_HUMAN	867.1947
IP6K1_HUMAN	536.88892
RT27_HUMAN	19319.211
ELMO1_HUMAN	2353.4185
FIG4_HUMAN	689.80048
F168A_HUMAN	330.58734
AP3S1_HUMAN	13522.835
TSC1_HUMAN	2948.1719
UBXN4_HUMAN	4761.0757
PHF3_HUMAN	1280.9609
NDRG1_HUMAN	3874.3369
HS105_HUMAN	24188.373
SEPT8_HUMAN	7556.1245
CNOT9_HUMAN	4520.7271

LGAT1_HUMAN	1028.3578
TBCD5_HUMAN	3688.354
ZN592_HUMAN	811.99829
JADE3_HUMAN	881.4668
MY18A_HUMAN	1091.3494
LAR4B_HUMAN	3705.6335
GCN1_HUMAN	20319.996
HMHA1_HUMAN	178.54189
PRP16_HUMAN	3226.0544
NU205_HUMAN	74193.672
ANS1A_HUMAN	1592.3429
PXDN_HUMAN	13779.103
FAN_HUMAN	766.93402
GPI8_HUMAN	2130.3208
RT31_HUMAN	8726.1963
AKAP1_HUMAN	192.09004
ALG3_HUMAN	1153.3904
NEUG_HUMAN	345.09982
AN32B_HUMAN	67581.875
NECT2_HUMAN	10978.859
PGTA_HUMAN	11141.069
TFG_HUMAN	12887.515
HTRA1_HUMAN	19220.387
ARC1A_HUMAN	8632.7656
TF2H4_HUMAN	1548.068
RREB1_HUMAN	373.27496
HDAC2_HUMAN	10721.445
STAM1_HUMAN	2745.7107
REQU_HUMAN	2748.363
SC65_HUMAN	9040.5049
CBP_HUMAN	519.94861
KAT6A_HUMAN	281.57623
SYMPK_HUMAN	5245.687
RBP56_HUMAN	2409.8215
GOGA1_HUMAN	929.50842
GGH_HUMAN	8118.7065
NRCAM_HUMAN	5506.583
RPGR_HUMAN	541.18335
SHIP1_HUMAN	4775.1255
DDX17_HUMAN	89788.828
KIFA3_HUMAN	1186.2867
CASPA_HUMAN	1601.6808
APBB2_HUMAN	1477.4281
RAD50_HUMAN	13424.064
CELF1_HUMAN	8684.2793
OSTF1_HUMAN	7581.1631
ARHG1_HUMAN	11942.924
XPF_HUMAN	852.91718
UFD1_HUMAN	25634.264
GSLG1_HUMAN	17287.99

RENT1_HUMAN	11099.397
CSN5_HUMAN	15674.454
GPKOW_HUMAN	6888.4341
SMRC1_HUMAN	3144.958
SMRD2_HUMAN	3637.1343
RAB8B_HUMAN	20500.688
BAD_HUMAN	290.54538
FUBP2_HUMAN	15054.119
GCDH_HUMAN	10209.885
RIT1_HUMAN	500.20511
PEX13_HUMAN	446.69623
TNPO1_HUMAN	36363.137
ARHG2_HUMAN	23093.031
NEP1_HUMAN	9846.3359
CLP1_HUMAN	2567.9382
GLMN_HUMAN	5870.1055
UBP13_HUMAN	2424.2903
DVL3_HUMAN	1411.4545
USP9X_HUMAN	10464.479
UBP7_HUMAN	11329.421
CUL5_HUMAN	5272.4697
STMN2_HUMAN	48976.195
VPP1_HUMAN	3308.3396
LPP_HUMAN	9712.0752
RBPMS_HUMAN	1089.0873
EXT2_HUMAN	959.67023
MED12_HUMAN	751.27252
TP4A1_HUMAN	91.324219
KPBB_HUMAN	3348.2759
SCAM4_HUMAN	3243.0168
HPS3_HUMAN	1277.2533
SMCE1_HUMAN	5239.4956
CAVN3_HUMAN	10532.578
RIFK_HUMAN	660.57703
MYDGF_HUMAN	31745.814
TM40L_HUMAN	1185.9642
YIPF5_HUMAN	2037.6136
PIGT_HUMAN	4685.7827
IGSF8_HUMAN	1047.1959
RL36L_HUMAN	5419.8354
RAB24_HUMAN	669.72894
OSBP2_HUMAN	1289.7214
ZN622_HUMAN	2440.1008
RRF2M_HUMAN	1899.0898
UB2E3_HUMAN	12673.326
5NT3B_HUMAN	4003.7185
WBP2_HUMAN	2842.7749
PSMG2_HUMAN	14123.541
NCLN_HUMAN	14711.463
MUL1_HUMAN	521.2962

MRTFA_HUMAN	379.15817
LFG3_HUMAN	3937.5879
ERGI1_HUMAN	11955.353
UTP4_HUMAN	1801.7386
FAKD4_HUMAN	4787.3164
MARC2_HUMAN	3247.019
F162A_HUMAN	9781.8652
CCD47_HUMAN	7488.0605
RM24_HUMAN	6710.1064
EXOC4_HUMAN	9926.4111
MGN2_HUMAN	38614.035
P33MX_HUMAN	961.33929
RFT1_HUMAN	2756.6189
ISOC2_HUMAN	2557.8101
NTAN1_HUMAN	1423.3677
FERM2_HUMAN	13600.517
FUBP1_HUMAN	39586.742
TTC17_HUMAN	690.08325
LRC59_HUMAN	81999.836
VTI1A_HUMAN	406.25311
ESAM_HUMAN	7772.8936
PBIP1_HUMAN	6278.4326
MCUR1_HUMAN	1061.1041
K1143_HUMAN	1197.7992
VP33A_HUMAN	3920.4656
FKB10_HUMAN	16443.516
TTC28_HUMAN	2264.2341
EXOS8_HUMAN	2544.4897
ZN428_HUMAN	635.19489
SH3K1_HUMAN	3158.3591
SKA1_HUMAN	508.57516
RNF25_HUMAN	1709.3729
AIDA_HUMAN	8976.3018
PINX1_HUMAN	873.53845
ARL8A_HUMAN	8501.2695
OTUL_HUMAN	2406.9646
CHCH1_HUMAN	1927.4702
PPWD1_HUMAN	5528.1816
CC127_HUMAN	1501.84
UPP_HUMAN	648.3689
TAM41_HUMAN	1787.4672
MOB3A_HUMAN	2220.5344
DOC10_HUMAN	2087.9131
ATG2B_HUMAN	996.98401
LENG1_HUMAN	1334.1693
TBC20_HUMAN	748.60144
EFHD2_HUMAN	33687.742
GALM_HUMAN	1963.8188
SYTL4_HUMAN	3391.1106
P5CR2_HUMAN	7143.8369

CSTOS_HUMAN	2093.542
DCPS_HUMAN	14681.233
PP14B_HUMAN	3323.7964
INT12_HUMAN	901.54718
NSUN4_HUMAN	1269.8157
RHDF1_HUMAN	535.74298
CTHR1_HUMAN	5228.582
ACSF2_HUMAN	1628.7711
ISOC1_HUMAN	6246.2788
GCC1_HUMAN	738.47021
FWCH2_HUMAN	3758.6685
ASTRA_HUMAN	1574.0892
HAUS1_HUMAN	396.81628
FAF2_HUMAN	10749.064
CC124_HUMAN	7679.7334
FXRD1_HUMAN	4672.4326
OPTN_HUMAN	2764.9556
AP2M1_HUMAN	46656.742
GCP3_HUMAN	3149.6875
S7A6O_HUMAN	1079.4268
KCD12_HUMAN	52229.984
RCN3_HUMAN	1694.235
NMD3_HUMAN	5362.6689
REPS1_HUMAN	1180.1541
TIM14_HUMAN	4259.5542
RMD1_HUMAN	6741.2471
NUD16_HUMAN	7615.311
ESS2_HUMAN	1383.3413
CMBL_HUMAN	3379.9009
MSI2H_HUMAN	3162.2449
SNR40_HUMAN	8012.2949
RMC1_HUMAN	1634.3871
RM38_HUMAN	8603.5469
RSPRY_HUMAN	554.8045
ERLEC_HUMAN	3261.8323
RRFM_HUMAN	6332.1958
SPDLY_HUMAN	1153.2592
ELP4_HUMAN	2903.4351
SIR1_HUMAN	662.77057
YIPF6_HUMAN	1158.9988
SEH1_HUMAN	3954.6533
TCAL4_HUMAN	1903.4277
KBP_HUMAN	14344.982
GNA1_HUMAN	10344.063
F120B_HUMAN	2060.1306
KTI12_HUMAN	1074.301
RT24_HUMAN	5532.5254
RM53_HUMAN	1634.0009
T3HPD_HUMAN	6590.0571
RNF31_HUMAN	1888.6807

DAZP1_HUMAN	14961.469
SGTB_HUMAN	1525.1571
SAAL1_HUMAN	2426.8477
MITOK_HUMAN	1035.0865
SGF29_HUMAN	878.64526
RRP36_HUMAN	724.42706
C1GLC_HUMAN	1378.2629
RBM33_HUMAN	2294.5598
DTBP1_HUMAN	2685.6333
DNJA3_HUMAN	5195.5146
TMA16_HUMAN	5718.3057
MB12A_HUMAN	3827.4033
PTCD3_HUMAN	6970.5815
MMAB_HUMAN	4462.9312
CYFP2_HUMAN	254.06807
GIMA5_HUMAN	820.32623
TRI11_HUMAN	1784.6309
CCD97_HUMAN	919.34906
CNRP1_HUMAN	12802.878
EDC3_HUMAN	4307.3003
DYL2_HUMAN	6588.2705
CPNE2_HUMAN	5985.8081
S10AG_HUMAN	4284.9883
SIPA1_HUMAN	3521.0083
SCRN2_HUMAN	1444.4115
THOC1_HUMAN	6037.5381
OTUB1_HUMAN	25595.803
TRM61_HUMAN	878.2702
HMCES_HUMAN	306.96234
CHMP6_HUMAN	3690.7466
BICD1_HUMAN	581.07782
PGM2_HUMAN	27860.488
IMP4_HUMAN	3824.9668
CERS2_HUMAN	6384.9512
DUS3L_HUMAN	1078.3966
LTV1_HUMAN	3765.1133
SDSL_HUMAN	729.91705
RM48_HUMAN	2862.4436
PLPP_HUMAN	1465.0741
AURKB_HUMAN	699.90131
DCNL1_HUMAN	2876.2134
FAH2A_HUMAN	4432.9854
SMRD1_HUMAN	1718.9178
TOE1_HUMAN	2485.2244
CDA7L_HUMAN	576.26447
RUSF1_HUMAN	1559.87
DDX27_HUMAN	6088.1846
BORC6_HUMAN	1024.8619
SYMM_HUMAN	297.39001
A7L3B_HUMAN	3325.2466

MTNB_HUMAN	1949.4771
ZC21A_HUMAN	923.5387
LIN37_HUMAN	200.04146
S41A3_HUMAN	217.75554
SNF8_HUMAN	2578.0142
ZCCHL_HUMAN	2544.8105
P121A_HUMAN	195.06348
TONSL_HUMAN	573.66071
PDLI5_HUMAN	38364.309
CREL1_HUMAN	2523.6082
ERO1A_HUMAN	24199.148
PRR11_HUMAN	1089.6689
FMC1_HUMAN	2295.9878
SAHH3_HUMAN	12147.189
DOCK6_HUMAN	6671.5908
MED30_HUMAN	1094.6816
PGAM5_HUMAN	14353.76
INT4_HUMAN	2367.3879
DDRGK_HUMAN	5627.5493
DHTK1_HUMAN	961.70563
SCLY_HUMAN	2670.8381
FUBP3_HUMAN	16954.445
SPF45_HUMAN	5215.8398
RCC1L_HUMAN	1580.6207
SYNM_HUMAN	1412.381
SUCB2_HUMAN	11538.694
LRCH3_HUMAN	3866.9177
GMPPA_HUMAN	7535.4785
HPDL_HUMAN	1470.1099
ABHEB_HUMAN	2235.27
NGLY1_HUMAN	1438.2236
SC22A_HUMAN	5655.2749
ATPMK_HUMAN	12873.13
NSL1_HUMAN	1256.6466
PAWR_HUMAN	4330.6426
MET2A_HUMAN	389.84973
THOC3_HUMAN	5223.813
ITCH_HUMAN	3283.991
TXD15_HUMAN	908.64923
KIRR1_HUMAN	2281.2139
LRIG1_HUMAN	730.92993
COG3_HUMAN	1956.0298
CK5P3_HUMAN	7359.2061
VPS39_HUMAN	3518.3875
VPS50_HUMAN	2358.1882
VCIP1_HUMAN	3782.4648
SPTCS_HUMAN	599.68311
ELMO2_HUMAN	7492.0449
TMX3_HUMAN	11987.941
DCAF5_HUMAN	2045.4928

CHAP1_HUMAN	2659.2424
LMBL3_HUMAN	3372.7676
ZFP91_HUMAN	2194.4561
PCD16_HUMAN	2191.825
CLMN_HUMAN	5655.5469
PDLI2_HUMAN	452.35739
FACR2_HUMAN	4019.8132
BT3L4_HUMAN	7731.8613
RN170_HUMAN	710.26886
ANCHR_HUMAN	1945.2925
S35E1_HUMAN	1137.326
UBP47_HUMAN	5104.8916
ZN521_HUMAN	462.40887
CLP1L_HUMAN	3254.7366
TOPK_HUMAN	4717.8496
ARL5B_HUMAN	950.02112
DNJC1_HUMAN	1598.6941
SCYL1_HUMAN	6474.5869
Z512B_HUMAN	723.4212
LRAT2_HUMAN	766.66711
EXOC2_HUMAN	7283.6904
CNDP2_HUMAN	45617.691
EHMT2_HUMAN	1596.2665
ZFR_HUMAN	3807.6321
F210B_HUMAN	699.02686
B3GT6_HUMAN	1643.1842
EP400_HUMAN	932.84882
SNX27_HUMAN	3558.9822
KI16B_HUMAN	260.02176
IFT74_HUMAN	612.67609
TRI47_HUMAN	4130.6162
CNO6L_HUMAN	2933.531
DHRS1_HUMAN	444.86502
DJC30_HUMAN	585.38861
UB2E2_HUMAN	146.45671
CAR19_HUMAN	2699.8882
PRRC1_HUMAN	6169.1704
FGD4_HUMAN	3663.1064
FXL18_HUMAN	3897.1167
ZN512_HUMAN	3240.0334
NSE2_HUMAN	524.80713
NSE3_HUMAN	257.13177
HS12B_HUMAN	5496.5474
YTDC1_HUMAN	2229.3909
CCD43_HUMAN	1528.8457
COG8_HUMAN	2439.9883
CKLF3_HUMAN	3650.8533
DAA10_HUMAN	2779.3508
NOL4L_HUMAN	5410.9404
MBOA7_HUMAN	11435.741

DOCK7_HUMAN	8356.1641
SFXN2_HUMAN	621.89044
ZN830_HUMAN	973.0271
F210A_HUMAN	2168.2769
FRMD6_HUMAN	680.88647
NSUN5_HUMAN	263.39487
RPR1A_HUMAN	3946.7844
AGAP3_HUMAN	1597.5048
ARAP1_HUMAN	3446.9309
IPO9_HUMAN	17314.65
MIA2_HUMAN	1404.2822
DCBD2_HUMAN	818.05786
ARHGH_HUMAN	1892.5038
INP4A_HUMAN	792.76123
RBM14_HUMAN	20246.498
ZN363_HUMAN	378.10464
KLHL5_HUMAN	882.09863
UHRF2_HUMAN	5118.1543
NED4L_HUMAN	3410.1389
QKI_HUMAN	15688.577
LENG8_HUMAN	416.5466
FMNL2_HUMAN	2173.0134
PUS7_HUMAN	6263.1855
F111A_HUMAN	1064.9982
TPPC9_HUMAN	634.90973
TRNT1_HUMAN	4727.3374
SMG1_HUMAN	1202.8915
ALS2_HUMAN	882.73187
TM237_HUMAN	996.21362
ALKB3_HUMAN	349.72931
RHG07_HUMAN	686.20935
PP1RA_HUMAN	4891.2905
S38A2_HUMAN	592.84003
UIF_HUMAN	2403.4934
MTMR9_HUMAN	1986.9004
VPS35_HUMAN	28078.115
PURB_HUMAN	2295.1184
XPO6_HUMAN	2299.6038
HHIP_HUMAN	33645.352
MAGI1_HUMAN	1371.9811
SPAG5_HUMAN	810.11816
NACC1_HUMAN	4570.917
SNX18_HUMAN	2098.8965
PASK_HUMAN	382.95236
CIC_HUMAN	1293.9404
UIMC1_HUMAN	400.43887
VP13A_HUMAN	2529.3564
MED15_HUMAN	1886.6079
EFGM_HUMAN	9578.3994
ERGI2_HUMAN	3652.2061

MCCA_HUMAN	1882.6395
KKCC2_HUMAN	815.93463
TGS1_HUMAN	664.7699
NUDC1_HUMAN	12042.228
ERBIN_HUMAN	2071.2256
GCP6_HUMAN	2023.187
GCP5_HUMAN	756.31812
UBP28_HUMAN	787.34119
FNBP1_HUMAN	4598.4404
PRPK_HUMAN	5200.6895
PIGS_HUMAN	5443.0059
WRIP1_HUMAN	3897.1592
RANB9_HUMAN	2719.8494
CLCC1_HUMAN	2327.3386
MYADM_HUMAN	6440.8638
NEB2_HUMAN	1697.3422
SRPK1_HUMAN	4913.7939
SMC6_HUMAN	1194.2305
KCD15_HUMAN	1008.1151
STRBP_HUMAN	14095.348
TM209_HUMAN	3140.2883
GPX7_HUMAN	1793.7094
CK5P2_HUMAN	301.44641
CP2S1_HUMAN	1295.3468
IWS1_HUMAN	8156.2905
SIN3A_HUMAN	1491.4471
OSBL9_HUMAN	5701.1274
CRBN_HUMAN	888.70239
INT14_HUMAN	1310.5417
AEDO_HUMAN	2886.1943
CK5P1_HUMAN	802.93652
RSF1_HUMAN	443.18707
RBM15_HUMAN	3681.4182
PP16B_HUMAN	346.80035
RUFY1_HUMAN	4609.834
MINT_HUMAN	559.68402
PNKP_HUMAN	5802.0391
MMS19_HUMAN	10629.934
UHRF1_HUMAN	4560.1729
NIBA2_HUMAN	29780.174
YMEL1_HUMAN	6622.0059
RMD3_HUMAN	4863.8296
HTF4_HUMAN	750.56635
CYH2_HUMAN	1364.7821
TBCB_HUMAN	14513.006
PSB7_HUMAN	22514.063
CNN2_HUMAN	13521.698
SEC62_HUMAN	6532.2441
PCY2_HUMAN	5967.9678
CDC5L_HUMAN	14884.626

PSMD1_HUMAN	27928.025
SDF2_HUMAN	1651.8628
PFD5_HUMAN	2062.3005
RING2_HUMAN	2017.5623
PARK7_HUMAN	14890.216
S1PR3_HUMAN	708.33575
GA2L1_HUMAN	336.91379
EYA3_HUMAN	689.22559
NEUR1_HUMAN	6035.7588
SORT_HUMAN	251.59126
VAT1_HUMAN	111982.54
LGMN_HUMAN	2740.5845
PLIN2_HUMAN	462.4603
DNJC2_HUMAN	4260.9116
MPP8_HUMAN	728.93549
NUP88_HUMAN	8768.6064
PKP4_HUMAN	331.29053
PI3R4_HUMAN	4547.0615
P2RX4_HUMAN	2619.8257
POP1_HUMAN	4349.8428
S10AD_HUMAN	27268.422
SCAFB_HUMAN	1654.12
TSNAX_HUMAN	5573.1572
SPS2_HUMAN	2099.0583
EIF3C_HUMAN	25972.352
TTC1_HUMAN	789.51422
DNJC7_HUMAN	10182.743
PHB2_HUMAN	88679.906
CSN8_HUMAN	2164.1621
PRP18_HUMAN	661.1983
CHP1_HUMAN	5390.394
KIF2C_HUMAN	2065.4915
RGPD5_HUMAN	214.40546
MGLL_HUMAN	3346.2512
ATX2_HUMAN	3112.7544
DOK1_HUMAN	736.75018
METH_HUMAN	8941.0117
HCD2_HUMAN	32487.85
COCA1_HUMAN	4266.9512
SMAD5_HUMAN	1440.4689
SGMR1_HUMAN	4971.2441
ROAA_HUMAN	15822.078
NP1L4_HUMAN	27372.832
MGST2_HUMAN	38763.367
SNAG_HUMAN	4804.3315
PI51A_HUMAN	1028.7238
ABCA3_HUMAN	1297.1761
M3K3_HUMAN	1055.4135
ATP5S_HUMAN	1198.5836
MIPEP_HUMAN	3908.0999

ACON_HUMAN	21496.445
TM9S2_HUMAN	6853.6895
S29A1_HUMAN	8746.1631
TS101_HUMAN	8153.7349
CPNE1_HUMAN	18283.063
TCPH_HUMAN	46302.832
MYD88_HUMAN	5673.2998
EBP2_HUMAN	8153.2446
ANM1_HUMAN	24518.195
BAG1_HUMAN	1644.9541
ATF6B_HUMAN	1407.3247
SH3G1_HUMAN	26709.883
TEP1_HUMAN	7470.5962
VRK1_HUMAN	10312.129
GDF15_HUMAN	8338.1719
AKAP9_HUMAN	10428.555
NIPS1_HUMAN	13394.538
CND3_HUMAN	6689.2349
ARP5L_HUMAN	13207.48
MICU1_HUMAN	980.19836
CG025_HUMAN	635.28583
F118B_HUMAN	1899.2811
SIN1_HUMAN	725.24011
RBM4B_HUMAN	15640.049
ZFY21_HUMAN	426.85916
DDX50_HUMAN	4686.8252
RM34_HUMAN	2060.8164
RNZ2_HUMAN	6860.0347
TRIR_HUMAN	3676.3296
GRWD1_HUMAN	5399.9243
MACD1_HUMAN	1572.3134
TCF25_HUMAN	3066.9524
CMS1_HUMAN	4268.0825
KLDC3_HUMAN	730.85229
ECSIT_HUMAN	2384.3049
MEP50_HUMAN	16550.701
CYBC1_HUMAN	1072.2228
VKOR1_HUMAN	2456.9714
DPH2_HUMAN	1675.4207
TBA1C_HUMAN	11971.713
SELS_HUMAN	2029.3429
APOL2_HUMAN	5148.063
MBB1A_HUMAN	14013.571
FERM1_HUMAN	889.6004
MGME1_HUMAN	604.56183
GORS1_HUMAN	1276.0919
FYCO1_HUMAN	1558.8185
ACBD6_HUMAN	3531.3604
COR1B_HUMAN	35178.32
TXD17_HUMAN	14475.216

BUD13_HUMAN	1188.4996
CPPED_HUMAN	20484.365
VPS25_HUMAN	2973.2493
RM45_HUMAN	12462.465
CG050_HUMAN	8492.9443
TIRR_HUMAN	2397.6899
CAB45_HUMAN	8389.0811
PDD2L_HUMAN	944.5592
PAAF1_HUMAN	3844.5635
PYM1_HUMAN	3762.5872
MIC25_HUMAN	777.14337
ADPGK_HUMAN	4835.8394
GPTC1_HUMAN	696.56073
RIOK1_HUMAN	1674.3589
LLPH_HUMAN	2813.6704
SLD5_HUMAN	1585.9413
UTP23_HUMAN	494.69391
PELO_HUMAN	5836.4224
PXL2A_HUMAN	74750.172
TRI56_HUMAN	1354.6722
ERP44_HUMAN	51535.395
LXN_HUMAN	16687.254
NOL10_HUMAN	1284.1493
NTPCR_HUMAN	6580.7915
TIM29_HUMAN	5667.1196
TACO1_HUMAN	2154.1956
GCP2_HUMAN	5259.9683
ESYT1_HUMAN	28260.611
UBAC1_HUMAN	1271.6044
YIPF4_HUMAN	2200.8789
PHAF1_HUMAN	363.11392
SEN34_HUMAN	1739.6115
CNPY3_HUMAN	1654.0308
MTG1_HUMAN	2382.3826
ALG1_HUMAN	3477.0103
LIMD2_HUMAN	846.66852
HAUS8_HUMAN	248.97229
INP5K_HUMAN	2685.6509
PSMG3_HUMAN	3927.6382
CSN4_HUMAN	11977.39
SYT11_HUMAN	868.11285
WAC_HUMAN	552.08954
DIDO1_HUMAN	3210.7124
MTA3_HUMAN	5139.3428
RBM42_HUMAN	9530.7139
DCTN5_HUMAN	2455.0806
MCMBP_HUMAN	4715.4033
AASD1_HUMAN	4174.2896
DCNL5_HUMAN	8026.2124
AN32E_HUMAN	6407.894

LRRC1_HUMAN	921.4585
P4K2A_HUMAN	2606.1096
TMM43_HUMAN	24925.264
FSD1_HUMAN	1112.0844
TBCD_HUMAN	9567.5723
NDC1_HUMAN	1491.9069
HGH1_HUMAN	1554.776
DHRS4_HUMAN	4037.3845
LMF2_HUMAN	4614.4209
NDUF3_HUMAN	6012.3491
MMTA2_HUMAN	1595.2831
TMM70_HUMAN	4201.2036
MED18_HUMAN	1689.1576
ISCA1_HUMAN	800.92474
TBB6_HUMAN	118339.36
PAXX_HUMAN	1427.0818
RPC3_HUMAN	2066.5979
HNRL1_HUMAN	16815.193
MSTO1_HUMAN	5878.1143
PHF23_HUMAN	2100.5012
PDC10_HUMAN	13041.382
RPP25_HUMAN	1936.0333
DERL1_HUMAN	18259.76
HTAI2_HUMAN	6909.0806
DDX23_HUMAN	10619.151
TCAB1_HUMAN	293.18884
MIC26_HUMAN	4222.1797
DHRS6_HUMAN	7206.8442
MTNA_HUMAN	4435.563
WDR18_HUMAN	6884.5723
VAMP8_HUMAN	783.38171
THUM3_HUMAN	2137.0413
MTND_HUMAN	6000.4702
MECR_HUMAN	2443.7688
EMC6_HUMAN	1623.8293
NTM1A_HUMAN	3384.3975
KTNB1_HUMAN	577.8609
LST8_HUMAN	1386.415
ASHWN_HUMAN	1271.6703
TM109_HUMAN	49803.336
PBDC1_HUMAN	4155.3594
PTSS2_HUMAN	4200.6606
NOC4L_HUMAN	4274.7646
UT14A_HUMAN	3026.1006
DUS23_HUMAN	5474.5898
TMED9_HUMAN	40987.203
TM147_HUMAN	4813.2661
NUP58_HUMAN	4294.1055
SELO_HUMAN	1986.6105
DPCD_HUMAN	2529.0134

GNL3_HUMAN	10933.828
AFG2B_HUMAN	5247.7119
RIOK2_HUMAN	1384.2129
TR61B_HUMAN	212.88815
TMUB1_HUMAN	1976.2365
TIM21_HUMAN	2986.1033
KIFC1_HUMAN	710.79938
NUP85_HUMAN	7349.125
ELOV1_HUMAN	1990.0549
KATL1_HUMAN	1180.8145
HIRP3_HUMAN	910.59991
IFT27_HUMAN	2157.9661
YJU2_HUMAN	2365.8013
NUDT9_HUMAN	5085.3374
SYTM_HUMAN	3674.5444
THIC_HUMAN	22057.475
REPI1_HUMAN	477.64581
RBM4_HUMAN	3528.1226
FUND2_HUMAN	1086.2148
RPAP1_HUMAN	2665.4292
SF3B5_HUMAN	1859.1506
SFXN3_HUMAN	15707.55
CHID1_HUMAN	2433.0171
PAPOG_HUMAN	1570.0116
CAR10_HUMAN	507.60287
NADAP_HUMAN	6319.0146
CDK19_HUMAN	590.39795
LS14B_HUMAN	1570.2764
TPSNR_HUMAN	796.55603
JAM3_HUMAN	7321.5591
CARD6_HUMAN	1373.8767
BTBD2_HUMAN	459.40665
OSB11_HUMAN	1775.3545
OSB10_HUMAN	4189.7544
RFIP5_HUMAN	2972.21
TB10A_HUMAN	317.33304
C1QT5_HUMAN	3561.5815
NAA15_HUMAN	19852.586
B2L13_HUMAN	799.4234
CAR11_HUMAN	1133.8475
S12A9_HUMAN	1316.9198
SRRT_HUMAN	21245.826
TGT_HUMAN	4794.7573
AP1M1_HUMAN	16365.391
NUSAP_HUMAN	478.44498
HDHD5_HUMAN	3619.1025
FACD2_HUMAN	526.81427
MAK16_HUMAN	3261.6111
HDAC8_HUMAN	239.20119
RTF2_HUMAN	1142.8026

CHM4A_HUMAN	2896.5144
EIF2A_HUMAN	15967.901
PDIP3_HUMAN	4213.0352
K1671_HUMAN	1728.2202
SHAN3_HUMAN	11671.906
GNB1L_HUMAN	1901.037
FUT8_HUMAN	1018.5596
RM32_HUMAN	1445.4838
RM20_HUMAN	4604.6606
RM13_HUMAN	9103.3408
RM09_HUMAN	10085.911
RM04_HUMAN	4812.105
RM01_HUMAN	8063.3267
MK67I_HUMAN	2965.9329
HYCCI_HUMAN	1539.6115
YTHD1_HUMAN	2123.6016
HELZ2_HUMAN	860.56079
HOIL1_HUMAN	2387.043
RT26_HUMAN	5745.4399
NEUL_HUMAN	12124.444
CEP41_HUMAN	3906.895
SETD2_HUMAN	635.81061
PANK2_HUMAN	603.71344
DOCK9_HUMAN	11353.271
FRMD8_HUMAN	4960.2437
CNTP3_HUMAN	1040.8539
NSD3_HUMAN	1543.9182
NUF2_HUMAN	3131.7844
RM37_HUMAN	10468.197
GTPB4_HUMAN	7231.6099
ASPC1_HUMAN	1975.1803
OSBL8_HUMAN	2548.8247
UACA_HUMAN	2914.8457
RAB34_HUMAN	5201.4771
DPH1_HUMAN	1131.6862
WDR11_HUMAN	4034.2407
REN3B_HUMAN	2121.9287
CRNL1_HUMAN	3687.6418
TBL1R_HUMAN	3545.0483
UBL5_HUMAN	3608.6453
PP12C_HUMAN	2983.9006
KPCD2_HUMAN	5240.7417
EDEM3_HUMAN	3020.085
NIBA1_HUMAN	574.58325
UBXN6_HUMAN	4098.7666
TM6S1_HUMAN	392.53424
UCK2_HUMAN	6709.8208
SN_HUMAN	1835.8033
API5_HUMAN	23743.48
NLRP1_HUMAN	1160.6343

TRIM5_HUMAN	1504.7495
TRIM4_HUMAN	52212.207
FTO_HUMAN	6331.4438
ZDHC5_HUMAN	648.65234
TNG6_HUMAN	1416.0997
TB182_HUMAN	7457.0625
UBE2O_HUMAN	6389.2275
ZY11B_HUMAN	2344.6123
TANC1_HUMAN	5599.7461
EPT1_HUMAN	570.64392
XPO4_HUMAN	7956.4116
LNP_HUMAN	1143.1666
CEP44_HUMAN	169.1306
TTYH3_HUMAN	9547.4141
KLHL4_HUMAN	2081.0796
MTMRC_HUMAN	812.73987
WDR33_HUMAN	5806.9961
WDR12_HUMAN	5584.4551
YIPF3_HUMAN	5345.8853
TINAL_HUMAN	2370.1863
ADSSP_HUMAN	6781.0137
PITH1_HUMAN	4430.2998
DERL2_HUMAN	7069.8779
COMD5_HUMAN	2304.093
SENP6_HUMAN	203.95023
REXO4_HUMAN	2371.4287
DDX24_HUMAN	4485.2891
RPA49_HUMAN	3399.9646
SKI8_HUMAN	7922.6963
SLIRP_HUMAN	6927.2334
SRR_HUMAN	1459.91
NIF3L_HUMAN	3755.5393
EGLN1_HUMAN	4772.5981
MCLN1_HUMAN	563.62531
PIP30_HUMAN	3431.009
MFF_HUMAN	1066.1484
NAA50_HUMAN	8540.5811
UBA5_HUMAN	6532.9756
MKRN2_HUMAN	1660.3745
T126A_HUMAN	3505.4998
PAIP1_HUMAN	11371.866
CLPB_HUMAN	4467.8481
RB33B_HUMAN	3827.2021
LSG1_HUMAN	6089.731
F107B_HUMAN	6921.853
NAT10_HUMAN	15358.106
COMD4_HUMAN	1967.4395
KLC2_HUMAN	2823.2275
ILKAP_HUMAN	5720.6689
XRN2_HUMAN	15935.025

TOLIP_HUMAN	5019.2144
SP130_HUMAN	720.29059
NSRP1_HUMAN	187.79677
INT2_HUMAN	1259.1997
RGAP1_HUMAN	666.45654
PAR12_HUMAN	2553.6909
CSTFT_HUMAN	1199.0574
5NT3A_HUMAN	1982.9048
CYRIA_HUMAN	16755.682
TM222_HUMAN	847.31262
HDHD2_HUMAN	2526.5313
GATA_HUMAN	1986.8895
DDX47_HUMAN	6915.9829
MAGT1_HUMAN	6691.5186
RAB1B_HUMAN	56390.738
RM18_HUMAN	5868.6099
TSYL1_HUMAN	567.80487
TM168_HUMAN	677.83649
LMA2L_HUMAN	8008.7417
SMG9_HUMAN	1493.427
F234A_HUMAN	235.39679
OSBL5_HUMAN	788.30994
TDIF1_HUMAN	1716.0574
SIL1_HUMAN	1491.8688
METL9_HUMAN	858.4342
APC1_HUMAN	4670.6494
I2BPL_HUMAN	869.0321
CYTM1_HUMAN	1207.33
RPC6_HUMAN	2098.1147
NUCKS_HUMAN	6122.9912
TMX4_HUMAN	7181.8735
KI13A_HUMAN	19567.107
ASCC2_HUMAN	6079.8735
RBNS5_HUMAN	611.46454
ISCU_HUMAN	2791.0796
OSBL2_HUMAN	1137.3768
ATG5_HUMAN	3758.1235
WDR13_HUMAN	2361.7107
EPN3_HUMAN	1933.5438
EHD4_HUMAN	94453.141
VP33B_HUMAN	3478.8479
VPS16_HUMAN	7987.355
VPS11_HUMAN	5251.4663
SH3L3_HUMAN	7784.7012
GAN_HUMAN	805.65967
S2532_HUMAN	1490.0586
TARA_HUMAN	7578.6997
EPC1_HUMAN	282.75101
SLK_HUMAN	10938.064
PPIL3_HUMAN	576.70776

S38A1_HUMAN	754.04327
PDCL3_HUMAN	6077.9473
TAOK3_HUMAN	5912.5713
RBGPR_HUMAN	8703.8291
ADNP_HUMAN	2447.7231
DPH5_HUMAN	2122.5439
DHX36_HUMAN	5931.2656
IPYR2_HUMAN	7797.8335
SPNS1_HUMAN	4595.959
RM46_HUMAN	2152.9895
S12A5_HUMAN	452.58893
PARL_HUMAN	1150.6119
PININ_HUMAN	10923.679
TM245_HUMAN	1047.691
FOXP1_HUMAN	1559.3798
BACD3_HUMAN	4108.4922
MOD5_HUMAN	364.34341
CK068_HUMAN	6692.9004
GHITM_HUMAN	5657.6182
BOLA2_HUMAN	7012.5977
TXNIP_HUMAN	1791.0454
TMX1_HUMAN	24613.891
NELFA_HUMAN	3605.738
GCP60_HUMAN	8556.7627
BORG4_HUMAN	937.13129
PTN23_HUMAN	8056.9946
SEM6B_HUMAN	5281.6104
UN45A_HUMAN	16480.766
MFSD1_HUMAN	1832.0457
DNJC5_HUMAN	1042.9542
DSN1_HUMAN	579.01941
CHM4B_HUMAN	18761.627
RWDD1_HUMAN	2499.5999
CUED2_HUMAN	678.2019
FXL15_HUMAN	636.26563
FN3K_HUMAN	2332.8862
OFUT1_HUMAN	7884.0352
PIGU_HUMAN	4747.7461
TOR3A_HUMAN	3643.4536
WNK1_HUMAN	3877.2932
AMPB_HUMAN	20962.422
GLP3L_HUMAN	5302.2593
GOLP3_HUMAN	4273.3013
OSGL1_HUMAN	1880.01
GAPR1_HUMAN	4913.585
TRABD_HUMAN	2026.9675
MTG2_HUMAN	95.838493
SENP3_HUMAN	4518.2407
OSBL3_HUMAN	4209.7583
SMRCD_HUMAN	2837.6631

EHD1_HUMAN	21623.041
CAPAM_HUMAN	889.62018
ESF1_HUMAN	659.41803
ALG2_HUMAN	4061.0889
HEAT1_HUMAN	7470.9116
SG196_HUMAN	694.89557
RABE2_HUMAN	1883.8685
TFB2M_HUMAN	3572.0366
ZCHC4_HUMAN	1708.0391
STEEP_HUMAN	233.41679
CIA2A_HUMAN	1377.0541
DHX35_HUMAN	1673.4955
F124B_HUMAN	4435.5938
HAUS4_HUMAN	3532.9465
CC134_HUMAN	2295.7288
CCD86_HUMAN	3388.022
REEP4_HUMAN	2136.9863
SOX17_HUMAN	525.20972
OPA3_HUMAN	1566.1403
CIAO3_HUMAN	2117.9478
DHX33_HUMAN	569.08246
NOL6_HUMAN	3790.7402
WDCP_HUMAN	518.07904
YTDC2_HUMAN	3412.9675
AZI2_HUMAN	328.70599
RPAP3_HUMAN	3779.709
BCAS3_HUMAN	345.45505
ALG9_HUMAN	3206.5996
LDAH_HUMAN	2024.9843
RIOX1_HUMAN	3271.0085
WDR55_HUMAN	2536.6995
RANB3_HUMAN	6808.6963
DCTP1_HUMAN	162.21907
PEAK1_HUMAN	1711.2424
RPF2_HUMAN	3083.2964
SMYD3_HUMAN	3229.5601
AAMDC_HUMAN	2227.8474
DOCK5_HUMAN	947.66565
WDR26_HUMAN	3330.5039
TDRD3_HUMAN	2590.0825
CH033_HUMAN	1739.5582
AT133_HUMAN	613.62164
MET17_HUMAN	886.50378
SFR19_HUMAN	1779.4451
NRDE2_HUMAN	1026.5037
PGES2_HUMAN	6265.4429
PHAX_HUMAN	2775.7649
UBE2Z_HUMAN	5963.1372
ACAD9_HUMAN	6375.4136
NT5D2_HUMAN	10593.718

PKRI1_HUMAN	1252.4027
CAAP1_HUMAN	1389.2811
NOL11_HUMAN	1170.9254
DDX31_HUMAN	732.94501
TMT1A_HUMAN	2991.384
PAAT_HUMAN	1714.3945
MMRN2_HUMAN	15407.058
MINY3_HUMAN	2342.26
EVA1A_HUMAN	539.63947
AKTIP_HUMAN	1077.752
PKHF2_HUMAN	1698.3319
ANKZ1_HUMAN	2305.8359
GORS2_HUMAN	3810.1907
ZWILC_HUMAN	2036.4205
JUPI2_HUMAN	3040.96
GHC1_HUMAN	5657.7598
PPIP2_HUMAN	3159.7864
MED20_HUMAN	1866.3263
WDR76_HUMAN	233.4705
QTRT2_HUMAN	1547.223
ARP8_HUMAN	1397.8416
ARMT1_HUMAN	4870.8457
PANK3_HUMAN	515.19623
CNO10_HUMAN	1552.6323
LRC40_HUMAN	19190.82
EHMT1_HUMAN	2577.1313
SFXN1_HUMAN	23803.973
SPE39_HUMAN	1650.8025
COG4_HUMAN	7792.5625
ARP5_HUMAN	829.47241
VP37B_HUMAN	675.20105
RM44_HUMAN	8584.9092
L2HDH_HUMAN	1783.7164
CSN7B_HUMAN	3150.1233
NHEJ1_HUMAN	695.64838
S61A2_HUMAN	13354.938
FKRP_HUMAN	746.89844
ELP3_HUMAN	6844.6157
RPF1_HUMAN	1298.7466
RPA2_HUMAN	1370.4156
ZMAT3_HUMAN	443.49149
UCK1_HUMAN	853.71222
KT3K_HUMAN	1842.2333
TBC17_HUMAN	1120.4523
SYCM_HUMAN	4274.4609
S52A2_HUMAN	2319.0015
PPCS_HUMAN	12508.208
UBTD1_HUMAN	2780.989
WDR41_HUMAN	766.23309
FBRS_HUMAN	567.00098

NMNA1_HUMAN	1441.4915
NJMU_HUMAN	1763.7512
SIAE_HUMAN	880.08173
PKHA5_HUMAN	793.37262
SMUF2_HUMAN	2332.551
RENT2_HUMAN	3774.7366
GBB4_HUMAN	1723.207
XPO5_HUMAN	7778.4189
GRPE1_HUMAN	14815.72
MYG1_HUMAN	11610.007
PKHA3_HUMAN	408.66647
PKHA1_HUMAN	1815.4705
RISC_HUMAN	4015.4827
NET4_HUMAN	7774.3335
CYBP_HUMAN	32821.887
RRAGC_HUMAN	9694.9307
ZFYV1_HUMAN	1097.4011
IF122_HUMAN	464.95438
RDH14_HUMAN	4201.2324
PARVB_HUMAN	10228.812
TENS1_HUMAN	3278.9341
PLRKT_HUMAN	2981.8967
NMRL1_HUMAN	1757.6342
VEZA_HUMAN	673.96594
SPC25_HUMAN	2079.8987
TAF9B_HUMAN	939.61243
S38AA_HUMAN	1892.6846
EKI1_HUMAN	1647.0428
AGRL4_HUMAN	8280.5908
TM165_HUMAN	4959.3672
TPC_HUMAN	1098.4813
EMAL4_HUMAN	5719.1938
MRM3_HUMAN	2838.5217
GLOD4_HUMAN	48001.965
NEK6_HUMAN	1172.9391
MCCB_HUMAN	3496.1619
NCOA5_HUMAN	44867.363
EPG5_HUMAN	1100.2643
MOV10_HUMAN	7800.0776
MET14_HUMAN	1187.325
GBA2_HUMAN	3103.5217
CWC22_HUMAN	2164.8989
RAVR2_HUMAN	994.06805
CHD8_HUMAN	1076.8336
E41L5_HUMAN	1641.6224
GPN1_HUMAN	4292.854
SDF2L_HUMAN	4196.9707
KC1G1_HUMAN	3627.1064
SYF1_HUMAN	4697.7832
PREB_HUMAN	4298.2852

SRA1_HUMAN	1529.8286
AT131_HUMAN	5094.2095
GOPC_HUMAN	1972.548
RM47_HUMAN	5846.9272
LYRM4_HUMAN	2341.3552
SPCS_HUMAN	1402.3782
CHM1A_HUMAN	7904.0093
TM9S3_HUMAN	11978.447
MYO10_HUMAN	5552.8652
APMAP_HUMAN	48659.324
WDR6_HUMAN	5179.3945
TRXR2_HUMAN	9673.8848
ABCB6_HUMAN	548.40314
ARFG3_HUMAN	5172.1973
ZCC17_HUMAN	960.10431
RAB18_HUMAN	15250.959
PALMD_HUMAN	9829.1377
SSU72_HUMAN	2343.9434
VTA1_HUMAN	20940.357
SYSM_HUMAN	3618.4519
RT30_HUMAN	5649.2598
DLRB1_HUMAN	7173.1709
EMC7_HUMAN	7683.709
ENY2_HUMAN	1025.2294
EXOS4_HUMAN	4414.2744
UBE2T_HUMAN	3137.0833
NOP10_HUMAN	4628.6543
OSGEP_HUMAN	4774.7949
DMAP1_HUMAN	1048.3918
PCD12_HUMAN	806.70917
INO1_HUMAN	7801.062
BRD7_HUMAN	333.12286
DCP1A_HUMAN	4123.2446
ACO13_HUMAN	8781.9854
MED4_HUMAN	876.2381
TIDC1_HUMAN	218.88792
RIC8A_HUMAN	20217.57
C1QR1_HUMAN	34160.137
LUC7L_HUMAN	8153.4517
ESM1_HUMAN	478.59039
LZTL1_HUMAN	1898.1704
RM40_HUMAN	5583.7842
SSF1_HUMAN	2626.6755
PLCB1_HUMAN	1386.4662
TIGAR_HUMAN	14330.583
CL004_HUMAN	925.3786
RTN4_HUMAN	17112.246
RPR1B_HUMAN	7074.7715
XPP3_HUMAN	2335.7061
NIT2_HUMAN	7178.4268

AVEN_HUMAN	603.25403
INCE_HUMAN	631.07562
EXOS5_HUMAN	2093.8325
EXOS3_HUMAN	5694.1104
KI13B_HUMAN	4410.9229
ANLN_HUMAN	2652.6843
XPP1_HUMAN	13196.576
GEPH_HUMAN	4498.6626
BIN3_HUMAN	3227.0076
SAS10_HUMAN	1733.2786
STAR7_HUMAN	1257.1262
BIRC6_HUMAN	4466.4282
PDLI7_HUMAN	18497.688
ACSA_HUMAN	9787.958
DBLOH_HUMAN	3971.8918
DDX21_HUMAN	21361.885
SAR1A_HUMAN	28472.977
SIAS_HUMAN	30348.314
SHLB2_HUMAN	3738.8562
ASH1L_HUMAN	17249.713
EI2BG_HUMAN	8883.248
MBNL1_HUMAN	11905.636
S17A5_HUMAN	3254.2249
4ET_HUMAN	1053.261
CHSTC_HUMAN	1986.12
ST7_HUMAN	1313.4279
PICK1_HUMAN	1056.4159
SH2B1_HUMAN	1373.1675
PYRG2_HUMAN	6734.9907
DPOE3_HUMAN	3178.3076
CHRC1_HUMAN	2586.7727
AAAS_HUMAN	6498.5903
SNRK_HUMAN	3588.8542
ABCBA_HUMAN	2512.4187
BAZ1A_HUMAN	1459.1848
STRN4_HUMAN	4202.4937
ADPPT_HUMAN	9118.4922
METL5_HUMAN	2131.2615
OSTC_HUMAN	22437.797
COXM2_HUMAN	336.89023
PLS4_HUMAN	2191.8701
RNC_HUMAN	499.32489
SNX15_HUMAN	857.36908
HEBP1_HUMAN	2830.001
ABC3C_HUMAN	1216.7687
VPS45_HUMAN	2671.1826
PNO1_HUMAN	2369.9192
RM17_HUMAN	10207.929
PHP14_HUMAN	403.41953
SERC1_HUMAN	829.67554

RHG35_HUMAN	3236.4265
F1142_HUMAN	2283.5637
PLS3_HUMAN	3388.1907
PLCC_HUMAN	2432.3345
HELLS_HUMAN	1781.5167
C1GLT_HUMAN	2109.4812
LTBP3_HUMAN	390.74484
TOM22_HUMAN	4461.6445
LANC2_HUMAN	3643.6697
KIF15_HUMAN	1115.9667
TM7S3_HUMAN	1516.6797
HOME3_HUMAN	6399.644
SYFB_HUMAN	25846.471
SYIM_HUMAN	13483.502
SLX9_HUMAN	2777.665
KLC4_HUMAN	2132.0496
DIAP3_HUMAN	2008.3907
BMP2K_HUMAN	690.90924
ATG3_HUMAN	4998.7852
SIR3_HUMAN	822.32727
PDS5B_HUMAN	4965.2227
SMC4_HUMAN	29543.354
MA2C1_HUMAN	6953.5015
SAC1_HUMAN	8186.6235
DEPP1_HUMAN	946.19586
OLA1_HUMAN	32010.473
CUTC_HUMAN	933.07367
ZF64B_HUMAN	1021.1507
ECHD1_HUMAN	9209.2959
RBM12_HUMAN	85977.844
TB22B_HUMAN	1830.2994
MDN1_HUMAN	8284.5781
ACS2L_HUMAN	1075.2045
ZCHC3_HUMAN	936.00861
DECR2_HUMAN	4470.2236
ABHDA_HUMAN	2865.7661
T11L1_HUMAN	2169.5652
STAU2_HUMAN	5761.8242
SHFL_HUMAN	3399.0505
DDX28_HUMAN	1659.928
T106B_HUMAN	2118.3486
LIN7C_HUMAN	1731.4921
PLCE_HUMAN	4689.4395
TXLNG_HUMAN	1414.8704
SPS2L_HUMAN	2153.3049
UFSP2_HUMAN	2187.2644
ABCF3_HUMAN	7164.2861
CYRIB_HUMAN	10917.053
MITOS_HUMAN	1459.5663
DD19A_HUMAN	19743.439

GIMA4_HUMAN	25933.309
TYDP1_HUMAN	2157.6167
POTE1_HUMAN	681.13623
TBC23_HUMAN	3916.5544
DCA13_HUMAN	1826.4929
IMP3_HUMAN	5289.0654
MRGBP_HUMAN	875.70673
TYW1_HUMAN	486.04999
EXOC1_HUMAN	10356.967
PCMD2_HUMAN	450.4429
INT9_HUMAN	1832.2386
CC50A_HUMAN	15218.686
UQCC1_HUMAN	2433.8494
SEP11_HUMAN	22825.764
S38A7_HUMAN	15801.868
MED17_HUMAN	13033.726
PARVA_HUMAN	8603.0557
UBP40_HUMAN	789.48834
PANK4_HUMAN	10146.094
FBX28_HUMAN	403.78198
TBC13_HUMAN	6434.0342
EXD2_HUMAN	2292.4302
DJC11_HUMAN	6010.2563
INT7_HUMAN	13742.753
FANCI_HUMAN	989.20941
ATD3A_HUMAN	36110.066
ARL8B_HUMAN	12396.804
DJC17_HUMAN	4798.9624
INT13_HUMAN	3165.9585
RIC8B_HUMAN	926.30884
GNL3L_HUMAN	1528.9283
DDX18_HUMAN	6539.9492
ASF1B_HUMAN	2280.2556
KLH11_HUMAN	1473.272
INT10_HUMAN	3111.699
RT18A_HUMAN	5923.2378
PNPO_HUMAN	823.42548
ARMC1_HUMAN	4264.2061
RPC5_HUMAN	552.38318
SDA1_HUMAN	3665.2083
PAPD1_HUMAN	4218.5498
NLE1_HUMAN	2978.5618
NECP2_HUMAN	12223.275
RPC2_HUMAN	4676.4712
RBM28_HUMAN	3563.5208
ANO10_HUMAN	8072.0615
RBM22_HUMAN	3111.3931
DMAC2_HUMAN	244.47978
WDR70_HUMAN	1546.4976
TMM51_HUMAN	316.00116

ARGL1_HUMAN	4490.105
SLTM_HUMAN	3343.511
FKB14_HUMAN	890.81488
RMND1_HUMAN	2848.561
PK1IP_HUMAN	1651.217
HIF1N_HUMAN	1498.4462
OXSM_HUMAN	7727.4712
RM22_HUMAN	15574.096
CZIB_HUMAN	14748.34
BABA1_HUMAN	4016.8765
CLN6_HUMAN	136.73923
ASB6_HUMAN	2311.5361
THG1_HUMAN	1644.6208
HPF1_HUMAN	2608.1736
IRAK4_HUMAN	2203.7141
UCKL1_HUMAN	2605.3252
TM160_HUMAN	3129.906
NALP2_HUMAN	3842.875
F120C_HUMAN	367.53726
TSAP1_HUMAN	1131.4088
NDUBB_HUMAN	3203.1367
RM16_HUMAN	9041.6104
NHP2_HUMAN	7488.0669
OCAD1_HUMAN	8430.7256
ADPRS_HUMAN	12017.847
MARH5_HUMAN	4593.9717
LYAR_HUMAN	4855.7549
T161A_HUMAN	653.05591
IMPA3_HUMAN	4139.9624
MIC19_HUMAN	12106.737
DUS2L_HUMAN	233.50015
CKLF6_HUMAN	1584.9735
SIR5_HUMAN	1291.4641
MIOS_HUMAN	1155.2703
MTMRA_HUMAN	4757.4185
NSMA3_HUMAN	4238.4521
CWC25_HUMAN	1901.7521
TEX10_HUMAN	4551.6836
DCA16_HUMAN	1565.7792
THUM1_HUMAN	3399.6577
P4HTM_HUMAN	330.67276
TOR4A_HUMAN	3747.9426
TRM1_HUMAN	3877.6414
GDAP2_HUMAN	1727.7533
NDE1_HUMAN	1418.0273
BABA2_HUMAN	1528.5491
QPCTL_HUMAN	2233.2617
ARL15_HUMAN	2898.5305
KCTD5_HUMAN	1368.7334
CARF_HUMAN	3303.6465

DJB12_HUMAN	2720.0313
NSE4A_HUMAN	655.15607
GAR1_HUMAN	6527.8013
STAB1_HUMAN	9926.8066
PP4R2_HUMAN	1479.3904
DPP3_HUMAN	20939.576
AATF_HUMAN	948.1897
TBA8_HUMAN	4438.8667
DDX56_HUMAN	2611.4788
SPHK1_HUMAN	2088.9451
TE2IP_HUMAN	168.69995
ABI2_HUMAN	2844.2456
BCLF1_HUMAN	11994.689
UTP6_HUMAN	1535.5397
TAB2_HUMAN	152.00771
RM39_HUMAN	7879.7681
M3K20_HUMAN	2915.0605
FKB11_HUMAN	12524.306
TMOD3_HUMAN	12635.872
BET1L_HUMAN	2059.8416
ELOV5_HUMAN	2807.5347
KBRS2_HUMAN	1724.3914
UGGG2_HUMAN	397.22318
UGGG1_HUMAN	27683.951
CDK12_HUMAN	2147.1257
FAKD2_HUMAN	4461.8193
TECR_HUMAN	6560.3223
ERAP1_HUMAN	14121.058
ARP10_HUMAN	4103.9512
USE1_HUMAN	2254.084
CISD1_HUMAN	2860.3943
GGA3_HUMAN	2029.7943
TLS1_HUMAN	1245.6628
F120A_HUMAN	8161.1816
GDE1_HUMAN	174.29399
SMAL1_HUMAN	1290.8518
GLTP_HUMAN	2653.2146
SPG21_HUMAN	3050.5432
RM35_HUMAN	1918.1066
UBIP1_HUMAN	1192.9418
IF2B1_HUMAN	17861.963
SACS_HUMAN	2007.1993
MTCH1_HUMAN	9064.2822
NUDT4_HUMAN	7904.4233
HPBP1_HUMAN	8609.2666
MAT2B_HUMAN	27809.074
MYOF_HUMAN	44292.039
ITSN2_HUMAN	2868.8425
NOP53_HUMAN	994.1936
EHD3_HUMAN	51820.848

EHD2_HUMAN	80591.133
ARHGC_HUMAN	3033.4824
CNOT2_HUMAN	4317.1431
SPN90_HUMAN	1729.2134
PD1L1_HUMAN	1269.6604
OGFR_HUMAN	7958.4292
LMCD1_HUMAN	2162.1912
CRIM1_HUMAN	2540.7222
SELN_HUMAN	4137.4795
PALS2_HUMAN	3199.7483
CHMP5_HUMAN	6461.3281
CWC15_HUMAN	2830.2563
RM15_HUMAN	9542.041
THYN1_HUMAN	8731.7734
TAP26_HUMAN	333.34857
NDUF4_HUMAN	3314.9858
HACD3_HUMAN	12518.255
EMC3_HUMAN	3170.3606
NDUAD_HUMAN	7810.896
PDP1_HUMAN	5379.2959
KCMF1_HUMAN	1141.9973
RAI14_HUMAN	9453.5557
VAPA_HUMAN	15710.329
RM27_HUMAN	3495.3926
TMEM9_HUMAN	915.33606
SENP1_HUMAN	784.02167
CXXC1_HUMAN	386.44949
SH3B4_HUMAN	6260.5039
SEP10_HUMAN	10833.342
GMIP_HUMAN	948.80841
PHRF1_HUMAN	5087.8828
POGK_HUMAN	927.92688
DAPLE_HUMAN	235.59126
RHG23_HUMAN	1144.7192
VPS18_HUMAN	10251.809
RCC2_HUMAN	40025.063
RELCH_HUMAN	1258.551
DIP2B_HUMAN	4267.5879
JCAD_HUMAN	2953.4282
SLAI2_HUMAN	555.82074
TEN3_HUMAN	902.12274
UBP36_HUMAN	634.82599
BCCIP_HUMAN	13586.554
STK26_HUMAN	1691.2488
ARMX1_HUMAN	3000.0869
ABI3_HUMAN	6622.1904
FPRP_HUMAN	14562.665
CT2NL_HUMAN	6313.2153
TM181_HUMAN	2489.7542
IBTK_HUMAN	704.23395

HTR5B_HUMAN	1572.9213
ZNFX1_HUMAN	1299.4578
CHPF2_HUMAN	2943.6597
PCD10_HUMAN	663.58813
RRBP1_HUMAN	37114.262
CPSF2_HUMAN	5319.4087
KLHL9_HUMAN	2502.7327
SYLC_HUMAN	27303.41
MYEF2_HUMAN	12276.646
KLH42_HUMAN	727.8017
E2AK4_HUMAN	4584.5552
WDR35_HUMAN	714.64221
RBM27_HUMAN	2569.7705
KANL3_HUMAN	1151.3214
HECW2_HUMAN	2550.3667
FRM4A_HUMAN	1025.5813
ANFY1_HUMAN	5958.6685
SUCB1_HUMAN	8173.3306
GMPR2_HUMAN	6627.2915
STX18_HUMAN	795.05322
DPM3_HUMAN	940.19989
IMPCT_HUMAN	1568.0773
UVRAG_HUMAN	2529.0029
ATX10_HUMAN	11542.278
MBD2_HUMAN	2890.2131
NCDN_HUMAN	2250.8274
TFP11_HUMAN	1201.8625
EP15R_HUMAN	9069.1064
MYO1A_HUMAN	8740.2568
ORC3_HUMAN	1736.5945
SAE1_HUMAN	11870.123
COPG2_HUMAN	10069.606
PI4KB_HUMAN	875.50543
MRC2_HUMAN	1340.6104
COMD3_HUMAN	1672.025
GBG12_HUMAN	2202.0635
RBL2A_HUMAN	1507.6277
MTRR_HUMAN	434.31738
UXT_HUMAN	1130.2281
ASH2L_HUMAN	2249.6753
DHCR7_HUMAN	22778.314
TR10D_HUMAN	517.25769
HDAC6_HUMAN	2538.083
SPAST_HUMAN	3669.0649
DKK3_HUMAN	491.9675
TRMB_HUMAN	6781.2197
GULP1_HUMAN	1477.1666
VPS29_HUMAN	16179.647
EIF3K_HUMAN	20323.605
EXTL2_HUMAN	1103.9664

GRHPR_HUMAN	14731.995
CATZ_HUMAN	22154.576
KS6B2_HUMAN	1476.1279
DJB11_HUMAN	11554.876
RNF14_HUMAN	2290.6409
SAE2_HUMAN	17910.51
MO4L1_HUMAN	1631.0812
NXF1_HUMAN	7431.8643
SE1L1_HUMAN	1334.4753
B4GT7_HUMAN	1654.7823
PEF1_HUMAN	4155.2959
ZMYM2_HUMAN	1271.8237
CSN7A_HUMAN	7763.4087
DIC_HUMAN	6458.7549
AASS_HUMAN	8094.2827
CLIP2_HUMAN	1330.2957
QCR9_HUMAN	13366.057
MTFP1_HUMAN	2048.158
ZO2_HUMAN	23693.531
DNJB4_HUMAN	17364.016
ST17A_HUMAN	305.99472
CFDP1_HUMAN	4235.1431
DAXX_HUMAN	745.44934
TRM7_HUMAN	2676.9551
VTI1B_HUMAN	2838.0117
STK39_HUMAN	2481.0515
ADDG_HUMAN	3604.5178
LRWD1_HUMAN	2407.0266
NPS3A_HUMAN	5423.3379
CGBP1_HUMAN	3622.4617
PISD_HUMAN	895.33105
ABCF2_HUMAN	13584.061
TES_HUMAN	6118.1841
AAKG2_HUMAN	4254.7461
GCP4_HUMAN	1763.1443
SYWM_HUMAN	1546.657
LIMD1_HUMAN	800.34607
SEC63_HUMAN	10274.975
GTR6_HUMAN	999.57471
Z3H7B_HUMAN	5937.0972
NDRG3_HUMAN	9379.9395
ARMX3_HUMAN	5183.7168
SWP70_HUMAN	20510.213
SUN2_HUMAN	9739.1533
RLP24_HUMAN	1846.7881
LTOR3_HUMAN	4610.7495
LIMA1_HUMAN	18741.258
AFF4_HUMAN	7961.103
SRP68_HUMAN	12529.336
CHRD1_HUMAN	12642.191

TBK1_HUMAN	4583.3594
SEPT9_HUMAN	15566.203
PCYOX_HUMAN	10903.434
DDX20_HUMAN	5797.6006
SHPK_HUMAN	3831.4028
DPP2_HUMAN	4239.3506
CEIP2_HUMAN	4661.2544
UBP25_HUMAN	2962.9314
BAP29_HUMAN	4172.8149
NB5R1_HUMAN	6421.3315
S30BP_HUMAN	3138.5186
ZNHI2_HUMAN	10482.509
PFD2_HUMAN	9940.8271
GPN3_HUMAN	1559.5245
PUF60_HUMAN	21221.492
NRBP_HUMAN	5101.2632
ENOPH_HUMAN	7848.8945
FEZ2_HUMAN	847.16785
NDUAC_HUMAN	7680.7998
EI2BD_HUMAN	12789.182
VATH_HUMAN	6408.6411
PRAF1_HUMAN	2306.186
IPO11_HUMAN	1765.2128
TR112_HUMAN	1306.6792
DACH1_HUMAN	1258.3801
XPO7_HUMAN	7014.2114
LCMT1_HUMAN	4032.3557
VPS51_HUMAN	4399.6924
BAZ2A_HUMAN	412.63245
BAZ1B_HUMAN	3575.0247
ATIF1_HUMAN	1201.2377
KAD3_HUMAN	21194.535
LCAP_HUMAN	8887.8184
MBD1_HUMAN	445.38007
CNOT7_HUMAN	2699.4104
RABX5_HUMAN	3484.6714
MSRA_HUMAN	2267.4478
NAGK_HUMAN	30574.979
HACL1_HUMAN	3662.1372
TRM6_HUMAN	1573.5568
SH3L2_HUMAN	1949.1914
NGAP_HUMAN	824.79224
TSR3_HUMAN	1388.4059
S2513_HUMAN	16682.922
DBNL_HUMAN	11932.354
DDX41_HUMAN	6297.7568
DCTN4_HUMAN	7310.3569
CDC23_HUMAN	3942.2288
APC7_HUMAN	5471.6187
APC5_HUMAN	962.9563

APC4_HUMAN	2532.5342
ANC2_HUMAN	1988.0452
GGA2_HUMAN	5046.498
GGA1_HUMAN	2644.6841
STML2_HUMAN	15598.256
NAGPA_HUMAN	520.94501
VPS28_HUMAN	8077.4971
LSM7_HUMAN	35358.641
DBR1_HUMAN	3847.4829
TASOR_HUMAN	400.15872
JUPI1_HUMAN	565.6004
FBX3_HUMAN	1384.3639
AKA11_HUMAN	450.74582
FBW1B_HUMAN	1060.7013
GMEB2_HUMAN	1522.0831
MRT4_HUMAN	15487.533
TNIK_HUMAN	1328.7389
CPSF3_HUMAN	5539.3691
DP13A_HUMAN	5470.1694
BORG2_HUMAN	842.73865
TLK1_HUMAN	793.68896
GPTC8_HUMAN	1767.5457
PARP4_HUMAN	9572.0879
NXT1_HUMAN	806.08362
NUDT5_HUMAN	13933.595
RCOR1_HUMAN	1221.4209
PPCT_HUMAN	2146.8718
MA1B1_HUMAN	1956.2916
RALY_HUMAN	27615.125
TF3C4_HUMAN	4710.7886
ERG28_HUMAN	2786.5164
PACN3_HUMAN	938.76868
ACAD8_HUMAN	1519.9553
ANGL2_HUMAN	5077.4263
ACINU_HUMAN	12602.4
AMFR_HUMAN	3965.2878
AGO2_HUMAN	4191.0938
NUP50_HUMAN	7969.5962
CDV3_HUMAN	18424.99
CNO11_HUMAN	1380.1649
INT6_HUMAN	2360.2395
BAG5_HUMAN	2628.6357
AGO1_HUMAN	4407.4375
RAB21_HUMAN	8741.25
RB22A_HUMAN	5372.2344
TPC2L_HUMAN	2055.1401
ZN346_HUMAN	2348.0493
PSME2_HUMAN	15201.021
TAOK2_HUMAN	2624.9409
MKLN1_HUMAN	3486.3755

DNPEP_HUMAN	7886.7891
RAB23_HUMAN	2318.406
MCTS1_HUMAN	14320.52
MTUS1_HUMAN	410.39722
WWC3_HUMAN	880.86395
PALD_HUMAN	5088.0977
S39AA_HUMAN	1335.0865
INO80_HUMAN	292.48926
CCPG1_HUMAN	847.08459
KDIS_HUMAN	888.60779
ASAP1_HUMAN	4258.9209
MRTFB_HUMAN	1766.3433
ANR50_HUMAN	4433.4839
MED23_HUMAN	2721.3096
SHRM4_HUMAN	3506.0999
YETS2_HUMAN	1092.899
NDRG4_HUMAN	603.74304
TBC24_HUMAN	2153.7124
ISY1_HUMAN	1165.1481
TMCC3_HUMAN	3102.9395
HECD1_HUMAN	4555.0752
ZMYD8_HUMAN	1885.0015
CIZ1_HUMAN	310.14572
COR1C_HUMAN	20088.744
TPX2_HUMAN	1569.8633
ABT1_HUMAN	2494.9766
NOB1_HUMAN	6186.5337
AKP8L_HUMAN	3536.8479
MAFF_HUMAN	902.00635
ASC_HUMAN	5353.0498
TMCO1_HUMAN	10970.347
FZR1_HUMAN	351.17715
APC10_HUMAN	900.21729
MYO6_HUMAN	6090.4448
DD19B_HUMAN	1586.2922
PPT2_HUMAN	2645.9143
PRP19_HUMAN	25228.313
SUFU_HUMAN	1219.4065
BOK_HUMAN	2976.5388
NOL7_HUMAN	3529.3545
SNX12_HUMAN	10204.67
SYNRG_HUMAN	1300.5077
VPS4A_HUMAN	8929.9063
G3BP2_HUMAN	11287.54
CHIP_HUMAN	4257.6509
PACN2_HUMAN	10549.824
MAGD2_HUMAN	20197.033
SNX7_HUMAN	2156.5842
SNX6_HUMAN	7817.3115
DUS12_HUMAN	4787.7485

STX8_HUMAN	1848.3099
SSRG_HUMAN	6188.0313
PSD13_HUMAN	36197.164
FAF1_HUMAN	7065.8623
EPCR_HUMAN	19555.625
PPIE_HUMAN	3438.3796
DIM1_HUMAN	5072.689
TIM_HUMAN	999.83569
CSN3_HUMAN	9486.2051
MINP1_HUMAN	3230.4827
NOVA2_HUMAN	7607.8564
WDR3_HUMAN	4047.5947
TTF2_HUMAN	687.07202
NSF1C_HUMAN	8815.2217
COG5_HUMAN	3100.5725
S12A4_HUMAN	6344.4131
AP4E1_HUMAN	344.71545
MACF1_HUMAN	38327.039
CP131_HUMAN	927.36597
SCAF8_HUMAN	2319.7729
PP6R1_HUMAN	1970.0895
TRI33_HUMAN	3085.5603
PHF8_HUMAN	1514.386
TNR6B_HUMAN	830.49438
PLCL2_HUMAN	683.79565
SMG5_HUMAN	656.99768
EXOC7_HUMAN	9852.5498
ZC3H4_HUMAN	191.84468
UBP24_HUMAN	3964.2341
TBD2B_HUMAN	1826.2985
SMAG1_HUMAN	1214.1398
FOXJ3_HUMAN	1049.1946
CBPC1_HUMAN	701.41449
DICER_HUMAN	1008.0495
XCT_HUMAN	962.18799
MARE3_HUMAN	1358.255
SHOC2_HUMAN	4837.2148
SRRM2_HUMAN	15714.562
PA2G4_HUMAN	79411.273
SPG7_HUMAN	1289.6222
BAIP2_HUMAN	817.12299
GAB2_HUMAN	386.30386
SMC3_HUMAN	7327.7622
CHM2B_HUMAN	18923.037
SH2B3_HUMAN	1236.7354
ZN148_HUMAN	306.5162
MTMR6_HUMAN	4356.7559
JAG2_HUMAN	873.13428
NIP7_HUMAN	1817.5261
GLCNE_HUMAN	2973.0039

RTRAF_HUMAN	11229.108
RUVB2_HUMAN	33543.813
CDYL_HUMAN	645.66058
PIN4_HUMAN	6948.7241
AKT3_HUMAN	1373.0989
FACE2_HUMAN	1086.9946
CHKB_HUMAN	711.44086
EIF3L_HUMAN	54197.488
PLAP_HUMAN	11102.363
RUVB1_HUMAN	50832.586
NUDC_HUMAN	29358.709
BCS1_HUMAN	3027.3074
VDAC3_HUMAN	24078.48
COF2_HUMAN	21205.389
ERGI3_HUMAN	2658.3755
SYFA_HUMAN	28874.818
SC5A6_HUMAN	635.55865
RT33_HUMAN	5418.042
ASF1A_HUMAN	2804.0537
DRG1_HUMAN	16386.803
TPPC4_HUMAN	3533.5723
NCKP1_HUMAN	12502.6
CNPY2_HUMAN	10799.519
EXOG_HUMAN	856.0332
EXC6B_HUMAN	2697.1785
PLAK2_HUMAN	15654.511
CARD8_HUMAN	2541.7976
AT11B_HUMAN	333.55521
OFUT2_HUMAN	7247.2622
DLGP4_HUMAN	2492.6736
ST38L_HUMAN	2484.4954
SAC2_HUMAN	2695.6829
FND3A_HUMAN	4431.4146
NISCH_HUMAN	1644.9749
FYV1_HUMAN	1068.8982
WDR37_HUMAN	2162.1443
E41L3_HUMAN	18739.277
AMOL2_HUMAN	157.01611
SIK3_HUMAN	403.07651
KDM2A_HUMAN	1338.54
RRP44_HUMAN	6175.0288
TPPC8_HUMAN	1988.9924
LRCH1_HUMAN	5435.2778
RCL1_HUMAN	3906.1152
GSTK1_HUMAN	10488.204
LTOR2_HUMAN	2105.4531
RT28_HUMAN	10023.505
COA3_HUMAN	4156.0469
DDX52_HUMAN	2886.8936
RT17_HUMAN	5035.1797

RT07_HUMAN	7414.7173
CRYL1_HUMAN	3369.4429
TMA7_HUMAN	6394.2563
PDIP2_HUMAN	9184.8926
AP3M1_HUMAN	12359.779
MAN1_HUMAN	4886.7363
CHSP1_HUMAN	1614.7657
COG6_HUMAN	2532.1763
TR150_HUMAN	17323.746
WBP11_HUMAN	4847.4033
TDRKH_HUMAN	654.92712
MED16_HUMAN	1821.5619
NOP58_HUMAN	11859.232
GIT1_HUMAN	6351.1318
ZN281_HUMAN	522.4314
AR2BP_HUMAN	3854.9834
TMM98_HUMAN	472.14777
SGT1_HUMAN	25354.039
SYYM_HUMAN	7830.2876
NAGA_HUMAN	3883.0547
ACOT9_HUMAN	24365.16
AAR2_HUMAN	1070.6367
NOSIP_HUMAN	4098.3447
DEOC_HUMAN	5678.8252
MEMO1_HUMAN	5687.9575
TMX2_HUMAN	4989.6763
FCF1_HUMAN	1395.8615
LSM2_HUMAN	6034.4995
SHLB1_HUMAN	11587.641
CIA30_HUMAN	2306.8103
CAB39_HUMAN	14215.8
LC7L2_HUMAN	14103.757
UB2J1_HUMAN	6439.5332
RBMX2_HUMAN	1110.0791
DHRS7_HUMAN	4404.5103
RT02_HUMAN	10800.566
UTP11_HUMAN	256.62286
PHOCN_HUMAN	10946.549
RRP7A_HUMAN	1816.1628
SBDS_HUMAN	15135.39
TMED5_HUMAN	9272.8252
EXOS1_HUMAN	3204.5813
TMED7_HUMAN	5051.4111
SF3B6_HUMAN	11454.621
RM11_HUMAN	11759.275
ORN_HUMAN	17935.863
RRP15_HUMAN	3270.2729
WASC3_HUMAN	1489.6144
NOP16_HUMAN	6789.9819
TPRKB_HUMAN	4378.5254

PPIL1_HUMAN	3505.1133
UFC1_HUMAN	5665.6772
CIA2B_HUMAN	252.13031
RT16_HUMAN	4196.5845
RT18C_HUMAN	1476.8813
FIS1_HUMAN	11332.275
TIM16_HUMAN	2277.3687
KAD6_HUMAN	4113.625
RT23_HUMAN	11431.522
GOT1B_HUMAN	4193.9521
HDGR3_HUMAN	3110.887
PTH2_HUMAN	7752.6323
CHMP3_HUMAN	49017.66
STRAP_HUMAN	18922.215
RTCB_HUMAN	45989.715
FBX7_HUMAN	4190.4321
RAP2C_HUMAN	1860.4667
STA13_HUMAN	2723.8215
RBGP1_HUMAN	1791.9211
TMED3_HUMAN	1085.2504
T22D4_HUMAN	1685.5143
DOP2_HUMAN	526.41199
ZN330_HUMAN	727.91968
R3HC1_HUMAN	1219.2705
NOC2L_HUMAN	7753.2954
RL36_HUMAN	64032.742
CCDC9_HUMAN	2427.1396
CHTOP_HUMAN	2462.0554
SAMH1_HUMAN	8170.1841
SKAP_HUMAN	858.34155
HBS1L_HUMAN	4663.5278
AAKB1_HUMAN	1093.5492
WIPI4_HUMAN	205.3573
DMXL1_HUMAN	1568.4243
VPP2_HUMAN	3070.7478
TLN1_HUMAN	92446.109
KIF3A_HUMAN	1850.9077
TRRAP_HUMAN	2510.2083
MTCL1_HUMAN	492.13904
DCAF1_HUMAN	594.60669
TCAF1_HUMAN	6807.5215
RBM19_HUMAN	937.41095
DAAM1_HUMAN	501.14224
PLXD1_HUMAN	18170.342
HECD4_HUMAN	266.22253
WAC2C_HUMAN	404.98715
UBP15_HUMAN	6398.1084
FARP1_HUMAN	1562.777
MARF1_HUMAN	721.81293
C170B_HUMAN	758.8559

RPGF2_HUMAN	9243.377
MYO5A_HUMAN	3927.6804
DTNA_HUMAN	407.53198
LOXL2_HUMAN	6261.5728
CRBG1_HUMAN	2910.051
TRAF6_HUMAN	2000.8711
M4K5_HUMAN	3168.5437
HYOU1_HUMAN	28483.855
ATG4B_HUMAN	5758.1611
TBL2_HUMAN	3813.9595
WIPI2_HUMAN	3055.4993
TELO2_HUMAN	2594.0161
LAS1L_HUMAN	6099.9038
AFG32_HUMAN	8873.707
KLF12_HUMAN	412.92401
ARI1_HUMAN	5515.2085
LSM4_HUMAN	25032.766
RN114_HUMAN	2946.4744
SAM50_HUMAN	14432.792
PRC2C_HUMAN	19453.863
OARD1_HUMAN	7689.999
IFT25_HUMAN	1398.748
PPME1_HUMAN	10612.709
RBM7_HUMAN	2347.8318
TIM22_HUMAN	1139.5984
NUB1_HUMAN	2243.8926
YTHD2_HUMAN	3175.6028
CTDP1_HUMAN	3300.6714
PAXB1_HUMAN	1010.4158
NDK7_HUMAN	2838.0667
SP16H_HUMAN	16974.988
UTP18_HUMAN	2751.3083
UCHL5_HUMAN	17333.18
CD2AP_HUMAN	11113.668
VATD_HUMAN	5735.3389
TNPO3_HUMAN	6326.1646
SRPRB_HUMAN	11493.294
CERT_HUMAN	1598.2985
GMPPB_HUMAN	17107.764
FADS3_HUMAN	944.85596
TF3C5_HUMAN	6285.9985
TF3C3_HUMAN	3579.2532
TPPC1_HUMAN	6084.4854
TRPV2_HUMAN	1274.3011
MRCKB_HUMAN	10937.781
RBM8A_HUMAN	18371.268
DJC15_HUMAN	917.00732
UBP16_HUMAN	1136.3905
TSSC4_HUMAN	1814.5662
ZN706_HUMAN	5443.9038

MAGD1_HUMAN	2685.2749
SNX14_HUMAN	375.61218
SNX11_HUMAN	961.75891
SNX9_HUMAN	11214.371
SNX8_HUMAN	1435.1802
SNX5_HUMAN	6602.6807
LIPG_HUMAN	1659.1708
NUBP2_HUMAN	6379.6357
PEX16_HUMAN	955.75055
LYVE1_HUMAN	5484.5894
BACE2_HUMAN	1778.7261
HEBP2_HUMAN	757.26617
HCFC2_HUMAN	866.35156
UBIA1_HUMAN	1340.1307
PUS1_HUMAN	4676.4014
LRRF2_HUMAN	4494.5303
FHOD1_HUMAN	7040.8975
SERC_HUMAN	21329.461
NCOR2_HUMAN	2272.0969
ORNT1_HUMAN	1425.2449
JAM1_HUMAN	16372.585
NPTN_HUMAN	1549.62
AGRG1_HUMAN	1110.1757
SPIN1_HUMAN	2820.4536
S12A7_HUMAN	7736.9443
ALG6_HUMAN	1587.3042
ALG5_HUMAN	8030.355
RT18B_HUMAN	4507.8428
COPG1_HUMAN	29432.047
AUP1_HUMAN	2733.5239
FKBP7_HUMAN	1712.9015
CLIC4_HUMAN	47521.098
NFS1_HUMAN	3218.2568
CFA20_HUMAN	9598.0977
TACC3_HUMAN	244.42647
SPCS1_HUMAN	5290.5513
SAR1B_HUMAN	5765.4541
AP4B1_HUMAN	967.76147
MTCH2_HUMAN	21306.813
SELK_HUMAN	1768.6885
BIG2_HUMAN	3123.5566
BIG1_HUMAN	4520.498
MD1L1_HUMAN	3448.1582
STK24_HUMAN	12850.699
5MP1_HUMAN	43845.074
COMDA_HUMAN	2867.5427
DC1L1_HUMAN	18950.514
EPN1_HUMAN	3614.6023
UBP3_HUMAN	19574.52
TX264_HUMAN	5644.1807

CABIN_HUMAN	658.00842
CEPT1_HUMAN	9281.3008
DNM3A_HUMAN	619.98273
OAS3_HUMAN	2239.2971
KAD5_HUMAN	17895.168
NEMO_HUMAN	3802.6611
IF2B2_HUMAN	23147.301
ZNT1_HUMAN	3127.7314
S4A7_HUMAN	3048.3789
NDUB9_HUMAN	13161.195
SQOR_HUMAN	2646.2844
ROBO1_HUMAN	833.66248
STON1_HUMAN	665.82397
NCOA3_HUMAN	634.96808
NUMBL_HUMAN	1659.8477
M3K4_HUMAN	1734.9276
DDX49_HUMAN	1073.9187
CAN7_HUMAN	906.78284
WASF2_HUMAN	10434.783
SCC4_HUMAN	2013.7933
MORC2_HUMAN	929.59631
NS1BP_HUMAN	1716.6335
S23IP_HUMAN	16072.944
GAL3B_HUMAN	4468.6426
IFT56_HUMAN	2552.6008
UBA6_HUMAN	16844.234
ESYT2_HUMAN	14710.5
SHOT1_HUMAN	2711.2744
TM223_HUMAN	1724.4995
RHG10_HUMAN	2825.8496
HACL2_HUMAN	7757.3804
SPD2B_HUMAN	11433.829
NBAS_HUMAN	2531.6238
TYW5_HUMAN	370.8284
DEND3_HUMAN	1228.676
VWA8_HUMAN	1814.9907
SBNO1_HUMAN	2151.6709
GTPBA_HUMAN	1427.5754
WDR91_HUMAN	885.25323
DEN11_HUMAN	674.41241
PDES1_HUMAN	717.8241
TPC13_HUMAN	1195.8505
CNOT1_HUMAN	9471.3418
MP3B2_HUMAN	23017.842
PGP_HUMAN	9335.0498
RIMC1_HUMAN	5154.3481
TCAF2_HUMAN	6400.1675
SMHD1_HUMAN	5031.0752
CAST2_HUMAN	2102.0779
U119B_HUMAN	2401.2776

MET15_HUMAN	753.21466
CC85C_HUMAN	404.04123
RTL8C_HUMAN	3226.8989
ZSWM8_HUMAN	901.70251
P121C_HUMAN	1954.9529
P20L1_HUMAN	238.76212
RUXGL_HUMAN	13922.684
ELAP2_HUMAN	1536.0389
NUD19_HUMAN	2733.8743
MI4GD_HUMAN	1085.432
CALM1_HUMAN	3431.811
MYO9A_HUMAN	494.22943
GPHRA_HUMAN	1363.6661
WASH3_HUMAN	7531.4297
MCRI1_HUMAN	713.45386
NACAM_HUMAN	54709.305
CK098_HUMAN	4060.5679
ASURF_HUMAN	788.65698
MIDUO_HUMAN	2634.585
DNS2A_HUMAN	4299.1182
ADAS_HUMAN	16136.15
KIF2A_HUMAN	9003.8018
DX39A_HUMAN	66072.102
PDLI1_HUMAN	9685.3438
BACH_HUMAN	46782.371
MYO1C_HUMAN	31092.164
SNP23_HUMAN	8230.0303
HAX1_HUMAN	1497.7723
AIP_HUMAN	7716.5283
GTPB1_HUMAN	4814.708
LEG9_HUMAN	515.38446
STXB3_HUMAN	8198.1982
AP4M1_HUMAN	2079.4636
ARVC_HUMAN	6299.7847
SMAP_HUMAN	1629.4629
AP3B1_HUMAN	14041.009
APBB1_HUMAN	1784.1959
LEG8_HUMAN	1269.5409
NDUS8_HUMAN	629.29657
IKBE_HUMAN	1477.6169
PSD11_HUMAN	39160.523
PSD12_HUMAN	26968.246
PSMD9_HUMAN	3137.144
MEN1_HUMAN	679.61115
GET1_HUMAN	2956.1892
PGRC1_HUMAN	1343.4133
SPT5H_HUMAN	3986.7415
TAF4_HUMAN	228.25563
DFFA_HUMAN	1592.4746
HIP1_HUMAN	2555.1423

CLIC1_HUMAN	194302.58
EIF3F_HUMAN	25472.654
WWP2_HUMAN	594.45264
ODPX_HUMAN	4261.522
QSOX1_HUMAN	3165.5203
DCTN6_HUMAN	1509.0487
ACATN_HUMAN	4208.0698
WASL_HUMAN	6236.3022
IPO5_HUMAN	41879.387
RPOM_HUMAN	503.26898
EF2K_HUMAN	3756.0732
SAP18_HUMAN	10606.579
EMAL1_HUMAN	2493.425
IF2B3_HUMAN	36831.18
DNM1L_HUMAN	40501.617
RTCA_HUMAN	7443.1465
P3C2A_HUMAN	5967.521
GOLI4_HUMAN	2189.9917
AGRIN_HUMAN	2700.8945
PLOD2_HUMAN	38988.004
EXOC5_HUMAN	9487.1436
BT3A1_HUMAN	647.62964
NDUA4_HUMAN	5797.3735
PSDE_HUMAN	42907.105
BIN1_HUMAN	4021.7537
CLD5_HUMAN	4128.2837
IMA4_HUMAN	26380.697
STK25_HUMAN	3042.3811
KRIT1_HUMAN	293.19324
VMA5A_HUMAN	1400.1963
PESC_HUMAN	6206.1558
SDCB1_HUMAN	41557.555
PITM1_HUMAN	440.77515
MPP10_HUMAN	2209.8994
NOP56_HUMAN	3262.6484
DDX3X_HUMAN	60952.414
MFNG_HUMAN	491.5506
PODXL_HUMAN	35489.551
CCN1_HUMAN	47807.168
PIR_HUMAN	36975.129
IMA3_HUMAN	6141.0093
TRI38_HUMAN	623.66888
NFIB_HUMAN	9835.6855
PPP6_HUMAN	8763.1035
NDKM_HUMAN	2103.9983
P3C2B_HUMAN	1020.4486
MA2B1_HUMAN	2613.2004
PDXK_HUMAN	4903.4009
SCD_HUMAN	4423.8433
ARI1A_HUMAN	2493.5588

NEMP1_HUMAN	951.17255
TXND9_HUMAN	3423.7786
DPYL4_HUMAN	28499.574
TRAD1_HUMAN	1380.0782
COX7R_HUMAN	330.27393
UBFD1_HUMAN	5721.2261
CTRO_HUMAN	1728.9003
COPE_HUMAN	16306.269
BORG1_HUMAN	1716.1425
AP3D1_HUMAN	12200.85
CCS_HUMAN	1575.5598
ABLM1_HUMAN	4170.6924
DVL2_HUMAN	1193.2902
CHD1_HUMAN	2831.3906
CHD2_HUMAN	2693.3945
GOSR2_HUMAN	4362.8687
TOR1A_HUMAN	5914.96
TOR1B_HUMAN	4109.7358
STX16_HUMAN	2319.0869
ADA10_HUMAN	9510.6924
ABCD4_HUMAN	1465.3732
EI24_HUMAN	4069.5847
P5I11_HUMAN	2992.2358
KMT2D_HUMAN	234.15622
APAF_HUMAN	2166.8662
RIOK3_HUMAN	1139.6643
MP2K7_HUMAN	474.12421
ACOT8_HUMAN	1747.9287
CDIPT_HUMAN	42195.879
PDCD5_HUMAN	2827.1069
ANM5_HUMAN	18869.877
NHRF1_HUMAN	4695.3052
CHK1_HUMAN	990.72461
TR10B_HUMAN	6120.3091
TPP1_HUMAN	18024.838
TCRG1_HUMAN	16291.554
NDC80_HUMAN	2264.2571
KIF3C_HUMAN	172.14803
NRP1_HUMAN	14180.423
TNPO2_HUMAN	2735.6829
UN13B_HUMAN	853.8056
TR10C_HUMAN	286.46185
RPC1_HUMAN	2155.333
TSN4_HUMAN	424.32654
PSA7_HUMAN	98033.18
SCAM3_HUMAN	4790.7773
BCKD_HUMAN	2499.6155
MGST3_HUMAN	6363.3599
TX1B3_HUMAN	10414.412
GIPC1_HUMAN	6728.2798

IKKB_HUMAN	2461.2195
RGS12_HUMAN	1262.6003
TIM23_HUMAN	2355.5535
HAT1_HUMAN	11631.208
CSKP_HUMAN	2409.6763
PLD2_HUMAN	1251.5088
QCR8_HUMAN	16388.416
ML12B_HUMAN	64307.57
HGS_HUMAN	6308.0381
RAB7L_HUMAN	491.00766
VP26C_HUMAN	1775.7869
MYPT1_HUMAN	11407.918
GAK_HUMAN	5248.9458
HNRDL_HUMAN	16051.161
XPO1_HUMAN	33141.16
BTAF1_HUMAN	1281.7064
ARHGA_HUMAN	3024.4729
ZN609_HUMAN	1367.8086
SPTN2_HUMAN	1020.0316
MAST4_HUMAN	1371.0358
SC16A_HUMAN	6717.8574
PLXB2_HUMAN	2079.9497
KHNYN_HUMAN	340.48422
TCPR2_HUMAN	821.84277
SR140_HUMAN	24408.131
SET1A_HUMAN	612.58911
N4BP3_HUMAN	469.75961
SYNJ2_HUMAN	3832.2542
SYNEM_HUMAN	6462.9165
GRRE1_HUMAN	1379.9766
KIF3B_HUMAN	1527.3802
PUR4_HUMAN	23882.168
ANR28_HUMAN	1436.465
ARHGB_HUMAN	405.06824
MRPP3_HUMAN	1599.2911
IKKA_HUMAN	2000.3649
NPC1_HUMAN	3824.2612
DEGS1_HUMAN	2806.1519
ANGP2_HUMAN	6297.522
SCAM1_HUMAN	4759.1597
SCAM2_HUMAN	2421.6633
ARC1B_HUMAN	40465.141
ARPC2_HUMAN	44860.84
ARPC3_HUMAN	46537.211
RPAC1_HUMAN	8120.0996
PLS1_HUMAN	1038.9615
TIF1A_HUMAN	485.83371
PGRC2_HUMAN	10311.62
RGL2_HUMAN	2049.29
WDR46_HUMAN	410.66589

NKRF_HUMAN	1658.5537
GNPAT_HUMAN	2682.0994
LAMA5_HUMAN	1177.3143
ZN185_HUMAN	6695.7261
CASC3_HUMAN	488.98569
RT12_HUMAN	2072.2671
ACOX3_HUMAN	2334.4241
RER1_HUMAN	17660.424
SURF4_HUMAN	23408.082
MK13_HUMAN	2110.3384
SPTC1_HUMAN	6800.8193
SPTC2_HUMAN	9926.3193
OGT1_HUMAN	6126.0156
PMM2_HUMAN	13645.127
TM9S1_HUMAN	2018.4564
TRI18_HUMAN	1231.7479
HMGB3_HUMAN	25465.223
PPM1G_HUMAN	9819.5234
SHIP2_HUMAN	4419.1807
EIF3D_HUMAN	24783.695
EIF3H_HUMAN	32436.328
HDAC3_HUMAN	1593.6749
NVL_HUMAN	1468.7228
BCAT2_HUMAN	8004.7188
IPO8_HUMAN	5033.6826
STX7_HUMAN	5970.3286
MOT4_HUMAN	13580.948
MRP4_HUMAN	2203.9629
RPA34_HUMAN	1031.9427
P4HA2_HUMAN	5389.1904
CAN5_HUMAN	583.21136
YKT6_HUMAN	44906.086
NUP42_HUMAN	1309.0662
ARPC5_HUMAN	38749.609
MAFG_HUMAN	124.73845
PDPK1_HUMAN	1149.7922
TPSN_HUMAN	12297.789
R113A_HUMAN	445.79272
KDM6A_HUMAN	851.56427
DHX15_HUMAN	33695.254
MCES_HUMAN	6226.1821
ZZEF1_HUMAN	3246.8445
ASAP2_HUMAN	1407.9734
FLRT2_HUMAN	1021.4991
TTI1_HUMAN	1500.1549
RRP8_HUMAN	1744.5326
PJA2_HUMAN	694.67249
CYB5B_HUMAN	1906.9757
PRP4_HUMAN	7281.4297
SERA_HUMAN	26693.531

NDUS4_HUMAN	3804.3667
DC1L2_HUMAN	12200.152
PSMD3_HUMAN	42910.547
RFOX2_HUMAN	2036.754
PAPS1_HUMAN	15714.237
ZW10_HUMAN	7041.894
B4GT5_HUMAN	318.87598
SNUT1_HUMAN	7431.9502
GPAA1_HUMAN	6663.0371
DAPK3_HUMAN	6214.7036
TGFI1_HUMAN	1933.5153
AP5Z1_HUMAN	826.68787
CTIF_HUMAN	2988.833
MTSS1_HUMAN	642.16248
VIP2_HUMAN	1886.1265
M3K7_HUMAN	671.20752
MCA3_HUMAN	25191.127
RIPK2_HUMAN	2062.3745
HNRPR_HUMAN	59193.152
PRPF3_HUMAN	8549.5859
TXNL1_HUMAN	49819.414
TPD54_HUMAN	20771.748
EMC8_HUMAN	4455.0361
ERI3_HUMAN	2770.6262
SYNJ1_HUMAN	1621.4327
FIBP_HUMAN	1518.9198
IF4G3_HUMAN	24886.156
PPIH_HUMAN	5098.188
HTRA2_HUMAN	3230.5933
MS18B_HUMAN	200.37938
ARK72_HUMAN	13581.838
E41L2_HUMAN	12500.327
TGON2_HUMAN	932.94128
B4GA1_HUMAN	967.32806
WIPF1_HUMAN	1813.9125
AT8B1_HUMAN	1413.952
AKA10_HUMAN	410.01791
DENR_HUMAN	14586.795
XPOT_HUMAN	17153.721
DNPH1_HUMAN	10330.482
TIM44_HUMAN	9847.9463
TPPC3_HUMAN	16989.063
CHM2A_HUMAN	9266.9141
NCK2_HUMAN	2375.7917
TSN6_HUMAN	2354.1873
PLRG1_HUMAN	6107.0439
PRC1_HUMAN	1578.0629
RGS10_HUMAN	1989.6028
ZN207_HUMAN	2229.5442
NDUB5_HUMAN	5392.1143

NDUB3_HUMAN	9906.7217
NDUA2_HUMAN	3153.9524
LDB2_HUMAN	2555.5083
GET3_HUMAN	24773.863
BUB1_HUMAN	801.3891
BUB3_HUMAN	28853.135
ST1B1_HUMAN	8373.7627
ACTN4_HUMAN	59928.367
BUD23_HUMAN	847.57007
HTSF1_HUMAN	2832.1638
ERD23_HUMAN	2241.4868
AP1G1_HUMAN	12166.582
STX6_HUMAN	1831.0811
SNG2_HUMAN	4228.0244
SGTA_HUMAN	2315.7241
ENSA_HUMAN	7208.6182
MCAT_HUMAN	7453.4111
SYNC_HUMAN	37610.832
MYO1B_HUMAN	6267.9971
PM34_HUMAN	655.97992
CPSF5_HUMAN	16518.236
LANC1_HUMAN	11266.702
STRN_HUMAN	5443.5254
U3IP2_HUMAN	3476.5088
SCO2_HUMAN	1133.7357
CF410_HUMAN	276.21844
AKAP8_HUMAN	2797.9597
GTPB6_HUMAN	901.77399
G6PT1_HUMAN	1292.1051
IDH3B_HUMAN	8391.9668
NRDC_HUMAN	12564.435
CALU_HUMAN	17924.127
EDIL3_HUMAN	4554.5527
ATP9B_HUMAN	779.64063
SAHH2_HUMAN	1531.1266
KIF1C_HUMAN	3501.2268
EXTL3_HUMAN	645.24573
ORC5_HUMAN	1086.147
NDUS5_HUMAN	12048.302
PDE6D_HUMAN	4073.9358
ORC4_HUMAN	833.64825
PEX1_HUMAN	1417.7372
RAD21_HUMAN	5962.144
DHX16_HUMAN	4332.4014
GMFG_HUMAN	3831.7939
3BP5_HUMAN	2870.783
AGRB3_HUMAN	1519.5728
MED14_HUMAN	12740.826
KPRB_HUMAN	6682.5425
SMCA5_HUMAN	5797.2817

JIP4_HUMAN	91729.547
NUAK1_HUMAN	478.50458
NPA1P_HUMAN	748.09094
SI1L3_HUMAN	288.23444
ZC3H1_HUMAN	312.35959
AQR_HUMAN	7850.0522
OPA1_HUMAN	6518.3359
ZEB2_HUMAN	1304.0684
GANP_HUMAN	1902.6
PCDGC_HUMAN	1153.2385
PI51C_HUMAN	3334.8911
KIF1B_HUMAN	2831.1968
MABP1_HUMAN	961.45056
KDM1A_HUMAN	5655.7773
TBCD4_HUMAN	9290.6748
FADS1_HUMAN	2245.9253
GSDME_HUMAN	8000.3813
EVI5_HUMAN	342.10968
NRP2_HUMAN	7297.084
MA1A2_HUMAN	3335.4492
MPZL2_HUMAN	3992.9509
ACSL4_HUMAN	10764.716
SNX3_HUMAN	31114.523
OGA_HUMAN	7250.9702
VINEX_HUMAN	3796.5999
HNRPQ_HUMAN	41026.242
PRP17_HUMAN	854.07813
RNBP6_HUMAN	32674.926
NEMF_HUMAN	6296.1475
GMDS_HUMAN	4790.8364
NMT2_HUMAN	5043.8296
CCNT1_HUMAN	1741.4246
BUB1B_HUMAN	1458.571
PLOD3_HUMAN	24790.814
IF4E2_HUMAN	5723.0518
DIAP1_HUMAN	72100.914
TSN3_HUMAN	7555.0713
EXOC3_HUMAN	10999.44
PLIN3_HUMAN	21065.934
ANM3_HUMAN	1210.2017
IMA7_HUMAN	8622.8018
UGDH_HUMAN	38035.18
TPST2_HUMAN	2293.8572
LPXN_HUMAN	1069.4365
CTND1_HUMAN	41210.074
ICMT_HUMAN	5468.543
EIF1B_HUMAN	2091.7578
SNX2_HUMAN	17773.596
DPM1_HUMAN	16955.289
USO1_HUMAN	21148.863

RT14_HUMAN	1588.8292
TOM1_HUMAN	3651.8342
H2B1K_HUMAN	120852.28
CCD22_HUMAN	4255.5352
PQBP1_HUMAN	3113.0181
TI17B_HUMAN	783.90198
PRAF2_HUMAN	9291.5391
DKC1_HUMAN	8497.1797
IF2P_HUMAN	30945.172
EDF1_HUMAN	23435.588
KIN17_HUMAN	1680.4463
DIAP2_HUMAN	7476.0142
DNJA2_HUMAN	8426.8555
BRD4_HUMAN	3306.3794
HUS1_HUMAN	712.42719
PFD1_HUMAN	1783.9598
PP1RB_HUMAN	1913.5861
NBN_HUMAN	11645.728
MCE1_HUMAN	1656.7664
ABCB7_HUMAN	2694.0168
SRGP2_HUMAN	1745.4418
PLXA2_HUMAN	6321.8105
XYLK_HUMAN	1076.4025
WDR1_HUMAN	106638.38
ATP9A_HUMAN	3109.0032
N4BP1_HUMAN	851.63049
ROCK2_HUMAN	7857.2236
CLAP2_HUMAN	3316.6665
PTCD1_HUMAN	369.65546
CPNE3_HUMAN	17408.51
ATG13_HUMAN	1529.1587
HIP1R_HUMAN	10616.253
BRE1B_HUMAN	4912.7007
ZC11A_HUMAN	2994.7334
CLU_HUMAN	7514.4678
CAND2_HUMAN	2016.6046
T22D2_HUMAN	334.6044
DJC13_HUMAN	7706.772
PHAR2_HUMAN	1271.1178
PP6R2_HUMAN	1429.2317
ATS4_HUMAN	1894.8881
CNOT3_HUMAN	5334.0791
ANR17_HUMAN	4420.6953
SIN3B_HUMAN	617.51935
DNJB6_HUMAN	3135.9426
COQ9_HUMAN	1281.9728
GGCT_HUMAN	6744.7964
NDUS7_HUMAN	12107.489
NDUS2_HUMAN	10819.063
ZPR1_HUMAN	3220.7288

DUS11_HUMAN	435.22324
NIPS2_HUMAN	10433.871
HMMR_HUMAN	373.45993
PDCD6_HUMAN	13605.762
TBCA_HUMAN	18914.822
VATG1_HUMAN	11963.506
VPS4B_HUMAN	63824.914
MPU1_HUMAN	21123.096
H2AY_HUMAN	1284.9852
SH3L1_HUMAN	1726.1969
FLNB_HUMAN	123160.42
NCOR1_HUMAN	6310.938
PEX14_HUMAN	4869.1992
TRIM3_HUMAN	3678.6067
CISY_HUMAN	109470.16
SPAG7_HUMAN	8478.7568
SC22B_HUMAN	39050.898
PR40A_HUMAN	11916.132
TACC1_HUMAN	8651.1611
NDK6_HUMAN	1341.4017
CDC45_HUMAN	865.51794
LRCH4_HUMAN	519.07434
MTX2_HUMAN	1773.525
VP26A_HUMAN	36243.309
MPPB_HUMAN	12032.41
MED24_HUMAN	1051.135
KTNA1_HUMAN	2698.4741
PSIP1_HUMAN	1370.9312
ERLN1_HUMAN	7154.2729
NDUS3_HUMAN	19441.639
SRS10_HUMAN	4589.834
CLN5_HUMAN	1769.2
CLD11_HUMAN	1338.693
ECI2_HUMAN	13540.238
KHDR3_HUMAN	758.89307
EED_HUMAN	2131.8032
BAF_HUMAN	3530.2825
SF3B1_HUMAN	25356
CSDE1_HUMAN	19246.211
WBP4_HUMAN	1517.4263
PRKRA_HUMAN	4602.7974
ITA10_HUMAN	2291.6199
KS6A5_HUMAN	4205.4585
MED6_HUMAN	379.16605
MYCB2_HUMAN	2108.9929
KBL_HUMAN	1533.5645
NPM3_HUMAN	3759.5327
LYPA1_HUMAN	13059.183
ERAL1_HUMAN	5581.1108
U520_HUMAN	17076.301

MTU1_HUMAN	1390.6144
TIPRL_HUMAN	9939.3369
OFD1_HUMAN	1847.0807
KS6A4_HUMAN	1604.4507
SURF6_HUMAN	1024.5406
PPM1B_HUMAN	2961.5469
UTP20_HUMAN	4889.4961
NU155_HUMAN	11757.696
XRP2_HUMAN	7877.0669
WDHD1_HUMAN	3395.7312
CRTAP_HUMAN	17227.635
S2512_HUMAN	11407.61
RENR_HUMAN	2075.6372
RNH2A_HUMAN	2102.7249
CD123_HUMAN	3168.1008
BCAR3_HUMAN	555.83966
POP7_HUMAN	2188.0991
RPP40_HUMAN	1307.8846
EIF3G_HUMAN	24164.914
EIF3J_HUMAN	20229.148
CBR3_HUMAN	13336.259
PSD10_HUMAN	3620.1445
AP1G2_HUMAN	2701.0588
FACE1_HUMAN	13761.771
IDHC_HUMAN	51783.633
GATB_HUMAN	1152.8085
SCO1_HUMAN	7048.4839
ATRN_HUMAN	757.19482
RBBP9_HUMAN	8554.2695
STAM2_HUMAN	4737.5742
DGAT1_HUMAN	679.70734
CCNK_HUMAN	3961.0469
PRAF3_HUMAN	58936.66
DYSF_HUMAN	25717.646
PIAS1_HUMAN	1107.3286
SPF27_HUMAN	757.45953
DCTN3_HUMAN	13471.489
DNJC8_HUMAN	14953.709
SPF30_HUMAN	3610.4766
RAD17_HUMAN	215.3795
ATP5H_HUMAN	4682.3105
TSN9_HUMAN	2558.8452
FLOT1_HUMAN	5907.3657
TRIO_HUMAN	4128.585
ATP5L_HUMAN	36977.105
MPDZ_HUMAN	2524.2297
CBPD_HUMAN	9147.4902
GLRX3_HUMAN	51232.902
RL1D1_HUMAN	6941.0688
WFS1_HUMAN	7204.9092

CLPX_HUMAN	5233.6021
S14L2_HUMAN	4299.5552
SYUG_HUMAN	1051.2031
CIAO1_HUMAN	3422.9673
SRP72_HUMAN	11446.723
DDAH1_HUMAN	10802.776
RECQ5_HUMAN	764.22882
RMP_HUMAN	1472.4478
B3GA3_HUMAN	1009.6856
MTA2_HUMAN	12227.223
AL1A2_HUMAN	6089.1694
STK10_HUMAN	6903.8389
KPCD3_HUMAN	1950.2693
GFPT2_HUMAN	1991.2811
SLIT2_HUMAN	3198.1392
LTN1_HUMAN	2289.53
TOM70_HUMAN	8646.6475
PKHG5_HUMAN	936.36475
IPO13_HUMAN	1127.4185
DDHD2_HUMAN	1114.7997
MYO1D_HUMAN	8697.9063
TOX4_HUMAN	3142.4399
MICA2_HUMAN	3002.5171
SC24D_HUMAN	17824.008
FCSD2_HUMAN	472.03488
UFL1_HUMAN	11161.668
SRBS2_HUMAN	7957.4736
SASH1_HUMAN	3630.2759
UBXN7_HUMAN	5514.4746
KLH18_HUMAN	355.78998
SUN1_HUMAN	965.79016
PLPHP_HUMAN	8176.5835
ERLN2_HUMAN	12659.186
PRP6_HUMAN	12972.769
PCF11_HUMAN	741.87848
FRYL_HUMAN	8072.2168
NFAT5_HUMAN	292.14761
ENDD1_HUMAN	1192.5065
GLCE_HUMAN	5082.2925
GLSK_HUMAN	22653.984
HAUS5_HUMAN	811.86304
ABLM3_HUMAN	1283.8374
FBX21_HUMAN	687.16534
MTCL2_HUMAN	1656.1373
UBP19_HUMAN	1088.684
WDR47_HUMAN	1381.1816
AP2A2_HUMAN	22585.355
SC31A_HUMAN	42013.652
ARHGF_HUMAN	9005.0869
HEXI1_HUMAN	1032.7599

RPP14_HUMAN	1771.7974
CCNB2_HUMAN	91.761017
YIF1A_HUMAN	5203.0591
UBR5_HUMAN	2184.6272
AGFG2_HUMAN	2974.1873
PRS23_HUMAN	3615.8489
SCAF4_HUMAN	1984.172
NDUB6_HUMAN	6700.6592
MFN2_HUMAN	2188.405
SPN1_HUMAN	1021.2525
UBE4B_HUMAN	5289.2339
ZFPL1_HUMAN	4756.2422
ELP1_HUMAN	5181.7666
NDUB4_HUMAN	5582.4712
NDUB8_HUMAN	6230.4946
NDUA7_HUMAN	4218.1372
VAMP5_HUMAN	1220.181
RTN3_HUMAN	9667.0957
LETM1_HUMAN	14106.78
EPN2_HUMAN	3006.9238
ZRAB2_HUMAN	7485.1411
SNX4_HUMAN	3844.1865
LC7L3_HUMAN	3990.9473
KI20A_HUMAN	753.78149
APOL3_HUMAN	679.18317
KIF4A_HUMAN	4612.2358
MTMR5_HUMAN	1220.634
GOSR1_HUMAN	8324.5039
KAT7_HUMAN	1233.5255
ATE1_HUMAN	1611.7206
VAPB_HUMAN	4712.104
SNAPN_HUMAN	2758.7788
MPZL1_HUMAN	2244.7483
NDUC2_HUMAN	8761.0137
NDUAA_HUMAN	13603.062
FKBP9_HUMAN	13341.068
CELF2_HUMAN	1164.5781
6PGL_HUMAN	5516.4727
PAPS2_HUMAN	24208.529
SMC2_HUMAN	11356.031
ATG7_HUMAN	7232.4253
TACC2_HUMAN	1337.9214
TRI16_HUMAN	3577.7361
SYFM_HUMAN	1315.0793
ZBT7A_HUMAN	170.40826
LYPA2_HUMAN	4491.1372
IPO7_HUMAN	36776.73
ARI2_HUMAN	1224.3961
SLU7_HUMAN	724.84143
AGM1_HUMAN	16366.031

MOCS3_HUMAN	850.57178
CD2B2_HUMAN	2740.7961
SVIL_HUMAN	6519.0054
AHSA1_HUMAN	36388.051
PARN_HUMAN	2593.9382
PSMG1_HUMAN	11010.814
SGPL1_HUMAN	3192.51
CNEP1_HUMAN	3168.6558
NSA2_HUMAN	1012.825
G6PE_HUMAN	8479.7695
SC24A_HUMAN	7731.2251
SC24B_HUMAN	7914.209
AGRL2_HUMAN	1679.7609
NADK_HUMAN	820.32819
TYDP2_HUMAN	1344.5421
MPC2_HUMAN	2021.4548
ETHE1_HUMAN	7960.3584
ACSL3_HUMAN	13367.029
RPA1_HUMAN	2973.3464
YETS4_HUMAN	793.12482
ZBT11_HUMAN	289.71863
CNOT4_HUMAN	1492.0591
STABP_HUMAN	1305.1366
CPSF4_HUMAN	1717.8082
NFAC1_HUMAN	835.53796
ASML_HUMAN	5736.4824
CDS2_HUMAN	836.91888
PPR3D_HUMAN	853.31244
BRD1_HUMAN	422.55453
RPP29_HUMAN	1222.0903
HERC2_HUMAN	877.30469
RAB3D_HUMAN	2051.2129
SNP29_HUMAN	4769.5146
OXSR1_HUMAN	5925.6899
GGPPS_HUMAN	1426.6527
HS74L_HUMAN	7173.8276
PTBP3_HUMAN	12198.629
TBCD8_HUMAN	521.73895
IL33_HUMAN	642.42676
STR3N_HUMAN	2773.7861
LSM8_HUMAN	966.47229
AP2A1_HUMAN	28039.459
WIZ_HUMAN	1724.4446
RIGI_HUMAN	6302.9194
STAU1_HUMAN	14621.004
TTC4_HUMAN	4528.626
TM50A_HUMAN	2376.3379
CAVN2_HUMAN	42658.789
BAG2_HUMAN	11209.475
BAG3_HUMAN	1992.0145

M4K4_HUMAN	10636.084
DCMC_HUMAN	476.06512
QORL1_HUMAN	1550.1758
AIFM1_HUMAN	18386.902
EMAL2_HUMAN	995.67932
LATS1_HUMAN	1187.0082
NUD14_HUMAN	4483.5107
TSN15_HUMAN	1119.1263
BPNT1_HUMAN	6031.5044
FADS2_HUMAN	24417.98
DDAH2_HUMAN	481.04617
ABHGA_HUMAN	3172.7981
TXD12_HUMAN	280.00482
ECD_HUMAN	4217.0649
RECK_HUMAN	2389.3914
MBD3_HUMAN	2410.0688
TOP3B_HUMAN	448.18036
NUDT3_HUMAN	3441.4482
BCL10_HUMAN	4811.5449
NDUBA_HUMAN	13741.941
CLPT1_HUMAN	6504.4883
ZBED1_HUMAN	800.45563
MOC2B_HUMAN	1042.582
TOM40_HUMAN	19529.436
PX11B_HUMAN	3432.3315
PAK4_HUMAN	5892.7847
APBA3_HUMAN	477.16345
ACL6A_HUMAN	9846.5254
NSD2_HUMAN	755.26251
LDHA_HUMAN	276362.16
AL1A1_HUMAN	18385.193
DHE3_HUMAN	41177.652
DYR_HUMAN	13532.967
NB5R3_HUMAN	56864.336
GSHR_HUMAN	16578.639
COX1_HUMAN	810.33215
COX2_HUMAN	77013.805
PNPH_HUMAN	66762.109
HPRT_HUMAN	26719.035
AATM_HUMAN	52738.953
ABL1_HUMAN	1029.9448
PGK1_HUMAN	164945.31
KAD1_HUMAN	28070.092
UROK_HUMAN	614.63702
TPA_HUMAN	434.03461
ADA_HUMAN	3382.1877
A1AT_HUMAN	26342.334
A2MG_HUMAN	327.17508
CYTC_HUMAN	3749.3196
RASN_HUMAN	26859.207

RASH_HUMAN	3088.2119
RASK_HUMAN	3063.7407
PDGFB_HUMAN	495.24603
LDLR_HUMAN	3060.2246
TGFB1_HUMAN	3711.115
HLAB_HUMAN	5458.7397
HLAH_HUMAN	65434.859
CO1A1_HUMAN	2127.4351
CO4A1_HUMAN	761.07855
LMNA_HUMAN	143771.78
APOA1_HUMAN	3158.5261
FINC_HUMAN	28554.26
FETUA_HUMAN	1547.9863
ALBU_HUMAN	89530.039
TFR1_HUMAN	15877.21
FRIL_HUMAN	16702.602
FRIH_HUMAN	12377.72
MT2_HUMAN	8556.7021
NU1M_HUMAN	5358.043
NU4M_HUMAN	2251.406
NU5M_HUMAN	3116.1272
MMP1_HUMAN	6878.2314
HMDH_HUMAN	266.84961
CATA_HUMAN	7902.124
RAF1_HUMAN	1693.6843
GBA1_HUMAN	15482.665
ALDOA_HUMAN	284012.06
CYTB_HUMAN	5452.27
ANXA1_HUMAN	188631.92
APOB_HUMAN	1489.3705
PRIO_HUMAN	1374.1697
SODM_HUMAN	3167.3184
OAT_HUMAN	24587.105
KITH_HUMAN	2742.3525
VWF_HUMAN	77837.313
TBB4A_HUMAN	80204.391
G3P_HUMAN	447942.75
ARLY_HUMAN	7139.48
HLAA_HUMAN	41306.707
CPNS1_HUMAN	25350.904
MT1E_HUMAN	134.00432
HSPB1_HUMAN	132126.92
TYSY_HUMAN	9804.3184
RPN1_HUMAN	73927.008
RPN2_HUMAN	55459.133
GNAI2_HUMAN	30867.137
H2A1B_HUMAN	90611.797
B3A2_HUMAN	1070.6798
AT1A1_HUMAN	49066.883
AT1B1_HUMAN	8668.9443

A4_HUMAN	6844.4985
ALDH2_HUMAN	30362.455
ITB3_HUMAN	7021.519
HMGN1_HUMAN	730.69
PAI2_HUMAN	10293.52
PAI1_HUMAN	62049.707
ADT2_HUMAN	196999.17
ISG15_HUMAN	5262.0444
PCCA_HUMAN	3128.0112
PCCB_HUMAN	4727.7178
IF2A_HUMAN	54645.473
HMGN2_HUMAN	452.76315
ICAM1_HUMAN	5993.1763
RLA2_HUMAN	5392.6729
RLA0_HUMAN	175591.69
JUN_HUMAN	1296.677
RPC4_HUMAN	1657.4628
LA_HUMAN	33353.207
ITB1_HUMAN	103206.98
K1C18_HUMAN	48969.383
CO5A2_HUMAN	2138.9663
DCUP_HUMAN	28536.203
FYN_HUMAN	567.86212
AGAL_HUMAN	1145.326
GELS_HUMAN	11868.028
RB_HUMAN	2345.4648
PTMA_HUMAN	4081.3691
CDK1_HUMAN	29409.283
ATPB_HUMAN	181751.88
S10A9_HUMAN	1190.8127
S10A6_HUMAN	10456.442
IF4E_HUMAN	17182.25
ENOA_HUMAN	610737.81
PYGL_HUMAN	8699.6904
G6PI_HUMAN	64757.125
DPOLB_HUMAN	610.53143
NPM_HUMAN	60064.258
TPM3_HUMAN	58384.891
ITAV_HUMAN	15629.549
HEXA_HUMAN	15217.717
HYEP_HUMAN	10794.562
LDHB_HUMAN	203626.77
CENPB_HUMAN	1007.5602
GPX1_HUMAN	15371.357
PDIA1_HUMAN	160528.77
H10_HUMAN	14317.637
ACYP1_HUMAN	3677.425
FES_HUMAN	1163.6174
CATD_HUMAN	70982.297
ANXA2_HUMAN	401304.38

CAN1_HUMAN	28555.814
TBB5_HUMAN	501343.84
SAP_HUMAN	5485.7378
HEXB_HUMAN	17037.111
CATL1_HUMAN	1114.9225
PROF1_HUMAN	259132.3
PMGE_HUMAN	3927.071
APT_HUMAN	57821.156
SYEP_HUMAN	21194.881
CATB_HUMAN	72089.305
HS90A_HUMAN	147744.28
GALT_HUMAN	506.2616
HNRPC_HUMAN	99217.047
LAMB1_HUMAN	25436.201
YES_HUMAN	15139.182
LYN_HUMAN	2858.3506
FUMH_HUMAN	29849.066
ERCC1_HUMAN	534.24438
TSP1_HUMAN	358831.44
SP1_HUMAN	921.15601
IGF1R_HUMAN	1241.8528
CO1A2_HUMAN	662.71289
ANXA6_HUMAN	99851.578
RHOC_HUMAN	85209.672
DAF_HUMAN	5983.728
4F2_HUMAN	14550.838
BGLR_HUMAN	1349.501
PFKAM_HUMAN	10915.712
HS90B_HUMAN	725146.94
SRPRA_HUMAN	11801.939
ASNS_HUMAN	4445.5371
MMP2_HUMAN	5288.9536
HEM3_HUMAN	2456.981
NEP_HUMAN	582.03571
MGP_HUMAN	5138.6602
ODPA_HUMAN	29207.896
CO4A2_HUMAN	6040.0938
CY1_HUMAN	15791.964
RU2B_HUMAN	7747.3833
MET_HUMAN	3630.4492
RU17_HUMAN	46318.645
ITA5_HUMAN	66638.703
NFIC_HUMAN	1263.8628
VIME_HUMAN	1317269
RS17_HUMAN	36197.855
GNAI3_HUMAN	4118.9268
ANXA5_HUMAN	439125.06
RSSA_HUMAN	114590.54
CD63_HUMAN	5967.1206
RM03_HUMAN	5630.689

SNRPA_HUMAN	19697.002
ENOG_HUMAN	10628.902
THIK_HUMAN	6377.0918
SRP19_HUMAN	5498.9307
GSTP1_HUMAN	135933.72
RU1C_HUMAN	4397.0532
LEG1_HUMAN	30742.666
DHPR_HUMAN	9156.2383
HMGB1_HUMAN	73829.5
SPRC_HUMAN	3250.9016
TPM1_HUMAN	59965.047
CLCA_HUMAN	1947.5333
CLCB_HUMAN	1116.2185
ANXA4_HUMAN	14746.716
CN37_HUMAN	12509.333
HMOX1_HUMAN	4903.3125
DLDH_HUMAN	17055.379
ROA1_HUMAN	137826.78
RU2A_HUMAN	22419.244
COX6C_HUMAN	17160.623
TACD2_HUMAN	3103.7871
PARP1_HUMAN	37670.242
DPOLA_HUMAN	12689.87
UCHL1_HUMAN	8878.5566
LKHA4_HUMAN	35382.652
ALDOC_HUMAN	23519.236
H2AZ_HUMAN	3484.5361
H2A1_HUMAN	885.43079
MYZAP_HUMAN	469.75162
GRL1A_HUMAN	592.84967
GST2_HUMAN	4311.7695
POTEJ_HUMAN	11751.104
LIMS3_HUMAN	3304.9072
HS71A_HUMAN	88699.438
ERPG3_HUMAN	1552.5098
TBA3C_HUMAN	52321.156
ZTRF1_HUMAN	850.8736
RAP2A_HUMAN	8479.0938
SRGN_HUMAN	1005.5443
RO60_HUMAN	10788.145
LYAG_HUMAN	5351.6582
RRAS_HUMAN	11102.299
HLAC_HUMAN	1645.9794
ARAF_HUMAN	1696.0419
H14_HUMAN	353373.28
ODP2_HUMAN	22713.148
PTPRF_HUMAN	4515.334
COT1_HUMAN	4535.4912
THIO_HUMAN	4342.2568
COX5B_HUMAN	1274.4722

PPGB_HUMAN	8364.1758
MGST1_HUMAN	10801.557
KAP0_HUMAN	16410.092
TFPI1_HUMAN	1848.3192
ESTD_HUMAN	20002.229
CH60_HUMAN	141947.16
CLUS_HUMAN	5918.0186
BIP_HUMAN	252987.31
LAMC1_HUMAN	20947.055
PPAL_HUMAN	7252.4541
MTAP2_HUMAN	3830.0352
HSP7C_HUMAN	244134.11
GTR1_HUMAN	3998.1772
GTR3_HUMAN	2850.2595
EPB41_HUMAN	24165.588
UMPS_HUMAN	13582.648
ODPB_HUMAN	15390.098
ODB2_HUMAN	1598.261
PYGB_HUMAN	14768.42
PYGM_HUMAN	32490.182
RALA_HUMAN	33150.637
RALB_HUMAN	58339.363
BCR_HUMAN	4791.6372
LAMP1_HUMAN	84835.211
ERG_HUMAN	6353.9033
ACADM_HUMAN	14356.357
TOP1_HUMAN	37285.742
TOP2A_HUMAN	285.23938
G6PD_HUMAN	66390.758
UBL4A_HUMAN	937.65863
PYC_HUMAN	3697.8416
C1TC_HUMAN	53556.289
MPRI_HUMAN	4889.626
ADHX_HUMAN	35702.793
CDK4_HUMAN	2221.6682
PRPS2_HUMAN	20569.91
PABP1_HUMAN	114503.39
PCNA_HUMAN	27517.893
HARS1_HUMAN	36505.684
CO6A1_HUMAN	1360.4901
ADT1_HUMAN	234125.44
ADT3_HUMAN	85997.578
IMDH2_HUMAN	38878.184
TPR_HUMAN	14709.576
ANXA3_HUMAN	51904.699
ODBA_HUMAN	6074.3945
ACTN1_HUMAN	98552.5
ACE_HUMAN	15760.677
SRC_HUMAN	3434.2815
PEPD_HUMAN	6984.1343

XRCC6_HUMAN	95963.086
XRCC5_HUMAN	58325.984
UNG_HUMAN	966.35913
COX41_HUMAN	58606.285
HEM1_HUMAN	728.08905
LAMP2_HUMAN	51789.383
RINI_HUMAN	43735.238
ICAM2_HUMAN	49800.965
EF2_HUMAN	187491.69
PDIA4_HUMAN	122885.87
P4HA1_HUMAN	8787.7324
TCTP_HUMAN	14439.328
HEM2_HUMAN	5419.2251
HLAE_HUMAN	1320.7235
PLST_HUMAN	144573.52
ACPH_HUMAN	16697.203
ETFA_HUMAN	26854.727
GYS1_HUMAN	4760.2974
KAP2_HUMAN	7338.2295
T2FB_HUMAN	4430.2168
CD59_HUMAN	15458.929
MTDC_HUMAN	4507.8672
MIF_HUMAN	29979.174
CD99_HUMAN	1947.6268
GLU2B_HUMAN	65260.586
HCLS1_HUMAN	6057.2178
FPPS_HUMAN	63926.699
CX7A2_HUMAN	15834.817
NID1_HUMAN	16721.605
AK1A1_HUMAN	26933.137
KPYM_HUMAN	453083.16
ENPL_HUMAN	353101.78
CCNB1_HUMAN	1397.6921
MYL6B_HUMAN	9144.5166
RSMB_HUMAN	43101.18
IDE_HUMAN	20502.65
PO2F1_HUMAN	532.86945
HNRPL_HUMAN	18389.783
SYDC_HUMAN	38106.102
ETS1_HUMAN	1438.9286
PLAK_HUMAN	23856.498
QCR7_HUMAN	1114.1005
BRAF_HUMAN	4061.5173
FABP4_HUMAN	25823.721
GLNA_HUMAN	519.02069
ALDR_HUMAN	46898.004
AMPN_HUMAN	49508.758
PVR_HUMAN	17255.336
RAC2_HUMAN	20676.588
ERF3A_HUMAN	13032.252

ARSA_HUMAN	1473.9585
B4GT1_HUMAN	1979.7817
EZRI_HUMAN	38227.328
UCHL3_HUMAN	5022.021
FOSL1_HUMAN	708.31537
FOSL2_HUMAN	257.65909
MCP_HUMAN	4471.3315
NDKA_HUMAN	29037.215
NQO1_HUMAN	23934.361
GNS_HUMAN	45424.223
PHKG2_HUMAN	2138.637
ARSB_HUMAN	2001.9135
RS2_HUMAN	149721.59
ITF2_HUMAN	1030.6749
DESP_HUMAN	1061.6613
RFA2_HUMAN	7837.7437
TIMP2_HUMAN	4122.9746
CD44_HUMAN	9736.0303
CBR1_HUMAN	22881.875
ACADS_HUMAN	2039.0155
CREB1_HUMAN	3362.6589
BGAL_HUMAN	10184.401
PECA1_HUMAN	69069.141
PP2BB_HUMAN	5577.5801
NCK1_HUMAN	10272.495
GCFC2_HUMAN	963.7301
H15_HUMAN	206612.89
H13_HUMAN	30135.336
H12_HUMAN	35235.059
NCPR_HUMAN	11289.073
FER_HUMAN	378.98511
AT2A2_HUMAN	39722.488
PLCG2_HUMAN	7823.6948
FAAA_HUMAN	5687.3091
STMN1_HUMAN	74150.953
YBOX3_HUMAN	14301.558
ZNF22_HUMAN	1412.0472
ZNF24_HUMAN	371.12015
NAGAB_HUMAN	3845.2429
HMGA1_HUMAN	38494.941
TMM11_HUMAN	4474.3682
AATC_HUMAN	22008.74
KPCA_HUMAN	5290.5845
ITA2_HUMAN	19255.355
CXA1_HUMAN	1434.1969
ASM_HUMAN	1067.2051
UBF1_HUMAN	10891.781
JUND_HUMAN	649.40997
TAL1_HUMAN	926.25024
ATF7_HUMAN	108.10201

NDUB7_HUMAN	5468.0513
KAPCA_HUMAN	13360.885
CAN2_HUMAN	42658.043
PTN2_HUMAN	1948.0505
PYRG1_HUMAN	16398.572
EGLN_HUMAN	48395.148
DDX5_HUMAN	82167.938
PFKAL_HUMAN	48365.688
VGFR1_HUMAN	11565.915
PRS6A_HUMAN	45130.746
TCPA_HUMAN	62917.191
PTN1_HUMAN	18645.172
ERCC2_HUMAN	3988.5125
RL35A_HUMAN	76628.328
ITB5_HUMAN	7842.7896
ARF4_HUMAN	107533.48
RL7_HUMAN	179082.22
VINC_HUMAN	79798.641
PTPRA_HUMAN	2652.5037
ARY1_HUMAN	1504.8458
SON_HUMAN	8107.1392
NELFE_HUMAN	4610.8501
RL17_HUMAN	97389.063
PGAM1_HUMAN	81436.531
RCC1_HUMAN	22127.004
ATF1_HUMAN	903.05914
ATF6A_HUMAN	584.02826
DNLI1_HUMAN	7003.8726
XRCC1_HUMAN	4503.5879
AMD_HUMAN	982.50482
CADH2_HUMAN	12381.799
PLCG1_HUMAN	11434.098
NUCL_HUMAN	165180.09
HXK1_HUMAN	49559.16
RPB3_HUMAN	2133.28
RPAB1_HUMAN	4838.856
NDUV2_HUMAN	3819.5964
ERCC3_HUMAN	2522.4893
RO52_HUMAN	5558.8511
E2AK2_HUMAN	10972.529
SPEE_HUMAN	48496.25
SL9A1_HUMAN	1416.959
CSK22_HUMAN	6974.6621
NFKB1_HUMAN	4735.7285
AT2B1_HUMAN	5881.7852
IF2B_HUMAN	23038.336
ANXA7_HUMAN	21922.613
TBP_HUMAN	352.32263
BTF3_HUMAN	5828.5244
RAB3B_HUMAN	2004.2919

RAB4A_HUMAN	4046.7876
RAB5A_HUMAN	57676.438
RAB6A_HUMAN	39659.773
MSH3_HUMAN	1254.8945
ANPRB_HUMAN	485.06537
PSB1_HUMAN	38606.957
MPRD_HUMAN	4852.231
LMNB1_HUMAN	67851.734
ICAL_HUMAN	15497.595
IMDH1_HUMAN	5372.1313
CO5A1_HUMAN	1994.2399
NEBU_HUMAN	1405.9019
ASPG_HUMAN	2412.5151
RASA1_HUMAN	6384.8286
PTMS_HUMAN	745.51648
CD11B_HUMAN	9130.0791
GSTM3_HUMAN	1736.7367
VATB2_HUMAN	12639.174
VATC1_HUMAN	4783.356
CSRP1_HUMAN	62311.055
FLNA_HUMAN	243659.23
NF1_HUMAN	610.07959
AOFA_HUMAN	1587.427
ACOHC_HUMAN	22729.074
S1PR1_HUMAN	8490.8672
SYT1_HUMAN	1372.1155
5NTD_HUMAN	14339.871
TAF1_HUMAN	207.58118
MK_HUMAN	1820.3666
VDAC1_HUMAN	87815.125
PGS1_HUMAN	13323.935
SDHB_HUMAN	20045.715
CD9_HUMAN	19736.104
COMT_HUMAN	5795.1558
TGM2_HUMAN	348884.91
MUTA_HUMAN	8666.416
OSBP1_HUMAN	7660.707
PIMT_HUMAN	2127.3005
FBRL_HUMAN	12936.19
PUR2_HUMAN	24475.604
PUR6_HUMAN	46450.762
SCP2_HUMAN	22668.041
UBA1_HUMAN	69630.523
NDKB_HUMAN	126880.27
ADRO_HUMAN	12535.548
ROA2_HUMAN	129182.35
RFX1_HUMAN	895.9856
CBL_HUMAN	4567.4077
IBP4_HUMAN	1062.8502
KAPCB_HUMAN	1582.3395

QCR2_HUMAN	19707.611
HEMH_HUMAN	4371.8848
XPA_HUMAN	697.27277
TCEA1_HUMAN	12554.472
PGH1_HUMAN	670.02417
ITA6_HUMAN	20350.889
SFPQ_HUMAN	86660.297
TBG1_HUMAN	13106.305
PPIB_HUMAN	153263.47
MAOM_HUMAN	15222.096
SYWC_HUMAN	29549.488
RS3_HUMAN	209672.25
KS6B1_HUMAN	3098.1365
JAK1_HUMAN	5480.8159
PTPRB_HUMAN	13126.255
PTPRE_HUMAN	7573.6885
PTPRG_HUMAN	1294.5524
SP100_HUMAN	2656.6873
CRCM_HUMAN	434.2388
TNFL4_HUMAN	7476.2725
SAHH_HUMAN	83815.82
COF1_HUMAN	586159.19
IF4B_HUMAN	17351.609
HAP40_HUMAN	1985.4801
AT2B4_HUMAN	8642.5039
DGKA_HUMAN	4702.2109
CPT2_HUMAN	3384.9963
KTHY_HUMAN	20310.758
RIR1_HUMAN	15646.453
CCND1_HUMAN	960.60614
RAE1_HUMAN	1279.3309
ERD21_HUMAN	1397.006
COT2_HUMAN	2313.115
EF1B_HUMAN	15500.949
AT5F1_HUMAN	19471.775
PPAC_HUMAN	13632.503
KPCL_HUMAN	3693.4473
THIL_HUMAN	27423.246
MYL9_HUMAN	1464.9716
RPB1_HUMAN	37768.5
CDK2_HUMAN	10976.918
ARBK1_HUMAN	4496.4966
PAR1_HUMAN	1588.6245
MCM3_HUMAN	46235.242
THTM_HUMAN	7370.1528
RS12_HUMAN	26383.051
BRD2_HUMAN	1244.3556
TNR6_HUMAN	139.99019
TYY1_HUMAN	2195.5977
DNJB1_HUMAN	9227.2012

DNJB2_HUMAN	654.32361
ATPA_HUMAN	216540.36
PSA1_HUMAN	97756.398
PSA2_HUMAN	25770
PSA3_HUMAN	39444.68
PSA4_HUMAN	81164.172
TNR5_HUMAN	1901.1812
ITA3_HUMAN	26690.691
PTX3_HUMAN	2254.8621
MOES_HUMAN	142785.5
DDX6_HUMAN	17727.469
DNMT1_HUMAN	8478.1299
U2AF2_HUMAN	41830.805
RL13_HUMAN	122626.97
RAE2_HUMAN	771.76825
IVD_HUMAN	1673.5735
MGAT1_HUMAN	4344.1123
HMGB2_HUMAN	21543.889
PTBP1_HUMAN	65846.688
SYTC_HUMAN	31379.861
SYVC_HUMAN	23048.941
EF1G_HUMAN	130137.44
FKBP2_HUMAN	16921.68
IL3RA_HUMAN	1141.1208
STOM_HUMAN	33681.813
KAD4_HUMAN	1716.929
1433T_HUMAN	59260.852
MK03_HUMAN	16909.611
MARK3_HUMAN	746.54297
RL10_HUMAN	44539.473
CO8A1_HUMAN	2233.0222
RFA1_HUMAN	17823.732
APEX1_HUMAN	31830.777
CD82_HUMAN	1209.8412
DCK_HUMAN	2959.6765
PYR1_HUMAN	35715.129
CALR_HUMAN	193251.94
MAP4_HUMAN	11087.22
CALX_HUMAN	122920.43
P85A_HUMAN	1525.9473
IP3KB_HUMAN	1081.9662
PSB8_HUMAN	3898.1472
PSB9_HUMAN	4863.0615
PSA5_HUMAN	30642.213
PSB4_HUMAN	20631.906
PSB6_HUMAN	43803.332
PSB5_HUMAN	35100.137
GSTM2_HUMAN	1025.3303
ABCD3_HUMAN	7730.2358
ITPI2_HUMAN	4167.1157

LYOX_HUMAN	2526.2354
NDUS1_HUMAN	17659.488
DPOD1_HUMAN	11592.604
SMCA1_HUMAN	1066.7626
MK01_HUMAN	51024.961
RXRB_HUMAN	1249.41
ERCC5_HUMAN	1016.8108
GRN_HUMAN	4285.1582
PTPRM_HUMAN	1204.9523
AMPL_HUMAN	37067.035
CD34_HUMAN	5673.0986
T2EA_HUMAN	2993.5093
T2EB_HUMAN	3898.7231
TPP2_HUMAN	13878.236
IMPA1_HUMAN	6824.7124
CCN2_HUMAN	29980.697
EPHA2_HUMAN	13131.43
EPHB2_HUMAN	12488.573
SHC1_HUMAN	5024.5313
3MG_HUMAN	6648.3286
TKT_HUMAN	138790.25
CASP1_HUMAN	678.11737
NOS3_HUMAN	4204.2124
RBMS1_HUMAN	4627.4409
PML_HUMAN	10611.957
TYK2_HUMAN	1374.6906
EF1D_HUMAN	22629.428
MARCS_HUMAN	41394.527
GNA11_HUMAN	17191.086
AL4A1_HUMAN	4213.9487
ERP29_HUMAN	29995.951
PRDX6_HUMAN	141627.3
BLVRB_HUMAN	7456.4692
PRDX5_HUMAN	27309.777
PRDX3_HUMAN	23008.619
RL12_HUMAN	112779.38
ECHM_HUMAN	14824.208
KCY_HUMAN	26452.508
PEBP1_HUMAN	30181.23
PDIA3_HUMAN	165797.47
2AAA_HUMAN	63035.313
2AAB_HUMAN	4997.2944
CDC27_HUMAN	4742.5508
PPIF_HUMAN	12481.347
NKTR_HUMAN	1282.9032
NMT1_HUMAN	22145.51
HMOX2_HUMAN	16636.695
PURA2_HUMAN	66582.859
UFO_HUMAN	2559.3196
AMRP_HUMAN	5497.6499

TSPO_HUMAN	11078.35
PUR8_HUMAN	17932.479
CLIP1_HUMAN	5798.5459
SORCN_HUMAN	4550.7588
ILEU_HUMAN	6475.229
CTR1_HUMAN	4103.2539
AL1B1_HUMAN	6928.3086
RPB2_HUMAN	8378.1475
SDHA_HUMAN	11509.906
COR1A_HUMAN	1003.3621
GDIA_HUMAN	21372.582
METK2_HUMAN	44267.496
KAP3_HUMAN	10303.737
RIR2_HUMAN	13025.328
TIA1_HUMAN	17802.967
SC6A6_HUMAN	440.72247
DNJA1_HUMAN	13948.522
AKT1_HUMAN	2741.4985
QCR1_HUMAN	88522.766
3HIDH_HUMAN	5092.5063
PUR9_HUMAN	33197.777
HNRH3_HUMAN	5552.2002
HNRH1_HUMAN	57819.012
1433B_HUMAN	174187.08
STIP1_HUMAN	122003.63
S10AB_HUMAN	17868.898
PRDX2_HUMAN	49011.879
ARRB2_HUMAN	726.33307
GLPK_HUMAN	1138.52
DCTD_HUMAN	7524.4077
P5CR1_HUMAN	5869.6504
GBP1_HUMAN	4451.7446
GBP2_HUMAN	4612.5469
ELF1_HUMAN	1146.7428
TF2H1_HUMAN	1168.7849
STX2_HUMAN	440.24872
RL9_HUMAN	79325.211
ACSL1_HUMAN	4452.1245
CADH5_HUMAN	30522.021
KINH_HUMAN	35957.754
DUT_HUMAN	7241.8032
MRP1_HUMAN	3979.1626
ABCD1_HUMAN	856.08276
TTK_HUMAN	372.32047
MCM4_HUMAN	12407.355
MCM5_HUMAN	32433.793
MCM7_HUMAN	23184.078
GALNS_HUMAN	360.39893
GLYC_HUMAN	5398.2173
GLYM_HUMAN	59371.355

HS71L_HUMAN	1855.8326
HSP74_HUMAN	34877.172
GRK5_HUMAN	128.43695
MPI_HUMAN	3920.0356
GPC1_HUMAN	480.41098
PROF2_HUMAN	19487.246
CTNA1_HUMAN	55170.379
CTNB1_HUMAN	49227.195
PHB1_HUMAN	121449.93
SPB6_HUMAN	24988.234
MERL_HUMAN	3385.3521
RADI_HUMAN	425129.59
RFA3_HUMAN	1902.6868
RFC4_HUMAN	8161.687
RFC2_HUMAN	8519.4619
RFC1_HUMAN	3897.7341
RL22_HUMAN	130449.09
T2FA_HUMAN	7509.1475
SPRE_HUMAN	6877.5615
HRH1_HUMAN	235.82732
CBS_HUMAN	730.06305
FBN1_HUMAN	3213.8845
FBN2_HUMAN	283.20303
GDE_HUMAN	4945.1694
MYH9_HUMAN	224768.06
MYH10_HUMAN	198674.38
TIE1_HUMAN	17926.566
COPB2_HUMAN	47608.535
SOAT1_HUMAN	2667.1516
ADDA_HUMAN	7986.3286
BASI_HUMAN	59026.02
FUS_HUMAN	62293.383
NU214_HUMAN	7219.4634
DEK_HUMAN	22814.52
SOX18_HUMAN	2036.5066
MYH11_HUMAN	65512.309
PPM1A_HUMAN	5342.4321
HMGCL_HUMAN	4604.0024
VGFR3_HUMAN	9284.5449
VGFR2_HUMAN	4061.7383
PRS7_HUMAN	61191.875
GGT5_HUMAN	2384.2583
ARL2_HUMAN	11100.563
ARL3_HUMAN	25248.979
MP2K2_HUMAN	17354.842
ATPG_HUMAN	40860.777
VATE1_HUMAN	8161.9507
HEM6_HUMAN	11822.081
RL4_HUMAN	154295.63
8ODP_HUMAN	1315.7467

LONM_HUMAN	12621.245
PGM1_HUMAN	9981.2813
PP1G_HUMAN	9918.1357
GNL1_HUMAN	32393.02
ODO2_HUMAN	22670.816
GMPR1_HUMAN	3665.0625
GPX4_HUMAN	11568.813
ACVL1_HUMAN	3162.5974
DHB2_HUMAN	1072.007
SRP14_HUMAN	24934.115
TGFR2_HUMAN	4222.0044
NUP62_HUMAN	471.56339
HPCL1_HUMAN	7003.561
FDFT_HUMAN	10701.715
ZEB1_HUMAN	1955.3652
TAGL2_HUMAN	41691.48
TALDO_HUMAN	116449.91
SYUA_HUMAN	3244.1187
ETFB_HUMAN	19523.031
RBMX_HUMAN	15055.801
COIL_HUMAN	2321.6826
VKGC_HUMAN	2683.2751
LICH_HUMAN	4027.9543
VATA_HUMAN	19676.891
GRP75_HUMAN	125030.61
IF4A3_HUMAN	30071.496
SMBP2_HUMAN	800.38336
CDN1A_HUMAN	1003.2487
RS19_HUMAN	100188.38
RL3_HUMAN	43747.359
COIA1_HUMAN	31997.48
OST48_HUMAN	95166.289
AN32A_HUMAN	38561.984
FEN1_HUMAN	34033.824
CUX1_HUMAN	500.03812
CAPG_HUMAN	23230.379
CAP2_HUMAN	4023.4465
IL6RB_HUMAN	6402.7417
TXLNA_HUMAN	9276.082
TCPZ_HUMAN	110590.36
NNMT_HUMAN	40219.199
PSB10_HUMAN	2431.3748
PBX2_HUMAN	1633.8324
RL13A_HUMAN	131163.52
ARL1_HUMAN	24702.354
MLH1_HUMAN	10750.232
STAT3_HUMAN	9043.9746
UBP8_HUMAN	5973.2842
MDHC_HUMAN	80200.141
MDHM_HUMAN	125855.48

RFC5_HUMAN	11102.717
RFC3_HUMAN	4644.1021
ECHA_HUMAN	37430.363
IF2G_HUMAN	39337.211
ETV3_HUMAN	359.70901
CETN2_HUMAN	1847.5833
ETV6_HUMAN	2539.4414
EIF2D_HUMAN	4799.9287
BUD31_HUMAN	2850.1729
UBA7_HUMAN	2015.6145
NAA10_HUMAN	17296.732
KDM5C_HUMAN	300.19388
CSK_HUMAN	10482.427
GARS_HUMAN	23071.227
SYIC_HUMAN	51846.789
S19A1_HUMAN	1438.5892
EIF1_HUMAN	4520.9111
KPCI_HUMAN	447.45441
ELK3_HUMAN	1084.0717
ACTY_HUMAN	27290.854
ECI1_HUMAN	7600.2495
LAP2A_HUMAN	7799.46
LAP2B_HUMAN	98976.594
STAT1_HUMAN	21788.109
STAT6_HUMAN	6520.5479
STA5A_HUMAN	7395.3892
MTREX_HUMAN	12167.612
AK1C3_HUMAN	3536.6892
PK3CA_HUMAN	1751.6013
PK3CB_HUMAN	1079.6199
MTOR_HUMAN	10358.368
PI4KA_HUMAN	4710.7354
EPS15_HUMAN	8129.1694
CASP3_HUMAN	20720.006
CASP2_HUMAN	552.94891
RS27_HUMAN	10366.143
ABL2_HUMAN	634.20142
HELZ_HUMAN	1615.1757
CNDD3_HUMAN	1516.639
RBM34_HUMAN	7752.8794
LPPRC_HUMAN	43936.648
THIM_HUMAN	12069.981
RL35_HUMAN	61474.926
PCP_HUMAN	25193.01
HD_HUMAN	4736.1333
ECE1_HUMAN	15311.042
MTHR_HUMAN	759.13953
SATT_HUMAN	1398.8016
LIS1_HUMAN	21606.68
MUC18_HUMAN	103660.07

CACP_HUMAN	4895.3062
MATR3_HUMAN	29877.945
MSH2_HUMAN	13282.729
GRK6_HUMAN	2509.7717
GPDM_HUMAN	10870.295
SSRA_HUMAN	32058.232
PTN9_HUMAN	2288.5613
RANG_HUMAN	51744.609
NAMPT_HUMAN	93104.172
PRS6B_HUMAN	31103.574
EFTS_HUMAN	7422.2842
PPIC_HUMAN	8204.2969
VDAC2_HUMAN	83533.117
ACDSB_HUMAN	3626.498
CBX5_HUMAN	2069.0259
UBP5_HUMAN	37743.996
MK08_HUMAN	1607.9247
MP2K4_HUMAN	5947.458
KI67_HUMAN	2420.4426
KPB2_HUMAN	1371.7751
KPB1_HUMAN	509.83105
RAGP1_HUMAN	23565.051
RECQ1_HUMAN	26122.945
NOP2_HUMAN	8096.0703
ATRX_HUMAN	2213.7598
CRK_HUMAN	6879.4189
CRKL_HUMAN	5957.1602
IF2M_HUMAN	3172.698
BAG6_HUMAN	3895.063
NSF_HUMAN	21683.389
NOTC1_HUMAN	3325.4941
MP2K3_HUMAN	5886.9722
BRCC3_HUMAN	2294.3027
RL27A_HUMAN	186484.42
RL5_HUMAN	58786.793
RL21_HUMAN	40034.426
RL28_HUMAN	46390.93
RS9_HUMAN	211083.3
RS5_HUMAN	112566.97
RS10_HUMAN	72141.359
MAP1B_HUMAN	25159.895
GNPI1_HUMAN	15420.433
NEDD4_HUMAN	3051.0789
UTRN_HUMAN	11204.164
IQGA1_HUMAN	163991.09
GLYG_HUMAN	15972.204
STT3A_HUMAN	48576.605
PA24A_HUMAN	1979.0374
CAZA2_HUMAN	28028.766
CAPZB_HUMAN	43555.266

IF1AX_HUMAN	8848.8857
AL1A3_HUMAN	429.48096
SYQ_HUMAN	31633.91
RL29_HUMAN	33737.5
LEG7_HUMAN	241.08423
TISD_HUMAN	918.23474
UCRI_HUMAN	17167.521
ATPO_HUMAN	56282.125
LIMS1_HUMAN	17224.354
GLIP1_HUMAN	509.60425
PPCE_HUMAN	20628.65
MAOX_HUMAN	18432.484
IREB2_HUMAN	533.22937
TFPI2_HUMAN	441.99124
RFX5_HUMAN	4558.4409
PI42A_HUMAN	4824.9766
COPD_HUMAN	48334.266
LSS_HUMAN	22954.367
PP2BC_HUMAN	1259.9514
GSH1_HUMAN	3111.6611
GSH0_HUMAN	9910.9932
CD151_HUMAN	7814.1748
TPC10_HUMAN	764.22504
PSMD8_HUMAN	38588.82
PRC2A_HUMAN	2518.1941
GSHB_HUMAN	16436.176
TCPE_HUMAN	97974.789
PTSS1_HUMAN	5444.2095
NEST_HUMAN	48609.605
HSP13_HUMAN	9616.4365
KC1A_HUMAN	10043.628
KC1D_HUMAN	4387.8916
IDHP_HUMAN	76030.273
PK3CG_HUMAN	2346.4189
PIPNB_HUMAN	16738.99
DPOD2_HUMAN	5918.168
MRP_HUMAN	17591.57
PAXI_HUMAN	4690.356
CAMLG_HUMAN	568.68207
NR2C2_HUMAN	875.98187
MAPK2_HUMAN	2139.0891
DNSL1_HUMAN	1122.1676
AL9A1_HUMAN	13720.315
RL34_HUMAN	57906.18
RPIA_HUMAN	3436.4451
LMAN1_HUMAN	22439.16
NRAM2_HUMAN	945.23413
NASP_HUMAN	28029.924
FAS_HUMAN	69559.938
FNTA_HUMAN	10864.643

FNTB_HUMAN	4460.5029
DHYS_HUMAN	2275.7012
TCPG_HUMAN	79885.047
RM19_HUMAN	8066.5669
ARRB1_HUMAN	7313.6514
EFTU_HUMAN	84384.977
AL7A1_HUMAN	24117.393
UB2R1_HUMAN	4133.7388
INPP_HUMAN	4347.647
CENPF_HUMAN	123.71323
SRP09_HUMAN	48284.621
PCY1A_HUMAN	10822.236
SYAC_HUMAN	24284.377
SYCC_HUMAN	17712.945
SYHM_HUMAN	6562.1104
SYSC_HUMAN	30090.264
PPM1F_HUMAN	25151.184
MA2A2_HUMAN	1907.6765
PRI1_HUMAN	2392.9104
PRI2_HUMAN	1600.3817
CASP4_HUMAN	2415.5308
KC1E_HUMAN	307.15497
PSB3_HUMAN	30784.971
PSB2_HUMAN	28566.936
MCM2_HUMAN	18871.076
ACADV_HUMAN	12590.854
YLPM1_HUMAN	7258.0483
VPS41_HUMAN	1767.2758
TMEDA_HUMAN	40623.297
RBM25_HUMAN	11103.91
NUMB_HUMAN	6280.604
CLK3_HUMAN	990.73499
PSN1_HUMAN	1587.4761
EI2BB_HUMAN	5438.1377
HINT1_HUMAN	4894.3823
NU153_HUMAN	6820.167
RBP2_HUMAN	12922.489
RGS19_HUMAN	861.46808
RGS3_HUMAN	2883.2747
TSC2_HUMAN	2658.7903
NDUV1_HUMAN	12006.455
GSK3A_HUMAN	1945.7234
GSK3B_HUMAN	5980.9287
TAF6_HUMAN	820.19574
5NTC_HUMAN	2906.5696
SPS1_HUMAN	6365.9087
MTHFS_HUMAN	1608.2523
GUAA_HUMAN	21146.24
DNLI3_HUMAN	5200.333
DNLI4_HUMAN	1910.6865

MRE11_HUMAN	6648.4023
ENTP1_HUMAN	1156.3184
GNAQ_HUMAN	8600.2031
IDH3A_HUMAN	12548.14
ST1A1_HUMAN	2016.8595
MMP14_HUMAN	22257.055
PPOX_HUMAN	2908.6606
GDIB_HUMAN	75837.477
EMD_HUMAN	8648.0078
CPT1A_HUMAN	9511.3086
S26A2_HUMAN	999.13965
SPB8_HUMAN	13038.448
SPB9_HUMAN	56248.637
SERPH_HUMAN	101968.41
PDLI4_HUMAN	7729.3574
F10A1_HUMAN	90399.945
PEX5_HUMAN	1568.6445
ERF_HUMAN	1541.0758
VASP_HUMAN	7779.8721
DYN2_HUMAN	28380.496
MAP2_HUMAN	5864.6523
AP4A_HUMAN	3947.679
CDK7_HUMAN	4055.4858
KNTC1_HUMAN	2607.2576
RASF2_HUMAN	3218.9338
CDK9_HUMAN	4494.2202
LRBA_HUMAN	5259.8232
BCAM_HUMAN	5400.1484
PPT1_HUMAN	4838.5938
RL14_HUMAN	128448.16
TCPQ_HUMAN	169734.47
TCPD_HUMAN	45498.324
ANX11_HUMAN	24762.809
PAPOA_HUMAN	8096.8462
FXR1_HUMAN	18389.465
FXR2_HUMAN	14860.141
RAB5C_HUMAN	29670.18
RAB7A_HUMAN	81641.008
RAB9A_HUMAN	4208.8345
RAB13_HUMAN	12669.718
RB27A_HUMAN	8287.7373
PLCD1_HUMAN	1158.4485
RT29_HUMAN	5558.4541
DUS3_HUMAN	6491.2148
SMCA2_HUMAN	432.21896
SMCA4_HUMAN	6994.1265
IDH3G_HUMAN	4567.855
GALK1_HUMAN	14894.059
SSRD_HUMAN	39705.34
BAP31_HUMAN	16093.173

TPMT_HUMAN	3188.8137
MECP2_HUMAN	992.70752
HCFC1_HUMAN	16295.322
IRAK1_HUMAN	1076.9807
CAV2_HUMAN	1081.863
AL3A2_HUMAN	4430.9663
DHB4_HUMAN	18545.52
PSMD7_HUMAN	22270.186
SUOX_HUMAN	2844.0461
SPHM_HUMAN	7177.8057
STA5B_HUMAN	1902.6898
UBP11_HUMAN	1699.2509
CLCN7_HUMAN	1788.8629
VAMP7_HUMAN	2213.1067
KS6A3_HUMAN	6304.3857
BMX_HUMAN	8500.6592
AFF1_HUMAN	201.42476
HDGF_HUMAN	20161.713
CCNH_HUMAN	870.57983
MAT1_HUMAN	1273.8621
NEK3_HUMAN	997.19141
UB2E1_HUMAN	1809.0311
NDUA8_HUMAN	7333.8618
ROA3_HUMAN	30205.98
6PGD_HUMAN	112921.66
HNRPM_HUMAN	101470.68
IMA1_HUMAN	31452.221
IMA5_HUMAN	5135.4888
NCBP2_HUMAN	2534.4077
GDS1_HUMAN	12631.549
RPAB3_HUMAN	3208.7241
MP2K6_HUMAN	2719.9512
GDIR1_HUMAN	71803.969
GDIR2_HUMAN	81449.906
AGFG1_HUMAN	4147.6411
HNRPF_HUMAN	15301.21
STAT2_HUMAN	2833.1235
MSH6_HUMAN	7644.3501
KIF11_HUMAN	3938.3523
VAV2_HUMAN	5285.5464
ZN143_HUMAN	821.38446
RBM5_HUMAN	3976.1907
RIDA_HUMAN	452.83249
SPSY_HUMAN	26005.988
HXK2_HUMAN	25232.879
EFNB2_HUMAN	572.21497
RM12_HUMAN	28272.725
DGKQ_HUMAN	148.40396
NDST1_HUMAN	984.3407
THOP1_HUMAN	13052.53

CAZA1_HUMAN	100262.43
HMGA2_HUMAN	808.61884
CRIP2_HUMAN	42246.395
NUP98_HUMAN	8028.6313
BIEA_HUMAN	28990.885
TXTP_HUMAN	16609.566
PPP5_HUMAN	10981.726
PLK1_HUMAN	2320.0422
DAPK1_HUMAN	1807.5681
ARFP2_HUMAN	3045.1541
ARFP1_HUMAN	6265.0737
NUBP1_HUMAN	5581.4819
ACLY_HUMAN	83789.406
MAP11_HUMAN	6344.4214
SUCA_HUMAN	9598.3301
MVD1_HUMAN	5195.0679
PGTB1_HUMAN	2844.2063
PGTB2_HUMAN	6182.9189
COPB_HUMAN	32019.502
COPA_HUMAN	56928.828
CATC_HUMAN	11006.857
LIMK1_HUMAN	6415.061
LIMK2_HUMAN	367.8085
CLH2_HUMAN	398.26248
AP3M2_HUMAN	1491.0709
AP2S1_HUMAN	11156.872
CCHL_HUMAN	4973.7158
MK12_HUMAN	2594.406
PTTG_HUMAN	2982.6089
SMTN_HUMAN	2222.7185
MOT1_HUMAN	8718.3701
IST1_HUMAN	10978.169
SC24C_HUMAN	20762.957
TCP4_HUMAN	47072.145
DPOG1_HUMAN	1296.3247
ICLN_HUMAN	9259.1328
SYRC_HUMAN	36799.164
HIRA_HUMAN	1591.1472
ATX3_HUMAN	3029.2654
ATN1_HUMAN	798.01453
PMS1_HUMAN	474.30408
PMS2_HUMAN	462.04813
CA2D1_HUMAN	1847.5582
SYYC_HUMAN	38279.402
UBP14_HUMAN	22275.131
AAKG1_HUMAN	4635.1563
HSP72_HUMAN	18545.074
BCAT1_HUMAN	25339.852
AT1B3_HUMAN	28288.57
RD23B_HUMAN	1395.3599

EPHB4_HUMAN	3760.9521
ANAG_HUMAN	2584.2698
KAD2_HUMAN	6029.0571
P5CS_HUMAN	18663.049
SNAA_HUMAN	20852.34
IF5_HUMAN	22421.227
S12A2_HUMAN	5572.21
PSMD4_HUMAN	22540.58
DRG2_HUMAN	7792.2754
XPO2_HUMAN	176233.67
TERA_HUMAN	173422.53
MFAP1_HUMAN	1105.9053
ECHB_HUMAN	60802.801
MANF_HUMAN	17275.527
AFAD_HUMAN	5325.2935
ELL_HUMAN	870.17334
BRPF1_HUMAN	1741.6389
NP1L1_HUMAN	52946.707
CASP7_HUMAN	8295.9014
CASP6_HUMAN	5773.4355
ADK_HUMAN	12840.734
DSRAD_HUMAN	13470.986
LAMB2_HUMAN	4267.8022
CAD13_HUMAN	4329.8979
TPD52_HUMAN	164.43486
SEC13_HUMAN	13804.211
NH2L1_HUMAN	6539.8125
PSA_HUMAN	32132.971
ALR_HUMAN	302.58298
HNRH2_HUMAN	12449.094
SCOT1_HUMAN	13990.486
EIF3B_HUMAN	21944.41
FCGRN_HUMAN	8243.1025
BID_HUMAN	2146.9746
ATPK_HUMAN	17641.393
RRP1_HUMAN	3755.2268
SYMC_HUMAN	33855.531
ARP19_HUMAN	6329.7646
AP1S2_HUMAN	4836.6016
ATP68_HUMAN	8926.3691
ATP5E_HUMAN	5739.1528
ATP5I_HUMAN	23341.863
HDAC4_HUMAN	1514.1652
IF6_HUMAN	32456.537
CTBP2_HUMAN	22875.15
NDUA6_HUMAN	9804.0215
PEX3_HUMAN	1273.3027
DHB7_HUMAN	2395.6912
BCAR1_HUMAN	10472.325
STX17_HUMAN	2307.7488

CF298_HUMAN	765.44019
WDR4_HUMAN	1613.0414
TMM33_HUMAN	32212.244
GEMI4_HUMAN	3537.3396
CORO7_HUMAN	6462.4199
NU107_HUMAN	10218.641
GSDMD_HUMAN	10822.739
SELB_HUMAN	3055.5684
SESN2_HUMAN	386.64853
ANTR2_HUMAN	1346.9667
MTPN_HUMAN	530.57623
ARPC4_HUMAN	118504.27
CD81_HUMAN	7430.9976
SC61G_HUMAN	34374.098
TPIS_HUMAN	147789.08
EIF3E_HUMAN	31475.596
SC61B_HUMAN	21726.732
PTEN_HUMAN	1035.2344
PP4C_HUMAN	8784.417
GBRL2_HUMAN	9958.7803
ROMO1_HUMAN	3289.7937
MYL6_HUMAN	7511.2769
ACTB_HUMAN	1705306.9
IF4A1_HUMAN	182634.73
RS20_HUMAN	111621.84
PRPS1_HUMAN	5930.5713
PSA6_HUMAN	50658.711
S10AA_HUMAN	47561.285
CDC42_HUMAN	21197.363
DEST_HUMAN	17599.008
GMFB_HUMAN	4048.4187
RAB8A_HUMAN	4238.418
SPCS3_HUMAN	18818.391
SRP54_HUMAN	10376.395
RAB4B_HUMAN	2708.7024
RAB2A_HUMAN	33714.273
RAB5B_HUMAN	7287.8203
RAB10_HUMAN	7383.939
UB2D3_HUMAN	17032.84
UBC12_HUMAN	17105.123
UBE2K_HUMAN	19875.715
UBE2N_HUMAN	26555.688
RAB14_HUMAN	22649.953
ARP3_HUMAN	74538.766
ARP2_HUMAN	60557.563
ACTZ_HUMAN	23449.934
CSN2_HUMAN	13970.73
ABCE1_HUMAN	26938.377
RAP1B_HUMAN	38757.16
RAP2B_HUMAN	3695.1016

MAX_HUMAN	1553.191
RS3A_HUMAN	95404.023
RL26_HUMAN	122111.5
PSME3_HUMAN	12046.904
RL15_HUMAN	168995.34
RL27_HUMAN	184235.02
VA0D1_HUMAN	11543.01
RL37A_HUMAN	7238.6567
RHOA_HUMAN	13502.348
RND3_HUMAN	744.9101
NAA20_HUMAN	2177.7083
CH10_HUMAN	10505.749
S61A1_HUMAN	28800.738
PFD3_HUMAN	13376.58
STXB1_HUMAN	7581.813
B2MG_HUMAN	1312.8335
DAD1_HUMAN	26234.043
NPC2_HUMAN	5415.7202
COPZ1_HUMAN	22732.889
RL37_HUMAN	530.83685
SUMO2_HUMAN	11822.751
DCAF7_HUMAN	3157.1511
WDR5_HUMAN	3260.2356
AP1S1_HUMAN	6818.9097
NTF2_HUMAN	5945.0186
HNRPK_HUMAN	168411.69
1433G_HUMAN	52871.77
UBP46_HUMAN	1232.9475
RRAS2_HUMAN	14159.643
RS7_HUMAN	72957.766
PP1A_HUMAN	27969.438
PP1B_HUMAN	27166.662
PRS4_HUMAN	42843.848
PRS8_HUMAN	51974.953
RS8_HUMAN	140786.8
RS15A_HUMAN	111310.47
RS16_HUMAN	211294.55
UB2G1_HUMAN	1938.158
UBE2H_HUMAN	4944.5967
1433E_HUMAN	94722.609
RS14_HUMAN	150545.41
RS23_HUMAN	53840.234
RS18_HUMAN	217425.55
RS29_HUMAN	22597.24
RS13_HUMAN	106420.02
RS11_HUMAN	40830.137
RUXE_HUMAN	15253.225
RUXF_HUMAN	7535.1455
LSM3_HUMAN	3258.0989
LSM6_HUMAN	9895.1113

SMD1_HUMAN	12633.998
SMD2_HUMAN	19009.498
SMD3_HUMAN	47814.418
TYB4_HUMAN	7389.7061
ARF6_HUMAN	22230.816
PRS10_HUMAN	28431.07
SELT_HUMAN	4271.7109
TBPL1_HUMAN	808.94165
RL7A_HUMAN	110921.77
RPB7_HUMAN	2734.2048
RB11A_HUMAN	73027.844
ERF1_HUMAN	23101.619
CNBP_HUMAN	5489.6958
YPEL5_HUMAN	843.86841
RS4X_HUMAN	163326.17
PP2AB_HUMAN	35011.883
ACTA_HUMAN	340203.81
RHOB_HUMAN	6079.2915
RL23A_HUMAN	163531.53
RS6_HUMAN	193414.02
H4_HUMAN	240546.52
RAB1A_HUMAN	9888.873
RAN_HUMAN	266466.53
RL23_HUMAN	93022.852
RAP1A_HUMAN	5528.8711
RS15_HUMAN	25058.424
RS24_HUMAN	51473.402
RS25_HUMAN	206264.06
RS26_HUMAN	59259.328
RS28_HUMAN	27610.549
RS30_HUMAN	58308.402
GBB1_HUMAN	11168.245
RBX1_HUMAN	14611.379
GBB2_HUMAN	46490.922
RL30_HUMAN	78988.453
RL39_HUMAN	28302.129
RL31_HUMAN	98766.344
RL10A_HUMAN	70253.961
RL32_HUMAN	34159.184
RL11_HUMAN	81332.914
RL8_HUMAN	88965.891
PPIA_HUMAN	124352.63
FKB1A_HUMAN	26657.645
RS27A_HUMAN	96298.359
GRB2_HUMAN	7221.7891
TRA2B_HUMAN	8533.1006
RAC1_HUMAN	69730.758
AP2B1_HUMAN	29367.988
VAMP2_HUMAN	9620.8994
GNAS2_HUMAN	13720.167

GNAI1_HUMAN	765.07733
1433Z_HUMAN	221773.3
2ABA_HUMAN	33978.547
SUMO1_HUMAN	14195.06
DYL1_HUMAN	8478.1572
DYLT1_HUMAN	5692.8188
RL38_HUMAN	50432.934
SKP1_HUMAN	1952.5874
GBG5_HUMAN	928.98663
RS21_HUMAN	11103.729
IF5A1_HUMAN	70074.914
RACK1_HUMAN	161774.97
SPT4H_HUMAN	2038.9751
UBC9_HUMAN	15441.567
TYB10_HUMAN	5812.3818
PP2AA_HUMAN	2334.5593
YBOX1_HUMAN	41430.988
SC11A_HUMAN	47124.625
CSK2B_HUMAN	6419.229
TPM4_HUMAN	81720.523
UB2L3_HUMAN	39424.832
EF1A1_HUMAN	1093660.4
TBA1B_HUMAN	959115.63
TBA4A_HUMAN	970.18347
TBB4B_HUMAN	288097.69
CSK21_HUMAN	10860.153
PA1B2_HUMAN	8939.041
H31_HUMAN	27232.285
HBB_HUMAN	1638.1327
HBA_HUMAN	1662.9519
CXAR_HUMAN	2170.4099
F193A_HUMAN	3071.7559
NOP14_HUMAN	1857.2153
IGBP1_HUMAN	6423.3008
SHPS1_HUMAN	1268.1028
SERB_HUMAN	8810.7646
RBM6_HUMAN	3241.8608
IF4G2_HUMAN	30483.59
RPP38_HUMAN	2595.5371
RPP30_HUMAN	6460.9443
GTF2I_HUMAN	30160.922
PI42B_HUMAN	1933.4865
CNTP1_HUMAN	3221.6699
SRPK2_HUMAN	3552.7732
TCPB_HUMAN	78822.156
S35A2_HUMAN	1264.8622
RAE1L_HUMAN	8527.7139
GSTO1_HUMAN	144829.13
PRKDC_HUMAN	50322.629
ADA17_HUMAN	2878.8601

BL1S1_HUMAN	1367.9498
SRPX_HUMAN	3543.1619
ARGI2_HUMAN	909.18573
NTH_HUMAN	1578.4675
IN35_HUMAN	991.51666
NUCB2_HUMAN	3691.3918
BASP1_HUMAN	7489.1768
DCD_HUMAN	4708.0952
TMF1_HUMAN	1914.7852
RT22_HUMAN	9512.0801
RT25_HUMAN	2227.1216
RT10_HUMAN	4122.1753
RT35_HUMAN	2160.5012
RT05_HUMAN	3107.3308
KGD4_HUMAN	261.71469
RT11_HUMAN	6438.5806
RT15_HUMAN	6738.2676
RT21_HUMAN	4891.6909
RT34_HUMAN	18734.91
RT06_HUMAN	3053.606
RT09_HUMAN	4737.1948
SARNP_HUMAN	24530.41
LACTB_HUMAN	2385.4978
COG7_HUMAN	3613.7603
RL24_HUMAN	95318.406
TXN4A_HUMAN	3113.9666
RL36A_HUMAN	16153.77
CBX1_HUMAN	3516.4788
SMAD3_HUMAN	4590.6279
ARF1_HUMAN	108177
ARF5_HUMAN	13353.846
ERH_HUMAN	2405.1897
RHOG_HUMAN	9636.0322
RL19_HUMAN	93287.586
SERF2_HUMAN	2752.554
SRSF3_HUMAN	81245.648
FOXK1_HUMAN	5618.854
CCZ1B_HUMAN	3319.3689
DAB2_HUMAN	13617.792
PGBM_HUMAN	78463.727
XIAP_HUMAN	1358.7372
EFNB1_HUMAN	4183.5361
RBM10_HUMAN	5478.7974
RBM3_HUMAN	10723.234
AT2C1_HUMAN	2657.9102
AT11A_HUMAN	1495.1449
CYC_HUMAN	67367.586
EM55_HUMAN	3268.0166
TFAM_HUMAN	10586.258
PIPNA_HUMAN	10683.358

S25A3_HUMAN	127836.27
VIGLN_HUMAN	36214.055
TF2B_HUMAN	2428.6787
CDK6_HUMAN	834.13098
CDK5_HUMAN	5172.1201
CDK17_HUMAN	8340.9307
PURA_HUMAN	4897.5039
BORG5_HUMAN	3627.1057
CLH1_HUMAN	91797.984
HSF1_HUMAN	963.3429
NFKB2_HUMAN	2416.5549
FKBP3_HUMAN	44262.738
REEP5_HUMAN	12272.992
DHSO_HUMAN	7710.6855
HNRPU_HUMAN	75562.352
U2AF1_HUMAN	11825.87
SPTB2_HUMAN	52012.199
TIAR_HUMAN	9362.8682
SET_HUMAN	109876.94
SRSF2_HUMAN	32884.652
FOXK2_HUMAN	1486.7809
RELB_HUMAN	407.26215
GALK2_HUMAN	4412.5972
AMPD2_HUMAN	9752.5439
FABP5_HUMAN	75223.672
CAP1_HUMAN	67624.133
FLI1_HUMAN	6554.5093
HMCS1_HUMAN	13029.521
IFM3_HUMAN	10592.019
LAT1_HUMAN	1707.4463
NC2B_HUMAN	3099.7637
EXOSX_HUMAN	3987.219
OTUD4_HUMAN	766.02753
PFKAP_HUMAN	49507.094
XPC_HUMAN	1241.1392
EWS_HUMAN	34147.652
CDR2_HUMAN	2228.4631
OCRL_HUMAN	2768.302
PLCB3_HUMAN	9990.8037
TAGL_HUMAN	5638.6367
AK17A_HUMAN	2648.1482
PYRD_HUMAN	560.85773
ODO1_HUMAN	18012.824
CENPE_HUMAN	175.25465
KIF23_HUMAN	789.36084
MMSA_HUMAN	6341.708
NRG1_HUMAN	495.87881
CP27A_HUMAN	999.14636
DSG1_HUMAN	713.87433
SP3_HUMAN	321.73917

H11_HUMAN	1949.8966
RL18A_HUMAN	79429.531
MP2K1_HUMAN	14608.963
TIE2_HUMAN	2541.1829
FKBP4_HUMAN	22337.053
PLOD1_HUMAN	17983.186
NUCB1_HUMAN	7041.6953
RL6_HUMAN	245714.16
TOP2B_HUMAN	1772.5941
AKA12_HUMAN	20734.957
M2OM_HUMAN	19994.832
DYST_HUMAN	3164.9485
ENL_HUMAN	1722.0537
MECOM_HUMAN	1169.2695
GNA12_HUMAN	451.72418
CAV1_HUMAN	92873.289
ACY1_HUMAN	2218.9988
TNAP2_HUMAN	1770.9902
LMNB2_HUMAN	22579.678
PTPS_HUMAN	887.46094
UPAR_HUMAN	637.05182
KIME_HUMAN	6660.8667
ERCC6_HUMAN	139.01277
TAP1_HUMAN	3555.2976
TAP2_HUMAN	1689.6351
CEBPZ_HUMAN	3302.522
ZNF92_HUMAN	830.35095
TF65_HUMAN	8489.4932
UBXN1_HUMAN	10637.228
GLGB_HUMAN	23825.729
IF4G1_HUMAN	28464.146
NOTC2_HUMAN	943.27985
TLE1_HUMAN	2121.5261
TLE3_HUMAN	1550.2848
LGUL_HUMAN	24099.211
ACVR1_HUMAN	687.65228
SSBP_HUMAN	14731.805
1433F_HUMAN	53806.715
PLP2_HUMAN	16640.74
CSTF1_HUMAN	8152.3916
UBE3A_HUMAN	6836.6655
DYN1_HUMAN	1731.2058
PTN12_HUMAN	7423.0547
FAK1_HUMAN	10465.332
SRS11_HUMAN	138563.03
KPCD_HUMAN	2292.5901
CALD1_HUMAN	38885.391
FOLC_HUMAN	1660.5901
CTSL2_HUMAN	733.9884
PTN11_HUMAN	9614.5469

KDSR_HUMAN	4377.77
PUR1_HUMAN	8849.042
GFPT1_HUMAN	18378.084
EXOS9_HUMAN	3147.5369
PSME1_HUMAN	43027.559
SUH_HUMAN	1090.8658
MEF2C_HUMAN	399.16476
APLP2_HUMAN	6751.709
GABPA_HUMAN	3160.3848
RING1_HUMAN	498.61221
FMR1_HUMAN	1080.0403
PRDX1_HUMAN	180450.14
RL18_HUMAN	135966.72
C1QBP_HUMAN	30117.344
CKAP4_HUMAN	122641.79
ZO1_HUMAN	19262.119
TISB_HUMAN	1208.5011
SPAG1_HUMAN	1219.4908
KHDR1_HUMAN	30862.307
BAX_HUMAN	17840.908
B2CL1_HUMAN	5396.6455
MCL1_HUMAN	459.24612
DPOE1_HUMAN	4769.813
KLC1_HUMAN	21837.229
SOS1_HUMAN	1392.0549
SRSF1_HUMAN	81264.469
RHG01_HUMAN	15899.22
TLE5_HUMAN	1449.5059
SRSF4_HUMAN	6343.8535
PCDH1_HUMAN	7737.397
TGM3_HUMAN	194.20097
PP2BA_HUMAN	6294.8413
DHX9_HUMAN	54343.262
QOR_HUMAN	9303.9541
GOGA3_HUMAN	3168.5107
GOGA2_HUMAN	4190.4448
ECHP_HUMAN	1110.0425
MFGM_HUMAN	3757.5779
PDE4D_HUMAN	797.12756
DSC1_HUMAN	1432.3918
HDHD1_HUMAN	2853.9812
TICN1_HUMAN	1124.2246
CD47_HUMAN	14659.982
PPID_HUMAN	15454.738
SSRP1_HUMAN	23204.637
CAMP2_HUMAN	3312.1096
SPIR1_HUMAN	859.70111
SLFN5_HUMAN	2853.071
VAC14_HUMAN	6125.5493
NSUN2_HUMAN	17427.059

DMWD_HUMAN	321.00192
RBBP4_HUMAN	55400.129
NCBP1_HUMAN	14660.765
MGT5A_HUMAN	618.89203
EP300_HUMAN	3934.9221
AHNK_HUMAN	117786.06
FCHO2_HUMAN	1022.8669
ELP6_HUMAN	972.72498
HSP7E_HUMAN	6346.3813
SCRN3_HUMAN	4053.3628
CGNL1_HUMAN	4888.27
LTOR4_HUMAN	6473.0903
NEXN_HUMAN	2389.2183
MGAT2_HUMAN	5938.9219
GALT2_HUMAN	7312.9253
GALT1_HUMAN	9447.5996
AP1B1_HUMAN	34947.918
CPSF1_HUMAN	7895.104
BST2_HUMAN	1244.5225
MPPA_HUMAN	7879.1646
SIA4A_HUMAN	1092.2131
SCRN1_HUMAN	9551.5635
WASC5_HUMAN	6696.647
NU160_HUMAN	9340.5537
SCAP_HUMAN	2862.9331
FOXO1_HUMAN	690.06677
TBL3_HUMAN	2090.9985
TF3C1_HUMAN	2451.3557
TWF1_HUMAN	15203.212
ASPH_HUMAN	13455.049
TFCP2_HUMAN	2889.239
AKP13_HUMAN	2687.1479
FBLN3_HUMAN	27448.959
SNF5_HUMAN	3817.3716
BPTF_HUMAN	997.76184
CDC20_HUMAN	1458.4471
FSTL1_HUMAN	7500.6426
STX4_HUMAN	2333.5273
GRSF1_HUMAN	6423.4443
M4K2_HUMAN	1832.1661
MERTK_HUMAN	3306.9685
SFSWA_HUMAN	1428.4733
CHD3_HUMAN	894.82635
SF3A3_HUMAN	22398.373
DPYD_HUMAN	933.39771
TP53B_HUMAN	5327.1025
TM115_HUMAN	1560.4634
IFRD2_HUMAN	685.40881
TRI26_HUMAN	1468.39
AIMP1_HUMAN	15291.806

ILF2_HUMAN	76800.195
ILF3_HUMAN	88473.969
LMAN2_HUMAN	54194.906
TRAP1_HUMAN	373731.16
TRAF2_HUMAN	3142.8762
DLG1_HUMAN	3206.5774
MYO1E_HUMAN	10468.232
PP1R8_HUMAN	4103.2163
TP4A2_HUMAN	4703.0767
ABR_HUMAN	4547.4419
NPRL3_HUMAN	895.69275
SEC20_HUMAN	6030.9497
BNIP2_HUMAN	963.65521
NFX1_HUMAN	132.633
CSTF3_HUMAN	6638.5854
TSN31_HUMAN	1681.2192
ECH1_HUMAN	10893.167
RHG05_HUMAN	747.4278
STRN3_HUMAN	5966.5781
CDC16_HUMAN	3309.6931
STK4_HUMAN	4281.1694
FLII_HUMAN	26255.939
TRI32_HUMAN	761.07648
COASY_HUMAN	6487.4556
RM28_HUMAN	7208.0903
ACACA_HUMAN	13812.104
CSN1_HUMAN	14635.797
UBP4_HUMAN	3461.5042
CAF1A_HUMAN	482.64941
CAF1B_HUMAN	1086.1072
RED_HUMAN	9111.5283
MTAP_HUMAN	9113.5313
AAPK1_HUMAN	5090.8359
LIPA1_HUMAN	2155.7224
CACO2_HUMAN	749.05957
EI2BE_HUMAN	17408.521
TADBP_HUMAN	15782.687
ROA0_HUMAN	14789.911
AIMP2_HUMAN	13210.478
FADD_HUMAN	4509.54
PRDX4_HUMAN	38938.008
MP2K5_HUMAN	630.88043
MK07_HUMAN	858.86554
PAK2_HUMAN	16776.102
CBX3_HUMAN	22967.492
STK3_HUMAN	1437.0291
STX5_HUMAN	3839.5183
PSMD2_HUMAN	34234.012
MMRN1_HUMAN	13039.137
DDX10_HUMAN	783.86591

DNJC3_HUMAN	22046.631
SBP1_HUMAN	7852.6475
NDK3_HUMAN	3735.7241
SRSF9_HUMAN	9793.7344
SRSF5_HUMAN	7066.1704
SRSF6_HUMAN	59803.316
MD2L1_HUMAN	8157.0278
TIF1B_HUMAN	32051.424
SEM3F_HUMAN	340.76175
STX3_HUMAN	1842.566
G3BP1_HUMAN	35775.406
NMI_HUMAN	5105.2856
PTK7_HUMAN	3952.1987
PABP4_HUMAN	28667.18
ATM_HUMAN	1879.4994
GRB10_HUMAN	3192.6013
MTA1_HUMAN	2845.5876
EIF3I_HUMAN	19562.188
PPIL2_HUMAN	1095.4664
2A5G_HUMAN	3031.8464
CTBP1_HUMAN	6178.7837
PHLP_HUMAN	6484.8647
PLD1_HUMAN	4431.1772
TARB1_HUMAN	658.52692
UB2V1_HUMAN	31636.469
RM49_HUMAN	1689.7778
DC1I2_HUMAN	24022.135
ORC2_HUMAN	1691.8079
ILK_HUMAN	30667.775
NNTM_HUMAN	23216.324
SNTB2_HUMAN	14266.521
XRCC4_HUMAN	3864.9172
PPIG_HUMAN	6714.3179
TCOF_HUMAN	4940.3794
U119A_HUMAN	702.68457
SF3B2_HUMAN	20283.188
OS9_HUMAN	2365.3931
GOGA4_HUMAN	2557.6584
HAP28_HUMAN	17343.943
ADAM9_HUMAN	9652.1787
ADA15_HUMAN	4624.8306
TMED1_HUMAN	5281.5664
FKBP5_HUMAN	23985.129
MYO9B_HUMAN	5243.1543
ROCK1_HUMAN	8877.9746
NFAC2_HUMAN	1191.4733
TOP3A_HUMAN	683.96216
SMAD4_HUMAN	3591.8101
VPP3_HUMAN	5235.2319
BIRC2_HUMAN	813.04492

PICAL_HUMAN	10941.464
MTM1_HUMAN	2801.6206
SQSTM_HUMAN	6210.2065
MTX1_HUMAN	4291.2588
TBB3_HUMAN	11334.51
ASAH1_HUMAN	450.97968
PRP4B_HUMAN	2400.4946
PIN1_HUMAN	5662.8931
ATR_HUMAN	1907.4961
4EBP1_HUMAN	805.48169
RIPK1_HUMAN	1922.9495
HDAC1_HUMAN	18172.547
KCC2G_HUMAN	2972.3218
KCC2D_HUMAN	4807.7568
DCTN2_HUMAN	6221.1167
PKD2_HUMAN	389.89252
ULA1_HUMAN	8734.7949
ITPK1_HUMAN	1630.6372
SNW1_HUMAN	7319.2627
DGKZ_HUMAN	4527.4712
STIM1_HUMAN	2744.3628
GRAP_HUMAN	3263.561
TRA2A_HUMAN	2324.9431
SNX1_HUMAN	13540.462
KRR1_HUMAN	3825.4731
PWP1_HUMAN	4736.4355
MTMR1_HUMAN	1179.0481
MTMR2_HUMAN	5988.8931
CUL1_HUMAN	5716.7256
CUL2_HUMAN	11466.023
CUL3_HUMAN	8118.833
CUL4A_HUMAN	4872.6577
CUL4B_HUMAN	14317.399
ASPP2_HUMAN	334.42584
DYR1A_HUMAN	756.17847
FCL_HUMAN	15055.22
RAB31_HUMAN	200.21843
RAB32_HUMAN	9411.582
FHL1_HUMAN	11026.582
FHL3_HUMAN	1518.5275
RIN1_HUMAN	370.72998
AAMP_HUMAN	1987.0272
MOGS_HUMAN	35172.797
CD166_HUMAN	5868.9873
THOC5_HUMAN	2666.2983
SHRM2_HUMAN	1407.0782
SPTN1_HUMAN	33126.461
NOG2_HUMAN	3675.4849
AUHM_HUMAN	735.72894
DX39B_HUMAN	10526.45

BLMH_HUMAN	27368.875
EXOS2_HUMAN	4001.6567
BMPR2_HUMAN	1718.3136
TBB2A_HUMAN	183021.81
TF2H2_HUMAN	1832.9209
TF2H3_HUMAN	1843.7117
BYST_HUMAN	7881.623
RPGF1_HUMAN	2790.9255
IDI1_HUMAN	10119.824
PEBB_HUMAN	6904.749
NFYC_HUMAN	955.53479
CDK13_HUMAN	1160.4708
CKAP5_HUMAN	26652.254
CIRBP_HUMAN	6680.5098
KCC1A_HUMAN	1253.1844
COTL1_HUMAN	31835.967
HNRPD_HUMAN	138183.66
SCRB2_HUMAN	31775.287
NID2_HUMAN	1053.7452
LRP8_HUMAN	311.06012
DAG1_HUMAN	4299.4395
VEZF1_HUMAN	112.8673
BOP1_HUMAN	3656.2112
UBE4A_HUMAN	4120.5449
SEPT6_HUMAN	5502.2549
KEAP1_HUMAN	1623.5736
URB2_HUMAN	1975.197
DHX34_HUMAN	726.8512
MORC3_HUMAN	1222.5209
SAFB2_HUMAN	7136.2578
EIF3A_HUMAN	42937.746
ARHG7_HUMAN	6896.4614
EFR3A_HUMAN	5244.2036
UBP2L_HUMAN	15504.67
SCRIB_HUMAN	6215.9648
GIT2_HUMAN	3774.7002
SREC_HUMAN	3886.437
MLEC_HUMAN	23007.49
TTL12_HUMAN	10472.888
DPOA2_HUMAN	1948.6106
DOCK1_HUMAN	8000.1499
TFDP1_HUMAN	2230.8999
FHL2_HUMAN	19674.697
DPYL3_HUMAN	54437.047
ICT1_HUMAN	5221.1646
ZMYM3_HUMAN	489.16232
DCTN1_HUMAN	14670.154
DYHC1_HUMAN	76321.539
EI2BA_HUMAN	8568.0957
IF4A2_HUMAN	7580.3203

ELOA1_HUMAN	1528.5447
SRC8_HUMAN	19255.225
NUCG_HUMAN	3179.2241
FLOT2_HUMAN	4382.6855
RCN2_HUMAN	15001.296
TRI25_HUMAN	21062.92
FLNC_HUMAN	49883.305
FKBP8_HUMAN	5474.1982
FA50A_HUMAN	5873.1035
FRG1_HUMAN	2486.6082
GNA13_HUMAN	52871.379
GAMT_HUMAN	429.25708
GALE_HUMAN	10284.928
LRC32_HUMAN	870.60144
CAPR1_HUMAN	20232.563
BECN1_HUMAN	989.14246
RBM39_HUMAN	16499.514
HLTF_HUMAN	1386.2141
ERG1_HUMAN	3113.5525
PDIA5_HUMAN	13267.849
KPRA_HUMAN	9395.8691
DHX8_HUMAN	2071.0413
MCM6_HUMAN	18515.137
ITPR2_HUMAN	6160.4482
ITPR3_HUMAN	7010.7095
ITPR1_HUMAN	614.29517
RASA3_HUMAN	2707.1672
PLSI_HUMAN	1938.4993
IRF3_HUMAN	3953.2139
LAGE3_HUMAN	1140.9087
BLTP2_HUMAN	483.0751
TRIPC_HUMAN	5389.8921
PUM1_HUMAN	4089.8367
MDC1_HUMAN	177.8261
EPN4_HUMAN	10224.729
KANK1_HUMAN	3145.5227
SMC1A_HUMAN	57514.926
RRP1B_HUMAN	4725.3779
DIP2A_HUMAN	6834.4922
RRP5_HUMAN	3276.7559
PSF1_HUMAN	2452.5034
BMS1_HUMAN	2233.7422
LPIN1_HUMAN	2218.9475
UBP10_HUMAN	10711.138
MESD_HUMAN	10849.172
GANAB_HUMAN	61328.941
RFTN1_HUMAN	5191.5991
MFS10_HUMAN	2354.6194
2A5D_HUMAN	12370.396
LBR_HUMAN	19077.818

COG2_HUMAN	1049.1465
MVP_HUMAN	80095.656
LTBP1_HUMAN	1234.5565
LTBP2_HUMAN	2095.5073
CBX2_HUMAN	265.13943
GOGB1_HUMAN	4031.4417
CASP8_HUMAN	2050.2854
NAA30_HUMAN	2595.9883
KIF22_HUMAN	462.05331
MEF2D_HUMAN	1709.0804
CHD4_HUMAN	17806.553
LASP1_HUMAN	21707.545
PTGR1_HUMAN	9779.6406
NC2A_HUMAN	4692.4688
NFAC4_HUMAN	640.28503
ZN638_HUMAN	4087.303
IMB1_HUMAN	56779.902
NOLC1_HUMAN	2208.543
NUMA1_HUMAN	10787.983
PSME4_HUMAN	7057.4702
CUL7_HUMAN	2179.3613
SLMAP_HUMAN	919.41376
GAPD1_HUMAN	10359.117
NAA25_HUMAN	8499.9375
MELT_HUMAN	1603.3296
CND2_HUMAN	5921.6514
PAF15_HUMAN	862.47961
SPCS2_HUMAN	20405.76
EMC2_HUMAN	5061.874
FL2D_HUMAN	1457.1204
PSMD6_HUMAN	16796.057
HERP1_HUMAN	183.44466
MD2BP_HUMAN	492.23053
MO4L2_HUMAN	2760.9822
ABRX2_HUMAN	2146.0012
SEPT2_HUMAN	43763.953
SART3_HUMAN	14846.345
CND1_HUMAN	12467.749
SUZ12_HUMAN	816.40601
EXOS7_HUMAN	3940.8481
TNIP1_HUMAN	1321.1217
U5S1_HUMAN	68644.109
SYLM_HUMAN	2758.0139
R3HD1_HUMAN	1494.4691
TRAM2_HUMAN	1480.5522
SNX17_HUMAN	4410.5151
AR6P1_HUMAN	3448.0291
RB3GP_HUMAN	3866.5295
S39AE_HUMAN	2543.1255
SYK_HUMAN	38870.055

SETB1_HUMAN	1075.7225
LRC14_HUMAN	1220.2568
RRS1_HUMAN	4082.7405
DPOD3_HUMAN	1128.0646
IF4H_HUMAN	31491.701
ACAP2_HUMAN	4308.6338
KIF14_HUMAN	410.07211
BRD3_HUMAN	778.14575
WDR43_HUMAN	1343.6367
ACOX1_HUMAN	6876.0376
OXA1L_HUMAN	6732.5137
EEA1_HUMAN	13640.295
PDIA6_HUMAN	133925.06
PA1B3_HUMAN	5268.4702
PDK3_HUMAN	845.38641
PEA15_HUMAN	2364.3855
EBP_HUMAN	13106.705
PMVK_HUMAN	6434.1074
KPCD1_HUMAN	685.7229
PLEC_HUMAN	65095.797
PCM1_HUMAN	2676.1265
NOMO1_HUMAN	26140.014
PON2_HUMAN	10338.642
2A5A_HUMAN	4010.2422
IPYR_HUMAN	49080.969
TEBP_HUMAN	18689.967
STK38_HUMAN	2980.2212
NONO_HUMAN	59688.258
PTPA_HUMAN	25544.27
PTPRK_HUMAN	3274.6003
PWP2_HUMAN	2358.4431
RABE1_HUMAN	1906.6017
RAB35_HUMAN	7152.7339
RNPS1_HUMAN	5011.6982
RBBP5_HUMAN	2874.8733
RCN1_HUMAN	19826.545
RBP1_HUMAN	496.57108
L2GL1_HUMAN	1804.142
TTF1_HUMAN	6498.5996
TMED2_HUMAN	7778.2104
PCBP1_HUMAN	181042
PCBP2_HUMAN	169694.7
ELOC_HUMAN	4060.9338
ELOB_HUMAN	2766.2302
RHEB_HUMAN	12042.574
UBE3C_HUMAN	4699.4312
TOM20_HUMAN	8872.8652
MTFR1_HUMAN	455.98724
DHC24_HUMAN	13298.408
SF3B3_HUMAN	24765.229

PUM3_HUMAN	1951.0293
DLGP5_HUMAN	3273.1741
RSU1_HUMAN	50904.332
CNN3_HUMAN	26540.676
KS6A1_HUMAN	907.36121
SAFB1_HUMAN	5806.0801
SF3A2_HUMAN	5264.4258
RBMS2_HUMAN	5210.2632
PP1R7_HUMAN	15363.644
SC23A_HUMAN	22584.664
SC23B_HUMAN	4121.2544
CYH1_HUMAN	4895.834
SF3A1_HUMAN	12136.12
SKI2_HUMAN	8480.0635
SURF1_HUMAN	303.70432
TAF7_HUMAN	1345.7694
TERF2_HUMAN	1527.298
MARE2_HUMAN	12419.685
TGIF1_HUMAN	245.58084
NHRF2_HUMAN	3681.6936
TRADD_HUMAN	2090.6265
TRAM1_HUMAN	10026.567
TSN_HUMAN	11378.137
TRBP2_HUMAN	1317.1005
SF01_HUMAN	15521.578
CIP4_HUMAN	7303.6206
TRIPB_HUMAN	3406.5229
PCH2_HUMAN	9711.6045
MED1_HUMAN	2056.2397
TRIP4_HUMAN	3143.959
JHD2C_HUMAN	443.80737
IKBB_HUMAN	2075.6104
TRIP6_HUMAN	5337.1509
PTN14_HUMAN	2456.0693
MARE1_HUMAN	42720.156
ELAV1_HUMAN	45610.316
NSDHL_HUMAN	17857.447
MYLK_HUMAN	3521.321
TAB1_HUMAN	3445.6914
HERC1_HUMAN	2420.3982
AAAT_HUMAN	9495.7881
MLF2_HUMAN	1086.8302
TOM34_HUMAN	7195.4326
SMAD2_HUMAN	4368.9502
SMAD1_HUMAN	19141.037
MSMO1_HUMAN	6176.478
ITSN1_HUMAN	2275.1968
TBCE_HUMAN	4530.3345
TBCC_HUMAN	4199.3354
UB2V2_HUMAN	1858.6697

STK11_HUMAN	686.47394
STXB2_HUMAN	5624.0249
VAMP3_HUMAN	3786.177
NEDD8_HUMAN	4238.5576
VAS1_HUMAN	4793.8828
VPS72_HUMAN	558.3609
RB11B_HUMAN	16642.99
ZYX_HUMAN	4193.5054
ETFD_HUMAN	1632.0995
SEPT7_HUMAN	32376.238
ADRM1_HUMAN	15625.742
CCDC6_HUMAN	4833.6147
UAP1_HUMAN	20611.35
E2F4_HUMAN	479.32822
IBP7_HUMAN	8745.7354
PDCD2_HUMAN	484.81906
LAMA4_HUMAN	15546.808
PSMD5_HUMAN	25960.049
PKN1_HUMAN	8387.3643
PKN2_HUMAN	7668.8438
TAF12_HUMAN	1083.1151
CSRP2_HUMAN	1678.91
DDB1_HUMAN	39899.813
2A5E_HUMAN	4871.0288
MK14_HUMAN	5868.8838
RM23_HUMAN	4476.3525
CDC37_HUMAN	33515.07
DPYL2_HUMAN	68668.375
SYPL1_HUMAN	13373.422
RBBP7_HUMAN	9244.4551
M3K11_HUMAN	1177.5177
TAF9_HUMAN	1790.6851
CALRL_HUMAN	8316.2979
BAK_HUMAN	819.97461
SRSF7_HUMAN	39112.703
CPSF6_HUMAN	17689.154
SMN_HUMAN	3211.2979
DREB_HUMAN	10981.392
NRF1_HUMAN	1935.6346
FSCN1_HUMAN	158037.7
IF16_HUMAN	33330.703
DECR_HUMAN	30118.916
MA2A1_HUMAN	7655.978
NDUA5_HUMAN	2072.4543
CEGT_HUMAN	959.25183
CLPP_HUMAN	4090.2668
THTR_HUMAN	16416.463
UBE2S_HUMAN	472.94254
KAT1_HUMAN	835.32452
KGUA_HUMAN	4170.5039

GLO2_HUMAN	8602.1455
NDUA9_HUMAN	11920.444
MAON_HUMAN	1912.4363
PCKGM_HUMAN	3934.197
UPP1_HUMAN	1920.5291
DDR2_HUMAN	2769.8184
HCDH_HUMAN	12352.576
CP51A_HUMAN	17548.221
UGPA_HUMAN	30266.857
F263_HUMAN	2015.1877
TRXR1_HUMAN	42862.551
MIC60_HUMAN	18052.99
ECSCR_HUMAN	4574.3638
KNOP1_HUMAN	1407.5754
HNRL2_HUMAN	15545.133
T132A_HUMAN	589.19037
INF2_HUMAN	6781.4224
PDS5A_HUMAN	7539.7739
CL16A_HUMAN	1818.0238
KIF7_HUMAN	498.2529
RHG31_HUMAN	3294.5032
MTHSD_HUMAN	4809.6299
AAK1_HUMAN	4670.7104
WASC4_HUMAN	4762.5737
ERC6L_HUMAN	1382.9119
TSR1_HUMAN	8099.8809
RGPA2_HUMAN	1072.5061
D19L1_HUMAN	5326.4604
IAH1_HUMAN	1186.6313
ALG11_HUMAN	2517.1946
QRIC1_HUMAN	3834.8125
SMU1_HUMAN	10877.339
CTU2_HUMAN	8859.1338
PGGHG_HUMAN	165.07817
LRRF1_HUMAN	4880.0635
PGPS1_HUMAN	386.39801
CR021_HUMAN	23.216099
P3H1_HUMAN	20405.756
TRM5_HUMAN	4097.1406
EMAL3_HUMAN	4619.3828
RPA43_HUMAN	2362.4211
MA7D1_HUMAN	2832.8979
UAP1L_HUMAN	12148.227
S2535_HUMAN	222.71263
CHD9_HUMAN	3705.0989
TKFC_HUMAN	3557.0857
LSM12_HUMAN	7127.1787
HSDL1_HUMAN	3694.5977
AL1L2_HUMAN	866.146
GNPTA_HUMAN	388.64423

GRDN_HUMAN	1736.6381
RABL6_HUMAN	5808.2412
TIM50_HUMAN	6729.4126
PAR14_HUMAN	3376.0403
FUT11_HUMAN	1385.3132
GLYR1_HUMAN	8300.7188
CE022_HUMAN	1832.6703
C2CD3_HUMAN	186.97217
VP26B_HUMAN	3994.8088
LARP7_HUMAN	3049.9597
NAKD2_HUMAN	983.09875
KCD21_HUMAN	2516.3188
GXLT1_HUMAN	549.13318
ACSF3_HUMAN	1587.8409
PPCEL_HUMAN	2515.4277
TB10B_HUMAN	3302.5417
TPRN_HUMAN	280.48734
ANO6_HUMAN	3930.856
FIL1L_HUMAN	1907.068
GRAP1_HUMAN	3340.1765
ZNG1F_HUMAN	1178.0648
MIX23_HUMAN	4185.8496
MBRL_HUMAN	1384.9706
FA98B_HUMAN	6585.3535
RHG29_HUMAN	7815.2554
PDCD4_HUMAN	10180.087
FND3B_HUMAN	6286.29
CRTC2_HUMAN	436.86914
GPAT3_HUMAN	523.85321
CEP55_HUMAN	2677.9885
NCBP3_HUMAN	1780.6206
QORX_HUMAN	20921.932
NACHO_HUMAN	3522.6277
PHLA2_HUMAN	1740.9102
PDLI3_HUMAN	880.56604
PAR10_HUMAN	575.15002
DHB12_HUMAN	24412.305
GLE1_HUMAN	3026.4885
UBP39_HUMAN	7174.8389
KLH22_HUMAN	1122.6503
AGK_HUMAN	13065.487
SETMR_HUMAN	401.2587
LACB2_HUMAN	2046.6924
P5CR3_HUMAN	2785.5039
EIPR1_HUMAN	5596.2778
BOREA_HUMAN	277.91956
COBL1_HUMAN	1050.1028
H1BP3_HUMAN	3664.7764
CYBR1_HUMAN	670.38434
WDR81_HUMAN	3018.0117

ACTBL_HUMAN	274898.69
CCD93_HUMAN	3832.6145
OCAD2_HUMAN	9413.0273
PKHG4_HUMAN	775.2251
HS904_HUMAN	982.18677
DCAF6_HUMAN	431.40521
TM41B_HUMAN	812.26404
SGMR2_HUMAN	11511.29
YIF1B_HUMAN	2822.2212
ZN326_HUMAN	3250.2781
NOM1_HUMAN	1018.7792
RIPL1_HUMAN	2332.249
DJC21_HUMAN	1398.3777
TP8L3_HUMAN	3792.9507
HERC4_HUMAN	7360.1768
PIGG_HUMAN	1321.2732
PP6R3_HUMAN	5734.4438
DDX6L_HUMAN	304.36655
RABL3_HUMAN	23891.576
FA76B_HUMAN	286.19257
COQ5_HUMAN	579.4743
SH319_HUMAN	3615.9502
NHSL2_HUMAN	2381.2217
EMC4_HUMAN	3729.665
SYEM_HUMAN	1344.2954
CC038_HUMAN	918.49182
EFMT2_HUMAN	404.60068
TGO1_HUMAN	2111.2527
PREP_HUMAN	13028.948
WDR44_HUMAN	3018.22
PRC2B_HUMAN	3968.2063
TJAP1_HUMAN	1109.6495
RRP12_HUMAN	7232.147
COA6_HUMAN	475.41327
TOIP1_HUMAN	14405.088
CEP78_HUMAN	373.33005
SYAM_HUMAN	4406.3057
TOR2A_HUMAN	2553.6699
PCID2_HUMAN	3415.2944
HABP4_HUMAN	1700.1665
S27A3_HUMAN	1264.144
SAMD9_HUMAN	1770.3711
P4R3B_HUMAN	2382.4995
WIPI3_HUMAN	1859.4435
WIPI1_HUMAN	1124.8312
EOGT_HUMAN	11744.712
SMAG2_HUMAN	633.1059
TBCEL_HUMAN	3503.9163
TDIF2_HUMAN	887.01379
RBG1L_HUMAN	2025.2231

TTC38_HUMAN	1311.6328
COX20_HUMAN	3943.6858
EXOS6_HUMAN	5249.4492
TM201_HUMAN	2120.7498
ATAT_HUMAN	465.02893
SNP47_HUMAN	281.05655
NU188_HUMAN	6015.8975
HP1B3_HUMAN	34408.113
SYVM_HUMAN	847.71912
CE170_HUMAN	5013.8101
ARH_HUMAN	1688.2953
ODR4_HUMAN	2656.0085
NOL9_HUMAN	3458.1023
NHSL1_HUMAN	1434.7224
HYI_HUMAN	475.44763
TPRGL_HUMAN	928.97156
C2D1B_HUMAN	1587.2428
FBP1L_HUMAN	17630.969
SYRM_HUMAN	991.61871
THEM4_HUMAN	1250.2601
CHCH9_HUMAN	602.19727
FKB15_HUMAN	12003.482
ZC3HD_HUMAN	1331.9877
CI114_HUMAN	1249.4353
ARMD3_HUMAN	712.08649
CSCL2_HUMAN	1406.6357
GPTC4_HUMAN	481.74149
CAF17_HUMAN	1625.5023
HECD3_HUMAN	4998.9277
GT253_HUMAN	12473.117
UBR4_HUMAN	11468.229
STXB5_HUMAN	1403.0317
RHG21_HUMAN	1275.1364
CAMP1_HUMAN	1250.9221
RM02_HUMAN	3316.3186
UBAP2_HUMAN	4147.0654
QNG1_HUMAN	2464.803
KPRP_HUMAN	892.66608
ZN618_HUMAN	3057.0596
ACBD5_HUMAN	5917.811
RBM26_HUMAN	3571.7825
ATD3B_HUMAN	1715.4778
WLS_HUMAN	5618.8086
INT11_HUMAN	3780.7849
CPTP_HUMAN	477.88992
DCAF8_HUMAN	4501.7534
CODA1_HUMAN	713.00067
TUT4_HUMAN	2394.6768
FRY_HUMAN	2142.6345
RNH2B_HUMAN	1294.1014

ATPF1_HUMAN	2150.8643
RC3H1_HUMAN	943.21881
DDI2_HUMAN	3974.1128
THMS2_HUMAN	636.89294
NDUF5_HUMAN	1061.1664
NT5D1_HUMAN	3118.7275
BIG3_HUMAN	1214.2128
VP13D_HUMAN	437.22986
CROCC_HUMAN	1161.9576
LYRM7_HUMAN	1561.5007
RAIN_HUMAN	23946.926
RIF1_HUMAN	1749.4141
VPS53_HUMAN	3695.8225
STRP1_HUMAN	4360.9609
MRCKA_HUMAN	5671.7681
RPRD2_HUMAN	973.23944
RN220_HUMAN	513.35529
PR38B_HUMAN	4988.7036
BRE1A_HUMAN	3520.5156
MYOME_HUMAN	475.57144
ZN318_HUMAN	906.64563
F1711_HUMAN	974.2948
DIK1B_HUMAN	3854.6165
LRCH2_HUMAN	1197.1022
CDKAL_HUMAN	4369.3735
BROX_HUMAN	6073.2397
FOCAD_HUMAN	7524.1772
RSBN1_HUMAN	277.3085
DAB2P_HUMAN	727.70905
LYPL1_HUMAN	7594.9692
PEAR1_HUMAN	2002.8252
ECM29_HUMAN	16098.431
TUT7_HUMAN	836.0434
SHE_HUMAN	742.78577
DEN4C_HUMAN	3626.9399
NAA35_HUMAN	4710.5464
ZMYM4_HUMAN	2508.7002
FHI2A_HUMAN	4571.6528
ZDH20_HUMAN	2500.54
SPRY7_HUMAN	1518.323
FBXW9_HUMAN	207.97511
CD276_HUMAN	3200.1091
RN213_HUMAN	6136.8003
TENS2_HUMAN	2198.4602
KANK2_HUMAN	770.60895
WAC2A_HUMAN	961.60309
LPCT4_HUMAN	1222.8854
STEA3_HUMAN	1777.3572
F91A1_HUMAN	3705.5266
TBC9B_HUMAN	7215.4648

MAP1S_HUMAN	9869.7305
2ABD_HUMAN	3771.0376
AR6P4_HUMAN	4375.7935
A16L1_HUMAN	2690.5186
HGNAT_HUMAN	2445.948
ARID2_HUMAN	563.86377
UTP25_HUMAN	1779.0647
GL8D1_HUMAN	1463.0203
SE1L3_HUMAN	965.56738
TENS3_HUMAN	3142.1458
HAUS3_HUMAN	595.21875
SPT2_HUMAN	492.43604
MBLC2_HUMAN	1828.6613
CRBG3_HUMAN	661.57391
INT3_HUMAN	4635.3096
RHG17_HUMAN	19846.652
NCAS2_HUMAN	2803.824
C19L1_HUMAN	10751.383
VIR_HUMAN	3366.3228
CYTSA_HUMAN	4507.8325
HEAT6_HUMAN	1001.8331
ANR40_HUMAN	786.42035
WWC2_HUMAN	1614.4158
SCAR3_HUMAN	1152.2218
ATLA3_HUMAN	19501.219
RL7L_HUMAN	7226.0591
NXN_HUMAN	2968.7559
TTC19_HUMAN	591.56281
IQEC1_HUMAN	2775.0586
CPIN1_HUMAN	9347.9238
ZFAN6_HUMAN	1103.6449
SMYD5_HUMAN	2259.8506
OTU7B_HUMAN	685.00903
THOC7_HUMAN	1191.7802
NADE_HUMAN	3006.1792
ELP2_HUMAN	2775.322
LTOR1_HUMAN	1906.8536
DRS7B_HUMAN	2165.0017
TWF2_HUMAN	15223.411
CNDH2_HUMAN	481.89017
K0930_HUMAN	859.42102
TNG2_HUMAN	8266.2686
P4R3A_HUMAN	3963.1387
RAB12_HUMAN	23125.461
DEN5A_HUMAN	240.47377
SDE2_HUMAN	148.56821
K2C80_HUMAN	746.68005
NIPBL_HUMAN	586.57141
SCMC2_HUMAN	3852.269
PDE12_HUMAN	7197.8027

NAA16_HUMAN	1558.3971
DE10B_HUMAN	3389.3777
RHBD2_HUMAN	1830.2073
SCMC1_HUMAN	29376.074
REEP3_HUMAN	759.5968
RETST_HUMAN	3218.7258
RINT1_HUMAN	1107.2589
TM214_HUMAN	10197.804
HIBCH_HUMAN	8564.4219
RWDD4_HUMAN	1565.3568
NEPRO_HUMAN	1361.759
ARMC6_HUMAN	4406.7695
TTI2_HUMAN	1719.3787
ANR54_HUMAN	2615.4277
ZNT6_HUMAN	1227.2924
TAPT1_HUMAN	19491.398
KANK3_HUMAN	1669.2988
JMJD6_HUMAN	5627.0356
PPR18_HUMAN	8417.9863
CAVN1_HUMAN	115409.73
ZCHC8_HUMAN	2968.2246
DHX57_HUMAN	3765.1355
ERAP2_HUMAN	13653.021
MBOA5_HUMAN	3677.4248
CDC73_HUMAN	6282.7954
RM14_HUMAN	3236.1223
S27A4_HUMAN	7137.332
C2D1A_HUMAN	4171.6553
TATD1_HUMAN	3128.365
MET2B_HUMAN	1597.3438
MSD2_HUMAN	802.26312
TAF2_HUMAN	1833.0786
CH082_HUMAN	1003.1578
MED27_HUMAN	867.57971
EDC4_HUMAN	18220.768
CEP85_HUMAN	258.27304
ANM9_HUMAN	500.61987
PRP8_HUMAN	13582.271
FBX42_HUMAN	420.39297
SCYL2_HUMAN	4363.1016
TTC27_HUMAN	4505.4077
SFXN4_HUMAN	986.70502
GOLM2_HUMAN	2577.136
NFRKB_HUMAN	654.73651
FAHD1_HUMAN	12655.845
RL22L_HUMAN	10233.212
PKN3_HUMAN	1457.6367
ALKB5_HUMAN	962.24585
PDXD1_HUMAN	7051.4487
MEAK7_HUMAN	3876.0244

INT5_HUMAN	2954.4563
GIMA6_HUMAN	703.93829
S27A1_HUMAN	1219.9314
PGM2L_HUMAN	10056.261
CTR9_HUMAN	4848.7998
AAGAB_HUMAN	2187.8237
TMM88_HUMAN	547.36731
PSRC1_HUMAN	368.64124
SKI3_HUMAN	5417.3252
ULK3_HUMAN	1447.5256
SYDM_HUMAN	9676.1865
TMM65_HUMAN	4869.0034
IN80C_HUMAN	1334.408
FBX38_HUMAN	1450.317
NCEH1_HUMAN	10099.615
FIGL1_HUMAN	864.25354
TRI65_HUMAN	1539.9836
RHDF2_HUMAN	795.98743
MDEAS_HUMAN	1602.3418
BRAT1_HUMAN	3909.0811
WDR59_HUMAN	1204.5582
ZC3HE_HUMAN	1642.9244
CC137_HUMAN	882.24823
OGFD3_HUMAN	1095.1648
TXD11_HUMAN	221.79259
LARP1_HUMAN	13254.729
TMED8_HUMAN	2472.5452
ZNT9_HUMAN	740.2099
TRAF7_HUMAN	1190.4944
RICTR_HUMAN	4062.7717
WDR74_HUMAN	698.92462
ATRAP_HUMAN	2289.5044
SMRD3_HUMAN	3034.5754
C1TM_HUMAN	21274.641
AFTIN_HUMAN	428.88449
FIP1_HUMAN	6144.5137
CRTC3_HUMAN	252.0666
CRTC1_HUMAN	580.37158
CP20A_HUMAN	3456.2266
PLGT2_HUMAN	3578.2683
TM205_HUMAN	8890.3916
LRSM1_HUMAN	2932.5256
GAK1B_HUMAN	3972.7957
LCLT1_HUMAN	2598.0051
ABRX1_HUMAN	169.29071
CWC27_HUMAN	1531.5634
CREL2_HUMAN	1978.6715
WDR82_HUMAN	5723.7607
MIC27_HUMAN	1772.8103
FAT4_HUMAN	1034.0867

DPP8_HUMAN	888.93713
MCAF1_HUMAN	2363.1865
RBP10_HUMAN	1681.0974
PACS1_HUMAN	6752.5024
MPRIP_HUMAN	6707.8589
RBMS3_HUMAN	2933.3938
PNCB_HUMAN	5063.6475
DNMBP_HUMAN	4791.0127
HACD2_HUMAN	882.52338
B3GLT_HUMAN	1422.4899
GGYF2_HUMAN	8304.3281
CD109_HUMAN	10693.827
THADA_HUMAN	1814.0767
HSDL2_HUMAN	4192.4927
KAT3_HUMAN	7188.0449
PPR21_HUMAN	1720.4984
THSD4_HUMAN	948.14795
SYNE3_HUMAN	1205.562
ZN574_HUMAN	482.92047
NFXL1_HUMAN	398.84854
NBEL2_HUMAN	3766.3779
FGD5_HUMAN	4631.1895
QSOX2_HUMAN	2833.0703
SRCAP_HUMAN	400.09314
RIPR1_HUMAN	4480.8936
YJ005_HUMAN	6461.4731
ARHGI_HUMAN	1074.2047
TBCD9_HUMAN	327.34634
UBR3_HUMAN	393.53549
TMPPE_HUMAN	522.53125
CRACD_HUMAN	603.88379
CCD9B_HUMAN	522.95569
NUD18_HUMAN	427.2662
PAXI1_HUMAN	528.88934
STXB4_HUMAN	181.32045
TMTC3_HUMAN	5837.7549
VP13C_HUMAN	11343.608
UBP34_HUMAN	850.00592
RAPH1_HUMAN	3047.7146
MOB2_HUMAN	4985.6587
IKIP_HUMAN	24076.303
PREX2_HUMAN	1469.626
F10C1_HUMAN	1540.8712
UB2R2_HUMAN	6652.2119
MD13L_HUMAN	321.10458
LARP4_HUMAN	7102.5752
MED25_HUMAN	1727.09
RS27L_HUMAN	63230.594
HAKAI_HUMAN	1091.5774
INT8_HUMAN	3934.2415

BT2A1_HUMAN	406.04111
SPT6H_HUMAN	3523.2219
SND1_HUMAN	84791.141
MARK2_HUMAN	1681.8428
COX15_HUMAN	1357.5249
DDX46_HUMAN	13719.396
RUFY3_HUMAN	689.70648
TM10C_HUMAN	9338.626
5MP2_HUMAN	25512.93
MON1B_HUMAN	1703.2202
LRC8D_HUMAN	356.01364
ASGL1_HUMAN	1375.0947
KCTD9_HUMAN	6146.8047
DHX30_HUMAN	8948.8691
EIF3M_HUMAN	16444.924
MEPCE_HUMAN	3298.3425
ARMX2_HUMAN	1196.6775
SYPM_HUMAN	427.88873
RSRC2_HUMAN	1523.906
RRAGA_HUMAN	4704.4648
CYFP1_HUMAN	27056.943
NDUF7_HUMAN	2867.231
GET4_HUMAN	8678.7051
CSN6_HUMAN	21048.844
PCAT2_HUMAN	18679.928
ENOF1_HUMAN	1909.9319
MAEA_HUMAN	1739.0319
EPMIP_HUMAN	1855.5461
DHX32_HUMAN	379.35577
TAOK1_HUMAN	3615.9197
FAKD5_HUMAN	1888.7712
MOB1B_HUMAN	14716.041
KDM3B_HUMAN	4167.2969
CHM1B_HUMAN	5279.1938
RIR2B_HUMAN	3758.2351
HS2ST_HUMAN	5514.7656
STRAA_HUMAN	1605.5621
MICA3_HUMAN	1401.8285
DYM_HUMAN	1772.8087
PHF5A_HUMAN	2343.4541
PXL2C_HUMAN	1894.0676
APTX_HUMAN	2242.8174
ERMP1_HUMAN	4562.2847
GRIN1_HUMAN	192.87875
TRM1L_HUMAN	2244.8105
ZCCHV_HUMAN	9330.7363
RM21_HUMAN	4809.4766
EFL1_HUMAN	4005.248
SETX_HUMAN	712.88672
TPC11_HUMAN	2030.0331

KANL1_HUMAN	173.86888
NUP54_HUMAN	8950.418
ATG9A_HUMAN	1697.8439
GLUCM_HUMAN	5996.8872
CC186_HUMAN	922.13171
ARMC9_HUMAN	2095.5391
VP35L_HUMAN	6068.7695
POGZ_HUMAN	3529.0212
ZFY16_HUMAN	847.96832
MON2_HUMAN	3612.5293
MYCPP_HUMAN	1175.1611
MYH14_HUMAN	9318.0322
NUFP2_HUMAN	4753.6011
SZRD1_HUMAN	1560.4609
MAVS_HUMAN	973.3125
CLAP1_HUMAN	6274.2056
DHX29_HUMAN	4636.7324
NPHP3_HUMAN	626.39331
LRP10_HUMAN	1871.4087
HAUS6_HUMAN	1667.8285
PLGT3_HUMAN	6978.7905
TT21B_HUMAN	2334.1453
HEAT3_HUMAN	5688.8315
KI21A_HUMAN	484.44861
HDGR2_HUMAN	10044.637
DCXR_HUMAN	7465.9897
BRAP_HUMAN	2108.8765
EMSY_HUMAN	759.15405
GOGA7_HUMAN	2279.9836
RHG22_HUMAN	517.87817
WAPL_HUMAN	1722.9603
I2BP2_HUMAN	798.4071
RBBP6_HUMAN	2307.978
SH3R1_HUMAN	987.40698
GRASP_HUMAN	1822.0601
FRMD5_HUMAN	459.19916
SEN54_HUMAN	421.74966
PTAR1_HUMAN	313.55048
ARPIN_HUMAN	3629.6089
TCPR1_HUMAN	925.69037
RABEK_HUMAN	1159.017
MTEF4_HUMAN	502.10492
HUWE1_HUMAN	14150.731
YTHD3_HUMAN	9649.498
CTU1_HUMAN	3130.6292
UB2Q1_HUMAN	5931.3374
RM55_HUMAN	10505.54
TMED4_HUMAN	3320.8779
RM10_HUMAN	1105.9897
COXM1_HUMAN	4713.7246

CENPV_HUMAN	1561.5605
SLN11_HUMAN	4584.3857
MEGF8_HUMAN	495.09595
T179B_HUMAN	825.88104
GALT7_HUMAN	6401.5757
SCD5_HUMAN	2871.2837
PHLB2_HUMAN	2990.0376
AGRG6_HUMAN	1876.9421
DHDDS_HUMAN	786.36157
GLT10_HUMAN	576.53253
TPC6B_HUMAN	1016.4025
PP4P1_HUMAN	1440.3425
CLC14_HUMAN	14344.071
PATL1_HUMAN	1037.3655
DPP9_HUMAN	7041.8364
SYVN1_HUMAN	2052.55
TRPT1_HUMAN	642.32452
SETD3_HUMAN	6230.6743
TTC7B_HUMAN	1269.4159
ACOT1_HUMAN	4969.9331
NOP9_HUMAN	2749.2661
PABP2_HUMAN	19030.775
MTA70_HUMAN	2482.1013
PB1_HUMAN	3422.5173
YRDC_HUMAN	1714.0994
PRP39_HUMAN	2312.2429
LYRIC_HUMAN	13683.537
ZN598_HUMAN	3914.0349
GPAT4_HUMAN	1111.6649
KTN1_HUMAN	8249.501
EST1A_HUMAN	1710.2748
NLRX1_HUMAN	1901.4269
BCL9L_HUMAN	443.89468
PHLB1_HUMAN	7670.5503
UBP48_HUMAN	6696.7588
CAPS2_HUMAN	546.77258
URP2_HUMAN	22807.738
NAA40_HUMAN	4176.8486
NT5D3_HUMAN	684.95551
AACS_HUMAN	8428.5078
LUZP1_HUMAN	6628.4287
THOC4_HUMAN	23440.164
GP180_HUMAN	576.40613
FHI2B_HUMAN	3094.8003
IQGA3_HUMAN	3873.1519
ZCH18_HUMAN	1911.4889
VPS36_HUMAN	2128.5867
CAND1_HUMAN	36897.691
RETR3_HUMAN	2820.5383
HOOK3_HUMAN	7059.3311

SESD1_HUMAN	1067.3551
COMD7_HUMAN	1474.7715
MON1A_HUMAN	718.18927
AMZ2_HUMAN	1295.9762
THOC6_HUMAN	2356.6384
MET16_HUMAN	1150.9559
PARG_HUMAN	623.0896
LIPB1_HUMAN	8598.3184
LONP2_HUMAN	1046.4854
ZC3C1_HUMAN	3160.0679
CHD1L_HUMAN	8246.2861
FCSD1_HUMAN	171.63438
CCD25_HUMAN	8194.2246
PRSR2_HUMAN	777.16479
STING_HUMAN	7911.1631
AROS_HUMAN	672.36072
CDR2L_HUMAN	147.69519
RLGPB_HUMAN	1369.2181
RGPS2_HUMAN	507.75073
CARM1_HUMAN	8526.6533
NIT1_HUMAN	4958.8257
COMD2_HUMAN	4493.7764
HTR5A_HUMAN	1368.2666
CNDG2_HUMAN	883.40094
PPR3B_HUMAN	186.99536
FBX11_HUMAN	199.38116
ANKL2_HUMAN	1718.8202
MEX3D_HUMAN	1740.0062
DGKH_HUMAN	282.85471
DDX42_HUMAN	15836.332
SPAS2_HUMAN	2540.7925
VRK2_HUMAN	2763.5339
CACL1_HUMAN	645.92059
NDUAB_HUMAN	8854.9883
DAAF5_HUMAN	8991.5479
PTH_HUMAN	977.36023
STX12_HUMAN	11497.67
P66A_HUMAN	3219.1218
CPNE8_HUMAN	24180.854
GPSM1_HUMAN	902.07202
RAB43_HUMAN	1118.3586
C2CD5_HUMAN	1787.2872
MIB1_HUMAN	719.49738
PRAG1_HUMAN	1543.4075
HPS6_HUMAN	1583.7299
I2BP1_HUMAN	1995.4009
RB6I2_HUMAN	4708.9624
RIOX2_HUMAN	1858.4165
ZDH13_HUMAN	7540.3286
ZDH17_HUMAN	450.52573

CRLF3_HUMAN	1767.1119
TPPC5_HUMAN	4778.4912
ARMC8_HUMAN	2839.3496
RELL1_HUMAN	1144.1503
T126B_HUMAN	1356.6
PLD3_HUMAN	15696.431
HID1_HUMAN	1883.4821
ANKY2_HUMAN	1506.4508
ERI1_HUMAN	1422.9945
VRK3_HUMAN	681.74573
LIX1L_HUMAN	2395.2258
NUDC3_HUMAN	4745.3574
AHNK2_HUMAN	19260.271
FMNL3_HUMAN	6660.1299
MMAA_HUMAN	1871.3895
M4K3_HUMAN	196.16023
NAV3_HUMAN	830.03656
P3H3_HUMAN	11529.625
CCD50_HUMAN	9176.5527
FABD_HUMAN	1812.856
KSR1_HUMAN	1489.902
CEP97_HUMAN	1750.7605
NNRD_HUMAN	1410.326
WDR75_HUMAN	684.57581
MFN1_HUMAN	2112.4395
CTL2_HUMAN	5215.9556
IPRI_HUMAN	1366.0769
WDFY1_HUMAN	4461.0361
MA7D3_HUMAN	2374.9517
CC117_HUMAN	320.80539
NXP20_HUMAN	7489.7046
DCNL3_HUMAN	368.82382
DEN6A_HUMAN	985.48267
MGAP_HUMAN	459.36108
GCC2_HUMAN	2859.3625
Z3H7A_HUMAN	1886.4967
PHF6_HUMAN	6838.4663
ARCH_HUMAN	801.09229
LRC8A_HUMAN	5796.9292
SULF2_HUMAN	2672.4495
UBR1_HUMAN	1336.796
UBR2_HUMAN	1453.1458
RHG12_HUMAN	688.08557
CHERP_HUMAN	8325.1504
CDAN1_HUMAN	633.67651
ANKH1_HUMAN	2643.166
SUGP1_HUMAN	2242.4651
SUGP2_HUMAN	26403.443
UEVLD_HUMAN	2146.9927
CCAR1_HUMAN	10679.535

DHX40_HUMAN	722.47131
DJC10_HUMAN	10620.499
NELFD_HUMAN	5562.5278
MIRO2_HUMAN	5700.5889
MIRO1_HUMAN	5090.2222
SIR2_HUMAN	3467.2454
PHC2_HUMAN	1142.8553
BAP18_HUMAN	6186.7808
RM41_HUMAN	8631.4043
NRM_HUMAN	5154.0908
GPAM1_HUMAN	814.62976
PARP9_HUMAN	1087.3782
RB12B_HUMAN	2943.3381
S35F2_HUMAN	1263.7281
RPAP2_HUMAN	331.96246
PLPL6_HUMAN	6871.0146
SMC5_HUMAN	1012.9313
DDX60_HUMAN	840.4726
CMIP_HUMAN	514.33557
MILK2_HUMAN	582.94092
DHX37_HUMAN	2357.1401
KBTB2_HUMAN	3567.741
RAVR1_HUMAN	5463.7417
SPB1_HUMAN	5575.1064
TM192_HUMAN	1648.0162
M21D2_HUMAN	1110.1125
SRRM1_HUMAN	4375.7979
SMAP1_HUMAN	3773.5576
DI3L2_HUMAN	3531.5962
SUV3_HUMAN	5095.0117
ERF3B_HUMAN	7108.0464
ZZZ3_HUMAN	323.72015
EXOC8_HUMAN	8280.2881
CJ067_HUMAN	12913.85
TRI22_HUMAN	5348.5522
THNS1_HUMAN	613.80988
K2013_HUMAN	4256.7651
MICU2_HUMAN	1589.0199
AN13A_HUMAN	3456.2021
PHAR4_HUMAN	1965.045
TRM2A_HUMAN	2976.6062
RUSD2_HUMAN	612.32074
ELMD2_HUMAN	2256.8467
A16A1_HUMAN	7009.271
K319L_HUMAN	2100.6677
AGRF5_HUMAN	1816.0708
XRN1_HUMAN	4270.9434
PELP1_HUMAN	13655.196
ABI1_HUMAN	6374.1616
WDFY3_HUMAN	3919.1243

SELH_HUMAN	7500.2544
VKORL_HUMAN	2495.4968
SPART_HUMAN	8138.8223
TTC5_HUMAN	1409.6639
MIER1_HUMAN	2318.47
RPTOR_HUMAN	1574.525
ORML3_HUMAN	4233.666
CCAR2_HUMAN	51005.281
NDUF2_HUMAN	7942.9746
VPS52_HUMAN	5949.7031
NUP93_HUMAN	24504.664
S11IP_HUMAN	1076.4901
ZN687_HUMAN	649.43958
CMTR1_HUMAN	4165.6909
LRC47_HUMAN	15435.483
DOCK4_HUMAN	7677.9551
CAH13_HUMAN	1521.2313
ARG28_HUMAN	9439.3994
INT1_HUMAN	36602.316
H2B3B_HUMAN	6073.9214
RHG24_HUMAN	3227.9934
ARM10_HUMAN	3117.7085
ABD12_HUMAN	3730.3464
CLASR_HUMAN	494.40234
FA43A_HUMAN	2986.4668
TM256_HUMAN	2016.7725
PIAS4_HUMAN	285.13361
GPD1L_HUMAN	3867.3438
S35F6_HUMAN	2441.3159
RHG18_HUMAN	21710.766
ASCC3_HUMAN	9672.4277
MILK1_HUMAN	917.76233
VPS8_HUMAN	2846.4526
PALS1_HUMAN	620.25208
STAG2_HUMAN	3108.6157
SYNPO_HUMAN	7885.793
FNBP4_HUMAN	1578.082
GUF1_HUMAN	241.32864
RYBP_HUMAN	368.95749
GALT4_HUMAN	2749.6614
MINK1_HUMAN	1224.691
MESH1_HUMAN	1249.8171
PTGR3_HUMAN	2288.0625
CBR4_HUMAN	673.19824
EMC5_HUMAN	3207.8723
TM199_HUMAN	2988.1477
FBXL6_HUMAN	198.12692
OGFD1_HUMAN	2070.0383
AFAP1_HUMAN	3535.2781
OXR1_HUMAN	1265.6814

ZGPAT_HUMAN	1210.1643
CAHM5_HUMAN	1814.3459
SRBD1_HUMAN	1098.1798
WDTC1_HUMAN	2501.4663
NKAP_HUMAN	1120.0679
MACOI_HUMAN	550.55188
SH2D3_HUMAN	3519.2363
ARRD1_HUMAN	856.58704
DHRSX_HUMAN	1318.1172
CISD2_HUMAN	9574.1895
RP25L_HUMAN	2033.6438
ATPF2_HUMAN	1240.2925
TTC9C_HUMAN	1775.5483
JAGN1_HUMAN	3753.1438
RNF10_HUMAN	663.28387
RFLB_HUMAN	2222.9932
LZTR1_HUMAN	529.39435
COMD1_HUMAN	3227.8713
CPSF7_HUMAN	13492.932
S15A4_HUMAN	679.21826
MYCT1_HUMAN	12050.063
ATL1_HUMAN	230.69528
ARFG2_HUMAN	2985.2153
OTU6B_HUMAN	3269.802
FITM2_HUMAN	2416.8157
CA052_HUMAN	1304.7303
EFNMT_HUMAN	2259.4526
AR6P6_HUMAN	1234.2971
ARFG1_HUMAN	1320.7922
SIR6_HUMAN	573.31805
EMC1_HUMAN	13936.892
PAF1_HUMAN	2716.7271
CCYL1_HUMAN	1203.505
UBR7_HUMAN	3595.3718
DDX51_HUMAN	1529.4882
F241A_HUMAN	5886.4922
S2529_HUMAN	3565.9792
ENAH_HUMAN	14377.707
DCBD1_HUMAN	913.55884
GPT11_HUMAN	1272.3242
RM43_HUMAN	6585.9219
RLIG1_HUMAN	2513.8914
CS047_HUMAN	1003.1184
ASCC1_HUMAN	3602.9631
LRC57_HUMAN	7079.6992
EIF1A_HUMAN	894.66925
KRI1_HUMAN	1607.1147
CC71L_HUMAN	1000.6511
ZN579_HUMAN	1888.5674
PR38A_HUMAN	2524.3142

MLKL_HUMAN	4946.9819
GALD1_HUMAN	3236.2163
ANR52_HUMAN	670.42761
AT11C_HUMAN	829.51202
AFG2A_HUMAN	4161.0786
NHLC2_HUMAN	7574.8076
AVL9_HUMAN	1921.8414
S43A3_HUMAN	5590.4302
XXLT1_HUMAN	7965.6621
GOLM1_HUMAN	797.83435
GT251_HUMAN	27763.404
SUMF2_HUMAN	3568.6675
SIDT2_HUMAN	555.99609
SUMF1_HUMAN	4096.2744
PGLT1_HUMAN	3231.0522
UBAC2_HUMAN	2230.4575
PCYXL_HUMAN	1531.3096
TM87A_HUMAN	2599.9895
RDH13_HUMAN	3453.2859
TTC13_HUMAN	1325.4629
DHB11_HUMAN	9689.2383
TXND5_HUMAN	271312.38
SPC24_HUMAN	1302.6093
ATAD1_HUMAN	2231.1614
SCPDL_HUMAN	19329.336
UXS1_HUMAN	1119.9685
RN149_HUMAN	1270.7454
RETR2_HUMAN	965.20398
SERB1_HUMAN	10581.285
LEMD2_HUMAN	6096.1602
NOA1_HUMAN	1459.2338
NECP1_HUMAN	2709.3474
FA98A_HUMAN	4529.0161
PAG15_HUMAN	1463.1248
MTMRE_HUMAN	1765.7682
NF2IP_HUMAN	3990.3428
DGLB_HUMAN	1218.8372
CHSTE_HUMAN	4369.9634
GALT6_HUMAN	2294.9043
RN169_HUMAN	752.10791
PDPR_HUMAN	4929.8125
NNRE_HUMAN	2843.1724
SMG8_HUMAN	1294.754
RN214_HUMAN	2734.7332
LS14A_HUMAN	3219.9634
GIMA8_HUMAN	17359.473
CCNY_HUMAN	3599.2056
MROH1_HUMAN	3944.2122
PAPD5_HUMAN	1289.7611
PEPL1_HUMAN	3263.8967

EHBP1_HUMAN	1418.4077
RB15B_HUMAN	2138.884
SIA7C_HUMAN	1893.5063
TNR6A_HUMAN	1542.3142
PHC3_HUMAN	399.83221
DIK2A_HUMAN	3698.4165
CNNM3_HUMAN	724.15552
ABCF1_HUMAN	12809.239
MCU_HUMAN	2089.8481
PK3C3_HUMAN	6876.9966
GSTCD_HUMAN	422.36536
SRFB1_HUMAN	1112.1191
NGDN_HUMAN	1205.7451
SHCBP_HUMAN	1289.6958
PDZD8_HUMAN	620.51843
DP13B_HUMAN	2427.3003
ZNT7_HUMAN	8641.04
NAV1_HUMAN	1425.0148
PPHLN_HUMAN	278.19067
WDR19_HUMAN	929.2124
FBX22_HUMAN	4625.0278
PCAT1_HUMAN	5634.4858
ZMIZ2_HUMAN	1370.1954
SYNE1_HUMAN	23292.914
UBP32_HUMAN	492.22989
BD1L1_HUMAN	875.48462
ARI1B_HUMAN	1370.678
FAD1_HUMAN	5849.2939
FLCN_HUMAN	2095.1289
NUP43_HUMAN	6418.7266
NUP37_HUMAN	8874.457
NUP35_HUMAN	3171.4795
ENASE_HUMAN	439.53616
RAI3_HUMAN	526.48523
TOIP2_HUMAN	7255.4287
ABHDB_HUMAN	1996.2153
NEUA_HUMAN	6120.5015
TSN14_HUMAN	3805.5806
TTL_HUMAN	483.00137
SVIP_HUMAN	1543.1064
ATLA2_HUMAN	1655.8319
MSPD2_HUMAN	1676.0522
PLBL2_HUMAN	13436.828
RASF8_HUMAN	668.29156
DDX55_HUMAN	533.7627
MFSD8_HUMAN	270.27664
TDRD7_HUMAN	1207.1215
GIMA7_HUMAN	6490.793
NEDD1_HUMAN	2076.8225
NCOA7_HUMAN	2224.6179

THOC2_HUMAN	4196.2139
WDR36_HUMAN	2889.4651
PPTC7_HUMAN	591.06061
RP9_HUMAN	666.04327
VANG1_HUMAN	990.32123
SNIP1_HUMAN	383.01529
G45IP_HUMAN	3110.5999
WDR48_HUMAN	3262.2478
EXOC6_HUMAN	6413.2622
SMRC2_HUMAN	4744.9956
NPL4_HUMAN	8447.4033
CX038_HUMAN	1473.937
SPT20_HUMAN	1842.6459
NUBPL_HUMAN	917.80377
ABHD4_HUMAN	1124.292
FBX30_HUMAN	1080.5729
S35B2_HUMAN	7420.9019
PUM2_HUMAN	2588.7905
TIP_HUMAN	1373.9493
GOGA5_HUMAN	1501.1057
KLDC4_HUMAN	1078.2285
SHKB1_HUMAN	2577.501
UBA3_HUMAN	28876.262
PXL2B_HUMAN	1131.5885
DJB14_HUMAN	617.40912
KISHA_HUMAN	17981.842
PI42C_HUMAN	1065.9844
WDR20_HUMAN	460.47391
TBC15_HUMAN	8699.5596
RDH11_HUMAN	15971.011
LRC20_HUMAN	1087.0204
IFI44_HUMAN	265.58456
RM30_HUMAN	731.18176
ZFAN1_HUMAN	707.66492
CIP2A_HUMAN	3692.1736
P4K2B_HUMAN	864.47394
STT3B_HUMAN	31027.381
PNPT1_HUMAN	9875.0195
SPP2B_HUMAN	1111.3529
HM13_HUMAN	47443.512
PREX1_HUMAN	4602.9819
URGCP_HUMAN	2054.3718
BICD2_HUMAN	3971.6594
NEK9_HUMAN	5478.8682
PKHO2_HUMAN	3208.5696
DTX3L_HUMAN	1187.6859
DDX54_HUMAN	3676.4294
DLG5_HUMAN	288.24271
BRX1_HUMAN	3960.375
GNPI2_HUMAN	3077.7178

LRC8C_HUMAN	17286.008
NEK7_HUMAN	2941.6873
RBCC1_HUMAN	526.56842
MICA1_HUMAN	4109.7588
ELP5_HUMAN	754.87744
SSH3_HUMAN	1322.7615
S3TC1_HUMAN	487.33527
TBCK_HUMAN	1194.0128
DTD1_HUMAN	5074.5166
DCA11_HUMAN	999.46399
UTP15_HUMAN	1009.232
GPX8_HUMAN	22102.791
AF1L1_HUMAN	754.21533
SH3R3_HUMAN	5148.2305
TP4AP_HUMAN	661.39331
CE192_HUMAN	736.68427
GEMI5_HUMAN	5642.7734
PIGO_HUMAN	170.14282
RPGF6_HUMAN	465.70648
SMCR8_HUMAN	420.23526
PARD3_HUMAN	1738.0272
IPO4_HUMAN	13324.778
UBP33_HUMAN	392.05743
PNISR_HUMAN	1543.7031
PP4R1_HUMAN	6394.7432
UBS3B_HUMAN	1289.4489
GIPC2_HUMAN	545.51245
WIPF2_HUMAN	881.17462
ABHD5_HUMAN	1025.1464
SETD7_HUMAN	4928.6548
NOC3L_HUMAN	4465.2383
SCRB1_HUMAN	19763.943
COG1_HUMAN	2702.1792
SCFD2_HUMAN	2443.2825
SMAP2_HUMAN	1541.8485
ZC3HF_HUMAN	14793.636
PPIL4_HUMAN	4083.1545
TF3C2_HUMAN	1910.1893
TB22A_HUMAN	3453.8943
PHF10_HUMAN	1210.9956
RAB2B_HUMAN	3384.8601
CCD12_HUMAN	952.30286
IASPP_HUMAN	1034.5035
ARB2A_HUMAN	2411.3674
CHUR_HUMAN	2372.4353
TGFA1_HUMAN	1737.0048
TM263_HUMAN	5563.2305
HDAC7_HUMAN	5795.7539
STYX_HUMAN	1522.9502
PTPM1_HUMAN	618.29486

NU133_HUMAN	8850.375
PDC6I_HUMAN	51174.477
FBLI1_HUMAN	15186.617
CATIN_HUMAN	1132.2928
CHAC2_HUMAN	1837.1769
CHMP7_HUMAN	4896.6401
THEM6_HUMAN	30777.305
NAT14_HUMAN	4392.9678
BCL7C_HUMAN	4291.459
MITD1_HUMAN	629.27179
CTF18_HUMAN	619.64374
LEO1_HUMAN	3499.7971
DCAKD_HUMAN	1950.5848
NUDC2_HUMAN	3582.7769
SNR27_HUMAN	1974.9016
TFB1M_HUMAN	7531.8647
STAG1_HUMAN	1227.7535
SCFD1_HUMAN	10736.094
TP8L1_HUMAN	2597.5425
CANT1_HUMAN	2793.6301
TPC12_HUMAN	1504.7278
HNRLL_HUMAN	4545.9429
FACR1_HUMAN	13934.1
UBCP1_HUMAN	6617.0552
SEN15_HUMAN	731.83179
PCNP_HUMAN	6321.4844
DNJA4_HUMAN	1568.3593
SPRY4_HUMAN	5575.1206
MAIP1_HUMAN	3251.7837
TRUB1_HUMAN	1738.7732
LMO7_HUMAN	1526.3127
CTL1_HUMAN	1728.2064
CKAP2_HUMAN	864.95129
ATX2L_HUMAN	13016.228
ARAP3_HUMAN	10656.33
GIMA1_HUMAN	8428.2207
PRP31_HUMAN	10433.215
NELFB_HUMAN	4698.9492
PALLD_HUMAN	13653.726
RUFY2_HUMAN	1690.1639
SREK1_HUMAN	3376.6216
GEMI6_HUMAN	1571.5714
CSKI2_HUMAN	2792.8853
ATRIP_HUMAN	593.40552
PSPC1_HUMAN	14882.412
MADD_HUMAN	546.04846
SYNE2_HUMAN	9823.4775
P66B_HUMAN	2386.8948
DNJC9_HUMAN	16797.186
BRI3B_HUMAN	1534.9873

CTBL1_HUMAN	13939.737
ATG4A_HUMAN	3003.6228
RIN2_HUMAN	972.84399
ELYS_HUMAN	2302.1172
DGCR8_HUMAN	996.04742
ROBO4_HUMAN	2421.3655
OVCA2_HUMAN	1918.6862
IRGQ_HUMAN	2199.9604
DDB2_HUMAN	10087.967
CLC2B_HUMAN	1879.6143
DDX1_HUMAN	25600.059
S14L1_HUMAN	4829.0244
PIEZ1_HUMAN	1296.7146
FAM3C_HUMAN	5178.1235
H1X_HUMAN	13760.785
PSMF1_HUMAN	6620.6157
GBF1_HUMAN	9389.4883
SMG7_HUMAN	377.55386
RTF1_HUMAN	6899.269
NICA_HUMAN	14409.067
SNX19_HUMAN	346.12167
TM9S4_HUMAN	9182.8047
TM131_HUMAN	1012.1624
IP6K1_HUMAN	970.13129
RT27_HUMAN	17605.543
ELMO1_HUMAN	2860.1875
FIG4_HUMAN	720.09967
AP3S1_HUMAN	13073.948
TSC1_HUMAN	4679.4248
UBXN4_HUMAN	5038.3472
PHF3_HUMAN	1303.583
NDRG1_HUMAN	3426.7522
HS105_HUMAN	23458.49
SEPT8_HUMAN	8183.8325
CNOT9_HUMAN	4839.2192
LGAT1_HUMAN	1165.1954
TBCD5_HUMAN	3866.6978
ZN592_HUMAN	891.70776
JADE3_HUMAN	1015.2752
MY18A_HUMAN	1315.0101
LAR4B_HUMAN	3613.3806
GCN1_HUMAN	21289.297
HMHA1_HUMAN	169.48917
PRP16_HUMAN	2866.0366
NU205_HUMAN	72220.133
ANS1A_HUMAN	2136.9739
PXDN_HUMAN	17443.947
FAN_HUMAN	1017.2823
GPI8_HUMAN	2468.6899
RT31_HUMAN	8314.8135

AKAP1_HUMAN	160.46965
ALG3_HUMAN	970.86041
NEUG_HUMAN	420.67227
AN32B_HUMAN	66250.203
NECT2_HUMAN	12280.836
PGTA_HUMAN	10728.218
TFG_HUMAN	12223.421
HTRA1_HUMAN	14959.605
ARC1A_HUMAN	8414.6729
TF2H4_HUMAN	1406.3673
RREB1_HUMAN	432.34976
HDAC2_HUMAN	9730.1084
STAM1_HUMAN	2148.6792
REQU_HUMAN	2037.8114
SC65_HUMAN	8998.7383
CBP_HUMAN	278.82339
KAT6A_HUMAN	179.61342
SYMPK_HUMAN	5221.9355
RBP56_HUMAN	1773.4603
GOGA1_HUMAN	1007.3752
GGH_HUMAN	8840.415
NRCAM_HUMAN	5526.146
RPGR_HUMAN	797.10266
SHIP1_HUMAN	4552.1553
DDX17_HUMAN	85764.063
KIFA3_HUMAN	1593.293
CASPA_HUMAN	2714.6626
APBB2_HUMAN	1380.28
RAD50_HUMAN	12950.486
CELF1_HUMAN	7015.627
OSTF1_HUMAN	6541.0566
ARHG1_HUMAN	12646.908
XPF_HUMAN	650.13477
UFD1_HUMAN	26315.701
GSLG1_HUMAN	18798.602
RENT1_HUMAN	11348.977
CSN5_HUMAN	15375.206
GPKOW_HUMAN	6047.7832
SMRC1_HUMAN	2521.8591
SMRD2_HUMAN	3193.7395
RAB8B_HUMAN	21076.926
BAD_HUMAN	473.69797
FUBP2_HUMAN	16208.888
GCDH_HUMAN	10177.597
RIT1_HUMAN	590.36731
PEX13_HUMAN	566.29541
TNPO1_HUMAN	34071.766
ARHG2_HUMAN	24594.268
NEP1_HUMAN	5405.5293
CLP1_HUMAN	3412.7588

GLMN_HUMAN	6129.3608
UBP13_HUMAN	2203.8606
DVL3_HUMAN	805.91565
USP9X_HUMAN	11087.201
UBP7_HUMAN	11781.772
CUL5_HUMAN	4994.9385
STMN2_HUMAN	64132.637
VPP1_HUMAN	2909.9424
LPP_HUMAN	10235.081
RBPMS_HUMAN	764.49182
EXT2_HUMAN	1100.42
MED12_HUMAN	782.66266
TP4A1_HUMAN	161.57877
KPBB_HUMAN	3213.5654
SCAM4_HUMAN	3381.7415
HPS3_HUMAN	1252.7738
SMCE1_HUMAN	4425.4106
CAVN3_HUMAN	10119.684
MYDGF_HUMAN	35038.625
TM40L_HUMAN	1307.0133
YIPF5_HUMAN	2117.2747
PIGT_HUMAN	5019.1187
IGSF8_HUMAN	1014.5695
RL36L_HUMAN	6125.3765
RAB24_HUMAN	229.80421
OSBP2_HUMAN	1552.4136
ZN622_HUMAN	2790.342
RRF2M_HUMAN	2149.772
UB2E3_HUMAN	13902.393
5NT3B_HUMAN	3264.9229
WBP2_HUMAN	2598.457
PSMG2_HUMAN	15144.977
NCLN_HUMAN	16094.283
MUL1_HUMAN	659.11639
MRTFA_HUMAN	402.11664
LFG3_HUMAN	3464.4907
ERGI1_HUMAN	12328.739
UTP4_HUMAN	722.04095
FAKD4_HUMAN	4441.478
MARC2_HUMAN	3342.3586
F162A_HUMAN	9192.8174
CCD47_HUMAN	8038.4727
RM24_HUMAN	6611.293
EXOC4_HUMAN	10501.396
MGN2_HUMAN	33730.859
P33MX_HUMAN	855.72235
RFT1_HUMAN	2880.989
ISOC2_HUMAN	2011.9316
NTAN1_HUMAN	1792.7972
FERM2_HUMAN	15379.98

FUBP1_HUMAN	49849.609
TTC17_HUMAN	623.23126
LRC59_HUMAN	83365.398
VTI1A_HUMAN	302.93329
ESAM_HUMAN	6583.3647
PBIP1_HUMAN	5801.3506
MCUR1_HUMAN	269.71616
K1143_HUMAN	1275.1287
VP33A_HUMAN	3734.6362
FKB10_HUMAN	15133.254
TTC28_HUMAN	2775.6978
EXOS8_HUMAN	2177.8733
ZN428_HUMAN	453.00864
SH3K1_HUMAN	2456.6187
SKA1_HUMAN	330.92413
RNF25_HUMAN	1623.8577
AIDA_HUMAN	7746.7241
PINX1_HUMAN	752.06689
ARL8A_HUMAN	8674.4258
OTUL_HUMAN	2652.301
CHCH1_HUMAN	631.75311
PPWD1_HUMAN	5301.4614
UPP_HUMAN	251.91304
TAM41_HUMAN	2552.741
MOB3A_HUMAN	2695.5024
DOC10_HUMAN	1669.2062
ATG2B_HUMAN	875.15942
LENG1_HUMAN	1228.024
TBC20_HUMAN	539.49548
EFHD2_HUMAN	30505.469
GALM_HUMAN	2513.0115
SYTL4_HUMAN	3467.4963
P5CR2_HUMAN	7992.4053
CSTOS_HUMAN	2044.9396
DCPS_HUMAN	15525.794
PP14B_HUMAN	2539.6675
INT12_HUMAN	683.45068
NSUN4_HUMAN	1829.519
RHDF1_HUMAN	373.92499
CTHR1_HUMAN	4029.554
ACSF2_HUMAN	1840.641
ISOC1_HUMAN	6960.9253
GCC1_HUMAN	613.88135
FWCH2_HUMAN	3697.8589
ASTRA_HUMAN	2116.5825
HAUS1_HUMAN	271.14227
FAF2_HUMAN	11210.973
CC124_HUMAN	5656.2212
FXRD1_HUMAN	4773.728
OPTN_HUMAN	2850.7983

AP2M1_HUMAN	47124.277
GCP3_HUMAN	3225.9756
S7A6O_HUMAN	980.57898
KCD12_HUMAN	59701.934
RCN3_HUMAN	374.80969
NMD3_HUMAN	6392.73
REPS1_HUMAN	800.21619
TIM14_HUMAN	4012.9797
RMD1_HUMAN	7323.3306
NUD16_HUMAN	7711.4443
ESS2_HUMAN	1519.8037
CMBL_HUMAN	3080.0288
MSI2H_HUMAN	2806.3308
SNR40_HUMAN	7024.9194
RMC1_HUMAN	1667.5417
RM38_HUMAN	7478.6206
RSPRY_HUMAN	544.11938
ERLEC_HUMAN	3164.4741
RRFM_HUMAN	6416.9185
SPDLY_HUMAN	1030.2128
ELP4_HUMAN	3050.1514
SIR1_HUMAN	656.4967
YIPF6_HUMAN	899.15759
SEH1_HUMAN	4137.8066
TCAL4_HUMAN	2490.7202
KBP_HUMAN	13238.015
GNA1_HUMAN	9704.6387
F120B_HUMAN	2281.5652
KTI12_HUMAN	1027.551
RT24_HUMAN	3651.6675
RM53_HUMAN	1613.2548
T3HPD_HUMAN	6938.6147
RNF31_HUMAN	1760.3022
DAZP1_HUMAN	13050.408
SGTB_HUMAN	786.46399
SAAL1_HUMAN	2234.124
MITOK_HUMAN	1183.2609
SGF29_HUMAN	1241.7135
RRP36_HUMAN	668.11218
C1GLC_HUMAN	2500.1272
RBM33_HUMAN	2641.6553
DTBP1_HUMAN	2012.9304
DNJA3_HUMAN	5273.4312
TMA16_HUMAN	5143.1294
MB12A_HUMAN	3565.9392
PTCD3_HUMAN	7450.4424
MMAB_HUMAN	4695.4595
CYFP2_HUMAN	393.59128
GIMA5_HUMAN	1165.699
TRI11_HUMAN	1297.7659

CCD97_HUMAN	810.87488
CNRP1_HUMAN	11374.432
EDC3_HUMAN	4679.3384
DYL2_HUMAN	7460.0317
CPNE2_HUMAN	6475.4912
S10AG_HUMAN	4827.4004
SIPA1_HUMAN	3945.2395
SCRN2_HUMAN	1609.1309
THOC1_HUMAN	5013.7656
OTUB1_HUMAN	27273.824
TRM61_HUMAN	790.84326
HMCES_HUMAN	288.89673
CHMP6_HUMAN	4550.4854
BICD1_HUMAN	661.09973
PGM2_HUMAN	29689.248
IMP4_HUMAN	2516.2546
CERS2_HUMAN	6138.7261
DUS3L_HUMAN	695.91309
LTV1_HUMAN	3473.8435
SDSL_HUMAN	739.43225
RM48_HUMAN	2577.074
PLPP_HUMAN	1917.3016
AURKB_HUMAN	389.43103
DCNL1_HUMAN	3245.9653
FAH2A_HUMAN	4875.4902
SMRD1_HUMAN	1510.1898
TOE1_HUMAN	2523.0894
RUSF1_HUMAN	1856.6388
DDX27_HUMAN	3896.7864
BORC6_HUMAN	981.86072
SYMM_HUMAN	834.34125
A7L3B_HUMAN	3056.0107
GWL_HUMAN	193.19099
MTNB_HUMAN	2030.6791
ZC21A_HUMAN	605.42859
LIN37_HUMAN	567.75085
S41A3_HUMAN	344.21979
SNF8_HUMAN	2436.1052
ZCCHL_HUMAN	2242.4387
P121A_HUMAN	172.26764
TONSL_HUMAN	853.55975
PDLI5_HUMAN	28487.768
CREL1_HUMAN	2006.23
ERO1A_HUMAN	24428.688
PRR11_HUMAN	307.6571
FMC1_HUMAN	2603.0684
SAHH3_HUMAN	11846.771
DOCK6_HUMAN	6740.8423
MED30_HUMAN	978.20593
PGAM5_HUMAN	13808.806

INT4_HUMAN	2635.4468
DDRGK_HUMAN	5197.1763
DHTK1_HUMAN	1185.5481
SCLY_HUMAN	2864.4795
FUBP3_HUMAN	16578.691
SPF45_HUMAN	4356.7446
RCC1L_HUMAN	1165.0925
SYNM_HUMAN	1566.3549
SUCB2_HUMAN	12594.821
LRCH3_HUMAN	4099.2241
GMPPA_HUMAN	7666.7593
HPDL_HUMAN	1407.392
ABHEB_HUMAN	2428.9866
NGLY1_HUMAN	1355.0211
SC22A_HUMAN	693.55304
ATPMK_HUMAN	14094.72
NSL1_HUMAN	780.57654
PAWR_HUMAN	4232.5903
MET2A_HUMAN	342.48959
THOC3_HUMAN	4135.4561
ITCH_HUMAN	3197.3555
TXD15_HUMAN	902.63129
KIRR1_HUMAN	2214.4709
LRIG1_HUMAN	1115.6218
COG3_HUMAN	2327.3596
CK5P3_HUMAN	7902.4209
VPS39_HUMAN	3730.657
VPS50_HUMAN	2420.8672
VCIP1_HUMAN	3878.5186
SPTCS_HUMAN	318.34668
ELMO2_HUMAN	8037.8281
TMX3_HUMAN	12734.17
DCAF5_HUMAN	2106.3186
CHAP1_HUMAN	2232.9485
LMBL3_HUMAN	3867.9697
ZFP91_HUMAN	615.23615
PCD16_HUMAN	2303.5198
CLMN_HUMAN	9257.2061
PDLI2_HUMAN	169.66783
FACR2_HUMAN	4694.8745
BT3L4_HUMAN	8418.1162
RN170_HUMAN	640.05597
ANCHR_HUMAN	1339.3729
S35E1_HUMAN	1574.2148
UBP47_HUMAN	4930.042
ZN521_HUMAN	387.3092
CLP1L_HUMAN	3439.3657
TOPK_HUMAN	3684.2952
ARL5B_HUMAN	818.31903
DNJC1_HUMAN	1352.8245

SCYL1_HUMAN	6489.6113
Z512B_HUMAN	816.65619
LRAT2_HUMAN	529.03314
EXOC2_HUMAN	8169.0327
CNDP2_HUMAN	49067.762
EHMT2_HUMAN	632.51929
ZFR_HUMAN	2706.5952
F210B_HUMAN	758.17688
B3GT6_HUMAN	1360.6764
EP400_HUMAN	1043.2822
SNX27_HUMAN	4504.9629
KI16B_HUMAN	389.1424
IFT74_HUMAN	490.50467
TRI47_HUMAN	4560.0908
CNO6L_HUMAN	3398.1335
DHRS1_HUMAN	449.54395
DJC30_HUMAN	387.53235
UB2E2_HUMAN	98.646919
CAR19_HUMAN	3456.0061
PRRC1_HUMAN	6929.1685
FGD4_HUMAN	3721.4609
FXL18_HUMAN	4471.6143
ZN512_HUMAN	2681.8625
NSE2_HUMAN	1309.4432
NSE3_HUMAN	268.02698
PCMD1_HUMAN	176.9248
HS12B_HUMAN	5681.3354
YTDC1_HUMAN	1930.2656
CCD43_HUMAN	143.59248
COG8_HUMAN	2784.1409
CKLF3_HUMAN	4589.207
DAA10_HUMAN	2700.8784
NOL4L_HUMAN	4774.9243
MBOA7_HUMAN	12115.836
DOCK7_HUMAN	8014.6309
ZN830_HUMAN	323.71988
F210A_HUMAN	1822.306
FRMD6_HUMAN	841.26373
NSUN5_HUMAN	414.67511
RPR1A_HUMAN	3232.074
AGAP3_HUMAN	1733.5563
ARAP1_HUMAN	3310.0762
IPO9_HUMAN	18383.549
MIA2_HUMAN	1529.7571
DCBD2_HUMAN	1057.9785
ARHGH_HUMAN	2171.3484
INP4A_HUMAN	825.46924
RBM14_HUMAN	19802.688
ZN363_HUMAN	478.35577
KLHL5_HUMAN	684.58636

UHRF2_HUMAN	4820.1621
NED4L_HUMAN	3149.4185
QKI_HUMAN	14885.9
LENG8_HUMAN	718.93701
FMNL2_HUMAN	1035.1874
PUS7_HUMAN	6132.7642
F111A_HUMAN	906.68512
TPPC9_HUMAN	832.88263
TRNT1_HUMAN	4191.7363
SMG1_HUMAN	1122.4449
ALS2_HUMAN	1095.4458
TM237_HUMAN	366.98581
ALKB3_HUMAN	322.15277
RHG07_HUMAN	410.0015
PP1RA_HUMAN	4007.0857
S38A2_HUMAN	892.7876
UIF_HUMAN	2069.7397
MTMR9_HUMAN	2285.2312
VPS35_HUMAN	29566.465
PURB_HUMAN	3260.1204
XPO6_HUMAN	2198.4473
HHIP_HUMAN	39316.012
MAGI1_HUMAN	1464.0172
SPAG5_HUMAN	960.19061
NACC1_HUMAN	5239.6104
SNX18_HUMAN	2551.8403
PASK_HUMAN	399.99869
CIC_HUMAN	1027.5594
UIMC1_HUMAN	473.92795
VP13A_HUMAN	2676.8689
MED15_HUMAN	2544.5686
EFGM_HUMAN	10016.511
ERGI2_HUMAN	3554.9639
MCCA_HUMAN	1890.799
KKCC2_HUMAN	943.12555
TGS1_HUMAN	1099.6981
NUDC1_HUMAN	12704.616
ERBIN_HUMAN	1895.4757
GCP6_HUMAN	1940.8314
GCP5_HUMAN	496.53629
UBP28_HUMAN	732.05853
FNBP1_HUMAN	4579.1299
PRPK_HUMAN	5258.8623
PIGS_HUMAN	6272.1636
WRIP1_HUMAN	3815.9636
RANB9_HUMAN	2459.2227
CLCC1_HUMAN	2368.064
MYADM_HUMAN	6816.8008
NEB2_HUMAN	1900.6766
SRPK1_HUMAN	4646.562

SMC6_HUMAN	1165.6517
KCD15_HUMAN	1217.7081
STRBP_HUMAN	19219.066
TM209_HUMAN	3394.3633
GPX7_HUMAN	1261.4108
CP2S1_HUMAN	1272.7211
IWS1_HUMAN	8382.752
SIN3A_HUMAN	1190.6033
OSBL9_HUMAN	5512.6616
CRBN_HUMAN	885.69745
INT14_HUMAN	1859.2058
AEDO_HUMAN	2709.9668
CK5P1_HUMAN	861.5506
RSF1_HUMAN	212.79761
RBM15_HUMAN	3265.1768
PP16B_HUMAN	517.64111
RUFY1_HUMAN	4685.752
MINT_HUMAN	276.58234
PNKP_HUMAN	6227.8662
MMS19_HUMAN	10746.198
UHRF1_HUMAN	3793.6516
NIBA2_HUMAN	31259.432
YMEL1_HUMAN	6650.7642
RMD3_HUMAN	4899.0996
HTF4_HUMAN	809.70544
CYH2_HUMAN	997.57574
TBCB_HUMAN	14945.204
PSB7_HUMAN	22432.184
CNN2_HUMAN	14105.075
SEC62_HUMAN	4830.5239
PCY2_HUMAN	6448.8467
CDC5L_HUMAN	14023.813
PSMD1_HUMAN	29089.438
SDF2_HUMAN	1912.5879
PFD5_HUMAN	1537.0195
RING2_HUMAN	1733.7275
PARK7_HUMAN	13230.521
S1PR3_HUMAN	233.34727
GA2L1_HUMAN	375.65451
EYA3_HUMAN	941.75867
NEUR1_HUMAN	7238.2163
SORT_HUMAN	383.79602
VAT1_HUMAN	118862.56
LGMN_HUMAN	3667.0427
PLIN2_HUMAN	404.87619
DNJC2_HUMAN	4779.5732
MPP8_HUMAN	663.11163
NUP88_HUMAN	9250.5264
PKP4_HUMAN	259.41818
PI3R4_HUMAN	4632.2949

P2RX4_HUMAN	2698.8774
POP1_HUMAN	5166.2124
S10AD_HUMAN	29813.518
SCAFB_HUMAN	1035.6741
TSNAX_HUMAN	6255.9263
SPS2_HUMAN	2141.0403
EIF3C_HUMAN	25565.281
TTC1_HUMAN	937.02588
DNJC7_HUMAN	9924.3193
PHB2_HUMAN	88173.766
CSN8_HUMAN	1426.2834
PRP18_HUMAN	312.45209
CHP1_HUMAN	4842.146
KIF2C_HUMAN	2009.4458
RGPD5_HUMAN	174.07848
MGLL_HUMAN	3486.4221
ATX2_HUMAN	2963.3
DOK1_HUMAN	872.09766
METH_HUMAN	9133.0293
HCD2_HUMAN	34850.551
COCA1_HUMAN	4589.0913
SMAD5_HUMAN	1233.4614
SGMR1_HUMAN	4884.0322
ROAA_HUMAN	11980.948
NP1L4_HUMAN	21125.475
MGST2_HUMAN	55589.969
SNAG_HUMAN	5156.9688
PI51A_HUMAN	750.85773
ABCA3_HUMAN	1439.958
M3K3_HUMAN	1301.1726
ATP5S_HUMAN	1511.012
MIPEP_HUMAN	3387.3713
ACON_HUMAN	21822.633
TM9S2_HUMAN	7506.1548
S29A1_HUMAN	8897.5566
TS101_HUMAN	7553.6079
CPNE1_HUMAN	20141.258
TCPH_HUMAN	45741.895
MYD88_HUMAN	6308.6279
EBP2_HUMAN	3301.9268
ANM1_HUMAN	24481.186
BAG1_HUMAN	1119.459
ATF6B_HUMAN	1556.952
SH3G1_HUMAN	27043.877
TEP1_HUMAN	6793.7373
VRK1_HUMAN	11199.5
GDF15_HUMAN	6373.4033
AKAP9_HUMAN	9775.6016
NIPS1_HUMAN	13343.287
CND3_HUMAN	6639.2188

ARP5L_HUMAN	13776.601
MICU1_HUMAN	753.32013
CG025_HUMAN	886.74323
F118B_HUMAN	2326.4573
SIN1_HUMAN	292.01743
RBM4B_HUMAN	14316.922
DDX50_HUMAN	3756.6816
RM34_HUMAN	2766.9824
RNZ2_HUMAN	6481.6035
TRIR_HUMAN	2198.0928
GRWD1_HUMAN	5610.0811
MACD1_HUMAN	1381.8888
TCF25_HUMAN	3293.8306
CMS1_HUMAN	3555.6467
KLDC3_HUMAN	967.69403
ECSIT_HUMAN	2450.7727
MEP50_HUMAN	17045.605
CYBC1_HUMAN	995.24951
VKOR1_HUMAN	3053.5786
DPH2_HUMAN	1445.6588
TBA1C_HUMAN	13347.722
SELS_HUMAN	2410.7478
APOL2_HUMAN	4699.9204
MBB1A_HUMAN	14141.542
FERM1_HUMAN	293.17258
MGME1_HUMAN	669.45483
GORS1_HUMAN	2757.3506
FYCO1_HUMAN	1388.1641
ACBD6_HUMAN	2226.7249
COR1B_HUMAN	32676.459
TXD17_HUMAN	11386.537
BUD13_HUMAN	918.58099
CPPED_HUMAN	21055.189
VPS25_HUMAN	3038.6821
RM45_HUMAN	11847.609
CG050_HUMAN	8342.6436
TIRR_HUMAN	1941.2686
CAB45_HUMAN	7514.3032
PDD2L_HUMAN	964.47235
PAAF1_HUMAN	4533.7227
PYM1_HUMAN	3452.7041
MIC25_HUMAN	785.56421
ADPGK_HUMAN	5844.7681
GPTC1_HUMAN	502.49878
RIOK1_HUMAN	1278.5189
LLPH_HUMAN	2546.6074
SLD5_HUMAN	1352.4312
UTP23_HUMAN	362.37408
PELO_HUMAN	5600.6709
PXL2A_HUMAN	83293.375

TRI56_HUMAN	1192.7024
ERP44_HUMAN	49683.5
LXN_HUMAN	20195.137
NOL10_HUMAN	1143.1766
NTPCR_HUMAN	6972.0581
TIM29_HUMAN	4610.5923
TACO1_HUMAN	2743.0554
GCP2_HUMAN	4965.2168
ESYT1_HUMAN	29070.836
UBAC1_HUMAN	1145.8911
YIPF4_HUMAN	1468.3634
PHAF1_HUMAN	1129.3129
SEN34_HUMAN	1480.3442
CNPY3_HUMAN	878.76294
MTG1_HUMAN	2201.7878
ALG1_HUMAN	3963.6504
LIMD2_HUMAN	744.37341
HAUS8_HUMAN	830.80078
INP5K_HUMAN	2887.4766
PSMG3_HUMAN	3768.2144
CSN4_HUMAN	12481.474
SYT11_HUMAN	1038.9734
WAC_HUMAN	396.06158
DIDO1_HUMAN	2500.0283
MTA3_HUMAN	5197.0264
RBM42_HUMAN	7492.6699
DCTN5_HUMAN	2344.0728
MCMBP_HUMAN	4481.0264
AASD1_HUMAN	3941.1304
DCNL5_HUMAN	6540.2969
AN32E_HUMAN	6645.2227
LRRC1_HUMAN	1074.4067
P4K2A_HUMAN	2971.2239
TMM43_HUMAN	24966.68
FSD1_HUMAN	1064.2455
TBCD_HUMAN	9874.1553
NDC1_HUMAN	1742.3179
DHRS4_HUMAN	4171.7305
LMF2_HUMAN	4984.876
NDUF3_HUMAN	6831.623
MMTA2_HUMAN	1897.364
TMM70_HUMAN	4857.8696
MED18_HUMAN	1690.9609
ISCA1_HUMAN	895.30914
TBB6_HUMAN	124506.61
PAXX_HUMAN	1259.5723
RPC3_HUMAN	2216.2263
HNRL1_HUMAN	16626.947
MSTO1_HUMAN	6527.4644
PHF23_HUMAN	1226.6498

PDC10_HUMAN	14065.646
RPP25_HUMAN	2338.7397
DERL1_HUMAN	17426.264
HTAI2_HUMAN	6526.1406
DDX23_HUMAN	9768.6289
TCAB1_HUMAN	1008.1089
MIC26_HUMAN	3926.0972
DHRS6_HUMAN	7460.3247
MTNA_HUMAN	4786.8535
WDR18_HUMAN	6526.2002
VAMP8_HUMAN	1044.3904
THUM3_HUMAN	1939.9521
MTND_HUMAN	5069.0132
MECR_HUMAN	2524.3877
EMC6_HUMAN	5761.5352
NTM1A_HUMAN	2940.1023
KTNB1_HUMAN	552.01282
LST8_HUMAN	1494.7054
ASHWN_HUMAN	1555.3414
TM109_HUMAN	47470.34
PBDC1_HUMAN	3370.3892
PTSS2_HUMAN	2642.5662
NOC4L_HUMAN	2413.8706
UT14A_HUMAN	2663.4983
DUS23_HUMAN	4426.458
TMED9_HUMAN	41936.918
TM147_HUMAN	3024.2793
NUP58_HUMAN	4391.5908
SELO_HUMAN	2685.2297
DPCD_HUMAN	1584.8926
GNL3_HUMAN	9916.6055
AFG2B_HUMAN	5281.6768
RIOK2_HUMAN	1305.2881
TR61B_HUMAN	217.11726
TMUB1_HUMAN	1421.069
TIM21_HUMAN	3051.582
KIFC1_HUMAN	614.55768
NUP85_HUMAN	7169.8237
ELOV1_HUMAN	2136.4807
KATL1_HUMAN	1021.5979
HIRP3_HUMAN	930.29431
IFT27_HUMAN	2238.5596
YJU2_HUMAN	1834.2246
NUDT9_HUMAN	5288.3091
SYTM_HUMAN	3378.4299
THIC_HUMAN	24973.945
REPI1_HUMAN	654.08429
RBM4_HUMAN	2635.4473
FUND2_HUMAN	1236.422
RPAP1_HUMAN	2547.5823

SF3B5_HUMAN	462.01596
SFXN3_HUMAN	17568.688
CHID1_HUMAN	2627.895
PAPOG_HUMAN	1056.748
CAR10_HUMAN	489.15604
NADAP_HUMAN	6359.355
CDK19_HUMAN	581.67548
LS14B_HUMAN	1452.2196
TPSNR_HUMAN	1058.8308
JAM3_HUMAN	7838.1948
CARD6_HUMAN	1284.2645
BTBD2_HUMAN	675.82172
SGPP1_HUMAN	233.53722
OSB11_HUMAN	1726.7516
OSB10_HUMAN	3603.2869
RFIP5_HUMAN	2499.374
TB10A_HUMAN	206.25581
C1QT5_HUMAN	4264.6528
NAA15_HUMAN	19442.814
KLF16_HUMAN	46.383812
B2L13_HUMAN	763.43292
CAR11_HUMAN	950.37689
S12A9_HUMAN	1269.7787
SRRT_HUMAN	21121.758
TGT_HUMAN	4865.6567
AP1M1_HUMAN	16733.5
NUSAP_HUMAN	403.91846
HDHD5_HUMAN	4012.4111
FACD2_HUMAN	453.44171
MAK16_HUMAN	2205.8391
HDAC8_HUMAN	465.51169
RTF2_HUMAN	1067.3395
CHM4A_HUMAN	3332.2219
EIF2A_HUMAN	16428.002
PDIP3_HUMAN	3970.0903
K1671_HUMAN	1281.1318
SHAN3_HUMAN	10387.193
GNB1L_HUMAN	1773.3457
FUT8_HUMAN	975.86816
RM32_HUMAN	1260.0768
RM20_HUMAN	4859.0762
RM13_HUMAN	8920.377
RM09_HUMAN	9715.2305
RM04_HUMAN	4829.8091
RM01_HUMAN	8028.939
MK67I_HUMAN	1621.5697
HYCCI_HUMAN	1319.181
YTHD1_HUMAN	1861.2053
HELZ2_HUMAN	784.09479
HOIL1_HUMAN	2794.8972

RT26_HUMAN	5318.7461
NEUL_HUMAN	11416.294
CEP41_HUMAN	3367.9907
SETD2_HUMAN	513.66888
PANK2_HUMAN	562.62811
DOCK9_HUMAN	9259.127
FRMD8_HUMAN	3072.5598
CNTP3_HUMAN	1247.2566
NSD3_HUMAN	1777.3809
NUF2_HUMAN	3239.5544
RM37_HUMAN	10128.038
GTPB4_HUMAN	5638.5376
ASPC1_HUMAN	1920.0541
OSBL8_HUMAN	2649.228
UACA_HUMAN	3159.5693
RAB34_HUMAN	5883.8984
DPH1_HUMAN	1084.9912
WDR11_HUMAN	3878.4805
REN3B_HUMAN	1949.8035
CRNL1_HUMAN	3611.2034
TBL1R_HUMAN	4155.4946
UBL5_HUMAN	3931.8328
PP12C_HUMAN	2612.6987
KPCD2_HUMAN	6073.0039
EDEM3_HUMAN	3349.9309
NIBA1_HUMAN	509.20309
UBXN6_HUMAN	3683.1575
TM6S1_HUMAN	942.4566
UCK2_HUMAN	5782.0156
SN_HUMAN	1882.99
API5_HUMAN	25463.961
NLRP1_HUMAN	788.85681
TRIM5_HUMAN	1935.0698
TRIM4_HUMAN	54008.801
FTO_HUMAN	6480.4941
ZDHC5_HUMAN	379.7832
TNG6_HUMAN	1408.0376
TB182_HUMAN	5408.3481
UBE2O_HUMAN	6763.8149
ZY11B_HUMAN	1636.3531
TANC1_HUMAN	5856.0024
EPT1_HUMAN	1518.9418
XPO4_HUMAN	8510.0684
LNP_HUMAN	1360.1979
CEP44_HUMAN	269.3046
TTYH3_HUMAN	8747.5996
KLHL4_HUMAN	2003.4302
MTMRC_HUMAN	846.18585
WDR33_HUMAN	5307.9233
WDR12_HUMAN	3748.2722

YIPF3_HUMAN	4956.8486
TINAL_HUMAN	2944.3608
ADSSP_HUMAN	8375.9512
PITH1_HUMAN	4699.5698
DERL2_HUMAN	7425.0684
COMD5_HUMAN	2361.3782
SENP6_HUMAN	208.62122
REXO4_HUMAN	1804.8669
DDX24_HUMAN	4562.1396
RPA49_HUMAN	2617.196
SKI8_HUMAN	7725.1953
SLIRP_HUMAN	8146.9131
SRR_HUMAN	1377.7262
NIF3L_HUMAN	3752.3374
EGLN1_HUMAN	5907.4492
MCLN1_HUMAN	1583.4774
PIP30_HUMAN	3316.0693
MFF_HUMAN	1118.135
NAA50_HUMAN	9695.665
UBA5_HUMAN	6617.1079
MKRN2_HUMAN	2205.8613
T126A_HUMAN	4000.7375
PAIP1_HUMAN	11687.918
CLPB_HUMAN	4423.4199
RB33B_HUMAN	3154.1768
LSG1_HUMAN	5734.229
F107B_HUMAN	6517.1001
NAT10_HUMAN	11582.977
COMD4_HUMAN	2775.1287
KLC2_HUMAN	2490.9189
ILKAP_HUMAN	6010.5376
XRN2_HUMAN	16063.151
TOLIP_HUMAN	4312.585
SP130_HUMAN	757.1405
BRD8_HUMAN	648.31531
NSRP1_HUMAN	261.86255
INT2_HUMAN	1103.0486
RGAP1_HUMAN	529.00476
PAR12_HUMAN	2730.7769
CSTFT_HUMAN	1229.3593
5NT3A_HUMAN	2956.2974
CYRIA_HUMAN	18463.637
TM222_HUMAN	696.7309
HDHD2_HUMAN	2627.6965
GATA_HUMAN	2024.5588
DDX47_HUMAN	5443.0303
MAGT1_HUMAN	6603.3911
RAB1B_HUMAN	53661.934
RM18_HUMAN	6304.2227
TSYL1_HUMAN	921.64185

TM168_HUMAN	1011.2641
LMA2L_HUMAN	7520.5347
SMG9_HUMAN	1816.4879
F234A_HUMAN	357.95175
OSBL5_HUMAN	694.73956
TDIF1_HUMAN	863.18573
SIL1_HUMAN	1250.626
METL9_HUMAN	476.00995
APC1_HUMAN	5266.7007
CYTM1_HUMAN	1351.8383
RPC6_HUMAN	2645.1272
NUCKS_HUMAN	2967.4402
TMX4_HUMAN	5871.4272
KI13A_HUMAN	22674.336
ASCC2_HUMAN	6505.7988
RBNS5_HUMAN	397.23782
ISCU_HUMAN	3060.7224
OSBL2_HUMAN	1738.1531
ATG5_HUMAN	4173.2935
WDR13_HUMAN	2487.4546
EPN3_HUMAN	1942.1073
EHD4_HUMAN	91928.953
VP33B_HUMAN	4122.4004
VPS16_HUMAN	7877.8232
VPS11_HUMAN	5012.8267
SH3L3_HUMAN	11711.912
GAN_HUMAN	535.9375
S2532_HUMAN	1384.8409
TARA_HUMAN	6875.4106
SLK_HUMAN	10276.206
PPIL3_HUMAN	423.47867
S38A1_HUMAN	570.03125
PDCL3_HUMAN	5226.9966
TAOK3_HUMAN	5353.2183
RBGPR_HUMAN	8918.6016
ADNP_HUMAN	2186.5798
DPH5_HUMAN	1941.6976
DHX36_HUMAN	6064.1958
IPYR2_HUMAN	7987.1641
SPNS1_HUMAN	4226.25
RM46_HUMAN	2719.6875
S12A5_HUMAN	669.84558
PARL_HUMAN	1163.074
PININ_HUMAN	6780.9287
TM245_HUMAN	1371.9573
FOXP1_HUMAN	1765.5255
BACD3_HUMAN	3613.7112
MOD5_HUMAN	428.88528
CK068_HUMAN	6372.104
GHITM_HUMAN	5761.5063

BOLA2_HUMAN	6412.3413
MMAD_HUMAN	678.93292
TXNIP_HUMAN	630.29169
TMX1_HUMAN	18013.354
NELFA_HUMAN	2783.8486
GCP60_HUMAN	8485.1045
BORG4_HUMAN	840.90765
PTN23_HUMAN	8773.542
SEM6B_HUMAN	6449.3418
UN45A_HUMAN	17026.717
MFSD1_HUMAN	1939.092
DNJC5_HUMAN	559.76105
DSN1_HUMAN	663.33759
CHM4B_HUMAN	20757.443
RWDD1_HUMAN	3368.165
CUED2_HUMAN	449.43555
FXL15_HUMAN	412.52792
FN3K_HUMAN	2698.218
OFUT1_HUMAN	7663.688
PIGU_HUMAN	4859.4946
TOR3A_HUMAN	4342.5371
WNK1_HUMAN	3664.2231
AMPB_HUMAN	22296.496
GLP3L_HUMAN	5110.5542
GOLP3_HUMAN	4044.9595
OSGL1_HUMAN	2479.2163
SIA7D_HUMAN	548.52484
GAPR1_HUMAN	4055.6882
TRABD_HUMAN	2324.0708
MTG2_HUMAN	58.685604
SENP3_HUMAN	3871.79
OSBL3_HUMAN	4341.9399
SMRCD_HUMAN	2659.1328
EHD1_HUMAN	21177.889
CAPAM_HUMAN	783.65723
ESF1_HUMAN	375.90051
ALG2_HUMAN	3530.7058
HEAT1_HUMAN	4941.3022
SG196_HUMAN	847.11835
RABE2_HUMAN	1003.1473
TFB2M_HUMAN	3350.0984
ZCHC4_HUMAN	656.42377
STEEP_HUMAN	288.80505
CIA2A_HUMAN	1677.2472
DHX35_HUMAN	1039.2919
F124B_HUMAN	3907.4775
HAUS4_HUMAN	2686.1006
CC134_HUMAN	4378.9082
CCD86_HUMAN	2541.3384
REEP4_HUMAN	1914.5039

SOX17_HUMAN	734.62378
OPA3_HUMAN	2175.8489
CIAO3_HUMAN	1847.865
DHX33_HUMAN	1063.9586
NOL6_HUMAN	2609.3774
WDCP_HUMAN	663.21912
YTDC2_HUMAN	3044.3406
AZI2_HUMAN	129.33583
RPAP3_HUMAN	2915.5581
BCAS3_HUMAN	610.63391
ALG9_HUMAN	2998.2349
LDAH_HUMAN	1891.2324
RIOX1_HUMAN	3157.5217
WDR55_HUMAN	3269.6089
RANB3_HUMAN	5746.832
PEAK1_HUMAN	1809.0737
RPF2_HUMAN	1542.2275
SMYD3_HUMAN	3785.2949
AAMDC_HUMAN	1823.8267
DOCK5_HUMAN	1200.7128
WDR26_HUMAN	3540.968
TDRD3_HUMAN	2579.4497
CH033_HUMAN	1592.5879
MET17_HUMAN	490.79474
SFR19_HUMAN	1557.5806
NRDE2_HUMAN	1117.0138
PGES2_HUMAN	6520.4155
PHAX_HUMAN	2831.1387
UBE2Z_HUMAN	6259.5396
ACAD9_HUMAN	6084.6475
NT5D2_HUMAN	10776.603
PKRI1_HUMAN	2667.0115
CAAP1_HUMAN	1181.6451
NOL11_HUMAN	740.01019
DDX31_HUMAN	394.36179
TMT1A_HUMAN	3416.7786
PAAT_HUMAN	2108.6399
MMRN2_HUMAN	13819.266
MINY3_HUMAN	1930.474
EVA1A_HUMAN	1734.13
AKTIP_HUMAN	406.99002
PKHF2_HUMAN	1736.0089
ANKZ1_HUMAN	2117.8801
GORS2_HUMAN	3297.4087
ZWILC_HUMAN	1836.1272
JUPI2_HUMAN	1682.8474
GHC1_HUMAN	6241.0757
PPIP2_HUMAN	2571.5149
MED20_HUMAN	2062.2173
WDR76_HUMAN	195.12444

QTRT2_HUMAN	1285.3416
ARP8_HUMAN	1069.142
ARMT1_HUMAN	4347.853
PANK3_HUMAN	249.73199
CNO10_HUMAN	1331.3256
LRC40_HUMAN	18371.172
EHMT1_HUMAN	2042.7014
SFXN1_HUMAN	26069.682
SPE39_HUMAN	1661.9373
COG4_HUMAN	7344.1753
ARP5_HUMAN	705.41229
VP37B_HUMAN	660.10083
RM44_HUMAN	8384.4053
L2HDH_HUMAN	1541.6183
CSN7B_HUMAN	3791.2346
NHEJ1_HUMAN	682.01544
S61A2_HUMAN	12883.562
FKRP_HUMAN	937.49744
ELP3_HUMAN	6312.0503
RPF1_HUMAN	576.99176
RPA2_HUMAN	1379.4283
UCK1_HUMAN	537.1153
KT3K_HUMAN	1777.9194
TBC17_HUMAN	1500.0823
SYCM_HUMAN	4305.9238
S52A2_HUMAN	1847.3619
PPCS_HUMAN	11516.811
UBTD1_HUMAN	2185.2473
WDR41_HUMAN	289.23346
FBRS_HUMAN	432.16885
NMNA1_HUMAN	1759.8423
NJMU_HUMAN	1771.5856
SIAE_HUMAN	1257.9863
PKHA5_HUMAN	741.76068
SMUF2_HUMAN	1733.7831
RENT2_HUMAN	4282.7622
GBB4_HUMAN	1514.1525
XPO5_HUMAN	7582.022
GRPE1_HUMAN	13919.303
MYG1_HUMAN	11386.558
PKHA3_HUMAN	413.27975
PKHA1_HUMAN	1696.8162
RISC_HUMAN	3961.282
NET4_HUMAN	7922.0596
CYBP_HUMAN	31538.9
RRAGC_HUMAN	9721.9043
ZFYV1_HUMAN	1574.9535
IF122_HUMAN	645.02136
RDH14_HUMAN	4222.0601
PARVB_HUMAN	11777.475

TENS1_HUMAN	3543.9548
PLRKT_HUMAN	2824.8784
NMRL1_HUMAN	2406.3218
VEZA_HUMAN	918.07629
SPC25_HUMAN	2097.4602
TAF9B_HUMAN	574.70197
S38AA_HUMAN	2550.7305
EKI1_HUMAN	1645.3528
AGRL4_HUMAN	8423.8105
TM165_HUMAN	5425.6943
TPC_HUMAN	1030.1501
EMAL4_HUMAN	5647.8569
MRM3_HUMAN	2948.4412
GLOD4_HUMAN	45512.816
NEK6_HUMAN	811.04181
MCCB_HUMAN	3713.8679
NCOA5_HUMAN	32617.717
EPG5_HUMAN	676.15424
MOV10_HUMAN	8282.1523
MET14_HUMAN	2151.2476
GBA2_HUMAN	3082.4331
CWC22_HUMAN	1896.4626
RAVR2_HUMAN	1201.5865
CHD8_HUMAN	853.04926
E41L5_HUMAN	1317.2777
GPN1_HUMAN	4391.5293
SDF2L_HUMAN	4584.7529
KC1G1_HUMAN	3561.49
SYF1_HUMAN	5026.4502
PREB_HUMAN	5997.2236
SRA1_HUMAN	1510.7087
AT131_HUMAN	5113.6211
GOPC_HUMAN	2118.6201
RM47_HUMAN	5689.3667
LYRM4_HUMAN	3092.1917
SPCS_HUMAN	1982.5636
CHM1A_HUMAN	8517.4248
TM9S3_HUMAN	11773.82
MYO10_HUMAN	5673.7583
APMAP_HUMAN	48895.754
WDR6_HUMAN	4977.3652
TRXR2_HUMAN	12642.993
ABCB6_HUMAN	379.05597
ARFG3_HUMAN	4664.2427
ZCC17_HUMAN	843.6203
RAB18_HUMAN	15057.677
PALMD_HUMAN	11128.069
SSU72_HUMAN	2766.2686
VTA1_HUMAN	18504.121
SYSM_HUMAN	4030.7065

RT30_HUMAN	5546.0439
DLRB1_HUMAN	4169.0688
EMC7_HUMAN	8160.8579
ENY2_HUMAN	2996.1531
EXOS4_HUMAN	3663.3542
UBE2T_HUMAN	3242.209
NOP10_HUMAN	2807.5002
OSGEP_HUMAN	5741.7202
DMAP1_HUMAN	956.73578
PCD12_HUMAN	510.87289
INO1_HUMAN	7077.5049
BRD7_HUMAN	404.133
DCP1A_HUMAN	3741.8301
ACO13_HUMAN	9426.0244
MED4_HUMAN	789.09308
TIDC1_HUMAN	258.0484
RIC8A_HUMAN	21713.748
C1QR1_HUMAN	33207.008
LUC7L_HUMAN	8473.5332
ESM1_HUMAN	547.0744
LZTL1_HUMAN	1749.5792
RM40_HUMAN	5098.6567
SSF1_HUMAN	1362.1677
PLCB1_HUMAN	1662.5999
TIGAR_HUMAN	15250.615
CL004_HUMAN	834.38184
RTN4_HUMAN	16643.365
RPR1B_HUMAN	7053.1279
XPP3_HUMAN	2289.4651
NIT2_HUMAN	6974.6929
AVEN_HUMAN	536.68341
INCE_HUMAN	233.80176
EXOS5_HUMAN	2003.6445
EXOS3_HUMAN	4542.8013
KI13B_HUMAN	5152.4243
ANLN_HUMAN	2153.0212
XPP1_HUMAN	13625.355
GEPH_HUMAN	4512.7183
BIN3_HUMAN	2639.4343
SAS10_HUMAN	1282.1725
STAR7_HUMAN	1185.4625
BIRC6_HUMAN	4324.5605
PDLI7_HUMAN	16696.691
ACSA_HUMAN	10134.469
DBLOH_HUMAN	2800.1753
DDX21_HUMAN	10321.604
SAR1A_HUMAN	27339.492
SIAS_HUMAN	37151.359
SHLB2_HUMAN	3899.5122
ASH1L_HUMAN	11458.94

EI2BG_HUMAN	10219.073
MBNL1_HUMAN	9739.8184
S17A5_HUMAN	3999.7368
4ET_HUMAN	1192.2107
CHSTC_HUMAN	952.40436
ST7_HUMAN	975.80511
PICK1_HUMAN	980.91779
SH2B1_HUMAN	1489.7609
PYRG2_HUMAN	6232.4653
DPOE3_HUMAN	2427.8696
CHRC1_HUMAN	2361.2566
AAAS_HUMAN	6603.8984
SNRK_HUMAN	3368.063
ABCBA_HUMAN	3146.3328
BAZ1A_HUMAN	1106.4507
STRN4_HUMAN	4261.2236
ADPPT_HUMAN	10502.582
METL5_HUMAN	2722.7847
OSTC_HUMAN	18668.959
COXM2_HUMAN	333.84851
PLS4_HUMAN	2121.1296
RNC_HUMAN	613.11096
SNX15_HUMAN	554.26544
HEBP1_HUMAN	1936.2344
ABC3C_HUMAN	1722.9741
VPS45_HUMAN	2709.5442
PNO1_HUMAN	2085.9307
RM17_HUMAN	9806.6133
PHP14_HUMAN	331.69553
SERC1_HUMAN	719.75
RHG35_HUMAN	3249.6077
F1142_HUMAN	2351.6919
PLS3_HUMAN	3215.3633
PLCD_HUMAN	1235.4349
PLCC_HUMAN	3267.356
HELLS_HUMAN	1677.9607
C1GLT_HUMAN	1823.4237
LTBP3_HUMAN	468.88553
TOM22_HUMAN	4117.7671
LANC2_HUMAN	4255.6636
KIF15_HUMAN	1176.5325
TM7S3_HUMAN	1266.1025
HOME3_HUMAN	5685.2583
SYFB_HUMAN	25425.232
SYIM_HUMAN	13949.549
SLX9_HUMAN	2866.6563
SAMN1_HUMAN	572.3938
KLC4_HUMAN	2038.0118
DIAP3_HUMAN	2003.5248
BMP2K_HUMAN	789.19385

ATG3_HUMAN	4017.0803
SIR3_HUMAN	359.15234
PDS5B_HUMAN	5399.6074
SMC4_HUMAN	32650.389
MA2C1_HUMAN	7414.772
SAC1_HUMAN	9184.1084
DEPP1_HUMAN	1039.8571
OLA1_HUMAN	32549.762
CUTC_HUMAN	1169.0458
ZF64B_HUMAN	891.94263
ECHD1_HUMAN	10138.051
RBM12_HUMAN	90288.75
TB22B_HUMAN	1863.062
MDN1_HUMAN	7684.438
ACS2L_HUMAN	1354.0142
ZCHC3_HUMAN	1029.3341
DECR2_HUMAN	5340.1362
ABHDA_HUMAN	2853.1245
T11L1_HUMAN	2013.7708
STAU2_HUMAN	5135.1265
SHFL_HUMAN	2659.0442
DDX28_HUMAN	1004.7108
T106B_HUMAN	2113.7368
LIN7C_HUMAN	1026.603
PLCE_HUMAN	4373.3457
TXLNG_HUMAN	1619.1016
SPS2L_HUMAN	2012.8392
UFSP2_HUMAN	2397.8167
ABCF3_HUMAN	7544.4082
CYRIB_HUMAN	11685.087
MITOS_HUMAN	1832.923
DD19A_HUMAN	19582.848
GIMA4_HUMAN	34061.461
TYDP1_HUMAN	2511.2537
POTE1_HUMAN	626.07831
TBC23_HUMAN	4364.5732
DCA13_HUMAN	1123.4641
IMP3_HUMAN	3370.6001
MRGBP_HUMAN	1601.0374
TYW1_HUMAN	683.04468
EXOC1_HUMAN	10074.656
PCMD2_HUMAN	1106.2482
INT9_HUMAN	2948.5728
CC50A_HUMAN	13592.941
UQCC1_HUMAN	2551.0635
SEP11_HUMAN	23921.873
S38A7_HUMAN	15140.211
MED17_HUMAN	15637.397
PARVA_HUMAN	8954.9385
UBP40_HUMAN	577.40662

PANK4_HUMAN	10100.718
FBX28_HUMAN	468.80115
TBC13_HUMAN	6267.2734
EXD2_HUMAN	2261.9036
DJC11_HUMAN	5684.2764
INT7_HUMAN	13486.055
FANCI_HUMAN	918.07373
ATD3A_HUMAN	36751.477
ARL8B_HUMAN	10728.658
DJC17_HUMAN	5323.0244
INT13_HUMAN	3380.9963
RIC8B_HUMAN	1061.0591
GNL3L_HUMAN	1394.561
DDX18_HUMAN	4275.8145
ASF1B_HUMAN	2005.6757
KLH11_HUMAN	1167.2416
INT10_HUMAN	3373.5569
RT18A_HUMAN	4725.9985
PNPO_HUMAN	1918.8134
ARMC1_HUMAN	4391.9312
RPC5_HUMAN	801.61664
SDA1_HUMAN	3832.2554
PAPD1_HUMAN	4849.4102
NLE1_HUMAN	2376.6018
NECP2_HUMAN	12653.756
RPC2_HUMAN	4328.4976
RBM28_HUMAN	2246.9514
ANO10_HUMAN	8345.9756
RBM22_HUMAN	2626.1389
DMAC2_HUMAN	192.39291
WDR70_HUMAN	1388.6987
TMM51_HUMAN	232.31377
ARGL1_HUMAN	3738.7507
IFT57_HUMAN	466.23932
SLTM_HUMAN	2439.7683
FKB14_HUMAN	1382.1583
RMND1_HUMAN	2702.0276
PK1IP_HUMAN	1483.1252
HIF1N_HUMAN	1487.9731
OXSM_HUMAN	7838.9932
RM22_HUMAN	15088.37
CZIB_HUMAN	15109.112
BABA1_HUMAN	3570.2869
CLN6_HUMAN	610.61737
ASB6_HUMAN	2951.0088
THG1_HUMAN	1187.2682
HPF1_HUMAN	2724.8269
IRAK4_HUMAN	2680.2004
UCKL1_HUMAN	3110.7275
TM160_HUMAN	3604.4468

NALP2_HUMAN	3689.5271
F120C_HUMAN	892.32892
TSAP1_HUMAN	775.89398
NDUBB_HUMAN	2038.7468
RM16_HUMAN	9533.3271
NHP2_HUMAN	4804.8735
OCAD1_HUMAN	6159.0547
ADPRS_HUMAN	11871.836
MARH5_HUMAN	4656.8281
LYAR_HUMAN	5455.1479
T161A_HUMAN	617.88464
IMPA3_HUMAN	4485.8369
MIC19_HUMAN	11128.355
DUS2L_HUMAN	189.41579
CKLF6_HUMAN	923.29535
SIR5_HUMAN	1536.5931
MIOS_HUMAN	1207.017
MTMRA_HUMAN	5159.3423
NSMA3_HUMAN	4121.5737
CWC25_HUMAN	1438.6232
TEX10_HUMAN	4459.8984
DCA16_HUMAN	2081.5896
THUM1_HUMAN	3878.1267
P4HTM_HUMAN	335.62805
TOR4A_HUMAN	3561.1858
TRM1_HUMAN	3826.6338
GDAP2_HUMAN	1188.0822
NDE1_HUMAN	1264.6083
BABA2_HUMAN	1679.4097
QPCTL_HUMAN	2278.2327
ARL15_HUMAN	2647.4724
KCTD5_HUMAN	1140.3413
CARF_HUMAN	3238.2009
DJB12_HUMAN	2321.271
ALKB4_HUMAN	1171.4747
NSE4A_HUMAN	897.07825
GAR1_HUMAN	3235.1003
STAB1_HUMAN	9981.7588
PP4R2_HUMAN	1499.4253
DPP3_HUMAN	23012.428
AATF_HUMAN	746.53204
TBA8_HUMAN	4175.1436
DDX56_HUMAN	1782.0247
SPHK1_HUMAN	2263.5498
TE2IP_HUMAN	298.62323
ABI2_HUMAN	2978.9592
BCLF1_HUMAN	12311.102
UTP6_HUMAN	850.76074
TAB2_HUMAN	98.608963
RM39_HUMAN	7808.5942

M3K20_HUMAN	2447.1331
FKB11_HUMAN	11144.588
TMOD3_HUMAN	12013.749
BET1L_HUMAN	2110.0496
ELOV5_HUMAN	2720.2529
KBRS2_HUMAN	1243.5198
UGGG2_HUMAN	512.33221
UGGG1_HUMAN	28425.414
CDK12_HUMAN	2002.9673
FAKD2_HUMAN	4888.2573
TECR_HUMAN	6581.2388
ERAP1_HUMAN	14204.869
ARP10_HUMAN	4670.8975
USE1_HUMAN	1948.8468
CISD1_HUMAN	2075.5933
GGA3_HUMAN	2260.2852
TLS1_HUMAN	930.00171
F120A_HUMAN	8273.5039
SMAL1_HUMAN	1428.8665
GLTP_HUMAN	2295.4282
SPG21_HUMAN	4093.6599
RM35_HUMAN	5323.8647
UBIP1_HUMAN	882.9707
IF2B1_HUMAN	17617.514
SACS_HUMAN	2168.199
MTCH1_HUMAN	8004.6226
NUDT4_HUMAN	9060.6162
HPBP1_HUMAN	8394.334
MAT2B_HUMAN	29504.008
MYOF_HUMAN	45171.301
ITSN2_HUMAN	3075.9182
NOP53_HUMAN	992.22034
EHD3_HUMAN	50409.07
EHD2_HUMAN	90150.047
ARHGC_HUMAN	2579.2004
CNOT2_HUMAN	4334.7407
SPN90_HUMAN	1922.1714
PD1L1_HUMAN	1631.7546
CALL5_HUMAN	1053.0413
OGFR_HUMAN	6598.9063
LMCD1_HUMAN	1072.5317
CRIM1_HUMAN	2424.4888
SELN_HUMAN	4403.0913
PALS2_HUMAN	3528.2219
CHMP5_HUMAN	6996.521
CWC15_HUMAN	1762.6475
RM15_HUMAN	10214.648
THYN1_HUMAN	9970.2598
TAP26_HUMAN	234.73006
NDUF4_HUMAN	3573.9856

HACD3_HUMAN	11838.017
EMC3_HUMAN	3315.1985
NDUAD_HUMAN	8757.6465
PDP1_HUMAN	5552.4229
KCMF1_HUMAN	1222.3492
RAI14_HUMAN	9359.7871
VAPA_HUMAN	11526.219
RM27_HUMAN	4387.0947
TMEM9_HUMAN	1087.0072
SENP1_HUMAN	877.08643
CXXC1_HUMAN	274.8949
SH3B4_HUMAN	6902.144
SEP10_HUMAN	11858.305
GMIP_HUMAN	981.06531
PHRF1_HUMAN	3048.3176
POGK_HUMAN	1224.4888
DAPLE_HUMAN	224.57025
RHG23_HUMAN	1447.0646
VPS18_HUMAN	10287.537
RCC2_HUMAN	41442.262
RELCH_HUMAN	1247.3661
DIP2B_HUMAN	4839.9526
JCAD_HUMAN	2585.9021
SLAI2_HUMAN	527.44958
TEN3_HUMAN	494.29868
UBP36_HUMAN	860.30853
BCCIP_HUMAN	14192.966
STK26_HUMAN	1142.4725
ARMX1_HUMAN	3336.665
ABI3_HUMAN	6511.0894
FPRP_HUMAN	14906.274
CT2NL_HUMAN	6090.6587
TM181_HUMAN	2702.7588
IBTK_HUMAN	384.46594
HTR5B_HUMAN	1588.1658
ZNFX1_HUMAN	1205.2043
CHPF2_HUMAN	2058.6538
PCD10_HUMAN	529.17194
RRBP1_HUMAN	38441.539
CPSF2_HUMAN	4477.6382
KLHL9_HUMAN	2535.1848
SYLC_HUMAN	27544.488
MYEF2_HUMAN	11389.111
KLH42_HUMAN	349.34164
E2AK4_HUMAN	4477.4316
RBM27_HUMAN	2438.5422
HECW2_HUMAN	2938.5007
FRM4A_HUMAN	1060.9467
ANFY1_HUMAN	5520.0752
RERE_HUMAN	1011.6447

SUCB1_HUMAN	8907.5996
GMPR2_HUMAN	6936.3232
STX18_HUMAN	788.94928
DPM3_HUMAN	981.11761
IMPCT_HUMAN	1475.8488
UVRAG_HUMAN	2730.9551
ATX10_HUMAN	11875.428
MBD2_HUMAN	3230.9043
NCDN_HUMAN	2738.7461
TFP11_HUMAN	1205.4825
EP15R_HUMAN	9978.3311
MYO1A_HUMAN	8338.1475
ORC3_HUMAN	1705.7949
SAE1_HUMAN	12285.431
COPG2_HUMAN	10546.136
PI4KB_HUMAN	685.32458
MRC2_HUMAN	1440.7694
COMD3_HUMAN	1745.5056
GBG12_HUMAN	1758.1259
RBL2A_HUMAN	1199.8956
MTRR_HUMAN	711.11194
UXT_HUMAN	1424.4958
ASH2L_HUMAN	2602.0486
DHCR7_HUMAN	22938.076
TR10D_HUMAN	721.46375
HDAC6_HUMAN	2215.3496
SPAST_HUMAN	4137.752
DKK3_HUMAN	761.84888
TRMB_HUMAN	8088.1494
GULP1_HUMAN	1860.3719
VPS29_HUMAN	17541.895
EIF3K_HUMAN	20040.564
EXTL2_HUMAN	1078.6653
GRHPR_HUMAN	16318.222
CATZ_HUMAN	23354.096
KS6B2_HUMAN	1800.7975
DJB11_HUMAN	14425.033
RNF14_HUMAN	2349.479
SAE2_HUMAN	18509.588
MO4L1_HUMAN	1530.0964
NXF1_HUMAN	7573.0303
SE1L1_HUMAN	1316.4578
B4GT7_HUMAN	2464.0049
PEF1_HUMAN	3726.2344
ZMYM2_HUMAN	898.24396
CSN7A_HUMAN	8253.4697
DIC_HUMAN	6339.0518
AASS_HUMAN	8603.1494
CLIP2_HUMAN	1279.6165
QCR9_HUMAN	15736.953

MTFP1_HUMAN	2375.2737
ZO2_HUMAN	19956.564
DNJB4_HUMAN	21579.357
ST17A_HUMAN	1042.4371
CFDP1_HUMAN	4192.8369
DAXX_HUMAN	1018.6751
TRM7_HUMAN	2206.4236
VTI1B_HUMAN	3859.4746
STK39_HUMAN	3088.4443
ADDG_HUMAN	3508.6394
LRWD1_HUMAN	2846.7808
NPS3A_HUMAN	8875.4434
CGBP1_HUMAN	3652.283
PISD_HUMAN	628.69006
ABCF2_HUMAN	12647.124
TES_HUMAN	6133.3135
AAKG2_HUMAN	4530.6333
GCP4_HUMAN	3232.2993
KDM5B_HUMAN	155.68329
SYWM_HUMAN	1304.0071
LIMD1_HUMAN	745.4751
SEC63_HUMAN	11505.438
GTR6_HUMAN	794.75507
Z3H7B_HUMAN	6087.7734
NDRG3_HUMAN	7929.0093
ARMX3_HUMAN	4946.5566
SWP70_HUMAN	21494.045
SUN2_HUMAN	9295.9883
RLP24_HUMAN	378.67123
LTOR3_HUMAN	5221.9385
LIMA1_HUMAN	11979.676
AFF4_HUMAN	6353.1885
SRP68_HUMAN	11909.161
CHRD1_HUMAN	12996.039
TBK1_HUMAN	4422.2529
SEPT9_HUMAN	15923.099
PCYOX_HUMAN	11618.165
DDX20_HUMAN	5254.8804
SHPK_HUMAN	5440.4849
DPP2_HUMAN	3753.5515
CEIP2_HUMAN	4537.6987
UBP25_HUMAN	3377.4316
BAP29_HUMAN	4439.7744
NB5R1_HUMAN	6426.4888
S30BP_HUMAN	2888.2588
ZNHI2_HUMAN	10743.327
PFD2_HUMAN	11465.101
GPN3_HUMAN	365.59152
PUF60_HUMAN	21223.457
NRBP_HUMAN	5487.8491

ENOPH_HUMAN	8193.4678
FEZ2_HUMAN	1374.4741
NDUAC_HUMAN	7985.7871
EI2BD_HUMAN	12660.078
VATH_HUMAN	5866.52
PRAF1_HUMAN	3253.6462
IPO11_HUMAN	2102.5417
TR112_HUMAN	408.33527
DACH1_HUMAN	1168.8225
XPO7_HUMAN	6886.3921
LCMT1_HUMAN	4532.3872
VPS51_HUMAN	3654.5764
BAZ2A_HUMAN	289.1561
BAZ1B_HUMAN	2178.2983
ATIF1_HUMAN	1031.0082
KAD3_HUMAN	21411.438
LCAP_HUMAN	8691.3945
MBD1_HUMAN	340.81778
CNOT7_HUMAN	2277.6084
RABX5_HUMAN	3046.0928
MSRA_HUMAN	2641.1958
NAGK_HUMAN	30379.354
HACL1_HUMAN	4127.2734
TRM6_HUMAN	1436.8628
SH3L2_HUMAN	1194.1794
NGAP_HUMAN	771.77283
TSR3_HUMAN	971.57037
S2513_HUMAN	17829.209
DBNL_HUMAN	10770.542
DDX41_HUMAN	5789.1353
DCTN4_HUMAN	8239.8076
CDC23_HUMAN	3793.0081
APC7_HUMAN	4877.6938
APC5_HUMAN	912.047
APC4_HUMAN	1945.6643
ANC2_HUMAN	2400.2773
GGA2_HUMAN	4275.7188
GGA1_HUMAN	2358.5007
STML2_HUMAN	14910.021
NAGPA_HUMAN	727.71228
VPS28_HUMAN	6774.707
LSM7_HUMAN	1479.3269
DBR1_HUMAN	3517.6331
TASOR_HUMAN	479.22729
JUPI1_HUMAN	348.02408
FBX3_HUMAN	640.22827
AKA11_HUMAN	487.11227
FBW1B_HUMAN	525.70959
GMEB2_HUMAN	704.5462
MRT4_HUMAN	11634.892

TNIK_HUMAN	1286.8683
CPSF3_HUMAN	5329.3311
DP13A_HUMAN	5973.0107
BORG2_HUMAN	694.00201
TLK1_HUMAN	528.55981
GPTC8_HUMAN	1619.937
PARP4_HUMAN	8794.6914
NXT1_HUMAN	475.06009
NUDT5_HUMAN	13236.451
RCOR1_HUMAN	1334.1588
PPCT_HUMAN	1160.5372
MA1B1_HUMAN	1875.4207
RALY_HUMAN	15459.75
TF3C4_HUMAN	3941.0159
ERG28_HUMAN	3501.1279
PACN3_HUMAN	1142.3135
ACAD8_HUMAN	1595.2727
ANGL2_HUMAN	5051.231
ACINU_HUMAN	6812.7974
AMFR_HUMAN	3606.4868
AGO2_HUMAN	4528.6958
NUP50_HUMAN	7674.6528
CDV3_HUMAN	14371.64
CNO11_HUMAN	1451.1647
INT6_HUMAN	1960.3118
BAG5_HUMAN	2769.3169
AGO1_HUMAN	4472.6099
RAB21_HUMAN	9089.625
RB22A_HUMAN	5580.8735
TPC2L_HUMAN	2536.97
ZN346_HUMAN	2640.5664
PSME2_HUMAN	17260.525
TAOK2_HUMAN	2384.7188
MKLN1_HUMAN	3734.6687
DNPEP_HUMAN	8105.6006
RAB23_HUMAN	2748.5381
MCTS1_HUMAN	14460.48
MTUS1_HUMAN	560.35297
WWC3_HUMAN	769.64087
PALD_HUMAN	6552.4839
S39AA_HUMAN	1344.0352
INO80_HUMAN	766.24377
CCPG1_HUMAN	721.61493
KDIS_HUMAN	938.22498
ASAP1_HUMAN	3960.3125
MRTFB_HUMAN	1790.2506
ANR50_HUMAN	4304.5532
MED23_HUMAN	2972.2205
SHRM4_HUMAN	3183.7405
YETS2_HUMAN	945.06641

NDRG4_HUMAN	427.31747
TBC24_HUMAN	1593.4021
ISY1_HUMAN	1113.2913
TMCC3_HUMAN	3509.3767
HECD1_HUMAN	4683.9595
ZMYD8_HUMAN	1846.354
CIZ1_HUMAN	180.52245
COR1C_HUMAN	14216.034
TPX2_HUMAN	1407.8619
ABT1_HUMAN	1960.5782
NOB1_HUMAN	6137.4604
AKP8L_HUMAN	3660.8479
MAFF_HUMAN	616.20587
ASC_HUMAN	4724.4336
TMCO1_HUMAN	11412.988
FZR1_HUMAN	186.55449
APC10_HUMAN	1098.447
MYO6_HUMAN	7504.5073
DD19B_HUMAN	1004.6018
PPT2_HUMAN	2108.7971
PRP19_HUMAN	21749.525
SUFU_HUMAN	955.49536
NOL7_HUMAN	1675.5757
SNX12_HUMAN	10251.982
SYNRG_HUMAN	609.83966
VPS4A_HUMAN	8962.4902
G3BP2_HUMAN	9467.4932
CHIP_HUMAN	4391.1719
PACN2_HUMAN	10746.268
MAGD2_HUMAN	18209.791
SNX7_HUMAN	1608.0377
SNX6_HUMAN	8340.8672
DUS12_HUMAN	5013.7021
STX8_HUMAN	1706.0004
SSRG_HUMAN	5079.5
PSD13_HUMAN	34669.938
FAF1_HUMAN	6194.02
EPCR_HUMAN	19450.064
PPIE_HUMAN	2818.541
DIM1_HUMAN	4520.5303
CSN3_HUMAN	9804.9043
MINP1_HUMAN	3249.0398
NOVA2_HUMAN	9013.8672
WDR3_HUMAN	2563.3281
TTF2_HUMAN	638.45538
NSF1C_HUMAN	9633.5791
COG5_HUMAN	2967.0996
S12A4_HUMAN	6078.8589
AP4E1_HUMAN	232.16376
MACF1_HUMAN	38189.859

CP131_HUMAN	938.29944
SCAF8_HUMAN	2351.0073
PP6R1_HUMAN	2695.0107
TRI33_HUMAN	2830.9287
PHF8_HUMAN	1135.4242
TNR6B_HUMAN	738.16254
PLCL2_HUMAN	578.78137
SMG5_HUMAN	1258.5361
EXOC7_HUMAN	11133.288
ZC3H4_HUMAN	117.53719
UBP24_HUMAN	3875.2651
TBD2B_HUMAN	2081.2346
SMAG1_HUMAN	1258.8798
FOXJ3_HUMAN	1319.1486
CBPC1_HUMAN	782.32159
DICER_HUMAN	1329.8342
XCT_HUMAN	947.55688
MARE3_HUMAN	2082.6997
SHOC2_HUMAN	4475.937
SRRM2_HUMAN	9201.6006
MGT4B_HUMAN	823.70142
PA2G4_HUMAN	78499.961
SPG7_HUMAN	1404.2408
BAIP2_HUMAN	759.97455
GAB2_HUMAN	734.82416
SMC3_HUMAN	6422.2124
CHM2B_HUMAN	11580.577
SH2B3_HUMAN	1460.8607
ZN148_HUMAN	202.67224
MTMR6_HUMAN	4655.6826
JAG2_HUMAN	1142.5996
NIP7_HUMAN	1345.6193
GLCNE_HUMAN	2766.188
RTRAF_HUMAN	11764.152
RUVB2_HUMAN	32648.607
CDYL_HUMAN	490.6026
PIN4_HUMAN	6617.7847
AKT3_HUMAN	1328.7635
FACE2_HUMAN	4367.4736
CHKB_HUMAN	703.84912
EIF3L_HUMAN	55139.859
PLAP_HUMAN	12297.405
RUVB1_HUMAN	48958.898
NUDC_HUMAN	30703.029
BCS1_HUMAN	2962.3196
VDAC3_HUMAN	22544.025
COF2_HUMAN	19570.65
ERGI3_HUMAN	2389.3474
SYFA_HUMAN	27780.232
SC5A6_HUMAN	822.38159

RT33_HUMAN	5208.6636
ASF1A_HUMAN	3202.8049
DRG1_HUMAN	15110.251
TPPC4_HUMAN	3958.6917
NCKP1_HUMAN	12762.431
CNPY2_HUMAN	8613.6533
EXOG_HUMAN	963.34039
EXC6B_HUMAN	2589.3169
PLAK2_HUMAN	14391.237
CARD8_HUMAN	2046.5001
AT11B_HUMAN	299.36594
OFUT2_HUMAN	6420.8564
DJC16_HUMAN	644.80139
DLGP4_HUMAN	2070.7156
ST38L_HUMAN	2641.1228
SAC2_HUMAN	2875.4463
FND3A_HUMAN	4234.0127
NISCH_HUMAN	1172.0358
FYV1_HUMAN	1150.9176
WDR37_HUMAN	2205.9531
E41L3_HUMAN	18256.91
AMOL2_HUMAN	281.15955
SIK3_HUMAN	295.22043
KDM2A_HUMAN	1048.3792
RRP44_HUMAN	6185.292
TPPC8_HUMAN	2161.0378
LRCH1_HUMAN	4474.5371
RCL1_HUMAN	2839.6819
GSTK1_HUMAN	9054.0547
LTOR2_HUMAN	1669.502
RT28_HUMAN	10083.896
COA3_HUMAN	4749.4717
DDX52_HUMAN	1986.2715
RT17_HUMAN	5924.9204
RT07_HUMAN	8216.8516
CRYL1_HUMAN	3653.4622
TMA7_HUMAN	6105.9404
PDIP2_HUMAN	10044.132
AP3M1_HUMAN	13511.997
MAN1_HUMAN	4369.084
CHSP1_HUMAN	2070.7883
COG6_HUMAN	2425.3352
TR150_HUMAN	17356.465
WBP11_HUMAN	3738.7288
TDRKH_HUMAN	851.95233
MED16_HUMAN	1861.0536
NOP58_HUMAN	7397.6401
GIT1_HUMAN	6849.0913
ZN281_HUMAN	749.47388
AR2BP_HUMAN	3580.0884

TMM98_HUMAN	829.74377
SGT1_HUMAN	27179.541
SYYM_HUMAN	7380.7583
NAGA_HUMAN	3320.9285
ACOT9_HUMAN	28093.449
AAR2_HUMAN	1456.575
NOSIP_HUMAN	3851.8015
DEOC_HUMAN	5435.7334
MEMO1_HUMAN	5671.3452
TMX2_HUMAN	5576.6069
FCF1_HUMAN	782.51898
LSM2_HUMAN	5526.584
SHLB1_HUMAN	12492.726
CIA30_HUMAN	1707.0343
CAB39_HUMAN	15262.111
LC7L2_HUMAN	13680.627
UB2J1_HUMAN	5978.1943
RBMX2_HUMAN	679.10754
DHRS7_HUMAN	5008.5498
RT02_HUMAN	10253.932
UTP11_HUMAN	188.19151
PHOCN_HUMAN	12339.769
RRP7A_HUMAN	956.99683
SBDS_HUMAN	16893.494
TMED5_HUMAN	10377.161
EXOS1_HUMAN	2516.5295
TMED7_HUMAN	5012.0269
SF3B6_HUMAN	10185.966
RM11_HUMAN	11737.332
ORN_HUMAN	16072.535
RRP15_HUMAN	2042.214
WASC3_HUMAN	1442.2595
NOP16_HUMAN	4927.7573
TPRKB_HUMAN	4118.6108
PPIL1_HUMAN	3632.5859
UFC1_HUMAN	6196.4458
CIA2B_HUMAN	506.68845
RT16_HUMAN	4578.3813
RT18C_HUMAN	1491.3468
FIS1_HUMAN	11540.772
TIM16_HUMAN	2348.1614
KAD6_HUMAN	3698.0801
RT23_HUMAN	10806.93
GOT1B_HUMAN	3871.8931
HDGR3_HUMAN	2802.3801
PTH2_HUMAN	7003.771
CHMP3_HUMAN	50166.758
STRAP_HUMAN	18822.9
RTCB_HUMAN	47191.527
FBX7_HUMAN	3816.1731

RAP2C_HUMAN	1668.7214
STA13_HUMAN	2735.1018
RBGP1_HUMAN	1600.3688
TMED3_HUMAN	1104.5052
T22D4_HUMAN	799.05676
DOP2_HUMAN	744.14417
ZN330_HUMAN	582.73309
R3HC1_HUMAN	1094.6006
NOC2L_HUMAN	7897.5239
RL36_HUMAN	44710.066
CCDC9_HUMAN	2183.2117
CHTOP_HUMAN	2053.8435
SAMH1_HUMAN	8534.3867
SKAP_HUMAN	527.6991
HBS1L_HUMAN	4626.8481
AAKB1_HUMAN	1661.5917
WIPI4_HUMAN	434.5441
DMXL1_HUMAN	1087.5214
VPP2_HUMAN	2767.5308
TLN1_HUMAN	97898.617
KIF3A_HUMAN	1831.0009
TRRAP_HUMAN	2278.6775
MTCL1_HUMAN	641.44092
DCAF1_HUMAN	664.75317
TCAF1_HUMAN	7315.564
RBM19_HUMAN	472.08429
DAAM1_HUMAN	439.73477
PLXD1_HUMAN	19984.951
HECD4_HUMAN	316.17035
WAC2C_HUMAN	263.96088
UBP15_HUMAN	6429.23
FARP1_HUMAN	1384.0514
MARF1_HUMAN	570.61169
C170B_HUMAN	681.09052
RPGF2_HUMAN	9368.9473
MYO5A_HUMAN	6614.1113
DTNA_HUMAN	476.52704
LOXL2_HUMAN	6070.8594
CRBG1_HUMAN	2832.9199
TRAF6_HUMAN	558.58929
M4K5_HUMAN	3390.0723
HYOU1_HUMAN	31652.082
ATG4B_HUMAN	5844.2617
TBL2_HUMAN	4202.7476
WIPI2_HUMAN	3466.655
TELO2_HUMAN	2516.1506
LAS1L_HUMAN	6086.8198
AFG32_HUMAN	9700.3887
KLF12_HUMAN	198.56517
ARI1_HUMAN	5981.4854

LSM4_HUMAN	24375.111
RN114_HUMAN	2905.6968
SAM50_HUMAN	14599.195
PRC2C_HUMAN	16477.432
OARD1_HUMAN	7218.1245
IFT25_HUMAN	2456.5137
PPME1_HUMAN	10692.521
RBM7_HUMAN	1499.8052
TIM22_HUMAN	1613.5115
NUB1_HUMAN	2288.4907
YTHD2_HUMAN	2791.4746
CTDP1_HUMAN	3550.501
PAXB1_HUMAN	1116.1381
NDK7_HUMAN	2474.4216
SP16H_HUMAN	13347.162
UTP18_HUMAN	1406.8551
UCHL5_HUMAN	17887.381
CD2AP_HUMAN	11066.9
VATD_HUMAN	5114.5425
TNPO3_HUMAN	6104.6655
SRPRB_HUMAN	11069.962
ORC6_HUMAN	206.41017
CERT_HUMAN	1431.9891
GMPPB_HUMAN	18511.795
FADS3_HUMAN	1131.7797
TF3C5_HUMAN	6884.6738
TF3C3_HUMAN	3586.9502
TPPC1_HUMAN	5774.0811
TRPV2_HUMAN	804.91937
MRCKB_HUMAN	11761.466
RBM8A_HUMAN	15633.148
DJC15_HUMAN	796.7558
UBP16_HUMAN	1173.049
TSSC4_HUMAN	2390.2866
ZN706_HUMAN	3517.6526
MAGD1_HUMAN	4013.9089
SNX14_HUMAN	947.59467
SNX11_HUMAN	978.71094
SNX9_HUMAN	12426.461
SNX8_HUMAN	1530.833
SNX5_HUMAN	6321.77
LIPG_HUMAN	1816.5411
NUBP2_HUMAN	6517.6108
PEX16_HUMAN	1033.7904
LYVE1_HUMAN	5663.3569
BACE2_HUMAN	2056.5186
HEBP2_HUMAN	585.0957
HCFC2_HUMAN	757.1264
UBIA1_HUMAN	340.71893
PUS1_HUMAN	5478.8286

LRRF2_HUMAN	3882.0322
FHOD1_HUMAN	7002.3906
SERC_HUMAN	20726.484
NCOR2_HUMAN	1829.8162
ORNT1_HUMAN	1544.6398
JAM1_HUMAN	17360.98
NPTN_HUMAN	1521.9452
AGRG1_HUMAN	1300.0173
SPIN1_HUMAN	2195.8311
S12A7_HUMAN	8567.9648
ALG6_HUMAN	1898.9199
ALG5_HUMAN	7546.2456
RT18B_HUMAN	4199.9287
COPG1_HUMAN	30727.803
AUP1_HUMAN	2717.5466
FKBP7_HUMAN	1895.6497
CLIC4_HUMAN	49412.457
NFS1_HUMAN	5453.2661
CFA20_HUMAN	9142.5693
TACC3_HUMAN	237.4962
SPCS1_HUMAN	6996.895
SAR1B_HUMAN	5003.0679
AP4B1_HUMAN	595.06128
MTCH2_HUMAN	22282.447
SELK_HUMAN	1440.4502
BIG2_HUMAN	3648.3542
BIG1_HUMAN	4346.6792
MD1L1_HUMAN	3267.8435
STK24_HUMAN	15367.773
5MP1_HUMAN	44663.117
COMDA_HUMAN	3662.8452
DC1L1_HUMAN	20371.754
EPN1_HUMAN	3973.4602
UBP3_HUMAN	16504.105
TX264_HUMAN	5431.2109
CEPT1_HUMAN	8657.8213
DNM3A_HUMAN	541.98578
OAS3_HUMAN	2265.4678
KAD5_HUMAN	18179.053
NEMO_HUMAN	4087.5056
IF2B2_HUMAN	22829.721
ZNT1_HUMAN	2880.4312
S4A7_HUMAN	2701.249
NDUB9_HUMAN	14025.793
SQOR_HUMAN	3353.2043
ROBO1_HUMAN	610.12335
STON1_HUMAN	1032.0016
NCOA3_HUMAN	472.97925
NUMBL_HUMAN	1357.9021
M3K4_HUMAN	1305.6328

DDX49_HUMAN	1168.5558
CAN7_HUMAN	1193.9067
WASF2_HUMAN	9692
SCC4_HUMAN	2510.3708
MORC2_HUMAN	1189.8734
NS1BP_HUMAN	1527.2679
S23IP_HUMAN	16682.701
