# Supplementary material for: Proteomic Analysis of Endothelial Activation Induced by Adult Angiostrongylus vasorum Homogenate: Insights into Vascular Remodeling and Hemostatic Imbalance
Source: Animals (Basel). 2026 Mar 15;16(6):926. doi: 10.3390/ani16060926 (PMC13023303; doi:10.3390/ani16060926)
Supplement: Supplementary file 1 [file animals-16-00926-s001.zip › Supplmentary Table S6.pdf]

Supplementary Table S6: Proteins identified in the

R.Condition	R.Replicate	PG.ProteinAccess
Control	1	A0A0B4J2D5
Control	1	A0AVF1
Control	1	A0AVT1
Control	1	A0FGR8
Control	1	A0MZ66
Control	1	A0PJW6
Control	1	A1A4S6
Control	1	A1L0T0
Control	1	A1X283
Control	1	A2RRP1
Control	1	A2RUC4
Control	1	A2RUS2
Control	1	A3KMH1
Control	1	A3KN83
Control	1	A4D1E9
Control	1	A4D1P6
Control	1	A5PLL7
Control	1	A5PLN9
Control	1	A5YKK6
Control	1	A6NCE7
Control	1	A6NDG6
Control	1	A6NDU8
Control	1	A6NED2
Control	1	A6NFQ2
Control	1	A6NHR9
Control	1	A6NHX0
Control	1	A6NIH7
Control	1	A6NJ78
Control	1	A6NKD9
Control	1	A6ZKI3
Control	1	A7E2V4
Control	1	A8CG34
Control	1	A8MW92
Control	1	A8MWD9
Control	1	A8MWY0
Control	1	A8MXV4
Control	1	A9UHW6
Control	1	P0DP23
Control	1	B2RTY4
Control	1	B7ZAQ6
Control	1	C4AMC7
Control	1	C9JLW8
Control	1	E9PAV3
Control	1	E9PRG8
Control	1	L0R819
Control	1	L0R8F8
Control	1	O00115
Control	1	O00116

Control	1	O00139
Control	1	O00148
Control	1	O00151
Control	1	O00154
Control	1	O00159
Control	1	O00161
Control	1	O00165
Control	1	O00170
Control	1	O00178
Control	1	O00182
Control	1	O00186
Control	1	O00189
Control	1	O00192
Control	1	O00193
Control	1	O00203
Control	1	O00213
Control	1	O00214
Control	1	O00217
Control	1	O00221
Control	1	O00231
Control	1	O00232
Control	1	O00233
Control	1	O00255
Control	1	O00258
Control	1	O00264
Control	1	O00267
Control	1	O00268
Control	1	O00273
Control	1	O00291
Control	1	O00299
Control	1	O00303
Control	1	O00308
Control	1	O00330
Control	1	O00391
Control	1	O00399
Control	1	O00400
Control	1	O00401
Control	1	O00410
Control	1	O00411
Control	1	O00418
Control	1	O00422
Control	1	O00423
Control	1	O00425
Control	1	O00429
Control	1	O00442
Control	1	O00443
Control	1	O00461
Control	1	O00468
Control	1	O00469
Control	1	O00471

Control	1	O00481
Control	1	O00483
Control	1	O00487
Control	1	O00499
Control	1	O00501
Control	1	O00505
Control	1	O00506
Control	1	O00522
Control	1	O00534
Control	1	O00541
Control	1	O00560
Control	1	O00562
Control	1	O00566
Control	1	O00567
Control	1	O00571
Control	1	O00587
Control	1	O00592
Control	1	O00622
Control	1	O00625
Control	1	O00629
Control	1	O00635
Control	1	O00712
Control	1	O00743
Control	1	O00746
Control	1	O00750
Control	1	O00754
Control	1	O00764
Control	1	O00767
Control	1	O14497
Control	1	O14524
Control	1	O14530
Control	1	O14531
Control	1	O14545
Control	1	O14562
Control	1	O14578
Control	1	O14579
Control	1	O14613
Control	1	O14617
Control	1	O14618
Control	1	O14639
Control	1	O14640
Control	1	O14641
Control	1	O14646
Control	1	O14647
Control	1	O14653
Control	1	O14656
Control	1	O14657
Control	1	O14662
Control	1	O14672
Control	1	O14678

Control	1	O14681
Control	1	O14683
Control	1	O14686
Control	1	O14727
Control	1	O14730
Control	1	O14733
Control	1	O14734
Control	1	O14735
Control	1	O14737
Control	1	O14744
Control	1	O14745
Control	1	O14757
Control	1	O14763
Control	1	O14773
Control	1	O14776
Control	1	O14777
Control	1	O14786
Control	1	O14787
Control	1	O14795
Control	1	O14798
Control	1	O14802
Control	1	O14817
Control	1	O14818
Control	1	O14828
Control	1	O14874
Control	1	O14880
Control	1	O14907
Control	1	O14908
Control	1	O14920
Control	1	O14924
Control	1	O14925
Control	1	O14929
Control	1	O14936
Control	1	O14939
Control	1	O14949
Control	1	O14950
Control	1	O14964
Control	1	O14966
Control	1	O14972
Control	1	O14974
Control	1	O14976
Control	1	O14979
Control	1	O14980
Control	1	O14981
Control	1	O15013
Control	1	O15014
Control	1	O15020
Control	1	O15021
Control	1	O15027
Control	1	O15031

Control	1	O15037
Control	1	O15040
Control	1	O15042
Control	1	O15047
Control	1	O15049
Control	1	O15056
Control	1	O15061
Control	1	O15063
Control	1	O15066
Control	1	O15067
Control	1	O15084
Control	1	O15085
Control	1	O15091
Control	1	O15111
Control	1	O15118
Control	1	O15121
Control	1	O15123
Control	1	O15126
Control	1	O15127
Control	1	O15143
Control	1	O15144
Control	1	O15145
Control	1	O15160
Control	1	O15162
Control	1	O15164
Control	1	O15173
Control	1	O15211
Control	1	O15213
Control	1	O15226
Control	1	O15228
Control	1	O15230
Control	1	O15231
Control	1	O15234
Control	1	O15235
Control	1	O15254
Control	1	O15258
Control	1	O15260
Control	1	O15269
Control	1	O15270
Control	1	O15294
Control	1	O15305
Control	1	O15321
Control	1	O15344
Control	1	O15347
Control	1	O15355
Control	1	O15357
Control	1	O15371
Control	1	O15372
Control	1	O15379
Control	1	O15381

Control	1	O15382
Control	1	O15397
Control	1	O15400
Control	1	O15427
Control	1	O15439
Control	1	O15446
Control	1	O15460
Control	1	O15484
Control	1	O15498
Control	1	O15504
Control	1	O15511
Control	1	O15519
Control	1	O15525
Control	1	O15530
Control	1	O15533
Control	1	O15541
Control	1	O43143
Control	1	O43148
Control	1	O43149
Control	1	O43150
Control	1	O43155
Control	1	O43156
Control	1	O43159
Control	1	O43164
Control	1	O43169
Control	1	O43172
Control	1	O43175
Control	1	O43181
Control	1	O43237
Control	1	O43242
Control	1	O43251
Control	1	O43252
Control	1	O43264
Control	1	O43286
Control	1	O43290
Control	1	O43292
Control	1	O43293
Control	1	O43294
Control	1	O43299
Control	1	O43310
Control	1	O43312
Control	1	O43314
Control	1	O43318
Control	1	O43324
Control	1	O43353
Control	1	O43390
Control	1	O43395
Control	1	O43396
Control	1	O43399
Control	1	O43402

Control	1	O43414
Control	1	O43426
Control	1	O43427
Control	1	O43432
Control	1	O43447
Control	1	O43464
Control	1	O43482
Control	1	O43488
Control	1	O43491
Control	1	O43493
Control	1	O43505
Control	1	O43516
Control	1	O43520
Control	1	O43572
Control	1	O43583
Control	1	O43592
Control	1	O43598
Control	1	O43615
Control	1	O43617
Control	1	O43633
Control	1	O43639
Control	1	O43657
Control	1	O43660
Control	1	O43663
Control	1	O43665
Control	1	O43670
Control	1	O43674
Control	1	O43676
Control	1	O43678
Control	1	O43679
Control	1	O43681
Control	1	O43683
Control	1	O43684
Control	1	O43704
Control	1	O43707
Control	1	O43709
Control	1	O43719
Control	1	O43731
Control	1	O43747
Control	1	O43752
Control	1	O43760
Control	1	O43765
Control	1	O43768
Control	1	O43772
Control	1	O43776
Control	1	O43795
Control	1	O43808
Control	1	O43809
Control	1	O43813
Control	1	O43815

Control	1	O43818
Control	1	O43819
Control	1	O43822
Control	1	O43823
Control	1	O43824
Control	1	O43826
Control	1	O43837
Control	1	O43847
Control	1	O43852
Control	1	O43854
Control	1	O43861
Control	1	O43865
Control	1	O43896
Control	1	O43909
Control	1	O43913
Control	1	O43920
Control	1	O43924
Control	1	O43929
Control	1	O43933
Control	1	O60216
Control	1	O60231
Control	1	O60234
Control	1	O60239
Control	1	O60244
Control	1	O60256
Control	1	O60264
Control	1	O60271
Control	1	O60285
Control	1	O60287
Control	1	O60292
Control	1	O60293
Control	1	O60306
Control	1	O60313
Control	1	O60318
Control	1	O60330
Control	1	O60331
Control	1	O60333
Control	1	O60336
Control	1	O60341
Control	1	O60343
Control	1	O60427
Control	1	O60443
Control	1	O60462
Control	1	O60476
Control	1	O60487
Control	1	O60488
Control	1	O60493
Control	1	O60502
Control	1	O60504
Control	1	O60506

Control	1	O60508
Control	1	O60518
Control	1	O60524
Control	1	O60547
Control	1	O60551
Control	1	O60563
Control	1	O60566
Control	1	O60568
Control	1	O60573
Control	1	O60610
Control	1	O60637
Control	1	O60645
Control	1	O60664
Control	1	O60678
Control	1	O60684
Control	1	O60701
Control	1	O60704
Control	1	O60711
Control	1	O60716
Control	1	O60725
Control	1	O60749
Control	1	O60762
Control	1	O60763
Control	1	O60783
Control	1	O60784
Control	1	O60814
Control	1	O60826
Control	1	O60828
Control	1	O60830
Control	1	O60831
Control	1	O60832
Control	1	O60841
Control	1	O60869
Control	1	O60870
Control	1	O60879
Control	1	O60884
Control	1	O60885
Control	1	O60921
Control	1	O60925
Control	1	O60927
Control	1	O60934
Control	1	O60942
Control	1	O75027
Control	1	O75044
Control	1	O75051
Control	1	O75063
Control	1	O75083
Control	1	O75110
Control	1	O75113
Control	1	O75116

Control	1	O75122
Control	1	O75127
Control	1	O75131
Control	1	O75143
Control	1	O75146
Control	1	O75150
Control	1	O75151
Control	1	O75152
Control	1	O75153
Control	1	O75155
Control	1	O75157
Control	1	O75165
Control	1	O75167
Control	1	O75170
Control	1	O75173
Control	1	O75175
Control	1	O75179
Control	1	O75182
Control	1	O75190
Control	1	O75208
Control	1	O75223
Control	1	O75251
Control	1	O75306
Control	1	O75312
Control	1	O75319
Control	1	O75323
Control	1	O75330
Control	1	O75340
Control	1	O75347
Control	1	O75348
Control	1	O75351
Control	1	O75352
Control	1	O75367
Control	1	O75368
Control	1	O75369
Control	1	O75376
Control	1	O75381
Control	1	O75382
Control	1	O75390
Control	1	O75391
Control	1	O75396
Control	1	O75400
Control	1	O75410
Control	1	O75414
Control	1	O75419
Control	1	O75427
Control	1	O75431
Control	1	O75436
Control	1	O75439
Control	1	O75448

Control	1	O75449
Control	1	O75475
Control	1	O75477
Control	1	O75489
Control	1	O75494
Control	1	O75503
Control	1	O75508
Control	1	O75521
Control	1	O75525
Control	1	O75530
Control	1	O75531
Control	1	O75533
Control	1	O75534
Control	1	O75554
Control	1	O75569
Control	1	O75578
Control	1	O75582
Control	1	O75586
Control	1	O75592
Control	1	O75600
Control	1	O75608
Control	1	O75616
Control	1	O75643
Control	1	O75648
Control	1	O75663
Control	1	O75665
Control	1	O75676
Control	1	O75683
Control	1	O75688
Control	1	O75691
Control	1	O75694
Control	1	O75695
Control	1	O75717
Control	1	O75718
Control	1	O75746
Control	1	O75787
Control	1	O75792
Control	1	O75794
Control	1	O75815
Control	1	O75817
Control	1	O75818
Control	1	O75821
Control	1	O75822
Control	1	O75828
Control	1	O75832
Control	1	O75843
Control	1	O75844
Control	1	O75874
Control	1	O75879
Control	1	O75880

Control	1	O75882
Control	1	O75884
Control	1	O75886
Control	1	O75907
Control	1	O75909
Control	1	O75915
Control	1	O75923
Control	1	O75925
Control	1	O75934
Control	1	O75935
Control	1	O75937
Control	1	O75940
Control	1	O75947
Control	1	O75954
Control	1	O75955
Control	1	O75962
Control	1	O75964
Control	1	O75970
Control	1	O75976
Control	1	O76003
Control	1	O76021
Control	1	O76024
Control	1	O76031
Control	1	O76054
Control	1	O76070
Control	1	O76071
Control	1	O76094
Control	1	O94760
Control	1	O94762
Control	1	O94763
Control	1	O94766
Control	1	O94776
Control	1	O94788
Control	1	O94804
Control	1	O94806
Control	1	O94808
Control	1	O94813
Control	1	O94822
Control	1	O94826
Control	1	O94827
Control	1	O94829
Control	1	O94830
Control	1	O94832
Control	1	O94842
Control	1	O94851
Control	1	O94855
Control	1	O94868
Control	1	O94874
Control	1	O94875
Control	1	O94885

Control	1	O94888
Control	1	O94889
Control	1	O94901
Control	1	O94903
Control	1	O94905
Control	1	O94906
Control	1	O94913
Control	1	O94915
Control	1	O94916
Control	1	O94919
Control	1	O94923
Control	1	O94925
Control	1	O94927
Control	1	O94929
Control	1	O94952
Control	1	O94953
Control	1	O94964
Control	1	O94966
Control	1	O94967
Control	1	O94973
Control	1	O94979
Control	1	O94989
Control	1	O94992
Control	1	O95059
Control	1	O95070
Control	1	O95071
Control	1	O95081
Control	1	O95084
Control	1	O95104
Control	1	O95139
Control	1	O95140
Control	1	O95149
Control	1	O95155
Control	1	O95159
Control	1	O95163
Control	1	O95168
Control	1	O95169
Control	1	O95182
Control	1	O95183
Control	1	O95197
Control	1	O95202
Control	1	O95208
Control	1	O95218
Control	1	O95219
Control	1	O95232
Control	1	O95235
Control	1	O95236
Control	1	O95239
Control	1	O95243
Control	1	O95248

Control	1	O95249
Control	1	O95251
Control	1	O95260
Control	1	O95292
Control	1	O95295
Control	1	O95297
Control	1	O95298
Control	1	O95299
Control	1	O95302
Control	1	O95319
Control	1	O95336
Control	1	O95340
Control	1	O95347
Control	1	O95352
Control	1	O95359
Control	1	O95361
Control	1	O95363
Control	1	O95365
Control	1	O95372
Control	1	O95373
Control	1	O95376
Control	1	O95391
Control	1	O95394
Control	1	O95396
Control	1	O95400
Control	1	O95425
Control	1	O95433
Control	1	O95453
Control	1	O95456
Control	1	O95470
Control	1	O95476
Control	1	O95478
Control	1	O95479
Control	1	O95486
Control	1	O95487
Control	1	O95490
Control	1	O95544
Control	1	O95551
Control	1	O95563
Control	1	O95571
Control	1	O95573
Control	1	O95602
Control	1	O95619
Control	1	O95625
Control	1	O95628
Control	1	O95630
Control	1	O95639
Control	1	O95644
Control	1	O95671
Control	1	O95674

Control	1	O95685
Control	1	O95696
Control	1	O95707
Control	1	O95714
Control	1	O95716
Control	1	O95721
Control	1	O95747
Control	1	O95749
Control	1	O95757
Control	1	O95758
Control	1	O95772
Control	1	O95777
Control	1	O95782
Control	1	O95785
Control	1	O95786
Control	1	O95793
Control	1	O95801
Control	1	O95807
Control	1	O95810
Control	1	O95816
Control	1	O95817
Control	1	O95819
Control	1	O95822
Control	1	O95825
Control	1	O95831
Control	1	O95834
Control	1	O95835
Control	1	O95848
Control	1	O95858
Control	1	O95861
Control	1	O95864
Control	1	O95865
Control	1	O95870
Control	1	O95881
Control	1	O95905
Control	1	O95983
Control	1	O95985
Control	1	O95989
Control	1	O95999
Control	1	O96000
Control	1	O96005
Control	1	O96006
Control	1	O96007
Control	1	O96008
Control	1	O96011
Control	1	O96013
Control	1	O96018
Control	1	O96019
Control	1	O96028
Control	1	P00338

Control	1	P00352
Control	1	P00367
Control	1	P00374
Control	1	P00387
Control	1	P00390
Control	1	P00395
Control	1	P00403
Control	1	P00491
Control	1	P00492
Control	1	P00505
Control	1	P00519
Control	1	P00558
Control	1	P00568
Control	1	P00749
Control	1	P00750
Control	1	P00813
Control	1	P01009
Control	1	P01023
Control	1	P01034
Control	1	P01111
Control	1	P01112
Control	1	P01116
Control	1	P01127
Control	1	P01130
Control	1	P01137
Control	1	P01889
Control	1	P01893
Control	1	P02452
Control	1	P02462
Control	1	P02545
Control	1	P02647
Control	1	P02751
Control	1	P02765
Control	1	P02768
Control	1	P02786
Control	1	P02792
Control	1	P02794
Control	1	P02795
Control	1	P03886
Control	1	P03905
Control	1	P03915
Control	1	P03956
Control	1	P04035
Control	1	P04040
Control	1	P04049
Control	1	P04062
Control	1	P04075
Control	1	P04080
Control	1	P04083
Control	1	P04114

Control	1	P04156
Control	1	P04179
Control	1	P04181
Control	1	P04183
Control	1	P04275
Control	1	P04406
Control	1	P04424
Control	1	P04439
Control	1	P04632
Control	1	P04637
Control	1	P04732
Control	1	P04792
Control	1	P04818
Control	1	P04843
Control	1	P04844
Control	1	P04899
Control	1	P04908
Control	1	P04920
Control	1	P05023
Control	1	P05026
Control	1	P05067
Control	1	P05091
Control	1	P05106
Control	1	P05109
Control	1	P05114
Control	1	P05120
Control	1	P05121
Control	1	P05141
Control	1	P05161
Control	1	P05165
Control	1	P05166
Control	1	P05198
Control	1	P05204
Control	1	P05362
Control	1	P05387
Control	1	P05388
Control	1	P05412
Control	1	P05423
Control	1	P05455
Control	1	P05556
Control	1	P05783
Control	1	P05997
Control	1	P06132
Control	1	P06241
Control	1	P06280
Control	1	P06396
Control	1	P06400
Control	1	P06454
Control	1	P06493
Control	1	P06576

Control	1	P06703
Control	1	P06730
Control	1	P06733
Control	1	P06737
Control	1	P06744
Control	1	P06746
Control	1	P06748
Control	1	P06753
Control	1	P06756
Control	1	P06865
Control	1	P07099
Control	1	P07195
Control	1	P07199
Control	1	P07203
Control	1	P07237
Control	1	P07305
Control	1	P07311
Control	1	P07332
Control	1	P07339
Control	1	P07355
Control	1	P07384
Control	1	P07437
Control	1	P07602
Control	1	P07686
Control	1	P07711
Control	1	P07737
Control	1	P07738
Control	1	P07741
Control	1	P07814
Control	1	P07858
Control	1	P07900
Control	1	P07902
Control	1	P07910
Control	1	P07942
Control	1	P07947
Control	1	P07948
Control	1	P07954
Control	1	P07992
Control	1	P07996
Control	1	P08047
Control	1	P08069
Control	1	P08123
Control	1	P08133
Control	1	P08134
Control	1	P08174
Control	1	P08195
Control	1	P08236
Control	1	P08237
Control	1	P08238
Control	1	P08240

Control	1	P08243
Control	1	P08253
Control	1	P08397
Control	1	P08473
Control	1	P08493
Control	1	P08559
Control	1	P08572
Control	1	P08574
Control	1	P08579
Control	1	P08581
Control	1	P08621
Control	1	P08648
Control	1	P08651
Control	1	P08670
Control	1	P08708
Control	1	P08754
Control	1	P08758
Control	1	P08865
Control	1	P08962
Control	1	P09001
Control	1	P09012
Control	1	P09104
Control	1	P09110
Control	1	P09132
Control	1	P09211
Control	1	P09234
Control	1	P09382
Control	1	P09417
Control	1	P09429
Control	1	P09486
Control	1	P09493
Control	1	P09496
Control	1	P09497
Control	1	P09525
Control	1	P09543
Control	1	P09601
Control	1	P09622
Control	1	P09651
Control	1	P09661
Control	1	P09669
Control	1	P09758
Control	1	P09874
Control	1	P09884
Control	1	P09936
Control	1	P09960
Control	1	P09972
Control	1	P0C0S5
Control	1	P0C0S8
Control	1	P0CAP2
Control	1	P0CG29

Control	1	P0CG39
Control	1	P0CW19
Control	1	P0DMV8
Control	1	P0DP91
Control	1	P0DPH7
Control	1	P0DTL6
Control	1	P10114
Control	1	P10124
Control	1	P10155
Control	1	P10253
Control	1	P10301
Control	1	P10321
Control	1	P10398
Control	1	P10412
Control	1	P10515
Control	1	P10586
Control	1	P10589
Control	1	P10599
Control	1	P10606
Control	1	P10619
Control	1	P10620
Control	1	P10644
Control	1	P10646
Control	1	P10768
Control	1	P10809
Control	1	P10909
Control	1	P11021
Control	1	P11047
Control	1	P11117
Control	1	P11137
Control	1	P11142
Control	1	P11166
Control	1	P11169
Control	1	P11171
Control	1	P11172
Control	1	P11177
Control	1	P11182
Control	1	P11216
Control	1	P11217
Control	1	P11233
Control	1	P11234
Control	1	P11274
Control	1	P11279
Control	1	P11308
Control	1	P11310
Control	1	P11387
Control	1	P11388
Control	1	P11413
Control	1	P11441
Control	1	P11498

Control	1	P11586
Control	1	P11717
Control	1	P11766
Control	1	P11802
Control	1	P11908
Control	1	P11940
Control	1	P12004
Control	1	P12081
Control	1	P12109
Control	1	P12111
Control	1	P12235
Control	1	P12236
Control	1	P12268
Control	1	P12270
Control	1	P12429
Control	1	P12694
Control	1	P12814
Control	1	P12821
Control	1	P12931
Control	1	P12955
Control	1	P12956
Control	1	P13010
Control	1	P13051
Control	1	P13073
Control	1	P13196
Control	1	P13473
Control	1	P13489
Control	1	P13598
Control	1	P13639
Control	1	P13667
Control	1	P13674
Control	1	P13693
Control	1	P13716
Control	1	P13747
Control	1	P13797
Control	1	P13798
Control	1	P13804
Control	1	P13807
Control	1	P13861
Control	1	P13984
Control	1	P13987
Control	1	P13995
Control	1	P14174
Control	1	P14209
Control	1	P14314
Control	1	P14317
Control	1	P14324
Control	1	P14406
Control	1	P14543
Control	1	P14550

Control	1	P14618
Control	1	P14625
Control	1	P14635
Control	1	P14649
Control	1	P14678
Control	1	P14735
Control	1	P14859
Control	1	P14866
Control	1	P14868
Control	1	P14921
Control	1	P14923
Control	1	P14927
Control	1	P15056
Control	1	P15090
Control	1	P15104
Control	1	P15121
Control	1	P15144
Control	1	P15151
Control	1	P15153
Control	1	P15170
Control	1	P15289
Control	1	P15291
Control	1	P15311
Control	1	P15374
Control	1	P15407
Control	1	P15408
Control	1	P15529
Control	1	P15531
Control	1	P15559
Control	1	P15586
Control	1	P15735
Control	1	P15848
Control	1	P15880
Control	1	P15884
Control	1	P15924
Control	1	P15927
Control	1	P16035
Control	1	P16070
Control	1	P16152
Control	1	P16219
Control	1	P16220
Control	1	P16278
Control	1	P16284
Control	1	P16298
Control	1	P16333
Control	1	P16383
Control	1	P16401
Control	1	P16402
Control	1	P16403
Control	1	P16435

Control	1	P16591
Control	1	P16615
Control	1	P16885
Control	1	P16930
Control	1	P16949
Control	1	P16989
Control	1	P17026
Control	1	P17028
Control	1	P17050
Control	1	P17096
Control	1	P17152
Control	1	P17174
Control	1	P17252
Control	1	P17301
Control	1	P17302
Control	1	P17405
Control	1	P17480
Control	1	P17535
Control	1	P17542
Control	1	P17568
Control	1	P17612
Control	1	P17655
Control	1	P17706
Control	1	P17812
Control	1	P17813
Control	1	P17844
Control	1	P17858
Control	1	P17948
Control	1	P17980
Control	1	P17987
Control	1	P18031
Control	1	P18074
Control	1	P18077
Control	1	P18084
Control	1	P18085
Control	1	P18124
Control	1	P18206
Control	1	P18433
Control	1	P18440
Control	1	P18583
Control	1	P18615
Control	1	P18621
Control	1	P18669
Control	1	P18754
Control	1	P18846
Control	1	P18850
Control	1	P18858
Control	1	P18887
Control	1	P19021
Control	1	P19022

Control	1	P19174
Control	1	P19338
Control	1	P19367
Control	1	P19387
Control	1	P19388
Control	1	P19404
Control	1	P19447
Control	1	P19474
Control	1	P19525
Control	1	P19623
Control	1	P19634
Control	1	P19784
Control	1	P19838
Control	1	P20020
Control	1	P20042
Control	1	P20073
Control	1	P20248
Control	1	P20290
Control	1	P20337
Control	1	P20338
Control	1	P20339
Control	1	P20340
Control	1	P20585
Control	1	P20594
Control	1	P20618
Control	1	P20645
Control	1	P20674
Control	1	P20700
Control	1	P20810
Control	1	P20839
Control	1	P20908
Control	1	P20929
Control	1	P20933
Control	1	P20936
Control	1	P21127
Control	1	P21266
Control	1	P21281
Control	1	P21283
Control	1	P21291
Control	1	P21333
Control	1	P21359
Control	1	P21397
Control	1	P21399
Control	1	P21453
Control	1	P21579
Control	1	P21589
Control	1	P21675
Control	1	P21741
Control	1	P21796
Control	1	P21810

Control	1	P21912
Control	1	P21926
Control	1	P21964
Control	1	P21980
Control	1	P22033
Control	1	P22059
Control	1	P22061
Control	1	P22087
Control	1	P22102
Control	1	P22234
Control	1	P22307
Control	1	P22314
Control	1	P22392
Control	1	P22570
Control	1	P22626
Control	1	P22670
Control	1	P22681
Control	1	P22692
Control	1	P22694
Control	1	P22695
Control	1	P22830
Control	1	P23025
Control	1	P23193
Control	1	P23219
Control	1	P23229
Control	1	P23246
Control	1	P23258
Control	1	P23284
Control	1	P23368
Control	1	P23381
Control	1	P23396
Control	1	P23443
Control	1	P23458
Control	1	P23467
Control	1	P23469
Control	1	P23470
Control	1	P23497
Control	1	P23508
Control	1	P23510
Control	1	P23526
Control	1	P23528
Control	1	P23588
Control	1	P23610
Control	1	P23634
Control	1	P23743
Control	1	P23786
Control	1	P23919
Control	1	P23921
Control	1	P24385
Control	1	P24386

Control	1	P24390
Control	1	P24468
Control	1	P24534
Control	1	P24539
Control	1	P24666
Control	1	P24723
Control	1	P24752
Control	1	P24844
Control	1	P24928
Control	1	P24941
Control	1	P25098
Control	1	P25116
Control	1	P25205
Control	1	P25325
Control	1	P25398
Control	1	P25440
Control	1	P25490
Control	1	P25685
Control	1	P25686
Control	1	P25705
Control	1	P25774
Control	1	P25786
Control	1	P25787
Control	1	P25788
Control	1	P25789
Control	1	P25942
Control	1	P26006
Control	1	P26022
Control	1	P26038
Control	1	P26196
Control	1	P26358
Control	1	P26368
Control	1	P26373
Control	1	P26374
Control	1	P26440
Control	1	P26572
Control	1	P26583
Control	1	P26599
Control	1	P26639
Control	1	P26640
Control	1	P26641
Control	1	P26885
Control	1	P26951
Control	1	P27105
Control	1	P27144
Control	1	P27348
Control	1	P27361
Control	1	P27448
Control	1	P27635
Control	1	P27694

Control	1	P27695
Control	1	P27701
Control	1	P27707
Control	1	P27708
Control	1	P27797
Control	1	P27816
Control	1	P27824
Control	1	P27986
Control	1	P27987
Control	1	P28062
Control	1	P28065
Control	1	P28066
Control	1	P28070
Control	1	P28072
Control	1	P28074
Control	1	P28161
Control	1	P28288
Control	1	P28290
Control	1	P28300
Control	1	P28331
Control	1	P28340
Control	1	P28370
Control	1	P28482
Control	1	P28702
Control	1	P28715
Control	1	P28799
Control	1	P28827
Control	1	P28838
Control	1	P28906
Control	1	P29083
Control	1	P29084
Control	1	P29144
Control	1	P29218
Control	1	P29279
Control	1	P29317
Control	1	P29323
Control	1	P29353
Control	1	P29372
Control	1	P29401
Control	1	P29466
Control	1	P29474
Control	1	P29558
Control	1	P29590
Control	1	P29597
Control	1	P29692
Control	1	P29966
Control	1	P29992
Control	1	P30038
Control	1	P30040
Control	1	P30041

Control	1	P30043
Control	1	P30044
Control	1	P30048
Control	1	P30050
Control	1	P30084
Control	1	P30085
Control	1	P30086
Control	1	P30101
Control	1	P30153
Control	1	P30154
Control	1	P30260
Control	1	P30405
Control	1	P30414
Control	1	P30419
Control	1	P30519
Control	1	P30520
Control	1	P30530
Control	1	P30533
Control	1	P30536
Control	1	P30566
Control	1	P30622
Control	1	P30626
Control	1	P30740
Control	1	P30825
Control	1	P30837
Control	1	P30876
Control	1	P31040
Control	1	P31146
Control	1	P31150
Control	1	P31153
Control	1	P31323
Control	1	P31350
Control	1	P31431
Control	1	P31483
Control	1	P31641
Control	1	P31689
Control	1	P31749
Control	1	P31930
Control	1	P31937
Control	1	P31939
Control	1	P31942
Control	1	P31943
Control	1	P31944
Control	1	P31946
Control	1	P31948
Control	1	P31949
Control	1	P32119
Control	1	P32121
Control	1	P32189
Control	1	P32321

Control	1	P32322
Control	1	P32455
Control	1	P32456
Control	1	P32519
Control	1	P32780
Control	1	P32856
Control	1	P32969
Control	1	P33121
Control	1	P33151
Control	1	P33176
Control	1	P33316
Control	1	P33527
Control	1	P33897
Control	1	P33981
Control	1	P33991
Control	1	P33992
Control	1	P33993
Control	1	P34059
Control	1	P34896
Control	1	P34897
Control	1	P34931
Control	1	P34932
Control	1	P34949
Control	1	P35052
Control	1	P35080
Control	1	P35221
Control	1	P35222
Control	1	P35232
Control	1	P35237
Control	1	P35240
Control	1	P35241
Control	1	P35244
Control	1	P35249
Control	1	P35250
Control	1	P35251
Control	1	P35268
Control	1	P35269
Control	1	P35270
Control	1	P35520
Control	1	P35555
Control	1	P35556
Control	1	P35573
Control	1	P35579
Control	1	P35580
Control	1	P35590
Control	1	P35606
Control	1	P35610
Control	1	P35611
Control	1	P35613
Control	1	P35637

Control	1	P35658
Control	1	P35659
Control	1	P35713
Control	1	P35749
Control	1	P35813
Control	1	P35914
Control	1	P35968
Control	1	P35998
Control	1	P36269
Control	1	P36404
Control	1	P36405
Control	1	P36507
Control	1	P36542
Control	1	P36543
Control	1	P36551
Control	1	P36578
Control	1	P36639
Control	1	P36776
Control	1	P36871
Control	1	P36873
Control	1	P36915
Control	1	P36957
Control	1	P36959
Control	1	P36969
Control	1	P37023
Control	1	P37059
Control	1	P37108
Control	1	P37173
Control	1	P37198
Control	1	P37235
Control	1	P37268
Control	1	P37275
Control	1	P37802
Control	1	P37837
Control	1	P37840
Control	1	P38117
Control	1	P38159
Control	1	P38432
Control	1	P38435
Control	1	P38571
Control	1	P38606
Control	1	P38646
Control	1	P38919
Control	1	P38936
Control	1	P39019
Control	1	P39023
Control	1	P39060
Control	1	P39656
Control	1	P39687
Control	1	P39748

Control	1	P39880
Control	1	P40121
Control	1	P40123
Control	1	P40189
Control	1	P40222
Control	1	P40227
Control	1	P40261
Control	1	P40306
Control	1	P40425
Control	1	P40429
Control	1	P40616
Control	1	P40692
Control	1	P40763
Control	1	P40818
Control	1	P40925
Control	1	P40926
Control	1	P40937
Control	1	P40938
Control	1	P40939
Control	1	P41091
Control	1	P41162
Control	1	P41208
Control	1	P41212
Control	1	P41214
Control	1	P41223
Control	1	P41226
Control	1	P41227
Control	1	P41229
Control	1	P41240
Control	1	P41250
Control	1	P41252
Control	1	P41440
Control	1	P41567
Control	1	P41743
Control	1	P41970
Control	1	P42025
Control	1	P42126
Control	1	P42166
Control	1	P42167
Control	1	P42224
Control	1	P42226
Control	1	P42229
Control	1	P42285
Control	1	P42330
Control	1	P42336
Control	1	P42338
Control	1	P42345
Control	1	P42356
Control	1	P42566
Control	1	P42574

Control	1	P42575
Control	1	P42677
Control	1	P42684
Control	1	P42694
Control	1	P42695
Control	1	P42696
Control	1	P42704
Control	1	P42765
Control	1	P42766
Control	1	P42785
Control	1	P42858
Control	1	P42892
Control	1	P42898
Control	1	P43007
Control	1	P43034
Control	1	P43121
Control	1	P43155
Control	1	P43243
Control	1	P43246
Control	1	P43250
Control	1	P43304
Control	1	P43307
Control	1	P43378
Control	1	P43487
Control	1	P43490
Control	1	P43686
Control	1	P43897
Control	1	P45877
Control	1	P45880
Control	1	P45954
Control	1	P45973
Control	1	P45974
Control	1	P45983
Control	1	P45985
Control	1	P46013
Control	1	P46019
Control	1	P46020
Control	1	P46060
Control	1	P46063
Control	1	P46087
Control	1	P46100
Control	1	P46108
Control	1	P46109
Control	1	P46199
Control	1	P46379
Control	1	P46459
Control	1	P46531
Control	1	P46734
Control	1	P46736
Control	1	P46776

Control	1	P46777
Control	1	P46778
Control	1	P46779
Control	1	P46781
Control	1	P46782
Control	1	P46783
Control	1	P46821
Control	1	P46926
Control	1	P46934
Control	1	P46939
Control	1	P46940
Control	1	P46976
Control	1	P46977
Control	1	P47712
Control	1	P47755
Control	1	P47756
Control	1	P47813
Control	1	P47895
Control	1	P47897
Control	1	P47914
Control	1	P47974
Control	1	P47985
Control	1	P48047
Control	1	P48059
Control	1	P48060
Control	1	P48147
Control	1	P48163
Control	1	P48200
Control	1	P48307
Control	1	P48382
Control	1	P48426
Control	1	P48444
Control	1	P48449
Control	1	P48454
Control	1	P48506
Control	1	P48507
Control	1	P48509
Control	1	P48553
Control	1	P48556
Control	1	P48634
Control	1	P48637
Control	1	P48643
Control	1	P48651
Control	1	P48681
Control	1	P48723
Control	1	P48729
Control	1	P48730
Control	1	P48735
Control	1	P48736
Control	1	P48739

Control	1	P49005
Control	1	P49006
Control	1	P49023
Control	1	P49069
Control	1	P49116
Control	1	P49137
Control	1	P49184
Control	1	P49189
Control	1	P49207
Control	1	P49247
Control	1	P49257
Control	1	P49281
Control	1	P49321
Control	1	P49327
Control	1	P49354
Control	1	P49356
Control	1	P49366
Control	1	P49368
Control	1	P49406
Control	1	P49407
Control	1	P49411
Control	1	P49419
Control	1	P49427
Control	1	P49441
Control	1	P49454
Control	1	P49458
Control	1	P49585
Control	1	P49588
Control	1	P49589
Control	1	P49590
Control	1	P49591
Control	1	P49593
Control	1	P49641
Control	1	P49642
Control	1	P49643
Control	1	P49662
Control	1	P49674
Control	1	P49720
Control	1	P49721
Control	1	P49736
Control	1	P49748
Control	1	P49750
Control	1	P49754
Control	1	P49755
Control	1	P49756
Control	1	P49757
Control	1	P49761
Control	1	P49768
Control	1	P49770
Control	1	P49773

Control	1	P49790
Control	1	P49792
Control	1	P49795
Control	1	P49796
Control	1	P49810
Control	1	P49815
Control	1	P49821
Control	1	P49840
Control	1	P49841
Control	1	P49848
Control	1	P49902
Control	1	P49903
Control	1	P49914
Control	1	P49915
Control	1	P49916
Control	1	P49917
Control	1	P49959
Control	1	P50148
Control	1	P50213
Control	1	P50225
Control	1	P50281
Control	1	P50336
Control	1	P50395
Control	1	P50402
Control	1	P50416
Control	1	P50443
Control	1	P50452
Control	1	P50453
Control	1	P50454
Control	1	P50479
Control	1	P50502
Control	1	P50542
Control	1	P50548
Control	1	P50552
Control	1	P50570
Control	1	P50579
Control	1	P50583
Control	1	P50613
Control	1	P50748
Control	1	P50749
Control	1	P50750
Control	1	P50851
Control	1	P50895
Control	1	P50897
Control	1	P50914
Control	1	P50990
Control	1	P50991
Control	1	P50995
Control	1	P51003
Control	1	P51114

Control	1	P51116
Control	1	P51148
Control	1	P51149
Control	1	P51151
Control	1	P51153
Control	1	P51159
Control	1	P51178
Control	1	P51398
Control	1	P51452
Control	1	P51531
Control	1	P51532
Control	1	P51553
Control	1	P51570
Control	1	P51571
Control	1	P51572
Control	1	P51580
Control	1	P51608
Control	1	P51610
Control	1	P51617
Control	1	P51636
Control	1	P51648
Control	1	P51659
Control	1	P51665
Control	1	P51687
Control	1	P51688
Control	1	P51692
Control	1	P51784
Control	1	P51798
Control	1	P51809
Control	1	P51812
Control	1	P51813
Control	1	P51858
Control	1	P51946
Control	1	P51948
Control	1	P51956
Control	1	P51965
Control	1	P51970
Control	1	P51991
Control	1	P52209
Control	1	P52272
Control	1	P52292
Control	1	P52294
Control	1	P52298
Control	1	P52306
Control	1	P52434
Control	1	P52564
Control	1	P52565
Control	1	P52566
Control	1	P52594
Control	1	P52597

Control	1	P52630
Control	1	P52701
Control	1	P52732
Control	1	P52735
Control	1	P52747
Control	1	P52756
Control	1	P52758
Control	1	P52788
Control	1	P52789
Control	1	P52815
Control	1	P52824
Control	1	P52848
Control	1	P52888
Control	1	P52907
Control	1	P52926
Control	1	P52943
Control	1	P52948
Control	1	P53004
Control	1	P53007
Control	1	P53041
Control	1	P53350
Control	1	P53355
Control	1	P53365
Control	1	P53367
Control	1	P53384
Control	1	P53396
Control	1	P53582
Control	1	P53597
Control	1	P53602
Control	1	P53609
Control	1	P53611
Control	1	P53618
Control	1	P53621
Control	1	P53634
Control	1	P53667
Control	1	P53675
Control	1	P53677
Control	1	P53680
Control	1	P53701
Control	1	P53778
Control	1	P53801
Control	1	P53814
Control	1	P53985
Control	1	P53990
Control	1	P53992
Control	1	P53999
Control	1	P54098
Control	1	P54105
Control	1	P54136
Control	1	P54198

Control	1	P54252
Control	1	P54259
Control	1	P54277
Control	1	P54278
Control	1	P54289
Control	1	P54577
Control	1	P54578
Control	1	P54619
Control	1	P54652
Control	1	P54687
Control	1	P54709
Control	1	P54727
Control	1	P54760
Control	1	P54802
Control	1	P54819
Control	1	P54886
Control	1	P54920
Control	1	P55010
Control	1	P55011
Control	1	P55036
Control	1	P55039
Control	1	P55060
Control	1	P55072
Control	1	P55081
Control	1	P55084
Control	1	P55145
Control	1	P55196
Control	1	P55199
Control	1	P55209
Control	1	P55210
Control	1	P55212
Control	1	P55263
Control	1	P55265
Control	1	P55268
Control	1	P55290
Control	1	P55735
Control	1	P55769
Control	1	P55786
Control	1	P55789
Control	1	P55795
Control	1	P55809
Control	1	P55884
Control	1	P55899
Control	1	P55957
Control	1	P56134
Control	1	P56182
Control	1	P56192
Control	1	P56211
Control	1	P56377
Control	1	P56378

Control	1	P56381
Control	1	P56385
Control	1	P56524
Control	1	P56537
Control	1	P56545
Control	1	P56556
Control	1	P56589
Control	1	P56937
Control	1	P56945
Control	1	P56962
Control	1	P57076
Control	1	P57081
Control	1	P57088
Control	1	P57678
Control	1	P57737
Control	1	P57740
Control	1	P57764
Control	1	P57772
Control	1	P58004
Control	1	P58335
Control	1	P58546
Control	1	P59998
Control	1	P60033
Control	1	P60059
Control	1	P60174
Control	1	P60228
Control	1	P60468
Control	1	P60484
Control	1	P60510
Control	1	P60520
Control	1	P60602
Control	1	P60660
Control	1	P60709
Control	1	P60842
Control	1	P60866
Control	1	P60891
Control	1	P60900
Control	1	P60903
Control	1	P60953
Control	1	P60981
Control	1	P60983
Control	1	P61006
Control	1	P61009
Control	1	P61011
Control	1	P61018
Control	1	P61019
Control	1	P61020
Control	1	P61026
Control	1	P61077
Control	1	P61081

Control	1	P61086
Control	1	P61088
Control	1	P61106
Control	1	P61158
Control	1	P61160
Control	1	P61163
Control	1	P61201
Control	1	P61221
Control	1	P61224
Control	1	P61225
Control	1	P61244
Control	1	P61247
Control	1	P61254
Control	1	P61289
Control	1	P61313
Control	1	P61353
Control	1	P61421
Control	1	P61513
Control	1	P61586
Control	1	P61587
Control	1	P61599
Control	1	P61604
Control	1	P61619
Control	1	P61758
Control	1	P61764
Control	1	P61769
Control	1	P61803
Control	1	P61916
Control	1	P61923
Control	1	P61956
Control	1	P61962
Control	1	P61964
Control	1	P61966
Control	1	P61970
Control	1	P61978
Control	1	P61981
Control	1	P62068
Control	1	P62070
Control	1	P62081
Control	1	P62136
Control	1	P62140
Control	1	P62191
Control	1	P62195
Control	1	P62241
Control	1	P62244
Control	1	P62249
Control	1	P62253
Control	1	P62256
Control	1	P62258
Control	1	P62263

Control	1	P62266
Control	1	P62269
Control	1	P62273
Control	1	P62277
Control	1	P62280
Control	1	P62304
Control	1	P62306
Control	1	P62310
Control	1	P62312
Control	1	P62314
Control	1	P62316
Control	1	P62318
Control	1	P62328
Control	1	P62330
Control	1	P62333
Control	1	P62341
Control	1	P62380
Control	1	P62424
Control	1	P62487
Control	1	P62491
Control	1	P62495
Control	1	P62633
Control	1	P62699
Control	1	P62701
Control	1	P62714
Control	1	P62736
Control	1	P62745
Control	1	P62750
Control	1	P62753
Control	1	P62805
Control	1	P62820
Control	1	P62826
Control	1	P62829
Control	1	P62834
Control	1	P62841
Control	1	P62847
Control	1	P62851
Control	1	P62854
Control	1	P62857
Control	1	P62861
Control	1	P62873
Control	1	P62877
Control	1	P62879
Control	1	P62888
Control	1	P62891
Control	1	P62899
Control	1	P62906
Control	1	P62910
Control	1	P62913
Control	1	P62917

Control	1	P62937
Control	1	P62942
Control	1	P62979
Control	1	P62993
Control	1	P62995
Control	1	P63000
Control	1	P63010
Control	1	P63027
Control	1	P63092
Control	1	P63096
Control	1	P63104
Control	1	P63151
Control	1	P63165
Control	1	P63167
Control	1	P63172
Control	1	P63173
Control	1	P63208
Control	1	P63220
Control	1	P63241
Control	1	P63244
Control	1	P63272
Control	1	P63279
Control	1	P63313
Control	1	P67775
Control	1	P67809
Control	1	P67812
Control	1	P67870
Control	1	P67936
Control	1	P68036
Control	1	P68104
Control	1	P68363
Control	1	P68366
Control	1	P68371
Control	1	P68400
Control	1	P68402
Control	1	P68431
Control	1	P68871
Control	1	P69905
Control	1	P78310
Control	1	P78312
Control	1	P78316
Control	1	P78318
Control	1	P78324
Control	1	P78330
Control	1	P78332
Control	1	P78344
Control	1	P78345
Control	1	P78346
Control	1	P78347
Control	1	P78356

Control	1	P78357
Control	1	P78362
Control	1	P78371
Control	1	P78381
Control	1	P78406
Control	1	P78417
Control	1	P78527
Control	1	P78536
Control	1	P78537
Control	1	P78539
Control	1	P78540
Control	1	P78549
Control	1	P80217
Control	1	P80303
Control	1	P80723
Control	1	P81605
Control	1	P82094
Control	1	P82650
Control	1	P82663
Control	1	P82664
Control	1	P82673
Control	1	P82675
Control	1	P82909
Control	1	P82912
Control	1	P82914
Control	1	P82921
Control	1	P82930
Control	1	P82932
Control	1	P82933
Control	1	P82979
Control	1	P83111
Control	1	P83436
Control	1	P83731
Control	1	P83876
Control	1	P83881
Control	1	P83916
Control	1	P84022
Control	1	P84077
Control	1	P84085
Control	1	P84090
Control	1	P84095
Control	1	P84098
Control	1	P84101
Control	1	P84103
Control	1	P85037
Control	1	P86790
Control	1	P98082
Control	1	P98160
Control	1	P98170
Control	1	P98172

Control	1	P98175
Control	1	P98179
Control	1	P98194
Control	1	P98196
Control	1	P99999
Control	1	Q00013
Control	1	Q00059
Control	1	Q00169
Control	1	Q00325
Control	1	Q00341
Control	1	Q00403
Control	1	Q00534
Control	1	Q00535
Control	1	Q00537
Control	1	Q00577
Control	1	Q00587
Control	1	Q00610
Control	1	Q00613
Control	1	Q00653
Control	1	Q00688
Control	1	Q00765
Control	1	Q00796
Control	1	Q00839
Control	1	Q01081
Control	1	Q01082
Control	1	Q01085
Control	1	Q01105
Control	1	Q01130
Control	1	Q01167
Control	1	Q01201
Control	1	Q01415
Control	1	Q01433
Control	1	Q01469
Control	1	Q01518
Control	1	Q01543
Control	1	Q01581
Control	1	Q01628
Control	1	Q01650
Control	1	Q01658
Control	1	Q01780
Control	1	Q01804
Control	1	Q01813
Control	1	Q01831
Control	1	Q01844
Control	1	Q01850
Control	1	Q01968
Control	1	Q01970
Control	1	Q01995
Control	1	Q02040
Control	1	Q02127

Control	1	Q02218
Control	1	Q02224
Control	1	Q02241
Control	1	Q02252
Control	1	Q02297
Control	1	Q02318
Control	1	Q02413
Control	1	Q02447
Control	1	Q02539
Control	1	Q02543
Control	1	Q02750
Control	1	Q02763
Control	1	Q02790
Control	1	Q02809
Control	1	Q02818
Control	1	Q02878
Control	1	Q02880
Control	1	Q02952
Control	1	Q02978
Control	1	Q03001
Control	1	Q03111
Control	1	Q03112
Control	1	Q03113
Control	1	Q03135
Control	1	Q03154
Control	1	Q03169
Control	1	Q03252
Control	1	Q03393
Control	1	Q03405
Control	1	Q03426
Control	1	Q03468
Control	1	Q03518
Control	1	Q03519
Control	1	Q03701
Control	1	Q03936
Control	1	Q04206
Control	1	Q04323
Control	1	Q04446
Control	1	Q04637
Control	1	Q04721
Control	1	Q04724
Control	1	Q04726
Control	1	Q04760
Control	1	Q04771
Control	1	Q04837
Control	1	Q04864
Control	1	Q04917
Control	1	Q04941
Control	1	Q05048
Control	1	Q05086

Control	1	Q05193
Control	1	Q05209
Control	1	Q05397
Control	1	Q05519
Control	1	Q05655
Control	1	Q05682
Control	1	Q05932
Control	1	Q05D32
Control	1	Q06124
Control	1	Q06136
Control	1	Q06203
Control	1	Q06210
Control	1	Q06265
Control	1	Q06323
Control	1	Q06330
Control	1	Q06413
Control	1	Q06481
Control	1	Q06546
Control	1	Q06587
Control	1	Q06787
Control	1	Q06830
Control	1	Q07020
Control	1	Q07021
Control	1	Q07065
Control	1	Q07157
Control	1	Q07352
Control	1	Q07617
Control	1	Q07666
Control	1	Q07812
Control	1	Q07817
Control	1	Q07820
Control	1	Q07864
Control	1	Q07866
Control	1	Q07889
Control	1	Q07955
Control	1	Q07960
Control	1	Q08170
Control	1	Q08174
Control	1	Q08209
Control	1	Q08211
Control	1	Q08257
Control	1	Q08378
Control	1	Q08379
Control	1	Q08426
Control	1	Q08431
Control	1	Q08499
Control	1	Q08554
Control	1	Q08623
Control	1	Q08629
Control	1	Q08722

Control	1	Q08752
Control	1	Q08945
Control	1	Q08AD1
Control	1	Q08AE8
Control	1	Q08AF3
Control	1	Q08AM6
Control	1	Q08J23
Control	1	Q09019
Control	1	Q09028
Control	1	Q09161
Control	1	Q09328
Control	1	Q09472
Control	1	Q09666
Control	1	Q0JRZ9
Control	1	Q0PNE2
Control	1	Q0VDF9
Control	1	Q0VDG4
Control	1	Q0VF96
Control	1	Q0VGL1
Control	1	Q0ZGT2
Control	1	Q10469
Control	1	Q10471
Control	1	Q10472
Control	1	Q10567
Control	1	Q10570
Control	1	Q10589
Control	1	Q10713
Control	1	Q11201
Control	1	Q12765
Control	1	Q12768
Control	1	Q12769
Control	1	Q12770
Control	1	Q12778
Control	1	Q12788
Control	1	Q12789
Control	1	Q12792
Control	1	Q12797
Control	1	Q12800
Control	1	Q12802
Control	1	Q12805
Control	1	Q12824
Control	1	Q12830
Control	1	Q12834
Control	1	Q12841
Control	1	Q12846
Control	1	Q12849
Control	1	Q12851
Control	1	Q12866
Control	1	Q12872
Control	1	Q12873

Control	1	Q12874
Control	1	Q12888
Control	1	Q12893
Control	1	Q12894
Control	1	Q12899
Control	1	Q12904
Control	1	Q12905
Control	1	Q12906
Control	1	Q12907
Control	1	Q12931
Control	1	Q12933
Control	1	Q12959
Control	1	Q12965
Control	1	Q12972
Control	1	Q12974
Control	1	Q12979
Control	1	Q12980
Control	1	Q12981
Control	1	Q12982
Control	1	Q12986
Control	1	Q12996
Control	1	Q12999
Control	1	Q13011
Control	1	Q13017
Control	1	Q13033
Control	1	Q13042
Control	1	Q13043
Control	1	Q13045
Control	1	Q13049
Control	1	Q13057
Control	1	Q13084
Control	1	Q13085
Control	1	Q13098
Control	1	Q13107
Control	1	Q13111
Control	1	Q13112
Control	1	Q13123
Control	1	Q13126
Control	1	Q13131
Control	1	Q13136
Control	1	Q13137
Control	1	Q13144
Control	1	Q13148
Control	1	Q13151
Control	1	Q13155
Control	1	Q13158
Control	1	Q13162
Control	1	Q13164
Control	1	Q13177
Control	1	Q13185

Control	1	Q13188
Control	1	Q13190
Control	1	Q13200
Control	1	Q13201
Control	1	Q13206
Control	1	Q13217
Control	1	Q13228
Control	1	Q13232
Control	1	Q13242
Control	1	Q13243
Control	1	Q13247
Control	1	Q13257
Control	1	Q13263
Control	1	Q13275
Control	1	Q13277
Control	1	Q13283
Control	1	Q13287
Control	1	Q13308
Control	1	Q13310
Control	1	Q13315
Control	1	Q13322
Control	1	Q13325
Control	1	Q13330
Control	1	Q13347
Control	1	Q13356
Control	1	Q13362
Control	1	Q13363
Control	1	Q13371
Control	1	Q13393
Control	1	Q13395
Control	1	Q13404
Control	1	Q13405
Control	1	Q13409
Control	1	Q13416
Control	1	Q13418
Control	1	Q13423
Control	1	Q13425
Control	1	Q13426
Control	1	Q13427
Control	1	Q13428
Control	1	Q13432
Control	1	Q13435
Control	1	Q13438
Control	1	Q13439
Control	1	Q13442
Control	1	Q13443
Control	1	Q13444
Control	1	Q13445
Control	1	Q13451
Control	1	Q13459

Control	1	Q13464
Control	1	Q13469
Control	1	Q13472
Control	1	Q13485
Control	1	Q13488
Control	1	Q13490
Control	1	Q13492
Control	1	Q13496
Control	1	Q13501
Control	1	Q13505
Control	1	Q13509
Control	1	Q13510
Control	1	Q13523
Control	1	Q13526
Control	1	Q13535
Control	1	Q13541
Control	1	Q13546
Control	1	Q13547
Control	1	Q13555
Control	1	Q13557
Control	1	Q13561
Control	1	Q13563
Control	1	Q13564
Control	1	Q13572
Control	1	Q13573
Control	1	Q13574
Control	1	Q13586
Control	1	Q13588
Control	1	Q13595
Control	1	Q13596
Control	1	Q13601
Control	1	Q13610
Control	1	Q13613
Control	1	Q13614
Control	1	Q13616
Control	1	Q13617
Control	1	Q13618
Control	1	Q13619
Control	1	Q13620
Control	1	Q13625
Control	1	Q13627
Control	1	Q13630
Control	1	Q13636
Control	1	Q13637
Control	1	Q13641
Control	1	Q13642
Control	1	Q13643
Control	1	Q13671
Control	1	Q13685
Control	1	Q13724

Control	1	Q13740
Control	1	Q13769
Control	1	Q13796
Control	1	Q13813
Control	1	Q13823
Control	1	Q13825
Control	1	Q13835
Control	1	Q13838
Control	1	Q13867
Control	1	Q13868
Control	1	Q13873
Control	1	Q13885
Control	1	Q13888
Control	1	Q13889
Control	1	Q13895
Control	1	Q13905
Control	1	Q13907
Control	1	Q13951
Control	1	Q13952
Control	1	Q14004
Control	1	Q14008
Control	1	Q14011
Control	1	Q14012
Control	1	Q14019
Control	1	Q14103
Control	1	Q14108
Control	1	Q14112
Control	1	Q14114
Control	1	Q14118
Control	1	Q14119
Control	1	Q14137
Control	1	Q14139
Control	1	Q14141
Control	1	Q14145
Control	1	Q14146
Control	1	Q14147
Control	1	Q14149
Control	1	Q14151
Control	1	Q14152
Control	1	Q14155
Control	1	Q14156
Control	1	Q14157
Control	1	Q14160
Control	1	Q14161
Control	1	Q14162
Control	1	Q14165
Control	1	Q14166
Control	1	Q14181
Control	1	Q14185
Control	1	Q14186

Control	1	Q14192
Control	1	Q14195
Control	1	Q14197
Control	1	Q14202
Control	1	Q14203
Control	1	Q14204
Control	1	Q14232
Control	1	Q14240
Control	1	Q14241
Control	1	Q14247
Control	1	Q14249
Control	1	Q14254
Control	1	Q14257
Control	1	Q14258
Control	1	Q14315
Control	1	Q14318
Control	1	Q14320
Control	1	Q14331
Control	1	Q14344
Control	1	Q14353
Control	1	Q14376
Control	1	Q14392
Control	1	Q14444
Control	1	Q14457
Control	1	Q14498
Control	1	Q14511
Control	1	Q14527
Control	1	Q14534
Control	1	Q14554
Control	1	Q14558
Control	1	Q14562
Control	1	Q14566
Control	1	Q14571
Control	1	Q14573
Control	1	Q14643
Control	1	Q14644
Control	1	Q14651
Control	1	Q14653
Control	1	Q14657
Control	1	Q14667
Control	1	Q14669
Control	1	Q14671
Control	1	Q14676
Control	1	Q14677
Control	1	Q14678
Control	1	Q14683
Control	1	Q14684
Control	1	Q14689
Control	1	Q14690
Control	1	Q14691

Control	1	Q14692
Control	1	Q14693
Control	1	Q14694
Control	1	Q14696
Control	1	Q14697
Control	1	Q14699
Control	1	Q14728
Control	1	Q14738
Control	1	Q14739
Control	1	Q14746
Control	1	Q14764
Control	1	Q14766
Control	1	Q14767
Control	1	Q14781
Control	1	Q14789
Control	1	Q14790
Control	1	Q147X3
Control	1	Q14807
Control	1	Q14814
Control	1	Q14839
Control	1	Q14847
Control	1	Q14914
Control	1	Q14919
Control	1	Q14934
Control	1	Q14966
Control	1	Q14974
Control	1	Q14978
Control	1	Q14980
Control	1	Q14997
Control	1	Q14999
Control	1	Q14BN4
Control	1	Q14C86
Control	1	Q14CX7
Control	1	Q14D04
Control	1	Q15003
Control	1	Q15004
Control	1	Q15005
Control	1	Q15006
Control	1	Q15007
Control	1	Q15008
Control	1	Q15013
Control	1	Q15014
Control	1	Q15018
Control	1	Q15019
Control	1	Q15020
Control	1	Q15021
Control	1	Q15022
Control	1	Q15024
Control	1	Q15025
Control	1	Q15029

Control	1	Q15031
Control	1	Q15032
Control	1	Q15035
Control	1	Q15036
Control	1	Q15041
Control	1	Q15042
Control	1	Q15043
Control	1	Q15046
Control	1	Q15047
Control	1	Q15048
Control	1	Q15050
Control	1	Q15054
Control	1	Q15056
Control	1	Q15057
Control	1	Q15058
Control	1	Q15059
Control	1	Q15061
Control	1	Q15067
Control	1	Q15070
Control	1	Q15075
Control	1	Q15084
Control	1	Q15102
Control	1	Q15120
Control	1	Q15121
Control	1	Q15125
Control	1	Q15126
Control	1	Q15139
Control	1	Q15149
Control	1	Q15154
Control	1	Q15155
Control	1	Q15165
Control	1	Q15172
Control	1	Q15181
Control	1	Q15185
Control	1	Q15208
Control	1	Q15233
Control	1	Q15257
Control	1	Q15262
Control	1	Q15269
Control	1	Q15276
Control	1	Q15286
Control	1	Q15287
Control	1	Q15291
Control	1	Q15293
Control	1	Q15311
Control	1	Q15334
Control	1	Q15345
Control	1	Q15361
Control	1	Q15363
Control	1	Q15365

Control	1	Q15366
Control	1	Q15369
Control	1	Q15370
Control	1	Q15382
Control	1	Q15386
Control	1	Q15388
Control	1	Q15390
Control	1	Q15392
Control	1	Q15393
Control	1	Q15397
Control	1	Q15398
Control	1	Q15404
Control	1	Q15417
Control	1	Q15418
Control	1	Q15424
Control	1	Q15428
Control	1	Q15434
Control	1	Q15435
Control	1	Q15436
Control	1	Q15437
Control	1	Q15438
Control	1	Q15459
Control	1	Q15477
Control	1	Q15517
Control	1	Q15526
Control	1	Q15542
Control	1	Q15545
Control	1	Q15554
Control	1	Q15555
Control	1	Q15599
Control	1	Q15628
Control	1	Q15629
Control	1	Q15631
Control	1	Q15633
Control	1	Q15637
Control	1	Q15642
Control	1	Q15643
Control	1	Q15645
Control	1	Q15648
Control	1	Q15650
Control	1	Q15651
Control	1	Q15652
Control	1	Q15653
Control	1	Q15654
Control	1	Q15678
Control	1	Q15691
Control	1	Q15717
Control	1	Q15738
Control	1	Q15742
Control	1	Q15746

Control	1	Q15750
Control	1	Q15751
Control	1	Q15758
Control	1	Q15773
Control	1	Q15785
Control	1	Q15796
Control	1	Q15797
Control	1	Q15800
Control	1	Q15811
Control	1	Q15813
Control	1	Q15814
Control	1	Q15819
Control	1	Q15831
Control	1	Q15833
Control	1	Q15836
Control	1	Q15843
Control	1	Q15904
Control	1	Q15906
Control	1	Q15907
Control	1	Q15942
Control	1	Q16134
Control	1	Q16181
Control	1	Q16186
Control	1	Q16204
Control	1	Q16222
Control	1	Q16254
Control	1	Q16270
Control	1	Q16342
Control	1	Q16363
Control	1	Q16401
Control	1	Q16512
Control	1	Q16513
Control	1	Q16527
Control	1	Q16531
Control	1	Q16537
Control	1	Q16539
Control	1	Q16540
Control	1	Q16543
Control	1	Q16555
Control	1	Q16563
Control	1	Q16576
Control	1	Q16584
Control	1	Q16594
Control	1	Q16602
Control	1	Q16611
Control	1	Q16629
Control	1	Q16630
Control	1	Q16637
Control	1	Q16643
Control	1	Q16656

Control	1	Q16658
Control	1	Q16666
Control	1	Q16698
Control	1	Q16706
Control	1	Q16718
Control	1	Q16739
Control	1	Q16740
Control	1	Q16762
Control	1	Q16763
Control	1	Q16773
Control	1	Q16774
Control	1	Q16775
Control	1	Q16795
Control	1	Q16798
Control	1	Q16822
Control	1	Q16831
Control	1	Q16832
Control	1	Q16836
Control	1	Q16850
Control	1	Q16851
Control	1	Q16875
Control	1	Q16881
Control	1	Q16891
Control	1	Q19T08
Control	1	Q1ED39
Control	1	Q1KMD3
Control	1	Q24JP5
Control	1	Q27J81
Control	1	Q29RF7
Control	1	Q2KHT3
Control	1	Q2M1P5
Control	1	Q2M1Z3
Control	1	Q2M296
Control	1	Q2M2I8
Control	1	Q2M389
Control	1	Q2NKX8
Control	1	Q2NL82
Control	1	Q2PPJ7
Control	1	Q2PZI1
Control	1	Q2TAA2
Control	1	Q2TAA5
Control	1	Q2TAL8
Control	1	Q2TAY7
Control	1	Q2VPK5
Control	1	Q32M88
Control	1	Q32MZ4
Control	1	Q32P28
Control	1	Q32P41
Control	1	Q32P44
Control	1	Q3B726

Control	1	Q3KQU3
Control	1	Q3KQV9
Control	1	Q3L8U1
Control	1	Q3LXA3
Control	1	Q3MHD2
Control	1	Q3SXM5
Control	1	Q3SY69
Control	1	Q3T906
Control	1	Q3V6T2
Control	1	Q3YEC7
Control	1	Q3ZCQ8
Control	1	Q460N5
Control	1	Q495W5
Control	1	Q49A26
Control	1	Q49AR2
Control	1	Q4AC94
Control	1	Q4G0F5
Control	1	Q4G0J3
Control	1	Q4G0N4
Control	1	Q4G0X4
Control	1	Q4G148
Control	1	Q4G176
Control	1	Q4J6C6
Control	1	Q4KMP7
Control	1	Q4KMQ1
Control	1	Q4KMQ2
Control	1	Q4L180
Control	1	Q4V328
Control	1	Q4V339
Control	1	Q4VC31
Control	1	Q4ZIN3
Control	1	Q52LJ0
Control	1	Q52LW3
Control	1	Q53EL6
Control	1	Q53EP0
Control	1	Q53ET0
Control	1	Q53EU6
Control	1	Q53EZ4
Control	1	Q53F19
Control	1	Q53FA7
Control	1	Q53FP2
Control	1	Q53GA4
Control	1	Q53GG5
Control	1	Q53GL7
Control	1	Q53GQ0
Control	1	Q53GS7
Control	1	Q53GS9
Control	1	Q53GT1
Control	1	Q53H12
Control	1	Q53H82

Control	1	Q53H96
Control	1	Q53HC9
Control	1	Q53HL2
Control	1	Q53SF7
Control	1	Q53T59
Control	1	Q53TN4
Control	1	Q562E7
Control	1	Q562R1
Control	1	Q567U6
Control	1	Q56VL3
Control	1	Q58EX7
Control	1	Q58FG1
Control	1	Q58WW2
Control	1	Q5BJD5
Control	1	Q5BJF2
Control	1	Q5BJH7
Control	1	Q5BKZ1
Control	1	Q5C9Z4
Control	1	Q5EBL4
Control	1	Q5F1R6
Control	1	Q5GJ75
Control	1	Q5GLZ8
Control	1	Q5H8A4
Control	1	Q5H9R7
Control	1	Q5HYI8
Control	1	Q5HYJ3
Control	1	Q5HYK3
Control	1	Q5HYK7
Control	1	Q5HYW2
Control	1	Q5J8M3
Control	1	Q5JPH6
Control	1	Q5JPI3
Control	1	Q5JPI9
Control	1	Q5JRA6
Control	1	Q5JRX3
Control	1	Q5JSH3
Control	1	Q5JSZ5
Control	1	Q5JTD0
Control	1	Q5JTH9
Control	1	Q5JTJ3
Control	1	Q5JTV8
Control	1	Q5JTW2
Control	1	Q5JTZ9
Control	1	Q5JU69
Control	1	Q5JVF3
Control	1	Q5JVS0
Control	1	Q5K4L6
Control	1	Q5K651
Control	1	Q5MIZ7
Control	1	Q5MNZ6

Control	1	Q5MNZ9
Control	1	Q5NDL2
Control	1	Q5PRF9
Control	1	Q5QJ74
Control	1	Q5QJE6
Control	1	Q5R372
Control	1	Q5R3I4
Control	1	Q5RI15
Control	1	Q5RKV6
Control	1	Q5SNT2
Control	1	Q5SQI0
Control	1	Q5SQN1
Control	1	Q5SRE5
Control	1	Q5SSJ5
Control	1	Q5ST30
Control	1	Q5SW79
Control	1	Q5SW96
Control	1	Q5SWX8
Control	1	Q5SY16
Control	1	Q5SYE7
Control	1	Q5T013
Control	1	Q5T0D9
Control	1	Q5T0F9
Control	1	Q5T0N5
Control	1	Q5T160
Control	1	Q5T1C6
Control	1	Q5T1J5
Control	1	Q5T1M5
Control	1	Q5T200
Control	1	Q5T280
Control	1	Q5T2E6
Control	1	Q5T3F8
Control	1	Q5T3I0
Control	1	Q5T440
Control	1	Q5T447
Control	1	Q5T4B2
Control	1	Q5T4S7
Control	1	Q5T5C0
Control	1	Q5T5U3
Control	1	Q5T5Y3
Control	1	Q5T653
Control	1	Q5T6F2
Control	1	Q5T6V5
Control	1	Q5T749
Control	1	Q5T7W0
Control	1	Q5T8D3
Control	1	Q5T8P6
Control	1	Q5T9A4
Control	1	Q5T9L3
Control	1	Q5TA45

Control	1	Q5TA50
Control	1	Q5TAQ9
Control	1	Q5TAT6
Control	1	Q5TAX3
Control	1	Q5TBA9
Control	1	Q5TBB1
Control	1	Q5TC12
Control	1	Q5TC82
Control	1	Q5TDH0
Control	1	Q5TEJ8
Control	1	Q5TEU4
Control	1	Q5TFE4
Control	1	Q5TH69
Control	1	Q5THJ4
Control	1	Q5TZA2
Control	1	Q5U5X0
Control	1	Q5U651
Control	1	Q5UIP0
Control	1	Q5VIR6
Control	1	Q5VSL9
Control	1	Q5VT25
Control	1	Q5VT52
Control	1	Q5VTB9
Control	1	Q5VTL8
Control	1	Q5VTR2
Control	1	Q5VU43
Control	1	Q5VUA4
Control	1	Q5VUB5
Control	1	Q5VUD6
Control	1	Q5VUJ6
Control	1	Q5VV42
Control	1	Q5VW32
Control	1	Q5VW36
Control	1	Q5VWQ0
Control	1	Q5VWQ8
Control	1	Q5VWZ2
Control	1	Q5VY43
Control	1	Q5VYK3
Control	1	Q5VYS8
Control	1	Q5VZ18
Control	1	Q5VZ89
Control	1	Q5VZE5
Control	1	Q5VZL5
Control	1	Q5W0V3
Control	1	Q5W0Z9
Control	1	Q5W111
Control	1	Q5XUX1
Control	1	Q5ZPR3
Control	1	Q63HN8
Control	1	Q63HR2

Control	1	Q63ZY3
Control	1	Q641Q2
Control	1	Q643R3
Control	1	Q658P3
Control	1	Q658Y4
Control	1	Q66K14
Control	1	Q66K74
Control	1	Q66LE6
Control	1	Q66PJ3
Control	1	Q676U5
Control	1	Q68CP4
Control	1	Q68CP9
Control	1	Q68CQ4
Control	1	Q68CQ7
Control	1	Q68CR1
Control	1	Q68CZ2
Control	1	Q68CZ6
Control	1	Q68D10
Control	1	Q68D91
Control	1	Q68DQ2
Control	1	Q68E01
Control	1	Q68EM7
Control	1	Q69YN2
Control	1	Q69YN4
Control	1	Q69YQ0
Control	1	Q6AI08
Control	1	Q6AI12
Control	1	Q6AWC2
Control	1	Q6DD88
Control	1	Q6DKI1
Control	1	Q6DKJ4
Control	1	Q6DKK2
Control	1	Q6DN90
Control	1	Q6FI81
Control	1	Q6FIF0
Control	1	Q6GMV2
Control	1	Q6GQQ9
Control	1	Q6I9Y2
Control	1	Q6IA69
Control	1	Q6IA86
Control	1	Q6IAA8
Control	1	Q6IAN0
Control	1	Q6IBS0
Control	1	Q6IBW4
Control	1	Q6ICL3
Control	1	Q6IN85
Control	1	Q6IQ22
Control	1	Q6IQ26
Control	1	Q6KB66
Control	1	Q6KC79

Control	1	Q6KCM7
Control	1	Q6L8Q7
Control	1	Q6N069
Control	1	Q6NSW5
Control	1	Q6NTF9
Control	1	Q6NUK1
Control	1	Q6NUK4
Control	1	Q6NUM9
Control	1	Q6NUQ1
Control	1	Q6NUQ4
Control	1	Q6NVY1
Control	1	Q6NW29
Control	1	Q6NW34
Control	1	Q6NXE6
Control	1	Q6NXR4
Control	1	Q6NXT1
Control	1	Q6NXT4
Control	1	Q6NXT6
Control	1	Q6NY19
Control	1	Q6NYC1
Control	1	Q6NYC8
Control	1	Q6NZI2
Control	1	Q6NZY4
Control	1	Q6P158
Control	1	Q6P179
Control	1	Q6P1A2
Control	1	Q6P1J9
Control	1	Q6P1L8
Control	1	Q6P1M0
Control	1	Q6P1N0
Control	1	Q6P1N9
Control	1	Q6P1Q9
Control	1	Q6P1R3
Control	1	Q6P1X5
Control	1	Q6P1X6
Control	1	Q6P2C8
Control	1	Q6P2E9
Control	1	Q6P2H3
Control	1	Q6P2P2
Control	1	Q6P2Q9
Control	1	Q6P3S6
Control	1	Q6P3W7
Control	1	Q6P3X3
Control	1	Q6P4A7
Control	1	Q6P4E1
Control	1	Q6P4R8
Control	1	Q6P587
Control	1	Q6P5R6
Control	1	Q6P5Z2
Control	1	Q6P6C2

Control	1	Q6P996
Control	1	Q6P9B6
Control	1	Q6P9B9
Control	1	Q6P9H5
Control	1	Q6PCB7
Control	1	Q6PCE3
Control	1	Q6PD62
Control	1	Q6PD74
Control	1	Q6PGN9
Control	1	Q6PGP7
Control	1	Q6PHR2
Control	1	Q6PI48
Control	1	Q6PI78
Control	1	Q6PI98
Control	1	Q6PIJ6
Control	1	Q6PIU2
Control	1	Q6PIW4
Control	1	Q6PJ69
Control	1	Q6PJF5
Control	1	Q6PJG2
Control	1	Q6PJG6
Control	1	Q6PJI9
Control	1	Q6PJT7
Control	1	Q6PK04
Control	1	Q6PK18
Control	1	Q6PKC3
Control	1	Q6PKG0
Control	1	Q6PL18
Control	1	Q6PL24
Control	1	Q6PML9
Control	1	Q6Q0C0
Control	1	Q6R327
Control	1	Q6RFH5
Control	1	Q6RW13
Control	1	Q6STE5
Control	1	Q6UB35
Control	1	Q6ULP2
Control	1	Q6UN15
Control	1	Q6UUV7
Control	1	Q6UUV9
Control	1	Q6UW02
Control	1	Q6UW63
Control	1	Q6UW68
Control	1	Q6UWE0
Control	1	Q6UWH4
Control	1	Q6UWP7
Control	1	Q6UWZ7
Control	1	Q6UX04
Control	1	Q6UXH1
Control	1	Q6UXN9

Control	1	Q6UXV4
Control	1	Q6V0I7
Control	1	Q6V1X1
Control	1	Q6VMQ6
Control	1	Q6VN20
Control	1	Q6VY07
Control	1	Q6WCQ1
Control	1	Q6XE24
Control	1	Q6XQN6
Control	1	Q6XZF7
Control	1	Q6Y1H2
Control	1	Q6Y288
Control	1	Q6Y7W6
Control	1	Q6YHK3
Control	1	Q6YHU6
Control	1	Q6YN16
Control	1	Q6YP21
Control	1	Q6ZMI0
Control	1	Q6ZMP0
Control	1	Q6ZMZ3
Control	1	Q6ZN55
Control	1	Q6ZNB6
Control	1	Q6ZNJ1
Control	1	Q6ZNL6
Control	1	Q6ZRP7
Control	1	Q6ZRS2
Control	1	Q6ZS17
Control	1	Q6ZSR9
Control	1	Q6ZSZ5
Control	1	Q6ZT07
Control	1	Q6ZT12
Control	1	Q6ZT21
Control	1	Q6ZU35
Control	1	Q6ZUT6
Control	1	Q6ZVK8
Control	1	Q6ZW31
Control	1	Q6ZW49
Control	1	Q6ZXV5
Control	1	Q709C8
Control	1	Q70CQ2
Control	1	Q70E73
Control	1	Q70IA6
Control	1	Q70UQ0
Control	1	Q70Z35
Control	1	Q70Z53
Control	1	Q712K3
Control	1	Q71F56
Control	1	Q71RC2
Control	1	Q71SY5
Control	1	Q71UM5

Control	1	Q75N03
Control	1	Q75QN2
Control	1	Q7KYR7
Control	1	Q7KZ85
Control	1	Q7KZF4
Control	1	Q7KZI7
Control	1	Q7KZN9
Control	1	Q7L014
Control	1	Q7L099
Control	1	Q7L0Y3
Control	1	Q7L1Q6
Control	1	Q7L1V2
Control	1	Q7L1W4
Control	1	Q7L266
Control	1	Q7L273
Control	1	Q7L2E3
Control	1	Q7L2H7
Control	1	Q7L2J0
Control	1	Q7L311
Control	1	Q7L3T8
Control	1	Q7L4I2
Control	1	Q7L523
Control	1	Q7L576
Control	1	Q7L592
Control	1	Q7L5D6
Control	1	Q7L5N1
Control	1	Q7L5N7
Control	1	Q7L5Y1
Control	1	Q7L5Y9
Control	1	Q7L775
Control	1	Q7L7V1
Control	1	Q7L7X3
Control	1	Q7L8L6
Control	1	Q7L9L4
Control	1	Q7LBC6
Control	1	Q7LBR1
Control	1	Q7LG56
Control	1	Q7LGA3
Control	1	Q7RTN6
Control	1	Q7RTP6
Control	1	Q7RTS9
Control	1	Q7RTT2
Control	1	Q7RTV0
Control	1	Q7RTV5
Control	1	Q7Z2E3
Control	1	Q7Z2K6
Control	1	Q7Z2K8
Control	1	Q7Z2T5
Control	1	Q7Z2W4
Control	1	Q7Z2W9

Control	1	Q7Z2Z2
Control	1	Q7Z333
Control	1	Q7Z392
Control	1	Q7Z3B4
Control	1	Q7Z3C6
Control	1	Q7Z3D6
Control	1	Q7Z3E2
Control	1	Q7Z3E5
Control	1	Q7Z3J2
Control	1	Q7Z3K3
Control	1	Q7Z3T8
Control	1	Q7Z3U7
Control	1	Q7Z401
Control	1	Q7Z406
Control	1	Q7Z417
Control	1	Q7Z422
Control	1	Q7Z434
Control	1	Q7Z460
Control	1	Q7Z478
Control	1	Q7Z494
Control	1	Q7Z4F1
Control	1	Q7Z4H7
Control	1	Q7Z4H8
Control	1	Q7Z4L5
Control	1	Q7Z4Q2
Control	1	Q7Z4S6
Control	1	Q7Z4V5
Control	1	Q7Z4W1
Control	1	Q7Z569
Control	1	Q7Z589
Control	1	Q7Z5G4
Control	1	Q7Z5H3
Control	1	Q7Z5K2
Control	1	Q7Z5L9
Control	1	Q7Z6E9
Control	1	Q7Z6J0
Control	1	Q7Z6J2
Control	1	Q7Z6J6
Control	1	Q7Z6J9
Control	1	Q7Z6K3
Control	1	Q7Z6K5
Control	1	Q7Z6L1
Control	1	Q7Z6M1
Control	1	Q7Z6M4
Control	1	Q7Z6Z7
Control	1	Q7Z739
Control	1	Q7Z7A3
Control	1	Q7Z7C8
Control	1	Q7Z7E8
Control	1	Q7Z7F7

Control	1	Q7Z7H5
Control	1	Q7Z7H8
Control	1	Q7Z7K0
Control	1	Q7Z7K6
Control	1	Q7Z7L1
Control	1	Q7Z7M0
Control	1	Q7Z7N9
Control	1	Q86SF2
Control	1	Q86SK9
Control	1	Q86SQ0
Control	1	Q86SQ4
Control	1	Q86SQ9
Control	1	Q86SR1
Control	1	Q86SZ2
Control	1	Q86T03
Control	1	Q86T13
Control	1	Q86T24
Control	1	Q86TB9
Control	1	Q86TI2
Control	1	Q86TM6
Control	1	Q86TU7
Control	1	Q86TV6
Control	1	Q86TX2
Control	1	Q86U38
Control	1	Q86U42
Control	1	Q86U44
Control	1	Q86U86
Control	1	Q86U90
Control	1	Q86UA1
Control	1	Q86UE4
Control	1	Q86UK7
Control	1	Q86UL3
Control	1	Q86UP2
Control	1	Q86US8
Control	1	Q86UT6
Control	1	Q86UU1
Control	1	Q86UV5
Control	1	Q86UW7
Control	1	Q86UX7
Control	1	Q86UY8
Control	1	Q86V21
Control	1	Q86V48
Control	1	Q86V81
Control	1	Q86V85
Control	1	Q86V87
Control	1	Q86VI3
Control	1	Q86VM9
Control	1	Q86VN1
Control	1	Q86VP6
Control	1	Q86VR2

Control	1	Q86VS8
Control	1	Q86VW0
Control	1	Q86VX2
Control	1	Q86VX9
Control	1	Q86W34
Control	1	Q86W42
Control	1	Q86W50
Control	1	Q86W56
Control	1	Q86W92
Control	1	Q86WA8
Control	1	Q86WB0
Control	1	Q86WJ1
Control	1	Q86WN1
Control	1	Q86WR0
Control	1	Q86WR7
Control	1	Q86WV6
Control	1	Q86WX3
Control	1	Q86X02
Control	1	Q86X10
Control	1	Q86X27
Control	1	Q86X55
Control	1	Q86X76
Control	1	Q86X83
Control	1	Q86XA9
Control	1	Q86XI2
Control	1	Q86XI6
Control	1	Q86XK2
Control	1	Q86XL3
Control	1	Q86XN8
Control	1	Q86XP1
Control	1	Q86XP3
Control	1	Q86XZ4
Control	1	Q86Y07
Control	1	Q86Y37
Control	1	Q86Y39
Control	1	Q86Y56
Control	1	Q86Y79
Control	1	Q86Y82
Control	1	Q86YP4
Control	1	Q86YQ8
Control	1	Q86YR5
Control	1	Q86YS6
Control	1	Q86YS7
Control	1	Q86YT6
Control	1	Q86YV5
Control	1	Q86YV9
Control	1	Q8IU81
Control	1	Q8IUD2
Control	1	Q8IUF8
Control	1	Q8IUH4

Control	1	Q8IUH5
Control	1	Q8IUI8
Control	1	Q8IUR0
Control	1	Q8IUR7
Control	1	Q8IUW5
Control	1	Q8IV08
Control	1	Q8IV36
Control	1	Q8IV38
Control	1	Q8IV48
Control	1	Q8IV63
Control	1	Q8IVB5
Control	1	Q8IVD9
Control	1	Q8IVF2
Control	1	Q8IVF7
Control	1	Q8IVH4
Control	1	Q8IVH8
Control	1	Q8IVL0
Control	1	Q8IVL6
Control	1	Q8IVM0
Control	1	Q8IVS2
Control	1	Q8IVT5
Control	1	Q8IW35
Control	1	Q8IW45
Control	1	Q8IWA0
Control	1	Q8IWA4
Control	1	Q8IWA5
Control	1	Q8IWB1
Control	1	Q8IWB7
Control	1	Q8IWC1
Control	1	Q8IWE2
Control	1	Q8IWE4
Control	1	Q8IWF6
Control	1	Q8IWI9
Control	1	Q8IWJ2
Control	1	Q8IWR0
Control	1	Q8IWS0
Control	1	Q8IWT0
Control	1	Q8IWT6
Control	1	Q8IWU5
Control	1	Q8IWV7
Control	1	Q8IWV8
Control	1	Q8IWW6
Control	1	Q8IWX8
Control	1	Q8IWY9
Control	1	Q8IWZ3
Control	1	Q8IWZ8
Control	1	Q8IX01
Control	1	Q8IX04
Control	1	Q8IX12
Control	1	Q8IX18

Control	1	Q8IXB1
Control	1	Q8IXH7
Control	1	Q8IXI1
Control	1	Q8IXI2
Control	1	Q8IXJ6
Control	1	Q8IXK0
Control	1	Q8IXM2
Control	1	Q8IXM3
Control	1	Q8IXM6
Control	1	Q8IXQ4
Control	1	Q8IXQ6
Control	1	Q8IXT5
Control	1	Q8IXU6
Control	1	Q8IXW5
Control	1	Q8IY17
Control	1	Q8IY18
Control	1	Q8IY21
Control	1	Q8IY33
Control	1	Q8IY37
Control	1	Q8IY47
Control	1	Q8IY67
Control	1	Q8IY81
Control	1	Q8IY95
Control	1	Q8IYA6
Control	1	Q8IYB1
Control	1	Q8IYB3
Control	1	Q8IYB5
Control	1	Q8IYB7
Control	1	Q8IYB8
Control	1	Q8IYD1
Control	1	Q8IYI6
Control	1	Q8IYJ2
Control	1	Q8IYM9
Control	1	Q8IYQ7
Control	1	Q8IYS2
Control	1	Q8IYU8
Control	1	Q8IZ07
Control	1	Q8IZ21
Control	1	Q8IZ69
Control	1	Q8IZ73
Control	1	Q8IZ81
Control	1	Q8IZ83
Control	1	Q8IZA0
Control	1	Q8IZF2
Control	1	Q8IZH2
Control	1	Q8IZL8
Control	1	Q8IZP0
Control	1	Q8IZQ1
Control	1	Q8IZQ5
Control	1	Q8N0U8

Control	1	Q8N0X7
Control	1	Q8N0Z6
Control	1	Q8N108
Control	1	Q8N122
Control	1	Q8N138
Control	1	Q8N163
Control	1	Q8N183
Control	1	Q8N1B4
Control	1	Q8N1F7
Control	1	Q8N1F8
Control	1	Q8N1G0
Control	1	Q8N1G2
Control	1	Q8N1G4
Control	1	Q8N1I0
Control	1	Q8N1Q1
Control	1	Q8N1W1
Control	1	Q8N201
Control	1	Q8N257
Control	1	Q8N264
Control	1	Q8N2F6
Control	1	Q8N2K0
Control	1	Q8N2M8
Control	1	Q8N2R8
Control	1	Q8N2U0
Control	1	Q8N335
Control	1	Q8N357
Control	1	Q8N392
Control	1	Q8N3C0
Control	1	Q8N3F8
Control	1	Q8N3P4
Control	1	Q8N3R9
Control	1	Q8N3U4
Control	1	Q8N3V7
Control	1	Q8N3X1
Control	1	Q8N442
Control	1	Q8N488
Control	1	Q8N4A0
Control	1	Q8N4C8
Control	1	Q8N4P3
Control	1	Q8N4Q0
Control	1	Q8N4T8
Control	1	Q8N4V1
Control	1	Q8N511
Control	1	Q8N531
Control	1	Q8N543
Control	1	Q8N556
Control	1	Q8N567
Control	1	Q8N573
Control	1	Q8N5A5
Control	1	Q8N5C1

Control	1	Q8N5C6
Control	1	Q8N5D0
Control	1	Q8N5F7
Control	1	Q8N5G2
Control	1	Q8N5H7
Control	1	Q8N5I2
Control	1	Q8N5I4
Control	1	Q8N5K1
Control	1	Q8N5L8
Control	1	Q8N5M1
Control	1	Q8N5M4
Control	1	Q8N5M9
Control	1	Q8N5U6
Control	1	Q8N5W9
Control	1	Q8N653
Control	1	Q8N668
Control	1	Q8N684
Control	1	Q8N697
Control	1	Q8N699
Control	1	Q8N6G6
Control	1	Q8N6H7
Control	1	Q8N6M0
Control	1	Q8N6M3
Control	1	Q8N6N3
Control	1	Q8N6R0
Control	1	Q8N6S5
Control	1	Q8N6T3
Control	1	Q8N6T7
Control	1	Q8N755
Control	1	Q8N766
Control	1	Q8N7H5
Control	1	Q8N7R7
Control	1	Q8N806
Control	1	Q8N8A6
Control	1	Q8N8J7
Control	1	Q8N8R3
Control	1	Q8N8S7
Control	1	Q8N8Z6
Control	1	Q8N954
Control	1	Q8N983
Control	1	Q8N999
Control	1	Q8N9F7
Control	1	Q8N9M1
Control	1	Q8N9N2
Control	1	Q8N9N7
Control	1	Q8N9N8
Control	1	Q8N9T8
Control	1	Q8N9Z2
Control	1	Q8NAF0
Control	1	Q8NAV1

Control	1	Q8NB16
Control	1	Q8NB37
Control	1	Q8NB46
Control	1	Q8NB49
Control	1	Q8NB90
Control	1	Q8NBF2
Control	1	Q8NBF6
Control	1	Q8NBI5
Control	1	Q8NBI6
Control	1	Q8NBJ4
Control	1	Q8NBJ5
Control	1	Q8NBJ7
Control	1	Q8NBJ9
Control	1	Q8NBK3
Control	1	Q8NBL1
Control	1	Q8NBM4
Control	1	Q8NBM8
Control	1	Q8NBN3
Control	1	Q8NBN7
Control	1	Q8NBP0
Control	1	Q8NBQ5
Control	1	Q8NBS9
Control	1	Q8NBT2
Control	1	Q8NBU5
Control	1	Q8NBX0
Control	1	Q8NBZ7
Control	1	Q8NC42
Control	1	Q8NC44
Control	1	Q8NC51
Control	1	Q8NC56
Control	1	Q8NC60
Control	1	Q8NC96
Control	1	Q8NCA5
Control	1	Q8NCC3
Control	1	Q8NCE2
Control	1	Q8NCF5
Control	1	Q8NCG7
Control	1	Q8NCH0
Control	1	Q8NCL4
Control	1	Q8NCN4
Control	1	Q8NCN5
Control	1	Q8NCW5
Control	1	Q8ND04
Control	1	Q8ND24
Control	1	Q8ND56
Control	1	Q8ND71
Control	1	Q8ND76
Control	1	Q8NDA8
Control	1	Q8NDF8
Control	1	Q8NDH3

Control	1	Q8NDI1
Control	1	Q8NDT2
Control	1	Q8NDV1
Control	1	Q8NDV7
Control	1	Q8NDX5
Control	1	Q8NDZ4
Control	1	Q8NE01
Control	1	Q8NE71
Control	1	Q8NE86
Control	1	Q8NEB9
Control	1	Q8NEC7
Control	1	Q8NEF9
Control	1	Q8NEJ9
Control	1	Q8NEM2
Control	1	Q8NEN9
Control	1	Q8NEU8
Control	1	Q8NEW0
Control	1	Q8NEY1
Control	1	Q8NEY8
Control	1	Q8NEZ3
Control	1	Q8NEZ5
Control	1	Q8NF37
Control	1	Q8NF64
Control	1	Q8NF91
Control	1	Q8NFA0
Control	1	Q8NFC6
Control	1	Q8NFD5
Control	1	Q8NFF5
Control	1	Q8NFG4
Control	1	Q8NFH3
Control	1	Q8NFH4
Control	1	Q8NFH5
Control	1	Q8NFI3
Control	1	Q8NFJ5
Control	1	Q8NFQ8
Control	1	Q8NFV4
Control	1	Q8NFW8
Control	1	Q8NG11
Control	1	Q8NG68
Control	1	Q8NHG7
Control	1	Q8NHH9
Control	1	Q8NHP6
Control	1	Q8NHP8
Control	1	Q8NHQ8
Control	1	Q8NHQ9
Control	1	Q8NHS3
Control	1	Q8NHU6
Control	1	Q8NHV1
Control	1	Q8NHV4
Control	1	Q8NI08

Control	1	Q8NI27
Control	1	Q8NI36
Control	1	Q8NI37
Control	1	Q8TA86
Control	1	Q8TAA9
Control	1	Q8TAD8
Control	1	Q8TAE8
Control	1	Q8TAF3
Control	1	Q8TAG9
Control	1	Q8TAQ2
Control	1	Q8TAT6
Control	1	Q8TB03
Control	1	Q8TB22
Control	1	Q8TB37
Control	1	Q8TB40
Control	1	Q8TB52
Control	1	Q8TB61
Control	1	Q8TB72
Control	1	Q8TB96
Control	1	Q8TBA6
Control	1	Q8TBB5
Control	1	Q8TBC3
Control	1	Q8TBC4
Control	1	Q8TBF2
Control	1	Q8TBM8
Control	1	Q8TBQ9
Control	1	Q8TBX8
Control	1	Q8TBZ3
Control	1	Q8TC07
Control	1	Q8TC12
Control	1	Q8TCA0
Control	1	Q8TCB0
Control	1	Q8TCC3
Control	1	Q8TCF1
Control	1	Q8TCG1
Control	1	Q8TCG2
Control	1	Q8TCJ2
Control	1	Q8TCS8
Control	1	Q8TCT7
Control	1	Q8TCT9
Control	1	Q8TCU6
Control	1	Q8TCY9
Control	1	Q8TD16
Control	1	Q8TD19
Control	1	Q8TD55
Control	1	Q8TDB6
Control	1	Q8TDD1
Control	1	Q8TDM6
Control	1	Q8TDN6
Control	1	Q8TDQ7

Control	1	Q8TDW0
Control	1	Q8TDX7
Control	1	Q8TDY2
Control	1	Q8TDZ2
Control	1	Q8TE02
Control	1	Q8TE77
Control	1	Q8TE82
Control	1	Q8TEA7
Control	1	Q8TEA8
Control	1	Q8TEB1
Control	1	Q8TED0
Control	1	Q8TED1
Control	1	Q8TED9
Control	1	Q8TEJ3
Control	1	Q8TEL6
Control	1	Q8TEP8
Control	1	Q8TEQ6
Control	1	Q8TEQ8
Control	1	Q8TEU7
Control	1	Q8TEV9
Control	1	Q8TEW0
Control	1	Q8TEX9
Control	1	Q8TEY7
Control	1	Q8TF01
Control	1	Q8TF05
Control	1	Q8TF42
Control	1	Q8TF74
Control	1	Q8WTS1
Control	1	Q8WTS6
Control	1	Q8WTT2
Control	1	Q8WTV0
Control	1	Q8WTW3
Control	1	Q8WU76
Control	1	Q8WU79
Control	1	Q8WU90
Control	1	Q8WUA2
Control	1	Q8WUA4
Control	1	Q8WUA7
Control	1	Q8WUB8
Control	1	Q8WUD1
Control	1	Q8WUD4
Control	1	Q8WUF5
Control	1	Q8WUF8
Control	1	Q8WUH1
Control	1	Q8WUH2
Control	1	Q8WUH6
Control	1	Q8WUI4
Control	1	Q8WUK0
Control	1	Q8WUM0
Control	1	Q8WUM4

Control	1	Q8WUP2
Control	1	Q8WUQ7
Control	1	Q8WUX2
Control	1	Q8WUX9
Control	1	Q8WUY1
Control	1	Q8WUY8
Control	1	Q8WUZ0
Control	1	Q8WV22
Control	1	Q8WV92
Control	1	Q8WVB6
Control	1	Q8WVC0
Control	1	Q8WVC6
Control	1	Q8WVJ2
Control	1	Q8WVK2
Control	1	Q8WVM0
Control	1	Q8WVM7
Control	1	Q8WVM8
Control	1	Q8WVP5
Control	1	Q8WVQ1
Control	1	Q8WVT3
Control	1	Q8WVV9
Control	1	Q8WVX9
Control	1	Q8WVY7
Control	1	Q8WW01
Control	1	Q8WW12
Control	1	Q8WW22
Control	1	Q8WW59
Control	1	Q8WWC4
Control	1	Q8WWH5
Control	1	Q8WWI1
Control	1	Q8WWI5
Control	1	Q8WWK9
Control	1	Q8WWM7
Control	1	Q8WWN8
Control	1	Q8WWP7
Control	1	Q8WWQ0
Control	1	Q8WWY3
Control	1	Q8WX92
Control	1	Q8WX93
Control	1	Q8WXA3
Control	1	Q8WXA9
Control	1	Q8WXE0
Control	1	Q8WXE1
Control	1	Q8WXF1
Control	1	Q8WXG6
Control	1	Q8WXH0
Control	1	Q8WXI9
Control	1	Q8WXX5
Control	1	Q8WY22
Control	1	Q8WYA6

Control	1	Q8WYN0
Control	1	Q8WYP3
Control	1	Q8WYP5
Control	1	Q8WYQ5
Control	1	Q8WZ75
Control	1	Q8WZ82
Control	1	Q8WZA9
Control	1	Q92466
Control	1	Q92478
Control	1	Q92499
Control	1	Q92503
Control	1	Q92508
Control	1	Q92520
Control	1	Q92522
Control	1	Q92530
Control	1	Q92538
Control	1	Q92541
Control	1	Q92542
Control	1	Q92543
Control	1	Q92544
Control	1	Q92545
Control	1	Q92551
Control	1	Q92552
Control	1	Q92556
Control	1	Q92562
Control	1	Q92567
Control	1	Q92572
Control	1	Q92574
Control	1	Q92575
Control	1	Q92576
Control	1	Q92597
Control	1	Q92598
Control	1	Q92599
Control	1	Q92600
Control	1	Q92604
Control	1	Q92609
Control	1	Q92610
Control	1	Q92613
Control	1	Q92614
Control	1	Q92615
Control	1	Q92616
Control	1	Q92619
Control	1	Q92620
Control	1	Q92621
Control	1	Q92625
Control	1	Q92626
Control	1	Q92636
Control	1	Q92643
Control	1	Q92665
Control	1	Q92667

Control	1	Q92685
Control	1	Q92686
Control	1	Q92688
Control	1	Q92692
Control	1	Q92696
Control	1	Q92734
Control	1	Q92743
Control	1	Q92747
Control	1	Q92759
Control	1	Q92766
Control	1	Q92769
Control	1	Q92783
Control	1	Q92785
Control	1	Q92791
Control	1	Q92793
Control	1	Q92794
Control	1	Q92797
Control	1	Q92804
Control	1	Q92805
Control	1	Q92820
Control	1	Q92823
Control	1	Q92834
Control	1	Q92835
Control	1	Q92841
Control	1	Q92845
Control	1	Q92851
Control	1	Q92870
Control	1	Q92878
Control	1	Q92879
Control	1	Q92882
Control	1	Q92888
Control	1	Q92889
Control	1	Q92890
Control	1	Q92896
Control	1	Q92900
Control	1	Q92905
Control	1	Q92917
Control	1	Q92922
Control	1	Q92925
Control	1	Q92930
Control	1	Q92934
Control	1	Q92945
Control	1	Q92947
Control	1	Q92963
Control	1	Q92968
Control	1	Q92973
Control	1	Q92974
Control	1	Q92979
Control	1	Q92989
Control	1	Q92990

Control	1	Q92995
Control	1	Q92997
Control	1	Q93008
Control	1	Q93009
Control	1	Q93034
Control	1	Q93045
Control	1	Q93050
Control	1	Q93052
Control	1	Q93062
Control	1	Q93063
Control	1	Q93074
Control	1	Q93096
Control	1	Q93100
Control	1	Q969E2
Control	1	Q969F9
Control	1	Q969G3
Control	1	Q969G5
Control	1	Q969G6
Control	1	Q969H8
Control	1	Q969M3
Control	1	Q969N2
Control	1	Q969P0
Control	1	Q969Q0
Control	1	Q969Q5
Control	1	Q969R2
Control	1	Q969S3
Control	1	Q969S9
Control	1	Q969T4
Control	1	Q969T7
Control	1	Q969T9
Control	1	Q969U7
Control	1	Q969V3
Control	1	Q969V5
Control	1	Q969V6
Control	1	Q969X1
Control	1	Q969X5
Control	1	Q969X6
Control	1	Q969Z0
Control	1	Q969Z3
Control	1	Q96A26
Control	1	Q96A33
Control	1	Q96A35
Control	1	Q96A65
Control	1	Q96A72
Control	1	Q96A73
Control	1	Q96AA3
Control	1	Q96AB3
Control	1	Q96AB6
Control	1	Q96AC1
Control	1	Q96AE4

Control	1	Q96AE7
Control	1	Q96AG4
Control	1	Q96AJ9
Control	1	Q96AP7
Control	1	Q96AQ6
Control	1	Q96AQ8
Control	1	Q96AT1
Control	1	Q96AX1
Control	1	Q96AY3
Control	1	Q96AY4
Control	1	Q96B26
Control	1	Q96B54
Control	1	Q96B97
Control	1	Q96BD8
Control	1	Q96BF6
Control	1	Q96BH1
Control	1	Q96BJ3
Control	1	Q96BK5
Control	1	Q96BM9
Control	1	Q96BN8
Control	1	Q96BP2
Control	1	Q96BP3
Control	1	Q96BQ5
Control	1	Q96BR5
Control	1	Q96BW1
Control	1	Q96BW9
Control	1	Q96BX8
Control	1	Q96BY6
Control	1	Q96BY7
Control	1	Q96BZ8
Control	1	Q96BZ9
Control	1	Q96C19
Control	1	Q96C23
Control	1	Q96C24
Control	1	Q96C36
Control	1	Q96C57
Control	1	Q96C86
Control	1	Q96C90
Control	1	Q96CB8
Control	1	Q96CB9
Control	1	Q96CC6
Control	1	Q96CG8
Control	1	Q96CM8
Control	1	Q96CN7
Control	1	Q96CN9
Control	1	Q96CP2
Control	1	Q96CP6
Control	1	Q96CS2
Control	1	Q96CS3
Control	1	Q96CT7

Control	1	Q96CU9
Control	1	Q96CV9
Control	1	Q96CW1
Control	1	Q96CW5
Control	1	Q96CW6
Control	1	Q96CX2
Control	1	Q96D15
Control	1	Q96D46
Control	1	Q96D71
Control	1	Q96DA6
Control	1	Q96DB5
Control	1	Q96DE0
Control	1	Q96DF8
Control	1	Q96DG6
Control	1	Q96DH6
Control	1	Q96DI7
Control	1	Q96DM3
Control	1	Q96DV4
Control	1	Q96DX4
Control	1	Q96DZ1
Control	1	Q96E11
Control	1	Q96E29
Control	1	Q96EA4
Control	1	Q96EB1
Control	1	Q96EB6
Control	1	Q96EC8
Control	1	Q96EE3
Control	1	Q96EI5
Control	1	Q96EK5
Control	1	Q96EK6
Control	1	Q96EK7
Control	1	Q96EK9
Control	1	Q96EL2
Control	1	Q96EL3
Control	1	Q96EM0
Control	1	Q96EP0
Control	1	Q96EP5
Control	1	Q96EQ0
Control	1	Q96ER3
Control	1	Q96ER9
Control	1	Q96ES7
Control	1	Q96EU6
Control	1	Q96EU7
Control	1	Q96EV2
Control	1	Q96EV8
Control	1	Q96EY1
Control	1	Q96EY4
Control	1	Q96EY5
Control	1	Q96EY7
Control	1	Q96EY8

Control	1	Q96F07
Control	1	Q96F44
Control	1	Q96F63
Control	1	Q96F85
Control	1	Q96F86
Control	1	Q96FJ2
Control	1	Q96FN4
Control	1	Q96FQ6
Control	1	Q96FS4
Control	1	Q96FV2
Control	1	Q96FV9
Control	1	Q96FW1
Control	1	Q96FX7
Control	1	Q96FZ2
Control	1	Q96FZ7
Control	1	Q96G01
Control	1	Q96G03
Control	1	Q96G21
Control	1	Q96G23
Control	1	Q96G46
Control	1	Q96GA3
Control	1	Q96GA7
Control	1	Q96GC5
Control	1	Q96GD0
Control	1	Q96GD4
Control	1	Q96GG9
Control	1	Q96GK7
Control	1	Q96GM5
Control	1	Q96GM8
Control	1	Q96GN5
Control	1	Q96GQ5
Control	1	Q96GQ7
Control	1	Q96GS4
Control	1	Q96GW9
Control	1	Q96GX2
Control	1	Q96GX5
Control	1	Q96GX9
Control	1	Q96GY0
Control	1	Q96GY3
Control	1	Q96H20
Control	1	Q96H79
Control	1	Q96HA1
Control	1	Q96HA7
Control	1	Q96HC4
Control	1	Q96HD1
Control	1	Q96HE7
Control	1	Q96HE9
Control	1	Q96HJ9
Control	1	Q96HN2
Control	1	Q96HP0

Control	1	Q96HR3
Control	1	Q96HS1
Control	1	Q96HW7
Control	1	Q96HY6
Control	1	Q96HY7
Control	1	Q96I15
Control	1	Q96I24
Control	1	Q96I25
Control	1	Q96I51
Control	1	Q96I59
Control	1	Q96I99
Control	1	Q96II8
Control	1	Q96IJ6
Control	1	Q96IR7
Control	1	Q96IU4
Control	1	Q96IV0
Control	1	Q96IW7
Control	1	Q96IX5
Control	1	Q96IY1
Control	1	Q96IZ0
Control	1	Q96IZ6
Control	1	Q96J01
Control	1	Q96J02
Control	1	Q96J42
Control	1	Q96J84
Control	1	Q96JB2
Control	1	Q96JB5
Control	1	Q96JC1
Control	1	Q96JG6
Control	1	Q96JH7
Control	1	Q96JI7
Control	1	Q96JJ3
Control	1	Q96JJ7
Control	1	Q96JK2
Control	1	Q96JM3
Control	1	Q96JM7
Control	1	Q96JP5
Control	1	Q96JQ0
Control	1	Q96JQ2
Control	1	Q96JY6
Control	1	Q96K12
Control	1	Q96K17
Control	1	Q96K19
Control	1	Q96K21
Control	1	Q96K37
Control	1	Q96K76
Control	1	Q96K83
Control	1	Q96KA5
Control	1	Q96KB5
Control	1	Q96KC2

Control	1	Q96KC8
Control	1	Q96KG9
Control	1	Q96KM6
Control	1	Q96KN1
Control	1	Q96KP1
Control	1	Q96KP4
Control	1	Q96KQ7
Control	1	Q96KR1
Control	1	Q96KR6
Control	1	Q96L58
Control	1	Q96L91
Control	1	Q96L92
Control	1	Q96LB3
Control	1	Q96LD4
Control	1	Q96LI5
Control	1	Q96LJ7
Control	1	Q96LL9
Control	1	Q96LR5
Control	1	Q96LW7
Control	1	Q96M27
Control	1	Q96M96
Control	1	Q96ME1
Control	1	Q96ME7
Control	1	Q96MF7
Control	1	Q96MG7
Control	1	Q96MG8
Control	1	Q96MM6
Control	1	Q96MU7
Control	1	Q96MW1
Control	1	Q96MW5
Control	1	Q96MX0
Control	1	Q96MX6
Control	1	Q96MY1
Control	1	Q96N66
Control	1	Q96N67
Control	1	Q96NB2
Control	1	Q96NB3
Control	1	Q96ND0
Control	1	Q96NE9
Control	1	Q96P11
Control	1	Q96P16
Control	1	Q96P47
Control	1	Q96P48
Control	1	Q96P70
Control	1	Q96PC5
Control	1	Q96PD2
Control	1	Q96PE2
Control	1	Q96PE3
Control	1	Q96PK6
Control	1	Q96PM5

Control	1	Q96PQ7
Control	1	Q96PU4
Control	1	Q96PU5
Control	1	Q96PU8
Control	1	Q96PY5
Control	1	Q96PZ0
Control	1	Q96PZ2
Control	1	Q96Q05
Control	1	Q96Q06
Control	1	Q96Q11
Control	1	Q96Q15
Control	1	Q96Q42
Control	1	Q96Q45
Control	1	Q96Q83
Control	1	Q96QB1
Control	1	Q96QC0
Control	1	Q96QD8
Control	1	Q96QD9
Control	1	Q96QG7
Control	1	Q96QK1
Control	1	Q96QR8
Control	1	Q96QU8
Control	1	Q96QV1
Control	1	Q96QZ7
Control	1	Q96R06
Control	1	Q96RE7
Control	1	Q96RF0
Control	1	Q96RG2
Control	1	Q96RK0
Control	1	Q96RL1
Control	1	Q96RL7
Control	1	Q96RN5
Control	1	Q96RP9
Control	1	Q96RQ1
Control	1	Q96RQ3
Control	1	Q96RR4
Control	1	Q96RS0
Control	1	Q96RS6
Control	1	Q96RT1
Control	1	Q96RT7
Control	1	Q96RU2
Control	1	Q96RU3
Control	1	Q96S44
Control	1	Q96S52
Control	1	Q96S55
Control	1	Q96S59
Control	1	Q96S66
Control	1	Q96S97
Control	1	Q96SB3
Control	1	Q96SB4

Control	1	Q96SB8
Control	1	Q96SI1
Control	1	Q96SI9
Control	1	Q96SK2
Control	1	Q96SL4
Control	1	Q96SN8
Control	1	Q96SQ9
Control	1	Q96ST2
Control	1	Q96ST3
Control	1	Q96SU4
Control	1	Q96SW2
Control	1	Q96SY0
Control	1	Q96SZ5
Control	1	Q96SZ6
Control	1	Q96T23
Control	1	Q96T37
Control	1	Q96T49
Control	1	Q96T51
Control	1	Q96T58
Control	1	Q96T60
Control	1	Q96T76
Control	1	Q96T88
Control	1	Q96TA1
Control	1	Q96TA2
Control	1	Q96TC7
Control	1	Q99081
Control	1	Q99418
Control	1	Q99426
Control	1	Q99436
Control	1	Q99439
Control	1	Q99442
Control	1	Q99447
Control	1	Q99459
Control	1	Q99460
Control	1	Q99470
Control	1	Q99471
Control	1	Q99496
Control	1	Q99497
Control	1	Q99501
Control	1	Q99504
Control	1	Q99519
Control	1	Q99523
Control	1	Q99536
Control	1	Q99538
Control	1	Q99541
Control	1	Q99543
Control	1	Q99549
Control	1	Q99567
Control	1	Q99569
Control	1	Q99570

Control	1	Q99571
Control	1	Q99575
Control	1	Q99584
Control	1	Q99590
Control	1	Q99598
Control	1	Q99611
Control	1	Q99613
Control	1	Q99614
Control	1	Q99615
Control	1	Q99623
Control	1	Q99627
Control	1	Q99633
Control	1	Q99653
Control	1	Q99661
Control	1	Q99685
Control	1	Q99700
Control	1	Q99704
Control	1	Q99707
Control	1	Q99714
Control	1	Q99715
Control	1	Q99717
Control	1	Q99720
Control	1	Q99729
Control	1	Q99733
Control	1	Q99735
Control	1	Q99747
Control	1	Q99755
Control	1	Q99758
Control	1	Q99759
Control	1	Q99766
Control	1	Q99797
Control	1	Q99798
Control	1	Q99805
Control	1	Q99808
Control	1	Q99816
Control	1	Q99829
Control	1	Q99832
Control	1	Q99836
Control	1	Q99848
Control	1	Q99873
Control	1	Q99933
Control	1	Q99941
Control	1	Q99958
Control	1	Q99961
Control	1	Q99973
Control	1	Q99986
Control	1	Q99988
Control	1	Q99996
Control	1	Q9BPW8
Control	1	Q9BPX3

Control	1	Q9BPX5
Control	1	Q9BPX6
Control	1	Q9BPX7
Control	1	Q9BPY3
Control	1	Q9BPZ7
Control	1	Q9BQ04
Control	1	Q9BQ24
Control	1	Q9BQ39
Control	1	Q9BQ48
Control	1	Q9BQ52
Control	1	Q9BQ61
Control	1	Q9BQ67
Control	1	Q9BQ69
Control	1	Q9BQ70
Control	1	Q9BQ75
Control	1	Q9BQ90
Control	1	Q9BQ95
Control	1	Q9BQA1
Control	1	Q9BQA9
Control	1	Q9BQB6
Control	1	Q9BQC3
Control	1	Q9BQE3
Control	1	Q9BQE4
Control	1	Q9BQE5
Control	1	Q9BQG0
Control	1	Q9BQP7
Control	1	Q9BQQ3
Control	1	Q9BQS8
Control	1	Q9BR61
Control	1	Q9BR76
Control	1	Q9BRA2
Control	1	Q9BRD0
Control	1	Q9BRF8
Control	1	Q9BRG1
Control	1	Q9BRJ2
Control	1	Q9BRJ6
Control	1	Q9BRJ7
Control	1	Q9BRK5
Control	1	Q9BRP1
Control	1	Q9BRP4
Control	1	Q9BRP8
Control	1	Q9BRQ6
Control	1	Q9BRR6
Control	1	Q9BRR8
Control	1	Q9BRS2
Control	1	Q9BRT6
Control	1	Q9BRT9
Control	1	Q9BRU9
Control	1	Q9BRX2
Control	1	Q9BRX8

Control	1	Q9BRZ2
Control	1	Q9BS26
Control	1	Q9BS40
Control	1	Q9BSC4
Control	1	Q9BSD7
Control	1	Q9BSF4
Control	1	Q9BSH4
Control	1	Q9BSJ2
Control	1	Q9BSJ8
Control	1	Q9BSL1
Control	1	Q9BSR8
Control	1	Q9BSV6
Control	1	Q9BT09
Control	1	Q9BT17
Control	1	Q9BT22
Control	1	Q9BT23
Control	1	Q9BT25
Control	1	Q9BT40
Control	1	Q9BT73
Control	1	Q9BT78
Control	1	Q9BT88
Control	1	Q9BTA9
Control	1	Q9BTC0
Control	1	Q9BTC8
Control	1	Q9BTD8
Control	1	Q9BTE1
Control	1	Q9BTE3
Control	1	Q9BTE6
Control	1	Q9BTE7
Control	1	Q9BTT0
Control	1	Q9BTT6
Control	1	Q9BTU6
Control	1	Q9BTV4
Control	1	Q9BTV5
Control	1	Q9BTW9
Control	1	Q9BTX1
Control	1	Q9BTY7
Control	1	Q9BTZ2
Control	1	Q9BU23
Control	1	Q9BU61
Control	1	Q9BU76
Control	1	Q9BUB7
Control	1	Q9BUE0
Control	1	Q9BUE6
Control	1	Q9BUF5
Control	1	Q9BUH6
Control	1	Q9BUI4
Control	1	Q9BUJ2
Control	1	Q9BUK6
Control	1	Q9BUL5

Control	1	Q9BUL8
Control	1	Q9BUL9
Control	1	Q9BUN8
Control	1	Q9BUP3
Control	1	Q9BUQ8
Control	1	Q9BUR4
Control	1	Q9BUR5
Control	1	Q9BUT1
Control	1	Q9BV20
Control	1	Q9BV38
Control	1	Q9BV44
Control	1	Q9BV57
Control	1	Q9BV79
Control	1	Q9BV81
Control	1	Q9BV86
Control	1	Q9BV94
Control	1	Q9BVA0
Control	1	Q9BVC4
Control	1	Q9BVC5
Control	1	Q9BVC6
Control	1	Q9BVG4
Control	1	Q9BVG9
Control	1	Q9BVI4
Control	1	Q9BVJ6
Control	1	Q9BVJ7
Control	1	Q9BVK6
Control	1	Q9BVK8
Control	1	Q9BVL2
Control	1	Q9BVL4
Control	1	Q9BVM2
Control	1	Q9BVP2
Control	1	Q9BVQ7
Control	1	Q9BVS4
Control	1	Q9BVS5
Control	1	Q9BVT8
Control	1	Q9BVV7
Control	1	Q9BW19
Control	1	Q9BW27
Control	1	Q9BW60
Control	1	Q9BW62
Control	1	Q9BW71
Control	1	Q9BW83
Control	1	Q9BW85
Control	1	Q9BW91
Control	1	Q9BW92
Control	1	Q9BWD1
Control	1	Q9BWE0
Control	1	Q9BWF3
Control	1	Q9BWH2
Control	1	Q9BWH6

Control	1	Q9BWJ5
Control	1	Q9BWM7
Control	1	Q9BWS9
Control	1	Q9BWT3
Control	1	Q9BWT7
Control	1	Q9BWU0
Control	1	Q9BWU1
Control	1	Q9BX40
Control	1	Q9BX59
Control	1	Q9BX67
Control	1	Q9BX69
Control	1	Q9BX70
Control	1	Q9BX95
Control	1	Q9BXB4
Control	1	Q9BXB5
Control	1	Q9BXF6
Control	1	Q9BXJ0
Control	1	Q9BXJ9
Control	1	Q9BXK1
Control	1	Q9BXK5
Control	1	Q9BXL7
Control	1	Q9BXP2
Control	1	Q9BXP5
Control	1	Q9BXR0
Control	1	Q9BXS5
Control	1	Q9BXS6
Control	1	Q9BXW7
Control	1	Q9BXW9
Control	1	Q9BXY0
Control	1	Q9BY41
Control	1	Q9BY42
Control	1	Q9BY43
Control	1	Q9BY44
Control	1	Q9BY77
Control	1	Q9BY89
Control	1	Q9BYB0
Control	1	Q9BYB4
Control	1	Q9BYC5
Control	1	Q9BYC8
Control	1	Q9BYC9
Control	1	Q9BYD1
Control	1	Q9BYD2
Control	1	Q9BYD3
Control	1	Q9BYD6
Control	1	Q9BYG3
Control	1	Q9BYI3
Control	1	Q9BYJ9
Control	1	Q9BYK8
Control	1	Q9BYM8
Control	1	Q9BYN8

Control	1	Q9BYT8
Control	1	Q9BYV8
Control	1	Q9BYW2
Control	1	Q9BZ23
Control	1	Q9BZ29
Control	1	Q9BZ67
Control	1	Q9BZ76
Control	1	Q9BZ95
Control	1	Q9BZD4
Control	1	Q9BZE1
Control	1	Q9BZE4
Control	1	Q9BZE9
Control	1	Q9BZF1
Control	1	Q9BZF9
Control	1	Q9BZG1
Control	1	Q9BZG8
Control	1	Q9BZH6
Control	1	Q9BZI7
Control	1	Q9BZJ0
Control	1	Q9BZK7
Control	1	Q9BZL1
Control	1	Q9BZL4
Control	1	Q9BZL6
Control	1	Q9BZQ6
Control	1	Q9BZQ8
Control	1	Q9BZV1
Control	1	Q9BZW5
Control	1	Q9BZX2
Control	1	Q9BZZ2
Control	1	Q9BZZ5
Control	1	Q9C000
Control	1	Q9C035
Control	1	Q9C037
Control	1	Q9C0B1
Control	1	Q9C0B5
Control	1	Q9C0B7
Control	1	Q9C0C2
Control	1	Q9C0C9
Control	1	Q9C0D3
Control	1	Q9C0D5
Control	1	Q9C0D9
Control	1	Q9C0E2
Control	1	Q9C0E8
Control	1	Q9C0F1
Control	1	Q9C0H2
Control	1	Q9C0H6
Control	1	Q9C0I1
Control	1	Q9C0J8
Control	1	Q9GZL7
Control	1	Q9GZM5

Control	1	Q9GZM7
Control	1	Q9GZN8
Control	1	Q9GZP4
Control	1	Q9GZP9
Control	1	Q9GZQ3
Control	1	Q9GZR2
Control	1	Q9GZR7
Control	1	Q9GZS1
Control	1	Q9GZS3
Control	1	Q9GZT3
Control	1	Q9GZT4
Control	1	Q9GZT8
Control	1	Q9GZT9
Control	1	Q9GZU1
Control	1	Q9GZU8
Control	1	Q9GZY8
Control	1	Q9GZZ1
Control	1	Q9GZZ9
Control	1	Q9H000
Control	1	Q9H061
Control	1	Q9H074
Control	1	Q9H078
Control	1	Q9H082
Control	1	Q9H089
Control	1	Q9H098
Control	1	Q9H0A0
Control	1	Q9H0A8
Control	1	Q9H0B6
Control	1	Q9H0C8
Control	1	Q9H0D6
Control	1	Q9H0E2
Control	1	Q9H0E3
Control	1	Q9H0E9
Control	1	Q9H0G5
Control	1	Q9H0H0
Control	1	Q9H0H5
Control	1	Q9H0J9
Control	1	Q9H0L4
Control	1	Q9H0P0
Control	1	Q9H0Q0
Control	1	Q9H0R3
Control	1	Q9H0R4
Control	1	Q9H0R6
Control	1	Q9H0S4
Control	1	Q9H0U3
Control	1	Q9H0U4
Control	1	Q9H0U6
Control	1	Q9H0U9
Control	1	Q9H0V1
Control	1	Q9H0V9

Control	1	Q9H0W8
Control	1	Q9H0X9
Control	1	Q9H147
Control	1	Q9H173
Control	1	Q9H1A3
Control	1	Q9H1A4
Control	1	Q9H1B7
Control	1	Q9H1C7
Control	1	Q9H1D9
Control	1	Q9H1E3
Control	1	Q9H1E5
Control	1	Q9H1H9
Control	1	Q9H1I8
Control	1	Q9H1K0
Control	1	Q9H1K1
Control	1	Q9H1P3
Control	1	Q9H1Y0
Control	1	Q9H1Z4
Control	1	Q9H201
Control	1	Q9H223
Control	1	Q9H267
Control	1	Q9H269
Control	1	Q9H270
Control	1	Q9H299
Control	1	Q9H2C0
Control	1	Q9H2D1
Control	1	Q9H2D6
Control	1	Q9H2F5
Control	1	Q9H2G2
Control	1	Q9H2H8
Control	1	Q9H2H9
Control	1	Q9H2J4
Control	1	Q9H2K8
Control	1	Q9H2M9
Control	1	Q9H2P0
Control	1	Q9H2P9
Control	1	Q9H2U1
Control	1	Q9H2U2
Control	1	Q9H2V7
Control	1	Q9H2W6
Control	1	Q9H300
Control	1	Q9H307
Control	1	Q9H330
Control	1	Q9H334
Control	1	Q9H3F6
Control	1	Q9H3H1
Control	1	Q9H3H3
Control	1	Q9H3K2
Control	1	Q9H3K6
Control	1	Q9H3L0

Control	1	Q9H3N1
Control	1	Q9H3P2
Control	1	Q9H3P7
Control	1	Q9H3Q1
Control	1	Q9H3S7
Control	1	Q9H3T3
Control	1	Q9H3U1
Control	1	Q9H3U5
Control	1	Q9H3Z4
Control	1	Q9H444
Control	1	Q9H446
Control	1	Q9H467
Control	1	Q9H469
Control	1	Q9H479
Control	1	Q9H488
Control	1	Q9H490
Control	1	Q9H497
Control	1	Q9H4A3
Control	1	Q9H4A4
Control	1	Q9H4A5
Control	1	Q9H4A6
Control	1	Q9H4B0
Control	1	Q9H4F1
Control	1	Q9H4G4
Control	1	Q9H4I3
Control	1	Q9H4L4
Control	1	Q9H4L5
Control	1	Q9H4L7
Control	1	Q9H4M9
Control	1	Q9H4Z3
Control	1	Q9H501
Control	1	Q9H553
Control	1	Q9H583
Control	1	Q9H5K3
Control	1	Q9H5N1
Control	1	Q9H5Q4
Control	1	Q9H5U6
Control	1	Q9H5V9
Control	1	Q9H5X1
Control	1	Q9H5Z1
Control	1	Q9H5Z6
Control	1	Q9H6D7
Control	1	Q9H6E4
Control	1	Q9H6F5
Control	1	Q9H6H4
Control	1	Q9H6I2
Control	1	Q9H6K4
Control	1	Q9H6Q4
Control	1	Q9H6R0
Control	1	Q9H6R4

Control	1	Q9H6R7
Control	1	Q9H6S0
Control	1	Q9H6T3
Control	1	Q9H6U6
Control	1	Q9H6U8
Control	1	Q9H6V9
Control	1	Q9H6W3
Control	1	Q9H6Y2
Control	1	Q9H6Z4
Control	1	Q9H773
Control	1	Q9H792
Control	1	Q9H7B2
Control	1	Q9H7B4
Control	1	Q9H7C9
Control	1	Q9H7D0
Control	1	Q9H7D7
Control	1	Q9H7E2
Control	1	Q9H7E9
Control	1	Q9H7F0
Control	1	Q9H7H0
Control	1	Q9H7N4
Control	1	Q9H7Z3
Control	1	Q9H7Z7
Control	1	Q9H814
Control	1	Q9H832
Control	1	Q9H845
Control	1	Q9H857
Control	1	Q9H875
Control	1	Q9H8G2
Control	1	Q9H8H0
Control	1	Q9H8H2
Control	1	Q9H8H3
Control	1	Q9H8K7
Control	1	Q9H8L6
Control	1	Q9H8M7
Control	1	Q9H8M9
Control	1	Q9H8T0
Control	1	Q9H8W4
Control	1	Q9H8Y5
Control	1	Q9H8Y8
Control	1	Q9H900
Control	1	Q9H910
Control	1	Q9H936
Control	1	Q9H939
Control	1	Q9H944
Control	1	Q9H967
Control	1	Q9H974
Control	1	Q9H981
Control	1	Q9H993
Control	1	Q9H999

Control	1	Q9H9A5
Control	1	Q9H9A6
Control	1	Q9H9B1
Control	1	Q9H9B4
Control	1	Q9H9C1
Control	1	Q9H9E3
Control	1	Q9H9F9
Control	1	Q9H9H4
Control	1	Q9H9J2
Control	1	Q9H9P8
Control	1	Q9H9Q2
Control	1	Q9H9Q4
Control	1	Q9H9S3
Control	1	Q9H9S5
Control	1	Q9H9T3
Control	1	Q9H9Y2
Control	1	Q9H9Y6
Control	1	Q9HA38
Control	1	Q9HA47
Control	1	Q9HA64
Control	1	Q9HA65
Control	1	Q9HA77
Control	1	Q9HAB3
Control	1	Q9HAB8
Control	1	Q9HAC8
Control	1	Q9HAD4
Control	1	Q9HAN9
Control	1	Q9HAS0
Control	1	Q9HAT2
Control	1	Q9HAU0
Control	1	Q9HAU4
Control	1	Q9HAU5
Control	1	Q9HAV0
Control	1	Q9HAV4
Control	1	Q9HAV7
Control	1	Q9HB07
Control	1	Q9HB20
Control	1	Q9HB21
Control	1	Q9HB40
Control	1	Q9HB63
Control	1	Q9HB71
Control	1	Q9HB90
Control	1	Q9HBF4
Control	1	Q9HBG6
Control	1	Q9HBH5
Control	1	Q9HBI1
Control	1	Q9HBL0
Control	1	Q9HBL7
Control	1	Q9HBL8
Control	1	Q9HBM0

Control	1	Q9HBM1
Control	1	Q9HBM6
Control	1	Q9HBR0
Control	1	Q9HBU6
Control	1	Q9HBW9
Control	1	Q9HC07
Control	1	Q9HC21
Control	1	Q9HC35
Control	1	Q9HC36
Control	1	Q9HC38
Control	1	Q9HC98
Control	1	Q9HCC0
Control	1	Q9HCD5
Control	1	Q9HCE0
Control	1	Q9HCE1
Control	1	Q9HCE5
Control	1	Q9HCG7
Control	1	Q9HCG8
Control	1	Q9HCJ3
Control	1	Q9HCK8
Control	1	Q9HCM4
Control	1	Q9HCN4
Control	1	Q9HCN8
Control	1	Q9HCP0
Control	1	Q9HCS7
Control	1	Q9HCU5
Control	1	Q9HD15
Control	1	Q9HD20
Control	1	Q9HD26
Control	1	Q9HD33
Control	1	Q9HD34
Control	1	Q9HD40
Control	1	Q9HD42
Control	1	Q9HD45
Control	1	Q9HD67
Control	1	Q9HDC9
Control	1	Q9NNW5
Control	1	Q9NNW7
Control	1	Q9NP58
Control	1	Q9NP61
Control	1	Q9NP64
Control	1	Q9NP72
Control	1	Q9NP74
Control	1	Q9NP77
Control	1	Q9NP79
Control	1	Q9NP81
Control	1	Q9NP92
Control	1	Q9NP97
Control	1	Q9NPA0
Control	1	Q9NPA8

Control	1	Q9NPD3
Control	1	Q9NPD8
Control	1	Q9NPE3
Control	1	Q9NPF4
Control	1	Q9NPF5
Control	1	Q9NPG4
Control	1	Q9NPH2
Control	1	Q9NPI1
Control	1	Q9NPI6
Control	1	Q9NPJ3
Control	1	Q9NPJ6
Control	1	Q9NPL8
Control	1	Q9NPQ8
Control	1	Q9NPY3
Control	1	Q9NQ29
Control	1	Q9NQ30
Control	1	Q9NQ48
Control	1	Q9NQ50
Control	1	Q9NQ55
Control	1	Q9NQ66
Control	1	Q9NQ88
Control	1	Q9NQ89
Control	1	Q9NQC3
Control	1	Q9NQG5
Control	1	Q9NQH7
Control	1	Q9NQR4
Control	1	Q9NQS1
Control	1	Q9NQS7
Control	1	Q9NQT4
Control	1	Q9NQT5
Control	1	Q9NQT8
Control	1	Q9NQW6
Control	1	Q9NQW7
Control	1	Q9NQX3
Control	1	Q9NQY0
Control	1	Q9NQZ2
Control	1	Q9NQZ5
Control	1	Q9NR09
Control	1	Q9NR12
Control	1	Q9NR19
Control	1	Q9NR28
Control	1	Q9NR30
Control	1	Q9NR31
Control	1	Q9NR45
Control	1	Q9NR46
Control	1	Q9NR48
Control	1	Q9NR50
Control	1	Q9NR56
Control	1	Q9NRA2
Control	1	Q9NRA8

Control	1	Q9NRB3
Control	1	Q9NRC1
Control	1	Q9NRD5
Control	1	Q9NRF2
Control	1	Q9NRF8
Control	1	Q9NRF9
Control	1	Q9NRG0
Control	1	Q9NRG9
Control	1	Q9NRH2
Control	1	Q9NRK6
Control	1	Q9NRL2
Control	1	Q9NRL3
Control	1	Q9NRN7
Control	1	Q9NRN9
Control	1	Q9NRP0
Control	1	Q9NRP2
Control	1	Q9NRQ2
Control	1	Q9NRS6
Control	1	Q9NRV9
Control	1	Q9NRW3
Control	1	Q9NRW7
Control	1	Q9NRX1
Control	1	Q9NRX2
Control	1	Q9NRX4
Control	1	Q9NRX5
Control	1	Q9NRY4
Control	1	Q9NRY5
Control	1	Q9NRY6
Control	1	Q9NRZ5
Control	1	Q9NRZ7
Control	1	Q9NRZ9
Control	1	Q9NS00
Control	1	Q9NS15
Control	1	Q9NS69
Control	1	Q9NS86
Control	1	Q9NS87
Control	1	Q9NS93
Control	1	Q9NSC5
Control	1	Q9NSD9
Control	1	Q9NSE4
Control	1	Q9NSI2
Control	1	Q9NSI8
Control	1	Q9NSK0
Control	1	Q9NSV4
Control	1	Q9NSY1
Control	1	Q9NT62
Control	1	Q9NTI5
Control	1	Q9NTJ3
Control	1	Q9NTJ4
Control	1	Q9NTJ5

Control	1	Q9NTK1
Control	1	Q9NTK5
Control	1	Q9NTM9
Control	1	Q9NTW7
Control	1	Q9NTX5
Control	1	Q9NTZ6
Control	1	Q9NU19
Control	1	Q9NU22
Control	1	Q9NUB1
Control	1	Q9NUD5
Control	1	Q9NUI1
Control	1	Q9NUJ1
Control	1	Q9NUJ3
Control	1	Q9NUL3
Control	1	Q9NUL5
Control	1	Q9NUL7
Control	1	Q9NUM4
Control	1	Q9NUP9
Control	1	Q9NUQ2
Control	1	Q9NUQ3
Control	1	Q9NUQ6
Control	1	Q9NUQ7
Control	1	Q9NUQ8
Control	1	Q9NUQ9
Control	1	Q9NUT2
Control	1	Q9NUU7
Control	1	Q9NUV9
Control	1	Q9NUW8
Control	1	Q9NUX5
Control	1	Q9NUY8
Control	1	Q9NV06
Control	1	Q9NV31
Control	1	Q9NV70
Control	1	Q9NV79
Control	1	Q9NV88
Control	1	Q9NV96
Control	1	Q9NVA1
Control	1	Q9NVA2
Control	1	Q9NVC3
Control	1	Q9NVC6
Control	1	Q9NVD7
Control	1	Q9NVE5
Control	1	Q9NVE7
Control	1	Q9NVF7
Control	1	Q9NVG8
Control	1	Q9NVH0
Control	1	Q9NVH1
Control	1	Q9NVH2
Control	1	Q9NVI1
Control	1	Q9NVI7

Control	1	Q9NVJ2
Control	1	Q9NVM6
Control	1	Q9NVM9
Control	1	Q9NVN3
Control	1	Q9NVN8
Control	1	Q9NVP1
Control	1	Q9NVP2
Control	1	Q9NVR0
Control	1	Q9NVR2
Control	1	Q9NVS2
Control	1	Q9NVS9
Control	1	Q9NVT9
Control	1	Q9NVU0
Control	1	Q9NVU7
Control	1	Q9NVV4
Control	1	Q9NVX2
Control	1	Q9NVZ3
Control	1	Q9NW08
Control	1	Q9NW13
Control	1	Q9NW15
Control	1	Q9NW64
Control	1	Q9NW82
Control	1	Q9NWB6
Control	1	Q9NWB7
Control	1	Q9NWH9
Control	1	Q9NWM8
Control	1	Q9NWS8
Control	1	Q9NWT1
Control	1	Q9NWT6
Control	1	Q9NWU1
Control	1	Q9NWU5
Control	1	Q9NWV4
Control	1	Q9NWV8
Control	1	Q9NWW5
Control	1	Q9NWX5
Control	1	Q9NWX6
Control	1	Q9NWY4
Control	1	Q9NWZ3
Control	1	Q9NWZ5
Control	1	Q9NX00
Control	1	Q9NX02
Control	1	Q9NX05
Control	1	Q9NX07
Control	1	Q9NX14
Control	1	Q9NX20
Control	1	Q9NX24
Control	1	Q9NX40
Control	1	Q9NX46
Control	1	Q9NX47
Control	1	Q9NX58

Control	1	Q9NX61
Control	1	Q9NX62
Control	1	Q9NX63
Control	1	Q9NX76
Control	1	Q9NXA8
Control	1	Q9NXC5
Control	1	Q9NXD2
Control	1	Q9NXE4
Control	1	Q9NXE8
Control	1	Q9NXF1
Control	1	Q9NXF7
Control	1	Q9NXG2
Control	1	Q9NXG6
Control	1	Q9NXH8
Control	1	Q9NXH9
Control	1	Q9NXN4
Control	1	Q9NXR1
Control	1	Q9NXR7
Control	1	Q9NXS2
Control	1	Q9NXU5
Control	1	Q9NXV2
Control	1	Q9NXV6
Control	1	Q9NXW2
Control	1	Q9NXW9
Control	1	Q9NXX6
Control	1	Q9NY12
Control	1	Q9NY15
Control	1	Q9NY27
Control	1	Q9NY33
Control	1	Q9NY61
Control	1	Q9NY65
Control	1	Q9NY93
Control	1	Q9NYA1
Control	1	Q9NYB0
Control	1	Q9NYB9
Control	1	Q9NYF8
Control	1	Q9NYH9
Control	1	Q9NYJ8
Control	1	Q9NYK5
Control	1	Q9NYL2
Control	1	Q9NYL4
Control	1	Q9NYL9
Control	1	Q9NYM9
Control	1	Q9NYP7
Control	1	Q9NYR9
Control	1	Q9NYU1
Control	1	Q9NYU2
Control	1	Q9NYV4
Control	1	Q9NYY8
Control	1	Q9NZ01

Control	1	Q9NZ08
Control	1	Q9NZ32
Control	1	Q9NZ43
Control	1	Q9NZ45
Control	1	Q9NZ52
Control	1	Q9NZ63
Control	1	Q9NZB2
Control	1	Q9NZC3
Control	1	Q9NZC9
Control	1	Q9NZD2
Control	1	Q9NZD8
Control	1	Q9NZE8
Control	1	Q9NZI7
Control	1	Q9NZI8
Control	1	Q9NZJ4
Control	1	Q9NZJ7
Control	1	Q9NZJ9
Control	1	Q9NZL4
Control	1	Q9NZL9
Control	1	Q9NZM1
Control	1	Q9NZM3
Control	1	Q9NZM5
Control	1	Q9NZN3
Control	1	Q9NZN4
Control	1	Q9NZN5
Control	1	Q9NZN8
Control	1	Q9NZQ3
Control	1	Q9NZQ7
Control	1	Q9NZT1
Control	1	Q9NZT2
Control	1	Q9NZU5
Control	1	Q9NZV1
Control	1	Q9NZV5
Control	1	Q9NZW5
Control	1	Q9NZZ3
Control	1	Q9P013
Control	1	Q9P015
Control	1	Q9P016
Control	1	Q9P031
Control	1	Q9P032
Control	1	Q9P035
Control	1	Q9P0I2
Control	1	Q9P0J0
Control	1	Q9P0J1
Control	1	Q9P0J7
Control	1	Q9P0K7
Control	1	Q9P0L0
Control	1	Q9P0M9
Control	1	Q9P0T7
Control	1	Q9P0U3

Control	1	Q9P0U4
Control	1	Q9P0V3
Control	1	Q9P0V9
Control	1	Q9P107
Control	1	Q9P1Y6
Control	1	Q9P215
Control	1	Q9P219
Control	1	Q9P227
Control	1	Q9P253
Control	1	Q9P258
Control	1	Q9P260
Control	1	Q9P265
Control	1	Q9P266
Control	1	Q9P270
Control	1	Q9P273
Control	1	Q9P275
Control	1	Q9P287
Control	1	Q9P289
Control	1	Q9P291
Control	1	Q9P2A4
Control	1	Q9P2B2
Control	1	Q9P2B4
Control	1	Q9P2C4
Control	1	Q9P2D0
Control	1	Q9P2D3
Control	1	Q9P2E3
Control	1	Q9P2E5
Control	1	Q9P2E7
Control	1	Q9P2E9
Control	1	Q9P2I0
Control	1	Q9P2J3
Control	1	Q9P2J5
Control	1	Q9P2K5
Control	1	Q9P2K6
Control	1	Q9P2K8
Control	1	Q9P2L0
Control	1	Q9P2N5
Control	1	Q9P2N6
Control	1	Q9P2P5
Control	1	Q9P2Q2
Control	1	Q9P2R3
Control	1	Q9P2R6
Control	1	Q9P2R7
Control	1	Q9P2T1
Control	1	Q9P2W9
Control	1	Q9P2X0
Control	1	Q9P2X3
Control	1	Q9P2Y5
Control	1	Q9UBB4
Control	1	Q9UBB5

Control	1	Q9UBB6
Control	1	Q9UBB9
Control	1	Q9UBC2
Control	1	Q9UBC5
Control	1	Q9UBD5
Control	1	Q9UBE0
Control	1	Q9UBF2
Control	1	Q9UBF8
Control	1	Q9UBG0
Control	1	Q9UBI1
Control	1	Q9UBI6
Control	1	Q9UBK7
Control	1	Q9UBK8
Control	1	Q9UBK9
Control	1	Q9UBL3
Control	1	Q9UBM7
Control	1	Q9UBN6
Control	1	Q9UBN7
Control	1	Q9UBP0
Control	1	Q9UBP6
Control	1	Q9UBP9
Control	1	Q9UBQ0
Control	1	Q9UBQ5
Control	1	Q9UBQ6
Control	1	Q9UBQ7
Control	1	Q9UBR2
Control	1	Q9UBS0
Control	1	Q9UBS4
Control	1	Q9UBS8
Control	1	Q9UBT2
Control	1	Q9UBU8
Control	1	Q9UBU9
Control	1	Q9UBV2
Control	1	Q9UBV7
Control	1	Q9UBV8
Control	1	Q9UBW7
Control	1	Q9UBW8
Control	1	Q9UBX3
Control	1	Q9UDR5
Control	1	Q9UDT6
Control	1	Q9UDW1
Control	1	Q9UDX5
Control	1	Q9UDY2
Control	1	Q9UDY4
Control	1	Q9UEE5
Control	1	Q9UEE9
Control	1	Q9UER7
Control	1	Q9UET6
Control	1	Q9UEU0
Control	1	Q9UEW8

Control	1	Q9UEY8
Control	1	Q9UFC0
Control	1	Q9UFN0
Control	1	Q9UFW8
Control	1	Q9UG56
Control	1	Q9UG63
Control	1	Q9UGI8
Control	1	Q9UGJ0
Control	1	Q9UGJ1
Control	1	Q9UGL1
Control	1	Q9UGM6
Control	1	Q9UGP4
Control	1	Q9UGP8
Control	1	Q9UGQ3
Control	1	Q9UGR2
Control	1	Q9UGV2
Control	1	Q9UH62
Control	1	Q9UH65
Control	1	Q9UH99
Control	1	Q9UHA3
Control	1	Q9UHA4
Control	1	Q9UHB6
Control	1	Q9UHB7
Control	1	Q9UHB9
Control	1	Q9UHD1
Control	1	Q9UHD2
Control	1	Q9UHD8
Control	1	Q9UHG3
Control	1	Q9UHI6
Control	1	Q9UHJ6
Control	1	Q9UHL4
Control	1	Q9UHN6
Control	1	Q9UHP3
Control	1	Q9UHQ4
Control	1	Q9UHQ9
Control	1	Q9UHR5
Control	1	Q9UHR6
Control	1	Q9UHV9
Control	1	Q9UHW5
Control	1	Q9UHX1
Control	1	Q9UHY1
Control	1	Q9UHY7
Control	1	Q9UHY8
Control	1	Q9UI09
Control	1	Q9UI10
Control	1	Q9UI12
Control	1	Q9UI14
Control	1	Q9UI26
Control	1	Q9UI30
Control	1	Q9UI36

Control	1	Q9UIA9
Control	1	Q9UIC8
Control	1	Q9UID3
Control	1	Q9UIF8
Control	1	Q9UIF9
Control	1	Q9UIG0
Control	1	Q9UIJ7
Control	1	Q9UIQ6
Control	1	Q9UIS9
Control	1	Q9UIV1
Control	1	Q9UJ41
Control	1	Q9UJ68
Control	1	Q9UJ70
Control	1	Q9UJ83
Control	1	Q9UJA5
Control	1	Q9UJC5
Control	1	Q9UJF2
Control	1	Q9UJK0
Control	1	Q9UJS0
Control	1	Q9UJU6
Control	1	Q9UJV9
Control	1	Q9UJW0
Control	1	Q9UJX2
Control	1	Q9UJX3
Control	1	Q9UJX4
Control	1	Q9UJX5
Control	1	Q9UJX6
Control	1	Q9UJY4
Control	1	Q9UJY5
Control	1	Q9UJZ1
Control	1	Q9UK23
Control	1	Q9UK41
Control	1	Q9UK45
Control	1	Q9UK59
Control	1	Q9UK61
Control	1	Q9UK76
Control	1	Q9UK99
Control	1	Q9UKA4
Control	1	Q9UKB1
Control	1	Q9UKD1
Control	1	Q9UKD2
Control	1	Q9UKE5
Control	1	Q9UKF6
Control	1	Q9UKG1
Control	1	Q9UKI8
Control	1	Q9UKJ3
Control	1	Q9UKK3
Control	1	Q9UKK6
Control	1	Q9UKK9
Control	1	Q9UKL0

Control	1	Q9UKL6
Control	1	Q9UKM7
Control	1	Q9UKM9
Control	1	Q9UKN8
Control	1	Q9UKR5
Control	1	Q9UKS6
Control	1	Q9UKU7
Control	1	Q9UKU9
Control	1	Q9UKV3
Control	1	Q9UKV5
Control	1	Q9UKV8
Control	1	Q9UKX7
Control	1	Q9UKY7
Control	1	Q9UKZ1
Control	1	Q9UL03
Control	1	Q9UL15
Control	1	Q9UL18
Control	1	Q9UL25
Control	1	Q9UL26
Control	1	Q9UL33
Control	1	Q9UL40
Control	1	Q9UL46
Control	1	Q9UL54
Control	1	Q9UL63
Control	1	Q9ULA0
Control	1	Q9ULC3
Control	1	Q9ULC4
Control	1	Q9ULD2
Control	1	Q9ULE0
Control	1	Q9ULE6
Control	1	Q9ULF5
Control	1	Q9ULG1
Control	1	Q9ULG6
Control	1	Q9ULH0
Control	1	Q9ULH1
Control	1	Q9ULH7
Control	1	Q9ULJ7
Control	1	Q9ULK4
Control	1	Q9ULL8
Control	1	Q9ULM3
Control	1	Q9ULP0
Control	1	Q9ULP9
Control	1	Q9ULR0
Control	1	Q9ULS5
Control	1	Q9ULT8
Control	1	Q9ULU4
Control	1	Q9ULV3
Control	1	Q9ULV4
Control	1	Q9ULW0
Control	1	Q9ULW3

Control	1	Q9ULX3
Control	1	Q9ULX6
Control	1	Q9ULX9
Control	1	Q9ULZ3
Control	1	Q9UM00
Control	1	Q9UM11
Control	1	Q9UM13
Control	1	Q9UM54
Control	1	Q9UMR2
Control	1	Q9UMR5
Control	1	Q9UMS4
Control	1	Q9UMX3
Control	1	Q9UMY1
Control	1	Q9UMY4
Control	1	Q9UMZ2
Control	1	Q9UN37
Control	1	Q9UN86
Control	1	Q9UNE7
Control	1	Q9UNF0
Control	1	Q9UNF1
Control	1	Q9UNH6
Control	1	Q9UNH7
Control	1	Q9UNI6
Control	1	Q9UNK0
Control	1	Q9UNL2
Control	1	Q9UNM6
Control	1	Q9UNN5
Control	1	Q9UNN8
Control	1	Q9UNP9
Control	1	Q9UNQ2
Control	1	Q9UNS1
Control	1	Q9UNS2
Control	1	Q9UNW1
Control	1	Q9UNW9
Control	1	Q9UNX4
Control	1	Q9UNY4
Control	1	Q9UNZ2
Control	1	Q9UP83
Control	1	Q9UP95
Control	1	Q9UPM8
Control	1	Q9UPN3
Control	1	Q9UPN4
Control	1	Q9UPN6
Control	1	Q9UPN7
Control	1	Q9UPN9
Control	1	Q9UPP1
Control	1	Q9UPQ9
Control	1	Q9UPR0
Control	1	Q9UPR3
Control	1	Q9UPT5

Control	1	Q9UPT8
Control	1	Q9UPU5
Control	1	Q9UPU7
Control	1	Q9UPU9
Control	1	Q9UPW0
Control	1	Q9UPW5
Control	1	Q9UPY3
Control	1	Q9UPY5
Control	1	Q9UPY8
Control	1	Q9UQ13
Control	1	Q9UQ35
Control	1	Q9UQ53
Control	1	Q9UQ80
Control	1	Q9UQ90
Control	1	Q9UQB8
Control	1	Q9UQC2
Control	1	Q9UQE7
Control	1	Q9UQN3
Control	1	Q9UQQ2
Control	1	Q9UQR1
Control	1	Q9Y217
Control	1	Q9Y219
Control	1	Q9Y221
Control	1	Q9Y223
Control	1	Q9Y224
Control	1	Q9Y230
Control	1	Q9Y232
Control	1	Q9Y237
Control	1	Q9Y243
Control	1	Q9Y256
Control	1	Q9Y259
Control	1	Q9Y262
Control	1	Q9Y263
Control	1	Q9Y265
Control	1	Q9Y266
Control	1	Q9Y276
Control	1	Q9Y277
Control	1	Q9Y281
Control	1	Q9Y282
Control	1	Q9Y285
Control	1	Q9Y289
Control	1	Q9Y291
Control	1	Q9Y294
Control	1	Q9Y295
Control	1	Q9Y296
Control	1	Q9Y2A7
Control	1	Q9Y2B0
Control	1	Q9Y2C4
Control	1	Q9Y2D4
Control	1	Q9Y2D5

Control	1	Q9Y2G2
Control	1	Q9Y2G3
Control	1	Q9Y2G5
Control	1	Q9Y2G8
Control	1	Q9Y2H0
Control	1	Q9Y2H1
Control	1	Q9Y2H2
Control	1	Q9Y2H6
Control	1	Q9Y2I1
Control	1	Q9Y2I7
Control	1	Q9Y2I8
Control	1	Q9Y2J2
Control	1	Q9Y2J4
Control	1	Q9Y2K2
Control	1	Q9Y2K7
Control	1	Q9Y2L1
Control	1	Q9Y2L5
Control	1	Q9Y2L9
Control	1	Q9Y2P8
Control	1	Q9Y2Q3
Control	1	Q9Y2Q5
Control	1	Q9Y2Q9
Control	1	Q9Y2R0
Control	1	Q9Y2R4
Control	1	Q9Y2R5
Control	1	Q9Y2R9
Control	1	Q9Y2S2
Control	1	Q9Y2S6
Control	1	Q9Y2S7
Control	1	Q9Y2T2
Control	1	Q9Y2U8
Control	1	Q9Y2V2
Control	1	Q9Y2V7
Control	1	Q9Y2W1
Control	1	Q9Y2W2
Control	1	Q9Y2W6
Control	1	Q9Y2X0
Control	1	Q9Y2X3
Control	1	Q9Y2X7
Control	1	Q9Y2X9
Control	1	Q9Y2Y0
Control	1	Q9Y2Y6
Control	1	Q9Y2Z0
Control	1	Q9Y2Z4
Control	1	Q9Y303
Control	1	Q9Y305
Control	1	Q9Y312
Control	1	Q9Y314
Control	1	Q9Y315
Control	1	Q9Y316

Control	1	Q9Y320
Control	1	Q9Y324
Control	1	Q9Y333
Control	1	Q9Y371
Control	1	Q9Y375
Control	1	Q9Y376
Control	1	Q9Y383
Control	1	Q9Y385
Control	1	Q9Y388
Control	1	Q9Y394
Control	1	Q9Y399
Control	1	Q9Y3A2
Control	1	Q9Y3A3
Control	1	Q9Y3A4
Control	1	Q9Y3A5
Control	1	Q9Y3A6
Control	1	Q9Y3B2
Control	1	Q9Y3B3
Control	1	Q9Y3B4
Control	1	Q9Y3B7
Control	1	Q9Y3B8
Control	1	Q9Y3B9
Control	1	Q9Y3C0
Control	1	Q9Y3C1
Control	1	Q9Y3C4
Control	1	Q9Y3C6
Control	1	Q9Y3C8
Control	1	Q9Y3D3
Control	1	Q9Y3D5
Control	1	Q9Y3D6
Control	1	Q9Y3D7
Control	1	Q9Y3D8
Control	1	Q9Y3D9
Control	1	Q9Y3E0
Control	1	Q9Y3E1
Control	1	Q9Y3E5
Control	1	Q9Y3E7
Control	1	Q9Y3F4
Control	1	Q9Y3I0
Control	1	Q9Y3I1
Control	1	Q9Y3L5
Control	1	Q9Y3M8
Control	1	Q9Y3P9
Control	1	Q9Y3Q3
Control	1	Q9Y3Q8
Control	1	Q9Y3S2
Control	1	Q9Y3T6
Control	1	Q9Y3T9
Control	1	Q9Y3U8
Control	1	Q9Y3X0

Control	1	Q9Y3Y2
Control	1	Q9Y3Z3
Control	1	Q9Y448
Control	1	Q9Y450
Control	1	Q9Y478
Control	1	Q9Y484
Control	1	Q9Y485
Control	1	Q9Y487
Control	1	Q9Y490
Control	1	Q9Y496
Control	1	Q9Y4A5
Control	1	Q9Y4B5
Control	1	Q9Y4B6
Control	1	Q9Y4C2
Control	1	Q9Y4C8
Control	1	Q9Y4D1
Control	1	Q9Y4D7
Control	1	Q9Y4D8
Control	1	Q9Y4E1
Control	1	Q9Y4E6
Control	1	Q9Y4E8
Control	1	Q9Y4F1
Control	1	Q9Y4F3
Control	1	Q9Y4F5
Control	1	Q9Y4G8
Control	1	Q9Y4I1
Control	1	Q9Y4J8
Control	1	Q9Y4K0
Control	1	Q9Y4K1
Control	1	Q9Y4K3
Control	1	Q9Y4K4
Control	1	Q9Y4L1
Control	1	Q9Y4P1
Control	1	Q9Y4P3
Control	1	Q9Y4P8
Control	1	Q9Y4R8
Control	1	Q9Y4W2
Control	1	Q9Y4W6
Control	1	Q9Y4X4
Control	1	Q9Y4X5
Control	1	Q9Y4Z0
Control	1	Q9Y508
Control	1	Q9Y512
Control	1	Q9Y520
Control	1	Q9Y530
Control	1	Q9Y547
Control	1	Q9Y570
Control	1	Q9Y580
Control	1	Q9Y584
Control	1	Q9Y5A7

Control	1	Q9Y5A9
Control	1	Q9Y5B0
Control	1	Q9Y5B6
Control	1	Q9Y5B8
Control	1	Q9Y5B9
Control	1	Q9Y5J1
Control	1	Q9Y5K5
Control	1	Q9Y5K6
Control	1	Q9Y5K8
Control	1	Q9Y5L0
Control	1	Q9Y5M8
Control	1	Q9Y5N6
Control	1	Q9Y5P4
Control	1	Q9Y5P6
Control	1	Q9Y5Q0
Control	1	Q9Y5Q8
Control	1	Q9Y5Q9
Control	1	Q9Y5R8
Control	1	Q9Y5S1
Control	1	Q9Y5S2
Control	1	Q9Y5S9
Control	1	Q9Y5T4
Control	1	Q9Y5T5
Control	1	Q9Y5U2
Control	1	Q9Y5V0
Control	1	Q9Y5V3
Control	1	Q9Y5W7
Control	1	Q9Y5W9
Control	1	Q9Y5X1
Control	1	Q9Y5X2
Control	1	Q9Y5X3
Control	1	Q9Y5X9
Control	1	Q9Y5Y2
Control	1	Q9Y5Y5
Control	1	Q9Y5Y7
Control	1	Q9Y5Z0
Control	1	Q9Y5Z7
Control	1	Q9Y5Z9
Control	1	Q9Y606
Control	1	Q9Y608
Control	1	Q9Y613
Control	1	Q9Y617
Control	1	Q9Y618
Control	1	Q9Y619
Control	1	Q9Y624
Control	1	Q9Y639
Control	1	Q9Y653
Control	1	Q9Y657
Control	1	Q9Y666
Control	1	Q9Y672

Control	1	Q9Y673
Control	1	Q9Y676
Control	1	Q9Y678
Control	1	Q9Y679
Control	1	Q9Y680
Control	1	Q9Y696
Control	1	Q9Y697
Control	1	Q9Y6A4
Control	1	Q9Y6A5
Control	1	Q9Y6A9
Control	1	Q9Y6B6
Control	1	Q9Y6B7
Control	1	Q9Y6C9
Control	1	Q9Y6D0
Control	1	Q9Y6D5
Control	1	Q9Y6D6
Control	1	Q9Y6D9
Control	1	Q9Y6E0
Control	1	Q9Y6E2
Control	1	Q9Y6G5
Control	1	Q9Y6G9
Control	1	Q9Y6I3
Control	1	Q9Y6I4
Control	1	Q9Y6I9
Control	1	Q9Y6J0
Control	1	Q9Y6K0
Control	1	Q9Y6K1
Control	1	Q9Y6K5
Control	1	Q9Y6K8
Control	1	Q9Y6K9
Control	1	Q9Y6M1
Control	1	Q9Y6M5
Control	1	Q9Y6M7
Control	1	Q9Y6M9
Control	1	Q9Y6N5
Control	1	Q9Y6N7
Control	1	Q9Y6Q2
Control	1	Q9Y6Q9
Control	1	Q9Y6R0
Control	1	Q9Y6R4
Control	1	Q9Y6V7
Control	1	Q9Y6W3
Control	1	Q9Y6W5
Control	1	Q9Y6X2
Control	1	Q9Y6X3
Control	1	Q9Y6X9
Control	1	Q9Y6Y0
Control	1	Q9Y6Y8
Control	2	A0A0B4J2D5
Control	2	A0AVF1

Control	2	A0AVT1
Control	2	A0FGR8
Control	2	A0JNW5
Control	2	A0MZ66
Control	2	A0PJW6
Control	2	A1A4S6
Control	2	A1L0T0
Control	2	A1X283
Control	2	A2RRP1
Control	2	A2RUC4
Control	2	A2RUS2
Control	2	A3KMH1
Control	2	A3KN83
Control	2	A4D1E9
Control	2	A4D1P6
Control	2	A4D1U4
Control	2	A5PLL7
Control	2	A5PLN9
Control	2	A5YKK6
Control	2	A6NCE7
Control	2	A6NDG6
Control	2	A6NDU8
Control	2	A6NED2
Control	2	A6NFQ2
Control	2	A6NHR9
Control	2	A6NHX0
Control	2	A6NIH7
Control	2	A6NJ78
Control	2	A6NKD9
Control	2	A6ZKI3
Control	2	A7E2V4
Control	2	A8CG34
Control	2	A8MW92
Control	2	A8MWD9
Control	2	A8MWY0
Control	2	A8MXV4
Control	2	A9UHW6
Control	2	P0DP23
Control	2	B2RTY4
Control	2	B7ZAQ6
Control	2	C4AMC7
Control	2	C9JLW8
Control	2	E9PAV3
Control	2	E9PRG8
Control	2	L0R819
Control	2	L0R8F8
Control	2	O00115
Control	2	O00116
Control	2	O00139
Control	2	O00148

Control	2	O00151
Control	2	O00154
Control	2	O00159
Control	2	O00161
Control	2	O00165
Control	2	O00170
Control	2	O00178
Control	2	O00182
Control	2	O00186
Control	2	O00189
Control	2	O00192
Control	2	O00193
Control	2	O00203
Control	2	O00213
Control	2	O00214
Control	2	O00217
Control	2	O00221
Control	2	O00231
Control	2	O00232
Control	2	O00233
Control	2	O00255
Control	2	O00258
Control	2	O00264
Control	2	O00267
Control	2	O00268
Control	2	O00273
Control	2	O00291
Control	2	O00299
Control	2	O00303
Control	2	O00308
Control	2	O00330
Control	2	O00391
Control	2	O00399
Control	2	O00400
Control	2	O00401
Control	2	O00410
Control	2	O00411
Control	2	O00418
Control	2	O00422
Control	2	O00423
Control	2	O00425
Control	2	O00429
Control	2	O00442
Control	2	O00443
Control	2	O00461
Control	2	O00468
Control	2	O00469
Control	2	O00471
Control	2	O00481
Control	2	O00483

Control	2	O00487
Control	2	O00499
Control	2	O00501
Control	2	O00505
Control	2	O00506
Control	2	O00522
Control	2	O00534
Control	2	O00541
Control	2	O00560
Control	2	O00562
Control	2	O00566
Control	2	O00567
Control	2	O00571
Control	2	O00587
Control	2	O00592
Control	2	O00622
Control	2	O00625
Control	2	O00629
Control	2	O00635
Control	2	O00712
Control	2	O00743
Control	2	O00746
Control	2	O00750
Control	2	O00754
Control	2	O00764
Control	2	O00767
Control	2	O14497
Control	2	O14524
Control	2	O14530
Control	2	O14531
Control	2	O14545
Control	2	O14548
Control	2	O14562
Control	2	O14578
Control	2	O14579
Control	2	O14613
Control	2	O14617
Control	2	O14618
Control	2	O14639
Control	2	O14640
Control	2	O14641
Control	2	O14646
Control	2	O14647
Control	2	O14653
Control	2	O14656
Control	2	O14657
Control	2	O14662
Control	2	O14672
Control	2	O14678
Control	2	O14681

Control	2	O14683
Control	2	O14686
Control	2	O14727
Control	2	O14730
Control	2	O14733
Control	2	O14734
Control	2	O14735
Control	2	O14737
Control	2	O14744
Control	2	O14745
Control	2	O14757
Control	2	O14763
Control	2	O14773
Control	2	O14776
Control	2	O14777
Control	2	O14782
Control	2	O14786
Control	2	O14787
Control	2	O14795
Control	2	O14798
Control	2	O14802
Control	2	O14817
Control	2	O14818
Control	2	O14828
Control	2	O14874
Control	2	O14880
Control	2	O14907
Control	2	O14908
Control	2	O14920
Control	2	O14924
Control	2	O14925
Control	2	O14929
Control	2	O14936
Control	2	O14939
Control	2	O14949
Control	2	O14950
Control	2	O14964
Control	2	O14966
Control	2	O14972
Control	2	O14974
Control	2	O14976
Control	2	O14979
Control	2	O14980
Control	2	O14981
Control	2	O15013
Control	2	O15014
Control	2	O15020
Control	2	O15021
Control	2	O15027
Control	2	O15031

Control	2	O15037
Control	2	O15040
Control	2	O15042
Control	2	O15047
Control	2	O15049
Control	2	O15056
Control	2	O15061
Control	2	O15063
Control	2	O15066
Control	2	O15067
Control	2	O15084
Control	2	O15085
Control	2	O15091
Control	2	O15111
Control	2	O15118
Control	2	O15121
Control	2	O15123
Control	2	O15126
Control	2	O15127
Control	2	O15143
Control	2	O15144
Control	2	O15145
Control	2	O15160
Control	2	O15162
Control	2	O15164
Control	2	O15173
Control	2	O15211
Control	2	O15213
Control	2	O15226
Control	2	O15228
Control	2	O15230
Control	2	O15231
Control	2	O15234
Control	2	O15235
Control	2	O15254
Control	2	O15258
Control	2	O15260
Control	2	O15264
Control	2	O15269
Control	2	O15270
Control	2	O15294
Control	2	O15305
Control	2	O15321
Control	2	O15344
Control	2	O15347
Control	2	O15355
Control	2	O15357
Control	2	O15371
Control	2	O15372
Control	2	O15379

Control	2	O15381
Control	2	O15382
Control	2	O15397
Control	2	O15400
Control	2	O15427
Control	2	O15439
Control	2	O15446
Control	2	O15460
Control	2	O15484
Control	2	O15498
Control	2	O15504
Control	2	O15511
Control	2	O15519
Control	2	O15525
Control	2	O15530
Control	2	O15533
Control	2	O15541
Control	2	O15550
Control	2	O43143
Control	2	O43148
Control	2	O43149
Control	2	O43150
Control	2	O43155
Control	2	O43156
Control	2	O43159
Control	2	O43164
Control	2	O43169
Control	2	O43172
Control	2	O43175
Control	2	O43181
Control	2	O43237
Control	2	O43242
Control	2	O43251
Control	2	O43252
Control	2	O43264
Control	2	O43290
Control	2	O43292
Control	2	O43293
Control	2	O43294
Control	2	O43299
Control	2	O43310
Control	2	O43312
Control	2	O43314
Control	2	O43318
Control	2	O43324
Control	2	O43353
Control	2	O43390
Control	2	O43395
Control	2	O43396
Control	2	O43399

Control	2	O43402
Control	2	O43414
Control	2	O43426
Control	2	O43427
Control	2	O43432
Control	2	O43447
Control	2	O43464
Control	2	O43482
Control	2	O43488
Control	2	O43491
Control	2	O43493
Control	2	O43505
Control	2	O43516
Control	2	O43520
Control	2	O43572
Control	2	O43583
Control	2	O43592
Control	2	O43598
Control	2	O43615
Control	2	O43617
Control	2	O43633
Control	2	O43639
Control	2	O43657
Control	2	O43660
Control	2	O43663
Control	2	O43665
Control	2	O43670
Control	2	O43674
Control	2	O43676
Control	2	O43678
Control	2	O43679
Control	2	O43681
Control	2	O43683
Control	2	O43684
Control	2	O43704
Control	2	O43707
Control	2	O43709
Control	2	O43719
Control	2	O43731
Control	2	O43747
Control	2	O43752
Control	2	O43760
Control	2	O43765
Control	2	O43768
Control	2	O43772
Control	2	O43776
Control	2	O43795
Control	2	O43808
Control	2	O43809
Control	2	O43813

Control	2	O43815
Control	2	O43818
Control	2	O43819
Control	2	O43822
Control	2	O43823
Control	2	O43824
Control	2	O43826
Control	2	O43837
Control	2	O43847
Control	2	O43852
Control	2	O43854
Control	2	O43861
Control	2	O43865
Control	2	O43896
Control	2	O43909
Control	2	O43913
Control	2	O43920
Control	2	O43924
Control	2	O43929
Control	2	O43933
Control	2	O60216
Control	2	O60231
Control	2	O60234
Control	2	O60239
Control	2	O60244
Control	2	O60245
Control	2	O60256
Control	2	O60264
Control	2	O60271
Control	2	O60285
Control	2	O60287
Control	2	O60292
Control	2	O60293
Control	2	O60306
Control	2	O60313
Control	2	O60315
Control	2	O60318
Control	2	O60330
Control	2	O60331
Control	2	O60333
Control	2	O60336
Control	2	O60341
Control	2	O60343
Control	2	O60427
Control	2	O60443
Control	2	O60447
Control	2	O60462
Control	2	O60476
Control	2	O60487
Control	2	O60488

Control	2	O60493
Control	2	O60502
Control	2	O60504
Control	2	O60506
Control	2	O60508
Control	2	O60518
Control	2	O60524
Control	2	O60547
Control	2	O60551
Control	2	O60563
Control	2	O60566
Control	2	O60568
Control	2	O60573
Control	2	O60610
Control	2	O60637
Control	2	O60645
Control	2	O60664
Control	2	O60678
Control	2	O60684
Control	2	O60701
Control	2	O60704
Control	2	O60711
Control	2	O60716
Control	2	O60725
Control	2	O60749
Control	2	O60762
Control	2	O60763
Control	2	O60783
Control	2	O60784
Control	2	O60814
Control	2	O60826
Control	2	O60828
Control	2	O60830
Control	2	O60831
Control	2	O60832
Control	2	O60841
Control	2	O60869
Control	2	O60870
Control	2	O60879
Control	2	O60884
Control	2	O60885
Control	2	O60921
Control	2	O60925
Control	2	O60927
Control	2	O60934
Control	2	O60942
Control	2	O75027
Control	2	O75044
Control	2	O75051
Control	2	O75063

Control	2	O75083
Control	2	O75110
Control	2	O75113
Control	2	O75116
Control	2	O75122
Control	2	O75127
Control	2	O75131
Control	2	O75143
Control	2	O75146
Control	2	O75150
Control	2	O75151
Control	2	O75152
Control	2	O75153
Control	2	O75155
Control	2	O75157
Control	2	O75165
Control	2	O75167
Control	2	O75170
Control	2	O75173
Control	2	O75175
Control	2	O75179
Control	2	O75182
Control	2	O75190
Control	2	O75208
Control	2	O75223
Control	2	O75251
Control	2	O75306
Control	2	O75312
Control	2	O75319
Control	2	O75323
Control	2	O75330
Control	2	O75340
Control	2	O75347
Control	2	O75348
Control	2	O75351
Control	2	O75352
Control	2	O75367
Control	2	O75368
Control	2	O75369
Control	2	O75376
Control	2	O75381
Control	2	O75382
Control	2	O75390
Control	2	O75391
Control	2	O75396
Control	2	O75400
Control	2	O75410
Control	2	O75414
Control	2	O75419
Control	2	O75427

Control	2	O75431
Control	2	O75436
Control	2	O75439
Control	2	O75448
Control	2	O75449
Control	2	O75475
Control	2	O75477
Control	2	O75489
Control	2	O75494
Control	2	O75503
Control	2	O75508
Control	2	O75521
Control	2	O75530
Control	2	O75531
Control	2	O75533
Control	2	O75534
Control	2	O75554
Control	2	O75569
Control	2	O75578
Control	2	O75582
Control	2	O75586
Control	2	O75592
Control	2	O75600
Control	2	O75608
Control	2	O75616
Control	2	O75643
Control	2	O75648
Control	2	O75663
Control	2	O75665
Control	2	O75676
Control	2	O75683
Control	2	O75688
Control	2	O75691
Control	2	O75694
Control	2	O75695
Control	2	O75717
Control	2	O75718
Control	2	O75746
Control	2	O75787
Control	2	O75792
Control	2	O75794
Control	2	O75815
Control	2	O75817
Control	2	O75818
Control	2	O75821
Control	2	O75822
Control	2	O75828
Control	2	O75832
Control	2	O75843
Control	2	O75844

Control	2	O75874
Control	2	O75879
Control	2	O75880
Control	2	O75882
Control	2	O75884
Control	2	O75886
Control	2	O75907
Control	2	O75909
Control	2	O75915
Control	2	O75923
Control	2	O75925
Control	2	O75934
Control	2	O75935
Control	2	O75937
Control	2	O75940
Control	2	O75943
Control	2	O75947
Control	2	O75954
Control	2	O75955
Control	2	O75962
Control	2	O75964
Control	2	O75970
Control	2	O75976
Control	2	O76003
Control	2	O76021
Control	2	O76024
Control	2	O76031
Control	2	O76054
Control	2	O76070
Control	2	O76071
Control	2	O76094
Control	2	O94760
Control	2	O94762
Control	2	O94763
Control	2	O94766
Control	2	O94776
Control	2	O94788
Control	2	O94804
Control	2	O94806
Control	2	O94808
Control	2	O94813
Control	2	O94822
Control	2	O94826
Control	2	O94827
Control	2	O94829
Control	2	O94830
Control	2	O94832
Control	2	O94842
Control	2	O94851
Control	2	O94855

Control	2	O94868
Control	2	O94874
Control	2	O94875
Control	2	O94885
Control	2	O94888
Control	2	O94889
Control	2	O94901
Control	2	O94903
Control	2	O94905
Control	2	O94906
Control	2	O94913
Control	2	O94915
Control	2	O94916
Control	2	O94919
Control	2	O94923
Control	2	O94925
Control	2	O94927
Control	2	O94929
Control	2	O94952
Control	2	O94953
Control	2	O94964
Control	2	O94966
Control	2	O94967
Control	2	O94973
Control	2	O94979
Control	2	O94989
Control	2	O94992
Control	2	O95059
Control	2	O95067
Control	2	O95070
Control	2	O95071
Control	2	O95081
Control	2	O95084
Control	2	O95104
Control	2	O95139
Control	2	O95140
Control	2	O95149
Control	2	O95155
Control	2	O95159
Control	2	O95163
Control	2	O95168
Control	2	O95169
Control	2	O95182
Control	2	O95183
Control	2	O95197
Control	2	O95202
Control	2	O95208
Control	2	O95218
Control	2	O95219
Control	2	O95232

Control	2	O95235
Control	2	O95236
Control	2	O95239
Control	2	O95243
Control	2	O95248
Control	2	O95249
Control	2	O95251
Control	2	O95260
Control	2	O95292
Control	2	O95295
Control	2	O95297
Control	2	O95298
Control	2	O95299
Control	2	O95302
Control	2	O95319
Control	2	O95336
Control	2	O95340
Control	2	O95347
Control	2	O95352
Control	2	O95359
Control	2	O95361
Control	2	O95363
Control	2	O95365
Control	2	O95372
Control	2	O95373
Control	2	O95376
Control	2	O95391
Control	2	O95394
Control	2	O95396
Control	2	O95400
Control	2	O95425
Control	2	O95433
Control	2	O95453
Control	2	O95456
Control	2	O95470
Control	2	O95476
Control	2	O95478
Control	2	O95479
Control	2	O95486
Control	2	O95487
Control	2	O95490
Control	2	O95544
Control	2	O95551
Control	2	O95563
Control	2	O95571
Control	2	O95573
Control	2	O95602
Control	2	O95619
Control	2	O95625
Control	2	O95628

Control	2	O95630
Control	2	O95639
Control	2	O95644
Control	2	O95671
Control	2	O95674
Control	2	O95685
Control	2	O95707
Control	2	O95714
Control	2	O95716
Control	2	O95721
Control	2	O95747
Control	2	O95749
Control	2	O95757
Control	2	O95758
Control	2	O95759
Control	2	O95772
Control	2	O95777
Control	2	O95782
Control	2	O95785
Control	2	O95786
Control	2	O95793
Control	2	O95801
Control	2	O95807
Control	2	O95810
Control	2	O95816
Control	2	O95817
Control	2	O95819
Control	2	O95822
Control	2	O95825
Control	2	O95831
Control	2	O95834
Control	2	O95835
Control	2	O95848
Control	2	O95858
Control	2	O95861
Control	2	O95864
Control	2	O95865
Control	2	O95870
Control	2	O95881
Control	2	O95905
Control	2	O95983
Control	2	O95985
Control	2	O95989
Control	2	O95999
Control	2	O96000
Control	2	O96005
Control	2	O96006
Control	2	O96007
Control	2	O96008
Control	2	O96011

Control	2	O96013
Control	2	O96018
Control	2	O96019
Control	2	O96028
Control	2	P00338
Control	2	P00352
Control	2	P00367
Control	2	P00374
Control	2	P00387
Control	2	P00390
Control	2	P00403
Control	2	P00491
Control	2	P00492
Control	2	P00505
Control	2	P00519
Control	2	P00558
Control	2	P00568
Control	2	P00749
Control	2	P00750
Control	2	P00813
Control	2	P01023
Control	2	P01034
Control	2	P01111
Control	2	P01112
Control	2	P01116
Control	2	P01127
Control	2	P01130
Control	2	P01137
Control	2	P01889
Control	2	P01893
Control	2	P02452
Control	2	P02462
Control	2	P02545
Control	2	P02647
Control	2	P02751
Control	2	P02765
Control	2	P02768
Control	2	P02786
Control	2	P02792
Control	2	P02794
Control	2	P02795
Control	2	P03886
Control	2	P03905
Control	2	P03915
Control	2	P03956
Control	2	P04035
Control	2	P04040
Control	2	P04049
Control	2	P04062
Control	2	P04075

Control	2	P04080
Control	2	P04083
Control	2	P04114
Control	2	P04156
Control	2	P04179
Control	2	P04181
Control	2	P04183
Control	2	P04275
Control	2	P04350
Control	2	P04406
Control	2	P04424
Control	2	P04439
Control	2	P04632
Control	2	P04637
Control	2	P04732
Control	2	P04792
Control	2	P04818
Control	2	P04843
Control	2	P04844
Control	2	P04899
Control	2	P04908
Control	2	P04920
Control	2	P05023
Control	2	P05026
Control	2	P05067
Control	2	P05091
Control	2	P05106
Control	2	P05109
Control	2	P05114
Control	2	P05120
Control	2	P05121
Control	2	P05141
Control	2	P05161
Control	2	P05165
Control	2	P05166
Control	2	P05198
Control	2	P05204
Control	2	P05362
Control	2	P05387
Control	2	P05388
Control	2	P05412
Control	2	P05423
Control	2	P05455
Control	2	P05556
Control	2	P05783
Control	2	P05997
Control	2	P06132
Control	2	P06241
Control	2	P06280
Control	2	P06396

Control	2	P06400
Control	2	P06454
Control	2	P06493
Control	2	P06576
Control	2	P06702
Control	2	P06703
Control	2	P06730
Control	2	P06733
Control	2	P06737
Control	2	P06744
Control	2	P06746
Control	2	P06748
Control	2	P06753
Control	2	P06756
Control	2	P06865
Control	2	P07099
Control	2	P07195
Control	2	P07199
Control	2	P07203
Control	2	P07237
Control	2	P07305
Control	2	P07311
Control	2	P07332
Control	2	P07339
Control	2	P07355
Control	2	P07384
Control	2	P07437
Control	2	P07602
Control	2	P07686
Control	2	P07711
Control	2	P07737
Control	2	P07738
Control	2	P07741
Control	2	P07814
Control	2	P07858
Control	2	P07900
Control	2	P07902
Control	2	P07910
Control	2	P07942
Control	2	P07947
Control	2	P07948
Control	2	P07954
Control	2	P07992
Control	2	P07996
Control	2	P08047
Control	2	P08069
Control	2	P08123
Control	2	P08133
Control	2	P08134
Control	2	P08174

Control	2	P08195
Control	2	P08236
Control	2	P08237
Control	2	P08238
Control	2	P08240
Control	2	P08243
Control	2	P08253
Control	2	P08397
Control	2	P08473
Control	2	P08493
Control	2	P08559
Control	2	P08572
Control	2	P08574
Control	2	P08579
Control	2	P08581
Control	2	P08621
Control	2	P08648
Control	2	P08651
Control	2	P08670
Control	2	P08708
Control	2	P08754
Control	2	P08758
Control	2	P08865
Control	2	P08962
Control	2	P09001
Control	2	P09012
Control	2	P09104
Control	2	P09110
Control	2	P09132
Control	2	P09211
Control	2	P09234
Control	2	P09382
Control	2	P09417
Control	2	P09429
Control	2	P09486
Control	2	P09493
Control	2	P09496
Control	2	P09497
Control	2	P09525
Control	2	P09543
Control	2	P09601
Control	2	P09622
Control	2	P09651
Control	2	P09661
Control	2	P09669
Control	2	P09758
Control	2	P09874
Control	2	P09884
Control	2	P09936
Control	2	P09960

Control	2	P09972
Control	2	P0C0S5
Control	2	P0C0S8
Control	2	P0CAP2
Control	2	P0CG29
Control	2	P0CG39
Control	2	P0CW19
Control	2	P0DMV8
Control	2	P0DP91
Control	2	P0DPH7
Control	2	P0DTL6
Control	2	P10114
Control	2	P10124
Control	2	P10155
Control	2	P10253
Control	2	P10301
Control	2	P10321
Control	2	P10398
Control	2	P10412
Control	2	P10515
Control	2	P10586
Control	2	P10589
Control	2	P10599
Control	2	P10606
Control	2	P10619
Control	2	P10620
Control	2	P10644
Control	2	P10646
Control	2	P10768
Control	2	P10809
Control	2	P10909
Control	2	P11021
Control	2	P11047
Control	2	P11117
Control	2	P11137
Control	2	P11142
Control	2	P11166
Control	2	P11169
Control	2	P11171
Control	2	P11172
Control	2	P11177
Control	2	P11182
Control	2	P11216
Control	2	P11217
Control	2	P11233
Control	2	P11234
Control	2	P11274
Control	2	P11279
Control	2	P11308
Control	2	P11310

Control	2	P11387
Control	2	P11388
Control	2	P11413
Control	2	P11441
Control	2	P11498
Control	2	P11586
Control	2	P11717
Control	2	P11766
Control	2	P11802
Control	2	P11908
Control	2	P11940
Control	2	P12004
Control	2	P12081
Control	2	P12109
Control	2	P12111
Control	2	P12235
Control	2	P12236
Control	2	P12268
Control	2	P12270
Control	2	P12429
Control	2	P12694
Control	2	P12814
Control	2	P12821
Control	2	P12931
Control	2	P12955
Control	2	P12956
Control	2	P13010
Control	2	P13051
Control	2	P13073
Control	2	P13196
Control	2	P13473
Control	2	P13489
Control	2	P13598
Control	2	P13639
Control	2	P13667
Control	2	P13674
Control	2	P13693
Control	2	P13716
Control	2	P13747
Control	2	P13797
Control	2	P13798
Control	2	P13804
Control	2	P13807
Control	2	P13861
Control	2	P13984
Control	2	P13987
Control	2	P13995
Control	2	P14174
Control	2	P14209
Control	2	P14314

Control	2	P14317
Control	2	P14324
Control	2	P14406
Control	2	P14543
Control	2	P14550
Control	2	P14618
Control	2	P14625
Control	2	P14635
Control	2	P14649
Control	2	P14678
Control	2	P14735
Control	2	P14859
Control	2	P14866
Control	2	P14868
Control	2	P14921
Control	2	P14923
Control	2	P14927
Control	2	P15056
Control	2	P15090
Control	2	P15104
Control	2	P15121
Control	2	P15144
Control	2	P15151
Control	2	P15153
Control	2	P15170
Control	2	P15289
Control	2	P15291
Control	2	P15311
Control	2	P15374
Control	2	P15407
Control	2	P15408
Control	2	P15529
Control	2	P15531
Control	2	P15559
Control	2	P15586
Control	2	P15735
Control	2	P15848
Control	2	P15880
Control	2	P15884
Control	2	P15924
Control	2	P15927
Control	2	P16035
Control	2	P16070
Control	2	P16152
Control	2	P16219
Control	2	P16220
Control	2	P16278
Control	2	P16284
Control	2	P16298
Control	2	P16333

Control	2	P16383
Control	2	P16401
Control	2	P16402
Control	2	P16403
Control	2	P16435
Control	2	P16591
Control	2	P16615
Control	2	P16885
Control	2	P16930
Control	2	P16949
Control	2	P16989
Control	2	P17026
Control	2	P17028
Control	2	P17050
Control	2	P17096
Control	2	P17152
Control	2	P17174
Control	2	P17252
Control	2	P17301
Control	2	P17302
Control	2	P17405
Control	2	P17480
Control	2	P17535
Control	2	P17542
Control	2	P17544
Control	2	P17568
Control	2	P17612
Control	2	P17655
Control	2	P17706
Control	2	P17812
Control	2	P17813
Control	2	P17844
Control	2	P17858
Control	2	P17948
Control	2	P17980
Control	2	P17987
Control	2	P18031
Control	2	P18074
Control	2	P18077
Control	2	P18084
Control	2	P18085
Control	2	P18124
Control	2	P18206
Control	2	P18433
Control	2	P18440
Control	2	P18583
Control	2	P18615
Control	2	P18621
Control	2	P18669
Control	2	P18754

Control	2	P18846
Control	2	P18850
Control	2	P18858
Control	2	P18887
Control	2	P19021
Control	2	P19022
Control	2	P19174
Control	2	P19338
Control	2	P19367
Control	2	P19387
Control	2	P19388
Control	2	P19404
Control	2	P19447
Control	2	P19474
Control	2	P19525
Control	2	P19623
Control	2	P19634
Control	2	P19784
Control	2	P19838
Control	2	P20020
Control	2	P20042
Control	2	P20073
Control	2	P20248
Control	2	P20290
Control	2	P20337
Control	2	P20338
Control	2	P20339
Control	2	P20340
Control	2	P20585
Control	2	P20594
Control	2	P20618
Control	2	P20645
Control	2	P20674
Control	2	P20700
Control	2	P20810
Control	2	P20839
Control	2	P20908
Control	2	P20929
Control	2	P20933
Control	2	P20936
Control	2	P20962
Control	2	P21127
Control	2	P21266
Control	2	P21281
Control	2	P21283
Control	2	P21291
Control	2	P21333
Control	2	P21359
Control	2	P21397
Control	2	P21399

Control	2	P21453
Control	2	P21579
Control	2	P21589
Control	2	P21675
Control	2	P21741
Control	2	P21796
Control	2	P21810
Control	2	P21912
Control	2	P21926
Control	2	P21964
Control	2	P21980
Control	2	P22033
Control	2	P22059
Control	2	P22061
Control	2	P22087
Control	2	P22102
Control	2	P22234
Control	2	P22307
Control	2	P22314
Control	2	P22392
Control	2	P22528
Control	2	P22570
Control	2	P22626
Control	2	P22670
Control	2	P22681
Control	2	P22692
Control	2	P22694
Control	2	P22695
Control	2	P22830
Control	2	P23193
Control	2	P23219
Control	2	P23229
Control	2	P23246
Control	2	P23258
Control	2	P23284
Control	2	P23368
Control	2	P23381
Control	2	P23396
Control	2	P23443
Control	2	P23458
Control	2	P23467
Control	2	P23469
Control	2	P23470
Control	2	P23497
Control	2	P23508
Control	2	P23510
Control	2	P23526
Control	2	P23528
Control	2	P23588
Control	2	P23610

Control	2	P23634
Control	2	P23743
Control	2	P23786
Control	2	P23919
Control	2	P23921
Control	2	P24385
Control	2	P24386
Control	2	P24390
Control	2	P24468
Control	2	P24534
Control	2	P24539
Control	2	P24666
Control	2	P24723
Control	2	P24752
Control	2	P24844
Control	2	P24928
Control	2	P24941
Control	2	P25098
Control	2	P25116
Control	2	P25205
Control	2	P25325
Control	2	P25398
Control	2	P25440
Control	2	P25445
Control	2	P25490
Control	2	P25685
Control	2	P25686
Control	2	P25705
Control	2	P25774
Control	2	P25786
Control	2	P25787
Control	2	P25788
Control	2	P25789
Control	2	P25942
Control	2	P26006
Control	2	P26022
Control	2	P26038
Control	2	P26196
Control	2	P26358
Control	2	P26368
Control	2	P26373
Control	2	P26374
Control	2	P26440
Control	2	P26572
Control	2	P26583
Control	2	P26599
Control	2	P26639
Control	2	P26640
Control	2	P26641
Control	2	P26885

Control	2	P26951
Control	2	P27105
Control	2	P27144
Control	2	P27348
Control	2	P27361
Control	2	P27448
Control	2	P27635
Control	2	P27658
Control	2	P27694
Control	2	P27695
Control	2	P27701
Control	2	P27707
Control	2	P27708
Control	2	P27797
Control	2	P27816
Control	2	P27824
Control	2	P27986
Control	2	P27987
Control	2	P28062
Control	2	P28065
Control	2	P28066
Control	2	P28070
Control	2	P28072
Control	2	P28074
Control	2	P28161
Control	2	P28288
Control	2	P28290
Control	2	P28300
Control	2	P28331
Control	2	P28340
Control	2	P28370
Control	2	P28482
Control	2	P28702
Control	2	P28715
Control	2	P28799
Control	2	P28827
Control	2	P28838
Control	2	P28906
Control	2	P29083
Control	2	P29084
Control	2	P29144
Control	2	P29218
Control	2	P29279
Control	2	P29317
Control	2	P29323
Control	2	P29353
Control	2	P29372
Control	2	P29401
Control	2	P29466
Control	2	P29474

Control	2	P29558
Control	2	P29590
Control	2	P29597
Control	2	P29692
Control	2	P29966
Control	2	P29992
Control	2	P30038
Control	2	P30040
Control	2	P30041
Control	2	P30043
Control	2	P30044
Control	2	P30048
Control	2	P30050
Control	2	P30084
Control	2	P30085
Control	2	P30086
Control	2	P30101
Control	2	P30153
Control	2	P30154
Control	2	P30260
Control	2	P30281
Control	2	P30405
Control	2	P30414
Control	2	P30419
Control	2	P30519
Control	2	P30520
Control	2	P30530
Control	2	P30533
Control	2	P30536
Control	2	P30566
Control	2	P30622
Control	2	P30626
Control	2	P30740
Control	2	P30825
Control	2	P30837
Control	2	P30876
Control	2	P31040
Control	2	P31146
Control	2	P31150
Control	2	P31151
Control	2	P31153
Control	2	P31323
Control	2	P31350
Control	2	P31431
Control	2	P31483
Control	2	P31641
Control	2	P31689
Control	2	P31749
Control	2	P31930
Control	2	P31937

Control	2	P31939
Control	2	P31942
Control	2	P31943
Control	2	P31944
Control	2	P31946
Control	2	P31948
Control	2	P31949
Control	2	P32119
Control	2	P32121
Control	2	P32189
Control	2	P32321
Control	2	P32322
Control	2	P32455
Control	2	P32456
Control	2	P32519
Control	2	P32780
Control	2	P32856
Control	2	P32969
Control	2	P33121
Control	2	P33151
Control	2	P33176
Control	2	P33316
Control	2	P33527
Control	2	P33897
Control	2	P33981
Control	2	P33991
Control	2	P33992
Control	2	P33993
Control	2	P34059
Control	2	P34896
Control	2	P34897
Control	2	P34931
Control	2	P34932
Control	2	P34947
Control	2	P34949
Control	2	P35052
Control	2	P35080
Control	2	P35221
Control	2	P35222
Control	2	P35232
Control	2	P35237
Control	2	P35240
Control	2	P35241
Control	2	P35244
Control	2	P35249
Control	2	P35250
Control	2	P35251
Control	2	P35268
Control	2	P35269
Control	2	P35270

Control	2	P35367
Control	2	P35520
Control	2	P35555
Control	2	P35556
Control	2	P35573
Control	2	P35579
Control	2	P35580
Control	2	P35590
Control	2	P35606
Control	2	P35610
Control	2	P35611
Control	2	P35613
Control	2	P35637
Control	2	P35658
Control	2	P35659
Control	2	P35713
Control	2	P35749
Control	2	P35813
Control	2	P35914
Control	2	P35916
Control	2	P35968
Control	2	P35998
Control	2	P36269
Control	2	P36404
Control	2	P36405
Control	2	P36507
Control	2	P36542
Control	2	P36543
Control	2	P36551
Control	2	P36578
Control	2	P36639
Control	2	P36776
Control	2	P36871
Control	2	P36873
Control	2	P36915
Control	2	P36957
Control	2	P36959
Control	2	P36969
Control	2	P37023
Control	2	P37059
Control	2	P37108
Control	2	P37173
Control	2	P37198
Control	2	P37235
Control	2	P37268
Control	2	P37275
Control	2	P37802
Control	2	P37837
Control	2	P37840
Control	2	P38117

Control	2	P38159
Control	2	P38432
Control	2	P38435
Control	2	P38571
Control	2	P38606
Control	2	P38646
Control	2	P38919
Control	2	P38935
Control	2	P38936
Control	2	P39019
Control	2	P39023
Control	2	P39060
Control	2	P39656
Control	2	P39687
Control	2	P39748
Control	2	P39880
Control	2	P40121
Control	2	P40123
Control	2	P40189
Control	2	P40222
Control	2	P40227
Control	2	P40261
Control	2	P40306
Control	2	P40425
Control	2	P40429
Control	2	P40616
Control	2	P40692
Control	2	P40763
Control	2	P40818
Control	2	P40925
Control	2	P40926
Control	2	P40937
Control	2	P40938
Control	2	P40939
Control	2	P41091
Control	2	P41162
Control	2	P41208
Control	2	P41212
Control	2	P41214
Control	2	P41223
Control	2	P41226
Control	2	P41227
Control	2	P41229
Control	2	P41240
Control	2	P41250
Control	2	P41252
Control	2	P41440
Control	2	P41567
Control	2	P41743
Control	2	P41970

Control	2	P42025
Control	2	P42126
Control	2	P42166
Control	2	P42167
Control	2	P42224
Control	2	P42226
Control	2	P42229
Control	2	P42285
Control	2	P42330
Control	2	P42336
Control	2	P42338
Control	2	P42345
Control	2	P42356
Control	2	P42566
Control	2	P42574
Control	2	P42575
Control	2	P42677
Control	2	P42684
Control	2	P42694
Control	2	P42695
Control	2	P42696
Control	2	P42704
Control	2	P42765
Control	2	P42766
Control	2	P42785
Control	2	P42858
Control	2	P42892
Control	2	P42898
Control	2	P43007
Control	2	P43034
Control	2	P43121
Control	2	P43155
Control	2	P43243
Control	2	P43246
Control	2	P43250
Control	2	P43304
Control	2	P43307
Control	2	P43378
Control	2	P43487
Control	2	P43490
Control	2	P43686
Control	2	P43897
Control	2	P45877
Control	2	P45880
Control	2	P45954
Control	2	P45973
Control	2	P45974
Control	2	P45983
Control	2	P45985
Control	2	P46013

Control	2	P46019
Control	2	P46020
Control	2	P46060
Control	2	P46063
Control	2	P46087
Control	2	P46100
Control	2	P46108
Control	2	P46109
Control	2	P46199
Control	2	P46379
Control	2	P46459
Control	2	P46531
Control	2	P46734
Control	2	P46736
Control	2	P46776
Control	2	P46777
Control	2	P46778
Control	2	P46779
Control	2	P46781
Control	2	P46782
Control	2	P46783
Control	2	P46821
Control	2	P46926
Control	2	P46934
Control	2	P46939
Control	2	P46940
Control	2	P46976
Control	2	P46977
Control	2	P47712
Control	2	P47755
Control	2	P47756
Control	2	P47813
Control	2	P47895
Control	2	P47897
Control	2	P47914
Control	2	P47929
Control	2	P47974
Control	2	P47985
Control	2	P48047
Control	2	P48059
Control	2	P48060
Control	2	P48147
Control	2	P48163
Control	2	P48200
Control	2	P48307
Control	2	P48382
Control	2	P48426
Control	2	P48444
Control	2	P48449
Control	2	P48454

Control	2	P48506
Control	2	P48507
Control	2	P48509
Control	2	P48553
Control	2	P48556
Control	2	P48634
Control	2	P48637
Control	2	P48643
Control	2	P48651
Control	2	P48681
Control	2	P48723
Control	2	P48729
Control	2	P48730
Control	2	P48735
Control	2	P48736
Control	2	P48739
Control	2	P49005
Control	2	P49006
Control	2	P49023
Control	2	P49069
Control	2	P49116
Control	2	P49137
Control	2	P49184
Control	2	P49189
Control	2	P49207
Control	2	P49247
Control	2	P49257
Control	2	P49281
Control	2	P49321
Control	2	P49327
Control	2	P49354
Control	2	P49356
Control	2	P49366
Control	2	P49368
Control	2	P49406
Control	2	P49407
Control	2	P49411
Control	2	P49419
Control	2	P49427
Control	2	P49441
Control	2	P49454
Control	2	P49458
Control	2	P49585
Control	2	P49588
Control	2	P49589
Control	2	P49590
Control	2	P49591
Control	2	P49593
Control	2	P49641
Control	2	P49642

Control	2	P49643
Control	2	P49662
Control	2	P49674
Control	2	P49720
Control	2	P49721
Control	2	P49736
Control	2	P49748
Control	2	P49750
Control	2	P49754
Control	2	P49755
Control	2	P49756
Control	2	P49757
Control	2	P49761
Control	2	P49768
Control	2	P49770
Control	2	P49773
Control	2	P49790
Control	2	P49792
Control	2	P49795
Control	2	P49796
Control	2	P49810
Control	2	P49815
Control	2	P49821
Control	2	P49840
Control	2	P49841
Control	2	P49848
Control	2	P49902
Control	2	P49903
Control	2	P49914
Control	2	P49915
Control	2	P49916
Control	2	P49917
Control	2	P49959
Control	2	P50148
Control	2	P50213
Control	2	P50225
Control	2	P50281
Control	2	P50336
Control	2	P50395
Control	2	P50402
Control	2	P50416
Control	2	P50443
Control	2	P50452
Control	2	P50453
Control	2	P50454
Control	2	P50479
Control	2	P50502
Control	2	P50542
Control	2	P50548
Control	2	P50552

Control	2	P50570
Control	2	P50579
Control	2	P50583
Control	2	P50613
Control	2	P50748
Control	2	P50749
Control	2	P50750
Control	2	P50851
Control	2	P50895
Control	2	P50897
Control	2	P50914
Control	2	P50990
Control	2	P50991
Control	2	P50995
Control	2	P51003
Control	2	P51114
Control	2	P51116
Control	2	P51148
Control	2	P51149
Control	2	P51151
Control	2	P51153
Control	2	P51159
Control	2	P51178
Control	2	P51398
Control	2	P51452
Control	2	P51531
Control	2	P51532
Control	2	P51553
Control	2	P51570
Control	2	P51571
Control	2	P51572
Control	2	P51580
Control	2	P51608
Control	2	P51610
Control	2	P51617
Control	2	P51636
Control	2	P51648
Control	2	P51659
Control	2	P51665
Control	2	P51687
Control	2	P51688
Control	2	P51692
Control	2	P51784
Control	2	P51798
Control	2	P51809
Control	2	P51812
Control	2	P51813
Control	2	P51858
Control	2	P51946
Control	2	P51948

Control	2	P51956
Control	2	P51965
Control	2	P51970
Control	2	P51991
Control	2	P52209
Control	2	P52272
Control	2	P52292
Control	2	P52294
Control	2	P52298
Control	2	P52306
Control	2	P52434
Control	2	P52564
Control	2	P52565
Control	2	P52566
Control	2	P52594
Control	2	P52597
Control	2	P52630
Control	2	P52701
Control	2	P52732
Control	2	P52735
Control	2	P52747
Control	2	P52756
Control	2	P52758
Control	2	P52788
Control	2	P52789
Control	2	P52799
Control	2	P52815
Control	2	P52824
Control	2	P52848
Control	2	P52888
Control	2	P52907
Control	2	P52926
Control	2	P52943
Control	2	P52948
Control	2	P53004
Control	2	P53007
Control	2	P53041
Control	2	P53350
Control	2	P53355
Control	2	P53365
Control	2	P53367
Control	2	P53384
Control	2	P53396
Control	2	P53582
Control	2	P53597
Control	2	P53602
Control	2	P53609
Control	2	P53611
Control	2	P53618
Control	2	P53621

Control	2	P53634
Control	2	P53667
Control	2	P53671
Control	2	P53675
Control	2	P53677
Control	2	P53680
Control	2	P53701
Control	2	P53778
Control	2	P53801
Control	2	P53814
Control	2	P53985
Control	2	P53990
Control	2	P53992
Control	2	P53999
Control	2	P54098
Control	2	P54105
Control	2	P54136
Control	2	P54198
Control	2	P54252
Control	2	P54259
Control	2	P54277
Control	2	P54278
Control	2	P54289
Control	2	P54577
Control	2	P54578
Control	2	P54619
Control	2	P54652
Control	2	P54687
Control	2	P54709
Control	2	P54727
Control	2	P54760
Control	2	P54802
Control	2	P54819
Control	2	P54886
Control	2	P54920
Control	2	P55010
Control	2	P55011
Control	2	P55036
Control	2	P55039
Control	2	P55060
Control	2	P55072
Control	2	P55081
Control	2	P55084
Control	2	P55145
Control	2	P55196
Control	2	P55199
Control	2	P55201
Control	2	P55209
Control	2	P55210
Control	2	P55212

Control	2	P55263
Control	2	P55265
Control	2	P55268
Control	2	P55290
Control	2	P55735
Control	2	P55769
Control	2	P55786
Control	2	P55789
Control	2	P55795
Control	2	P55809
Control	2	P55884
Control	2	P55899
Control	2	P55957
Control	2	P56134
Control	2	P56182
Control	2	P56192
Control	2	P56211
Control	2	P56377
Control	2	P56378
Control	2	P56381
Control	2	P56385
Control	2	P56524
Control	2	P56537
Control	2	P56545
Control	2	P56556
Control	2	P56589
Control	2	P56937
Control	2	P56945
Control	2	P56962
Control	2	P57076
Control	2	P57081
Control	2	P57088
Control	2	P57678
Control	2	P57737
Control	2	P57740
Control	2	P57764
Control	2	P57772
Control	2	P58004
Control	2	P58335
Control	2	P58546
Control	2	P59998
Control	2	P60033
Control	2	P60059
Control	2	P60174
Control	2	P60228
Control	2	P60468
Control	2	P60484
Control	2	P60510
Control	2	P60520
Control	2	P60602

Control	2	P60660
Control	2	P60709
Control	2	P60842
Control	2	P60866
Control	2	P60891
Control	2	P60900
Control	2	P60903
Control	2	P60953
Control	2	P60981
Control	2	P60983
Control	2	P61006
Control	2	P61009
Control	2	P61011
Control	2	P61018
Control	2	P61019
Control	2	P61020
Control	2	P61026
Control	2	P61077
Control	2	P61081
Control	2	P61086
Control	2	P61088
Control	2	P61106
Control	2	P61158
Control	2	P61160
Control	2	P61163
Control	2	P61201
Control	2	P61221
Control	2	P61224
Control	2	P61225
Control	2	P61244
Control	2	P61247
Control	2	P61254
Control	2	P61289
Control	2	P61313
Control	2	P61353
Control	2	P61421
Control	2	P61513
Control	2	P61586
Control	2	P61587
Control	2	P61599
Control	2	P61604
Control	2	P61619
Control	2	P61626
Control	2	P61758
Control	2	P61764
Control	2	P61769
Control	2	P61803
Control	2	P61916
Control	2	P61923
Control	2	P61927

Control	2	P61956
Control	2	P61962
Control	2	P61964
Control	2	P61966
Control	2	P61970
Control	2	P61978
Control	2	P61981
Control	2	P62068
Control	2	P62070
Control	2	P62081
Control	2	P62136
Control	2	P62140
Control	2	P62191
Control	2	P62195
Control	2	P62241
Control	2	P62244
Control	2	P62249
Control	2	P62253
Control	2	P62256
Control	2	P62258
Control	2	P62263
Control	2	P62266
Control	2	P62269
Control	2	P62273
Control	2	P62277
Control	2	P62280
Control	2	P62304
Control	2	P62306
Control	2	P62310
Control	2	P62312
Control	2	P62314
Control	2	P62316
Control	2	P62318
Control	2	P62328
Control	2	P62330
Control	2	P62333
Control	2	P62341
Control	2	P62380
Control	2	P62424
Control	2	P62487
Control	2	P62491
Control	2	P62495
Control	2	P62633
Control	2	P62699
Control	2	P62701
Control	2	P62714
Control	2	P62736
Control	2	P62745
Control	2	P62750
Control	2	P62753

Control	2	P62805
Control	2	P62820
Control	2	P62826
Control	2	P62829
Control	2	P62834
Control	2	P62841
Control	2	P62847
Control	2	P62851
Control	2	P62854
Control	2	P62857
Control	2	P62861
Control	2	P62873
Control	2	P62877
Control	2	P62879
Control	2	P62888
Control	2	P62891
Control	2	P62899
Control	2	P62906
Control	2	P62910
Control	2	P62913
Control	2	P62917
Control	2	P62937
Control	2	P62942
Control	2	P62979
Control	2	P62993
Control	2	P62995
Control	2	P63000
Control	2	P63010
Control	2	P63027
Control	2	P63092
Control	2	P63096
Control	2	P63104
Control	2	P63151
Control	2	P63165
Control	2	P63167
Control	2	P63172
Control	2	P63173
Control	2	P63208
Control	2	P63218
Control	2	P63220
Control	2	P63241
Control	2	P63244
Control	2	P63272
Control	2	P63279
Control	2	P63313
Control	2	P67775
Control	2	P67809
Control	2	P67812
Control	2	P67870
Control	2	P67936

Control	2	P68036
Control	2	P68104
Control	2	P68363
Control	2	P68366
Control	2	P68371
Control	2	P68400
Control	2	P68402
Control	2	P68431
Control	2	P68871
Control	2	P69905
Control	2	P78310
Control	2	P78312
Control	2	P78316
Control	2	P78318
Control	2	P78324
Control	2	P78330
Control	2	P78332
Control	2	P78344
Control	2	P78345
Control	2	P78346
Control	2	P78347
Control	2	P78356
Control	2	P78357
Control	2	P78362
Control	2	P78371
Control	2	P78381
Control	2	P78406
Control	2	P78417
Control	2	P78527
Control	2	P78536
Control	2	P78537
Control	2	P78539
Control	2	P78540
Control	2	P78549
Control	2	P80217
Control	2	P80303
Control	2	P80723
Control	2	P81605
Control	2	P82094
Control	2	P82650
Control	2	P82663
Control	2	P82664
Control	2	P82673
Control	2	P82675
Control	2	P82912
Control	2	P82914
Control	2	P82921
Control	2	P82930
Control	2	P82932
Control	2	P82933

Control	2	P82979
Control	2	P83111
Control	2	P83436
Control	2	P83731
Control	2	P83876
Control	2	P83881
Control	2	P83916
Control	2	P84022
Control	2	P84077
Control	2	P84085
Control	2	P84090
Control	2	P84095
Control	2	P84098
Control	2	P84101
Control	2	P84103
Control	2	P85037
Control	2	P86790
Control	2	P98082
Control	2	P98160
Control	2	P98170
Control	2	P98172
Control	2	P98175
Control	2	P98179
Control	2	P98194
Control	2	P98196
Control	2	P99999
Control	2	Q00013
Control	2	Q00059
Control	2	Q00169
Control	2	Q00325
Control	2	Q00341
Control	2	Q00403
Control	2	Q00534
Control	2	Q00535
Control	2	Q00537
Control	2	Q00577
Control	2	Q00587
Control	2	Q00610
Control	2	Q00613
Control	2	Q00653
Control	2	Q00688
Control	2	Q00765
Control	2	Q00796
Control	2	Q00839
Control	2	Q01081
Control	2	Q01082
Control	2	Q01085
Control	2	Q01105
Control	2	Q01130
Control	2	Q01167

Control	2	Q01201
Control	2	Q01415
Control	2	Q01433
Control	2	Q01469
Control	2	Q01518
Control	2	Q01543
Control	2	Q01581
Control	2	Q01628
Control	2	Q01650
Control	2	Q01658
Control	2	Q01780
Control	2	Q01804
Control	2	Q01813
Control	2	Q01831
Control	2	Q01844
Control	2	Q01850
Control	2	Q01968
Control	2	Q01970
Control	2	Q01995
Control	2	Q02040
Control	2	Q02127
Control	2	Q02218
Control	2	Q02224
Control	2	Q02241
Control	2	Q02252
Control	2	Q02297
Control	2	Q02318
Control	2	Q02413
Control	2	Q02447
Control	2	Q02539
Control	2	Q02543
Control	2	Q02750
Control	2	Q02763
Control	2	Q02790
Control	2	Q02809
Control	2	Q02818
Control	2	Q02878
Control	2	Q02880
Control	2	Q02952
Control	2	Q02978
Control	2	Q03001
Control	2	Q03111
Control	2	Q03112
Control	2	Q03113
Control	2	Q03135
Control	2	Q03154
Control	2	Q03169
Control	2	Q03252
Control	2	Q03393
Control	2	Q03405

Control	2	Q03426
Control	2	Q03468
Control	2	Q03518
Control	2	Q03519
Control	2	Q03701
Control	2	Q03936
Control	2	Q04206
Control	2	Q04323
Control	2	Q04446
Control	2	Q04637
Control	2	Q04656
Control	2	Q04721
Control	2	Q04724
Control	2	Q04726
Control	2	Q04760
Control	2	Q04771
Control	2	Q04837
Control	2	Q04864
Control	2	Q04917
Control	2	Q04941
Control	2	Q05048
Control	2	Q05086
Control	2	Q05193
Control	2	Q05209
Control	2	Q05397
Control	2	Q05519
Control	2	Q05655
Control	2	Q05682
Control	2	Q05932
Control	2	Q05D32
Control	2	Q06124
Control	2	Q06136
Control	2	Q06203
Control	2	Q06210
Control	2	Q06265
Control	2	Q06323
Control	2	Q06330
Control	2	Q06413
Control	2	Q06481
Control	2	Q06546
Control	2	Q06587
Control	2	Q06787
Control	2	Q06830
Control	2	Q07020
Control	2	Q07021
Control	2	Q07065
Control	2	Q07157
Control	2	Q07352
Control	2	Q07617
Control	2	Q07666

Control	2	Q07812
Control	2	Q07817
Control	2	Q07820
Control	2	Q07864
Control	2	Q07866
Control	2	Q07889
Control	2	Q07955
Control	2	Q07960
Control	2	Q08117
Control	2	Q08170
Control	2	Q08174
Control	2	Q08209
Control	2	Q08211
Control	2	Q08257
Control	2	Q08378
Control	2	Q08379
Control	2	Q08426
Control	2	Q08431
Control	2	Q08499
Control	2	Q08554
Control	2	Q08623
Control	2	Q08629
Control	2	Q08722
Control	2	Q08752
Control	2	Q08945
Control	2	Q08AD1
Control	2	Q08AE8
Control	2	Q08AF3
Control	2	Q08AM6
Control	2	Q08J23
Control	2	Q09019
Control	2	Q09028
Control	2	Q09161
Control	2	Q09328
Control	2	Q09472
Control	2	Q09666
Control	2	Q0JRZ9
Control	2	Q0PNE2
Control	2	Q0VDF9
Control	2	Q0VDG4
Control	2	Q0VF96
Control	2	Q0VGL1
Control	2	Q0ZGT2
Control	2	Q10469
Control	2	Q10471
Control	2	Q10472
Control	2	Q10567
Control	2	Q10570
Control	2	Q10589
Control	2	Q10713

Control	2	Q11201
Control	2	Q12765
Control	2	Q12768
Control	2	Q12769
Control	2	Q12770
Control	2	Q12778
Control	2	Q12788
Control	2	Q12789
Control	2	Q12792
Control	2	Q12797
Control	2	Q12800
Control	2	Q12802
Control	2	Q12805
Control	2	Q12824
Control	2	Q12830
Control	2	Q12834
Control	2	Q12841
Control	2	Q12846
Control	2	Q12849
Control	2	Q12851
Control	2	Q12866
Control	2	Q12872
Control	2	Q12873
Control	2	Q12874
Control	2	Q12882
Control	2	Q12888
Control	2	Q12893
Control	2	Q12894
Control	2	Q12899
Control	2	Q12904
Control	2	Q12905
Control	2	Q12906
Control	2	Q12907
Control	2	Q12931
Control	2	Q12933
Control	2	Q12959
Control	2	Q12965
Control	2	Q12972
Control	2	Q12974
Control	2	Q12979
Control	2	Q12980
Control	2	Q12981
Control	2	Q12982
Control	2	Q12986
Control	2	Q12996
Control	2	Q12999
Control	2	Q13011
Control	2	Q13017
Control	2	Q13033
Control	2	Q13042

Control	2	Q13043
Control	2	Q13045
Control	2	Q13049
Control	2	Q13057
Control	2	Q13084
Control	2	Q13085
Control	2	Q13098
Control	2	Q13107
Control	2	Q13111
Control	2	Q13112
Control	2	Q13123
Control	2	Q13126
Control	2	Q13131
Control	2	Q13136
Control	2	Q13137
Control	2	Q13144
Control	2	Q13148
Control	2	Q13151
Control	2	Q13155
Control	2	Q13158
Control	2	Q13162
Control	2	Q13163
Control	2	Q13164
Control	2	Q13177
Control	2	Q13185
Control	2	Q13188
Control	2	Q13190
Control	2	Q13200
Control	2	Q13201
Control	2	Q13206
Control	2	Q13217
Control	2	Q13228
Control	2	Q13232
Control	2	Q13242
Control	2	Q13243
Control	2	Q13247
Control	2	Q13257
Control	2	Q13263
Control	2	Q13275
Control	2	Q13277
Control	2	Q13283
Control	2	Q13287
Control	2	Q13308
Control	2	Q13310
Control	2	Q13315
Control	2	Q13322
Control	2	Q13330
Control	2	Q13347
Control	2	Q13356
Control	2	Q13362

Control	2	Q13363
Control	2	Q13371
Control	2	Q13393
Control	2	Q13395
Control	2	Q13404
Control	2	Q13405
Control	2	Q13409
Control	2	Q13416
Control	2	Q13418
Control	2	Q13423
Control	2	Q13425
Control	2	Q13426
Control	2	Q13427
Control	2	Q13428
Control	2	Q13432
Control	2	Q13435
Control	2	Q13438
Control	2	Q13439
Control	2	Q13442
Control	2	Q13443
Control	2	Q13444
Control	2	Q13445
Control	2	Q13451
Control	2	Q13459
Control	2	Q13464
Control	2	Q13469
Control	2	Q13472
Control	2	Q13485
Control	2	Q13488
Control	2	Q13490
Control	2	Q13492
Control	2	Q13496
Control	2	Q13501
Control	2	Q13505
Control	2	Q13509
Control	2	Q13510
Control	2	Q13523
Control	2	Q13526
Control	2	Q13535
Control	2	Q13546
Control	2	Q13547
Control	2	Q13555
Control	2	Q13557
Control	2	Q13561
Control	2	Q13563
Control	2	Q13564
Control	2	Q13572
Control	2	Q13573
Control	2	Q13574
Control	2	Q13586

Control	2	Q13588
Control	2	Q13595
Control	2	Q13596
Control	2	Q13601
Control	2	Q13610
Control	2	Q13613
Control	2	Q13614
Control	2	Q13616
Control	2	Q13617
Control	2	Q13618
Control	2	Q13619
Control	2	Q13620
Control	2	Q13625
Control	2	Q13627
Control	2	Q13630
Control	2	Q13637
Control	2	Q13641
Control	2	Q13642
Control	2	Q13643
Control	2	Q13671
Control	2	Q13685
Control	2	Q13724
Control	2	Q13740
Control	2	Q13769
Control	2	Q13796
Control	2	Q13813
Control	2	Q13823
Control	2	Q13825
Control	2	Q13835
Control	2	Q13838
Control	2	Q13867
Control	2	Q13868
Control	2	Q13873
Control	2	Q13885
Control	2	Q13888
Control	2	Q13889
Control	2	Q13895
Control	2	Q13905
Control	2	Q13907
Control	2	Q13951
Control	2	Q13952
Control	2	Q14004
Control	2	Q14008
Control	2	Q14011
Control	2	Q14012
Control	2	Q14019
Control	2	Q14103
Control	2	Q14108
Control	2	Q14112
Control	2	Q14114

Control	2	Q14118
Control	2	Q14119
Control	2	Q14137
Control	2	Q14139
Control	2	Q14141
Control	2	Q14145
Control	2	Q14146
Control	2	Q14147
Control	2	Q14149
Control	2	Q14151
Control	2	Q14152
Control	2	Q14155
Control	2	Q14156
Control	2	Q14157
Control	2	Q14160
Control	2	Q14161
Control	2	Q14162
Control	2	Q14165
Control	2	Q14166
Control	2	Q14181
Control	2	Q14185
Control	2	Q14186
Control	2	Q14192
Control	2	Q14195
Control	2	Q14197
Control	2	Q14202
Control	2	Q14203
Control	2	Q14204
Control	2	Q14232
Control	2	Q14240
Control	2	Q14241
Control	2	Q14247
Control	2	Q14249
Control	2	Q14254
Control	2	Q14257
Control	2	Q14258
Control	2	Q14315
Control	2	Q14318
Control	2	Q14320
Control	2	Q14331
Control	2	Q14344
Control	2	Q14353
Control	2	Q14376
Control	2	Q14392
Control	2	Q14444
Control	2	Q14457
Control	2	Q14498
Control	2	Q14511
Control	2	Q14527
Control	2	Q14534

Control	2	Q14554
Control	2	Q14558
Control	2	Q14562
Control	2	Q14566
Control	2	Q14571
Control	2	Q14573
Control	2	Q14643
Control	2	Q14644
Control	2	Q14651
Control	2	Q14653
Control	2	Q14657
Control	2	Q14667
Control	2	Q14669
Control	2	Q14671
Control	2	Q14676
Control	2	Q14677
Control	2	Q14678
Control	2	Q14683
Control	2	Q14684
Control	2	Q14689
Control	2	Q14690
Control	2	Q14691
Control	2	Q14692
Control	2	Q14693
Control	2	Q14694
Control	2	Q14696
Control	2	Q14697
Control	2	Q14699
Control	2	Q14728
Control	2	Q14738
Control	2	Q14739
Control	2	Q14746
Control	2	Q14764
Control	2	Q14766
Control	2	Q14767
Control	2	Q14781
Control	2	Q14789
Control	2	Q14790
Control	2	Q147X3
Control	2	Q14807
Control	2	Q14814
Control	2	Q14839
Control	2	Q14847
Control	2	Q14914
Control	2	Q14919
Control	2	Q14934
Control	2	Q14966
Control	2	Q14974
Control	2	Q14978
Control	2	Q14980

Control	2	Q14997
Control	2	Q14999
Control	2	Q14BN4
Control	2	Q14C86
Control	2	Q14CX7
Control	2	Q14D04
Control	2	Q15003
Control	2	Q15004
Control	2	Q15005
Control	2	Q15006
Control	2	Q15007
Control	2	Q15008
Control	2	Q15011
Control	2	Q15013
Control	2	Q15014
Control	2	Q15018
Control	2	Q15019
Control	2	Q15020
Control	2	Q15021
Control	2	Q15022
Control	2	Q15024
Control	2	Q15025
Control	2	Q15029
Control	2	Q15031
Control	2	Q15032
Control	2	Q15035
Control	2	Q15036
Control	2	Q15041
Control	2	Q15042
Control	2	Q15043
Control	2	Q15046
Control	2	Q15047
Control	2	Q15048
Control	2	Q15050
Control	2	Q15054
Control	2	Q15056
Control	2	Q15057
Control	2	Q15058
Control	2	Q15059
Control	2	Q15061
Control	2	Q15067
Control	2	Q15070
Control	2	Q15075
Control	2	Q15084
Control	2	Q15102
Control	2	Q15120
Control	2	Q15121
Control	2	Q15125
Control	2	Q15126
Control	2	Q15139

Control	2	Q15149
Control	2	Q15154
Control	2	Q15155
Control	2	Q15165
Control	2	Q15172
Control	2	Q15181
Control	2	Q15185
Control	2	Q15208
Control	2	Q15233
Control	2	Q15257
Control	2	Q15262
Control	2	Q15269
Control	2	Q15276
Control	2	Q15286
Control	2	Q15287
Control	2	Q15291
Control	2	Q15293
Control	2	Q15311
Control	2	Q15334
Control	2	Q15345
Control	2	Q15361
Control	2	Q15363
Control	2	Q15365
Control	2	Q15366
Control	2	Q15369
Control	2	Q15370
Control	2	Q15382
Control	2	Q15386
Control	2	Q15388
Control	2	Q15390
Control	2	Q15392
Control	2	Q15393
Control	2	Q15397
Control	2	Q15398
Control	2	Q15404
Control	2	Q15417
Control	2	Q15418
Control	2	Q15424
Control	2	Q15428
Control	2	Q15434
Control	2	Q15435
Control	2	Q15436
Control	2	Q15437
Control	2	Q15438
Control	2	Q15459
Control	2	Q15477
Control	2	Q15517
Control	2	Q15526
Control	2	Q15542
Control	2	Q15545

Control	2	Q15554
Control	2	Q15555
Control	2	Q15583
Control	2	Q15599
Control	2	Q15628
Control	2	Q15629
Control	2	Q15631
Control	2	Q15633
Control	2	Q15637
Control	2	Q15642
Control	2	Q15643
Control	2	Q15645
Control	2	Q15648
Control	2	Q15650
Control	2	Q15651
Control	2	Q15652
Control	2	Q15653
Control	2	Q15654
Control	2	Q15678
Control	2	Q15691
Control	2	Q15717
Control	2	Q15738
Control	2	Q15742
Control	2	Q15746
Control	2	Q15750
Control	2	Q15751
Control	2	Q15758
Control	2	Q15773
Control	2	Q15785
Control	2	Q15796
Control	2	Q15797
Control	2	Q15800
Control	2	Q15811
Control	2	Q15813
Control	2	Q15814
Control	2	Q15819
Control	2	Q15831
Control	2	Q15833
Control	2	Q15836
Control	2	Q15843
Control	2	Q15904
Control	2	Q15906
Control	2	Q15907
Control	2	Q15942
Control	2	Q16134
Control	2	Q16181
Control	2	Q16186
Control	2	Q16204
Control	2	Q16222
Control	2	Q16254

Control	2	Q16270
Control	2	Q16342
Control	2	Q16363
Control	2	Q16401
Control	2	Q16512
Control	2	Q16513
Control	2	Q16514
Control	2	Q16527
Control	2	Q16531
Control	2	Q16537
Control	2	Q16539
Control	2	Q16540
Control	2	Q16543
Control	2	Q16555
Control	2	Q16563
Control	2	Q16576
Control	2	Q16584
Control	2	Q16594
Control	2	Q16602
Control	2	Q16611
Control	2	Q16629
Control	2	Q16630
Control	2	Q16637
Control	2	Q16643
Control	2	Q16656
Control	2	Q16658
Control	2	Q16666
Control	2	Q16698
Control	2	Q16706
Control	2	Q16718
Control	2	Q16739
Control	2	Q16740
Control	2	Q16762
Control	2	Q16763
Control	2	Q16773
Control	2	Q16774
Control	2	Q16775
Control	2	Q16795
Control	2	Q16798
Control	2	Q16822
Control	2	Q16831
Control	2	Q16832
Control	2	Q16836
Control	2	Q16850
Control	2	Q16851
Control	2	Q16875
Control	2	Q16881
Control	2	Q16891
Control	2	Q19T08
Control	2	Q1ED39

Control	2	Q1KMD3
Control	2	Q24JP5
Control	2	Q27J81
Control	2	Q29RF7
Control	2	Q2KHT3
Control	2	Q2M1P5
Control	2	Q2M1Z3
Control	2	Q2M296
Control	2	Q2M2I8
Control	2	Q2M389
Control	2	Q2NKX8
Control	2	Q2NL82
Control	2	Q2PPJ7
Control	2	Q2PZI1
Control	2	Q2TAA2
Control	2	Q2TAA5
Control	2	Q2TAL8
Control	2	Q2TAY7
Control	2	Q2VPK5
Control	2	Q32M88
Control	2	Q32MZ4
Control	2	Q32NB8
Control	2	Q32NC0
Control	2	Q32P28
Control	2	Q32P41
Control	2	Q32P44
Control	2	Q3B726
Control	2	Q3KQU3
Control	2	Q3KQV9
Control	2	Q3L8U1
Control	2	Q3LXA3
Control	2	Q3MHD2
Control	2	Q3SXM5
Control	2	Q3SY69
Control	2	Q3T906
Control	2	Q3V6T2
Control	2	Q3YEC7
Control	2	Q3ZCQ8
Control	2	Q460N5
Control	2	Q495W5
Control	2	Q49A26
Control	2	Q49AR2
Control	2	Q4AC94
Control	2	Q4G0F5
Control	2	Q4G0J3
Control	2	Q4G0N4
Control	2	Q4G0X4
Control	2	Q4G148
Control	2	Q4G176
Control	2	Q4J6C6

Control	2	Q4KMP7
Control	2	Q4KMQ1
Control	2	Q4KMQ2
Control	2	Q4L180
Control	2	Q4V328
Control	2	Q4V339
Control	2	Q4VC31
Control	2	Q4ZIN3
Control	2	Q52LJ0
Control	2	Q52LW3
Control	2	Q53EL6
Control	2	Q53EP0
Control	2	Q53ET0
Control	2	Q53EU6
Control	2	Q53EZ4
Control	2	Q53F19
Control	2	Q53FA7
Control	2	Q53FP2
Control	2	Q53GA4
Control	2	Q53GG5
Control	2	Q53GL7
Control	2	Q53GQ0
Control	2	Q53GS7
Control	2	Q53GS9
Control	2	Q53GT1
Control	2	Q53H12
Control	2	Q53H47
Control	2	Q53H82
Control	2	Q53H96
Control	2	Q53HC9
Control	2	Q53HL2
Control	2	Q53SF7
Control	2	Q53T59
Control	2	Q53TN4
Control	2	Q562E7
Control	2	Q562R1
Control	2	Q567U6
Control	2	Q56VL3
Control	2	Q58EX7
Control	2	Q58FG1
Control	2	Q58WW2
Control	2	Q5BJD5
Control	2	Q5BJF2
Control	2	Q5BJH7
Control	2	Q5BKZ1
Control	2	Q5C9Z4
Control	2	Q5EBL4
Control	2	Q5F1R6
Control	2	Q5GJ75
Control	2	Q5GLZ8

Control	2	Q5H8A4
Control	2	Q5H9R7
Control	2	Q5HYI8
Control	2	Q5HYJ3
Control	2	Q5HYK7
Control	2	Q5HYW2
Control	2	Q5J8M3
Control	2	Q5JPH6
Control	2	Q5JPI3
Control	2	Q5JPI9
Control	2	Q5JRA6
Control	2	Q5JRX3
Control	2	Q5JSH3
Control	2	Q5JSZ5
Control	2	Q5JTD0
Control	2	Q5JTH9
Control	2	Q5JTJ3
Control	2	Q5JTV8
Control	2	Q5JTW2
Control	2	Q5JTZ9
Control	2	Q5JU69
Control	2	Q5JVF3
Control	2	Q5JVS0
Control	2	Q5K4L6
Control	2	Q5K651
Control	2	Q5MIZ7
Control	2	Q5MNZ6
Control	2	Q5MNZ9
Control	2	Q5NDL2
Control	2	Q5PRF9
Control	2	Q5QJ74
Control	2	Q5QJE6
Control	2	Q5R372
Control	2	Q5R3I4
Control	2	Q5RI15
Control	2	Q5RKV6
Control	2	Q5SNT2
Control	2	Q5SQI0
Control	2	Q5SQN1
Control	2	Q5SRE5
Control	2	Q5SSJ5
Control	2	Q5ST30
Control	2	Q5SW79
Control	2	Q5SW96
Control	2	Q5SWX8
Control	2	Q5SY16
Control	2	Q5SYE7
Control	2	Q5T013
Control	2	Q5T0D9
Control	2	Q5T0F9

Control	2	Q5T0N5
Control	2	Q5T160
Control	2	Q5T1C6
Control	2	Q5T1J5
Control	2	Q5T1M5
Control	2	Q5T200
Control	2	Q5T280
Control	2	Q5T2E6
Control	2	Q5T3F8
Control	2	Q5T3I0
Control	2	Q5T440
Control	2	Q5T447
Control	2	Q5T4B2
Control	2	Q5T4S7
Control	2	Q5T5C0
Control	2	Q5T5U3
Control	2	Q5T5Y3
Control	2	Q5T653
Control	2	Q5T6F2
Control	2	Q5T6V5
Control	2	Q5T749
Control	2	Q5T7W0
Control	2	Q5T8D3
Control	2	Q5T8P6
Control	2	Q5T9A4
Control	2	Q5T9L3
Control	2	Q5TA45
Control	2	Q5TA50
Control	2	Q5TAQ9
Control	2	Q5TAT6
Control	2	Q5TAX3
Control	2	Q5TBA9
Control	2	Q5TBB1
Control	2	Q5TC12
Control	2	Q5TC82
Control	2	Q5TDH0
Control	2	Q5TEU4
Control	2	Q5TFE4
Control	2	Q5TH69
Control	2	Q5THJ4
Control	2	Q5TZA2
Control	2	Q5U5X0
Control	2	Q5U651
Control	2	Q5UIP0
Control	2	Q5VIR6
Control	2	Q5VSL9
Control	2	Q5VT25
Control	2	Q5VT52
Control	2	Q5VTB9
Control	2	Q5VTL8

Control	2	Q5VTR2
Control	2	Q5VU43
Control	2	Q5VUA4
Control	2	Q5VUB5
Control	2	Q5VUD6
Control	2	Q5VUJ6
Control	2	Q5VV42
Control	2	Q5VW32
Control	2	Q5VW36
Control	2	Q5VWQ0
Control	2	Q5VWQ8
Control	2	Q5VWZ2
Control	2	Q5VY43
Control	2	Q5VYK3
Control	2	Q5VYS8
Control	2	Q5VZ18
Control	2	Q5VZ89
Control	2	Q5VZE5
Control	2	Q5VZL5
Control	2	Q5W0V3
Control	2	Q5W0Z9
Control	2	Q5W111
Control	2	Q5XUX1
Control	2	Q5ZPR3
Control	2	Q63HN8
Control	2	Q63HR2
Control	2	Q63ZY3
Control	2	Q641Q2
Control	2	Q643R3
Control	2	Q658P3
Control	2	Q658Y4
Control	2	Q66K14
Control	2	Q66K74
Control	2	Q66LE6
Control	2	Q66PJ3
Control	2	Q676U5
Control	2	Q68CP4
Control	2	Q68CP9
Control	2	Q68CQ4
Control	2	Q68CQ7
Control	2	Q68CR1
Control	2	Q68CZ2
Control	2	Q68CZ6
Control	2	Q68D10
Control	2	Q68D91
Control	2	Q68DQ2
Control	2	Q68E01
Control	2	Q68EM7
Control	2	Q69YL0
Control	2	Q69YN2

Control	2	Q69YN4
Control	2	Q69YQ0
Control	2	Q6AI08
Control	2	Q6AI12
Control	2	Q6AWC2
Control	2	Q6DD88
Control	2	Q6DKI1
Control	2	Q6DKJ4
Control	2	Q6DKK2
Control	2	Q6DN90
Control	2	Q6FI81
Control	2	Q6FIF0
Control	2	Q6GMV2
Control	2	Q6GQQ9
Control	2	Q6I9Y2
Control	2	Q6IA69
Control	2	Q6IA86
Control	2	Q6IAA8
Control	2	Q6IAN0
Control	2	Q6IBS0
Control	2	Q6IBW4
Control	2	Q6ICG6
Control	2	Q6ICL3
Control	2	Q6IN85
Control	2	Q6IQ22
Control	2	Q6IQ26
Control	2	Q6IQ49
Control	2	Q6KB66
Control	2	Q6KC79
Control	2	Q6KCM7
Control	2	Q6L8Q7
Control	2	Q6N069
Control	2	Q6NSW5
Control	2	Q6NTF9
Control	2	Q6NUK1
Control	2	Q6NUK4
Control	2	Q6NUM9
Control	2	Q6NUQ1
Control	2	Q6NUQ4
Control	2	Q6NVY1
Control	2	Q6NW29
Control	2	Q6NW34
Control	2	Q6NXE6
Control	2	Q6NXR4
Control	2	Q6NXT1
Control	2	Q6NXT4
Control	2	Q6NXT6
Control	2	Q6NY19
Control	2	Q6NYC1
Control	2	Q6NYC8

Control	2	Q6NZI2
Control	2	Q6NZY4
Control	2	Q6P158
Control	2	Q6P179
Control	2	Q6P1A2
Control	2	Q6P1J9
Control	2	Q6P1L8
Control	2	Q6P1M0
Control	2	Q6P1N0
Control	2	Q6P1N9
Control	2	Q6P1Q9
Control	2	Q6P1R3
Control	2	Q6P1X5
Control	2	Q6P1X6
Control	2	Q6P2C8
Control	2	Q6P2E9
Control	2	Q6P2H3
Control	2	Q6P2P2
Control	2	Q6P2Q9
Control	2	Q6P3S6
Control	2	Q6P3W7
Control	2	Q6P3X3
Control	2	Q6P4A7
Control	2	Q6P4E1
Control	2	Q6P4R8
Control	2	Q6P587
Control	2	Q6P5R6
Control	2	Q6P5Z2
Control	2	Q6P6C2
Control	2	Q6P996
Control	2	Q6P9B6
Control	2	Q6P9B9
Control	2	Q6P9H5
Control	2	Q6PCB7
Control	2	Q6PCE3
Control	2	Q6PD62
Control	2	Q6PD74
Control	2	Q6PEY1
Control	2	Q6PGP7
Control	2	Q6PHR2
Control	2	Q6PI48
Control	2	Q6PI78
Control	2	Q6PI98
Control	2	Q6PIJ6
Control	2	Q6PIU2
Control	2	Q6PIW4
Control	2	Q6PJ69
Control	2	Q6PJF5
Control	2	Q6PJG2
Control	2	Q6PJG6

Control	2	Q6PJI9
Control	2	Q6PJT7
Control	2	Q6PK04
Control	2	Q6PK18
Control	2	Q6PKC3
Control	2	Q6PKG0
Control	2	Q6PL18
Control	2	Q6PL24
Control	2	Q6PML9
Control	2	Q6Q0C0
Control	2	Q6R327
Control	2	Q6RFH5
Control	2	Q6RW13
Control	2	Q6STE5
Control	2	Q6UB35
Control	2	Q6ULP2
Control	2	Q6UN15
Control	2	Q6UUV7
Control	2	Q6UUV9
Control	2	Q6UW02
Control	2	Q6UW63
Control	2	Q6UW68
Control	2	Q6UWE0
Control	2	Q6UWH4
Control	2	Q6UWP7
Control	2	Q6UX04
Control	2	Q6UXH1
Control	2	Q6UXN9
Control	2	Q6UXV4
Control	2	Q6V0I7
Control	2	Q6V1X1
Control	2	Q6VMQ6
Control	2	Q6VN20
Control	2	Q6VY07
Control	2	Q6WCQ1
Control	2	Q6XE24
Control	2	Q6XQN6
Control	2	Q6XZF7
Control	2	Q6Y1H2
Control	2	Q6Y288
Control	2	Q6Y7W6
Control	2	Q6YHK3
Control	2	Q6YHU6
Control	2	Q6YN16
Control	2	Q6YP21
Control	2	Q6ZMI0
Control	2	Q6ZMP0
Control	2	Q6ZMZ3
Control	2	Q6ZN55
Control	2	Q6ZNB6

Control	2	Q6ZNJ1
Control	2	Q6ZNL6
Control	2	Q6ZRP7
Control	2	Q6ZRS2
Control	2	Q6ZS17
Control	2	Q6ZSR9
Control	2	Q6ZSZ5
Control	2	Q6ZT07
Control	2	Q6ZT12
Control	2	Q6ZT21
Control	2	Q6ZU35
Control	2	Q6ZUT6
Control	2	Q6ZVK8
Control	2	Q6ZW49
Control	2	Q6ZWJ1
Control	2	Q6ZXV5
Control	2	Q709C8
Control	2	Q70CQ2
Control	2	Q70E73
Control	2	Q70IA6
Control	2	Q70UQ0
Control	2	Q70Z35
Control	2	Q70Z53
Control	2	Q712K3
Control	2	Q71RC2
Control	2	Q71SY5
Control	2	Q71UM5
Control	2	Q75N03
Control	2	Q75QN2
Control	2	Q7KYR7
Control	2	Q7KZ85
Control	2	Q7KZF4
Control	2	Q7KZI7
Control	2	Q7KZN9
Control	2	Q7L014
Control	2	Q7L099
Control	2	Q7L0Y3
Control	2	Q7L1Q6
Control	2	Q7L1V2
Control	2	Q7L1W4
Control	2	Q7L266
Control	2	Q7L273
Control	2	Q7L2E3
Control	2	Q7L2H7
Control	2	Q7L2J0
Control	2	Q7L311
Control	2	Q7L3T8
Control	2	Q7L4I2
Control	2	Q7L523
Control	2	Q7L576

Control	2	Q7L592
Control	2	Q7L5D6
Control	2	Q7L5N1
Control	2	Q7L5N7
Control	2	Q7L5Y1
Control	2	Q7L5Y9
Control	2	Q7L775
Control	2	Q7L7V1
Control	2	Q7L7X3
Control	2	Q7L8L6
Control	2	Q7L9L4
Control	2	Q7LBC6
Control	2	Q7LBR1
Control	2	Q7LG56
Control	2	Q7LGA3
Control	2	Q7RTN6
Control	2	Q7RTP6
Control	2	Q7RTS9
Control	2	Q7RTT2
Control	2	Q7RTV0
Control	2	Q7RTV5
Control	2	Q7Z2E3
Control	2	Q7Z2K6
Control	2	Q7Z2K8
Control	2	Q7Z2T5
Control	2	Q7Z2W4
Control	2	Q7Z2W9
Control	2	Q7Z2Z2
Control	2	Q7Z333
Control	2	Q7Z392
Control	2	Q7Z3B3
Control	2	Q7Z3B4
Control	2	Q7Z3C6
Control	2	Q7Z3D6
Control	2	Q7Z3E2
Control	2	Q7Z3E5
Control	2	Q7Z3J2
Control	2	Q7Z3K3
Control	2	Q7Z3T8
Control	2	Q7Z3U7
Control	2	Q7Z401
Control	2	Q7Z406
Control	2	Q7Z417
Control	2	Q7Z422
Control	2	Q7Z434
Control	2	Q7Z460
Control	2	Q7Z478
Control	2	Q7Z4F1
Control	2	Q7Z4H7
Control	2	Q7Z4H8

Control	2	Q7Z4L5
Control	2	Q7Z4Q2
Control	2	Q7Z4S6
Control	2	Q7Z4V5
Control	2	Q7Z4W1
Control	2	Q7Z569
Control	2	Q7Z589
Control	2	Q7Z5G4
Control	2	Q7Z5H3
Control	2	Q7Z5K2
Control	2	Q7Z5L9
Control	2	Q7Z6E9
Control	2	Q7Z6J0
Control	2	Q7Z6J2
Control	2	Q7Z6J6
Control	2	Q7Z6J9
Control	2	Q7Z6K3
Control	2	Q7Z6K5
Control	2	Q7Z6L1
Control	2	Q7Z6M1
Control	2	Q7Z6M4
Control	2	Q7Z6Z7
Control	2	Q7Z739
Control	2	Q7Z7A3
Control	2	Q7Z7C8
Control	2	Q7Z7E8
Control	2	Q7Z7F7
Control	2	Q7Z7H5
Control	2	Q7Z7H8
Control	2	Q7Z7K0
Control	2	Q7Z7K6
Control	2	Q7Z7L1
Control	2	Q7Z7M0
Control	2	Q7Z7N9
Control	2	Q86SF2
Control	2	Q86SK9
Control	2	Q86SQ0
Control	2	Q86SQ4
Control	2	Q86SQ9
Control	2	Q86SR1
Control	2	Q86SZ2
Control	2	Q86T03
Control	2	Q86T13
Control	2	Q86T24
Control	2	Q86TB9
Control	2	Q86TI2
Control	2	Q86TM6
Control	2	Q86TN4
Control	2	Q86TU7
Control	2	Q86TV6

Control	2	Q86TX2
Control	2	Q86U38
Control	2	Q86U42
Control	2	Q86U44
Control	2	Q86U86
Control	2	Q86U90
Control	2	Q86UA1
Control	2	Q86UE4
Control	2	Q86UK7
Control	2	Q86UL3
Control	2	Q86UP2
Control	2	Q86US8
Control	2	Q86UT6
Control	2	Q86UU0
Control	2	Q86UU1
Control	2	Q86UV5
Control	2	Q86UW7
Control	2	Q86UX7
Control	2	Q86UY6
Control	2	Q86UY8
Control	2	Q86V21
Control	2	Q86V48
Control	2	Q86V81
Control	2	Q86V85
Control	2	Q86V87
Control	2	Q86VI3
Control	2	Q86VM9
Control	2	Q86VN1
Control	2	Q86VP6
Control	2	Q86VR2
Control	2	Q86VS8
Control	2	Q86VW0
Control	2	Q86VX2
Control	2	Q86VX9
Control	2	Q86W34
Control	2	Q86W42
Control	2	Q86W50
Control	2	Q86W56
Control	2	Q86W92
Control	2	Q86WA8
Control	2	Q86WB0
Control	2	Q86WJ1
Control	2	Q86WN1
Control	2	Q86WR0
Control	2	Q86WR7
Control	2	Q86WV6
Control	2	Q86WX3
Control	2	Q86X02
Control	2	Q86X10
Control	2	Q86X27

Control	2	Q86X55
Control	2	Q86X76
Control	2	Q86X83
Control	2	Q86XA9
Control	2	Q86XI2
Control	2	Q86XI6
Control	2	Q86XK2
Control	2	Q86XL3
Control	2	Q86XN8
Control	2	Q86XP1
Control	2	Q86XP3
Control	2	Q86XZ4
Control	2	Q86Y07
Control	2	Q86Y37
Control	2	Q86Y39
Control	2	Q86Y56
Control	2	Q86Y79
Control	2	Q86Y82
Control	2	Q86YP4
Control	2	Q86YQ8
Control	2	Q86YR5
Control	2	Q86YS6
Control	2	Q86YS7
Control	2	Q86YT6
Control	2	Q86YV5
Control	2	Q86YV9
Control	2	Q8IU81
Control	2	Q8IUD2
Control	2	Q8IUF8
Control	2	Q8IUH4
Control	2	Q8IUH5
Control	2	Q8IUI8
Control	2	Q8IUR0
Control	2	Q8IUR7
Control	2	Q8IUW5
Control	2	Q8IUX1
Control	2	Q8IV08
Control	2	Q8IV36
Control	2	Q8IV38
Control	2	Q8IV48
Control	2	Q8IV63
Control	2	Q8IVB5
Control	2	Q8IVD9
Control	2	Q8IVF2
Control	2	Q8IVF7
Control	2	Q8IVH4
Control	2	Q8IVH8
Control	2	Q8IVL0
Control	2	Q8IVL6
Control	2	Q8IVM0

Control	2	Q8IVS2
Control	2	Q8IVT5
Control	2	Q8IW35
Control	2	Q8IW45
Control	2	Q8IWA0
Control	2	Q8IWA4
Control	2	Q8IWA5
Control	2	Q8IWB1
Control	2	Q8IWB7
Control	2	Q8IWC1
Control	2	Q8IWD4
Control	2	Q8IWE2
Control	2	Q8IWE4
Control	2	Q8IWF6
Control	2	Q8IWI9
Control	2	Q8IWJ2
Control	2	Q8IWR0
Control	2	Q8IWS0
Control	2	Q8IWT0
Control	2	Q8IWT6
Control	2	Q8IWU5
Control	2	Q8IWV7
Control	2	Q8IWV8
Control	2	Q8IWW6
Control	2	Q8IWX8
Control	2	Q8IWY9
Control	2	Q8IWZ3
Control	2	Q8IWZ8
Control	2	Q8IX01
Control	2	Q8IX04
Control	2	Q8IX12
Control	2	Q8IX18
Control	2	Q8IXB1
Control	2	Q8IXH7
Control	2	Q8IXI1
Control	2	Q8IXI2
Control	2	Q8IXJ6
Control	2	Q8IXK0
Control	2	Q8IXM2
Control	2	Q8IXM3
Control	2	Q8IXM6
Control	2	Q8IXQ4
Control	2	Q8IXQ6
Control	2	Q8IXT5
Control	2	Q8IXU6
Control	2	Q8IXW5
Control	2	Q8IY17
Control	2	Q8IY18
Control	2	Q8IY21
Control	2	Q8IY22

Control	2	Q8IY33
Control	2	Q8IY37
Control	2	Q8IY47
Control	2	Q8IY67
Control	2	Q8IY81
Control	2	Q8IY95
Control	2	Q8IYB1
Control	2	Q8IYB3
Control	2	Q8IYB5
Control	2	Q8IYB7
Control	2	Q8IYB8
Control	2	Q8IYD1
Control	2	Q8IYH5
Control	2	Q8IYI6
Control	2	Q8IYJ2
Control	2	Q8IYM9
Control	2	Q8IYQ7
Control	2	Q8IYS2
Control	2	Q8IYU8
Control	2	Q8IZ07
Control	2	Q8IZ21
Control	2	Q8IZ69
Control	2	Q8IZ73
Control	2	Q8IZ81
Control	2	Q8IZ83
Control	2	Q8IZA0
Control	2	Q8IZF2
Control	2	Q8IZH2
Control	2	Q8IZL8
Control	2	Q8IZP0
Control	2	Q8IZQ1
Control	2	Q8IZQ5
Control	2	Q8N0U8
Control	2	Q8N0X7
Control	2	Q8N0Z6
Control	2	Q8N108
Control	2	Q8N122
Control	2	Q8N138
Control	2	Q8N163
Control	2	Q8N183
Control	2	Q8N1B4
Control	2	Q8N1F7
Control	2	Q8N1F8
Control	2	Q8N1G0
Control	2	Q8N1G2
Control	2	Q8N1G4
Control	2	Q8N1I0
Control	2	Q8N1Q1
Control	2	Q8N1W1
Control	2	Q8N201

Control	2	Q8N257
Control	2	Q8N264
Control	2	Q8N2F6
Control	2	Q8N2K0
Control	2	Q8N2M8
Control	2	Q8N2R8
Control	2	Q8N2U0
Control	2	Q8N2W9
Control	2	Q8N335
Control	2	Q8N357
Control	2	Q8N392
Control	2	Q8N3C0
Control	2	Q8N3F8
Control	2	Q8N3P4
Control	2	Q8N3R9
Control	2	Q8N3U4
Control	2	Q8N3V7
Control	2	Q8N3X1
Control	2	Q8N442
Control	2	Q8N488
Control	2	Q8N4A0
Control	2	Q8N4C8
Control	2	Q8N4P3
Control	2	Q8N4Q0
Control	2	Q8N4T8
Control	2	Q8N4V1
Control	2	Q8N511
Control	2	Q8N531
Control	2	Q8N543
Control	2	Q8N556
Control	2	Q8N567
Control	2	Q8N573
Control	2	Q8N5A5
Control	2	Q8N5C1
Control	2	Q8N5C6
Control	2	Q8N5D0
Control	2	Q8N5F7
Control	2	Q8N5G2
Control	2	Q8N5H7
Control	2	Q8N5I2
Control	2	Q8N5I4
Control	2	Q8N5K1
Control	2	Q8N5L8
Control	2	Q8N5M1
Control	2	Q8N5M4
Control	2	Q8N5M9
Control	2	Q8N5U6
Control	2	Q8N5W9
Control	2	Q8N653
Control	2	Q8N668

Control	2	Q8N684
Control	2	Q8N697
Control	2	Q8N699
Control	2	Q8N6G6
Control	2	Q8N6H7
Control	2	Q8N6M0
Control	2	Q8N6M3
Control	2	Q8N6N3
Control	2	Q8N6R0
Control	2	Q8N6S5
Control	2	Q8N6T3
Control	2	Q8N6T7
Control	2	Q8N755
Control	2	Q8N766
Control	2	Q8N7H5
Control	2	Q8N7R7
Control	2	Q8N806
Control	2	Q8N8A6
Control	2	Q8N8J7
Control	2	Q8N8R3
Control	2	Q8N8S7
Control	2	Q8N8Z6
Control	2	Q8N954
Control	2	Q8N983
Control	2	Q8N999
Control	2	Q8N9F7
Control	2	Q8N9M1
Control	2	Q8N9N2
Control	2	Q8N9N7
Control	2	Q8N9N8
Control	2	Q8N9T8
Control	2	Q8N9Z2
Control	2	Q8NAF0
Control	2	Q8NAV1
Control	2	Q8NB16
Control	2	Q8NB37
Control	2	Q8NB46
Control	2	Q8NB49
Control	2	Q8NB90
Control	2	Q8NBF2
Control	2	Q8NBF6
Control	2	Q8NBI5
Control	2	Q8NBI6
Control	2	Q8NBJ4
Control	2	Q8NBJ5
Control	2	Q8NBJ7
Control	2	Q8NBJ9
Control	2	Q8NBK3
Control	2	Q8NBL1
Control	2	Q8NBM4

Control	2	Q8NBM8
Control	2	Q8NBN3
Control	2	Q8NBN7
Control	2	Q8NBP0
Control	2	Q8NBQ5
Control	2	Q8NBS9
Control	2	Q8NBT2
Control	2	Q8NBU5
Control	2	Q8NBX0
Control	2	Q8NBZ7
Control	2	Q8NC42
Control	2	Q8NC44
Control	2	Q8NC51
Control	2	Q8NC56
Control	2	Q8NC60
Control	2	Q8NC96
Control	2	Q8NCA5
Control	2	Q8NCC3
Control	2	Q8NCE2
Control	2	Q8NCF5
Control	2	Q8NCG7
Control	2	Q8NCH0
Control	2	Q8NCL4
Control	2	Q8NCN4
Control	2	Q8NCN5
Control	2	Q8NCW5
Control	2	Q8ND04
Control	2	Q8ND24
Control	2	Q8ND56
Control	2	Q8ND71
Control	2	Q8ND76
Control	2	Q8NDA8
Control	2	Q8NDF8
Control	2	Q8NDH3
Control	2	Q8NDI1
Control	2	Q8NDT2
Control	2	Q8NDV1
Control	2	Q8NDV7
Control	2	Q8NDX5
Control	2	Q8NDZ4
Control	2	Q8NE01
Control	2	Q8NE71
Control	2	Q8NE86
Control	2	Q8NEB9
Control	2	Q8NEC7
Control	2	Q8NEF9
Control	2	Q8NEJ9
Control	2	Q8NEM2
Control	2	Q8NEN9
Control	2	Q8NEU8

Control	2	Q8NEW0
Control	2	Q8NEY1
Control	2	Q8NEY8
Control	2	Q8NEZ3
Control	2	Q8NEZ5
Control	2	Q8NF37
Control	2	Q8NF64
Control	2	Q8NF91
Control	2	Q8NFA0
Control	2	Q8NFC6
Control	2	Q8NFD5
Control	2	Q8NFF5
Control	2	Q8NFG4
Control	2	Q8NFH3
Control	2	Q8NFH4
Control	2	Q8NFH5
Control	2	Q8NFI3
Control	2	Q8NFJ5
Control	2	Q8NFQ8
Control	2	Q8NFV4
Control	2	Q8NFW8
Control	2	Q8NG11
Control	2	Q8NG68
Control	2	Q8NHG7
Control	2	Q8NHH9
Control	2	Q8NHP6
Control	2	Q8NHP8
Control	2	Q8NHQ8
Control	2	Q8NHQ9
Control	2	Q8NHS3
Control	2	Q8NHU6
Control	2	Q8NHV1
Control	2	Q8NHV4
Control	2	Q8NI08
Control	2	Q8NI27
Control	2	Q8NI36
Control	2	Q8NI37
Control	2	Q8TA86
Control	2	Q8TAA9
Control	2	Q8TAD8
Control	2	Q8TAE8
Control	2	Q8TAF3
Control	2	Q8TAG9
Control	2	Q8TAQ2
Control	2	Q8TAT6
Control	2	Q8TB03
Control	2	Q8TB22
Control	2	Q8TB37
Control	2	Q8TB40
Control	2	Q8TB52

Control	2	Q8TB61
Control	2	Q8TB72
Control	2	Q8TB96
Control	2	Q8TBA6
Control	2	Q8TBB5
Control	2	Q8TBC3
Control	2	Q8TBC4
Control	2	Q8TBF2
Control	2	Q8TBM8
Control	2	Q8TBQ9
Control	2	Q8TBX8
Control	2	Q8TBZ3
Control	2	Q8TC07
Control	2	Q8TC12
Control	2	Q8TCA0
Control	2	Q8TCB0
Control	2	Q8TCC3
Control	2	Q8TCF1
Control	2	Q8TCG1
Control	2	Q8TCG2
Control	2	Q8TCJ2
Control	2	Q8TCS8
Control	2	Q8TCT7
Control	2	Q8TCT9
Control	2	Q8TCU6
Control	2	Q8TCY9
Control	2	Q8TD16
Control	2	Q8TD19
Control	2	Q8TD55
Control	2	Q8TDB6
Control	2	Q8TDD1
Control	2	Q8TDM6
Control	2	Q8TDN6
Control	2	Q8TDQ7
Control	2	Q8TDW0
Control	2	Q8TDX7
Control	2	Q8TDY2
Control	2	Q8TDZ2
Control	2	Q8TE02
Control	2	Q8TE77
Control	2	Q8TE82
Control	2	Q8TEA7
Control	2	Q8TEA8
Control	2	Q8TEB1
Control	2	Q8TED0
Control	2	Q8TED1
Control	2	Q8TED9
Control	2	Q8TEJ3
Control	2	Q8TEL6
Control	2	Q8TEP8

Control	2	Q8TEQ6
Control	2	Q8TEQ8
Control	2	Q8TEU7
Control	2	Q8TEV9
Control	2	Q8TEW0
Control	2	Q8TEX9
Control	2	Q8TEY7
Control	2	Q8TF01
Control	2	Q8TF05
Control	2	Q8TF42
Control	2	Q8TF74
Control	2	Q8WTS1
Control	2	Q8WTS6
Control	2	Q8WTT2
Control	2	Q8WTV0
Control	2	Q8WTW3
Control	2	Q8WU76
Control	2	Q8WU79
Control	2	Q8WU90
Control	2	Q8WUA2
Control	2	Q8WUA4
Control	2	Q8WUA7
Control	2	Q8WUB8
Control	2	Q8WUD1
Control	2	Q8WUD4
Control	2	Q8WUF5
Control	2	Q8WUF8
Control	2	Q8WUH1
Control	2	Q8WUH2
Control	2	Q8WUH6
Control	2	Q8WUI4
Control	2	Q8WUJ0
Control	2	Q8WUK0
Control	2	Q8WUM0
Control	2	Q8WUM4
Control	2	Q8WUM9
Control	2	Q8WUP2
Control	2	Q8WUQ7
Control	2	Q8WUX2
Control	2	Q8WUX9
Control	2	Q8WUY1
Control	2	Q8WUY8
Control	2	Q8WUZ0
Control	2	Q8WV22
Control	2	Q8WV92
Control	2	Q8WVB6
Control	2	Q8WVC0
Control	2	Q8WVC6
Control	2	Q8WVJ2
Control	2	Q8WVK2

Control	2	Q8WVM0
Control	2	Q8WVM7
Control	2	Q8WVM8
Control	2	Q8WVP5
Control	2	Q8WVQ1
Control	2	Q8WVT3
Control	2	Q8WVV9
Control	2	Q8WVX9
Control	2	Q8WVY7
Control	2	Q8WW01
Control	2	Q8WW12
Control	2	Q8WW22
Control	2	Q8WW59
Control	2	Q8WWC4
Control	2	Q8WWH5
Control	2	Q8WWI1
Control	2	Q8WWI5
Control	2	Q8WWK9
Control	2	Q8WWM7
Control	2	Q8WWN8
Control	2	Q8WWP7
Control	2	Q8WWQ0
Control	2	Q8WWY3
Control	2	Q8WX92
Control	2	Q8WX93
Control	2	Q8WXA3
Control	2	Q8WXA9
Control	2	Q8WXD5
Control	2	Q8WXE0
Control	2	Q8WXF1
Control	2	Q8WXG6
Control	2	Q8WXH0
Control	2	Q8WXI9
Control	2	Q8WXW3
Control	2	Q8WXX5
Control	2	Q8WY22
Control	2	Q8WYA6
Control	2	Q8WYN0
Control	2	Q8WYP3
Control	2	Q8WYP5
Control	2	Q8WYQ5
Control	2	Q8WZ42
Control	2	Q8WZ75
Control	2	Q8WZ82
Control	2	Q8WZA9
Control	2	Q92466
Control	2	Q92478
Control	2	Q92499
Control	2	Q92503
Control	2	Q92508

Control	2	Q92520
Control	2	Q92522
Control	2	Q92530
Control	2	Q92538
Control	2	Q92540
Control	2	Q92541
Control	2	Q92542
Control	2	Q92543
Control	2	Q92544
Control	2	Q92545
Control	2	Q92551
Control	2	Q92552
Control	2	Q92556
Control	2	Q92567
Control	2	Q92572
Control	2	Q92574
Control	2	Q92575
Control	2	Q92576
Control	2	Q92597
Control	2	Q92598
Control	2	Q92599
Control	2	Q92600
Control	2	Q92604
Control	2	Q92609
Control	2	Q92610
Control	2	Q92613
Control	2	Q92614
Control	2	Q92615
Control	2	Q92616
Control	2	Q92619
Control	2	Q92620
Control	2	Q92621
Control	2	Q92625
Control	2	Q92626
Control	2	Q92636
Control	2	Q92643
Control	2	Q92665
Control	2	Q92667
Control	2	Q92685
Control	2	Q92686
Control	2	Q92688
Control	2	Q92692
Control	2	Q92696
Control	2	Q92734
Control	2	Q92743
Control	2	Q92747
Control	2	Q92759
Control	2	Q92766
Control	2	Q92769
Control	2	Q92783

Control	2	Q92785
Control	2	Q92791
Control	2	Q92793
Control	2	Q92794
Control	2	Q92797
Control	2	Q92804
Control	2	Q92805
Control	2	Q92820
Control	2	Q92823
Control	2	Q92834
Control	2	Q92835
Control	2	Q92841
Control	2	Q92845
Control	2	Q92851
Control	2	Q92870
Control	2	Q92878
Control	2	Q92879
Control	2	Q92882
Control	2	Q92888
Control	2	Q92889
Control	2	Q92890
Control	2	Q92896
Control	2	Q92900
Control	2	Q92905
Control	2	Q92917
Control	2	Q92922
Control	2	Q92925
Control	2	Q92930
Control	2	Q92934
Control	2	Q92945
Control	2	Q92947
Control	2	Q92963
Control	2	Q92968
Control	2	Q92973
Control	2	Q92974
Control	2	Q92979
Control	2	Q92989
Control	2	Q92990
Control	2	Q92995
Control	2	Q93008
Control	2	Q93009
Control	2	Q93034
Control	2	Q93045
Control	2	Q93050
Control	2	Q93052
Control	2	Q93062
Control	2	Q93063
Control	2	Q93074
Control	2	Q93096
Control	2	Q93100

Control	2	Q969E2
Control	2	Q969F9
Control	2	Q969G3
Control	2	Q969G5
Control	2	Q969G6
Control	2	Q969H8
Control	2	Q969M1
Control	2	Q969M3
Control	2	Q969N2
Control	2	Q969P0
Control	2	Q969Q0
Control	2	Q969Q5
Control	2	Q969R2
Control	2	Q969S3
Control	2	Q969S9
Control	2	Q969T4
Control	2	Q969T7
Control	2	Q969T9
Control	2	Q969U7
Control	2	Q969V3
Control	2	Q969V5
Control	2	Q969V6
Control	2	Q969X1
Control	2	Q969X5
Control	2	Q969X6
Control	2	Q969Z0
Control	2	Q969Z3
Control	2	Q96A26
Control	2	Q96A33
Control	2	Q96A35
Control	2	Q96A65
Control	2	Q96A72
Control	2	Q96A73
Control	2	Q96AA3
Control	2	Q96AB3
Control	2	Q96AB6
Control	2	Q96AC1
Control	2	Q96AE4
Control	2	Q96AE7
Control	2	Q96AG4
Control	2	Q96AJ9
Control	2	Q96AP7
Control	2	Q96AQ6
Control	2	Q96AQ8
Control	2	Q96AT1
Control	2	Q96AX1
Control	2	Q96AY3
Control	2	Q96AY4
Control	2	Q96B26
Control	2	Q96B54

Control	2	Q96B97
Control	2	Q96BD8
Control	2	Q96BH1
Control	2	Q96BJ3
Control	2	Q96BK5
Control	2	Q96BM9
Control	2	Q96BN8
Control	2	Q96BP2
Control	2	Q96BP3
Control	2	Q96BQ5
Control	2	Q96BR5
Control	2	Q96BW1
Control	2	Q96BW9
Control	2	Q96BX8
Control	2	Q96BY6
Control	2	Q96BY7
Control	2	Q96BZ8
Control	2	Q96BZ9
Control	2	Q96C19
Control	2	Q96C23
Control	2	Q96C24
Control	2	Q96C36
Control	2	Q96C57
Control	2	Q96C86
Control	2	Q96C90
Control	2	Q96CB8
Control	2	Q96CB9
Control	2	Q96CG8
Control	2	Q96CM8
Control	2	Q96CN7
Control	2	Q96CN9
Control	2	Q96CP2
Control	2	Q96CP6
Control	2	Q96CS2
Control	2	Q96CS3
Control	2	Q96CT7
Control	2	Q96CU9
Control	2	Q96CV9
Control	2	Q96CW1
Control	2	Q96CW5
Control	2	Q96CW6
Control	2	Q96CX2
Control	2	Q96D15
Control	2	Q96D46
Control	2	Q96D71
Control	2	Q96DA6
Control	2	Q96DB5
Control	2	Q96DE0
Control	2	Q96DF8
Control	2	Q96DG6

Control	2	Q96DH6
Control	2	Q96DI7
Control	2	Q96DM3
Control	2	Q96DV4
Control	2	Q96DX4
Control	2	Q96DZ1
Control	2	Q96E11
Control	2	Q96E29
Control	2	Q96EA4
Control	2	Q96EB1
Control	2	Q96EB6
Control	2	Q96EC8
Control	2	Q96EE3
Control	2	Q96EI5
Control	2	Q96EK5
Control	2	Q96EK6
Control	2	Q96EK7
Control	2	Q96EK9
Control	2	Q96EL2
Control	2	Q96EL3
Control	2	Q96EM0
Control	2	Q96EP0
Control	2	Q96EP5
Control	2	Q96EQ0
Control	2	Q96ER3
Control	2	Q96ER9
Control	2	Q96ES7
Control	2	Q96EU6
Control	2	Q96EU7
Control	2	Q96EV2
Control	2	Q96EV8
Control	2	Q96EY1
Control	2	Q96EY4
Control	2	Q96EY5
Control	2	Q96EY7
Control	2	Q96EY8
Control	2	Q96F07
Control	2	Q96F15
Control	2	Q96F44
Control	2	Q96F63
Control	2	Q96F85
Control	2	Q96F86
Control	2	Q96FJ2
Control	2	Q96FN4
Control	2	Q96FQ6
Control	2	Q96FS4
Control	2	Q96FV2
Control	2	Q96FV9
Control	2	Q96FW1
Control	2	Q96FX7

Control	2	Q96FZ2
Control	2	Q96FZ7
Control	2	Q96G01
Control	2	Q96G03
Control	2	Q96G21
Control	2	Q96G23
Control	2	Q96G46
Control	2	Q96GA3
Control	2	Q96GA7
Control	2	Q96GC5
Control	2	Q96GD0
Control	2	Q96GD4
Control	2	Q96GG9
Control	2	Q96GK7
Control	2	Q96GM5
Control	2	Q96GM8
Control	2	Q96GN5
Control	2	Q96GQ5
Control	2	Q96GQ7
Control	2	Q96GS4
Control	2	Q96GW9
Control	2	Q96GX2
Control	2	Q96GX5
Control	2	Q96GX9
Control	2	Q96GY0
Control	2	Q96GY3
Control	2	Q96GZ6
Control	2	Q96H20
Control	2	Q96H79
Control	2	Q96HA1
Control	2	Q96HA7
Control	2	Q96HC4
Control	2	Q96HD1
Control	2	Q96HE7
Control	2	Q96HE9
Control	2	Q96HJ9
Control	2	Q96HN2
Control	2	Q96HP0
Control	2	Q96HR3
Control	2	Q96HS1
Control	2	Q96HW7
Control	2	Q96HY6
Control	2	Q96HY7
Control	2	Q96I15
Control	2	Q96I24
Control	2	Q96I25
Control	2	Q96I51
Control	2	Q96I59
Control	2	Q96I99
Control	2	Q96II8

Control	2	Q96IJ6
Control	2	Q96IR7
Control	2	Q96IU4
Control	2	Q96IV0
Control	2	Q96IW7
Control	2	Q96IX5
Control	2	Q96IY1
Control	2	Q96IZ0
Control	2	Q96IZ6
Control	2	Q96J01
Control	2	Q96J02
Control	2	Q96J42
Control	2	Q96J84
Control	2	Q96JA1
Control	2	Q96JB2
Control	2	Q96JB5
Control	2	Q96JC1
Control	2	Q96JG6
Control	2	Q96JH7
Control	2	Q96JJ3
Control	2	Q96JJ7
Control	2	Q96JK2
Control	2	Q96JM3
Control	2	Q96JM7
Control	2	Q96JP5
Control	2	Q96JQ0
Control	2	Q96JQ2
Control	2	Q96JY6
Control	2	Q96K12
Control	2	Q96K17
Control	2	Q96K19
Control	2	Q96K21
Control	2	Q96K37
Control	2	Q96K76
Control	2	Q96K83
Control	2	Q96KA5
Control	2	Q96KB5
Control	2	Q96KC2
Control	2	Q96KC8
Control	2	Q96KG9
Control	2	Q96KM6
Control	2	Q96KN1
Control	2	Q96KP1
Control	2	Q96KP4
Control	2	Q96KQ7
Control	2	Q96KR1
Control	2	Q96KR6
Control	2	Q96L58
Control	2	Q96L91
Control	2	Q96L92

Control	2	Q96L93
Control	2	Q96LB3
Control	2	Q96LD4
Control	2	Q96LI5
Control	2	Q96LJ7
Control	2	Q96LL9
Control	2	Q96LR5
Control	2	Q96LW7
Control	2	Q96M27
Control	2	Q96M96
Control	2	Q96ME1
Control	2	Q96ME7
Control	2	Q96MF7
Control	2	Q96MG7
Control	2	Q96MG8
Control	2	Q96MM6
Control	2	Q96MU7
Control	2	Q96MW1
Control	2	Q96MW5
Control	2	Q96MX0
Control	2	Q96MX6
Control	2	Q96MY1
Control	2	Q96N66
Control	2	Q96N67
Control	2	Q96NB2
Control	2	Q96NB3
Control	2	Q96ND0
Control	2	Q96NE9
Control	2	Q96P11
Control	2	Q96P16
Control	2	Q96P47
Control	2	Q96P48
Control	2	Q96P70
Control	2	Q96PC5
Control	2	Q96PD2
Control	2	Q96PE2
Control	2	Q96PE3
Control	2	Q96PK6
Control	2	Q96PM5
Control	2	Q96PQ7
Control	2	Q96PU4
Control	2	Q96PU5
Control	2	Q96PU8
Control	2	Q96PV6
Control	2	Q96PY5
Control	2	Q96PZ0
Control	2	Q96PZ2
Control	2	Q96Q05
Control	2	Q96Q06
Control	2	Q96Q11

Control	2	Q96Q15
Control	2	Q96Q42
Control	2	Q96Q45
Control	2	Q96Q83
Control	2	Q96QB1
Control	2	Q96QC0
Control	2	Q96QD8
Control	2	Q96QD9
Control	2	Q96QG7
Control	2	Q96QK1
Control	2	Q96QR8
Control	2	Q96QU8
Control	2	Q96QV1
Control	2	Q96QZ7
Control	2	Q96R06
Control	2	Q96RE7
Control	2	Q96RF0
Control	2	Q96RG2
Control	2	Q96RK0
Control	2	Q96RL1
Control	2	Q96RL7
Control	2	Q96RN5
Control	2	Q96RP9
Control	2	Q96RQ1
Control	2	Q96RQ3
Control	2	Q96RR4
Control	2	Q96RS0
Control	2	Q96RS6
Control	2	Q96RT1
Control	2	Q96RT7
Control	2	Q96RT8
Control	2	Q96RU2
Control	2	Q96RU3
Control	2	Q96S44
Control	2	Q96S52
Control	2	Q96S55
Control	2	Q96S59
Control	2	Q96S66
Control	2	Q96S97
Control	2	Q96SB3
Control	2	Q96SB4
Control	2	Q96SB8
Control	2	Q96SI1
Control	2	Q96SI9
Control	2	Q96SK2
Control	2	Q96SL4
Control	2	Q96SN8
Control	2	Q96SQ9
Control	2	Q96ST2
Control	2	Q96ST3

Control	2	Q96SU4
Control	2	Q96SW2
Control	2	Q96SY0
Control	2	Q96SZ5
Control	2	Q96SZ6
Control	2	Q96T23
Control	2	Q96T37
Control	2	Q96T49
Control	2	Q96T51
Control	2	Q96T58
Control	2	Q96T60
Control	2	Q96T76
Control	2	Q96T88
Control	2	Q96TA1
Control	2	Q96TA2
Control	2	Q96TC7
Control	2	Q99081
Control	2	Q99426
Control	2	Q99436
Control	2	Q99439
Control	2	Q99442
Control	2	Q99447
Control	2	Q99459
Control	2	Q99460
Control	2	Q99470
Control	2	Q99471
Control	2	Q99496
Control	2	Q99497
Control	2	Q99500
Control	2	Q99501
Control	2	Q99504
Control	2	Q99519
Control	2	Q99536
Control	2	Q99538
Control	2	Q99541
Control	2	Q99543
Control	2	Q99549
Control	2	Q99567
Control	2	Q99569
Control	2	Q99570
Control	2	Q99571
Control	2	Q99575
Control	2	Q99584
Control	2	Q99590
Control	2	Q99598
Control	2	Q99611
Control	2	Q99613
Control	2	Q99614
Control	2	Q99615
Control	2	Q99623

Control	2	Q99627
Control	2	Q99633
Control	2	Q99653
Control	2	Q99661
Control	2	Q99666
Control	2	Q99685
Control	2	Q99700
Control	2	Q99704
Control	2	Q99707
Control	2	Q99714
Control	2	Q99715
Control	2	Q99717
Control	2	Q99720
Control	2	Q99729
Control	2	Q99733
Control	2	Q99735
Control	2	Q99747
Control	2	Q99755
Control	2	Q99758
Control	2	Q99759
Control	2	Q99766
Control	2	Q99797
Control	2	Q99798
Control	2	Q99805
Control	2	Q99808
Control	2	Q99816
Control	2	Q99829
Control	2	Q99832
Control	2	Q99836
Control	2	Q99848
Control	2	Q99873
Control	2	Q99933
Control	2	Q99941
Control	2	Q99958
Control	2	Q99961
Control	2	Q99973
Control	2	Q99986
Control	2	Q99988
Control	2	Q99996
Control	2	Q9BPW8
Control	2	Q9BPX3
Control	2	Q9BPX5
Control	2	Q9BPX6
Control	2	Q9BPX7
Control	2	Q9BPY3
Control	2	Q9BPZ7
Control	2	Q9BQ04
Control	2	Q9BQ24
Control	2	Q9BQ39
Control	2	Q9BQ48

Control	2	Q9BQ52
Control	2	Q9BQ61
Control	2	Q9BQ67
Control	2	Q9BQ69
Control	2	Q9BQ70
Control	2	Q9BQ75
Control	2	Q9BQ90
Control	2	Q9BQ95
Control	2	Q9BQA1
Control	2	Q9BQA9
Control	2	Q9BQB6
Control	2	Q9BQC3
Control	2	Q9BQE3
Control	2	Q9BQE4
Control	2	Q9BQE5
Control	2	Q9BQG0
Control	2	Q9BQL6
Control	2	Q9BQP7
Control	2	Q9BQQ3
Control	2	Q9BQS8
Control	2	Q9BR61
Control	2	Q9BR76
Control	2	Q9BRA2
Control	2	Q9BRD0
Control	2	Q9BRF8
Control	2	Q9BRG1
Control	2	Q9BRJ2
Control	2	Q9BRJ6
Control	2	Q9BRJ7
Control	2	Q9BRK5
Control	2	Q9BRP1
Control	2	Q9BRP4
Control	2	Q9BRP8
Control	2	Q9BRQ6
Control	2	Q9BRR6
Control	2	Q9BRR8
Control	2	Q9BRS2
Control	2	Q9BRT6
Control	2	Q9BRT9
Control	2	Q9BRU9
Control	2	Q9BRX2
Control	2	Q9BRX8
Control	2	Q9BRZ2
Control	2	Q9BS26
Control	2	Q9BS40
Control	2	Q9BSC4
Control	2	Q9BSD7
Control	2	Q9BSF4
Control	2	Q9BSH4
Control	2	Q9BSJ2

Control	2	Q9BSJ8
Control	2	Q9BSL1
Control	2	Q9BSR8
Control	2	Q9BSU1
Control	2	Q9BSV6
Control	2	Q9BT09
Control	2	Q9BT17
Control	2	Q9BT22
Control	2	Q9BT23
Control	2	Q9BT25
Control	2	Q9BT40
Control	2	Q9BT73
Control	2	Q9BT78
Control	2	Q9BT88
Control	2	Q9BTA9
Control	2	Q9BTC0
Control	2	Q9BTC8
Control	2	Q9BTD8
Control	2	Q9BTE1
Control	2	Q9BTE3
Control	2	Q9BTE6
Control	2	Q9BTE7
Control	2	Q9BTT0
Control	2	Q9BTT6
Control	2	Q9BTU6
Control	2	Q9BTV4
Control	2	Q9BTV5
Control	2	Q9BTW9
Control	2	Q9BTX1
Control	2	Q9BTY7
Control	2	Q9BTZ2
Control	2	Q9BU23
Control	2	Q9BU61
Control	2	Q9BU76
Control	2	Q9BUB7
Control	2	Q9BUE0
Control	2	Q9BUE6
Control	2	Q9BUF5
Control	2	Q9BUH6
Control	2	Q9BUI4
Control	2	Q9BUJ2
Control	2	Q9BUK6
Control	2	Q9BUL5
Control	2	Q9BUL8
Control	2	Q9BUL9
Control	2	Q9BUN8
Control	2	Q9BUP3
Control	2	Q9BUQ8
Control	2	Q9BUR4
Control	2	Q9BUR5

Control	2	Q9BUT1
Control	2	Q9BV20
Control	2	Q9BV38
Control	2	Q9BV40
Control	2	Q9BV44
Control	2	Q9BV57
Control	2	Q9BV79
Control	2	Q9BV81
Control	2	Q9BV86
Control	2	Q9BV94
Control	2	Q9BVA0
Control	2	Q9BVC4
Control	2	Q9BVC5
Control	2	Q9BVC6
Control	2	Q9BVG4
Control	2	Q9BVG9
Control	2	Q9BVI4
Control	2	Q9BVJ6
Control	2	Q9BVJ7
Control	2	Q9BVK6
Control	2	Q9BVK8
Control	2	Q9BVL2
Control	2	Q9BVL4
Control	2	Q9BVM2
Control	2	Q9BVP2
Control	2	Q9BVQ7
Control	2	Q9BVS4
Control	2	Q9BVS5
Control	2	Q9BVT8
Control	2	Q9BVV7
Control	2	Q9BW19
Control	2	Q9BW27
Control	2	Q9BW60
Control	2	Q9BW62
Control	2	Q9BW71
Control	2	Q9BW83
Control	2	Q9BW85
Control	2	Q9BW91
Control	2	Q9BW92
Control	2	Q9BWD1
Control	2	Q9BWE0
Control	2	Q9BWF3
Control	2	Q9BWH2
Control	2	Q9BWH6
Control	2	Q9BWJ5
Control	2	Q9BWM7
Control	2	Q9BWN1
Control	2	Q9BWS9
Control	2	Q9BWT3
Control	2	Q9BWT7

Control	2	Q9BWU0
Control	2	Q9BWU1
Control	2	Q9BX40
Control	2	Q9BX59
Control	2	Q9BX67
Control	2	Q9BX69
Control	2	Q9BX70
Control	2	Q9BX95
Control	2	Q9BXB4
Control	2	Q9BXB5
Control	2	Q9BXF6
Control	2	Q9BXI6
Control	2	Q9BXJ0
Control	2	Q9BXJ9
Control	2	Q9BXK1
Control	2	Q9BXK5
Control	2	Q9BXL7
Control	2	Q9BXP2
Control	2	Q9BXP5
Control	2	Q9BXR0
Control	2	Q9BXS5
Control	2	Q9BXS6
Control	2	Q9BXW7
Control	2	Q9BXW9
Control	2	Q9BXY0
Control	2	Q9BY41
Control	2	Q9BY42
Control	2	Q9BY43
Control	2	Q9BY44
Control	2	Q9BY77
Control	2	Q9BY89
Control	2	Q9BYB0
Control	2	Q9BYB4
Control	2	Q9BYC5
Control	2	Q9BYC8
Control	2	Q9BYC9
Control	2	Q9BYD1
Control	2	Q9BYD2
Control	2	Q9BYD3
Control	2	Q9BYD6
Control	2	Q9BYG3
Control	2	Q9BYI3
Control	2	Q9BYK8
Control	2	Q9BYM8
Control	2	Q9BYN8
Control	2	Q9BYT8
Control	2	Q9BYV8
Control	2	Q9BYW2
Control	2	Q9BZ23
Control	2	Q9BZ29

Control	2	Q9BZ67
Control	2	Q9BZ76
Control	2	Q9BZ95
Control	2	Q9BZD4
Control	2	Q9BZE1
Control	2	Q9BZE4
Control	2	Q9BZE9
Control	2	Q9BZF1
Control	2	Q9BZF9
Control	2	Q9BZG1
Control	2	Q9BZG8
Control	2	Q9BZH6
Control	2	Q9BZI7
Control	2	Q9BZJ0
Control	2	Q9BZK7
Control	2	Q9BZL1
Control	2	Q9BZL4
Control	2	Q9BZL6
Control	2	Q9BZQ6
Control	2	Q9BZQ8
Control	2	Q9BZV1
Control	2	Q9BZW5
Control	2	Q9BZX2
Control	2	Q9BZZ2
Control	2	Q9BZZ5
Control	2	Q9C000
Control	2	Q9C035
Control	2	Q9C037
Control	2	Q9C0B1
Control	2	Q9C0B5
Control	2	Q9C0B7
Control	2	Q9C0C2
Control	2	Q9C0C9
Control	2	Q9C0D3
Control	2	Q9C0D5
Control	2	Q9C0D9
Control	2	Q9C0E2
Control	2	Q9C0E8
Control	2	Q9C0F1
Control	2	Q9C0H2
Control	2	Q9C0H6
Control	2	Q9C0I1
Control	2	Q9C0J8
Control	2	Q9GZL7
Control	2	Q9GZM5
Control	2	Q9GZM7
Control	2	Q9GZN8
Control	2	Q9GZP4
Control	2	Q9GZP9
Control	2	Q9GZQ3

Control	2	Q9GZR1
Control	2	Q9GZR2
Control	2	Q9GZR7
Control	2	Q9GZS1
Control	2	Q9GZS3
Control	2	Q9GZT3
Control	2	Q9GZT4
Control	2	Q9GZT8
Control	2	Q9GZT9
Control	2	Q9GZU1
Control	2	Q9GZU8
Control	2	Q9GZY8
Control	2	Q9GZZ1
Control	2	Q9GZZ8
Control	2	Q9GZZ9
Control	2	Q9H000
Control	2	Q9H061
Control	2	Q9H074
Control	2	Q9H078
Control	2	Q9H082
Control	2	Q9H089
Control	2	Q9H098
Control	2	Q9H0A0
Control	2	Q9H0A8
Control	2	Q9H0B6
Control	2	Q9H0C8
Control	2	Q9H0D6
Control	2	Q9H0E2
Control	2	Q9H0E3
Control	2	Q9H0E9
Control	2	Q9H0G5
Control	2	Q9H0H0
Control	2	Q9H0H5
Control	2	Q9H0J9
Control	2	Q9H0L4
Control	2	Q9H0P0
Control	2	Q9H0Q0
Control	2	Q9H0R3
Control	2	Q9H0R4
Control	2	Q9H0R6
Control	2	Q9H0S4
Control	2	Q9H0U3
Control	2	Q9H0U4
Control	2	Q9H0U6
Control	2	Q9H0U9
Control	2	Q9H0V1
Control	2	Q9H0V9
Control	2	Q9H0W8
Control	2	Q9H0X4
Control	2	Q9H0X9

Control	2	Q9H147
Control	2	Q9H173
Control	2	Q9H1A3
Control	2	Q9H1A4
Control	2	Q9H1B7
Control	2	Q9H1C7
Control	2	Q9H1D9
Control	2	Q9H1E3
Control	2	Q9H1E5
Control	2	Q9H1H9
Control	2	Q9H1I8
Control	2	Q9H1K0
Control	2	Q9H1K1
Control	2	Q9H1P3
Control	2	Q9H1Y0
Control	2	Q9H1Z4
Control	2	Q9H201
Control	2	Q9H223
Control	2	Q9H267
Control	2	Q9H269
Control	2	Q9H270
Control	2	Q9H299
Control	2	Q9H2C0
Control	2	Q9H2D1
Control	2	Q9H2D6
Control	2	Q9H2G2
Control	2	Q9H2H8
Control	2	Q9H2H9
Control	2	Q9H2J4
Control	2	Q9H2K8
Control	2	Q9H2M9
Control	2	Q9H2P0
Control	2	Q9H2P9
Control	2	Q9H2U1
Control	2	Q9H2U2
Control	2	Q9H2V7
Control	2	Q9H2W6
Control	2	Q9H2X9
Control	2	Q9H300
Control	2	Q9H307
Control	2	Q9H330
Control	2	Q9H334
Control	2	Q9H3F6
Control	2	Q9H3H1
Control	2	Q9H3H3
Control	2	Q9H3K2
Control	2	Q9H3K6
Control	2	Q9H3L0
Control	2	Q9H3M7
Control	2	Q9H3N1

Control	2	Q9H3P2
Control	2	Q9H3P7
Control	2	Q9H3Q1
Control	2	Q9H3S7
Control	2	Q9H3T3
Control	2	Q9H3U1
Control	2	Q9H3U5
Control	2	Q9H3Z4
Control	2	Q9H410
Control	2	Q9H444
Control	2	Q9H446
Control	2	Q9H467
Control	2	Q9H469
Control	2	Q9H479
Control	2	Q9H488
Control	2	Q9H490
Control	2	Q9H497
Control	2	Q9H4A3
Control	2	Q9H4A4
Control	2	Q9H4A5
Control	2	Q9H4A6
Control	2	Q9H4B0
Control	2	Q9H4F1
Control	2	Q9H4G4
Control	2	Q9H4I3
Control	2	Q9H4K7
Control	2	Q9H4L4
Control	2	Q9H4L5
Control	2	Q9H4L7
Control	2	Q9H4M9
Control	2	Q9H4Z3
Control	2	Q9H501
Control	2	Q9H553
Control	2	Q9H583
Control	2	Q9H5K3
Control	2	Q9H5N1
Control	2	Q9H5Q4
Control	2	Q9H5U6
Control	2	Q9H5V9
Control	2	Q9H5X1
Control	2	Q9H5Z1
Control	2	Q9H5Z6
Control	2	Q9H6D7
Control	2	Q9H6E4
Control	2	Q9H6F5
Control	2	Q9H6H4
Control	2	Q9H6I2
Control	2	Q9H6K4
Control	2	Q9H6Q4
Control	2	Q9H6R0

Control	2	Q9H6R4
Control	2	Q9H6R7
Control	2	Q9H6S0
Control	2	Q9H6S1
Control	2	Q9H6T3
Control	2	Q9H6U6
Control	2	Q9H6U8
Control	2	Q9H6V9
Control	2	Q9H6W3
Control	2	Q9H6Y2
Control	2	Q9H6Z4
Control	2	Q9H773
Control	2	Q9H792
Control	2	Q9H7B2
Control	2	Q9H7B4
Control	2	Q9H7C9
Control	2	Q9H7D0
Control	2	Q9H7D7
Control	2	Q9H7E2
Control	2	Q9H7E9
Control	2	Q9H7F0
Control	2	Q9H7H0
Control	2	Q9H7N4
Control	2	Q9H7Z3
Control	2	Q9H7Z7
Control	2	Q9H814
Control	2	Q9H832
Control	2	Q9H845
Control	2	Q9H857
Control	2	Q9H875
Control	2	Q9H8G2
Control	2	Q9H8H0
Control	2	Q9H8H2
Control	2	Q9H8H3
Control	2	Q9H8K7
Control	2	Q9H8L6
Control	2	Q9H8M7
Control	2	Q9H8M9
Control	2	Q9H8T0
Control	2	Q9H8W4
Control	2	Q9H8Y5
Control	2	Q9H8Y8
Control	2	Q9H900
Control	2	Q9H910
Control	2	Q9H936
Control	2	Q9H939
Control	2	Q9H944
Control	2	Q9H967
Control	2	Q9H974
Control	2	Q9H981

Control	2	Q9H993
Control	2	Q9H999
Control	2	Q9H9A5
Control	2	Q9H9A6
Control	2	Q9H9B1
Control	2	Q9H9B4
Control	2	Q9H9C1
Control	2	Q9H9E3
Control	2	Q9H9F9
Control	2	Q9H9H4
Control	2	Q9H9J2
Control	2	Q9H9P8
Control	2	Q9H9Q2
Control	2	Q9H9Q4
Control	2	Q9H9S3
Control	2	Q9H9S5
Control	2	Q9H9T3
Control	2	Q9H9Y2
Control	2	Q9H9Y6
Control	2	Q9HA38
Control	2	Q9HA47
Control	2	Q9HA64
Control	2	Q9HA65
Control	2	Q9HA77
Control	2	Q9HAB3
Control	2	Q9HAB8
Control	2	Q9HAC8
Control	2	Q9HAD4
Control	2	Q9HAN9
Control	2	Q9HAS0
Control	2	Q9HAT2
Control	2	Q9HAU0
Control	2	Q9HAU4
Control	2	Q9HAU5
Control	2	Q9HAV0
Control	2	Q9HAV4
Control	2	Q9HAV7
Control	2	Q9HB07
Control	2	Q9HB20
Control	2	Q9HB21
Control	2	Q9HB40
Control	2	Q9HB63
Control	2	Q9HB71
Control	2	Q9HB90
Control	2	Q9HBF4
Control	2	Q9HBG6
Control	2	Q9HBH5
Control	2	Q9HBI1
Control	2	Q9HBL0
Control	2	Q9HBL7

Control	2	Q9HBL8
Control	2	Q9HBM0
Control	2	Q9HBM1
Control	2	Q9HBM6
Control	2	Q9HBR0
Control	2	Q9HBU6
Control	2	Q9HBW9
Control	2	Q9HC07
Control	2	Q9HC21
Control	2	Q9HC35
Control	2	Q9HC36
Control	2	Q9HC38
Control	2	Q9HC98
Control	2	Q9HCC0
Control	2	Q9HCD5
Control	2	Q9HCE0
Control	2	Q9HCE1
Control	2	Q9HCE5
Control	2	Q9HCG7
Control	2	Q9HCG8
Control	2	Q9HCJ3
Control	2	Q9HCK8
Control	2	Q9HCM4
Control	2	Q9HCN4
Control	2	Q9HCN8
Control	2	Q9HCP0
Control	2	Q9HCS7
Control	2	Q9HCU5
Control	2	Q9HD15
Control	2	Q9HD20
Control	2	Q9HD26
Control	2	Q9HD33
Control	2	Q9HD34
Control	2	Q9HD40
Control	2	Q9HD42
Control	2	Q9HD45
Control	2	Q9HD67
Control	2	Q9HDC9
Control	2	Q9NNW5
Control	2	Q9NNW7
Control	2	Q9NP58
Control	2	Q9NP61
Control	2	Q9NP64
Control	2	Q9NP72
Control	2	Q9NP74
Control	2	Q9NP77
Control	2	Q9NP79
Control	2	Q9NP81
Control	2	Q9NP92
Control	2	Q9NP97

Control	2	Q9NPA0
Control	2	Q9NPA8
Control	2	Q9NPD3
Control	2	Q9NPD8
Control	2	Q9NPE3
Control	2	Q9NPF4
Control	2	Q9NPF5
Control	2	Q9NPG4
Control	2	Q9NPH2
Control	2	Q9NPI1
Control	2	Q9NPI6
Control	2	Q9NPJ3
Control	2	Q9NPJ6
Control	2	Q9NPL8
Control	2	Q9NPQ8
Control	2	Q9NPY3
Control	2	Q9NQ29
Control	2	Q9NQ30
Control	2	Q9NQ48
Control	2	Q9NQ50
Control	2	Q9NQ55
Control	2	Q9NQ66
Control	2	Q9NQ88
Control	2	Q9NQC3
Control	2	Q9NQG5
Control	2	Q9NQH7
Control	2	Q9NQR4
Control	2	Q9NQS1
Control	2	Q9NQS7
Control	2	Q9NQT4
Control	2	Q9NQT5
Control	2	Q9NQT8
Control	2	Q9NQW6
Control	2	Q9NQW7
Control	2	Q9NQX3
Control	2	Q9NQY0
Control	2	Q9NQZ2
Control	2	Q9NQZ5
Control	2	Q9NR09
Control	2	Q9NR12
Control	2	Q9NR19
Control	2	Q9NR28
Control	2	Q9NR30
Control	2	Q9NR31
Control	2	Q9NR45
Control	2	Q9NR46
Control	2	Q9NR48
Control	2	Q9NR50
Control	2	Q9NR56
Control	2	Q9NRA2

Control	2	Q9NRA8
Control	2	Q9NRB3
Control	2	Q9NRC1
Control	2	Q9NRD5
Control	2	Q9NRF2
Control	2	Q9NRF8
Control	2	Q9NRF9
Control	2	Q9NRG0
Control	2	Q9NRG9
Control	2	Q9NRH2
Control	2	Q9NRK6
Control	2	Q9NRL2
Control	2	Q9NRL3
Control	2	Q9NRN7
Control	2	Q9NRN9
Control	2	Q9NRP0
Control	2	Q9NRP2
Control	2	Q9NRQ2
Control	2	Q9NRR4
Control	2	Q9NRS6
Control	2	Q9NRV9
Control	2	Q9NRW3
Control	2	Q9NRW7
Control	2	Q9NRX1
Control	2	Q9NRX2
Control	2	Q9NRX4
Control	2	Q9NRX5
Control	2	Q9NRY4
Control	2	Q9NRY5
Control	2	Q9NRY6
Control	2	Q9NRZ5
Control	2	Q9NRZ7
Control	2	Q9NRZ9
Control	2	Q9NS00
Control	2	Q9NS15
Control	2	Q9NS69
Control	2	Q9NS86
Control	2	Q9NS87
Control	2	Q9NS93
Control	2	Q9NSC5
Control	2	Q9NSD9
Control	2	Q9NSE4
Control	2	Q9NSI2
Control	2	Q9NSI8
Control	2	Q9NSK0
Control	2	Q9NSV4
Control	2	Q9NSY1
Control	2	Q9NT62
Control	2	Q9NTG7
Control	2	Q9NTI5

Control	2	Q9NTJ3
Control	2	Q9NTJ4
Control	2	Q9NTJ5
Control	2	Q9NTK1
Control	2	Q9NTK5
Control	2	Q9NTM9
Control	2	Q9NTW7
Control	2	Q9NTX5
Control	2	Q9NTZ6
Control	2	Q9NU19
Control	2	Q9NU22
Control	2	Q9NUB1
Control	2	Q9NUD5
Control	2	Q9NUI1
Control	2	Q9NUJ1
Control	2	Q9NUJ3
Control	2	Q9NUL3
Control	2	Q9NUL5
Control	2	Q9NUL7
Control	2	Q9NUM4
Control	2	Q9NUP9
Control	2	Q9NUQ2
Control	2	Q9NUQ3
Control	2	Q9NUQ6
Control	2	Q9NUQ7
Control	2	Q9NUQ8
Control	2	Q9NUQ9
Control	2	Q9NUT2
Control	2	Q9NUU7
Control	2	Q9NUV9
Control	2	Q9NUW8
Control	2	Q9NUY8
Control	2	Q9NV06
Control	2	Q9NV31
Control	2	Q9NV56
Control	2	Q9NV66
Control	2	Q9NV70
Control	2	Q9NV79
Control	2	Q9NV88
Control	2	Q9NV96
Control	2	Q9NVA1
Control	2	Q9NVA2
Control	2	Q9NVC3
Control	2	Q9NVC6
Control	2	Q9NVD7
Control	2	Q9NVE5
Control	2	Q9NVE7
Control	2	Q9NVF7
Control	2	Q9NVG8
Control	2	Q9NVH0

Control	2	Q9NVH1
Control	2	Q9NVH2
Control	2	Q9NVI1
Control	2	Q9NVI7
Control	2	Q9NVJ2
Control	2	Q9NVM6
Control	2	Q9NVM9
Control	2	Q9NVN3
Control	2	Q9NVN8
Control	2	Q9NVP1
Control	2	Q9NVP2
Control	2	Q9NVR0
Control	2	Q9NVR2
Control	2	Q9NVS2
Control	2	Q9NVS9
Control	2	Q9NVT9
Control	2	Q9NVU0
Control	2	Q9NVU7
Control	2	Q9NVV4
Control	2	Q9NVX2
Control	2	Q9NVZ3
Control	2	Q9NW08
Control	2	Q9NW13
Control	2	Q9NW15
Control	2	Q9NW64
Control	2	Q9NW82
Control	2	Q9NW97
Control	2	Q9NWB6
Control	2	Q9NWH9
Control	2	Q9NWM8
Control	2	Q9NWS8
Control	2	Q9NWT1
Control	2	Q9NWT6
Control	2	Q9NWU1
Control	2	Q9NWU5
Control	2	Q9NWV4
Control	2	Q9NWV8
Control	2	Q9NWW5
Control	2	Q9NWX5
Control	2	Q9NWX6
Control	2	Q9NWY4
Control	2	Q9NWZ3
Control	2	Q9NWZ5
Control	2	Q9NX00
Control	2	Q9NX02
Control	2	Q9NX05
Control	2	Q9NX07
Control	2	Q9NX14
Control	2	Q9NX20
Control	2	Q9NX24

Control	2	Q9NX40
Control	2	Q9NX46
Control	2	Q9NX47
Control	2	Q9NX58
Control	2	Q9NX61
Control	2	Q9NX62
Control	2	Q9NX63
Control	2	Q9NX74
Control	2	Q9NX76
Control	2	Q9NXA8
Control	2	Q9NXC5
Control	2	Q9NXD2
Control	2	Q9NXE4
Control	2	Q9NXE8
Control	2	Q9NXF1
Control	2	Q9NXF7
Control	2	Q9NXG2
Control	2	Q9NXG6
Control	2	Q9NXH8
Control	2	Q9NXH9
Control	2	Q9NXN4
Control	2	Q9NXR1
Control	2	Q9NXR7
Control	2	Q9NXS2
Control	2	Q9NXU5
Control	2	Q9NXV2
Control	2	Q9NXV6
Control	2	Q9NXW2
Control	2	Q9NXW9
Control	2	Q9NXX6
Control	2	Q9NY12
Control	2	Q9NY15
Control	2	Q9NY27
Control	2	Q9NY33
Control	2	Q9NY61
Control	2	Q9NY65
Control	2	Q9NY93
Control	2	Q9NYA1
Control	2	Q9NYB0
Control	2	Q9NYB9
Control	2	Q9NYF8
Control	2	Q9NYH9
Control	2	Q9NYJ8
Control	2	Q9NYK5
Control	2	Q9NYL2
Control	2	Q9NYL4
Control	2	Q9NYL9
Control	2	Q9NYM9
Control	2	Q9NYP7
Control	2	Q9NYR9

Control	2	Q9NYU1
Control	2	Q9NYU2
Control	2	Q9NYV4
Control	2	Q9NYY8
Control	2	Q9NZ01
Control	2	Q9NZ08
Control	2	Q9NZ32
Control	2	Q9NZ43
Control	2	Q9NZ45
Control	2	Q9NZ52
Control	2	Q9NZ63
Control	2	Q9NZB2
Control	2	Q9NZC3
Control	2	Q9NZC9
Control	2	Q9NZD2
Control	2	Q9NZD8
Control	2	Q9NZE8
Control	2	Q9NZI7
Control	2	Q9NZI8
Control	2	Q9NZJ4
Control	2	Q9NZJ7
Control	2	Q9NZJ9
Control	2	Q9NZL4
Control	2	Q9NZL9
Control	2	Q9NZM1
Control	2	Q9NZM3
Control	2	Q9NZM5
Control	2	Q9NZN3
Control	2	Q9NZN4
Control	2	Q9NZN5
Control	2	Q9NZN8
Control	2	Q9NZQ3
Control	2	Q9NZQ7
Control	2	Q9NZT1
Control	2	Q9NZT2
Control	2	Q9NZU5
Control	2	Q9NZV1
Control	2	Q9NZV5
Control	2	Q9NZW5
Control	2	Q9NZZ3
Control	2	Q9P013
Control	2	Q9P015
Control	2	Q9P016
Control	2	Q9P031
Control	2	Q9P032
Control	2	Q9P035
Control	2	Q9P0I2
Control	2	Q9P0J0
Control	2	Q9P0J1
Control	2	Q9P0J7

Control	2	Q9P0K7
Control	2	Q9P0L0
Control	2	Q9P0M9
Control	2	Q9P0T7
Control	2	Q9P0U3
Control	2	Q9P0U4
Control	2	Q9P0V3
Control	2	Q9P0V9
Control	2	Q9P107
Control	2	Q9P1Y6
Control	2	Q9P215
Control	2	Q9P219
Control	2	Q9P227
Control	2	Q9P253
Control	2	Q9P258
Control	2	Q9P260
Control	2	Q9P265
Control	2	Q9P266
Control	2	Q9P270
Control	2	Q9P273
Control	2	Q9P275
Control	2	Q9P287
Control	2	Q9P289
Control	2	Q9P291
Control	2	Q9P2A4
Control	2	Q9P2B2
Control	2	Q9P2B4
Control	2	Q9P2C4
Control	2	Q9P2D0
Control	2	Q9P2D3
Control	2	Q9P2E3
Control	2	Q9P2E5
Control	2	Q9P2E7
Control	2	Q9P2E9
Control	2	Q9P2I0
Control	2	Q9P2J3
Control	2	Q9P2J5
Control	2	Q9P2K5
Control	2	Q9P2K6
Control	2	Q9P2K8
Control	2	Q9P2L0
Control	2	Q9P2N5
Control	2	Q9P2N6
Control	2	Q9P2P5
Control	2	Q9P2Q2
Control	2	Q9P2R3
Control	2	Q9P2R7
Control	2	Q9P2T1
Control	2	Q9P2W9
Control	2	Q9P2X0

Control	2	Q9P2X3
Control	2	Q9P2Y5
Control	2	Q9UBB4
Control	2	Q9UBB5
Control	2	Q9UBB6
Control	2	Q9UBB9
Control	2	Q9UBC2
Control	2	Q9UBC5
Control	2	Q9UBD5
Control	2	Q9UBE0
Control	2	Q9UBF2
Control	2	Q9UBF8
Control	2	Q9UBG0
Control	2	Q9UBI1
Control	2	Q9UBI6
Control	2	Q9UBK7
Control	2	Q9UBK8
Control	2	Q9UBK9
Control	2	Q9UBL3
Control	2	Q9UBM7
Control	2	Q9UBN6
Control	2	Q9UBN7
Control	2	Q9UBP0
Control	2	Q9UBP4
Control	2	Q9UBP6
Control	2	Q9UBP9
Control	2	Q9UBQ0
Control	2	Q9UBQ5
Control	2	Q9UBQ6
Control	2	Q9UBQ7
Control	2	Q9UBR2
Control	2	Q9UBS0
Control	2	Q9UBS4
Control	2	Q9UBS8
Control	2	Q9UBT2
Control	2	Q9UBU8
Control	2	Q9UBU9
Control	2	Q9UBV2
Control	2	Q9UBV7
Control	2	Q9UBV8
Control	2	Q9UBW7
Control	2	Q9UBW8
Control	2	Q9UBX3
Control	2	Q9UDR5
Control	2	Q9UDT6
Control	2	Q9UDW1
Control	2	Q9UDX5
Control	2	Q9UDY2
Control	2	Q9UDY4
Control	2	Q9UEE5

Control	2	Q9UEE9
Control	2	Q9UER7
Control	2	Q9UET6
Control	2	Q9UEU0
Control	2	Q9UEW8
Control	2	Q9UEY8
Control	2	Q9UFC0
Control	2	Q9UFN0
Control	2	Q9UFW8
Control	2	Q9UG56
Control	2	Q9UG63
Control	2	Q9UGI8
Control	2	Q9UGJ0
Control	2	Q9UGJ1
Control	2	Q9UGL1
Control	2	Q9UGM6
Control	2	Q9UGP4
Control	2	Q9UGP8
Control	2	Q9UGQ3
Control	2	Q9UGR2
Control	2	Q9UGV2
Control	2	Q9UH62
Control	2	Q9UH65
Control	2	Q9UH99
Control	2	Q9UHA3
Control	2	Q9UHA4
Control	2	Q9UHB6
Control	2	Q9UHB7
Control	2	Q9UHB9
Control	2	Q9UHD1
Control	2	Q9UHD2
Control	2	Q9UHD8
Control	2	Q9UHG3
Control	2	Q9UHI6
Control	2	Q9UHJ6
Control	2	Q9UHL4
Control	2	Q9UHN6
Control	2	Q9UHP3
Control	2	Q9UHQ4
Control	2	Q9UHQ9
Control	2	Q9UHR5
Control	2	Q9UHR6
Control	2	Q9UHV9
Control	2	Q9UHW5
Control	2	Q9UHX1
Control	2	Q9UHY1
Control	2	Q9UHY7
Control	2	Q9UHY8
Control	2	Q9UI09
Control	2	Q9UI10

Control	2	Q9UI12
Control	2	Q9UI14
Control	2	Q9UI26
Control	2	Q9UI30
Control	2	Q9UI36
Control	2	Q9UIA9
Control	2	Q9UIC8
Control	2	Q9UID3
Control	2	Q9UIF8
Control	2	Q9UIF9
Control	2	Q9UIG0
Control	2	Q9UII2
Control	2	Q9UIJ7
Control	2	Q9UIQ6
Control	2	Q9UIS9
Control	2	Q9UIV1
Control	2	Q9UJ41
Control	2	Q9UJ68
Control	2	Q9UJ70
Control	2	Q9UJ83
Control	2	Q9UJA5
Control	2	Q9UJC5
Control	2	Q9UJF2
Control	2	Q9UJK0
Control	2	Q9UJS0
Control	2	Q9UJU6
Control	2	Q9UJV9
Control	2	Q9UJW0
Control	2	Q9UJX2
Control	2	Q9UJX3
Control	2	Q9UJX4
Control	2	Q9UJX5
Control	2	Q9UJX6
Control	2	Q9UJY4
Control	2	Q9UJY5
Control	2	Q9UJZ1
Control	2	Q9UK23
Control	2	Q9UK41
Control	2	Q9UK45
Control	2	Q9UK59
Control	2	Q9UK61
Control	2	Q9UK76
Control	2	Q9UK99
Control	2	Q9UKA4
Control	2	Q9UKB1
Control	2	Q9UKD1
Control	2	Q9UKD2
Control	2	Q9UKE5
Control	2	Q9UKF6
Control	2	Q9UKG1

Control	2	Q9UKI2
Control	2	Q9UKI8
Control	2	Q9UKJ3
Control	2	Q9UKK3
Control	2	Q9UKK6
Control	2	Q9UKK9
Control	2	Q9UKL0
Control	2	Q9UKL6
Control	2	Q9UKM7
Control	2	Q9UKM9
Control	2	Q9UKN8
Control	2	Q9UKR5
Control	2	Q9UKS6
Control	2	Q9UKU7
Control	2	Q9UKU9
Control	2	Q9UKV3
Control	2	Q9UKV5
Control	2	Q9UKV8
Control	2	Q9UKX7
Control	2	Q9UKY7
Control	2	Q9UKZ1
Control	2	Q9UL03
Control	2	Q9UL15
Control	2	Q9UL18
Control	2	Q9UL25
Control	2	Q9UL26
Control	2	Q9UL33
Control	2	Q9UL40
Control	2	Q9UL46
Control	2	Q9UL54
Control	2	Q9UL63
Control	2	Q9ULA0
Control	2	Q9ULC3
Control	2	Q9ULC4
Control	2	Q9ULD2
Control	2	Q9ULE0
Control	2	Q9ULE6
Control	2	Q9ULF5
Control	2	Q9ULG1
Control	2	Q9ULG6
Control	2	Q9ULH0
Control	2	Q9ULH1
Control	2	Q9ULH7
Control	2	Q9ULJ7
Control	2	Q9ULK4
Control	2	Q9ULL8
Control	2	Q9ULM3
Control	2	Q9ULP0
Control	2	Q9ULP9
Control	2	Q9ULR0

Control	2	Q9ULS5
Control	2	Q9ULT8
Control	2	Q9ULU4
Control	2	Q9ULV3
Control	2	Q9ULV4
Control	2	Q9ULW0
Control	2	Q9ULW3
Control	2	Q9ULX3
Control	2	Q9ULX6
Control	2	Q9ULX9
Control	2	Q9ULZ3
Control	2	Q9UM00
Control	2	Q9UM11
Control	2	Q9UM13
Control	2	Q9UM54
Control	2	Q9UMR2
Control	2	Q9UMR5
Control	2	Q9UMS4
Control	2	Q9UMX1
Control	2	Q9UMX3
Control	2	Q9UMY1
Control	2	Q9UMY4
Control	2	Q9UMZ2
Control	2	Q9UN37
Control	2	Q9UN86
Control	2	Q9UNE7
Control	2	Q9UNF0
Control	2	Q9UNF1
Control	2	Q9UNH6
Control	2	Q9UNH7
Control	2	Q9UNI6
Control	2	Q9UNK0
Control	2	Q9UNL2
Control	2	Q9UNM6
Control	2	Q9UNN5
Control	2	Q9UNN8
Control	2	Q9UNP9
Control	2	Q9UNQ2
Control	2	Q9UNS2
Control	2	Q9UNW1
Control	2	Q9UNW9
Control	2	Q9UNX4
Control	2	Q9UNY4
Control	2	Q9UNZ2
Control	2	Q9UP83
Control	2	Q9UP95
Control	2	Q9UPM8
Control	2	Q9UPN3
Control	2	Q9UPN4
Control	2	Q9UPN6

Control	2	Q9UPN7
Control	2	Q9UPN9
Control	2	Q9UPP1
Control	2	Q9UPQ9
Control	2	Q9UPR0
Control	2	Q9UPR3
Control	2	Q9UPT5
Control	2	Q9UPT8
Control	2	Q9UPU5
Control	2	Q9UPU7
Control	2	Q9UPU9
Control	2	Q9UPW0
Control	2	Q9UPW5
Control	2	Q9UPY3
Control	2	Q9UPY5
Control	2	Q9UPY8
Control	2	Q9UQ13
Control	2	Q9UQ35
Control	2	Q9UQ53
Control	2	Q9UQ80
Control	2	Q9UQ90
Control	2	Q9UQB8
Control	2	Q9UQC2
Control	2	Q9UQE7
Control	2	Q9UQN3
Control	2	Q9UQQ2
Control	2	Q9UQR1
Control	2	Q9Y217
Control	2	Q9Y219
Control	2	Q9Y221
Control	2	Q9Y223
Control	2	Q9Y224
Control	2	Q9Y230
Control	2	Q9Y232
Control	2	Q9Y237
Control	2	Q9Y243
Control	2	Q9Y256
Control	2	Q9Y259
Control	2	Q9Y262
Control	2	Q9Y263
Control	2	Q9Y265
Control	2	Q9Y266
Control	2	Q9Y276
Control	2	Q9Y277
Control	2	Q9Y281
Control	2	Q9Y282
Control	2	Q9Y285
Control	2	Q9Y289
Control	2	Q9Y291
Control	2	Q9Y294

Control	2	Q9Y295
Control	2	Q9Y296
Control	2	Q9Y2A7
Control	2	Q9Y2B0
Control	2	Q9Y2C4
Control	2	Q9Y2D4
Control	2	Q9Y2D5
Control	2	Q9Y2G2
Control	2	Q9Y2G3
Control	2	Q9Y2G5
Control	2	Q9Y2G8
Control	2	Q9Y2H0
Control	2	Q9Y2H1
Control	2	Q9Y2H2
Control	2	Q9Y2H6
Control	2	Q9Y2I1
Control	2	Q9Y2I7
Control	2	Q9Y2I8
Control	2	Q9Y2J2
Control	2	Q9Y2J4
Control	2	Q9Y2K2
Control	2	Q9Y2K7
Control	2	Q9Y2L1
Control	2	Q9Y2L5
Control	2	Q9Y2L9
Control	2	Q9Y2P8
Control	2	Q9Y2Q3
Control	2	Q9Y2Q5
Control	2	Q9Y2Q9
Control	2	Q9Y2R0
Control	2	Q9Y2R4
Control	2	Q9Y2R5
Control	2	Q9Y2R9
Control	2	Q9Y2S2
Control	2	Q9Y2S6
Control	2	Q9Y2S7
Control	2	Q9Y2T2
Control	2	Q9Y2U8
Control	2	Q9Y2V2
Control	2	Q9Y2V7
Control	2	Q9Y2W1
Control	2	Q9Y2W2
Control	2	Q9Y2W6
Control	2	Q9Y2X0
Control	2	Q9Y2X3
Control	2	Q9Y2X7
Control	2	Q9Y2X9
Control	2	Q9Y2Y0
Control	2	Q9Y2Y6
Control	2	Q9Y2Z0

Control	2	Q9Y2Z4
Control	2	Q9Y303
Control	2	Q9Y305
Control	2	Q9Y312
Control	2	Q9Y314
Control	2	Q9Y315
Control	2	Q9Y316
Control	2	Q9Y320
Control	2	Q9Y324
Control	2	Q9Y333
Control	2	Q9Y371
Control	2	Q9Y375
Control	2	Q9Y376
Control	2	Q9Y383
Control	2	Q9Y385
Control	2	Q9Y388
Control	2	Q9Y394
Control	2	Q9Y399
Control	2	Q9Y3A2
Control	2	Q9Y3A3
Control	2	Q9Y3A4
Control	2	Q9Y3A5
Control	2	Q9Y3A6
Control	2	Q9Y3B2
Control	2	Q9Y3B3
Control	2	Q9Y3B4
Control	2	Q9Y3B7
Control	2	Q9Y3B8
Control	2	Q9Y3B9
Control	2	Q9Y3C0
Control	2	Q9Y3C1
Control	2	Q9Y3C4
Control	2	Q9Y3C6
Control	2	Q9Y3C8
Control	2	Q9Y3D0
Control	2	Q9Y3D3
Control	2	Q9Y3D5
Control	2	Q9Y3D6
Control	2	Q9Y3D7
Control	2	Q9Y3D8
Control	2	Q9Y3D9
Control	2	Q9Y3E0
Control	2	Q9Y3E1
Control	2	Q9Y3E5
Control	2	Q9Y3E7
Control	2	Q9Y3F4
Control	2	Q9Y3I0
Control	2	Q9Y3I1
Control	2	Q9Y3L5
Control	2	Q9Y3M8

Control	2	Q9Y3P9
Control	2	Q9Y3Q3
Control	2	Q9Y3Q8
Control	2	Q9Y3R5
Control	2	Q9Y3S2
Control	2	Q9Y3T6
Control	2	Q9Y3T9
Control	2	Q9Y3U8
Control	2	Q9Y3X0
Control	2	Q9Y3Y2
Control	2	Q9Y3Z3
Control	2	Q9Y448
Control	2	Q9Y450
Control	2	Q9Y478
Control	2	Q9Y484
Control	2	Q9Y485
Control	2	Q9Y487
Control	2	Q9Y490
Control	2	Q9Y496
Control	2	Q9Y4A5
Control	2	Q9Y4B5
Control	2	Q9Y4B6
Control	2	Q9Y4C2
Control	2	Q9Y4C8
Control	2	Q9Y4D1
Control	2	Q9Y4D7
Control	2	Q9Y4D8
Control	2	Q9Y4E6
Control	2	Q9Y4E8
Control	2	Q9Y4F1
Control	2	Q9Y4F3
Control	2	Q9Y4F5
Control	2	Q9Y4G8
Control	2	Q9Y4I1
Control	2	Q9Y4J8
Control	2	Q9Y4K0
Control	2	Q9Y4K1
Control	2	Q9Y4K3
Control	2	Q9Y4K4
Control	2	Q9Y4L1
Control	2	Q9Y4P1
Control	2	Q9Y4P3
Control	2	Q9Y4P8
Control	2	Q9Y4R8
Control	2	Q9Y4W2
Control	2	Q9Y4W6
Control	2	Q9Y4X4
Control	2	Q9Y4X5
Control	2	Q9Y4Z0
Control	2	Q9Y508

Control	2	Q9Y512
Control	2	Q9Y520
Control	2	Q9Y530
Control	2	Q9Y547
Control	2	Q9Y570
Control	2	Q9Y580
Control	2	Q9Y584
Control	2	Q9Y5A7
Control	2	Q9Y5A9
Control	2	Q9Y5B0
Control	2	Q9Y5B6
Control	2	Q9Y5B8
Control	2	Q9Y5B9
Control	2	Q9Y5J1
Control	2	Q9Y5K5
Control	2	Q9Y5K6
Control	2	Q9Y5K8
Control	2	Q9Y5L0
Control	2	Q9Y5M8
Control	2	Q9Y5N6
Control	2	Q9Y5P4
Control	2	Q9Y5P6
Control	2	Q9Y5Q0
Control	2	Q9Y5Q8
Control	2	Q9Y5Q9
Control	2	Q9Y5R8
Control	2	Q9Y5S1
Control	2	Q9Y5S2
Control	2	Q9Y5S9
Control	2	Q9Y5T4
Control	2	Q9Y5T5
Control	2	Q9Y5U2
Control	2	Q9Y5V0
Control	2	Q9Y5V3
Control	2	Q9Y5W7
Control	2	Q9Y5W9
Control	2	Q9Y5X1
Control	2	Q9Y5X2
Control	2	Q9Y5X3
Control	2	Q9Y5X9
Control	2	Q9Y5Y2
Control	2	Q9Y5Y5
Control	2	Q9Y5Y7
Control	2	Q9Y5Z0
Control	2	Q9Y5Z4
Control	2	Q9Y5Z7
Control	2	Q9Y5Z9
Control	2	Q9Y606
Control	2	Q9Y608
Control	2	Q9Y613

Control	2	Q9Y617
Control	2	Q9Y618
Control	2	Q9Y619
Control	2	Q9Y624
Control	2	Q9Y639
Control	2	Q9Y653
Control	2	Q9Y657
Control	2	Q9Y666
Control	2	Q9Y672
Control	2	Q9Y673
Control	2	Q9Y676
Control	2	Q9Y678
Control	2	Q9Y679
Control	2	Q9Y680
Control	2	Q9Y696
Control	2	Q9Y697
Control	2	Q9Y6A4
Control	2	Q9Y6A5
Control	2	Q9Y6A9
Control	2	Q9Y6B6
Control	2	Q9Y6B7
Control	2	Q9Y6C9
Control	2	Q9Y6D0
Control	2	Q9Y6D5
Control	2	Q9Y6D6
Control	2	Q9Y6D9
Control	2	Q9Y6E0
Control	2	Q9Y6E2
Control	2	Q9Y6G5
Control	2	Q9Y6G9
Control	2	Q9Y6I3
Control	2	Q9Y6I4
Control	2	Q9Y6I9
Control	2	Q9Y6K0
Control	2	Q9Y6K1
Control	2	Q9Y6K5
Control	2	Q9Y6K8
Control	2	Q9Y6K9
Control	2	Q9Y6M1
Control	2	Q9Y6M5
Control	2	Q9Y6M7
Control	2	Q9Y6M9
Control	2	Q9Y6N5
Control	2	Q9Y6N7
Control	2	Q9Y6Q2
Control	2	Q9Y6Q9
Control	2	Q9Y6R0
Control	2	Q9Y6R4
Control	2	Q9Y6V7
Control	2	Q9Y6W3

Control	2	Q9Y6W5
Control	2	Q9Y6X2
Control	2	Q9Y6X3
Control	2	Q9Y6X9
Control	2	Q9Y6Y0
Control	2	Q9Y6Y8
Control	3	A0A0B4J2D5
Control	3	A0AVF1
Control	3	A0AVT1
Control	3	A0FGR8
Control	3	A0JNW5
Control	3	A0MZ66
Control	3	A0PJW6
Control	3	A1A4S6
Control	3	A1L0T0
Control	3	A1X283
Control	3	A2RRP1
Control	3	A2RUC4
Control	3	A2RUS2
Control	3	A3KMH1
Control	3	A3KN83
Control	3	A4D1E9
Control	3	A4D1P6
Control	3	A4D1U4
Control	3	A5PLL7
Control	3	A5PLN9
Control	3	A5YKK6
Control	3	A6NCE7
Control	3	A6NDG6
Control	3	A6NDU8
Control	3	A6NED2
Control	3	A6NFQ2
Control	3	A6NHR9
Control	3	A6NHX0
Control	3	A6NIH7
Control	3	A6NJ78
Control	3	A6NKD9
Control	3	A6ZKI3
Control	3	A7E2V4
Control	3	A8CG34
Control	3	A8MW92
Control	3	A8MWD9
Control	3	A8MWY0
Control	3	A8MXV4
Control	3	A9UHW6
Control	3	P0DP23
Control	3	B7ZAQ6
Control	3	C4AMC7
Control	3	C9JLW8
Control	3	E9PAV3

Control	3	E9PRG8
Control	3	L0R819
Control	3	L0R8F8
Control	3	O00115
Control	3	O00116
Control	3	O00139
Control	3	O00148
Control	3	O00151
Control	3	O00154
Control	3	O00159
Control	3	O00161
Control	3	O00165
Control	3	O00170
Control	3	O00178
Control	3	O00182
Control	3	O00186
Control	3	O00189
Control	3	O00192
Control	3	O00193
Control	3	O00203
Control	3	O00213
Control	3	O00214
Control	3	O00217
Control	3	O00221
Control	3	O00231
Control	3	O00232
Control	3	O00233
Control	3	O00255
Control	3	O00258
Control	3	O00264
Control	3	O00267
Control	3	O00268
Control	3	O00273
Control	3	O00291
Control	3	O00299
Control	3	O00303
Control	3	O00308
Control	3	O00330
Control	3	O00391
Control	3	O00399
Control	3	O00400
Control	3	O00401
Control	3	O00410
Control	3	O00411
Control	3	O00418
Control	3	O00422
Control	3	O00423
Control	3	O00425
Control	3	O00429
Control	3	O00442

Control	3	O00443
Control	3	O00461
Control	3	O00468
Control	3	O00469
Control	3	O00471
Control	3	O00481
Control	3	O00483
Control	3	O00487
Control	3	O00499
Control	3	O00501
Control	3	O00505
Control	3	O00506
Control	3	O00522
Control	3	O00534
Control	3	O00541
Control	3	O00560
Control	3	O00562
Control	3	O00566
Control	3	O00567
Control	3	O00571
Control	3	O00587
Control	3	O00592
Control	3	O00622
Control	3	O00625
Control	3	O00629
Control	3	O00635
Control	3	O00712
Control	3	O00743
Control	3	O00746
Control	3	O00754
Control	3	O00764
Control	3	O00767
Control	3	O14497
Control	3	O14524
Control	3	O14530
Control	3	O14531
Control	3	O14545
Control	3	O14548
Control	3	O14562
Control	3	O14578
Control	3	O14579
Control	3	O14613
Control	3	O14617
Control	3	O14618
Control	3	O14639
Control	3	O14641
Control	3	O14646
Control	3	O14647
Control	3	O14653
Control	3	O14656

Control	3	O14657
Control	3	O14662
Control	3	O14672
Control	3	O14678
Control	3	O14681
Control	3	O14683
Control	3	O14686
Control	3	O14727
Control	3	O14730
Control	3	O14733
Control	3	O14734
Control	3	O14735
Control	3	O14737
Control	3	O14744
Control	3	O14745
Control	3	O14757
Control	3	O14763
Control	3	O14773
Control	3	O14776
Control	3	O14777
Control	3	O14786
Control	3	O14787
Control	3	O14795
Control	3	O14798
Control	3	O14802
Control	3	O14817
Control	3	O14818
Control	3	O14828
Control	3	O14874
Control	3	O14880
Control	3	O14907
Control	3	O14908
Control	3	O14920
Control	3	O14924
Control	3	O14925
Control	3	O14929
Control	3	O14936
Control	3	O14939
Control	3	O14949
Control	3	O14950
Control	3	O14964
Control	3	O14966
Control	3	O14972
Control	3	O14974
Control	3	O14976
Control	3	O14979
Control	3	O14980
Control	3	O14981
Control	3	O15013
Control	3	O15014

Control	3	O15020
Control	3	O15021
Control	3	O15027
Control	3	O15031
Control	3	O15037
Control	3	O15040
Control	3	O15042
Control	3	O15047
Control	3	O15049
Control	3	O15056
Control	3	O15061
Control	3	O15063
Control	3	O15066
Control	3	O15067
Control	3	O15084
Control	3	O15085
Control	3	O15091
Control	3	O15111
Control	3	O15118
Control	3	O15121
Control	3	O15123
Control	3	O15126
Control	3	O15127
Control	3	O15143
Control	3	O15144
Control	3	O15145
Control	3	O15160
Control	3	O15162
Control	3	O15164
Control	3	O15173
Control	3	O15211
Control	3	O15213
Control	3	O15226
Control	3	O15228
Control	3	O15230
Control	3	O15231
Control	3	O15234
Control	3	O15235
Control	3	O15254
Control	3	O15258
Control	3	O15260
Control	3	O15264
Control	3	O15269
Control	3	O15270
Control	3	O15294
Control	3	O15305
Control	3	O15321
Control	3	O15344
Control	3	O15347
Control	3	O15355

Control	3	O15357
Control	3	O15371
Control	3	O15372
Control	3	O15379
Control	3	O15381
Control	3	O15382
Control	3	O15397
Control	3	O15400
Control	3	O15427
Control	3	O15439
Control	3	O15446
Control	3	O15460
Control	3	O15484
Control	3	O15498
Control	3	O15504
Control	3	O15511
Control	3	O15519
Control	3	O15525
Control	3	O15530
Control	3	O15533
Control	3	O15541
Control	3	O15550
Control	3	O43143
Control	3	O43148
Control	3	O43149
Control	3	O43150
Control	3	O43155
Control	3	O43156
Control	3	O43159
Control	3	O43164
Control	3	O43169
Control	3	O43172
Control	3	O43175
Control	3	O43181
Control	3	O43237
Control	3	O43242
Control	3	O43251
Control	3	O43252
Control	3	O43264
Control	3	O43286
Control	3	O43290
Control	3	O43292
Control	3	O43293
Control	3	O43294
Control	3	O43299
Control	3	O43310
Control	3	O43312
Control	3	O43314
Control	3	O43318
Control	3	O43324

Control	3	O43353
Control	3	O43390
Control	3	O43395
Control	3	O43396
Control	3	O43399
Control	3	O43402
Control	3	O43414
Control	3	O43426
Control	3	O43427
Control	3	O43432
Control	3	O43447
Control	3	O43464
Control	3	O43482
Control	3	O43488
Control	3	O43491
Control	3	O43493
Control	3	O43505
Control	3	O43516
Control	3	O43520
Control	3	O43572
Control	3	O43583
Control	3	O43592
Control	3	O43598
Control	3	O43615
Control	3	O43617
Control	3	O43633
Control	3	O43639
Control	3	O43657
Control	3	O43660
Control	3	O43663
Control	3	O43665
Control	3	O43670
Control	3	O43674
Control	3	O43676
Control	3	O43678
Control	3	O43679
Control	3	O43681
Control	3	O43683
Control	3	O43684
Control	3	O43704
Control	3	O43707
Control	3	O43709
Control	3	O43719
Control	3	O43731
Control	3	O43747
Control	3	O43752
Control	3	O43760
Control	3	O43765
Control	3	O43768
Control	3	O43772

Control	3	O43776
Control	3	O43795
Control	3	O43808
Control	3	O43809
Control	3	O43813
Control	3	O43815
Control	3	O43818
Control	3	O43819
Control	3	O43822
Control	3	O43823
Control	3	O43824
Control	3	O43826
Control	3	O43837
Control	3	O43847
Control	3	O43852
Control	3	O43854
Control	3	O43865
Control	3	O43896
Control	3	O43909
Control	3	O43913
Control	3	O43920
Control	3	O43924
Control	3	O43929
Control	3	O43933
Control	3	O60216
Control	3	O60231
Control	3	O60234
Control	3	O60239
Control	3	O60244
Control	3	O60256
Control	3	O60264
Control	3	O60271
Control	3	O60285
Control	3	O60287
Control	3	O60292
Control	3	O60293
Control	3	O60306
Control	3	O60313
Control	3	O60315
Control	3	O60318
Control	3	O60330
Control	3	O60331
Control	3	O60333
Control	3	O60336
Control	3	O60341
Control	3	O60343
Control	3	O60427
Control	3	O60443
Control	3	O60447
Control	3	O60462

Control	3	O60476
Control	3	O60487
Control	3	O60488
Control	3	O60493
Control	3	O60502
Control	3	O60504
Control	3	O60506
Control	3	O60508
Control	3	O60518
Control	3	O60524
Control	3	O60547
Control	3	O60551
Control	3	O60563
Control	3	O60566
Control	3	O60568
Control	3	O60573
Control	3	O60610
Control	3	O60637
Control	3	O60645
Control	3	O60664
Control	3	O60678
Control	3	O60684
Control	3	O60701
Control	3	O60704
Control	3	O60711
Control	3	O60716
Control	3	O60725
Control	3	O60739
Control	3	O60749
Control	3	O60762
Control	3	O60763
Control	3	O60783
Control	3	O60784
Control	3	O60814
Control	3	O60826
Control	3	O60828
Control	3	O60830
Control	3	O60831
Control	3	O60832
Control	3	O60841
Control	3	O60869
Control	3	O60870
Control	3	O60879
Control	3	O60884
Control	3	O60885
Control	3	O60921
Control	3	O60925
Control	3	O60927
Control	3	O60934
Control	3	O60942

Control	3	O75027
Control	3	O75044
Control	3	O75051
Control	3	O75063
Control	3	O75083
Control	3	O75110
Control	3	O75113
Control	3	O75116
Control	3	O75122
Control	3	O75127
Control	3	O75131
Control	3	O75143
Control	3	O75146
Control	3	O75150
Control	3	O75151
Control	3	O75152
Control	3	O75153
Control	3	O75155
Control	3	O75157
Control	3	O75165
Control	3	O75167
Control	3	O75170
Control	3	O75173
Control	3	O75175
Control	3	O75179
Control	3	O75182
Control	3	O75190
Control	3	O75208
Control	3	O75223
Control	3	O75251
Control	3	O75306
Control	3	O75312
Control	3	O75319
Control	3	O75323
Control	3	O75330
Control	3	O75340
Control	3	O75347
Control	3	O75348
Control	3	O75351
Control	3	O75352
Control	3	O75367
Control	3	O75368
Control	3	O75369
Control	3	O75376
Control	3	O75381
Control	3	O75382
Control	3	O75390
Control	3	O75391
Control	3	O75396
Control	3	O75400

Control	3	O75410
Control	3	O75414
Control	3	O75419
Control	3	O75427
Control	3	O75431
Control	3	O75436
Control	3	O75439
Control	3	O75448
Control	3	O75449
Control	3	O75475
Control	3	O75477
Control	3	O75489
Control	3	O75494
Control	3	O75503
Control	3	O75508
Control	3	O75521
Control	3	O75525
Control	3	O75530
Control	3	O75531
Control	3	O75533
Control	3	O75534
Control	3	O75554
Control	3	O75569
Control	3	O75578
Control	3	O75582
Control	3	O75586
Control	3	O75592
Control	3	O75600
Control	3	O75608
Control	3	O75616
Control	3	O75643
Control	3	O75648
Control	3	O75663
Control	3	O75665
Control	3	O75676
Control	3	O75683
Control	3	O75688
Control	3	O75691
Control	3	O75694
Control	3	O75695
Control	3	O75717
Control	3	O75718
Control	3	O75746
Control	3	O75787
Control	3	O75792
Control	3	O75794
Control	3	O75815
Control	3	O75817
Control	3	O75818
Control	3	O75821

Control	3	O75822
Control	3	O75828
Control	3	O75832
Control	3	O75843
Control	3	O75844
Control	3	O75874
Control	3	O75879
Control	3	O75880
Control	3	O75882
Control	3	O75884
Control	3	O75886
Control	3	O75907
Control	3	O75909
Control	3	O75915
Control	3	O75923
Control	3	O75925
Control	3	O75934
Control	3	O75935
Control	3	O75937
Control	3	O75940
Control	3	O75943
Control	3	O75947
Control	3	O75954
Control	3	O75955
Control	3	O75962
Control	3	O75964
Control	3	O75970
Control	3	O75976
Control	3	O76003
Control	3	O76021
Control	3	O76024
Control	3	O76031
Control	3	O76054
Control	3	O76070
Control	3	O76071
Control	3	O76094
Control	3	O94760
Control	3	O94763
Control	3	O94766
Control	3	O94776
Control	3	O94788
Control	3	O94804
Control	3	O94806
Control	3	O94808
Control	3	O94813
Control	3	O94822
Control	3	O94826
Control	3	O94827
Control	3	O94829
Control	3	O94830

Control	3	O94832
Control	3	O94842
Control	3	O94851
Control	3	O94855
Control	3	O94868
Control	3	O94874
Control	3	O94875
Control	3	O94885
Control	3	O94888
Control	3	O94889
Control	3	O94901
Control	3	O94903
Control	3	O94905
Control	3	O94906
Control	3	O94913
Control	3	O94915
Control	3	O94916
Control	3	O94919
Control	3	O94923
Control	3	O94925
Control	3	O94927
Control	3	O94929
Control	3	O94952
Control	3	O94953
Control	3	O94964
Control	3	O94966
Control	3	O94967
Control	3	O94973
Control	3	O94979
Control	3	O94989
Control	3	O94992
Control	3	O95059
Control	3	O95067
Control	3	O95070
Control	3	O95071
Control	3	O95081
Control	3	O95084
Control	3	O95104
Control	3	O95139
Control	3	O95140
Control	3	O95149
Control	3	O95155
Control	3	O95159
Control	3	O95163
Control	3	O95168
Control	3	O95169
Control	3	O95182
Control	3	O95183
Control	3	O95197
Control	3	O95202

Control	3	O95208
Control	3	O95218
Control	3	O95219
Control	3	O95232
Control	3	O95235
Control	3	O95236
Control	3	O95239
Control	3	O95243
Control	3	O95248
Control	3	O95249
Control	3	O95251
Control	3	O95260
Control	3	O95292
Control	3	O95295
Control	3	O95297
Control	3	O95298
Control	3	O95299
Control	3	O95302
Control	3	O95319
Control	3	O95336
Control	3	O95340
Control	3	O95347
Control	3	O95352
Control	3	O95359
Control	3	O95361
Control	3	O95363
Control	3	O95365
Control	3	O95372
Control	3	O95373
Control	3	O95376
Control	3	O95391
Control	3	O95394
Control	3	O95396
Control	3	O95400
Control	3	O95425
Control	3	O95433
Control	3	O95453
Control	3	O95456
Control	3	O95470
Control	3	O95476
Control	3	O95478
Control	3	O95479
Control	3	O95486
Control	3	O95487
Control	3	O95490
Control	3	O95544
Control	3	O95551
Control	3	O95563
Control	3	O95571
Control	3	O95573

Control	3	O95602
Control	3	O95619
Control	3	O95625
Control	3	O95628
Control	3	O95630
Control	3	O95639
Control	3	O95644
Control	3	O95671
Control	3	O95674
Control	3	O95685
Control	3	O95696
Control	3	O95707
Control	3	O95714
Control	3	O95716
Control	3	O95721
Control	3	O95747
Control	3	O95749
Control	3	O95757
Control	3	O95758
Control	3	O95759
Control	3	O95772
Control	3	O95777
Control	3	O95782
Control	3	O95785
Control	3	O95786
Control	3	O95793
Control	3	O95801
Control	3	O95807
Control	3	O95810
Control	3	O95816
Control	3	O95817
Control	3	O95819
Control	3	O95822
Control	3	O95825
Control	3	O95831
Control	3	O95834
Control	3	O95835
Control	3	O95848
Control	3	O95858
Control	3	O95861
Control	3	O95864
Control	3	O95865
Control	3	O95870
Control	3	O95881
Control	3	O95905
Control	3	O95980
Control	3	O95983
Control	3	O95985
Control	3	O95989
Control	3	O95999

Control	3	O96000
Control	3	O96005
Control	3	O96006
Control	3	O96007
Control	3	O96008
Control	3	O96011
Control	3	O96013
Control	3	O96019
Control	3	O96028
Control	3	P00338
Control	3	P00352
Control	3	P00367
Control	3	P00374
Control	3	P00387
Control	3	P00390
Control	3	P00403
Control	3	P00491
Control	3	P00492
Control	3	P00505
Control	3	P00519
Control	3	P00558
Control	3	P00568
Control	3	P00749
Control	3	P00750
Control	3	P00813
Control	3	P01034
Control	3	P01111
Control	3	P01112
Control	3	P01116
Control	3	P01127
Control	3	P01130
Control	3	P01137
Control	3	P01889
Control	3	P01893
Control	3	P02452
Control	3	P02462
Control	3	P02545
Control	3	P02647
Control	3	P02751
Control	3	P02765
Control	3	P02768
Control	3	P02786
Control	3	P02792
Control	3	P02794
Control	3	P02795
Control	3	P03886
Control	3	P03905
Control	3	P03915
Control	3	P03956
Control	3	P04035

Control	3	P04040
Control	3	P04049
Control	3	P04062
Control	3	P04075
Control	3	P04080
Control	3	P04083
Control	3	P04114
Control	3	P04156
Control	3	P04179
Control	3	P04181
Control	3	P04183
Control	3	P04275
Control	3	P04350
Control	3	P04406
Control	3	P04424
Control	3	P04439
Control	3	P04632
Control	3	P04732
Control	3	P04792
Control	3	P04818
Control	3	P04843
Control	3	P04844
Control	3	P04899
Control	3	P04908
Control	3	P04920
Control	3	P05023
Control	3	P05026
Control	3	P05067
Control	3	P05091
Control	3	P05106
Control	3	P05109
Control	3	P05114
Control	3	P05120
Control	3	P05121
Control	3	P05141
Control	3	P05161
Control	3	P05165
Control	3	P05166
Control	3	P05198
Control	3	P05204
Control	3	P05362
Control	3	P05387
Control	3	P05388
Control	3	P05412
Control	3	P05423
Control	3	P05455
Control	3	P05556
Control	3	P05783
Control	3	P05997
Control	3	P06132

Control	3	P06241
Control	3	P06280
Control	3	P06396
Control	3	P06400
Control	3	P06454
Control	3	P06493
Control	3	P06576
Control	3	P06702
Control	3	P06703
Control	3	P06730
Control	3	P06733
Control	3	P06737
Control	3	P06744
Control	3	P06746
Control	3	P06748
Control	3	P06753
Control	3	P06756
Control	3	P06865
Control	3	P07099
Control	3	P07195
Control	3	P07199
Control	3	P07203
Control	3	P07237
Control	3	P07305
Control	3	P07311
Control	3	P07332
Control	3	P07339
Control	3	P07355
Control	3	P07384
Control	3	P07437
Control	3	P07602
Control	3	P07686
Control	3	P07711
Control	3	P07737
Control	3	P07738
Control	3	P07741
Control	3	P07814
Control	3	P07858
Control	3	P07900
Control	3	P07902
Control	3	P07910
Control	3	P07942
Control	3	P07947
Control	3	P07948
Control	3	P07954
Control	3	P07992
Control	3	P07996
Control	3	P08047
Control	3	P08069
Control	3	P08123

Control	3	P08133
Control	3	P08134
Control	3	P08174
Control	3	P08195
Control	3	P08236
Control	3	P08237
Control	3	P08238
Control	3	P08240
Control	3	P08243
Control	3	P08253
Control	3	P08397
Control	3	P08473
Control	3	P08493
Control	3	P08559
Control	3	P08572
Control	3	P08574
Control	3	P08579
Control	3	P08581
Control	3	P08621
Control	3	P08648
Control	3	P08651
Control	3	P08670
Control	3	P08708
Control	3	P08754
Control	3	P08758
Control	3	P08865
Control	3	P08962
Control	3	P09001
Control	3	P09012
Control	3	P09104
Control	3	P09110
Control	3	P09132
Control	3	P09211
Control	3	P09234
Control	3	P09382
Control	3	P09417
Control	3	P09429
Control	3	P09486
Control	3	P09493
Control	3	P09496
Control	3	P09497
Control	3	P09525
Control	3	P09543
Control	3	P09601
Control	3	P09622
Control	3	P09651
Control	3	P09661
Control	3	P09669
Control	3	P09758
Control	3	P09874

Control	3	P09884
Control	3	P09936
Control	3	P09960
Control	3	P09972
Control	3	P0C0S5
Control	3	P0C0S8
Control	3	P0CAP1
Control	3	P0CAP2
Control	3	P0CG29
Control	3	P0CG39
Control	3	P0CW19
Control	3	P0DMV8
Control	3	P0DP91
Control	3	P0DPH7
Control	3	P0DTL6
Control	3	P10114
Control	3	P10124
Control	3	P10155
Control	3	P10253
Control	3	P10301
Control	3	P10321
Control	3	P10398
Control	3	P10412
Control	3	P10515
Control	3	P10586
Control	3	P10589
Control	3	P10599
Control	3	P10606
Control	3	P10619
Control	3	P10620
Control	3	P10644
Control	3	P10646
Control	3	P10768
Control	3	P10809
Control	3	P10909
Control	3	P11021
Control	3	P11047
Control	3	P11117
Control	3	P11137
Control	3	P11142
Control	3	P11166
Control	3	P11169
Control	3	P11171
Control	3	P11172
Control	3	P11177
Control	3	P11182
Control	3	P11216
Control	3	P11217
Control	3	P11233
Control	3	P11234

Control	3	P11274
Control	3	P11279
Control	3	P11308
Control	3	P11310
Control	3	P11387
Control	3	P11388
Control	3	P11413
Control	3	P11441
Control	3	P11498
Control	3	P11586
Control	3	P11717
Control	3	P11766
Control	3	P11802
Control	3	P11908
Control	3	P11940
Control	3	P12004
Control	3	P12081
Control	3	P12109
Control	3	P12111
Control	3	P12235
Control	3	P12236
Control	3	P12268
Control	3	P12270
Control	3	P12429
Control	3	P12694
Control	3	P12814
Control	3	P12821
Control	3	P12931
Control	3	P12955
Control	3	P12956
Control	3	P13010
Control	3	P13051
Control	3	P13073
Control	3	P13196
Control	3	P13473
Control	3	P13489
Control	3	P13598
Control	3	P13639
Control	3	P13667
Control	3	P13674
Control	3	P13693
Control	3	P13716
Control	3	P13747
Control	3	P13797
Control	3	P13798
Control	3	P13804
Control	3	P13807
Control	3	P13861
Control	3	P13984
Control	3	P13987

Control	3	P13995
Control	3	P14174
Control	3	P14209
Control	3	P14314
Control	3	P14317
Control	3	P14324
Control	3	P14406
Control	3	P14543
Control	3	P14550
Control	3	P14618
Control	3	P14625
Control	3	P14635
Control	3	P14649
Control	3	P14678
Control	3	P14735
Control	3	P14859
Control	3	P14866
Control	3	P14868
Control	3	P14921
Control	3	P14923
Control	3	P14927
Control	3	P15056
Control	3	P15090
Control	3	P15104
Control	3	P15121
Control	3	P15144
Control	3	P15151
Control	3	P15153
Control	3	P15170
Control	3	P15289
Control	3	P15291
Control	3	P15311
Control	3	P15374
Control	3	P15407
Control	3	P15408
Control	3	P15529
Control	3	P15531
Control	3	P15559
Control	3	P15586
Control	3	P15735
Control	3	P15848
Control	3	P15880
Control	3	P15884
Control	3	P15924
Control	3	P15927
Control	3	P16035
Control	3	P16070
Control	3	P16152
Control	3	P16219
Control	3	P16220

Control	3	P16278
Control	3	P16284
Control	3	P16298
Control	3	P16333
Control	3	P16383
Control	3	P16401
Control	3	P16402
Control	3	P16403
Control	3	P16435
Control	3	P16591
Control	3	P16615
Control	3	P16885
Control	3	P16930
Control	3	P16949
Control	3	P16989
Control	3	P17026
Control	3	P17028
Control	3	P17050
Control	3	P17096
Control	3	P17152
Control	3	P17174
Control	3	P17252
Control	3	P17301
Control	3	P17302
Control	3	P17405
Control	3	P17480
Control	3	P17535
Control	3	P17542
Control	3	P17544
Control	3	P17568
Control	3	P17612
Control	3	P17655
Control	3	P17706
Control	3	P17812
Control	3	P17813
Control	3	P17844
Control	3	P17858
Control	3	P17948
Control	3	P17980
Control	3	P17987
Control	3	P18031
Control	3	P18074
Control	3	P18077
Control	3	P18084
Control	3	P18085
Control	3	P18124
Control	3	P18206
Control	3	P18433
Control	3	P18440
Control	3	P18583

Control	3	P18615
Control	3	P18621
Control	3	P18669
Control	3	P18754
Control	3	P18846
Control	3	P18850
Control	3	P18858
Control	3	P18887
Control	3	P19022
Control	3	P19174
Control	3	P19338
Control	3	P19367
Control	3	P19387
Control	3	P19388
Control	3	P19404
Control	3	P19447
Control	3	P19474
Control	3	P19525
Control	3	P19623
Control	3	P19634
Control	3	P19784
Control	3	P19838
Control	3	P20020
Control	3	P20042
Control	3	P20073
Control	3	P20248
Control	3	P20290
Control	3	P20337
Control	3	P20338
Control	3	P20339
Control	3	P20340
Control	3	P20585
Control	3	P20594
Control	3	P20618
Control	3	P20645
Control	3	P20700
Control	3	P20810
Control	3	P20839
Control	3	P20908
Control	3	P20929
Control	3	P20933
Control	3	P20936
Control	3	P20962
Control	3	P21127
Control	3	P21266
Control	3	P21281
Control	3	P21283
Control	3	P21291
Control	3	P21333
Control	3	P21359

Control	3	P21397
Control	3	P21399
Control	3	P21453
Control	3	P21579
Control	3	P21589
Control	3	P21675
Control	3	P21741
Control	3	P21796
Control	3	P21810
Control	3	P21912
Control	3	P21926
Control	3	P21964
Control	3	P21980
Control	3	P22033
Control	3	P22059
Control	3	P22061
Control	3	P22087
Control	3	P22102
Control	3	P22234
Control	3	P22307
Control	3	P22314
Control	3	P22392
Control	3	P22528
Control	3	P22570
Control	3	P22626
Control	3	P22670
Control	3	P22681
Control	3	P22692
Control	3	P22694
Control	3	P22695
Control	3	P22830
Control	3	P23025
Control	3	P23193
Control	3	P23219
Control	3	P23229
Control	3	P23246
Control	3	P23258
Control	3	P23284
Control	3	P23368
Control	3	P23381
Control	3	P23396
Control	3	P23443
Control	3	P23458
Control	3	P23467
Control	3	P23469
Control	3	P23470
Control	3	P23497
Control	3	P23508
Control	3	P23510
Control	3	P23526

Control	3	P23528
Control	3	P23588
Control	3	P23610
Control	3	P23634
Control	3	P23743
Control	3	P23786
Control	3	P23919
Control	3	P23921
Control	3	P24385
Control	3	P24386
Control	3	P24390
Control	3	P24468
Control	3	P24534
Control	3	P24539
Control	3	P24666
Control	3	P24723
Control	3	P24752
Control	3	P24928
Control	3	P24941
Control	3	P25098
Control	3	P25116
Control	3	P25205
Control	3	P25325
Control	3	P25398
Control	3	P25440
Control	3	P25490
Control	3	P25685
Control	3	P25686
Control	3	P25705
Control	3	P25774
Control	3	P25786
Control	3	P25787
Control	3	P25788
Control	3	P25789
Control	3	P25942
Control	3	P26006
Control	3	P26022
Control	3	P26038
Control	3	P26196
Control	3	P26358
Control	3	P26368
Control	3	P26373
Control	3	P26374
Control	3	P26440
Control	3	P26572
Control	3	P26583
Control	3	P26599
Control	3	P26639
Control	3	P26640
Control	3	P26641

Control	3	P26885
Control	3	P26951
Control	3	P27105
Control	3	P27144
Control	3	P27348
Control	3	P27361
Control	3	P27448
Control	3	P27635
Control	3	P27658
Control	3	P27694
Control	3	P27695
Control	3	P27701
Control	3	P27707
Control	3	P27708
Control	3	P27797
Control	3	P27816
Control	3	P27824
Control	3	P27986
Control	3	P27987
Control	3	P28062
Control	3	P28065
Control	3	P28066
Control	3	P28070
Control	3	P28072
Control	3	P28074
Control	3	P28161
Control	3	P28288
Control	3	P28290
Control	3	P28300
Control	3	P28331
Control	3	P28340
Control	3	P28370
Control	3	P28482
Control	3	P28702
Control	3	P28715
Control	3	P28799
Control	3	P28827
Control	3	P28838
Control	3	P28906
Control	3	P29083
Control	3	P29084
Control	3	P29144
Control	3	P29218
Control	3	P29279
Control	3	P29317
Control	3	P29323
Control	3	P29353
Control	3	P29372
Control	3	P29401
Control	3	P29466

Control	3	P29474
Control	3	P29558
Control	3	P29590
Control	3	P29597
Control	3	P29692
Control	3	P29966
Control	3	P29992
Control	3	P30038
Control	3	P30040
Control	3	P30041
Control	3	P30043
Control	3	P30044
Control	3	P30048
Control	3	P30050
Control	3	P30084
Control	3	P30085
Control	3	P30086
Control	3	P30101
Control	3	P30153
Control	3	P30154
Control	3	P30260
Control	3	P30281
Control	3	P30405
Control	3	P30414
Control	3	P30419
Control	3	P30519
Control	3	P30520
Control	3	P30530
Control	3	P30533
Control	3	P30536
Control	3	P30566
Control	3	P30622
Control	3	P30626
Control	3	P30740
Control	3	P30825
Control	3	P30837
Control	3	P30876
Control	3	P31040
Control	3	P31146
Control	3	P31150
Control	3	P31151
Control	3	P31153
Control	3	P31323
Control	3	P31350
Control	3	P31431
Control	3	P31483
Control	3	P31641
Control	3	P31689
Control	3	P31749
Control	3	P31930

Control	3	P31937
Control	3	P31939
Control	3	P31942
Control	3	P31943
Control	3	P31944
Control	3	P31946
Control	3	P31948
Control	3	P31949
Control	3	P32119
Control	3	P32121
Control	3	P32189
Control	3	P32321
Control	3	P32322
Control	3	P32455
Control	3	P32456
Control	3	P32519
Control	3	P32780
Control	3	P32856
Control	3	P32969
Control	3	P33121
Control	3	P33151
Control	3	P33176
Control	3	P33316
Control	3	P33527
Control	3	P33897
Control	3	P33981
Control	3	P33991
Control	3	P33992
Control	3	P33993
Control	3	P34059
Control	3	P34896
Control	3	P34897
Control	3	P34931
Control	3	P34932
Control	3	P34949
Control	3	P35052
Control	3	P35080
Control	3	P35221
Control	3	P35222
Control	3	P35232
Control	3	P35237
Control	3	P35240
Control	3	P35241
Control	3	P35244
Control	3	P35249
Control	3	P35250
Control	3	P35251
Control	3	P35268
Control	3	P35269
Control	3	P35270

Control	3	P35367
Control	3	P35520
Control	3	P35555
Control	3	P35556
Control	3	P35573
Control	3	P35579
Control	3	P35580
Control	3	P35590
Control	3	P35606
Control	3	P35610
Control	3	P35611
Control	3	P35613
Control	3	P35637
Control	3	P35658
Control	3	P35659
Control	3	P35713
Control	3	P35749
Control	3	P35813
Control	3	P35914
Control	3	P35916
Control	3	P35968
Control	3	P35998
Control	3	P36269
Control	3	P36404
Control	3	P36405
Control	3	P36507
Control	3	P36542
Control	3	P36543
Control	3	P36551
Control	3	P36578
Control	3	P36639
Control	3	P36776
Control	3	P36871
Control	3	P36873
Control	3	P36915
Control	3	P36957
Control	3	P36959
Control	3	P36969
Control	3	P37023
Control	3	P37059
Control	3	P37108
Control	3	P37173
Control	3	P37198
Control	3	P37235
Control	3	P37268
Control	3	P37275
Control	3	P37802
Control	3	P37837
Control	3	P37840
Control	3	P38117

Control	3	P38159
Control	3	P38432
Control	3	P38435
Control	3	P38571
Control	3	P38606
Control	3	P38646
Control	3	P38919
Control	3	P38935
Control	3	P38936
Control	3	P39019
Control	3	P39023
Control	3	P39060
Control	3	P39656
Control	3	P39687
Control	3	P39748
Control	3	P39880
Control	3	P40121
Control	3	P40123
Control	3	P40189
Control	3	P40222
Control	3	P40227
Control	3	P40261
Control	3	P40306
Control	3	P40425
Control	3	P40429
Control	3	P40616
Control	3	P40692
Control	3	P40763
Control	3	P40818
Control	3	P40925
Control	3	P40926
Control	3	P40937
Control	3	P40938
Control	3	P40939
Control	3	P41091
Control	3	P41162
Control	3	P41208
Control	3	P41212
Control	3	P41214
Control	3	P41223
Control	3	P41226
Control	3	P41227
Control	3	P41229
Control	3	P41240
Control	3	P41250
Control	3	P41252
Control	3	P41440
Control	3	P41567
Control	3	P41743
Control	3	P41970

Control	3	P42025
Control	3	P42126
Control	3	P42166
Control	3	P42167
Control	3	P42224
Control	3	P42226
Control	3	P42229
Control	3	P42285
Control	3	P42330
Control	3	P42336
Control	3	P42338
Control	3	P42345
Control	3	P42356
Control	3	P42566
Control	3	P42574
Control	3	P42575
Control	3	P42677
Control	3	P42684
Control	3	P42694
Control	3	P42695
Control	3	P42696
Control	3	P42704
Control	3	P42765
Control	3	P42766
Control	3	P42785
Control	3	P42858
Control	3	P42892
Control	3	P42898
Control	3	P43007
Control	3	P43034
Control	3	P43121
Control	3	P43155
Control	3	P43243
Control	3	P43246
Control	3	P43250
Control	3	P43304
Control	3	P43307
Control	3	P43378
Control	3	P43487
Control	3	P43490
Control	3	P43686
Control	3	P43897
Control	3	P45877
Control	3	P45880
Control	3	P45954
Control	3	P45973
Control	3	P45974
Control	3	P45983
Control	3	P45985
Control	3	P46013

Control	3	P46019
Control	3	P46020
Control	3	P46060
Control	3	P46063
Control	3	P46087
Control	3	P46100
Control	3	P46108
Control	3	P46109
Control	3	P46199
Control	3	P46379
Control	3	P46459
Control	3	P46531
Control	3	P46734
Control	3	P46736
Control	3	P46776
Control	3	P46777
Control	3	P46778
Control	3	P46779
Control	3	P46781
Control	3	P46782
Control	3	P46783
Control	3	P46821
Control	3	P46926
Control	3	P46934
Control	3	P46939
Control	3	P46940
Control	3	P46976
Control	3	P46977
Control	3	P47712
Control	3	P47755
Control	3	P47756
Control	3	P47813
Control	3	P47895
Control	3	P47897
Control	3	P47914
Control	3	P47929
Control	3	P47974
Control	3	P47985
Control	3	P48047
Control	3	P48059
Control	3	P48060
Control	3	P48147
Control	3	P48163
Control	3	P48200
Control	3	P48307
Control	3	P48382
Control	3	P48426
Control	3	P48444
Control	3	P48449
Control	3	P48454

Control	3	P48506
Control	3	P48507
Control	3	P48509
Control	3	P48553
Control	3	P48556
Control	3	P48634
Control	3	P48637
Control	3	P48643
Control	3	P48651
Control	3	P48681
Control	3	P48723
Control	3	P48729
Control	3	P48730
Control	3	P48735
Control	3	P48736
Control	3	P48739
Control	3	P49005
Control	3	P49006
Control	3	P49023
Control	3	P49069
Control	3	P49116
Control	3	P49137
Control	3	P49184
Control	3	P49189
Control	3	P49207
Control	3	P49247
Control	3	P49257
Control	3	P49281
Control	3	P49321
Control	3	P49327
Control	3	P49354
Control	3	P49356
Control	3	P49366
Control	3	P49368
Control	3	P49406
Control	3	P49407
Control	3	P49411
Control	3	P49419
Control	3	P49427
Control	3	P49441
Control	3	P49454
Control	3	P49458
Control	3	P49585
Control	3	P49588
Control	3	P49589
Control	3	P49590
Control	3	P49591
Control	3	P49593
Control	3	P49641
Control	3	P49642

Control	3	P49643
Control	3	P49662
Control	3	P49674
Control	3	P49720
Control	3	P49721
Control	3	P49736
Control	3	P49748
Control	3	P49750
Control	3	P49754
Control	3	P49755
Control	3	P49756
Control	3	P49757
Control	3	P49761
Control	3	P49768
Control	3	P49770
Control	3	P49773
Control	3	P49790
Control	3	P49792
Control	3	P49795
Control	3	P49796
Control	3	P49810
Control	3	P49815
Control	3	P49821
Control	3	P49840
Control	3	P49841
Control	3	P49848
Control	3	P49902
Control	3	P49903
Control	3	P49915
Control	3	P49916
Control	3	P49917
Control	3	P49959
Control	3	P49961
Control	3	P50148
Control	3	P50213
Control	3	P50225
Control	3	P50281
Control	3	P50336
Control	3	P50395
Control	3	P50402
Control	3	P50416
Control	3	P50443
Control	3	P50452
Control	3	P50453
Control	3	P50454
Control	3	P50479
Control	3	P50502
Control	3	P50542
Control	3	P50548
Control	3	P50552

Control	3	P50570
Control	3	P50579
Control	3	P50583
Control	3	P50613
Control	3	P50748
Control	3	P50749
Control	3	P50750
Control	3	P50851
Control	3	P50895
Control	3	P50897
Control	3	P50914
Control	3	P50990
Control	3	P50991
Control	3	P50995
Control	3	P51003
Control	3	P51114
Control	3	P51116
Control	3	P51148
Control	3	P51149
Control	3	P51151
Control	3	P51153
Control	3	P51159
Control	3	P51178
Control	3	P51398
Control	3	P51452
Control	3	P51531
Control	3	P51532
Control	3	P51553
Control	3	P51570
Control	3	P51571
Control	3	P51572
Control	3	P51580
Control	3	P51608
Control	3	P51610
Control	3	P51617
Control	3	P51636
Control	3	P51648
Control	3	P51659
Control	3	P51665
Control	3	P51687
Control	3	P51688
Control	3	P51692
Control	3	P51784
Control	3	P51798
Control	3	P51809
Control	3	P51812
Control	3	P51813
Control	3	P51825
Control	3	P51858
Control	3	P51946

Control	3	P51948
Control	3	P51956
Control	3	P51965
Control	3	P51970
Control	3	P51991
Control	3	P52209
Control	3	P52272
Control	3	P52292
Control	3	P52294
Control	3	P52298
Control	3	P52306
Control	3	P52434
Control	3	P52564
Control	3	P52565
Control	3	P52566
Control	3	P52594
Control	3	P52597
Control	3	P52630
Control	3	P52701
Control	3	P52732
Control	3	P52735
Control	3	P52747
Control	3	P52756
Control	3	P52758
Control	3	P52788
Control	3	P52789
Control	3	P52799
Control	3	P52815
Control	3	P52824
Control	3	P52848
Control	3	P52888
Control	3	P52907
Control	3	P52926
Control	3	P52943
Control	3	P52948
Control	3	P53004
Control	3	P53007
Control	3	P53041
Control	3	P53350
Control	3	P53355
Control	3	P53365
Control	3	P53367
Control	3	P53384
Control	3	P53396
Control	3	P53582
Control	3	P53597
Control	3	P53602
Control	3	P53609
Control	3	P53611
Control	3	P53618

Control	3	P53621
Control	3	P53634
Control	3	P53667
Control	3	P53671
Control	3	P53675
Control	3	P53677
Control	3	P53680
Control	3	P53701
Control	3	P53778
Control	3	P53801
Control	3	P53814
Control	3	P53985
Control	3	P53990
Control	3	P53992
Control	3	P53999
Control	3	P54098
Control	3	P54105
Control	3	P54136
Control	3	P54198
Control	3	P54252
Control	3	P54259
Control	3	P54277
Control	3	P54278
Control	3	P54289
Control	3	P54577
Control	3	P54578
Control	3	P54619
Control	3	P54652
Control	3	P54687
Control	3	P54709
Control	3	P54727
Control	3	P54760
Control	3	P54802
Control	3	P54819
Control	3	P54886
Control	3	P54920
Control	3	P55010
Control	3	P55011
Control	3	P55036
Control	3	P55039
Control	3	P55060
Control	3	P55072
Control	3	P55081
Control	3	P55084
Control	3	P55145
Control	3	P55196
Control	3	P55199
Control	3	P55201
Control	3	P55209
Control	3	P55210

Control	3	P55212
Control	3	P55263
Control	3	P55265
Control	3	P55268
Control	3	P55290
Control	3	P55327
Control	3	P55735
Control	3	P55769
Control	3	P55786
Control	3	P55789
Control	3	P55795
Control	3	P55809
Control	3	P55884
Control	3	P55899
Control	3	P55957
Control	3	P56134
Control	3	P56182
Control	3	P56192
Control	3	P56211
Control	3	P56377
Control	3	P56378
Control	3	P56381
Control	3	P56385
Control	3	P56524
Control	3	P56537
Control	3	P56545
Control	3	P56556
Control	3	P56589
Control	3	P56937
Control	3	P56945
Control	3	P56962
Control	3	P57076
Control	3	P57081
Control	3	P57088
Control	3	P57678
Control	3	P57737
Control	3	P57740
Control	3	P57764
Control	3	P57772
Control	3	P58004
Control	3	P58335
Control	3	P58546
Control	3	P59998
Control	3	P60033
Control	3	P60059
Control	3	P60174
Control	3	P60228
Control	3	P60468
Control	3	P60484
Control	3	P60510

Control	3	P60520
Control	3	P60602
Control	3	P60660
Control	3	P60709
Control	3	P60842
Control	3	P60866
Control	3	P60891
Control	3	P60900
Control	3	P60903
Control	3	P60953
Control	3	P60981
Control	3	P60983
Control	3	P61006
Control	3	P61009
Control	3	P61011
Control	3	P61018
Control	3	P61019
Control	3	P61020
Control	3	P61026
Control	3	P61077
Control	3	P61081
Control	3	P61086
Control	3	P61088
Control	3	P61106
Control	3	P61158
Control	3	P61160
Control	3	P61163
Control	3	P61201
Control	3	P61221
Control	3	P61224
Control	3	P61225
Control	3	P61244
Control	3	P61247
Control	3	P61254
Control	3	P61289
Control	3	P61313
Control	3	P61353
Control	3	P61421
Control	3	P61513
Control	3	P61586
Control	3	P61587
Control	3	P61599
Control	3	P61604
Control	3	P61619
Control	3	P61626
Control	3	P61758
Control	3	P61764
Control	3	P61769
Control	3	P61803
Control	3	P61916

Control	3	P61923
Control	3	P61927
Control	3	P61956
Control	3	P61962
Control	3	P61964
Control	3	P61966
Control	3	P61970
Control	3	P61978
Control	3	P61981
Control	3	P62068
Control	3	P62070
Control	3	P62081
Control	3	P62136
Control	3	P62140
Control	3	P62191
Control	3	P62195
Control	3	P62241
Control	3	P62244
Control	3	P62249
Control	3	P62253
Control	3	P62256
Control	3	P62258
Control	3	P62263
Control	3	P62266
Control	3	P62269
Control	3	P62273
Control	3	P62277
Control	3	P62280
Control	3	P62304
Control	3	P62306
Control	3	P62310
Control	3	P62312
Control	3	P62314
Control	3	P62316
Control	3	P62318
Control	3	P62328
Control	3	P62330
Control	3	P62333
Control	3	P62341
Control	3	P62380
Control	3	P62424
Control	3	P62487
Control	3	P62491
Control	3	P62495
Control	3	P62633
Control	3	P62699
Control	3	P62701
Control	3	P62714
Control	3	P62736
Control	3	P62745

Control	3	P62750
Control	3	P62753
Control	3	P62805
Control	3	P62820
Control	3	P62826
Control	3	P62829
Control	3	P62834
Control	3	P62841
Control	3	P62847
Control	3	P62851
Control	3	P62854
Control	3	P62857
Control	3	P62861
Control	3	P62873
Control	3	P62877
Control	3	P62879
Control	3	P62888
Control	3	P62891
Control	3	P62899
Control	3	P62906
Control	3	P62910
Control	3	P62913
Control	3	P62917
Control	3	P62937
Control	3	P62942
Control	3	P62979
Control	3	P62993
Control	3	P62995
Control	3	P63000
Control	3	P63010
Control	3	P63027
Control	3	P63092
Control	3	P63096
Control	3	P63104
Control	3	P63151
Control	3	P63165
Control	3	P63167
Control	3	P63172
Control	3	P63173
Control	3	P63208
Control	3	P63218
Control	3	P63220
Control	3	P63241
Control	3	P63244
Control	3	P63272
Control	3	P63279
Control	3	P63313
Control	3	P67775
Control	3	P67809
Control	3	P67812

Control	3	P67870
Control	3	P67936
Control	3	P68036
Control	3	P68104
Control	3	P68363
Control	3	P68366
Control	3	P68371
Control	3	P68400
Control	3	P68402
Control	3	P68431
Control	3	P68871
Control	3	P69905
Control	3	P78310
Control	3	P78312
Control	3	P78316
Control	3	P78318
Control	3	P78324
Control	3	P78330
Control	3	P78332
Control	3	P78344
Control	3	P78345
Control	3	P78346
Control	3	P78347
Control	3	P78356
Control	3	P78357
Control	3	P78362
Control	3	P78371
Control	3	P78381
Control	3	P78406
Control	3	P78417
Control	3	P78527
Control	3	P78536
Control	3	P78537
Control	3	P78539
Control	3	P78540
Control	3	P78549
Control	3	P80217
Control	3	P80303
Control	3	P80723
Control	3	P81605
Control	3	P82094
Control	3	P82650
Control	3	P82663
Control	3	P82664
Control	3	P82673
Control	3	P82675
Control	3	P82909
Control	3	P82912
Control	3	P82914
Control	3	P82921

Control	3	P82930
Control	3	P82932
Control	3	P82933
Control	3	P82979
Control	3	P83111
Control	3	P83436
Control	3	P83731
Control	3	P83876
Control	3	P83881
Control	3	P83916
Control	3	P84022
Control	3	P84077
Control	3	P84085
Control	3	P84090
Control	3	P84095
Control	3	P84098
Control	3	P84101
Control	3	P84103
Control	3	P85037
Control	3	P86790
Control	3	P98082
Control	3	P98160
Control	3	P98170
Control	3	P98172
Control	3	P98175
Control	3	P98179
Control	3	P98194
Control	3	P98196
Control	3	P99999
Control	3	Q00013
Control	3	Q00059
Control	3	Q00169
Control	3	Q00325
Control	3	Q00341
Control	3	Q00403
Control	3	Q00534
Control	3	Q00535
Control	3	Q00537
Control	3	Q00577
Control	3	Q00587
Control	3	Q00610
Control	3	Q00613
Control	3	Q00653
Control	3	Q00688
Control	3	Q00765
Control	3	Q00796
Control	3	Q00839
Control	3	Q01081
Control	3	Q01082
Control	3	Q01085

Control	3	Q01105
Control	3	Q01130
Control	3	Q01167
Control	3	Q01201
Control	3	Q01415
Control	3	Q01433
Control	3	Q01469
Control	3	Q01518
Control	3	Q01543
Control	3	Q01581
Control	3	Q01628
Control	3	Q01650
Control	3	Q01658
Control	3	Q01780
Control	3	Q01804
Control	3	Q01813
Control	3	Q01831
Control	3	Q01844
Control	3	Q01850
Control	3	Q01968
Control	3	Q01970
Control	3	Q01995
Control	3	Q02040
Control	3	Q02127
Control	3	Q02218
Control	3	Q02224
Control	3	Q02241
Control	3	Q02252
Control	3	Q02297
Control	3	Q02318
Control	3	Q02413
Control	3	Q02447
Control	3	Q02539
Control	3	Q02543
Control	3	Q02750
Control	3	Q02763
Control	3	Q02790
Control	3	Q02809
Control	3	Q02818
Control	3	Q02878
Control	3	Q02880
Control	3	Q02952
Control	3	Q02978
Control	3	Q03001
Control	3	Q03111
Control	3	Q03112
Control	3	Q03113
Control	3	Q03135
Control	3	Q03154
Control	3	Q03169

Control	3	Q03252
Control	3	Q03393
Control	3	Q03405
Control	3	Q03426
Control	3	Q03468
Control	3	Q03518
Control	3	Q03519
Control	3	Q03701
Control	3	Q03936
Control	3	Q04206
Control	3	Q04323
Control	3	Q04446
Control	3	Q04637
Control	3	Q04656
Control	3	Q04721
Control	3	Q04724
Control	3	Q04726
Control	3	Q04760
Control	3	Q04771
Control	3	Q04837
Control	3	Q04864
Control	3	Q04917
Control	3	Q04941
Control	3	Q05048
Control	3	Q05086
Control	3	Q05193
Control	3	Q05209
Control	3	Q05397
Control	3	Q05519
Control	3	Q05655
Control	3	Q05682
Control	3	Q05932
Control	3	Q05D32
Control	3	Q06124
Control	3	Q06136
Control	3	Q06203
Control	3	Q06210
Control	3	Q06265
Control	3	Q06323
Control	3	Q06330
Control	3	Q06413
Control	3	Q06481
Control	3	Q06546
Control	3	Q06587
Control	3	Q06787
Control	3	Q06830
Control	3	Q07020
Control	3	Q07021
Control	3	Q07065
Control	3	Q07157

Control	3	Q07352
Control	3	Q07617
Control	3	Q07666
Control	3	Q07812
Control	3	Q07817
Control	3	Q07820
Control	3	Q07864
Control	3	Q07866
Control	3	Q07889
Control	3	Q07955
Control	3	Q07960
Control	3	Q08117
Control	3	Q08170
Control	3	Q08174
Control	3	Q08188
Control	3	Q08209
Control	3	Q08211
Control	3	Q08257
Control	3	Q08378
Control	3	Q08379
Control	3	Q08426
Control	3	Q08431
Control	3	Q08499
Control	3	Q08554
Control	3	Q08623
Control	3	Q08629
Control	3	Q08722
Control	3	Q08752
Control	3	Q08945
Control	3	Q08AD1
Control	3	Q08AE8
Control	3	Q08AF3
Control	3	Q08AM6
Control	3	Q08J23
Control	3	Q09019
Control	3	Q09028
Control	3	Q09161
Control	3	Q09328
Control	3	Q09472
Control	3	Q09666
Control	3	Q0JRZ9
Control	3	Q0PNE2
Control	3	Q0VDF9
Control	3	Q0VDG4
Control	3	Q0VF96
Control	3	Q0VGL1
Control	3	Q0ZGT2
Control	3	Q10469
Control	3	Q10471
Control	3	Q10472

Control	3	Q10567
Control	3	Q10570
Control	3	Q10589
Control	3	Q10713
Control	3	Q11201
Control	3	Q12765
Control	3	Q12768
Control	3	Q12769
Control	3	Q12770
Control	3	Q12778
Control	3	Q12788
Control	3	Q12789
Control	3	Q12792
Control	3	Q12797
Control	3	Q12800
Control	3	Q12802
Control	3	Q12805
Control	3	Q12824
Control	3	Q12830
Control	3	Q12834
Control	3	Q12841
Control	3	Q12846
Control	3	Q12849
Control	3	Q12851
Control	3	Q12866
Control	3	Q12872
Control	3	Q12873
Control	3	Q12874
Control	3	Q12888
Control	3	Q12893
Control	3	Q12894
Control	3	Q12899
Control	3	Q12904
Control	3	Q12905
Control	3	Q12906
Control	3	Q12907
Control	3	Q12931
Control	3	Q12933
Control	3	Q12959
Control	3	Q12965
Control	3	Q12972
Control	3	Q12974
Control	3	Q12979
Control	3	Q12980
Control	3	Q12981
Control	3	Q12982
Control	3	Q12986
Control	3	Q12996
Control	3	Q12999
Control	3	Q13011

Control	3	Q13017
Control	3	Q13033
Control	3	Q13042
Control	3	Q13043
Control	3	Q13045
Control	3	Q13049
Control	3	Q13057
Control	3	Q13084
Control	3	Q13085
Control	3	Q13098
Control	3	Q13107
Control	3	Q13111
Control	3	Q13112
Control	3	Q13123
Control	3	Q13126
Control	3	Q13131
Control	3	Q13136
Control	3	Q13144
Control	3	Q13148
Control	3	Q13151
Control	3	Q13155
Control	3	Q13158
Control	3	Q13162
Control	3	Q13163
Control	3	Q13177
Control	3	Q13185
Control	3	Q13188
Control	3	Q13190
Control	3	Q13200
Control	3	Q13201
Control	3	Q13206
Control	3	Q13217
Control	3	Q13228
Control	3	Q13232
Control	3	Q13242
Control	3	Q13243
Control	3	Q13247
Control	3	Q13257
Control	3	Q13263
Control	3	Q13275
Control	3	Q13277
Control	3	Q13283
Control	3	Q13287
Control	3	Q13308
Control	3	Q13310
Control	3	Q13315
Control	3	Q13322
Control	3	Q13325
Control	3	Q13330
Control	3	Q13347

Control	3	Q13356
Control	3	Q13362
Control	3	Q13363
Control	3	Q13371
Control	3	Q13393
Control	3	Q13395
Control	3	Q13404
Control	3	Q13405
Control	3	Q13409
Control	3	Q13416
Control	3	Q13418
Control	3	Q13423
Control	3	Q13425
Control	3	Q13426
Control	3	Q13427
Control	3	Q13428
Control	3	Q13432
Control	3	Q13435
Control	3	Q13438
Control	3	Q13439
Control	3	Q13442
Control	3	Q13443
Control	3	Q13444
Control	3	Q13445
Control	3	Q13451
Control	3	Q13459
Control	3	Q13464
Control	3	Q13469
Control	3	Q13472
Control	3	Q13485
Control	3	Q13488
Control	3	Q13490
Control	3	Q13492
Control	3	Q13496
Control	3	Q13501
Control	3	Q13505
Control	3	Q13509
Control	3	Q13510
Control	3	Q13523
Control	3	Q13526
Control	3	Q13535
Control	3	Q13541
Control	3	Q13546
Control	3	Q13547
Control	3	Q13555
Control	3	Q13557
Control	3	Q13561
Control	3	Q13563
Control	3	Q13564
Control	3	Q13572

Control	3	Q13573
Control	3	Q13574
Control	3	Q13586
Control	3	Q13588
Control	3	Q13595
Control	3	Q13596
Control	3	Q13601
Control	3	Q13610
Control	3	Q13613
Control	3	Q13614
Control	3	Q13616
Control	3	Q13617
Control	3	Q13618
Control	3	Q13619
Control	3	Q13620
Control	3	Q13625
Control	3	Q13627
Control	3	Q13630
Control	3	Q13637
Control	3	Q13641
Control	3	Q13642
Control	3	Q13643
Control	3	Q13671
Control	3	Q13685
Control	3	Q13724
Control	3	Q13740
Control	3	Q13769
Control	3	Q13796
Control	3	Q13813
Control	3	Q13823
Control	3	Q13825
Control	3	Q13835
Control	3	Q13838
Control	3	Q13867
Control	3	Q13868
Control	3	Q13873
Control	3	Q13885
Control	3	Q13888
Control	3	Q13889
Control	3	Q13895
Control	3	Q13905
Control	3	Q13907
Control	3	Q13951
Control	3	Q13952
Control	3	Q14004
Control	3	Q14008
Control	3	Q14011
Control	3	Q14012
Control	3	Q14019
Control	3	Q14103

Control	3	Q14108
Control	3	Q14112
Control	3	Q14114
Control	3	Q14118
Control	3	Q14119
Control	3	Q14137
Control	3	Q14139
Control	3	Q14141
Control	3	Q14145
Control	3	Q14146
Control	3	Q14147
Control	3	Q14149
Control	3	Q14151
Control	3	Q14152
Control	3	Q14155
Control	3	Q14156
Control	3	Q14157
Control	3	Q14160
Control	3	Q14161
Control	3	Q14162
Control	3	Q14165
Control	3	Q14166
Control	3	Q14181
Control	3	Q14185
Control	3	Q14186
Control	3	Q14192
Control	3	Q14195
Control	3	Q14197
Control	3	Q14202
Control	3	Q14203
Control	3	Q14204
Control	3	Q14232
Control	3	Q14240
Control	3	Q14241
Control	3	Q14247
Control	3	Q14249
Control	3	Q14254
Control	3	Q14257
Control	3	Q14258
Control	3	Q14315
Control	3	Q14318
Control	3	Q14320
Control	3	Q14331
Control	3	Q14344
Control	3	Q14353
Control	3	Q14376
Control	3	Q14392
Control	3	Q14444
Control	3	Q14457
Control	3	Q14498

Control	3	Q14511
Control	3	Q14527
Control	3	Q14534
Control	3	Q14554
Control	3	Q14558
Control	3	Q14562
Control	3	Q14566
Control	3	Q14571
Control	3	Q14573
Control	3	Q14643
Control	3	Q14644
Control	3	Q14651
Control	3	Q14653
Control	3	Q14657
Control	3	Q14667
Control	3	Q14669
Control	3	Q14671
Control	3	Q14676
Control	3	Q14677
Control	3	Q14678
Control	3	Q14683
Control	3	Q14684
Control	3	Q14689
Control	3	Q14690
Control	3	Q14691
Control	3	Q14692
Control	3	Q14693
Control	3	Q14694
Control	3	Q14696
Control	3	Q14697
Control	3	Q14699
Control	3	Q14728
Control	3	Q14738
Control	3	Q14739
Control	3	Q14746
Control	3	Q14764
Control	3	Q14766
Control	3	Q14767
Control	3	Q14781
Control	3	Q14789
Control	3	Q14790
Control	3	Q147X3
Control	3	Q14807
Control	3	Q14814
Control	3	Q14839
Control	3	Q14847
Control	3	Q14914
Control	3	Q14919
Control	3	Q14934
Control	3	Q14966

Control	3	Q14974
Control	3	Q14978
Control	3	Q14980
Control	3	Q14997
Control	3	Q14999
Control	3	Q14BN4
Control	3	Q14C86
Control	3	Q14CX7
Control	3	Q14D04
Control	3	Q15003
Control	3	Q15004
Control	3	Q15005
Control	3	Q15006
Control	3	Q15007
Control	3	Q15008
Control	3	Q15011
Control	3	Q15013
Control	3	Q15014
Control	3	Q15018
Control	3	Q15019
Control	3	Q15020
Control	3	Q15021
Control	3	Q15022
Control	3	Q15024
Control	3	Q15025
Control	3	Q15029
Control	3	Q15031
Control	3	Q15032
Control	3	Q15035
Control	3	Q15036
Control	3	Q15041
Control	3	Q15042
Control	3	Q15043
Control	3	Q15046
Control	3	Q15047
Control	3	Q15048
Control	3	Q15050
Control	3	Q15054
Control	3	Q15056
Control	3	Q15057
Control	3	Q15058
Control	3	Q15059
Control	3	Q15061
Control	3	Q15067
Control	3	Q15070
Control	3	Q15075
Control	3	Q15084
Control	3	Q15102
Control	3	Q15120
Control	3	Q15121

Control	3	Q15125
Control	3	Q15126
Control	3	Q15139
Control	3	Q15149
Control	3	Q15154
Control	3	Q15155
Control	3	Q15165
Control	3	Q15172
Control	3	Q15181
Control	3	Q15185
Control	3	Q15208
Control	3	Q15233
Control	3	Q15257
Control	3	Q15262
Control	3	Q15269
Control	3	Q15276
Control	3	Q15286
Control	3	Q15287
Control	3	Q15291
Control	3	Q15293
Control	3	Q15311
Control	3	Q15334
Control	3	Q15345
Control	3	Q15361
Control	3	Q15363
Control	3	Q15365
Control	3	Q15366
Control	3	Q15369
Control	3	Q15370
Control	3	Q15382
Control	3	Q15386
Control	3	Q15388
Control	3	Q15390
Control	3	Q15392
Control	3	Q15393
Control	3	Q15397
Control	3	Q15398
Control	3	Q15404
Control	3	Q15417
Control	3	Q15418
Control	3	Q15424
Control	3	Q15428
Control	3	Q15434
Control	3	Q15435
Control	3	Q15436
Control	3	Q15437
Control	3	Q15438
Control	3	Q15459
Control	3	Q15477
Control	3	Q15517

Control	3	Q15526
Control	3	Q15542
Control	3	Q15545
Control	3	Q15554
Control	3	Q15555
Control	3	Q15583
Control	3	Q15599
Control	3	Q15628
Control	3	Q15629
Control	3	Q15631
Control	3	Q15633
Control	3	Q15637
Control	3	Q15642
Control	3	Q15643
Control	3	Q15645
Control	3	Q15648
Control	3	Q15650
Control	3	Q15651
Control	3	Q15652
Control	3	Q15653
Control	3	Q15654
Control	3	Q15678
Control	3	Q15691
Control	3	Q15717
Control	3	Q15738
Control	3	Q15742
Control	3	Q15746
Control	3	Q15750
Control	3	Q15751
Control	3	Q15758
Control	3	Q15773
Control	3	Q15785
Control	3	Q15796
Control	3	Q15797
Control	3	Q15800
Control	3	Q15811
Control	3	Q15813
Control	3	Q15814
Control	3	Q15819
Control	3	Q15831
Control	3	Q15833
Control	3	Q15836
Control	3	Q15843
Control	3	Q15904
Control	3	Q15906
Control	3	Q15907
Control	3	Q15942
Control	3	Q16134
Control	3	Q16181
Control	3	Q16186

Control	3	Q16204
Control	3	Q16222
Control	3	Q16254
Control	3	Q16270
Control	3	Q16342
Control	3	Q16363
Control	3	Q16401
Control	3	Q16512
Control	3	Q16513
Control	3	Q16514
Control	3	Q16527
Control	3	Q16531
Control	3	Q16537
Control	3	Q16539
Control	3	Q16540
Control	3	Q16543
Control	3	Q16555
Control	3	Q16563
Control	3	Q16576
Control	3	Q16584
Control	3	Q16594
Control	3	Q16602
Control	3	Q16611
Control	3	Q16629
Control	3	Q16630
Control	3	Q16637
Control	3	Q16643
Control	3	Q16656
Control	3	Q16658
Control	3	Q16666
Control	3	Q16698
Control	3	Q16706
Control	3	Q16718
Control	3	Q16739
Control	3	Q16740
Control	3	Q16762
Control	3	Q16763
Control	3	Q16773
Control	3	Q16774
Control	3	Q16775
Control	3	Q16795
Control	3	Q16798
Control	3	Q16822
Control	3	Q16831
Control	3	Q16832
Control	3	Q16836
Control	3	Q16850
Control	3	Q16851
Control	3	Q16875
Control	3	Q16881

Control	3	Q16891
Control	3	Q19T08
Control	3	Q1ED39
Control	3	Q1KMD3
Control	3	Q24JP5
Control	3	Q27J81
Control	3	Q29RF7
Control	3	Q2KHT3
Control	3	Q2M1P5
Control	3	Q2M1Z3
Control	3	Q2M296
Control	3	Q2M2I8
Control	3	Q2M389
Control	3	Q2NKX8
Control	3	Q2NL82
Control	3	Q2PPJ7
Control	3	Q2PZI1
Control	3	Q2TAA2
Control	3	Q2TAA5
Control	3	Q2TAL8
Control	3	Q2TAY7
Control	3	Q2VPK5
Control	3	Q32M88
Control	3	Q32MZ4
Control	3	Q32NB8
Control	3	Q32NC0
Control	3	Q32P28
Control	3	Q32P41
Control	3	Q32P44
Control	3	Q3B726
Control	3	Q3KQU3
Control	3	Q3KQV9
Control	3	Q3L8U1
Control	3	Q3LXA3
Control	3	Q3MHD2
Control	3	Q3SXM5
Control	3	Q3SY69
Control	3	Q3T906
Control	3	Q3V6T2
Control	3	Q3YEC7
Control	3	Q3ZCQ8
Control	3	Q460N5
Control	3	Q495W5
Control	3	Q49A26
Control	3	Q49AR2
Control	3	Q4AC94
Control	3	Q4G0F5
Control	3	Q4G0J3
Control	3	Q4G0N4
Control	3	Q4G0X4

Control	3	Q4G148
Control	3	Q4G176
Control	3	Q4J6C6
Control	3	Q4KMP7
Control	3	Q4KMQ1
Control	3	Q4KMQ2
Control	3	Q4L180
Control	3	Q4V328
Control	3	Q4V339
Control	3	Q4VC31
Control	3	Q4ZIN3
Control	3	Q52LJ0
Control	3	Q52LW3
Control	3	Q53EL6
Control	3	Q53EP0
Control	3	Q53ET0
Control	3	Q53EU6
Control	3	Q53EZ4
Control	3	Q53F19
Control	3	Q53FA7
Control	3	Q53FP2
Control	3	Q53GA4
Control	3	Q53GG5
Control	3	Q53GL7
Control	3	Q53GQ0
Control	3	Q53GS7
Control	3	Q53GS9
Control	3	Q53GT1
Control	3	Q53H12
Control	3	Q53H47
Control	3	Q53H82
Control	3	Q53H96
Control	3	Q53HC9
Control	3	Q53HL2
Control	3	Q53SF7
Control	3	Q53T59
Control	3	Q53TN4
Control	3	Q562E7
Control	3	Q562R1
Control	3	Q567U6
Control	3	Q56VL3
Control	3	Q58EX7
Control	3	Q58FG1
Control	3	Q58WW2
Control	3	Q5BJD5
Control	3	Q5BJF2
Control	3	Q5BJH7
Control	3	Q5BKZ1
Control	3	Q5C9Z4
Control	3	Q5EBL4

Control	3	Q5F1R6
Control	3	Q5GJ75
Control	3	Q5GLZ8
Control	3	Q5H8A4
Control	3	Q5H9R7
Control	3	Q5HYI8
Control	3	Q5HYJ3
Control	3	Q5HYK3
Control	3	Q5HYK7
Control	3	Q5HYW2
Control	3	Q5J8M3
Control	3	Q5JPH6
Control	3	Q5JPI3
Control	3	Q5JPI9
Control	3	Q5JRA6
Control	3	Q5JRX3
Control	3	Q5JSH3
Control	3	Q5JSZ5
Control	3	Q5JTD0
Control	3	Q5JTH9
Control	3	Q5JTJ3
Control	3	Q5JTV8
Control	3	Q5JTW2
Control	3	Q5JTZ9
Control	3	Q5JU69
Control	3	Q5JVF3
Control	3	Q5JVS0
Control	3	Q5K4L6
Control	3	Q5K651
Control	3	Q5MIZ7
Control	3	Q5MNZ6
Control	3	Q5MNZ9
Control	3	Q5NDL2
Control	3	Q5PRF9
Control	3	Q5QJ74
Control	3	Q5QJE6
Control	3	Q5R372
Control	3	Q5R3I4
Control	3	Q5RI15
Control	3	Q5RKV6
Control	3	Q5SNT2
Control	3	Q5SQI0
Control	3	Q5SQN1
Control	3	Q5SRE5
Control	3	Q5SSJ5
Control	3	Q5ST30
Control	3	Q5SW79
Control	3	Q5SW96
Control	3	Q5SWX8
Control	3	Q5SY16

Control	3	Q5SYE7
Control	3	Q5T013
Control	3	Q5T0D9
Control	3	Q5T0F9
Control	3	Q5T0N5
Control	3	Q5T160
Control	3	Q5T1C6
Control	3	Q5T1J5
Control	3	Q5T1M5
Control	3	Q5T200
Control	3	Q5T280
Control	3	Q5T2E6
Control	3	Q5T3F8
Control	3	Q5T3I0
Control	3	Q5T440
Control	3	Q5T447
Control	3	Q5T4B2
Control	3	Q5T4S7
Control	3	Q5T5C0
Control	3	Q5T5U3
Control	3	Q5T5Y3
Control	3	Q5T653
Control	3	Q5T6F2
Control	3	Q5T6V5
Control	3	Q5T749
Control	3	Q5T7W0
Control	3	Q5T8D3
Control	3	Q5T8P6
Control	3	Q5T9A4
Control	3	Q5T9L3
Control	3	Q5TA45
Control	3	Q5TA50
Control	3	Q5TAQ9
Control	3	Q5TAT6
Control	3	Q5TAX3
Control	3	Q5TBA9
Control	3	Q5TBB1
Control	3	Q5TC12
Control	3	Q5TC82
Control	3	Q5TDH0
Control	3	Q5TEJ8
Control	3	Q5TEU4
Control	3	Q5TFE4
Control	3	Q5TH69
Control	3	Q5THJ4
Control	3	Q5TZA2
Control	3	Q5U5X0
Control	3	Q5U651
Control	3	Q5UIP0
Control	3	Q5VIR6

Control	3	Q5VSL9
Control	3	Q5VT25
Control	3	Q5VT52
Control	3	Q5VTB9
Control	3	Q5VTL8
Control	3	Q5VTR2
Control	3	Q5VU43
Control	3	Q5VUA4
Control	3	Q5VUB5
Control	3	Q5VUD6
Control	3	Q5VUJ6
Control	3	Q5VV42
Control	3	Q5VW32
Control	3	Q5VW36
Control	3	Q5VWQ0
Control	3	Q5VWQ8
Control	3	Q5VWZ2
Control	3	Q5VY43
Control	3	Q5VYK3
Control	3	Q5VYS8
Control	3	Q5VZ18
Control	3	Q5VZ89
Control	3	Q5VZE5
Control	3	Q5VZL5
Control	3	Q5W0V3
Control	3	Q5W0Z9
Control	3	Q5W111
Control	3	Q5XUX1
Control	3	Q5ZPR3
Control	3	Q63HN8
Control	3	Q63HR2
Control	3	Q63ZY3
Control	3	Q641Q2
Control	3	Q643R3
Control	3	Q658P3
Control	3	Q658Y4
Control	3	Q66K14
Control	3	Q66K74
Control	3	Q66LE6
Control	3	Q66PJ3
Control	3	Q676U5
Control	3	Q68CP4
Control	3	Q68CP9
Control	3	Q68CQ4
Control	3	Q68CQ7
Control	3	Q68CR1
Control	3	Q68CZ2
Control	3	Q68CZ6
Control	3	Q68D10
Control	3	Q68D91

Control	3	Q68DQ2
Control	3	Q68E01
Control	3	Q68EM7
Control	3	Q69YL0
Control	3	Q69YN2
Control	3	Q69YN4
Control	3	Q69YQ0
Control	3	Q6AI08
Control	3	Q6AI12
Control	3	Q6AWC2
Control	3	Q6AZY7
Control	3	Q6DD88
Control	3	Q6DKI1
Control	3	Q6DKJ4
Control	3	Q6DKK2
Control	3	Q6DN90
Control	3	Q6FI81
Control	3	Q6FIF0
Control	3	Q6GMV2
Control	3	Q6GQQ9
Control	3	Q6I9Y2
Control	3	Q6IA69
Control	3	Q6IA86
Control	3	Q6IAA8
Control	3	Q6IAN0
Control	3	Q6IBS0
Control	3	Q6IBW4
Control	3	Q6ICG6
Control	3	Q6ICL3
Control	3	Q6IN85
Control	3	Q6IQ22
Control	3	Q6IQ49
Control	3	Q6KB66
Control	3	Q6KC79
Control	3	Q6KCM7
Control	3	Q6L8Q7
Control	3	Q6N069
Control	3	Q6NSW5
Control	3	Q6NTF9
Control	3	Q6NUK1
Control	3	Q6NUK4
Control	3	Q6NUM9
Control	3	Q6NUQ1
Control	3	Q6NUQ4
Control	3	Q6NVY1
Control	3	Q6NW29
Control	3	Q6NW34
Control	3	Q6NXE6
Control	3	Q6NXR4
Control	3	Q6NXT1

Control	3	Q6NXT6
Control	3	Q6NY19
Control	3	Q6NYC1
Control	3	Q6NYC8
Control	3	Q6NZI2
Control	3	Q6NZY4
Control	3	Q6P158
Control	3	Q6P179
Control	3	Q6P1A2
Control	3	Q6P1J9
Control	3	Q6P1L8
Control	3	Q6P1M0
Control	3	Q6P1N0
Control	3	Q6P1N9
Control	3	Q6P1Q9
Control	3	Q6P1R3
Control	3	Q6P1X5
Control	3	Q6P1X6
Control	3	Q6P2C8
Control	3	Q6P2E9
Control	3	Q6P2H3
Control	3	Q6P2P2
Control	3	Q6P2Q9
Control	3	Q6P3S6
Control	3	Q6P3W7
Control	3	Q6P3X3
Control	3	Q6P4A7
Control	3	Q6P4E1
Control	3	Q6P4R8
Control	3	Q6P587
Control	3	Q6P5R6
Control	3	Q6P5Z2
Control	3	Q6P6C2
Control	3	Q6P996
Control	3	Q6P9B6
Control	3	Q6P9B9
Control	3	Q6P9H5
Control	3	Q6PCB7
Control	3	Q6PCE3
Control	3	Q6PD62
Control	3	Q6PD74
Control	3	Q6PEY1
Control	3	Q6PGN9
Control	3	Q6PGP7
Control	3	Q6PHR2
Control	3	Q6PI48
Control	3	Q6PI78
Control	3	Q6PI98
Control	3	Q6PIJ6
Control	3	Q6PIU2

Control	3	Q6PIW4
Control	3	Q6PJ69
Control	3	Q6PJF5
Control	3	Q6PJG2
Control	3	Q6PJG6
Control	3	Q6PJI9
Control	3	Q6PJT7
Control	3	Q6PK04
Control	3	Q6PK18
Control	3	Q6PKC3
Control	3	Q6PKG0
Control	3	Q6PL18
Control	3	Q6PL24
Control	3	Q6PML9
Control	3	Q6Q0C0
Control	3	Q6R327
Control	3	Q6RFH5
Control	3	Q6RW13
Control	3	Q6STE5
Control	3	Q6UB35
Control	3	Q6ULP2
Control	3	Q6UN15
Control	3	Q6UUV7
Control	3	Q6UUV9
Control	3	Q6UW02
Control	3	Q6UW63
Control	3	Q6UW68
Control	3	Q6UWE0
Control	3	Q6UWH4
Control	3	Q6UWP7
Control	3	Q6UWZ7
Control	3	Q6UX04
Control	3	Q6UXH1
Control	3	Q6UXN9
Control	3	Q6UXV4
Control	3	Q6V0I7
Control	3	Q6V1X1
Control	3	Q6VMQ6
Control	3	Q6VN20
Control	3	Q6VY07
Control	3	Q6WCQ1
Control	3	Q6XE24
Control	3	Q6XQN6
Control	3	Q6XZF7
Control	3	Q6Y1H2
Control	3	Q6Y288
Control	3	Q6Y7W6
Control	3	Q6YHK3
Control	3	Q6YHU6
Control	3	Q6YN16

Control	3	Q6YP21
Control	3	Q6ZMI0
Control	3	Q6ZMP0
Control	3	Q6ZMZ3
Control	3	Q6ZN55
Control	3	Q6ZNB6
Control	3	Q6ZNJ1
Control	3	Q6ZNL6
Control	3	Q6ZRP7
Control	3	Q6ZRS2
Control	3	Q6ZS17
Control	3	Q6ZSR9
Control	3	Q6ZSZ5
Control	3	Q6ZT12
Control	3	Q6ZT21
Control	3	Q6ZU35
Control	3	Q6ZUT6
Control	3	Q6ZVK8
Control	3	Q6ZW49
Control	3	Q6ZXV5
Control	3	Q709C8
Control	3	Q70CQ2
Control	3	Q70E73
Control	3	Q70IA6
Control	3	Q70UQ0
Control	3	Q70Z35
Control	3	Q70Z53
Control	3	Q712K3
Control	3	Q71F56
Control	3	Q71RC2
Control	3	Q71SY5
Control	3	Q71UM5
Control	3	Q75N03
Control	3	Q75QN2
Control	3	Q7KYR7
Control	3	Q7KZ85
Control	3	Q7KZF4
Control	3	Q7KZI7
Control	3	Q7KZN9
Control	3	Q7L014
Control	3	Q7L099
Control	3	Q7L0Y3
Control	3	Q7L1Q6
Control	3	Q7L1V2
Control	3	Q7L266
Control	3	Q7L273
Control	3	Q7L2E3
Control	3	Q7L2H7
Control	3	Q7L2J0
Control	3	Q7L311

Control	3	Q7L3T8
Control	3	Q7L4I2
Control	3	Q7L523
Control	3	Q7L576
Control	3	Q7L592
Control	3	Q7L5D6
Control	3	Q7L5N1
Control	3	Q7L5N7
Control	3	Q7L5Y1
Control	3	Q7L5Y9
Control	3	Q7L775
Control	3	Q7L7V1
Control	3	Q7L7X3
Control	3	Q7L8L6
Control	3	Q7L9L4
Control	3	Q7LBC6
Control	3	Q7LBR1
Control	3	Q7LG56
Control	3	Q7LGA3
Control	3	Q7RTN6
Control	3	Q7RTP6
Control	3	Q7RTS9
Control	3	Q7RTT2
Control	3	Q7RTV0
Control	3	Q7RTV5
Control	3	Q7Z2E3
Control	3	Q7Z2K6
Control	3	Q7Z2K8
Control	3	Q7Z2T5
Control	3	Q7Z2W4
Control	3	Q7Z2W9
Control	3	Q7Z2Z2
Control	3	Q7Z333
Control	3	Q7Z392
Control	3	Q7Z3B3
Control	3	Q7Z3B4
Control	3	Q7Z3C6
Control	3	Q7Z3D6
Control	3	Q7Z3E2
Control	3	Q7Z3E5
Control	3	Q7Z3J2
Control	3	Q7Z3K3
Control	3	Q7Z3T8
Control	3	Q7Z3U7
Control	3	Q7Z401
Control	3	Q7Z406
Control	3	Q7Z417
Control	3	Q7Z422
Control	3	Q7Z434
Control	3	Q7Z460

Control	3	Q7Z478
Control	3	Q7Z494
Control	3	Q7Z4F1
Control	3	Q7Z4H7
Control	3	Q7Z4H8
Control	3	Q7Z4L5
Control	3	Q7Z4Q2
Control	3	Q7Z4S6
Control	3	Q7Z4V5
Control	3	Q7Z4W1
Control	3	Q7Z569
Control	3	Q7Z589
Control	3	Q7Z5G4
Control	3	Q7Z5H3
Control	3	Q7Z5K2
Control	3	Q7Z5L9
Control	3	Q7Z6E9
Control	3	Q7Z6J0
Control	3	Q7Z6J2
Control	3	Q7Z6J6
Control	3	Q7Z6J9
Control	3	Q7Z6K3
Control	3	Q7Z6K5
Control	3	Q7Z6L1
Control	3	Q7Z6M1
Control	3	Q7Z6M4
Control	3	Q7Z6Z7
Control	3	Q7Z739
Control	3	Q7Z7A3
Control	3	Q7Z7C8
Control	3	Q7Z7E8
Control	3	Q7Z7F7
Control	3	Q7Z7H5
Control	3	Q7Z7K0
Control	3	Q7Z7K6
Control	3	Q7Z7L1
Control	3	Q7Z7M0
Control	3	Q7Z7N9
Control	3	Q86SF2
Control	3	Q86SK9
Control	3	Q86SQ0
Control	3	Q86SQ4
Control	3	Q86SQ9
Control	3	Q86SR1
Control	3	Q86SZ2
Control	3	Q86T03
Control	3	Q86T13
Control	3	Q86TB9
Control	3	Q86TI2
Control	3	Q86TM6

Control	3	Q86TN4
Control	3	Q86TU7
Control	3	Q86TV6
Control	3	Q86TX2
Control	3	Q86U38
Control	3	Q86U42
Control	3	Q86U44
Control	3	Q86U86
Control	3	Q86U90
Control	3	Q86UA1
Control	3	Q86UE4
Control	3	Q86UK7
Control	3	Q86UL3
Control	3	Q86UP2
Control	3	Q86US8
Control	3	Q86UT6
Control	3	Q86UU0
Control	3	Q86UU1
Control	3	Q86UV5
Control	3	Q86UW7
Control	3	Q86UX7
Control	3	Q86UY6
Control	3	Q86UY8
Control	3	Q86V21
Control	3	Q86V48
Control	3	Q86V81
Control	3	Q86V85
Control	3	Q86V87
Control	3	Q86VI3
Control	3	Q86VM9
Control	3	Q86VN1
Control	3	Q86VP6
Control	3	Q86VR2
Control	3	Q86VS8
Control	3	Q86VW0
Control	3	Q86VX2
Control	3	Q86VX9
Control	3	Q86W34
Control	3	Q86W42
Control	3	Q86W50
Control	3	Q86W56
Control	3	Q86W92
Control	3	Q86WA8
Control	3	Q86WB0
Control	3	Q86WJ1
Control	3	Q86WN1
Control	3	Q86WR0
Control	3	Q86WR7
Control	3	Q86WV6
Control	3	Q86WX3

Control	3	Q86X02
Control	3	Q86X10
Control	3	Q86X27
Control	3	Q86X55
Control	3	Q86X76
Control	3	Q86X83
Control	3	Q86XA9
Control	3	Q86XI2
Control	3	Q86XK2
Control	3	Q86XL3
Control	3	Q86XN8
Control	3	Q86XP1
Control	3	Q86XP3
Control	3	Q86XZ4
Control	3	Q86Y07
Control	3	Q86Y37
Control	3	Q86Y39
Control	3	Q86Y56
Control	3	Q86Y79
Control	3	Q86Y82
Control	3	Q86YP4
Control	3	Q86YQ8
Control	3	Q86YR5
Control	3	Q86YS6
Control	3	Q86YS7
Control	3	Q86YT6
Control	3	Q86YV5
Control	3	Q86YV9
Control	3	Q8IU81
Control	3	Q8IUD2
Control	3	Q8IUF8
Control	3	Q8IUH4
Control	3	Q8IUH5
Control	3	Q8IUI8
Control	3	Q8IUR0
Control	3	Q8IUR7
Control	3	Q8IUW5
Control	3	Q8IUX1
Control	3	Q8IV08
Control	3	Q8IV36
Control	3	Q8IV38
Control	3	Q8IV48
Control	3	Q8IV63
Control	3	Q8IVB5
Control	3	Q8IVD9
Control	3	Q8IVF2
Control	3	Q8IVF7
Control	3	Q8IVH4
Control	3	Q8IVH8
Control	3	Q8IVL0

Control	3	Q8IVL6
Control	3	Q8IVM0
Control	3	Q8IVS2
Control	3	Q8IVT5
Control	3	Q8IW35
Control	3	Q8IW45
Control	3	Q8IWA0
Control	3	Q8IWA4
Control	3	Q8IWA5
Control	3	Q8IWB1
Control	3	Q8IWB7
Control	3	Q8IWC1
Control	3	Q8IWD4
Control	3	Q8IWE2
Control	3	Q8IWE4
Control	3	Q8IWF6
Control	3	Q8IWI9
Control	3	Q8IWJ2
Control	3	Q8IWR0
Control	3	Q8IWS0
Control	3	Q8IWT0
Control	3	Q8IWT6
Control	3	Q8IWU5
Control	3	Q8IWV7
Control	3	Q8IWV8
Control	3	Q8IWW6
Control	3	Q8IWX8
Control	3	Q8IWY9
Control	3	Q8IWZ3
Control	3	Q8IWZ8
Control	3	Q8IX01
Control	3	Q8IX04
Control	3	Q8IX12
Control	3	Q8IX18
Control	3	Q8IXB1
Control	3	Q8IXH7
Control	3	Q8IXI1
Control	3	Q8IXI2
Control	3	Q8IXJ6
Control	3	Q8IXK0
Control	3	Q8IXM2
Control	3	Q8IXM3
Control	3	Q8IXM6
Control	3	Q8IXQ4
Control	3	Q8IXQ6
Control	3	Q8IXT5
Control	3	Q8IXU6
Control	3	Q8IXW5
Control	3	Q8IY17
Control	3	Q8IY18

Control	3	Q8IY21
Control	3	Q8IY22
Control	3	Q8IY33
Control	3	Q8IY37
Control	3	Q8IY47
Control	3	Q8IY67
Control	3	Q8IY81
Control	3	Q8IY95
Control	3	Q8IYB1
Control	3	Q8IYB3
Control	3	Q8IYB5
Control	3	Q8IYB7
Control	3	Q8IYB8
Control	3	Q8IYD1
Control	3	Q8IYH5
Control	3	Q8IYI6
Control	3	Q8IYJ2
Control	3	Q8IYM9
Control	3	Q8IYQ7
Control	3	Q8IYS2
Control	3	Q8IYU8
Control	3	Q8IZ07
Control	3	Q8IZ21
Control	3	Q8IZ69
Control	3	Q8IZ73
Control	3	Q8IZ81
Control	3	Q8IZ83
Control	3	Q8IZA0
Control	3	Q8IZF2
Control	3	Q8IZH2
Control	3	Q8IZL8
Control	3	Q8IZP0
Control	3	Q8IZQ1
Control	3	Q8IZQ5
Control	3	Q8N0U8
Control	3	Q8N0X7
Control	3	Q8N0Z6
Control	3	Q8N108
Control	3	Q8N122
Control	3	Q8N138
Control	3	Q8N163
Control	3	Q8N183
Control	3	Q8N1B4
Control	3	Q8N1F7
Control	3	Q8N1F8
Control	3	Q8N1G0
Control	3	Q8N1G2
Control	3	Q8N1G4
Control	3	Q8N1I0
Control	3	Q8N1Q1

Control	3	Q8N1W1
Control	3	Q8N201
Control	3	Q8N257
Control	3	Q8N264
Control	3	Q8N2F6
Control	3	Q8N2K0
Control	3	Q8N2M8
Control	3	Q8N2R8
Control	3	Q8N2U0
Control	3	Q8N2W9
Control	3	Q8N335
Control	3	Q8N357
Control	3	Q8N392
Control	3	Q8N3C0
Control	3	Q8N3F8
Control	3	Q8N3P4
Control	3	Q8N3R9
Control	3	Q8N3U4
Control	3	Q8N3V7
Control	3	Q8N3X1
Control	3	Q8N442
Control	3	Q8N488
Control	3	Q8N4A0
Control	3	Q8N4C8
Control	3	Q8N4P3
Control	3	Q8N4Q0
Control	3	Q8N4V1
Control	3	Q8N511
Control	3	Q8N531
Control	3	Q8N543
Control	3	Q8N556
Control	3	Q8N567
Control	3	Q8N573
Control	3	Q8N5A5
Control	3	Q8N5C1
Control	3	Q8N5C6
Control	3	Q8N5D0
Control	3	Q8N5F7
Control	3	Q8N5G2
Control	3	Q8N5H7
Control	3	Q8N5I2
Control	3	Q8N5I4
Control	3	Q8N5K1
Control	3	Q8N5L8
Control	3	Q8N5M1
Control	3	Q8N5M4
Control	3	Q8N5M9
Control	3	Q8N5U6
Control	3	Q8N5W9
Control	3	Q8N653

Control	3	Q8N668
Control	3	Q8N684
Control	3	Q8N697
Control	3	Q8N699
Control	3	Q8N6G6
Control	3	Q8N6H7
Control	3	Q8N6M0
Control	3	Q8N6M3
Control	3	Q8N6N3
Control	3	Q8N6R0
Control	3	Q8N6S5
Control	3	Q8N6T3
Control	3	Q8N6T7
Control	3	Q8N755
Control	3	Q8N766
Control	3	Q8N7H5
Control	3	Q8N7R7
Control	3	Q8N806
Control	3	Q8N8A6
Control	3	Q8N8J7
Control	3	Q8N8R3
Control	3	Q8N8S7
Control	3	Q8N8Z6
Control	3	Q8N954
Control	3	Q8N983
Control	3	Q8N999
Control	3	Q8N9F7
Control	3	Q8N9M1
Control	3	Q8N9N2
Control	3	Q8N9N7
Control	3	Q8N9N8
Control	3	Q8N9T8
Control	3	Q8N9Z2
Control	3	Q8NAF0
Control	3	Q8NAV1
Control	3	Q8NB16
Control	3	Q8NB37
Control	3	Q8NB46
Control	3	Q8NB49
Control	3	Q8NB90
Control	3	Q8NBF2
Control	3	Q8NBF6
Control	3	Q8NBI5
Control	3	Q8NBI6
Control	3	Q8NBJ4
Control	3	Q8NBJ5
Control	3	Q8NBJ7
Control	3	Q8NBJ9
Control	3	Q8NBK3
Control	3	Q8NBL1

Control	3	Q8NBM4
Control	3	Q8NBM8
Control	3	Q8NBN3
Control	3	Q8NBN7
Control	3	Q8NBP0
Control	3	Q8NBQ5
Control	3	Q8NBS9
Control	3	Q8NBT2
Control	3	Q8NBU5
Control	3	Q8NBX0
Control	3	Q8NBZ7
Control	3	Q8NC42
Control	3	Q8NC44
Control	3	Q8NC51
Control	3	Q8NC56
Control	3	Q8NC60
Control	3	Q8NC96
Control	3	Q8NCA5
Control	3	Q8NCC3
Control	3	Q8NCE2
Control	3	Q8NCF5
Control	3	Q8NCG7
Control	3	Q8NCH0
Control	3	Q8NCL4
Control	3	Q8NCN4
Control	3	Q8NCN5
Control	3	Q8NCW5
Control	3	Q8ND04
Control	3	Q8ND24
Control	3	Q8ND56
Control	3	Q8ND71
Control	3	Q8ND76
Control	3	Q8NDA8
Control	3	Q8NDF8
Control	3	Q8NDH3
Control	3	Q8NDI1
Control	3	Q8NDT2
Control	3	Q8NDV1
Control	3	Q8NDV7
Control	3	Q8NDX5
Control	3	Q8NDZ4
Control	3	Q8NE01
Control	3	Q8NE71
Control	3	Q8NE86
Control	3	Q8NEB9
Control	3	Q8NEC7
Control	3	Q8NEF9
Control	3	Q8NEJ9
Control	3	Q8NEM2
Control	3	Q8NEN9

Control	3	Q8NEU8
Control	3	Q8NEW0
Control	3	Q8NEY1
Control	3	Q8NEY8
Control	3	Q8NEZ3
Control	3	Q8NEZ5
Control	3	Q8NF37
Control	3	Q8NF64
Control	3	Q8NF91
Control	3	Q8NFA0
Control	3	Q8NFC6
Control	3	Q8NFD5
Control	3	Q8NFF5
Control	3	Q8NFG4
Control	3	Q8NFH3
Control	3	Q8NFH4
Control	3	Q8NFH5
Control	3	Q8NFI3
Control	3	Q8NFJ5
Control	3	Q8NFQ8
Control	3	Q8NFV4
Control	3	Q8NFW8
Control	3	Q8NFZ0
Control	3	Q8NG11
Control	3	Q8NG68
Control	3	Q8NHG7
Control	3	Q8NHH9
Control	3	Q8NHP6
Control	3	Q8NHP8
Control	3	Q8NHQ8
Control	3	Q8NHQ9
Control	3	Q8NHS3
Control	3	Q8NHU6
Control	3	Q8NHV1
Control	3	Q8NHV4
Control	3	Q8NI08
Control	3	Q8NI27
Control	3	Q8NI36
Control	3	Q8NI37
Control	3	Q8TA86
Control	3	Q8TAA9
Control	3	Q8TAD8
Control	3	Q8TAE8
Control	3	Q8TAF3
Control	3	Q8TAG9
Control	3	Q8TAQ2
Control	3	Q8TAT6
Control	3	Q8TB03
Control	3	Q8TB22
Control	3	Q8TB40

Control	3	Q8TB52
Control	3	Q8TB61
Control	3	Q8TB72
Control	3	Q8TB96
Control	3	Q8TBA6
Control	3	Q8TBB5
Control	3	Q8TBC3
Control	3	Q8TBC4
Control	3	Q8TBF2
Control	3	Q8TBM8
Control	3	Q8TBQ9
Control	3	Q8TBX8
Control	3	Q8TBZ3
Control	3	Q8TC07
Control	3	Q8TC12
Control	3	Q8TCA0
Control	3	Q8TCB0
Control	3	Q8TCC3
Control	3	Q8TCF1
Control	3	Q8TCG1
Control	3	Q8TCG2
Control	3	Q8TCJ2
Control	3	Q8TCS8
Control	3	Q8TCT7
Control	3	Q8TCT9
Control	3	Q8TCU6
Control	3	Q8TCY9
Control	3	Q8TD16
Control	3	Q8TD19
Control	3	Q8TD55
Control	3	Q8TDB6
Control	3	Q8TDD1
Control	3	Q8TDM6
Control	3	Q8TDN6
Control	3	Q8TDQ7
Control	3	Q8TDW0
Control	3	Q8TDX7
Control	3	Q8TDY2
Control	3	Q8TDZ2
Control	3	Q8TE02
Control	3	Q8TE77
Control	3	Q8TE82
Control	3	Q8TEA7
Control	3	Q8TEA8
Control	3	Q8TEB1
Control	3	Q8TED0
Control	3	Q8TED1
Control	3	Q8TED9
Control	3	Q8TEJ3
Control	3	Q8TEL6

Control	3	Q8TEP8
Control	3	Q8TEQ6
Control	3	Q8TEQ8
Control	3	Q8TEU7
Control	3	Q8TEV9
Control	3	Q8TEW0
Control	3	Q8TEX9
Control	3	Q8TEY7
Control	3	Q8TF01
Control	3	Q8TF05
Control	3	Q8TF42
Control	3	Q8TF74
Control	3	Q8WTS1
Control	3	Q8WTS6
Control	3	Q8WTT2
Control	3	Q8WTV0
Control	3	Q8WTW3
Control	3	Q8WU76
Control	3	Q8WU79
Control	3	Q8WU90
Control	3	Q8WUA2
Control	3	Q8WUA4
Control	3	Q8WUA7
Control	3	Q8WUB8
Control	3	Q8WUD1
Control	3	Q8WUD4
Control	3	Q8WUF5
Control	3	Q8WUF8
Control	3	Q8WUH1
Control	3	Q8WUH2
Control	3	Q8WUH6
Control	3	Q8WUI4
Control	3	Q8WUK0
Control	3	Q8WUM0
Control	3	Q8WUM4
Control	3	Q8WUM9
Control	3	Q8WUP2
Control	3	Q8WUQ7
Control	3	Q8WUX2
Control	3	Q8WUX9
Control	3	Q8WUY1
Control	3	Q8WUY8
Control	3	Q8WUZ0
Control	3	Q8WV22
Control	3	Q8WV92
Control	3	Q8WVB6
Control	3	Q8WVC0
Control	3	Q8WVC6
Control	3	Q8WVJ2
Control	3	Q8WVK2

Control	3	Q8WVM0
Control	3	Q8WVM7
Control	3	Q8WVM8
Control	3	Q8WVP5
Control	3	Q8WVQ1
Control	3	Q8WVT3
Control	3	Q8WVV9
Control	3	Q8WVX9
Control	3	Q8WVY7
Control	3	Q8WW01
Control	3	Q8WW12
Control	3	Q8WW22
Control	3	Q8WW59
Control	3	Q8WWC4
Control	3	Q8WWH5
Control	3	Q8WWI1
Control	3	Q8WWI5
Control	3	Q8WWK9
Control	3	Q8WWM7
Control	3	Q8WWN8
Control	3	Q8WWP7
Control	3	Q8WWY3
Control	3	Q8WX92
Control	3	Q8WX93
Control	3	Q8WXA3
Control	3	Q8WXA9
Control	3	Q8WXD5
Control	3	Q8WXE0
Control	3	Q8WXE1
Control	3	Q8WXF1
Control	3	Q8WXG6
Control	3	Q8WXH0
Control	3	Q8WXI9
Control	3	Q8WXW3
Control	3	Q8WXX5
Control	3	Q8WY22
Control	3	Q8WYA6
Control	3	Q8WYN0
Control	3	Q8WYP3
Control	3	Q8WYP5
Control	3	Q8WZ42
Control	3	Q8WZ75
Control	3	Q8WZ82
Control	3	Q8WZA9
Control	3	Q92466
Control	3	Q92478
Control	3	Q92499
Control	3	Q92503
Control	3	Q92508
Control	3	Q92520

Control	3	Q92522
Control	3	Q92530
Control	3	Q92538
Control	3	Q92540
Control	3	Q92541
Control	3	Q92542
Control	3	Q92543
Control	3	Q92544
Control	3	Q92545
Control	3	Q92551
Control	3	Q92552
Control	3	Q92556
Control	3	Q92562
Control	3	Q92567
Control	3	Q92572
Control	3	Q92574
Control	3	Q92575
Control	3	Q92576
Control	3	Q92597
Control	3	Q92598
Control	3	Q92599
Control	3	Q92600
Control	3	Q92609
Control	3	Q92610
Control	3	Q92613
Control	3	Q92614
Control	3	Q92615
Control	3	Q92616
Control	3	Q92619
Control	3	Q92620
Control	3	Q92621
Control	3	Q92625
Control	3	Q92626
Control	3	Q92636
Control	3	Q92643
Control	3	Q92665
Control	3	Q92667
Control	3	Q92685
Control	3	Q92686
Control	3	Q92688
Control	3	Q92692
Control	3	Q92696
Control	3	Q92734
Control	3	Q92743
Control	3	Q92747
Control	3	Q92759
Control	3	Q92766
Control	3	Q92769
Control	3	Q92783
Control	3	Q92785

Control	3	Q92791
Control	3	Q92793
Control	3	Q92794
Control	3	Q92797
Control	3	Q92804
Control	3	Q92805
Control	3	Q92820
Control	3	Q92823
Control	3	Q92834
Control	3	Q92835
Control	3	Q92841
Control	3	Q92845
Control	3	Q92851
Control	3	Q92870
Control	3	Q92878
Control	3	Q92879
Control	3	Q92882
Control	3	Q92888
Control	3	Q92889
Control	3	Q92890
Control	3	Q92896
Control	3	Q92900
Control	3	Q92905
Control	3	Q92917
Control	3	Q92922
Control	3	Q92925
Control	3	Q92930
Control	3	Q92934
Control	3	Q92945
Control	3	Q92947
Control	3	Q92963
Control	3	Q92968
Control	3	Q92973
Control	3	Q92974
Control	3	Q92979
Control	3	Q92989
Control	3	Q92990
Control	3	Q92995
Control	3	Q92997
Control	3	Q93008
Control	3	Q93009
Control	3	Q93034
Control	3	Q93045
Control	3	Q93050
Control	3	Q93052
Control	3	Q93062
Control	3	Q93063
Control	3	Q93074
Control	3	Q93096
Control	3	Q93100

Control	3	Q969E2
Control	3	Q969F9
Control	3	Q969G3
Control	3	Q969G5
Control	3	Q969G6
Control	3	Q969H8
Control	3	Q969M1
Control	3	Q969M3
Control	3	Q969N2
Control	3	Q969P0
Control	3	Q969Q0
Control	3	Q969Q5
Control	3	Q969R2
Control	3	Q969S3
Control	3	Q969S9
Control	3	Q969T4
Control	3	Q969T7
Control	3	Q969T9
Control	3	Q969U7
Control	3	Q969V3
Control	3	Q969V5
Control	3	Q969V6
Control	3	Q969X1
Control	3	Q969X5
Control	3	Q969X6
Control	3	Q969Z0
Control	3	Q969Z3
Control	3	Q96A26
Control	3	Q96A33
Control	3	Q96A35
Control	3	Q96A65
Control	3	Q96A72
Control	3	Q96A73
Control	3	Q96AA3
Control	3	Q96AB3
Control	3	Q96AB6
Control	3	Q96AC1
Control	3	Q96AE4
Control	3	Q96AE7
Control	3	Q96AG4
Control	3	Q96AJ9
Control	3	Q96AP7
Control	3	Q96AQ6
Control	3	Q96AQ8
Control	3	Q96AT1
Control	3	Q96AX1
Control	3	Q96AY3
Control	3	Q96AY4
Control	3	Q96B26
Control	3	Q96B54

Control	3	Q96B97
Control	3	Q96BD8
Control	3	Q96BF6
Control	3	Q96BH1
Control	3	Q96BJ3
Control	3	Q96BK5
Control	3	Q96BM9
Control	3	Q96BN8
Control	3	Q96BP2
Control	3	Q96BP3
Control	3	Q96BQ5
Control	3	Q96BR5
Control	3	Q96BW1
Control	3	Q96BW9
Control	3	Q96BX8
Control	3	Q96BY6
Control	3	Q96BY7
Control	3	Q96BZ8
Control	3	Q96BZ9
Control	3	Q96C19
Control	3	Q96C23
Control	3	Q96C24
Control	3	Q96C36
Control	3	Q96C57
Control	3	Q96C86
Control	3	Q96C90
Control	3	Q96CB8
Control	3	Q96CB9
Control	3	Q96CC6
Control	3	Q96CG8
Control	3	Q96CM8
Control	3	Q96CN7
Control	3	Q96CN9
Control	3	Q96CP2
Control	3	Q96CP6
Control	3	Q96CS2
Control	3	Q96CS3
Control	3	Q96CT7
Control	3	Q96CU9
Control	3	Q96CV9
Control	3	Q96CW1
Control	3	Q96CW5
Control	3	Q96CW6
Control	3	Q96CX2
Control	3	Q96D15
Control	3	Q96D46
Control	3	Q96D71
Control	3	Q96DA6
Control	3	Q96DB5
Control	3	Q96DE0

Control	3	Q96DF8
Control	3	Q96DG6
Control	3	Q96DH6
Control	3	Q96DI7
Control	3	Q96DM3
Control	3	Q96DV4
Control	3	Q96DX4
Control	3	Q96DZ1
Control	3	Q96E11
Control	3	Q96E29
Control	3	Q96EA4
Control	3	Q96EB1
Control	3	Q96EB6
Control	3	Q96EC8
Control	3	Q96EE3
Control	3	Q96EI5
Control	3	Q96EK5
Control	3	Q96EK6
Control	3	Q96EK7
Control	3	Q96EK9
Control	3	Q96EL2
Control	3	Q96EL3
Control	3	Q96EM0
Control	3	Q96EP0
Control	3	Q96EP5
Control	3	Q96EQ0
Control	3	Q96ER3
Control	3	Q96ER9
Control	3	Q96ES7
Control	3	Q96EU6
Control	3	Q96EU7
Control	3	Q96EV2
Control	3	Q96EV8
Control	3	Q96EY1
Control	3	Q96EY4
Control	3	Q96EY5
Control	3	Q96EY7
Control	3	Q96EY8
Control	3	Q96F07
Control	3	Q96F15
Control	3	Q96F44
Control	3	Q96F63
Control	3	Q96F85
Control	3	Q96F86
Control	3	Q96FJ2
Control	3	Q96FN4
Control	3	Q96FQ6
Control	3	Q96FS4
Control	3	Q96FV2
Control	3	Q96FV9

Control	3	Q96FW1
Control	3	Q96FX7
Control	3	Q96FZ2
Control	3	Q96FZ7
Control	3	Q96G01
Control	3	Q96G03
Control	3	Q96G21
Control	3	Q96G23
Control	3	Q96G46
Control	3	Q96GA3
Control	3	Q96GA7
Control	3	Q96GC5
Control	3	Q96GD0
Control	3	Q96GD4
Control	3	Q96GG9
Control	3	Q96GK7
Control	3	Q96GM5
Control	3	Q96GM8
Control	3	Q96GN5
Control	3	Q96GQ5
Control	3	Q96GQ7
Control	3	Q96GS4
Control	3	Q96GW9
Control	3	Q96GX2
Control	3	Q96GX5
Control	3	Q96GX9
Control	3	Q96GY0
Control	3	Q96GY3
Control	3	Q96GZ6
Control	3	Q96H20
Control	3	Q96H79
Control	3	Q96HA1
Control	3	Q96HA7
Control	3	Q96HC4
Control	3	Q96HD1
Control	3	Q96HE7
Control	3	Q96HE9
Control	3	Q96HJ9
Control	3	Q96HN2
Control	3	Q96HP0
Control	3	Q96HR3
Control	3	Q96HS1
Control	3	Q96HW7
Control	3	Q96HY6
Control	3	Q96HY7
Control	3	Q96I15
Control	3	Q96I24
Control	3	Q96I25
Control	3	Q96I51
Control	3	Q96I59

Control	3	Q96I99
Control	3	Q96II8
Control	3	Q96IJ6
Control	3	Q96IR7
Control	3	Q96IU4
Control	3	Q96IV0
Control	3	Q96IW7
Control	3	Q96IX5
Control	3	Q96IY1
Control	3	Q96IZ0
Control	3	Q96IZ6
Control	3	Q96J01
Control	3	Q96J02
Control	3	Q96J42
Control	3	Q96J84
Control	3	Q96JA1
Control	3	Q96JB2
Control	3	Q96JB5
Control	3	Q96JC1
Control	3	Q96JG6
Control	3	Q96JH7
Control	3	Q96JI7
Control	3	Q96JJ3
Control	3	Q96JJ7
Control	3	Q96JK2
Control	3	Q96JM3
Control	3	Q96JM7
Control	3	Q96JP5
Control	3	Q96JQ0
Control	3	Q96JQ2
Control	3	Q96JY6
Control	3	Q96K12
Control	3	Q96K17
Control	3	Q96K19
Control	3	Q96K37
Control	3	Q96K76
Control	3	Q96K83
Control	3	Q96KA5
Control	3	Q96KB5
Control	3	Q96KC2
Control	3	Q96KC8
Control	3	Q96KG9
Control	3	Q96KM6
Control	3	Q96KN1
Control	3	Q96KP1
Control	3	Q96KP4
Control	3	Q96KQ7
Control	3	Q96KR1
Control	3	Q96KR6
Control	3	Q96L58

Control	3	Q96L91
Control	3	Q96L92
Control	3	Q96L93
Control	3	Q96LB3
Control	3	Q96LD4
Control	3	Q96LI5
Control	3	Q96LJ7
Control	3	Q96LL9
Control	3	Q96LR5
Control	3	Q96LW7
Control	3	Q96M27
Control	3	Q96M96
Control	3	Q96ME1
Control	3	Q96ME7
Control	3	Q96MF7
Control	3	Q96MG7
Control	3	Q96MG8
Control	3	Q96MM6
Control	3	Q96MU7
Control	3	Q96MW1
Control	3	Q96MW5
Control	3	Q96MX0
Control	3	Q96MX6
Control	3	Q96MY1
Control	3	Q96N66
Control	3	Q96N67
Control	3	Q96NB2
Control	3	Q96NB3
Control	3	Q96ND0
Control	3	Q96NE9
Control	3	Q96P11
Control	3	Q96P16
Control	3	Q96P47
Control	3	Q96P48
Control	3	Q96P70
Control	3	Q96PC5
Control	3	Q96PD2
Control	3	Q96PE2
Control	3	Q96PE3
Control	3	Q96PK6
Control	3	Q96PQ7
Control	3	Q96PU4
Control	3	Q96PU5
Control	3	Q96PU8
Control	3	Q96PY5
Control	3	Q96PZ0
Control	3	Q96PZ2
Control	3	Q96Q05
Control	3	Q96Q11
Control	3	Q96Q15

Control	3	Q96Q42
Control	3	Q96Q45
Control	3	Q96Q83
Control	3	Q96QB1
Control	3	Q96QC0
Control	3	Q96QD8
Control	3	Q96QD9
Control	3	Q96QG7
Control	3	Q96QK1
Control	3	Q96QR8
Control	3	Q96QU8
Control	3	Q96QV1
Control	3	Q96QZ7
Control	3	Q96R06
Control	3	Q96RE7
Control	3	Q96RF0
Control	3	Q96RG2
Control	3	Q96RK0
Control	3	Q96RL1
Control	3	Q96RL7
Control	3	Q96RN5
Control	3	Q96RP9
Control	3	Q96RQ1
Control	3	Q96RQ3
Control	3	Q96RR4
Control	3	Q96RS0
Control	3	Q96RS6
Control	3	Q96RT1
Control	3	Q96RT7
Control	3	Q96RT8
Control	3	Q96RU2
Control	3	Q96RU3
Control	3	Q96S44
Control	3	Q96S52
Control	3	Q96S55
Control	3	Q96S59
Control	3	Q96S66
Control	3	Q96S97
Control	3	Q96SB3
Control	3	Q96SB4
Control	3	Q96SB8
Control	3	Q96SI1
Control	3	Q96SI9
Control	3	Q96SK2
Control	3	Q96SL4
Control	3	Q96SQ9
Control	3	Q96ST2
Control	3	Q96ST3
Control	3	Q96SU4
Control	3	Q96SW2

Control	3	Q96SY0
Control	3	Q96SZ5
Control	3	Q96SZ6
Control	3	Q96T23
Control	3	Q96T37
Control	3	Q96T51
Control	3	Q96T58
Control	3	Q96T60
Control	3	Q96T76
Control	3	Q96T88
Control	3	Q96TA1
Control	3	Q96TA2
Control	3	Q96TC7
Control	3	Q99081
Control	3	Q99418
Control	3	Q99426
Control	3	Q99436
Control	3	Q99439
Control	3	Q99442
Control	3	Q99447
Control	3	Q99459
Control	3	Q99460
Control	3	Q99470
Control	3	Q99471
Control	3	Q99496
Control	3	Q99497
Control	3	Q99501
Control	3	Q99504
Control	3	Q99519
Control	3	Q99523
Control	3	Q99536
Control	3	Q99538
Control	3	Q99541
Control	3	Q99543
Control	3	Q99549
Control	3	Q99567
Control	3	Q99569
Control	3	Q99570
Control	3	Q99571
Control	3	Q99575
Control	3	Q99584
Control	3	Q99590
Control	3	Q99598
Control	3	Q99611
Control	3	Q99613
Control	3	Q99614
Control	3	Q99615
Control	3	Q99623
Control	3	Q99627
Control	3	Q99633

Control	3	Q99653
Control	3	Q99661
Control	3	Q99666
Control	3	Q99685
Control	3	Q99700
Control	3	Q99704
Control	3	Q99707
Control	3	Q99714
Control	3	Q99715
Control	3	Q99717
Control	3	Q99720
Control	3	Q99729
Control	3	Q99733
Control	3	Q99735
Control	3	Q99747
Control	3	Q99755
Control	3	Q99758
Control	3	Q99759
Control	3	Q99766
Control	3	Q99797
Control	3	Q99798
Control	3	Q99805
Control	3	Q99808
Control	3	Q99816
Control	3	Q99829
Control	3	Q99832
Control	3	Q99836
Control	3	Q99848
Control	3	Q99873
Control	3	Q99933
Control	3	Q99941
Control	3	Q99958
Control	3	Q99961
Control	3	Q99973
Control	3	Q99986
Control	3	Q99988
Control	3	Q99996
Control	3	Q9BPW8
Control	3	Q9BPX3
Control	3	Q9BPX5
Control	3	Q9BPX6
Control	3	Q9BPX7
Control	3	Q9BPY3
Control	3	Q9BQ04
Control	3	Q9BQ24
Control	3	Q9BQ39
Control	3	Q9BQ48
Control	3	Q9BQ52
Control	3	Q9BQ61
Control	3	Q9BQ67

Control	3	Q9BQ69
Control	3	Q9BQ70
Control	3	Q9BQ75
Control	3	Q9BQ90
Control	3	Q9BQ95
Control	3	Q9BQA1
Control	3	Q9BQA9
Control	3	Q9BQB6
Control	3	Q9BQC3
Control	3	Q9BQE3
Control	3	Q9BQE4
Control	3	Q9BQE5
Control	3	Q9BQG0
Control	3	Q9BQP7
Control	3	Q9BQQ3
Control	3	Q9BQS8
Control	3	Q9BR61
Control	3	Q9BR76
Control	3	Q9BRA2
Control	3	Q9BRD0
Control	3	Q9BRF8
Control	3	Q9BRG1
Control	3	Q9BRJ2
Control	3	Q9BRJ6
Control	3	Q9BRJ7
Control	3	Q9BRK5
Control	3	Q9BRP1
Control	3	Q9BRP4
Control	3	Q9BRP8
Control	3	Q9BRQ6
Control	3	Q9BRR6
Control	3	Q9BRR8
Control	3	Q9BRS2
Control	3	Q9BRT6
Control	3	Q9BRT9
Control	3	Q9BRU9
Control	3	Q9BRX2
Control	3	Q9BRX8
Control	3	Q9BRZ2
Control	3	Q9BS26
Control	3	Q9BS40
Control	3	Q9BSC4
Control	3	Q9BSD7
Control	3	Q9BSF4
Control	3	Q9BSH4
Control	3	Q9BSJ2
Control	3	Q9BSJ8
Control	3	Q9BSL1
Control	3	Q9BSR8
Control	3	Q9BSU1

Control	3	Q9BSV6
Control	3	Q9BT09
Control	3	Q9BT17
Control	3	Q9BT22
Control	3	Q9BT23
Control	3	Q9BT25
Control	3	Q9BT40
Control	3	Q9BT73
Control	3	Q9BT78
Control	3	Q9BT88
Control	3	Q9BTA9
Control	3	Q9BTC0
Control	3	Q9BTC8
Control	3	Q9BTD8
Control	3	Q9BTE1
Control	3	Q9BTE3
Control	3	Q9BTE6
Control	3	Q9BTE7
Control	3	Q9BTT0
Control	3	Q9BTT6
Control	3	Q9BTU6
Control	3	Q9BTV4
Control	3	Q9BTV5
Control	3	Q9BTW9
Control	3	Q9BTX1
Control	3	Q9BTY7
Control	3	Q9BTZ2
Control	3	Q9BU23
Control	3	Q9BU61
Control	3	Q9BU76
Control	3	Q9BUB7
Control	3	Q9BUE0
Control	3	Q9BUE6
Control	3	Q9BUF5
Control	3	Q9BUH6
Control	3	Q9BUI4
Control	3	Q9BUJ2
Control	3	Q9BUK6
Control	3	Q9BUL5
Control	3	Q9BUL8
Control	3	Q9BUL9
Control	3	Q9BUN8
Control	3	Q9BUP3
Control	3	Q9BUQ8
Control	3	Q9BUR4
Control	3	Q9BUR5
Control	3	Q9BUT1
Control	3	Q9BV20
Control	3	Q9BV38
Control	3	Q9BV40

Control	3	Q9BV44
Control	3	Q9BV57
Control	3	Q9BV79
Control	3	Q9BV81
Control	3	Q9BV86
Control	3	Q9BVA0
Control	3	Q9BVC4
Control	3	Q9BVC5
Control	3	Q9BVC6
Control	3	Q9BVG4
Control	3	Q9BVG9
Control	3	Q9BVI4
Control	3	Q9BVJ6
Control	3	Q9BVJ7
Control	3	Q9BVK6
Control	3	Q9BVK8
Control	3	Q9BVL2
Control	3	Q9BVL4
Control	3	Q9BVM2
Control	3	Q9BVP2
Control	3	Q9BVQ7
Control	3	Q9BVS4
Control	3	Q9BVS5
Control	3	Q9BVT8
Control	3	Q9BVV7
Control	3	Q9BW19
Control	3	Q9BW27
Control	3	Q9BW60
Control	3	Q9BW62
Control	3	Q9BW71
Control	3	Q9BW83
Control	3	Q9BW85
Control	3	Q9BW91
Control	3	Q9BW92
Control	3	Q9BWD1
Control	3	Q9BWE0
Control	3	Q9BWF3
Control	3	Q9BWH2
Control	3	Q9BWH6
Control	3	Q9BWJ5
Control	3	Q9BWM7
Control	3	Q9BWN1
Control	3	Q9BWS9
Control	3	Q9BWT3
Control	3	Q9BWT7
Control	3	Q9BWU0
Control	3	Q9BWU1
Control	3	Q9BX40
Control	3	Q9BX59
Control	3	Q9BX67

Control	3	Q9BX69
Control	3	Q9BX70
Control	3	Q9BX95
Control	3	Q9BXB4
Control	3	Q9BXB5
Control	3	Q9BXF6
Control	3	Q9BXI6
Control	3	Q9BXJ0
Control	3	Q9BXJ9
Control	3	Q9BXK1
Control	3	Q9BXK5
Control	3	Q9BXL7
Control	3	Q9BXP2
Control	3	Q9BXP5
Control	3	Q9BXR0
Control	3	Q9BXS5
Control	3	Q9BXS6
Control	3	Q9BXW7
Control	3	Q9BXW9
Control	3	Q9BXY0
Control	3	Q9BY41
Control	3	Q9BY42
Control	3	Q9BY43
Control	3	Q9BY44
Control	3	Q9BY77
Control	3	Q9BY89
Control	3	Q9BYB0
Control	3	Q9BYB4
Control	3	Q9BYC5
Control	3	Q9BYC8
Control	3	Q9BYC9
Control	3	Q9BYD1
Control	3	Q9BYD2
Control	3	Q9BYD3
Control	3	Q9BYD6
Control	3	Q9BYG3
Control	3	Q9BYI3
Control	3	Q9BYJ9
Control	3	Q9BYK8
Control	3	Q9BYM8
Control	3	Q9BYN8
Control	3	Q9BYT8
Control	3	Q9BYV8
Control	3	Q9BYW2
Control	3	Q9BZ23
Control	3	Q9BZ29
Control	3	Q9BZ67
Control	3	Q9BZ76
Control	3	Q9BZ95
Control	3	Q9BZD4

Control	3	Q9BZE1
Control	3	Q9BZE4
Control	3	Q9BZE9
Control	3	Q9BZF1
Control	3	Q9BZF9
Control	3	Q9BZG1
Control	3	Q9BZG8
Control	3	Q9BZH6
Control	3	Q9BZI7
Control	3	Q9BZJ0
Control	3	Q9BZK7
Control	3	Q9BZL1
Control	3	Q9BZL4
Control	3	Q9BZL6
Control	3	Q9BZQ6
Control	3	Q9BZQ8
Control	3	Q9BZV1
Control	3	Q9BZW5
Control	3	Q9BZX2
Control	3	Q9BZZ2
Control	3	Q9BZZ5
Control	3	Q9C000
Control	3	Q9C035
Control	3	Q9C037
Control	3	Q9C0B1
Control	3	Q9C0B5
Control	3	Q9C0B7
Control	3	Q9C0C2
Control	3	Q9C0C9
Control	3	Q9C0D3
Control	3	Q9C0D5
Control	3	Q9C0D9
Control	3	Q9C0E2
Control	3	Q9C0E8
Control	3	Q9C0F1
Control	3	Q9C0H2
Control	3	Q9C0H6
Control	3	Q9C0I1
Control	3	Q9C0J8
Control	3	Q9GZL7
Control	3	Q9GZM5
Control	3	Q9GZM7
Control	3	Q9GZN8
Control	3	Q9GZP4
Control	3	Q9GZP9
Control	3	Q9GZQ3
Control	3	Q9GZR1
Control	3	Q9GZR2
Control	3	Q9GZR7
Control	3	Q9GZS1

Control	3	Q9GZS3
Control	3	Q9GZT3
Control	3	Q9GZT4
Control	3	Q9GZT8
Control	3	Q9GZT9
Control	3	Q9GZU1
Control	3	Q9GZU8
Control	3	Q9GZY8
Control	3	Q9GZZ1
Control	3	Q9GZZ9
Control	3	Q9H000
Control	3	Q9H061
Control	3	Q9H074
Control	3	Q9H078
Control	3	Q9H082
Control	3	Q9H089
Control	3	Q9H098
Control	3	Q9H0A0
Control	3	Q9H0A8
Control	3	Q9H0B6
Control	3	Q9H0C8
Control	3	Q9H0D6
Control	3	Q9H0E2
Control	3	Q9H0E3
Control	3	Q9H0E9
Control	3	Q9H0G5
Control	3	Q9H0H0
Control	3	Q9H0H5
Control	3	Q9H0J9
Control	3	Q9H0L4
Control	3	Q9H0P0
Control	3	Q9H0Q0
Control	3	Q9H0R3
Control	3	Q9H0R4
Control	3	Q9H0R6
Control	3	Q9H0S4
Control	3	Q9H0U3
Control	3	Q9H0U4
Control	3	Q9H0U6
Control	3	Q9H0U9
Control	3	Q9H0V1
Control	3	Q9H0V9
Control	3	Q9H0W8
Control	3	Q9H0X4
Control	3	Q9H0X9
Control	3	Q9H147
Control	3	Q9H173
Control	3	Q9H1A3
Control	3	Q9H1A4
Control	3	Q9H1B7

Control	3	Q9H1C7
Control	3	Q9H1D9
Control	3	Q9H1E3
Control	3	Q9H1E5
Control	3	Q9H1H9
Control	3	Q9H1I8
Control	3	Q9H1K0
Control	3	Q9H1K1
Control	3	Q9H1P3
Control	3	Q9H1Y0
Control	3	Q9H1Z4
Control	3	Q9H201
Control	3	Q9H223
Control	3	Q9H267
Control	3	Q9H269
Control	3	Q9H270
Control	3	Q9H299
Control	3	Q9H2C0
Control	3	Q9H2D1
Control	3	Q9H2D6
Control	3	Q9H2F5
Control	3	Q9H2G2
Control	3	Q9H2H8
Control	3	Q9H2H9
Control	3	Q9H2J4
Control	3	Q9H2K8
Control	3	Q9H2M9
Control	3	Q9H2P0
Control	3	Q9H2P9
Control	3	Q9H2U1
Control	3	Q9H2U2
Control	3	Q9H2V7
Control	3	Q9H2W6
Control	3	Q9H2X9
Control	3	Q9H300
Control	3	Q9H307
Control	3	Q9H330
Control	3	Q9H334
Control	3	Q9H3F6
Control	3	Q9H3H3
Control	3	Q9H3K2
Control	3	Q9H3K6
Control	3	Q9H3L0
Control	3	Q9H3M7
Control	3	Q9H3N1
Control	3	Q9H3P2
Control	3	Q9H3P7
Control	3	Q9H3Q1
Control	3	Q9H3S7
Control	3	Q9H3T3

Control	3	Q9H3U1
Control	3	Q9H3U5
Control	3	Q9H3Z4
Control	3	Q9H410
Control	3	Q9H444
Control	3	Q9H446
Control	3	Q9H467
Control	3	Q9H479
Control	3	Q9H488
Control	3	Q9H490
Control	3	Q9H497
Control	3	Q9H4A3
Control	3	Q9H4A4
Control	3	Q9H4A5
Control	3	Q9H4A6
Control	3	Q9H4B0
Control	3	Q9H4F1
Control	3	Q9H4G4
Control	3	Q9H4I3
Control	3	Q9H4K7
Control	3	Q9H4L4
Control	3	Q9H4L5
Control	3	Q9H4L7
Control	3	Q9H4M9
Control	3	Q9H4Z3
Control	3	Q9H501
Control	3	Q9H553
Control	3	Q9H583
Control	3	Q9H5K3
Control	3	Q9H5N1
Control	3	Q9H5Q4
Control	3	Q9H5U6
Control	3	Q9H5V9
Control	3	Q9H5X1
Control	3	Q9H5Z1
Control	3	Q9H5Z6
Control	3	Q9H6D7
Control	3	Q9H6E4
Control	3	Q9H6F5
Control	3	Q9H6H4
Control	3	Q9H6I2
Control	3	Q9H6K4
Control	3	Q9H6Q4
Control	3	Q9H6R0
Control	3	Q9H6R4
Control	3	Q9H6R7
Control	3	Q9H6S0
Control	3	Q9H6S1
Control	3	Q9H6T3
Control	3	Q9H6U6

Control	3	Q9H6U8
Control	3	Q9H6V9
Control	3	Q9H6W3
Control	3	Q9H6Y2
Control	3	Q9H6Z4
Control	3	Q9H792
Control	3	Q9H7B2
Control	3	Q9H7B4
Control	3	Q9H7C9
Control	3	Q9H7D0
Control	3	Q9H7D7
Control	3	Q9H7E2
Control	3	Q9H7E9
Control	3	Q9H7F0
Control	3	Q9H7H0
Control	3	Q9H7N4
Control	3	Q9H7Z3
Control	3	Q9H7Z7
Control	3	Q9H814
Control	3	Q9H832
Control	3	Q9H845
Control	3	Q9H857
Control	3	Q9H875
Control	3	Q9H8G2
Control	3	Q9H8H0
Control	3	Q9H8H2
Control	3	Q9H8H3
Control	3	Q9H8K7
Control	3	Q9H8L6
Control	3	Q9H8M7
Control	3	Q9H8M9
Control	3	Q9H8T0
Control	3	Q9H8W4
Control	3	Q9H8Y5
Control	3	Q9H8Y8
Control	3	Q9H900
Control	3	Q9H910
Control	3	Q9H936
Control	3	Q9H939
Control	3	Q9H944
Control	3	Q9H967
Control	3	Q9H974
Control	3	Q9H981
Control	3	Q9H993
Control	3	Q9H999
Control	3	Q9H9A5
Control	3	Q9H9A6
Control	3	Q9H9B1
Control	3	Q9H9B4
Control	3	Q9H9C1

Control	3	Q9H9E3
Control	3	Q9H9F9
Control	3	Q9H9H4
Control	3	Q9H9J2
Control	3	Q9H9P8
Control	3	Q9H9Q2
Control	3	Q9H9Q4
Control	3	Q9H9S3
Control	3	Q9H9S5
Control	3	Q9H9T3
Control	3	Q9H9Y2
Control	3	Q9H9Y6
Control	3	Q9HA38
Control	3	Q9HA47
Control	3	Q9HA64
Control	3	Q9HA65
Control	3	Q9HA77
Control	3	Q9HAB3
Control	3	Q9HAB8
Control	3	Q9HAC8
Control	3	Q9HAD4
Control	3	Q9HAN9
Control	3	Q9HAS0
Control	3	Q9HAT2
Control	3	Q9HAU0
Control	3	Q9HAU4
Control	3	Q9HAU5
Control	3	Q9HAV0
Control	3	Q9HAV4
Control	3	Q9HAV7
Control	3	Q9HB07
Control	3	Q9HB20
Control	3	Q9HB21
Control	3	Q9HB40
Control	3	Q9HB63
Control	3	Q9HB71
Control	3	Q9HB90
Control	3	Q9HBF4
Control	3	Q9HBG6
Control	3	Q9HBH5
Control	3	Q9HBI1
Control	3	Q9HBL0
Control	3	Q9HBL7
Control	3	Q9HBL8
Control	3	Q9HBM0
Control	3	Q9HBM1
Control	3	Q9HBM6
Control	3	Q9HBR0
Control	3	Q9HBU6
Control	3	Q9HBW9

Control	3	Q9HC07
Control	3	Q9HC21
Control	3	Q9HC35
Control	3	Q9HC36
Control	3	Q9HC38
Control	3	Q9HC98
Control	3	Q9HCC0
Control	3	Q9HCD5
Control	3	Q9HCE0
Control	3	Q9HCE1
Control	3	Q9HCE5
Control	3	Q9HCG7
Control	3	Q9HCG8
Control	3	Q9HCJ3
Control	3	Q9HCK8
Control	3	Q9HCM4
Control	3	Q9HCN4
Control	3	Q9HCN8
Control	3	Q9HCP0
Control	3	Q9HCS7
Control	3	Q9HCU5
Control	3	Q9HD15
Control	3	Q9HD20
Control	3	Q9HD26
Control	3	Q9HD33
Control	3	Q9HD34
Control	3	Q9HD40
Control	3	Q9HD42
Control	3	Q9HD45
Control	3	Q9HD67
Control	3	Q9HDC9
Control	3	Q9NNW5
Control	3	Q9NNW7
Control	3	Q9NP58
Control	3	Q9NP61
Control	3	Q9NP64
Control	3	Q9NP72
Control	3	Q9NP74
Control	3	Q9NP77
Control	3	Q9NP79
Control	3	Q9NP81
Control	3	Q9NP92
Control	3	Q9NP97
Control	3	Q9NPA0
Control	3	Q9NPA8
Control	3	Q9NPD3
Control	3	Q9NPD8
Control	3	Q9NPE3
Control	3	Q9NPF4
Control	3	Q9NPF5

Control	3	Q9NPG4
Control	3	Q9NPH2
Control	3	Q9NPI1
Control	3	Q9NPI6
Control	3	Q9NPJ3
Control	3	Q9NPJ6
Control	3	Q9NPL8
Control	3	Q9NPQ8
Control	3	Q9NPY3
Control	3	Q9NQ29
Control	3	Q9NQ30
Control	3	Q9NQ48
Control	3	Q9NQ50
Control	3	Q9NQ55
Control	3	Q9NQ66
Control	3	Q9NQ88
Control	3	Q9NQ89
Control	3	Q9NQC3
Control	3	Q9NQG5
Control	3	Q9NQH7
Control	3	Q9NQR4
Control	3	Q9NQS1
Control	3	Q9NQS7
Control	3	Q9NQT4
Control	3	Q9NQT5
Control	3	Q9NQT8
Control	3	Q9NQW6
Control	3	Q9NQW7
Control	3	Q9NQX3
Control	3	Q9NQY0
Control	3	Q9NQZ2
Control	3	Q9NQZ5
Control	3	Q9NR09
Control	3	Q9NR12
Control	3	Q9NR19
Control	3	Q9NR28
Control	3	Q9NR30
Control	3	Q9NR31
Control	3	Q9NR45
Control	3	Q9NR46
Control	3	Q9NR48
Control	3	Q9NR50
Control	3	Q9NR56
Control	3	Q9NRA2
Control	3	Q9NRA8
Control	3	Q9NRB3
Control	3	Q9NRC1
Control	3	Q9NRD5
Control	3	Q9NRF2
Control	3	Q9NRF8

Control	3	Q9NRF9
Control	3	Q9NRG0
Control	3	Q9NRG9
Control	3	Q9NRH2
Control	3	Q9NRK6
Control	3	Q9NRL2
Control	3	Q9NRL3
Control	3	Q9NRN7
Control	3	Q9NRN9
Control	3	Q9NRP0
Control	3	Q9NRP2
Control	3	Q9NRQ2
Control	3	Q9NRR4
Control	3	Q9NRS6
Control	3	Q9NRV9
Control	3	Q9NRW3
Control	3	Q9NRW7
Control	3	Q9NRX1
Control	3	Q9NRX2
Control	3	Q9NRX4
Control	3	Q9NRX5
Control	3	Q9NRY4
Control	3	Q9NRY5
Control	3	Q9NRY6
Control	3	Q9NRZ5
Control	3	Q9NRZ7
Control	3	Q9NRZ9
Control	3	Q9NS00
Control	3	Q9NS15
Control	3	Q9NS69
Control	3	Q9NS86
Control	3	Q9NS87
Control	3	Q9NS93
Control	3	Q9NSC5
Control	3	Q9NSD9
Control	3	Q9NSE4
Control	3	Q9NSI2
Control	3	Q9NSI8
Control	3	Q9NSK0
Control	3	Q9NSV4
Control	3	Q9NSY1
Control	3	Q9NT62
Control	3	Q9NTG7
Control	3	Q9NTI5
Control	3	Q9NTJ3
Control	3	Q9NTJ4
Control	3	Q9NTJ5
Control	3	Q9NTK1
Control	3	Q9NTK5
Control	3	Q9NTM9

Control	3	Q9NTW7
Control	3	Q9NTX5
Control	3	Q9NTZ6
Control	3	Q9NU19
Control	3	Q9NU22
Control	3	Q9NUB1
Control	3	Q9NUD5
Control	3	Q9NUI1
Control	3	Q9NUJ1
Control	3	Q9NUJ3
Control	3	Q9NUL3
Control	3	Q9NUL7
Control	3	Q9NUM4
Control	3	Q9NUP9
Control	3	Q9NUQ2
Control	3	Q9NUQ3
Control	3	Q9NUQ6
Control	3	Q9NUQ7
Control	3	Q9NUQ8
Control	3	Q9NUQ9
Control	3	Q9NUT2
Control	3	Q9NUU7
Control	3	Q9NUV9
Control	3	Q9NUW8
Control	3	Q9NUX5
Control	3	Q9NUY8
Control	3	Q9NV06
Control	3	Q9NV31
Control	3	Q9NV56
Control	3	Q9NV66
Control	3	Q9NV70
Control	3	Q9NV79
Control	3	Q9NV88
Control	3	Q9NV96
Control	3	Q9NVA1
Control	3	Q9NVA2
Control	3	Q9NVC3
Control	3	Q9NVC6
Control	3	Q9NVD7
Control	3	Q9NVE5
Control	3	Q9NVE7
Control	3	Q9NVF7
Control	3	Q9NVG8
Control	3	Q9NVH0
Control	3	Q9NVH1
Control	3	Q9NVH2
Control	3	Q9NVI1
Control	3	Q9NVI7
Control	3	Q9NVJ2
Control	3	Q9NVM6

Control	3	Q9NVM9
Control	3	Q9NVN3
Control	3	Q9NVN8
Control	3	Q9NVP1
Control	3	Q9NVP2
Control	3	Q9NVR0
Control	3	Q9NVR2
Control	3	Q9NVS2
Control	3	Q9NVS9
Control	3	Q9NVT9
Control	3	Q9NVU0
Control	3	Q9NVU7
Control	3	Q9NVV4
Control	3	Q9NVX2
Control	3	Q9NVZ3
Control	3	Q9NW08
Control	3	Q9NW13
Control	3	Q9NW15
Control	3	Q9NW64
Control	3	Q9NW81
Control	3	Q9NW82
Control	3	Q9NW97
Control	3	Q9NWB6
Control	3	Q9NWB7
Control	3	Q9NWH9
Control	3	Q9NWM8
Control	3	Q9NWS8
Control	3	Q9NWT1
Control	3	Q9NWT6
Control	3	Q9NWU1
Control	3	Q9NWU5
Control	3	Q9NWV4
Control	3	Q9NWV8
Control	3	Q9NWW5
Control	3	Q9NWX5
Control	3	Q9NWX6
Control	3	Q9NWY4
Control	3	Q9NWZ3
Control	3	Q9NWZ5
Control	3	Q9NX00
Control	3	Q9NX02
Control	3	Q9NX05
Control	3	Q9NX07
Control	3	Q9NX14
Control	3	Q9NX20
Control	3	Q9NX24
Control	3	Q9NX40
Control	3	Q9NX46
Control	3	Q9NX47
Control	3	Q9NX58

Control	3	Q9NX61
Control	3	Q9NX62
Control	3	Q9NX63
Control	3	Q9NX74
Control	3	Q9NX76
Control	3	Q9NXA8
Control	3	Q9NXC5
Control	3	Q9NXD2
Control	3	Q9NXE4
Control	3	Q9NXE8
Control	3	Q9NXF1
Control	3	Q9NXF7
Control	3	Q9NXG2
Control	3	Q9NXG6
Control	3	Q9NXH8
Control	3	Q9NXH9
Control	3	Q9NXN4
Control	3	Q9NXR1
Control	3	Q9NXR7
Control	3	Q9NXS2
Control	3	Q9NXU5
Control	3	Q9NXV2
Control	3	Q9NXV6
Control	3	Q9NXW2
Control	3	Q9NXW9
Control	3	Q9NXX6
Control	3	Q9NY12
Control	3	Q9NY15
Control	3	Q9NY27
Control	3	Q9NY33
Control	3	Q9NY61
Control	3	Q9NY65
Control	3	Q9NY93
Control	3	Q9NYA1
Control	3	Q9NYB0
Control	3	Q9NYB9
Control	3	Q9NYF8
Control	3	Q9NYH9
Control	3	Q9NYJ8
Control	3	Q9NYK5
Control	3	Q9NYL2
Control	3	Q9NYL4
Control	3	Q9NYL9
Control	3	Q9NYM9
Control	3	Q9NYP7
Control	3	Q9NYR9
Control	3	Q9NYU1
Control	3	Q9NYU2
Control	3	Q9NYV4
Control	3	Q9NYY8

Control	3	Q9NZ01
Control	3	Q9NZ08
Control	3	Q9NZ32
Control	3	Q9NZ43
Control	3	Q9NZ45
Control	3	Q9NZ52
Control	3	Q9NZ63
Control	3	Q9NZB2
Control	3	Q9NZC3
Control	3	Q9NZC9
Control	3	Q9NZD2
Control	3	Q9NZD8
Control	3	Q9NZE8
Control	3	Q9NZI7
Control	3	Q9NZI8
Control	3	Q9NZJ4
Control	3	Q9NZJ7
Control	3	Q9NZJ9
Control	3	Q9NZL4
Control	3	Q9NZL9
Control	3	Q9NZM1
Control	3	Q9NZM3
Control	3	Q9NZM5
Control	3	Q9NZN3
Control	3	Q9NZN4
Control	3	Q9NZN5
Control	3	Q9NZN8
Control	3	Q9NZQ3
Control	3	Q9NZQ7
Control	3	Q9NZT1
Control	3	Q9NZT2
Control	3	Q9NZU5
Control	3	Q9NZV1
Control	3	Q9NZV5
Control	3	Q9NZW5
Control	3	Q9NZZ3
Control	3	Q9P013
Control	3	Q9P015
Control	3	Q9P016
Control	3	Q9P031
Control	3	Q9P032
Control	3	Q9P035
Control	3	Q9P0I2
Control	3	Q9P0J0
Control	3	Q9P0J1
Control	3	Q9P0J7
Control	3	Q9P0K7
Control	3	Q9P0L0
Control	3	Q9P0M9
Control	3	Q9P0T7

Control	3	Q9P0U3
Control	3	Q9P0U4
Control	3	Q9P0V3
Control	3	Q9P0V9
Control	3	Q9P107
Control	3	Q9P1Y6
Control	3	Q9P215
Control	3	Q9P219
Control	3	Q9P227
Control	3	Q9P253
Control	3	Q9P258
Control	3	Q9P260
Control	3	Q9P265
Control	3	Q9P266
Control	3	Q9P270
Control	3	Q9P273
Control	3	Q9P275
Control	3	Q9P287
Control	3	Q9P289
Control	3	Q9P291
Control	3	Q9P2A4
Control	3	Q9P2B2
Control	3	Q9P2B4
Control	3	Q9P2C4
Control	3	Q9P2D3
Control	3	Q9P2E3
Control	3	Q9P2E5
Control	3	Q9P2E7
Control	3	Q9P2E9
Control	3	Q9P2I0
Control	3	Q9P2J3
Control	3	Q9P2J5
Control	3	Q9P2K5
Control	3	Q9P2K6
Control	3	Q9P2K8
Control	3	Q9P2L0
Control	3	Q9P2N5
Control	3	Q9P2N6
Control	3	Q9P2P5
Control	3	Q9P2Q2
Control	3	Q9P2R3
Control	3	Q9P2R6
Control	3	Q9P2R7
Control	3	Q9P2T1
Control	3	Q9P2W9
Control	3	Q9P2X0
Control	3	Q9P2X3
Control	3	Q9P2Y5
Control	3	Q9UBB4
Control	3	Q9UBB5

Control	3	Q9UBB6
Control	3	Q9UBB9
Control	3	Q9UBC2
Control	3	Q9UBC5
Control	3	Q9UBD5
Control	3	Q9UBE0
Control	3	Q9UBF2
Control	3	Q9UBF8
Control	3	Q9UBG0
Control	3	Q9UBI1
Control	3	Q9UBI6
Control	3	Q9UBK7
Control	3	Q9UBK8
Control	3	Q9UBK9
Control	3	Q9UBL3
Control	3	Q9UBM7
Control	3	Q9UBN6
Control	3	Q9UBN7
Control	3	Q9UBP0
Control	3	Q9UBP4
Control	3	Q9UBP6
Control	3	Q9UBP9
Control	3	Q9UBQ0
Control	3	Q9UBQ5
Control	3	Q9UBQ6
Control	3	Q9UBQ7
Control	3	Q9UBR2
Control	3	Q9UBS0
Control	3	Q9UBS4
Control	3	Q9UBS8
Control	3	Q9UBT2
Control	3	Q9UBU8
Control	3	Q9UBU9
Control	3	Q9UBV2
Control	3	Q9UBV7
Control	3	Q9UBV8
Control	3	Q9UBW7
Control	3	Q9UBW8
Control	3	Q9UBX3
Control	3	Q9UDR5
Control	3	Q9UDT6
Control	3	Q9UDW1
Control	3	Q9UDX5
Control	3	Q9UDY2
Control	3	Q9UDY4
Control	3	Q9UEE5
Control	3	Q9UEE9
Control	3	Q9UER7
Control	3	Q9UET6
Control	3	Q9UEU0

Control	3	Q9UEW8
Control	3	Q9UEY8
Control	3	Q9UFC0
Control	3	Q9UFN0
Control	3	Q9UFW8
Control	3	Q9UG56
Control	3	Q9UG63
Control	3	Q9UGI8
Control	3	Q9UGJ0
Control	3	Q9UGJ1
Control	3	Q9UGL1
Control	3	Q9UGM6
Control	3	Q9UGP4
Control	3	Q9UGP8
Control	3	Q9UGQ3
Control	3	Q9UGR2
Control	3	Q9UGV2
Control	3	Q9UH62
Control	3	Q9UH65
Control	3	Q9UH99
Control	3	Q9UHA3
Control	3	Q9UHA4
Control	3	Q9UHB6
Control	3	Q9UHB7
Control	3	Q9UHB9
Control	3	Q9UHD1
Control	3	Q9UHD2
Control	3	Q9UHD8
Control	3	Q9UHG3
Control	3	Q9UHI6
Control	3	Q9UHJ6
Control	3	Q9UHL4
Control	3	Q9UHN6
Control	3	Q9UHP3
Control	3	Q9UHQ4
Control	3	Q9UHQ9
Control	3	Q9UHR5
Control	3	Q9UHR6
Control	3	Q9UHV9
Control	3	Q9UHW5
Control	3	Q9UHX1
Control	3	Q9UHY1
Control	3	Q9UHY7
Control	3	Q9UHY8
Control	3	Q9UI09
Control	3	Q9UI10
Control	3	Q9UI12
Control	3	Q9UI14
Control	3	Q9UI26
Control	3	Q9UI30

Control	3	Q9UI36
Control	3	Q9UIA9
Control	3	Q9UIC8
Control	3	Q9UID3
Control	3	Q9UIF8
Control	3	Q9UIF9
Control	3	Q9UIG0
Control	3	Q9UII2
Control	3	Q9UIJ7
Control	3	Q9UIQ6
Control	3	Q9UIS9
Control	3	Q9UIV1
Control	3	Q9UJ41
Control	3	Q9UJ68
Control	3	Q9UJ70
Control	3	Q9UJ83
Control	3	Q9UJA5
Control	3	Q9UJC5
Control	3	Q9UJF2
Control	3	Q9UJK0
Control	3	Q9UJS0
Control	3	Q9UJU6
Control	3	Q9UJV9
Control	3	Q9UJW0
Control	3	Q9UJX2
Control	3	Q9UJX3
Control	3	Q9UJX4
Control	3	Q9UJX5
Control	3	Q9UJX6
Control	3	Q9UJY4
Control	3	Q9UJY5
Control	3	Q9UJZ1
Control	3	Q9UK23
Control	3	Q9UK41
Control	3	Q9UK45
Control	3	Q9UK59
Control	3	Q9UK61
Control	3	Q9UK76
Control	3	Q9UK99
Control	3	Q9UKA4
Control	3	Q9UKB1
Control	3	Q9UKD1
Control	3	Q9UKD2
Control	3	Q9UKE5
Control	3	Q9UKF6
Control	3	Q9UKG1
Control	3	Q9UKI2
Control	3	Q9UKI8
Control	3	Q9UKJ3
Control	3	Q9UKK3

Control	3	Q9UKK6
Control	3	Q9UKK9
Control	3	Q9UKL0
Control	3	Q9UKL6
Control	3	Q9UKM7
Control	3	Q9UKM9
Control	3	Q9UKN8
Control	3	Q9UKR5
Control	3	Q9UKS6
Control	3	Q9UKU7
Control	3	Q9UKU9
Control	3	Q9UKV3
Control	3	Q9UKV5
Control	3	Q9UKV8
Control	3	Q9UKX7
Control	3	Q9UKY7
Control	3	Q9UKZ1
Control	3	Q9UL03
Control	3	Q9UL15
Control	3	Q9UL18
Control	3	Q9UL25
Control	3	Q9UL26
Control	3	Q9UL33
Control	3	Q9UL40
Control	3	Q9UL46
Control	3	Q9UL54
Control	3	Q9UL63
Control	3	Q9ULA0
Control	3	Q9ULC3
Control	3	Q9ULC4
Control	3	Q9ULD2
Control	3	Q9ULE0
Control	3	Q9ULE6
Control	3	Q9ULF5
Control	3	Q9ULG1
Control	3	Q9ULG6
Control	3	Q9ULH0
Control	3	Q9ULH1
Control	3	Q9ULH7
Control	3	Q9ULJ7
Control	3	Q9ULK4
Control	3	Q9ULL8
Control	3	Q9ULM3
Control	3	Q9ULP0
Control	3	Q9ULP9
Control	3	Q9ULR0
Control	3	Q9ULS5
Control	3	Q9ULT8
Control	3	Q9ULU4
Control	3	Q9ULV3

Control	3	Q9ULV4
Control	3	Q9ULW0
Control	3	Q9ULW3
Control	3	Q9ULX3
Control	3	Q9ULX6
Control	3	Q9ULX9
Control	3	Q9ULZ3
Control	3	Q9UM00
Control	3	Q9UM13
Control	3	Q9UM54
Control	3	Q9UMR2
Control	3	Q9UMR5
Control	3	Q9UMS4
Control	3	Q9UMX1
Control	3	Q9UMX3
Control	3	Q9UMY1
Control	3	Q9UMY4
Control	3	Q9UMZ2
Control	3	Q9UN37
Control	3	Q9UN86
Control	3	Q9UNE7
Control	3	Q9UNF0
Control	3	Q9UNF1
Control	3	Q9UNH6
Control	3	Q9UNH7
Control	3	Q9UNI6
Control	3	Q9UNK0
Control	3	Q9UNL2
Control	3	Q9UNM6
Control	3	Q9UNN5
Control	3	Q9UNN8
Control	3	Q9UNP9
Control	3	Q9UNQ2
Control	3	Q9UNS2
Control	3	Q9UNW1
Control	3	Q9UNW9
Control	3	Q9UNX4
Control	3	Q9UNY4
Control	3	Q9UNZ2
Control	3	Q9UP83
Control	3	Q9UP95
Control	3	Q9UPM8
Control	3	Q9UPN3
Control	3	Q9UPN4
Control	3	Q9UPN6
Control	3	Q9UPN7
Control	3	Q9UPN9
Control	3	Q9UPP1
Control	3	Q9UPQ9
Control	3	Q9UPR0

Control	3	Q9UPR3
Control	3	Q9UPT5
Control	3	Q9UPT8
Control	3	Q9UPU5
Control	3	Q9UPU7
Control	3	Q9UPU9
Control	3	Q9UPW0
Control	3	Q9UPW5
Control	3	Q9UPY3
Control	3	Q9UPY5
Control	3	Q9UPY8
Control	3	Q9UQ13
Control	3	Q9UQ35
Control	3	Q9UQ53
Control	3	Q9UQ80
Control	3	Q9UQ90
Control	3	Q9UQB8
Control	3	Q9UQC2
Control	3	Q9UQE7
Control	3	Q9UQN3
Control	3	Q9UQQ2
Control	3	Q9UQR1
Control	3	Q9Y217
Control	3	Q9Y219
Control	3	Q9Y221
Control	3	Q9Y223
Control	3	Q9Y224
Control	3	Q9Y230
Control	3	Q9Y232
Control	3	Q9Y237
Control	3	Q9Y243
Control	3	Q9Y256
Control	3	Q9Y259
Control	3	Q9Y262
Control	3	Q9Y263
Control	3	Q9Y265
Control	3	Q9Y266
Control	3	Q9Y276
Control	3	Q9Y277
Control	3	Q9Y281
Control	3	Q9Y282
Control	3	Q9Y285
Control	3	Q9Y289
Control	3	Q9Y291
Control	3	Q9Y294
Control	3	Q9Y295
Control	3	Q9Y296
Control	3	Q9Y2A7
Control	3	Q9Y2B0
Control	3	Q9Y2C4

Control	3	Q9Y2D4
Control	3	Q9Y2D5
Control	3	Q9Y2G2
Control	3	Q9Y2G3
Control	3	Q9Y2G5
Control	3	Q9Y2G8
Control	3	Q9Y2H0
Control	3	Q9Y2H1
Control	3	Q9Y2H2
Control	3	Q9Y2H6
Control	3	Q9Y2I1
Control	3	Q9Y2I7
Control	3	Q9Y2I8
Control	3	Q9Y2J2
Control	3	Q9Y2J4
Control	3	Q9Y2K2
Control	3	Q9Y2K7
Control	3	Q9Y2L1
Control	3	Q9Y2L5
Control	3	Q9Y2L9
Control	3	Q9Y2P8
Control	3	Q9Y2Q3
Control	3	Q9Y2Q5
Control	3	Q9Y2Q9
Control	3	Q9Y2R0
Control	3	Q9Y2R4
Control	3	Q9Y2R5
Control	3	Q9Y2R9
Control	3	Q9Y2S2
Control	3	Q9Y2S6
Control	3	Q9Y2S7
Control	3	Q9Y2T2
Control	3	Q9Y2U8
Control	3	Q9Y2V2
Control	3	Q9Y2V7
Control	3	Q9Y2W1
Control	3	Q9Y2W2
Control	3	Q9Y2W6
Control	3	Q9Y2X0
Control	3	Q9Y2X3
Control	3	Q9Y2X7
Control	3	Q9Y2X9
Control	3	Q9Y2Y0
Control	3	Q9Y2Z0
Control	3	Q9Y2Z4
Control	3	Q9Y303
Control	3	Q9Y305
Control	3	Q9Y312
Control	3	Q9Y314
Control	3	Q9Y315

Control	3	Q9Y316
Control	3	Q9Y320
Control	3	Q9Y324
Control	3	Q9Y333
Control	3	Q9Y371
Control	3	Q9Y375
Control	3	Q9Y376
Control	3	Q9Y383
Control	3	Q9Y385
Control	3	Q9Y388
Control	3	Q9Y394
Control	3	Q9Y399
Control	3	Q9Y3A2
Control	3	Q9Y3A3
Control	3	Q9Y3A4
Control	3	Q9Y3A5
Control	3	Q9Y3A6
Control	3	Q9Y3B2
Control	3	Q9Y3B3
Control	3	Q9Y3B4
Control	3	Q9Y3B7
Control	3	Q9Y3B8
Control	3	Q9Y3B9
Control	3	Q9Y3C0
Control	3	Q9Y3C1
Control	3	Q9Y3C4
Control	3	Q9Y3C6
Control	3	Q9Y3C8
Control	3	Q9Y3D0
Control	3	Q9Y3D3
Control	3	Q9Y3D5
Control	3	Q9Y3D6
Control	3	Q9Y3D7
Control	3	Q9Y3D8
Control	3	Q9Y3D9
Control	3	Q9Y3E0
Control	3	Q9Y3E1
Control	3	Q9Y3E5
Control	3	Q9Y3E7
Control	3	Q9Y3F4
Control	3	Q9Y3I0
Control	3	Q9Y3I1
Control	3	Q9Y3L5
Control	3	Q9Y3M8
Control	3	Q9Y3P9
Control	3	Q9Y3Q3
Control	3	Q9Y3Q8
Control	3	Q9Y3R5
Control	3	Q9Y3S2
Control	3	Q9Y3T6

Control	3	Q9Y3T9
Control	3	Q9Y3U8
Control	3	Q9Y3X0
Control	3	Q9Y3Y2
Control	3	Q9Y3Z3
Control	3	Q9Y448
Control	3	Q9Y450
Control	3	Q9Y478
Control	3	Q9Y484
Control	3	Q9Y485
Control	3	Q9Y487
Control	3	Q9Y490
Control	3	Q9Y496
Control	3	Q9Y4A5
Control	3	Q9Y4B5
Control	3	Q9Y4B6
Control	3	Q9Y4C2
Control	3	Q9Y4C8
Control	3	Q9Y4D1
Control	3	Q9Y4D7
Control	3	Q9Y4D8
Control	3	Q9Y4E1
Control	3	Q9Y4E6
Control	3	Q9Y4E8
Control	3	Q9Y4F1
Control	3	Q9Y4F3
Control	3	Q9Y4F5
Control	3	Q9Y4G8
Control	3	Q9Y4I1
Control	3	Q9Y4J8
Control	3	Q9Y4K0
Control	3	Q9Y4K1
Control	3	Q9Y4K3
Control	3	Q9Y4K4
Control	3	Q9Y4L1
Control	3	Q9Y4P1
Control	3	Q9Y4P3
Control	3	Q9Y4P8
Control	3	Q9Y4R8
Control	3	Q9Y4W2
Control	3	Q9Y4W6
Control	3	Q9Y4X4
Control	3	Q9Y4X5
Control	3	Q9Y4Z0
Control	3	Q9Y508
Control	3	Q9Y512
Control	3	Q9Y520
Control	3	Q9Y530
Control	3	Q9Y547
Control	3	Q9Y570

Control	3	Q9Y580
Control	3	Q9Y584
Control	3	Q9Y5A7
Control	3	Q9Y5A9
Control	3	Q9Y5B0
Control	3	Q9Y5B6
Control	3	Q9Y5B8
Control	3	Q9Y5B9
Control	3	Q9Y5J1
Control	3	Q9Y5K5
Control	3	Q9Y5K6
Control	3	Q9Y5K8
Control	3	Q9Y5L0
Control	3	Q9Y5M8
Control	3	Q9Y5N6
Control	3	Q9Y5P4
Control	3	Q9Y5P6
Control	3	Q9Y5Q0
Control	3	Q9Y5Q8
Control	3	Q9Y5Q9
Control	3	Q9Y5R8
Control	3	Q9Y5S1
Control	3	Q9Y5S2
Control	3	Q9Y5S9
Control	3	Q9Y5T4
Control	3	Q9Y5T5
Control	3	Q9Y5U2
Control	3	Q9Y5V0
Control	3	Q9Y5V3
Control	3	Q9Y5W7
Control	3	Q9Y5W9
Control	3	Q9Y5X1
Control	3	Q9Y5X2
Control	3	Q9Y5X3
Control	3	Q9Y5X9
Control	3	Q9Y5Y2
Control	3	Q9Y5Y5
Control	3	Q9Y5Y7
Control	3	Q9Y5Z0
Control	3	Q9Y5Z4
Control	3	Q9Y5Z7
Control	3	Q9Y5Z9
Control	3	Q9Y606
Control	3	Q9Y608
Control	3	Q9Y613
Control	3	Q9Y617
Control	3	Q9Y618
Control	3	Q9Y619
Control	3	Q9Y624
Control	3	Q9Y639

Control	3	Q9Y653
Control	3	Q9Y657
Control	3	Q9Y666
Control	3	Q9Y672
Control	3	Q9Y673
Control	3	Q9Y676
Control	3	Q9Y678
Control	3	Q9Y679
Control	3	Q9Y680
Control	3	Q9Y696
Control	3	Q9Y697
Control	3	Q9Y6A4
Control	3	Q9Y6A5
Control	3	Q9Y6A9
Control	3	Q9Y6B6
Control	3	Q9Y6B7
Control	3	Q9Y6C9
Control	3	Q9Y6D0
Control	3	Q9Y6D5
Control	3	Q9Y6D6
Control	3	Q9Y6D9
Control	3	Q9Y6E0
Control	3	Q9Y6E2
Control	3	Q9Y6G5
Control	3	Q9Y6G9
Control	3	Q9Y6I3
Control	3	Q9Y6I4
Control	3	Q9Y6I9
Control	3	Q9Y6J0
Control	3	Q9Y6K0
Control	3	Q9Y6K1
Control	3	Q9Y6K5
Control	3	Q9Y6K8
Control	3	Q9Y6K9
Control	3	Q9Y6M1
Control	3	Q9Y6M5
Control	3	Q9Y6M7
Control	3	Q9Y6M9
Control	3	Q9Y6N5
Control	3	Q9Y6N7
Control	3	Q9Y6Q9
Control	3	Q9Y6R0
Control	3	Q9Y6R4
Control	3	Q9Y6V7
Control	3	Q9Y6W3
Control	3	Q9Y6W5
Control	3	Q9Y6X2
Control	3	Q9Y6X3
Control	3	Q9Y6X9
Control	3	Q9Y6Y0

Control	3	Q9Y6Y8

supernatants of the three replicates of cells treated used as a control group.

PG.ProteinDescriptions
Putative glutamine amidotransferase-like class 1 domain-containing protein 3B, mitochondrial
Intraflagellar transport protein 56
Ubiquitin-like modifier-activating enzyme 6
Extended synaptotagmin-2
Shootin-1
Transmembrane protein 223
Rho GTPase-activating protein 10
2-hydroxyacyl-CoA lyase 2
SH3 and PX domain-containing protein 2B
NBAS subunit of NRZ tethering complex
tRNA wybutosine-synthesizing protein 5
DENN domain-containing protein 3
von Willebrand factor A domain-containing protein 8
Protein strawberry notch homolog 1
GTP-binding protein 10
WD repeat-containing protein 91
Plasmanylethanolamine desaturase 1
Trafficking protein particle complex subunit 13
CCR4-NOT transcription complex subunit 1
Microtubule-associated proteins 1A/1B light chain 3 beta 2
Glycerol-3-phosphate phosphatase
RAB7A-interacting MON1-CCZ1 complex subunit 1
RCC1 domain-containing protein 1
TRPM8 channel-associated factor 2
Structural maintenance of chromosomes flexible hinge domain-containing protein 1
Cytosolic arginine sensor for mTORC1 subunit 2
Protein unc-119 homolog B
12S rRNA N4-methylcytidine (m4C) methyltransferase
Coiled-coil domain-containing protein 85C
Retrotransposon Gag-like protein 8C
Zinc finger SWIM domain-containing protein 8
Nuclear envelope pore membrane protein POM 121C
PHD finger protein 20-like protein 1
Putative small nuclear ribonucleoprotein G-like protein 15
Endosome/lysosome-associated apoptosis and autophagy regulator family member 2
Acyl-coenzyme A diphosphatase NUDT19
MIF4G domain-containing protein
Calmodulin-1
Unconventional myosin-IXa
Golgi pH regulator A
Putative WAS protein family homolog 3
Mapk-regulated corepressor-interacting protein 1
Nascent polypeptide-associated complex subunit alpha, muscle-specific form
Uncharacterized protein C11orf98
ASNSD1 upstream open reading frame protein
Mitochondrial ribosome and complex I assembly factor AltMIEF1
Deoxyribonuclease-2-alpha
Alkyldihydroxyacetonephosphate synthase, peroxisomal

Kinesin-like protein KIF2A
ATP-dependent RNA helicase DDX39A
PDZ and LIM domain protein 1
Cytosolic acyl coenzyme A thioester hydrolase
Unconventional myosin-Ic
Synaptosomal-associated protein 23
HCLS1-associated protein X-1
AH receptor-interacting protein
GTP-binding protein 1
Galectin-9
Syntaxin-binding protein 3
AP-4 complex subunit mu-1
Splicing regulator ARVCF
Small acidic protein
AP-3 complex subunit beta-1
Amyloid beta precursor protein binding family B member 1
Galectin-8
NADH dehydrogenase [ubiquinone] iron-sulfur protein 8, mitochondrial
NF-kappa-B inhibitor epsilon
26S proteasome non-ATPase regulatory subunit 11
26S proteasome non-ATPase regulatory subunit 12
26S proteasome non-ATPase regulatory subunit 9
Menin
Guided entry of tail-anchored proteins factor 1
Membrane-associated progesterone receptor component 1
Transcription elongation factor SPT5
Transcription initiation factor TFIID subunit 4
DNA fragmentation factor subunit alpha
Huntingtin-interacting protein 1
Chloride intracellular channel protein 1
Eukaryotic translation initiation factor 3 subunit F
NEDD4-like E3 ubiquitin-protein ligase WWP2
Pyruvate dehydrogenase protein X component, mitochondrial
Sulfhydryl oxidase 1
Dynactin subunit 6
Acetyl-coenzyme A transporter 1
Actin nucleation-promoting factor WASL
Importin-5
DNA-directed RNA polymerase, mitochondrial
Eukaryotic elongation factor 2 kinase
Histone deacetylase complex subunit SAP18
Echinoderm microtubule-associated protein-like 1
Insulin-like growth factor 2 mRNA-binding protein 3
Dynamin-1-like protein
RNA 3'-terminal phosphate cyclase
Phosphatidylinositol 4-phosphate 3-kinase C2 domain-containing subunit alpha
Golgi integral membrane protein 4
Agrin
Procollagen-lysine,2-oxoglutarate 5-dioxygenase 2
Exocyst complex component 5

Butyrophilin subfamily 3 member A1
Cytochrome c oxidase subunit NDUFA4
26S proteasome non-ATPase regulatory subunit 14
Myc box-dependent-interacting protein 1
Claudin-5
Importin subunit alpha-4
Serine/threonine-protein kinase 25
Krev interaction trapped protein 1
von Willebrand factor A domain-containing protein 5A
Pescadillo homolog
Syntenin-1
Membrane-associated phosphatidylinositol transfer protein 1
U3 small nucleolar ribonucleoprotein protein MPP10
Nucleolar protein 56
ATP-dependent RNA helicase DDX3X
Beta-1,3-N-acetylglucosaminyltransferase manic fringe
Podocalyxin
CCN family member 1
Pirin
Importin subunit alpha-3
E3 ubiquitin-protein ligase TRIM38
Nuclear factor 1 B-type
Serine/threonine-protein phosphatase 6 catalytic subunit
Nucleoside diphosphate kinase, mitochondrial
Phosphatidylinositol 4-phosphate 3-kinase C2 domain-containing subunit beta
Lysosomal alpha-mannosidase
Pyridoxal kinase
Stearoyl-CoA desaturase
AT-rich interactive domain-containing protein 1A
Nuclear envelope integral membrane protein 1
Thioredoxin domain-containing protein 9
Dihydropyrimidinase-related protein 4
TRAF-type zinc finger domain-containing protein 1
Ubiquitin domain-containing protein UBFD1
Citron Rho-interacting kinase
Coatomer subunit epsilon
Cdc42 effector protein 2
AP-3 complex subunit delta-1
Copper chaperone for superoxide dismutase
Actin-binding LIM protein 1
Segment polarity protein dishevelled homolog DVL-1
Segment polarity protein dishevelled homolog DVL-2
Chromodomain-helicase-DNA-binding protein 1
Chromodomain-helicase-DNA-binding protein 2
Golgi SNAP receptor complex member 2
Torsin-1A
Torsin-1B
Syntaxin-16
Disintegrin and metalloproteinase domain-containing protein 10
Lysosomal cobalamin transporter ABCD4

Etoposide-induced protein 2.4 homolog
Tumor protein p53-inducible protein 11
Histone-lysine N-methyltransferase 2D
Apoptotic protease-activating factor 1
Serine/threonine-protein kinase RIO3
Dual specificity mitogen-activated protein kinase kinase 7
Acyl-coenzyme A thioesterase 8
CDP-diacylglycerol--inositol 3-phosphatidyltransferase
Programmed cell death protein 5
Protein arginine N-methyltransferase 5
Na(+)/H(+) exchange regulatory cofactor NHE-RF1
Serine/threonine-protein kinase Chk1
Tumor necrosis factor receptor superfamily member 10B
Tripeptidyl-peptidase 1
Transcription elongation regulator 1
Kinetochore protein NDC80 homolog
Neuropilin-1
Transportin-2
Protein unc-13 homolog B
Tumor necrosis factor receptor superfamily member 10C
DNA-directed RNA polymerase III subunit RPC1
Tetraspanin-4
Proteasome subunit alpha type-7
Secretory carrier-associated membrane protein 3
Branched-chain alpha-ketoacid dehydrogenase kinase
Glutathione S-transferase 3, mitochondrial
Tax1-binding protein 3
PDZ domain-containing protein GIPC1
Inhibitor of nuclear factor kappa-B kinase subunit beta
Regulator of G-protein signaling 12
Mitochondrial import inner membrane translocase subunit Tim23
Histone acetyltransferase type B catalytic subunit
Peripheral plasma membrane protein CASK
Phospholipase D2
Cytochrome b-c1 complex subunit 8
Myosin regulatory light chain 12B
Hepatocyte growth factor-regulated tyrosine kinase substrate
Ras-related protein Rab-7L1
Vacuolar protein sorting-associated protein 26C
Protein phosphatase 1 regulatory subunit 12A
Cyclin-G-associated kinase
Heterogeneous nuclear ribonucleoprotein D-like
Exportin-1
TATA-binding protein-associated factor 172
Rho guanine nucleotide exchange factor 10
Zinc finger protein 609
Spectrin beta chain, non-erythrocytic 2
Microtubule-associated serine/threonine-protein kinase 4
Protein transport protein Sec16A
Plexin-B2

Protein KHNYN
Tectonin beta-propeller repeat-containing protein 2
U2 snRNP-associated SURP motif-containing protein
Histone-lysine N-methyltransferase SETD1A
NEDD4-binding protein 3
Synaptojanin-2
Synemin
Granule associated Rac and RHOG effector protein 1
Kinesin-like protein KIF3B
Phosphoribosylformylglycinamidine synthase
Serine/threonine-protein phosphatase 6 regulatory ankyrin repeat subunit A
Rho guanine nucleotide exchange factor 11
Mitochondrial ribonuclease P catalytic subunit
Inhibitor of nuclear factor kappa-B kinase subunit alpha
NPC intracellular cholesterol transporter 1
Sphingolipid delta(4)-desaturase DES1
Angiopoietin-2
Secretory carrier-associated membrane protein 1
Secretory carrier-associated membrane protein 2
Actin-related protein 2/3 complex subunit 1B
Actin-related protein 2/3 complex subunit 2
Actin-related protein 2/3 complex subunit 3
DNA-directed RNA polymerases I and III subunit RPAC1
Phospholipid scramblase 1
Transcription intermediary factor 1-alpha
Membrane-associated progesterone receptor component 2
Ral guanine nucleotide dissociation stimulator-like 2
WD repeat-containing protein 46
NF-kappa-B-repressing factor
Dihydroxyacetone phosphate acyltransferase
Laminin subunit alpha-5
Zinc finger protein 185
Protein CASC3
Small ribosomal subunit protein uS12m
Peroxisomal acyl-coenzyme A oxidase 3
Protein RER1
Surfeit locus protein 4
Serine palmitoyltransferase 1
Serine palmitoyltransferase 2
UDP-N-acetylglucosamine--peptide N-acetylglucosaminyltransferase 110 kDa subunit
Phosphomannomutase 2
Transmembrane 9 superfamily member 1
E3 ubiquitin-protein ligase Midline-1
High mobility group protein B3
Protein phosphatase 1G
Phosphatidylinositol 3,4,5-trisphosphate 5-phosphatase 2
Eukaryotic translation initiation factor 3 subunit D
Eukaryotic translation initiation factor 3 subunit H
Histone deacetylase 3
Nuclear valosin-containing protein-like

Branched-chain-amino-acid aminotransferase, mitochondrial
Importin-8
Syntaxin-7
Monocarboxylate transporter 4
ATP-binding cassette sub-family C member 4
DNA-directed RNA polymerase I subunit RPA34
Prolyl 4-hydroxylase subunit alpha-2
Calpain-5
Synaptobrevin homolog YKT6
Nucleoporin NUP42
Actin-related protein 2/3 complex subunit 5
CASP8 and FADD-like apoptosis regulator
Transcription factor MafG
3-phosphoinositide-dependent protein kinase 1
Tapasin
E3 ubiquitin-protein ligase RNF113A
ATP-dependent RNA helicase DHX15
mRNA cap guanine-N7 methyltransferase
Zinc finger ZZ-type and EF-hand domain-containing protein 1
Arf-GAP with SH3 domain, ANK repeat and PH domain-containing protein 2
Leucine-rich repeat transmembrane protein FLRT2
TELO2-interacting protein 1 homolog
Ribosomal RNA-processing protein 8
E3 ubiquitin-protein ligase Praja-2
Cytochrome b5 type B
U4/U6 small nuclear ribonucleoprotein Prp4
D-3-phosphoglycerate dehydrogenase
NADH dehydrogenase [ubiquinone] iron-sulfur protein 4, mitochondrial
Cytoplasmic dynein 1 light intermediate chain 2
26S proteasome non-ATPase regulatory subunit 3
RNA binding protein fox-1 homolog 2
Bifunctional 3'-phosphoadenosine 5'-phosphosulfate synthase 1
Centromere/kinetochore protein zw10 homolog
Beta-1,4-galactosyltransferase 5
U4/U6.U5 tri-snRNP-associated protein 1
Glycosylphosphatidylinositol anchor attachment 1 protein
Death-associated protein kinase 3
Transforming growth factor beta-1-induced transcript 1 protein
AP-5 complex subunit zeta-1
CBP80/20-dependent translation initiation factor
Protein MTSS 1
Inositol hexakisphosphate and diphosphoinositol-pentakisphosphate kinase 2
Mitogen-activated protein kinase kinase kinase 7
Eukaryotic translation elongation factor 1 epsilon-1
Receptor-interacting serine/threonine-protein kinase 2
Heterogeneous nuclear ribonucleoprotein R
U4/U6 small nuclear ribonucleoprotein Prp3
Thioredoxin-like protein 1
Tumor protein D54
ER membrane protein complex subunit 8

ERI1 exoribonuclease 3
Synaptojanin-1
Acidic fibroblast growth factor intracellular-binding protein
Eukaryotic translation initiation factor 4 gamma 3
Peptidyl-prolyl cis-trans isomerase H
Serine protease HTRA2, mitochondrial
Protein Mis18-beta
Aflatoxin B1 aldehyde reductase member 2
Band 4.1-like protein 2
Trans-Golgi network integral membrane protein 2
Beta-1,4-glucuronyltransferase 1
WAS/WASL-interacting protein family member 1
Phospholipid-transporting ATPase IC
A-kinase anchor protein 10, mitochondrial
Density-regulated protein
Exportin-T
5-hydroxymethyl-dUMP N-hydrolase
Mitochondrial import inner membrane translocase subunit TIM44
Trafficking protein particle complex subunit 3
Charged multivesicular body protein 2a
Cytoplasmic protein NCK2
Tetraspanin-6
Pleiotropic regulator 1
Protein regulator of cytokinesis 1
Regulator of G-protein signaling 10
BUB3-interacting and GLEBS motif-containing protein ZNF207
NADH dehydrogenase [ubiquinone] 1 beta subcomplex subunit 5, mitochondrial
NADH dehydrogenase [ubiquinone] 1 beta subcomplex subunit 3
NADH dehydrogenase [ubiquinone] 1 alpha subcomplex subunit 2
LIM domain-binding protein 2
ATPase GET3
Mitotic checkpoint serine/threonine-protein kinase BUB1
Mitotic checkpoint protein BUB3
Sulfotransferase 1B1
Alpha-actinin-4
Probable 18S rRNA (guanine-N(7))-methyltransferase
17S U2 SnRNP complex component HTATSF1
ER lumen protein-retaining receptor 3
AP-1 complex subunit gamma-1
Syntaxin-6
Synaptogyrin-2
Small glutamine-rich tetratricopeptide repeat-containing protein alpha
Alpha-endosulfine
Mitochondrial carnitine/acylcarnitine carrier protein
Asparagine--tRNA ligase, cytoplasmic
Unconventional myosin-Ib
Peroxisomal membrane protein PMP34
Cleavage and polyadenylation specificity factor subunit 5
Glutathione S-transferase LANCL1
Striatin

U3 small nucleolar RNA-interacting protein 2
Protein SCO2 homolog, mitochondrial
Cilia- and flagella-associated protein 410
A-kinase anchor protein 8
Putative GTP-binding protein 6
Glucose-6-phosphate exchanger SLC37A4
Isocitrate dehydrogenase [NAD] subunit beta, mitochondrial
Nardilysin
Calumenin
EGF-like repeat and discoidin I-like domain-containing protein 3
Probable phospholipid-transporting ATPase IIB
S-adenosylhomocysteine hydrolase-like protein 1
Kinesin-like protein KIF1C
Exostosin-like 3
Origin recognition complex subunit 5
NADH dehydrogenase [ubiquinone] iron-sulfur protein 5
Retinal rod rhodopsin-sensitive cGMP 3',5'-cyclic phosphodiesterase subunit delta
Origin recognition complex subunit 4
Peroxisomal ATPase PEX1
Double-strand-break repair protein rad21 homolog
Pre-mRNA-splicing factor ATP-dependent RNA helicase DHX16
Glia maturation factor gamma
SH3 domain-binding protein 5
Mediator of RNA polymerase II transcription subunit 14
Phosphoribosyl pyrophosphate synthase-associated protein 2
SWI/SNF-related matrix-associated actin-dependent regulator of chromatin subfamily A member 5
C-Jun-amino-terminal kinase-interacting protein 4
NUAK family SNF1-like kinase 1
Nucleolar pre-ribosomal-associated protein 1
Signal-induced proliferation-associated 1-like protein 3
Zinc finger C3H1 domain-containing protein
RNA helicase aquarius
Dynamin-like 120 kDa protein, mitochondrial
Germinal-center associated nuclear protein
Protocadherin gamma-A12
Phosphatidylinositol 4-phosphate 5-kinase type-1 gamma
Kinesin-like protein KIF1B
Mitogen-activated protein kinase-binding protein 1
Lysine-specific histone demethylase 1A
TBC1 domain family member 4
Acyl-CoA (8-3)-desaturase
Gasdermin-E
Neuropilin-2
Mannosyl-oligosaccharide 1,2-alpha-mannosidase IB
Myelin protein zero-like protein 2
Long-chain-fatty-acid--CoA ligase 4
Sorting nexin-3
Protein O-GlcNAcase
Vinexin
Heterogeneous nuclear ribonucleoprotein Q

Pre-mRNA-processing factor 17
Ran-binding protein 6
Ribosome quality control complex subunit NEMF
GDP-mannose 4,6 dehydratase
Glycylpeptide N-tetradecanoyltransferase 2
Cyclin-T1
Mitotic checkpoint serine/threonine-protein kinase BUB1 beta
Multifunctional procollagen lysine hydroxylase and glycosyltransferase LH3
Eukaryotic translation initiation factor 4E type 2
Protein diaphanous homolog 1
Tetraspanin-3
Exocyst complex component 3
Perilipin-3
Protein arginine N-methyltransferase 3
Importin subunit alpha-7
UDP-glucose 6-dehydrogenase
Protein-tyrosine sulfotransferase 2
Leupaxin
Catenin delta-1
Protein-S-isoprenylcysteine O-methyltransferase
Sorting nexin-2
Dolichol-phosphate mannosyltransferase subunit 1
General vesicular transport factor p115
Small ribosomal subunit protein uS14m
Target of Myb1 membrane trafficking protein
Histone H2B type 1-K
Coiled-coil domain-containing protein 22
Polyglutamine-binding protein 1
Mitochondrial import inner membrane translocase subunit Tim17-B
PRA1 family protein 2
H/ACA ribonucleoprotein complex subunit DKC1
Eukaryotic translation initiation factor 5B
Endothelial differentiation-related factor 1
DNA/RNA-binding protein KIN17
Protein diaphanous homolog 2
DnaJ homolog subfamily A member 2
Bromodomain-containing protein 4
Checkpoint protein HUS1
Prefoldin subunit 1
E3 ubiquitin-protein ligase PPP1R11
Nibrin
mRNA-capping enzyme
Iron-sulfur clusters transporter ABCB7, mitochondrial
SLIT-ROBO Rho GTPase-activating protein 2
Plexin-A2
Glycosaminoglycan xylosylkinase
WD repeat-containing protein 1
Probable phospholipid-transporting ATPase IIA
NEDD4-binding protein 1
Rho-associated protein kinase 2

CLIP-associating protein 2
Pentatricopeptide repeat-containing protein 1, mitochondrial
Copine-3
Autophagy-related protein 13
Huntingtin-interacting protein 1-related protein
E3 ubiquitin-protein ligase BRE1B
Lysine-specific demethylase PHF2
Zinc finger CCCH domain-containing protein 11A
Clustered mitochondria protein homolog
Cullin-associated NEDD8-dissociated protein 2
TSC22 domain family protein 2
DnaJ homolog subfamily C member 13
Phosphatase and actin regulator 2
Serine/threonine-protein phosphatase 6 regulatory subunit 2
A disintegrin and metalloproteinase with thrombospondin motifs 4
CCR4-NOT transcription complex subunit 3
Ankyrin repeat domain-containing protein 17
Paired amphipathic helix protein Sin3b
DnaJ homolog subfamily B member 6
Ubiquinone biosynthesis protein COQ9, mitochondrial
Gamma-glutamylcyclotransferase
NADH dehydrogenase [ubiquinone] iron-sulfur protein 7, mitochondrial
NADH dehydrogenase [ubiquinone] iron-sulfur protein 2, mitochondrial
Zinc finger protein ZPR1
RNA/RNP complex-1-interacting phosphatase
Protein NipSnap homolog 2
Hyaluronan mediated motility receptor
Programmed cell death protein 6
Tubulin-specific chaperone A
V-type proton ATPase subunit G 1
Vacuolar protein sorting-associated protein 4B
Mannose-P-dolichol utilization defect 1 protein
Core histone macro-H2A.1
Adapter SH3BGRL
Filamin-B
Nuclear receptor corepressor 1
Peroxisomal membrane protein PEX14
Tripartite motif-containing protein 3
Citrate synthase, mitochondrial
Sperm-associated antigen 7
Vesicle-trafficking protein SEC22b
Pre-mRNA-processing factor 40 homolog A
Transforming acidic coiled-coil-containing protein 1
Nucleoside diphosphate kinase 6
Cell division control protein 45 homolog
Leucine-rich repeat and calponin homology domain-containing protein 4
Metaxin-2
Vacuolar protein sorting-associated protein 26A
Mitochondrial-processing peptidase subunit beta
Mediator of RNA polymerase II transcription subunit 24

Katanin p60 ATPase-containing subunit A1
PC4 and SFRS1-interacting protein
Erlin-1
NADH dehydrogenase [ubiquinone] iron-sulfur protein 3, mitochondrial
Serine/arginine-rich splicing factor 10
Ceroid-lipofuscinosis neuronal protein 5
Claudin-11
Enoyl-CoA delta isomerase 2
KH domain-containing, RNA-binding, signal transduction-associated protein 3
Polycomb protein EED
Barrier-to-autointegration factor
Splicing factor 3B subunit 1
Cold shock domain-containing protein E1
WW domain-binding protein 4
Interferon-inducible double-stranded RNA-dependent protein kinase activator A
Integrin alpha-10
Ribosomal protein S6 kinase alpha-5
Mediator of RNA polymerase II transcription subunit 6
E3 ubiquitin-protein ligase MYCBP2
2-amino-3-ketobutyrate coenzyme A ligase, mitochondrial
Acyl-protein thioesterase 1
GTPase Era, mitochondrial
U5 small nuclear ribonucleoprotein 200 kDa helicase
Mitochondrial tRNA-specific 2-thiouridylase 1
TIP41-like protein
Centriole and centriolar satellite protein OFD1
Ribosomal protein S6 kinase alpha-4
Surfeit locus protein 6
Protein phosphatase 1B
Small subunit processome component 20 homolog
Nuclear pore complex protein Nup155
Protein XRP2
WD repeat and HMG-box DNA-binding protein 1
Cartilage-associated protein
Electrogenic aspartate/glutamate antiporter SLC25A12, mitochondrial
Renin receptor
Ribonuclease H2 subunit A
Translation initiation factor eIF2 assembly protein
Breast cancer anti-estrogen resistance protein 3
Ribonuclease P protein subunit p20
Ribonuclease P protein subunit p40
Eukaryotic translation initiation factor 3 subunit G
Eukaryotic translation initiation factor 3 subunit J
Carbonyl reductase [NADPH] 3
26S proteasome non-ATPase regulatory subunit 10
AP-1 complex subunit gamma-like 2
CAAX prenyl protease 1 homolog
Isocitrate dehydrogenase [NADP] cytoplasmic
Glutamyl-tRNA(Gln) amidotransferase subunit B, mitochondrial
Protein SCO1 homolog, mitochondrial

Attractin
Serine hydrolase RBBP9
Signal transducing adapter molecule 2
Diacylglycerol O-acyltransferase 1
Cyclin-K
PRA1 family protein 3
Dysferlin
E3 SUMO-protein ligase PIAS1
Pre-mRNA-splicing factor SPF27
Dynactin subunit 3
DnaJ homolog subfamily C member 8
Survival of motor neuron-related-splicing factor 30
ATP synthase subunit d, mitochondrial
Tetraspanin-9
Flotillin-1
Triple functional domain protein
ATP synthase subunit g, mitochondrial
Multiple PDZ domain protein
Carboxypeptidase D
Glutaredoxin-3
Ribosomal L1 domain-containing protein 1
Wolframin
ATP-dependent Clp protease ATP-binding subunit clpX-like, mitochondrial
SEC14-like protein 2
Gamma-synuclein
Probable cytosolic iron-sulfur protein assembly protein CIAO1
Signal recognition particle subunit SRP72
N(G),N(G)-dimethylarginine dimethylaminohydrolase 1
ATP-dependent DNA helicase Q5
Unconventional prefoldin RPB5 interactor 1
Galactosylgalactosylxylosylprotein 3-beta-glucuronosyltransferase 3
Metastasis-associated protein MTA2
Retinal dehydrogenase 2
Serine/threonine-protein kinase 10
Serine/threonine-protein kinase D3
Glutamine--fructose-6-phosphate aminotransferase [isomerizing] 2
Slit homolog 2 protein
E3 ubiquitin-protein ligase listerin
Mitochondrial import receptor subunit TOM70
Pleckstrin homology domain-containing family G member 5
Importin-13
Phospholipase DDHD2
Unconventional myosin-Id
TOX high mobility group box family member 4
[F-actin]-monooxygenase MICAL2
Protein transport protein Sec24D
F-BAR and double SH3 domains protein 2
E3 UFM1-protein ligase 1
Sorbin and SH3 domain-containing protein 2
SAM and SH3 domain-containing protein 1

UBX domain-containing protein 7
Kelch-like protein 18
SUN domain-containing protein 1
Pyridoxal phosphate homeostasis protein
Erlin-2
Pre-mRNA-processing factor 6
Pre-mRNA cleavage complex 2 protein Pcf11
Protein furry homolog-like
Nuclear factor of activated T-cells 5
Endonuclease domain-containing 1 protein
D-glucuronyl C5-epimerase
Glutaminase kidney isoform, mitochondrial
HAUS augmin-like complex subunit 5
Actin-binding LIM protein 3
F-box only protein 21
Lysine-specific demethylase 4B
Microtubule cross-linking factor 2
Ubiquitin carboxyl-terminal hydrolase 19
WD repeat-containing protein 47
AP-2 complex subunit alpha-2
Protein transport protein Sec31A
Rho guanine nucleotide exchange factor 15
Protein HEXIM1
Ribonuclease P protein subunit p14
Protein YIF1A
E3 ubiquitin-protein ligase UBR5
Arf-GAP domain and FG repeat-containing protein 2
Serine protease 23
SR-related and CTD-associated factor 4
NADH dehydrogenase [ubiquinone] 1 beta subcomplex subunit 6
Mitofusin-2
Snurportin-1
Ubiquitin conjugation factor E4 B
Zinc finger protein-like 1
Elongator complex protein 1
NADH dehydrogenase [ubiquinone] 1 beta subcomplex subunit 4
NADH dehydrogenase [ubiquinone] 1 beta subcomplex subunit 8, mitochondrial
NADH dehydrogenase [ubiquinone] 1 alpha subcomplex subunit 7
Vesicle-associated membrane protein 5
Reticulon-3
Mitochondrial proton/calcium exchanger protein
Epsin-2
Zinc finger Ran-binding domain-containing protein 2
Sorting nexin-4
Luc7-like protein 3
Kinesin-like protein KIF20A
Apolipoprotein L3
Chromosome-associated kinesin KIF4A
Methyl-CpG-binding domain protein 4
Myotubularin-related protein 5

Golgi SNAP receptor complex member 1
Histone acetyltransferase KAT7
Arginyl-tRNA--protein transferase 1
Vesicle-associated membrane protein-associated protein B/C
SNARE-associated protein Snapin
Myelin protein zero-like protein 1
NADH dehydrogenase [ubiquinone] 1 subunit C2
NADH dehydrogenase [ubiquinone] 1 alpha subcomplex subunit 10, mitochondrial
Peptidyl-prolyl cis-trans isomerase FKBP9
CUGBP Elav-like family member 2
6-phosphogluconolactonase
Bifunctional 3'-phosphoadenosine 5'-phosphosulfate synthase 2
Structural maintenance of chromosomes protein 2
Ubiquitin-like modifier-activating enzyme ATG7
Transforming acidic coiled-coil-containing protein 2
Tripartite motif-containing protein 16
Phenylalanine--tRNA ligase, mitochondrial
Zinc finger and BTB domain-containing protein 7A
Acyl-protein thioesterase 2
Importin-7
E3 ubiquitin-protein ligase ARIH2
Pre-mRNA-splicing factor SLU7
Phosphoacetylglucosamine mutase
Adenylyltransferase and sulfurtransferase MOCS3
CD2 antigen cytoplasmic tail-binding protein 2
Supervillin
Activator of 90 kDa heat shock protein ATPase homolog 1
Poly(A)-specific ribonuclease PARN
Proteasome assembly chaperone 1
Sphingosine-1-phosphate lyase 1
CTD nuclear envelope phosphatase 1
Ribosome biogenesis protein NSA2 homolog
GDH/6PGL endoplasmic bifunctional protein
Protein transport protein Sec24A
Protein transport protein Sec24B
Adhesion G protein-coupled receptor L2
NAD kinase
Tyrosyl-DNA phosphodiesterase 2
Mitochondrial pyruvate carrier 2
Persulfide dioxygenase ETHE1, mitochondrial
Fatty acid CoA ligase Acsl3
DNA-directed RNA polymerase I subunit RPA1
YEATS domain-containing protein 4
Zinc finger and BTB domain-containing protein 11
CCR4-NOT transcription complex subunit 4
STAM-binding protein
Cleavage and polyadenylation specificity factor subunit 4
Nuclear factor of activated T-cells, cytoplasmic 1
Probable bifunctional dTTP/UTP pyrophosphatase/methyltransferase protein
Phosphatidate cytidylyltransferase 2

Protein phosphatase 1 regulatory subunit 3D
Bromodomain-containing protein 1
Ribonuclease P protein subunit p29
E3 ubiquitin-protein ligase HERC2
Ras-related protein Rab-3D
Synaptosomal-associated protein 29
Serine/threonine-protein kinase OSR1
Geranylgeranyl pyrophosphate synthase
Heat shock 70 kDa protein 4L
Polypyrimidine tract-binding protein 3
STARD3 N-terminal-like protein
U6 snRNA-associated Sm-like protein LSm8
AP-2 complex subunit alpha-1
Protein Wiz
Antiviral innate immune response receptor RIG-I
Double-stranded RNA-binding protein Staufen homolog 1
Tetratricopeptide repeat protein 4
Transmembrane protein 50A
Caveolae-associated protein 2
BAG family molecular chaperone regulator 2
BAG family molecular chaperone regulator 3
Mitogen-activated protein kinase kinase kinase kinase 4
Malonyl-CoA decarboxylase, mitochondrial
Quinone oxidoreductase-like protein 1
Apoptosis-inducing factor 1, mitochondrial
Echinoderm microtubule-associated protein-like 2
Serine/threonine-protein kinase LATS1
Uridine diphosphate glucose pyrophosphatase NUDT14
Tetraspanin-15
3'(2'),5'-bisphosphate nucleotidase 1
Acyl-CoA 6-desaturase
Putative hydrolase DDAH2
Phosphatidylserine lipase ABHD16A
Thioredoxin domain-containing protein 12
Protein ecdysoneless homolog
Methyl-CpG-binding domain protein 3
DNA topoisomerase 3-beta-1
Diphosphoinositol polyphosphate phosphohydrolase 1
B-cell lymphoma/leukemia 10
NADH dehydrogenase [ubiquinone] 1 beta subcomplex subunit 10
Putative lipid scramblase CLPTM1
E3 SUMO-protein ligase ZBED1
Molybdopterin synthase catalytic subunit
Mitochondrial import receptor subunit TOM40 homolog
Peroxisomal membrane protein 11B
Serine/threonine-protein kinase PAK 4
Amyloid-beta A4 precursor protein-binding family A member 3
Actin-like protein 6A
Histone-lysine N-methyltransferase NSD2
L-lactate dehydrogenase A chain

Aldehyde dehydrogenase 1A1
Glutamate dehydrogenase 1, mitochondrial
Dihydrofolate reductase
NADH-cytochrome b5 reductase 3
Glutathione reductase, mitochondrial
Cytochrome c oxidase subunit 1
Cytochrome c oxidase subunit 2
Purine nucleoside phosphorylase
Hypoxanthine-guanine phosphoribosyltransferase
Aspartate aminotransferase, mitochondrial
Tyrosine-protein kinase ABL1
Phosphoglycerate kinase 1
Adenylate kinase isoenzyme 1
Urokinase-type plasminogen activator
Tissue-type plasminogen activator
Adenosine deaminase
Alpha-1-antitrypsin
Alpha-2-macroglobulin
Cystatin-C
GTPase NRas
GTPase HRas
GTPase KRas
Platelet-derived growth factor subunit B
Low-density lipoprotein receptor
Transforming growth factor beta-1 proprotein
HLA class I histocompatibility antigen, B alpha chain
Putative HLA class I histocompatibility antigen, alpha chain H
Collagen alpha-1(I) chain
Collagen alpha-1(IV) chain
Prelamin-A/C
Apolipoprotein A-I
Fibronectin
Alpha-2-HS-glycoprotein
Albumin
Transferrin receptor protein 1
Ferritin light chain
Ferritin heavy chain
Metallothionein-2
NADH-ubiquinone oxidoreductase chain 1
NADH-ubiquinone oxidoreductase chain 4
NADH-ubiquinone oxidoreductase chain 5
Interstitial collagenase
3-hydroxy-3-methylglutaryl-coenzyme A reductase
Catalase
RAF proto-oncogene serine/threonine-protein kinase
Lysosomal acid glucosylceramidase
Fructose-bisphosphate aldolase A
Cystatin-B
Annexin A1
Apolipoprotein B-100

Major prion protein
Superoxide dismutase [Mn], mitochondrial
Ornithine aminotransferase, mitochondrial
Thymidine kinase, cytosolic
von Willebrand factor
Glyceraldehyde-3-phosphate dehydrogenase
Argininosuccinate lyase
HLA class I histocompatibility antigen, A alpha chain
Calpain small subunit 1
Cellular tumor antigen p53
Metallothionein-1E
Heat shock protein beta-1
Thymidylate synthase
Dolichyl-diphosphooligosaccharide--protein glycosyltransferase subunit 1
Dolichyl-diphosphooligosaccharide--protein glycosyltransferase subunit 2
Guanine nucleotide-binding protein G(i) subunit alpha-2
Histone H2A type 1-B/E
Anion exchange protein 2
Sodium/potassium-transporting ATPase subunit alpha-1
Sodium/potassium-transporting ATPase subunit beta-1
Amyloid-beta precursor protein
Aldehyde dehydrogenase, mitochondrial
Integrin beta-3
Protein S100-A8
Non-histone chromosomal protein HMG-14
Plasminogen activator inhibitor 2
Plasminogen activator inhibitor 1
ADP/ATP translocase 2
Ubiquitin-like protein ISG15
Propionyl-CoA carboxylase alpha chain, mitochondrial
Propionyl-CoA carboxylase beta chain, mitochondrial
Eukaryotic translation initiation factor 2 subunit 1
Non-histone chromosomal protein HMG-17
Intercellular adhesion molecule 1
Large ribosomal subunit protein P2
Large ribosomal subunit protein uL10
Transcription factor Jun
DNA-directed RNA polymerase III subunit RPC4
Lupus La protein
Integrin beta-1
Keratin, type I cytoskeletal 18
Collagen alpha-2(V) chain
Uroporphyrinogen decarboxylase
Tyrosine-protein kinase Fyn
Alpha-galactosidase A
Gelsolin
Retinoblastoma-associated protein
Prothymosin alpha
Cyclin-dependent kinase 1
ATP synthase subunit beta, mitochondrial

Protein S100-A6
Eukaryotic translation initiation factor 4E
Alpha-enolase
Glycogen phosphorylase, liver form
Glucose-6-phosphate isomerase
DNA polymerase beta
Nucleophosmin
Tropomyosin alpha-3 chain
Integrin alpha-V
Beta-hexosaminidase subunit alpha
Epoxide hydrolase 1
L-lactate dehydrogenase B chain
Major centromere autoantigen B
Glutathione peroxidase 1
Protein disulfide-isomerase
Histone H1.0
Acylphosphatase-1
Tyrosine-protein kinase Fes/Fps
Cathepsin D
Annexin A2
Calpain-1 catalytic subunit
Tubulin beta chain
Prosaposin
Beta-hexosaminidase subunit beta
Procathepsin L
Profilin-1
Bisphosphoglycerate mutase
Adenine phosphoribosyltransferase
Bifunctional glutamate/proline--tRNA ligase
Cathepsin B
Heat shock protein HSP 90-alpha
Galactose-1-phosphate uridylyltransferase
Heterogeneous nuclear ribonucleoproteins C1/C2
Laminin subunit beta-1
Tyrosine-protein kinase Yes
Tyrosine-protein kinase Lyn
Fumarate hydratase, mitochondrial
DNA excision repair protein ERCC-1
Thrombospondin-1
Transcription factor Sp1
Insulin-like growth factor 1 receptor
Collagen alpha-2(I) chain
Annexin A6
Rho-related GTP-binding protein RhoC
Complement decay-accelerating factor
Amino acid transporter heavy chain SLC3A2
Beta-glucuronidase
ATP-dependent 6-phosphofructokinase, muscle type
Heat shock protein HSP 90-beta
Signal recognition particle receptor subunit alpha

Asparagine synthetase [glutamine-hydrolyzing]
72 kDa type IV collagenase
Porphobilinogen deaminase
Neprilysin
Matrix Gla protein
Pyruvate dehydrogenase E1 component subunit alpha, somatic form, mitochondrial
Collagen alpha-2(IV) chain
Cytochrome c1, heme protein, mitochondrial
U2 small nuclear ribonucleoprotein B''
Hepatocyte growth factor receptor
U1 small nuclear ribonucleoprotein 70 kDa
Integrin alpha-5
Nuclear factor 1 C-type
Vimentin
Small ribosomal subunit protein eS17
Guanine nucleotide-binding protein G(i) subunit alpha-3
Annexin A5
Small ribosomal subunit protein uS2
CD63 antigen
Large ribosomal subunit protein uL3m
U1 small nuclear ribonucleoprotein A
Gamma-enolase
3-ketoacyl-CoA thiolase, peroxisomal
Signal recognition particle 19 kDa protein
Glutathione S-transferase P
U1 small nuclear ribonucleoprotein C
Galectin-1
Dihydropteridine reductase
High mobility group protein B1
SPARC
Tropomyosin alpha-1 chain
Clathrin light chain A
Clathrin light chain B
Annexin A4
2',3'-cyclic-nucleotide 3'-phosphodiesterase
Heme oxygenase 1
Dihydrolipoyl dehydrogenase, mitochondrial
Heterogeneous nuclear ribonucleoprotein A1
U2 small nuclear ribonucleoprotein A'
Cytochrome c oxidase subunit 6C
Tumor-associated calcium signal transducer 2
Poly [ADP-ribose] polymerase 1
DNA polymerase alpha catalytic subunit
Ubiquitin carboxyl-terminal hydrolase isozyme L1
Leukotriene A-4 hydrolase
Fructose-bisphosphate aldolase C
Histone H2A.Z
Histone H2A type 1
DNA-directed RNA polymerase II subunit GRINL1A
Glutathione S-transferase theta-2

POTE ankyrin domain family member J
LIM and senescent cell antigen-like-containing domain protein 3
Heat shock 70 kDa protein 1A
Chimeric ERCC6-PGBD3 protein
Tubulin alpha-3C chain
Zinc finger TRAF-type-containing protein 1
Ras-related protein Rap-2a
Serglycin
RNA-binding protein RO60
Lysosomal alpha-glucosidase
Ras-related protein R-Ras
HLA class I histocompatibility antigen, C alpha chain
Serine/threonine-protein kinase A-Raf
Histone H1.4
Dihydrolipoyllysine-residue acetyltransferase component of pyruvate dehydrogenase complex, mitochond
Receptor-type tyrosine-protein phosphatase F
COUP transcription factor 1
Thioredoxin
Cytochrome c oxidase subunit 5B, mitochondrial
Lysosomal protective protein
Microsomal glutathione S-transferase 1
cAMP-dependent protein kinase type I-alpha regulatory subunit
Tissue factor pathway inhibitor
S-formylglutathione hydrolase
60 kDa heat shock protein, mitochondrial
Clusterin
Endoplasmic reticulum chaperone BiP
Laminin subunit gamma-1
Lysosomal acid phosphatase
Microtubule-associated protein 2
Heat shock cognate 71 kDa protein
Solute carrier family 2, facilitated glucose transporter member 1
Solute carrier family 2, facilitated glucose transporter member 3
Protein 4.1
Uridine 5'-monophosphate synthase
Pyruvate dehydrogenase E1 component subunit beta, mitochondrial
Lipoamide acyltransferase component of branched-chain alpha-keto acid dehydrogenase complex, mitoch
Glycogen phosphorylase, brain form
Glycogen phosphorylase, muscle form
Ras-related protein Ral-A
Ras-related protein Ral-B
Breakpoint cluster region protein
Lysosome-associated membrane glycoprotein 1
Transcriptional regulator ERG
Medium-chain specific acyl-CoA dehydrogenase, mitochondrial
DNA topoisomerase 1
DNA topoisomerase 2-alpha
Glucose-6-phosphate 1-dehydrogenase
Ubiquitin-like protein 4A
Pyruvate carboxylase, mitochondrial

C-1-tetrahydrofolate synthase, cytoplasmic
Cation-independent mannose-6-phosphate receptor
Alcohol dehydrogenase class-3
Cyclin-dependent kinase 4
Ribose-phosphate pyrophosphokinase 2
Polyadenylate-binding protein 1
Proliferating cell nuclear antigen
Histidine--tRNA ligase, cytoplasmic
Collagen alpha-1(VI) chain
Collagen alpha-3(VI) chain
ADP/ATP translocase 1
ADP/ATP translocase 3
Inosine-5'-monophosphate dehydrogenase 2
Nucleoprotein TPR
Annexin A3
2-oxoisovalerate dehydrogenase subunit alpha, mitochondrial
Alpha-actinin-1
Angiotensin-converting enzyme
Proto-oncogene tyrosine-protein kinase Src
Xaa-Pro dipeptidase
X-ray repair cross-complementing protein 6
X-ray repair cross-complementing protein 5
Uracil-DNA glycosylase
Cytochrome c oxidase subunit 4 isoform 1, mitochondrial
5-aminolevulinate synthase, non-specific, mitochondrial
Lysosome-associated membrane glycoprotein 2
Ribonuclease inhibitor
Intercellular adhesion molecule 2
Elongation factor 2
Protein disulfide-isomerase A4
Prolyl 4-hydroxylase subunit alpha-1
Translationally-controlled tumor protein
Delta-aminolevulinic acid dehydratase
HLA class I histocompatibility antigen, alpha chain E
Plastin-3
Acylamino-acid-releasing enzyme
Electron transfer flavoprotein subunit alpha, mitochondrial
Glycogen [starch] synthase, muscle
cAMP-dependent protein kinase type II-alpha regulatory subunit
General transcription factor IIF subunit 2
CD59 glycoprotein
Bifunctional methylenetetrahydrofolate dehydrogenase/cyclohydrolase, mitochondrial
Macrophage migration inhibitory factor
CD99 antigen
Glucosidase 2 subunit beta
Hematopoietic lineage cell-specific protein
Farnesyl pyrophosphate synthase
Cytochrome c oxidase subunit 7A2, mitochondrial
Nidogen-1
Aldo-keto reductase family 1 member A1

Pyruvate kinase PKM
Endoplasmin
G2/mitotic-specific cyclin-B1
Myosin light chain 6B
Small nuclear ribonucleoprotein-associated proteins B and B'
Insulin-degrading enzyme
POU domain, class 2, transcription factor 1
Heterogeneous nuclear ribonucleoprotein L
Aspartate--tRNA ligase, cytoplasmic
Protein C-ets-1
Junction plakoglobin
Cytochrome b-c1 complex subunit 7
Serine/threonine-protein kinase B-raf
Fatty acid-binding protein, adipocyte
Glutamine synthetase
Aldo-keto reductase family 1 member B1
Aminopeptidase N
Poliovirus receptor
Ras-related C3 botulinum toxin substrate 2
Eukaryotic peptide chain release factor GTP-binding subunit ERF3A
Arylsulfatase A
Beta-1,4-galactosyltransferase 1
Ezrin
Ubiquitin carboxyl-terminal hydrolase isozyme L3
Fos-related antigen 1
Fos-related antigen 2
Membrane cofactor protein
Nucleoside diphosphate kinase A
NAD(P)H dehydrogenase [quinone] 1
N-acetylglucosamine-6-sulfatase
Phosphorylase b kinase gamma catalytic chain, liver/testis isoform
Arylsulfatase B
Small ribosomal subunit protein uS5
Transcription factor 4
Desmoplakin
Replication protein A 32 kDa subunit
Metalloproteinase inhibitor 2
CD44 antigen
Carbonyl reductase [NADPH] 1
Short-chain specific acyl-CoA dehydrogenase, mitochondrial
Cyclic AMP-responsive element-binding protein 1
Beta-galactosidase
Platelet endothelial cell adhesion molecule
Serine/threonine-protein phosphatase 2B catalytic subunit beta isoform
SH2/SH3 adapter protein NCK1
Intron Large complex component GCFC2
Histone H1.5
Histone H1.3
Histone H1.2
NADPH--cytochrome P450 reductase

Tyrosine-protein kinase Fer
Sarcoplasmic/endoplasmic reticulum calcium ATPase 2
1-phosphatidylinositol 4,5-bisphosphate phosphodiesterase gamma-2
Fumarylacetoacetase
Stathmin
Y-box-binding protein 3
Zinc finger protein 22
Zinc finger protein 24
Alpha-N-acetylgalactosaminidase
High mobility group protein HMG-I/HMG-Y
Transmembrane protein 11, mitochondrial
Aspartate aminotransferase, cytoplasmic
Protein kinase C alpha type
Integrin alpha-2
Gap junction alpha-1 protein
Sphingomyelin phosphodiesterase
Nucleolar transcription factor 1
Transcription factor JunD
T-cell acute lymphocytic leukemia protein 1
NADH dehydrogenase [ubiquinone] 1 beta subcomplex subunit 7
cAMP-dependent protein kinase catalytic subunit alpha
Calpain-2 catalytic subunit
Tyrosine-protein phosphatase non-receptor type 2
CTP synthase 1
Endoglin
Probable ATP-dependent RNA helicase DDX5
ATP-dependent 6-phosphofructokinase, liver type
Vascular endothelial growth factor receptor 1
26S proteasome regulatory subunit 6A
T-complex protein 1 subunit alpha
Tyrosine-protein phosphatase non-receptor type 1
General transcription and DNA repair factor IIH helicase subunit XPD
Large ribosomal subunit protein eL33
Integrin beta-5
ADP-ribosylation factor 4
Large ribosomal subunit protein uL30
Vinculin
Receptor-type tyrosine-protein phosphatase alpha
Arylamine N-acetyltransferase 1
Protein SON
Negative elongation factor E
Large ribosomal subunit protein uL22
Phosphoglycerate mutase 1
Regulator of chromosome condensation
Cyclic AMP-dependent transcription factor ATF-1
Cyclic AMP-dependent transcription factor ATF-6 alpha
DNA ligase 1
DNA repair protein XRCC1
Peptidyl-glycine alpha-amidating monooxygenase
Cadherin-2

1-phosphatidylinositol 4,5-bisphosphate phosphodiesterase gamma-1
Nucleolin
Hexokinase-1
DNA-directed RNA polymerase II subunit RPB3
DNA-directed RNA polymerases I, II, and III subunit RPABC1
NADH dehydrogenase [ubiquinone] flavoprotein 2, mitochondrial
General transcription and DNA repair factor IIH helicase subunit XPB
E3 ubiquitin-protein ligase TRIM21
Interferon-induced, double-stranded RNA-activated protein kinase
Spermidine synthase
Sodium/hydrogen exchanger 1
Casein kinase II subunit alpha'
Nuclear factor NF-kappa-B p105 subunit
Plasma membrane calcium-transporting ATPase 1
Eukaryotic translation initiation factor 2 subunit 2
Annexin A7
Cyclin-A2
Transcription factor BTF3
Ras-related protein Rab-3B
Ras-related protein Rab-4A
Ras-related protein Rab-5A
Ras-related protein Rab-6A
DNA mismatch repair protein Msh3
Atrial natriuretic peptide receptor 2
Proteasome subunit beta type-1
Cation-dependent mannose-6-phosphate receptor
Cytochrome c oxidase subunit 5A, mitochondrial
Lamin-B1
Calpastatin
Inosine-5'-monophosphate dehydrogenase 1
Collagen alpha-1(V) chain
Nebulin
N(4)-(beta-N-acetylglucosaminyl)-L-asparaginase
Ras GTPase-activating protein 1
Cyclin-dependent kinase 11B
Glutathione S-transferase Mu 3
V-type proton ATPase subunit B, brain isoform
V-type proton ATPase subunit C 1
Cysteine and glycine-rich protein 1
Filamin-A
Neurofibromin
Amine oxidase [flavin-containing] A
Cytoplasmic aconitate hydratase
Sphingosine 1-phosphate receptor 1
Synaptotagmin-1
5'-nucleotidase
Transcription initiation factor TFIID subunit 1
Midkine
Voltage-dependent anion-selective channel protein 1
Biglycan

Succinate dehydrogenase [ubiquinone] iron-sulfur subunit, mitochondrial
CD9 antigen
Catechol O-methyltransferase
Protein-glutamine gamma-glutamyltransferase 2
Methylmalonyl-CoA mutase, mitochondrial
Oxysterol-binding protein 1
Protein-L-isoaspartate(D-aspartate) O-methyltransferase
rRNA 2'-O-methyltransferase fibrillarin
Trifunctional purine biosynthetic protein adenosine-3
Bifunctional phosphoribosylaminoimidazole carboxylase/phosphoribosylaminoimidazole succinocarboxam
Sterol carrier protein 2
Ubiquitin-like modifier-activating enzyme 1
Nucleoside diphosphate kinase B
NADPH:adrenodoxin oxidoreductase, mitochondrial
Heterogeneous nuclear ribonucleoproteins A2/B1
MHC class II regulatory factor RFX1
E3 ubiquitin-protein ligase CBL
Insulin-like growth factor-binding protein 4
cAMP-dependent protein kinase catalytic subunit beta
Cytochrome b-c1 complex subunit 2, mitochondrial
Ferrochelatase, mitochondrial
DNA repair protein complementing XP-A cells
Transcription elongation factor A protein 1
Prostaglandin G/H synthase 1
Integrin alpha-6
Splicing factor, proline- and glutamine-rich
Tubulin gamma-1 chain
Peptidyl-prolyl cis-trans isomerase B
NAD-dependent malic enzyme, mitochondrial
Tryptophan--tRNA ligase, cytoplasmic
Small ribosomal subunit protein uS3
Ribosomal protein S6 kinase beta-1
Tyrosine-protein kinase JAK1
Receptor-type tyrosine-protein phosphatase beta
Receptor-type tyrosine-protein phosphatase epsilon
Receptor-type tyrosine-protein phosphatase gamma
Nuclear autoantigen Sp-100
Colorectal mutant cancer protein
Tumor necrosis factor ligand superfamily member 4
Adenosylhomocysteinase
Cofilin-1
Eukaryotic translation initiation factor 4B
40-kDa huntingtin-associated protein
Plasma membrane calcium-transporting ATPase 4
Diacylglycerol kinase alpha
Carnitine O-palmitoyltransferase 2, mitochondrial
Thymidylate kinase
Ribonucleoside-diphosphate reductase large subunit
G1/S-specific cyclin-D1
Rab proteins geranylgeranyltransferase component A 1

ER lumen protein-retaining receptor 1
COUP transcription factor 2
Elongation factor 1-beta
ATP synthase F(0) complex subunit B1, mitochondrial
Low molecular weight phosphotyrosine protein phosphatase
Protein kinase C eta type
Acetyl-CoA acetyltransferase, mitochondrial
Myosin regulatory light polypeptide 9
DNA-directed RNA polymerase II subunit RPB1
Cyclin-dependent kinase 2
Beta-adrenergic receptor kinase 1
Proteinase-activated receptor 1
DNA replication licensing factor MCM3
3-mercaptopyruvate sulfurtransferase
Small ribosomal subunit protein eS12
Bromodomain-containing protein 2
Transcriptional repressor protein YY1
DnaJ homolog subfamily B member 1
DnaJ homolog subfamily B member 2
ATP synthase subunit alpha, mitochondrial
Cathepsin S
Proteasome subunit alpha type-1
Proteasome subunit alpha type-2
Proteasome subunit alpha type-3
Proteasome subunit alpha type-4
Tumor necrosis factor receptor superfamily member 5
Integrin alpha-3
Pentraxin-related protein PTX3
Moesin
Probable ATP-dependent RNA helicase DDX6
DNA (cytosine-5)-methyltransferase 1
Splicing factor U2AF 65 kDa subunit
Large ribosomal subunit protein eL13
Rab proteins geranylgeranyltransferase component A 2
Isovaleryl-CoA dehydrogenase, mitochondrial
Alpha-1,3-mannosyl-glycoprotein 2-beta-N-acetylglucosaminyltransferase
High mobility group protein B2
Polypyrimidine tract-binding protein 1
Threonine--tRNA ligase 1, cytoplasmic
Valine--tRNA ligase
Elongation factor 1-gamma
Peptidyl-prolyl cis-trans isomerase FKBP2
Interleukin-3 receptor subunit alpha
Stomatin
Adenylate kinase 4, mitochondrial
14-3-3 protein theta
Mitogen-activated protein kinase 3
MAP/microtubule affinity-regulating kinase 3
Large ribosomal subunit protein uL16
Replication protein A 70 kDa DNA-binding subunit

DNA-(apurinic or apyrimidinic site) endonuclease
CD82 antigen
Deoxycytidine kinase
Multifunctional protein CAD
Calreticulin
Microtubule-associated protein 4
Calnexin
Phosphatidylinositol 3-kinase regulatory subunit alpha
Inositol-trisphosphate 3-kinase B
Proteasome subunit beta type-8
Proteasome subunit beta type-9
Proteasome subunit alpha type-5
Proteasome subunit beta type-4
Proteasome subunit beta type-6
Proteasome subunit beta type-5
Glutathione S-transferase Mu 2
ATP-binding cassette sub-family D member 3
Protein ITPRID2
Protein-lysine 6-oxidase
NADH-ubiquinone oxidoreductase 75 kDa subunit, mitochondrial
DNA polymerase delta catalytic subunit
Probable global transcription activator SNF2L1
Mitogen-activated protein kinase 1
Retinoic acid receptor RXR-beta
DNA excision repair protein ERCC-5
Progranulin
Receptor-type tyrosine-protein phosphatase mu
Cytosol aminopeptidase
Hematopoietic progenitor cell antigen CD34
General transcription factor IIE subunit 1
Transcription initiation factor IIE subunit beta
Tripeptidyl-peptidase 2
Inositol monophosphatase 1
CCN family member 2
Ephrin type-A receptor 2
Ephrin type-B receptor 2
SHC-transforming protein 1
DNA-3-methyladenine glycosylase
Transketolase
Caspase-1
Nitric oxide synthase 3
RNA-binding motif, single-stranded-interacting protein 1
Protein PML
Non-receptor tyrosine-protein kinase TYK2
Elongation factor 1-delta
Myristoylated alanine-rich C-kinase substrate
Guanine nucleotide-binding protein subunit alpha-11
Delta-1-pyrroline-5-carboxylate dehydrogenase, mitochondrial
Endoplasmic reticulum resident protein 29
Peroxiredoxin-6

Flavin reductase (NADPH)
Peroxiredoxin-5, mitochondrial
Thioredoxin-dependent peroxide reductase, mitochondrial
Large ribosomal subunit protein uL11
Enoyl-CoA hydratase, mitochondrial
UMP-CMP kinase
Phosphatidylethanolamine-binding protein 1
Protein disulfide-isomerase A3
Serine/threonine-protein phosphatase 2A 65 kDa regulatory subunit A alpha isoform
Serine/threonine-protein phosphatase 2A 65 kDa regulatory subunit A beta isoform
Cell division cycle protein 27 homolog
Peptidyl-prolyl cis-trans isomerase F, mitochondrial
NK-tumor recognition protein
Glycylpeptide N-tetradecanoyltransferase 1
Heme oxygenase 2
Adenylosuccinate synthetase isozyme 2
Tyrosine-protein kinase receptor UFO
Alpha-2-macroglobulin receptor-associated protein
Translocator protein
Adenylosuccinate lyase
CAP-Gly domain-containing linker protein 1
Sorcin
Leukocyte elastase inhibitor
High affinity cationic amino acid transporter 1
Aldehyde dehydrogenase X, mitochondrial
DNA-directed RNA polymerase II subunit RPB2
Succinate dehydrogenase [ubiquinone] flavoprotein subunit, mitochondrial
Coronin-1A
Rab GDP dissociation inhibitor alpha
S-adenosylmethionine synthase isoform type-2
cAMP-dependent protein kinase type II-beta regulatory subunit
Ribonucleoside-diphosphate reductase subunit M2
Syndecan-4
Cytotoxic granule associated RNA binding protein TIA1
Sodium- and chloride-dependent taurine transporter
DnaJ homolog subfamily A member 1
RAC-alpha serine/threonine-protein kinase
Cytochrome b-c1 complex subunit 1, mitochondrial
3-hydroxyisobutyrate dehydrogenase, mitochondrial
Bifunctional purine biosynthesis protein ATIC
Heterogeneous nuclear ribonucleoprotein H3
Heterogeneous nuclear ribonucleoprotein H
Caspase-14
14-3-3 protein beta/alpha
Stress-induced-phosphoprotein 1
Protein S100-A11
Peroxiredoxin-2
Beta-arrestin-2
Glycerol kinase
Deoxycytidylate deaminase

Pyrroline-5-carboxylate reductase 1, mitochondrial
Guanylate-binding protein 1
Guanylate-binding protein 2
ETS-related transcription factor Elf-1
General transcription factor IIH subunit 1
Syntaxin-2
Large ribosomal subunit protein uL6
Long-chain-fatty-acid--CoA ligase 1
Cadherin-5
Kinesin-1 heavy chain
Deoxyuridine 5'-triphosphate nucleotidohydrolase, mitochondrial
Multidrug resistance-associated protein 1
ATP-binding cassette sub-family D member 1
Dual specificity protein kinase TTK
DNA replication licensing factor MCM4
DNA replication licensing factor MCM5
DNA replication licensing factor MCM7
N-acetylgalactosamine-6-sulfatase
Serine hydroxymethyltransferase, cytosolic
Serine hydroxymethyltransferase, mitochondrial
Heat shock 70 kDa protein 1-like
Heat shock 70 kDa protein 4
Mannose-6-phosphate isomerase
Glypican-1
Profilin-2
Catenin alpha-1
Catenin beta-1
Prohibitin 1
Serpin B6
Merlin
Radixin
Replication protein A 14 kDa subunit
Replication factor C subunit 4
Replication factor C subunit 2
Replication factor C subunit 1
Large ribosomal subunit protein eL22
General transcription factor IIF subunit 1
Sepiapterin reductase
Cystathionine beta-synthase
Fibrillin-1
Fibrillin-2
Glycogen debranching enzyme
Myosin-9
Myosin-10
Tyrosine-protein kinase receptor Tie-1
Coatomer subunit beta'
Sterol O-acyltransferase 1
Alpha-adducin
Basigin
RNA-binding protein FUS

Nuclear pore complex protein Nup214
Protein DEK
Transcription factor SOX-18
Myosin-11
Protein phosphatase 1A
Hydroxymethylglutaryl-CoA lyase, mitochondrial
Vascular endothelial growth factor receptor 2
26S proteasome regulatory subunit 7
Glutathione hydrolase 5 proenzyme
ADP-ribosylation factor-like protein 2
ADP-ribosylation factor-like protein 3
Dual specificity mitogen-activated protein kinase kinase 2
ATP synthase subunit gamma, mitochondrial
V-type proton ATPase subunit E 1
Oxygen-dependent coproporphyrinogen-III oxidase, mitochondrial
Large ribosomal subunit protein uL4
Oxidized purine nucleoside triphosphate hydrolase
Lon protease homolog, mitochondrial
Phosphoglucomutase-1
Serine/threonine-protein phosphatase PP1-gamma catalytic subunit
Guanine nucleotide-binding protein-like 1
Dihydrolipoyllysine-residue succinyltransferase component of 2-oxoglutarate dehydrogenase complex, mit
GMP reductase 1
Phospholipid hydroperoxide glutathione peroxidase GPX4
Serine/threonine-protein kinase receptor R3
17-beta-hydroxysteroid dehydrogenase type 2
Signal recognition particle 14 kDa protein
TGF-beta receptor type-2
Nuclear pore glycoprotein p62
Hippocalcin-like protein 1
Squalene synthase
Zinc finger E-box-binding homeobox 1
Transgelin-2
Transaldolase
Alpha-synuclein
Electron transfer flavoprotein subunit beta
RNA-binding motif protein, X chromosome
Coilin
Vitamin K-dependent gamma-carboxylase
Lysosomal acid lipase/cholesteryl ester hydrolase
V-type proton ATPase catalytic subunit A
Stress-70 protein, mitochondrial
Eukaryotic initiation factor 4A-III
Cyclin-dependent kinase inhibitor 1
Small ribosomal subunit protein eS19
Large ribosomal subunit protein uL3
Collagen alpha-1(XVIII) chain
Dolichyl-diphosphooligosaccharide--protein glycosyltransferase 48 kDa subunit
Acidic leucine-rich nuclear phosphoprotein 32 family member A
Flap endonuclease 1

Homeobox protein cut-like 1
Macrophage-capping protein
Adenylyl cyclase-associated protein 2
Interleukin-6 receptor subunit beta
Alpha-taxilin
T-complex protein 1 subunit zeta
Nicotinamide N-methyltransferase
Proteasome subunit beta type-10
Pre-B-cell leukemia transcription factor 2
Large ribosomal subunit protein uL13
ADP-ribosylation factor-like protein 1
DNA mismatch repair protein Mlh1
Signal transducer and activator of transcription 3
Ubiquitin carboxyl-terminal hydrolase 8
Malate dehydrogenase, cytoplasmic
Malate dehydrogenase, mitochondrial
Replication factor C subunit 5
Replication factor C subunit 3
Trifunctional enzyme subunit alpha, mitochondrial
Eukaryotic translation initiation factor 2 subunit 3
ETS translocation variant 3
Centrin-2
Transcription factor ETV6
Eukaryotic translation initiation factor 2D
Protein BUD31 homolog
Ubiquitin-like modifier-activating enzyme 7
N-alpha-acetyltransferase 10
Lysine-specific demethylase 5C
Tyrosine-protein kinase CSK
Glycine--tRNA ligase
Isoleucine--tRNA ligase, cytoplasmic
Reduced folate transporter
Eukaryotic translation initiation factor 1
Protein kinase C iota type
ETS domain-containing protein Elk-3
Beta-centractin
Enoyl-CoA delta isomerase 1, mitochondrial
Lamina-associated polypeptide 2, isoform alpha
Lamina-associated polypeptide 2, isoforms beta/gamma
Signal transducer and activator of transcription 1-alpha/beta
Signal transducer and activator of transcription 6
Signal transducer and activator of transcription 5A
Exosome RNA helicase MTR4
Aldo-keto reductase family 1 member C3
Phosphatidylinositol 4,5-bisphosphate 3-kinase catalytic subunit alpha isoform
Phosphatidylinositol 4,5-bisphosphate 3-kinase catalytic subunit beta isoform
Serine/threonine-protein kinase mTOR
Phosphatidylinositol 4-kinase alpha
Epidermal growth factor receptor substrate 15
Caspase-3

Caspase-2
Small ribosomal subunit protein eS27
Tyrosine-protein kinase ABL2
Probable helicase with zinc finger domain
Condensin-2 complex subunit D3
RNA-binding protein 34
Leucine-rich PPR motif-containing protein, mitochondrial
3-ketoacyl-CoA thiolase, mitochondrial
Large ribosomal subunit protein uL29
Lysosomal Pro-X carboxypeptidase
Huntingtin
Endothelin-converting enzyme 1
Methylenetetrahydrofolate reductase (NADPH)
Neutral amino acid transporter A
Platelet-activating factor acetylhydrolase IB subunit beta
Cell surface glycoprotein MUC18
Carnitine O-acetyltransferase
Matrin-3
DNA mismatch repair protein Msh2
G protein-coupled receptor kinase 6
Glycerol-3-phosphate dehydrogenase, mitochondrial
Translocon-associated protein subunit alpha
Tyrosine-protein phosphatase non-receptor type 9
Ran-specific GTPase-activating protein
Nicotinamide phosphoribosyltransferase
26S proteasome regulatory subunit 6B
Elongation factor Ts, mitochondrial
Peptidyl-prolyl cis-trans isomerase C
Voltage-dependent anion-selective channel protein 2
Short/branched chain specific acyl-CoA dehydrogenase, mitochondrial
Chromobox protein homolog 5
Ubiquitin carboxyl-terminal hydrolase 5
Mitogen-activated protein kinase 8
Dual specificity mitogen-activated protein kinase kinase 4
Proliferation marker protein Ki-67
Phosphorylase b kinase regulatory subunit alpha, liver isoform
Phosphorylase b kinase regulatory subunit alpha, skeletal muscle isoform
Ran GTPase-activating protein 1
ATP-dependent DNA helicase Q1
Probable 28S rRNA (cytosine(4447)-C(5))-methyltransferase
Transcriptional regulator ATRX
Adapter molecule crk
Crk-like protein
Translation initiation factor IF-2, mitochondrial
Large proline-rich protein BAG6
Vesicle-fusing ATPase
Neurogenic locus notch homolog protein 1
Dual specificity mitogen-activated protein kinase kinase 3
Lys-63-specific deubiquitinase BRCC36
Large ribosomal subunit protein uL15

Large ribosomal subunit protein uL18
Large ribosomal subunit protein eL21
Large ribosomal subunit protein eL28
Small ribosomal subunit protein uS4
Small ribosomal subunit protein uS7
Small ribosomal subunit protein eS10
Microtubule-associated protein 1B
Glucosamine-6-phosphate isomerase 1
E3 ubiquitin-protein ligase NEDD4
Utrophin
Ras GTPase-activating-like protein IQGAP1
Glycogenin-1
Dolichyl-diphosphooligosaccharide--protein glycosyltransferase subunit STT3A
Cytosolic phospholipase A2
F-actin-capping protein subunit alpha-2
F-actin-capping protein subunit beta
Eukaryotic translation initiation factor 1A, X-chromosomal
Retinaldehyde dehydrogenase 3
Glutamine--tRNA ligase
Large ribosomal subunit protein eL29
mRNA decay activator protein ZFP36L2
Cytochrome b-c1 complex subunit Rieske, mitochondrial
ATP synthase subunit O, mitochondrial
LIM and senescent cell antigen-like-containing domain protein 1
Glioma pathogenesis-related protein 1
Prolyl endopeptidase
NADP-dependent malic enzyme
Iron-responsive element-binding protein 2
Tissue factor pathway inhibitor 2
DNA-binding protein RFX5
Phosphatidylinositol 5-phosphate 4-kinase type-2 alpha
Coatomer subunit delta
Lanosterol synthase
Serine/threonine-protein phosphatase 2B catalytic subunit gamma isoform
Glutamate--cysteine ligase catalytic subunit
Glutamate--cysteine ligase regulatory subunit
CD151 antigen
Trafficking protein particle complex subunit 10
26S proteasome non-ATPase regulatory subunit 8
Protein PRRC2A
Glutathione synthetase
T-complex protein 1 subunit epsilon
Phosphatidylserine synthase 1
Nestin
Heat shock 70 kDa protein 13
Casein kinase I isoform alpha
Casein kinase I isoform delta
Isocitrate dehydrogenase [NADP], mitochondrial
Phosphatidylinositol 4,5-bisphosphate 3-kinase catalytic subunit gamma isoform
Phosphatidylinositol transfer protein beta isoform

DNA polymerase delta subunit 2
MARCKS-related protein
Paxillin
Guided entry of tail-anchored proteins factor CAMLG
Nuclear receptor subfamily 2 group C member 2
MAP kinase-activated protein kinase 2
Deoxyribonuclease-1-like 1
4-trimethylaminobutyraldehyde dehydrogenase
Large ribosomal subunit protein eL34
Ribose-5-phosphate isomerase
Protein ERGIC-53
Natural resistance-associated macrophage protein 2
Nuclear autoantigenic sperm protein
Fatty acid synthase
Protein farnesyltransferase/geranylgeranyltransferase type-1 subunit alpha
Protein farnesyltransferase subunit beta
Deoxyhypusine synthase
T-complex protein 1 subunit gamma
Large ribosomal subunit protein bL19m
Beta-arrestin-1
Elongation factor Tu, mitochondrial
Alpha-aminoadipic semialdehyde dehydrogenase
Ubiquitin-conjugating enzyme E2 R1
Inositol polyphosphate 1-phosphatase
Centromere protein F
Signal recognition particle 9 kDa protein
Choline-phosphate cytidylyltransferase A
Alanine--tRNA ligase, cytoplasmic
Cysteine--tRNA ligase, cytoplasmic
Histidine--tRNA ligase, mitochondrial
Serine--tRNA ligase, cytoplasmic
Protein phosphatase 1F
Alpha-mannosidase 2x
DNA primase small subunit
DNA primase large subunit
Caspase-4
Casein kinase I isoform epsilon
Proteasome subunit beta type-3
Proteasome subunit beta type-2
DNA replication licensing factor MCM2
Very long-chain specific acyl-CoA dehydrogenase, mitochondrial
YLP motif-containing protein 1
Vacuolar protein sorting-associated protein 41 homolog
Transmembrane emp24 domain-containing protein 10
RNA-binding protein 25
Protein numb homolog
Dual specificity protein kinase CLK3
Presenilin-1
Translation initiation factor eIF2B subunit beta
Adenosine 5'-monophosphoramidase HINT1

Nuclear pore complex protein Nup153
E3 SUMO-protein ligase RanBP2
Regulator of G-protein signaling 19
Regulator of G-protein signaling 3
Presenilin-2
Tuberin
NADH dehydrogenase [ubiquinone] flavoprotein 1, mitochondrial
Glycogen synthase kinase-3 alpha
Glycogen synthase kinase-3 beta
Transcription initiation factor TFIID subunit 6
Cytosolic purine 5'-nucleotidase
Selenide, water dikinase 1
5-formyltetrahydrofolate cyclo-ligase
GMP synthase [glutamine-hydrolyzing]
DNA ligase 3
DNA ligase 4
Double-strand break repair protein MRE11
Guanine nucleotide-binding protein G(q) subunit alpha
Isocitrate dehydrogenase [NAD] subunit alpha, mitochondrial
Sulfotransferase 1A1
Matrix metalloproteinase-14
Protoporphyrinogen oxidase
Rab GDP dissociation inhibitor beta
Emerin
Carnitine O-palmitoyltransferase 1, liver isoform
Sulfate transporter
Serpin B8
Serpin B9
Serpin H1
PDZ and LIM domain protein 4
Hsc70-interacting protein
Peroxisomal targeting signal 1 receptor
ETS domain-containing transcription factor ERF
Vasodilator-stimulated phosphoprotein
Dynamin-2
Methionine aminopeptidase 2
Bis(5'-nucleosyl)-tetraphosphatase [asymmetrical]
Cyclin-dependent kinase 7
Kinetochore-associated protein 1
Ras association domain-containing protein 2
Cyclin-dependent kinase 9
Lipopolysaccharide-responsive and beige-like anchor protein
Basal cell adhesion molecule
Palmitoyl-protein thioesterase 1
Large ribosomal subunit protein eL14
T-complex protein 1 subunit theta
T-complex protein 1 subunit delta
Annexin A11
Poly(A) polymerase alpha
RNA-binding protein FXR1

RNA-binding protein FXR2
Ras-related protein Rab-5C
Ras-related protein Rab-7a
Ras-related protein Rab-9A
Ras-related protein Rab-13
Ras-related protein Rab-27A
1-phosphatidylinositol 4,5-bisphosphate phosphodiesterase delta-1
Small ribosomal subunit protein mS29
Dual specificity protein phosphatase 3
Probable global transcription activator SNF2L2
Transcription activator BRG1
Isocitrate dehydrogenase [NAD] subunit gamma, mitochondrial
Galactokinase
Translocon-associated protein subunit delta
B-cell receptor-associated protein 31
Thiopurine S-methyltransferase
Methyl-CpG-binding protein 2
Host cell factor 1
Interleukin-1 receptor-associated kinase 1
Caveolin-2
Aldehyde dehydrogenase family 3 member A2
Peroxisomal multifunctional enzyme type 2
26S proteasome non-ATPase regulatory subunit 7
Sulfite oxidase, mitochondrial
N-sulphoglucosamine sulphohydrolase
Signal transducer and activator of transcription 5B
Ubiquitin carboxyl-terminal hydrolase 11
H(+)/Cl(-) exchange transporter 7
Vesicle-associated membrane protein 7
Ribosomal protein S6 kinase alpha-3
Cytoplasmic tyrosine-protein kinase BMX
Hepatoma-derived growth factor
Cyclin-H
CDK-activating kinase assembly factor MAT1
Serine/threonine-protein kinase Nek3
Ubiquitin-conjugating enzyme E2 E1
NADH dehydrogenase [ubiquinone] 1 alpha subcomplex subunit 8
Heterogeneous nuclear ribonucleoprotein A3
6-phosphogluconate dehydrogenase, decarboxylating
Heterogeneous nuclear ribonucleoprotein M
Importin subunit alpha-1
Importin subunit alpha-5
Nuclear cap-binding protein subunit 2
Rap1 GTPase-GDP dissociation stimulator 1
DNA-directed RNA polymerases I, II, and III subunit RPABC3
Dual specificity mitogen-activated protein kinase kinase 6
Rho GDP-dissociation inhibitor 1
Rho GDP-dissociation inhibitor 2
Arf-GAP domain and FG repeat-containing protein 1
Heterogeneous nuclear ribonucleoprotein F

Signal transducer and activator of transcription 2
DNA mismatch repair protein Msh6
Kinesin-like protein KIF11
Guanine nucleotide exchange factor VAV2
Zinc finger protein 143
RNA-binding protein 5
2-iminobutanoate/2-iminopropanoate deaminase
Spermine synthase
Hexokinase-2
Large ribosomal subunit protein bL12m
Diacylglycerol kinase theta
Bifunctional heparan sulfate N-deacetylase/N-sulfotransferase 1
Thimet oligopeptidase
F-actin-capping protein subunit alpha-1
High mobility group protein HMGI-C
Cysteine-rich protein 2
Nuclear pore complex protein Nup98-Nup96
Biliverdin reductase A
Tricarboxylate transport protein, mitochondrial
Serine/threonine-protein phosphatase 5
Serine/threonine-protein kinase PLK1
Death-associated protein kinase 1
Arfaptin-2
Arfaptin-1
Cytosolic Fe-S cluster assembly factor NUBP1
ATP-citrate synthase
Methionine aminopeptidase 1
Succinate--CoA ligase [ADP/GDP-forming] subunit alpha, mitochondrial
Diphosphomevalonate decarboxylase
Geranylgeranyl transferase type-1 subunit beta
Geranylgeranyl transferase type-2 subunit beta
Coatomer subunit beta
Coatomer subunit alpha
Dipeptidyl peptidase 1
LIM domain kinase 1
Clathrin heavy chain 2
AP-3 complex subunit mu-2
AP-2 complex subunit sigma
Holocytochrome c-type synthase
Mitogen-activated protein kinase 12
Pituitary tumor-transforming gene 1 protein-interacting protein
Smoothelin
Monocarboxylate transporter 1
IST1 homolog
Protein transport protein Sec24C
Activated RNA polymerase II transcriptional coactivator p15
DNA polymerase subunit gamma-1
Methylosome subunit pICln
Arginine--tRNA ligase, cytoplasmic
Protein HIRA

Ataxin-3
Atrophin-1
PMS1 protein homolog 1
Mismatch repair endonuclease PMS2
Voltage-dependent calcium channel subunit alpha-2/delta-1
Tyrosine--tRNA ligase, cytoplasmic
Ubiquitin carboxyl-terminal hydrolase 14
5'-AMP-activated protein kinase subunit gamma-1
Heat shock-related 70 kDa protein 2
Branched-chain-amino-acid aminotransferase, cytosolic
Sodium/potassium-transporting ATPase subunit beta-3
UV excision repair protein RAD23 homolog B
Ephrin type-B receptor 4
Alpha-N-acetylglucosaminidase
Adenylate kinase 2, mitochondrial
Delta-1-pyrroline-5-carboxylate synthase
Alpha-soluble NSF attachment protein
Eukaryotic translation initiation factor 5
Solute carrier family 12 member 2
26S proteasome non-ATPase regulatory subunit 4
Developmentally-regulated GTP-binding protein 2
Exportin-2
Transitional endoplasmic reticulum ATPase
Microfibrillar-associated protein 1
Trifunctional enzyme subunit beta, mitochondrial
Mesencephalic astrocyte-derived neurotrophic factor
Afadin
RNA polymerase II elongation factor ELL
Nucleosome assembly protein 1-like 1
Caspase-7
Caspase-6
Adenosine kinase
Double-stranded RNA-specific adenosine deaminase
Laminin subunit beta-2
Cadherin-13
Protein SEC13 homolog
NHP2-like protein 1
Puromycin-sensitive aminopeptidase
FAD-linked sulfhydryl oxidase ALR
Heterogeneous nuclear ribonucleoprotein H2
Succinyl-CoA:3-ketoacid coenzyme A transferase 1, mitochondrial
Eukaryotic translation initiation factor 3 subunit B
IgG receptor FcRn large subunit p51
BH3-interacting domain death agonist
ATP synthase subunit f, mitochondrial
Ribosomal RNA processing protein 1 homolog A
Methionine--tRNA ligase, cytoplasmic
cAMP-regulated phosphoprotein 19
AP-1 complex subunit sigma-2
ATP synthase subunit ATP5MJ, mitochondrial

ATP synthase subunit epsilon, mitochondrial
ATP synthase subunit e, mitochondrial
Histone deacetylase 4
Eukaryotic translation initiation factor 6
C-terminal-binding protein 2
NADH dehydrogenase [ubiquinone] 1 alpha subcomplex subunit 6
Peroxisomal biogenesis factor 3
3-keto-steroid reductase/17-beta-hydroxysteroid dehydrogenase 7
Breast cancer anti-estrogen resistance protein 1
Syntaxin-17
Cilia- and flagella-associated protein 298
tRNA (guanine-N(7)-)-methyltransferase non-catalytic subunit WDR4
Transmembrane protein 33
Gem-associated protein 4
Coronin-7
Nuclear pore complex protein Nup107
Gasdermin-D
Selenocysteine-specific elongation factor
Sestrin-2
Anthrax toxin receptor 2
Myotrophin
Actin-related protein 2/3 complex subunit 4
CD81 antigen
Protein transport protein Sec61 subunit gamma
Triosephosphate isomerase
Eukaryotic translation initiation factor 3 subunit E
Protein transport protein Sec61 subunit beta
Phosphatidylinositol 3,4,5-trisphosphate 3-phosphatase and dual-specificity protein phosphatase PTEN
Serine/threonine-protein phosphatase 4 catalytic subunit
Gamma-aminobutyric acid receptor-associated protein-like 2
Reactive oxygen species modulator 1
Myosin light polypeptide 6
Actin, cytoplasmic 1
Eukaryotic initiation factor 4A-I
Small ribosomal subunit protein uS10
Ribose-phosphate pyrophosphokinase 1
Proteasome subunit alpha type-6
Protein S100-A10
Cell division control protein 42 homolog
Destrin
Glia maturation factor beta
Ras-related protein Rab-8A
Signal peptidase complex subunit 3
Signal recognition particle subunit SRP54
Ras-related protein Rab-4B
Ras-related protein Rab-2A
Ras-related protein Rab-5B
Ras-related protein Rab-10
Ubiquitin-conjugating enzyme E2 D3
NEDD8-conjugating enzyme Ubc12

Ubiquitin-conjugating enzyme E2 K
Ubiquitin-conjugating enzyme E2 N
Ras-related protein Rab-14
Actin-related protein 3
Actin-related protein 2
Alpha-centractin
COP9 signalosome complex subunit 2
ATP-binding cassette sub-family E member 1
Ras-related protein Rap-1b
Ras-related protein Rap-2b
Protein max
Small ribosomal subunit protein eS1
Large ribosomal subunit protein uL24
Proteasome activator complex subunit 3
Large ribosomal subunit protein eL15
Large ribosomal subunit protein eL27
V-type proton ATPase subunit d 1
Large ribosomal subunit protein eL43
Transforming protein RhoA
Rho-related GTP-binding protein RhoE
N-alpha-acetyltransferase 20
10 kDa heat shock protein, mitochondrial
Protein transport protein Sec61 subunit alpha isoform 1
Prefoldin subunit 3
Syntaxin-binding protein 1
Beta-2-microglobulin
Dolichyl-diphosphooligosaccharide--protein glycosyltransferase subunit DAD1
NPC intracellular cholesterol transporter 2
Coatomer subunit zeta-1
Small ubiquitin-related modifier 2
DDB1- and CUL4-associated factor 7
WD repeat-containing protein 5
AP-1 complex subunit sigma-1A
Nuclear transport factor 2
Heterogeneous nuclear ribonucleoprotein K
14-3-3 protein gamma
Ubiquitin carboxyl-terminal hydrolase 46
Ras-related protein R-Ras2
Small ribosomal subunit protein eS7
Serine/threonine-protein phosphatase PP1-alpha catalytic subunit
Serine/threonine-protein phosphatase PP1-beta catalytic subunit
26S proteasome regulatory subunit 4
26S proteasome regulatory subunit 8
Small ribosomal subunit protein eS8
Small ribosomal subunit protein uS8
Small ribosomal subunit protein uS9
Ubiquitin-conjugating enzyme E2 G1
Ubiquitin-conjugating enzyme E2 H
14-3-3 protein epsilon
Small ribosomal subunit protein uS11

Small ribosomal subunit protein uS12
Small ribosomal subunit protein uS13
Small ribosomal subunit protein uS14
Small ribosomal subunit protein uS15
Small ribosomal subunit protein uS17
Small nuclear ribonucleoprotein E
Small nuclear ribonucleoprotein F
U6 snRNA-associated Sm-like protein LSm3
U6 snRNA-associated Sm-like protein LSm6
Small nuclear ribonucleoprotein Sm D1
Small nuclear ribonucleoprotein Sm D2
Small nuclear ribonucleoprotein Sm D3
Thymosin beta-4
ADP-ribosylation factor 6
26S proteasome regulatory subunit 10B
Thioredoxin reductase-like selenoprotein T
TATA box-binding protein-like 1
Large ribosomal subunit protein eL8
DNA-directed RNA polymerase II subunit RPB7
Ras-related protein Rab-11A
Eukaryotic peptide chain release factor subunit 1
CCHC-type zinc finger nucleic acid binding protein
Protein yippee-like 5
Small ribosomal subunit protein eS4, X isoform
Serine/threonine-protein phosphatase 2A catalytic subunit beta isoform
Actin, aortic smooth muscle
Rho-related GTP-binding protein RhoB
Large ribosomal subunit protein uL23
Small ribosomal subunit protein eS6
Histone H4
Ras-related protein Rab-1A
GTP-binding nuclear protein Ran
Large ribosomal subunit protein uL14
Ras-related protein Rap-1A
Small ribosomal subunit protein uS19
Small ribosomal subunit protein eS24
Small ribosomal subunit protein eS25
Small ribosomal subunit protein eS26
Small ribosomal subunit protein eS28
Ubiquitin-like FUBI-ribosomal protein eS30 fusion protein
Guanine nucleotide-binding protein G(I)/G(S)/G(T) subunit beta-1
E3 ubiquitin-protein ligase RBX1
Guanine nucleotide-binding protein G(I)/G(S)/G(T) subunit beta-2
Large ribosomal subunit protein eL30
Large ribosomal subunit protein eL39
Large ribosomal subunit protein eL31
Large ribosomal subunit protein uL1
Large ribosomal subunit protein eL32
Large ribosomal subunit protein uL5
Large ribosomal subunit protein uL2

Peptidyl-prolyl cis-trans isomerase A
Peptidyl-prolyl cis-trans isomerase FKBP1A
Ubiquitin-ribosomal protein eS31 fusion protein
Growth factor receptor-bound protein 2
Transformer-2 protein homolog beta
Ras-related C3 botulinum toxin substrate 1
AP-2 complex subunit beta
Vesicle-associated membrane protein 2
Guanine nucleotide-binding protein G(s) subunit alpha isoforms short
Guanine nucleotide-binding protein G(i) subunit alpha-1
14-3-3 protein zeta/delta
Serine/threonine-protein phosphatase 2A 55 kDa regulatory subunit B alpha isoform
Small ubiquitin-related modifier 1
Dynein light chain 1, cytoplasmic
Dynein light chain Tctex-type 1
Large ribosomal subunit protein eL38
S-phase kinase-associated protein 1
Small ribosomal subunit protein eS21
Eukaryotic translation initiation factor 5A-1
Small ribosomal subunit protein RACK1
Transcription elongation factor SPT4
SUMO-conjugating enzyme UBC9
Thymosin beta-10
Serine/threonine-protein phosphatase 2A catalytic subunit alpha isoform
Y-box-binding protein 1
Signal peptidase complex catalytic subunit SEC11A
Casein kinase II subunit beta
Tropomyosin alpha-4 chain
Ubiquitin-conjugating enzyme E2 L3
Elongation factor 1-alpha 1
Tubulin alpha-1B chain
Tubulin alpha-4A chain
Tubulin beta-4B chain
Casein kinase II subunit alpha
Platelet-activating factor acetylhydrolase IB subunit alpha2
Histone H3.1
Hemoglobin subunit beta
Hemoglobin subunit alpha
Coxsackievirus and adenovirus receptor
Protein FAM193A
Nucleolar protein 14
Immunoglobulin-binding protein 1
Tyrosine-protein phosphatase non-receptor type substrate 1
Phosphoserine phosphatase
RNA-binding protein 6
Eukaryotic translation initiation factor 4 gamma 2
Ribonuclease P protein subunit p38
Ribonuclease P protein subunit p30
General transcription factor II-I
Phosphatidylinositol 5-phosphate 4-kinase type-2 beta

Contactin-associated protein 1
SRSF protein kinase 2
T-complex protein 1 subunit beta
UDP-galactose translocator
mRNA export factor RAE1
Glutathione S-transferase omega-1
DNA-dependent protein kinase catalytic subunit
Disintegrin and metalloproteinase domain-containing protein 17
Biogenesis of lysosome-related organelles complex 1 subunit 1
Sushi repeat-containing protein SRPX
Arginase-2, mitochondrial
Endonuclease III-like protein 1
Interferon-induced 35 kDa protein
Nucleobindin-2
Brain acid soluble protein 1
Dermcidin
TATA element modulatory factor
Small ribosomal subunit protein mS22
Small ribosomal subunit protein mS25
Small ribosomal subunit protein uS10m
Small ribosomal subunit protein mS35
Small ribosomal subunit protein uS5m
Alpha-ketoglutarate dehydrogenase component 4
Small ribosomal subunit protein uS11m
Small ribosomal subunit protein uS15m
Small ribosomal subunit protein bS21m
Small ribosomal subunit protein mS34
Small ribosomal subunit protein bS6m
Small ribosomal subunit protein uS9m
SAP domain-containing ribonucleoprotein
Serine beta-lactamase-like protein LACTB, mitochondrial
Conserved oligomeric Golgi complex subunit 7
Large ribosomal subunit protein eL24
Thioredoxin-like protein 4A
Large ribosomal subunit protein eL42
Chromobox protein homolog 1
Mothers against decapentaplegic homolog 3
ADP-ribosylation factor 1
ADP-ribosylation factor 5
Enhancer of rudimentary homolog
Rho-related GTP-binding protein RhoG
Large ribosomal subunit protein eL19
Small EDRK-rich factor 2
Serine/arginine-rich splicing factor 3
Forkhead box protein K1
Vacuolar fusion protein CCZ1 homolog B
Disabled homolog 2
Basement membrane-specific heparan sulfate proteoglycan core protein
E3 ubiquitin-protein ligase XIAP
Ephrin-B1

RNA-binding protein 10
RNA-binding protein 3
Calcium-transporting ATPase type 2C member 1
Phospholipid-transporting ATPase IH
Cytochrome c
55 kDa erythrocyte membrane protein
Transcription factor A, mitochondrial
Phosphatidylinositol transfer protein alpha isoform
Solute carrier family 25 member 3
Vigilin
Transcription initiation factor IIB
Cyclin-dependent kinase 6
Cyclin-dependent kinase 5
Cyclin-dependent kinase 17
Transcriptional activator protein Pur-alpha
Cdc42 effector protein 1
Clathrin heavy chain 1
Heat shock factor protein 1
Nuclear factor NF-kappa-B p100 subunit
Peptidyl-prolyl cis-trans isomerase FKBP3
Receptor expression-enhancing protein 5
Sorbitol dehydrogenase
Heterogeneous nuclear ribonucleoprotein U
Splicing factor U2AF 35 kDa subunit
Spectrin beta chain, non-erythrocytic 1
Nucleolysin TIAR
Protein SET
Serine/arginine-rich splicing factor 2
Forkhead box protein K2
Transcription factor RelB
N-acetylgalactosamine kinase
AMP deaminase 2
Fatty acid-binding protein 5
Adenylyl cyclase-associated protein 1
Friend leukemia integration 1 transcription factor
Hydroxymethylglutaryl-CoA synthase, cytoplasmic
Interferon-induced transmembrane protein 3
Large neutral amino acids transporter small subunit 1
Protein Dr1
Exosome complex component 10
OTU domain-containing protein 4
ATP-dependent 6-phosphofructokinase, platelet type
DNA repair protein complementing XP-C cells
RNA-binding protein EWS
Cerebellar degeneration-related protein 2
Inositol polyphosphate 5-phosphatase OCRL
1-phosphatidylinositol 4,5-bisphosphate phosphodiesterase beta-3
Transgelin
A-kinase anchor protein 17A
Dihydroorotate dehydrogenase (quinone), mitochondrial

2-oxoglutarate dehydrogenase complex component E1
Centromere-associated protein E
Kinesin-like protein KIF23
Methylmalonate-semialdehyde/malonate-semialdehyde dehydrogenase [acylating], mitochondrial
Pro-neuregulin-1, membrane-bound isoform
Sterol 26-hydroxylase, mitochondrial
Desmoglein-1
Transcription factor Sp3
Histone H1.1
Large ribosomal subunit protein eL20
Dual specificity mitogen-activated protein kinase kinase 1
Angiopoietin-1 receptor
Peptidyl-prolyl cis-trans isomerase FKBP4
Procollagen-lysine,2-oxoglutarate 5-dioxygenase 1
Nucleobindin-1
Large ribosomal subunit protein eL6
DNA topoisomerase 2-beta
A-kinase anchor protein 12
Mitochondrial 2-oxoglutarate/malate carrier protein
Dystonin
Protein ENL
Histone-lysine N-methyltransferase MECOM
Guanine nucleotide-binding protein subunit alpha-12
Caveolin-1
Aminoacylase-1
Tumor necrosis factor alpha-induced protein 2
Lamin-B2
6-pyruvoyl tetrahydrobiopterin synthase
Urokinase plasminogen activator surface receptor
Mevalonate kinase
DNA excision repair protein ERCC-6
Antigen peptide transporter 1
Antigen peptide transporter 2
CCAAT/enhancer-binding protein zeta
Zinc finger protein 92
Transcription factor p65
UBX domain-containing protein 1
1,4-alpha-glucan-branching enzyme
Eukaryotic translation initiation factor 4 gamma 1
Neurogenic locus notch homolog protein 2
Transducin-like enhancer protein 1
Transducin-like enhancer protein 3
Lactoylglutathione lyase
Activin receptor type-1
Single-stranded DNA-binding protein, mitochondrial
Proto-oncogene c-Rel
14-3-3 protein eta
Proteolipid protein 2
Cleavage stimulation factor subunit 1
Ubiquitin-protein ligase E3A

Dynamin-1
Tyrosine-protein phosphatase non-receptor type 12
Focal adhesion kinase 1
Serine/arginine-rich splicing factor 11
Protein kinase C delta type
Caldesmon
Folylpolyglutamate synthase, mitochondrial
CTD small phosphatase-like protein 2
Tyrosine-protein phosphatase non-receptor type 11
3-ketodihydrosphingosine reductase
Amidophosphoribosyltransferase
Glutamine--fructose-6-phosphate aminotransferase [isomerizing] 1
Exosome complex component RRP45
Proteasome activator complex subunit 1
Recombining binding protein suppressor of hairless
Myocyte-specific enhancer factor 2C
Amyloid beta precursor like protein 2
GA-binding protein alpha chain
E3 ubiquitin-protein ligase RING1
Fragile X messenger ribonucleoprotein 1
Peroxiredoxin-1
Large ribosomal subunit protein eL18
Complement component 1 Q subcomponent-binding protein, mitochondrial
Cytoskeleton-associated protein 4
Tight junction protein ZO-1
mRNA decay activator protein ZFP36L1
Sperm-associated antigen 1
KH domain-containing, RNA-binding, signal transduction-associated protein 1
Apoptosis regulator BAX
Bcl-2-like protein 1
Induced myeloid leukemia cell differentiation protein Mcl-1
DNA polymerase epsilon catalytic subunit A
Kinesin light chain 1
Son of sevenless homolog 1
Serine/arginine-rich splicing factor 1
Rho GTPase-activating protein 1
Serine/arginine-rich splicing factor 4
Protocadherin-1
Protein phosphatase 3 catalytic subunit alpha
ATP-dependent RNA helicase A
Quinone oxidoreductase
Golgin subfamily A member 3
Golgin subfamily A member 2
Peroxisomal bifunctional enzyme
Lactadherin
3',5'-cyclic-AMP phosphodiesterase 4D
Desmocollin-1
Pseudouridine-5'-phosphatase
Testican-1
Leukocyte surface antigen CD47

Peptidyl-prolyl cis-trans isomerase D
FACT complex subunit SSRP1
Calmodulin-regulated spectrin-associated protein 2
Protein spire homolog 1
Schlafen family member 5
Protein VAC14 homolog
RNA cytosine C(5)-methyltransferase NSUN2
Dystrophia myotonica WD repeat-containing protein
Histone-binding protein RBBP4
Nuclear cap-binding protein subunit 1
Alpha-1,6-mannosylglycoprotein 6-beta-N-acetylglucosaminyltransferase A
Histone acetyltransferase p300
Neuroblast differentiation-associated protein AHNAK
F-BAR domain only protein 2
Elongator complex protein 6
Heat shock 70 kDa protein 14
Secernin-3
Cingulin-like protein 1
Ragulator complex protein LAMTOR4
Nexilin
Alpha-1,6-mannosyl-glycoprotein 2-beta-N-acetylglucosaminyltransferase
Polypeptide N-acetylgalactosaminyltransferase 2
Polypeptide N-acetylgalactosaminyltransferase 1
AP-1 complex subunit beta-1
Cleavage and polyadenylation specificity factor subunit 1
Bone marrow stromal antigen 2
Mitochondrial-processing peptidase subunit alpha
CMP-N-acetylneuraminate-beta-galactosamide-alpha-2,3-sialyltransferase 1
Secernin-1
WASH complex subunit 5
Nuclear pore complex protein Nup160
Sterol regulatory element-binding protein cleavage-activating protein
Forkhead box protein O1
Transducin beta-like protein 3
General transcription factor 3C polypeptide 1
Twinfilin-1
Aspartyl/asparaginyl beta-hydroxylase
Alpha-globin transcription factor CP2
A-kinase anchor protein 13
EGF-containing fibulin-like extracellular matrix protein 1
SWI/SNF-related matrix-associated actin-dependent regulator of chromatin subfamily B member 1
Nucleosome-remodeling factor subunit BPTF
Cell division cycle protein 20 homolog
Follistatin-related protein 1
Syntaxin-4
G-rich sequence factor 1
Mitogen-activated protein kinase kinase kinase kinase 2
Tyrosine-protein kinase Mer
Splicing factor, suppressor of white-apricot homolog
Chromodomain-helicase-DNA-binding protein 3

Splicing factor 3A subunit 3
TP53-binding protein 1
Transmembrane protein 115
Interferon-related developmental regulator 2
Tripartite motif-containing protein 26
Aminoacyl tRNA synthase complex-interacting multifunctional protein 1
Interleukin enhancer-binding factor 2
Interleukin enhancer-binding factor 3
Vesicular integral-membrane protein VIP36
Heat shock protein 75 kDa, mitochondrial
TNF receptor-associated factor 2
Disks large homolog 1
Unconventional myosin-Ie
Nuclear inhibitor of protein phosphatase 1
Protein tyrosine phosphatase type IVA 2
Active breakpoint cluster region-related protein
GATOR1 complex protein NPRL3
Vesicle transport protein SEC20
BCL2/adenovirus E1B 19 kDa protein-interacting protein 2
Transcriptional repressor NF-X1
Cleavage stimulation factor subunit 3
Tetraspanin-31
Delta(3,5)-Delta(2,4)-dienoyl-CoA isomerase, mitochondrial
Rho GTPase-activating protein 5
Striatin-3
Cell division cycle protein 16 homolog
Serine/threonine-protein kinase 4
Protein flightless-1 homolog
E3 ubiquitin-protein ligase TRIM32
Bifunctional coenzyme A synthase
Large ribosomal subunit protein bL28m
Acetyl-CoA carboxylase 1
COP9 signalosome complex subunit 1
Ubiquitin carboxyl-terminal hydrolase 4
Chromatin assembly factor 1 subunit A
Chromatin assembly factor 1 subunit B
Protein Red
S-methyl-5'-thioadenosine phosphorylase
5'-AMP-activated protein kinase catalytic subunit alpha-1
Liprin-alpha-1
Calcium-binding and coiled-coil domain-containing protein 2
Translation initiation factor eIF2B subunit epsilon
TAR DNA-binding protein 43
Heterogeneous nuclear ribonucleoprotein A0
Aminoacyl tRNA synthase complex-interacting multifunctional protein 2
FAS-associated death domain protein
Peroxiredoxin-4
Mitogen-activated protein kinase 7
Serine/threonine-protein kinase PAK 2
Chromobox protein homolog 3

Serine/threonine-protein kinase 3
Syntaxin-5
26S proteasome non-ATPase regulatory subunit 2
Multimerin-1
Probable ATP-dependent RNA helicase DDX10
DnaJ homolog subfamily C member 3
Methanethiol oxidase
Nucleoside diphosphate kinase 3
Serine/arginine-rich splicing factor 9
Serine/arginine-rich splicing factor 5
Serine/arginine-rich splicing factor 6
Mitotic spindle assembly checkpoint protein MAD2A
Transcription intermediary factor 1-beta
Semaphorin-3F
Syntaxin-3
Ras GTPase-activating protein-binding protein 1
N-myc-interactor
Inactive tyrosine-protein kinase 7
Polyadenylate-binding protein 4
Serine-protein kinase ATM
Growth factor receptor-bound protein 10
Interferon-induced protein with tetratricopeptide repeats 5
Metastasis-associated protein MTA1
Eukaryotic translation initiation factor 3 subunit I
RING-type E3 ubiquitin-protein ligase PPIL2
Serine/threonine-protein phosphatase 2A 56 kDa regulatory subunit gamma isoform
C-terminal-binding protein 1
Phosducin-like protein
Phospholipase D1
Probable methyltransferase TARBP1
Ubiquitin-conjugating enzyme E2 variant 1
Large ribosomal subunit protein mL49
Cytoplasmic dynein 1 intermediate chain 2
Origin recognition complex subunit 2
Integrin-linked protein kinase
NAD(P) transhydrogenase, mitochondrial
Beta-2-syntrophin
DNA repair protein XRCC4
Peptidyl-prolyl cis-trans isomerase G
Treacle protein
Protein unc-119 homolog A
Splicing factor 3B subunit 2
Protein OS-9
Golgin subfamily A member 4
28 kDa heat- and acid-stable phosphoprotein
Disintegrin and metalloproteinase domain-containing protein 9
Disintegrin and metalloproteinase domain-containing protein 15
Transmembrane emp24 domain-containing protein 1
Peptidyl-prolyl cis-trans isomerase FKBP5
Unconventional myosin-IXb

Rho-associated protein kinase 1
Nuclear factor of activated T-cells, cytoplasmic 2
DNA topoisomerase 3-alpha
Mothers against decapentaplegic homolog 4
V-type proton ATPase 116 kDa subunit a 3
Baculoviral IAP repeat-containing protein 2
Phosphatidylinositol-binding clathrin assembly protein
Myotubularin
Sequestosome-1
Metaxin-1
Tubulin beta-3 chain
Acid ceramidase
Serine/threonine-protein kinase PRP4 homolog
Peptidyl-prolyl cis-trans isomerase NIMA-interacting 1
Serine/threonine-protein kinase ATR
Eukaryotic translation initiation factor 4E-binding protein 1
Receptor-interacting serine/threonine-protein kinase 1
Histone deacetylase 1
Calcium/calmodulin-dependent protein kinase type II subunit gamma
Calcium/calmodulin-dependent protein kinase type II subunit delta
Dynactin subunit 2
Polycystin-2
NEDD8-activating enzyme E1 regulatory subunit
Inositol-tetrakisphosphate 1-kinase
SNW domain-containing protein 1
Diacylglycerol kinase zeta
Stromal interaction molecule 1
GRB2-related adapter protein
Transformer-2 protein homolog alpha
Sorting nexin-1
KRR1 small subunit processome component homolog
Periodic tryptophan protein 1 homolog
Myotubularin-related protein 1
Myotubularin-related protein 2
Cullin-1
Cullin-2
Cullin-3
Cullin-4A
Cullin-4B
Apoptosis-stimulating of p53 protein 2
Dual specificity tyrosine-phosphorylation-regulated kinase 1A
GDP-L-fucose synthase
Ras-related protein Rab-31
Ras-related protein Rab-32
Trophoblast glycoprotein
Four and a half LIM domains protein 1
Four and a half LIM domains protein 3
Ras and Rab interactor 1
Angio-associated migratory cell protein
Mannosyl-oligosaccharide glucosidase

CD166 antigen
THO complex subunit 5 homolog
Protein Shroom2
Spectrin alpha chain, non-erythrocytic 1
Nucleolar GTP-binding protein 2
Methylglutaconyl-CoA hydratase, mitochondrial
Plakophilin-1
Spliceosome RNA helicase DDX39B
Bleomycin hydrolase
Exosome complex component RRP4
Bone morphogenetic protein receptor type-2
Tubulin beta-2A chain
General transcription factor IIH subunit 2
General transcription factor IIH subunit 3
Bystin
Rap guanine nucleotide exchange factor 1
Isopentenyl-diphosphate Delta-isomerase 1
Core-binding factor subunit beta
Nuclear transcription factor Y subunit gamma
Cyclin-dependent kinase 13
Cytoskeleton-associated protein 5
Cold-inducible RNA-binding protein
Calcium/calmodulin-dependent protein kinase type 1
Coactosin-like protein
Heterogeneous nuclear ribonucleoprotein D0
Lysosome membrane protein 2
Nidogen-2
Low-density lipoprotein receptor-related protein 8
Dystroglycan 1
Vascular endothelial zinc finger 1
Ribosome biogenesis protein BOP1
Ubiquitin conjugation factor E4 A
Septin-6
Kelch-like ECH-associated protein 1
Unhealthy ribosome biogenesis protein 2 homolog
Probable ATP-dependent RNA helicase DHX34
MORC family CW-type zinc finger protein 3
Scaffold attachment factor B2
Eukaryotic translation initiation factor 3 subunit A
Rho guanine nucleotide exchange factor 7
Protein EFR3 homolog A
Ubiquitin-associated protein 2-like
Protein scribble homolog
ARF GTPase-activating protein GIT2
Scavenger receptor class F member 1
Malectin
Tubulin--tyrosine ligase-like protein 12
DNA polymerase alpha subunit B
Dedicator of cytokinesis protein 1
Transcription factor Dp-1

Four and a half LIM domains protein 2
Dihydropyrimidinase-related protein 3
Large ribosomal subunit protein mL62
Zinc finger MYM-type protein 3
Dynactin subunit 1
Cytoplasmic dynein 1 heavy chain 1
Translation initiation factor eIF2B subunit alpha
Eukaryotic initiation factor 4A-II
Elongin-A
Src substrate cortactin
Endonuclease G, mitochondrial
Flotillin-2
Reticulocalbin-2
E3 ubiquitin/ISG15 ligase TRIM25
Filamin-C
Peptidyl-prolyl cis-trans isomerase FKBP8
Protein FAM50A
Protein FRG1
Guanine nucleotide-binding protein subunit alpha-13
Guanidinoacetate N-methyltransferase
UDP-glucose 4-epimerase
Transforming growth factor beta activator LRRC32
Caprin-1
Beclin-1
RNA-binding protein 39
Enhancer of filamentation 1
Helicase-like transcription factor
Squalene monooxygenase
Protein disulfide-isomerase A5
Phosphoribosyl pyrophosphate synthase-associated protein 1
ATP-dependent RNA helicase DHX8
DNA replication licensing factor MCM6
Inositol 1,4,5-trisphosphate receptor type 2
Inositol 1,4,5-trisphosphate receptor type 3
Inositol 1,4,5-trisphosphate receptor type 1
Ras GTPase-activating protein 3
Plastin-1
Interferon regulatory factor 3
EKC/KEOPS complex subunit LAGE3
Bridge-like lipid transfer protein family member 2
E3 ubiquitin-protein ligase TRIP12
Pumilio homolog 1
Mediator of DNA damage checkpoint protein 1
Clathrin interactor 1
KN motif and ankyrin repeat domain-containing protein 1
Structural maintenance of chromosomes protein 1A
Ribosomal RNA processing protein 1 homolog B
Disco-interacting protein 2 homolog A
Protein RRP5 homolog
DNA replication complex GINS protein PSF1

Ribosome biogenesis protein BMS1 homolog
Phosphatidate phosphatase LPIN1
Ubiquitin carboxyl-terminal hydrolase 10
LRP chaperone MESD
Neutral alpha-glucosidase AB
Raftlin
Major facilitator superfamily domain-containing protein 10
Serine/threonine-protein phosphatase 2A 56 kDa regulatory subunit delta isoform
Delta(14)-sterol reductase LBR
Conserved oligomeric Golgi complex subunit 2
Major vault protein
Latent-transforming growth factor beta-binding protein 1
Latent-transforming growth factor beta-binding protein 2
Chromobox protein homolog 2
Golgin subfamily B member 1
Caspase-8
N-alpha-acetyltransferase 30
Kinesin-like protein KIF22
Myocyte-specific enhancer factor 2D
Chromodomain-helicase-DNA-binding protein 4
LIM and SH3 domain protein 1
Prostaglandin reductase 1
Dr1-associated corepressor
Nuclear factor of activated T-cells, cytoplasmic 4
Zinc finger protein 638
Importin subunit beta-1
Nucleolar and coiled-body phosphoprotein 1
Nuclear mitotic apparatus protein 1
Proteasome activator complex subunit 4
Cullin-7
Sarcolemmal membrane-associated protein
GTPase-activating protein and VPS9 domain-containing protein 1
N-alpha-acetyltransferase 25, NatB auxiliary subunit
Ventricular zone-expressed PH domain-containing protein homolog 1
Condensin complex subunit 2
PCNA-associated factor
Signal peptidase complex subunit 2
ER membrane protein complex subunit 2
Pre-mRNA-splicing regulator WTAP
26S proteasome non-ATPase regulatory subunit 6
MAD2L1-binding protein
Mortality factor 4-like protein 2
BRISC complex subunit Abraxas 2
Septin-2
Squamous cell carcinoma antigen recognized by T-cells 3
Condensin complex subunit 1
Polycomb protein SUZ12
Exosome complex component RRP42
TNFAIP3-interacting protein 1
116 kDa U5 small nuclear ribonucleoprotein component

Leucine--tRNA ligase, mitochondrial
R3H domain-containing protein 1
Translocating chain-associated membrane protein 2
Sorting nexin-17
ADP-ribosylation factor-like protein 6-interacting protein 1
Rab3 GTPase-activating protein catalytic subunit
Metal cation symporter ZIP14
Lysine--tRNA ligase
Histone-lysine N-methyltransferase SETDB1
Leucine-rich repeat-containing protein 14
Ribosome biogenesis regulatory protein homolog
DNA polymerase delta subunit 3
Eukaryotic translation initiation factor 4H
Arf-GAP with coiled-coil, ANK repeat and PH domain-containing protein 2
Kinesin-like protein KIF14
Bromodomain-containing protein 3
WD repeat-containing protein 43
Peroxisomal acyl-coenzyme A oxidase 1
Mitochondrial inner membrane protein OXA1L
Early endosome antigen 1
Protein disulfide-isomerase A6
Platelet-activating factor acetylhydrolase IB subunit alpha1
[Pyruvate dehydrogenase (acetyl-transferring)] kinase isozyme 3, mitochondrial
Astrocytic phosphoprotein PEA-15
3-beta-hydroxysteroid-Delta(8),Delta(7)-isomerase
Phosphomevalonate kinase
Serine/threonine-protein kinase D1
Plectin
Pericentriolar material 1 protein
BOS complex subunit NOMO1
Serum paraoxonase/arylesterase 2
Serine/threonine-protein phosphatase 2A 56 kDa regulatory subunit alpha isoform
Inorganic pyrophosphatase
Prostaglandin E synthase 3
Serine/threonine-protein kinase 38
Non-POU domain-containing octamer-binding protein
Serine/threonine-protein phosphatase 2A activator
Receptor-type tyrosine-protein phosphatase kappa
Periodic tryptophan protein 2 homolog
Rab GTPase-binding effector protein 1
Ras-related protein Rab-35
RNA-binding protein with serine-rich domain 1
Retinoblastoma-binding protein 5
Reticulocalbin-1
RalA-binding protein 1
Lethal(2) giant larvae protein homolog 1
Leucine-rich repeat-containing protein 41
Transcription termination factor 1
Transmembrane emp24 domain-containing protein 2
Poly(rC)-binding protein 1

Poly(rC)-binding protein 2
Elongin-C
Elongin-B
GTP-binding protein Rheb
Ubiquitin-protein ligase E3C
Mitochondrial import receptor subunit TOM20 homolog
Mitochondrial fission regulator 1
Delta(24)-sterol reductase
Splicing factor 3B subunit 3
Pumilio homolog 3
Disks large-associated protein 5
Ras suppressor protein 1
Calponin-3
Ribosomal protein S6 kinase alpha-1
Scaffold attachment factor B1
Splicing factor 3A subunit 2
RNA-binding motif, single-stranded-interacting protein 2
Protein phosphatase 1 regulatory subunit 7
Protein transport protein Sec23A
Protein transport protein Sec23B
Cytohesin-1
Splicing factor 3A subunit 1
Superkiller complex protein 2
Corneodesmosin
Surfeit locus protein 1
Transcription initiation factor TFIID subunit 5
Transcription initiation factor TFIID subunit 7
Telomeric repeat-binding factor 2
Microtubule-associated protein RP/EB family member 2
Na(+)/H(+) exchange regulatory cofactor NHE-RF2
Tumor necrosis factor receptor type 1-associated DEATH domain protein
Translocating chain-associated membrane protein 1
Translin
RISC-loading complex subunit TARBP2
Splicing factor 1
Cdc42-interacting protein 4
Thyroid receptor-interacting protein 11
Pachytene checkpoint protein 2 homolog
Mediator of RNA polymerase II transcription subunit 1
Activating signal cointegrator 1
High mobility group nucleosome-binding domain-containing protein 3
Probable JmjC domain-containing histone demethylation protein 2C
NF-kappa-B inhibitor beta
Thyroid receptor-interacting protein 6
Tyrosine-protein phosphatase non-receptor type 14
Microtubule-associated protein RP/EB family member 1
ELAV-like protein 1
Sterol-4-alpha-carboxylate 3-dehydrogenase, decarboxylating
NGFI-A-binding protein 2
Myosin light chain kinase, smooth muscle

TGF-beta-activated kinase 1 and MAP3K7-binding protein 1
Probable E3 ubiquitin-protein ligase HERC1
Neutral amino acid transporter B(0)
Myeloid leukemia factor 2
Mitochondrial import receptor subunit TOM34
Mothers against decapentaplegic homolog 2
Mothers against decapentaplegic homolog 1
Methylsterol monooxygenase 1
Intersectin-1
Tubulin-specific chaperone E
Tubulin-specific chaperone C
Ubiquitin-conjugating enzyme E2 variant 2
Serine/threonine-protein kinase STK11
Syntaxin-binding protein 2
Vesicle-associated membrane protein 3
NEDD8
V-type proton ATPase subunit S1
Vacuolar protein sorting-associated protein 72 homolog
Ras-related protein Rab-11B
Zyxin
Electron transfer flavoprotein-ubiquinone oxidoreductase, mitochondrial
Septin-7
Proteasomal ubiquitin receptor ADRM1
Coiled-coil domain-containing protein 6
UDP-N-acetylhexosamine pyrophosphorylase
Transcription factor E2F4
Insulin-like growth factor-binding protein 7
Programmed cell death protein 2
Laminin subunit alpha-4
26S proteasome non-ATPase regulatory subunit 5
Serine/threonine-protein kinase N1
Serine/threonine-protein kinase N2
Cysteine and glycine-rich protein 2
DNA damage-binding protein 1
Serine/threonine-protein phosphatase 2A 56 kDa regulatory subunit epsilon isoform
Mitogen-activated protein kinase 14
Large ribosomal subunit protein uL23m
Hsp90 co-chaperone Cdc37
Dihydropyrimidinase-related protein 2
Synaptophysin-like protein 1
Histone-binding protein RBBP7
Mitogen-activated protein kinase kinase kinase 11
Transcription initiation factor TFIID subunit 9
Calcitonin gene-related peptide type 1 receptor
Bcl-2 homologous antagonist/killer
Serine/arginine-rich splicing factor 7
Cleavage and polyadenylation specificity factor subunit 6
Survival motor neuron protein
Drebrin
Nuclear respiratory factor 1

Fascin
Gamma-interferon-inducible protein 16
2,4-dienoyl-CoA reductase [(3E)-enoyl-CoA-producing], mitochondrial
Alpha-mannosidase 2
NADH dehydrogenase [ubiquinone] 1 alpha subcomplex subunit 5
Ceramide glucosyltransferase
ATP-dependent Clp protease proteolytic subunit, mitochondrial
Thiosulfate sulfurtransferase
Ubiquitin-conjugating enzyme E2 S
Kynurenine--oxoglutarate transaminase 1
Guanylate kinase
Hydroxyacylglutathione hydrolase, mitochondrial
NADH dehydrogenase [ubiquinone] 1 alpha subcomplex subunit 9, mitochondrial
NADP-dependent malic enzyme, mitochondrial
Phosphoenolpyruvate carboxykinase [GTP], mitochondrial
Uridine phosphorylase 1
Discoidin domain-containing receptor 2
Hydroxyacyl-coenzyme A dehydrogenase, mitochondrial
Lanosterol 14-alpha demethylase
UTP--glucose-1-phosphate uridylyltransferase
6-phosphofructo-2-kinase/fructose-2,6-bisphosphatase 3
Thioredoxin reductase 1, cytoplasmic
MICOS complex subunit MIC60
Endothelial cell-specific chemotaxis regulator
Lysine-rich nucleolar protein 1
Heterogeneous nuclear ribonucleoprotein U-like protein 2
Transmembrane protein 132A
Inverted formin-2
Sister chromatid cohesion protein PDS5 homolog A
Protein CLEC16A
Kinesin-like protein KIF7
Rho GTPase-activating protein 31
Methenyltetrahydrofolate synthase domain-containing protein
AP2-associated protein kinase 1
WASH complex subunit 4
DNA excision repair protein ERCC-6-like
Pre-rRNA-processing protein TSR1 homolog
Ral GTPase-activating protein subunit alpha-2
Protein C-mannosyl-transferase DPY19L1
Isoamyl acetate-hydrolyzing esterase 1 homolog
GDP-Man:Man(3)GlcNAc(2)-PP-Dol alpha-1,2-mannosyltransferase
Transcriptional regulator QRICH1
WD40 repeat-containing protein SMU1
Cytoplasmic tRNA 2-thiolation protein 2
Protein-glucosylgalactosylhydroxylysine glucosidase
Leucine-rich repeat flightless-interacting protein 1
Prolyl 3-hydroxylase 1
tRNA (guanine(37)-N1)-methyltransferase
Echinoderm microtubule-associated protein-like 3
DNA-directed RNA polymerase I subunit RPA43

MAP7 domain-containing protein 1
UDP-N-acetylhexosamine pyrophosphorylase-like protein 1
Chromodomain-helicase-DNA-binding protein 9
Triokinase/FMN cyclase
Protein LSM12
Inactive hydroxysteroid dehydrogenase-like protein 1
Mitochondrial 10-formyltetrahydrofolate dehydrogenase
N-acetylglucosamine-1-phosphotransferase subunits alpha/beta
Girdin
Rab-like protein 6
Mitochondrial import inner membrane translocase subunit TIM50
Protein mono-ADP-ribosyltransferase PARP14
Alpha-(1,3)-fucosyltransferase 11
Cytokine-like nuclear factor N-PAC
UPF0489 protein C5orf22
C2 domain-containing protein 3
Vacuolar protein sorting-associated protein 26B
La-related protein 7
NAD kinase 2, mitochondrial
BTB/POZ domain-containing protein KCTD21
Glucoside xylosyltransferase 1
Malonate--CoA ligase ACSF3, mitochondrial
Prolyl endopeptidase-like
TBC1 domain family member 10B
Taperin
Anoctamin-6
Filamin A-interacting protein 1-like
GRIP1-associated protein 1
Zinc-regulated GTPase metalloprotein activator 1F
Protein MIX23
Membralin
Protein FAM98B
Rho GTPase-activating protein 29
Programmed cell death protein 4
Fibronectin type III domain-containing protein 3B
CREB-regulated transcription coactivator 2
Glycerol-3-phosphate acyltransferase 3
Centrosomal protein of 55 kDa
Nuclear cap-binding protein subunit 3
Quinone oxidoreductase PIG3
Novel acetylcholine receptor chaperone
Pleckstrin homology-like domain family A member 2
PDZ and LIM domain protein 3
Protein mono-ADP-ribosyltransferase PARP10
Very-long-chain 3-oxoacyl-CoA reductase
mRNA export factor GLE1
Ubiquitin carboxyl-terminal hydrolase 39
Kelch-like protein 22
Acylglycerol kinase, mitochondrial
Endoribonuclease LACTB2

Pyrroline-5-carboxylate reductase 3
EARP and GARP complex-interacting protein 1
Borealin
Cordon-bleu protein-like 1
HCLS1-binding protein 3
Plasma membrane ascorbate-dependent reductase CYBRD1
WD repeat-containing protein 81
Beta-actin-like protein 2
Coiled-coil domain-containing protein 93
OCIA domain-containing protein 2
Puratrophin-1
Putative heat shock protein HSP 90-alpha A4
DDB1- and CUL4-associated factor 6
Transmembrane protein 41B
Sigma intracellular receptor 2
Protein YIF1B
DBIRD complex subunit ZNF326
Nucleolar MIF4G domain-containing protein 1
RILP-like protein 1
DnaJ homolog subfamily C member 21
Tumor necrosis factor alpha-induced protein 8-like protein 3
Probable E3 ubiquitin-protein ligase HERC4
GPI ethanolamine phosphate transferase 2
Serine/threonine-protein phosphatase 6 regulatory subunit 3
Rab-like protein 3
Protein FAM76B
2-methoxy-6-polyprenyl-1,4-benzoquinol methylase, mitochondrial
SH3 domain-containing protein 19
NHS-like protein 2
ER membrane protein complex subunit 4
Nondiscriminating glutamyl-tRNA synthetase EARS2, mitochondrial
Uncharacterized protein C3orf38
EEF1A lysine methyltransferase 2
Transport and Golgi organization protein 1 homolog
Presequence protease, mitochondrial
WD repeat-containing protein 44
Protein PRRC2B
Tight junction-associated protein 1
RRP12-like protein
Cytochrome c oxidase assembly factor 6 homolog
Torsin-1A-interacting protein 1
Centrosomal protein of 78 kDa
Alanine--tRNA ligase, mitochondrial
Torsin-2A
PCI domain-containing protein 2
Intracellular hyaluronan-binding protein 4
Long-chain fatty acid transport protein 3
Sterile alpha motif domain-containing protein 9
Serine/threonine-protein phosphatase 4 regulatory subunit 3B
WD repeat domain phosphoinositide-interacting protein 3

WD repeat domain phosphoinositide-interacting protein 1
EGF domain-specific O-linked N-acetylglucosamine transferase
Protein Smaug homolog 2
Tubulin-specific chaperone cofactor E-like protein
Deoxynucleotidyltransferase terminal-interacting protein 2
Rab GTPase-activating protein 1-like
Tetratricopeptide repeat protein 38
Cytochrome c oxidase assembly protein COX20, mitochondrial
Exosome complex component MTR3
Transmembrane protein 201
Alpha-tubulin N-acetyltransferase 1
Synaptosomal-associated protein 47
Nucleoporin NUP188
Heterochromatin protein 1-binding protein 3
Valine--tRNA ligase, mitochondrial
Centrosomal protein of 170 kDa
Low density lipoprotein receptor adapter protein 1
Protein odr-4 homolog
Polynucleotide 5'-hydroxyl-kinase NOL9
NHS-like protein 1
Putative hydroxypyruvate isomerase
Tumor protein p63-regulated gene 1-like protein
Coiled-coil and C2 domain-containing protein 1B
Formin-binding protein 1-like
Probable arginine--tRNA ligase, mitochondrial
Acyl-coenzyme A thioesterase THEM4
Putative coiled-coil-helix-coiled-coil-helix domain-containing protein CHCHD2P9, mitochondrial
FK506-binding protein 15
Zinc finger CCCH domain-containing protein 13
Putative methyltransferase C9orf114
Armadillo-like helical domain-containing protein 3
CSC1-like protein 2
G patch domain-containing protein 4
Putative transferase CAF17, mitochondrial
E3 ubiquitin-protein ligase HECTD3
Inactive glycosyltransferase 25 family member 3
E3 ubiquitin-protein ligase UBR4
Syntaxin-binding protein 5
Rho GTPase-activating protein 21
Calmodulin-regulated spectrin-associated protein 1
Large ribosomal subunit protein uL2m
Ubiquitin-associated protein 2
Queuosine 5'-phosphate N-glycosylase/hydrolase
Keratinocyte proline-rich protein
Zinc finger protein 618
Acyl-CoA-binding domain-containing protein 5
RNA-binding protein 26
ATPase family AAA domain-containing protein 3B
Protein wntless homolog
Integrator complex subunit 11

Ceramide-1-phosphate transfer protein
DDB1- and CUL4-associated factor 8
Collagen alpha-1(XIII) chain
Terminal uridylyltransferase 4
Protein furry homolog
Ribonuclease H2 subunit B
ATP synthase mitochondrial F1 complex assembly factor 1
Roquin-1
Protein DDI1 homolog 2
Protein THEMIS2
Arginine-hydroxylase NDUFAF5, mitochondrial
5'-nucleotidase domain-containing protein 1
Brefeldin A-inhibited guanine nucleotide-exchange protein 3
Intermembrane lipid transfer protein VPS13D
Rootletin
Complex III assembly factor LYRM7
Ras-interacting protein 1
Telomere-associated protein RIF1
Vacuolar protein sorting-associated protein 53 homolog
Striatin-interacting protein 1
Serine/threonine-protein kinase MRCK alpha
Regulation of nuclear pre-mRNA domain-containing protein 2
E3 ubiquitin-protein ligase RNF220
Pre-mRNA-splicing factor 38B
E3 ubiquitin-protein ligase BRE1A
Myomegalin
Zinc finger protein 318
Protein FAM171A1
Divergent protein kinase domain 1B
Leucine-rich repeat and calponin homology domain-containing protein 2
Threonylcarbamoyladenosine tRNA methylthiotransferase
BRO1 domain-containing protein BROX
Focadhesin
Lysine-specific demethylase 9
Disabled homolog 2-interacting protein
Lysophospholipase-like protein 1
Platelet endothelial aggregation receptor 1
Proteasome adapter and scaffold protein ECM29
Terminal uridylyltransferase 7
SH2 domain-containing adapter protein E
DENN domain-containing protein 4C
N-alpha-acetyltransferase 35, NatC auxiliary subunit
Zinc finger MYM-type protein 4
FHF complex subunit HOOK interacting protein 2A
Palmitoyltransferase ZDHHC20
SPRY domain-containing protein 7
F-box/WD repeat-containing protein 9
CD276 antigen
E3 ubiquitin-protein ligase RNF213
Tensin-2

KN motif and ankyrin repeat domain-containing protein 2
WASH complex subunit 2A
Lysophospholipid acyltransferase LPCAT4
Metalloreductase STEAP3
Protein FAM91A1
TBC1 domain family member 9B
Microtubule-associated protein 1S
Serine/threonine-protein phosphatase 2A 55 kDa regulatory subunit B delta isoform
ADP-ribosylation factor-like protein 6-interacting protein 4
Autophagy-related protein 16-1
Heparan-alpha-glucosaminide N-acetyltransferase
AT-rich interactive domain-containing protein 2
U3 small nucleolar RNA-associated protein 25 homolog
Glycosyltransferase 8 domain-containing protein 1
Protein sel-1 homolog 3
Tensin-3
HAUS augmin-like complex subunit 3
Protein SPT2 homolog
Acyl-coenzyme A thioesterase MBLAC2
Very large A-kinase anchor protein
Integrator complex subunit 3
Rho GTPase-activating protein 17
CWF19-like protein 1
Protein virilizer homolog
Cytospin-A
HEAT repeat-containing protein 6
Ankyrin repeat domain-containing protein 40
Protein WWC2
Atlastin-3
Ribosomal protein uL30-like
Nucleoredoxin
Tetratricopeptide repeat protein 19, mitochondrial
IQ motif and SEC7 domain-containing protein 1
Anamorsin
AN1-type zinc finger protein 6
Histone-lysine N-trimethyltransferase SMYD5
OTU domain-containing protein 7B
THO complex subunit 7 homolog
Glutamine-dependent NAD(+) synthetase
Elongator complex protein 2
Ragulator complex protein LAMTOR1
Dehydrogenase/reductase SDR family member 7B
Twinfilin-2
Condensin-2 complex subunit H2
Transport and Golgi organization protein 2 homolog
Serine/threonine-protein phosphatase 4 regulatory subunit 3A
Ras-related protein Rab-12
DENN domain-containing protein 5A
Keratin, type II cytoskeletal 80
Nipped-B-like protein

Mitochondrial adenyl nucleotide antiporter SLC25A25
2',5'-phosphodiesterase 12
N-alpha-acetyltransferase 16, NatA auxiliary subunit
Putative DENN domain-containing protein 10 B
Rhomboid domain-containing protein 2
Mitochondrial adenyl nucleotide antiporter SLC25A24
Receptor expression-enhancing protein 3
All-trans-retinol 13,14-reductase
RAD50-interacting protein 1
Transmembrane protein 214
3-hydroxyisobutyryl-CoA hydrolase, mitochondrial
RWD domain-containing protein 4
Nucleolus and neural progenitor protein
Armadillo repeat-containing protein 6
TELO2-interacting protein 2
Ankyrin repeat domain-containing protein 54
Zinc transporter 6
Transmembrane anterior posterior transformation protein 1 homolog
KN motif and ankyrin repeat domain-containing protein 3
Bifunctional arginine demethylase and lysyl-hydroxylase JMJD6
Phostensin
Caveolae-associated protein 1
Zinc finger CCHC domain-containing protein 8
Putative ATP-dependent RNA helicase DHX57
Endoplasmic reticulum aminopeptidase 2
Lysophospholipid acyltransferase 5
Parafibromin
Large ribosomal subunit protein uL14m
Long-chain fatty acid transport protein 4
Coiled-coil and C2 domain-containing protein 1A
Deoxyribonuclease TATDN1
tRNA N(3)-methylcytidine methyltransferase METTL2B
Myb/SANT-like DNA-binding domain-containing protein 2
Transcription initiation factor TFIID subunit 2
UPF0598 protein C8orf82
Mediator of RNA polymerase II transcription subunit 27
Enhancer of mRNA-decapping protein 4
Centrosomal protein of 85 kDa
Protein arginine N-methyltransferase 9
Pre-mRNA-processing-splicing factor 8
F-box only protein 42
SCY1-like protein 2
Tetratricopeptide repeat protein 27
Sideroflexin-4
Protein GOLM2
Nuclear factor related to kappa-B-binding protein
Acylpyruvase FAHD1, mitochondrial
Ribosomal protein eL22-like
Serine/threonine-protein kinase N3
RNA demethylase ALKBH5

Pyridoxal-dependent decarboxylase domain-containing protein 1
MTOR-associated protein MEAK7
Integrator complex subunit 5
GTPase IMAP family member 6
Long-chain fatty acid transport protein 1
Glucose 1,6-bisphosphate synthase
RNA polymerase-associated protein CTR9 homolog
Alpha- and gamma-adaptin-binding protein p34
Proline/serine-rich coiled-coil protein 1
Superkiller complex protein 3
Serine/threonine-protein kinase ULK3
Aspartate--tRNA ligase, mitochondrial
Transmembrane protein 65
INO80 complex subunit C
F-box only protein 38
Neutral cholesterol ester hydrolase 1
Fidgetin-like protein 1
E3 ubiquitin-protein ligase TRIM65
Inactive rhomboid protein 2
Mitotic deacetylase-associated SANT domain protein
BRCA1-associated ATM activator 1
GATOR2 complex protein WDR59
Zinc finger CCCH domain-containing protein 14
Coiled-coil domain-containing protein 137
2-oxoglutarate and iron-dependent oxygenase domain-containing protein 3
Thioredoxin domain-containing protein 11
La-related protein 1
ATPase family AAA domain-containing protein 2
Protein TMED8
Proton-coupled zinc antiporter SLC30A9, mitochondrial
E3 ubiquitin-protein ligase TRAF7
Rapamycin-insensitive companion of mTOR
WD repeat-containing protein 74
Type-1 angiotensin II receptor-associated protein
SWI/SNF-related matrix-associated actin-dependent regulator of chromatin subfamily D member 3
Monofunctional C1-tetrahydrofolate synthase, mitochondrial
Aftiphilin
Pre-mRNA 3'-end-processing factor FIP1
CREB-regulated transcription coactivator 3
CREB-regulated transcription coactivator 1
Cytochrome P450 20A1
Protein O-glucosyltransferase 2
Transmembrane protein 205
E3 ubiquitin-protein ligase LRSAM1
Golgi-associated kinase 1B
Lysocardiolipin acyltransferase 1
BRCA1-A complex subunit Abraxas 1
Spliceosome-associated protein CWC27 homolog
Protein disulfide isomerase CRELD2
WD repeat-containing protein 82

MICOS complex subunit MIC27
Protocadherin Fat 4
Dipeptidyl peptidase 8
Activating transcription factor 7-interacting protein 1
Ran-binding protein 10
Phosphofurin acidic cluster sorting protein 1
Myosin phosphatase Rho-interacting protein
RNA-binding motif, single-stranded-interacting protein 3
Nicotinate phosphoribosyltransferase
Dynamin-binding protein
Very-long-chain (3R)-3-hydroxyacyl-CoA dehydratase 2
Beta-1,3-glucosyltransferase
GRB10-interacting GYF protein 2
CD109 antigen
tRNA (32-2'-O)-methyltransferase regulator THADA
Hydroxysteroid dehydrogenase-like protein 2
Kynurenine--oxoglutarate transaminase 3
Protein phosphatase 1 regulatory subunit 21
Thrombospondin type-1 domain-containing protein 4
Nesprin-3
Zinc finger protein 574
NF-X1-type zinc finger protein NFXL1
Neurobeachin-like protein 2
FYVE, RhoGEF and PH domain-containing protein 5
Sulfhydryl oxidase 2
Helicase SRCAP
Rho family-interacting cell polarization regulator 1
Uncharacterized protein FLJ45252
Rho guanine nucleotide exchange factor 18
TBC1 domain family member 9
E3 ubiquitin-protein ligase UBR3
Transmembrane protein with metallophosphoesterase domain
Capping protein-inhibiting regulator of actin dynamics
Coiled-coil domain-containing protein 9B
8-oxo-dGDP phosphatase NUDT18
Rho GTPase-activating protein SYDE1
PAX-interacting protein 1
Protein O-mannosyl-transferase TMTC3
Intermembrane lipid transfer protein VPS13C
Ubiquitin carboxyl-terminal hydrolase 34
Ras-associated and pleckstrin homology domains-containing protein 1
MOB kinase activator 2
Inhibitor of nuclear factor kappa-B kinase-interacting protein
Phosphatidylinositol 3,4,5-trisphosphate-dependent Rac exchanger 2 protein
Protein FRA10AC1
Ubiquitin-conjugating enzyme E2 R2
Mediator of RNA polymerase II transcription subunit 13-like
La-related protein 4
Mediator of RNA polymerase II transcription subunit 25
Ribosomal protein eS27-like

E3 ubiquitin-protein ligase Hakai
Integrator complex subunit 8
Butyrophilin subfamily 2 member A1
Transcription elongation factor SPT6
Staphylococcal nuclease domain-containing protein 1
Serine/threonine-protein kinase MARK2
Cytochrome c oxidase assembly protein COX15 homolog
Probable ATP-dependent RNA helicase DDX46
Protein RUFY3
tRNA methyltransferase 10 homolog C
eIF5-mimic protein 2
Vacuolar fusion protein MON1 homolog B
Volume-regulated anion channel subunit LRRC8D
Isoaspartyl peptidase/L-asparaginase
BTB/POZ domain-containing protein KCTD9
ATP-dependent RNA helicase DHX30
Eukaryotic translation initiation factor 3 subunit M
7SK snRNA methylphosphate capping enzyme
Armadillo repeat-containing X-linked protein 2
Probable proline--tRNA ligase, mitochondrial
Arginine/serine-rich coiled-coil protein 2
Ras-related GTP-binding protein A
Cytoplasmic FMR1-interacting protein 1
Protein arginine methyltransferase NDUFAF7, mitochondrial
Golgi to ER traffic protein 4 homolog
COP9 signalosome complex subunit 6
Lysophosphatidylcholine acyltransferase 2
Mitochondrial enolase superfamily member 1
E3 ubiquitin-protein transferase MAEA
EPM2A-interacting protein 1
Putative pre-mRNA-splicing factor ATP-dependent RNA helicase DHX32
Serine/threonine-protein kinase TAO1
FAST kinase domain-containing protein 5, mitochondrial
MOB kinase activator 1B
Lysine-specific demethylase 3B
Charged multivesicular body protein 1b
Ribonucleoside-diphosphate reductase subunit M2 B
Heparan sulfate 2-O-sulfotransferase 1
STE20-related kinase adapter protein alpha
[F-actin]-monooxygenase MICAL3
Dymeclin
Keratin, type II cytoskeletal 78
PHD finger-like domain-containing protein 5A
Peroxiredoxin-like 2C
Aprataxin
Endoplasmic reticulum metallopeptidase 1
G protein-regulated inducer of neurite outgrowth 1
TRMT1-like protein
Zinc finger CCCH-type antiviral protein 1
Large ribosomal subunit protein bL21m

Elongation factor-like GTPase 1
Probable helicase senataxin
Trafficking protein particle complex subunit 11
Nucleoporin p54
Autophagy-related protein 9A
D-glutamate cyclase, mitochondrial
Coiled-coil domain-containing protein 186
LisH domain-containing protein ARMC9
VPS35 endosomal protein-sorting factor-like
Pogo transposable element with ZNF domain
Zinc finger FYVE domain-containing protein 16
Protein MON2 homolog
C-myc promoter-binding protein
Myosin-14
FMR1-interacting protein NUFIP2
SUZ domain-containing protein 1
Mitochondrial antiviral-signaling protein
CLIP-associating protein 1
ATP-dependent RNA helicase DHX29
Nephrocystin-3
Low-density lipoprotein receptor-related protein 10
HAUS augmin-like complex subunit 6
Protein O-glucosyltransferase 3
Tetratricopeptide repeat protein 21B
HEAT repeat-containing protein 3
Kinesin-like protein KIF21A
Hepatoma-derived growth factor-related protein 2
L-xylulose reductase
BRCA1-associated protein
BRCA2-interacting transcriptional repressor EMSY
Golgin subfamily A member 7
Rho GTPase-activating protein 22
Wings apart-like protein homolog
Interferon regulatory factor 2-binding protein 2
E3 ubiquitin-protein ligase RBBP6
E3 ubiquitin-protein ligase SH3RF1
Protein TAMALIN
FERM domain-containing protein 5
tRNA-splicing endonuclease subunit Sen54
Protein prenyltransferase alpha subunit repeat-containing protein 1
Arpin
Tectonin beta-propeller repeat-containing protein 1
Rab9 effector protein with kelch motifs
Transcription termination factor 4, mitochondrial
E3 ubiquitin-protein ligase HUWE1
YTH domain-containing family protein 3
Cytoplasmic tRNA 2-thiolation protein 1
Transcription initiation factor TFIID subunit 8
Ubiquitin-conjugating enzyme E2 Q1
Large ribosomal subunit protein mL55

Transmembrane emp24 domain-containing protein 4
Large ribosomal subunit protein uL10m
COX assembly mitochondrial protein homolog
Centromere protein V
Schlafen family member 11
Multiple epidermal growth factor-like domains protein 8
Transmembrane protein 179B
N-acetylgalactosaminyltransferase 7
Stearoyl-CoA desaturase 5
Pleckstrin homology-like domain family B member 2
Adhesion G-protein coupled receptor G6
Dehydrodolichyl diphosphate synthase complex subunit DHDDS
Polypeptide N-acetylgalactosaminyltransferase 10
Trafficking protein particle complex subunit 6B
Type 1 phosphatidylinositol 4,5-bisphosphate 4-phosphatase
C-type lectin domain family 14 member A
Transcriptional regulator Kaiso
Protein PAT1 homolog 1
Dipeptidyl peptidase 9
E3 ubiquitin-protein ligase synoviolin
Actin-histidine N-methyltransferase
Tetratricopeptide repeat protein 7B
Acyl-coenzyme A thioesterase 1
Nucleolar protein 9
Polyadenylate-binding protein 2
N6-adenosine-methyltransferase catalytic subunit
Protein polybromo-1
Threonylcarbamoyl-AMP synthase
Pre-mRNA-processing factor 39
Protein LYRIC
E3 ubiquitin-protein ligase ZNF598
Glycerol-3-phosphate acyltransferase 4
Kinectin
Telomerase-binding protein EST1A
NLR family member X1
Pleckstrin homology-like domain family B member 1
Ubiquitin carboxyl-terminal hydrolase 48
Calcium-dependent secretion activator 2
Fermitin family homolog 3
5'-nucleotidase domain-containing protein 3
Acetoacetyl-CoA synthetase
Leucine zipper protein 1
THO complex subunit 4
Integral membrane protein GPR180
FHF complex subunit HOOK-interacting protein 2B
Ras GTPase-activating-like protein IQGAP3
Zinc finger CCCH domain-containing protein 18
Vacuolar protein-sorting-associated protein 36
Cullin-associated NEDD8-dissociated protein 1
Reticulophagy regulator 3

Protein Hook homolog 3
SEC14 domain and spectrin repeat-containing protein 1
COMM domain-containing protein 7
Vacuolar fusion protein MON1 homolog A
Archaemetzincin-2
THO complex subunit 6 homolog
RNA N6-adenosine-methyltransferase METTL16
Poly(ADP-ribose) glycohydrolase
Liprin-beta-1
Lon protease homolog 2, peroxisomal
Zinc finger C3HC-type protein 1
Chromodomain-helicase-DNA-binding protein 1-like
F-BAR and double SH3 domains protein 1
Coiled-coil domain-containing protein 25
Proline and serine-rich protein 2
Stimulator of interferon genes protein
Active regulator of SIRT1
Cerebellar degeneration-related protein 2-like
Ral GTPase-activating protein subunit beta
Ras-specific guanine nucleotide-releasing factor RalGPS2
Histone-arginine methyltransferase CARM1
Deaminated glutathione amidase
COMM domain-containing protein 2
HEAT repeat-containing protein 5A
Condensin-2 complex subunit G2
Protein phosphatase 1 regulatory subunit 3B
F-box only protein 11
Ankyrin repeat and LEM domain-containing protein 2
RNA-binding protein MEX3D
Diacylglycerol kinase eta
ATP-dependent RNA helicase DDX42
Spermatogenesis-associated serine-rich protein 2
Serine/threonine-protein kinase VRK2
CDK2-associated and cullin domain-containing protein 1
NADH dehydrogenase [ubiquinone] 1 alpha subcomplex subunit 11
Dynein axonemal assembly factor 5
Peptidyl-tRNA hydrolase
Syntaxin-12
Transcriptional repressor p66-alpha
Copine-8
G-protein-signaling modulator 1
Ras-related protein Rab-43
C2 domain-containing protein 5
E3 ubiquitin-protein ligase MIB1
Inactive tyrosine-protein kinase PRAG1
BLOC-2 complex member HPS6
Interferon regulatory factor 2-binding protein 1
ELKS/Rab6-interacting/CAST family member 1
Ribosomal oxygenase 2
Palmitoyltransferase ZDHHC13

Palmitoyltransferase ZDHHC17
Cytokine receptor-like factor 3
Trafficking protein particle complex subunit 5
Armadillo repeat-containing protein 8
RELT-like protein 1
5'-3' exonuclease PLD3
Protein HID1
Ankyrin repeat and MYND domain-containing protein 2
3'-5' exoribonuclease 1
Inactive serine/threonine-protein kinase VRK3
LIX1-like protein
NudC domain-containing protein 3
Protein AHNAK2
Formin-like protein 3
Methylmalonic aciduria type A protein, mitochondrial
Mitogen-activated protein kinase kinase kinase kinase 3
Neuron navigator 3
Prolyl 3-hydroxylase 3
Coiled-coil domain-containing protein 50
Malonyl-CoA-acyl carrier protein transacylase, mitochondrial
Kinase suppressor of Ras 1
Centrosomal protein of 97 kDa
ATP-dependent (S)-NAD(P)H-hydrate dehydratase
WD repeat-containing protein 75
Mitofusin-1
Choline transporter-like protein 2
Inositol 1,4,5-trisphosphate receptor-interacting protein
WD repeat and FYVE domain-containing protein 1
MAP7 domain-containing protein 3
Protein NOXP20
DCN1-like protein 3
Protein DENND6A
MAX gene-associated protein
GRIP and coiled-coil domain-containing protein 2
Zinc finger CCCH domain-containing protein 7A
PHD finger protein 6
Protein archease
Volume-regulated anion channel subunit LRRC8A
Extracellular sulfatase Sulf-2
E3 ubiquitin-protein ligase UBR1
E3 ubiquitin-protein ligase UBR2
Rho GTPase-activating protein 12
Calcium homeostasis endoplasmic reticulum protein
Codanin-1
Ankyrin repeat and KH domain-containing protein 1
SURP and G-patch domain-containing protein 1
SURP and G-patch domain-containing protein 2
Ubiquitin-conjugating enzyme E2 variant 3
Cell division cycle and apoptosis regulator protein 1
Probable ATP-dependent RNA helicase DHX40

DnaJ homolog subfamily C member 10
Negative elongation factor C/D
Mitochondrial Rho GTPase 2
Mitochondrial Rho GTPase 1
NAD-dependent protein deacetylase sirtuin-2
Polyhomeotic-like protein 2
Chromatin complexes subunit BAP18
Large ribosomal subunit protein mL41
Nurim
GPALPP motifs-containing protein 1
Protein mono-ADP-ribosyltransferase PARP9
RNA-binding protein 12B
Solute carrier family 35 member F2
Putative RNA polymerase II subunit B1 CTD phosphatase RPAP2
Patatin-like phospholipase domain-containing protein 6
Structural maintenance of chromosomes protein 5
Probable ATP-dependent RNA helicase DDX60
MICAL-like protein 2
Probable ATP-dependent RNA helicase DHX37
Kelch repeat and BTB domain-containing protein 2
Ribonucleoprotein PTB-binding 1
pre-rRNA 2'-O-ribose RNA methyltransferase FTSJ3
Transmembrane protein 192
Cytoskeleton-associated protein 2-like
Nucleotidyltransferase MB21D2
Serine/arginine repetitive matrix protein 1
Stromal membrane-associated protein 1
DIS3-like exonuclease 2
ATP-dependent RNA helicase SUPV3L1, mitochondrial
Eukaryotic peptide chain release factor GTP-binding subunit ERF3B
Exocyst complex component 8
Uncharacterized protein C10orf67, mitochondrial
E3 ubiquitin-protein ligase TRIM22
Threonine synthase-like 1
Uncharacterized protein KIAA2013
Calcium uptake protein 2, mitochondrial
Ankyrin repeat domain-containing protein 13A
Phosphatase and actin regulator 4
tRNA (uracil-5-)-methyltransferase homolog A
Pseudouridylate synthase RPUSD2
ELMO domain-containing protein 2
Aldehyde dehydrogenase family 16 member A1
Dyslexia-associated protein KIAA0319-like protein
Adhesion G protein-coupled receptor F5
5'-3' exoribonuclease 1
Proline-, glutamic acid- and leucine-rich protein 1
Abl interactor 1
WD repeat and FYVE domain-containing protein 3
Selenoprotein H
Vitamin K epoxide reductase complex subunit 1-like protein 1

Spartin
Tetratricopeptide repeat protein 5
Mesoderm induction early response protein 1
Regulatory-associated protein of mTOR
ORM1-like protein 3
Cell cycle and apoptosis regulator protein 2
NADH dehydrogenase [ubiquinone] 1 alpha subcomplex assembly factor 2
Vacuolar protein sorting-associated protein 52 homolog
Nuclear pore complex protein Nup93
Serine/threonine-protein kinase 11-interacting protein
Zinc finger protein 687
Cap-specific mRNA (nucleoside-2'-O-)-methyltransferase 1
Leucine-rich repeat-containing protein 47
Dedicator of cytokinesis protein 4
Carbonic anhydrase 13
Rho guanine nucleotide exchange factor 28
Integrator complex subunit 1
Histone H2B type 3-B
Rho GTPase-activating protein 24
Armadillo repeat-containing protein 10
Lysophosphatidylserine lipase ABHD12
CLK4-associating serine/arginine rich protein
Protein FAM43A
Transmembrane protein 256
Glycerol-3-phosphate dehydrogenase 1-like protein
Solute carrier family 35 member F6
Rho GTPase-activating protein 18
Activating signal cointegrator 1 complex subunit 3
MICAL-like protein 1
Vacuolar protein sorting-associated protein 8 homolog
Protein PALS1
Cohesin subunit SA-2
Synaptopodin
Formin-binding protein 4
Translation factor GUF1, mitochondrial
RING1 and YY1-binding protein
Polypeptide N-acetylgalactosaminyltransferase 4
Misshapen-like kinase 1
Guanosine-3',5'-bis(diphosphate) 3'-pyrophosphohydrolase MESH1
Prostaglandin reductase 3
3-oxoacyl-[acyl-carrier-protein] reductase
ER membrane protein complex subunit 5
Transmembrane protein 199
F-box/LRR-repeat protein 6
Prolyl 3-hydroxylase OGFOD1
Actin filament-associated protein 1
Zinc finger CCHC domain-containing protein 9
Oxidation resistance protein 1
Zinc finger CCCH-type with G patch domain-containing protein
Calcium homeostasis modulator protein 5

S1 RNA-binding domain-containing protein 1
WD and tetratricopeptide repeats protein 1
NF-kappa-B-activating protein
Macoilin
SH2 domain-containing protein 3C
Arrestin domain-containing protein 1
Dehydrogenase/reductase SDR family member on chromosome X
CDGSH iron-sulfur domain-containing protein 2
Ribonuclease P protein subunit p25-like protein
ATP synthase mitochondrial F1 complex assembly factor 2
Tetratricopeptide repeat protein 9C
Protein jagunal homolog 1
E3 ubiquitin-protein ligase RNF10
Refilin-B
Leucine-zipper-like transcriptional regulator 1
COMM domain-containing protein 1
Cleavage and polyadenylation specificity factor subunit 7
Solute carrier family 15 member 4
Myc target protein 1
ADAMTS-like protein 1
ADP-ribosylation factor GTPase-activating protein 2
Deubiquitinase OTUD6B
Acyl-coenzyme A diphosphatase FITM2
UPF0690 protein C1orf52
eEF1A lysine and N-terminal methyltransferase
ADP-ribosylation factor-like protein 6-interacting protein 6
ADP-ribosylation factor GTPase-activating protein 1
NAD-dependent protein deacylase sirtuin-6
Solute carrier family 66 member 3
ER membrane protein complex subunit 1
RNA polymerase II-associated factor 1 homolog
Cyclin-Y-like protein 1
Putative E3 ubiquitin-protein ligase UBR7
ATP-dependent RNA helicase DDX51
Uncharacterized protein FAM241A
Mitochondrial basic amino acids transporter
Protein enabled homolog
Discoidin, CUB and LCCL domain-containing protein 1
G patch domain-containing protein 11
Large ribosomal subunit protein mL43
RNA ligase 1
Lysophospholipase D GDPD1
Uncharacterized protein C19orf47
Activating signal cointegrator 1 complex subunit 1
Leucine-rich repeat-containing protein 57
Probable RNA-binding protein EIF1AD
Protein KRI1 homolog
Coiled-coil domain-containing protein 71L
Zinc finger protein 579
Pre-mRNA-splicing factor 38A

Mixed lineage kinase domain-like protein
Glutamine amidotransferase-like class 1 domain-containing protein 1
Serine/threonine-protein phosphatase 6 regulatory ankyrin repeat subunit C
Phospholipid-transporting ATPase IG
ATPase family gene 2 protein homolog A
NHL repeat-containing protein 2
Late secretory pathway protein AVL9 homolog
Equilibrative nucleobase transporter 1
Xyloside xylosyltransferase 1
Golgi membrane protein 1
Procollagen galactosyltransferase 1
Inactive C-alpha-formylglycine-generating enzyme 2
SID1 transmembrane family member 2
Formylglycine-generating enzyme
Protein O-glucosyltransferase 1
Ubiquitin-associated domain-containing protein 2
Prenylcysteine oxidase-like
Transmembrane protein 87A
Retinol dehydrogenase 13
Tetratricopeptide repeat protein 13
Estradiol 17-beta-dehydrogenase 11
Thioredoxin domain-containing protein 5
Kinetochore protein Spc24
Outer mitochondrial transmembrane helix translocase
Saccharopine dehydrogenase-like oxidoreductase
UDP-glucuronic acid decarboxylase 1
E3 ubiquitin-protein ligase RNF149
Reticulophagy regulator 2
SERPINE1 mRNA-binding protein 1
LEM domain-containing protein 2
Nitric oxide-associated protein 1
Adaptin ear-binding coat-associated protein 1
Protein FAM98A
Phospholipase A2 group XV
Myotubularin-related protein 14
NFATC2-interacting protein
Diacylglycerol lipase-beta
Carbohydrate sulfotransferase 14
Polypeptide N-acetylgalactosaminyltransferase 6
E3 ubiquitin-protein ligase RNF169
Pyruvate dehydrogenase phosphatase regulatory subunit, mitochondrial
NAD(P)H-hydrate epimerase
Nonsense-mediated mRNA decay factor SMG8
RING finger protein 214
Protein LSM14 homolog A
GTPase IMAP family member 8
Cyclin-Y
Maestro heat-like repeat-containing protein family member 1
Terminal nucleotidyltransferase 4B
Probable aminopeptidase NPEPL1

EH domain-binding protein 1
Putative RNA-binding protein 15B
Alpha-N-acetylgalactosaminide alpha-2,6-sialyltransferase 3
Trinucleotide repeat-containing gene 6A protein
Polyhomeotic-like protein 3
Divergent protein kinase domain 2A
Metal transporter CNNM3
ATP-binding cassette sub-family F member 1
Calcium uniporter protein, mitochondrial
Phosphatidylinositol 3-kinase catalytic subunit type 3
Glutathione S-transferase C-terminal domain-containing protein
Serum response factor-binding protein 1
Neuroguidin
SHC SH2 domain-binding protein 1
PDZ domain-containing protein 8
DCC-interacting protein 13-beta
Zinc transporter 7
Neuron navigator 1
Periphilin-1
WD repeat-containing protein 19
F-box only protein 22
Lysophosphatidylcholine acyltransferase 1
Zinc finger MIZ domain-containing protein 2
Nesprin-1
Ubiquitin carboxyl-terminal hydrolase 32
Biorientation of chromosomes in cell division protein 1-like 1
AT-rich interactive domain-containing protein 1B
FAD synthase
Folliculin
Nucleoporin Nup43
Nucleoporin Nup37
Nucleoporin NUP35
Cytosolic endo-beta-N-acetylglucosaminidase
Retinoic acid-induced protein 3
Torsin-1A-interacting protein 2
sn-1-specific diacylglycerol lipase ABHD11
N-acylneuraminate cytidylyltransferase
Tetraspanin-14
Tubulin--tyrosine ligase
Small VCP/p97-interacting protein
Atlastin-2
Motile sperm domain-containing protein 2
Putative phospholipase B-like 2
Ras association domain-containing protein 8
ATP-dependent RNA helicase DDX55
Major facilitator superfamily domain-containing protein 8
Tudor domain-containing protein 7
GTPase IMAP family member 7
Protein NEDD1
Nuclear receptor coactivator 7

THO complex subunit 2
WD repeat-containing protein 36
Protein phosphatase PTC7 homolog
Retinitis pigmentosa 9 protein
Vang-like protein 1
Smad nuclear-interacting protein 1
Large ribosomal subunit protein mL64
WD repeat-containing protein 48
Exocyst complex component 6
SWI/SNF complex subunit SMARCC2
Nuclear protein localization protein 4 homolog
Uncharacterized protein CXorf38
Spermatogenesis-associated protein 20
Iron-sulfur cluster transfer protein NUBPL
(Lyso)-N-acylphosphatidylethanolamine lipase
F-box only protein 30
Adenosine 3'-phospho 5'-phosphosulfate transporter 1
Pumilio homolog 2
T-cell immunomodulatory protein
Golgin subfamily A member 5
Kelch domain-containing protein 4
SH3KBP1-binding protein 1
NEDD8-activating enzyme E1 catalytic subunit
Prostamide/prostaglandin F synthase
DnaJ homolog subfamily B member 14
Protein kish-A
Phosphatidylinositol 5-phosphate 4-kinase type-2 gamma
WD repeat-containing protein 20
TBC1 domain family member 15
Retinol dehydrogenase 11
Leucine-rich repeat-containing protein 20
Interferon-induced protein 44
Large ribosomal subunit protein uL30m
AN1-type zinc finger protein 1
Protein CIP2A
Phosphatidylinositol 4-kinase type 2-beta
Dolichyl-diphosphooligosaccharide--protein glycosyltransferase subunit STT3B
Polyribonucleotide nucleotidyltransferase 1, mitochondrial
Signal peptide peptidase-like 2B
Minor histocompatibility antigen H13
Phosphatidylinositol 3,4,5-trisphosphate-dependent Rac exchanger 1 protein
Up-regulator of cell proliferation
Protein bicaudal D homolog 2
Serine/threonine-protein kinase Nek9
Pleckstrin homology domain-containing family O member 2
E3 ubiquitin-protein ligase DTX3L
ATP-dependent RNA helicase DDX54
Disks large homolog 5
Ribosome biogenesis protein BRX1 homolog
Glucosamine-6-phosphate isomerase 2

Volume-regulated anion channel subunit LRRC8C
Serine/threonine-protein kinase Nek7
RB1-inducible coiled-coil protein 1
[F-actin]-monooxygenase MICAL1
Elongator complex protein 5
Protein phosphatase Slingshot homolog 3
SH3 domain and tetratricopeptide repeat-containing protein 1
TBC domain-containing protein kinase-like protein
D-aminoacyl-tRNA deacylase 1
DDB1- and CUL4-associated factor 11
U3 small nucleolar RNA-associated protein 15 homolog
Probable glutathione peroxidase 8
Actin filament-associated protein 1-like 1
E3 ubiquitin-protein ligase SH3RF3
Short transient receptor potential channel 4-associated protein
Centrosomal protein of 192 kDa
Gem-associated protein 5
GPI ethanolamine phosphate transferase 3
Rap guanine nucleotide exchange factor 6
Guanine nucleotide exchange protein SMCR8
Partitioning defective 3 homolog
Importin-4
Ubiquitin carboxyl-terminal hydrolase 33
Arginine/serine-rich protein PNISR
Serine/threonine-protein phosphatase 4 regulatory subunit 1
Ubiquitin-associated and SH3 domain-containing protein B
WAS/WASL-interacting protein family member 2
1-acylglycerol-3-phosphate O-acyltransferase ABHD5
Histone-lysine N-methyltransferase SETD7
Nucleolar complex protein 3 homolog
Scavenger receptor class B member 1
Conserved oligomeric Golgi complex subunit 1
Sec1 family domain-containing protein 2
Stromal membrane-associated protein 2
Zinc finger CCCH domain-containing protein 15
Peptidyl-prolyl cis-trans isomerase-like 4
General transcription factor 3C polypeptide 2
TBC1 domain family member 22A
PHD finger protein 10
Ras-related protein Rab-2B
Coiled-coil domain-containing protein 12
RelA-associated inhibitor
Cotranscriptional regulator ARB2A
Protein Churchill
Transforming growth factor-beta receptor-associated protein 1
Transmembrane protein 263
Histone deacetylase 7
Phosphatidylglycerophosphatase and protein-tyrosine phosphatase 1
Nuclear pore complex protein Nup133
Programmed cell death 6-interacting protein

Filamin-binding LIM protein 1
Splicing factor Cactin
Glutathione-specific gamma-glutamylcyclotransferase 2
Charged multivesicular body protein 7
Protein THEM6
Probable N-acetyltransferase 14
B-cell CLL/lymphoma 7 protein family member C
Non-structural maintenance of chromosomes element 1 homolog
MIT domain-containing protein 1
Chromosome transmission fidelity protein 18 homolog
RNA polymerase-associated protein LEO1
Dephospho-CoA kinase domain-containing protein
NudC domain-containing protein 2
U4/U6.U5 small nuclear ribonucleoprotein 27 kDa protein
Dimethyladenosine transferase 1, mitochondrial
Cohesin subunit SA-1
Sec1 family domain-containing protein 1
Tumor necrosis factor alpha-induced protein 8-like protein 1
Soluble calcium-activated nucleotidase 1
Trafficking protein particle complex subunit 12
Heterogeneous nuclear ribonucleoprotein L-like
Fatty acyl-CoA reductase 1
Ubiquitin-like domain-containing CTD phosphatase 1
tRNA-splicing endonuclease subunit Sen15
PEST proteolytic signal-containing nuclear protein
DnaJ homolog subfamily A member 4
SPRY domain-containing protein 4
m-AAA protease-interacting protein 1, mitochondrial
Pseudouridylate synthase TRUB1
LIM domain only protein 7
Choline transporter-like protein 1
Cytoskeleton-associated protein 2
Ataxin-2-like protein
Arf-GAP with Rho-GAP domain, ANK repeat and PH domain-containing protein 3
GTPase IMAP family member 1
PH-interacting protein
U4/U6 small nuclear ribonucleoprotein Prp31
Negative elongation factor B
Palladin
RUN and FYVE domain-containing protein 2
Splicing regulatory glutamine/lysine-rich protein 1
Caskin-2
ATR-interacting protein
Paraspeckle component 1
MAP kinase-activating death domain protein
Nesprin-2
Transcriptional repressor p66-beta
DnaJ homolog subfamily C member 9
BRI3-binding protein
Beta-catenin-like protein 1

Cysteine protease ATG4A
Ras and Rab interactor 2
Protein ELYS
Microprocessor complex subunit DGCR8
Roundabout homolog 4
Esterase OVCA2
Immunity-related GTPase family Q protein
DNA damage-binding protein 2
C-type lectin domain family 2 member B
ATP-dependent RNA helicase DDX1
SEC14-like protein 1
Piezo-type mechanosensitive ion channel component 1
Protein FAM3C
Histone H1.10
Proteasome inhibitor PI31 subunit
Golgi-specific brefeldin A-resistance guanine nucleotide exchange factor 1
RNA polymerase-associated protein RTF1 homolog
Nicastrin
Sorting nexin-19
Transmembrane 9 superfamily member 4
Transmembrane protein 131
Inositol hexakisphosphate kinase 1
Small ribosomal subunit protein mS27
Engulfment and cell motility protein 1
Polyphosphoinositide phosphatase
Protein FAM168A
AP-3 complex subunit sigma-1
Hamartin
UBX domain-containing protein 4
PHD finger protein 3
Protein NDRG1
Heat shock protein 105 kDa
Septin-8
CCR4-NOT transcription complex subunit 9
Acyl-CoA:lysophosphatidylglycerol acyltransferase 1
TBC1 domain family member 5
Zinc finger protein 592
Protein Jade-3
Unconventional myosin-XVIIIa
La-related protein 4B
Stalled ribosome sensor GCN1
Rho GTPase-activating protein 45
Pre-mRNA-splicing factor ATP-dependent RNA helicase PRP16
Nuclear pore complex protein Nup205
Ankyrin repeat and SAM domain-containing protein 1A
Peroxidasin homolog
Protein FAN
GPI-anchor transamidase
Small ribosomal subunit protein mS31
A-kinase anchor protein 1, mitochondrial

Dol-P-Man:Man(5)GlcNAc(2)-PP-Dol alpha-1,3-mannosyltransferase
Neurogranin
Acidic leucine-rich nuclear phosphoprotein 32 family member B
Nectin-2
Geranylgeranyl transferase type-2 subunit alpha
Protein TFG
Serine protease HTRA1
Actin-related protein 2/3 complex subunit 1A
General transcription factor IIH subunit 4
Ras-responsive element-binding protein 1
Histone deacetylase 2
Signal transducing adapter molecule 1
Zinc finger protein ubi-d4
Endoplasmic reticulum protein SC65
CREB-binding protein
Histone acetyltransferase KAT6A
Symplekin
TATA-binding protein-associated factor 2N
Golgin subfamily A member 1
Gamma-glutamyl hydrolase
Neuronal cell adhesion molecule
X-linked retinitis pigmentosa GTPase regulator
Phosphatidylinositol 3,4,5-trisphosphate 5-phosphatase 1
Probable ATP-dependent RNA helicase DDX17
Kinesin-associated protein 3
Caspase-10
Amyloid beta precursor protein binding family B member 2
DNA repair protein RAD50
CUGBP Elav-like family member 1
Osteoclast-stimulating factor 1
Rho guanine nucleotide exchange factor 1
DNA repair endonuclease XPF
Ubiquitin recognition factor in ER-associated degradation protein 1
Golgi apparatus protein 1
Regulator of nonsense transcripts 1
COP9 signalosome complex subunit 5
G-patch domain and KOW motifs-containing protein
SWI/SNF complex subunit SMARCC1
SWI/SNF-related matrix-associated actin-dependent regulator of chromatin subfamily D member 2
Ras-related protein Rab-8B
Bcl2-associated agonist of cell death
Far upstream element-binding protein 2
Glutaryl-CoA dehydrogenase, mitochondrial
GTP-binding protein Rit1
Peroxisomal membrane protein PEX13
Transportin-1
Rho guanine nucleotide exchange factor 2
Ribosomal RNA small subunit methyltransferase NEP1
Polyribonucleotide 5'-hydroxyl-kinase Clp1
Glomulin

Ubiquitin carboxyl-terminal hydrolase 13
Segment polarity protein dishevelled homolog DVL-3
Probable ubiquitin carboxyl-terminal hydrolase FAF-X
Ubiquitin carboxyl-terminal hydrolase 7
Cullin-5
Stathmin-2
V-type proton ATPase 116 kDa subunit a 1
Lipoma-preferred partner
RNA-binding protein with multiple splicing
Exostosin-2
Mediator of RNA polymerase II transcription subunit 12
Protein tyrosine phosphatase type IVA 1
Phosphorylase b kinase regulatory subunit beta
Secretory carrier-associated membrane protein 4
BLOC-2 complex member HPS3
SWI/SNF-related matrix-associated actin-dependent regulator of chromatin subfamily E member 1
Caveolae-associated protein 3
Riboflavin kinase
Myeloid-derived growth factor
Protein YIPF5
GPI transamidase component PIG-T
Immunoglobulin superfamily member 8
Ribosomal protein eL42-like
Ras-related protein Rab-24
Oxysterol-binding protein 2
Cytoplasmic 60S subunit biogenesis factor ZNF622
Ribosome-releasing factor 2, mitochondrial
Ubiquitin-conjugating enzyme E2 E3
7-methylguanosine phosphate-specific 5'-nucleotidase
WW domain-binding protein 2
Proteasome assembly chaperone 2
BOS complex subunit NCLN
Mitochondrial ubiquitin ligase activator of NFKB 1
Myocardin-related transcription factor A
Protein lifeguard 3
Endoplasmic reticulum-Golgi intermediate compartment protein 1
U3 small nucleolar RNA-associated protein 4 homolog
FAST kinase domain-containing protein 4
Mitochondrial amidoxime reducing component 2
Protein FAM162A
PAT complex subunit CCDC47
Large ribosomal subunit protein uL24m
Exocyst complex component 4
Protein mago nashi homolog 2
Putative monooxygenase p33MONOX
Protein RFT1 homolog
Isochorismatase domain-containing protein 2
Protein N-terminal asparagine amidohydrolase
Fermitin family homolog 2
Far upstream element-binding protein 1

Tetratricopeptide repeat protein 17
Leucine-rich repeat-containing protein 59
Vesicle transport through interaction with t-SNAREs homolog 1A
Endothelial cell-selective adhesion molecule
Pre-B-cell leukemia transcription factor-interacting protein 1
Mitochondrial calcium uniporter regulator 1
Uncharacterized protein KIAA1143
Vacuolar protein sorting-associated protein 33A
Peptidyl-prolyl cis-trans isomerase FKBP10
Tetratricopeptide repeat protein 28
Exosome complex component RRP43
Zinc finger protein 428
SH3 domain-containing kinase-binding protein 1
Spindle and kinetochore-associated protein 1
Nucleus accumbens-associated protein 2
E3 ubiquitin-protein ligase RNF25
Axin interactor, dorsalization-associated protein
PIN2/TERF1-interacting telomerase inhibitor 1
ADP-ribosylation factor-like protein 8A
Ubiquitin thioesterase otulin
Small ribosomal subunit protein mS37
Peptidylprolyl isomerase domain and WD repeat-containing protein 1
Coiled-coil domain-containing protein 127
Cytochrome c oxidase assembly factor 7
Uracil phosphoribosyltransferase homolog
Phosphatidate cytidylyltransferase, mitochondrial
MOB kinase activator 3A
Dedicator of cytokinesis protein 10
Autophagy-related protein 2 homolog B
Leukocyte receptor cluster member 1
TBC1 domain family member 20
EF-hand domain-containing protein D2
Galactose mutarotase
Synaptotagmin-like protein 4
Pyrroline-5-carboxylate reductase 2
Protein CUSTOS
m7GpppX diphosphatase
Protein phosphatase 1 regulatory subunit 14B
Integrator complex subunit 12
5-methylcytosine rRNA methyltransferase NSUN4
Inactive rhomboid protein 1
Collagen triple helix repeat-containing protein 1
Medium-chain acyl-CoA ligase ACSF2, mitochondrial
Isochorismatase domain-containing protein 1
GRIP and coiled-coil domain-containing protein 1
FLYWCH family member 2
Protein Aster-A
HAUS augmin-like complex subunit 1
FAS-associated factor 2
Coiled-coil domain-containing protein 124

FAD-dependent oxidoreductase domain-containing protein 1
Optineurin
AP-2 complex subunit mu
Gamma-tubulin complex component 3
Probable RNA polymerase II nuclear localization protein SLC7A6OS
BTB/POZ domain-containing protein KCTD12
Reticulocalbin-3
60S ribosomal export protein NMD3
RalBP1-associated Eps domain-containing protein 1
Mitochondrial import inner membrane translocase subunit TIM14
Regulator of microtubule dynamics protein 1
U8 snoRNA-decapping enzyme
Splicing factor ESS-2 homolog
Carboxymethylenebutenolidase homolog
RNA-binding protein Musashi homolog 2
U5 small nuclear ribonucleoprotein 40 kDa protein
Regulator of MON1-CCZ1 complex
Large ribosomal subunit protein mL38
RING finger and SPRY domain-containing protein 1
Endoplasmic reticulum lectin 1
Ribosome-recycling factor, mitochondrial
Transcription termination factor 3, mitochondrial
Protein Spindly
Elongator complex protein 4
NAD-dependent protein deacetylase sirtuin-1
Protein YIPF6
Nucleoporin SEH1
Transcription elongation factor A protein-like 4
KIF-binding protein
Glucosamine 6-phosphate N-acetyltransferase
Constitutive coactivator of peroxisome proliferator-activated receptor gamma
Protein KTI12 homolog
Small ribosomal subunit protein uS3m
Large ribosomal subunit protein mL53
Trans-3-hydroxy-L-proline dehydratase
E3 ubiquitin-protein ligase RNF31
DAZ-associated protein 1
Small glutamine-rich tetratricopeptide repeat-containing protein beta
Protein SAAL1
Mitochondrial potassium channel
SAGA-associated factor 29
Ribosomal RNA processing protein 36 homolog
C1GALT1-specific chaperone 1
RNA-binding protein 33
Dysbindin
DnaJ homolog subfamily A member 3, mitochondrial
Translation machinery-associated protein 16
Multivesicular body subunit 12A
Small ribosomal subunit protein mS39
Corrinoid adenosyltransferase MMAB

Cytoplasmic FMR1-interacting protein 2
E3 ubiquitin-protein ligase TRIM11
Coiled-coil domain-containing protein 97
CB1 cannabinoid receptor-interacting protein 1
Enhancer of mRNA-decapping protein 3
Dynein light chain 2, cytoplasmic
Copine-2
Protein S100-A16
Signal-induced proliferation-associated protein 1
Secernin-2
THO complex subunit 1
Ubiquitin thioesterase OTUB1
tRNA (adenine(58)-N(1))-methyltransferase catalytic subunit TRMT61A
Abasic site processing protein HMCES
Charged multivesicular body protein 6
Protein bicaudal D homolog 1
Phosphopentomutase
U3 small nucleolar ribonucleoprotein protein IMP4
Ceramide synthase 2
tRNA-dihydrouridine(47) synthase [NAD(P)(+)]-like
Protein LTV1 homolog
Serine dehydratase-like
Large ribosomal subunit protein mL48
Chronophin
Aurora kinase B
DCN1-like protein 1
Fumarylacetoacetate hydrolase domain-containing protein 2A
SWI/SNF-related matrix-associated actin-dependent regulator of chromatin subfamily D member 1
Target of EGR1 protein 1
Cell division cycle-associated 7-like protein
RUS family member 1
Probable ATP-dependent RNA helicase DDX27
BLOC-1-related complex subunit 6
Methionine--tRNA ligase, mitochondrial
Ataxin-7-like protein 3B
Serine/threonine-protein kinase greatwall
Methylthioribulose-1-phosphate dehydratase
Zinc finger C2HC domain-containing protein 1A
Protein lin-37 homolog
Vacuolar-sorting protein SNF8
Zinc finger CCCH-type antiviral protein 1-like
Nuclear envelope pore membrane protein POM 121
Tonsoku-like protein
PDZ and LIM domain protein 5
Protein disulfide isomerase CRELD1
ERO1-like protein alpha
Proline-rich protein 11
Protein FMC1 homolog
Adenosylhomocysteinase 3
Dedicator of cytokinesis protein 6

Mediator of RNA polymerase II transcription subunit 30
Serine/threonine-protein phosphatase PGAM5, mitochondrial
Integrator complex subunit 4
DDRGK domain-containing protein 1
2-oxoadipate dehydrogenase complex component E1
Selenocysteine lyase
Far upstream element-binding protein 3
Splicing factor 45
RCC1-like G exchanging factor-like protein
Asparaginyl-tRNA synthetase
Succinate--CoA ligase [GDP-forming] subunit beta, mitochondrial
DISP complex protein LRCH3
Mannose-1-phosphate guanyltransferase alpha
4-hydroxyphenylpyruvate dioxygenase-like protein
Putative protein-lysine deacylase ABHD14B
Peptide-N(4)-(N-acetyl-beta-glucosaminyl)asparagine amidase
Vesicle-trafficking protein SEC22a
ATP synthase membrane subunit K, mitochondrial
Kinetochore-associated protein NSL1 homolog
PRKC apoptosis WT1 regulator protein
tRNA N(3)-methylcytidine methyltransferase METTL2A
THO complex subunit 3
E3 ubiquitin-protein ligase Itchy homolog
Thioredoxin domain-containing protein 15
Kin of IRRE-like protein 1
Conserved oligomeric Golgi complex subunit 3
CDK5 regulatory subunit-associated protein 3
Vam6/Vps39-like protein
Syndetin
Deubiquitinating protein VCPIP1
Spatacsin
Engulfment and cell motility protein 2
Protein disulfide-isomerase TMX3
DDB1- and CUL4-associated factor 5
Chromosome alignment-maintaining phosphoprotein 1
Lethal(3)malignant brain tumor-like protein 3
E3 ubiquitin-protein ligase ZFP91
Protocadherin-16
Calmin
PDZ and LIM domain protein 2
Fatty acyl-CoA reductase 2
Transcription factor BTF3 homolog 4
E3 ubiquitin-protein ligase RNF170
Abscission/NoCut checkpoint regulator
Solute carrier family 35 member E1
Ubiquitin carboxyl-terminal hydrolase 47
Zinc finger protein 521
Lipid scramblase CLPTM1L
Lymphokine-activated killer T-cell-originated protein kinase
ADP-ribosylation factor-like protein 5B

DnaJ homolog subfamily C member 1
N-terminal kinase-like protein
Zinc finger protein 512B
Protein LRATD2
Exocyst complex component 2
Cytosolic non-specific dipeptidase
Histone-lysine N-methyltransferase EHMT2
Zinc finger RNA-binding protein
Protein FAM210B, mitochondrial
Beta-1,3-galactosyltransferase 6
E1A-binding protein p400
Sorting nexin-27
Intraflagellar transport protein 74 homolog
E3 ubiquitin-protein ligase TRIM47
CCR4-NOT transcription complex subunit 6-like
Dehydrogenase/reductase SDR family member 1
DnaJ homolog subfamily C member 30, mitochondrial
Ubiquitin-conjugating enzyme E2 E2
Caspase recruitment domain-containing protein 19
Protein PRRC1
FYVE, RhoGEF and PH domain-containing protein 4
F-box/LRR-repeat protein 18
Zinc finger protein 512
E3 SUMO-protein ligase NSE2
Non-structural maintenance of chromosomes element 3 homolog
Protein-L-isoaspartate O-methyltransferase domain-containing protein 1
Heat shock 70 kDa protein 12B
YTH domain-containing protein 1
Coiled-coil domain-containing protein 43
Conserved oligomeric Golgi complex subunit 8
CKLF-like MARVEL transmembrane domain-containing protein 3
Dynein axonemal assembly factor 10
Nucleolar protein 4-like
Lysophospholipid acyltransferase 7
Dedicator of cytokinesis protein 7
Sideroflexin-2
Zinc finger protein 830
Protein FAM210A
FERM domain-containing protein 6
28S rRNA (cytosine-C(5))-methyltransferase
Regulation of nuclear pre-mRNA domain-containing protein 1A
Arf-GAP with GTPase, ANK repeat and PH domain-containing protein 3
Arf-GAP with Rho-GAP domain, ANK repeat and PH domain-containing protein 1
Importin-9
Melanoma inhibitory activity protein 2
Discoidin, CUB and LCCL domain-containing protein 2
Rho guanine nucleotide exchange factor 17
Inositol polyphosphate-4-phosphatase type I A
RNA-binding protein 14
RING finger and CHY zinc finger domain-containing protein 1

Kelch-like protein 5
E3 ubiquitin-protein ligase UHRF2
E3 ubiquitin-protein ligase NEDD4-like
KH domain-containing RNA-binding protein QKI
Formin-like protein 2
Pseudouridylate synthase 7 homolog
Serine protease FAM111A
Trafficking protein particle complex subunit 9
Perilipin-4
CCA tRNA nucleotidyltransferase 1, mitochondrial
Serine/threonine-protein kinase SMG1
Alsin
Transmembrane protein 237
Alpha-ketoglutarate-dependent dioxygenase alkB homolog 3
Rho GTPase-activating protein 7
Serine/threonine-protein phosphatase 1 regulatory subunit 10
Sodium-coupled neutral amino acid symporter 2
UAP56-interacting factor
Myotubularin-related protein 9
Vacuolar protein sorting-associated protein 35
Transcriptional activator protein Pur-beta
Exportin-6
Hedgehog-interacting protein
Membrane-associated guanylate kinase, WW and PDZ domain-containing protein 1
Sperm-associated antigen 5
Nucleus accumbens-associated protein 1
Sorting nexin-18
PAS domain-containing serine/threonine-protein kinase
Protein capicua homolog
BRCA1-A complex subunit RAP80
Intermembrane lipid transfer protein VPS13A
Mediator of RNA polymerase II transcription subunit 15
Elongation factor G, mitochondrial
Endoplasmic reticulum-Golgi intermediate compartment protein 2
Methylcrotonoyl-CoA carboxylase subunit alpha, mitochondrial
Calcium/calmodulin-dependent protein kinase kinase 2
Trimethylguanosine synthase
NudC domain-containing protein 1
Erbin
Gamma-tubulin complex component 6
Ubiquitin carboxyl-terminal hydrolase 28
Formin-binding protein 1
EKC/KEOPS complex subunit TP53RK
GPI transamidase component PIG-S
ATPase WRNIP1
Ran-binding protein 9
Chloride channel CLIC-like protein 1
Myeloid-associated differentiation marker
Neurabin-2
SRSF protein kinase 1

Structural maintenance of chromosomes protein 6
BTB/POZ domain-containing protein KCTD15
Spermatid perinuclear RNA-binding protein
Transmembrane protein 209
Glutathione peroxidase 7
CDK5 regulatory subunit-associated protein 2
Cytochrome P450 2S1
Protein IWS1 homolog
Paired amphipathic helix protein Sin3a
Oxysterol-binding protein-related protein 9
Protein cereblon
Integrator complex subunit 14
2-aminoethanethiol dioxygenase
Mitochondrial tRNA methylthiotransferase CDK5RAP1
Remodeling and spacing factor 1
RNA-binding protein 15
Protein phosphatase 1 regulatory inhibitor subunit 16B
RUN and FYVE domain-containing protein 1
Msx2-interacting protein
Bifunctional polynucleotide phosphatase/kinase
MMS19 nucleotide excision repair protein homolog
E3 ubiquitin-protein ligase UHRF1
Protein Niban 2
ATP-dependent zinc metalloprotease YME1L1
Regulator of microtubule dynamics protein 3
Transcription factor 12
Cytohesin-2
Tubulin-folding cofactor B
Proteasome subunit beta type-7
Calponin-2
Translocation protein SEC62
Ethanolamine-phosphate cytidylyltransferase
Cell division cycle 5-like protein
26S proteasome non-ATPase regulatory subunit 1
Stromal cell-derived factor 2
Prefoldin subunit 5
E3 ubiquitin-protein ligase RING2
Parkinson disease protein 7
GAS2-like protein 1
Eyes absent homolog 3
Sialidase-1
Sortilin
Synaptic vesicle membrane protein VAT-1 homolog
Legumain
Perilipin-2
DnaJ homolog subfamily C member 2
M-phase phosphoprotein 8
Nuclear pore complex protein Nup88
Plakophilin-4
Phosphoinositide 3-kinase regulatory subunit 4

P2X purinoceptor 4
Ribonucleases P/MRP protein subunit POP1
Protein S100-A13
Protein SCAF11
Translin-associated protein X
Selenide, water dikinase 2
Eukaryotic translation initiation factor 3 subunit C
Tetratricopeptide repeat protein 1
DnaJ homolog subfamily C member 7
Prohibitin-2
COP9 signalosome complex subunit 8
Pre-mRNA-splicing factor 18
Calcineurin B homologous protein 1
Kinesin-like protein KIF2C
Monoglyceride lipase
Ataxin-2
Docking protein 1
Methionine synthase
3-hydroxyacyl-CoA dehydrogenase type-2
Collagen alpha-1(XII) chain
Mothers against decapentaplegic homolog 5
Sigma non-opioid intracellular receptor 1
Heterogeneous nuclear ribonucleoprotein A/B
Nucleosome assembly protein 1-like 4
Microsomal glutathione S-transferase 2
Gamma-soluble NSF attachment protein
Phosphatidylinositol 4-phosphate 5-kinase type-1 alpha
Phospholipid-transporting ATPase ABCA3
Mitogen-activated protein kinase kinase kinase 3
ATP synthase subunit s, mitochondrial
Mitochondrial intermediate peptidase
Aconitate hydratase, mitochondrial
Transmembrane 9 superfamily member 2
Equilibrative nucleoside transporter 1
Tumor susceptibility gene 101 protein
Copine-1
T-complex protein 1 subunit eta
Myeloid differentiation primary response protein MyD88
Probable rRNA-processing protein EBP2
Protein arginine N-methyltransferase 1
BAG family molecular chaperone regulator 1
Cyclic AMP-dependent transcription factor ATF-6 beta
Forkhead box protein C2
Endophilin-A2
Telomerase protein component 1
Serine/threonine-protein kinase VRK1
Growth/differentiation factor 15
A-kinase anchor protein 9
Protein NipSnap homolog 1
Condensin complex subunit 3

Actin-related protein 2/3 complex subunit 5-like protein
Calcium uptake protein 1, mitochondrial
UPF0415 protein C7orf25
Protein FAM118B
Target of rapamycin complex 2 subunit MAPKAP1
RNA-binding protein 4B
Zinc finger FYVE domain-containing protein 21
ATP-dependent RNA helicase DDX50
Large ribosomal subunit protein bL34m
Zinc phosphodiesterase ELAC protein 2
Telomerase RNA component interacting RNase
Glutamate-rich WD repeat-containing protein 1
ADP-ribose glycohydrolase MACROD1
Ribosome quality control complex subunit TCF25
Protein CMSS1
Kelch domain-containing protein 3
Evolutionarily conserved signaling intermediate in Toll pathway, mitochondrial
Methylosome protein WDR77
Cytochrome b-245 chaperone 1
Vitamin K epoxide reductase complex subunit 1
2-(3-amino-3-carboxypropyl)histidine synthase subunit 2
Tubulin alpha-1C chain
Selenoprotein S
Apolipoprotein L2
Myb-binding protein 1A
Mitochondrial genome maintenance exonuclease 1
Golgi reassembly-stacking protein 1
FYVE and coiled-coil domain-containing protein 1
Acyl-CoA-binding domain-containing protein 6
Coronin-1B
Thioredoxin domain-containing protein 17
BUD13 homolog
Serine/threonine-protein phosphatase CPPED1
Vacuolar protein-sorting-associated protein 25
Large ribosomal subunit protein mL45
Uncharacterized protein C7orf50
Tudor-interacting repair regulator protein
45 kDa calcium-binding protein
Programmed cell death protein 2-like
Proteasomal ATPase-associated factor 1
Partner of Y14 and mago
MICOS complex subunit MIC25
ADP-dependent glucokinase
G patch domain-containing protein 1
Serine/threonine-protein kinase RIO1
Protein LLP homolog
DNA replication complex GINS protein SLD5
rRNA-processing protein UTP23 homolog
Protein pelota homolog
Peroxiredoxin-like 2A

E3 ubiquitin-protein ligase TRIM56
Endoplasmic reticulum resident protein 44
Latexin
Nucleolar protein 10
Cancer-related nucleoside-triphosphatase
Mitochondrial import inner membrane translocase subunit Tim29
Translational activator of cytochrome c oxidase 1
Gamma-tubulin complex component 2
Extended synaptotagmin-1
Ubiquitin-associated domain-containing protein 1
Protein YIPF4
tRNA-splicing endonuclease subunit Sen34
Protein canopy homolog 3
Mitochondrial ribosome-associated GTPase 1
Chitobiosyldiphosphodolichol beta-mannosyltransferase
LIM domain-containing protein 2
HAUS augmin-like complex subunit 8
Inositol polyphosphate 5-phosphatase K
Proteasome assembly chaperone 3
COP9 signalosome complex subunit 4
Synaptotagmin-11
WW domain-containing adapter protein with coiled-coil
Death-inducer obliterator 1
Metastasis-associated protein MTA3
RNA-binding protein 42
Dynactin subunit 5
Mini-chromosome maintenance complex-binding protein
Alanyl-tRNA editing protein Aarsd1
DCN1-like protein 5
Acidic leucine-rich nuclear phosphoprotein 32 family member E
Leucine-rich repeat-containing protein 1
Phosphatidylinositol 4-kinase type 2-alpha
Transmembrane protein 43
Fibronectin type III and SPRY domain-containing protein 1
Tubulin-specific chaperone D
Nucleoporin NDC1
Protein HGH1 homolog
Dehydrogenase/reductase SDR family member 4
Lipase maturation factor 2
NADH dehydrogenase [ubiquinone] 1 alpha subcomplex assembly factor 3
Multiple myeloma tumor-associated protein 2
Transmembrane protein 70, mitochondrial
Mediator of RNA polymerase II transcription subunit 18
Iron-sulfur cluster assembly 1 homolog, mitochondrial
Tubulin beta-6 chain
Protein PAXX
DNA-directed RNA polymerase III subunit RPC3
Heterogeneous nuclear ribonucleoprotein U-like protein 1
Protein misato homolog 1
PHD finger protein 23

Programmed cell death protein 10
Ribonuclease P protein subunit p25
Derlin-1
Oxidoreductase HTATIP2
Probable ATP-dependent RNA helicase DDX23
Telomerase Cajal body protein 1
MICOS complex subunit MIC26
Dehydrogenase/reductase SDR family member 6
Methylthioribose-1-phosphate isomerase
WD repeat-containing protein 18
tRNA (guanine(6)-N2)-methyltransferase THUMP3
Acireductone dioxygenase
Enoyl-[acyl-carrier-protein] reductase, mitochondrial
ER membrane protein complex subunit 6
N-terminal Xaa-Pro-Lys N-methyltransferase 1
ER degradation-enhancing alpha-mannosidase-like protein 2
Katanin p80 WD40 repeat-containing subunit B1
Target of rapamycin complex subunit LST8
Ashwin
Voltage-gated monoatomic cation channel TMEM109
Protein PBDC1
Phosphatidylserine synthase 2
Nucleolar complex protein 4 homolog
U3 small nucleolar RNA-associated protein 14 homolog A
Dual specificity protein phosphatase 23
Transmembrane emp24 domain-containing protein 9
BOS complex subunit TMEM147
Nucleoporin p58/p45
Protein adenylyltransferase SelO, mitochondrial
Protein DPCD
Guanine nucleotide-binding protein-like 3
ATPase family gene 2 protein homolog B
Serine/threonine-protein kinase RIO2
tRNA (adenine(58)-N(1))-methyltransferase, mitochondrial
Transmembrane and ubiquitin-like domain-containing protein 1
Mitochondrial import inner membrane translocase subunit Tim21
Kinesin-like protein KIFC1
Nuclear pore complex protein Nup85
Very long chain fatty acid elongase 1
Katanin p60 ATPase-containing subunit A-like 1
HIRA-interacting protein 3
Intraflagellar transport protein 27 homolog
Splicing factor YJU2
ADP-ribose pyrophosphatase, mitochondrial
Threonine--tRNA ligase, mitochondrial
Acetyl-CoA acetyltransferase, cytosolic
Replication initiator 1
RNA-binding protein 4
FUN14 domain-containing protein 2
RNA polymerase II-associated protein 1

Splicing factor 3B subunit 5
Sideroflexin-3
Chitinase domain-containing protein 1
Poly(A) polymerase gamma
Caspase recruitment domain-containing protein 10
Kanadaptin
Cyclin-dependent kinase 19
Protein LSM14 homolog B
Tapasin-related protein
Junctional adhesion molecule C
Caspase recruitment domain-containing protein 6
BTB/POZ domain-containing protein 2
Sphingosine-1-phosphate phosphatase 1
Oxysterol-binding protein-related protein 11
Oxysterol-binding protein-related protein 10
Rab11 family-interacting protein 5
Complement C1q tumor necrosis factor-related protein 5
N-alpha-acetyltransferase 15, NatA auxiliary subunit
Krueppel-like factor 16
Bcl-2-like protein 13
Caspase recruitment domain-containing protein 11
Solute carrier family 12 member 9
Serrate RNA effector molecule homolog
Queuine tRNA-ribosyltransferase catalytic subunit 1
AP-1 complex subunit mu-1
Nucleolar and spindle-associated protein 1
Haloacid dehalogenase-like hydrolase domain-containing 5
Fanconi anemia group D2 protein
Protein MAK16 homolog
Histone deacetylase 8
Replication termination factor 2
Charged multivesicular body protein 4a
Eukaryotic translation initiation factor 2A
Polymerase delta-interacting protein 3
Uncharacterized protein KIAA1671
SH3 and multiple ankyrin repeat domains protein 3
Guanine nucleotide-binding protein subunit beta-like protein 1
Alpha-(1,6)-fucosyltransferase
Large ribosomal subunit protein bL32m
Large ribosomal subunit protein bL20m
Large ribosomal subunit protein uL13m
Large ribosomal subunit protein bL9m
Large ribosomal subunit protein uL4m
Large ribosomal subunit protein uL1m
MKI67 FHA domain-interacting nucleolar phosphoprotein
Hyccin
YTH domain-containing family protein 1
3'-5' exoribonuclease HELZ2
RanBP-type and C3HC4-type zinc finger-containing protein 1
Small ribosomal subunit protein mS26

Neurolysin, mitochondrial
Centrosomal protein of 41 kDa
Histone-lysine N-methyltransferase SETD2
Pantothenate kinase 2, mitochondrial
Dedicator of cytokinesis protein 9
FERM domain-containing protein 8
Contactin-associated protein-like 3
Histone-lysine N-methyltransferase NSD3
Kinetochore protein Nuf2
Large ribosomal subunit protein mL37
GTP-binding protein 4
Tether containing UBX domain for GLUT4
Oxysterol-binding protein-related protein 8
Uveal autoantigen with coiled-coil domains and ankyrin repeats
Ras-related protein Rab-34
2-(3-amino-3-carboxypropyl)histidine synthase subunit 1
WD repeat-containing protein 11
Regulator of nonsense transcripts 3B
Crooked neck-like protein 1
F-box-like/WD repeat-containing protein TBL1XR1
Ubiquitin-like protein 5
Protein phosphatase 1 regulatory subunit 12C
Serine/threonine-protein kinase D2
ER degradation-enhancing alpha-mannosidase-like protein 3
Protein Niban 1
UBX domain-containing protein 6
Transmembrane 6 superfamily member 1
Uridine-cytidine kinase 2
Sialoadhesin
Apoptosis inhibitor 5
NACHT, LRR and PYD domains-containing protein 1
Tripartite motif-containing protein 5
E3 ubiquitin-protein ligase TRIM4
Alpha-ketoglutarate-dependent dioxygenase FTO
Palmitoyltransferase ZDHHC5
Transport and Golgi organization protein 6 homolog
182 kDa tankyrase-1-binding protein
(E3-independent) E2 ubiquitin-conjugating enzyme
Protein zyg-11 homolog B
Protein TANC1
Ethanolaminephosphotransferase 1
Exportin-4
Endoplasmic reticulum junction formation protein lunapark
Centrosomal protein of 44 kDa
Protein tweety homolog 3
Kelch-like protein 4
Myotubularin-related protein 12
pre-mRNA 3' end processing protein WDR33
Ribosome biogenesis protein WDR12
Protein YIPF3

Tubulointerstitial nephritis antigen-like
Adipose-secreted signaling protein
PITH domain-containing protein 1
Derlin-2
COMM domain-containing protein 5
RNA exonuclease 4
ATP-dependent RNA helicase DDX24
DNA-directed RNA polymerase I subunit RPA49
Superkiller complex protein 8
SRA stem-loop-interacting RNA-binding protein, mitochondrial
Serine racemase
NIF3-like protein 1
Egl nine homolog 1
Mucolipin-1
PSME3-interacting protein
Mitochondrial fission factor
N-alpha-acetyltransferase 50
Ubiquitin-like modifier-activating enzyme 5
E3 ubiquitin-protein ligase makorin-2
Transmembrane protein 126A
Polyadenylate-binding protein-interacting protein 1
Mitochondrial disaggregase
Ras-related protein Rab-33B
Large subunit GTPase 1 homolog
Protein FAM107B
RNA cytidine acetyltransferase
COMM domain-containing protein 4
Kinesin light chain 2
Integrin-linked kinase-associated serine/threonine phosphatase 2C
5'-3' exoribonuclease 2
Toll-interacting protein
Histone deacetylase complex subunit SAP130
Bromodomain-containing protein 8
Nuclear speckle splicing regulatory protein 1
Integrator complex subunit 2
Rac GTPase-activating protein 1
Protein mono-ADP-ribosyltransferase PARP12
Cleavage stimulation factor subunit 2 tau variant
Cytosolic 5'-nucleotidase 3A
CYFIP-related Rac1 interactor A
Transmembrane protein 222
Haloacid dehalogenase-like hydrolase domain-containing protein 2
Glutamyl-tRNA(Gln) amidotransferase subunit A, mitochondrial
Probable ATP-dependent RNA helicase DDX47
Magnesium transporter protein 1
Ras-related protein Rab-1B
Large ribosomal subunit protein uL18m
Testis-specific Y-encoded-like protein 1
Transmembrane protein 168
VIP36-like protein

Nonsense-mediated mRNA decay factor SMG9
Oxysterol-binding protein-related protein 5
Deoxynucleotidyltransferase terminal-interacting protein 1
Nucleotide exchange factor SIL1
Protein-L-histidine N-pros-methyltransferase
Anaphase-promoting complex subunit 1
Probable E3 ubiquitin-protein ligase IRF2BPL
Cysteine-rich and transmembrane domain-containing protein 1
DNA-directed RNA polymerase III subunit RPC6
Nuclear ubiquitous casein and cyclin-dependent kinase substrate 1
Thioredoxin-related transmembrane protein 4
Kinesin-like protein KIF13A
Activating signal cointegrator 1 complex subunit 2
Rabenosyn-5
Iron-sulfur cluster assembly enzyme ISCU
Oxysterol-binding protein-related protein 2
Autophagy protein 5
WD repeat-containing protein 13
Epsin-3
EH domain-containing protein 4
Vacuolar protein sorting-associated protein 33B
Vacuolar protein sorting-associated protein 16 homolog
Vacuolar protein sorting-associated protein 11 homolog
SH3 domain-binding glutamic acid-rich-like protein 3
Gigaxonin
Solute carrier family 25 member 32
TRIO and F-actin-binding protein
Enhancer of polycomb homolog 1
STE20-like serine/threonine-protein kinase
Peptidyl-prolyl cis-trans isomerase-like 3
Sodium-coupled neutral amino acid symporter 1
Phosducin-like protein 3
Serine/threonine-protein kinase TAO3
Rab3 GTPase-activating protein non-catalytic subunit
Activity-dependent neuroprotector homeobox protein
Diphthine methyl ester synthase
ATP-dependent DNA/RNA helicase DHX36
Inorganic pyrophosphatase 2, mitochondrial
Protein spinster homolog 1
Large ribosomal subunit protein mL46
Presenilin-associated rhomboid-like protein, mitochondrial
Pinin
Transmembrane protein 245
Forkhead box protein P1
BTB/POZ domain-containing adapter for CUL3-mediated RhoA degradation protein 3
tRNA dimethylallyltransferase
UPF0696 protein C11orf68
Growth hormone-inducible transmembrane protein
BolA-like protein 2
Cobalamin trafficking protein CblD

Thioredoxin-related transmembrane protein 1
Negative elongation factor A
Golgi resident protein GCP60
Cdc42 effector protein 4
Tyrosine-protein phosphatase non-receptor type 23
Semaphorin-6B
Protein unc-45 homolog A
Major facilitator superfamily domain-containing protein 1
DnaJ homolog subfamily C member 5
Charged multivesicular body protein 4b
RWD domain-containing protein 1
CUE domain-containing protein 2
F-box/LRR-repeat protein 15
Fructosamine-3-kinase
GDP-fucose protein O-fucosyltransferase 1
Phosphatidylinositol glycan anchor biosynthesis class U protein
Torsin-3A
Serine/threonine-protein kinase WNK1
Aminopeptidase B
Golgi phosphoprotein 3-like
Golgi phosphoprotein 3
tRNA N6-adenosine threonylcarbamoyltransferase, mitochondrial
Alpha-N-acetyl-neuraminyl-2,3-beta-galactosyl-1,3-N-acetyl-galactosaminide alpha-2,6-sialyltransferase
Golgi-associated plant pathogenesis-related protein 1
TraB domain-containing protein
Sentrin-specific protease 3
Oxysterol-binding protein-related protein 3
SWI/SNF-related matrix-associated actin-dependent regulator of chromatin subfamily A containing DEAD/[illegible]
EH domain-containing protein 1
mRNA (2'-O-methyladenosine-N(6)-)-methyltransferase
ESF1 homolog
Alpha-1,3/1,6-mannosyltransferase ALG2
HEAT repeat-containing protein 1
Protein O-mannose kinase
Rab GTPase-binding effector protein 2
Dimethyladenosine transferase 2, mitochondrial
rRNA N6-adenosine-methyltransferase ZCCHC4
STING ER exit protein
Cytosolic iron-sulfur assembly component 2A
Probable ATP-dependent RNA helicase DHX35
Protein FAM124B
HAUS augmin-like complex subunit 4
Coiled-coil domain-containing protein 134
Coiled-coil domain-containing protein 86
Receptor expression-enhancing protein 4
Transcription factor SOX-17
Optic atrophy 3 protein
Cytosolic iron-sulfur assembly component 3
ATP-dependent RNA helicase DHX33
Nucleolar protein 6

WD repeat and coiled-coil-containing protein
3'-5' RNA helicase YTHDC2
RNA polymerase II-associated protein 3
BCAS3 microtubule associated cell migration factor
Alpha-1,2-mannosyltransferase ALG9
Lipid droplet-associated hydrolase
Ribosomal oxygenase 1
WD repeat-containing protein 55
Ran-binding protein 3
dCTP pyrophosphatase 1
Inactive tyrosine-protein kinase PEAK1
Ribosome production factor 2 homolog
Histone-lysine N-methyltransferase SMYD3
Mth938 domain-containing protein
Dedicator of cytokinesis protein 5
WD repeat-containing protein 26
Tudor domain-containing protein 3
UPF0488 protein C8orf33
Polyamine-transporting ATPase 13A3
Methyltransferase-like protein 17, mitochondrial
Splicing factor, arginine/serine-rich 19
Nuclear exosome regulator NRDE2
Prostaglandin E synthase 2
Phosphorylated adapter RNA export protein
Ubiquitin-conjugating enzyme E2 Z
Complex I assembly factor ACAD9, mitochondrial
5'-nucleotidase domain-containing protein 2
PRKR-interacting protein 1
Caspase activity and apoptosis inhibitor 1
Nucleolar protein 11
Probable ATP-dependent RNA helicase DDX31
Thiol S-methyltransferase TMT1A
ATPase PAAT
Multimerin-2
Ubiquitin carboxyl-terminal hydrolase MINDY-3
Protein eva-1 homolog A
AKT-interacting protein
Pleckstrin homology domain-containing family F member 2
tRNA endonuclease ANKZF1
Golgi reassembly-stacking protein 2
Protein zwilch homolog
Jupiter microtubule associated homolog 2
Mitochondrial glutamate carrier 1
Proline-serine-threonine phosphatase-interacting protein 2
Mediator of RNA polymerase II transcription subunit 20
WD repeat-containing protein 76
Queuine tRNA-ribosyltransferase accessory subunit 2
Actin-related protein 8
Damage-control phosphatase ARMT1
Pantothenate kinase 3

CCR4-NOT transcription complex subunit 10
Leucine-rich repeat-containing protein 40
Histone-lysine N-methyltransferase EHMT1
Sideroflexin-1
Spermatogenesis-defective protein 39 homolog
Conserved oligomeric Golgi complex subunit 4
Actin-related protein 5
Vacuolar protein sorting-associated protein 37B
Large ribosomal subunit protein mL44
L-2-hydroxyglutarate dehydrogenase, mitochondrial
COP9 signalosome complex subunit 7b
Non-homologous end-joining factor 1
Protein transport protein Sec61 subunit alpha isoform 2
Ribitol 5-phosphate transferase FKRP
Elongator complex protein 3
Ribosome production factor 1
DNA-directed RNA polymerase I subunit RPA2
Zinc finger matrin-type protein 3
Uridine-cytidine kinase 1
Ketosamine-3-kinase
TBC1 domain family member 17
Probable cysteine--tRNA ligase, mitochondrial
Solute carrier family 52, riboflavin transporter, member 2
Phosphopantothenate--cysteine ligase
Ubiquitin domain-containing protein 1
WD repeat-containing protein 41
Nicotinamide/nicotinic acid mononucleotide adenylyltransferase 1
Protein Njmu-R1
Sialate O-acetylesterase
Pleckstrin homology domain-containing family A member 5
E3 ubiquitin-protein ligase SMURF2
Regulator of nonsense transcripts 2
Guanine nucleotide-binding protein subunit beta-4
Exportin-5
GrpE protein homolog 1, mitochondrial
MYG1 exonuclease
Pleckstrin homology domain-containing family A member 3
Pleckstrin homology domain-containing family A member 1
Retinoid-inducible serine carboxypeptidase
Netrin-4
Calcyclin-binding protein
Ras-related GTP-binding protein C
Zinc finger FYVE domain-containing protein 1
Intraflagellar transport protein 122 homolog
Retinol dehydrogenase 14
Beta-parvin
Tensin-1
Plasminogen receptor (KT)
NmrA-like family domain-containing protein 1
Vezatin

Kinetochore protein Spc25
Transcription initiation factor TFIID subunit 9B
Solute carrier family 38 member 10
Ethanolamine kinase 1
Adhesion G protein-coupled receptor L4
Putative divalent cation/proton antiporter TMEM165
Mitochondrial thiamine pyrophosphate carrier
Echinoderm microtubule-associated protein-like 4
rRNA methyltransferase 3, mitochondrial
Glyoxalase domain-containing protein 4
Serine/threonine-protein kinase Nek6
Methylcrotonoyl-CoA carboxylase beta chain, mitochondrial
Nuclear receptor coactivator 5
Ectopic P granules protein 5 homolog
Helicase MOV-10
N6-adenosine-methyltransferase non-catalytic subunit
Non-lysosomal glucosylceramidase
Pre-mRNA-splicing factor CWC22 homolog
Ribonucleoprotein PTB-binding 2
Chromodomain-helicase-DNA-binding protein 8
Band 4.1-like protein 5
GPN-loop GTPase 1
Stromal cell-derived factor 2-like protein 1
Casein kinase I isoform gamma-1
Pre-mRNA-splicing factor SYF1
Prolactin regulatory element-binding protein
Steroid receptor RNA activator 1
Endoplasmic reticulum transmembrane helix translocase
Golgi-associated PDZ and coiled-coil motif-containing protein
Large ribosomal subunit protein uL29m
LYR motif-containing protein 4
O-phosphoseryl-tRNA(Sec) selenium transferase
Charged multivesicular body protein 1a
Transmembrane 9 superfamily member 3
Unconventional myosin-X
Adipocyte plasma membrane-associated protein
tRNA (34-2'-O)-methyltransferase regulator WDR6
Thioredoxin reductase 2, mitochondrial
ATP-binding cassette sub-family B member 6
ADP-ribosylation factor GTPase-activating protein 3
Zinc finger CCHC domain-containing protein 17
Ras-related protein Rab-18
Palmdelphin
RNA polymerase II subunit A C-terminal domain phosphatase SSU72
Vacuolar protein sorting-associated protein VTA1 homolog
Serine--tRNA ligase, mitochondrial
Large ribosomal subunit protein mL65
Dynein light chain roadblock-type 1
ER membrane protein complex subunit 7
Transcription and mRNA export factor ENY2

Exosome complex component RRP41
Ubiquitin-conjugating enzyme E2 T
H/ACA ribonucleoprotein complex subunit 3
tRNA N6-adenosine threonylcarbamoyltransferase
DNA methyltransferase 1-associated protein 1
Protocadherin-12
Inositol-3-phosphate synthase 1
Bromodomain-containing protein 7
mRNA-decapping enzyme 1A
Acyl-coenzyme A thioesterase 13
Mediator of RNA polymerase II transcription subunit 4
Complex I assembly factor TIMMDC1, mitochondrial
Synembryn-A
Complement component C1q receptor
Putative RNA-binding protein Luc7-like 1
Endothelial cell-specific molecule 1
Leucine zipper transcription factor-like protein 1
Large ribosomal subunit protein mL40
Suppressor of SWI4 1 homolog
1-phosphatidylinositol 4,5-bisphosphate phosphodiesterase beta-1
Fructose-2,6-bisphosphatase TIGAR
Protein C12orf4
Reticulon-4
Regulation of nuclear pre-mRNA domain-containing protein 1B
Xaa-Pro aminopeptidase 3
Omega-amidase NIT2
Cell death regulator Aven
Inner centromere protein
Exosome complex component RRP46
Exosome complex component RRP40
Kinesin-like protein KIF13B
Anillin
Xaa-Pro aminopeptidase 1
Gephyrin
Bridging integrator 3
Something about silencing protein 10
StAR-related lipid transfer protein 7, mitochondrial
Baculoviral IAP repeat-containing protein 6
PDZ and LIM domain protein 7
Acetyl-coenzyme A synthetase, cytoplasmic
Diablo IAP-binding mitochondrial protein
Nucleolar RNA helicase 2
GTP-binding protein SAR1a
Sialic acid synthase
Endophilin-B2
Histone-lysine N-methyltransferase ASH1L
Translation initiation factor eIF2B subunit gamma
Muscleblind-like protein 1
Sialin
Eukaryotic translation initiation factor 4E transporter

Carbohydrate sulfotransferase 12
Suppressor of tumorigenicity 7 protein
PRKCA-binding protein
SH2B adapter protein 1
CTP synthase 2
DNA polymerase epsilon subunit 3
Chromatin accessibility complex protein 1
Aladin
SNF-related serine/threonine-protein kinase
ATP-binding cassette sub-family B member 10, mitochondrial
Bromodomain adjacent to zinc finger domain protein 1A
Striatin-4
L-aminoadipate-semialdehyde dehydrogenase-phosphopantetheinyl transferase
rRNA N6-adenosine-methyltransferase METTL5
Oligosaccharyltransferase complex subunit OSTC
COX assembly mitochondrial protein 2 homolog
Phospholipid scramblase 4
Sorting nexin-15
Heme-binding protein 1
DNA dC->dU-editing enzyme APOBEC-3C
Vacuolar protein sorting-associated protein 45
RNA-binding protein PNO1
Large ribosomal subunit protein bL17m
14 kDa phosphohistidine phosphatase
Serine incorporator 1
Rho GTPase-activating protein 35
Protein FAM114A2
Phospholipid scramblase 3
1-acyl-sn-glycerol-3-phosphate acyltransferase delta
1-acyl-sn-glycerol-3-phosphate acyltransferase gamma
Lymphoid-specific helicase
Glycoprotein-N-acetylgalactosamine 3-beta-galactosyltransferase 1
Latent-transforming growth factor beta-binding protein 3
Mitochondrial import receptor subunit TOM22 homolog
LanC-like protein 2
Kinesin-like protein KIF15
Transmembrane 7 superfamily member 3
Homer protein homolog 3
Phenylalanine--tRNA ligase beta subunit
Isoleucine--tRNA ligase, mitochondrial
Ribosome biogenesis protein SLX9 homolog
SAM domain-containing protein SAMSN-1
Kinesin light chain 4
Protein diaphanous homolog 3
BMP-2-inducible protein kinase
Ubiquitin-like-conjugating enzyme ATG3
Sister chromatid cohesion protein PDS5 homolog B
Structural maintenance of chromosomes protein 4
Alpha-mannosidase 2C1
Phosphatidylinositol-3-phosphatase SAC1

Protein DEPP1
Obg-like ATPase 1
Copper homeostasis protein cutC homolog
Zinc finger protein 64
Ethylmalonyl-CoA decarboxylase
RNA-binding protein 12
TBC1 domain family member 22B
Midasin
Acetyl-coenzyme A synthetase 2-like, mitochondrial
Zinc finger CCHC domain-containing protein 3
Peroxisomal 2,4-dienoyl-CoA reductase [(3E)-enoyl-CoA-producing]
Palmitoyl-protein thioesterase ABHD10, mitochondrial
T-complex protein 11-like protein 1
Double-stranded RNA-binding protein Staufen homolog 2
Shiftless antiviral inhibitor of ribosomal frameshifting protein
Probable ATP-dependent RNA helicase DDX28
Transmembrane protein 106B
Protein lin-7 homolog C
1-acyl-sn-glycerol-3-phosphate acyltransferase epsilon
Gamma-taxilin
SPATS2-like protein
Ufm1-specific protease 2
ATP-binding cassette sub-family F member 3
CYFIP-related Rac1 interactor B
Mitochondrial potassium channel ATP-binding subunit
ATP-dependent RNA helicase DDX19A
GTPase IMAP family member 4
Tyrosyl-DNA phosphodiesterase 1
Protection of telomeres protein 1
TBC1 domain family member 23
DDB1- and CUL4-associated factor 13
U3 small nucleolar ribonucleoprotein protein IMP3
Exocyst complex component 1
Protein-L-isoaspartate O-methyltransferase domain-containing protein 2
Integrator complex subunit 9
Cell cycle control protein 50A
Ubiquinol-cytochrome c reductase complex assembly factor 1
Septin-11
Sodium-coupled neutral amino acid transporter 7
Mediator of RNA polymerase II transcription subunit 17
Alpha-parvin
Ubiquitin carboxyl-terminal hydrolase 40
4'-phosphopantetheine phosphatase
F-box only protein 28
TBC1 domain family member 13
Exonuclease 3'-5' domain-containing protein 2
DnaJ homolog subfamily C member 11
Integrator complex subunit 7
Fanconi anemia group I protein
ATPase family AAA domain-containing protein 3A

ADP-ribosylation factor-like protein 8B
DnaJ homolog subfamily C member 17
Integrator complex subunit 13
Synembryn-B
Guanine nucleotide-binding protein-like 3-like protein
ATP-dependent RNA helicase DDX18
Histone chaperone ASF1B
Kelch-like protein 11
Integrator complex subunit 10
Large ribosomal subunit protein mL66
Pyridoxine-5'-phosphate oxidase
Armadillo repeat-containing protein 1
DNA-directed RNA polymerase III subunit RPC5
Protein SDA1 homolog
Poly(A) RNA polymerase, mitochondrial
Notchless protein homolog 1
Adaptin ear-binding coat-associated protein 2
DNA-directed RNA polymerase III subunit RPC2
RNA-binding protein 28
Anoctamin-10
Pre-mRNA-splicing factor RBM22
WD repeat-containing protein 70
Arginine and glutamate-rich protein 1
Intraflagellar transport protein 57 homolog
SAFB-like transcription modulator
Peptidyl-prolyl cis-trans isomerase FKBP14
Required for meiotic nuclear division protein 1 homolog
p21-activated protein kinase-interacting protein 1
Hypoxia-inducible factor 1-alpha inhibitor
3-oxoacyl-[acyl-carrier-protein] synthase, mitochondrial
Large ribosomal subunit protein uL22m
CXXC motif containing zinc binding protein
BRISC and BRCA1-A complex member 1
Ceroid-lipofuscinosis neuronal protein 6
Ankyrin repeat and SOCS box protein 6
Probable tRNA(His) guanylyltransferase
Histone PARylation factor 1
Interleukin-1 receptor-associated kinase 4
Uridine-cytidine kinase-like 1
Transmembrane protein 160
NACHT, LRR and PYD domains-containing protein 2
Constitutive coactivator of PPAR-gamma-like protein 2
tRNA selenocysteine 1-associated protein 1
NADH dehydrogenase [ubiquinone] 1 beta subcomplex subunit 11, mitochondrial
Large ribosomal subunit protein uL16m
H/ACA ribonucleoprotein complex subunit 2
OCIA domain-containing protein 1
ADP-ribosylhydrolase ARH3
E3 ubiquitin-protein ligase MARCHF5
Cell growth-regulating nucleolar protein

Transmembrane protein 161A
Golgi-resident adenosine 3',5'-bisphosphate 3'-phosphatase
MICOS complex subunit MIC19
CKLF-like MARVEL transmembrane domain-containing protein 6
NAD-dependent protein deacylase sirtuin-5, mitochondrial
GATOR2 complex protein MIOS
Myotubularin-related protein 10
Sphingomyelin phosphodiesterase 4
Pre-mRNA-splicing factor CWC25 homolog
Testis-expressed protein 10
DDB1- and CUL4-associated factor 16
THUMP domain-containing protein 1
Transmembrane prolyl 4-hydroxylase
Torsin-4A
tRNA (guanine(26)-N(2))-dimethyltransferase
Ganglioside-induced differentiation-associated protein 2
Nuclear distribution protein nudE homolog 1
BRISC and BRCA1-A complex member 2
Glutaminyl-peptide cyclotransferase-like protein
ADP-ribosylation factor-like protein 15
BTB/POZ domain-containing protein KCTD5
CDKN2A-interacting protein
DnaJ homolog subfamily B member 12
Alpha-ketoglutarate-dependent dioxygenase alkB homolog 4
Non-structural maintenance of chromosomes element 4 homolog A
H/ACA ribonucleoprotein complex subunit 1
Stabilin-1
Serine/threonine-protein phosphatase 4 regulatory subunit 2
Dipeptidyl peptidase 3
Protein AATF
Tubulin alpha-8 chain
Probable ATP-dependent RNA helicase DDX56
Sphingosine kinase 1
Telomeric repeat-binding factor 2-interacting protein 1
Abl interactor 2
Bcl-2-associated transcription factor 1
U3 small nucleolar RNA-associated protein 6 homolog
TGF-beta-activated kinase 1 and MAP3K7-binding protein 2
Large ribosomal subunit protein mL39
Mitogen-activated protein kinase kinase kinase 20
Peptidyl-prolyl cis-trans isomerase FKBP11
Tropomodulin-3
BET1-like protein
Very long chain fatty acid elongase 5
NF-kappa-B inhibitor-interacting Ras-like protein 2
UDP-glucose:glycoprotein glucosyltransferase 2
UDP-glucose:glycoprotein glucosyltransferase 1
Cyclin-dependent kinase 12
FAST kinase domain-containing protein 2, mitochondrial
Very-long-chain enoyl-CoA reductase

Endoplasmic reticulum aminopeptidase 1
Actin-related protein 10
Vesicle transport protein USE1
CDGSH iron-sulfur domain-containing protein 1
ADP-ribosylation factor-binding protein GGA3
Splicing factor C9orf78
Constitutive coactivator of PPAR-gamma-like protein 1
Glycerophosphodiester phosphodiesterase 1
SWI/SNF-related matrix-associated actin-dependent regulator of chromatin subfamily A-like protein 1
Glycolipid transfer protein
Maspardin
Large ribosomal subunit protein bL35m
Upstream-binding protein 1
Insulin-like growth factor 2 mRNA-binding protein 1
Sacsin
Mitochondrial carrier homolog 1
Diphosphoinositol polyphosphate phosphohydrolase 2
Hsp70-binding protein 1
Methionine adenosyltransferase 2 subunit beta
Myoferlin
Intersectin-2
Ribosome biogenesis protein NOP53
EH domain-containing protein 3
EH domain-containing protein 2
Rho guanine nucleotide exchange factor 12
CCR4-NOT transcription complex subunit 2
NCK-interacting protein with SH3 domain
Programmed cell death 1 ligand 1
Calmodulin-like protein 5
Opioid growth factor receptor
LIM and cysteine-rich domains protein 1
Cysteine-rich motor neuron 1 protein
Selenoprotein N
Protein PALS2
Charged multivesicular body protein 5
Spliceosome-associated protein CWC15 homolog
Large ribosomal subunit protein uL15m
Thymocyte nuclear protein 1
Thyroid transcription factor 1-associated protein 26
NADH dehydrogenase [ubiquinone] 1 alpha subcomplex assembly factor 4
Very-long-chain (3R)-3-hydroxyacyl-CoA dehydratase 3
ER membrane protein complex subunit 3
NADH dehydrogenase [ubiquinone] 1 alpha subcomplex subunit 13
[Pyruvate dehydrogenase [acetyl-transferring]]-phosphatase 1, mitochondrial
E3 ubiquitin-protein ligase KCMF1
Ankycorbin
Vesicle-associated membrane protein-associated protein A
Large ribosomal subunit protein bL27m
Proton-transporting V-type ATPase complex assembly regulator TMEM9
Sentrin-specific protease 1

CXXC-type zinc finger protein 1
SH3 domain-binding protein 4
Septin-10
GEM-interacting protein
PHD and RING finger domain-containing protein 1
Pogo transposable element with KRAB domain
Protein Daple
Rho GTPase-activating protein 23
Vacuolar protein sorting-associated protein 18 homolog
Protein RCC2
RAB11-binding protein RELCH
Disco-interacting protein 2 homolog B
Junctional cadherin 5-associated protein
SLAIN motif-containing protein 2
Teneurin-3
Ubiquitin carboxyl-terminal hydrolase 36
BRCA2 and CDKN1A-interacting protein
Serine/threonine-protein kinase 26
Armadillo repeat-containing X-linked protein 1
ABI gene family member 3
Prostaglandin F2 receptor negative regulator
CTTNBP2 N-terminal-like protein
Transmembrane protein 181
Inhibitor of Bruton tyrosine kinase
HEAT repeat-containing protein 5B
NFX1-type zinc finger-containing protein 1
Chondroitin sulfate glucuronyltransferase
Protocadherin-10
Ribosome-binding protein 1
Cleavage and polyadenylation specificity factor subunit 2
Kelch-like protein 9
Leucine--tRNA ligase, cytoplasmic
Myelin expression factor 2
Kelch-like protein 42
eIF-2-alpha kinase GCN2
WD repeat-containing protein 35
RNA-binding protein 27
KAT8 regulatory NSL complex subunit 3
E3 ubiquitin-protein ligase HECW2
FERM domain-containing protein 4A
Rabankyrin-5
Arginine-glutamic acid dipeptide repeats protein
Succinate--CoA ligase [ADP-forming] subunit beta, mitochondrial
GMP reductase 2
Syntaxin-18
Dolichol-phosphate mannosyltransferase subunit 3
Protein IMPACT
UV radiation resistance-associated gene protein
Ataxin-10
Methyl-CpG-binding domain protein 2

Neurochondrin
Tuftelin-interacting protein 11
Epidermal growth factor receptor substrate 15-like 1
Unconventional myosin-Ia
Origin recognition complex subunit 3
SUMO-activating enzyme subunit 1
Coatomer subunit gamma-2
Phosphatidylinositol 4-kinase beta
C-type mannose receptor 2
COMM domain-containing protein 3
Guanine nucleotide-binding protein G(I)/G(S)/G(O) subunit gamma-12
Rab-like protein 2A
Methionine synthase reductase
Protein UXT
Set1/Ash2 histone methyltransferase complex subunit ASH2
7-dehydrocholesterol reductase
Tumor necrosis factor receptor superfamily member 10D
Histone deacetylase 6
Spastin
tRNA (guanine-N(7)-)-methyltransferase
PTB domain-containing engulfment adapter protein 1
Vacuolar protein sorting-associated protein 29
Eukaryotic translation initiation factor 3 subunit K
Exostosin-like 2
Glyoxylate reductase/hydroxypyruvate reductase
Cathepsin Z
Ribosomal protein S6 kinase beta-2
DnaJ homolog subfamily B member 11
E3 ubiquitin-protein ligase RNF14
SUMO-activating enzyme subunit 2
Mortality factor 4-like protein 1
Nuclear RNA export factor 1
Protein sel-1 homolog 1
Beta-1,4-galactosyltransferase 7
Peflin
Zinc finger MYM-type protein 2
COP9 signalosome complex subunit 7a
Mitochondrial dicarboxylate carrier
Alpha-aminoadipic semialdehyde synthase, mitochondrial
CAP-Gly domain-containing linker protein 2
Cytochrome b-c1 complex subunit 9
Mitochondrial fission process protein 1
Tight junction protein ZO-2
DnaJ homolog subfamily B member 4
Serine/threonine-protein kinase 17A
Craniofacial development protein 1
Death domain-associated protein 6
tRNA (cytidine(32)/guanosine(34)-2'-O)-methyltransferase
Vesicle transport through interaction with t-SNAREs homolog 1B
STE20/SPS1-related proline-alanine-rich protein kinase

Gamma-adducin
Leucine-rich repeat and WD repeat-containing protein 1
Protein NipSnap homolog 3A
CGG triplet repeat-binding protein 1
Phosphatidylserine decarboxylase proenzyme, mitochondrial
ATP-binding cassette sub-family F member 2
Testin
5'-AMP-activated protein kinase subunit gamma-2
Gamma-tubulin complex component 4
Lysine-specific demethylase 5B
Tryptophan--tRNA ligase, mitochondrial
LIM domain-containing protein 1
Translocation protein SEC63 homolog
Solute carrier family 2, facilitated glucose transporter member 6
Zinc finger CCCH domain-containing protein 7B
Protein NDRG3
Armadillo repeat-containing X-linked protein 3
Switch-associated protein 70
SUN domain-containing protein 2
Probable ribosome biogenesis protein RLP24
Ragulator complex protein LAMTOR3
LIM domain and actin-binding protein 1
AF4/FMR2 family member 4
Signal recognition particle subunit SRP68
Cysteine and histidine-rich domain-containing protein 1
Serine/threonine-protein kinase TBK1
Septin-9
Prenylcysteine oxidase 1
Probable ATP-dependent RNA helicase DDX20
Sedoheptulokinase
Dipeptidyl peptidase 2
Inactive cell surface hyaluronidase CEMIP2
Ubiquitin carboxyl-terminal hydrolase 25
B-cell receptor-associated protein 29
NADH-cytochrome b5 reductase 1
SAP30-binding protein
Zinc finger HIT domain-containing protein 2
Prefoldin subunit 2
GPN-loop GTPase 3
Poly(U)-binding-splicing factor PUF60
Nuclear receptor-binding protein
Enolase-phosphatase E1
Fasciculation and elongation protein zeta-2
NADH dehydrogenase [ubiquinone] 1 alpha subcomplex subunit 12
Translation initiation factor eIF2B subunit delta
V-type proton ATPase subunit H
Prenylated Rab acceptor protein 1
Importin-11
Multifunctional methyltransferase subunit TRM112-like protein
Dachshund homolog 1

Exportin-7
Leucine carboxyl methyltransferase 1
Vacuolar protein sorting-associated protein 51 homolog
Bromodomain adjacent to zinc finger domain protein 2B
Bromodomain adjacent to zinc finger domain protein 2A
Tyrosine-protein kinase BAZ1B
GTP:AMP phosphotransferase AK3, mitochondrial
Leucyl-cystinyl aminopeptidase
Methyl-CpG-binding domain protein 1
CCR4-NOT transcription complex subunit 7
Rab5 GDP/GTP exchange factor
Mitochondrial peptide methionine sulfoxide reductase
N-acetyl-D-glucosamine kinase
2-hydroxyacyl-CoA lyase 1
tRNA (adenine(58)-N(1))-methyltransferase non-catalytic subunit TRM6
SH3 domain-binding glutamic acid-rich-like protein 2
Ras GTPase-activating protein nGAP
18S rRNA aminocarboxypropyltransferase
Electrogenic aspartate/glutamate antiporter SLC25A13, mitochondrial
Drebrin-like protein
Probable ATP-dependent RNA helicase DDX41
Dynactin subunit 4
Cell division cycle protein 23 homolog
Anaphase-promoting complex subunit 7
Anaphase-promoting complex subunit 5
Anaphase-promoting complex subunit 4
Anaphase-promoting complex subunit 2
ADP-ribosylation factor-binding protein GGA2
ADP-ribosylation factor-binding protein GGA1
Stomatin-like protein 2, mitochondrial
N-acetylglucosamine-1-phosphodiester alpha-N-acetylglucosaminidase
Vacuolar protein sorting-associated protein 28 homolog
U6 snRNA-associated Sm-like protein LSm7
Lariat debranching enzyme
Protein TASOR
Jupiter microtubule associated homolog 1
F-box only protein 3
A-kinase anchor protein 11
F-box/WD repeat-containing protein 11
Glucocorticoid modulatory element-binding protein 2
mRNA turnover protein 4 homolog
TRAF2 and NCK-interacting protein kinase
Cleavage and polyadenylation specificity factor subunit 3
DCC-interacting protein 13-alpha
Serine/threonine-protein kinase tousled-like 1
G patch domain-containing protein 8
Protein mono-ADP-ribosyltransferase PARP4
NTF2-related export protein 1
ADP-sugar pyrophosphatase
REST corepressor 1

Phosphatidylcholine transfer protein
Endoplasmic reticulum mannosyl-oligosaccharide 1,2-alpha-mannosidase
RNA-binding protein Raly
General transcription factor 3C polypeptide 4
Ergosterol biosynthetic protein 28 homolog
Protein kinase C and casein kinase substrate in neurons protein 3
Isobutyryl-CoA dehydrogenase, mitochondrial
Angiopoietin-related protein 2
Apoptotic chromatin condensation inducer in the nucleus
E3 ubiquitin-protein ligase AMFR
Protein argonaute-2
Nuclear pore complex protein Nup50
Protein CDV3 homolog
CCR4-NOT transcription complex subunit 11
Integrator complex subunit 6
BAG family molecular chaperone regulator 5
Protein argonaute-1
Ras-related protein Rab-21
Ras-related protein Rab-22A
Trafficking protein particle complex subunit 2-like protein
Zinc finger protein 346
Proteasome activator complex subunit 2
Serine/threonine-protein kinase TAO2
Muskelin
Aspartyl aminopeptidase
Ras-related protein Rab-23
Malignant T-cell-amplified sequence 1
Microtubule-associated tumor suppressor 1
Protein WWC3
Paladin
Zinc transporter ZIP10
Chromatin-remodeling ATPase INO80
Cell cycle progression protein 1
Kinase D-interacting substrate of 220 kDa
Arf-GAP with SH3 domain, ANK repeat and PH domain-containing protein 1
Myocardin-related transcription factor B
Ankyrin repeat domain-containing protein 50
Mediator of RNA polymerase II transcription subunit 23
Protein Shroom4
YEATS domain-containing protein 2
Protein NDRG4
TBC1 domain family member 24
Pre-mRNA-splicing factor ISY1 homolog
Transmembrane and coiled-coil domain protein 3
E3 ubiquitin-protein ligase HECTD1
MYND-type zinc finger-containing chromatin reader ZMYND8
Cip1-interacting zinc finger protein
Coronin-1C
Targeting protein for Xklp2
Activator of basal transcription 1

RNA-binding protein NOB1
A-kinase anchor protein 8-like
Transcription factor MafF
Apoptosis-associated speck-like protein containing a CARD
Calcium load-activated calcium channel
Fizzy-related protein homolog
Anaphase-promoting complex subunit 10
Unconventional myosin-VI
ATP-dependent RNA helicase DDX19B
Lysosomal thioesterase PPT2
Pre-mRNA-processing factor 19
Bcl-2-related ovarian killer protein
Nucleolar protein 7
Sorting nexin-12
Synergin gamma
Vacuolar protein sorting-associated protein 4A
Ras GTPase-activating protein-binding protein 2
E3 ubiquitin-protein ligase CHIP
Protein kinase C and casein kinase substrate in neurons protein 2
Melanoma-associated antigen D2
Sorting nexin-7
Sorting nexin-6
Dual specificity protein phosphatase 12
Syntaxin-8
Translocon-associated protein subunit gamma
26S proteasome non-ATPase regulatory subunit 13
FAS-associated factor 1
Endothelial protein C receptor
Peptidyl-prolyl cis-trans isomerase E
Probable dimethyladenosine transferase
Protein timeless homolog
COP9 signalosome complex subunit 3
Multiple inositol polyphosphate phosphatase 1
RNA-binding protein Nova-2
WD repeat-containing protein 3
Transcription termination factor 2
NSFL1 cofactor p47
Conserved oligomeric Golgi complex subunit 5
Solute carrier family 12 member 4
AP-4 complex subunit epsilon-1
Microtubule-actin cross-linking factor 1, isoforms 1/2/3/4/5
Centrosomal protein of 131 kDa
SR-related and CTD-associated factor 8
Serine/threonine-protein phosphatase 6 regulatory subunit 1
E3 ubiquitin-protein ligase TRIM33
Histone lysine demethylase PHF8
Trinucleotide repeat-containing gene 6B protein
Inactive phospholipase C-like protein 2
Nonsense-mediated mRNA decay factor SMG5
Exocyst complex component 7

Zinc finger CCCH domain-containing protein 4
Ubiquitin carboxyl-terminal hydrolase 24
TBC1 domain family member 2B
Protein Smaug homolog 1
Forkhead box protein J3
Cytosolic carboxypeptidase 1
Endoribonuclease Dicer
Cystine/glutamate transporter
Microtubule-associated protein RP/EB family member 3
Leucine-rich repeat protein SHOC-2
Serine/arginine repetitive matrix protein 2
Alpha-1,3-mannosyl-glycoprotein 4-beta-N-acetylglucosaminyltransferase B
Proliferation-associated protein 2G4
Paraplegin
Brain-specific angiogenesis inhibitor 1-associated protein 2
GRB2-associated-binding protein 2
Structural maintenance of chromosomes protein 3
Charged multivesicular body protein 2b
SH2B adapter protein 3
Zinc finger protein 148
Myotubularin-related protein 6
Protein jagged-2
60S ribosome subunit biogenesis protein NIP7 homolog
Bifunctional UDP-N-acetylglucosamine 2-epimerase/N-acetylmannosamine kinase
RNA transcription, translation and transport factor protein
RuvB-like 2
Chromodomain Y-like protein
Peptidyl-prolyl cis-trans isomerase NIMA-interacting 4
RAC-gamma serine/threonine-protein kinase
CAAX prenyl protease 2
Choline/ethanolamine kinase
Eukaryotic translation initiation factor 3 subunit L
Phospholipase A-2-activating protein
RuvB-like 1
Nuclear migration protein nudC
Mitochondrial chaperone BCS1
Voltage-dependent anion-selective channel protein 3
Cofilin-2
Endoplasmic reticulum-Golgi intermediate compartment protein 3
Phenylalanine--tRNA ligase alpha subunit
Sodium-dependent multivitamin transporter
Small ribosomal subunit protein mS33
Histone chaperone ASF1A
Developmentally-regulated GTP-binding protein 1
Trafficking protein particle complex subunit 4
Nck-associated protein 1
Protein canopy homolog 2
Nuclease EXOG, mitochondrial
Exocyst complex component 6B
PALM2-AKAP2 fusion protein

Caspase recruitment domain-containing protein 8
Phospholipid-transporting ATPase IF
GDP-fucose protein O-fucosyltransferase 2
DnaJ homolog subfamily C member 16
Disks large-associated protein 4
Serine/threonine-protein kinase 38-like
Phosphatidylinositide phosphatase SAC2
Fibronectin type-III domain-containing protein 3A
Nischarin
1-phosphatidylinositol 3-phosphate 5-kinase
WD repeat-containing protein 37
Band 4.1-like protein 3
Angiomotin-like protein 2
Serine/threonine-protein kinase SIK3
Lysine-specific demethylase 2A
Exosome complex exonuclease RRP44
Trafficking protein particle complex subunit 8
Leucine-rich repeat and calponin homology domain-containing protein 1
RNA 3'-terminal phosphate cyclase-like protein
Glutathione S-transferase kappa 1
Ragulator complex protein LAMTOR2
Small ribosomal subunit protein bS1m
Cytochrome c oxidase assembly factor 3 homolog, mitochondrial
Probable ATP-dependent RNA helicase DDX52
Small ribosomal subunit protein uS17m
Small ribosomal subunit protein uS7m
Lambda-crystallin homolog
Translation machinery-associated protein 7
Polymerase delta-interacting protein 2
AP-3 complex subunit mu-1
Inner nuclear membrane protein Man1
Calcium-regulated heat-stable protein 1
Conserved oligomeric Golgi complex subunit 6
Thyroid hormone receptor-associated protein 3
WW domain-binding protein 11
Tudor and KH domain-containing protein
Mediator of RNA polymerase II transcription subunit 16
Nucleolar protein 58
ARF GTPase-activating protein GIT1
Zinc finger protein 281
ADP-ribosylation factor-like protein 2-binding protein
Transmembrane protein 98
Protein SGT1 homolog
Tyrosine--tRNA ligase, mitochondrial
N-acetylglucosamine-6-phosphate deacetylase
Acyl-coenzyme A thioesterase 9, mitochondrial
Protein AAR2 homolog
Nitric oxide synthase-interacting protein
Deoxyribose-phosphate aldolase
Protein MEMO1

Thioredoxin-related transmembrane protein 2
rRNA-processing protein FCF1 homolog
U6 snRNA-associated Sm-like protein LSm2
Endophilin-B1
Complex I intermediate-associated protein 30, mitochondrial
Calcium-binding protein 39
Putative RNA-binding protein Luc7-like 2
Ubiquitin-conjugating enzyme E2 J1
RNA-binding motif protein, X-linked 2
Dehydrogenase/reductase SDR family member 7
Small ribosomal subunit protein uS2m
Probable U3 small nucleolar RNA-associated protein 11
MOB-like protein phocein
Ribosomal RNA-processing protein 7 homolog A
Ribosome maturation protein SBDS
Transmembrane emp24 domain-containing protein 5
Exosome complex component CSL4
Transmembrane emp24 domain-containing protein 7
Splicing factor 3B subunit 6
Large ribosomal subunit protein uL11m
Oligoribonuclease, mitochondrial
RRP15-like protein
WASH complex subunit 3
Nucleolar protein 16
EKC/KEOPS complex subunit TPRKB
Peptidyl-prolyl cis-trans isomerase-like 1
Ubiquitin-fold modifier-conjugating enzyme 1
Small ribosomal subunit protein bS16m
Small ribosomal subunit protein bS18m
Mitochondrial fission 1 protein
Mitochondrial import inner membrane translocase subunit TIM16
Adenylate kinase isoenzyme 6
Small ribosomal subunit protein mS23
Vesicle transport protein GOT1B
Hepatoma-derived growth factor-related protein 3
Peptidyl-tRNA hydrolase 2, mitochondrial
Charged multivesicular body protein 3
Serine-threonine kinase receptor-associated protein
RNA-splicing ligase RtcB homolog
F-box only protein 7
Ras-related protein Rap-2c
StAR-related lipid transfer protein 13
Rab GTPase-activating protein 1
Transmembrane emp24 domain-containing protein 3
TSC22 domain family protein 4
Zinc finger protein 330
R3H and coiled-coil domain-containing protein 1
Nucleolar complex protein 2 homolog
Large ribosomal subunit protein eL36
Coiled-coil domain-containing protein 9

Chromatin target of PRMT1 protein
Deoxynucleoside triphosphate triphosphohydrolase SAMHD1
Small kinetochore-associated protein
HBS1-like protein
5'-AMP-activated protein kinase subunit beta-1
WD repeat domain phosphoinositide-interacting protein 4
DmX-like protein 1
V-type proton ATPase 116 kDa subunit a 2
Talin-1
Kinesin-like protein KIF3A
Transformation/transcription domain-associated protein
Microtubule cross-linking factor 1
DDB1- and CUL4-associated factor 1
TRPM8 channel-associated factor 1
Probable RNA-binding protein 19
Disheveled-associated activator of morphogenesis 1
Plexin-D1
Probable E3 ubiquitin-protein ligase HECTD4
WASH complex subunit 2C
WD repeat-containing protein 7
Ubiquitin carboxyl-terminal hydrolase 15
FERM, ARHGEF and pleckstrin domain-containing protein 1
Meiosis regulator and mRNA stability factor 1
Centrosomal protein of 170 kDa protein B
Rap guanine nucleotide exchange factor 2
Unconventional myosin-Va
Dystrobrevin alpha
Lysyl oxidase homolog 2
Beta/gamma crystallin domain-containing protein 1
TNF receptor-associated factor 6
Mitogen-activated protein kinase kinase kinase kinase 5
Hypoxia up-regulated protein 1
Cysteine protease ATG4B
Transducin beta-like protein 2
WD repeat domain phosphoinositide-interacting protein 2
Telomere length regulation protein TEL2 homolog
Ribosomal biogenesis protein LAS1L
AFG3-like protein 2
Krueppel-like factor 12
E3 ubiquitin-protein ligase ARIH1
U6 snRNA-associated Sm-like protein LSm4
E3 ubiquitin-protein ligase RNF114
Sorting and assembly machinery component 50 homolog
Protein PRRC2C
ADP-ribose glycohydrolase OARD1
Intraflagellar transport protein 25 homolog
Protein phosphatase methylesterase 1
RNA-binding protein 7
Mitochondrial import inner membrane translocase subunit Tim22
NEDD8 ultimate buster 1

YTH domain-containing family protein 2
RNA polymerase II subunit A C-terminal domain phosphatase
PAX3- and PAX7-binding protein 1
Nucleoside diphosphate kinase 7
FACT complex subunit SPT16
U3 small nucleolar RNA-associated protein 18 homolog
Ubiquitin carboxyl-terminal hydrolase isozyme L5
CD2-associated protein
V-type proton ATPase subunit D
Transportin-3
Signal recognition particle receptor subunit beta
Origin recognition complex subunit 6
Ceramide transfer protein
Mannose-1-phosphate guanyltransferase beta
Fatty acid desaturase 3
General transcription factor 3C polypeptide 5
General transcription factor 3C polypeptide 3
Trafficking protein particle complex subunit 1
Transient receptor potential cation channel subfamily V member 2
Serine/threonine-protein kinase MRCK beta
RNA-binding protein 8A
DnaJ homolog subfamily C member 15
Ubiquitin carboxyl-terminal hydrolase 16
U5 small nuclear ribonucleoprotein TSSC4
Zinc finger protein 706
Melanoma-associated antigen D1
Sorting nexin-14
Sorting nexin-11
Sorting nexin-9
Sorting nexin-8
Sorting nexin-5
Endothelial lipase
Cytosolic Fe-S cluster assembly factor NUBP2
Peroxisomal membrane protein PEX16
Lymphatic vessel endothelial hyaluronic acid receptor 1
Beta-secretase 2
Host cell factor 2
UbiA prenyltransferase domain-containing protein 1
Pseudouridylate synthase 1 homolog
Leucine-rich repeat flightless-interacting protein 2
FH1/FH2 domain-containing protein 1
Phosphoserine aminotransferase
Nuclear receptor corepressor 2
Mitochondrial ornithine transporter 1
Junctional adhesion molecule A
Neuroplastin
Adhesion G-protein coupled receptor G1
Spindlin-1
Solute carrier family 12 member 7
Dolichyl pyrophosphate Man9GlcNAc2 alpha-1,3-glucosyltransferase

Dolichyl-phosphate beta-glucosyltransferase
Small ribosomal subunit protein mS40
Coatomer subunit gamma-1
Lipid droplet-regulating VLDL assembly factor AUP1
Peptidyl-prolyl cis-trans isomerase FKBP7
Chloride intracellular channel protein 4
Cysteine desulfurase
Cilia- and flagella-associated protein 20
Transforming acidic coiled-coil-containing protein 3
Signal peptidase complex subunit 1
GTP-binding protein SAR1b
AP-4 complex subunit beta-1
Mitochondrial carrier homolog 2
Selenoprotein K
Brefeldin A-inhibited guanine nucleotide-exchange protein 2
Brefeldin A-inhibited guanine nucleotide-exchange protein 1
Mitotic spindle assembly checkpoint protein MAD1
Serine/threonine-protein kinase 24
eIF5-mimic protein 1
COMM domain-containing protein 10
Cytoplasmic dynein 1 light intermediate chain 1
Epsin-1
Ubiquitin carboxyl-terminal hydrolase 3
Testis-expressed protein 264
Calcineurin-binding protein cabin-1
Choline/ethanolaminephosphotransferase 1
DNA (cytosine-5)-methyltransferase 3A
2'-5'-oligoadenylate synthase 3
Adenylate kinase isoenzyme 5
NF-kappa-B essential modulator
Insulin-like growth factor 2 mRNA-binding protein 2
Proton-coupled zinc antiporter SLC30A1
Sodium bicarbonate cotransporter 3
NADH dehydrogenase [ubiquinone] 1 beta subcomplex subunit 9
Sulfide:quinone oxidoreductase, mitochondrial
Roundabout homolog 1
Stonin-1
Nuclear receptor coactivator 3
Numb-like protein
Mitogen-activated protein kinase kinase kinase 4
Probable ATP-dependent RNA helicase DDX49
Calpain-7
Actin-binding protein WASF2
E3 SUMO-protein ligase PIAS3
MAU2 chromatid cohesion factor homolog
ATPase MORC2
Influenza virus NS1A-binding protein
SEC23-interacting protein
Putative glutamine amidotransferase-like class 1 domain-containing protein 3B, mitochondrial
Intraflagellar transport protein 56

Ubiquitin-like modifier-activating enzyme 6
Extended synaptotagmin-2
Bridge-like lipid transfer protein family member 3B
Shootin-1
Transmembrane protein 223
Rho GTPase-activating protein 10
2-hydroxyacyl-CoA lyase 2
SH3 and PX domain-containing protein 2B
NBAS subunit of NRZ tethering complex
tRNA wybutosine-synthesizing protein 5
DENN domain-containing protein 3
von Willebrand factor A domain-containing protein 8
Protein strawberry notch homolog 1
GTP-binding protein 10
WD repeat-containing protein 91
DENN domain-containing protein 11
Plasmanylethanolamine desaturase 1
Trafficking protein particle complex subunit 13
CCR4-NOT transcription complex subunit 1
Microtubule-associated proteins 1A/1B light chain 3 beta 2
Glycerol-3-phosphate phosphatase
RAB7A-interacting MON1-CCZ1 complex subunit 1
RCC1 domain-containing protein 1
TRPM8 channel-associated factor 2
Structural maintenance of chromosomes flexible hinge domain-containing protein 1
Cytosolic arginine sensor for mTORC1 subunit 2
Protein unc-119 homolog B
12S rRNA N4-methylcytidine (m4C) methyltransferase
Coiled-coil domain-containing protein 85C
Retrotransposon Gag-like protein 8C
Zinc finger SWIM domain-containing protein 8
Nuclear envelope pore membrane protein POM 121C
PHD finger protein 20-like protein 1
Putative small nuclear ribonucleoprotein G-like protein 15
Endosome/lysosome-associated apoptosis and autophagy regulator family member 2
Acyl-coenzyme A diphosphatase NUDT19
MIF4G domain-containing protein
Calmodulin-1
Unconventional myosin-IXa
Golgi pH regulator A
Putative WAS protein family homolog 3
Mapk-regulated corepressor-interacting protein 1
Nascent polypeptide-associated complex subunit alpha, muscle-specific form
Uncharacterized protein C11orf98
ASNSD1 upstream open reading frame protein
Mitochondrial ribosome and complex I assembly factor AltMIEF1
Deoxyribonuclease-2-alpha
Alkyldihydroxyacetonephosphate synthase, peroxisomal
Kinesin-like protein KIF2A
ATP-dependent RNA helicase DDX39A

PDZ and LIM domain protein 1
Cytosolic acyl coenzyme A thioester hydrolase
Unconventional myosin-Ic
Synaptosomal-associated protein 23
HCLS1-associated protein X-1
AH receptor-interacting protein
GTP-binding protein 1
Galectin-9
Syntaxin-binding protein 3
AP-4 complex subunit mu-1
Splicing regulator ARVCF
Small acidic protein
AP-3 complex subunit beta-1
Amyloid beta precursor protein binding family B member 1
Galectin-8
NADH dehydrogenase [ubiquinone] iron-sulfur protein 8, mitochondrial
NF-kappa-B inhibitor epsilon
26S proteasome non-ATPase regulatory subunit 11
26S proteasome non-ATPase regulatory subunit 12
26S proteasome non-ATPase regulatory subunit 9
Menin
Guided entry of tail-anchored proteins factor 1
Membrane-associated progesterone receptor component 1
Transcription elongation factor SPT5
Transcription initiation factor TFIID subunit 4
DNA fragmentation factor subunit alpha
Huntingtin-interacting protein 1
Chloride intracellular channel protein 1
Eukaryotic translation initiation factor 3 subunit F
NEDD4-like E3 ubiquitin-protein ligase WWP2
Pyruvate dehydrogenase protein X component, mitochondrial
Sulfhydryl oxidase 1
Dynactin subunit 6
Acetyl-coenzyme A transporter 1
Actin nucleation-promoting factor WASL
Importin-5
DNA-directed RNA polymerase, mitochondrial
Eukaryotic elongation factor 2 kinase
Histone deacetylase complex subunit SAP18
Echinoderm microtubule-associated protein-like 1
Insulin-like growth factor 2 mRNA-binding protein 3
Dynamin-1-like protein
RNA 3'-terminal phosphate cyclase
Phosphatidylinositol 4-phosphate 3-kinase C2 domain-containing subunit alpha
Golgi integral membrane protein 4
Agrin
Procollagen-lysine,2-oxoglutarate 5-dioxygenase 2
Exocyst complex component 5
Butyrophilin subfamily 3 member A1
Cytochrome c oxidase subunit NDUFA4

26S proteasome non-ATPase regulatory subunit 14
Myc box-dependent-interacting protein 1
Claudin-5
Importin subunit alpha-4
Serine/threonine-protein kinase 25
Krev interaction trapped protein 1
von Willebrand factor A domain-containing protein 5A
Pescadillo homolog
Syntenin-1
Membrane-associated phosphatidylinositol transfer protein 1
U3 small nucleolar ribonucleoprotein protein MPP10
Nucleolar protein 56
ATP-dependent RNA helicase DDX3X
Beta-1,3-N-acetylglucosaminyltransferase manic fringe
Podocalyxin
CCN family member 1
Pirin
Importin subunit alpha-3
E3 ubiquitin-protein ligase TRIM38
Nuclear factor 1 B-type
Serine/threonine-protein phosphatase 6 catalytic subunit
Nucleoside diphosphate kinase, mitochondrial
Phosphatidylinositol 4-phosphate 3-kinase C2 domain-containing subunit beta
Lysosomal alpha-mannosidase
Pyridoxal kinase
Stearoyl-CoA desaturase
AT-rich interactive domain-containing protein 1A
Nuclear envelope integral membrane protein 1
Thioredoxin domain-containing protein 9
Dihydropyrimidinase-related protein 4
TRAF-type zinc finger domain-containing protein 1
Cytochrome c oxidase subunit 7A-related protein, mitochondrial
Ubiquitin domain-containing protein UBFD1
Citron Rho-interacting kinase
Coatomer subunit epsilon
Cdc42 effector protein 2
AP-3 complex subunit delta-1
Copper chaperone for superoxide dismutase
Actin-binding LIM protein 1
Segment polarity protein dishevelled homolog DVL-1
Segment polarity protein dishevelled homolog DVL-2
Chromodomain-helicase-DNA-binding protein 1
Chromodomain-helicase-DNA-binding protein 2
Golgi SNAP receptor complex member 2
Torsin-1A
Torsin-1B
Syntaxin-16
Disintegrin and metalloproteinase domain-containing protein 10
Lysosomal cobalamin transporter ABCD4
Etoposide-induced protein 2.4 homolog

Tumor protein p53-inducible protein 11
Histone-lysine N-methyltransferase 2D
Apoptotic protease-activating factor 1
Serine/threonine-protein kinase RIO3
Dual specificity mitogen-activated protein kinase kinase 7
Acyl-coenzyme A thioesterase 8
CDP-diacylglycerol--inositol 3-phosphatidyltransferase
Programmed cell death protein 5
Protein arginine N-methyltransferase 5
Na(+)/H(+) exchange regulatory cofactor NHE-RF1
Serine/threonine-protein kinase Chk1
Tumor necrosis factor receptor superfamily member 10B
Tripeptidyl-peptidase 1
Transcription elongation regulator 1
Kinetochore protein NDC80 homolog
Kinesin-like protein KIF3C
Neuropilin-1
Transportin-2
Protein unc-13 homolog B
Tumor necrosis factor receptor superfamily member 10C
DNA-directed RNA polymerase III subunit RPC1
Tetraspanin-4
Proteasome subunit alpha type-7
Secretory carrier-associated membrane protein 3
Branched-chain alpha-ketoacid dehydrogenase kinase
Glutathione S-transferase 3, mitochondrial
Tax1-binding protein 3
PDZ domain-containing protein GIPC1
Inhibitor of nuclear factor kappa-B kinase subunit beta
Regulator of G-protein signaling 12
Mitochondrial import inner membrane translocase subunit Tim23
Histone acetyltransferase type B catalytic subunit
Peripheral plasma membrane protein CASK
Phospholipase D2
Cytochrome b-c1 complex subunit 8
Myosin regulatory light chain 12B
Hepatocyte growth factor-regulated tyrosine kinase substrate
Ras-related protein Rab-7L1
Vacuolar protein sorting-associated protein 26C
Protein phosphatase 1 regulatory subunit 12A
Cyclin-G-associated kinase
Heterogeneous nuclear ribonucleoprotein D-like
Exportin-1
TATA-binding protein-associated factor 172
Rho guanine nucleotide exchange factor 10
Zinc finger protein 609
Spectrin beta chain, non-erythrocytic 2
Microtubule-associated serine/threonine-protein kinase 4
Protein transport protein Sec16A
Plexin-B2

Protein KHNYN
Tectonin beta-propeller repeat-containing protein 2
U2 snRNP-associated SURP motif-containing protein
Histone-lysine N-methyltransferase SETD1A
NEDD4-binding protein 3
Synaptojanin-2
Synemin
Granule associated Rac and RHOG effector protein 1
Kinesin-like protein KIF3B
Phosphoribosylformylglycinamidine synthase
Serine/threonine-protein phosphatase 6 regulatory ankyrin repeat subunit A
Rho guanine nucleotide exchange factor 11
Mitochondrial ribonuclease P catalytic subunit
Inhibitor of nuclear factor kappa-B kinase subunit alpha
NPC intracellular cholesterol transporter 1
Sphingolipid delta(4)-desaturase DES1
Angiopoietin-2
Secretory carrier-associated membrane protein 1
Secretory carrier-associated membrane protein 2
Actin-related protein 2/3 complex subunit 1B
Actin-related protein 2/3 complex subunit 2
Actin-related protein 2/3 complex subunit 3
DNA-directed RNA polymerases I and III subunit RPAC1
Phospholipid scramblase 1
Transcription intermediary factor 1-alpha
Membrane-associated progesterone receptor component 2
Ral guanine nucleotide dissociation stimulator-like 2
WD repeat-containing protein 46
NF-kappa-B-repressing factor
Dihydroxyacetone phosphate acyltransferase
Laminin subunit alpha-5
Zinc finger protein 185
Protein CASC3
Small ribosomal subunit protein uS12m
Peroxisomal acyl-coenzyme A oxidase 3
Protein RER1
Surfeit locus protein 4
Mitogen-activated protein kinase 13
Serine palmitoyltransferase 1
Serine palmitoyltransferase 2
UDP-N-acetylglucosamine--peptide N-acetylglucosaminyltransferase 110 kDa subunit
Phosphomannomutase 2
Transmembrane 9 superfamily member 1
E3 ubiquitin-protein ligase Midline-1
High mobility group protein B3
Protein phosphatase 1G
Phosphatidylinositol 3,4,5-trisphosphate 5-phosphatase 2
Eukaryotic translation initiation factor 3 subunit D
Eukaryotic translation initiation factor 3 subunit H
Histone deacetylase 3

Nuclear valosin-containing protein-like
Branched-chain-amino-acid aminotransferase, mitochondrial
Importin-8
Syntaxin-7
Monocarboxylate transporter 4
ATP-binding cassette sub-family C member 4
DNA-directed RNA polymerase I subunit RPA34
Prolyl 4-hydroxylase subunit alpha-2
Calpain-5
Synaptobrevin homolog YKT6
Nucleoporin NUP42
Actin-related protein 2/3 complex subunit 5
CASP8 and FADD-like apoptosis regulator
Transcription factor MafG
3-phosphoinositide-dependent protein kinase 1
Tapasin
E3 ubiquitin-protein ligase RNF113A
Lysine-specific demethylase 6A
ATP-dependent RNA helicase DHX15
mRNA cap guanine-N7 methyltransferase
Zinc finger ZZ-type and EF-hand domain-containing protein 1
Arf-GAP with SH3 domain, ANK repeat and PH domain-containing protein 2
Leucine-rich repeat transmembrane protein FLRT2
TELO2-interacting protein 1 homolog
Ribosomal RNA-processing protein 8
E3 ubiquitin-protein ligase Praja-2
Cytochrome b5 type B
U4/U6 small nuclear ribonucleoprotein Prp4
D-3-phosphoglycerate dehydrogenase
NADH dehydrogenase [ubiquinone] iron-sulfur protein 4, mitochondrial
Cytoplasmic dynein 1 light intermediate chain 2
26S proteasome non-ATPase regulatory subunit 3
RNA binding protein fox-1 homolog 2
Bifunctional 3'-phosphoadenosine 5'-phosphosulfate synthase 1
Centromere/kinetochore protein zw10 homolog
U4/U6.U5 tri-snRNP-associated protein 1
Glycosylphosphatidylinositol anchor attachment 1 protein
Death-associated protein kinase 3
Transforming growth factor beta-1-induced transcript 1 protein
AP-5 complex subunit zeta-1
CBP80/20-dependent translation initiation factor
Protein MTSS 1
Inositol hexakisphosphate and diphosphoinositol-pentakisphosphate kinase 2
Mitogen-activated protein kinase kinase kinase 7
Eukaryotic translation elongation factor 1 epsilon-1
Receptor-interacting serine/threonine-protein kinase 2
Heterogeneous nuclear ribonucleoprotein R
U4/U6 small nuclear ribonucleoprotein Prp3
Thioredoxin-like protein 1
Tumor protein D54

ER membrane protein complex subunit 8
ERI1 exoribonuclease 3
Synaptojanin-1
Acidic fibroblast growth factor intracellular-binding protein
Eukaryotic translation initiation factor 4 gamma 3
Peptidyl-prolyl cis-trans isomerase H
Serine protease HTRA2, mitochondrial
Protein Mis18-beta
Aflatoxin B1 aldehyde reductase member 2
Band 4.1-like protein 2
Trans-Golgi network integral membrane protein 2
Beta-1,4-glucuronyltransferase 1
WAS/WASL-interacting protein family member 1
Phospholipid-transporting ATPase IC
A-kinase anchor protein 10, mitochondrial
Density-regulated protein
Exportin-T
5-hydroxymethyl-dUMP N-hydrolase
Mitochondrial import inner membrane translocase subunit TIM44
Trafficking protein particle complex subunit 3
Charged multivesicular body protein 2a
Cytoplasmic protein NCK2
Tetraspanin-6
Pleiotropic regulator 1
Protein regulator of cytokinesis 1
Regulator of G-protein signaling 10
BUB3-interacting and GLEBS motif-containing protein ZNF207
NADH dehydrogenase [ubiquinone] 1 beta subcomplex subunit 5, mitochondrial
NADH dehydrogenase [ubiquinone] 1 beta subcomplex subunit 3
NADH dehydrogenase [ubiquinone] 1 alpha subcomplex subunit 2
LIM domain-binding protein 2
ATPase GET3
Mitotic checkpoint serine/threonine-protein kinase BUB1
Mitotic checkpoint protein BUB3
Sulfotransferase 1B1
Alpha-actinin-4
Probable 18S rRNA (guanine-N(7))-methyltransferase
17S U2 SnRNP complex component HTATSF1
ER lumen protein-retaining receptor 3
AP-1 complex subunit gamma-1
Syntaxin-6
Synaptogyrin-2
Small glutamine-rich tetratricopeptide repeat-containing protein alpha
Alpha-endosulfine
Mitochondrial carnitine/acylcarnitine carrier protein
Asparagine--tRNA ligase, cytoplasmic
Unconventional myosin-Ib
Peroxisomal membrane protein PMP34
Cleavage and polyadenylation specificity factor subunit 5
Glutathione S-transferase LANCL1

Striatin
U3 small nucleolar RNA-interacting protein 2
Protein SCO2 homolog, mitochondrial
Cilia- and flagella-associated protein 410
A-kinase anchor protein 8
Putative GTP-binding protein 6
Glucose-6-phosphate exchanger SLC37A4
Isocitrate dehydrogenase [NAD] subunit beta, mitochondrial
Nardilysin
Calumenin
EGF-like repeat and discoidin I-like domain-containing protein 3
Probable phospholipid-transporting ATPase IIB
S-adenosylhomocysteine hydrolase-like protein 1
Kinesin-like protein KIF1C
Exostosin-like 3
Origin recognition complex subunit 5
NADH dehydrogenase [ubiquinone] iron-sulfur protein 5
Retinal rod rhodopsin-sensitive cGMP 3',5'-cyclic phosphodiesterase subunit delta
Origin recognition complex subunit 4
Peroxisomal ATPase PEX1
Double-strand-break repair protein rad21 homolog
Pre-mRNA-splicing factor ATP-dependent RNA helicase DHX16
Glia maturation factor gamma
SH3 domain-binding protein 5
Mediator of RNA polymerase II transcription subunit 14
Protocadherin-7
Phosphoribosyl pyrophosphate synthase-associated protein 2
SWI/SNF-related matrix-associated actin-dependent regulator of chromatin subfamily A member 5
C-Jun-amino-terminal kinase-interacting protein 4
NUAK family SNF1-like kinase 1
Nucleolar pre-ribosomal-associated protein 1
Signal-induced proliferation-associated 1-like protein 3
Zinc finger C3H1 domain-containing protein
RNA helicase aquarius
Dynamin-like 120 kDa protein, mitochondrial
Zinc finger E-box-binding homeobox 2
Germinal-center associated nuclear protein
Protocadherin gamma-A12
Phosphatidylinositol 4-phosphate 5-kinase type-1 gamma
Kinesin-like protein KIF1B
Mitogen-activated protein kinase-binding protein 1
Lysine-specific histone demethylase 1A
TBC1 domain family member 4
Acyl-CoA (8-3)-desaturase
Gasdermin-E
Ecotropic viral integration site 5 protein homolog
Neuropilin-2
Mannosyl-oligosaccharide 1,2-alpha-mannosidase IB
Myelin protein zero-like protein 2
Long-chain-fatty-acid--CoA ligase 4

Sorting nexin-3
Protein O-GlcNAcase
Vinexin
Heterogeneous nuclear ribonucleoprotein Q
Pre-mRNA-processing factor 17
Ran-binding protein 6
Ribosome quality control complex subunit NEMF
GDP-mannose 4,6 dehydratase
Glycylpeptide N-tetradecanoyltransferase 2
Cyclin-T1
Mitotic checkpoint serine/threonine-protein kinase BUB1 beta
Multifunctional procollagen lysine hydroxylase and glycosyltransferase LH3
Eukaryotic translation initiation factor 4E type 2
Protein diaphanous homolog 1
Tetraspanin-3
Exocyst complex component 3
Perilipin-3
Protein arginine N-methyltransferase 3
Importin subunit alpha-7
UDP-glucose 6-dehydrogenase
Protein-tyrosine sulfotransferase 2
Leupaxin
Catenin delta-1
Protein-S-isoprenylcysteine O-methyltransferase
Sorting nexin-2
Dolichol-phosphate mannosyltransferase subunit 1
General vesicular transport factor p115
Small ribosomal subunit protein uS14m
Target of Myb1 membrane trafficking protein
Histone H2B type 1-K
Coiled-coil domain-containing protein 22
Polyglutamine-binding protein 1
Mitochondrial import inner membrane translocase subunit Tim17-B
PRA1 family protein 2
H/ACA ribonucleoprotein complex subunit DKC1
Eukaryotic translation initiation factor 5B
Endothelial differentiation-related factor 1
DNA/RNA-binding protein KIN17
Protein diaphanous homolog 2
DnaJ homolog subfamily A member 2
Bromodomain-containing protein 4
Checkpoint protein HUS1
Prefoldin subunit 1
E3 ubiquitin-protein ligase PPP1R11
Nibrin
mRNA-capping enzyme
Iron-sulfur clusters transporter ABCB7, mitochondrial
SLIT-ROBO Rho GTPase-activating protein 2
Plexin-A2
Glycosaminoglycan xylosylkinase

WD repeat-containing protein 1
Probable phospholipid-transporting ATPase IIA
NEDD4-binding protein 1
Rho-associated protein kinase 2
CLIP-associating protein 2
Pentatricopeptide repeat-containing protein 1, mitochondrial
Copine-3
Autophagy-related protein 13
Huntingtin-interacting protein 1-related protein
E3 ubiquitin-protein ligase BRE1B
Lysine-specific demethylase PHF2
Zinc finger CCCH domain-containing protein 11A
Clustered mitochondria protein homolog
Cullin-associated NEDD8-dissociated protein 2
TSC22 domain family protein 2
DnaJ homolog subfamily C member 13
Phosphatase and actin regulator 2
Serine/threonine-protein phosphatase 6 regulatory subunit 2
A disintegrin and metalloproteinase with thrombospondin motifs 4
CCR4-NOT transcription complex subunit 3
Ankyrin repeat domain-containing protein 17
Paired amphipathic helix protein Sin3b
DnaJ homolog subfamily B member 6
Ubiquinone biosynthesis protein COQ9, mitochondrial
Gamma-glutamylcyclotransferase
NADH dehydrogenase [ubiquinone] iron-sulfur protein 7, mitochondrial
NADH dehydrogenase [ubiquinone] iron-sulfur protein 2, mitochondrial
Zinc finger protein ZPR1
RNA/RNP complex-1-interacting phosphatase
Protein NipSnap homolog 2
Hyaluronan mediated motility receptor
Programmed cell death protein 6
Tubulin-specific chaperone A
V-type proton ATPase subunit G 1
Vacuolar protein sorting-associated protein 4B
Mannose-P-dolichol utilization defect 1 protein
Core histone macro-H2A.1
Adapter SH3BGRL
Filamin-B
Nuclear receptor corepressor 1
Peroxisomal membrane protein PEX14
Tripartite motif-containing protein 3
Citrate synthase, mitochondrial
Sperm-associated antigen 7
Vesicle-trafficking protein SEC22b
Pre-mRNA-processing factor 40 homolog A
Transforming acidic coiled-coil-containing protein 1
Nucleoside diphosphate kinase 6
Cell division control protein 45 homolog
Leucine-rich repeat and calponin homology domain-containing protein 4

Metaxin-2
Vacuolar protein sorting-associated protein 26A
Mitochondrial-processing peptidase subunit beta
Mediator of RNA polymerase II transcription subunit 24
Katanin p60 ATPase-containing subunit A1
PC4 and SFRS1-interacting protein
Erlin-1
NADH dehydrogenase [ubiquinone] iron-sulfur protein 3, mitochondrial
Serine/arginine-rich splicing factor 10
Ceroid-lipofuscinosis neuronal protein 5
Claudin-11
Enoyl-CoA delta isomerase 2
Polycomb protein EED
Barrier-to-autointegration factor
Splicing factor 3B subunit 1
Cold shock domain-containing protein E1
WW domain-binding protein 4
Interferon-inducible double-stranded RNA-dependent protein kinase activator A
Integrin alpha-10
Ribosomal protein S6 kinase alpha-5
Mediator of RNA polymerase II transcription subunit 6
E3 ubiquitin-protein ligase MYCBP2
2-amino-3-ketobutyrate coenzyme A ligase, mitochondrial
Acyl-protein thioesterase 1
GTPase Era, mitochondrial
U5 small nuclear ribonucleoprotein 200 kDa helicase
Mitochondrial tRNA-specific 2-thiouridylase 1
TIP41-like protein
Centriole and centriolar satellite protein OFD1
Ribosomal protein S6 kinase alpha-4
Surfeit locus protein 6
Protein phosphatase 1B
Small subunit processome component 20 homolog
Nuclear pore complex protein Nup155
Protein XRP2
WD repeat and HMG-box DNA-binding protein 1
Cartilage-associated protein
Electrogenic aspartate/glutamate antiporter SLC25A12, mitochondrial
Renin receptor
Ribonuclease H2 subunit A
Translation initiation factor eIF2 assembly protein
Breast cancer anti-estrogen resistance protein 3
Ribonuclease P protein subunit p20
Ribonuclease P protein subunit p40
Eukaryotic translation initiation factor 3 subunit G
Eukaryotic translation initiation factor 3 subunit J
Carbonyl reductase [NADPH] 3
26S proteasome non-ATPase regulatory subunit 10
AP-1 complex subunit gamma-like 2
CAAX prenyl protease 1 homolog

Isocitrate dehydrogenase [NADP] cytoplasmic
Glutamyl-tRNA(Gln) amidotransferase subunit B, mitochondrial
Protein SCO1 homolog, mitochondrial
Attractin
Serine hydrolase RBBP9
Signal transducing adapter molecule 2
Diacylglycerol O-acyltransferase 1
Cyclin-K
PRA1 family protein 3
Dysferlin
E3 SUMO-protein ligase PIAS1
Pre-mRNA-splicing factor SPF27
Dynactin subunit 3
DnaJ homolog subfamily C member 8
Survival of motor neuron-related-splicing factor 30
Cell cycle checkpoint protein RAD17
ATP synthase subunit d, mitochondrial
Tetraspanin-9
Flotillin-1
Triple functional domain protein
ATP synthase subunit g, mitochondrial
Multiple PDZ domain protein
Carboxypeptidase D
Glutaredoxin-3
Ribosomal L1 domain-containing protein 1
Wolframin
ATP-dependent Clp protease ATP-binding subunit clpX-like, mitochondrial
SEC14-like protein 2
Gamma-synuclein
Probable cytosolic iron-sulfur protein assembly protein CIAO1
Signal recognition particle subunit SRP72
N(G),N(G)-dimethylarginine dimethylaminohydrolase 1
ATP-dependent DNA helicase Q5
Unconventional prefoldin RPB5 interactor 1
Galactosylgalactosylxylosylprotein 3-beta-glucuronosyltransferase 3
Metastasis-associated protein MTA2
Retinal dehydrogenase 2
Serine/threonine-protein kinase 10
Serine/threonine-protein kinase D3
Glutamine--fructose-6-phosphate aminotransferase [isomerizing] 2
Slit homolog 2 protein
E3 ubiquitin-protein ligase listerin
Mitochondrial import receptor subunit TOM70
Pleckstrin homology domain-containing family G member 5
Importin-13
Phospholipase DDHD2
Unconventional myosin-Id
TOX high mobility group box family member 4
[F-actin]-monooxygenase MICAL2
Protein transport protein Sec24D

F-BAR and double SH3 domains protein 2
E3 UFM1-protein ligase 1
Sorbin and SH3 domain-containing protein 2
SAM and SH3 domain-containing protein 1
UBX domain-containing protein 7
Kelch-like protein 18
SUN domain-containing protein 1
Pyridoxal phosphate homeostasis protein
Erlin-2
Pre-mRNA-processing factor 6
Pre-mRNA cleavage complex 2 protein Pcf11
Protein furry homolog-like
Nuclear factor of activated T-cells 5
Endonuclease domain-containing 1 protein
D-glucuronyl C5-epimerase
Glutaminase kidney isoform, mitochondrial
HAUS augmin-like complex subunit 5
Actin-binding LIM protein 3
F-box only protein 21
Lysine-specific demethylase 4B
Microtubule cross-linking factor 2
Ubiquitin carboxyl-terminal hydrolase 19
WD repeat-containing protein 47
AP-2 complex subunit alpha-2
Protein transport protein Sec31A
Rho guanine nucleotide exchange factor 15
Protein HEXIM1
Ribonuclease P protein subunit p14
G2/mitotic-specific cyclin-B2
Protein YIF1A
E3 ubiquitin-protein ligase UBR5
Arf-GAP domain and FG repeat-containing protein 2
Serine protease 23
SR-related and CTD-associated factor 4
NADH dehydrogenase [ubiquinone] 1 beta subcomplex subunit 6
Mitofusin-2
Snurportin-1
Ubiquitin conjugation factor E4 B
Zinc finger protein-like 1
Elongator complex protein 1
NADH dehydrogenase [ubiquinone] 1 beta subcomplex subunit 4
NADH dehydrogenase [ubiquinone] 1 beta subcomplex subunit 8, mitochondrial
NADH dehydrogenase [ubiquinone] 1 alpha subcomplex subunit 7
Vesicle-associated membrane protein 5
Reticulon-3
Mitochondrial proton/calcium exchanger protein
Epsin-2
Zinc finger Ran-binding domain-containing protein 2
Sorting nexin-4
Luc7-like protein 3

Kinesin-like protein KIF20A
Apolipoprotein L3
Chromosome-associated kinesin KIF4A
Methyl-CpG-binding domain protein 4
Myotubularin-related protein 5
Golgi SNAP receptor complex member 1
Histone acetyltransferase KAT7
Arginyl-tRNA--protein transferase 1
Vesicle-associated membrane protein-associated protein B/C
SNARE-associated protein Snapin
Myelin protein zero-like protein 1
NADH dehydrogenase [ubiquinone] 1 subunit C2
NADH dehydrogenase [ubiquinone] 1 alpha subcomplex subunit 10, mitochondrial
Peptidyl-prolyl cis-trans isomerase FKBP9
CUGBP Elav-like family member 2
6-phosphogluconolactonase
Bifunctional 3'-phosphoadenosine 5'-phosphosulfate synthase 2
Structural maintenance of chromosomes protein 2
Ubiquitin-like modifier-activating enzyme ATG7
Transforming acidic coiled-coil-containing protein 2
Tripartite motif-containing protein 16
Phenylalanine--tRNA ligase, mitochondrial
Zinc finger and BTB domain-containing protein 7A
Acyl-protein thioesterase 2
Importin-7
E3 ubiquitin-protein ligase ARIH2
Pre-mRNA-splicing factor SLU7
Phosphoacetylglucosamine mutase
Adenylyltransferase and sulfurtransferase MOCS3
CD2 antigen cytoplasmic tail-binding protein 2
Supervillin
Activator of 90 kDa heat shock protein ATPase homolog 1
Poly(A)-specific ribonuclease PARN
Proteasome assembly chaperone 1
Sphingosine-1-phosphate lyase 1
CTD nuclear envelope phosphatase 1
Ribosome biogenesis protein NSA2 homolog
GDH/6PGL endoplasmic bifunctional protein
Protein transport protein Sec24A
Protein transport protein Sec24B
Adhesion G protein-coupled receptor L2
NAD kinase
Tyrosyl-DNA phosphodiesterase 2
Mitochondrial pyruvate carrier 2
Persulfide dioxygenase ETHE1, mitochondrial
Fatty acid CoA ligase Acsl3
DNA-directed RNA polymerase I subunit RPA1
YEATS domain-containing protein 4
Zinc finger and BTB domain-containing protein 11
CCR4-NOT transcription complex subunit 4

STAM-binding protein
Cleavage and polyadenylation specificity factor subunit 4
Nuclear factor of activated T-cells, cytoplasmic 1
Probable bifunctional dTTP/UTP pyrophosphatase/methyltransferase protein
Phosphatidate cytidylyltransferase 2
Protein phosphatase 1 regulatory subunit 3D
Ribonuclease P protein subunit p29
E3 ubiquitin-protein ligase HERC2
Ras-related protein Rab-3D
Synaptosomal-associated protein 29
Serine/threonine-protein kinase OSR1
Geranylgeranyl pyrophosphate synthase
Heat shock 70 kDa protein 4L
Polypyrimidine tract-binding protein 3
TBC1 domain family member 8
STARD3 N-terminal-like protein
U6 snRNA-associated Sm-like protein LSm8
AP-2 complex subunit alpha-1
Protein Wiz
Antiviral innate immune response receptor RIG-I
Double-stranded RNA-binding protein Staufen homolog 1
Tetratricopeptide repeat protein 4
Transmembrane protein 50A
Caveolae-associated protein 2
BAG family molecular chaperone regulator 2
BAG family molecular chaperone regulator 3
Mitogen-activated protein kinase kinase kinase kinase 4
Malonyl-CoA decarboxylase, mitochondrial
Quinone oxidoreductase-like protein 1
Apoptosis-inducing factor 1, mitochondrial
Echinoderm microtubule-associated protein-like 2
Serine/threonine-protein kinase LATS1
Uridine diphosphate glucose pyrophosphatase NUDT14
Tetraspanin-15
3'(2'),5'-bisphosphate nucleotidase 1
Acyl-CoA 6-desaturase
Putative hydrolase DDAH2
Phosphatidylserine lipase ABHD16A
Thioredoxin domain-containing protein 12
Protein ecdysoneless homolog
Methyl-CpG-binding domain protein 3
DNA topoisomerase 3-beta-1
Diphosphoinositol polyphosphate phosphohydrolase 1
B-cell lymphoma/leukemia 10
NADH dehydrogenase [ubiquinone] 1 beta subcomplex subunit 10
Putative lipid scramblase CLPTM1
E3 SUMO-protein ligase ZBED1
Molybdopterin synthase catalytic subunit
Mitochondrial import receptor subunit TOM40 homolog
Peroxisomal membrane protein 11B

Serine/threonine-protein kinase PAK 4
Amyloid-beta A4 precursor protein-binding family A member 3
Actin-like protein 6A
Histone-lysine N-methyltransferase NSD2
L-lactate dehydrogenase A chain
Aldehyde dehydrogenase 1A1
Glutamate dehydrogenase 1, mitochondrial
Dihydrofolate reductase
NADH-cytochrome b5 reductase 3
Glutathione reductase, mitochondrial
Cytochrome c oxidase subunit 2
Purine nucleoside phosphorylase
Hypoxanthine-guanine phosphoribosyltransferase
Aspartate aminotransferase, mitochondrial
Tyrosine-protein kinase ABL1
Phosphoglycerate kinase 1
Adenylate kinase isoenzyme 1
Urokinase-type plasminogen activator
Tissue-type plasminogen activator
Adenosine deaminase
Alpha-2-macroglobulin
Cystatin-C
GTPase NRas
GTPase HRas
GTPase KRas
Platelet-derived growth factor subunit B
Low-density lipoprotein receptor
Transforming growth factor beta-1 proprotein
HLA class I histocompatibility antigen, B alpha chain
Putative HLA class I histocompatibility antigen, alpha chain H
Collagen alpha-1(I) chain
Collagen alpha-1(IV) chain
Prelamin-A/C
Apolipoprotein A-I
Fibronectin
Alpha-2-HS-glycoprotein
Albumin
Transferrin receptor protein 1
Ferritin light chain
Ferritin heavy chain
Metallothionein-2
NADH-ubiquinone oxidoreductase chain 1
NADH-ubiquinone oxidoreductase chain 4
NADH-ubiquinone oxidoreductase chain 5
Interstitial collagenase
3-hydroxy-3-methylglutaryl-coenzyme A reductase
Catalase
RAF proto-oncogene serine/threonine-protein kinase
Lysosomal acid glucosylceramidase
Fructose-bisphosphate aldolase A

Cystatin-B
Annexin A1
Apolipoprotein B-100
Major prion protein
Superoxide dismutase [Mn], mitochondrial
Ornithine aminotransferase, mitochondrial
Thymidine kinase, cytosolic
von Willebrand factor
Tubulin beta-4A chain
Glyceraldehyde-3-phosphate dehydrogenase
Argininosuccinate lyase
HLA class I histocompatibility antigen, A alpha chain
Calpain small subunit 1
Cellular tumor antigen p53
Metallothionein-1E
Heat shock protein beta-1
Thymidylate synthase
Dolichyl-diphosphooligosaccharide--protein glycosyltransferase subunit 1
Dolichyl-diphosphooligosaccharide--protein glycosyltransferase subunit 2
Guanine nucleotide-binding protein G(i) subunit alpha-2
Histone H2A type 1-B/E
Anion exchange protein 2
Sodium/potassium-transporting ATPase subunit alpha-1
Sodium/potassium-transporting ATPase subunit beta-1
Amyloid-beta precursor protein
Aldehyde dehydrogenase, mitochondrial
Integrin beta-3
Protein S100-A8
Non-histone chromosomal protein HMG-14
Plasminogen activator inhibitor 2
Plasminogen activator inhibitor 1
ADP/ATP translocase 2
Ubiquitin-like protein ISG15
Propionyl-CoA carboxylase alpha chain, mitochondrial
Propionyl-CoA carboxylase beta chain, mitochondrial
Eukaryotic translation initiation factor 2 subunit 1
Non-histone chromosomal protein HMG-17
Intercellular adhesion molecule 1
Large ribosomal subunit protein P2
Large ribosomal subunit protein uL10
Transcription factor Jun
DNA-directed RNA polymerase III subunit RPC4
Lupus La protein
Integrin beta-1
Keratin, type I cytoskeletal 18
Collagen alpha-2(V) chain
Uroporphyrinogen decarboxylase
Tyrosine-protein kinase Fyn
Alpha-galactosidase A
Gelsolin

Retinoblastoma-associated protein
Prothymosin alpha
Cyclin-dependent kinase 1
ATP synthase subunit beta, mitochondrial
Protein S100-A9
Protein S100-A6
Eukaryotic translation initiation factor 4E
Alpha-enolase
Glycogen phosphorylase, liver form
Glucose-6-phosphate isomerase
DNA polymerase beta
Nucleophosmin
Tropomyosin alpha-3 chain
Integrin alpha-V
Beta-hexosaminidase subunit alpha
Epoxide hydrolase 1
L-lactate dehydrogenase B chain
Major centromere autoantigen B
Glutathione peroxidase 1
Protein disulfide-isomerase
Histone H1.0
Acylphosphatase-1
Tyrosine-protein kinase Fes/Fps
Cathepsin D
Annexin A2
Calpain-1 catalytic subunit
Tubulin beta chain
Prosaposin
Beta-hexosaminidase subunit beta
Procathepsin L
Profilin-1
Bisphosphoglycerate mutase
Adenine phosphoribosyltransferase
Bifunctional glutamate/proline--tRNA ligase
Cathepsin B
Heat shock protein HSP 90-alpha
Galactose-1-phosphate uridylyltransferase
Heterogeneous nuclear ribonucleoproteins C1/C2
Laminin subunit beta-1
Tyrosine-protein kinase Yes
Tyrosine-protein kinase Lyn
Fumarate hydratase, mitochondrial
DNA excision repair protein ERCC-1
Thrombospondin-1
Transcription factor Sp1
Insulin-like growth factor 1 receptor
Collagen alpha-2(I) chain
Annexin A6
Rho-related GTP-binding protein RhoC
Complement decay-accelerating factor

Amino acid transporter heavy chain SLC3A2
Beta-glucuronidase
ATP-dependent 6-phosphofructokinase, muscle type
Heat shock protein HSP 90-beta
Signal recognition particle receptor subunit alpha
Asparagine synthetase [glutamine-hydrolyzing]
72 kDa type IV collagenase
Porphobilinogen deaminase
Neprilysin
Matrix Gla protein
Pyruvate dehydrogenase E1 component subunit alpha, somatic form, mitochondrial
Collagen alpha-2(IV) chain
Cytochrome c1, heme protein, mitochondrial
U2 small nuclear ribonucleoprotein B''
Hepatocyte growth factor receptor
U1 small nuclear ribonucleoprotein 70 kDa
Integrin alpha-5
Nuclear factor 1 C-type
Vimentin
Small ribosomal subunit protein eS17
Guanine nucleotide-binding protein G(i) subunit alpha-3
Annexin A5
Small ribosomal subunit protein uS2
CD63 antigen
Large ribosomal subunit protein uL3m
U1 small nuclear ribonucleoprotein A
Gamma-enolase
3-ketoacyl-CoA thiolase, peroxisomal
Signal recognition particle 19 kDa protein
Glutathione S-transferase P
U1 small nuclear ribonucleoprotein C
Galectin-1
Dihydropteridine reductase
High mobility group protein B1
SPARC
Tropomyosin alpha-1 chain
Clathrin light chain A
Clathrin light chain B
Annexin A4
2',3'-cyclic-nucleotide 3'-phosphodiesterase
Heme oxygenase 1
Dihydrolipoyl dehydrogenase, mitochondrial
Heterogeneous nuclear ribonucleoprotein A1
U2 small nuclear ribonucleoprotein A'
Cytochrome c oxidase subunit 6C
Tumor-associated calcium signal transducer 2
Poly [ADP-ribose] polymerase 1
DNA polymerase alpha catalytic subunit
Ubiquitin carboxyl-terminal hydrolase isozyme L1
Leukotriene A-4 hydrolase

Fructose-bisphosphate aldolase C
Histone H2A.Z
Histone H2A type 1
DNA-directed RNA polymerase II subunit GRINL1A
Glutathione S-transferase theta-2
POTE ankyrin domain family member J
LIM and senescent cell antigen-like-containing domain protein 3
Heat shock 70 kDa protein 1A
Chimeric ERCC6-PGBD3 protein
Tubulin alpha-3C chain
Zinc finger TRAF-type-containing protein 1
Ras-related protein Rap-2a
Serglycin
RNA-binding protein RO60
Lysosomal alpha-glucosidase
Ras-related protein R-Ras
HLA class I histocompatibility antigen, C alpha chain
Serine/threonine-protein kinase A-Raf
Histone H1.4
Dihydrolipoyllysine-residue acetyltransferase component of pyruvate dehydrogenase complex, mitochond
Receptor-type tyrosine-protein phosphatase F
COUP transcription factor 1
Thioredoxin
Cytochrome c oxidase subunit 5B, mitochondrial
Lysosomal protective protein
Microsomal glutathione S-transferase 1
cAMP-dependent protein kinase type I-alpha regulatory subunit
Tissue factor pathway inhibitor
S-formylglutathione hydrolase
60 kDa heat shock protein, mitochondrial
Clusterin
Endoplasmic reticulum chaperone BiP
Laminin subunit gamma-1
Lysosomal acid phosphatase
Microtubule-associated protein 2
Heat shock cognate 71 kDa protein
Solute carrier family 2, facilitated glucose transporter member 1
Solute carrier family 2, facilitated glucose transporter member 3
Protein 4.1
Uridine 5'-monophosphate synthase
Pyruvate dehydrogenase E1 component subunit beta, mitochondrial
Lipoamide acyltransferase component of branched-chain alpha-keto acid dehydrogenase complex, mitoch
Glycogen phosphorylase, brain form
Glycogen phosphorylase, muscle form
Ras-related protein Ral-A
Ras-related protein Ral-B
Breakpoint cluster region protein
Lysosome-associated membrane glycoprotein 1
Transcriptional regulator ERG
Medium-chain specific acyl-CoA dehydrogenase, mitochondrial

DNA topoisomerase 1
DNA topoisomerase 2-alpha
Glucose-6-phosphate 1-dehydrogenase
Ubiquitin-like protein 4A
Pyruvate carboxylase, mitochondrial
C-1-tetrahydrofolate synthase, cytoplasmic
Cation-independent mannose-6-phosphate receptor
Alcohol dehydrogenase class-3
Cyclin-dependent kinase 4
Ribose-phosphate pyrophosphokinase 2
Polyadenylate-binding protein 1
Proliferating cell nuclear antigen
Histidine--tRNA ligase, cytoplasmic
Collagen alpha-1(VI) chain
Collagen alpha-3(VI) chain
ADP/ATP translocase 1
ADP/ATP translocase 3
Inosine-5'-monophosphate dehydrogenase 2
Nucleoprotein TPR
Annexin A3
2-oxoisovalerate dehydrogenase subunit alpha, mitochondrial
Alpha-actinin-1
Angiotensin-converting enzyme
Proto-oncogene tyrosine-protein kinase Src
Xaa-Pro dipeptidase
X-ray repair cross-complementing protein 6
X-ray repair cross-complementing protein 5
Uracil-DNA glycosylase
Cytochrome c oxidase subunit 4 isoform 1, mitochondrial
5-aminolevulinate synthase, non-specific, mitochondrial
Lysosome-associated membrane glycoprotein 2
Ribonuclease inhibitor
Intercellular adhesion molecule 2
Elongation factor 2
Protein disulfide-isomerase A4
Prolyl 4-hydroxylase subunit alpha-1
Translationally-controlled tumor protein
Delta-aminolevulinic acid dehydratase
HLA class I histocompatibility antigen, alpha chain E
Plastin-3
Acylamino-acid-releasing enzyme
Electron transfer flavoprotein subunit alpha, mitochondrial
Glycogen [starch] synthase, muscle
cAMP-dependent protein kinase type II-alpha regulatory subunit
General transcription factor IIF subunit 2
CD59 glycoprotein
Bifunctional methylenetetrahydrofolate dehydrogenase/cyclohydrolase, mitochondrial
Macrophage migration inhibitory factor
CD99 antigen
Glucosidase 2 subunit beta

Hematopoietic lineage cell-specific protein
Farnesyl pyrophosphate synthase
Cytochrome c oxidase subunit 7A2, mitochondrial
Nidogen-1
Aldo-keto reductase family 1 member A1
Pyruvate kinase PKM
Endoplasmin
G2/mitotic-specific cyclin-B1
Myosin light chain 6B
Small nuclear ribonucleoprotein-associated proteins B and B'
Insulin-degrading enzyme
POU domain, class 2, transcription factor 1
Heterogeneous nuclear ribonucleoprotein L
Aspartate--tRNA ligase, cytoplasmic
Protein C-ets-1
Junction plakoglobin
Cytochrome b-c1 complex subunit 7
Serine/threonine-protein kinase B-raf
Fatty acid-binding protein, adipocyte
Glutamine synthetase
Aldo-keto reductase family 1 member B1
Aminopeptidase N
Poliovirus receptor
Ras-related C3 botulinum toxin substrate 2
Eukaryotic peptide chain release factor GTP-binding subunit ERF3A
Arylsulfatase A
Beta-1,4-galactosyltransferase 1
Ezrin
Ubiquitin carboxyl-terminal hydrolase isozyme L3
Fos-related antigen 1
Fos-related antigen 2
Membrane cofactor protein
Nucleoside diphosphate kinase A
NAD(P)H dehydrogenase [quinone] 1
N-acetylglucosamine-6-sulfatase
Phosphorylase b kinase gamma catalytic chain, liver/testis isoform
Arylsulfatase B
Small ribosomal subunit protein uS5
Transcription factor 4
Desmoplakin
Replication protein A 32 kDa subunit
Metalloproteinase inhibitor 2
CD44 antigen
Carbonyl reductase [NADPH] 1
Short-chain specific acyl-CoA dehydrogenase, mitochondrial
Cyclic AMP-responsive element-binding protein 1
Beta-galactosidase
Platelet endothelial cell adhesion molecule
Serine/threonine-protein phosphatase 2B catalytic subunit beta isoform
SH2/SH3 adapter protein NCK1

Intron Large complex component GCFC2
Histone H1.5
Histone H1.3
Histone H1.2
NADPH--cytochrome P450 reductase
Tyrosine-protein kinase Fer
Sarcoplasmic/endoplasmic reticulum calcium ATPase 2
1-phosphatidylinositol 4,5-bisphosphate phosphodiesterase gamma-2
Fumarylacetoacetase
Stathmin
Y-box-binding protein 3
Zinc finger protein 22
Zinc finger protein 24
Alpha-N-acetylgalactosaminidase
High mobility group protein HMG-I/HMG-Y
Transmembrane protein 11, mitochondrial
Aspartate aminotransferase, cytoplasmic
Protein kinase C alpha type
Integrin alpha-2
Gap junction alpha-1 protein
Sphingomyelin phosphodiesterase
Nucleolar transcription factor 1
Transcription factor JunD
T-cell acute lymphocytic leukemia protein 1
Cyclic AMP-dependent transcription factor ATF-7
NADH dehydrogenase [ubiquinone] 1 beta subcomplex subunit 7
cAMP-dependent protein kinase catalytic subunit alpha
Calpain-2 catalytic subunit
Tyrosine-protein phosphatase non-receptor type 2
CTP synthase 1
Endoglin
Probable ATP-dependent RNA helicase DDX5
ATP-dependent 6-phosphofructokinase, liver type
Vascular endothelial growth factor receptor 1
26S proteasome regulatory subunit 6A
T-complex protein 1 subunit alpha
Tyrosine-protein phosphatase non-receptor type 1
General transcription and DNA repair factor IIH helicase subunit XPD
Large ribosomal subunit protein eL33
Integrin beta-5
ADP-ribosylation factor 4
Large ribosomal subunit protein uL30
Vinculin
Receptor-type tyrosine-protein phosphatase alpha
Arylamine N-acetyltransferase 1
Protein SON
Negative elongation factor E
Large ribosomal subunit protein uL22
Phosphoglycerate mutase 1
Regulator of chromosome condensation

Cyclic AMP-dependent transcription factor ATF-1
Cyclic AMP-dependent transcription factor ATF-6 alpha
DNA ligase 1
DNA repair protein XRCC1
Peptidyl-glycine alpha-amidating monooxygenase
Cadherin-2
1-phosphatidylinositol 4,5-bisphosphate phosphodiesterase gamma-1
Nucleolin
Hexokinase-1
DNA-directed RNA polymerase II subunit RPB3
DNA-directed RNA polymerases I, II, and III subunit RPABC1
NADH dehydrogenase [ubiquinone] flavoprotein 2, mitochondrial
General transcription and DNA repair factor IIH helicase subunit XPB
E3 ubiquitin-protein ligase TRIM21
Interferon-induced, double-stranded RNA-activated protein kinase
Spermidine synthase
Sodium/hydrogen exchanger 1
Casein kinase II subunit alpha'
Nuclear factor NF-kappa-B p105 subunit
Plasma membrane calcium-transporting ATPase 1
Eukaryotic translation initiation factor 2 subunit 2
Annexin A7
Cyclin-A2
Transcription factor BTF3
Ras-related protein Rab-3B
Ras-related protein Rab-4A
Ras-related protein Rab-5A
Ras-related protein Rab-6A
DNA mismatch repair protein Msh3
Atrial natriuretic peptide receptor 2
Proteasome subunit beta type-1
Cation-dependent mannose-6-phosphate receptor
Cytochrome c oxidase subunit 5A, mitochondrial
Lamin-B1
Calpastatin
Inosine-5'-monophosphate dehydrogenase 1
Collagen alpha-1(V) chain
Nebulin
N(4)-(beta-N-acetylglucosaminyl)-L-asparaginase
Ras GTPase-activating protein 1
Parathymosin
Cyclin-dependent kinase 11B
Glutathione S-transferase Mu 3
V-type proton ATPase subunit B, brain isoform
V-type proton ATPase subunit C 1
Cysteine and glycine-rich protein 1
Filamin-A
Neurofibromin
Amine oxidase [flavin-containing] A
Cytoplasmic aconitate hydratase

Sphingosine 1-phosphate receptor 1
Synaptotagmin-1
5'-nucleotidase
Transcription initiation factor TFIID subunit 1
Midkine
Voltage-dependent anion-selective channel protein 1
Biglycan
Succinate dehydrogenase [ubiquinone] iron-sulfur subunit, mitochondrial
CD9 antigen
Catechol O-methyltransferase
Protein-glutamine gamma-glutamyltransferase 2
Methylmalonyl-CoA mutase, mitochondrial
Oxysterol-binding protein 1
Protein-L-isoaspartate(D-aspartate) O-methyltransferase
rRNA 2'-O-methyltransferase fibrillarin
Trifunctional purine biosynthetic protein adenosine-3
Bifunctional phosphoribosylaminoimidazole carboxylase/phosphoribosylaminoimidazole succinocarboxam
Sterol carrier protein 2
Ubiquitin-like modifier-activating enzyme 1
Nucleoside diphosphate kinase B
Cornifin-B
NADPH:adrenodoxin oxidoreductase, mitochondrial
Heterogeneous nuclear ribonucleoproteins A2/B1
MHC class II regulatory factor RFX1
E3 ubiquitin-protein ligase CBL
Insulin-like growth factor-binding protein 4
cAMP-dependent protein kinase catalytic subunit beta
Cytochrome b-c1 complex subunit 2, mitochondrial
Ferrochelatase, mitochondrial
Transcription elongation factor A protein 1
Prostaglandin G/H synthase 1
Integrin alpha-6
Splicing factor, proline- and glutamine-rich
Tubulin gamma-1 chain
Peptidyl-prolyl cis-trans isomerase B
NAD-dependent malic enzyme, mitochondrial
Tryptophan--tRNA ligase, cytoplasmic
Small ribosomal subunit protein uS3
Ribosomal protein S6 kinase beta-1
Tyrosine-protein kinase JAK1
Receptor-type tyrosine-protein phosphatase beta
Receptor-type tyrosine-protein phosphatase epsilon
Receptor-type tyrosine-protein phosphatase gamma
Nuclear autoantigen Sp-100
Colorectal mutant cancer protein
Tumor necrosis factor ligand superfamily member 4
Adenosylhomocysteinase
Cofilin-1
Eukaryotic translation initiation factor 4B
40-kDa huntingtin-associated protein

Plasma membrane calcium-transporting ATPase 4
Diacylglycerol kinase alpha
Carnitine O-palmitoyltransferase 2, mitochondrial
Thymidylate kinase
Ribonucleoside-diphosphate reductase large subunit
G1/S-specific cyclin-D1
Rab proteins geranylgeranyltransferase component A 1
ER lumen protein-retaining receptor 1
COUP transcription factor 2
Elongation factor 1-beta
ATP synthase F(0) complex subunit B1, mitochondrial
Low molecular weight phosphotyrosine protein phosphatase
Protein kinase C eta type
Acetyl-CoA acetyltransferase, mitochondrial
Myosin regulatory light polypeptide 9
DNA-directed RNA polymerase II subunit RPB1
Cyclin-dependent kinase 2
Beta-adrenergic receptor kinase 1
Proteinase-activated receptor 1
DNA replication licensing factor MCM3
3-mercaptopyruvate sulfurtransferase
Small ribosomal subunit protein eS12
Bromodomain-containing protein 2
Tumor necrosis factor receptor superfamily member 6
Transcriptional repressor protein YY1
DnaJ homolog subfamily B member 1
DnaJ homolog subfamily B member 2
ATP synthase subunit alpha, mitochondrial
Cathepsin S
Proteasome subunit alpha type-1
Proteasome subunit alpha type-2
Proteasome subunit alpha type-3
Proteasome subunit alpha type-4
Tumor necrosis factor receptor superfamily member 5
Integrin alpha-3
Pentraxin-related protein PTX3
Moesin
Probable ATP-dependent RNA helicase DDX6
DNA (cytosine-5)-methyltransferase 1
Splicing factor U2AF 65 kDa subunit
Large ribosomal subunit protein eL13
Rab proteins geranylgeranyltransferase component A 2
Isovaleryl-CoA dehydrogenase, mitochondrial
Alpha-1,3-mannosyl-glycoprotein 2-beta-N-acetylglucosaminyltransferase
High mobility group protein B2
Polypyrimidine tract-binding protein 1
Threonine--tRNA ligase 1, cytoplasmic
Valine--tRNA ligase
Elongation factor 1-gamma
Peptidyl-prolyl cis-trans isomerase FKBP2

Interleukin-3 receptor subunit alpha
Stomatin
Adenylate kinase 4, mitochondrial
14-3-3 protein theta
Mitogen-activated protein kinase 3
MAP/microtubule affinity-regulating kinase 3
Large ribosomal subunit protein uL16
Collagen alpha-1(VIII) chain
Replication protein A 70 kDa DNA-binding subunit
DNA-(apurinic or apyrimidinic site) endonuclease
CD82 antigen
Deoxycytidine kinase
Multifunctional protein CAD
Calreticulin
Microtubule-associated protein 4
Calnexin
Phosphatidylinositol 3-kinase regulatory subunit alpha
Inositol-trisphosphate 3-kinase B
Proteasome subunit beta type-8
Proteasome subunit beta type-9
Proteasome subunit alpha type-5
Proteasome subunit beta type-4
Proteasome subunit beta type-6
Proteasome subunit beta type-5
Glutathione S-transferase Mu 2
ATP-binding cassette sub-family D member 3
Protein ITPRID2
Protein-lysine 6-oxidase
NADH-ubiquinone oxidoreductase 75 kDa subunit, mitochondrial
DNA polymerase delta catalytic subunit
Probable global transcription activator SNF2L1
Mitogen-activated protein kinase 1
Retinoic acid receptor RXR-beta
DNA excision repair protein ERCC-5
Progranulin
Receptor-type tyrosine-protein phosphatase mu
Cytosol aminopeptidase
Hematopoietic progenitor cell antigen CD34
General transcription factor IIE subunit 1
Transcription initiation factor IIE subunit beta
Tripeptidyl-peptidase 2
Inositol monophosphatase 1
CCN family member 2
Ephrin type-A receptor 2
Ephrin type-B receptor 2
SHC-transforming protein 1
DNA-3-methyladenine glycosylase
Transketolase
Caspase-1
Nitric oxide synthase 3

RNA-binding motif, single-stranded-interacting protein 1
Protein PML
Non-receptor tyrosine-protein kinase TYK2
Elongation factor 1-delta
Myristoylated alanine-rich C-kinase substrate
Guanine nucleotide-binding protein subunit alpha-11
Delta-1-pyrroline-5-carboxylate dehydrogenase, mitochondrial
Endoplasmic reticulum resident protein 29
Peroxiredoxin-6
Flavin reductase (NADPH)
Peroxiredoxin-5, mitochondrial
Thioredoxin-dependent peroxide reductase, mitochondrial
Large ribosomal subunit protein uL11
Enoyl-CoA hydratase, mitochondrial
UMP-CMP kinase
Phosphatidylethanolamine-binding protein 1
Protein disulfide-isomerase A3
Serine/threonine-protein phosphatase 2A 65 kDa regulatory subunit A alpha isoform
Serine/threonine-protein phosphatase 2A 65 kDa regulatory subunit A beta isoform
Cell division cycle protein 27 homolog
G1/S-specific cyclin-D3
Peptidyl-prolyl cis-trans isomerase F, mitochondrial
NK-tumor recognition protein
Glycylpeptide N-tetradecanoyltransferase 1
Heme oxygenase 2
Adenylosuccinate synthetase isozyme 2
Tyrosine-protein kinase receptor UFO
Alpha-2-macroglobulin receptor-associated protein
Translocator protein
Adenylosuccinate lyase
CAP-Gly domain-containing linker protein 1
Sorcin
Leukocyte elastase inhibitor
High affinity cationic amino acid transporter 1
Aldehyde dehydrogenase X, mitochondrial
DNA-directed RNA polymerase II subunit RPB2
Succinate dehydrogenase [ubiquinone] flavoprotein subunit, mitochondrial
Coronin-1A
Rab GDP dissociation inhibitor alpha
Protein S100-A7
S-adenosylmethionine synthase isoform type-2
cAMP-dependent protein kinase type II-beta regulatory subunit
Ribonucleoside-diphosphate reductase subunit M2
Syndecan-4
Cytotoxic granule associated RNA binding protein TIA1
Sodium- and chloride-dependent taurine transporter
DnaJ homolog subfamily A member 1
RAC-alpha serine/threonine-protein kinase
Cytochrome b-c1 complex subunit 1, mitochondrial
3-hydroxyisobutyrate dehydrogenase, mitochondrial

Bifunctional purine biosynthesis protein ATIC
Heterogeneous nuclear ribonucleoprotein H3
Heterogeneous nuclear ribonucleoprotein H
Caspase-14
14-3-3 protein beta/alpha
Stress-induced-phosphoprotein 1
Protein S100-A11
Peroxiredoxin-2
Beta-arrestin-2
Glycerol kinase
Deoxycytidylate deaminase
Pyrroline-5-carboxylate reductase 1, mitochondrial
Guanylate-binding protein 1
Guanylate-binding protein 2
ETS-related transcription factor Elf-1
General transcription factor IIH subunit 1
Syntaxin-2
Large ribosomal subunit protein uL6
Long-chain-fatty-acid--CoA ligase 1
Cadherin-5
Kinesin-1 heavy chain
Deoxyuridine 5'-triphosphate nucleotidohydrolase, mitochondrial
Multidrug resistance-associated protein 1
ATP-binding cassette sub-family D member 1
Dual specificity protein kinase TTK
DNA replication licensing factor MCM4
DNA replication licensing factor MCM5
DNA replication licensing factor MCM7
N-acetylgalactosamine-6-sulfatase
Serine hydroxymethyltransferase, cytosolic
Serine hydroxymethyltransferase, mitochondrial
Heat shock 70 kDa protein 1-like
Heat shock 70 kDa protein 4
G protein-coupled receptor kinase 5
Mannose-6-phosphate isomerase
Glypican-1
Profilin-2
Catenin alpha-1
Catenin beta-1
Prohibitin 1
Serpin B6
Merlin
Radixin
Replication protein A 14 kDa subunit
Replication factor C subunit 4
Replication factor C subunit 2
Replication factor C subunit 1
Large ribosomal subunit protein eL22
General transcription factor IIF subunit 1
Sepiapterin reductase

Histamine H1 receptor
Cystathionine beta-synthase
Fibrillin-1
Fibrillin-2
Glycogen debranching enzyme
Myosin-9
Myosin-10
Tyrosine-protein kinase receptor Tie-1
Coatomer subunit beta'
Sterol O-acyltransferase 1
Alpha-adducin
Basigin
RNA-binding protein FUS
Nuclear pore complex protein Nup214
Protein DEK
Transcription factor SOX-18
Myosin-11
Protein phosphatase 1A
Hydroxymethylglutaryl-CoA lyase, mitochondrial
Vascular endothelial growth factor receptor 3
Vascular endothelial growth factor receptor 2
26S proteasome regulatory subunit 7
Glutathione hydrolase 5 proenzyme
ADP-ribosylation factor-like protein 2
ADP-ribosylation factor-like protein 3
Dual specificity mitogen-activated protein kinase kinase 2
ATP synthase subunit gamma, mitochondrial
V-type proton ATPase subunit E 1
Oxygen-dependent coproporphyrinogen-III oxidase, mitochondrial
Large ribosomal subunit protein uL4
Oxidized purine nucleoside triphosphate hydrolase
Lon protease homolog, mitochondrial
Phosphoglucomutase-1
Serine/threonine-protein phosphatase PP1-gamma catalytic subunit
Guanine nucleotide-binding protein-like 1
Dihydrolipoyllysine-residue succinyltransferase component of 2-oxoglutarate dehydrogenase complex, mit
GMP reductase 1
Phospholipid hydroperoxide glutathione peroxidase GPX4
Serine/threonine-protein kinase receptor R3
17-beta-hydroxysteroid dehydrogenase type 2
Signal recognition particle 14 kDa protein
TGF-beta receptor type-2
Nuclear pore glycoprotein p62
Hippocalcin-like protein 1
Squalene synthase
Zinc finger E-box-binding homeobox 1
Transgelin-2
Transaldolase
Alpha-synuclein
Electron transfer flavoprotein subunit beta

RNA-binding motif protein, X chromosome
Coilin
Vitamin K-dependent gamma-carboxylase
Lysosomal acid lipase/cholesteryl ester hydrolase
V-type proton ATPase catalytic subunit A
Stress-70 protein, mitochondrial
Eukaryotic initiation factor 4A-III
DNA-binding protein SMUBP-2
Cyclin-dependent kinase inhibitor 1
Small ribosomal subunit protein eS19
Large ribosomal subunit protein uL3
Collagen alpha-1(XVIII) chain
Dolichyl-diphosphooligosaccharide--protein glycosyltransferase 48 kDa subunit
Acidic leucine-rich nuclear phosphoprotein 32 family member A
Flap endonuclease 1
Homeobox protein cut-like 1
Macrophage-capping protein
Adenylyl cyclase-associated protein 2
Interleukin-6 receptor subunit beta
Alpha-taxilin
T-complex protein 1 subunit zeta
Nicotinamide N-methyltransferase
Proteasome subunit beta type-10
Pre-B-cell leukemia transcription factor 2
Large ribosomal subunit protein uL13
ADP-ribosylation factor-like protein 1
DNA mismatch repair protein Mlh1
Signal transducer and activator of transcription 3
Ubiquitin carboxyl-terminal hydrolase 8
Malate dehydrogenase, cytoplasmic
Malate dehydrogenase, mitochondrial
Replication factor C subunit 5
Replication factor C subunit 3
Trifunctional enzyme subunit alpha, mitochondrial
Eukaryotic translation initiation factor 2 subunit 3
ETS translocation variant 3
Centrin-2
Transcription factor ETV6
Eukaryotic translation initiation factor 2D
Protein BUD31 homolog
Ubiquitin-like modifier-activating enzyme 7
N-alpha-acetyltransferase 10
Lysine-specific demethylase 5C
Tyrosine-protein kinase CSK
Glycine--tRNA ligase
Isoleucine--tRNA ligase, cytoplasmic
Reduced folate transporter
Eukaryotic translation initiation factor 1
Protein kinase C iota type
ETS domain-containing protein Elk-3

Beta-centractin
Enoyl-CoA delta isomerase 1, mitochondrial
Lamina-associated polypeptide 2, isoform alpha
Lamina-associated polypeptide 2, isoforms beta/gamma
Signal transducer and activator of transcription 1-alpha/beta
Signal transducer and activator of transcription 6
Signal transducer and activator of transcription 5A
Exosome RNA helicase MTR4
Aldo-keto reductase family 1 member C3
Phosphatidylinositol 4,5-bisphosphate 3-kinase catalytic subunit alpha isoform
Phosphatidylinositol 4,5-bisphosphate 3-kinase catalytic subunit beta isoform
Serine/threonine-protein kinase mTOR
Phosphatidylinositol 4-kinase alpha
Epidermal growth factor receptor substrate 15
Caspase-3
Caspase-2
Small ribosomal subunit protein eS27
Tyrosine-protein kinase ABL2
Probable helicase with zinc finger domain
Condensin-2 complex subunit D3
RNA-binding protein 34
Leucine-rich PPR motif-containing protein, mitochondrial
3-ketoacyl-CoA thiolase, mitochondrial
Large ribosomal subunit protein uL29
Lysosomal Pro-X carboxypeptidase
Huntingtin
Endothelin-converting enzyme 1
Methylenetetrahydrofolate reductase (NADPH)
Neutral amino acid transporter A
Platelet-activating factor acetylhydrolase IB subunit beta
Cell surface glycoprotein MUC18
Carnitine O-acetyltransferase
Matrin-3
DNA mismatch repair protein Msh2
G protein-coupled receptor kinase 6
Glycerol-3-phosphate dehydrogenase, mitochondrial
Translocon-associated protein subunit alpha
Tyrosine-protein phosphatase non-receptor type 9
Ran-specific GTPase-activating protein
Nicotinamide phosphoribosyltransferase
26S proteasome regulatory subunit 6B
Elongation factor Ts, mitochondrial
Peptidyl-prolyl cis-trans isomerase C
Voltage-dependent anion-selective channel protein 2
Short/branched chain specific acyl-CoA dehydrogenase, mitochondrial
Chromobox protein homolog 5
Ubiquitin carboxyl-terminal hydrolase 5
Mitogen-activated protein kinase 8
Dual specificity mitogen-activated protein kinase kinase 4
Proliferation marker protein Ki-67

Phosphorylase b kinase regulatory subunit alpha, liver isoform
Phosphorylase b kinase regulatory subunit alpha, skeletal muscle isoform
Ran GTPase-activating protein 1
ATP-dependent DNA helicase Q1
Probable 28S rRNA (cytosine(4447)-C(5))-methyltransferase
Transcriptional regulator ATRX
Adapter molecule crk
Crk-like protein
Translation initiation factor IF-2, mitochondrial
Large proline-rich protein BAG6
Vesicle-fusing ATPase
Neurogenic locus notch homolog protein 1
Dual specificity mitogen-activated protein kinase kinase 3
Lys-63-specific deubiquitinase BRCC36
Large ribosomal subunit protein uL15
Large ribosomal subunit protein uL18
Large ribosomal subunit protein eL21
Large ribosomal subunit protein eL28
Small ribosomal subunit protein uS4
Small ribosomal subunit protein uS7
Small ribosomal subunit protein eS10
Microtubule-associated protein 1B
Glucosamine-6-phosphate isomerase 1
E3 ubiquitin-protein ligase NEDD4
Utrophin
Ras GTPase-activating-like protein IQGAP1
Glycogenin-1
Dolichyl-diphosphooligosaccharide--protein glycosyltransferase subunit STT3A
Cytosolic phospholipase A2
F-actin-capping protein subunit alpha-2
F-actin-capping protein subunit beta
Eukaryotic translation initiation factor 1A, X-chromosomal
Retinaldehyde dehydrogenase 3
Glutamine--tRNA ligase
Large ribosomal subunit protein eL29
Galectin-7
mRNA decay activator protein ZFP36L2
Cytochrome b-c1 complex subunit Rieske, mitochondrial
ATP synthase subunit O, mitochondrial
LIM and senescent cell antigen-like-containing domain protein 1
Glioma pathogenesis-related protein 1
Prolyl endopeptidase
NADP-dependent malic enzyme
Iron-responsive element-binding protein 2
Tissue factor pathway inhibitor 2
DNA-binding protein RFX5
Phosphatidylinositol 5-phosphate 4-kinase type-2 alpha
Coatomer subunit delta
Lanosterol synthase
Serine/threonine-protein phosphatase 2B catalytic subunit gamma isoform

Glutamate--cysteine ligase catalytic subunit
Glutamate--cysteine ligase regulatory subunit
CD151 antigen
Trafficking protein particle complex subunit 10
26S proteasome non-ATPase regulatory subunit 8
Protein PRRC2A
Glutathione synthetase
T-complex protein 1 subunit epsilon
Phosphatidylserine synthase 1
Nestin
Heat shock 70 kDa protein 13
Casein kinase I isoform alpha
Casein kinase I isoform delta
Isocitrate dehydrogenase [NADP], mitochondrial
Phosphatidylinositol 4,5-bisphosphate 3-kinase catalytic subunit gamma isoform
Phosphatidylinositol transfer protein beta isoform
DNA polymerase delta subunit 2
MARCKS-related protein
Paxillin
Guided entry of tail-anchored proteins factor CAMLG
Nuclear receptor subfamily 2 group C member 2
MAP kinase-activated protein kinase 2
Deoxyribonuclease-1-like 1
4-trimethylaminobutyraldehyde dehydrogenase
Large ribosomal subunit protein eL34
Ribose-5-phosphate isomerase
Protein ERGIC-53
Natural resistance-associated macrophage protein 2
Nuclear autoantigenic sperm protein
Fatty acid synthase
Protein farnesyltransferase/geranylgeranyltransferase type-1 subunit alpha
Protein farnesyltransferase subunit beta
Deoxyhypusine synthase
T-complex protein 1 subunit gamma
Large ribosomal subunit protein bL19m
Beta-arrestin-1
Elongation factor Tu, mitochondrial
Alpha-aminoadipic semialdehyde dehydrogenase
Ubiquitin-conjugating enzyme E2 R1
Inositol polyphosphate 1-phosphatase
Centromere protein F
Signal recognition particle 9 kDa protein
Choline-phosphate cytidylyltransferase A
Alanine--tRNA ligase, cytoplasmic
Cysteine--tRNA ligase, cytoplasmic
Histidine--tRNA ligase, mitochondrial
Serine--tRNA ligase, cytoplasmic
Protein phosphatase 1F
Alpha-mannosidase 2x
DNA primase small subunit

DNA primase large subunit
Caspase-4
Casein kinase I isoform epsilon
Proteasome subunit beta type-3
Proteasome subunit beta type-2
DNA replication licensing factor MCM2
Very long-chain specific acyl-CoA dehydrogenase, mitochondrial
YLP motif-containing protein 1
Vacuolar protein sorting-associated protein 41 homolog
Transmembrane emp24 domain-containing protein 10
RNA-binding protein 25
Protein numb homolog
Dual specificity protein kinase CLK3
Presenilin-1
Translation initiation factor eIF2B subunit beta
Adenosine 5'-monophosphoramidase HINT1
Nuclear pore complex protein Nup153
E3 SUMO-protein ligase RanBP2
Regulator of G-protein signaling 19
Regulator of G-protein signaling 3
Presenilin-2
Tuberin
NADH dehydrogenase [ubiquinone] flavoprotein 1, mitochondrial
Glycogen synthase kinase-3 alpha
Glycogen synthase kinase-3 beta
Transcription initiation factor TFIID subunit 6
Cytosolic purine 5'-nucleotidase
Selenide, water dikinase 1
5-formyltetrahydrofolate cyclo-ligase
GMP synthase [glutamine-hydrolyzing]
DNA ligase 3
DNA ligase 4
Double-strand break repair protein MRE11
Guanine nucleotide-binding protein G(q) subunit alpha
Isocitrate dehydrogenase [NAD] subunit alpha, mitochondrial
Sulfotransferase 1A1
Matrix metalloproteinase-14
Protoporphyrinogen oxidase
Rab GDP dissociation inhibitor beta
Emerin
Carnitine O-palmitoyltransferase 1, liver isoform
Sulfate transporter
Serpin B8
Serpin B9
Serpin H1
PDZ and LIM domain protein 4
Hsc70-interacting protein
Peroxisomal targeting signal 1 receptor
ETS domain-containing transcription factor ERF
Vasodilator-stimulated phosphoprotein

Dynamin-2
Methionine aminopeptidase 2
Bis(5'-nucleosyl)-tetraphosphatase [asymmetrical]
Cyclin-dependent kinase 7
Kinetochore-associated protein 1
Ras association domain-containing protein 2
Cyclin-dependent kinase 9
Lipopolysaccharide-responsive and beige-like anchor protein
Basal cell adhesion molecule
Palmitoyl-protein thioesterase 1
Large ribosomal subunit protein eL14
T-complex protein 1 subunit theta
T-complex protein 1 subunit delta
Annexin A11
Poly(A) polymerase alpha
RNA-binding protein FXR1
RNA-binding protein FXR2
Ras-related protein Rab-5C
Ras-related protein Rab-7a
Ras-related protein Rab-9A
Ras-related protein Rab-13
Ras-related protein Rab-27A
1-phosphatidylinositol 4,5-bisphosphate phosphodiesterase delta-1
Small ribosomal subunit protein mS29
Dual specificity protein phosphatase 3
Probable global transcription activator SNF2L2
Transcription activator BRG1
Isocitrate dehydrogenase [NAD] subunit gamma, mitochondrial
Galactokinase
Translocon-associated protein subunit delta
B-cell receptor-associated protein 31
Thiopurine S-methyltransferase
Methyl-CpG-binding protein 2
Host cell factor 1
Interleukin-1 receptor-associated kinase 1
Caveolin-2
Aldehyde dehydrogenase family 3 member A2
Peroxisomal multifunctional enzyme type 2
26S proteasome non-ATPase regulatory subunit 7
Sulfite oxidase, mitochondrial
N-sulphoglucosamine sulphohydrolase
Signal transducer and activator of transcription 5B
Ubiquitin carboxyl-terminal hydrolase 11
H(+)/Cl(-) exchange transporter 7
Vesicle-associated membrane protein 7
Ribosomal protein S6 kinase alpha-3
Cytoplasmic tyrosine-protein kinase BMX
Hepatoma-derived growth factor
Cyclin-H
CDK-activating kinase assembly factor MAT1

Serine/threonine-protein kinase Nek3
Ubiquitin-conjugating enzyme E2 E1
NADH dehydrogenase [ubiquinone] 1 alpha subcomplex subunit 8
Heterogeneous nuclear ribonucleoprotein A3
6-phosphogluconate dehydrogenase, decarboxylating
Heterogeneous nuclear ribonucleoprotein M
Importin subunit alpha-1
Importin subunit alpha-5
Nuclear cap-binding protein subunit 2
Rap1 GTPase-GDP dissociation stimulator 1
DNA-directed RNA polymerases I, II, and III subunit RPABC3
Dual specificity mitogen-activated protein kinase kinase 6
Rho GDP-dissociation inhibitor 1
Rho GDP-dissociation inhibitor 2
Arf-GAP domain and FG repeat-containing protein 1
Heterogeneous nuclear ribonucleoprotein F
Signal transducer and activator of transcription 2
DNA mismatch repair protein Msh6
Kinesin-like protein KIF11
Guanine nucleotide exchange factor VAV2
Zinc finger protein 143
RNA-binding protein 5
2-iminobutanoate/2-iminopropanoate deaminase
Spermine synthase
Hexokinase-2
Ephrin-B2
Large ribosomal subunit protein bL12m
Diacylglycerol kinase theta
Bifunctional heparan sulfate N-deacetylase/N-sulfotransferase 1
Thimet oligopeptidase
F-actin-capping protein subunit alpha-1
High mobility group protein HMGI-C
Cysteine-rich protein 2
Nuclear pore complex protein Nup98-Nup96
Biliverdin reductase A
Tricarboxylate transport protein, mitochondrial
Serine/threonine-protein phosphatase 5
Serine/threonine-protein kinase PLK1
Death-associated protein kinase 1
Arfaptin-2
Arfaptin-1
Cytosolic Fe-S cluster assembly factor NUBP1
ATP-citrate synthase
Methionine aminopeptidase 1
Succinate--CoA ligase [ADP/GDP-forming] subunit alpha, mitochondrial
Diphosphomevalonate decarboxylase
Geranylgeranyl transferase type-1 subunit beta
Geranylgeranyl transferase type-2 subunit beta
Coatomer subunit beta
Coatomer subunit alpha

Dipeptidyl peptidase 1
LIM domain kinase 1
LIM domain kinase 2
Clathrin heavy chain 2
AP-3 complex subunit mu-2
AP-2 complex subunit sigma
Holocytochrome c-type synthase
Mitogen-activated protein kinase 12
Pituitary tumor-transforming gene 1 protein-interacting protein
Smoothelin
Monocarboxylate transporter 1
IST1 homolog
Protein transport protein Sec24C
Activated RNA polymerase II transcriptional coactivator p15
DNA polymerase subunit gamma-1
Methylosome subunit pICln
Arginine--tRNA ligase, cytoplasmic
Protein HIRA
Ataxin-3
Atrophin-1
PMS1 protein homolog 1
Mismatch repair endonuclease PMS2
Voltage-dependent calcium channel subunit alpha-2/delta-1
Tyrosine--tRNA ligase, cytoplasmic
Ubiquitin carboxyl-terminal hydrolase 14
5'-AMP-activated protein kinase subunit gamma-1
Heat shock-related 70 kDa protein 2
Branched-chain-amino-acid aminotransferase, cytosolic
Sodium/potassium-transporting ATPase subunit beta-3
UV excision repair protein RAD23 homolog B
Ephrin type-B receptor 4
Alpha-N-acetylglucosaminidase
Adenylate kinase 2, mitochondrial
Delta-1-pyrroline-5-carboxylate synthase
Alpha-soluble NSF attachment protein
Eukaryotic translation initiation factor 5
Solute carrier family 12 member 2
26S proteasome non-ATPase regulatory subunit 4
Developmentally-regulated GTP-binding protein 2
Exportin-2
Transitional endoplasmic reticulum ATPase
Microfibrillar-associated protein 1
Trifunctional enzyme subunit beta, mitochondrial
Mesencephalic astrocyte-derived neurotrophic factor
Afadin
RNA polymerase II elongation factor ELL
Peregrin
Nucleosome assembly protein 1-like 1
Caspase-7
Caspase-6

Adenosine kinase
Double-stranded RNA-specific adenosine deaminase
Laminin subunit beta-2
Cadherin-13
Protein SEC13 homolog
NHP2-like protein 1
Puromycin-sensitive aminopeptidase
FAD-linked sulfhydryl oxidase ALR
Heterogeneous nuclear ribonucleoprotein H2
Succinyl-CoA:3-ketoacid coenzyme A transferase 1, mitochondrial
Eukaryotic translation initiation factor 3 subunit B
IgG receptor FcRn large subunit p51
BH3-interacting domain death agonist
ATP synthase subunit f, mitochondrial
Ribosomal RNA processing protein 1 homolog A
Methionine--tRNA ligase, cytoplasmic
cAMP-regulated phosphoprotein 19
AP-1 complex subunit sigma-2
ATP synthase subunit ATP5MJ, mitochondrial
ATP synthase subunit epsilon, mitochondrial
ATP synthase subunit e, mitochondrial
Histone deacetylase 4
Eukaryotic translation initiation factor 6
C-terminal-binding protein 2
NADH dehydrogenase [ubiquinone] 1 alpha subcomplex subunit 6
Peroxisomal biogenesis factor 3
3-keto-steroid reductase/17-beta-hydroxysteroid dehydrogenase 7
Breast cancer anti-estrogen resistance protein 1
Syntaxin-17
Cilia- and flagella-associated protein 298
tRNA (guanine-N(7)-)-methyltransferase non-catalytic subunit WDR4
Transmembrane protein 33
Gem-associated protein 4
Coronin-7
Nuclear pore complex protein Nup107
Gasdermin-D
Selenocysteine-specific elongation factor
Sestrin-2
Anthrax toxin receptor 2
Myotrophin
Actin-related protein 2/3 complex subunit 4
CD81 antigen
Protein transport protein Sec61 subunit gamma
Triosephosphate isomerase
Eukaryotic translation initiation factor 3 subunit E
Protein transport protein Sec61 subunit beta
Phosphatidylinositol 3,4,5-trisphosphate 3-phosphatase and dual-specificity protein phosphatase PTEN
Serine/threonine-protein phosphatase 4 catalytic subunit
Gamma-aminobutyric acid receptor-associated protein-like 2
Reactive oxygen species modulator 1

Myosin light polypeptide 6
Actin, cytoplasmic 1
Eukaryotic initiation factor 4A-I
Small ribosomal subunit protein uS10
Ribose-phosphate pyrophosphokinase 1
Proteasome subunit alpha type-6
Protein S100-A10
Cell division control protein 42 homolog
Destrin
Glia maturation factor beta
Ras-related protein Rab-8A
Signal peptidase complex subunit 3
Signal recognition particle subunit SRP54
Ras-related protein Rab-4B
Ras-related protein Rab-2A
Ras-related protein Rab-5B
Ras-related protein Rab-10
Ubiquitin-conjugating enzyme E2 D3
NEDD8-conjugating enzyme Ubc12
Ubiquitin-conjugating enzyme E2 K
Ubiquitin-conjugating enzyme E2 N
Ras-related protein Rab-14
Actin-related protein 3
Actin-related protein 2
Alpha-centractin
COP9 signalosome complex subunit 2
ATP-binding cassette sub-family E member 1
Ras-related protein Rap-1b
Ras-related protein Rap-2b
Protein max
Small ribosomal subunit protein eS1
Large ribosomal subunit protein uL24
Proteasome activator complex subunit 3
Large ribosomal subunit protein eL15
Large ribosomal subunit protein eL27
V-type proton ATPase subunit d 1
Large ribosomal subunit protein eL43
Transforming protein RhoA
Rho-related GTP-binding protein RhoE
N-alpha-acetyltransferase 20
10 kDa heat shock protein, mitochondrial
Protein transport protein Sec61 subunit alpha isoform 1
Lysozyme C
Prefoldin subunit 3
Syntaxin-binding protein 1
Beta-2-microglobulin
Dolichyl-diphosphooligosaccharide--protein glycosyltransferase subunit DAD1
NPC intracellular cholesterol transporter 2
Coatomer subunit zeta-1
Large ribosomal subunit protein eL37

Small ubiquitin-related modifier 2
DDB1- and CUL4-associated factor 7
WD repeat-containing protein 5
AP-1 complex subunit sigma-1A
Nuclear transport factor 2
Heterogeneous nuclear ribonucleoprotein K
14-3-3 protein gamma
Ubiquitin carboxyl-terminal hydrolase 46
Ras-related protein R-Ras2
Small ribosomal subunit protein eS7
Serine/threonine-protein phosphatase PP1-alpha catalytic subunit
Serine/threonine-protein phosphatase PP1-beta catalytic subunit
26S proteasome regulatory subunit 4
26S proteasome regulatory subunit 8
Small ribosomal subunit protein eS8
Small ribosomal subunit protein uS8
Small ribosomal subunit protein uS9
Ubiquitin-conjugating enzyme E2 G1
Ubiquitin-conjugating enzyme E2 H
14-3-3 protein epsilon
Small ribosomal subunit protein uS11
Small ribosomal subunit protein uS12
Small ribosomal subunit protein uS13
Small ribosomal subunit protein uS14
Small ribosomal subunit protein uS15
Small ribosomal subunit protein uS17
Small nuclear ribonucleoprotein E
Small nuclear ribonucleoprotein F
U6 snRNA-associated Sm-like protein LSm3
U6 snRNA-associated Sm-like protein LSm6
Small nuclear ribonucleoprotein Sm D1
Small nuclear ribonucleoprotein Sm D2
Small nuclear ribonucleoprotein Sm D3
Thymosin beta-4
ADP-ribosylation factor 6
26S proteasome regulatory subunit 10B
Thioredoxin reductase-like selenoprotein T
TATA box-binding protein-like 1
Large ribosomal subunit protein eL8
DNA-directed RNA polymerase II subunit RPB7
Ras-related protein Rab-11A
Eukaryotic peptide chain release factor subunit 1
CCHC-type zinc finger nucleic acid binding protein
Protein yippee-like 5
Small ribosomal subunit protein eS4, X isoform
Serine/threonine-protein phosphatase 2A catalytic subunit beta isoform
Actin, aortic smooth muscle
Rho-related GTP-binding protein RhoB
Large ribosomal subunit protein uL23
Small ribosomal subunit protein eS6

Histone H4
Ras-related protein Rab-1A
GTP-binding nuclear protein Ran
Large ribosomal subunit protein uL14
Ras-related protein Rap-1A
Small ribosomal subunit protein uS19
Small ribosomal subunit protein eS24
Small ribosomal subunit protein eS25
Small ribosomal subunit protein eS26
Small ribosomal subunit protein eS28
Ubiquitin-like FUBI-ribosomal protein eS30 fusion protein
Guanine nucleotide-binding protein G(I)/G(S)/G(T) subunit beta-1
E3 ubiquitin-protein ligase RBX1
Guanine nucleotide-binding protein G(I)/G(S)/G(T) subunit beta-2
Large ribosomal subunit protein eL30
Large ribosomal subunit protein eL39
Large ribosomal subunit protein eL31
Large ribosomal subunit protein uL1
Large ribosomal subunit protein eL32
Large ribosomal subunit protein uL5
Large ribosomal subunit protein uL2
Peptidyl-prolyl cis-trans isomerase A
Peptidyl-prolyl cis-trans isomerase FKBP1A
Ubiquitin-ribosomal protein eS31 fusion protein
Growth factor receptor-bound protein 2
Transformer-2 protein homolog beta
Ras-related C3 botulinum toxin substrate 1
AP-2 complex subunit beta
Vesicle-associated membrane protein 2
Guanine nucleotide-binding protein G(s) subunit alpha isoforms short
Guanine nucleotide-binding protein G(i) subunit alpha-1
14-3-3 protein zeta/delta
Serine/threonine-protein phosphatase 2A 55 kDa regulatory subunit B alpha isoform
Small ubiquitin-related modifier 1
Dynein light chain 1, cytoplasmic
Dynein light chain Tctex-type 1
Large ribosomal subunit protein eL38
S-phase kinase-associated protein 1
Guanine nucleotide-binding protein G(I)/G(S)/G(O) subunit gamma-5
Small ribosomal subunit protein eS21
Eukaryotic translation initiation factor 5A-1
Small ribosomal subunit protein RACK1
Transcription elongation factor SPT4
SUMO-conjugating enzyme UBC9
Thymosin beta-10
Serine/threonine-protein phosphatase 2A catalytic subunit alpha isoform
Y-box-binding protein 1
Signal peptidase complex catalytic subunit SEC11A
Casein kinase II subunit beta
Tropomyosin alpha-4 chain

Ubiquitin-conjugating enzyme E2 L3
Elongation factor 1-alpha 1
Tubulin alpha-1B chain
Tubulin alpha-4A chain
Tubulin beta-4B chain
Casein kinase II subunit alpha
Platelet-activating factor acetylhydrolase IB subunit alpha2
Histone H3.1
Hemoglobin subunit beta
Hemoglobin subunit alpha
Coxsackievirus and adenovirus receptor
Protein FAM193A
Nucleolar protein 14
Immunoglobulin-binding protein 1
Tyrosine-protein phosphatase non-receptor type substrate 1
Phosphoserine phosphatase
RNA-binding protein 6
Eukaryotic translation initiation factor 4 gamma 2
Ribonuclease P protein subunit p38
Ribonuclease P protein subunit p30
General transcription factor II-I
Phosphatidylinositol 5-phosphate 4-kinase type-2 beta
Contactin-associated protein 1
SRSF protein kinase 2
T-complex protein 1 subunit beta
UDP-galactose translocator
mRNA export factor RAE1
Glutathione S-transferase omega-1
DNA-dependent protein kinase catalytic subunit
Disintegrin and metalloproteinase domain-containing protein 17
Biogenesis of lysosome-related organelles complex 1 subunit 1
Sushi repeat-containing protein SRPX
Arginase-2, mitochondrial
Endonuclease III-like protein 1
Interferon-induced 35 kDa protein
Nucleobindin-2
Brain acid soluble protein 1
Dermcidin
TATA element modulatory factor
Small ribosomal subunit protein mS22
Small ribosomal subunit protein mS25
Small ribosomal subunit protein uS10m
Small ribosomal subunit protein mS35
Small ribosomal subunit protein uS5m
Small ribosomal subunit protein uS11m
Small ribosomal subunit protein uS15m
Small ribosomal subunit protein bS21m
Small ribosomal subunit protein mS34
Small ribosomal subunit protein bS6m
Small ribosomal subunit protein uS9m

SAP domain-containing ribonucleoprotein
Serine beta-lactamase-like protein LACTB, mitochondrial
Conserved oligomeric Golgi complex subunit 7
Large ribosomal subunit protein eL24
Thioredoxin-like protein 4A
Large ribosomal subunit protein eL42
Chromobox protein homolog 1
Mothers against decapentaplegic homolog 3
ADP-ribosylation factor 1
ADP-ribosylation factor 5
Enhancer of rudimentary homolog
Rho-related GTP-binding protein RhoG
Large ribosomal subunit protein eL19
Small EDRK-rich factor 2
Serine/arginine-rich splicing factor 3
Forkhead box protein K1
Vacuolar fusion protein CCZ1 homolog B
Disabled homolog 2
Basement membrane-specific heparan sulfate proteoglycan core protein
E3 ubiquitin-protein ligase XIAP
Ephrin-B1
RNA-binding protein 10
RNA-binding protein 3
Calcium-transporting ATPase type 2C member 1
Phospholipid-transporting ATPase IH
Cytochrome c
55 kDa erythrocyte membrane protein
Transcription factor A, mitochondrial
Phosphatidylinositol transfer protein alpha isoform
Solute carrier family 25 member 3
Vigilin
Transcription initiation factor IIB
Cyclin-dependent kinase 6
Cyclin-dependent kinase 5
Cyclin-dependent kinase 17
Transcriptional activator protein Pur-alpha
Cdc42 effector protein 1
Clathrin heavy chain 1
Heat shock factor protein 1
Nuclear factor NF-kappa-B p100 subunit
Peptidyl-prolyl cis-trans isomerase FKBP3
Receptor expression-enhancing protein 5
Sorbitol dehydrogenase
Heterogeneous nuclear ribonucleoprotein U
Splicing factor U2AF 35 kDa subunit
Spectrin beta chain, non-erythrocytic 1
Nucleolysin TIAR
Protein SET
Serine/arginine-rich splicing factor 2
Forkhead box protein K2

Transcription factor RelB
N-acetylgalactosamine kinase
AMP deaminase 2
Fatty acid-binding protein 5
Adenylyl cyclase-associated protein 1
Friend leukemia integration 1 transcription factor
Hydroxymethylglutaryl-CoA synthase, cytoplasmic
Interferon-induced transmembrane protein 3
Large neutral amino acids transporter small subunit 1
Protein Dr1
Exosome complex component 10
OTU domain-containing protein 4
ATP-dependent 6-phosphofructokinase, platelet type
DNA repair protein complementing XP-C cells
RNA-binding protein EWS
Cerebellar degeneration-related protein 2
Inositol polyphosphate 5-phosphatase OCRL
1-phosphatidylinositol 4,5-bisphosphate phosphodiesterase beta-3
Transgelin
A-kinase anchor protein 17A
Dihydroorotate dehydrogenase (quinone), mitochondrial
2-oxoglutarate dehydrogenase complex component E1
Centromere-associated protein E
Kinesin-like protein KIF23
Methylmalonate-semialdehyde/malonate-semialdehyde dehydrogenase [acylating], mitochondrial
Pro-neuregulin-1, membrane-bound isoform
Sterol 26-hydroxylase, mitochondrial
Desmoglein-1
Transcription factor Sp3
Histone H1.1
Large ribosomal subunit protein eL20
Dual specificity mitogen-activated protein kinase kinase 1
Angiopoietin-1 receptor
Peptidyl-prolyl cis-trans isomerase FKBP4
Procollagen-lysine,2-oxoglutarate 5-dioxygenase 1
Nucleobindin-1
Large ribosomal subunit protein eL6
DNA topoisomerase 2-beta
A-kinase anchor protein 12
Mitochondrial 2-oxoglutarate/malate carrier protein
Dystonin
Protein ENL
Histone-lysine N-methyltransferase MECOM
Guanine nucleotide-binding protein subunit alpha-12
Caveolin-1
Aminoacylase-1
Tumor necrosis factor alpha-induced protein 2
Lamin-B2
6-pyruvoyl tetrahydrobiopterin synthase
Urokinase plasminogen activator surface receptor

Mevalonate kinase
DNA excision repair protein ERCC-6
Antigen peptide transporter 1
Antigen peptide transporter 2
CCAAT/enhancer-binding protein zeta
Zinc finger protein 92
Transcription factor p65
UBX domain-containing protein 1
1,4-alpha-glucan-branching enzyme
Eukaryotic translation initiation factor 4 gamma 1
Copper-transporting ATPase 1
Neurogenic locus notch homolog protein 2
Transducin-like enhancer protein 1
Transducin-like enhancer protein 3
Lactoylglutathione lyase
Activin receptor type-1
Single-stranded DNA-binding protein, mitochondrial
Proto-oncogene c-Rel
14-3-3 protein eta
Proteolipid protein 2
Cleavage stimulation factor subunit 1
Ubiquitin-protein ligase E3A
Dynamin-1
Tyrosine-protein phosphatase non-receptor type 12
Focal adhesion kinase 1
Serine/arginine-rich splicing factor 11
Protein kinase C delta type
Caldesmon
Folylpolyglutamate synthase, mitochondrial
CTD small phosphatase-like protein 2
Tyrosine-protein phosphatase non-receptor type 11
3-ketodihydrosphingosine reductase
Amidophosphoribosyltransferase
Glutamine--fructose-6-phosphate aminotransferase [isomerizing] 1
Exosome complex component RRP45
Proteasome activator complex subunit 1
Recombining binding protein suppressor of hairless
Myocyte-specific enhancer factor 2C
Amyloid beta precursor like protein 2
GA-binding protein alpha chain
E3 ubiquitin-protein ligase RING1
Fragile X messenger ribonucleoprotein 1
Peroxiredoxin-1
Large ribosomal subunit protein eL18
Complement component 1 Q subcomponent-binding protein, mitochondrial
Cytoskeleton-associated protein 4
Tight junction protein ZO-1
mRNA decay activator protein ZFP36L1
Sperm-associated antigen 1
KH domain-containing, RNA-binding, signal transduction-associated protein 1

Apoptosis regulator BAX
Bcl-2-like protein 1
Induced myeloid leukemia cell differentiation protein Mcl-1
DNA polymerase epsilon catalytic subunit A
Kinesin light chain 1
Son of sevenless homolog 1
Serine/arginine-rich splicing factor 1
Rho GTPase-activating protein 1
TLE family member 5
Serine/arginine-rich splicing factor 4
Protocadherin-1
Protein phosphatase 3 catalytic subunit alpha
ATP-dependent RNA helicase A
Quinone oxidoreductase
Golgin subfamily A member 3
Golgin subfamily A member 2
Peroxisomal bifunctional enzyme
Lactadherin
3',5'-cyclic-AMP phosphodiesterase 4D
Desmocollin-1
Pseudouridine-5'-phosphatase
Testican-1
Leukocyte surface antigen CD47
Peptidyl-prolyl cis-trans isomerase D
FACT complex subunit SSRP1
Calmodulin-regulated spectrin-associated protein 2
Protein spire homolog 1
Schlafen family member 5
Protein VAC14 homolog
RNA cytosine C(5)-methyltransferase NSUN2
Dystrophia myotonica WD repeat-containing protein
Histone-binding protein RBBP4
Nuclear cap-binding protein subunit 1
Alpha-1,6-mannosylglycoprotein 6-beta-N-acetylglucosaminyltransferase A
Histone acetyltransferase p300
Neuroblast differentiation-associated protein AHNAK
F-BAR domain only protein 2
Elongator complex protein 6
Heat shock 70 kDa protein 14
Secernin-3
Cingulin-like protein 1
Ragulator complex protein LAMTOR4
Nexilin
Alpha-1,6-mannosyl-glycoprotein 2-beta-N-acetylglucosaminyltransferase
Polypeptide N-acetylgalactosaminyltransferase 2
Polypeptide N-acetylgalactosaminyltransferase 1
AP-1 complex subunit beta-1
Cleavage and polyadenylation specificity factor subunit 1
Bone marrow stromal antigen 2
Mitochondrial-processing peptidase subunit alpha

CMP-N-acetylneuraminate-beta-galactosamide-alpha-2,3-sialyltransferase 1
Secernin-1
WASH complex subunit 5
Nuclear pore complex protein Nup160
Sterol regulatory element-binding protein cleavage-activating protein
Forkhead box protein O1
Transducin beta-like protein 3
General transcription factor 3C polypeptide 1
Twinfilin-1
Aspartyl/asparaginyl beta-hydroxylase
Alpha-globin transcription factor CP2
A-kinase anchor protein 13
EGF-containing fibulin-like extracellular matrix protein 1
SWI/SNF-related matrix-associated actin-dependent regulator of chromatin subfamily B member 1
Nucleosome-remodeling factor subunit BPTF
Cell division cycle protein 20 homolog
Follistatin-related protein 1
Syntaxin-4
G-rich sequence factor 1
Mitogen-activated protein kinase kinase kinase kinase 2
Tyrosine-protein kinase Mer
Splicing factor, suppressor of white-apricot homolog
Chromodomain-helicase-DNA-binding protein 3
Splicing factor 3A subunit 3
Dihydropyrimidine dehydrogenase [NADP(+)]
TP53-binding protein 1
Transmembrane protein 115
Interferon-related developmental regulator 2
Tripartite motif-containing protein 26
Aminoacyl tRNA synthase complex-interacting multifunctional protein 1
Interleukin enhancer-binding factor 2
Interleukin enhancer-binding factor 3
Vesicular integral-membrane protein VIP36
Heat shock protein 75 kDa, mitochondrial
TNF receptor-associated factor 2
Disks large homolog 1
Unconventional myosin-Ie
Nuclear inhibitor of protein phosphatase 1
Protein tyrosine phosphatase type IVA 2
Active breakpoint cluster region-related protein
GATOR1 complex protein NPRL3
Vesicle transport protein SEC20
BCL2/adenovirus E1B 19 kDa protein-interacting protein 2
Transcriptional repressor NF-X1
Cleavage stimulation factor subunit 3
Tetraspanin-31
Delta(3,5)-Delta(2,4)-dienoyl-CoA isomerase, mitochondrial
Rho GTPase-activating protein 5
Striatin-3
Cell division cycle protein 16 homolog

Serine/threonine-protein kinase 4
Protein flightless-1 homolog
E3 ubiquitin-protein ligase TRIM32
Bifunctional coenzyme A synthase
Large ribosomal subunit protein bL28m
Acetyl-CoA carboxylase 1
COP9 signalosome complex subunit 1
Ubiquitin carboxyl-terminal hydrolase 4
Chromatin assembly factor 1 subunit A
Chromatin assembly factor 1 subunit B
Protein Red
S-methyl-5'-thioadenosine phosphorylase
5'-AMP-activated protein kinase catalytic subunit alpha-1
Liprin-alpha-1
Calcium-binding and coiled-coil domain-containing protein 2
Translation initiation factor eIF2B subunit epsilon
TAR DNA-binding protein 43
Heterogeneous nuclear ribonucleoprotein A0
Aminoacyl tRNA synthase complex-interacting multifunctional protein 2
FAS-associated death domain protein
Peroxiredoxin-4
Dual specificity mitogen-activated protein kinase kinase 5
Mitogen-activated protein kinase 7
Serine/threonine-protein kinase PAK 2
Chromobox protein homolog 3
Serine/threonine-protein kinase 3
Syntaxin-5
26S proteasome non-ATPase regulatory subunit 2
Multimerin-1
Probable ATP-dependent RNA helicase DDX10
DnaJ homolog subfamily C member 3
Methanethiol oxidase
Nucleoside diphosphate kinase 3
Serine/arginine-rich splicing factor 9
Serine/arginine-rich splicing factor 5
Serine/arginine-rich splicing factor 6
Mitotic spindle assembly checkpoint protein MAD2A
Transcription intermediary factor 1-beta
Semaphorin-3F
Syntaxin-3
Ras GTPase-activating protein-binding protein 1
N-myc-interactor
Inactive tyrosine-protein kinase 7
Polyadenylate-binding protein 4
Serine-protein kinase ATM
Growth factor receptor-bound protein 10
Metastasis-associated protein MTA1
Eukaryotic translation initiation factor 3 subunit I
RING-type E3 ubiquitin-protein ligase PPIL2
Serine/threonine-protein phosphatase 2A 56 kDa regulatory subunit gamma isoform

C-terminal-binding protein 1
Phosducin-like protein
Phospholipase D1
Probable methyltransferase TARBP1
Ubiquitin-conjugating enzyme E2 variant 1
Large ribosomal subunit protein mL49
Cytoplasmic dynein 1 intermediate chain 2
Origin recognition complex subunit 2
Integrin-linked protein kinase
NAD(P) transhydrogenase, mitochondrial
Beta-2-syntrophin
DNA repair protein XRCC4
Peptidyl-prolyl cis-trans isomerase G
Treacle protein
Protein unc-119 homolog A
Splicing factor 3B subunit 2
Protein OS-9
Golgin subfamily A member 4
28 kDa heat- and acid-stable phosphoprotein
Disintegrin and metalloproteinase domain-containing protein 9
Disintegrin and metalloproteinase domain-containing protein 15
Transmembrane emp24 domain-containing protein 1
Peptidyl-prolyl cis-trans isomerase FKBP5
Unconventional myosin-IXb
Rho-associated protein kinase 1
Nuclear factor of activated T-cells, cytoplasmic 2
DNA topoisomerase 3-alpha
Mothers against decapentaplegic homolog 4
V-type proton ATPase 116 kDa subunit a 3
Baculoviral IAP repeat-containing protein 2
Phosphatidylinositol-binding clathrin assembly protein
Myotubularin
Sequestosome-1
Metaxin-1
Tubulin beta-3 chain
Acid ceramidase
Serine/threonine-protein kinase PRP4 homolog
Peptidyl-prolyl cis-trans isomerase NIMA-interacting 1
Serine/threonine-protein kinase ATR
Receptor-interacting serine/threonine-protein kinase 1
Histone deacetylase 1
Calcium/calmodulin-dependent protein kinase type II subunit gamma
Calcium/calmodulin-dependent protein kinase type II subunit delta
Dynactin subunit 2
Polycystin-2
NEDD8-activating enzyme E1 regulatory subunit
Inositol-tetrakisphosphate 1-kinase
SNW domain-containing protein 1
Diacylglycerol kinase zeta
Stromal interaction molecule 1

GRB2-related adapter protein
Transformer-2 protein homolog alpha
Sorting nexin-1
KRR1 small subunit processome component homolog
Periodic tryptophan protein 1 homolog
Myotubularin-related protein 1
Myotubularin-related protein 2
Cullin-1
Cullin-2
Cullin-3
Cullin-4A
Cullin-4B
Apoptosis-stimulating of p53 protein 2
Dual specificity tyrosine-phosphorylation-regulated kinase 1A
GDP-L-fucose synthase
Ras-related protein Rab-32
Trophoblast glycoprotein
Four and a half LIM domains protein 1
Four and a half LIM domains protein 3
Ras and Rab interactor 1
Angio-associated migratory cell protein
Mannosyl-oligosaccharide glucosidase
CD166 antigen
THO complex subunit 5 homolog
Protein Shroom2
Spectrin alpha chain, non-erythrocytic 1
Nucleolar GTP-binding protein 2
Methylglutaconyl-CoA hydratase, mitochondrial
Plakophilin-1
Spliceosome RNA helicase DDX39B
Bleomycin hydrolase
Exosome complex component RRP4
Bone morphogenetic protein receptor type-2
Tubulin beta-2A chain
General transcription factor IIH subunit 2
General transcription factor IIH subunit 3
Bystin
Rap guanine nucleotide exchange factor 1
Isopentenyl-diphosphate Delta-isomerase 1
Core-binding factor subunit beta
Nuclear transcription factor Y subunit gamma
Cyclin-dependent kinase 13
Cytoskeleton-associated protein 5
Cold-inducible RNA-binding protein
Calcium/calmodulin-dependent protein kinase type 1
Coactosin-like protein
Heterogeneous nuclear ribonucleoprotein D0
Lysosome membrane protein 2
Nidogen-2
Low-density lipoprotein receptor-related protein 8

Dystroglycan 1
Vascular endothelial zinc finger 1
Ribosome biogenesis protein BOP1
Ubiquitin conjugation factor E4 A
Septin-6
Kelch-like ECH-associated protein 1
Unhealthy ribosome biogenesis protein 2 homolog
Probable ATP-dependent RNA helicase DHX34
MORC family CW-type zinc finger protein 3
Scaffold attachment factor B2
Eukaryotic translation initiation factor 3 subunit A
Rho guanine nucleotide exchange factor 7
Protein EFR3 homolog A
Ubiquitin-associated protein 2-like
Protein scribble homolog
ARF GTPase-activating protein GIT2
Scavenger receptor class F member 1
Malectin
Tubulin--tyrosine ligase-like protein 12
DNA polymerase alpha subunit B
Dedicator of cytokinesis protein 1
Transcription factor Dp-1
Four and a half LIM domains protein 2
Dihydropyrimidinase-related protein 3
Large ribosomal subunit protein mL62
Zinc finger MYM-type protein 3
Dynactin subunit 1
Cytoplasmic dynein 1 heavy chain 1
Translation initiation factor eIF2B subunit alpha
Eukaryotic initiation factor 4A-II
Elongin-A
Src substrate cortactin
Endonuclease G, mitochondrial
Flotillin-2
Reticulocalbin-2
E3 ubiquitin/ISG15 ligase TRIM25
Filamin-C
Peptidyl-prolyl cis-trans isomerase FKBP8
Protein FAM50A
Protein FRG1
Guanine nucleotide-binding protein subunit alpha-13
Guanidinoacetate N-methyltransferase
UDP-glucose 4-epimerase
Transforming growth factor beta activator LRRC32
Caprin-1
Beclin-1
RNA-binding protein 39
Enhancer of filamentation 1
Helicase-like transcription factor
Squalene monooxygenase

Protein disulfide-isomerase A5
Phosphoribosyl pyrophosphate synthase-associated protein 1
ATP-dependent RNA helicase DHX8
DNA replication licensing factor MCM6
Inositol 1,4,5-trisphosphate receptor type 2
Inositol 1,4,5-trisphosphate receptor type 3
Inositol 1,4,5-trisphosphate receptor type 1
Ras GTPase-activating protein 3
Plastin-1
Interferon regulatory factor 3
EKC/KEOPS complex subunit LAGE3
Bridge-like lipid transfer protein family member 2
E3 ubiquitin-protein ligase TRIP12
Pumilio homolog 1
Mediator of DNA damage checkpoint protein 1
Clathrin interactor 1
KN motif and ankyrin repeat domain-containing protein 1
Structural maintenance of chromosomes protein 1A
Ribosomal RNA processing protein 1 homolog B
Disco-interacting protein 2 homolog A
Protein RRP5 homolog
DNA replication complex GINS protein PSF1
Ribosome biogenesis protein BMS1 homolog
Phosphatidate phosphatase LPIN1
Ubiquitin carboxyl-terminal hydrolase 10
LRP chaperone MESD
Neutral alpha-glucosidase AB
Raftlin
Major facilitator superfamily domain-containing protein 10
Serine/threonine-protein phosphatase 2A 56 kDa regulatory subunit delta isoform
Delta(14)-sterol reductase LBR
Conserved oligomeric Golgi complex subunit 2
Major vault protein
Latent-transforming growth factor beta-binding protein 1
Latent-transforming growth factor beta-binding protein 2
Chromobox protein homolog 2
Golgin subfamily B member 1
Caspase-8
N-alpha-acetyltransferase 30
Kinesin-like protein KIF22
Myocyte-specific enhancer factor 2D
Chromodomain-helicase-DNA-binding protein 4
LIM and SH3 domain protein 1
Prostaglandin reductase 1
Dr1-associated corepressor
Nuclear factor of activated T-cells, cytoplasmic 4
Zinc finger protein 638
Importin subunit beta-1
Nucleolar and coiled-body phosphoprotein 1
Nuclear mitotic apparatus protein 1

Proteasome activator complex subunit 4
Cullin-7
Sarcolemmal membrane-associated protein
GTPase-activating protein and VPS9 domain-containing protein 1
N-alpha-acetyltransferase 25, NatB auxiliary subunit
Ventricular zone-expressed PH domain-containing protein homolog 1
Condensin complex subunit 2
PCNA-associated factor
Signal peptidase complex subunit 2
ER membrane protein complex subunit 2
Pre-mRNA-splicing regulator WTAP
26S proteasome non-ATPase regulatory subunit 6
Homocysteine-responsive endoplasmic reticulum-resident ubiquitin-like domain member 1 protein
MAD2L1-binding protein
Mortality factor 4-like protein 2
BRISC complex subunit Abraxas 2
Septin-2
Squamous cell carcinoma antigen recognized by T-cells 3
Condensin complex subunit 1
Polycomb protein SUZ12
Exosome complex component RRP42
TNFAIP3-interacting protein 1
116 kDa U5 small nuclear ribonucleoprotein component
Leucine--tRNA ligase, mitochondrial
R3H domain-containing protein 1
Translocating chain-associated membrane protein 2
Sorting nexin-17
ADP-ribosylation factor-like protein 6-interacting protein 1
Rab3 GTPase-activating protein catalytic subunit
Metal cation symporter ZIP14
Lysine--tRNA ligase
Histone-lysine N-methyltransferase SETDB1
Leucine-rich repeat-containing protein 14
Ribosome biogenesis regulatory protein homolog
DNA polymerase delta subunit 3
Eukaryotic translation initiation factor 4H
Arf-GAP with coiled-coil, ANK repeat and PH domain-containing protein 2
Kinesin-like protein KIF14
Bromodomain-containing protein 3
WD repeat-containing protein 43
Peroxisomal acyl-coenzyme A oxidase 1
Mitochondrial inner membrane protein OXA1L
Early endosome antigen 1
Protein disulfide-isomerase A6
Platelet-activating factor acetylhydrolase IB subunit alpha1
[Pyruvate dehydrogenase (acetyl-transferring)] kinase isozyme 3, mitochondrial
Astrocytic phosphoprotein PEA-15
3-beta-hydroxysteroid-Delta(8),Delta(7)-isomerase
Phosphomevalonate kinase
Serine/threonine-protein kinase D1

Plectin
Pericentriolar material 1 protein
BOS complex subunit NOMO1
Serum paraoxonase/arylesterase 2
Serine/threonine-protein phosphatase 2A 56 kDa regulatory subunit alpha isoform
Inorganic pyrophosphatase
Prostaglandin E synthase 3
Serine/threonine-protein kinase 38
Non-POU domain-containing octamer-binding protein
Serine/threonine-protein phosphatase 2A activator
Receptor-type tyrosine-protein phosphatase kappa
Periodic tryptophan protein 2 homolog
Rab GTPase-binding effector protein 1
Ras-related protein Rab-35
RNA-binding protein with serine-rich domain 1
Retinoblastoma-binding protein 5
Reticulocalbin-1
RalA-binding protein 1
Lethal(2) giant larvae protein homolog 1
Leucine-rich repeat-containing protein 41
Transcription termination factor 1
Transmembrane emp24 domain-containing protein 2
Poly(rC)-binding protein 1
Poly(rC)-binding protein 2
Elongin-C
Elongin-B
GTP-binding protein Rheb
Ubiquitin-protein ligase E3C
Mitochondrial import receptor subunit TOM20 homolog
Mitochondrial fission regulator 1
Delta(24)-sterol reductase
Splicing factor 3B subunit 3
Pumilio homolog 3
Disks large-associated protein 5
Ras suppressor protein 1
Calponin-3
Ribosomal protein S6 kinase alpha-1
Scaffold attachment factor B1
Splicing factor 3A subunit 2
RNA-binding motif, single-stranded-interacting protein 2
Protein phosphatase 1 regulatory subunit 7
Protein transport protein Sec23A
Protein transport protein Sec23B
Cytohesin-1
Splicing factor 3A subunit 1
Superkiller complex protein 2
Corneodesmosin
Surfeit locus protein 1
Transcription initiation factor TFIID subunit 5
Transcription initiation factor TFIID subunit 7

Telomeric repeat-binding factor 2
Microtubule-associated protein RP/EB family member 2
Homeobox protein TGIF1
Na(+)/H(+) exchange regulatory cofactor NHE-RF2
Tumor necrosis factor receptor type 1-associated DEATH domain protein
Translocating chain-associated membrane protein 1
Translin
RISC-loading complex subunit TARBP2
Splicing factor 1
Cdc42-interacting protein 4
Thyroid receptor-interacting protein 11
Pachytene checkpoint protein 2 homolog
Mediator of RNA polymerase II transcription subunit 1
Activating signal cointegrator 1
High mobility group nucleosome-binding domain-containing protein 3
Probable JmjC domain-containing histone demethylation protein 2C
NF-kappa-B inhibitor beta
Thyroid receptor-interacting protein 6
Tyrosine-protein phosphatase non-receptor type 14
Microtubule-associated protein RP/EB family member 1
ELAV-like protein 1
Sterol-4-alpha-carboxylate 3-dehydrogenase, decarboxylating
NGFI-A-binding protein 2
Myosin light chain kinase, smooth muscle
TGF-beta-activated kinase 1 and MAP3K7-binding protein 1
Probable E3 ubiquitin-protein ligase HERC1
Neutral amino acid transporter B(0)
Myeloid leukemia factor 2
Mitochondrial import receptor subunit TOM34
Mothers against decapentaplegic homolog 2
Mothers against decapentaplegic homolog 1
Methylsterol monooxygenase 1
Intersectin-1
Tubulin-specific chaperone E
Tubulin-specific chaperone C
Ubiquitin-conjugating enzyme E2 variant 2
Serine/threonine-protein kinase STK11
Syntaxin-binding protein 2
Vesicle-associated membrane protein 3
NEDD8
V-type proton ATPase subunit S1
Vacuolar protein sorting-associated protein 72 homolog
Ras-related protein Rab-11B
Zyxin
Electron transfer flavoprotein-ubiquinone oxidoreductase, mitochondrial
Septin-7
Proteasomal ubiquitin receptor ADRM1
Coiled-coil domain-containing protein 6
UDP-N-acetylhexosamine pyrophosphorylase
Transcription factor E2F4

Insulin-like growth factor-binding protein 7
Programmed cell death protein 2
Laminin subunit alpha-4
26S proteasome non-ATPase regulatory subunit 5
Serine/threonine-protein kinase N1
Serine/threonine-protein kinase N2
Transcription initiation factor TFIID subunit 12
Cysteine and glycine-rich protein 2
DNA damage-binding protein 1
Serine/threonine-protein phosphatase 2A 56 kDa regulatory subunit epsilon isoform
Mitogen-activated protein kinase 14
Large ribosomal subunit protein uL23m
Hsp90 co-chaperone Cdc37
Dihydropyrimidinase-related protein 2
Synaptophysin-like protein 1
Histone-binding protein RBBP7
Mitogen-activated protein kinase kinase kinase 11
Transcription initiation factor TFIID subunit 9
Calcitonin gene-related peptide type 1 receptor
Bcl-2 homologous antagonist/killer
Serine/arginine-rich splicing factor 7
Cleavage and polyadenylation specificity factor subunit 6
Survival motor neuron protein
Drebrin
Nuclear respiratory factor 1
Fascin
Gamma-interferon-inducible protein 16
2,4-dienoyl-CoA reductase [(3E)-enoyl-CoA-producing], mitochondrial
Alpha-mannosidase 2
NADH dehydrogenase [ubiquinone] 1 alpha subcomplex subunit 5
Ceramide glucosyltransferase
ATP-dependent Clp protease proteolytic subunit, mitochondrial
Thiosulfate sulfurtransferase
Ubiquitin-conjugating enzyme E2 S
Kynurenine--oxoglutarate transaminase 1
Guanylate kinase
Hydroxyacylglutathione hydrolase, mitochondrial
NADH dehydrogenase [ubiquinone] 1 alpha subcomplex subunit 9, mitochondrial
NADP-dependent malic enzyme, mitochondrial
Phosphoenolpyruvate carboxykinase [GTP], mitochondrial
Uridine phosphorylase 1
Discoidin domain-containing receptor 2
Hydroxyacyl-coenzyme A dehydrogenase, mitochondrial
Lanosterol 14-alpha demethylase
UTP--glucose-1-phosphate uridylyltransferase
6-phosphofructo-2-kinase/fructose-2,6-bisphosphatase 3
Thioredoxin reductase 1, cytoplasmic
MICOS complex subunit MIC60
Endothelial cell-specific chemotaxis regulator
Lysine-rich nucleolar protein 1

Heterogeneous nuclear ribonucleoprotein U-like protein 2
Transmembrane protein 132A
Inverted formin-2
Sister chromatid cohesion protein PDS5 homolog A
Protein CLEC16A
Kinesin-like protein KIF7
Rho GTPase-activating protein 31
Methenyltetrahydrofolate synthase domain-containing protein
AP2-associated protein kinase 1
WASH complex subunit 4
DNA excision repair protein ERCC-6-like
Pre-rRNA-processing protein TSR1 homolog
Ral GTPase-activating protein subunit alpha-2
Protein C-mannosyl-transferase DPY19L1
Isoamyl acetate-hydrolyzing esterase 1 homolog
GDP-Man:Man(3)GlcNAc(2)-PP-Dol alpha-1,2-mannosyltransferase
Transcriptional regulator QRICH1
WD40 repeat-containing protein SMU1
Cytoplasmic tRNA 2-thiolation protein 2
Protein-glucosylgalactosylhydroxylysine glucosidase
Leucine-rich repeat flightless-interacting protein 1
CDP-diacylglycerol--glycerol-3-phosphate 3-phosphatidyltransferase, mitochondrial
UPF0711 protein C18orf21
Prolyl 3-hydroxylase 1
tRNA (guanine(37)-N1)-methyltransferase
Echinoderm microtubule-associated protein-like 3
DNA-directed RNA polymerase I subunit RPA43
MAP7 domain-containing protein 1
UDP-N-acetylhexosamine pyrophosphorylase-like protein 1
Chromodomain-helicase-DNA-binding protein 9
Triokinase/FMN cyclase
Protein LSM12
Inactive hydroxysteroid dehydrogenase-like protein 1
Mitochondrial 10-formyltetrahydrofolate dehydrogenase
N-acetylglucosamine-1-phosphotransferase subunits alpha/beta
Girdin
Rab-like protein 6
Mitochondrial import inner membrane translocase subunit TIM50
Protein mono-ADP-ribosyltransferase PARP14
Alpha-(1,3)-fucosyltransferase 11
Cytokine-like nuclear factor N-PAC
UPF0489 protein C5orf22
C2 domain-containing protein 3
Vacuolar protein sorting-associated protein 26B
La-related protein 7
NAD kinase 2, mitochondrial
BTB/POZ domain-containing protein KCTD21
Glucoside xylosyltransferase 1
Malonate--CoA ligase ACSF3, mitochondrial
Prolyl endopeptidase-like

TBC1 domain family member 10B
Taperin
Anoctamin-6
Filamin A-interacting protein 1-like
GRIP1-associated protein 1
Zinc-regulated GTPase metalloprotein activator 1F
Protein MIX23
Membralin
Protein FAM98B
Rho GTPase-activating protein 29
Programmed cell death protein 4
Fibronectin type III domain-containing protein 3B
CREB-regulated transcription coactivator 2
Glycerol-3-phosphate acyltransferase 3
Centrosomal protein of 55 kDa
Nuclear cap-binding protein subunit 3
Quinone oxidoreductase PIG3
Novel acetylcholine receptor chaperone
Pleckstrin homology-like domain family A member 2
PDZ and LIM domain protein 3
Protein mono-ADP-ribosyltransferase PARP10
Very-long-chain 3-oxoacyl-CoA reductase
mRNA export factor GLE1
Ubiquitin carboxyl-terminal hydrolase 39
Kelch-like protein 22
Acylglycerol kinase, mitochondrial
Histone-lysine N-methyltransferase SETMAR
Endoribonuclease LACTB2
Pyrroline-5-carboxylate reductase 3
EARP and GARP complex-interacting protein 1
Borealin
Cordon-bleu protein-like 1
HCLS1-binding protein 3
Plasma membrane ascorbate-dependent reductase CYBRD1
WD repeat-containing protein 81
Beta-actin-like protein 2
Coiled-coil domain-containing protein 93
OCIA domain-containing protein 2
Puratrophin-1
Putative heat shock protein HSP 90-alpha A4
DDB1- and CUL4-associated factor 6
Transmembrane protein 41B
Sigma intracellular receptor 2
Protein YIF1B
DBIRD complex subunit ZNF326
Nucleolar MIF4G domain-containing protein 1
RILP-like protein 1
DnaJ homolog subfamily C member 21
Tumor necrosis factor alpha-induced protein 8-like protein 3
Probable E3 ubiquitin-protein ligase HERC4

GPI ethanolamine phosphate transferase 2
Serine/threonine-protein phosphatase 6 regulatory subunit 3
Rab-like protein 3
Protein FAM76B
SH3 domain-containing protein 19
NHS-like protein 2
ER membrane protein complex subunit 4
Nondiscriminating glutamyl-tRNA synthetase EARS2, mitochondrial
Uncharacterized protein C3orf38
EEF1A lysine methyltransferase 2
Transport and Golgi organization protein 1 homolog
Presequence protease, mitochondrial
WD repeat-containing protein 44
Protein PRRC2B
Tight junction-associated protein 1
RRP12-like protein
Cytochrome c oxidase assembly factor 6 homolog
Torsin-1A-interacting protein 1
Centrosomal protein of 78 kDa
Alanine--tRNA ligase, mitochondrial
Torsin-2A
PCI domain-containing protein 2
Intracellular hyaluronan-binding protein 4
Long-chain fatty acid transport protein 3
Sterile alpha motif domain-containing protein 9
Serine/threonine-protein phosphatase 4 regulatory subunit 3B
WD repeat domain phosphoinositide-interacting protein 3
WD repeat domain phosphoinositide-interacting protein 1
EGF domain-specific O-linked N-acetylglucosamine transferase
Protein Smaug homolog 2
Tubulin-specific chaperone cofactor E-like protein
Deoxynucleotidyltransferase terminal-interacting protein 2
Rab GTPase-activating protein 1-like
Tetratricopeptide repeat protein 38
Cytochrome c oxidase assembly protein COX20, mitochondrial
Exosome complex component MTR3
Transmembrane protein 201
Alpha-tubulin N-acetyltransferase 1
Synaptosomal-associated protein 47
Nucleoporin NUP188
Heterochromatin protein 1-binding protein 3
Valine--tRNA ligase, mitochondrial
Centrosomal protein of 170 kDa
Low density lipoprotein receptor adapter protein 1
Protein odr-4 homolog
Polynucleotide 5'-hydroxyl-kinase NOL9
NHS-like protein 1
Putative hydroxypyruvate isomerase
Tumor protein p63-regulated gene 1-like protein
Coiled-coil and C2 domain-containing protein 1B

Formin-binding protein 1-like
Probable arginine--tRNA ligase, mitochondrial
Acyl-coenzyme A thioesterase THEM4
Putative coiled-coil-helix-coiled-coil-helix domain-containing protein CHCHD2P9, mitochondrial
FK506-binding protein 15
Zinc finger CCCH domain-containing protein 13
Putative methyltransferase C9orf114
Armadillo-like helical domain-containing protein 3
CSC1-like protein 2
G patch domain-containing protein 4
Putative transferase CAF17, mitochondrial
E3 ubiquitin-protein ligase HECTD3
Inactive glycosyltransferase 25 family member 3
E3 ubiquitin-protein ligase UBR4
Syntaxin-binding protein 5
Rho GTPase-activating protein 21
Calmodulin-regulated spectrin-associated protein 1
Large ribosomal subunit protein uL2m
Ubiquitin-associated protein 2
Queuosine 5'-phosphate N-glycosylase/hydrolase
Keratinocyte proline-rich protein
Zinc finger protein 618
Acyl-CoA-binding domain-containing protein 5
RNA-binding protein 26
ATPase family AAA domain-containing protein 3B
Protein wntless homolog
Integrator complex subunit 11
Ceramide-1-phosphate transfer protein
DDB1- and CUL4-associated factor 8
Collagen alpha-1(XIII) chain
Terminal uridylyltransferase 4
Protein furry homolog
Ribonuclease H2 subunit B
ATP synthase mitochondrial F1 complex assembly factor 1
Roquin-1
Protein DDI1 homolog 2
Arginine-hydroxylase NDUFAF5, mitochondrial
5'-nucleotidase domain-containing protein 1
Brefeldin A-inhibited guanine nucleotide-exchange protein 3
Intermembrane lipid transfer protein VPS13D
Rootletin
Complex III assembly factor LYRM7
Ras-interacting protein 1
Telomere-associated protein RIF1
Vacuolar protein sorting-associated protein 53 homolog
Striatin-interacting protein 1
Serine/threonine-protein kinase MRCK alpha
Regulation of nuclear pre-mRNA domain-containing protein 2
E3 ubiquitin-protein ligase RNF220
Pre-mRNA-splicing factor 38B

E3 ubiquitin-protein ligase BRE1A
Myomegalin
Zinc finger protein 318
Protein FAM171A1
Divergent protein kinase domain 1B
Leucine-rich repeat and calponin homology domain-containing protein 2
Threonylcarbamoyladenosine tRNA methylthiotransferase
BRO1 domain-containing protein BROX
Focadhesin
Lysine-specific demethylase 9
Disabled homolog 2-interacting protein
Lysophospholipase-like protein 1
Platelet endothelial aggregation receptor 1
Proteasome adapter and scaffold protein ECM29
Terminal uridylyltransferase 7
SH2 domain-containing adapter protein E
DENN domain-containing protein 4C
N-alpha-acetyltransferase 35, NatC auxiliary subunit
Zinc finger MYM-type protein 4
FHF complex subunit HOOK interacting protein 2A
Palmitoyltransferase ZDHHC20
SPRY domain-containing protein 7
F-box/WD repeat-containing protein 9
CD276 antigen
E3 ubiquitin-protein ligase RNF213
Tensin-2
KN motif and ankyrin repeat domain-containing protein 2
WASH complex subunit 2A
Lysophospholipid acyltransferase LPCAT4
Metalloreductase STEAP3
Protein FAM91A1
TBC1 domain family member 9B
Microtubule-associated protein 1S
Serine/threonine-protein phosphatase 2A 55 kDa regulatory subunit B delta isoform
ADP-ribosylation factor-like protein 6-interacting protein 4
Autophagy-related protein 16-1
Heparan-alpha-glucosaminide N-acetyltransferase
AT-rich interactive domain-containing protein 2
U3 small nucleolar RNA-associated protein 25 homolog
Glycosyltransferase 8 domain-containing protein 1
Protein sel-1 homolog 3
Tensin-3
HAUS augmin-like complex subunit 3
Protein SPT2 homolog
Acyl-coenzyme A thioesterase MBLAC2
Very large A-kinase anchor protein
Integrator complex subunit 3
Rho GTPase-activating protein 17
Protein NCBP2AS2
CWF19-like protein 1

Protein virilizer homolog
Cytospin-A
HEAT repeat-containing protein 6
Ankyrin repeat domain-containing protein 40
Protein WWC2
Atlastin-3
Ribosomal protein uL30-like
Nucleoredoxin
Tetratricopeptide repeat protein 19, mitochondrial
IQ motif and SEC7 domain-containing protein 1
Anamorsin
AN1-type zinc finger protein 6
Histone-lysine N-trimethyltransferase SMYD5
OTU domain-containing protein 7B
THO complex subunit 7 homolog
Glutamine-dependent NAD(+) synthetase
Elongator complex protein 2
Ragulator complex protein LAMTOR1
Dehydrogenase/reductase SDR family member 7B
Twinfilin-2
Condensin-2 complex subunit H2
Uncharacterized protein KIAA0930
Transport and Golgi organization protein 2 homolog
Serine/threonine-protein phosphatase 4 regulatory subunit 3A
Ras-related protein Rab-12
DENN domain-containing protein 5A
Splicing regulator SDE2
Keratin, type II cytoskeletal 80
Nipped-B-like protein
Mitochondrial adenyl nucleotide antiporter SLC25A25
2',5'-phosphodiesterase 12
N-alpha-acetyltransferase 16, NatA auxiliary subunit
Putative DENN domain-containing protein 10 B
Rhomboid domain-containing protein 2
Mitochondrial adenyl nucleotide antiporter SLC25A24
Receptor expression-enhancing protein 3
All-trans-retinol 13,14-reductase
RAD50-interacting protein 1
Transmembrane protein 214
3-hydroxyisobutyryl-CoA hydrolase, mitochondrial
RWD domain-containing protein 4
Nucleolus and neural progenitor protein
Armadillo repeat-containing protein 6
TELO2-interacting protein 2
Ankyrin repeat domain-containing protein 54
Zinc transporter 6
Transmembrane anterior posterior transformation protein 1 homolog
KN motif and ankyrin repeat domain-containing protein 3
Bifunctional arginine demethylase and lysyl-hydroxylase JMJD6
Phostensin

Caveolae-associated protein 1
Zinc finger CCHC domain-containing protein 8
Putative ATP-dependent RNA helicase DHX57
Endoplasmic reticulum aminopeptidase 2
Lysophospholipid acyltransferase 5
Parafibromin
Large ribosomal subunit protein uL14m
Long-chain fatty acid transport protein 4
Coiled-coil and C2 domain-containing protein 1A
Deoxyribonuclease TATDN1
tRNA N(3)-methylcytidine methyltransferase METTL2B
Myb/SANT-like DNA-binding domain-containing protein 2
Transcription initiation factor TFIID subunit 2
UPF0598 protein C8orf82
Mediator of RNA polymerase II transcription subunit 27
Enhancer of mRNA-decapping protein 4
Centrosomal protein of 85 kDa
Protein arginine N-methyltransferase 9
Pre-mRNA-processing-splicing factor 8
F-box only protein 42
SCY1-like protein 2
Tetratricopeptide repeat protein 27
Sideroflexin-4
Protein GOLM2
Nuclear factor related to kappa-B-binding protein
Acylpyruvase FAHD1, mitochondrial
Ribosomal protein eL22-like
Serine/threonine-protein kinase N3
RNA demethylase ALKBH5
Pyridoxal-dependent decarboxylase domain-containing protein 1
MTOR-associated protein MEAK7
Integrator complex subunit 5
GTPase IMAP family member 6
Long-chain fatty acid transport protein 1
Glucose 1,6-bisphosphate synthase
RNA polymerase-associated protein CTR9 homolog
Alpha- and gamma-adaptin-binding protein p34
Transmembrane protein 88
Superkiller complex protein 3
Serine/threonine-protein kinase ULK3
Aspartate--tRNA ligase, mitochondrial
Transmembrane protein 65
INO80 complex subunit C
F-box only protein 38
Neutral cholesterol ester hydrolase 1
Fidgetin-like protein 1
E3 ubiquitin-protein ligase TRIM65
Inactive rhomboid protein 2
Mitotic deacetylase-associated SANT domain protein
BRCA1-associated ATM activator 1

GATOR2 complex protein WDR59
Zinc finger CCCH domain-containing protein 14
Coiled-coil domain-containing protein 137
2-oxoglutarate and iron-dependent oxygenase domain-containing protein 3
Thioredoxin domain-containing protein 11
La-related protein 1
ATPase family AAA domain-containing protein 2
Protein TMED8
Proton-coupled zinc antiporter SLC30A9, mitochondrial
E3 ubiquitin-protein ligase TRAF7
Rapamycin-insensitive companion of mTOR
WD repeat-containing protein 74
Type-1 angiotensin II receptor-associated protein
SWI/SNF-related matrix-associated actin-dependent regulator of chromatin subfamily D member 3
Monofunctional C1-tetrahydrofolate synthase, mitochondrial
Aftiphilin
Pre-mRNA 3'-end-processing factor FIP1
CREB-regulated transcription coactivator 3
CREB-regulated transcription coactivator 1
Cytochrome P450 20A1
Protein O-glucosyltransferase 2
Transmembrane protein 205
E3 ubiquitin-protein ligase LRSAM1
Golgi-associated kinase 1B
Lysocardiolipin acyltransferase 1
Spliceosome-associated protein CWC27 homolog
Protein disulfide isomerase CRELD2
WD repeat-containing protein 82
MICOS complex subunit MIC27
Protocadherin Fat 4
Dipeptidyl peptidase 8
Activating transcription factor 7-interacting protein 1
Ran-binding protein 10
Phosphofurin acidic cluster sorting protein 1
Myosin phosphatase Rho-interacting protein
RNA-binding motif, single-stranded-interacting protein 3
Nicotinate phosphoribosyltransferase
Dynamin-binding protein
Very-long-chain (3R)-3-hydroxyacyl-CoA dehydratase 2
Beta-1,3-glucosyltransferase
GRB10-interacting GYF protein 2
CD109 antigen
tRNA (32-2'-O)-methyltransferase regulator THADA
Hydroxysteroid dehydrogenase-like protein 2
Kynurenine--oxoglutarate transaminase 3
Protein phosphatase 1 regulatory subunit 21
Thrombospondin type-1 domain-containing protein 4
Nesprin-3
Zinc finger protein 574
NF-X1-type zinc finger protein NFXL1

Neurobeachin-like protein 2
FYVE, RhoGEF and PH domain-containing protein 5
Sulfhydryl oxidase 2
Helicase SRCAP
Rho family-interacting cell polarization regulator 1
Uncharacterized protein FLJ45252
Rho guanine nucleotide exchange factor 18
TBC1 domain family member 9
E3 ubiquitin-protein ligase UBR3
Transmembrane protein with metallophosphoesterase domain
Capping protein-inhibiting regulator of actin dynamics
Coiled-coil domain-containing protein 9B
8-oxo-dGDP phosphatase NUDT18
PAX-interacting protein 1
Syntaxin-binding protein 4
Protein O-mannosyl-transferase TMTC3
Intermembrane lipid transfer protein VPS13C
Ubiquitin carboxyl-terminal hydrolase 34
Ras-associated and pleckstrin homology domains-containing protein 1
MOB kinase activator 2
Inhibitor of nuclear factor kappa-B kinase-interacting protein
Phosphatidylinositol 3,4,5-trisphosphate-dependent Rac exchanger 2 protein
Protein FRA10AC1
Ubiquitin-conjugating enzyme E2 R2
La-related protein 4
Mediator of RNA polymerase II transcription subunit 25
Ribosomal protein eS27-like
E3 ubiquitin-protein ligase Hakai
Integrator complex subunit 8
Butyrophilin subfamily 2 member A1
Transcription elongation factor SPT6
Staphylococcal nuclease domain-containing protein 1
Serine/threonine-protein kinase MARK2
Cytochrome c oxidase assembly protein COX15 homolog
Probable ATP-dependent RNA helicase DDX46
Protein RUFY3
tRNA methyltransferase 10 homolog C
eIF5-mimic protein 2
Vacuolar fusion protein MON1 homolog B
Volume-regulated anion channel subunit LRRC8D
Isoaspartyl peptidase/L-asparaginase
BTB/POZ domain-containing protein KCTD9
ATP-dependent RNA helicase DHX30
Eukaryotic translation initiation factor 3 subunit M
7SK snRNA methylphosphate capping enzyme
Armadillo repeat-containing X-linked protein 2
Probable proline--tRNA ligase, mitochondrial
Arginine/serine-rich coiled-coil protein 2
Ras-related GTP-binding protein A
Cytoplasmic FMR1-interacting protein 1

Protein arginine methyltransferase NDUFAF7, mitochondrial
Golgi to ER traffic protein 4 homolog
COP9 signalosome complex subunit 6
Lysophosphatidylcholine acyltransferase 2
Mitochondrial enolase superfamily member 1
E3 ubiquitin-protein transferase MAEA
EPM2A-interacting protein 1
Putative pre-mRNA-splicing factor ATP-dependent RNA helicase DHX32
Serine/threonine-protein kinase TAO1
FAST kinase domain-containing protein 5, mitochondrial
MOB kinase activator 1B
Lysine-specific demethylase 3B
Charged multivesicular body protein 1b
Ribonucleoside-diphosphate reductase subunit M2 B
Heparan sulfate 2-O-sulfotransferase 1
STE20-related kinase adapter protein alpha
[F-actin]-monooxygenase MICAL3
Dymeclin
TKeratin, type II cytoskeletal 78
PHD finger-like domain-containing protein 5A
Peroxiredoxin-like 2C
Aprataxin
Endoplasmic reticulum metallopeptidase 1
G protein-regulated inducer of neurite outgrowth 1
TRMT1-like protein
Zinc finger CCCH-type antiviral protein 1
Large ribosomal subunit protein bL21m
Elongation factor-like GTPase 1
Probable helicase senataxin
Trafficking protein particle complex subunit 11
KAT8 regulatory NSL complex subunit 1
Nucleoporin p54
Autophagy-related protein 9A
D-glutamate cyclase, mitochondrial
Coiled-coil domain-containing protein 186
LisH domain-containing protein ARMC9
VPS35 endosomal protein-sorting factor-like
Pogo transposable element with ZNF domain
Zinc finger FYVE domain-containing protein 16
Protein MON2 homolog
C-myc promoter-binding protein
Myosin-14
FMR1-interacting protein NUFIP2
SUZ domain-containing protein 1
Mitochondrial antiviral-signaling protein
CLIP-associating protein 1
ATP-dependent RNA helicase DHX29
Low-density lipoprotein receptor-related protein 10
HAUS augmin-like complex subunit 6
Protein O-glucosyltransferase 3

Tetratricopeptide repeat protein 21B
HEAT repeat-containing protein 3
Kinesin-like protein KIF21A
Hepatoma-derived growth factor-related protein 2
L-xylulose reductase
BRCA1-associated protein
BRCA2-interacting transcriptional repressor EMSY
Golgin subfamily A member 7
Rho GTPase-activating protein 22
Wings apart-like protein homolog
Interferon regulatory factor 2-binding protein 2
E3 ubiquitin-protein ligase RBBP6
E3 ubiquitin-protein ligase SH3RF1
Protein TAMALIN
FERM domain-containing protein 5
tRNA-splicing endonuclease subunit Sen54
Protein prenyltransferase alpha subunit repeat-containing protein 1
Arpin
Tectonin beta-propeller repeat-containing protein 1
Rab9 effector protein with kelch motifs
Transcription termination factor 4, mitochondrial
E3 ubiquitin-protein ligase HUWE1
YTH domain-containing family protein 3
Cytoplasmic tRNA 2-thiolation protein 1
Transcription initiation factor TFIID subunit 8
Ubiquitin-conjugating enzyme E2 Q1
Large ribosomal subunit protein mL55
Transmembrane emp24 domain-containing protein 4
Large ribosomal subunit protein uL10m
COX assembly mitochondrial protein homolog
Centromere protein V
Schlafen family member 11
Multiple epidermal growth factor-like domains protein 8
Transmembrane protein 179B
N-acetylgalactosaminyltransferase 7
Stearoyl-CoA desaturase 5
Pleckstrin homology-like domain family B member 2
Adhesion G-protein coupled receptor G6
Dehydrodolichyl diphosphate synthase complex subunit DHDDS
Polypeptide N-acetylgalactosaminyltransferase 10
Trafficking protein particle complex subunit 6B
Type 1 phosphatidylinositol 4,5-bisphosphate 4-phosphatase
C-type lectin domain family 14 member A
Transcriptional regulator Kaiso
Protein PAT1 homolog 1
Dipeptidyl peptidase 9
E3 ubiquitin-protein ligase synoviolin
tRNA 2'-phosphotransferase 1
Actin-histidine N-methyltransferase
Tetratricopeptide repeat protein 7B

Acyl-coenzyme A thioesterase 1
Nucleolar protein 9
Polyadenylate-binding protein 2
N6-adenosine-methyltransferase catalytic subunit
Protein polybromo-1
Threonylcarbamoyl-AMP synthase
Pre-mRNA-processing factor 39
Protein LYRIC
E3 ubiquitin-protein ligase ZNF598
Glycerol-3-phosphate acyltransferase 4
Kinectin
Telomerase-binding protein EST1A
NLR family member X1
B-cell CLL/lymphoma 9-like protein
Pleckstrin homology-like domain family B member 1
Ubiquitin carboxyl-terminal hydrolase 48
Calcium-dependent secretion activator 2
Fermitin family homolog 3
N-alpha-acetyltransferase 40
5'-nucleotidase domain-containing protein 3
Acetoacetyl-CoA synthetase
Leucine zipper protein 1
THO complex subunit 4
Integral membrane protein GPR180
FHF complex subunit HOOK-interacting protein 2B
Ras GTPase-activating-like protein IQGAP3
Zinc finger CCCH domain-containing protein 18
Vacuolar protein-sorting-associated protein 36
Cullin-associated NEDD8-dissociated protein 1
Reticulophagy regulator 3
Protein Hook homolog 3
SEC14 domain and spectrin repeat-containing protein 1
COMM domain-containing protein 7
Vacuolar fusion protein MON1 homolog A
Archaemetzincin-2
THO complex subunit 6 homolog
RNA N6-adenosine-methyltransferase METTL16
Poly(ADP-ribose) glycohydrolase
Liprin-beta-1
Lon protease homolog 2, peroxisomal
Zinc finger C3HC-type protein 1
Chromodomain-helicase-DNA-binding protein 1-like
F-BAR and double SH3 domains protein 1
Coiled-coil domain-containing protein 25
Proline and serine-rich protein 2
Stimulator of interferon genes protein
Active regulator of SIRT1
Cerebellar degeneration-related protein 2-like
Ral GTPase-activating protein subunit beta
Ras-specific guanine nucleotide-releasing factor RalGPS2

Histone-arginine methyltransferase CARM1
Deaminated glutathione amidase
COMM domain-containing protein 2
HEAT repeat-containing protein 5A
Condensin-2 complex subunit G2
Protein phosphatase 1 regulatory subunit 3B
F-box only protein 11
Ankyrin repeat and LEM domain-containing protein 2
RNA-binding protein MEX3D
Diacylglycerol kinase eta
ATP-dependent RNA helicase DDX42
Spermatogenesis-associated serine-rich protein 2
Serine/threonine-protein kinase VRK2
CDK2-associated and cullin domain-containing protein 1
NADH dehydrogenase [ubiquinone] 1 alpha subcomplex subunit 11
Dynein axonemal assembly factor 5
Peptidyl-tRNA hydrolase
Syntaxin-12
Transcriptional repressor p66-alpha
Copine-8
G-protein-signaling modulator 1
Ras-related protein Rab-43
C2 domain-containing protein 5
E3 ubiquitin-protein ligase MIB1
Inactive tyrosine-protein kinase PRAG1
BLOC-2 complex member HPS6
Interferon regulatory factor 2-binding protein 1
ELKS/Rab6-interacting/CAST family member 1
Ribosomal oxygenase 2
Palmitoyltransferase ZDHHC13
Palmitoyltransferase ZDHHC17
Cytokine receptor-like factor 3
Trafficking protein particle complex subunit 5
Armadillo repeat-containing protein 8
RELT-like protein 1
Complex I assembly factor TMEM126B, mitochondrial
5'-3' exonuclease PLD3
Protein HID1
Ankyrin repeat and MYND domain-containing protein 2
3'-5' exoribonuclease 1
Inactive serine/threonine-protein kinase VRK3
LIX1-like protein
NudC domain-containing protein 3
Protein AHNAK2
Formin-like protein 3
Methylmalonic aciduria type A protein, mitochondrial
Mitogen-activated protein kinase kinase kinase kinase 3
Neuron navigator 3
Prolyl 3-hydroxylase 3
Coiled-coil domain-containing protein 50

Malonyl-CoA-acyl carrier protein transacylase, mitochondrial
Kinase suppressor of Ras 1
Centrosomal protein of 97 kDa
ATP-dependent (S)-NAD(P)H-hydrate dehydratase
WD repeat-containing protein 75
Mitofusin-1
Choline transporter-like protein 2
Inositol 1,4,5-trisphosphate receptor-interacting protein
WD repeat and FYVE domain-containing protein 1
MAP7 domain-containing protein 3
Coiled-coil domain-containing protein 117
Protein NOXP20
DCN1-like protein 3
Protein DENND6A
MAX gene-associated protein
GRIP and coiled-coil domain-containing protein 2
Zinc finger CCCH domain-containing protein 7A
PHD finger protein 6
Protein archease
Volume-regulated anion channel subunit LRRC8A
Extracellular sulfatase Sulf-2
E3 ubiquitin-protein ligase UBR1
E3 ubiquitin-protein ligase UBR2
Rho GTPase-activating protein 12
Calcium homeostasis endoplasmic reticulum protein
Codanin-1
Ankyrin repeat and KH domain-containing protein 1
SURP and G-patch domain-containing protein 1
SURP and G-patch domain-containing protein 2
Ubiquitin-conjugating enzyme E2 variant 3
Cell division cycle and apoptosis regulator protein 1
Probable ATP-dependent RNA helicase DHX40
DnaJ homolog subfamily C member 10
Negative elongation factor C/D
Mitochondrial Rho GTPase 2
Mitochondrial Rho GTPase 1
NAD-dependent protein deacetylase sirtuin-2
Polyhomeotic-like protein 2
Chromatin complexes subunit BAP18
Large ribosomal subunit protein mL41
Nurim
GPALPP motifs-containing protein 1
Protein mono-ADP-ribosyltransferase PARP9
RNA-binding protein 12B
Solute carrier family 35 member F2
Putative RNA polymerase II subunit B1 CTD phosphatase RPAP2
Patatin-like phospholipase domain-containing protein 6
Structural maintenance of chromosomes protein 5
Probable ATP-dependent RNA helicase DDX60
C-Maf-inducing protein

MICAL-like protein 2
Probable ATP-dependent RNA helicase DHX37
Kelch repeat and BTB domain-containing protein 2
Ribonucleoprotein PTB-binding 1
pre-rRNA 2'-O-ribose RNA methyltransferase FTSJ3
Transmembrane protein 192
Nucleotidyltransferase MB21D2
Serine/arginine repetitive matrix protein 1
Stromal membrane-associated protein 1
DIS3-like exonuclease 2
ATP-dependent RNA helicase SUPV3L1, mitochondrial
Eukaryotic peptide chain release factor GTP-binding subunit ERF3B
ZZ-type zinc finger-containing protein 3
Exocyst complex component 8
Uncharacterized protein C10orf67, mitochondrial
E3 ubiquitin-protein ligase TRIM22
Threonine synthase-like 1
Uncharacterized protein KIAA2013
Calcium uptake protein 2, mitochondrial
Ankyrin repeat domain-containing protein 13A
Phosphatase and actin regulator 4
tRNA (uracil-5-)-methyltransferase homolog A
Pseudouridylate synthase RPUSD2
ELMO domain-containing protein 2
Aldehyde dehydrogenase family 16 member A1
Dyslexia-associated protein KIAA0319-like protein
Adhesion G protein-coupled receptor F5
5'-3' exoribonuclease 1
Proline-, glutamic acid- and leucine-rich protein 1
Abl interactor 1
WD repeat and FYVE domain-containing protein 3
Selenoprotein H
Vitamin K epoxide reductase complex subunit 1-like protein 1
Spartin
Tetratricopeptide repeat protein 5
Mesoderm induction early response protein 1
Regulatory-associated protein of mTOR
ORM1-like protein 3
Cell cycle and apoptosis regulator protein 2
NADH dehydrogenase [ubiquinone] 1 alpha subcomplex assembly factor 2
Vacuolar protein sorting-associated protein 52 homolog
Nuclear pore complex protein Nup93
Serine/threonine-protein kinase 11-interacting protein
Zinc finger protein 687
Cap-specific mRNA (nucleoside-2'-O-)-methyltransferase 1
Leucine-rich repeat-containing protein 47
Dedicator of cytokinesis protein 4
Carbonic anhydrase 13
Rho guanine nucleotide exchange factor 28
Integrator complex subunit 1

Histone H2B type 3-B
Rho GTPase-activating protein 24
Armadillo repeat-containing protein 10
Lysophosphatidylserine lipase ABHD12
CLK4-associating serine/arginine rich protein
Protein FAM43A
Transmembrane protein 256
E3 SUMO-protein ligase PIAS4
Glycerol-3-phosphate dehydrogenase 1-like protein
Solute carrier family 35 member F6
Rho GTPase-activating protein 18
Activating signal cointegrator 1 complex subunit 3
MICAL-like protein 1
Vacuolar protein sorting-associated protein 8 homolog
Protein PALS1
Cohesin subunit SA-2
Synaptopodin
Formin-binding protein 4
Translation factor GUF1, mitochondrial
RING1 and YY1-binding protein
Polypeptide N-acetylgalactosaminyltransferase 4
Misshapen-like kinase 1
Guanosine-3',5'-bis(diphosphate) 3'-pyrophosphohydrolase MESH1
Prostaglandin reductase 3
3-oxoacyl-[acyl-carrier-protein] reductase
ER membrane protein complex subunit 5
Transmembrane protein 199
F-box/LRR-repeat protein 6
Prolyl 3-hydroxylase OGFOD1
Actin filament-associated protein 1
Zinc finger CCHC domain-containing protein 9
Oxidation resistance protein 1
Zinc finger CCCH-type with G patch domain-containing protein
Calcium homeostasis modulator protein 5
S1 RNA-binding domain-containing protein 1
WD and tetratricopeptide repeats protein 1
NF-kappa-B-activating protein
Macoilin
SH2 domain-containing protein 3C
Arrestin domain-containing protein 1
Dehydrogenase/reductase SDR family member on chromosome X
CDGSH iron-sulfur domain-containing protein 2
Ribonuclease P protein subunit p25-like protein
ATP synthase mitochondrial F1 complex assembly factor 2
Tetratricopeptide repeat protein 9C
Protein jagunal homolog 1
E3 ubiquitin-protein ligase RNF10
Refilin-B
Leucine-zipper-like transcriptional regulator 1
COMM domain-containing protein 1

Cleavage and polyadenylation specificity factor subunit 7
Solute carrier family 15 member 4
Myc target protein 1
ADAMTS-like protein 1
ADP-ribosylation factor GTPase-activating protein 2
Deubiquitinase OTUD6B
Acyl-coenzyme A diphosphatase FITM2
UPF0690 protein C1orf52
eEF1A lysine and N-terminal methyltransferase
ADP-ribosylation factor-like protein 6-interacting protein 6
ADP-ribosylation factor GTPase-activating protein 1
NAD-dependent protein deacylase sirtuin-6
Solute carrier family 66 member 3
ER membrane protein complex subunit 1
RNA polymerase II-associated factor 1 homolog
Cyclin-Y-like protein 1
Putative E3 ubiquitin-protein ligase UBR7
ATP-dependent RNA helicase DDX51
Uncharacterized protein FAM241A
Mitochondrial basic amino acids transporter
Protein enabled homolog
Discoidin, CUB and LCCL domain-containing protein 1
G patch domain-containing protein 11
Large ribosomal subunit protein mL43
RNA ligase 1
Lysophospholipase D GDPD1
Uncharacterized protein C19orf47
Activating signal cointegrator 1 complex subunit 1
Leucine-rich repeat-containing protein 57
Probable RNA-binding protein EIF1AD
Protein KRI1 homolog
Coiled-coil domain-containing protein 71L
Zinc finger protein 579
Pre-mRNA-splicing factor 38A
Mixed lineage kinase domain-like protein
Glutamine amidotransferase-like class 1 domain-containing protein 1
Serine/threonine-protein phosphatase 6 regulatory ankyrin repeat subunit C
Phospholipid-transporting ATPase IG
ATPase family gene 2 protein homolog A
NHL repeat-containing protein 2
Late secretory pathway protein AVL9 homolog
Equilibrative nucleobase transporter 1
Xyloside xylosyltransferase 1
Golgi membrane protein 1
Procollagen galactosyltransferase 1
Inactive C-alpha-formylglycine-generating enzyme 2
SID1 transmembrane family member 2
Formylglycine-generating enzyme
Protein O-glucosyltransferase 1
Ubiquitin-associated domain-containing protein 2

Prenylcysteine oxidase-like
Transmembrane protein 87A
Retinol dehydrogenase 13
Tetratricopeptide repeat protein 13
Estradiol 17-beta-dehydrogenase 11
Thioredoxin domain-containing protein 5
Kinetochore protein Spc24
Outer mitochondrial transmembrane helix translocase
Saccharopine dehydrogenase-like oxidoreductase
UDP-glucuronic acid decarboxylase 1
E3 ubiquitin-protein ligase RNF149
Reticulophagy regulator 2
SERPINE1 mRNA-binding protein 1
LEM domain-containing protein 2
Nitric oxide-associated protein 1
Adaptin ear-binding coat-associated protein 1
Protein FAM98A
Phospholipase A2 group XV
Myotubularin-related protein 14
NFATC2-interacting protein
Diacylglycerol lipase-beta
Carbohydrate sulfotransferase 14
Polypeptide N-acetylgalactosaminyltransferase 6
E3 ubiquitin-protein ligase RNF169
Pyruvate dehydrogenase phosphatase regulatory subunit, mitochondrial
NAD(P)H-hydrate epimerase
Nonsense-mediated mRNA decay factor SMG8
RING finger protein 214
Protein LSM14 homolog A
GTPase IMAP family member 8
Cyclin-Y
Maestro heat-like repeat-containing protein family member 1
Terminal nucleotidyltransferase 4B
Probable aminopeptidase NPEPL1
EH domain-binding protein 1
Putative RNA-binding protein 15B
Alpha-N-acetylgalactosaminide alpha-2,6-sialyltransferase 3
Trinucleotide repeat-containing gene 6A protein
Polyhomeotic-like protein 3
Divergent protein kinase domain 2A
Metal transporter CNNM3
ATP-binding cassette sub-family F member 1
Calcium uniporter protein, mitochondrial
Phosphatidylinositol 3-kinase catalytic subunit type 3
Glutathione S-transferase C-terminal domain-containing protein
Serum response factor-binding protein 1
Neuroguidin
SHC SH2 domain-binding protein 1
PDZ domain-containing protein 8
DCC-interacting protein 13-beta

Zinc transporter 7
Neuron navigator 1
Periphilin-1
WD repeat-containing protein 19
F-box only protein 22
Lysophosphatidylcholine acyltransferase 1
Zinc finger MIZ domain-containing protein 2
Nesprin-1
Ubiquitin carboxyl-terminal hydrolase 32
Biorientation of chromosomes in cell division protein 1-like 1
AT-rich interactive domain-containing protein 1B
FAD synthase
Folliculin
Nucleoporin Nup43
Nucleoporin Nup37
Nucleoporin NUP35
Cytosolic endo-beta-N-acetylglucosaminidase
Retinoic acid-induced protein 3
Torsin-1A-interacting protein 2
sn-1-specific diacylglycerol lipase ABHD11
N-acylneuraminate cytidylyltransferase
Tetraspanin-14
Tubulin--tyrosine ligase
Small VCP/p97-interacting protein
Atlastin-2
Motile sperm domain-containing protein 2
Putative phospholipase B-like 2
Ras association domain-containing protein 8
ATP-dependent RNA helicase DDX55
Major facilitator superfamily domain-containing protein 8
Tudor domain-containing protein 7
GTPase IMAP family member 7
Protein NEDD1
Nuclear receptor coactivator 7
THO complex subunit 2
WD repeat-containing protein 36
Protein phosphatase PTC7 homolog
Retinitis pigmentosa 9 protein
Vang-like protein 1
Smad nuclear-interacting protein 1
Large ribosomal subunit protein mL64
WD repeat-containing protein 48
Exocyst complex component 6
SWI/SNF complex subunit SMARCC2
Nuclear protein localization protein 4 homolog
Uncharacterized protein CXorf38
Spermatogenesis-associated protein 20
Iron-sulfur cluster transfer protein NUBPL
(Lyso)-N-acylphosphatidylethanolamine lipase
F-box only protein 30

Adenosine 3'-phospho 5'-phosphosulfate transporter 1
Pumilio homolog 2
T-cell immunomodulatory protein
Golgin subfamily A member 5
Kelch domain-containing protein 4
SH3KBP1-binding protein 1
NEDD8-activating enzyme E1 catalytic subunit
Prostamide/prostaglandin F synthase
DnaJ homolog subfamily B member 14
Protein kish-A
Phosphatidylinositol 5-phosphate 4-kinase type-2 gamma
WD repeat-containing protein 20
TBC1 domain family member 15
Retinol dehydrogenase 11
Leucine-rich repeat-containing protein 20
Interferon-induced protein 44
Large ribosomal subunit protein uL30m
AN1-type zinc finger protein 1
Protein CIP2A
Phosphatidylinositol 4-kinase type 2-beta
Dolichyl-diphosphooligosaccharide--protein glycosyltransferase subunit STT3B
Polyribonucleotide nucleotidyltransferase 1, mitochondrial
Signal peptide peptidase-like 2B
Minor histocompatibility antigen H13
Phosphatidylinositol 3,4,5-trisphosphate-dependent Rac exchanger 1 protein
Up-regulator of cell proliferation
Protein bicaudal D homolog 2
Serine/threonine-protein kinase Nek9
Pleckstrin homology domain-containing family O member 2
E3 ubiquitin-protein ligase DTX3L
ATP-dependent RNA helicase DDX54
Disks large homolog 5
Ribosome biogenesis protein BRX1 homolog
Glucosamine-6-phosphate isomerase 2
Volume-regulated anion channel subunit LRRC8C
Serine/threonine-protein kinase Nek7
RB1-inducible coiled-coil protein 1
[F-actin]-monooxygenase MICAL1
Elongator complex protein 5
Protein phosphatase Slingshot homolog 3
SH3 domain and tetratricopeptide repeat-containing protein 1
TBC domain-containing protein kinase-like protein
D-aminoacyl-tRNA deacylase 1
DDB1- and CUL4-associated factor 11
U3 small nucleolar RNA-associated protein 15 homolog
Probable glutathione peroxidase 8
Actin filament-associated protein 1-like 1
E3 ubiquitin-protein ligase SH3RF3
Short transient receptor potential channel 4-associated protein
Centrosomal protein of 192 kDa

Gem-associated protein 5
GPI ethanolamine phosphate transferase 3
Rap guanine nucleotide exchange factor 6
Guanine nucleotide exchange protein SMCR8
Partitioning defective 3 homolog
Importin-4
Ubiquitin carboxyl-terminal hydrolase 33
Arginine/serine-rich protein PNISR
Serine/threonine-protein phosphatase 4 regulatory subunit 1
Ubiquitin-associated and SH3 domain-containing protein B
WAS/WASL-interacting protein family member 2
1-acylglycerol-3-phosphate O-acyltransferase ABHD5
Histone-lysine N-methyltransferase SETD7
Nucleolar complex protein 3 homolog
Scavenger receptor class B member 1
Conserved oligomeric Golgi complex subunit 1
Sec1 family domain-containing protein 2
Stromal membrane-associated protein 2
Zinc finger CCCH domain-containing protein 15
Peptidyl-prolyl cis-trans isomerase-like 4
General transcription factor 3C polypeptide 2
TBC1 domain family member 22A
PHD finger protein 10
Ras-related protein Rab-2B
Coiled-coil domain-containing protein 12
RelA-associated inhibitor
Cotranscriptional regulator ARB2A
Protein Churchill
Transforming growth factor-beta receptor-associated protein 1
Transmembrane protein 263
Histone deacetylase 7
Serine/threonine/tyrosine-interacting protein
Phosphatidylglycerophosphatase and protein-tyrosine phosphatase 1
Nuclear pore complex protein Nup133
Programmed cell death 6-interacting protein
Sodium-dependent phosphate transporter 1
Filamin-binding LIM protein 1
Splicing factor Cactin
Glutathione-specific gamma-glutamylcyclotransferase 2
Charged multivesicular body protein 7
Protein THEM6
Probable N-acetyltransferase 14
B-cell CLL/lymphoma 7 protein family member C
Non-structural maintenance of chromosomes element 1 homolog
MIT domain-containing protein 1
Chromosome transmission fidelity protein 18 homolog
RNA polymerase-associated protein LEO1
Dephospho-CoA kinase domain-containing protein
NudC domain-containing protein 2
U4/U6.U5 small nuclear ribonucleoprotein 27 kDa protein

Dimethyladenosine transferase 1, mitochondrial
Cohesin subunit SA-1
Sec1 family domain-containing protein 1
Tumor necrosis factor alpha-induced protein 8-like protein 1
Soluble calcium-activated nucleotidase 1
Trafficking protein particle complex subunit 12
Heterogeneous nuclear ribonucleoprotein L-like
Fatty acyl-CoA reductase 1
Ubiquitin-like domain-containing CTD phosphatase 1
tRNA-splicing endonuclease subunit Sen15
PEST proteolytic signal-containing nuclear protein
DnaJ homolog subfamily A member 4
SPRY domain-containing protein 4
m-AAA protease-interacting protein 1, mitochondrial
Pseudouridylate synthase TRUB1
LIM domain only protein 7
Choline transporter-like protein 1
Cytoskeleton-associated protein 2
Ataxin-2-like protein
Arf-GAP with Rho-GAP domain, ANK repeat and PH domain-containing protein 3
GTPase IMAP family member 1
PH-interacting protein
U4/U6 small nuclear ribonucleoprotein Prp31
Negative elongation factor B
Palladin
RUN and FYVE domain-containing protein 2
Splicing regulatory glutamine/lysine-rich protein 1
Gem-associated protein 6
Caskin-2
Paraspeckle component 1
MAP kinase-activating death domain protein
Nesprin-2
Transcriptional repressor p66-beta
Progesterone-induced-blocking factor 1
DnaJ homolog subfamily C member 9
BRI3-binding protein
Beta-catenin-like protein 1
Cysteine protease ATG4A
Ras and Rab interactor 2
Protein ELYS
Microprocessor complex subunit DGCR8
Titin
Roundabout homolog 4
Esterase OVCA2
Immunity-related GTPase family Q protein
DNA damage-binding protein 2
C-type lectin domain family 2 member B
ATP-dependent RNA helicase DDX1
SEC14-like protein 1
Piezo-type mechanosensitive ion channel component 1

Protein FAM3C
Histone H1.10
Proteasome inhibitor PI31 subunit
Golgi-specific brefeldin A-resistance guanine nucleotide exchange factor 1
Nonsense-mediated mRNA decay factor SMG7
RNA polymerase-associated protein RTF1 homolog
Nicastrin
Sorting nexin-19
Transmembrane 9 superfamily member 4
Transmembrane protein 131
Inositol hexakisphosphate kinase 1
Small ribosomal subunit protein mS27
Engulfment and cell motility protein 1
Protein FAM168A
AP-3 complex subunit sigma-1
Hamartin
UBX domain-containing protein 4
PHD finger protein 3
Protein NDRG1
Heat shock protein 105 kDa
Septin-8
CCR4-NOT transcription complex subunit 9
Acyl-CoA:lysophosphatidylglycerol acyltransferase 1
TBC1 domain family member 5
Zinc finger protein 592
Protein Jade-3
Unconventional myosin-XVIIIa
La-related protein 4B
Stalled ribosome sensor GCN1
Rho GTPase-activating protein 45
Pre-mRNA-splicing factor ATP-dependent RNA helicase PRP16
Nuclear pore complex protein Nup205
Ankyrin repeat and SAM domain-containing protein 1A
Peroxidasin homolog
Protein FAN
GPI-anchor transamidase
Small ribosomal subunit protein mS31
A-kinase anchor protein 1, mitochondrial
Dol-P-Man:Man(5)GlcNAc(2)-PP-Dol alpha-1,3-mannosyltransferase
Neurogranin
Acidic leucine-rich nuclear phosphoprotein 32 family member B
Nectin-2
Geranylgeranyl transferase type-2 subunit alpha
Protein TFG
Serine protease HTRA1
Actin-related protein 2/3 complex subunit 1A
General transcription factor IIH subunit 4
Ras-responsive element-binding protein 1
Histone deacetylase 2
Signal transducing adapter molecule 1

Zinc finger protein ubi-d4
Endoplasmic reticulum protein SC65
CREB-binding protein
Histone acetyltransferase KAT6A
Symplekin
TATA-binding protein-associated factor 2N
Golgin subfamily A member 1
Gamma-glutamyl hydrolase
Neuronal cell adhesion molecule
X-linked retinitis pigmentosa GTPase regulator
Phosphatidylinositol 3,4,5-trisphosphate 5-phosphatase 1
Probable ATP-dependent RNA helicase DDX17
Kinesin-associated protein 3
Caspase-10
Amyloid beta precursor protein binding family B member 2
DNA repair protein RAD50
CUGBP Elav-like family member 1
Osteoclast-stimulating factor 1
Rho guanine nucleotide exchange factor 1
DNA repair endonuclease XPF
Ubiquitin recognition factor in ER-associated degradation protein 1
Golgi apparatus protein 1
Regulator of nonsense transcripts 1
COP9 signalosome complex subunit 5
G-patch domain and KOW motifs-containing protein
SWI/SNF complex subunit SMARCC1
SWI/SNF-related matrix-associated actin-dependent regulator of chromatin subfamily D member 2
Ras-related protein Rab-8B
Bcl2-associated agonist of cell death
Far upstream element-binding protein 2
Glutaryl-CoA dehydrogenase, mitochondrial
GTP-binding protein Rit1
Peroxisomal membrane protein PEX13
Transportin-1
Rho guanine nucleotide exchange factor 2
Ribosomal RNA small subunit methyltransferase NEP1
Polyribonucleotide 5'-hydroxyl-kinase Clp1
Glomulin
Ubiquitin carboxyl-terminal hydrolase 13
Probable ubiquitin carboxyl-terminal hydrolase FAF-X
Ubiquitin carboxyl-terminal hydrolase 7
Cullin-5
Stathmin-2
V-type proton ATPase 116 kDa subunit a 1
Lipoma-preferred partner
RNA-binding protein with multiple splicing
Exostosin-2
Mediator of RNA polymerase II transcription subunit 12
Protein tyrosine phosphatase type IVA 1
Phosphorylase b kinase regulatory subunit beta

Secretory carrier-associated membrane protein 4
BLOC-2 complex member HPS3
SWI/SNF-related matrix-associated actin-dependent regulator of chromatin subfamily E member 1
Caveolae-associated protein 3
Riboflavin kinase
Myeloid-derived growth factor
Mitochondrial import receptor subunit TOM40B
Protein YIPF5
GPI transamidase component PIG-T
Immunoglobulin superfamily member 8
Ribosomal protein eL42-like
Ras-related protein Rab-24
Oxysterol-binding protein 2
Cytoplasmic 60S subunit biogenesis factor ZNF622
Ribosome-releasing factor 2, mitochondrial
Ubiquitin-conjugating enzyme E2 E3
7-methylguanosine phosphate-specific 5'-nucleotidase
WW domain-binding protein 2
Proteasome assembly chaperone 2
BOS complex subunit NCLN
Mitochondrial ubiquitin ligase activator of NFKB 1
Myocardin-related transcription factor A
Protein lifeguard 3
Endoplasmic reticulum-Golgi intermediate compartment protein 1
U3 small nucleolar RNA-associated protein 4 homolog
FAST kinase domain-containing protein 4
Mitochondrial amidoxime reducing component 2
Protein FAM162A
PAT complex subunit CCDC47
Large ribosomal subunit protein uL24m
Exocyst complex component 4
Protein mago nashi homolog 2
Putative monooxygenase p33MONOX
Protein RFT1 homolog
Isochorismatase domain-containing protein 2
Protein N-terminal asparagine amidohydrolase
Fermitin family homolog 2
Far upstream element-binding protein 1
Tetratricopeptide repeat protein 17
Leucine-rich repeat-containing protein 59
Vesicle transport through interaction with t-SNAREs homolog 1A
Endothelial cell-selective adhesion molecule
Pre-B-cell leukemia transcription factor-interacting protein 1
Mitochondrial calcium uniporter regulator 1
Uncharacterized protein KIAA1143
Vacuolar protein sorting-associated protein 33A
Peptidyl-prolyl cis-trans isomerase FKBP10
Tetratricopeptide repeat protein 28
Exosome complex component RRP43
Zinc finger protein 428

SH3 domain-containing kinase-binding protein 1
Spindle and kinetochore-associated protein 1
E3 ubiquitin-protein ligase RNF25
Axin interactor, dorsalization-associated protein
PIN2/TERF1-interacting telomerase inhibitor 1
ADP-ribosylation factor-like protein 8A
Ubiquitin thioesterase otulin
Small ribosomal subunit protein mS37
Peptidylprolyl isomerase domain and WD repeat-containing protein 1
Coiled-coil domain-containing protein 127
Cytochrome c oxidase assembly factor 7
Uracil phosphoribosyltransferase homolog
Phosphatidate cytidylyltransferase, mitochondrial
MOB kinase activator 3A
Dedicator of cytokinesis protein 10
Autophagy-related protein 2 homolog B
Leukocyte receptor cluster member 1
TBC1 domain family member 20
EF-hand domain-containing protein D2
Galactose mutarotase
Synaptotagmin-like protein 4
Pyrroline-5-carboxylate reductase 2
Protein CUSTOS
m7GpppX diphosphatase
Protein phosphatase 1 regulatory subunit 14B
Integrator complex subunit 12
5-methylcytosine rRNA methyltransferase NSUN4
Collagen triple helix repeat-containing protein 1
Medium-chain acyl-CoA ligase ACSF2, mitochondrial
Isochorismatase domain-containing protein 1
GRIP and coiled-coil domain-containing protein 1
FLYWCH family member 2
Protein Aster-A
HAUS augmin-like complex subunit 1
FAS-associated factor 2
Coiled-coil domain-containing protein 124
FAD-dependent oxidoreductase domain-containing protein 1
Optineurin
AP-2 complex subunit mu
Gamma-tubulin complex component 3
Probable RNA polymerase II nuclear localization protein SLC7A6OS
BTB/POZ domain-containing protein KCTD12
Reticulocalbin-3
60S ribosomal export protein NMD3
RalBP1-associated Eps domain-containing protein 1
Mitochondrial import inner membrane translocase subunit TIM14
Regulator of microtubule dynamics protein 1
U8 snoRNA-decapping enzyme
Splicing factor ESS-2 homolog
Carboxymethylenebutenolidase homolog

RNA-binding protein Musashi homolog 2
U5 small nuclear ribonucleoprotein 40 kDa protein
Regulator of MON1-CCZ1 complex
Large ribosomal subunit protein mL38
RING finger and SPRY domain-containing protein 1
Endoplasmic reticulum lectin 1
Ribosome-recycling factor, mitochondrial
Transcription termination factor 3, mitochondrial
Protein Spindly
Elongator complex protein 4
NAD-dependent protein deacetylase sirtuin-1
Protein YIPF6
Nucleoporin SEH1
Transcription elongation factor A protein-like 4
KIF-binding protein
Glucosamine 6-phosphate N-acetyltransferase
Constitutive coactivator of peroxisome proliferator-activated receptor gamma
Protein KTI12 homolog
Small ribosomal subunit protein uS3m
Large ribosomal subunit protein mL53
Trans-3-hydroxy-L-proline dehydratase
E3 ubiquitin-protein ligase RNF31
DAZ-associated protein 1
Small glutamine-rich tetratricopeptide repeat-containing protein beta
Protein SAAL1
Mitochondrial potassium channel
SAGA-associated factor 29
Ribosomal RNA processing protein 36 homolog
C1GALT1-specific chaperone 1
RNA-binding protein 33
Dysbindin
DnaJ homolog subfamily A member 3, mitochondrial
Translation machinery-associated protein 16
Multivesicular body subunit 12A
Small ribosomal subunit protein mS39
Corrinoid adenosyltransferase MMAB
Cytoplasmic FMR1-interacting protein 2
GTPase IMAP family member 5
E3 ubiquitin-protein ligase TRIM11
Coiled-coil domain-containing protein 97
CB1 cannabinoid receptor-interacting protein 1
Enhancer of mRNA-decapping protein 3
Dynein light chain 2, cytoplasmic
Copine-2
Protein S100-A16
Signal-induced proliferation-associated protein 1
Secernin-2
THO complex subunit 1
Ubiquitin thioesterase OTUB1
tRNA (adenine(58)-N(1))-methyltransferase catalytic subunit TRMT61A

Abasic site processing protein HMCES
Charged multivesicular body protein 6
Protein bicaudal D homolog 1
Phosphopentomutase
U3 small nucleolar ribonucleoprotein protein IMP4
Ceramide synthase 2
tRNA-dihydrouridine(47) synthase [NAD(P)(+)]-like
Protein LTV1 homolog
Serine dehydratase-like
Large ribosomal subunit protein mL48
Chronophin
Aurora kinase B
DCN1-like protein 1
Fumarylacetoacetate hydrolase domain-containing protein 2A
SWI/SNF-related matrix-associated actin-dependent regulator of chromatin subfamily D member 1
Target of EGR1 protein 1
Cell division cycle-associated 7-like protein
RUS family member 1
Probable ATP-dependent RNA helicase DDX27
BLOC-1-related complex subunit 6
Methionine--tRNA ligase, mitochondrial
Ataxin-7-like protein 3B
Serine/threonine-protein kinase greatwall
Methylthioribulose-1-phosphate dehydratase
Zinc finger C2HC domain-containing protein 1A
Protein lin-37 homolog
Solute carrier family 41 member 3
Vacuolar-sorting protein SNF8
Zinc finger CCCH-type antiviral protein 1-like
Nuclear envelope pore membrane protein POM 121
Tonsoku-like protein
PDZ and LIM domain protein 5
Protein disulfide isomerase CRELD1
ERO1-like protein alpha
Proline-rich protein 11
Protein FMC1 homolog
Adenosylhomocysteinase 3
Dedicator of cytokinesis protein 6
Mediator of RNA polymerase II transcription subunit 30
Serine/threonine-protein phosphatase PGAM5, mitochondrial
Integrator complex subunit 4
DDRGK domain-containing protein 1
2-oxoadipate dehydrogenase complex component E1
Selenocysteine lyase
Far upstream element-binding protein 3
Splicing factor 45
RCC1-like G exchanging factor-like protein
Asparaginyl-tRNA synthetase
Succinate--CoA ligase [GDP-forming] subunit beta, mitochondrial
DISP complex protein LRCH3

Mannose-1-phosphate guanyltransferase alpha
4-hydroxyphenylpyruvate dioxygenase-like protein
Putative protein-lysine deacylase ABHD14B
Peptide-N(4)-(N-acetyl-beta-glucosaminyl)asparagine amidase
Vesicle-trafficking protein SEC22a
ATP synthase membrane subunit K, mitochondrial
Kinetochore-associated protein NSL1 homolog
PRKC apoptosis WT1 regulator protein
tRNA N(3)-methylcytidine methyltransferase METTL2A
THO complex subunit 3
E3 ubiquitin-protein ligase Itchy homolog
Thioredoxin domain-containing protein 15
Kin of IRRE-like protein 1
Leucine-rich repeats and immunoglobulin-like domains protein 1
Conserved oligomeric Golgi complex subunit 3
CDK5 regulatory subunit-associated protein 3
Vam6/Vps39-like protein
Syndetin
Deubiquitinating protein VCPIP1
Engulfment and cell motility protein 2
Protein disulfide-isomerase TMX3
DDB1- and CUL4-associated factor 5
Chromosome alignment-maintaining phosphoprotein 1
Lethal(3)malignant brain tumor-like protein 3
E3 ubiquitin-protein ligase ZFP91
Protocadherin-16
Calmin
PDZ and LIM domain protein 2
Fatty acyl-CoA reductase 2
Transcription factor BTF3 homolog 4
E3 ubiquitin-protein ligase RNF170
Abscission/NoCut checkpoint regulator
Solute carrier family 35 member E1
Ubiquitin carboxyl-terminal hydrolase 47
Zinc finger protein 521
Lipid scramblase CLPTM1L
Lymphokine-activated killer T-cell-originated protein kinase
ADP-ribosylation factor-like protein 5B
DnaJ homolog subfamily C member 1
N-terminal kinase-like protein
Zinc finger protein 512B
Protein LRATD2
Exocyst complex component 2
Cytosolic non-specific dipeptidase
Histone-lysine N-methyltransferase EHMT2
Zinc finger RNA-binding protein
Protein FAM210B, mitochondrial
Beta-1,3-galactosyltransferase 6
E1A-binding protein p400
Sorting nexin-27

Kinesin-like protein KIF16B
Intraflagellar transport protein 74 homolog
E3 ubiquitin-protein ligase TRIM47
CCR4-NOT transcription complex subunit 6-like
Dehydrogenase/reductase SDR family member 1
DnaJ homolog subfamily C member 30, mitochondrial
Ubiquitin-conjugating enzyme E2 E2
Caspase recruitment domain-containing protein 19
Protein PRRC1
FYVE, RhoGEF and PH domain-containing protein 4
F-box/LRR-repeat protein 18
Zinc finger protein 512
E3 SUMO-protein ligase NSE2
Non-structural maintenance of chromosomes element 3 homolog
Protein-L-isoaspartate O-methyltransferase domain-containing protein 1
Heat shock 70 kDa protein 12B
YTH domain-containing protein 1
Coiled-coil domain-containing protein 43
Conserved oligomeric Golgi complex subunit 8
CKLF-like MARVEL transmembrane domain-containing protein 3
Dynein axonemal assembly factor 10
Nucleolar protein 4-like
Lysophospholipid acyltransferase 7
Dedicator of cytokinesis protein 7
Sideroflexin-2
Zinc finger protein 830
Protein FAM210A
FERM domain-containing protein 6
28S rRNA (cytosine-C(5))-methyltransferase
Regulation of nuclear pre-mRNA domain-containing protein 1A
Arf-GAP with GTPase, ANK repeat and PH domain-containing protein 3
Arf-GAP with Rho-GAP domain, ANK repeat and PH domain-containing protein 1
Importin-9
Melanoma inhibitory activity protein 2
Discoidin, CUB and LCCL domain-containing protein 2
Rho guanine nucleotide exchange factor 17
Inositol polyphosphate-4-phosphatase type I A
RNA-binding protein 14
RING finger and CHY zinc finger domain-containing protein 1
Kelch-like protein 5
E3 ubiquitin-protein ligase UHRF2
E3 ubiquitin-protein ligase NEDD4-like
KH domain-containing RNA-binding protein QKI
Leukocyte receptor cluster member 8
Formin-like protein 2
Pseudouridylate synthase 7 homolog
Serine protease FAM111A
Trafficking protein particle complex subunit 9
Perilipin-4
CCA tRNA nucleotidyltransferase 1, mitochondrial

Serine/threonine-protein kinase SMG1
Alsin
Transmembrane protein 237
Alpha-ketoglutarate-dependent dioxygenase alkB homolog 3
Rho GTPase-activating protein 7
Serine/threonine-protein phosphatase 1 regulatory subunit 10
Sodium-coupled neutral amino acid symporter 2
UAP56-interacting factor
Myotubularin-related protein 9
Vacuolar protein sorting-associated protein 35
Transcriptional activator protein Pur-beta
Exportin-6
Hedgehog-interacting protein
Membrane-associated guanylate kinase, WW and PDZ domain-containing protein 1
Sperm-associated antigen 5
Nucleus accumbens-associated protein 1
Sorting nexin-18
PAS domain-containing serine/threonine-protein kinase
Protein capicua homolog
BRCA1-A complex subunit RAP80
Intermembrane lipid transfer protein VPS13A
Mediator of RNA polymerase II transcription subunit 15
Elongation factor G, mitochondrial
Endoplasmic reticulum-Golgi intermediate compartment protein 2
Methylcrotonoyl-CoA carboxylase subunit alpha, mitochondrial
Calcium/calmodulin-dependent protein kinase kinase 2
Trimethylguanosine synthase
NudC domain-containing protein 1
Erbin
Gamma-tubulin complex component 6
Gamma-tubulin complex component 5
Ubiquitin carboxyl-terminal hydrolase 28
Formin-binding protein 1
EKC/KEOPS complex subunit TP53RK
GPI transamidase component PIG-S
ATPase WRNIP1
Ran-binding protein 9
Chloride channel CLIC-like protein 1
Myeloid-associated differentiation marker
Neurabin-2
SRSF protein kinase 1
Structural maintenance of chromosomes protein 6
BTB/POZ domain-containing protein KCTD15
Spermatid perinuclear RNA-binding protein
Transmembrane protein 209
Glutathione peroxidase 7
CDK5 regulatory subunit-associated protein 2
Cytochrome P450 2S1
Protein IWS1 homolog
Paired amphipathic helix protein Sin3a

Oxysterol-binding protein-related protein 9
Protein cereblon
Integrator complex subunit 14
2-aminoethanethiol dioxygenase
Mitochondrial tRNA methylthiotransferase CDK5RAP1
Remodeling and spacing factor 1
RNA-binding protein 15
Protein phosphatase 1 regulatory inhibitor subunit 16B
RUN and FYVE domain-containing protein 1
Msx2-interacting protein
Bifunctional polynucleotide phosphatase/kinase
MMS19 nucleotide excision repair protein homolog
E3 ubiquitin-protein ligase UHRF1
Protein Niban 2
ATP-dependent zinc metalloprotease YME1L1
Regulator of microtubule dynamics protein 3
Transcription factor 12
Tubulin-folding cofactor B
Proteasome subunit beta type-7
Calponin-2
Translocation protein SEC62
Ethanolamine-phosphate cytidylyltransferase
Cell division cycle 5-like protein
26S proteasome non-ATPase regulatory subunit 1
Stromal cell-derived factor 2
Prefoldin subunit 5
E3 ubiquitin-protein ligase RING2
Parkinson disease protein 7
Sphingosine 1-phosphate receptor 3
GAS2-like protein 1
Eyes absent homolog 3
Sialidase-1
Synaptic vesicle membrane protein VAT-1 homolog
Legumain
Perilipin-2
DnaJ homolog subfamily C member 2
M-phase phosphoprotein 8
Nuclear pore complex protein Nup88
Plakophilin-4
Phosphoinositide 3-kinase regulatory subunit 4
P2X purinoceptor 4
Ribonucleases P/MRP protein subunit POP1
Protein S100-A13
Protein SCAF11
Translin-associated protein X
Selenide, water dikinase 2
Eukaryotic translation initiation factor 3 subunit C
Tetratricopeptide repeat protein 1
DnaJ homolog subfamily C member 7
Prohibitin-2

COP9 signalosome complex subunit 8
Pre-mRNA-splicing factor 18
Calcineurin B homologous protein 1
Kinesin-like protein KIF2C
RANBP2-like and GRIP domain-containing protein 5/6
Monoglyceride lipase
Ataxin-2
Docking protein 1
Methionine synthase
3-hydroxyacyl-CoA dehydrogenase type-2
Collagen alpha-1(XII) chain
Mothers against decapentaplegic homolog 5
Sigma non-opioid intracellular receptor 1
Heterogeneous nuclear ribonucleoprotein A/B
Nucleosome assembly protein 1-like 4
Microsomal glutathione S-transferase 2
Gamma-soluble NSF attachment protein
Phosphatidylinositol 4-phosphate 5-kinase type-1 alpha
Phospholipid-transporting ATPase ABCA3
Mitogen-activated protein kinase kinase kinase 3
ATP synthase subunit s, mitochondrial
Mitochondrial intermediate peptidase
Aconitate hydratase, mitochondrial
Transmembrane 9 superfamily member 2
Equilibrative nucleoside transporter 1
Tumor susceptibility gene 101 protein
Copine-1
T-complex protein 1 subunit eta
Myeloid differentiation primary response protein MyD88
Probable rRNA-processing protein EBP2
Protein arginine N-methyltransferase 1
BAG family molecular chaperone regulator 1
Cyclic AMP-dependent transcription factor ATF-6 beta
Forkhead box protein C2
Endophilin-A2
Telomerase protein component 1
Serine/threonine-protein kinase VRK1
Growth/differentiation factor 15
A-kinase anchor protein 9
Protein NipSnap homolog 1
Condensin complex subunit 3
Actin-related protein 2/3 complex subunit 5-like protein
Calcium uptake protein 1, mitochondrial
UPF0415 protein C7orf25
Protein FAM118B
Target of rapamycin complex 2 subunit MAPKAP1
RNA-binding protein 4B
Zinc finger FYVE domain-containing protein 21
ATP-dependent RNA helicase DDX50
Large ribosomal subunit protein bL34m

Zinc phosphodiesterase ELAC protein 2
Telomerase RNA component interacting RNase
Glutamate-rich WD repeat-containing protein 1
ADP-ribose glycohydrolase MACROD1
Ribosome quality control complex subunit TCF25
Protein CMSS1
Kelch domain-containing protein 3
Evolutionarily conserved signaling intermediate in Toll pathway, mitochondrial
Methylosome protein WDR77
Cytochrome b-245 chaperone 1
Vitamin K epoxide reductase complex subunit 1
2-(3-amino-3-carboxypropyl)histidine synthase subunit 2
Tubulin alpha-1C chain
Selenoprotein S
Apolipoprotein L2
Myb-binding protein 1A
Fermitin family homolog 1
Mitochondrial genome maintenance exonuclease 1
Golgi reassembly-stacking protein 1
FYVE and coiled-coil domain-containing protein 1
Acyl-CoA-binding domain-containing protein 6
Coronin-1B
Thioredoxin domain-containing protein 17
BUD13 homolog
Serine/threonine-protein phosphatase CPPED1
Vacuolar protein-sorting-associated protein 25
Large ribosomal subunit protein mL45
Uncharacterized protein C7orf50
Tudor-interacting repair regulator protein
45 kDa calcium-binding protein
Programmed cell death protein 2-like
Proteasomal ATPase-associated factor 1
Partner of Y14 and mago
MICOS complex subunit MIC25
ADP-dependent glucokinase
G patch domain-containing protein 1
Serine/threonine-protein kinase RIO1
Protein LLP homolog
DNA replication complex GINS protein SLD5
rRNA-processing protein UTP23 homolog
Protein pelota homolog
Peroxiredoxin-like 2A
E3 ubiquitin-protein ligase TRIM56
Endoplasmic reticulum resident protein 44
Latexin
Nucleolar protein 10
Cancer-related nucleoside-triphosphatase
Mitochondrial import inner membrane translocase subunit Tim29
Translational activator of cytochrome c oxidase 1
Gamma-tubulin complex component 2

Extended synaptotagmin-1
Ubiquitin-associated domain-containing protein 1
Protein YIPF4
Phagosome assembly factor 1
tRNA-splicing endonuclease subunit Sen34
Protein canopy homolog 3
Mitochondrial ribosome-associated GTPase 1
Chitobiosyldiphosphodolichol beta-mannosyltransferase
LIM domain-containing protein 2
HAUS augmin-like complex subunit 8
Inositol polyphosphate 5-phosphatase K
Proteasome assembly chaperone 3
COP9 signalosome complex subunit 4
Synaptotagmin-11
WW domain-containing adapter protein with coiled-coil
Death-inducer obliterator 1
Metastasis-associated protein MTA3
RNA-binding protein 42
Dynactin subunit 5
Mini-chromosome maintenance complex-binding protein
Alanyl-tRNA editing protein Aarsd1
DCN1-like protein 5
Acidic leucine-rich nuclear phosphoprotein 32 family member E
Leucine-rich repeat-containing protein 1
Phosphatidylinositol 4-kinase type 2-alpha
Transmembrane protein 43
Fibronectin type III and SPRY domain-containing protein 1
Tubulin-specific chaperone D
Nucleoporin NDC1
Protein HGH1 homolog
Dehydrogenase/reductase SDR family member 4
Lipase maturation factor 2
NADH dehydrogenase [ubiquinone] 1 alpha subcomplex assembly factor 3
Multiple myeloma tumor-associated protein 2
Transmembrane protein 70, mitochondrial
Mediator of RNA polymerase II transcription subunit 18
Iron-sulfur cluster assembly 1 homolog, mitochondrial
Tubulin beta-6 chain
Protein PAXX
DNA-directed RNA polymerase III subunit RPC3
Heterogeneous nuclear ribonucleoprotein U-like protein 1
Protein misato homolog 1
PHD finger protein 23
Programmed cell death protein 10
Ribonuclease P protein subunit p25
Derlin-1
Oxidoreductase HTATIP2
Probable ATP-dependent RNA helicase DDX23
Telomerase Cajal body protein 1
MICOS complex subunit MIC26

Dehydrogenase/reductase SDR family member 6
Methylthioribose-1-phosphate isomerase
WD repeat-containing protein 18
Vesicle-associated membrane protein 8
tRNA (guanine(6)-N2)-methyltransferase THUMP3
Acireductone dioxygenase
Enoyl-[acyl-carrier-protein] reductase, mitochondrial
ER membrane protein complex subunit 6
N-terminal Xaa-Pro-Lys N-methyltransferase 1
ER degradation-enhancing alpha-mannosidase-like protein 2
Katanin p80 WD40 repeat-containing subunit B1
Target of rapamycin complex subunit LST8
Ashwin
Voltage-gated monoatomic cation channel TMEM109
Protein PBDC1
Phosphatidylserine synthase 2
Nucleolar complex protein 4 homolog
U3 small nucleolar RNA-associated protein 14 homolog A
Dual specificity protein phosphatase 23
Transmembrane emp24 domain-containing protein 9
BOS complex subunit TMEM147
Nucleoporin p58/p45
Protein adenylyltransferase SelO, mitochondrial
Protein DPCD
Guanine nucleotide-binding protein-like 3
ATPase family gene 2 protein homolog B
Serine/threonine-protein kinase RIO2
tRNA (adenine(58)-N(1))-methyltransferase, mitochondrial
Transmembrane and ubiquitin-like domain-containing protein 1
Mitochondrial import inner membrane translocase subunit Tim21
Kinesin-like protein KIFC1
Nuclear pore complex protein Nup85
Very long chain fatty acid elongase 1
Katanin p60 ATPase-containing subunit A-like 1
HIRA-interacting protein 3
Intraflagellar transport protein 27 homolog
Splicing factor YJU2
ADP-ribose pyrophosphatase, mitochondrial
Threonine--tRNA ligase, mitochondrial
Acetyl-CoA acetyltransferase, cytosolic
Replication initiator 1
RNA-binding protein 4
FUN14 domain-containing protein 2
RNA polymerase II-associated protein 1
Splicing factor 3B subunit 5
Sideroflexin-3
Proline-rich protein 14
Chitinase domain-containing protein 1
Poly(A) polymerase gamma
Caspase recruitment domain-containing protein 10

Kanadaptin
Cyclin-dependent kinase 19
Protein LSM14 homolog B
Tapasin-related protein
Junctional adhesion molecule C
Caspase recruitment domain-containing protein 6
BTB/POZ domain-containing protein 2
Sphingosine-1-phosphate phosphatase 1
Oxysterol-binding protein-related protein 11
Oxysterol-binding protein-related protein 10
Rab11 family-interacting protein 5
TBC1 domain family member 10A
Complement C1q tumor necrosis factor-related protein 5
N-alpha-acetyltransferase 15, NatA auxiliary subunit
Krueppel-like factor 16
Bcl-2-like protein 13
Caspase recruitment domain-containing protein 11
Solute carrier family 12 member 9
Serrate RNA effector molecule homolog
Queuine tRNA-ribosyltransferase catalytic subunit 1
AP-1 complex subunit mu-1
Nucleolar and spindle-associated protein 1
Haloacid dehalogenase-like hydrolase domain-containing 5
Fanconi anemia group D2 protein
Protein MAK16 homolog
Histone deacetylase 8
Replication termination factor 2
Charged multivesicular body protein 4a
Eukaryotic translation initiation factor 2A
Polymerase delta-interacting protein 3
Uncharacterized protein KIAA1671
SH3 and multiple ankyrin repeat domains protein 3
Guanine nucleotide-binding protein subunit beta-like protein 1
Alpha-(1,6)-fucosyltransferase
Large ribosomal subunit protein bL32m
Large ribosomal subunit protein bL20m
Large ribosomal subunit protein uL13m
Large ribosomal subunit protein bL9m
Large ribosomal subunit protein uL4m
Large ribosomal subunit protein uL1m
MKI67 FHA domain-interacting nucleolar phosphoprotein
Hyccin
3'-5' exoribonuclease HELZ2
RanBP-type and C3HC4-type zinc finger-containing protein 1
Small ribosomal subunit protein mS26
Neurolysin, mitochondrial
Centrosomal protein of 41 kDa
Histone-lysine N-methyltransferase SETD2
Pantothenate kinase 2, mitochondrial
Dedicator of cytokinesis protein 9

FERM domain-containing protein 8
Contactin-associated protein-like 3
Histone-lysine N-methyltransferase NSD3
Kinetochore protein Nuf2
Large ribosomal subunit protein mL37
GTP-binding protein 4
Tether containing UBX domain for GLUT4
Oxysterol-binding protein-related protein 8
Uveal autoantigen with coiled-coil domains and ankyrin repeats
Ras-related protein Rab-34
2-(3-amino-3-carboxypropyl)histidine synthase subunit 1
WD repeat-containing protein 11
Regulator of nonsense transcripts 3B
Crooked neck-like protein 1
F-box-like/WD repeat-containing protein TBL1XR1
Ubiquitin-like protein 5
Protein phosphatase 1 regulatory subunit 12C
Serine/threonine-protein kinase D2
ER degradation-enhancing alpha-mannosidase-like protein 3
Protein Niban 1
UBX domain-containing protein 6
Transmembrane 6 superfamily member 1
Uridine-cytidine kinase 2
Sialoadhesin
Apoptosis inhibitor 5
NACHT, LRR and PYD domains-containing protein 1
Tripartite motif-containing protein 5
E3 ubiquitin-protein ligase TRIM4
Alpha-ketoglutarate-dependent dioxygenase FTO
Palmitoyltransferase ZDHHC5
Transport and Golgi organization protein 6 homolog
182 kDa tankyrase-1-binding protein
(E3-independent) E2 ubiquitin-conjugating enzyme
Protein zyg-11 homolog B
Protein TANC1
Ethanolaminephosphotransferase 1
Exportin-4
Endoplasmic reticulum junction formation protein lunapark
Centrosomal protein of 44 kDa
Protein tweety homolog 3
Kelch-like protein 4
Myotubularin-related protein 12
pre-mRNA 3' end processing protein WDR33
Ribosome biogenesis protein WDR12
Protein YIPF3
Tubulointerstitial nephritis antigen-like
Adipose-secreted signaling protein
PITH domain-containing protein 1
Derlin-2
COMM domain-containing protein 5

Sentrin-specific protease 6
RNA exonuclease 4
ATP-dependent RNA helicase DDX24
DNA-directed RNA polymerase I subunit RPA49
Superkiller complex protein 8
SRA stem-loop-interacting RNA-binding protein, mitochondrial
Serine racemase
NIF3-like protein 1
Egl nine homolog 1
Mucolipin-1
PSME3-interacting protein
Mitochondrial fission factor
N-alpha-acetyltransferase 50
Extracellular glycoprotein lacritin
Ubiquitin-like modifier-activating enzyme 5
E3 ubiquitin-protein ligase makorin-2
Transmembrane protein 126A
Polyadenylate-binding protein-interacting protein 1
Mitochondrial disaggregase
Ras-related protein Rab-33B
Large subunit GTPase 1 homolog
Protein FAM107B
RNA cytidine acetyltransferase
COMM domain-containing protein 4
Kinesin light chain 2
Integrin-linked kinase-associated serine/threonine phosphatase 2C
5'-3' exoribonuclease 2
Toll-interacting protein
Histone deacetylase complex subunit SAP130
Bromodomain-containing protein 8
Nuclear speckle splicing regulatory protein 1
Integrator complex subunit 2
Rac GTPase-activating protein 1
Protein mono-ADP-ribosyltransferase PARP12
Cleavage stimulation factor subunit 2 tau variant
Cytosolic 5'-nucleotidase 3A
CYFIP-related Rac1 interactor A
Transmembrane protein 222
Haloacid dehalogenase-like hydrolase domain-containing protein 2
Glutamyl-tRNA(Gln) amidotransferase subunit A, mitochondrial
Probable ATP-dependent RNA helicase DDX47
Magnesium transporter protein 1
Ras-related protein Rab-1B
Large ribosomal subunit protein uL18m
Testis-specific Y-encoded-like protein 1
Transmembrane protein 168
VIP36-like protein
Nonsense-mediated mRNA decay factor SMG9
Protein FAM234A
Oxysterol-binding protein-related protein 5

Deoxynucleotidyltransferase terminal-interacting protein 1
Nucleotide exchange factor SIL1
Protein-L-histidine N-pros-methyltransferase
Anaphase-promoting complex subunit 1
Probable E3 ubiquitin-protein ligase IRF2BPL
Cysteine-rich and transmembrane domain-containing protein 1
DNA-directed RNA polymerase III subunit RPC6
Nuclear ubiquitous casein and cyclin-dependent kinase substrate 1
Thioredoxin-related transmembrane protein 4
Kinesin-like protein KIF13A
Activating signal cointegrator 1 complex subunit 2
Rabenosyn-5
Iron-sulfur cluster assembly enzyme ISCU
Oxysterol-binding protein-related protein 2
Autophagy protein 5
WD repeat-containing protein 13
Epsin-3
EH domain-containing protein 4
Vacuolar protein sorting-associated protein 33B
Vacuolar protein sorting-associated protein 16 homolog
Vacuolar protein sorting-associated protein 11 homolog
SH3 domain-binding glutamic acid-rich-like protein 3
Gigaxonin
Solute carrier family 25 member 32
TRIO and F-actin-binding protein
STE20-like serine/threonine-protein kinase
Peptidyl-prolyl cis-trans isomerase-like 3
Sodium-coupled neutral amino acid symporter 1
Phosducin-like protein 3
Serine/threonine-protein kinase TAO3
Rab3 GTPase-activating protein non-catalytic subunit
Activity-dependent neuroprotector homeobox protein
Diphthine methyl ester synthase
ATP-dependent DNA/RNA helicase DHX36
Inorganic pyrophosphatase 2, mitochondrial
Protein spinster homolog 1
Large ribosomal subunit protein mL46
Solute carrier family 12 member 5
Presenilin-associated rhomboid-like protein, mitochondrial
Pinin
Transmembrane protein 245
Forkhead box protein P1
BTB/POZ domain-containing adapter for CUL3-mediated RhoA degradation protein 3
tRNA dimethylallyltransferase
UPF0696 protein C11orf68
Growth hormone-inducible transmembrane protein
BolA-like protein 2
Cobalamin trafficking protein CblD
Thioredoxin-interacting protein
Thioredoxin-related transmembrane protein 1

Negative elongation factor A
Golgi resident protein GCP60
Cdc42 effector protein 4
Tyrosine-protein phosphatase non-receptor type 23
Semaphorin-6B
Protein unc-45 homolog A
Major facilitator superfamily domain-containing protein 1
DnaJ homolog subfamily C member 5
Kinetochore-associated protein DSN1 homolog
Charged multivesicular body protein 4b
RWD domain-containing protein 1
CUE domain-containing protein 2
F-box/LRR-repeat protein 15
Fructosamine-3-kinase
GDP-fucose protein O-fucosyltransferase 1
Phosphatidylinositol glycan anchor biosynthesis class U protein
Torsin-3A
Serine/threonine-protein kinase WNK1
Aminopeptidase B
Golgi phosphoprotein 3-like
Golgi phosphoprotein 3
tRNA N6-adenosine threonylcarbamoyltransferase, mitochondrial
Alpha-N-acetyl-neuraminyl-2,3-beta-galactosyl-1,3-N-acetyl-galactosaminide alpha-2,6-sialyltransferase
Golgi-associated plant pathogenesis-related protein 1
TraB domain-containing protein
Mitochondrial ribosome-associated GTPase 2
Sentrin-specific protease 3
Oxysterol-binding protein-related protein 3
SWI/SNF-related matrix-associated actin-dependent regulator of chromatin subfamily A containing DEAD/H
EH domain-containing protein 1
mRNA (2'-O-methyladenosine-N(6)-)-methyltransferase
ESF1 homolog
Alpha-1,3/1,6-mannosyltransferase ALG2
HEAT repeat-containing protein 1
Protein O-mannose kinase
Rab GTPase-binding effector protein 2
Dimethyladenosine transferase 2, mitochondrial
rRNA N6-adenosine-methyltransferase ZCCHC4
STING ER exit protein
Cytosolic iron-sulfur assembly component 2A
Probable ATP-dependent RNA helicase DHX35
Protein FAM124B
HAUS augmin-like complex subunit 4
Coiled-coil domain-containing protein 134
Coiled-coil domain-containing protein 86
Receptor expression-enhancing protein 4
Transcription factor SOX-17
Optic atrophy 3 protein
Cytosolic iron-sulfur assembly component 3
ATP-dependent RNA helicase DHX33

Nucleolar protein 6
WD repeat and coiled-coil-containing protein
3'-5' RNA helicase YTHDC2
5-azacytidine-induced protein 2
RNA polymerase II-associated protein 3
BCAS3 microtubule associated cell migration factor
Alpha-1,2-mannosyltransferase ALG9
Lipid droplet-associated hydrolase
Ribosomal oxygenase 1
WD repeat-containing protein 55
Ran-binding protein 3
dCTP pyrophosphatase 1
Inactive tyrosine-protein kinase PEAK1
Ribosome production factor 2 homolog
Histone-lysine N-methyltransferase SMYD3
Mth938 domain-containing protein
Dedicator of cytokinesis protein 5
WD repeat-containing protein 26
Tudor domain-containing protein 3
UPF0488 protein C8orf33
Polyamine-transporting ATPase 13A3
Methyltransferase-like protein 17, mitochondrial
Splicing factor, arginine/serine-rich 19
Nuclear exosome regulator NRDE2
Prostaglandin E synthase 2
Phosphorylated adapter RNA export protein
Ubiquitin-conjugating enzyme E2 Z
Complex I assembly factor ACAD9, mitochondrial
5'-nucleotidase domain-containing protein 2
PRKR-interacting protein 1
Caspase activity and apoptosis inhibitor 1
Nucleolar protein 11
Probable ATP-dependent RNA helicase DDX31
Thiol S-methyltransferase TMT1A
ATPase PAAT
Multimerin-2
Ubiquitin carboxyl-terminal hydrolase MINDY-3
Protein eva-1 homolog A
AKT-interacting protein
Pleckstrin homology domain-containing family F member 2
tRNA endonuclease ANKZF1
Golgi reassembly-stacking protein 2
Protein zwilch homolog
Jupiter microtubule associated homolog 2
Mitochondrial glutamate carrier 1
Proline-serine-threonine phosphatase-interacting protein 2
Mediator of RNA polymerase II transcription subunit 20
WD repeat-containing protein 76
Queuine tRNA-ribosyltransferase accessory subunit 2
Actin-related protein 8

Damage-control phosphatase ARMT1
Pantothenate kinase 3
CCR4-NOT transcription complex subunit 10
Leucine-rich repeat-containing protein 40
Histone-lysine N-methyltransferase EHMT1
Sideroflexin-1
Spermatogenesis-defective protein 39 homolog
Conserved oligomeric Golgi complex subunit 4
Actin-related protein 5
Vacuolar protein sorting-associated protein 37B
Large ribosomal subunit protein mL44
L-2-hydroxyglutarate dehydrogenase, mitochondrial
COP9 signalosome complex subunit 7b
Non-homologous end-joining factor 1
Protein transport protein Sec61 subunit alpha isoform 2
Ribitol 5-phosphate transferase FKRP
Elongator complex protein 3
Ribosome production factor 1
DNA-directed RNA polymerase I subunit RPA2
Zinc finger matrin-type protein 3
Uridine-cytidine kinase 1
Ketosamine-3-kinase
TBC1 domain family member 17
Probable cysteine--tRNA ligase, mitochondrial
Solute carrier family 52, riboflavin transporter, member 2
Phosphopantothenate--cysteine ligase
Ubiquitin domain-containing protein 1
WD repeat-containing protein 41
Nicotinamide/nicotinic acid mononucleotide adenylyltransferase 1
Protein Njmu-R1
Sialate O-acetylesterase
Pleckstrin homology domain-containing family A member 5
E3 ubiquitin-protein ligase SMURF2
Regulator of nonsense transcripts 2
Guanine nucleotide-binding protein subunit beta-4
Exportin-5
GrpE protein homolog 1, mitochondrial
MYG1 exonuclease
Pleckstrin homology domain-containing family A member 3
Pleckstrin homology domain-containing family A member 1
Retinoid-inducible serine carboxypeptidase
Netrin-4
Calcyclin-binding protein
Ras-related GTP-binding protein C
Zinc finger FYVE domain-containing protein 1
Intraflagellar transport protein 122 homolog
Retinol dehydrogenase 14
Beta-parvin
Tensin-1
Plasminogen receptor (KT)

NmrA-like family domain-containing protein 1
Vezatin
Kinetochore protein Spc25
Transcription initiation factor TFIID subunit 9B
Solute carrier family 38 member 10
Ethanolamine kinase 1
Adhesion G protein-coupled receptor L4
Putative divalent cation/proton antiporter TMEM165
Mitochondrial thiamine pyrophosphate carrier
Echinoderm microtubule-associated protein-like 4
rRNA methyltransferase 3, mitochondrial
Glyoxalase domain-containing protein 4
Serine/threonine-protein kinase Nek6
Methylcrotonoyl-CoA carboxylase beta chain, mitochondrial
Nuclear receptor coactivator 5
Ectopic P granules protein 5 homolog
Helicase MOV-10
N6-adenosine-methyltransferase non-catalytic subunit
Non-lysosomal glucosylceramidase
Pre-mRNA-splicing factor CWC22 homolog
Ribonucleoprotein PTB-binding 2
Chromodomain-helicase-DNA-binding protein 8
Band 4.1-like protein 5
GPN-loop GTPase 1
Stromal cell-derived factor 2-like protein 1
Casein kinase I isoform gamma-1
Pre-mRNA-splicing factor SYF1
Prolactin regulatory element-binding protein
Steroid receptor RNA activator 1
Endoplasmic reticulum transmembrane helix translocase
Golgi-associated PDZ and coiled-coil motif-containing protein
Large ribosomal subunit protein uL29m
LYR motif-containing protein 4
O-phosphoseryl-tRNA(Sec) selenium transferase
Charged multivesicular body protein 1a
Transmembrane 9 superfamily member 3
Unconventional myosin-X
Adipocyte plasma membrane-associated protein
tRNA (34-2'-O)-methyltransferase regulator WDR6
Thioredoxin reductase 2, mitochondrial
ATP-binding cassette sub-family B member 6
ADP-ribosylation factor GTPase-activating protein 3
Zinc finger CCHC domain-containing protein 17
Ras-related protein Rab-18
Palmdelphin
RNA polymerase II subunit A C-terminal domain phosphatase SSU72
Vacuolar protein sorting-associated protein VTA1 homolog
Serine--tRNA ligase, mitochondrial
Large ribosomal subunit protein mL65
Dynein light chain roadblock-type 1

ER membrane protein complex subunit 7
Transcription and mRNA export factor ENY2
Exosome complex component RRP41
Ubiquitin-conjugating enzyme E2 T
H/ACA ribonucleoprotein complex subunit 3
tRNA N6-adenosine threonylcarbamoyltransferase
DNA methyltransferase 1-associated protein 1
Protocadherin-12
Inositol-3-phosphate synthase 1
Bromodomain-containing protein 7
mRNA-decapping enzyme 1A
Acyl-coenzyme A thioesterase 13
Mediator of RNA polymerase II transcription subunit 4
Complex I assembly factor TIMMDC1, mitochondrial
Synembryn-A
Complement component C1q receptor
Putative RNA-binding protein Luc7-like 1
Endothelial cell-specific molecule 1
Leucine zipper transcription factor-like protein 1
Large ribosomal subunit protein mL40
Suppressor of SWI4 1 homolog
1-phosphatidylinositol 4,5-bisphosphate phosphodiesterase beta-1
Fructose-2,6-bisphosphatase TIGAR
Reticulon-4
Regulation of nuclear pre-mRNA domain-containing protein 1B
Xaa-Pro aminopeptidase 3
Omega-amidase NIT2
Cell death regulator Aven
Inner centromere protein
Exosome complex component RRP46
Exosome complex component RRP40
Kinesin-like protein KIF13B
Anillin
Xaa-Pro aminopeptidase 1
Gephyrin
Bridging integrator 3
Something about silencing protein 10
StAR-related lipid transfer protein 7, mitochondrial
Baculoviral IAP repeat-containing protein 6
PDZ and LIM domain protein 7
Acetyl-coenzyme A synthetase, cytoplasmic
Diablo IAP-binding mitochondrial protein
Nucleolar RNA helicase 2
GTP-binding protein SAR1a
Sialic acid synthase
Endophilin-B2
Histone-lysine N-methyltransferase ASH1L
Translation initiation factor eIF2B subunit gamma
Muscleblind-like protein 1
Sialin

Eukaryotic translation initiation factor 4E transporter
Carbohydrate sulfotransferase 12
Suppressor of tumorigenicity 7 protein
PRKCA-binding protein
SH2B adapter protein 1
CTP synthase 2
DNA polymerase epsilon subunit 3
Chromatin accessibility complex protein 1
Aladin
SNF-related serine/threonine-protein kinase
ATP-binding cassette sub-family B member 10, mitochondrial
Bromodomain adjacent to zinc finger domain protein 1A
Striatin-4
L-aminoadipate-semialdehyde dehydrogenase-phosphopantetheinyl transferase
rRNA N6-adenosine-methyltransferase METTL5
Oligosaccharyltransferase complex subunit OSTC
COX assembly mitochondrial protein 2 homolog
Phospholipid scramblase 4
Ribonuclease 3
Sorting nexin-15
Heme-binding protein 1
DNA dC->dU-editing enzyme APOBEC-3C
Vacuolar protein sorting-associated protein 45
RNA-binding protein PNO1
Large ribosomal subunit protein bL17m
14 kDa phosphohistidine phosphatase
Serine incorporator 1
Rho GTPase-activating protein 35
Protein FAM114A2
Phospholipid scramblase 3
1-acyl-sn-glycerol-3-phosphate acyltransferase delta
1-acyl-sn-glycerol-3-phosphate acyltransferase gamma
Lymphoid-specific helicase
Glycoprotein-N-acetylgalactosamine 3-beta-galactosyltransferase 1
Latent-transforming growth factor beta-binding protein 3
Mitochondrial import receptor subunit TOM22 homolog
LanC-like protein 2
Kinesin-like protein KIF15
Transmembrane 7 superfamily member 3
Homer protein homolog 3
Phenylalanine--tRNA ligase beta subunit
Isoleucine--tRNA ligase, mitochondrial
Ribosome biogenesis protein SLX9 homolog
SAM domain-containing protein SAMSN-1
Kinesin light chain 4
Protein diaphanous homolog 3
BMP-2-inducible protein kinase
Ubiquitin-like-conjugating enzyme ATG3
NAD-dependent protein deacetylase sirtuin-3, mitochondrial
Sister chromatid cohesion protein PDS5 homolog B

Structural maintenance of chromosomes protein 4
Alpha-mannosidase 2C1
Phosphatidylinositol-3-phosphatase SAC1
Protein DEPP1
Obg-like ATPase 1
Copper homeostasis protein cutC homolog
Zinc finger protein 64
Ethylmalonyl-CoA decarboxylase
RNA-binding protein 12
TBC1 domain family member 22B
Midasin
Acetyl-coenzyme A synthetase 2-like, mitochondrial
Zinc finger CCHC domain-containing protein 3
Peroxisomal 2,4-dienoyl-CoA reductase [(3E)-enoyl-CoA-producing]
Palmitoyl-protein thioesterase ABHD10, mitochondrial
T-complex protein 11-like protein 1
Double-stranded RNA-binding protein Staufen homolog 2
Shiftless antiviral inhibitor of ribosomal frameshifting protein
Probable ATP-dependent RNA helicase DDX28
Transmembrane protein 106B
Protein lin-7 homolog C
1-acyl-sn-glycerol-3-phosphate acyltransferase epsilon
Gamma-taxilin
SPATS2-like protein
Ufm1-specific protease 2
ATP-binding cassette sub-family F member 3
CYFIP-related Rac1 interactor B
Mitochondrial potassium channel ATP-binding subunit
ATP-dependent RNA helicase DDX19A
GTPase IMAP family member 4
Tyrosyl-DNA phosphodiesterase 1
TBC1 domain family member 23
DDB1- and CUL4-associated factor 13
U3 small nucleolar ribonucleoprotein protein IMP3
MRG/MORF4L-binding protein
S-adenosyl-L-methionine-dependent tRNA 4-demethylwyosine synthase TYW1
Exocyst complex component 1
Protein-L-isoaspartate O-methyltransferase domain-containing protein 2
Integrator complex subunit 9
Cell cycle control protein 50A
Ubiquinol-cytochrome c reductase complex assembly factor 1
Septin-11
Sodium-coupled neutral amino acid transporter 7
Mediator of RNA polymerase II transcription subunit 17
Alpha-parvin
Ubiquitin carboxyl-terminal hydrolase 40
4'-phosphopantetheine phosphatase
F-box only protein 28
TBC1 domain family member 13
Exonuclease 3'-5' domain-containing protein 2

DnaJ homolog subfamily C member 11
Integrator complex subunit 7
Fanconi anemia group I protein
ATPase family AAA domain-containing protein 3A
ADP-ribosylation factor-like protein 8B
DnaJ homolog subfamily C member 17
Integrator complex subunit 13
Synembryn-B
Guanine nucleotide-binding protein-like 3-like protein
ATP-dependent RNA helicase DDX18
Histone chaperone ASF1B
Kelch-like protein 11
Integrator complex subunit 10
Large ribosomal subunit protein mL66
Pyridoxine-5'-phosphate oxidase
Armadillo repeat-containing protein 1
DNA-directed RNA polymerase III subunit RPC5
Protein SDA1 homolog
Poly(A) RNA polymerase, mitochondrial
Notchless protein homolog 1
Adaptin ear-binding coat-associated protein 2
DNA-directed RNA polymerase III subunit RPC2
RNA-binding protein 28
Anoctamin-10
Pre-mRNA-splicing factor RBM22
WD repeat-containing protein 70
Transmembrane protein 51
Arginine and glutamate-rich protein 1
SAFB-like transcription modulator
Peptidyl-prolyl cis-trans isomerase FKBP14
Required for meiotic nuclear division protein 1 homolog
p21-activated protein kinase-interacting protein 1
Hypoxia-inducible factor 1-alpha inhibitor
3-oxoacyl-[acyl-carrier-protein] synthase, mitochondrial
Large ribosomal subunit protein uL22m
CXXC motif containing zinc binding protein
BRISC and BRCA1-A complex member 1
Ceroid-lipofuscinosis neuronal protein 6
Ankyrin repeat and SOCS box protein 6
Probable tRNA(His) guanylyltransferase
Histone PARylation factor 1
Interleukin-1 receptor-associated kinase 4
Uridine-cytidine kinase-like 1
Transmembrane protein 160
NACHT, LRR and PYD domains-containing protein 2
Constitutive coactivator of PPAR-gamma-like protein 2
tRNA selenocysteine 1-associated protein 1
NADH dehydrogenase [ubiquinone] 1 beta subcomplex subunit 11, mitochondrial
Large ribosomal subunit protein uL16m
H/ACA ribonucleoprotein complex subunit 2

OCIA domain-containing protein 1
ADP-ribosylhydrolase ARH3
E3 ubiquitin-protein ligase MARCHF5
Cell growth-regulating nucleolar protein
Transmembrane protein 161A
Golgi-resident adenosine 3',5'-bisphosphate 3'-phosphatase
MICOS complex subunit MIC19
tRNA-dihydrouridine(20) synthase [NAD(P)+]-like
CKLF-like MARVEL transmembrane domain-containing protein 6
NAD-dependent protein deacylase sirtuin-5, mitochondrial
GATOR2 complex protein MIOS
Myotubularin-related protein 10
Sphingomyelin phosphodiesterase 4
Pre-mRNA-splicing factor CWC25 homolog
Testis-expressed protein 10
DDB1- and CUL4-associated factor 16
THUMP domain-containing protein 1
Transmembrane prolyl 4-hydroxylase
Torsin-4A
tRNA (guanine(26)-N(2))-dimethyltransferase
Ganglioside-induced differentiation-associated protein 2
Nuclear distribution protein nudE homolog 1
BRISC and BRCA1-A complex member 2
Glutaminyl-peptide cyclotransferase-like protein
ADP-ribosylation factor-like protein 15
BTB/POZ domain-containing protein KCTD5
CDKN2A-interacting protein
DnaJ homolog subfamily B member 12
Alpha-ketoglutarate-dependent dioxygenase alkB homolog 4
Non-structural maintenance of chromosomes element 4 homolog A
H/ACA ribonucleoprotein complex subunit 1
Stabilin-1
Serine/threonine-protein phosphatase 4 regulatory subunit 2
Dipeptidyl peptidase 3
Protein AATF
Tubulin alpha-8 chain
Probable ATP-dependent RNA helicase DDX56
Sphingosine kinase 1
Telomeric repeat-binding factor 2-interacting protein 1
Abl interactor 2
Bcl-2-associated transcription factor 1
U3 small nucleolar RNA-associated protein 6 homolog
TGF-beta-activated kinase 1 and MAP3K7-binding protein 2
Large ribosomal subunit protein mL39
Mitogen-activated protein kinase kinase kinase 20
Peptidyl-prolyl cis-trans isomerase FKBP11
Tropomodulin-3
BET1-like protein
Very long chain fatty acid elongase 5
NF-kappa-B inhibitor-interacting Ras-like protein 2

UDP-glucose:glycoprotein glucosyltransferase 2
UDP-glucose:glycoprotein glucosyltransferase 1
Cyclin-dependent kinase 12
FAST kinase domain-containing protein 2, mitochondrial
Very-long-chain enoyl-CoA reductase
Endoplasmic reticulum aminopeptidase 1
Actin-related protein 10
Vesicle transport protein USE1
CDGSH iron-sulfur domain-containing protein 1
ADP-ribosylation factor-binding protein GGA3
Splicing factor C9orf78
Constitutive coactivator of PPAR-gamma-like protein 1
Glycerophosphodiester phosphodiesterase 1
SWI/SNF-related matrix-associated actin-dependent regulator of chromatin subfamily A-like protein 1
Glycolipid transfer protein
Maspardin
Large ribosomal subunit protein bL35m
Upstream-binding protein 1
Insulin-like growth factor 2 mRNA-binding protein 1
Sacsin
Mitochondrial carrier homolog 1
Diphosphoinositol polyphosphate phosphohydrolase 2
Hsp70-binding protein 1
Methionine adenosyltransferase 2 subunit beta
Myoferlin
Intersectin-2
Ribosome biogenesis protein NOP53
EH domain-containing protein 3
EH domain-containing protein 2
Rho guanine nucleotide exchange factor 12
CCR4-NOT transcription complex subunit 2
NCK-interacting protein with SH3 domain
Programmed cell death 1 ligand 1
Calmodulin-like protein 5
Opioid growth factor receptor
LIM and cysteine-rich domains protein 1
Cysteine-rich motor neuron 1 protein
Selenoprotein N
Protein PALS2
Charged multivesicular body protein 5
Spliceosome-associated protein CWC15 homolog
Large ribosomal subunit protein uL15m
Thymocyte nuclear protein 1
Thyroid transcription factor 1-associated protein 26
NADH dehydrogenase [ubiquinone] 1 alpha subcomplex assembly factor 4
Very-long-chain (3R)-3-hydroxyacyl-CoA dehydratase 3
ER membrane protein complex subunit 3
NADH dehydrogenase [ubiquinone] 1 alpha subcomplex subunit 13
[Pyruvate dehydrogenase [acetyl-transferring]]-phosphatase 1, mitochondrial
E3 ubiquitin-protein ligase KCMF1

Ankycorbin
Vesicle-associated membrane protein-associated protein A
Large ribosomal subunit protein bL27m
Proton-transporting V-type ATPase complex assembly regulator TMEM9
Sentrin-specific protease 1
CXXC-type zinc finger protein 1
SH3 domain-binding protein 4
Septin-10
GEM-interacting protein
PHD and RING finger domain-containing protein 1
Pogo transposable element with KRAB domain
Protein Daple
Rho GTPase-activating protein 23
Vacuolar protein sorting-associated protein 18 homolog
Protein RCC2
RAB11-binding protein RELCH
Disco-interacting protein 2 homolog B
Junctional cadherin 5-associated protein
SLAIN motif-containing protein 2
Teneurin-3
Ubiquitin carboxyl-terminal hydrolase 36
BRCA2 and CDKN1A-interacting protein
Serine/threonine-protein kinase 26
Armadillo repeat-containing X-linked protein 1
ABI gene family member 3
Prostaglandin F2 receptor negative regulator
CTTNBP2 N-terminal-like protein
Transmembrane protein 181
Inhibitor of Bruton tyrosine kinase
HEAT repeat-containing protein 5B
NFX1-type zinc finger-containing protein 1
Chondroitin sulfate glucuronyltransferase
Protocadherin-10
Ribosome-binding protein 1
Cleavage and polyadenylation specificity factor subunit 2
Kelch-like protein 9
Leucine--tRNA ligase, cytoplasmic
Myelin expression factor 2
Kelch-like protein 42
eIF-2-alpha kinase GCN2
WD repeat-containing protein 35
RNA-binding protein 27
KAT8 regulatory NSL complex subunit 3
E3 ubiquitin-protein ligase HECW2
FERM domain-containing protein 4A
Rabankyrin-5
Succinate--CoA ligase [ADP-forming] subunit beta, mitochondrial
GMP reductase 2
Syntaxin-18
Dolichol-phosphate mannosyltransferase subunit 3

Protein IMPACT
UV radiation resistance-associated gene protein
Ataxin-10
Methyl-CpG-binding domain protein 2
Neurochondrin
Tuftelin-interacting protein 11
Epidermal growth factor receptor substrate 15-like 1
Unconventional myosin-Ia
Origin recognition complex subunit 3
SUMO-activating enzyme subunit 1
Coatomer subunit gamma-2
Phosphatidylinositol 4-kinase beta
C-type mannose receptor 2
COMM domain-containing protein 3
Guanine nucleotide-binding protein G(I)/G(S)/G(O) subunit gamma-12
Rab-like protein 2A
Methionine synthase reductase
Protein UXT
Set1/Ash2 histone methyltransferase complex subunit ASH2
7-dehydrocholesterol reductase
Tumor necrosis factor receptor superfamily member 10D
Histone deacetylase 6
Spastin
Dickkopf-related protein 3
tRNA (guanine-N(7)-)-methyltransferase
PTB domain-containing engulfment adapter protein 1
Vacuolar protein sorting-associated protein 29
Eukaryotic translation initiation factor 3 subunit K
Exostosin-like 2
Glyoxylate reductase/hydroxypyruvate reductase
Cathepsin Z
Ribosomal protein S6 kinase beta-2
DnaJ homolog subfamily B member 11
E3 ubiquitin-protein ligase RNF14
SUMO-activating enzyme subunit 2
Mortality factor 4-like protein 1
Nuclear RNA export factor 1
Protein sel-1 homolog 1
Beta-1,4-galactosyltransferase 7
Peflin
Zinc finger MYM-type protein 2
COP9 signalosome complex subunit 7a
Mitochondrial dicarboxylate carrier
Alpha-aminoadipic semialdehyde synthase, mitochondrial
CAP-Gly domain-containing linker protein 2
Cytochrome b-c1 complex subunit 9
Mitochondrial fission process protein 1
Tight junction protein ZO-2
DnaJ homolog subfamily B member 4
Serine/threonine-protein kinase 17A

Craniofacial development protein 1
Death domain-associated protein 6
tRNA (cytidine(32)/guanosine(34)-2'-O)-methyltransferase
Vesicle transport through interaction with t-SNAREs homolog 1B
STE20/SPS1-related proline-alanine-rich protein kinase
Gamma-adducin
Leucine-rich repeat and WD repeat-containing protein 1
Protein NipSnap homolog 3A
CGG triplet repeat-binding protein 1
Phosphatidylserine decarboxylase proenzyme, mitochondrial
ATP-binding cassette sub-family F member 2
Testin
5'-AMP-activated protein kinase subunit gamma-2
Gamma-tubulin complex component 4
Lysine-specific demethylase 5B
Tryptophan--tRNA ligase, mitochondrial
LIM domain-containing protein 1
Translocation protein SEC63 homolog
Solute carrier family 2, facilitated glucose transporter member 6
Zinc finger CCCH domain-containing protein 7B
Protein NDRG3
Armadillo repeat-containing X-linked protein 3
Switch-associated protein 70
SUN domain-containing protein 2
Probable ribosome biogenesis protein RLP24
Ragulator complex protein LAMTOR3
LIM domain and actin-binding protein 1
AF4/FMR2 family member 4
Signal recognition particle subunit SRP68
Cysteine and histidine-rich domain-containing protein 1
Serine/threonine-protein kinase TBK1
Septin-9
Prenylcysteine oxidase 1
Probable ATP-dependent RNA helicase DDX20
Sedoheptulokinase
Dipeptidyl peptidase 2
Inactive cell surface hyaluronidase CEMIP2
Ubiquitin carboxyl-terminal hydrolase 25
B-cell receptor-associated protein 29
NADH-cytochrome b5 reductase 1
SAP30-binding protein
Zinc finger HIT domain-containing protein 2
Prefoldin subunit 2
GPN-loop GTPase 3
Poly(U)-binding-splicing factor PUF60
Nuclear receptor-binding protein
Enolase-phosphatase E1
Fasciculation and elongation protein zeta-2
NADH dehydrogenase [ubiquinone] 1 alpha subcomplex subunit 12
Translation initiation factor eIF2B subunit delta

V-type proton ATPase subunit H
Prenylated Rab acceptor protein 1
Importin-11
Multifunctional methyltransferase subunit TRM112-like protein
Dachshund homolog 1
Exportin-7
Leucine carboxyl methyltransferase 1
Vacuolar protein sorting-associated protein 51 homolog
Bromodomain adjacent to zinc finger domain protein 2B
Bromodomain adjacent to zinc finger domain protein 2A
Tyrosine-protein kinase BAZ1B
ATPase inhibitor, mitochondrial
GTP:AMP phosphotransferase AK3, mitochondrial
Leucyl-cystinyl aminopeptidase
Methyl-CpG-binding domain protein 1
CCR4-NOT transcription complex subunit 7
Rab5 GDP/GTP exchange factor
Mitochondrial peptide methionine sulfoxide reductase
N-acetyl-D-glucosamine kinase
2-hydroxyacyl-CoA lyase 1
tRNA (adenine(58)-N(1))-methyltransferase non-catalytic subunit TRM6
SH3 domain-binding glutamic acid-rich-like protein 2
Ras GTPase-activating protein nGAP
18S rRNA aminocarboxypropyltransferase
Electrogenic aspartate/glutamate antiporter SLC25A13, mitochondrial
Drebrin-like protein
Probable ATP-dependent RNA helicase DDX41
Dynactin subunit 4
Cell division cycle protein 23 homolog
Anaphase-promoting complex subunit 7
Anaphase-promoting complex subunit 5
Anaphase-promoting complex subunit 4
Anaphase-promoting complex subunit 2
ADP-ribosylation factor-binding protein GGA2
ADP-ribosylation factor-binding protein GGA1
Stomatin-like protein 2, mitochondrial
N-acetylglucosamine-1-phosphodiester alpha-N-acetylglucosaminidase
Vacuolar protein sorting-associated protein 28 homolog
U6 snRNA-associated Sm-like protein LSm7
Lariat debranching enzyme
Protein TASOR
Jupiter microtubule associated homolog 1
F-box only protein 3
A-kinase anchor protein 11
F-box/WD repeat-containing protein 11
Glucocorticoid modulatory element-binding protein 2
mRNA turnover protein 4 homolog
TRAF2 and NCK-interacting protein kinase
Cleavage and polyadenylation specificity factor subunit 3
DCC-interacting protein 13-alpha

Cdc42 effector protein 3
Serine/threonine-protein kinase tousled-like 1
G patch domain-containing protein 8
Protein mono-ADP-ribosyltransferase PARP4
NTF2-related export protein 1
ADP-sugar pyrophosphatase
REST corepressor 1
Phosphatidylcholine transfer protein
Endoplasmic reticulum mannosyl-oligosaccharide 1,2-alpha-mannosidase
RNA-binding protein Raly
General transcription factor 3C polypeptide 4
Ergosterol biosynthetic protein 28 homolog
Protein kinase C and casein kinase substrate in neurons protein 3
Isobutyryl-CoA dehydrogenase, mitochondrial
Angiopoietin-related protein 2
Apoptotic chromatin condensation inducer in the nucleus
E3 ubiquitin-protein ligase AMFR
Protein argonaute-2
Nuclear pore complex protein Nup50
Protein CDV3 homolog
CCR4-NOT transcription complex subunit 11
Integrator complex subunit 6
BAG family molecular chaperone regulator 5
Protein argonaute-1
Ras-related protein Rab-21
Ras-related protein Rab-22A
Trafficking protein particle complex subunit 2-like protein
Zinc finger protein 346
Proteasome activator complex subunit 2
Serine/threonine-protein kinase TAO2
Muskelin
Aspartyl aminopeptidase
Ras-related protein Rab-23
Malignant T-cell-amplified sequence 1
Microtubule-associated tumor suppressor 1
Protein WWC3
Paladin
Zinc transporter ZIP10
Chromatin-remodeling ATPase INO80
Cell cycle progression protein 1
Kinase D-interacting substrate of 220 kDa
Arf-GAP with SH3 domain, ANK repeat and PH domain-containing protein 1
Myocardin-related transcription factor B
Ankyrin repeat domain-containing protein 50
Mediator of RNA polymerase II transcription subunit 23
Protein Shroom4
YEATS domain-containing protein 2
Protein NDRG4
TBC1 domain family member 24
Pre-mRNA-splicing factor ISY1 homolog

Transmembrane and coiled-coil domain protein 3
E3 ubiquitin-protein ligase HECTD1
MYND-type zinc finger-containing chromatin reader ZMYND8
Cip1-interacting zinc finger protein
Coronin-1C
Targeting protein for Xklp2
Activator of basal transcription 1
RNA-binding protein NOB1
A-kinase anchor protein 8-like
Transcription factor MafF
Apoptosis-associated speck-like protein containing a CARD
Calcium load-activated calcium channel
Fizzy-related protein homolog
Anaphase-promoting complex subunit 10
Unconventional myosin-VI
ATP-dependent RNA helicase DDX19B
Lysosomal thioesterase PPT2
Pre-mRNA-processing factor 19
Suppressor of fused homolog
Bcl-2-related ovarian killer protein
Nucleolar protein 7
Sorting nexin-12
Synergin gamma
Vacuolar protein sorting-associated protein 4A
Ras GTPase-activating protein-binding protein 2
E3 ubiquitin-protein ligase CHIP
Protein kinase C and casein kinase substrate in neurons protein 2
Melanoma-associated antigen D2
Sorting nexin-7
Sorting nexin-6
Dual specificity protein phosphatase 12
Syntaxin-8
Translocon-associated protein subunit gamma
26S proteasome non-ATPase regulatory subunit 13
FAS-associated factor 1
Endothelial protein C receptor
Peptidyl-prolyl cis-trans isomerase E
Probable dimethyladenosine transferase
COP9 signalosome complex subunit 3
Multiple inositol polyphosphate phosphatase 1
RNA-binding protein Nova-2
WD repeat-containing protein 3
Transcription termination factor 2
NSFL1 cofactor p47
Conserved oligomeric Golgi complex subunit 5
Solute carrier family 12 member 4
AP-4 complex subunit epsilon-1
Microtubule-actin cross-linking factor 1, isoforms 1/2/3/4/5
Centrosomal protein of 131 kDa
SR-related and CTD-associated factor 8

Serine/threonine-protein phosphatase 6 regulatory subunit 1
E3 ubiquitin-protein ligase TRIM33
Histone lysine demethylase PHF8
Trinucleotide repeat-containing gene 6B protein
Inactive phospholipase C-like protein 2
Nonsense-mediated mRNA decay factor SMG5
Exocyst complex component 7
Zinc finger CCCH domain-containing protein 4
Ubiquitin carboxyl-terminal hydrolase 24
TBC1 domain family member 2B
Protein Smaug homolog 1
Forkhead box protein J3
Cytosolic carboxypeptidase 1
Endoribonuclease Dicer
Cystine/glutamate transporter
Microtubule-associated protein RP/EB family member 3
Leucine-rich repeat protein SHOC-2
Serine/arginine repetitive matrix protein 2
Alpha-1,3-mannosyl-glycoprotein 4-beta-N-acetylglucosaminyltransferase B
Proliferation-associated protein 2G4
Paraplegin
Brain-specific angiogenesis inhibitor 1-associated protein 2
GRB2-associated-binding protein 2
Structural maintenance of chromosomes protein 3
Charged multivesicular body protein 2b
SH2B adapter protein 3
Zinc finger protein 148
Myotubularin-related protein 6
Protein jagged-2
60S ribosome subunit biogenesis protein NIP7 homolog
Bifunctional UDP-N-acetylglucosamine 2-epimerase/N-acetylmannosamine kinase
RNA transcription, translation and transport factor protein
RuvB-like 2
Chromodomain Y-like protein
Peptidyl-prolyl cis-trans isomerase NIMA-interacting 4
RAC-gamma serine/threonine-protein kinase
CAAX prenyl protease 2
Choline/ethanolamine kinase
Eukaryotic translation initiation factor 3 subunit L
Phospholipase A-2-activating protein
RuvB-like 1
Nuclear migration protein nudC
Mitochondrial chaperone BCS1
Voltage-dependent anion-selective channel protein 3
Cofilin-2
Endoplasmic reticulum-Golgi intermediate compartment protein 3
Phenylalanine--tRNA ligase alpha subunit
Sodium-dependent multivitamin transporter
Small ribosomal subunit protein mS33
Histone chaperone ASF1A

Developmentally-regulated GTP-binding protein 1
Trafficking protein particle complex subunit 4
Nck-associated protein 1
Protein canopy homolog 2
Nuclease EXOG, mitochondrial
Exocyst complex component 6B
PALM2-AKAP2 fusion protein
Caspase recruitment domain-containing protein 8
Phospholipid-transporting ATPase IF
GDP-fucose protein O-fucosyltransferase 2
DnaJ homolog subfamily C member 16
Disks large-associated protein 4
Serine/threonine-protein kinase 38-like
Phosphatidylinositide phosphatase SAC2
Fibronectin type-III domain-containing protein 3A
Nischarin
1-phosphatidylinositol 3-phosphate 5-kinase
WD repeat-containing protein 37
Band 4.1-like protein 3
Angiomotin-like protein 2
Serine/threonine-protein kinase SIK3
Lysine-specific demethylase 2A
Exosome complex exonuclease RRP44
Trafficking protein particle complex subunit 8
Leucine-rich repeat and calponin homology domain-containing protein 1
RNA 3'-terminal phosphate cyclase-like protein
Glutathione S-transferase kappa 1
Ragulator complex protein LAMTOR2
Small ribosomal subunit protein bS1m
Cytochrome c oxidase assembly factor 3 homolog, mitochondrial
Probable ATP-dependent RNA helicase DDX52
Small ribosomal subunit protein uS17m
Small ribosomal subunit protein uS7m
Lambda-crystallin homolog
Translation machinery-associated protein 7
Polymerase delta-interacting protein 2
AP-3 complex subunit mu-1
Inner nuclear membrane protein Man1
Calcium-regulated heat-stable protein 1
Conserved oligomeric Golgi complex subunit 6
Thyroid hormone receptor-associated protein 3
WW domain-binding protein 11
Tudor and KH domain-containing protein
Mediator of RNA polymerase II transcription subunit 16
Nucleolar protein 58
ARF GTPase-activating protein GIT1
Zinc finger protein 281
ADP-ribosylation factor-like protein 2-binding protein
Transmembrane protein 98
Protein SGT1 homolog

Tyrosine--tRNA ligase, mitochondrial
N-acetylglucosamine-6-phosphate deacetylase
Acyl-coenzyme A thioesterase 9, mitochondrial
Protein AAR2 homolog
Nitric oxide synthase-interacting protein
Deoxyribose-phosphate aldolase
Protein MEMO1
Thioredoxin-related transmembrane protein 2
rRNA-processing protein FCF1 homolog
U6 snRNA-associated Sm-like protein LSm2
Endophilin-B1
Complex I intermediate-associated protein 30, mitochondrial
Calcium-binding protein 39
Putative RNA-binding protein Luc7-like 2
Ubiquitin-conjugating enzyme E2 J1
RNA-binding motif protein, X-linked 2
Dehydrogenase/reductase SDR family member 7
Small ribosomal subunit protein uS2m
Probable U3 small nucleolar RNA-associated protein 11
MOB-like protein phocein
Ribosomal RNA-processing protein 7 homolog A
Ribosome maturation protein SBDS
Transmembrane emp24 domain-containing protein 5
Exosome complex component CSL4
Transmembrane emp24 domain-containing protein 7
Splicing factor 3B subunit 6
Large ribosomal subunit protein uL11m
Oligoribonuclease, mitochondrial
RRP15-like protein
WASH complex subunit 3
Nucleolar protein 16
EKC/KEOPS complex subunit TPRKB
Peptidyl-prolyl cis-trans isomerase-like 1
Ubiquitin-fold modifier-conjugating enzyme 1
Cytosolic iron-sulfur assembly component 2B
Small ribosomal subunit protein bS16m
Small ribosomal subunit protein bS18m
Mitochondrial fission 1 protein
Mitochondrial import inner membrane translocase subunit TIM16
Adenylate kinase isoenzyme 6
Small ribosomal subunit protein mS23
Vesicle transport protein GOT1B
Hepatoma-derived growth factor-related protein 3
Peptidyl-tRNA hydrolase 2, mitochondrial
Charged multivesicular body protein 3
Serine-threonine kinase receptor-associated protein
RNA-splicing ligase RtcB homolog
F-box only protein 7
Ras-related protein Rap-2c
StAR-related lipid transfer protein 13

Rab GTPase-activating protein 1
Transmembrane emp24 domain-containing protein 3
TSC22 domain family protein 4
Protein dopey-2
Zinc finger protein 330
R3H and coiled-coil domain-containing protein 1
Nucleolar complex protein 2 homolog
Large ribosomal subunit protein eL36
Coiled-coil domain-containing protein 9
Chromatin target of PRMT1 protein
Deoxynucleoside triphosphate triphosphohydrolase SAMHD1
Small kinetochore-associated protein
HBS1-like protein
5'-AMP-activated protein kinase subunit beta-1
WD repeat domain phosphoinositide-interacting protein 4
DmX-like protein 1
V-type proton ATPase 116 kDa subunit a 2
Talin-1
Kinesin-like protein KIF3A
Transformation/transcription domain-associated protein
Microtubule cross-linking factor 1
DDB1- and CUL4-associated factor 1
TRPM8 channel-associated factor 1
Probable RNA-binding protein 19
Disheveled-associated activator of morphogenesis 1
Plexin-D1
Probable E3 ubiquitin-protein ligase HECTD4
WD repeat-containing protein 7
Ubiquitin carboxyl-terminal hydrolase 15
FERM, ARHGEF and pleckstrin domain-containing protein 1
Meiosis regulator and mRNA stability factor 1
Centrosomal protein of 170 kDa protein B
Rap guanine nucleotide exchange factor 2
Unconventional myosin-Va
Dystrobrevin alpha
Lysyl oxidase homolog 2
Beta/gamma crystallin domain-containing protein 1
TNF receptor-associated factor 6
Mitogen-activated protein kinase kinase kinase kinase 5
Hypoxia up-regulated protein 1
Cysteine protease ATG4B
Transducin beta-like protein 2
WD repeat domain phosphoinositide-interacting protein 2
Telomere length regulation protein TEL2 homolog
Ribosomal biogenesis protein LAS1L
AFG3-like protein 2
Krueppel-like factor 12
E3 ubiquitin-protein ligase ARIH1
U6 snRNA-associated Sm-like protein LSm4
E3 ubiquitin-protein ligase RNF114

Sorting and assembly machinery component 50 homolog
Protein PRRC2C
ADP-ribose glycohydrolase OARD1
Intraflagellar transport protein 25 homolog
Protein phosphatase methylesterase 1
RNA-binding protein 7
Mitochondrial import inner membrane translocase subunit Tim22
NEDD8 ultimate buster 1
YTH domain-containing family protein 2
RNA polymerase II subunit A C-terminal domain phosphatase
PAX3- and PAX7-binding protein 1
Nucleoside diphosphate kinase 7
FACT complex subunit SPT16
U3 small nucleolar RNA-associated protein 18 homolog
Ubiquitin carboxyl-terminal hydrolase isozyme L5
CD2-associated protein
V-type proton ATPase subunit D
Transportin-3
Signal recognition particle receptor subunit beta
Origin recognition complex subunit 6
Ceramide transfer protein
Mannose-1-phosphate guanyltransferase beta
Fatty acid desaturase 3
General transcription factor 3C polypeptide 5
General transcription factor 3C polypeptide 3
Trafficking protein particle complex subunit 1
Transient receptor potential cation channel subfamily V member 2
Serine/threonine-protein kinase MRCK beta
RNA-binding protein 8A
DnaJ homolog subfamily C member 15
Ubiquitin carboxyl-terminal hydrolase 16
U5 small nuclear ribonucleoprotein TSSC4
Zinc finger protein 706
Melanoma-associated antigen D1
Sorting nexin-14
Sorting nexin-11
Sorting nexin-9
Sorting nexin-8
Sorting nexin-5
Endothelial lipase
Cytosolic Fe-S cluster assembly factor NUBP2
Peroxisomal membrane protein PEX16
Lymphatic vessel endothelial hyaluronic acid receptor 1
Beta-secretase 2
Heme-binding protein 2
Host cell factor 2
UbiA prenyltransferase domain-containing protein 1
Pseudouridylate synthase 1 homolog
Leucine-rich repeat flightless-interacting protein 2
FH1/FH2 domain-containing protein 1

Phosphoserine aminotransferase
Nuclear receptor corepressor 2
Mitochondrial ornithine transporter 1
Junctional adhesion molecule A
Neuroplastin
Adhesion G-protein coupled receptor G1
Spindlin-1
Solute carrier family 12 member 7
Dolichyl pyrophosphate Man9GlcNAc2 alpha-1,3-glucosyltransferase
Dolichyl-phosphate beta-glucosyltransferase
Small ribosomal subunit protein mS40
Coatomer subunit gamma-1
Lipid droplet-regulating VLDL assembly factor AUP1
Peptidyl-prolyl cis-trans isomerase FKBP7
Chloride intracellular channel protein 4
Cysteine desulfurase
Cilia- and flagella-associated protein 20
Transforming acidic coiled-coil-containing protein 3
Signal peptidase complex subunit 1
GTP-binding protein SAR1b
AP-4 complex subunit beta-1
Mitochondrial carrier homolog 2
Selenoprotein K
Brefeldin A-inhibited guanine nucleotide-exchange protein 2
Brefeldin A-inhibited guanine nucleotide-exchange protein 1
Mitotic spindle assembly checkpoint protein MAD1
Serine/threonine-protein kinase 24
eIF5-mimic protein 1
COMM domain-containing protein 10
Cytoplasmic dynein 1 light intermediate chain 1
Epsin-1
Ubiquitin carboxyl-terminal hydrolase 3
Testis-expressed protein 264
Choline/ethanolaminephosphotransferase 1
DNA (cytosine-5)-methyltransferase 3A
2'-5'-oligoadenylate synthase 3
Adenylate kinase isoenzyme 5
NF-kappa-B essential modulator
Insulin-like growth factor 2 mRNA-binding protein 2
Proton-coupled zinc antiporter SLC30A1
Sodium bicarbonate cotransporter 3
NADH dehydrogenase [ubiquinone] 1 beta subcomplex subunit 9
Sulfide:quinone oxidoreductase, mitochondrial
Roundabout homolog 1
Stonin-1
Nuclear receptor coactivator 3
Numb-like protein
Mitogen-activated protein kinase kinase kinase 4
Probable ATP-dependent RNA helicase DDX49
Calpain-7

Actin-binding protein WASF2
E3 SUMO-protein ligase PIAS3
MAU2 chromatid cohesion factor homolog
ATPase MORC2
Influenza virus NS1A-binding protein
SEC23-interacting protein
Putative glutamine amidotransferase-like class 1 domain-containing protein 3B, mitochondrial
Intraflagellar transport protein 56
Ubiquitin-like modifier-activating enzyme 6
Extended synaptotagmin-2
Bridge-like lipid transfer protein family member 3B
Shootin-1
Transmembrane protein 223
Rho GTPase-activating protein 10
2-hydroxyacyl-CoA lyase 2
SH3 and PX domain-containing protein 2B
NBAS subunit of NRZ tethering complex
tRNA wybutosine-synthesizing protein 5
DENN domain-containing protein 3
von Willebrand factor A domain-containing protein 8
Protein strawberry notch homolog 1
GTP-binding protein 10
WD repeat-containing protein 91
DENN domain-containing protein 11
Plasmanylethanolamine desaturase 1
Trafficking protein particle complex subunit 13
CCR4-NOT transcription complex subunit 1
Microtubule-associated proteins 1A/1B light chain 3 beta 2
Glycerol-3-phosphate phosphatase
RAB7A-interacting MON1-CCZ1 complex subunit 1
RCC1 domain-containing protein 1
TRPM8 channel-associated factor 2
Structural maintenance of chromosomes flexible hinge domain-containing protein 1
Cytosolic arginine sensor for mTORC1 subunit 2
Protein unc-119 homolog B
12S rRNA N4-methylcytidine (m4C) methyltransferase
Coiled-coil domain-containing protein 85C
Retrotransposon Gag-like protein 8C
Zinc finger SWIM domain-containing protein 8
Nuclear envelope pore membrane protein POM 121C
PHD finger protein 20-like protein 1
Putative small nuclear ribonucleoprotein G-like protein 15
Endosome/lysosome-associated apoptosis and autophagy regulator family member 2
Acyl-coenzyme A diphosphatase NUDT19
MIF4G domain-containing protein
Calmodulin-1
Golgi pH regulator A
Putative WAS protein family homolog 3
Mapk-regulated corepressor-interacting protein 1
Nascent polypeptide-associated complex subunit alpha, muscle-specific form

Uncharacterized protein C11orf98
ASNSD1 upstream open reading frame protein
Mitochondrial ribosome and complex I assembly factor AltMIEF1
Deoxyribonuclease-2-alpha
Alkyldihydroxyacetonephosphate synthase, peroxisomal
Kinesin-like protein KIF2A
ATP-dependent RNA helicase DDX39A
PDZ and LIM domain protein 1
Cytosolic acyl coenzyme A thioester hydrolase
Unconventional myosin-Ic
Synaptosomal-associated protein 23
HCLS1-associated protein X-1
AH receptor-interacting protein
GTP-binding protein 1
Galectin-9
Syntaxin-binding protein 3
AP-4 complex subunit mu-1
Splicing regulator ARVCF
Small acidic protein
AP-3 complex subunit beta-1
Amyloid beta precursor protein binding family B member 1
Galectin-8
NADH dehydrogenase [ubiquinone] iron-sulfur protein 8, mitochondrial
NF-kappa-B inhibitor epsilon
26S proteasome non-ATPase regulatory subunit 11
26S proteasome non-ATPase regulatory subunit 12
26S proteasome non-ATPase regulatory subunit 9
Menin
Guided entry of tail-anchored proteins factor 1
Membrane-associated progesterone receptor component 1
Transcription elongation factor SPT5
Transcription initiation factor TFIID subunit 4
DNA fragmentation factor subunit alpha
Huntingtin-interacting protein 1
Chloride intracellular channel protein 1
Eukaryotic translation initiation factor 3 subunit F
NEDD4-like E3 ubiquitin-protein ligase WWP2
Pyruvate dehydrogenase protein X component, mitochondrial
Sulfhydryl oxidase 1
Dynactin subunit 6
Acetyl-coenzyme A transporter 1
Actin nucleation-promoting factor WASL
Importin-5
DNA-directed RNA polymerase, mitochondrial
Eukaryotic elongation factor 2 kinase
Histone deacetylase complex subunit SAP18
Echinoderm microtubule-associated protein-like 1
Insulin-like growth factor 2 mRNA-binding protein 3
Dynamin-1-like protein
RNA 3'-terminal phosphate cyclase

Phosphatidylinositol 4-phosphate 3-kinase C2 domain-containing subunit alpha
Golgi integral membrane protein 4
Agrin
Procollagen-lysine,2-oxoglutarate 5-dioxygenase 2
Exocyst complex component 5
Butyrophilin subfamily 3 member A1
Cytochrome c oxidase subunit NDUFA4
26S proteasome non-ATPase regulatory subunit 14
Myc box-dependent-interacting protein 1
Claudin-5
Importin subunit alpha-4
Serine/threonine-protein kinase 25
Krev interaction trapped protein 1
von Willebrand factor A domain-containing protein 5A
Pescadillo homolog
Syntenin-1
Membrane-associated phosphatidylinositol transfer protein 1
U3 small nucleolar ribonucleoprotein protein MPP10
Nucleolar protein 56
ATP-dependent RNA helicase DDX3X
Beta-1,3-N-acetylglucosaminyltransferase manic fringe
Podocalyxin
CCN family member 1
Pirin
Importin subunit alpha-3
E3 ubiquitin-protein ligase TRIM38
Nuclear factor 1 B-type
Serine/threonine-protein phosphatase 6 catalytic subunit
Nucleoside diphosphate kinase, mitochondrial
Lysosomal alpha-mannosidase
Pyridoxal kinase
Stearoyl-CoA desaturase
AT-rich interactive domain-containing protein 1A
Nuclear envelope integral membrane protein 1
Thioredoxin domain-containing protein 9
Dihydropyrimidinase-related protein 4
TRAF-type zinc finger domain-containing protein 1
Cytochrome c oxidase subunit 7A-related protein, mitochondrial
Ubiquitin domain-containing protein UBFD1
Citron Rho-interacting kinase
Coatomer subunit epsilon
Cdc42 effector protein 2
AP-3 complex subunit delta-1
Copper chaperone for superoxide dismutase
Actin-binding LIM protein 1
Segment polarity protein dishevelled homolog DVL-2
Chromodomain-helicase-DNA-binding protein 1
Chromodomain-helicase-DNA-binding protein 2
Golgi SNAP receptor complex member 2
Torsin-1A

Torsin-1B
Syntaxin-16
Disintegrin and metalloproteinase domain-containing protein 10
Lysosomal cobalamin transporter ABCD4
Etoposide-induced protein 2.4 homolog
Tumor protein p53-inducible protein 11
Histone-lysine N-methyltransferase 2D
Apoptotic protease-activating factor 1
Serine/threonine-protein kinase RIO3
Dual specificity mitogen-activated protein kinase kinase 7
Acyl-coenzyme A thioesterase 8
CDP-diacylglycerol--inositol 3-phosphatidyltransferase
Programmed cell death protein 5
Protein arginine N-methyltransferase 5
Na(+)/H(+) exchange regulatory cofactor NHE-RF1
Serine/threonine-protein kinase Chk1
Tumor necrosis factor receptor superfamily member 10B
Tripeptidyl-peptidase 1
Transcription elongation regulator 1
Kinetochore protein NDC80 homolog
Neuropilin-1
Transportin-2
Protein unc-13 homolog B
Tumor necrosis factor receptor superfamily member 10C
DNA-directed RNA polymerase III subunit RPC1
Tetraspanin-4
Proteasome subunit alpha type-7
Secretory carrier-associated membrane protein 3
Branched-chain alpha-ketoacid dehydrogenase kinase
Glutathione S-transferase 3, mitochondrial
Tax1-binding protein 3
PDZ domain-containing protein GIPC1
Inhibitor of nuclear factor kappa-B kinase subunit beta
Regulator of G-protein signaling 12
Mitochondrial import inner membrane translocase subunit Tim23
Histone acetyltransferase type B catalytic subunit
Peripheral plasma membrane protein CASK
Phospholipase D2
Cytochrome b-c1 complex subunit 8
Myosin regulatory light chain 12B
Hepatocyte growth factor-regulated tyrosine kinase substrate
Ras-related protein Rab-7L1
Vacuolar protein sorting-associated protein 26C
Protein phosphatase 1 regulatory subunit 12A
Cyclin-G-associated kinase
Heterogeneous nuclear ribonucleoprotein D-like
Exportin-1
TATA-binding protein-associated factor 172
Rho guanine nucleotide exchange factor 10
Zinc finger protein 609

Spectrin beta chain, non-erythrocytic 2
Microtubule-associated serine/threonine-protein kinase 4
Protein transport protein Sec16A
Plexin-B2
Protein KHNYN
Tectonin beta-propeller repeat-containing protein 2
U2 snRNP-associated SURP motif-containing protein
Histone-lysine N-methyltransferase SETD1A
NEDD4-binding protein 3
Synaptojanin-2
Synemin
Granule associated Rac and RHOG effector protein 1
Kinesin-like protein KIF3B
Phosphoribosylformylglycinamidine synthase
Serine/threonine-protein phosphatase 6 regulatory ankyrin repeat subunit A
Rho guanine nucleotide exchange factor 11
Mitochondrial ribonuclease P catalytic subunit
Inhibitor of nuclear factor kappa-B kinase subunit alpha
NPC intracellular cholesterol transporter 1
Sphingolipid delta(4)-desaturase DES1
Angiopoietin-2
Secretory carrier-associated membrane protein 1
Secretory carrier-associated membrane protein 2
Actin-related protein 2/3 complex subunit 1B
Actin-related protein 2/3 complex subunit 2
Actin-related protein 2/3 complex subunit 3
DNA-directed RNA polymerases I and III subunit RPAC1
Phospholipid scramblase 1
Transcription intermediary factor 1-alpha
Membrane-associated progesterone receptor component 2
Ral guanine nucleotide dissociation stimulator-like 2
WD repeat-containing protein 46
NF-kappa-B-repressing factor
Dihydroxyacetone phosphate acyltransferase
Laminin subunit alpha-5
Zinc finger protein 185
Protein CASC3
Small ribosomal subunit protein uS12m
Peroxisomal acyl-coenzyme A oxidase 3
Protein RER1
Surfeit locus protein 4
Mitogen-activated protein kinase 13
Serine palmitoyltransferase 1
Serine palmitoyltransferase 2
UDP-N-acetylglucosamine--peptide N-acetylglucosaminyltransferase 110 kDa subunit
Phosphomannomutase 2
Transmembrane 9 superfamily member 1
E3 ubiquitin-protein ligase Midline-1
High mobility group protein B3
Protein phosphatase 1G

Phosphatidylinositol 3,4,5-trisphosphate 5-phosphatase 2
Eukaryotic translation initiation factor 3 subunit D
Eukaryotic translation initiation factor 3 subunit H
Histone deacetylase 3
Nuclear valosin-containing protein-like
Branched-chain-amino-acid aminotransferase, mitochondrial
Importin-8
Syntaxin-7
Monocarboxylate transporter 4
ATP-binding cassette sub-family C member 4
DNA-directed RNA polymerase I subunit RPA34
Prolyl 4-hydroxylase subunit alpha-2
Calpain-5
Synaptobrevin homolog YKT6
Nucleoporin NUP42
Actin-related protein 2/3 complex subunit 5
CASP8 and FADD-like apoptosis regulator
Transcription factor MafG
3-phosphoinositide-dependent protein kinase 1
Tapasin
E3 ubiquitin-protein ligase RNF113A
Lysine-specific demethylase 6A
ATP-dependent RNA helicase DHX15
mRNA cap guanine-N7 methyltransferase
Zinc finger ZZ-type and EF-hand domain-containing protein 1
Arf-GAP with SH3 domain, ANK repeat and PH domain-containing protein 2
Leucine-rich repeat transmembrane protein FLRT2
TELO2-interacting protein 1 homolog
Ribosomal RNA-processing protein 8
E3 ubiquitin-protein ligase Praja-2
Cytochrome b5 type B
U4/U6 small nuclear ribonucleoprotein Prp4
D-3-phosphoglycerate dehydrogenase
NADH dehydrogenase [ubiquinone] iron-sulfur protein 4, mitochondrial
Cytoplasmic dynein 1 light intermediate chain 2
26S proteasome non-ATPase regulatory subunit 3
RNA binding protein fox-1 homolog 2
Bifunctional 3'-phosphoadenosine 5'-phosphosulfate synthase 1
Centromere/kinetochore protein zw10 homolog
Beta-1,4-galactosyltransferase 5
U4/U6.U5 tri-snRNP-associated protein 1
Glycosylphosphatidylinositol anchor attachment 1 protein
Death-associated protein kinase 3
Transforming growth factor beta-1-induced transcript 1 protein
AP-5 complex subunit zeta-1
CBP80/20-dependent translation initiation factor
Protein MTSS 1
Inositol hexakisphosphate and diphosphoinositol-pentakisphosphate kinase 2
Mitogen-activated protein kinase kinase kinase 7
Eukaryotic translation elongation factor 1 epsilon-1

Receptor-interacting serine/threonine-protein kinase 2
Heterogeneous nuclear ribonucleoprotein R
U4/U6 small nuclear ribonucleoprotein Prp3
Thioredoxin-like protein 1
Tumor protein D54
ER membrane protein complex subunit 8
ERI1 exoribonuclease 3
Synaptojanin-1
Acidic fibroblast growth factor intracellular-binding protein
Eukaryotic translation initiation factor 4 gamma 3
Peptidyl-prolyl cis-trans isomerase H
Serine protease HTRA2, mitochondrial
Protein Mis18-beta
Aflatoxin B1 aldehyde reductase member 2
Band 4.1-like protein 2
Trans-Golgi network integral membrane protein 2
Beta-1,4-glucuronyltransferase 1
WAS/WASL-interacting protein family member 1
Phospholipid-transporting ATPase IC
A-kinase anchor protein 10, mitochondrial
Density-regulated protein
Exportin-T
5-hydroxymethyl-dUMP N-hydrolase
Mitochondrial import inner membrane translocase subunit TIM44
Trafficking protein particle complex subunit 3
Charged multivesicular body protein 2a
Cytoplasmic protein NCK2
Tetraspanin-6
Pleiotropic regulator 1
Protein regulator of cytokinesis 1
Regulator of G-protein signaling 10
BUB3-interacting and GLEBS motif-containing protein ZNF207
NADH dehydrogenase [ubiquinone] 1 beta subcomplex subunit 5, mitochondrial
NADH dehydrogenase [ubiquinone] 1 beta subcomplex subunit 3
NADH dehydrogenase [ubiquinone] 1 alpha subcomplex subunit 2
LIM domain-binding protein 2
ATPase GET3
Mitotic checkpoint serine/threonine-protein kinase BUB1
Mitotic checkpoint protein BUB3
Sulfotransferase 1B1
Alpha-actinin-4
Probable 18S rRNA (guanine-N(7))-methyltransferase
17S U2 SnRNP complex component HTATSF1
ER lumen protein-retaining receptor 3
AP-1 complex subunit gamma-1
Syntaxin-6
Synaptogyrin-2
Small glutamine-rich tetratricopeptide repeat-containing protein alpha
Alpha-endosulfine
Mitochondrial carnitine/acylcarnitine carrier protein

Asparagine--tRNA ligase, cytoplasmic
Unconventional myosin-Ib
Peroxisomal membrane protein PMP34
Cleavage and polyadenylation specificity factor subunit 5
Glutathione S-transferase LANCL1
Striatin
U3 small nucleolar RNA-interacting protein 2
Protein SCO2 homolog, mitochondrial
Cilia- and flagella-associated protein 410
A-kinase anchor protein 8
Putative GTP-binding protein 6
Glucose-6-phosphate exchanger SLC37A4
Isocitrate dehydrogenase [NAD] subunit beta, mitochondrial
Nardilysin
Calumenin
EGF-like repeat and discoidin I-like domain-containing protein 3
S-adenosylhomocysteine hydrolase-like protein 1
Kinesin-like protein KIF1C
Exostosin-like 3
Origin recognition complex subunit 5
NADH dehydrogenase [ubiquinone] iron-sulfur protein 5
Retinal rod rhodopsin-sensitive cGMP 3',5'-cyclic phosphodiesterase subunit delta
Origin recognition complex subunit 4
Peroxisomal ATPase PEX1
Double-strand-break repair protein rad21 homolog
Pre-mRNA-splicing factor ATP-dependent RNA helicase DHX16
Glia maturation factor gamma
SH3 domain-binding protein 5
Mediator of RNA polymerase II transcription subunit 14
Phosphoribosyl pyrophosphate synthase-associated protein 2
SWI/SNF-related matrix-associated actin-dependent regulator of chromatin subfamily A member 5
C-Jun-amino-terminal kinase-interacting protein 4
NUAK family SNF1-like kinase 1
Nucleolar pre-ribosomal-associated protein 1
Signal-induced proliferation-associated 1-like protein 3
Zinc finger C3H1 domain-containing protein
RNA helicase aquarius
Dynamin-like 120 kDa protein, mitochondrial
Zinc finger E-box-binding homeobox 2
Germinal-center associated nuclear protein
Protocadherin gamma-A12
Phosphatidylinositol 4-phosphate 5-kinase type-1 gamma
Kinesin-like protein KIF1B
Mitogen-activated protein kinase-binding protein 1
Lysine-specific histone demethylase 1A
TBC1 domain family member 4
Acyl-CoA (8-3)-desaturase
Gasdermin-E
Ecotropic viral integration site 5 protein homolog
Neuropilin-2

Mannosyl-oligosaccharide 1,2-alpha-mannosidase IB
Myelin protein zero-like protein 2
Long-chain-fatty-acid--CoA ligase 4
Sorting nexin-3
Protein O-GlcNAcase
Vinexin
Heterogeneous nuclear ribonucleoprotein Q
Pre-mRNA-processing factor 17
Ran-binding protein 6
Ribosome quality control complex subunit NEMF
GDP-mannose 4,6 dehydratase
Glycylpeptide N-tetradecanoyltransferase 2
Cyclin-T1
Mitotic checkpoint serine/threonine-protein kinase BUB1 beta
Multifunctional procollagen lysine hydroxylase and glycosyltransferase LH3
Eukaryotic translation initiation factor 4E type 2
Protein diaphanous homolog 1
Tetraspanin-3
Exocyst complex component 3
Perilipin-3
Protein arginine N-methyltransferase 3
Importin subunit alpha-7
UDP-glucose 6-dehydrogenase
Protein-tyrosine sulfotransferase 2
Leupaxin
Catenin delta-1
Protein-S-isoprenylcysteine O-methyltransferase
Eukaryotic translation initiation factor 1b
Sorting nexin-2
Dolichol-phosphate mannosyltransferase subunit 1
General vesicular transport factor p115
Small ribosomal subunit protein uS14m
Target of Myb1 membrane trafficking protein
Histone H2B type 1-K
Coiled-coil domain-containing protein 22
Polyglutamine-binding protein 1
Mitochondrial import inner membrane translocase subunit Tim17-B
PRA1 family protein 2
H/ACA ribonucleoprotein complex subunit DKC1
Eukaryotic translation initiation factor 5B
Endothelial differentiation-related factor 1
DNA/RNA-binding protein KIN17
Protein diaphanous homolog 2
DnaJ homolog subfamily A member 2
Bromodomain-containing protein 4
Checkpoint protein HUS1
Prefoldin subunit 1
E3 ubiquitin-protein ligase PPP1R11
Nibrin
mRNA-capping enzyme

Iron-sulfur clusters transporter ABCB7, mitochondrial
SLIT-ROBO Rho GTPase-activating protein 2
Plexin-A2
Glycosaminoglycan xylosylkinase
WD repeat-containing protein 1
Probable phospholipid-transporting ATPase IIA
NEDD4-binding protein 1
Rho-associated protein kinase 2
CLIP-associating protein 2
Pentatricopeptide repeat-containing protein 1, mitochondrial
Copine-3
Autophagy-related protein 13
Huntingtin-interacting protein 1-related protein
E3 ubiquitin-protein ligase BRE1B
Lysine-specific demethylase PHF2
Zinc finger CCCH domain-containing protein 11A
Clustered mitochondria protein homolog
Cullin-associated NEDD8-dissociated protein 2
TSC22 domain family protein 2
DnaJ homolog subfamily C member 13
Phosphatase and actin regulator 2
Serine/threonine-protein phosphatase 6 regulatory subunit 2
A disintegrin and metalloproteinase with thrombospondin motifs 4
CCR4-NOT transcription complex subunit 3
Ankyrin repeat domain-containing protein 17
Paired amphipathic helix protein Sin3b
DnaJ homolog subfamily B member 6
Ubiquinone biosynthesis protein COQ9, mitochondrial
Gamma-glutamylcyclotransferase
NADH dehydrogenase [ubiquinone] iron-sulfur protein 7, mitochondrial
NADH dehydrogenase [ubiquinone] iron-sulfur protein 2, mitochondrial
Zinc finger protein ZPR1
RNA/RNP complex-1-interacting phosphatase
Protein NipSnap homolog 2
Hyaluronan mediated motility receptor
Programmed cell death protein 6
Tubulin-specific chaperone A
V-type proton ATPase subunit G 1
Vacuolar protein sorting-associated protein 4B
Mannose-P-dolichol utilization defect 1 protein
Core histone macro-H2A.1
Adapter SH3BGRL
Filamin-B
Nuclear receptor corepressor 1
Peroxisomal membrane protein PEX14
Tripartite motif-containing protein 3
Citrate synthase, mitochondrial
Sperm-associated antigen 7
Vesicle-trafficking protein SEC22b
Pre-mRNA-processing factor 40 homolog A

Transforming acidic coiled-coil-containing protein 1
Nucleoside diphosphate kinase 6
Cell division control protein 45 homolog
Leucine-rich repeat and calponin homology domain-containing protein 4
Metaxin-2
Vacuolar protein sorting-associated protein 26A
Mitochondrial-processing peptidase subunit beta
Mediator of RNA polymerase II transcription subunit 24
Katanin p60 ATPase-containing subunit A1
PC4 and SFRS1-interacting protein
Erlin-1
NADH dehydrogenase [ubiquinone] iron-sulfur protein 3, mitochondrial
Serine/arginine-rich splicing factor 10
Ceroid-lipofuscinosis neuronal protein 5
Claudin-11
Enoyl-CoA delta isomerase 2
KH domain-containing, RNA-binding, signal transduction-associated protein 3
Polycomb protein EED
Barrier-to-autointegration factor
Splicing factor 3B subunit 1
Cold shock domain-containing protein E1
WW domain-binding protein 4
Interferon-inducible double-stranded RNA-dependent protein kinase activator A
Integrin alpha-10
Ribosomal protein S6 kinase alpha-5
Mediator of RNA polymerase II transcription subunit 6
E3 ubiquitin-protein ligase MYCBP2
2-amino-3-ketobutyrate coenzyme A ligase, mitochondrial
Acyl-protein thioesterase 1
GTPase Era, mitochondrial
U5 small nuclear ribonucleoprotein 200 kDa helicase
Mitochondrial tRNA-specific 2-thiouridylase 1
TIP41-like protein
Centriole and centriolar satellite protein OFD1
Ribosomal protein S6 kinase alpha-4
Surfeit locus protein 6
Protein phosphatase 1B
Small subunit processome component 20 homolog
Nuclear pore complex protein Nup155
Protein XRP2
WD repeat and HMG-box DNA-binding protein 1
Cartilage-associated protein
Electrogenic aspartate/glutamate antiporter SLC25A12, mitochondrial
Renin receptor
Ribonuclease H2 subunit A
Translation initiation factor eIF2 assembly protein
Breast cancer anti-estrogen resistance protein 3
Ribonuclease P protein subunit p20
Ribonuclease P protein subunit p40
Eukaryotic translation initiation factor 3 subunit G

Eukaryotic translation initiation factor 3 subunit J
Carbonyl reductase [NADPH] 3
26S proteasome non-ATPase regulatory subunit 10
AP-1 complex subunit gamma-like 2
CAAX prenyl protease 1 homolog
Isocitrate dehydrogenase [NADP] cytoplasmic
Glutamyl-tRNA(Gln) amidotransferase subunit B, mitochondrial
Protein SCO1 homolog, mitochondrial
Attractin
Serine hydrolase RBBP9
Signal transducing adapter molecule 2
Diacylglycerol O-acyltransferase 1
Cyclin-K
PRA1 family protein 3
Dysferlin
E3 SUMO-protein ligase PIAS1
Pre-mRNA-splicing factor SPF27
Dynactin subunit 3
DnaJ homolog subfamily C member 8
Survival of motor neuron-related-splicing factor 30
Cell cycle checkpoint protein RAD17
ATP synthase subunit d, mitochondrial
Tetraspanin-9
Flotillin-1
Triple functional domain protein
ATP synthase subunit g, mitochondrial
Multiple PDZ domain protein
Carboxypeptidase D
Glutaredoxin-3
Ribosomal L1 domain-containing protein 1
Wolframin
ATP-dependent Clp protease ATP-binding subunit clpX-like, mitochondrial
SEC14-like protein 2
Gamma-synuclein
Probable cytosolic iron-sulfur protein assembly protein CIAO1
Signal recognition particle subunit SRP72
N(G),N(G)-dimethylarginine dimethylaminohydrolase 1
Unconventional prefoldin RPB5 interactor 1
Galactosylgalactosylxylosylprotein 3-beta-glucuronosyltransferase 3
Metastasis-associated protein MTA2
Retinal dehydrogenase 2
Serine/threonine-protein kinase 10
Serine/threonine-protein kinase D3
Glutamine--fructose-6-phosphate aminotransferase [isomerizing] 2
Slit homolog 2 protein
E3 ubiquitin-protein ligase listerin
Mitochondrial import receptor subunit TOM70
Pleckstrin homology domain-containing family G member 5
Importin-13
Phospholipase DDHD2

Unconventional myosin-Id
TOX high mobility group box family member 4
[F-actin]-monooxygenase MICAL2
Protein transport protein Sec24D
F-BAR and double SH3 domains protein 2
E3 UFM1-protein ligase 1
Sorbin and SH3 domain-containing protein 2
SAM and SH3 domain-containing protein 1
UBX domain-containing protein 7
Kelch-like protein 18
SUN domain-containing protein 1
Pyridoxal phosphate homeostasis protein
Erlin-2
Pre-mRNA-processing factor 6
Pre-mRNA cleavage complex 2 protein Pcf11
Protein furry homolog-like
Nuclear factor of activated T-cells 5
Endonuclease domain-containing 1 protein
D-glucuronyl C5-epimerase
Glutaminase kidney isoform, mitochondrial
HAUS augmin-like complex subunit 5
Actin-binding LIM protein 3
F-box only protein 21
Lysine-specific demethylase 4B
Microtubule cross-linking factor 2
Ubiquitin carboxyl-terminal hydrolase 19
WD repeat-containing protein 47
AP-2 complex subunit alpha-2
Protein transport protein Sec31A
Rho guanine nucleotide exchange factor 15
Protein HEXIM1
Ribonuclease P protein subunit p14
G2/mitotic-specific cyclin-B2
Protein YIF1A
E3 ubiquitin-protein ligase UBR5
Arf-GAP domain and FG repeat-containing protein 2
Serine protease 23
SR-related and CTD-associated factor 4
NADH dehydrogenase [ubiquinone] 1 beta subcomplex subunit 6
Mitofusin-2
Snurportin-1
Ubiquitin conjugation factor E4 B
Zinc finger protein-like 1
Elongator complex protein 1
NADH dehydrogenase [ubiquinone] 1 beta subcomplex subunit 4
NADH dehydrogenase [ubiquinone] 1 beta subcomplex subunit 8, mitochondrial
NADH dehydrogenase [ubiquinone] 1 alpha subcomplex subunit 7
Vesicle-associated membrane protein 5
Reticulon-3
Mitochondrial proton/calcium exchanger protein

Epsin-2
Zinc finger Ran-binding domain-containing protein 2
Sorting nexin-4
Luc7-like protein 3
Kinesin-like protein KIF20A
Apolipoprotein L3
Chromosome-associated kinesin KIF4A
Methyl-CpG-binding domain protein 4
Myotubularin-related protein 5
Golgi SNAP receptor complex member 1
Histone acetyltransferase KAT7
Arginyl-tRNA--protein transferase 1
Vesicle-associated membrane protein-associated protein B/C
SNARE-associated protein Snapin
Myelin protein zero-like protein 1
NADH dehydrogenase [ubiquinone] 1 subunit C2
NADH dehydrogenase [ubiquinone] 1 alpha subcomplex subunit 10, mitochondrial
Peptidyl-prolyl cis-trans isomerase FKBP9
CUGBP Elav-like family member 2
6-phosphogluconolactonase
Bifunctional 3'-phosphoadenosine 5'-phosphosulfate synthase 2
Structural maintenance of chromosomes protein 2
Ubiquitin-like modifier-activating enzyme ATG7
Transforming acidic coiled-coil-containing protein 2
Tripartite motif-containing protein 16
Phenylalanine--tRNA ligase, mitochondrial
Zinc finger and BTB domain-containing protein 7A
Acyl-protein thioesterase 2
Importin-7
E3 ubiquitin-protein ligase ARIH2
Pre-mRNA-splicing factor SLU7
Phosphoacetylglucosamine mutase
Adenylyltransferase and sulfurtransferase MOCS3
CD2 antigen cytoplasmic tail-binding protein 2
Supervillin
Activator of 90 kDa heat shock protein ATPase homolog 1
Poly(A)-specific ribonuclease PARN
Proteasome assembly chaperone 1
Sphingosine-1-phosphate lyase 1
CTD nuclear envelope phosphatase 1
Ribosome biogenesis protein NSA2 homolog
GDH/6PGL endoplasmic bifunctional protein
Protein transport protein Sec24A
Protein transport protein Sec24B
Adhesion G protein-coupled receptor L2
NAD kinase
Tyrosyl-DNA phosphodiesterase 2
Mitochondrial pyruvate carrier 2
Persulfide dioxygenase ETHE1, mitochondrial
Fatty acid CoA ligase Acsl3

DNA-directed RNA polymerase I subunit RPA1
YEATS domain-containing protein 4
Zinc finger and BTB domain-containing protein 11
CCR4-NOT transcription complex subunit 4
STAM-binding protein
Cleavage and polyadenylation specificity factor subunit 4
Nuclear factor of activated T-cells, cytoplasmic 1
Probable bifunctional dTTP/UTP pyrophosphatase/methyltransferase protein
Phosphatidate cytidylyltransferase 2
Protein phosphatase 1 regulatory subunit 3D
Bromodomain-containing protein 1
Ribonuclease P protein subunit p29
E3 ubiquitin-protein ligase HERC2
Ras-related protein Rab-3D
Synaptosomal-associated protein 29
Serine/threonine-protein kinase OSR1
Geranylgeranyl pyrophosphate synthase
Heat shock 70 kDa protein 4L
Polypyrimidine tract-binding protein 3
TBC1 domain family member 8
STARD3 N-terminal-like protein
U6 snRNA-associated Sm-like protein LSm8
AP-2 complex subunit alpha-1
Protein Wiz
Antiviral innate immune response receptor RIG-I
Double-stranded RNA-binding protein Staufen homolog 1
Tetratricopeptide repeat protein 4
Transmembrane protein 50A
Caveolae-associated protein 2
BAG family molecular chaperone regulator 2
BAG family molecular chaperone regulator 3
Mitogen-activated protein kinase kinase kinase kinase 4
Malonyl-CoA decarboxylase, mitochondrial
Quinone oxidoreductase-like protein 1
Apoptosis-inducing factor 1, mitochondrial
Echinoderm microtubule-associated protein-like 2
Serine/threonine-protein kinase LATS1
Uridine diphosphate glucose pyrophosphatase NUDT14
Tetraspanin-15
3'(2'),5'-bisphosphate nucleotidase 1
Acyl-CoA 6-desaturase
Putative hydrolase DDAH2
Phosphatidylserine lipase ABHD16A
Thioredoxin domain-containing protein 12
Protein ecdysoneless homolog
Reversion-inducing cysteine-rich protein with Kazal motifs
Methyl-CpG-binding domain protein 3
DNA topoisomerase 3-beta-1
Diphosphoinositol polyphosphate phosphohydrolase 1
B-cell lymphoma/leukemia 10

NADH dehydrogenase [ubiquinone] 1 beta subcomplex subunit 10
Putative lipid scramblase CLPTM1
E3 SUMO-protein ligase ZBED1
Molybdopterin synthase catalytic subunit
Mitochondrial import receptor subunit TOM40 homolog
Peroxisomal membrane protein 11B
Serine/threonine-protein kinase PAK 4
Actin-like protein 6A
Histone-lysine N-methyltransferase NSD2
L-lactate dehydrogenase A chain
Aldehyde dehydrogenase 1A1
Glutamate dehydrogenase 1, mitochondrial
Dihydrofolate reductase
NADH-cytochrome b5 reductase 3
Glutathione reductase, mitochondrial
Cytochrome c oxidase subunit 2
Purine nucleoside phosphorylase
Hypoxanthine-guanine phosphoribosyltransferase
Aspartate aminotransferase, mitochondrial
Tyrosine-protein kinase ABL1
Phosphoglycerate kinase 1
Adenylate kinase isoenzyme 1
Urokinase-type plasminogen activator
Tissue-type plasminogen activator
Adenosine deaminase
Cystatin-C
GTPase NRas
GTPase HRas
GTPase KRas
Platelet-derived growth factor subunit B
Low-density lipoprotein receptor
Transforming growth factor beta-1 proprotein
HLA class I histocompatibility antigen, B alpha chain
Putative HLA class I histocompatibility antigen, alpha chain H
Collagen alpha-1(I) chain
Collagen alpha-1(IV) chain
Prelamin-A/C
Apolipoprotein A-I
Fibronectin
Alpha-2-HS-glycoprotein
Albumin
Transferrin receptor protein 1
Ferritin light chain
Ferritin heavy chain
Metallothionein-2
NADH-ubiquinone oxidoreductase chain 1
NADH-ubiquinone oxidoreductase chain 4
NADH-ubiquinone oxidoreductase chain 5
Interstitial collagenase
3-hydroxy-3-methylglutaryl-coenzyme A reductase

Catalase
RAF proto-oncogene serine/threonine-protein kinase
Lysosomal acid glucosylceramidase
Fructose-bisphosphate aldolase A
Cystatin-B
Annexin A1
Apolipoprotein B-100
Major prion protein
Superoxide dismutase [Mn], mitochondrial
Ornithine aminotransferase, mitochondrial
Thymidine kinase, cytosolic
von Willebrand factor
Tubulin beta-4A chain
Glyceraldehyde-3-phosphate dehydrogenase
Argininosuccinate lyase
HLA class I histocompatibility antigen, A alpha chain
Calpain small subunit 1
Metallothionein-1E
Heat shock protein beta-1
Thymidylate synthase
Dolichyl-diphosphooligosaccharide--protein glycosyltransferase subunit 1
Dolichyl-diphosphooligosaccharide--protein glycosyltransferase subunit 2
Guanine nucleotide-binding protein G(i) subunit alpha-2
Histone H2A type 1-B/E
Anion exchange protein 2
Sodium/potassium-transporting ATPase subunit alpha-1
Sodium/potassium-transporting ATPase subunit beta-1
Amyloid-beta precursor protein
Aldehyde dehydrogenase, mitochondrial
Integrin beta-3
Protein S100-A8
Non-histone chromosomal protein HMG-14
Plasminogen activator inhibitor 2
Plasminogen activator inhibitor 1
ADP/ATP translocase 2
Ubiquitin-like protein ISG15
Propionyl-CoA carboxylase alpha chain, mitochondrial
Propionyl-CoA carboxylase beta chain, mitochondrial
Eukaryotic translation initiation factor 2 subunit 1
Non-histone chromosomal protein HMG-17
Intercellular adhesion molecule 1
Large ribosomal subunit protein P2
Large ribosomal subunit protein uL10
Transcription factor Jun
DNA-directed RNA polymerase III subunit RPC4
Lupus La protein
Integrin beta-1
Keratin, type I cytoskeletal 18
Collagen alpha-2(V) chain
Uroporphyrinogen decarboxylase

Tyrosine-protein kinase Fyn
Alpha-galactosidase A
Gelsolin
Retinoblastoma-associated protein
Prothymosin alpha
Cyclin-dependent kinase 1
ATP synthase subunit beta, mitochondrial
Protein S100-A9
Protein S100-A6
Eukaryotic translation initiation factor 4E
Alpha-enolase
Glycogen phosphorylase, liver form
Glucose-6-phosphate isomerase
DNA polymerase beta
Nucleophosmin
Tropomyosin alpha-3 chain
Integrin alpha-V
Beta-hexosaminidase subunit alpha
Epoxide hydrolase 1
L-lactate dehydrogenase B chain
Major centromere autoantigen B
Glutathione peroxidase 1
Protein disulfide-isomerase
Histone H1.0
Acylphosphatase-1
Tyrosine-protein kinase Fes/Fps
Cathepsin D
Annexin A2
Calpain-1 catalytic subunit
Tubulin beta chain
Prosaposin
Beta-hexosaminidase subunit beta
Procathepsin L
Profilin-1
Bisphosphoglycerate mutase
Adenine phosphoribosyltransferase
Bifunctional glutamate/proline--tRNA ligase
Cathepsin B
Heat shock protein HSP 90-alpha
Galactose-1-phosphate uridylyltransferase
Heterogeneous nuclear ribonucleoproteins C1/C2
Laminin subunit beta-1
Tyrosine-protein kinase Yes
Tyrosine-protein kinase Lyn
Fumarate hydratase, mitochondrial
DNA excision repair protein ERCC-1
Thrombospondin-1
Transcription factor Sp1
Insulin-like growth factor 1 receptor
Collagen alpha-2(I) chain

Annexin A6
Rho-related GTP-binding protein RhoC
Complement decay-accelerating factor
Amino acid transporter heavy chain SLC3A2
Beta-glucuronidase
ATP-dependent 6-phosphofructokinase, muscle type
Heat shock protein HSP 90-beta
Signal recognition particle receptor subunit alpha
Asparagine synthetase [glutamine-hydrolyzing]
72 kDa type IV collagenase
Porphobilinogen deaminase
Neprilysin
Matrix Gla protein
Pyruvate dehydrogenase E1 component subunit alpha, somatic form, mitochondrial
Collagen alpha-2(IV) chain
Cytochrome c1, heme protein, mitochondrial
U2 small nuclear ribonucleoprotein B''
Hepatocyte growth factor receptor
U1 small nuclear ribonucleoprotein 70 kDa
Integrin alpha-5
Nuclear factor 1 C-type
Vimentin
Small ribosomal subunit protein eS17
Guanine nucleotide-binding protein G(i) subunit alpha-3
Annexin A5
Small ribosomal subunit protein uS2
CD63 antigen
Large ribosomal subunit protein uL3m
U1 small nuclear ribonucleoprotein A
Gamma-enolase
3-ketoacyl-CoA thiolase, peroxisomal
Signal recognition particle 19 kDa protein
Glutathione S-transferase P
U1 small nuclear ribonucleoprotein C
Galectin-1
Dihydropteridine reductase
High mobility group protein B1
SPARC
Tropomyosin alpha-1 chain
Clathrin light chain A
Clathrin light chain B
Annexin A4
2',3'-cyclic-nucleotide 3'-phosphodiesterase
Heme oxygenase 1
Dihydrolipoyl dehydrogenase, mitochondrial
Heterogeneous nuclear ribonucleoprotein A1
U2 small nuclear ribonucleoprotein A'
Cytochrome c oxidase subunit 6C
Tumor-associated calcium signal transducer 2
Poly [ADP-ribose] polymerase 1

DNA polymerase alpha catalytic subunit
Ubiquitin carboxyl-terminal hydrolase isozyme L1
Leukotriene A-4 hydrolase
Fructose-bisphosphate aldolase C
Histone H2A.Z
Histone H2A type 1
Myocardial zonula adherens protein
DNA-directed RNA polymerase II subunit GRINL1A
Glutathione S-transferase theta-2
POTE ankyrin domain family member J
LIM and senescent cell antigen-like-containing domain protein 3
Heat shock 70 kDa protein 1A
Chimeric ERCC6-PGBD3 protein
Tubulin alpha-3C chain
Zinc finger TRAF-type-containing protein 1
Ras-related protein Rap-2a
Serglycin
RNA-binding protein RO60
Lysosomal alpha-glucosidase
Ras-related protein R-Ras
HLA class I histocompatibility antigen, C alpha chain
Serine/threonine-protein kinase A-Raf
Histone H1.4
Dihydrolipoyllysine-residue acetyltransferase component of pyruvate dehydrogenase complex, mitochond
Receptor-type tyrosine-protein phosphatase F
COUP transcription factor 1
Thioredoxin
Cytochrome c oxidase subunit 5B, mitochondrial
Lysosomal protective protein
Microsomal glutathione S-transferase 1
cAMP-dependent protein kinase type I-alpha regulatory subunit
Tissue factor pathway inhibitor
S-formylglutathione hydrolase
60 kDa heat shock protein, mitochondrial
Clusterin
Endoplasmic reticulum chaperone BiP
Laminin subunit gamma-1
Lysosomal acid phosphatase
Microtubule-associated protein 2
Heat shock cognate 71 kDa protein
Solute carrier family 2, facilitated glucose transporter member 1
Solute carrier family 2, facilitated glucose transporter member 3
Protein 4.1
Uridine 5'-monophosphate synthase
Pyruvate dehydrogenase E1 component subunit beta, mitochondrial
Lipoamide acyltransferase component of branched-chain alpha-keto acid dehydrogenase complex, mitoch
Glycogen phosphorylase, brain form
Glycogen phosphorylase, muscle form
Ras-related protein Ral-A
Ras-related protein Ral-B

Breakpoint cluster region protein
Lysosome-associated membrane glycoprotein 1
Transcriptional regulator ERG
Medium-chain specific acyl-CoA dehydrogenase, mitochondrial
DNA topoisomerase 1
DNA topoisomerase 2-alpha
Glucose-6-phosphate 1-dehydrogenase
Ubiquitin-like protein 4A
Pyruvate carboxylase, mitochondrial
C-1-tetrahydrofolate synthase, cytoplasmic
Cation-independent mannose-6-phosphate receptor
Alcohol dehydrogenase class-3
Cyclin-dependent kinase 4
Ribose-phosphate pyrophosphokinase 2
Polyadenylate-binding protein 1
Proliferating cell nuclear antigen
Histidine--tRNA ligase, cytoplasmic
Collagen alpha-1(VI) chain
Collagen alpha-3(VI) chain
ADP/ATP translocase 1
ADP/ATP translocase 3
Inosine-5'-monophosphate dehydrogenase 2
Nucleoprotein TPR
Annexin A3
2-oxoisovalerate dehydrogenase subunit alpha, mitochondrial
Alpha-actinin-1
Angiotensin-converting enzyme
Proto-oncogene tyrosine-protein kinase Src
Xaa-Pro dipeptidase
X-ray repair cross-complementing protein 6
X-ray repair cross-complementing protein 5
Uracil-DNA glycosylase
Cytochrome c oxidase subunit 4 isoform 1, mitochondrial
5-aminolevulinate synthase, non-specific, mitochondrial
Lysosome-associated membrane glycoprotein 2
Ribonuclease inhibitor
Intercellular adhesion molecule 2
Elongation factor 2
Protein disulfide-isomerase A4
Prolyl 4-hydroxylase subunit alpha-1
Translationally-controlled tumor protein
Delta-aminolevulinic acid dehydratase
HLA class I histocompatibility antigen, alpha chain E
Plastin-3
Acylamino-acid-releasing enzyme
Electron transfer flavoprotein subunit alpha, mitochondrial
Glycogen [starch] synthase, muscle
cAMP-dependent protein kinase type II-alpha regulatory subunit
General transcription factor IIF subunit 2
CD59 glycoprotein

Bifunctional methylenetetrahydrofolate dehydrogenase/cyclohydrolase, mitochondrial
Macrophage migration inhibitory factor
CD99 antigen
Glucosidase 2 subunit beta
Hematopoietic lineage cell-specific protein
Farnesyl pyrophosphate synthase
Cytochrome c oxidase subunit 7A2, mitochondrial
Nidogen-1
Aldo-keto reductase family 1 member A1
Pyruvate kinase PKM
Endoplasmin
G2/mitotic-specific cyclin-B1
Myosin light chain 6B
Small nuclear ribonucleoprotein-associated proteins B and B'
Insulin-degrading enzyme
POU domain, class 2, transcription factor 1
Heterogeneous nuclear ribonucleoprotein L
Aspartate--tRNA ligase, cytoplasmic
Protein C-ets-1
Junction plakoglobin
Cytochrome b-c1 complex subunit 7
Serine/threonine-protein kinase B-raf
Fatty acid-binding protein, adipocyte
Glutamine synthetase
Aldo-keto reductase family 1 member B1
Aminopeptidase N
Poliovirus receptor
Ras-related C3 botulinum toxin substrate 2
Eukaryotic peptide chain release factor GTP-binding subunit ERF3A
Arylsulfatase A
Beta-1,4-galactosyltransferase 1
Ezrin
Ubiquitin carboxyl-terminal hydrolase isozyme L3
Fos-related antigen 1
Fos-related antigen 2
Membrane cofactor protein
Nucleoside diphosphate kinase A
NAD(P)H dehydrogenase [quinone] 1
N-acetylglucosamine-6-sulfatase
Phosphorylase b kinase gamma catalytic chain, liver/testis isoform
Arylsulfatase B
Small ribosomal subunit protein uS5
Transcription factor 4
Desmoplakin
Replication protein A 32 kDa subunit
Metalloproteinase inhibitor 2
CD44 antigen
Carbonyl reductase [NADPH] 1
Short-chain specific acyl-CoA dehydrogenase, mitochondrial
Cyclic AMP-responsive element-binding protein 1

Beta-galactosidase
Platelet endothelial cell adhesion molecule
Serine/threonine-protein phosphatase 2B catalytic subunit beta isoform
SH2/SH3 adapter protein NCK1
Intron Large complex component GCFC2
Histone H1.5
Histone H1.3
Histone H1.2
NADPH--cytochrome P450 reductase
Tyrosine-protein kinase Fer
Sarcoplasmic/endoplasmic reticulum calcium ATPase 2
1-phosphatidylinositol 4,5-bisphosphate phosphodiesterase gamma-2
Fumarylacetoacetase
Stathmin
Y-box-binding protein 3
Zinc finger protein 22
Zinc finger protein 24
Alpha-N-acetylgalactosaminidase
High mobility group protein HMG-I/HMG-Y
Transmembrane protein 11, mitochondrial
Aspartate aminotransferase, cytoplasmic
Protein kinase C alpha type
Integrin alpha-2
Gap junction alpha-1 protein
Sphingomyelin phosphodiesterase
Nucleolar transcription factor 1
Transcription factor JunD
T-cell acute lymphocytic leukemia protein 1
Cyclic AMP-dependent transcription factor ATF-7
NADH dehydrogenase [ubiquinone] 1 beta subcomplex subunit 7
cAMP-dependent protein kinase catalytic subunit alpha
Calpain-2 catalytic subunit
Tyrosine-protein phosphatase non-receptor type 2
CTP synthase 1
Endoglin
Probable ATP-dependent RNA helicase DDX5
ATP-dependent 6-phosphofructokinase, liver type
Vascular endothelial growth factor receptor 1
26S proteasome regulatory subunit 6A
T-complex protein 1 subunit alpha
Tyrosine-protein phosphatase non-receptor type 1
General transcription and DNA repair factor IIH helicase subunit XPD
Large ribosomal subunit protein eL33
Integrin beta-5
ADP-ribosylation factor 4
Large ribosomal subunit protein uL30
Vinculin
Receptor-type tyrosine-protein phosphatase alpha
Arylamine N-acetyltransferase 1
Protein SON

Negative elongation factor E
Large ribosomal subunit protein uL22
Phosphoglycerate mutase 1
Regulator of chromosome condensation
Cyclic AMP-dependent transcription factor ATF-1
Cyclic AMP-dependent transcription factor ATF-6 alpha
DNA ligase 1
DNA repair protein XRCC1
Cadherin-2
1-phosphatidylinositol 4,5-bisphosphate phosphodiesterase gamma-1
Nucleolin
Hexokinase-1
DNA-directed RNA polymerase II subunit RPB3
DNA-directed RNA polymerases I, II, and III subunit RPABC1
NADH dehydrogenase [ubiquinone] flavoprotein 2, mitochondrial
General transcription and DNA repair factor IIH helicase subunit XPB
E3 ubiquitin-protein ligase TRIM21
Interferon-induced, double-stranded RNA-activated protein kinase
Spermidine synthase
Sodium/hydrogen exchanger 1
Casein kinase II subunit alpha'
Nuclear factor NF-kappa-B p105 subunit
Plasma membrane calcium-transporting ATPase 1
Eukaryotic translation initiation factor 2 subunit 2
Annexin A7
Cyclin-A2
Transcription factor BTF3
Ras-related protein Rab-3B
Ras-related protein Rab-4A
Ras-related protein Rab-5A
Ras-related protein Rab-6A
DNA mismatch repair protein Msh3
Atrial natriuretic peptide receptor 2
Proteasome subunit beta type-1
Cation-dependent mannose-6-phosphate receptor
Lamin-B1
Calpastatin
Inosine-5'-monophosphate dehydrogenase 1
Collagen alpha-1(V) chain
Nebulin
N(4)-(beta-N-acetylglucosaminyl)-L-asparaginase
Ras GTPase-activating protein 1
Parathymosin
Cyclin-dependent kinase 11B
Glutathione S-transferase Mu 3
V-type proton ATPase subunit B, brain isoform
V-type proton ATPase subunit C 1
Cysteine and glycine-rich protein 1
Filamin-A
Neurofibromin

Amine oxidase [flavin-containing] A
Cytoplasmic aconitate hydratase
Sphingosine 1-phosphate receptor 1
Synaptotagmin-1
5'-nucleotidase
Transcription initiation factor TFIID subunit 1
Midkine
Voltage-dependent anion-selective channel protein 1
Biglycan
Succinate dehydrogenase [ubiquinone] iron-sulfur subunit, mitochondrial
CD9 antigen
Catechol O-methyltransferase
Protein-glutamine gamma-glutamyltransferase 2
Methylmalonyl-CoA mutase, mitochondrial
Oxysterol-binding protein 1
Protein-L-isoaspartate(D-aspartate) O-methyltransferase
rRNA 2'-O-methyltransferase fibrillarin
Trifunctional purine biosynthetic protein adenosine-3
Bifunctional phosphoribosylaminoimidazole carboxylase/phosphoribosylaminoimidazole succinocarboxam
Sterol carrier protein 2
Ubiquitin-like modifier-activating enzyme 1
Nucleoside diphosphate kinase B
Cornifin-B
NADPH:adrenodoxin oxidoreductase, mitochondrial
Heterogeneous nuclear ribonucleoproteins A2/B1
MHC class II regulatory factor RFX1
E3 ubiquitin-protein ligase CBL
Insulin-like growth factor-binding protein 4
cAMP-dependent protein kinase catalytic subunit beta
Cytochrome b-c1 complex subunit 2, mitochondrial
Ferrochelatase, mitochondrial
DNA repair protein complementing XP-A cells
Transcription elongation factor A protein 1
Prostaglandin G/H synthase 1
Integrin alpha-6
Splicing factor, proline- and glutamine-rich
Tubulin gamma-1 chain
Peptidyl-prolyl cis-trans isomerase B
NAD-dependent malic enzyme, mitochondrial
Tryptophan--tRNA ligase, cytoplasmic
Small ribosomal subunit protein uS3
Ribosomal protein S6 kinase beta-1
Tyrosine-protein kinase JAK1
Receptor-type tyrosine-protein phosphatase beta
Receptor-type tyrosine-protein phosphatase epsilon
Receptor-type tyrosine-protein phosphatase gamma
Nuclear autoantigen Sp-100
Colorectal mutant cancer protein
Tumor necrosis factor ligand superfamily member 4
Adenosylhomocysteinase

Cofilin-1
Eukaryotic translation initiation factor 4B
40-kDa huntingtin-associated protein
Plasma membrane calcium-transporting ATPase 4
Diacylglycerol kinase alpha
Carnitine O-palmitoyltransferase 2, mitochondrial
Thymidylate kinase
Ribonucleoside-diphosphate reductase large subunit
G1/S-specific cyclin-D1
Rab proteins geranylgeranyltransferase component A 1
ER lumen protein-retaining receptor 1
COUP transcription factor 2
Elongation factor 1-beta
ATP synthase F(0) complex subunit B1, mitochondrial
Low molecular weight phosphotyrosine protein phosphatase
Protein kinase C eta type
Acetyl-CoA acetyltransferase, mitochondrial
DNA-directed RNA polymerase II subunit RPB1
Cyclin-dependent kinase 2
Beta-adrenergic receptor kinase 1
Proteinase-activated receptor 1
DNA replication licensing factor MCM3
3-mercaptopyruvate sulfurtransferase
Small ribosomal subunit protein eS12
Bromodomain-containing protein 2
Transcriptional repressor protein YY1
DnaJ homolog subfamily B member 1
DnaJ homolog subfamily B member 2
ATP synthase subunit alpha, mitochondrial
Cathepsin S
Proteasome subunit alpha type-1
Proteasome subunit alpha type-2
Proteasome subunit alpha type-3
Proteasome subunit alpha type-4
Tumor necrosis factor receptor superfamily member 5
Integrin alpha-3
Pentraxin-related protein PTX3
Moesin
Probable ATP-dependent RNA helicase DDX6
DNA (cytosine-5)-methyltransferase 1
Splicing factor U2AF 65 kDa subunit
Large ribosomal subunit protein eL13
Rab proteins geranylgeranyltransferase component A 2
Isovaleryl-CoA dehydrogenase, mitochondrial
Alpha-1,3-mannosyl-glycoprotein 2-beta-N-acetylglucosaminyltransferase
High mobility group protein B2
Polypyrimidine tract-binding protein 1
Threonine--tRNA ligase 1, cytoplasmic
Valine--tRNA ligase
Elongation factor 1-gamma

Peptidyl-prolyl cis-trans isomerase FKBP2
Interleukin-3 receptor subunit alpha
Stomatin
Adenylate kinase 4, mitochondrial
14-3-3 protein theta
Mitogen-activated protein kinase 3
MAP/microtubule affinity-regulating kinase 3
Large ribosomal subunit protein uL16
Collagen alpha-1(VIII) chain
Replication protein A 70 kDa DNA-binding subunit
DNA-(apurinic or apyrimidinic site) endonuclease
CD82 antigen
Deoxycytidine kinase
Multifunctional protein CAD
Calreticulin
Microtubule-associated protein 4
Calnexin
Phosphatidylinositol 3-kinase regulatory subunit alpha
Inositol-trisphosphate 3-kinase B
Proteasome subunit beta type-8
Proteasome subunit beta type-9
Proteasome subunit alpha type-5
Proteasome subunit beta type-4
Proteasome subunit beta type-6
Proteasome subunit beta type-5
Glutathione S-transferase Mu 2
ATP-binding cassette sub-family D member 3
Protein ITPRID2
Protein-lysine 6-oxidase
NADH-ubiquinone oxidoreductase 75 kDa subunit, mitochondrial
DNA polymerase delta catalytic subunit
Probable global transcription activator SNF2L1
Mitogen-activated protein kinase 1
Retinoic acid receptor RXR-beta
DNA excision repair protein ERCC-5
Progranulin
Receptor-type tyrosine-protein phosphatase mu
Cytosol aminopeptidase
Hematopoietic progenitor cell antigen CD34
General transcription factor IIE subunit 1
Transcription initiation factor IIE subunit beta
Tripeptidyl-peptidase 2
Inositol monophosphatase 1
CCN family member 2
Ephrin type-A receptor 2
Ephrin type-B receptor 2
SHC-transforming protein 1
DNA-3-methyladenine glycosylase
Transketolase
Caspase-1

Nitric oxide synthase 3
RNA-binding motif, single-stranded-interacting protein 1
Protein PML
Non-receptor tyrosine-protein kinase TYK2
Elongation factor 1-delta
Myristoylated alanine-rich C-kinase substrate
Guanine nucleotide-binding protein subunit alpha-11
Delta-1-pyrroline-5-carboxylate dehydrogenase, mitochondrial
Endoplasmic reticulum resident protein 29
Peroxiredoxin-6
Flavin reductase (NADPH)
Peroxiredoxin-5, mitochondrial
Thioredoxin-dependent peroxide reductase, mitochondrial
Large ribosomal subunit protein uL11
Enoyl-CoA hydratase, mitochondrial
UMP-CMP kinase
Phosphatidylethanolamine-binding protein 1
Protein disulfide-isomerase A3
Serine/threonine-protein phosphatase 2A 65 kDa regulatory subunit A alpha isoform
Serine/threonine-protein phosphatase 2A 65 kDa regulatory subunit A beta isoform
Cell division cycle protein 27 homolog
G1/S-specific cyclin-D3
Peptidyl-prolyl cis-trans isomerase F, mitochondrial
NK-tumor recognition protein
Glycylpeptide N-tetradecanoyltransferase 1
Heme oxygenase 2
Adenylosuccinate synthetase isozyme 2
Tyrosine-protein kinase receptor UFO
Alpha-2-macroglobulin receptor-associated protein
Translocator protein
Adenylosuccinate lyase
CAP-Gly domain-containing linker protein 1
Sorcin
Leukocyte elastase inhibitor
High affinity cationic amino acid transporter 1
Aldehyde dehydrogenase X, mitochondrial
DNA-directed RNA polymerase II subunit RPB2
Succinate dehydrogenase [ubiquinone] flavoprotein subunit, mitochondrial
Coronin-1A
Rab GDP dissociation inhibitor alpha
Protein S100-A7
S-adenosylmethionine synthase isoform type-2
cAMP-dependent protein kinase type II-beta regulatory subunit
Ribonucleoside-diphosphate reductase subunit M2
Syndecan-4
Cytotoxic granule associated RNA binding protein TIA1
Sodium- and chloride-dependent taurine transporter
DnaJ homolog subfamily A member 1
RAC-alpha serine/threonine-protein kinase
Cytochrome b-c1 complex subunit 1, mitochondrial

3-hydroxyisobutyrate dehydrogenase, mitochondrial
Bifunctional purine biosynthesis protein ATIC
Heterogeneous nuclear ribonucleoprotein H3
Heterogeneous nuclear ribonucleoprotein H
Caspase-14
14-3-3 protein beta/alpha
Stress-induced-phosphoprotein 1
Protein S100-A11
Peroxiredoxin-2
Beta-arrestin-2
Glycerol kinase
Deoxycytidylate deaminase
Pyrroline-5-carboxylate reductase 1, mitochondrial
Guanylate-binding protein 1
Guanylate-binding protein 2
ETS-related transcription factor Elf-1
General transcription factor IIH subunit 1
Syntaxin-2
Large ribosomal subunit protein uL6
Long-chain-fatty-acid--CoA ligase 1
Cadherin-5
Kinesin-1 heavy chain
Deoxyuridine 5'-triphosphate nucleotidohydrolase, mitochondrial
Multidrug resistance-associated protein 1
ATP-binding cassette sub-family D member 1
Dual specificity protein kinase TTK
DNA replication licensing factor MCM4
DNA replication licensing factor MCM5
DNA replication licensing factor MCM7
N-acetylgalactosamine-6-sulfatase
Serine hydroxymethyltransferase, cytosolic
Serine hydroxymethyltransferase, mitochondrial
Heat shock 70 kDa protein 1-like
Heat shock 70 kDa protein 4
Mannose-6-phosphate isomerase
Glypican-1
Profilin-2
Catenin alpha-1
Catenin beta-1
Prohibitin 1
Serpin B6
Merlin
Radixin
Replication protein A 14 kDa subunit
Replication factor C subunit 4
Replication factor C subunit 2
Replication factor C subunit 1
Large ribosomal subunit protein eL22
General transcription factor IIF subunit 1
Sepiapterin reductase

Histamine H1 receptor
Cystathionine beta-synthase
Fibrillin-1
Fibrillin-2
Glycogen debranching enzyme
Myosin-9
Myosin-10
Tyrosine-protein kinase receptor Tie-1
Coatomer subunit beta'
Sterol O-acyltransferase 1
Alpha-adducin
Basigin
RNA-binding protein FUS
Nuclear pore complex protein Nup214
Protein DEK
Transcription factor SOX-18
Myosin-11
Protein phosphatase 1A
Hydroxymethylglutaryl-CoA lyase, mitochondrial
Vascular endothelial growth factor receptor 3
Vascular endothelial growth factor receptor 2
26S proteasome regulatory subunit 7
Glutathione hydrolase 5 proenzyme
ADP-ribosylation factor-like protein 2
ADP-ribosylation factor-like protein 3
Dual specificity mitogen-activated protein kinase kinase 2
ATP synthase subunit gamma, mitochondrial
V-type proton ATPase subunit E 1
Oxygen-dependent coproporphyrinogen-III oxidase, mitochondrial
Large ribosomal subunit protein uL4
Oxidized purine nucleoside triphosphate hydrolase
Lon protease homolog, mitochondrial
Phosphoglucomutase-1
Serine/threonine-protein phosphatase PP1-gamma catalytic subunit
Guanine nucleotide-binding protein-like 1
Dihydrolipoyllysine-residue succinyltransferase component of 2-oxoglutarate dehydrogenase complex, mit
GMP reductase 1
Phospholipid hydroperoxide glutathione peroxidase GPX4
Serine/threonine-protein kinase receptor R3
17-beta-hydroxysteroid dehydrogenase type 2
Signal recognition particle 14 kDa protein
TGF-beta receptor type-2
Nuclear pore glycoprotein p62
Hippocalcin-like protein 1
Squalene synthase
Zinc finger E-box-binding homeobox 1
Transgelin-2
Transaldolase
Alpha-synuclein
Electron transfer flavoprotein subunit beta

RNA-binding motif protein, X chromosome
Coilin
Vitamin K-dependent gamma-carboxylase
Lysosomal acid lipase/cholesteryl ester hydrolase
V-type proton ATPase catalytic subunit A
Stress-70 protein, mitochondrial
Eukaryotic initiation factor 4A-III
DNA-binding protein SMUBP-2
Cyclin-dependent kinase inhibitor 1
Small ribosomal subunit protein eS19
Large ribosomal subunit protein uL3
Collagen alpha-1(XVIII) chain
Dolichyl-diphosphooligosaccharide--protein glycosyltransferase 48 kDa subunit
Acidic leucine-rich nuclear phosphoprotein 32 family member A
Flap endonuclease 1
Homeobox protein cut-like 1
Macrophage-capping protein
Adenylyl cyclase-associated protein 2
Interleukin-6 receptor subunit beta
Alpha-taxilin
T-complex protein 1 subunit zeta
Nicotinamide N-methyltransferase
Proteasome subunit beta type-10
Pre-B-cell leukemia transcription factor 2
Large ribosomal subunit protein uL13
ADP-ribosylation factor-like protein 1
DNA mismatch repair protein Mlh1
Signal transducer and activator of transcription 3
Ubiquitin carboxyl-terminal hydrolase 8
Malate dehydrogenase, cytoplasmic
Malate dehydrogenase, mitochondrial
Replication factor C subunit 5
Replication factor C subunit 3
Trifunctional enzyme subunit alpha, mitochondrial
Eukaryotic translation initiation factor 2 subunit 3
ETS translocation variant 3
Centrin-2
Transcription factor ETV6
Eukaryotic translation initiation factor 2D
Protein BUD31 homolog
Ubiquitin-like modifier-activating enzyme 7
N-alpha-acetyltransferase 10
Lysine-specific demethylase 5C
Tyrosine-protein kinase CSK
Glycine--tRNA ligase
Isoleucine--tRNA ligase, cytoplasmic
Reduced folate transporter
Eukaryotic translation initiation factor 1
Protein kinase C iota type
ETS domain-containing protein Elk-3

Beta-centractin
Enoyl-CoA delta isomerase 1, mitochondrial
Lamina-associated polypeptide 2, isoform alpha
Lamina-associated polypeptide 2, isoforms beta/gamma
Signal transducer and activator of transcription 1-alpha/beta
Signal transducer and activator of transcription 6
Signal transducer and activator of transcription 5A
Exosome RNA helicase MTR4
Aldo-keto reductase family 1 member C3
Phosphatidylinositol 4,5-bisphosphate 3-kinase catalytic subunit alpha isoform
Phosphatidylinositol 4,5-bisphosphate 3-kinase catalytic subunit beta isoform
Serine/threonine-protein kinase mTOR
Phosphatidylinositol 4-kinase alpha
Epidermal growth factor receptor substrate 15
Caspase-3
Caspase-2
Small ribosomal subunit protein eS27
Tyrosine-protein kinase ABL2
Probable helicase with zinc finger domain
Condensin-2 complex subunit D3
RNA-binding protein 34
Leucine-rich PPR motif-containing protein, mitochondrial
3-ketoacyl-CoA thiolase, mitochondrial
Large ribosomal subunit protein uL29
Lysosomal Pro-X carboxypeptidase
Huntingtin
Endothelin-converting enzyme 1
Methylenetetrahydrofolate reductase (NADPH)
Neutral amino acid transporter A
Platelet-activating factor acetylhydrolase IB subunit beta
Cell surface glycoprotein MUC18
Carnitine O-acetyltransferase
Matrin-3
DNA mismatch repair protein Msh2
G protein-coupled receptor kinase 6
Glycerol-3-phosphate dehydrogenase, mitochondrial
Translocon-associated protein subunit alpha
Tyrosine-protein phosphatase non-receptor type 9
Ran-specific GTPase-activating protein
Nicotinamide phosphoribosyltransferase
26S proteasome regulatory subunit 6B
Elongation factor Ts, mitochondrial
Peptidyl-prolyl cis-trans isomerase C
Voltage-dependent anion-selective channel protein 2
Short/branched chain specific acyl-CoA dehydrogenase, mitochondrial
Chromobox protein homolog 5
Ubiquitin carboxyl-terminal hydrolase 5
Mitogen-activated protein kinase 8
Dual specificity mitogen-activated protein kinase kinase 4
Proliferation marker protein Ki-67

Phosphorylase b kinase regulatory subunit alpha, liver isoform
Phosphorylase b kinase regulatory subunit alpha, skeletal muscle isoform
Ran GTPase-activating protein 1
ATP-dependent DNA helicase Q1
Probable 28S rRNA (cytosine(4447)-C(5))-methyltransferase
Transcriptional regulator ATRX
Adapter molecule crk
Crk-like protein
Translation initiation factor IF-2, mitochondrial
Large proline-rich protein BAG6
Vesicle-fusing ATPase
Neurogenic locus notch homolog protein 1
Dual specificity mitogen-activated protein kinase kinase 3
Lys-63-specific deubiquitinase BRCC36
Large ribosomal subunit protein uL15
Large ribosomal subunit protein uL18
Large ribosomal subunit protein eL21
Large ribosomal subunit protein eL28
Small ribosomal subunit protein uS4
Small ribosomal subunit protein uS7
Small ribosomal subunit protein eS10
Microtubule-associated protein 1B
Glucosamine-6-phosphate isomerase 1
E3 ubiquitin-protein ligase NEDD4
Utrophin
Ras GTPase-activating-like protein IQGAP1
Glycogenin-1
Dolichyl-diphosphooligosaccharide--protein glycosyltransferase subunit STT3A
Cytosolic phospholipase A2
F-actin-capping protein subunit alpha-2
F-actin-capping protein subunit beta
Eukaryotic translation initiation factor 1A, X-chromosomal
Retinaldehyde dehydrogenase 3
Glutamine--tRNA ligase
Large ribosomal subunit protein eL29
Galectin-7
mRNA decay activator protein ZFP36L2
Cytochrome b-c1 complex subunit Rieske, mitochondrial
ATP synthase subunit O, mitochondrial
LIM and senescent cell antigen-like-containing domain protein 1
Glioma pathogenesis-related protein 1
Prolyl endopeptidase
NADP-dependent malic enzyme
Iron-responsive element-binding protein 2
Tissue factor pathway inhibitor 2
DNA-binding protein RFX5
Phosphatidylinositol 5-phosphate 4-kinase type-2 alpha
Coatomer subunit delta
Lanosterol synthase
Serine/threonine-protein phosphatase 2B catalytic subunit gamma isoform

Glutamate--cysteine ligase catalytic subunit
Glutamate--cysteine ligase regulatory subunit
CD151 antigen
Trafficking protein particle complex subunit 10
26S proteasome non-ATPase regulatory subunit 8
Protein PRRC2A
Glutathione synthetase
T-complex protein 1 subunit epsilon
Phosphatidylserine synthase 1
Nestin
Heat shock 70 kDa protein 13
Casein kinase I isoform alpha
Casein kinase I isoform delta
Isocitrate dehydrogenase [NADP], mitochondrial
Phosphatidylinositol 4,5-bisphosphate 3-kinase catalytic subunit gamma isoform
Phosphatidylinositol transfer protein beta isoform
DNA polymerase delta subunit 2
MARCKS-related protein
Paxillin
Guided entry of tail-anchored proteins factor CAMLG
Nuclear receptor subfamily 2 group C member 2
MAP kinase-activated protein kinase 2
Deoxyribonuclease-1-like 1
4-trimethylaminobutyraldehyde dehydrogenase
Large ribosomal subunit protein eL34
Ribose-5-phosphate isomerase
Protein ERGIC-53
Natural resistance-associated macrophage protein 2
Nuclear autoantigenic sperm protein
Fatty acid synthase
Protein farnesyltransferase/geranylgeranyltransferase type-1 subunit alpha
Protein farnesyltransferase subunit beta
Deoxyhypusine synthase
T-complex protein 1 subunit gamma
Large ribosomal subunit protein bL19m
Beta-arrestin-1
Elongation factor Tu, mitochondrial
Alpha-aminoadipic semialdehyde dehydrogenase
Ubiquitin-conjugating enzyme E2 R1
Inositol polyphosphate 1-phosphatase
Centromere protein F
Signal recognition particle 9 kDa protein
Choline-phosphate cytidylyltransferase A
Alanine--tRNA ligase, cytoplasmic
Cysteine--tRNA ligase, cytoplasmic
Histidine--tRNA ligase, mitochondrial
Serine--tRNA ligase, cytoplasmic
Protein phosphatase 1F
Alpha-mannosidase 2x
DNA primase small subunit

DNA primase large subunit
Caspase-4
Casein kinase I isoform epsilon
Proteasome subunit beta type-3
Proteasome subunit beta type-2
DNA replication licensing factor MCM2
Very long-chain specific acyl-CoA dehydrogenase, mitochondrial
YLP motif-containing protein 1
Vacuolar protein sorting-associated protein 41 homolog
Transmembrane emp24 domain-containing protein 10
RNA-binding protein 25
Protein numb homolog
Dual specificity protein kinase CLK3
Presenilin-1
Translation initiation factor eIF2B subunit beta
Adenosine 5'-monophosphoramidase HINT1
Nuclear pore complex protein Nup153
E3 SUMO-protein ligase RanBP2
Regulator of G-protein signaling 19
Regulator of G-protein signaling 3
Presenilin-2
Tuberin
NADH dehydrogenase [ubiquinone] flavoprotein 1, mitochondrial
Glycogen synthase kinase-3 alpha
Glycogen synthase kinase-3 beta
Transcription initiation factor TFIID subunit 6
Cytosolic purine 5'-nucleotidase
Selenide, water dikinase 1
GMP synthase [glutamine-hydrolyzing]
DNA ligase 3
DNA ligase 4
Double-strand break repair protein MRE11
Ectonucleoside triphosphate diphosphohydrolase 1
Guanine nucleotide-binding protein G(q) subunit alpha
Isocitrate dehydrogenase [NAD] subunit alpha, mitochondrial
Sulfotransferase 1A1
Matrix metalloproteinase-14
Protoporphyrinogen oxidase
Rab GDP dissociation inhibitor beta
Emerin
Carnitine O-palmitoyltransferase 1, liver isoform
Sulfate transporter
Serpin B8
Serpin B9
Serpin H1
PDZ and LIM domain protein 4
Hsc70-interacting protein
Peroxisomal targeting signal 1 receptor
ETS domain-containing transcription factor ERF
Vasodilator-stimulated phosphoprotein

Dynamin-2
Methionine aminopeptidase 2
Bis(5'-nucleosyl)-tetraphosphatase [asymmetrical]
Cyclin-dependent kinase 7
Kinetochore-associated protein 1
Ras association domain-containing protein 2
Cyclin-dependent kinase 9
Lipopolysaccharide-responsive and beige-like anchor protein
Basal cell adhesion molecule
Palmitoyl-protein thioesterase 1
Large ribosomal subunit protein eL14
T-complex protein 1 subunit theta
T-complex protein 1 subunit delta
Annexin A11
Poly(A) polymerase alpha
RNA-binding protein FXR1
RNA-binding protein FXR2
Ras-related protein Rab-5C
Ras-related protein Rab-7a
Ras-related protein Rab-9A
Ras-related protein Rab-13
Ras-related protein Rab-27A
1-phosphatidylinositol 4,5-bisphosphate phosphodiesterase delta-1
Small ribosomal subunit protein mS29
Dual specificity protein phosphatase 3
Probable global transcription activator SNF2L2
Transcription activator BRG1
Isocitrate dehydrogenase [NAD] subunit gamma, mitochondrial
Galactokinase
Translocon-associated protein subunit delta
B-cell receptor-associated protein 31
Thiopurine S-methyltransferase
Methyl-CpG-binding protein 2
Host cell factor 1
Interleukin-1 receptor-associated kinase 1
Caveolin-2
Aldehyde dehydrogenase family 3 member A2
Peroxisomal multifunctional enzyme type 2
26S proteasome non-ATPase regulatory subunit 7
Sulfite oxidase, mitochondrial
N-sulphoglucosamine sulphohydrolase
Signal transducer and activator of transcription 5B
Ubiquitin carboxyl-terminal hydrolase 11
H(+)/Cl(-) exchange transporter 7
Vesicle-associated membrane protein 7
Ribosomal protein S6 kinase alpha-3
Cytoplasmic tyrosine-protein kinase BMX
AF4/FMR2 family member 1
Hepatoma-derived growth factor
Cyclin-H

CDK-activating kinase assembly factor MAT1
Serine/threonine-protein kinase Nek3
Ubiquitin-conjugating enzyme E2 E1
NADH dehydrogenase [ubiquinone] 1 alpha subcomplex subunit 8
Heterogeneous nuclear ribonucleoprotein A3
6-phosphogluconate dehydrogenase, decarboxylating
Heterogeneous nuclear ribonucleoprotein M
Importin subunit alpha-1
Importin subunit alpha-5
Nuclear cap-binding protein subunit 2
Rap1 GTPase-GDP dissociation stimulator 1
DNA-directed RNA polymerases I, II, and III subunit RPABC3
Dual specificity mitogen-activated protein kinase kinase 6
Rho GDP-dissociation inhibitor 1
Rho GDP-dissociation inhibitor 2
Arf-GAP domain and FG repeat-containing protein 1
Heterogeneous nuclear ribonucleoprotein F
Signal transducer and activator of transcription 2
DNA mismatch repair protein Msh6
Kinesin-like protein KIF11
Guanine nucleotide exchange factor VAV2
Zinc finger protein 143
RNA-binding protein 5
2-iminobutanoate/2-iminopropanoate deaminase
Spermine synthase
Hexokinase-2
Ephrin-B2
Large ribosomal subunit protein bL12m
Diacylglycerol kinase theta
Bifunctional heparan sulfate N-deacetylase/N-sulfotransferase 1
Thimet oligopeptidase
F-actin-capping protein subunit alpha-1
High mobility group protein HMGI-C
Cysteine-rich protein 2
Nuclear pore complex protein Nup98-Nup96
Biliverdin reductase A
Tricarboxylate transport protein, mitochondrial
Serine/threonine-protein phosphatase 5
Serine/threonine-protein kinase PLK1
Death-associated protein kinase 1
Arfaptin-2
Arfaptin-1
Cytosolic Fe-S cluster assembly factor NUBP1
ATP-citrate synthase
Methionine aminopeptidase 1
Succinate--CoA ligase [ADP/GDP-forming] subunit alpha, mitochondrial
Diphosphomevalonate decarboxylase
Geranylgeranyl transferase type-1 subunit beta
Geranylgeranyl transferase type-2 subunit beta
Coatomer subunit beta

Coatomer subunit alpha
Dipeptidyl peptidase 1
LIM domain kinase 1
LIM domain kinase 2
Clathrin heavy chain 2
AP-3 complex subunit mu-2
AP-2 complex subunit sigma
Holocytochrome c-type synthase
Mitogen-activated protein kinase 12
Pituitary tumor-transforming gene 1 protein-interacting protein
Smoothelin
Monocarboxylate transporter 1
IST1 homolog
Protein transport protein Sec24C
Activated RNA polymerase II transcriptional coactivator p15
DNA polymerase subunit gamma-1
Methylosome subunit pICln
Arginine--tRNA ligase, cytoplasmic
Protein HIRA
Ataxin-3
Atrophin-1
PMS1 protein homolog 1
Mismatch repair endonuclease PMS2
Voltage-dependent calcium channel subunit alpha-2/delta-1
Tyrosine--tRNA ligase, cytoplasmic
Ubiquitin carboxyl-terminal hydrolase 14
5'-AMP-activated protein kinase subunit gamma-1
Heat shock-related 70 kDa protein 2
Branched-chain-amino-acid aminotransferase, cytosolic
Sodium/potassium-transporting ATPase subunit beta-3
UV excision repair protein RAD23 homolog B
Ephrin type-B receptor 4
Alpha-N-acetylglucosaminidase
Adenylate kinase 2, mitochondrial
Delta-1-pyrroline-5-carboxylate synthase
Alpha-soluble NSF attachment protein
Eukaryotic translation initiation factor 5
Solute carrier family 12 member 2
26S proteasome non-ATPase regulatory subunit 4
Developmentally-regulated GTP-binding protein 2
Exportin-2
Transitional endoplasmic reticulum ATPase
Microfibrillar-associated protein 1
Trifunctional enzyme subunit beta, mitochondrial
Mesencephalic astrocyte-derived neurotrophic factor
Afadin
RNA polymerase II elongation factor ELL
Peregrin
Nucleosome assembly protein 1-like 1
Caspase-7

Caspase-6
Adenosine kinase
Double-stranded RNA-specific adenosine deaminase
Laminin subunit beta-2
Cadherin-13
Tumor protein D52
Protein SEC13 homolog
NHP2-like protein 1
Puromycin-sensitive aminopeptidase
FAD-linked sulfhydryl oxidase ALR
Heterogeneous nuclear ribonucleoprotein H2
Succinyl-CoA:3-ketoacid coenzyme A transferase 1, mitochondrial
Eukaryotic translation initiation factor 3 subunit B
IgG receptor FcRn large subunit p51
BH3-interacting domain death agonist
ATP synthase subunit f, mitochondrial
Ribosomal RNA processing protein 1 homolog A
Methionine--tRNA ligase, cytoplasmic
cAMP-regulated phosphoprotein 19
AP-1 complex subunit sigma-2
ATP synthase subunit ATP5MJ, mitochondrial
ATP synthase subunit epsilon, mitochondrial
ATP synthase subunit e, mitochondrial
Histone deacetylase 4
Eukaryotic translation initiation factor 6
C-terminal-binding protein 2
NADH dehydrogenase [ubiquinone] 1 alpha subcomplex subunit 6
Peroxisomal biogenesis factor 3
3-keto-steroid reductase/17-beta-hydroxysteroid dehydrogenase 7
Breast cancer anti-estrogen resistance protein 1
Syntaxin-17
Cilia- and flagella-associated protein 298
tRNA (guanine-N(7)-)-methyltransferase non-catalytic subunit WDR4
Transmembrane protein 33
Gem-associated protein 4
Coronin-7
Nuclear pore complex protein Nup107
Gasdermin-D
Selenocysteine-specific elongation factor
Sestrin-2
Anthrax toxin receptor 2
Myotrophin
Actin-related protein 2/3 complex subunit 4
CD81 antigen
Protein transport protein Sec61 subunit gamma
Triosephosphate isomerase
Eukaryotic translation initiation factor 3 subunit E
Protein transport protein Sec61 subunit beta
Phosphatidylinositol 3,4,5-trisphosphate 3-phosphatase and dual-specificity protein phosphatase PTEN
Serine/threonine-protein phosphatase 4 catalytic subunit

Gamma-aminobutyric acid receptor-associated protein-like 2
Reactive oxygen species modulator 1
Myosin light polypeptide 6
Actin, cytoplasmic 1
Eukaryotic initiation factor 4A-I
Small ribosomal subunit protein uS10
Ribose-phosphate pyrophosphokinase 1
Proteasome subunit alpha type-6
Protein S100-A10
Cell division control protein 42 homolog
Destrin
Glia maturation factor beta
Ras-related protein Rab-8A
Signal peptidase complex subunit 3
Signal recognition particle subunit SRP54
Ras-related protein Rab-4B
Ras-related protein Rab-2A
Ras-related protein Rab-5B
Ras-related protein Rab-10
Ubiquitin-conjugating enzyme E2 D3
NEDD8-conjugating enzyme Ubc12
Ubiquitin-conjugating enzyme E2 K
Ubiquitin-conjugating enzyme E2 N
Ras-related protein Rab-14
Actin-related protein 3
Actin-related protein 2
Alpha-centractin
COP9 signalosome complex subunit 2
ATP-binding cassette sub-family E member 1
Ras-related protein Rap-1b
Ras-related protein Rap-2b
Protein max
Small ribosomal subunit protein eS1
Large ribosomal subunit protein uL24
Proteasome activator complex subunit 3
Large ribosomal subunit protein eL15
Large ribosomal subunit protein eL27
V-type proton ATPase subunit d 1
Large ribosomal subunit protein eL43
Transforming protein RhoA
Rho-related GTP-binding protein RhoE
N-alpha-acetyltransferase 20
10 kDa heat shock protein, mitochondrial
Protein transport protein Sec61 subunit alpha isoform 1
Lysozyme C
Prefoldin subunit 3
Syntaxin-binding protein 1
Beta-2-microglobulin
Dolichyl-diphosphooligosaccharide--protein glycosyltransferase subunit DAD1
NPC intracellular cholesterol transporter 2

Coatomer subunit zeta-1
Large ribosomal subunit protein eL37
Small ubiquitin-related modifier 2
DDB1- and CUL4-associated factor 7
WD repeat-containing protein 5
AP-1 complex subunit sigma-1A
Nuclear transport factor 2
Heterogeneous nuclear ribonucleoprotein K
14-3-3 protein gamma
Ubiquitin carboxyl-terminal hydrolase 46
Ras-related protein R-Ras2
Small ribosomal subunit protein eS7
Serine/threonine-protein phosphatase PP1-alpha catalytic subunit
Serine/threonine-protein phosphatase PP1-beta catalytic subunit
26S proteasome regulatory subunit 4
26S proteasome regulatory subunit 8
Small ribosomal subunit protein eS8
Small ribosomal subunit protein uS8
Small ribosomal subunit protein uS9
Ubiquitin-conjugating enzyme E2 G1
Ubiquitin-conjugating enzyme E2 H
14-3-3 protein epsilon
Small ribosomal subunit protein uS11
Small ribosomal subunit protein uS12
Small ribosomal subunit protein uS13
Small ribosomal subunit protein uS14
Small ribosomal subunit protein uS15
Small ribosomal subunit protein uS17
Small nuclear ribonucleoprotein E
Small nuclear ribonucleoprotein F
U6 snRNA-associated Sm-like protein LSm3
U6 snRNA-associated Sm-like protein LSm6
Small nuclear ribonucleoprotein Sm D1
Small nuclear ribonucleoprotein Sm D2
Small nuclear ribonucleoprotein Sm D3
Thymosin beta-4
ADP-ribosylation factor 6
26S proteasome regulatory subunit 10B
Thioredoxin reductase-like selenoprotein T
TATA box-binding protein-like 1
Large ribosomal subunit protein eL8
DNA-directed RNA polymerase II subunit RPB7
Ras-related protein Rab-11A
Eukaryotic peptide chain release factor subunit 1
CCHC-type zinc finger nucleic acid binding protein
Protein yippee-like 5
Small ribosomal subunit protein eS4, X isoform
Serine/threonine-protein phosphatase 2A catalytic subunit beta isoform
Actin, aortic smooth muscle
Rho-related GTP-binding protein RhoB

Large ribosomal subunit protein uL23
Small ribosomal subunit protein eS6
Histone H4
Ras-related protein Rab-1A
GTP-binding nuclear protein Ran
Large ribosomal subunit protein uL14
Ras-related protein Rap-1A
Small ribosomal subunit protein uS19
Small ribosomal subunit protein eS24
Small ribosomal subunit protein eS25
Small ribosomal subunit protein eS26
Small ribosomal subunit protein eS28
Ubiquitin-like FUBI-ribosomal protein eS30 fusion protein
Guanine nucleotide-binding protein G(I)/G(S)/G(T) subunit beta-1
E3 ubiquitin-protein ligase RBX1
Guanine nucleotide-binding protein G(I)/G(S)/G(T) subunit beta-2
Large ribosomal subunit protein eL30
Large ribosomal subunit protein eL39
Large ribosomal subunit protein eL31
Large ribosomal subunit protein uL1
Large ribosomal subunit protein eL32
Large ribosomal subunit protein uL5
Large ribosomal subunit protein uL2
Peptidyl-prolyl cis-trans isomerase A
Peptidyl-prolyl cis-trans isomerase FKBP1A
Ubiquitin-ribosomal protein eS31 fusion protein
Growth factor receptor-bound protein 2
Transformer-2 protein homolog beta
Ras-related C3 botulinum toxin substrate 1
AP-2 complex subunit beta
Vesicle-associated membrane protein 2
Guanine nucleotide-binding protein G(s) subunit alpha isoforms short
Guanine nucleotide-binding protein G(i) subunit alpha-1
14-3-3 protein zeta/delta
Serine/threonine-protein phosphatase 2A 55 kDa regulatory subunit B alpha isoform
Small ubiquitin-related modifier 1
Dynein light chain 1, cytoplasmic
Dynein light chain Tctex-type 1
Large ribosomal subunit protein eL38
S-phase kinase-associated protein 1
Guanine nucleotide-binding protein G(I)/G(S)/G(O) subunit gamma-5
Small ribosomal subunit protein eS21
Eukaryotic translation initiation factor 5A-1
Small ribosomal subunit protein RACK1
Transcription elongation factor SPT4
SUMO-conjugating enzyme UBC9
Thymosin beta-10
Serine/threonine-protein phosphatase 2A catalytic subunit alpha isoform
Y-box-binding protein 1
Signal peptidase complex catalytic subunit SEC11A

Casein kinase II subunit beta
Tropomyosin alpha-4 chain
Ubiquitin-conjugating enzyme E2 L3
Elongation factor 1-alpha 1
Tubulin alpha-1B chain
Tubulin alpha-4A chain
Tubulin beta-4B chain
Casein kinase II subunit alpha
Platelet-activating factor acetylhydrolase IB subunit alpha2
Histone H3.1
Hemoglobin subunit beta
Hemoglobin subunit alpha
Coxsackievirus and adenovirus receptor
Protein FAM193A
Nucleolar protein 14
Immunoglobulin-binding protein 1
Tyrosine-protein phosphatase non-receptor type substrate 1
Phosphoserine phosphatase
RNA-binding protein 6
Eukaryotic translation initiation factor 4 gamma 2
Ribonuclease P protein subunit p38
Ribonuclease P protein subunit p30
General transcription factor II-I
Phosphatidylinositol 5-phosphate 4-kinase type-2 beta
Contactin-associated protein 1
SRSF protein kinase 2
T-complex protein 1 subunit beta
UDP-galactose translocator
mRNA export factor RAE1
Glutathione S-transferase omega-1
DNA-dependent protein kinase catalytic subunit
Disintegrin and metalloproteinase domain-containing protein 17
Biogenesis of lysosome-related organelles complex 1 subunit 1
Sushi repeat-containing protein SRPX
Arginase-2, mitochondrial
Endonuclease III-like protein 1
Interferon-induced 35 kDa protein
Nucleobindin-2
Brain acid soluble protein 1
Dermcidin
TATA element modulatory factor
Small ribosomal subunit protein mS22
Small ribosomal subunit protein mS25
Small ribosomal subunit protein uS10m
Small ribosomal subunit protein mS35
Small ribosomal subunit protein uS5m
Alpha-ketoglutarate dehydrogenase component 4
Small ribosomal subunit protein uS11m
Small ribosomal subunit protein uS15m
Small ribosomal subunit protein bS21m

Small ribosomal subunit protein mS34
Small ribosomal subunit protein bS6m
Small ribosomal subunit protein uS9m
SAP domain-containing ribonucleoprotein
Serine beta-lactamase-like protein LACTB, mitochondrial
Conserved oligomeric Golgi complex subunit 7
Large ribosomal subunit protein eL24
Thioredoxin-like protein 4A
Large ribosomal subunit protein eL42
Chromobox protein homolog 1
Mothers against decapentaplegic homolog 3
ADP-ribosylation factor 1
ADP-ribosylation factor 5
Enhancer of rudimentary homolog
Rho-related GTP-binding protein RhoG
Large ribosomal subunit protein eL19
Small EDRK-rich factor 2
Serine/arginine-rich splicing factor 3
Forkhead box protein K1
Vacuolar fusion protein CCZ1 homolog B
Disabled homolog 2
Basement membrane-specific heparan sulfate proteoglycan core protein
E3 ubiquitin-protein ligase XIAP
Ephrin-B1
RNA-binding protein 10
RNA-binding protein 3
Calcium-transporting ATPase type 2C member 1
Phospholipid-transporting ATPase IH
Cytochrome c
55 kDa erythrocyte membrane protein
Transcription factor A, mitochondrial
Phosphatidylinositol transfer protein alpha isoform
Solute carrier family 25 member 3
Vigilin
Transcription initiation factor IIB
Cyclin-dependent kinase 6
Cyclin-dependent kinase 5
Cyclin-dependent kinase 17
Transcriptional activator protein Pur-alpha
Cdc42 effector protein 1
Clathrin heavy chain 1
Heat shock factor protein 1
Nuclear factor NF-kappa-B p100 subunit
Peptidyl-prolyl cis-trans isomerase FKBP3
Receptor expression-enhancing protein 5
Sorbitol dehydrogenase
Heterogeneous nuclear ribonucleoprotein U
Splicing factor U2AF 35 kDa subunit
Spectrin beta chain, non-erythrocytic 1
Nucleolysin TIAR

Protein SET
Serine/arginine-rich splicing factor 2
Forkhead box protein K2
Transcription factor RelB
N-acetylgalactosamine kinase
AMP deaminase 2
Fatty acid-binding protein 5
Adenylyl cyclase-associated protein 1
Friend leukemia integration 1 transcription factor
Hydroxymethylglutaryl-CoA synthase, cytoplasmic
Interferon-induced transmembrane protein 3
Large neutral amino acids transporter small subunit 1
Protein Dr1
Exosome complex component 10
OTU domain-containing protein 4
ATP-dependent 6-phosphofructokinase, platelet type
DNA repair protein complementing XP-C cells
RNA-binding protein EWS
Cerebellar degeneration-related protein 2
Inositol polyphosphate 5-phosphatase OCRL
1-phosphatidylinositol 4,5-bisphosphate phosphodiesterase beta-3
Transgelin
A-kinase anchor protein 17A
Dihydroorotate dehydrogenase (quinone), mitochondrial
2-oxoglutarate dehydrogenase complex component E1
Centromere-associated protein E
Kinesin-like protein KIF23
Methylmalonate-semialdehyde/malonate-semialdehyde dehydrogenase [acylating], mitochondrial
Pro-neuregulin-1, membrane-bound isoform
Sterol 26-hydroxylase, mitochondrial
Desmoglein-1
Transcription factor Sp3
Histone H1.1
Large ribosomal subunit protein eL20
Dual specificity mitogen-activated protein kinase kinase 1
Angiopoietin-1 receptor
Peptidyl-prolyl cis-trans isomerase FKBP4
Procollagen-lysine,2-oxoglutarate 5-dioxygenase 1
Nucleobindin-1
Large ribosomal subunit protein eL6
DNA topoisomerase 2-beta
A-kinase anchor protein 12
Mitochondrial 2-oxoglutarate/malate carrier protein
Dystonin
Protein ENL
Histone-lysine N-methyltransferase MECOM
Guanine nucleotide-binding protein subunit alpha-12
Caveolin-1
Aminoacylase-1
Tumor necrosis factor alpha-induced protein 2

Lamin-B2
6-pyruvoyl tetrahydrobiopterin synthase
Urokinase plasminogen activator surface receptor
Mevalonate kinase
DNA excision repair protein ERCC-6
Antigen peptide transporter 1
Antigen peptide transporter 2
CCAAT/enhancer-binding protein zeta
Zinc finger protein 92
Transcription factor p65
UBX domain-containing protein 1
1,4-alpha-glucan-branching enzyme
Eukaryotic translation initiation factor 4 gamma 1
Copper-transporting ATPase 1
Neurogenic locus notch homolog protein 2
Transducin-like enhancer protein 1
Transducin-like enhancer protein 3
Lactoylglutathione lyase
Activin receptor type-1
Single-stranded DNA-binding protein, mitochondrial
Proto-oncogene c-Rel
14-3-3 protein eta
Proteolipid protein 2
Cleavage stimulation factor subunit 1
Ubiquitin-protein ligase E3A
Dynamin-1
Tyrosine-protein phosphatase non-receptor type 12
Focal adhesion kinase 1
Serine/arginine-rich splicing factor 11
Protein kinase C delta type
Caldesmon
Folylpolyglutamate synthase, mitochondrial
CTD small phosphatase-like protein 2
Tyrosine-protein phosphatase non-receptor type 11
3-ketodihydrosphingosine reductase
Amidophosphoribosyltransferase
Glutamine--fructose-6-phosphate aminotransferase [isomerizing] 1
Exosome complex component RRP45
Proteasome activator complex subunit 1
Recombining binding protein suppressor of hairless
Myocyte-specific enhancer factor 2C
Amyloid beta precursor like protein 2
GA-binding protein alpha chain
E3 ubiquitin-protein ligase RING1
Fragile X messenger ribonucleoprotein 1
Peroxiredoxin-1
Large ribosomal subunit protein eL18
Complement component 1 Q subcomponent-binding protein, mitochondrial
Cytoskeleton-associated protein 4
Tight junction protein ZO-1

mRNA decay activator protein ZFP36L1
Sperm-associated antigen 1
KH domain-containing, RNA-binding, signal transduction-associated protein 1
Apoptosis regulator BAX
Bcl-2-like protein 1
Induced myeloid leukemia cell differentiation protein Mcl-1
DNA polymerase epsilon catalytic subunit A
Kinesin light chain 1
Son of sevenless homolog 1
Serine/arginine-rich splicing factor 1
Rho GTPase-activating protein 1
TLE family member 5
Serine/arginine-rich splicing factor 4
Protocadherin-1
Protein-glutamine gamma-glutamyltransferase E
Protein phosphatase 3 catalytic subunit alpha
ATP-dependent RNA helicase A
Quinone oxidoreductase
Golgin subfamily A member 3
Golgin subfamily A member 2
Peroxisomal bifunctional enzyme
Lactadherin
3',5'-cyclic-AMP phosphodiesterase 4D
Desmocollin-1
Pseudouridine-5'-phosphatase
Testican-1
Leukocyte surface antigen CD47
Peptidyl-prolyl cis-trans isomerase D
FACT complex subunit SSRP1
Calmodulin-regulated spectrin-associated protein 2
Protein spire homolog 1
Schlafen family member 5
Protein VAC14 homolog
RNA cytosine C(5)-methyltransferase NSUN2
Dystrophia myotonica WD repeat-containing protein
Histone-binding protein RBBP4
Nuclear cap-binding protein subunit 1
Alpha-1,6-mannosylglycoprotein 6-beta-N-acetylglucosaminyltransferase A
Histone acetyltransferase p300
Neuroblast differentiation-associated protein AHNAK
F-BAR domain only protein 2
Elongator complex protein 6
Heat shock 70 kDa protein 14
Secernin-3
Cingulin-like protein 1
Ragulator complex protein LAMTOR4
Nexilin
Alpha-1,6-mannosyl-glycoprotein 2-beta-N-acetylglucosaminyltransferase
Polypeptide N-acetylgalactosaminyltransferase 2
Polypeptide N-acetylgalactosaminyltransferase 1

AP-1 complex subunit beta-1
Cleavage and polyadenylation specificity factor subunit 1
Bone marrow stromal antigen 2
Mitochondrial-processing peptidase subunit alpha
CMP-N-acetylneuraminate-beta-galactosamide-alpha-2,3-sialyltransferase 1
Secernin-1
WASH complex subunit 5
Nuclear pore complex protein Nup160
Sterol regulatory element-binding protein cleavage-activating protein
Forkhead box protein O1
Transducin beta-like protein 3
General transcription factor 3C polypeptide 1
Twinfilin-1
Aspartyl/asparaginyl beta-hydroxylase
Alpha-globin transcription factor CP2
A-kinase anchor protein 13
EGF-containing fibulin-like extracellular matrix protein 1
SWI/SNF-related matrix-associated actin-dependent regulator of chromatin subfamily B member 1
Nucleosome-remodeling factor subunit BPTF
Cell division cycle protein 20 homolog
Follistatin-related protein 1
Syntaxin-4
G-rich sequence factor 1
Mitogen-activated protein kinase kinase kinase kinase 2
Tyrosine-protein kinase Mer
Splicing factor, suppressor of white-apricot homolog
Chromodomain-helicase-DNA-binding protein 3
Splicing factor 3A subunit 3
TP53-binding protein 1
Transmembrane protein 115
Interferon-related developmental regulator 2
Tripartite motif-containing protein 26
Aminoacyl tRNA synthase complex-interacting multifunctional protein 1
Interleukin enhancer-binding factor 2
Interleukin enhancer-binding factor 3
Vesicular integral-membrane protein VIP36
Heat shock protein 75 kDa, mitochondrial
TNF receptor-associated factor 2
Disks large homolog 1
Unconventional myosin-Ie
Nuclear inhibitor of protein phosphatase 1
Protein tyrosine phosphatase type IVA 2
Active breakpoint cluster region-related protein
GATOR1 complex protein NPRL3
Vesicle transport protein SEC20
BCL2/adenovirus E1B 19 kDa protein-interacting protein 2
Transcriptional repressor NF-X1
Cleavage stimulation factor subunit 3
Tetraspanin-31
Delta(3,5)-Delta(2,4)-dienoyl-CoA isomerase, mitochondrial

Rho GTPase-activating protein 5
Striatin-3
Cell division cycle protein 16 homolog
Serine/threonine-protein kinase 4
Protein flightless-1 homolog
E3 ubiquitin-protein ligase TRIM32
Bifunctional coenzyme A synthase
Large ribosomal subunit protein bL28m
Acetyl-CoA carboxylase 1
COP9 signalosome complex subunit 1
Ubiquitin carboxyl-terminal hydrolase 4
Chromatin assembly factor 1 subunit A
Chromatin assembly factor 1 subunit B
Protein Red
S-methyl-5'-thioadenosine phosphorylase
5'-AMP-activated protein kinase catalytic subunit alpha-1
Liprin-alpha-1
Translation initiation factor eIF2B subunit epsilon
TAR DNA-binding protein 43
Heterogeneous nuclear ribonucleoprotein A0
Aminoacyl tRNA synthase complex-interacting multifunctional protein 2
FAS-associated death domain protein
Peroxiredoxin-4
Dual specificity mitogen-activated protein kinase kinase 5
Serine/threonine-protein kinase PAK 2
Chromobox protein homolog 3
Serine/threonine-protein kinase 3
Syntaxin-5
26S proteasome non-ATPase regulatory subunit 2
Multimerin-1
Probable ATP-dependent RNA helicase DDX10
DnaJ homolog subfamily C member 3
Methanethiol oxidase
Nucleoside diphosphate kinase 3
Serine/arginine-rich splicing factor 9
Serine/arginine-rich splicing factor 5
Serine/arginine-rich splicing factor 6
Mitotic spindle assembly checkpoint protein MAD2A
Transcription intermediary factor 1-beta
Semaphorin-3F
Syntaxin-3
Ras GTPase-activating protein-binding protein 1
N-myc-interactor
Inactive tyrosine-protein kinase 7
Polyadenylate-binding protein 4
Serine-protein kinase ATM
Growth factor receptor-bound protein 10
Interferon-induced protein with tetratricopeptide repeats 5
Metastasis-associated protein MTA1
Eukaryotic translation initiation factor 3 subunit I

RING-type E3 ubiquitin-protein ligase PPIL2
Serine/threonine-protein phosphatase 2A 56 kDa regulatory subunit gamma isoform
C-terminal-binding protein 1
Phosducin-like protein
Phospholipase D1
Probable methyltransferase TARBP1
Ubiquitin-conjugating enzyme E2 variant 1
Large ribosomal subunit protein mL49
Cytoplasmic dynein 1 intermediate chain 2
Origin recognition complex subunit 2
Integrin-linked protein kinase
NAD(P) transhydrogenase, mitochondrial
Beta-2-syntrophin
DNA repair protein XRCC4
Peptidyl-prolyl cis-trans isomerase G
Treacle protein
Protein unc-119 homolog A
Splicing factor 3B subunit 2
Protein OS-9
Golgin subfamily A member 4
28 kDa heat- and acid-stable phosphoprotein
Disintegrin and metalloproteinase domain-containing protein 9
Disintegrin and metalloproteinase domain-containing protein 15
Transmembrane emp24 domain-containing protein 1
Peptidyl-prolyl cis-trans isomerase FKBP5
Unconventional myosin-IXb
Rho-associated protein kinase 1
Nuclear factor of activated T-cells, cytoplasmic 2
DNA topoisomerase 3-alpha
Mothers against decapentaplegic homolog 4
V-type proton ATPase 116 kDa subunit a 3
Baculoviral IAP repeat-containing protein 2
Phosphatidylinositol-binding clathrin assembly protein
Myotubularin
Sequestosome-1
Metaxin-1
Tubulin beta-3 chain
Acid ceramidase
Serine/threonine-protein kinase PRP4 homolog
Peptidyl-prolyl cis-trans isomerase NIMA-interacting 1
Serine/threonine-protein kinase ATR
Eukaryotic translation initiation factor 4E-binding protein 1
Receptor-interacting serine/threonine-protein kinase 1
Histone deacetylase 1
Calcium/calmodulin-dependent protein kinase type II subunit gamma
Calcium/calmodulin-dependent protein kinase type II subunit delta
Dynactin subunit 2
Polycystin-2
NEDD8-activating enzyme E1 regulatory subunit
Inositol-tetrakisphosphate 1-kinase

SNW domain-containing protein 1
Diacylglycerol kinase zeta
Stromal interaction molecule 1
GRB2-related adapter protein
Transformer-2 protein homolog alpha
Sorting nexin-1
KRR1 small subunit processome component homolog
Periodic tryptophan protein 1 homolog
Myotubularin-related protein 1
Myotubularin-related protein 2
Cullin-1
Cullin-2
Cullin-3
Cullin-4A
Cullin-4B
Apoptosis-stimulating of p53 protein 2
Dual specificity tyrosine-phosphorylation-regulated kinase 1A
GDP-L-fucose synthase
Ras-related protein Rab-32
Trophoblast glycoprotein
Four and a half LIM domains protein 1
Four and a half LIM domains protein 3
Ras and Rab interactor 1
Angio-associated migratory cell protein
Mannosyl-oligosaccharide glucosidase
CD166 antigen
THO complex subunit 5 homolog
Protein Shroom2
Spectrin alpha chain, non-erythrocytic 1
Nucleolar GTP-binding protein 2
Methylglutaconyl-CoA hydratase, mitochondrial
Plakophilin-1
Spliceosome RNA helicase DDX39B
Bleomycin hydrolase
Exosome complex component RRP4
Bone morphogenetic protein receptor type-2
Tubulin beta-2A chain
General transcription factor IIH subunit 2
General transcription factor IIH subunit 3
Bystin
Rap guanine nucleotide exchange factor 1
Isopentenyl-diphosphate Delta-isomerase 1
Core-binding factor subunit beta
Nuclear transcription factor Y subunit gamma
Cyclin-dependent kinase 13
Cytoskeleton-associated protein 5
Cold-inducible RNA-binding protein
Calcium/calmodulin-dependent protein kinase type 1
Coactosin-like protein
Heterogeneous nuclear ribonucleoprotein D0

Lysosome membrane protein 2
Nidogen-2
Low-density lipoprotein receptor-related protein 8
Dystroglycan 1
Vascular endothelial zinc finger 1
Ribosome biogenesis protein BOP1
Ubiquitin conjugation factor E4 A
Septin-6
Kelch-like ECH-associated protein 1
Unhealthy ribosome biogenesis protein 2 homolog
Probable ATP-dependent RNA helicase DHX34
MORC family CW-type zinc finger protein 3
Scaffold attachment factor B2
Eukaryotic translation initiation factor 3 subunit A
Rho guanine nucleotide exchange factor 7
Protein EFR3 homolog A
Ubiquitin-associated protein 2-like
Protein scribble homolog
ARF GTPase-activating protein GIT2
Scavenger receptor class F member 1
Malectin
Tubulin--tyrosine ligase-like protein 12
DNA polymerase alpha subunit B
Dedicator of cytokinesis protein 1
Transcription factor Dp-1
Four and a half LIM domains protein 2
Dihydropyrimidinase-related protein 3
Large ribosomal subunit protein mL62
Zinc finger MYM-type protein 3
Dynactin subunit 1
Cytoplasmic dynein 1 heavy chain 1
Translation initiation factor eIF2B subunit alpha
Eukaryotic initiation factor 4A-II
Elongin-A
Src substrate cortactin
Endonuclease G, mitochondrial
Flotillin-2
Reticulocalbin-2
E3 ubiquitin/ISG15 ligase TRIM25
Filamin-C
Peptidyl-prolyl cis-trans isomerase FKBP8
Protein FAM50A
Protein FRG1
Guanine nucleotide-binding protein subunit alpha-13
Guanidinoacetate N-methyltransferase
UDP-glucose 4-epimerase
Transforming growth factor beta activator LRRC32
Caprin-1
Beclin-1
RNA-binding protein 39

Enhancer of filamentation 1
Helicase-like transcription factor
Squalene monooxygenase
Protein disulfide-isomerase A5
Phosphoribosyl pyrophosphate synthase-associated protein 1
ATP-dependent RNA helicase DHX8
DNA replication licensing factor MCM6
Inositol 1,4,5-trisphosphate receptor type 2
Inositol 1,4,5-trisphosphate receptor type 3
Inositol 1,4,5-trisphosphate receptor type 1
Ras GTPase-activating protein 3
Plastin-1
Interferon regulatory factor 3
EKC/KEOPS complex subunit LAGE3
Bridge-like lipid transfer protein family member 2
E3 ubiquitin-protein ligase TRIP12
Pumilio homolog 1
Mediator of DNA damage checkpoint protein 1
Clathrin interactor 1
KN motif and ankyrin repeat domain-containing protein 1
Structural maintenance of chromosomes protein 1A
Ribosomal RNA processing protein 1 homolog B
Disco-interacting protein 2 homolog A
Protein RRP5 homolog
DNA replication complex GINS protein PSF1
Ribosome biogenesis protein BMS1 homolog
Phosphatidate phosphatase LPIN1
Ubiquitin carboxyl-terminal hydrolase 10
LRP chaperone MESD
Neutral alpha-glucosidase AB
Raftlin
Major facilitator superfamily domain-containing protein 10
Serine/threonine-protein phosphatase 2A 56 kDa regulatory subunit delta isoform
Delta(14)-sterol reductase LBR
Conserved oligomeric Golgi complex subunit 2
Major vault protein
Latent-transforming growth factor beta-binding protein 1
Latent-transforming growth factor beta-binding protein 2
Chromobox protein homolog 2
Golgin subfamily B member 1
Caspase-8
N-alpha-acetyltransferase 30
Kinesin-like protein KIF22
Myocyte-specific enhancer factor 2D
Chromodomain-helicase-DNA-binding protein 4
LIM and SH3 domain protein 1
Prostaglandin reductase 1
Dr1-associated corepressor
Nuclear factor of activated T-cells, cytoplasmic 4
Zinc finger protein 638

Importin subunit beta-1
Nucleolar and coiled-body phosphoprotein 1
Nuclear mitotic apparatus protein 1
Proteasome activator complex subunit 4
Cullin-7
Sarcolemmal membrane-associated protein
GTPase-activating protein and VPS9 domain-containing protein 1
N-alpha-acetyltransferase 25, NatB auxiliary subunit
Ventricular zone-expressed PH domain-containing protein homolog 1
Condensin complex subunit 2
PCNA-associated factor
Signal peptidase complex subunit 2
ER membrane protein complex subunit 2
Pre-mRNA-splicing regulator WTAP
26S proteasome non-ATPase regulatory subunit 6
Homocysteine-responsive endoplasmic reticulum-resident ubiquitin-like domain member 1 protein
MAD2L1-binding protein
Mortality factor 4-like protein 2
BRISC complex subunit Abraxas 2
Septin-2
Squamous cell carcinoma antigen recognized by T-cells 3
Condensin complex subunit 1
Polycomb protein SUZ12
Exosome complex component RRP42
TNFAIP3-interacting protein 1
116 kDa U5 small nuclear ribonucleoprotein component
Leucine--tRNA ligase, mitochondrial
R3H domain-containing protein 1
Translocating chain-associated membrane protein 2
Sorting nexin-17
ADP-ribosylation factor-like protein 6-interacting protein 1
Rab3 GTPase-activating protein catalytic subunit
Metal cation symporter ZIP14
Lysine--tRNA ligase
Histone-lysine N-methyltransferase SETDB1
Leucine-rich repeat-containing protein 14
Ribosome biogenesis regulatory protein homolog
DNA polymerase delta subunit 3
Eukaryotic translation initiation factor 4H
Arf-GAP with coiled-coil, ANK repeat and PH domain-containing protein 2
Kinesin-like protein KIF14
Bromodomain-containing protein 3
WD repeat-containing protein 43
Peroxisomal acyl-coenzyme A oxidase 1
Mitochondrial inner membrane protein OXA1L
Early endosome antigen 1
Protein disulfide-isomerase A6
Platelet-activating factor acetylhydrolase IB subunit alpha1
[Pyruvate dehydrogenase (acetyl-transferring)] kinase isozyme 3, mitochondrial
Astrocytic phosphoprotein PEA-15

3-beta-hydroxysteroid-Delta(8),Delta(7)-isomerase
Phosphomevalonate kinase
Serine/threonine-protein kinase D1
Plectin
Pericentriolar material 1 protein
BOS complex subunit NOMO1
Serum paraoxonase/arylesterase 2
Serine/threonine-protein phosphatase 2A 56 kDa regulatory subunit alpha isoform
Inorganic pyrophosphatase
Prostaglandin E synthase 3
Serine/threonine-protein kinase 38
Non-POU domain-containing octamer-binding protein
Serine/threonine-protein phosphatase 2A activator
Receptor-type tyrosine-protein phosphatase kappa
Periodic tryptophan protein 2 homolog
Rab GTPase-binding effector protein 1
Ras-related protein Rab-35
RNA-binding protein with serine-rich domain 1
Retinoblastoma-binding protein 5
Reticulocalbin-1
RalA-binding protein 1
Lethal(2) giant larvae protein homolog 1
Leucine-rich repeat-containing protein 41
Transcription termination factor 1
Transmembrane emp24 domain-containing protein 2
Poly(rC)-binding protein 1
Poly(rC)-binding protein 2
Elongin-C
Elongin-B
GTP-binding protein Rheb
Ubiquitin-protein ligase E3C
Mitochondrial import receptor subunit TOM20 homolog
Mitochondrial fission regulator 1
Delta(24)-sterol reductase
Splicing factor 3B subunit 3
Pumilio homolog 3
Disks large-associated protein 5
Ras suppressor protein 1
Calponin-3
Ribosomal protein S6 kinase alpha-1
Scaffold attachment factor B1
Splicing factor 3A subunit 2
RNA-binding motif, single-stranded-interacting protein 2
Protein phosphatase 1 regulatory subunit 7
Protein transport protein Sec23A
Protein transport protein Sec23B
Cytohesin-1
Splicing factor 3A subunit 1
Superkiller complex protein 2
Corneodesmosin

Surfeit locus protein 1
Transcription initiation factor TFIID subunit 5
Transcription initiation factor TFIID subunit 7
Telomeric repeat-binding factor 2
Microtubule-associated protein RP/EB family member 2
Homeobox protein TGIF1
Na(+)/H(+) exchange regulatory cofactor NHE-RF2
Tumor necrosis factor receptor type 1-associated DEATH domain protein
Translocating chain-associated membrane protein 1
Translin
RISC-loading complex subunit TARBP2
Splicing factor 1
Cdc42-interacting protein 4
Thyroid receptor-interacting protein 11
Pachytene checkpoint protein 2 homolog
Mediator of RNA polymerase II transcription subunit 1
Activating signal cointegrator 1
High mobility group nucleosome-binding domain-containing protein 3
Probable JmjC domain-containing histone demethylation protein 2C
NF-kappa-B inhibitor beta
Thyroid receptor-interacting protein 6
Tyrosine-protein phosphatase non-receptor type 14
Microtubule-associated protein RP/EB family member 1
ELAV-like protein 1
Sterol-4-alpha-carboxylate 3-dehydrogenase, decarboxylating
NGFI-A-binding protein 2
Myosin light chain kinase, smooth muscle
TGF-beta-activated kinase 1 and MAP3K7-binding protein 1
Probable E3 ubiquitin-protein ligase HERC1
Neutral amino acid transporter B(0)
Myeloid leukemia factor 2
Mitochondrial import receptor subunit TOM34
Mothers against decapentaplegic homolog 2
Mothers against decapentaplegic homolog 1
Methylsterol monooxygenase 1
Intersectin-1
Tubulin-specific chaperone E
Tubulin-specific chaperone C
Ubiquitin-conjugating enzyme E2 variant 2
Serine/threonine-protein kinase STK11
Syntaxin-binding protein 2
Vesicle-associated membrane protein 3
NEDD8
V-type proton ATPase subunit S1
Vacuolar protein sorting-associated protein 72 homolog
Ras-related protein Rab-11B
Zyxin
Electron transfer flavoprotein-ubiquinone oxidoreductase, mitochondrial
Septin-7
Proteasomal ubiquitin receptor ADRM1

Coiled-coil domain-containing protein 6
UDP-N-acetylhexosamine pyrophosphorylase
Transcription factor E2F4
Insulin-like growth factor-binding protein 7
Programmed cell death protein 2
Laminin subunit alpha-4
26S proteasome non-ATPase regulatory subunit 5
Serine/threonine-protein kinase N1
Serine/threonine-protein kinase N2
Transcription initiation factor TFIID subunit 12
Cysteine and glycine-rich protein 2
DNA damage-binding protein 1
Serine/threonine-protein phosphatase 2A 56 kDa regulatory subunit epsilon isoform
Mitogen-activated protein kinase 14
Large ribosomal subunit protein uL23m
Hsp90 co-chaperone Cdc37
Dihydropyrimidinase-related protein 2
Synaptophysin-like protein 1
Histone-binding protein RBBP7
Mitogen-activated protein kinase kinase kinase 11
Transcription initiation factor TFIID subunit 9
Calcitonin gene-related peptide type 1 receptor
Bcl-2 homologous antagonist/killer
Serine/arginine-rich splicing factor 7
Cleavage and polyadenylation specificity factor subunit 6
Survival motor neuron protein
Drebrin
Nuclear respiratory factor 1
Fascin
Gamma-interferon-inducible protein 16
2,4-dienoyl-CoA reductase [(3E)-enoyl-CoA-producing], mitochondrial
Alpha-mannosidase 2
NADH dehydrogenase [ubiquinone] 1 alpha subcomplex subunit 5
Ceramide glucosyltransferase
ATP-dependent Clp protease proteolytic subunit, mitochondrial
Thiosulfate sulfurtransferase
Ubiquitin-conjugating enzyme E2 S
Kynurenine--oxoglutarate transaminase 1
Guanylate kinase
Hydroxyacylglutathione hydrolase, mitochondrial
NADH dehydrogenase [ubiquinone] 1 alpha subcomplex subunit 9, mitochondrial
NADP-dependent malic enzyme, mitochondrial
Phosphoenolpyruvate carboxykinase [GTP], mitochondrial
Uridine phosphorylase 1
Discoidin domain-containing receptor 2
Hydroxyacyl-coenzyme A dehydrogenase, mitochondrial
Lanosterol 14-alpha demethylase
UTP--glucose-1-phosphate uridylyltransferase
6-phosphofructo-2-kinase/fructose-2,6-bisphosphatase 3
Thioredoxin reductase 1, cytoplasmic

MICOS complex subunit MIC60
Endothelial cell-specific chemotaxis regulator
Lysine-rich nucleolar protein 1
Heterogeneous nuclear ribonucleoprotein U-like protein 2
Transmembrane protein 132A
Inverted formin-2
Sister chromatid cohesion protein PDS5 homolog A
Protein CLEC16A
Kinesin-like protein KIF7
Rho GTPase-activating protein 31
Methenyltetrahydrofolate synthase domain-containing protein
AP2-associated protein kinase 1
WASH complex subunit 4
DNA excision repair protein ERCC-6-like
Pre-rRNA-processing protein TSR1 homolog
Ral GTPase-activating protein subunit alpha-2
Protein C-mannosyl-transferase DPY19L1
Isoamyl acetate-hydrolyzing esterase 1 homolog
GDP-Man:Man(3)GlcNAc(2)-PP-Dol alpha-1,2-mannosyltransferase
Transcriptional regulator QRICH1
WD40 repeat-containing protein SMU1
Cytoplasmic tRNA 2-thiolation protein 2
Protein-glucosylgalactosylhydroxylysine glucosidase
Leucine-rich repeat flightless-interacting protein 1
CDP-diacylglycerol--glycerol-3-phosphate 3-phosphatidyltransferase, mitochondrial
UPF0711 protein C18orf21
Prolyl 3-hydroxylase 1
tRNA (guanine(37)-N1)-methyltransferase
Echinoderm microtubule-associated protein-like 3
DNA-directed RNA polymerase I subunit RPA43
MAP7 domain-containing protein 1
UDP-N-acetylhexosamine pyrophosphorylase-like protein 1
Chromodomain-helicase-DNA-binding protein 9
Triokinase/FMN cyclase
Protein LSM12
Inactive hydroxysteroid dehydrogenase-like protein 1
Mitochondrial 10-formyltetrahydrofolate dehydrogenase
N-acetylglucosamine-1-phosphotransferase subunits alpha/beta
Girdin
Rab-like protein 6
Mitochondrial import inner membrane translocase subunit TIM50
Protein mono-ADP-ribosyltransferase PARP14
Alpha-(1,3)-fucosyltransferase 11
Cytokine-like nuclear factor N-PAC
UPF0489 protein C5orf22
C2 domain-containing protein 3
Vacuolar protein sorting-associated protein 26B
La-related protein 7
NAD kinase 2, mitochondrial
BTB/POZ domain-containing protein KCTD21

Glucoside xylosyltransferase 1
Malonate--CoA ligase ACSF3, mitochondrial
Prolyl endopeptidase-like
TBC1 domain family member 10B
Taperin
Anoctamin-6
Filamin A-interacting protein 1-like
GRIP1-associated protein 1
Zinc-regulated GTPase metalloprotein activator 1F
Protein MIX23
Membralin
Protein FAM98B
Rho GTPase-activating protein 29
Programmed cell death protein 4
Fibronectin type III domain-containing protein 3B
CREB-regulated transcription coactivator 2
Glycerol-3-phosphate acyltransferase 3
Centrosomal protein of 55 kDa
Nuclear cap-binding protein subunit 3
Quinone oxidoreductase PIG3
Novel acetylcholine receptor chaperone
Pleckstrin homology-like domain family A member 2
PDZ and LIM domain protein 3
Protein mono-ADP-ribosyltransferase PARP10
Very-long-chain 3-oxoacyl-CoA reductase
mRNA export factor GLE1
Ubiquitin carboxyl-terminal hydrolase 39
Kelch-like protein 22
Acylglycerol kinase, mitochondrial
Histone-lysine N-methyltransferase SETMAR
Endoribonuclease LACTB2
Pyrroline-5-carboxylate reductase 3
EARP and GARP complex-interacting protein 1
Borealin
Cordon-bleu protein-like 1
HCLS1-binding protein 3
Plasma membrane ascorbate-dependent reductase CYBRD1
WD repeat-containing protein 81
Beta-actin-like protein 2
Coiled-coil domain-containing protein 93
OCIA domain-containing protein 2
Puratrophin-1
Putative heat shock protein HSP 90-alpha A4
DDB1- and CUL4-associated factor 6
Transmembrane protein 41B
Sigma intracellular receptor 2
Protein YIF1B
DBIRD complex subunit ZNF326
Nucleolar MIF4G domain-containing protein 1
RILP-like protein 1

DnaJ homolog subfamily C member 21
Tumor necrosis factor alpha-induced protein 8-like protein 3
Probable E3 ubiquitin-protein ligase HERC4
GPI ethanolamine phosphate transferase 2
Serine/threonine-protein phosphatase 6 regulatory subunit 3
Rab-like protein 3
Protein FAM76B
2-methoxy-6-polyprenyl-1,4-benzoquinol methylase, mitochondrial
SH3 domain-containing protein 19
NHS-like protein 2
ER membrane protein complex subunit 4
Nondiscriminating glutamyl-tRNA synthetase EARS2, mitochondrial
Uncharacterized protein C3orf38
EEF1A lysine methyltransferase 2
Transport and Golgi organization protein 1 homolog
Presequence protease, mitochondrial
WD repeat-containing protein 44
Protein PRRC2B
Tight junction-associated protein 1
RRP12-like protein
Cytochrome c oxidase assembly factor 6 homolog
Torsin-1A-interacting protein 1
Centrosomal protein of 78 kDa
Alanine--tRNA ligase, mitochondrial
Torsin-2A
PCI domain-containing protein 2
Intracellular hyaluronan-binding protein 4
Long-chain fatty acid transport protein 3
Sterile alpha motif domain-containing protein 9
Serine/threonine-protein phosphatase 4 regulatory subunit 3B
WD repeat domain phosphoinositide-interacting protein 3
WD repeat domain phosphoinositide-interacting protein 1
EGF domain-specific O-linked N-acetylglucosamine transferase
Protein Smaug homolog 2
Tubulin-specific chaperone cofactor E-like protein
Deoxynucleotidyltransferase terminal-interacting protein 2
Rab GTPase-activating protein 1-like
Tetratricopeptide repeat protein 38
Cytochrome c oxidase assembly protein COX20, mitochondrial
Exosome complex component MTR3
Transmembrane protein 201
Alpha-tubulin N-acetyltransferase 1
Synaptosomal-associated protein 47
Nucleoporin NUP188
Heterochromatin protein 1-binding protein 3
Valine--tRNA ligase, mitochondrial
Centrosomal protein of 170 kDa
Low density lipoprotein receptor adapter protein 1
Protein odr-4 homolog
Polynucleotide 5'-hydroxyl-kinase NOL9

NHS-like protein 1
Putative hydroxypyruvate isomerase
Tumor protein p63-regulated gene 1-like protein
Coiled-coil and C2 domain-containing protein 1B
Formin-binding protein 1-like
Probable arginine--tRNA ligase, mitochondrial
Acyl-coenzyme A thioesterase THEM4
Putative coiled-coil-helix-coiled-coil-helix domain-containing protein CHCHD2P9, mitochondrial
FK506-binding protein 15
Zinc finger CCCH domain-containing protein 13
Putative methyltransferase C9orf114
Armadillo-like helical domain-containing protein 3
CSC1-like protein 2
G patch domain-containing protein 4
Putative transferase CAF17, mitochondrial
E3 ubiquitin-protein ligase HECTD3
Inactive glycosyltransferase 25 family member 3
E3 ubiquitin-protein ligase UBR4
Syntaxin-binding protein 5
Rho GTPase-activating protein 21
Calmodulin-regulated spectrin-associated protein 1
Large ribosomal subunit protein uL2m
Ubiquitin-associated protein 2
Queuosine 5'-phosphate N-glycosylase/hydrolase
Keratinocyte proline-rich protein
Zinc finger protein 618
Acyl-CoA-binding domain-containing protein 5
RNA-binding protein 26
ATPase family AAA domain-containing protein 3B
Protein wntless homolog
Integrator complex subunit 11
Ceramide-1-phosphate transfer protein
DDB1- and CUL4-associated factor 8
Collagen alpha-1(XIII) chain
Terminal uridylyltransferase 4
Protein furry homolog
Ribonuclease H2 subunit B
ATP synthase mitochondrial F1 complex assembly factor 1
Roquin-1
Protein DDI1 homolog 2
Protein THEMIS2
Arginine-hydroxylase NDUFAF5, mitochondrial
5'-nucleotidase domain-containing protein 1
Brefeldin A-inhibited guanine nucleotide-exchange protein 3
Intermembrane lipid transfer protein VPS13D
Rootletin
Complex III assembly factor LYRM7
Ras-interacting protein 1
Telomere-associated protein RIF1
Vacuolar protein sorting-associated protein 53 homolog

Striatin-interacting protein 1
Serine/threonine-protein kinase MRCK alpha
Regulation of nuclear pre-mRNA domain-containing protein 2
E3 ubiquitin-protein ligase RNF220
Pre-mRNA-splicing factor 38B
E3 ubiquitin-protein ligase BRE1A
Myomegalin
Zinc finger protein 318
Protein FAM171A1
Divergent protein kinase domain 1B
Leucine-rich repeat and calponin homology domain-containing protein 2
Threonylcarbamoyladenosine tRNA methylthiotransferase
BRO1 domain-containing protein BROX
Focadhesin
Lysine-specific demethylase 9
Disabled homolog 2-interacting protein
Lysophospholipase-like protein 1
Platelet endothelial aggregation receptor 1
Proteasome adapter and scaffold protein ECM29
Terminal uridylyltransferase 7
SH2 domain-containing adapter protein E
DENN domain-containing protein 4C
N-alpha-acetyltransferase 35, NatC auxiliary subunit
Zinc finger MYM-type protein 4
FHF complex subunit HOOK interacting protein 2A
Palmitoyltransferase ZDHHC20
SPRY domain-containing protein 7
F-box/WD repeat-containing protein 9
CD276 antigen
E3 ubiquitin-protein ligase RNF213
Tensin-2
KN motif and ankyrin repeat domain-containing protein 2
WASH complex subunit 2A
Lysophospholipid acyltransferase LPCAT4
Metalloreductase STEAP3
Protein FAM91A1
TBC1 domain family member 9B
Microtubule-associated protein 1S
Serine/threonine-protein phosphatase 2A 55 kDa regulatory subunit B delta isoform
ADP-ribosylation factor-like protein 6-interacting protein 4
Autophagy-related protein 16-1
Heparan-alpha-glucosaminide N-acetyltransferase
AT-rich interactive domain-containing protein 2
U3 small nucleolar RNA-associated protein 25 homolog
Glycosyltransferase 8 domain-containing protein 1
Protein sel-1 homolog 3
Tensin-3
HAUS augmin-like complex subunit 3
Protein SPT2 homolog
Acyl-coenzyme A thioesterase MBLAC2

Very large A-kinase anchor protein
Integrator complex subunit 3
Rho GTPase-activating protein 17
Protein NCBP2AS2
CWF19-like protein 1
Protein virilizer homolog
Cytospin-A
HEAT repeat-containing protein 6
Ankyrin repeat domain-containing protein 40
Protein WWC2
Scavenger receptor class A member 3
Atlastin-3
Ribosomal protein uL30-like
Nucleoredoxin
Tetratricopeptide repeat protein 19, mitochondrial
IQ motif and SEC7 domain-containing protein 1
Anamorsin
AN1-type zinc finger protein 6
Histone-lysine N-trimethyltransferase SMYD5
OTU domain-containing protein 7B
THO complex subunit 7 homolog
Glutamine-dependent NAD(+) synthetase
Elongator complex protein 2
Ragulator complex protein LAMTOR1
Dehydrogenase/reductase SDR family member 7B
Twinfilin-2
Condensin-2 complex subunit H2
Uncharacterized protein KIAA0930
Transport and Golgi organization protein 2 homolog
Serine/threonine-protein phosphatase 4 regulatory subunit 3A
Ras-related protein Rab-12
Splicing regulator SDE2
Keratin, type II cytoskeletal 80
Nipped-B-like protein
Mitochondrial adenyl nucleotide antiporter SLC25A25
2',5'-phosphodiesterase 12
N-alpha-acetyltransferase 16, NatA auxiliary subunit
Putative DENN domain-containing protein 10 B
Rhomboid domain-containing protein 2
Mitochondrial adenyl nucleotide antiporter SLC25A24
Receptor expression-enhancing protein 3
All-trans-retinol 13,14-reductase
RAD50-interacting protein 1
Transmembrane protein 214
3-hydroxyisobutyryl-CoA hydrolase, mitochondrial
RWD domain-containing protein 4
Nucleolus and neural progenitor protein
Armadillo repeat-containing protein 6
TELO2-interacting protein 2
Ankyrin repeat domain-containing protein 54

Transmembrane anterior posterior transformation protein 1 homolog
KN motif and ankyrin repeat domain-containing protein 3
Bifunctional arginine demethylase and lysyl-hydroxylase JMJD6
Phostensin
Caveolae-associated protein 1
Zinc finger CCHC domain-containing protein 8
Putative ATP-dependent RNA helicase DHX57
Endoplasmic reticulum aminopeptidase 2
Lysophospholipid acyltransferase 5
Parafibromin
Large ribosomal subunit protein uL14m
Long-chain fatty acid transport protein 4
Coiled-coil and C2 domain-containing protein 1A
Deoxyribonuclease TATDN1
tRNA N(3)-methylcytidine methyltransferase METTL2B
Myb/SANT-like DNA-binding domain-containing protein 2
Transcription initiation factor TFIID subunit 2
UPF0598 protein C8orf82
Mediator of RNA polymerase II transcription subunit 27
Enhancer of mRNA-decapping protein 4
Centrosomal protein of 85 kDa
Protein arginine N-methyltransferase 9
Pre-mRNA-processing-splicing factor 8
F-box only protein 42
SCY1-like protein 2
Tetratricopeptide repeat protein 27
Sideroflexin-4
Protein GOLM2
Nuclear factor related to kappa-B-binding protein
Acylpyruvase FAHD1, mitochondrial
Ribosomal protein eL22-like
Serine/threonine-protein kinase N3
RNA demethylase ALKBH5
Pyridoxal-dependent decarboxylase domain-containing protein 1
MTOR-associated protein MEAK7
Integrator complex subunit 5
GTPase IMAP family member 6
Long-chain fatty acid transport protein 1
Glucose 1,6-bisphosphate synthase
RNA polymerase-associated protein CTR9 homolog
Alpha- and gamma-adaptin-binding protein p34
Transmembrane protein 88
Proline/serine-rich coiled-coil protein 1
Superkiller complex protein 3
Serine/threonine-protein kinase ULK3
Aspartate--tRNA ligase, mitochondrial
Transmembrane protein 65
INO80 complex subunit C
F-box only protein 38
Neutral cholesterol ester hydrolase 1

Fidgetin-like protein 1
E3 ubiquitin-protein ligase TRIM65
Inactive rhomboid protein 2
Mitotic deacetylase-associated SANT domain protein
BRCA1-associated ATM activator 1
GATOR2 complex protein WDR59
Zinc finger CCCH domain-containing protein 14
Coiled-coil domain-containing protein 137
2-oxoglutarate and iron-dependent oxygenase domain-containing protein 3
Thioredoxin domain-containing protein 11
La-related protein 1
ATPase family AAA domain-containing protein 2
Protein TMED8
Proton-coupled zinc antiporter SLC30A9, mitochondrial
E3 ubiquitin-protein ligase TRAF7
Rapamycin-insensitive companion of mTOR
WD repeat-containing protein 74
Type-1 angiotensin II receptor-associated protein
SWI/SNF-related matrix-associated actin-dependent regulator of chromatin subfamily D member 3
Monofunctional C1-tetrahydrofolate synthase, mitochondrial
Aftiphilin
Pre-mRNA 3'-end-processing factor FIP1
CREB-regulated transcription coactivator 3
CREB-regulated transcription coactivator 1
Cytochrome P450 20A1
Protein O-glucosyltransferase 2
Transmembrane protein 205
E3 ubiquitin-protein ligase LRSAM1
Golgi-associated kinase 1B
Lysocardiolipin acyltransferase 1
BRCA1-A complex subunit Abraxas 1
Spliceosome-associated protein CWC27 homolog
Protein disulfide isomerase CRELD2
WD repeat-containing protein 82
MICOS complex subunit MIC27
Protocadherin Fat 4
Dipeptidyl peptidase 8
Activating transcription factor 7-interacting protein 1
Ran-binding protein 10
Phosphofurin acidic cluster sorting protein 1
Myosin phosphatase Rho-interacting protein
RNA-binding motif, single-stranded-interacting protein 3
Nicotinate phosphoribosyltransferase
Dynamin-binding protein
Very-long-chain (3R)-3-hydroxyacyl-CoA dehydratase 2
Beta-1,3-glucosyltransferase
GRB10-interacting GYF protein 2
CD109 antigen
tRNA (32-2'-O)-methyltransferase regulator THADA
Hydroxysteroid dehydrogenase-like protein 2

Kynurenine--oxoglutarate transaminase 3
Protein phosphatase 1 regulatory subunit 21
Thrombospondin type-1 domain-containing protein 4
Nesprin-3
Zinc finger protein 574
NF-X1-type zinc finger protein NFXL1
Neurobeachin-like protein 2
FYVE, RhoGEF and PH domain-containing protein 5
Sulfhydryl oxidase 2
Helicase SRCAP
Rho family-interacting cell polarization regulator 1
Uncharacterized protein FLJ45252
Rho guanine nucleotide exchange factor 18
E3 ubiquitin-protein ligase UBR3
Transmembrane protein with metallophosphoesterase domain
Capping protein-inhibiting regulator of actin dynamics
Coiled-coil domain-containing protein 9B
8-oxo-dGDP phosphatase NUDT18
PAX-interacting protein 1
Protein O-mannosyl-transferase TMTC3
Intermembrane lipid transfer protein VPS13C
Ubiquitin carboxyl-terminal hydrolase 34
Ras-associated and pleckstrin homology domains-containing protein 1
MOB kinase activator 2
Inhibitor of nuclear factor kappa-B kinase-interacting protein
Phosphatidylinositol 3,4,5-trisphosphate-dependent Rac exchanger 2 protein
Protein FRA10AC1
Ubiquitin-conjugating enzyme E2 R2
Mediator of RNA polymerase II transcription subunit 13-like
La-related protein 4
Mediator of RNA polymerase II transcription subunit 25
Ribosomal protein eS27-like
E3 ubiquitin-protein ligase Hakai
Integrator complex subunit 8
Butyrophilin subfamily 2 member A1
Transcription elongation factor SPT6
Staphylococcal nuclease domain-containing protein 1
Serine/threonine-protein kinase MARK2
Cytochrome c oxidase assembly protein COX15 homolog
Probable ATP-dependent RNA helicase DDX46
Protein RUFY3
tRNA methyltransferase 10 homolog C
eIF5-mimic protein 2
Vacuolar fusion protein MON1 homolog B
Isoaspartyl peptidase/L-asparaginase
BTB/POZ domain-containing protein KCTD9
ATP-dependent RNA helicase DHX30
Eukaryotic translation initiation factor 3 subunit M
7SK snRNA methylphosphate capping enzyme
Armadillo repeat-containing X-linked protein 2

Probable proline--tRNA ligase, mitochondrial
Arginine/serine-rich coiled-coil protein 2
Ras-related GTP-binding protein A
Cytoplasmic FMR1-interacting protein 1
Protein arginine methyltransferase NDUFAF7, mitochondrial
Golgi to ER traffic protein 4 homolog
COP9 signalosome complex subunit 6
Lysophosphatidylcholine acyltransferase 2
Mitochondrial enolase superfamily member 1
E3 ubiquitin-protein transferase MAEA
EPM2A-interacting protein 1
Putative pre-mRNA-splicing factor ATP-dependent RNA helicase DHX32
Serine/threonine-protein kinase TAO1
FAST kinase domain-containing protein 5, mitochondrial
MOB kinase activator 1B
Lysine-specific demethylase 3B
Charged multivesicular body protein 1b
Ribonucleoside-diphosphate reductase subunit M2 B
Heparan sulfate 2-O-sulfotransferase 1
STE20-related kinase adapter protein alpha
[F-actin]-monooxygenase MICAL3
Dymeclin
Keratin, type II cytoskeletal 78
PHD finger-like domain-containing protein 5A
Peroxiredoxin-like 2C
Aprataxin
Endoplasmic reticulum metallopeptidase 1
G protein-regulated inducer of neurite outgrowth 1
TRMT1-like protein
Zinc finger CCCH-type antiviral protein 1
Large ribosomal subunit protein bL21m
Elongation factor-like GTPase 1
Probable helicase senataxin
Trafficking protein particle complex subunit 11
KAT8 regulatory NSL complex subunit 1
Nucleoporin p54
Autophagy-related protein 9A
D-glutamate cyclase, mitochondrial
Coiled-coil domain-containing protein 186
LisH domain-containing protein ARMC9
VPS35 endosomal protein-sorting factor-like
Pogo transposable element with ZNF domain
Zinc finger FYVE domain-containing protein 16
Protein MON2 homolog
C-myc promoter-binding protein
Myosin-14
FMR1-interacting protein NUFIP2
SUZ domain-containing protein 1
Mitochondrial antiviral-signaling protein
CLIP-associating protein 1

ATP-dependent RNA helicase DHX29
Nephrocystin-3
Low-density lipoprotein receptor-related protein 10
HAUS augmin-like complex subunit 6
Protein O-glucosyltransferase 3
Tetratricopeptide repeat protein 21B
HEAT repeat-containing protein 3
Kinesin-like protein KIF21A
Hepatoma-derived growth factor-related protein 2
L-xylulose reductase
BRCA1-associated protein
BRCA2-interacting transcriptional repressor EMSY
Golgin subfamily A member 7
Rho GTPase-activating protein 22
Wings apart-like protein homolog
Interferon regulatory factor 2-binding protein 2
E3 ubiquitin-protein ligase RBBP6
E3 ubiquitin-protein ligase SH3RF1
Protein TAMALIN
FERM domain-containing protein 5
tRNA-splicing endonuclease subunit Sen54
Protein prenyltransferase alpha subunit repeat-containing protein 1
Arpin
Tectonin beta-propeller repeat-containing protein 1
Rab9 effector protein with kelch motifs
Transcription termination factor 4, mitochondrial
E3 ubiquitin-protein ligase HUWE1
YTH domain-containing family protein 3
Cytoplasmic tRNA 2-thiolation protein 1
Transcription initiation factor TFIID subunit 8
Ubiquitin-conjugating enzyme E2 Q1
Large ribosomal subunit protein mL55
Transmembrane emp24 domain-containing protein 4
COX assembly mitochondrial protein homolog
Centromere protein V
Schlafen family member 11
Multiple epidermal growth factor-like domains protein 8
Transmembrane protein 179B
N-acetylgalactosaminyltransferase 7
Stearoyl-CoA desaturase 5
Pleckstrin homology-like domain family B member 2
Adhesion G-protein coupled receptor G6
Dehydrodolichyl diphosphate synthase complex subunit DHDDS
Polypeptide N-acetylgalactosaminyltransferase 10
Trafficking protein particle complex subunit 6B
Type 1 phosphatidylinositol 4,5-bisphosphate 4-phosphatase
C-type lectin domain family 14 member A
Protein PAT1 homolog 1
Dipeptidyl peptidase 9
E3 ubiquitin-protein ligase synoviolin

tRNA 2'-phosphotransferase 1
Actin-histidine N-methyltransferase
Tetratricopeptide repeat protein 7B
Acyl-coenzyme A thioesterase 1
Nucleolar protein 9
Polyadenylate-binding protein 2
N6-adenosine-methyltransferase catalytic subunit
Protein polybromo-1
Threonylcarbamoyl-AMP synthase
Pre-mRNA-processing factor 39
Protein LYRIC
E3 ubiquitin-protein ligase ZNF598
Glycerol-3-phosphate acyltransferase 4
Kinectin
Telomerase-binding protein EST1A
NLR family member X1
B-cell CLL/lymphoma 9-like protein
Pleckstrin homology-like domain family B member 1
Ubiquitin carboxyl-terminal hydrolase 48
Calcium-dependent secretion activator 2
Fermitin family homolog 3
N-alpha-acetyltransferase 40
5'-nucleotidase domain-containing protein 3
Acetoacetyl-CoA synthetase
Leucine zipper protein 1
THO complex subunit 4
Integral membrane protein GPR180
FHF complex subunit HOOK-interacting protein 2B
Ras GTPase-activating-like protein IQGAP3
Zinc finger CCCH domain-containing protein 18
Vacuolar protein-sorting-associated protein 36
Cullin-associated NEDD8-dissociated protein 1
Reticulophagy regulator 3
Protein Hook homolog 3
SEC14 domain and spectrin repeat-containing protein 1
COMM domain-containing protein 7
Vacuolar fusion protein MON1 homolog A
Archaemetzincin-2
THO complex subunit 6 homolog
RNA N6-adenosine-methyltransferase METTL16
Poly(ADP-ribose) glycohydrolase
Liprin-beta-1
Lon protease homolog 2, peroxisomal
Zinc finger C3HC-type protein 1
Chromodomain-helicase-DNA-binding protein 1-like
F-BAR and double SH3 domains protein 1
Coiled-coil domain-containing protein 25
Proline and serine-rich protein 2
Stimulator of interferon genes protein
Active regulator of SIRT1

Cerebellar degeneration-related protein 2-like
Ral GTPase-activating protein subunit beta
Ras-specific guanine nucleotide-releasing factor RalGPS2
Histone-arginine methyltransferase CARM1
Deaminated glutathione amidase
COMM domain-containing protein 2
HEAT repeat-containing protein 5A
Condensin-2 complex subunit G2
F-box only protein 11
Ankyrin repeat and LEM domain-containing protein 2
RNA-binding protein MEX3D
Diacylglycerol kinase eta
ATP-dependent RNA helicase DDX42
Spermatogenesis-associated serine-rich protein 2
Serine/threonine-protein kinase VRK2
CDK2-associated and cullin domain-containing protein 1
NADH dehydrogenase [ubiquinone] 1 alpha subcomplex subunit 11
Dynein axonemal assembly factor 5
Peptidyl-tRNA hydrolase
Syntaxin-12
Transcriptional repressor p66-alpha
Copine-8
G-protein-signaling modulator 1
Ras-related protein Rab-43
C2 domain-containing protein 5
E3 ubiquitin-protein ligase MIB1
Inactive tyrosine-protein kinase PRAG1
BLOC-2 complex member HPS6
Interferon regulatory factor 2-binding protein 1
ELKS/Rab6-interacting/CAST family member 1
Ribosomal oxygenase 2
Palmitoyltransferase ZDHHC13
Palmitoyltransferase ZDHHC17
Cytokine receptor-like factor 3
Trafficking protein particle complex subunit 5
Armadillo repeat-containing protein 8
RELT-like protein 1
Complex I assembly factor TMEM126B, mitochondrial
5'-3' exonuclease PLD3
Protein HID1
Ankyrin repeat and MYND domain-containing protein 2
3'-5' exoribonuclease 1
Inactive serine/threonine-protein kinase VRK3
LIX1-like protein
NudC domain-containing protein 3
Protein AHNAK2
Formin-like protein 3
Methylmalonic aciduria type A protein, mitochondrial
Mitogen-activated protein kinase kinase kinase kinase 3
Neuron navigator 3

Prolyl 3-hydroxylase 3
Coiled-coil domain-containing protein 50
Malonyl-CoA-acyl carrier protein transacylase, mitochondrial
Kinase suppressor of Ras 1
Centrosomal protein of 97 kDa
ATP-dependent (S)-NAD(P)H-hydrate dehydratase
WD repeat-containing protein 75
Mitofusin-1
Choline transporter-like protein 2
Inositol 1,4,5-trisphosphate receptor-interacting protein
WD repeat and FYVE domain-containing protein 1
MAP7 domain-containing protein 3
Coiled-coil domain-containing protein 117
Protein NOXP20
DCN1-like protein 3
Protein DENND6A
MAX gene-associated protein
GRIP and coiled-coil domain-containing protein 2
Zinc finger CCCH domain-containing protein 7A
PHD finger protein 6
Protein archease
Volume-regulated anion channel subunit LRRC8A
Extracellular sulfatase Sulf-2
E3 ubiquitin-protein ligase UBR1
E3 ubiquitin-protein ligase UBR2
Rho GTPase-activating protein 12
Calcium homeostasis endoplasmic reticulum protein
Codanin-1
Ankyrin repeat and KH domain-containing protein 1
SURP and G-patch domain-containing protein 1
SURP and G-patch domain-containing protein 2
Ubiquitin-conjugating enzyme E2 variant 3
Cell division cycle and apoptosis regulator protein 1
Probable ATP-dependent RNA helicase DHX40
DnaJ homolog subfamily C member 10
Negative elongation factor C/D
Mitochondrial Rho GTPase 2
Mitochondrial Rho GTPase 1
NAD-dependent protein deacetylase sirtuin-2
Polyhomeotic-like protein 2
Chromatin complexes subunit BAP18
Large ribosomal subunit protein mL41
Nurim
GPALPP motifs-containing protein 1
Protein mono-ADP-ribosyltransferase PARP9
RNA-binding protein 12B
Solute carrier family 35 member F2
Putative RNA polymerase II subunit B1 CTD phosphatase RPAP2
Patatin-like phospholipase domain-containing protein 6
Structural maintenance of chromosomes protein 5

Probable ATP-dependent RNA helicase DDX60
C-Maf-inducing protein
MICAL-like protein 2
Probable ATP-dependent RNA helicase DHX37
Kelch repeat and BTB domain-containing protein 2
Ribonucleoprotein PTB-binding 1
pre-rRNA 2'-O-ribose RNA methyltransferase FTSJ3
Transmembrane protein 192
Nucleotidyltransferase MB21D2
Serine/arginine repetitive matrix protein 1
Stromal membrane-associated protein 1
DIS3-like exonuclease 2
ATP-dependent RNA helicase SUPV3L1, mitochondrial
Eukaryotic peptide chain release factor GTP-binding subunit ERF3B
ZZ-type zinc finger-containing protein 3
Exocyst complex component 8
Uncharacterized protein C10orf67, mitochondrial
E3 ubiquitin-protein ligase TRIM22
Threonine synthase-like 1
Uncharacterized protein KIAA2013
Calcium uptake protein 2, mitochondrial
Ankyrin repeat domain-containing protein 13A
Phosphatase and actin regulator 4
tRNA (uracil-5-)-methyltransferase homolog A
Pseudouridylate synthase RPUSD2
ELMO domain-containing protein 2
Aldehyde dehydrogenase family 16 member A1
Dyslexia-associated protein KIAA0319-like protein
Adhesion G protein-coupled receptor F5
5'-3' exoribonuclease 1
Proline-, glutamic acid- and leucine-rich protein 1
Abl interactor 1
WD repeat and FYVE domain-containing protein 3
Selenoprotein H
Vitamin K epoxide reductase complex subunit 1-like protein 1
Spartin
Tetratricopeptide repeat protein 5
Mesoderm induction early response protein 1
Regulatory-associated protein of mTOR
ORM1-like protein 3
Cell cycle and apoptosis regulator protein 2
NADH dehydrogenase [ubiquinone] 1 alpha subcomplex assembly factor 2
Vacuolar protein sorting-associated protein 52 homolog
Nuclear pore complex protein Nup93
Serine/threonine-protein kinase 11-interacting protein
Zinc finger protein 687
Cap-specific mRNA (nucleoside-2'-O-)-methyltransferase 1
Leucine-rich repeat-containing protein 47
Dedicator of cytokinesis protein 4
Carbonic anhydrase 13

Rho guanine nucleotide exchange factor 28
Integrator complex subunit 1
Histone H2B type 3-B
Rho GTPase-activating protein 24
Armadillo repeat-containing protein 10
Lysophosphatidylserine lipase ABHD12
CLK4-associating serine/arginine rich protein
Protein FAM43A
Transmembrane protein 256
E3 SUMO-protein ligase PIAS4
Glycerol-3-phosphate dehydrogenase 1-like protein
Solute carrier family 35 member F6
Rho GTPase-activating protein 18
Activating signal cointegrator 1 complex subunit 3
MICAL-like protein 1
Vacuolar protein sorting-associated protein 8 homolog
Protein PALS1
Cohesin subunit SA-2
Synaptopodin
Formin-binding protein 4
Translation factor GUF1, mitochondrial
RING1 and YY1-binding protein
Polypeptide N-acetylgalactosaminyltransferase 4
Misshapen-like kinase 1
Guanosine-3',5'-bis(diphosphate) 3'-pyrophosphohydrolase MESH1
Prostaglandin reductase 3
ER membrane protein complex subunit 5
Transmembrane protein 199
F-box/LRR-repeat protein 6
Prolyl 3-hydroxylase OGFOD1
Actin filament-associated protein 1
Zinc finger CCHC domain-containing protein 9
Oxidation resistance protein 1
Zinc finger CCCH-type with G patch domain-containing protein
Calcium homeostasis modulator protein 5
S1 RNA-binding domain-containing protein 1
WD and tetratricopeptide repeats protein 1
NF-kappa-B-activating protein
Macoilin
SH2 domain-containing protein 3C
Arrestin domain-containing protein 1
Dehydrogenase/reductase SDR family member on chromosome X
CDGSH iron-sulfur domain-containing protein 2
Ribonuclease P protein subunit p25-like protein
ATP synthase mitochondrial F1 complex assembly factor 2
Tetratricopeptide repeat protein 9C
Protein jagunal homolog 1
E3 ubiquitin-protein ligase RNF10
Refilin-B
Leucine-zipper-like transcriptional regulator 1

COMM domain-containing protein 1
Cleavage and polyadenylation specificity factor subunit 7
Solute carrier family 15 member 4
Myc target protein 1
ADAMTS-like protein 1
ADP-ribosylation factor GTPase-activating protein 2
Deubiquitinase OTUD6B
Acyl-coenzyme A diphosphatase FITM2
UPF0690 protein C1orf52
eEF1A lysine and N-terminal methyltransferase
ADP-ribosylation factor-like protein 6-interacting protein 6
ADP-ribosylation factor GTPase-activating protein 1
NAD-dependent protein deacylase sirtuin-6
Solute carrier family 66 member 3
ER membrane protein complex subunit 1
RNA polymerase II-associated factor 1 homolog
Cyclin-Y-like protein 1
Putative E3 ubiquitin-protein ligase UBR7
ATP-dependent RNA helicase DDX51
Uncharacterized protein FAM241A
Mitochondrial basic amino acids transporter
Protein enabled homolog
Discoidin, CUB and LCCL domain-containing protein 1
G patch domain-containing protein 11
Large ribosomal subunit protein mL43
RNA ligase 1
Lysophospholipase D GDPD1
Uncharacterized protein C19orf47
Activating signal cointegrator 1 complex subunit 1
Leucine-rich repeat-containing protein 57
Probable RNA-binding protein EIF1AD
Protein KRI1 homolog
Coiled-coil domain-containing protein 71L
Zinc finger protein 579
Pre-mRNA-splicing factor 38A
Mixed lineage kinase domain-like protein
Glutamine amidotransferase-like class 1 domain-containing protein 1
Serine/threonine-protein phosphatase 6 regulatory ankyrin repeat subunit C
Phospholipid-transporting ATPase IG
ATPase family gene 2 protein homolog A
NHL repeat-containing protein 2
Late secretory pathway protein AVL9 homolog
Equilibrative nucleobase transporter 1
Xyloside xylosyltransferase 1
Golgi membrane protein 1
Procollagen galactosyltransferase 1
Inactive C-alpha-formylglycine-generating enzyme 2
SID1 transmembrane family member 2
Formylglycine-generating enzyme
Protein O-glucosyltransferase 1

Ubiquitin-associated domain-containing protein 2
Prenylcysteine oxidase-like
Transmembrane protein 87A
Retinol dehydrogenase 13
Tetratricopeptide repeat protein 13
Estradiol 17-beta-dehydrogenase 11
Thioredoxin domain-containing protein 5
Kinetochore protein Spc24
Outer mitochondrial transmembrane helix translocase
Saccharopine dehydrogenase-like oxidoreductase
UDP-glucuronic acid decarboxylase 1
E3 ubiquitin-protein ligase RNF149
Reticulophagy regulator 2
SERPINE1 mRNA-binding protein 1
LEM domain-containing protein 2
Nitric oxide-associated protein 1
Adaptin ear-binding coat-associated protein 1
Protein FAM98A
Phospholipase A2 group XV
Myotubularin-related protein 14
NFATC2-interacting protein
Diacylglycerol lipase-beta
Carbohydrate sulfotransferase 14
Polypeptide N-acetylgalactosaminyltransferase 6
E3 ubiquitin-protein ligase RNF169
Pyruvate dehydrogenase phosphatase regulatory subunit, mitochondrial
NAD(P)H-hydrate epimerase
Nonsense-mediated mRNA decay factor SMG8
RING finger protein 214
Protein LSM14 homolog A
GTPase IMAP family member 8
Cyclin-Y
Maestro heat-like repeat-containing protein family member 1
Terminal nucleotidyltransferase 4B
Probable aminopeptidase NPEPL1
EH domain-binding protein 1
Putative RNA-binding protein 15B
Alpha-N-acetylgalactosaminide alpha-2,6-sialyltransferase 3
Trinucleotide repeat-containing gene 6A protein
Polyhomeotic-like protein 3
Divergent protein kinase domain 2A
Metal transporter CNNM3
ATP-binding cassette sub-family F member 1
Calcium uniporter protein, mitochondrial
Phosphatidylinositol 3-kinase catalytic subunit type 3
Glutathione S-transferase C-terminal domain-containing protein
Serum response factor-binding protein 1
Neuroguidin
SHC SH2 domain-binding protein 1
PDZ domain-containing protein 8

DCC-interacting protein 13-beta
Zinc transporter 7
Neuron navigator 1
Periphilin-1
WD repeat-containing protein 19
F-box only protein 22
Lysophosphatidylcholine acyltransferase 1
Zinc finger MIZ domain-containing protein 2
Nesprin-1
Ubiquitin carboxyl-terminal hydrolase 32
Biorientation of chromosomes in cell division protein 1-like 1
AT-rich interactive domain-containing protein 1B
FAD synthase
Folliculin
Nucleoporin Nup43
Nucleoporin Nup37
Nucleoporin NUP35
Cytosolic endo-beta-N-acetylglucosaminidase
Retinoic acid-induced protein 3
Torsin-1A-interacting protein 2
sn-1-specific diacylglycerol lipase ABHD11
N-acylneuraminate cytidylyltransferase
F-box DNA helicase 1
Tetraspanin-14
Tubulin--tyrosine ligase
Small VCP/p97-interacting protein
Atlastin-2
Motile sperm domain-containing protein 2
Putative phospholipase B-like 2
Ras association domain-containing protein 8
ATP-dependent RNA helicase DDX55
Major facilitator superfamily domain-containing protein 8
Tudor domain-containing protein 7
GTPase IMAP family member 7
Protein NEDD1
Nuclear receptor coactivator 7
THO complex subunit 2
WD repeat-containing protein 36
Protein phosphatase PTC7 homolog
Retinitis pigmentosa 9 protein
Vang-like protein 1
Smad nuclear-interacting protein 1
Large ribosomal subunit protein mL64
WD repeat-containing protein 48
Exocyst complex component 6
SWI/SNF complex subunit SMARCC2
Nuclear protein localization protein 4 homolog
Uncharacterized protein CXorf38
Spermatogenesis-associated protein 20
(Lyso)-N-acylphosphatidylethanolamine lipase

F-box only protein 30
Adenosine 3'-phospho 5'-phosphosulfate transporter 1
Pumilio homolog 2
T-cell immunomodulatory protein
Golgin subfamily A member 5
Kelch domain-containing protein 4
SH3KBP1-binding protein 1
NEDD8-activating enzyme E1 catalytic subunit
Prostamide/prostaglandin F synthase
DnaJ homolog subfamily B member 14
Protein kish-A
Phosphatidylinositol 5-phosphate 4-kinase type-2 gamma
WD repeat-containing protein 20
TBC1 domain family member 15
Retinol dehydrogenase 11
Leucine-rich repeat-containing protein 20
Interferon-induced protein 44
Large ribosomal subunit protein uL30m
AN1-type zinc finger protein 1
Protein CIP2A
Phosphatidylinositol 4-kinase type 2-beta
Dolichyl-diphosphooligosaccharide--protein glycosyltransferase subunit STT3B
Polyribonucleotide nucleotidyltransferase 1, mitochondrial
Signal peptide peptidase-like 2B
Minor histocompatibility antigen H13
Phosphatidylinositol 3,4,5-trisphosphate-dependent Rac exchanger 1 protein
Up-regulator of cell proliferation
Protein bicaudal D homolog 2
Serine/threonine-protein kinase Nek9
Pleckstrin homology domain-containing family O member 2
E3 ubiquitin-protein ligase DTX3L
ATP-dependent RNA helicase DDX54
Disks large homolog 5
Ribosome biogenesis protein BRX1 homolog
Glucosamine-6-phosphate isomerase 2
Volume-regulated anion channel subunit LRRC8C
Serine/threonine-protein kinase Nek7
RB1-inducible coiled-coil protein 1
[F-actin]-monooxygenase MICAL1
Elongator complex protein 5
Protein phosphatase Slingshot homolog 3
SH3 domain and tetratricopeptide repeat-containing protein 1
TBC domain-containing protein kinase-like protein
D-aminoacyl-tRNA deacylase 1
DDB1- and CUL4-associated factor 11
U3 small nucleolar RNA-associated protein 15 homolog
Probable glutathione peroxidase 8
Actin filament-associated protein 1-like 1
E3 ubiquitin-protein ligase SH3RF3
Short transient receptor potential channel 4-associated protein

Centrosomal protein of 192 kDa
Gem-associated protein 5
GPI ethanolamine phosphate transferase 3
Rap guanine nucleotide exchange factor 6
Guanine nucleotide exchange protein SMCR8
Partitioning defective 3 homolog
Importin-4
Ubiquitin carboxyl-terminal hydrolase 33
Arginine/serine-rich protein PNISR
Serine/threonine-protein phosphatase 4 regulatory subunit 1
Ubiquitin-associated and SH3 domain-containing protein B
WAS/WASL-interacting protein family member 2
1-acylglycerol-3-phosphate O-acyltransferase ABHD5
Histone-lysine N-methyltransferase SETD7
Nucleolar complex protein 3 homolog
Scavenger receptor class B member 1
Conserved oligomeric Golgi complex subunit 1
Sec1 family domain-containing protein 2
Stromal membrane-associated protein 2
Zinc finger CCCH domain-containing protein 15
Peptidyl-prolyl cis-trans isomerase-like 4
General transcription factor 3C polypeptide 2
TBC1 domain family member 22A
PHD finger protein 10
Ras-related protein Rab-2B
Coiled-coil domain-containing protein 12
RelA-associated inhibitor
Cotranscriptional regulator ARB2A
Protein Churchill
Transforming growth factor-beta receptor-associated protein 1
Transmembrane protein 263
Histone deacetylase 7
Phosphatidylglycerophosphatase and protein-tyrosine phosphatase 1
Nuclear pore complex protein Nup133
Programmed cell death 6-interacting protein
Sodium-dependent phosphate transporter 1
Filamin-binding LIM protein 1
Splicing factor Cactin
Glutathione-specific gamma-glutamylcyclotransferase 2
Charged multivesicular body protein 7
Protein THEM6
Probable N-acetyltransferase 14
B-cell CLL/lymphoma 7 protein family member C
Non-structural maintenance of chromosomes element 1 homolog
MIT domain-containing protein 1
Chromosome transmission fidelity protein 18 homolog
RNA polymerase-associated protein LEO1
Dephospho-CoA kinase domain-containing protein
NudC domain-containing protein 2
U4/U6.U5 small nuclear ribonucleoprotein 27 kDa protein

Dimethyladenosine transferase 1, mitochondrial
Cohesin subunit SA-1
Sec1 family domain-containing protein 1
Tumor necrosis factor alpha-induced protein 8-like protein 1
Soluble calcium-activated nucleotidase 1
Trafficking protein particle complex subunit 12
Heterogeneous nuclear ribonucleoprotein L-like
Fatty acyl-CoA reductase 1
Ubiquitin-like domain-containing CTD phosphatase 1
tRNA-splicing endonuclease subunit Sen15
PEST proteolytic signal-containing nuclear protein
DnaJ homolog subfamily A member 4
SPRY domain-containing protein 4
m-AAA protease-interacting protein 1, mitochondrial
Pseudouridylate synthase TRUB1
LIM domain only protein 7
Choline transporter-like protein 1
Cytoskeleton-associated protein 2
Ataxin-2-like protein
Arf-GAP with Rho-GAP domain, ANK repeat and PH domain-containing protein 3
GTPase IMAP family member 1
U4/U6 small nuclear ribonucleoprotein Prp31
Negative elongation factor B
Palladin
RUN and FYVE domain-containing protein 2
Splicing regulatory glutamine/lysine-rich protein 1
Gem-associated protein 6
Caskin-2
ATR-interacting protein
Paraspeckle component 1
MAP kinase-activating death domain protein
Nesprin-2
Transcriptional repressor p66-beta
Progesterone-induced-blocking factor 1
DnaJ homolog subfamily C member 9
BRI3-binding protein
Beta-catenin-like protein 1
Cysteine protease ATG4A
Ras and Rab interactor 2
Protein ELYS
Titin
Roundabout homolog 4
Esterase OVCA2
Immunity-related GTPase family Q protein
DNA damage-binding protein 2
C-type lectin domain family 2 member B
ATP-dependent RNA helicase DDX1
SEC14-like protein 1
Piezo-type mechanosensitive ion channel component 1
Protein FAM3C

Histone H1.10
Proteasome inhibitor PI31 subunit
Golgi-specific brefeldin A-resistance guanine nucleotide exchange factor 1
Nonsense-mediated mRNA decay factor SMG7
RNA polymerase-associated protein RTF1 homolog
Nicastrin
Sorting nexin-19
Transmembrane 9 superfamily member 4
Transmembrane protein 131
Inositol hexakisphosphate kinase 1
Small ribosomal subunit protein mS27
Engulfment and cell motility protein 1
Polyphosphoinositide phosphatase
Protein FAM168A
AP-3 complex subunit sigma-1
Hamartin
UBX domain-containing protein 4
PHD finger protein 3
Protein NDRG1
Heat shock protein 105 kDa
Septin-8
CCR4-NOT transcription complex subunit 9
TBC1 domain family member 5
Zinc finger protein 592
Protein Jade-3
Unconventional myosin-XVIIIa
La-related protein 4B
Stalled ribosome sensor GCN1
Rho GTPase-activating protein 45
Pre-mRNA-splicing factor ATP-dependent RNA helicase PRP16
Nuclear pore complex protein Nup205
Ankyrin repeat and SAM domain-containing protein 1A
Peroxidasin homolog
Protein FAN
GPI-anchor transamidase
Small ribosomal subunit protein mS31
A-kinase anchor protein 1, mitochondrial
Dol-P-Man:Man(5)GlcNAc(2)-PP-Dol alpha-1,3-mannosyltransferase
Neurogranin
Acidic leucine-rich nuclear phosphoprotein 32 family member B
Nectin-2
Geranylgeranyl transferase type-2 subunit alpha
Protein TFG
Serine protease HTRA1
Actin-related protein 2/3 complex subunit 1A
General transcription factor IIH subunit 4
Ras-responsive element-binding protein 1
Histone deacetylase 2
Signal transducing adapter molecule 1
Zinc finger protein ubi-d4

Endoplasmic reticulum protein SC65
CREB-binding protein
Histone acetyltransferase KAT6A
Symplekin
TATA-binding protein-associated factor 2N
Golgin subfamily A member 1
Gamma-glutamyl hydrolase
Neuronal cell adhesion molecule
X-linked retinitis pigmentosa GTPase regulator
Phosphatidylinositol 3,4,5-trisphosphate 5-phosphatase 1
Probable ATP-dependent RNA helicase DDX17
Kinesin-associated protein 3
Caspase-10
Amyloid beta precursor protein binding family B member 2
DNA repair protein RAD50
CUGBP Elav-like family member 1
Osteoclast-stimulating factor 1
Rho guanine nucleotide exchange factor 1
DNA repair endonuclease XPF
Ubiquitin recognition factor in ER-associated degradation protein 1
Golgi apparatus protein 1
Regulator of nonsense transcripts 1
COP9 signalosome complex subunit 5
G-patch domain and KOW motifs-containing protein
SWI/SNF complex subunit SMARCC1
SWI/SNF-related matrix-associated actin-dependent regulator of chromatin subfamily D member 2
Ras-related protein Rab-8B
Bcl2-associated agonist of cell death
Far upstream element-binding protein 2
Glutaryl-CoA dehydrogenase, mitochondrial
GTP-binding protein Rit1
Peroxisomal membrane protein PEX13
Transportin-1
Rho guanine nucleotide exchange factor 2
Ribosomal RNA small subunit methyltransferase NEP1
Polyribonucleotide 5'-hydroxyl-kinase Clp1
Glomulin
Ubiquitin carboxyl-terminal hydrolase 13
Segment polarity protein dishevelled homolog DVL-3
Probable ubiquitin carboxyl-terminal hydrolase FAF-X
Ubiquitin carboxyl-terminal hydrolase 7
Cullin-5
Stathmin-2
V-type proton ATPase 116 kDa subunit a 1
Lipoma-preferred partner
RNA-binding protein with multiple splicing
Exostosin-2
Mediator of RNA polymerase II transcription subunit 12
Protein tyrosine phosphatase type IVA 1
Phosphorylase b kinase regulatory subunit beta

Secretory carrier-associated membrane protein 4
BLOC-2 complex member HPS3
SWI/SNF-related matrix-associated actin-dependent regulator of chromatin subfamily E member 1
Caveolae-associated protein 3
Riboflavin kinase
Myeloid-derived growth factor
Mitochondrial import receptor subunit TOM40B
Protein YIPF5
GPI transamidase component PIG-T
Immunoglobulin superfamily member 8
Ribosomal protein eL42-like
Ras-related protein Rab-24
Oxysterol-binding protein 2
Cytoplasmic 60S subunit biogenesis factor ZNF622
Ribosome-releasing factor 2, mitochondrial
Ubiquitin-conjugating enzyme E2 E3
7-methylguanosine phosphate-specific 5'-nucleotidase
WW domain-binding protein 2
Proteasome assembly chaperone 2
BOS complex subunit NCLN
Mitochondrial ubiquitin ligase activator of NFKB 1
Myocardin-related transcription factor A
Protein lifeguard 3
Endoplasmic reticulum-Golgi intermediate compartment protein 1
U3 small nucleolar RNA-associated protein 4 homolog
FAST kinase domain-containing protein 4
Mitochondrial amidoxime reducing component 2
Protein FAM162A
PAT complex subunit CCDC47
Large ribosomal subunit protein uL24m
Exocyst complex component 4
Protein mago nashi homolog 2
Putative monooxygenase p33MONOX
Protein RFT1 homolog
Isochorismatase domain-containing protein 2
Protein N-terminal asparagine amidohydrolase
Fermitin family homolog 2
Far upstream element-binding protein 1
Tetratricopeptide repeat protein 17
Leucine-rich repeat-containing protein 59
Vesicle transport through interaction with t-SNAREs homolog 1A
Endothelial cell-selective adhesion molecule
Pre-B-cell leukemia transcription factor-interacting protein 1
Mitochondrial calcium uniporter regulator 1
Uncharacterized protein KIAA1143
Vacuolar protein sorting-associated protein 33A
Peptidyl-prolyl cis-trans isomerase FKBP10
Tetratricopeptide repeat protein 28
Exosome complex component RRP43
Zinc finger protein 428

SH3 domain-containing kinase-binding protein 1
Spindle and kinetochore-associated protein 1
Nucleus accumbens-associated protein 2
E3 ubiquitin-protein ligase RNF25
Axin interactor, dorsalization-associated protein
PIN2/TERF1-interacting telomerase inhibitor 1
ADP-ribosylation factor-like protein 8A
Ubiquitin thioesterase otulin
Small ribosomal subunit protein mS37
Peptidylprolyl isomerase domain and WD repeat-containing protein 1
Coiled-coil domain-containing protein 127
Cytochrome c oxidase assembly factor 7
Uracil phosphoribosyltransferase homolog
Phosphatidate cytidylyltransferase, mitochondrial
MOB kinase activator 3A
Dedicator of cytokinesis protein 10
Autophagy-related protein 2 homolog B
Leukocyte receptor cluster member 1
TBC1 domain family member 20
EF-hand domain-containing protein D2
Galactose mutarotase
Synaptotagmin-like protein 4
Pyrroline-5-carboxylate reductase 2
Protein CUSTOS
m7GpppX diphosphatase
Protein phosphatase 1 regulatory subunit 14B
Integrator complex subunit 12
5-methylcytosine rRNA methyltransferase NSUN4
Inactive rhomboid protein 1
Collagen triple helix repeat-containing protein 1
Medium-chain acyl-CoA ligase ACSF2, mitochondrial
Isochorismatase domain-containing protein 1
GRIP and coiled-coil domain-containing protein 1
FLYWCH family member 2
Protein Aster-A
HAUS augmin-like complex subunit 1
FAS-associated factor 2
Coiled-coil domain-containing protein 124
FAD-dependent oxidoreductase domain-containing protein 1
Optineurin
AP-2 complex subunit mu
Gamma-tubulin complex component 3
Probable RNA polymerase II nuclear localization protein SLC7A6OS
BTB/POZ domain-containing protein KCTD12
Reticulocalbin-3
60S ribosomal export protein NMD3
RalBP1-associated Eps domain-containing protein 1
Mitochondrial import inner membrane translocase subunit TIM14
Regulator of microtubule dynamics protein 1
U8 snoRNA-decapping enzyme

Splicing factor ESS-2 homolog
Carboxymethylenebutenolidase homolog
RNA-binding protein Musashi homolog 2
U5 small nuclear ribonucleoprotein 40 kDa protein
Regulator of MON1-CCZ1 complex
Large ribosomal subunit protein mL38
RING finger and SPRY domain-containing protein 1
Endoplasmic reticulum lectin 1
Ribosome-recycling factor, mitochondrial
Transcription termination factor 3, mitochondrial
Protein Spindly
Elongator complex protein 4
NAD-dependent protein deacetylase sirtuin-1
Protein YIPF6
Nucleoporin SEH1
Transcription elongation factor A protein-like 4
KIF-binding protein
Glucosamine 6-phosphate N-acetyltransferase
Constitutive coactivator of peroxisome proliferator-activated receptor gamma
Protein KTI12 homolog
Small ribosomal subunit protein uS3m
Large ribosomal subunit protein mL53
Trans-3-hydroxy-L-proline dehydratase
E3 ubiquitin-protein ligase RNF31
DAZ-associated protein 1
Small glutamine-rich tetratricopeptide repeat-containing protein beta
Protein SAAL1
Mitochondrial potassium channel
SAGA-associated factor 29
Ribosomal RNA processing protein 36 homolog
C1GALT1-specific chaperone 1
RNA-binding protein 33
Dysbindin
DnaJ homolog subfamily A member 3, mitochondrial
Translation machinery-associated protein 16
Multivesicular body subunit 12A
Small ribosomal subunit protein mS39
Corrinoid adenosyltransferase MMAB
Cytoplasmic FMR1-interacting protein 2
GTPase IMAP family member 5
E3 ubiquitin-protein ligase TRIM11
Coiled-coil domain-containing protein 97
CB1 cannabinoid receptor-interacting protein 1
Enhancer of mRNA-decapping protein 3
Dynein light chain 2, cytoplasmic
Copine-2
Protein S100-A16
Signal-induced proliferation-associated protein 1
Secernin-2
THO complex subunit 1

Ubiquitin thioesterase OTUB1
tRNA (adenine(58)-N(1))-methyltransferase catalytic subunit TRMT61A
Abasic site processing protein HMCES
Charged multivesicular body protein 6
Protein bicaudal D homolog 1
Phosphopentomutase
U3 small nucleolar ribonucleoprotein protein IMP4
Ceramide synthase 2
tRNA-dihydrouridine(47) synthase [NAD(P)(+)]-like
Protein LTV1 homolog
Serine dehydratase-like
Large ribosomal subunit protein mL48
Chronophin
Aurora kinase B
DCN1-like protein 1
Fumarylacetoacetate hydrolase domain-containing protein 2A
SWI/SNF-related matrix-associated actin-dependent regulator of chromatin subfamily D member 1
Target of EGR1 protein 1
Cell division cycle-associated 7-like protein
RUS family member 1
Probable ATP-dependent RNA helicase DDX27
BLOC-1-related complex subunit 6
Methionine--tRNA ligase, mitochondrial
Ataxin-7-like protein 3B
Serine/threonine-protein kinase greatwall
Methylthioribulose-1-phosphate dehydratase
Zinc finger C2HC domain-containing protein 1A
Protein lin-37 homolog
Solute carrier family 41 member 3
Vacuolar-sorting protein SNF8
Zinc finger CCCH-type antiviral protein 1-like
Nuclear envelope pore membrane protein POM 121
Tonsoku-like protein
PDZ and LIM domain protein 5
Protein disulfide isomerase CRELD1
ERO1-like protein alpha
Proline-rich protein 11
Protein FMC1 homolog
Adenosylhomocysteinase 3
Dedicator of cytokinesis protein 6
Mediator of RNA polymerase II transcription subunit 30
Serine/threonine-protein phosphatase PGAM5, mitochondrial
Integrator complex subunit 4
DDRGK domain-containing protein 1
2-oxoadipate dehydrogenase complex component E1
Selenocysteine lyase
Far upstream element-binding protein 3
Splicing factor 45
RCC1-like G exchanging factor-like protein
Asparaginyl-tRNA synthetase

Succinate--CoA ligase [GDP-forming] subunit beta, mitochondrial
DISP complex protein LRCH3
Mannose-1-phosphate guanyltransferase alpha
4-hydroxyphenylpyruvate dioxygenase-like protein
Putative protein-lysine deacylase ABHD14B
Peptide-N(4)-(N-acetyl-beta-glucosaminyl)asparagine amidase
Vesicle-trafficking protein SEC22a
ATP synthase membrane subunit K, mitochondrial
Kinetochore-associated protein NSL1 homolog
PRKC apoptosis WT1 regulator protein
tRNA N(3)-methylcytidine methyltransferase METTL2A
THO complex subunit 3
E3 ubiquitin-protein ligase Itchy homolog
Thioredoxin domain-containing protein 15
Kin of IRRE-like protein 1
Leucine-rich repeats and immunoglobulin-like domains protein 1
Conserved oligomeric Golgi complex subunit 3
CDK5 regulatory subunit-associated protein 3
Vam6/Vps39-like protein
Syndetin
Deubiquitinating protein VCPIP1
Spatacsin
Engulfment and cell motility protein 2
Protein disulfide-isomerase TMX3
DDB1- and CUL4-associated factor 5
Chromosome alignment-maintaining phosphoprotein 1
Lethal(3)malignant brain tumor-like protein 3
E3 ubiquitin-protein ligase ZFP91
Protocadherin-16
Calmin
PDZ and LIM domain protein 2
Fatty acyl-CoA reductase 2
Transcription factor BTF3 homolog 4
E3 ubiquitin-protein ligase RNF170
Solute carrier family 35 member E1
Ubiquitin carboxyl-terminal hydrolase 47
Zinc finger protein 521
Lipid scramblase CLPTM1L
Lymphokine-activated killer T-cell-originated protein kinase
ADP-ribosylation factor-like protein 5B
DnaJ homolog subfamily C member 1
N-terminal kinase-like protein
Zinc finger protein 512B
Protein LRATD2
Exocyst complex component 2
Cytosolic non-specific dipeptidase
Histone-lysine N-methyltransferase EHMT2
Zinc finger RNA-binding protein
Protein FAM210B, mitochondrial
Beta-1,3-galactosyltransferase 6

E1A-binding protein p400
Sorting nexin-27
Kinesin-like protein KIF16B
Intraflagellar transport protein 74 homolog
E3 ubiquitin-protein ligase TRIM47
CCR4-NOT transcription complex subunit 6-like
Dehydrogenase/reductase SDR family member 1
DnaJ homolog subfamily C member 30, mitochondrial
Ubiquitin-conjugating enzyme E2 E2
Caspase recruitment domain-containing protein 19
Protein PRRC1
FYVE, RhoGEF and PH domain-containing protein 4
F-box/LRR-repeat protein 18
Zinc finger protein 512
E3 SUMO-protein ligase NSE2
Non-structural maintenance of chromosomes element 3 homolog
Protein-L-isoaspartate O-methyltransferase domain-containing protein 1
Heat shock 70 kDa protein 12B
YTH domain-containing protein 1
Coiled-coil domain-containing protein 43
Conserved oligomeric Golgi complex subunit 8
CKLF-like MARVEL transmembrane domain-containing protein 3
Dynein axonemal assembly factor 10
Nucleolar protein 4-like
Lysophospholipid acyltransferase 7
Dedicator of cytokinesis protein 7
Sideroflexin-2
Zinc finger protein 830
Protein FAM210A
FERM domain-containing protein 6
28S rRNA (cytosine-C(5))-methyltransferase
Regulation of nuclear pre-mRNA domain-containing protein 1A
Arf-GAP with GTPase, ANK repeat and PH domain-containing protein 3
Arf-GAP with Rho-GAP domain, ANK repeat and PH domain-containing protein 1
Importin-9
Melanoma inhibitory activity protein 2
Discoidin, CUB and LCCL domain-containing protein 2
Rho guanine nucleotide exchange factor 17
Inositol polyphosphate-4-phosphatase type I A
RNA-binding protein 14
Kelch-like protein 5
E3 ubiquitin-protein ligase UHRF2
E3 ubiquitin-protein ligase NEDD4-like
KH domain-containing RNA-binding protein QKI
Formin-like protein 2
Pseudouridylate synthase 7 homolog
Serine protease FAM111A
Trafficking protein particle complex subunit 9
CCA tRNA nucleotidyltransferase 1, mitochondrial
Serine/threonine-protein kinase SMG1

Alsin
Transmembrane protein 237
Alpha-ketoglutarate-dependent dioxygenase alkB homolog 3
Rho GTPase-activating protein 7
Serine/threonine-protein phosphatase 1 regulatory subunit 10
Sodium-coupled neutral amino acid symporter 2
UAP56-interacting factor
Myotubularin-related protein 9
Vacuolar protein sorting-associated protein 35
Transcriptional activator protein Pur-beta
Exportin-6
Hedgehog-interacting protein
Membrane-associated guanylate kinase, WW and PDZ domain-containing protein 1
Sperm-associated antigen 5
Nucleus accumbens-associated protein 1
Sorting nexin-18
PAS domain-containing serine/threonine-protein kinase
Protein capicua homolog
BRCA1-A complex subunit RAP80
Intermembrane lipid transfer protein VPS13A
Mediator of RNA polymerase II transcription subunit 15
Elongation factor G, mitochondrial
Endoplasmic reticulum-Golgi intermediate compartment protein 2
Methylcrotonoyl-CoA carboxylase subunit alpha, mitochondrial
Calcium/calmodulin-dependent protein kinase kinase 2
Trimethylguanosine synthase
NudC domain-containing protein 1
Erbin
Gamma-tubulin complex component 6
Gamma-tubulin complex component 5
Ubiquitin carboxyl-terminal hydrolase 28
Formin-binding protein 1
EKC/KEOPS complex subunit TP53RK
GPI transamidase component PIG-S
ATPase WRNIP1
Ran-binding protein 9
Chloride channel CLIC-like protein 1
Myeloid-associated differentiation marker
Neurabin-2
SRSF protein kinase 1
Structural maintenance of chromosomes protein 6
BTB/POZ domain-containing protein KCTD15
Spermatid perinuclear RNA-binding protein
Transmembrane protein 209
Glutathione peroxidase 7
Cytochrome P450 2S1
Protein IWS1 homolog
Paired amphipathic helix protein Sin3a
Oxysterol-binding protein-related protein 9
Protein cereblon

Integrator complex subunit 14
2-aminoethanethiol dioxygenase
Mitochondrial tRNA methylthiotransferase CDK5RAP1
Remodeling and spacing factor 1
RNA-binding protein 15
RUN and FYVE domain-containing protein 1
Msx2-interacting protein
Bifunctional polynucleotide phosphatase/kinase
MMS19 nucleotide excision repair protein homolog
E3 ubiquitin-protein ligase UHRF1
Protein Niban 2
ATP-dependent zinc metalloprotease YME1L1
Regulator of microtubule dynamics protein 3
Transcription factor 12
Cytohesin-2
Tubulin-folding cofactor B
Proteasome subunit beta type-7
Calponin-2
Translocation protein SEC62
Ethanolamine-phosphate cytidylyltransferase
Cell division cycle 5-like protein
26S proteasome non-ATPase regulatory subunit 1
Stromal cell-derived factor 2
Prefoldin subunit 5
E3 ubiquitin-protein ligase RING2
Parkinson disease protein 7
GAS2-like protein 1
Eyes absent homolog 3
Sialidase-1
Sortilin
Synaptic vesicle membrane protein VAT-1 homolog
Legumain
Perilipin-2
DnaJ homolog subfamily C member 2
M-phase phosphoprotein 8
Nuclear pore complex protein Nup88
Plakophilin-4
Phosphoinositide 3-kinase regulatory subunit 4
P2X purinoceptor 4
Ribonucleases P/MRP protein subunit POP1
Protein S100-A13
Protein SCAF11
Translin-associated protein X
Selenide, water dikinase 2
Eukaryotic translation initiation factor 3 subunit C
Tetratricopeptide repeat protein 1
DnaJ homolog subfamily C member 7
Prohibitin-2
COP9 signalosome complex subunit 8
Pre-mRNA-splicing factor 18

Calcineurin B homologous protein 1
Kinesin-like protein KIF2C
RANBP2-like and GRIP domain-containing protein 5/6
Monoglyceride lipase
Ataxin-2
Docking protein 1
Methionine synthase
3-hydroxyacyl-CoA dehydrogenase type-2
Collagen alpha-1(XII) chain
Mothers against decapentaplegic homolog 5
Sigma non-opioid intracellular receptor 1
Heterogeneous nuclear ribonucleoprotein A/B
Nucleosome assembly protein 1-like 4
Microsomal glutathione S-transferase 2
Gamma-soluble NSF attachment protein
Phosphatidylinositol 4-phosphate 5-kinase type-1 alpha
Phospholipid-transporting ATPase ABCA3
Mitogen-activated protein kinase kinase kinase 3
ATP synthase subunit s, mitochondrial
Mitochondrial intermediate peptidase
Aconitate hydratase, mitochondrial
Transmembrane 9 superfamily member 2
Equilibrative nucleoside transporter 1
Tumor susceptibility gene 101 protein
Copine-1
T-complex protein 1 subunit eta
Myeloid differentiation primary response protein MyD88
Probable rRNA-processing protein EBP2
Protein arginine N-methyltransferase 1
BAG family molecular chaperone regulator 1
Cyclic AMP-dependent transcription factor ATF-6 beta
Forkhead box protein C2
Endophilin-A2
Telomerase protein component 1
Serine/threonine-protein kinase VRK1
Growth/differentiation factor 15
A-kinase anchor protein 9
Protein NipSnap homolog 1
Condensin complex subunit 3
Actin-related protein 2/3 complex subunit 5-like protein
Calcium uptake protein 1, mitochondrial
UPF0415 protein C7orf25
Protein FAM118B
RNA-binding protein 4B
Zinc finger FYVE domain-containing protein 21
ATP-dependent RNA helicase DDX50
Large ribosomal subunit protein bL34m
Zinc phosphodiesterase ELAC protein 2
Telomerase RNA component interacting RNase
Glutamate-rich WD repeat-containing protein 1

ADP-ribose glycohydrolase MACROD1
Ribosome quality control complex subunit TCF25
Protein CMSS1
Kelch domain-containing protein 3
Evolutionarily conserved signaling intermediate in Toll pathway, mitochondrial
Methylosome protein WDR77
Cytochrome b-245 chaperone 1
Vitamin K epoxide reductase complex subunit 1
2-(3-amino-3-carboxypropyl)histidine synthase subunit 2
Tubulin alpha-1C chain
Selenoprotein S
Apolipoprotein L2
Myb-binding protein 1A
Mitochondrial genome maintenance exonuclease 1
Golgi reassembly-stacking protein 1
FYVE and coiled-coil domain-containing protein 1
Acyl-CoA-binding domain-containing protein 6
Coronin-1B
Thioredoxin domain-containing protein 17
BUD13 homolog
Serine/threonine-protein phosphatase CPPED1
Vacuolar protein-sorting-associated protein 25
Large ribosomal subunit protein mL45
Uncharacterized protein C7orf50
Tudor-interacting repair regulator protein
45 kDa calcium-binding protein
Programmed cell death protein 2-like
Proteasomal ATPase-associated factor 1
Partner of Y14 and mago
MICOS complex subunit MIC25
ADP-dependent glucokinase
G patch domain-containing protein 1
Serine/threonine-protein kinase RIO1
Protein LLP homolog
DNA replication complex GINS protein SLD5
rRNA-processing protein UTP23 homolog
Protein pelota homolog
Peroxiredoxin-like 2A
E3 ubiquitin-protein ligase TRIM56
Endoplasmic reticulum resident protein 44
Latexin
Nucleolar protein 10
Cancer-related nucleoside-triphosphatase
Mitochondrial import inner membrane translocase subunit Tim29
Translational activator of cytochrome c oxidase 1
Gamma-tubulin complex component 2
Extended synaptotagmin-1
Ubiquitin-associated domain-containing protein 1
Protein YIPF4
Phagosome assembly factor 1

tRNA-splicing endonuclease subunit Sen34
Protein canopy homolog 3
Mitochondrial ribosome-associated GTPase 1
Chitobiosyldiphosphodolichol beta-mannosyltransferase
LIM domain-containing protein 2
HAUS augmin-like complex subunit 8
Inositol polyphosphate 5-phosphatase K
Proteasome assembly chaperone 3
COP9 signalosome complex subunit 4
Synaptotagmin-11
WW domain-containing adapter protein with coiled-coil
Death-inducer obliterator 1
Metastasis-associated protein MTA3
RNA-binding protein 42
Dynactin subunit 5
Mini-chromosome maintenance complex-binding protein
Alanyl-tRNA editing protein Aarsd1
DCN1-like protein 5
Acidic leucine-rich nuclear phosphoprotein 32 family member E
Leucine-rich repeat-containing protein 1
Phosphatidylinositol 4-kinase type 2-alpha
Transmembrane protein 43
Fibronectin type III and SPRY domain-containing protein 1
Tubulin-specific chaperone D
Nucleoporin NDC1
Protein HGH1 homolog
Dehydrogenase/reductase SDR family member 4
Lipase maturation factor 2
NADH dehydrogenase [ubiquinone] 1 alpha subcomplex assembly factor 3
Multiple myeloma tumor-associated protein 2
Transmembrane protein 70, mitochondrial
Mediator of RNA polymerase II transcription subunit 18
Iron-sulfur cluster assembly 1 homolog, mitochondrial
Tubulin beta-6 chain
Protein PAXX
DNA-directed RNA polymerase III subunit RPC3
Heterogeneous nuclear ribonucleoprotein U-like protein 1
Protein misato homolog 1
PHD finger protein 23
Programmed cell death protein 10
Ribonuclease P protein subunit p25
Derlin-1
Oxidoreductase HTATIP2
Probable ATP-dependent RNA helicase DDX23
Telomerase Cajal body protein 1
MICOS complex subunit MIC26
Dehydrogenase/reductase SDR family member 6
Methylthioribose-1-phosphate isomerase
WD repeat-containing protein 18
Vesicle-associated membrane protein 8

tRNA (guanine(6)-N2)-methyltransferase THUMP3
Acireductone dioxygenase
Enoyl-[acyl-carrier-protein] reductase, mitochondrial
ER membrane protein complex subunit 6
N-terminal Xaa-Pro-Lys N-methyltransferase 1
Katanin p80 WD40 repeat-containing subunit B1
Target of rapamycin complex subunit LST8
Ashwin
Voltage-gated monoatomic cation channel TMEM109
Protein PBDC1
Phosphatidylserine synthase 2
Nucleolar complex protein 4 homolog
U3 small nucleolar RNA-associated protein 14 homolog A
Dual specificity protein phosphatase 23
Transmembrane emp24 domain-containing protein 9
BOS complex subunit TMEM147
Nucleoporin p58/p45
Protein adenylyltransferase SelO, mitochondrial
Protein DPCD
Guanine nucleotide-binding protein-like 3
ATPase family gene 2 protein homolog B
Serine/threonine-protein kinase RIO2
tRNA (adenine(58)-N(1))-methyltransferase, mitochondrial
Transmembrane and ubiquitin-like domain-containing protein 1
Mitochondrial import inner membrane translocase subunit Tim21
Kinesin-like protein KIFC1
Nuclear pore complex protein Nup85
Very long chain fatty acid elongase 1
Katanin p60 ATPase-containing subunit A-like 1
HIRA-interacting protein 3
Intraflagellar transport protein 27 homolog
Splicing factor YJU2
ADP-ribose pyrophosphatase, mitochondrial
Threonine--tRNA ligase, mitochondrial
Acetyl-CoA acetyltransferase, cytosolic
Replication initiator 1
RNA-binding protein 4
FUN14 domain-containing protein 2
RNA polymerase II-associated protein 1
Splicing factor 3B subunit 5
Sideroflexin-3
Proline-rich protein 14
Chitinase domain-containing protein 1
Poly(A) polymerase gamma
Caspase recruitment domain-containing protein 10
Kanadaptin
Cyclin-dependent kinase 19
Protein LSM14 homolog B
Tapasin-related protein
Junctional adhesion molecule C

Caspase recruitment domain-containing protein 6
BTB/POZ domain-containing protein 2
Sphingosine-1-phosphate phosphatase 1
Oxysterol-binding protein-related protein 11
Oxysterol-binding protein-related protein 10
Rab11 family-interacting protein 5
TBC1 domain family member 10A
Complement C1q tumor necrosis factor-related protein 5
N-alpha-acetyltransferase 15, NatA auxiliary subunit
Krueppel-like factor 16
Bcl-2-like protein 13
Caspase recruitment domain-containing protein 11
Solute carrier family 12 member 9
Serrate RNA effector molecule homolog
Queuine tRNA-ribosyltransferase catalytic subunit 1
AP-1 complex subunit mu-1
Nucleolar and spindle-associated protein 1
Haloacid dehalogenase-like hydrolase domain-containing 5
Fanconi anemia group D2 protein
Protein MAK16 homolog
Histone deacetylase 8
Replication termination factor 2
Charged multivesicular body protein 4a
Eukaryotic translation initiation factor 2A
Polymerase delta-interacting protein 3
Uncharacterized protein KIAA1671
SH3 and multiple ankyrin repeat domains protein 3
Guanine nucleotide-binding protein subunit beta-like protein 1
Alpha-(1,6)-fucosyltransferase
Large ribosomal subunit protein bL32m
Large ribosomal subunit protein bL20m
Large ribosomal subunit protein uL13m
Large ribosomal subunit protein bL9m
Large ribosomal subunit protein uL4m
Large ribosomal subunit protein uL1m
MKI67 FHA domain-interacting nucleolar phosphoprotein
Hyccin
YTH domain-containing family protein 1
3'-5' exoribonuclease HELZ2
RanBP-type and C3HC4-type zinc finger-containing protein 1
Small ribosomal subunit protein mS26
Neurolysin, mitochondrial
Centrosomal protein of 41 kDa
Histone-lysine N-methyltransferase SETD2
Pantothenate kinase 2, mitochondrial
Dedicator of cytokinesis protein 9
FERM domain-containing protein 8
Contactin-associated protein-like 3
Histone-lysine N-methyltransferase NSD3
Kinetochore protein Nuf2

Large ribosomal subunit protein mL37
GTP-binding protein 4
Tether containing UBX domain for GLUT4
Oxysterol-binding protein-related protein 8
Uveal autoantigen with coiled-coil domains and ankyrin repeats
Ras-related protein Rab-34
2-(3-amino-3-carboxypropyl)histidine synthase subunit 1
WD repeat-containing protein 11
Regulator of nonsense transcripts 3B
Crooked neck-like protein 1
F-box-like/WD repeat-containing protein TBL1XR1
Ubiquitin-like protein 5
Protein phosphatase 1 regulatory subunit 12C
Serine/threonine-protein kinase D2
ER degradation-enhancing alpha-mannosidase-like protein 3
Protein Niban 1
UBX domain-containing protein 6
Transmembrane 6 superfamily member 1
Uridine-cytidine kinase 2
Sialoadhesin
Apoptosis inhibitor 5
NACHT, LRR and PYD domains-containing protein 1
Tripartite motif-containing protein 5
E3 ubiquitin-protein ligase TRIM4
Alpha-ketoglutarate-dependent dioxygenase FTO
Palmitoyltransferase ZDHHC5
Transport and Golgi organization protein 6 homolog
182 kDa tankyrase-1-binding protein
(E3-independent) E2 ubiquitin-conjugating enzyme
Protein zyg-11 homolog B
Protein TANC1
Ethanolaminephosphotransferase 1
Exportin-4
Endoplasmic reticulum junction formation protein lunapark
Centrosomal protein of 44 kDa
Protein tweety homolog 3
Kelch-like protein 4
Myotubularin-related protein 12
pre-mRNA 3' end processing protein WDR33
Ribosome biogenesis protein WDR12
Protein YIPF3
Tubulointerstitial nephritis antigen-like
Adipose-secreted signaling protein
PITH domain-containing protein 1
Derlin-2
COMM domain-containing protein 5
Sentrin-specific protease 6
RNA exonuclease 4
ATP-dependent RNA helicase DDX24
DNA-directed RNA polymerase I subunit RPA49

Superkiller complex protein 8
SRA stem-loop-interacting RNA-binding protein, mitochondrial
Serine racemase
NIF3-like protein 1
Egl nine homolog 1
Mucolipin-1
PSME3-interacting protein
Mitochondrial fission factor
N-alpha-acetyltransferase 50
Ubiquitin-like modifier-activating enzyme 5
E3 ubiquitin-protein ligase makorin-2
Transmembrane protein 126A
Polyadenylate-binding protein-interacting protein 1
Mitochondrial disaggregase
Ras-related protein Rab-33B
Large subunit GTPase 1 homolog
Protein FAM107B
RNA cytidine acetyltransferase
COMM domain-containing protein 4
Kinesin light chain 2
Integrin-linked kinase-associated serine/threonine phosphatase 2C
5'-3' exoribonuclease 2
Toll-interacting protein
Histone deacetylase complex subunit SAP130
Bromodomain-containing protein 8
Nuclear speckle splicing regulatory protein 1
Integrator complex subunit 2
Rac GTPase-activating protein 1
Protein mono-ADP-ribosyltransferase PARP12
Cleavage stimulation factor subunit 2 tau variant
Cytosolic 5'-nucleotidase 3A
CYFIP-related Rac1 interactor A
Transmembrane protein 222
Haloacid dehalogenase-like hydrolase domain-containing protein 2
Glutamyl-tRNA(Gln) amidotransferase subunit A, mitochondrial
Probable ATP-dependent RNA helicase DDX47
Magnesium transporter protein 1
Ras-related protein Rab-1B
Large ribosomal subunit protein uL18m
Testis-specific Y-encoded-like protein 1
Transmembrane protein 168
VIP36-like protein
Nonsense-mediated mRNA decay factor SMG9
Protein FAM234A
Oxysterol-binding protein-related protein 5
Deoxynucleotidyltransferase terminal-interacting protein 1
Nucleotide exchange factor SIL1
Protein-L-histidine N-pros-methyltransferase
Anaphase-promoting complex subunit 1
Probable E3 ubiquitin-protein ligase IRF2BPL

Cysteine-rich and transmembrane domain-containing protein 1
DNA-directed RNA polymerase III subunit RPC6
Nuclear ubiquitous casein and cyclin-dependent kinase substrate 1
Thioredoxin-related transmembrane protein 4
Kinesin-like protein KIF13A
Activating signal cointegrator 1 complex subunit 2
Rabenosyn-5
Iron-sulfur cluster assembly enzyme ISCU
Oxysterol-binding protein-related protein 2
Autophagy protein 5
WD repeat-containing protein 13
Epsin-3
EH domain-containing protein 4
Vacuolar protein sorting-associated protein 33B
Vacuolar protein sorting-associated protein 16 homolog
Vacuolar protein sorting-associated protein 11 homolog
SH3 domain-binding glutamic acid-rich-like protein 3
Gigaxonin
Solute carrier family 25 member 32
TRIO and F-actin-binding protein
Enhancer of polycomb homolog 1
STE20-like serine/threonine-protein kinase
Peptidyl-prolyl cis-trans isomerase-like 3
Sodium-coupled neutral amino acid symporter 1
Phosducin-like protein 3
Serine/threonine-protein kinase TAO3
Rab3 GTPase-activating protein non-catalytic subunit
Activity-dependent neuroprotector homeobox protein
Diphthine methyl ester synthase
ATP-dependent DNA/RNA helicase DHX36
Inorganic pyrophosphatase 2, mitochondrial
Protein spinster homolog 1
Large ribosomal subunit protein mL46
Solute carrier family 12 member 5
Presenilin-associated rhomboid-like protein, mitochondrial
Pinin
Transmembrane protein 245
Forkhead box protein P1
BTB/POZ domain-containing adapter for CUL3-mediated RhoA degradation protein 3
UPF0696 protein C11orf68
Growth hormone-inducible transmembrane protein
BolA-like protein 2
Cobalamin trafficking protein CblD
Thioredoxin-interacting protein
Thioredoxin-related transmembrane protein 1
Negative elongation factor A
Golgi resident protein GCP60
Cdc42 effector protein 4
Tyrosine-protein phosphatase non-receptor type 23
Semaphorin-6B

Protein unc-45 homolog A
Major facilitator superfamily domain-containing protein 1
DnaJ homolog subfamily C member 5
Kinetochore-associated protein DSN1 homolog
Charged multivesicular body protein 4b
RWD domain-containing protein 1
CUE domain-containing protein 2
Fructosamine-3-kinase
GDP-fucose protein O-fucosyltransferase 1
Phosphatidylinositol glycan anchor biosynthesis class U protein
Torsin-3A
Serine/threonine-protein kinase WNK1
Aminopeptidase B
Golgi phosphoprotein 3-like
Golgi phosphoprotein 3
tRNA N6-adenosine threonylcarbamoyltransferase, mitochondrial
Alpha-N-acetyl-neuraminyl-2,3-beta-galactosyl-1,3-N-acetyl-galactosaminide alpha-2,6-sialyltransferase
Golgi-associated plant pathogenesis-related protein 1
TraB domain-containing protein
Mitochondrial ribosome-associated GTPase 2
Sentrin-specific protease 3
Oxysterol-binding protein-related protein 3
SWI/SNF-related matrix-associated actin-dependent regulator of chromatin subfamily A containing DEAD/H
EH domain-containing protein 1
mRNA (2'-O-methyladenosine-N(6)-)-methyltransferase
ESF1 homolog
Alpha-1,3/1,6-mannosyltransferase ALG2
HEAT repeat-containing protein 1
Protein O-mannose kinase
Rab GTPase-binding effector protein 2
Dimethyladenosine transferase 2, mitochondrial
rRNA N6-adenosine-methyltransferase ZCCHC4
STING ER exit protein
Cytosolic iron-sulfur assembly component 2A
Probable ATP-dependent RNA helicase DHX35
Protein FAM124B
HAUS augmin-like complex subunit 4
Coiled-coil domain-containing protein 134
Coiled-coil domain-containing protein 86
Receptor expression-enhancing protein 4
Transcription factor SOX-17
Optic atrophy 3 protein
Cytosolic iron-sulfur assembly component 3
ATP-dependent RNA helicase DHX33
Nucleolar protein 6
WD repeat and coiled-coil-containing protein
3'-5' RNA helicase YTHDC2
5-azacytidine-induced protein 2
RNA polymerase II-associated protein 3
BCAS3 microtubule associated cell migration factor

Alpha-1,2-mannosyltransferase ALG9
Lipid droplet-associated hydrolase
Ribosomal oxygenase 1
WD repeat-containing protein 55
Ran-binding protein 3
Inactive tyrosine-protein kinase PEAK1
Ribosome production factor 2 homolog
Histone-lysine N-methyltransferase SMYD3
Mth938 domain-containing protein
Dedicator of cytokinesis protein 5
WD repeat-containing protein 26
Tudor domain-containing protein 3
UPF0488 protein C8orf33
Polyamine-transporting ATPase 13A3
Methyltransferase-like protein 17, mitochondrial
Splicing factor, arginine/serine-rich 19
Nuclear exosome regulator NRDE2
Prostaglandin E synthase 2
Phosphorylated adapter RNA export protein
Ubiquitin-conjugating enzyme E2 Z
Complex I assembly factor ACAD9, mitochondrial
5'-nucleotidase domain-containing protein 2
PRKR-interacting protein 1
Caspase activity and apoptosis inhibitor 1
Nucleolar protein 11
Probable ATP-dependent RNA helicase DDX31
Thiol S-methyltransferase TMT1A
ATPase PAAT
Multimerin-2
Ubiquitin carboxyl-terminal hydrolase MINDY-3
Protein eva-1 homolog A
AKT-interacting protein
Pleckstrin homology domain-containing family F member 2
tRNA endonuclease ANKZF1
Golgi reassembly-stacking protein 2
Protein zwilch homolog
Jupiter microtubule associated homolog 2
Mitochondrial glutamate carrier 1
Proline-serine-threonine phosphatase-interacting protein 2
Mediator of RNA polymerase II transcription subunit 20
WD repeat-containing protein 76
Queuine tRNA-ribosyltransferase accessory subunit 2
Actin-related protein 8
Damage-control phosphatase ARMT1
Pantothenate kinase 3
CCR4-NOT transcription complex subunit 10
Leucine-rich repeat-containing protein 40
Histone-lysine N-methyltransferase EHMT1
Sideroflexin-1
Spermatogenesis-defective protein 39 homolog

Conserved oligomeric Golgi complex subunit 4
Actin-related protein 5
Vacuolar protein sorting-associated protein 37B
Large ribosomal subunit protein mL44
L-2-hydroxyglutarate dehydrogenase, mitochondrial
COP9 signalosome complex subunit 7b
Non-homologous end-joining factor 1
Protein transport protein Sec61 subunit alpha isoform 2
Ribitol 5-phosphate transferase FKRP
Elongator complex protein 3
Ribosome production factor 1
DNA-directed RNA polymerase I subunit RPA2
Zinc finger matrin-type protein 3
Uridine-cytidine kinase 1
Ketosamine-3-kinase
TBC1 domain family member 17
Probable cysteine--tRNA ligase, mitochondrial
Solute carrier family 52, riboflavin transporter, member 2
Phosphopantothenate--cysteine ligase
Ubiquitin domain-containing protein 1
WD repeat-containing protein 41
Nicotinamide/nicotinic acid mononucleotide adenylyltransferase 1
Protein Njmu-R1
Sialate O-acetylesterase
Pleckstrin homology domain-containing family A member 5
E3 ubiquitin-protein ligase SMURF2
Regulator of nonsense transcripts 2
Guanine nucleotide-binding protein subunit beta-4
Exportin-5
GrpE protein homolog 1, mitochondrial
MYG1 exonuclease
Pleckstrin homology domain-containing family A member 3
Pleckstrin homology domain-containing family A member 1
Retinoid-inducible serine carboxypeptidase
Netrin-4
Calcyclin-binding protein
Ras-related GTP-binding protein C
Zinc finger FYVE domain-containing protein 1
Intraflagellar transport protein 122 homolog
Retinol dehydrogenase 14
Beta-parvin
Tensin-1
Plasminogen receptor (KT)
NmrA-like family domain-containing protein 1
Vezatin
Kinetochore protein Spc25
Transcription initiation factor TFIID subunit 9B
Solute carrier family 38 member 10
Ethanolamine kinase 1
Adhesion G protein-coupled receptor L4

Putative divalent cation/proton antiporter TMEM165
Mitochondrial thiamine pyrophosphate carrier
Echinoderm microtubule-associated protein-like 4
rRNA methyltransferase 3, mitochondrial
Glyoxalase domain-containing protein 4
Serine/threonine-protein kinase Nek6
Methylcrotonoyl-CoA carboxylase beta chain, mitochondrial
Nuclear receptor coactivator 5
Ectopic P granules protein 5 homolog
Helicase MOV-10
N6-adenosine-methyltransferase non-catalytic subunit
Non-lysosomal glucosylceramidase
Pre-mRNA-splicing factor CWC22 homolog
Ribonucleoprotein PTB-binding 2
Chromodomain-helicase-DNA-binding protein 8
Band 4.1-like protein 5
GPN-loop GTPase 1
Stromal cell-derived factor 2-like protein 1
Casein kinase I isoform gamma-1
Pre-mRNA-splicing factor SYF1
Prolactin regulatory element-binding protein
Steroid receptor RNA activator 1
Endoplasmic reticulum transmembrane helix translocase
Golgi-associated PDZ and coiled-coil motif-containing protein
Large ribosomal subunit protein uL29m
LYR motif-containing protein 4
O-phosphoseryl-tRNA(Sec) selenium transferase
Charged multivesicular body protein 1a
Transmembrane 9 superfamily member 3
Unconventional myosin-X
Adipocyte plasma membrane-associated protein
tRNA (34-2'-O)-methyltransferase regulator WDR6
Thioredoxin reductase 2, mitochondrial
ATP-binding cassette sub-family B member 6
ADP-ribosylation factor GTPase-activating protein 3
Zinc finger CCHC domain-containing protein 17
Ras-related protein Rab-18
Palmdelphin
RNA polymerase II subunit A C-terminal domain phosphatase SSU72
Vacuolar protein sorting-associated protein VTA1 homolog
Serine--tRNA ligase, mitochondrial
Large ribosomal subunit protein mL65
Dynein light chain roadblock-type 1
ER membrane protein complex subunit 7
Transcription and mRNA export factor ENY2
Exosome complex component RRP41
Ubiquitin-conjugating enzyme E2 T
H/ACA ribonucleoprotein complex subunit 3
tRNA N6-adenosine threonylcarbamoyltransferase
DNA methyltransferase 1-associated protein 1

Protocadherin-12
Inositol-3-phosphate synthase 1
Bromodomain-containing protein 7
mRNA-decapping enzyme 1A
Acyl-coenzyme A thioesterase 13
Mediator of RNA polymerase II transcription subunit 4
Complex I assembly factor TIMMDC1, mitochondrial
Synembryn-A
Complement component C1q receptor
Putative RNA-binding protein Luc7-like 1
Endothelial cell-specific molecule 1
Leucine zipper transcription factor-like protein 1
Large ribosomal subunit protein mL40
Suppressor of SWI4 1 homolog
1-phosphatidylinositol 4,5-bisphosphate phosphodiesterase beta-1
Fructose-2,6-bisphosphatase TIGAR
Protein C12orf4
Reticulon-4
Regulation of nuclear pre-mRNA domain-containing protein 1B
Xaa-Pro aminopeptidase 3
Omega-amidase NIT2
Cell death regulator Aven
Inner centromere protein
Exosome complex component RRP46
Exosome complex component RRP40
Kinesin-like protein KIF13B
Anillin
Xaa-Pro aminopeptidase 1
Gephyrin
Bridging integrator 3
Something about silencing protein 10
StAR-related lipid transfer protein 7, mitochondrial
Baculoviral IAP repeat-containing protein 6
PDZ and LIM domain protein 7
Acetyl-coenzyme A synthetase, cytoplasmic
Diablo IAP-binding mitochondrial protein
Nucleolar RNA helicase 2
GTP-binding protein SAR1a
Sialic acid synthase
Endophilin-B2
Histone-lysine N-methyltransferase ASH1L
Translation initiation factor eIF2B subunit gamma
Muscleblind-like protein 1
Sialin
Eukaryotic translation initiation factor 4E transporter
Carbohydrate sulfotransferase 12
Suppressor of tumorigenicity 7 protein
PRKCA-binding protein
SH2B adapter protein 1
CTP synthase 2

DNA polymerase epsilon subunit 3
Chromatin accessibility complex protein 1
Aladin
SNF-related serine/threonine-protein kinase
ATP-binding cassette sub-family B member 10, mitochondrial
Bromodomain adjacent to zinc finger domain protein 1A
Striatin-4
L-aminoadipate-semialdehyde dehydrogenase-phosphopantetheinyl transferase
rRNA N6-adenosine-methyltransferase METTL5
Oligosaccharyltransferase complex subunit OSTC
COX assembly mitochondrial protein 2 homolog
Phospholipid scramblase 4
Ribonuclease 3
Sorting nexin-15
Heme-binding protein 1
DNA dC->dU-editing enzyme APOBEC-3C
Vacuolar protein sorting-associated protein 45
RNA-binding protein PNO1
Large ribosomal subunit protein bL17m
14 kDa phosphohistidine phosphatase
Serine incorporator 1
Rho GTPase-activating protein 35
Protein FAM114A2
Phospholipid scramblase 3
1-acyl-sn-glycerol-3-phosphate acyltransferase delta
1-acyl-sn-glycerol-3-phosphate acyltransferase gamma
Lymphoid-specific helicase
Glycoprotein-N-acetylgalactosamine 3-beta-galactosyltransferase 1
Latent-transforming growth factor beta-binding protein 3
Mitochondrial import receptor subunit TOM22 homolog
LanC-like protein 2
Kinesin-like protein KIF15
Transmembrane 7 superfamily member 3
Homer protein homolog 3
Phenylalanine--tRNA ligase beta subunit
Isoleucine--tRNA ligase, mitochondrial
Ribosome biogenesis protein SLX9 homolog
SAM domain-containing protein SAMSN-1
Kinesin light chain 4
Protein diaphanous homolog 3
BMP-2-inducible protein kinase
Ubiquitin-like-conjugating enzyme ATG3
NAD-dependent protein deacetylase sirtuin-3, mitochondrial
Sister chromatid cohesion protein PDS5 homolog B
Structural maintenance of chromosomes protein 4
Alpha-mannosidase 2C1
Phosphatidylinositol-3-phosphatase SAC1
Protein DEPP1
Obg-like ATPase 1
Copper homeostasis protein cutC homolog

Zinc finger protein 64
Ethylmalonyl-CoA decarboxylase
RNA-binding protein 12
TBC1 domain family member 22B
Midasin
Acetyl-coenzyme A synthetase 2-like, mitochondrial
Zinc finger CCHC domain-containing protein 3
Peroxisomal 2,4-dienoyl-CoA reductase [(3E)-enoyl-CoA-producing]
Palmitoyl-protein thioesterase ABHD10, mitochondrial
T-complex protein 11-like protein 1
Double-stranded RNA-binding protein Staufen homolog 2
Probable ATP-dependent RNA helicase DDX28
Transmembrane protein 106B
Protein lin-7 homolog C
1-acyl-sn-glycerol-3-phosphate acyltransferase epsilon
Gamma-taxilin
SPATS2-like protein
Ufm1-specific protease 2
ATP-binding cassette sub-family F member 3
CYFIP-related Rac1 interactor B
Mitochondrial potassium channel ATP-binding subunit
ATP-dependent RNA helicase DDX19A
GTPase IMAP family member 4
Tyrosyl-DNA phosphodiesterase 1
Protection of telomeres protein 1
TBC1 domain family member 23
DDB1- and CUL4-associated factor 13
U3 small nucleolar ribonucleoprotein protein IMP3
MRG/MORF4L-binding protein
S-adenosyl-L-methionine-dependent tRNA 4-demethylwyosine synthase TYW1
Exocyst complex component 1
Protein-L-isoaspartate O-methyltransferase domain-containing protein 2
Integrator complex subunit 9
Cell cycle control protein 50A
Ubiquinol-cytochrome c reductase complex assembly factor 1
Septin-11
Sodium-coupled neutral amino acid transporter 7
Mediator of RNA polymerase II transcription subunit 17
Alpha-parvin
Ubiquitin carboxyl-terminal hydrolase 40
4'-phosphopantetheine phosphatase
F-box only protein 28
TBC1 domain family member 13
Exonuclease 3'-5' domain-containing protein 2
DnaJ homolog subfamily C member 11
Integrator complex subunit 7
Fanconi anemia group I protein
ATPase family AAA domain-containing protein 3A
ADP-ribosylation factor-like protein 8B
DnaJ homolog subfamily C member 17

Integrator complex subunit 13
Synembryn-B
Guanine nucleotide-binding protein-like 3-like protein
ATP-dependent RNA helicase DDX18
Histone chaperone ASF1B
Kelch-like protein 11
Integrator complex subunit 10
Large ribosomal subunit protein mL66
Pyridoxine-5'-phosphate oxidase
Armadillo repeat-containing protein 1
DNA-directed RNA polymerase III subunit RPC5
Protein SDA1 homolog
Poly(A) RNA polymerase, mitochondrial
Notchless protein homolog 1
Adaptin ear-binding coat-associated protein 2
DNA-directed RNA polymerase III subunit RPC2
RNA-binding protein 28
Anoctamin-10
Pre-mRNA-splicing factor RBM22
Distal membrane-arm assembly complex protein 2
WD repeat-containing protein 70
Transmembrane protein 51
Arginine and glutamate-rich protein 1
Intraflagellar transport protein 57 homolog
SAFB-like transcription modulator
Peptidyl-prolyl cis-trans isomerase FKBP14
Required for meiotic nuclear division protein 1 homolog
p21-activated protein kinase-interacting protein 1
Hypoxia-inducible factor 1-alpha inhibitor
3-oxoacyl-[acyl-carrier-protein] synthase, mitochondrial
Large ribosomal subunit protein uL22m
CXXC motif containing zinc binding protein
BRISC and BRCA1-A complex member 1
Ceroid-lipofuscinosis neuronal protein 6
Ankyrin repeat and SOCS box protein 6
Probable tRNA(His) guanylyltransferase
Histone PARylation factor 1
Interleukin-1 receptor-associated kinase 4
Uridine-cytidine kinase-like 1
Transmembrane protein 160
NACHT, LRR and PYD domains-containing protein 2
Constitutive coactivator of PPAR-gamma-like protein 2
tRNA selenocysteine 1-associated protein 1
NADH dehydrogenase [ubiquinone] 1 beta subcomplex subunit 11, mitochondrial
Large ribosomal subunit protein uL16m
H/ACA ribonucleoprotein complex subunit 2
OCIA domain-containing protein 1
ADP-ribosylhydrolase ARH3
E3 ubiquitin-protein ligase MARCHF5
Cell growth-regulating nucleolar protein

Transmembrane protein 161A
Golgi-resident adenosine 3',5'-bisphosphate 3'-phosphatase
MICOS complex subunit MIC19
tRNA-dihydrouridine(20) synthase [NAD(P)+]-like
CKLF-like MARVEL transmembrane domain-containing protein 6
NAD-dependent protein deacylase sirtuin-5, mitochondrial
GATOR2 complex protein MIOS
Myotubularin-related protein 10
Sphingomyelin phosphodiesterase 4
Pre-mRNA-splicing factor CWC25 homolog
Testis-expressed protein 10
DDB1- and CUL4-associated factor 16
THUMP domain-containing protein 1
Transmembrane prolyl 4-hydroxylase
Torsin-4A
tRNA (guanine(26)-N(2))-dimethyltransferase
Ganglioside-induced differentiation-associated protein 2
Nuclear distribution protein nudE homolog 1
BRISC and BRCA1-A complex member 2
Glutaminyl-peptide cyclotransferase-like protein
ADP-ribosylation factor-like protein 15
BTB/POZ domain-containing protein KCTD5
CDKN2A-interacting protein
DnaJ homolog subfamily B member 12
Alpha-ketoglutarate-dependent dioxygenase alkB homolog 4
Non-structural maintenance of chromosomes element 4 homolog A
H/ACA ribonucleoprotein complex subunit 1
Stabilin-1
Serine/threonine-protein phosphatase 4 regulatory subunit 2
Dipeptidyl peptidase 3
Protein AATF
Tubulin alpha-8 chain
Probable ATP-dependent RNA helicase DDX56
Sphingosine kinase 1
Telomeric repeat-binding factor 2-interacting protein 1
Abl interactor 2
Bcl-2-associated transcription factor 1
U3 small nucleolar RNA-associated protein 6 homolog
TGF-beta-activated kinase 1 and MAP3K7-binding protein 2
Large ribosomal subunit protein mL39
Mitogen-activated protein kinase kinase kinase 20
Peptidyl-prolyl cis-trans isomerase FKBP11
Tropomodulin-3
BET1-like protein
Very long chain fatty acid elongase 5
NF-kappa-B inhibitor-interacting Ras-like protein 2
UDP-glucose:glycoprotein glucosyltransferase 2
UDP-glucose:glycoprotein glucosyltransferase 1
Cyclin-dependent kinase 12
FAST kinase domain-containing protein 2, mitochondrial

Very-long-chain enoyl-CoA reductase
Endoplasmic reticulum aminopeptidase 1
Actin-related protein 10
Vesicle transport protein USE1
CDGSH iron-sulfur domain-containing protein 1
ADP-ribosylation factor-binding protein GGA3
Splicing factor C9orf78
Constitutive coactivator of PPAR-gamma-like protein 1
Glycerophosphodiester phosphodiesterase 1
SWI/SNF-related matrix-associated actin-dependent regulator of chromatin subfamily A-like protein 1
Glycolipid transfer protein
Maspardin
Large ribosomal subunit protein bL35m
Upstream-binding protein 1
Insulin-like growth factor 2 mRNA-binding protein 1
Sacsin
Mitochondrial carrier homolog 1
Diphosphoinositol polyphosphate phosphohydrolase 2
Hsp70-binding protein 1
Methionine adenosyltransferase 2 subunit beta
Myoferlin
Intersectin-2
Ribosome biogenesis protein NOP53
EH domain-containing protein 3
EH domain-containing protein 2
Rho guanine nucleotide exchange factor 12
CCR4-NOT transcription complex subunit 2
NCK-interacting protein with SH3 domain
Programmed cell death 1 ligand 1
Calmodulin-like protein 5
Opioid growth factor receptor
LIM and cysteine-rich domains protein 1
Cysteine-rich motor neuron 1 protein
Selenoprotein N
Protein PALS2
Charged multivesicular body protein 5
Spliceosome-associated protein CWC15 homolog
Large ribosomal subunit protein uL15m
Thymocyte nuclear protein 1
Thyroid transcription factor 1-associated protein 26
NADH dehydrogenase [ubiquinone] 1 alpha subcomplex assembly factor 4
Very-long-chain (3R)-3-hydroxyacyl-CoA dehydratase 3
ER membrane protein complex subunit 3
NADH dehydrogenase [ubiquinone] 1 alpha subcomplex subunit 13
[Pyruvate dehydrogenase [acetyl-transferring]]-phosphatase 1, mitochondrial
E3 ubiquitin-protein ligase KCMF1
Ankycorbin
Vesicle-associated membrane protein-associated protein A
Large ribosomal subunit protein bL27m
Proton-transporting V-type ATPase complex assembly regulator TMEM9

Sentrin-specific protease 1
CXXC-type zinc finger protein 1
SH3 domain-binding protein 4
Septin-10
GEM-interacting protein
PHD and RING finger domain-containing protein 1
Pogo transposable element with KRAB domain
Protein Daple
Rho GTPase-activating protein 23
Vacuolar protein sorting-associated protein 18 homolog
Protein RCC2
RAB11-binding protein RELCH
Disco-interacting protein 2 homolog B
Junctional cadherin 5-associated protein
SLAIN motif-containing protein 2
Teneurin-3
Ubiquitin carboxyl-terminal hydrolase 36
BRCA2 and CDKN1A-interacting protein
Serine/threonine-protein kinase 26
Armadillo repeat-containing X-linked protein 1
ABI gene family member 3
Prostaglandin F2 receptor negative regulator
CTTNBP2 N-terminal-like protein
Transmembrane protein 181
HEAT repeat-containing protein 5B
NFX1-type zinc finger-containing protein 1
Chondroitin sulfate glucuronyltransferase
Protocadherin-10
Ribosome-binding protein 1
Cleavage and polyadenylation specificity factor subunit 2
Kelch-like protein 9
Leucine--tRNA ligase, cytoplasmic
Myelin expression factor 2
Kelch-like protein 42
eIF-2-alpha kinase GCN2
WD repeat-containing protein 35
RNA-binding protein 27
KAT8 regulatory NSL complex subunit 3
E3 ubiquitin-protein ligase HECW2
FERM domain-containing protein 4A
Rabankyrin-5
Arginine-glutamic acid dipeptide repeats protein
Succinate--CoA ligase [ADP-forming] subunit beta, mitochondrial
GMP reductase 2
Syntaxin-18
Dolichol-phosphate mannosyltransferase subunit 3
Protein IMPACT
UV radiation resistance-associated gene protein
Ataxin-10
Methyl-CpG-binding domain protein 2

Neurochondrin
Tuftelin-interacting protein 11
Epidermal growth factor receptor substrate 15-like 1
Unconventional myosin-Ia
Origin recognition complex subunit 3
SUMO-activating enzyme subunit 1
Coatomer subunit gamma-2
Phosphatidylinositol 4-kinase beta
C-type mannose receptor 2
COMM domain-containing protein 3
Guanine nucleotide-binding protein G(I)/G(S)/G(O) subunit gamma-12
Rab-like protein 2A
Methionine synthase reductase
Protein UXT
Set1/Ash2 histone methyltransferase complex subunit ASH2
7-dehydrocholesterol reductase
Tumor necrosis factor receptor superfamily member 10D
Histone deacetylase 6
Spastin
Dickkopf-related protein 3
tRNA (guanine-N(7)-)-methyltransferase
PTB domain-containing engulfment adapter protein 1
Vacuolar protein sorting-associated protein 29
Eukaryotic translation initiation factor 3 subunit K
Exostosin-like 2
Glyoxylate reductase/hydroxypyruvate reductase
Cathepsin Z
Ribosomal protein S6 kinase beta-2
DnaJ homolog subfamily B member 11
E3 ubiquitin-protein ligase RNF14
SUMO-activating enzyme subunit 2
Mortality factor 4-like protein 1
Nuclear RNA export factor 1
Protein sel-1 homolog 1
Beta-1,4-galactosyltransferase 7
Peflin
Zinc finger MYM-type protein 2
COP9 signalosome complex subunit 7a
Mitochondrial dicarboxylate carrier
Alpha-aminoadipic semialdehyde synthase, mitochondrial
CAP-Gly domain-containing linker protein 2
Cytochrome b-c1 complex subunit 9
Mitochondrial fission process protein 1
Tight junction protein ZO-2
DnaJ homolog subfamily B member 4
Serine/threonine-protein kinase 17A
Craniofacial development protein 1
Death domain-associated protein 6
tRNA (cytidine(32)/guanosine(34)-2'-O)-methyltransferase
Vesicle transport through interaction with t-SNAREs homolog 1B

STE20/SPS1-related proline-alanine-rich protein kinase
Gamma-adducin
Leucine-rich repeat and WD repeat-containing protein 1
Protein NipSnap homolog 3A
CGG triplet repeat-binding protein 1
Phosphatidylserine decarboxylase proenzyme, mitochondrial
ATP-binding cassette sub-family F member 2
Testin
5'-AMP-activated protein kinase subunit gamma-2
Gamma-tubulin complex component 4
Lysine-specific demethylase 5B
Tryptophan--tRNA ligase, mitochondrial
LIM domain-containing protein 1
Translocation protein SEC63 homolog
Solute carrier family 2, facilitated glucose transporter member 6
Zinc finger CCCH domain-containing protein 7B
Protein NDRG3
Armadillo repeat-containing X-linked protein 3
Switch-associated protein 70
SUN domain-containing protein 2
Probable ribosome biogenesis protein RLP24
Ragulator complex protein LAMTOR3
LIM domain and actin-binding protein 1
AF4/FMR2 family member 4
Signal recognition particle subunit SRP68
Cysteine and histidine-rich domain-containing protein 1
Serine/threonine-protein kinase TBK1
Septin-9
Prenylcysteine oxidase 1
Probable ATP-dependent RNA helicase DDX20
Sedoheptulokinase
Dipeptidyl peptidase 2
Inactive cell surface hyaluronidase CEMIP2
Ubiquitin carboxyl-terminal hydrolase 25
B-cell receptor-associated protein 29
NADH-cytochrome b5 reductase 1
SAP30-binding protein
Zinc finger HIT domain-containing protein 2
Prefoldin subunit 2
GPN-loop GTPase 3
Poly(U)-binding-splicing factor PUF60
Nuclear receptor-binding protein
Enolase-phosphatase E1
Fasciculation and elongation protein zeta-2
NADH dehydrogenase [ubiquinone] 1 alpha subcomplex subunit 12
Translation initiation factor eIF2B subunit delta
V-type proton ATPase subunit H
Prenylated Rab acceptor protein 1
Importin-11
Multifunctional methyltransferase subunit TRM112-like protein

Dachshund homolog 1
Exportin-7
Leucine carboxyl methyltransferase 1
Vacuolar protein sorting-associated protein 51 homolog
Bromodomain adjacent to zinc finger domain protein 2B
Bromodomain adjacent to zinc finger domain protein 2A
Tyrosine-protein kinase BAZ1B
ATPase inhibitor, mitochondrial
GTP:AMP phosphotransferase AK3, mitochondrial
Leucyl-cystinyl aminopeptidase
Methyl-CpG-binding domain protein 1
CCR4-NOT transcription complex subunit 7
Rab5 GDP/GTP exchange factor
Mitochondrial peptide methionine sulfoxide reductase
N-acetyl-D-glucosamine kinase
2-hydroxyacyl-CoA lyase 1
tRNA (adenine(58)-N(1))-methyltransferase non-catalytic subunit TRM6
SH3 domain-binding glutamic acid-rich-like protein 2
Ras GTPase-activating protein nGAP
18S rRNA aminocarboxypropyltransferase
Electrogenic aspartate/glutamate antiporter SLC25A13, mitochondrial
Drebrin-like protein
Probable ATP-dependent RNA helicase DDX41
Dynactin subunit 4
Cell division cycle protein 23 homolog
Anaphase-promoting complex subunit 7
Anaphase-promoting complex subunit 5
Anaphase-promoting complex subunit 4
Anaphase-promoting complex subunit 2
ADP-ribosylation factor-binding protein GGA2
ADP-ribosylation factor-binding protein GGA1
Stomatin-like protein 2, mitochondrial
N-acetylglucosamine-1-phosphodiester alpha-N-acetylglucosaminidase
Vacuolar protein sorting-associated protein 28 homolog
U6 snRNA-associated Sm-like protein LSm7
Lariat debranching enzyme
Protein TASOR
Jupiter microtubule associated homolog 1
F-box only protein 3
A-kinase anchor protein 11
F-box/WD repeat-containing protein 11
Glucocorticoid modulatory element-binding protein 2
mRNA turnover protein 4 homolog
TRAF2 and NCK-interacting protein kinase
Cleavage and polyadenylation specificity factor subunit 3
DCC-interacting protein 13-alpha
Cdc42 effector protein 3
Serine/threonine-protein kinase tousled-like 1
G patch domain-containing protein 8
Protein mono-ADP-ribosyltransferase PARP4

NTF2-related export protein 1
ADP-sugar pyrophosphatase
REST corepressor 1
Phosphatidylcholine transfer protein
Endoplasmic reticulum mannosyl-oligosaccharide 1,2-alpha-mannosidase
RNA-binding protein Raly
General transcription factor 3C polypeptide 4
Ergosterol biosynthetic protein 28 homolog
Protein kinase C and casein kinase substrate in neurons protein 3
Isobutyryl-CoA dehydrogenase, mitochondrial
Angiopoietin-related protein 2
Apoptotic chromatin condensation inducer in the nucleus
E3 ubiquitin-protein ligase AMFR
Protein argonaute-2
Nuclear pore complex protein Nup50
Protein CDV3 homolog
CCR4-NOT transcription complex subunit 11
Integrator complex subunit 6
BAG family molecular chaperone regulator 5
Protein argonaute-1
Ras-related protein Rab-21
Ras-related protein Rab-22A
Trafficking protein particle complex subunit 2-like protein
Zinc finger protein 346
Proteasome activator complex subunit 2
Serine/threonine-protein kinase TAO2
Muskelin
Aspartyl aminopeptidase
Ras-related protein Rab-23
Malignant T-cell-amplified sequence 1
Microtubule-associated tumor suppressor 1
Protein WWC3
Paladin
Zinc transporter ZIP10
Chromatin-remodeling ATPase INO80
Cell cycle progression protein 1
Kinase D-interacting substrate of 220 kDa
Arf-GAP with SH3 domain, ANK repeat and PH domain-containing protein 1
Myocardin-related transcription factor B
Ankyrin repeat domain-containing protein 50
Mediator of RNA polymerase II transcription subunit 23
Protein Shroom4
YEATS domain-containing protein 2
Protein NDRG4
TBC1 domain family member 24
Pre-mRNA-splicing factor ISY1 homolog
Transmembrane and coiled-coil domain protein 3
E3 ubiquitin-protein ligase HECTD1
MYND-type zinc finger-containing chromatin reader ZMYND8
Cip1-interacting zinc finger protein

Coronin-1C
Targeting protein for Xklp2
Activator of basal transcription 1
RNA-binding protein NOB1
A-kinase anchor protein 8-like
Transcription factor MafF
Apoptosis-associated speck-like protein containing a CARD
Calcium load-activated calcium channel
Anaphase-promoting complex subunit 10
Unconventional myosin-VI
ATP-dependent RNA helicase DDX19B
Lysosomal thioesterase PPT2
Pre-mRNA-processing factor 19
Suppressor of fused homolog
Bcl-2-related ovarian killer protein
Nucleolar protein 7
Sorting nexin-12
Synergin gamma
Vacuolar protein sorting-associated protein 4A
Ras GTPase-activating protein-binding protein 2
E3 ubiquitin-protein ligase CHIP
Protein kinase C and casein kinase substrate in neurons protein 2
Melanoma-associated antigen D2
Sorting nexin-7
Sorting nexin-6
Dual specificity protein phosphatase 12
Syntaxin-8
Translocon-associated protein subunit gamma
26S proteasome non-ATPase regulatory subunit 13
FAS-associated factor 1
Endothelial protein C receptor
Peptidyl-prolyl cis-trans isomerase E
Probable dimethyladenosine transferase
COP9 signalosome complex subunit 3
Multiple inositol polyphosphate phosphatase 1
RNA-binding protein Nova-2
WD repeat-containing protein 3
Transcription termination factor 2
NSFL1 cofactor p47
Conserved oligomeric Golgi complex subunit 5
Solute carrier family 12 member 4
AP-4 complex subunit epsilon-1
Microtubule-actin cross-linking factor 1, isoforms 1/2/3/4/5
Centrosomal protein of 131 kDa
SR-related and CTD-associated factor 8
Serine/threonine-protein phosphatase 6 regulatory subunit 1
E3 ubiquitin-protein ligase TRIM33
Histone lysine demethylase PHF8
Trinucleotide repeat-containing gene 6B protein
Inactive phospholipase C-like protein 2

Nonsense-mediated mRNA decay factor SMG5
Exocyst complex component 7
Zinc finger CCCH domain-containing protein 4
Ubiquitin carboxyl-terminal hydrolase 24
TBC1 domain family member 2B
Protein Smaug homolog 1
Forkhead box protein J3
Cytosolic carboxypeptidase 1
Endoribonuclease Dicer
Cystine/glutamate transporter
Microtubule-associated protein RP/EB family member 3
Leucine-rich repeat protein SHOC-2
Serine/arginine repetitive matrix protein 2
Alpha-1,3-mannosyl-glycoprotein 4-beta-N-acetylglucosaminyltransferase B
Proliferation-associated protein 2G4
Paraplegin
Brain-specific angiogenesis inhibitor 1-associated protein 2
GRB2-associated-binding protein 2
Structural maintenance of chromosomes protein 3
Charged multivesicular body protein 2b
SH2B adapter protein 3
Zinc finger protein 148
Myotubularin-related protein 6
Protein jagged-2
60S ribosome subunit biogenesis protein NIP7 homolog
Bifunctional UDP-N-acetylglucosamine 2-epimerase/N-acetylmannosamine kinase
RNA transcription, translation and transport factor protein
RuvB-like 2
Chromodomain Y-like protein
Peptidyl-prolyl cis-trans isomerase NIMA-interacting 4
RAC-gamma serine/threonine-protein kinase
CAAX prenyl protease 2
Choline/ethanolamine kinase
Eukaryotic translation initiation factor 3 subunit L
Phospholipase A-2-activating protein
RuvB-like 1
Nuclear migration protein nudC
Mitochondrial chaperone BCS1
Voltage-dependent anion-selective channel protein 3
Cofilin-2
Endoplasmic reticulum-Golgi intermediate compartment protein 3
Phenylalanine--tRNA ligase alpha subunit
Sodium-dependent multivitamin transporter
Small ribosomal subunit protein mS33
Histone chaperone ASF1A
Developmentally-regulated GTP-binding protein 1
Trafficking protein particle complex subunit 4
Nck-associated protein 1
Protein canopy homolog 2
Nuclease EXOG, mitochondrial

Exocyst complex component 6B
PALM2-AKAP2 fusion protein
Caspase recruitment domain-containing protein 8
Phospholipid-transporting ATPase IF
GDP-fucose protein O-fucosyltransferase 2
DnaJ homolog subfamily C member 16
Disks large-associated protein 4
Serine/threonine-protein kinase 38-like
Phosphatidylinositide phosphatase SAC2
Fibronectin type-III domain-containing protein 3A
Nischarin
1-phosphatidylinositol 3-phosphate 5-kinase
WD repeat-containing protein 37
Band 4.1-like protein 3
Angiomotin-like protein 2
Serine/threonine-protein kinase SIK3
Lysine-specific demethylase 2A
Exosome complex exonuclease RRP44
Trafficking protein particle complex subunit 8
Leucine-rich repeat and calponin homology domain-containing protein 1
RNA 3'-terminal phosphate cyclase-like protein
Glutathione S-transferase kappa 1
Ragulator complex protein LAMTOR2
Small ribosomal subunit protein bS1m
Cytochrome c oxidase assembly factor 3 homolog, mitochondrial
Probable ATP-dependent RNA helicase DDX52
Small ribosomal subunit protein uS17m
Small ribosomal subunit protein uS7m
Lambda-crystallin homolog
Translation machinery-associated protein 7
Polymerase delta-interacting protein 2
AP-3 complex subunit mu-1
Inner nuclear membrane protein Man1
Calcium-regulated heat-stable protein 1
Conserved oligomeric Golgi complex subunit 6
Thyroid hormone receptor-associated protein 3
WW domain-binding protein 11
Tudor and KH domain-containing protein
Mediator of RNA polymerase II transcription subunit 16
Nucleolar protein 58
ARF GTPase-activating protein GIT1
Zinc finger protein 281
ADP-ribosylation factor-like protein 2-binding protein
Protein SGT1 homolog
Tyrosine--tRNA ligase, mitochondrial
N-acetylglucosamine-6-phosphate deacetylase
Acyl-coenzyme A thioesterase 9, mitochondrial
Protein AAR2 homolog
Nitric oxide synthase-interacting protein
Deoxyribose-phosphate aldolase

Protein MEMO1
Thioredoxin-related transmembrane protein 2
rRNA-processing protein FCF1 homolog
U6 snRNA-associated Sm-like protein LSm2
Endophilin-B1
Complex I intermediate-associated protein 30, mitochondrial
Calcium-binding protein 39
Putative RNA-binding protein Luc7-like 2
Ubiquitin-conjugating enzyme E2 J1
RNA-binding motif protein, X-linked 2
Dehydrogenase/reductase SDR family member 7
Small ribosomal subunit protein uS2m
Probable U3 small nucleolar RNA-associated protein 11
MOB-like protein phocein
Ribosomal RNA-processing protein 7 homolog A
Ribosome maturation protein SBDS
Transmembrane emp24 domain-containing protein 5
Exosome complex component CSL4
Transmembrane emp24 domain-containing protein 7
Splicing factor 3B subunit 6
Large ribosomal subunit protein uL11m
Oligoribonuclease, mitochondrial
RRP15-like protein
WASH complex subunit 3
Nucleolar protein 16
EKC/KEOPS complex subunit TPRKB
Peptidyl-prolyl cis-trans isomerase-like 1
Ubiquitin-fold modifier-conjugating enzyme 1
Cytosolic iron-sulfur assembly component 2B
Small ribosomal subunit protein bS16m
Small ribosomal subunit protein bS18m
Mitochondrial fission 1 protein
Mitochondrial import inner membrane translocase subunit TIM16
Adenylate kinase isoenzyme 6
Small ribosomal subunit protein mS23
Vesicle transport protein GOT1B
Hepatoma-derived growth factor-related protein 3
Peptidyl-tRNA hydrolase 2, mitochondrial
Charged multivesicular body protein 3
Serine-threonine kinase receptor-associated protein
RNA-splicing ligase RtcB homolog
F-box only protein 7
Ras-related protein Rap-2c
StAR-related lipid transfer protein 13
Rab GTPase-activating protein 1
Transmembrane emp24 domain-containing protein 3
TSC22 domain family protein 4
Protein dopey-2
Zinc finger protein 330
R3H and coiled-coil domain-containing protein 1

Nucleolar complex protein 2 homolog
Large ribosomal subunit protein eL36
Coiled-coil domain-containing protein 9
Chromatin target of PRMT1 protein
Deoxynucleoside triphosphate triphosphohydrolase SAMHD1
Small kinetochore-associated protein
HBS1-like protein
5'-AMP-activated protein kinase subunit beta-1
WD repeat domain phosphoinositide-interacting protein 4
DmX-like protein 1
V-type proton ATPase 116 kDa subunit a 2
Talin-1
Kinesin-like protein KIF3A
Transformation/transcription domain-associated protein
Microtubule cross-linking factor 1
DDB1- and CUL4-associated factor 1
TRPM8 channel-associated factor 1
Probable RNA-binding protein 19
Disheveled-associated activator of morphogenesis 1
Plexin-D1
Probable E3 ubiquitin-protein ligase HECTD4
WASH complex subunit 2C
WD repeat-containing protein 7
Ubiquitin carboxyl-terminal hydrolase 15
FERM, ARHGEF and pleckstrin domain-containing protein 1
Meiosis regulator and mRNA stability factor 1
Centrosomal protein of 170 kDa protein B
Rap guanine nucleotide exchange factor 2
Unconventional myosin-Va
Dystrobrevin alpha
Lysyl oxidase homolog 2
Beta/gamma crystallin domain-containing protein 1
TNF receptor-associated factor 6
Mitogen-activated protein kinase kinase kinase kinase 5
Hypoxia up-regulated protein 1
Cysteine protease ATG4B
Transducin beta-like protein 2
WD repeat domain phosphoinositide-interacting protein 2
Telomere length regulation protein TEL2 homolog
Ribosomal biogenesis protein LAS1L
AFG3-like protein 2
Krueppel-like factor 12
E3 ubiquitin-protein ligase ARIH1
U6 snRNA-associated Sm-like protein LSm4
E3 ubiquitin-protein ligase RNF114
Sorting and assembly machinery component 50 homolog
Protein PRRC2C
ADP-ribose glycohydrolase OARD1
Intraflagellar transport protein 25 homolog
Protein phosphatase methylesterase 1

RNA-binding protein 7
Mitochondrial import inner membrane translocase subunit Tim22
NEDD8 ultimate buster 1
YTH domain-containing family protein 2
RNA polymerase II subunit A C-terminal domain phosphatase
PAX3- and PAX7-binding protein 1
Nucleoside diphosphate kinase 7
FACT complex subunit SPT16
U3 small nucleolar RNA-associated protein 18 homolog
Ubiquitin carboxyl-terminal hydrolase isozyme L5
CD2-associated protein
V-type proton ATPase subunit D
Transportin-3
Signal recognition particle receptor subunit beta
Origin recognition complex subunit 6
Ceramide transfer protein
Mannose-1-phosphate guanyltransferase beta
Fatty acid desaturase 3
General transcription factor 3C polypeptide 5
General transcription factor 3C polypeptide 3
Trafficking protein particle complex subunit 1
Transient receptor potential cation channel subfamily V member 2
Serine/threonine-protein kinase MRCK beta
RNA-binding protein 8A
DnaJ homolog subfamily C member 15
Ubiquitin carboxyl-terminal hydrolase 16
U5 small nuclear ribonucleoprotein TSSC4
Zinc finger protein 706
Melanoma-associated antigen D1
Sorting nexin-14
Sorting nexin-11
Sorting nexin-9
Sorting nexin-8
Sorting nexin-5
Endothelial lipase
Cytosolic Fe-S cluster assembly factor NUBP2
Peroxisomal membrane protein PEX16
Lymphatic vessel endothelial hyaluronic acid receptor 1
Beta-secretase 2
Heme-binding protein 2
Host cell factor 2
UbiA prenyltransferase domain-containing protein 1
Pseudouridylate synthase 1 homolog
Leucine-rich repeat flightless-interacting protein 2
FH1/FH2 domain-containing protein 1
Phosphoserine aminotransferase
Nuclear receptor corepressor 2
Mitochondrial ornithine transporter 1
Junctional adhesion molecule A
Neuroplastin

Adhesion G-protein coupled receptor G1
Spindlin-1
Solute carrier family 12 member 7
Dolichyl pyrophosphate Man9GlcNAc2 alpha-1,3-glucosyltransferase
Dolichyl-phosphate beta-glucosyltransferase
Small ribosomal subunit protein mS40
Coatomer subunit gamma-1
Lipid droplet-regulating VLDL assembly factor AUP1
Peptidyl-prolyl cis-trans isomerase FKBP7
Chloride intracellular channel protein 4
Cysteine desulfurase
Cilia- and flagella-associated protein 20
Transforming acidic coiled-coil-containing protein 3
Signal peptidase complex subunit 1
GTP-binding protein SAR1b
AP-4 complex subunit beta-1
Mitochondrial carrier homolog 2
Selenoprotein K
Brefeldin A-inhibited guanine nucleotide-exchange protein 2
Brefeldin A-inhibited guanine nucleotide-exchange protein 1
Mitotic spindle assembly checkpoint protein MAD1
Serine/threonine-protein kinase 24
eIF5-mimic protein 1
COMM domain-containing protein 10
Cytoplasmic dynein 1 light intermediate chain 1
Epsin-1
Ubiquitin carboxyl-terminal hydrolase 3
Testis-expressed protein 264
Calcineurin-binding protein cabin-1
Choline/ethanolaminephosphotransferase 1
DNA (cytosine-5)-methyltransferase 3A
2'-5'-oligoadenylate synthase 3
Adenylate kinase isoenzyme 5
NF-kappa-B essential modulator
Insulin-like growth factor 2 mRNA-binding protein 2
Proton-coupled zinc antiporter SLC30A1
Sodium bicarbonate cotransporter 3
NADH dehydrogenase [ubiquinone] 1 beta subcomplex subunit 9
Sulfide:quinone oxidoreductase, mitochondrial
Roundabout homolog 1
Nuclear receptor coactivator 3
Numb-like protein
Mitogen-activated protein kinase kinase kinase 4
Probable ATP-dependent RNA helicase DDX49
Calpain-7
Actin-binding protein WASF2
E3 SUMO-protein ligase PIAS3
MAU2 chromatid cohesion factor homolog
ATPase MORC2
Influenza virus NS1A-binding protein

SEC23-interacting protein

PG.ProteinNames	PG.Quantity
GAL3B_HUMAN	3936.16504
IFT56_HUMAN	1551.27954
UBA6_HUMAN	17694.8379
ESYT2_HUMAN	13826.1797
SHOT1_HUMAN	2253.54834
TM223_HUMAN	1222.14551
RHG10_HUMAN	3906.13257
HACL2_HUMAN	6407.50537
SPD2B_HUMAN	10480.543
NBAS_HUMAN	2293.9248
TYW5_HUMAN	869.28186
DEND3_HUMAN	1286.65723
VWA8_HUMAN	1835.74146
SBNO1_HUMAN	2040.49304
GTPBA_HUMAN	1263.03394
WDR91_HUMAN	533.309509
PDES1_HUMAN	1181.42419
TPC13_HUMAN	1222.45825
CNOT1_HUMAN	8281.13477
MP3B2_HUMAN	31477.7031
PGP_HUMAN	8859.17871
RIMC1_HUMAN	1380.35376
RCCD1_HUMAN	2977.66162
TCAF2_HUMAN	9773.42773
SMHD1_HUMAN	5830.91309
CAST2_HUMAN	487.13266
U119B_HUMAN	4795.44629
MET15_HUMAN	791.979553
CC85C_HUMAN	330.751953
RTL8C_HUMAN	2466.9563
ZSWM8_HUMAN	796.449951
P121C_HUMAN	2638.15454
P20L1_HUMAN	207.723969
RUXGL_HUMAN	6050.20166
ELAP2_HUMAN	266.371613
NUD19_HUMAN	1643.62573
MI4GD_HUMAN	1032.53699
CALM1_HUMAN	3160.1814
MYO9A_HUMAN	257.383911
GPHRA_HUMAN	1591.61194
WASH3_HUMAN	6677.55176
MCRI1_HUMAN	388.29303
NACAM_HUMAN	64603.2734
CK098_HUMAN	2861.68994
ASURF_HUMAN	746.482178
MIDUO_HUMAN	3760.80933
DNS2A_HUMAN	2735.21826
ADAS_HUMAN	12758.0479

KIF2A_HUMAN	8596.11719
DX39A_HUMAN	92207.625
PDLI1_HUMAN	11588.6328
BACH_HUMAN	48867.2383
MYO1C_HUMAN	29571.3301
SNP23_HUMAN	7806.729
HAX1_HUMAN	1390.11035
AIP_HUMAN	6353.05713
GTPB1_HUMAN	4919.35596
LEG9_HUMAN	340.369507
STXB3_HUMAN	7593.03125
AP4M1_HUMAN	1373.53943
ARVC_HUMAN	3567.47314
SMAP_HUMAN	1757.87354
AP3B1_HUMAN	12749.5674
APBB1_HUMAN	1605.06885
LEG8_HUMAN	809.584717
NDUS8_HUMAN	893.128723
IKBE_HUMAN	1112.97729
PSD11_HUMAN	41589.6641
PSD12_HUMAN	28550.8965
PSMD9_HUMAN	2819.85596
MEN1_HUMAN	605.369446
GET1_HUMAN	2397.02832
PGRC1_HUMAN	1396.76904
SPT5H_HUMAN	4011.09668
TAF4_HUMAN	244.481232
DFFA_HUMAN	1924.64746
HIP1_HUMAN	1680.58521
CLIC1_HUMAN	212952.328
EIF3F_HUMAN	22859.584
WWP2_HUMAN	618.396057
ODPX_HUMAN	4217.3208
QSOX1_HUMAN	3861.25806
DCTN6_HUMAN	1352.50281
ACATN_HUMAN	4011.65771
WASL_HUMAN	3708.8728
IPO5_HUMAN	39502.1094
RPOM_HUMAN	838.375244
EF2K_HUMAN	3130.24023
SAP18_HUMAN	11056.2129
EMAL1_HUMAN	2819.99268
IF2B3_HUMAN	33634.6094
DNM1L_HUMAN	44339.1133
RTCA_HUMAN	4949.49414
P3C2A_HUMAN	5412.92139
GOLI4_HUMAN	2512.34961
AGRIN_HUMAN	1615.47131
PLOD2_HUMAN	41638.3789
EXOC5_HUMAN	8658.64648

BT3A1_HUMAN	399.173035
NDUA4_HUMAN	5088.33838
PSDE_HUMAN	43184.9336
BIN1_HUMAN	3954.2373
CLD5_HUMAN	1778.38867
IMA4_HUMAN	25352.0137
STK25_HUMAN	3535.03516
KRIT1_HUMAN	544.624329
VMA5A_HUMAN	835.693115
PESC_HUMAN	9799.34961
SDCB1_HUMAN	41224.9453
PITM1_HUMAN	281.341522
MPP10_HUMAN	3640.50415
NOP56_HUMAN	7503.46875
DDX3X_HUMAN	64747.8477
MFNG_HUMAN	192.434753
PODXL_HUMAN	34975.8242
CCN1_HUMAN	52007.1094
PIR_HUMAN	27363.5293
IMA3_HUMAN	5684.26611
TRI38_HUMAN	495.334961
NFIB_HUMAN	4719.10938
PPP6_HUMAN	8859.29004
NDKM_HUMAN	1727.12415
P3C2B_HUMAN	528.841919
MA2B1_HUMAN	2395.59521
PDXK_HUMAN	5504.97363
SCD_HUMAN	6926.63867
ARI1A_HUMAN	2832.68457
NEMP1_HUMAN	1504.66187
TXND9_HUMAN	4399.17041
DPYL4_HUMAN	39804.6563
TRAD1_HUMAN	1286.88257
UBFD1_HUMAN	4957.39697
CTRO_HUMAN	1421.02454
COPE_HUMAN	17570.123
BORG1_HUMAN	317.024109
AP3D1_HUMAN	11318.4961
CCS_HUMAN	964.686951
ABLM1_HUMAN	3027.55518
DVL1_HUMAN	469.376343
DVL2_HUMAN	642.865295
CHD1_HUMAN	2858.31738
CHD2_HUMAN	2752.77344
GOSR2_HUMAN	5241.0498
TOR1A_HUMAN	5182.68701
TOR1B_HUMAN	3608.48584
STX16_HUMAN	2291.69604
ADA10_HUMAN	8219.22852
ABCD4_HUMAN	824.735168

EI24_HUMAN	4069.89258
P5I11_HUMAN	1944.18628
KMT2D_HUMAN	259.700378
APAF_HUMAN	2583.04004
RIOK3_HUMAN	726.910645
MP2K7_HUMAN	487.515564
ACOT8_HUMAN	1857.0813
CDIPT_HUMAN	32380.2461
PDCD5_HUMAN	3426.16553
ANM5_HUMAN	18373.5762
NHRF1_HUMAN	6300.54053
CHK1_HUMAN	1217.84949
TR10B_HUMAN	4235.86182
TPP1_HUMAN	15633.0684
TCRG1_HUMAN	17157.9961
NDC80_HUMAN	2352.90723
NRP1_HUMAN	10520.2178
TNPO2_HUMAN	2735.96631
UN13B_HUMAN	933.08667
TR10C_HUMAN	168.521774
RPC1_HUMAN	2043.90991
TSN4_HUMAN	245.335342
PSA7_HUMAN	100045.57
SCAM3_HUMAN	4855.97998
BCKD_HUMAN	2575.19263
MGST3_HUMAN	9360.51563
TX1B3_HUMAN	9036.78516
GIPC1_HUMAN	6267.62451
IKKB_HUMAN	2746.61108
RGS12_HUMAN	951.864014
TIM23_HUMAN	1531.26331
HAT1_HUMAN	8525.96094
CSKP_HUMAN	2132.2124
PLD2_HUMAN	920.948792
QCR8_HUMAN	12788.6777
ML12B_HUMAN	83306.0625
HGS_HUMAN	6943.3042
RAB7L_HUMAN	511.138855
VP26C_HUMAN	1307.46191
MYPT1_HUMAN	10281.6455
GAK_HUMAN	4992.39697
HNRDL_HUMAN	27735.9004
XPO1_HUMAN	28347.5762
BTAF1_HUMAN	1438.95044
ARHGA_HUMAN	3852.25513
ZN609_HUMAN	1243.84595
SPTN2_HUMAN	795.938416
MAST4_HUMAN	1425.55884
SC16A_HUMAN	6730.68701
PLXB2_HUMAN	2332.07788

KHNYN_HUMAN	411.196106
TCPR2_HUMAN	642.500977
SR140_HUMAN	26630.7031
SET1A_HUMAN	632.145935
N4BP3_HUMAN	225.151657
SYNJ2_HUMAN	4624.74854
SYNEM_HUMAN	5202.82471
GRRE1_HUMAN	1054.84839
KIF3B_HUMAN	1381.95874
PUR4_HUMAN	22188.4277
ANR28_HUMAN	1581.27832
ARHGB_HUMAN	379.506378
MRPP3_HUMAN	1774.59595
IKKA_HUMAN	1541.83496
NPC1_HUMAN	3765.72559
DEGS1_HUMAN	2840.3103
ANGP2_HUMAN	2799.68896
SCAM1_HUMAN	3944.57788
SCAM2_HUMAN	2026.50598
ARC1B_HUMAN	36476.2188
ARPC2_HUMAN	43298.7344
ARPC3_HUMAN	43246.6563
RPAC1_HUMAN	9732.4541
PLS1_HUMAN	753.803894
TIF1A_HUMAN	304.358368
PGRC2_HUMAN	9658.74805
RGL2_HUMAN	770.358154
WDR46_HUMAN	1126.95422
NKRF_HUMAN	2531.38599
GNPAT_HUMAN	2071.72021
LAMA5_HUMAN	820.060669
ZN185_HUMAN	12082.9707
CASC3_HUMAN	372.740601
RT12_HUMAN	2318.72241
ACOX3_HUMAN	1656.01929
RER1_HUMAN	15585.6074
SURF4_HUMAN	22075.5742
SPTC1_HUMAN	7195.12939
SPTC2_HUMAN	7973.69922
OGT1_HUMAN	7172.6626
PMM2_HUMAN	14347.042
TM9S1_HUMAN	1960.83704
TRI18_HUMAN	285.948669
HMGB3_HUMAN	22498.5488
PPM1G_HUMAN	11448.9092
SHIP2_HUMAN	4375.77295
EIF3D_HUMAN	25521.4961
EIF3H_HUMAN	30506.2012
HDAC3_HUMAN	1146.62781
NVL_HUMAN	1603.21741

BCAT2_HUMAN	9208.5127
IPO8_HUMAN	5375.68896
STX7_HUMAN	6104.35889
MOT4_HUMAN	15488.373
MRP4_HUMAN	1998.19067
RPA34_HUMAN	2096.5437
P4HA2_HUMAN	6563.90479
CAN5_HUMAN	598.708313
YKT6_HUMAN	48351.9883
NUP42_HUMAN	495.207306
ARPC5_HUMAN	38877.1602
CFLAR_HUMAN	707.646912
MAFG_HUMAN	354.789276
PDPK1_HUMAN	991.725037
TPSN_HUMAN	10178.0684
R113A_HUMAN	775.487671
DHX15_HUMAN	33802.793
MCES_HUMAN	5695.89648
ZZEF1_HUMAN	3301.84546
ASAP2_HUMAN	1085.48206
FLRT2_HUMAN	891.406311
TTI1_HUMAN	1422.67407
RRP8_HUMAN	2468.51929
PJA2_HUMAN	365.08847
CYB5B_HUMAN	1385.2229
PRP4_HUMAN	7902.45752
SERA_HUMAN	18779.3984
NDUS4_HUMAN	3284.79199
DC1L2_HUMAN	14588.8496
PSMD3_HUMAN	41572.6172
RFOX2_HUMAN	3111.60278
PAPS1_HUMAN	15974.3887
ZW10_HUMAN	6515.86572
B4GT5_HUMAN	213.587677
SNUT1_HUMAN	8034.8374
GPAA1_HUMAN	6358.23584
DAPK3_HUMAN	6644.25244
TGFI1_HUMAN	3008.61255
AP5Z1_HUMAN	612.17041
CTIF_HUMAN	3283.87598
MTSS1_HUMAN	656.308228
VIP2_HUMAN	928.52124
M3K7_HUMAN	597.85199
MCA3_HUMAN	23112.0254
RIPK2_HUMAN	2077.49683
HNRPR_HUMAN	84188.7266
PRPF3_HUMAN	7961.8877
TXNL1_HUMAN	49316.9258
TPD54_HUMAN	21500.8828
EMC8_HUMAN	5195.3623

ERI3_HUMAN	2308.08643
SYNJ1_HUMAN	1203.29675
FIBP_HUMAN	1740.25305
IF4G3_HUMAN	24446.4395
PPIH_HUMAN	5087.84619
HTRA2_HUMAN	3931.39917
MS18B_HUMAN	301.009552
ARK72_HUMAN	11478.9541
E41L2_HUMAN	13648.0654
TGON2_HUMAN	1086.17957
B4GA1_HUMAN	1103.5835
WIPF1_HUMAN	2124.76367
AT8B1_HUMAN	1305.21472
AKA10_HUMAN	416.236084
DENR_HUMAN	20490.5059
XPOT_HUMAN	16259.8838
DNPH1_HUMAN	9243.66406
TIM44_HUMAN	8604.56055
TPPC3_HUMAN	15761.5889
CHM2A_HUMAN	8598.63086
NCK2_HUMAN	2200.99658
TSN6_HUMAN	1838.67456
PLRG1_HUMAN	6527.6626
PRC1_HUMAN	2509.7561
RGS10_HUMAN	2733.28809
ZN207_HUMAN	1901.6283
NDUB5_HUMAN	5786.10498
NDUB3_HUMAN	9758.7666
NDUA2_HUMAN	1991.73169
LDB2_HUMAN	1479.49902
GET3_HUMAN	24724.918
BUB1_HUMAN	390.175354
BUB3_HUMAN	26913.5391
ST1B1_HUMAN	8739.60059
ACTN4_HUMAN	70066.7266
BUD23_HUMAN	673.038879
HTSF1_HUMAN	2443.40771
ERD23_HUMAN	2216.57373
AP1G1_HUMAN	13496.207
STX6_HUMAN	1441.61816
SNG2_HUMAN	4404.57031
SGTA_HUMAN	2828.98438
ENSA_HUMAN	7586.7417
MCAT_HUMAN	8707.51367
SYNC_HUMAN	40082.5469
MYO1B_HUMAN	6491.42236
PM34_HUMAN	1769.95435
CPSF5_HUMAN	14878.2393
LANC1_HUMAN	10158.1494
STRN_HUMAN	5462.18457

U3IP2_HUMAN	6880.95801
SCO2_HUMAN	858.077454
CF410_HUMAN	141.111679
AKAP8_HUMAN	2627.35718
GTPB6_HUMAN	1007.55627
G6PT1_HUMAN	2024.65234
IDH3B_HUMAN	7244.49951
NRDC_HUMAN	13440.8398
CALU_HUMAN	19245.2656
EDIL3_HUMAN	7040.15479
ATP9B_HUMAN	1012.31287
SAHH2_HUMAN	2726.33276
KIF1C_HUMAN	3194.38306
EXTL3_HUMAN	741.704529
ORC5_HUMAN	1048.43616
NDUS5_HUMAN	12420.8057
PDE6D_HUMAN	3597.2478
ORC4_HUMAN	1091.40393
PEX1_HUMAN	703.710693
RAD21_HUMAN	9141.87793
DHX16_HUMAN	4448.43066
GMFG_HUMAN	2800.89355
3BP5_HUMAN	2737.22803
MED14_HUMAN	13033.4844
KPRB_HUMAN	4877.2959
SMCA5_HUMAN	7725.27588
JIP4_HUMAN	89776.8047
NUAK1_HUMAN	316.478119
NPA1P_HUMAN	1268.96704
SI1L3_HUMAN	211.188309
ZC3H1_HUMAN	293.465668
AQR_HUMAN	7316.71729
OPA1_HUMAN	6247.12549
GANP_HUMAN	1683.41125
PCDGC_HUMAN	840.44043
PI51C_HUMAN	4138.58936
KIF1B_HUMAN	2788.42798
MABP1_HUMAN	801.660645
KDM1A_HUMAN	5369.08154
TBCD4_HUMAN	8384.96387
FADS1_HUMAN	3556.64136
GSDME_HUMAN	7782.07617
NRP2_HUMAN	6512.3623
MA1A2_HUMAN	1039.68335
MPZL2_HUMAN	2118.70093
ACSL4_HUMAN	12911.6777
SNX3_HUMAN	29891.4434
OGA_HUMAN	6382.48926
VINEX_HUMAN	3668.34204
HNRPQ_HUMAN	45179.5625

PRP17_HUMAN	1143.35657
RNBP6_HUMAN	36423.5547
NEMF_HUMAN	6104.40186
GMDS_HUMAN	3123.24219
NMT2_HUMAN	5291.54102
CCNT1_HUMAN	1819.1106
BUB1B_HUMAN	1298.09875
PLOD3_HUMAN	22085.6914
IF4E2_HUMAN	4674.71045
DIAP1_HUMAN	64805.2852
TSN3_HUMAN	9815.60742
EXOC3_HUMAN	10277.7354
PLIN3_HUMAN	18910.2402
ANM3_HUMAN	978.63092
IMA7_HUMAN	9300.64355
UGDH_HUMAN	44207.5117
TPST2_HUMAN	1703.21179
LPXN_HUMAN	1667.47388
CTND1_HUMAN	34538.8438
ICMT_HUMAN	4475.63428
SNX2_HUMAN	17120.9551
DPM1_HUMAN	14997.5449
USO1_HUMAN	20363.2871
RT14_HUMAN	1633.28174
TOM1_HUMAN	4166.09863
H2B1K_HUMAN	96751.3906
CCD22_HUMAN	3945.26782
PQBP1_HUMAN	6156.31494
TI17B_HUMAN	519.434021
PRAF2_HUMAN	8848.69727
DKC1_HUMAN	11551.4463
IF2P_HUMAN	34546.6484
EDF1_HUMAN	21650.9395
KIN17_HUMAN	547.00708
DIAP2_HUMAN	6767.97998
DNJA2_HUMAN	8807.16992
BRD4_HUMAN	4661.5542
HUS1_HUMAN	219.998169
PFD1_HUMAN	2292.07739
PP1RB_HUMAN	2204.14771
NBN_HUMAN	10155.9258
MCE1_HUMAN	1233.52454
ABCB7_HUMAN	2218.40405
SRGP2_HUMAN	1854.7583
PLXA2_HUMAN	9872.92285
XYLK_HUMAN	722.928955
WDR1_HUMAN	108695.898
ATP9A_HUMAN	2196.99268
N4BP1_HUMAN	816.992676
ROCK2_HUMAN	7027.56299

CLAP2_HUMAN	3748.31372
PTCD1_HUMAN	950.275696
CPNE3_HUMAN	19357.7363
ATG13_HUMAN	712.575806
HIP1R_HUMAN	8022.01904
BRE1B_HUMAN	4980.24512
PHF2_HUMAN	428.603607
ZC11A_HUMAN	3325.48779
CLU_HUMAN	9271.79883
CAND2_HUMAN	761.841797
T22D2_HUMAN	454.55365
DJC13_HUMAN	6823.12402
PHAR2_HUMAN	894.641907
PP6R2_HUMAN	1627.29126
ATS4_HUMAN	1722.59546
CNOT3_HUMAN	5082.32275
ANR17_HUMAN	5902.90234
SIN3B_HUMAN	768.779724
DNJB6_HUMAN	3001.90405
COQ9_HUMAN	1267.16174
GGCT_HUMAN	6636.5332
NDUS7_HUMAN	10176.7402
NDUS2_HUMAN	10245.1279
ZPR1_HUMAN	3197.25684
DUS11_HUMAN	737.12561
NIPS2_HUMAN	7314.17383
HMMR_HUMAN	537.099792
PDCD6_HUMAN	12507.877
TBCA_HUMAN	21227.3223
VATG1_HUMAN	15415.2686
VPS4B_HUMAN	52753.2227
MPU1_HUMAN	1106.35742
H2AY_HUMAN	1264.65234
SH3L1_HUMAN	1248.82849
FLNB_HUMAN	123161.313
NCOR1_HUMAN	4540.61768
PEX14_HUMAN	3937.62817
TRIM3_HUMAN	3550.64771
CISY_HUMAN	117224.859
SPAG7_HUMAN	9086.1709
SC22B_HUMAN	42057.4063
PR40A_HUMAN	10432.1934
TACC1_HUMAN	7922.38037
NDK6_HUMAN	1398.7124
CDC45_HUMAN	1050.979
LRCH4_HUMAN	556.684326
MTX2_HUMAN	1423.26599
VP26A_HUMAN	36123.4531
MPPB_HUMAN	11590.3096
MED24_HUMAN	885.79187

KTNA1_HUMAN	2701.30737
PSIP1_HUMAN	2631.46899
ERLN1_HUMAN	5499.80371
NDUS3_HUMAN	12144.0645
SRS10_HUMAN	8022.91748
CLN5_HUMAN	2244.23267
CLD11_HUMAN	1182.94177
ECI2_HUMAN	15706.3174
KHDR3_HUMAN	1465.88281
EED_HUMAN	1955.77185
BAF_HUMAN	2360.24829
SF3B1_HUMAN	29484.3379
CSDE1_HUMAN	19662.043
WBP4_HUMAN	1518.55005
PRKRA_HUMAN	4213.9126
ITA10_HUMAN	1033.03992
KS6A5_HUMAN	1935.16809
MED6_HUMAN	383.821045
MYCB2_HUMAN	1896.07507
KBL_HUMAN	1552.14417
LYPA1_HUMAN	10637.9512
ERAL1_HUMAN	5260.48975
U520_HUMAN	18887.1191
MTU1_HUMAN	1413.71069
TIPRL_HUMAN	11308.9033
OFD1_HUMAN	2014.98352
KS6A4_HUMAN	1041.16663
SURF6_HUMAN	1151.10767
PPM1B_HUMAN	3023.67432
UTP20_HUMAN	5672.89697
NU155_HUMAN	10995.9668
XRP2_HUMAN	7716.32422
WDHD1_HUMAN	3536.64575
CRTAP_HUMAN	17278.9492
S2512_HUMAN	10500.375
RENR_HUMAN	2820.85718
RNH2A_HUMAN	1770.50256
CD123_HUMAN	3399.67407
BCAR3_HUMAN	569.414307
POP7_HUMAN	3055.52661
RPP40_HUMAN	1339.53503
EIF3G_HUMAN	21661.3262
EIF3J_HUMAN	23307.9219
CBR3_HUMAN	12687.1494
PSD10_HUMAN	3525.26074
AP1G2_HUMAN	1129.18982
FACE1_HUMAN	13280.0332
IDHC_HUMAN	46017.1094
GATB_HUMAN	966.948608
SCO1_HUMAN	5354.36523

ATRN_HUMAN	649.709961
RBBP9_HUMAN	7666.82666
STAM2_HUMAN	5130.86035
DGAT1_HUMAN	230.24855
CCNK_HUMAN	4421.68213
PRAF3_HUMAN	39928.4414
DYSF_HUMAN	18309.8613
PIAS1_HUMAN	1896.76746
SPF27_HUMAN	899.664124
DCTN3_HUMAN	14826.9346
DNJC8_HUMAN	11960.6992
SPF30_HUMAN	3870.20068
ATP5H_HUMAN	3653.79761
TSN9_HUMAN	4567.25293
FLOT1_HUMAN	6327.33398
TRIO_HUMAN	4483.0332
ATP5L_HUMAN	31571.373
MPDZ_HUMAN	2153.81787
CBPD_HUMAN	7236.25146
GLRX3_HUMAN	55764.6914
RL1D1_HUMAN	20323.2539
WFS1_HUMAN	5597.64893
CLPX_HUMAN	5533.24023
S14L2_HUMAN	3953.1084
SYUG_HUMAN	1420.48767
CIAO1_HUMAN	3287.66431
SRP72_HUMAN	11598.6504
DDAH1_HUMAN	11651.6113
RECQ5_HUMAN	750.294189
RMP_HUMAN	1261.23169
B3GA3_HUMAN	707.911499
MTA2_HUMAN	12341.3389
AL1A2_HUMAN	4939.09668
STK10_HUMAN	5601.29492
KPCD3_HUMAN	872.705933
GFPT2_HUMAN	1846.8717
SLIT2_HUMAN	1993.50317
LTN1_HUMAN	2726.97754
TOM70_HUMAN	7970.28516
PKHG5_HUMAN	1174.50317
IPO13_HUMAN	807.470459
DDHD2_HUMAN	671.493835
MYO1D_HUMAN	7443.60547
TOX4_HUMAN	2417.73779
MICA2_HUMAN	2613.87988
SC24D_HUMAN	16454.2734
FCSD2_HUMAN	630.113342
UFL1_HUMAN	9343.37305
SRBS2_HUMAN	7032.46045
SASH1_HUMAN	4228.53418

UBXN7_HUMAN	4433.16943
KLH18_HUMAN	849.290649
SUN1_HUMAN	1779.21362
PLPHP_HUMAN	9022.06152
ERLN2_HUMAN	12770.6602
PRP6_HUMAN	13178.125
PCF11_HUMAN	773.615967
FRYL_HUMAN	6228.32764
NFAT5_HUMAN	252.141525
ENDD1_HUMAN	1818.72131
GLCE_HUMAN	6649.33887
GLSK_HUMAN	23463.293
HAUS5_HUMAN	743.365784
ABLM3_HUMAN	592.869568
FBX21_HUMAN	726.595093
KDM4B_HUMAN	343.399445
MTCL2_HUMAN	1559.16162
UBP19_HUMAN	1113.48389
WDR47_HUMAN	2551.97974
AP2A2_HUMAN	21002.7813
SC31A_HUMAN	43654.6406
ARHGF_HUMAN	7999.6875
HEXI1_HUMAN	1396.51868
RPP14_HUMAN	390.746338
YIF1A_HUMAN	4018.66895
UBR5_HUMAN	2272.08984
AGFG2_HUMAN	2049.07446
PRS23_HUMAN	4823.7041
SCAF4_HUMAN	2433.77588
NDUB6_HUMAN	4588.83252
MFN2_HUMAN	2215.19165
SPN1_HUMAN	1038.37134
UBE4B_HUMAN	5050.16748
ZFPL1_HUMAN	4010.90698
ELP1_HUMAN	4561.11523
NDUB4_HUMAN	4387.08594
NDUB8_HUMAN	5455.24902
NDUA7_HUMAN	3358.14038
VAMP5_HUMAN	1083.95825
RTN3_HUMAN	10224.292
LETM1_HUMAN	13559.6055
EPN2_HUMAN	2821.62061
ZRAB2_HUMAN	7970.78613
SNX4_HUMAN	3536.30371
LC7L3_HUMAN	4473.26465
KI20A_HUMAN	1255.56824
APOL3_HUMAN	520.681885
KIF4A_HUMAN	4514.70117
MBD4_HUMAN	256.811707
MTMR5_HUMAN	1139.66919

GOSR1_HUMAN	8420.12109
KAT7_HUMAN	673.800842
ATE1_HUMAN	1626.28735
VAPB_HUMAN	5843.27002
SNAPN_HUMAN	2594.94141
MPZL1_HUMAN	1882.35034
NDUC2_HUMAN	9438.22266
NDUAA_HUMAN	9460.24023
FKBP9_HUMAN	11814.8223
CELF2_HUMAN	790.032898
6PGL_HUMAN	5364.08252
PAPS2_HUMAN	26298.957
SMC2_HUMAN	12315.915
ATG7_HUMAN	6846.21094
TACC2_HUMAN	2061.05518
TRI16_HUMAN	2939.9248
SYFM_HUMAN	1250.50366
ZBT7A_HUMAN	720.822998
LYPA2_HUMAN	4952.12158
IPO7_HUMAN	36358.4063
ARI2_HUMAN	1286.71167
SLU7_HUMAN	1059.39697
AGM1_HUMAN	18289.4844
MOCS3_HUMAN	964.513428
CD2B2_HUMAN	3085.02515
SVIL_HUMAN	5893.69189
AHSA1_HUMAN	40171.5898
PARN_HUMAN	3025.81543
PSMG1_HUMAN	10065.5264
SGPL1_HUMAN	3142.31763
CNEP1_HUMAN	925.377563
NSA2_HUMAN	4453.85693
G6PE_HUMAN	7579.34863
SC24A_HUMAN	8529.62598
SC24B_HUMAN	7540.22412
AGRL2_HUMAN	1235.24023
NADK_HUMAN	814.713867
TYDP2_HUMAN	1420.27087
MPC2_HUMAN	2131.5415
ETHE1_HUMAN	7154.74854
ACSL3_HUMAN	12735.2598
RPA1_HUMAN	3343.04175
YETS4_HUMAN	1077.89392
ZBT11_HUMAN	536.873291
CNOT4_HUMAN	900.236023
STABP_HUMAN	2045.83789
CPSF4_HUMAN	2005.8042
NFAC1_HUMAN	705.920532
ASML_HUMAN	5248.96094
CDS2_HUMAN	595.533691

PPR3D_HUMAN	1008.6347
BRD1_HUMAN	999.335938
RPP29_HUMAN	697.412903
HERC2_HUMAN	1128.80078
RAB3D_HUMAN	1643.96143
SNP29_HUMAN	4032.04028
OXSR1_HUMAN	5931.4751
GGPPS_HUMAN	1501.57483
HS74L_HUMAN	7138.90918
PTBP3_HUMAN	13813.5488
STR3N_HUMAN	3502.34546
LSM8_HUMAN	423.550781
AP2A1_HUMAN	27833.3262
WIZ_HUMAN	1800.04041
RIGI_HUMAN	7330.57764
STAU1_HUMAN	14886.6699
TTC4_HUMAN	4507.05859
TM50A_HUMAN	2326.24341
CAVN2_HUMAN	29482.582
BAG2_HUMAN	10779.1191
BAG3_HUMAN	3874.43701
M4K4_HUMAN	9863.93848
DCMC_HUMAN	262.320831
QORL1_HUMAN	1846.00745
AIFM1_HUMAN	15551.4688
EMAL2_HUMAN	1246.94971
LATS1_HUMAN	1838.5625
NUD14_HUMAN	2243.89966
TSN15_HUMAN	859.486938
BPNT1_HUMAN	6975.52246
FADS2_HUMAN	33152.7969
DDAH2_HUMAN	906.336609
ABHGA_HUMAN	3005.04517
TXD12_HUMAN	1039.37415
ECD_HUMAN	4792.02344
MBD3_HUMAN	1302.47095
TOP3B_HUMAN	819.790466
NUDT3_HUMAN	2665.97778
BCL10_HUMAN	5251.57324
NDUBA_HUMAN	12592.7803
CLPT1_HUMAN	6173.25146
ZBED1_HUMAN	398.667603
MOC2B_HUMAN	828.941406
TOM40_HUMAN	18032.1426
PX11B_HUMAN	2798.26245
PAK4_HUMAN	6971.48633
APBA3_HUMAN	505.931213
ACL6A_HUMAN	10195.2813
NSD2_HUMAN	675.808655
LDHA_HUMAN	292022.438

AL1A1_HUMAN	8894.14844
DHE3_HUMAN	39391.7695
DYR_HUMAN	11134.6816
NB5R3_HUMAN	40615.4883
GSHR_HUMAN	19332.6367
COX1_HUMAN	1214.25952
COX2_HUMAN	58511.293
PNPH_HUMAN	63430.8125
HPRT_HUMAN	28057.2012
AATM_HUMAN	56536.4375
ABL1_HUMAN	608.792908
PGK1_HUMAN	172879.406
KAD1_HUMAN	28593.2344
UROK_HUMAN	1746.74072
TPA_HUMAN	648.841187
ADA_HUMAN	2907.90063
A1AT_HUMAN	38177.0117
A2MG_HUMAN	361.696655
CYTC_HUMAN	5726.00635
RASN_HUMAN	29369.5664
RASH_HUMAN	3433.33545
RASK_HUMAN	3202.32129
PDGFB_HUMAN	1689.53503
LDLR_HUMAN	5057.31641
TGFB1_HUMAN	3356.46948
HLAB_HUMAN	3198.38843
HLAH_HUMAN	38383.0156
CO1A1_HUMAN	1077.37769
CO4A1_HUMAN	876.846802
LMNA_HUMAN	200172.375
APOA1_HUMAN	5752.50244
FINC_HUMAN	23403.3184
FETUA_HUMAN	2040.02759
ALBU_HUMAN	237256.813
TFR1_HUMAN	40205.6602
FRIL_HUMAN	4554.63721
FRIH_HUMAN	3406.13745
MT2_HUMAN	18780.918
NU1M_HUMAN	1218.13367
NU4M_HUMAN	1199.98975
NU5M_HUMAN	2221.39966
MMP1_HUMAN	11461.1787
HMDH_HUMAN	220.235962
CATA_HUMAN	6082.64648
RAF1_HUMAN	1741.50085
GBA1_HUMAN	10635.8193
ALDOA_HUMAN	281322.25
CYTB_HUMAN	5259.57861
ANXA1_HUMAN	184639.563
APOB_HUMAN	2849.04297

PRIO_HUMAN	1335.50952
SODM_HUMAN	608.847351
OAT_HUMAN	32548.3184
KITH_HUMAN	3722.1333
VWF_HUMAN	37732.8164
G3P_HUMAN	419369.063
ARLY_HUMAN	6621.76953
HLAA_HUMAN	35508.4141
CPNS1_HUMAN	21422.5449
P53_HUMAN	133.325775
MT1E_HUMAN	181.014771
HSPB1_HUMAN	107159.68
TYSY_HUMAN	16468.6563
RPN1_HUMAN	72819.3906
RPN2_HUMAN	44923.293
GNAI2_HUMAN	35348.9883
H2A1B_HUMAN	69561.2578
B3A2_HUMAN	1690.36975
AT1A1_HUMAN	53524.2461
AT1B1_HUMAN	12306.7578
A4_HUMAN	5272.45068
ALDH2_HUMAN	26463.0273
ITB3_HUMAN	6002.87207
S10A8_HUMAN	186.206741
HMGN1_HUMAN	948.851746
PAI2_HUMAN	17020.1797
PAI1_HUMAN	115676.641
ADT2_HUMAN	234787.406
ISG15_HUMAN	6161.78369
PCCA_HUMAN	2854.22778
PCCB_HUMAN	3330.19507
IF2A_HUMAN	57611.3945
HMGN2_HUMAN	471.776031
ICAM1_HUMAN	10328.5322
RLA2_HUMAN	5350.76367
RLA0_HUMAN	181407.922
JUN_HUMAN	1896.48096
RPC4_HUMAN	1205.93884
LA_HUMAN	30638.8477
ITB1_HUMAN	115603.055
K1C18_HUMAN	63314.8086
CO5A2_HUMAN	1312.59692
DCUP_HUMAN	17397.834
FYN_HUMAN	438.643677
AGAL_HUMAN	849.926575
GELS_HUMAN	7487.5791
RB_HUMAN	2268.45142
PTMA_HUMAN	3163.53662
CDK1_HUMAN	31951.2813
ATPB_HUMAN	163670.906

S10A6_HUMAN	10243.1953
IF4E_HUMAN	17445.1914
ENOA_HUMAN	515909.375
PYGL_HUMAN	8755.93555
G6PI_HUMAN	61391.6992
DPOLB_HUMAN	514.687622
NPM_HUMAN	65963.9453
TPM3_HUMAN	61700.8359
ITAV_HUMAN	18445.1582
HEXA_HUMAN	9587.37598
HYEP_HUMAN	9023.875
LDHB_HUMAN	204574.313
CENPB_HUMAN	1177.93445
GPX1_HUMAN	18042.707
PDIA1_HUMAN	164041.719
H10_HUMAN	16773.9668
ACYP1_HUMAN	4898.67871
FES_HUMAN	643.254944
CATD_HUMAN	59931.3672
ANXA2_HUMAN	414209.375
CAN1_HUMAN	28418.1816
TBB5_HUMAN	56610.707
SAP_HUMAN	4821.20752
HEXB_HUMAN	15580.0723
CATL1_HUMAN	1818.85242
PROF1_HUMAN	257233.922
PMGE_HUMAN	4556.49658
APT_HUMAN	56488.1641
SYEP_HUMAN	21391.0605
CATB_HUMAN	84604.8047
HS90A_HUMAN	144865.5
GALT_HUMAN	881.188599
HNRPC_HUMAN	128718.211
LAMB1_HUMAN	17794.0547
YES_HUMAN	17466.2422
LYN_HUMAN	2871.56055
FUMH_HUMAN	30148.1738
ERCC1_HUMAN	297.058746
TSP1_HUMAN	129923.211
SP1_HUMAN	1076.80835
IGF1R_HUMAN	1184.60559
CO1A2_HUMAN	583.618042
ANXA6_HUMAN	91368.7734
RHOC_HUMAN	92433.7734
DAF_HUMAN	9447.60352
4F2_HUMAN	18632.5527
BGLR_HUMAN	467.167267
PFKAM_HUMAN	9340.16992
HS90B_HUMAN	725313.5
SRPRA_HUMAN	12066.1729

ASNS_HUMAN	5270.78174
MMP2_HUMAN	6836.70557
HEM3_HUMAN	1679.2876
NEP_HUMAN	378.798187
MGP_HUMAN	3051.32178
ODPA_HUMAN	30799.4766
CO4A2_HUMAN	6862.19775
CY1_HUMAN	12806.625
RU2B_HUMAN	8294.18945
MET_HUMAN	2610.70923
RU17_HUMAN	49977.1641
ITA5_HUMAN	79976.2266
NFIC_HUMAN	1091.60254
VIME_HUMAN	1443545.5
RS17_HUMAN	35971.3359
GNAI3_HUMAN	8285.16699
ANXA5_HUMAN	414309.094
RSSA_HUMAN	116952.859
CD63_HUMAN	2951.60107
RM03_HUMAN	5071.96973
SNRPA_HUMAN	23874.7852
ENOG_HUMAN	11799.0625
THIK_HUMAN	6101.60498
SRP19_HUMAN	5733.43848
GSTP1_HUMAN	129154.164
RU1C_HUMAN	5119.79541
LEG1_HUMAN	31473.8887
DHPR_HUMAN	8794.33203
HMGB1_HUMAN	57800.0586
SPRC_HUMAN	4206.25391
TPM1_HUMAN	69644.7813
CLCA_HUMAN	2836.07129
CLCB_HUMAN	367.349457
ANXA4_HUMAN	13971.6309
CN37_HUMAN	12565.8926
HMOX1_HUMAN	9544.32617
DLDH_HUMAN	12118.626
ROA1_HUMAN	157870.031
RU2A_HUMAN	27202.1543
COX6C_HUMAN	16021.0576
TACD2_HUMAN	2586.32129
PARP1_HUMAN	36600.6563
DPOLA_HUMAN	13353.2148
UCHL1_HUMAN	11439.5039
LKHA4_HUMAN	26685.3418
ALDOC_HUMAN	13660.3154
H2AZ_HUMAN	2928.13086
H2A1_HUMAN	711.546448
GRL1A_HUMAN	1118.82922
GST2_HUMAN	4068.66504

POTEJ_HUMAN	8947.19336
LIMS3_HUMAN	4300.97852
HS71A_HUMAN	85944.5469
ERPG3_HUMAN	1359.03906
TBA3C_HUMAN	42581.5742
ZTRF1_HUMAN	742.699158
RAP2A_HUMAN	7455.33203
SRGN_HUMAN	2372.16846
RO60_HUMAN	9856.22363
LYAG_HUMAN	3048.56616
RRAS_HUMAN	12779.3955
HLAC_HUMAN	1136.14478
ARAF_HUMAN	2116.64014
H14_HUMAN	311095.344
ODP2_HUMAN	23301.1152
PTPRF_HUMAN	4099.85645
COT1_HUMAN	4386.35059
THIO_HUMAN	4056.71948
COX5B_HUMAN	935.940186
PPGB_HUMAN	6640.14258
MGST1_HUMAN	13682.0254
KAP0_HUMAN	18903.8594
TFPI1_HUMAN	1082.203
ESTD_HUMAN	20315.1367
CH60_HUMAN	139557.25
CLUS_HUMAN	1615.21021
BIP_HUMAN	241612.859
LAMC1_HUMAN	12064.7383
PPAL_HUMAN	4905.10645
MTAP2_HUMAN	3087.29736
HSP7C_HUMAN	267944.469
GTR1_HUMAN	5360.08008
GTR3_HUMAN	2531.11084
EPB41_HUMAN	21719.0039
UMPS_HUMAN	13608.6191
ODPB_HUMAN	15224.4814
ODB2_HUMAN	1305.23962
PYGB_HUMAN	16799.5527
PYGM_HUMAN	35485.1094
RALA_HUMAN	27071.2051
RALB_HUMAN	44511.6328
BCR_HUMAN	4269.47266
LAMP1_HUMAN	69817.3828
ERG_HUMAN	5473.06787
ACADM_HUMAN	9415.30078
TOP1_HUMAN	45371.7188
TOP2A_HUMAN	462.285645
G6PD_HUMAN	72432.3594
UBL4A_HUMAN	1198.34155
PYC_HUMAN	2600.03198

C1TC_HUMAN	53272.918
MPRI_HUMAN	4418.95557
ADHX_HUMAN	32699.125
CDK4_HUMAN	3127.30151
PRPS2_HUMAN	22715.0391
PABP1_HUMAN	113499.773
PCNA_HUMAN	30706.5781
HARS1_HUMAN	41104.8516
CO6A1_HUMAN	2755.21436
CO6A3_HUMAN	2077.85522
ADT1_HUMAN	240151.5
ADT3_HUMAN	97834.3047
IMDH2_HUMAN	39777.0586
TPR_HUMAN	13980.7109
ANXA3_HUMAN	45656.9258
ODBA_HUMAN	4210.36328
ACTN1_HUMAN	113042.625
ACE_HUMAN	5243.64746
SRC_HUMAN	4580.95508
PEPD_HUMAN	9048.65234
XRCC6_HUMAN	79306.0391
XRCC5_HUMAN	55351.3945
UNG_HUMAN	1139.24878
COX41_HUMAN	56870.9063
HEM1_HUMAN	891.086182
LAMP2_HUMAN	51019.6406
RINI_HUMAN	46693.043
ICAM2_HUMAN	47562.0938
EF2_HUMAN	200098.547
PDIA4_HUMAN	112290.82
P4HA1_HUMAN	9593.13672
TCTP_HUMAN	14891.3408
HEM2_HUMAN	4747.06689
HLAE_HUMAN	692.163879
PLST_HUMAN	148807.5
ACPH_HUMAN	17209.1426
ETFA_HUMAN	22674.4414
GYS1_HUMAN	5460.51758
KAP2_HUMAN	7560.82373
T2FB_HUMAN	5053.03271
CD59_HUMAN	23206.459
MTDC_HUMAN	7131.0293
MIF_HUMAN	32930.043
CD99_HUMAN	2122.52905
GLU2B_HUMAN	70164.0938
HCLS1_HUMAN	7360.86279
FPPS_HUMAN	55693.2813
CX7A2_HUMAN	15866.7051
NID1_HUMAN	6585.75684
AK1A1_HUMAN	25949.4414

KPYM_HUMAN	455199.313
ENPL_HUMAN	296404.281
CCNB1_HUMAN	1848.35266
MYL6B_HUMAN	12553.0332
RSMB_HUMAN	48695.7578
IDE_HUMAN	19313.0977
PO2F1_HUMAN	588.083252
HNRPL_HUMAN	27238.7656
SYDC_HUMAN	34913.918
ETS1_HUMAN	1298.29285
PLAK_HUMAN	14899.6123
QCR7_HUMAN	1164.1886
BRAF_HUMAN	4867.7583
FABP4_HUMAN	14399.7295
GLNA_HUMAN	933.103333
ALDR_HUMAN	44020.5078
AMPN_HUMAN	72626.6406
PVR_HUMAN	22683.7129
RAC2_HUMAN	23427.1641
ERF3A_HUMAN	15939.584
ARSA_HUMAN	663.248657
B4GT1_HUMAN	1262.36914
EZRI_HUMAN	38110.2539
UCHL3_HUMAN	5871.79834
FOSL1_HUMAN	1636.64734
FOSL2_HUMAN	281.329773
MCP_HUMAN	5185.76953
NDKA_HUMAN	30609.6328
NQO1_HUMAN	15371.1016
GNS_HUMAN	36554.0156
PHKG2_HUMAN	1808.8385
ARSB_HUMAN	1228.62927
RS2_HUMAN	156199.266
ITF2_HUMAN	990.203125
DESP_HUMAN	882.499207
RFA2_HUMAN	6093.56299
TIMP2_HUMAN	4670.31104
CD44_HUMAN	15923.6221
CBR1_HUMAN	25225.5
ACADS_HUMAN	1174.25574
CREB1_HUMAN	2827.32397
BGAL_HUMAN	9003.94238
PECA1_HUMAN	51449.8516
PP2BB_HUMAN	5274.08008
NCK1_HUMAN	8189.26611
GCFC2_HUMAN	889.299011
H15_HUMAN	312911.344
H13_HUMAN	31773.5723
H12_HUMAN	45023.5859
NCPR_HUMAN	10588.8506

FER_HUMAN	384.624054
AT2A2_HUMAN	41275.7539
PLCG2_HUMAN	4451.82715
FAAA_HUMAN	3924.14014
STMN1_HUMAN	53953.082
YBOX3_HUMAN	16637.9414
ZNF22_HUMAN	1103.72961
ZNF24_HUMAN	566.388184
NAGAB_HUMAN	3017.10132
HMGA1_HUMAN	53533.8477
TMM11_HUMAN	3356.95947
AATC_HUMAN	18493.5176
KPCA_HUMAN	5859.31055
ITA2_HUMAN	25410.0254
CXA1_HUMAN	1619.61133
ASM_HUMAN	1126.66638
UBF1_HUMAN	9187.50879
JUND_HUMAN	730.232971
TAL1_HUMAN	756.410828
NDUB7_HUMAN	4635.39453
KAPCA_HUMAN	12630.9316
CAN2_HUMAN	42414.0625
PTN2_HUMAN	2211.02661
PYRG1_HUMAN	16450.4902
EGLN_HUMAN	62538.6094
DDX5_HUMAN	90611.5547
PFKAL_HUMAN	44383.8906
VGFR1_HUMAN	6958.59521
PRS6A_HUMAN	39568.1719
TCPA_HUMAN	65804.0703
PTN1_HUMAN	22686.3223
ERCC2_HUMAN	3770.26685
RL35A_HUMAN	69840.8984
ITB5_HUMAN	6196.19873
ARF4_HUMAN	129822.188
RL7_HUMAN	166499.531
VINC_HUMAN	79627.7734
PTPRA_HUMAN	1372.62
ARY1_HUMAN	1428.4314
SON_HUMAN	10890.1328
NELFE_HUMAN	4056.73486
RL17_HUMAN	98893.4141
PGAM1_HUMAN	80875.1406
RCC1_HUMAN	24983.0156
ATF1_HUMAN	749.582764
ATF6A_HUMAN	396.583771
DNLI1_HUMAN	5875.05078
XRCC1_HUMAN	2934.23438
AMD_HUMAN	631.646057
CADH2_HUMAN	13826.8076

PLCG1_HUMAN	10233.9785
NUCL_HUMAN	174000.625
HXK1_HUMAN	59319.4883
RPB3_HUMAN	1452.14355
RPAB1_HUMAN	5255.27539
NDUV2_HUMAN	1277.61011
ERCC3_HUMAN	2007.04126
RO52_HUMAN	5599.49658
E2AK2_HUMAN	9967.33789
SPEE_HUMAN	53755.5859
SL9A1_HUMAN	1369.27026
CSK22_HUMAN	6783.03174
NFKB1_HUMAN	4392.86768
AT2B1_HUMAN	4299.73877
IF2B_HUMAN	24395.0391
ANXA7_HUMAN	20950.0059
CCNA2_HUMAN	814.807068
BTF3_HUMAN	7667.82764
RAB3B_HUMAN	3611.69922
RAB4A_HUMAN	3111.78979
RAB5A_HUMAN	70693.0781
RAB6A_HUMAN	41472.8047
MSH3_HUMAN	1783.94421
ANPRB_HUMAN	683.96283
PSB1_HUMAN	40992.6055
MPRD_HUMAN	5235.07129
COX5A_HUMAN	170.245499
LMNB1_HUMAN	89668.0938
ICAL_HUMAN	18800.0156
IMDH1_HUMAN	5579.86572
CO5A1_HUMAN	1152.79517
NEBU_HUMAN	1245.90393
ASPG_HUMAN	1857.39258
RASA1_HUMAN	5135.30273
CD11B_HUMAN	9463.38867
GSTM3_HUMAN	2147.10034
VATB2_HUMAN	13936.5469
VATC1_HUMAN	5138.1709
CSRP1_HUMAN	76984.5781
FLNA_HUMAN	266047.969
NF1_HUMAN	373.890472
AOFA_HUMAN	1325.56055
ACOHC_HUMAN	27362.4277
S1PR1_HUMAN	7890.68213
SYT1_HUMAN	1453.27991
5NTD_HUMAN	13415.1055
TAF1_HUMAN	139.135803
MK_HUMAN	656.48938
VDAC1_HUMAN	95108.9297
PGS1_HUMAN	3470.22192

SDHB_HUMAN	11789.4512
CD9_HUMAN	23634.248
COMT_HUMAN	5048.28613
TGM2_HUMAN	284973.281
MUTA_HUMAN	8313.37109
OSBP1_HUMAN	6248.60156
PIMT_HUMAN	2410.59546
FBRL_HUMAN	27300.7441
PUR2_HUMAN	26348.4902
PUR6_HUMAN	54774.418
SCP2_HUMAN	21950.1719
UBA1_HUMAN	64664.1992
NDKB_HUMAN	127364.43
ADRO_HUMAN	11475.3174
ROA2_HUMAN	223772.234
RFX1_HUMAN	1030.4707
CBL_HUMAN	3936.0105
IBP4_HUMAN	992.230042
KAPCB_HUMAN	1717.51904
QCR2_HUMAN	16247.749
HEMH_HUMAN	3101.46875
XPA_HUMAN	332.132629
TCEA1_HUMAN	13349.6992
PGH1_HUMAN	955.429199
ITA6_HUMAN	21442.6543
SFPQ_HUMAN	91043.375
TBG1_HUMAN	11916
PPIB_HUMAN	134522.656
MAOM_HUMAN	13717.3086
SYWC_HUMAN	20761.709
RS3_HUMAN	209635.797
KS6B1_HUMAN	3212.35547
JAK1_HUMAN	6290.22559
PTPRB_HUMAN	12093.6182
PTPRE_HUMAN	5114.39502
PTPRG_HUMAN	1052.90686
SP100_HUMAN	8796.90527
CRCM_HUMAN	637.103699
TNFL4_HUMAN	12472.9902
SAHH_HUMAN	84408.0938
COF1_HUMAN	657602.938
IF4B_HUMAN	23920.8066
HAP40_HUMAN	1526.77002
AT2B4_HUMAN	6409.48096
DGKA_HUMAN	3999.55273
CPT2_HUMAN	3025.15601
KTHY_HUMAN	20255.043
RIR1_HUMAN	16040.5615
CCND1_HUMAN	2159.52563
RAE1_HUMAN	1360.30176

ERD21_HUMAN	1088.047
COT2_HUMAN	2123.89844
EF1B_HUMAN	19101.8047
AT5F1_HUMAN	17660.0371
PPAC_HUMAN	17447.2246
KPCL_HUMAN	3175.32129
THIL_HUMAN	26310.082
MYL9_HUMAN	198.770859
RPB1_HUMAN	31692.5918
CDK2_HUMAN	10737.7412
ARBK1_HUMAN	3448.8667
PAR1_HUMAN	983.114197
MCM3_HUMAN	46119.75
THTM_HUMAN	7250.77783
RS12_HUMAN	30368.5156
BRD2_HUMAN	1839.55896
TYY1_HUMAN	2440.08594
DNJB1_HUMAN	9949.1543
DNJB2_HUMAN	402.335632
ATPA_HUMAN	205345.75
CATS_HUMAN	1727.84924
PSA1_HUMAN	102563.773
PSA2_HUMAN	29973.9746
PSA3_HUMAN	43662.1758
PSA4_HUMAN	83696.8828
TNR5_HUMAN	655.221436
ITA3_HUMAN	20621.5625
PTX3_HUMAN	3482.95117
MOES_HUMAN	141446.078
DDX6_HUMAN	17614.8555
DNMT1_HUMAN	9287.88574
U2AF2_HUMAN	52400.2734
RL13_HUMAN	114464.445
RAE2_HUMAN	812.59198
IVD_HUMAN	1358.00146
MGAT1_HUMAN	3484.69653
HMGB2_HUMAN	14914.8057
PTBP1_HUMAN	58016.4258
SYTC_HUMAN	36690.0898
SYVC_HUMAN	23440.6855
EF1G_HUMAN	144416.188
FKBP2_HUMAN	14060.8223
IL3RA_HUMAN	1998.45435
STOM_HUMAN	33057.2539
KAD4_HUMAN	1441.6842
1433T_HUMAN	51269.1641
MK03_HUMAN	15573.0039
MARK3_HUMAN	615.57074
RL10_HUMAN	44663.2188
RFA1_HUMAN	13997.1104

APEX1_HUMAN	26253.4863
CD82_HUMAN	1222.53845
DCK_HUMAN	3679.48071
PYR1_HUMAN	37025.6797
CALR_HUMAN	172595.781
MAP4_HUMAN	26502.4414
CALX_HUMAN	115133.617
P85A_HUMAN	1442.81628
IP3KB_HUMAN	866.57782
PSB8_HUMAN	2975.01953
PSB9_HUMAN	3480.02539
PSA5_HUMAN	30379.6445
PSB4_HUMAN	18571.4336
PSB6_HUMAN	34387.7031
PSB5_HUMAN	37931.7227
GSTM2_HUMAN	796.286621
ABCD3_HUMAN	5994.2876
ITPI2_HUMAN	5632.7168
LYOX_HUMAN	2512.44409
NDUS1_HUMAN	13214.3564
DPOD1_HUMAN	9356.47461
SMCA1_HUMAN	808.323364
MK01_HUMAN	46782.0742
RXRB_HUMAN	952.471313
ERCC5_HUMAN	852.163696
GRN_HUMAN	3266.08569
PTPRM_HUMAN	665.89032
AMPL_HUMAN	25161.7773
CD34_HUMAN	1647.50269
T2EA_HUMAN	2762.24097
T2EB_HUMAN	2865.52954
TPP2_HUMAN	13587.2441
IMPA1_HUMAN	6372.66016
CCN2_HUMAN	24551.3809
EPHA2_HUMAN	23174.9082
EPHB2_HUMAN	13448.2031
SHC1_HUMAN	5210.76709
3MG_HUMAN	6042.48633
TKT_HUMAN	133631.578
CASP1_HUMAN	331.60788
NOS3_HUMAN	2787.12012
RBMS1_HUMAN	4515.27197
PML_HUMAN	9029.46582
TYK2_HUMAN	1043.60303
EF1D_HUMAN	42751.6523
MARCS_HUMAN	42424.3555
GNA11_HUMAN	16904.6191
AL4A1_HUMAN	3334.46313
ERP29_HUMAN	29020.8555
PRDX6_HUMAN	147998.5

BLVRB_HUMAN	8566.37695
PRDX5_HUMAN	24444.2715
PRDX3_HUMAN	21680.3945
RL12_HUMAN	123892.148
ECHM_HUMAN	14861.6563
KCY_HUMAN	27296.707
PEBP1_HUMAN	28624.6348
PDIA3_HUMAN	138747.938
2AAA_HUMAN	64108.1992
2AAB_HUMAN	4948.46484
CDC27_HUMAN	4817.10889
PPIF_HUMAN	11828.3057
NKTR_HUMAN	1540.31689
NMT1_HUMAN	17859.1719
HMOX2_HUMAN	15544.291
PURA2_HUMAN	63501.8125
UFO_HUMAN	3504.04541
AMRP_HUMAN	5909.59814
TSPO_HUMAN	10610.2422
PUR8_HUMAN	17357.332
CLIP1_HUMAN	7047.7959
SORCN_HUMAN	3583.05127
ILEU_HUMAN	4222.41992
CTR1_HUMAN	5170.8208
AL1B1_HUMAN	7491.58447
RPB2_HUMAN	8784.52148
SDHA_HUMAN	8940.49609
COR1A_HUMAN	1641.66003
GDIA_HUMAN	20070.625
METK2_HUMAN	51566.5352
KAP3_HUMAN	8506.5752
RIR2_HUMAN	14695.9072
SDC4_HUMAN	214.311737
TIA1_HUMAN	17330.2227
SC6A6_HUMAN	895.118042
DNJA1_HUMAN	15160.4375
AKT1_HUMAN	3044.02783
QCR1_HUMAN	73239.0234
3HIDH_HUMAN	5060.9126
PUR9_HUMAN	30350.3281
HNRH3_HUMAN	7812.79541
HNRH1_HUMAN	70377.4297
CASPE_HUMAN	1312.8623
1433B_HUMAN	172769.625
STIP1_HUMAN	118315.297
S10AB_HUMAN	16565.3496
PRDX2_HUMAN	42101.2578
ARRB2_HUMAN	690.959412
GLPK_HUMAN	1048.07825
DCTD_HUMAN	7278.22461

P5CR1_HUMAN	5597.53662
GBP1_HUMAN	3519.67578
GBP2_HUMAN	2996.34302
ELF1_HUMAN	1271.97742
TF2H1_HUMAN	1044.79199
STX2_HUMAN	373.079132
RL9_HUMAN	96762.2031
ACSL1_HUMAN	5342.60449
CADH5_HUMAN	27769.002
KINH_HUMAN	34742.0117
DUT_HUMAN	7226.49756
MRP1_HUMAN	4759.97217
ABCD1_HUMAN	710.621277
TTK_HUMAN	1041.62122
MCM4_HUMAN	13187.8691
MCM5_HUMAN	30289.3223
MCM7_HUMAN	21418.9297
GALNS_HUMAN	418.618866
GLYC_HUMAN	4367.91846
GLYM_HUMAN	67230.8359
HS71L_HUMAN	1191.20837
HSP74_HUMAN	36122.7734
MPI_HUMAN	2483.03101
GPC1_HUMAN	735.689209
PROF2_HUMAN	21787.5547
CTNA1_HUMAN	49728.5313
CTNB1_HUMAN	48036.9609
PHB1_HUMAN	104120.398
SPB6_HUMAN	20032.9746
MERL_HUMAN	3818.92212
RADI_HUMAN	374880.719
RFA3_HUMAN	1213.54211
RFC4_HUMAN	7319.67139
RFC2_HUMAN	7119.61768
RFC1_HUMAN	3731.78809
RL22_HUMAN	134112.5
T2FA_HUMAN	7763.98633
SPRE_HUMAN	4964.66455
CBS_HUMAN	678.328735
FBN1_HUMAN	2408.58032
FBN2_HUMAN	370.781128
GDE_HUMAN	4044.09106
MYH9_HUMAN	182128.344
MYH10_HUMAN	103442.5
TIE1_HUMAN	12229.5361
COPB2_HUMAN	49248.6445
SOAT1_HUMAN	3450.89258
ADDA_HUMAN	6892.646
BASI_HUMAN	60831.6211
FUS_HUMAN	67342.1953

NU214_HUMAN	6682.72461
DEK_HUMAN	26498.4551
SOX18_HUMAN	169.199203
MYH11_HUMAN	60494.4336
PPM1A_HUMAN	5105.36426
HMGCL_HUMAN	3288.02588
VGFR2_HUMAN	3337.14429
PRS7_HUMAN	65877.9297
GGT5_HUMAN	818.000793
ARL2_HUMAN	10273.3203
ARL3_HUMAN	23718.3926
MP2K2_HUMAN	14566.4111
ATPG_HUMAN	45752.6172
VATE1_HUMAN	10077.7256
HEM6_HUMAN	11560.3262
RL4_HUMAN	160265.422
8ODP_HUMAN	1523.61768
LONM_HUMAN	13023.334
PGM1_HUMAN	8678.68945
PP1G_HUMAN	10801.2725
GNL1_HUMAN	28917.0098
ODO2_HUMAN	23361.9355
GMPR1_HUMAN	3160.8606
GPX4_HUMAN	8941.34277
ACVL1_HUMAN	3087.61426
DHB2_HUMAN	372.391968
SRP14_HUMAN	22880.0352
TGFR2_HUMAN	3804.16113
NUP62_HUMAN	889.332642
HPCL1_HUMAN	7681.7627
FDFT_HUMAN	12109.6582
ZEB1_HUMAN	2058.75439
TAGL2_HUMAN	50730.7617
TALDO_HUMAN	120464.055
SYUA_HUMAN	4651.85889
ETFB_HUMAN	18031.0293
RBMX_HUMAN	18980.4844
COIL_HUMAN	2698.59985
VKGC_HUMAN	1988.93298
LICH_HUMAN	353.710358
VATA_HUMAN	23195.3418
GRP75_HUMAN	134242.656
IF4A3_HUMAN	34598.2109
CDN1A_HUMAN	2177.27466
RS19_HUMAN	119175.414
RL3_HUMAN	43957.6797
COIA1_HUMAN	19527.5352
OST48_HUMAN	105066.375
AN32A_HUMAN	30478.8867
FEN1_HUMAN	33159.3359

CUX1_HUMAN	329.831573
CAPG_HUMAN	20088.9844
CAP2_HUMAN	3570.55371
IL6RB_HUMAN	6068.25293
TXLNA_HUMAN	9711.11426
TCPZ_HUMAN	113518.766
NNMT_HUMAN	39860.8516
PSB10_HUMAN	1466.54736
PBX2_HUMAN	1019.79712
RL13A_HUMAN	133388.984
ARL1_HUMAN	24575.6074
MLH1_HUMAN	8778.54297
STAT3_HUMAN	7900.28857
UBP8_HUMAN	6496.7417
MDHC_HUMAN	68653.8438
MDHM_HUMAN	123231.781
RFC5_HUMAN	10686.7861
RFC3_HUMAN	4358.26465
ECHA_HUMAN	34820.8477
IF2G_HUMAN	39518.5938
ETV3_HUMAN	188.264023
CETN2_HUMAN	1336.47351
ETV6_HUMAN	1994.81396
EIF2D_HUMAN	4732.41699
BUD31_HUMAN	1188.61426
UBA7_HUMAN	651.257568
NAA10_HUMAN	18051.2461
KDM5C_HUMAN	340.598602
CSK_HUMAN	10899.0322
GARS_HUMAN	24818.7773
SYIC_HUMAN	49970.6328
S19A1_HUMAN	1517.87708
EIF1_HUMAN	4081.81323
KPCI_HUMAN	653.977295
ELK3_HUMAN	688.614929
ACTY_HUMAN	23074.9121
ECI1_HUMAN	6507.80908
LAP2A_HUMAN	10600.4053
LAP2B_HUMAN	93298.7734
STAT1_HUMAN	17259.7266
STAT6_HUMAN	5194.45605
STA5A_HUMAN	5818.05859
MTREX_HUMAN	13517.1914
AK1C3_HUMAN	4377.16553
PK3CA_HUMAN	1262.82727
PK3CB_HUMAN	1799.80396
MTOR_HUMAN	9060.02441
PI4KA_HUMAN	3730.82544
EPS15_HUMAN	7758.87939
CASP3_HUMAN	24499.793

CASP2_HUMAN	1599.58044
RS27_HUMAN	9339.83887
ABL2_HUMAN	925.520264
HELZ_HUMAN	1033.68042
CNDD3_HUMAN	1044.41406
RBM34_HUMAN	7818.33398
LPPRC_HUMAN	47940.1797
THIM_HUMAN	10539.5557
RL35_HUMAN	42947.9922
PCP_HUMAN	16615.9805
HD_HUMAN	3219.27905
ECE1_HUMAN	11663.2617
MTHR_HUMAN	309.349335
SATT_HUMAN	1069.8374
LIS1_HUMAN	19754.8652
MUC18_HUMAN	104770.078
CACP_HUMAN	3800.99414
MATR3_HUMAN	36237.2461
MSH2_HUMAN	12043.2422
GRK6_HUMAN	1155.76208
GPDM_HUMAN	8167.56396
SSRA_HUMAN	35206.8047
PTN9_HUMAN	1845.95703
RANG_HUMAN	51688.6602
NAMPT_HUMAN	91455.3828
PRS6B_HUMAN	31881.3008
EFTS_HUMAN	7423.35938
PPIC_HUMAN	10495.9268
VDAC2_HUMAN	99754.8047
ACDSB_HUMAN	3161.51147
CBX5_HUMAN	4390.92432
UBP5_HUMAN	36192.207
MK08_HUMAN	1373.35718
MP2K4_HUMAN	2896.37231
KI67_HUMAN	5726.69531
KPB2_HUMAN	387.779388
KPB1_HUMAN	1362.80676
RAGP1_HUMAN	28828.9258
RECQ1_HUMAN	21341.2832
NOP2_HUMAN	17839.834
ATRX_HUMAN	2263.73242
CRK_HUMAN	6554.02393
CRKL_HUMAN	7082.27197
IF2M_HUMAN	3171.26978
BAG6_HUMAN	5852.60205
NSF_HUMAN	21796.8926
NOTC1_HUMAN	2661.41626
MP2K3_HUMAN	5311.9209
BRCC3_HUMAN	2127.55249
RL27A_HUMAN	196929.484

RL5_HUMAN	52357.1719
RL21_HUMAN	46914.5586
RL28_HUMAN	44027.1992
RS9_HUMAN	233040.484
RS5_HUMAN	109762.969
RS10_HUMAN	70944.2109
MAP1B_HUMAN	34209.3828
GNPI1_HUMAN	14689.4893
NEDD4_HUMAN	2362.20557
UTRN_HUMAN	10018.0313
IQGA1_HUMAN	163709.031
GLYG_HUMAN	13446.4668
STT3A_HUMAN	48342.9609
PA24A_HUMAN	2661.20972
CAZA2_HUMAN	24190.7793
CAPZB_HUMAN	40164.7461
IF1AX_HUMAN	5387.94434
AL1A3_HUMAN	506.478058
SYQ_HUMAN	31543.8242
RL29_HUMAN	42044.7148
TISD_HUMAN	524.355896
UCRI_HUMAN	14314.5771
ATPO_HUMAN	54875.1719
LIMS1_HUMAN	14270.2051
GLIP1_HUMAN	985.685364
PPCE_HUMAN	21806.2305
MAOX_HUMAN	19439.2051
IREB2_HUMAN	2125.43799
TFPI2_HUMAN	723.989075
RFX5_HUMAN	2501.44263
PI42A_HUMAN	5031.08203
COPD_HUMAN	52673.3828
LSS_HUMAN	22487.3281
PP2BC_HUMAN	551.388855
GSH1_HUMAN	2106.28491
GSH0_HUMAN	13250.9805
CD151_HUMAN	12452.0957
TPC10_HUMAN	876.856079
PSMD8_HUMAN	36673.332
PRC2A_HUMAN	3168.01416
GSHB_HUMAN	15487.2129
TCPE_HUMAN	96665.7813
PTSS1_HUMAN	5088.49023
NEST_HUMAN	109888.93
HSP13_HUMAN	8019.06641
KC1A_HUMAN	10870.9111
KC1D_HUMAN	4321.55859
IDHP_HUMAN	63411.5391
PK3CG_HUMAN	2087.8457
PIPNB_HUMAN	18647.8262

DPOD2_HUMAN	4707.34473
MRP_HUMAN	13540.4395
PAXI_HUMAN	5061.10352
CAMLG_HUMAN	702.850159
NR2C2_HUMAN	923.122375
MAPK2_HUMAN	1847.8335
DNSL1_HUMAN	1479.69067
AL9A1_HUMAN	12493.6729
RL34_HUMAN	61377.7031
RPIA_HUMAN	2935.21021
LMAN1_HUMAN	18689.6914
NRAM2_HUMAN	828.494141
NASP_HUMAN	25529.4551
FAS_HUMAN	70057.0703
FNTA_HUMAN	10568.4443
FNTB_HUMAN	4698.80908
DHYS_HUMAN	2425.26245
TCPG_HUMAN	84647.8281
RM19_HUMAN	8203.90527
ARRB1_HUMAN	8296.27734
EFTU_HUMAN	90147.4453
AL7A1_HUMAN	18267.4375
UB2R1_HUMAN	4112.5083
INPP_HUMAN	4214.00049
CENPF_HUMAN	283.490784
SRP09_HUMAN	44434.0391
PCY1A_HUMAN	10532.833
SYAC_HUMAN	23604.6172
SYCC_HUMAN	15762.9854
SYHM_HUMAN	5970.24561
SYSC_HUMAN	30600.2715
PPM1F_HUMAN	22731.3066
MA2A2_HUMAN	1827.21973
PRI1_HUMAN	2044.30811
PRI2_HUMAN	1529.54919
CASP4_HUMAN	3048.34595
KC1E_HUMAN	621.194946
PSB3_HUMAN	31434.3438
PSB2_HUMAN	26263.2324
MCM2_HUMAN	18793.168
ACADV_HUMAN	8812.18164
YLPM1_HUMAN	6205.96777
VPS41_HUMAN	1159.21069
TMEDA_HUMAN	36973.6758
RBM25_HUMAN	10899.9736
NUMB_HUMAN	7304.97852
CLK3_HUMAN	1306.58252
PSN1_HUMAN	2135.9248
EI2BB_HUMAN	6252.52393
HINT1_HUMAN	5820.58545

NU153_HUMAN	6758.65625
RBP2_HUMAN	12284.1543
RGS19_HUMAN	1625.23889
RGS3_HUMAN	2084.38599
PSN2_HUMAN	379.199463
TSC2_HUMAN	3399.43091
NDUV1_HUMAN	8860.08594
GSK3A_HUMAN	2198.22998
GSK3B_HUMAN	7409.50977
TAF6_HUMAN	1000.0426
5NTC_HUMAN	2897.06592
SPS1_HUMAN	6157.85791
MTHFS_HUMAN	964.519104
GUAA_HUMAN	21984.4023
DNLI3_HUMAN	4737.78711
DNLI4_HUMAN	2513.67627
MRE11_HUMAN	5995.99609
GNAQ_HUMAN	7535.97217
IDH3A_HUMAN	12640.2168
ST1A1_HUMAN	1007.00909
MMP14_HUMAN	26703.2266
PPOX_HUMAN	1777.5166
GDIB_HUMAN	70080.2578
EMD_HUMAN	7627.07959
CPT1A_HUMAN	6536.43457
S26A2_HUMAN	883.197754
SPB8_HUMAN	13924.2246
SPB9_HUMAN	58266.2578
SERPH_HUMAN	93486.3828
PDLI4_HUMAN	10869.0654
F10A1_HUMAN	77027.7344
PEX5_HUMAN	1154.30261
ERF_HUMAN	1085.63281
VASP_HUMAN	10168.4307
DYN2_HUMAN	24866.1992
MAP2_HUMAN	4591.30176
AP4A_HUMAN	4142.43799
CDK7_HUMAN	4389.63525
KNTC1_HUMAN	2318.49365
RASF2_HUMAN	1894.87805
CDK9_HUMAN	4039.99048
LRBA_HUMAN	4196.93115
BCAM_HUMAN	3105.91919
PPT1_HUMAN	5003.20801
RL14_HUMAN	130026.891
TCPQ_HUMAN	181837.484
TCPD_HUMAN	49224.4961
ANX11_HUMAN	22465.9961
PAPOA_HUMAN	7354.2251
FXR1_HUMAN	19687.1309

FXR2_HUMAN	16037.5713
RAB5C_HUMAN	28332.1504
RAB7A_HUMAN	99059.0391
RAB9A_HUMAN	3221.46167
RAB13_HUMAN	11097.0107
RB27A_HUMAN	6694.90186
PLCD1_HUMAN	511.431274
RT29_HUMAN	6099.36768
DUS3_HUMAN	7457.94629
SMCA2_HUMAN	643.863586
SMCA4_HUMAN	7943.85498
IDH3G_HUMAN	2923.28711
GALK1_HUMAN	13652.3594
SSRD_HUMAN	43254.6602
BAP31_HUMAN	17710.9668
TPMT_HUMAN	3417.09546
MECP2_HUMAN	930.081726
HCFC1_HUMAN	15042.9033
IRAK1_HUMAN	938.316345
CAV2_HUMAN	1128.08105
AL3A2_HUMAN	3709.59717
DHB4_HUMAN	16210.9023
PSMD7_HUMAN	20760.4121
SUOX_HUMAN	2268.06543
SPHM_HUMAN	5588.73145
STA5B_HUMAN	1799.00806
UBP11_HUMAN	1292.92981
CLCN7_HUMAN	2193.15942
VAMP7_HUMAN	2486.66382
KS6A3_HUMAN	6102.89648
BMX_HUMAN	5987.58105
HDGF_HUMAN	15323.9463
CCNH_HUMAN	697.825256
MAT1_HUMAN	1433.76917
NEK3_HUMAN	629.974548
UB2E1_HUMAN	2695.96606
NDUA8_HUMAN	6383.59863
ROA3_HUMAN	42897.5039
6PGD_HUMAN	126234.391
HNRPM_HUMAN	92147.5859
IMA1_HUMAN	32369.8457
IMA5_HUMAN	4830.14697
NCBP2_HUMAN	3766.98486
GDS1_HUMAN	13525.7744
RPAB3_HUMAN	3379.90576
MP2K6_HUMAN	1821.09314
GDIR1_HUMAN	68361.4297
GDIR2_HUMAN	69371.6875
AGFG1_HUMAN	3567.81812
HNRPF_HUMAN	15812.4268

STAT2_HUMAN	2841.61499
MSH6_HUMAN	8744.40918
KIF11_HUMAN	5265.61035
VAV2_HUMAN	6739.40918
ZN143_HUMAN	868.55835
RBM5_HUMAN	4069.08374
RIDA_HUMAN	998.745056
SPSY_HUMAN	27249.4902
HXK2_HUMAN	33853.9492
RM12_HUMAN	15778.8818
DGKQ_HUMAN	184.483826
NDST1_HUMAN	936.858948
THOP1_HUMAN	13491.9063
CAZA1_HUMAN	100110.414
HMGA2_HUMAN	1335.27283
CRIP2_HUMAN	39214.6641
NUP98_HUMAN	7628.37305
BIEA_HUMAN	25941.5625
TXTP_HUMAN	16041.8887
PPP5_HUMAN	10878.7998
PLK1_HUMAN	3021.23877
DAPK1_HUMAN	1248.48718
ARFP2_HUMAN	2612.08447
ARFP1_HUMAN	6773.14893
NUBP1_HUMAN	3774.71118
ACLY_HUMAN	87190.3906
MAP11_HUMAN	9270.73926
SUCA_HUMAN	10445.9365
MVD1_HUMAN	5061.77295
PGTB1_HUMAN	2975.36548
PGTB2_HUMAN	5229.29004
COPB_HUMAN	29230.1367
COPA_HUMAN	58764.8281
CATC_HUMAN	7761.62744
LIMK1_HUMAN	7113.30762
CLH2_HUMAN	446.164612
AP3M2_HUMAN	903.612427
AP2S1_HUMAN	10624.8467
CCHL_HUMAN	6657.13086
MK12_HUMAN	1748.09045
PTTG_HUMAN	4593.35059
SMTN_HUMAN	3387.01563
MOT1_HUMAN	7530.48291
IST1_HUMAN	10632.3223
SC24C_HUMAN	19421.1055
TCP4_HUMAN	50683.7539
DPOG1_HUMAN	1381.56226
ICLN_HUMAN	8072.45557
SYRC_HUMAN	35724.5938
HIRA_HUMAN	1829.4917

ATX3_HUMAN	2690.11133
ATN1_HUMAN	994.004578
PMS1_HUMAN	323.612213
PMS2_HUMAN	579.259521
CA2D1_HUMAN	2115.13892
SYYC_HUMAN	39121.5703
UBP14_HUMAN	21620.8516
AAKG1_HUMAN	4233.53223
HSP72_HUMAN	8327.93652
BCAT1_HUMAN	23360.0371
AT1B3_HUMAN	26523.4219
RD23B_HUMAN	1407.79053
EPHB4_HUMAN	2935.85205
ANAG_HUMAN	1720.52686
KAD2_HUMAN	6276.08545
P5CS_HUMAN	19109.0977
SNAA_HUMAN	22605.8809
IF5_HUMAN	21238.043
S12A2_HUMAN	7311.85352
PSMD4_HUMAN	22134.4043
DRG2_HUMAN	7849.0542
XPO2_HUMAN	164017.969
TERA_HUMAN	148953.297
MFAP1_HUMAN	2009.74829
ECHB_HUMAN	56754.2891
MANF_HUMAN	16279.6533
AFAD_HUMAN	4259.2085
ELL_HUMAN	1103.88037
NP1L1_HUMAN	61366.8359
CASP7_HUMAN	6461.52344
CASP6_HUMAN	4310.42627
ADK_HUMAN	14013.168
DSRAD_HUMAN	12639.9824
LAMB2_HUMAN	3500.37769
CAD13_HUMAN	2229.46558
SEC13_HUMAN	17010.8457
NH2L1_HUMAN	12129.0859
PSA_HUMAN	31750.4844
ALR_HUMAN	518.537476
HNRH2_HUMAN	11095.5117
SCOT1_HUMAN	12393.3174
EIF3B_HUMAN	22498.4355
FCGRN_HUMAN	4852.90381
BID_HUMAN	1999.69385
ATPK_HUMAN	14268.2041
RRP1_HUMAN	6491.93701
SYMC_HUMAN	34151.7383
ARP19_HUMAN	6319.70654
AP1S2_HUMAN	3905.74023
ATP68_HUMAN	9614.81152

ATP5E_HUMAN	5303.76807
ATP5I_HUMAN	23603.3887
HDAC4_HUMAN	1513.6687
IF6_HUMAN	37675.3477
CTBP2_HUMAN	22376.9727
NDUA6_HUMAN	9488.02734
PEX3_HUMAN	278.052002
DHB7_HUMAN	1658.79968
BCAR1_HUMAN	8526.59766
STX17_HUMAN	1130.94861
CF298_HUMAN	644.294861
WDR4_HUMAN	2037.66479
TMM33_HUMAN	37381.3906
GEMI4_HUMAN	3913.19556
CORO7_HUMAN	5742.81934
NU107_HUMAN	9311.04199
GSDMD_HUMAN	8129.34961
SELB_HUMAN	2357.89771
SESN2_HUMAN	711.471069
ANTR2_HUMAN	1800.8446
MTPN_HUMAN	992.402283
ARPC4_HUMAN	117868.313
CD81_HUMAN	11004.8945
SC61G_HUMAN	2310.48853
TPIS_HUMAN	150634.859
EIF3E_HUMAN	33809.8672
SC61B_HUMAN	27063.8418
PTEN_HUMAN	401.864594
PP4C_HUMAN	9472.48242
GBRL2_HUMAN	14953.8418
ROMO1_HUMAN	3240.75952
MYL6_HUMAN	8302.00488
ACTB_HUMAN	1696413.75
IF4A1_HUMAN	194905.531
RS20_HUMAN	100736.68
PRPS1_HUMAN	6350.16748
PSA6_HUMAN	51266.5703
S10AA_HUMAN	25642.5352
CDC42_HUMAN	16539.5117
DEST_HUMAN	17831.7832
GMFB_HUMAN	3659.91406
RAB8A_HUMAN	4133.51318
SPCS3_HUMAN	19181.4355
SRP54_HUMAN	11353.4287
RAB4B_HUMAN	1716.03711
RAB2A_HUMAN	36771.2969
RAB5B_HUMAN	6445.83496
RAB10_HUMAN	6977.50781
UB2D3_HUMAN	15620.1582
UBC12_HUMAN	20255.668

UBE2K_HUMAN	24289.582
UBE2N_HUMAN	26373.0293
RAB14_HUMAN	24884.4043
ARP3_HUMAN	65871.2344
ARP2_HUMAN	54223.8789
ACTZ_HUMAN	20895.2441
CSN2_HUMAN	14526.0078
ABCE1_HUMAN	26836.7383
RAP1B_HUMAN	30891.9355
RAP2B_HUMAN	4531.71094
MAX_HUMAN	1691.46985
RS3A_HUMAN	103640.547
RL26_HUMAN	135620.906
PSME3_HUMAN	11177.4473
RL15_HUMAN	144346.313
RL27_HUMAN	187256.313
VA0D1_HUMAN	15830.6357
RL37A_HUMAN	9527.95117
RHOA_HUMAN	15651.459
RND3_HUMAN	922.919556
NAA20_HUMAN	2743.29736
CH10_HUMAN	11088.9775
S61A1_HUMAN	22672.8457
PFD3_HUMAN	9748.38965
STXB1_HUMAN	7665.21338
B2MG_HUMAN	547.291504
DAD1_HUMAN	22790.8965
NPC2_HUMAN	4733.7627
COPZ1_HUMAN	25654.3438
SUMO2_HUMAN	18752.9805
DCAF7_HUMAN	3080.04077
WDR5_HUMAN	2857.00391
AP1S1_HUMAN	8662.31543
NTF2_HUMAN	3755.1084
HNRPK_HUMAN	175116.828
1433G_HUMAN	54347.7031
UBP46_HUMAN	1695.38818
RRAS2_HUMAN	17256.6621
RS7_HUMAN	40976.5625
PP1A_HUMAN	22014.2578
PP1B_HUMAN	22912.3066
PRS4_HUMAN	41390.5781
PRS8_HUMAN	49960.2891
RS8_HUMAN	149527.813
RS15A_HUMAN	109588.891
RS16_HUMAN	204276.594
UB2G1_HUMAN	1670.94373
UBE2H_HUMAN	4836.53223
1433E_HUMAN	78542.5469
RS14_HUMAN	168224.203

RS23_HUMAN	51964.375
RS18_HUMAN	224863.453
RS29_HUMAN	24932.9609
RS13_HUMAN	106998.695
RS11_HUMAN	45722.1445
RUXE_HUMAN	12806.5137
RUXF_HUMAN	7748.57666
LSM3_HUMAN	2605.56421
LSM6_HUMAN	8707.4668
SMD1_HUMAN	17091.3516
SMD2_HUMAN	25842.4453
SMD3_HUMAN	51299.1094
TYB4_HUMAN	3981.53589
ARF6_HUMAN	20852.4629
PRS10_HUMAN	29403.2988
SELT_HUMAN	4172.146
TBPL1_HUMAN	147.953796
RL7A_HUMAN	120284.867
RPB7_HUMAN	2689.14648
RB11A_HUMAN	68815.4922
ERF1_HUMAN	27556.0449
CNBP_HUMAN	5925.9585
YPEL5_HUMAN	710.570129
RS4X_HUMAN	151997.625
PP2AB_HUMAN	33888.0391
ACTA_HUMAN	366883.938
RHOB_HUMAN	6426.8125
RL23A_HUMAN	145643.781
RS6_HUMAN	215296.266
H4_HUMAN	145490.594
RAB1A_HUMAN	9398.83105
RAN_HUMAN	293250.938
RL23_HUMAN	96938.6172
RAP1A_HUMAN	4417.76514
RS15_HUMAN	23757.9414
RS24_HUMAN	46510.2383
RS25_HUMAN	215617.438
RS26_HUMAN	60245.4766
RS28_HUMAN	33736.6875
RS30_HUMAN	51499.5156
GBB1_HUMAN	14074.1943
RBX1_HUMAN	11713.0361
GBB2_HUMAN	52174.9805
RL30_HUMAN	93423.2109
RL39_HUMAN	30235.6211
RL31_HUMAN	97512.6328
RL10A_HUMAN	81892.4844
RL32_HUMAN	33395.4414
RL11_HUMAN	84685.5625
RL8_HUMAN	82277.5938

PPIA_HUMAN	143063.375
FKB1A_HUMAN	30856.6855
RS27A_HUMAN	138222.313
GRB2_HUMAN	9534.60352
TRA2B_HUMAN	14685.9854
RAC1_HUMAN	73187.5391
AP2B1_HUMAN	30114.8594
VAMP2_HUMAN	9431.70313
GNAS2_HUMAN	14067.0635
GNAI1_HUMAN	689.290405
1433Z_HUMAN	211976.922
2ABA_HUMAN	31578.3086
SUMO1_HUMAN	19402.3242
DYL1_HUMAN	9655.80957
DYLT1_HUMAN	4087.30151
RL38_HUMAN	51743.6797
SKP1_HUMAN	3381.81616
RS21_HUMAN	13489.6826
IF5A1_HUMAN	74242.1797
RACK1_HUMAN	178825.203
SPT4H_HUMAN	2263.18848
UBC9_HUMAN	16211.5449
TYB10_HUMAN	4160.77197
PP2AA_HUMAN	3602.7168
YBOX1_HUMAN	37107.9531
SC11A_HUMAN	43419.3789
CSK2B_HUMAN	6648.78906
TPM4_HUMAN	86558.875
UB2L3_HUMAN	27949.0684
EF1A1_HUMAN	1265286.88
TBA1B_HUMAN	965781.75
TBA4A_HUMAN	598.529968
TBB4B_HUMAN	277365.063
CSK21_HUMAN	9998.16309
PA1B2_HUMAN	1265.26416
H31_HUMAN	11688.874
HBB_HUMAN	23569.9922
HBA_HUMAN	29320.6953
CXAR_HUMAN	1470.96594
F193A_HUMAN	2158.05762
NOP14_HUMAN	3603.54761
IGBP1_HUMAN	6414.45947
SHPS1_HUMAN	1139.29858
SERB_HUMAN	10316.252
RBM6_HUMAN	3480.52563
IF4G2_HUMAN	33976.4805
RPP38_HUMAN	2638.94067
RPP30_HUMAN	6743.23926
GTF2I_HUMAN	26137.9609
PI42B_HUMAN	1651.60327

CNTP1_HUMAN	4446.52441
SRPK2_HUMAN	2890.7041
TCPB_HUMAN	81543.9922
S35A2_HUMAN	513.935364
RAE1L_HUMAN	7897.79102
GSTO1_HUMAN	160769.766
PRKDC_HUMAN	46137.2305
ADA17_HUMAN	2310.98755
BL1S1_HUMAN	1338.64563
SRPX_HUMAN	2257.16406
ARGI2_HUMAN	1191.276
NTH_HUMAN	668.964783
IN35_HUMAN	608.9104
NUCB2_HUMAN	1750.18372
BASP1_HUMAN	11650.8564
DCD_HUMAN	252.450241
TMF1_HUMAN	2199.86938
RT22_HUMAN	9057.29688
RT25_HUMAN	1855.69031
RT10_HUMAN	4587.23047
RT35_HUMAN	1825.33569
RT05_HUMAN	3938.53369
KGD4_HUMAN	188.121445
RT11_HUMAN	4150.17627
RT15_HUMAN	7507.34766
RT21_HUMAN	3164.25708
RT34_HUMAN	17609.4316
RT06_HUMAN	4749.25537
RT09_HUMAN	4429.29785
SARNP_HUMAN	28047.9844
LACTB_HUMAN	2698.81836
COG7_HUMAN	4717.31396
RL24_HUMAN	100979.977
TXN4A_HUMAN	3046.52246
RL36A_HUMAN	15290.1367
CBX1_HUMAN	8765.39648
SMAD3_HUMAN	5041.19678
ARF1_HUMAN	108480.672
ARF5_HUMAN	9349.45508
ERH_HUMAN	1449.26147
RHOG_HUMAN	9967.88477
RL19_HUMAN	76261.7188
SERF2_HUMAN	2861.14282
SRSF3_HUMAN	97922.9531
FOXK1_HUMAN	6040.46582
CCZ1B_HUMAN	3087.80249
DAB2_HUMAN	13773.6084
PGBM_HUMAN	37417.625
XIAP_HUMAN	1185.2804
EFNB1_HUMAN	3286.94067

RBM10_HUMAN	5527.62891
RBM3_HUMAN	16307.4902
AT2C1_HUMAN	2552.62866
AT11A_HUMAN	1127.50073
CYC_HUMAN	49467.8984
EM55_HUMAN	2566.59912
TFAM_HUMAN	10537.043
PIPNA_HUMAN	8059.30127
S25A3_HUMAN	104951.594
VIGLN_HUMAN	33723.8125
TF2B_HUMAN	2101.7124
CDK6_HUMAN	391.421082
CDK5_HUMAN	3211.479
CDK17_HUMAN	9133.81641
PURA_HUMAN	4871.92529
BORG5_HUMAN	5264.29199
CLH1_HUMAN	91629.4375
HSF1_HUMAN	1512.00989
NFKB2_HUMAN	2344.75415
FKBP3_HUMAN	47216.6953
REEP5_HUMAN	11115.8496
DHSO_HUMAN	9405.02734
HNRPU_HUMAN	85372.6953
U2AF1_HUMAN	13989.8447
SPTB2_HUMAN	54230.7813
TIAR_HUMAN	9500.27148
SET_HUMAN	83058.3906
SRSF2_HUMAN	34726.3789
FOXK2_HUMAN	1144.73901
RELB_HUMAN	432.61557
GALK2_HUMAN	4049.98706
AMPD2_HUMAN	9703.25293
FABP5_HUMAN	89564.2344
CAP1_HUMAN	74748.2656
FLI1_HUMAN	2770.73901
HMCS1_HUMAN	15562.0527
IFM3_HUMAN	6240.37549
LAT1_HUMAN	2830.61157
NC2B_HUMAN	2263.30542
EXOSX_HUMAN	4366.10791
OTUD4_HUMAN	937.052979
PFKAP_HUMAN	48000.3789
XPC_HUMAN	913.165039
EWS_HUMAN	36045.1875
CDR2_HUMAN	4327.91846
OCRL_HUMAN	2495.88232
PLCB3_HUMAN	9358.39453
TAGL_HUMAN	5363.54102
AK17A_HUMAN	1804.02039
PYRD_HUMAN	254.031601

ODO1_HUMAN	16381.8984
CENPE_HUMAN	346.303314
KIF23_HUMAN	1033.26331
MMSA_HUMAN	3330.51025
NRG1_HUMAN	635.121216
CP27A_HUMAN	448.991058
DSG1_HUMAN	766.30188
SP3_HUMAN	578.689575
H11_HUMAN	1699.88269
RL18A_HUMAN	93483.5391
MP2K1_HUMAN	13371.6895
TIE2_HUMAN	2071.7627
FKBP4_HUMAN	25446.0684
PLOD1_HUMAN	17708.6719
NUCB1_HUMAN	7481.70605
RL6_HUMAN	258541.938
TOP2B_HUMAN	1743.14197
AKA12_HUMAN	40860.2891
M2OM_HUMAN	17396.2598
DYST_HUMAN	3191.84204
ENL_HUMAN	1509.48157
MECOM_HUMAN	1104.16479
GNA12_HUMAN	1056.29175
CAV1_HUMAN	73251.8672
ACY1_HUMAN	1474.72742
TNAP2_HUMAN	955.583557
LMNB2_HUMAN	31321.3691
PTPS_HUMAN	2471.32886
UPAR_HUMAN	1186.94104
KIME_HUMAN	5966.20557
ERCC6_HUMAN	230.001541
TAP1_HUMAN	2564.22803
TAP2_HUMAN	1606.9613
CEBPZ_HUMAN	4016.16089
ZNF92_HUMAN	1590.66199
TF65_HUMAN	9173.86328
UBXN1_HUMAN	10019.877
GLGB_HUMAN	24724.3457
IF4G1_HUMAN	31474.4258
NOTC2_HUMAN	1164.94495
TLE1_HUMAN	2180.29785
TLE3_HUMAN	1637.92981
LGUL_HUMAN	32602.4805
ACVR1_HUMAN	299.248535
SSBP_HUMAN	15688.2617
REL_HUMAN	769.033508
1433F_HUMAN	50474.707
PLP2_HUMAN	26749.5664
CSTF1_HUMAN	7809.35254
UBE3A_HUMAN	7031.84863

DYN1_HUMAN	819.153503
PTN12_HUMAN	5232.16797
FAK1_HUMAN	10134.375
SRS11_HUMAN	104799.344
KPCD_HUMAN	2908.21045
CALD1_HUMAN	40811.582
FOLC_HUMAN	3603.74121
CTSL2_HUMAN	960.990906
PTN11_HUMAN	8973.73633
KDSR_HUMAN	1951.91736
PUR1_HUMAN	7387.26758
GFPT1_HUMAN	19828.4844
EXOS9_HUMAN	4035.02295
PSME1_HUMAN	37744.5469
SUH_HUMAN	951.108521
MEF2C_HUMAN	363.221039
APLP2_HUMAN	6578.94287
GABPA_HUMAN	2420.18335
RING1_HUMAN	518.668701
FMR1_HUMAN	911.205811
PRDX1_HUMAN	198063.094
RL18_HUMAN	129426.211
C1QBP_HUMAN	32645.3086
CKAP4_HUMAN	115395.063
ZO1_HUMAN	14316.2275
TISB_HUMAN	1399.21716
SPAG1_HUMAN	999.035217
KHDR1_HUMAN	32535.7305
BAX_HUMAN	14820.1768
B2CL1_HUMAN	5981.22754
MCL1_HUMAN	1181.72815
DPOE1_HUMAN	3277.58594
KLC1_HUMAN	22260.707
SOS1_HUMAN	1247.25671
SRSF1_HUMAN	97813.8906
RHG01_HUMAN	15561.002
SRSF4_HUMAN	9007.8291
PCDH1_HUMAN	6554.53955
PP2BA_HUMAN	4639.01758
DHX9_HUMAN	64383.832
QOR_HUMAN	8383.70996
GOGA3_HUMAN	3157.87622
GOGA2_HUMAN	4192.39258
ECHP_HUMAN	676.684692
MFGM_HUMAN	3212.47827
PDE4D_HUMAN	968.572815
DSC1_HUMAN	1056.99707
HDHD1_HUMAN	2898.42261
TICN1_HUMAN	1837.65198
CD47_HUMAN	16499.5977

PPID_HUMAN	17030.8262
SSRP1_HUMAN	26098.5801
CAMP2_HUMAN	2739.17554
SPIR1_HUMAN	670.213074
SLFN5_HUMAN	3022.34741
VAC14_HUMAN	4735.45068
NSUN2_HUMAN	18499.1992
DMWD_HUMAN	149.918488
RBBP4_HUMAN	63728.5156
NCBP1_HUMAN	13642.2656
MGT5A_HUMAN	590.981079
EP300_HUMAN	3103.54248
AHNK_HUMAN	124661.938
FCHO2_HUMAN	1569.23584
ELP6_HUMAN	1331.29321
HSP7E_HUMAN	6913.89844
SCRN3_HUMAN	1108.97107
CGNL1_HUMAN	4122.86963
LTOR4_HUMAN	3666.83179
NEXN_HUMAN	4245.28662
MGAT2_HUMAN	6556.7334
GALT2_HUMAN	8457.79199
GALT1_HUMAN	10215.5635
AP1B1_HUMAN	37562.5898
CPSF1_HUMAN	8065.11377
BST2_HUMAN	342.465637
MPPA_HUMAN	8752.17969
SIA4A_HUMAN	615.823669
SCRN1_HUMAN	7779.48975
WASC5_HUMAN	6438.75244
NU160_HUMAN	7540.90137
SCAP_HUMAN	2614.85864
FOXO1_HUMAN	454.156433
TBL3_HUMAN	3222.58325
TF3C1_HUMAN	2634.2207
TWF1_HUMAN	16751.498
ASPH_HUMAN	12657.6328
TFCP2_HUMAN	2628.47681
AKP13_HUMAN	2343.89282
FBLN3_HUMAN	28460.6738
SNF5_HUMAN	3786.90405
BPTF_HUMAN	1232.41541
CDC20_HUMAN	2069.24048
FSTL1_HUMAN	9145.3877
STX4_HUMAN	2029.82092
GRSF1_HUMAN	9465.99805
M4K2_HUMAN	850.114868
MERTK_HUMAN	3140.23267
SFSWA_HUMAN	1305.97205
CHD3_HUMAN	766.454956

SF3A3_HUMAN	24305.5762
TP53B_HUMAN	6564.04688
TM115_HUMAN	2004.14795
IFRD2_HUMAN	1922.89941
TRI26_HUMAN	1298.30383
AIMP1_HUMAN	14124.2051
ILF2_HUMAN	78609.2813
ILF3_HUMAN	84421.2344
LMAN2_HUMAN	47054.1914
TRAP1_HUMAN	388336.875
TRAF2_HUMAN	4378.13232
DLG1_HUMAN	2617.9624
MYO1E_HUMAN	9432.3457
PP1R8_HUMAN	5121.33545
TP4A2_HUMAN	4637.52686
ABR_HUMAN	4711.75635
NPRL3_HUMAN	699.90509
SEC20_HUMAN	5042.59521
BNIP2_HUMAN	1035.59949
NFX1_HUMAN	238.292664
CSTF3_HUMAN	6345.69043
TSN31_HUMAN	2002.1416
ECH1_HUMAN	7717.90625
RHG05_HUMAN	922.190552
STRN3_HUMAN	4949.2207
CDC16_HUMAN	3102.69922
STK4_HUMAN	2850.67407
FLII_HUMAN	23896.7813
TRI32_HUMAN	1426.96216
COASY_HUMAN	3771.98975
RM28_HUMAN	4673.24316
ACACA_HUMAN	13189.5137
CSN1_HUMAN	15237.8027
UBP4_HUMAN	3628.55127
CAF1A_HUMAN	496.116333
CAF1B_HUMAN	200.829819
RED_HUMAN	10488.9023
MTAP_HUMAN	11191.627
AAPK1_HUMAN	3986.34448
LIPA1_HUMAN	2064.69092
CACO2_HUMAN	481.233795
EI2BE_HUMAN	19659.2617
TADBP_HUMAN	16097.2275
ROA0_HUMAN	18483.502
AIMP2_HUMAN	10568.5674
FADD_HUMAN	4984.22314
PRDX4_HUMAN	45178.8867
MK07_HUMAN	373.029205
PAK2_HUMAN	13498
CBX3_HUMAN	25131.8828

STK3_HUMAN	766.916931
STX5_HUMAN	3553.16553
PSMD2_HUMAN	37502.1563
MMRN1_HUMAN	5073.20313
DDX10_HUMAN	1147.25012
DNJC3_HUMAN	21131.2168
SBP1_HUMAN	3054.22656
NDK3_HUMAN	3277.16357
SRSF9_HUMAN	19846.459
SRSF5_HUMAN	5255.96533
SRSF6_HUMAN	75911.6797
MD2L1_HUMAN	11319.9092
TIF1B_HUMAN	35509.7305
SEM3F_HUMAN	429.107941
STX3_HUMAN	1581.5376
G3BP1_HUMAN	38835.7227
NMI_HUMAN	3451.96826
PTK7_HUMAN	2924.56909
PABP4_HUMAN	30150.9492
ATM_HUMAN	1218.84839
GRB10_HUMAN	2676.92847
IFIT5_HUMAN	939.694153
MTA1_HUMAN	3480.75537
EIF3I_HUMAN	19482.3887
PPIL2_HUMAN	1140.37549
2A5G_HUMAN	1200.3811
CTBP1_HUMAN	5729.50146
PHLP_HUMAN	5915.83203
PLD1_HUMAN	2802.13721
TARB1_HUMAN	368.371063
UB2V1_HUMAN	37789.8984
RM49_HUMAN	971.817688
DC1I2_HUMAN	21934.4629
ORC2_HUMAN	1050.65771
ILK_HUMAN	28935.7559
NNTM_HUMAN	19764.4277
SNTB2_HUMAN	15509.251
XRCC4_HUMAN	4301.57959
PPIG_HUMAN	7174.71924
TCOF_HUMAN	12432.5488
U119A_HUMAN	961.492371
SF3B2_HUMAN	23010.7656
OS9_HUMAN	1599.30322
GOGA4_HUMAN	2555.41309
HAP28_HUMAN	18295.2402
ADAM9_HUMAN	11889.7949
ADA15_HUMAN	3388.7937
TMED1_HUMAN	3505.31201
FKBP5_HUMAN	22811.6406
MYO9B_HUMAN	5280.61328

ROCK1_HUMAN	7702.51367
NFAC2_HUMAN	670.514282
TOP3A_HUMAN	1162.68738
SMAD4_HUMAN	4550.05176
VPP3_HUMAN	6010.1167
BIRC2_HUMAN	809.187439
PICAL_HUMAN	9316.16602
MTM1_HUMAN	2886.22656
SQSTM_HUMAN	6703.87891
MTX1_HUMAN	3672.76563
TBB3_HUMAN	2674.92603
ASAH1_HUMAN	197.349731
PRP4B_HUMAN	3302.89673
PIN1_HUMAN	7814.31934
ATR_HUMAN	1516.29541
4EBP1_HUMAN	397.526398
RIPK1_HUMAN	1356.71082
HDAC1_HUMAN	17715.8066
KCC2G_HUMAN	4316.94287
KCC2D_HUMAN	4172.10059
DCTN2_HUMAN	6172.11768
PKD2_HUMAN	306.43399
ULA1_HUMAN	8428.99219
ITPK1_HUMAN	1385.4751
SNW1_HUMAN	8824.37793
DGKZ_HUMAN	3055.17163
STIM1_HUMAN	2604.86548
GRAP_HUMAN	3262.03198
TRA2A_HUMAN	4617.54932
SNX1_HUMAN	11935.6465
KRR1_HUMAN	5519.32764
PWP1_HUMAN	5273.1875
MTMR1_HUMAN	1248.28711
MTMR2_HUMAN	5309.54785
CUL1_HUMAN	5512.24854
CUL2_HUMAN	12030.0771
CUL3_HUMAN	6605.44189
CUL4A_HUMAN	4673.90332
CUL4B_HUMAN	16855.1641
ASPP2_HUMAN	365.099426
DYR1A_HUMAN	751.423767
FCL_HUMAN	13145.9844
RAB31_HUMAN	158.985916
RAB32_HUMAN	10939.1309
TPBG_HUMAN	1454.31873
FHL1_HUMAN	11454.5449
FHL3_HUMAN	2375.30225
RIN1_HUMAN	660.450195
AAMP_HUMAN	3000.87769
MOGS_HUMAN	32426.6035

CD166_HUMAN	6485.11914
THOC5_HUMAN	2457.98022
SHRM2_HUMAN	1148.55334
SPTN1_HUMAN	33008.207
NOG2_HUMAN	4444.64502
AUHM_HUMAN	794.29834
PKP1_HUMAN	181.197693
DX39B_HUMAN	14601.0723
BLMH_HUMAN	24854.832
EXOS2_HUMAN	4653.64502
BMPR2_HUMAN	1789.37891
TBB2A_HUMAN	152954.281
TF2H2_HUMAN	2524.20239
TF2H3_HUMAN	2441.36499
BYST_HUMAN	11794.5322
RPGF1_HUMAN	2291.14014
IDI1_HUMAN	10129.3145
PEBB_HUMAN	9047.13379
NFYC_HUMAN	733.106079
CDK13_HUMAN	1339.85095
CKAP5_HUMAN	28188.875
CIRBP_HUMAN	5127.9126
KCC1A_HUMAN	1589.38757
COTL1_HUMAN	35201.8828
HNRPD_HUMAN	148147.859
SCRB2_HUMAN	27310.5703
NID2_HUMAN	796.056396
LRP8_HUMAN	805.099854
DAG1_HUMAN	3575.09985
VEZF1_HUMAN	311.789398
BOP1_HUMAN	8241.44824
UBE4A_HUMAN	3568.88672
SEPT6_HUMAN	4604.07715
KEAP1_HUMAN	2321.6084
URB2_HUMAN	1915.80701
DHX34_HUMAN	601.278992
MORC3_HUMAN	1306.58093
SAFB2_HUMAN	10850.8965
EIF3A_HUMAN	44002.1953
ARHG7_HUMAN	6809.87891
EFR3A_HUMAN	4684.48535
UBP2L_HUMAN	17112.2949
SCRIB_HUMAN	5669.02051
GIT2_HUMAN	2932.75781
SREC_HUMAN	3291.50171
MLEC_HUMAN	21290.3105
TTL12_HUMAN	9840.1377
DPOA2_HUMAN	2430.46973
DOCK1_HUMAN	5211.69336
TFDP1_HUMAN	2115.4707

FHL2_HUMAN	21976.793
DPYL3_HUMAN	57491.7344
ICT1_HUMAN	8650.79102
ZMYM3_HUMAN	435.173615
DCTN1_HUMAN	14050.1133
DYHC1_HUMAN	72171.8984
EI2BA_HUMAN	8935.99414
IF4A2_HUMAN	8794.65332
ELOA1_HUMAN	1791.48035
SRC8_HUMAN	19374.4004
NUCG_HUMAN	3049.41699
FLOT2_HUMAN	3706.43311
RCN2_HUMAN	11159.9385
TRI25_HUMAN	16519.6074
FLNC_HUMAN	54363.875
FKBP8_HUMAN	4671.57715
FA50A_HUMAN	6114.30029
FRG1_HUMAN	2197.70557
GNA13_HUMAN	48478.3984
GAMT_HUMAN	1389.22229
GALE_HUMAN	9526.99414
LRC32_HUMAN	334.995422
CAPR1_HUMAN	21513.0586
BECN1_HUMAN	889.494324
RBM39_HUMAN	17380.3379
CASL_HUMAN	579.523376
HLTF_HUMAN	1276.9054
ERG1_HUMAN	2971.14014
PDIA5_HUMAN	13068.2559
KPRA_HUMAN	7906.1665
DHX8_HUMAN	2362.03687
MCM6_HUMAN	19038.2383
ITPR2_HUMAN	4468.28662
ITPR3_HUMAN	5079.521
ITPR1_HUMAN	576.370911
RASA3_HUMAN	3263.30493
PLSI_HUMAN	2675.7334
IRF3_HUMAN	3577.78809
LAGE3_HUMAN	896.184509
BLTP2_HUMAN	446.681458
TRIPC_HUMAN	5491.33984
PUM1_HUMAN	4395.26611
MDC1_HUMAN	590.230713
EPN4_HUMAN	11675.1914
KANK1_HUMAN	4167.99414
SMC1A_HUMAN	69132.5703
RRP1B_HUMAN	5257.71094
DIP2A_HUMAN	7011.30225
RRP5_HUMAN	8081.38086
PSF1_HUMAN	2769.8147

BMS1_HUMAN	4759.1665
LPIN1_HUMAN	2276.91284
UBP10_HUMAN	10448.0791
MESD_HUMAN	10406.4971
GANAB_HUMAN	55977.293
RFTN1_HUMAN	3959.76978
MFS10_HUMAN	1825.47766
2A5D_HUMAN	13193.1426
LBR_HUMAN	16568.5059
COG2_HUMAN	669.625122
MVP_HUMAN	88898.8516
LTBP1_HUMAN	930.403198
LTBP2_HUMAN	2405.79224
CBX2_HUMAN	234.386963
GOGB1_HUMAN	3487.11475
CASP8_HUMAN	2429.00024
NAA30_HUMAN	2592.17969
KIF22_HUMAN	250.525131
MEF2D_HUMAN	1526.35071
CHD4_HUMAN	17590.1855
LASP1_HUMAN	40088.5117
PTGR1_HUMAN	10943.876
NC2A_HUMAN	4858.13281
NFAC4_HUMAN	345.415466
ZN638_HUMAN	4292.896
IMB1_HUMAN	54144.9102
NOLC1_HUMAN	5240.01318
NUMA1_HUMAN	10452.2041
PSME4_HUMAN	8409.3125
CUL7_HUMAN	1290.26038
SLMAP_HUMAN	785.700378
GAPD1_HUMAN	10850.3389
NAA25_HUMAN	7713.40137
MELT_HUMAN	1870.11963
CND2_HUMAN	6495.61328
PAF15_HUMAN	344.941681
SPCS2_HUMAN	19578.9453
EMC2_HUMAN	5477.62109
FL2D_HUMAN	1242.39795
PSMD6_HUMAN	16226.5635
MD2BP_HUMAN	1930.32227
MO4L2_HUMAN	4651.73975
ABRX2_HUMAN	2432.63818
SEPT2_HUMAN	40177.2734
SART3_HUMAN	13843.1865
CND1_HUMAN	12625.4131
SUZ12_HUMAN	1143.95581
EXOS7_HUMAN	3767.677
TNIP1_HUMAN	1113.97668
U5S1_HUMAN	71206.3047

SYLM_HUMAN	3585.08789
R3HD1_HUMAN	1555.48108
TRAM2_HUMAN	1286.43262
SNX17_HUMAN	4442.85986
AR6P1_HUMAN	5640.83008
RB3GP_HUMAN	4297.17969
S39AE_HUMAN	3199.67432
SYK_HUMAN	36405.5625
SETB1_HUMAN	978.115234
LRC14_HUMAN	713.347961
RRS1_HUMAN	8401.14746
DPOD3_HUMAN	2276.78027
IF4H_HUMAN	31464.2617
ACAP2_HUMAN	4369.84912
KIF14_HUMAN	365.111908
BRD3_HUMAN	1515.41638
WDR43_HUMAN	4394.23975
ACOX1_HUMAN	7160.56689
OXA1L_HUMAN	8128.90869
EEA1_HUMAN	13882.9404
PDIA6_HUMAN	99015.5859
PA1B3_HUMAN	4507.58691
PDK3_HUMAN	673.615784
PEA15_HUMAN	3449.79541
EBP_HUMAN	12926.0273
PMVK_HUMAN	5823.93018
KPCD1_HUMAN	225.524506
PLEC_HUMAN	73594.8438
PCM1_HUMAN	2496.36938
NOMO1_HUMAN	27031.4805
PON2_HUMAN	6958.45068
2A5A_HUMAN	1541.70459
IPYR_HUMAN	50235.8516
TEBP_HUMAN	17622.8457
STK38_HUMAN	2622.99072
NONO_HUMAN	54517.5977
PTPA_HUMAN	22588.416
PTPRK_HUMAN	3985.38818
PWP2_HUMAN	4709.84912
RABE1_HUMAN	1408.10925
RAB35_HUMAN	7821.73682
RNPS1_HUMAN	7356.86768
RBBP5_HUMAN	2500.45898
RCN1_HUMAN	20113.7559
RBP1_HUMAN	438.866852
L2GL1_HUMAN	2855.64258
LRC41_HUMAN	809.926697
TTF1_HUMAN	7418.05176
TMED2_HUMAN	5730.03906
PCBP1_HUMAN	160618.234

PCBP2_HUMAN	162656.844
ELOC_HUMAN	3314.5498
ELOB_HUMAN	2132.35913
RHEB_HUMAN	12014.5498
UBE3C_HUMAN	4023.7373
TOM20_HUMAN	9379.92578
MTFR1_HUMAN	673.81958
DHC24_HUMAN	17570.6797
SF3B3_HUMAN	27242.7695
PUM3_HUMAN	4546.61475
DLGP5_HUMAN	3847.47168
RSU1_HUMAN	42267.2813
CNN3_HUMAN	21191.623
KS6A1_HUMAN	853.060181
SAFB1_HUMAN	9638.52148
SF3A2_HUMAN	6809.20947
RBMS2_HUMAN	6286.36523
PP1R7_HUMAN	15634.2441
SC23A_HUMAN	21431.0918
SC23B_HUMAN	4457.2207
CYH1_HUMAN	4294.29785
SF3A1_HUMAN	15059.4072
SKI2_HUMAN	7645.6499
CDSN_HUMAN	223.715134
SURF1_HUMAN	1457.10413
TAF5_HUMAN	385.88089
TAF7_HUMAN	1858.94519
TERF2_HUMAN	1454.1571
MARE2_HUMAN	12417.9834
NHRF2_HUMAN	3601.07251
TRADD_HUMAN	2757.73779
TRAM1_HUMAN	9365.28711
TSN_HUMAN	10628.0049
TRBP2_HUMAN	1284.90527
SF01_HUMAN	15945.5967
CIP4_HUMAN	7308.0918
TRIPB_HUMAN	2906.25122
PCH2_HUMAN	9503.20801
MED1_HUMAN	1704.47009
TRIP4_HUMAN	4061.13672
HMGN3_HUMAN	162.821487
JHD2C_HUMAN	342.034149
IKBB_HUMAN	2196.52295
TRIP6_HUMAN	4959.31396
PTN14_HUMAN	2333.48999
MARE1_HUMAN	52266.3398
ELAV1_HUMAN	55465.8984
NSDHL_HUMAN	16266.5703
NAB2_HUMAN	237.584961
MYLK_HUMAN	2234.02539

TAB1_HUMAN	3654.86475
HERC1_HUMAN	1905.99438
AAAT_HUMAN	9042.08105
MLF2_HUMAN	756.687622
TOM34_HUMAN	6090.93457
SMAD2_HUMAN	4776.28955
SMAD1_HUMAN	18334.5449
MSMO1_HUMAN	7320.69629
ITSN1_HUMAN	2442.83862
TBCE_HUMAN	4501.25244
TBCC_HUMAN	5670.86523
UB2V2_HUMAN	1736.76526
STK11_HUMAN	1011.33386
STXB2_HUMAN	4607.40771
VAMP3_HUMAN	5541.0835
NEDD8_HUMAN	6195.24316
VAS1_HUMAN	5894.51611
VPS72_HUMAN	1093.15796
RB11B_HUMAN	14777.7686
ZYX_HUMAN	7908.65381
ETFD_HUMAN	1433.59241
SEPT7_HUMAN	31248.6992
ADRM1_HUMAN	18100.7793
CCDC6_HUMAN	5461.84473
UAP1_HUMAN	22990.5449
E2F4_HUMAN	680.4729
IBP7_HUMAN	10308.1855
PDCD2_HUMAN	890.85022
LAMA4_HUMAN	8569.12988
PSMD5_HUMAN	22482.793
PKN1_HUMAN	7460.99756
PKN2_HUMAN	7988.38574
CSRP2_HUMAN	1358.48108
DDB1_HUMAN	41263.7188
2A5E_HUMAN	4902.27588
MK14_HUMAN	5005.20898
RM23_HUMAN	1100.44006
CDC37_HUMAN	34202.4727
DPYL2_HUMAN	63100.6328
SYPL1_HUMAN	11863.25
RBBP7_HUMAN	8603.76855
M3K11_HUMAN	747.383301
TAF9_HUMAN	3001.2373
CALRL_HUMAN	5634.66406
BAK_HUMAN	526.485413
SRSF7_HUMAN	38429.3828
CPSF6_HUMAN	18500.2129
SMN_HUMAN	3183.75146
DREB_HUMAN	15599.0186
NRF1_HUMAN	2133.68335

FSCN1_HUMAN	142629.219
IF16_HUMAN	30559.9492
DECR_HUMAN	26435.7324
MA2A1_HUMAN	7581.90137
NDUA5_HUMAN	1474.49585
CEGT_HUMAN	1533.18896
CLPP_HUMAN	3788.00977
THTR_HUMAN	11211.4658
UBE2S_HUMAN	1722.9032
KAT1_HUMAN	760.926453
KGUA_HUMAN	2754.30542
GLO2_HUMAN	8100.88232
NDUA9_HUMAN	7607.54492
MAON_HUMAN	2269.48975
PCKGM_HUMAN	2574.23364
UPP1_HUMAN	1943.90881
DDR2_HUMAN	2186.33643
HCDH_HUMAN	10423.4072
CP51A_HUMAN	17841.668
UGPA_HUMAN	29419.8086
F263_HUMAN	1753.64612
TRXR1_HUMAN	52640.3164
MIC60_HUMAN	17666.9707
ECSCR_HUMAN	2440.72656
KNOP1_HUMAN	1144.25928
HNRL2_HUMAN	17534.0176
T132A_HUMAN	197.907043
INF2_HUMAN	5946.06689
PDS5A_HUMAN	7181.2959
CL16A_HUMAN	1046.13586
KIF7_HUMAN	525.71814
RHG31_HUMAN	2789.94092
MTHSD_HUMAN	735.99176
AAK1_HUMAN	4140.89551
WASC4_HUMAN	4654.05225
ERC6L_HUMAN	1804.95361
TSR1_HUMAN	7001.5376
RGPA2_HUMAN	996.618286
D19L1_HUMAN	5319.36182
IAH1_HUMAN	1324.47144
ALG11_HUMAN	1794.15295
QRIC1_HUMAN	3618.69849
SMU1_HUMAN	12122.5889
CTU2_HUMAN	7841.64941
PGGHG_HUMAN	816.229065
LRRF1_HUMAN	4887.37549
P3H1_HUMAN	17449.6777
TRM5_HUMAN	3232.82056
EMAL3_HUMAN	3790.97461
RPA43_HUMAN	2603.57129

MA7D1_HUMAN	3409.03906
UAP1L_HUMAN	11082.3691
CHD9_HUMAN	4528.94482
TKFC_HUMAN	1718.98938
LSM12_HUMAN	7807.11572
HSDL1_HUMAN	5462.8208
AL1L2_HUMAN	788.147522
GNPTA_HUMAN	728.675598
GRDN_HUMAN	1734.29138
RABL6_HUMAN	6765.71631
TIM50_HUMAN	5625.33105
PAR14_HUMAN	2371.20581
FUT11_HUMAN	532.128479
GLYR1_HUMAN	7413.41943
CE022_HUMAN	1882.33423
C2CD3_HUMAN	115.742615
VP26B_HUMAN	5159.31348
LARP7_HUMAN	3026.50684
NAKD2_HUMAN	791.26886
KCD21_HUMAN	2027.95654
GXLT1_HUMAN	605.681702
ACSF3_HUMAN	1100.68408
PPCEL_HUMAN	2928.57617
TB10B_HUMAN	2743.84204
TPRN_HUMAN	273.970825
ANO6_HUMAN	4004.55078
FIL1L_HUMAN	2079.65869
GRAP1_HUMAN	2543.39014
ZNG1F_HUMAN	966.946167
MIX23_HUMAN	3253.77539
MBRL_HUMAN	1309.75964
FA98B_HUMAN	6109.44385
RHG29_HUMAN	7545.92578
PDCD4_HUMAN	6594.21582
FND3B_HUMAN	7152.4126
CRTC2_HUMAN	246.781143
GPAT3_HUMAN	1328.71423
CEP55_HUMAN	2623.22583
NCBP3_HUMAN	2746.53784
QORX_HUMAN	19158.584
NACHO_HUMAN	2606.06982
PHLA2_HUMAN	2267.30859
PDLI3_HUMAN	599.928833
PAR10_HUMAN	702.591125
DHB12_HUMAN	24037.3164
GLE1_HUMAN	2866.07275
UBP39_HUMAN	5810.25049
KLH22_HUMAN	1368.52991
AGK_HUMAN	11288.8008
LACB2_HUMAN	1914.4873

P5CR3_HUMAN	2876.54932
EIPR1_HUMAN	4631.94238
BOREA_HUMAN	416.281281
COBL1_HUMAN	1043.07507
H1BP3_HUMAN	3481.30737
CYBR1_HUMAN	1053.9884
WDR81_HUMAN	2977.72559
ACTBL_HUMAN	253422.375
CCD93_HUMAN	3729.56689
OCAD2_HUMAN	11947.1152
PKHG4_HUMAN	1005.70416
HS904_HUMAN	319.365784
DCAF6_HUMAN	628.747314
TM41B_HUMAN	365.782532
SGMR2_HUMAN	9444.33496
YIF1B_HUMAN	3407.73779
ZN326_HUMAN	4164.52539
NOM1_HUMAN	1583.13831
RIPL1_HUMAN	1335.30737
DJC21_HUMAN	1378.25378
TP8L3_HUMAN	5629.22559
HERC4_HUMAN	6925.16992
PIGG_HUMAN	931.499146
PP6R3_HUMAN	6298.62695
RABL3_HUMAN	22865.3574
FA76B_HUMAN	352.46933
COQ5_HUMAN	440.865753
SH319_HUMAN	3603.6167
NHSL2_HUMAN	1840.80273
EMC4_HUMAN	3969.2334
SYEM_HUMAN	2280.61108
CC038_HUMAN	702.568848
EFMT2_HUMAN	476.635254
TGO1_HUMAN	1933.84143
PREP_HUMAN	13119.75
WDR44_HUMAN	2947.68311
PRC2B_HUMAN	3255.02002
TJAP1_HUMAN	1316.28735
RRP12_HUMAN	11572.6162
COA6_HUMAN	593.187378
TOIP1_HUMAN	14328.3564
CEP78_HUMAN	253.737411
SYAM_HUMAN	3791.60718
TOR2A_HUMAN	494.874695
PCID2_HUMAN	3543.41748
HABP4_HUMAN	1267.97363
S27A3_HUMAN	540.801514
SAMD9_HUMAN	1320.01636
P4R3B_HUMAN	2607.74609
WIPI3_HUMAN	1980.30164

WIPI1_HUMAN	1146.00562
EOGT_HUMAN	9645.55566
SMAG2_HUMAN	577.739197
TBCEL_HUMAN	2539.51367
TDIF2_HUMAN	2285.88794
RBG1L_HUMAN	1981.88892
TTC38_HUMAN	1021.34045
COX20_HUMAN	4275.72021
EXOS6_HUMAN	5695.04883
TM201_HUMAN	3052.60669
ATAT_HUMAN	397.340057
SNP47_HUMAN	714.580627
NU188_HUMAN	5778.21533
HP1B3_HUMAN	38066.7734
SYVM_HUMAN	566.125061
CE170_HUMAN	7470.67529
ARH_HUMAN	669.089722
ODR4_HUMAN	3485.73706
NOL9_HUMAN	3838.42676
NHSL1_HUMAN	2346.78296
HYI_HUMAN	533.171387
TPRGL_HUMAN	758.687439
C2D1B_HUMAN	1590.41724
FBP1L_HUMAN	15082.25
SYRM_HUMAN	680.904602
THEM4_HUMAN	1399.22314
CHCH9_HUMAN	732.910095
FKB15_HUMAN	13590.5625
ZC3HD_HUMAN	1961.72363
CI114_HUMAN	1528.04517
ARMD3_HUMAN	670.49408
CSCL2_HUMAN	2133.25317
GPTC4_HUMAN	313.66748
CAF17_HUMAN	1956.54065
HECD3_HUMAN	3770.20703
GT253_HUMAN	1140.70532
UBR4_HUMAN	11783.5537
STXB5_HUMAN	1381.95459
RHG21_HUMAN	1304.78088
CAMP1_HUMAN	1376.32947
RM02_HUMAN	3922.0437
UBAP2_HUMAN	4541.91895
QNG1_HUMAN	1147.86816
KPRP_HUMAN	1065.59668
ZN618_HUMAN	3144.90015
ACBD5_HUMAN	5881.03125
RBM26_HUMAN	4076.84619
ATD3B_HUMAN	2292.50781
WLS_HUMAN	7469.77441
INT11_HUMAN	2220.69141

CPTP_HUMAN	473.380157
DCAF8_HUMAN	3484.66748
CODA1_HUMAN	903.5625
TUT4_HUMAN	8796.64941
FRY_HUMAN	719.169373
RNH2B_HUMAN	1111.49353
ATPF1_HUMAN	1060.91882
RC3H1_HUMAN	951.496582
DDI2_HUMAN	4231.7334
THMS2_HUMAN	293.389862
NDUF5_HUMAN	1035.86353
NT5D1_HUMAN	2990.82153
BIG3_HUMAN	1133.9635
VP13D_HUMAN	534.560059
CROCC_HUMAN	927.427979
LYRM7_HUMAN	1593.979
RAIN_HUMAN	21507.2129
RIF1_HUMAN	2614.99536
VPS53_HUMAN	4720.35889
STRP1_HUMAN	3995.53198
MRCKA_HUMAN	6228.82031
RPRD2_HUMAN	1003.72156
RN220_HUMAN	287.440125
PR38B_HUMAN	3838.65845
BRE1A_HUMAN	3634.72949
MYOME_HUMAN	725.043823
ZN318_HUMAN	588.824402
F1711_HUMAN	1474.65186
DIK1B_HUMAN	3039.37012
LRCH2_HUMAN	1092.71411
CDKAL_HUMAN	4281.7959
BROX_HUMAN	4572.93994
FOCAD_HUMAN	7220.5835
RSBN1_HUMAN	223.140137
DAB2P_HUMAN	478.569092
LYPL1_HUMAN	6158.51807
PEAR1_HUMAN	1673.80237
ECM29_HUMAN	14597.6084
TUT7_HUMAN	843.269287
SHE_HUMAN	567.542114
DEN4C_HUMAN	2331.40576
NAA35_HUMAN	4420.91211
ZMYM4_HUMAN	2706.14502
FHI2A_HUMAN	4092.1543
ZDH20_HUMAN	2695.08691
SPRY7_HUMAN	946.660706
FBXW9_HUMAN	300.767639
CD276_HUMAN	2426.22656
RN213_HUMAN	4378.31982
TENS2_HUMAN	1317.92017

KANK2_HUMAN	623.859192
WAC2A_HUMAN	1685.81873
LPCT4_HUMAN	731.944153
STEA3_HUMAN	1633.25378
F91A1_HUMAN	4322.12256
TBC9B_HUMAN	6422.00342
MAP1S_HUMAN	9729.31543
2ABD_HUMAN	2681.80664
AR6P4_HUMAN	5006.86475
A16L1_HUMAN	2702.26196
HGNAT_HUMAN	1553.50757
ARID2_HUMAN	527.229614
UTP25_HUMAN	2283.59302
GL8D1_HUMAN	1346.33618
SE1L3_HUMAN	423.468719
TENS3_HUMAN	4583.25488
HAUS3_HUMAN	789.452759
SPT2_HUMAN	1558.88538
MBLC2_HUMAN	1704.61267
CRBG3_HUMAN	697.469849
INT3_HUMAN	3855.51099
RHG17_HUMAN	17381.3633
C19L1_HUMAN	8306.81934
VIR_HUMAN	3942.16699
CYTSA_HUMAN	5159.18848
HEAT6_HUMAN	748.348083
ANR40_HUMAN	226.674454
WWC2_HUMAN	1953.98779
ATLA3_HUMAN	20203.0273
RL7L_HUMAN	21318.1445
NXN_HUMAN	686.602539
TTC19_HUMAN	1025.87659
IQEC1_HUMAN	856.810303
CPIN1_HUMAN	12213.7979
ZFAN6_HUMAN	1414.82996
SMYD5_HUMAN	2609.16943
OTU7B_HUMAN	915.733093
THOC7_HUMAN	1304.59082
NADE_HUMAN	2926.18506
ELP2_HUMAN	2634.29175
LTOR1_HUMAN	2521.83252
DRS7B_HUMAN	1574.93616
TWF2_HUMAN	15913.9473
CNDH2_HUMAN	518.72644
TNG2_HUMAN	7735.64453
P4R3A_HUMAN	3249.6582
RAB12_HUMAN	23640.1289
DEN5A_HUMAN	83.0271835
K2C80_HUMAN	802.527893
NIPBL_HUMAN	488.121094

SCMC2_HUMAN	3428.07227
PDE12_HUMAN	6439.19678
NAA16_HUMAN	2116.46948
DE10B_HUMAN	4563.19287
RHBD2_HUMAN	369.214996
SCMC1_HUMAN	29461.8887
REEP3_HUMAN	1026.63171
RETST_HUMAN	2814.76489
RINT1_HUMAN	1501.33044
TM214_HUMAN	10886.8281
HIBCH_HUMAN	5323.14209
RWDD4_HUMAN	2709.80322
NEPRO_HUMAN	1091.29651
ARMC6_HUMAN	4748.01514
TTI2_HUMAN	1709.69043
ANR54_HUMAN	2162.28882
ZNT6_HUMAN	1705.49646
TAPT1_HUMAN	13675.8096
KANK3_HUMAN	1309.38232
JMJD6_HUMAN	6354.45654
PPR18_HUMAN	8274.25195
CAVN1_HUMAN	91582.8828
ZCHC8_HUMAN	2563.52417
DHX57_HUMAN	2599.9873
ERAP2_HUMAN	11129.5088
MBOA5_HUMAN	2389.86865
CDC73_HUMAN	6085.45703
RM14_HUMAN	2177.70166
S27A4_HUMAN	6052.33447
C2D1A_HUMAN	3740.26221
TATD1_HUMAN	2732.7644
MET2B_HUMAN	1073.89355
MSD2_HUMAN	784.8302
TAF2_HUMAN	1636.25769
CH082_HUMAN	650.072021
MED27_HUMAN	919.16333
EDC4_HUMAN	15581.2637
CEP85_HUMAN	509.46759
ANM9_HUMAN	192.916626
PRP8_HUMAN	13534.8643
FBX42_HUMAN	866.375854
SCYL2_HUMAN	4319.92432
TTC27_HUMAN	4594.61768
SFXN4_HUMAN	1123.01147
GOLM2_HUMAN	4005.11328
NFRKB_HUMAN	662.553833
FAHD1_HUMAN	10717.2998
RL22L_HUMAN	12006.2061
PKN3_HUMAN	992.248657
ALKB5_HUMAN	541.432861

PDXD1_HUMAN	6123.29297
MEAK7_HUMAN	3833.71655
INT5_HUMAN	2149.22217
GIMA6_HUMAN	688.665588
S27A1_HUMAN	880.906921
PGM2L_HUMAN	10068.9424
CTR9_HUMAN	5215.37598
AAGAB_HUMAN	2022.65869
PSRC1_HUMAN	107.998627
SKI3_HUMAN	5005.45752
ULK3_HUMAN	1311.21863
SYDM_HUMAN	8022.62451
TMM65_HUMAN	5731.0542
IN80C_HUMAN	1229.89551
FBX38_HUMAN	1945.38818
NCEH1_HUMAN	11958.8682
FIGL1_HUMAN	788.420227
TRI65_HUMAN	1575.59961
RHDF2_HUMAN	649.237671
MDEAS_HUMAN	1891.76147
BRAT1_HUMAN	2794.38843
WDR59_HUMAN	334.300934
ZC3HE_HUMAN	2027.89941
CC137_HUMAN	1242.10046
OGFD3_HUMAN	569.888184
TXD11_HUMAN	314.53418
LARP1_HUMAN	14129.75
ATAD2_HUMAN	314.22226
TMED8_HUMAN	2389.98877
ZNT9_HUMAN	374.773285
TRAF7_HUMAN	1031.47229
RICTR_HUMAN	3434.52197
WDR74_HUMAN	1918.12854
ATRAP_HUMAN	1473.11633
SMRD3_HUMAN	1297.92334
C1TM_HUMAN	22364.9492
AFTIN_HUMAN	485.406586
FIP1_HUMAN	6766.32666
CRTC3_HUMAN	402.633301
CRTC1_HUMAN	947.728943
CP20A_HUMAN	3324.66138
PLGT2_HUMAN	3495.8916
TM205_HUMAN	6651.81006
LRSM1_HUMAN	2749.95776
GAK1B_HUMAN	2015.02197
LCLT1_HUMAN	2274.38623
ABRX1_HUMAN	56.5461044
CWC27_HUMAN	1634.26453
CREL2_HUMAN	1647.18103
WDR82_HUMAN	3272.44165

MIC27_HUMAN	1487.9834
FAT4_HUMAN	594.755554
DPP8_HUMAN	567.886719
MCAF1_HUMAN	1844.34277
RBP10_HUMAN	1274.46484
PACS1_HUMAN	6281.30273
MPRIP_HUMAN	5775.21729
RBMS3_HUMAN	2763.20068
PNCB_HUMAN	4184.94141
DNMBP_HUMAN	5710.28076
HACD2_HUMAN	1249.24304
B3GLT_HUMAN	1962.51648
GGYF2_HUMAN	7693.85303
CD109_HUMAN	8662.73535
THADA_HUMAN	1552.19702
HSDL2_HUMAN	4037.47632
KAT3_HUMAN	7227.26416
PPR21_HUMAN	1007.02765
THSD4_HUMAN	681.856934
SYNE3_HUMAN	1379.21716
ZN574_HUMAN	429.67868
NFXL1_HUMAN	342.499023
NBEL2_HUMAN	2642.03784
FGD5_HUMAN	3597.94995
QSOX2_HUMAN	1787.3999
SRCAP_HUMAN	461.919098
RIPR1_HUMAN	4407.45947
YJ005_HUMAN	6149.42529
ARHGI_HUMAN	1478.98132
TBCD9_HUMAN	219.132858
UBR3_HUMAN	585.931213
TMPPE_HUMAN	1520.18481
CRACD_HUMAN	307.250275
CCD9B_HUMAN	526.095764
NUD18_HUMAN	444.846283
SYDE1_HUMAN	211.518555
PAXI1_HUMAN	475.17981
TMTC3_HUMAN	4846.61279
VP13C_HUMAN	9915.65332
UBP34_HUMAN	695.444275
RAPH1_HUMAN	2631.99146
MOB2_HUMAN	4591.95801
IKIP_HUMAN	25351.3379
PREX2_HUMAN	738.995911
F10C1_HUMAN	2445.17847
UB2R2_HUMAN	6053.53271
MD13L_HUMAN	347.910339
LARP4_HUMAN	7840.75098
MED25_HUMAN	1325.80127
RS27L_HUMAN	53914.1641

HAKAI_HUMAN	1360.51489
INT8_HUMAN	4184.86572
BT2A1_HUMAN	609.513611
SPT6H_HUMAN	3907.99902
SND1_HUMAN	86291.6875
MARK2_HUMAN	1336.26636
COX15_HUMAN	1383.85291
DDX46_HUMAN	13222.3711
RUFY3_HUMAN	722.528503
TM10C_HUMAN	9369.68945
5MP2_HUMAN	28697.6055
MON1B_HUMAN	911.848511
LRC8D_HUMAN	373.95285
ASGL1_HUMAN	1119.33972
KCTD9_HUMAN	1900.03186
DHX30_HUMAN	9513.02148
EIF3M_HUMAN	16602.123
MEPCE_HUMAN	3217.83374
ARMX2_HUMAN	1098.57922
SYPM_HUMAN	333.506287
RSRC2_HUMAN	1913.51135
RRAGA_HUMAN	5168.89111
CYFP1_HUMAN	24915.1836
NDUF7_HUMAN	2384.67334
GET4_HUMAN	8959.00879
CSN6_HUMAN	21091.3691
PCAT2_HUMAN	14678.3848
ENOF1_HUMAN	1592.48938
MAEA_HUMAN	1757.48083
EPMIP_HUMAN	1585.23596
DHX32_HUMAN	417.093842
TAOK1_HUMAN	3693.89087
FAKD5_HUMAN	2086.16431
MOB1B_HUMAN	12785.0781
KDM3B_HUMAN	4205.66455
CHM1B_HUMAN	4009.88525
RIR2B_HUMAN	4301.46875
HS2ST_HUMAN	6271.4751
STRAA_HUMAN	1677.41248
MICA3_HUMAN	1178.74829
DYM_HUMAN	1600.03687
K2C78_HUMAN	351.073364
PHF5A_HUMAN	2194.49585
PXL2C_HUMAN	1434.56665
APTX_HUMAN	2063.34717
ERMP1_HUMAN	3045.16382
GRIN1_HUMAN	312.90329
TRM1L_HUMAN	2111.11255
ZCCHV_HUMAN	9484.53027
RM21_HUMAN	5453.60693

EFL1_HUMAN	4430.23047
SETX_HUMAN	602.259216
TPC11_HUMAN	2116.47485
NUP54_HUMAN	9006.125
ATG9A_HUMAN	1206.11145
GLUCM_HUMAN	3027.15063
CC186_HUMAN	575.527161
ARMC9_HUMAN	2588.7627
VP35L_HUMAN	5952.93311
POGZ_HUMAN	2873.59839
ZFY16_HUMAN	983.013
MON2_HUMAN	3909.00586
MYCPP_HUMAN	606.576721
MYH14_HUMAN	18917.1602
NUFP2_HUMAN	5040.85791
SZRD1_HUMAN	933.206726
MAVS_HUMAN	1132.14648
CLAP1_HUMAN	5395.16406
DHX29_HUMAN	5255.32031
NPHP3_HUMAN	226.242493
LRP10_HUMAN	1520.71399
HAUS6_HUMAN	1613.19812
PLGT3_HUMAN	2299.01587
TT21B_HUMAN	878.581482
HEAT3_HUMAN	7365.27881
KI21A_HUMAN	625.195862
HDGR2_HUMAN	9779.08691
DCXR_HUMAN	6655.72949
BRAP_HUMAN	1936.83765
EMSY_HUMAN	773.726257
GOGA7_HUMAN	2316.24536
RHG22_HUMAN	739.848694
WAPL_HUMAN	1600.76233
I2BP2_HUMAN	1243.71545
RBBP6_HUMAN	2239.70313
SH3R1_HUMAN	767.230408
GRASP_HUMAN	1665.64563
FRMD5_HUMAN	388.911499
SEN54_HUMAN	361.629456
PTAR1_HUMAN	816.920837
ARPIN_HUMAN	3039.67212
TCPR1_HUMAN	982.999207
RABEK_HUMAN	1166.10217
MTEF4_HUMAN	592.178711
HUWE1_HUMAN	12654.3301
YTHD3_HUMAN	11568.5156
CTU1_HUMAN	3935.23804
TAF8_HUMAN	942.919189
UB2Q1_HUMAN	5016.63867
RM55_HUMAN	9830.44629

TMED4_HUMAN	3274.72314
RM10_HUMAN	557.8573
COXM1_HUMAN	5333.8833
CENPV_HUMAN	2899.35107
SLN11_HUMAN	4243.33008
MEGF8_HUMAN	545.357361
T179B_HUMAN	736.281494
GALT7_HUMAN	7628.32666
SCD5_HUMAN	2967.05542
PHLB2_HUMAN	2300.03345
AGRG6_HUMAN	1984.78833
DHDDS_HUMAN	1669.80347
GLT10_HUMAN	707.509644
TPC6B_HUMAN	762.660767
PP4P1_HUMAN	2555.46753
CLC14_HUMAN	11096.9912
KAISO_HUMAN	832.523071
PATL1_HUMAN	833.875977
DPP9_HUMAN	7142.22949
SYVN1_HUMAN	1495.11401
SETD3_HUMAN	6450.37061
TTC7B_HUMAN	1519.97046
ACOT1_HUMAN	3467.89917
NOP9_HUMAN	2647.03784
PABP2_HUMAN	22162.5
MTA70_HUMAN	2183.896
PB1_HUMAN	3673.90234
YRDC_HUMAN	2254.16187
PRP39_HUMAN	2036.47522
LYRIC_HUMAN	14808.7324
ZN598_HUMAN	4991.28857
GPAT4_HUMAN	929.92749
KTN1_HUMAN	7415.62695
EST1A_HUMAN	1182.93628
NLRX1_HUMAN	691.735229
PHLB1_HUMAN	9906.59766
UBP48_HUMAN	5163.71533
CAPS2_HUMAN	310.430145
URP2_HUMAN	27492.2754
NT5D3_HUMAN	405.000336
AACS_HUMAN	7846.62842
LUZP1_HUMAN	7089.27246
THOC4_HUMAN	23885.7422
GP180_HUMAN	851.104675
FHI2B_HUMAN	2509.49731
IQGA3_HUMAN	3571.25562
ZCH18_HUMAN	1926.82336
VPS36_HUMAN	2101.34033
CAND1_HUMAN	32718.8457
RETR3_HUMAN	2993.69897

HOOK3_HUMAN	5908.10596
SESD1_HUMAN	618.610657
COMD7_HUMAN	1020.0874
MON1A_HUMAN	578.614136
AMZ2_HUMAN	599.742432
THOC6_HUMAN	1804.17676
MET16_HUMAN	1288.9895
PARG_HUMAN	418.638306
LIPB1_HUMAN	7860.64404
LONP2_HUMAN	861.67981
ZC3C1_HUMAN	3582.00806
CHD1L_HUMAN	6304.84521
FCSD1_HUMAN	208.942291
CCD25_HUMAN	6978.24561
PRSR2_HUMAN	312.942505
STING_HUMAN	6519.15186
AROS_HUMAN	1408.71985
CDR2L_HUMAN	111.202362
RLGPB_HUMAN	804.394165
RGPS2_HUMAN	381.525726
CARM1_HUMAN	9116.65137
NIT1_HUMAN	5586.30908
COMD2_HUMAN	3066.0603
HTR5A_HUMAN	1225.61096
CNDG2_HUMAN	710.444763
PPR3B_HUMAN	239.468246
FBX11_HUMAN	120.854088
ANKL2_HUMAN	2060.65015
MEX3D_HUMAN	1676.93896
DGKH_HUMAN	276.136871
DDX42_HUMAN	15300.5303
SPAS2_HUMAN	3113.31812
VRK2_HUMAN	2692.7417
CACL1_HUMAN	223.336838
NDUAB_HUMAN	6358.80469
DAAF5_HUMAN	7166.57275
PTH_HUMAN	1014.86987
STX12_HUMAN	12150.3857
P66A_HUMAN	3733.66357
CPNE8_HUMAN	21455.5723
GPSM1_HUMAN	1110.60718
RAB43_HUMAN	1159.26379
C2CD5_HUMAN	1591.17554
MIB1_HUMAN	731.587891
PRAG1_HUMAN	1419.39673
HPS6_HUMAN	1642.63989
I2BP1_HUMAN	1716.71521
RB6I2_HUMAN	4321.82471
RIOX2_HUMAN	2077.49365
ZDH13_HUMAN	9349.72559

ZDH17_HUMAN	1107.43457
CRLF3_HUMAN	1524.67419
TPPC5_HUMAN	6194.35449
ARMC8_HUMAN	2427.36621
RELL1_HUMAN	838.408325
PLD3_HUMAN	18479.1465
HID1_HUMAN	260.571381
ANKY2_HUMAN	974.893555
ERI1_HUMAN	695.73053
VRK3_HUMAN	597.116333
LIX1L_HUMAN	994.002502
NUDC3_HUMAN	4701.86084
AHNK2_HUMAN	25591.4023
FMNL3_HUMAN	5609.24854
MMAA_HUMAN	1337.2998
M4K3_HUMAN	234.13707
NAV3_HUMAN	2694.22705
P3H3_HUMAN	9967.28418
CCD50_HUMAN	8410.18555
FABD_HUMAN	2020.59558
KSR1_HUMAN	989.949829
CEP97_HUMAN	756.835205
NNRD_HUMAN	912.30127
WDR75_HUMAN	3325.61206
MFN1_HUMAN	1897.47815
CTL2_HUMAN	2835.15137
IPRI_HUMAN	1620.75415
WDFY1_HUMAN	4845.52295
MA7D3_HUMAN	2965.54858
NXP20_HUMAN	7377.39941
DCNL3_HUMAN	384.377838
DEN6A_HUMAN	533.614014
MGAP_HUMAN	442.309021
GCC2_HUMAN	2849.04224
Z3H7A_HUMAN	2193.07959
PHF6_HUMAN	6566.1123
ARCH_HUMAN	847.505859
LRC8A_HUMAN	5698.00586
SULF2_HUMAN	3800.74805
UBR1_HUMAN	1678.29578
UBR2_HUMAN	1291.09766
RHG12_HUMAN	1016.3067
CHERP_HUMAN	8935.13184
CDAN1_HUMAN	477.102386
ANKH1_HUMAN	2043.84033
SUGP1_HUMAN	1952.35999
SUGP2_HUMAN	24330.8223
UEVLD_HUMAN	1872.53894
CCAR1_HUMAN	9709.18164
DHX40_HUMAN	622.64032

DJC10_HUMAN	9368.4873
NELFD_HUMAN	5198.47705
MIRO2_HUMAN	4125.60791
MIRO1_HUMAN	3845.64429
SIR2_HUMAN	2735.98853
PHC2_HUMAN	842.848328
BAP18_HUMAN	4815.50537
RM41_HUMAN	7749.1875
NRM_HUMAN	5715.05762
GPAM1_HUMAN	792.529236
PARP9_HUMAN	448.261749
RB12B_HUMAN	3531.09277
S35F2_HUMAN	1714.66101
RPAP2_HUMAN	383.447174
PLPL6_HUMAN	6070.11621
SMC5_HUMAN	1213.75012
DDX60_HUMAN	414.903778
MILK2_HUMAN	566.854797
DHX37_HUMAN	2910.83496
KBTB2_HUMAN	2793.66577
RAVR1_HUMAN	4976.43994
SPB1_HUMAN	8555.58789
TM192_HUMAN	1158.6792
CKP2L_HUMAN	565.90979
M21D2_HUMAN	2106.81836
SRRM1_HUMAN	5021.88037
SMAP1_HUMAN	3165.49805
DI3L2_HUMAN	3367.88208
SUV3_HUMAN	4380.95801
ERF3B_HUMAN	7695.65479
EXOC8_HUMAN	6664.79199
CJ067_HUMAN	10900.1953
TRI22_HUMAN	5023.29102
THNS1_HUMAN	433.542053
K2013_HUMAN	3053.12109
MICU2_HUMAN	705.608032
AN13A_HUMAN	1561.28699
PHAR4_HUMAN	1548.78516
TRM2A_HUMAN	2903.02637
RUSD2_HUMAN	1031.38379
ELMD2_HUMAN	2105.72998
A16A1_HUMAN	5626.05566
K319L_HUMAN	1236.94019
AGRF5_HUMAN	1880.94043
XRN1_HUMAN	4954.2832
PELP1_HUMAN	12444.3438
ABI1_HUMAN	6589.77344
WDFY3_HUMAN	4483.65625
SELH_HUMAN	6839.25
VKORL_HUMAN	2239.39795

SPART_HUMAN	8730.83301
TTC5_HUMAN	1639.01746
MIER1_HUMAN	2382.75806
RPTOR_HUMAN	1755.58374
ORML3_HUMAN	3551.00391
CCAR2_HUMAN	50817.9414
NDUF2_HUMAN	7752.97461
VPS52_HUMAN	5641.3418
NUP93_HUMAN	23368.498
S11IP_HUMAN	2704.31909
ZN687_HUMAN	408.63443
CMTR1_HUMAN	3898.18701
LRC47_HUMAN	13078.0449
DOCK4_HUMAN	7401.04248
CAH13_HUMAN	1762.06335
ARG28_HUMAN	9689.63965
INT1_HUMAN	30794.1445
H2B3B_HUMAN	11564.6445
RHG24_HUMAN	3574.30493
ARM10_HUMAN	2759.28369
ABD12_HUMAN	2412.43262
CLASR_HUMAN	616.385925
FA43A_HUMAN	2468.35815
TM256_HUMAN	286.828949
GPD1L_HUMAN	3117.89063
S35F6_HUMAN	2228.51025
RHG18_HUMAN	19331.0762
ASCC3_HUMAN	10194.4639
MILK1_HUMAN	970.562134
VPS8_HUMAN	2181.20728
PALS1_HUMAN	756.342163
STAG2_HUMAN	3139.58423
SYNPO_HUMAN	8741.67578
FNBP4_HUMAN	1101.79504
GUF1_HUMAN	489.935883
RYBP_HUMAN	506.325195
GALT4_HUMAN	4726.06445
MINK1_HUMAN	1078.46252
MESH1_HUMAN	859.07373
PTGR3_HUMAN	2318.23218
CBR4_HUMAN	283.362549
EMC5_HUMAN	2095.05322
TM199_HUMAN	2519.80981
FBXL6_HUMAN	181.764908
OGFD1_HUMAN	2012.755
AFAP1_HUMAN	4963.43359
ZCHC9_HUMAN	267.750244
OXR1_HUMAN	835.637207
ZGPAT_HUMAN	883.191467
CAHM5_HUMAN	1301.38708

SRBD1_HUMAN	1058.35461
WDTC1_HUMAN	3724.92163
NKAP_HUMAN	765.914612
MACOI_HUMAN	450.701691
SH2D3_HUMAN	3841.89771
ARRD1_HUMAN	697.049805
DHRSX_HUMAN	1308.95776
CISD2_HUMAN	7959.88037
RP25L_HUMAN	2229.87988
ATPF2_HUMAN	1675.73303
TTC9C_HUMAN	2284.20239
JAGN1_HUMAN	3743.78833
RNF10_HUMAN	501.838593
RFLB_HUMAN	1704.91919
LZTR1_HUMAN	263.884705
COMD1_HUMAN	2534.02417
CPSF7_HUMAN	12517.8057
S15A4_HUMAN	740.24707
MYCT1_HUMAN	11111.0283
ATL1_HUMAN	299.029816
ARFG2_HUMAN	4155.07568
OTU6B_HUMAN	4096.12988
FITM2_HUMAN	1537.63367
CA052_HUMAN	1274.22852
EFNMT_HUMAN	2255.07739
AR6P6_HUMAN	1544.66614
ARFG1_HUMAN	2681.2251
SIR6_HUMAN	1495.03687
S66A3_HUMAN	966.117737
EMC1_HUMAN	14165.3545
PAF1_HUMAN	2211.41943
CCYL1_HUMAN	1398.85669
UBR7_HUMAN	3469.79639
DDX51_HUMAN	2587.33447
F241A_HUMAN	4561.35107
S2529_HUMAN	2551.11353
ENAH_HUMAN	15824.8447
DCBD1_HUMAN	696.360046
GPT11_HUMAN	1506.61426
RM43_HUMAN	7178.70166
RLIG1_HUMAN	3398.13037
GDPD1_HUMAN	454.43222
CS047_HUMAN	1173.45447
ASCC1_HUMAN	3462.50928
LRC57_HUMAN	5959.81055
EIF1A_HUMAN	932.52594
KRI1_HUMAN	1539.40442
CC71L_HUMAN	1240.71106
ZN579_HUMAN	1850.00183
PR38A_HUMAN	3478.55444

MLKL_HUMAN	4976.69043
GALD1_HUMAN	2583.37964
ANR52_HUMAN	723.131287
AT11C_HUMAN	913.342346
AFG2A_HUMAN	4763.32666
NHLC2_HUMAN	6607.1123
AVL9_HUMAN	2105.30005
S43A3_HUMAN	3505.25708
XXLT1_HUMAN	6015.4541
GOLM1_HUMAN	832.20874
GT251_HUMAN	25869.4629
SUMF2_HUMAN	2229.59155
SIDT2_HUMAN	788.834045
SUMF1_HUMAN	2655.55322
PGLT1_HUMAN	2601.39868
UBAC2_HUMAN	2351.02344
PCYXL_HUMAN	1320.68359
TM87A_HUMAN	2662.93677
RDH13_HUMAN	2504.72534
TTC13_HUMAN	1062.29712
DHB11_HUMAN	6698.72754
TXND5_HUMAN	250851.781
SPC24_HUMAN	1225.51282
ATAD1_HUMAN	2571.31543
SCPDL_HUMAN	15423.8154
UXS1_HUMAN	1011.16138
RN149_HUMAN	1425.0437
RETR2_HUMAN	701.33783
SERB1_HUMAN	17282.3848
LEMD2_HUMAN	5879.39502
NOA1_HUMAN	1669.86255
NECP1_HUMAN	3099.57251
FA98A_HUMAN	5500.14258
PAG15_HUMAN	1384.22534
MTMRE_HUMAN	659.18457
NF2IP_HUMAN	2855.6626
DGLB_HUMAN	818.392395
CHSTE_HUMAN	4004.33936
GALT6_HUMAN	2554.9458
RN169_HUMAN	565.71637
PDPR_HUMAN	3809.82056
NNRE_HUMAN	2688.69507
SMG8_HUMAN	1393.72412
RN214_HUMAN	2492.58643
LS14A_HUMAN	4103.82617
GIMA8_HUMAN	5876.17725
CCNY_HUMAN	3143.20361
MROH1_HUMAN	1810.80127
PAPD5_HUMAN	1415.35486
PEPL1_HUMAN	2093.77539

EHBP1_HUMAN	1203.15601
RB15B_HUMAN	1848.58179
SIA7C_HUMAN	1194.427
TNR6A_HUMAN	1225.47131
PHC3_HUMAN	217.503494
DIK2A_HUMAN	3876.39111
CNNM3_HUMAN	783.009583
ABCF1_HUMAN	13603.04
MCU_HUMAN	1676.37659
PK3C3_HUMAN	5345.97949
GSTCD_HUMAN	262.462952
SRFB1_HUMAN	1601.85632
NGDN_HUMAN	1935.71082
SHCBP_HUMAN	1738.35095
PDZD8_HUMAN	648.107788
DP13B_HUMAN	1998.69177
ZNT7_HUMAN	9536.78711
NAV1_HUMAN	1767.45703
PPHLN_HUMAN	960.446472
WDR19_HUMAN	331.257996
FBX22_HUMAN	4974.2583
PCAT1_HUMAN	4058.32104
ZMIZ2_HUMAN	1827.21155
SYNE1_HUMAN	28358.2441
UBP32_HUMAN	762.655457
BD1L1_HUMAN	1059.40344
ARI1B_HUMAN	786.176086
FAD1_HUMAN	4146.22314
FLCN_HUMAN	1563.099
NUP43_HUMAN	5710.48682
NUP37_HUMAN	6968.98682
NUP35_HUMAN	3282.2583
ENASE_HUMAN	451.541229
RAI3_HUMAN	1159.64282
TOIP2_HUMAN	6320.61426
ABHDB_HUMAN	2633.72681
NEUA_HUMAN	7553.77734
TSN14_HUMAN	4540.13086
TTL_HUMAN	363.866455
SVIP_HUMAN	1329.9873
ATLA2_HUMAN	1716.48572
MSPD2_HUMAN	1429.83984
PLBL2_HUMAN	10596.8018
RASF8_HUMAN	722.796265
DDX55_HUMAN	630.367371
MFSD8_HUMAN	229.164871
TDRD7_HUMAN	802.972412
GIMA7_HUMAN	2632.34937
NEDD1_HUMAN	1853.08545
NCOA7_HUMAN	1692.3031

THOC2_HUMAN	4956.14502
WDR36_HUMAN	8696.14648
PPTC7_HUMAN	546.446716
RP9_HUMAN	597.48175
VANG1_HUMAN	663.419678
SNIP1_HUMAN	484.143707
G45IP_HUMAN	3590.89624
WDR48_HUMAN	1523.76587
EXOC6_HUMAN	5441.28271
SMRC2_HUMAN	5468.14795
NPL4_HUMAN	7926.96045
CX038_HUMAN	1884.96924
SPT20_HUMAN	1236.80212
NUBPL_HUMAN	734.627563
ABHD4_HUMAN	371.239624
FBX30_HUMAN	1454.09119
S35B2_HUMAN	8991.01758
PUM2_HUMAN	1964.14966
TIP_HUMAN	873.397705
GOGA5_HUMAN	1426.71863
KLDC4_HUMAN	776.463379
SHKB1_HUMAN	3152.95337
UBA3_HUMAN	27355.1289
PXL2B_HUMAN	546.742554
DJB14_HUMAN	930.882446
KISHA_HUMAN	1563.06824
PI42C_HUMAN	1316.05029
WDR20_HUMAN	533.955017
TBC15_HUMAN	7279.05273
RDH11_HUMAN	16816.832
LRC20_HUMAN	778.7005
IFI44_HUMAN	212.108719
RM30_HUMAN	968.002075
ZFAN1_HUMAN	905.135498
CIP2A_HUMAN	4829.70801
P4K2B_HUMAN	770.927307
STT3B_HUMAN	32593.9102
PNPT1_HUMAN	10014.4063
SPP2B_HUMAN	1268.75854
HM13_HUMAN	45400.0195
PREX1_HUMAN	3102.91846
URGCP_HUMAN	983.844421
BICD2_HUMAN	4800.74414
NEK9_HUMAN	4867.98779
PKHO2_HUMAN	3510.62061
DTX3L_HUMAN	724.541321
DDX54_HUMAN	4285.104
DLG5_HUMAN	582.822876
BRX1_HUMAN	8709.56738
GNPI2_HUMAN	3484.16333

LRC8C_HUMAN	13191.7715
NEK7_HUMAN	3989.24097
RBCC1_HUMAN	470.968842
MICA1_HUMAN	4089.55664
ELP5_HUMAN	2556.75952
SSH3_HUMAN	1160.17712
S3TC1_HUMAN	279.927551
TBCK_HUMAN	742.622925
DTD1_HUMAN	5268.22705
DCA11_HUMAN	949.533691
UTP15_HUMAN	1842.69519
GPX8_HUMAN	23745.1641
AF1L1_HUMAN	368.252594
SH3R3_HUMAN	3137.6394
TP4AP_HUMAN	1313.00208
CE192_HUMAN	509.671326
GEMI5_HUMAN	5646.82227
PIGO_HUMAN	291.693024
RPGF6_HUMAN	484.088684
SMCR8_HUMAN	502.725616
PARD3_HUMAN	1594.30994
IPO4_HUMAN	13666.1709
UBP33_HUMAN	566.908447
PNISR_HUMAN	2295.64722
PP4R1_HUMAN	5917.87207
UBS3B_HUMAN	1400.09387
WIPF2_HUMAN	847.492249
ABHD5_HUMAN	339.741852
SETD7_HUMAN	5688.78613
NOC3L_HUMAN	5273.33789
SCRB1_HUMAN	20409.957
COG1_HUMAN	2836.13794
SCFD2_HUMAN	2634.23901
SMAP2_HUMAN	1729.56348
ZC3HF_HUMAN	15979.7441
PPIL4_HUMAN	3718.98975
TF3C2_HUMAN	1859.27478
TB22A_HUMAN	3216.06323
PHF10_HUMAN	1387.34839
RAB2B_HUMAN	3351.67847
CCD12_HUMAN	1537.96326
IASPP_HUMAN	1146.19714
ARB2A_HUMAN	2079.8877
CHUR_HUMAN	2684.29907
TGFA1_HUMAN	785.914001
TM263_HUMAN	3634.65015
HDAC7_HUMAN	4676.27832
PTPM1_HUMAN	493.151306
NU133_HUMAN	7938.47607
PDC6I_HUMAN	53238.1211

FBLI1_HUMAN	18356.3008
CATIN_HUMAN	1099.69812
CHAC2_HUMAN	2218.22168
CHMP7_HUMAN	4195.08057
THEM6_HUMAN	21660.7227
NAT14_HUMAN	4374.81982
BCL7C_HUMAN	2929.5293
NSE1_HUMAN	664.867004
MITD1_HUMAN	541.545654
CTF18_HUMAN	669.646057
LEO1_HUMAN	3189.87524
DCAKD_HUMAN	1153.1842
NUDC2_HUMAN	4658.83691
SNR27_HUMAN	2918.01855
TFB1M_HUMAN	8952.07715
STAG1_HUMAN	1917.40259
SCFD1_HUMAN	8871.42676
TP8L1_HUMAN	2652.37573
CANT1_HUMAN	2626.2981
TPC12_HUMAN	1544.479
HNRLL_HUMAN	4190.57813
FACR1_HUMAN	13991.3643
UBCP1_HUMAN	6895.55957
SEN15_HUMAN	694.633789
PCNP_HUMAN	8288.54395
DNJA4_HUMAN	240.60788
SPRY4_HUMAN	5980.56104
MAIP1_HUMAN	2392.05859
TRUB1_HUMAN	1770.0022
LMO7_HUMAN	2159.76733
CTL1_HUMAN	1243.68994
CKAP2_HUMAN	989.120789
ATX2L_HUMAN	13249.4521
ARAP3_HUMAN	8633.33984
GIMA1_HUMAN	4407.30566
PHIP_HUMAN	258.686584
PRP31_HUMAN	9676.37891
NELFB_HUMAN	2708.24683
PALLD_HUMAN	15583.7227
RUFY2_HUMAN	1278.74207
SREK1_HUMAN	3003.79907
CSKI2_HUMAN	1904.17334
ATRIP_HUMAN	468.389282
PSPC1_HUMAN	16441.1465
MADD_HUMAN	487.232025
SYNE2_HUMAN	7037.13574
P66B_HUMAN	2566.78467
DNJC9_HUMAN	17367.1777
BRI3B_HUMAN	1237.41406
CTBL1_HUMAN	11127.2646

ATG4A_HUMAN	2926.29858
RIN2_HUMAN	755.112244
ELYS_HUMAN	3139.44971
DGCR8_HUMAN	772.405334
ROBO4_HUMAN	3774.53345
OVCA2_HUMAN	2546.75586
IRGQ_HUMAN	765.097717
DDB2_HUMAN	7714.91357
CLC2B_HUMAN	1602.03748
DDX1_HUMAN	26667.8262
S14L1_HUMAN	6470.146
PIEZ1_HUMAN	1136.36694
FAM3C_HUMAN	6078.9082
H1X_HUMAN	9901.9502
PSMF1_HUMAN	5560.12549
GBF1_HUMAN	9186.93359
RTF1_HUMAN	6954.27393
NICA_HUMAN	10711.1318
SNX19_HUMAN	573.454773
TM9S4_HUMAN	8341.94141
TM131_HUMAN	712.396973
IP6K1_HUMAN	682.211975
RT27_HUMAN	15673.7168
ELMO1_HUMAN	1690.15051
FIG4_HUMAN	582.970337
F168A_HUMAN	251.821884
AP3S1_HUMAN	14610.7021
TSC1_HUMAN	4888.86768
UBXN4_HUMAN	2992.30908
PHF3_HUMAN	1298.5459
NDRG1_HUMAN	3825.37671
HS105_HUMAN	30185.8477
SEPT8_HUMAN	7446.4834
CNOT9_HUMAN	4788.76953
LGAT1_HUMAN	556.117126
TBCD5_HUMAN	3484.45093
ZN592_HUMAN	454.314819
JADE3_HUMAN	761.804688
MY18A_HUMAN	1465.78577
LAR4B_HUMAN	3489.45239
GCN1_HUMAN	22671.832
HMHA1_HUMAN	137.998779
PRP16_HUMAN	2465.073
NU205_HUMAN	72251.0234
ANS1A_HUMAN	993.43219
PXDN_HUMAN	8739.90137
FAN_HUMAN	778.649414
GPI8_HUMAN	803.129944
RT31_HUMAN	8928.50488
AKAP1_HUMAN	242.086288

ALG3_HUMAN	750.286133
NEUG_HUMAN	375.285614
AN32B_HUMAN	59110.7969
NECT2_HUMAN	8812.83789
PGTA_HUMAN	10770.4502
TFG_HUMAN	12758.6104
HTRA1_HUMAN	18023.1543
ARC1A_HUMAN	8718.25098
TF2H4_HUMAN	875.723877
RREB1_HUMAN	291.563538
HDAC2_HUMAN	9710.18457
STAM1_HUMAN	2201.09839
REQU_HUMAN	2549.35522
SC65_HUMAN	8734.38184
CBP_HUMAN	512.112854
KAT6A_HUMAN	249.567734
SYMPK_HUMAN	4895.28418
RBP56_HUMAN	1179.95667
GOGA1_HUMAN	955.700928
GGH_HUMAN	5483.62109
NRCAM_HUMAN	7654.11865
RPGR_HUMAN	521.946045
SHIP1_HUMAN	4696.26123
DDX17_HUMAN	86736.5547
KIFA3_HUMAN	1588.94604
CASPA_HUMAN	1549.41492
APBB2_HUMAN	1108.9679
RAD50_HUMAN	11657.9854
CELF1_HUMAN	8989.69629
OSTF1_HUMAN	5833.02148
ARHG1_HUMAN	12292.8594
XPF_HUMAN	608.338135
UFD1_HUMAN	28661.5137
GSLG1_HUMAN	17392.4395
RENT1_HUMAN	11207.6611
CSN5_HUMAN	15809.7754
GPKOW_HUMAN	6069.93652
SMRC1_HUMAN	3684.76025
SMRD2_HUMAN	3209.20483
RAB8B_HUMAN	20917.4258
BAD_HUMAN	256.689545
FUBP2_HUMAN	19849.2832
GCDH_HUMAN	7905.82715
RIT1_HUMAN	356.139374
PEX13_HUMAN	717.708374
TNPO1_HUMAN	34189.4844
ARHG2_HUMAN	19247.4824
NEP1_HUMAN	12143.0205
CLP1_HUMAN	3574.83984
GLMN_HUMAN	5665.37158

UBP13_HUMAN	2077.28955
DVL3_HUMAN	1194.62952
USP9X_HUMAN	9799.09863
UBP7_HUMAN	10293.7188
CUL5_HUMAN	5123.28027
STMN2_HUMAN	49137.3984
VPP1_HUMAN	3066.24097
LPP_HUMAN	8909.64941
RBPMS_HUMAN	725.382751
EXT2_HUMAN	685.108154
MED12_HUMAN	648.938416
TP4A1_HUMAN	417.979523
KPBB_HUMAN	2640.52563
SCAM4_HUMAN	2111.52612
HPS3_HUMAN	892.499268
SMCE1_HUMAN	5617.3457
CAVN3_HUMAN	12836.0938
RIFK_HUMAN	496.593201
MYDGF_HUMAN	36319.5156
YIPF5_HUMAN	2289.79102
PIGT_HUMAN	4554.56006
IGSF8_HUMAN	524.345398
RL36L_HUMAN	2587.69409
RAB24_HUMAN	665.408203
OSBP2_HUMAN	370.647614
ZN622_HUMAN	3138.9939
RRF2M_HUMAN	2573.72339
UB2E3_HUMAN	11599.9043
5NT3B_HUMAN	3833.97119
WBP2_HUMAN	3387.17847
PSMG2_HUMAN	15906.4102
NCLN_HUMAN	16355.2188
MUL1_HUMAN	535.288391
MRTFA_HUMAN	294.211212
LFG3_HUMAN	5498.38867
ERGI1_HUMAN	11632.501
UTP4_HUMAN	2350.10522
FAKD4_HUMAN	5098.98535
MARC2_HUMAN	2624.63281
F162A_HUMAN	9383.90723
CCD47_HUMAN	7804.97266
RM24_HUMAN	5685.73926
EXOC4_HUMAN	9222.31641
MGN2_HUMAN	38338.1328
P33MX_HUMAN	675.700134
RFT1_HUMAN	2647.63281
ISOC2_HUMAN	716.520874
NTAN1_HUMAN	1324.78503
FERM2_HUMAN	14731.2871
FUBP1_HUMAN	51652.5039

TTC17_HUMAN	700.576904
LRC59_HUMAN	95828.7344
VTI1A_HUMAN	219.814438
ESAM_HUMAN	4978.56494
PBIP1_HUMAN	5591.4292
MCUR1_HUMAN	978.493286
K1143_HUMAN	888.740295
VP33A_HUMAN	3611.44556
FKB10_HUMAN	15932.25
TTC28_HUMAN	1880.0376
EXOS8_HUMAN	2552.26709
ZN428_HUMAN	372.491852
SH3K1_HUMAN	4028.35132
SKA1_HUMAN	541.209961
NACC2_HUMAN	786.803223
RNF25_HUMAN	1061.052
AIDA_HUMAN	8705.7998
PINX1_HUMAN	925.538696
ARL8A_HUMAN	7170.36182
OTUL_HUMAN	2371.72852
CHCH1_HUMAN	2356.08325
PPWD1_HUMAN	6183.71387
CC127_HUMAN	781.186462
COA7_HUMAN	1567.93701
UPP_HUMAN	217.437897
TAM41_HUMAN	1482.12036
MOB3A_HUMAN	1857.43933
DOC10_HUMAN	2735.66382
ATG2B_HUMAN	1199.50281
LENG1_HUMAN	890.820374
TBC20_HUMAN	581.550293
EFHD2_HUMAN	34996.3047
GALM_HUMAN	1450.36914
SYTL4_HUMAN	2668.74146
P5CR2_HUMAN	6466.14746
CSTOS_HUMAN	2479.90967
DCPS_HUMAN	12345.499
PP14B_HUMAN	3674.40796
INT12_HUMAN	873.319519
NSUN4_HUMAN	2157.97998
RHDF1_HUMAN	303.194794
CTHR1_HUMAN	2582.61694
ACSF2_HUMAN	631.614868
ISOC1_HUMAN	8009.68066
GCC1_HUMAN	580.114746
FWCH2_HUMAN	3720.14795
ASTRA_HUMAN	1657.57361
HAUS1_HUMAN	162.498825
FAF2_HUMAN	11499.791
CC124_HUMAN	7099.20752

FXRD1_HUMAN	4208.75928
OPTN_HUMAN	2919.15942
AP2M1_HUMAN	44545.1836
GCP3_HUMAN	3007.39136
S7A6O_HUMAN	1542.86572
KCD12_HUMAN	37207.2383
RCN3_HUMAN	392.259674
NMD3_HUMAN	5594.99268
REPS1_HUMAN	989.975952
TIM14_HUMAN	3572.57959
RMD1_HUMAN	7801.00488
NUD16_HUMAN	5002.0542
ESS2_HUMAN	1329.45923
CMBL_HUMAN	2133.23218
MSI2H_HUMAN	2957.07886
SNR40_HUMAN	8121.10986
RMC1_HUMAN	1451.36438
RM38_HUMAN	8636.67676
RSPRY_HUMAN	431.555817
ERLEC_HUMAN	3007.96362
RRFM_HUMAN	5157.99023
MTEF3_HUMAN	1191.13696
SPDLY_HUMAN	2036.06567
ELP4_HUMAN	2536.65234
SIR1_HUMAN	622.647827
YIPF6_HUMAN	1356.79138
SEH1_HUMAN	3861.34619
TCAL4_HUMAN	1976.63123
KBP_HUMAN	15982.1611
GNA1_HUMAN	12134.7939
F120B_HUMAN	1741.41113
KTI12_HUMAN	1289.91138
RT24_HUMAN	3841.31152
RM53_HUMAN	931.419128
T3HPD_HUMAN	7443.64844
RNF31_HUMAN	1844.229
DAZP1_HUMAN	17653.2109
SGTB_HUMAN	670.918579
SAAL1_HUMAN	1782.86719
MITOK_HUMAN	1412.44592
SGF29_HUMAN	864.218872
RRP36_HUMAN	1366.96631
C1GLC_HUMAN	1594.46497
RBM33_HUMAN	1972.19434
DTBP1_HUMAN	1326.81873
DNJA3_HUMAN	5044.3877
TMA16_HUMAN	5262.07178
MB12A_HUMAN	3553.35547
PTCD3_HUMAN	6980.5791
MMAB_HUMAN	3467.30859

CYFP2_HUMAN	369.477386
TRI11_HUMAN	1335.7135
CCD97_HUMAN	637.078674
CNRP1_HUMAN	10451.4873
EDC3_HUMAN	3850.84448
DYL2_HUMAN	8811.46094
CPNE2_HUMAN	4000.25854
S10AG_HUMAN	4941.31982
SIPA1_HUMAN	3358.37524
SCRN2_HUMAN	1024.75989
THOC1_HUMAN	4976.0791
OTUB1_HUMAN	26395.9141
TRM61_HUMAN	768.91748
HMCES_HUMAN	405.393311
CHMP6_HUMAN	4802.80322
BICD1_HUMAN	355.984711
PGM2_HUMAN	29972.3301
IMP4_HUMAN	3988.02441
CERS2_HUMAN	5771.65869
DUS3L_HUMAN	840.674438
LTV1_HUMAN	3934.55371
SDSL_HUMAN	447.480072
RM48_HUMAN	3309.48926
PLPP_HUMAN	1260.33704
AURKB_HUMAN	691.080994
DCNL1_HUMAN	2474.16064
FAH2A_HUMAN	4317.3418
SMRD1_HUMAN	1926.06409
TOE1_HUMAN	2362.20166
CDA7L_HUMAN	252.088516
RUSF1_HUMAN	1452.15845
DDX27_HUMAN	8190.31689
BORC6_HUMAN	1062.0387
SYMM_HUMAN	1229.22083
A7L3B_HUMAN	2376.16577
GWL_HUMAN	414.237579
MTNB_HUMAN	1932.64087
ZC21A_HUMAN	466.811981
LIN37_HUMAN	245.874496
SNF8_HUMAN	2457.01001
ZCCHL_HUMAN	2452.54443
P121A_HUMAN	261.738525
TONSL_HUMAN	736.547546
PDLI5_HUMAN	41747.8281
CREL1_HUMAN	986.124512
ERO1A_HUMAN	24166.7363
PRR11_HUMAN	789.870667
FMC1_HUMAN	2928.48145
SAHH3_HUMAN	13667.5361
DOCK6_HUMAN	5814.4209

MED30_HUMAN	202.244949
PGAM5_HUMAN	14873.0566
INT4_HUMAN	2093.93994
DDRGK_HUMAN	3714.45703
DHTK1_HUMAN	1074.96533
SCLY_HUMAN	1487.27881
FUBP3_HUMAN	17789.5684
SPF45_HUMAN	4687.21777
RCC1L_HUMAN	1456.75317
SYNM_HUMAN	1473.58459
SUCB2_HUMAN	11618.6758
LRCH3_HUMAN	3746.45093
GMPPA_HUMAN	6761.72266
HPDL_HUMAN	1130.5105
ABHEB_HUMAN	1750.65588
NGLY1_HUMAN	2344.87866
SC22A_HUMAN	2210.78125
ATPMK_HUMAN	14057.1455
NSL1_HUMAN	869.321472
PAWR_HUMAN	4146.63916
MET2A_HUMAN	546.029175
THOC3_HUMAN	5702.91846
ITCH_HUMAN	2971.05005
TXD15_HUMAN	426.615051
KIRR1_HUMAN	2324.74243
COG3_HUMAN	1959.00818
CK5P3_HUMAN	7123.76563
VPS39_HUMAN	3538.17896
VPS50_HUMAN	1982.08875
VCIP1_HUMAN	3429.20361
SPTCS_HUMAN	534.045898
ELMO2_HUMAN	6364.99707
TMX3_HUMAN	14730.6504
DCAF5_HUMAN	1902.70276
CHAP1_HUMAN	2363.0542
LMBL3_HUMAN	3017.75781
ZFP91_HUMAN	568.281189
PCD16_HUMAN	972.587952
CLMN_HUMAN	18028.2734
PDLI2_HUMAN	587.728455
FACR2_HUMAN	4995.45264
BT3L4_HUMAN	11513.666
RN170_HUMAN	561.994812
ANCHR_HUMAN	928.700623
S35E1_HUMAN	1146.28149
UBP47_HUMAN	4812.07666
ZN521_HUMAN	324.609619
CLP1L_HUMAN	1980.96338
TOPK_HUMAN	6162.68604
ARL5B_HUMAN	796.36676

DNJC1_HUMAN	1582.85193
SCYL1_HUMAN	5356.05762
Z512B_HUMAN	190.129944
LRAT2_HUMAN	512.012329
EXOC2_HUMAN	7163.09082
CNDP2_HUMAN	42401.9766
EHMT2_HUMAN	1463.45361
ZFR_HUMAN	3612.48779
F210B_HUMAN	1809.33215
B3GT6_HUMAN	1106.13379
EP400_HUMAN	765.462891
SNX27_HUMAN	3715.78882
IFT74_HUMAN	583.770264
TRI47_HUMAN	3900.44971
CNO6L_HUMAN	2529.88916
DHRS1_HUMAN	454.695251
DJC30_HUMAN	627.478516
UB2E2_HUMAN	308.863861
CAR19_HUMAN	2806.43018
PRRC1_HUMAN	6299.27686
FGD4_HUMAN	3781.11938
FXL18_HUMAN	3408.93896
ZN512_HUMAN	2374.86035
NSE2_HUMAN	1476.63013
NSE3_HUMAN	196.549759
PCMD1_HUMAN	368.619141
HS12B_HUMAN	3811.55444
YTDC1_HUMAN	2289.29395
CCD43_HUMAN	2306.42432
COG8_HUMAN	2142.19141
CKLF3_HUMAN	3529.43481
DAA10_HUMAN	2856.02026
NOL4L_HUMAN	6244.43994
MBOA7_HUMAN	11661.6738
DOCK7_HUMAN	9475.73242
SFXN2_HUMAN	724.217285
ZN830_HUMAN	517.245483
F210A_HUMAN	511.766907
FRMD6_HUMAN	743.587158
NSUN5_HUMAN	233.621994
RPR1A_HUMAN	2871.49487
AGAP3_HUMAN	1419.27417
ARAP1_HUMAN	2848.23193
IPO9_HUMAN	17026.2988
MIA2_HUMAN	1539.57703
DCBD2_HUMAN	1521.85901
ARHGH_HUMAN	1166.39307
INP4A_HUMAN	601.464783
RBM14_HUMAN	19241.2188
ZN363_HUMAN	439.77597

KLHL5_HUMAN	984.804321
UHRF2_HUMAN	4491.03418
NED4L_HUMAN	3127.43994
QKI_HUMAN	12770.3877
FMNL2_HUMAN	1419.02856
PUS7_HUMAN	5526.20215
F111A_HUMAN	921.966248
TPPC9_HUMAN	710.315979
PLIN4_HUMAN	1750.01062
TRNT1_HUMAN	3705.75659
SMG1_HUMAN	1092.14697
ALS2_HUMAN	679.584778
TM237_HUMAN	734.106873
ALKB3_HUMAN	409.611511
RHG07_HUMAN	870.262329
PP1RA_HUMAN	3592.40869
S38A2_HUMAN	1568.75024
UIF_HUMAN	2793.95874
MTMR9_HUMAN	1909.625
VPS35_HUMAN	29081.2754
PURB_HUMAN	2651.59326
XPO6_HUMAN	2044.22119
HHIP_HUMAN	39951.1367
MAGI1_HUMAN	1026.40417
SPAG5_HUMAN	1065.07996
NACC1_HUMAN	4978.59521
SNX18_HUMAN	2288.50366
PASK_HUMAN	325.398041
CIC_HUMAN	2607.0166
UIMC1_HUMAN	562.862305
VP13A_HUMAN	1756.23828
MED15_HUMAN	2411.5564
EFGM_HUMAN	10042.5918
ERGI2_HUMAN	551.798462
MCCA_HUMAN	1700.61609
KKCC2_HUMAN	440.450684
TGS1_HUMAN	385.185791
NUDC1_HUMAN	12514.4814
ERBIN_HUMAN	1333.3667
GCP6_HUMAN	1648.83948
UBP28_HUMAN	681.265015
FNBP1_HUMAN	3594.77197
PRPK_HUMAN	4692.09229
PIGS_HUMAN	5516.13477
WRIP1_HUMAN	1626.61816
RANB9_HUMAN	2069.80371
CLCC1_HUMAN	2756.43237
MYADM_HUMAN	7245.06006
NEB2_HUMAN	1463.55737
SRPK1_HUMAN	4509.04346

SMC6_HUMAN	2136.6626
KCD15_HUMAN	626.743469
STRBP_HUMAN	8998.66113
TM209_HUMAN	2572.50293
GPX7_HUMAN	336.271484
CK5P2_HUMAN	642.865906
CP2S1_HUMAN	735.124084
IWS1_HUMAN	6884.47754
SIN3A_HUMAN	1280.96619
OSBL9_HUMAN	5382.65234
CRBN_HUMAN	868.629089
INT14_HUMAN	1633.65063
AEDO_HUMAN	2678.99316
CK5P1_HUMAN	924.972412
RSF1_HUMAN	282.448639
RBM15_HUMAN	2958.5
PP16B_HUMAN	389.884186
RUFY1_HUMAN	4123.45215
MINT_HUMAN	732.759277
PNKP_HUMAN	5603.55762
MMS19_HUMAN	9665.77637
UHRF1_HUMAN	6163.60889
NIBA2_HUMAN	32579.6172
YMEL1_HUMAN	6413.87402
RMD3_HUMAN	4327.61719
HTF4_HUMAN	1204.10974
CYH2_HUMAN	817.602051
TBCB_HUMAN	13588.0596
PSB7_HUMAN	23760.3418
CNN2_HUMAN	15357.3086
SEC62_HUMAN	7695.40137
PCY2_HUMAN	4689.62061
CDC5L_HUMAN	13844.9424
PSMD1_HUMAN	28775.5449
SDF2_HUMAN	2866.39258
PFD5_HUMAN	1265.79175
RING2_HUMAN	3349.20605
PARK7_HUMAN	18815.8887
GA2L1_HUMAN	205.308182
EYA3_HUMAN	616.691467
NEUR1_HUMAN	6368.23633
SORT_HUMAN	430.81311
VAT1_HUMAN	118098.07
LGMN_HUMAN	876.217712
PLIN2_HUMAN	131.921524
DNJC2_HUMAN	4363.04834
MPP8_HUMAN	934.504639
NUP88_HUMAN	8582.6709
PKP4_HUMAN	465.364227
PI3R4_HUMAN	3694.6106

P2RX4_HUMAN	2759.40112
POP1_HUMAN	5333.67432
S10AD_HUMAN	31517.7969
SCAFB_HUMAN	1453.72644
TSNAX_HUMAN	5815.01611
SPS2_HUMAN	1919.35364
EIF3C_HUMAN	25591.0156
TTC1_HUMAN	778.262207
DNJC7_HUMAN	10282.7539
PHB2_HUMAN	82511.4297
CSN8_HUMAN	2147.22388
PRP18_HUMAN	592.663391
CHP1_HUMAN	4588.50781
KIF2C_HUMAN	2388.26855
MGLL_HUMAN	2653.32227
ATX2_HUMAN	2923.97925
DOK1_HUMAN	224.088654
METH_HUMAN	8697.21875
HCD2_HUMAN	33613.9063
COCA1_HUMAN	1969.0708
SMAD5_HUMAN	1018.53809
SGMR1_HUMAN	4860.375
ROAA_HUMAN	10896.2188
NP1L4_HUMAN	27201.0566
MGST2_HUMAN	28973.5273
SNAG_HUMAN	6456.37451
PI51A_HUMAN	1657.03125
ABCA3_HUMAN	988.01239
M3K3_HUMAN	348.723236
ATP5S_HUMAN	1669.17102
MIPEP_HUMAN	3378.87207
ACON_HUMAN	14574.8955
TM9S2_HUMAN	7243.23926
S29A1_HUMAN	8284.86523
TS101_HUMAN	7670.89551
CPNE1_HUMAN	17774.8613
TCPH_HUMAN	50834.3242
MYD88_HUMAN	6034.7334
EBP2_HUMAN	9788.87012
ANM1_HUMAN	22435.6641
BAG1_HUMAN	733.940552
ATF6B_HUMAN	690.075562
FOXC2_HUMAN	476.757263
SH3G1_HUMAN	28068.5156
TEP1_HUMAN	5962.22363
VRK1_HUMAN	10977.7627
GDF15_HUMAN	18057.3145
AKAP9_HUMAN	10489.9805
NIPS1_HUMAN	12134.8975
CND3_HUMAN	7034.14355

ARP5L_HUMAN	12923.5352
MICU1_HUMAN	912.571777
CG025_HUMAN	688.86615
F118B_HUMAN	1326.0415
SIN1_HUMAN	1433.74585
RBM4B_HUMAN	17220.7695
ZFY21_HUMAN	1587.25525
DDX50_HUMAN	5269.02539
RM34_HUMAN	2799.28516
RNZ2_HUMAN	6446.7124
TRIR_HUMAN	3910.77905
GRWD1_HUMAN	6364.56104
MACD1_HUMAN	2473.27905
TCF25_HUMAN	2842.53467
CMS1_HUMAN	4601.0835
KLDC3_HUMAN	995.189636
ECSIT_HUMAN	1959.8269
MEP50_HUMAN	17265.7012
CYBC1_HUMAN	1261.14746
VKOR1_HUMAN	3366.18066
DPH2_HUMAN	737.175293
TBA1C_HUMAN	16357.5986
SELS_HUMAN	2934.13086
APOL2_HUMAN	3488.16406
MBB1A_HUMAN	16145.6035
MGME1_HUMAN	605.6698
GORS1_HUMAN	1435.6239
FYCO1_HUMAN	1081.39258
ACBD6_HUMAN	3023.81299
COR1B_HUMAN	31349.8203
TXD17_HUMAN	12722.4766
BUD13_HUMAN	1030.43774
CPPED_HUMAN	19781.377
VPS25_HUMAN	2130.83618
RM45_HUMAN	11327.1348
CG050_HUMAN	8146.45361
TIRR_HUMAN	2144.76538
CAB45_HUMAN	7077.83887
PDD2L_HUMAN	920.761475
PAAF1_HUMAN	4368.85059
PYM1_HUMAN	3053.84009
MIC25_HUMAN	764.037964
ADPGK_HUMAN	4752.3916
GPTC1_HUMAN	122.252945
RIOK1_HUMAN	1423.64319
LLPH_HUMAN	2791.7981
SLD5_HUMAN	936.77594
UTP23_HUMAN	262.582764
PELO_HUMAN	5587.9585
PXL2A_HUMAN	32712.998

TRI56_HUMAN	1196.15613
ERP44_HUMAN	50314.7109
LXN_HUMAN	10755.7383
NOL10_HUMAN	2049.42432
NTPCR_HUMAN	5961.61816
TIM29_HUMAN	4490.09082
TACO1_HUMAN	2436.50781
GCP2_HUMAN	5465.54346
ESYT1_HUMAN	27363.7891
UBAC1_HUMAN	1220.81641
YIPF4_HUMAN	2530.65063
SEN34_HUMAN	2021.08667
CNPY3_HUMAN	1143.45825
MTG1_HUMAN	1648.54919
ALG1_HUMAN	2707.14063
LIMD2_HUMAN	268.33902
HAUS8_HUMAN	1059.69006
INP5K_HUMAN	1812.67676
PSMG3_HUMAN	3844.99365
CSN4_HUMAN	11499.3467
SYT11_HUMAN	1360.39758
WAC_HUMAN	573.835876
DIDO1_HUMAN	2312.09326
MTA3_HUMAN	4125.67139
RBM42_HUMAN	8385.09277
DCTN5_HUMAN	1821.01526
MCMBP_HUMAN	5089.56055
AASD1_HUMAN	3866.8811
DCNL5_HUMAN	6494.89746
AN32E_HUMAN	5853.4834
LRRC1_HUMAN	1427.59607
P4K2A_HUMAN	2827.02881
TMM43_HUMAN	25626.4473
FSD1_HUMAN	976.373047
TBCD_HUMAN	8244.50098
NDC1_HUMAN	1475.90259
HGH1_HUMAN	819.678711
DHRS4_HUMAN	4028.2854
LMF2_HUMAN	4561.63379
NDUF3_HUMAN	2067.95483
MMTA2_HUMAN	1326.42993
TMM70_HUMAN	4229.68066
MED18_HUMAN	1039.45264
ISCA1_HUMAN	245.319473
TBB6_HUMAN	115949.336
PAXX_HUMAN	1178.6189
RPC3_HUMAN	1596.18213
HNRL1_HUMAN	16433.1406
MSTO1_HUMAN	4742.32471
PHF23_HUMAN	1684.10974

PDC10_HUMAN	15288.0254
RPP25_HUMAN	1801.01343
DERL1_HUMAN	16811.4707
HTAI2_HUMAN	6222.59521
DDX23_HUMAN	9825.87109
TCAB1_HUMAN	700.342224
MIC26_HUMAN	3551.74097
DHRS6_HUMAN	6919.23779
MTNA_HUMAN	4583.09814
WDR18_HUMAN	6446.82764
THUM3_HUMAN	1727.80457
MTND_HUMAN	4850.65088
MECR_HUMAN	2047.5791
EMC6_HUMAN	5850.74902
NTM1A_HUMAN	2215.27856
EDEM2_HUMAN	426.30188
KTNB1_HUMAN	340.648621
LST8_HUMAN	1459.97681
ASHWN_HUMAN	833.538696
TM109_HUMAN	50116.9141
PBDC1_HUMAN	4144.28369
PTSS2_HUMAN	1936.04651
NOC4L_HUMAN	5190.60107
UT14A_HUMAN	4509.34375
DUS23_HUMAN	4466.90771
TMED9_HUMAN	39452.0859
TM147_HUMAN	1700.99573
NUP58_HUMAN	3401.46704
SELO_HUMAN	2610.86499
DPCD_HUMAN	2249.21045
GNL3_HUMAN	12850.8262
AFG2B_HUMAN	5726.84033
RIOK2_HUMAN	1133.12427
TR61B_HUMAN	304.239014
TMUB1_HUMAN	1797.33423
TIM21_HUMAN	3015.79321
KIFC1_HUMAN	903.671082
NUP85_HUMAN	8437.50586
ELOV1_HUMAN	2495.12695
KATL1_HUMAN	1501.17651
HIRP3_HUMAN	848.411682
IFT27_HUMAN	2186.83203
YJU2_HUMAN	1529.82715
NUDT9_HUMAN	4374.20654
SYTM_HUMAN	2465.26392
THIC_HUMAN	21909.5273
REPI1_HUMAN	626.546204
RBM4_HUMAN	3425.46313
FUND2_HUMAN	1264.17285
RPAP1_HUMAN	2164.11011

SF3B5_HUMAN	1537.04114
SFXN3_HUMAN	15162.4443
CHID1_HUMAN	2058.31934
PAPOG_HUMAN	1217.71301
CAR10_HUMAN	330.491882
NADAP_HUMAN	6100.0127
CDK19_HUMAN	702.331238
LS14B_HUMAN	1535.10449
TPSNR_HUMAN	785.691528
JAM3_HUMAN	7919.26904
CARD6_HUMAN	1158.58484
BTBD2_HUMAN	769.175415
SGPP1_HUMAN	258.442566
OSB11_HUMAN	1696.81152
OSB10_HUMAN	3375.70923
RFIP5_HUMAN	2089.35083
C1QT5_HUMAN	3267.67139
NAA15_HUMAN	22555.3105
KLF16_HUMAN	317.935913
B2L13_HUMAN	833.383423
CAR11_HUMAN	1210.79688
S12A9_HUMAN	1213.2594
SRRT_HUMAN	19290.2148
TGT_HUMAN	5243.0625
AP1M1_HUMAN	16328.6357
NUSAP_HUMAN	300.981537
HDHD5_HUMAN	4246.1748
FACD2_HUMAN	525.337097
MAK16_HUMAN	3319.01343
HDAC8_HUMAN	405.773102
RTF2_HUMAN	982.687195
CHM4A_HUMAN	2966.50635
EIF2A_HUMAN	17948.1563
PDIP3_HUMAN	4036.66821
K1671_HUMAN	1227.27722
SHAN3_HUMAN	8182.59961
GNB1L_HUMAN	1729.18115
FUT8_HUMAN	1334.47449
RM32_HUMAN	1377.17944
RM20_HUMAN	4667.61523
RM13_HUMAN	8805.10352
RM09_HUMAN	9433.27539
RM04_HUMAN	4713.66992
RM01_HUMAN	8115.32861
MK67I_HUMAN	4358.43115
HYCCI_HUMAN	1742.11951
YTHD1_HUMAN	887.781921
HELZ2_HUMAN	664.895569
HOIL1_HUMAN	2327.04932
RT26_HUMAN	5123.62305

NEUL_HUMAN	10991.5566
CEP41_HUMAN	2930.23071
SETD2_HUMAN	721.438293
PANK2_HUMAN	540.801086
DOCK9_HUMAN	9653.12793
FRMD8_HUMAN	4200.03174
CNTP3_HUMAN	1412.63953
NSD3_HUMAN	2315.45239
NUF2_HUMAN	3693.90039
RM37_HUMAN	10626.1914
GTPB4_HUMAN	11107.0439
ASPC1_HUMAN	2027.32178
OSBL8_HUMAN	2098.85791
UACA_HUMAN	2305.92065
RAB34_HUMAN	5264.86768
DPH1_HUMAN	903.46936
WDR11_HUMAN	3901.88428
REN3B_HUMAN	2026.38672
CRNL1_HUMAN	3636.08008
TBL1R_HUMAN	3542.3269
UBL5_HUMAN	4752.07373
PP12C_HUMAN	2327.4248
KPCD2_HUMAN	4294.11328
EDEM3_HUMAN	3476.97949
NIBA1_HUMAN	367.915283
UBXN6_HUMAN	4333.43311
TM6S1_HUMAN	886.150635
UCK2_HUMAN	7188.58105
SN_HUMAN	970.33374
API5_HUMAN	24212.7539
NLRP1_HUMAN	784.523193
TRIM5_HUMAN	1733.49438
TRIM4_HUMAN	65892.1641
FTO_HUMAN	5671.90088
ZDHC5_HUMAN	291.022034
TNG6_HUMAN	1425.97107
TB182_HUMAN	5265.16504
UBE2O_HUMAN	6264.61377
ZY11B_HUMAN	2180.29907
TANC1_HUMAN	5216.08838
EPT1_HUMAN	1296.16663
XPO4_HUMAN	6502.97705
LNP_HUMAN	728.671631
CEP44_HUMAN	445.43866
TTYH3_HUMAN	8198.82715
KLHL4_HUMAN	1857.29016
MTMRC_HUMAN	584.788574
WDR33_HUMAN	5168.52881
WDR12_HUMAN	7621.31055
YIPF3_HUMAN	5304.59766

TINAL_HUMAN	1602.25293
ADSSP_HUMAN	7414.68359
PITH1_HUMAN	3739.33862
DERL2_HUMAN	6982.04639
COMD5_HUMAN	1855.09961
REXO4_HUMAN	2651.78882
DDX24_HUMAN	5407.34131
RPA49_HUMAN	3755.71558
SKI8_HUMAN	7909.40186
SLIRP_HUMAN	8346.67969
SRR_HUMAN	979.679077
NIF3L_HUMAN	2115.5271
EGLN1_HUMAN	4077.41284
MCLN1_HUMAN	864.907593
PIP30_HUMAN	3620.16846
MFF_HUMAN	575.296082
NAA50_HUMAN	9521.3916
UBA5_HUMAN	6199.58301
MKRN2_HUMAN	1499.19336
T126A_HUMAN	3608.62695
PAIP1_HUMAN	11337.6553
CLPB_HUMAN	4817.3623
RB33B_HUMAN	4449.63037
LSG1_HUMAN	6218.89893
F107B_HUMAN	8166.2124
NAT10_HUMAN	17055.6211
COMD4_HUMAN	1216.01685
KLC2_HUMAN	2794.04907
ILKAP_HUMAN	6165.9873
XRN2_HUMAN	12338.1182
TOLIP_HUMAN	3989.0332
SP130_HUMAN	857.867798
BRD8_HUMAN	882.186462
NSRP1_HUMAN	198.176163
INT2_HUMAN	975.374023
RGAP1_HUMAN	400.745514
PAR12_HUMAN	2111.7207
CSTFT_HUMAN	1055.22852
5NT3A_HUMAN	1997.32849
CYRIA_HUMAN	14424.2207
TM222_HUMAN	1083.0719
HDHD2_HUMAN	2303.99438
GATA_HUMAN	1758.63977
DDX47_HUMAN	7889.50195
MAGT1_HUMAN	7211.84619
RAB1B_HUMAN	56985.168
RM18_HUMAN	7007.72266
TSYL1_HUMAN	1115.99963
TM168_HUMAN	723.751282
LMA2L_HUMAN	7300.229

SMG9_HUMAN	1670.21997
OSBL5_HUMAN	792.196777
TDIF1_HUMAN	1865.96191
SIL1_HUMAN	1562.55164
METL9_HUMAN	732.947449
APC1_HUMAN	4166.77588
I2BPL_HUMAN	961.647034
CYTM1_HUMAN	979.556213
RPC6_HUMAN	2111.3252
NUCKS_HUMAN	5068.75732
TMX4_HUMAN	2850.63257
KI13A_HUMAN	21406.502
ASCC2_HUMAN	6419.74658
RBNS5_HUMAN	457.488708
ISCU_HUMAN	2924.06885
OSBL2_HUMAN	1299.39526
ATG5_HUMAN	3465.7561
WDR13_HUMAN	2768.72363
EPN3_HUMAN	448.720642
EHD4_HUMAN	94392.2031
VP33B_HUMAN	2805.4751
VPS16_HUMAN	7379.87402
VPS11_HUMAN	4245.23682
SH3L3_HUMAN	9257.71777
GAN_HUMAN	603.622498
S2532_HUMAN	1580.67297
TARA_HUMAN	6197.87988
EPC1_HUMAN	194.859894
SLK_HUMAN	11581.6064
PPIL3_HUMAN	1391.28918
S38A1_HUMAN	1228.00037
PDCL3_HUMAN	6458.9541
TAOK3_HUMAN	6009.89404
RBGPR_HUMAN	9626.90332
ADNP_HUMAN	1669.27112
DPH5_HUMAN	2388.79785
DHX36_HUMAN	6525.42529
IPYR2_HUMAN	6750.91699
SPNS1_HUMAN	3980.3667
RM46_HUMAN	1626.45496
PARL_HUMAN	282.759552
PININ_HUMAN	7991.95068
TM245_HUMAN	1120.41638
FOXP1_HUMAN	1040.43958
BACD3_HUMAN	2092.35571
MOD5_HUMAN	445.824585
CK068_HUMAN	6373.53955
GHITM_HUMAN	6598.86035
BOLA2_HUMAN	6148.68066
MMAD_HUMAN	1506.63452

TMX1_HUMAN	22878.8574
NELFA_HUMAN	3210.74756
GCP60_HUMAN	8549.03613
BORG4_HUMAN	1178.15491
PTN23_HUMAN	7514.68799
SEM6B_HUMAN	4001.00757
UN45A_HUMAN	19332.3223
MFSD1_HUMAN	1479.46521
DNJC5_HUMAN	953.956177
CHM4B_HUMAN	18294.0254
RWDD1_HUMAN	2576.88818
CUED2_HUMAN	371.150482
FXL15_HUMAN	838.37738
FN3K_HUMAN	1108.74255
OFUT1_HUMAN	8724.30664
PIGU_HUMAN	4495.13818
TOR3A_HUMAN	2648.93799
WNK1_HUMAN	3416.2417
AMPB_HUMAN	18499.0293
GLP3L_HUMAN	4863.49463
GOLP3_HUMAN	3691.03394
OSGL1_HUMAN	1715.38074
SIA7D_HUMAN	1084.91064
GAPR1_HUMAN	5061.17871
TRABD_HUMAN	2318.90405
SENP3_HUMAN	3909.40527
OSBL3_HUMAN	4257.06104
SMRCD_HUMAN	1929.48645
EHD1_HUMAN	21233.4063
CAPAM_HUMAN	708.124451
ESF1_HUMAN	1091.94666
ALG2_HUMAN	3673.90991
HEAT1_HUMAN	8865.83398
SG196_HUMAN	570.486389
RABE2_HUMAN	1949.20752
TFB2M_HUMAN	3788.69604
ZCHC4_HUMAN	1522.07422
STEEP_HUMAN	401.268036
CIA2A_HUMAN	1708.41589
DHX35_HUMAN	719.726563
F124B_HUMAN	2534.36084
HAUS4_HUMAN	2981.40576
CC134_HUMAN	2994.06763
CCD86_HUMAN	3984.16406
REEP4_HUMAN	1897.86548
SOX17_HUMAN	962.482483
OPA3_HUMAN	1793.2627
CIAO3_HUMAN	892.827271
DHX33_HUMAN	744.937683
NOL6_HUMAN	3932.98389

WDCP_HUMAN	395.64328
YTDC2_HUMAN	2990.85522
RPAP3_HUMAN	3697.54834
BCAS3_HUMAN	473.402252
ALG9_HUMAN	3022.99146
LDAH_HUMAN	1242.46973
RIOX1_HUMAN	3337.4209
WDR55_HUMAN	2485.14429
RANB3_HUMAN	7255.59961
DCTP1_HUMAN	510.015656
PEAK1_HUMAN	1754.51794
RPF2_HUMAN	3351.55591
SMYD3_HUMAN	3910.95459
AAMDC_HUMAN	1479.88281
DOCK5_HUMAN	1155.83716
WDR26_HUMAN	2899.6355
TDRD3_HUMAN	1616.01233
CH033_HUMAN	2308.59839
AT133_HUMAN	407.834564
MET17_HUMAN	489.843964
SFR19_HUMAN	2026.6604
NRDE2_HUMAN	543.86969
PGES2_HUMAN	6556.47607
PHAX_HUMAN	2830.59058
UBE2Z_HUMAN	5920.375
ACAD9_HUMAN	5795.33203
NT5D2_HUMAN	9598.91699
PKRI1_HUMAN	1970.16248
CAAP1_HUMAN	1605.42932
NOL11_HUMAN	1511.01453
DDX31_HUMAN	848.550171
TMT1A_HUMAN	1962.01184
PAAT_HUMAN	1716.57959
MMRN2_HUMAN	11958.1621
MINY3_HUMAN	1069.13843
EVA1A_HUMAN	2203.05884
AKTIP_HUMAN	757.492493
PKHF2_HUMAN	287.076355
ANKZ1_HUMAN	2303.18481
GORS2_HUMAN	2871.89453
ZWILC_HUMAN	1119.00439
JUPI2_HUMAN	2689.34448
GHC1_HUMAN	6456.73145
PPIP2_HUMAN	3345.10522
MED20_HUMAN	1463.10718
WDR76_HUMAN	308.178253
QTRT2_HUMAN	1716.35583
ARP8_HUMAN	1214.85559
ARMT1_HUMAN	4325.08057
PANK3_HUMAN	551.9328

CNO10_HUMAN	1564.94666
LRC40_HUMAN	16969.2695
EHMT1_HUMAN	2070.86206
SFXN1_HUMAN	25626.5605
SPE39_HUMAN	1472.59583
COG4_HUMAN	7346.85303
ARP5_HUMAN	987.797241
VP37B_HUMAN	411.774841
RM44_HUMAN	7914.35107
L2HDH_HUMAN	667.543762
CSN7B_HUMAN	3147.46875
NHEJ1_HUMAN	628.573303
S61A2_HUMAN	12702.3428
FKRP_HUMAN	851.672729
ELP3_HUMAN	5361.40625
RPF1_HUMAN	2311.1792
RPA2_HUMAN	2033.60803
ZMAT3_HUMAN	290.565186
UCK1_HUMAN	925.749329
KT3K_HUMAN	1404.11682
TBC17_HUMAN	1443.28052
SYCM_HUMAN	3960.98877
S52A2_HUMAN	850.953613
PPCS_HUMAN	8942.29395
UBTD1_HUMAN	2456.83765
WDR41_HUMAN	475.703339
NMNA1_HUMAN	1282.06738
NJMU_HUMAN	1525.37537
SIAE_HUMAN	263.687988
PKHA5_HUMAN	874.694641
SMUF2_HUMAN	2743.47656
RENT2_HUMAN	2880.57251
GBB4_HUMAN	1737.17871
XPO5_HUMAN	6769.8877
GRPE1_HUMAN	17748.2305
MYG1_HUMAN	10093.29
PKHA3_HUMAN	443.65683
PKHA1_HUMAN	1478.62317
RISC_HUMAN	3215.91919
NET4_HUMAN	4307.24707
CYBP_HUMAN	35480.8711
RRAGC_HUMAN	8107.72803
ZFYV1_HUMAN	1035.83215
IF122_HUMAN	996.338501
RDH14_HUMAN	3983.36719
PARVB_HUMAN	12700.1611
TENS1_HUMAN	2698.45483
PLRKT_HUMAN	1912.48816
NMRL1_HUMAN	2136.01904
VEZA_HUMAN	846.30188

SPC25_HUMAN	2066.93994
TAF9B_HUMAN	605.052673
S38AA_HUMAN	1450.81018
EKI1_HUMAN	2797.6189
AGRL4_HUMAN	9704.80273
TM165_HUMAN	5301.62988
TPC_HUMAN	1340.18958
EMAL4_HUMAN	5426.26709
MRM3_HUMAN	3121.95557
GLOD4_HUMAN	43250.4961
NEK6_HUMAN	1390.6189
MCCB_HUMAN	2395.40894
NCOA5_HUMAN	46423.4492
EPG5_HUMAN	898.536865
MOV10_HUMAN	6597.21484
MET14_HUMAN	1573.48865
GBA2_HUMAN	2833.66211
CWC22_HUMAN	1850.12402
RAVR2_HUMAN	588.759949
CHD8_HUMAN	894.342529
E41L5_HUMAN	1478.12061
GPN1_HUMAN	3960.62866
SDF2L_HUMAN	4163.87158
KC1G1_HUMAN	3456.22778
SYF1_HUMAN	4339.55225
PREB_HUMAN	3454.72559
SRA1_HUMAN	2139.77734
AT131_HUMAN	5316.93359
GOPC_HUMAN	1875.32849
RM47_HUMAN	4623.61328
LYRM4_HUMAN	2258.75464
SPCS_HUMAN	743.445618
CHM1A_HUMAN	7237.40088
TM9S3_HUMAN	12943.6172
MYO10_HUMAN	5131.56885
APMAP_HUMAN	49129.6289
WDR6_HUMAN	5259.62793
TRXR2_HUMAN	9249.47852
ABCB6_HUMAN	1571.474
ARFG3_HUMAN	5278.65674
ZCC17_HUMAN	621.826355
RAB18_HUMAN	17442.416
PALMD_HUMAN	9388.22363
SSU72_HUMAN	2406.34668
VTA1_HUMAN	22171.0684
SYSM_HUMAN	3368.71826
RT30_HUMAN	5810.15137
DLRB1_HUMAN	4400.72754
EMC7_HUMAN	8132.84961
ENY2_HUMAN	1752.81946

EXOS4_HUMAN	4060.55615
UBE2T_HUMAN	3858.49634
NOP10_HUMAN	4229.58301
OSGEP_HUMAN	4882.65283
DMAP1_HUMAN	899.446289
PCD12_HUMAN	717.676758
INO1_HUMAN	6005.33008
BRD7_HUMAN	847.319519
DCP1A_HUMAN	4483.35693
ACO13_HUMAN	8304.94531
MED4_HUMAN	571.829895
TIDC1_HUMAN	192.131165
RIC8A_HUMAN	20726.0703
C1QR1_HUMAN	28127.875
LUC7L_HUMAN	7911.40723
ESM1_HUMAN	457.688599
LZTL1_HUMAN	2238.02563
RM40_HUMAN	4198.75488
SSF1_HUMAN	3457.04077
PLCB1_HUMAN	761.588135
TIGAR_HUMAN	15019.8877
CL004_HUMAN	750.093689
RTN4_HUMAN	19563.1406
RPR1B_HUMAN	7020.23584
XPP3_HUMAN	1947.65283
NIT2_HUMAN	7863.7251
AVEN_HUMAN	857.978943
INCE_HUMAN	534.396057
EXOS5_HUMAN	2993.99121
EXOS3_HUMAN	5177.74365
KI13B_HUMAN	4119.14648
ANLN_HUMAN	3039.47827
XPP1_HUMAN	12205.207
GEPH_HUMAN	4123.75342
BIN3_HUMAN	2473.13672
SAS10_HUMAN	2260.5542
STAR7_HUMAN	1573.96753
BIRC6_HUMAN	3805.07495
PDLI7_HUMAN	21128.2383
ACSA_HUMAN	9086.1377
DBLOH_HUMAN	2681.93164
DDX21_HUMAN	26235.1387
SAR1A_HUMAN	25683.1914
SIAS_HUMAN	31243.9531
SHLB2_HUMAN	3162.34521
ASH1L_HUMAN	21657.3242
EI2BG_HUMAN	10337.2715
MBNL1_HUMAN	10922.4795
S17A5_HUMAN	2977.2876
4ET_HUMAN	1026.04102

CHSTC_HUMAN	1292.46472
ST7_HUMAN	433.634338
PICK1_HUMAN	456.597015
SH2B1_HUMAN	1381.43872
PYRG2_HUMAN	6033.39502
DPOE3_HUMAN	2916.49536
CHRC1_HUMAN	2465.60547
AAAS_HUMAN	6503.03027
SNRK_HUMAN	3170.17456
ABCBA_HUMAN	2191.21387
BAZ1A_HUMAN	1318.65906
STRN4_HUMAN	5251.91992
ADPPT_HUMAN	10127.4375
METL5_HUMAN	2472.83423
OSTC_HUMAN	18336.9688
COXM2_HUMAN	1689.09827
PLS4_HUMAN	1000.28076
SNX15_HUMAN	858.896606
HEBP1_HUMAN	2379.56763
ABC3C_HUMAN	2789.58936
VPS45_HUMAN	2460.47949
PNO1_HUMAN	3074.33691
RM17_HUMAN	11954.4346
PHP14_HUMAN	205.936935
SERC1_HUMAN	930.284729
RHG35_HUMAN	2616.3623
F1142_HUMAN	2550.34473
PLS3_HUMAN	2695.85132
PLCD_HUMAN	805.23938
PLCC_HUMAN	2993.7334
HELLS_HUMAN	1482.11462
C1GLT_HUMAN	2178.89282
LTBP3_HUMAN	213.548599
TOM22_HUMAN	3030.74146
LANC2_HUMAN	6483.26855
KIF15_HUMAN	947.221741
TM7S3_HUMAN	389.113251
HOME3_HUMAN	4465.06201
SYFB_HUMAN	27067.0488
SYIM_HUMAN	12137.6924
SLX9_HUMAN	4177.32227
SAMN1_HUMAN	1433.42505
KLC4_HUMAN	1784.08716
DIAP3_HUMAN	2343.39429
BMP2K_HUMAN	591.068298
ATG3_HUMAN	4278.97021
PDS5B_HUMAN	4494.36426
SMC4_HUMAN	31730.6289
MA2C1_HUMAN	6437.4043
SAC1_HUMAN	7995.92529

DEPP1_HUMAN	1103.03674
OLA1_HUMAN	34833.9883
CUTC_HUMAN	871.185486
ZF64B_HUMAN	924.540161
ECHD1_HUMAN	9416.76855
RBM12_HUMAN	96447.2813
TB22B_HUMAN	1182.96753
MDN1_HUMAN	8914.32031
ACS2L_HUMAN	1140.62134
ZCHC3_HUMAN	119.536514
DECR2_HUMAN	4896.20947
ABHDA_HUMAN	2471.46533
T11L1_HUMAN	925.298767
STAU2_HUMAN	5060.79932
SHFL_HUMAN	706.356689
DDX28_HUMAN	1567.35217
T106B_HUMAN	3005.32104
LIN7C_HUMAN	1837.66565
PLCE_HUMAN	4751.23877
TXLNG_HUMAN	1555.80798
SPS2L_HUMAN	2295.64526
UFSP2_HUMAN	2838.42383
ABCF3_HUMAN	7202.22607
CYRIB_HUMAN	10626.5654
MITOS_HUMAN	2695.13501
DD19A_HUMAN	16959.043
GIMA4_HUMAN	16533.3164
TYDP1_HUMAN	1692.72644
POTE1_HUMAN	292.434906
TBC23_HUMAN	4281.76953
DCA13_HUMAN	3423.61841
IMP3_HUMAN	4997.60254
EXOC1_HUMAN	10022.7832
PCMD2_HUMAN	774.246704
INT9_HUMAN	1937.9314
CC50A_HUMAN	10286.1904
UQCC1_HUMAN	2136.85303
SEP11_HUMAN	22712.8867
S38A7_HUMAN	12462.7715
MED17_HUMAN	8594.44434
PARVA_HUMAN	9133.89063
UBP40_HUMAN	339.731781
PANK4_HUMAN	11190.1846
FBX28_HUMAN	523.189636
TBC13_HUMAN	6037.93359
EXD2_HUMAN	2023.57556
DJC11_HUMAN	5149.20264
INT7_HUMAN	11381.5215
FANCI_HUMAN	962.943237
ATD3A_HUMAN	35566.2969

ARL8B_HUMAN	2269.3291
DJC17_HUMAN	4265.1875
INT13_HUMAN	2983.74658
RIC8B_HUMAN	496.196472
GNL3L_HUMAN	1692.24475
DDX18_HUMAN	8136.16357
ASF1B_HUMAN	1683.36902
KLH11_HUMAN	603.278015
INT10_HUMAN	2003.18433
RT18A_HUMAN	4870.58936
PNPO_HUMAN	1647.12695
ARMC1_HUMAN	4035.16016
RPC5_HUMAN	770.771729
SDA1_HUMAN	4066.03052
PAPD1_HUMAN	4117.13135
NLE1_HUMAN	3959.74878
NECP2_HUMAN	10077.7148
RPC2_HUMAN	4315.94238
RBM28_HUMAN	3679.63354
ANO10_HUMAN	9152.83203
RBM22_HUMAN	3864.51587
WDR70_HUMAN	1577.50525
ARGL1_HUMAN	4079.92578
IFT57_HUMAN	262.587738
SLTM_HUMAN	3369.37158
FKB14_HUMAN	2974.20703
RMND1_HUMAN	2104.95459
PK1IP_HUMAN	2698.39209
HIF1N_HUMAN	1557.04285
OXSM_HUMAN	4289.62402
RM22_HUMAN	16519.2207
CZIB_HUMAN	16819.6484
BABA1_HUMAN	3647.84058
CLN6_HUMAN	1117.74902
ASB6_HUMAN	2088.50513
THG1_HUMAN	648.554871
HPF1_HUMAN	1942.55859
IRAK4_HUMAN	1517.31335
UCKL1_HUMAN	2735.24487
TM160_HUMAN	3106.98535
NALP2_HUMAN	3913.28857
F120C_HUMAN	472.49469
TSAP1_HUMAN	1147.00562
NDUBB_HUMAN	1965.79065
RM16_HUMAN	8208.34277
NHP2_HUMAN	6917.02539
OCAD1_HUMAN	7912.1416
ADPRS_HUMAN	10211.0088
MARH5_HUMAN	4016.57837
LYAR_HUMAN	5925.89502

T161A_HUMAN	395.597198
IMPA3_HUMAN	4663.19678
MIC19_HUMAN	9855.51367
CKLF6_HUMAN	1479.93604
SIR5_HUMAN	1204.65564
MIOS_HUMAN	991.690613
MTMRA_HUMAN	3553.44019
NSMA3_HUMAN	3742.86572
CWC25_HUMAN	1964.03137
TEX10_HUMAN	3772.20483
DCA16_HUMAN	1587.92688
THUM1_HUMAN	4456.36719
P4HTM_HUMAN	249.695618
TOR4A_HUMAN	2978.13599
TRM1_HUMAN	4332.78857
GDAP2_HUMAN	1457.17065
NDE1_HUMAN	1585.22791
BABA2_HUMAN	817.711792
QPCTL_HUMAN	2079.55933
ARL15_HUMAN	1670.37732
KCTD5_HUMAN	2100.68286
CARF_HUMAN	3680.34375
DJB12_HUMAN	3288.23584
ALKB4_HUMAN	1121.76294
NSE4A_HUMAN	220.997192
GAR1_HUMAN	5351.28027
STAB1_HUMAN	8463.0127
PP4R2_HUMAN	1730.54004
DPP3_HUMAN	20575.9316
AATF_HUMAN	1440.24768
TBA8_HUMAN	4516.07324
DDX56_HUMAN	3531.57544
SPHK1_HUMAN	2189.8645
TE2IP_HUMAN	222.309921
ABI2_HUMAN	2188.20459
BCLF1_HUMAN	12315.9951
UTP6_HUMAN	1804.49707
TAB2_HUMAN	224.828903
RM39_HUMAN	8262.83984
M3K20_HUMAN	2734.37158
FKB11_HUMAN	13721.96
TMOD3_HUMAN	12033.7324
BET1L_HUMAN	2053.52173
ELOV5_HUMAN	3201.47437
KBRS2_HUMAN	1288.48181
UGGG2_HUMAN	686.957214
UGGG1_HUMAN	24021.832
CDK12_HUMAN	2265.86035
FAKD2_HUMAN	3521.56958
TECR_HUMAN	7017.67969

ERAP1_HUMAN	12432.6553
ARP10_HUMAN	3858.45654
USE1_HUMAN	1599.53589
CISD1_HUMAN	1961.2832
GGA3_HUMAN	604.340332
TLS1_HUMAN	979.159546
F120A_HUMAN	8051.86816
GDE1_HUMAN	796.088745
SMAL1_HUMAN	1077.49695
GLTP_HUMAN	1638.77966
SPG21_HUMAN	4154.60059
RM35_HUMAN	2952.41235
UBIP1_HUMAN	1004.69788
IF2B1_HUMAN	17678.3086
SACS_HUMAN	2098.65259
MTCH1_HUMAN	6377.40381
NUDT4_HUMAN	8045.729
HPBP1_HUMAN	8296.78516
MAT2B_HUMAN	30027.1641
MYOF_HUMAN	41886.8125
ITSN2_HUMAN	2642.52026
NOP53_HUMAN	1251.42322
EHD3_HUMAN	46700.3203
EHD2_HUMAN	60737.2188
ARHGC_HUMAN	2917.19238
CNOT2_HUMAN	3464.51245
SPN90_HUMAN	1496.11426
PD1L1_HUMAN	1200.67212
CALL5_HUMAN	415.602936
OGFR_HUMAN	7609.49512
LMCD1_HUMAN	886.36084
CRIM1_HUMAN	2311.39697
SELN_HUMAN	3537.41602
PALS2_HUMAN	3194.24658
CHMP5_HUMAN	5296.46533
CWC15_HUMAN	2250.38037
RM15_HUMAN	8412.80371
THYN1_HUMAN	7897.36768
TAP26_HUMAN	558.870911
NDUF4_HUMAN	3630.40698
HACD3_HUMAN	11197.0879
EMC3_HUMAN	3313.07178
NDUAD_HUMAN	6498.58691
PDP1_HUMAN	4567.67578
KCMF1_HUMAN	1644.24915
RAI14_HUMAN	8906.41797
VAPA_HUMAN	14049.0723
RM27_HUMAN	3443.0813
TMEM9_HUMAN	1192.96448
SENP1_HUMAN	431.111603

CXXC1_HUMAN	168.105438
SH3B4_HUMAN	7184.81982
SEP10_HUMAN	11565.9775
GMIP_HUMAN	1087.95032
PHRF1_HUMAN	2966.9541
POGK_HUMAN	630.798035
DAPLE_HUMAN	154.492798
RHG23_HUMAN	1240.74805
VPS18_HUMAN	7926.43945
RCC2_HUMAN	35808.6563
RELCH_HUMAN	1195.0824
DIP2B_HUMAN	4591.14014
JCAD_HUMAN	2293.37622
SLAI2_HUMAN	688.394287
TEN3_HUMAN	924.305786
UBP36_HUMAN	575.555237
BCCIP_HUMAN	14029.5771
STK26_HUMAN	1148.86658
ARMX1_HUMAN	2169.50488
ABI3_HUMAN	6600.77539
FPRP_HUMAN	11225.9707
CT2NL_HUMAN	6165.82666
TM181_HUMAN	3097.96753
IBTK_HUMAN	1079.01782
HTR5B_HUMAN	1749.15015
ZNFX1_HUMAN	1392.88269
CHPF2_HUMAN	2493.45483
PCD10_HUMAN	974.641113
RRBP1_HUMAN	35579.3828
CPSF2_HUMAN	5117.47217
KLHL9_HUMAN	2748.45386
SYLC_HUMAN	25341.8516
MYEF2_HUMAN	8930.2207
KLH42_HUMAN	375.371307
E2AK4_HUMAN	4373.36572
WDR35_HUMAN	762.370911
RBM27_HUMAN	2442.17554
KANL3_HUMAN	524.401001
HECW2_HUMAN	2244.5896
FRM4A_HUMAN	980.756165
ANFY1_HUMAN	5062.49023
RERE_HUMAN	176.8423
SUCB1_HUMAN	8690.9873
GMPR2_HUMAN	6977.03906
STX18_HUMAN	499.640503
DPM3_HUMAN	790.667297
IMPCT_HUMAN	1760.92395
UVRAG_HUMAN	2560.52173
ATX10_HUMAN	10105.3955
MBD2_HUMAN	3543.24878

NCDN_HUMAN	1350.39685
TFP11_HUMAN	1319.98035
EP15R_HUMAN	11491.8066
MYO1A_HUMAN	5736.06348
ORC3_HUMAN	1480.81677
SAE1_HUMAN	12704.708
COPG2_HUMAN	8760.81348
PI4KB_HUMAN	985.891235
MRC2_HUMAN	1034.61011
COMD3_HUMAN	954.08252
GBG12_HUMAN	1992.495
RBL2A_HUMAN	2727.84546
MTRR_HUMAN	550.574097
UXT_HUMAN	1024.35718
ASH2L_HUMAN	1921.22595
DHCR7_HUMAN	25035.3809
TR10D_HUMAN	421.583527
HDAC6_HUMAN	1958.66882
SPAST_HUMAN	2751.15771
TRMB_HUMAN	5957.67285
GULP1_HUMAN	1112.6012
VPS29_HUMAN	12544.1992
EIF3K_HUMAN	18457.168
EXTL2_HUMAN	1915.29651
GRHPR_HUMAN	13515.0322
CATZ_HUMAN	19542.1699
KS6B2_HUMAN	1532.77344
DJB11_HUMAN	12261.998
RNF14_HUMAN	2483.15723
SAE2_HUMAN	17755.1602
MO4L1_HUMAN	1562.56384
NXF1_HUMAN	6795.86572
SE1L1_HUMAN	1694.0697
B4GT7_HUMAN	2228.17627
PEF1_HUMAN	1812.10059
ZMYM2_HUMAN	973.399109
CSN7A_HUMAN	7766.56445
DIC_HUMAN	7205.64209
AASS_HUMAN	6855.63379
CLIP2_HUMAN	1687.03748
QCR9_HUMAN	10036.0059
MTFP1_HUMAN	2083.26636
ZO2_HUMAN	22017.3457
DNJB4_HUMAN	15456.0674
ST17A_HUMAN	1035.28064
CFDP1_HUMAN	3966.92261
DAXX_HUMAN	1271.36279
TRM7_HUMAN	2545.0105
VTI1B_HUMAN	2627.62012
STK39_HUMAN	3186.48315

ADDG_HUMAN	3071.38672
LRWD1_HUMAN	2325.03906
NPS3A_HUMAN	6861.56885
CGBP1_HUMAN	3103.96924
PISD_HUMAN	768.059814
ABCF2_HUMAN	14037.6777
TES_HUMAN	5221.03906
AAKG2_HUMAN	3303.02124
GCP4_HUMAN	2793.45166
KDM5B_HUMAN	208.236313
SYWM_HUMAN	1058.09521
LIMD1_HUMAN	849.540344
SEC63_HUMAN	11526.3975
GTR6_HUMAN	626.552246
Z3H7B_HUMAN	6733.92871
NDRG3_HUMAN	7394.83838
ARMX3_HUMAN	5044.14893
SWP70_HUMAN	22604.3809
SUN2_HUMAN	9990.8877
RLP24_HUMAN	1858.61182
LTOR3_HUMAN	2891.97144
LIMA1_HUMAN	17442.3066
AFF4_HUMAN	7093.76611
SRP68_HUMAN	12706.1113
CHRD1_HUMAN	13987.417
TBK1_HUMAN	4365.41504
SEPT9_HUMAN	15533.8818
PCYOX_HUMAN	7904.31299
DDX20_HUMAN	5270.86621
SHPK_HUMAN	4197.2417
DPP2_HUMAN	2033.92236
CEIP2_HUMAN	3447.55859
UBP25_HUMAN	2954.25
BAP29_HUMAN	4337.08203
NB5R1_HUMAN	6109.51807
S30BP_HUMAN	3847.0708
ZNHI2_HUMAN	8633.1709
PFD2_HUMAN	11197.373
GPN3_HUMAN	1673.83618
PUF60_HUMAN	21419.0703
NRBP_HUMAN	5190.53564
ENOPH_HUMAN	8326.4707
FEZ2_HUMAN	1912.15955
NDUAC_HUMAN	3431.5083
EI2BD_HUMAN	13687.2324
VATH_HUMAN	6144.59814
PRAF1_HUMAN	2244.86694
IPO11_HUMAN	2148.7373
TR112_HUMAN	159.609451
DACH1_HUMAN	408.27182

XPO7_HUMAN	6248.42285
LCMT1_HUMAN	4065.37402
VPS51_HUMAN	4918.11816
BAZ2B_HUMAN	351.896088
BAZ2A_HUMAN	554.765869
BAZ1B_HUMAN	3753.17773
KAD3_HUMAN	17015.2656
LCAP_HUMAN	8548.87891
MBD1_HUMAN	464.248291
CNOT7_HUMAN	2112.1958
RABX5_HUMAN	3320.14575
MSRA_HUMAN	2962.89868
NAGK_HUMAN	25813.1973
HACL1_HUMAN	3528.75977
TRM6_HUMAN	1635.46973
SH3L2_HUMAN	2307.16968
NGAP_HUMAN	659.093445
TSR3_HUMAN	1163.82263
S2513_HUMAN	15394.0449
DBNL_HUMAN	11486.3467
DDX41_HUMAN	6147.88525
DCTN4_HUMAN	7845.7915
CDC23_HUMAN	3660.15625
APC7_HUMAN	4734.98096
APC5_HUMAN	1036.85059
APC4_HUMAN	1973.28308
ANC2_HUMAN	1926.74841
GGA2_HUMAN	4621.34863
GGA1_HUMAN	2823.77979
STML2_HUMAN	15265.3262
NAGPA_HUMAN	819.003296
VPS28_HUMAN	6538.35693
LSM7_HUMAN	1029.95422
DBR1_HUMAN	3218.729
TASOR_HUMAN	458.799469
JUPI1_HUMAN	554.153564
FBX3_HUMAN	974.365356
AKA11_HUMAN	617.384399
FBW1B_HUMAN	1024.16455
GMEB2_HUMAN	841.14032
MRT4_HUMAN	18832.4043
TNIK_HUMAN	1173.80066
CPSF3_HUMAN	5387.23975
DP13A_HUMAN	4810.46582
TLK1_HUMAN	850.790894
GPTC8_HUMAN	1079.98108
PARP4_HUMAN	9643.18262
NXT1_HUMAN	577.501099
NUDT5_HUMAN	11460.5723
RCOR1_HUMAN	1153.1095

PPCT_HUMAN	1177.05737
MA1B1_HUMAN	1348.65454
RALY_HUMAN	18028.584
TF3C4_HUMAN	5088.51855
ERG28_HUMAN	2220.41357
PACN3_HUMAN	1274.56738
ACAD8_HUMAN	1332.18542
ANGL2_HUMAN	3017.50952
ACINU_HUMAN	13145.6787
AMFR_HUMAN	2958.46216
AGO2_HUMAN	3796.74023
NUP50_HUMAN	8039.93555
CDV3_HUMAN	21464.9609
CNO11_HUMAN	766.964783
INT6_HUMAN	1437.08752
BAG5_HUMAN	2677.59082
AGO1_HUMAN	3999.93115
RAB21_HUMAN	9451.78516
RB22A_HUMAN	5230.47559
TPC2L_HUMAN	2250.77905
ZN346_HUMAN	2352.76343
PSME2_HUMAN	11426.3574
TAOK2_HUMAN	2206.62476
MKLN1_HUMAN	3532.19824
DNPEP_HUMAN	6220.54736
RAB23_HUMAN	3931.55713
MCTS1_HUMAN	14788.5371
MTUS1_HUMAN	314.326935
WWC3_HUMAN	901.910278
PALD_HUMAN	1687.66296
S39AA_HUMAN	1146.21753
INO80_HUMAN	416.981812
CCPG1_HUMAN	961.3974
KDIS_HUMAN	556.748108
ASAP1_HUMAN	3563.21484
MRTFB_HUMAN	1210.79236
ANR50_HUMAN	5236.06396
MED23_HUMAN	2464.07983
SHRM4_HUMAN	3173.07153
YETS2_HUMAN	971.901917
NDRG4_HUMAN	1219.08606
TBC24_HUMAN	2121.39355
ISY1_HUMAN	873.52301
TMCC3_HUMAN	1691.33093
HECD1_HUMAN	4715.87744
ZMYD8_HUMAN	2155.93628
CIZ1_HUMAN	321.3237
COR1C_HUMAN	18765.7168
TPX2_HUMAN	1715.01367
ABT1_HUMAN	2153.43164

NOB1_HUMAN	5753.90576
AKP8L_HUMAN	3799.48779
MAFF_HUMAN	1350.37256
ASC_HUMAN	4733.16309
TMCO1_HUMAN	4194.85547
FZR1_HUMAN	235.759003
APC10_HUMAN	861.594727
MYO6_HUMAN	5784.89941
DD19B_HUMAN	1582.40576
PPT2_HUMAN	2481.45215
PRP19_HUMAN	23847.0098
BOK_HUMAN	2447.16919
NOL7_HUMAN	5116.76416
SNX12_HUMAN	9728.83594
SYNRG_HUMAN	636.295532
VPS4A_HUMAN	9044.97754
G3BP2_HUMAN	10847.8086
CHIP_HUMAN	5169.21484
PACN2_HUMAN	10699.5703
MAGD2_HUMAN	18347.7598
SNX7_HUMAN	1763.84998
SNX6_HUMAN	7653.90479
DUS12_HUMAN	4144.14941
STX8_HUMAN	386.51178
SSRG_HUMAN	5849.4043
PSD13_HUMAN	37310.2891
FAF1_HUMAN	6345.2915
EPCR_HUMAN	20964.8164
PPIE_HUMAN	4346.74805
DIM1_HUMAN	6376.6792
TIM_HUMAN	822.970459
CSN3_HUMAN	10195.0527
MINP1_HUMAN	3057.09985
NOVA2_HUMAN	7376.10059
WDR3_HUMAN	5034.91357
TTF2_HUMAN	318.493713
NSF1C_HUMAN	11209.5547
COG5_HUMAN	2925.84766
S12A4_HUMAN	7249.83691
AP4E1_HUMAN	292.169495
MACF1_HUMAN	42372.5078
CP131_HUMAN	1205.33472
SCAF8_HUMAN	2378.1853
PP6R1_HUMAN	2305.86548
TRI33_HUMAN	3383.8103
PHF8_HUMAN	569.657898
TNR6B_HUMAN	780.765808
PLCL2_HUMAN	381.937347
SMG5_HUMAN	1095.88525
EXOC7_HUMAN	9099.63965

ZC3H4_HUMAN	161.499374
UBP24_HUMAN	3236.47705
TBD2B_HUMAN	1297.33325
SMAG1_HUMAN	1004.44073
FOXJ3_HUMAN	1580.6344
CBPC1_HUMAN	409.279755
DICER_HUMAN	1323.30261
XCT_HUMAN	1871.95972
MARE3_HUMAN	1510.48657
SHOC2_HUMAN	4555.69824
SRRM2_HUMAN	13466.3115
MGT4B_HUMAN	1394.5094
PA2G4_HUMAN	82458.6641
SPG7_HUMAN	1253.92517
BAIP2_HUMAN	468.211395
GAB2_HUMAN	596.027344
SMC3_HUMAN	7406.05176
CHM2B_HUMAN	11931.0469
SH2B3_HUMAN	1069.81677
ZN148_HUMAN	343.585022
MTMR6_HUMAN	4941.875
JAG2_HUMAN	1377.00061
NIP7_HUMAN	3919.28857
GLCNE_HUMAN	3066.46875
RTRAF_HUMAN	11845.626
RUVB2_HUMAN	31110.8027
CDYL_HUMAN	605.44696
PIN4_HUMAN	5673.0918
AKT3_HUMAN	1729.14221
FACE2_HUMAN	1181.80444
CHKB_HUMAN	502.399719
EIF3L_HUMAN	58746.5078
PLAP_HUMAN	10971.8896
RUVB1_HUMAN	51667.7344
NUDC_HUMAN	32300.2793
BCS1_HUMAN	2816.20313
VDAC3_HUMAN	21344.4395
COF2_HUMAN	20040.3809
ERGI3_HUMAN	2567.45288
SYFA_HUMAN	27118.5
SC5A6_HUMAN	751.517456
RT33_HUMAN	4290.54248
ASF1A_HUMAN	3041.21362
DRG1_HUMAN	14283.5479
TPPC4_HUMAN	3457.68921
NCKP1_HUMAN	11357.0654
CNPY2_HUMAN	6059.55859
EXOG_HUMAN	715.056091
EXC6B_HUMAN	3136.45728
PLAK2_HUMAN	15489.3975

CARD8_HUMAN	1774.36206
AT11B_HUMAN	288.233765
OFUT2_HUMAN	7137.69922
DJC16_HUMAN	943.953186
DLGP4_HUMAN	3223.99878
ST38L_HUMAN	2171.10986
SAC2_HUMAN	2592.17676
FND3A_HUMAN	4119.0166
NISCH_HUMAN	1219.30713
FYV1_HUMAN	1102.78918
WDR37_HUMAN	1624.27698
E41L3_HUMAN	22780.2227
AMOL2_HUMAN	292.045258
SIK3_HUMAN	289.106506
KDM2A_HUMAN	1697.72205
RRP44_HUMAN	6428.00342
TPPC8_HUMAN	1787.23096
LRCH1_HUMAN	3101.61328
RCL1_HUMAN	5474.05225
GSTK1_HUMAN	7381.19141
LTOR2_HUMAN	1443.44409
RT28_HUMAN	8658.59863
COA3_HUMAN	4409.9751
DDX52_HUMAN	1703.15479
RT17_HUMAN	6181.95215
RT07_HUMAN	9516.79004
CRYL1_HUMAN	2968.5293
TMA7_HUMAN	4990.66602
PDIP2_HUMAN	8279.91797
AP3M1_HUMAN	14090.0928
MAN1_HUMAN	4251.11963
CHSP1_HUMAN	1332.19873
COG6_HUMAN	2593.92847
TR150_HUMAN	16457.9023
WBP11_HUMAN	4457.80078
TDRKH_HUMAN	544.008545
MED16_HUMAN	544.596863
NOP58_HUMAN	16843.8281
GIT1_HUMAN	5477.07373
ZN281_HUMAN	734.822083
AR2BP_HUMAN	3142.87744
TMM98_HUMAN	601.322144
SGT1_HUMAN	29712.9766
SYYM_HUMAN	6088.34473
NAGA_HUMAN	2150.93042
ACOT9_HUMAN	26747.2441
AAR2_HUMAN	1355.30017
NOSIP_HUMAN	5657.40967
DEOC_HUMAN	4199.93506
MEMO1_HUMAN	6163.72705

TMX2_HUMAN	5057.10107
FCF1_HUMAN	1652.9342
LSM2_HUMAN	5109.78369
SHLB1_HUMAN	11075.1045
CIA30_HUMAN	2325.552
CAB39_HUMAN	15003.3242
LC7L2_HUMAN	12686.8232
UB2J1_HUMAN	5465.79688
RBMX2_HUMAN	482.174713
DHRS7_HUMAN	3832.52563
RT02_HUMAN	10294.9111
UTP11_HUMAN	692.971558
PHOCN_HUMAN	11401.1777
RRP7A_HUMAN	1816.50757
SBDS_HUMAN	18056.4922
TMED5_HUMAN	10096.9453
EXOS1_HUMAN	3503.03345
TMED7_HUMAN	4530.71289
SF3B6_HUMAN	13534.7061
RM11_HUMAN	11912.8281
ORN_HUMAN	17083.123
RRP15_HUMAN	6655.99072
WASC3_HUMAN	1110.72595
NOP16_HUMAN	7037.18213
TPRKB_HUMAN	3760.10791
PPIL1_HUMAN	3533.71387
UFC1_HUMAN	5298.74756
RT16_HUMAN	5516.65381
RT18C_HUMAN	1243.0564
FIS1_HUMAN	1867.40674
TIM16_HUMAN	2795.3374
KAD6_HUMAN	6170.43848
RT23_HUMAN	12002.71
GOT1B_HUMAN	2476.85376
HDGR3_HUMAN	2996.98462
PTH2_HUMAN	7985.51318
CHMP3_HUMAN	40572.2695
STRAP_HUMAN	21387.5664
RTCB_HUMAN	47654.6328
FBX7_HUMAN	3351.18652
RAP2C_HUMAN	1518.08057
STA13_HUMAN	3594.74561
RBGP1_HUMAN	1931.85266
TMED3_HUMAN	1075.21375
T22D4_HUMAN	1262.66711
ZN330_HUMAN	944.016846
R3HC1_HUMAN	807.661438
NOC2L_HUMAN	9957.88281
RL36_HUMAN	55832.9414
CCDC9_HUMAN	1988.20166

CHTOP_HUMAN	1842.98853
SAMH1_HUMAN	6368.05908
SKAP_HUMAN	1068.51782
HBS1L_HUMAN	4717.30908
AAKB1_HUMAN	919.884033
WIPI4_HUMAN	433.25946
DMXL1_HUMAN	755.70636
VPP2_HUMAN	2842.80591
TLN1_HUMAN	95657.1016
KIF3A_HUMAN	1737.8606
TRRAP_HUMAN	2187.00146
MTCL1_HUMAN	666.678101
DCAF1_HUMAN	569.284973
TCAF1_HUMAN	7313.96387
RBM19_HUMAN	813.53479
DAAM1_HUMAN	401.156677
PLXD1_HUMAN	15185.793
HECD4_HUMAN	592.199341
WAC2C_HUMAN	357.456482
WDR7_HUMAN	322.451965
UBP15_HUMAN	7168.67041
FARP1_HUMAN	1099.63123
MARF1_HUMAN	688.166138
C170B_HUMAN	654.419373
RPGF2_HUMAN	6559.10596
MYO5A_HUMAN	5025.10352
DTNA_HUMAN	860.761841
LOXL2_HUMAN	5906.74365
CRBG1_HUMAN	1020.03088
TRAF6_HUMAN	2561.98975
M4K5_HUMAN	3685.81396
HYOU1_HUMAN	28825.8906
ATG4B_HUMAN	6759.63672
TBL2_HUMAN	4548.23291
WIPI2_HUMAN	3020.95117
TELO2_HUMAN	2506.25854
LAS1L_HUMAN	5605.62988
AFG32_HUMAN	8149.48486
KLF12_HUMAN	161.364639
ARI1_HUMAN	6742.64502
LSM4_HUMAN	23887.502
RN114_HUMAN	3242.90527
SAM50_HUMAN	15534.7383
PRC2C_HUMAN	17809.9453
OARD1_HUMAN	7463.33398
IFT25_HUMAN	1693.59302
PPME1_HUMAN	11107.3828
RBM7_HUMAN	1702.97168
TIM22_HUMAN	1359.06653
NUB1_HUMAN	1841.17151

YTHD2_HUMAN	3577.62109
CTDP1_HUMAN	2991.41284
PAXB1_HUMAN	991.338196
NDK7_HUMAN	2634.0249
SP16H_HUMAN	14874.3066
UTP18_HUMAN	3641.19067
UCHL5_HUMAN	18824.8027
CD2AP_HUMAN	10872.8926
VATD_HUMAN	7094.01611
TNPO3_HUMAN	5651.08008
SRPRB_HUMAN	12449.9971
ORC6_HUMAN	554.305725
CERT_HUMAN	1552.52734
GMPPB_HUMAN	17527.7207
FADS3_HUMAN	1294.4021
TF3C5_HUMAN	5553.38916
TF3C3_HUMAN	3412.6582
TPPC1_HUMAN	5369.96582
TRPV2_HUMAN	523.659119
MRCKB_HUMAN	10159.5693
RBM8A_HUMAN	18245.834
DJC15_HUMAN	1237.27222
UBP16_HUMAN	1069.26318
TSSC4_HUMAN	3380.7146
ZN706_HUMAN	3538.76196
MAGD1_HUMAN	3515.50659
SNX14_HUMAN	651.127625
SNX11_HUMAN	1023.11255
SNX9_HUMAN	11278.8809
SNX8_HUMAN	1035.82898
SNX5_HUMAN	5979.2915
LIPG_HUMAN	1480.66187
NUBP2_HUMAN	5954.8374
PEX16_HUMAN	1203.38489
LYVE1_HUMAN	3106.3313
BACE2_HUMAN	862.885193
HCFC2_HUMAN	935.398743
UBIA1_HUMAN	3485.11255
PUS1_HUMAN	4780.19531
LRRF2_HUMAN	4320.29541
FHOD1_HUMAN	6992.84619
SERC_HUMAN	21562.8496
NCOR2_HUMAN	2588.93652
ORNT1_HUMAN	1636.98804
JAM1_HUMAN	15013.3584
NPTN_HUMAN	1433.62439
AGRG1_HUMAN	404.128693
SPIN1_HUMAN	2173.7168
S12A7_HUMAN	6632.27197
ALG6_HUMAN	852.704895

ALG5_HUMAN	7330.8374
RT18B_HUMAN	5145.21045
COPG1_HUMAN	31142.4297
AUP1_HUMAN	2379.1189
FKBP7_HUMAN	1101.48914
CLIC4_HUMAN	58045.2383
NFS1_HUMAN	3884.60278
CFA20_HUMAN	9105.97559
TACC3_HUMAN	551.291382
SPCS1_HUMAN	6474.57959
SAR1B_HUMAN	4581.74316
AP4B1_HUMAN	762.086975
MTCH2_HUMAN	19675.9551
SELK_HUMAN	510.132507
BIG2_HUMAN	2938.85547
BIG1_HUMAN	3868.27856
MD1L1_HUMAN	2945.20313
STK24_HUMAN	12027.1875
5MP1_HUMAN	43715.4609
COMDA_HUMAN	1701.24573
DC1L1_HUMAN	18379.0039
EPN1_HUMAN	2850.10352
UBP3_HUMAN	20176.0469
TX264_HUMAN	5479.76758
CABIN_HUMAN	661.313354
CEPT1_HUMAN	8231.93848
DNM3A_HUMAN	646.508362
OAS3_HUMAN	1026.84631
KAD5_HUMAN	14080.8311
NEMO_HUMAN	4543.96729
IF2B2_HUMAN	21920.8125
ZNT1_HUMAN	2876.27441
S4A7_HUMAN	1802.40491
NDUB9_HUMAN	11477.4463
SQOR_HUMAN	2501.9248
ROBO1_HUMAN	471.670929
STON1_HUMAN	219.245728
NCOA3_HUMAN	424.397675
NUMBL_HUMAN	2252.28955
M3K4_HUMAN	1188.62549
DDX49_HUMAN	1808.56848
CAN7_HUMAN	965.139038
WASF2_HUMAN	10091.3818
PIAS3_HUMAN	333.661621
SCC4_HUMAN	1793.6925
MORC2_HUMAN	990.72998
NS1BP_HUMAN	1678.93018
S23IP_HUMAN	15627.5664
GAL3B_HUMAN	3491.77783
IFT56_HUMAN	2744.62012

UBA6_HUMAN	18109.2305
ESYT2_HUMAN	14395.3105
BLT3B_HUMAN	150.472778
SHOT1_HUMAN	2450.28271
TM223_HUMAN	355.453033
RHG10_HUMAN	2970.7793
HACL2_HUMAN	7904.18799
SPD2B_HUMAN	11434.0674
NBAS_HUMAN	2539.06079
TYW5_HUMAN	208.692383
DEND3_HUMAN	1494.02637
VWA8_HUMAN	2217.09131
SBNO1_HUMAN	2282.73096
GTPBA_HUMAN	1328.54858
WDR91_HUMAN	596.173828
DEN11_HUMAN	247.532822
PDES1_HUMAN	575.931946
TPC13_HUMAN	1449.91333
CNOT1_HUMAN	8433.76172
MP3B2_HUMAN	30562.5508
PGP_HUMAN	8076.01172
RIMC1_HUMAN	961.027466
RCCD1_HUMAN	3547.61475
TCAF2_HUMAN	8420.69727
SMHD1_HUMAN	5932.14844
CAST2_HUMAN	698.234558
U119B_HUMAN	4967.14111
MET15_HUMAN	659.144714
CC85C_HUMAN	649.550171
RTL8C_HUMAN	3211.27319
ZSWM8_HUMAN	890.854675
P121C_HUMAN	2599.41162
P20L1_HUMAN	441.65451
RUXGL_HUMAN	15497.3945
ELAP2_HUMAN	1231.39001
NUD19_HUMAN	2615.09937
MI4GD_HUMAN	602.720764
CALM1_HUMAN	4314.1499
MYO9A_HUMAN	276.202698
GPHRA_HUMAN	1321.50146
WASH3_HUMAN	6210.51074
MCRI1_HUMAN	1768.65662
NACAM_HUMAN	61049.3711
CK098_HUMAN	3074.13354
ASURF_HUMAN	975.080444
MIDUO_HUMAN	3211.41968
DNS2A_HUMAN	3408.65479
ADAS_HUMAN	13865.627
KIF2A_HUMAN	7836.75488
DX39A_HUMAN	84607.2031

PDLI1_HUMAN	8793.35156
BACH_HUMAN	47266.9102
MYO1C_HUMAN	31568.6875
SNP23_HUMAN	9121.55566
HAX1_HUMAN	1778.07239
AIP_HUMAN	6959.14844
GTPB1_HUMAN	4560.14648
LEG9_HUMAN	582.581177
STXB3_HUMAN	8256.25879
AP4M1_HUMAN	2944.5481
ARVC_HUMAN	4353.78662
SMAP_HUMAN	1074.24451
AP3B1_HUMAN	13343.7373
APBB1_HUMAN	1638.31372
LEG8_HUMAN	856.779419
NDUS8_HUMAN	818.545471
IKBE_HUMAN	1023.88251
PSD11_HUMAN	39481.707
PSD12_HUMAN	26411.1836
PSMD9_HUMAN	3096.44751
MEN1_HUMAN	565.641724
GET1_HUMAN	2251.93311
PGRC1_HUMAN	953.257935
SPT5H_HUMAN	4799.95557
TAF4_HUMAN	319.522156
DFFA_HUMAN	1529.89709
HIP1_HUMAN	1846.62695
CLIC1_HUMAN	213451.688
EIF3F_HUMAN	24344.3574
WWP2_HUMAN	810.314575
ODPX_HUMAN	4029.61279
QSOX1_HUMAN	4172.67188
DCTN6_HUMAN	1299.59033
ACATN_HUMAN	4076.93188
WASL_HUMAN	4684.91455
IPO5_HUMAN	42444.5469
RPOM_HUMAN	951.063293
EF2K_HUMAN	3255.55518
SAP18_HUMAN	11304.084
EMAL1_HUMAN	2414.22266
IF2B3_HUMAN	36514.168
DNM1L_HUMAN	40338.168
RTCA_HUMAN	6296.46387
P3C2A_HUMAN	5452.50928
GOLI4_HUMAN	2371.84839
AGRIN_HUMAN	1921.29529
PLOD2_HUMAN	44084.8555
EXOC5_HUMAN	8272.30469
BT3A1_HUMAN	717.940063
NDUA4_HUMAN	5136.4502

PSDE_HUMAN	42300.1719
BIN1_HUMAN	3598.271
CLD5_HUMAN	2419.27441
IMA4_HUMAN	24392.7363
STK25_HUMAN	2969.58301
KRIT1_HUMAN	363.25766
VMA5A_HUMAN	845.443298
PESC_HUMAN	10255.2832
SDCB1_HUMAN	44812.8516
PITM1_HUMAN	479.711243
MPP10_HUMAN	5020.99072
NOP56_HUMAN	7222.80029
DDX3X_HUMAN	69991.6797
MFNG_HUMAN	362.909027
PODXL_HUMAN	35738.707
CCN1_HUMAN	58047.5391
PIR_HUMAN	29896.7813
IMA3_HUMAN	6428.67383
TRI38_HUMAN	697.519836
NFIB_HUMAN	4613.66064
PPP6_HUMAN	9309.38086
NDKM_HUMAN	2188.46753
P3C2B_HUMAN	418.264465
MA2B1_HUMAN	1802.12012
PDXK_HUMAN	3474.55615
SCD_HUMAN	5113.99316
ARI1A_HUMAN	2739.98047
NEMP1_HUMAN	663.676025
TXND9_HUMAN	3923.2688
DPYL4_HUMAN	39604.3516
TRAD1_HUMAN	1409.328
COX7R_HUMAN	784.178833
UBFD1_HUMAN	4697.74463
CTRO_HUMAN	2050.61304
COPE_HUMAN	16798.3105
BORG1_HUMAN	3031.39917
AP3D1_HUMAN	11370.9111
CCS_HUMAN	1026.85181
ABLM1_HUMAN	3524.06787
DVL1_HUMAN	296.186462
DVL2_HUMAN	1315.93225
CHD1_HUMAN	3344.41357
CHD2_HUMAN	4631.73975
GOSR2_HUMAN	5216.46191
TOR1A_HUMAN	5326.42334
TOR1B_HUMAN	3432.43213
STX16_HUMAN	2209.00952
ADA10_HUMAN	8972.38867
ABCD4_HUMAN	1367.82458
EI24_HUMAN	3501.01709

P5I11_HUMAN	2435.96411
KMT2D_HUMAN	198.062057
APAF_HUMAN	2700.73657
RIOK3_HUMAN	832.680603
MP2K7_HUMAN	442.770813
ACOT8_HUMAN	2061.1665
CDIPT_HUMAN	34476.8672
PDCD5_HUMAN	2537.60571
ANM5_HUMAN	17930.8027
NHRF1_HUMAN	4735.68848
CHK1_HUMAN	1302.28748
TR10B_HUMAN	5979.77148
TPP1_HUMAN	13464.0713
TCRG1_HUMAN	17093.2168
NDC80_HUMAN	2616.61401
KIF3C_HUMAN	298.05191
NRP1_HUMAN	12891.0273
TNPO2_HUMAN	3304.02002
UN13B_HUMAN	880.695923
TR10C_HUMAN	235.41394
RPC1_HUMAN	1871.55872
TSN4_HUMAN	370.963318
PSA7_HUMAN	94058.5547
SCAM3_HUMAN	5036.87646
BCKD_HUMAN	2535.9856
MGST3_HUMAN	7488.61475
TX1B3_HUMAN	9372.7959
GIPC1_HUMAN	7018.96094
IKKB_HUMAN	2653.31665
RGS12_HUMAN	1973.11572
TIM23_HUMAN	2258.18896
HAT1_HUMAN	11453.8613
CSKP_HUMAN	2489.63696
PLD2_HUMAN	962.873413
QCR8_HUMAN	12676.2139
ML12B_HUMAN	77866.625
HGS_HUMAN	7887.3252
RAB7L_HUMAN	739.179443
VP26C_HUMAN	1193.24036
MYPT1_HUMAN	11263.2119
GAK_HUMAN	5460.40723
HNRDL_HUMAN	26699.0352
XPO1_HUMAN	28313.0781
BTAF1_HUMAN	1707.86902
ARHGA_HUMAN	3438.01831
ZN609_HUMAN	1175.93555
SPTN2_HUMAN	1206.14905
MAST4_HUMAN	1275.19141
SC16A_HUMAN	6958.53076
PLXB2_HUMAN	2649.47583

KHNYN_HUMAN	448.963837
TCPR2_HUMAN	396.432343
SR140_HUMAN	26057.707
SET1A_HUMAN	859.46991
N4BP3_HUMAN	262.88739
SYNJ2_HUMAN	4701.46045
SYNEM_HUMAN	5975.87402
GRRE1_HUMAN	1023.70673
KIF3B_HUMAN	1898.6333
PUR4_HUMAN	23704.9863
ANR28_HUMAN	1740.5719
ARHGB_HUMAN	549.289795
MRPP3_HUMAN	1161.8335
IKKA_HUMAN	1672.30139
NPC1_HUMAN	4036.09131
DEGS1_HUMAN	2937.354
ANGP2_HUMAN	3398.99878
SCAM1_HUMAN	5259.95313
SCAM2_HUMAN	1977.77039
ARC1B_HUMAN	37510.6758
ARPC2_HUMAN	38350.5
ARPC3_HUMAN	47106.5313
RPAC1_HUMAN	9472.71191
PLS1_HUMAN	1354.71558
TIF1A_HUMAN	449.807648
PGRC2_HUMAN	11448.0732
RGL2_HUMAN	1654.60498
WDR46_HUMAN	1428.65796
NKRF_HUMAN	2039.03918
GNPAT_HUMAN	2317.37988
LAMA5_HUMAN	1122.42151
ZN185_HUMAN	12479.3574
CASC3_HUMAN	536.847656
RT12_HUMAN	2649.14087
ACOX3_HUMAN	1929.68982
RER1_HUMAN	16064.4229
SURF4_HUMAN	18870.7813
MK13_HUMAN	973.080505
SPTC1_HUMAN	7100.43896
SPTC2_HUMAN	8694.26953
OGT1_HUMAN	7885.4707
PMM2_HUMAN	14449.165
TM9S1_HUMAN	2174.90161
TRI18_HUMAN	1121.13843
HMGB3_HUMAN	18788.3086
PPM1G_HUMAN	13363.0537
SHIP2_HUMAN	4843.88184
EIF3D_HUMAN	27222.9238
EIF3H_HUMAN	30770.0039
HDAC3_HUMAN	1214.06396

NVL_HUMAN	1427.60046
BCAT2_HUMAN	7909.4585
IPO8_HUMAN	4748.1875
STX7_HUMAN	7114.52832
MOT4_HUMAN	15127.3906
MRP4_HUMAN	2242.40088
RPA34_HUMAN	2182.37061
P4HA2_HUMAN	6856.70459
CAN5_HUMAN	729.31311
YKT6_HUMAN	44687.5547
NUP42_HUMAN	557.049927
ARPC5_HUMAN	36550.0391
CFLAR_HUMAN	487.039795
MAFG_HUMAN	282.487366
PDPK1_HUMAN	1356.9303
TPSN_HUMAN	10703.416
R113A_HUMAN	840.329041
KDM6A_HUMAN	848.989075
DHX15_HUMAN	33849.2188
MCES_HUMAN	5539.61914
ZZEF1_HUMAN	3041.85498
ASAP2_HUMAN	915.695068
FLRT2_HUMAN	1368.15552
TTI1_HUMAN	1424.45227
RRP8_HUMAN	2614.16943
PJA2_HUMAN	373.601532
CYB5B_HUMAN	996.272888
PRP4_HUMAN	8333.15918
SERA_HUMAN	18233.1719
NDUS4_HUMAN	2864.16943
DC1L2_HUMAN	12139.6504
PSMD3_HUMAN	40540.9414
RFOX2_HUMAN	2692.02808
PAPS1_HUMAN	15330.7324
ZW10_HUMAN	6476.12793
SNUT1_HUMAN	7381.77393
GPAA1_HUMAN	5279.56104
DAPK3_HUMAN	6784.76318
TGFI1_HUMAN	2672.87207
AP5Z1_HUMAN	739.2323
CTIF_HUMAN	3711.23828
MTSS1_HUMAN	487.741913
VIP2_HUMAN	1576.19824
M3K7_HUMAN	636.978027
MCA3_HUMAN	23678.1133
RIPK2_HUMAN	2440.08325
HNRPR_HUMAN	87464.625
PRPF3_HUMAN	8257.56543
TXNL1_HUMAN	51113.8984
TPD54_HUMAN	18182.9414

EMC8_HUMAN	4023.82251
ERI3_HUMAN	2094.96973
SYNJ1_HUMAN	1725.68396
FIBP_HUMAN	2081.40332
IF4G3_HUMAN	25043.6836
PPIH_HUMAN	4895.29102
HTRA2_HUMAN	3200.82373
MS18B_HUMAN	228.25856
ARK72_HUMAN	11917.2803
E41L2_HUMAN	12547.0498
TGON2_HUMAN	1092.95056
B4GA1_HUMAN	1028.25403
WIPF1_HUMAN	2172.93408
AT8B1_HUMAN	1287.71704
AKA10_HUMAN	423.795685
DENR_HUMAN	17616.0879
XPOT_HUMAN	15637.4521
DNPH1_HUMAN	9542.62598
TIM44_HUMAN	9890.22949
TPPC3_HUMAN	14946.5928
CHM2A_HUMAN	8781.9707
NCK2_HUMAN	2460.50269
TSN6_HUMAN	1088.79395
PLRG1_HUMAN	7076.71094
PRC1_HUMAN	1793.46655
RGS10_HUMAN	2666.47144
ZN207_HUMAN	2764.92432
NDUB5_HUMAN	5072.58203
NDUB3_HUMAN	8945.44727
NDUA2_HUMAN	1436.59473
LDB2_HUMAN	1257.77124
GET3_HUMAN	24375.9375
BUB1_HUMAN	470.835022
BUB3_HUMAN	28104.2598
ST1B1_HUMAN	7393.44971
ACTN4_HUMAN	70386.0547
BUD23_HUMAN	925.833435
HTSF1_HUMAN	2772.98608
ERD23_HUMAN	2660.32373
AP1G1_HUMAN	12407.5674
STX6_HUMAN	972.041992
SNG2_HUMAN	4236.58398
SGTA_HUMAN	2534.48413
ENSA_HUMAN	7593.05713
MCAT_HUMAN	7768.6665
SYNC_HUMAN	37408.8125
MYO1B_HUMAN	6404.7373
PM34_HUMAN	1354.4707
CPSF5_HUMAN	15851.1221
LANC1_HUMAN	9327.5293

STRN_HUMAN	5958.12158
U3IP2_HUMAN	6638.52734
SCO2_HUMAN	975.8797
CF410_HUMAN	112.752533
AKAP8_HUMAN	2889.09644
GTPB6_HUMAN	1051.23291
G6PT1_HUMAN	1221.5647
IDH3B_HUMAN	7208.89941
NRDC_HUMAN	12431.5908
CALU_HUMAN	16543.2285
EDIL3_HUMAN	7354.92041
ATP9B_HUMAN	618.802551
SAHH2_HUMAN	2133.37695
KIF1C_HUMAN	3245.01563
EXTL3_HUMAN	759.026245
ORC5_HUMAN	1162.52356
NDUS5_HUMAN	11975.1328
PDE6D_HUMAN	3365.12769
ORC4_HUMAN	1061.08557
PEX1_HUMAN	896.023682
RAD21_HUMAN	7236.94775
DHX16_HUMAN	4351.18359
GMFG_HUMAN	2149.24805
3BP5_HUMAN	3500.03369
MED14_HUMAN	12698.75
PCDH7_HUMAN	157.136002
KPRB_HUMAN	6553.16113
SMCA5_HUMAN	8078.68115
JIP4_HUMAN	88452.9766
NUAK1_HUMAN	301.648651
NPA1P_HUMAN	1223.67419
SI1L3_HUMAN	291.378052
ZC3H1_HUMAN	223.961975
AQR_HUMAN	7529.90381
OPA1_HUMAN	5940.15088
ZEB2_HUMAN	393.627319
GANP_HUMAN	1868.37207
PCDGC_HUMAN	1211.56494
PI51C_HUMAN	3071.15454
KIF1B_HUMAN	2736.14551
MABP1_HUMAN	671.773743
KDM1A_HUMAN	6424.86523
TBCD4_HUMAN	8325.93457
FADS1_HUMAN	2991.84009
GSDME_HUMAN	7763.00244
EVI5_HUMAN	295.091614
NRP2_HUMAN	7131.3501
MA1A2_HUMAN	3070.15674
MPZL2_HUMAN	3564.59985
ACSL4_HUMAN	12550.5938

SNX3_HUMAN	28581.002
OGA_HUMAN	6983.26123
VINEX_HUMAN	3297.22729
HNRPQ_HUMAN	43693.8672
PRP17_HUMAN	2009.50061
RNBP6_HUMAN	34852.1211
NEMF_HUMAN	6115.25684
GMDS_HUMAN	4511.89258
NMT2_HUMAN	5027.02002
CCNT1_HUMAN	1759.77429
BUB1B_HUMAN	1926.21985
PLOD3_HUMAN	24639.3223
IF4E2_HUMAN	5084.57617
DIAP1_HUMAN	60687.3398
TSN3_HUMAN	4713.96045
EXOC3_HUMAN	10099.5771
PLIN3_HUMAN	17775.5352
ANM3_HUMAN	1849.14368
IMA7_HUMAN	11192.6084
UGDH_HUMAN	40993.668
TPST2_HUMAN	1644.53699
LPXN_HUMAN	1930.29346
CTND1_HUMAN	39162.0469
ICMT_HUMAN	4508.91602
SNX2_HUMAN	15416.8447
DPM1_HUMAN	14617.9297
USO1_HUMAN	18565.4238
RT14_HUMAN	2363.53491
TOM1_HUMAN	3374.71265
H2B1K_HUMAN	90639
CCD22_HUMAN	4057.49536
PQBP1_HUMAN	5924.73877
TI17B_HUMAN	693.97229
PRAF2_HUMAN	10129.1094
DKC1_HUMAN	11237.9932
IF2P_HUMAN	32471.2109
EDF1_HUMAN	24131.0293
KIN17_HUMAN	1030.22668
DIAP2_HUMAN	7220.04932
DNJA2_HUMAN	8447.91992
BRD4_HUMAN	4389.06543
HUS1_HUMAN	991.276489
PFD1_HUMAN	1686.73206
PP1RB_HUMAN	2115.3252
NBN_HUMAN	9965.35742
MCE1_HUMAN	1576.64844
ABCB7_HUMAN	2567.88745
SRGP2_HUMAN	1774.91943
PLXA2_HUMAN	9417.70508
XYLK_HUMAN	810.089783

WDR1_HUMAN	106837.938
ATP9A_HUMAN	1711.98157
N4BP1_HUMAN	733.084778
ROCK2_HUMAN	7549.97754
CLAP2_HUMAN	3388.78882
PTCD1_HUMAN	747.624878
CPNE3_HUMAN	19440.3223
ATG13_HUMAN	1129.17651
HIP1R_HUMAN	8159.1499
BRE1B_HUMAN	4677.98389
PHF2_HUMAN	576.901428
ZC11A_HUMAN	3383.01953
CLU_HUMAN	9057.18555
CAND2_HUMAN	1051.63147
T22D2_HUMAN	431.333832
DJC13_HUMAN	6970.4751
PHAR2_HUMAN	1007.48529
PP6R2_HUMAN	1657.74316
ATS4_HUMAN	2326.89966
CNOT3_HUMAN	5396.22021
ANR17_HUMAN	5637.26221
SIN3B_HUMAN	752.556396
DNJB6_HUMAN	3440.3186
COQ9_HUMAN	1248.24548
GGCT_HUMAN	6464.91992
NDUS7_HUMAN	11221.1035
NDUS2_HUMAN	9933.95313
ZPR1_HUMAN	2553.23486
DUS11_HUMAN	850.190247
NIPS2_HUMAN	8910.48828
HMMR_HUMAN	557.246216
PDCD6_HUMAN	12228.3525
TBCA_HUMAN	16883.4727
VATG1_HUMAN	15937.3203
VPS4B_HUMAN	64353.3242
MPU1_HUMAN	10959.499
H2AY_HUMAN	1021.02313
SH3L1_HUMAN	1340.08264
FLNB_HUMAN	129803.367
NCOR1_HUMAN	6293.37061
PEX14_HUMAN	3953.93994
TRIM3_HUMAN	3980.04492
CISY_HUMAN	119084.359
SPAG7_HUMAN	10640.9023
SC22B_HUMAN	40172.3242
PR40A_HUMAN	11651.417
TACC1_HUMAN	8174.84521
NDK6_HUMAN	1624.97766
CDC45_HUMAN	979.53009
LRCH4_HUMAN	705.419678

MTX2_HUMAN	1988.67419
VP26A_HUMAN	34136.168
MPPB_HUMAN	12561.6426
MED24_HUMAN	907.426575
KTNA1_HUMAN	2183.83423
PSIP1_HUMAN	2014.40149
ERLN1_HUMAN	6021.52344
NDUS3_HUMAN	15847.5186
SRS10_HUMAN	6998.95557
CLN5_HUMAN	2931.61084
CLD11_HUMAN	1722.92322
ECI2_HUMAN	16080.6299
EED_HUMAN	2715.16504
BAF_HUMAN	3004.25415
SF3B1_HUMAN	29225.5762
CSDE1_HUMAN	20137.9199
WBP4_HUMAN	1330.44177
PRKRA_HUMAN	5228.05957
ITA10_HUMAN	1047.01599
KS6A5_HUMAN	1832.37756
MED6_HUMAN	422.730011
MYCB2_HUMAN	1574.02869
KBL_HUMAN	1498.09827
LYPA1_HUMAN	9791.38379
ERAL1_HUMAN	5220.64111
U520_HUMAN	18778.2891
MTU1_HUMAN	1392.46802
TIPRL_HUMAN	9927.68359
OFD1_HUMAN	1533.44116
KS6A4_HUMAN	1611.38391
SURF6_HUMAN	1605.34888
PPM1B_HUMAN	2520.8479
UTP20_HUMAN	6269.01123
NU155_HUMAN	10523.3545
XRP2_HUMAN	7851.61719
WDHD1_HUMAN	4068.66504
CRTAP_HUMAN	19199.9902
S2512_HUMAN	11240.0566
RENR_HUMAN	3284.54248
RNH2A_HUMAN	1582.57251
CD123_HUMAN	3453.15283
BCAR3_HUMAN	719.069153
POP7_HUMAN	1868.97375
RPP40_HUMAN	1169.73352
EIF3G_HUMAN	21611.9668
EIF3J_HUMAN	21105.7539
CBR3_HUMAN	12790.2471
PSD10_HUMAN	3495.26563
AP1G2_HUMAN	1370.35461
FACE1_HUMAN	12088.8008

IDHC_HUMAN	46832.0352
GATB_HUMAN	977.989319
SCO1_HUMAN	7810.56348
ATRN_HUMAN	884.629395
RBBP9_HUMAN	7027.33789
STAM2_HUMAN	4732.97559
DGAT1_HUMAN	502.380585
CCNK_HUMAN	4366.14355
PRAF3_HUMAN	47407.0156
DYSF_HUMAN	19982.4766
PIAS1_HUMAN	655.180725
SPF27_HUMAN	694.284058
DCTN3_HUMAN	14051.0889
DNJC8_HUMAN	13429.2402
SPF30_HUMAN	4029.42871
RAD17_HUMAN	227.793655
ATP5H_HUMAN	4503.71045
TSN9_HUMAN	1949.08557
FLOT1_HUMAN	6556.85498
TRIO_HUMAN	5041.35742
ATP5L_HUMAN	36047.6172
MPDZ_HUMAN	1985.9325
CBPD_HUMAN	7758.47461
GLRX3_HUMAN	51307.4414
RL1D1_HUMAN	21035.332
WFS1_HUMAN	5740.65088
CLPX_HUMAN	5531.56592
S14L2_HUMAN	4135.94385
SYUG_HUMAN	988.99762
CIAO1_HUMAN	3345.85547
SRP72_HUMAN	11048.9756
DDAH1_HUMAN	8945.41797
RECQ5_HUMAN	1128.48608
RMP_HUMAN	1535.27295
B3GA3_HUMAN	780.221313
MTA2_HUMAN	13374.6855
AL1A2_HUMAN	4499.39502
STK10_HUMAN	5766.9541
KPCD3_HUMAN	1677.40686
GFPT2_HUMAN	1338.40466
SLIT2_HUMAN	1762.42957
LTN1_HUMAN	2554.02075
TOM70_HUMAN	7291.13525
PKHG5_HUMAN	1160.96948
IPO13_HUMAN	1082.23547
DDHD2_HUMAN	599.337463
MYO1D_HUMAN	7529.67041
TOX4_HUMAN	2556.90332
MICA2_HUMAN	2944.06567
SC24D_HUMAN	16831.8242

FCSD2_HUMAN	535.503052
UFL1_HUMAN	10766.2744
SRBS2_HUMAN	6711.75098
SASH1_HUMAN	4353.59277
UBXN7_HUMAN	3943.97021
KLH18_HUMAN	866.477905
SUN1_HUMAN	1628.02539
PLPHP_HUMAN	8255.61133
ERLN2_HUMAN	12415.3125
PRP6_HUMAN	13750.1572
PCF11_HUMAN	595.676941
FRYL_HUMAN	6577.3877
NFAT5_HUMAN	235.056107
ENDD1_HUMAN	1349.98254
GLCE_HUMAN	7075.32324
GLSK_HUMAN	24307.1777
HAUS5_HUMAN	545.934753
ABLM3_HUMAN	588.553101
FBX21_HUMAN	550.351257
KDM4B_HUMAN	328.738342
MTCL2_HUMAN	2342.51978
UBP19_HUMAN	1236.44263
WDR47_HUMAN	2164.17847
AP2A2_HUMAN	20602.9629
SC31A_HUMAN	42569.1836
ARHGF_HUMAN	7997.61572
HEXI1_HUMAN	1937.41895
RPP14_HUMAN	537.124084
CCNB2_HUMAN	239.168259
YIF1A_HUMAN	4785.10449
UBR5_HUMAN	2320.42334
AGFG2_HUMAN	1899.25366
PRS23_HUMAN	5389.07764
SCAF4_HUMAN	2251.19043
NDUB6_HUMAN	6520.19434
MFN2_HUMAN	2069.56689
SPN1_HUMAN	1332.87878
UBE4B_HUMAN	5502.20557
ZFPL1_HUMAN	4914.50977
ELP1_HUMAN	5028.83252
NDUB4_HUMAN	5227.76855
NDUB8_HUMAN	6140.81738
NDUA7_HUMAN	4219.73291
VAMP5_HUMAN	1248.68201
RTN3_HUMAN	9989.55664
LETM1_HUMAN	15019.7773
EPN2_HUMAN	2767.16943
ZRAB2_HUMAN	7554.13965
SNX4_HUMAN	3333.91309
LC7L3_HUMAN	4155.18262

KI20A_HUMAN	1106.37891
APOL3_HUMAN	351.234344
KIF4A_HUMAN	5285.30762
MBD4_HUMAN	333.785492
MTMR5_HUMAN	1271.81848
GOSR1_HUMAN	9220.28223
KAT7_HUMAN	1075.45532
ATE1_HUMAN	1550.11462
VAPB_HUMAN	5013.93018
SNAPN_HUMAN	2519.27441
MPZL1_HUMAN	3061.98486
NDUC2_HUMAN	8974.3252
NDUAA_HUMAN	12018.8086
FKBP9_HUMAN	11161.0127
CELF2_HUMAN	1595.50781
6PGL_HUMAN	4445.2998
PAPS2_HUMAN	26846.3262
SMC2_HUMAN	11447.8428
ATG7_HUMAN	6488.25049
TACC2_HUMAN	1405.10352
TRI16_HUMAN	2343.35278
SYFM_HUMAN	1109.5531
ZBT7A_HUMAN	1111.53186
LYPA2_HUMAN	5053.18213
IPO7_HUMAN	38906.8711
ARI2_HUMAN	1191.76428
SLU7_HUMAN	1272.5249
AGM1_HUMAN	16369.6396
MOCS3_HUMAN	878.558838
CD2B2_HUMAN	2755.76782
SVIL_HUMAN	6004.21436
AHSA1_HUMAN	37684.8633
PARN_HUMAN	3108.7229
PSMG1_HUMAN	11650.1387
SGPL1_HUMAN	3035.35132
CNEP1_HUMAN	3080.36987
NSA2_HUMAN	3650.11206
G6PE_HUMAN	7364.12939
SC24A_HUMAN	8911.08789
SC24B_HUMAN	7048.37939
AGRL2_HUMAN	1729.39307
NADK_HUMAN	766.435181
TYDP2_HUMAN	970.555298
MPC2_HUMAN	1686.24622
ETHE1_HUMAN	7798.59619
ACSL3_HUMAN	13293.6113
RPA1_HUMAN	3890.05859
YETS4_HUMAN	915.760132
ZBT11_HUMAN	505.313568
CNOT4_HUMAN	991.598816

STABP_HUMAN	1653.10059
CPSF4_HUMAN	1957.26135
NFAC1_HUMAN	483.769928
ASML_HUMAN	5281.08203
CDS2_HUMAN	624.947449
PPR3D_HUMAN	1057.38574
RPP29_HUMAN	885.241882
HERC2_HUMAN	1132.36963
RAB3D_HUMAN	1755.10986
SNP29_HUMAN	4174.71729
OXSR1_HUMAN	5977.66846
GGPPS_HUMAN	1566.45801
HS74L_HUMAN	7051.50195
PTBP3_HUMAN	13681.6504
TBCD8_HUMAN	882.362793
STR3N_HUMAN	2799.60938
LSM8_HUMAN	447.816833
AP2A1_HUMAN	28898.8867
WIZ_HUMAN	1711.32178
RIGI_HUMAN	7694.32324
STAU1_HUMAN	14366.7441
TTC4_HUMAN	5087.43555
TM50A_HUMAN	2978.85571
CAVN2_HUMAN	23340.7012
BAG2_HUMAN	10700.1475
BAG3_HUMAN	2919.07813
M4K4_HUMAN	10386.8418
DCMC_HUMAN	293.25592
QORL1_HUMAN	1585.19507
AIFM1_HUMAN	15042.0264
EMAL2_HUMAN	1152.16187
LATS1_HUMAN	2022.05042
NUD14_HUMAN	4121.74512
TSN15_HUMAN	1252.88916
BPNT1_HUMAN	7334.49121
FADS2_HUMAN	30938.6426
DDAH2_HUMAN	307.558868
ABHGA_HUMAN	2711.50342
TXD12_HUMAN	1482.26086
ECD_HUMAN	4961.19775
MBD3_HUMAN	1875.03174
TOP3B_HUMAN	607.28717
NUDT3_HUMAN	2980.24316
BCL10_HUMAN	3972.45239
NDUBA_HUMAN	12849.4785
CLPT1_HUMAN	7827.14111
ZBED1_HUMAN	329.706207
MOC2B_HUMAN	750.333496
TOM40_HUMAN	20447.1504
PX11B_HUMAN	3064.61475

PAK4_HUMAN	7101.4707
APBA3_HUMAN	825.27356
ACL6A_HUMAN	8295.15625
NSD2_HUMAN	1048.72437
LDHA_HUMAN	283209.719
AL1A1_HUMAN	9373.56055
DHE3_HUMAN	41006.5391
DYR_HUMAN	11272.9102
NB5R3_HUMAN	52781.5742
GSHR_HUMAN	18785.0762
COX2_HUMAN	64469.1211
PNPH_HUMAN	64363.4063
HPRT_HUMAN	26371.2402
AATM_HUMAN	62583.6523
ABL1_HUMAN	659.265869
PGK1_HUMAN	159211.984
KAD1_HUMAN	28878.1797
UROK_HUMAN	1990.30774
TPA_HUMAN	636.390442
ADA_HUMAN	2609.979
A2MG_HUMAN	106.288651
CYTC_HUMAN	6267.05127
RASN_HUMAN	29855.6992
RASH_HUMAN	3557.7124
RASK_HUMAN	2416.85815
PDGFB_HUMAN	1141.27588
LDLR_HUMAN	4804.7959
TGFB1_HUMAN	3553.46436
HLAB_HUMAN	3228.60327
HLAH_HUMAN	46049.4258
CO1A1_HUMAN	1842.073
CO4A1_HUMAN	1289.22058
LMNA_HUMAN	195455.297
APOA1_HUMAN	2501.87329
FINC_HUMAN	32756.291
FETUA_HUMAN	3838.1626
ALBU_HUMAN	114432.352
TFR1_HUMAN	39869.7148
FRIL_HUMAN	6939.07813
FRIH_HUMAN	4072.11841
MT2_HUMAN	17957.7656
NU1M_HUMAN	5962.38525
NU4M_HUMAN	2278.99805
NU5M_HUMAN	2049.16016
MMP1_HUMAN	14682.7285
HMDH_HUMAN	185.788635
CATA_HUMAN	6392.18799
RAF1_HUMAN	1621.15771
GBA1_HUMAN	12108.3457
ALDOA_HUMAN	289446.781

CYTB_HUMAN	4999.53809
ANXA1_HUMAN	192359.844
APOB_HUMAN	3586.46338
PRIO_HUMAN	1556.97327
SODM_HUMAN	2218.71191
OAT_HUMAN	31012.1309
KITH_HUMAN	3856.48438
VWF_HUMAN	39069.1211
TBB4A_HUMAN	75911.25
G3P_HUMAN	461331.188
ARLY_HUMAN	7150.81738
HLAA_HUMAN	35681.6992
CPNS1_HUMAN	18061.6855
P53_HUMAN	283.104431
MT1E_HUMAN	203.308365
HSPB1_HUMAN	102756.602
TYSY_HUMAN	13253.3115
RPN1_HUMAN	72426.5469
RPN2_HUMAN	47933.3164
GNAI2_HUMAN	39897.2188
H2A1B_HUMAN	56499.8438
B3A2_HUMAN	2025.38184
AT1A1_HUMAN	55599.3008
AT1B1_HUMAN	14007.8174
A4_HUMAN	5955.46484
ALDH2_HUMAN	27383.9883
ITB3_HUMAN	8793.90527
S10A8_HUMAN	327.76709
HMGN1_HUMAN	723.040955
PAI2_HUMAN	16048.3506
PAI1_HUMAN	132724.031
ADT2_HUMAN	239623.375
ISG15_HUMAN	4743.79102
PCCA_HUMAN	3032.22388
PCCB_HUMAN	3887.78101
IF2A_HUMAN	56479.1719
HMGN2_HUMAN	452.787476
ICAM1_HUMAN	12009.4883
RLA2_HUMAN	6445.1333
RLA0_HUMAN	176369.797
JUN_HUMAN	1837.16541
RPC4_HUMAN	1945.72424
LA_HUMAN	29677.1484
ITB1_HUMAN	116318.234
K1C18_HUMAN	61441.9375
CO5A2_HUMAN	1357.8739
DCUP_HUMAN	25631.1289
FYN_HUMAN	516.634766
AGAL_HUMAN	936.769104
GELS_HUMAN	8228.37305

RB_HUMAN	2121.16479
PTMA_HUMAN	2866.69043
CDK1_HUMAN	29329.8906
ATPB_HUMAN	168393.703
S10A9_HUMAN	1161.3313
S10A6_HUMAN	10754.6572
IF4E_HUMAN	17546.668
ENOA_HUMAN	581589.5
PYGL_HUMAN	9411.21875
G6PI_HUMAN	63951.5078
DPOLB_HUMAN	311.193176
NPM_HUMAN	66835.7266
TPM3_HUMAN	58067.6055
ITAV_HUMAN	19294.8457
HEXA_HUMAN	9688.88574
HYEP_HUMAN	9850.22949
LDHB_HUMAN	199870.5
CENPB_HUMAN	993.479675
GPX1_HUMAN	13020.4277
PDIA1_HUMAN	154347.688
H10_HUMAN	16413.4082
ACYP1_HUMAN	3297.03589
FES_HUMAN	894.704956
CATD_HUMAN	58196.6992
ANXA2_HUMAN	390883.344
CAN1_HUMAN	28463.7598
TBB5_HUMAN	357282.344
SAP_HUMAN	4838.93555
HEXB_HUMAN	14866.8018
CATL1_HUMAN	2065.98462
PROF1_HUMAN	261991.328
PMGE_HUMAN	4074.42773
APT_HUMAN	58149.7734
SYEP_HUMAN	21300.9355
CATB_HUMAN	76326.25
HS90A_HUMAN	144801.719
GALT_HUMAN	1609.19116
HNRPC_HUMAN	132320.297
LAMB1_HUMAN	18618.8223
YES_HUMAN	18964.3828
LYN_HUMAN	2974.73169
FUMH_HUMAN	28894.666
ERCC1_HUMAN	273.245392
TSP1_HUMAN	140324.641
SP1_HUMAN	1009.96191
IGF1R_HUMAN	1197.02234
CO1A2_HUMAN	890.90509
ANXA6_HUMAN	92390.7344
RHOC_HUMAN	94645.4922
DAF_HUMAN	10616.1494

4F2_HUMAN	20102.2344
BGLR_HUMAN	1013.17981
PFKAM_HUMAN	9723.00098
HS90B_HUMAN	719850.063
SRPRA_HUMAN	12036.2295
ASNS_HUMAN	5568.50439
MMP2_HUMAN	8683.14648
HEM3_HUMAN	1742.03381
NEP_HUMAN	617.445557
MGP_HUMAN	2470.79883
ODPA_HUMAN	32107.5176
CO4A2_HUMAN	8861.7998
CY1_HUMAN	11386.8799
RU2B_HUMAN	8129.99121
MET_HUMAN	3161.28687
RU17_HUMAN	53332.1367
ITA5_HUMAN	86867.2891
NFIC_HUMAN	560.799927
VIME_HUMAN	1465728.5
RS17_HUMAN	37594.5352
GNAI3_HUMAN	11940.3281
ANXA5_HUMAN	435638.313
RSSA_HUMAN	104951.633
CD63_HUMAN	2989.13184
RM03_HUMAN	5760.58643
SNRPA_HUMAN	22987.832
ENOG_HUMAN	9807.11914
THIK_HUMAN	5855.3252
SRP19_HUMAN	6292.00146
GSTP1_HUMAN	126442.992
RU1C_HUMAN	6937.42676
LEG1_HUMAN	40670.2031
DHPR_HUMAN	8510.5957
HMGB1_HUMAN	56057.8594
SPRC_HUMAN	3340.50562
TPM1_HUMAN	56272.0625
CLCA_HUMAN	2345.0498
CLCB_HUMAN	630.817871
ANXA4_HUMAN	14441.0029
CN37_HUMAN	10939.9639
HMOX1_HUMAN	10289.9248
DLDH_HUMAN	15022.6738
ROA1_HUMAN	184535.281
RU2A_HUMAN	25347.9766
COX6C_HUMAN	16313.582
TACD2_HUMAN	2813.58594
PARP1_HUMAN	33775.6211
DPOLA_HUMAN	12183.8223
UCHL1_HUMAN	9019.08398
LKHA4_HUMAN	27132.3652

ALDOC_HUMAN	17581.9258
H2AZ_HUMAN	3371.9563
H2A1_HUMAN	797.533569
GRL1A_HUMAN	474.285767
GST2_HUMAN	3484.05859
POTEJ_HUMAN	7005.77051
LIMS3_HUMAN	4746.87402
HS71A_HUMAN	85661.5938
ERPG3_HUMAN	1476.98083
TBA3C_HUMAN	59759.4727
ZTRF1_HUMAN	596.850342
RAP2A_HUMAN	9419.49609
SRGN_HUMAN	2437.60181
RO60_HUMAN	10665.5898
LYAG_HUMAN	3085.42041
RRAS_HUMAN	12403.3516
HLAC_HUMAN	1316.72888
ARAF_HUMAN	1929.15417
H14_HUMAN	285883.031
ODP2_HUMAN	22341.5078
PTPRF_HUMAN	4573.97168
COT1_HUMAN	4103.56494
THIO_HUMAN	3991.04126
COX5B_HUMAN	1175.95337
PPGB_HUMAN	6654.20166
MGST1_HUMAN	12480.7471
KAP0_HUMAN	18511.2871
TFPI1_HUMAN	1273.17175
ESTD_HUMAN	18090.9063
CH60_HUMAN	137279.125
CLUS_HUMAN	2306.98877
BIP_HUMAN	252680
LAMC1_HUMAN	12469.4502
PPAL_HUMAN	5192.04004
MTAP2_HUMAN	2895.28271
HSP7C_HUMAN	267182.281
GTR1_HUMAN	5025.22461
GTR3_HUMAN	2622.47632
EPB41_HUMAN	21484.9863
UMPS_HUMAN	12440.582
ODPB_HUMAN	14471.4902
ODB2_HUMAN	1321.2998
PYGB_HUMAN	15698.2285
PYGM_HUMAN	34442.3359
RALA_HUMAN	30245.9395
RALB_HUMAN	41028.0117
BCR_HUMAN	4423.85449
LAMP1_HUMAN	71944.8203
ERG_HUMAN	6269.90088
ACADM_HUMAN	10142.7725

TOP1_HUMAN	44116.0195
TOP2A_HUMAN	667.149536
G6PD_HUMAN	69832.8984
UBL4A_HUMAN	1153.66455
PYC_HUMAN	2695.29688
C1TC_HUMAN	51324.1953
MPRI_HUMAN	5261.07178
ADHX_HUMAN	32983.1445
CDK4_HUMAN	3460.521
PRPS2_HUMAN	20328.3965
PABP1_HUMAN	125021.977
PCNA_HUMAN	29595.834
HARS1_HUMAN	40705.1289
CO6A1_HUMAN	1746.59204
CO6A3_HUMAN	408.505981
ADT1_HUMAN	227151.234
ADT3_HUMAN	89394.7578
IMDH2_HUMAN	45627.8359
TPR_HUMAN	13447.3184
ANXA3_HUMAN	45204.4961
ODBA_HUMAN	3856.14063
ACTN1_HUMAN	111899.031
ACE_HUMAN	5793.5293
SRC_HUMAN	4780.46826
PEPD_HUMAN	8065.27637
XRCC6_HUMAN	81454.8281
XRCC5_HUMAN	53025.4141
UNG_HUMAN	1143.93311
COX41_HUMAN	51563.918
HEM1_HUMAN	1998.17603
LAMP2_HUMAN	54273.3359
RINI_HUMAN	50724.1914
ICAM2_HUMAN	50790.0195
EF2_HUMAN	201643.938
PDIA4_HUMAN	112093.094
P4HA1_HUMAN	10308.0771
TCTP_HUMAN	13972.7979
HEM2_HUMAN	4883.44922
HLAE_HUMAN	659.299805
PLST_HUMAN	144869.906
ACPH_HUMAN	16405.9199
ETFA_HUMAN	22581.748
GYS1_HUMAN	5917.95459
KAP2_HUMAN	7570.54834
T2FB_HUMAN	4487.6167
CD59_HUMAN	27024.0781
MTDC_HUMAN	7039.95313
MIF_HUMAN	31780.9668
CD99_HUMAN	1552.93982
GLU2B_HUMAN	71870.8125

HCLS1_HUMAN	7537.01514
FPPS_HUMAN	58195.6836
CX7A2_HUMAN	13516.2539
NID1_HUMAN	6941.04199
AK1A1_HUMAN	25071.291
KPYM_HUMAN	473996.594
ENPL_HUMAN	310593.781
CCNB1_HUMAN	2507.70215
MYL6B_HUMAN	9290.83203
RSMB_HUMAN	52827.1836
IDE_HUMAN	19643.5547
PO2F1_HUMAN	439.069519
HNRPL_HUMAN	26483.0508
SYDC_HUMAN	33771.1758
ETS1_HUMAN	1159.14648
PLAK_HUMAN	15053.0498
QCR7_HUMAN	952.466125
BRAF_HUMAN	3340.0105
FABP4_HUMAN	14339.7256
GLNA_HUMAN	504.409637
ALDR_HUMAN	43964.3164
AMPN_HUMAN	83080.0156
PVR_HUMAN	23311.1816
RAC2_HUMAN	25232.9883
ERF3A_HUMAN	15197.6016
ARSA_HUMAN	490.3479
B4GT1_HUMAN	1925.375
EZRI_HUMAN	36901.8672
UCHL3_HUMAN	4662.85547
FOSL1_HUMAN	974.976135
FOSL2_HUMAN	302.935455
MCP_HUMAN	5517.55225
NDKA_HUMAN	27193.8223
NQO1_HUMAN	16085.0605
GNS_HUMAN	36554.7813
PHKG2_HUMAN	1520.82288
ARSB_HUMAN	1395.07971
RS2_HUMAN	151131.984
ITF2_HUMAN	896.926392
DESP_HUMAN	1043.83008
RFA2_HUMAN	6559.96094
TIMP2_HUMAN	6999.5127
CD44_HUMAN	16347.2168
CBR1_HUMAN	23561.8242
ACADS_HUMAN	1147.02246
CREB1_HUMAN	3118.59985
BGAL_HUMAN	8072.92236
PECA1_HUMAN	51320.4063
PP2BB_HUMAN	5485.88818
NCK1_HUMAN	9433.2666

GCFC2_HUMAN	863.750183
H15_HUMAN	281780.094
H13_HUMAN	31875.459
H12_HUMAN	45040.3281
NCPR_HUMAN	10773.5439
FER_HUMAN	473.106537
AT2A2_HUMAN	40563.5859
PLCG2_HUMAN	4882.99756
FAAA_HUMAN	4979.86914
STMN1_HUMAN	49941.1094
YBOX3_HUMAN	13884.2529
ZNF22_HUMAN	883.749878
ZNF24_HUMAN	506.251099
NAGAB_HUMAN	2907.93652
HMGA1_HUMAN	47198.5195
TMM11_HUMAN	4221.10645
AATC_HUMAN	18045.3867
KPCA_HUMAN	6195.56543
ITA2_HUMAN	26991.9141
CXA1_HUMAN	2407.64722
ASM_HUMAN	1592.52625
UBF1_HUMAN	9560.81543
JUND_HUMAN	985.162109
TAL1_HUMAN	457.663239
ATF7_HUMAN	210.068436
NDUB7_HUMAN	4263.78857
KAPCA_HUMAN	10849.8584
CAN2_HUMAN	45518.0078
PTN2_HUMAN	2192.12158
PYRG1_HUMAN	18092.8027
EGLN_HUMAN	69344.3516
DDX5_HUMAN	92796.8203
PFKAL_HUMAN	45032.4141
VGFR1_HUMAN	6107.20264
PRS6A_HUMAN	39336.9805
TCPA_HUMAN	65573.3984
PTN1_HUMAN	21014.6934
ERCC2_HUMAN	3677.34375
RL35A_HUMAN	73260.0156
ITB5_HUMAN	7293.1167
ARF4_HUMAN	109772.086
RL7_HUMAN	184702.703
VINC_HUMAN	78577.0078
PTPRA_HUMAN	1618.72949
ARY1_HUMAN	1266.91248
SON_HUMAN	10417.4834
NELFE_HUMAN	4484.77051
RL17_HUMAN	99179.3125
PGAM1_HUMAN	79684.2266
RCC1_HUMAN	25616.4258

ATF1_HUMAN	859.876831
ATF6A_HUMAN	576.200623
DNLI1_HUMAN	7466.5166
XRCC1_HUMAN	3723.09277
AMD_HUMAN	1471.56067
CADH2_HUMAN	14137.458
PLCG1_HUMAN	10838.6992
NUCL_HUMAN	157803.781
HXK1_HUMAN	58774.4766
RPB3_HUMAN	1823.91992
RPAB1_HUMAN	5398.45313
NDUV2_HUMAN	2588.71338
ERCC3_HUMAN	2300.31348
RO52_HUMAN	5705.29053
E2AK2_HUMAN	9259.31348
SPEE_HUMAN	47273.8047
SL9A1_HUMAN	1584.26282
CSK22_HUMAN	7688.42676
NFKB1_HUMAN	4519.79736
AT2B1_HUMAN	4435.85156
IF2B_HUMAN	25362.6387
ANXA7_HUMAN	22555.7598
CCNA2_HUMAN	926.852051
BTF3_HUMAN	6683.19141
RAB3B_HUMAN	3957.88525
RAB4A_HUMAN	3544.54248
RAB5A_HUMAN	58181.9297
RAB6A_HUMAN	37117.2109
MSH3_HUMAN	1390.17456
ANPRB_HUMAN	398.203918
PSB1_HUMAN	39884.2578
MPRD_HUMAN	5567.45557
COX5A_HUMAN	484.543274
LMNB1_HUMAN	84917.5703
ICAL_HUMAN	17228.3887
IMDH1_HUMAN	6207.74023
CO5A1_HUMAN	2148.80566
NEBU_HUMAN	2353.22852
ASPG_HUMAN	1581.65808
RASA1_HUMAN	5574.69971
PTMS_HUMAN	424.337463
CD11B_HUMAN	9343.42383
GSTM3_HUMAN	1907.01746
VATB2_HUMAN	14014.8164
VATC1_HUMAN	4966.57764
CSRP1_HUMAN	78523.0703
FLNA_HUMAN	268895.688
NF1_HUMAN	573.558228
AOFA_HUMAN	1235.6748
ACOHC_HUMAN	27892.7129

S1PR1_HUMAN	6151.32422
SYT1_HUMAN	1275.13867
5NTD_HUMAN	16836.0176
TAF1_HUMAN	196.880493
MK_HUMAN	345.885651
VDAC1_HUMAN	98923.8203
PGS1_HUMAN	4389.22656
SDHB_HUMAN	15226.6025
CD9_HUMAN	25304.2344
COMT_HUMAN	5734.729
TGM2_HUMAN	296179.25
MUTA_HUMAN	6771.31982
OSBP1_HUMAN	7137.30908
PIMT_HUMAN	2567.37988
FBRL_HUMAN	25774.1484
PUR2_HUMAN	24597.4961
PUR6_HUMAN	50868.6992
SCP2_HUMAN	20527.2324
UBA1_HUMAN	64696.3516
NDKB_HUMAN	123396.172
SPR1B_HUMAN	126.980362
ADRO_HUMAN	12092.2539
ROA2_HUMAN	219343.016
RFX1_HUMAN	811.554443
CBL_HUMAN	4315.86182
IBP4_HUMAN	862.791199
KAPCB_HUMAN	1620.1449
QCR2_HUMAN	15858.1582
HEMH_HUMAN	3408.6582
TCEA1_HUMAN	12455.9736
PGH1_HUMAN	1333.07996
ITA6_HUMAN	22667.0371
SFPQ_HUMAN	96418.6328
TBG1_HUMAN	12050.0488
PPIB_HUMAN	141268.828
MAOM_HUMAN	13981.0566
SYWC_HUMAN	23940.0059
RS3_HUMAN	210621.375
KS6B1_HUMAN	3343.19116
JAK1_HUMAN	6606.53516
PTPRB_HUMAN	12404.5869
PTPRE_HUMAN	5765.85205
PTPRG_HUMAN	1169.16479
SP100_HUMAN	7378.57715
CRCM_HUMAN	759.88208
TNFL4_HUMAN	10091.8574
SAHH_HUMAN	86984.0234
COF1_HUMAN	575008.25
IF4B_HUMAN	24056.248
HAP40_HUMAN	1145.50342

AT2B4_HUMAN	6692.77051
DGKA_HUMAN	4237.45459
CPT2_HUMAN	3238.64209
KTHY_HUMAN	19809.3438
RIR1_HUMAN	15240.9141
CCND1_HUMAN	2353.3291
RAE1_HUMAN	1597.99902
ERD21_HUMAN	1112.34241
COT2_HUMAN	2328.00244
EF1B_HUMAN	17997.5645
AT5F1_HUMAN	16324.3398
PPAC_HUMAN	15183.5977
KPCL_HUMAN	3027.28589
THIL_HUMAN	26803.3457
MYL9_HUMAN	571.644958
RPB1_HUMAN	35593.9297
CDK2_HUMAN	10060.8145
ARBK1_HUMAN	3660.89966
PAR1_HUMAN	1565.44543
MCM3_HUMAN	44500.8086
THTM_HUMAN	5458.58789
RS12_HUMAN	35434.8125
BRD2_HUMAN	1663.29175
TNR6_HUMAN	29.3170242
TYY1_HUMAN	1805.21936
DNJB1_HUMAN	11095.4014
DNJB2_HUMAN	415.714661
ATPA_HUMAN	210931.828
CATS_HUMAN	1067.27234
PSA1_HUMAN	97759.1875
PSA2_HUMAN	25155.9102
PSA3_HUMAN	39404.0625
PSA4_HUMAN	77630.1094
TNR5_HUMAN	841.965698
ITA3_HUMAN	22020.2559
PTX3_HUMAN	3880.31836
MOES_HUMAN	147578.219
DDX6_HUMAN	18502.1133
DNMT1_HUMAN	9497.31641
U2AF2_HUMAN	48488.2617
RL13_HUMAN	127020.57
RAE2_HUMAN	938.271851
IVD_HUMAN	1321.66956
MGAT1_HUMAN	4293.54297
HMGB2_HUMAN	14024.3643
PTBP1_HUMAN	58551.7383
SYTC_HUMAN	35520.5469
SYVC_HUMAN	23461.9121
EF1G_HUMAN	137660.859
FKBP2_HUMAN	15303.0332

IL3RA_HUMAN	985.653381
STOM_HUMAN	36945.2188
KAD4_HUMAN	1625.48035
1433T_HUMAN	51835.7148
MK03_HUMAN	14333.1934
MARK3_HUMAN	808.089417
RL10_HUMAN	49433.8047
CO8A1_HUMAN	884.403687
RFA1_HUMAN	14548.2734
APEX1_HUMAN	26511.8672
CD82_HUMAN	1282.44348
DCK_HUMAN	3072.71875
PYR1_HUMAN	36368.7148
CALR_HUMAN	179609.266
MAP4_HUMAN	22923.3535
CALX_HUMAN	112596.813
P85A_HUMAN	1384.698
IP3KB_HUMAN	939.942749
PSB8_HUMAN	3078.00684
PSB9_HUMAN	3227.16113
PSA5_HUMAN	23875.9102
PSB4_HUMAN	16517.6348
PSB6_HUMAN	39909.3828
PSB5_HUMAN	35341.8633
GSTM2_HUMAN	885.086182
ABCD3_HUMAN	6592.51221
ITPI2_HUMAN	4946.08545
LYOX_HUMAN	2688.79102
NDUS1_HUMAN	14894.3105
DPOD1_HUMAN	10412.4854
SMCA1_HUMAN	1075.78564
MK01_HUMAN	43582.0938
RXRB_HUMAN	1057.03662
ERCC5_HUMAN	1063.14368
GRN_HUMAN	4563.75635
PTPRM_HUMAN	742.014038
AMPL_HUMAN	26023.0547
CD34_HUMAN	908.336426
T2EA_HUMAN	2562.18188
T2EB_HUMAN	2887.82056
TPP2_HUMAN	13600.6113
IMPA1_HUMAN	6322.2583
CCN2_HUMAN	28337.2188
EPHA2_HUMAN	23053.1523
EPHB2_HUMAN	16882.5156
SHC1_HUMAN	5183.28369
3MG_HUMAN	6062.49219
TKT_HUMAN	137810.641
CASP1_HUMAN	433.934814
NOS3_HUMAN	3687.99707

RBMS1_HUMAN	5397.83154
PML_HUMAN	9534.29785
TYK2_HUMAN	1369.68066
EF1D_HUMAN	38067.543
MARCS_HUMAN	34975.6563
GNA11_HUMAN	16539.543
AL4A1_HUMAN	3240.07593
ERP29_HUMAN	28318.8887
PRDX6_HUMAN	131514.891
BLVRB_HUMAN	8139.96338
PRDX5_HUMAN	23504.4316
PRDX3_HUMAN	21777.4453
RL12_HUMAN	125991.563
ECHM_HUMAN	12778.3154
KCY_HUMAN	25827.5566
PEBP1_HUMAN	24005.4063
PDIA3_HUMAN	148490.031
2AAA_HUMAN	63193.9258
2AAB_HUMAN	5210.87451
CDC27_HUMAN	5479.95703
CCND3_HUMAN	588.295898
PPIF_HUMAN	14335.8633
NKTR_HUMAN	1603.44861
NMT1_HUMAN	17286.3164
HMOX2_HUMAN	16329.0947
PURA2_HUMAN	63163.0781
UFO_HUMAN	3282.35693
AMRP_HUMAN	5985.61328
TSPO_HUMAN	9046.81934
PUR8_HUMAN	17603.4844
CLIP1_HUMAN	6663.62207
SORCN_HUMAN	3606.00342
ILEU_HUMAN	5003.00146
CTR1_HUMAN	5921.67139
AL1B1_HUMAN	7367.19629
RPB2_HUMAN	8751.65039
SDHA_HUMAN	8916.46484
COR1A_HUMAN	2003.57788
GDIA_HUMAN	20199.8652
S10A7_HUMAN	332.935364
METK2_HUMAN	49018.6094
KAP3_HUMAN	8468.57422
RIR2_HUMAN	14722.0518
SDC4_HUMAN	846.806763
TIA1_HUMAN	17732.0781
SC6A6_HUMAN	793.429688
DNJA1_HUMAN	17096.6094
AKT1_HUMAN	3238.22876
QCR1_HUMAN	77338.9766
3HIDH_HUMAN	4007.72192

PUR9_HUMAN	32314.498
HNRH3_HUMAN	10041.0576
HNRH1_HUMAN	70857.3359
CASPE_HUMAN	1513.56433
1433B_HUMAN	154242.547
STIP1_HUMAN	120435.828
S10AB_HUMAN	10739.0098
PRDX2_HUMAN	42109.9219
ARRB2_HUMAN	326.253693
GLPK_HUMAN	841.730103
DCTD_HUMAN	5682.51904
P5CR1_HUMAN	5086.50879
GBP1_HUMAN	3181.60693
GBP2_HUMAN	2245.06909
ELF1_HUMAN	1140.25281
TF2H1_HUMAN	700.033875
STX2_HUMAN	262.779541
RL9_HUMAN	84951.6953
ACSL1_HUMAN	5388.39795
CADH5_HUMAN	24917.7832
KINH_HUMAN	35675.0273
DUT_HUMAN	7285.44385
MRP1_HUMAN	4938.94189
ABCD1_HUMAN	723.05011
TTK_HUMAN	1239.25464
MCM4_HUMAN	12191.3818
MCM5_HUMAN	30259.8613
MCM7_HUMAN	21639.5449
GALNS_HUMAN	486.677277
GLYC_HUMAN	4334.1333
GLYM_HUMAN	63687.4023
HS71L_HUMAN	1546.72839
HSP74_HUMAN	36998.5508
GRK5_HUMAN	92.6834106
MPI_HUMAN	1927.72302
GPC1_HUMAN	1503.35669
PROF2_HUMAN	20585.8066
CTNA1_HUMAN	52712.1836
CTNB1_HUMAN	49287.6758
PHB1_HUMAN	105849.281
SPB6_HUMAN	20887.9102
MERL_HUMAN	3605.62744
RADI_HUMAN	390933.75
RFA3_HUMAN	1670.61865
RFC4_HUMAN	7641.59229
RFC2_HUMAN	8272.61621
RFC1_HUMAN	3662.57275
RL22_HUMAN	141456.953
T2FA_HUMAN	7819.10449
SPRE_HUMAN	6811.93555

HRH1_HUMAN	584.115784
CBS_HUMAN	933.996155
FBN1_HUMAN	1879.20105
FBN2_HUMAN	231.139053
GDE_HUMAN	3890.68091
MYH9_HUMAN	195399.922
MYH10_HUMAN	116238.414
TIE1_HUMAN	11880.2656
COPB2_HUMAN	45402.1992
SOAT1_HUMAN	3673.71802
ADDA_HUMAN	6117.53809
BASI_HUMAN	60654.1445
FUS_HUMAN	71477.8828
NU214_HUMAN	6980.44385
DEK_HUMAN	23936.8008
SOX18_HUMAN	756.948547
MYH11_HUMAN	57087.1797
PPM1A_HUMAN	5335.57129
HMGCL_HUMAN	4023.66968
VGFR3_HUMAN	3216.70166
VGFR2_HUMAN	3673.64404
PRS7_HUMAN	63440.457
GGT5_HUMAN	1062.79199
ARL2_HUMAN	8879.21094
ARL3_HUMAN	22532.8809
MP2K2_HUMAN	14814.6436
ATPG_HUMAN	42953.1367
VATE1_HUMAN	10488.1914
HEM6_HUMAN	13099.292
RL4_HUMAN	157630.234
8ODP_HUMAN	866.065125
LONM_HUMAN	13169.5547
PGM1_HUMAN	8792.94434
PP1G_HUMAN	12255.2354
GNL1_HUMAN	28522.584
ODO2_HUMAN	26287.6543
GMPR1_HUMAN	3405.68628
GPX4_HUMAN	10548.1133
ACVL1_HUMAN	3313.00024
DHB2_HUMAN	808.425842
SRP14_HUMAN	21468.1172
TGFR2_HUMAN	4059.71704
NUP62_HUMAN	632.941345
HPCL1_HUMAN	6283.70459
FDFT_HUMAN	12767.7256
ZEB1_HUMAN	1538.1134
TAGL2_HUMAN	46815.6328
TALDO_HUMAN	115226.125
SYUA_HUMAN	3563.69604
ETFB_HUMAN	15984.2578

RBMX_HUMAN	32080.2715
COIL_HUMAN	2340.23926
VKGC_HUMAN	2692.48413
LICH_HUMAN	1131.72949
VATA_HUMAN	21895.8359
GRP75_HUMAN	132943.328
IF4A3_HUMAN	33327.8438
SMBP2_HUMAN	448.684875
CDN1A_HUMAN	2374.80225
RS19_HUMAN	105628.617
RL3_HUMAN	46683.9336
COIA1_HUMAN	23395.9844
OST48_HUMAN	99457.4766
AN32A_HUMAN	30420.9688
FEN1_HUMAN	33551.2617
CUX1_HUMAN	442.976501
CAPG_HUMAN	18672.4375
CAP2_HUMAN	3642.72803
IL6RB_HUMAN	6792.42432
TXLNA_HUMAN	9513.22559
TCPZ_HUMAN	104130.438
NNMT_HUMAN	38535.4414
PSB10_HUMAN	1405.13611
PBX2_HUMAN	1019.95422
RL13A_HUMAN	141131.547
ARL1_HUMAN	22988.3965
MLH1_HUMAN	8127.61182
STAT3_HUMAN	8450.93164
UBP8_HUMAN	5469.78369
MDHC_HUMAN	75545.6953
MDHM_HUMAN	133329.734
RFC5_HUMAN	10190.502
RFC3_HUMAN	4303.51953
ECHA_HUMAN	36318.1992
IF2G_HUMAN	39257.0234
ETV3_HUMAN	283.363098
CETN2_HUMAN	1445.46265
ETV6_HUMAN	2058.75122
EIF2D_HUMAN	5170.021
BUD31_HUMAN	4483.22559
UBA7_HUMAN	1275.99158
NAA10_HUMAN	17594.7832
KDM5C_HUMAN	370.071075
CSK_HUMAN	11185.2705
GARS_HUMAN	25097.3926
SYIC_HUMAN	48623.957
S19A1_HUMAN	2055.46729
EIF1_HUMAN	4457.57959
KPCI_HUMAN	837.892761
ELK3_HUMAN	451.972839

ACTY_HUMAN	25393.5371
ECI1_HUMAN	5707.60498
LAP2A_HUMAN	9217.97168
LAP2B_HUMAN	90698.5391
STAT1_HUMAN	17254.8555
STAT6_HUMAN	5091.72559
STA5A_HUMAN	5894.68652
MTREX_HUMAN	13721.5098
AK1C3_HUMAN	4502.19971
PK3CA_HUMAN	1841.64783
PK3CB_HUMAN	1268.79321
MTOR_HUMAN	9546.44727
PI4KA_HUMAN	4074.37598
EPS15_HUMAN	7655.92773
CASP3_HUMAN	23883.2188
CASP2_HUMAN	1134.48169
RS27_HUMAN	12484.8477
ABL2_HUMAN	701.001221
HELZ_HUMAN	1285.88281
CNDD3_HUMAN	1625.27063
RBM34_HUMAN	8724.65527
LPPRC_HUMAN	49519.9609
THIM_HUMAN	10294.1289
RL35_HUMAN	49583.875
PCP_HUMAN	15772.2471
HD_HUMAN	3719.05518
ECE1_HUMAN	13260.8213
MTHR_HUMAN	506.359955
SATT_HUMAN	1091.74487
LIS1_HUMAN	20644.6641
MUC18_HUMAN	113729.242
CACP_HUMAN	3879.69312
MATR3_HUMAN	36549.082
MSH2_HUMAN	11572.4697
GRK6_HUMAN	1296.3053
GPDM_HUMAN	9603.17285
SSRA_HUMAN	34205.5977
PTN9_HUMAN	2049.8252
RANG_HUMAN	52196.8398
NAMPT_HUMAN	87284.8594
PRS6B_HUMAN	29069.6875
EFTS_HUMAN	7983.98682
PPIC_HUMAN	10748.1943
VDAC2_HUMAN	97994.8125
ACDSB_HUMAN	2794.7373
CBX5_HUMAN	4001.43555
UBP5_HUMAN	34736.9609
MK08_HUMAN	1312.66846
MP2K4_HUMAN	4869.40186
KI67_HUMAN	5565.71045

KPB2_HUMAN	268.827484
KPB1_HUMAN	770.684631
RAGP1_HUMAN	26651.4746
RECQ1_HUMAN	20582.7051
NOP2_HUMAN	16924.502
ATRX_HUMAN	2037.91431
CRK_HUMAN	6487.45166
CRKL_HUMAN	6542.71143
IF2M_HUMAN	3009.1001
BAG6_HUMAN	5404.7041
NSF_HUMAN	20105.4277
NOTC1_HUMAN	3100.95874
MP2K3_HUMAN	6441.62939
BRCC3_HUMAN	2234.20459
RL27A_HUMAN	216084
RL5_HUMAN	54419.3125
RL21_HUMAN	46423.3438
RL28_HUMAN	49959.9297
RS9_HUMAN	205794.891
RS5_HUMAN	117742.781
RS10_HUMAN	83921.0781
MAP1B_HUMAN	33100.9961
GNPI1_HUMAN	13684.5195
NEDD4_HUMAN	3318.08691
UTRN_HUMAN	9868.9873
IQGA1_HUMAN	161242.172
GLYG_HUMAN	15617.168
STT3A_HUMAN	50290.5078
PA24A_HUMAN	2611.50269
CAZA2_HUMAN	23101.4316
CAPZB_HUMAN	41263.2578
IF1AX_HUMAN	8020.62842
AL1A3_HUMAN	763.875183
SYQ_HUMAN	29322.5527
RL29_HUMAN	36488.582
LEG7_HUMAN	626.460388
TISD_HUMAN	780.284607
UCRI_HUMAN	13273.6992
ATPO_HUMAN	56958.7461
LIMS1_HUMAN	16910.4492
GLIP1_HUMAN	1180.01282
PPCE_HUMAN	21061.0859
MAOX_HUMAN	18354.5293
IREB2_HUMAN	1977.04065
TFPI2_HUMAN	1201.55542
RFX5_HUMAN	6062.19238
PI42A_HUMAN	5590.57813
COPD_HUMAN	47251.2383
LSS_HUMAN	24433.957
PP2BC_HUMAN	936.933655

GSH1_HUMAN	2705.40283
GSH0_HUMAN	12892.9199
CD151_HUMAN	10557.2383
TPC10_HUMAN	1122.85352
PSMD8_HUMAN	36961.9688
PRC2A_HUMAN	3126.81934
GSHB_HUMAN	15918.5371
TCPE_HUMAN	97903.1016
PTSS1_HUMAN	4929.24023
NEST_HUMAN	104408.477
HSP13_HUMAN	8417.08691
KC1A_HUMAN	11287.3975
KC1D_HUMAN	4481.21826
IDHP_HUMAN	65092.1211
PK3CG_HUMAN	2875.31006
PIPNB_HUMAN	17721.8125
DPOD2_HUMAN	4886.31006
MRP_HUMAN	10721.501
PAXI_HUMAN	5660.2334
CAMLG_HUMAN	405.197754
NR2C2_HUMAN	848.233154
MAPK2_HUMAN	2219.47046
DNSL1_HUMAN	2344.93091
AL9A1_HUMAN	12754.8926
RL34_HUMAN	59930.1406
RPIA_HUMAN	2551.77466
LMAN1_HUMAN	19270.8594
NRAM2_HUMAN	1314.24487
NASP_HUMAN	20188.8125
FAS_HUMAN	69921.6719
FNTA_HUMAN	10703.5771
FNTB_HUMAN	4028.49609
DHYS_HUMAN	2779.86035
TCPG_HUMAN	83183.2969
RM19_HUMAN	9412.72754
ARRB1_HUMAN	6687.15381
EFTU_HUMAN	80546.3281
AL7A1_HUMAN	19219.9258
UB2R1_HUMAN	5786.64453
INPP_HUMAN	3852.96875
CENPF_HUMAN	384.02478
SRP09_HUMAN	42913.9609
PCY1A_HUMAN	11198.416
SYAC_HUMAN	23155.5039
SYCC_HUMAN	17527.6016
SYHM_HUMAN	6057.43945
SYSC_HUMAN	29466.084
PPM1F_HUMAN	21732.1504
MA2A2_HUMAN	1732.69336
PRI1_HUMAN	2482.34058

PRI2_HUMAN	1540.65881
CASP4_HUMAN	2894.30908
KC1E_HUMAN	973.427673
PSB3_HUMAN	31199.0195
PSB2_HUMAN	31141.8535
MCM2_HUMAN	18641.8965
ACADV_HUMAN	9281.91406
YLPM1_HUMAN	6974.43262
VPS41_HUMAN	1223.17725
TMEDA_HUMAN	40333.0039
RBM25_HUMAN	10300.2227
NUMB_HUMAN	6786.76758
CLK3_HUMAN	746.151733
PSN1_HUMAN	1518.21777
EI2BB_HUMAN	6009.05713
HINT1_HUMAN	6105.92383
NU153_HUMAN	7859.93457
RBP2_HUMAN	12911.0254
RGS19_HUMAN	1073.77039
RGS3_HUMAN	2361.65283
PSN2_HUMAN	315.09024
TSC2_HUMAN	2848.84619
NDUV1_HUMAN	9650.74219
GSK3A_HUMAN	2624.4707
GSK3B_HUMAN	6973.15039
TAF6_HUMAN	1041.01392
5NTC_HUMAN	3301.36108
SPS1_HUMAN	6508.84668
MTHFS_HUMAN	492.882172
GUAA_HUMAN	20832.459
DNLI3_HUMAN	4698.35986
DNLI4_HUMAN	2133.33301
MRE11_HUMAN	6343.96436
GNAQ_HUMAN	8292.77539
IDH3A_HUMAN	12695.9629
ST1A1_HUMAN	1428.80029
MMP14_HUMAN	29646.8965
PPOX_HUMAN	1572.23669
GDIB_HUMAN	71644.6016
EMD_HUMAN	7958.25977
CPT1A_HUMAN	7931.03369
S26A2_HUMAN	1122.18481
SPB8_HUMAN	15460.3066
SPB9_HUMAN	53141.5391
SERPH_HUMAN	90889.4609
PDLI4_HUMAN	10768.4043
F10A1_HUMAN	88939.4688
PEX5_HUMAN	1222.00867
ERF_HUMAN	1261.34143
VASP_HUMAN	9801.51855

DYN2_HUMAN	24496.1543
MAP2_HUMAN	4397.24023
AP4A_HUMAN	4549.31201
CDK7_HUMAN	4849.30273
KNTC1_HUMAN	2658.83374
RASF2_HUMAN	2529.60962
CDK9_HUMAN	4625.71875
LRBA_HUMAN	3894.25586
BCAM_HUMAN	3209.16406
PPT1_HUMAN	3867.33911
RL14_HUMAN	140554.328
TCPQ_HUMAN	174809.938
TCPD_HUMAN	47385.8359
ANX11_HUMAN	22877.8906
PAPOA_HUMAN	7457.64746
FXR1_HUMAN	18616.5527
FXR2_HUMAN	14354.5693
RAB5C_HUMAN	26012.0938
RAB7A_HUMAN	90314.2031
RAB9A_HUMAN	4261.8374
RAB13_HUMAN	10610.374
RB27A_HUMAN	7123.69971
PLCD1_HUMAN	1296.474
RT29_HUMAN	6103.48779
DUS3_HUMAN	5638.75732
SMCA2_HUMAN	499.136963
SMCA4_HUMAN	7869.7832
IDH3G_HUMAN	3633.82617
GALK1_HUMAN	13833.667
SSRD_HUMAN	43758.7734
BAP31_HUMAN	16725.2012
TPMT_HUMAN	3642.26392
MECP2_HUMAN	714.151123
HCFC1_HUMAN	14899.7246
IRAK1_HUMAN	1261.89685
CAV2_HUMAN	1057.37646
AL3A2_HUMAN	4152.69238
DHB4_HUMAN	16079.0166
PSMD7_HUMAN	20636.4648
SUOX_HUMAN	2283.36792
SPHM_HUMAN	5806.03467
STA5B_HUMAN	1685.58557
UBP11_HUMAN	783.827942
CLCN7_HUMAN	2172.74634
VAMP7_HUMAN	2282.62427
KS6A3_HUMAN	6053.89941
BMX_HUMAN	5811.0625
HDGF_HUMAN	18767.5254
CCNH_HUMAN	978.366089
MAT1_HUMAN	1322.95593

NEK3_HUMAN	720.223328
UB2E1_HUMAN	2797.74023
NDUA8_HUMAN	6056.28516
ROA3_HUMAN	50145.8125
6PGD_HUMAN	121464.742
HNRPM_HUMAN	94683.0234
IMA1_HUMAN	35374.6758
IMA5_HUMAN	4525.32666
NCBP2_HUMAN	2834.5249
GDS1_HUMAN	12742.8096
RPAB3_HUMAN	3479.67944
MP2K6_HUMAN	1710.55835
GDIR1_HUMAN	68168.8672
GDIR2_HUMAN	72865.7578
AGFG1_HUMAN	4195.03223
HNRPF_HUMAN	17312.2012
STAT2_HUMAN	2871.99365
MSH6_HUMAN	7710.19336
KIF11_HUMAN	4703.59277
VAV2_HUMAN	6408.49951
ZN143_HUMAN	1072.62463
RBM5_HUMAN	4608.95313
RIDA_HUMAN	463.658142
SPSY_HUMAN	24626.2734
HXK2_HUMAN	36939.707
EFNB2_HUMAN	500.711731
RM12_HUMAN	10494.8789
DGKQ_HUMAN	135.582367
NDST1_HUMAN	1630.22864
THOP1_HUMAN	13982.5684
CAZA1_HUMAN	95916.0469
HMGA2_HUMAN	1232.07117
CRIP2_HUMAN	40304.043
NUP98_HUMAN	8087.35742
BIEA_HUMAN	26826.8105
TXTP_HUMAN	15587.2695
PPP5_HUMAN	11402.3672
PLK1_HUMAN	3245.44336
DAPK1_HUMAN	1389.84619
ARFP2_HUMAN	2112.30493
ARFP1_HUMAN	5403.03613
NUBP1_HUMAN	4849.23633
ACLY_HUMAN	82781.3672
MAP11_HUMAN	8617.12988
SUCA_HUMAN	11502.1914
MVD1_HUMAN	6271.51514
PGTB1_HUMAN	2594.66333
PGTB2_HUMAN	7033.25098
COPB_HUMAN	30498.0801
COPA_HUMAN	56858.7227

CATC_HUMAN	6164.59229
LIMK1_HUMAN	8079.0957
LIMK2_HUMAN	403.778168
CLH2_HUMAN	242.07399
AP3M2_HUMAN	337.214661
AP2S1_HUMAN	11011.4824
CCHL_HUMAN	5876.45166
MK12_HUMAN	2773.84814
PTTG_HUMAN	3670.12183
SMTN_HUMAN	3582.59644
MOT1_HUMAN	8838.13379
IST1_HUMAN	10180.9258
SC24C_HUMAN	18377.8379
TCP4_HUMAN	38764.7656
DPOG1_HUMAN	1231.37683
ICLN_HUMAN	10596.9121
SYRC_HUMAN	37461.5859
HIRA_HUMAN	2185.30713
ATX3_HUMAN	2330.76196
ATN1_HUMAN	1838.02112
PMS1_HUMAN	452.01416
PMS2_HUMAN	421.219543
CA2D1_HUMAN	2297.27393
SYYC_HUMAN	39257.0078
UBP14_HUMAN	22425.5137
AAKG1_HUMAN	3793.36719
HSP72_HUMAN	9748.13086
BCAT1_HUMAN	23049.7891
AT1B3_HUMAN	26332.6133
RD23B_HUMAN	1177.927
EPHB4_HUMAN	3475.7207
ANAG_HUMAN	1359.77527
KAD2_HUMAN	5603.54102
P5CS_HUMAN	18577.8184
SNAA_HUMAN	23046.9648
IF5_HUMAN	23290.9375
S12A2_HUMAN	7398.04883
PSMD4_HUMAN	22013.9395
DRG2_HUMAN	7385.80713
XPO2_HUMAN	164008.094
TERA_HUMAN	161948.453
MFAP1_HUMAN	1746.28296
ECHB_HUMAN	57101.8359
MANF_HUMAN	15953.2256
AFAD_HUMAN	4431.87207
ELL_HUMAN	1220.36353
BRPF1_HUMAN	1556.88867
NP1L1_HUMAN	57215.7539
CASP7_HUMAN	6686.34961
CASP6_HUMAN	4135.01807

ADK_HUMAN	12173.2373
DSRAD_HUMAN	12796.8643
LAMB2_HUMAN	3722.59131
CAD13_HUMAN	2917.6123
SEC13_HUMAN	13797.7363
NH2L1_HUMAN	11391.3926
PSA_HUMAN	32549.8945
ALR_HUMAN	444.2146
HNRH2_HUMAN	14279.2734
SCOT1_HUMAN	12383.0352
EIF3B_HUMAN	22116.0957
FCGRN_HUMAN	5848.86621
BID_HUMAN	2332.23853
ATPK_HUMAN	18883.6816
RRP1_HUMAN	6556.33496
SYMC_HUMAN	33074.5625
ARP19_HUMAN	5608.24463
AP1S2_HUMAN	3994.39624
ATP68_HUMAN	9278.02051
ATP5E_HUMAN	5966.6084
ATP5I_HUMAN	24268.5254
HDAC4_HUMAN	1564.70313
IF6_HUMAN	38137.9375
CTBP2_HUMAN	19840.252
NDUA6_HUMAN	7868.65771
PEX3_HUMAN	1336.03381
DHB7_HUMAN	2092.28271
BCAR1_HUMAN	9826.64844
STX17_HUMAN	728.677246
CF298_HUMAN	956.384644
WDR4_HUMAN	1630.55957
TMM33_HUMAN	38229.6523
GEMI4_HUMAN	3698.80957
CORO7_HUMAN	6537.30762
NU107_HUMAN	9816.39551
GSDMD_HUMAN	9190.0625
SELB_HUMAN	2559.40283
SESN2_HUMAN	672.961548
ANTR2_HUMAN	1675.56042
MTPN_HUMAN	667.306213
ARPC4_HUMAN	117603.086
CD81_HUMAN	14575.1123
SC61G_HUMAN	26012.5117
TPIS_HUMAN	145835.781
EIF3E_HUMAN	32323.8887
SC61B_HUMAN	25807.8203
PTEN_HUMAN	452.038208
PP4C_HUMAN	7897.23193
GBRL2_HUMAN	13037.916
ROMO1_HUMAN	3742.95972

MYL6_HUMAN	7338.24219
ACTB_HUMAN	1732865.63
IF4A1_HUMAN	178970.453
RS20_HUMAN	101348.023
PRPS1_HUMAN	5625.58203
PSA6_HUMAN	49087.0898
S10AA_HUMAN	37486.293
CDC42_HUMAN	18532.2266
DEST_HUMAN	21185.5293
GMFB_HUMAN	3189.54785
RAB8A_HUMAN	3685.30371
SPCS3_HUMAN	20142.3828
SRP54_HUMAN	10265.1123
RAB4B_HUMAN	2115.18091
RAB2A_HUMAN	32695.6934
RAB5B_HUMAN	5940.64844
RAB10_HUMAN	6677.02441
UB2D3_HUMAN	16426.8984
UBC12_HUMAN	18977.6641
UBE2K_HUMAN	19415.2363
UBE2N_HUMAN	23290.2266
RAB14_HUMAN	23300.6113
ARP3_HUMAN	68639.4922
ARP2_HUMAN	52989.418
ACTZ_HUMAN	19563.9297
CSN2_HUMAN	13089.0518
ABCE1_HUMAN	26284.7188
RAP1B_HUMAN	33007.8203
RAP2B_HUMAN	4181.35449
MAX_HUMAN	1396.19238
RS3A_HUMAN	105331.641
RL26_HUMAN	131979.422
PSME3_HUMAN	10324.417
RL15_HUMAN	158951.375
RL27_HUMAN	184607.328
VA0D1_HUMAN	17182.5586
RL37A_HUMAN	11778.8721
RHOA_HUMAN	15544.9688
RND3_HUMAN	1273.43335
NAA20_HUMAN	2664.06274
CH10_HUMAN	9548.2666
S61A1_HUMAN	26633.2461
LYSC_HUMAN	1771.00952
PFD3_HUMAN	9434.93652
STXB1_HUMAN	6665.78076
B2MG_HUMAN	614.611877
DAD1_HUMAN	25365.3867
NPC2_HUMAN	3931.47485
COPZ1_HUMAN	24950.4141
RL37_HUMAN	490.4599

SUMO2_HUMAN	15012.1338
DCAF7_HUMAN	2712.44995
WDR5_HUMAN	3379.55664
AP1S1_HUMAN	7795.97705
NTF2_HUMAN	5351.66113
HNRPK_HUMAN	224797.438
1433G_HUMAN	56311.1289
UBP46_HUMAN	2392.99756
RRAS2_HUMAN	17205.6738
RS7_HUMAN	59313.9336
PP1A_HUMAN	25722.2266
PP1B_HUMAN	25692.6172
PRS4_HUMAN	41146.7734
PRS8_HUMAN	50934.6016
RS8_HUMAN	158148.578
RS15A_HUMAN	119806.852
RS16_HUMAN	222847.781
UB2G1_HUMAN	1703.96191
UBE2H_HUMAN	4421.48096
1433E_HUMAN	79846.8984
RS14_HUMAN	160251.703
RS23_HUMAN	51259.7344
RS18_HUMAN	235002.734
RS29_HUMAN	34068.5156
RS13_HUMAN	117382.008
RS11_HUMAN	43802.8945
RUXE_HUMAN	24703.1133
RUXF_HUMAN	7942.10059
LSM3_HUMAN	3069.66626
LSM6_HUMAN	9538.69922
SMD1_HUMAN	12851.6133
SMD2_HUMAN	26610.2148
SMD3_HUMAN	64362.0977
TYB4_HUMAN	6118.19238
ARF6_HUMAN	19385.3691
PRS10_HUMAN	28935.4102
SELT_HUMAN	4608.29492
TBPL1_HUMAN	1078.73584
RL7A_HUMAN	133936.938
RPB7_HUMAN	2566.89795
RB11A_HUMAN	66884.25
ERF1_HUMAN	23619.498
CNBP_HUMAN	7950.71484
YPEL5_HUMAN	962.692505
RS4X_HUMAN	165265.719
PP2AB_HUMAN	36548.2891
ACTA_HUMAN	337737.875
RHOB_HUMAN	6692.37061
RL23A_HUMAN	171443.063
RS6_HUMAN	204037.984

H4_HUMAN	152716.328
RAB1A_HUMAN	9194.19238
RAN_HUMAN	260386
RL23_HUMAN	98702.8125
RAP1A_HUMAN	4826.30127
RS15_HUMAN	29401.5898
RS24_HUMAN	44133.3477
RS25_HUMAN	205446.438
RS26_HUMAN	51082.8047
RS28_HUMAN	41553.3438
RS30_HUMAN	68786.9219
GBB1_HUMAN	18843.8008
RBX1_HUMAN	15220.6963
GBB2_HUMAN	51876.9844
RL30_HUMAN	103825.344
RL39_HUMAN	26386.3242
RL31_HUMAN	100760.953
RL10A_HUMAN	75969.6094
RL32_HUMAN	35777.0781
RL11_HUMAN	83945.1719
RL8_HUMAN	95907.2109
PPIA_HUMAN	116497.242
FKB1A_HUMAN	29576.7402
RS27A_HUMAN	124492.867
GRB2_HUMAN	7731.95264
TRA2B_HUMAN	12439.3154
RAC1_HUMAN	73617.9609
AP2B1_HUMAN	30334.7363
VAMP2_HUMAN	7672.021
GNAS2_HUMAN	12686.6709
GNAI1_HUMAN	1226.59338
1433Z_HUMAN	205923.266
2ABA_HUMAN	28621.2949
SUMO1_HUMAN	16626.332
DYL1_HUMAN	9019.2998
DYLT1_HUMAN	4192.33105
RL38_HUMAN	49765.5508
SKP1_HUMAN	2838.7998
GBG5_HUMAN	844.943542
RS21_HUMAN	13125.0205
IF5A1_HUMAN	67825.7813
RACK1_HUMAN	180586.797
SPT4H_HUMAN	2194.51807
UBC9_HUMAN	15454.0771
TYB10_HUMAN	4868.5332
PP2AA_HUMAN	3482.16724
YBOX1_HUMAN	35484.457
SC11A_HUMAN	44059.2383
CSK2B_HUMAN	6839.27783
TPM4_HUMAN	85636.6563

UB2L3_HUMAN	33281.832
EF1A1_HUMAN	1221695.88
TBA1B_HUMAN	943579.125
TBA4A_HUMAN	2852.34644
TBB4B_HUMAN	289111.438
CSK21_HUMAN	11011.3711
PA1B2_HUMAN	7047.23242
H31_HUMAN	17789.0449
HBB_HUMAN	3082.82788
HBA_HUMAN	10333.8652
CXAR_HUMAN	2350.51489
F193A_HUMAN	2887.98242
NOP14_HUMAN	3812.70044
IGBP1_HUMAN	5515.70215
SHPS1_HUMAN	1067.99707
SERB_HUMAN	9001.61914
RBM6_HUMAN	3216.9624
IF4G2_HUMAN	33777.0352
RPP38_HUMAN	2700.1416
RPP30_HUMAN	5893.67578
GTF2I_HUMAN	25979.3223
PI42B_HUMAN	1705.89941
CNTP1_HUMAN	5339.89795
SRPK2_HUMAN	2566.36768
TCPB_HUMAN	77481.7422
S35A2_HUMAN	1234.75342
RAE1L_HUMAN	10582.0293
GSTO1_HUMAN	142231.234
PRKDC_HUMAN	46311.457
ADA17_HUMAN	2482.32129
BL1S1_HUMAN	1048.49792
SRPX_HUMAN	1494.72986
ARGI2_HUMAN	985.005188
NTH_HUMAN	600.22821
IN35_HUMAN	788.122314
NUCB2_HUMAN	2522.64893
BASP1_HUMAN	10579.4434
DCD_HUMAN	2895.54175
TMF1_HUMAN	2286.82861
RT22_HUMAN	9061.33008
RT25_HUMAN	2956.53662
RT10_HUMAN	4328.84131
RT35_HUMAN	1995.68762
RT05_HUMAN	3363.49097
RT11_HUMAN	5600.78906
RT15_HUMAN	7489.03027
RT21_HUMAN	4243.729
RT34_HUMAN	19659.3047
RT06_HUMAN	4133.76855
RT09_HUMAN	5336.63477

SARNP_HUMAN	29970.2871
LACTB_HUMAN	2828.52002
COG7_HUMAN	3578.69897
RL24_HUMAN	96583.1875
TXN4A_HUMAN	2267.37964
RL36A_HUMAN	16099.8418
CBX1_HUMAN	4617.22998
SMAD3_HUMAN	5604.25586
ARF1_HUMAN	90666.8906
ARF5_HUMAN	10813.3203
ERH_HUMAN	1999.00928
RHOG_HUMAN	9013.17773
RL19_HUMAN	74714.7813
SERF2_HUMAN	3159.21753
SRSF3_HUMAN	95715.5
FOXK1_HUMAN	6346.41895
CCZ1B_HUMAN	3127.96313
DAB2_HUMAN	16105.8584
PGBM_HUMAN	43067.7969
XIAP_HUMAN	1284.03564
EFNB1_HUMAN	4767.1748
RBM10_HUMAN	5331.59863
RBM3_HUMAN	17867.3008
AT2C1_HUMAN	2592.93652
AT11A_HUMAN	1568.18958
CYC_HUMAN	55033.4883
EM55_HUMAN	3030.87842
TFAM_HUMAN	10582.1592
PIPNA_HUMAN	9588.1084
S25A3_HUMAN	115731.461
VIGLN_HUMAN	34181.0742
TF2B_HUMAN	2502.95898
CDK6_HUMAN	1888.28943
CDK5_HUMAN	3290.10498
CDK17_HUMAN	9756.35547
PURA_HUMAN	5539.97852
BORG5_HUMAN	5219.05518
CLH1_HUMAN	88937.1875
HSF1_HUMAN	960.947998
NFKB2_HUMAN	2584.12378
FKBP3_HUMAN	41022.1563
REEP5_HUMAN	13655.543
DHSO_HUMAN	8696.05176
HNRPU_HUMAN	88879.5938
U2AF1_HUMAN	12132.7939
SPTB2_HUMAN	54808.5156
TIAR_HUMAN	9592.86328
SET_HUMAN	97720.9922
SRSF2_HUMAN	34362.3359
FOXK2_HUMAN	1300.59412

RELB_HUMAN	579.143127
GALK2_HUMAN	5019.09473
AMPD2_HUMAN	9528.19336
FABP5_HUMAN	95485.8516
CAP1_HUMAN	78107.3359
FLI1_HUMAN	3788.052
HMCS1_HUMAN	15887.6563
IFM3_HUMAN	9632.74023
LAT1_HUMAN	2702.83057
NC2B_HUMAN	2255.82568
EXOSX_HUMAN	4367.84277
OTUD4_HUMAN	724.046387
PFKAP_HUMAN	48131.9453
XPC_HUMAN	1399.89185
EWS_HUMAN	33916.4844
CDR2_HUMAN	3830.14648
OCRL_HUMAN	2664.05933
PLCB3_HUMAN	9752.36328
TAGL_HUMAN	5215.15283
AK17A_HUMAN	2442.80029
PYRD_HUMAN	449.900604
ODO1_HUMAN	15424.3584
CENPE_HUMAN	234.498535
KIF23_HUMAN	1466.43884
MMSA_HUMAN	4045.74585
NRG1_HUMAN	499.802277
CP27A_HUMAN	701.032471
DSG1_HUMAN	867.167603
SP3_HUMAN	395.159668
H11_HUMAN	1333.98523
RL18A_HUMAN	78533.1484
MP2K1_HUMAN	14258.8721
TIE2_HUMAN	2096.64722
FKBP4_HUMAN	23960.6758
PLOD1_HUMAN	19086.1699
NUCB1_HUMAN	5250.58984
RL6_HUMAN	240409.234
TOP2B_HUMAN	2011.88416
AKA12_HUMAN	41301.9492
M2OM_HUMAN	17005.3652
DYST_HUMAN	3657.63696
ENL_HUMAN	2098.36108
MECOM_HUMAN	1281.80493
GNA12_HUMAN	1552.03149
CAV1_HUMAN	79761.5703
ACY1_HUMAN	1766.23853
TNAP2_HUMAN	2288.85938
LMNB2_HUMAN	29622.7598
PTPS_HUMAN	1838.38452
UPAR_HUMAN	1550.25635

KIME_HUMAN	5960.38965
ERCC6_HUMAN	133.717682
TAP1_HUMAN	2711.50122
TAP2_HUMAN	1657.51721
CEBPZ_HUMAN	4234.68311
ZNF92_HUMAN	966.367126
TF65_HUMAN	8737.80371
UBXN1_HUMAN	7824.97363
GLGB_HUMAN	25948.002
IF4G1_HUMAN	29706.9531
ATP7A_HUMAN	227.394058
NOTC2_HUMAN	1490.45825
TLE1_HUMAN	1829.59216
TLE3_HUMAN	1480.52722
LGUL_HUMAN	28386.1152
ACVR1_HUMAN	488.763458
SSBP_HUMAN	15319.8242
REL_HUMAN	577.638733
1433F_HUMAN	48967.5039
PLP2_HUMAN	41553.2031
CSTF1_HUMAN	7470.41748
UBE3A_HUMAN	6808.87158
DYN1_HUMAN	1034.32593
PTN12_HUMAN	5983.69678
FAK1_HUMAN	10812.0928
SRS11_HUMAN	115084.023
KPCD_HUMAN	2654.08813
CALD1_HUMAN	40506.1406
FOLC_HUMAN	2023.98413
CTSL2_HUMAN	1374.69946
PTN11_HUMAN	8856.15332
KDSR_HUMAN	2979.09741
PUR1_HUMAN	7250.54639
GFPT1_HUMAN	19944.4746
EXOS9_HUMAN	3005.91724
PSME1_HUMAN	36491.8516
SUH_HUMAN	918.669373
MEF2C_HUMAN	390.828918
APLP2_HUMAN	7405.54883
GABPA_HUMAN	2562.59546
RING1_HUMAN	379.072479
FMR1_HUMAN	844.634521
PRDX1_HUMAN	180221.219
RL18_HUMAN	136440.703
C1QBP_HUMAN	26516.1543
CKAP4_HUMAN	119718.766
ZO1_HUMAN	16270.1104
TISB_HUMAN	1631.28223
SPAG1_HUMAN	1393.42419
KHDR1_HUMAN	34076.2656

BAX_HUMAN	14447.4443
B2CL1_HUMAN	6018.979
MCL1_HUMAN	1091.2926
DPOE1_HUMAN	4376.24707
KLC1_HUMAN	20592.8574
SOS1_HUMAN	1111.72522
SRSF1_HUMAN	101236.469
RHG01_HUMAN	15979.4229
TLE5_HUMAN	222.879089
SRSF4_HUMAN	9213.22461
PCDH1_HUMAN	7109.02393
PP2BA_HUMAN	4855.43066
DHX9_HUMAN	63619.9961
QOR_HUMAN	7291.63281
GOGA3_HUMAN	3010.17432
GOGA2_HUMAN	3956.24463
ECHP_HUMAN	792.960205
MFGM_HUMAN	3676.07544
PDE4D_HUMAN	780.061035
DSC1_HUMAN	1542.57593
HDHD1_HUMAN	2548.24878
TICN1_HUMAN	1510.49634
CD47_HUMAN	16293.249
PPID_HUMAN	15645.2178
SSRP1_HUMAN	26135.7656
CAMP2_HUMAN	2776.91943
SPIR1_HUMAN	886.204224
SLFN5_HUMAN	3487.18726
VAC14_HUMAN	5822.29443
NSUN2_HUMAN	20172.4844
DMWD_HUMAN	461.253265
RBBP4_HUMAN	53944.5664
NCBP1_HUMAN	15234.4326
MGT5A_HUMAN	853.099426
EP300_HUMAN	2359.7627
AHNK_HUMAN	131475.391
FCHO2_HUMAN	1171.89734
ELP6_HUMAN	820.443237
HSP7E_HUMAN	6280.2041
SCRN3_HUMAN	1232.10999
CGNL1_HUMAN	4261.76025
LTOR4_HUMAN	4877.9834
NEXN_HUMAN	4102.4043
MGAT2_HUMAN	6555.32568
GALT2_HUMAN	8861.24023
GALT1_HUMAN	9113.61328
AP1B1_HUMAN	34347.8633
CPSF1_HUMAN	7655.18799
BST2_HUMAN	817.639404
MPPA_HUMAN	8222.5625

SIA4A_HUMAN	371.750854
SCRN1_HUMAN	9112.04102
WASC5_HUMAN	6842.26123
NU160_HUMAN	7727.80811
SCAP_HUMAN	2374.96484
FOXO1_HUMAN	579.52063
TBL3_HUMAN	4343.90674
TF3C1_HUMAN	2922.4397
TWF1_HUMAN	16520.2305
ASPH_HUMAN	12458.2803
TFCP2_HUMAN	2602.61035
AKP13_HUMAN	2640.64087
FBLN3_HUMAN	29692.4473
SNF5_HUMAN	4620.61572
BPTF_HUMAN	875.582458
CDC20_HUMAN	1680.23389
FSTL1_HUMAN	8990.56445
STX4_HUMAN	2142.77832
GRSF1_HUMAN	7337.69141
M4K2_HUMAN	1002.93939
MERTK_HUMAN	2835.71069
SFSWA_HUMAN	1145.45776
CHD3_HUMAN	795.011536
SF3A3_HUMAN	26313.3203
DPYD_HUMAN	217.202866
TP53B_HUMAN	6975.32031
TM115_HUMAN	1767.84399
IFRD2_HUMAN	942.556213
TRI26_HUMAN	1284.95874
AIMP1_HUMAN	15642.667
ILF2_HUMAN	79157.6172
ILF3_HUMAN	88245.3047
LMAN2_HUMAN	45524.9336
TRAP1_HUMAN	375457.156
TRAF2_HUMAN	3478.13135
DLG1_HUMAN	3207.20313
MYO1E_HUMAN	9364.53613
PP1R8_HUMAN	4991.33643
TP4A2_HUMAN	4478.57764
ABR_HUMAN	4267.30908
NPRL3_HUMAN	664.904358
SEC20_HUMAN	5140.59961
BNIP2_HUMAN	2980.55273
NFX1_HUMAN	279.780396
CSTF3_HUMAN	6493.46045
TSN31_HUMAN	2328.19238
ECH1_HUMAN	8497.80664
RHG05_HUMAN	840.790955
STRN3_HUMAN	5500.88721
CDC16_HUMAN	3360.30688

STK4_HUMAN	2876.45142
FLII_HUMAN	24954.4258
TRI32_HUMAN	1430.05286
COASY_HUMAN	4899.41406
RM28_HUMAN	5623.78125
ACACA_HUMAN	13800.3994
CSN1_HUMAN	15054.3213
UBP4_HUMAN	3794.12305
CAF1A_HUMAN	532.616638
CAF1B_HUMAN	1416.39734
RED_HUMAN	9695.04199
MTAP_HUMAN	10187.5566
AAPK1_HUMAN	4996.40283
LIPA1_HUMAN	2234.41895
CACO2_HUMAN	309.726837
EI2BE_HUMAN	16301.3271
TADBP_HUMAN	14470.7559
ROA0_HUMAN	23494.0566
AIMP2_HUMAN	12081.4736
FADD_HUMAN	4178.03662
PRDX4_HUMAN	47661.9414
MP2K5_HUMAN	292.508728
MK07_HUMAN	566.120667
PAK2_HUMAN	14335.2891
CBX3_HUMAN	24470.459
STK3_HUMAN	942.942139
STX5_HUMAN	3944.0188
PSMD2_HUMAN	37462.6953
MMRN1_HUMAN	6250.72607
DDX10_HUMAN	1137.5033
DNJC3_HUMAN	19954.0508
SBP1_HUMAN	3381.00562
NDK3_HUMAN	3284.16162
SRSF9_HUMAN	19578.2207
SRSF5_HUMAN	6059.98193
SRSF6_HUMAN	69143.5156
MD2L1_HUMAN	8964.83887
TIF1B_HUMAN	33172.957
SEM3F_HUMAN	357.502441
STX3_HUMAN	1439.23975
G3BP1_HUMAN	38633.2422
NMI_HUMAN	3249.11426
PTK7_HUMAN	2942.12817
PABP4_HUMAN	29542.3555
ATM_HUMAN	1300.18408
GRB10_HUMAN	3067.3772
MTA1_HUMAN	3342.00488
EIF3I_HUMAN	19479.1426
PPIL2_HUMAN	1022.7749
2A5G_HUMAN	1899.26172

CTBP1_HUMAN	5652.73584
PHLP_HUMAN	5914.24561
PLD1_HUMAN	3121.15308
TARB1_HUMAN	551.021179
UB2V1_HUMAN	36590.2422
RM49_HUMAN	519.213257
DC1I2_HUMAN	22613.4805
ORC2_HUMAN	1245.06616
ILK_HUMAN	31238.8535
NNTM_HUMAN	18645.8301
SNTB2_HUMAN	15486.7148
XRCC4_HUMAN	4558.92041
PPIG_HUMAN	8616.11035
TCOF_HUMAN	9296.14746
U119A_HUMAN	921.412109
SF3B2_HUMAN	23321.9375
OS9_HUMAN	1810.30774
GOGA4_HUMAN	2311.75293
HAP28_HUMAN	18750.6484
ADAM9_HUMAN	12830.3135
ADA15_HUMAN	4224.32324
TMED1_HUMAN	4145.62354
FKBP5_HUMAN	19492.0176
MYO9B_HUMAN	5499.69336
ROCK1_HUMAN	8112.71143
NFAC2_HUMAN	730.256897
TOP3A_HUMAN	622.014709
SMAD4_HUMAN	3571.04102
VPP3_HUMAN	6313.5415
BIRC2_HUMAN	668.997314
PICAL_HUMAN	11027.7402
MTM1_HUMAN	2015.60498
SQSTM_HUMAN	8765.95508
MTX1_HUMAN	3965.01392
TBB3_HUMAN	11162.9053
ASAH1_HUMAN	454.224457
PRP4B_HUMAN	2976.18701
PIN1_HUMAN	6521.01611
ATR_HUMAN	1709.86926
RIPK1_HUMAN	1571.70776
HDAC1_HUMAN	16221.9824
KCC2G_HUMAN	3936.25757
KCC2D_HUMAN	4909.0415
DCTN2_HUMAN	4859.43652
PKD2_HUMAN	415.434235
ULA1_HUMAN	8321.75879
ITPK1_HUMAN	1656.95825
SNW1_HUMAN	9298.5625
DGKZ_HUMAN	3772.14233
STIM1_HUMAN	2822.21484

GRAP_HUMAN	3152.64478
TRA2A_HUMAN	4788.61572
SNX1_HUMAN	13054.0537
KRR1_HUMAN	5533.31982
PWP1_HUMAN	5588.32373
MTMR1_HUMAN	1121.66003
MTMR2_HUMAN	5255.64795
CUL1_HUMAN	5647.26123
CUL2_HUMAN	11349.8486
CUL3_HUMAN	6847.83594
CUL4A_HUMAN	4361.52393
CUL4B_HUMAN	15429.7539
ASPP2_HUMAN	498.979919
DYR1A_HUMAN	883.399292
FCL_HUMAN	13667.4775
RAB32_HUMAN	10351.2139
TPBG_HUMAN	738.676819
FHL1_HUMAN	14557.1201
FHL3_HUMAN	2392.6853
RIN1_HUMAN	729.514954
AAMP_HUMAN	2893.18774
MOGS_HUMAN	33885.0117
CD166_HUMAN	6717.5166
THOC5_HUMAN	3786.68457
SHRM2_HUMAN	1225.30457
SPTN1_HUMAN	33915.1133
NOG2_HUMAN	4714.92432
AUHM_HUMAN	463.114685
PKP1_HUMAN	249.420547
DX39B_HUMAN	11969.4619
BLMH_HUMAN	24204.9258
EXOS2_HUMAN	4462.32666
BMPR2_HUMAN	2409.0979
TBB2A_HUMAN	156816.219
TF2H2_HUMAN	2353.60669
TF2H3_HUMAN	1441.41333
BYST_HUMAN	10718.1553
RPGF1_HUMAN	2630.15039
IDI1_HUMAN	8704.36621
PEBB_HUMAN	7707.35645
NFYC_HUMAN	1618.45007
CDK13_HUMAN	1316.52893
CKAP5_HUMAN	29771.6133
CIRBP_HUMAN	6773.70654
KCC1A_HUMAN	1484.42126
COTL1_HUMAN	31320.6621
HNRPD_HUMAN	156289.734
SCRB2_HUMAN	26462.3477
NID2_HUMAN	1187.95593
LRP8_HUMAN	405.425171

DAG1_HUMAN	3440.15356
VEZF1_HUMAN	151.324799
BOP1_HUMAN	8887.80469
UBE4A_HUMAN	4277.36768
SEPT6_HUMAN	5264.62695
KEAP1_HUMAN	1880.47766
URB2_HUMAN	2045.53296
DHX34_HUMAN	1567.10974
MORC3_HUMAN	1120.56714
SAFB2_HUMAN	12207.1006
EIF3A_HUMAN	45219.1563
ARHG7_HUMAN	7429.5332
EFR3A_HUMAN	3918.15674
UBP2L_HUMAN	18982.543
SCRIB_HUMAN	6466.89795
GIT2_HUMAN	3888.65234
SREC_HUMAN	4112.4873
MLEC_HUMAN	22305.5313
TTL12_HUMAN	9301.79199
DPOA2_HUMAN	2042.09082
DOCK1_HUMAN	5669.90625
TFDP1_HUMAN	2387.28638
FHL2_HUMAN	23783.1113
DPYL3_HUMAN	57323.8477
ICT1_HUMAN	6334.37207
ZMYM3_HUMAN	696.348938
DCTN1_HUMAN	14254.4668
DYHC1_HUMAN	73902.6641
EI2BA_HUMAN	8241.93555
IF4A2_HUMAN	7996.94043
ELOA1_HUMAN	1847.18286
SRC8_HUMAN	18990.3145
NUCG_HUMAN	3192.02734
FLOT2_HUMAN	3845.63965
RCN2_HUMAN	12169.8018
TRI25_HUMAN	17418.2051
FLNC_HUMAN	56972.5117
FKBP8_HUMAN	4813.19189
FA50A_HUMAN	5922.00391
FRG1_HUMAN	2400.41699
GNA13_HUMAN	51680.3398
GAMT_HUMAN	1160.40918
GALE_HUMAN	8915.92285
LRC32_HUMAN	454.682281
CAPR1_HUMAN	22678.2168
BECN1_HUMAN	620.132813
RBM39_HUMAN	16499.8262
CASL_HUMAN	1051.69263
HLTF_HUMAN	1887.74072
ERG1_HUMAN	2521.16748

PDIA5_HUMAN	12320.4873
KPRA_HUMAN	8096.50781
DHX8_HUMAN	2201.87646
MCM6_HUMAN	17680.7441
ITPR2_HUMAN	5826.00391
ITPR3_HUMAN	6230.76123
ITPR1_HUMAN	519.82312
RASA3_HUMAN	4320.41064
PLSI_HUMAN	2841.35254
IRF3_HUMAN	4133.49951
LAGE3_HUMAN	1315.78162
BLTP2_HUMAN	547.919495
TRIPC_HUMAN	5428.14502
PUM1_HUMAN	4591.47119
MDC1_HUMAN	528.675964
EPN4_HUMAN	10330.96
KANK1_HUMAN	2933.37988
SMC1A_HUMAN	66453.0391
RRP1B_HUMAN	6228.23193
DIP2A_HUMAN	6175.30078
RRP5_HUMAN	7884.29346
PSF1_HUMAN	2305.34619
BMS1_HUMAN	4669.41016
LPIN1_HUMAN	2551.38696
UBP10_HUMAN	11040.999
MESD_HUMAN	10440.5752
GANAB_HUMAN	58479.7227
RFTN1_HUMAN	4113.33545
MFS10_HUMAN	1860.74341
2A5D_HUMAN	13003.5146
LBR_HUMAN	15774.8682
COG2_HUMAN	936.879944
MVP_HUMAN	93257.2031
LTBP1_HUMAN	1016.12701
LTBP2_HUMAN	2666.12671
CBX2_HUMAN	256.834259
GOGB1_HUMAN	3557.64429
CASP8_HUMAN	1024.35425
NAA30_HUMAN	3076.65283
KIF22_HUMAN	862.775085
MEF2D_HUMAN	1409.52673
CHD4_HUMAN	16531.959
LASP1_HUMAN	40682.1563
PTGR1_HUMAN	11138.8008
NC2A_HUMAN	5461.73486
NFAC4_HUMAN	552.535278
ZN638_HUMAN	4238.24316
IMB1_HUMAN	51308.3125
NOLC1_HUMAN	5359.95801
NUMA1_HUMAN	10684.2891

PSME4_HUMAN	7869.03174
CUL7_HUMAN	1721.93823
SLMAP_HUMAN	917.068054
GAPD1_HUMAN	11556.5186
NAA25_HUMAN	8397.19434
MELT_HUMAN	1617.88232
CND2_HUMAN	6292.21338
PAF15_HUMAN	822.066467
SPCS2_HUMAN	21948.3945
EMC2_HUMAN	5035.74023
FL2D_HUMAN	1374.85596
PSMD6_HUMAN	15824.123
HERP1_HUMAN	139.544495
MD2BP_HUMAN	1735.14575
MO4L2_HUMAN	3933.22974
ABRX2_HUMAN	2191.40894
SEPT2_HUMAN	39754.9805
SART3_HUMAN	13499.4453
CND1_HUMAN	12316.1143
SUZ12_HUMAN	937.245972
EXOS7_HUMAN	3896.64673
TNIP1_HUMAN	1019.61102
U5S1_HUMAN	72316.8906
SYLM_HUMAN	3126.02905
R3HD1_HUMAN	1464.93152
TRAM2_HUMAN	1068.02051
SNX17_HUMAN	4706.4707
AR6P1_HUMAN	7667.79102
RB3GP_HUMAN	4609.20508
S39AE_HUMAN	2872.77393
SYK_HUMAN	36265.2422
SETB1_HUMAN	1094.18213
LRC14_HUMAN	971.230469
RRS1_HUMAN	8102.22314
DPOD3_HUMAN	1914.18115
IF4H_HUMAN	35748.3555
ACAP2_HUMAN	4397.76318
KIF14_HUMAN	433.599243
BRD3_HUMAN	1190.00024
WDR43_HUMAN	4386.11182
ACOX1_HUMAN	8209.50293
OXA1L_HUMAN	7425.31494
EEA1_HUMAN	12961.3418
PDIA6_HUMAN	117185.32
PA1B3_HUMAN	4453.7002
PDK3_HUMAN	739.817383
PEA15_HUMAN	2393.2063
EBP_HUMAN	11053.8984
PMVK_HUMAN	5433.00439
KPCD1_HUMAN	256.506927

PLEC_HUMAN	72182.5078
PCM1_HUMAN	2549.03418
NOMO1_HUMAN	27196.2598
PON2_HUMAN	7719.31787
2A5A_HUMAN	2780.83154
IPYR_HUMAN	46317.9961
TEBP_HUMAN	19528.4707
STK38_HUMAN	2703.43872
NONO_HUMAN	60850.0313
PTPA_HUMAN	21101.8574
PTPRK_HUMAN	4089.4519
PWP2_HUMAN	4727.07178
RABE1_HUMAN	1739.41357
RAB35_HUMAN	6069.12256
RNPS1_HUMAN	7040.46729
RBBP5_HUMAN	2646.11328
RCN1_HUMAN	18581.5078
RBP1_HUMAN	585.255981
L2GL1_HUMAN	2481.87646
LRC41_HUMAN	2152.85425
TTF1_HUMAN	9594.75195
TMED2_HUMAN	5769.73486
PCBP1_HUMAN	168005.938
PCBP2_HUMAN	163684.859
ELOC_HUMAN	4055.04248
ELOB_HUMAN	2412.86108
RHEB_HUMAN	11252.8975
UBE3C_HUMAN	4688.4585
TOM20_HUMAN	9538.59863
MTFR1_HUMAN	1243.74426
DHC24_HUMAN	18335.9199
SF3B3_HUMAN	27108.9668
PUM3_HUMAN	5456.37158
DLGP5_HUMAN	4046.77539
RSU1_HUMAN	42327.0859
CNN3_HUMAN	20480.2695
KS6A1_HUMAN	572.102966
SAFB1_HUMAN	9400.03418
SF3A2_HUMAN	6508.51123
RBMS2_HUMAN	7368.73779
PP1R7_HUMAN	15802.1611
SC23A_HUMAN	21537.9766
SC23B_HUMAN	4380.25488
CYH1_HUMAN	4179.23633
SF3A1_HUMAN	13550.2637
SKI2_HUMAN	8639.83008
CDSN_HUMAN	653.652832
SURF1_HUMAN	2281.23291
TAF5_HUMAN	488.866913
TAF7_HUMAN	1739.76208

TERF2_HUMAN	1185.93884
MARE2_HUMAN	12120.6367
TGIF1_HUMAN	231.728851
NHRF2_HUMAN	3733.42358
TRADD_HUMAN	2425.05225
TRAM1_HUMAN	9079.04297
TSN_HUMAN	9557.77441
TRBP2_HUMAN	1247.9209
SF01_HUMAN	16069.665
CIP4_HUMAN	6692.85254
TRIPB_HUMAN	2805.30615
PCH2_HUMAN	8785.27539
MED1_HUMAN	1615.08447
TRIP4_HUMAN	3828.28833
HMGN3_HUMAN	31.6242619
JHD2C_HUMAN	337.955322
IKBB_HUMAN	2115.46875
TRIP6_HUMAN	5122.26709
PTN14_HUMAN	2293.72485
MARE1_HUMAN	48275.7617
ELAV1_HUMAN	53915.1289
NSDHL_HUMAN	17731.543
NAB2_HUMAN	482.184601
MYLK_HUMAN	2420.0874
TAB1_HUMAN	3029.66504
HERC1_HUMAN	2136.55737
AAAT_HUMAN	13408.8643
MLF2_HUMAN	1410.65137
TOM34_HUMAN	7379.34229
SMAD2_HUMAN	4749.82422
SMAD1_HUMAN	18694.6914
MSMO1_HUMAN	6738.33936
ITSN1_HUMAN	2230.4729
TBCE_HUMAN	4843.21533
TBCC_HUMAN	4864.05664
UB2V2_HUMAN	1524.78308
STK11_HUMAN	516.486755
STXB2_HUMAN	4652.09082
VAMP3_HUMAN	4537.63672
NEDD8_HUMAN	3943.73584
VAS1_HUMAN	5567.43994
VPS72_HUMAN	1717.09216
RB11B_HUMAN	14855.1553
ZYX_HUMAN	8119.94287
ETFD_HUMAN	1033.85278
SEPT7_HUMAN	30516.666
ADRM1_HUMAN	19908.8086
CCDC6_HUMAN	5096.92236
UAP1_HUMAN	25042.873
E2F4_HUMAN	595.771729

IBP7_HUMAN	9912.24023
PDCD2_HUMAN	766.832214
LAMA4_HUMAN	8750.19043
PSMD5_HUMAN	23694.6426
PKN1_HUMAN	8256.00098
PKN2_HUMAN	6582.62207
TAF12_HUMAN	1100.30115
CSRP2_HUMAN	1228.32214
DDB1_HUMAN	37067.6719
2A5E_HUMAN	5062.88818
MK14_HUMAN	5680.35596
RM23_HUMAN	4448.79541
CDC37_HUMAN	34303.0313
DPYL2_HUMAN	63246.8477
SYPL1_HUMAN	11922.6533
RBBP7_HUMAN	7957.85449
M3K11_HUMAN	691.280884
TAF9_HUMAN	2457.72192
CALRL_HUMAN	5659.12891
BAK_HUMAN	848.152283
SRSF7_HUMAN	46272.6367
CPSF6_HUMAN	16479.5137
SMN_HUMAN	3586.43774
DREB_HUMAN	16571.5645
NRF1_HUMAN	1664.77466
FSCN1_HUMAN	137937.047
IF16_HUMAN	26238.6797
DECR_HUMAN	26950.8164
MA2A1_HUMAN	7151.87256
NDUA5_HUMAN	1407.0835
CEGT_HUMAN	1360.42676
CLPP_HUMAN	3703.74219
THTR_HUMAN	11554.6738
UBE2S_HUMAN	1625.94543
KAT1_HUMAN	638.882385
KGUA_HUMAN	3251.42578
GLO2_HUMAN	7555.90039
NDUA9_HUMAN	9569.0625
MAON_HUMAN	1574.9834
PCKGM_HUMAN	2812.06763
UPP1_HUMAN	1958.16736
DDR2_HUMAN	2027.39209
HCDH_HUMAN	11285.6455
CP51A_HUMAN	15479.3887
UGPA_HUMAN	28780.8867
F263_HUMAN	2053.78564
TRXR1_HUMAN	49028.3086
MIC60_HUMAN	17439.1094
ECSCR_HUMAN	3407.43945
KNOP1_HUMAN	1076.2439

HNRL2_HUMAN	17012.7598
T132A_HUMAN	398.451569
INF2_HUMAN	6810.0874
PDS5A_HUMAN	7294.97021
CL16A_HUMAN	842.053589
KIF7_HUMAN	522.777649
RHG31_HUMAN	2777.31006
MTHSD_HUMAN	5282.59131
AAK1_HUMAN	5122.7959
WASC4_HUMAN	4884.46387
ERC6L_HUMAN	1928.19067
TSR1_HUMAN	8937.45117
RGPA2_HUMAN	754.07312
D19L1_HUMAN	5213.24854
IAH1_HUMAN	1553.39063
ALG11_HUMAN	2407.0144
QRIC1_HUMAN	3658.30005
SMU1_HUMAN	13635.9131
CTU2_HUMAN	9186.30859
PGGHG_HUMAN	945.24115
LRRF1_HUMAN	5003.09717
PGPS1_HUMAN	650.611816
CR021_HUMAN	66.6152344
P3H1_HUMAN	19570.4141
TRM5_HUMAN	3178.88892
EMAL3_HUMAN	3586.5874
RPA43_HUMAN	3071.6228
MA7D1_HUMAN	3647.75806
UAP1L_HUMAN	12984.6592
CHD9_HUMAN	4014.9397
TKFC_HUMAN	2195.83911
LSM12_HUMAN	6616.82471
HSDL1_HUMAN	3828.52856
AL1L2_HUMAN	565.799622
GNPTA_HUMAN	1331.15344
GRDN_HUMAN	1810.34021
RABL6_HUMAN	6172.72559
TIM50_HUMAN	6498.07178
PAR14_HUMAN	2655.79272
FUT11_HUMAN	1024.67651
GLYR1_HUMAN	7455.18652
CE022_HUMAN	1802.42334
C2CD3_HUMAN	283.352936
VP26B_HUMAN	4224.02832
LARP7_HUMAN	2945.15308
NAKD2_HUMAN	921.032898
KCD21_HUMAN	1737.74841
GXLT1_HUMAN	362.355865
ACSF3_HUMAN	1459.23584
PPCEL_HUMAN	2682.54565

TB10B_HUMAN	3026.9375
TPRN_HUMAN	465.305481
ANO6_HUMAN	3983.07373
FIL1L_HUMAN	2086.42139
GRAP1_HUMAN	2925.3811
ZNG1F_HUMAN	870.985107
MIX23_HUMAN	4083.54028
MBRL_HUMAN	832.716614
FA98B_HUMAN	5922.67725
RHG29_HUMAN	8236.87988
PDCD4_HUMAN	6217.84473
FND3B_HUMAN	8077.43994
CRTC2_HUMAN	362.261658
GPAT3_HUMAN	715.720459
CEP55_HUMAN	2555.17065
NCBP3_HUMAN	2632.46729
QORX_HUMAN	20509.9805
NACHO_HUMAN	3339.3689
PHLA2_HUMAN	2994.91797
PDLI3_HUMAN	739.901184
PAR10_HUMAN	290.659424
DHB12_HUMAN	22920.1953
GLE1_HUMAN	3036.6228
UBP39_HUMAN	7092.10254
KLH22_HUMAN	1101.03516
AGK_HUMAN	10726.0381
SETMR_HUMAN	156.92424
LACB2_HUMAN	2020.56982
P5CR3_HUMAN	3542.41479
EIPR1_HUMAN	5874.23584
BOREA_HUMAN	662.980591
COBL1_HUMAN	963.638062
H1BP3_HUMAN	2860.16382
CYBR1_HUMAN	656.297119
WDR81_HUMAN	1908.58276
ACTBL_HUMAN	287692.344
CCD93_HUMAN	3575.51489
OCAD2_HUMAN	13830.6885
PKHG4_HUMAN	781.473206
HS904_HUMAN	1105.63074
DCAF6_HUMAN	683.033813
TM41B_HUMAN	969.583862
SGMR2_HUMAN	11357.5479
YIF1B_HUMAN	3427.32446
ZN326_HUMAN	4307.45313
NOM1_HUMAN	1319.73352
RIPL1_HUMAN	1671.32373
DJC21_HUMAN	1057.04846
TP8L3_HUMAN	5162.65234
HERC4_HUMAN	6765.00049

PIGG_HUMAN	1263.44446
PP6R3_HUMAN	5793.14063
RABL3_HUMAN	25178.75
FA76B_HUMAN	513.87616
SH319_HUMAN	3225.32251
NHSL2_HUMAN	2096.32935
EMC4_HUMAN	3618.82568
SYEM_HUMAN	1995.82861
CC038_HUMAN	881.911194
EFMT2_HUMAN	472.799225
TGO1_HUMAN	2085.73096
PREP_HUMAN	14354.9854
WDR44_HUMAN	3126.27637
PRC2B_HUMAN	3827.07813
TJAP1_HUMAN	1015.32074
RRP12_HUMAN	11546.7031
COA6_HUMAN	558.826294
TOIP1_HUMAN	14827.1426
CEP78_HUMAN	409.772644
SYAM_HUMAN	4246.78613
TOR2A_HUMAN	545.286804
PCID2_HUMAN	3840.56006
HABP4_HUMAN	1815.68738
S27A3_HUMAN	644.390442
SAMD9_HUMAN	1322.90698
P4R3B_HUMAN	2572.10107
WIPI3_HUMAN	1835.29114
WIPI1_HUMAN	841.380188
EOGT_HUMAN	13035.6904
SMAG2_HUMAN	331.509186
TBCEL_HUMAN	2506.82935
TDIF2_HUMAN	2229.05884
RBG1L_HUMAN	1932.7074
TTC38_HUMAN	1360.5376
COX20_HUMAN	5035.48242
EXOS6_HUMAN	5688.09229
TM201_HUMAN	1704.01563
ATAT_HUMAN	333.103729
SNP47_HUMAN	494.836975
NU188_HUMAN	6085.6958
HP1B3_HUMAN	35524.6172
SYVM_HUMAN	690.195862
CE170_HUMAN	7313.82568
ARH_HUMAN	895.943176
ODR4_HUMAN	3348.77368
NOL9_HUMAN	3926.73364
NHSL1_HUMAN	1974.54895
HYI_HUMAN	423.020233
TPRGL_HUMAN	1289.20593
C2D1B_HUMAN	1785.2738

FBP1L_HUMAN	13948.8789
SYRM_HUMAN	860.368469
THEM4_HUMAN	1322.4469
CHCH9_HUMAN	933.757202
FKB15_HUMAN	12152.4189
ZC3HD_HUMAN	2016.13269
CI114_HUMAN	1982.26318
ARMD3_HUMAN	593.463623
CSCL2_HUMAN	1780.4967
GPTC4_HUMAN	407.515198
CAF17_HUMAN	1934.3623
HECD3_HUMAN	3881.00806
GT253_HUMAN	13285.2998
UBR4_HUMAN	11194.3955
STXB5_HUMAN	1191.19226
RHG21_HUMAN	934.489746
CAMP1_HUMAN	1437.68713
RM02_HUMAN	3590.43726
UBAP2_HUMAN	5000.43652
QNG1_HUMAN	1367.33398
KPRP_HUMAN	2204.58301
ZN618_HUMAN	3374.85913
ACBD5_HUMAN	6605.31689
RBM26_HUMAN	3694.90747
ATD3B_HUMAN	2408.49194
WLS_HUMAN	6840.98438
INT11_HUMAN	3568.57153
CPTP_HUMAN	569.333313
DCAF8_HUMAN	4372.25781
CODA1_HUMAN	750.593811
TUT4_HUMAN	6461.06689
FRY_HUMAN	786.476501
RNH2B_HUMAN	1198.87537
ATPF1_HUMAN	1578.59888
RC3H1_HUMAN	762.882935
DDI2_HUMAN	3904.39038
NDUF5_HUMAN	1435.41101
NT5D1_HUMAN	3254.2832
BIG3_HUMAN	1163.93018
VP13D_HUMAN	342.991821
CROCC_HUMAN	1241.30908
LYRM7_HUMAN	1253.71863
RAIN_HUMAN	22473.584
RIF1_HUMAN	2553.24707
VPS53_HUMAN	4838.07178
STRP1_HUMAN	4014.35547
MRCKA_HUMAN	7521.89014
RPRD2_HUMAN	945.917236
RN220_HUMAN	392.214905
PR38B_HUMAN	4514.25684

BRE1A_HUMAN	3752.01904
MYOME_HUMAN	583.842346
ZN318_HUMAN	715.061523
F1711_HUMAN	978.632202
DIK1B_HUMAN	2467.41064
LRCH2_HUMAN	1156.25378
CDKAL_HUMAN	4079.4375
BROX_HUMAN	4994.65137
FOCAD_HUMAN	7401.49316
RSBN1_HUMAN	311.973328
DAB2P_HUMAN	805.324219
LYPL1_HUMAN	6972.44385
PEAR1_HUMAN	2456.47266
ECM29_HUMAN	15305.1152
TUT7_HUMAN	824.451111
SHE_HUMAN	575.549438
DEN4C_HUMAN	3140.53442
NAA35_HUMAN	4353.84863
ZMYM4_HUMAN	2016.14551
FHI2A_HUMAN	4432.18115
ZDH20_HUMAN	2603.5166
SPRY7_HUMAN	1479.09619
FBXW9_HUMAN	231.105362
CD276_HUMAN	2949.47363
RN213_HUMAN	4584.64404
TENS2_HUMAN	1687.06299
KANK2_HUMAN	592.570068
WAC2A_HUMAN	1581.80835
LPCT4_HUMAN	941.97998
STEA3_HUMAN	2502.02124
F91A1_HUMAN	4349.06006
TBC9B_HUMAN	6550.96387
MAP1S_HUMAN	10346.9424
2ABD_HUMAN	3584.38379
AR6P4_HUMAN	4121.52686
A16L1_HUMAN	3039.23096
HGNAT_HUMAN	2174.64771
ARID2_HUMAN	327.949402
UTP25_HUMAN	2300.01758
GL8D1_HUMAN	1382.85779
SE1L3_HUMAN	1038.57141
TENS3_HUMAN	4824.59814
HAUS3_HUMAN	907.262451
SPT2_HUMAN	1154.09546
MBLC2_HUMAN	2232.85303
CRBG3_HUMAN	860.475891
INT3_HUMAN	3948.54932
RHG17_HUMAN	17159.0156
NCAS2_HUMAN	2878.35547
C19L1_HUMAN	8748.27051

VIR_HUMAN	3135.94946
CYTSA_HUMAN	4301.67725
HEAT6_HUMAN	1375.95386
ANR40_HUMAN	401.791565
WWC2_HUMAN	1647.65271
ATLA3_HUMAN	19532.2188
RL7L_HUMAN	21192.5117
NXN_HUMAN	1395.4425
TTC19_HUMAN	421.449493
IQEC1_HUMAN	1315.79858
CPIN1_HUMAN	11019.7109
ZFAN6_HUMAN	1632.08142
SMYD5_HUMAN	2652.78345
OTU7B_HUMAN	522.864868
THOC7_HUMAN	1499.82288
NADE_HUMAN	2861.81665
ELP2_HUMAN	2473.08228
LTOR1_HUMAN	2812.43677
DRS7B_HUMAN	2088.11255
TWF2_HUMAN	16442.4941
CNDH2_HUMAN	365.943298
K0930_HUMAN	897.11499
TNG2_HUMAN	5719.47607
P4R3A_HUMAN	3219.84375
RAB12_HUMAN	24142.3887
DEN5A_HUMAN	198.936142
SDE2_HUMAN	275.953094
K2C80_HUMAN	887.406189
NIPBL_HUMAN	600.262329
SCMC2_HUMAN	3786.59668
PDE12_HUMAN	7413.99219
NAA16_HUMAN	1412.26465
DE10B_HUMAN	3161.49146
RHBD2_HUMAN	248.677444
SCMC1_HUMAN	30410.8398
REEP3_HUMAN	938.480286
RETST_HUMAN	2592.74707
RINT1_HUMAN	1362.67761
TM214_HUMAN	10684.1113
HIBCH_HUMAN	6105.42188
RWDD4_HUMAN	2921.58521
NEPRO_HUMAN	1349.0686
ARMC6_HUMAN	5262.14697
TTI2_HUMAN	1733.13208
ANR54_HUMAN	1825.68616
ZNT6_HUMAN	1189.40942
TAPT1_HUMAN	36164.3281
KANK3_HUMAN	1403.23279
JMJD6_HUMAN	5327.40479
PPR18_HUMAN	8859.12793

CAVN1_HUMAN	89154.6328
ZCHC8_HUMAN	3199.11279
DHX57_HUMAN	3330.48364
ERAP2_HUMAN	11643.2041
MBOA5_HUMAN	2738.39673
CDC73_HUMAN	6294.00684
RM14_HUMAN	3811.98218
S27A4_HUMAN	6494.79541
C2D1A_HUMAN	4225.49609
TATD1_HUMAN	3001.26709
MET2B_HUMAN	1179.77661
MSD2_HUMAN	584.960632
TAF2_HUMAN	1386.32654
CH082_HUMAN	1714.20959
MED27_HUMAN	1450.68445
EDC4_HUMAN	18092.3574
CEP85_HUMAN	491.899658
ANM9_HUMAN	190.141861
PRP8_HUMAN	14706.8584
FBX42_HUMAN	247.903168
SCYL2_HUMAN	3892.02979
TTC27_HUMAN	4483.05469
SFXN4_HUMAN	783.663818
GOLM2_HUMAN	3213.37744
NFRKB_HUMAN	548.001404
FAHD1_HUMAN	12089.5586
RL22L_HUMAN	9161.17188
PKN3_HUMAN	1039.50928
ALKB5_HUMAN	511.383484
PDXD1_HUMAN	7033.22852
MEAK7_HUMAN	3744.31104
INT5_HUMAN	2589.35815
GIMA6_HUMAN	875.468384
S27A1_HUMAN	556.805542
PGM2L_HUMAN	8673.84668
CTR9_HUMAN	5145.46387
AAGAB_HUMAN	1950.3031
TMM88_HUMAN	313.059998
SKI3_HUMAN	5278.57861
ULK3_HUMAN	1111.74548
SYDM_HUMAN	6889.51563
TMM65_HUMAN	4250.13477
IN80C_HUMAN	1568.66211
FBX38_HUMAN	1838.50659
NCEH1_HUMAN	11763.9766
FIGL1_HUMAN	671.287415
TRI65_HUMAN	1592.54272
RHDF2_HUMAN	862.718811
MDEAS_HUMAN	2063.49097
BRAT1_HUMAN	3297.38208

WDR59_HUMAN	438.262848
ZC3HE_HUMAN	2754.03735
CC137_HUMAN	1618.55237
OGFD3_HUMAN	444.838104
TXD11_HUMAN	355.549835
LARP1_HUMAN	16533.5703
ATAD2_HUMAN	360.535217
TMED8_HUMAN	2428.646
ZNT9_HUMAN	563.072876
TRAF7_HUMAN	885.972107
RICTR_HUMAN	3532.88965
WDR74_HUMAN	1970.99683
ATRAP_HUMAN	2009.48633
SMRD3_HUMAN	1553.7019
C1TM_HUMAN	25045.3652
AFTIN_HUMAN	531.183167
FIP1_HUMAN	6220.73779
CRTC3_HUMAN	418.774506
CRTC1_HUMAN	357.160736
CP20A_HUMAN	2224.58545
PLGT2_HUMAN	3392.21167
TM205_HUMAN	7513.39258
LRSM1_HUMAN	2235.39624
GAK1B_HUMAN	2096.60767
LCLT1_HUMAN	2531.31348
CWC27_HUMAN	1526.28784
CREL2_HUMAN	1494.56604
WDR82_HUMAN	4041.44312
MIC27_HUMAN	2148.44922
FAT4_HUMAN	772.527466
DPP8_HUMAN	599.298523
MCAF1_HUMAN	2000.80591
RBP10_HUMAN	1644.05774
PACS1_HUMAN	6005.05762
MPRIP_HUMAN	7183.59424
RBMS3_HUMAN	3481.16602
PNCB_HUMAN	4220.51855
DNMBP_HUMAN	5455.69824
HACD2_HUMAN	968.676147
B3GLT_HUMAN	1777.62207
GGYF2_HUMAN	8910.21191
CD109_HUMAN	8782.23633
THADA_HUMAN	1773.15649
HSDL2_HUMAN	4492.27881
KAT3_HUMAN	6623.93066
PPR21_HUMAN	1202.74109
THSD4_HUMAN	970.974487
SYNE3_HUMAN	1983.90295
ZN574_HUMAN	383.927307
NFXL1_HUMAN	369.743774

NBEL2_HUMAN	3029.43628
FGD5_HUMAN	3499.60059
QSOX2_HUMAN	2522.82446
SRCAP_HUMAN	187.8564
RIPR1_HUMAN	4771.83691
YJ005_HUMAN	7011.71582
ARHGI_HUMAN	1158.46277
TBCD9_HUMAN	239.10141
UBR3_HUMAN	595.883362
TMPPE_HUMAN	1398.38806
CRACD_HUMAN	355.594727
CCD9B_HUMAN	391.104462
NUD18_HUMAN	477.412659
PAXI1_HUMAN	938.972351
STXB4_HUMAN	183.681641
TMTC3_HUMAN	5439.08594
VP13C_HUMAN	9925.0625
UBP34_HUMAN	865.847778
RAPH1_HUMAN	2320.5376
MOB2_HUMAN	5068.86084
IKIP_HUMAN	25465.9922
PREX2_HUMAN	708.812988
F10C1_HUMAN	2485.18994
UB2R2_HUMAN	6390.41846
LARP4_HUMAN	9366.47266
MED25_HUMAN	1268.57983
RS27L_HUMAN	62651.8984
HAKAI_HUMAN	1384.14099
INT8_HUMAN	3533.0603
BT2A1_HUMAN	706.885132
SPT6H_HUMAN	3880.68921
SND1_HUMAN	85328.6875
MARK2_HUMAN	1734.89734
COX15_HUMAN	1552.28674
DDX46_HUMAN	12823.8652
RUFY3_HUMAN	613.470276
TM10C_HUMAN	9661.7793
5MP2_HUMAN	28864.4707
MON1B_HUMAN	942.57312
LRC8D_HUMAN	415.199341
ASGL1_HUMAN	1131.22339
KCTD9_HUMAN	3541.99487
DHX30_HUMAN	10370.7217
EIF3M_HUMAN	20830.6602
MEPCE_HUMAN	4211.96436
ARMX2_HUMAN	1555.099
SYPM_HUMAN	513.387451
RSRC2_HUMAN	1904.53528
RRAGA_HUMAN	5126.896
CYFP1_HUMAN	24843.0703

NDUF7_HUMAN	2432.18579
GET4_HUMAN	9738.62695
CSN6_HUMAN	20725.5527
PCAT2_HUMAN	14522.2246
ENOF1_HUMAN	1967.78418
MAEA_HUMAN	1940.77917
EPMIP_HUMAN	1380.30847
DHX32_HUMAN	428.881348
TAOK1_HUMAN	3414.04785
FAKD5_HUMAN	2682.83325
MOB1B_HUMAN	11785.5283
KDM3B_HUMAN	3489.62183
CHM1B_HUMAN	3941.99146
RIR2B_HUMAN	3224.25195
HS2ST_HUMAN	6178.0874
STRAA_HUMAN	1418.43372
MICA3_HUMAN	1372.86047
DYM_HUMAN	1643.6499
K2C78_HUMAN	330.838348
PHF5A_HUMAN	3782.34155
PXL2C_HUMAN	2777.79321
APTX_HUMAN	2767.53882
ERMP1_HUMAN	3424.14648
GRIN1_HUMAN	207.36232
TRM1L_HUMAN	2035.53528
ZCCHV_HUMAN	9531.34961
RM21_HUMAN	4289.479
EFL1_HUMAN	4411.32129
SETX_HUMAN	517.54126
TPC11_HUMAN	2376.23804
KANL1_HUMAN	244.333054
NUP54_HUMAN	8154.46582
ATG9A_HUMAN	1201.96277
GLUCM_HUMAN	3551.44873
CC186_HUMAN	758.050903
ARMC9_HUMAN	1749.8407
VP35L_HUMAN	6424.62695
POGZ_HUMAN	2965.40454
ZFY16_HUMAN	926.156982
MON2_HUMAN	3604.82739
MYCPP_HUMAN	857.884216
MYH14_HUMAN	7024.68457
NUFP2_HUMAN	5709.44678
SZRD1_HUMAN	1456.39111
MAVS_HUMAN	1668.39368
CLAP1_HUMAN	5864.56201
DHX29_HUMAN	5686.24219
LRP10_HUMAN	1739.37134
HAUS6_HUMAN	1799.89014
PLGT3_HUMAN	3392.79565

TT21B_HUMAN	1580.7417
HEAT3_HUMAN	8083.90039
KI21A_HUMAN	431.74884
HDGR2_HUMAN	8211.20898
DCXR_HUMAN	7041.81641
BRAP_HUMAN	2200.32056
EMSY_HUMAN	653.077759
GOGA7_HUMAN	2458.12402
RHG22_HUMAN	818.504944
WAPL_HUMAN	1298.14026
I2BP2_HUMAN	1287.39905
RBBP6_HUMAN	2302.04736
SH3R1_HUMAN	781.001404
GRASP_HUMAN	998.889526
FRMD5_HUMAN	786.895813
SEN54_HUMAN	385.773346
PTAR1_HUMAN	540.04187
ARPIN_HUMAN	3132.22168
TCPR1_HUMAN	1162.60168
RABEK_HUMAN	1296.4082
MTEF4_HUMAN	534.339966
HUWE1_HUMAN	12875.585
YTHD3_HUMAN	11514.3604
CTU1_HUMAN	3629.09619
TAF8_HUMAN	364.373108
UB2Q1_HUMAN	4518.18945
RM55_HUMAN	11856.1895
TMED4_HUMAN	3879.823
RM10_HUMAN	527.303528
COXM1_HUMAN	5219.02197
CENPV_HUMAN	2265.78784
SLN11_HUMAN	4200.28809
MEGF8_HUMAN	414.557678
T179B_HUMAN	1139.65356
GALT7_HUMAN	7331.77734
SCD5_HUMAN	2509.52539
PHLB2_HUMAN	2783.33765
AGRG6_HUMAN	2293.729
DHDDS_HUMAN	366.244293
GLT10_HUMAN	566.52533
TPC6B_HUMAN	845.916199
PP4P1_HUMAN	2733.30737
CLC14_HUMAN	12891.2031
KAISO_HUMAN	774.624084
PATL1_HUMAN	1319.40515
DPP9_HUMAN	6510.89697
SYVN1_HUMAN	1345.33728
TRPT1_HUMAN	711.937805
SETD3_HUMAN	6036.40381
TTC7B_HUMAN	1027.82141

ACOT1_HUMAN	3383.24438
NOP9_HUMAN	3389.0459
PABP2_HUMAN	19540.834
MTA70_HUMAN	2239.146
PB1_HUMAN	4103.34375
YRDC_HUMAN	2369.47363
PRP39_HUMAN	1917.15393
LYRIC_HUMAN	15077.9326
ZN598_HUMAN	4938.39746
GPAT4_HUMAN	1701.88562
KTN1_HUMAN	7360.17725
EST1A_HUMAN	1450.64136
NLRX1_HUMAN	870.344727
BCL9L_HUMAN	421.667328
PHLB1_HUMAN	9645.17969
UBP48_HUMAN	5685.20508
CAPS2_HUMAN	586.717712
URP2_HUMAN	29198.1348
NAA40_HUMAN	3942.96436
NT5D3_HUMAN	572.00061
AACS_HUMAN	8282.77734
LUZP1_HUMAN	7188.52734
THOC4_HUMAN	27744.0176
GP180_HUMAN	396.82016
FHI2B_HUMAN	2873.09326
IQGA3_HUMAN	3486.53809
ZCH18_HUMAN	2156.0769
VPS36_HUMAN	1769.25562
CAND1_HUMAN	34411.0625
RETR3_HUMAN	2912.86353
HOOK3_HUMAN	6034.62842
SESD1_HUMAN	527.658875
COMD7_HUMAN	1489.80225
MON1A_HUMAN	774.184753
AMZ2_HUMAN	954.33728
THOC6_HUMAN	2169.94263
MET16_HUMAN	1018.91504
PARG_HUMAN	572.171753
LIPB1_HUMAN	8863.29004
LONP2_HUMAN	1137.93567
ZC3C1_HUMAN	3576.18213
CHD1L_HUMAN	8272.35645
FCSD1_HUMAN	304.32608
CCD25_HUMAN	6530.05859
PRSR2_HUMAN	1071.64795
STING_HUMAN	6797.42236
AROS_HUMAN	1447.56262
CDR2L_HUMAN	155.341171
RLGPB_HUMAN	1087.8772
RGPS2_HUMAN	502.672791

CARM1_HUMAN	9058.4248
NIT1_HUMAN	4815.79395
COMD2_HUMAN	3895.50146
HTR5A_HUMAN	1252.67163
CNDG2_HUMAN	972.797241
PPR3B_HUMAN	152.936798
FBX11_HUMAN	285.155762
ANKL2_HUMAN	1916.8407
MEX3D_HUMAN	1827.49854
DGKH_HUMAN	188.94902
DDX42_HUMAN	15902.3555
SPAS2_HUMAN	3418.9978
VRK2_HUMAN	2883.27148
CACL1_HUMAN	243.845566
NDUAB_HUMAN	7346.66113
DAAF5_HUMAN	7875.75879
PTH_HUMAN	1100.21558
STX12_HUMAN	13433.0322
P66A_HUMAN	3903.94263
CPNE8_HUMAN	24064.5879
GPSM1_HUMAN	760.246521
RAB43_HUMAN	1650.74072
C2CD5_HUMAN	1408.02881
MIB1_HUMAN	584.303955
PRAG1_HUMAN	1273.3634
HPS6_HUMAN	1723.92139
I2BP1_HUMAN	1157.81128
RB6I2_HUMAN	3667.89526
RIOX2_HUMAN	2373.51343
ZDH13_HUMAN	8554.33008
ZDH17_HUMAN	630.311218
CRLF3_HUMAN	1373.9292
TPPC5_HUMAN	5707.33008
ARMC8_HUMAN	2581.55884
RELL1_HUMAN	1261.34375
T126B_HUMAN	1071.87341
PLD3_HUMAN	19183.459
HID1_HUMAN	482.195801
ANKY2_HUMAN	1209.46399
ERI1_HUMAN	1261.16724
VRK3_HUMAN	438.78479
LIX1L_HUMAN	1961.79736
NUDC3_HUMAN	4482.07324
AHNK2_HUMAN	26901.0508
FMNL3_HUMAN	5781.1333
MMAA_HUMAN	1515.8291
M4K3_HUMAN	459.73996
NAV3_HUMAN	2831.38525
P3H3_HUMAN	11525.9629
CCD50_HUMAN	9182.24414

FABD_HUMAN	2370.90356
KSR1_HUMAN	1334.14978
CEP97_HUMAN	1592.77454
NNRD_HUMAN	1608.99988
WDR75_HUMAN	2569.69043
MFN1_HUMAN	2518.53003
CTL2_HUMAN	3261.80786
IPRI_HUMAN	2252.61084
WDFY1_HUMAN	4988.85156
MA7D3_HUMAN	2710.33447
CC117_HUMAN	364.142395
NXP20_HUMAN	6785.10352
DCNL3_HUMAN	523.460449
DEN6A_HUMAN	773.126892
MGAP_HUMAN	507.55423
GCC2_HUMAN	2856.98413
Z3H7A_HUMAN	1557.44763
PHF6_HUMAN	8109.98633
ARCH_HUMAN	958.295105
LRC8A_HUMAN	5960.99316
SULF2_HUMAN	4662.76221
UBR1_HUMAN	1801.59863
UBR2_HUMAN	1551.57593
RHG12_HUMAN	1299.09082
CHERP_HUMAN	9551.70117
CDAN1_HUMAN	583.603577
ANKH1_HUMAN	2722.2417
SUGP1_HUMAN	1962.47839
SUGP2_HUMAN	21323.9277
UEVLD_HUMAN	2285.56445
CCAR1_HUMAN	9252.39063
DHX40_HUMAN	387.875702
DJC10_HUMAN	10200.1992
NELFD_HUMAN	4578.17334
MIRO2_HUMAN	4260.24316
MIRO1_HUMAN	4342.36084
SIR2_HUMAN	3024.75586
PHC2_HUMAN	851.472717
BAP18_HUMAN	4658.68799
RM41_HUMAN	9426.41504
NRM_HUMAN	5557.5415
GPAM1_HUMAN	1006.04456
PARP9_HUMAN	460.174469
RB12B_HUMAN	3488.21729
S35F2_HUMAN	1738.42273
RPAP2_HUMAN	279.936066
PLPL6_HUMAN	6283.57861
SMC5_HUMAN	1220.26514
DDX60_HUMAN	487.596222
CMIP_HUMAN	486.860352

MILK2_HUMAN	623.578308
DHX37_HUMAN	2551.34351
KBTB2_HUMAN	2814.40552
RAVR1_HUMAN	4585.29004
SPB1_HUMAN	9486.4043
TM192_HUMAN	1359.00085
M21D2_HUMAN	1549.6405
SRRM1_HUMAN	4685.91064
SMAP1_HUMAN	2889.70435
DI3L2_HUMAN	3579.34253
SUV3_HUMAN	4871.19092
ERF3B_HUMAN	7347.10596
ZZZ3_HUMAN	481.670929
EXOC8_HUMAN	7757.37061
CJ067_HUMAN	12674.0557
TRI22_HUMAN	4813.87305
THNS1_HUMAN	636.332947
K2013_HUMAN	3935.48584
MICU2_HUMAN	1147.37756
AN13A_HUMAN	2361.32593
PHAR4_HUMAN	1221.13049
TRM2A_HUMAN	2250.01855
RUSD2_HUMAN	753.928528
ELMD2_HUMAN	1889.37439
A16A1_HUMAN	5779.02832
K319L_HUMAN	1374.68445
AGRF5_HUMAN	1757.11584
XRN1_HUMAN	4920.61963
PELP1_HUMAN	14186.6318
ABI1_HUMAN	6087.87451
WDFY3_HUMAN	4277.62158
SELH_HUMAN	5310.33008
VKORL_HUMAN	2037.18713
SPART_HUMAN	9239.50293
TTC5_HUMAN	1376.72852
MIER1_HUMAN	1397.52637
RPTOR_HUMAN	2093.08789
ORML3_HUMAN	4129.39307
CCAR2_HUMAN	51509.1875
NDUF2_HUMAN	7864.56201
VPS52_HUMAN	5924.72021
NUP93_HUMAN	24387.6328
S11IP_HUMAN	836.927856
ZN687_HUMAN	601.638306
CMTR1_HUMAN	3626.08081
LRC47_HUMAN	13710.9893
DOCK4_HUMAN	7243.03613
CAH13_HUMAN	1804.54688
ARG28_HUMAN	8334.70996
INT1_HUMAN	31653.9727

H2B3B_HUMAN	4263.40234
RHG24_HUMAN	3650.81592
ARM10_HUMAN	3456.73804
ABD12_HUMAN	2680.21387
CLASR_HUMAN	559.515381
FA43A_HUMAN	2634.72974
TM256_HUMAN	1437.05688
PIAS4_HUMAN	399.698914
GPD1L_HUMAN	3699.49121
S35F6_HUMAN	2691.97144
RHG18_HUMAN	18683.3984
ASCC3_HUMAN	9778.19629
MILK1_HUMAN	728.971619
VPS8_HUMAN	2611.5603
PALS1_HUMAN	880.764587
STAG2_HUMAN	3707.24146
SYNPO_HUMAN	10381.9238
FNBP4_HUMAN	1481.55481
GUF1_HUMAN	427.289124
RYBP_HUMAN	515.225159
GALT4_HUMAN	3343.35376
MINK1_HUMAN	629.192627
MESH1_HUMAN	918.171265
PTGR3_HUMAN	2283.39795
CBR4_HUMAN	713.845337
EMC5_HUMAN	2799.37134
TM199_HUMAN	3293.80884
FBXL6_HUMAN	518.575256
OGFD1_HUMAN	2409.73828
AFAP1_HUMAN	5488.05176
ZCHC9_HUMAN	812.324097
OXR1_HUMAN	928.827026
ZGPAT_HUMAN	1392.47717
CAHM5_HUMAN	1550.28271
SRBD1_HUMAN	668.100464
WDTC1_HUMAN	2924.73901
NKAP_HUMAN	983.516418
MACOI_HUMAN	629.232239
SH2D3_HUMAN	3908.99829
ARRD1_HUMAN	891.199341
DHRSX_HUMAN	1355.36914
CISD2_HUMAN	8259.82324
RP25L_HUMAN	2279.22119
ATPF2_HUMAN	1280.02417
TTC9C_HUMAN	2212.07178
JAGN1_HUMAN	3701.14038
RNF10_HUMAN	858.353455
RFLB_HUMAN	1524.47241
LZTR1_HUMAN	534.206482
COMD1_HUMAN	2080.39429

CPSF7_HUMAN	13870.0664
S15A4_HUMAN	812.005798
MYCT1_HUMAN	11845.5303
ATL1_HUMAN	304.615906
ARFG2_HUMAN	5387.57471
OTU6B_HUMAN	3062.20605
FITM2_HUMAN	659.089417
CA052_HUMAN	1380.43188
EFNMT_HUMAN	2795.46509
AR6P6_HUMAN	1441.08569
ARFG1_HUMAN	2298.63501
SIR6_HUMAN	918.278137
S66A3_HUMAN	876.512024
EMC1_HUMAN	13454.5205
PAF1_HUMAN	3078.05054
CCYL1_HUMAN	1078.51001
UBR7_HUMAN	3240.9873
DDX51_HUMAN	2155.00049
F241A_HUMAN	5580.0293
S2529_HUMAN	2390.30615
ENAH_HUMAN	15143.5996
DCBD1_HUMAN	1646.53918
GPT11_HUMAN	996.521912
RM43_HUMAN	7026.92627
RLIG1_HUMAN	3290.55273
GDPD1_HUMAN	355.097717
CS047_HUMAN	800.451172
ASCC1_HUMAN	2990.43359
LRC57_HUMAN	6280.95264
EIF1A_HUMAN	885.375732
KRI1_HUMAN	1796.71033
CC71L_HUMAN	1461.85303
ZN579_HUMAN	2168.36816
PR38A_HUMAN	3412.42798
MLKL_HUMAN	5187.7666
GALD1_HUMAN	2428.34399
ANR52_HUMAN	1424.96875
AT11C_HUMAN	1309.73999
AFG2A_HUMAN	4678.40332
NHLC2_HUMAN	7411.6665
AVL9_HUMAN	1859.15527
S43A3_HUMAN	5966.51611
XXLT1_HUMAN	7732.89941
GOLM1_HUMAN	440.710938
GT251_HUMAN	28254.2363
SUMF2_HUMAN	2800.45996
SIDT2_HUMAN	696.001526
SUMF1_HUMAN	4420.56348
PGLT1_HUMAN	2755.42065
UBAC2_HUMAN	1657.30566

PCYXL_HUMAN	1305.45105
TM87A_HUMAN	1944.47485
RDH13_HUMAN	2346.96484
TTC13_HUMAN	1126.87854
DHB11_HUMAN	7227.96924
TXND5_HUMAN	227415.219
SPC24_HUMAN	1462.58179
ATAD1_HUMAN	2662.34473
SCPDL_HUMAN	16875.8477
UXS1_HUMAN	684.619751
RN149_HUMAN	2601.45776
RETR2_HUMAN	617.322205
SERB1_HUMAN	14624.2656
LEMD2_HUMAN	5591.104
NOA1_HUMAN	1729.25342
NECP1_HUMAN	2439.85913
FA98A_HUMAN	5081.38965
PAG15_HUMAN	1091.10779
MTMRE_HUMAN	987.104492
NF2IP_HUMAN	3131.16479
DGLB_HUMAN	917.761597
CHSTE_HUMAN	3956.51196
GALT6_HUMAN	2610.34839
RN169_HUMAN	358.38678
PDPR_HUMAN	3772.83862
NNRE_HUMAN	2187.88208
SMG8_HUMAN	1242.52869
RN214_HUMAN	2327.80981
LS14A_HUMAN	3230.85449
GIMA8_HUMAN	6640.96045
CCNY_HUMAN	3363.35718
MROH1_HUMAN	3988.04297
PAPD5_HUMAN	1601.5957
PEPL1_HUMAN	2761.7561
EHBP1_HUMAN	1414.75659
RB15B_HUMAN	2138.20776
SIA7C_HUMAN	1162.82935
TNR6A_HUMAN	1507.75269
PHC3_HUMAN	447.376831
DIK2A_HUMAN	4103.29248
CNNM3_HUMAN	563.500366
ABCF1_HUMAN	13624.6338
MCU_HUMAN	1656.1853
PK3C3_HUMAN	5931.01563
GSTCD_HUMAN	227.951233
SRFB1_HUMAN	1130.9978
NGDN_HUMAN	1928.24072
SHCBP_HUMAN	2280.72803
PDZD8_HUMAN	780.319885
DP13B_HUMAN	2531.73438

ZNT7_HUMAN	8616.49219
NAV1_HUMAN	1798.32483
PPHLN_HUMAN	620.531006
WDR19_HUMAN	616.854248
FBX22_HUMAN	4503.19629
PCAT1_HUMAN	4777.72656
ZMIZ2_HUMAN	1478.72083
SYNE1_HUMAN	26342.4785
UBP32_HUMAN	533.243042
BD1L1_HUMAN	1231.66882
ARI1B_HUMAN	1156.49646
FAD1_HUMAN	4611.9458
FLCN_HUMAN	1959.9071
NUP43_HUMAN	5160.02637
NUP37_HUMAN	8330.05273
NUP35_HUMAN	3757.29785
ENASE_HUMAN	461.043213
RAI3_HUMAN	1355.37549
TOIP2_HUMAN	6367.9585
ABHDB_HUMAN	2505.91479
NEUA_HUMAN	6005.6377
TSN14_HUMAN	4425.7749
TTL_HUMAN	320.340698
SVIP_HUMAN	1295.00391
ATLA2_HUMAN	1740.01807
MSPD2_HUMAN	876.243042
PLBL2_HUMAN	10891.3301
RASF8_HUMAN	622.176086
DDX55_HUMAN	561.344299
MFSD8_HUMAN	338.873596
TDRD7_HUMAN	789.419312
GIMA7_HUMAN	2344.81738
NEDD1_HUMAN	1537.19177
NCOA7_HUMAN	1899.78674
THOC2_HUMAN	5019.40479
WDR36_HUMAN	7580.43408
PPTC7_HUMAN	605.392883
RP9_HUMAN	710.288208
VANG1_HUMAN	854.38739
SNIP1_HUMAN	402.769073
G45IP_HUMAN	4363.79346
WDR48_HUMAN	2423.66626
EXOC6_HUMAN	6011.24463
SMRC2_HUMAN	5288.77686
NPL4_HUMAN	8070.13623
CX038_HUMAN	2034.24805
SPT20_HUMAN	1491.96521
NUBPL_HUMAN	1266.46289
ABHD4_HUMAN	892.795532
FBX30_HUMAN	1366.41833

S35B2_HUMAN	8355.84082
PUM2_HUMAN	1811.7533
TIP_HUMAN	781.332214
GOGA5_HUMAN	1626.6156
KLDC4_HUMAN	1057.82971
SHKB1_HUMAN	2341.58667
UBA3_HUMAN	27047.0391
PXL2B_HUMAN	651.984985
DJB14_HUMAN	751.33075
KISHA_HUMAN	1555.15576
PI42C_HUMAN	768.562256
WDR20_HUMAN	512.395508
TBC15_HUMAN	7444.91016
RDH11_HUMAN	15085.1621
LRC20_HUMAN	437.046204
IFI44_HUMAN	212.824982
RM30_HUMAN	1178.18347
ZFAN1_HUMAN	1696.53625
CIP2A_HUMAN	3798.28296
P4K2B_HUMAN	523.329712
STT3B_HUMAN	35852.8477
PNPT1_HUMAN	10367.7715
SPP2B_HUMAN	1008.43524
HM13_HUMAN	43279.2617
PREX1_HUMAN	3146.78516
URGCP_HUMAN	1909.58337
BICD2_HUMAN	4152.42676
NEK9_HUMAN	4834.93018
PKHO2_HUMAN	3451.68823
DTX3L_HUMAN	855.603333
DDX54_HUMAN	5102.40918
DLG5_HUMAN	663.065979
BRX1_HUMAN	9708.58789
GNPI2_HUMAN	2717.0769
LRC8C_HUMAN	14392.1396
NEK7_HUMAN	3338.01709
RBCC1_HUMAN	215.789627
MICA1_HUMAN	4315.83057
ELP5_HUMAN	1516.40771
SSH3_HUMAN	901.137024
S3TC1_HUMAN	340.601379
TBCK_HUMAN	627.889587
DTD1_HUMAN	4126.87354
DCA11_HUMAN	950.608154
UTP15_HUMAN	3457.75073
GPX8_HUMAN	20471.332
AF1L1_HUMAN	583.685059
SH3R3_HUMAN	4117.11621
TP4AP_HUMAN	900.189087
CE192_HUMAN	462.110657

GEMI5_HUMAN	6129.17578
PIGO_HUMAN	227.210388
RPGF6_HUMAN	371.573181
SMCR8_HUMAN	505.72998
PARD3_HUMAN	1304.62622
IPO4_HUMAN	13129.2744
UBP33_HUMAN	409.374939
PNISR_HUMAN	1290.12183
PP4R1_HUMAN	7105.45801
UBS3B_HUMAN	1778.74023
WIPF2_HUMAN	1292.44678
ABHD5_HUMAN	572.018005
SETD7_HUMAN	5687.31934
NOC3L_HUMAN	4480.92383
SCRB1_HUMAN	20207.8398
COG1_HUMAN	2411.1416
SCFD2_HUMAN	2655.37476
SMAP2_HUMAN	2046.70862
ZC3HF_HUMAN	15429.9834
PPIL4_HUMAN	4346.63477
TF3C2_HUMAN	2963.3623
TB22A_HUMAN	3285.64453
PHF10_HUMAN	1767.55176
RAB2B_HUMAN	3980.81104
CCD12_HUMAN	1723.62061
IASPP_HUMAN	2087.71948
ARB2A_HUMAN	2336.64453
CHUR_HUMAN	2718.5835
TGFA1_HUMAN	916.381775
TM263_HUMAN	4284.84229
HDAC7_HUMAN	5335.08203
STYX_HUMAN	119.134132
PTPM1_HUMAN	612.63916
NU133_HUMAN	8059.15381
PDC6I_HUMAN	54358.5078
S20A1_HUMAN	452.641296
FBLI1_HUMAN	19655.0273
CATIN_HUMAN	1148.07397
CHAC2_HUMAN	2018.46521
CHMP7_HUMAN	4116.25781
THEM6_HUMAN	24329.6563
NAT14_HUMAN	3491.91016
BCL7C_HUMAN	2366.42383
NSE1_HUMAN	894.504578
MITD1_HUMAN	502.605255
CTF18_HUMAN	557.40155
LEO1_HUMAN	2989.52612
DCAKD_HUMAN	1994.08704
NUDC2_HUMAN	4381.51758
SNR27_HUMAN	1114.38965

TFB1M_HUMAN	7430.60107
STAG1_HUMAN	1638.32434
SCFD1_HUMAN	9853.22949
TP8L1_HUMAN	3245.77026
CANT1_HUMAN	3129.39648
TPC12_HUMAN	1906.46704
HNRLL_HUMAN	3701.8728
FACR1_HUMAN	12669.1484
UBCP1_HUMAN	6454.94482
SEN15_HUMAN	1003.89636
PCNP_HUMAN	8113.74121
DNJA4_HUMAN	1156.93103
SPRY4_HUMAN	5327.44434
MAIP1_HUMAN	3061.15991
TRUB1_HUMAN	1923.0553
LMO7_HUMAN	1871.45435
CTL1_HUMAN	1854.6759
CKAP2_HUMAN	1049.74536
ATX2L_HUMAN	14922.3545
ARAP3_HUMAN	9053.28613
GIMA1_HUMAN	4685.61768
PHIP_HUMAN	156.911575
PRP31_HUMAN	9781.28223
NELFB_HUMAN	3190.53613
PALLD_HUMAN	15511.2832
RUFY2_HUMAN	1389.26306
SREK1_HUMAN	3074.01758
GEMI6_HUMAN	1291.69653
CSKI2_HUMAN	1727.13794
PSPC1_HUMAN	16849.0664
MADD_HUMAN	282.669189
SYNE2_HUMAN	6790.30615
P66B_HUMAN	3177.12891
PIBF1_HUMAN	644.624268
DNJC9_HUMAN	16481.6855
BRI3B_HUMAN	1831.8562
CTBL1_HUMAN	12749.9561
ATG4A_HUMAN	3287.74585
RIN2_HUMAN	1081.72375
ELYS_HUMAN	3395.80981
DGCR8_HUMAN	526.746277
TITIN_HUMAN	583.805481
ROBO4_HUMAN	3530.7251
OVCA2_HUMAN	2714.28564
IRGQ_HUMAN	1829.89722
DDB2_HUMAN	8439.56641
CLC2B_HUMAN	1785.27136
DDX1_HUMAN	24601.0645
S14L1_HUMAN	5905.71924
PIEZ1_HUMAN	1611.76465

FAM3C_HUMAN	5246.03369
H1X_HUMAN	9745.25684
PSMF1_HUMAN	7238.62988
GBF1_HUMAN	9143.15039
SMG7_HUMAN	80.7962799
RTF1_HUMAN	5824.04736
NICA_HUMAN	11975.4189
SNX19_HUMAN	559.359619
TM9S4_HUMAN	10168.3203
TM131_HUMAN	834.539612
IP6K1_HUMAN	753.840576
RT27_HUMAN	16514.5918
ELMO1_HUMAN	1707.87964
F168A_HUMAN	330.418793
AP3S1_HUMAN	14074.9697
TSC1_HUMAN	3237.39746
UBXN4_HUMAN	3031.15601
PHF3_HUMAN	699.130432
NDRG1_HUMAN	3823.25537
HS105_HUMAN	28311.9609
SEPT8_HUMAN	7229.8418
CNOT9_HUMAN	5089.25732
LGAT1_HUMAN	665.964172
TBCD5_HUMAN	3245.84644
ZN592_HUMAN	957.266052
JADE3_HUMAN	722.311462
MY18A_HUMAN	959.520203
LAR4B_HUMAN	4002.18164
GCN1_HUMAN	22501.8809
HMHA1_HUMAN	141.28418
PRP16_HUMAN	2496.66382
NU205_HUMAN	69981.9375
ANS1A_HUMAN	1261.16284
PXDN_HUMAN	11126.0596
FAN_HUMAN	840.49762
GPI8_HUMAN	1744.03931
RT31_HUMAN	9314.80078
AKAP1_HUMAN	182.560791
ALG3_HUMAN	1167.30603
NEUG_HUMAN	479.418854
AN32B_HUMAN	62508.1016
NECT2_HUMAN	10855.9287
PGTA_HUMAN	11408.5508
TFG_HUMAN	11141.6709
HTRA1_HUMAN	21852.4063
ARC1A_HUMAN	9409.26172
TF2H4_HUMAN	1152.44678
RREB1_HUMAN	429.758179
HDAC2_HUMAN	11956.2871
STAM1_HUMAN	1794.07825

REQU_HUMAN	2970.25732
SC65_HUMAN	9761.26758
CBP_HUMAN	434.7052
KAT6A_HUMAN	241.919922
SYMPK_HUMAN	4461.65625
RBP56_HUMAN	2500.48877
GOGA1_HUMAN	741.385986
GGH_HUMAN	5018.57178
NRCAM_HUMAN	7892.43066
RPGR_HUMAN	635.776245
SHIP1_HUMAN	4582.68018
DDX17_HUMAN	91359.4766
KIFA3_HUMAN	2391.16455
CASPA_HUMAN	1492.95764
APBB2_HUMAN	1451.47107
RAD50_HUMAN	10809.376
CELF1_HUMAN	10199.5645
OSTF1_HUMAN	4023.5498
ARHG1_HUMAN	11944.8799
XPF_HUMAN	846.712891
UFD1_HUMAN	27114.0195
GSLG1_HUMAN	18482.5762
RENT1_HUMAN	10711.8408
CSN5_HUMAN	13504.3223
GPKOW_HUMAN	6171.35107
SMRC1_HUMAN	3926.3186
SMRD2_HUMAN	3819.02026
RAB8B_HUMAN	18222.373
BAD_HUMAN	642.800476
FUBP2_HUMAN	13768.3008
GCDH_HUMAN	9142.92969
RIT1_HUMAN	938.252197
PEX13_HUMAN	600.138611
TNPO1_HUMAN	39142.7695
ARHG2_HUMAN	20212.4258
NEP1_HUMAN	12168.7529
CLP1_HUMAN	2686.96606
GLMN_HUMAN	6004.82031
UBP13_HUMAN	1907.70166
USP9X_HUMAN	9818.33691
UBP7_HUMAN	10739.9385
CUL5_HUMAN	4685.51855
STMN2_HUMAN	45167.4297
VPP1_HUMAN	3454.45361
LPP_HUMAN	9787.67383
RBPMS_HUMAN	723.229614
EXT2_HUMAN	674.930237
MED12_HUMAN	928.289246
TP4A1_HUMAN	318.498138
KPBB_HUMAN	3343.63794

SCAM4_HUMAN	3292.99854
HPS3_HUMAN	1370.36145
SMCE1_HUMAN	4958.82715
CAVN3_HUMAN	11852.5781
RIFK_HUMAN	377.177673
MYDGF_HUMAN	31260.543
TM40L_HUMAN	1310.255
YIPF5_HUMAN	2517.42114
PIGT_HUMAN	4759.63965
IGSF8_HUMAN	1082.17273
RL36L_HUMAN	6475.9668
RAB24_HUMAN	731.579529
OSBP2_HUMAN	1392.73865
ZN622_HUMAN	2431.30151
RRF2M_HUMAN	2373.57227
UB2E3_HUMAN	12151.3242
5NT3B_HUMAN	3567.62793
WBP2_HUMAN	2738.00171
PSMG2_HUMAN	15769.5459
NCLN_HUMAN	17479.7773
MUL1_HUMAN	892.905701
MRTFA_HUMAN	393.201385
LFG3_HUMAN	4674.09229
ERGI1_HUMAN	11330.2246
UTP4_HUMAN	2495.78589
FAKD4_HUMAN	4915.39209
MARC2_HUMAN	2916.58936
F162A_HUMAN	9879.81543
CCD47_HUMAN	8569.96777
RM24_HUMAN	6103.50977
EXOC4_HUMAN	9798.4834
MGN2_HUMAN	37660.5547
P33MX_HUMAN	825.088318
RFT1_HUMAN	3224.92407
ISOC2_HUMAN	858.940369
NTAN1_HUMAN	1471.53821
FERM2_HUMAN	13153.0898
FUBP1_HUMAN	45614.4844
TTC17_HUMAN	950.894348
LRC59_HUMAN	96628.5469
VTI1A_HUMAN	348.310364
ESAM_HUMAN	6179.64746
PBIP1_HUMAN	6273.75
MCUR1_HUMAN	937.831299
K1143_HUMAN	878.626526
VP33A_HUMAN	3745.91016
FKB10_HUMAN	15938.915
TTC28_HUMAN	2086.8396
EXOS8_HUMAN	2416.8894
ZN428_HUMAN	812.269836

SH3K1_HUMAN	4385.27881
SKA1_HUMAN	659.781738
RNF25_HUMAN	1522.13184
AIDA_HUMAN	8469.0127
PINX1_HUMAN	938.410034
ARL8A_HUMAN	7890.87646
OTUL_HUMAN	2761.48657
CHCH1_HUMAN	2073.36719
PPWD1_HUMAN	5136.91016
CC127_HUMAN	2082.84351
COA7_HUMAN	1102.63477
UPP_HUMAN	288.091187
TAM41_HUMAN	2525.27441
MOB3A_HUMAN	2029.19556
DOC10_HUMAN	2764.9646
ATG2B_HUMAN	1023.11298
LENG1_HUMAN	1093.47705
TBC20_HUMAN	461.051788
EFHD2_HUMAN	37529.457
GALM_HUMAN	1548.87048
SYTL4_HUMAN	2453.91235
P5CR2_HUMAN	6707.61084
CSTOS_HUMAN	2163.60913
DCPS_HUMAN	12644.082
PP14B_HUMAN	3076.79907
INT12_HUMAN	1132.41748
NSUN4_HUMAN	2313.74463
CTHR1_HUMAN	3323.74121
ACSF2_HUMAN	1289.98145
ISOC1_HUMAN	6685.13379
GCC1_HUMAN	569.527161
FWCH2_HUMAN	2922.74097
ASTRA_HUMAN	1531.33105
HAUS1_HUMAN	290.389771
FAF2_HUMAN	12342.9648
CC124_HUMAN	6820.07227
FXRD1_HUMAN	4400.39697
OPTN_HUMAN	2927.40454
AP2M1_HUMAN	45796.5234
GCP3_HUMAN	2826.94019
S7A6O_HUMAN	1542.02161
KCD12_HUMAN	40745.8555
RCN3_HUMAN	1334.01697
NMD3_HUMAN	6015.57324
REPS1_HUMAN	927.320679
TIM14_HUMAN	3367.9834
RMD1_HUMAN	6334.94775
NUD16_HUMAN	3775.95239
ESS2_HUMAN	1260.94739
CMBL_HUMAN	3066.32178

MSI2H_HUMAN	2488.27197
SNR40_HUMAN	7902.91992
RMC1_HUMAN	907.387695
RM38_HUMAN	9981.68066
RSPRY_HUMAN	395.00766
ERLEC_HUMAN	3446.44385
RRFM_HUMAN	5573.74316
MTEF3_HUMAN	439.471283
SPDLY_HUMAN	1556.98157
ELP4_HUMAN	2388.00488
SIR1_HUMAN	443.766266
YIPF6_HUMAN	1301.35046
SEH1_HUMAN	3692.3728
TCAL4_HUMAN	1494.77698
KBP_HUMAN	15491.4395
GNA1_HUMAN	12080.2871
F120B_HUMAN	2097.43994
KTI12_HUMAN	1398.92114
RT24_HUMAN	3955.97144
RM53_HUMAN	1919.52942
T3HPD_HUMAN	7879.21094
RNF31_HUMAN	1835.61536
DAZP1_HUMAN	18640.7559
SGTB_HUMAN	610.630188
SAAL1_HUMAN	3181.30322
MITOK_HUMAN	1538.63062
SGF29_HUMAN	1031.92285
RRP36_HUMAN	1169.75818
C1GLC_HUMAN	785.954895
RBM33_HUMAN	2148.32861
DTBP1_HUMAN	1788.27966
DNJA3_HUMAN	6872.45166
TMA16_HUMAN	4672.4165
MB12A_HUMAN	3569.18921
PTCD3_HUMAN	7159.08398
MMAB_HUMAN	3686.14063
CYFP2_HUMAN	396.763184
GIMA5_HUMAN	265.525879
TRI11_HUMAN	884.420288
CCD97_HUMAN	317.051422
CNRP1_HUMAN	9990.39648
EDC3_HUMAN	5554.46631
DYL2_HUMAN	7132.69531
CPNE2_HUMAN	4450.99756
S10AG_HUMAN	4110.88965
SIPA1_HUMAN	4026.01636
SCRN2_HUMAN	879.499512
THOC1_HUMAN	5133.03613
OTUB1_HUMAN	25671.1641
TRM61_HUMAN	766.458679

HMCES_HUMAN	202.624527
CHMP6_HUMAN	3751.87402
BICD1_HUMAN	409.648224
PGM2_HUMAN	27830.5977
IMP4_HUMAN	4856.72705
CERS2_HUMAN	6485.11084
DUS3L_HUMAN	920.440125
LTV1_HUMAN	3752.46509
SDSL_HUMAN	695.197998
RM48_HUMAN	2650.43384
PLPP_HUMAN	1246.94299
AURKB_HUMAN	812.958984
DCNL1_HUMAN	3008.91284
FAH2A_HUMAN	3827.19824
SMRD1_HUMAN	1752.74329
TOE1_HUMAN	2271.021
CDA7L_HUMAN	1173.78772
RUSF1_HUMAN	1437.31055
DDX27_HUMAN	8610.84668
BORC6_HUMAN	1081.83362
SYMM_HUMAN	1348.7406
A7L3B_HUMAN	1951.45508
GWL_HUMAN	219.920837
MTNB_HUMAN	2091.60571
ZC21A_HUMAN	724.258118
LIN37_HUMAN	236.168839
S41A3_HUMAN	204.334351
SNF8_HUMAN	2136.93115
ZCCHL_HUMAN	2549.52466
P121A_HUMAN	206.874985
TONSL_HUMAN	708.936768
PDLI5_HUMAN	42034.1563
CREL1_HUMAN	1671.16199
ERO1A_HUMAN	24338.5371
PRR11_HUMAN	1110.27722
FMC1_HUMAN	3020.18506
SAHH3_HUMAN	9905.30762
DOCK6_HUMAN	5932.56494
MED30_HUMAN	622.922119
PGAM5_HUMAN	14465.583
INT4_HUMAN	1957.16187
DDRGK_HUMAN	3202.06689
DHTK1_HUMAN	1094.5708
SCLY_HUMAN	2539.70068
FUBP3_HUMAN	17068.8379
SPF45_HUMAN	4790.19824
RCC1L_HUMAN	1733.98828
SYNM_HUMAN	1140.49329
SUCB2_HUMAN	11582.4219
LRCH3_HUMAN	3401.41382

GMPPA_HUMAN	7417.06885
HPDL_HUMAN	1471.95361
ABHEB_HUMAN	1516.02307
NGLY1_HUMAN	1656.12451
SC22A_HUMAN	4489.27246
ATPMK_HUMAN	12648.8975
NSL1_HUMAN	1317.1499
PAWR_HUMAN	4566.35352
MET2A_HUMAN	396.490234
THOC3_HUMAN	4534.60938
ITCH_HUMAN	3005.46362
TXD15_HUMAN	893.063293
KIRR1_HUMAN	2234.58057
LRIG1_HUMAN	1289.99097
COG3_HUMAN	2258.12891
CK5P3_HUMAN	6820.52002
VPS39_HUMAN	3522.78638
VPS50_HUMAN	2428.35132
VCIP1_HUMAN	3477.54199
ELMO2_HUMAN	6642.14648
TMX3_HUMAN	12109.8965
DCAF5_HUMAN	1812.53796
CHAP1_HUMAN	2188.12402
LMBL3_HUMAN	3033.18335
ZFP91_HUMAN	1038.4696
PCD16_HUMAN	1248.83948
CLMN_HUMAN	12858.9629
PDLI2_HUMAN	619.428406
FACR2_HUMAN	5522.82861
BT3L4_HUMAN	8177.99268
RN170_HUMAN	856.285461
ANCHR_HUMAN	1583.90759
S35E1_HUMAN	1125.99561
UBP47_HUMAN	4739.84033
ZN521_HUMAN	362.602722
CLP1L_HUMAN	2964.71313
TOPK_HUMAN	5515.97754
ARL5B_HUMAN	500.407867
DNJC1_HUMAN	1652.87207
SCYL1_HUMAN	6254.22021
Z512B_HUMAN	834.613464
LRAT2_HUMAN	598.485291
EXOC2_HUMAN	7322.60449
CNDP2_HUMAN	45620.7422
EHMT2_HUMAN	1335.38647
ZFR_HUMAN	4314.28564
F210B_HUMAN	287.51474
B3GT6_HUMAN	1372.31628
EP400_HUMAN	932.491333
SNX27_HUMAN	4314.82666

KI16B_HUMAN	219.954346
IFT74_HUMAN	796.465149
TRI47_HUMAN	4327.9082
CNO6L_HUMAN	4297.06152
DHRS1_HUMAN	557.838867
DJC30_HUMAN	436.11261
UB2E2_HUMAN	176.629532
CAR19_HUMAN	2669.95435
PRRC1_HUMAN	7148.77637
FGD4_HUMAN	3641.95752
FXL18_HUMAN	3337.4126
ZN512_HUMAN	2497.52026
NSE2_HUMAN	902.347229
NSE3_HUMAN	160.741409
PCMD1_HUMAN	364.801727
HS12B_HUMAN	4471.75244
YTDC1_HUMAN	2210.43848
CCD43_HUMAN	1476.95239
COG8_HUMAN	2512.6626
CKLF3_HUMAN	3242.7688
DAA10_HUMAN	2547.0542
NOL4L_HUMAN	6744.61279
MBOA7_HUMAN	10916.4492
DOCK7_HUMAN	9709.42285
SFXN2_HUMAN	980.493591
ZN830_HUMAN	330.613312
F210A_HUMAN	1662.17969
FRMD6_HUMAN	599.036499
NSUN5_HUMAN	381.028778
RPR1A_HUMAN	2878.40503
AGAP3_HUMAN	1493.63733
ARAP1_HUMAN	3318.57983
IPO9_HUMAN	15481.4902
MIA2_HUMAN	1404.70398
DCBD2_HUMAN	1354.16663
ARHGH_HUMAN	1210.39172
INP4A_HUMAN	727.517944
RBM14_HUMAN	19434.3047
ZN363_HUMAN	346.203278
KLHL5_HUMAN	798.074036
UHRF2_HUMAN	4265.25293
NED4L_HUMAN	3070.00903
QKI_HUMAN	15080.6445
LENG8_HUMAN	912.996582
FMNL2_HUMAN	1524.71387
PUS7_HUMAN	5521.0835
F111A_HUMAN	614.769653
TPPC9_HUMAN	672.339233
PLIN4_HUMAN	172.515854
TRNT1_HUMAN	3788.62378

SMG1_HUMAN	1076.72424
ALS2_HUMAN	749.625732
TM237_HUMAN	1110.65503
ALKB3_HUMAN	521.943298
RHG07_HUMAN	782.607544
PP1RA_HUMAN	3831.34717
S38A2_HUMAN	1458.09351
UIF_HUMAN	2625.26025
MTMR9_HUMAN	2532.52563
VPS35_HUMAN	28305.3887
PURB_HUMAN	3054.40479
XPO6_HUMAN	2487.42944
HHIP_HUMAN	41577.3984
MAGI1_HUMAN	875.387085
SPAG5_HUMAN	956.420044
NACC1_HUMAN	5431.85791
SNX18_HUMAN	2547.89063
PASK_HUMAN	370.750916
CIC_HUMAN	1519.68848
UIMC1_HUMAN	413.584839
VP13A_HUMAN	2039.42529
MED15_HUMAN	2396.80103
EFGM_HUMAN	10308.3281
ERGI2_HUMAN	2328.36597
MCCA_HUMAN	1517.05164
KKCC2_HUMAN	598.289978
TGS1_HUMAN	799.537048
NUDC1_HUMAN	12323.3945
ERBIN_HUMAN	1804.2074
GCP6_HUMAN	1723.14685
GCP5_HUMAN	1319.12549
UBP28_HUMAN	669.29248
FNBP1_HUMAN	4030.85352
PRPK_HUMAN	4962.10889
PIGS_HUMAN	5555.98145
WRIP1_HUMAN	3101.24121
RANB9_HUMAN	2316.8772
CLCC1_HUMAN	2523.65771
MYADM_HUMAN	8346.84082
NEB2_HUMAN	1759.32654
SRPK1_HUMAN	4678.41943
SMC6_HUMAN	1561.69824
KCD15_HUMAN	643.848389
STRBP_HUMAN	15415.21
TM209_HUMAN	2645
GPX7_HUMAN	1241.62378
CK5P2_HUMAN	465.732727
CP2S1_HUMAN	854.723145
IWS1_HUMAN	7389.0415
SIN3A_HUMAN	984.97937

OSBL9_HUMAN	5720.32764
CRBN_HUMAN	888.185852
INT14_HUMAN	1413.35291
AEDO_HUMAN	2221.04761
CK5P1_HUMAN	678.156006
RSF1_HUMAN	280.936462
RBM15_HUMAN	3220.02051
PP16B_HUMAN	237.83699
RUFY1_HUMAN	4268.40088
MINT_HUMAN	645.141113
PNKP_HUMAN	5219.58154
MMS19_HUMAN	10262.4082
UHRF1_HUMAN	6117.43018
NIBA2_HUMAN	33328.8828
YMEL1_HUMAN	6652.58057
RMD3_HUMAN	4450.03125
HTF4_HUMAN	1115.25952
TBCB_HUMAN	13887.8799
PSB7_HUMAN	20771.6074
CNN2_HUMAN	15490.3613
SEC62_HUMAN	8804.71289
PCY2_HUMAN	5169.17432
CDC5L_HUMAN	14351.9658
PSMD1_HUMAN	28227.2832
SDF2_HUMAN	2069.229
PFD5_HUMAN	1808.21716
RING2_HUMAN	2276.19165
PARK7_HUMAN	12056.7217
S1PR3_HUMAN	370.210236
GA2L1_HUMAN	239.419754
EYA3_HUMAN	505.085114
NEUR1_HUMAN	5789.43506
VAT1_HUMAN	119251.891
LGMN_HUMAN	2148.0105
PLIN2_HUMAN	465.100189
DNJC2_HUMAN	4763.24854
MPP8_HUMAN	773.825195
NUP88_HUMAN	9172.3623
PKP4_HUMAN	233.026581
PI3R4_HUMAN	3851.03003
P2RX4_HUMAN	2947.32837
POP1_HUMAN	5836.92041
S10AD_HUMAN	26474.6914
SCAFB_HUMAN	1334.82886
TSNAX_HUMAN	5355.62891
SPS2_HUMAN	1581.91333
EIF3C_HUMAN	26293.7969
TTC1_HUMAN	495.400085
DNJC7_HUMAN	10400.8613
PHB2_HUMAN	85136.125

CSN8_HUMAN	1693.30249
PRP18_HUMAN	767.415649
CHP1_HUMAN	4297.56885
KIF2C_HUMAN	2609.64648
RGPD5_HUMAN	251.094574
MGLL_HUMAN	3198.43457
ATX2_HUMAN	2828.8208
DOK1_HUMAN	456.043793
METH_HUMAN	9117.68457
HCD2_HUMAN	33062.375
COCA1_HUMAN	2106.95239
SMAD5_HUMAN	1372.66992
SGMR1_HUMAN	4001.36353
ROAA_HUMAN	15329.7783
NP1L4_HUMAN	25326.0527
MGST2_HUMAN	27910.9727
SNAG_HUMAN	5548.09473
PI51A_HUMAN	1329.66443
ABCA3_HUMAN	907.268372
M3K3_HUMAN	1175.10254
ATP5S_HUMAN	1002.36322
MIPEP_HUMAN	3702.44824
ACON_HUMAN	14256.4434
TM9S2_HUMAN	6567.67676
S29A1_HUMAN	10837.9199
TS101_HUMAN	7885.10449
CPNE1_HUMAN	17231.8672
TCPH_HUMAN	48177.4648
MYD88_HUMAN	5851.99023
EBP2_HUMAN	10542.2676
ANM1_HUMAN	23285.1289
BAG1_HUMAN	338.679688
ATF6B_HUMAN	630.794495
FOXC2_HUMAN	575.327881
SH3G1_HUMAN	28095.0352
TEP1_HUMAN	7761.41309
VRK1_HUMAN	10953.8906
GDF15_HUMAN	19808.4824
AKAP9_HUMAN	10026.5938
NIPS1_HUMAN	11531.2383
CND3_HUMAN	7207.24658
ARP5L_HUMAN	11197.8984
MICU1_HUMAN	818.362183
CG025_HUMAN	766.324768
F118B_HUMAN	1890.00208
SIN1_HUMAN	1565.78589
RBM4B_HUMAN	16816.8086
ZFY21_HUMAN	1389.27307
DDX50_HUMAN	5678.55127
RM34_HUMAN	2548.51709

RNZ2_HUMAN	7612.19092
TRIR_HUMAN	3619.28052
GRWD1_HUMAN	6760.37891
MACD1_HUMAN	830.212646
TCF25_HUMAN	3403.16479
CMS1_HUMAN	3862.31348
KLDC3_HUMAN	963.285828
ECSIT_HUMAN	1956.23926
MEP50_HUMAN	17531.7598
CYBC1_HUMAN	1305.61707
VKOR1_HUMAN	4096.81934
DPH2_HUMAN	1692.6366
TBA1C_HUMAN	19611.2637
SELS_HUMAN	2716.96631
APOL2_HUMAN	3900.49658
MBB1A_HUMAN	17397.5527
FERM1_HUMAN	940.72229
MGME1_HUMAN	454.792572
GORS1_HUMAN	3373.38379
FYCO1_HUMAN	1282.21375
ACBD6_HUMAN	2690.46704
COR1B_HUMAN	32886.6758
TXD17_HUMAN	11882.2871
BUD13_HUMAN	902.769165
CPPED_HUMAN	16821.2422
VPS25_HUMAN	2709.15332
RM45_HUMAN	13100.29
CG050_HUMAN	8936.16797
TIRR_HUMAN	2062.6814
CAB45_HUMAN	7189.33838
PDD2L_HUMAN	447.015503
PAAF1_HUMAN	5180.46387
PYM1_HUMAN	3570.6521
MIC25_HUMAN	963.699524
ADPGK_HUMAN	5842.21582
GPTC1_HUMAN	443.859131
RIOK1_HUMAN	2011.66211
LLPH_HUMAN	3054.37427
SLD5_HUMAN	698.340454
UTP23_HUMAN	435.752014
PELO_HUMAN	6235.45117
PXL2A_HUMAN	63852.6094
TRI56_HUMAN	1238.94812
ERP44_HUMAN	54018.6289
LXN_HUMAN	12023.2939
NOL10_HUMAN	2045.78687
NTPCR_HUMAN	4851.05176
TIM29_HUMAN	4535.59668
TACO1_HUMAN	2388.26489
GCP2_HUMAN	5247.38379

ESYT1_HUMAN	30181.8203
UBAC1_HUMAN	1181.19946
YIPF4_HUMAN	2638.98657
PHAF1_HUMAN	670.436646
SEN34_HUMAN	1619.49377
CNPY3_HUMAN	1593.401
MTG1_HUMAN	2685.54297
ALG1_HUMAN	3622.20068
LIMD2_HUMAN	1034.84778
HAUS8_HUMAN	789.257324
INP5K_HUMAN	1985.06506
PSMG3_HUMAN	3307.00269
CSN4_HUMAN	12006.0488
SYT11_HUMAN	1228.52429
WAC_HUMAN	510.573639
DIDO1_HUMAN	2933.0332
MTA3_HUMAN	5057.96631
RBM42_HUMAN	9729.30859
DCTN5_HUMAN	2190.13818
MCMBP_HUMAN	4959.8042
AASD1_HUMAN	4072.85181
DCNL5_HUMAN	6234.19287
AN32E_HUMAN	6266.75879
LRRC1_HUMAN	647.551392
P4K2A_HUMAN	2523.96753
TMM43_HUMAN	24439.8574
FSD1_HUMAN	1245.4032
TBCD_HUMAN	8125.12451
NDC1_HUMAN	1579.7301
HGH1_HUMAN	423.228638
DHRS4_HUMAN	3869.83398
LMF2_HUMAN	4820.52246
NDUF3_HUMAN	5278.46973
MMTA2_HUMAN	1241.13953
TMM70_HUMAN	4509.0127
MED18_HUMAN	1466.26782
ISCA1_HUMAN	1111.67163
TBB6_HUMAN	122923.406
PAXX_HUMAN	1193.76294
RPC3_HUMAN	1997.78003
HNRL1_HUMAN	15962.9629
MSTO1_HUMAN	5380.18457
PHF23_HUMAN	1477.07019
PDC10_HUMAN	13004.0664
RPP25_HUMAN	1980.03748
DERL1_HUMAN	17715.8906
HTAI2_HUMAN	5075.76367
DDX23_HUMAN	9725.79395
TCAB1_HUMAN	579.823425
MIC26_HUMAN	4244.62158

DHRS6_HUMAN	6115.01563
MTNA_HUMAN	4458.48145
WDR18_HUMAN	5976.22852
VAMP8_HUMAN	683.572632
THUM3_HUMAN	1921.2605
MTND_HUMAN	4978.57715
MECR_HUMAN	2215.09277
EMC6_HUMAN	5759.11816
NTM1A_HUMAN	3248.40112
EDEM2_HUMAN	643.770203
KTNB1_HUMAN	583.273438
LST8_HUMAN	1307.86438
ASHWN_HUMAN	1262.46204
TM109_HUMAN	64188.0391
PBDC1_HUMAN	3042.57275
PTSS2_HUMAN	3009.32935
NOC4L_HUMAN	5245.16602
UT14A_HUMAN	4606.73975
DUS23_HUMAN	3996.17944
TMED9_HUMAN	41158.8789
TM147_HUMAN	3473.62646
NUP58_HUMAN	2962.28174
SELO_HUMAN	2234.27563
DPCD_HUMAN	1936.25757
GNL3_HUMAN	14919.9863
AFG2B_HUMAN	6457.81592
RIOK2_HUMAN	1407.01038
TR61B_HUMAN	126.955521
TMUB1_HUMAN	1288.45386
TIM21_HUMAN	3176.28979
KIFC1_HUMAN	745.73822
NUP85_HUMAN	7226.80762
ELOV1_HUMAN	2587.50171
KATL1_HUMAN	1172.44434
HIRP3_HUMAN	150.864166
IFT27_HUMAN	1716.38184
YJU2_HUMAN	2416.54297
NUDT9_HUMAN	4759.51709
SYTM_HUMAN	3408.45239
THIC_HUMAN	22827.5508
REPI1_HUMAN	462.91803
RBM4_HUMAN	3619.40454
FUND2_HUMAN	1173.50854
RPAP1_HUMAN	1968.44214
SF3B5_HUMAN	876.53894
SFXN3_HUMAN	16234.9785
PRR14_HUMAN	2326.03442
CHID1_HUMAN	2662.23047
PAPOG_HUMAN	1477.17456
CAR10_HUMAN	336.613342

NADAP_HUMAN	6319.19141
CDK19_HUMAN	589.402893
LS14B_HUMAN	1268.89709
TPSNR_HUMAN	483.061157
JAM3_HUMAN	8024.10986
CARD6_HUMAN	1478.8031
BTBD2_HUMAN	538.665894
SGPP1_HUMAN	179.633392
OSB11_HUMAN	1733.30701
OSB10_HUMAN	3015.07666
RFIP5_HUMAN	2344.6228
TB10A_HUMAN	186.133347
C1QT5_HUMAN	3568.3457
NAA15_HUMAN	21666.1738
KLF16_HUMAN	58.9909554
B2L13_HUMAN	507.627808
CAR11_HUMAN	1132.36926
S12A9_HUMAN	1212.06458
SRRT_HUMAN	21337.6133
TGT_HUMAN	5036.17041
AP1M1_HUMAN	16390.3672
NUSAP_HUMAN	530.33252
HDHD5_HUMAN	3636.84155
FACD2_HUMAN	527.545715
MAK16_HUMAN	3897.60767
HDAC8_HUMAN	276.640625
RTF2_HUMAN	1345.24976
CHM4A_HUMAN	2932.71729
EIF2A_HUMAN	17825.2266
PDIP3_HUMAN	4583.24902
K1671_HUMAN	1491.74243
SHAN3_HUMAN	8906.30957
GNB1L_HUMAN	2035.93823
FUT8_HUMAN	1282.32117
RM32_HUMAN	1238.89648
RM20_HUMAN	4881.19434
RM13_HUMAN	9832.07129
RM09_HUMAN	10301.9814
RM04_HUMAN	5251.05225
RM01_HUMAN	7775.88916
MK67I_HUMAN	4123.42285
HYCCI_HUMAN	1988.57849
HELZ2_HUMAN	636.3302
HOIL1_HUMAN	1745.12769
RT26_HUMAN	5058.95264
NEUL_HUMAN	10835.7139
CEP41_HUMAN	2221.03198
SETD2_HUMAN	783.126221
PANK2_HUMAN	563.99054
DOCK9_HUMAN	9784.58203

FRMD8_HUMAN	4273.52344
CNTP3_HUMAN	1687.22522
NSD3_HUMAN	1441.01428
NUF2_HUMAN	3193.11133
RM37_HUMAN	11193.5254
GTPB4_HUMAN	12371.251
ASPC1_HUMAN	2276.7478
OSBL8_HUMAN	2658.38623
UACA_HUMAN	2573.90723
RAB34_HUMAN	4628.0625
DPH1_HUMAN	993.231812
WDR11_HUMAN	4132.65137
REN3B_HUMAN	1744.55579
CRNL1_HUMAN	3862.39233
TBL1R_HUMAN	3749.89453
UBL5_HUMAN	4205.10107
PP12C_HUMAN	2544.98242
KPCD2_HUMAN	4633.23779
EDEM3_HUMAN	3326.2373
NIBA1_HUMAN	403.887482
UBXN6_HUMAN	4437.99072
TM6S1_HUMAN	573.068359
UCK2_HUMAN	7315.49121
SN_HUMAN	1303.33459
API5_HUMAN	23241.9824
NLRP1_HUMAN	815.763855
TRIM5_HUMAN	1698.03064
TRIM4_HUMAN	49839.8984
FTO_HUMAN	5805.31592
ZDHC5_HUMAN	677.965393
TNG6_HUMAN	1444.90515
TB182_HUMAN	5341.71826
UBE2O_HUMAN	7055.56787
ZY11B_HUMAN	1456.2876
TANC1_HUMAN	5436.5752
EPT1_HUMAN	2399.9292
XPO4_HUMAN	8021.97559
LNP_HUMAN	1555.07996
CEP44_HUMAN	539.242615
TTYH3_HUMAN	9491.40723
KLHL4_HUMAN	2092.37793
MTMRC_HUMAN	862.63916
WDR33_HUMAN	5904.71191
WDR12_HUMAN	7528.0166
YIPF3_HUMAN	5410.53125
TINAL_HUMAN	2365.83252
ADSSP_HUMAN	7554.27344
PITH1_HUMAN	4226.38916
DERL2_HUMAN	7540.70264
COMD5_HUMAN	2270.5376

SENP6_HUMAN	226.084274
REXO4_HUMAN	2481.54126
DDX24_HUMAN	5638.58301
RPA49_HUMAN	4009.18066
SKI8_HUMAN	7433.90918
SLIRP_HUMAN	8090.41016
SRR_HUMAN	1101.45557
NIF3L_HUMAN	901.433655
EGLN1_HUMAN	5081.85498
MCLN1_HUMAN	684.760864
PIP30_HUMAN	3717.39941
MFF_HUMAN	922.017578
NAA50_HUMAN	9777.39746
LACRT_HUMAN	847.383057
UBA5_HUMAN	6517.91504
MKRN2_HUMAN	2364.77319
T126A_HUMAN	3840.04834
PAIP1_HUMAN	12306.4814
CLPB_HUMAN	4571.22217
RB33B_HUMAN	3803.4834
LSG1_HUMAN	6781.49902
F107B_HUMAN	8012.50244
NAT10_HUMAN	17978.3926
COMD4_HUMAN	2021.41162
KLC2_HUMAN	2674.92749
ILKAP_HUMAN	5672.58105
XRN2_HUMAN	15488.1357
TOLIP_HUMAN	4077.0249
SP130_HUMAN	710.495361
BRD8_HUMAN	757.71814
NSRP1_HUMAN	256.848541
INT2_HUMAN	880.483459
RGAP1_HUMAN	748.781494
PAR12_HUMAN	2249.23389
CSTFT_HUMAN	1217.16223
5NT3A_HUMAN	2177.14819
CYRIA_HUMAN	13614.7764
TM222_HUMAN	891.65033
HDHD2_HUMAN	1975.22314
GATA_HUMAN	2817.42041
DDX47_HUMAN	8126.92285
MAGT1_HUMAN	7524.33887
RAB1B_HUMAN	54066.6563
RM18_HUMAN	7131.35742
TSYL1_HUMAN	1101.6283
TM168_HUMAN	741.156128
LMA2L_HUMAN	6698.98096
SMG9_HUMAN	2193.94214
F234A_HUMAN	312.530609
OSBL5_HUMAN	819.481384

TDIF1_HUMAN	1121.86206
SIL1_HUMAN	1282.41467
METL9_HUMAN	1182.86841
APC1_HUMAN	5182.2627
I2BPL_HUMAN	1454.6283
CYTM1_HUMAN	1247.25281
RPC6_HUMAN	1936.14807
NUCKS_HUMAN	3712.57935
TMX4_HUMAN	5295.99365
KI13A_HUMAN	12221.0654
ASCC2_HUMAN	6518.51074
RBNS5_HUMAN	749.926575
ISCU_HUMAN	3098.45825
OSBL2_HUMAN	1582.31543
ATG5_HUMAN	4061.28613
WDR13_HUMAN	2486.21973
EPN3_HUMAN	800.335327
EHD4_HUMAN	95117.25
VP33B_HUMAN	3386.62231
VPS16_HUMAN	6681.9043
VPS11_HUMAN	4809.85938
SH3L3_HUMAN	11403.7041
GAN_HUMAN	552.847595
S2532_HUMAN	1630.25464
TARA_HUMAN	6380.17676
SLK_HUMAN	10342.9365
PPIL3_HUMAN	744.066772
S38A1_HUMAN	1860.58228
PDCL3_HUMAN	5949.05127
TAOK3_HUMAN	6657.0835
RBGPR_HUMAN	9002.02246
ADNP_HUMAN	2413.91675
DPH5_HUMAN	2246.49878
DHX36_HUMAN	7035.89355
IPYR2_HUMAN	7557.2168
SPNS1_HUMAN	4350.75098
RM46_HUMAN	2044.41345
S12A5_HUMAN	463.309937
PARL_HUMAN	1304.33142
PININ_HUMAN	8527
TM245_HUMAN	1217.99133
FOXP1_HUMAN	1559.62817
BACD3_HUMAN	4547.80957
MOD5_HUMAN	1301.7616
CK068_HUMAN	5681.81592
GHITM_HUMAN	6767.34229
BOLA2_HUMAN	5326.25977
MMAD_HUMAN	1494.73328
TXNIP_HUMAN	158.863739
TMX1_HUMAN	24446.8574

NELFA_HUMAN	3250.63867
GCP60_HUMAN	9344.19824
BORG4_HUMAN	1089.51917
PTN23_HUMAN	7221.76514
SEM6B_HUMAN	5143.99561
UN45A_HUMAN	18686.9355
MFSD1_HUMAN	1879.91724
DNJC5_HUMAN	984.618408
DSN1_HUMAN	300.756744
CHM4B_HUMAN	17866.043
RWDD1_HUMAN	2119.20117
CUED2_HUMAN	520.013367
FXL15_HUMAN	547.135254
FN3K_HUMAN	2059.04468
OFUT1_HUMAN	8988.72363
PIGU_HUMAN	3941.19531
TOR3A_HUMAN	3290.29858
WNK1_HUMAN	2986.92822
AMPB_HUMAN	20338.0117
GLP3L_HUMAN	4743.27783
GOLP3_HUMAN	3703.48901
OSGL1_HUMAN	1648.64722
SIA7D_HUMAN	973.943604
GAPR1_HUMAN	5591.61572
TRABD_HUMAN	2227.86621
MTG2_HUMAN	70.0795441
SENP3_HUMAN	4075.41479
OSBL3_HUMAN	5038.71484
SMRCD_HUMAN	2123.29468
EHD1_HUMAN	22257.8906
CAPAM_HUMAN	522.047913
ESF1_HUMAN	1082.99609
ALG2_HUMAN	3869.61182
HEAT1_HUMAN	9911.80176
SG196_HUMAN	490.056213
RABE2_HUMAN	1325.39941
TFB2M_HUMAN	4029.56006
ZCHC4_HUMAN	1751.72021
STEEP_HUMAN	587.389038
CIA2A_HUMAN	1304.89551
DHX35_HUMAN	760.470825
F124B_HUMAN	2385.74268
HAUS4_HUMAN	2418.49365
CC134_HUMAN	1722.39429
CCD86_HUMAN	3594.46045
REEP4_HUMAN	2036.38818
SOX17_HUMAN	932.432922
OPA3_HUMAN	2310.05249
CIAO3_HUMAN	1504.42615
DHX33_HUMAN	1504.44141

NOL6_HUMAN	4318.32715
WDCP_HUMAN	510.688293
YTDC2_HUMAN	2915.12109
AZI2_HUMAN	152.52916
RPAP3_HUMAN	3232.92456
BCAS3_HUMAN	173.70108
ALG9_HUMAN	3019.20776
LDAH_HUMAN	1605.30566
RIOX1_HUMAN	2806.79199
WDR55_HUMAN	2845.84766
RANB3_HUMAN	6861.15674
DCTP1_HUMAN	560.342224
PEAK1_HUMAN	1695.12341
RPF2_HUMAN	3068.30005
SMYD3_HUMAN	4207.80713
AAMDC_HUMAN	1350.45715
DOCK5_HUMAN	893.045471
WDR26_HUMAN	3075.44336
TDRD3_HUMAN	2357.01343
CH033_HUMAN	1934.41907
AT133_HUMAN	730.865967
MET17_HUMAN	392.81604
SFR19_HUMAN	1337.77039
NRDE2_HUMAN	606.099121
PGES2_HUMAN	6952.64258
PHAX_HUMAN	2436.76807
UBE2Z_HUMAN	5178.32227
ACAD9_HUMAN	5916.85352
NT5D2_HUMAN	9235.21777
PKRI1_HUMAN	2556.29395
CAAP1_HUMAN	1261.07947
NOL11_HUMAN	1662.12256
DDX31_HUMAN	1068.05176
TMT1A_HUMAN	2608.74219
PAAT_HUMAN	1890.02759
MMRN2_HUMAN	12593.3721
MINY3_HUMAN	2022.8678
EVA1A_HUMAN	2395.17725
AKTIP_HUMAN	351.351349
PKHF2_HUMAN	1752.48071
ANKZ1_HUMAN	2087.05981
GORS2_HUMAN	3101.74512
ZWILC_HUMAN	1940.44971
JUPI2_HUMAN	2784.01147
GHC1_HUMAN	6278.82227
PPIP2_HUMAN	3125.66455
MED20_HUMAN	2011.16895
WDR76_HUMAN	313.224792
QTRT2_HUMAN	1736.11853
ARP8_HUMAN	1453.66772

ARMT1_HUMAN	4067.62915
PANK3_HUMAN	422.052887
CNO10_HUMAN	1589.61914
LRC40_HUMAN	16831.0156
EHMT1_HUMAN	2572.36548
SFXN1_HUMAN	26112.4453
SPE39_HUMAN	1557.38196
COG4_HUMAN	7786.15381
ARP5_HUMAN	696.077942
VP37B_HUMAN	693.046814
RM44_HUMAN	8004.71338
L2HDH_HUMAN	1040.33167
CSN7B_HUMAN	2576.45093
NHEJ1_HUMAN	523.682495
S61A2_HUMAN	14018.584
FKRP_HUMAN	937.002136
ELP3_HUMAN	6257.01514
RPF1_HUMAN	2274.49976
RPA2_HUMAN	1821.62
ZMAT3_HUMAN	403.010834
UCK1_HUMAN	348.010223
KT3K_HUMAN	1507.87317
TBC17_HUMAN	1347.4248
SYCM_HUMAN	4596.52051
S52A2_HUMAN	2333.21851
PPCS_HUMAN	9543.36523
UBTD1_HUMAN	2233.25488
WDR41_HUMAN	900.983398
NMNA1_HUMAN	1275.87585
NJMU_HUMAN	1359.30261
SIAE_HUMAN	325.274323
PKHA5_HUMAN	857.361694
SMUF2_HUMAN	2917.18115
RENT2_HUMAN	4231.02783
GBB4_HUMAN	2229.88721
XPO5_HUMAN	6729.9751
GRPE1_HUMAN	14988.0186
MYG1_HUMAN	10817.7637
PKHA3_HUMAN	423.012115
PKHA1_HUMAN	1487.88696
RISC_HUMAN	3094.26221
NET4_HUMAN	3907.979
CYBP_HUMAN	31042.1699
RRAGC_HUMAN	8549.11914
ZFYV1_HUMAN	919.54718
IF122_HUMAN	765.215515
RDH14_HUMAN	3860.35083
PARVB_HUMAN	12254.1309
TENS1_HUMAN	2940.67163
PLRKT_HUMAN	2455.62354

NMRL1_HUMAN	1743.48584
VEZA_HUMAN	566.104309
SPC25_HUMAN	2071.46313
TAF9B_HUMAN	677.887573
S38AA_HUMAN	1520.91162
EKI1_HUMAN	2195.99854
AGRL4_HUMAN	9445.16406
TM165_HUMAN	5471.5415
TPC_HUMAN	420.562469
EMAL4_HUMAN	4936.75244
MRM3_HUMAN	2834.65674
GLOD4_HUMAN	44583.6602
NEK6_HUMAN	2079.38428
MCCB_HUMAN	2789.15063
NCOA5_HUMAN	43703.4063
EPG5_HUMAN	1291.77124
MOV10_HUMAN	6179.9209
MET14_HUMAN	2021.56702
GBA2_HUMAN	2674.70996
CWC22_HUMAN	1514.0968
RAVR2_HUMAN	818.363892
CHD8_HUMAN	1297.7832
E41L5_HUMAN	1243.03247
GPN1_HUMAN	4268.56152
SDF2L_HUMAN	3995.8584
KC1G1_HUMAN	3387.01294
SYF1_HUMAN	4205.34521
PREB_HUMAN	3963.23267
SRA1_HUMAN	1575.78333
AT131_HUMAN	5472.87549
GOPC_HUMAN	1549.13245
RM47_HUMAN	5302.14795
LYRM4_HUMAN	2217.92603
SPCS_HUMAN	1271.55823
CHM1A_HUMAN	7468.9458
TM9S3_HUMAN	13721.0781
MYO10_HUMAN	4886.25
APMAP_HUMAN	48799.0391
WDR6_HUMAN	5911.93896
TRXR2_HUMAN	11767.0566
ABCB6_HUMAN	729.541321
ARFG3_HUMAN	4249.50293
ZCC17_HUMAN	954.727051
RAB18_HUMAN	17238.4824
PALMD_HUMAN	7972.30469
SSU72_HUMAN	2597.17041
VTA1_HUMAN	20802.8926
SYSM_HUMAN	3659.4585
RT30_HUMAN	6116.26758
DLRB1_HUMAN	4025.3457

EMC7_HUMAN	8316.25879
ENY2_HUMAN	2812.57202
EXOS4_HUMAN	3689.22021
UBE2T_HUMAN	4004.09985
NOP10_HUMAN	3955.11108
OSGEP_HUMAN	5072.42529
DMAP1_HUMAN	781.95813
PCD12_HUMAN	487.041504
INO1_HUMAN	6214.24609
BRD7_HUMAN	828.686829
DCP1A_HUMAN	4966.49072
ACO13_HUMAN	8343.26465
MED4_HUMAN	625.161804
TIDC1_HUMAN	230.855484
RIC8A_HUMAN	20611.6191
C1QR1_HUMAN	33537.0391
LUC7L_HUMAN	8355.7998
ESM1_HUMAN	990.03302
LZTL1_HUMAN	1747.81201
RM40_HUMAN	3532.37573
SSF1_HUMAN	3414.35376
PLCB1_HUMAN	924.759033
TIGAR_HUMAN	15898.2891
RTN4_HUMAN	18712.418
RPR1B_HUMAN	7059.53857
XPP3_HUMAN	2325.33032
NIT2_HUMAN	6598.37793
AVEN_HUMAN	767.598511
INCE_HUMAN	1007.90802
EXOS5_HUMAN	2131.18188
EXOS3_HUMAN	5312.3374
KI13B_HUMAN	5025.3457
ANLN_HUMAN	3805.05396
XPP1_HUMAN	12706.9902
GEPH_HUMAN	3913.89233
BIN3_HUMAN	2742.27808
SAS10_HUMAN	2139.02979
STAR7_HUMAN	1403.61682
BIRC6_HUMAN	4187.01709
PDLI7_HUMAN	26329.8281
ACSA_HUMAN	9050.21582
DBLOH_HUMAN	2122.53003
DDX21_HUMAN	26089.9219
SAR1A_HUMAN	24316.4492
SIAS_HUMAN	32704.0117
SHLB2_HUMAN	3575.33203
ASH1L_HUMAN	17171.8262
EI2BG_HUMAN	10068.8965
MBNL1_HUMAN	11915.8457
S17A5_HUMAN	2957.92334

4ET_HUMAN	1031.81775
CHSTC_HUMAN	1684.2605
ST7_HUMAN	1629.37341
PICK1_HUMAN	731.329956
SH2B1_HUMAN	1855.53369
PYRG2_HUMAN	5652.30225
DPOE3_HUMAN	2375.14819
CHRC1_HUMAN	2421.58496
AAAS_HUMAN	7100.71484
SNRK_HUMAN	3128.14795
ABCBA_HUMAN	2638.95996
BAZ1A_HUMAN	1544.29907
STRN4_HUMAN	4167.39551
ADPPT_HUMAN	9850.05469
METL5_HUMAN	2109.67017
OSTC_HUMAN	22027.0781
COXM2_HUMAN	530.983032
PLS4_HUMAN	1682.13989
RNC_HUMAN	506.822632
SNX15_HUMAN	630.348267
HEBP1_HUMAN	2050.14136
ABC3C_HUMAN	2751.4104
VPS45_HUMAN	2723.8291
PNO1_HUMAN	2932.79199
RM17_HUMAN	11588.8174
PHP14_HUMAN	208.330948
SERC1_HUMAN	865.532288
RHG35_HUMAN	2829.01074
F1142_HUMAN	1837.46655
PLS3_HUMAN	2551.98071
PLCD_HUMAN	575.493164
PLCC_HUMAN	2642.85156
HELLS_HUMAN	2033.21289
C1GLT_HUMAN	2324.80933
LTBP3_HUMAN	354.638428
TOM22_HUMAN	3495.40283
LANC2_HUMAN	4914.32568
KIF15_HUMAN	860.267456
TM7S3_HUMAN	1558.12915
HOME3_HUMAN	5124.48926
SYFB_HUMAN	27607.8691
SYIM_HUMAN	12468.2607
SLX9_HUMAN	3614.82739
SAMN1_HUMAN	615.361633
KLC4_HUMAN	1818.87683
DIAP3_HUMAN	2269.37158
BMP2K_HUMAN	792.877625
ATG3_HUMAN	3595.14282
SIR3_HUMAN	546.945007
PDS5B_HUMAN	4801.14697

SMC4_HUMAN	30483.5352
MA2C1_HUMAN	6275.81934
SAC1_HUMAN	7778.26709
DEPP1_HUMAN	904.93042
OLA1_HUMAN	30780.7656
CUTC_HUMAN	1129.42896
ZF64B_HUMAN	1242.02173
ECHD1_HUMAN	10103.9063
RBM12_HUMAN	85321.5938
TB22B_HUMAN	1379.85718
MDN1_HUMAN	9097.8252
ACS2L_HUMAN	790.313232
ZCHC3_HUMAN	810.955994
DECR2_HUMAN	5034.21094
ABHDA_HUMAN	2432.23193
T11L1_HUMAN	1644.66565
STAU2_HUMAN	5426.47314
SHFL_HUMAN	734.230164
DDX28_HUMAN	1549.5741
T106B_HUMAN	2408.00244
LIN7C_HUMAN	1661.54907
PLCE_HUMAN	4606.3877
TXLNG_HUMAN	1843.45593
SPS2L_HUMAN	2800.94678
UFSP2_HUMAN	2469.76831
ABCF3_HUMAN	7448.33691
CYRIB_HUMAN	10282.3496
MITOS_HUMAN	2329.54224
DD19A_HUMAN	17878.6016
GIMA4_HUMAN	18348.6426
TYDP1_HUMAN	2058.48389
TBC23_HUMAN	3956.60132
DCA13_HUMAN	2812.14063
IMP3_HUMAN	5374.33154
MRGBP_HUMAN	1121.61169
TYW1_HUMAN	485.827576
EXOC1_HUMAN	9259.38965
PCMD2_HUMAN	443.573883
INT9_HUMAN	2526.62671
CC50A_HUMAN	10320.7051
UQCC1_HUMAN	2564.22046
SEP11_HUMAN	21910.6328
S38A7_HUMAN	13033.752
MED17_HUMAN	12022.2627
PARVA_HUMAN	9277.65918
UBP40_HUMAN	462.548615
PANK4_HUMAN	10658.5186
FBX28_HUMAN	415.282074
TBC13_HUMAN	6232.3208
EXD2_HUMAN	2247.20166

DJC11_HUMAN	6405.91162
INT7_HUMAN	11795.2461
FANCI_HUMAN	867.562805
ATD3A_HUMAN	37189.6211
ARL8B_HUMAN	6406.5083
DJC17_HUMAN	4413.18994
INT13_HUMAN	3034.10913
RIC8B_HUMAN	920.549255
GNL3L_HUMAN	1754.39014
DDX18_HUMAN	10258.8193
ASF1B_HUMAN	2356.13428
KLH11_HUMAN	866.855835
INT10_HUMAN	1963.39075
RT18A_HUMAN	6508.16064
PNPO_HUMAN	980.403259
ARMC1_HUMAN	4042.49658
RPC5_HUMAN	853.804199
SDA1_HUMAN	4312.29785
PAPD1_HUMAN	4744.33594
NLE1_HUMAN	3623.94238
NECP2_HUMAN	11283.9658
RPC2_HUMAN	4438.43213
RBM28_HUMAN	4048.85156
ANO10_HUMAN	9106.96875
RBM22_HUMAN	2988.09619
WDR70_HUMAN	1536.8291
TMM51_HUMAN	315.709137
ARGL1_HUMAN	3317.48242
SLTM_HUMAN	3749.22485
FKB14_HUMAN	1245.89978
RMND1_HUMAN	3087.7207
PK1IP_HUMAN	2837.55078
HIF1N_HUMAN	1295.03894
OXSM_HUMAN	7286.80615
RM22_HUMAN	15720.5439
CZIB_HUMAN	13930.5693
BABA1_HUMAN	3587.82544
CLN6_HUMAN	351.724304
ASB6_HUMAN	3044.80566
THG1_HUMAN	556.570435
HPF1_HUMAN	1891.41284
IRAK4_HUMAN	1941.89539
UCKL1_HUMAN	2571.8667
TM160_HUMAN	3128.5459
NALP2_HUMAN	4066.95313
F120C_HUMAN	1037.69543
TSAP1_HUMAN	563.138672
NDUBB_HUMAN	1867.78479
RM16_HUMAN	8912.25
NHP2_HUMAN	6401.03516

OCAD1_HUMAN	7205.33594
ADPRS_HUMAN	10069.8096
MARH5_HUMAN	3809.51636
LYAR_HUMAN	5065.50928
T161A_HUMAN	819.200806
IMPA3_HUMAN	4877.61719
MIC19_HUMAN	11216.9688
DUS2L_HUMAN	191.22261
CKLF6_HUMAN	588.267517
SIR5_HUMAN	1136.12024
MIOS_HUMAN	888.462341
MTMRA_HUMAN	3645.44189
NSMA3_HUMAN	3888.56763
CWC25_HUMAN	1715.69775
TEX10_HUMAN	4094.63599
DCA16_HUMAN	1635.229
THUM1_HUMAN	4612.07178
P4HTM_HUMAN	290.046722
TOR4A_HUMAN	3504.57764
TRM1_HUMAN	4461.48486
GDAP2_HUMAN	734.537598
NDE1_HUMAN	1294.82361
BABA2_HUMAN	1113.21692
QPCTL_HUMAN	2368.48071
ARL15_HUMAN	2015.03198
KCTD5_HUMAN	2096.29028
CARF_HUMAN	2650.37134
DJB12_HUMAN	2718.94678
ALKB4_HUMAN	876.708313
NSE4A_HUMAN	504.165527
GAR1_HUMAN	5771.1123
STAB1_HUMAN	9271.39063
PP4R2_HUMAN	1677.90405
DPP3_HUMAN	22795.4297
AATF_HUMAN	1283.9032
TBA8_HUMAN	4521.4707
DDX56_HUMAN	3200.43042
SPHK1_HUMAN	2874.57739
TE2IP_HUMAN	281.728973
ABI2_HUMAN	2614.75146
BCLF1_HUMAN	11171.3057
UTP6_HUMAN	2198.15381
TAB2_HUMAN	130.706909
RM39_HUMAN	7252.17969
M3K20_HUMAN	2487.71436
FKB11_HUMAN	12713.7959
TMOD3_HUMAN	8896.60254
BET1L_HUMAN	2053.26953
ELOV5_HUMAN	2755.02368
KBRS2_HUMAN	1469.00159

UGGG2_HUMAN	672.791748
UGGG1_HUMAN	25394.1445
CDK12_HUMAN	2824.18994
FAKD2_HUMAN	4017.09888
TECR_HUMAN	6653.79736
ERAP1_HUMAN	12713.9102
ARP10_HUMAN	3867.52686
USE1_HUMAN	1470.56409
CISD1_HUMAN	2073.46045
GGA3_HUMAN	1671.93591
TLS1_HUMAN	848.25354
F120A_HUMAN	9139.92969
GDE1_HUMAN	826.072815
SMAL1_HUMAN	1216.1925
GLTP_HUMAN	2107.24048
SPG21_HUMAN	3813.09424
RM35_HUMAN	2956.9873
UBIP1_HUMAN	960.488953
IF2B1_HUMAN	17516.5898
SACS_HUMAN	2325.03247
MTCH1_HUMAN	6920.01758
NUDT4_HUMAN	7331.93701
HPBP1_HUMAN	8095.44238
MAT2B_HUMAN	29002.7754
MYOF_HUMAN	44587.2656
ITSN2_HUMAN	3036.64282
NOP53_HUMAN	1599.91479
EHD3_HUMAN	44894.0898
EHD2_HUMAN	63223.3086
ARHGC_HUMAN	2976.11426
CNOT2_HUMAN	4960.41357
SPN90_HUMAN	1398.46826
PD1L1_HUMAN	1488.32764
CALL5_HUMAN	888.914551
OGFR_HUMAN	6005.29883
LMCD1_HUMAN	931.934143
CRIM1_HUMAN	2289.67896
SELN_HUMAN	4317.30225
PALS2_HUMAN	3917.41431
CHMP5_HUMAN	5761.42725
CWC15_HUMAN	2236.84839
RM15_HUMAN	10290.3848
THYN1_HUMAN	7376.91846
TAP26_HUMAN	245.89212
NDUF4_HUMAN	3200.27637
HACD3_HUMAN	11295.1777
EMC3_HUMAN	2934.24854
NDUAD_HUMAN	7052.85791
PDP1_HUMAN	4825.16016
KCMF1_HUMAN	1343.78442

RAI14_HUMAN	8970.57813
VAPA_HUMAN	15048.9238
RM27_HUMAN	4566.50488
TMEM9_HUMAN	1123.06104
SENP1_HUMAN	1243.85889
CXXC1_HUMAN	291.835907
SH3B4_HUMAN	7693.73779
SEP10_HUMAN	10489.915
GMIP_HUMAN	1175.51221
PHRF1_HUMAN	4141.20801
POGK_HUMAN	1086.88293
DAPLE_HUMAN	225.82283
RHG23_HUMAN	888.784302
VPS18_HUMAN	8881.12207
RCC2_HUMAN	39925.9063
RELCH_HUMAN	788.164001
DIP2B_HUMAN	4822.36865
JCAD_HUMAN	2095.70459
SLAI2_HUMAN	569.256714
TEN3_HUMAN	1315.71094
UBP36_HUMAN	663.963867
BCCIP_HUMAN	13546.4326
STK26_HUMAN	1226.63721
ARMX1_HUMAN	2713.27002
ABI3_HUMAN	7054.271
FPRP_HUMAN	12710.4883
CT2NL_HUMAN	5595.42285
TM181_HUMAN	1996.14563
IBTK_HUMAN	186.094635
HTR5B_HUMAN	1810.245
ZNFX1_HUMAN	1643.76343
CHPF2_HUMAN	3195.84058
PCD10_HUMAN	439.55957
RRBP1_HUMAN	34981.6328
CPSF2_HUMAN	4976.59717
KLHL9_HUMAN	2796.90747
SYLC_HUMAN	26699.4316
MYEF2_HUMAN	9812.25391
KLH42_HUMAN	662.369141
E2AK4_HUMAN	4024.60229
WDR35_HUMAN	749.253723
RBM27_HUMAN	2641.1394
KANL3_HUMAN	939.362427
HECW2_HUMAN	2692.47461
FRM4A_HUMAN	1084.70764
ANFY1_HUMAN	5562.64746
SUCB1_HUMAN	8313.12695
GMPR2_HUMAN	5968.14453
STX18_HUMAN	949.354797
DPM3_HUMAN	892.595276

IMPCT_HUMAN	1301.9137
UVRAG_HUMAN	2342.03613
ATX10_HUMAN	11471.7627
MBD2_HUMAN	3534.68481
NCDN_HUMAN	2420.75781
TFP11_HUMAN	1098.64612
EP15R_HUMAN	9704.49121
MYO1A_HUMAN	6486.67188
ORC3_HUMAN	1658.33862
SAE1_HUMAN	12375.374
COPG2_HUMAN	8329.00293
PI4KB_HUMAN	842.972351
MRC2_HUMAN	1177.46716
COMD3_HUMAN	1185.49292
GBG12_HUMAN	2493.83398
RBL2A_HUMAN	2039.04846
MTRR_HUMAN	471.922546
UXT_HUMAN	899.644104
ASH2L_HUMAN	2771.71411
DHCR7_HUMAN	23587.2793
TR10D_HUMAN	790.967712
HDAC6_HUMAN	1891.24011
SPAST_HUMAN	3247.85864
DKK3_HUMAN	745.414246
TRMB_HUMAN	6227.90527
GULP1_HUMAN	1474.62891
VPS29_HUMAN	14470.6211
EIF3K_HUMAN	21757.9277
EXTL2_HUMAN	1781.28467
GRHPR_HUMAN	13343.8291
CATZ_HUMAN	17471.9961
KS6B2_HUMAN	1078.5271
DJB11_HUMAN	11920.5947
RNF14_HUMAN	2508.65698
SAE2_HUMAN	18021.4453
MO4L1_HUMAN	1591.02002
NXF1_HUMAN	7656.68262
SE1L1_HUMAN	1384.69897
B4GT7_HUMAN	2207.08472
PEF1_HUMAN	2716.87109
ZMYM2_HUMAN	1035.76147
CSN7A_HUMAN	7416.88135
DIC_HUMAN	7289.19238
AASS_HUMAN	6490.89941
CLIP2_HUMAN	1325.74097
QCR9_HUMAN	9905.50391
MTFP1_HUMAN	2191.56128
ZO2_HUMAN	21995.3691
DNJB4_HUMAN	14541.585
ST17A_HUMAN	800.259338

CFDP1_HUMAN	3437.65088
DAXX_HUMAN	992.382935
TRM7_HUMAN	2962.69189
VTI1B_HUMAN	2588.05078
STK39_HUMAN	2562.69116
ADDG_HUMAN	2867.07593
LRWD1_HUMAN	2777.36377
NPS3A_HUMAN	7646.7168
CGBP1_HUMAN	3491.95044
PISD_HUMAN	285.51181
ABCF2_HUMAN	13855.1387
TES_HUMAN	6467.63965
AAKG2_HUMAN	3543.54077
GCP4_HUMAN	2884.51074
KDM5B_HUMAN	186.945404
SYWM_HUMAN	1154.02429
LIMD1_HUMAN	1255.65466
SEC63_HUMAN	10604.9277
GTR6_HUMAN	829.481506
Z3H7B_HUMAN	6350.19824
NDRG3_HUMAN	7492.96533
ARMX3_HUMAN	5518.88232
SWP70_HUMAN	20524.1855
SUN2_HUMAN	9221.19824
RLP24_HUMAN	2399.03271
LTOR3_HUMAN	1761.85022
LIMA1_HUMAN	17478.8867
AFF4_HUMAN	7526.05811
SRP68_HUMAN	11885.2139
CHRD1_HUMAN	15482.7559
TBK1_HUMAN	4575.17529
SEPT9_HUMAN	14641.8145
PCYOX_HUMAN	8039.09326
DDX20_HUMAN	6201.02734
SHPK_HUMAN	3953.34546
DPP2_HUMAN	2770.58936
CEIP2_HUMAN	3927.05615
UBP25_HUMAN	3042.06738
BAP29_HUMAN	4260.11279
NB5R1_HUMAN	6211.81592
S30BP_HUMAN	3236.03052
ZNHI2_HUMAN	10158.8779
PFD2_HUMAN	8439.90723
GPN3_HUMAN	1821.82361
PUF60_HUMAN	19782.8848
NRBP_HUMAN	5380.23291
ENOPH_HUMAN	8066.30225
FEZ2_HUMAN	901.964478
NDUAC_HUMAN	5326.17822
EI2BD_HUMAN	12641.9824

VATH_HUMAN	6070.31641
PRAF1_HUMAN	2656.14771
IPO11_HUMAN	2281.3457
TR112_HUMAN	535.345337
DACH1_HUMAN	328.974579
XPO7_HUMAN	6178.64844
LCMT1_HUMAN	3645.69165
VPS51_HUMAN	4239.21338
BAZ2B_HUMAN	751.025574
BAZ2A_HUMAN	580.058655
BAZ1B_HUMAN	4330.78711
ATIF1_HUMAN	1026.58203
KAD3_HUMAN	18066.7988
LCAP_HUMAN	8693.65625
MBD1_HUMAN	545.178223
CNOT7_HUMAN	2266.99609
RABX5_HUMAN	3203.10474
MSRA_HUMAN	2078.19556
NAGK_HUMAN	27170.6152
HACL1_HUMAN	3424.53882
TRM6_HUMAN	1612.11975
SH3L2_HUMAN	2102.12744
NGAP_HUMAN	698.711548
TSR3_HUMAN	1180.60364
S2513_HUMAN	15205.7988
DBNL_HUMAN	12231.3662
DDX41_HUMAN	5750.58008
DCTN4_HUMAN	7745.32129
CDC23_HUMAN	3630.40527
APC7_HUMAN	5163.02783
APC5_HUMAN	1041.9873
APC4_HUMAN	1877.92322
ANC2_HUMAN	2321.70752
GGA2_HUMAN	5607.9873
GGA1_HUMAN	2444.54883
STML2_HUMAN	14576.9951
NAGPA_HUMAN	550.784973
VPS28_HUMAN	7374.10938
LSM7_HUMAN	1245.72705
DBR1_HUMAN	3250.25146
TASOR_HUMAN	310.197876
JUPI1_HUMAN	505.120972
FBX3_HUMAN	1307.03809
AKA11_HUMAN	353.152435
FBW1B_HUMAN	1074.48999
GMEB2_HUMAN	1252.5199
MRT4_HUMAN	18909.2813
TNIK_HUMAN	1447.76758
CPSF3_HUMAN	5620.33984
DP13A_HUMAN	4941.84619

BORG2_HUMAN	1009.11987
TLK1_HUMAN	792.676819
GPTC8_HUMAN	1536.58679
PARP4_HUMAN	10370.4639
NXT1_HUMAN	915.670471
NUDT5_HUMAN	10640.3232
RCOR1_HUMAN	1063.9502
PPCT_HUMAN	1875.43701
MA1B1_HUMAN	1637.65698
RALY_HUMAN	19542.6094
TF3C4_HUMAN	4827.45117
ERG28_HUMAN	2117.72778
PACN3_HUMAN	1086.57605
ACAD8_HUMAN	1421.07166
ANGL2_HUMAN	2621.96313
ACINU_HUMAN	11936.4795
AMFR_HUMAN	3721.62695
AGO2_HUMAN	4816.54736
NUP50_HUMAN	8285.02344
CDV3_HUMAN	20282.6309
CNO11_HUMAN	1023.50159
INT6_HUMAN	1900.46997
BAG5_HUMAN	3121.39722
AGO1_HUMAN	4184.70166
RAB21_HUMAN	8894.9502
RB22A_HUMAN	4981.65283
TPC2L_HUMAN	2067.63135
ZN346_HUMAN	2523.42578
PSME2_HUMAN	11063.3018
TAOK2_HUMAN	2741.45776
MKLN1_HUMAN	3825.9939
DNPEP_HUMAN	6937.04688
RAB23_HUMAN	3871.69385
MCTS1_HUMAN	16150.8896
MTUS1_HUMAN	381.824677
WWC3_HUMAN	502.997314
PALD_HUMAN	2921.47192
S39AA_HUMAN	2460.56128
INO80_HUMAN	579.602844
CCPG1_HUMAN	1070.00378
KDIS_HUMAN	798.985168
ASAP1_HUMAN	3694.18457
MRTFB_HUMAN	1159.59399
ANR50_HUMAN	5063.83447
MED23_HUMAN	2245.82495
SHRM4_HUMAN	3059.32861
YETS2_HUMAN	1154.09277
NDRG4_HUMAN	1206.42261
TBC24_HUMAN	2107.69482
ISY1_HUMAN	1000.51141

TMCC3_HUMAN	1919.90649
HECD1_HUMAN	4856.979
ZMYD8_HUMAN	1889.07324
CIZ1_HUMAN	386.819031
COR1C_HUMAN	21536.9453
TPX2_HUMAN	1925.60144
ABT1_HUMAN	2306.31567
NOB1_HUMAN	6406.32275
AKP8L_HUMAN	4027.06079
MAFF_HUMAN	1362.52954
ASC_HUMAN	4242.11621
TMCO1_HUMAN	11067.916
FZR1_HUMAN	273.15213
APC10_HUMAN	1290.37854
MYO6_HUMAN	5397.88721
DD19B_HUMAN	1928.51416
PPT2_HUMAN	1772.90613
PRP19_HUMAN	23622.377
SUFU_HUMAN	678.285645
BOK_HUMAN	2647.56079
NOL7_HUMAN	3365.92554
SNX12_HUMAN	9547.64844
SYNRG_HUMAN	737.456116
VPS4A_HUMAN	8766.63184
G3BP2_HUMAN	11314.7861
CHIP_HUMAN	5822.62207
PACN2_HUMAN	9885.2002
MAGD2_HUMAN	18281.9922
SNX7_HUMAN	1724.63647
SNX6_HUMAN	7673.58252
DUS12_HUMAN	5071.43848
STX8_HUMAN	1054.90564
SSRG_HUMAN	5874.38672
PSD13_HUMAN	36034.2148
FAF1_HUMAN	6563.54883
EPCR_HUMAN	21640.2422
PPIE_HUMAN	1570.56079
DIM1_HUMAN	5209.75537
CSN3_HUMAN	10486.752
MINP1_HUMAN	3170.08716
NOVA2_HUMAN	6390.69385
WDR3_HUMAN	5812.7793
TTF2_HUMAN	311.582336
NSF1C_HUMAN	8402.39551
COG5_HUMAN	2862.81348
S12A4_HUMAN	7131.57471
AP4E1_HUMAN	286.493713
MACF1_HUMAN	42107.8477
CP131_HUMAN	1029.32776
SCAF8_HUMAN	2465.52466

PP6R1_HUMAN	2746.76758
TRI33_HUMAN	3064.35767
PHF8_HUMAN	1211.54102
TNR6B_HUMAN	732.639404
PLCL2_HUMAN	637.552246
SMG5_HUMAN	1188.4624
EXOC7_HUMAN	9694.08594
ZC3H4_HUMAN	149.958115
UBP24_HUMAN	3462.33984
TBD2B_HUMAN	1810.13879
SMAG1_HUMAN	1260.12244
FOXJ3_HUMAN	1055.4729
CBPC1_HUMAN	705.956177
DICER_HUMAN	866.190674
XCT_HUMAN	2318.84717
MARE3_HUMAN	1488.01514
SHOC2_HUMAN	4451.41016
SRRM2_HUMAN	11594.5898
MGT4B_HUMAN	306.24176
PA2G4_HUMAN	79075.8672
SPG7_HUMAN	1355.65259
BAIP2_HUMAN	730.097717
GAB2_HUMAN	283.682648
SMC3_HUMAN	7069.4126
CHM2B_HUMAN	13766.583
SH2B3_HUMAN	1111.14673
ZN148_HUMAN	313.150055
MTMR6_HUMAN	3844.41455
JAG2_HUMAN	1488.93127
NIP7_HUMAN	3900.9939
GLCNE_HUMAN	3347.81616
RTRAF_HUMAN	10725.7041
RUVB2_HUMAN	30268.8555
CDYL_HUMAN	681.78479
PIN4_HUMAN	5818.28857
AKT3_HUMAN	1393.73828
FACE2_HUMAN	832.215637
CHKB_HUMAN	390.403351
EIF3L_HUMAN	54128.6367
PLAP_HUMAN	12284.9492
RUVB1_HUMAN	49651.0391
NUDC_HUMAN	28917.6406
BCS1_HUMAN	2741.26514
VDAC3_HUMAN	24152.6777
COF2_HUMAN	22845.0195
ERGI3_HUMAN	3898.97607
SYFA_HUMAN	24843.7461
SC5A6_HUMAN	1500.14832
RT33_HUMAN	5456.61035
ASF1A_HUMAN	2324.44604

DRG1_HUMAN	13982.4238
TPPC4_HUMAN	3657.40137
NCKP1_HUMAN	11798.9473
CNPY2_HUMAN	6456.90674
EXOG_HUMAN	1059.16943
EXC6B_HUMAN	2883.97461
PLAK2_HUMAN	17292.3223
CARD8_HUMAN	2356.06836
AT11B_HUMAN	257.214325
OFUT2_HUMAN	5610.95068
DJC16_HUMAN	548.205811
DLGP4_HUMAN	2737.96533
ST38L_HUMAN	2618.5293
SAC2_HUMAN	2266.58276
FND3A_HUMAN	4600.61035
NISCH_HUMAN	1330.68811
FYV1_HUMAN	989.3255
WDR37_HUMAN	2146.03198
E41L3_HUMAN	22162.9121
AMOL2_HUMAN	170.869843
SIK3_HUMAN	200.002502
KDM2A_HUMAN	1495.8241
RRP44_HUMAN	6883.9082
TPPC8_HUMAN	2021.51172
LRCH1_HUMAN	4574.06396
RCL1_HUMAN	5865.65771
GSTK1_HUMAN	7707.89209
LTOR2_HUMAN	2032.78394
RT28_HUMAN	10643.3389
COA3_HUMAN	4442.14258
DDX52_HUMAN	2590.46704
RT17_HUMAN	5027.96484
RT07_HUMAN	8701.64844
CRYL1_HUMAN	3067.26465
TMA7_HUMAN	5914.17139
PDIP2_HUMAN	10081.4932
AP3M1_HUMAN	13856.29
MAN1_HUMAN	4293.33984
CHSP1_HUMAN	911.54248
COG6_HUMAN	2044.55029
TR150_HUMAN	15858.8799
WBP11_HUMAN	4179.86865
TDRKH_HUMAN	694.152222
MED16_HUMAN	1037.13135
NOP58_HUMAN	15339.9736
GIT1_HUMAN	6202.24951
ZN281_HUMAN	160.769989
AR2BP_HUMAN	3452.32495
TMM98_HUMAN	776.509521
SGT1_HUMAN	27120.9902

SYYM_HUMAN	6686.13965
NAGA_HUMAN	3802.01782
ACOT9_HUMAN	24269.832
AAR2_HUMAN	1221.25745
NOSIP_HUMAN	5553.34424
DEOC_HUMAN	4801.45752
MEMO1_HUMAN	5905.90869
TMX2_HUMAN	5603.38232
FCF1_HUMAN	1825.11841
LSM2_HUMAN	4407.06348
SHLB1_HUMAN	10465.96
CIA30_HUMAN	693.19397
CAB39_HUMAN	14071.3193
LC7L2_HUMAN	12544.5488
UB2J1_HUMAN	5899.47461
RBMX2_HUMAN	680.477234
DHRS7_HUMAN	5045.13477
RT02_HUMAN	11289.6309
UTP11_HUMAN	916.302917
PHOCN_HUMAN	12942.1328
RRP7A_HUMAN	2217.20654
SBDS_HUMAN	15953.1729
TMED5_HUMAN	8903.38965
EXOS1_HUMAN	3666.34741
TMED7_HUMAN	4965.35645
SF3B6_HUMAN	12114.583
RM11_HUMAN	11038.1221
ORN_HUMAN	17537.0801
RRP15_HUMAN	5299.8291
WASC3_HUMAN	1079.33325
NOP16_HUMAN	7093.64209
TPRKB_HUMAN	3707.36841
PPIL1_HUMAN	2976.66919
UFC1_HUMAN	5070.19043
CIA2B_HUMAN	535.02063
RT16_HUMAN	4933.52686
RT18C_HUMAN	727.505493
FIS1_HUMAN	1509.255
TIM16_HUMAN	2664.61987
KAD6_HUMAN	4338.99072
RT23_HUMAN	12466.3154
GOT1B_HUMAN	2976.89624
HDGR3_HUMAN	2655.80103
PTH2_HUMAN	8424.2207
CHMP3_HUMAN	43431.7383
STRAP_HUMAN	18918.0723
RTCB_HUMAN	44713.5547
FBX7_HUMAN	3631.89478
RAP2C_HUMAN	1829.21472
STA13_HUMAN	3281.56177

RBGP1_HUMAN	1586.11047
TMED3_HUMAN	1133.30444
T22D4_HUMAN	1245.77124
DOP2_HUMAN	339.488495
ZN330_HUMAN	803.99884
R3HC1_HUMAN	1090.20544
NOC2L_HUMAN	9438.84668
RL36_HUMAN	59438.9063
CCDC9_HUMAN	1829.12146
CHTOP_HUMAN	2267.12622
SAMH1_HUMAN	6230.32031
SKAP_HUMAN	953.650757
HBS1L_HUMAN	4628.69922
AAKB1_HUMAN	988.158936
WIPI4_HUMAN	517.53009
DMXL1_HUMAN	878.230103
VPP2_HUMAN	2976.68066
TLN1_HUMAN	94322.8594
KIF3A_HUMAN	1654.39734
TRRAP_HUMAN	2565.50903
MTCL1_HUMAN	857.019226
DCAF1_HUMAN	702.056091
TCAF1_HUMAN	8093.37012
RBM19_HUMAN	1172.09106
DAAM1_HUMAN	553.519531
PLXD1_HUMAN	17038.2109
HECD4_HUMAN	481.689514
WDR7_HUMAN	338.366028
UBP15_HUMAN	7589.90723
FARP1_HUMAN	1449.61243
MARF1_HUMAN	612.339233
C170B_HUMAN	749.981995
RPGF2_HUMAN	9058.88086
MYO5A_HUMAN	5364.69287
DTNA_HUMAN	1158.3667
LOXL2_HUMAN	6335.11572
CRBG1_HUMAN	1373.9325
TRAF6_HUMAN	2139.02637
M4K5_HUMAN	3625.23267
HYOU1_HUMAN	28836.9121
ATG4B_HUMAN	6991.64063
TBL2_HUMAN	4439.1123
WIPI2_HUMAN	3047.84009
TELO2_HUMAN	2656.76831
LAS1L_HUMAN	6607.80078
AFG32_HUMAN	9014.94629
KLF12_HUMAN	283.294403
ARI1_HUMAN	5633.31641
LSM4_HUMAN	26436.2266
RN114_HUMAN	3278.57813

SAM50_HUMAN	16117.5537
PRC2C_HUMAN	19735.623
OARD1_HUMAN	8433.6543
IFT25_HUMAN	713.411621
PPME1_HUMAN	9206.76367
RBM7_HUMAN	2067.99585
TIM22_HUMAN	1604.43848
NUB1_HUMAN	1598.33044
YTHD2_HUMAN	2983.38403
CTDP1_HUMAN	3318.79468
PAXB1_HUMAN	1157.91443
NDK7_HUMAN	3196.2041
SP16H_HUMAN	14898.3975
UTP18_HUMAN	4118.38086
UCHL5_HUMAN	17393.459
CD2AP_HUMAN	11010.8369
VATD_HUMAN	6005.01074
TNPO3_HUMAN	6458.17822
SRPRB_HUMAN	13484.1279
ORC6_HUMAN	592.351074
CERT_HUMAN	1225.83582
GMPPB_HUMAN	17001.0098
FADS3_HUMAN	1256.22644
TF3C5_HUMAN	6313.55566
TF3C3_HUMAN	3617.57446
TPPC1_HUMAN	5508.98389
TRPV2_HUMAN	837.604675
MRCKB_HUMAN	11016.0049
RBM8A_HUMAN	16006.0967
DJC15_HUMAN	819.758362
UBP16_HUMAN	1418.24719
TSSC4_HUMAN	1802.04956
ZN706_HUMAN	4732.60107
MAGD1_HUMAN	3281.95508
SNX14_HUMAN	553.418518
SNX11_HUMAN	1101.15198
SNX9_HUMAN	11277.5918
SNX8_HUMAN	1385.37134
SNX5_HUMAN	6117.67627
LIPG_HUMAN	2111.00269
NUBP2_HUMAN	6006.88623
PEX16_HUMAN	906.067627
LYVE1_HUMAN	2403.52441
BACE2_HUMAN	1354.10034
HEBP2_HUMAN	498.033386
HCFC2_HUMAN	755.015503
UBIA1_HUMAN	1707.3866
PUS1_HUMAN	5029.76904
LRRF2_HUMAN	3531.20874
FHOD1_HUMAN	7476.08057

SERC_HUMAN	20341.9473
NCOR2_HUMAN	2311.87085
ORNT1_HUMAN	1292.48962
JAM1_HUMAN	16464.8711
NPTN_HUMAN	1654.17554
AGRG1_HUMAN	1076.03479
SPIN1_HUMAN	2302.53809
S12A7_HUMAN	9679.35645
ALG6_HUMAN	1393.62292
ALG5_HUMAN	7965.47949
RT18B_HUMAN	4731.5459
COPG1_HUMAN	31543.4512
AUP1_HUMAN	3023.24219
FKBP7_HUMAN	1552.07544
CLIC4_HUMAN	49277.7344
NFS1_HUMAN	3672.2959
CFA20_HUMAN	9766.71289
TACC3_HUMAN	565.693542
SPCS1_HUMAN	6185.61914
SAR1B_HUMAN	5227.9502
AP4B1_HUMAN	1028.94824
MTCH2_HUMAN	23625.6309
SELK_HUMAN	805.600403
BIG2_HUMAN	3586.85156
BIG1_HUMAN	4443.81006
MD1L1_HUMAN	3122.40405
STK24_HUMAN	13039.6738
5MP1_HUMAN	42345.3047
COMDA_HUMAN	2886.01221
DC1L1_HUMAN	19184.9258
EPN1_HUMAN	3435.52759
UBP3_HUMAN	18603.918
TX264_HUMAN	6140.17725
CEPT1_HUMAN	8268.39551
DNM3A_HUMAN	762.626221
OAS3_HUMAN	1532.48486
KAD5_HUMAN	11957.1533
NEMO_HUMAN	3913.11548
IF2B2_HUMAN	22435.752
ZNT1_HUMAN	3572.09473
S4A7_HUMAN	2370.9646
NDUB9_HUMAN	15025.7559
SQOR_HUMAN	2507.0564
ROBO1_HUMAN	697.028259
STON1_HUMAN	419.663391
NCOA3_HUMAN	530.43042
NUMBL_HUMAN	2177.67798
M3K4_HUMAN	1202.90881
DDX49_HUMAN	1599.93347
CAN7_HUMAN	1199.00525

WASF2_HUMAN	10433.2461
PIAS3_HUMAN	360.965332
SCC4_HUMAN	1479.9259
MORC2_HUMAN	969.707642
NS1BP_HUMAN	1958.61938
S23IP_HUMAN	16721.168
GAL3B_HUMAN	3592.32739
IFT56_HUMAN	3799.21729
UBA6_HUMAN	17528.7793
ESYT2_HUMAN	13593.3008
BLT3B_HUMAN	88.2252808
SHOT1_HUMAN	2647.4895
TM223_HUMAN	1075.95105
RHG10_HUMAN	3330.14404
HACL2_HUMAN	7405.50049
SPD2B_HUMAN	12101.2988
NBAS_HUMAN	2338.15454
TYW5_HUMAN	1044.4646
DEND3_HUMAN	1554.84631
VWA8_HUMAN	2155.87939
SBNO1_HUMAN	2196.91479
GTPBA_HUMAN	1271.59241
WDR91_HUMAN	603.289917
DEN11_HUMAN	422.671722
PDES1_HUMAN	773.203735
TPC13_HUMAN	1471.81494
CNOT1_HUMAN	8615.59375
MP3B2_HUMAN	30852.8477
PGP_HUMAN	7937.97119
RIMC1_HUMAN	1013.69116
RCCD1_HUMAN	774.909119
TCAF2_HUMAN	8683.70508
SMHD1_HUMAN	5885.54395
CAST2_HUMAN	2042.9364
U119B_HUMAN	4454.10938
MET15_HUMAN	497.491302
CC85C_HUMAN	668.729492
RTL8C_HUMAN	2857.35229
ZSWM8_HUMAN	876.168884
P121C_HUMAN	2909.16016
P20L1_HUMAN	606.953857
RUXGL_HUMAN	14877.373
ELAP2_HUMAN	643.750366
NUD19_HUMAN	2606.80078
MI4GD_HUMAN	831.27124
CALM1_HUMAN	3170.03418
GPHRA_HUMAN	1542.71948
WASH3_HUMAN	7086.85791
MCRI1_HUMAN	300.304993
NACAM_HUMAN	58039.6016

CK098_HUMAN	4420.97998
ASURF_HUMAN	827.336304
MIDUO_HUMAN	3333.76172
DNS2A_HUMAN	3384.87329
ADAS_HUMAN	13186.8867
KIF2A_HUMAN	8760.05566
DX39A_HUMAN	79116.7969
PDLI1_HUMAN	7921.24854
BACH_HUMAN	46577.7109
MYO1C_HUMAN	30901.3438
SNP23_HUMAN	9516.52734
HAX1_HUMAN	1895.02161
AIP_HUMAN	7066.31104
GTPB1_HUMAN	4598.19482
LEG9_HUMAN	245.042892
STXB3_HUMAN	8118.68799
AP4M1_HUMAN	1909.68542
ARVC_HUMAN	4354.28564
SMAP_HUMAN	1110.56921
AP3B1_HUMAN	13166.4619
APBB1_HUMAN	1565.8407
LEG8_HUMAN	1166.21509
NDUS8_HUMAN	489.090118
IKBE_HUMAN	1276.8988
PSD11_HUMAN	37875.7539
PSD12_HUMAN	26933.7988
PSMD9_HUMAN	2881.52515
MEN1_HUMAN	479.569763
GET1_HUMAN	2735.44873
PGRC1_HUMAN	1501.06372
SPT5H_HUMAN	4036.49512
TAF4_HUMAN	227.490662
DFFA_HUMAN	1593.93823
HIP1_HUMAN	2019.06396
CLIC1_HUMAN	203344.766
EIF3F_HUMAN	26234.4219
WWP2_HUMAN	728.784912
ODPX_HUMAN	3687.21802
QSOX1_HUMAN	3512.35425
DCTN6_HUMAN	1337.49939
ACATN_HUMAN	4490.04053
WASL_HUMAN	5324.74609
IPO5_HUMAN	40921.5078
RPOM_HUMAN	864.102112
EF2K_HUMAN	3046.25366
SAP18_HUMAN	11615.1094
EMAL1_HUMAN	2797.84253
IF2B3_HUMAN	37057.1016
DNM1L_HUMAN	41162.4844
RTCA_HUMAN	6120.20264

P3C2A_HUMAN	5532.86963
GOLI4_HUMAN	2533.34351
AGRIN_HUMAN	1730.15125
PLOD2_HUMAN	42445.8555
EXOC5_HUMAN	8282.50781
BT3A1_HUMAN	399.949341
NDUA4_HUMAN	5002.72021
PSDE_HUMAN	42578.7148
BIN1_HUMAN	3719.55835
CLD5_HUMAN	2182.79468
IMA4_HUMAN	23442.6602
STK25_HUMAN	3533.90283
KRIT1_HUMAN	419.508728
VMA5A_HUMAN	843.828186
PESC_HUMAN	8863.18066
SDCB1_HUMAN	44526.0156
PITM1_HUMAN	442.170471
MPP10_HUMAN	4174.91162
NOP56_HUMAN	6246.02148
DDX3X_HUMAN	70335.3047
MFNG_HUMAN	199.113647
PODXL_HUMAN	42938.1094
CCN1_HUMAN	45095.6289
PIR_HUMAN	30154.6719
IMA3_HUMAN	6577.7373
TRI38_HUMAN	609.205505
NFIB_HUMAN	5434.19775
PPP6_HUMAN	8794.75879
NDKM_HUMAN	2153.25269
MA2B1_HUMAN	2398.1123
PDXK_HUMAN	2285.90063
SCD_HUMAN	4578.53027
ARI1A_HUMAN	2700.90234
NEMP1_HUMAN	863.404907
TXND9_HUMAN	4064.15137
DPYL4_HUMAN	39830.7109
TRAD1_HUMAN	1410.49097
COX7R_HUMAN	667.612244
UBFD1_HUMAN	5380.48535
CTRO_HUMAN	1778.04077
COPE_HUMAN	16782.8828
BORG1_HUMAN	2725.62451
AP3D1_HUMAN	12123.9326
CCS_HUMAN	956.547363
ABLM1_HUMAN	3557.55737
DVL2_HUMAN	1146.5835
CHD1_HUMAN	3543.62036
CHD2_HUMAN	3968.92358
GOSR2_HUMAN	5061.8833
TOR1A_HUMAN	5937.54785

TOR1B_HUMAN	3254.90381
STX16_HUMAN	2291.3833
ADA10_HUMAN	9732.21582
ABCD4_HUMAN	1358.12634
EI24_HUMAN	4595.64307
P5I11_HUMAN	2057.84326
KMT2D_HUMAN	232.738327
APAF_HUMAN	2265.0166
RIOK3_HUMAN	496.956635
MP2K7_HUMAN	608.638611
ACOT8_HUMAN	1612.96228
CDIPT_HUMAN	34240.2109
PDCD5_HUMAN	2342.90234
ANM5_HUMAN	18625.7305
NHRF1_HUMAN	4190.28223
CHK1_HUMAN	1270.28369
TR10B_HUMAN	5045.30469
TPP1_HUMAN	12180.1797
TCRG1_HUMAN	16866.4746
NDC80_HUMAN	2339.06714
NRP1_HUMAN	13929.1201
TNPO2_HUMAN	2697.01587
UN13B_HUMAN	807.305237
TR10C_HUMAN	199.516037
RPC1_HUMAN	1912.4939
TSN4_HUMAN	395.591461
PSA7_HUMAN	92217.625
SCAM3_HUMAN	4901.5874
BCKD_HUMAN	2307.47363
MGST3_HUMAN	6875.36035
TX1B3_HUMAN	10810.3779
GIPC1_HUMAN	6715.62451
IKKB_HUMAN	2377.96387
RGS12_HUMAN	1661.70203
TIM23_HUMAN	1820.69897
HAT1_HUMAN	9236.59961
CSKP_HUMAN	3160.99194
PLD2_HUMAN	1161.04639
QCR8_HUMAN	11562.8887
ML12B_HUMAN	89316.7969
HGS_HUMAN	6881.77881
RAB7L_HUMAN	751.71521
VP26C_HUMAN	1527.96204
MYPT1_HUMAN	11549.834
GAK_HUMAN	5778.83691
HNRDL_HUMAN	31709.6426
XPO1_HUMAN	27441.2344
BTAF1_HUMAN	1597.63171
ARHGA_HUMAN	3627.43384
ZN609_HUMAN	1324.37305

SPTN2_HUMAN	1015.14716
MAST4_HUMAN	1527.10876
SC16A_HUMAN	6789.54004
PLXB2_HUMAN	2351.21313
KHNYN_HUMAN	520.599915
TCPR2_HUMAN	541.346802
SR140_HUMAN	23886.6289
SET1A_HUMAN	782.461426
N4BP3_HUMAN	364.780334
SYNJ2_HUMAN	4555.32715
SYNEM_HUMAN	6063.95801
GRRE1_HUMAN	1031.58765
KIF3B_HUMAN	1394.84619
PUR4_HUMAN	22905.3848
ANR28_HUMAN	1623.74866
ARHGB_HUMAN	481.417847
MRPP3_HUMAN	901.831604
IKKA_HUMAN	1708.51514
NPC1_HUMAN	4104.92725
DEGS1_HUMAN	2565.37622
ANGP2_HUMAN	2740.63696
SCAM1_HUMAN	5092.646
SCAM2_HUMAN	2361.1272
ARC1B_HUMAN	37842.6133
ARPC2_HUMAN	38356.5313
ARPC3_HUMAN	46692.6328
RPAC1_HUMAN	9607.92676
PLS1_HUMAN	1027.75403
TIF1A_HUMAN	558.224915
PGRC2_HUMAN	10757.7988
RGL2_HUMAN	1342.93152
WDR46_HUMAN	1265.0658
NKRF_HUMAN	2333.62109
GNPAT_HUMAN	2529.71509
LAMA5_HUMAN	1088.37781
ZN185_HUMAN	11621.7891
CASC3_HUMAN	656.13031
RT12_HUMAN	2420.16479
ACOX3_HUMAN	2116.93774
RER1_HUMAN	14488.2354
SURF4_HUMAN	21421.4766
MK13_HUMAN	1584.39941
SPTC1_HUMAN	6367.75879
SPTC2_HUMAN	8285.20313
OGT1_HUMAN	6785.2002
PMM2_HUMAN	14930.0908
TM9S1_HUMAN	2569.46777
TRI18_HUMAN	1029.19092
HMGB3_HUMAN	20418.3789
PPM1G_HUMAN	12799.6611

SHIP2_HUMAN	5753.34326
EIF3D_HUMAN	26250.2969
EIF3H_HUMAN	29976.4824
HDAC3_HUMAN	1547.77783
NVL_HUMAN	1395.30139
BCAT2_HUMAN	8050.66162
IPO8_HUMAN	5058.08447
STX7_HUMAN	6882.74219
MOT4_HUMAN	14674.8105
MRP4_HUMAN	2413.60376
RPA34_HUMAN	2477.81445
P4HA2_HUMAN	6778.30566
CAN5_HUMAN	506.627014
YKT6_HUMAN	46024.8711
NUP42_HUMAN	1457.7356
ARPC5_HUMAN	37381.3672
CFLAR_HUMAN	644.721924
MAFG_HUMAN	509.833252
PDPK1_HUMAN	1446.48535
TPSN_HUMAN	9780.95703
R113A_HUMAN	798.701172
KDM6A_HUMAN	735.557129
DHX15_HUMAN	33923.2188
MCES_HUMAN	6422.11279
ZZEF1_HUMAN	3259.5249
ASAP2_HUMAN	1076.42285
FLRT2_HUMAN	885.804138
TTI1_HUMAN	907.380066
RRP8_HUMAN	2548.20532
PJA2_HUMAN	525.38031
CYB5B_HUMAN	1033.36169
PRP4_HUMAN	7543.71436
SERA_HUMAN	18653.8398
NDUS4_HUMAN	2478.99658
DC1L2_HUMAN	12468.1484
PSMD3_HUMAN	40109.7305
RFOX2_HUMAN	3077.72363
PAPS1_HUMAN	16055.2695
ZW10_HUMAN	6578.38867
B4GT5_HUMAN	183.883347
SNUT1_HUMAN	7890.76807
GPAA1_HUMAN	6983.45508
DAPK3_HUMAN	7140.28369
TGFI1_HUMAN	2584.32861
AP5Z1_HUMAN	767.362488
CTIF_HUMAN	3453.03564
MTSS1_HUMAN	457.641785
VIP2_HUMAN	1712.5636
M3K7_HUMAN	635.267639
MCA3_HUMAN	24576.0996

RIPK2_HUMAN	2540.88257
HNRPR_HUMAN	87987.125
PRPF3_HUMAN	8595.88867
TXNL1_HUMAN	48892.832
TPD54_HUMAN	18196.0371
EMC8_HUMAN	5062.29492
ERI3_HUMAN	2388.94507
SYNJ1_HUMAN	1518.71753
FIBP_HUMAN	2067.37524
IF4G3_HUMAN	26972.2637
PPIH_HUMAN	5263.36426
HTRA2_HUMAN	1739.56909
MS18B_HUMAN	317.528809
ARK72_HUMAN	11304.0791
E41L2_HUMAN	12574.9512
TGON2_HUMAN	988.140564
B4GA1_HUMAN	1036.57324
WIPF1_HUMAN	2297.51855
AT8B1_HUMAN	996.903687
AKA10_HUMAN	243.922409
DENR_HUMAN	15806.6943
XPOT_HUMAN	14613.167
DNPH1_HUMAN	9024.79102
TIM44_HUMAN	9719.97363
TPPC3_HUMAN	15757.0049
CHM2A_HUMAN	9273.07813
NCK2_HUMAN	2030.76367
TSN6_HUMAN	1434.33691
PLRG1_HUMAN	7766.80518
PRC1_HUMAN	1938.41541
RGS10_HUMAN	3040.88281
ZN207_HUMAN	2324.68579
NDUB5_HUMAN	5487.74902
NDUB3_HUMAN	8909.62012
NDUA2_HUMAN	2382.53418
LDB2_HUMAN	1038.48804
GET3_HUMAN	23555.7148
BUB1_HUMAN	483.384003
BUB3_HUMAN	28459.043
ST1B1_HUMAN	7855.47754
ACTN4_HUMAN	70545.5078
BUD23_HUMAN	1229.70129
HTSF1_HUMAN	2378.01392
ERD23_HUMAN	2324.25732
AP1G1_HUMAN	11616.5088
STX6_HUMAN	1039.22473
SNG2_HUMAN	3269.49194
SGTA_HUMAN	2192.66846
ENSA_HUMAN	6217.32373
MCAT_HUMAN	6937.3833

SYNC_HUMAN	38900.9766
MYO1B_HUMAN	6304.18262
PM34_HUMAN	1405.07837
CPSF5_HUMAN	15853.6396
LANC1_HUMAN	9459.42383
STRN_HUMAN	6149.55371
U3IP2_HUMAN	5510.04102
SCO2_HUMAN	1140.32349
CF410_HUMAN	254.900864
AKAP8_HUMAN	2870.21021
GTPB6_HUMAN	632.347595
G6PT1_HUMAN	1128.29688
IDH3B_HUMAN	7424.39502
NRDC_HUMAN	12473.5801
CALU_HUMAN	18245.7891
EDIL3_HUMAN	7358.48535
SAHH2_HUMAN	2447.34595
KIF1C_HUMAN	3087.51782
EXTL3_HUMAN	725.200439
ORC5_HUMAN	1225.19214
NDUS5_HUMAN	13422.0684
PDE6D_HUMAN	3244.68262
ORC4_HUMAN	1169.93066
PEX1_HUMAN	1457.90454
RAD21_HUMAN	7673.13525
DHX16_HUMAN	4405.51172
GMFG_HUMAN	2060.62915
3BP5_HUMAN	2665.3042
MED14_HUMAN	13488.5479
KPRB_HUMAN	6788.94385
SMCA5_HUMAN	7338.47803
JIP4_HUMAN	91122.1953
NUAK1_HUMAN	362.799133
NPA1P_HUMAN	1118.2345
SI1L3_HUMAN	206.734268
ZC3H1_HUMAN	226.563385
AQR_HUMAN	7311.66016
OPA1_HUMAN	6088.96729
ZEB2_HUMAN	479.750916
GANP_HUMAN	2081.875
PCDGC_HUMAN	1135.99255
PI51C_HUMAN	3626.99268
KIF1B_HUMAN	2650.94849
MABP1_HUMAN	1773.34485
KDM1A_HUMAN	4911.30908
TBCD4_HUMAN	8198.49316
FADS1_HUMAN	2707.27197
GSDME_HUMAN	7307.86768
EVI5_HUMAN	281.856781
NRP2_HUMAN	7285.67334

MA1A2_HUMAN	3278.46997
MPZL2_HUMAN	3101.14404
ACSL4_HUMAN	12217.0166
SNX3_HUMAN	29742.3379
OGA_HUMAN	7112.40137
VINEX_HUMAN	3320.49683
HNRPQ_HUMAN	44268.2422
PRP17_HUMAN	1260.88867
RNBP6_HUMAN	39073.3984
NEMF_HUMAN	6059.9585
GMDS_HUMAN	4103.42432
NMT2_HUMAN	5657.10596
CCNT1_HUMAN	1675.97546
BUB1B_HUMAN	1691.14978
PLOD3_HUMAN	24258.7402
IF4E2_HUMAN	5477.80811
DIAP1_HUMAN	62401.5391
TSN3_HUMAN	10839.7822
EXOC3_HUMAN	9954.63184
PLIN3_HUMAN	19588.0879
ANM3_HUMAN	1689.6825
IMA7_HUMAN	10167.6162
UGDH_HUMAN	42182.4492
TPST2_HUMAN	1539.19763
LPXN_HUMAN	2264.91187
CTND1_HUMAN	39927.6992
ICMT_HUMAN	4501.50586
EIF1B_HUMAN	725.448181
SNX2_HUMAN	16614.9082
DPM1_HUMAN	14639.7256
USO1_HUMAN	19174.2793
RT14_HUMAN	2126.30762
TOM1_HUMAN	3590.73438
H2B1K_HUMAN	86408.7734
CCD22_HUMAN	4256.92139
PQBP1_HUMAN	7247.58936
TI17B_HUMAN	571.109619
PRAF2_HUMAN	8399.30176
DKC1_HUMAN	10527.8105
IF2P_HUMAN	33501.0273
EDF1_HUMAN	24026.377
KIN17_HUMAN	1531.93506
DIAP2_HUMAN	7010.36328
DNJA2_HUMAN	9402.84277
BRD4_HUMAN	3889.30029
HUS1_HUMAN	872.782654
PFD1_HUMAN	1187.46484
PP1RB_HUMAN	2152.98926
NBN_HUMAN	10670.9697
MCE1_HUMAN	963.801453

ABCB7_HUMAN	2882.35742
SRGP2_HUMAN	1813.34009
PLXA2_HUMAN	9676.24707
XYLK_HUMAN	648.378479
WDR1_HUMAN	100290.797
ATP9A_HUMAN	2711.31763
N4BP1_HUMAN	727.37146
ROCK2_HUMAN	7510.79199
CLAP2_HUMAN	3092.93433
PTCD1_HUMAN	677.331238
CPNE3_HUMAN	18731.5293
ATG13_HUMAN	1117.12061
HIP1R_HUMAN	8442.02344
BRE1B_HUMAN	4808.84424
PHF2_HUMAN	493.19986
ZC11A_HUMAN	3229.68042
CLU_HUMAN	9365.16992
CAND2_HUMAN	1333.53748
T22D2_HUMAN	320.421326
DJC13_HUMAN	7324.53711
PHAR2_HUMAN	1037.15198
PP6R2_HUMAN	1665.22839
ATS4_HUMAN	1736.83032
CNOT3_HUMAN	5973.30469
ANR17_HUMAN	5818.50049
SIN3B_HUMAN	783.801331
DNJB6_HUMAN	3322.41431
COQ9_HUMAN	1250.78381
GGCT_HUMAN	6721.1665
NDUS7_HUMAN	11149.7158
NDUS2_HUMAN	9975.50293
ZPR1_HUMAN	2740.375
DUS11_HUMAN	1044.30151
NIPS2_HUMAN	8026.95459
HMMR_HUMAN	463.812561
PDCD6_HUMAN	12594.667
TBCA_HUMAN	15099.8477
VATG1_HUMAN	14892.2012
VPS4B_HUMAN	55060.6875
MPU1_HUMAN	18872.1406
H2AY_HUMAN	938.137085
SH3L1_HUMAN	1036.61694
FLNB_HUMAN	124791.398
NCOR1_HUMAN	6916.97656
PEX14_HUMAN	4398.59082
TRIM3_HUMAN	3774.70386
CISY_HUMAN	112229.148
SPAG7_HUMAN	9790.71289
SC22B_HUMAN	40222.1172
PR40A_HUMAN	12071.8154

TACC1_HUMAN	7832.86865
NDK6_HUMAN	1327.74194
CDC45_HUMAN	1057.63867
LRCH4_HUMAN	574.209656
MTX2_HUMAN	1933.29358
VP26A_HUMAN	35151.8906
MPPB_HUMAN	11857.3105
MED24_HUMAN	1065.64905
KTNA1_HUMAN	2127.15063
PSIP1_HUMAN	2522.354
ERLN1_HUMAN	6353.3418
NDUS3_HUMAN	17378.1621
SRS10_HUMAN	7597.14551
CLN5_HUMAN	2008.60339
CLD11_HUMAN	1864.0957
ECI2_HUMAN	15882.2607
KHDR3_HUMAN	1386.48083
EED_HUMAN	2733.90479
BAF_HUMAN	2643.30811
SF3B1_HUMAN	29992.1953
CSDE1_HUMAN	22007.2813
WBP4_HUMAN	908.547668
PRKRA_HUMAN	5229.46582
ITA10_HUMAN	1464.64185
KS6A5_HUMAN	2361.2168
MED6_HUMAN	425.928375
MYCB2_HUMAN	1720.10217
KBL_HUMAN	1609.9458
LYPA1_HUMAN	10099.5664
ERAL1_HUMAN	5700.70459
U520_HUMAN	18339.8086
MTU1_HUMAN	1574.53601
TIPRL_HUMAN	9683.78027
OFD1_HUMAN	1969.03918
KS6A4_HUMAN	1567.22681
SURF6_HUMAN	1421.52881
PPM1B_HUMAN	2104.08936
UTP20_HUMAN	6150.13428
NU155_HUMAN	10924.2275
XRP2_HUMAN	7370.94775
WDHD1_HUMAN	3857.42798
CRTAP_HUMAN	17407.1133
S2512_HUMAN	11166.0303
RENR_HUMAN	2694.66211
RNH2A_HUMAN	1732.56531
CD123_HUMAN	3756.63647
BCAR3_HUMAN	776.739868
POP7_HUMAN	1676.76282
RPP40_HUMAN	1113.62036
EIF3G_HUMAN	23963.2344

EIF3J_HUMAN	20790.1992
CBR3_HUMAN	12258.1533
PSD10_HUMAN	3359.84229
AP1G2_HUMAN	1066.09961
FACE1_HUMAN	13101.1025
IDHC_HUMAN	46689.0039
GATB_HUMAN	1601.78821
SCO1_HUMAN	7111.09277
ATRN_HUMAN	711.849182
RBBP9_HUMAN	6664.02002
STAM2_HUMAN	4656.18701
DGAT1_HUMAN	728.884155
CCNK_HUMAN	4823.90479
PRAF3_HUMAN	54573.3906
DYSF_HUMAN	19985.8809
PIAS1_HUMAN	489.778687
SPF27_HUMAN	962.551819
DCTN3_HUMAN	12753.2568
DNJC8_HUMAN	13793.4434
SPF30_HUMAN	3829.39795
RAD17_HUMAN	122.725128
ATP5H_HUMAN	3064
TSN9_HUMAN	1599.2478
FLOT1_HUMAN	6164.34766
TRIO_HUMAN	4924.40137
ATP5L_HUMAN	35128.0859
MPDZ_HUMAN	2160.06519
CBPD_HUMAN	7670.07324
GLRX3_HUMAN	47938.8125
RL1D1_HUMAN	18233.1738
WFS1_HUMAN	5742.00537
CLPX_HUMAN	5516.1084
S14L2_HUMAN	3922.68823
SYUG_HUMAN	502.300964
CIAO1_HUMAN	3345.51025
SRP72_HUMAN	11268.4404
DDAH1_HUMAN	8738.89941
RMP_HUMAN	1490.17334
B3GA3_HUMAN	1133.43616
MTA2_HUMAN	13101.8154
AL1A2_HUMAN	4512.55322
STK10_HUMAN	5196.05957
KPCD3_HUMAN	1839.58594
GFPT2_HUMAN	1321.89917
SLIT2_HUMAN	1489.43787
LTN1_HUMAN	2604.34009
TOM70_HUMAN	7616.77393
PKHG5_HUMAN	1549.63879
IPO13_HUMAN	1110.94861
DDHD2_HUMAN	658.833923

MYO1D_HUMAN	7571.80811
TOX4_HUMAN	2998.33594
MICA2_HUMAN	3008.10278
SC24D_HUMAN	16039.8066
FCSD2_HUMAN	592.333374
UFL1_HUMAN	9633.55859
SRBS2_HUMAN	7345.0957
SASH1_HUMAN	4111.69873
UBXN7_HUMAN	4206.88086
KLH18_HUMAN	939.209229
SUN1_HUMAN	1601.56763
PLPHP_HUMAN	7267.69531
ERLN2_HUMAN	11464.8613
PRP6_HUMAN	13204.248
PCF11_HUMAN	675.874878
FRYL_HUMAN	6342.80176
NFAT5_HUMAN	152.662872
ENDD1_HUMAN	1129.72729
GLCE_HUMAN	6342.27295
GLSK_HUMAN	24698.9492
HAUS5_HUMAN	760.008118
ABLM3_HUMAN	1457.88269
FBX21_HUMAN	795.161133
KDM4B_HUMAN	370.070587
MTCL2_HUMAN	2245.32153
UBP19_HUMAN	1127.0199
WDR47_HUMAN	2278.43604
AP2A2_HUMAN	19553.6895
SC31A_HUMAN	42781.2344
ARHGF_HUMAN	8502.43652
HEXI1_HUMAN	1394.552
RPP14_HUMAN	1420.30481
CCNB2_HUMAN	203.0345
YIF1A_HUMAN	4869.62012
UBR5_HUMAN	1946.30457
AGFG2_HUMAN	2200.02246
PRS23_HUMAN	4665.41748
SCAF4_HUMAN	2577.52734
NDUB6_HUMAN	6431.7207
MFN2_HUMAN	2193.44238
SPN1_HUMAN	1196.57715
UBE4B_HUMAN	5459.95752
ZFPL1_HUMAN	4038.68701
ELP1_HUMAN	5220.72754
NDUB4_HUMAN	5280.73096
NDUB8_HUMAN	5099.59668
NDUA7_HUMAN	3229.95654
VAMP5_HUMAN	1222.11304
RTN3_HUMAN	9049.85645
LETM1_HUMAN	14567.8115

EPN2_HUMAN	2919.20337
ZRAB2_HUMAN	7872.50391
SNX4_HUMAN	3778.7561
LC7L3_HUMAN	3932.97632
KI20A_HUMAN	1246.05029
APOL3_HUMAN	249.732071
KIF4A_HUMAN	5198.72412
MBD4_HUMAN	310.003357
MTMR5_HUMAN	1221.75464
GOSR1_HUMAN	15735.2588
KAT7_HUMAN	1149.94141
ATE1_HUMAN	1617.10291
VAPB_HUMAN	5082.67188
SNAPN_HUMAN	2489.07837
MPZL1_HUMAN	2886.3606
NDUC2_HUMAN	7133.34473
NDUAA_HUMAN	12244.1104
FKBP9_HUMAN	11541.6631
CELF2_HUMAN	1322.60437
6PGL_HUMAN	5255.02441
PAPS2_HUMAN	25324.3398
SMC2_HUMAN	11112.8789
ATG7_HUMAN	6628.70654
TACC2_HUMAN	1366.43518
TRI16_HUMAN	2958.89185
SYFM_HUMAN	1282.547
ZBT7A_HUMAN	1086.94629
LYPA2_HUMAN	4520.41748
IPO7_HUMAN	37619.0781
ARI2_HUMAN	946.560669
SLU7_HUMAN	965.046997
AGM1_HUMAN	16802.1465
MOCS3_HUMAN	911.947571
CD2B2_HUMAN	2373.21387
SVIL_HUMAN	6236.99756
AHSA1_HUMAN	39337.5938
PARN_HUMAN	2563.44458
PSMG1_HUMAN	11346.7686
SGPL1_HUMAN	3394.17798
CNEP1_HUMAN	2821.16748
NSA2_HUMAN	3343.62817
G6PE_HUMAN	7757.30566
SC24A_HUMAN	8634.9834
SC24B_HUMAN	7629.61377
AGRL2_HUMAN	1467.39734
NADK_HUMAN	680.634949
TYDP2_HUMAN	1534.04919
MPC2_HUMAN	1698.95056
ETHE1_HUMAN	6541.01611
ACSL3_HUMAN	13179.9609

RPA1_HUMAN	3655.5498
YETS4_HUMAN	541.548279
ZBT11_HUMAN	416.009369
CNOT4_HUMAN	1411.21094
STABP_HUMAN	1500.52295
CPSF4_HUMAN	1995.29053
NFAC1_HUMAN	861.988586
ASML_HUMAN	5178.56201
CDS2_HUMAN	847.810181
PPR3D_HUMAN	907.70343
BRD1_HUMAN	979.190796
RPP29_HUMAN	627.444763
HERC2_HUMAN	965.731384
RAB3D_HUMAN	1581.2052
SNP29_HUMAN	4298.94531
OXSR1_HUMAN	5957.15234
GGPPS_HUMAN	1359.71741
HS74L_HUMAN	7944.604
PTBP3_HUMAN	12152.8711
TBCD8_HUMAN	907.94812
STR3N_HUMAN	3306.66089
LSM8_HUMAN	980.917175
AP2A1_HUMAN	27578.4648
WIZ_HUMAN	1840.37866
RIGI_HUMAN	7127.81934
STAU1_HUMAN	15322.2139
TTC4_HUMAN	4861.70898
TM50A_HUMAN	1980.05054
CAVN2_HUMAN	22468.873
BAG2_HUMAN	13065.9053
BAG3_HUMAN	2957.23511
M4K4_HUMAN	9925.97852
DCMC_HUMAN	164.860489
QORL1_HUMAN	1377.34473
AIFM1_HUMAN	16531.2539
EMAL2_HUMAN	1018.90796
LATS1_HUMAN	1758.78235
NUD14_HUMAN	3346.14917
TSN15_HUMAN	824.327881
BPNT1_HUMAN	6771.59424
FADS2_HUMAN	31211.9863
DDAH2_HUMAN	343.215973
ABHGA_HUMAN	2829.8623
TXD12_HUMAN	954.823242
ECD_HUMAN	5164.7417
RECK_HUMAN	20747.2344
MBD3_HUMAN	1240.33459
TOP3B_HUMAN	319.512512
NUDT3_HUMAN	2927.67896
BCL10_HUMAN	5239.29053

NDUBA_HUMAN	11930.4482
CLPT1_HUMAN	6873.3208
ZBED1_HUMAN	791.247009
MOC2B_HUMAN	1082.01599
TOM40_HUMAN	19074.6094
PX11B_HUMAN	2883.61963
PAK4_HUMAN	6678.18555
ACL6A_HUMAN	9354.36035
NSD2_HUMAN	1193.83887
LDHA_HUMAN	291982.906
AL1A1_HUMAN	8227.07227
DHE3_HUMAN	38971.9766
DYR_HUMAN	11259.501
NB5R3_HUMAN	51686.1172
GSHR_HUMAN	18411.2813
COX2_HUMAN	65297.2617
PNPH_HUMAN	72418.8281
HPRT_HUMAN	24629.1191
AATM_HUMAN	56399.2539
ABL1_HUMAN	714.004456
PGK1_HUMAN	163180.313
KAD1_HUMAN	26116.5566
UROK_HUMAN	1625.25354
TPA_HUMAN	488.708862
ADA_HUMAN	2975.86548
CYTC_HUMAN	7436.03516
RASN_HUMAN	30209.3164
RASH_HUMAN	3217.7168
RASK_HUMAN	2405.73047
PDGFB_HUMAN	1280.15869
LDLR_HUMAN	4321.92969
TGFB1_HUMAN	3584.79004
HLAB_HUMAN	2588.95166
HLAH_HUMAN	49029.5586
CO1A1_HUMAN	1827.30847
CO4A1_HUMAN	1179.60583
LMNA_HUMAN	177647.656
APOA1_HUMAN	2223.67798
FINC_HUMAN	29644.373
FETUA_HUMAN	1195.45154
ALBU_HUMAN	66121.4922
TFR1_HUMAN	37951.0703
FRIL_HUMAN	8270.07129
FRIH_HUMAN	3530.75146
MT2_HUMAN	14074.8984
NU1M_HUMAN	7198.6626
NU4M_HUMAN	1910.1803
NU5M_HUMAN	2149.82837
MMP1_HUMAN	13510.7139
HMDH_HUMAN	329.362183

CATA_HUMAN	6110.96045
RAF1_HUMAN	1222.24329
GBA1_HUMAN	11391.5371
ALDOA_HUMAN	292686.844
CYTB_HUMAN	4335.76855
ANXA1_HUMAN	194269.656
APOB_HUMAN	3499.43677
PRIO_HUMAN	1821.6123
SODM_HUMAN	2569.63159
OAT_HUMAN	32581.498
KITH_HUMAN	3007.21851
VWF_HUMAN	36038.3477
TBB4A_HUMAN	71351.4531
G3P_HUMAN	474270.813
ARLY_HUMAN	6380.7583
HLAA_HUMAN	34451.1914
CPNS1_HUMAN	19382.9824
MT1E_HUMAN	202.261063
HSPB1_HUMAN	109478.234
TYSY_HUMAN	13291.7432
RPN1_HUMAN	72435.5938
RPN2_HUMAN	46355.9297
GNAI2_HUMAN	32835.8008
H2A1B_HUMAN	64595.8438
B3A2_HUMAN	1767.64758
AT1A1_HUMAN	54326.9688
AT1B1_HUMAN	13575.2695
A4_HUMAN	6689.80371
ALDH2_HUMAN	26575.7598
ITB3_HUMAN	7745.68506
S10A8_HUMAN	7403.39453
HMGN1_HUMAN	754.849182
PAI2_HUMAN	16446.0762
PAI1_HUMAN	124142.164
ADT2_HUMAN	213994.578
ISG15_HUMAN	4456.25195
PCCA_HUMAN	2755.47852
PCCB_HUMAN	3864.51904
IF2A_HUMAN	58737.0078
HMGN2_HUMAN	692.251831
ICAM1_HUMAN	10488.5107
RLA2_HUMAN	5120.7417
RLA0_HUMAN	183087.922
JUN_HUMAN	1952.88745
RPC4_HUMAN	1671.21863
LA_HUMAN	31239.3301
ITB1_HUMAN	113579.367
K1C18_HUMAN	57365.5859
CO5A2_HUMAN	1481.74597
DCUP_HUMAN	28310.2559

FYN_HUMAN	339.389374
AGAL_HUMAN	441.951447
GELS_HUMAN	7716.48291
RB_HUMAN	2303.15039
PTMA_HUMAN	2840.23975
CDK1_HUMAN	30147.3477
ATPB_HUMAN	173419.219
S10A9_HUMAN	15811.916
S10A6_HUMAN	10406.624
IF4E_HUMAN	16581.166
ENOA_HUMAN	569954.688
PYGL_HUMAN	9029.50586
G6PI_HUMAN	65468.2773
DPOLB_HUMAN	463.961212
NPM_HUMAN	66873.2656
TPM3_HUMAN	52893.1953
ITAV_HUMAN	18428.1816
HEXA_HUMAN	9357.10254
HYEP_HUMAN	10090.043
LDHB_HUMAN	193912.813
CENPB_HUMAN	1106.02368
GPX1_HUMAN	13157.1953
PDIA1_HUMAN	151060.438
H10_HUMAN	14987.791
ACYP1_HUMAN	3194.76172
FES_HUMAN	954.784424
CATD_HUMAN	61317.6602
ANXA2_HUMAN	389725.375
CAN1_HUMAN	27298.3945
TBB5_HUMAN	304799.563
SAP_HUMAN	4373.45361
HEXB_HUMAN	14924.0313
CATL1_HUMAN	1606.74524
PROF1_HUMAN	265163.281
PMGE_HUMAN	4043.49927
APT_HUMAN	57438.6875
SYEP_HUMAN	21587.4238
CATB_HUMAN	77404.6563
HS90A_HUMAN	149643.594
GALT_HUMAN	921.350708
HNRPC_HUMAN	139905.875
LAMB1_HUMAN	18659.3926
YES_HUMAN	18682.8516
LYN_HUMAN	3068.46582
FUMH_HUMAN	28109.5547
ERCC1_HUMAN	548.603455
TSP1_HUMAN	137097
SP1_HUMAN	844.88562
IGF1R_HUMAN	1185.15454
CO1A2_HUMAN	747.759094

ANXA6_HUMAN	92001.8359
RHOC_HUMAN	90429.3828
DAF_HUMAN	9360.06348
4F2_HUMAN	18603.7891
BGLR_HUMAN	882.19574
PFKAM_HUMAN	9386.2959
HS90B_HUMAN	725460.938
SRPRA_HUMAN	12020.5313
ASNS_HUMAN	5119.48389
MMP2_HUMAN	7476.03662
HEM3_HUMAN	1650.60559
NEP_HUMAN	629.536377
MGP_HUMAN	2345.573
ODPA_HUMAN	30277.9258
CO4A2_HUMAN	8812.61719
CY1_HUMAN	13078.1533
RU2B_HUMAN	7318.11914
MET_HUMAN	3310.49023
RU17_HUMAN	50476.1055
ITA5_HUMAN	83544.6719
NFIC_HUMAN	834.130066
VIME_HUMAN	1443631.25
RS17_HUMAN	37728.8242
GNAI3_HUMAN	8491.55469
ANXA5_HUMAN	429573.281
RSSA_HUMAN	114711.195
CD63_HUMAN	4349.64209
RM03_HUMAN	5811.39941
SNRPA_HUMAN	23520.5605
ENOG_HUMAN	12841.3623
THIK_HUMAN	6132.06494
SRP19_HUMAN	5877.75146
GSTP1_HUMAN	126837.375
RU1C_HUMAN	6624.18848
LEG1_HUMAN	32930.0391
DHPR_HUMAN	8512.68457
HMGB1_HUMAN	59559.9297
SPRC_HUMAN	2139.10498
TPM1_HUMAN	61332.7305
CLCA_HUMAN	2304.80225
CLCB_HUMAN	599.220337
ANXA4_HUMAN	14243.5869
CN37_HUMAN	10753.333
HMOX1_HUMAN	10035.3984
DLDH_HUMAN	12535.207
ROA1_HUMAN	169733.656
RU2A_HUMAN	25329.2871
COX6C_HUMAN	13141.6904
TACD2_HUMAN	2781.90698
PARP1_HUMAN	34829.7266

DPOLA_HUMAN	12266.5605
UCHL1_HUMAN	6626.84814
LKHA4_HUMAN	25876.9609
ALDOC_HUMAN	21555.9355
H2AZ_HUMAN	4560.1543
H2A1_HUMAN	685.387695
MYZAP_HUMAN	223.268188
GRL1A_HUMAN	954.407715
GST2_HUMAN	3775.08887
POTEJ_HUMAN	11659.6748
LIMS3_HUMAN	3820.24048
HS71A_HUMAN	88263.6563
ERPG3_HUMAN	1677.68665
TBA3C_HUMAN	53586.25
ZTRF1_HUMAN	824.329529
RAP2A_HUMAN	8186.06445
SRGN_HUMAN	2584.49609
RO60_HUMAN	10463.6279
LYAG_HUMAN	3016.41431
RRAS_HUMAN	11981.5791
HLAC_HUMAN	1089.49792
ARAF_HUMAN	1761.18555
H14_HUMAN	297671.531
ODP2_HUMAN	22990.127
PTPRF_HUMAN	4173.06738
COT1_HUMAN	4782.88525
THIO_HUMAN	4618.44043
COX5B_HUMAN	884.766479
PPGB_HUMAN	6317.94238
MGST1_HUMAN	11610.8711
KAP0_HUMAN	17834.9492
TFPI1_HUMAN	1058.02759
ESTD_HUMAN	17758.6035
CH60_HUMAN	143416.641
CLUS_HUMAN	2184.88647
BIP_HUMAN	243707.672
LAMC1_HUMAN	12535.9707
PPAL_HUMAN	4502.72217
MTAP2_HUMAN	3141.4104
HSP7C_HUMAN	267963.406
GTR1_HUMAN	5609.75098
GTR3_HUMAN	2668.09888
EPB41_HUMAN	20497.1289
UMPS_HUMAN	13868.752
ODPB_HUMAN	15968.3105
ODB2_HUMAN	1383.8396
PYGB_HUMAN	14765.0957
PYGM_HUMAN	33198.5156
RALA_HUMAN	29380.9609
RALB_HUMAN	43752.4023

BCR_HUMAN	4347.41455
LAMP1_HUMAN	70275.6563
ERG_HUMAN	5983.01709
ACADM_HUMAN	10179.8164
TOP1_HUMAN	41714.1406
TOP2A_HUMAN	689.00592
G6PD_HUMAN	71036.9141
UBL4A_HUMAN	1067.2926
PYC_HUMAN	3255.76147
C1TC_HUMAN	51796.793
MPRI_HUMAN	4887.98145
ADHX_HUMAN	33299.9141
CDK4_HUMAN	3164.92065
PRPS2_HUMAN	17764.4336
PABP1_HUMAN	123924.352
PCNA_HUMAN	29577.3633
HARS1_HUMAN	38514.5625
CO6A1_HUMAN	1244.15576
CO6A3_HUMAN	293.050415
ADT1_HUMAN	224744.703
ADT3_HUMAN	81950.2813
IMDH2_HUMAN	42262.6094
TPR_HUMAN	13806.4307
ANXA3_HUMAN	43132.5273
ODBA_HUMAN	4129.78369
ACTN1_HUMAN	111785.617
ACE_HUMAN	5398.68066
SRC_HUMAN	4305.125
PEPD_HUMAN	6719.88037
XRCC6_HUMAN	85911.1563
XRCC5_HUMAN	50331.1172
UNG_HUMAN	989.422546
COX41_HUMAN	50439.6328
HEM1_HUMAN	1335.18188
LAMP2_HUMAN	50995.6445
RINI_HUMAN	45039.4258
ICAM2_HUMAN	48479.9727
EF2_HUMAN	199752.203
PDIA4_HUMAN	110775.32
P4HA1_HUMAN	9560.24219
TCTP_HUMAN	12275.9883
HEM2_HUMAN	4845.20166
HLAE_HUMAN	757.438049
PLST_HUMAN	143566.328
ACPH_HUMAN	17206.7578
ETFA_HUMAN	21955.5938
GYS1_HUMAN	5528.58594
KAP2_HUMAN	7607.38135
T2FB_HUMAN	4278.62451
CD59_HUMAN	25322.6133

MTDC_HUMAN	7029.27002
MIF_HUMAN	34661.1523
CD99_HUMAN	1461.57629
GLU2B_HUMAN	65426.1836
HCLS1_HUMAN	8052.54199
FPPS_HUMAN	60704.4609
CX7A2_HUMAN	14990.3789
NID1_HUMAN	5986.23438
AK1A1_HUMAN	25686.8945
KPYM_HUMAN	481477.594
ENPL_HUMAN	312998.938
CCNB1_HUMAN	2718.59229
MYL6B_HUMAN	7786.58691
RSMB_HUMAN	48006.7109
IDE_HUMAN	19307.0781
PO2F1_HUMAN	502.487823
HNRPL_HUMAN	24259.166
SYDC_HUMAN	34112.5039
ETS1_HUMAN	1286.96484
PLAK_HUMAN	19947.0313
QCR7_HUMAN	679.769226
BRAF_HUMAN	3142.09961
FABP4_HUMAN	13453.4375
GLNA_HUMAN	594.692566
ALDR_HUMAN	43801.7031
AMPN_HUMAN	80064.1563
PVR_HUMAN	23336.3008
RAC2_HUMAN	25476.3672
ERF3A_HUMAN	15322.3311
ARSA_HUMAN	822.520386
B4GT1_HUMAN	1745.22546
EZRI_HUMAN	35924.8828
UCHL3_HUMAN	4887.69238
FOSL1_HUMAN	1012.83209
FOSL2_HUMAN	335.973633
MCP_HUMAN	4892.4668
NDKA_HUMAN	28488.7988
NQO1_HUMAN	17032.9707
GNS_HUMAN	37733.9219
PHKG2_HUMAN	1319.80627
ARSB_HUMAN	1555.76929
RS2_HUMAN	152052.469
ITF2_HUMAN	1506.51025
DESP_HUMAN	4477.33447
RFA2_HUMAN	6039.9834
TIMP2_HUMAN	5820.10547
CD44_HUMAN	15606.7012
CBR1_HUMAN	23697.8008
ACADS_HUMAN	1268.75671
CREB1_HUMAN	2591.09985

BGAL_HUMAN	8398.84766
PECA1_HUMAN	50766.8281
PP2BB_HUMAN	6119.12842
NCK1_HUMAN	9136.52246
GCFC2_HUMAN	1120.97888
H15_HUMAN	244343.969
H13_HUMAN	31437.6836
H12_HUMAN	33072.0781
NCPR_HUMAN	10581.0645
FER_HUMAN	285.593018
AT2A2_HUMAN	38131.3789
PLCG2_HUMAN	4165.03809
FAAA_HUMAN	4825.42529
STMN1_HUMAN	42075.0234
YBOX3_HUMAN	18264.5488
ZNF22_HUMAN	823.774536
ZNF24_HUMAN	199.469269
NAGAB_HUMAN	2889.59839
HMGA1_HUMAN	55361.3242
TMM11_HUMAN	3869.00781
AATC_HUMAN	19080.4473
KPCA_HUMAN	6843.78369
ITA2_HUMAN	26072.3945
CXA1_HUMAN	2073.52319
ASM_HUMAN	2654.93726
UBF1_HUMAN	8398.34863
JUND_HUMAN	597.977722
TAL1_HUMAN	770.384155
ATF7_HUMAN	197.097519
NDUB7_HUMAN	4522.81738
KAPCA_HUMAN	13058.4063
CAN2_HUMAN	42748.8008
PTN2_HUMAN	1875.38684
PYRG1_HUMAN	18051.8359
EGLN_HUMAN	67937.3281
DDX5_HUMAN	91631.2422
PFKAL_HUMAN	45406.6328
VGFR1_HUMAN	7458.26904
PRS6A_HUMAN	41067.8047
TCPA_HUMAN	68274.75
PTN1_HUMAN	21544.6563
ERCC2_HUMAN	3395.86938
RL35A_HUMAN	74411.375
ITB5_HUMAN	6631.51514
ARF4_HUMAN	112801.703
RL7_HUMAN	180135.313
VINC_HUMAN	83100
PTPRA_HUMAN	1995.55579
ARY1_HUMAN	1042.86707
SON_HUMAN	10228.3809

NELFE_HUMAN	4554.25879
RL17_HUMAN	90608.8906
PGAM1_HUMAN	81404.8203
RCC1_HUMAN	24317.6602
ATF1_HUMAN	967.496338
ATF6A_HUMAN	537.977173
DNLI1_HUMAN	7229.82471
XRCC1_HUMAN	3723.15845
CADH2_HUMAN	14009.5654
PLCG1_HUMAN	10450.2979
NUCL_HUMAN	155398.203
HXK1_HUMAN	55550.5625
RPB3_HUMAN	1823.17468
RPAB1_HUMAN	5287.12012
NDUV2_HUMAN	2537.86816
ERCC3_HUMAN	2815.42993
RO52_HUMAN	6511.55664
E2AK2_HUMAN	9180.66797
SPEE_HUMAN	50512.9258
SL9A1_HUMAN	1559.75793
CSK22_HUMAN	7703.84766
NFKB1_HUMAN	4675.28369
AT2B1_HUMAN	4457.42139
IF2B_HUMAN	24726.3457
ANXA7_HUMAN	21333.4414
CCNA2_HUMAN	1064.09717
BTF3_HUMAN	6497.17041
RAB3B_HUMAN	3754.31201
RAB4A_HUMAN	3868.26147
RAB5A_HUMAN	58627.5742
RAB6A_HUMAN	38991.1055
MSH3_HUMAN	1431.24194
ANPRB_HUMAN	498.112183
PSB1_HUMAN	37407.4375
MPRD_HUMAN	5863.9082
LMNB1_HUMAN	76352.9922
ICAL_HUMAN	17989.873
IMDH1_HUMAN	5364.27393
CO5A1_HUMAN	1836.12769
NEBU_HUMAN	2163.05737
ASPG_HUMAN	1616.59241
RASA1_HUMAN	5704.10498
PTMS_HUMAN	814.709106
CD11B_HUMAN	9115.96875
GSTM3_HUMAN	1857.26636
VATB2_HUMAN	14490.5791
VATC1_HUMAN	4595.08398
CSRP1_HUMAN	77611.5703
FLNA_HUMAN	257078.938
NF1_HUMAN	455.327423

AOFA_HUMAN	875.338989
ACOHC_HUMAN	27522.6113
S1PR1_HUMAN	5622.37598
SYT1_HUMAN	1078.46777
5NTD_HUMAN	15725.9082
TAF1_HUMAN	274.894135
MK_HUMAN	522.958496
VDAC1_HUMAN	91888.3516
PGS1_HUMAN	4265.78906
SDHB_HUMAN	14527.6387
CD9_HUMAN	23828.8984
COMT_HUMAN	5367.81689
TGM2_HUMAN	296833.281
MUTA_HUMAN	6621.76904
OSBP1_HUMAN	7100.07422
PIMT_HUMAN	1841.51575
FBRL_HUMAN	21574.0645
PUR2_HUMAN	25040.0293
PUR6_HUMAN	50752.0273
SCP2_HUMAN	19945.2754
UBA1_HUMAN	65795.6875
NDKB_HUMAN	128150.602
SPR1B_HUMAN	279.000305
ADRO_HUMAN	11451.3037
ROA2_HUMAN	217579.938
RFX1_HUMAN	702.859192
CBL_HUMAN	4038.85547
IBP4_HUMAN	776.965942
KAPCB_HUMAN	1790.0575
QCR2_HUMAN	15350.7588
HEMH_HUMAN	3012.32593
XPA_HUMAN	382.087616
TCEA1_HUMAN	13389.7988
PGH1_HUMAN	1052.17529
ITA6_HUMAN	22474.5781
SFPQ_HUMAN	97558.5391
TBG1_HUMAN	13006.8828
PPIB_HUMAN	143719.297
MAOM_HUMAN	13659.0469
SYWC_HUMAN	22034.4824
RS3_HUMAN	220584.672
KS6B1_HUMAN	3710.54224
JAK1_HUMAN	6898.36621
PTPRB_HUMAN	12547.8154
PTPRE_HUMAN	5863.43066
PTPRG_HUMAN	1158.96558
SP100_HUMAN	5843.08398
CRCM_HUMAN	745.878906
TNFL4_HUMAN	8960.65137
SAHH_HUMAN	86278.7422

COF1_HUMAN	597087.75
IF4B_HUMAN	24882.0156
HAP40_HUMAN	1939.94836
AT2B4_HUMAN	6714.24561
DGKA_HUMAN	4366.11035
CPT2_HUMAN	3075.32568
KTHY_HUMAN	20351.0703
RIR1_HUMAN	15828.4922
CCND1_HUMAN	1679.71545
RAE1_HUMAN	2273.96265
ERD21_HUMAN	1172.0907
COT2_HUMAN	2731.61816
EF1B_HUMAN	15380.2207
AT5F1_HUMAN	16439.4883
PPAC_HUMAN	15353.0996
KPCL_HUMAN	3345.77124
THIL_HUMAN	25465.4824
RPB1_HUMAN	32391.252
CDK2_HUMAN	11113.5391
ARBK1_HUMAN	3888.80176
PAR1_HUMAN	1647.26062
MCM3_HUMAN	44659.1211
THTM_HUMAN	6017.08252
RS12_HUMAN	30014.8887
BRD2_HUMAN	1645.27051
TYY1_HUMAN	2327.1189
DNJB1_HUMAN	11164.2451
DNJB2_HUMAN	509.671082
ATPA_HUMAN	202847.656
CATS_HUMAN	975.245361
PSA1_HUMAN	94716.9453
PSA2_HUMAN	25631.2832
PSA3_HUMAN	36494.5938
PSA4_HUMAN	77083.7344
TNR5_HUMAN	593.16748
ITA3_HUMAN	20466.1855
PTX3_HUMAN	3886.11572
MOES_HUMAN	140105
DDX6_HUMAN	17829.7363
DNMT1_HUMAN	9220.72559
U2AF2_HUMAN	51955.2695
RL13_HUMAN	125924.953
RAE2_HUMAN	1174.89954
IVD_HUMAN	1396.03357
MGAT1_HUMAN	4258.27539
HMGB2_HUMAN	15597.8945
PTBP1_HUMAN	62137.9102
SYTC_HUMAN	36497.3633
SYVC_HUMAN	22903.8926
EF1G_HUMAN	143972.688

FKBP2_HUMAN	17648.8496
IL3RA_HUMAN	1208.35413
STOM_HUMAN	35409.4258
KAD4_HUMAN	1697.17126
1433T_HUMAN	52984.4375
MK03_HUMAN	13372.4404
MARK3_HUMAN	720.126953
RL10_HUMAN	48565.2109
CO8A1_HUMAN	803.588135
RFA1_HUMAN	14300.9619
APEX1_HUMAN	28527.373
CD82_HUMAN	1008.47418
DCK_HUMAN	3755.26782
PYR1_HUMAN	36516.9102
CALR_HUMAN	178831.391
MAP4_HUMAN	23090.5488
CALX_HUMAN	116030.195
P85A_HUMAN	1351.3064
IP3KB_HUMAN	1090.85034
PSB8_HUMAN	2688.67896
PSB9_HUMAN	3184.01782
PSA5_HUMAN	26108.2441
PSB4_HUMAN	16587.1328
PSB6_HUMAN	40781.668
PSB5_HUMAN	37230.9609
GSTM2_HUMAN	811.269165
ABCD3_HUMAN	5932.75195
ITPI2_HUMAN	4809.99707
LYOX_HUMAN	2194.77271
NDUS1_HUMAN	14172.3818
DPOD1_HUMAN	10077.0361
SMCA1_HUMAN	1125.27966
MK01_HUMAN	44814.1172
RXRB_HUMAN	1010.05432
ERCC5_HUMAN	1057.12415
GRN_HUMAN	4632.12695
PTPRM_HUMAN	794.271606
AMPL_HUMAN	25078.8945
CD34_HUMAN	1742.05981
T2EA_HUMAN	2929.00146
T2EB_HUMAN	3224.9104
TPP2_HUMAN	13295.6035
IMPA1_HUMAN	6853.50537
CCN2_HUMAN	21384.2773
EPHA2_HUMAN	21686.1328
EPHB2_HUMAN	14932.1348
SHC1_HUMAN	5580.63672
3MG_HUMAN	6199.98975
TKT_HUMAN	135982.484
CASP1_HUMAN	561.570496

NOS3_HUMAN	3265.00952
RBMS1_HUMAN	4939.78174
PML_HUMAN	9370.10938
TYK2_HUMAN	1410.5105
EF1D_HUMAN	38316.9336
MARCS_HUMAN	33618.2227
GNA11_HUMAN	17229.748
AL4A1_HUMAN	2860.80322
ERP29_HUMAN	24405.0723
PRDX6_HUMAN	137054.891
BLVRB_HUMAN	8793.34668
PRDX5_HUMAN	23692.9805
PRDX3_HUMAN	21048.2949
RL12_HUMAN	127669.211
ECHM_HUMAN	13172.2393
KCY_HUMAN	25073.4238
PEBP1_HUMAN	23525.3887
PDIA3_HUMAN	142953.531
2AAA_HUMAN	62523.3984
2AAB_HUMAN	5331.93945
CDC27_HUMAN	4637.26855
CCND3_HUMAN	375.481506
PPIF_HUMAN	11308.7217
NKTR_HUMAN	1345.26306
NMT1_HUMAN	19960.834
HMOX2_HUMAN	16253.8359
PURA2_HUMAN	63110.0234
UFO_HUMAN	3697.47534
AMRP_HUMAN	5673.68408
TSPO_HUMAN	8368.9873
PUR8_HUMAN	17455.498
CLIP1_HUMAN	6306.76514
SORCN_HUMAN	3219.70215
ILEU_HUMAN	4760.29639
CTR1_HUMAN	5545.83057
AL1B1_HUMAN	7772.854
RPB2_HUMAN	8614.61523
SDHA_HUMAN	7981.42676
COR1A_HUMAN	1762.3313
GDIA_HUMAN	19871.543
S10A7_HUMAN	3310.05859
METK2_HUMAN	50729.2344
KAP3_HUMAN	8081.18115
RIR2_HUMAN	16205.2314
SDC4_HUMAN	1012.87585
TIA1_HUMAN	18506.0566
SC6A6_HUMAN	719.488953
DNJA1_HUMAN	16090.6289
AKT1_HUMAN	3115.13623
QCR1_HUMAN	76497.0391

3HIDH_HUMAN	4296.4043
PUR9_HUMAN	32542.2715
HNRH3_HUMAN	10088.9443
HNRH1_HUMAN	69187.5469
CASPE_HUMAN	10823.9844
1433B_HUMAN	158792.453
STIP1_HUMAN	123045.508
S10AB_HUMAN	9770.16699
PRDX2_HUMAN	43439.5156
ARRB2_HUMAN	758.698608
GLPK_HUMAN	1374.78088
DCTD_HUMAN	6779.99365
P5CR1_HUMAN	5264.16455
GBP1_HUMAN	3395.55005
GBP2_HUMAN	2739.00317
ELF1_HUMAN	1194.52075
TF2H1_HUMAN	945.052979
STX2_HUMAN	312.407257
RL9_HUMAN	81957.1875
ACSL1_HUMAN	5281.72998
CADH5_HUMAN	24723.0898
KINH_HUMAN	34741.5469
DUT_HUMAN	6749.95313
MRP1_HUMAN	5138.96729
ABCD1_HUMAN	611.653198
TTK_HUMAN	1252.24194
MCM4_HUMAN	12477.2686
MCM5_HUMAN	31623.1211
MCM7_HUMAN	20821.3867
GALNS_HUMAN	415.668488
GLYC_HUMAN	4453.24902
GLYM_HUMAN	60903.9102
HS71L_HUMAN	1843.88989
HSP74_HUMAN	37421.1523
MPI_HUMAN	2046.50635
GPC1_HUMAN	1288.54724
PROF2_HUMAN	19649.2305
CTNA1_HUMAN	51846.4414
CTNB1_HUMAN	48495.6797
PHB1_HUMAN	106558.039
SPB6_HUMAN	19540.5
MERL_HUMAN	4048.78564
RADI_HUMAN	377236.281
RFA3_HUMAN	1755.98523
RFC4_HUMAN	8182.15381
RFC2_HUMAN	8233.61035
RFC1_HUMAN	4121.354
RL22_HUMAN	142402.156
T2FA_HUMAN	7447.48389
SPRE_HUMAN	6725.0083

HRH1_HUMAN	714.693054
CBS_HUMAN	992.043823
FBN1_HUMAN	1791.80652
FBN2_HUMAN	323.378448
GDE_HUMAN	4100.93701
MYH9_HUMAN	195590.25
MYH10_HUMAN	119259.117
TIE1_HUMAN	12266.874
COPB2_HUMAN	47225.0039
SOAT1_HUMAN	2745.61157
ADDA_HUMAN	6164.83984
BASI_HUMAN	58242.1992
FUS_HUMAN	71707.1172
NU214_HUMAN	7425.12451
DEK_HUMAN	24414.9043
SOX18_HUMAN	1070.76758
MYH11_HUMAN	52449.4609
PPM1A_HUMAN	4944.49268
HMGCL_HUMAN	3499.3916
VGFR3_HUMAN	2729.10962
VGFR2_HUMAN	3687.96289
PRS7_HUMAN	64015.8555
GGT5_HUMAN	843.927979
ARL2_HUMAN	10271.5361
ARL3_HUMAN	23080.377
MP2K2_HUMAN	15514.3135
ATPG_HUMAN	42605.5195
VATE1_HUMAN	10267.8311
HEM6_HUMAN	12431.4297
RL4_HUMAN	157418.594
8ODP_HUMAN	1099.32837
LONM_HUMAN	13106.9785
PGM1_HUMAN	8227.71289
PP1G_HUMAN	10887.3555
GNL1_HUMAN	27240.2266
ODO2_HUMAN	24487.4727
GMPR1_HUMAN	3381.15015
GPX4_HUMAN	11217.7051
ACVL1_HUMAN	3156.69116
DHB2_HUMAN	965.605164
SRP14_HUMAN	21487.6797
TGFR2_HUMAN	4303.24902
NUP62_HUMAN	953.769897
HPCL1_HUMAN	6619.06641
FDFT_HUMAN	12732.4238
ZEB1_HUMAN	1907.35645
TAGL2_HUMAN	46661.0859
TALDO_HUMAN	116656.93
SYUA_HUMAN	3343.02979
ETFB_HUMAN	17635.4375

RBMX_HUMAN	30457
COIL_HUMAN	2534.18945
VKGC_HUMAN	2691.55493
LICH_HUMAN	710.374817
VATA_HUMAN	21942.4766
GRP75_HUMAN	135401.906
IF4A3_HUMAN	34450.8906
SMBP2_HUMAN	511.766663
CDN1A_HUMAN	2156.22607
RS19_HUMAN	112793.906
RL3_HUMAN	45952.25
COIA1_HUMAN	21566.1582
OST48_HUMAN	95562.6641
AN32A_HUMAN	30722.7617
FEN1_HUMAN	34949.3086
CUX1_HUMAN	349.210571
CAPG_HUMAN	19717.9102
CAP2_HUMAN	3647.95508
IL6RB_HUMAN	6723.18896
TXLNA_HUMAN	9687.6748
TCPZ_HUMAN	108310.813
NNMT_HUMAN	36075.7773
PSB10_HUMAN	1463.19275
PBX2_HUMAN	906.878479
RL13A_HUMAN	142653.609
ARL1_HUMAN	19591.4668
MLH1_HUMAN	9210.49805
STAT3_HUMAN	8155.37695
UBP8_HUMAN	6360.2002
MDHC_HUMAN	77789.3203
MDHM_HUMAN	123559.117
RFC5_HUMAN	9185.27051
RFC3_HUMAN	4455.39844
ECHA_HUMAN	34052.7695
IF2G_HUMAN	39025.0078
ETV3_HUMAN	496.086639
CETN2_HUMAN	1426.07434
ETV6_HUMAN	2030.18701
EIF2D_HUMAN	5115.62256
BUD31_HUMAN	3902.6145
UBA7_HUMAN	1742.27905
NAA10_HUMAN	17562.6484
KDM5C_HUMAN	412.090088
CSK_HUMAN	10571.0752
GARS_HUMAN	24735.9512
SYIC_HUMAN	50165.8281
S19A1_HUMAN	1758.37842
EIF1_HUMAN	4195.70508
KPCI_HUMAN	775.931641
ELK3_HUMAN	935.137878

ACTY_HUMAN	25527.1152
ECI1_HUMAN	6214.12207
LAP2A_HUMAN	9801.9248
LAP2B_HUMAN	90004.0156
STAT1_HUMAN	16556.8594
STAT6_HUMAN	4892.5542
STA5A_HUMAN	6471.48242
MTREX_HUMAN	13503.124
AK1C3_HUMAN	3681.11011
PK3CA_HUMAN	1870.16199
PK3CB_HUMAN	2621.12671
MTOR_HUMAN	9957.55762
PI4KA_HUMAN	4017.77148
EPS15_HUMAN	7271.07471
CASP3_HUMAN	22472.8965
CASP2_HUMAN	809.863586
RS27_HUMAN	9291.03809
ABL2_HUMAN	626.010437
HELZ_HUMAN	1529.401
CNDD3_HUMAN	1990.06396
RBM34_HUMAN	8095.48975
LPPRC_HUMAN	47469.9336
THIM_HUMAN	10797.7461
RL35_HUMAN	46514.7852
PCP_HUMAN	16555.2539
HD_HUMAN	3410.03491
ECE1_HUMAN	12894.5479
MTHR_HUMAN	493.792267
SATT_HUMAN	1724.82495
LIS1_HUMAN	19202.9375
MUC18_HUMAN	113125.313
CACP_HUMAN	4558.09521
MATR3_HUMAN	35879.293
MSH2_HUMAN	12379.2969
GRK6_HUMAN	1308.65613
GPDM_HUMAN	9737.03613
SSRA_HUMAN	34518.4922
PTN9_HUMAN	1747.68958
RANG_HUMAN	48202.4805
NAMPT_HUMAN	85029.2578
PRS6B_HUMAN	30667.5645
EFTS_HUMAN	8491.99512
PPIC_HUMAN	9593.09668
VDAC2_HUMAN	92390.375
ACDSB_HUMAN	2896.39478
CBX5_HUMAN	3834.51392
UBP5_HUMAN	36014.0586
MK08_HUMAN	1477.72375
MP2K4_HUMAN	5039.12891
KI67_HUMAN	5483.34277

KPB2_HUMAN	1016.83301
KPB1_HUMAN	1172.62097
RAGP1_HUMAN	26931.3477
RECQ1_HUMAN	22144.8535
NOP2_HUMAN	15881.3115
ATRX_HUMAN	2441.29272
CRK_HUMAN	6552.74463
CRKL_HUMAN	6491.50537
IF2M_HUMAN	2938.7854
BAG6_HUMAN	5757.04932
NSF_HUMAN	20349.918
NOTC1_HUMAN	2572.83447
MP2K3_HUMAN	6062.33691
BRCC3_HUMAN	2167.70508
RL27A_HUMAN	225192.078
RL5_HUMAN	59306.5078
RL21_HUMAN	43579.5195
RL28_HUMAN	46727.293
RS9_HUMAN	218661.297
RS5_HUMAN	116002.406
RS10_HUMAN	80710.5781
MAP1B_HUMAN	35020.3125
GNPI1_HUMAN	14821.4922
NEDD4_HUMAN	3336.97485
UTRN_HUMAN	9905.56836
IQGA1_HUMAN	166873.609
GLYG_HUMAN	14271.3584
STT3A_HUMAN	46166.5391
PA24A_HUMAN	2383.3208
CAZA2_HUMAN	23316.7656
CAPZB_HUMAN	41291.1719
IF1AX_HUMAN	7982.42676
AL1A3_HUMAN	966.057556
SYQ_HUMAN	29789.2461
RL29_HUMAN	35791.3047
LEG7_HUMAN	1554.15771
TISD_HUMAN	921.91748
UCRI_HUMAN	12985.3984
ATPO_HUMAN	53451.3125
LIMS1_HUMAN	16267.5781
GLIP1_HUMAN	1551.94006
PPCE_HUMAN	20579.8984
MAOX_HUMAN	18276.7012
IREB2_HUMAN	2284.65283
TFPI2_HUMAN	993.630981
RFX5_HUMAN	5001.36475
PI42A_HUMAN	5336.67578
COPD_HUMAN	47137.957
LSS_HUMAN	23728.4453
PP2BC_HUMAN	789.093689

GSH1_HUMAN	2383.39819
GSH0_HUMAN	10485.6338
CD151_HUMAN	10328.1318
TPC10_HUMAN	1015.47961
PSMD8_HUMAN	35639.1445
PRC2A_HUMAN	3300.48657
GSHB_HUMAN	15544.4639
TCPE_HUMAN	97309.3516
PTSS1_HUMAN	5656.73486
NEST_HUMAN	106330.766
HSP13_HUMAN	8936.625
KC1A_HUMAN	11079.2822
KC1D_HUMAN	4506.47705
IDHP_HUMAN	64173.3281
PK3CG_HUMAN	1925.76685
PIPNB_HUMAN	17482.9824
DPOD2_HUMAN	4745.46875
MRP_HUMAN	10156.2266
PAXI_HUMAN	5631.35938
CAMLG_HUMAN	541.402039
NR2C2_HUMAN	891.679504
MAPK2_HUMAN	2350.42285
DNSL1_HUMAN	1821.72778
AL9A1_HUMAN	12663.9297
RL34_HUMAN	56678.6484
RPIA_HUMAN	2720.78271
LMAN1_HUMAN	18932.0352
NRAM2_HUMAN	1300.94727
NASP_HUMAN	20471.8125
FAS_HUMAN	69976.0938
FNTA_HUMAN	10268.5928
FNTB_HUMAN	3689.21582
DHYS_HUMAN	2857.63843
TCPG_HUMAN	84445.1719
RM19_HUMAN	9898.51367
ARRB1_HUMAN	6604.00439
EFTU_HUMAN	80481.3672
AL7A1_HUMAN	18780.3379
UB2R1_HUMAN	5066.15332
INPP_HUMAN	3639.87842
CENPF_HUMAN	398.10968
SRP09_HUMAN	44024.5234
PCY1A_HUMAN	10253.3008
SYAC_HUMAN	22825.1094
SYCC_HUMAN	17181.5078
SYHM_HUMAN	6139.76904
SYSC_HUMAN	30324.2559
PPM1F_HUMAN	22608.3555
MA2A2_HUMAN	2443.08643
PRI1_HUMAN	3198.60059

PRI2_HUMAN	1324.08081
CASP4_HUMAN	2659.19775
KC1E_HUMAN	947.778564
PSB3_HUMAN	32043.1934
PSB2_HUMAN	28239.0586
MCM2_HUMAN	19091.5332
ACADV_HUMAN	9090.04688
YLPM1_HUMAN	7052.81104
VPS41_HUMAN	1534.2113
TMEDA_HUMAN	37164.8711
RBM25_HUMAN	10225.7314
NUMB_HUMAN	6487.05225
CLK3_HUMAN	1025.16187
PSN1_HUMAN	1500.12219
EI2BB_HUMAN	6249.05078
HINT1_HUMAN	5620.33594
NU153_HUMAN	7219.56787
RBP2_HUMAN	12603.8438
RGS19_HUMAN	677.899536
RGS3_HUMAN	2345.60327
PSN2_HUMAN	430.878662
TSC2_HUMAN	2680.2395
NDUV1_HUMAN	8865.07715
GSK3A_HUMAN	2096.08813
GSK3B_HUMAN	7094.87207
TAF6_HUMAN	1043.54517
5NTC_HUMAN	2700.09351
SPS1_HUMAN	5478.41064
GUAA_HUMAN	21664.5156
DNLI3_HUMAN	5075.9707
DNLI4_HUMAN	2128.21191
MRE11_HUMAN	6188.29102
ENTP1_HUMAN	463.585266
GNAQ_HUMAN	8011.38379
IDH3A_HUMAN	11932.124
ST1A1_HUMAN	1387.20422
MMP14_HUMAN	29728.5254
PPOX_HUMAN	1574.82397
GDIB_HUMAN	73157.7031
EMD_HUMAN	7618.66357
CPT1A_HUMAN	7113.98926
S26A2_HUMAN	1063.77283
SPB8_HUMAN	14288.168
SPB9_HUMAN	53220.2969
SERPH_HUMAN	92174.75
PDLI4_HUMAN	10639.8252
F10A1_HUMAN	87845
PEX5_HUMAN	1288.83215
ERF_HUMAN	1611.51025
VASP_HUMAN	8835.19141

DYN2_HUMAN	24162.1367
MAP2_HUMAN	4164.39258
AP4A_HUMAN	3755.63843
CDK7_HUMAN	4571.0874
KNTC1_HUMAN	2564.50684
RASF2_HUMAN	2878.2644
CDK9_HUMAN	4149.7998
LRBA_HUMAN	4116.0835
BCAM_HUMAN	3629.77075
PPT1_HUMAN	3444.7356
RL14_HUMAN	140356.672
TCPQ_HUMAN	175109.344
TCPD_HUMAN	48164.1406
ANX11_HUMAN	23136.8203
PAPOA_HUMAN	7970.8208
FXR1_HUMAN	18157.9863
FXR2_HUMAN	14964.7109
RAB5C_HUMAN	28992.2246
RAB7A_HUMAN	91943.4063
RAB9A_HUMAN	4045.37915
RAB13_HUMAN	10560.0117
RB27A_HUMAN	6784.68652
PLCD1_HUMAN	1203.22534
RT29_HUMAN	5919.59082
DUS3_HUMAN	5793.92578
SMCA2_HUMAN	402.857758
SMCA4_HUMAN	7496.39648
IDH3G_HUMAN	3320.10522
GALK1_HUMAN	13847.1406
SSRD_HUMAN	39349.8672
BAP31_HUMAN	17262.584
TPMT_HUMAN	4432.75537
MECP2_HUMAN	544.808655
HCFC1_HUMAN	15750.5635
IRAK1_HUMAN	1468.50769
CAV2_HUMAN	1123.66663
AL3A2_HUMAN	3859.83105
DHB4_HUMAN	16421.8008
PSMD7_HUMAN	20286.3809
SUOX_HUMAN	2437.26953
SPHM_HUMAN	6816.91992
STA5B_HUMAN	1590.97656
UBP11_HUMAN	2054.89014
CLCN7_HUMAN	2401.97827
VAMP7_HUMAN	2499.69971
KS6A3_HUMAN	6201.97021
BMX_HUMAN	5931.49072
AFF1_HUMAN	330.518463
HDGF_HUMAN	18659.1484
CCNH_HUMAN	887.676758

MAT1_HUMAN	1119.14563
NEK3_HUMAN	777.315491
UB2E1_HUMAN	2104.51782
NDUA8_HUMAN	6050.9541
ROA3_HUMAN	49544.4766
6PGD_HUMAN	122907.656
HNRPM_HUMAN	96824.8906
IMA1_HUMAN	35602.7891
IMA5_HUMAN	5039.44922
NCBP2_HUMAN	2921.05542
GDS1_HUMAN	12848.168
RPAB3_HUMAN	3794.81226
MP2K6_HUMAN	1733.21704
GDIR1_HUMAN	73244.5547
GDIR2_HUMAN	76282.8203
AGFG1_HUMAN	4404.70654
HNRPF_HUMAN	16850.0898
STAT2_HUMAN	2496.96167
MSH6_HUMAN	7473.28076
KIF11_HUMAN	5243.48291
VAV2_HUMAN	4861.64453
ZN143_HUMAN	444.340149
RBM5_HUMAN	4283.875
RIDA_HUMAN	558.016724
SPSY_HUMAN	23363.3789
HXK2_HUMAN	35335.9414
EFNB2_HUMAN	504.388794
RM12_HUMAN	22629.7363
DGKQ_HUMAN	159.086136
NDST1_HUMAN	1319.19421
THOP1_HUMAN	12569.3604
CAZA1_HUMAN	101302.703
HMGA2_HUMAN	1386.19495
CRIP2_HUMAN	40331.0313
NUP98_HUMAN	8195.58203
BIEA_HUMAN	24446.1465
TXTP_HUMAN	14880.6387
PPP5_HUMAN	10279.917
PLK1_HUMAN	3270.95044
DAPK1_HUMAN	1237.63171
ARFP2_HUMAN	3481.88647
ARFP1_HUMAN	5970.41553
NUBP1_HUMAN	4034.52979
ACLY_HUMAN	84104.0938
MAP11_HUMAN	8055.54932
SUCA_HUMAN	11053.4082
MVD1_HUMAN	5562.3877
PGTB1_HUMAN	2782.5542
PGTB2_HUMAN	7520.2583
COPB_HUMAN	30340.3262

COPA_HUMAN	56487.3789
CATC_HUMAN	6394.40137
LIMK1_HUMAN	9971.92285
LIMK2_HUMAN	628.454956
CLH2_HUMAN	538.71875
AP3M2_HUMAN	976.085571
AP2S1_HUMAN	10628.7861
CCHL_HUMAN	6022.64063
MK12_HUMAN	1343.96606
PTTG_HUMAN	3916.60229
SMTN_HUMAN	3359.98071
MOT1_HUMAN	9288.20508
IST1_HUMAN	10849.374
SC24C_HUMAN	18534.709
TCP4_HUMAN	44651.9102
DPOG1_HUMAN	1522.56567
ICLN_HUMAN	11181.5791
SYRC_HUMAN	37599.1211
HIRA_HUMAN	1290.40247
ATX3_HUMAN	2563.11938
ATN1_HUMAN	1654.14636
PMS1_HUMAN	777.208801
PMS2_HUMAN	493.651184
CA2D1_HUMAN	2112.30981
SYYC_HUMAN	37501.0469
UBP14_HUMAN	22673.8789
AAKG1_HUMAN	3440.22656
HSP72_HUMAN	11395.9082
BCAT1_HUMAN	24269.0098
AT1B3_HUMAN	27409.5898
RD23B_HUMAN	1027.19653
EPHB4_HUMAN	3232.70239
ANAG_HUMAN	1756.45422
KAD2_HUMAN	5172.87549
P5CS_HUMAN	18634.0313
SNAA_HUMAN	21959.625
IF5_HUMAN	21872.7246
S12A2_HUMAN	7590.27148
PSMD4_HUMAN	20717.0117
DRG2_HUMAN	7988.6709
XPO2_HUMAN	166441.734
TERA_HUMAN	163791.328
MFAP1_HUMAN	1723.47156
ECHB_HUMAN	54669.1094
MANF_HUMAN	16365.1885
AFAD_HUMAN	4264.35205
ELL_HUMAN	1067.15552
BRPF1_HUMAN	1443.86157
NP1L1_HUMAN	53795.3867
CASP7_HUMAN	7097.44775

CASP6_HUMAN	4879.53662
ADK_HUMAN	13956.2666
DSRAD_HUMAN	13093.6172
LAMB2_HUMAN	4022.01904
CAD13_HUMAN	3459.61328
TPD52_HUMAN	143.824387
SEC13_HUMAN	14947.3223
NH2L1_HUMAN	10084.1523
PSA_HUMAN	32240.8828
ALR_HUMAN	250.045105
HNRH2_HUMAN	13756.5078
SCOT1_HUMAN	12153.585
EIF3B_HUMAN	22594.1816
FCGRN_HUMAN	5712.30225
BID_HUMAN	2089.94507
ATPK_HUMAN	20836.4297
RRP1_HUMAN	6286.67822
SYMC_HUMAN	33120.5195
ARP19_HUMAN	6115.48096
AP1S2_HUMAN	4695.53418
ATP68_HUMAN	8043.95557
ATP5E_HUMAN	5879.81348
ATP5I_HUMAN	23415.1367
HDAC4_HUMAN	1632.41589
IF6_HUMAN	36724.8008
CTBP2_HUMAN	21628.6797
NDUA6_HUMAN	8402.78223
PEX3_HUMAN	622.458069
DHB7_HUMAN	1129.47546
BCAR1_HUMAN	9650.50879
STX17_HUMAN	1544.4137
CF298_HUMAN	843.729736
WDR4_HUMAN	1369.90149
TMM33_HUMAN	34890.4609
GEMI4_HUMAN	3596.1521
CORO7_HUMAN	6156.70654
NU107_HUMAN	10079.7578
GSDMD_HUMAN	8096.56543
SELB_HUMAN	2264.7417
SESN2_HUMAN	1005.53186
ANTR2_HUMAN	1733.94226
MTPN_HUMAN	458.715454
ARPC4_HUMAN	109341.57
CD81_HUMAN	10855.4365
SC61G_HUMAN	40079.0508
TPIS_HUMAN	140130.813
EIF3E_HUMAN	33592.8633
SC61B_HUMAN	27864.3223
PTEN_HUMAN	542.170044
PP4C_HUMAN	7721.12646

GBRL2_HUMAN	12387.0625
ROMO1_HUMAN	3384.36963
MYL6_HUMAN	5633.60889
ACTB_HUMAN	1865388.38
IF4A1_HUMAN	184071.078
RS20_HUMAN	107822.414
PRPS1_HUMAN	5915.47998
PSA6_HUMAN	49968.6094
S10AA_HUMAN	35137.2773
CDC42_HUMAN	18468.4648
DEST_HUMAN	17749.3828
GMFB_HUMAN	2881.72827
RAB8A_HUMAN	4050.8396
SPCS3_HUMAN	18317.0273
SRP54_HUMAN	10161.1035
RAB4B_HUMAN	2220.58276
RAB2A_HUMAN	33950.4414
RAB5B_HUMAN	6402.61572
RAB10_HUMAN	6790.2124
UB2D3_HUMAN	16183.5234
UBC12_HUMAN	17419.8867
UBE2K_HUMAN	19754.2813
UBE2N_HUMAN	24123.4492
RAB14_HUMAN	23762.5156
ARP3_HUMAN	69577.0859
ARP2_HUMAN	57726.8477
ACTZ_HUMAN	20626.1406
CSN2_HUMAN	13228.6914
ABCE1_HUMAN	26558.6934
RAP1B_HUMAN	35099.168
RAP2B_HUMAN	4443.34277
MAX_HUMAN	1468.28308
RS3A_HUMAN	104466.555
RL26_HUMAN	131649.688
PSME3_HUMAN	11804.1582
RL15_HUMAN	160858.484
RL27_HUMAN	184541.797
VA0D1_HUMAN	16852.0977
RL37A_HUMAN	9559.50684
RHOA_HUMAN	13678.8652
RND3_HUMAN	1101.76501
NAA20_HUMAN	2841.31616
CH10_HUMAN	8243.3291
S61A1_HUMAN	28602.8633
LYSC_HUMAN	209.851456
PFD3_HUMAN	10984.917
STXB1_HUMAN	7072.50439
B2MG_HUMAN	602.185303
DAD1_HUMAN	26865.5879
NPC2_HUMAN	4122.2373

COPZ1_HUMAN	23406.459
RL37_HUMAN	731.100037
SUMO2_HUMAN	20658.2363
DCAF7_HUMAN	2902.49023
WDR5_HUMAN	3485.64771
AP1S1_HUMAN	7758.38721
NTF2_HUMAN	6190.51709
HNRPK_HUMAN	217121.266
1433G_HUMAN	53385.9375
UBP46_HUMAN	1556.09497
RRAS2_HUMAN	17045.8848
RS7_HUMAN	56840.2383
PP1A_HUMAN	25017.8242
PP1B_HUMAN	24848.1133
PRS4_HUMAN	41649.0273
PRS8_HUMAN	54686.1641
RS8_HUMAN	150897.922
RS15A_HUMAN	120266.945
RS16_HUMAN	223620.672
UB2G1_HUMAN	1664.36731
UBE2H_HUMAN	4841.43018
1433E_HUMAN	90632.0234
RS14_HUMAN	150477.219
RS23_HUMAN	47409.3477
RS18_HUMAN	243735.438
RS29_HUMAN	33575.2656
RS13_HUMAN	113745.875
RS11_HUMAN	44491.3555
RUXE_HUMAN	25529.1445
RUXF_HUMAN	7832.93164
LSM3_HUMAN	2213.51489
LSM6_HUMAN	9420.21094
SMD1_HUMAN	14432.8633
SMD2_HUMAN	22945.0293
SMD3_HUMAN	53442.7695
TYB4_HUMAN	4139.21143
ARF6_HUMAN	20299.1523
PRS10_HUMAN	28175.9395
SELT_HUMAN	4504.69531
TBPL1_HUMAN	973.141418
RL7A_HUMAN	118628.906
RPB7_HUMAN	2733.25659
RB11A_HUMAN	63437.1641
ERF1_HUMAN	23255.0059
CNBP_HUMAN	7534.66992
YPEL5_HUMAN	1003.31976
RS4X_HUMAN	154861.484
PP2AB_HUMAN	34297.2422
ACTA_HUMAN	354443.188
RHOB_HUMAN	6491.47363

RL23A_HUMAN	164724.484
RS6_HUMAN	214301.156
H4_HUMAN	136088.234
RAB1A_HUMAN	9436.09375
RAN_HUMAN	265573.344
RL23_HUMAN	102720.359
RAP1A_HUMAN	5571.46436
RS15_HUMAN	27199.959
RS24_HUMAN	49183.1758
RS25_HUMAN	208379.234
RS26_HUMAN	52618.8789
RS28_HUMAN	48021.418
RS30_HUMAN	56332.3398
GBB1_HUMAN	14094.6992
RBX1_HUMAN	14223.1777
GBB2_HUMAN	52187.6289
RL30_HUMAN	91968.9844
RL39_HUMAN	30370.1914
RL31_HUMAN	105499.898
RL10A_HUMAN	73866.3438
RL32_HUMAN	36198.6641
RL11_HUMAN	82857.4063
RL8_HUMAN	98029.5234
PPIA_HUMAN	118806.859
FKB1A_HUMAN	25190.9648
RS27A_HUMAN	117557.859
GRB2_HUMAN	7108.70752
TRA2B_HUMAN	12156.9824
RAC1_HUMAN	73569.3438
AP2B1_HUMAN	29918.4004
VAMP2_HUMAN	8475.43262
GNAS2_HUMAN	13718.2295
GNAI1_HUMAN	813.359131
1433Z_HUMAN	208218.234
2ABA_HUMAN	28553.9629
SUMO1_HUMAN	14805.7031
DYL1_HUMAN	10234.1494
DYLT1_HUMAN	4274.92822
RL38_HUMAN	49341.7695
SKP1_HUMAN	2726.14526
GBG5_HUMAN	1289.71936
RS21_HUMAN	11757.3965
IF5A1_HUMAN	73255.4375
RACK1_HUMAN	178801.109
SPT4H_HUMAN	2169.94434
UBC9_HUMAN	14480.9229
TYB10_HUMAN	3743.625
PP2AA_HUMAN	3153.99756
YBOX1_HUMAN	39723.2461
SC11A_HUMAN	44108.0547

CSK2B_HUMAN	7121.00586
TPM4_HUMAN	79798.3281
UB2L3_HUMAN	31137.1328
EF1A1_HUMAN	1195501.25
TBA1B_HUMAN	978806.625
TBA4A_HUMAN	4337.42822
TBB4B_HUMAN	289835.625
CSK21_HUMAN	10135.5234
PA1B2_HUMAN	7154.87939
H31_HUMAN	16033.0908
HBB_HUMAN	3268.96191
HBA_HUMAN	6197.64453
CXAR_HUMAN	2051.54004
F193A_HUMAN	2626.61279
NOP14_HUMAN	3331.77075
IGBP1_HUMAN	5993.34033
SHPS1_HUMAN	983.614258
SERB_HUMAN	8806.15039
RBM6_HUMAN	3582.96216
IF4G2_HUMAN	33055.6875
RPP38_HUMAN	3079.0354
RPP30_HUMAN	5132.1665
GTF2I_HUMAN	26988.6191
PI42B_HUMAN	1557.52527
CNTP1_HUMAN	5022.27295
SRPK2_HUMAN	3182.75269
TCPB_HUMAN	79513.1406
S35A2_HUMAN	1084.94678
RAE1L_HUMAN	9299.58496
GSTO1_HUMAN	134764.297
PRKDC_HUMAN	44687.0156
ADA17_HUMAN	2576.23291
BL1S1_HUMAN	1277.01331
SRPX_HUMAN	2342.70044
ARGI2_HUMAN	1331.48645
NTH_HUMAN	538.586304
IN35_HUMAN	565.233276
NUCB2_HUMAN	2402.26392
BASP1_HUMAN	9000.58008
DCD_HUMAN	3701.40894
TMF1_HUMAN	2051.64722
RT22_HUMAN	9715.38965
RT25_HUMAN	2177.39258
RT10_HUMAN	4604.31104
RT35_HUMAN	2133.14429
RT05_HUMAN	3617.09863
KGD4_HUMAN	271.780945
RT11_HUMAN	6202.37256
RT15_HUMAN	7445.73535
RT21_HUMAN	3700.59155

RT34_HUMAN	19183.3711
RT06_HUMAN	3877.88037
RT09_HUMAN	5603.53613
SARNP_HUMAN	29004.1719
LACTB_HUMAN	2550.04053
COG7_HUMAN	3765.5835
RL24_HUMAN	100846.531
TXN4A_HUMAN	3654.67334
RL36A_HUMAN	16790.3164
CBX1_HUMAN	3378.37036
SMAD3_HUMAN	5300.146
ARF1_HUMAN	101117.305
ARF5_HUMAN	11301.4697
ERH_HUMAN	1928.1239
RHOG_HUMAN	9130.51563
RL19_HUMAN	75720.2656
SERF2_HUMAN	3432.23071
SRSF3_HUMAN	97880.4297
FOXK1_HUMAN	5828.92236
CCZ1B_HUMAN	3149.39648
DAB2_HUMAN	15182.2334
PGBM_HUMAN	39828.5
XIAP_HUMAN	1139.54456
EFNB1_HUMAN	4646.95654
RBM10_HUMAN	5335.9873
RBM3_HUMAN	16763.3867
AT2C1_HUMAN	2718.75928
AT11A_HUMAN	1637.56592
CYC_HUMAN	49816.9063
EM55_HUMAN	2908.71729
TFAM_HUMAN	9918.97656
PIPNA_HUMAN	9739.01758
S25A3_HUMAN	109910.727
VIGLN_HUMAN	35223.6016
TF2B_HUMAN	2853.64331
CDK6_HUMAN	1545.88184
CDK5_HUMAN	3817.76367
CDK17_HUMAN	9121.35352
PURA_HUMAN	5406.38037
BORG5_HUMAN	5321.92969
CLH1_HUMAN	87504.75
HSF1_HUMAN	587.350647
NFKB2_HUMAN	2554.82935
FKBP3_HUMAN	38728.0078
REEP5_HUMAN	12868.4951
DHSO_HUMAN	8762.50879
HNRPU_HUMAN	83261.7266
U2AF1_HUMAN	12078.8457
SPTB2_HUMAN	56274.9609
TIAR_HUMAN	8603.14941

SET_HUMAN	95426.125
SRSF2_HUMAN	33295.5703
FOXK2_HUMAN	1818.22876
RELB_HUMAN	667.005066
GALK2_HUMAN	4715.72461
AMPD2_HUMAN	8996.74414
FABP5_HUMAN	84992.0938
CAP1_HUMAN	73299.1094
FLI1_HUMAN	3964.52295
HMCS1_HUMAN	14874.2051
IFM3_HUMAN	8571.39453
LAT1_HUMAN	2669.36353
NC2B_HUMAN	2277.87305
EXOSX_HUMAN	4398.15576
OTUD4_HUMAN	820.927307
PFKAP_HUMAN	45737.0234
XPC_HUMAN	1529.74915
EWS_HUMAN	33925.7188
CDR2_HUMAN	3653.03638
OCRL_HUMAN	2810.8811
PLCB3_HUMAN	9826.16895
TAGL_HUMAN	4469.70801
AK17A_HUMAN	2829.41724
PYRD_HUMAN	553.94696
ODO1_HUMAN	15781.96
CENPE_HUMAN	419.37262
KIF23_HUMAN	1035.29553
MMSA_HUMAN	3289.94189
NRG1_HUMAN	303.837311
CP27A_HUMAN	891.246582
DSG1_HUMAN	5006.20557
SP3_HUMAN	274.60672
H11_HUMAN	1365.43347
RL18A_HUMAN	84011.4375
MP2K1_HUMAN	14306.6465
TIE2_HUMAN	1921.81152
FKBP4_HUMAN	23894.0742
PLOD1_HUMAN	17620.084
NUCB1_HUMAN	4219.82764
RL6_HUMAN	243680.344
TOP2B_HUMAN	2039.04834
AKA12_HUMAN	39027.4336
M2OM_HUMAN	17156.584
DYST_HUMAN	3441.63794
ENL_HUMAN	1473.91736
MECOM_HUMAN	767.874756
GNA12_HUMAN	1366.62854
CAV1_HUMAN	77958.1641
ACY1_HUMAN	1685.151
TNAP2_HUMAN	1434.95715

LMNB2_HUMAN	28355.3047
PTPS_HUMAN	3062.61646
UPAR_HUMAN	1424.62976
KIME_HUMAN	5892.31934
ERCC6_HUMAN	113.314911
TAP1_HUMAN	2887.60278
TAP2_HUMAN	1613.10291
CEBPZ_HUMAN	4095.81763
ZNF92_HUMAN	1383.50403
TF65_HUMAN	8345.2334
UBXN1_HUMAN	9254.4082
GLGB_HUMAN	25658.9805
IF4G1_HUMAN	29631.4531
ATP7A_HUMAN	217.221237
NOTC2_HUMAN	1204.77222
TLE1_HUMAN	2039.50916
TLE3_HUMAN	1366.20386
LGUL_HUMAN	29421.832
ACVR1_HUMAN	479.452362
SSBP_HUMAN	15166.2256
REL_HUMAN	741.716125
1433F_HUMAN	52688.4414
PLP2_HUMAN	34224.4023
CSTF1_HUMAN	7257.56396
UBE3A_HUMAN	6360.22852
DYN1_HUMAN	1687.12354
PTN12_HUMAN	6655.67529
FAK1_HUMAN	10468.4063
SRS11_HUMAN	124222.648
KPCD_HUMAN	2918.23218
CALD1_HUMAN	41761.25
FOLC_HUMAN	1462.74585
CTSL2_HUMAN	1337.50342
PTN11_HUMAN	8340.52637
KDSR_HUMAN	4242.65527
PUR1_HUMAN	7289.86279
GFPT1_HUMAN	20728.0352
EXOS9_HUMAN	4175.5752
PSME1_HUMAN	36953.6172
SUH_HUMAN	883.066162
MEF2C_HUMAN	459.04129
APLP2_HUMAN	6660.87891
GABPA_HUMAN	2629.88037
RING1_HUMAN	547.582153
FMR1_HUMAN	751.099976
PRDX1_HUMAN	184320.797
RL18_HUMAN	137047.266
C1QBP_HUMAN	29024.5566
CKAP4_HUMAN	121404.086
ZO1_HUMAN	15634.168

TISB_HUMAN	1154.21228
SPAG1_HUMAN	1283.94543
KHDR1_HUMAN	33850.0664
BAX_HUMAN	16001.3799
B2CL1_HUMAN	6084.04395
MCL1_HUMAN	1657.06067
DPOE1_HUMAN	3925.76904
KLC1_HUMAN	21948.4434
SOS1_HUMAN	1640.79553
SRSF1_HUMAN	89957.8516
RHG01_HUMAN	15679.1602
TLE5_HUMAN	1037.23816
SRSF4_HUMAN	8860.3418
PCDH1_HUMAN	7279.84033
TGM3_HUMAN	1248.95056
PP2BA_HUMAN	4790.96924
DHX9_HUMAN	61895.3711
QOR_HUMAN	7967.43066
GOGA3_HUMAN	3189.63794
GOGA2_HUMAN	3628.80176
ECHP_HUMAN	975.058838
MFGM_HUMAN	5016.33789
PDE4D_HUMAN	842.050964
DSC1_HUMAN	3388.354
HDHD1_HUMAN	2736.51343
TICN1_HUMAN	1371.48438
CD47_HUMAN	16424.8398
PPID_HUMAN	15790.8125
SSRP1_HUMAN	26243.1055
CAMP2_HUMAN	3477.4978
SPIR1_HUMAN	762.569153
SLFN5_HUMAN	3491.28882
VAC14_HUMAN	5911.43604
NSUN2_HUMAN	19458.6563
DMWD_HUMAN	414.208649
RBBP4_HUMAN	58405.8086
NCBP1_HUMAN	14033.9063
MGT5A_HUMAN	665.006226
EP300_HUMAN	2698.01489
AHNK_HUMAN	131228.422
FCHO2_HUMAN	1576.18054
ELP6_HUMAN	1168.31836
HSP7E_HUMAN	6329.97168
SCRN3_HUMAN	2739.97168
CGNL1_HUMAN	4957.45508
LTOR4_HUMAN	4946.47363
NEXN_HUMAN	3892.91504
MGAT2_HUMAN	6226.64893
GALT2_HUMAN	8449.15527
GALT1_HUMAN	8856.82617

AP1B1_HUMAN	32597.3574
CPSF1_HUMAN	7860.23438
BST2_HUMAN	671.887085
MPPA_HUMAN	7736.54443
SIA4A_HUMAN	666.245667
SCRN1_HUMAN	8384.69824
WASC5_HUMAN	7051.28857
NU160_HUMAN	7808.24658
SCAP_HUMAN	2426.39893
FOXO1_HUMAN	556.559753
TBL3_HUMAN	3801.82813
TF3C1_HUMAN	2515.28271
TWF1_HUMAN	16762.1523
ASPH_HUMAN	12529.5957
TFCP2_HUMAN	2236.86841
AKP13_HUMAN	2178.57495
FBLN3_HUMAN	24522.0371
SNF5_HUMAN	4419.78906
BPTF_HUMAN	868.507324
CDC20_HUMAN	1871.71484
FSTL1_HUMAN	6793.66846
STX4_HUMAN	2008.07788
GRSF1_HUMAN	8238.51074
M4K2_HUMAN	1070.09851
MERTK_HUMAN	3913.63501
SFSWA_HUMAN	1264.83618
CHD3_HUMAN	694.314758
SF3A3_HUMAN	25556.6934
TP53B_HUMAN	6097.29004
TM115_HUMAN	2271.84204
IFRD2_HUMAN	1694.60291
TRI26_HUMAN	1490.25037
AIMP1_HUMAN	15625.6953
ILF2_HUMAN	74961.6016
ILF3_HUMAN	90212.8672
LMAN2_HUMAN	45292.3242
TRAP1_HUMAN	382334.688
TRAF2_HUMAN	2889.93579
DLG1_HUMAN	3736.47461
MYO1E_HUMAN	8676.49707
PP1R8_HUMAN	4265.11523
TP4A2_HUMAN	4449.3584
ABR_HUMAN	4298.52441
NPRL3_HUMAN	759.948486
SEC20_HUMAN	5230.14014
BNIP2_HUMAN	1708.22546
NFX1_HUMAN	184.981232
CSTF3_HUMAN	6677.02344
TSN31_HUMAN	1174.323
ECH1_HUMAN	8037.1543

RHG05_HUMAN	1066.1095
STRN3_HUMAN	4876.71582
CDC16_HUMAN	3266.60107
STK4_HUMAN	3674.3335
FLII_HUMAN	24980.5508
TRI32_HUMAN	1353.42029
COASY_HUMAN	4455.29883
RM28_HUMAN	6529.75244
ACACA_HUMAN	13807.498
CSN1_HUMAN	14764.4883
UBP4_HUMAN	3647.84766
CAF1A_HUMAN	564.384399
CAF1B_HUMAN	1118.58472
RED_HUMAN	10865.3857
MTAP_HUMAN	10734.1426
AAPK1_HUMAN	4560.00195
LIPA1_HUMAN	2403.59888
EI2BE_HUMAN	18812.8125
TADBP_HUMAN	16024.1826
ROA0_HUMAN	20632.9785
AIMP2_HUMAN	12662.5859
FADD_HUMAN	5797.63574
PRDX4_HUMAN	42056.1211
MP2K5_HUMAN	415.006409
PAK2_HUMAN	14966.5605
CBX3_HUMAN	23219.3125
STK3_HUMAN	1282.25366
STX5_HUMAN	3445.32837
PSMD2_HUMAN	37188.8008
MMRN1_HUMAN	6338.30664
DDX10_HUMAN	1203.19287
DNJC3_HUMAN	21369.6191
SBP1_HUMAN	3452.75
NDK3_HUMAN	3853.16431
SRSF9_HUMAN	17598.541
SRSF5_HUMAN	6609.34766
SRSF6_HUMAN	69371.4141
MD2L1_HUMAN	9407.0625
TIF1B_HUMAN	34452.1563
SEM3F_HUMAN	444.889099
STX3_HUMAN	1213.82104
G3BP1_HUMAN	41613.0234
NMI_HUMAN	3683.75635
PTK7_HUMAN	2743.78638
PABP4_HUMAN	29911.4766
ATM_HUMAN	1445.44971
GRB10_HUMAN	2827.83789
IFIT5_HUMAN	458.456848
MTA1_HUMAN	3506.82788
EIF3I_HUMAN	18537.7188

PPIL2_HUMAN	1156.56689
2A5G_HUMAN	2882.82983
CTBP1_HUMAN	5032.45068
PHLP_HUMAN	5195.53418
PLD1_HUMAN	3009.91626
TARB1_HUMAN	465.804779
UB2V1_HUMAN	36842.1328
RM49_HUMAN	405.562286
DC1I2_HUMAN	23391.832
ORC2_HUMAN	1481.55457
ILK_HUMAN	33324.2383
NNTM_HUMAN	18565.4199
SNTB2_HUMAN	16005.7744
XRCC4_HUMAN	3570.85156
PPIG_HUMAN	7965.88965
TCOF_HUMAN	8870.25684
U119A_HUMAN	873.957153
SF3B2_HUMAN	25140.6602
OS9_HUMAN	2162.10303
GOGA4_HUMAN	2621.5625
HAP28_HUMAN	19222.3574
ADAM9_HUMAN	11846.877
ADA15_HUMAN	4328.02441
TMED1_HUMAN	3954.27832
FKBP5_HUMAN	19463.4375
MYO9B_HUMAN	5407.40137
ROCK1_HUMAN	8556.58105
NFAC2_HUMAN	906.810425
TOP3A_HUMAN	924.660583
SMAD4_HUMAN	3705.31201
VPP3_HUMAN	6900.98193
BIRC2_HUMAN	876.326355
PICAL_HUMAN	11472.1719
MTM1_HUMAN	1983.7583
SQSTM_HUMAN	8978.90234
MTX1_HUMAN	5091.98486
TBB3_HUMAN	13520.1152
ASAH1_HUMAN	374.441833
PRP4B_HUMAN	2860.13574
PIN1_HUMAN	6211.27197
ATR_HUMAN	1883.68286
4EBP1_HUMAN	501.013947
RIPK1_HUMAN	1568.79919
HDAC1_HUMAN	17529.5742
KCC2G_HUMAN	4303.23535
KCC2D_HUMAN	4723.11719
DCTN2_HUMAN	4521.31396
PKD2_HUMAN	414.319183
ULA1_HUMAN	8810.5957
ITPK1_HUMAN	1330.66943

SNW1_HUMAN	9374.63086
DGKZ_HUMAN	4230.28125
STIM1_HUMAN	2917.07593
GRAP_HUMAN	3530.13647
TRA2A_HUMAN	4108.92578
SNX1_HUMAN	11738.6113
KRR1_HUMAN	5666.94531
PWP1_HUMAN	5176.90576
MTMR1_HUMAN	1344.16931
MTMR2_HUMAN	5165.60791
CUL1_HUMAN	5221.5835
CUL2_HUMAN	10687.6094
CUL3_HUMAN	7118.47852
CUL4A_HUMAN	4223.22803
CUL4B_HUMAN	15667.9707
ASPP2_HUMAN	558.078796
DYR1A_HUMAN	390.086365
FCL_HUMAN	14679.6406
RAB32_HUMAN	9298.35645
TPBG_HUMAN	1598.24426
FHL1_HUMAN	12129.5537
FHL3_HUMAN	2138.40796
RIN1_HUMAN	598.57782
AAMP_HUMAN	2837.26904
MOGS_HUMAN	32805.4844
CD166_HUMAN	5905.45117
THOC5_HUMAN	3456.20044
SHRM2_HUMAN	1384.54297
SPTN1_HUMAN	35174.1523
NOG2_HUMAN	4441.65723
AUHM_HUMAN	1017.92383
PKP1_HUMAN	693.477661
DX39B_HUMAN	12086.8818
BLMH_HUMAN	24523.5391
EXOS2_HUMAN	4893.80566
BMPR2_HUMAN	2320.72119
TBB2A_HUMAN	180383.234
TF2H2_HUMAN	1694.70972
TF2H3_HUMAN	2164.75073
BYST_HUMAN	10733.1523
RPGF1_HUMAN	2512.28247
IDI1_HUMAN	10082.2852
PEBB_HUMAN	7957.94678
NFYC_HUMAN	1041.96509
CDK13_HUMAN	1385.05212
CKAP5_HUMAN	28334.0508
CIRBP_HUMAN	6926.68701
KCC1A_HUMAN	2020.60571
COTL1_HUMAN	32041.2813
HNRPD_HUMAN	156592.375

SCRB2_HUMAN	26453.2578
NID2_HUMAN	755.797913
LRP8_HUMAN	231.552231
DAG1_HUMAN	3657.76147
VEZF1_HUMAN	157.913895
BOP1_HUMAN	7458.81055
UBE4A_HUMAN	3874.78735
SEPT6_HUMAN	5438.75732
KEAP1_HUMAN	1539.44299
URB2_HUMAN	1777.63293
DHX34_HUMAN	1168.25964
MORC3_HUMAN	1258.47412
SAFB2_HUMAN	11316.2695
EIF3A_HUMAN	45556.7617
ARHG7_HUMAN	7389.21777
EFR3A_HUMAN	4671.84521
UBP2L_HUMAN	19127.5684
SCRIB_HUMAN	6615.9917
GIT2_HUMAN	3671.44824
SREC_HUMAN	4056.31323
MLEC_HUMAN	17217.8926
TTL12_HUMAN	9957.25
DPOA2_HUMAN	1961.7854
DOCK1_HUMAN	5761.27734
TFDP1_HUMAN	1936.7207
FHL2_HUMAN	20283.4238
DPYL3_HUMAN	58423.0977
ICT1_HUMAN	6572.69629
ZMYM3_HUMAN	768.599121
DCTN1_HUMAN	14225.8271
DYHC1_HUMAN	72907.5234
EI2BA_HUMAN	8241.14258
IF4A2_HUMAN	7915.73926
ELOA1_HUMAN	1495.74072
SRC8_HUMAN	19143.6641
NUCG_HUMAN	3221.57568
FLOT2_HUMAN	3978.63599
RCN2_HUMAN	13450.9297
TRI25_HUMAN	17594.3164
FLNC_HUMAN	54204.1875
FKBP8_HUMAN	5394.2832
FA50A_HUMAN	5549.83252
FRG1_HUMAN	2317.36597
GNA13_HUMAN	50817.8438
GAMT_HUMAN	1298.15405
GALE_HUMAN	8175.59961
LRC32_HUMAN	301.409302
CAPR1_HUMAN	23407.5859
BECN1_HUMAN	740.293884
RBM39_HUMAN	15764.5049

CASL_HUMAN	1134.58777
HLTF_HUMAN	1217.85938
ERG1_HUMAN	2515.22461
PDIA5_HUMAN	12621.9131
KPRA_HUMAN	8689.97266
DHX8_HUMAN	2284.73779
MCM6_HUMAN	17796.623
ITPR2_HUMAN	5012.77637
ITPR3_HUMAN	6159.60156
ITPR1_HUMAN	680.837341
RASA3_HUMAN	4128.30615
PLSI_HUMAN	1225.30896
IRF3_HUMAN	4034.46802
LAGE3_HUMAN	1018.97418
BLTP2_HUMAN	504.369965
TRIPC_HUMAN	5536.33105
PUM1_HUMAN	4734.2583
MDC1_HUMAN	611.961487
EPN4_HUMAN	10453.3389
KANK1_HUMAN	3165.03662
SMC1A_HUMAN	62119.3281
RRP1B_HUMAN	6500.51123
DIP2A_HUMAN	6904.28809
RRP5_HUMAN	6535.69922
PSF1_HUMAN	2199.22754
BMS1_HUMAN	3770.57764
LPIN1_HUMAN	2140.11914
UBP10_HUMAN	11377.29
MESD_HUMAN	11042.7031
GANAB_HUMAN	55669.3047
RFTN1_HUMAN	3795.27148
MFS10_HUMAN	2028.25037
2A5D_HUMAN	11268.9443
LBR_HUMAN	15650.9736
COG2_HUMAN	858.086121
MVP_HUMAN	93624.2891
LTBP1_HUMAN	1041.00037
LTBP2_HUMAN	2555.52954
CBX2_HUMAN	272.67746
GOGB1_HUMAN	3953.51294
CASP8_HUMAN	1616.27405
NAA30_HUMAN	3233.80249
KIF22_HUMAN	1287.24426
MEF2D_HUMAN	1571.62061
CHD4_HUMAN	17126.5645
LASP1_HUMAN	41569.3047
PTGR1_HUMAN	9994.90625
NC2A_HUMAN	5624.24219
NFAC4_HUMAN	483.190796
ZN638_HUMAN	4240.69629

IMB1_HUMAN	51185.2695
NOLC1_HUMAN	4234.67871
NUMA1_HUMAN	10675.1943
PSME4_HUMAN	8467.7998
CUL7_HUMAN	1674.58728
SLMAP_HUMAN	1131.02039
GAPD1_HUMAN	11132.208
NAA25_HUMAN	8956.78516
MELT_HUMAN	1488.99707
CND2_HUMAN	6739.04395
PAF15_HUMAN	868.692261
SPCS2_HUMAN	20839.873
EMC2_HUMAN	5093.21289
FL2D_HUMAN	1305.60718
PSMD6_HUMAN	16043.0254
HERP1_HUMAN	243.297882
MD2BP_HUMAN	1489.59814
MO4L2_HUMAN	4432.9292
ABRX2_HUMAN	2335.87549
SEPT2_HUMAN	40468.0781
SART3_HUMAN	13600.0771
CND1_HUMAN	13009.8574
SUZ12_HUMAN	866.395264
EXOS7_HUMAN	4267.01416
TNIP1_HUMAN	1162.03137
U5S1_HUMAN	70494.1328
SYLM_HUMAN	3072.97559
R3HD1_HUMAN	1558.04102
TRAM2_HUMAN	1178.6416
SNX17_HUMAN	4815.91553
AR6P1_HUMAN	5632.55127
RB3GP_HUMAN	4178.33643
S39AE_HUMAN	2757.91138
SYK_HUMAN	38128.3672
SETB1_HUMAN	1127.70154
LRC14_HUMAN	931.89093
RRS1_HUMAN	8567.20605
DPOD3_HUMAN	1958.85559
IF4H_HUMAN	39012.1641
ACAP2_HUMAN	4414.3418
KIF14_HUMAN	527.368835
BRD3_HUMAN	1243.86902
WDR43_HUMAN	3423.81909
ACOX1_HUMAN	7795.83838
OXA1L_HUMAN	7370.68848
EEA1_HUMAN	13781.9873
PDIA6_HUMAN	113930.109
PA1B3_HUMAN	4259.32666
PDK3_HUMAN	840.500122
PEA15_HUMAN	1974.03333

EBP_HUMAN	10134.3828
PMVK_HUMAN	6127.99756
KPCD1_HUMAN	688.461548
PLEC_HUMAN	70314.3047
PCM1_HUMAN	2646.4729
NOMO1_HUMAN	27004.2754
PON2_HUMAN	8052.64795
2A5A_HUMAN	2594.0769
IPYR_HUMAN	47901.3711
TEBP_HUMAN	19079.8555
STK38_HUMAN	2860.77783
NONO_HUMAN	63597.5195
PTPA_HUMAN	21849.3086
PTPRK_HUMAN	4076.97656
PWP2_HUMAN	4527.00928
RABE1_HUMAN	1871.37305
RAB35_HUMAN	6140.52148
RNPS1_HUMAN	6198.1333
RBBP5_HUMAN	2844.71533
RCN1_HUMAN	16768.2266
RBP1_HUMAN	426.972504
L2GL1_HUMAN	2335.29858
LRC41_HUMAN	786.455627
TTF1_HUMAN	8499.50586
TMED2_HUMAN	5895.50684
PCBP1_HUMAN	167903.672
PCBP2_HUMAN	154054.266
ELOC_HUMAN	3683.97266
ELOB_HUMAN	2759.28638
RHEB_HUMAN	11432.4453
UBE3C_HUMAN	4359.77588
TOM20_HUMAN	10280.8486
MTFR1_HUMAN	1474.29016
DHC24_HUMAN	16727.4316
SF3B3_HUMAN	26669.0293
PUM3_HUMAN	5670.66162
DLGP5_HUMAN	3924.66162
RSU1_HUMAN	45198.5859
CNN3_HUMAN	20996.334
KS6A1_HUMAN	539.908386
SAFB1_HUMAN	8888.81445
SF3A2_HUMAN	5818.31641
RBMS2_HUMAN	7840.08838
PP1R7_HUMAN	16062.7529
SC23A_HUMAN	21377.8906
SC23B_HUMAN	4285.36182
CYH1_HUMAN	4849.31055
SF3A1_HUMAN	14014.0713
SKI2_HUMAN	9028.5293
CDSN_HUMAN	2082.49146

SURF1_HUMAN	747.818726
TAF5_HUMAN	180.771515
TAF7_HUMAN	1725.18567
TERF2_HUMAN	933.090759
MARE2_HUMAN	12001.2725
TGIF1_HUMAN	118.568214
NHRF2_HUMAN	3583.28613
TRADD_HUMAN	1794.89783
TRAM1_HUMAN	7844.99805
TSN_HUMAN	10169.4688
TRBP2_HUMAN	1138.25793
SF01_HUMAN	16504.0527
CIP4_HUMAN	6811.44287
TRIPB_HUMAN	2852.68042
PCH2_HUMAN	9163.09961
MED1_HUMAN	1766.94348
TRIP4_HUMAN	3858.1626
HMGN3_HUMAN	45.2150574
JHD2C_HUMAN	312.477539
IKBB_HUMAN	2108.91675
TRIP6_HUMAN	5302.67822
PTN14_HUMAN	2609.29883
MARE1_HUMAN	51062.8047
ELAV1_HUMAN	51927.8008
NSDHL_HUMAN	19386.8203
NAB2_HUMAN	235.858978
MYLK_HUMAN	2814.7627
TAB1_HUMAN	3127.2832
HERC1_HUMAN	2285.60791
AAAT_HUMAN	12713.4277
MLF2_HUMAN	1181.06885
TOM34_HUMAN	7742.51953
SMAD2_HUMAN	4559.13867
SMAD1_HUMAN	18249.834
MSMO1_HUMAN	6375.1543
ITSN1_HUMAN	2289.89893
TBCE_HUMAN	4484.54834
TBCC_HUMAN	4097.35303
UB2V2_HUMAN	1961.46802
STK11_HUMAN	360.064423
STXB2_HUMAN	4347.91406
VAMP3_HUMAN	3929.89722
NEDD8_HUMAN	4106.76611
VAS1_HUMAN	5301.78955
VPS72_HUMAN	1568.79785
RB11B_HUMAN	17202.7402
ZYX_HUMAN	6778.99756
ETFD_HUMAN	1298.81238
SEPT7_HUMAN	33031.0977
ADRM1_HUMAN	18985.1113

CCDC6_HUMAN	5131.43848
UAP1_HUMAN	24941.9707
E2F4_HUMAN	532.691833
IBP7_HUMAN	8787.47559
PDCD2_HUMAN	906.567383
LAMA4_HUMAN	8899.03809
PSMD5_HUMAN	22566.6172
PKN1_HUMAN	8677.6709
PKN2_HUMAN	7315.96338
TAF12_HUMAN	758.730591
CSRP2_HUMAN	1482.76538
DDB1_HUMAN	38319.0742
2A5E_HUMAN	4596.05225
MK14_HUMAN	5367.26709
RM23_HUMAN	4241.14355
CDC37_HUMAN	34593.6719
DPYL2_HUMAN	61565.9648
SYPL1_HUMAN	12079.1514
RBBP7_HUMAN	8262.03906
M3K11_HUMAN	893.616089
TAF9_HUMAN	2595.27686
CALRL_HUMAN	5564.12158
BAK_HUMAN	494.338409
SRSF7_HUMAN	47040.9258
CPSF6_HUMAN	15830.0254
SMN_HUMAN	2876.35791
DREB_HUMAN	15913.0352
NRF1_HUMAN	2257.82031
FSCN1_HUMAN	136501.375
IF16_HUMAN	26710.5508
DECR_HUMAN	26374.7363
MA2A1_HUMAN	7386.90234
NDUA5_HUMAN	1613.75024
CEGT_HUMAN	888.172791
CLPP_HUMAN	4054.4873
THTR_HUMAN	12623.6055
UBE2S_HUMAN	1535.43494
KAT1_HUMAN	790.04303
KGUA_HUMAN	3163.90015
GLO2_HUMAN	9175.8916
NDUA9_HUMAN	10448.3203
MAON_HUMAN	1115.005
PCKGM_HUMAN	2617.99902
UPP1_HUMAN	1723.10559
DDR2_HUMAN	2046.90173
HCDH_HUMAN	10162.7637
CP51A_HUMAN	16705.4785
UGPA_HUMAN	28288.8965
F263_HUMAN	2169.3606
TRXR1_HUMAN	52220.5938

MIC60_HUMAN	16991.4531
ECSCR_HUMAN	2986.08228
KNOP1_HUMAN	1981.91504
HNRL2_HUMAN	16697.2461
T132A_HUMAN	240.216904
INF2_HUMAN	6674.42627
PDS5A_HUMAN	7415.75391
CL16A_HUMAN	1624.19116
KIF7_HUMAN	536.75116
RHG31_HUMAN	2976.02539
MTHSD_HUMAN	3696.31079
AAK1_HUMAN	5072.57471
WASC4_HUMAN	4815.2168
ERC6L_HUMAN	2010.4314
TSR1_HUMAN	8593.93848
RGPA2_HUMAN	860.006348
D19L1_HUMAN	4722.95068
IAH1_HUMAN	1659.14917
ALG11_HUMAN	2366.31592
QRIC1_HUMAN	3360.23047
SMU1_HUMAN	13254.1826
CTU2_HUMAN	8951.14551
PGGHG_HUMAN	336.878571
LRRF1_HUMAN	5144.08984
PGPS1_HUMAN	750.750488
CR021_HUMAN	48.7646751
P3H1_HUMAN	19097.3691
TRM5_HUMAN	3167.51074
EMAL3_HUMAN	3920.31665
RPA43_HUMAN	2735.58032
MA7D1_HUMAN	3562.57129
UAP1L_HUMAN	12240.0654
CHD9_HUMAN	3658.89526
TKFC_HUMAN	2572.04761
LSM12_HUMAN	6569.18066
HSDL1_HUMAN	2718.09546
AL1L2_HUMAN	430.126984
GNPTA_HUMAN	425.89917
GRDN_HUMAN	1520.04053
RABL6_HUMAN	6635.23047
TIM50_HUMAN	6186.97754
PAR14_HUMAN	2486.72559
FUT11_HUMAN	859.439636
GLYR1_HUMAN	7171.41064
CE022_HUMAN	1807.95593
C2CD3_HUMAN	140.789932
VP26B_HUMAN	3608.31958
LARP7_HUMAN	3218.698
NAKD2_HUMAN	1107.94971
KCD21_HUMAN	1669.09961

GXLT1_HUMAN	437.655853
ACSF3_HUMAN	1470.68884
PPCEL_HUMAN	2625.96509
TB10B_HUMAN	2983.90991
TPRN_HUMAN	142.31929
ANO6_HUMAN	3222.15845
FIL1L_HUMAN	2255.89282
GRAP1_HUMAN	2888.79834
ZNG1F_HUMAN	1488.25964
MIX23_HUMAN	3891.31812
MBRL_HUMAN	1321.77527
FA98B_HUMAN	5998.93164
RHG29_HUMAN	8073.88672
PDCD4_HUMAN	7176.67139
FND3B_HUMAN	7769.44873
CRTC2_HUMAN	546.18811
GPAT3_HUMAN	1252.97375
CEP55_HUMAN	2891.64844
NCBP3_HUMAN	2412.28809
QORX_HUMAN	19529.5254
NACHO_HUMAN	2842.05347
PHLA2_HUMAN	694.068542
PDLI3_HUMAN	654.694641
PAR10_HUMAN	572.0224
DHB12_HUMAN	23766.8418
GLE1_HUMAN	3005.19458
UBP39_HUMAN	6855.44482
KLH22_HUMAN	1364.00159
AGK_HUMAN	10665.9473
SETMR_HUMAN	383.089294
LACB2_HUMAN	1908.25269
P5CR3_HUMAN	3031.05591
EIPR1_HUMAN	5100.87891
BOREA_HUMAN	434.526581
COBL1_HUMAN	1072.5675
H1BP3_HUMAN	3567.41943
CYBR1_HUMAN	489.359802
WDR81_HUMAN	2887.27783
ACTBL_HUMAN	248725.891
CCD93_HUMAN	3945.18823
OCAD2_HUMAN	13111.3574
PKHG4_HUMAN	1086.93799
HS904_HUMAN	1448.43823
DCAF6_HUMAN	478.653015
TM41B_HUMAN	995.823181
SGMR2_HUMAN	11896.6006
YIF1B_HUMAN	3373.18994
ZN326_HUMAN	4324.18848
NOM1_HUMAN	1310.81116
RIPL1_HUMAN	2296.58643

DJC21_HUMAN	1466.07654
TP8L3_HUMAN	5548.35254
HERC4_HUMAN	7344.65186
PIGG_HUMAN	960.853394
PP6R3_HUMAN	5594.86475
RABL3_HUMAN	23589.5156
FA76B_HUMAN	291.673553
COQ5_HUMAN	687.830444
SH319_HUMAN	3387.90601
NHSL2_HUMAN	2094.51929
EMC4_HUMAN	3788.30347
SYEM_HUMAN	2356.02466
CC038_HUMAN	1049.12
EFMT2_HUMAN	626.432556
TGO1_HUMAN	2115.84033
PREP_HUMAN	14020.5156
WDR44_HUMAN	3351.49585
PRC2B_HUMAN	4493.2749
TJAP1_HUMAN	1059.87805
RRP12_HUMAN	11037.4043
COA6_HUMAN	292.685913
TOIP1_HUMAN	15421.3281
CEP78_HUMAN	590.23114
SYAM_HUMAN	4184.91797
TOR2A_HUMAN	468.18576
PCID2_HUMAN	3549.06714
HABP4_HUMAN	1745.7583
S27A3_HUMAN	821.79718
SAMD9_HUMAN	1294.50757
P4R3B_HUMAN	2314.79785
WIPI3_HUMAN	2143.71631
WIPI1_HUMAN	870.959351
EOGT_HUMAN	11407.4609
SMAG2_HUMAN	716.351563
TBCEL_HUMAN	2839.63672
TDIF2_HUMAN	1863.63892
RBG1L_HUMAN	1701.24951
TTC38_HUMAN	1377.16223
COX20_HUMAN	4049.30005
EXOS6_HUMAN	5561.44189
TM201_HUMAN	2245.66187
ATAT_HUMAN	420.633728
SNP47_HUMAN	859.570129
NU188_HUMAN	6607.86719
HP1B3_HUMAN	34587.0547
SYVM_HUMAN	597.985229
CE170_HUMAN	7377.57715
ARH_HUMAN	1119.84094
ODR4_HUMAN	3234.91431
NOL9_HUMAN	3431.62622

NHSL1_HUMAN	1940.6698
HYI_HUMAN	335.63031
TPRGL_HUMAN	874.012146
C2D1B_HUMAN	1747.33459
FBP1L_HUMAN	13965.2627
SYRM_HUMAN	977.774963
THEM4_HUMAN	883.674805
CHCH9_HUMAN	1035.25647
FKB15_HUMAN	12946.4463
ZC3HD_HUMAN	2014.0802
CI114_HUMAN	1649.98083
ARMD3_HUMAN	471.172028
CSCL2_HUMAN	1598.86938
GPTC4_HUMAN	488.634308
CAF17_HUMAN	2219.48169
HECD3_HUMAN	4764.74805
GT253_HUMAN	10662.4365
UBR4_HUMAN	11121.4238
STXB5_HUMAN	1298.05969
RHG21_HUMAN	1174.99048
CAMP1_HUMAN	1529.36487
RM02_HUMAN	3330.63208
UBAP2_HUMAN	4881.86279
QNG1_HUMAN	1555.80371
KPRP_HUMAN	2989.25049
ZN618_HUMAN	3398.05933
ACBD5_HUMAN	6459.96924
RBM26_HUMAN	3601.28101
ATD3B_HUMAN	2281.26074
WLS_HUMAN	7645.37842
INT11_HUMAN	3130.73511
CPTP_HUMAN	691.042603
DCAF8_HUMAN	4454.36377
CODA1_HUMAN	661.355713
TUT4_HUMAN	10742.3906
FRY_HUMAN	1264.10034
RNH2B_HUMAN	1131.74121
ATPF1_HUMAN	2354.30615
RC3H1_HUMAN	865.244324
DDI2_HUMAN	4107.92822
THMS2_HUMAN	327.694733
NDUF5_HUMAN	1209.90576
NT5D1_HUMAN	3052.69971
BIG3_HUMAN	1319.23523
VP13D_HUMAN	513.927917
CROCC_HUMAN	1453.6792
LYRM7_HUMAN	1560.40979
RAIN_HUMAN	23389.3574
RIF1_HUMAN	2440.76758
VPS53_HUMAN	4246.1665

STRP1_HUMAN	4565.78271
MRCKA_HUMAN	6947.48584
RPRD2_HUMAN	1024.11646
RN220_HUMAN	537.421936
PR38B_HUMAN	4658.36523
BRE1A_HUMAN	4090.49072
MYOME_HUMAN	460.009216
ZN318_HUMAN	854.983643
F1711_HUMAN	1273.97327
DIK1B_HUMAN	3036.92383
LRCH2_HUMAN	1402.20569
CDKAL_HUMAN	4996.00488
BROX_HUMAN	5526.15186
FOCAD_HUMAN	7106.23193
RSBN1_HUMAN	281.311737
DAB2P_HUMAN	780.947998
LYPL1_HUMAN	6557.62695
PEAR1_HUMAN	1942.81982
ECM29_HUMAN	15461.2979
TUT7_HUMAN	1119.99768
SHE_HUMAN	445.852325
DEN4C_HUMAN	3029.3623
NAA35_HUMAN	4327.52246
ZMYM4_HUMAN	2105.82788
FHI2A_HUMAN	4169.54199
ZDH20_HUMAN	2547.39136
SPRY7_HUMAN	2052.9397
FBXW9_HUMAN	195.091125
CD276_HUMAN	3112.35352
RN213_HUMAN	4708.33545
TENS2_HUMAN	1459.28308
KANK2_HUMAN	602.957397
WAC2A_HUMAN	1578.6073
LPCT4_HUMAN	1235.15491
STEA3_HUMAN	1937.44873
F91A1_HUMAN	4015.82178
TBC9B_HUMAN	6327.7749
MAP1S_HUMAN	10747.2344
2ABD_HUMAN	3253.97632
AR6P4_HUMAN	4347.33447
A16L1_HUMAN	3361.17163
HGNAT_HUMAN	1989.08826
ARID2_HUMAN	877.610779
UTP25_HUMAN	2316.30298
GL8D1_HUMAN	1154.76062
SE1L3_HUMAN	1031.31372
TENS3_HUMAN	4801.57813
HAUS3_HUMAN	791.226624
SPT2_HUMAN	1121.16968
MBLC2_HUMAN	1761.62793

CRBG3_HUMAN	351.851379
INT3_HUMAN	3787.91333
RHG17_HUMAN	17424.5469
NCAS2_HUMAN	2764.18945
C19L1_HUMAN	10129.502
VIR_HUMAN	3324.08154
CYTSA_HUMAN	4916.05713
HEAT6_HUMAN	960.196533
ANR40_HUMAN	415.160858
WWC2_HUMAN	1643.00415
SCAR3_HUMAN	253.078842
ATLA3_HUMAN	18345.3379
RL7L_HUMAN	18450.5098
NXN_HUMAN	1835.7738
TTC19_HUMAN	1066.19434
IQEC1_HUMAN	2989.45215
CPIN1_HUMAN	10012.3057
ZFAN6_HUMAN	1783.03687
SMYD5_HUMAN	2423.94873
OTU7B_HUMAN	713.071777
THOC7_HUMAN	1679.77698
NADE_HUMAN	2846.25195
ELP2_HUMAN	2673.63916
LTOR1_HUMAN	2429.03979
DRS7B_HUMAN	1860.224
TWF2_HUMAN	16252.4561
CNDH2_HUMAN	630.390747
K0930_HUMAN	782.531982
TNG2_HUMAN	6892.78271
P4R3A_HUMAN	3910.33252
RAB12_HUMAN	22746.6426
SDE2_HUMAN	144.432724
K2C80_HUMAN	1429.33813
NIPBL_HUMAN	474.892761
SCMC2_HUMAN	3404.06274
PDE12_HUMAN	7616.30273
NAA16_HUMAN	1744.19153
DE10B_HUMAN	4061.73901
RHBD2_HUMAN	1664.14294
SCMC1_HUMAN	30032.9043
REEP3_HUMAN	836.897705
RETST_HUMAN	2738.45947
RINT1_HUMAN	1135.02856
TM214_HUMAN	10271.9688
HIBCH_HUMAN	6305.18652
RWDD4_HUMAN	2194.76416
NEPRO_HUMAN	1312.22009
ARMC6_HUMAN	4888.28418
TTI2_HUMAN	1649.55603
ANR54_HUMAN	616.520508

TAPT1_HUMAN	28777.0703
KANK3_HUMAN	1267.34778
JMJD6_HUMAN	4143.78711
PPR18_HUMAN	8891.61523
CAVN1_HUMAN	87553.1406
ZCHC8_HUMAN	3331.20874
DHX57_HUMAN	3381.9939
ERAP2_HUMAN	11518.0869
MBOA5_HUMAN	2882.63794
CDC73_HUMAN	6506.93652
RM14_HUMAN	4093.7002
S27A4_HUMAN	6810.52441
C2D1A_HUMAN	4000.44971
TATD1_HUMAN	2428.44556
MET2B_HUMAN	1303.94519
MSD2_HUMAN	713.472351
TAF2_HUMAN	1245.3562
CH082_HUMAN	1082.97485
MED27_HUMAN	948.236694
EDC4_HUMAN	18199.6738
CEP85_HUMAN	715.194763
ANM9_HUMAN	445.176208
PRP8_HUMAN	14173.9023
FBX42_HUMAN	1112.47607
SCYL2_HUMAN	4080.72949
TTC27_HUMAN	4646.88525
SFXN4_HUMAN	919.510315
GOLM2_HUMAN	2842.33154
NFRKB_HUMAN	698.87085
FAHD1_HUMAN	11917.0605
RL22L_HUMAN	10460.9189
PKN3_HUMAN	1151.22278
ALKB5_HUMAN	803.272888
PDXD1_HUMAN	7048.17578
MEAK7_HUMAN	3706.302
INT5_HUMAN	2398.98633
GIMA6_HUMAN	650.143982
S27A1_HUMAN	760.846802
PGM2L_HUMAN	8082.00195
CTR9_HUMAN	5156.1709
AAGAB_HUMAN	1493.44568
TMM88_HUMAN	263.510895
PSRC1_HUMAN	107.610985
SKI3_HUMAN	5377.96875
ULK3_HUMAN	1399.12854
SYDM_HUMAN	7682.79883
TMM65_HUMAN	5438.9751
IN80C_HUMAN	1953.10376
FBX38_HUMAN	1825.12854
NCEH1_HUMAN	11130.0527

FIGL1_HUMAN	603.251526
TRI65_HUMAN	1817.2179
RHDF2_HUMAN	950.930054
MDEAS_HUMAN	2617.87207
BRAT1_HUMAN	3524.68018
WDR59_HUMAN	1466.57532
ZC3HE_HUMAN	2227.41992
CC137_HUMAN	1678.05908
OGFD3_HUMAN	797.516724
TXD11_HUMAN	220.114365
LARP1_HUMAN	16928.3828
ATAD2_HUMAN	228.265427
TMED8_HUMAN	2474.73511
ZNT9_HUMAN	756.312256
TRAF7_HUMAN	1255.95898
RICTR_HUMAN	2722.55103
WDR74_HUMAN	1953.03943
ATRAP_HUMAN	1389.46497
SMRD3_HUMAN	1245.02075
C1TM_HUMAN	24992.1094
AFTIN_HUMAN	765.179443
FIP1_HUMAN	6783.63721
CRTC3_HUMAN	443.548706
CRTC1_HUMAN	530.674072
CP20A_HUMAN	3232.8479
PLGT2_HUMAN	3492.81543
TM205_HUMAN	6290.61768
LRSM1_HUMAN	2531.93628
GAK1B_HUMAN	2694.12061
LCLT1_HUMAN	2538.1123
ABRX1_HUMAN	173.35704
CWC27_HUMAN	1752.45276
CREL2_HUMAN	1461.37073
WDR82_HUMAN	3697.10522
MIC27_HUMAN	2315.23926
FAT4_HUMAN	855.088257
DPP8_HUMAN	679.029724
MCAF1_HUMAN	1939.44641
RBP10_HUMAN	1695.06116
PACS1_HUMAN	6575.17725
MPRIP_HUMAN	7493.58643
RBMS3_HUMAN	2822.11816
PNCB_HUMAN	3175.30396
DNMBP_HUMAN	5279.89648
HACD2_HUMAN	899.920715
B3GLT_HUMAN	1447.81714
GGYF2_HUMAN	8857.69824
CD109_HUMAN	8632.55762
THADA_HUMAN	1846.45728
HSDL2_HUMAN	4139.8457

KAT3_HUMAN	6833.7168
PPR21_HUMAN	1245.88916
THSD4_HUMAN	953.878052
SYNE3_HUMAN	1659.59875
ZN574_HUMAN	522.845703
NFXL1_HUMAN	359.814423
NBEL2_HUMAN	2779.7522
FGD5_HUMAN	3830.45337
QSOX2_HUMAN	2572.16724
SRCAP_HUMAN	434.513641
RIPR1_HUMAN	4859.66211
YJ005_HUMAN	6829.32764
ARHGI_HUMAN	953.427124
UBR3_HUMAN	736.506531
TMPPE_HUMAN	1240.8811
CRACD_HUMAN	311.388031
CCD9B_HUMAN	419.031586
NUD18_HUMAN	430.343933
PAXI1_HUMAN	880.01178
TMTC3_HUMAN	5476.91357
VP13C_HUMAN	9281.66309
UBP34_HUMAN	600.143616
RAPH1_HUMAN	2690.71509
MOB2_HUMAN	4769.5708
IKIP_HUMAN	25862.2598
PREX2_HUMAN	894.486206
F10C1_HUMAN	1425.1781
UB2R2_HUMAN	5863.5708
MD13L_HUMAN	556.664307
LARP4_HUMAN	9519.0293
MED25_HUMAN	1460.94995
RS27L_HUMAN	59892.0859
HAKAI_HUMAN	1304.29578
INT8_HUMAN	3591.58252
BT2A1_HUMAN	357.200562
SPT6H_HUMAN	4181.05078
SND1_HUMAN	87602.8594
MARK2_HUMAN	1835.71106
COX15_HUMAN	1705.30664
DDX46_HUMAN	13065.8809
RUFY3_HUMAN	770.479858
TM10C_HUMAN	9580.28809
5MP2_HUMAN	26903.9922
MON1B_HUMAN	1134.74475
ASGL1_HUMAN	1079.71362
KCTD9_HUMAN	6571.55029
DHX30_HUMAN	9489.84082
EIF3M_HUMAN	19209.4609
MEPCE_HUMAN	3663.01709
ARMX2_HUMAN	1451.28015

SYPM_HUMAN	485.372437
RSRC2_HUMAN	1641.54492
RRAGA_HUMAN	4208.34473
CYFP1_HUMAN	24338.2363
NDUF7_HUMAN	2390.78394
GET4_HUMAN	9752.4502
CSN6_HUMAN	19826.791
PCAT2_HUMAN	13465.084
ENOF1_HUMAN	1772.86487
MAEA_HUMAN	1999.71082
EPMIP_HUMAN	1599.30786
DHX32_HUMAN	386.248657
TAOK1_HUMAN	2881.7439
FAKD5_HUMAN	3194.98438
MOB1B_HUMAN	12165.5947
KDM3B_HUMAN	4100.76807
CHM1B_HUMAN	4987.06348
RIR2B_HUMAN	3722.21753
HS2ST_HUMAN	5648.99902
STRAA_HUMAN	1521.15027
MICA3_HUMAN	1521.90698
DYM_HUMAN	1622.58728
K2C78_HUMAN	747.852051
PHF5A_HUMAN	3298.91992
PXL2C_HUMAN	3955.5791
APTX_HUMAN	2193.63696
ERMP1_HUMAN	3215.34619
GRIN1_HUMAN	252.327789
TRM1L_HUMAN	2342.4812
ZCCHV_HUMAN	8935.09961
RM21_HUMAN	4837.47119
EFL1_HUMAN	4564.07617
SETX_HUMAN	575.849243
TPC11_HUMAN	2338.17261
KANL1_HUMAN	471.824738
NUP54_HUMAN	8663.56445
ATG9A_HUMAN	1103.9043
GLUCM_HUMAN	3937.56152
CC186_HUMAN	574.768311
ARMC9_HUMAN	2299.29663
VP35L_HUMAN	5729.53125
POGZ_HUMAN	3690.2981
ZFY16_HUMAN	1127.79968
MON2_HUMAN	3626.6582
MYCPP_HUMAN	654.257324
MYH14_HUMAN	7994.33984
NUFP2_HUMAN	5446.45459
SZRD1_HUMAN	1569.33716
MAVS_HUMAN	1794.65588
CLAP1_HUMAN	6297.74268

DHX29_HUMAN	5631.70459
NPHP3_HUMAN	402.564728
LRP10_HUMAN	1963.52649
HAUS6_HUMAN	1725.47681
PLGT3_HUMAN	4854.67236
TT21B_HUMAN	1417.2124
HEAT3_HUMAN	7100.73584
KI21A_HUMAN	468.993317
HDGR2_HUMAN	9262.58496
DCXR_HUMAN	6028.8916
BRAP_HUMAN	2139.43164
EMSY_HUMAN	537.644165
GOGA7_HUMAN	2436.80322
RHG22_HUMAN	1175.61279
WAPL_HUMAN	1282.8811
I2BP2_HUMAN	1167.56787
RBBP6_HUMAN	2134.45703
SH3R1_HUMAN	1108.23376
GRASP_HUMAN	889.266541
FRMD5_HUMAN	747.714783
SEN54_HUMAN	459.251587
PTAR1_HUMAN	395.547516
ARPIN_HUMAN	2881.74487
TCPR1_HUMAN	1038.52466
RABEK_HUMAN	1419.72705
MTEF4_HUMAN	505.055542
HUWE1_HUMAN	12578.6348
YTHD3_HUMAN	11365.5811
CTU1_HUMAN	3173.23657
TAF8_HUMAN	329.203491
UB2Q1_HUMAN	5109.51221
RM55_HUMAN	10135.3887
TMED4_HUMAN	3827.64331
COXM1_HUMAN	4774.54443
CENPV_HUMAN	2120.33911
SLN11_HUMAN	4168.71484
MEGF8_HUMAN	494.74707
T179B_HUMAN	922.013794
GALT7_HUMAN	6302.32422
SCD5_HUMAN	2423.5542
PHLB2_HUMAN	3016.76782
AGRG6_HUMAN	1867.91418
DHDDS_HUMAN	398.473297
GLT10_HUMAN	657.753357
TPC6B_HUMAN	1182.96899
PP4P1_HUMAN	2251.23877
CLC14_HUMAN	11145.3457
PATL1_HUMAN	1299.93481
DPP9_HUMAN	6285.70557
SYVN1_HUMAN	1392.74011

TRPT1_HUMAN	512.313049
SETD3_HUMAN	6590.74951
TTC7B_HUMAN	1042.78259
ACOT1_HUMAN	3249.12866
NOP9_HUMAN	2836.67603
PABP2_HUMAN	21335.8574
MTA70_HUMAN	2108.67017
PB1_HUMAN	3406.74878
YRDC_HUMAN	2349.50903
PRP39_HUMAN	1721.03943
LYRIC_HUMAN	15560.1396
ZN598_HUMAN	4947.7793
GPAT4_HUMAN	1867.37463
KTN1_HUMAN	7366.14404
EST1A_HUMAN	1826.46118
NLRX1_HUMAN	1102.40979
BCL9L_HUMAN	387.267609
PHLB1_HUMAN	10130.0234
UBP48_HUMAN	6328.8374
CAPS2_HUMAN	541.231445
URP2_HUMAN	28484.9805
NAA40_HUMAN	4451.40479
NT5D3_HUMAN	845.946045
AACS_HUMAN	7880.45801
LUZP1_HUMAN	7459.93652
THOC4_HUMAN	26179.9453
GP180_HUMAN	743.124146
FHI2B_HUMAN	3020.16577
IQGA3_HUMAN	3990.2251
ZCH18_HUMAN	2445.99756
VPS36_HUMAN	2038.23291
CAND1_HUMAN	34459.1836
RETR3_HUMAN	2609.75488
HOOK3_HUMAN	6283.74316
SESD1_HUMAN	1020.36639
COMD7_HUMAN	1505.09277
MON1A_HUMAN	326.628784
AMZ2_HUMAN	1121.04956
THOC6_HUMAN	2266.60596
MET16_HUMAN	1011.71106
PARG_HUMAN	605.015625
LIPB1_HUMAN	9095.38379
LONP2_HUMAN	769.037354
ZC3C1_HUMAN	3279.23193
CHD1L_HUMAN	8633.49707
FCSD1_HUMAN	235.744614
CCD25_HUMAN	6559.33838
PRSR2_HUMAN	1368.80115
STING_HUMAN	7502.94385
AROS_HUMAN	1661.87708

CDR2L_HUMAN	142.853699
RLGPB_HUMAN	1315.44055
RGPS2_HUMAN	420.822479
CARM1_HUMAN	7535.98633
NIT1_HUMAN	4304.57568
COMD2_HUMAN	3760.11206
HTR5A_HUMAN	1094.72168
CNDG2_HUMAN	1130.51196
FBX11_HUMAN	285.513489
ANKL2_HUMAN	2175.36938
MEX3D_HUMAN	1875.43384
DGKH_HUMAN	372.004517
DDX42_HUMAN	17270.0898
SPAS2_HUMAN	3625.62378
VRK2_HUMAN	2854.78369
CACL1_HUMAN	337.300415
NDUAB_HUMAN	8015.88818
DAAF5_HUMAN	7558.74268
PTH_HUMAN	1301.05774
STX12_HUMAN	13192.5986
P66A_HUMAN	4242.104
CPNE8_HUMAN	20959.8477
GPSM1_HUMAN	884
RAB43_HUMAN	1938.14453
C2CD5_HUMAN	1453.90405
MIB1_HUMAN	684.486267
PRAG1_HUMAN	1535.47961
HPS6_HUMAN	1466.48242
I2BP1_HUMAN	796.79425
RB6I2_HUMAN	3785.71338
RIOX2_HUMAN	2125.73022
ZDH13_HUMAN	9330.3418
ZDH17_HUMAN	586.650452
CRLF3_HUMAN	1893.55627
TPPC5_HUMAN	5568.62207
ARMC8_HUMAN	2672.17236
RELL1_HUMAN	714.04718
T126B_HUMAN	1330.03491
PLD3_HUMAN	19572.3398
HID1_HUMAN	996.799377
ANKY2_HUMAN	856.306763
ERI1_HUMAN	1026.92273
VRK3_HUMAN	541.811279
LIX1L_HUMAN	2602.14331
NUDC3_HUMAN	4759.39551
AHNK2_HUMAN	27905.6738
FMNL3_HUMAN	6080.99561
MMAA_HUMAN	1576.4856
M4K3_HUMAN	580.160095
NAV3_HUMAN	2391.46948

P3H3_HUMAN	10364.4277
CCD50_HUMAN	9407.49512
FABD_HUMAN	1941.03357
KSR1_HUMAN	1677.14063
CEP97_HUMAN	970.317505
NNRD_HUMAN	1116.77063
WDR75_HUMAN	2273.02222
MFN1_HUMAN	2241.95386
CTL2_HUMAN	3328.09644
IPRI_HUMAN	1842.05017
WDFY1_HUMAN	4622.22803
MA7D3_HUMAN	2839.07861
CC117_HUMAN	387.915131
NXP20_HUMAN	6758.18213
DCNL3_HUMAN	484.134338
DEN6A_HUMAN	810.758484
MGAP_HUMAN	411.431183
GCC2_HUMAN	3272.05835
Z3H7A_HUMAN	1981.07214
PHF6_HUMAN	8046.50684
ARCH_HUMAN	689.443298
LRC8A_HUMAN	5841.13867
SULF2_HUMAN	3749.4856
UBR1_HUMAN	1645.01306
UBR2_HUMAN	1372.08569
RHG12_HUMAN	1197.15149
CHERP_HUMAN	9793.55859
CDAN1_HUMAN	553.430603
ANKH1_HUMAN	2585.18506
SUGP1_HUMAN	1800.57117
SUGP2_HUMAN	23824.5605
UEVLD_HUMAN	1803.2074
CCAR1_HUMAN	9421.62305
DHX40_HUMAN	787.299866
DJC10_HUMAN	9711.65039
NELFD_HUMAN	5135.2334
MIRO2_HUMAN	4931.48145
MIRO1_HUMAN	4525.229
SIR2_HUMAN	2670.80444
PHC2_HUMAN	997.768433
BAP18_HUMAN	4553.58203
RM41_HUMAN	10081.5215
NRM_HUMAN	5059.44141
GPAM1_HUMAN	1482.15039
PARP9_HUMAN	558.87323
RB12B_HUMAN	3695.2041
S35F2_HUMAN	1605.23694
RPAP2_HUMAN	179.502182
PLPL6_HUMAN	5717.46143
SMC5_HUMAN	1166.47949

DDX60_HUMAN	345.983063
CMIP_HUMAN	394.358582
MILK2_HUMAN	526.71814
DHX37_HUMAN	2420.74707
KBTB2_HUMAN	3464.2002
RAVR1_HUMAN	4889.1123
SPB1_HUMAN	9244.94727
TM192_HUMAN	1382.33447
M21D2_HUMAN	1365.12854
SRRM1_HUMAN	4776.65674
SMAP1_HUMAN	2802.63428
DI3L2_HUMAN	3219.69482
SUV3_HUMAN	5062.55664
ERF3B_HUMAN	7068.32422
ZZZ3_HUMAN	202.03421
EXOC8_HUMAN	7618.2041
CJ067_HUMAN	10283.0059
TRI22_HUMAN	4556.64697
THNS1_HUMAN	646.537354
K2013_HUMAN	3500.10913
MICU2_HUMAN	1020.78784
AN13A_HUMAN	3078.08374
PHAR4_HUMAN	1652.52539
TRM2A_HUMAN	2624.99268
RUSD2_HUMAN	1056.245
ELMD2_HUMAN	2346.93823
A16A1_HUMAN	5368.0332
K319L_HUMAN	1472.80713
AGRF5_HUMAN	1894.20996
XRN1_HUMAN	4879.31982
PELP1_HUMAN	13282.7354
ABI1_HUMAN	6264.59326
WDFY3_HUMAN	4633.53906
SELH_HUMAN	5817.59961
VKORL_HUMAN	1801.33325
SPART_HUMAN	9135.82715
TTC5_HUMAN	1410.80945
MIER1_HUMAN	1582.76843
RPTOR_HUMAN	1928.22217
ORML3_HUMAN	3926.34912
CCAR2_HUMAN	50953.6172
NDUF2_HUMAN	7623.89014
VPS52_HUMAN	5626.70605
NUP93_HUMAN	23952.9824
S11IP_HUMAN	887.72876
ZN687_HUMAN	602.847046
CMTR1_HUMAN	4333.06201
LRC47_HUMAN	13802.5869
DOCK4_HUMAN	7305.18604
CAH13_HUMAN	1812.16187

ARG28_HUMAN	8391.53516
INT1_HUMAN	33199.9648
H2B3B_HUMAN	2737.84155
RHG24_HUMAN	3874.62964
ARM10_HUMAN	3416.26245
ABD12_HUMAN	2577.03687
CLASR_HUMAN	338.001007
FA43A_HUMAN	2518.29932
TM256_HUMAN	1861.37866
PIAS4_HUMAN	1305.38794
GPD1L_HUMAN	3585.92041
S35F6_HUMAN	2268.91162
RHG18_HUMAN	19372.5234
ASCC3_HUMAN	9617.06641
MILK1_HUMAN	1097.17664
VPS8_HUMAN	2618.85938
PALS1_HUMAN	1148.45239
STAG2_HUMAN	3414.83911
SYNPO_HUMAN	9878.98926
FNBP4_HUMAN	1414.13245
GUF1_HUMAN	282.720947
RYBP_HUMAN	563.181396
GALT4_HUMAN	4305.02393
MINK1_HUMAN	1414.00098
MESH1_HUMAN	1191.03247
PTGR3_HUMAN	2382.08838
EMC5_HUMAN	3075.58862
TM199_HUMAN	3444.02002
FBXL6_HUMAN	509.503571
OGFD1_HUMAN	1951.75098
AFAP1_HUMAN	5081.41211
ZCHC9_HUMAN	587.548889
OXR1_HUMAN	794.737854
ZGPAT_HUMAN	1293.77148
CAHM5_HUMAN	1251.06018
SRBD1_HUMAN	972.370972
WDTC1_HUMAN	3314.87915
NKAP_HUMAN	969.72229
MACOI_HUMAN	653.950867
SH2D3_HUMAN	3896.6936
ARRD1_HUMAN	827.790344
DHRSX_HUMAN	1683.72253
CISD2_HUMAN	7252.37305
RP25L_HUMAN	1903.27844
ATPF2_HUMAN	1147.39734
TTC9C_HUMAN	1718.23352
JAGN1_HUMAN	3845.89209
RNF10_HUMAN	313.730377
RFLB_HUMAN	1916.20886
LZTR1_HUMAN	640.802124

COMD1_HUMAN	2843.95874
CPSF7_HUMAN	13112.8896
S15A4_HUMAN	318.523529
MYCT1_HUMAN	13700.1318
ATL1_HUMAN	300.178314
ARFG2_HUMAN	5253.40625
OTU6B_HUMAN	3146.34961
FITM2_HUMAN	2292.62817
CA052_HUMAN	526.784668
EFNMT_HUMAN	2828.91113
AR6P6_HUMAN	2096.22388
ARFG1_HUMAN	2309.36401
SIR6_HUMAN	1035.51685
S66A3_HUMAN	423.7677
EMC1_HUMAN	14468.3398
PAF1_HUMAN	3159.21655
CCYL1_HUMAN	1593.63049
UBR7_HUMAN	3183.52271
DDX51_HUMAN	2194.38135
F241A_HUMAN	5841.48096
S2529_HUMAN	2588.62183
ENAH_HUMAN	14626.6084
DCBD1_HUMAN	1085.13464
GPT11_HUMAN	1530.82983
RM43_HUMAN	6723.33594
RLIG1_HUMAN	2995.29028
GDPD1_HUMAN	633.31665
CS047_HUMAN	1205.75806
ASCC1_HUMAN	3641.31128
LRC57_HUMAN	7051.55762
EIF1A_HUMAN	1078.18555
KRI1_HUMAN	1710.84558
CC71L_HUMAN	1414.56433
ZN579_HUMAN	2223.29639
PR38A_HUMAN	3756.35889
MLKL_HUMAN	5128.9082
GALD1_HUMAN	2714.94531
ANR52_HUMAN	1098.1925
AT11C_HUMAN	1479.24268
AFG2A_HUMAN	4491.69629
NHLC2_HUMAN	7970.90527
AVL9_HUMAN	1577.51233
S43A3_HUMAN	5040.35498
XXLT1_HUMAN	8310.56934
GOLM1_HUMAN	752.976257
GT251_HUMAN	28078.1777
SUMF2_HUMAN	2597.85132
SIDT2_HUMAN	544.346741
SUMF1_HUMAN	5041.26074
PGLT1_HUMAN	2857.21899

UBAC2_HUMAN	1806.05042
PCYXL_HUMAN	834.471802
TM87A_HUMAN	1869.30127
RDH13_HUMAN	2738.83008
TTC13_HUMAN	1117.28308
DHB11_HUMAN	8499.24414
TXND5_HUMAN	217386.188
SPC24_HUMAN	1631.45996
ATAD1_HUMAN	2214.56323
SCPDL_HUMAN	17194.5059
UXS1_HUMAN	1125.37329
RN149_HUMAN	2217.59985
RETR2_HUMAN	1025.24512
SERB1_HUMAN	15681.6377
LEMD2_HUMAN	6474.01465
NOA1_HUMAN	1897.44983
NECP1_HUMAN	3227.70386
FA98A_HUMAN	5054.29346
PAG15_HUMAN	1362.72815
MTMRE_HUMAN	1025.14172
NF2IP_HUMAN	3920.84399
DGLB_HUMAN	919.567078
CHSTE_HUMAN	4414.20459
GALT6_HUMAN	2918.95996
RN169_HUMAN	875.123596
PDPR_HUMAN	3504.92334
NNRE_HUMAN	2340.63794
SMG8_HUMAN	1372.48328
RN214_HUMAN	2542.85938
LS14A_HUMAN	4412.8335
GIMA8_HUMAN	7226.52637
CCNY_HUMAN	3343.49731
MROH1_HUMAN	4105.84082
PAPD5_HUMAN	1568.2218
PEPL1_HUMAN	2651.01636
EHBP1_HUMAN	1571.36365
RB15B_HUMAN	2447.66626
SIA7C_HUMAN	1345.30701
TNR6A_HUMAN	1747.30237
PHC3_HUMAN	694.25354
DIK2A_HUMAN	3775.99292
CNNM3_HUMAN	611.342834
ABCF1_HUMAN	14042.8184
MCU_HUMAN	1764.9657
PK3C3_HUMAN	6272.31104
GSTCD_HUMAN	302.310181
SRFB1_HUMAN	1576.98877
NGDN_HUMAN	1704.54236
SHCBP_HUMAN	1488.31653
PDZD8_HUMAN	533.7948

DP13B_HUMAN	2045.8396
ZNT7_HUMAN	8722.25586
NAV1_HUMAN	1848.33691
PPHLN_HUMAN	713.080261
WDR19_HUMAN	397.141052
FBX22_HUMAN	5333.34814
PCAT1_HUMAN	4278.48926
ZMIZ2_HUMAN	1566.63574
SYNE1_HUMAN	24050.5918
UBP32_HUMAN	742.758301
BD1L1_HUMAN	1141.51086
ARI1B_HUMAN	1096.03601
FAD1_HUMAN	4009.13306
FLCN_HUMAN	1764.71362
NUP43_HUMAN	5950.91455
NUP37_HUMAN	7954.6665
NUP35_HUMAN	3866.98291
ENASE_HUMAN	362.863678
RAI3_HUMAN	1162.0968
TOIP2_HUMAN	6678.96826
ABHDB_HUMAN	2556.01221
NEUA_HUMAN	6958.69824
FBH1_HUMAN	682.10022
TSN14_HUMAN	5151.16602
TTL_HUMAN	237.32579
SVIP_HUMAN	1917.69836
ATLA2_HUMAN	1494.23938
MSPD2_HUMAN	1549.51465
PLBL2_HUMAN	10257.3252
RASF8_HUMAN	890.653381
DDX55_HUMAN	597.160095
MFSD8_HUMAN	378.090973
TDRD7_HUMAN	737.463745
GIMA7_HUMAN	3246.33398
NEDD1_HUMAN	1706.38892
NCOA7_HUMAN	1958.1001
THOC2_HUMAN	4466.77295
WDR36_HUMAN	6135.8623
PPTC7_HUMAN	680.012756
RP9_HUMAN	826.981262
VANG1_HUMAN	874.276245
SNIP1_HUMAN	537.679749
G45IP_HUMAN	4309.64453
WDR48_HUMAN	2505.2373
EXOC6_HUMAN	5949.15723
SMRC2_HUMAN	4910.05225
NPL4_HUMAN	8394.53027
CX038_HUMAN	2533.31396
SPT20_HUMAN	1711.26807
ABHD4_HUMAN	617.596069

FBX30_HUMAN	1419.61584
S35B2_HUMAN	8314.93164
PUM2_HUMAN	2123.04102
TIP_HUMAN	1265.42615
GOGA5_HUMAN	1088.02539
KLDC4_HUMAN	617.31311
SHKB1_HUMAN	2995.2876
UBA3_HUMAN	29748.8594
PXL2B_HUMAN	422.750732
DJB14_HUMAN	659.415833
KISHA_HUMAN	1536.10852
PI42C_HUMAN	1424.07007
WDR20_HUMAN	385.961487
TBC15_HUMAN	7804.39795
RDH11_HUMAN	14050.1738
LRC20_HUMAN	893.010559
IFI44_HUMAN	299.620636
RM30_HUMAN	873.169495
ZFAN1_HUMAN	855.357544
CIP2A_HUMAN	4662.97559
P4K2B_HUMAN	389.460602
STT3B_HUMAN	32343.6094
PNPT1_HUMAN	10730.2178
SPP2B_HUMAN	1432.92993
HM13_HUMAN	41765.8789
PREX1_HUMAN	3080.7478
URGCP_HUMAN	1366.46375
BICD2_HUMAN	4936.71143
NEK9_HUMAN	4831.78662
PKHO2_HUMAN	3804.01831
DTX3L_HUMAN	741.614746
DDX54_HUMAN	5354.69629
DLG5_HUMAN	715.173523
BRX1_HUMAN	8327.39063
GNPI2_HUMAN	3263.7981
LRC8C_HUMAN	15535.1465
NEK7_HUMAN	3485.23779
RBCC1_HUMAN	384.907959
MICA1_HUMAN	3798.06079
ELP5_HUMAN	2398.48071
SSH3_HUMAN	1274.49963
S3TC1_HUMAN	422.725098
TBCK_HUMAN	650.965881
DTD1_HUMAN	5103.46582
DCA11_HUMAN	576.789429
UTP15_HUMAN	2919.26563
GPX8_HUMAN	21422.5625
AF1L1_HUMAN	699.064148
SH3R3_HUMAN	10373.9971
TP4AP_HUMAN	848.373291

CE192_HUMAN	676.845276
GEMI5_HUMAN	6073.6626
PIGO_HUMAN	356.987366
RPGF6_HUMAN	397.74765
SMCR8_HUMAN	628.881531
PARD3_HUMAN	1390.22144
IPO4_HUMAN	14151.1367
UBP33_HUMAN	427.347687
PNISR_HUMAN	1576.17957
PP4R1_HUMAN	7240.15723
UBS3B_HUMAN	1375.20911
WIPF2_HUMAN	1431.69971
ABHD5_HUMAN	1364.00305
SETD7_HUMAN	5540.09814
NOC3L_HUMAN	4617.28711
SCRB1_HUMAN	19328.4707
COG1_HUMAN	2597.0564
SCFD2_HUMAN	2478.30688
SMAP2_HUMAN	2173.0144
ZC3HF_HUMAN	15247.165
PPIL4_HUMAN	4404.72656
TF3C2_HUMAN	2411.03955
TB22A_HUMAN	2997.60327
PHF10_HUMAN	1157.62341
RAB2B_HUMAN	4761.50635
CCD12_HUMAN	1176.04919
IASPP_HUMAN	2338.27173
ARB2A_HUMAN	1818.55298
CHUR_HUMAN	2761.40698
TGFA1_HUMAN	1025.85608
TM263_HUMAN	4746.58594
HDAC7_HUMAN	5009.25195
PTPM1_HUMAN	453.389465
NU133_HUMAN	8621.65918
PDC6I_HUMAN	54249.5859
S20A1_HUMAN	623.535889
FBLI1_HUMAN	20162.5547
CATIN_HUMAN	1499.31201
CHAC2_HUMAN	1759.10645
CHMP7_HUMAN	5113.79688
THEM6_HUMAN	27038.5488
NAT14_HUMAN	4638.06885
BCL7C_HUMAN	3410.39063
NSE1_HUMAN	936.566833
MITD1_HUMAN	674.441101
CTF18_HUMAN	595.881042
LEO1_HUMAN	3533.89648
DCAKD_HUMAN	773.003906
NUDC2_HUMAN	3249.98804
SNR27_HUMAN	2891.3623

TFB1M_HUMAN	8805.32031
STAG1_HUMAN	1572.1427
SCFD1_HUMAN	10300.6914
TP8L1_HUMAN	2894.05078
CANT1_HUMAN	2957.99023
TPC12_HUMAN	1511.38745
HNRLL_HUMAN	3887.61621
FACR1_HUMAN	12852.8564
UBCP1_HUMAN	6530.78223
SEN15_HUMAN	710.899414
PCNP_HUMAN	7447.52832
DNJA4_HUMAN	1651.02466
SPRY4_HUMAN	5156.89502
MAIP1_HUMAN	2550.94507
TRUB1_HUMAN	1991.80957
LMO7_HUMAN	2026.12122
CTL1_HUMAN	1955.57666
CKAP2_HUMAN	1184.56055
ATX2L_HUMAN	15026.4434
ARAP3_HUMAN	9267.91504
GIMA1_HUMAN	4372.89697
PRP31_HUMAN	9755.98828
NELFB_HUMAN	3441.05249
PALLD_HUMAN	15899.1963
RUFY2_HUMAN	1517.54919
SREK1_HUMAN	3266.30762
GEMI6_HUMAN	657.100342
CSKI2_HUMAN	1872.76733
ATRIP_HUMAN	419.776428
PSPC1_HUMAN	16600.1465
MADD_HUMAN	350.23349
SYNE2_HUMAN	6945.4292
P66B_HUMAN	2918.18579
PIBF1_HUMAN	613.668579
DNJC9_HUMAN	16067.0684
BRI3B_HUMAN	1727.88135
CTBL1_HUMAN	12940.3018
ATG4A_HUMAN	3721.10132
RIN2_HUMAN	920.859985
ELYS_HUMAN	3450.39404
TITIN_HUMAN	131.192047
ROBO4_HUMAN	3613.13501
OVCA2_HUMAN	2434.61597
IRGQ_HUMAN	1796.69336
DDB2_HUMAN	9408.56738
CLC2B_HUMAN	1577.12085
DDX1_HUMAN	24751.6348
S14L1_HUMAN	6718.23975
PIEZ1_HUMAN	1355.14185
FAM3C_HUMAN	5406.83252

H1X_HUMAN	9526.1416
PSMF1_HUMAN	7607.95947
GBF1_HUMAN	9419.15039
SMG7_HUMAN	263.566895
RTF1_HUMAN	5974.97461
NICA_HUMAN	12285.4385
SNX19_HUMAN	1044.40283
TM9S4_HUMAN	9018.72754
TM131_HUMAN	764.782532
IP6K1_HUMAN	216.309479
RT27_HUMAN	18449.1758
ELMO1_HUMAN	1925.40955
FIG4_HUMAN	354.360077
F168A_HUMAN	366.451202
AP3S1_HUMAN	12348.0176
TSC1_HUMAN	2713.49146
UBXN4_HUMAN	3574.93896
PHF3_HUMAN	1239.66321
NDRG1_HUMAN	3090.99927
HS105_HUMAN	27920.8809
SEPT8_HUMAN	6890.10938
CNOT9_HUMAN	4704.80225
TBCD5_HUMAN	3686.72339
ZN592_HUMAN	720.698242
JADE3_HUMAN	971.244385
MY18A_HUMAN	1141.63782
LAR4B_HUMAN	4032.92065
GCN1_HUMAN	22621.3047
HMHA1_HUMAN	182.835373
PRP16_HUMAN	2965.44849
NU205_HUMAN	67440.1953
ANS1A_HUMAN	1006.98273
PXDN_HUMAN	10480.2607
FAN_HUMAN	806.322205
GPI8_HUMAN	1590.95215
RT31_HUMAN	9237.46387
AKAP1_HUMAN	291.586975
ALG3_HUMAN	1094.46997
NEUG_HUMAN	528.531921
AN32B_HUMAN	61265.543
NECT2_HUMAN	9684.63379
PGTA_HUMAN	11289.5166
TFG_HUMAN	12172.126
HTRA1_HUMAN	17522.6895
ARC1A_HUMAN	8951.95898
TF2H4_HUMAN	1153.07349
RREB1_HUMAN	504.715942
HDAC2_HUMAN	11554.4961
STAM1_HUMAN	2093.10156
REQU_HUMAN	2927.33618

SC65_HUMAN	8821.99316
CBP_HUMAN	499.991333
KAT6A_HUMAN	254.606598
SYMPK_HUMAN	4741.74902
RBP56_HUMAN	1483.03149
GOGA1_HUMAN	688.893982
GGH_HUMAN	4611.7915
NRCAM_HUMAN	8855.10742
RPGR_HUMAN	542.893127
SHIP1_HUMAN	4908.73096
DDX17_HUMAN	87963.7578
KIFA3_HUMAN	2182.48999
CASPA_HUMAN	1483.87756
APBB2_HUMAN	1276.42773
RAD50_HUMAN	11843.2813
CELF1_HUMAN	9306.74609
OSTF1_HUMAN	4036.37988
ARHG1_HUMAN	12153.6396
XPF_HUMAN	636.751587
UFD1_HUMAN	27371.6953
GSLG1_HUMAN	16818.4082
RENT1_HUMAN	11107.4336
CSN5_HUMAN	14248.1553
GPKOW_HUMAN	5829.4292
SMRC1_HUMAN	3735.33301
SMRD2_HUMAN	3606.70776
RAB8B_HUMAN	18941.8984
BAD_HUMAN	577.060303
FUBP2_HUMAN	13358.8447
GCDH_HUMAN	8063.62939
RIT1_HUMAN	1140.29614
PEX13_HUMAN	633.717163
TNPO1_HUMAN	36878.6992
ARHG2_HUMAN	18386.9414
NEP1_HUMAN	10138.6035
CLP1_HUMAN	3202.19336
GLMN_HUMAN	5812.87451
UBP13_HUMAN	2025.38708
DVL3_HUMAN	800.5354
USP9X_HUMAN	9394.73926
UBP7_HUMAN	10580.7568
CUL5_HUMAN	4859.80566
STMN2_HUMAN	39175.1445
VPP1_HUMAN	3537.37549
LPP_HUMAN	10403.3203
RBPMS_HUMAN	944.379517
EXT2_HUMAN	908.791138
MED12_HUMAN	883.472229
TP4A1_HUMAN	671.568726
KPBB_HUMAN	2713.83862

SCAM4_HUMAN	3638.8457
HPS3_HUMAN	997.189209
SMCE1_HUMAN	5132.78809
CAVN3_HUMAN	12120.7021
RIFK_HUMAN	421.624756
MYDGF_HUMAN	34481.4961
TM40L_HUMAN	1431.89709
YIPF5_HUMAN	2408.10303
PIGT_HUMAN	4278.39648
IGSF8_HUMAN	433.885712
RL36L_HUMAN	6003.61182
RAB24_HUMAN	314.151672
OSBP2_HUMAN	1486.15454
ZN622_HUMAN	2528.9585
RRF2M_HUMAN	1910.8468
UB2E3_HUMAN	10508.4951
5NT3B_HUMAN	4202.77686
WBP2_HUMAN	2912.05347
PSMG2_HUMAN	14978.6885
NCLN_HUMAN	16572.9238
MUL1_HUMAN	439.67157
MRTFA_HUMAN	600.261841
LFG3_HUMAN	5031.65723
ERGI1_HUMAN	10771.4355
UTP4_HUMAN	1828.32397
FAKD4_HUMAN	4989.71094
MARC2_HUMAN	2892.71313
F162A_HUMAN	9924.7832
CCD47_HUMAN	8320.52051
RM24_HUMAN	6421.59326
EXOC4_HUMAN	9519.14648
MGN2_HUMAN	33974.0469
P33MX_HUMAN	921.643921
RFT1_HUMAN	3620.44653
ISOC2_HUMAN	1512.74463
NTAN1_HUMAN	1486.29382
FERM2_HUMAN	13454.5596
FUBP1_HUMAN	43180.7461
TTC17_HUMAN	783.000183
LRC59_HUMAN	95610.1719
VTI1A_HUMAN	404.964111
ESAM_HUMAN	6140.4126
PBIP1_HUMAN	5863.625
MCUR1_HUMAN	676.360352
K1143_HUMAN	984.341431
VP33A_HUMAN	3877.80127
FKB10_HUMAN	16920.3574
TTC28_HUMAN	2050.50903
EXOS8_HUMAN	2649.40234
ZN428_HUMAN	866.103821

SH3K1_HUMAN	3899.57715
SKA1_HUMAN	637.158936
NACC2_HUMAN	676.342163
RNF25_HUMAN	1485.54797
AIDA_HUMAN	7827.48926
PINX1_HUMAN	875.447937
ARL8A_HUMAN	8166.87207
OTUL_HUMAN	2351.20264
CHCH1_HUMAN	2317.98706
PPWD1_HUMAN	5471.99756
CC127_HUMAN	628.847656
COA7_HUMAN	2333.03516
UPP_HUMAN	460.944489
TAM41_HUMAN	2709.58887
MOB3A_HUMAN	2358.9939
DOC10_HUMAN	2938.2688
ATG2B_HUMAN	1588.57629
LENG1_HUMAN	981.527161
TBC20_HUMAN	782.904602
EFHD2_HUMAN	36099.5664
GALM_HUMAN	1109.95947
SYTL4_HUMAN	2565.92285
P5CR2_HUMAN	6320.79297
CSTOS_HUMAN	2548.81128
DCPS_HUMAN	13639.0068
PP14B_HUMAN	2816.70898
INT12_HUMAN	1137.36951
NSUN4_HUMAN	2429.78687
RHDF1_HUMAN	581.291626
CTHR1_HUMAN	3467.42554
ACSF2_HUMAN	1025.39648
ISOC1_HUMAN	7136.79688
GCC1_HUMAN	660.524536
FWCH2_HUMAN	3376.90698
ASTRA_HUMAN	1769.66602
HAUS1_HUMAN	577.605408
FAF2_HUMAN	11881.0391
CC124_HUMAN	6875.01367
FXRD1_HUMAN	4203.70166
OPTN_HUMAN	2634.42139
AP2M1_HUMAN	46202.1797
GCP3_HUMAN	3038.12744
S7A6O_HUMAN	997.860901
KCD12_HUMAN	38379.9258
RCN3_HUMAN	876.012634
NMD3_HUMAN	6202.53662
REPS1_HUMAN	1114.88672
TIM14_HUMAN	3128.2605
RMD1_HUMAN	5922.79346
NUD16_HUMAN	5676.15283

ESS2_HUMAN	1561.49048
CMBL_HUMAN	2840.47461
MSI2H_HUMAN	2677.15796
SNR40_HUMAN	8349.3457
RMC1_HUMAN	1702.83826
RM38_HUMAN	8885.63184
RSPRY_HUMAN	537.189514
ERLEC_HUMAN	3305.90967
RRFM_HUMAN	5799.32813
MTEF3_HUMAN	5169.05566
SPDLY_HUMAN	1708.34314
ELP4_HUMAN	2656.88477
SIR1_HUMAN	829.823242
YIPF6_HUMAN	1414.51416
SEH1_HUMAN	4518.79199
TCAL4_HUMAN	1967.12817
KBP_HUMAN	15753.8418
GNA1_HUMAN	11192.5527
F120B_HUMAN	2107.7373
KTI12_HUMAN	1258.03491
RT24_HUMAN	5181.23584
RM53_HUMAN	1745.1311
T3HPD_HUMAN	7772.67627
RNF31_HUMAN	1435.24841
DAZP1_HUMAN	16238.8301
SGTB_HUMAN	674.205322
SAAL1_HUMAN	2107.90308
MITOK_HUMAN	1120.58179
SGF29_HUMAN	1065.05945
RRP36_HUMAN	912.67627
C1GLC_HUMAN	1326.39941
RBM33_HUMAN	2152.72949
DTBP1_HUMAN	3367.4646
DNJA3_HUMAN	5667.33154
TMA16_HUMAN	5121.75195
MB12A_HUMAN	3774.89722
PTCD3_HUMAN	7251.90918
MMAB_HUMAN	3951.22437
CYFP2_HUMAN	421.180115
GIMA5_HUMAN	315.912781
TRI11_HUMAN	1409.47705
CCD97_HUMAN	594.028931
CNRP1_HUMAN	10071.1045
EDC3_HUMAN	4773.23877
DYL2_HUMAN	5904.14404
CPNE2_HUMAN	4563.81494
S10AG_HUMAN	4702.02197
SIPA1_HUMAN	3930.74487
SCRN2_HUMAN	977.33075
THOC1_HUMAN	5608.96143

OTUB1_HUMAN	25955.7695
TRM61_HUMAN	765.760254
HMCES_HUMAN	308.836365
CHMP6_HUMAN	3528.78418
BICD1_HUMAN	315.319794
PGM2_HUMAN	28788.3613
IMP4_HUMAN	3983.24487
CERS2_HUMAN	6705.05127
DUS3L_HUMAN	671.852478
LTV1_HUMAN	3882.9895
SDSL_HUMAN	737.512024
RM48_HUMAN	2727.70923
PLPP_HUMAN	1666.35254
AURKB_HUMAN	756.834412
DCNL1_HUMAN	2957.34668
FAH2A_HUMAN	4110.78076
SMRD1_HUMAN	1376.22107
TOE1_HUMAN	2438.70166
CDA7L_HUMAN	884.131775
RUSF1_HUMAN	1300.7384
DDX27_HUMAN	7813.71631
BORC6_HUMAN	1143.86938
SYMM_HUMAN	354.649475
A7L3B_HUMAN	549.618591
GWL_HUMAN	336.124847
MTNB_HUMAN	1919.58862
ZC21A_HUMAN	864.740112
LIN37_HUMAN	389.692993
S41A3_HUMAN	209.781403
SNF8_HUMAN	1850.36975
ZCCHL_HUMAN	2789.7688
P121A_HUMAN	232.684998
TONSL_HUMAN	503.938477
PDLI5_HUMAN	39657.793
CREL1_HUMAN	1700.56128
ERO1A_HUMAN	22938.0059
PRR11_HUMAN	1425.96899
FMC1_HUMAN	2564.49927
SAHH3_HUMAN	11347.6455
DOCK6_HUMAN	5739.40723
MED30_HUMAN	639.257507
PGAM5_HUMAN	14748.0684
INT4_HUMAN	2201.20947
DDRGK_HUMAN	4657.02783
DHTK1_HUMAN	799.273254
SCLY_HUMAN	2430.17261
FUBP3_HUMAN	17340.3184
SPF45_HUMAN	4798.27979
RCC1L_HUMAN	1747.39612
SYNM_HUMAN	1632.98889

SUCB2_HUMAN	11504.8574
LRCH3_HUMAN	4025.94507
GMPPA_HUMAN	7481.90381
HPDL_HUMAN	1251.90442
ABHEB_HUMAN	1785.4613
NGLY1_HUMAN	2025.19287
SC22A_HUMAN	5604.63721
ATPMK_HUMAN	12860.9707
NSL1_HUMAN	1106.38965
PAWR_HUMAN	4217.95361
MET2A_HUMAN	422.003082
THOC3_HUMAN	5093.97266
ITCH_HUMAN	3380.46973
TXD15_HUMAN	775.258972
KIRR1_HUMAN	2122.32983
LRIG1_HUMAN	570.635803
COG3_HUMAN	2057.84814
CK5P3_HUMAN	7133.8457
VPS39_HUMAN	3340.60596
VPS50_HUMAN	2333.07007
VCIP1_HUMAN	3701.03491
SPTCS_HUMAN	631.021545
ELMO2_HUMAN	6661.4751
TMX3_HUMAN	13000.8438
DCAF5_HUMAN	1791.58435
CHAP1_HUMAN	2331.56543
LMBL3_HUMAN	3285.32471
ZFP91_HUMAN	1045.48999
PCD16_HUMAN	1104.27869
CLMN_HUMAN	14368.8398
PDLI2_HUMAN	567.342346
FACR2_HUMAN	4815.76221
BT3L4_HUMAN	8839.78711
RN170_HUMAN	661.386047
S35E1_HUMAN	1679.21777
UBP47_HUMAN	4808.65625
ZN521_HUMAN	275.199554
CLP1L_HUMAN	2651.77539
TOPK_HUMAN	4909.99023
ARL5B_HUMAN	346.425079
DNJC1_HUMAN	1829.73364
SCYL1_HUMAN	5974.43701
Z512B_HUMAN	797.811829
LRAT2_HUMAN	694.98761
EXOC2_HUMAN	7058.93555
CNDP2_HUMAN	45747.2852
EHMT2_HUMAN	1671.84656
ZFR_HUMAN	4140.84863
F210B_HUMAN	645.612671
B3GT6_HUMAN	1608.50867

EP400_HUMAN	1098.20374
SNX27_HUMAN	4023.4729
KI16B_HUMAN	248.911057
IFT74_HUMAN	599.17749
TRI47_HUMAN	4207.34033
CNO6L_HUMAN	3308.44336
DHRS1_HUMAN	432.184967
DJC30_HUMAN	973.429932
UB2E2_HUMAN	98.7395248
CAR19_HUMAN	2838.01709
PRRC1_HUMAN	5966.64795
FGD4_HUMAN	3927.57007
FXL18_HUMAN	4050.67358
ZN512_HUMAN	2841.61182
NSE2_HUMAN	820.837463
NSE3_HUMAN	309.524323
PCMD1_HUMAN	396.225159
HS12B_HUMAN	4063.69116
YTDC1_HUMAN	2032.94092
CCD43_HUMAN	1146.87524
COG8_HUMAN	2149.91968
CKLF3_HUMAN	3415.13623
DAA10_HUMAN	2806.80469
NOL4L_HUMAN	5679.10449
MBOA7_HUMAN	10345.1914
DOCK7_HUMAN	9549.49023
SFXN2_HUMAN	1840.98474
ZN830_HUMAN	590.158813
F210A_HUMAN	334.982544
FRMD6_HUMAN	1022.4129
NSUN5_HUMAN	297.636414
RPR1A_HUMAN	3669.5188
AGAP3_HUMAN	1411.71448
ARAP1_HUMAN	3367.43872
IPO9_HUMAN	15826.0967
MIA2_HUMAN	1542.19971
DCBD2_HUMAN	1290.66443
ARHGH_HUMAN	1264.09973
INP4A_HUMAN	568.072388
RBM14_HUMAN	19976.8477
KLHL5_HUMAN	800.614319
UHRF2_HUMAN	4734.0376
NED4L_HUMAN	3510.15894
QKI_HUMAN	14616.2217
FMNL2_HUMAN	1543.93958
PUS7_HUMAN	5401.93164
F111A_HUMAN	822.210876
TPPC9_HUMAN	766.12738
TRNT1_HUMAN	3875.93408
SMG1_HUMAN	1057.66882

ALS2_HUMAN	1056.42871
TM237_HUMAN	1179.26965
ALKB3_HUMAN	238.87204
RHG07_HUMAN	924.731201
PP1RA_HUMAN	3956.81812
S38A2_HUMAN	960.762085
UIF_HUMAN	2572.03784
MTMR9_HUMAN	2356.39624
VPS35_HUMAN	27042.1914
PURB_HUMAN	2871.0874
XPO6_HUMAN	2377.77563
HHIP_HUMAN	36705.207
MAGI1_HUMAN	937.147827
SPAG5_HUMAN	1161.02063
NACC1_HUMAN	5313.88867
SNX18_HUMAN	2061.3479
PASK_HUMAN	364.097992
CIC_HUMAN	1670.94153
UIMC1_HUMAN	445.838623
VP13A_HUMAN	2293.76343
MED15_HUMAN	2675.04639
EFGM_HUMAN	10676.624
ERGI2_HUMAN	1709.55835
MCCA_HUMAN	1416.98389
KKCC2_HUMAN	667.394775
TGS1_HUMAN	751.465149
NUDC1_HUMAN	12280.4121
ERBIN_HUMAN	1850.45374
GCP6_HUMAN	1908.68335
GCP5_HUMAN	697.905701
UBP28_HUMAN	779.110779
FNBP1_HUMAN	3619.9397
PRPK_HUMAN	5277.57959
PIGS_HUMAN	5622.82275
WRIP1_HUMAN	3056.79834
RANB9_HUMAN	2538.2749
CLCC1_HUMAN	2557.12109
MYADM_HUMAN	6851.375
NEB2_HUMAN	1845.46118
SRPK1_HUMAN	4247.49805
SMC6_HUMAN	1448.34644
KCD15_HUMAN	729.644775
STRBP_HUMAN	18868.5352
TM209_HUMAN	3011.77563
GPX7_HUMAN	1097.81763
CP2S1_HUMAN	978.468018
IWS1_HUMAN	7444.75928
SIN3A_HUMAN	1180.59595
OSBL9_HUMAN	5702.55859
CRBN_HUMAN	736.373413

INT14_HUMAN	1247.271
AEDO_HUMAN	3076.90601
CK5P1_HUMAN	664.64978
RSF1_HUMAN	351.472778
RBM15_HUMAN	3214.14551
RUFY1_HUMAN	4071.18042
MINT_HUMAN	604.625549
PNKP_HUMAN	5184.2124
MMS19_HUMAN	10291.7178
UHRF1_HUMAN	5542.92871
NIBA2_HUMAN	33701.7266
YMEL1_HUMAN	7611.22461
RMD3_HUMAN	4839.56201
HTF4_HUMAN	827.261597
CYH2_HUMAN	568.146423
TBCB_HUMAN	14416.1162
PSB7_HUMAN	21445.9863
CNN2_HUMAN	16189.8447
SEC62_HUMAN	6952.68457
PCY2_HUMAN	5131.71973
CDC5L_HUMAN	13739.1172
PSMD1_HUMAN	29501.8789
SDF2_HUMAN	2311.06616
PFD5_HUMAN	1161.77319
RING2_HUMAN	1996.08923
PARK7_HUMAN	13509.3984
GA2L1_HUMAN	257.948456
EYA3_HUMAN	619.464905
NEUR1_HUMAN	5584.5083
SORT_HUMAN	181.748718
VAT1_HUMAN	116180.828
LGMN_HUMAN	2456.27222
PLIN2_HUMAN	465.824036
DNJC2_HUMAN	4810.51025
MPP8_HUMAN	484.769806
NUP88_HUMAN	9035.12695
PKP4_HUMAN	245.502106
PI3R4_HUMAN	3718.99121
P2RX4_HUMAN	2774.28247
POP1_HUMAN	6249.11621
S10AD_HUMAN	27743.623
SCAFB_HUMAN	1542.12244
TSNAX_HUMAN	5416.85303
SPS2_HUMAN	1974.43994
EIF3C_HUMAN	27056.1777
TTC1_HUMAN	563.23877
DNJC7_HUMAN	11314.168
PHB2_HUMAN	87295.2188
CSN8_HUMAN	1455.75671
PRP18_HUMAN	752.530945

CHP1_HUMAN	3566.57031
KIF2C_HUMAN	2848.80249
RGPD5_HUMAN	277.30188
MGLL_HUMAN	3314.0188
ATX2_HUMAN	3032.95898
DOK1_HUMAN	337.759918
METH_HUMAN	9113.55176
HCD2_HUMAN	32385.373
COCA1_HUMAN	2013.60742
SMAD5_HUMAN	1454.67615
SGMR1_HUMAN	4598.69873
ROAA_HUMAN	14485.0107
NP1L4_HUMAN	25461.6738
MGST2_HUMAN	26852.0508
SNAG_HUMAN	6008.66797
PI51A_HUMAN	1322.35364
ABCA3_HUMAN	1269.05042
M3K3_HUMAN	1097.59961
ATP5S_HUMAN	1340.92651
MIPEP_HUMAN	3515.85522
ACON_HUMAN	14985.5791
TM9S2_HUMAN	6703.27588
S29A1_HUMAN	9826.35742
TS101_HUMAN	8363.4502
CPNE1_HUMAN	17205.7813
TCPH_HUMAN	47171.2656
MYD88_HUMAN	5974.9375
EBP2_HUMAN	8877.42285
ANM1_HUMAN	23479.1055
BAG1_HUMAN	1170.57703
ATF6B_HUMAN	1152.81946
FOXC2_HUMAN	450.017059
SH3G1_HUMAN	27984.4629
TEP1_HUMAN	6894.46924
VRK1_HUMAN	12240.0254
GDF15_HUMAN	17264.2168
AKAP9_HUMAN	9469.09961
NIPS1_HUMAN	10914.0039
CND3_HUMAN	6789.8042
ARP5L_HUMAN	10932.79
MICU1_HUMAN	741.727905
CG025_HUMAN	870.105225
F118B_HUMAN	1843.65344
RBM4B_HUMAN	16787.4902
ZFY21_HUMAN	1848.08179
DDX50_HUMAN	5302.48047
RM34_HUMAN	3195.43726
RNZ2_HUMAN	7446.9873
TRIR_HUMAN	3721.43701
GRWD1_HUMAN	7157.9624

MACD1_HUMAN	721.633057
TCF25_HUMAN	2863.33423
CMS1_HUMAN	3559.84888
KLDC3_HUMAN	1130.19885
ECSIT_HUMAN	2373.3125
MEP50_HUMAN	17944.6191
CYBC1_HUMAN	1109.6792
VKOR1_HUMAN	3325.77954
DPH2_HUMAN	1614.48315
TBA1C_HUMAN	18654.9824
SELS_HUMAN	2729.13843
APOL2_HUMAN	4647.21826
MBB1A_HUMAN	17297.4082
MGME1_HUMAN	627.837219
GORS1_HUMAN	769.264954
FYCO1_HUMAN	1466.6864
ACBD6_HUMAN	2752.52905
COR1B_HUMAN	32467.0605
TXD17_HUMAN	10561.8203
BUD13_HUMAN	776.40094
CPPED_HUMAN	16616.8457
VPS25_HUMAN	2855.18384
RM45_HUMAN	12330.5752
CG050_HUMAN	9158.87695
TIRR_HUMAN	2613.65991
CAB45_HUMAN	7082.60303
PDD2L_HUMAN	982.375854
PAAF1_HUMAN	4119.44629
PYM1_HUMAN	3596.90063
MIC25_HUMAN	871.222534
ADPGK_HUMAN	5266.20557
GPTC1_HUMAN	389.457764
RIOK1_HUMAN	1549.63635
LLPH_HUMAN	3158.13696
SLD5_HUMAN	551.688965
UTP23_HUMAN	388.362946
PELO_HUMAN	5951.72998
PXL2A_HUMAN	62952.707
TRI56_HUMAN	1199.35156
ERP44_HUMAN	51106.8203
LXN_HUMAN	14522.1113
NOL10_HUMAN	2269.67358
NTPCR_HUMAN	5692.38965
TIM29_HUMAN	5223.29248
TACO1_HUMAN	2778.46851
GCP2_HUMAN	4820.05664
ESYT1_HUMAN	29081.2852
UBAC1_HUMAN	1310.56909
YIPF4_HUMAN	2748.62354
PHAF1_HUMAN	884.921021

SEN34_HUMAN	1325.35535
CNPY3_HUMAN	1360.57654
MTG1_HUMAN	2461.30322
ALG1_HUMAN	3667.13257
LIMD2_HUMAN	869.270935
HAUS8_HUMAN	491.09668
INP5K_HUMAN	2305.54126
PSMG3_HUMAN	3065.79761
CSN4_HUMAN	12056.6895
SYT11_HUMAN	1015.15802
WAC_HUMAN	473.610901
DIDO1_HUMAN	2993.33301
MTA3_HUMAN	4834.65869
RBM42_HUMAN	9192.90723
DCTN5_HUMAN	1847.39441
MCMBP_HUMAN	4691.14258
AASD1_HUMAN	4018.11719
DCNL5_HUMAN	5949.10547
AN32E_HUMAN	6042.83789
LRRC1_HUMAN	610.51532
P4K2A_HUMAN	2593.08716
TMM43_HUMAN	25173.123
FSD1_HUMAN	1026.92517
TBCD_HUMAN	8549.6875
NDC1_HUMAN	1475.14514
HGH1_HUMAN	1240.80261
DHRS4_HUMAN	3368.25562
LMF2_HUMAN	5050.85107
NDUF3_HUMAN	5620.40137
MMTA2_HUMAN	902.41925
TMM70_HUMAN	4031.66797
MED18_HUMAN	1180.15576
ISCA1_HUMAN	922.75
TBB6_HUMAN	123145.664
PAXX_HUMAN	1104.31836
RPC3_HUMAN	2079.55933
HNRL1_HUMAN	15991.8965
MSTO1_HUMAN	5477.1665
PHF23_HUMAN	1594.3335
PDC10_HUMAN	13221.4453
RPP25_HUMAN	1539.70264
DERL1_HUMAN	17759.8809
HTAI2_HUMAN	4949.83154
DDX23_HUMAN	9834.27539
TCAB1_HUMAN	956.93988
MIC26_HUMAN	5230.84473
DHRS6_HUMAN	6476.26855
MTNA_HUMAN	4468.47021
WDR18_HUMAN	6151.8877
VAMP8_HUMAN	920.42926

THUM3_HUMAN	2001.25427
MTND_HUMAN	4985.26807
MECR_HUMAN	2128.16528
EMC6_HUMAN	6830.89063
NTM1A_HUMAN	3134.13843
KTNB1_HUMAN	471.141174
LST8_HUMAN	1646.82935
ASHWN_HUMAN	1366.4845
TM109_HUMAN	64302.0313
PBDC1_HUMAN	2984.64063
PTSS2_HUMAN	3444.66455
NOC4L_HUMAN	5052.55859
UT14A_HUMAN	4380.07422
DUS23_HUMAN	4471.4082
TMED9_HUMAN	41914.8047
TM147_HUMAN	3788.68164
NUP58_HUMAN	3823.47021
SELO_HUMAN	2636.17065
DPCD_HUMAN	1820.26343
GNL3_HUMAN	14922.3369
AFG2B_HUMAN	6179.24365
RIOK2_HUMAN	1656.61084
TR61B_HUMAN	222.278397
TMUB1_HUMAN	1881.3053
TIM21_HUMAN	2962.61035
KIFC1_HUMAN	799.020996
NUP85_HUMAN	6649.29492
ELOV1_HUMAN	1823.73877
KATL1_HUMAN	1224.3252
HIRP3_HUMAN	370.405609
IFT27_HUMAN	1976.4137
YJU2_HUMAN	2317.89111
NUDT9_HUMAN	4468.56445
SYTM_HUMAN	4201.2168
THIC_HUMAN	22442.1348
REPI1_HUMAN	648.416138
RBM4_HUMAN	3624.7063
FUND2_HUMAN	1017.47968
RPAP1_HUMAN	2576.06738
SF3B5_HUMAN	1622.21375
SFXN3_HUMAN	16293.834
PRR14_HUMAN	948.142273
CHID1_HUMAN	2422.11353
PAPOG_HUMAN	915.238586
CAR10_HUMAN	310.384705
NADAP_HUMAN	5968.69873
CDK19_HUMAN	576.151062
LS14B_HUMAN	1310.60901
TPSNR_HUMAN	670.854004
JAM3_HUMAN	7531.65088

CARD6_HUMAN	1437.29651
BTBD2_HUMAN	820.062866
SGPP1_HUMAN	126.794182
OSB11_HUMAN	1735.74219
OSB10_HUMAN	3665.13599
RFIP5_HUMAN	2141.37256
TB10A_HUMAN	232.503311
C1QT5_HUMAN	3942.97974
NAA15_HUMAN	21315.3008
KLF16_HUMAN	27.0282402
B2L13_HUMAN	706.404053
CAR11_HUMAN	1299.09595
S12A9_HUMAN	1295.36621
SRRT_HUMAN	21170.1875
TGT_HUMAN	5945.9502
AP1M1_HUMAN	15238.5576
NUSAP_HUMAN	600.642517
HDHD5_HUMAN	3537.71094
FACD2_HUMAN	502.176117
MAK16_HUMAN	3061.58276
HDAC8_HUMAN	278.520172
RTF2_HUMAN	1334.48999
CHM4A_HUMAN	2817.00293
EIF2A_HUMAN	16948.5332
PDIP3_HUMAN	4562.49658
K1671_HUMAN	1606.43213
SHAN3_HUMAN	8950.38281
GNB1L_HUMAN	1870.33203
FUT8_HUMAN	1213.56934
RM32_HUMAN	1135.88623
RM20_HUMAN	4865.74512
RM13_HUMAN	10044.6963
RM09_HUMAN	9998.69141
RM04_HUMAN	5188.11475
RM01_HUMAN	8370.17871
MK67I_HUMAN	4054.65259
HYCCI_HUMAN	1380.95837
YTHD1_HUMAN	880.919678
HELZ2_HUMAN	627.517822
HOIL1_HUMAN	2174.7605
RT26_HUMAN	5757.95313
NEUL_HUMAN	10302.6768
CEP41_HUMAN	3270.22266
SETD2_HUMAN	630.497437
PANK2_HUMAN	766.582947
DOCK9_HUMAN	9837.34863
FRMD8_HUMAN	4163.48096
CNTP3_HUMAN	1597.16138
NSD3_HUMAN	1352.93408
NUF2_HUMAN	3561.51074

RM37_HUMAN	10540.9824
GTPB4_HUMAN	11276.9561
ASPC1_HUMAN	2146.26636
OSBL8_HUMAN	2471.03638
UACA_HUMAN	2635.7373
RAB34_HUMAN	4640.90869
DPH1_HUMAN	978.911621
WDR11_HUMAN	4174.99268
REN3B_HUMAN	1804.78833
CRNL1_HUMAN	3528.30371
TBL1R_HUMAN	3466.87109
UBL5_HUMAN	4833.24902
PP12C_HUMAN	2696.0686
KPCD2_HUMAN	4759.86523
EDEM3_HUMAN	2928.45288
NIBA1_HUMAN	237.626007
UBXN6_HUMAN	3930.17163
TM6S1_HUMAN	430.315491
UCK2_HUMAN	7122.27002
SN_HUMAN	904.97583
API5_HUMAN	23403.0176
NLRP1_HUMAN	699.647644
TRIM5_HUMAN	1363.0813
TRIM4_HUMAN	48675.8945
FTO_HUMAN	5473.19775
ZDHC5_HUMAN	689.639709
TNG6_HUMAN	1710.50525
TB182_HUMAN	5535.71338
UBE2O_HUMAN	6377.7915
ZY11B_HUMAN	1422.99609
TANC1_HUMAN	5135.87549
EPT1_HUMAN	1852.88684
XPO4_HUMAN	7487.13428
LNP_HUMAN	1223.68201
CEP44_HUMAN	263.486816
TTYH3_HUMAN	10571.708
KLHL4_HUMAN	1991.5188
MTMRC_HUMAN	575.016968
WDR33_HUMAN	6257.50293
WDR12_HUMAN	7186.15967
YIPF3_HUMAN	5255.71143
TINAL_HUMAN	2084.82153
ADSSP_HUMAN	8165.98438
PITH1_HUMAN	3933.70313
DERL2_HUMAN	6639.25537
COMD5_HUMAN	1752.03979
SENP6_HUMAN	198.990555
REXO4_HUMAN	2190.9375
DDX24_HUMAN	5712.93115
RPA49_HUMAN	3947.57202

SKI8_HUMAN	8006.86865
SLIRP_HUMAN	8604.77148
SRR_HUMAN	1528.44849
NIF3L_HUMAN	2649.04053
EGLN1_HUMAN	4776.39404
MCLN1_HUMAN	788.338623
PIP30_HUMAN	3671.09497
MFF_HUMAN	957.712097
NAA50_HUMAN	9819.82129
UBA5_HUMAN	6952.83594
MKRN2_HUMAN	1821.95215
T126A_HUMAN	3571.78052
PAIP1_HUMAN	12357.1924
CLPB_HUMAN	4988.00146
RB33B_HUMAN	3861.11182
LSG1_HUMAN	6599.13525
F107B_HUMAN	7491.35645
NAT10_HUMAN	17357.3438
COMD4_HUMAN	2678.03955
KLC2_HUMAN	2916.52368
ILKAP_HUMAN	5367.12305
XRN2_HUMAN	16242.3408
TOLIP_HUMAN	4851.81885
SP130_HUMAN	663.577515
BRD8_HUMAN	1128.84302
NSRP1_HUMAN	270.65921
INT2_HUMAN	997.936707
RGAP1_HUMAN	876.412659
PAR12_HUMAN	2220.26099
CSTFT_HUMAN	961.990601
5NT3A_HUMAN	3054.96313
CYRIA_HUMAN	13966.0039
TM222_HUMAN	894.495178
HDHD2_HUMAN	2154.57983
GATA_HUMAN	2168.30518
DDX47_HUMAN	7872.67627
MAGT1_HUMAN	7028.31445
RAB1B_HUMAN	55133.9023
RM18_HUMAN	6643.53223
TSYL1_HUMAN	1032.49597
TM168_HUMAN	573.342224
LMA2L_HUMAN	7300.70117
SMG9_HUMAN	1959.25635
F234A_HUMAN	199.210815
OSBL5_HUMAN	857.524902
TDIF1_HUMAN	1214.81152
SIL1_HUMAN	1151.21069
METL9_HUMAN	1447.43933
APC1_HUMAN	4220.85205
I2BPL_HUMAN	787.953735

CYTM1_HUMAN	356.121674
RPC6_HUMAN	2332.80859
NUCKS_HUMAN	3170.75903
TMX4_HUMAN	4802.50049
KI13A_HUMAN	16642.5215
ASCC2_HUMAN	7473.30615
RBNS5_HUMAN	741.248474
ISCU_HUMAN	3260.88257
OSBL2_HUMAN	1538.2782
ATG5_HUMAN	3691.42236
WDR13_HUMAN	2880.42139
EPN3_HUMAN	1329.65149
EHD4_HUMAN	92131.2656
VP33B_HUMAN	3488.54272
VPS16_HUMAN	7411.15039
VPS11_HUMAN	4663.09375
SH3L3_HUMAN	13562.6426
GAN_HUMAN	965.986145
S2532_HUMAN	1535.47961
TARA_HUMAN	6518.82178
EPC1_HUMAN	150.827499
SLK_HUMAN	10259.0742
PPIL3_HUMAN	621.175598
S38A1_HUMAN	1834.09155
PDCL3_HUMAN	6382.16846
TAOK3_HUMAN	6049.43799
RBGPR_HUMAN	9901.03516
ADNP_HUMAN	2253.11646
DPH5_HUMAN	2240.70459
DHX36_HUMAN	7641.91553
IPYR2_HUMAN	7555.07373
SPNS1_HUMAN	4646.16602
RM46_HUMAN	2162.48193
S12A5_HUMAN	788.590332
PARL_HUMAN	1151.31104
PININ_HUMAN	8764.69336
TM245_HUMAN	1140.31885
FOXP1_HUMAN	1279.0575
BACD3_HUMAN	4106.24219
CK068_HUMAN	5465.59424
GHITM_HUMAN	7004.86133
BOLA2_HUMAN	5945.39648
MMAD_HUMAN	1027.66516
TXNIP_HUMAN	408.207153
TMX1_HUMAN	23940.7871
NELFA_HUMAN	3045.21655
GCP60_HUMAN	9177.75781
BORG4_HUMAN	827.528748
PTN23_HUMAN	7665.82422
SEM6B_HUMAN	4830.29395

UN45A_HUMAN	18413.8691
MFSD1_HUMAN	2071.62622
DNJC5_HUMAN	705.57196
DSN1_HUMAN	262.180054
CHM4B_HUMAN	17476.4121
RWDD1_HUMAN	2061.55176
CUED2_HUMAN	549.607788
FN3K_HUMAN	1642.36731
OFUT1_HUMAN	8375.55176
PIGU_HUMAN	4348.20654
TOR3A_HUMAN	3310.69458
WNK1_HUMAN	3511.68359
AMPB_HUMAN	18575.7031
GLP3L_HUMAN	4947.05615
GOLP3_HUMAN	3959.50439
OSGL1_HUMAN	1862.45557
SIA7D_HUMAN	1304.20239
GAPR1_HUMAN	5310.5957
TRABD_HUMAN	2073.40552
MTG2_HUMAN	150.608643
SENP3_HUMAN	3745.12451
OSBL3_HUMAN	4818.58057
SMRCD_HUMAN	2722.96411
EHD1_HUMAN	22099.5137
CAPAM_HUMAN	532.199524
ESF1_HUMAN	836.028015
ALG2_HUMAN	3672.53076
HEAT1_HUMAN	8861.77246
SG196_HUMAN	623.09137
RABE2_HUMAN	1732.36499
TFB2M_HUMAN	4410.85498
ZCHC4_HUMAN	912.039368
STEEP_HUMAN	456.631714
CIA2A_HUMAN	1490.67346
DHX35_HUMAN	1429.37903
F124B_HUMAN	3417.83008
HAUS4_HUMAN	2663.20044
CC134_HUMAN	3760.26123
CCD86_HUMAN	4362.40967
REEP4_HUMAN	2368.37573
SOX17_HUMAN	946.387329
OPA3_HUMAN	2328.54419
CIAO3_HUMAN	1333.63013
DHX33_HUMAN	1449.8197
NOL6_HUMAN	3820.27686
WDCP_HUMAN	593.909912
YTDC2_HUMAN	3045.85791
AZI2_HUMAN	211.817596
RPAP3_HUMAN	3017.75024
BCAS3_HUMAN	468.704376

ALG9_HUMAN	2688.44141
LDAH_HUMAN	1918.01355
RIOX1_HUMAN	3132.43359
WDR55_HUMAN	2685.5979
RANB3_HUMAN	6566.45752
PEAK1_HUMAN	1781.0614
RPF2_HUMAN	3149.6687
SMYD3_HUMAN	3363.87158
AAMDC_HUMAN	1257.83057
DOCK5_HUMAN	1204.78442
WDR26_HUMAN	3458.69019
TDRD3_HUMAN	2248.68213
CH033_HUMAN	1667.93311
AT133_HUMAN	874.198853
MET17_HUMAN	544.471558
SFR19_HUMAN	1753.28516
NRDE2_HUMAN	967.951111
PGES2_HUMAN	6232.28955
PHAX_HUMAN	2738.26538
UBE2Z_HUMAN	5930.0957
ACAD9_HUMAN	5749.05518
NT5D2_HUMAN	9436.61621
PKRI1_HUMAN	1607.32275
CAAP1_HUMAN	1460.0011
NOL11_HUMAN	1600.10596
DDX31_HUMAN	796.550293
TMT1A_HUMAN	2357.91479
PAAT_HUMAN	2599.44214
MMRN2_HUMAN	12040.7607
MINY3_HUMAN	2285.29907
EVA1A_HUMAN	2393.04883
AKTIP_HUMAN	140.797623
PKHF2_HUMAN	1483.21631
ANKZ1_HUMAN	2277.88062
GORS2_HUMAN	3358.25513
ZWILC_HUMAN	1771.71082
JUPI2_HUMAN	2699.60938
GHC1_HUMAN	6531.55273
PPIP2_HUMAN	3444.32886
MED20_HUMAN	1717.97607
WDR76_HUMAN	242.40242
QTRT2_HUMAN	1810.59412
ARP8_HUMAN	1233.48474
ARMT1_HUMAN	4368.60547
PANK3_HUMAN	528.578247
CNO10_HUMAN	1607.50171
LRC40_HUMAN	17346.0039
EHMT1_HUMAN	2366.31104
SFXN1_HUMAN	26332.3457
SPE39_HUMAN	1382.198

COG4_HUMAN	7736.29199
ARP5_HUMAN	569.450684
VP37B_HUMAN	489.888275
RM44_HUMAN	8759.63672
L2HDH_HUMAN	758.980225
CSN7B_HUMAN	3177.58057
NHEJ1_HUMAN	907.032837
S61A2_HUMAN	13685.5928
FKRP_HUMAN	1019.57263
ELP3_HUMAN	5817.87598
RPF1_HUMAN	2430.58862
RPA2_HUMAN	1755.29272
ZMAT3_HUMAN	626.816772
UCK1_HUMAN	882.752441
KT3K_HUMAN	1603.75623
TBC17_HUMAN	1101.23596
SYCM_HUMAN	4227.63135
S52A2_HUMAN	1925.46094
PPCS_HUMAN	10298.5664
UBTD1_HUMAN	2379.2561
WDR41_HUMAN	774.115234
NMNA1_HUMAN	1600.83472
NJMU_HUMAN	1350.57727
SIAE_HUMAN	317.343781
PKHA5_HUMAN	763.817017
SMUF2_HUMAN	2871.23242
RENT2_HUMAN	3406.90942
GBB4_HUMAN	1888.31873
XPO5_HUMAN	7105.3833
GRPE1_HUMAN	15411.4258
MYG1_HUMAN	10390.5996
PKHA3_HUMAN	409.461395
PKHA1_HUMAN	1712.50598
RISC_HUMAN	3606.43604
NET4_HUMAN	3759.60254
CYBP_HUMAN	32504.8477
RRAGC_HUMAN	8006.4126
ZFYV1_HUMAN	1366.59912
IF122_HUMAN	727.873535
RDH14_HUMAN	4320.74219
PARVB_HUMAN	12821.5967
TENS1_HUMAN	3157.57983
PLRKT_HUMAN	2164.927
NMRL1_HUMAN	1755.58887
VEZA_HUMAN	515.676941
SPC25_HUMAN	1994.39465
TAF9B_HUMAN	940.671265
S38AA_HUMAN	1607.51758
EKI1_HUMAN	1897.07202
AGRL4_HUMAN	10041.4639

TM165_HUMAN	5545.58887
TPC_HUMAN	878.762695
EMAL4_HUMAN	5202.06641
MRM3_HUMAN	2754.48926
GLOD4_HUMAN	43432.8086
NEK6_HUMAN	826.738159
MCCB_HUMAN	2619.08057
NCOA5_HUMAN	43114.2852
EPG5_HUMAN	1423.73389
MOV10_HUMAN	6528.72852
MET14_HUMAN	1585.36816
GBA2_HUMAN	2752.8291
CWC22_HUMAN	1424.08093
RAVR2_HUMAN	759.585632
CHD8_HUMAN	1099.24902
E41L5_HUMAN	1658.95996
GPN1_HUMAN	3984.67627
SDF2L_HUMAN	4581.72607
KC1G1_HUMAN	3462.94946
SYF1_HUMAN	4414.94385
PREB_HUMAN	4161.08594
SRA1_HUMAN	1565.61829
AT131_HUMAN	5200.1333
GOPC_HUMAN	1742.61914
RM47_HUMAN	6283.54053
LYRM4_HUMAN	2449.90967
SPCS_HUMAN	294.321198
CHM1A_HUMAN	8231.08594
TM9S3_HUMAN	12066.0771
MYO10_HUMAN	5004.36816
APMAP_HUMAN	47503.1836
WDR6_HUMAN	5528.33643
TRXR2_HUMAN	10916.2559
ABCB6_HUMAN	1070.41602
ARFG3_HUMAN	5014.71826
ZCC17_HUMAN	910.240784
RAB18_HUMAN	17788.627
PALMD_HUMAN	7483.67188
SSU72_HUMAN	2523.62964
VTA1_HUMAN	19936.3066
SYSM_HUMAN	3658.24292
RT30_HUMAN	5686.57178
DLRB1_HUMAN	3677.58472
EMC7_HUMAN	7654.47559
ENY2_HUMAN	1241.35034
EXOS4_HUMAN	3998.37988
UBE2T_HUMAN	3896.48584
NOP10_HUMAN	4371.73682
OSGEP_HUMAN	4740.26563
DMAP1_HUMAN	919.87561

PCD12_HUMAN	624.965271
INO1_HUMAN	6277.09717
BRD7_HUMAN	713.916382
DCP1A_HUMAN	4626.03271
ACO13_HUMAN	9370.68652
MED4_HUMAN	834.860046
TIDC1_HUMAN	193.171463
RIC8A_HUMAN	20946.8164
C1QR1_HUMAN	30912.334
LUC7L_HUMAN	8031.33447
ESM1_HUMAN	859.991699
LZTL1_HUMAN	1830.83398
RM40_HUMAN	4732.76611
SSF1_HUMAN	3215.59424
PLCB1_HUMAN	1182.13489
TIGAR_HUMAN	15951.792
CL004_HUMAN	1023.58459
RTN4_HUMAN	15873.415
RPR1B_HUMAN	6768.57031
XPP3_HUMAN	2204.76099
NIT2_HUMAN	6573.95996
AVEN_HUMAN	728.062622
INCE_HUMAN	1133.8562
EXOS5_HUMAN	2066.42017
EXOS3_HUMAN	5203.83057
KI13B_HUMAN	5064.33252
ANLN_HUMAN	3371.61523
XPP1_HUMAN	12311.5342
GEPH_HUMAN	4664.06543
BIN3_HUMAN	3077.09717
SAS10_HUMAN	2139.66235
STAR7_HUMAN	1151.50293
BIRC6_HUMAN	4254.54346
PDLI7_HUMAN	24128.3555
ACSA_HUMAN	9405.49316
DBLOH_HUMAN	2103.07153
DDX21_HUMAN	24168.4863
SAR1A_HUMAN	26125.959
SIAS_HUMAN	32977.7813
SHLB2_HUMAN	3769.37915
ASH1L_HUMAN	16697.8359
EI2BG_HUMAN	9878.24121
MBNL1_HUMAN	10853.3467
S17A5_HUMAN	3058.84839
4ET_HUMAN	1101.67395
CHSTC_HUMAN	1897.96179
ST7_HUMAN	1079.49878
PICK1_HUMAN	762.348755
SH2B1_HUMAN	1013.34973
PYRG2_HUMAN	5434.91309

DPOE3_HUMAN	2498.72852
CHRC1_HUMAN	2090.85693
AAAS_HUMAN	6635.91162
SNRK_HUMAN	3403.72729
ABCBA_HUMAN	2598.23755
BAZ1A_HUMAN	1587.74133
STRN4_HUMAN	4360.50342
ADPPT_HUMAN	10417.9756
METL5_HUMAN	2583.78491
OSTC_HUMAN	20541.0586
COXM2_HUMAN	379.150787
PLS4_HUMAN	1571.65173
RNC_HUMAN	578.270447
SNX15_HUMAN	890.125183
HEBP1_HUMAN	2075.56323
ABC3C_HUMAN	2199.82227
VPS45_HUMAN	2458.16455
PNO1_HUMAN	2561.08325
RM17_HUMAN	11919.8555
PHP14_HUMAN	160.936127
SERC1_HUMAN	968.917542
RHG35_HUMAN	2693.39063
F1142_HUMAN	2311.50317
PLS3_HUMAN	2629.40869
PLCD_HUMAN	789.296326
PLCC_HUMAN	2728.1543
HELLS_HUMAN	1744.18958
C1GLT_HUMAN	1979.34778
LTBP3_HUMAN	374.635071
TOM22_HUMAN	4030.22852
LANC2_HUMAN	3854.69019
KIF15_HUMAN	1064.58997
TM7S3_HUMAN	1350.8855
HOME3_HUMAN	5258.99756
SYFB_HUMAN	27074.7656
SYIM_HUMAN	13157.8037
SLX9_HUMAN	3981.77979
SAMN1_HUMAN	352.789001
KLC4_HUMAN	1577.49353
DIAP3_HUMAN	2041.49377
BMP2K_HUMAN	1238.65466
ATG3_HUMAN	3363.0061
SIR3_HUMAN	307.237305
PDS5B_HUMAN	4814.86816
SMC4_HUMAN	32098.3359
MA2C1_HUMAN	6025.79297
SAC1_HUMAN	7498.56396
DEPP1_HUMAN	1131.39819
OLA1_HUMAN	32856.4805
CUTC_HUMAN	1126.20166

ZF64B_HUMAN	334.849213
ECHD1_HUMAN	9738.9873
RBM12_HUMAN	87865.2188
TB22B_HUMAN	1296.67432
MDN1_HUMAN	8325.8916
ACS2L_HUMAN	1239.39258
ZCHC3_HUMAN	788.215088
DECR2_HUMAN	4959.17236
ABHDA_HUMAN	2730.54834
T11L1_HUMAN	1635.43323
STAU2_HUMAN	4774.1792
DDX28_HUMAN	1690.0929
T106B_HUMAN	2565.97998
LIN7C_HUMAN	1732.91931
PLCE_HUMAN	4332.78662
TXLNG_HUMAN	1569.59839
SPS2L_HUMAN	2542.06396
UFSP2_HUMAN	2458.89014
ABCF3_HUMAN	7057.17627
CYRIB_HUMAN	9791.78906
MITOS_HUMAN	2910.54834
DD19A_HUMAN	17776.2148
GIMA4_HUMAN	17654.3047
TYDP1_HUMAN	2064.93628
POTE1_HUMAN	874.02002
TBC23_HUMAN	3959.14966
DCA13_HUMAN	2701.04663
IMP3_HUMAN	5485.02539
MRGBP_HUMAN	1173.37048
TYW1_HUMAN	426.885895
EXOC1_HUMAN	9532.6709
PCMD2_HUMAN	652.646912
INT9_HUMAN	2174.49072
CC50A_HUMAN	10062.9512
UQCC1_HUMAN	2137.69238
SEP11_HUMAN	22413.8516
S38A7_HUMAN	14180.8398
MED17_HUMAN	10022.8164
PARVA_HUMAN	8357.03906
UBP40_HUMAN	285.714081
PANK4_HUMAN	9376.69238
FBX28_HUMAN	674.112549
TBC13_HUMAN	6317.97705
EXD2_HUMAN	1688.56201
DJC11_HUMAN	6339.88232
INT7_HUMAN	13678.8496
FANCI_HUMAN	872.151184
ATD3A_HUMAN	38592.8359
ARL8B_HUMAN	5068.82813
DJC17_HUMAN	4201.75195

INT13_HUMAN	3110.37329
RIC8B_HUMAN	259.904968
GNL3L_HUMAN	1742.10229
DDX18_HUMAN	8034.31934
ASF1B_HUMAN	2248.9541
KLH11_HUMAN	844.846863
INT10_HUMAN	2328.66602
RT18A_HUMAN	6475.35645
PNPO_HUMAN	2334.07227
ARMC1_HUMAN	3831.77466
RPC5_HUMAN	634.703918
SDA1_HUMAN	4111.56885
PAPD1_HUMAN	4481.42627
NLE1_HUMAN	3281.09424
NECP2_HUMAN	12216.7158
RPC2_HUMAN	3859.66992
RBM28_HUMAN	4071.00659
ANO10_HUMAN	9122.52148
RBM22_HUMAN	2906.52197
DMAC2_HUMAN	293.196838
WDR70_HUMAN	1623.02087
TMM51_HUMAN	180.525162
ARGL1_HUMAN	3506.45264
IFT57_HUMAN	136.680634
SLTM_HUMAN	3555.34302
FKB14_HUMAN	1355.29114
RMND1_HUMAN	2522.12158
PK1IP_HUMAN	1992.62549
HIF1N_HUMAN	1271.32043
OXSM_HUMAN	7883.2793
RM22_HUMAN	15457.6406
CZIB_HUMAN	10901.085
BABA1_HUMAN	3220.1355
CLN6_HUMAN	491.21817
ASB6_HUMAN	2350.37378
THG1_HUMAN	1084.2218
HPF1_HUMAN	2325.81616
IRAK4_HUMAN	1937.91663
UCKL1_HUMAN	1732.93933
TM160_HUMAN	3113.8877
NALP2_HUMAN	4248.93164
F120C_HUMAN	789.796326
TSAP1_HUMAN	720.694397
NDUBB_HUMAN	1911.56726
RM16_HUMAN	9572.51367
NHP2_HUMAN	6363.26221
OCAD1_HUMAN	7194.1792
ADPRS_HUMAN	9812.6582
MARH5_HUMAN	4360.08301
LYAR_HUMAN	5128.07031

T161A_HUMAN	552.695984
IMPA3_HUMAN	4699.229
MIC19_HUMAN	10861.0762
DUS2L_HUMAN	226.237976
CKLF6_HUMAN	1109.75281
SIR5_HUMAN	964.685425
MIOS_HUMAN	1008.32245
MTMRA_HUMAN	3611.02783
NSMA3_HUMAN	3732.17529
CWC25_HUMAN	1587.74341
TEX10_HUMAN	4216.95117
DCA16_HUMAN	1501.34473
THUM1_HUMAN	4100.61719
P4HTM_HUMAN	321.947266
TOR4A_HUMAN	3289.41968
TRM1_HUMAN	4335.41113
GDAP2_HUMAN	1476.88879
NDE1_HUMAN	1245.63538
BABA2_HUMAN	1301.46521
QPCTL_HUMAN	2386.11816
ARL15_HUMAN	2148.72241
KCTD5_HUMAN	2050.427
CARF_HUMAN	2808.5293
DJB12_HUMAN	2492.43066
ALKB4_HUMAN	1404.50134
NSE4A_HUMAN	261.521149
GAR1_HUMAN	5641.33643
STAB1_HUMAN	8145.53711
PP4R2_HUMAN	1485.89856
DPP3_HUMAN	22954.5918
AATF_HUMAN	1151.35669
TBA8_HUMAN	4110.79785
DDX56_HUMAN	2993.18164
SPHK1_HUMAN	2823.26465
TE2IP_HUMAN	161.721619
ABI2_HUMAN	2475.90918
BCLF1_HUMAN	11506.375
UTP6_HUMAN	1383.48376
TAB2_HUMAN	149.622971
RM39_HUMAN	8316.00098
M3K20_HUMAN	2448.37354
FKB11_HUMAN	12697.1738
TMOD3_HUMAN	9165.05957
BET1L_HUMAN	2136.24292
ELOV5_HUMAN	2851.87769
KBRS2_HUMAN	1583.22791
UGGG2_HUMAN	659.044434
UGGG1_HUMAN	24451.8652
CDK12_HUMAN	2396.76172
FAKD2_HUMAN	3979.70215

TECR_HUMAN	6378.57764
ERAP1_HUMAN	12221.4014
ARP10_HUMAN	4155.22217
USE1_HUMAN	2278.02075
CISD1_HUMAN	2173.25049
GGA3_HUMAN	1416.20508
TLS1_HUMAN	979.973694
F120A_HUMAN	8657.75879
GDE1_HUMAN	790.923645
SMAL1_HUMAN	1406.0365
GLTP_HUMAN	2232.93774
SPG21_HUMAN	3723.37061
RM35_HUMAN	5608.42627
UBIP1_HUMAN	565.907227
IF2B1_HUMAN	18004.248
SACS_HUMAN	2194.59277
MTCH1_HUMAN	7445.56104
NUDT4_HUMAN	7076.99707
HPBP1_HUMAN	8958.41406
MAT2B_HUMAN	32057.0469
MYOF_HUMAN	44226.3398
ITSN2_HUMAN	2945.74268
NOP53_HUMAN	1506.39685
EHD3_HUMAN	44802.957
EHD2_HUMAN	61409.1133
ARHGC_HUMAN	2980.91309
CNOT2_HUMAN	3742.95654
SPN90_HUMAN	1707.08423
PD1L1_HUMAN	1447.75708
CALL5_HUMAN	15642.6592
OGFR_HUMAN	6650.64307
LMCD1_HUMAN	779.774841
CRIM1_HUMAN	2521.91309
SELN_HUMAN	4270.76025
PALS2_HUMAN	3788.81885
CHMP5_HUMAN	5726.62207
CWC15_HUMAN	2380.86304
RM15_HUMAN	10070.4395
THYN1_HUMAN	7215.02979
TAP26_HUMAN	396.318695
NDUF4_HUMAN	3147.42676
HACD3_HUMAN	10904.043
EMC3_HUMAN	3454.71313
NDUAD_HUMAN	7500.80811
PDP1_HUMAN	5097.14844
KCMF1_HUMAN	1266.27954
RAI14_HUMAN	9517.36816
VAPA_HUMAN	16094.1973
RM27_HUMAN	3738.36548
TMEM9_HUMAN	1244.4978

SENP1_HUMAN	1279.84473
CXXC1_HUMAN	333.220093
SH3B4_HUMAN	7386.01855
SEP10_HUMAN	11452.7451
GMIP_HUMAN	1084.74097
PHRF1_HUMAN	4870.56836
POGK_HUMAN	1094.72058
DAPLE_HUMAN	201.968155
RHG23_HUMAN	1025.63037
VPS18_HUMAN	9432.4082
RCC2_HUMAN	38770.25
RELCH_HUMAN	1249.94885
DIP2B_HUMAN	5084.43555
JCAD_HUMAN	2184.01343
SLAI2_HUMAN	587.24231
TEN3_HUMAN	1751.25647
UBP36_HUMAN	617.971863
BCCIP_HUMAN	13914.793
STK26_HUMAN	1445.58191
ARMX1_HUMAN	2829.45361
ABI3_HUMAN	7013.98145
FPRP_HUMAN	12965.7217
CT2NL_HUMAN	5679.65039
TM181_HUMAN	2209.26221
HTR5B_HUMAN	1707.41101
ZNFX1_HUMAN	1669.93298
CHPF2_HUMAN	2650.73169
PCD10_HUMAN	567.253784
RRBP1_HUMAN	36035.7695
CPSF2_HUMAN	4754.92871
KLHL9_HUMAN	2855.15308
SYLC_HUMAN	26584.3789
MYEF2_HUMAN	10828.3252
KLH42_HUMAN	620.721802
E2AK4_HUMAN	4088.2312
WDR35_HUMAN	1005.25568
RBM27_HUMAN	2276.71558
KANL3_HUMAN	1046.50793
HECW2_HUMAN	2722.8335
FRM4A_HUMAN	1107.04688
ANFY1_HUMAN	5507.29102
RERE_HUMAN	745.257263
SUCB1_HUMAN	7933.95361
GMPR2_HUMAN	5689.50146
STX18_HUMAN	739.208008
DPM3_HUMAN	752.392578
IMPCT_HUMAN	1432.82715
UVRAG_HUMAN	2431.66187
ATX10_HUMAN	12549.1201
MBD2_HUMAN	3804.36523

NCDN_HUMAN	2605.51611
TFP11_HUMAN	1179.54187
EP15R_HUMAN	10532.5508
MYO1A_HUMAN	6362.11035
ORC3_HUMAN	1650.04968
SAE1_HUMAN	12176.6738
COPG2_HUMAN	8124.16602
PI4KB_HUMAN	999.41394
MRC2_HUMAN	1036.68274
COMD3_HUMAN	1209.34875
GBG12_HUMAN	2268.95142
RBL2A_HUMAN	966.268005
MTRR_HUMAN	162.830414
UXT_HUMAN	1495.20459
ASH2L_HUMAN	2188.6416
DHCR7_HUMAN	22935.3945
TR10D_HUMAN	1026.45728
HDAC6_HUMAN	1686.53894
SPAST_HUMAN	3277.24243
DKK3_HUMAN	962.744995
TRMB_HUMAN	6464.48828
GULP1_HUMAN	1241.79932
VPS29_HUMAN	13482.5557
EIF3K_HUMAN	19266.2324
EXTL2_HUMAN	1585.58411
GRHPR_HUMAN	14155.4863
CATZ_HUMAN	17981.1992
KS6B2_HUMAN	889.447327
DJB11_HUMAN	13246.4512
RNF14_HUMAN	2626.89844
SAE2_HUMAN	19365.4063
MO4L1_HUMAN	1704.25037
NXF1_HUMAN	7295.18359
SE1L1_HUMAN	1498.84021
B4GT7_HUMAN	2403.04468
PEF1_HUMAN	2979.29102
ZMYM2_HUMAN	1001.17096
CSN7A_HUMAN	7738.68018
DIC_HUMAN	5859.10449
AASS_HUMAN	6979.10498
CLIP2_HUMAN	1372.99268
QCR9_HUMAN	10373.1602
MTFP1_HUMAN	2102.47852
ZO2_HUMAN	23116.6719
DNJB4_HUMAN	14758.3457
ST17A_HUMAN	1036.03491
CFDP1_HUMAN	3757.92554
DAXX_HUMAN	908.66333
TRM7_HUMAN	3186.64429
VTI1B_HUMAN	2973.35669

STK39_HUMAN	2702.34155
ADDG_HUMAN	2689.10229
LRWD1_HUMAN	2753.01685
NPS3A_HUMAN	6354.68896
CGBP1_HUMAN	3409.35132
PISD_HUMAN	917.55658
ABCF2_HUMAN	14133.667
TES_HUMAN	6316.87207
AAKG2_HUMAN	3650.07544
GCP4_HUMAN	2698.93823
KDM5B_HUMAN	263.508881
SYWM_HUMAN	1257.00684
LIMD1_HUMAN	1217.73413
SEC63_HUMAN	10272.0166
GTR6_HUMAN	1001.35095
Z3H7B_HUMAN	6631.96289
NDRG3_HUMAN	9373.5166
ARMX3_HUMAN	5530.97607
SWP70_HUMAN	19967.9238
SUN2_HUMAN	8669.90918
RLP24_HUMAN	2085.05615
LTOR3_HUMAN	6221.87842
LIMA1_HUMAN	17592.5352
AFF4_HUMAN	7305.04004
SRP68_HUMAN	11782.8613
CHRD1_HUMAN	14128.6953
TBK1_HUMAN	4976.82129
SEPT9_HUMAN	14912.124
PCYOX_HUMAN	8713.4082
DDX20_HUMAN	6052.09473
SHPK_HUMAN	4244.6875
DPP2_HUMAN	3224.08618
CEIP2_HUMAN	4156.39258
UBP25_HUMAN	3174.47144
BAP29_HUMAN	4420.61475
NB5R1_HUMAN	5704.9502
S30BP_HUMAN	3320.521
ZNHI2_HUMAN	10869.748
PFD2_HUMAN	7893.1001
GPN3_HUMAN	860.552246
PUF60_HUMAN	21251.1602
NRBP_HUMAN	5325.35938
ENOPH_HUMAN	7150.2334
FEZ2_HUMAN	1543.2373
NDUAC_HUMAN	5244.50586
EI2BD_HUMAN	13764.1035
VATH_HUMAN	6093.65088
PRAF1_HUMAN	3065.66797
IPO11_HUMAN	1900.07922
TR112_HUMAN	215.34285

DACH1_HUMAN	419.719452
XPO7_HUMAN	6427.08447
LCMT1_HUMAN	4047.47461
VPS51_HUMAN	3831.89502
BAZ2B_HUMAN	611.620239
BAZ2A_HUMAN	521.590332
BAZ1B_HUMAN	3977.36108
ATIF1_HUMAN	917.509338
KAD3_HUMAN	17190.1621
LCAP_HUMAN	8540.08984
MBD1_HUMAN	425.598602
CNOT7_HUMAN	2356.94702
RABX5_HUMAN	3552.37061
MSRA_HUMAN	2071.07764
NAGK_HUMAN	26688.668
HACL1_HUMAN	3122.38452
TRM6_HUMAN	1797.74585
SH3L2_HUMAN	1951.97192
NGAP_HUMAN	755.275391
TSR3_HUMAN	1580.03992
S2513_HUMAN	15678.9053
DBNL_HUMAN	12387.7715
DDX41_HUMAN	5621.69336
DCTN4_HUMAN	7467.91895
CDC23_HUMAN	3579.33032
APC7_HUMAN	4734.229
APC5_HUMAN	988.588623
APC4_HUMAN	1885.94531
ANC2_HUMAN	2424.47046
GGA2_HUMAN	5456.51855
GGA1_HUMAN	2392.44531
STML2_HUMAN	14927.3223
NAGPA_HUMAN	1046.44312
VPS28_HUMAN	7458.53955
LSM7_HUMAN	3676.49487
DBR1_HUMAN	3550.44434
TASOR_HUMAN	471.957733
JUPI1_HUMAN	481.791016
FBX3_HUMAN	1201.91077
AKA11_HUMAN	442.220001
FBW1B_HUMAN	993.629883
GMEB2_HUMAN	1337.32227
MRT4_HUMAN	16956.6816
TNIK_HUMAN	1526.14502
CPSF3_HUMAN	5630.14209
DP13A_HUMAN	5013.08789
BORG2_HUMAN	735.154175
TLK1_HUMAN	880.091614
GPTC8_HUMAN	1670.89063
PARP4_HUMAN	10369.4746

NXT1_HUMAN	598.658508
NUDT5_HUMAN	11074.9971
RCOR1_HUMAN	1280.02722
PPCT_HUMAN	762.189148
MA1B1_HUMAN	2179.74268
RALY_HUMAN	18945.9629
TF3C4_HUMAN	4965.30908
ERG28_HUMAN	2140.76587
PACN3_HUMAN	1107.71082
ACAD8_HUMAN	1889.96045
ANGL2_HUMAN	2544.22534
ACINU_HUMAN	10590.0908
AMFR_HUMAN	4200.00293
AGO2_HUMAN	4564.13867
NUP50_HUMAN	8512.90723
CDV3_HUMAN	20588.0957
CNO11_HUMAN	984.741699
INT6_HUMAN	2310.97681
BAG5_HUMAN	2747.84326
AGO1_HUMAN	4120.7002
RAB21_HUMAN	7505.20068
RB22A_HUMAN	5424.20313
TPC2L_HUMAN	2184.61035
ZN346_HUMAN	2338.7627
PSME2_HUMAN	12419.7432
TAOK2_HUMAN	2446.01782
MKLN1_HUMAN	4744.15186
DNPEP_HUMAN	7211.79932
RAB23_HUMAN	3945.57544
MCTS1_HUMAN	14764.7461
MTUS1_HUMAN	298.528748
WWC3_HUMAN	510.781982
PALD_HUMAN	2763.74878
S39AA_HUMAN	1194.32788
INO80_HUMAN	532.236938
CCPG1_HUMAN	1312.22119
KDIS_HUMAN	846.618347
ASAP1_HUMAN	4445.08301
MRTFB_HUMAN	1096.27734
ANR50_HUMAN	4971.47461
MED23_HUMAN	2556.06934
SHRM4_HUMAN	3456.60864
YETS2_HUMAN	1325.62561
NDRG4_HUMAN	448.376068
TBC24_HUMAN	2288.52588
ISY1_HUMAN	837.79834
TMCC3_HUMAN	2029.34045
HECD1_HUMAN	4527.51563
ZMYD8_HUMAN	2222.38208
CIZ1_HUMAN	356.418793

COR1C_HUMAN	19840.4082
TPX2_HUMAN	1709.65137
ABT1_HUMAN	2356.23682
NOB1_HUMAN	6111.19287
AKP8L_HUMAN	4285.72363
MAFF_HUMAN	1900.82764
ASC_HUMAN	4231.96143
TMCO1_HUMAN	11246.9971
APC10_HUMAN	1514.7865
MYO6_HUMAN	5576.90088
DD19B_HUMAN	1272.49121
PPT2_HUMAN	1732.82178
PRP19_HUMAN	21877.3086
SUFU_HUMAN	1818.44128
BOK_HUMAN	2483.46851
NOL7_HUMAN	3852.73438
SNX12_HUMAN	10024.7773
SYNRG_HUMAN	859.485535
VPS4A_HUMAN	8750.34375
G3BP2_HUMAN	11428.9844
CHIP_HUMAN	5382.37402
PACN2_HUMAN	10806.5576
MAGD2_HUMAN	17147.6641
SNX7_HUMAN	2218.12695
SNX6_HUMAN	7596.44531
DUS12_HUMAN	4846.12207
STX8_HUMAN	1421.44556
SSRG_HUMAN	7237.44971
PSD13_HUMAN	34696.6016
FAF1_HUMAN	6587
EPCR_HUMAN	18895.0977
PPIE_HUMAN	2017.62561
DIM1_HUMAN	4608.51465
CSN3_HUMAN	9722.19141
MINP1_HUMAN	3447.67285
NOVA2_HUMAN	6935.85938
WDR3_HUMAN	4990.62939
TTF2_HUMAN	321.669189
NSF1C_HUMAN	7670.09766
COG5_HUMAN	3132.7439
S12A4_HUMAN	7022.06006
AP4E1_HUMAN	344.343231
MACF1_HUMAN	42748.5703
CP131_HUMAN	1054.57922
SCAF8_HUMAN	2607.62378
PP6R1_HUMAN	2774.20239
TRI33_HUMAN	3085.91431
PHF8_HUMAN	986.613037
TNR6B_HUMAN	764.394348
PLCL2_HUMAN	597.777283

SMG5_HUMAN	1141.01685
EXOC7_HUMAN	9655.65918
ZC3H4_HUMAN	173.764328
UBP24_HUMAN	3370.04639
TBD2B_HUMAN	1705.33362
SMAG1_HUMAN	1342.60242
FOXJ3_HUMAN	1260.1051
CBPC1_HUMAN	670.304749
DICER_HUMAN	1120.97522
XCT_HUMAN	2908.22656
MARE3_HUMAN	1369.81921
SHOC2_HUMAN	4571.63818
SRRM2_HUMAN	11861.0537
MGT4B_HUMAN	1345.57898
PA2G4_HUMAN	81442.1328
SPG7_HUMAN	1382.2002
BAIP2_HUMAN	881.740051
GAB2_HUMAN	684.403625
SMC3_HUMAN	6624.5
CHM2B_HUMAN	15839.8447
SH2B3_HUMAN	1160.54932
ZN148_HUMAN	305.871277
MTMR6_HUMAN	4477.6499
JAG2_HUMAN	1534.1239
NIP7_HUMAN	2938.13013
GLCNE_HUMAN	3159.01758
RTRAF_HUMAN	10317.0371
RUVB2_HUMAN	32051.0195
CDYL_HUMAN	615.486572
PIN4_HUMAN	5827.03857
AKT3_HUMAN	1648.34351
FACE2_HUMAN	2998.33838
CHKB_HUMAN	768.439636
EIF3L_HUMAN	54221.5273
PLAP_HUMAN	11695.835
RUVB1_HUMAN	49440.1641
NUDC_HUMAN	31204.5469
BCS1_HUMAN	2769.14917
VDAC3_HUMAN	22418.0391
COF2_HUMAN	23450.1797
ERGI3_HUMAN	3689.24414
SYFA_HUMAN	27059.9648
SC5A6_HUMAN	2457.54102
RT33_HUMAN	5698.53223
ASF1A_HUMAN	2438.09033
DRG1_HUMAN	14577.9482
TPPC4_HUMAN	3686.61548
NCKP1_HUMAN	11377.7471
CNPY2_HUMAN	6016.76318
EXOG_HUMAN	869.349915

EXC6B_HUMAN	3342.9563
PLAK2_HUMAN	16827.1445
CARD8_HUMAN	1864.5697
AT11B_HUMAN	234.823349
OFUT2_HUMAN	7187.77588
DJC16_HUMAN	528.180237
DLGP4_HUMAN	2587.05176
ST38L_HUMAN	2039.99048
SAC2_HUMAN	2377.45752
FND3A_HUMAN	4271.5166
NISCH_HUMAN	1697.91418
FYV1_HUMAN	939.846313
WDR37_HUMAN	1990.79736
E41L3_HUMAN	21632.7031
AMOL2_HUMAN	411.395752
SIK3_HUMAN	285.24472
KDM2A_HUMAN	1390.20508
RRP44_HUMAN	6614.17041
TPPC8_HUMAN	1951.12952
LRCH1_HUMAN	4915.64404
RCL1_HUMAN	4923.9668
GSTK1_HUMAN	8318.8125
LTOR2_HUMAN	1874.62891
RT28_HUMAN	10951.5859
COA3_HUMAN	4230.90479
DDX52_HUMAN	2578.27148
RT17_HUMAN	5769.21875
RT07_HUMAN	8993.44922
CRYL1_HUMAN	3149.65576
TMA7_HUMAN	6247.32813
PDIP2_HUMAN	9525.13867
AP3M1_HUMAN	14347.6162
MAN1_HUMAN	4416.30469
CHSP1_HUMAN	1428.4187
COG6_HUMAN	2107.59399
TR150_HUMAN	16727.9922
WBP11_HUMAN	4348.26807
TDRKH_HUMAN	538.772949
MED16_HUMAN	1648.07788
NOP58_HUMAN	14170.5479
GIT1_HUMAN	5828.23389
ZN281_HUMAN	262.485962
AR2BP_HUMAN	3190.92114
SGT1_HUMAN	26574.1113
SYYM_HUMAN	7299.75293
NAGA_HUMAN	3346.8855
ACOT9_HUMAN	24822.2813
AAR2_HUMAN	943.983704
NOSIP_HUMAN	5221.18115
DEOC_HUMAN	4681.32617

MEMO1_HUMAN	5468.16113
TMX2_HUMAN	5459.81885
FCF1_HUMAN	1860.10254
LSM2_HUMAN	4723.79785
SHLB1_HUMAN	10488.5371
CIA30_HUMAN	1564.40271
CAB39_HUMAN	13361.7549
LC7L2_HUMAN	12366.4434
UB2J1_HUMAN	6183.21143
RBMX2_HUMAN	519.383972
DHRS7_HUMAN	5188.78027
RT02_HUMAN	11386.791
UTP11_HUMAN	628.872925
PHOCN_HUMAN	10045.5947
RRP7A_HUMAN	1660.62305
SBDS_HUMAN	16911.2637
TMED5_HUMAN	8888.86133
EXOS1_HUMAN	3432.42432
TMED7_HUMAN	4942.86719
SF3B6_HUMAN	11974.0664
RM11_HUMAN	11571.4707
ORN_HUMAN	19219.6992
RRP15_HUMAN	4801.44043
WASC3_HUMAN	1549.31042
NOP16_HUMAN	6609.95605
TPRKB_HUMAN	3955.14966
PPIL1_HUMAN	3439.24023
UFC1_HUMAN	4648.32031
CIA2B_HUMAN	631.828308
RT16_HUMAN	4480.12354
RT18C_HUMAN	1196.73877
FIS1_HUMAN	1245.17444
TIM16_HUMAN	2680.96362
KAD6_HUMAN	4288.9668
RT23_HUMAN	12281.0557
GOT1B_HUMAN	3072.00854
HDGR3_HUMAN	3263.66895
PTH2_HUMAN	7923.42725
CHMP3_HUMAN	48404.9023
STRAP_HUMAN	19830.7051
RTCB_HUMAN	46434.9883
FBX7_HUMAN	3510.32495
RAP2C_HUMAN	1524.12488
STA13_HUMAN	3302.6665
RBGP1_HUMAN	1693.39355
TMED3_HUMAN	962.51001
T22D4_HUMAN	1233.71692
DOP2_HUMAN	406.340149
ZN330_HUMAN	648.907227
R3HC1_HUMAN	1136.43738

NOC2L_HUMAN	8139.57617
RL36_HUMAN	62130.1523
CCDC9_HUMAN	2093.146
CHTOP_HUMAN	2139.52905
SAMH1_HUMAN	5625.5498
SKAP_HUMAN	1222.37573
HBS1L_HUMAN	4851.18457
AAKB1_HUMAN	990.904785
WIPI4_HUMAN	413.984741
DMXL1_HUMAN	519.239502
VPP2_HUMAN	3070.2522
TLN1_HUMAN	94413.5078
KIF3A_HUMAN	2094.823
TRRAP_HUMAN	2412.19043
MTCL1_HUMAN	673.308777
DCAF1_HUMAN	772.363098
TCAF1_HUMAN	7550.49707
RBM19_HUMAN	1011.9599
DAAM1_HUMAN	481.070648
PLXD1_HUMAN	17501.8262
HECD4_HUMAN	609.124451
WAC2C_HUMAN	433.843903
WDR7_HUMAN	402.022461
UBP15_HUMAN	6991.53174
FARP1_HUMAN	1771.47803
MARF1_HUMAN	702.281372
C170B_HUMAN	830.491455
RPGF2_HUMAN	8617.625
MYO5A_HUMAN	5521.41016
DTNA_HUMAN	760.243164
LOXL2_HUMAN	5818.44385
CRBG1_HUMAN	973.818787
TRAF6_HUMAN	2410.93677
M4K5_HUMAN	3636.75
HYOU1_HUMAN	28458.0195
ATG4B_HUMAN	7164.65186
TBL2_HUMAN	4669.62549
WIPI2_HUMAN	2666.78369
TELO2_HUMAN	2680.41846
LAS1L_HUMAN	6358.91895
AFG32_HUMAN	8938.68457
KLF12_HUMAN	203.348587
ARI1_HUMAN	5601.75635
LSM4_HUMAN	24281.1152
RN114_HUMAN	3120.70874
SAM50_HUMAN	15440.2793
PRC2C_HUMAN	19986.2148
OARD1_HUMAN	7410.20557
IFT25_HUMAN	1120.10608
PPME1_HUMAN	10586.748

RBM7_HUMAN	1931.84546
TIM22_HUMAN	1675.97632
NUB1_HUMAN	1824.53662
YTHD2_HUMAN	3409.88306
CTDP1_HUMAN	3262.00146
PAXB1_HUMAN	1184.77454
NDK7_HUMAN	2654.33569
SP16H_HUMAN	14579.0664
UTP18_HUMAN	3337.85034
UCHL5_HUMAN	17356.9941
CD2AP_HUMAN	11679.9424
VATD_HUMAN	6566.19189
TNPO3_HUMAN	5991.00244
SRPRB_HUMAN	12338.7705
ORC6_HUMAN	796.265137
CERT_HUMAN	1358.49805
GMPPB_HUMAN	15898.2236
FADS3_HUMAN	965.951355
TF3C5_HUMAN	6280.29639
TF3C3_HUMAN	3532.97729
TPPC1_HUMAN	4585.87451
TRPV2_HUMAN	669.245605
MRCKB_HUMAN	10017.8174
RBM8A_HUMAN	16452.9492
DJC15_HUMAN	1471.49878
UBP16_HUMAN	1303.59644
TSSC4_HUMAN	1653.91492
ZN706_HUMAN	4386.28564
MAGD1_HUMAN	3292.52588
SNX14_HUMAN	535.66449
SNX11_HUMAN	923.033691
SNX9_HUMAN	11038.5674
SNX8_HUMAN	1301.44446
SNX5_HUMAN	5969.13184
LIPG_HUMAN	1952.98242
NUBP2_HUMAN	6097.33984
PEX16_HUMAN	651.89856
LYVE1_HUMAN	2459.71118
BACE2_HUMAN	1563.5354
HEBP2_HUMAN	402.347412
HCFC2_HUMAN	744.662537
UBIA1_HUMAN	667.094299
PUS1_HUMAN	4470.08447
LRRF2_HUMAN	4321.73779
FHOD1_HUMAN	7583.75488
SERC_HUMAN	20148.3633
NCOR2_HUMAN	2310.41797
ORNT1_HUMAN	980.877319
JAM1_HUMAN	15254.3564
NPTN_HUMAN	1284.5896

AGRG1_HUMAN	981.716614
SPIN1_HUMAN	2621.58154
S12A7_HUMAN	10328.4766
ALG6_HUMAN	602.428711
ALG5_HUMAN	8442.58203
RT18B_HUMAN	4445.31445
COPG1_HUMAN	30518.0195
AUP1_HUMAN	3745.19434
FKBP7_HUMAN	1467.96594
CLIC4_HUMAN	47877.418
NFS1_HUMAN	4398.29248
CFA20_HUMAN	9238.77734
TACC3_HUMAN	587.827637
SPCS1_HUMAN	6592.52393
SAR1B_HUMAN	5637.8999
AP4B1_HUMAN	935.317993
MTCH2_HUMAN	22908.3516
SELK_HUMAN	1634.54297
BIG2_HUMAN	3547.05542
BIG1_HUMAN	4105.73096
MD1L1_HUMAN	3192.34692
STK24_HUMAN	14624.876
5MP1_HUMAN	41689.8242
COMDA_HUMAN	2341.54834
DC1L1_HUMAN	19950.4805
EPN1_HUMAN	3342.10718
UBP3_HUMAN	16523.6973
TX264_HUMAN	5551.05762
CABIN_HUMAN	165.599792
CEPT1_HUMAN	7859.40234
DNM3A_HUMAN	630.40863
OAS3_HUMAN	1516.07568
KAD5_HUMAN	14809.7266
NEMO_HUMAN	3656.20483
IF2B2_HUMAN	23434.0762
ZNT1_HUMAN	3250.5979
S4A7_HUMAN	2409.21851
NDUB9_HUMAN	13302.4219
SQOR_HUMAN	2316.01514
ROBO1_HUMAN	367.441376
NCOA3_HUMAN	558.79541
NUMBL_HUMAN	2009.84851
M3K4_HUMAN	1043.93237
DDX49_HUMAN	1718.20459
CAN7_HUMAN	1107.68518
WASF2_HUMAN	10113.5674
PIAS3_HUMAN	282.890747
SCC4_HUMAN	1509.19482
MORC2_HUMAN	1270.67065
NS1BP_HUMAN	1618.41516

S23IP_HUMAN	16904.7715
