# Supplementary material for: Proteomic Analysis of Endothelial Activation Induced by Adult Angiostrongylus vasorum Homogenate: Insights into Vascular Remodeling and Hemostatic Imbalance
Source: Animals (Basel). 2026 Mar 15;16(6):926. doi: 10.3390/ani16060926 (PMC13023303; doi:10.3390/ani16060926)
Supplement: Supplementary file 1 [file animals-16-00926-s001.zip › Supplmentary Table S7.pdf]

Supplementary Table S7: Dysregulated proteins in cell supernatants and lysates.

Upregulated Proteins in Cell Supernatants	Upregulated Proteins in Cell Lysates
TIE1	PTMA
H1-10	H1-10
GSN	GSN
FABP4	FABP4
TFPI	TFPI
CLU	CLU
MDK	MDK
ACE	ACE
GASK1B	GASK1B
VWF	VWF
BGN	BGN
ALDH1A1	ALDH1A1
NID1	NID1
MMRN1	MMRN1
HSPG2	HSPG2
H3C1	H3C1
PXDN	PXDN
ANGPT2	ANGPT2
HEXA	HEXA
FLT1	FLT1
FBN1	FBN1
H2AC4	H2AC4
S100A9	LRRC32
S100A8	ANGPTL2
IQGAP1	DGLUCY
WARS1	RIMOC1
H1-3	TMCC3
YWHAG	B2M
SPARC	BICD1
RNH1	ST3GAL1
SPTBN1	LAMA4
H1-5	CARD10
PNP	ADGRB3
EZR	TKFC
YWHAQ	CAVIN2
PRCP	HLA-B
IDH2	LAMC1
TIMP1	GGT5
NME2	PALD1
IFI16	DPYD
OTUB1	RNGTT
TPI1	NFIB
UBA1	LYVE1
VARS1	GIMAP7
TALDO1	GIPC2
RDX	TMEM167A
PGAM1	THG1L
GSTO1	CNPY2

KPNB1	CRYBG1
CFL1	BACE2
ACTN4	FCGRT
API5	CLDN5
AK1	FTL
MSN	APOL3
RPS10	COMMD3
CCN1	MED30
CAVIN1	IFI35
VCP	DAGLB
NPEPPS	MRPL10
H2BC11	PLSCR4
COL4A2	USP40
GSTP1	CNOT11
HTRA1	FIS1
RPS20	PCYOX1L
PRKDC	SIGLEC1
CLIC4	LDLRAP1
SRP9	HEBP2
XPO1	KLHL11
HMGB1	ERCC1
SERPINB8	BST2
PLOD1	GIMAP1
PPP2R1A	CAMKK2
KCTD12	COL8A1
VPS35	AP1G2
CCT4	CTSC
FSCN1	MON1A
DHX9	EPN3
APEX1	NXN
ILF3	BCAM
ALCAM	STX17
SERPINB6	SIAE
FABP5	CD34
MDH1	DOP1B
HMGB2	ARL8B
RAC1	ITGA10
AP2B1	THBS1
PGK1	GIMAP8
CORO1B	LPGAT1
ACO1	ALKBH5
XRCC6	HSD17B2
CAND1	FAM124B
PRDX1	B4GALT5
BCAT1	ARHGEF17
CTSZ	AGA
WDR1	ANKS1A
DDB1	NQO1
GOT2	SELENBP1
MDH2	FIG4

FASN	FLT4
DIPK2B	PLCB1
TCN2	RPS6KA1
EIF3E	WDR48
CD93	SHFL
RPL10	FN3K
CYCS	KANK3
LTA4H	CARHSP1
C1QBP	ANKRD40
PSMA5	GBP2
PSME2	CYTH2
NAMPT	CD40
PRDX2	EIF1B
PGM1	ARVCF
MAPK1	ARSA
DDX5	CASP1
ANXA3	CDH13
LMNB1	HID1
LOX	NUDT16
ARRB1	STX6
EHD2	MTUS1
SUB1	TAL1
COLGALT1	MAP4K2
NME1	NSMCE4A
TPP1	STON1
SPTAN1	POGLUT3
FH	MYL9
TXNL1	CYP2S1
RHOC	DOK1
ECHS1	COL12A1
EHD1	GIMAP4
TUFM	TOR2A
H2AC21	GMFG
STIP1	THEMIS2
ARPC1B	LXN
CD59	GGH
PSME1	FAM210A
FDPS	GALM
CLIC1	SLC44A2
NUCKS1	ATXN7L3B
RPL18A	NUCB2
HSPE1	CD63
SRSF1	PPOX
NAGK	LDB2
MATR3	QNG1
TIMP2	CLTB
RAB11A	LIPA
COTL1	HLA-E
ARHGDIB	MPI
TIGAR	ISOC2

PROCR	GPX7
PRMT1	SMARCD3
PRDX6	PSMB9
U2AF2	ITFG1
LARS1	RPA3
NNMT	CCDC97
LAP3	TMEM88
CLSTN1	CYP27A1
BMP6	WDR19
MAGOH	PRXL2B
RPL38	PICK1
BUB3	MTMR14
NDUFS3	NDUFV2
RNASE1	DIABLO
TNPO1	EHHADH
PSMB6	ZNF281
ACAT2	GUSB
ARF4	SUMF2
SNRNP70	ERGIC2
ACTR2	SCRN3
PARVA	ENTPD1
STMN1	ALG6
IGFBP4	MAPK7
SNX1	ACSF2
SNRPA	CBR4
PCNA	MTHFR
RARS1	DCHS1
IPO5	MAPK13
CDC42	RIC8B
IMPDH2	NTHL1
HNRNPAB	BCL7C
PSMD12	SOD2
UQCRC1	NTN4
RPL36	TMX4
ARPC4	DPP7
TRAP1	TNFRSF10C
ARPC3	GAA
CAPN1	NIBAN1
DYSF	SCARA3
LIMS1	TECPR2
AEBP1	STYX
PFKL	OAS3
NUCB1	PARP9
PDCD6IP	SRPX
RBMX	BABAM2
	CIAO3
	TUBB
	MGP
	FTH1
	STX8

	TBC1D9
	NDUFA12
	NPM3
	AP3M2
	MMTAG2
	FLI1
	GRK6
	RPS6KA5
	NAGLU
	GINS4
	HIRIP3
	SULT1A1
	MYZAP
	AKTIP
	NIF3L1
	UBXN4
	ARL5B
	PRXL2A
	MAPK12
	PLCG2
	ALDH6A1
	MED16
	MGST2
	NDUFA2
	ZBED1
	KLF12
	DMXL1
	MRTFB
	TTC21B
	PI4K2B
	UBA7
	TBP
	GIMAP5
	MON1B
	TXNIP
	LSM7
	DHODH
	PAFAH1B2
	PLEKHF2
	ZEB2
	DHX35
	ATF6B
	BAG1
	PREX2
	ACADS
	ELK3
	FYN
	LRRC20
	MRPL12
	RGL2

	ATP5IF1
	GGA3
	CAND2
	DDX60L
	ZCCHC3
	RPP14
	KCTD15
	C12orf4
	SLC27A1
	ZFYVE19
	PSRC1
	PRMT9
	FBRS
	HSPA2
	PRKD1
	MFNG
	SLC27A3
	PIK3C2B
	LGMN
	FES
	AGTRAP
	MTHFS
	PHPT1
	GLA
	TLE5
	MPDU1
	PEF1
	DACH1
	PEX3
	NUP42
	L2HGDH
	KIAA0930
	S1PR3
	PPP2R5A
	ELAPOR2
	PIGK
	SCRN2
	NDUFAF3
	RHBDD2
	SLIT2
	DDX60
	PLS1
	SELENOK
	DENND5A
	IGHMBP2
	COX5A
	TMEM223
	TMEM256
	SOX18
	HMGN3

	PBX2
	MRPL49
	MRPL23
	KDM6A
	CRACD
	N4BP3
	DENND11
	YTHDF1
	IL33
	APBA3
	TTF2
	TGFBRAP1
	RHBDF1
	MBD3
	KCTD9
	EIF4EBP1
	TRMT112
	PPP1R16B
	DGCR8
	SEPSECS
	MACROH2A1
	IQSEC1
	XPA
	CYB5B
	TRPV2
	LSM8
	H2AC11
	H4C1
	LENG8
	ZZZ3
	PGS1
	LSM3
	TSPAN4
	CASTOR2
	FRY
	H2BC12
	TTL
	PHF2
	CRELD1
	TRPT1
	SGTB
	MAP4K3
	RPL37
	IBTK
	H2AZ1
	RERE
	ANKRD54
	TUBB4A
	FITM2
	H2BC26

	IFT57
	GPR180
	UPRT
	NLRX1
	FBH1
	TAPBPL
	GEMIN6
	ACVR1
	TOP2B
	MYO9A

Downregulated Proteins in Cell Supernatants	Downregulated Proteins in Cell Lysates
PTMA	TFRC
APOB	APOB
SERPINE1	SERPINE1
C3	SERPINB2
CACNA2D1	CEP85
ADAMTS13	ICAM1
COL1A1	EPHA2
MAP4	ST6GALNAC4
F13A1	KCTD5
MYO1C	NSA2
DCD	HMOX1
RNASE4	MT2A
COL1A2	REL
AHNAK	AKAP12
PHB1	FOXC2
CAD	MMP1
GSPT1	IREB2
SRGN	PPP2CA
PTPA	GTPBP4
VIM	GDF15
	SLC4A2
	UTP11
	S100A9
	S100A8
	WDR43
	CD81
	TAPT1
	SLC6A6
	CIC
	CENPE
	NIFK
	TTK
	DCAF13
	POLR1G
	LPXN
	RND3
	SPTY2D1
	CD44
	RPF1
	NDRG4
	PCMTD1
	SP100
	SLC38A1
	SIRT6
	ZBTB7A
	PTCD1
	UTP18
	KRT78

	NOL11
	NOC4L
	GDE1
	TNFSF4
	CCPG1
	VPS72
	TACC3
	RCCD1
	WDR36
	WDR12
	ZFYVE21
	GALT
	PPAN
	ORC6
	RRP15
	SPHK1
	WDR75
	RSL24D1
	NGLY1
	RPL7L1
	PIBF1
	PTX3
	PDCD11
	CCND3
	CENPF
	WDR46
	SPEN
	DNTTIP2
	WDR3
	SLC38A2
	BRIX1
	MKI67
	SMC6
	WDR74
	SLC25A17
	CASP2
	BRD7
	BAZ2B
	ARL6IP1
	UTP15
	RSL1D1
	DDX27
	ZBTB11
	ZNF185
	FOSL1
	DDX31
	PTP4A1
	PIP4P1
	CCDC43
	TENM3

	WDR7
	COL6A1
	BOP1
	LYZ
	CDSN
	ABCB6
	NIP7
	RCL1
	RPS19BP1
	SLC5A6
	COL6A3
	SRSF9
	GPRC5A
	HEATR1
	LACRT
	INCENP
	TP53
	UBIAD1
	DDX21
	UBE2S
	NOL10
	S100A7
	NOP58
	GPAT3
	GNAI3
	DDX18
	CASP14
	HECTD4
	CALML5
	GPC1
	ZNF24
	PRPF18
	FCF1
	PTS
	NOP2
	TRAF6
	PLIN4
	DCBLD2
	PDLIM2
	PKP1
	RIDA
	NOLC1
	STEEP1
	SLC7A6OS
	CCNA2
	FRA10AC1
	BRD1
	DTNA
	ATP13A3
	HBA1

	ANKRD52
	ABCB8
	HRH1
	AATF
	GNA12
	PWP2
	LGALS7
	COA7
	EBNA1BP2
	DCAF6
	HBB
	PLAUR
	BRD8
	CDKN1A
	CBX5
	MTERF3
	WDR45
	AHSG
	PLP2
	UGCG
	TFPI2
	BAZ2A
	PUM3
	NHSL1
	PEDS1
	MTG2
	DLG5
	DOCK10
	MCL1
	DCBLD1
	TGM3
	DNAJC30
	COL13A1
	KPRP
	GLIPR1
	PTGS1
	MAPKAP1
	DSG1
	PLAU
	SLC7A11
	DLC1
	VEZF1
	NOP14
	CHM
	EMG1
	SAMSN1
	POLRMT
	DVL1
	EXTL2
	DSP

	NOP56
	CLN6
	SUMO2
	NEDD9
	DDX49
	NOL7
	NAV3
	CMC2

