# Supplementary figures and images for: Proteomic Analysis of Endothelial Activation Induced by Adult Angiostrongylus vasorum Homogenate: Insights into Vascular Remodeling and Hemostatic Imbalance
Source: Animals (Basel). 2026 Mar 15;16(6):926. doi: 10.3390/ani16060926 (PMC13023303; doi:10.3390/ani16060926)

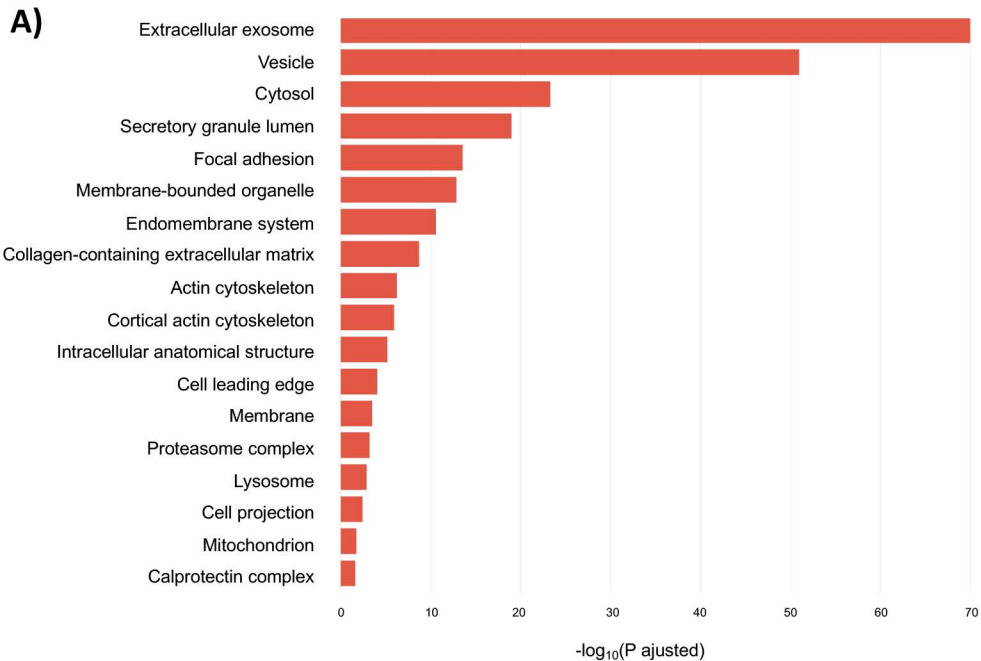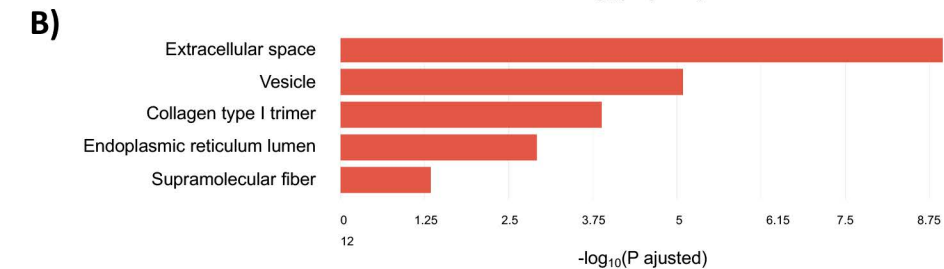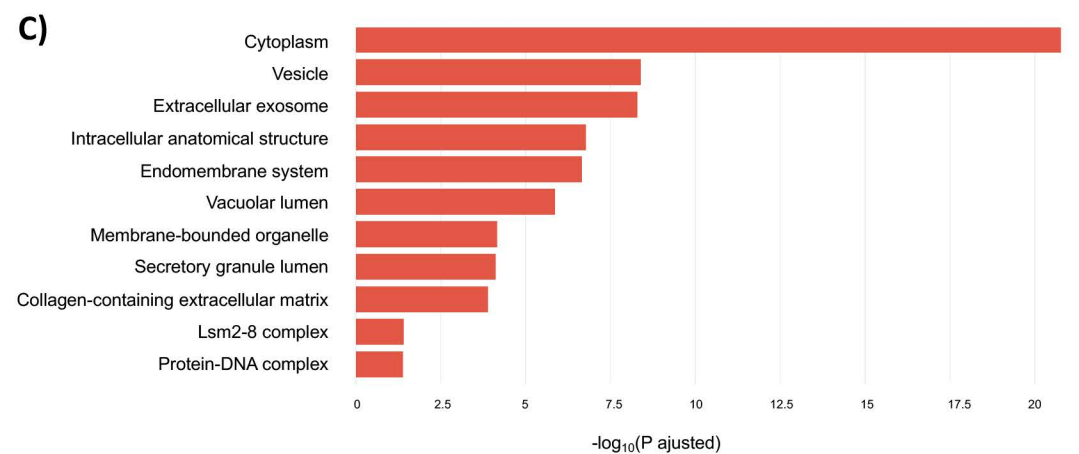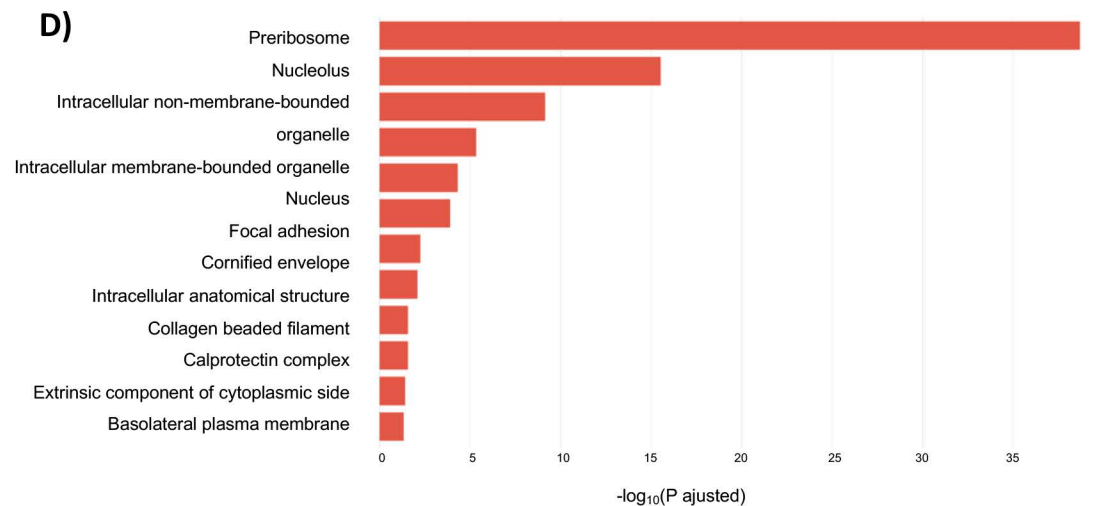

Supplement: Supplementary file 1 [file animals-16-00926-s001.zip › Supplmentary Figure S2.pdf]
